# Supplementary material for: Structural assessment of the full-length wild-type tumor suppressor protein p53 by mass spectrometry-guided computational modeling
Source: Sci Rep. 2023 May 25;13:8497. doi: 10.1038/s41598-023-35437-5 (PMC10213020; doi:10.1038/s41598-023-35437-5)
Supplement: Supplementary file 2 — Supplementary Table 1. [file 41598_2023_35437_MOESM2_ESM.pdf]

TableS1

ModelID	RefinementClass	Chains	Residue1	Residue2	Cross-Linker	Distance
2	template	AA	320	357	BS2G	27.94
2	template	DD	320	373	BS2G	33.81
2	template	DD	373	382	BS2G	26.58
2	template	DD	370	386	BS2G	39.57
2	template	DD	373	386	BS2G	37.35
2	template	AA	319	357	BS2G	28.49
2	template	AA	367	373	BS2G	16.24
2	template	DD	370	382	BS2G	31.42
2	template	CC	376	382	BS2G	16.89
2	template	DD	357	373	BS2G	35.47
2	template	AA	320	366	BS2G	36.84
2	template	AA	370	376	BS2G	18.15
2	template	AA	320	357	DBSU	27.94
2	template	AA	357	319	DBSU	28.49
2	template	DD	372	319	DBSU	34.45
2	template	DD	370	357	DBSU	30.65
2	template	DD	382	357	DBSU	54.06
2	template	AA	372	370	DBSU	6.48
2	template	DD	381	370	DBSU	29.74
2	template	AA	373	370	DBSU	8.98
2	template	DD	381	372	DBSU	26.23
2	template	DD	372	382	DBSU	28.52
2	template	DD	372	386	DBSU	38.42
2	template	DD	373	386	DBSU	37.35
2	template	CB	381	386	DBSU	76.94
2	template	BD;DB	357	357	DBSU	18.17
2	template	CB	373	319	DBSU	22.02
2	template	CB	372	357	DBSU	44.34
2	template	CB	381	357	DBSU	64.89
2	template	CB	373	357	DBSU	44.91
2	template	DA	372	370	DBSU	45.17
2	template	DA	373	370	DBSU	46.66

**2**	template	AD	382	373	DBSU	72.66
**3986**	allXL	AA	320	357	BS2G	24.2
**3986**	allXL	BB	320	373	BS2G	33.45
**3986**	allXL	DD	373	382	BS2G	17.05
**3986**	allXL	DD	370	386	BS2G	20.74
**3986**	allXL	DD	373	386	BS2G	23.09
**3986**	allXL	AA	319	357	BS2G	24.6
**3986**	allXL	AA	367	373	BS2G	13.66
**3986**	allXL	DD	370	382	BS2G	18.51
**3986**	allXL	CC	376	382	BS2G	13.8
**3986**	allXL	BB	357	373	BS2G	25.34
**3986**	allXL	AA	320	366	BS2G	25.06
**3986**	allXL	CC	370	376	BS2G	15.89
**3986**	allXL	AA	320	357	DBSU	24.2
**3986**	allXL	AA	357	319	DBSU	24.6
**3986**	allXL	BB	372	319	DBSU	37.64
**3986**	allXL	BB	370	357	DBSU	28.33
**3986**	allXL	DD	382	357	DBSU	33.49
**3986**	allXL	AA	372	370	DBSU	6.32
**3986**	allXL	DD	381	370	DBSU	20.52
**3986**	allXL	CC	373	370	DBSU	8.94
**3986**	allXL	DD	381	372	DBSU	20.36
**3986**	allXL	DD	372	382	DBSU	19.67
**3986**	allXL	DD	372	386	DBSU	24.46
**3986**	allXL	DD	373	386	DBSU	23.09
**3986**	allXL	BC	381	386	DBSU	37.1
**3986**	allXL	BD;DB	357	357	DBSU	18.54
**3986**	allXL	AD	373	319	DBSU	25.35
**3986**	allXL	BD	372	357	DBSU	37.07
**3986**	allXL	AC	381	357	DBSU	43.92
**3986**	allXL	BD	373	357	DBSU	37.12
**3986**	allXL	BC	372	370	DBSU	52.53
**3986**	allXL	BC	373	370	DBSU	52.25
**3986**	allXL	DB	382	373	DBSU	49.24

**3987**	allXL	CC	320	357	BS2G	18.46
**3987**	allXL	BB	320	373	BS2G	25.62
**3987**	allXL	CC	373	382	BS2G	18.11
**3987**	allXL	AA	370	386	BS2G	17.63
**3987**	allXL	AA	373	386	BS2G	20.05
**3987**	allXL	CC	319	357	BS2G	18.92
**3987**	allXL	AA	367	373	BS2G	13.26
**3987**	allXL	CC	370	382	BS2G	17.77
**3987**	allXL	BB	376	382	BS2G	13.28
**3987**	allXL	DD	357	373	BS2G	23.21
**3987**	allXL	AA	320	366	BS2G	26.28
**3987**	allXL	DD	370	376	BS2G	15.85
**3987**	allXL	CC	320	357	DBSU	18.46
**3987**	allXL	CC	357	319	DBSU	18.92
**3987**	allXL	BB	372	319	DBSU	29.18
**3987**	allXL	DD	370	357	DBSU	24.85
**3987**	allXL	DD	382	357	DBSU	25.7
**3987**	allXL	AA	372	370	DBSU	6.29
**3987**	allXL	DD	381	370	DBSU	18.99
**3987**	allXL	AA	373	370	DBSU	9.1
**3987**	allXL	CC	381	372	DBSU	19.09
**3987**	allXL	CC	372	382	DBSU	17.55
**3987**	allXL	AA	372	386	DBSU	19.67
**3987**	allXL	AA	373	386	DBSU	20.05
**3987**	allXL	DB	381	386	DBSU	58.77
**3987**	allXL	BD;DB	357	357	DBSU	18.99
**3987**	allXL	DA	373	319	DBSU	31.18
**3987**	allXL	DB	372	357	DBSU	29.31
**3987**	allXL	DB	381	357	DBSU	21.24
**3987**	allXL	DB	373	357	DBSU	25.63
**3987**	allXL	DB	372	370	DBSU	52.59
**3987**	allXL	DB	373	370	DBSU	49.03
**3987**	allXL	DB	382	373	DBSU	42.94
**3988**	allXL	AA	320	357	BS2G	25.51

**3988**	allXL	BB	320	373	BS2G	16.7
**3988**	allXL	BB	373	382	BS2G	14.94
**3988**	allXL	CC	370	386	BS2G	24.53
**3988**	allXL	BB	373	386	BS2G	23.06
**3988**	allXL	AA	319	357	BS2G	23.98
**3988**	allXL	AA	367	373	BS2G	12.95
**3988**	allXL	CC	370	382	BS2G	20.7
**3988**	allXL	BB	376	382	BS2G	13.32
**3988**	allXL	DD	357	373	BS2G	27.94
**3988**	allXL	BB	320	366	BS2G	19.19
**3988**	allXL	CC	370	376	BS2G	15.52
**3988**	allXL	AA	320	357	DBSU	25.51
**3988**	allXL	AA	357	319	DBSU	23.98
**3988**	allXL	BB	372	319	DBSU	17.51
**3988**	allXL	BB	370	357	DBSU	28.37
**3988**	allXL	BB	382	357	DBSU	26.74
**3988**	allXL	AA	372	370	DBSU	6.38
**3988**	allXL	CC	381	370	DBSU	22.44
**3988**	allXL	AA	373	370	DBSU	9.22
**3988**	allXL	BB	381	372	DBSU	19.01
**3988**	allXL	BB	372	382	DBSU	16.75
**3988**	allXL	BB	372	386	DBSU	23.05
**3988**	allXL	BB	373	386	DBSU	23.06
**3988**	allXL	BD	381	386	DBSU	38.9
**3988**	allXL	BD;DB	357	357	DBSU	19.0
**3988**	allXL	AD	373	319	DBSU	26.98
**3988**	allXL	DB	372	357	DBSU	39.49
**3988**	allXL	DB	381	357	DBSU	35.97
**3988**	allXL	DB	373	357	DBSU	36.65
**3988**	allXL	CB	372	370	DBSU	36.98
**3988**	allXL	DA	373	370	DBSU	37.54
**3988**	allXL	DB	382	373	DBSU	37.78
**3989**	allXL	AA	320	357	BS2G	23.08
**3989**	allXL	BB	320	373	BS2G	25.72

**3989**	allXL	CC	373	382	BS2G	19.76
**3989**	allXL	AA	370	386	BS2G	23.71
**3989**	allXL	CC	373	386	BS2G	24.69
**3989**	allXL	AA	319	357	BS2G	23.75
**3989**	allXL	AA	367	373	BS2G	13.11
**3989**	allXL	CC	370	382	BS2G	19.59
**3989**	allXL	CC	376	382	BS2G	13.95
**3989**	allXL	BB	357	373	BS2G	28.72
**3989**	allXL	AA	320	366	BS2G	26.34
**3989**	allXL	CC	370	376	BS2G	15.12
**3989**	allXL	AA	320	357	DBSU	23.08
**3989**	allXL	AA	357	319	DBSU	23.75
**3989**	allXL	BB	372	319	DBSU	29.69
**3989**	allXL	CC	370	357	DBSU	26.13
**3989**	allXL	BB	382	357	DBSU	20.7
**3989**	allXL	AA	372	370	DBSU	6.44
**3989**	allXL	CC	381	370	DBSU	19.06
**3989**	allXL	BB	373	370	DBSU	9.15
**3989**	allXL	DD	381	372	DBSU	19.53
**3989**	allXL	CC	372	382	DBSU	19.56
**3989**	allXL	CC	372	386	DBSU	23.31
**3989**	allXL	CC	373	386	DBSU	24.69
**3989**	allXL	AD	381	386	DBSU	37.7
**3989**	allXL	BD;DB	357	357	DBSU	17.87
**3989**	allXL	AD	373	319	DBSU	13.1
**3989**	allXL	CA	372	357	DBSU	32.83
**3989**	allXL	BD	381	357	DBSU	35.09
**3989**	allXL	CA	373	357	DBSU	31.34
**3989**	allXL	AD	372	370	DBSU	36.54
**3989**	allXL	AD	373	370	DBSU	33.92
**3989**	allXL	DA	382	373	DBSU	47.4
**3990**	allXL	CC	320	357	BS2G	21.97
**3990**	allXL	DD	320	373	BS2G	13.34
**3990**	allXL	DD	373	382	BS2G	17.99

**3990**	allXL	BB	370	386	BS2G	23.43
**3990**	allXL	BB	373	386	BS2G	25.92
**3990**	allXL	BB	319	357	BS2G	22.09
**3990**	allXL	BB	367	373	BS2G	11.56
**3990**	allXL	DD	370	382	BS2G	20.3
**3990**	allXL	CC	376	382	BS2G	13.83
**3990**	allXL	DD	357	373	BS2G	23.17
**3990**	allXL	AA	320	366	BS2G	23.94
**3990**	allXL	CC	370	376	BS2G	14.88
**3990**	allXL	CC	320	357	DBSU	21.97
**3990**	allXL	BB	357	319	DBSU	22.09
**3990**	allXL	DD	372	319	DBSU	13.26
**3990**	allXL	DD	370	357	DBSU	19.91
**3990**	allXL	AA	382	357	DBSU	38.44
**3990**	allXL	AA	372	370	DBSU	6.1
**3990**	allXL	DD	381	370	DBSU	19.43
**3990**	allXL	AA	373	370	DBSU	9.1
**3990**	allXL	DD	381	372	DBSU	17.09
**3990**	allXL	DD	372	382	DBSU	19.07
**3990**	allXL	BB	372	386	DBSU	26.7
**3990**	allXL	BB	373	386	DBSU	25.92
**3990**	allXL	AD	381	386	DBSU	32.08
**3990**	allXL	BD;DB	357	357	DBSU	19.42
**3990**	allXL	AD	373	319	DBSU	40.46
**3990**	allXL	DB	372	357	DBSU	38.98
**3990**	allXL	BD	381	357	DBSU	51.34
**3990**	allXL	DB	373	357	DBSU	40.82
**3990**	allXL	DA	372	370	DBSU	49.95
**3990**	allXL	AD	373	370	DBSU	51.67
**3990**	allXL	AD	382	373	DBSU	43.36
**3991**	allXL	AA	320	357	BS2G	23.12
**3991**	allXL	CC	320	373	BS2G	18.61
**3991**	allXL	AA	373	382	BS2G	14.12
**3991**	allXL	AA	370	386	BS2G	14.0

**3991**	allXL	AA	373	386	BS2G	13.34
**3991**	allXL	AA	319	357	BS2G	21.37
**3991**	allXL	AA	367	373	BS2G	12.17
**3991**	allXL	CC	370	382	BS2G	18.4
**3991**	allXL	AA	376	382	BS2G	13.46
**3991**	allXL	BB	357	373	BS2G	11.16
**3991**	allXL	AA	320	366	BS2G	23.33
**3991**	allXL	CC	370	376	BS2G	13.78
**3991**	allXL	AA	320	357	DBSU	23.12
**3991**	allXL	AA	357	319	DBSU	21.37
**3991**	allXL	CC	372	319	DBSU	19.66
**3991**	allXL	BB	370	357	DBSU	20.82
**3991**	allXL	BB	382	357	DBSU	22.1
**3991**	allXL	AA	372	370	DBSU	6.45
**3991**	allXL	CC	381	370	DBSU	17.26
**3991**	allXL	DD	373	370	DBSU	8.87
**3991**	allXL	DD	381	372	DBSU	17.22
**3991**	allXL	DD	372	382	DBSU	15.52
**3991**	allXL	AA	372	386	DBSU	14.72
**3991**	allXL	AA	373	386	DBSU	13.34
**3991**	allXL	BC	381	386	DBSU	39.49
**3991**	allXL	BD;DB	357	357	DBSU	19.13
**3991**	allXL	CB	373	319	DBSU	34.58
**3991**	allXL	BD	372	357	DBSU	31.41
**3991**	allXL	BD	381	357	DBSU	29.69
**3991**	allXL	BD	373	357	DBSU	27.96
**3991**	allXL	CB	372	370	DBSU	15.22
**3991**	allXL	CB	373	370	DBSU	18.72
**3991**	allXL	CB	382	373	DBSU	34.26
**3992**	allXL	AA	320	357	BS2G	23.75
**3992**	allXL	BB	320	373	BS2G	27.2
**3992**	allXL	CC	373	382	BS2G	17.17
**3992**	allXL	DD	370	386	BS2G	21.45
**3992**	allXL	DD	373	386	BS2G	21.01

**3992**	allXL	AA	319	357	BS2G	21.57
**3992**	allXL	AA	367	373	BS2G	11.95
**3992**	allXL	CC	370	382	BS2G	22.74
**3992**	allXL	BB	376	382	BS2G	13.78
**3992**	allXL	BB	357	373	BS2G	15.37
**3992**	allXL	DD	320	366	BS2G	16.98
**3992**	allXL	AA	370	376	BS2G	17.62
**3992**	allXL	AA	320	357	DBSU	23.75
**3992**	allXL	AA	357	319	DBSU	21.57
**3992**	allXL	DD	372	319	DBSU	25.84
**3992**	allXL	BB	370	357	DBSU	24.53
**3992**	allXL	BB	382	357	DBSU	23.3
**3992**	allXL	AA	372	370	DBSU	6.31
**3992**	allXL	DD	381	370	DBSU	23.29
**3992**	allXL	DD	373	370	DBSU	8.9
**3992**	allXL	DD	381	372	DBSU	19.09
**3992**	allXL	CC	372	382	DBSU	17.6
**3992**	allXL	DD	372	386	DBSU	18.97
**3992**	allXL	DD	373	386	DBSU	21.01
**3992**	allXL	DB	381	386	DBSU	45.76
**3992**	allXL	BD;DB	357	357	DBSU	18.55
**3992**	allXL	DA	373	319	DBSU	27.27
**3992**	allXL	BD	372	357	DBSU	32.64
**3992**	allXL	BD	381	357	DBSU	20.73
**3992**	allXL	BD	373	357	DBSU	29.54
**3992**	allXL	BC	372	370	DBSU	48.88
**3992**	allXL	BC	373	370	DBSU	47.09
**3992**	allXL	BD	382	373	DBSU	30.89
**3993**	allXL	AA	320	357	BS2G	26.29
**3993**	allXL	BB	320	373	BS2G	35.01
**3993**	allXL	DD	373	382	BS2G	15.73
**3993**	allXL	BB	370	386	BS2G	20.31
**3993**	allXL	BB	373	386	BS2G	18.75
**3993**	allXL	AA	319	357	BS2G	28.58

**3993**	allXL	AA	367	373	BS2G	14.05
**3993**	allXL	BB	370	382	BS2G	20.35
**3993**	allXL	BB	376	382	BS2G	13.2
**3993**	allXL	AA	357	373	BS2G	34.65
**3993**	allXL	CC	320	366	BS2G	32.96
**3993**	allXL	BB	370	376	BS2G	16.23
**3993**	allXL	AA	320	357	DBSU	26.29
**3993**	allXL	AA	357	319	DBSU	28.58
**3993**	allXL	BB	372	319	DBSU	37.07
**3993**	allXL	DD	370	357	DBSU	29.35
**3993**	allXL	AA	382	357	DBSU	36.89
**3993**	allXL	AA	372	370	DBSU	6.23
**3993**	allXL	DD	381	370	DBSU	21.34
**3993**	allXL	CC	373	370	DBSU	9.17
**3993**	allXL	DD	381	372	DBSU	14.93
**3993**	allXL	DD	372	382	DBSU	14.88
**3993**	allXL	BB	372	386	DBSU	18.65
**3993**	allXL	BB	373	386	DBSU	18.75
**3993**	allXL	BC	381	386	DBSU	28.46
**3993**	allXL	BD;DB	357	357	DBSU	19.01
**3993**	allXL	AD	373	319	DBSU	30.02
**3993**	allXL	CB	372	357	DBSU	43.7
**3993**	allXL	AC	381	357	DBSU	53.63
**3993**	allXL	CB	373	357	DBSU	45.59
**3993**	allXL	CB	372	370	DBSU	22.14
**3993**	allXL	CB	373	370	DBSU	21.57
**3993**	allXL	BC	382	373	DBSU	18.12
**3994**	allXL	AA	320	357	BS2G	18.78
**3994**	allXL	CC	320	373	BS2G	18.51
**3994**	allXL	AA	373	382	BS2G	16.51
**3994**	allXL	AA	370	386	BS2G	15.21
**3994**	allXL	AA	373	386	BS2G	20.88
**3994**	allXL	AA	319	357	BS2G	17.84
**3994**	allXL	AA	367	373	BS2G	11.16

**3994**	allXL	AA	370	382	BS2G	16.42
**3994**	allXL	BB	376	382	BS2G	13.89
**3994**	allXL	DD	357	373	BS2G	31.61
**3994**	allXL	DD	320	366	BS2G	28.28
**3994**	allXL	DD	370	376	BS2G	15.65
**3994**	allXL	AA	320	357	DBSU	18.78
**3994**	allXL	AA	357	319	DBSU	17.84
**3994**	allXL	CC	372	319	DBSU	16.65
**3994**	allXL	DD	370	357	DBSU	22.1
**3994**	allXL	AA	382	357	DBSU	20.86
**3994**	allXL	AA	372	370	DBSU	6.57
**3994**	allXL	AA	381	370	DBSU	18.49
**3994**	allXL	AA	373	370	DBSU	8.78
**3994**	allXL	AA	381	372	DBSU	19.15
**3994**	allXL	AA	372	382	DBSU	17.85
**3994**	allXL	AA	372	386	DBSU	20.15
**3994**	allXL	AA	373	386	DBSU	20.88
**3994**	allXL	CA	381	386	DBSU	65.64
**3994**	allXL	BD;DB	357	357	DBSU	18.57
**3994**	allXL	AD	373	319	DBSU	22.99
**3994**	allXL	CB	372	357	DBSU	45.47
**3994**	allXL	AC	381	357	DBSU	28.5
**3994**	allXL	AC	373	357	DBSU	45.09
**3994**	allXL	CB	372	370	DBSU	42.81
**3994**	allXL	BC	373	370	DBSU	41.36
**3994**	allXL	BC	382	373	DBSU	53.56
**3995**	allXL	CC	320	357	BS2G	16.86
**3995**	allXL	DD	320	373	BS2G	21.95
**3995**	allXL	BB	373	382	BS2G	11.95
**3995**	allXL	BB	370	386	BS2G	16.81
**3995**	allXL	BB	373	386	BS2G	20.91
**3995**	allXL	CC	319	357	BS2G	16.35
**3995**	allXL	AA	367	373	BS2G	12.11
**3995**	allXL	BB	370	382	BS2G	14.37

**3995**	allXL	BB	376	382	BS2G	12.63
**3995**	allXL	DD	357	373	BS2G	11.12
**3995**	allXL	CC	320	366	BS2G	8.85
**3995**	allXL	DD	370	376	BS2G	14.48
**3995**	allXL	CC	320	357	DBSU	16.86
**3995**	allXL	CC	357	319	DBSU	16.35
**3995**	allXL	CC	372	319	DBSU	23.08
**3995**	allXL	DD	370	357	DBSU	11.19
**3995**	allXL	DD	382	357	DBSU	15.2
**3995**	allXL	AA	372	370	DBSU	6.27
**3995**	allXL	BB	381	370	DBSU	18.04
**3995**	allXL	AA	373	370	DBSU	8.93
**3995**	allXL	BB	381	372	DBSU	17.12
**3995**	allXL	BB	372	382	DBSU	14.38
**3995**	allXL	BB	372	386	DBSU	21.4
**3995**	allXL	BB	373	386	DBSU	20.91
**3995**	allXL	BD	381	386	DBSU	46.55
**3995**	allXL	BD;DB	357	357	DBSU	17.18
**3995**	allXL	AD	373	319	DBSU	41.22
**3995**	allXL	DB	372	357	DBSU	27.64
**3995**	allXL	DB	381	357	DBSU	17.84
**3995**	allXL	DB	373	357	DBSU	28.08
**3995**	allXL	BD	372	370	DBSU	43.71
**3995**	allXL	BD	373	370	DBSU	44.47
**3995**	allXL	DB	382	373	DBSU	37.76
**3996**	allXL	AA	320	357	BS2G	24.41
**3996**	allXL	CC	320	373	BS2G	25.95
**3996**	allXL	AA	373	382	BS2G	21.04
**3996**	allXL	AA	370	386	BS2G	23.61
**3996**	allXL	AA	373	386	BS2G	22.78
**3996**	allXL	AA	319	357	BS2G	23.81
**3996**	allXL	AA	367	373	BS2G	13.33
**3996**	allXL	BB	370	382	BS2G	25.7
**3996**	allXL	AA	376	382	BS2G	15.28

**3996**	allXL	BB	357	373	BS2G	28.48
**3996**	allXL	CC	320	366	BS2G	27.96
**3996**	allXL	CC	370	376	BS2G	15.9
**3996**	allXL	AA	320	357	DBSU	24.41
**3996**	allXL	AA	357	319	DBSU	23.81
**3996**	allXL	CC	372	319	DBSU	22.1
**3996**	allXL	DD	370	357	DBSU	28.6
**3996**	allXL	CD	382	357	DBSU	49.5
**3996**	allXL	AA	372	370	DBSU	6.35
**3996**	allXL	BB	381	370	DBSU	24.19
**3996**	allXL	AA	373	370	DBSU	9.37
**3996**	allXL	DD	381	372	DBSU	20.22
**3996**	allXL	DD	372	382	DBSU	22.2
**3996**	allXL	AA	372	386	DBSU	22.4
**3996**	allXL	AA	373	386	DBSU	22.78
**3996**	allXL	BC	381	386	DBSU	58.5
**3996**	allXL	BD;DB	357	357	DBSU	19.29
**3996**	allXL	CB	373	319	DBSU	19.29
**3996**	allXL	CB	372	357	DBSU	31.94
**3996**	allXL	CB	381	357	DBSU	31.03
**3996**	allXL	CB	373	357	DBSU	29.44
**3996**	allXL	CB	372	370	DBSU	40.77
**3996**	allXL	CB	373	370	DBSU	38.74
**3996**	allXL	CB	382	373	DBSU	42.46
**3997**	allXL	AA	320	357	BS2G	22.0
**3997**	allXL	CC	320	373	BS2G	23.78
**3997**	allXL	CC	373	382	BS2G	17.62
**3997**	allXL	DD	370	386	BS2G	11.49
**3997**	allXL	DD	373	386	BS2G	18.07
**3997**	allXL	AA	319	357	BS2G	21.06
**3997**	allXL	AA	367	373	BS2G	13.34
**3997**	allXL	DD	370	382	BS2G	17.81
**3997**	allXL	CC	376	382	BS2G	12.37
**3997**	allXL	CC	357	373	BS2G	26.53

**3997**	allXL	AA	320	366	BS2G	16.54
**3997**	allXL	BB	370	376	BS2G	15.93
**3997**	allXL	AA	320	357	DBSU	22.0
**3997**	allXL	AA	357	319	DBSU	21.06
**3997**	allXL	CC	372	319	DBSU	20.47
**3997**	allXL	DD	370	357	DBSU	26.86
**3997**	allXL	DD	382	357	DBSU	26.33
**3997**	allXL	AA	372	370	DBSU	6.95
**3997**	allXL	DD	381	370	DBSU	18.03
**3997**	allXL	AA;DD	373	370	DBSU	9.17
**3997**	allXL	DD	381	372	DBSU	20.03
**3997**	allXL	DD	372	382	DBSU	21.03
**3997**	allXL	DD	372	386	DBSU	18.01
**3997**	allXL	DD	373	386	DBSU	18.07
**3997**	allXL	AD	381	386	DBSU	49.27
**3997**	allXL	BD;DB	357	357	DBSU	18.3
**3997**	allXL	AD	373	319	DBSU	25.78
**3997**	allXL	DA	372	357	DBSU	37.11
**3997**	allXL	CA	381	357	DBSU	36.88
**3997**	allXL	DA	373	357	DBSU	35.81
**3997**	allXL	DA	372	370	DBSU	12.84
**3997**	allXL	DA	373	370	DBSU	15.2
**3997**	allXL	DA	382	373	DBSU	38.7
**3998**	allXL	AA	320	357	BS2G	24.13
**3998**	allXL	DD	320	373	BS2G	5.07
**3998**	allXL	AA	373	382	BS2G	18.38
**3998**	allXL	AA	370	386	BS2G	26.86
**3998**	allXL	AA	373	386	BS2G	26.46
**3998**	allXL	AA	319	357	BS2G	23.61
**3998**	allXL	AA	367	373	BS2G	13.81
**3998**	allXL	BB	370	382	BS2G	21.11
**3998**	allXL	BB	376	382	BS2G	13.93
**3998**	allXL	DD	357	373	BS2G	28.89
**3998**	allXL	DD	320	366	BS2G	20.42

**3998**	allXL	BB	370	376	BS2G	16.3
**3998**	allXL	AA	320	357	DBSU	24.13
**3998**	allXL	AA	357	319	DBSU	23.61
**3998**	allXL	DD	372	319	DBSU	7.68
**3998**	allXL	DD	370	357	DBSU	27.13
**3998**	allXL	AA	382	357	DBSU	46.33
**3998**	allXL	DD	372	370	DBSU	6.55
**3998**	allXL	BB	381	370	DBSU	22.78
**3998**	allXL	AA	373	370	DBSU	9.31
**3998**	allXL	CC	381	372	DBSU	21.87
**3998**	allXL	CC	372	382	DBSU	20.62
**3998**	allXL	CC	372	386	DBSU	25.57
**3998**	allXL	AA	373	386	DBSU	26.46
**3998**	allXL	AD	381	386	DBSU	39.88
**3998**	allXL	BD;DB	357	357	DBSU	18.75
**3998**	allXL	BC	373	319	DBSU	28.63
**3998**	allXL	BD	372	357	DBSU	34.65
**3998**	allXL	BD	381	357	DBSU	50.09
**3998**	allXL	BD	373	357	DBSU	34.69
**3998**	allXL	AD	372	370	DBSU	46.38
**3998**	allXL	AD	373	370	DBSU	46.18
**3998**	allXL	AD	382	373	DBSU	40.04
**3999**	allXL	AA	320	357	BS2G	22.99
**3999**	allXL	BB	320	373	BS2G	27.42
**3999**	allXL	BB	373	382	BS2G	19.0
**3999**	allXL	DD	370	386	BS2G	25.35
**3999**	allXL	DD	373	386	BS2G	25.03
**3999**	allXL	AA	319	357	BS2G	21.6
**3999**	allXL	AA	367	373	BS2G	12.82
**3999**	allXL	DD	370	382	BS2G	20.61
**3999**	allXL	BB	376	382	BS2G	14.73
**3999**	allXL	AA	357	373	BS2G	31.1
**3999**	allXL	CC	320	366	BS2G	21.58
**3999**	allXL	CC	370	376	BS2G	15.71

**3999**	allXL	AA	320	357	DBSU	22.99
**3999**	allXL	AA	357	319	DBSU	21.6
**3999**	allXL	BB	372	319	DBSU	28.67
**3999**	allXL	CC	370	357	DBSU	27.38
**3999**	allXL	AA	382	357	DBSU	45.85
**3999**	allXL	AA	372	370	DBSU	6.27
**3999**	allXL	DD	381	370	DBSU	20.15
**3999**	allXL	AA	373	370	DBSU	9.08
**3999**	allXL	DD	381	372	DBSU	19.67
**3999**	allXL	DD	372	382	DBSU	20.65
**3999**	allXL	DD	372	386	DBSU	24.52
**3999**	allXL	DD	373	386	DBSU	25.03
**3999**	allXL	DA	381	386	DBSU	23.19
**3999**	allXL	BD;DB	357	357	DBSU	19.32
**3999**	allXL	AD	373	319	DBSU	23.47
**3999**	allXL	BC	372	357	DBSU	40.44
**3999**	allXL	AD	381	357	DBSU	36.08
**3999**	allXL	BC	373	357	DBSU	38.44
**3999**	allXL	BC	372	370	DBSU	46.8
**3999**	allXL	BC	373	370	DBSU	43.34
**3999**	allXL	DA	382	373	DBSU	37.63
**4000**	allXL	CC	320	357	BS2G	23.62
**4000**	allXL	AA	320	373	BS2G	21.09
**4000**	allXL	DD	373	382	BS2G	18.88
**4000**	allXL	DD	370	386	BS2G	22.64
**4000**	allXL	DD	373	386	BS2G	23.73
**4000**	allXL	CC	319	357	BS2G	23.94
**4000**	allXL	AA	367	373	BS2G	10.79
**4000**	allXL	DD	370	382	BS2G	21.75
**4000**	allXL	CC	376	382	BS2G	14.56
**4000**	allXL	AA	357	373	BS2G	32.36
**4000**	allXL	AA	320	366	BS2G	16.56
**4000**	allXL	CC	370	376	BS2G	16.17
**4000**	allXL	CC	320	357	DBSU	23.62

**4000**	allXL	CC	357	319	DBSU	23.94
**4000**	allXL	AA	372	319	DBSU	19.94
**4000**	allXL	DD	370	357	DBSU	31.47
**4000**	allXL	AA	382	357	DBSU	39.29
**4000**	allXL	CC	372	370	DBSU	6.62
**4000**	allXL	DD	381	370	DBSU	20.28
**4000**	allXL	AA	373	370	DBSU	8.74
**4000**	allXL	DD	381	372	DBSU	17.06
**4000**	allXL	DD	372	382	DBSU	19.03
**4000**	allXL	DD	372	386	DBSU	22.06
**4000**	allXL	DD	373	386	DBSU	23.73
**4000**	allXL	AD	381	386	DBSU	48.99
**4000**	allXL	BD;DB	357	357	DBSU	18.42
**4000**	allXL	AD	373	319	DBSU	25.35
**4000**	allXL	AD	372	357	DBSU	43.2
**4000**	allXL	AD	381	357	DBSU	47.26
**4000**	allXL	AD	373	357	DBSU	43.9
**4000**	allXL	AD	372	370	DBSU	42.18
**4000**	allXL	DA	373	370	DBSU	40.91
**4000**	allXL	AD	382	373	DBSU	35.97
**4001**	allXL	AA	320	357	BS2G	11.02
**4001**	allXL	DD	320	373	BS2G	14.67
**4001**	allXL	CC	373	382	BS2G	20.0
**4001**	allXL	DD	370	386	BS2G	20.48
**4001**	allXL	DD	373	386	BS2G	23.27
**4001**	allXL	AA	319	357	BS2G	8.36
**4001**	allXL	AA	367	373	BS2G	12.04
**4001**	allXL	DD	370	382	BS2G	19.63
**4001**	allXL	CC	376	382	BS2G	14.86
**4001**	allXL	BB	357	373	BS2G	24.17
**4001**	allXL	DD	320	366	BS2G	23.58
**4001**	allXL	BB	370	376	BS2G	15.7
**4001**	allXL	AA	320	357	DBSU	11.02
**4001**	allXL	AA	357	319	DBSU	8.36

**4001**	allXL	DD	372	319	DBSU	14.5
**4001**	allXL	DD	370	357	DBSU	25.7
**4001**	allXL	BB	382	357	DBSU	25.44
**4001**	allXL	AA	372	370	DBSU	6.28
**4001**	allXL	DD	381	370	DBSU	20.12
**4001**	allXL	AA	373	370	DBSU	9.09
**4001**	allXL	CC	381	372	DBSU	20.08
**4001**	allXL	CC	372	382	DBSU	19.6
**4001**	allXL	DD	372	386	DBSU	22.22
**4001**	allXL	DD	373	386	DBSU	23.27
**4001**	allXL	BC	381	386	DBSU	30.67
**4001**	allXL	BD;DB	357	357	DBSU	18.3
**4001**	allXL	CB	373	319	DBSU	21.62
**4001**	allXL	BD	372	357	DBSU	39.32
**4001**	allXL	AC	381	357	DBSU	40.62
**4001**	allXL	AC	373	357	DBSU	37.08
**4001**	allXL	CB	372	370	DBSU	49.47
**4001**	allXL	CB	373	370	DBSU	47.54
**4001**	allXL	BC	382	373	DBSU	36.45
**4002**	allXL	CC	320	357	BS2G	26.61
**4002**	allXL	DD	320	373	BS2G	15.76
**4002**	allXL	BB	373	382	BS2G	19.32
**4002**	allXL	DD	370	386	BS2G	20.1
**4002**	allXL	AA	373	386	BS2G	23.3
**4002**	allXL	AA	319	357	BS2G	27.44
**4002**	allXL	AA	367	373	BS2G	14.68
**4002**	allXL	BB	370	382	BS2G	19.76
**4002**	allXL	CC	376	382	BS2G	14.12
**4002**	allXL	BB	357	373	BS2G	33.62
**4002**	allXL	DD	320	366	BS2G	19.82
**4002**	allXL	CC	370	376	BS2G	16.31
**4002**	allXL	CC	320	357	DBSU	26.61
**4002**	allXL	AA	357	319	DBSU	27.44
**4002**	allXL	DD	372	319	DBSU	17.58

**4002**	allXL	DD	370	357	DBSU	30.62
**4002**	allXL	BB	382	357	DBSU	35.53
**4002**	allXL	AA	372	370	DBSU	6.11
**4002**	allXL	DD	381	370	DBSU	19.97
**4002**	allXL	AA	373	370	DBSU	9.2
**4002**	allXL	DD	381	372	DBSU	19.11
**4002**	allXL	DD	372	382	DBSU	20.23
**4002**	allXL	DD	372	386	DBSU	22.58
**4002**	allXL	AA	373	386	DBSU	23.3
**4002**	allXL	DA	381	386	DBSU	29.93
**4002**	allXL	BD;DB	357	357	DBSU	17.93
**4002**	allXL	AD	373	319	DBSU	33.82
**4002**	allXL	BD	372	357	DBSU	48.9
**4002**	allXL	BD	381	357	DBSU	50.53
**4002**	allXL	BD	373	357	DBSU	51.1
**4002**	allXL	AD	372	370	DBSU	37.91
**4002**	allXL	DA	373	370	DBSU	35.63
**4002**	allXL	AD	382	373	DBSU	34.74
**4003**	allXL	AA	320	357	BS2G	24.18
**4003**	allXL	CC	320	373	BS2G	21.49
**4003**	allXL	BB	373	382	BS2G	17.41
**4003**	allXL	BB	370	386	BS2G	20.34
**4003**	allXL	DD	373	386	BS2G	20.44
**4003**	allXL	AA	319	357	BS2G	24.17
**4003**	allXL	DD	367	373	BS2G	13.13
**4003**	allXL	BB	370	382	BS2G	18.58
**4003**	allXL	CC	376	382	BS2G	14.43
**4003**	allXL	DD	357	373	BS2G	24.58
**4003**	allXL	CC	320	366	BS2G	22.62
**4003**	allXL	CC	370	376	BS2G	15.69
**4003**	allXL	AA	320	357	DBSU	24.18
**4003**	allXL	AA	357	319	DBSU	24.17
**4003**	allXL	CC	372	319	DBSU	19.46
**4003**	allXL	BB	370	357	DBSU	28.26

**4003**	allXL	DD	382	357	DBSU	32.86
**4003**	allXL	AA	372	370	DBSU	6.17
**4003**	allXL	BB	381	370	DBSU	18.26
**4003**	allXL	DD	373	370	DBSU	8.85
**4003**	allXL	DD	381	372	DBSU	17.42
**4003**	allXL	DD	372	382	DBSU	18.73
**4003**	allXL	DD	372	386	DBSU	17.87
**4003**	allXL	DD	373	386	DBSU	20.44
**4003**	allXL	DB	381	386	DBSU	51.57
**4003**	allXL	BD;DB	357	357	DBSU	18.88
**4003**	allXL	AD	373	319	DBSU	37.97
**4003**	allXL	DB	372	357	DBSU	43.34
**4003**	allXL	DB	381	357	DBSU	41.9
**4003**	allXL	AC	373	357	DBSU	40.86
**4003**	allXL	DB	372	370	DBSU	61.24
**4003**	allXL	DB	373	370	DBSU	59.2
**4003**	allXL	DB	382	373	DBSU	61.69
**4004**	allXL	CC	320	357	BS2G	25.65
**4004**	allXL	BB	320	373	BS2G	18.77
**4004**	allXL	CC	373	382	BS2G	14.98
**4004**	allXL	CC	370	386	BS2G	22.1
**4004**	allXL	CC	373	386	BS2G	19.99
**4004**	allXL	BB	319	357	BS2G	28.72
**4004**	allXL	AA	367	373	BS2G	12.93
**4004**	allXL	DD	370	382	BS2G	19.74
**4004**	allXL	CC	376	382	BS2G	14.55
**4004**	allXL	BB	357	373	BS2G	21.76
**4004**	allXL	DD	320	366	BS2G	25.22
**4004**	allXL	BB	370	376	BS2G	16.59
**4004**	allXL	CC	320	357	DBSU	25.65
**4004**	allXL	BB	357	319	DBSU	28.72
**4004**	allXL	BB	372	319	DBSU	17.38
**4004**	allXL	CC	370	357	DBSU	25.82
**4004**	allXL	DD	382	357	DBSU	26.11

**4004**	allXL	AA	372	370	DBSU	6.23
**4004**	allXL	DD	381	370	DBSU	20.98
**4004**	allXL	AA	373	370	DBSU	9.07
**4004**	allXL	CC	381	372	DBSU	20.0
**4004**	allXL	CC	372	382	DBSU	18.05
**4004**	allXL	CC	372	386	DBSU	21.58
**4004**	allXL	CC	373	386	DBSU	19.99
**4004**	allXL	BD	381	386	DBSU	56.08
**4004**	allXL	BD;DB	357	357	DBSU	18.52
**4004**	allXL	AD	373	319	DBSU	36.3
**4004**	allXL	CA	372	357	DBSU	41.67
**4004**	allXL	DB	381	357	DBSU	41.93
**4004**	allXL	BD	373	357	DBSU	39.91
**4004**	allXL	CA	372	370	DBSU	56.28
**4004**	allXL	CA	373	370	DBSU	58.04
**4004**	allXL	AD	382	373	DBSU	56.19
**4005**	allXL	AA	320	357	BS2G	25.22
**4005**	allXL	DD	320	373	BS2G	15.89
**4005**	allXL	AA	373	382	BS2G	15.41
**4005**	allXL	AA	370	386	BS2G	19.68
**4005**	allXL	BB	373	386	BS2G	19.29
**4005**	allXL	AA	319	357	BS2G	27.68
**4005**	allXL	AA	367	373	BS2G	10.98
**4005**	allXL	AA	370	382	BS2G	17.5
**4005**	allXL	BB	376	382	BS2G	13.63
**4005**	allXL	AA	357	373	BS2G	27.21
**4005**	allXL	DD	320	366	BS2G	24.4
**4005**	allXL	CC	370	376	BS2G	16.94
**4005**	allXL	AA	320	357	DBSU	25.22
**4005**	allXL	AA	357	319	DBSU	27.68
**4005**	allXL	DD	372	319	DBSU	15.15
**4005**	allXL	DD	370	357	DBSU	30.16
**4005**	allXL	BB	382	357	DBSU	30.28
**4005**	allXL	AA	372	370	DBSU	6.87

**4005**	allXL	AA	381	370	DBSU	19.92
**4005**	allXL	DD	373	370	DBSU	9.09
**4005**	allXL	AA	381	372	DBSU	17.23
**4005**	allXL	AA	372	382	DBSU	15.15
**4005**	allXL	BB	372	386	DBSU	18.81
**4005**	allXL	BB	373	386	DBSU	19.29
**4005**	allXL	DA	381	386	DBSU	40.36
**4005**	allXL	BD;DB	357	357	DBSU	19.34
**4005**	allXL	DA	373	319	DBSU	32.8
**4005**	allXL	DA	372	357	DBSU	39.89
**4005**	allXL	DA	381	357	DBSU	42.23
**4005**	allXL	DA	373	357	DBSU	37.02
**4005**	allXL	DA	372	370	DBSU	39.14
**4005**	allXL	DA	373	370	DBSU	35.48
**4005**	allXL	DA	382	373	DBSU	28.17
**4006**	allXL	AA	320	357	BS2G	26.61
**4006**	allXL	CC	320	373	BS2G	26.78
**4006**	allXL	AA	373	382	BS2G	20.67
**4006**	allXL	AA	370	386	BS2G	25.22
**4006**	allXL	AA	373	386	BS2G	23.75
**4006**	allXL	AA	319	357	BS2G	26.39
**4006**	allXL	BB	367	373	BS2G	13.02
**4006**	allXL	DD	370	382	BS2G	20.81
**4006**	allXL	BB	376	382	BS2G	14.51
**4006**	allXL	DD	357	373	BS2G	33.2
**4006**	allXL	AA	320	366	BS2G	16.2
**4006**	allXL	CC	370	376	BS2G	14.27
**4006**	allXL	AA	320	357	DBSU	26.61
**4006**	allXL	AA	357	319	DBSU	26.39
**4006**	allXL	AA	372	319	DBSU	25.62
**4006**	allXL	DD	370	357	DBSU	29.01
**4006**	allXL	DD	382	357	DBSU	19.96
**4006**	allXL	AA	372	370	DBSU	6.31
**4006**	allXL	DD	381	370	DBSU	17.59

**4006**	allXL	AA	373	370	DBSU	8.96
**4006**	allXL	DD	381	372	DBSU	19.35
**4006**	allXL	DD	372	382	DBSU	23.05
**4006**	allXL	AA	372	386	DBSU	25.46
**4006**	allXL	AA	373	386	DBSU	23.75
**4006**	allXL	BC	381	386	DBSU	31.28
**4006**	allXL	BD;DB	357	357	DBSU	17.93
**4006**	allXL	CB	373	319	DBSU	30.74
**4006**	allXL	CB	372	357	DBSU	25.35
**4006**	allXL	DB	381	357	DBSU	36.05
**4006**	allXL	CB	373	357	DBSU	26.54
**4006**	allXL	CB	372	370	DBSU	21.16
**4006**	allXL	CB	373	370	DBSU	17.46
**4006**	allXL	CB	382	373	DBSU	21.9
**4007**	allXL	CC	320	357	BS2G	25.22
**4007**	allXL	BB	320	373	BS2G	18.05
**4007**	allXL	AA	373	382	BS2G	18.83
**4007**	allXL	DD	370	386	BS2G	21.3
**4007**	allXL	AA	373	386	BS2G	24.4
**4007**	allXL	AA	319	357	BS2G	25.78
**4007**	allXL	BB	367	373	BS2G	12.85
**4007**	allXL	DD	370	382	BS2G	18.71
**4007**	allXL	CC	376	382	BS2G	13.71
**4007**	allXL	BB	357	373	BS2G	22.06
**4007**	allXL	DD	320	366	BS2G	23.63
**4007**	allXL	DD	370	376	BS2G	15.66
**4007**	allXL	CC	320	357	DBSU	25.22
**4007**	allXL	AA	357	319	DBSU	25.78
**4007**	allXL	BB	372	319	DBSU	22.14
**4007**	allXL	BB	370	357	DBSU	27.65
**4007**	allXL	BB	382	357	DBSU	17.48
**4007**	allXL	AA	372	370	DBSU	6.27
**4007**	allXL	DD	381	370	DBSU	18.49
**4007**	allXL	AA	373	370	DBSU	9.08

**4007**	allXL	DD	381	372	DBSU	21.89
**4007**	allXL	AA	372	382	DBSU	21.14
**4007**	allXL	AA	372	386	DBSU	25.21
**4007**	allXL	AA	373	386	DBSU	24.4
**4007**	allXL	BD	381	386	DBSU	31.89
**4007**	allXL	BD;DB	357	357	DBSU	18.83
**4007**	allXL	DA	373	319	DBSU	21.75
**4007**	allXL	DA	372	357	DBSU	37.29
**4007**	allXL	DB	381	357	DBSU	24.11
**4007**	allXL	DA	373	357	DBSU	34.23
**4007**	allXL	DA	372	370	DBSU	15.93
**4007**	allXL	DA	373	370	DBSU	16.02
**4007**	allXL	AD	382	373	DBSU	31.33
**4008**	allXL	CC	320	357	BS2G	25.18
**4008**	allXL	BB	320	373	BS2G	30.27
**4008**	allXL	BB	373	382	BS2G	21.41
**4008**	allXL	BB	370	386	BS2G	23.17
**4008**	allXL	BB	373	386	BS2G	20.1
**4008**	allXL	AA	319	357	BS2G	26.0
**4008**	allXL	AA	367	373	BS2G	11.57
**4008**	allXL	DD	370	382	BS2G	26.1
**4008**	allXL	BB	376	382	BS2G	13.64
**4008**	allXL	BB	357	373	BS2G	21.19
**4008**	allXL	BB	320	366	BS2G	16.43
**4008**	allXL	CC	370	376	BS2G	15.89
**4008**	allXL	CC	320	357	DBSU	25.18
**4008**	allXL	AA	357	319	DBSU	26.0
**4008**	allXL	BB	372	319	DBSU	30.63
**4008**	allXL	BB	370	357	DBSU	19.19
**4008**	allXL	BB	382	357	DBSU	25.01
**4008**	allXL	CC	372	370	DBSU	6.55
**4008**	allXL	DD	381	370	DBSU	25.02
**4008**	allXL	AA	373	370	DBSU	9.27
**4008**	allXL	DD	381	372	DBSU	24.04

**4008**	allXL	BB	372	382	DBSU	25.14
**4008**	allXL	BB	372	386	DBSU	22.93
**4008**	allXL	BB	373	386	DBSU	20.1
**4008**	allXL	DB	381	386	DBSU	62.05
**4008**	allXL	BD;DB	357	357	DBSU	18.19
**4008**	allXL	CB	373	319	DBSU	18.74
**4008**	allXL	BD	372	357	DBSU	34.78
**4008**	allXL	BD	381	357	DBSU	35.09
**4008**	allXL	BD	373	357	DBSU	33.74
**4008**	allXL	CB	372	370	DBSU	43.65
**4008**	allXL	CB	373	370	DBSU	45.9
**4008**	allXL	BD	382	373	DBSU	54.15
**4009**	allXL	AA	320	357	BS2G	23.64
**4009**	allXL	BB	320	373	BS2G	28.31
**4009**	allXL	DD	373	382	BS2G	18.83
**4009**	allXL	DD	370	386	BS2G	27.47
**4009**	allXL	DD	373	386	BS2G	28.45
**4009**	allXL	AA	319	357	BS2G	23.16
**4009**	allXL	DD	367	373	BS2G	13.51
**4009**	allXL	DD	370	382	BS2G	18.15
**4009**	allXL	CC	376	382	BS2G	14.31
**4009**	allXL	CC	357	373	BS2G	23.03
**4009**	allXL	DD	320	366	BS2G	18.29
**4009**	allXL	DD	370	376	BS2G	14.38
**4009**	allXL	AA	320	357	DBSU	23.64
**4009**	allXL	AA	357	319	DBSU	23.16
**4009**	allXL	BB	372	319	DBSU	24.27
**4009**	allXL	CC	370	357	DBSU	24.22
**4009**	allXL	CC	382	357	DBSU	12.8
**4009**	allXL	AA	372	370	DBSU	6.17
**4009**	allXL	DD	381	370	DBSU	16.0
**4009**	allXL	AA	373	370	DBSU	8.98
**4009**	allXL	DD	381	372	DBSU	18.88
**4009**	allXL	DD	372	382	DBSU	20.45

**4009**	allXL	DD	372	386	DBSU	29.53
**4009**	allXL	DD	373	386	DBSU	28.45
**4009**	allXL	DB	381	386	DBSU	36.59
**4009**	allXL	BD;DB	357	357	DBSU	18.48
**4009**	allXL	AD	373	319	DBSU	11.44
**4009**	allXL	AC	372	357	DBSU	39.48
**4009**	allXL	CA	381	357	DBSU	22.77
**4009**	allXL	AD	373	357	DBSU	37.58
**4009**	allXL	AD	372	370	DBSU	43.53
**4009**	allXL	AD	373	370	DBSU	40.06
**4009**	allXL	AD	382	373	DBSU	39.91
**4010**	allXL	AA	320	357	BS2G	22.04
**4010**	allXL	BB	320	373	BS2G	24.35
**4010**	allXL	BB	373	382	BS2G	18.03
**4010**	allXL	DD	370	386	BS2G	20.03
**4010**	allXL	AA	373	386	BS2G	19.34
**4010**	allXL	AA	319	357	BS2G	21.16
**4010**	allXL	AA	367	373	BS2G	13.35
**4010**	allXL	CC	370	382	BS2G	20.88
**4010**	allXL	AA;BB	376	382	BS2G	14.39
**4010**	allXL	DD	357	373	BS2G	25.78
**4010**	allXL	BB	320	366	BS2G	29.03
**4010**	allXL	CC	370	376	BS2G	15.1
**4010**	allXL	AA	320	357	DBSU	22.04
**4010**	allXL	AA	357	319	DBSU	21.16
**4010**	allXL	BB;CC	372	319	DBSU	24.3
**4010**	allXL	DD	370	357	DBSU	27.98
**4010**	allXL	DD	382	357	DBSU	21.02
**4010**	allXL	AA	372	370	DBSU	6.83
**4010**	allXL	CC	381	370	DBSU	18.92
**4010**	allXL	AA	373	370	DBSU	9.22
**4010**	allXL	CC	381	372	DBSU	18.02
**4010**	allXL	BB	372	382	DBSU	19.93
**4010**	allXL	AA	372	386	DBSU	18.96

**4010**	allXL	AA	373	386	DBSU	19.34
**4010**	allXL	BC	381	386	DBSU	32.89
**4010**	allXL	BD;DB	357	357	DBSU	18.89
**4010**	allXL	AD	373	319	DBSU	31.1
**4010**	allXL	AC	372	357	DBSU	35.68
**4010**	allXL	DB	381	357	DBSU	26.24
**4010**	allXL	AC	373	357	DBSU	33.49
**4010**	allXL	BC	372	370	DBSU	36.8
**4010**	allXL	BC	373	370	DBSU	34.11
**4010**	allXL	CB	382	373	DBSU	38.35
**4011**	allXL	AA	320	357	BS2G	23.27
**4011**	allXL	CC	320	373	BS2G	16.02
**4011**	allXL	DD	373	382	BS2G	20.65
**4011**	allXL	CC	370	386	BS2G	28.0
**4011**	allXL	AA	373	386	BS2G	26.77
**4011**	allXL	AA	319	357	BS2G	20.21
**4011**	allXL	DD	367	373	BS2G	14.06
**4011**	allXL	CC	370	382	BS2G	23.13
**4011**	allXL	CC	376	382	BS2G	14.17
**4011**	allXL	BB	357	373	BS2G	28.59
**4011**	allXL	CC	320	366	BS2G	21.87
**4011**	allXL	BB	370	376	BS2G	16.04
**4011**	allXL	AA	320	357	DBSU	23.27
**4011**	allXL	AA	357	319	DBSU	20.21
**4011**	allXL	CC	372	319	DBSU	13.11
**4011**	allXL	BB	370	357	DBSU	28.64
**4011**	allXL	DD	382	357	DBSU	51.51
**4011**	allXL	AA	372	370	DBSU	6.07
**4011**	allXL	DD	381	370	DBSU	22.81
**4011**	allXL	AA	373	370	DBSU	9.13
**4011**	allXL	DD	381	372	DBSU	20.73
**4011**	allXL	DD	372	382	DBSU	22.09
**4011**	allXL	CC	372	386	DBSU	29.32
**4011**	allXL	AA	373	386	DBSU	26.77

**4011**	allXL	BC	381	386	DBSU	78.87
**4011**	allXL	BD;DB	357	357	DBSU	18.4
**4011**	allXL	AD	373	319	DBSU	44.78
**4011**	allXL	BD	372	357	DBSU	39.73
**4011**	allXL	DB	381	357	DBSU	61.88
**4011**	allXL	BD	373	357	DBSU	41.96
**4011**	allXL	BC	372	370	DBSU	61.46
**4011**	allXL	BC	373	370	DBSU	62.52
**4011**	allXL	DB	382	373	DBSU	71.77
**4012**	allXL	CC	320	357	BS2G	25.48
**4012**	allXL	CC	320	373	BS2G	33.97
**4012**	allXL	BB	373	382	BS2G	16.17
**4012**	allXL	BB	370	386	BS2G	23.21
**4012**	allXL	AA	373	386	BS2G	22.92
**4012**	allXL	AA	319	357	BS2G	26.6
**4012**	allXL	AA	367	373	BS2G	12.36
**4012**	allXL	BB	370	382	BS2G	17.55
**4012**	allXL	BB	376	382	BS2G	13.74
**4012**	allXL	DD	357	373	BS2G	27.89
**4012**	allXL	AA	320	366	BS2G	25.37
**4012**	allXL	CC	370	376	BS2G	15.76
**4012**	allXL	CC	320	357	DBSU	25.48
**4012**	allXL	AA	357	319	DBSU	26.6
**4012**	allXL	CC	372	319	DBSU	32.12
**4012**	allXL	DD	370	357	DBSU	22.01
**4012**	allXL	AA	382	357	DBSU	18.75
**4012**	allXL	AA	372	370	DBSU	6.37
**4012**	allXL	BB	381	370	DBSU	19.51
**4012**	allXL	DD	373	370	DBSU	8.98
**4012**	allXL	BB	381	372	DBSU	18.96
**4012**	allXL	BB	372	382	DBSU	18.04
**4012**	allXL	AA	372	386	DBSU	25.46
**4012**	allXL	AA	373	386	DBSU	22.92
**4012**	allXL	CB	381	386	DBSU	55.44

**4012**	allXL	BD;DB	357	357	DBSU	18.98
**4012**	allXL	CB	373	319	DBSU	23.71
**4012**	allXL	DB	372	357	DBSU	44.38
**4012**	allXL	AC	381	357	DBSU	37.68
**4012**	allXL	DB	373	357	DBSU	42.89
**4012**	allXL	CB	372	370	DBSU	32.39
**4012**	allXL	CB	373	370	DBSU	32.47
**4012**	allXL	BC	382	373	DBSU	44.88
**4013**	allXL	BB	320	357	BS2G	26.01
**4013**	allXL	DD	320	373	BS2G	30.63
**4013**	allXL	AA	373	382	BS2G	17.29
**4013**	allXL	AA	370	386	BS2G	25.08
**4013**	allXL	BB	373	386	BS2G	24.76
**4013**	allXL	BB	319	357	BS2G	29.27
**4013**	allXL	AA	367	373	BS2G	10.9
**4013**	allXL	AA	370	382	BS2G	20.96
**4013**	allXL	BB	376	382	BS2G	13.06
**4013**	allXL	BB	357	373	BS2G	25.33
**4013**	allXL	DD	320	366	BS2G	21.12
**4013**	allXL	CC	370	376	BS2G	14.59
**4013**	allXL	BB	320	357	DBSU	26.01
**4013**	allXL	BB	357	319	DBSU	29.27
**4013**	allXL	DD	372	319	DBSU	27.62
**4013**	allXL	DD	370	357	DBSU	30.16
**4013**	allXL	BB	382	357	DBSU	21.85
**4013**	allXL	AA	372	370	DBSU	6.24
**4013**	allXL	AA	381	370	DBSU	23.91
**4013**	allXL	AA	373	370	DBSU	8.98
**4013**	allXL	AA	381	372	DBSU	19.73
**4013**	allXL	AA	372	382	DBSU	17.42
**4013**	allXL	AA	372	386	DBSU	23.88
**4013**	allXL	BB	373	386	DBSU	24.76
**4013**	allXL	BC	381	386	DBSU	54.28
**4013**	allXL	BD;DB	357	357	DBSU	18.77

**4013**	allXL	AD	373	319	DBSU	16.22
**4013**	allXL	CB	372	357	DBSU	42.24
**4013**	allXL	BD	381	357	DBSU	33.23
**4013**	allXL	BD	373	357	DBSU	41.9
**4013**	allXL	AD	372	370	DBSU	37.15
**4013**	allXL	AD	373	370	DBSU	38.9
**4013**	allXL	CB	382	373	DBSU	54.42
**4014**	allXL	CC	320	357	BS2G	26.12
**4014**	allXL	DD	320	373	BS2G	27.09
**4014**	allXL	BB	373	382	BS2G	21.57
**4014**	allXL	BB	370	386	BS2G	30.79
**4014**	allXL	DD	373	386	BS2G	25.35
**4014**	allXL	AA	319	357	BS2G	25.69
**4014**	allXL	AA	367	373	BS2G	13.87
**4014**	allXL	BB	370	382	BS2G	24.58
**4014**	allXL	BB	376	382	BS2G	14.76
**4014**	allXL	BB	357	373	BS2G	18.45
**4014**	allXL	DD	320	366	BS2G	23.28
**4014**	allXL	DD	370	376	BS2G	13.27
**4014**	allXL	CC	320	357	DBSU	26.12
**4014**	allXL	AA	357	319	DBSU	25.69
**4014**	allXL	DD	372	319	DBSU	20.1
**4014**	allXL	BB	370	357	DBSU	24.48
**4014**	allXL	BB	382	357	DBSU	33.01
**4014**	allXL	AA	372	370	DBSU	6.22
**4014**	allXL	DD	381	370	DBSU	24.26
**4014**	allXL	AA	373	370	DBSU	9.08
**4014**	allXL	DD	381	372	DBSU	22.07
**4014**	allXL	BB	372	382	DBSU	23.95
**4014**	allXL	DD	372	386	DBSU	27.13
**4014**	allXL	DD	373	386	DBSU	25.35
**4014**	allXL	AD	381	386	DBSU	27.44
**4014**	allXL	BD;DB	357	357	DBSU	19.38
**4014**	allXL	DA	373	319	DBSU	19.71

**4014**	allXL	BD	372	357	DBSU	22.89
**4014**	allXL	BD	381	357	DBSU	19.57
**4014**	allXL	BD	373	357	DBSU	20.97
**4014**	allXL	DA	372	370	DBSU	39.77
**4014**	allXL	DA	373	370	DBSU	41.96
**4014**	allXL	BD	382	373	DBSU	43.99
**4015**	allXL	CC	320	357	BS2G	24.57
**4015**	allXL	DD	320	373	BS2G	20.95
**4015**	allXL	AA	373	382	BS2G	15.33
**4015**	allXL	AA	370	386	BS2G	15.57
**4015**	allXL	AA	373	386	BS2G	19.87
**4015**	allXL	AA	319	357	BS2G	26.85
**4015**	allXL	BB	367	373	BS2G	12.62
**4015**	allXL	AA	370	382	BS2G	15.54
**4015**	allXL	CC	376	382	BS2G	13.82
**4015**	allXL	BB	357	373	BS2G	21.97
**4015**	allXL	AA	320	366	BS2G	19.66
**4015**	allXL	DD	370	376	BS2G	17.27
**4015**	allXL	CC	320	357	DBSU	24.57
**4015**	allXL	AA	357	319	DBSU	26.85
**4015**	allXL	DD	372	319	DBSU	14.07
**4015**	allXL	DD	370	357	DBSU	29.21
**4015**	allXL	BB	382	357	DBSU	19.07
**4015**	allXL	AA	372	370	DBSU	6.1
**4015**	allXL	AA	381	370	DBSU	18.55
**4015**	allXL	AA	373	370	DBSU	9.19
**4015**	allXL	AA	381	372	DBSU	16.64
**4015**	allXL	AA	372	382	DBSU	14.6
**4015**	allXL	AA	372	386	DBSU	17.04
**4015**	allXL	AA	373	386	DBSU	19.87
**4015**	allXL	DA	381	386	DBSU	38.69
**4015**	allXL	BD;DB	357	357	DBSU	17.81
**4015**	allXL	AD	373	319	DBSU	24.02
**4015**	allXL	BD	372	357	DBSU	35.02

**4015**	allXL	BD	381	357	DBSU	32.77
**4015**	allXL	BD	373	357	DBSU	35.79
**4015**	allXL	DA	372	370	DBSU	23.83
**4015**	allXL	DA	373	370	DBSU	23.88
**4015**	allXL	AD	382	373	DBSU	19.23
**4016**	allXL	AA	320	357	BS2G	26.63
**4016**	allXL	CC	320	373	BS2G	14.97
**4016**	allXL	DD	373	382	BS2G	18.48
**4016**	allXL	DD	370	386	BS2G	22.87
**4016**	allXL	AA	373	386	BS2G	25.59
**4016**	allXL	AA	319	357	BS2G	26.78
**4016**	allXL	AA	367	373	BS2G	13.31
**4016**	allXL	DD	370	382	BS2G	13.79
**4016**	allXL	BB	376	382	BS2G	13.72
**4016**	allXL	DD	357	373	BS2G	21.22
**4016**	allXL	CC	320	366	BS2G	28.09
**4016**	allXL	DD	370	376	BS2G	13.98
**4016**	allXL	AA	320	357	DBSU	26.63
**4016**	allXL	AA	357	319	DBSU	26.78
**4016**	allXL	CC	372	319	DBSU	12.1
**4016**	allXL	CC	370	357	DBSU	23.28
**4016**	allXL	BB	382	357	DBSU	23.77
**4016**	allXL	AA	372	370	DBSU	6.15
**4016**	allXL	DD	381	370	DBSU	13.75
**4016**	allXL	AA	373	370	DBSU	9.23
**4016**	allXL	DD	381	372	DBSU	17.36
**4016**	allXL	DD	372	382	DBSU	17.6
**4016**	allXL	AA	372	386	DBSU	23.85
**4016**	allXL	AA	373	386	DBSU	25.59
**4016**	allXL	BC	381	386	DBSU	55.56
**4016**	allXL	BD;DB	357	357	DBSU	18.38
**4016**	allXL	DA	373	319	DBSU	30.83
**4016**	allXL	DB	372	357	DBSU	38.51
**4016**	allXL	BD	381	357	DBSU	34.18

**4016**	allXL	DB	373	357	DBSU	35.26
**4016**	allXL	BC	372	370	DBSU	45.8
**4016**	allXL	BC	373	370	DBSU	47.89
**4016**	allXL	CB	382	373	DBSU	50.4
**4017**	allXL	AA	320	357	BS2G	23.8
**4017**	allXL	DD	320	373	BS2G	22.24
**4017**	allXL	DD	373	382	BS2G	19.58
**4017**	allXL	DD	370	386	BS2G	20.69
**4017**	allXL	DD	373	386	BS2G	26.74
**4017**	allXL	AA	319	357	BS2G	22.64
**4017**	allXL	AA	367	373	BS2G	13.63
**4017**	allXL	DD	370	382	BS2G	15.62
**4017**	allXL	CC	376	382	BS2G	14.86
**4017**	allXL	BB	357	373	BS2G	16.04
**4017**	allXL	DD	320	366	BS2G	28.17
**4017**	allXL	DD	370	376	BS2G	15.81
**4017**	allXL	AA	320	357	DBSU	23.8
**4017**	allXL	AA	357	319	DBSU	22.64
**4017**	allXL	DD	372	319	DBSU	25.56
**4017**	allXL	BB	370	357	DBSU	24.67
**4017**	allXL	BB	382	357	DBSU	12.98
**4017**	allXL	AA	372	370	DBSU	6.11
**4017**	allXL	DD	381	370	DBSU	14.07
**4017**	allXL	AA	373	370	DBSU	9.16
**4017**	allXL	DD	381	372	DBSU	17.92
**4017**	allXL	DD	372	382	DBSU	20.58
**4017**	allXL	CC	372	386	DBSU	25.31
**4017**	allXL	DD	373	386	DBSU	26.74
**4017**	allXL	BD	381	386	DBSU	31.4
**4017**	allXL	BD;DB	357	357	DBSU	18.26
**4017**	allXL	BC	373	319	DBSU	32.28
**4017**	allXL	BD	372	357	DBSU	30.92
**4017**	allXL	BD	381	357	DBSU	14.52
**4017**	allXL	BD	373	357	DBSU	27.6

**4017**	allXL	DA	372	370	DBSU	45.91
**4017**	allXL	DA	373	370	DBSU	45.06
**4017**	allXL	BD	382	373	DBSU	42.15
**4018**	allXL	BB	320	357	BS2G	25.58
**4018**	allXL	AA	320	373	BS2G	20.16
**4018**	allXL	CC	373	382	BS2G	17.15
**4018**	allXL	CC	370	386	BS2G	21.49
**4018**	allXL	CC	373	386	BS2G	16.65
**4018**	allXL	BB	319	357	BS2G	27.1
**4018**	allXL	AA	367	373	BS2G	12.78
**4018**	allXL	DD	370	382	BS2G	23.78
**4018**	allXL	BB	376	382	BS2G	13.79
**4018**	allXL	BB	357	373	BS2G	22.69
**4018**	allXL	AA	320	366	BS2G	18.24
**4018**	allXL	DD	370	376	BS2G	16.33
**4018**	allXL	BB	320	357	DBSU	25.58
**4018**	allXL	BB	357	319	DBSU	27.1
**4018**	allXL	AA	372	319	DBSU	19.33
**4018**	allXL	DD	370	357	DBSU	24.82
**4018**	allXL	BB	382	357	DBSU	19.83
**4018**	allXL	CC	372	370	DBSU	6.48
**4018**	allXL	DD	381	370	DBSU	22.95
**4018**	allXL	DD	373	370	DBSU	9.2
**4018**	allXL	CC	381	372	DBSU	18.64
**4018**	allXL	BB	372	382	DBSU	18.2
**4018**	allXL	CC	372	386	DBSU	17.72
**4018**	allXL	CC	373	386	DBSU	16.65
**4018**	allXL	DB	381	386	DBSU	30.97
**4018**	allXL	BD;DB	357	357	DBSU	19.24
**4018**	allXL	AD	373	319	DBSU	22.93
**4018**	allXL	BD	372	357	DBSU	36.52
**4018**	allXL	AD	381	357	DBSU	21.6
**4018**	allXL	BD	373	357	DBSU	33.75
**4018**	allXL	CB	372	370	DBSU	31.29

**4018**	allXL	CB	373	370	DBSU	30.5
**4018**	allXL	CB	382	373	DBSU	24.9
**4019**	allXL	CC	320	357	BS2G	25.98
**4019**	allXL	BB	320	373	BS2G	17.75
**4019**	allXL	DD	373	382	BS2G	17.42
**4019**	allXL	BB	370	386	BS2G	26.14
**4019**	allXL	BB	373	386	BS2G	22.62
**4019**	allXL	BB	319	357	BS2G	26.37
**4019**	allXL	BB	367	373	BS2G	13.35
**4019**	allXL	DD	370	382	BS2G	22.87
**4019**	allXL	BB	376	382	BS2G	13.69
**4019**	allXL	BB	357	373	BS2G	23.45
**4019**	allXL	BB	320	366	BS2G	21.56
**4019**	allXL	CC	370	376	BS2G	16.95
**4019**	allXL	CC	320	357	DBSU	25.98
**4019**	allXL	BB	357	319	DBSU	26.37
**4019**	allXL	BB	372	319	DBSU	15.94
**4019**	allXL	DD	370	357	DBSU	27.12
**4019**	allXL	BB	382	357	DBSU	34.23
**4019**	allXL	AA	372	370	DBSU	6.01
**4019**	allXL	DD	381	370	DBSU	24.35
**4019**	allXL	DD	373	370	DBSU	9.06
**4019**	allXL	DD	381	372	DBSU	17.69
**4019**	allXL	DD	372	382	DBSU	16.47
**4019**	allXL	DD	372	386	DBSU	21.57
**4019**	allXL	BB	373	386	DBSU	22.62
**4019**	allXL	AD	381	386	DBSU	55.07
**4019**	allXL	BD;DB	357	357	DBSU	19.31
**4019**	allXL	CB	373	319	DBSU	33.17
**4019**	allXL	CB	372	357	DBSU	44.57
**4019**	allXL	BD	381	357	DBSU	44.07
**4019**	allXL	BD	373	357	DBSU	41.22
**4019**	allXL	CB	372	370	DBSU	29.29
**4019**	allXL	CB	373	370	DBSU	32.94

**4019**	allXL	DA	382	373	DBSU	42.1
**4020**	allXL	CC	320	357	BS2G	23.76
**4020**	allXL	AA	320	373	BS2G	39.16
**4020**	allXL	DD	373	382	BS2G	18.49
**4020**	allXL	CC	370	386	BS2G	24.91
**4020**	allXL	DD	373	386	BS2G	25.38
**4020**	allXL	AA	319	357	BS2G	24.57
**4020**	allXL	AA	367	373	BS2G	12.54
**4020**	allXL	CC	370	382	BS2G	17.24
**4020**	allXL	BB	376	382	BS2G	14.3
**4020**	allXL	BB	357	373	BS2G	20.39
**4020**	allXL	AA	320	366	BS2G	31.95
**4020**	allXL	CC	370	376	BS2G	14.85
**4020**	allXL	CC	320	357	DBSU	23.76
**4020**	allXL	AA	357	319	DBSU	24.57
**4020**	allXL	AA	372	319	DBSU	41.21
**4020**	allXL	BB	370	357	DBSU	27.49
**4020**	allXL	BB	382	357	DBSU	16.71
**4020**	allXL	AA	372	370	DBSU	6.36
**4020**	allXL	CC	381	370	DBSU	18.19
**4020**	allXL	DD	373	370	DBSU	8.9
**4020**	allXL	CC	381	372	DBSU	19.94
**4020**	allXL	CC	372	382	DBSU	19.77
**4020**	allXL	DD	372	386	DBSU	26.16
**4020**	allXL	DD	373	386	DBSU	25.38
**4020**	allXL	DB	381	386	DBSU	52.64
**4020**	allXL	BD;DB	357	357	DBSU	18.89
**4020**	allXL	CB	373	319	DBSU	36.84
**4020**	allXL	BD	372	357	DBSU	24.71
**4020**	allXL	AC	381	357	DBSU	21.65
**4020**	allXL	BD	373	357	DBSU	24.72
**4020**	allXL	BD	372	370	DBSU	44.44
**4020**	allXL	BD	373	370	DBSU	42.12
**4020**	allXL	BD	382	373	DBSU	37.83

**4021**	allXL	AA	320	357	BS2G	20.83
**4021**	allXL	BB	320	373	BS2G	26.9
**4021**	allXL	DD	373	382	BS2G	18.07
**4021**	allXL	DD	370	386	BS2G	28.92
**4021**	allXL	DD	373	386	BS2G	23.68
**4021**	allXL	AA	319	357	BS2G	18.53
**4021**	allXL	AA	367	373	BS2G	12.19
**4021**	allXL	DD	370	382	BS2G	22.79
**4021**	allXL	BB	376	382	BS2G	14.46
**4021**	allXL	DD	357	373	BS2G	20.13
**4021**	allXL	AA	320	366	BS2G	30.02
**4021**	allXL	DD	370	376	BS2G	16.78
**4021**	allXL	AA	320	357	DBSU	20.83
**4021**	allXL	AA	357	319	DBSU	18.53
**4021**	allXL	BB	372	319	DBSU	32.57
**4021**	allXL	DD	370	357	DBSU	22.31
**4021**	allXL	BB	382	357	DBSU	16.97
**4021**	allXL	AA	372	370	DBSU	6.48
**4021**	allXL	DD	381	370	DBSU	21.56
**4021**	allXL	CC	373	370	DBSU	9.03
**4021**	allXL	DD	381	372	DBSU	16.29
**4021**	allXL	DD	372	382	DBSU	17.43
**4021**	allXL	DD	372	386	DBSU	22.47
**4021**	allXL	DD	373	386	DBSU	23.68
**4021**	allXL	DB	381	386	DBSU	41.31
**4021**	allXL	BD;DB	357	357	DBSU	18.99
**4021**	allXL	AD	373	319	DBSU	38.63
**4021**	allXL	AC	372	357	DBSU	38.93
**4021**	allXL	AC	381	357	DBSU	19.92
**4021**	allXL	AC	373	357	DBSU	35.69
**4021**	allXL	AD	372	370	DBSU	66.24
**4021**	allXL	DB	373	370	DBSU	63.97
**4021**	allXL	AC	382	373	DBSU	43.1
**4022**	allXL	AA	320	357	BS2G	25.37

**4022**	allXL	AA	320	373	BS2G	18.77
**4022**	allXL	BB	373	382	BS2G	15.29
**4022**	allXL	BB	370	386	BS2G	15.74
**4022**	allXL	BB	373	386	BS2G	21.32
**4022**	allXL	AA	319	357	BS2G	23.94
**4022**	allXL	AA	367	373	BS2G	12.4
**4022**	allXL	BB	370	382	BS2G	14.45
**4022**	allXL	BB	376	382	BS2G	14.62
**4022**	allXL	AA	357	373	BS2G	24.62
**4022**	allXL	BB	320	366	BS2G	21.18
**4022**	allXL	BB	370	376	BS2G	14.27
**4022**	allXL	AA	320	357	DBSU	25.37
**4022**	allXL	AA	357	319	DBSU	23.94
**4022**	allXL	BB	372	319	DBSU	20.05
**4022**	allXL	CC	370	357	DBSU	26.74
**4022**	allXL	AA	382	357	DBSU	20.02
**4022**	allXL	AA	372	370	DBSU	6.52
**4022**	allXL	BB	381	370	DBSU	12.46
**4022**	allXL	AA	373	370	DBSU	9.25
**4022**	allXL	BB	381	372	DBSU	14.77
**4022**	allXL	BB	372	382	DBSU	16.08
**4022**	allXL	BB	372	386	DBSU	19.79
**4022**	allXL	BB	373	386	DBSU	21.32
**4022**	allXL	CB	381	386	DBSU	29.03
**4022**	allXL	BD;DB	357	357	DBSU	17.75
**4022**	allXL	CB	373	319	DBSU	20.35
**4022**	allXL	CB	372	357	DBSU	27.79
**4022**	allXL	AC	381	357	DBSU	31.75
**4022**	allXL	CB	373	357	DBSU	25.71
**4022**	allXL	BC	372	370	DBSU	15.31
**4022**	allXL	CB	373	370	DBSU	15.0
**4022**	allXL	CB	382	373	DBSU	22.55
**4023**	allXL	CC	320	357	BS2G	23.86
**4023**	allXL	BB	320	373	BS2G	31.24

**4023**	allXL	BB	373	382	BS2G	18.77
**4023**	allXL	CC	370	386	BS2G	23.59
**4023**	allXL	AA	373	386	BS2G	20.11
**4023**	allXL	CC	319	357	BS2G	23.49
**4023**	allXL	BB	367	373	BS2G	13.35
**4023**	allXL	BB	370	382	BS2G	22.92
**4023**	allXL	AA	376	382	BS2G	13.57
**4023**	allXL	BB	357	373	BS2G	30.88
**4023**	allXL	AA	320	366	BS2G	22.52
**4023**	allXL	CC	370	376	BS2G	15.09
**4023**	allXL	CC	320	357	DBSU	23.86
**4023**	allXL	CC	357	319	DBSU	23.49
**4023**	allXL	BB	372	319	DBSU	32.47
**4023**	allXL	BB	370	357	DBSU	22.8
**4023**	allXL	BB	382	357	DBSU	32.21
**4023**	allXL	AA	372	370	DBSU	6.25
**4023**	allXL	BB	381	370	DBSU	22.27
**4023**	allXL	AA	373	370	DBSU	9.31
**4023**	allXL	DD	381	372	DBSU	20.39
**4023**	allXL	BB	372	382	DBSU	21.4
**4023**	allXL	AA	372	386	DBSU	23.72
**4023**	allXL	AA	373	386	DBSU	20.11
**4023**	allXL	DB	381	386	DBSU	63.08
**4023**	allXL	BD;DB	357	357	DBSU	17.68
**4023**	allXL	CB	373	319	DBSU	14.9
**4023**	allXL	CB	372	357	DBSU	35.07
**4023**	allXL	AC	381	357	DBSU	36.83
**4023**	allXL	CB	373	357	DBSU	34.52
**4023**	allXL	CB	372	370	DBSU	21.85
**4023**	allXL	CB	373	370	DBSU	22.28
**4023**	allXL	BC	382	373	DBSU	44.84
**4024**	allXL	CC	320	357	BS2G	23.78
**4024**	allXL	DD	320	373	BS2G	16.17
**4024**	allXL	CC	373	382	BS2G	19.54

**4024**	allXL	AA	370	386	BS2G	19.19
**4024**	allXL	AA	373	386	BS2G	24.19
**4024**	allXL	CC	319	357	BS2G	22.69
**4024**	allXL	AA	367	373	BS2G	12.45
**4024**	allXL	AA	370	382	BS2G	21.26
**4024**	allXL	CC	376	382	BS2G	13.88
**4024**	allXL	CC	357	373	BS2G	23.06
**4024**	allXL	AA	320	366	BS2G	20.52
**4024**	allXL	CC	370	376	BS2G	14.74
**4024**	allXL	CC	320	357	DBSU	23.78
**4024**	allXL	CC	357	319	DBSU	22.69
**4024**	allXL	DD	372	319	DBSU	17.56
**4024**	allXL	DD	370	357	DBSU	25.12
**4024**	allXL	AA	382	357	DBSU	34.35
**4024**	allXL	AA	372	370	DBSU	5.97
**4024**	allXL	AA	381	370	DBSU	21.96
**4024**	allXL	AA	373	370	DBSU	9.22
**4024**	allXL	DD	381	372	DBSU	17.85
**4024**	allXL	AA	372	382	DBSU	20.43
**4024**	allXL	AA	372	386	DBSU	21.52
**4024**	allXL	AA	373	386	DBSU	24.19
**4024**	allXL	DA	381	386	DBSU	30.04
**4024**	allXL	BD;DB	357	357	DBSU	17.83
**4024**	allXL	DA	373	319	DBSU	25.49
**4024**	allXL	DA	372	357	DBSU	39.6
**4024**	allXL	AD	381	357	DBSU	37.04
**4024**	allXL	DA	373	357	DBSU	38.58
**4024**	allXL	DA	372	370	DBSU	32.74
**4024**	allXL	DA	373	370	DBSU	33.8
**4024**	allXL	AD	382	373	DBSU	12.6
**4025**	allXL	CC	320	357	BS2G	21.53
**4025**	allXL	CC	320	373	BS2G	17.48
**4025**	allXL	CC	373	382	BS2G	15.6
**4025**	allXL	CC	370	386	BS2G	24.86

**4025**	allXL	CC	373	386	BS2G	21.27
**4025**	allXL	CC	319	357	BS2G	23.36
**4025**	allXL	BB	367	373	BS2G	14.06
**4025**	allXL	DD	370	382	BS2G	23.23
**4025**	allXL	CC	376	382	BS2G	13.71
**4025**	allXL	BB	357	373	BS2G	28.58
**4025**	allXL	CC	320	366	BS2G	19.37
**4025**	allXL	CC	370	376	BS2G	16.95
**4025**	allXL	CC	320	357	DBSU	21.53
**4025**	allXL	CC	357	319	DBSU	23.36
**4025**	allXL	CC	372	319	DBSU	15.18
**4025**	allXL	DD	370	357	DBSU	30.35
**4025**	allXL	BB	382	357	DBSU	35.07
**4025**	allXL	AA	372	370	DBSU	6.11
**4025**	allXL	DD	381	370	DBSU	21.99
**4025**	allXL	DD	373	370	DBSU	9.25
**4025**	allXL	DD	381	372	DBSU	18.09
**4025**	allXL	DD	372	382	DBSU	18.95
**4025**	allXL	CC	372	386	DBSU	23.55
**4025**	allXL	CC	373	386	DBSU	21.27
**4025**	allXL	AD	381	386	DBSU	12.94
**4025**	allXL	BD;DB	357	357	DBSU	19.65
**4025**	allXL	AD	373	319	DBSU	26.16
**4025**	allXL	BD	372	357	DBSU	44.48
**4025**	allXL	CB	381	357	DBSU	41.93
**4025**	allXL	BD	373	357	DBSU	44.13
**4025**	allXL	AD	372	370	DBSU	18.14
**4025**	allXL	AD	373	370	DBSU	19.31
**4025**	allXL	DA	382	373	DBSU	13.69
**4026**	allXL	AA	320	357	BS2G	23.96
**4026**	allXL	DD	320	373	BS2G	16.11
**4026**	allXL	DD	373	382	BS2G	16.43
**4026**	allXL	DD	370	386	BS2G	25.24
**4026**	allXL	DD	373	386	BS2G	21.74

**4026**	allXL	AA	319	357	BS2G	24.38
**4026**	allXL	BB	367	373	BS2G	12.73
**4026**	allXL	DD	370	382	BS2G	20.21
**4026**	allXL	DD	376	382	BS2G	13.39
**4026**	allXL	BB	357	373	BS2G	24.54
**4026**	allXL	DD	320	366	BS2G	29.08
**4026**	allXL	BB	370	376	BS2G	15.25
**4026**	allXL	AA	320	357	DBSU	23.96
**4026**	allXL	AA	357	319	DBSU	24.38
**4026**	allXL	DD	372	319	DBSU	13.89
**4026**	allXL	DD	370	357	DBSU	23.26
**4026**	allXL	BB	382	357	DBSU	38.65
**4026**	allXL	CC	372	370	DBSU	6.93
**4026**	allXL	DD	381	370	DBSU	21.19
**4026**	allXL	BB	373	370	DBSU	9.06
**4026**	allXL	DD	381	372	DBSU	18.19
**4026**	allXL	DD	372	382	DBSU	18.1
**4026**	allXL	DD	372	386	DBSU	24.53
**4026**	allXL	DD	373	386	DBSU	21.74
**4026**	allXL	CB	381	386	DBSU	43.22
**4026**	allXL	BD;DB	357	357	DBSU	18.67
**4026**	allXL	CB	373	319	DBSU	13.98
**4026**	allXL	DA	372	357	DBSU	38.34
**4026**	allXL	CB	381	357	DBSU	47.82
**4026**	allXL	BD	373	357	DBSU	39.03
**4026**	allXL	DA	372	370	DBSU	39.53
**4026**	allXL	DA	373	370	DBSU	41.1
**4026**	allXL	AD	382	373	DBSU	46.67
**4027**	allXL	AA	320	357	BS2G	18.12
**4027**	allXL	CC	320	373	BS2G	19.77
**4027**	allXL	DD	373	382	BS2G	16.44
**4027**	allXL	AA	370	386	BS2G	19.22
**4027**	allXL	AA	373	386	BS2G	17.34
**4027**	allXL	AA	319	357	BS2G	21.49

**4027**	allXL	AA	367	373	BS2G	10.8
**4027**	allXL	CC	370	382	BS2G	16.7
**4027**	allXL	CC	376	382	BS2G	14.68
**4027**	allXL	BB	357	373	BS2G	24.18
**4027**	allXL	BB	320	366	BS2G	26.34
**4027**	allXL	CC	370	376	BS2G	13.06
**4027**	allXL	AA	320	357	DBSU	18.12
**4027**	allXL	AA	357	319	DBSU	21.49
**4027**	allXL	DD	372	319	DBSU	18.81
**4027**	allXL	BB	370	357	DBSU	25.12
**4027**	allXL	CC	382	357	DBSU	16.67
**4027**	allXL	AA	372	370	DBSU	6.51
**4027**	allXL	CC	381	370	DBSU	17.55
**4027**	allXL	AA	373	370	DBSU	9.0
**4027**	allXL	DD	381	372	DBSU	16.47
**4027**	allXL	DD	372	382	DBSU	15.08
**4027**	allXL	AA	372	386	DBSU	15.67
**4027**	allXL	AA	373	386	DBSU	17.34
**4027**	allXL	BD	381	386	DBSU	40.95
**4027**	allXL	BD;DB	357	357	DBSU	18.22
**4027**	allXL	DA	373	319	DBSU	26.89
**4027**	allXL	BD	372	357	DBSU	33.07
**4027**	allXL	CA	381	357	DBSU	34.65
**4027**	allXL	BD	373	357	DBSU	33.93
**4027**	allXL	AD	372	370	DBSU	50.07
**4027**	allXL	AD	373	370	DBSU	46.76
**4027**	allXL	DA	382	373	DBSU	51.79
**4028**	allXL	AA	320	357	BS2G	24.18
**4028**	allXL	CC	320	373	BS2G	15.16
**4028**	allXL	DD	373	382	BS2G	19.24
**4028**	allXL	DD	370	386	BS2G	20.39
**4028**	allXL	DD	373	386	BS2G	22.01
**4028**	allXL	AA	319	357	BS2G	25.6
**4028**	allXL	AA	367	373	BS2G	12.92

**4028**	allXL	DD	370	382	BS2G	22.76
**4028**	allXL	BB	376	382	BS2G	14.1
**4028**	allXL	AA	357	373	BS2G	15.35
**4028**	allXL	CC	320	366	BS2G	17.32
**4028**	allXL	CC	370	376	BS2G	16.99
**4028**	allXL	AA	320	357	DBSU	24.18
**4028**	allXL	AA	357	319	DBSU	25.6
**4028**	allXL	CC	372	319	DBSU	15.18
**4028**	allXL	AA	370	357	DBSU	23.52
**4028**	allXL	AA	382	357	DBSU	29.77
**4028**	allXL	AA	372	370	DBSU	6.16
**4028**	allXL	DD	381	370	DBSU	21.29
**4028**	allXL	CC	373	370	DBSU	9.08
**4028**	allXL	DD	381	372	DBSU	17.4
**4028**	allXL	DD	372	382	DBSU	19.5
**4028**	allXL	DD	372	386	DBSU	20.59
**4028**	allXL	DD	373	386	DBSU	22.01
**4028**	allXL	BC	381	386	DBSU	43.16
**4028**	allXL	BD;DB	357	357	DBSU	18.84
**4028**	allXL	DA	373	319	DBSU	27.99
**4028**	allXL	AC	372	357	DBSU	34.56
**4028**	allXL	CB	381	357	DBSU	25.89
**4028**	allXL	AC	373	357	DBSU	31.62
**4028**	allXL	DA	372	370	DBSU	52.6
**4028**	allXL	DA	373	370	DBSU	49.75
**4028**	allXL	CB	382	373	DBSU	38.35
**4029**	allXL	AA	320	357	BS2G	21.75
**4029**	allXL	DD	320	373	BS2G	23.41
**4029**	allXL	BB	373	382	BS2G	18.24
**4029**	allXL	CC	370	386	BS2G	16.65
**4029**	allXL	CC	373	386	BS2G	19.65
**4029**	allXL	AA	319	357	BS2G	20.99
**4029**	allXL	AA	367	373	BS2G	13.52
**4029**	allXL	BB	370	382	BS2G	19.69

**4029**	allXL	CC	376	382	BS2G	13.34
**4029**	allXL	CC	357	373	BS2G	15.23
**4029**	allXL	BB	320	366	BS2G	14.85
**4029**	allXL	CC	370	376	BS2G	16.55
**4029**	allXL	AA	320	357	DBSU	21.75
**4029**	allXL	AA	357	319	DBSU	20.99
**4029**	allXL	DD	372	319	DBSU	22.2
**4029**	allXL	CC	370	357	DBSU	13.88
**4029**	allXL	CC	382	357	DBSU	32.3
**4029**	allXL	AA	372	370	DBSU	6.13
**4029**	allXL	BB	381	370	DBSU	20.76
**4029**	allXL	DD	373	370	DBSU	9.08
**4029**	allXL	DD	381	372	DBSU	18.22
**4029**	allXL	DD	372	382	DBSU	18.03
**4029**	allXL	CC	372	386	DBSU	17.4
**4029**	allXL	CC	373	386	DBSU	19.65
**4029**	allXL	AD	381	386	DBSU	49.12
**4029**	allXL	BD;DB	357	357	DBSU	19.29
**4029**	allXL	AD	373	319	DBSU	31.83
**4029**	allXL	CA	372	357	DBSU	22.6
**4029**	allXL	CA	381	357	DBSU	34.64
**4029**	allXL	CA	373	357	DBSU	25.82
**4029**	allXL	DA	372	370	DBSU	41.99
**4029**	allXL	AC	373	370	DBSU	44.91
**4029**	allXL	DA	382	373	DBSU	43.78
**4030**	allXL	CC	320	357	BS2G	25.24
**4030**	allXL	BB	320	373	BS2G	25.09
**4030**	allXL	AA	373	382	BS2G	16.1
**4030**	allXL	AA	370	386	BS2G	24.18
**4030**	allXL	AA	373	386	BS2G	21.5
**4030**	allXL	CC	319	357	BS2G	28.13
**4030**	allXL	AA	367	373	BS2G	13.74
**4030**	allXL	AA	370	382	BS2G	22.53
**4030**	allXL	AA	376	382	BS2G	12.58

**4030**	allXL	BB	357	373	BS2G	31.83
**4030**	allXL	AA	320	366	BS2G	24.03
**4030**	allXL	CC	370	376	BS2G	15.47
**4030**	allXL	CC	320	357	DBSU	25.24
**4030**	allXL	CC	357	319	DBSU	28.13
**4030**	allXL	DD	372	319	DBSU	22.31
**4030**	allXL	DD	370	357	DBSU	26.59
**4030**	allXL	AA	382	357	DBSU	43.32
**4030**	allXL	AA	372	370	DBSU	6.48
**4030**	allXL	AA	381	370	DBSU	25.23
**4030**	allXL	AA	373	370	DBSU	9.22
**4030**	allXL	AA	381	372	DBSU	21.55
**4030**	allXL	AA	372	382	DBSU	19.58
**4030**	allXL	AA	372	386	DBSU	23.84
**4030**	allXL	AA	373	386	DBSU	21.5
**4030**	allXL	CB	381	386	DBSU	29.77
**4030**	allXL	BD;DB	357	357	DBSU	18.86
**4030**	allXL	BC	373	319	DBSU	26.14
**4030**	allXL	BC	372	357	DBSU	41.72
**4030**	allXL	BC	381	357	DBSU	44.14
**4030**	allXL	BC	373	357	DBSU	40.12
**4030**	allXL	DA	372	370	DBSU	29.89
**4030**	allXL	DA	373	370	DBSU	32.16
**4030**	allXL	BC	382	373	DBSU	27.72
**4031**	allXL	BB	320	357	BS2G	24.82
**4031**	allXL	BB	320	373	BS2G	14.82
**4031**	allXL	DD	373	382	BS2G	18.9
**4031**	allXL	DD	370	386	BS2G	23.28
**4031**	allXL	DD	373	386	BS2G	24.98
**4031**	allXL	AA	319	357	BS2G	23.71
**4031**	allXL	DD	367	373	BS2G	14.38
**4031**	allXL	DD	370	382	BS2G	21.41
**4031**	allXL	CC	376	382	BS2G	12.89
**4031**	allXL	BB	357	373	BS2G	25.43

**4031**	allXL	BB	320	366	BS2G	16.02
**4031**	allXL	DD	370	376	BS2G	17.17
**4031**	allXL	BB	320	357	DBSU	24.82
**4031**	allXL	AA	357	319	DBSU	23.71
**4031**	allXL	BB	372	319	DBSU	17.05
**4031**	allXL	BB	370	357	DBSU	24.2
**4031**	allXL	DD	382	357	DBSU	29.05
**4031**	allXL	AA	372	370	DBSU	6.21
**4031**	allXL	DD	381	370	DBSU	22.51
**4031**	allXL	AA	373	370	DBSU	9.21
**4031**	allXL	DD	381	372	DBSU	20.95
**4031**	allXL	DD	372	382	DBSU	21.0
**4031**	allXL	DD	372	386	DBSU	26.3
**4031**	allXL	DD	373	386	DBSU	24.98
**4031**	allXL	BD	381	386	DBSU	70.48
**4031**	allXL	BD;DB	357	357	DBSU	19.32
**4031**	allXL	DA	373	319	DBSU	26.78
**4031**	allXL	BD	372	357	DBSU	37.14
**4031**	allXL	BC	381	357	DBSU	39.59
**4031**	allXL	BD	373	357	DBSU	37.88
**4031**	allXL	BC	372	370	DBSU	40.01
**4031**	allXL	BC	373	370	DBSU	39.8
**4031**	allXL	DA	382	373	DBSU	51.02
**4032**	allXL	AA	320	357	BS2G	19.43
**4032**	allXL	DD	320	373	BS2G	17.24
**4032**	allXL	DD	373	382	BS2G	20.61
**4032**	allXL	DD	370	386	BS2G	24.36
**4032**	allXL	DD	373	386	BS2G	25.1
**4032**	allXL	AA	319	357	BS2G	17.55
**4032**	allXL	AA	367	373	BS2G	14.27
**4032**	allXL	CC	370	382	BS2G	21.94
**4032**	allXL	CC	376	382	BS2G	13.96
**4032**	allXL	BB	357	373	BS2G	26.19
**4032**	allXL	AA	320	366	BS2G	25.6

**4032**	allXL	CC	370	376	BS2G	16.02
**4032**	allXL	AA	320	357	DBSU	19.43
**4032**	allXL	AA	357	319	DBSU	17.55
**4032**	allXL	DD	372	319	DBSU	18.85
**4032**	allXL	BB	370	357	DBSU	25.76
**4032**	allXL	BB	382	357	DBSU	10.89
**4032**	allXL	CC	372	370	DBSU	6.54
**4032**	allXL	DD	381	370	DBSU	20.99
**4032**	allXL	AA	373	370	DBSU	9.26
**4032**	allXL	DD	381	372	DBSU	18.66
**4032**	allXL	CC	372	382	DBSU	20.79
**4032**	allXL	DD	372	386	DBSU	23.88
**4032**	allXL	DD	373	386	DBSU	25.1
**4032**	allXL	AD	381	386	DBSU	38.45
**4032**	allXL	BD;DB	357	357	DBSU	18.4
**4032**	allXL	AD	373	319	DBSU	18.36
**4032**	allXL	CB	372	357	DBSU	33.78
**4032**	allXL	BD	381	357	DBSU	31.38
**4032**	allXL	CB	373	357	DBSU	33.0
**4032**	allXL	BC	372	370	DBSU	16.58
**4032**	allXL	BC	373	370	DBSU	15.98
**4032**	allXL	BC	382	373	DBSU	25.74
**4033**	allXL	AA	320	357	BS2G	14.78
**4033**	allXL	BB	320	373	BS2G	18.68
**4033**	allXL	AA	373	382	BS2G	19.59
**4033**	allXL	DD	370	386	BS2G	23.0
**4033**	allXL	AA	373	386	BS2G	24.68
**4033**	allXL	AA	319	357	BS2G	13.34
**4033**	allXL	AA	367	373	BS2G	12.38
**4033**	allXL	DD	370	382	BS2G	15.76
**4033**	allXL	CC	376	382	BS2G	13.94
**4033**	allXL	BB	357	373	BS2G	18.85
**4033**	allXL	DD	320	366	BS2G	20.69
**4033**	allXL	DD	370	376	BS2G	14.75

**4033**	allXL	AA	320	357	DBSU	14.78
**4033**	allXL	AA	357	319	DBSU	13.34
**4033**	allXL	BB	372	319	DBSU	15.21
**4033**	allXL	BB	370	357	DBSU	27.52
**4033**	allXL	BB	382	357	DBSU	24.24
**4033**	allXL	AA	372	370	DBSU	6.3
**4033**	allXL	DD	381	370	DBSU	12.94
**4033**	allXL	AA	373	370	DBSU	8.93
**4033**	allXL	DD	381	372	DBSU	17.7
**4033**	allXL	AA	372	382	DBSU	20.83
**4033**	allXL	AA	372	386	DBSU	24.19
**4033**	allXL	AA	373	386	DBSU	24.68
**4033**	allXL	BC	381	386	DBSU	41.42
**4033**	allXL	BD;DB	357	357	DBSU	18.58
**4033**	allXL	AD	373	319	DBSU	32.09
**4033**	allXL	AC	372	357	DBSU	35.02
**4033**	allXL	BD	381	357	DBSU	29.94
**4033**	allXL	BD	373	357	DBSU	34.26
**4033**	allXL	CB	372	370	DBSU	24.12
**4033**	allXL	CB	373	370	DBSU	23.65
**4033**	allXL	BC	382	373	DBSU	38.92
**4034**	allXL	AA	320	357	BS2G	25.67
**4034**	allXL	DD	320	373	BS2G	13.89
**4034**	allXL	BB	373	382	BS2G	17.51
**4034**	allXL	DD	370	386	BS2G	15.15
**4034**	allXL	DD	373	386	BS2G	21.45
**4034**	allXL	AA	319	357	BS2G	26.19
**4034**	allXL	AA	367	373	BS2G	12.55
**4034**	allXL	DD	370	382	BS2G	18.55
**4034**	allXL	BB	376	382	BS2G	12.56
**4034**	allXL	BB	357	373	BS2G	26.51
**4034**	allXL	DD	320	366	BS2G	17.87
**4034**	allXL	CC	370	376	BS2G	14.66
**4034**	allXL	AA	320	357	DBSU	25.67

**4034**	allXL	AA	357	319	DBSU	26.19
**4034**	allXL	DD	372	319	DBSU	9.72
**4034**	allXL	DD	370	357	DBSU	29.67
**4034**	allXL	BB	382	357	DBSU	26.74
**4034**	allXL	DD	372	370	DBSU	6.46
**4034**	allXL	DD	381	370	DBSU	18.93
**4034**	allXL	AA	373	370	DBSU	9.36
**4034**	allXL	DD	381	372	DBSU	19.44
**4034**	allXL	DD	372	382	DBSU	20.3
**4034**	allXL	DD	372	386	DBSU	19.96
**4034**	allXL	DD	373	386	DBSU	21.45
**4034**	allXL	CB	381	386	DBSU	36.29
**4034**	allXL	BD;DB	357	357	DBSU	18.52
**4034**	allXL	CB	373	319	DBSU	27.26
**4034**	allXL	CB	372	357	DBSU	22.99
**4034**	allXL	CB	381	357	DBSU	39.26
**4034**	allXL	CB	373	357	DBSU	23.6
**4034**	allXL	DA	372	370	DBSU	23.96
**4034**	allXL	DA	373	370	DBSU	20.79
**4034**	allXL	BC	382	373	DBSU	38.26
**4035**	allXL	BB	320	357	BS2G	24.97
**4035**	allXL	DD	320	373	BS2G	21.43
**4035**	allXL	DD	373	382	BS2G	19.99
**4035**	allXL	DD	370	386	BS2G	20.28
**4035**	allXL	DD	373	386	BS2G	24.71
**4035**	allXL	AA	319	357	BS2G	25.48
**4035**	allXL	AA	367	373	BS2G	13.83
**4035**	allXL	DD	370	382	BS2G	20.18
**4035**	allXL	CC	376	382	BS2G	14.06
**4035**	allXL	DD	357	373	BS2G	33.32
**4035**	allXL	CC	320	366	BS2G	15.44
**4035**	allXL	CC	370	376	BS2G	16.06
**4035**	allXL	BB	320	357	DBSU	24.97
**4035**	allXL	AA	357	319	DBSU	25.48

**4035**	allXL	CC	372	319	DBSU	20.18
**4035**	allXL	DD	370	357	DBSU	23.9
**4035**	allXL	DD	382	357	DBSU	36.68
**4035**	allXL	AA	372	370	DBSU	6.19
**4035**	allXL	DD	381	370	DBSU	19.28
**4035**	allXL	CC	373	370	DBSU	9.04
**4035**	allXL	DD	381	372	DBSU	19.29
**4035**	allXL	AA	372	382	DBSU	19.9
**4035**	allXL	AA	372	386	DBSU	23.01
**4035**	allXL	DD	373	386	DBSU	24.71
**4035**	allXL	CB	381	386	DBSU	47.33
**4035**	allXL	BD;DB	357	357	DBSU	18.89
**4035**	allXL	AD	373	319	DBSU	24.36
**4035**	allXL	CB	372	357	DBSU	30.6
**4035**	allXL	CB	381	357	DBSU	45.86
**4035**	allXL	CB	373	357	DBSU	32.57
**4035**	allXL	CB	372	370	DBSU	23.11
**4035**	allXL	CB	373	370	DBSU	20.92
**4035**	allXL	CB	382	373	DBSU	40.59
**4036**	allXL	AA	320	357	BS2G	21.05
**4036**	allXL	DD	320	373	BS2G	19.28
**4036**	allXL	CC	373	382	BS2G	17.8
**4036**	allXL	CC	370	386	BS2G	26.41
**4036**	allXL	CC	373	386	BS2G	22.05
**4036**	allXL	AA	319	357	BS2G	18.75
**4036**	allXL	AA	367	373	BS2G	12.09
**4036**	allXL	CC	370	382	BS2G	23.75
**4036**	allXL	CC	376	382	BS2G	13.41
**4036**	allXL	BB	357	373	BS2G	32.63
**4036**	allXL	AA	320	366	BS2G	25.12
**4036**	allXL	CC	370	376	BS2G	15.11
**4036**	allXL	AA	320	357	DBSU	21.05
**4036**	allXL	AA	357	319	DBSU	18.75
**4036**	allXL	DD	372	319	DBSU	17.62

**4036**	allXL	DD	370	357	DBSU	26.21
**4036**	allXL	CC	382	357	DBSU	38.73
**4036**	allXL	AA	372	370	DBSU	6.4
**4036**	allXL	BB	381	370	DBSU	24.84
**4036**	allXL	CC	373	370	DBSU	9.16
**4036**	allXL	BB	381	372	DBSU	18.75
**4036**	allXL	BB	372	382	DBSU	20.49
**4036**	allXL	AA	372	386	DBSU	23.61
**4036**	allXL	CC	373	386	DBSU	22.05
**4036**	allXL	DA	381	386	DBSU	62.13
**4036**	allXL	BD;DB	357	357	DBSU	18.39
**4036**	allXL	AD	373	319	DBSU	18.08
**4036**	allXL	DA	372	357	DBSU	36.5
**4036**	allXL	DA	381	357	DBSU	46.75
**4036**	allXL	DA	373	357	DBSU	38.68
**4036**	allXL	DA	372	370	DBSU	24.31
**4036**	allXL	DA	373	370	DBSU	25.61
**4036**	allXL	DA	382	373	DBSU	45.57
**4037**	allXL	AA	320	357	BS2G	24.55
**4037**	allXL	DD	320	373	BS2G	23.29
**4037**	allXL	CC	373	382	BS2G	17.59
**4037**	allXL	DD	370	386	BS2G	20.19
**4037**	allXL	AA	373	386	BS2G	21.52
**4037**	allXL	AA	319	357	BS2G	23.0
**4037**	allXL	AA	367	373	BS2G	13.77
**4037**	allXL	DD	370	382	BS2G	17.49
**4037**	allXL	CC	376	382	BS2G	14.31
**4037**	allXL	AA	357	373	BS2G	32.64
**4037**	allXL	AA	320	366	BS2G	23.12
**4037**	allXL	CC	370	376	BS2G	16.82
**4037**	allXL	AA	320	357	DBSU	24.55
**4037**	allXL	AA	357	319	DBSU	23.0
**4037**	allXL	CC	372	319	DBSU	24.21
**4037**	allXL	DD	370	357	DBSU	29.36

**4037**	allXL	AA	382	357	DBSU	39.61
**4037**	allXL	AA	372	370	DBSU	6.14
**4037**	allXL	DD	381	370	DBSU	19.45
**4037**	allXL	AA	373	370	DBSU	9.25
**4037**	allXL	BB	381	372	DBSU	19.51
**4037**	allXL	DD	372	382	DBSU	18.87
**4037**	allXL	DD	372	386	DBSU	22.73
**4037**	allXL	AA	373	386	DBSU	21.52
**4037**	allXL	DA	381	386	DBSU	47.8
**4037**	allXL	BD;DB	357	357	DBSU	18.18
**4037**	allXL	DA	373	319	DBSU	31.5
**4037**	allXL	CB	372	357	DBSU	41.83
**4037**	allXL	DA	381	357	DBSU	30.19
**4037**	allXL	DA	373	357	DBSU	41.37
**4037**	allXL	BC	372	370	DBSU	29.33
**4037**	allXL	BC	373	370	DBSU	30.35
**4037**	allXL	DA	382	373	DBSU	37.89
**4038**	allXL	BB	320	357	BS2G	22.27
**4038**	allXL	BB	320	373	BS2G	7.54
**4038**	allXL	BB	373	382	BS2G	18.11
**4038**	allXL	CC	370	386	BS2G	26.32
**4038**	allXL	AA	373	386	BS2G	26.82
**4038**	allXL	BB	319	357	BS2G	24.92
**4038**	allXL	AA	367	373	BS2G	13.64
**4038**	allXL	BB	370	382	BS2G	23.01
**4038**	allXL	BB	376	382	BS2G	12.76
**4038**	allXL	BB	357	373	BS2G	27.17
**4038**	allXL	BB	320	366	BS2G	13.87
**4038**	allXL	CC	370	376	BS2G	15.34
**4038**	allXL	BB	320	357	DBSU	22.27
**4038**	allXL	BB	357	319	DBSU	24.92
**4038**	allXL	BB	372	319	DBSU	10.03
**4038**	allXL	CC	370	357	DBSU	28.2
**4038**	allXL	CC	382	357	DBSU	35.96

**4038**	allXL	AA	372	370	DBSU	6.16
**4038**	allXL	CC	381	370	DBSU	23.34
**4038**	allXL	AA	373	370	DBSU	9.09
**4038**	allXL	BB	381	372	DBSU	22.56
**4038**	allXL	BB	372	382	DBSU	21.35
**4038**	allXL	AA	372	386	DBSU	28.34
**4038**	allXL	AA	373	386	DBSU	26.82
**4038**	allXL	BC	381	386	DBSU	50.57
**4038**	allXL	BD;DB	357	357	DBSU	19.44
**4038**	allXL	AD	373	319	DBSU	17.43
**4038**	allXL	AD	372	357	DBSU	37.33
**4038**	allXL	BC	381	357	DBSU	30.17
**4038**	allXL	AD	373	357	DBSU	40.97
**4038**	allXL	DA	372	370	DBSU	34.88
**4038**	allXL	DA	373	370	DBSU	34.58
**4038**	allXL	BC	382	373	DBSU	49.0
**4039**	allXL	AA	320	357	BS2G	26.16
**4039**	allXL	BB	320	373	BS2G	23.2
**4039**	allXL	DD	373	382	BS2G	19.97
**4039**	allXL	DD	370	386	BS2G	21.22
**4039**	allXL	DD	373	386	BS2G	24.75
**4039**	allXL	AA	319	357	BS2G	28.12
**4039**	allXL	AA	367	373	BS2G	12.92
**4039**	allXL	DD	370	382	BS2G	20.74
**4039**	allXL	CC	376	382	BS2G	14.42
**4039**	allXL	DD	357	373	BS2G	32.86
**4039**	allXL	CC	320	366	BS2G	20.15
**4039**	allXL	BB	370	376	BS2G	17.13
**4039**	allXL	AA	320	357	DBSU	26.16
**4039**	allXL	AA	357	319	DBSU	28.12
**4039**	allXL	BB	372	319	DBSU	22.82
**4039**	allXL	BB	370	357	DBSU	30.97
**4039**	allXL	DD	382	357	DBSU	39.97
**4039**	allXL	AA	372	370	DBSU	6.2

**4039**	allXL	DD	381	370	DBSU	19.99
**4039**	allXL	DD	373	370	DBSU	9.04
**4039**	allXL	DD	381	372	DBSU	18.9
**4039**	allXL	CC	372	382	DBSU	20.48
**4039**	allXL	DD	372	386	DBSU	24.9
**4039**	allXL	DD	373	386	DBSU	24.75
**4039**	allXL	BC	381	386	DBSU	41.87
**4039**	allXL	BD;DB	357	357	DBSU	19.07
**4039**	allXL	CB	373	319	DBSU	28.9
**4039**	allXL	CB	372	357	DBSU	43.46
**4039**	allXL	CB	381	357	DBSU	46.63
**4039**	allXL	CB	373	357	DBSU	46.89
**4039**	allXL	CB	372	370	DBSU	13.65
**4039**	allXL	CB	373	370	DBSU	17.06
**4039**	allXL	BC	382	373	DBSU	16.58
**4040**	allXL	AA	320	357	BS2G	27.1
**4040**	allXL	CC	320	373	BS2G	19.16
**4040**	allXL	CC	373	382	BS2G	19.79
**4040**	allXL	CC	370	386	BS2G	23.11
**4040**	allXL	CC	373	386	BS2G	23.56
**4040**	allXL	AA	319	357	BS2G	26.52
**4040**	allXL	BB	367	373	BS2G	14.1
**4040**	allXL	CC	370	382	BS2G	22.16
**4040**	allXL	CC	376	382	BS2G	12.33
**4040**	allXL	BB	357	373	BS2G	20.63
**4040**	allXL	CC	320	366	BS2G	16.43
**4040**	allXL	CC	370	376	BS2G	16.03
**4040**	allXL	AA	320	357	DBSU	27.1
**4040**	allXL	AA	357	319	DBSU	26.52
**4040**	allXL	CC	372	319	DBSU	16.72
**4040**	allXL	CC	370	357	DBSU	22.76
**4040**	allXL	BB	382	357	DBSU	18.81
**4040**	allXL	AA	372	370	DBSU	5.81
**4040**	allXL	CC	381	370	DBSU	25.17

**4040**	allXL	AA	373	370	DBSU	9.23
**4040**	allXL	AA	381	372	DBSU	21.36
**4040**	allXL	CC	372	382	DBSU	19.56
**4040**	allXL	CC	372	386	DBSU	21.52
**4040**	allXL	CC	373	386	DBSU	23.56
**4040**	allXL	AD	381	386	DBSU	29.46
**4040**	allXL	BD;DB	357	357	DBSU	18.52
**4040**	allXL	AD	373	319	DBSU	37.06
**4040**	allXL	CA	372	357	DBSU	38.01
**4040**	allXL	BD	381	357	DBSU	25.59
**4040**	allXL	CA	373	357	DBSU	36.24
**4040**	allXL	DA	372	370	DBSU	40.12
**4040**	allXL	DA	373	370	DBSU	43.37
**4040**	allXL	AD	382	373	DBSU	31.96
**4041**	allXL	CC	320	357	BS2G	25.46
**4041**	allXL	BB	320	373	BS2G	23.45
**4041**	allXL	DD	373	382	BS2G	15.72
**4041**	allXL	DD	370	386	BS2G	13.63
**4041**	allXL	DD	373	386	BS2G	11.07
**4041**	allXL	AA	319	357	BS2G	25.45
**4041**	allXL	BB	367	373	BS2G	13.36
**4041**	allXL	DD	370	382	BS2G	19.13
**4041**	allXL	BB	376	382	BS2G	15.3
**4041**	allXL	BB	357	373	BS2G	17.76
**4041**	allXL	BB	320	366	BS2G	21.98
**4041**	allXL	CC	370	376	BS2G	15.33
**4041**	allXL	CC	320	357	DBSU	25.46
**4041**	allXL	AA	357	319	DBSU	25.45
**4041**	allXL	BB	372	319	DBSU	23.35
**4041**	allXL	BB	370	357	DBSU	21.66
**4041**	allXL	BB	382	357	DBSU	30.16
**4041**	allXL	AA	372	370	DBSU	6.07
**4041**	allXL	DD	381	370	DBSU	18.95
**4041**	allXL	CC	373	370	DBSU	9.0

**4041**	allXL	DD	381	372	DBSU	13.94
**4041**	allXL	DD	372	382	DBSU	14.15
**4041**	allXL	DD	372	386	DBSU	8.14
**4041**	allXL	DD	373	386	DBSU	11.07
**4041**	allXL	BC	381	386	DBSU	56.51
**4041**	allXL	BD;DB	357	357	DBSU	20.0
**4041**	allXL	CB	373	319	DBSU	36.37
**4041**	allXL	CA	372	357	DBSU	35.21
**4041**	allXL	CA	381	357	DBSU	37.15
**4041**	allXL	CA	373	357	DBSU	32.79
**4041**	allXL	DA	372	370	DBSU	42.82
**4041**	allXL	DA	373	370	DBSU	43.27
**4041**	allXL	CB	382	373	DBSU	48.16
**4042**	allXL	AA	320	357	BS2G	23.62
**4042**	allXL	DD	320	373	BS2G	21.81
**4042**	allXL	CC	373	382	BS2G	21.99
**4042**	allXL	CC	370	386	BS2G	18.55
**4042**	allXL	CC	373	386	BS2G	23.81
**4042**	allXL	AA	319	357	BS2G	22.09
**4042**	allXL	AA	367	373	BS2G	13.13
**4042**	allXL	CC	370	382	BS2G	21.6
**4042**	allXL	CC	376	382	BS2G	14.44
**4042**	allXL	DD	357	373	BS2G	22.84
**4042**	allXL	BB	320	366	BS2G	25.04
**4042**	allXL	CC	370	376	BS2G	15.05
**4042**	allXL	AA	320	357	DBSU	23.62
**4042**	allXL	AA	357	319	DBSU	22.09
**4042**	allXL	DD	372	319	DBSU	20.21
**4042**	allXL	DD	370	357	DBSU	23.23
**4042**	allXL	DD	382	357	DBSU	27.71
**4042**	allXL	AA	372	370	DBSU	6.28
**4042**	allXL	DD	381	370	DBSU	20.49
**4042**	allXL	AA	373	370	DBSU	9.21
**4042**	allXL	DD	381	372	DBSU	20.53

**4042**	allXL	CC	372	382	DBSU	23.21
**4042**	allXL	CC	372	386	DBSU	23.48
**4042**	allXL	CC	373	386	DBSU	23.81
**4042**	allXL	BC	381	386	DBSU	56.94
**4042**	allXL	BD;DB	357	357	DBSU	19.34
**4042**	allXL	CB	373	319	DBSU	36.07
**4042**	allXL	CB	372	357	DBSU	40.89
**4042**	allXL	DB	381	357	DBSU	40.1
**4042**	allXL	DB	373	357	DBSU	40.66
**4042**	allXL	CB	372	370	DBSU	22.32
**4042**	allXL	CB	373	370	DBSU	25.37
**4042**	allXL	CB	382	373	DBSU	39.52
**4043**	allXL	CC	320	357	BS2G	24.73
**4043**	allXL	DD	320	373	BS2G	17.19
**4043**	allXL	BB	373	382	BS2G	19.05
**4043**	allXL	CC	370	386	BS2G	25.53
**4043**	allXL	CC	373	386	BS2G	27.08
**4043**	allXL	CC	319	357	BS2G	26.87
**4043**	allXL	AA	367	373	BS2G	13.76
**4043**	allXL	CC	370	382	BS2G	18.75
**4043**	allXL	BB	376	382	BS2G	14.34
**4043**	allXL	CC	357	373	BS2G	15.56
**4043**	allXL	BB	320	366	BS2G	20.21
**4043**	allXL	CC	370	376	BS2G	14.45
**4043**	allXL	CC	320	357	DBSU	24.73
**4043**	allXL	CC	357	319	DBSU	26.87
**4043**	allXL	DD	372	319	DBSU	17.94
**4043**	allXL	DD	370	357	DBSU	18.64
**4043**	allXL	CC	382	357	DBSU	18.79
**4043**	allXL	AA	372	370	DBSU	6.02
**4043**	allXL	CC	381	370	DBSU	20.74
**4043**	allXL	AA	373	370	DBSU	9.2
**4043**	allXL	BB	381	372	DBSU	21.0
**4043**	allXL	BB	372	382	DBSU	20.81

**4043**	allXL	CC	372	386	DBSU	28.54
**4043**	allXL	CC	373	386	DBSU	27.08
**4043**	allXL	BD	381	386	DBSU	34.76
**4043**	allXL	BD;DB	357	357	DBSU	19.22
**4043**	allXL	CB	373	319	DBSU	26.82
**4043**	allXL	CA	372	357	DBSU	27.93
**4043**	allXL	BD	381	357	DBSU	26.27
**4043**	allXL	CA	373	357	DBSU	24.87
**4043**	allXL	DA	372	370	DBSU	36.68
**4043**	allXL	CB	373	370	DBSU	39.27
**4043**	allXL	BD	382	373	DBSU	52.86
**4044**	allXL	CC	320	357	BS2G	26.52
**4044**	allXL	BB	320	373	BS2G	24.73
**4044**	allXL	BB	373	382	BS2G	21.09
**4044**	allXL	BB	370	386	BS2G	24.35
**4044**	allXL	BB	373	386	BS2G	28.53
**4044**	allXL	AA	319	357	BS2G	26.12
**4044**	allXL	BB	367	373	BS2G	13.25
**4044**	allXL	BB	370	382	BS2G	21.11
**4044**	allXL	CC	376	382	BS2G	13.15
**4044**	allXL	BB	357	373	BS2G	24.83
**4044**	allXL	AA	320	366	BS2G	23.79
**4044**	allXL	CC	370	376	BS2G	15.67
**4044**	allXL	CC	320	357	DBSU	26.52
**4044**	allXL	AA	357	319	DBSU	26.12
**4044**	allXL	BB	372	319	DBSU	31.97
**4044**	allXL	DD	370	357	DBSU	29.45
**4044**	allXL	CC	382	357	DBSU	30.61
**4044**	allXL	AA	372	370	DBSU	6.07
**4044**	allXL	DD	381	370	DBSU	21.74
**4044**	allXL	AA	373	370	DBSU	9.26
**4044**	allXL	DD	381	372	DBSU	22.14
**4044**	allXL	BB	372	382	DBSU	23.04
**4044**	allXL	BB	372	386	DBSU	29.08

**4044**	allXL	BB	373	386	DBSU	28.53
**4044**	allXL	CB	381	386	DBSU	54.44
**4044**	allXL	BD;DB	357	357	DBSU	18.46
**4044**	allXL	AD	373	319	DBSU	36.66
**4044**	allXL	DB	372	357	DBSU	41.06
**4044**	allXL	BC	381	357	DBSU	34.39
**4044**	allXL	DB	373	357	DBSU	39.04
**4044**	allXL	DA	372	370	DBSU	46.56
**4044**	allXL	AD	373	370	DBSU	48.63
**4044**	allXL	BC	382	373	DBSU	48.92
**4045**	allXL	CC	320	357	BS2G	24.24
**4045**	allXL	BB	320	373	BS2G	23.46
**4045**	allXL	BB	373	382	BS2G	13.85
**4045**	allXL	BB	370	386	BS2G	20.23
**4045**	allXL	DD	373	386	BS2G	19.65
**4045**	allXL	CC	319	357	BS2G	27.93
**4045**	allXL	AA	367	373	BS2G	9.88
**4045**	allXL	BB	370	382	BS2G	15.68
**4045**	allXL	BB	376	382	BS2G	12.69
**4045**	allXL	BB	357	373	BS2G	21.53
**4045**	allXL	AA	320	366	BS2G	20.63
**4045**	allXL	BB	370	376	BS2G	17.54
**4045**	allXL	CC	320	357	DBSU	24.24
**4045**	allXL	CC	357	319	DBSU	27.93
**4045**	allXL	BB	372	319	DBSU	27.39
**4045**	allXL	BB	370	357	DBSU	25.01
**4045**	allXL	AA	382	357	DBSU	26.29
**4045**	allXL	CC	372	370	DBSU	6.8
**4045**	allXL	BB	381	370	DBSU	18.82
**4045**	allXL	AA	373	370	DBSU	9.02
**4045**	allXL	BB	381	372	DBSU	15.67
**4045**	allXL	BB	372	382	DBSU	13.7
**4045**	allXL	DD	372	386	DBSU	19.69
**4045**	allXL	DD	373	386	DBSU	19.65

**4045**	allXL	AD	381	386	DBSU	38.2
**4045**	allXL	BD;DB	357	357	DBSU	18.43
**4045**	allXL	BC	373	319	DBSU	34.98
**4045**	allXL	BD	372	357	DBSU	36.18
**4045**	allXL	AC	381	357	DBSU	44.54
**4045**	allXL	BD	373	357	DBSU	38.29
**4045**	allXL	DA	372	370	DBSU	27.37
**4045**	allXL	DA	373	370	DBSU	28.46
**4045**	allXL	DA	382	373	DBSU	25.53
**4046**	allXL	CC	320	357	BS2G	24.28
**4046**	allXL	DD	320	373	BS2G	23.0
**4046**	allXL	DD	373	382	BS2G	19.58
**4046**	allXL	DD	370	386	BS2G	26.72
**4046**	allXL	DD	373	386	BS2G	23.21
**4046**	allXL	CC	319	357	BS2G	25.74
**4046**	allXL	BB	367	373	BS2G	13.49
**4046**	allXL	CC	370	382	BS2G	25.24
**4046**	allXL	BB	376	382	BS2G	12.97
**4046**	allXL	DD	357	373	BS2G	29.62
**4046**	allXL	DD	320	366	BS2G	24.28
**4046**	allXL	CC	370	376	BS2G	15.68
**4046**	allXL	CC	320	357	DBSU	24.28
**4046**	allXL	CC	357	319	DBSU	25.74
**4046**	allXL	DD	372	319	DBSU	23.64
**4046**	allXL	DD	370	357	DBSU	22.82
**4046**	allXL	DD	382	357	DBSU	36.44
**4046**	allXL	AA	372	370	DBSU	5.87
**4046**	allXL	CC	381	370	DBSU	25.33
**4046**	allXL	AA	373	370	DBSU	9.13
**4046**	allXL	DD	381	372	DBSU	20.21
**4046**	allXL	DD	372	382	DBSU	19.25
**4046**	allXL	DD	372	386	DBSU	21.03
**4046**	allXL	DD	373	386	DBSU	23.21
**4046**	allXL	BD	381	386	DBSU	44.4

**4046**	allXL	BD;DB	357	357	DBSU	18.71
**4046**	allXL	CB	373	319	DBSU	9.06
**4046**	allXL	CB	372	357	DBSU	37.67
**4046**	allXL	DB	381	357	DBSU	48.99
**4046**	allXL	CB	373	357	DBSU	38.68
**4046**	allXL	BC	372	370	DBSU	41.22
**4046**	allXL	BC	373	370	DBSU	44.63
**4046**	allXL	DB	382	373	DBSU	42.81
**4047**	allXL	AA	320	357	BS2G	22.74
**4047**	allXL	BB	320	373	BS2G	21.93
**4047**	allXL	BB	373	382	BS2G	19.18
**4047**	allXL	DD	370	386	BS2G	26.49
**4047**	allXL	BB	373	386	BS2G	20.72
**4047**	allXL	AA	319	357	BS2G	20.64
**4047**	allXL	BB	367	373	BS2G	13.67
**4047**	allXL	DD	370	382	BS2G	23.3
**4047**	allXL	BB	376	382	BS2G	12.66
**4047**	allXL	BB	357	373	BS2G	27.76
**4047**	allXL	AA	320	366	BS2G	23.93
**4047**	allXL	CC	370	376	BS2G	16.34
**4047**	allXL	AA	320	357	DBSU	22.74
**4047**	allXL	AA	357	319	DBSU	20.64
**4047**	allXL	BB	372	319	DBSU	26.95
**4047**	allXL	DD	370	357	DBSU	28.33
**4047**	allXL	BB	382	357	DBSU	28.34
**4047**	allXL	AA	372	370	DBSU	6.3
**4047**	allXL	DD	381	370	DBSU	24.26
**4047**	allXL	CC	373	370	DBSU	9.41
**4047**	allXL	DD	381	372	DBSU	18.76
**4047**	allXL	DD	372	382	DBSU	18.71
**4047**	allXL	BB	372	386	DBSU	21.2
**4047**	allXL	BB	373	386	DBSU	20.72
**4047**	allXL	BD	381	386	DBSU	54.33
**4047**	allXL	BD;DB	357	357	DBSU	19.26

**4047**	allXL	AD	373	319	DBSU	41.53
**4047**	allXL	BD	372	357	DBSU	46.11
**4047**	allXL	BD	381	357	DBSU	43.11
**4047**	allXL	BD	373	357	DBSU	45.89
**4047**	allXL	CB	372	370	DBSU	43.48
**4047**	allXL	CB	373	370	DBSU	42.17
**4047**	allXL	CB	382	373	DBSU	44.73
**4048**	allXL	AA	320	357	BS2G	24.75
**4048**	allXL	BB	320	373	BS2G	13.27
**4048**	allXL	DD	373	382	BS2G	19.27
**4048**	allXL	CC	370	386	BS2G	26.64
**4048**	allXL	DD	373	386	BS2G	26.97
**4048**	allXL	AA	319	357	BS2G	25.41
**4048**	allXL	BB	367	373	BS2G	12.5
**4048**	allXL	CC	370	382	BS2G	22.25
**4048**	allXL	CC	376	382	BS2G	14.3
**4048**	allXL	DD	357	373	BS2G	23.53
**4048**	allXL	CC	320	366	BS2G	15.92
**4048**	allXL	CC	370	376	BS2G	14.4
**4048**	allXL	AA	320	357	DBSU	24.75
**4048**	allXL	AA	357	319	DBSU	25.41
**4048**	allXL	BB	372	319	DBSU	17.85
**4048**	allXL	DD	370	357	DBSU	28.05
**4048**	allXL	CC	382	357	DBSU	28.08
**4048**	allXL	AA	372	370	DBSU	6.21
**4048**	allXL	CC	381	370	DBSU	24.28
**4048**	allXL	AA	373	370	DBSU	8.99
**4048**	allXL	AA	381	372	DBSU	21.64
**4048**	allXL	AA	372	382	DBSU	20.5
**4048**	allXL	AA	372	386	DBSU	27.9
**4048**	allXL	DD	373	386	DBSU	26.97
**4048**	allXL	BC	381	386	DBSU	41.19
**4048**	allXL	BD;DB	357	357	DBSU	18.7
**4048**	allXL	AD	373	319	DBSU	42.09

**4048**	allXL	BD	372	357	DBSU	40.46
**4048**	allXL	DB	381	357	DBSU	43.1
**4048**	allXL	DB	373	357	DBSU	38.32
**4048**	allXL	DA	372	370	DBSU	58.46
**4048**	allXL	DB	373	370	DBSU	58.06
**4048**	allXL	BC	382	373	DBSU	47.09
**4049**	allXL	BB	320	357	BS2G	24.38
**4049**	allXL	CC	320	373	BS2G	17.7
**4049**	allXL	DD	373	382	BS2G	16.94
**4049**	allXL	DD	370	386	BS2G	18.05
**4049**	allXL	BB	373	386	BS2G	22.26
**4049**	allXL	AA	319	357	BS2G	24.29
**4049**	allXL	AA	367	373	BS2G	13.39
**4049**	allXL	DD	370	382	BS2G	14.26
**4049**	allXL	BB	376	382	BS2G	14.6
**4049**	allXL	CC	357	373	BS2G	25.35
**4049**	allXL	DD	320	366	BS2G	29.35
**4049**	allXL	CC	370	376	BS2G	15.27
**4049**	allXL	BB	320	357	DBSU	24.38
**4049**	allXL	AA	357	319	DBSU	24.29
**4049**	allXL	CC	372	319	DBSU	18.16
**4049**	allXL	CC	370	357	DBSU	24.02
**4049**	allXL	CC	382	357	DBSU	34.83
**4049**	allXL	AA	372	370	DBSU	6.27
**4049**	allXL	DD	381	370	DBSU	14.89
**4049**	allXL	DD	373	370	DBSU	9.27
**4049**	allXL	BB	381	372	DBSU	17.37
**4049**	allXL	DD	372	382	DBSU	18.31
**4049**	allXL	BB	372	386	DBSU	22.59
**4049**	allXL	BB	373	386	DBSU	22.26
**4049**	allXL	AD	381	386	DBSU	62.56
**4049**	allXL	BD;DB	357	357	DBSU	18.66
**4049**	allXL	AD	373	319	DBSU	29.92
**4049**	allXL	CA	372	357	DBSU	38.89

**4049**	allXL	CA	381	357	DBSU	54.93
**4049**	allXL	CA	373	357	DBSU	42.09
**4049**	allXL	CB	372	370	DBSU	46.83
**4049**	allXL	CB	373	370	DBSU	44.49
**4049**	allXL	DA	382	373	DBSU	49.57
**4050**	allXL	AA	320	357	BS2G	24.29
**4050**	allXL	DD	320	373	BS2G	18.96
**4050**	allXL	DD	373	382	BS2G	18.5
**4050**	allXL	DD	370	386	BS2G	22.48
**4050**	allXL	DD	373	386	BS2G	26.3
**4050**	allXL	AA	319	357	BS2G	23.55
**4050**	allXL	BB	367	373	BS2G	11.86
**4050**	allXL	DD	370	382	BS2G	15.99
**4050**	allXL	BB	376	382	BS2G	14.61
**4050**	allXL	BB	357	373	BS2G	22.69
**4050**	allXL	DD	320	366	BS2G	29.12
**4050**	allXL	CC	370	376	BS2G	12.87
**4050**	allXL	AA	320	357	DBSU	24.29
**4050**	allXL	AA	357	319	DBSU	23.55
**4050**	allXL	CC	372	319	DBSU	17.65
**4050**	allXL	DD	370	357	DBSU	26.55
**4050**	allXL	BB	382	357	DBSU	26.39
**4050**	allXL	AA	372	370	DBSU	6.17
**4050**	allXL	DD	381	370	DBSU	16.44
**4050**	allXL	AA	373	370	DBSU	9.09
**4050**	allXL	DD	381	372	DBSU	18.59
**4050**	allXL	DD	372	382	DBSU	18.74
**4050**	allXL	AA	372	386	DBSU	24.46
**4050**	allXL	DD	373	386	DBSU	26.3
**4050**	allXL	CB	381	386	DBSU	60.98
**4050**	allXL	BD;DB	357	357	DBSU	18.23
**4050**	allXL	DA	373	319	DBSU	27.73
**4050**	allXL	DA	372	357	DBSU	36.67
**4050**	allXL	BD	381	357	DBSU	33.73

**4050**	allXL	DA	373	357	DBSU	33.61
**4050**	allXL	CB	372	370	DBSU	27.01
**4050**	allXL	BC	373	370	DBSU	28.69
**4050**	allXL	BC	382	373	DBSU	39.78
**4051**	allXL	AA	320	357	BS2G	25.59
**4051**	allXL	BB	320	373	BS2G	9.39
**4051**	allXL	DD	373	382	BS2G	19.1
**4051**	allXL	DD	370	386	BS2G	21.05
**4051**	allXL	DD	373	386	BS2G	27.25
**4051**	allXL	AA	319	357	BS2G	26.51
**4051**	allXL	AA	367	373	BS2G	13.89
**4051**	allXL	DD	370	382	BS2G	16.79
**4051**	allXL	CC	376	382	BS2G	15.13
**4051**	allXL	BB	357	373	BS2G	23.81
**4051**	allXL	AA	320	366	BS2G	16.26
**4051**	allXL	BB	370	376	BS2G	14.59
**4051**	allXL	AA	320	357	DBSU	25.59
**4051**	allXL	AA	357	319	DBSU	26.51
**4051**	allXL	BB	372	319	DBSU	12.22
**4051**	allXL	BB	370	357	DBSU	26.87
**4051**	allXL	BB	382	357	DBSU	38.19
**4051**	allXL	AA	372	370	DBSU	6.08
**4051**	allXL	DD	381	370	DBSU	15.24
**4051**	allXL	AA	373	370	DBSU	9.07
**4051**	allXL	DD	381	372	DBSU	17.02
**4051**	allXL	DD	372	382	DBSU	19.55
**4051**	allXL	DD	372	386	DBSU	26.4
**4051**	allXL	DD	373	386	DBSU	27.25
**4051**	allXL	DA	381	386	DBSU	21.33
**4051**	allXL	BD;DB	357	357	DBSU	17.87
**4051**	allXL	DA	373	319	DBSU	30.26
**4051**	allXL	BD	372	357	DBSU	38.4
**4051**	allXL	BC	381	357	DBSU	35.49
**4051**	allXL	BD	373	357	DBSU	38.53

**4051**	allXL	CB	372	370	DBSU	25.14
**4051**	allXL	CB	373	370	DBSU	26.83
**4051**	allXL	AD	382	373	DBSU	28.67
**4052**	allXL	CC	320	357	BS2G	22.57
**4052**	allXL	BB	320	373	BS2G	16.74
**4052**	allXL	DD	373	382	BS2G	17.79
**4052**	allXL	CC	370	386	BS2G	23.71
**4052**	allXL	CC	373	386	BS2G	22.79
**4052**	allXL	CC	319	357	BS2G	24.95
**4052**	allXL	BB	367	373	BS2G	14.0
**4052**	allXL	BB	370	382	BS2G	17.39
**4052**	allXL	CC	376	382	BS2G	14.3
**4052**	allXL	BB	357	373	BS2G	22.07
**4052**	allXL	AA	320	366	BS2G	21.56
**4052**	allXL	CC	370	376	BS2G	15.48
**4052**	allXL	CC	320	357	DBSU	22.57
**4052**	allXL	CC	357	319	DBSU	24.95
**4052**	allXL	AA	372	319	DBSU	17.41
**4052**	allXL	BB	370	357	DBSU	28.56
**4052**	allXL	BB	382	357	DBSU	21.61
**4052**	allXL	AA	372	370	DBSU	6.13
**4052**	allXL	BB	381	370	DBSU	19.14
**4052**	allXL	AA	373	370	DBSU	9.16
**4052**	allXL	DD	381	372	DBSU	17.33
**4052**	allXL	DD	372	382	DBSU	18.93
**4052**	allXL	CC	372	386	DBSU	25.02
**4052**	allXL	CC	373	386	DBSU	22.79
**4052**	allXL	DA	381	386	DBSU	18.29
**4052**	allXL	BD;DB	357	357	DBSU	18.44
**4052**	allXL	DA	373	319	DBSU	20.57
**4052**	allXL	DA	372	357	DBSU	38.68
**4052**	allXL	BD	381	357	DBSU	37.25
**4052**	allXL	BD	373	357	DBSU	40.2
**4052**	allXL	DA	372	370	DBSU	21.12

**4052**	allXL	DA	373	370	DBSU	20.54
**4052**	allXL	AD	382	373	DBSU	22.61
**4053**	allXL	AA	320	357	BS2G	24.93
**4053**	allXL	BB	320	373	BS2G	20.51
**4053**	allXL	AA	373	382	BS2G	18.86
**4053**	allXL	AA	370	386	BS2G	23.72
**4053**	allXL	AA	373	386	BS2G	25.96
**4053**	allXL	AA	319	357	BS2G	24.06
**4053**	allXL	DD	367	373	BS2G	14.91
**4053**	allXL	AA	370	382	BS2G	20.67
**4053**	allXL	BB	376	382	BS2G	13.73
**4053**	allXL	BB	357	373	BS2G	30.78
**4053**	allXL	BB	320	366	BS2G	25.0
**4053**	allXL	AA	370	376	BS2G	16.67
**4053**	allXL	AA	320	357	DBSU	24.93
**4053**	allXL	AA	357	319	DBSU	24.06
**4053**	allXL	BB	372	319	DBSU	22.76
**4053**	allXL	DD	370	357	DBSU	27.41
**4053**	allXL	BB	382	357	DBSU	32.53
**4053**	allXL	AA	372	370	DBSU	6.52
**4053**	allXL	DD	381	370	DBSU	18.95
**4053**	allXL	AA	373	370	DBSU	8.48
**4053**	allXL	DD	381	372	DBSU	18.57
**4053**	allXL	AA	372	382	DBSU	21.05
**4053**	allXL	AA	372	386	DBSU	27.07
**4053**	allXL	AA	373	386	DBSU	25.96
**4053**	allXL	CB	381	386	DBSU	23.92
**4053**	allXL	BD;DB	357	357	DBSU	18.4
**4053**	allXL	CB	373	319	DBSU	14.33
**4053**	allXL	CB	372	357	DBSU	38.59
**4053**	allXL	CB	381	357	DBSU	36.37
**4053**	allXL	CB	373	357	DBSU	35.28
**4053**	allXL	DA	372	370	DBSU	30.86
**4053**	allXL	DA	373	370	DBSU	28.75

**4053**	allXL	CB	382	373	DBSU	13.33
**4054**	allXL	AA	320	357	BS2G	21.4
**4054**	allXL	BB	320	373	BS2G	18.3
**4054**	allXL	BB	373	382	BS2G	17.63
**4054**	allXL	CC	370	386	BS2G	23.8
**4054**	allXL	BB	373	386	BS2G	24.88
**4054**	allXL	AA	319	357	BS2G	19.73
**4054**	allXL	AA	367	373	BS2G	12.97
**4054**	allXL	CC	370	382	BS2G	20.45
**4054**	allXL	CC	376	382	BS2G	14.51
**4054**	allXL	DD	357	373	BS2G	22.64
**4054**	allXL	CC	320	366	BS2G	18.9
**4054**	allXL	CC	370	376	BS2G	15.75
**4054**	allXL	AA	320	357	DBSU	21.4
**4054**	allXL	AA	357	319	DBSU	19.73
**4054**	allXL	CC	372	319	DBSU	19.3
**4054**	allXL	BB	370	357	DBSU	23.96
**4054**	allXL	DD	382	357	DBSU	26.08
**4054**	allXL	AA	372	370	DBSU	6.29
**4054**	allXL	DD	381	370	DBSU	20.71
**4054**	allXL	AA	373	370	DBSU	9.11
**4054**	allXL	BB	381	372	DBSU	17.44
**4054**	allXL	BB	372	382	DBSU	19.4
**4054**	allXL	BB	372	386	DBSU	24.94
**4054**	allXL	BB	373	386	DBSU	24.88
**4054**	allXL	BD	381	386	DBSU	59.86
**4054**	allXL	BD;DB	357	357	DBSU	18.8
**4054**	allXL	AD	373	319	DBSU	33.41
**4054**	allXL	DB	372	357	DBSU	40.31
**4054**	allXL	BD	381	357	DBSU	41.35
**4054**	allXL	DB	373	357	DBSU	38.77
**4054**	allXL	CB	372	370	DBSU	55.98
**4054**	allXL	BD	373	370	DBSU	56.37
**4054**	allXL	DA	382	373	DBSU	49.61

**4055**	allXL	CC	320	357	BS2G	24.93
**4055**	allXL	AA	320	373	BS2G	22.71
**4055**	allXL	DD	373	382	BS2G	15.36
**4055**	allXL	DD	370	386	BS2G	14.07
**4055**	allXL	DD	373	386	BS2G	20.0
**4055**	allXL	CC	319	357	BS2G	26.86
**4055**	allXL	BB	367	373	BS2G	13.19
**4055**	allXL	DD	370	382	BS2G	10.53
**4055**	allXL	CC	376	382	BS2G	14.26
**4055**	allXL	BB	357	373	BS2G	23.56
**4055**	allXL	CC	320	366	BS2G	19.17
**4055**	allXL	DD	370	376	BS2G	16.14
**4055**	allXL	CC	320	357	DBSU	24.93
**4055**	allXL	CC	357	319	DBSU	26.86
**4055**	allXL	CC	372	319	DBSU	22.6
**4055**	allXL	CC	370	357	DBSU	22.11
**4055**	allXL	BB	382	357	DBSU	22.59
**4055**	allXL	AA	372	370	DBSU	6.2
**4055**	allXL	DD	381	370	DBSU	12.33
**4055**	allXL	DD	373	370	DBSU	9.19
**4055**	allXL	DD	381	372	DBSU	16.01
**4055**	allXL	DD	372	382	DBSU	15.12
**4055**	allXL	DD	372	386	DBSU	17.7
**4055**	allXL	DD	373	386	DBSU	20.0
**4055**	allXL	DA	381	386	DBSU	50.65
**4055**	allXL	BD;DB	357	357	DBSU	17.77
**4055**	allXL	AD	373	319	DBSU	23.51
**4055**	allXL	AD	372	357	DBSU	33.32
**4055**	allXL	BD	381	357	DBSU	26.46
**4055**	allXL	AD	373	357	DBSU	34.56
**4055**	allXL	AD	372	370	DBSU	36.07
**4055**	allXL	DA	373	370	DBSU	35.18
**4055**	allXL	DA	382	373	DBSU	37.66
**4056**	allXL	AA	320	357	BS2G	25.23

**4056**	allXL	DD	320	373	BS2G	19.92
**4056**	allXL	DD	373	382	BS2G	17.25
**4056**	allXL	DD	370	386	BS2G	19.31
**4056**	allXL	DD	373	386	BS2G	20.36
**4056**	allXL	AA	319	357	BS2G	26.2
**4056**	allXL	BB	367	373	BS2G	10.8
**4056**	allXL	DD	370	382	BS2G	20.89
**4056**	allXL	AA	376	382	BS2G	14.22
**4056**	allXL	DD	357	373	BS2G	25.21
**4056**	allXL	BB	320	366	BS2G	20.17
**4056**	allXL	CC	370	376	BS2G	13.86
**4056**	allXL	AA	320	357	DBSU	25.23
**4056**	allXL	AA	357	319	DBSU	26.2
**4056**	allXL	BB	372	319	DBSU	21.29
**4056**	allXL	DD	370	357	DBSU	18.34
**4056**	allXL	BB	382	357	DBSU	22.0
**4056**	allXL	AA	372	370	DBSU	6.37
**4056**	allXL	DD	381	370	DBSU	22.58
**4056**	allXL	AA	373	370	DBSU	9.26
**4056**	allXL	DD	381	372	DBSU	19.88
**4056**	allXL	DD	372	382	DBSU	19.19
**4056**	allXL	DD	372	386	DBSU	20.44
**4056**	allXL	DD	373	386	DBSU	20.36
**4056**	allXL	AD	381	386	DBSU	64.88
**4056**	allXL	BD;DB	357	357	DBSU	19.53
**4056**	allXL	BC	373	319	DBSU	32.0
**4056**	allXL	BC	372	357	DBSU	36.02
**4056**	allXL	BD	381	357	DBSU	44.23
**4056**	allXL	BC	373	357	DBSU	39.27
**4056**	allXL	CB	372	370	DBSU	22.79
**4056**	allXL	CB	373	370	DBSU	25.4
**4056**	allXL	BC	382	373	DBSU	46.23
**4057**	allXL	AA	320	357	BS2G	20.57
**4057**	allXL	BB	320	373	BS2G	17.23

**4057**	allXL	BB	373	382	BS2G	16.29
**4057**	allXL	BB	370	386	BS2G	21.75
**4057**	allXL	AA	373	386	BS2G	21.29
**4057**	allXL	AA	319	357	BS2G	23.75
**4057**	allXL	AA	367	373	BS2G	13.7
**4057**	allXL	BB	370	382	BS2G	19.76
**4057**	allXL	BB	376	382	BS2G	12.42
**4057**	allXL	BB	357	373	BS2G	22.25
**4057**	allXL	DD	320	366	BS2G	24.13
**4057**	allXL	BB	370	376	BS2G	16.69
**4057**	allXL	AA	320	357	DBSU	20.57
**4057**	allXL	AA	357	319	DBSU	23.75
**4057**	allXL	BB	372	319	DBSU	20.56
**4057**	allXL	BB	370	357	DBSU	18.77
**4057**	allXL	AA	382	357	DBSU	15.18
**4057**	allXL	AA	372	370	DBSU	6.32
**4057**	allXL	BB	381	370	DBSU	22.72
**4057**	allXL	AA	373	370	DBSU	9.15
**4057**	allXL	BB	381	372	DBSU	20.0
**4057**	allXL	BB	372	382	DBSU	17.43
**4057**	allXL	BB	372	386	DBSU	21.57
**4057**	allXL	AA	373	386	DBSU	21.29
**4057**	allXL	BC	381	386	DBSU	59.08
**4057**	allXL	BD;DB	357	357	DBSU	18.0
**4057**	allXL	AD	373	319	DBSU	37.2
**4057**	allXL	BD	372	357	DBSU	31.5
**4057**	allXL	AC	381	357	DBSU	13.83
**4057**	allXL	AC	373	357	DBSU	33.04
**4057**	allXL	BC	372	370	DBSU	50.98
**4057**	allXL	BC	373	370	DBSU	49.66
**4057**	allXL	AC	382	373	DBSU	53.66
**4058**	allXL	BB	320	357	BS2G	24.24
**4058**	allXL	AA	320	373	BS2G	16.73
**4058**	allXL	AA	373	382	BS2G	15.97

**4058**	allXL	AA	370	386	BS2G	26.32
**4058**	allXL	CC	373	386	BS2G	25.45
**4058**	allXL	BB	319	357	BS2G	24.65
**4058**	allXL	AA	367	373	BS2G	13.16
**4058**	allXL	AA	370	382	BS2G	19.38
**4058**	allXL	CC	376	382	BS2G	12.88
**4058**	allXL	BB	357	373	BS2G	22.9
**4058**	allXL	BB	320	366	BS2G	22.37
**4058**	allXL	CC	370	376	BS2G	14.57
**4058**	allXL	BB	320	357	DBSU	24.24
**4058**	allXL	BB	357	319	DBSU	24.65
**4058**	allXL	AA	372	319	DBSU	18.58
**4058**	allXL	BB	370	357	DBSU	28.13
**4058**	allXL	DD	382	357	DBSU	21.7
**4058**	allXL	AA	372	370	DBSU	6.34
**4058**	allXL	AA	381	370	DBSU	21.79
**4058**	allXL	DD	373	370	DBSU	8.78
**4058**	allXL	AA	381	372	DBSU	20.12
**4058**	allXL	AA	372	382	DBSU	18.56
**4058**	allXL	CC	372	386	DBSU	24.91
**4058**	allXL	CC	373	386	DBSU	25.45
**4058**	allXL	BD	381	386	DBSU	52.54
**4058**	allXL	BD;DB	357	357	DBSU	18.23
**4058**	allXL	AD	373	319	DBSU	32.54
**4058**	allXL	BD	372	357	DBSU	36.95
**4058**	allXL	DB	381	357	DBSU	31.6
**4058**	allXL	BD	373	357	DBSU	36.12
**4058**	allXL	DA	372	370	DBSU	48.55
**4058**	allXL	DA	373	370	DBSU	48.86
**4058**	allXL	DB	382	373	DBSU	40.9
**4059**	allXL	CC	320	357	BS2G	27.03
**4059**	allXL	CC	320	373	BS2G	13.51
**4059**	allXL	DD	373	382	BS2G	20.24
**4059**	allXL	DD	370	386	BS2G	22.45

**4059**	allXL	DD	373	386	BS2G	26.26
**4059**	allXL	AA	319	357	BS2G	26.64
**4059**	allXL	AA	367	373	BS2G	13.05
**4059**	allXL	DD	370	382	BS2G	19.99
**4059**	allXL	BB	376	382	BS2G	13.34
**4059**	allXL	BB	357	373	BS2G	20.67
**4059**	allXL	CC	320	366	BS2G	18.38
**4059**	allXL	CC	370	376	BS2G	16.02
**4059**	allXL	CC	320	357	DBSU	27.03
**4059**	allXL	AA	357	319	DBSU	26.64
**4059**	allXL	CC	372	319	DBSU	10.42
**4059**	allXL	DD	370	357	DBSU	23.65
**4059**	allXL	BB	382	357	DBSU	17.82
**4059**	allXL	AA	372	370	DBSU	6.2
**4059**	allXL	DD	381	370	DBSU	20.83
**4059**	allXL	AA	373	370	DBSU	9.16
**4059**	allXL	DD	381	372	DBSU	21.41
**4059**	allXL	DD	372	382	DBSU	21.81
**4059**	allXL	DD	372	386	DBSU	26.24
**4059**	allXL	DD	373	386	DBSU	26.26
**4059**	allXL	AD	381	386	DBSU	32.18
**4059**	allXL	BD;DB	357	357	DBSU	17.98
**4059**	allXL	AD	373	319	DBSU	25.8
**4059**	allXL	BD	372	357	DBSU	36.35
**4059**	allXL	BD	381	357	DBSU	26.36
**4059**	allXL	BD	373	357	DBSU	33.72
**4059**	allXL	AD	372	370	DBSU	54.5
**4059**	allXL	AD	373	370	DBSU	52.51
**4059**	allXL	BD	382	373	DBSU	36.78
**4060**	allXL	CC	320	357	BS2G	19.67
**4060**	allXL	BB	320	373	BS2G	21.21
**4060**	allXL	CC	373	382	BS2G	19.67
**4060**	allXL	DD	370	386	BS2G	20.01
**4060**	allXL	DD	373	386	BS2G	18.83

**4060**	allXL	CC	319	357	BS2G	22.62
**4060**	allXL	AA	367	373	BS2G	12.73
**4060**	allXL	CC	370	382	BS2G	16.26
**4060**	allXL	CC	376	382	BS2G	13.29
**4060**	allXL	BB	357	373	BS2G	24.76
**4060**	allXL	DD	320	366	BS2G	25.3
**4060**	allXL	CC	370	376	BS2G	13.05
**4060**	allXL	CC	320	357	DBSU	19.67
**4060**	allXL	CC	357	319	DBSU	22.62
**4060**	allXL	DD	372	319	DBSU	21.1
**4060**	allXL	BB	370	357	DBSU	26.76
**4060**	allXL	BB	382	357	DBSU	32.68
**4060**	allXL	AA	372	370	DBSU	6.18
**4060**	allXL	CC	381	370	DBSU	18.8
**4060**	allXL	AA	373	370	DBSU	9.12
**4060**	allXL	DD	381	372	DBSU	19.25
**4060**	allXL	DD	372	382	DBSU	19.93
**4060**	allXL	DD	372	386	DBSU	16.35
**4060**	allXL	DD	373	386	DBSU	18.83
**4060**	allXL	DA	381	386	DBSU	48.07
**4060**	allXL	BD;DB	357	357	DBSU	18.56
**4060**	allXL	DA	373	319	DBSU	32.94
**4060**	allXL	AC	372	357	DBSU	43.27
**4060**	allXL	AC	381	357	DBSU	37.9
**4060**	allXL	AC	373	357	DBSU	39.58
**4060**	allXL	DA	372	370	DBSU	45.76
**4060**	allXL	DA	373	370	DBSU	45.78
**4060**	allXL	DA	382	373	DBSU	37.8
**4061**	allXL	BB	320	357	BS2G	23.93
**4061**	allXL	DD	320	373	BS2G	19.14
**4061**	allXL	DD	373	382	BS2G	19.03
**4061**	allXL	DD	370	386	BS2G	13.64
**4061**	allXL	DD	373	386	BS2G	19.39
**4061**	allXL	BB	319	357	BS2G	24.88

**4061**	allXL	AA	367	373	BS2G	12.88
**4061**	allXL	DD	370	382	BS2G	18.3
**4061**	allXL	BB	376	382	BS2G	13.32
**4061**	allXL	DD	357	373	BS2G	27.52
**4061**	allXL	CC	320	366	BS2G	20.27
**4061**	allXL	BB	370	376	BS2G	16.28
**4061**	allXL	BB	320	357	DBSU	23.93
**4061**	allXL	BB	357	319	DBSU	24.88
**4061**	allXL	CC	372	319	DBSU	22.41
**4061**	allXL	DD	370	357	DBSU	21.08
**4061**	allXL	DD	382	357	DBSU	37.66
**4061**	allXL	AA	372	370	DBSU	6.46
**4061**	allXL	DD	381	370	DBSU	18.59
**4061**	allXL	AA	373	370	DBSU	8.93
**4061**	allXL	DD	381	372	DBSU	18.98
**4061**	allXL	DD	372	382	DBSU	19.53
**4061**	allXL	DD	372	386	DBSU	17.69
**4061**	allXL	DD	373	386	DBSU	19.39
**4061**	allXL	DA	381	386	DBSU	43.52
**4061**	allXL	BD;DB	357	357	DBSU	19.61
**4061**	allXL	DA	373	319	DBSU	25.07
**4061**	allXL	DA	372	357	DBSU	24.61
**4061**	allXL	DA	381	357	DBSU	33.95
**4061**	allXL	DA	373	357	DBSU	22.81
**4061**	allXL	CB	372	370	DBSU	28.25
**4061**	allXL	DA	373	370	DBSU	28.26
**4061**	allXL	DA	382	373	DBSU	26.65
**4062**	allXL	CC	320	357	BS2G	22.98
**4062**	allXL	DD	320	373	BS2G	21.03
**4062**	allXL	AA	373	382	BS2G	16.09
**4062**	allXL	DD	370	386	BS2G	18.28
**4062**	allXL	AA	373	386	BS2G	21.5
**4062**	allXL	CC	319	357	BS2G	23.85
**4062**	allXL	AA	367	373	BS2G	14.64

**4062**	allXL	AA	370	382	BS2G	20.36
**4062**	allXL	AA	376	382	BS2G	14.22
**4062**	allXL	BB	357	373	BS2G	25.93
**4062**	allXL	CC	320	366	BS2G	23.59
**4062**	allXL	BB	370	376	BS2G	16.32
**4062**	allXL	CC	320	357	DBSU	22.98
**4062**	allXL	CC	357	319	DBSU	23.85
**4062**	allXL	DD	372	319	DBSU	24.71
**4062**	allXL	BB	370	357	DBSU	25.43
**4062**	allXL	DD	382	357	DBSU	31.64
**4062**	allXL	AA	372	370	DBSU	6.32
**4062**	allXL	DD	381	370	DBSU	20.6
**4062**	allXL	AA	373	370	DBSU	9.2
**4062**	allXL	DD	381	372	DBSU	19.24
**4062**	allXL	AA	372	382	DBSU	18.86
**4062**	allXL	DD	372	386	DBSU	21.72
**4062**	allXL	AA	373	386	DBSU	21.5
**4062**	allXL	AD	381	386	DBSU	56.95
**4062**	allXL	BD;DB	357	357	DBSU	17.07
**4062**	allXL	AD	373	319	DBSU	32.77
**4062**	allXL	BD	372	357	DBSU	28.36
**4062**	allXL	CA	381	357	DBSU	47.72
**4062**	allXL	BD	373	357	DBSU	30.77
**4062**	allXL	AD	372	370	DBSU	34.55
**4062**	allXL	AD	373	370	DBSU	33.17
**4062**	allXL	AD	382	373	DBSU	38.06
**4063**	allXL	AA	320	357	BS2G	18.74
**4063**	allXL	DD	320	373	BS2G	27.76
**4063**	allXL	DD	373	382	BS2G	18.14
**4063**	allXL	DD	370	386	BS2G	8.33
**4063**	allXL	DD	373	386	BS2G	11.82
**4063**	allXL	AA	319	357	BS2G	16.01
**4063**	allXL	AA	367	373	BS2G	12.36
**4063**	allXL	DD	370	382	BS2G	18.57

**4063**	allXL	CC	376	382	BS2G	13.22
**4063**	allXL	DD	357	373	BS2G	32.47
**4063**	allXL	AA	320	366	BS2G	19.57
**4063**	allXL	CC	370	376	BS2G	16.28
**4063**	allXL	AA	320	357	DBSU	18.74
**4063**	allXL	AA	357	319	DBSU	16.01
**4063**	allXL	DD	372	319	DBSU	29.43
**4063**	allXL	DD	370	357	DBSU	29.94
**4063**	allXL	DD	382	357	DBSU	34.42
**4063**	allXL	AA	372	370	DBSU	6.59
**4063**	allXL	DD	381	370	DBSU	19.28
**4063**	allXL	AA	373	370	DBSU	9.14
**4063**	allXL	DD	381	372	DBSU	20.26
**4063**	allXL	DD	372	382	DBSU	20.53
**4063**	allXL	DD	372	386	DBSU	12.22
**4063**	allXL	DD	373	386	DBSU	11.82
**4063**	allXL	CB	381	386	DBSU	46.78
**4063**	allXL	BD;DB	357	357	DBSU	18.87
**4063**	allXL	AD	373	319	DBSU	45.24
**4063**	allXL	DB	372	357	DBSU	49.88
**4063**	allXL	DB	381	357	DBSU	54.98
**4063**	allXL	DB	373	357	DBSU	51.26
**4063**	allXL	CB	372	370	DBSU	50.04
**4063**	allXL	CB	373	370	DBSU	51.83
**4063**	allXL	CB	382	373	DBSU	49.85
**4064**	allXL	AA	320	357	BS2G	23.46
**4064**	allXL	AA	320	373	BS2G	29.48
**4064**	allXL	AA	373	382	BS2G	15.37
**4064**	allXL	DD	370	386	BS2G	17.68
**4064**	allXL	AA	373	386	BS2G	20.11
**4064**	allXL	AA	319	357	BS2G	22.63
**4064**	allXL	AA	367	373	BS2G	13.95
**4064**	allXL	DD	370	382	BS2G	18.46
**4064**	allXL	AA	376	382	BS2G	14.6

**4064**	allXL	DD	357	373	BS2G	30.84
**4064**	allXL	DD	320	366	BS2G	25.15
**4064**	allXL	CC	370	376	BS2G	15.48
**4064**	allXL	AA	320	357	DBSU	23.46
**4064**	allXL	AA	357	319	DBSU	22.63
**4064**	allXL	CC	372	319	DBSU	24.96
**4064**	allXL	DD	370	357	DBSU	25.25
**4064**	allXL	AA	382	357	DBSU	31.8
**4064**	allXL	AA	372	370	DBSU	6.1
**4064**	allXL	DD	381	370	DBSU	17.22
**4064**	allXL	AA	373	370	DBSU	9.12
**4064**	allXL	DD	381	372	DBSU	15.78
**4064**	allXL	DD	372	382	DBSU	17.31
**4064**	allXL	DD	372	386	DBSU	18.73
**4064**	allXL	AA	373	386	DBSU	20.11
**4064**	allXL	DA	381	386	DBSU	52.02
**4064**	allXL	BD;DB	357	357	DBSU	19.63
**4064**	allXL	DA	373	319	DBSU	28.79
**4064**	allXL	DB	372	357	DBSU	41.16
**4064**	allXL	DB	381	357	DBSU	33.97
**4064**	allXL	DB	373	357	DBSU	39.34
**4064**	allXL	DA	372	370	DBSU	32.66
**4064**	allXL	DA	373	370	DBSU	33.67
**4064**	allXL	AD	382	373	DBSU	38.92
**4065**	allXL	CC	320	357	BS2G	26.38
**4065**	allXL	BB	320	373	BS2G	14.34
**4065**	allXL	CC	373	382	BS2G	18.28
**4065**	allXL	AA	370	386	BS2G	24.57
**4065**	allXL	AA	373	386	BS2G	23.1
**4065**	allXL	AA	319	357	BS2G	27.92
**4065**	allXL	AA	367	373	BS2G	13.3
**4065**	allXL	DD	370	382	BS2G	22.51
**4065**	allXL	AA	376	382	BS2G	13.93
**4065**	allXL	DD	357	373	BS2G	30.63

**4065**	allXL	BB	320	366	BS2G	15.85
**4065**	allXL	DD	370	376	BS2G	17.34
**4065**	allXL	CC	320	357	DBSU	26.38
**4065**	allXL	AA	357	319	DBSU	27.92
**4065**	allXL	BB	372	319	DBSU	15.54
**4065**	allXL	BB	370	357	DBSU	23.68
**4065**	allXL	DD	382	357	DBSU	26.82
**4065**	allXL	AA	372	370	DBSU	6.31
**4065**	allXL	DD	381	370	DBSU	20.42
**4065**	allXL	AA	373	370	DBSU	9.23
**4065**	allXL	DD	381	372	DBSU	19.72
**4065**	allXL	CC	372	382	DBSU	21.5
**4065**	allXL	AA	372	386	DBSU	25.08
**4065**	allXL	AA	373	386	DBSU	23.1
**4065**	allXL	BD	381	386	DBSU	43.95
**4065**	allXL	BD;DB	357	357	DBSU	18.85
**4065**	allXL	CB	373	319	DBSU	33.46
**4065**	allXL	CB	372	357	DBSU	33.13
**4065**	allXL	DB	381	357	DBSU	26.82
**4065**	allXL	CB	373	357	DBSU	31.16
**4065**	allXL	BC	372	370	DBSU	36.21
**4065**	allXL	DB	373	370	DBSU	38.16
**4065**	allXL	DB	382	373	DBSU	31.35
**4066**	allXL	BB;CC	320	357	BS2G	25.72
**4066**	allXL	DD	320	373	BS2G	11.64
**4066**	allXL	CC	373	382	BS2G	17.31
**4066**	allXL	CC	370	386	BS2G	21.76
**4066**	allXL	BB	373	386	BS2G	19.45
**4066**	allXL	CC	319	357	BS2G	25.88
**4066**	allXL	AA	367	373	BS2G	13.45
**4066**	allXL	CC	370	382	BS2G	18.47
**4066**	allXL	CC	376	382	BS2G	13.19
**4066**	allXL	BB	357	373	BS2G	21.99
**4066**	allXL	AA	320	366	BS2G	22.2

**4066**	allXL	CC	370	376	BS2G	15.56
**4066**	allXL	BB;CC	320	357	DBSU	25.72
**4066**	allXL	CC	357	319	DBSU	25.88
**4066**	allXL	DD	372	319	DBSU	16.14
**4066**	allXL	BB	370	357	DBSU	24.05
**4066**	allXL	BB	382	357	DBSU	24.77
**4066**	allXL	AA	372	370	DBSU	6.63
**4066**	allXL	CC	381	370	DBSU	21.16
**4066**	allXL	AA	373	370	DBSU	9.26
**4066**	allXL	CC	381	372	DBSU	19.33
**4066**	allXL	CC	372	382	DBSU	16.78
**4066**	allXL	BB	372	386	DBSU	19.63
**4066**	allXL	BB	373	386	DBSU	19.45
**4066**	allXL	DA	381	386	DBSU	58.85
**4066**	allXL	BD;DB	357	357	DBSU	17.8
**4066**	allXL	CB	373	319	DBSU	37.29
**4066**	allXL	BD	372	357	DBSU	15.18
**4066**	allXL	BD	381	357	DBSU	28.42
**4066**	allXL	BD	373	357	DBSU	17.0
**4066**	allXL	BD	372	370	DBSU	36.21
**4066**	allXL	BD	373	370	DBSU	37.16
**4066**	allXL	DA	382	373	DBSU	49.79
**4067**	allXL	AA	320	357	BS2G	22.92
**4067**	allXL	DD	320	373	BS2G	23.61
**4067**	allXL	BB	373	382	BS2G	14.65
**4067**	allXL	DD	370	386	BS2G	19.15
**4067**	allXL	DD	373	386	BS2G	21.84
**4067**	allXL	AA	319	357	BS2G	21.32
**4067**	allXL	DD	367	373	BS2G	13.38
**4067**	allXL	BB	370	382	BS2G	17.06
**4067**	allXL	BB	376	382	BS2G	13.66
**4067**	allXL	BB	357	373	BS2G	29.32
**4067**	allXL	BB	320	366	BS2G	19.18
**4067**	allXL	CC	370	376	BS2G	15.87

**4067**	allXL	AA	320	357	DBSU	22.92
**4067**	allXL	AA	357	319	DBSU	21.32
**4067**	allXL	DD	372	319	DBSU	25.94
**4067**	allXL	BB	370	357	DBSU	19.79
**4067**	allXL	BB	382	357	DBSU	30.25
**4067**	allXL	AA	372	370	DBSU	6.2
**4067**	allXL	DD	381	370	DBSU	18.49
**4067**	allXL	AA	373	370	DBSU	8.98
**4067**	allXL	DD	381	372	DBSU	16.49
**4067**	allXL	BB	372	382	DBSU	15.89
**4067**	allXL	DD	372	386	DBSU	22.59
**4067**	allXL	DD	373	386	DBSU	21.84
**4067**	allXL	AD	381	386	DBSU	52.65
**4067**	allXL	BD;DB	357	357	DBSU	18.87
**4067**	allXL	AD	373	319	DBSU	30.08
**4067**	allXL	BD	372	357	DBSU	39.41
**4067**	allXL	DA	381	357	DBSU	40.3
**4067**	allXL	BD	373	357	DBSU	40.85
**4067**	allXL	DA	372	370	DBSU	39.24
**4067**	allXL	DA	373	370	DBSU	37.49
**4067**	allXL	DA	382	373	DBSU	27.31
**4068**	allXL	AA	320	357	BS2G	23.74
**4068**	allXL	CC	320	373	BS2G	14.85
**4068**	allXL	DD	373	382	BS2G	17.91
**4068**	allXL	DD	370	386	BS2G	17.39
**4068**	allXL	DD	373	386	BS2G	21.13
**4068**	allXL	AA	319	357	BS2G	21.76
**4068**	allXL	AA	367	373	BS2G	11.56
**4068**	allXL	CC	370	382	BS2G	16.36
**4068**	allXL	BB	376	382	BS2G	14.07
**4068**	allXL	CC	357	373	BS2G	22.03
**4068**	allXL	AA	320	366	BS2G	21.13
**4068**	allXL	CC	370	376	BS2G	11.31
**4068**	allXL	AA	320	357	DBSU	23.74

**4068**	allXL	AA	357	319	DBSU	21.76
**4068**	allXL	CC	372	319	DBSU	19.89
**4068**	allXL	CC	370	357	DBSU	23.45
**4068**	allXL	BB	382	357	DBSU	19.44
**4068**	allXL	AA	372	370	DBSU	6.19
**4068**	allXL	CC	381	370	DBSU	17.2
**4068**	allXL	AA	373	370	DBSU	8.88
**4068**	allXL	DD	381	372	DBSU	16.79
**4068**	allXL	DD	372	382	DBSU	18.2
**4068**	allXL	DD	372	386	DBSU	19.47
**4068**	allXL	DD	373	386	DBSU	21.13
**4068**	allXL	AD	381	386	DBSU	37.74
**4068**	allXL	BD;DB	357	357	DBSU	19.31
**4068**	allXL	DA	373	319	DBSU	13.92
**4068**	allXL	DA	372	357	DBSU	31.24
**4068**	allXL	BD	381	357	DBSU	40.44
**4068**	allXL	DA	373	357	DBSU	32.06
**4068**	allXL	CB	372	370	DBSU	18.77
**4068**	allXL	CB	373	370	DBSU	18.53
**4068**	allXL	DA	382	373	DBSU	22.16
**4069**	allXL	AA	320	357	BS2G	25.73
**4069**	allXL	CC	320	373	BS2G	14.19
**4069**	allXL	DD	373	382	BS2G	21.21
**4069**	allXL	DD	370	386	BS2G	25.05
**4069**	allXL	DD	373	386	BS2G	28.92
**4069**	allXL	AA	319	357	BS2G	24.6
**4069**	allXL	AA	367	373	BS2G	13.47
**4069**	allXL	DD	370	382	BS2G	18.02
**4069**	allXL	CC	376	382	BS2G	14.83
**4069**	allXL	DD	357	373	BS2G	18.96
**4069**	allXL	BB	320	366	BS2G	17.87
**4069**	allXL	DD	370	376	BS2G	13.58
**4069**	allXL	AA	320	357	DBSU	25.73
**4069**	allXL	AA	357	319	DBSU	24.6

**4069**	allXL	CC	372	319	DBSU	17.75
**4069**	allXL	DD	370	357	DBSU	23.28
**4069**	allXL	CC	382	357	DBSU	39.39
**4069**	allXL	AA	372	370	DBSU	6.24
**4069**	allXL	DD	381	370	DBSU	15.28
**4069**	allXL	AA	373	370	DBSU	9.12
**4069**	allXL	DD	381	372	DBSU	17.99
**4069**	allXL	DD	372	382	DBSU	21.23
**4069**	allXL	DD	372	386	DBSU	27.79
**4069**	allXL	DD	373	386	DBSU	28.92
**4069**	allXL	AD	381	386	DBSU	59.24
**4069**	allXL	BD;DB	357	357	DBSU	18.52
**4069**	allXL	DA	373	319	DBSU	29.42
**4069**	allXL	DB	372	357	DBSU	32.45
**4069**	allXL	DA	381	357	DBSU	37.34
**4069**	allXL	DB	373	357	DBSU	31.76
**4069**	allXL	BC	372	370	DBSU	27.61
**4069**	allXL	BC	373	370	DBSU	29.48
**4069**	allXL	DA	382	373	DBSU	47.31
**4070**	allXL	AA	320	357	BS2G	20.64
**4070**	allXL	CC	320	373	BS2G	27.17
**4070**	allXL	CC	373	382	BS2G	14.62
**4070**	allXL	CC	370	386	BS2G	18.15
**4070**	allXL	CC	373	386	BS2G	12.05
**4070**	allXL	CC	319	357	BS2G	23.72
**4070**	allXL	AA	367	373	BS2G	11.96
**4070**	allXL	AA	370	382	BS2G	19.12
**4070**	allXL	CC	376	382	BS2G	12.97
**4070**	allXL	AA	357	373	BS2G	20.85
**4070**	allXL	BB	320	366	BS2G	18.66
**4070**	allXL	CC	370	376	BS2G	16.08
**4070**	allXL	AA	320	357	DBSU	20.64
**4070**	allXL	CC	357	319	DBSU	23.72
**4070**	allXL	CC	372	319	DBSU	30.39

**4070**	allXL	DD	370	357	DBSU	19.1
**4070**	allXL	BB	382	357	DBSU	21.29
**4070**	allXL	AA	372	370	DBSU	6.36
**4070**	allXL	AA	381	370	DBSU	20.34
**4070**	allXL	DD	373	370	DBSU	8.81
**4070**	allXL	AA	381	372	DBSU	18.76
**4070**	allXL	CC	372	382	DBSU	18.26
**4070**	allXL	CC	372	386	DBSU	13.38
**4070**	allXL	CC	373	386	DBSU	12.05
**4070**	allXL	BC	381	386	DBSU	19.51
**4070**	allXL	BD;DB	357	357	DBSU	17.93
**4070**	allXL	CB	373	319	DBSU	29.63
**4070**	allXL	CB	372	357	DBSU	26.72
**4070**	allXL	BD	381	357	DBSU	36.01
**4070**	allXL	CB	373	357	DBSU	29.42
**4070**	allXL	CB	372	370	DBSU	21.87
**4070**	allXL	CB	373	370	DBSU	22.55
**4070**	allXL	BC	382	373	DBSU	21.49
**4071**	allXL	AA	320	357	BS2G	22.06
**4071**	allXL	DD	320	373	BS2G	27.09
**4071**	allXL	DD	373	382	BS2G	19.8
**4071**	allXL	DD	370	386	BS2G	25.27
**4071**	allXL	DD	373	386	BS2G	26.53
**4071**	allXL	AA	319	357	BS2G	24.06
**4071**	allXL	AA	367	373	BS2G	14.01
**4071**	allXL	DD	370	382	BS2G	21.37
**4071**	allXL	BB	376	382	BS2G	14.4
**4071**	allXL	BB	357	373	BS2G	33.33
**4071**	allXL	AA	320	366	BS2G	20.91
**4071**	allXL	CC	370	376	BS2G	16.25
**4071**	allXL	AA	320	357	DBSU	22.06
**4071**	allXL	AA	357	319	DBSU	24.06
**4071**	allXL	DD	372	319	DBSU	23.23
**4071**	allXL	DD	370	357	DBSU	28.08

**4071**	allXL	DD	382	357	DBSU	42.31
**4071**	allXL	AA	372	370	DBSU	6.05
**4071**	allXL	DD	381	370	DBSU	19.69
**4071**	allXL	AA	373	370	DBSU	9.18
**4071**	allXL	DD	381	372	DBSU	19.02
**4071**	allXL	DD	372	382	DBSU	21.66
**4071**	allXL	DD	372	386	DBSU	27.06
**4071**	allXL	DD	373	386	DBSU	26.53
**4071**	allXL	CB	381	386	DBSU	60.86
**4071**	allXL	BD;DB	357	357	DBSU	18.67
**4071**	allXL	BC	373	319	DBSU	24.62
**4071**	allXL	BC	372	357	DBSU	40.78
**4071**	allXL	CB	381	357	DBSU	37.78
**4071**	allXL	BC	373	357	DBSU	40.16
**4071**	allXL	CB	372	370	DBSU	17.38
**4071**	allXL	CB	373	370	DBSU	16.06
**4071**	allXL	CB	382	373	DBSU	32.65
**4072**	allXL	BB	320	357	BS2G	25.98
**4072**	allXL	AA	320	373	BS2G	28.22
**4072**	allXL	BB	373	382	BS2G	20.36
**4072**	allXL	BB	370	386	BS2G	27.71
**4072**	allXL	DD	373	386	BS2G	29.75
**4072**	allXL	CC	319	357	BS2G	26.41
**4072**	allXL	AA	367	373	BS2G	12.34
**4072**	allXL	BB	370	382	BS2G	18.16
**4072**	allXL	BB	376	382	BS2G	15.11
**4072**	allXL	BB	357	373	BS2G	21.96
**4072**	allXL	CC	320	366	BS2G	14.38
**4072**	allXL	BB	370	376	BS2G	14.22
**4072**	allXL	BB	320	357	DBSU	25.98
**4072**	allXL	CC	357	319	DBSU	26.41
**4072**	allXL	CC	372	319	DBSU	26.68
**4072**	allXL	DD	370	357	DBSU	24.71
**4072**	allXL	BB	382	357	DBSU	34.27

**4072**	allXL	AA	372	370	DBSU	6.28
**4072**	allXL	BB	381	370	DBSU	19.83
**4072**	allXL	AA	373	370	DBSU	8.92
**4072**	allXL	DD	381	372	DBSU	19.37
**4072**	allXL	BB;DD	372	382	DBSU	22.01
**4072**	allXL	DD	372	386	DBSU	29.18
**4072**	allXL	DD	373	386	DBSU	29.75
**4072**	allXL	BC	381	386	DBSU	51.49
**4072**	allXL	BD;DB	357	357	DBSU	18.6
**4072**	allXL	CB	373	319	DBSU	22.93
**4072**	allXL	BD	372	357	DBSU	32.38
**4072**	allXL	BD	381	357	DBSU	32.88
**4072**	allXL	BD	373	357	DBSU	29.26
**4072**	allXL	BC	372	370	DBSU	20.91
**4072**	allXL	BC	373	370	DBSU	24.42
**4072**	allXL	CB	382	373	DBSU	40.85
**4073**	allXL	AA	320	357	BS2G	24.76
**4073**	allXL	DD	320	373	BS2G	10.79
**4073**	allXL	BB	373	382	BS2G	16.99
**4073**	allXL	BB	370	386	BS2G	24.34
**4073**	allXL	CC	373	386	BS2G	21.07
**4073**	allXL	AA	319	357	BS2G	26.73
**4073**	allXL	AA	367	373	BS2G	11.66
**4073**	allXL	BB	370	382	BS2G	16.39
**4073**	allXL	BB	376	382	BS2G	13.55
**4073**	allXL	BB	357	373	BS2G	23.39
**4073**	allXL	AA	320	366	BS2G	24.55
**4073**	allXL	BB	370	376	BS2G	16.56
**4073**	allXL	AA	320	357	DBSU	24.76
**4073**	allXL	AA	357	319	DBSU	26.73
**4073**	allXL	DD	372	319	DBSU	15.76
**4073**	allXL	DD	370	357	DBSU	17.52
**4073**	allXL	BB	382	357	DBSU	19.39
**4073**	allXL	DD	372	370	DBSU	6.59

**4073**	allXL	BB	381	370	DBSU	18.37
**4073**	allXL	AA	373	370	DBSU	8.79
**4073**	allXL	BB	381	372	DBSU	19.54
**4073**	allXL	BB	372	382	DBSU	18.81
**4073**	allXL	CC	372	386	DBSU	21.18
**4073**	allXL	CC	373	386	DBSU	21.07
**4073**	allXL	BD	381	386	DBSU	46.02
**4073**	allXL	BD;DB	357	357	DBSU	18.45
**4073**	allXL	AD	373	319	DBSU	18.22
**4073**	allXL	BD	372	357	DBSU	28.45
**4073**	allXL	BD	381	357	DBSU	11.09
**4073**	allXL	BD	373	357	DBSU	25.28
**4073**	allXL	DA	372	370	DBSU	25.23
**4073**	allXL	DA	373	370	DBSU	23.21
**4073**	allXL	DA	382	373	DBSU	27.63
**4074**	allXL	CC	320	357	BS2G	23.41
**4074**	allXL	BB	320	373	BS2G	22.45
**4074**	allXL	BB	373	382	BS2G	21.16
**4074**	allXL	DD	370	386	BS2G	21.25
**4074**	allXL	CC	373	386	BS2G	28.8
**4074**	allXL	AA	319	357	BS2G	25.55
**4074**	allXL	BB	367	373	BS2G	13.12
**4074**	allXL	BB	370	382	BS2G	17.37
**4074**	allXL	CC	376	382	BS2G	14.44
**4074**	allXL	DD	357	373	BS2G	24.4
**4074**	allXL	AA	320	366	BS2G	17.91
**4074**	allXL	BB	370	376	BS2G	13.83
**4074**	allXL	CC	320	357	DBSU	23.41
**4074**	allXL	AA	357	319	DBSU	25.55
**4074**	allXL	BB	372	319	DBSU	19.53
**4074**	allXL	DD	370	357	DBSU	24.52
**4074**	allXL	DD	382	357	DBSU	12.85
**4074**	allXL	AA	372	370	DBSU	6.14
**4074**	allXL	DD	381	370	DBSU	16.06

**4074**	allXL	CC	373	370	DBSU	9.14
**4074**	allXL	DD	381	372	DBSU	20.63
**4074**	allXL	BB	372	382	DBSU	22.14
**4074**	allXL	DD	372	386	DBSU	28.11
**4074**	allXL	CC	373	386	DBSU	28.8
**4074**	allXL	DB	381	386	DBSU	34.0
**4074**	allXL	BD;DB	357	357	DBSU	18.17
**4074**	allXL	DA	373	319	DBSU	27.69
**4074**	allXL	DB	372	357	DBSU	36.23
**4074**	allXL	DB	381	357	DBSU	26.28
**4074**	allXL	DB	373	357	DBSU	32.82
**4074**	allXL	BC	372	370	DBSU	42.05
**4074**	allXL	BC	373	370	DBSU	45.24
**4074**	allXL	BD	382	373	DBSU	45.31
**4075**	allXL	AA	320	357	BS2G	24.96
**4075**	allXL	AA	320	373	BS2G	33.26
**4075**	allXL	CC	373	382	BS2G	18.54
**4075**	allXL	DD	370	386	BS2G	17.46
**4075**	allXL	DD	373	386	BS2G	22.07
**4075**	allXL	AA	319	357	BS2G	24.85
**4075**	allXL	BB	367	373	BS2G	13.03
**4075**	allXL	DD	370	382	BS2G	19.43
**4075**	allXL	CC	376	382	BS2G	13.5
**4075**	allXL	BB	357	373	BS2G	14.75
**4075**	allXL	BB	320	366	BS2G	30.54
**4075**	allXL	DD	370	376	BS2G	16.86
**4075**	allXL	AA	320	357	DBSU	24.96
**4075**	allXL	AA	357	319	DBSU	24.85
**4075**	allXL	AA	372	319	DBSU	34.56
**4075**	allXL	BB	370	357	DBSU	18.79
**4075**	allXL	BB	382	357	DBSU	14.89
**4075**	allXL	AA	372	370	DBSU	6.13
**4075**	allXL	DD	381	370	DBSU	19.42
**4075**	allXL	AA	373	370	DBSU	9.03

**4075**	allXL	DD	381	372	DBSU	20.65
**4075**	allXL	BB	372	382	DBSU	21.7
**4075**	allXL	DD	372	386	DBSU	21.52
**4075**	allXL	DD	373	386	DBSU	22.07
**4075**	allXL	DA	381	386	DBSU	45.33
**4075**	allXL	BD;DB	357	357	DBSU	19.02
**4075**	allXL	DA	373	319	DBSU	23.65
**4075**	allXL	BD	372	357	DBSU	22.75
**4075**	allXL	BD	381	357	DBSU	32.19
**4075**	allXL	BD	373	357	DBSU	24.75
**4075**	allXL	BD	372	370	DBSU	39.3
**4075**	allXL	BD	373	370	DBSU	41.8
**4075**	allXL	BD	382	373	DBSU	45.65
**4076**	allXL	DD	320	357	BS2G	28.31
**4076**	allXL	CC	320	373	BS2G	15.86
**4076**	allXL	DD	373	382	BS2G	18.61
**4076**	allXL	DD	370	386	BS2G	19.13
**4076**	allXL	DD	373	386	BS2G	21.87
**4076**	allXL	DD	319	357	BS2G	28.32
**4076**	allXL	AA	367	373	BS2G	10.68
**4076**	allXL	AA	370	382	BS2G	20.59
**4076**	allXL	CC	376	382	BS2G	14.64
**4076**	allXL	BB	357	373	BS2G	29.34
**4076**	allXL	AA	320	366	BS2G	19.95
**4076**	allXL	CC	370	376	BS2G	12.78
**4076**	allXL	DD	320	357	DBSU	28.31
**4076**	allXL	DD	357	319	DBSU	28.32
**4076**	allXL	CC	372	319	DBSU	16.12
**4076**	allXL	CC	370	357	DBSU	29.67
**4076**	allXL	AA	382	357	DBSU	40.83
**4076**	allXL	AA	372	370	DBSU	6.51
**4076**	allXL	AA	381	370	DBSU	19.95
**4076**	allXL	AA	373	370	DBSU	8.63
**4076**	allXL	DD	381	372	DBSU	20.59

**4076**	allXL	DD	372	382	DBSU	20.14
**4076**	allXL	DD	372	386	DBSU	21.6
**4076**	allXL	DD	373	386	DBSU	21.87
**4076**	allXL	DA	381	386	DBSU	29.25
**4076**	allXL	AC;CA	357	357	DBSU	18.87
**4076**	allXL	DA	373	319	DBSU	26.17
**4076**	allXL	CB	372	357	DBSU	44.17
**4076**	allXL	DA	381	357	DBSU	43.08
**4076**	allXL	CB	373	357	DBSU	44.63
**4076**	allXL	CB	372	370	DBSU	33.83
**4076**	allXL	CB	373	370	DBSU	35.51
**4076**	allXL	DA	382	373	DBSU	33.66
**4077**	allXL	AA	320	357	BS2G	24.92
**4077**	allXL	BB	320	373	BS2G	20.01
**4077**	allXL	AA	373	382	BS2G	17.49
**4077**	allXL	CC	370	386	BS2G	20.85
**4077**	allXL	CC	373	386	BS2G	19.46
**4077**	allXL	AA	319	357	BS2G	26.07
**4077**	allXL	BB	367	373	BS2G	14.74
**4077**	allXL	DD	370	382	BS2G	24.26
**4077**	allXL	CC	376	382	BS2G	13.55
**4077**	allXL	CC	357	373	BS2G	20.47
**4077**	allXL	BB	320	366	BS2G	26.7
**4077**	allXL	CC	370	376	BS2G	16.47
**4077**	allXL	AA	320	357	DBSU	24.92
**4077**	allXL	AA	357	319	DBSU	26.07
**4077**	allXL	BB	372	319	DBSU	23.22
**4077**	allXL	DD	370	357	DBSU	15.39
**4077**	allXL	DD	382	357	DBSU	30.67
**4077**	allXL	AA	372	370	DBSU	6.01
**4077**	allXL	DD	381	370	DBSU	23.65
**4077**	allXL	AA	373	370	DBSU	9.12
**4077**	allXL	DD	381	372	DBSU	19.43
**4077**	allXL	DD	372	382	DBSU	20.78

**4077**	allXL	CC	372	386	DBSU	17.63
**4077**	allXL	CC	373	386	DBSU	19.46
**4077**	allXL	DB	381	386	DBSU	26.25
**4077**	allXL	BD;DB	357	357	DBSU	18.66
**4077**	allXL	AD	373	319	DBSU	30.19
**4077**	allXL	DB	372	357	DBSU	13.35
**4077**	allXL	DB	381	357	DBSU	14.84
**4077**	allXL	DB	373	357	DBSU	15.06
**4077**	allXL	DB	372	370	DBSU	36.61
**4077**	allXL	DB	373	370	DBSU	36.39
**4077**	allXL	BD	382	373	DBSU	31.84
**4078**	allXL	AA	320	357	BS2G	22.18
**4078**	allXL	DD	320	373	BS2G	18.89
**4078**	allXL	BB	373	382	BS2G	15.04
**4078**	allXL	DD	370	386	BS2G	19.28
**4078**	allXL	BB	373	386	BS2G	23.31
**4078**	allXL	AA	319	357	BS2G	25.16
**4078**	allXL	AA	367	373	BS2G	12.64
**4078**	allXL	DD	370	382	BS2G	19.64
**4078**	allXL	BB	376	382	BS2G	13.29
**4078**	allXL	BB	357	373	BS2G	34.26
**4078**	allXL	DD	320	366	BS2G	17.64
**4078**	allXL	DD	370	376	BS2G	16.24
**4078**	allXL	AA	320	357	DBSU	22.18
**4078**	allXL	AA	357	319	DBSU	25.16
**4078**	allXL	DD	372	319	DBSU	17.03
**4078**	allXL	DD	370	357	DBSU	28.11
**4078**	allXL	DD	382	357	DBSU	30.14
**4078**	allXL	AA	372	370	DBSU	6.82
**4078**	allXL	DD	381	370	DBSU	18.9
**4078**	allXL	AA	373	370	DBSU	9.1
**4078**	allXL	BB	381	372	DBSU	19.16
**4078**	allXL	BB	372	382	DBSU	17.16
**4078**	allXL	BB	372	386	DBSU	23.75

**4078**	allXL	BB	373	386	DBSU	23.31
**4078**	allXL	CB	381	386	DBSU	50.78
**4078**	allXL	BD;DB	357	357	DBSU	18.25
**4078**	allXL	CB	373	319	DBSU	12.07
**4078**	allXL	CB	372	357	DBSU	28.0
**4078**	allXL	CB	381	357	DBSU	32.87
**4078**	allXL	CB	373	357	DBSU	29.04
**4078**	allXL	BC	372	370	DBSU	12.92
**4078**	allXL	BC	373	370	DBSU	13.51
**4078**	allXL	BC	382	373	DBSU	27.41
**4079**	allXL	CC	320	357	BS2G	21.6
**4079**	allXL	CC	320	373	BS2G	14.69
**4079**	allXL	BB	373	382	BS2G	17.02
**4079**	allXL	DD	370	386	BS2G	26.1
**4079**	allXL	AA	373	386	BS2G	25.28
**4079**	allXL	CC	319	357	BS2G	20.22
**4079**	allXL	AA	367	373	BS2G	14.26
**4079**	allXL	DD	370	382	BS2G	18.98
**4079**	allXL	BB	376	382	BS2G	13.08
**4079**	allXL	BB	357	373	BS2G	24.11
**4079**	allXL	CC	320	366	BS2G	17.3
**4079**	allXL	CC	370	376	BS2G	17.37
**4079**	allXL	CC	320	357	DBSU	21.6
**4079**	allXL	CC	357	319	DBSU	20.22
**4079**	allXL	CC	372	319	DBSU	15.84
**4079**	allXL	BB	370	357	DBSU	25.06
**4079**	allXL	BB	382	357	DBSU	13.8
**4079**	allXL	AA	372	370	DBSU	6.0
**4079**	allXL	DD	381	370	DBSU	17.12
**4079**	allXL	AA	373	370	DBSU	9.1
**4079**	allXL	DD	381	372	DBSU	15.41
**4079**	allXL	BB	372	382	DBSU	17.88
**4079**	allXL	BB	372	386	DBSU	24.63
**4079**	allXL	AA	373	386	DBSU	25.28

**4079**	allXL	AD	381	386	DBSU	20.96
**4079**	allXL	BD;DB	357	357	DBSU	19.0
**4079**	allXL	AD	373	319	DBSU	11.29
**4079**	allXL	BD	372	357	DBSU	36.18
**4079**	allXL	BD	381	357	DBSU	18.98
**4079**	allXL	BD	373	357	DBSU	34.62
**4079**	allXL	CB	372	370	DBSU	18.1
**4079**	allXL	CB	373	370	DBSU	19.3
**4079**	allXL	DA	382	373	DBSU	27.78
**4080**	allXL	AA	320	357	BS2G	27.51
**4080**	allXL	DD	320	373	BS2G	13.13
**4080**	allXL	BB	373	382	BS2G	18.97
**4080**	allXL	CC	370	386	BS2G	19.31
**4080**	allXL	CC	373	386	BS2G	25.84
**4080**	allXL	AA	319	357	BS2G	27.93
**4080**	allXL	AA	367	373	BS2G	13.11
**4080**	allXL	DD	370	382	BS2G	12.83
**4080**	allXL	BB	376	382	BS2G	13.19
**4080**	allXL	BB	357	373	BS2G	13.81
**4080**	allXL	DD	320	366	BS2G	22.28
**4080**	allXL	CC	370	376	BS2G	13.33
**4080**	allXL	AA	320	357	DBSU	27.51
**4080**	allXL	AA	357	319	DBSU	27.93
**4080**	allXL	DD	372	319	DBSU	7.92
**4080**	allXL	BB	370	357	DBSU	14.43
**4080**	allXL	BB	382	357	DBSU	7.35
**4080**	allXL	AA	372	370	DBSU	6.38
**4080**	allXL	DD	381	370	DBSU	10.1
**4080**	allXL	CC	373	370	DBSU	8.61
**4080**	allXL	DD	381	372	DBSU	15.71
**4080**	allXL	DD	372	382	DBSU	18.72
**4080**	allXL	CC	372	386	DBSU	25.73
**4080**	allXL	CC	373	386	DBSU	25.84
**4080**	allXL	DB	381	386	DBSU	38.57

**4080**	allXL	BD;DB	357	357	DBSU	17.58
**4080**	allXL	DA	373	319	DBSU	37.89
**4080**	allXL	BD	372	357	DBSU	33.03
**4080**	allXL	BD	381	357	DBSU	17.7
**4080**	allXL	BD	373	357	DBSU	31.02
**4080**	allXL	DA	372	370	DBSU	30.76
**4080**	allXL	DA	373	370	DBSU	28.62
**4080**	allXL	AD	382	373	DBSU	43.33
**4081**	allXL	CC	320	357	BS2G	22.65
**4081**	allXL	BB	320	373	BS2G	13.79
**4081**	allXL	BB	373	382	BS2G	17.09
**4081**	allXL	DD	370	386	BS2G	12.57
**4081**	allXL	DD	373	386	BS2G	19.21
**4081**	allXL	CC	319	357	BS2G	25.15
**4081**	allXL	AA	367	373	BS2G	12.83
**4081**	allXL	BB	370	382	BS2G	14.87
**4081**	allXL	BB	376	382	BS2G	14.04
**4081**	allXL	CC	357	373	BS2G	23.34
**4081**	allXL	AA	320	366	BS2G	22.53
**4081**	allXL	BB	370	376	BS2G	14.78
**4081**	allXL	CC	320	357	DBSU	22.65
**4081**	allXL	CC	357	319	DBSU	25.15
**4081**	allXL	BB	372	319	DBSU	16.84
**4081**	allXL	BB	370	357	DBSU	20.28
**4081**	allXL	CC	382	357	DBSU	18.4
**4081**	allXL	AA	372	370	DBSU	6.33
**4081**	allXL	BB	381	370	DBSU	13.77
**4081**	allXL	DD	373	370	DBSU	9.0
**4081**	allXL	BB	381	372	DBSU	17.02
**4081**	allXL	BB	372	382	DBSU	18.49
**4081**	allXL	DD	372	386	DBSU	17.99
**4081**	allXL	DD	373	386	DBSU	19.21
**4081**	allXL	CA	381	386	DBSU	45.67
**4081**	allXL	BD;DB	357	357	DBSU	18.56

**4081**	allXL	AD	373	319	DBSU	29.94
**4081**	allXL	CA	372	357	DBSU	35.54
**4081**	allXL	CA	381	357	DBSU	29.38
**4081**	allXL	CA	373	357	DBSU	32.26
**4081**	allXL	DA	372	370	DBSU	42.3
**4081**	allXL	DA	373	370	DBSU	39.63
**4081**	allXL	CB	382	373	DBSU	37.71
**4082**	allXL	CC	320	357	BS2G	25.15
**4082**	allXL	BB	320	373	BS2G	20.04
**4082**	allXL	AA	373	382	BS2G	19.39
**4082**	allXL	CC	370	386	BS2G	23.74
**4082**	allXL	AA	373	386	BS2G	26.11
**4082**	allXL	CC	319	357	BS2G	28.15
**4082**	allXL	BB	367	373	BS2G	11.74
**4082**	allXL	CC	370	382	BS2G	20.51
**4082**	allXL	AA	376	382	BS2G	15.14
**4082**	allXL	AA	357	373	BS2G	19.01
**4082**	allXL	CC	320	366	BS2G	23.87
**4082**	allXL	BB	370	376	BS2G	13.34
**4082**	allXL	CC	320	357	DBSU	25.15
**4082**	allXL	CC	357	319	DBSU	28.15
**4082**	allXL	CC	372	319	DBSU	16.79
**4082**	allXL	AA	370	357	DBSU	25.22
**4082**	allXL	BB	382	357	DBSU	23.15
**4082**	allXL	AA	372	370	DBSU	6.4
**4082**	allXL	BB	381	370	DBSU	19.69
**4082**	allXL	AA	373	370	DBSU	9.2
**4082**	allXL	CC	381	372	DBSU	19.69
**4082**	allXL	AA	372	382	DBSU	18.89
**4082**	allXL	AA	372	386	DBSU	23.94
**4082**	allXL	AA	373	386	DBSU	26.11
**4082**	allXL	CB	381	386	DBSU	23.42
**4082**	allXL	BD;DB	357	357	DBSU	19.09
**4082**	allXL	DA	373	319	DBSU	20.54

**4082**	allXL	AC	372	357	DBSU	33.85
**4082**	allXL	AC	381	357	DBSU	23.14
**4082**	allXL	AC	373	357	DBSU	32.3
**4082**	allXL	CB	372	370	DBSU	39.62
**4082**	allXL	CB	373	370	DBSU	38.33
**4082**	allXL	CB	382	373	DBSU	25.14
**4083**	allXL	CC	320	357	BS2G	24.1
**4083**	allXL	BB	320	373	BS2G	12.64
**4083**	allXL	DD	373	382	BS2G	16.79
**4083**	allXL	DD	370	386	BS2G	27.32
**4083**	allXL	DD	373	386	BS2G	25.59
**4083**	allXL	CC	319	357	BS2G	24.64
**4083**	allXL	AA	367	373	BS2G	15.45
**4083**	allXL	DD	370	382	BS2G	19.9
**4083**	allXL	DD	376	382	BS2G	14.48
**4083**	allXL	DD	357	373	BS2G	33.78
**4083**	allXL	BB	320	366	BS2G	16.51
**4083**	allXL	DD	370	376	BS2G	17.09
**4083**	allXL	CC	320	357	DBSU	24.1
**4083**	allXL	CC	357	319	DBSU	24.64
**4083**	allXL	BB	372	319	DBSU	10.3
**4083**	allXL	BB	370	357	DBSU	27.39
**4083**	allXL	BB	382	357	DBSU	46.25
**4083**	allXL	AA	372	370	DBSU	6.25
**4083**	allXL	DD	381	370	DBSU	18.28
**4083**	allXL	AA	373	370	DBSU	9.28
**4083**	allXL	DD	381	372	DBSU	17.24
**4083**	allXL	DD	372	382	DBSU	18.17
**4083**	allXL	DD	372	386	DBSU	25.68
**4083**	allXL	DD	373	386	DBSU	25.59
**4083**	allXL	AD	381	386	DBSU	47.62
**4083**	allXL	BD;DB	357	357	DBSU	17.97
**4083**	allXL	AD	373	319	DBSU	37.01
**4083**	allXL	BD	372	357	DBSU	46.83

**4083**	allXL	BC	381	357	DBSU	44.32
**4083**	allXL	BD	373	357	DBSU	50.32
**4083**	allXL	DA	372	370	DBSU	49.25
**4083**	allXL	DA	373	370	DBSU	45.48
**4083**	allXL	DA	382	373	DBSU	39.68
**4084**	allXL	CC	320	357	BS2G	25.65
**4084**	allXL	DD	320	373	BS2G	11.14
**4084**	allXL	DD	373	382	BS2G	15.76
**4084**	allXL	DD	370	386	BS2G	16.12
**4084**	allXL	DD	373	386	BS2G	12.23
**4084**	allXL	AA	319	357	BS2G	28.03
**4084**	allXL	AA	367	373	BS2G	12.94
**4084**	allXL	BB	370	382	BS2G	14.79
**4084**	allXL	CC	376	382	BS2G	13.59
**4084**	allXL	BB	357	373	BS2G	21.32
**4084**	allXL	AA	320	366	BS2G	12.32
**4084**	allXL	BB	370	376	BS2G	15.8
**4084**	allXL	CC	320	357	DBSU	25.65
**4084**	allXL	AA	357	319	DBSU	28.03
**4084**	allXL	DD	372	319	DBSU	11.67
**4084**	allXL	DD	370	357	DBSU	20.69
**4084**	allXL	DD	382	357	DBSU	32.08
**4084**	allXL	AA	372	370	DBSU	6.3
**4084**	allXL	BB	381	370	DBSU	17.99
**4084**	allXL	AA	373	370	DBSU	9.09
**4084**	allXL	DD	381	372	DBSU	14.18
**4084**	allXL	DD	372	382	DBSU	14.9
**4084**	allXL	DD	372	386	DBSU	11.17
**4084**	allXL	DD	373	386	DBSU	12.23
**4084**	allXL	CB	381	386	DBSU	30.83
**4084**	allXL	BD;DB	357	357	DBSU	18.81
**4084**	allXL	AD	373	319	DBSU	26.42
**4084**	allXL	DB	372	357	DBSU	39.06
**4084**	allXL	CB	381	357	DBSU	41.34

**4084**	allXL	DB	373	357	DBSU	37.67
**4084**	allXL	AD	372	370	DBSU	30.68
**4084**	allXL	AD	373	370	DBSU	30.86
**4084**	allXL	CB	382	373	DBSU	34.55
**4085**	allXL	AA	320	357	BS2G	23.97
**4085**	allXL	DD	320	373	BS2G	16.69
**4085**	allXL	AA	373	382	BS2G	20.23
**4085**	allXL	AA	370	386	BS2G	28.11
**4085**	allXL	AA	373	386	BS2G	27.0
**4085**	allXL	AA	319	357	BS2G	23.36
**4085**	allXL	BB	367	373	BS2G	12.73
**4085**	allXL	DD	370	382	BS2G	23.61
**4085**	allXL	AA	376	382	BS2G	15.04
**4085**	allXL	BB	357	373	BS2G	25.37
**4085**	allXL	DD	320	366	BS2G	24.66
**4085**	allXL	CC	370	376	BS2G	14.23
**4085**	allXL	AA	320	357	DBSU	23.97
**4085**	allXL	AA	357	319	DBSU	23.36
**4085**	allXL	DD	372	319	DBSU	19.99
**4085**	allXL	BB	370	357	DBSU	17.93
**4085**	allXL	BB	382	357	DBSU	34.78
**4085**	allXL	AA	372	370	DBSU	6.17
**4085**	allXL	CC	381	370	DBSU	22.36
**4085**	allXL	AA	373	370	DBSU	9.07
**4085**	allXL	DD	381	372	DBSU	19.5
**4085**	allXL	AA	372	382	DBSU	20.09
**4085**	allXL	AA	372	386	DBSU	25.19
**4085**	allXL	AA	373	386	DBSU	27.0
**4085**	allXL	BC	381	386	DBSU	38.19
**4085**	allXL	BD;DB	357	357	DBSU	19.01
**4085**	allXL	DA	373	319	DBSU	36.05
**4085**	allXL	AC	372	357	DBSU	36.81
**4085**	allXL	CB	381	357	DBSU	40.01
**4085**	allXL	AC	373	357	DBSU	39.13

**4085**	allXL	BC	372	370	DBSU	43.33
**4085**	allXL	CB	373	370	DBSU	42.52
**4085**	allXL	CB	382	373	DBSU	20.46
**4086**	allXL	AA	320	357	BS2G	23.64
**4086**	allXL	DD	320	373	BS2G	20.34
**4086**	allXL	AA	373	382	BS2G	20.02
**4086**	allXL	CC	370	386	BS2G	27.55
**4086**	allXL	AA	373	386	BS2G	26.52
**4086**	allXL	CC	319	357	BS2G	22.68
**4086**	allXL	BB	367	373	BS2G	13.05
**4086**	allXL	BB	370	382	BS2G	21.53
**4086**	allXL	AA	376	382	BS2G	14.03
**4086**	allXL	DD	357	373	BS2G	24.63
**4086**	allXL	DD	320	366	BS2G	14.13
**4086**	allXL	CC	370	376	BS2G	15.17
**4086**	allXL	AA	320	357	DBSU	23.64
**4086**	allXL	CC	357	319	DBSU	22.68
**4086**	allXL	DD	372	319	DBSU	22.42
**4086**	allXL	CC	370	357	DBSU	25.1
**4086**	allXL	BB	382	357	DBSU	35.78
**4086**	allXL	AA	372	370	DBSU	6.51
**4086**	allXL	BB	381	370	DBSU	21.31
**4086**	allXL	AA	373	370	DBSU	9.21
**4086**	allXL	DD	381	372	DBSU	20.2
**4086**	allXL	CC	372	382	DBSU	22.0
**4086**	allXL	AA	372	386	DBSU	28.24
**4086**	allXL	AA	373	386	DBSU	26.52
**4086**	allXL	AD	381	386	DBSU	27.31
**4086**	allXL	AC;CA	357	357	DBSU	19.78
**4086**	allXL	AD	373	319	DBSU	23.47
**4086**	allXL	CA	372	357	DBSU	37.91
**4086**	allXL	AC	381	357	DBSU	43.7
**4086**	allXL	AC	373	357	DBSU	37.02
**4086**	allXL	DA	372	370	DBSU	25.05

**4086**	allXL	DA	373	370	DBSU	25.61
**4086**	allXL	DA	382	373	DBSU	29.4
**4087**	allXL	BB	320	357	BS2G	27.34
**4087**	allXL	BB	320	373	BS2G	21.06
**4087**	allXL	AA	373	382	BS2G	19.14
**4087**	allXL	AA	370	386	BS2G	27.48
**4087**	allXL	AA	373	386	BS2G	23.37
**4087**	allXL	AA	319	357	BS2G	29.7
**4087**	allXL	BB	367	373	BS2G	11.79
**4087**	allXL	BB	370	382	BS2G	24.48
**4087**	allXL	CC	376	382	BS2G	13.62
**4087**	allXL	DD	357	373	BS2G	20.1
**4087**	allXL	BB	320	366	BS2G	19.44
**4087**	allXL	BB	370	376	BS2G	16.42
**4087**	allXL	BB	320	357	DBSU	27.34
**4087**	allXL	AA	357	319	DBSU	29.7
**4087**	allXL	BB	372	319	DBSU	17.21
**4087**	allXL	DD	370	357	DBSU	19.75
**4087**	allXL	BB	382	357	DBSU	26.3
**4087**	allXL	AA	372	370	DBSU	6.19
**4087**	allXL	BB	381	370	DBSU	23.86
**4087**	allXL	DD	373	370	DBSU	8.67
**4087**	allXL	DD	381	372	DBSU	20.73
**4087**	allXL	AA	372	382	DBSU	22.38
**4087**	allXL	AA	372	386	DBSU	26.12
**4087**	allXL	AA	373	386	DBSU	23.37
**4087**	allXL	BC	381	386	DBSU	50.19
**4087**	allXL	BD;DB	357	357	DBSU	19.6
**4087**	allXL	CB	373	319	DBSU	24.26
**4087**	allXL	BC	372	357	DBSU	34.32
**4087**	allXL	DB	381	357	DBSU	42.41
**4087**	allXL	BC	373	357	DBSU	37.51
**4087**	allXL	BC	372	370	DBSU	22.68
**4087**	allXL	BC	373	370	DBSU	23.64

**4087**	allXL	CB	382	373	DBSU	41.52
**4088**	allXL	AA	320	357	BS2G	21.09
**4088**	allXL	BB	320	373	BS2G	19.09
**4088**	allXL	BB	373	382	BS2G	16.53
**4088**	allXL	AA	370	386	BS2G	26.49
**4088**	allXL	DD	373	386	BS2G	26.5
**4088**	allXL	AA	319	357	BS2G	23.66
**4088**	allXL	AA	367	373	BS2G	12.71
**4088**	allXL	BB	370	382	BS2G	21.32
**4088**	allXL	BB	376	382	BS2G	13.18
**4088**	allXL	DD	357	373	BS2G	12.21
**4088**	allXL	CC	320	366	BS2G	26.86
**4088**	allXL	AA	370	376	BS2G	15.68
**4088**	allXL	AA	320	357	DBSU	21.09
**4088**	allXL	AA	357	319	DBSU	23.66
**4088**	allXL	CC	372	319	DBSU	23.08
**4088**	allXL	DD	370	357	DBSU	17.55
**4088**	allXL	BB	382	357	DBSU	10.66
**4088**	allXL	CC	372	370	DBSU	6.63
**4088**	allXL	DD	381	370	DBSU	21.88
**4088**	allXL	DD	373	370	DBSU	8.82
**4088**	allXL	BB	381	372	DBSU	19.71
**4088**	allXL	BB	372	382	DBSU	18.52
**4088**	allXL	CC	372	386	DBSU	27.77
**4088**	allXL	DD	373	386	DBSU	26.5
**4088**	allXL	DB	381	386	DBSU	25.67
**4088**	allXL	BD;DB	357	357	DBSU	18.8
**4088**	allXL	AD	373	319	DBSU	28.37
**4088**	allXL	DB	372	357	DBSU	18.16
**4088**	allXL	DB	381	357	DBSU	27.57
**4088**	allXL	DB	373	357	DBSU	17.04
**4088**	allXL	DB	372	370	DBSU	45.63
**4088**	allXL	DB	373	370	DBSU	44.97
**4088**	allXL	BD	382	373	DBSU	25.55

**4089**	allXL	AA	320	357	BS2G	25.31
**4089**	allXL	AA	320	373	BS2G	15.07
**4089**	allXL	DD	373	382	BS2G	17.18
**4089**	allXL	DD	370	386	BS2G	20.16
**4089**	allXL	DD	373	386	BS2G	24.95
**4089**	allXL	AA	319	357	BS2G	27.52
**4089**	allXL	DD	367	373	BS2G	13.34
**4089**	allXL	DD	370	382	BS2G	16.36
**4089**	allXL	AA	376	382	BS2G	15.2
**4089**	allXL	DD	357	373	BS2G	13.24
**4089**	allXL	BB	320	366	BS2G	19.83
**4089**	allXL	CC	370	376	BS2G	13.44
**4089**	allXL	AA	320	357	DBSU	25.31
**4089**	allXL	AA	357	319	DBSU	27.52
**4089**	allXL	BB	372	319	DBSU	18.85
**4089**	allXL	DD	370	357	DBSU	20.21
**4089**	allXL	DD	382	357	DBSU	16.2
**4089**	allXL	AA	372	370	DBSU	6.2
**4089**	allXL	DD	381	370	DBSU	16.16
**4089**	allXL	CC	373	370	DBSU	9.02
**4089**	allXL	DD	381	372	DBSU	17.34
**4089**	allXL	DD	372	382	DBSU	19.03
**4089**	allXL	DD	372	386	DBSU	25.4
**4089**	allXL	DD	373	386	DBSU	24.95
**4089**	allXL	AD	381	386	DBSU	23.43
**4089**	allXL	BD;DB	357	357	DBSU	18.8
**4089**	allXL	AD	373	319	DBSU	27.66
**4089**	allXL	DB	372	357	DBSU	27.57
**4089**	allXL	DB	381	357	DBSU	30.98
**4089**	allXL	DB	373	357	DBSU	24.92
**4089**	allXL	CB	372	370	DBSU	36.98
**4089**	allXL	CB	373	370	DBSU	36.23
**4089**	allXL	CB	382	373	DBSU	29.42
**4090**	allXL	AA	320	357	BS2G	22.91

**4090**	allXL	DD	320	373	BS2G	20.13
**4090**	allXL	DD	373	382	BS2G	14.71
**4090**	allXL	AA	370	386	BS2G	14.24
**4090**	allXL	AA	373	386	BS2G	21.12
**4090**	allXL	AA	319	357	BS2G	21.82
**4090**	allXL	AA	367	373	BS2G	11.0
**4090**	allXL	DD	370	382	BS2G	14.16
**4090**	allXL	CC	376	382	BS2G	14.42
**4090**	allXL	BB	357	373	BS2G	12.95
**4090**	allXL	AA	320	366	BS2G	24.55
**4090**	allXL	AA	370	376	BS2G	14.52
**4090**	allXL	AA	320	357	DBSU	22.91
**4090**	allXL	AA	357	319	DBSU	21.82
**4090**	allXL	DD	372	319	DBSU	23.71
**4090**	allXL	BB	370	357	DBSU	18.3
**4090**	allXL	BB	382	357	DBSU	18.26
**4090**	allXL	AA	372	370	DBSU	6.59
**4090**	allXL	DD	381	370	DBSU	14.5
**4090**	allXL	AA	373	370	DBSU	8.91
**4090**	allXL	DD	381	372	DBSU	14.38
**4090**	allXL	DD	372	382	DBSU	13.96
**4090**	allXL	AA	372	386	DBSU	19.22
**4090**	allXL	AA	373	386	DBSU	21.12
**4090**	allXL	CB	381	386	DBSU	26.13
**4090**	allXL	BD;DB	357	357	DBSU	17.67
**4090**	allXL	CB	373	319	DBSU	10.49
**4090**	allXL	BD	372	357	DBSU	20.46
**4090**	allXL	BD	381	357	DBSU	26.84
**4090**	allXL	BD	373	357	DBSU	17.74
**4090**	allXL	CB	372	370	DBSU	22.91
**4090**	allXL	CB	373	370	DBSU	20.27
**4090**	allXL	AD	382	373	DBSU	30.25
**4091**	allXL	AA	320	357	BS2G	26.89
**4091**	allXL	DD	320	373	BS2G	16.51

**4091**	allXL	DD	373	382	BS2G	18.87
**4091**	allXL	AA	370	386	BS2G	30.49
**4091**	allXL	AA	373	386	BS2G	26.87
**4091**	allXL	AA	319	357	BS2G	26.9
**4091**	allXL	AA	367	373	BS2G	12.78
**4091**	allXL	BB	370	382	BS2G	23.73
**4091**	allXL	BB	376	382	BS2G	14.38
**4091**	allXL	DD	357	373	BS2G	24.5
**4091**	allXL	BB	320	366	BS2G	18.02
**4091**	allXL	CC	370	376	BS2G	15.54
**4091**	allXL	AA	320	357	DBSU	26.89
**4091**	allXL	AA	357	319	DBSU	26.9
**4091**	allXL	BB	372	319	DBSU	18.34
**4091**	allXL	DD	370	357	DBSU	23.5
**4091**	allXL	AA	382	357	DBSU	31.57
**4091**	allXL	AA	372	370	DBSU	6.46
**4091**	allXL	DD	381	370	DBSU	25.23
**4091**	allXL	DD	373	370	DBSU	8.91
**4091**	allXL	DD	381	372	DBSU	18.12
**4091**	allXL	DD	372	382	DBSU	18.41
**4091**	allXL	DD	372	386	DBSU	26.81
**4091**	allXL	AA	373	386	DBSU	26.87
**4091**	allXL	BC	381	386	DBSU	41.17
**4091**	allXL	BD;DB	357	357	DBSU	18.53
**4091**	allXL	CB	373	319	DBSU	20.47
**4091**	allXL	CB	372	357	DBSU	31.8
**4091**	allXL	CB	381	357	DBSU	47.48
**4091**	allXL	CB	373	357	DBSU	32.85
**4091**	allXL	CB	372	370	DBSU	18.33
**4091**	allXL	CB	373	370	DBSU	19.72
**4091**	allXL	CB	382	373	DBSU	21.21
**4092**	allXL	CC	320	357	BS2G	25.41
**4092**	allXL	BB	320	373	BS2G	23.6
**4092**	allXL	BB	373	382	BS2G	21.24

**4092**	allXL	DD	370	386	BS2G	27.34
**4092**	allXL	DD	373	386	BS2G	28.08
**4092**	allXL	AA	319	357	BS2G	26.23
**4092**	allXL	AA	367	373	BS2G	13.34
**4092**	allXL	DD	370	382	BS2G	20.05
**4092**	allXL	CC	376	382	BS2G	14.39
**4092**	allXL	BB	357	373	BS2G	31.12
**4092**	allXL	AA	320	366	BS2G	28.98
**4092**	allXL	DD	370	376	BS2G	15.25
**4092**	allXL	CC	320	357	DBSU	25.41
**4092**	allXL	AA	357	319	DBSU	26.23
**4092**	allXL	BB	372	319	DBSU	26.2
**4092**	allXL	DD	370	357	DBSU	31.38
**4092**	allXL	DD	382	357	DBSU	35.05
**4092**	allXL	AA	372	370	DBSU	6.19
**4092**	allXL	DD	381	370	DBSU	19.04
**4092**	allXL	AA	373	370	DBSU	9.04
**4092**	allXL	DD	381	372	DBSU	22.0
**4092**	allXL	AA	372	382	DBSU	22.93
**4092**	allXL	DD	372	386	DBSU	29.13
**4092**	allXL	DD	373	386	DBSU	28.08
**4092**	allXL	BC	381	386	DBSU	72.97
**4092**	allXL	BD;DB	357	357	DBSU	18.67
**4092**	allXL	AD	373	319	DBSU	29.98
**4092**	allXL	AD	372	357	DBSU	45.98
**4092**	allXL	DB	381	357	DBSU	47.3
**4092**	allXL	AD	373	357	DBSU	48.94
**4092**	allXL	BC	372	370	DBSU	43.11
**4092**	allXL	BC	373	370	DBSU	40.94
**4092**	allXL	BC	382	373	DBSU	53.98
**4093**	allXL	CC	320	357	BS2G	25.67
**4093**	allXL	DD	320	373	BS2G	22.16
**4093**	allXL	CC	373	382	BS2G	19.84
**4093**	allXL	CC	370	386	BS2G	22.55

**4093**	allXL	DD	373	386	BS2G	28.69
**4093**	allXL	CC	319	357	BS2G	27.61
**4093**	allXL	AA	367	373	BS2G	12.48
**4093**	allXL	CC	370	382	BS2G	15.58
**4093**	allXL	CC	376	382	BS2G	14.79
**4093**	allXL	CC	357	373	BS2G	29.66
**4093**	allXL	AA	320	366	BS2G	16.22
**4093**	allXL	CC	370	376	BS2G	12.6
**4093**	allXL	CC	320	357	DBSU	25.67
**4093**	allXL	CC	357	319	DBSU	27.61
**4093**	allXL	DD	372	319	DBSU	25.34
**4093**	allXL	DD	370	357	DBSU	25.25
**4093**	allXL	CC	382	357	DBSU	31.38
**4093**	allXL	AA	372	370	DBSU	6.51
**4093**	allXL	CC	381	370	DBSU	17.14
**4093**	allXL	AA	373	370	DBSU	9.24
**4093**	allXL	DD	381	372	DBSU	19.35
**4093**	allXL	CC	372	382	DBSU	20.18
**4093**	allXL	DD	372	386	DBSU	27.98
**4093**	allXL	DD	373	386	DBSU	28.69
**4093**	allXL	AD	381	386	DBSU	53.18
**4093**	allXL	BD;DB	357	357	DBSU	18.37
**4093**	allXL	CB	373	319	DBSU	20.96
**4093**	allXL	CB	372	357	DBSU	45.37
**4093**	allXL	DA	381	357	DBSU	50.14
**4093**	allXL	CA	373	357	DBSU	47.22
**4093**	allXL	BC	372	370	DBSU	49.47
**4093**	allXL	BC	373	370	DBSU	52.93
**4093**	allXL	DA	382	373	DBSU	46.91
**4094**	allXL	AA	320	357	BS2G	21.47
**4094**	allXL	BB	320	373	BS2G	30.75
**4094**	allXL	DD	373	382	BS2G	18.29
**4094**	allXL	CC	370	386	BS2G	21.1
**4094**	allXL	DD	373	386	BS2G	17.2

**4094**	allXL	AA	319	357	BS2G	25.09
**4094**	allXL	DD	367	373	BS2G	13.15
**4094**	allXL	CC	370	382	BS2G	19.2
**4094**	allXL	CC	376	382	BS2G	13.01
**4094**	allXL	BB	357	373	BS2G	23.49
**4094**	allXL	AA	320	366	BS2G	24.72
**4094**	allXL	CC	370	376	BS2G	14.0
**4094**	allXL	AA	320	357	DBSU	21.47
**4094**	allXL	AA	357	319	DBSU	25.09
**4094**	allXL	DD	372	319	DBSU	31.02
**4094**	allXL	BB	370	357	DBSU	23.08
**4094**	allXL	BB	382	357	DBSU	25.46
**4094**	allXL	AA	372	370	DBSU	6.05
**4094**	allXL	CC	381	370	DBSU	18.87
**4094**	allXL	DD	373	370	DBSU	9.11
**4094**	allXL	DD	381	372	DBSU	19.5
**4094**	allXL	DD	372	382	DBSU	19.59
**4094**	allXL	DD	372	386	DBSU	16.24
**4094**	allXL	DD	373	386	DBSU	17.2
**4094**	allXL	DB	381	386	DBSU	53.53
**4094**	allXL	BD;DB	357	357	DBSU	19.55
**4094**	allXL	AD	373	319	DBSU	31.15
**4094**	allXL	BD	372	357	DBSU	37.45
**4094**	allXL	BD	381	357	DBSU	26.06
**4094**	allXL	BD	373	357	DBSU	34.66
**4094**	allXL	DA	372	370	DBSU	45.33
**4094**	allXL	AD;DA	373	370	DBSU	48.8
**4094**	allXL	BD	382	373	DBSU	43.01
**4095**	allXL	AA	320	357	BS2G	25.59
**4095**	allXL	AA	320	373	BS2G	12.36
**4095**	allXL	BB	373	382	BS2G	18.11
**4095**	allXL	DD	370	386	BS2G	25.77
**4095**	allXL	BB	373	386	BS2G	24.57
**4095**	allXL	AA	319	357	BS2G	23.47

**4095**	allXL	AA	367	373	BS2G	14.93
**4095**	allXL	DD	370	382	BS2G	22.02
**4095**	allXL	BB	376	382	BS2G	14.71
**4095**	allXL	DD	357	373	BS2G	17.54
**4095**	allXL	AA	320	366	BS2G	16.27
**4095**	allXL	CC	370	376	BS2G	15.29
**4095**	allXL	AA	320	357	DBSU	25.59
**4095**	allXL	AA	357	319	DBSU	23.47
**4095**	allXL	AA	372	319	DBSU	13.8
**4095**	allXL	DD	370	357	DBSU	15.45
**4095**	allXL	DD	382	357	DBSU	31.97
**4095**	allXL	AA	372	370	DBSU	6.12
**4095**	allXL	DD	381	370	DBSU	19.87
**4095**	allXL	CC	373	370	DBSU	9.36
**4095**	allXL	DD	381	372	DBSU	18.44
**4095**	allXL	BB	372	382	DBSU	19.58
**4095**	allXL	BB	372	386	DBSU	24.77
**4095**	allXL	BB	373	386	DBSU	24.57
**4095**	allXL	DA	381	386	DBSU	57.04
**4095**	allXL	BD;DB	357	357	DBSU	18.61
**4095**	allXL	BC	373	319	DBSU	23.1
**4095**	allXL	DB	372	357	DBSU	25.77
**4095**	allXL	DB	381	357	DBSU	30.9
**4095**	allXL	DB	373	357	DBSU	23.89
**4095**	allXL	BC	372	370	DBSU	44.26
**4095**	allXL	BC	373	370	DBSU	42.01
**4095**	allXL	BC	382	373	DBSU	52.93
**4096**	allXL	CC	320	357	BS2G	27.5
**4096**	allXL	DD	320	373	BS2G	19.22
**4096**	allXL	DD	373	382	BS2G	16.02
**4096**	allXL	AA	370	386	BS2G	17.07
**4096**	allXL	AA	373	386	BS2G	20.42
**4096**	allXL	DD	319	357	BS2G	27.99
**4096**	allXL	BB	367	373	BS2G	13.05

**4096**	allXL	DD	370	382	BS2G	10.42
**4096**	allXL	CC	376	382	BS2G	13.93
**4096**	allXL	CC	357	373	BS2G	23.05
**4096**	allXL	CC	320	366	BS2G	17.6
**4096**	allXL	DD	370	376	BS2G	15.61
**4096**	allXL	CC	320	357	DBSU	27.5
**4096**	allXL	DD	357	319	DBSU	27.99
**4096**	allXL	DD	372	319	DBSU	12.27
**4096**	allXL	CC	370	357	DBSU	15.68
**4096**	allXL	CC	382	357	DBSU	33.42
**4096**	allXL	AA	372	370	DBSU	6.18
**4096**	allXL	DD	381	370	DBSU	9.79
**4096**	allXL	AA	373	370	DBSU	9.11
**4096**	allXL	DD	381	372	DBSU	14.4
**4096**	allXL	CC	372	382	DBSU	15.69
**4096**	allXL	AA	372	386	DBSU	18.88
**4096**	allXL	AA	373	386	DBSU	20.42
**4096**	allXL	CB	381	386	DBSU	25.57
**4096**	allXL	BD;DB	357	357	DBSU	18.13
**4096**	allXL	AD	373	319	DBSU	19.3
**4096**	allXL	CB	372	357	DBSU	30.9
**4096**	allXL	CB	381	357	DBSU	33.99
**4096**	allXL	CB	373	357	DBSU	27.56
**4096**	allXL	DA	372	370	DBSU	19.31
**4096**	allXL	AD	373	370	DBSU	18.96
**4096**	allXL	DA	382	373	DBSU	22.88
**4097**	allXL	AA	320	357	BS2G	25.86
**4097**	allXL	BB	320	373	BS2G	17.52
**4097**	allXL	BB	373	382	BS2G	19.71
**4097**	allXL	BB	370	386	BS2G	27.09
**4097**	allXL	DD	373	386	BS2G	27.85
**4097**	allXL	AA	319	357	BS2G	24.89
**4097**	allXL	AA	367	373	BS2G	10.93
**4097**	allXL	BB	370	382	BS2G	19.9

**4097**	allXL	BB	376	382	BS2G	14.33
**4097**	allXL	AA	357	373	BS2G	24.91
**4097**	allXL	AA	320	366	BS2G	15.13
**4097**	allXL	CC	370	376	BS2G	14.99
**4097**	allXL	AA	320	357	DBSU	25.86
**4097**	allXL	AA	357	319	DBSU	24.89
**4097**	allXL	BB	372	319	DBSU	22.53
**4097**	allXL	DD	370	357	DBSU	25.05
**4097**	allXL	BB	382	357	DBSU	26.41
**4097**	allXL	AA	372	370	DBSU	6.32
**4097**	allXL	BB	381	370	DBSU	22.28
**4097**	allXL	DD	373	370	DBSU	9.08
**4097**	allXL	DD	381	372	DBSU	19.01
**4097**	allXL	DD	372	382	DBSU	19.86
**4097**	allXL	DD	372	386	DBSU	27.36
**4097**	allXL	DD	373	386	DBSU	27.85
**4097**	allXL	DA	381	386	DBSU	43.57
**4097**	allXL	BD;DB	357	357	DBSU	18.84
**4097**	allXL	AD	373	319	DBSU	40.27
**4097**	allXL	BD	372	357	DBSU	46.4
**4097**	allXL	BC	381	357	DBSU	40.04
**4097**	allXL	AC	373	357	DBSU	44.44
**4097**	allXL	BC	372	370	DBSU	44.26
**4097**	allXL	BC	373	370	DBSU	41.19
**4097**	allXL	BC	382	373	DBSU	40.23
**4098**	allXL	AA	320	357	BS2G	24.6
**4098**	allXL	DD	320	373	BS2G	13.55
**4098**	allXL	BB	373	382	BS2G	16.05
**4098**	allXL	CC	370	386	BS2G	22.34
**4098**	allXL	DD	373	386	BS2G	25.49
**4098**	allXL	AA	319	357	BS2G	23.07
**4098**	allXL	AA	367	373	BS2G	9.93
**4098**	allXL	CC	370	382	BS2G	13.85
**4098**	allXL	BB	376	382	BS2G	12.67

**4098**	allXL	AA	357	373	BS2G	28.4
**4098**	allXL	CC	320	366	BS2G	20.1
**4098**	allXL	CC	370	376	BS2G	11.05
**4098**	allXL	AA	320	357	DBSU	24.6
**4098**	allXL	AA	357	319	DBSU	23.07
**4098**	allXL	DD	372	319	DBSU	15.46
**4098**	allXL	DD	370	357	DBSU	27.84
**4098**	allXL	AA	382	357	DBSU	25.77
**4098**	allXL	CC	372	370	DBSU	6.7
**4098**	allXL	CC	381	370	DBSU	15.62
**4098**	allXL	AA	373	370	DBSU	8.7
**4098**	allXL	DD	381	372	DBSU	17.18
**4098**	allXL	DD	372	382	DBSU	17.11
**4098**	allXL	DD	372	386	DBSU	23.42
**4098**	allXL	DD	373	386	DBSU	25.49
**4098**	allXL	DA	381	386	DBSU	38.55
**4098**	allXL	BD;DB	357	357	DBSU	18.62
**4098**	allXL	AD	373	319	DBSU	23.38
**4098**	allXL	CB	372	357	DBSU	29.61
**4098**	allXL	CB	381	357	DBSU	32.82
**4098**	allXL	CB	373	357	DBSU	32.95
**4098**	allXL	AD	372	370	DBSU	24.59
**4098**	allXL	DA	373	370	DBSU	24.86
**4098**	allXL	CB	382	373	DBSU	32.63
**4099**	allXL	AA	320	357	BS2G	19.76
**4099**	allXL	BB	320	373	BS2G	18.97
**4099**	allXL	BB	373	382	BS2G	19.59
**4099**	allXL	DD	370	386	BS2G	18.29
**4099**	allXL	DD	373	386	BS2G	24.92
**4099**	allXL	AA	319	357	BS2G	18.36
**4099**	allXL	AA	367	373	BS2G	12.57
**4099**	allXL	DD	370	382	BS2G	17.45
**4099**	allXL	CC	376	382	BS2G	14.06
**4099**	allXL	CC	357	373	BS2G	15.87

4099	allXL	AA	320	366	BS2G	22.78
4099	allXL	CC	370	376	BS2G	16.69
4099	allXL	AA	320	357	DBSU	19.76
4099	allXL	AA	357	319	DBSU	18.36
4099	allXL	BB	372	319	DBSU	20.57
4099	allXL	CC	370	357	DBSU	14.54
4099	allXL	BB	382	357	DBSU	12.72
4099	allXL	AA	372	370	DBSU	6.53
4099	allXL	DD	381	370	DBSU	16.45
4099	allXL	AA	373	370	DBSU	9.39
4099	allXL	DD	381	372	DBSU	17.73
4099	allXL	DD	372	382	DBSU	19.85
4099	allXL	DD	372	386	DBSU	23.15
4099	allXL	DD	373	386	DBSU	24.92
4099	allXL	AC	381	386	DBSU	37.35
4099	allXL	BD;DB	357	357	DBSU	18.76
4099	allXL	BC	373	319	DBSU	39.31
4099	allXL	CA	372	357	DBSU	34.0
4099	allXL	CA	381	357	DBSU	21.4
4099	allXL	CA	373	357	DBSU	31.75
4099	allXL	BC	372	370	DBSU	46.0
4099	allXL	BC	373	370	DBSU	44.59
4099	allXL	CA	382	373	DBSU	37.05
4100	allXL	AA	320	357	BS2G	22.17
4100	allXL	DD	320	373	BS2G	12.08
4100	allXL	BB	373	382	BS2G	19.14
4100	allXL	DD	370	386	BS2G	22.21
4100	allXL	CC	373	386	BS2G	25.11
4100	allXL	AA	319	357	BS2G	19.34
4100	allXL	AA	367	373	BS2G	13.56
4100	allXL	DD	370	382	BS2G	16.6
4100	allXL	BB	376	382	BS2G	14.09
4100	allXL	DD	357	373	BS2G	33.58
4100	allXL	BB	320	366	BS2G	13.6

**4100**	allXL	DD	370	376	BS2G	15.7
**4100**	allXL	AA	320	357	DBSU	22.17
**4100**	allXL	AA	357	319	DBSU	19.34
**4100**	allXL	DD	372	319	DBSU	11.52
**4100**	allXL	DD	370	357	DBSU	29.58
**4100**	allXL	BB	382	357	DBSU	27.7
**4100**	allXL	CC	372	370	DBSU	6.8
**4100**	allXL	DD	381	370	DBSU	17.11
**4100**	allXL	AA	373	370	DBSU	9.25
**4100**	allXL	DD	381	372	DBSU	20.61
**4100**	allXL	DD	372	382	DBSU	21.05
**4100**	allXL	CC	372	386	DBSU	25.23
**4100**	allXL	CC	373	386	DBSU	25.11
**4100**	allXL	AD	381	386	DBSU	41.62
**4100**	allXL	BD;DB	357	357	DBSU	18.51
**4100**	allXL	AD	373	319	DBSU	29.91
**4100**	allXL	CA	372	357	DBSU	46.97
**4100**	allXL	CA	381	357	DBSU	44.23
**4100**	allXL	DA	373	357	DBSU	48.24
**4100**	allXL	AD	372	370	DBSU	44.29
**4100**	allXL	AD	373	370	DBSU	41.74
**4100**	allXL	AD	382	373	DBSU	30.95
**4101**	allXL	CC	320	357	BS2G	23.95
**4101**	allXL	CC	320	373	BS2G	21.51
**4101**	allXL	DD	373	382	BS2G	17.14
**4101**	allXL	AA	370	386	BS2G	19.64
**4101**	allXL	AA	373	386	BS2G	23.12
**4101**	allXL	CC	319	357	BS2G	27.4
**4101**	allXL	BB	367	373	BS2G	13.17
**4101**	allXL	AA	370	382	BS2G	20.94
**4101**	allXL	BB	376	382	BS2G	14.41
**4101**	allXL	CC	357	373	BS2G	20.42
**4101**	allXL	CC	320	366	BS2G	14.64
**4101**	allXL	CC	370	376	BS2G	15.95

**4101**	allXL	CC	320	357	DBSU	23.95
**4101**	allXL	CC	357	319	DBSU	27.4
**4101**	allXL	CC	372	319	DBSU	22.69
**4101**	allXL	CC	370	357	DBSU	24.4
**4101**	allXL	CC	382	357	DBSU	25.77
**4101**	allXL	AA	372	370	DBSU	6.2
**4101**	allXL	AA	381	370	DBSU	22.27
**4101**	allXL	AA	373	370	DBSU	9.04
**4101**	allXL	DD	381	372	DBSU	18.19
**4101**	allXL	DD	372	382	DBSU	17.62
**4101**	allXL	AA	372	386	DBSU	21.55
**4101**	allXL	AA	373	386	DBSU	23.12
**4101**	allXL	DA	381	386	DBSU	34.91
**4101**	allXL	BD;DB	357	357	DBSU	18.21
**4101**	allXL	AD	373	319	DBSU	18.09
**4101**	allXL	CA	372	357	DBSU	42.89
**4101**	allXL	CA	381	357	DBSU	38.9
**4101**	allXL	CA	373	357	DBSU	39.38
**4101**	allXL	AD	372	370	DBSU	35.73
**4101**	allXL	AD	373	370	DBSU	36.1
**4101**	allXL	AD	382	373	DBSU	26.84
**4102**	allXL	AA	320	357	BS2G	25.3
**4102**	allXL	CC	320	373	BS2G	29.86
**4102**	allXL	BB	373	382	BS2G	19.76
**4102**	allXL	CC	370	386	BS2G	22.37
**4102**	allXL	BB	373	386	BS2G	24.83
**4102**	allXL	AA	319	357	BS2G	25.34
**4102**	allXL	BB	367	373	BS2G	12.91
**4102**	allXL	CC	370	382	BS2G	20.06
**4102**	allXL	CC	376	382	BS2G	13.84
**4102**	allXL	AA	357	373	BS2G	26.3
**4102**	allXL	BB	320	366	BS2G	28.78
**4102**	allXL	CC	370	376	BS2G	14.57
**4102**	allXL	AA	320	357	DBSU	25.3

**4102**	allXL	AA	357	319	DBSU	25.34
**4102**	allXL	DD	372	319	DBSU	25.86
**4102**	allXL	BB	370	357	DBSU	29.24
**4102**	allXL	BB	382	357	DBSU	28.82
**4102**	allXL	AA	372	370	DBSU	6.25
**4102**	allXL	DD	381	370	DBSU	18.7
**4102**	allXL	AA	373	370	DBSU	9.13
**4102**	allXL	DD	381	372	DBSU	19.1
**4102**	allXL	DD	372	382	DBSU	21.67
**4102**	allXL	BB	372	386	DBSU	25.84
**4102**	allXL	BB	373	386	DBSU	24.83
**4102**	allXL	CB	381	386	DBSU	31.57
**4102**	allXL	BD;DB	357	357	DBSU	18.89
**4102**	allXL	CB	373	319	DBSU	21.6
**4102**	allXL	CB	372	357	DBSU	34.74
**4102**	allXL	AC	381	357	DBSU	42.94
**4102**	allXL	AC	373	357	DBSU	33.49
**4102**	allXL	CB	372	370	DBSU	15.79
**4102**	allXL	CB	373	370	DBSU	15.04
**4102**	allXL	CB	382	373	DBSU	31.54
**4103**	allXL	AA	320	357	BS2G	25.09
**4103**	allXL	BB	320	373	BS2G	8.77
**4103**	allXL	DD	373	382	BS2G	16.16
**4103**	allXL	CC	370	386	BS2G	17.39
**4103**	allXL	CC	373	386	BS2G	23.36
**4103**	allXL	AA	319	357	BS2G	24.82
**4103**	allXL	AA	367	373	BS2G	12.72
**4103**	allXL	DD	370	382	BS2G	13.15
**4103**	allXL	CC	376	382	BS2G	14.07
**4103**	allXL	DD	357	373	BS2G	27.59
**4103**	allXL	BB	320	366	BS2G	15.83
**4103**	allXL	DD	370	376	BS2G	14.86
**4103**	allXL	AA	320	357	DBSU	25.09
**4103**	allXL	AA	357	319	DBSU	24.82

**4103**	allXL	BB	372	319	DBSU	12.42
**4103**	allXL	BB	370	357	DBSU	28.14
**4103**	allXL	DD	382	357	DBSU	39.09
**4103**	allXL	AA	372	370	DBSU	6.27
**4103**	allXL	DD	381	370	DBSU	12.51
**4103**	allXL	AA	373	370	DBSU	9.22
**4103**	allXL	DD	381	372	DBSU	15.78
**4103**	allXL	DD	372	382	DBSU	15.77
**4103**	allXL	CC	372	386	DBSU	21.3
**4103**	allXL	CC	373	386	DBSU	23.36
**4103**	allXL	DA	381	386	DBSU	39.58
**4103**	allXL	BD;DB	357	357	DBSU	18.62
**4103**	allXL	DA	373	319	DBSU	33.83
**4103**	allXL	BD	372	357	DBSU	39.93
**4103**	allXL	DB	381	357	DBSU	48.77
**4103**	allXL	DB	373	357	DBSU	39.76
**4103**	allXL	DA	372	370	DBSU	50.81
**4103**	allXL	DA	373	370	DBSU	52.8
**4103**	allXL	AD	382	373	DBSU	39.83
**4104**	allXL	AA	320	357	BS2G	22.22
**4104**	allXL	BB	320	373	BS2G	25.74
**4104**	allXL	DD	373	382	BS2G	20.79
**4104**	allXL	DD	370	386	BS2G	14.18
**4104**	allXL	DD	373	386	BS2G	18.41
**4104**	allXL	AA	319	357	BS2G	25.64
**4104**	allXL	AA	367	373	BS2G	12.43
**4104**	allXL	DD	370	382	BS2G	21.78
**4104**	allXL	CC	376	382	BS2G	14.51
**4104**	allXL	BB	357	373	BS2G	32.18
**4104**	allXL	AA	320	366	BS2G	18.07
**4104**	allXL	CC	370	376	BS2G	13.27
**4104**	allXL	AA	320	357	DBSU	22.22
**4104**	allXL	AA	357	319	DBSU	25.64
**4104**	allXL	BB	372	319	DBSU	29.43

**4104**	allXL	DD	370	357	DBSU	25.87
**4104**	allXL	AA	382	357	DBSU	16.77
**4104**	allXL	AA	372	370	DBSU	6.46
**4104**	allXL	DD	381	370	DBSU	21.73
**4104**	allXL	CC	373	370	DBSU	9.15
**4104**	allXL	DD	381	372	DBSU	21.95
**4104**	allXL	DD	372	382	DBSU	23.12
**4104**	allXL	DD	372	386	DBSU	18.89
**4104**	allXL	DD	373	386	DBSU	18.41
**4104**	allXL	CA	381	386	DBSU	49.35
**4104**	allXL	BD;DB	357	357	DBSU	18.57
**4104**	allXL	DA	373	319	DBSU	33.86
**4104**	allXL	DB	372	357	DBSU	42.94
**4104**	allXL	AC	381	357	DBSU	32.17
**4104**	allXL	DB	373	357	DBSU	43.44
**4104**	allXL	AD	372	370	DBSU	59.46
**4104**	allXL	AD	373	370	DBSU	59.49
**4104**	allXL	AC	382	373	DBSU	50.27
**4105**	allXL	CC	320	357	BS2G	26.79
**4105**	allXL	CC	320	373	BS2G	22.2
**4105**	allXL	DD	373	382	BS2G	18.33
**4105**	allXL	DD	370	386	BS2G	22.59
**4105**	allXL	CC	373	386	BS2G	26.84
**4105**	allXL	AA	319	357	BS2G	25.62
**4105**	allXL	AA	367	373	BS2G	13.42
**4105**	allXL	DD	370	382	BS2G	14.87
**4105**	allXL	CC	376	382	BS2G	14.86
**4105**	allXL	CC	357	373	BS2G	37.9
**4105**	allXL	DD	320	366	BS2G	24.22
**4105**	allXL	CC	370	376	BS2G	13.45
**4105**	allXL	CC	320	357	DBSU	26.79
**4105**	allXL	AA	357	319	DBSU	25.62
**4105**	allXL	CC	372	319	DBSU	16.78
**4105**	allXL	DD	370	357	DBSU	30.99

**4105**	allXL	DD	382	357	DBSU	36.03
**4105**	allXL	AA	372	370	DBSU	6.23
**4105**	allXL	DD	381	370	DBSU	14.4
**4105**	allXL	AA	373	370	DBSU	9.01
**4105**	allXL	DD	381	372	DBSU	17.56
**4105**	allXL	DD	372	382	DBSU	19.07
**4105**	allXL	CC	372	386	DBSU	27.47
**4105**	allXL	CC	373	386	DBSU	26.84
**4105**	allXL	CB	381	386	DBSU	53.4
**4105**	allXL	BD;DB	357	357	DBSU	19.03
**4105**	allXL	BC	373	319	DBSU	26.36
**4105**	allXL	CB	372	357	DBSU	41.94
**4105**	allXL	CB	381	357	DBSU	49.44
**4105**	allXL	CB	373	357	DBSU	43.91
**4105**	allXL	CB	372	370	DBSU	26.94
**4105**	allXL	CB	373	370	DBSU	26.29
**4105**	allXL	DA	382	373	DBSU	42.49
**4106**	allXL	AA	320	357	BS2G	23.89
**4106**	allXL	BB	320	373	BS2G	16.43
**4106**	allXL	CC	373	382	BS2G	18.24
**4106**	allXL	DD	370	386	BS2G	22.22
**4106**	allXL	CC	373	386	BS2G	26.06
**4106**	allXL	AA	319	357	BS2G	23.58
**4106**	allXL	AA	367	373	BS2G	12.23
**4106**	allXL	DD	370	382	BS2G	17.21
**4106**	allXL	CC	376	382	BS2G	14.12
**4106**	allXL	BB	357	373	BS2G	21.29
**4106**	allXL	CC	320	366	BS2G	19.1
**4106**	allXL	DD	370	376	BS2G	16.46
**4106**	allXL	AA	320	357	DBSU	23.89
**4106**	allXL	AA	357	319	DBSU	23.58
**4106**	allXL	BB	372	319	DBSU	15.69
**4106**	allXL	BB	370	357	DBSU	20.32
**4106**	allXL	BB	382	357	DBSU	11.71

**4106**	allXL	AA	372	370	DBSU	6.3
**4106**	allXL	DD	381	370	DBSU	15.48
**4106**	allXL	AA	373	370	DBSU	9.18
**4106**	allXL	DD	381	372	DBSU	18.39
**4106**	allXL	CC	372	382	DBSU	20.44
**4106**	allXL	CC	372	386	DBSU	27.59
**4106**	allXL	CC	373	386	DBSU	26.06
**4106**	allXL	DB	381	386	DBSU	36.67
**4106**	allXL	BD;DB	357	357	DBSU	17.81
**4106**	allXL	AD	373	319	DBSU	32.55
**4106**	allXL	BD	372	357	DBSU	40.42
**4106**	allXL	BD	381	357	DBSU	27.7
**4106**	allXL	BD	373	357	DBSU	38.88
**4106**	allXL	CB	372	370	DBSU	34.58
**4106**	allXL	CB	373	370	DBSU	37.5
**4106**	allXL	BD	382	373	DBSU	56.1
**4107**	allXL	CC	320	357	BS2G	24.0
**4107**	allXL	BB	320	373	BS2G	33.62
**4107**	allXL	CC	373	382	BS2G	18.28
**4107**	allXL	CC	370	386	BS2G	19.82
**4107**	allXL	DD	373	386	BS2G	25.61
**4107**	allXL	CC	319	357	BS2G	22.79
**4107**	allXL	AA	367	373	BS2G	14.03
**4107**	allXL	CC	370	382	BS2G	14.79
**4107**	allXL	AA	376	382	BS2G	14.09
**4107**	allXL	BB	357	373	BS2G	31.72
**4107**	allXL	DD	320	366	BS2G	18.35
**4107**	allXL	CC	370	376	BS2G	15.15
**4107**	allXL	CC	320	357	DBSU	24.0
**4107**	allXL	CC	357	319	DBSU	22.79
**4107**	allXL	DD	372	319	DBSU	32.21
**4107**	allXL	BB;DD	370	357	DBSU	30.33
**4107**	allXL	CC	382	357	DBSU	18.97
**4107**	allXL	AA	372	370	DBSU	6.19

**4107**	allXL	CC	381	370	DBSU	17.28
**4107**	allXL	AA	373	370	DBSU	9.21
**4107**	allXL	CC	381	372	DBSU	19.95
**4107**	allXL	CC	372	382	DBSU	18.03
**4107**	allXL	CC	372	386	DBSU	24.51
**4107**	allXL	DD	373	386	DBSU	25.61
**4107**	allXL	DB	381	386	DBSU	53.95
**4107**	allXL	BD;DB	357	357	DBSU	18.32
**4107**	allXL	CB	373	319	DBSU	36.29
**4107**	allXL	BD	372	357	DBSU	48.69
**4107**	allXL	CA	381	357	DBSU	30.77
**4107**	allXL	BD	373	357	DBSU	48.93
**4107**	allXL	DA	372	370	DBSU	47.67
**4107**	allXL	DA	373	370	DBSU	50.49
**4107**	allXL	CB	382	373	DBSU	57.58
**4108**	allXL	CC	320	357	BS2G	25.39
**4108**	allXL	DD	320	373	BS2G	16.43
**4108**	allXL	DD	373	382	BS2G	16.72
**4108**	allXL	DD	370	386	BS2G	27.49
**4108**	allXL	DD	373	386	BS2G	26.7
**4108**	allXL	AA	319	357	BS2G	28.2
**4108**	allXL	BB	367	373	BS2G	12.33
**4108**	allXL	DD	370	382	BS2G	20.35
**4108**	allXL	BB	376	382	BS2G	15.07
**4108**	allXL	BB	357	373	BS2G	22.63
**4108**	allXL	BB	320	366	BS2G	15.64
**4108**	allXL	CC	370	376	BS2G	16.73
**4108**	allXL	CC	320	357	DBSU	25.39
**4108**	allXL	AA	357	319	DBSU	28.2
**4108**	allXL	DD	372	319	DBSU	16.29
**4108**	allXL	DD	370	357	DBSU	23.15
**4108**	allXL	BB	382	357	DBSU	27.77
**4108**	allXL	AA	372	370	DBSU	6.22
**4108**	allXL	DD	381	370	DBSU	18.71

**4108**	allXL	AA	373	370	DBSU	9.12
**4108**	allXL	DD	381	372	DBSU	14.65
**4108**	allXL	DD	372	382	DBSU	16.94
**4108**	allXL	DD	372	386	DBSU	26.28
**4108**	allXL	DD	373	386	DBSU	26.7
**4108**	allXL	BD	381	386	DBSU	64.53
**4108**	allXL	BD;DB	357	357	DBSU	18.68
**4108**	allXL	AD	373	319	DBSU	38.37
**4108**	allXL	CB	372	357	DBSU	40.03
**4108**	allXL	BD	381	357	DBSU	31.06
**4108**	allXL	BD	373	357	DBSU	38.56
**4108**	allXL	DA	372	370	DBSU	28.64
**4108**	allXL	DA	373	370	DBSU	28.56
**4108**	allXL	CB	382	373	DBSU	46.53
**4109**	allXL	AA	320	357	BS2G	13.07
**4109**	allXL	BB	320	373	BS2G	11.63
**4109**	allXL	BB	373	382	BS2G	20.72
**4109**	allXL	CC	370	386	BS2G	25.43
**4109**	allXL	BB	373	386	BS2G	28.72
**4109**	allXL	AA	319	357	BS2G	16.76
**4109**	allXL	BB	367	373	BS2G	12.79
**4109**	allXL	CC	370	382	BS2G	21.62
**4109**	allXL	CC	376	382	BS2G	14.39
**4109**	allXL	DD	357	373	BS2G	27.93
**4109**	allXL	BB	320	366	BS2G	16.25
**4109**	allXL	CC	370	376	BS2G	13.01
**4109**	allXL	AA	320	357	DBSU	13.07
**4109**	allXL	AA	357	319	DBSU	16.76
**4109**	allXL	BB	372	319	DBSU	7.12
**4109**	allXL	DD	370	357	DBSU	25.24
**4109**	allXL	CC	382	357	DBSU	40.99
**4109**	allXL	CC	372	370	DBSU	6.66
**4109**	allXL	CC	381	370	DBSU	21.21
**4109**	allXL	CC	373	370	DBSU	8.84

**4109**	allXL	CC	381	372	DBSU	22.99
**4109**	allXL	CC	372	382	DBSU	23.0
**4109**	allXL	CC	372	386	DBSU	27.44
**4109**	allXL	BB	373	386	DBSU	28.72
**4109**	allXL	BC	381	386	DBSU	55.11
**4109**	allXL	BD;DB	357	357	DBSU	18.28
**4109**	allXL	AD	373	319	DBSU	27.43
**4109**	allXL	BC	372	357	DBSU	42.8
**4109**	allXL	CA	381	357	DBSU	37.72
**4109**	allXL	BC	373	357	DBSU	42.56
**4109**	allXL	BC	372	370	DBSU	26.75
**4109**	allXL	BC	373	370	DBSU	27.32
**4109**	allXL	BC	382	373	DBSU	31.16
**4110**	allXL	CC	320	357	BS2G	26.09
**4110**	allXL	DD	320	373	BS2G	23.93
**4110**	allXL	BB	373	382	BS2G	20.02
**4110**	allXL	BB	370	386	BS2G	19.06
**4110**	allXL	BB	373	386	BS2G	20.3
**4110**	allXL	AA	319	357	BS2G	28.23
**4110**	allXL	AA	367	373	BS2G	12.72
**4110**	allXL	CC	370	382	BS2G	20.93
**4110**	allXL	BB	376	382	BS2G	13.78
**4110**	allXL	DD	357	373	BS2G	18.69
**4110**	allXL	BB	320	366	BS2G	23.7
**4110**	allXL	CC	370	376	BS2G	14.7
**4110**	allXL	CC	320	357	DBSU	26.09
**4110**	allXL	AA	357	319	DBSU	28.23
**4110**	allXL	DD	372	319	DBSU	28.47
**4110**	allXL	CC	370	357	DBSU	15.18
**4110**	allXL	DD	382	357	DBSU	14.63
**4110**	allXL	AA	372	370	DBSU	6.41
**4110**	allXL	CC	381	370	DBSU	22.7
**4110**	allXL	AA	373	370	DBSU	8.68
**4110**	allXL	DD	381	372	DBSU	18.73

**4110**	allXL	DD	372	382	DBSU	20.77
**4110**	allXL	DD	372	386	DBSU	21.85
**4110**	allXL	BB	373	386	DBSU	20.3
**4110**	allXL	DB	381	386	DBSU	25.39
**4110**	allXL	BD;DB	357	357	DBSU	18.6
**4110**	allXL	AD	373	319	DBSU	20.89
**4110**	allXL	CA	372	357	DBSU	8.81
**4110**	allXL	BD	381	357	DBSU	22.45
**4110**	allXL	CA	373	357	DBSU	11.73
**4110**	allXL	CA	372	370	DBSU	40.28
**4110**	allXL	CA	373	370	DBSU	40.96
**4110**	allXL	BD	382	373	DBSU	29.65
**4111**	allXL	CC	320	357	BS2G	24.76
**4111**	allXL	BB	320	373	BS2G	16.15
**4111**	allXL	BB	373	382	BS2G	20.35
**4111**	allXL	CC	370	386	BS2G	27.41
**4111**	allXL	AA	373	386	BS2G	27.87
**4111**	allXL	CC	319	357	BS2G	25.08
**4111**	allXL	AA	367	373	BS2G	13.12
**4111**	allXL	CC	370	382	BS2G	23.08
**4111**	allXL	BB	376	382	BS2G	14.46
**4111**	allXL	BB	357	373	BS2G	32.28
**4111**	allXL	AA	320	366	BS2G	23.0
**4111**	allXL	CC	370	376	BS2G	15.42
**4111**	allXL	CC	320	357	DBSU	24.76
**4111**	allXL	CC	357	319	DBSU	25.08
**4111**	allXL	BB	372	319	DBSU	19.2
**4111**	allXL	BB	370	357	DBSU	31.37
**4111**	allXL	BB	382	357	DBSU	28.16
**4111**	allXL	AA	372	370	DBSU	6.26
**4111**	allXL	DD	381	370	DBSU	21.99
**4111**	allXL	AA	373	370	DBSU	9.02
**4111**	allXL	DD	381	372	DBSU	21.19
**4111**	allXL	BB	372	382	DBSU	23.29

**4111**	allXL	AA	372	386	DBSU	28.33
**4111**	allXL	AA	373	386	DBSU	27.87
**4111**	allXL	CB	381	386	DBSU	23.73
**4111**	allXL	BD;DB	357	357	DBSU	19.0
**4111**	allXL	CB	373	319	DBSU	21.86
**4111**	allXL	CB	372	357	DBSU	39.78
**4111**	allXL	CB	381	357	DBSU	31.2
**4111**	allXL	CB	373	357	DBSU	37.46
**4111**	allXL	CB	372	370	DBSU	11.81
**4111**	allXL	CB	373	370	DBSU	8.15
**4111**	allXL	CB	382	373	DBSU	21.22
**4112**	allXL	AA	320	357	BS2G	23.7
**4112**	allXL	DD	320	373	BS2G	10.2
**4112**	allXL	AA	373	382	BS2G	17.08
**4112**	allXL	AA	370	386	BS2G	23.43
**4112**	allXL	AA	373	386	BS2G	23.17
**4112**	allXL	AA	319	357	BS2G	23.69
**4112**	allXL	AA	367	373	BS2G	12.46
**4112**	allXL	CC	370	382	BS2G	21.43
**4112**	allXL	AA	376	382	BS2G	12.76
**4112**	allXL	DD	357	373	BS2G	23.74
**4112**	allXL	AA	320	366	BS2G	15.74
**4112**	allXL	CC	370	376	BS2G	14.33
**4112**	allXL	AA	320	357	DBSU	23.7
**4112**	allXL	AA	357	319	DBSU	23.69
**4112**	allXL	DD	372	319	DBSU	13.27
**4112**	allXL	DD	370	357	DBSU	16.79
**4112**	allXL	CC	382	357	DBSU	24.23
**4112**	allXL	AA	372	370	DBSU	6.25
**4112**	allXL	DD	381	370	DBSU	23.13
**4112**	allXL	AA	373	370	DBSU	9.06
**4112**	allXL	AA	381	372	DBSU	22.16
**4112**	allXL	AA	372	382	DBSU	19.92
**4112**	allXL	AA	372	386	DBSU	24.85

**4112**	allXL	AA	373	386	DBSU	23.17
**4112**	allXL	BC	381	386	DBSU	55.28
**4112**	allXL	BD;DB	357	357	DBSU	18.98
**4112**	allXL	CB	373	319	DBSU	23.3
**4112**	allXL	DB	372	357	DBSU	36.26
**4112**	allXL	CA	381	357	DBSU	40.09
**4112**	allXL	DB	373	357	DBSU	39.46
**4112**	allXL	BC	372	370	DBSU	27.83
**4112**	allXL	BC	373	370	DBSU	25.34
**4112**	allXL	BC	382	373	DBSU	25.61
**4113**	allXL	AA	320	357	BS2G	21.12
**4113**	allXL	BB	320	373	BS2G	10.69
**4113**	allXL	DD	373	382	BS2G	16.27
**4113**	allXL	DD	370	386	BS2G	25.61
**4113**	allXL	DD	373	386	BS2G	22.18
**4113**	allXL	AA	319	357	BS2G	19.23
**4113**	allXL	DD	367	373	BS2G	13.39
**4113**	allXL	DD	370	382	BS2G	20.71
**4113**	allXL	CC	376	382	BS2G	15.45
**4113**	allXL	DD	357	373	BS2G	24.16
**4113**	allXL	BB	320	366	BS2G	14.56
**4113**	allXL	CC	370	376	BS2G	16.99
**4113**	allXL	AA	320	357	DBSU	21.12
**4113**	allXL	AA	357	319	DBSU	19.23
**4113**	allXL	BB	372	319	DBSU	11.98
**4113**	allXL	BB	370	357	DBSU	22.55
**4113**	allXL	DD	382	357	DBSU	40.15
**4113**	allXL	AA	372	370	DBSU	6.51
**4113**	allXL	DD	381	370	DBSU	21.78
**4113**	allXL	DD	373	370	DBSU	8.68
**4113**	allXL	DD	381	372	DBSU	15.54
**4113**	allXL	DD	372	382	DBSU	14.46
**4113**	allXL	DD	372	386	DBSU	19.64
**4113**	allXL	DD	373	386	DBSU	22.18

**4113**	allXL	DB	381	386	DBSU	31.58
**4113**	allXL	BD;DB	357	357	DBSU	19.13
**4113**	allXL	CB	373	319	DBSU	20.78
**4113**	allXL	CB	372	357	DBSU	22.2
**4113**	allXL	CB	381	357	DBSU	26.62
**4113**	allXL	CB	373	357	DBSU	20.3
**4113**	allXL	CB	372	370	DBSU	26.81
**4113**	allXL	DB	373	370	DBSU	27.92
**4113**	allXL	DB	382	373	DBSU	28.09
**4114**	allXL	CC	320	357	BS2G	23.95
**4114**	allXL	DD	320	373	BS2G	26.1
**4114**	allXL	DD	373	382	BS2G	21.32
**4114**	allXL	BB	370	386	BS2G	34.75
**4114**	allXL	BB	373	386	BS2G	26.74
**4114**	allXL	CC	319	357	BS2G	23.61
**4114**	allXL	AA	367	373	BS2G	11.1
**4114**	allXL	AA	370	382	BS2G	28.09
**4114**	allXL	BB	376	382	BS2G	15.0
**4114**	allXL	BB	357	373	BS2G	28.21
**4114**	allXL	AA	320	366	BS2G	19.06
**4114**	allXL	CC	370	376	BS2G	17.59
**4114**	allXL	CC	320	357	DBSU	23.95
**4114**	allXL	CC	357	319	DBSU	23.61
**4114**	allXL	DD	372	319	DBSU	26.49
**4114**	allXL	CC	370	357	DBSU	26.07
**4114**	allXL	AA	382	357	DBSU	43.04
**4114**	allXL	AA	372	370	DBSU	6.4
**4114**	allXL	DD	381	370	DBSU	25.75
**4114**	allXL	AA	373	370	DBSU	8.95
**4114**	allXL	DD	381	372	DBSU	18.57
**4114**	allXL	DD	372	382	DBSU	21.47
**4114**	allXL	BB	372	386	DBSU	28.11
**4114**	allXL	BB	373	386	DBSU	26.74
**4114**	allXL	DA	381	386	DBSU	57.68

**4114**	allXL	BD;DB	357	357	DBSU	18.73
**4114**	allXL	AD	373	319	DBSU	31.72
**4114**	allXL	CA	372	357	DBSU	28.06
**4114**	allXL	CA	381	357	DBSU	41.74
**4114**	allXL	CA	373	357	DBSU	29.38
**4114**	allXL	AD	372	370	DBSU	48.03
**4114**	allXL	AD	373	370	DBSU	47.95
**4114**	allXL	AD	382	373	DBSU	52.18
**4115**	allXL	CC	320	357	BS2G	23.71
**4115**	allXL	AA	320	373	BS2G	26.35
**4115**	allXL	AA	373	382	BS2G	18.41
**4115**	allXL	AA	370	386	BS2G	28.09
**4115**	allXL	AA	373	386	BS2G	24.19
**4115**	allXL	AA	319	357	BS2G	22.29
**4115**	allXL	AA	367	373	BS2G	13.29
**4115**	allXL	AA	370	382	BS2G	25.43
**4115**	allXL	CC	376	382	BS2G	13.88
**4115**	allXL	BB	357	373	BS2G	23.17
**4115**	allXL	AA	320	366	BS2G	14.55
**4115**	allXL	CC	370	376	BS2G	16.87
**4115**	allXL	CC	320	357	DBSU	23.71
**4115**	allXL	AA	357	319	DBSU	22.29
**4115**	allXL	AA	372	319	DBSU	22.1
**4115**	allXL	BB	370	357	DBSU	26.35
**4115**	allXL	BB	382	357	DBSU	39.31
**4115**	allXL	AA	372	370	DBSU	6.41
**4115**	allXL	AA	381	370	DBSU	24.8
**4115**	allXL	CC	373	370	DBSU	9.19
**4115**	allXL	DD	381	372	DBSU	20.02
**4115**	allXL	CC	372	382	DBSU	21.64
**4115**	allXL	AA	372	386	DBSU	26.6
**4115**	allXL	AA	373	386	DBSU	24.19
**4115**	allXL	BC	381	386	DBSU	24.24
**4115**	allXL	BD;DB	357	357	DBSU	19.25

**4115**	allXL	CB	373	319	DBSU	13.88
**4115**	allXL	BD	372	357	DBSU	35.55
**4115**	allXL	BD	381	357	DBSU	43.54
**4115**	allXL	BD	373	357	DBSU	35.48
**4115**	allXL	CB	372	370	DBSU	40.75
**4115**	allXL	CB	373	370	DBSU	40.76
**4115**	allXL	CB	382	373	DBSU	35.06
**4116**	allXL	AA	320	357	BS2G	24.97
**4116**	allXL	BB	320	373	BS2G	27.25
**4116**	allXL	BB	373	382	BS2G	18.97
**4116**	allXL	DD	370	386	BS2G	25.98
**4116**	allXL	DD	373	386	BS2G	25.16
**4116**	allXL	AA	319	357	BS2G	24.88
**4116**	allXL	AA	367	373	BS2G	13.29
**4116**	allXL	DD	370	382	BS2G	21.67
**4116**	allXL	BB	376	382	BS2G	13.5
**4116**	allXL	BB	357	373	BS2G	29.96
**4116**	allXL	BB	320	366	BS2G	23.26
**4116**	allXL	CC	370	376	BS2G	15.01
**4116**	allXL	AA	320	357	DBSU	24.97
**4116**	allXL	AA	357	319	DBSU	24.88
**4116**	allXL	BB	372	319	DBSU	23.18
**4116**	allXL	BB	370	357	DBSU	29.01
**4116**	allXL	AA	382	357	DBSU	33.44
**4116**	allXL	AA	372	370	DBSU	6.3
**4116**	allXL	DD	381	370	DBSU	19.88
**4116**	allXL	AA	373	370	DBSU	9.36
**4116**	allXL	DD	381	372	DBSU	17.49
**4116**	allXL	DD	372	382	DBSU	19.62
**4116**	allXL	DD	372	386	DBSU	23.65
**4116**	allXL	DD	373	386	DBSU	25.16
**4116**	allXL	BC	381	386	DBSU	31.99
**4116**	allXL	BD;DB	357	357	DBSU	18.24
**4116**	allXL	AD	373	319	DBSU	27.24

**4116**	allXL	DA	372	357	DBSU	44.2
**4116**	allXL	CB	381	357	DBSU	35.43
**4116**	allXL	DA	373	357	DBSU	43.77
**4116**	allXL	DA	372	370	DBSU	32.86
**4116**	allXL	DA	373	370	DBSU	29.98
**4116**	allXL	CB	382	373	DBSU	34.87
**4117**	allXL	AA	320	357	BS2G	24.68
**4117**	allXL	CC	320	373	BS2G	10.28
**4117**	allXL	CC	373	382	BS2G	15.41
**4117**	allXL	AA	370	386	BS2G	21.1
**4117**	allXL	CC	373	386	BS2G	17.71
**4117**	allXL	AA	319	357	BS2G	27.34
**4117**	allXL	AA	367	373	BS2G	12.22
**4117**	allXL	AA	370	382	BS2G	19.6
**4117**	allXL	BB	376	382	BS2G	13.35
**4117**	allXL	DD	357	373	BS2G	31.85
**4117**	allXL	AA	320	366	BS2G	14.65
**4117**	allXL	AA	370	376	BS2G	16.61
**4117**	allXL	AA	320	357	DBSU	24.68
**4117**	allXL	AA	357	319	DBSU	27.34
**4117**	allXL	CC	372	319	DBSU	9.38
**4117**	allXL	DD	370	357	DBSU	22.35
**4117**	allXL	AA	382	357	DBSU	37.01
**4117**	allXL	AA	372	370	DBSU	6.49
**4117**	allXL	AA	381	370	DBSU	17.85
**4117**	allXL	AA	373	370	DBSU	9.06
**4117**	allXL	AA	381	372	DBSU	18.14
**4117**	allXL	CC	372	382	DBSU	18.49
**4117**	allXL	CC	372	386	DBSU	19.7
**4117**	allXL	CC	373	386	DBSU	17.71
**4117**	allXL	BC	381	386	DBSU	60.26
**4117**	allXL	BD;DB	357	357	DBSU	17.18
**4117**	allXL	AD	373	319	DBSU	23.85
**4117**	allXL	DB	372	357	DBSU	43.44

**4117**	allXL	CB	381	357	DBSU	50.98
**4117**	allXL	AD	373	357	DBSU	46.94
**4117**	allXL	AD	372	370	DBSU	32.16
**4117**	allXL	AD	373	370	DBSU	34.5
**4117**	allXL	DA	382	373	DBSU	47.12
**4118**	allXL	AA	320	357	BS2G	24.06
**4118**	allXL	DD	320	373	BS2G	11.49
**4118**	allXL	CC	373	382	BS2G	19.64
**4118**	allXL	CC	370	386	BS2G	21.17
**4118**	allXL	CC	373	386	BS2G	24.18
**4118**	allXL	AA	319	357	BS2G	22.09
**4118**	allXL	AA	367	373	BS2G	13.67
**4118**	allXL	BB	370	382	BS2G	20.62
**4118**	allXL	CC	376	382	BS2G	13.54
**4118**	allXL	BB	357	373	BS2G	26.72
**4118**	allXL	BB	320	366	BS2G	20.71
**4118**	allXL	CC	370	376	BS2G	15.65
**4118**	allXL	AA	320	357	DBSU	24.06
**4118**	allXL	AA	357	319	DBSU	22.09
**4118**	allXL	DD	372	319	DBSU	9.71
**4118**	allXL	BB	370	357	DBSU	23.03
**4118**	allXL	BB	382	357	DBSU	35.4
**4118**	allXL	AA	372	370	DBSU	6.22
**4118**	allXL	BB	381	370	DBSU	23.03
**4118**	allXL	DD	373	370	DBSU	8.89
**4118**	allXL	DD	381	372	DBSU	20.92
**4118**	allXL	CC	372	382	DBSU	21.87
**4118**	allXL	DD	372	386	DBSU	24.31
**4118**	allXL	CC	373	386	DBSU	24.18
**4118**	allXL	DA	381	386	DBSU	31.78
**4118**	allXL	BD;DB	357	357	DBSU	19.18
**4118**	allXL	AD	373	319	DBSU	21.45
**4118**	allXL	CB	372	357	DBSU	22.02
**4118**	allXL	BD	381	357	DBSU	43.57

**4118**	allXL	CB	373	357	DBSU	23.89
**4118**	allXL	DA	372	370	DBSU	20.88
**4118**	allXL	DA	373	370	DBSU	17.76
**4118**	allXL	DA	382	373	DBSU	25.37
**4119**	allXL	AA	320	357	BS2G	25.6
**4119**	allXL	CC	320	373	BS2G	17.01
**4119**	allXL	BB	373	382	BS2G	15.44
**4119**	allXL	DD	370	386	BS2G	21.19
**4119**	allXL	DD	373	386	BS2G	22.29
**4119**	allXL	AA	319	357	BS2G	24.63
**4119**	allXL	BB	367	373	BS2G	13.81
**4119**	allXL	AA	370	382	BS2G	20.76
**4119**	allXL	BB	376	382	BS2G	13.59
**4119**	allXL	CC	357	373	BS2G	25.41
**4119**	allXL	DD	320	366	BS2G	27.45
**4119**	allXL	CC	370	376	BS2G	15.61
**4119**	allXL	AA	320	357	DBSU	25.6
**4119**	allXL	AA	357	319	DBSU	24.63
**4119**	allXL	CC	372	319	DBSU	20.63
**4119**	allXL	CC	370	357	DBSU	24.58
**4119**	allXL	CC	382	357	DBSU	26.92
**4119**	allXL	AA	372	370	DBSU	6.04
**4119**	allXL	AA	381	370	DBSU	22.44
**4119**	allXL	CC	373	370	DBSU	9.11
**4119**	allXL	BB	381	372	DBSU	16.4
**4119**	allXL	BB	372	382	DBSU	16.57
**4119**	allXL	DD	372	386	DBSU	20.77
**4119**	allXL	DD	373	386	DBSU	22.29
**4119**	allXL	CA	381	386	DBSU	39.19
**4119**	allXL	BD;DB	357	357	DBSU	19.39
**4119**	allXL	AD	373	319	DBSU	16.29
**4119**	allXL	AC	372	357	DBSU	42.74
**4119**	allXL	CA	381	357	DBSU	33.56
**4119**	allXL	AD	373	357	DBSU	39.6

**4119**	allXL	AD	372	370	DBSU	54.57
**4119**	allXL	CA	373	370	DBSU	51.41
**4119**	allXL	CA	382	373	DBSU	35.58
**4120**	allXL	AA	320	357	BS2G	22.93
**4120**	allXL	BB	320	373	BS2G	29.18
**4120**	allXL	AA	373	382	BS2G	18.57
**4120**	allXL	AA	370	386	BS2G	24.04
**4120**	allXL	AA	373	386	BS2G	25.45
**4120**	allXL	AA	319	357	BS2G	21.44
**4120**	allXL	AA	367	373	BS2G	12.85
**4120**	allXL	CC	370	382	BS2G	19.97
**4120**	allXL	BB	376	382	BS2G	14.24
**4120**	allXL	BB	357	373	BS2G	28.96
**4120**	allXL	BB	320	366	BS2G	17.63
**4120**	allXL	CC	370	376	BS2G	14.72
**4120**	allXL	AA	320	357	DBSU	22.93
**4120**	allXL	AA	357	319	DBSU	21.44
**4120**	allXL	BB	372	319	DBSU	27.82
**4120**	allXL	BB	370	357	DBSU	24.4
**4120**	allXL	BB	382	357	DBSU	34.12
**4120**	allXL	AA	372	370	DBSU	6.4
**4120**	allXL	CC	381	370	DBSU	22.02
**4120**	allXL	AA	373	370	DBSU	9.12
**4120**	allXL	DD	381	372	DBSU	17.15
**4120**	allXL	AA	372	382	DBSU	18.94
**4120**	allXL	AA	372	386	DBSU	25.43
**4120**	allXL	AA	373	386	DBSU	25.45
**4120**	allXL	DA	381	386	DBSU	51.31
**4120**	allXL	BD;DB	357	357	DBSU	18.71
**4120**	allXL	CB	373	319	DBSU	6.77
**4120**	allXL	CB	372	357	DBSU	31.77
**4120**	allXL	BD	381	357	DBSU	46.47
**4120**	allXL	CB	373	357	DBSU	32.71
**4120**	allXL	CB	372	370	DBSU	28.37

**4120**	allXL	CB	373	370	DBSU	27.44
**4120**	allXL	BC	382	373	DBSU	48.68
**4121**	allXL	AA	320	357	BS2G	24.99
**4121**	allXL	DD	320	373	BS2G	27.72
**4121**	allXL	DD	373	382	BS2G	21.62
**4121**	allXL	DD	370	386	BS2G	26.09
**4121**	allXL	DD	373	386	BS2G	24.3
**4121**	allXL	AA	319	357	BS2G	25.22
**4121**	allXL	AA	367	373	BS2G	13.34
**4121**	allXL	DD	370	382	BS2G	27.23
**4121**	allXL	CC	376	382	BS2G	15.44
**4121**	allXL	BB	357	373	BS2G	16.42
**4121**	allXL	BB	320	366	BS2G	16.09
**4121**	allXL	CC	370	376	BS2G	17.23
**4121**	allXL	AA	320	357	DBSU	24.99
**4121**	allXL	AA	357	319	DBSU	25.22
**4121**	allXL	DD	372	319	DBSU	26.85
**4121**	allXL	BB	370	357	DBSU	18.41
**4121**	allXL	BB	382	357	DBSU	35.63
**4121**	allXL	AA	372	370	DBSU	6.23
**4121**	allXL	DD	381	370	DBSU	27.54
**4121**	allXL	AA	373	370	DBSU	9.1
**4121**	allXL	DD	381	372	DBSU	22.9
**4121**	allXL	DD	372	382	DBSU	23.51
**4121**	allXL	DD	372	386	DBSU	24.67
**4121**	allXL	DD	373	386	DBSU	24.3
**4121**	allXL	DB	381	386	DBSU	39.35
**4121**	allXL	BD;DB	357	357	DBSU	18.3
**4121**	allXL	DA	373	319	DBSU	30.68
**4121**	allXL	BD	372	357	DBSU	26.33
**4121**	allXL	BD	381	357	DBSU	20.56
**4121**	allXL	BD	373	357	DBSU	23.5
**4121**	allXL	DA	372	370	DBSU	43.28
**4121**	allXL	DA	373	370	DBSU	40.18

**4121**	allXL	BD	382	373	DBSU	29.49
**4122**	allXL	AA	320	357	BS2G	26.84
**4122**	allXL	CC	320	373	BS2G	23.81
**4122**	allXL	DD	373	382	BS2G	20.52
**4122**	allXL	BB	370	386	BS2G	23.99
**4122**	allXL	AA	373	386	BS2G	22.04
**4122**	allXL	AA	319	357	BS2G	26.48
**4122**	allXL	AA	367	373	BS2G	13.31
**4122**	allXL	CC	370	382	BS2G	20.69
**4122**	allXL	CC	376	382	BS2G	14.58
**4122**	allXL	DD	357	373	BS2G	26.46
**4122**	allXL	AA	320	366	BS2G	20.29
**4122**	allXL	CC	370	376	BS2G	13.99
**4122**	allXL	AA	320	357	DBSU	26.84
**4122**	allXL	AA	357	319	DBSU	26.48
**4122**	allXL	AA	372	319	DBSU	24.0
**4122**	allXL	DD	370	357	DBSU	25.17
**4122**	allXL	DD	382	357	DBSU	24.12
**4122**	allXL	AA	372	370	DBSU	6.13
**4122**	allXL	CC	381	370	DBSU	21.02
**4122**	allXL	CC	373	370	DBSU	9.1
**4122**	allXL	DD	381	372	DBSU	20.07
**4122**	allXL	DD	372	382	DBSU	21.03
**4122**	allXL	AA	372	386	DBSU	20.98
**4122**	allXL	AA	373	386	DBSU	22.04
**4122**	allXL	DA	381	386	DBSU	48.77
**4122**	allXL	BD;DB	357	357	DBSU	18.66
**4122**	allXL	CB	373	319	DBSU	29.61
**4122**	allXL	CB	372	357	DBSU	21.87
**4122**	allXL	DB	381	357	DBSU	40.03
**4122**	allXL	CB	373	357	DBSU	22.47
**4122**	allXL	DA	372	370	DBSU	25.58
**4122**	allXL	CB	373	370	DBSU	28.13
**4122**	allXL	CB	382	373	DBSU	37.7

**4123**	allXL	CC	320	357	BS2G	22.71
**4123**	allXL	AA	320	373	BS2G	18.1
**4123**	allXL	BB	373	382	BS2G	19.21
**4123**	allXL	AA	370	386	BS2G	25.69
**4123**	allXL	AA	373	386	BS2G	21.77
**4123**	allXL	CC	319	357	BS2G	25.09
**4123**	allXL	AA	367	373	BS2G	13.06
**4123**	allXL	BB	370	382	BS2G	26.51
**4123**	allXL	BB	376	382	BS2G	13.11
**4123**	allXL	AA	357	373	BS2G	36.9
**4123**	allXL	AA	320	366	BS2G	18.16
**4123**	allXL	DD	370	376	BS2G	16.35
**4123**	allXL	CC	320	357	DBSU	22.71
**4123**	allXL	CC	357	319	DBSU	25.09
**4123**	allXL	AA	372	319	DBSU	18.75
**4123**	allXL	BB	370	357	DBSU	29.34
**4123**	allXL	AA	382	357	DBSU	39.57
**4123**	allXL	AA	372	370	DBSU	6.24
**4123**	allXL	DD	381	370	DBSU	25.22
**4123**	allXL	AA	373	370	DBSU	9.01
**4123**	allXL	CC	381	372	DBSU	23.11
**4123**	allXL	BB	372	382	DBSU	22.44
**4123**	allXL	AA	372	386	DBSU	24.56
**4123**	allXL	AA	373	386	DBSU	21.77
**4123**	allXL	DA	381	386	DBSU	67.58
**4123**	allXL	BD;DB	357	357	DBSU	19.04
**4123**	allXL	BC	373	319	DBSU	35.03
**4123**	allXL	AD	372	357	DBSU	47.53
**4123**	allXL	AC	381	357	DBSU	60.23
**4123**	allXL	AD	373	357	DBSU	50.57
**4123**	allXL	AD	372	370	DBSU	43.66
**4123**	allXL	DA	373	370	DBSU	44.52
**4123**	allXL	DA	382	373	DBSU	54.92
**4124**	allXL	AA	320	357	BS2G	24.2

**4124**	allXL	CC	320	373	BS2G	17.9
**4124**	allXL	BB	373	382	BS2G	19.78
**4124**	allXL	AA	370	386	BS2G	23.6
**4124**	allXL	AA	373	386	BS2G	23.16
**4124**	allXL	AA	319	357	BS2G	23.15
**4124**	allXL	AA	367	373	BS2G	14.8
**4124**	allXL	BB	370	382	BS2G	22.05
**4124**	allXL	BB	376	382	BS2G	14.51
**4124**	allXL	AA	357	373	BS2G	35.44
**4124**	allXL	BB	320	366	BS2G	20.05
**4124**	allXL	BB	370	376	BS2G	16.76
**4124**	allXL	AA	320	357	DBSU	24.2
**4124**	allXL	AA	357	319	DBSU	23.15
**4124**	allXL	CC	372	319	DBSU	14.49
**4124**	allXL	DD	370	357	DBSU	29.47
**4124**	allXL	AA	382	357	DBSU	36.29
**4124**	allXL	AA	372	370	DBSU	6.06
**4124**	allXL	BB	381	370	DBSU	23.05
**4124**	allXL	AA	373	370	DBSU	9.13
**4124**	allXL	DD	381	372	DBSU	22.28
**4124**	allXL	AA	372	382	DBSU	22.12
**4124**	allXL	AA	372	386	DBSU	23.46
**4124**	allXL	AA	373	386	DBSU	23.16
**4124**	allXL	CA	381	386	DBSU	66.99
**4124**	allXL	BD;DB	357	357	DBSU	18.27
**4124**	allXL	AD	373	319	DBSU	33.96
**4124**	allXL	DA	372	357	DBSU	47.25
**4124**	allXL	AC	381	357	DBSU	43.47
**4124**	allXL	DA	373	357	DBSU	48.95
**4124**	allXL	DA	372	370	DBSU	43.37
**4124**	allXL	DA	373	370	DBSU	44.38
**4124**	allXL	AC	382	373	DBSU	57.38
**4125**	allXL	CC	320	357	BS2G	26.78
**4125**	allXL	BB	320	373	BS2G	15.86

**4125**	allXL	AA	373	382	BS2G	17.15
**4125**	allXL	DD	370	386	BS2G	23.33
**4125**	allXL	AA	373	386	BS2G	23.69
**4125**	allXL	CC	319	357	BS2G	28.75
**4125**	allXL	AA	367	373	BS2G	12.59
**4125**	allXL	DD	370	382	BS2G	18.49
**4125**	allXL	AA	376	382	BS2G	14.28
**4125**	allXL	BB	357	373	BS2G	16.03
**4125**	allXL	BB	320	366	BS2G	25.84
**4125**	allXL	DD	370	376	BS2G	16.73
**4125**	allXL	CC	320	357	DBSU	26.78
**4125**	allXL	CC	357	319	DBSU	28.75
**4125**	allXL	BB	372	319	DBSU	19.23
**4125**	allXL	BB	370	357	DBSU	18.52
**4125**	allXL	BB	382	357	DBSU	23.49
**4125**	allXL	AA	372	370	DBSU	6.68
**4125**	allXL	DD	381	370	DBSU	16.26
**4125**	allXL	AA	373	370	DBSU	9.33
**4125**	allXL	DD	381	372	DBSU	17.67
**4125**	allXL	AA	372	382	DBSU	20.18
**4125**	allXL	AA	372	386	DBSU	25.43
**4125**	allXL	AA	373	386	DBSU	23.69
**4125**	allXL	BD	381	386	DBSU	53.12
**4125**	allXL	BD;DB	357	357	DBSU	19.18
**4125**	allXL	AD	373	319	DBSU	27.2
**4125**	allXL	BD	372	357	DBSU	34.68
**4125**	allXL	BD	381	357	DBSU	33.49
**4125**	allXL	BD	373	357	DBSU	34.51
**4125**	allXL	DA	372	370	DBSU	46.91
**4125**	allXL	DA	373	370	DBSU	46.97
**4125**	allXL	AD	382	373	DBSU	56.45
**4126**	allXL	AA	320	357	BS2G	21.0
**4126**	allXL	DD	320	373	BS2G	17.27
**4126**	allXL	BB	373	382	BS2G	18.09

**4126**	allXL	AA	370	386	BS2G	17.46
**4126**	allXL	AA	373	386	BS2G	20.72
**4126**	allXL	AA	319	357	BS2G	19.51
**4126**	allXL	AA	367	373	BS2G	14.34
**4126**	allXL	BB	370	382	BS2G	17.39
**4126**	allXL	CC	376	382	BS2G	13.7
**4126**	allXL	BB	357	373	BS2G	32.24
**4126**	allXL	CC	320	366	BS2G	23.86
**4126**	allXL	CC	370	376	BS2G	15.26
**4126**	allXL	AA	320	357	DBSU	21.0
**4126**	allXL	AA	357	319	DBSU	19.51
**4126**	allXL	DD	372	319	DBSU	13.96
**4126**	allXL	DD	370	357	DBSU	24.29
**4126**	allXL	BB	382	357	DBSU	27.44
**4126**	allXL	AA	372	370	DBSU	6.09
**4126**	allXL	DD	381	370	DBSU	18.56
**4126**	allXL	AA	373	370	DBSU	9.2
**4126**	allXL	DD	381	372	DBSU	17.74
**4126**	allXL	AA	372	382	DBSU	18.44
**4126**	allXL	AA	372	386	DBSU	18.81
**4126**	allXL	AA	373	386	DBSU	20.72
**4126**	allXL	DA	381	386	DBSU	36.67
**4126**	allXL	BD;DB	357	357	DBSU	18.79
**4126**	allXL	CB	373	319	DBSU	30.51
**4126**	allXL	CB	372	357	DBSU	35.28
**4126**	allXL	BD	381	357	DBSU	37.13
**4126**	allXL	CB	373	357	DBSU	36.04
**4126**	allXL	DA	372	370	DBSU	25.96
**4126**	allXL	DA	373	370	DBSU	23.56
**4126**	allXL	AD	382	373	DBSU	40.17
**4127**	allXL	AA	320	357	BS2G	21.6
**4127**	allXL	BB	320	373	BS2G	24.21
**4127**	allXL	DD	373	382	BS2G	20.56
**4127**	allXL	DD	370	386	BS2G	20.56

**4127**	allXL	DD	373	386	BS2G	24.32
**4127**	allXL	CC	319	357	BS2G	20.19
**4127**	allXL	AA	367	373	BS2G	14.26
**4127**	allXL	CC	370	382	BS2G	18.88
**4127**	allXL	CC	376	382	BS2G	14.28
**4127**	allXL	BB	357	373	BS2G	22.95
**4127**	allXL	AA	320	366	BS2G	19.26
**4127**	allXL	CC	370	376	BS2G	12.25
**4127**	allXL	AA	320	357	DBSU	21.6
**4127**	allXL	CC	357	319	DBSU	20.19
**4127**	allXL	CC	372	319	DBSU	27.23
**4127**	allXL	AA	370	357	DBSU	25.37
**4127**	allXL	BB	382	357	DBSU	18.49
**4127**	allXL	AA	372	370	DBSU	5.78
**4127**	allXL	CC	381	370	DBSU	16.54
**4127**	allXL	AA	373	370	DBSU	8.91
**4127**	allXL	CC	381	372	DBSU	20.52
**4127**	allXL	BB	372	382	DBSU	22.45
**4127**	allXL	DD	372	386	DBSU	24.95
**4127**	allXL	DD	373	386	DBSU	24.32
**4127**	allXL	BC	381	386	DBSU	45.27
**4127**	allXL	BD;DB	357	357	DBSU	19.89
**4127**	allXL	CB	373	319	DBSU	42.21
**4127**	allXL	AC	372	357	DBSU	41.29
**4127**	allXL	BD	381	357	DBSU	33.59
**4127**	allXL	AC	373	357	DBSU	39.6
**4127**	allXL	BC	372	370	DBSU	37.23
**4127**	allXL	BC	373	370	DBSU	35.73
**4127**	allXL	CB	382	373	DBSU	31.79
**4128**	allXL	AA	320	357	BS2G	26.02
**4128**	allXL	BB	320	373	BS2G	16.19
**4128**	allXL	DD	373	382	BS2G	19.53
**4128**	allXL	DD	370	386	BS2G	18.45
**4128**	allXL	DD	373	386	BS2G	23.67

**4128**	allXL	AA	319	357	BS2G	26.46
**4128**	allXL	AA	367	373	BS2G	8.3
**4128**	allXL	CC	370	382	BS2G	17.01
**4128**	allXL	CC	376	382	BS2G	14.57
**4128**	allXL	DD	357	373	BS2G	24.32
**4128**	allXL	BB	320	366	BS2G	20.06
**4128**	allXL	CC	370	376	BS2G	11.9
**4128**	allXL	AA	320	357	DBSU	26.02
**4128**	allXL	AA	357	319	DBSU	26.46
**4128**	allXL	BB	372	319	DBSU	14.62
**4128**	allXL	DD	370	357	DBSU	24.24
**4128**	allXL	BB	382	357	DBSU	26.6
**4128**	allXL	CC	372	370	DBSU	6.63
**4128**	allXL	CC	381	370	DBSU	19.35
**4128**	allXL	AA	373	370	DBSU	8.93
**4128**	allXL	DD	381	372	DBSU	18.45
**4128**	allXL	DD	372	382	DBSU	19.12
**4128**	allXL	DD	372	386	DBSU	21.91
**4128**	allXL	DD	373	386	DBSU	23.67
**4128**	allXL	DB	381	386	DBSU	43.99
**4128**	allXL	BD;DB	357	357	DBSU	18.77
**4128**	allXL	AD	373	319	DBSU	13.36
**4128**	allXL	DB	372	357	DBSU	33.28
**4128**	allXL	BD	381	357	DBSU	42.51
**4128**	allXL	DB	373	357	DBSU	31.16
**4128**	allXL	CB	372	370	DBSU	28.23
**4128**	allXL	CB	373	370	DBSU	31.98
**4128**	allXL	BD	382	373	DBSU	37.11
**4129**	allXL	CC	320	357	BS2G	22.18
**4129**	allXL	DD	320	373	BS2G	19.82
**4129**	allXL	BB	373	382	BS2G	20.31
**4129**	allXL	AA	370	386	BS2G	30.83
**4129**	allXL	AA	373	386	BS2G	25.67
**4129**	allXL	CC	319	357	BS2G	22.78

**4129**	allXL	BB	367	373	BS2G	14.48
**4129**	allXL	CC	370	382	BS2G	26.73
**4129**	allXL	BB	376	382	BS2G	13.66
**4129**	allXL	BB	357	373	BS2G	29.59
**4129**	allXL	AA	320	366	BS2G	19.27
**4129**	allXL	BB	370	376	BS2G	14.17
**4129**	allXL	CC	320	357	DBSU	22.18
**4129**	allXL	CC	357	319	DBSU	22.78
**4129**	allXL	AA	372	319	DBSU	20.45
**4129**	allXL	BB	370	357	DBSU	30.94
**4129**	allXL	BB	382	357	DBSU	34.23
**4129**	allXL	AA	372	370	DBSU	6.04
**4129**	allXL	BB	381	370	DBSU	24.85
**4129**	allXL	BB	373	370	DBSU	8.82
**4129**	allXL	BB	381	372	DBSU	23.11
**4129**	allXL	BB	372	382	DBSU	24.09
**4129**	allXL	AA	372	386	DBSU	28.61
**4129**	allXL	AA	373	386	DBSU	25.67
**4129**	allXL	DA	381	386	DBSU	41.01
**4129**	allXL	BD;DB	357	357	DBSU	20.06
**4129**	allXL	CB	373	319	DBSU	11.48
**4129**	allXL	AD	372	357	DBSU	33.51
**4129**	allXL	AD	381	357	DBSU	42.17
**4129**	allXL	AD	373	357	DBSU	35.11
**4129**	allXL	AD	372	370	DBSU	26.15
**4129**	allXL	AD	373	370	DBSU	24.64
**4129**	allXL	AD	382	373	DBSU	18.57
**4130**	allXL	CC	320	357	BS2G	23.8
**4130**	allXL	DD	320	373	BS2G	21.36
**4130**	allXL	BB	373	382	BS2G	19.9
**4130**	allXL	AA	370	386	BS2G	26.48
**4130**	allXL	AA	373	386	BS2G	23.84
**4130**	allXL	AA	319	357	BS2G	25.44
**4130**	allXL	BB	367	373	BS2G	11.37

**4130**	allXL	BB	370	382	BS2G	22.44
**4130**	allXL	BB	376	382	BS2G	14.03
**4130**	allXL	AA	357	373	BS2G	15.78
**4130**	allXL	DD	320	366	BS2G	28.56
**4130**	allXL	CC	370	376	BS2G	15.74
**4130**	allXL	CC	320	357	DBSU	23.8
**4130**	allXL	AA	357	319	DBSU	25.44
**4130**	allXL	DD	372	319	DBSU	24.46
**4130**	allXL	AA	370	357	DBSU	21.13
**4130**	allXL	AA	382	357	DBSU	32.07
**4130**	allXL	AA	372	370	DBSU	5.99
**4130**	allXL	CC	381	370	DBSU	23.86
**4130**	allXL	DD	373	370	DBSU	9.25
**4130**	allXL	DD	381	372	DBSU	19.1
**4130**	allXL	DD	372	382	DBSU	20.35
**4130**	allXL	AA	372	386	DBSU	23.27
**4130**	allXL	AA	373	386	DBSU	23.84
**4130**	allXL	CA	381	386	DBSU	43.42
**4130**	allXL	BD;DB	357	357	DBSU	18.67
**4130**	allXL	AD	373	319	DBSU	27.39
**4130**	allXL	AC	372	357	DBSU	23.07
**4130**	allXL	AC	381	357	DBSU	26.16
**4130**	allXL	AC	373	357	DBSU	21.73
**4130**	allXL	DA	372	370	DBSU	25.62
**4130**	allXL	DA	373	370	DBSU	22.26
**4130**	allXL	AC	382	373	DBSU	32.38
**4131**	allXL	CC	320	357	BS2G	18.66
**4131**	allXL	DD	320	373	BS2G	17.77
**4131**	allXL	DD	373	382	BS2G	20.49
**4131**	allXL	DD	370	386	BS2G	17.83
**4131**	allXL	DD	373	386	BS2G	22.43
**4131**	allXL	CC	319	357	BS2G	18.26
**4131**	allXL	AA	367	373	BS2G	11.48
**4131**	allXL	DD	370	382	BS2G	20.61

**4131**	allXL	BB	376	382	BS2G	15.19
**4131**	allXL	DD	357	373	BS2G	31.05
**4131**	allXL	AA	320	366	BS2G	22.0
**4131**	allXL	CC	370	376	BS2G	14.52
**4131**	allXL	CC	320	357	DBSU	18.66
**4131**	allXL	CC	357	319	DBSU	18.26
**4131**	allXL	DD	372	319	DBSU	13.97
**4131**	allXL	DD	370	357	DBSU	23.14
**4131**	allXL	CD	382	357	DBSU	36.34
**4131**	allXL	AA	372	370	DBSU	6.57
**4131**	allXL	DD	381	370	DBSU	20.27
**4131**	allXL	AA	373	370	DBSU	9.23
**4131**	allXL	DD	381	372	DBSU	18.7
**4131**	allXL	DD	372	382	DBSU	19.62
**4131**	allXL	DD	372	386	DBSU	19.81
**4131**	allXL	DD	373	386	DBSU	22.43
**4131**	allXL	AD	381	386	DBSU	50.22
**4131**	allXL	BD;DB	357	357	DBSU	18.66
**4131**	allXL	BC	373	319	DBSU	16.53
**4131**	allXL	BC	372	357	DBSU	20.77
**4131**	allXL	CB	381	357	DBSU	18.64
**4131**	allXL	BC	373	357	DBSU	22.11
**4131**	allXL	CB	372	370	DBSU	9.06
**4131**	allXL	CB	373	370	DBSU	12.68
**4131**	allXL	BC	382	373	DBSU	29.48
**4132**	allXL	CC	320	357	BS2G	23.86
**4132**	allXL	BB	320	373	BS2G	12.02
**4132**	allXL	BB	373	382	BS2G	19.45
**4132**	allXL	DD	370	386	BS2G	29.96
**4132**	allXL	AA	373	386	BS2G	29.17
**4132**	allXL	AA	319	357	BS2G	22.63
**4132**	allXL	BB	367	373	BS2G	14.5
**4132**	allXL	BB	370	382	BS2G	22.96
**4132**	allXL	BB	376	382	BS2G	14.55

**4132**	allXL	BB	357	373	BS2G	26.21
**4132**	allXL	CC	320	366	BS2G	17.61
**4132**	allXL	CC	370	376	BS2G	14.98
**4132**	allXL	CC	320	357	DBSU	23.86
**4132**	allXL	AA	357	319	DBSU	22.63
**4132**	allXL	CC	372	319	DBSU	12.13
**4132**	allXL	DD	370	357	DBSU	26.95
**4132**	allXL	AA	382	357	DBSU	36.32
**4132**	allXL	CC	372	370	DBSU	6.61
**4132**	allXL	BB	381	370	DBSU	23.93
**4132**	allXL	AA	373	370	DBSU	8.99
**4132**	allXL	DD	381	372	DBSU	19.02
**4132**	allXL	DD	372	382	DBSU	21.8
**4132**	allXL	DD	372	386	DBSU	27.35
**4132**	allXL	AA	373	386	DBSU	29.17
**4132**	allXL	BD	381	386	DBSU	33.57
**4132**	allXL	BD;DB	357	357	DBSU	18.03
**4132**	allXL	AD	373	319	DBSU	27.03
**4132**	allXL	AC	372	357	DBSU	38.06
**4132**	allXL	AC	381	357	DBSU	34.79
**4132**	allXL	AC	373	357	DBSU	36.17
**4132**	allXL	BD	372	370	DBSU	54.5
**4132**	allXL	BD	373	370	DBSU	53.35
**4132**	allXL	AC	382	373	DBSU	44.05
**4133**	allXL	CC	320	357	BS2G	20.28
**4133**	allXL	CC	320	373	BS2G	19.14
**4133**	allXL	CC	373	382	BS2G	18.71
**4133**	allXL	CC	370	386	BS2G	23.92
**4133**	allXL	AA	373	386	BS2G	28.34
**4133**	allXL	CC	319	357	BS2G	21.19
**4133**	allXL	DD	367	373	BS2G	13.84
**4133**	allXL	CC	370	382	BS2G	17.14
**4133**	allXL	CC	376	382	BS2G	15.05
**4133**	allXL	CC	357	373	BS2G	33.23

**4133**	allXL	CC	320	366	BS2G	16.53
**4133**	allXL	CC	370	376	BS2G	13.2
**4133**	allXL	CC	320	357	DBSU	20.28
**4133**	allXL	CC	357	319	DBSU	21.19
**4133**	allXL	CC	372	319	DBSU	18.56
**4133**	allXL	CC	370	357	DBSU	27.24
**4133**	allXL	CC	382	357	DBSU	30.83
**4133**	allXL	AA	372	370	DBSU	6.12
**4133**	allXL	CC	381	370	DBSU	18.6
**4133**	allXL	AA	373	370	DBSU	9.13
**4133**	allXL	AA	381	372	DBSU	17.76
**4133**	allXL	AA	372	382	DBSU	19.08
**4133**	allXL	AA	372	386	DBSU	25.36
**4133**	allXL	AA	373	386	DBSU	28.34
**4133**	allXL	DA	381	386	DBSU	19.97
**4133**	allXL	BD;DB	357	357	DBSU	18.05
**4133**	allXL	AD	373	319	DBSU	12.62
**4133**	allXL	AD	372	357	DBSU	25.34
**4133**	allXL	AD	381	357	DBSU	24.68
**4133**	allXL	AD	373	357	DBSU	22.62
**4133**	allXL	CB	372	370	DBSU	14.26
**4133**	allXL	CB	373	370	DBSU	11.61
**4133**	allXL	AD	382	373	DBSU	14.28
**4134**	allXL	CC	320	357	BS2G	25.61
**4134**	allXL	BB	320	373	BS2G	14.56
**4134**	allXL	AA	373	382	BS2G	17.54
**4134**	allXL	AA	370	386	BS2G	18.36
**4134**	allXL	BB	373	386	BS2G	20.75
**4134**	allXL	BB	319	357	BS2G	27.0
**4134**	allXL	AA	367	373	BS2G	12.5
**4134**	allXL	AA	370	382	BS2G	15.77
**4134**	allXL	CC	376	382	BS2G	14.59
**4134**	allXL	BB	357	373	BS2G	26.0
**4134**	allXL	DD	320	366	BS2G	20.11

**4134**	allXL	CC	370	376	BS2G	15.02
**4134**	allXL	CC	320	357	DBSU	25.61
**4134**	allXL	BB	357	319	DBSU	27.0
**4134**	allXL	BB	372	319	DBSU	13.33
**4134**	allXL	DD	370	357	DBSU	23.69
**4134**	allXL	CC	382	357	DBSU	10.36
**4134**	allXL	AA	372	370	DBSU	7.07
**4134**	allXL	AA	381	370	DBSU	17.23
**4134**	allXL	AA	373	370	DBSU	8.82
**4134**	allXL	AA	381	372	DBSU	18.98
**4134**	allXL	AA	372	382	DBSU	18.81
**4134**	allXL	BB	372	386	DBSU	23.77
**4134**	allXL	BB	373	386	DBSU	20.75
**4134**	allXL	AC	381	386	DBSU	18.05
**4134**	allXL	BD;DB	357	357	DBSU	18.65
**4134**	allXL	AD	373	319	DBSU	23.36
**4134**	allXL	BD	372	357	DBSU	41.27
**4134**	allXL	CA	381	357	DBSU	25.44
**4134**	allXL	AC	373	357	DBSU	40.99
**4134**	allXL	AD	372	370	DBSU	37.17
**4134**	allXL	AD	373	370	DBSU	36.38
**4134**	allXL	CA	382	373	DBSU	37.58
**4135**	allXL	AA	320	357	BS2G	23.51
**4135**	allXL	DD	320	373	BS2G	13.95
**4135**	allXL	DD	373	382	BS2G	13.94
**4135**	allXL	DD	370	386	BS2G	23.66
**4135**	allXL	DD	373	386	BS2G	22.19
**4135**	allXL	AA	319	357	BS2G	23.57
**4135**	allXL	BB	367	373	BS2G	13.82
**4135**	allXL	DD	370	382	BS2G	19.71
**4135**	allXL	CC	376	382	BS2G	14.1
**4135**	allXL	DD	357	373	BS2G	19.72
**4135**	allXL	CC	320	366	BS2G	21.68
**4135**	allXL	CC	370	376	BS2G	16.39

**4135**	allXL	AA	320	357	DBSU	23.51
**4135**	allXL	AA	357	319	DBSU	23.57
**4135**	allXL	DD	372	319	DBSU	16.18
**4135**	allXL	DD	370	357	DBSU	21.68
**4135**	allXL	DD	382	357	DBSU	29.66
**4135**	allXL	AA	372	370	DBSU	5.94
**4135**	allXL	DD	381	370	DBSU	20.07
**4135**	allXL	AA	373	370	DBSU	9.18
**4135**	allXL	DD	381	372	DBSU	14.01
**4135**	allXL	DD	372	382	DBSU	14.64
**4135**	allXL	DD	372	386	DBSU	21.18
**4135**	allXL	DD	373	386	DBSU	22.19
**4135**	allXL	DA	381	386	DBSU	72.84
**4135**	allXL	BD;DB	357	357	DBSU	18.84
**4135**	allXL	BC	373	319	DBSU	34.11
**4135**	allXL	DB	372	357	DBSU	40.27
**4135**	allXL	DB	381	357	DBSU	44.94
**4135**	allXL	DB	373	357	DBSU	37.47
**4135**	allXL	CB	372	370	DBSU	43.29
**4135**	allXL	CB	373	370	DBSU	45.19
**4135**	allXL	BC	382	373	DBSU	55.43
**4136**	allXL	CC	320	357	BS2G	24.15
**4136**	allXL	DD	320	373	BS2G	25.13
**4136**	allXL	BB	373	382	BS2G	16.46
**4136**	allXL	BB	370	386	BS2G	25.6
**4136**	allXL	BB	373	386	BS2G	24.37
**4136**	allXL	CC	319	357	BS2G	23.96
**4136**	allXL	BB	367	373	BS2G	14.05
**4136**	allXL	DD	370	382	BS2G	20.59
**4136**	allXL	BB	376	382	BS2G	12.68
**4136**	allXL	BB	357	373	BS2G	20.65
**4136**	allXL	BB	320	366	BS2G	21.18
**4136**	allXL	CC	370	376	BS2G	16.54
**4136**	allXL	CC	320	357	DBSU	24.15

**4136**	allXL	CC	357	319	DBSU	23.96
**4136**	allXL	DD	372	319	DBSU	28.45
**4136**	allXL	BB	370	357	DBSU	23.98
**4136**	allXL	BB	382	357	DBSU	30.2
**4136**	allXL	AA	372	370	DBSU	6.17
**4136**	allXL	DD	381	370	DBSU	20.63
**4136**	allXL	AA	373	370	DBSU	9.18
**4136**	allXL	DD	381	372	DBSU	15.14
**4136**	allXL	DD	372	382	DBSU	15.45
**4136**	allXL	DD	372	386	DBSU	24.82
**4136**	allXL	BB	373	386	DBSU	24.37
**4136**	allXL	DA	381	386	DBSU	64.91
**4136**	allXL	BD;DB	357	357	DBSU	18.58
**4136**	allXL	AD	373	319	DBSU	17.67
**4136**	allXL	BD	372	357	DBSU	36.9
**4136**	allXL	BD	381	357	DBSU	35.68
**4136**	allXL	BD	373	357	DBSU	34.87
**4136**	allXL	DA	372	370	DBSU	39.31
**4136**	allXL	DA	373	370	DBSU	36.32
**4136**	allXL	DA	382	373	DBSU	43.34
**4137**	allXL	AA	320	357	BS2G	17.68
**4137**	allXL	BB	320	373	BS2G	18.81
**4137**	allXL	CC	373	382	BS2G	18.83
**4137**	allXL	CC	370	386	BS2G	23.53
**4137**	allXL	CC	373	386	BS2G	21.62
**4137**	allXL	AA	319	357	BS2G	15.24
**4137**	allXL	BB	367	373	BS2G	11.45
**4137**	allXL	DD	370	382	BS2G	17.08
**4137**	allXL	BB	376	382	BS2G	15.37
**4137**	allXL	BB	357	373	BS2G	29.48
**4137**	allXL	BB	320	366	BS2G	24.65
**4137**	allXL	CC	370	376	BS2G	12.62
**4137**	allXL	AA	320	357	DBSU	17.68
**4137**	allXL	AA	357	319	DBSU	15.24

**4137**	allXL	BB	372	319	DBSU	19.05
**4137**	allXL	DD	370	357	DBSU	29.97
**4137**	allXL	DD	382	357	DBSU	19.87
**4137**	allXL	AA	372	370	DBSU	6.51
**4137**	allXL	DD	381	370	DBSU	17.92
**4137**	allXL	AA	373	370	DBSU	9.21
**4137**	allXL	DD	381	372	DBSU	20.16
**4137**	allXL	CC	372	382	DBSU	19.99
**4137**	allXL	CC	372	386	DBSU	23.33
**4137**	allXL	CC	373	386	DBSU	21.62
**4137**	allXL	DB	381	386	DBSU	29.01
**4137**	allXL	BD;DB	357	357	DBSU	19.05
**4137**	allXL	AC	373	319	DBSU	38.83
**4137**	allXL	AC	372	357	DBSU	34.48
**4137**	allXL	DB	381	357	DBSU	28.11
**4137**	allXL	AC	373	357	DBSU	32.52
**4137**	allXL	CB	372	370	DBSU	43.66
**4137**	allXL	CB	373	370	DBSU	47.15
**4137**	allXL	CA	382	373	DBSU	44.09
**4138**	allXL	CC	320	357	BS2G	23.11
**4138**	allXL	BB	320	373	BS2G	30.97
**4138**	allXL	CC	373	382	BS2G	15.17
**4138**	allXL	CC	370	386	BS2G	23.52
**4138**	allXL	CC	373	386	BS2G	19.68
**4138**	allXL	CC	319	357	BS2G	23.22
**4138**	allXL	AA	367	373	BS2G	13.62
**4138**	allXL	CC	370	382	BS2G	23.11
**4138**	allXL	CC	376	382	BS2G	13.91
**4138**	allXL	BB	357	373	BS2G	21.57
**4138**	allXL	BB	320	366	BS2G	22.95
**4138**	allXL	BB	370	376	BS2G	16.61
**4138**	allXL	CC	320	357	DBSU	23.11
**4138**	allXL	CC	357	319	DBSU	23.22
**4138**	allXL	BB	372	319	DBSU	29.95

**4138**	allXL	BB	370	357	DBSU	14.74
**4138**	allXL	BB	382	357	DBSU	23.79
**4138**	allXL	AA	372	370	DBSU	6.16
**4138**	allXL	BB	381	370	DBSU	24.96
**4138**	allXL	BB	373	370	DBSU	9.11
**4138**	allXL	CC	381	372	DBSU	18.92
**4138**	allXL	CC	372	382	DBSU	16.28
**4138**	allXL	CC	372	386	DBSU	18.91
**4138**	allXL	CC	373	386	DBSU	19.68
**4138**	allXL	BC	381	386	DBSU	53.4
**4138**	allXL	BD;DB	357	357	DBSU	18.68
**4138**	allXL	DA	373	319	DBSU	23.21
**4138**	allXL	BD	372	357	DBSU	34.44
**4138**	allXL	BD	381	357	DBSU	30.72
**4138**	allXL	BD	373	357	DBSU	33.52
**4138**	allXL	DA	372	370	DBSU	40.62
**4138**	allXL	CB	373	370	DBSU	42.12
**4138**	allXL	CB	382	373	DBSU	46.84
**4139**	allXL	AA	320	357	BS2G	25.35
**4139**	allXL	CC	320	373	BS2G	20.99
**4139**	allXL	AA	373	382	BS2G	20.66
**4139**	allXL	BB	370	386	BS2G	11.04
**4139**	allXL	BB	373	386	BS2G	13.53
**4139**	allXL	AA	319	357	BS2G	24.0
**4139**	allXL	AA	367	373	BS2G	11.57
**4139**	allXL	DD	370	382	BS2G	18.73
**4139**	allXL	CC	376	382	BS2G	15.07
**4139**	allXL	CC	357	373	BS2G	13.41
**4139**	allXL	AA	320	366	BS2G	16.75
**4139**	allXL	BB	370	376	BS2G	14.87
**4139**	allXL	AA	320	357	DBSU	25.35
**4139**	allXL	AA	357	319	DBSU	24.0
**4139**	allXL	CC	372	319	DBSU	20.38
**4139**	allXL	CC	370	357	DBSU	9.88

**4139**	allXL	DD	382	357	DBSU	20.02
**4139**	allXL	AA	372	370	DBSU	6.43
**4139**	allXL	DD	381	370	DBSU	16.68
**4139**	allXL	AA	373	370	DBSU	9.01
**4139**	allXL	DD	381	372	DBSU	19.54
**4139**	allXL	AA	372	382	DBSU	22.25
**4139**	allXL	BB	372	386	DBSU	14.75
**4139**	allXL	BB	373	386	DBSU	13.53
**4139**	allXL	DA	381	386	DBSU	40.85
**4139**	allXL	BD;DB	357	357	DBSU	18.22
**4139**	allXL	AD	373	319	DBSU	25.74
**4139**	allXL	CA	372	357	DBSU	29.23
**4139**	allXL	DB	381	357	DBSU	33.14
**4139**	allXL	CA	373	357	DBSU	28.0
**4139**	allXL	AD	372	370	DBSU	42.79
**4139**	allXL	AD	373	370	DBSU	39.72
**4139**	allXL	AD	382	373	DBSU	32.23
**4140**	allXL	CC	320	357	BS2G	24.19
**4140**	allXL	BB	320	373	BS2G	23.23
**4140**	allXL	CC	373	382	BS2G	18.99
**4140**	allXL	CC	370	386	BS2G	25.47
**4140**	allXL	AA	373	386	BS2G	24.56
**4140**	allXL	AA	319	357	BS2G	27.14
**4140**	allXL	AA	367	373	BS2G	13.91
**4140**	allXL	CC	370	382	BS2G	22.18
**4140**	allXL	CC	376	382	BS2G	14.41
**4140**	allXL	BB	357	373	BS2G	18.48
**4140**	allXL	AA	320	366	BS2G	22.97
**4140**	allXL	CC	370	376	BS2G	16.99
**4140**	allXL	CC	320	357	DBSU	24.19
**4140**	allXL	AA	357	319	DBSU	27.14
**4140**	allXL	BB	372	319	DBSU	27.45
**4140**	allXL	BB	370	357	DBSU	22.74
**4140**	allXL	BB	382	357	DBSU	26.79

**4140**	allXL	AA	372	370	DBSU	6.22
**4140**	allXL	DD	381	370	DBSU	23.07
**4140**	allXL	AA	373	370	DBSU	9.29
**4140**	allXL	CC	381	372	DBSU	20.39
**4140**	allXL	CC	372	382	DBSU	18.94
**4140**	allXL	CC	372	386	DBSU	23.97
**4140**	allXL	AA	373	386	DBSU	24.56
**4140**	allXL	CB	381	386	DBSU	39.28
**4140**	allXL	BD;DB	357	357	DBSU	19.19
**4140**	allXL	CB	373	319	DBSU	39.11
**4140**	allXL	BD	372	357	DBSU	32.63
**4140**	allXL	BD	381	357	DBSU	32.51
**4140**	allXL	BD	373	357	DBSU	31.26
**4140**	allXL	DA	372	370	DBSU	44.97
**4140**	allXL	DA	373	370	DBSU	44.2
**4140**	allXL	DA	382	373	DBSU	47.1
**4141**	allXL	CC	320	357	BS2G	23.07
**4141**	allXL	BB	320	373	BS2G	13.77
**4141**	allXL	DD	373	382	BS2G	17.35
**4141**	allXL	DD	370	386	BS2G	24.41
**4141**	allXL	DD	373	386	BS2G	22.13
**4141**	allXL	CC	319	357	BS2G	21.66
**4141**	allXL	DD	367	373	BS2G	14.05
**4141**	allXL	DD	370	382	BS2G	23.14
**4141**	allXL	CC	376	382	BS2G	13.59
**4141**	allXL	BB	357	373	BS2G	21.65
**4141**	allXL	BB	320	366	BS2G	21.77
**4141**	allXL	BB	370	376	BS2G	17.01
**4141**	allXL	CC	320	357	DBSU	23.07
**4141**	allXL	CC	357	319	DBSU	21.66
**4141**	allXL	BB	372	319	DBSU	16.97
**4141**	allXL	BB	370	357	DBSU	24.75
**4141**	allXL	AB	382	357	DBSU	26.02
**4141**	allXL	AA	372	370	DBSU	6.22

**4141**	allXL	BB	381	370	DBSU	24.04
**4141**	allXL	DD	373	370	DBSU	8.96
**4141**	allXL	DD	381	372	DBSU	18.0
**4141**	allXL	DD	372	382	DBSU	16.79
**4141**	allXL	DD	372	386	DBSU	19.63
**4141**	allXL	DD	373	386	DBSU	22.13
**4141**	allXL	BC	381	386	DBSU	27.06
**4141**	allXL	BD;DB	357	357	DBSU	18.99
**4141**	allXL	CB	373	319	DBSU	23.01
**4141**	allXL	AC	372	357	DBSU	34.38
**4141**	allXL	AD	381	357	DBSU	22.08
**4141**	allXL	AD	373	357	DBSU	35.08
**4141**	allXL	CB	372	370	DBSU	30.97
**4141**	allXL	CB	373	370	DBSU	27.91
**4141**	allXL	CB	382	373	DBSU	24.13
**4142**	allXL	AA	320	357	BS2G	28.55
**4142**	allXL	DD	320	373	BS2G	14.96
**4142**	allXL	BB	373	382	BS2G	16.55
**4142**	allXL	BB	370	386	BS2G	24.26
**4142**	allXL	BB	373	386	BS2G	20.06
**4142**	allXL	AA	319	357	BS2G	29.86
**4142**	allXL	AA	367	373	BS2G	15.39
**4142**	allXL	BB	370	382	BS2G	23.73
**4142**	allXL	CC	376	382	BS2G	14.11
**4142**	allXL	DD	357	373	BS2G	28.25
**4142**	allXL	CC	320	366	BS2G	19.76
**4142**	allXL	CC	370	376	BS2G	16.76
**4142**	allXL	AA	320	357	DBSU	28.55
**4142**	allXL	AA	357	319	DBSU	29.86
**4142**	allXL	DD	372	319	DBSU	15.85
**4142**	allXL	DD	370	357	DBSU	20.4
**4142**	allXL	CC	382	357	DBSU	44.27
**4142**	allXL	AA	372	370	DBSU	6.09
**4142**	allXL	BB	381	370	DBSU	26.27

**4142**	allXL	DD	373	370	DBSU	9.3
**4142**	allXL	DD	381	372	DBSU	19.86
**4142**	allXL	BB	372	382	DBSU	18.15
**4142**	allXL	BB	372	386	DBSU	19.31
**4142**	allXL	BB	373	386	DBSU	20.06
**4142**	allXL	CB	381	386	DBSU	43.2
**4142**	allXL	BD;DB	357	357	DBSU	18.0
**4142**	allXL	CB	373	319	DBSU	26.61
**4142**	allXL	CB	372	357	DBSU	34.93
**4142**	allXL	CB	381	357	DBSU	49.49
**4142**	allXL	CB	373	357	DBSU	38.38
**4142**	allXL	CB	372	370	DBSU	15.87
**4142**	allXL	CB	373	370	DBSU	17.01
**4142**	allXL	CB	382	373	DBSU	21.36
**4143**	allXL	AA	320	357	BS2G	27.38
**4143**	allXL	BB	320	373	BS2G	12.75
**4143**	allXL	BB	373	382	BS2G	17.48
**4143**	allXL	AA	370	386	BS2G	24.73
**4143**	allXL	AA	373	386	BS2G	18.47
**4143**	allXL	AA	319	357	BS2G	27.56
**4143**	allXL	DD	367	373	BS2G	14.61
**4143**	allXL	DD	370	382	BS2G	20.66
**4143**	allXL	BB	376	382	BS2G	12.65
**4143**	allXL	DD	357	373	BS2G	28.4
**4143**	allXL	BB	320	366	BS2G	15.58
**4143**	allXL	CC	370	376	BS2G	16.66
**4143**	allXL	AA	320	357	DBSU	27.38
**4143**	allXL	AA	357	319	DBSU	27.56
**4143**	allXL	BB	372	319	DBSU	12.73
**4143**	allXL	BB	370	357	DBSU	25.94
**4143**	allXL	AA	382	357	DBSU	41.08
**4143**	allXL	AA	372	370	DBSU	6.07
**4143**	allXL	DD	381	370	DBSU	19.23
**4143**	allXL	AA	373	370	DBSU	9.33

**4143**	allXL	DD	381	372	DBSU	15.66
**4143**	allXL	DD	372	382	DBSU	18.04
**4143**	allXL	AA	372	386	DBSU	20.92
**4143**	allXL	AA	373	386	DBSU	18.47
**4143**	allXL	AD	381	386	DBSU	22.98
**4143**	allXL	BD;DB	357	357	DBSU	19.5
**4143**	allXL	DA	373	319	DBSU	27.26
**4143**	allXL	DA	372	357	DBSU	38.47
**4143**	allXL	DA	381	357	DBSU	36.51
**4143**	allXL	DA	373	357	DBSU	34.77
**4143**	allXL	DA	372	370	DBSU	28.79
**4143**	allXL	DA	373	370	DBSU	27.95
**4143**	allXL	DA	382	373	DBSU	14.56
**4144**	allXL	BB	320	357	BS2G	24.86
**4144**	allXL	DD	320	373	BS2G	14.08
**4144**	allXL	DD	373	382	BS2G	18.77
**4144**	allXL	CC	370	386	BS2G	16.44
**4144**	allXL	DD	373	386	BS2G	20.74
**4144**	allXL	AA	319	357	BS2G	26.87
**4144**	allXL	BB	367	373	BS2G	12.35
**4144**	allXL	DD	370	382	BS2G	18.94
**4144**	allXL	CC	376	382	BS2G	12.28
**4144**	allXL	BB	357	373	BS2G	27.53
**4144**	allXL	BB	320	366	BS2G	18.4
**4144**	allXL	CC	370	376	BS2G	15.31
**4144**	allXL	BB	320	357	DBSU	24.86
**4144**	allXL	AA	357	319	DBSU	26.87
**4144**	allXL	DD	372	319	DBSU	16.7
**4144**	allXL	DD	370	357	DBSU	27.44
**4144**	allXL	BB	382	357	DBSU	22.98
**4144**	allXL	AA	372	370	DBSU	6.29
**4144**	allXL	DD	381	370	DBSU	19.24
**4144**	allXL	AA	373	370	DBSU	9.29
**4144**	allXL	DD	381	372	DBSU	18.48

**4144**	allXL	DD	372	382	DBSU	18.75
**4144**	allXL	DD	372	386	DBSU	19.63
**4144**	allXL	DD	373	386	DBSU	20.74
**4144**	allXL	DB	381	386	DBSU	61.86
**4144**	allXL	BD;DB	357	357	DBSU	18.42
**4144**	allXL	AD	373	319	DBSU	45.23
**4144**	allXL	BD	372	357	DBSU	46.17
**4144**	allXL	BD	381	357	DBSU	31.86
**4144**	allXL	BD	373	357	DBSU	43.99
**4144**	allXL	BC	372	370	DBSU	50.75
**4144**	allXL	BC	373	370	DBSU	52.27
**4144**	allXL	DA	382	373	DBSU	53.69
**4145**	allXL	AA	320	357	BS2G	17.69
**4145**	allXL	CC	320	373	BS2G	32.04
**4145**	allXL	BB	373	382	BS2G	19.11
**4145**	allXL	BB	370	386	BS2G	25.71
**4145**	allXL	DD	373	386	BS2G	24.65
**4145**	allXL	AA	319	357	BS2G	15.19
**4145**	allXL	AA	367	373	BS2G	14.31
**4145**	allXL	BB	370	382	BS2G	20.45
**4145**	allXL	BB	376	382	BS2G	13.95
**4145**	allXL	BB	357	373	BS2G	11.57
**4145**	allXL	CC	320	366	BS2G	17.12
**4145**	allXL	CC	370	376	BS2G	16.68
**4145**	allXL	AA	320	357	DBSU	17.69
**4145**	allXL	AA	357	319	DBSU	15.19
**4145**	allXL	CC	372	319	DBSU	29.54
**4145**	allXL	BB	370	357	DBSU	12.68
**4145**	allXL	BB	382	357	DBSU	15.62
**4145**	allXL	AA	372	370	DBSU	6.24
**4145**	allXL	BB	381	370	DBSU	21.38
**4145**	allXL	CC	373	370	DBSU	9.09
**4145**	allXL	BB	381	372	DBSU	20.98
**4145**	allXL	BB	372	382	DBSU	21.15

**4145**	allXL	DD	372	386	DBSU	26.68
**4145**	allXL	DD	373	386	DBSU	24.65
**4145**	allXL	DB	381	386	DBSU	31.97
**4145**	allXL	BD;DB	357	357	DBSU	18.53
**4145**	allXL	CB	373	319	DBSU	13.42
**4145**	allXL	BD	372	357	DBSU	18.98
**4145**	allXL	BD	381	357	DBSU	10.98
**4145**	allXL	BD	373	357	DBSU	15.72
**4145**	allXL	CB	372	370	DBSU	21.65
**4145**	allXL	CB	373	370	DBSU	18.64
**4145**	allXL	BD	382	373	DBSU	28.66
**4146**	allXL	AA	320	357	BS2G	23.35
**4146**	allXL	CC	320	373	BS2G	28.11
**4146**	allXL	AA	373	382	BS2G	18.64
**4146**	allXL	CC	370	386	BS2G	27.05
**4146**	allXL	AA	373	386	BS2G	25.95
**4146**	allXL	AA	319	357	BS2G	23.25
**4146**	allXL	AA	367	373	BS2G	11.32
**4146**	allXL	CC	370	382	BS2G	23.89
**4146**	allXL	CC	376	382	BS2G	15.0
**4146**	allXL	AA	357	373	BS2G	28.89
**4146**	allXL	BB	320	366	BS2G	29.23
**4146**	allXL	CC	370	376	BS2G	16.47
**4146**	allXL	AA	320	357	DBSU	23.35
**4146**	allXL	AA	357	319	DBSU	23.25
**4146**	allXL	CC	372	319	DBSU	25.59
**4146**	allXL	CC	370	357	DBSU	30.12
**4146**	allXL	BB	382	357	DBSU	39.35
**4146**	allXL	CC	372	370	DBSU	6.49
**4146**	allXL	AA	381	370	DBSU	24.51
**4146**	allXL	DD	373	370	DBSU	9.0
**4146**	allXL	BB	381	372	DBSU	20.94
**4146**	allXL	BB	372	382	DBSU	21.12
**4146**	allXL	CC	372	386	DBSU	28.37

**4146**	allXL	AA	373	386	DBSU	25.95
**4146**	allXL	DA	381	386	DBSU	45.67
**4146**	allXL	BD;DB	357	357	DBSU	18.82
**4146**	allXL	DA	373	319	DBSU	28.84
**4146**	allXL	DA	372	357	DBSU	41.8
**4146**	allXL	DA	381	357	DBSU	42.68
**4146**	allXL	DA	373	357	DBSU	42.1
**4146**	allXL	DA	372	370	DBSU	44.81
**4146**	allXL	AD	373	370	DBSU	41.61
**4146**	allXL	AD	382	373	DBSU	30.05
**4147**	allXL	CC	320	357	BS2G	25.56
**4147**	allXL	AA	320	373	BS2G	24.24
**4147**	allXL	AA	373	382	BS2G	18.03
**4147**	allXL	AA	370	386	BS2G	18.5
**4147**	allXL	AA	373	386	BS2G	23.91
**4147**	allXL	AA	319	357	BS2G	23.95
**4147**	allXL	AA	367	373	BS2G	12.55
**4147**	allXL	AA	370	382	BS2G	17.51
**4147**	allXL	BB	376	382	BS2G	14.15
**4147**	allXL	AA	357	373	BS2G	27.13
**4147**	allXL	DD	320	366	BS2G	24.66
**4147**	allXL	CC	370	376	BS2G	15.62
**4147**	allXL	CC	320	357	DBSU	25.56
**4147**	allXL	AA	357	319	DBSU	23.95
**4147**	allXL	AA	372	319	DBSU	24.13
**4147**	allXL	CC	370	357	DBSU	21.37
**4147**	allXL	AA	382	357	DBSU	19.7
**4147**	allXL	AA	372	370	DBSU	6.45
**4147**	allXL	AA	381	370	DBSU	17.72
**4147**	allXL	AA	373	370	DBSU	9.37
**4147**	allXL	AA	381	372	DBSU	18.57
**4147**	allXL	AA	372	382	DBSU	19.13
**4147**	allXL	AA	372	386	DBSU	23.23
**4147**	allXL	AA	373	386	DBSU	23.91

**4147**	allXL	AC	381	386	DBSU	57.98
**4147**	allXL	BD;DB	357	357	DBSU	19.26
**4147**	allXL	AD	373	319	DBSU	19.24
**4147**	allXL	CA	372	357	DBSU	30.63
**4147**	allXL	AC	381	357	DBSU	29.72
**4147**	allXL	CA	373	357	DBSU	33.99
**4147**	allXL	AD	372	370	DBSU	36.66
**4147**	allXL	AD	373	370	DBSU	37.0
**4147**	allXL	DA	382	373	DBSU	47.4
**4148**	allXL	AA	320	357	BS2G	24.05
**4148**	allXL	DD	320	373	BS2G	24.88
**4148**	allXL	DD	373	382	BS2G	22.05
**4148**	allXL	DD	370	386	BS2G	25.54
**4148**	allXL	DD	373	386	BS2G	29.16
**4148**	allXL	AA	319	357	BS2G	25.37
**4148**	allXL	BB	367	373	BS2G	13.46
**4148**	allXL	DD	370	382	BS2G	21.03
**4148**	allXL	CC	376	382	BS2G	15.06
**4148**	allXL	CC	357	373	BS2G	13.91
**4148**	allXL	AA	320	366	BS2G	25.71
**4148**	allXL	CC	370	376	BS2G	15.15
**4148**	allXL	AA	320	357	DBSU	24.05
**4148**	allXL	AA	357	319	DBSU	25.37
**4148**	allXL	DD	372	319	DBSU	24.5
**4148**	allXL	CC	370	357	DBSU	17.72
**4148**	allXL	CC	382	357	DBSU	17.56
**4148**	allXL	AA	372	370	DBSU	6.38
**4148**	allXL	DD	381	370	DBSU	20.49
**4148**	allXL	CC	373	370	DBSU	9.33
**4148**	allXL	DD	381	372	DBSU	21.14
**4148**	allXL	DD	372	382	DBSU	22.74
**4148**	allXL	DD	372	386	DBSU	28.68
**4148**	allXL	DD	373	386	DBSU	29.16
**4148**	allXL	BC	381	386	DBSU	50.07

**4148**	allXL	BD;DB	357	357	DBSU	19.34
**4148**	allXL	DA	373	319	DBSU	23.01
**4148**	allXL	CA	372	357	DBSU	29.8
**4148**	allXL	CA	381	357	DBSU	20.22
**4148**	allXL	CA	373	357	DBSU	26.36
**4148**	allXL	DA	372	370	DBSU	42.55
**4148**	allXL	DA	373	370	DBSU	39.39
**4148**	allXL	CB	382	373	DBSU	47.32
**4149**	allXL	AA	320	357	BS2G	24.35
**4149**	allXL	BB	320	373	BS2G	25.81
**4149**	allXL	DD	373	382	BS2G	14.98
**4149**	allXL	DD	370	386	BS2G	20.63
**4149**	allXL	CC	373	386	BS2G	19.6
**4149**	allXL	AA	319	357	BS2G	22.86
**4149**	allXL	BB	367	373	BS2G	13.06
**4149**	allXL	DD	370	382	BS2G	16.57
**4149**	allXL	CC	376	382	BS2G	13.97
**4149**	allXL	DD	357	373	BS2G	32.35
**4149**	allXL	BB	320	366	BS2G	15.62
**4149**	allXL	CC	370	376	BS2G	14.67
**4149**	allXL	AA	320	357	DBSU	24.35
**4149**	allXL	AA	357	319	DBSU	22.86
**4149**	allXL	BB	372	319	DBSU	22.69
**4149**	allXL	DD	370	357	DBSU	23.63
**4149**	allXL	DD	382	357	DBSU	38.18
**4149**	allXL	AA	372	370	DBSU	6.03
**4149**	allXL	DD	381	370	DBSU	18.49
**4149**	allXL	CC	373	370	DBSU	9.04
**4149**	allXL	DD	381	372	DBSU	17.28
**4149**	allXL	DD	372	382	DBSU	16.11
**4149**	allXL	CC	372	386	DBSU	21.39
**4149**	allXL	CC	373	386	DBSU	19.6
**4149**	allXL	CB	381	386	DBSU	66.38
**4149**	allXL	BD;DB	357	357	DBSU	18.77

**4149**	allXL	AD	373	319	DBSU	33.65
**4149**	allXL	DB	372	357	DBSU	46.57
**4149**	allXL	DB	381	357	DBSU	58.81
**4149**	allXL	DB	373	357	DBSU	49.53
**4149**	allXL	BC	372	370	DBSU	47.74
**4149**	allXL	BC	373	370	DBSU	48.27
**4149**	allXL	CB	382	373	DBSU	45.48
**4150**	allXL	AA	320	357	BS2G	23.43
**4150**	allXL	BB	320	373	BS2G	14.32
**4150**	allXL	AA	373	382	BS2G	19.19
**4150**	allXL	AA	370	386	BS2G	20.07
**4150**	allXL	AA	373	386	BS2G	19.32
**4150**	allXL	AA	319	357	BS2G	23.71
**4150**	allXL	AA	367	373	BS2G	11.95
**4150**	allXL	AA	370	382	BS2G	20.1
**4150**	allXL	CC	376	382	BS2G	15.36
**4150**	allXL	BB	357	373	BS2G	30.48
**4150**	allXL	BB	320	366	BS2G	15.66
**4150**	allXL	BB	370	376	BS2G	15.28
**4150**	allXL	AA	320	357	DBSU	23.43
**4150**	allXL	AA	357	319	DBSU	23.71
**4150**	allXL	BB	372	319	DBSU	16.93
**4150**	allXL	BB	370	357	DBSU	27.11
**4150**	allXL	AA	382	357	DBSU	51.29
**4150**	allXL	AA	372	370	DBSU	6.2
**4150**	allXL	AA	381	370	DBSU	22.5
**4150**	allXL	DD	373	370	DBSU	9.07
**4150**	allXL	AA	381	372	DBSU	18.99
**4150**	allXL	AA	372	382	DBSU	17.42
**4150**	allXL	AA	372	386	DBSU	18.2
**4150**	allXL	AA	373	386	DBSU	19.32
**4150**	allXL	AD	381	386	DBSU	44.13
**4150**	allXL	BD;DB	357	357	DBSU	18.61
**4150**	allXL	DA	373	319	DBSU	35.66

**4150**	allXL	BD	372	357	DBSU	41.54
**4150**	allXL	DA	381	357	DBSU	43.01
**4150**	allXL	BD	373	357	DBSU	44.87
**4150**	allXL	DA	372	370	DBSU	46.53
**4150**	allXL	DA	373	370	DBSU	42.79
**4150**	allXL	DA	382	373	DBSU	28.66
**4151**	allXL	AA	320	357	BS2G	25.68
**4151**	allXL	CC	320	373	BS2G	29.23
**4151**	allXL	DD	373	382	BS2G	18.14
**4151**	allXL	BB	370	386	BS2G	19.71
**4151**	allXL	BB	373	386	BS2G	16.08
**4151**	allXL	AA	319	357	BS2G	27.24
**4151**	allXL	AA	367	373	BS2G	13.09
**4151**	allXL	DD	370	382	BS2G	17.4
**4151**	allXL	BB	376	382	BS2G	14.26
**4151**	allXL	BB	357	373	BS2G	29.81
**4151**	allXL	AA	320	366	BS2G	24.95
**4151**	allXL	DD	370	376	BS2G	17.07
**4151**	allXL	AA	320	357	DBSU	25.68
**4151**	allXL	AA	357	319	DBSU	27.24
**4151**	allXL	CC	372	319	DBSU	30.03
**4151**	allXL	CC	370	357	DBSU	29.58
**4151**	allXL	BB	382	357	DBSU	19.83
**4151**	allXL	AA	372	370	DBSU	6.35
**4151**	allXL	DD	381	370	DBSU	16.52
**4151**	allXL	AA	373	370	DBSU	9.15
**4151**	allXL	DD	381	372	DBSU	17.05
**4151**	allXL	DD	372	382	DBSU	18.47
**4151**	allXL	BB	372	386	DBSU	17.87
**4151**	allXL	BB	373	386	DBSU	16.08
**4151**	allXL	CB	381	386	DBSU	36.66
**4151**	allXL	BD;DB	357	357	DBSU	18.21
**4151**	allXL	AD	373	319	DBSU	22.44
**4151**	allXL	CB	372	357	DBSU	23.5

**4151**	allXL	BD	381	357	DBSU	32.41
**4151**	allXL	CB	373	357	DBSU	22.43
**4151**	allXL	CB	372	370	DBSU	31.62
**4151**	allXL	CB	373	370	DBSU	30.33
**4151**	allXL	BC	382	373	DBSU	33.28
**4152**	allXL	CC	320	357	BS2G	24.72
**4152**	allXL	BB	320	373	BS2G	26.53
**4152**	allXL	CC	373	382	BS2G	21.18
**4152**	allXL	DD	370	386	BS2G	22.97
**4152**	allXL	DD	373	386	BS2G	22.63
**4152**	allXL	AA	319	357	BS2G	26.08
**4152**	allXL	DD	367	373	BS2G	14.41
**4152**	allXL	DD	370	382	BS2G	21.61
**4152**	allXL	CC	376	382	BS2G	14.69
**4152**	allXL	DD	357	373	BS2G	26.4
**4152**	allXL	CC	320	366	BS2G	10.09
**4152**	allXL	DD	370	376	BS2G	14.95
**4152**	allXL	CC	320	357	DBSU	24.72
**4152**	allXL	AA	357	319	DBSU	26.08
**4152**	allXL	CC	372	319	DBSU	25.0
**4152**	allXL	CC	370	357	DBSU	22.07
**4152**	allXL	BB	382	357	DBSU	35.95
**4152**	allXL	AA	372	370	DBSU	5.89
**4152**	allXL	DD	381	370	DBSU	21.27
**4152**	allXL	DD	373	370	DBSU	9.09
**4152**	allXL	DD	381	372	DBSU	22.3
**4152**	allXL	CC	372	382	DBSU	22.77
**4152**	allXL	DD	372	386	DBSU	22.77
**4152**	allXL	DD	373	386	DBSU	22.63
**4152**	allXL	DB	381	386	DBSU	26.59
**4152**	allXL	BD;DB	357	357	DBSU	19.1
**4152**	allXL	AD	373	319	DBSU	32.91
**4152**	allXL	DB	372	357	DBSU	29.32
**4152**	allXL	BD	381	357	DBSU	44.3

**4152**	allXL	DB	373	357	DBSU	30.85
**4152**	allXL	DB	372	370	DBSU	40.95
**4152**	allXL	DB	373	370	DBSU	41.48
**4152**	allXL	BD	382	373	DBSU	21.11
**4153**	allXL	AA	320	357	BS2G	21.89
**4153**	allXL	BB	320	373	BS2G	20.52
**4153**	allXL	DD	373	382	BS2G	18.37
**4153**	allXL	DD	370	386	BS2G	21.88
**4153**	allXL	DD	373	386	BS2G	23.24
**4153**	allXL	AA	319	357	BS2G	25.31
**4153**	allXL	AA	367	373	BS2G	12.69
**4153**	allXL	DD	370	382	BS2G	21.69
**4153**	allXL	CC	376	382	BS2G	14.74
**4153**	allXL	CC	357	373	BS2G	18.34
**4153**	allXL	CC	320	366	BS2G	13.46
**4153**	allXL	CC	370	376	BS2G	15.4
**4153**	allXL	AA	320	357	DBSU	21.89
**4153**	allXL	AA	357	319	DBSU	25.31
**4153**	allXL	BB	372	319	DBSU	16.69
**4153**	allXL	DD	370	357	DBSU	17.41
**4153**	allXL	DD	382	357	DBSU	34.84
**4153**	allXL	AA	372	370	DBSU	6.35
**4153**	allXL	DD	381	370	DBSU	23.23
**4153**	allXL	AA	373	370	DBSU	9.45
**4153**	allXL	DD	381	372	DBSU	21.28
**4153**	allXL	DD	372	382	DBSU	20.8
**4153**	allXL	DD	372	386	DBSU	24.08
**4153**	allXL	DD	373	386	DBSU	23.24
**4153**	allXL	CA	381	386	DBSU	34.55
**4153**	allXL	BD;DB	357	357	DBSU	19.31
**4153**	allXL	AD	373	319	DBSU	20.66
**4153**	allXL	CA	372	357	DBSU	30.23
**4153**	allXL	CA	381	357	DBSU	45.09
**4153**	allXL	CA	373	357	DBSU	27.85

**4153**	allXL	AD	372	370	DBSU	39.26
**4153**	allXL	AD	373	370	DBSU	39.02
**4153**	allXL	AC	382	373	DBSU	37.8
**4154**	allXL	CC	320	357	BS2G	21.64
**4154**	allXL	BB	320	373	BS2G	20.9
**4154**	allXL	DD	373	382	BS2G	16.97
**4154**	allXL	DD	370	386	BS2G	9.84
**4154**	allXL	DD	373	386	BS2G	16.68
**4154**	allXL	AA	319	357	BS2G	21.03
**4154**	allXL	AA	367	373	BS2G	14.53
**4154**	allXL	DD	370	382	BS2G	16.43
**4154**	allXL	CC	376	382	BS2G	15.25
**4154**	allXL	CC	357	373	BS2G	21.81
**4154**	allXL	DD	320	366	BS2G	18.88
**4154**	allXL	CC	370	376	BS2G	16.19
**4154**	allXL	CC	320	357	DBSU	21.64
**4154**	allXL	AA	357	319	DBSU	21.03
**4154**	allXL	BB	372	319	DBSU	18.15
**4154**	allXL	CC	370	357	DBSU	19.42
**4154**	allXL	CC	382	357	DBSU	11.49
**4154**	allXL	AA	372	370	DBSU	6.23
**4154**	allXL	DD	381	370	DBSU	17.04
**4154**	allXL	AA	373	370	DBSU	8.9
**4154**	allXL	DD	381	372	DBSU	17.21
**4154**	allXL	DD	372	382	DBSU	18.17
**4154**	allXL	DD	372	386	DBSU	15.97
**4154**	allXL	DD	373	386	DBSU	16.68
**4154**	allXL	CA	381	386	DBSU	48.12
**4154**	allXL	BD;DB	357	357	DBSU	18.15
**4154**	allXL	DA	373	319	DBSU	33.22
**4154**	allXL	CA	372	357	DBSU	33.14
**4154**	allXL	CA	381	357	DBSU	9.45
**4154**	allXL	CA	373	357	DBSU	31.1
**4154**	allXL	AC;DA	372	370	DBSU	44.16

**4154**	allXL	CA	373	370	DBSU	41.6
**4154**	allXL	AC	382	373	DBSU	33.98
**4155**	allXL	AA	320	357	BS2G	23.8
**4155**	allXL	CC	320	373	BS2G	20.62
**4155**	allXL	BB	373	382	BS2G	20.46
**4155**	allXL	BB	370	386	BS2G	30.52
**4155**	allXL	BB	373	386	BS2G	26.69
**4155**	allXL	CC	319	357	BS2G	26.43
**4155**	allXL	AA	367	373	BS2G	12.3
**4155**	allXL	BB	370	382	BS2G	25.36
**4155**	allXL	BB	376	382	BS2G	13.82
**4155**	allXL	CC	357	373	BS2G	34.15
**4155**	allXL	BB	320	366	BS2G	20.31
**4155**	allXL	CC	370	376	BS2G	16.36
**4155**	allXL	AA	320	357	DBSU	23.8
**4155**	allXL	CC	357	319	DBSU	26.43
**4155**	allXL	CC	372	319	DBSU	23.66
**4155**	allXL	DD	370	357	DBSU	30.22
**4155**	allXL	CC	382	357	DBSU	48.24
**4155**	allXL	AA	372	370	DBSU	6.36
**4155**	allXL	DD	381	370	DBSU	25.46
**4155**	allXL	DD	373	370	DBSU	8.7
**4155**	allXL	DD	381	372	DBSU	23.16
**4155**	allXL	AA	372	382	DBSU	22.54
**4155**	allXL	BB	372	386	DBSU	29.29
**4155**	allXL	BB	373	386	DBSU	26.69
**4155**	allXL	BC	381	386	DBSU	45.97
**4155**	allXL	BD;DB	357	357	DBSU	18.63
**4155**	allXL	CB	373	319	DBSU	36.95
**4155**	allXL	CB	372	357	DBSU	37.85
**4155**	allXL	AC	381	357	DBSU	59.46
**4155**	allXL	CB	373	357	DBSU	40.36
**4155**	allXL	BC	372	370	DBSU	16.81
**4155**	allXL	BC	373	370	DBSU	19.85

**4155**	allXL	CB	382	373	DBSU	33.47
**4156**	allXL	AA	320	357	BS2G	22.04
**4156**	allXL	DD	320	373	BS2G	17.78
**4156**	allXL	BB	373	382	BS2G	18.8
**4156**	allXL	DD	370	386	BS2G	16.2
**4156**	allXL	DD	373	386	BS2G	21.25
**4156**	allXL	AA	319	357	BS2G	20.08
**4156**	allXL	DD	367	373	BS2G	14.54
**4156**	allXL	DD	370	382	BS2G	21.16
**4156**	allXL	CC	376	382	BS2G	13.54
**4156**	allXL	AA	357	373	BS2G	18.73
**4156**	allXL	BB	320	366	BS2G	19.6
**4156**	allXL	BB	370	376	BS2G	17.11
**4156**	allXL	AA	320	357	DBSU	22.04
**4156**	allXL	AA	357	319	DBSU	20.08
**4156**	allXL	DD	372	319	DBSU	12.34
**4156**	allXL	AA	370	357	DBSU	13.24
**4156**	allXL	AA	382	357	DBSU	35.09
**4156**	allXL	AA	372	370	DBSU	5.76
**4156**	allXL	DD	381	370	DBSU	21.32
**4156**	allXL	AA	373	370	DBSU	9.24
**4156**	allXL	AA	381	372	DBSU	19.73
**4156**	allXL	BB	372	382	DBSU	21.4
**4156**	allXL	DD	372	386	DBSU	21.22
**4156**	allXL	DD	373	386	DBSU	21.25
**4156**	allXL	AC	381	386	DBSU	29.9
**4156**	allXL	BD;DB	357	357	DBSU	18.95
**4156**	allXL	DA	373	319	DBSU	27.24
**4156**	allXL	AC	372	357	DBSU	12.42
**4156**	allXL	AC	381	357	DBSU	27.06
**4156**	allXL	AC	373	357	DBSU	10.76
**4156**	allXL	AC	372	370	DBSU	33.61
**4156**	allXL	AC	373	370	DBSU	30.2
**4156**	allXL	AC	382	373	DBSU	30.09

**4157**	allXL	AA	320	357	BS2G	22.73
**4157**	allXL	BB	320	373	BS2G	25.46
**4157**	allXL	DD	373	382	BS2G	19.99
**4157**	allXL	DD	370	386	BS2G	24.0
**4157**	allXL	AA	373	386	BS2G	30.18
**4157**	allXL	AA	319	357	BS2G	21.46
**4157**	allXL	BB	367	373	BS2G	9.96
**4157**	allXL	DD	370	382	BS2G	14.48
**4157**	allXL	CC	376	382	BS2G	14.72
**4157**	allXL	BB	357	373	BS2G	26.42
**4157**	allXL	AA	320	366	BS2G	34.32
**4157**	allXL	DD	370	376	BS2G	13.88
**4157**	allXL	AA	320	357	DBSU	22.73
**4157**	allXL	AA	357	319	DBSU	21.46
**4157**	allXL	BB	372	319	DBSU	30.02
**4157**	allXL	DD	370	357	DBSU	27.36
**4157**	allXL	BB	382	357	DBSU	18.49
**4157**	allXL	AA	372	370	DBSU	6.17
**4157**	allXL	DD	381	370	DBSU	12.33
**4157**	allXL	AA	373	370	DBSU	9.13
**4157**	allXL	DD	381	372	DBSU	17.52
**4157**	allXL	DD	372	382	DBSU	20.2
**4157**	allXL	DD	372	386	DBSU	30.37
**4157**	allXL	AA	373	386	DBSU	30.18
**4157**	allXL	DA	381	386	DBSU	40.85
**4157**	allXL	BD;DB	357	357	DBSU	19.37
**4157**	allXL	AD	373	319	DBSU	35.32
**4157**	allXL	AC	372	357	DBSU	39.5
**4157**	allXL	BD	381	357	DBSU	32.71
**4157**	allXL	AC	373	357	DBSU	37.49
**4157**	allXL	AD	372	370	DBSU	65.35
**4157**	allXL	AD	373	370	DBSU	64.4
**4157**	allXL	DA	382	373	DBSU	53.89
**4158**	allXL	CC	320	357	BS2G	24.42

**4158**	allXL	BB	320	373	BS2G	13.86
**4158**	allXL	CC	373	382	BS2G	15.61
**4158**	allXL	BB	370	386	BS2G	18.87
**4158**	allXL	CC	373	386	BS2G	20.88
**4158**	allXL	AA	319	357	BS2G	25.93
**4158**	allXL	AA	367	373	BS2G	10.23
**4158**	allXL	BB	370	382	BS2G	15.96
**4158**	allXL	BB	376	382	BS2G	14.02
**4158**	allXL	BB	357	373	BS2G	24.31
**4158**	allXL	BB	320	366	BS2G	22.96
**4158**	allXL	CC	370	376	BS2G	15.74
**4158**	allXL	CC	320	357	DBSU	24.42
**4158**	allXL	AA	357	319	DBSU	25.93
**4158**	allXL	BB	372	319	DBSU	16.01
**4158**	allXL	BB	370	357	DBSU	17.65
**4158**	allXL	AA	382	357	DBSU	21.7
**4158**	allXL	AA	372	370	DBSU	6.65
**4158**	allXL	BB	381	370	DBSU	15.75
**4158**	allXL	AA	373	370	DBSU	8.89
**4158**	allXL	BB	381	372	DBSU	17.42
**4158**	allXL	CC	372	382	DBSU	18.27
**4158**	allXL	CC	372	386	DBSU	22.67
**4158**	allXL	CC	373	386	DBSU	20.88
**4158**	allXL	DB	381	386	DBSU	54.77
**4158**	allXL	BD;DB	357	357	DBSU	18.51
**4158**	allXL	CB	373	319	DBSU	22.07
**4158**	allXL	AC	372	357	DBSU	31.91
**4158**	allXL	AC	381	357	DBSU	30.49
**4158**	allXL	AC	373	357	DBSU	30.06
**4158**	allXL	BC	372	370	DBSU	31.02
**4158**	allXL	BC	373	370	DBSU	32.92
**4158**	allXL	CB	382	373	DBSU	36.18
**4159**	allXL	AA	320	357	BS2G	21.58
**4159**	allXL	CC	320	373	BS2G	21.31

**4159**	allXL	AA	373	382	BS2G	16.36
**4159**	allXL	AA	370	386	BS2G	18.43
**4159**	allXL	AA	373	386	BS2G	20.45
**4159**	allXL	AA	319	357	BS2G	18.52
**4159**	allXL	AA	367	373	BS2G	11.08
**4159**	allXL	CC	370	382	BS2G	18.76
**4159**	allXL	CC	376	382	BS2G	13.93
**4159**	allXL	BB	357	373	BS2G	15.19
**4159**	allXL	CC	320	366	BS2G	16.09
**4159**	allXL	CC	370	376	BS2G	13.42
**4159**	allXL	AA	320	357	DBSU	21.58
**4159**	allXL	AA	357	319	DBSU	18.52
**4159**	allXL	CC	372	319	DBSU	18.37
**4159**	allXL	BB	370	357	DBSU	21.39
**4159**	allXL	CC	382	357	DBSU	17.55
**4159**	allXL	AA	372	370	DBSU	6.49
**4159**	allXL	CC	381	370	DBSU	21.2
**4159**	allXL	AA	373	370	DBSU	9.35
**4159**	allXL	DD	381	372	DBSU	21.04
**4159**	allXL	AA	372	382	DBSU	18.67
**4159**	allXL	AA	372	386	DBSU	20.99
**4159**	allXL	AA	373	386	DBSU	20.45
**4159**	allXL	AD	381	386	DBSU	42.01
**4159**	allXL	BD;DB	357	357	DBSU	18.48
**4159**	allXL	AD	373	319	DBSU	16.21
**4159**	allXL	BD	372	357	DBSU	25.04
**4159**	allXL	BD	381	357	DBSU	24.15
**4159**	allXL	BD	373	357	DBSU	22.84
**4159**	allXL	AD	372	370	DBSU	34.88
**4159**	allXL	AD	373	370	DBSU	37.31
**4159**	allXL	AC	382	373	DBSU	31.24
**4160**	allXL	CC	320	357	BS2G	25.06
**4160**	allXL	BB	320	373	BS2G	25.91
**4160**	allXL	CC	373	382	BS2G	19.46

**4160**	allXL	CC	370	386	BS2G	29.55
**4160**	allXL	CC	373	386	BS2G	25.55
**4160**	allXL	CC	319	357	BS2G	26.12
**4160**	allXL	AA	367	373	BS2G	13.64
**4160**	allXL	CC	370	382	BS2G	24.96
**4160**	allXL	CC	376	382	BS2G	15.5
**4160**	allXL	AA	357	373	BS2G	26.98
**4160**	allXL	BB	320	366	BS2G	16.51
**4160**	allXL	CC	370	376	BS2G	17.04
**4160**	allXL	CC	320	357	DBSU	25.06
**4160**	allXL	CC	357	319	DBSU	26.12
**4160**	allXL	CC	372	319	DBSU	26.1
**4160**	allXL	BB	370	357	DBSU	27.43
**4160**	allXL	AA	382	357	DBSU	30.51
**4160**	allXL	AA	372	370	DBSU	6.39
**4160**	allXL	CC	381	370	DBSU	24.88
**4160**	allXL	AA	373	370	DBSU	9.04
**4160**	allXL	CC	381	372	DBSU	20.21
**4160**	allXL	CC	372	382	DBSU	19.86
**4160**	allXL	CC	372	386	DBSU	24.48
**4160**	allXL	CC	373	386	DBSU	25.55
**4160**	allXL	CA	381	386	DBSU	68.05
**4160**	allXL	BD;DB	357	357	DBSU	19.64
**4160**	allXL	AD	373	319	DBSU	23.21
**4160**	allXL	BD	372	357	DBSU	40.86
**4160**	allXL	AC	381	357	DBSU	28.59
**4160**	allXL	AC	373	357	DBSU	41.5
**4160**	allXL	AD	372	370	DBSU	54.78
**4160**	allXL	AD	373	370	DBSU	53.15
**4160**	allXL	DA	382	373	DBSU	55.27
**4161**	allXL	CC	320	357	BS2G	26.9
**4161**	allXL	BB	320	373	BS2G	13.32
**4161**	allXL	DD	373	382	BS2G	17.2
**4161**	allXL	DD	370	386	BS2G	20.57

**4161**	allXL	DD	373	386	BS2G	20.99
**4161**	allXL	DD	319	357	BS2G	29.96
**4161**	allXL	AA	367	373	BS2G	12.61
**4161**	allXL	DD	370	382	BS2G	14.95
**4161**	allXL	BB	376	382	BS2G	13.35
**4161**	allXL	BB	357	373	BS2G	27.17
**4161**	allXL	AA	320	366	BS2G	17.03
**4161**	allXL	DD	370	376	BS2G	14.21
**4161**	allXL	CC	320	357	DBSU	26.9
**4161**	allXL	DD	357	319	DBSU	29.96
**4161**	allXL	BB	372	319	DBSU	15.93
**4161**	allXL	DD	370	357	DBSU	27.67
**4161**	allXL	DD	382	357	DBSU	16.98
**4161**	allXL	AA	372	370	DBSU	6.26
**4161**	allXL	DD	381	370	DBSU	13.49
**4161**	allXL	AA	373	370	DBSU	9.07
**4161**	allXL	DD	381	372	DBSU	17.69
**4161**	allXL	DD	372	382	DBSU	18.32
**4161**	allXL	DD	372	386	DBSU	21.86
**4161**	allXL	DD	373	386	DBSU	20.99
**4161**	allXL	CB	381	386	DBSU	26.15
**4161**	allXL	BD;DB	357	357	DBSU	18.31
**4161**	allXL	AD	373	319	DBSU	32.56
**4161**	allXL	BD	372	357	DBSU	43.1
**4161**	allXL	DB	381	357	DBSU	28.92
**4161**	allXL	CA	373	357	DBSU	40.72
**4161**	allXL	DA	372	370	DBSU	51.13
**4161**	allXL	DA	373	370	DBSU	52.99
**4161**	allXL	DB	382	373	DBSU	46.72
**4162**	allXL	AA	320	357	BS2G	26.61
**4162**	allXL	AA	320	373	BS2G	23.24
**4162**	allXL	BB	373	382	BS2G	19.75
**4162**	allXL	DD	370	386	BS2G	21.67
**4162**	allXL	DD	373	386	BS2G	17.66

**4162**	allXL	DD	319	357	BS2G	27.37
**4162**	allXL	AA	367	373	BS2G	11.84
**4162**	allXL	BB	370	382	BS2G	22.39
**4162**	allXL	BB	376	382	BS2G	14.94
**4162**	allXL	BB	357	373	BS2G	28.08
**4162**	allXL	CC	320	366	BS2G	15.87
**4162**	allXL	CC	370	376	BS2G	13.11
**4162**	allXL	AA	320	357	DBSU	26.61
**4162**	allXL	DD	357	319	DBSU	27.37
**4162**	allXL	CC	372	319	DBSU	21.04
**4162**	allXL	DD	370	357	DBSU	31.26
**4162**	allXL	BB	382	357	DBSU	20.72
**4162**	allXL	AA	372	370	DBSU	6.53
**4162**	allXL	CC	381	370	DBSU	22.79
**4162**	allXL	DD	373	370	DBSU	9.0
**4162**	allXL	DD	381	372	DBSU	19.79
**4162**	allXL	DD	372	382	DBSU	20.43
**4162**	allXL	DD	372	386	DBSU	15.83
**4162**	allXL	DD	373	386	DBSU	17.66
**4162**	allXL	DA	381	386	DBSU	39.25
**4162**	allXL	BD;DB	357	357	DBSU	18.04
**4162**	allXL	AD	373	319	DBSU	23.8
**4162**	allXL	BC	372	357	DBSU	42.24
**4162**	allXL	BD	381	357	DBSU	32.2
**4162**	allXL	BC	373	357	DBSU	39.41
**4162**	allXL	BC	372	370	DBSU	40.54
**4162**	allXL	BC	373	370	DBSU	37.95
**4162**	allXL	AD	382	373	DBSU	34.72
**4163**	allXL	AA	320	357	BS2G	23.48
**4163**	allXL	BB	320	373	BS2G	32.67
**4163**	allXL	DD	373	382	BS2G	18.09
**4163**	allXL	DD	370	386	BS2G	17.69
**4163**	allXL	DD	373	386	BS2G	25.9
**4163**	allXL	AA	319	357	BS2G	22.02

**4163**	allXL	AA	367	373	BS2G	12.27
**4163**	allXL	DD	370	382	BS2G	11.11
**4163**	allXL	CC	376	382	BS2G	14.7
**4163**	allXL	DD	357	373	BS2G	23.71
**4163**	allXL	BB	320	366	BS2G	17.95
**4163**	allXL	DD	370	376	BS2G	13.74
**4163**	allXL	AA	320	357	DBSU	23.48
**4163**	allXL	AA	357	319	DBSU	22.02
**4163**	allXL	AA	372	319	DBSU	32.48
**4163**	allXL	DD	370	357	DBSU	26.68
**4163**	allXL	BB	382	357	DBSU	20.61
**4163**	allXL	AA	372	370	DBSU	6.23
**4163**	allXL	DD	381	370	DBSU	9.59
**4163**	allXL	AA	373	370	DBSU	9.12
**4163**	allXL	DD	381	372	DBSU	14.96
**4163**	allXL	DD	372	382	DBSU	17.09
**4163**	allXL	DD	372	386	DBSU	23.56
**4163**	allXL	DD	373	386	DBSU	25.9
**4163**	allXL	DA	381	386	DBSU	58.7
**4163**	allXL	BD;DB	357	357	DBSU	20.01
**4163**	allXL	DA	373	319	DBSU	27.3
**4163**	allXL	DA	372	357	DBSU	36.09
**4163**	allXL	AC	381	357	DBSU	32.88
**4163**	allXL	DB	373	357	DBSU	34.75
**4163**	allXL	DA	372	370	DBSU	50.24
**4163**	allXL	AD	373	370	DBSU	52.68
**4163**	allXL	AD	382	373	DBSU	53.05
**4164**	allXL	AA	320	357	BS2G	24.49
**4164**	allXL	CC	320	373	BS2G	12.64
**4164**	allXL	CC	373	382	BS2G	15.72
**4164**	allXL	CC	370	386	BS2G	19.78
**4164**	allXL	CC	373	386	BS2G	18.8
**4164**	allXL	DD	319	357	BS2G	23.44
**4164**	allXL	AA	367	373	BS2G	11.77

**4164**	allXL	CC	370	382	BS2G	20.54
**4164**	allXL	CC	376	382	BS2G	13.07
**4164**	allXL	BB	357	373	BS2G	22.64
**4164**	allXL	BB	320	366	BS2G	22.53
**4164**	allXL	DD	370	376	BS2G	17.23
**4164**	allXL	AA	320	357	DBSU	24.49
**4164**	allXL	DD	357	319	DBSU	23.44
**4164**	allXL	CC	372	319	DBSU	12.74
**4164**	allXL	CC	370	357	DBSU	25.26
**4164**	allXL	BB	382	357	DBSU	25.31
**4164**	allXL	CC	372	370	DBSU	6.43
**4164**	allXL	CC	381	370	DBSU	23.73
**4164**	allXL	AA	373	370	DBSU	9.11
**4164**	allXL	CC	381	372	DBSU	18.58
**4164**	allXL	CC	372	382	DBSU	15.56
**4164**	allXL	CC	372	386	DBSU	16.58
**4164**	allXL	CC	373	386	DBSU	18.8
**4164**	allXL	BC	381	386	DBSU	48.83
**4164**	allXL	BD;DB	357	357	DBSU	19.18
**4164**	allXL	AD	373	319	DBSU	30.87
**4164**	allXL	BD	372	357	DBSU	43.45
**4164**	allXL	BD	381	357	DBSU	38.54
**4164**	allXL	BD	373	357	DBSU	41.41
**4164**	allXL	BC	372	370	DBSU	35.39
**4164**	allXL	BC	373	370	DBSU	38.78
**4164**	allXL	CB	382	373	DBSU	43.18
**4165**	allXL	AA	320	357	BS2G	24.46
**4165**	allXL	CC	320	373	BS2G	23.77
**4165**	allXL	BB	373	382	BS2G	15.87
**4165**	allXL	DD	370	386	BS2G	14.2
**4165**	allXL	BB	373	386	BS2G	22.48
**4165**	allXL	AA	319	357	BS2G	24.07
**4165**	allXL	AA	367	373	BS2G	13.7
**4165**	allXL	DD	370	382	BS2G	12.6

**4165**	allXL	BB	376	382	BS2G	12.3
**4165**	allXL	CC	357	373	BS2G	24.95
**4165**	allXL	BB	320	366	BS2G	28.41
**4165**	allXL	CC	370	376	BS2G	14.87
**4165**	allXL	AA	320	357	DBSU	24.46
**4165**	allXL	AA	357	319	DBSU	24.07
**4165**	allXL	CC	372	319	DBSU	21.6
**4165**	allXL	DD	370	357	DBSU	25.14
**4165**	allXL	DD	382	357	DBSU	23.58
**4165**	allXL	AA	372	370	DBSU	6.16
**4165**	allXL	DD	381	370	DBSU	12.08
**4165**	allXL	CC	373	370	DBSU	8.99
**4165**	allXL	DD	381	372	DBSU	17.12
**4165**	allXL	BB	372	382	DBSU	18.56
**4165**	allXL	DD	372	386	DBSU	21.52
**4165**	allXL	BB	373	386	DBSU	22.48
**4165**	allXL	BD	381	386	DBSU	39.91
**4165**	allXL	BD;DB	357	357	DBSU	18.34
**4165**	allXL	AD	373	319	DBSU	27.23
**4165**	allXL	BD	372	357	DBSU	34.35
**4165**	allXL	BD	381	357	DBSU	28.53
**4165**	allXL	BD	373	357	DBSU	33.42
**4165**	allXL	AD	372	370	DBSU	50.2
**4165**	allXL	DA	373	370	DBSU	50.12
**4165**	allXL	BD	382	373	DBSU	39.24
**4166**	allXL	AA	320	357	BS2G	25.11
**4166**	allXL	CC	320	373	BS2G	15.59
**4166**	allXL	CC	373	382	BS2G	18.97
**4166**	allXL	CC	370	386	BS2G	14.63
**4166**	allXL	CC	373	386	BS2G	21.22
**4166**	allXL	AA	319	357	BS2G	25.73
**4166**	allXL	AA	367	373	BS2G	11.02
**4166**	allXL	DD	370	382	BS2G	17.73
**4166**	allXL	BB	376	382	BS2G	13.87

**4166**	allXL	CC	357	373	BS2G	32.8
**4166**	allXL	AA	320	366	BS2G	17.96
**4166**	allXL	DD	370	376	BS2G	15.35
**4166**	allXL	AA	320	357	DBSU	25.11
**4166**	allXL	AA	357	319	DBSU	25.73
**4166**	allXL	CC	372	319	DBSU	15.93
**4166**	allXL	CC	370	357	DBSU	28.31
**4166**	allXL	BB	382	357	DBSU	28.32
**4166**	allXL	CC	372	370	DBSU	6.74
**4166**	allXL	DD	381	370	DBSU	17.7
**4166**	allXL	AA	373	370	DBSU	8.94
**4166**	allXL	DD	381	372	DBSU	21.23
**4166**	allXL	CC	372	382	DBSU	19.47
**4166**	allXL	CC	372	386	DBSU	19.65
**4166**	allXL	CC	373	386	DBSU	21.22
**4166**	allXL	BD	381	386	DBSU	58.21
**4166**	allXL	BD;DB	357	357	DBSU	18.49
**4166**	allXL	AD	373	319	DBSU	40.42
**4166**	allXL	CB	372	357	DBSU	41.1
**4166**	allXL	BD	381	357	DBSU	39.5
**4166**	allXL	CB	373	357	DBSU	41.63
**4166**	allXL	CB	372	370	DBSU	49.54
**4166**	allXL	CB	373	370	DBSU	50.82
**4166**	allXL	BC	382	373	DBSU	60.71
**4167**	allXL	AA	320	357	BS2G	23.87
**4167**	allXL	CC	320	373	BS2G	21.54
**4167**	allXL	DD	373	382	BS2G	21.4
**4167**	allXL	DD	370	386	BS2G	21.76
**4167**	allXL	DD	373	386	BS2G	21.45
**4167**	allXL	AA	319	357	BS2G	25.6
**4167**	allXL	AA	367	373	BS2G	13.09
**4167**	allXL	BB	370	382	BS2G	24.34
**4167**	allXL	CC	376	382	BS2G	14.85
**4167**	allXL	CC	357	373	BS2G	13.96

**4167**	allXL	CC	320	366	BS2G	12.69
**4167**	allXL	AA	370	376	BS2G	16.71
**4167**	allXL	AA	320	357	DBSU	23.87
**4167**	allXL	AA	357	319	DBSU	25.6
**4167**	allXL	CC	372	319	DBSU	18.6
**4167**	allXL	CC	370	357	DBSU	15.24
**4167**	allXL	BB	382	357	DBSU	20.55
**4167**	allXL	CC	372	370	DBSU	6.52
**4167**	allXL	AA	381	370	DBSU	23.63
**4167**	allXL	AA	373	370	DBSU	8.86
**4167**	allXL	AA	381	372	DBSU	22.89
**4167**	allXL	DD	372	382	DBSU	24.15
**4167**	allXL	DD	372	386	DBSU	22.64
**4167**	allXL	DD	373	386	DBSU	21.45
**4167**	allXL	CA	381	386	DBSU	19.45
**4167**	allXL	BD;DB	357	357	DBSU	19.04
**4167**	allXL	AD	373	319	DBSU	41.86
**4167**	allXL	CA	372	357	DBSU	25.2
**4167**	allXL	CA	381	357	DBSU	17.74
**4167**	allXL	CA	373	357	DBSU	21.65
**4167**	allXL	CA	372	370	DBSU	55.55
**4167**	allXL	CA	373	370	DBSU	52.39
**4167**	allXL	CA	382	373	DBSU	40.99
**4168**	allXL	AA	320	357	BS2G	24.81
**4168**	allXL	DD	320	373	BS2G	23.57
**4168**	allXL	AA	373	382	BS2G	20.32
**4168**	allXL	AA	370	386	BS2G	22.25
**4168**	allXL	AA	373	386	BS2G	25.54
**4168**	allXL	AA	319	357	BS2G	23.57
**4168**	allXL	AA	367	373	BS2G	12.3
**4168**	allXL	CC	370	382	BS2G	19.53
**4168**	allXL	CC	376	382	BS2G	14.9
**4168**	allXL	BB	357	373	BS2G	27.6
**4168**	allXL	BB	320	366	BS2G	21.14

**4168**	allXL	CC	370	376	BS2G	14.1
**4168**	allXL	AA	320	357	DBSU	24.81
**4168**	allXL	AA	357	319	DBSU	23.57
**4168**	allXL	DD	372	319	DBSU	23.56
**4168**	allXL	BB	370	357	DBSU	25.33
**4168**	allXL	BB	382	357	DBSU	37.49
**4168**	allXL	AA	372	370	DBSU	6.25
**4168**	allXL	CC	381	370	DBSU	17.55
**4168**	allXL	DD	373	370	DBSU	8.96
**4168**	allXL	DD	381	372	DBSU	17.77
**4168**	allXL	AA	372	382	DBSU	19.68
**4168**	allXL	AA	372	386	DBSU	23.2
**4168**	allXL	AA	373	386	DBSU	25.54
**4168**	allXL	AD	381	386	DBSU	44.09
**4168**	allXL	BD;DB	357	357	DBSU	18.01
**4168**	allXL	AD	373	319	DBSU	17.98
**4168**	allXL	BD	372	357	DBSU	39.0
**4168**	allXL	BD	381	357	DBSU	45.7
**4168**	allXL	BD	373	357	DBSU	38.82
**4168**	allXL	AD	372	370	DBSU	49.06
**4168**	allXL	AD	373	370	DBSU	45.56
**4168**	allXL	AD	382	373	DBSU	40.91
**4169**	allXL	AA	320	357	BS2G	22.05
**4169**	allXL	DD	320	373	BS2G	22.49
**4169**	allXL	BB	373	382	BS2G	17.13
**4169**	allXL	AA	370	386	BS2G	25.58
**4169**	allXL	AA	373	386	BS2G	23.67
**4169**	allXL	AA	319	357	BS2G	19.71
**4169**	allXL	AA	367	373	BS2G	14.79
**4169**	allXL	AA	370	382	BS2G	23.7
**4169**	allXL	BB	376	382	BS2G	11.96
**4169**	allXL	BB	357	373	BS2G	30.68
**4169**	allXL	DD	320	366	BS2G	25.66
**4169**	allXL	AA	370	376	BS2G	17.11

**4169**	allXL	AA	320	357	DBSU	22.05
**4169**	allXL	AA	357	319	DBSU	19.71
**4169**	allXL	DD	372	319	DBSU	18.97
**4169**	allXL	DD	370	357	DBSU	25.95
**4169**	allXL	BB	382	357	DBSU	38.04
**4169**	allXL	AA	372	370	DBSU	6.25
**4169**	allXL	AA	381	370	DBSU	24.66
**4169**	allXL	AA	373	370	DBSU	9.16
**4169**	allXL	AA	381	372	DBSU	21.81
**4169**	allXL	BB	372	382	DBSU	20.18
**4169**	allXL	CC	372	386	DBSU	24.1
**4169**	allXL	AA	373	386	DBSU	23.67
**4169**	allXL	DA	381	386	DBSU	26.11
**4169**	allXL	BD;DB	357	357	DBSU	19.37
**4169**	allXL	DA	373	319	DBSU	15.32
**4169**	allXL	DA	372	357	DBSU	22.39
**4169**	allXL	DA	381	357	DBSU	29.19
**4169**	allXL	DA	373	357	DBSU	22.09
**4169**	allXL	DA	372	370	DBSU	28.61
**4169**	allXL	DA	373	370	DBSU	28.35
**4169**	allXL	DA	382	373	DBSU	19.61
**4170**	allXL	AA	320	357	BS2G	25.14
**4170**	allXL	BB	320	373	BS2G	16.01
**4170**	allXL	DD	373	382	BS2G	18.45
**4170**	allXL	DD	370	386	BS2G	20.18
**4170**	allXL	AA	373	386	BS2G	24.77
**4170**	allXL	AA	319	357	BS2G	23.7
**4170**	allXL	BB	367	373	BS2G	13.6
**4170**	allXL	DD	370	382	BS2G	16.0
**4170**	allXL	BB	376	382	BS2G	14.02
**4170**	allXL	DD	357	373	BS2G	26.17
**4170**	allXL	AA	320	366	BS2G	13.55
**4170**	allXL	BB	370	376	BS2G	16.27
**4170**	allXL	AA	320	357	DBSU	25.14

**4170**	allXL	AA	357	319	DBSU	23.7
**4170**	allXL	BB	372	319	DBSU	12.7
**4170**	allXL	BB	370	357	DBSU	24.88
**4170**	allXL	DD	382	357	DBSU	17.08
**4170**	allXL	AA	372	370	DBSU	6.21
**4170**	allXL	DD	381	370	DBSU	17.5
**4170**	allXL	AA	373	370	DBSU	9.12
**4170**	allXL	DD	381	372	DBSU	20.16
**4170**	allXL	DD	372	382	DBSU	20.01
**4170**	allXL	DD	372	386	DBSU	26.54
**4170**	allXL	AA	373	386	DBSU	24.77
**4170**	allXL	AD	381	386	DBSU	44.88
**4170**	allXL	BD;DB	357	357	DBSU	18.32
**4170**	allXL	CB	373	319	DBSU	16.08
**4170**	allXL	CB	372	357	DBSU	32.75
**4170**	allXL	DB	381	357	DBSU	27.68
**4170**	allXL	CB	373	357	DBSU	33.15
**4170**	allXL	DA	372	370	DBSU	23.16
**4170**	allXL	AD	373	370	DBSU	23.2
**4170**	allXL	CB	382	373	DBSU	34.53
**4171**	allXL	AA	320	357	BS2G	24.89
**4171**	allXL	CC	320	373	BS2G	21.38
**4171**	allXL	DD	373	382	BS2G	20.21
**4171**	allXL	BB	370	386	BS2G	17.74
**4171**	allXL	BB	373	386	BS2G	20.32
**4171**	allXL	AA	319	357	BS2G	24.29
**4171**	allXL	AA	367	373	BS2G	11.59
**4171**	allXL	DD	370	382	BS2G	21.92
**4171**	allXL	CC	376	382	BS2G	14.83
**4171**	allXL	AA	357	373	BS2G	29.68
**4171**	allXL	DD	320	366	BS2G	18.96
**4171**	allXL	CC	370	376	BS2G	15.84
**4171**	allXL	AA	320	357	DBSU	24.89
**4171**	allXL	AA	357	319	DBSU	24.29

**4171**	allXL	CC	372	319	DBSU	22.38
**4171**	allXL	DD	370	357	DBSU	25.13
**4171**	allXL	CC	382	357	DBSU	30.73
**4171**	allXL	AA	372	370	DBSU	6.35
**4171**	allXL	DD	381	370	DBSU	21.1
**4171**	allXL	AA	373	370	DBSU	9.04
**4171**	allXL	DD	381	372	DBSU	20.6
**4171**	allXL	DD	372	382	DBSU	21.38
**4171**	allXL	BB	372	386	DBSU	20.67
**4171**	allXL	BB	373	386	DBSU	20.32
**4171**	allXL	CA	381	386	DBSU	27.89
**4171**	allXL	BD;DB	357	357	DBSU	17.86
**4171**	allXL	AD	373	319	DBSU	23.38
**4171**	allXL	CB	372	357	DBSU	42.61
**4171**	allXL	AD	381	357	DBSU	29.07
**4171**	allXL	CB	373	357	DBSU	41.11
**4171**	allXL	BC	372	370	DBSU	22.91
**4171**	allXL	CB	373	370	DBSU	20.85
**4171**	allXL	CB	382	373	DBSU	26.26
**4172**	allXL	CC	320	357	BS2G	25.87
**4172**	allXL	BB	320	373	BS2G	24.43
**4172**	allXL	BB	373	382	BS2G	17.95
**4172**	allXL	AA	370	386	BS2G	25.6
**4172**	allXL	DD	373	386	BS2G	25.57
**4172**	allXL	CC	319	357	BS2G	24.43
**4172**	allXL	AA	367	373	BS2G	11.51
**4172**	allXL	AA	370	382	BS2G	22.2
**4172**	allXL	CC	376	382	BS2G	14.34
**4172**	allXL	AA	357	373	BS2G	31.64
**4172**	allXL	AA	320	366	BS2G	11.17
**4172**	allXL	DD	370	376	BS2G	16.56
**4172**	allXL	CC	320	357	DBSU	25.87
**4172**	allXL	CC	357	319	DBSU	24.43
**4172**	allXL	BB	372	319	DBSU	20.29

**4172**	allXL	BB	370	357	DBSU	28.64
**4172**	allXL	BB	382	357	DBSU	41.6
**4172**	allXL	AA	372	370	DBSU	6.37
**4172**	allXL	AA	381	370	DBSU	23.66
**4172**	allXL	AA	373	370	DBSU	9.07
**4172**	allXL	BB	381	372	DBSU	20.77
**4172**	allXL	BB	372	382	DBSU	19.82
**4172**	allXL	AA	372	386	DBSU	24.9
**4172**	allXL	DD	373	386	DBSU	25.57
**4172**	allXL	AD	381	386	DBSU	36.56
**4172**	allXL	BD;DB	357	357	DBSU	17.89
**4172**	allXL	CB	373	319	DBSU	31.53
**4172**	allXL	DA	372	357	DBSU	50.25
**4172**	allXL	AD	381	357	DBSU	60.77
**4172**	allXL	DA	373	357	DBSU	50.39
**4172**	allXL	CB	372	370	DBSU	37.71
**4172**	allXL	CB	373	370	DBSU	37.27
**4172**	allXL	AD	382	373	DBSU	30.48
**4173**	allXL	CC	320	357	BS2G	25.4
**4173**	allXL	AA	320	373	BS2G	25.25
**4173**	allXL	DD	373	382	BS2G	19.5
**4173**	allXL	DD	370	386	BS2G	25.67
**4173**	allXL	DD	373	386	BS2G	24.32
**4173**	allXL	AA	319	357	BS2G	27.06
**4173**	allXL	DD	367	373	BS2G	13.95
**4173**	allXL	BB	370	382	BS2G	23.76
**4173**	allXL	CC	376	382	BS2G	13.78
**4173**	allXL	CC	357	373	BS2G	18.37
**4173**	allXL	AA	320	366	BS2G	16.94
**4173**	allXL	BB	370	376	BS2G	16.02
**4173**	allXL	CC	320	357	DBSU	25.4
**4173**	allXL	AA	357	319	DBSU	27.06
**4173**	allXL	AA	372	319	DBSU	24.71
**4173**	allXL	CC	370	357	DBSU	24.0

**4173**	allXL	CC	382	357	DBSU	26.16
**4173**	allXL	AA	372	370	DBSU	6.1
**4173**	allXL	DD	381	370	DBSU	23.54
**4173**	allXL	DD	373	370	DBSU	9.03
**4173**	allXL	DD	381	372	DBSU	16.33
**4173**	allXL	DD	372	382	DBSU	17.93
**4173**	allXL	DD	372	386	DBSU	21.26
**4173**	allXL	DD	373	386	DBSU	24.32
**4173**	allXL	DA	381	386	DBSU	43.4
**4173**	allXL	BD;DB	357	357	DBSU	18.81
**4173**	allXL	AD	373	319	DBSU	35.53
**4173**	allXL	CA	372	357	DBSU	35.92
**4173**	allXL	CA	381	357	DBSU	22.91
**4173**	allXL	CA	373	357	DBSU	32.09
**4173**	allXL	AD	372	370	DBSU	39.44
**4173**	allXL	AD	373	370	DBSU	36.26
**4173**	allXL	AD	382	373	DBSU	42.86
**4174**	allXL	AA	320	357	BS2G	22.83
**4174**	allXL	DD	320	373	BS2G	25.2
**4174**	allXL	DD	373	382	BS2G	17.83
**4174**	allXL	DD	370	386	BS2G	24.49
**4174**	allXL	AA	373	386	BS2G	24.48
**4174**	allXL	AA	319	357	BS2G	21.05
**4174**	allXL	DD	367	373	BS2G	14.06
**4174**	allXL	DD	370	382	BS2G	21.98
**4174**	allXL	CC	376	382	BS2G	14.19
**4174**	allXL	DD	357	373	BS2G	30.82
**4174**	allXL	CC	320	366	BS2G	19.37
**4174**	allXL	BB	370	376	BS2G	17.3
**4174**	allXL	AA	320	357	DBSU	22.83
**4174**	allXL	AA	357	319	DBSU	21.05
**4174**	allXL	DD	372	319	DBSU	26.9
**4174**	allXL	CC	370	357	DBSU	26.08
**4174**	allXL	BA	382	357	DBSU	37.09

**4174**	allXL	AA	372	370	DBSU	6.0
**4174**	allXL	DD	381	370	DBSU	20.65
**4174**	allXL	DD	373	370	DBSU	9.14
**4174**	allXL	DD	381	372	DBSU	14.89
**4174**	allXL	DD	372	382	DBSU	16.74
**4174**	allXL	DD	372	386	DBSU	22.42
**4174**	allXL	AA	373	386	DBSU	24.48
**4174**	allXL	CA	381	386	DBSU	31.26
**4174**	allXL	BD;DB	357	357	DBSU	18.17
**4174**	allXL	BC	373	319	DBSU	18.22
**4174**	allXL	BC	372	357	DBSU	25.79
**4174**	allXL	BC	381	357	DBSU	25.81
**4174**	allXL	BC	373	357	DBSU	25.06
**4174**	allXL	BC	372	370	DBSU	34.91
**4174**	allXL	BC	373	370	DBSU	33.88
**4174**	allXL	CA	382	373	DBSU	32.18
**4175**	allXL	CC	320	357	BS2G	21.79
**4175**	allXL	BB	320	373	BS2G	15.9
**4175**	allXL	AA	373	382	BS2G	21.81
**4175**	allXL	CC	370	386	BS2G	28.1
**4175**	allXL	CC	373	386	BS2G	24.87
**4175**	allXL	CC	319	357	BS2G	22.48
**4175**	allXL	AA	367	373	BS2G	12.56
**4175**	allXL	CC	370	382	BS2G	27.71
**4175**	allXL	CC	376	382	BS2G	14.01
**4175**	allXL	BB	357	373	BS2G	28.42
**4175**	allXL	BB	320	366	BS2G	26.18
**4175**	allXL	BB;CC	370	376	BS2G	15.98
**4175**	allXL	CC	320	357	DBSU	21.79
**4175**	allXL	CC	357	319	DBSU	22.48
**4175**	allXL	BB	372	319	DBSU	18.16
**4175**	allXL	BB	370	357	DBSU	30.77
**4175**	allXL	BB	382	357	DBSU	36.66
**4175**	allXL	AA	372	370	DBSU	6.36

**4175**	allXL	CC	381	370	DBSU	26.66
**4175**	allXL	AA	373	370	DBSU	8.69
**4175**	allXL	DD	381	372	DBSU	23.62
**4175**	allXL	AA	372	382	DBSU	23.59
**4175**	allXL	CC	372	386	DBSU	25.61
**4175**	allXL	CC	373	386	DBSU	24.87
**4175**	allXL	CB	381	386	DBSU	61.98
**4175**	allXL	BD;DB	357	357	DBSU	18.27
**4175**	allXL	DA	373	319	DBSU	32.55
**4175**	allXL	BD	372	357	DBSU	45.3
**4175**	allXL	BC	381	357	DBSU	32.45
**4175**	allXL	BD	373	357	DBSU	46.11
**4175**	allXL	DA	372	370	DBSU	50.6
**4175**	allXL	AD	373	370	DBSU	53.65
**4175**	allXL	BC	382	373	DBSU	46.04
**4176**	allXL	CC	320	357	BS2G	24.06
**4176**	allXL	DD	320	373	BS2G	25.03
**4176**	allXL	CC	373	382	BS2G	20.99
**4176**	allXL	CC	370	386	BS2G	22.24
**4176**	allXL	DD	373	386	BS2G	26.46
**4176**	allXL	AA	319	357	BS2G	26.33
**4176**	allXL	BB	367	373	BS2G	13.61
**4176**	allXL	CC	370	382	BS2G	17.94
**4176**	allXL	CC	376	382	BS2G	14.76
**4176**	allXL	BB	357	373	BS2G	17.63
**4176**	allXL	AA	320	366	BS2G	25.8
**4176**	allXL	CC	370	376	BS2G	14.62
**4176**	allXL	CC	320	357	DBSU	24.06
**4176**	allXL	AA	357	319	DBSU	26.33
**4176**	allXL	DD	372	319	DBSU	26.9
**4176**	allXL	DD	370	357	DBSU	23.03
**4176**	allXL	CC	382	357	DBSU	22.05
**4176**	allXL	AA	372	370	DBSU	6.29
**4176**	allXL	CC	381	370	DBSU	19.99

**4176**	allXL	AA	373	370	DBSU	8.95
**4176**	allXL	CC	381	372	DBSU	22.6
**4176**	allXL	CC	372	382	DBSU	21.38
**4176**	allXL	CC	372	386	DBSU	28.03
**4176**	allXL	DD	373	386	DBSU	26.46
**4176**	allXL	DB	381	386	DBSU	54.13
**4176**	allXL	BD;DB	357	357	DBSU	18.03
**4176**	allXL	CB	373	319	DBSU	25.16
**4176**	allXL	BD	372	357	DBSU	36.79
**4176**	allXL	BD	381	357	DBSU	28.76
**4176**	allXL	BD	373	357	DBSU	33.74
**4176**	allXL	AD	372	370	DBSU	47.82
**4176**	allXL	AD	373	370	DBSU	48.54
**4176**	allXL	CB	382	373	DBSU	46.88
**4177**	allXL	CC	320	357	BS2G	25.88
**4177**	allXL	BB	320	373	BS2G	27.84
**4177**	allXL	BB	373	382	BS2G	16.71
**4177**	allXL	BB	370	386	BS2G	20.84
**4177**	allXL	AA	373	386	BS2G	25.95
**4177**	allXL	CC	319	357	BS2G	26.78
**4177**	allXL	AA	367	373	BS2G	14.54
**4177**	allXL	BB	370	382	BS2G	15.16
**4177**	allXL	CC	376	382	BS2G	14.76
**4177**	allXL	DD	357	373	BS2G	30.75
**4177**	allXL	DD	320	366	BS2G	25.2
**4177**	allXL	BB	370	376	BS2G	15.98
**4177**	allXL	CC	320	357	DBSU	25.88
**4177**	allXL	CC	357	319	DBSU	26.78
**4177**	allXL	BB	372	319	DBSU	30.05
**4177**	allXL	DD	370	357	DBSU	29.17
**4177**	allXL	AA	382	357	DBSU	28.71
**4177**	allXL	AA	372	370	DBSU	6.32
**4177**	allXL	BB	381	370	DBSU	14.42
**4177**	allXL	CC	373	370	DBSU	9.03

**4177**	allXL	BB	381	372	DBSU	16.24
**4177**	allXL	BB	372	382	DBSU	17.01
**4177**	allXL	BB	372	386	DBSU	25.49
**4177**	allXL	AA	373	386	DBSU	25.95
**4177**	allXL	AD	381	386	DBSU	60.53
**4177**	allXL	BD;DB	357	357	DBSU	17.74
**4177**	allXL	DA	373	319	DBSU	25.21
**4177**	allXL	BC	372	357	DBSU	43.28
**4177**	allXL	AD	381	357	DBSU	41.17
**4177**	allXL	DB	373	357	DBSU	41.47
**4177**	allXL	AD	372	370	DBSU	35.47
**4177**	allXL	AD	373	370	DBSU	34.72
**4177**	allXL	AD	382	373	DBSU	41.52
**4178**	allXL	AA	320	357	BS2G	24.46
**4178**	allXL	AA	320	373	BS2G	32.73
**4178**	allXL	AA	373	382	BS2G	16.38
**4178**	allXL	BB	370	386	BS2G	21.2
**4178**	allXL	AA	373	386	BS2G	22.34
**4178**	allXL	AA	319	357	BS2G	24.69
**4178**	allXL	AA	367	373	BS2G	13.19
**4178**	allXL	BB	370	382	BS2G	19.05
**4178**	allXL	BB	376	382	BS2G	14.17
**4178**	allXL	DD	357	373	BS2G	18.14
**4178**	allXL	AA	320	366	BS2G	22.03
**4178**	allXL	CC	370	376	BS2G	15.2
**4178**	allXL	AA	320	357	DBSU	24.46
**4178**	allXL	AA	357	319	DBSU	24.69
**4178**	allXL	AA	372	319	DBSU	31.61
**4178**	allXL	DD	370	357	DBSU	22.31
**4178**	allXL	DD	382	357	DBSU	23.33
**4178**	allXL	AA	372	370	DBSU	6.84
**4178**	allXL	BB	381	370	DBSU	21.13
**4178**	allXL	DD	373	370	DBSU	9.01
**4178**	allXL	DD	381	372	DBSU	18.49

**4178**	allXL	DD	372	382	DBSU	18.78
**4178**	allXL	BB	372	386	DBSU	24.29
**4178**	allXL	AA	373	386	DBSU	22.34
**4178**	allXL	DB	381	386	DBSU	36.13
**4178**	allXL	BD;DB	357	357	DBSU	18.45
**4178**	allXL	CB	373	319	DBSU	11.51
**4178**	allXL	AC	372	357	DBSU	30.24
**4178**	allXL	DB	381	357	DBSU	31.5
**4178**	allXL	AC	373	357	DBSU	29.43
**4178**	allXL	CB	372	370	DBSU	32.23
**4178**	allXL	BC	373	370	DBSU	34.8
**4178**	allXL	DA	382	373	DBSU	41.01
**4179**	allXL	CC	320	357	BS2G	18.88
**4179**	allXL	AA	320	373	BS2G	21.86
**4179**	allXL	BB	373	382	BS2G	17.5
**4179**	allXL	AA	370	386	BS2G	26.19
**4179**	allXL	BB	373	386	BS2G	26.43
**4179**	allXL	CC	319	357	BS2G	21.68
**4179**	allXL	BB	367	373	BS2G	12.89
**4179**	allXL	BB	370	382	BS2G	23.5
**4179**	allXL	BB	376	382	BS2G	14.02
**4179**	allXL	BB	357	373	BS2G	29.32
**4179**	allXL	AA	320	366	BS2G	22.25
**4179**	allXL	CC	370	376	BS2G	16.29
**4179**	allXL	CC	320	357	DBSU	18.88
**4179**	allXL	CC	357	319	DBSU	21.68
**4179**	allXL	AA	372	319	DBSU	23.51
**4179**	allXL	DD	370	357	DBSU	32.14
**4179**	allXL	BB	382	357	DBSU	16.54
**4179**	allXL	AA	372	370	DBSU	6.19
**4179**	allXL	AA	381	370	DBSU	23.03
**4179**	allXL	AA	373	370	DBSU	9.09
**4179**	allXL	BB	381	372	DBSU	18.94
**4179**	allXL	BB	372	382	DBSU	18.81

**4179**	allXL	AA	372	386	DBSU	26.44
**4179**	allXL	BB	373	386	DBSU	26.43
**4179**	allXL	AD	381	386	DBSU	22.56
**4179**	allXL	BD;DB	357	357	DBSU	19.3
**4179**	allXL	CB	373	319	DBSU	13.93
**4179**	allXL	AD	372	357	DBSU	34.5
**4179**	allXL	BD	381	357	DBSU	33.67
**4179**	allXL	AD	373	357	DBSU	36.43
**4179**	allXL	AD	372	370	DBSU	17.34
**4179**	allXL	AD	373	370	DBSU	16.25
**4179**	allXL	AD	382	373	DBSU	15.39
**4180**	allXL	AA	320	357	BS2G	22.51
**4180**	allXL	AA	320	373	BS2G	13.07
**4180**	allXL	AA	373	382	BS2G	17.62
**4180**	allXL	BB	370	386	BS2G	16.91
**4180**	allXL	AA	373	386	BS2G	22.97
**4180**	allXL	AA	319	357	BS2G	20.46
**4180**	allXL	AA	367	373	BS2G	10.42
**4180**	allXL	BB	370	382	BS2G	17.51
**4180**	allXL	CC	376	382	BS2G	13.35
**4180**	allXL	AA	357	373	BS2G	28.65
**4180**	allXL	AA	320	366	BS2G	17.2
**4180**	allXL	BB	370	376	BS2G	15.26
**4180**	allXL	AA	320	357	DBSU	22.51
**4180**	allXL	AA	357	319	DBSU	20.46
**4180**	allXL	AA	372	319	DBSU	13.77
**4180**	allXL	BB	370	357	DBSU	28.31
**4180**	allXL	AA	382	357	DBSU	40.43
**4180**	allXL	CC	372	370	DBSU	6.66
**4180**	allXL	AA	381	370	DBSU	15.32
**4180**	allXL	AA	373	370	DBSU	8.33
**4180**	allXL	AA	381	372	DBSU	17.79
**4180**	allXL	DD	372	382	DBSU	19.65
**4180**	allXL	BB	372	386	DBSU	23.08

**4180**	allXL	AA	373	386	DBSU	22.97
**4180**	allXL	CB	381	386	DBSU	30.03
**4180**	allXL	BD;DB	357	357	DBSU	19.0
**4180**	allXL	DA	373	319	DBSU	20.36
**4180**	allXL	BC	372	357	DBSU	23.64
**4180**	allXL	BC	381	357	DBSU	27.02
**4180**	allXL	BC	373	357	DBSU	20.02
**4180**	allXL	DA	372	370	DBSU	15.81
**4180**	allXL	DA	373	370	DBSU	14.35
**4180**	allXL	BC	382	373	DBSU	23.72
**4181**	allXL	AA	320	357	BS2G	25.66
**4181**	allXL	CC	320	373	BS2G	19.81
**4181**	allXL	BB	373	382	BS2G	18.59
**4181**	allXL	DD	370	386	BS2G	22.47
**4181**	allXL	AA	373	386	BS2G	23.63
**4181**	allXL	AA	319	357	BS2G	28.86
**4181**	allXL	AA	367	373	BS2G	13.01
**4181**	allXL	BB	370	382	BS2G	20.43
**4181**	allXL	BB	376	382	BS2G	13.2
**4181**	allXL	DD	357	373	BS2G	19.41
**4181**	allXL	CC	320	366	BS2G	16.67
**4181**	allXL	DD	370	376	BS2G	15.22
**4181**	allXL	AA	320	357	DBSU	25.66
**4181**	allXL	AA	357	319	DBSU	28.86
**4181**	allXL	CC	372	319	DBSU	17.59
**4181**	allXL	DD	370	357	DBSU	25.46
**4181**	allXL	DD	382	357	DBSU	18.11
**4181**	allXL	AA	372	370	DBSU	6.34
**4181**	allXL	DD	381	370	DBSU	19.82
**4181**	allXL	AA	373	370	DBSU	9.23
**4181**	allXL	BB	381	372	DBSU	21.78
**4181**	allXL	BB	372	382	DBSU	21.43
**4181**	allXL	AA	372	386	DBSU	22.3
**4181**	allXL	AA	373	386	DBSU	23.63

**4181**	allXL	AD	381	386	DBSU	42.04
**4181**	allXL	BD;DB	357	357	DBSU	18.77
**4181**	allXL	AD	373	319	DBSU	38.04
**4181**	allXL	DB	372	357	DBSU	40.9
**4181**	allXL	DB	381	357	DBSU	31.05
**4181**	allXL	DB	373	357	DBSU	37.35
**4181**	allXL	AD	372	370	DBSU	38.98
**4181**	allXL	AD	373	370	DBSU	42.25
**4181**	allXL	DA	382	373	DBSU	37.45
**4182**	allXL	AA	320	357	BS2G	26.41
**4182**	allXL	DD	320	373	BS2G	18.45
**4182**	allXL	AA	373	382	BS2G	19.45
**4182**	allXL	AA	370	386	BS2G	21.07
**4182**	allXL	CC	373	386	BS2G	23.56
**4182**	allXL	AA	319	357	BS2G	27.29
**4182**	allXL	AA	367	373	BS2G	13.3
**4182**	allXL	AA	370	382	BS2G	21.09
**4182**	allXL	CC	376	382	BS2G	13.52
**4182**	allXL	BB	357	373	BS2G	26.09
**4182**	allXL	BB	320	366	BS2G	24.02
**4182**	allXL	CC	370	376	BS2G	16.14
**4182**	allXL	AA	320	357	DBSU	26.41
**4182**	allXL	AA	357	319	DBSU	27.29
**4182**	allXL	DD	372	319	DBSU	18.79
**4182**	allXL	DD	370	357	DBSU	23.8
**4182**	allXL	BB	382	357	DBSU	36.93
**4182**	allXL	AA	372	370	DBSU	6.42
**4182**	allXL	AA	381	370	DBSU	21.76
**4182**	allXL	AA	373	370	DBSU	8.89
**4182**	allXL	DD	381	372	DBSU	20.38
**4182**	allXL	AA	372	382	DBSU	20.71
**4182**	allXL	CC	372	386	DBSU	23.97
**4182**	allXL	CC	373	386	DBSU	23.56
**4182**	allXL	DA	381	386	DBSU	59.91

**4182**	allXL	BD;DB	357	357	DBSU	18.8
**4182**	allXL	AD	373	319	DBSU	26.5
**4182**	allXL	BD	372	357	DBSU	43.98
**4182**	allXL	CA	381	357	DBSU	52.27
**4182**	allXL	BD	373	357	DBSU	44.4
**4182**	allXL	DA	372	370	DBSU	29.75
**4182**	allXL	DA	373	370	DBSU	27.29
**4182**	allXL	DA	382	373	DBSU	41.81
**4183**	allXL	AA	320	357	BS2G	25.96
**4183**	allXL	BB	320	373	BS2G	16.83
**4183**	allXL	CC	373	382	BS2G	16.98
**4183**	allXL	DD	370	386	BS2G	20.27
**4183**	allXL	DD	373	386	BS2G	22.45
**4183**	allXL	AA	319	357	BS2G	27.87
**4183**	allXL	DD	367	373	BS2G	12.51
**4183**	allXL	DD	370	382	BS2G	20.77
**4183**	allXL	CC	376	382	BS2G	14.78
**4183**	allXL	BB	357	373	BS2G	13.0
**4183**	allXL	DD	320	366	BS2G	21.77
**4183**	allXL	CC	370	376	BS2G	16.55
**4183**	allXL	AA	320	357	DBSU	25.96
**4183**	allXL	AA	357	319	DBSU	27.87
**4183**	allXL	BB	372	319	DBSU	18.66
**4183**	allXL	BB	370	357	DBSU	22.34
**4183**	allXL	BB	382	357	DBSU	24.71
**4183**	allXL	AA	372	370	DBSU	6.27
**4183**	allXL	DD	381	370	DBSU	20.37
**4183**	allXL	AA	373	370	DBSU	8.82
**4183**	allXL	DD	381	372	DBSU	15.96
**4183**	allXL	DD	372	382	DBSU	17.61
**4183**	allXL	DD	372	386	DBSU	21.23
**4183**	allXL	DD	373	386	DBSU	22.45
**4183**	allXL	AD	381	386	DBSU	39.21
**4183**	allXL	BD;DB	357	357	DBSU	18.12

**4183**	allXL	CB	373	319	DBSU	11.84
**4183**	allXL	BD	372	357	DBSU	33.51
**4183**	allXL	BD	381	357	DBSU	36.85
**4183**	allXL	BD	373	357	DBSU	30.66
**4183**	allXL	AD	372	370	DBSU	23.05
**4183**	allXL	AD	373	370	DBSU	26.03
**4183**	allXL	DA	382	373	DBSU	42.69
**4184**	allXL	AA	320	357	BS2G	25.8
**4184**	allXL	DD	320	373	BS2G	8.9
**4184**	allXL	AA	373	382	BS2G	17.27
**4184**	allXL	AA	370	386	BS2G	18.61
**4184**	allXL	AA	373	386	BS2G	16.85
**4184**	allXL	AA	319	357	BS2G	25.3
**4184**	allXL	AA	367	373	BS2G	13.06
**4184**	allXL	DD	370	382	BS2G	13.46
**4184**	allXL	CC	376	382	BS2G	14.12
**4184**	allXL	BB	357	373	BS2G	18.42
**4184**	allXL	AA	320	366	BS2G	20.49
**4184**	allXL	DD	370	376	BS2G	14.06
**4184**	allXL	AA	320	357	DBSU	25.8
**4184**	allXL	AA	357	319	DBSU	25.3
**4184**	allXL	DD	372	319	DBSU	10.86
**4184**	allXL	BB	370	357	DBSU	24.36
**4184**	allXL	BB	382	357	DBSU	21.79
**4184**	allXL	AA	372	370	DBSU	6.17
**4184**	allXL	DD	381	370	DBSU	14.39
**4184**	allXL	CC	373	370	DBSU	8.9
**4184**	allXL	AA	381	372	DBSU	18.51
**4184**	allXL	DD	372	382	DBSU	19.5
**4184**	allXL	AA	372	386	DBSU	18.46
**4184**	allXL	AA	373	386	DBSU	16.85
**4184**	allXL	DA	381	386	DBSU	22.77
**4184**	allXL	BD;DB	357	357	DBSU	19.23
**4184**	allXL	AD	373	319	DBSU	25.92

**4184**	allXL	BD	372	357	DBSU	19.21
**4184**	allXL	BD	381	357	DBSU	14.97
**4184**	allXL	BD	373	357	DBSU	16.23
**4184**	allXL	AD	372	370	DBSU	24.24
**4184**	allXL	AD	373	370	DBSU	21.02
**4184**	allXL	AD	382	373	DBSU	19.76
**4185**	allXL	CC	320	357	BS2G	24.92
**4185**	allXL	DD	320	373	BS2G	14.75
**4185**	allXL	CC	373	382	BS2G	19.82
**4185**	allXL	DD	370	386	BS2G	20.54
**4185**	allXL	AA	373	386	BS2G	22.33
**4185**	allXL	CC	319	357	BS2G	23.04
**4185**	allXL	BB	367	373	BS2G	13.91
**4185**	allXL	DD	370	382	BS2G	23.11
**4185**	allXL	CC	376	382	BS2G	13.89
**4185**	allXL	AA	357	373	BS2G	14.11
**4185**	allXL	DD	320	366	BS2G	22.25
**4185**	allXL	BB	370	376	BS2G	16.05
**4185**	allXL	CC	320	357	DBSU	24.92
**4185**	allXL	CC	357	319	DBSU	23.04
**4185**	allXL	DD	372	319	DBSU	16.95
**4185**	allXL	AA	370	357	DBSU	17.31
**4185**	allXL	AA	382	357	DBSU	20.78
**4185**	allXL	AA	372	370	DBSU	6.15
**4185**	allXL	DD	381	370	DBSU	22.42
**4185**	allXL	AA	373	370	DBSU	9.22
**4185**	allXL	DD	381	372	DBSU	22.19
**4185**	allXL	AA;CC	372	382	DBSU	23.36
**4185**	allXL	AA	372	386	DBSU	23.72
**4185**	allXL	AA	373	386	DBSU	22.33
**4185**	allXL	DB	381	386	DBSU	47.35
**4185**	allXL	BD;DB	357	357	DBSU	18.28
**4185**	allXL	AC	373	319	DBSU	39.77
**4185**	allXL	AC	372	357	DBSU	20.43

**4185**	allXL	AC	381	357	DBSU	23.26
**4185**	allXL	AC	373	357	DBSU	17.17
**4185**	allXL	AC	372	370	DBSU	49.67
**4185**	allXL	AC	373	370	DBSU	48.01
**4185**	allXL	BD	382	373	DBSU	47.92
**4186**	allXL	CC	320	357	BS2G	24.91
**4186**	allXL	DD	320	373	BS2G	17.75
**4186**	allXL	BB	373	382	BS2G	18.53
**4186**	allXL	BB	370	386	BS2G	25.01
**4186**	allXL	AA	373	386	BS2G	25.97
**4186**	allXL	CC	319	357	BS2G	26.82
**4186**	allXL	AA	367	373	BS2G	11.38
**4186**	allXL	BB	370	382	BS2G	17.51
**4186**	allXL	CC	376	382	BS2G	14.13
**4186**	allXL	AA	357	373	BS2G	28.32
**4186**	allXL	CC	320	366	BS2G	23.5
**4186**	allXL	BB	370	376	BS2G	15.81
**4186**	allXL	CC	320	357	DBSU	24.91
**4186**	allXL	CC	357	319	DBSU	26.82
**4186**	allXL	DD	372	319	DBSU	23.67
**4186**	allXL	DD	370	357	DBSU	28.27
**4186**	allXL	BB	382	357	DBSU	31.19
**4186**	allXL	CC	372	370	DBSU	6.55
**4186**	allXL	BB	381	370	DBSU	19.92
**4186**	allXL	AA	373	370	DBSU	9.16
**4186**	allXL	BB	381	372	DBSU	20.63
**4186**	allXL	BB	372	382	DBSU	19.38
**4186**	allXL	AA	372	386	DBSU	26.64
**4186**	allXL	AA	373	386	DBSU	25.97
**4186**	allXL	CB	381	386	DBSU	43.16
**4186**	allXL	BD;DB	357	357	DBSU	17.7
**4186**	allXL	AD	373	319	DBSU	24.75
**4186**	allXL	CB	372	357	DBSU	25.61
**4186**	allXL	CB	381	357	DBSU	34.35

**4186**	allXL	CB	373	357	DBSU	24.68
**4186**	allXL	DA	372	370	DBSU	37.97
**4186**	allXL	DA	373	370	DBSU	34.39
**4186**	allXL	CB	382	373	DBSU	38.71
**4187**	allXL	CC	320	357	BS2G	25.4
**4187**	allXL	DD	320	373	BS2G	24.8
**4187**	allXL	DD	373	382	BS2G	21.53
**4187**	allXL	DD	370	386	BS2G	28.7
**4187**	allXL	AA	373	386	BS2G	26.79
**4187**	allXL	AA	319	357	BS2G	24.43
**4187**	allXL	DD	367	373	BS2G	13.34
**4187**	allXL	DD	370	382	BS2G	17.96
**4187**	allXL	CC	376	382	BS2G	15.26
**4187**	allXL	BB	357	373	BS2G	17.37
**4187**	allXL	AA	320	366	BS2G	17.68
**4187**	allXL	DD	370	376	BS2G	15.67
**4187**	allXL	CC	320	357	DBSU	25.4
**4187**	allXL	AA	357	319	DBSU	24.43
**4187**	allXL	AA	372	319	DBSU	24.55
**4187**	allXL	BB	370	357	DBSU	24.96
**4187**	allXL	DD	382	357	DBSU	19.97
**4187**	allXL	AA	372	370	DBSU	6.03
**4187**	allXL	DD	381	370	DBSU	17.52
**4187**	allXL	AA	373	370	DBSU	9.11
**4187**	allXL	DD	381	372	DBSU	21.24
**4187**	allXL	DD	372	382	DBSU	22.75
**4187**	allXL	AA	372	386	DBSU	28.0
**4187**	allXL	AA	373	386	DBSU	26.79
**4187**	allXL	CB	381	386	DBSU	53.7
**4187**	allXL	BD;DB	357	357	DBSU	18.51
**4187**	allXL	AD	373	319	DBSU	27.15
**4187**	allXL	BD	372	357	DBSU	34.12
**4187**	allXL	DB	381	357	DBSU	31.17
**4187**	allXL	BD	373	357	DBSU	34.26

**4187**	allXL	DA	372	370	DBSU	26.37
**4187**	allXL	DA	373	370	DBSU	24.59
**4187**	allXL	AD	382	373	DBSU	37.81
**4188**	allXL	CC	320	357	BS2G	21.37
**4188**	allXL	BB	320	373	BS2G	21.67
**4188**	allXL	AA	373	382	BS2G	16.69
**4188**	allXL	AA	370	386	BS2G	20.12
**4188**	allXL	BB	373	386	BS2G	19.28
**4188**	allXL	CC	319	357	BS2G	20.9
**4188**	allXL	AA	367	373	BS2G	10.5
**4188**	allXL	AA	370	382	BS2G	20.07
**4188**	allXL	BB	376	382	BS2G	13.14
**4188**	allXL	CC	357	373	BS2G	23.47
**4188**	allXL	BB	320	366	BS2G	20.2
**4188**	allXL	CC	370	376	BS2G	16.59
**4188**	allXL	CC	320	357	DBSU	21.37
**4188**	allXL	CC	357	319	DBSU	20.9
**4188**	allXL	BB	372	319	DBSU	23.93
**4188**	allXL	BB	370	357	DBSU	23.26
**4188**	allXL	AA	382	357	DBSU	19.65
**4188**	allXL	AA	372	370	DBSU	6.47
**4188**	allXL	AA	381	370	DBSU	23.16
**4188**	allXL	AA	373	370	DBSU	8.88
**4188**	allXL	DD	381	372	DBSU	16.58
**4188**	allXL	DD	372	382	DBSU	19.04
**4188**	allXL	BB	372	386	DBSU	19.67
**4188**	allXL	BB	373	386	DBSU	19.28
**4188**	allXL	BC	381	386	DBSU	39.0
**4188**	allXL	BD;DB	357	357	DBSU	19.1
**4188**	allXL	AD	373	319	DBSU	14.46
**4188**	allXL	AD	372	357	DBSU	30.96
**4188**	allXL	CA	381	357	DBSU	19.15
**4188**	allXL	AD	373	357	DBSU	30.32
**4188**	allXL	AD	372	370	DBSU	32.65

**4188**	allXL	BC	373	370	DBSU	34.4
**4188**	allXL	CA	382	373	DBSU	35.99
**4189**	allXL	AA	320	357	BS2G	25.8
**4189**	allXL	BB	320	373	BS2G	25.34
**4189**	allXL	CC	373	382	BS2G	18.86
**4189**	allXL	CC	370	386	BS2G	29.09
**4189**	allXL	CC	373	386	BS2G	23.01
**4189**	allXL	AA	319	357	BS2G	28.95
**4189**	allXL	BB	367	373	BS2G	13.01
**4189**	allXL	DD	370	382	BS2G	20.4
**4189**	allXL	CC	376	382	BS2G	13.16
**4189**	allXL	DD	357	373	BS2G	28.45
**4189**	allXL	BB	320	366	BS2G	25.48
**4189**	allXL	DD	370	376	BS2G	14.8
**4189**	allXL	AA	320	357	DBSU	25.8
**4189**	allXL	AA	357	319	DBSU	28.95
**4189**	allXL	BB	372	319	DBSU	26.05
**4189**	allXL	DD	370	357	DBSU	28.31
**4189**	allXL	BB	382	357	DBSU	39.65
**4189**	allXL	AA	372	370	DBSU	6.02
**4189**	allXL	DD	381	370	DBSU	17.53
**4189**	allXL	AA	373	370	DBSU	9.11
**4189**	allXL	DD	381	372	DBSU	18.41
**4189**	allXL	CC	372	382	DBSU	20.38
**4189**	allXL	CC	372	386	DBSU	23.36
**4189**	allXL	CC	373	386	DBSU	23.01
**4189**	allXL	BC	381	386	DBSU	64.61
**4189**	allXL	BD;DB	357	357	DBSU	19.69
**4189**	allXL	AD	373	319	DBSU	33.77
**4189**	allXL	DB	372	357	DBSU	39.07
**4189**	allXL	CB	381	357	DBSU	53.55
**4189**	allXL	DB	373	357	DBSU	41.7
**4189**	allXL	CB	372	370	DBSU	31.78
**4189**	allXL	CB	373	370	DBSU	32.64

**4189**	allXL	CB	382	373	DBSU	34.68
**4190**	allXL	AA	320	357	BS2G	19.35
**4190**	allXL	BB	320	373	BS2G	18.25
**4190**	allXL	CC	373	382	BS2G	20.5
**4190**	allXL	CC	370	386	BS2G	26.24
**4190**	allXL	AA	373	386	BS2G	24.25
**4190**	allXL	AA	319	357	BS2G	19.13
**4190**	allXL	AA	367	373	BS2G	13.03
**4190**	allXL	CC	370	382	BS2G	17.71
**4190**	allXL	CC	376	382	BS2G	14.72
**4190**	allXL	BB	357	373	BS2G	32.04
**4190**	allXL	CC	320	366	BS2G	15.81
**4190**	allXL	CC	370	376	BS2G	12.33
**4190**	allXL	AA	320	357	DBSU	19.35
**4190**	allXL	AA	357	319	DBSU	19.13
**4190**	allXL	BB	372	319	DBSU	19.2
**4190**	allXL	BB	370	357	DBSU	29.12
**4190**	allXL	CC	382	357	DBSU	34.32
**4190**	allXL	AA	372	370	DBSU	6.33
**4190**	allXL	CC	381	370	DBSU	19.68
**4190**	allXL	AA	373	370	DBSU	9.14
**4190**	allXL	CC	381	372	DBSU	22.07
**4190**	allXL	CC	372	382	DBSU	21.1
**4190**	allXL	AA	372	386	DBSU	24.11
**4190**	allXL	AA	373	386	DBSU	24.25
**4190**	allXL	CB	381	386	DBSU	38.85
**4190**	allXL	BD;DB	357	357	DBSU	19.37
**4190**	allXL	AD	373	319	DBSU	23.75
**4190**	allXL	DA	372	357	DBSU	42.6
**4190**	allXL	BC	381	357	DBSU	43.79
**4190**	allXL	DA	373	357	DBSU	40.42
**4190**	allXL	DA	372	370	DBSU	24.01
**4190**	allXL	DA	373	370	DBSU	23.3
**4190**	allXL	DA	382	373	DBSU	48.76

**4191**	allXL	AA	320	357	BS2G	24.91
**4191**	allXL	BB	320	373	BS2G	15.58
**4191**	allXL	BB	373	382	BS2G	18.5
**4191**	allXL	DD	370	386	BS2G	19.35
**4191**	allXL	BB	373	386	BS2G	23.45
**4191**	allXL	AA	319	357	BS2G	22.86
**4191**	allXL	DD	367	373	BS2G	15.03
**4191**	allXL	DD	370	382	BS2G	15.86
**4191**	allXL	CC	376	382	BS2G	14.42
**4191**	allXL	BB	357	373	BS2G	28.94
**4191**	allXL	AA	320	366	BS2G	14.68
**4191**	allXL	DD	370	376	BS2G	16.13
**4191**	allXL	AA	320	357	DBSU	24.91
**4191**	allXL	AA	357	319	DBSU	22.86
**4191**	allXL	AA	372	319	DBSU	16.22
**4191**	allXL	BB	370	357	DBSU	30.3
**4191**	allXL	DD	382	357	DBSU	30.6
**4191**	allXL	AA	372	370	DBSU	6.16
**4191**	allXL	DD	381	370	DBSU	16.73
**4191**	allXL	AA	373	370	DBSU	9.24
**4191**	allXL	BB	381	372	DBSU	19.66
**4191**	allXL	DD	372	382	DBSU	20.65
**4191**	allXL	DD	372	386	DBSU	24.33
**4191**	allXL	BB	373	386	DBSU	23.45
**4191**	allXL	AD	381	386	DBSU	41.76
**4191**	allXL	BD;DB	357	357	DBSU	18.81
**4191**	allXL	DA	373	319	DBSU	33.85
**4191**	allXL	CB	372	357	DBSU	31.67
**4191**	allXL	DB	381	357	DBSU	34.76
**4191**	allXL	CB	373	357	DBSU	32.74
**4191**	allXL	CB	372	370	DBSU	15.68
**4191**	allXL	CB	373	370	DBSU	19.02
**4191**	allXL	AD	382	373	DBSU	34.75
**4192**	allXL	AA	320	357	BS2G	23.3

**4192**	allXL	DD	320	373	BS2G	15.76
**4192**	allXL	DD	373	382	BS2G	13.19
**4192**	allXL	DD	370	386	BS2G	15.67
**4192**	allXL	DD	373	386	BS2G	17.72
**4192**	allXL	AA	319	357	BS2G	24.76
**4192**	allXL	AA	367	373	BS2G	12.19
**4192**	allXL	DD	370	382	BS2G	14.66
**4192**	allXL	CC	376	382	BS2G	14.34
**4192**	allXL	BB	357	373	BS2G	21.91
**4192**	allXL	DD	320	366	BS2G	31.05
**4192**	allXL	BB	370	376	BS2G	14.0
**4192**	allXL	AA	320	357	DBSU	23.3
**4192**	allXL	AA	357	319	DBSU	24.76
**4192**	allXL	DD	372	319	DBSU	21.26
**4192**	allXL	DD	370	357	DBSU	24.93
**4192**	allXL	DD	382	357	DBSU	20.46
**4192**	allXL	AA	372	370	DBSU	6.41
**4192**	allXL	DD	381	370	DBSU	16.63
**4192**	allXL	AA	373	370	DBSU	9.0
**4192**	allXL	DD	381	372	DBSU	14.11
**4192**	allXL	DD	372	382	DBSU	12.07
**4192**	allXL	DD	372	386	DBSU	14.53
**4192**	allXL	DD	373	386	DBSU	17.72
**4192**	allXL	DB	381	386	DBSU	38.46
**4192**	allXL	BD;DB	357	357	DBSU	18.66
**4192**	allXL	BC	373	319	DBSU	29.14
**4192**	allXL	BD	372	357	DBSU	29.89
**4192**	allXL	DB	381	357	DBSU	34.58
**4192**	allXL	BD	373	357	DBSU	29.38
**4192**	allXL	CB	372	370	DBSU	49.77
**4192**	allXL	BD	373	370	DBSU	49.62
**4192**	allXL	DB	382	373	DBSU	43.9
**4193**	allXL	AA	320	357	BS2G	20.4
**4193**	allXL	BB	320	373	BS2G	18.76

**4193**	allXL	DD	373	382	BS2G	19.45
**4193**	allXL	DD	370	386	BS2G	26.17
**4193**	allXL	DD	373	386	BS2G	24.03
**4193**	allXL	AA	319	357	BS2G	20.88
**4193**	allXL	AA	367	373	BS2G	13.07
**4193**	allXL	DD	370	382	BS2G	22.78
**4193**	allXL	BB	376	382	BS2G	15.05
**4193**	allXL	BB	357	373	BS2G	18.24
**4193**	allXL	BB	320	366	BS2G	16.12
**4193**	allXL	CC	370	376	BS2G	16.29
**4193**	allXL	AA	320	357	DBSU	20.4
**4193**	allXL	AA	357	319	DBSU	20.88
**4193**	allXL	BB	372	319	DBSU	18.9
**4193**	allXL	DD	370	357	DBSU	24.87
**4193**	allXL	DD	382	357	DBSU	35.11
**4193**	allXL	AA	372	370	DBSU	6.17
**4193**	allXL	DD	381	370	DBSU	23.12
**4193**	allXL	DD	373	370	DBSU	8.94
**4193**	allXL	DD	381	372	DBSU	19.67
**4193**	allXL	DD	372	382	DBSU	20.03
**4193**	allXL	DD	372	386	DBSU	22.77
**4193**	allXL	DD	373	386	DBSU	24.03
**4193**	allXL	BD	381	386	DBSU	51.1
**4193**	allXL	BD;DB	357	357	DBSU	18.62
**4193**	allXL	AD	373	319	DBSU	27.2
**4193**	allXL	BD	372	357	DBSU	32.4
**4193**	allXL	BD	381	357	DBSU	34.61
**4193**	allXL	BD	373	357	DBSU	30.09
**4193**	allXL	BD	372	370	DBSU	44.15
**4193**	allXL	BD	373	370	DBSU	41.46
**4193**	allXL	BD	382	373	DBSU	35.69
**4194**	allXL	CC	320	357	BS2G	24.02
**4194**	allXL	CC	320	373	BS2G	31.08
**4194**	allXL	DD	373	382	BS2G	18.76

**4194**	allXL	DD	370	386	BS2G	25.73
**4194**	allXL	DD	373	386	BS2G	27.6
**4194**	allXL	CC	319	357	BS2G	26.42
**4194**	allXL	AA	367	373	BS2G	13.47
**4194**	allXL	DD	370	382	BS2G	20.63
**4194**	allXL	AA	376	382	BS2G	14.72
**4194**	allXL	BB	357	373	BS2G	19.53
**4194**	allXL	AA	320	366	BS2G	27.53
**4194**	allXL	BB	370	376	BS2G	16.32
**4194**	allXL	CC	320	357	DBSU	24.02
**4194**	allXL	CC	357	319	DBSU	26.42
**4194**	allXL	CC	372	319	DBSU	32.95
**4194**	allXL	DD	370	357	DBSU	25.14
**4194**	allXL	BB	382	357	DBSU	24.74
**4194**	allXL	AA	372	370	DBSU	6.1
**4194**	allXL	DD	381	370	DBSU	20.27
**4194**	allXL	AA	373	370	DBSU	9.09
**4194**	allXL	DD	381	372	DBSU	17.44
**4194**	allXL	DD	372	382	DBSU	18.97
**4194**	allXL	DD	372	386	DBSU	26.51
**4194**	allXL	DD	373	386	DBSU	27.6
**4194**	allXL	AD	381	386	DBSU	57.83
**4194**	allXL	BD;DB	357	357	DBSU	19.1
**4194**	allXL	DA	373	319	DBSU	25.75
**4194**	allXL	BD	372	357	DBSU	36.69
**4194**	allXL	BD	381	357	DBSU	40.51
**4194**	allXL	BD	373	357	DBSU	34.36
**4194**	allXL	DA	372	370	DBSU	43.88
**4194**	allXL	DA	373	370	DBSU	42.49
**4194**	allXL	DA	382	373	DBSU	44.88
**4195**	allXL	AA	320	357	BS2G	21.01
**4195**	allXL	DD	320	373	BS2G	16.65
**4195**	allXL	AA	373	382	BS2G	18.07
**4195**	allXL	AA	370	386	BS2G	13.57

**4195**	allXL	AA	373	386	BS2G	15.39
**4195**	allXL	AA	319	357	BS2G	24.53
**4195**	allXL	AA	367	373	BS2G	13.24
**4195**	allXL	BB	370	382	BS2G	18.85
**4195**	allXL	AA	376	382	BS2G	13.48
**4195**	allXL	DD	357	373	BS2G	20.76
**4195**	allXL	BB	320	366	BS2G	18.08
**4195**	allXL	CC	370	376	BS2G	15.29
**4195**	allXL	AA	320	357	DBSU	21.01
**4195**	allXL	AA	357	319	DBSU	24.53
**4195**	allXL	DD	372	319	DBSU	17.58
**4195**	allXL	DD	370	357	DBSU	21.83
**4195**	allXL	BB	382	357	DBSU	36.21
**4195**	allXL	AA	372	370	DBSU	6.33
**4195**	allXL	BB	381	370	DBSU	19.96
**4195**	allXL	AA	373	370	DBSU	9.27
**4195**	allXL	AA	381	372	DBSU	19.61
**4195**	allXL	AA	372	382	DBSU	18.17
**4195**	allXL	AA	372	386	DBSU	13.47
**4195**	allXL	AA	373	386	DBSU	15.39
**4195**	allXL	BC	381	386	DBSU	16.67
**4195**	allXL	BD;DB	357	357	DBSU	19.0
**4195**	allXL	CB	373	319	DBSU	15.0
**4195**	allXL	CB	372	357	DBSU	29.79
**4195**	allXL	DA	381	357	DBSU	26.0
**4195**	allXL	CB	373	357	DBSU	29.49
**4195**	allXL	CB	372	370	DBSU	28.71
**4195**	allXL	CB	373	370	DBSU	26.37
**4195**	allXL	DA	382	373	DBSU	22.13
**4196**	allXL	BB	320	357	BS2G	26.91
**4196**	allXL	CC	320	373	BS2G	14.89
**4196**	allXL	BB	373	382	BS2G	17.66
**4196**	allXL	AA	370	386	BS2G	28.74
**4196**	allXL	AA	373	386	BS2G	27.67

**4196**	allXL	AA	319	357	BS2G	27.16
**4196**	allXL	AA	367	373	BS2G	12.6
**4196**	allXL	BB	370	382	BS2G	20.39
**4196**	allXL	BB	376	382	BS2G	14.23
**4196**	allXL	CC	357	373	BS2G	33.15
**4196**	allXL	CC	320	366	BS2G	25.69
**4196**	allXL	CC	370	376	BS2G	15.52
**4196**	allXL	BB	320	357	DBSU	26.91
**4196**	allXL	AA	357	319	DBSU	27.16
**4196**	allXL	CC	372	319	DBSU	18.09
**4196**	allXL	CC	370	357	DBSU	27.61
**4196**	allXL	BB	382	357	DBSU	28.47
**4196**	allXL	AA	372	370	DBSU	6.27
**4196**	allXL	BB	381	370	DBSU	21.83
**4196**	allXL	AA	373	370	DBSU	9.24
**4196**	allXL	AA	381	372	DBSU	19.74
**4196**	allXL	AA	372	382	DBSU	18.76
**4196**	allXL	AA	372	386	DBSU	25.87
**4196**	allXL	AA	373	386	DBSU	27.67
**4196**	allXL	CB	381	386	DBSU	60.12
**4196**	allXL	BD;DB	357	357	DBSU	19.0
**4196**	allXL	AD	373	319	DBSU	36.88
**4196**	allXL	CA	372	357	DBSU	49.21
**4196**	allXL	BD	381	357	DBSU	44.98
**4196**	allXL	CB	373	357	DBSU	49.73
**4196**	allXL	DA	372	370	DBSU	40.68
**4196**	allXL	DA	373	370	DBSU	40.6
**4196**	allXL	AD	382	373	DBSU	55.64
**4197**	allXL	AA	320	357	BS2G	23.69
**4197**	allXL	DD	320	373	BS2G	21.2
**4197**	allXL	BB	373	382	BS2G	15.49
**4197**	allXL	DD	370	386	BS2G	15.8
**4197**	allXL	DD	373	386	BS2G	21.36
**4197**	allXL	AA	319	357	BS2G	22.42

**4197**	allXL	BB	367	373	BS2G	14.04
**4197**	allXL	DD	370	382	BS2G	15.88
**4197**	allXL	BB	376	382	BS2G	12.95
**4197**	allXL	BB	357	373	BS2G	25.33
**4197**	allXL	CC	320	366	BS2G	13.26
**4197**	allXL	CC	370	376	BS2G	17.08
**4197**	allXL	AA	320	357	DBSU	23.69
**4197**	allXL	AA	357	319	DBSU	22.42
**4197**	allXL	DD	372	319	DBSU	20.58
**4197**	allXL	CC	370	357	DBSU	21.71
**4197**	allXL	BB	382	357	DBSU	20.64
**4197**	allXL	AA	372	370	DBSU	6.12
**4197**	allXL	DD	381	370	DBSU	15.43
**4197**	allXL	AA	373	370	DBSU	9.07
**4197**	allXL	DD	381	372	DBSU	12.14
**4197**	allXL	DD	372	382	DBSU	13.61
**4197**	allXL	DD	372	386	DBSU	18.17
**4197**	allXL	DD	373	386	DBSU	21.36
**4197**	allXL	DA	381	386	DBSU	55.73
**4197**	allXL	BD;DB	357	357	DBSU	18.01
**4197**	allXL	AD	373	319	DBSU	28.91
**4197**	allXL	CA	372	357	DBSU	31.14
**4197**	allXL	CA	381	357	DBSU	30.9
**4197**	allXL	CA	373	357	DBSU	30.28
**4197**	allXL	DA	372	370	DBSU	36.02
**4197**	allXL	DA	373	370	DBSU	33.2
**4197**	allXL	CA	382	373	DBSU	39.36
**4198**	allXL	AA	320	357	BS2G	24.55
**4198**	allXL	BB	320	373	BS2G	28.51
**4198**	allXL	DD	373	382	BS2G	18.79
**4198**	allXL	DD	370	386	BS2G	14.87
**4198**	allXL	DD	373	386	BS2G	24.14
**4198**	allXL	AA	319	357	BS2G	27.31
**4198**	allXL	BB	367	373	BS2G	12.98

**4198**	allXL	DD	370	382	BS2G	13.39
**4198**	allXL	AA	376	382	BS2G	14.71
**4198**	allXL	BB	357	373	BS2G	23.57
**4198**	allXL	CC	320	366	BS2G	26.76
**4198**	allXL	DD	370	376	BS2G	15.25
**4198**	allXL	AA	320	357	DBSU	24.55
**4198**	allXL	AA	357	319	DBSU	27.31
**4198**	allXL	BB	372	319	DBSU	29.13
**4198**	allXL	BB	370	357	DBSU	29.14
**4198**	allXL	DD	382	357	DBSU	25.83
**4198**	allXL	AA	372	370	DBSU	6.09
**4198**	allXL	DD	381	370	DBSU	13.15
**4198**	allXL	AA	373	370	DBSU	9.11
**4198**	allXL	DD	381	372	DBSU	16.83
**4198**	allXL	DD	372	382	DBSU	18.37
**4198**	allXL	DD	372	386	DBSU	22.11
**4198**	allXL	DD	373	386	DBSU	24.14
**4198**	allXL	BD	381	386	DBSU	55.36
**4198**	allXL	BD;DB	357	357	DBSU	18.21
**4198**	allXL	AD	373	319	DBSU	27.85
**4198**	allXL	BD	372	357	DBSU	40.91
**4198**	allXL	DB	381	357	DBSU	36.32
**4198**	allXL	BD	373	357	DBSU	40.21
**4198**	allXL	DA	372	370	DBSU	45.87
**4198**	allXL	DA	373	370	DBSU	44.93
**4198**	allXL	DB	382	373	DBSU	51.62
**4199**	allXL	AA	320	357	BS2G	19.24
**4199**	allXL	CC	320	373	BS2G	16.9
**4199**	allXL	AA	373	382	BS2G	17.12
**4199**	allXL	AA	370	386	BS2G	18.3
**4199**	allXL	AA	373	386	BS2G	16.55
**4199**	allXL	AA	319	357	BS2G	16.36
**4199**	allXL	AA	367	373	BS2G	13.91
**4199**	allXL	AA	370	382	BS2G	20.76

**4199**	allXL	AA	376	382	BS2G	14.56
**4199**	allXL	CC	357	373	BS2G	26.62
**4199**	allXL	BB	320	366	BS2G	23.43
**4199**	allXL	CC	370	376	BS2G	15.1
**4199**	allXL	AA	320	357	DBSU	19.24
**4199**	allXL	AA	357	319	DBSU	16.36
**4199**	allXL	CC	372	319	DBSU	11.95
**4199**	allXL	CC	370	357	DBSU	25.76
**4199**	allXL	CC	382	357	DBSU	32.14
**4199**	allXL	AA	372	370	DBSU	6.15
**4199**	allXL	AA	381	370	DBSU	23.49
**4199**	allXL	DD	373	370	DBSU	9.16
**4199**	allXL	AA	381	372	DBSU	20.26
**4199**	allXL	AA	372	382	DBSU	18.24
**4199**	allXL	AA	372	386	DBSU	17.86
**4199**	allXL	AA	373	386	DBSU	16.55
**4199**	allXL	CA	381	386	DBSU	47.05
**4199**	allXL	BD;DB	357	357	DBSU	18.38
**4199**	allXL	AD	373	319	DBSU	23.29
**4199**	allXL	AC	372	357	DBSU	44.76
**4199**	allXL	AC	381	357	DBSU	37.23
**4199**	allXL	CA	373	357	DBSU	45.26
**4199**	allXL	AD	372	370	DBSU	43.56
**4199**	allXL	AD	373	370	DBSU	45.37
**4199**	allXL	AC	382	373	DBSU	39.51
**4200**	allXL	AA	320	357	BS2G	23.47
**4200**	allXL	CC	320	373	BS2G	26.09
**4200**	allXL	BB	373	382	BS2G	19.62
**4200**	allXL	DD	370	386	BS2G	16.9
**4200**	allXL	DD	373	386	BS2G	20.99
**4200**	allXL	CC	319	357	BS2G	24.58
**4200**	allXL	AA	367	373	BS2G	12.91
**4200**	allXL	DD	370	382	BS2G	20.96
**4200**	allXL	BB	376	382	BS2G	14.31

**4200**	allXL	BB	357	373	BS2G	29.8
**4200**	allXL	CC	320	366	BS2G	23.83
**4200**	allXL	CC	370	376	BS2G	15.92
**4200**	allXL	AA	320	357	DBSU	23.47
**4200**	allXL	CC	357	319	DBSU	24.58
**4200**	allXL	CC	372	319	DBSU	29.08
**4200**	allXL	CC	370	357	DBSU	27.85
**4200**	allXL	BB	382	357	DBSU	22.05
**4200**	allXL	AA	372	370	DBSU	6.23
**4200**	allXL	DD	381	370	DBSU	20.4
**4200**	allXL	AA	373	370	DBSU	9.01
**4200**	allXL	DD	381	372	DBSU	20.93
**4200**	allXL	BB	372	382	DBSU	20.91
**4200**	allXL	DD	372	386	DBSU	20.86
**4200**	allXL	DD	373	386	DBSU	20.99
**4200**	allXL	BC	381	386	DBSU	19.11
**4200**	allXL	BD;DB	357	357	DBSU	18.46
**4200**	allXL	CB	373	319	DBSU	25.86
**4200**	allXL	CB	372	357	DBSU	24.63
**4200**	allXL	CB	381	357	DBSU	37.33
**4200**	allXL	CB	373	357	DBSU	25.78
**4200**	allXL	CB	372	370	DBSU	18.85
**4200**	allXL	CB	373	370	DBSU	15.1
**4200**	allXL	CB	382	373	DBSU	13.91
**4201**	allXL	AA	320	357	BS2G	26.72
**4201**	allXL	DD	320	373	BS2G	20.43
**4201**	allXL	CC	373	382	BS2G	15.54
**4201**	allXL	BB	370	386	BS2G	22.16
**4201**	allXL	CC	373	386	BS2G	20.6
**4201**	allXL	CC	319	357	BS2G	28.63
**4201**	allXL	AA	367	373	BS2G	13.28
**4201**	allXL	DD	370	382	BS2G	21.0
**4201**	allXL	CC	376	382	BS2G	13.79
**4201**	allXL	BB	357	373	BS2G	20.52

**4201**	allXL	BB	320	366	BS2G	17.85
**4201**	allXL	CC	370	376	BS2G	17.51
**4201**	allXL	AA	320	357	DBSU	26.72
**4201**	allXL	CC	357	319	DBSU	28.63
**4201**	allXL	DD	372	319	DBSU	17.09
**4201**	allXL	BB	370	357	DBSU	21.62
**4201**	allXL	BB	382	357	DBSU	38.41
**4201**	allXL	AA	372	370	DBSU	6.31
**4201**	allXL	DD	381	370	DBSU	21.42
**4201**	allXL	AA	373	370	DBSU	9.22
**4201**	allXL	CC	381	372	DBSU	18.48
**4201**	allXL	CC	372	382	DBSU	16.23
**4201**	allXL	CC	372	386	DBSU	18.96
**4201**	allXL	CC	373	386	DBSU	20.6
**4201**	allXL	BC	381	386	DBSU	47.79
**4201**	allXL	BD;DB	357	357	DBSU	18.76
**4201**	allXL	CB	373	319	DBSU	21.17
**4201**	allXL	CB	372	357	DBSU	25.74
**4201**	allXL	CB	381	357	DBSU	22.92
**4201**	allXL	CB	373	357	DBSU	22.34
**4201**	allXL	CB	372	370	DBSU	27.11
**4201**	allXL	CB	373	370	DBSU	26.01
**4201**	allXL	CB	382	373	DBSU	32.05
**4202**	allXL	CC	320	357	BS2G	23.03
**4202**	allXL	AA	320	373	BS2G	19.2
**4202**	allXL	DD	373	382	BS2G	17.45
**4202**	allXL	DD	370	386	BS2G	16.24
**4202**	allXL	DD	373	386	BS2G	18.26
**4202**	allXL	AA	319	357	BS2G	22.52
**4202**	allXL	AA	367	373	BS2G	13.3
**4202**	allXL	DD	370	382	BS2G	16.57
**4202**	allXL	AA	376	382	BS2G	15.45
**4202**	allXL	AA	357	373	BS2G	34.27
**4202**	allXL	BB	320	366	BS2G	15.29

**4202**	allXL	CC	370	376	BS2G	16.27
**4202**	allXL	CC	320	357	DBSU	23.03
**4202**	allXL	AA	357	319	DBSU	22.52
**4202**	allXL	AA	372	319	DBSU	14.86
**4202**	allXL	AA	370	357	DBSU	26.16
**4202**	allXL	BB	382	357	DBSU	40.76
**4202**	allXL	AA	372	370	DBSU	6.41
**4202**	allXL	DD	381	370	DBSU	18.41
**4202**	allXL	AA	373	370	DBSU	9.12
**4202**	allXL	DD	381	372	DBSU	18.16
**4202**	allXL	DD	372	382	DBSU	17.63
**4202**	allXL	DD	372	386	DBSU	16.33
**4202**	allXL	DD	373	386	DBSU	18.26
**4202**	allXL	BC	381	386	DBSU	31.83
**4202**	allXL	BD;DB	357	357	DBSU	19.17
**4202**	allXL	CB	373	319	DBSU	12.38
**4202**	allXL	CB	372	357	DBSU	43.09
**4202**	allXL	CB	381	357	DBSU	48.6
**4202**	allXL	CB	373	357	DBSU	41.1
**4202**	allXL	CB	372	370	DBSU	23.58
**4202**	allXL	CB	373	370	DBSU	19.82
**4202**	allXL	CB	382	373	DBSU	16.3
**4203**	allXL	CC	320	357	BS2G	23.81
**4203**	allXL	DD	320	373	BS2G	21.61
**4203**	allXL	CC	373	382	BS2G	12.1
**4203**	allXL	CC	370	386	BS2G	18.38
**4203**	allXL	CC	373	386	BS2G	16.92
**4203**	allXL	CC	319	357	BS2G	26.0
**4203**	allXL	AA	367	373	BS2G	9.23
**4203**	allXL	CC	370	382	BS2G	19.45
**4203**	allXL	CC	376	382	BS2G	13.6
**4203**	allXL	AA	357	373	BS2G	22.75
**4203**	allXL	CC	320	366	BS2G	24.27
**4203**	allXL	DD	370	376	BS2G	14.16

**4203**	allXL	CC	320	357	DBSU	23.81
**4203**	allXL	CC	357	319	DBSU	26.0
**4203**	allXL	DD	372	319	DBSU	28.16
**4203**	allXL	DD	370	357	DBSU	26.42
**4203**	allXL	DD	382	357	DBSU	9.7
**4203**	allXL	CC	372	370	DBSU	6.76
**4203**	allXL	CC	381	370	DBSU	22.73
**4203**	allXL	AA	373	370	DBSU	8.99
**4203**	allXL	CC	381	372	DBSU	17.59
**4203**	allXL	CC	372	382	DBSU	14.65
**4203**	allXL	CC	372	386	DBSU	16.6
**4203**	allXL	CC	373	386	DBSU	16.92
**4203**	allXL	BD	381	386	DBSU	64.55
**4203**	allXL	BD;DB	357	357	DBSU	18.43
**4203**	allXL	AD	373	319	DBSU	23.78
**4203**	allXL	AC	372	357	DBSU	39.9
**4203**	allXL	DB	381	357	DBSU	23.98
**4203**	allXL	AC	373	357	DBSU	38.05
**4203**	allXL	AD	372	370	DBSU	49.03
**4203**	allXL	DA	373	370	DBSU	46.15
**4203**	allXL	DA	382	373	DBSU	43.14
**4204**	allXL	AA	320	357	BS2G	19.76
**4204**	allXL	DD	320	373	BS2G	20.11
**4204**	allXL	AA	373	382	BS2G	17.77
**4204**	allXL	AA	370	386	BS2G	20.87
**4204**	allXL	AA	373	386	BS2G	21.1
**4204**	allXL	AA	319	357	BS2G	17.6
**4204**	allXL	AA	367	373	BS2G	14.02
**4204**	allXL	AA	370	382	BS2G	22.28
**4204**	allXL	BB	376	382	BS2G	14.04
**4204**	allXL	BB	357	373	BS2G	35.64
**4204**	allXL	CC	320	366	BS2G	19.92
**4204**	allXL	CC	370	376	BS2G	17.21
**4204**	allXL	AA	320	357	DBSU	19.76

**4204**	allXL	AA	357	319	DBSU	17.6
**4204**	allXL	DD	372	319	DBSU	20.37
**4204**	allXL	DD	370	357	DBSU	27.24
**4204**	allXL	BB	382	357	DBSU	39.13
**4204**	allXL	AA	372	370	DBSU	6.08
**4204**	allXL	AA	381	370	DBSU	22.98
**4204**	allXL	AA	373	370	DBSU	9.09
**4204**	allXL	BB	381	372	DBSU	18.9
**4204**	allXL	AA	372	382	DBSU	19.48
**4204**	allXL	AA	372	386	DBSU	20.67
**4204**	allXL	AA	373	386	DBSU	21.1
**4204**	allXL	DA	381	386	DBSU	33.79
**4204**	allXL	BD;DB	357	357	DBSU	18.07
**4204**	allXL	DA	373	319	DBSU	37.8
**4204**	allXL	DA	372	357	DBSU	45.96
**4204**	allXL	DA	381	357	DBSU	48.72
**4204**	allXL	DA	373	357	DBSU	45.44
**4204**	allXL	DA	372	370	DBSU	30.95
**4204**	allXL	DA	373	370	DBSU	28.24
**4204**	allXL	DA	382	373	DBSU	26.59
**4205**	allXL	AA	320	357	BS2G	25.23
**4205**	allXL	DD	320	373	BS2G	20.74
**4205**	allXL	BB	373	382	BS2G	15.86
**4205**	allXL	BB	370	386	BS2G	24.15
**4205**	allXL	BB	373	386	BS2G	25.87
**4205**	allXL	AA	319	357	BS2G	24.1
**4205**	allXL	DD	367	373	BS2G	13.88
**4205**	allXL	BB	370	382	BS2G	17.92
**4205**	allXL	BB	376	382	BS2G	13.29
**4205**	allXL	DD	357	373	BS2G	30.84
**4205**	allXL	AA	320	366	BS2G	24.47
**4205**	allXL	CC	370	376	BS2G	16.32
**4205**	allXL	AA	320	357	DBSU	25.23
**4205**	allXL	AA	357	319	DBSU	24.1

**4205**	allXL	DD	372	319	DBSU	22.44
**4205**	allXL	DD	370	357	DBSU	26.14
**4205**	allXL	BB	382	357	DBSU	21.0
**4205**	allXL	AA	372	370	DBSU	6.02
**4205**	allXL	BB	381	370	DBSU	18.32
**4205**	allXL	CC	373	370	DBSU	9.02
**4205**	allXL	DD	381	372	DBSU	14.41
**4205**	allXL	DD	372	382	DBSU	16.9
**4205**	allXL	DD	372	386	DBSU	23.56
**4205**	allXL	BB	373	386	DBSU	25.87
**4205**	allXL	AD	381	386	DBSU	41.8
**4205**	allXL	BD;DB	357	357	DBSU	17.94
**4205**	allXL	DA	373	319	DBSU	24.03
**4205**	allXL	DA	372	357	DBSU	35.0
**4205**	allXL	BD	381	357	DBSU	25.9
**4205**	allXL	DA	373	357	DBSU	34.09
**4205**	allXL	DA	372	370	DBSU	18.18
**4205**	allXL	DA	373	370	DBSU	15.14
**4205**	allXL	DA	382	373	DBSU	23.01
**4206**	allXL	AA	320	357	BS2G	24.38
**4206**	allXL	DD	320	373	BS2G	23.47
**4206**	allXL	CC	373	382	BS2G	18.72
**4206**	allXL	CC	370	386	BS2G	21.22
**4206**	allXL	AA	373	386	BS2G	24.03
**4206**	allXL	AA	319	357	BS2G	24.41
**4206**	allXL	AA	367	373	BS2G	14.94
**4206**	allXL	DD	370	382	BS2G	15.33
**4206**	allXL	CC	376	382	BS2G	14.01
**4206**	allXL	DD	357	373	BS2G	16.37
**4206**	allXL	BB	320	366	BS2G	15.11
**4206**	allXL	BB	370	376	BS2G	15.16
**4206**	allXL	AA	320	357	DBSU	24.38
**4206**	allXL	AA	357	319	DBSU	24.41
**4206**	allXL	BB	372	319	DBSU	24.01

**4206**	allXL	DD	370	357	DBSU	12.66
**4206**	allXL	CC	382	357	DBSU	19.67
**4206**	allXL	AA	372	370	DBSU	6.09
**4206**	allXL	DD	381	370	DBSU	15.29
**4206**	allXL	BB	373	370	DBSU	9.1
**4206**	allXL	DD	381	372	DBSU	17.04
**4206**	allXL	CC	372	382	DBSU	18.28
**4206**	allXL	AA	372	386	DBSU	21.43
**4206**	allXL	AA	373	386	DBSU	24.03
**4206**	allXL	DA	381	386	DBSU	43.17
**4206**	allXL	BD;DB	357	357	DBSU	19.13
**4206**	allXL	AD	373	319	DBSU	24.45
**4206**	allXL	CA	372	357	DBSU	15.84
**4206**	allXL	CA	381	357	DBSU	28.58
**4206**	allXL	CA	373	357	DBSU	14.18
**4206**	allXL	DA	372	370	DBSU	37.76
**4206**	allXL	DA	373	370	DBSU	37.67
**4206**	allXL	AD	382	373	DBSU	41.2
**4207**	allXL	AA	320	357	BS2G	26.92
**4207**	allXL	BB	320	373	BS2G	29.11
**4207**	allXL	AA	373	382	BS2G	20.85
**4207**	allXL	DD	370	386	BS2G	23.11
**4207**	allXL	DD	373	386	BS2G	26.9
**4207**	allXL	AA	319	357	BS2G	28.64
**4207**	allXL	AA	367	373	BS2G	11.5
**4207**	allXL	BB	370	382	BS2G	20.97
**4207**	allXL	CC	376	382	BS2G	14.82
**4207**	allXL	BB	357	373	BS2G	22.0
**4207**	allXL	BB	320	366	BS2G	20.02
**4207**	allXL	BB	370	376	BS2G	15.66
**4207**	allXL	AA	320	357	DBSU	26.92
**4207**	allXL	AA	357	319	DBSU	28.64
**4207**	allXL	BB	372	319	DBSU	29.38
**4207**	allXL	DD	370	357	DBSU	18.47

**4207**	allXL	BB	382	357	DBSU	31.72
**4207**	allXL	AA	372	370	DBSU	6.35
**4207**	allXL	BB	381	370	DBSU	21.22
**4207**	allXL	AA	373	370	DBSU	9.13
**4207**	allXL	AA	381	372	DBSU	21.49
**4207**	allXL	AA	372	382	DBSU	19.86
**4207**	allXL	AA	372	386	DBSU	26.37
**4207**	allXL	DD	373	386	DBSU	26.9
**4207**	allXL	BD	381	386	DBSU	43.2
**4207**	allXL	BD;DB	357	357	DBSU	19.01
**4207**	allXL	CB	373	319	DBSU	26.65
**4207**	allXL	DB	372	357	DBSU	31.3
**4207**	allXL	BD	381	357	DBSU	35.23
**4207**	allXL	BD	373	357	DBSU	31.31
**4207**	allXL	BD	372	370	DBSU	40.41
**4207**	allXL	BD	373	370	DBSU	38.54
**4207**	allXL	DB	382	373	DBSU	39.74
**4208**	allXL	AA	320	357	BS2G	24.81
**4208**	allXL	BB	320	373	BS2G	14.53
**4208**	allXL	CC	373	382	BS2G	19.04
**4208**	allXL	BB	370	386	BS2G	18.25
**4208**	allXL	BB	373	386	BS2G	20.22
**4208**	allXL	AA	319	357	BS2G	23.98
**4208**	allXL	BB	367	373	BS2G	14.22
**4208**	allXL	DD	370	382	BS2G	21.92
**4208**	allXL	BB	376	382	BS2G	14.2
**4208**	allXL	CC	357	373	BS2G	28.66
**4208**	allXL	CC	320	366	BS2G	11.62
**4208**	allXL	BB	370	376	BS2G	16.25
**4208**	allXL	AA	320	357	DBSU	24.81
**4208**	allXL	AA	357	319	DBSU	23.98
**4208**	allXL	BB	372	319	DBSU	17.7
**4208**	allXL	DD	370	357	DBSU	21.46
**4208**	allXL	DD	382	357	DBSU	33.09

**4208**	allXL	AA	372	370	DBSU	5.79
**4208**	allXL	DD	381	370	DBSU	21.05
**4208**	allXL	AA	373	370	DBSU	9.12
**4208**	allXL	DD	381	372	DBSU	19.43
**4208**	allXL	DD	372	382	DBSU	20.99
**4208**	allXL	BB	372	386	DBSU	21.77
**4208**	allXL	BB	373	386	DBSU	20.22
**4208**	allXL	BC	381	386	DBSU	63.69
**4208**	allXL	BD;DB	357	357	DBSU	18.24
**4208**	allXL	AD	373	319	DBSU	28.61
**4208**	allXL	DB	372	357	DBSU	33.74
**4208**	allXL	DB	381	357	DBSU	39.35
**4208**	allXL	DB	373	357	DBSU	37.33
**4208**	allXL	AD	372	370	DBSU	38.91
**4208**	allXL	AD	373	370	DBSU	39.71
**4208**	allXL	AD	382	373	DBSU	42.45
**4209**	allXL	CC	320	357	BS2G	19.91
**4209**	allXL	BB	320	373	BS2G	14.63
**4209**	allXL	DD	373	382	BS2G	19.66
**4209**	allXL	BB	370	386	BS2G	30.48
**4209**	allXL	DD	373	386	BS2G	26.22
**4209**	allXL	CC	319	357	BS2G	20.0
**4209**	allXL	AA	367	373	BS2G	14.09
**4209**	allXL	DD	370	382	BS2G	26.85
**4209**	allXL	CC	376	382	BS2G	14.31
**4209**	allXL	BB	357	373	BS2G	22.31
**4209**	allXL	AA	320	366	BS2G	19.79
**4209**	allXL	CC	370	376	BS2G	16.1
**4209**	allXL	CC	320	357	DBSU	19.91
**4209**	allXL	CC	357	319	DBSU	20.0
**4209**	allXL	BB	372	319	DBSU	14.98
**4209**	allXL	DD	370	357	DBSU	20.97
**4209**	allXL	BB	382	357	DBSU	35.0
**4209**	allXL	AA	372	370	DBSU	6.02

**4209**	allXL	DD	381	370	DBSU	24.87
**4209**	allXL	AA	373	370	DBSU	9.28
**4209**	allXL	DD	381	372	DBSU	20.44
**4209**	allXL	DD	372	382	DBSU	23.03
**4209**	allXL	DD	372	386	DBSU	28.88
**4209**	allXL	DD	373	386	DBSU	26.22
**4209**	allXL	AD	381	386	DBSU	67.3
**4209**	allXL	BD;DB	357	357	DBSU	18.79
**4209**	allXL	AD	373	319	DBSU	27.32
**4209**	allXL	BC	372	357	DBSU	37.56
**4209**	allXL	BD	381	357	DBSU	50.03
**4209**	allXL	BC	373	357	DBSU	39.83
**4209**	allXL	DA	372	370	DBSU	33.17
**4209**	allXL	DA	373	370	DBSU	33.72
**4209**	allXL	BC	382	373	DBSU	51.01
**4210**	allXL	AA	320	357	BS2G	23.57
**4210**	allXL	DD	320	373	BS2G	26.55
**4210**	allXL	DD	373	382	BS2G	18.72
**4210**	allXL	DD	370	386	BS2G	16.52
**4210**	allXL	DD	373	386	BS2G	19.99
**4210**	allXL	AA	319	357	BS2G	26.7
**4210**	allXL	AA	367	373	BS2G	13.48
**4210**	allXL	DD	370	382	BS2G	13.25
**4210**	allXL	CC	376	382	BS2G	13.55
**4210**	allXL	BB	357	373	BS2G	29.41
**4210**	allXL	CC	320	366	BS2G	25.7
**4210**	allXL	DD	370	376	BS2G	14.67
**4210**	allXL	AA	320	357	DBSU	23.57
**4210**	allXL	AA	357	319	DBSU	26.7
**4210**	allXL	DD	372	319	DBSU	26.77
**4210**	allXL	BB	370	357	DBSU	25.35
**4210**	allXL	DD	382	357	DBSU	40.45
**4210**	allXL	AA	372	370	DBSU	6.15
**4210**	allXL	DD	381	370	DBSU	13.78

**4210**	allXL	AA	373	370	DBSU	9.05
**4210**	allXL	DD	381	372	DBSU	18.24
**4210**	allXL	DD	372	382	DBSU	18.38
**4210**	allXL	DD	372	386	DBSU	19.02
**4210**	allXL	DD	373	386	DBSU	19.99
**4210**	allXL	BC	381	386	DBSU	31.92
**4210**	allXL	BD;DB	357	357	DBSU	17.69
**4210**	allXL	CB	373	319	DBSU	17.03
**4210**	allXL	CB	372	357	DBSU	34.43
**4210**	allXL	BD	381	357	DBSU	45.47
**4210**	allXL	CB	373	357	DBSU	36.9
**4210**	allXL	DA	372	370	DBSU	41.13
**4210**	allXL	DA	373	370	DBSU	38.44
**4210**	allXL	CB	382	373	DBSU	35.43
**4211**	allXL	AA	320	357	BS2G	23.99
**4211**	allXL	BB	320	373	BS2G	24.58
**4211**	allXL	BB	373	382	BS2G	16.75
**4211**	allXL	AA	370	386	BS2G	27.3
**4211**	allXL	BB	373	386	BS2G	27.51
**4211**	allXL	AA	319	357	BS2G	24.24
**4211**	allXL	BB	367	373	BS2G	12.53
**4211**	allXL	AA	370	382	BS2G	20.95
**4211**	allXL	BB	376	382	BS2G	13.24
**4211**	allXL	AA	357	373	BS2G	27.68
**4211**	allXL	DD	320	366	BS2G	29.51
**4211**	allXL	CC	370	376	BS2G	15.43
**4211**	allXL	AA	320	357	DBSU	23.99
**4211**	allXL	AA	357	319	DBSU	24.24
**4211**	allXL	BB	372	319	DBSU	29.44
**4211**	allXL	AA	370	357	DBSU	26.54
**4211**	allXL	BB	382	357	DBSU	29.82
**4211**	allXL	AA	372	370	DBSU	6.26
**4211**	allXL	AA	381	370	DBSU	22.7
**4211**	allXL	AA	373	370	DBSU	8.72

**4211**	allXL	BB	381	372	DBSU	20.16
**4211**	allXL	BB	372	382	DBSU	18.58
**4211**	allXL	BB	372	386	DBSU	28.62
**4211**	allXL	BB	373	386	DBSU	27.51
**4211**	allXL	AD	381	386	DBSU	67.59
**4211**	allXL	BD;DB	357	357	DBSU	17.74
**4211**	allXL	AD	373	319	DBSU	18.66
**4211**	allXL	AC	372	357	DBSU	29.15
**4211**	allXL	AD	381	357	DBSU	46.33
**4211**	allXL	AC	373	357	DBSU	31.08
**4211**	allXL	AD	372	370	DBSU	51.79
**4211**	allXL	BC	373	370	DBSU	48.81
**4211**	allXL	DA	382	373	DBSU	62.44
**4212**	allXL	CC	320	357	BS2G	23.27
**4212**	allXL	AA	320	373	BS2G	17.3
**4212**	allXL	BB	373	382	BS2G	17.33
**4212**	allXL	AA	370	386	BS2G	23.89
**4212**	allXL	DD	373	386	BS2G	21.5
**4212**	allXL	CC	319	357	BS2G	23.02
**4212**	allXL	AA	367	373	BS2G	12.72
**4212**	allXL	BB	370	382	BS2G	20.47
**4212**	allXL	BB	376	382	BS2G	13.9
**4212**	allXL	BB	357	373	BS2G	20.3
**4212**	allXL	AA	320	366	BS2G	19.76
**4212**	allXL	CC	370	376	BS2G	16.38
**4212**	allXL	CC	320	357	DBSU	23.27
**4212**	allXL	CC	357	319	DBSU	23.02
**4212**	allXL	AA	372	319	DBSU	18.48
**4212**	allXL	BB	370	357	DBSU	18.82
**4212**	allXL	DD	382	357	DBSU	28.41
**4212**	allXL	AA	372	370	DBSU	6.24
**4212**	allXL	BB	381	370	DBSU	20.52
**4212**	allXL	AA	373	370	DBSU	9.12
**4212**	allXL	AA	381	372	DBSU	17.9

**4212**	allXL	BB	372	382	DBSU	18.3
**4212**	allXL	DD	372	386	DBSU	22.12
**4212**	allXL	DD	373	386	DBSU	21.5
**4212**	allXL	DA	381	386	DBSU	67.12
**4212**	allXL	BD;DB	357	357	DBSU	17.6
**4212**	allXL	AD	373	319	DBSU	33.05
**4212**	allXL	BD	372	357	DBSU	28.14
**4212**	allXL	DB	381	357	DBSU	34.42
**4212**	allXL	BD	373	357	DBSU	30.19
**4212**	allXL	AD	372	370	DBSU	54.93
**4212**	allXL	DB	373	370	DBSU	56.68
**4212**	allXL	DB	382	373	DBSU	49.35
**4213**	allXL	AA	320	357	BS2G	23.31
**4213**	allXL	DD	320	373	BS2G	21.07
**4213**	allXL	DD	373	382	BS2G	15.4
**4213**	allXL	DD	370	386	BS2G	23.31
**4213**	allXL	CC	373	386	BS2G	24.17
**4213**	allXL	AA	319	357	BS2G	21.52
**4213**	allXL	BB	367	373	BS2G	10.58
**4213**	allXL	DD	370	382	BS2G	15.24
**4213**	allXL	BB	376	382	BS2G	14.52
**4213**	allXL	DD	357	373	BS2G	27.09
**4213**	allXL	BB	320	366	BS2G	31.32
**4213**	allXL	BB	370	376	BS2G	15.06
**4213**	allXL	AA	320	357	DBSU	23.31
**4213**	allXL	AA	357	319	DBSU	21.52
**4213**	allXL	DD	372	319	DBSU	23.29
**4213**	allXL	DD	370	357	DBSU	24.58
**4213**	allXL	BB	382	357	DBSU	32.94
**4213**	allXL	AA	372	370	DBSU	6.02
**4213**	allXL	DD	381	370	DBSU	15.51
**4213**	allXL	BB;DD	373	370	DBSU	9.15
**4213**	allXL	DD	381	372	DBSU	14.84
**4213**	allXL	DD	372	382	DBSU	14.22

**4213**	allXL	DD	372	386	DBSU	22.53
**4213**	allXL	CC	373	386	DBSU	24.17
**4213**	allXL	CB	381	386	DBSU	36.73
**4213**	allXL	BD;DB	357	357	DBSU	18.57
**4213**	allXL	CB	373	319	DBSU	19.23
**4213**	allXL	DA	372	357	DBSU	41.88
**4213**	allXL	DA	381	357	DBSU	28.32
**4213**	allXL	DA	373	357	DBSU	40.67
**4213**	allXL	CB	372	370	DBSU	32.88
**4213**	allXL	CB	373	370	DBSU	33.6
**4213**	allXL	BC	382	373	DBSU	17.13
**4214**	allXL	AA	320	357	BS2G	22.89
**4214**	allXL	DD	320	373	BS2G	18.28
**4214**	allXL	DD	373	382	BS2G	15.7
**4214**	allXL	DD	370	386	BS2G	11.53
**4214**	allXL	DD	373	386	BS2G	15.84
**4214**	allXL	AA	319	357	BS2G	20.71
**4214**	allXL	DD	367	373	BS2G	13.6
**4214**	allXL	DD	370	382	BS2G	17.2
**4214**	allXL	BB	376	382	BS2G	15.05
**4214**	allXL	AA	357	373	BS2G	31.31
**4214**	allXL	DD	320	366	BS2G	21.77
**4214**	allXL	CC	370	376	BS2G	15.26
**4214**	allXL	AA	320	357	DBSU	22.89
**4214**	allXL	AA	357	319	DBSU	20.71
**4214**	allXL	DD	372	319	DBSU	22.12
**4214**	allXL	BB	370	357	DBSU	29.18
**4214**	allXL	AA	382	357	DBSU	23.58
**4214**	allXL	AA	372	370	DBSU	6.46
**4214**	allXL	DD	381	370	DBSU	17.62
**4214**	allXL	BB	373	370	DBSU	8.77
**4214**	allXL	DD	381	372	DBSU	14.73
**4214**	allXL	DD	372	382	DBSU	15.5
**4214**	allXL	DD	372	386	DBSU	13.26

**4214**	allXL	DD	373	386	DBSU	15.84
**4214**	allXL	AC	381	386	DBSU	46.06
**4214**	allXL	BD;DB	357	357	DBSU	18.04
**4214**	allXL	AC	373	319	DBSU	32.4
**4214**	allXL	AC	372	357	DBSU	28.75
**4214**	allXL	AC	381	357	DBSU	13.58
**4214**	allXL	AC	373	357	DBSU	26.16
**4214**	allXL	AC	372	370	DBSU	48.79
**4214**	allXL	AC	373	370	DBSU	45.59
**4214**	allXL	AC	382	373	DBSU	43.84
**4215**	allXL	CC	320	357	BS2G	23.4
**4215**	allXL	DD	320	373	BS2G	23.57
**4215**	allXL	BB	373	382	BS2G	16.04
**4215**	allXL	DD	370	386	BS2G	24.45
**4215**	allXL	BB	373	386	BS2G	25.0
**4215**	allXL	AA	319	357	BS2G	23.25
**4215**	allXL	AA	367	373	BS2G	12.72
**4215**	allXL	DD	370	382	BS2G	21.58
**4215**	allXL	BB	376	382	BS2G	13.7
**4215**	allXL	BB	357	373	BS2G	28.89
**4215**	allXL	AA	320	366	BS2G	26.18
**4215**	allXL	CC	370	376	BS2G	17.24
**4215**	allXL	CC	320	357	DBSU	23.4
**4215**	allXL	AA	357	319	DBSU	23.25
**4215**	allXL	DD	372	319	DBSU	23.63
**4215**	allXL	DD	370	357	DBSU	22.89
**4215**	allXL	BB	382	357	DBSU	21.78
**4215**	allXL	AA	372	370	DBSU	6.24
**4215**	allXL	DD	381	370	DBSU	19.65
**4215**	allXL	AA	373	370	DBSU	9.17
**4215**	allXL	BB	381	372	DBSU	18.53
**4215**	allXL	BB	372	382	DBSU	18.35
**4215**	allXL	BB	372	386	DBSU	26.07
**4215**	allXL	BB	373	386	DBSU	25.0

**4215**	allXL	DB	381	386	DBSU	43.09
**4215**	allXL	BD;DB	357	357	DBSU	18.11
**4215**	allXL	BC	373	319	DBSU	20.53
**4215**	allXL	BC	372	357	DBSU	38.18
**4215**	allXL	BD	381	357	DBSU	33.82
**4215**	allXL	BC	373	357	DBSU	36.41
**4215**	allXL	BC	372	370	DBSU	39.96
**4215**	allXL	BC	373	370	DBSU	40.76
**4215**	allXL	BD	382	373	DBSU	50.22
**4216**	allXL	AA	320	357	BS2G	25.54
**4216**	allXL	DD	320	373	BS2G	15.74
**4216**	allXL	DD	373	382	BS2G	18.5
**4216**	allXL	DD	370	386	BS2G	19.14
**4216**	allXL	BB	373	386	BS2G	25.26
**4216**	allXL	AA	319	357	BS2G	24.46
**4216**	allXL	BB	367	373	BS2G	12.85
**4216**	allXL	DD	370	382	BS2G	13.39
**4216**	allXL	CC	376	382	BS2G	14.9
**4216**	allXL	BB	357	373	BS2G	24.06
**4216**	allXL	AA	320	366	BS2G	18.42
**4216**	allXL	CC;DD	370	376	BS2G	15.91
**4216**	allXL	AA	320	357	DBSU	25.54
**4216**	allXL	AA	357	319	DBSU	24.46
**4216**	allXL	DD	372	319	DBSU	12.79
**4216**	allXL	BB	370	357	DBSU	27.93
**4216**	allXL	BB	382	357	DBSU	15.72
**4216**	allXL	AA	372	370	DBSU	5.97
**4216**	allXL	DD	381	370	DBSU	11.91
**4216**	allXL	AA	373	370	DBSU	9.11
**4216**	allXL	DD	381	372	DBSU	16.1
**4216**	allXL	DD	372	382	DBSU	18.73
**4216**	allXL	DD	372	386	DBSU	26.22
**4216**	allXL	BB	373	386	DBSU	25.26
**4216**	allXL	CB	381	386	DBSU	18.4

**4216**	allXL	BD;DB	357	357	DBSU	18.34
**4216**	allXL	DA	373	319	DBSU	32.72
**4216**	allXL	DA	372	357	DBSU	39.15
**4216**	allXL	CB	381	357	DBSU	34.45
**4216**	allXL	DA	373	357	DBSU	36.13
**4216**	allXL	DA	372	370	DBSU	21.74
**4216**	allXL	DA	373	370	DBSU	18.47
**4216**	allXL	CB	382	373	DBSU	21.03
**4217**	allXL	AA	320	357	BS2G	24.71
**4217**	allXL	AA	320	373	BS2G	25.87
**4217**	allXL	DD	373	382	BS2G	15.63
**4217**	allXL	CC	370	386	BS2G	22.61
**4217**	allXL	AA	373	386	BS2G	22.47
**4217**	allXL	AA	319	357	BS2G	25.52
**4217**	allXL	DD	367	373	BS2G	12.48
**4217**	allXL	DD	370	382	BS2G	18.22
**4217**	allXL	CC	376	382	BS2G	14.19
**4217**	allXL	BB	357	373	BS2G	26.02
**4217**	allXL	AA	320	366	BS2G	13.55
**4217**	allXL	CC	370	376	BS2G	13.75
**4217**	allXL	AA	320	357	DBSU	24.71
**4217**	allXL	AA	357	319	DBSU	25.52
**4217**	allXL	AA	372	319	DBSU	24.42
**4217**	allXL	BB	370	357	DBSU	20.61
**4217**	allXL	DD	382	357	DBSU	16.79
**4217**	allXL	AA	372	370	DBSU	6.28
**4217**	allXL	DD	381	370	DBSU	19.22
**4217**	allXL	DD	373	370	DBSU	8.72
**4217**	allXL	DD	381	372	DBSU	18.81
**4217**	allXL	DD	372	382	DBSU	18.61
**4217**	allXL	AA	372	386	DBSU	25.22
**4217**	allXL	AA	373	386	DBSU	22.47
**4217**	allXL	BD	381	386	DBSU	14.21
**4217**	allXL	BD;DB	357	357	DBSU	19.04

**4217**	allXL	AD	373	319	DBSU	21.28
**4217**	allXL	BD	372	357	DBSU	36.43
**4217**	allXL	BD	381	357	DBSU	27.08
**4217**	allXL	BD	373	357	DBSU	35.22
**4217**	allXL	BD	372	370	DBSU	46.1
**4217**	allXL	BD	373	370	DBSU	43.99
**4217**	allXL	DB	382	373	DBSU	30.83
**4218**	allXL	AA	320	357	BS2G	23.54
**4218**	allXL	BB	320	373	BS2G	24.46
**4218**	allXL	DD	373	382	BS2G	16.66
**4218**	allXL	AA	370	386	BS2G	24.44
**4218**	allXL	DD	373	386	BS2G	23.25
**4218**	allXL	AA	319	357	BS2G	21.84
**4218**	allXL	AA	367	373	BS2G	12.4
**4218**	allXL	DD	370	382	BS2G	21.45
**4218**	allXL	CC	376	382	BS2G	14.68
**4218**	allXL	BB	357	373	BS2G	25.44
**4218**	allXL	AA	320	366	BS2G	18.14
**4218**	allXL	CC	370	376	BS2G	16.86
**4218**	allXL	AA	320	357	DBSU	23.54
**4218**	allXL	AA	357	319	DBSU	21.84
**4218**	allXL	CC	372	319	DBSU	26.61
**4218**	allXL	BB	370	357	DBSU	27.74
**4218**	allXL	BB	382	357	DBSU	11.18
**4218**	allXL	AA	372	370	DBSU	6.31
**4218**	allXL	DD	381	370	DBSU	22.75
**4218**	allXL	AA	373	370	DBSU	8.9
**4218**	allXL	DD	381	372	DBSU	17.0
**4218**	allXL	DD	372	382	DBSU	15.99
**4218**	allXL	DD	372	386	DBSU	20.83
**4218**	allXL	DD	373	386	DBSU	23.25
**4218**	allXL	BC	381	386	DBSU	44.52
**4218**	allXL	BD;DB	357	357	DBSU	19.01
**4218**	allXL	CB	373	319	DBSU	32.6

**4218**	allXL	CB	372	357	DBSU	43.76
**4218**	allXL	BD	381	357	DBSU	26.3
**4218**	allXL	BD	373	357	DBSU	42.86
**4218**	allXL	CB	372	370	DBSU	31.18
**4218**	allXL	CB	373	370	DBSU	32.16
**4218**	allXL	CB	382	373	DBSU	36.98
**4219**	allXL	AA	320	357	BS2G	25.09
**4219**	allXL	CC	320	373	BS2G	22.94
**4219**	allXL	BB;CC	373	382	BS2G	17.26
**4219**	allXL	DD	370	386	BS2G	23.64
**4219**	allXL	DD	373	386	BS2G	23.8
**4219**	allXL	AA	319	357	BS2G	24.95
**4219**	allXL	AA	367	373	BS2G	12.73
**4219**	allXL	DD	370	382	BS2G	22.11
**4219**	allXL	BB	376	382	BS2G	14.24
**4219**	allXL	CC	357	373	BS2G	29.47
**4219**	allXL	AA	320	366	BS2G	25.86
**4219**	allXL	DD	370	376	BS2G	16.4
**4219**	allXL	AA	320	357	DBSU	25.09
**4219**	allXL	AA	357	319	DBSU	24.95
**4219**	allXL	CC	372	319	DBSU	21.57
**4219**	allXL	DD	370	357	DBSU	26.89
**4219**	allXL	BB	382	357	DBSU	22.65
**4219**	allXL	AA	372	370	DBSU	6.34
**4219**	allXL	DD	381	370	DBSU	21.21
**4219**	allXL	CC	373	370	DBSU	9.22
**4219**	allXL	BB	381	372	DBSU	18.85
**4219**	allXL	BB	372	382	DBSU	17.03
**4219**	allXL	BB	372	386	DBSU	24.04
**4219**	allXL	DD	373	386	DBSU	23.8
**4219**	allXL	CA	381	386	DBSU	45.34
**4219**	allXL	BD;DB	357	357	DBSU	18.81
**4219**	allXL	AD	373	319	DBSU	20.37
**4219**	allXL	AC	372	357	DBSU	40.14

**4219**	allXL	BD	381	357	DBSU	39.51
**4219**	allXL	AC	373	357	DBSU	40.19
**4219**	allXL	CB	372	370	DBSU	44.39
**4219**	allXL	CB	373	370	DBSU	47.11
**4219**	allXL	CA	382	373	DBSU	59.35
**4220**	allXL	AA	320	357	BS2G	23.79
**4220**	allXL	AA	320	373	BS2G	16.36
**4220**	allXL	CC	373	382	BS2G	17.07
**4220**	allXL	DD	370	386	BS2G	23.77
**4220**	allXL	DD	373	386	BS2G	21.04
**4220**	allXL	AA	319	357	BS2G	26.56
**4220**	allXL	BB	367	373	BS2G	10.55
**4220**	allXL	CC	370	382	BS2G	18.29
**4220**	allXL	CC	376	382	BS2G	14.25
**4220**	allXL	CC	357	373	BS2G	27.74
**4220**	allXL	AA	320	366	BS2G	21.56
**4220**	allXL	CC	370	376	BS2G	12.03
**4220**	allXL	AA	320	357	DBSU	23.79
**4220**	allXL	AA	357	319	DBSU	26.56
**4220**	allXL	AA	372	319	DBSU	19.36
**4220**	allXL	CC	370	357	DBSU	22.93
**4220**	allXL	BB	382	357	DBSU	18.66
**4220**	allXL	AA	372	370	DBSU	6.36
**4220**	allXL	CC	381	370	DBSU	19.12
**4220**	allXL	AA	373	370	DBSU	8.96
**4220**	allXL	DD	381	372	DBSU	20.64
**4220**	allXL	CC	372	382	DBSU	19.07
**4220**	allXL	DD	372	386	DBSU	21.8
**4220**	allXL	DD	373	386	DBSU	21.04
**4220**	allXL	AD	381	386	DBSU	48.78
**4220**	allXL	BD;DB	357	357	DBSU	17.94
**4220**	allXL	AD	373	319	DBSU	23.13
**4220**	allXL	AD	372	357	DBSU	30.92
**4220**	allXL	CA	381	357	DBSU	36.16

**4220**	allXL	AD	373	357	DBSU	33.95
**4220**	allXL	AD	372	370	DBSU	24.53
**4220**	allXL	AD	373	370	DBSU	24.79
**4220**	allXL	AD	382	373	DBSU	25.9
**4221**	allXL	CC	320	357	BS2G	22.91
**4221**	allXL	DD	320	373	BS2G	22.81
**4221**	allXL	BB	373	382	BS2G	19.74
**4221**	allXL	BB	370	386	BS2G	19.0
**4221**	allXL	BB	373	386	BS2G	24.08
**4221**	allXL	AA	319	357	BS2G	20.76
**4221**	allXL	BB	367	373	BS2G	12.57
**4221**	allXL	BB	370	382	BS2G	18.35
**4221**	allXL	BB	376	382	BS2G	14.34
**4221**	allXL	BB	357	373	BS2G	28.79
**4221**	allXL	AA	320	366	BS2G	25.78
**4221**	allXL	BB	370	376	BS2G	14.67
**4221**	allXL	CC	320	357	DBSU	22.91
**4221**	allXL	AA	357	319	DBSU	20.76
**4221**	allXL	DD	372	319	DBSU	26.34
**4221**	allXL	BB	370	357	DBSU	26.36
**4221**	allXL	BB	382	357	DBSU	37.16
**4221**	allXL	AA	372	370	DBSU	6.57
**4221**	allXL	BB	381	370	DBSU	20.69
**4221**	allXL	AA	373	370	DBSU	7.69
**4221**	allXL	DD	381	372	DBSU	19.81
**4221**	allXL	BB	372	382	DBSU	21.44
**4221**	allXL	BB	372	386	DBSU	24.0
**4221**	allXL	BB	373	386	DBSU	24.08
**4221**	allXL	AD	381	386	DBSU	62.36
**4221**	allXL	BD;DB	357	357	DBSU	18.86
**4221**	allXL	AD	373	319	DBSU	39.94
**4221**	allXL	BD	372	357	DBSU	41.58
**4221**	allXL	BD	381	357	DBSU	54.1
**4221**	allXL	BD	373	357	DBSU	44.97

**4221**	allXL	DA	372	370	DBSU	52.09
**4221**	allXL	DA	373	370	DBSU	48.32
**4221**	allXL	DA	382	373	DBSU	51.38
**4222**	allXL	AA	320	357	BS2G	22.97
**4222**	allXL	CC	320	373	BS2G	15.87
**4222**	allXL	AA	373	382	BS2G	17.78
**4222**	allXL	AA	370	386	BS2G	26.17
**4222**	allXL	AA	373	386	BS2G	25.55
**4222**	allXL	AA	319	357	BS2G	22.38
**4222**	allXL	AA	367	373	BS2G	11.84
**4222**	allXL	DD	370	382	BS2G	19.41
**4222**	allXL	BB	376	382	BS2G	14.13
**4222**	allXL	CC	357	373	BS2G	32.96
**4222**	allXL	CC	320	366	BS2G	27.85
**4222**	allXL	CC	370	376	BS2G	15.71
**4222**	allXL	AA	320	357	DBSU	22.97
**4222**	allXL	AA	357	319	DBSU	22.38
**4222**	allXL	CC	372	319	DBSU	19.14
**4222**	allXL	BB	370	357	DBSU	26.98
**4222**	allXL	DD	382	357	DBSU	46.7
**4222**	allXL	AA	372	370	DBSU	6.37
**4222**	allXL	DD	381	370	DBSU	19.84
**4222**	allXL	AA	373	370	DBSU	8.79
**4222**	allXL	DD	381	372	DBSU	18.13
**4222**	allXL	DD	372	382	DBSU	18.2
**4222**	allXL	AA	372	386	DBSU	24.91
**4222**	allXL	AA	373	386	DBSU	25.55
**4222**	allXL	BC	381	386	DBSU	31.86
**4222**	allXL	BD;DB	357	357	DBSU	19.14
**4222**	allXL	AD	373	319	DBSU	31.35
**4222**	allXL	BD	372	357	DBSU	33.78
**4222**	allXL	CB	381	357	DBSU	51.47
**4222**	allXL	BD	373	357	DBSU	37.13
**4222**	allXL	CB	372	370	DBSU	36.59

**4222**	allXL	CB	373	370	DBSU	35.86
**4222**	allXL	BC	382	373	DBSU	42.28
**4223**	allXL	CC	320	357	BS2G	25.26
**4223**	allXL	BB	320	373	BS2G	18.97
**4223**	allXL	CC	373	382	BS2G	18.45
**4223**	allXL	CC	370	386	BS2G	15.9
**4223**	allXL	CC	373	386	BS2G	19.36
**4223**	allXL	AA	319	357	BS2G	25.42
**4223**	allXL	BB	367	373	BS2G	12.3
**4223**	allXL	CC	370	382	BS2G	20.09
**4223**	allXL	CC	376	382	BS2G	13.56
**4223**	allXL	BB	357	373	BS2G	20.28
**4223**	allXL	AA	320	366	BS2G	20.72
**4223**	allXL	CC	370	376	BS2G	14.89
**4223**	allXL	CC	320	357	DBSU	25.26
**4223**	allXL	AA	357	319	DBSU	25.42
**4223**	allXL	DD	372	319	DBSU	18.59
**4223**	allXL	DD	370	357	DBSU	26.05
**4223**	allXL	BB	382	357	DBSU	21.14
**4223**	allXL	AA	372	370	DBSU	6.5
**4223**	allXL	DD	381	370	DBSU	19.28
**4223**	allXL	AA	373	370	DBSU	9.25
**4223**	allXL	DD	381	372	DBSU	18.46
**4223**	allXL	CC	372	382	DBSU	20.38
**4223**	allXL	CC	372	386	DBSU	19.1
**4223**	allXL	CC	373	386	DBSU	19.36
**4223**	allXL	CB	381	386	DBSU	34.89
**4223**	allXL	BD;DB	357	357	DBSU	18.57
**4223**	allXL	CB	373	319	DBSU	20.02
**4223**	allXL	DA	372	357	DBSU	27.59
**4223**	allXL	BD	381	357	DBSU	34.54
**4223**	allXL	DA	373	357	DBSU	28.47
**4223**	allXL	CB	372	370	DBSU	19.47
**4223**	allXL	CB	373	370	DBSU	16.89

**4223**	allXL	BC	382	373	DBSU	27.6
**4224**	allXL	AA	320	357	BS2G	24.69
**4224**	allXL	CC	320	373	BS2G	17.71
**4224**	allXL	DD	373	382	BS2G	20.7
**4224**	allXL	DD	370	386	BS2G	15.21
**4224**	allXL	DD	373	386	BS2G	20.26
**4224**	allXL	AA	319	357	BS2G	26.02
**4224**	allXL	BB	367	373	BS2G	12.28
**4224**	allXL	DD	370	382	BS2G	20.71
**4224**	allXL	CC	376	382	BS2G	14.77
**4224**	allXL	BB	357	373	BS2G	27.59
**4224**	allXL	AA	320	366	BS2G	22.89
**4224**	allXL	CC	370	376	BS2G	13.1
**4224**	allXL	AA	320	357	DBSU	24.69
**4224**	allXL	AA	357	319	DBSU	26.02
**4224**	allXL	CC	372	319	DBSU	19.46
**4224**	allXL	DD	370	357	DBSU	22.19
**4224**	allXL	CC	382	357	DBSU	35.67
**4224**	allXL	AA	372	370	DBSU	6.06
**4224**	allXL	DD	381	370	DBSU	20.52
**4224**	allXL	AA	373	370	DBSU	9.32
**4224**	allXL	DD	381	372	DBSU	21.46
**4224**	allXL	DD	372	382	DBSU	22.68
**4224**	allXL	DD	372	386	DBSU	20.33
**4224**	allXL	DD	373	386	DBSU	20.26
**4224**	allXL	AD	381	386	DBSU	59.03
**4224**	allXL	BD;DB	357	357	DBSU	18.37
**4224**	allXL	AD	373	319	DBSU	31.31
**4224**	allXL	BD	372	357	DBSU	40.06
**4224**	allXL	CB	381	357	DBSU	40.12
**4224**	allXL	BD	373	357	DBSU	38.18
**4224**	allXL	DA	372	370	DBSU	41.07
**4224**	allXL	DA	373	370	DBSU	41.61
**4224**	allXL	CB	382	373	DBSU	45.4

**4225**	allXL	CC	320	357	BS2G	26.19
**4225**	allXL	DD	320	373	BS2G	24.5
**4225**	allXL	BB	373	382	BS2G	14.68
**4225**	allXL	AA	370	386	BS2G	23.11
**4225**	allXL	CC	373	386	BS2G	21.65
**4225**	allXL	CC	319	357	BS2G	26.54
**4225**	allXL	BB	367	373	BS2G	14.73
**4225**	allXL	DD	370	382	BS2G	22.32
**4225**	allXL	BB	376	382	BS2G	12.11
**4225**	allXL	DD	357	373	BS2G	32.56
**4225**	allXL	AA	320	366	BS2G	20.55
**4225**	allXL	CC	370	376	BS2G	16.49
**4225**	allXL	CC	320	357	DBSU	26.19
**4225**	allXL	CC	357	319	DBSU	26.54
**4225**	allXL	DD	372	319	DBSU	26.46
**4225**	allXL	DD	370	357	DBSU	24.12
**4225**	allXL	BB	382	357	DBSU	32.32
**4225**	allXL	AA	372	370	DBSU	6.19
**4225**	allXL	DD	381	370	DBSU	21.4
**4225**	allXL	AA	373	370	DBSU	9.21
**4225**	allXL	BB	381	372	DBSU	19.31
**4225**	allXL	BB	372	382	DBSU	16.71
**4225**	allXL	BB	372	386	DBSU	23.01
**4225**	allXL	CC	373	386	DBSU	21.65
**4225**	allXL	BC	381	386	DBSU	34.42
**4225**	allXL	BD;DB	357	357	DBSU	18.97
**4225**	allXL	CB	373	319	DBSU	19.77
**4225**	allXL	CB	372	357	DBSU	45.25
**4225**	allXL	BD	381	357	DBSU	52.57
**4225**	allXL	CB	373	357	DBSU	47.8
**4225**	allXL	BC	372	370	DBSU	47.23
**4225**	allXL	BC	373	370	DBSU	45.11
**4225**	allXL	BC	382	373	DBSU	32.76
**4226**	allXL	CC	320	357	BS2G	22.9

**4226**	allXL	BB	320	373	BS2G	24.47
**4226**	allXL	BB	373	382	BS2G	15.43
**4226**	allXL	BB	370	386	BS2G	21.92
**4226**	allXL	BB	373	386	BS2G	17.75
**4226**	allXL	CC	319	357	BS2G	24.82
**4226**	allXL	AA	367	373	BS2G	12.84
**4226**	allXL	DD	370	382	BS2G	21.64
**4226**	allXL	BB	376	382	BS2G	13.45
**4226**	allXL	AA	357	373	BS2G	30.37
**4226**	allXL	BB	320	366	BS2G	16.32
**4226**	allXL	CC	370	376	BS2G	17.13
**4226**	allXL	CC	320	357	DBSU	22.9
**4226**	allXL	CC	357	319	DBSU	24.82
**4226**	allXL	CC	372	319	DBSU	21.74
**4226**	allXL	BB	370	357	DBSU	26.51
**4226**	allXL	AA	382	357	DBSU	26.63
**4226**	allXL	AA	372	370	DBSU	6.33
**4226**	allXL	DD	381	370	DBSU	22.2
**4226**	allXL	AA	373	370	DBSU	9.29
**4226**	allXL	DD	381	372	DBSU	18.47
**4226**	allXL	BB	372	382	DBSU	16.72
**4226**	allXL	BB	372	386	DBSU	16.35
**4226**	allXL	BB	373	386	DBSU	17.75
**4226**	allXL	CB	381	386	DBSU	16.37
**4226**	allXL	BD;DB	357	357	DBSU	19.59
**4226**	allXL	AD	373	319	DBSU	22.64
**4226**	allXL	CB	372	357	DBSU	28.25
**4226**	allXL	AC	381	357	DBSU	36.29
**4226**	allXL	CB	373	357	DBSU	28.7
**4226**	allXL	CB	372	370	DBSU	29.18
**4226**	allXL	CB	373	370	DBSU	28.06
**4226**	allXL	CB	382	373	DBSU	28.37
**4227**	allXL	CC	320	357	BS2G	24.28
**4227**	allXL	DD	320	373	BS2G	21.36

**4227**	allXL	DD	373	382	BS2G	18.92
**4227**	allXL	CC	370	386	BS2G	32.08
**4227**	allXL	DD	373	386	BS2G	26.16
**4227**	allXL	AA	319	357	BS2G	23.3
**4227**	allXL	AA	367	373	BS2G	14.57
**4227**	allXL	DD	370	382	BS2G	22.89
**4227**	allXL	CC	376	382	BS2G	14.73
**4227**	allXL	BB;DD	357	373	BS2G	24.15
**4227**	allXL	CC	320	366	BS2G	23.7
**4227**	allXL	DD	370	376	BS2G	16.28
**4227**	allXL	CC	320	357	DBSU	24.28
**4227**	allXL	AA	357	319	DBSU	23.3
**4227**	allXL	DD	372	319	DBSU	20.3
**4227**	allXL	DD	370	357	DBSU	19.07
**4227**	allXL	BB	382	357	DBSU	38.35
**4227**	allXL	AA	372	370	DBSU	5.95
**4227**	allXL	DD	381	370	DBSU	21.75
**4227**	allXL	AA	373	370	DBSU	9.08
**4227**	allXL	DD	381	372	DBSU	19.53
**4227**	allXL	DD	372	382	DBSU	20.06
**4227**	allXL	DD	372	386	DBSU	27.81
**4227**	allXL	DD	373	386	DBSU	26.16
**4227**	allXL	AD	381	386	DBSU	32.14
**4227**	allXL	BD;DB	357	357	DBSU	17.89
**4227**	allXL	AD	373	319	DBSU	17.82
**4227**	allXL	AC	372	357	DBSU	26.87
**4227**	allXL	DA	381	357	DBSU	36.42
**4227**	allXL	AC	373	357	DBSU	28.59
**4227**	allXL	DA	372	370	DBSU	29.75
**4227**	allXL	DA	373	370	DBSU	27.85
**4227**	allXL	DA	382	373	DBSU	33.52
**4228**	allXL	CC	320	357	BS2G	26.57
**4228**	allXL	AA	320	373	BS2G	16.69
**4228**	allXL	CC	373	382	BS2G	16.83

**4228**	allXL	CC	370	386	BS2G	22.59
**4228**	allXL	CC	373	386	BS2G	19.48
**4228**	allXL	AA	319	357	BS2G	26.96
**4228**	allXL	BB	367	373	BS2G	13.05
**4228**	allXL	DD	370	382	BS2G	18.26
**4228**	allXL	CC	376	382	BS2G	13.87
**4228**	allXL	BB	357	373	BS2G	22.82
**4228**	allXL	AA	320	366	BS2G	21.08
**4228**	allXL	DD	370	376	BS2G	16.09
**4228**	allXL	CC	320	357	DBSU	26.57
**4228**	allXL	AA	357	319	DBSU	26.96
**4228**	allXL	AA	372	319	DBSU	19.81
**4228**	allXL	BB	370	357	DBSU	21.87
**4228**	allXL	BB	382	357	DBSU	17.63
**4228**	allXL	AA	372	370	DBSU	6.12
**4228**	allXL	DD	381	370	DBSU	16.46
**4228**	allXL	CC	373	370	DBSU	9.02
**4228**	allXL	CC	381	372	DBSU	18.93
**4228**	allXL	CC	372	382	DBSU	17.64
**4228**	allXL	CC	372	386	DBSU	17.68
**4228**	allXL	CC	373	386	DBSU	19.48
**4228**	allXL	BD	381	386	DBSU	28.09
**4228**	allXL	BD;DB	357	357	DBSU	17.99
**4228**	allXL	AD	373	319	DBSU	30.17
**4228**	allXL	BC	372	357	DBSU	36.27
**4228**	allXL	DB	381	357	DBSU	28.98
**4228**	allXL	BD	373	357	DBSU	38.44
**4228**	allXL	BC	372	370	DBSU	32.69
**4228**	allXL	BC	373	370	DBSU	33.89
**4228**	allXL	CB	382	373	DBSU	33.72
**4229**	allXL	CC	320	357	BS2G	23.23
**4229**	allXL	BB	320	373	BS2G	28.33
**4229**	allXL	BB	373	382	BS2G	21.29
**4229**	allXL	BB	370	386	BS2G	31.29

**4229**	allXL	BB	373	386	BS2G	28.81
**4229**	allXL	CC	319	357	BS2G	23.16
**4229**	allXL	BB	367	373	BS2G	13.61
**4229**	allXL	BB	370	382	BS2G	26.84
**4229**	allXL	BB	376	382	BS2G	14.57
**4229**	allXL	DD	357	373	BS2G	33.94
**4229**	allXL	AA	320	366	BS2G	21.8
**4229**	allXL	CC	370	376	BS2G	15.02
**4229**	allXL	CC	320	357	DBSU	23.23
**4229**	allXL	CC	357	319	DBSU	23.16
**4229**	allXL	BB	372	319	DBSU	27.41
**4229**	allXL	DD	370	357	DBSU	28.77
**4229**	allXL	BB	382	357	DBSU	29.02
**4229**	allXL	AA	372	370	DBSU	5.85
**4229**	allXL	CC	381	370	DBSU	27.3
**4229**	allXL	AA	373	370	DBSU	9.15
**4229**	allXL	DD	381	372	DBSU	22.95
**4229**	allXL	BB	372	382	DBSU	23.33
**4229**	allXL	BB	372	386	DBSU	30.15
**4229**	allXL	BB	373	386	DBSU	28.81
**4229**	allXL	DB	381	386	DBSU	57.58
**4229**	allXL	BD;DB	357	357	DBSU	19.29
**4229**	allXL	CB	373	319	DBSU	15.03
**4229**	allXL	CB	372	357	DBSU	41.74
**4229**	allXL	CA	381	357	DBSU	38.35
**4229**	allXL	DB	373	357	DBSU	42.46
**4229**	allXL	CB	372	370	DBSU	28.06
**4229**	allXL	CB	373	370	DBSU	31.0
**4229**	allXL	BC	382	373	DBSU	45.21
**4230**	allXL	AA	320	357	BS2G	22.59
**4230**	allXL	BB	320	373	BS2G	17.15
**4230**	allXL	CC	373	382	BS2G	20.57
**4230**	allXL	BB	370	386	BS2G	29.46
**4230**	allXL	BB	373	386	BS2G	29.92

**4230**	allXL	AA	319	357	BS2G	20.23
**4230**	allXL	AA	367	373	BS2G	14.03
**4230**	allXL	BB	370	382	BS2G	22.87
**4230**	allXL	CC	376	382	BS2G	14.47
**4230**	allXL	BB	357	373	BS2G	16.76
**4230**	allXL	BB	320	366	BS2G	18.08
**4230**	allXL	CC	370	376	BS2G	16.17
**4230**	allXL	AA	320	357	DBSU	22.59
**4230**	allXL	AA	357	319	DBSU	20.23
**4230**	allXL	BB	372	319	DBSU	19.15
**4230**	allXL	BB	370	357	DBSU	24.11
**4230**	allXL	BB	382	357	DBSU	35.64
**4230**	allXL	AA	372	370	DBSU	6.06
**4230**	allXL	BB	381	370	DBSU	22.03
**4230**	allXL	AA	373	370	DBSU	9.16
**4230**	allXL	BB	381	372	DBSU	21.77
**4230**	allXL	BB	372	382	DBSU	23.39
**4230**	allXL	BB	372	386	DBSU	31.73
**4230**	allXL	BB	373	386	DBSU	29.92
**4230**	allXL	BC	381	386	DBSU	49.67
**4230**	allXL	BD;DB	357	357	DBSU	18.45
**4230**	allXL	CB	373	319	DBSU	28.53
**4230**	allXL	CB	372	357	DBSU	32.43
**4230**	allXL	CB	381	357	DBSU	31.6
**4230**	allXL	CB	373	357	DBSU	30.98
**4230**	allXL	BC	372	370	DBSU	38.54
**4230**	allXL	CB	373	370	DBSU	36.76
**4230**	allXL	CB	382	373	DBSU	31.15
**4231**	allXL	AA	320	357	BS2G	25.53
**4231**	allXL	AA	320	373	BS2G	28.67
**4231**	allXL	AA	373	382	BS2G	19.31
**4231**	allXL	DD	370	386	BS2G	22.2
**4231**	allXL	DD	373	386	BS2G	24.3
**4231**	allXL	AA	319	357	BS2G	24.66

**4231**	allXL	AA	367	373	BS2G	13.9
**4231**	allXL	DD	370	382	BS2G	20.25
**4231**	allXL	AA	376	382	BS2G	15.41
**4231**	allXL	CC	357	373	BS2G	13.96
**4231**	allXL	AA	320	366	BS2G	16.97
**4231**	allXL	CC	370	376	BS2G	16.63
**4231**	allXL	AA	320	357	DBSU	25.53
**4231**	allXL	AA	357	319	DBSU	24.66
**4231**	allXL	AA	372	319	DBSU	26.8
**4231**	allXL	CC	370	357	DBSU	14.75
**4231**	allXL	CC	382	357	DBSU	29.02
**4231**	allXL	AA	372	370	DBSU	6.29
**4231**	allXL	DD	381	370	DBSU	18.19
**4231**	allXL	AA	373	370	DBSU	9.18
**4231**	allXL	DD	381	372	DBSU	16.31
**4231**	allXL	DD	372	382	DBSU	19.18
**4231**	allXL	DD	372	386	DBSU	21.61
**4231**	allXL	DD	373	386	DBSU	24.3
**4231**	allXL	CA	381	386	DBSU	24.93
**4231**	allXL	BD;DB	357	357	DBSU	18.57
**4231**	allXL	AD	373	319	DBSU	39.58
**4231**	allXL	CA	372	357	DBSU	23.92
**4231**	allXL	CA	381	357	DBSU	19.5
**4231**	allXL	CA	373	357	DBSU	20.09
**4231**	allXL	DA	372	370	DBSU	40.2
**4231**	allXL	DA	373	370	DBSU	43.58
**4231**	allXL	CA	382	373	DBSU	41.67
**4232**	allXL	CC	320	357	BS2G	24.09
**4232**	allXL	AA	320	373	BS2G	22.22
**4232**	allXL	CC	373	382	BS2G	14.62
**4232**	allXL	CC	370	386	BS2G	22.78
**4232**	allXL	CC	373	386	BS2G	18.35
**4232**	allXL	CC	319	357	BS2G	26.3
**4232**	allXL	BB	367	373	BS2G	11.2

**4232**	allXL	DD	370	382	BS2G	23.24
**4232**	allXL	CC	376	382	BS2G	14.19
**4232**	allXL	BB	357	373	BS2G	30.17
**4232**	allXL	DD	320	366	BS2G	17.91
**4232**	allXL	BB	370	376	BS2G	16.94
**4232**	allXL	CC	320	357	DBSU	24.09
**4232**	allXL	CC	357	319	DBSU	26.3
**4232**	allXL	AA	372	319	DBSU	23.02
**4232**	allXL	CC	370	357	DBSU	26.89
**4232**	allXL	AA	382	357	DBSU	22.39
**4232**	allXL	AA	372	370	DBSU	6.4
**4232**	allXL	DD	381	370	DBSU	21.59
**4232**	allXL	AA	373	370	DBSU	9.15
**4232**	allXL	CC	381	372	DBSU	18.73
**4232**	allXL	CC	372	382	DBSU	16.88
**4232**	allXL	CC	372	386	DBSU	17.8
**4232**	allXL	CC	373	386	DBSU	18.35
**4232**	allXL	CA	381	386	DBSU	63.08
**4232**	allXL	BD;DB	357	357	DBSU	17.98
**4232**	allXL	AD	373	319	DBSU	25.48
**4232**	allXL	CA	372	357	DBSU	39.05
**4232**	allXL	AD	381	357	DBSU	27.77
**4232**	allXL	CA	373	357	DBSU	38.92
**4232**	allXL	AD	372	370	DBSU	45.73
**4232**	allXL	AD	373	370	DBSU	42.95
**4232**	allXL	AD	382	373	DBSU	43.41
**4233**	allXL	CC	320	357	BS2G	24.74
**4233**	allXL	BB	320	373	BS2G	23.72
**4233**	allXL	AA	373	382	BS2G	14.28
**4233**	allXL	AA	370	386	BS2G	13.28
**4233**	allXL	AA	373	386	BS2G	10.79
**4233**	allXL	CC	319	357	BS2G	25.13
**4233**	allXL	BB	367	373	BS2G	13.99
**4233**	allXL	CC	370	382	BS2G	18.36

**4233**	allXL	AA	376	382	BS2G	12.49
**4233**	allXL	CC	357	373	BS2G	32.78
**4233**	allXL	AA	320	366	BS2G	19.89
**4233**	allXL	CC	370	376	BS2G	13.03
**4233**	allXL	CC	320	357	DBSU	24.74
**4233**	allXL	CC	357	319	DBSU	25.13
**4233**	allXL	BB	372	319	DBSU	20.23
**4233**	allXL	DD	370	357	DBSU	28.51
**4233**	allXL	BB	382	357	DBSU	36.61
**4233**	allXL	AA	372	370	DBSU	6.09
**4233**	allXL	CC	381	370	DBSU	19.85
**4233**	allXL	CC	373	370	DBSU	9.05
**4233**	allXL	AA	381	372	DBSU	19.92
**4233**	allXL	AA	372	382	DBSU	17.55
**4233**	allXL	AA	372	386	DBSU	12.03
**4233**	allXL	AA	373	386	DBSU	10.79
**4233**	allXL	BC	381	386	DBSU	51.3
**4233**	allXL	BD;DB	357	357	DBSU	17.71
**4233**	allXL	CB	373	319	DBSU	18.12
**4233**	allXL	CB	372	357	DBSU	30.19
**4233**	allXL	DB	381	357	DBSU	43.44
**4233**	allXL	CB	373	357	DBSU	33.52
**4233**	allXL	CB	372	370	DBSU	19.75
**4233**	allXL	CB	373	370	DBSU	21.08
**4233**	allXL	CB	382	373	DBSU	27.76
**4234**	allXL	CC	320	357	BS2G	25.57
**4234**	allXL	BB	320	373	BS2G	25.52
**4234**	allXL	BB	373	382	BS2G	18.16
**4234**	allXL	CC	370	386	BS2G	19.72
**4234**	allXL	BB	373	386	BS2G	24.09
**4234**	allXL	AA	319	357	BS2G	25.63
**4234**	allXL	AA	367	373	BS2G	12.5
**4234**	allXL	CC	370	382	BS2G	13.41
**4234**	allXL	BB	376	382	BS2G	14.63

**4234**	allXL	BB	357	373	BS2G	26.45
**4234**	allXL	AA	320	366	BS2G	21.98
**4234**	allXL	CC	370	376	BS2G	11.97
**4234**	allXL	CC	320	357	DBSU	25.57
**4234**	allXL	AA	357	319	DBSU	25.63
**4234**	allXL	BB	372	319	DBSU	27.97
**4234**	allXL	DD	370	357	DBSU	22.83
**4234**	allXL	BB	382	357	DBSU	30.14
**4234**	allXL	AA	372	370	DBSU	6.42
**4234**	allXL	CC	381	370	DBSU	14.96
**4234**	allXL	AA	373	370	DBSU	9.09
**4234**	allXL	CC	381	372	DBSU	18.1
**4234**	allXL	CC	372	382	DBSU	17.74
**4234**	allXL	CC	372	386	DBSU	26.05
**4234**	allXL	BB	373	386	DBSU	24.09
**4234**	allXL	BC	381	386	DBSU	47.22
**4234**	allXL	BD;DB	357	357	DBSU	18.46
**4234**	allXL	AD	373	319	DBSU	11.44
**4234**	allXL	AD	372	357	DBSU	36.56
**4234**	allXL	AC	381	357	DBSU	32.99
**4234**	allXL	AD	373	357	DBSU	35.14
**4234**	allXL	AD	372	370	DBSU	39.45
**4234**	allXL	AD	373	370	DBSU	40.08
**4234**	allXL	CB	382	373	DBSU	48.52
**4235**	allXL	CC	320	357	BS2G	25.43
**4235**	allXL	CC	320	373	BS2G	25.73
**4235**	allXL	BB	373	382	BS2G	18.43
**4235**	allXL	DD	370	386	BS2G	25.48
**4235**	allXL	BB	373	386	BS2G	26.87
**4235**	allXL	CC	319	357	BS2G	27.96
**4235**	allXL	AA	367	373	BS2G	13.19
**4235**	allXL	BB	370	382	BS2G	21.41
**4235**	allXL	BB	376	382	BS2G	12.88
**4235**	allXL	DD	357	373	BS2G	27.36

**4235**	allXL	BB	320	366	BS2G	18.25
**4235**	allXL	DD	370	376	BS2G	16.43
**4235**	allXL	CC	320	357	DBSU	25.43
**4235**	allXL	CC	357	319	DBSU	27.96
**4235**	allXL	CC	372	319	DBSU	23.42
**4235**	allXL	DD	370	357	DBSU	21.42
**4235**	allXL	DD	382	357	DBSU	32.35
**4235**	allXL	DD	372	370	DBSU	6.56
**4235**	allXL	DD	381	370	DBSU	18.85
**4235**	allXL	AA	373	370	DBSU	9.29
**4235**	allXL	DD	381	372	DBSU	19.89
**4235**	allXL	BB	372	382	DBSU	21.13
**4235**	allXL	AA	372	386	DBSU	27.68
**4235**	allXL	BB	373	386	DBSU	26.87
**4235**	allXL	BD	381	386	DBSU	66.25
**4235**	allXL	AC;CA	357	357	DBSU	19.16
**4235**	allXL	DA	373	319	DBSU	19.33
**4235**	allXL	DB	372	357	DBSU	35.08
**4235**	allXL	DB	381	357	DBSU	32.26
**4235**	allXL	DA	373	357	DBSU	36.31
**4235**	allXL	DA	372	370	DBSU	35.92
**4235**	allXL	DA	373	370	DBSU	33.58
**4235**	allXL	CB	382	373	DBSU	49.39
**4236**	allXL	AA	320	357	BS2G	27.7
**4236**	allXL	BB	320	373	BS2G	17.91
**4236**	allXL	BB	373	382	BS2G	13.08
**4236**	allXL	BB	370	386	BS2G	20.09
**4236**	allXL	BB	373	386	BS2G	14.51
**4236**	allXL	AA	319	357	BS2G	29.15
**4236**	allXL	AA	367	373	BS2G	13.4
**4236**	allXL	BB	370	382	BS2G	21.63
**4236**	allXL	BB	376	382	BS2G	12.25
**4236**	allXL	CC	357	373	BS2G	22.05
**4236**	allXL	AA	320	366	BS2G	21.2

**4236**	allXL	CC	370	376	BS2G	14.54
**4236**	allXL	AA	320	357	DBSU	27.7
**4236**	allXL	AA	357	319	DBSU	29.15
**4236**	allXL	CC	372	319	DBSU	21.1
**4236**	allXL	DD	370	357	DBSU	25.52
**4236**	allXL	BB	382	357	DBSU	22.71
**4236**	allXL	AA	372	370	DBSU	6.28
**4236**	allXL	CC	381	370	DBSU	23.94
**4236**	allXL	AA	373	370	DBSU	9.06
**4236**	allXL	BB	381	372	DBSU	17.59
**4236**	allXL	BB	372	382	DBSU	14.32
**4236**	allXL	BB	372	386	DBSU	13.98
**4236**	allXL	BB	373	386	DBSU	14.51
**4236**	allXL	BD	381	386	DBSU	40.59
**4236**	allXL	BD;DB	357	357	DBSU	19.5
**4236**	allXL	CB	373	319	DBSU	25.5
**4236**	allXL	CB	372	357	DBSU	33.77
**4236**	allXL	DB	381	357	DBSU	32.62
**4236**	allXL	CB	373	357	DBSU	33.5
**4236**	allXL	CB	372	370	DBSU	13.63
**4236**	allXL	CB	373	370	DBSU	16.66
**4236**	allXL	BC	382	373	DBSU	35.32
**4237**	allXL	CC	320	357	BS2G	22.86
**4237**	allXL	DD	320	373	BS2G	19.16
**4237**	allXL	AA	373	382	BS2G	18.23
**4237**	allXL	AA	370	386	BS2G	29.11
**4237**	allXL	AA	373	386	BS2G	26.05
**4237**	allXL	CC	319	357	BS2G	23.98
**4237**	allXL	BB	367	373	BS2G	13.76
**4237**	allXL	DD	370	382	BS2G	24.32
**4237**	allXL	CC	376	382	BS2G	14.15
**4237**	allXL	CC	357	373	BS2G	27.42
**4237**	allXL	DD	320	366	BS2G	17.42
**4237**	allXL	CC	370	376	BS2G	16.1

**4237**	allXL	CC	320	357	DBSU	22.86
**4237**	allXL	CC	357	319	DBSU	23.98
**4237**	allXL	DD	372	319	DBSU	19.77
**4237**	allXL	CC	370	357	DBSU	25.61
**4237**	allXL	BB	382	357	DBSU	23.04
**4237**	allXL	AA	372	370	DBSU	6.5
**4237**	allXL	DD	381	370	DBSU	21.48
**4237**	allXL	AA	373	370	DBSU	9.11
**4237**	allXL	DD	381	372	DBSU	19.31
**4237**	allXL	AA	372	382	DBSU	21.54
**4237**	allXL	AA	372	386	DBSU	28.49
**4237**	allXL	AA	373	386	DBSU	26.05
**4237**	allXL	DA	381	386	DBSU	69.58
**4237**	allXL	BD;DB	357	357	DBSU	18.59
**4237**	allXL	BC	373	319	DBSU	40.26
**4237**	allXL	BC	372	357	DBSU	44.45
**4237**	allXL	BD	381	357	DBSU	45.18
**4237**	allXL	CA	373	357	DBSU	43.45
**4237**	allXL	BC	372	370	DBSU	44.95
**4237**	allXL	BC	373	370	DBSU	47.56
**4237**	allXL	AD	382	373	DBSU	64.02
**4238**	allXL	CC	320	357	BS2G	23.16
**4238**	allXL	DD	320	373	BS2G	9.42
**4238**	allXL	BB	373	382	BS2G	20.74
**4238**	allXL	DD	370	386	BS2G	28.01
**4238**	allXL	AA	373	386	BS2G	27.07
**4238**	allXL	CC	319	357	BS2G	23.61
**4238**	allXL	BB	367	373	BS2G	10.68
**4238**	allXL	BB	370	382	BS2G	22.01
**4238**	allXL	BB	376	382	BS2G	15.01
**4238**	allXL	BB	357	373	BS2G	29.45
**4238**	allXL	DD	320	366	BS2G	20.72
**4238**	allXL	BB	370	376	BS2G	15.37
**4238**	allXL	CC	320	357	DBSU	23.16

**4238**	allXL	CC	357	319	DBSU	23.61
**4238**	allXL	DD	372	319	DBSU	10.12
**4238**	allXL	DD	370	357	DBSU	27.85
**4238**	allXL	DC	382	357	DBSU	34.86
**4238**	allXL	AA	372	370	DBSU	6.4
**4238**	allXL	BB	381	370	DBSU	21.55
**4238**	allXL	CC	373	370	DBSU	9.21
**4238**	allXL	DD	381	372	DBSU	21.85
**4238**	allXL	BB	372	382	DBSU	22.72
**4238**	allXL	DD	372	386	DBSU	29.9
**4238**	allXL	AA	373	386	DBSU	27.07
**4238**	allXL	BD	381	386	DBSU	38.29
**4238**	allXL	BD;DB	357	357	DBSU	18.54
**4238**	allXL	BC	373	319	DBSU	26.66
**4238**	allXL	BD	372	357	DBSU	40.04
**4238**	allXL	DA	381	357	DBSU	32.76
**4238**	allXL	BD	373	357	DBSU	39.48
**4238**	allXL	CB	372	370	DBSU	28.87
**4238**	allXL	CB	373	370	DBSU	29.26
**4238**	allXL	AD	382	373	DBSU	34.95
**4239**	allXL	CC	320	357	BS2G	25.53
**4239**	allXL	CC	320	373	BS2G	9.96
**4239**	allXL	CC	373	382	BS2G	21.32
**4239**	allXL	CC	370	386	BS2G	24.47
**4239**	allXL	CC	373	386	BS2G	24.9
**4239**	allXL	AA	319	357	BS2G	24.99
**4239**	allXL	AA	367	373	BS2G	14.43
**4239**	allXL	CC	370	382	BS2G	23.34
**4239**	allXL	CC	376	382	BS2G	14.15
**4239**	allXL	BB	357	373	BS2G	27.66
**4239**	allXL	CC	320	366	BS2G	16.86
**4239**	allXL	CC	370	376	BS2G	15.85
**4239**	allXL	CC	320	357	DBSU	25.53
**4239**	allXL	AA	357	319	DBSU	24.99

**4239**	allXL	CC	372	319	DBSU	9.94
**4239**	allXL	CC	370	357	DBSU	29.04
**4239**	allXL	CC	382	357	DBSU	24.28
**4239**	allXL	AA	372	370	DBSU	6.12
**4239**	allXL	CC	381	370	DBSU	23.44
**4239**	allXL	AA	373	370	DBSU	9.23
**4239**	allXL	CC	381	372	DBSU	22.6
**4239**	allXL	CC	372	382	DBSU	22.05
**4239**	allXL	CC	372	386	DBSU	23.9
**4239**	allXL	CC	373	386	DBSU	24.9
**4239**	allXL	DA	381	386	DBSU	43.97
**4239**	allXL	BD;DB	357	357	DBSU	18.74
**4239**	allXL	DA	373	319	DBSU	19.11
**4239**	allXL	DA	372	357	DBSU	35.77
**4239**	allXL	CA	381	357	DBSU	34.57
**4239**	allXL	DA	373	357	DBSU	39.41
**4239**	allXL	DA	372	370	DBSU	19.42
**4239**	allXL	DA	373	370	DBSU	20.16
**4239**	allXL	DA	382	373	DBSU	21.69
**4240**	allXL	CC	320	357	BS2G	21.71
**4240**	allXL	DD	320	373	BS2G	28.4
**4240**	allXL	BB	373	382	BS2G	19.96
**4240**	allXL	BB	370	386	BS2G	14.32
**4240**	allXL	BB	373	386	BS2G	12.64
**4240**	allXL	AA	319	357	BS2G	20.76
**4240**	allXL	AA	367	373	BS2G	11.66
**4240**	allXL	CC	370	382	BS2G	21.99
**4240**	allXL	BB	376	382	BS2G	13.6
**4240**	allXL	BB	357	373	BS2G	24.47
**4240**	allXL	DD	320	366	BS2G	25.35
**4240**	allXL	CC	370	376	BS2G	15.08
**4240**	allXL	CC	320	357	DBSU	21.71
**4240**	allXL	AA	357	319	DBSU	20.76
**4240**	allXL	DD	372	319	DBSU	26.61

**4240**	allXL	DD	370	357	DBSU	29.32
**4240**	allXL	BB	382	357	DBSU	25.86
**4240**	allXL	AA	372	370	DBSU	6.24
**4240**	allXL	CC	381	370	DBSU	23.99
**4240**	allXL	AA	373	370	DBSU	9.01
**4240**	allXL	DD	381	372	DBSU	17.76
**4240**	allXL	DD	372	382	DBSU	20.13
**4240**	allXL	BB	372	386	DBSU	14.5
**4240**	allXL	BB	373	386	DBSU	12.64
**4240**	allXL	BC	381	386	DBSU	53.94
**4240**	allXL	BD;DB	357	357	DBSU	18.24
**4240**	allXL	DA	373	319	DBSU	22.64
**4240**	allXL	DA	372	357	DBSU	35.54
**4240**	allXL	DA	381	357	DBSU	32.39
**4240**	allXL	DA	373	357	DBSU	32.51
**4240**	allXL	DA	372	370	DBSU	38.43
**4240**	allXL	DA	373	370	DBSU	38.9
**4240**	allXL	DA	382	373	DBSU	33.69
**4241**	allXL	AA	320	357	BS2G	22.57
**4241**	allXL	DD	320	373	BS2G	16.42
**4241**	allXL	CC	373	382	BS2G	18.98
**4241**	allXL	CC	370	386	BS2G	22.4
**4241**	allXL	CC	373	386	BS2G	25.65
**4241**	allXL	AA	319	357	BS2G	21.9
**4241**	allXL	DD	367	373	BS2G	13.2
**4241**	allXL	CC	370	382	BS2G	19.36
**4241**	allXL	CC	376	382	BS2G	14.92
**4241**	allXL	DD	357	373	BS2G	26.85
**4241**	allXL	DD	320	366	BS2G	24.85
**4241**	allXL	CC	370	376	BS2G	14.57
**4241**	allXL	AA	320	357	DBSU	22.57
**4241**	allXL	AA	357	319	DBSU	21.9
**4241**	allXL	DD	372	319	DBSU	18.8
**4241**	allXL	DD	370	357	DBSU	24.8

**4241**	allXL	BB	382	357	DBSU	19.23
**4241**	allXL	AA	372	370	DBSU	6.19
**4241**	allXL	CC	381	370	DBSU	21.19
**4241**	allXL	CC	373	370	DBSU	8.97
**4241**	allXL	DD	381	372	DBSU	18.88
**4241**	allXL	DD	372	382	DBSU	20.17
**4241**	allXL	DD	372	386	DBSU	24.86
**4241**	allXL	CC	373	386	DBSU	25.65
**4241**	allXL	BC	381	386	DBSU	35.47
**4241**	allXL	BD;DB	357	357	DBSU	18.44
**4241**	allXL	CB	373	319	DBSU	20.2
**4241**	allXL	DA	372	357	DBSU	27.55
**4241**	allXL	DA	381	357	DBSU	31.95
**4241**	allXL	DA	373	357	DBSU	27.76
**4241**	allXL	CB	372	370	DBSU	27.46
**4241**	allXL	CB	373	370	DBSU	26.53
**4241**	allXL	DA	382	373	DBSU	29.55
**4242**	allXL	CC	320	357	BS2G	24.03
**4242**	allXL	AA	320	373	BS2G	21.17
**4242**	allXL	DD	373	382	BS2G	18.54
**4242**	allXL	DD	370	386	BS2G	29.43
**4242**	allXL	DD	373	386	BS2G	26.61
**4242**	allXL	CC	319	357	BS2G	23.18
**4242**	allXL	DD	367	373	BS2G	13.59
**4242**	allXL	DD	370	382	BS2G	22.87
**4242**	allXL	CC	376	382	BS2G	14.75
**4242**	allXL	DD	357	373	BS2G	17.86
**4242**	allXL	BB	320	366	BS2G	20.7
**4242**	allXL	CC	370	376	BS2G	17.09
**4242**	allXL	CC	320	357	DBSU	24.03
**4242**	allXL	CC	357	319	DBSU	23.18
**4242**	allXL	AA	372	319	DBSU	22.83
**4242**	allXL	DD	370	357	DBSU	15.12
**4242**	allXL	BB	382	357	DBSU	27.72

**4242**	allXL	AA	372	370	DBSU	6.3
**4242**	allXL	DD	381	370	DBSU	23.56
**4242**	allXL	AA	373	370	DBSU	9.09
**4242**	allXL	DD	381	372	DBSU	17.38
**4242**	allXL	DD	372	382	DBSU	17.11
**4242**	allXL	DD	372	386	DBSU	24.29
**4242**	allXL	DD	373	386	DBSU	26.61
**4242**	allXL	DB	381	386	DBSU	63.69
**4242**	allXL	BD;DB	357	357	DBSU	17.96
**4242**	allXL	AD	373	319	DBSU	27.92
**4242**	allXL	DB	372	357	DBSU	28.85
**4242**	allXL	BD	381	357	DBSU	32.2
**4242**	allXL	DB	373	357	DBSU	25.41
**4242**	allXL	DA	372	370	DBSU	40.68
**4242**	allXL	DA	373	370	DBSU	41.83
**4242**	allXL	BD	382	373	DBSU	46.12
**4243**	allXL	AA	320	357	BS2G	25.52
**4243**	allXL	DD	320	373	BS2G	26.97
**4243**	allXL	DD	373	382	BS2G	16.8
**4243**	allXL	DD	370	386	BS2G	19.45
**4243**	allXL	DD	373	386	BS2G	20.49
**4243**	allXL	AA	319	357	BS2G	24.97
**4243**	allXL	AA	367	373	BS2G	12.52
**4243**	allXL	DD	370	382	BS2G	20.78
**4243**	allXL	CC	376	382	BS2G	14.54
**4243**	allXL	DD	357	373	BS2G	27.43
**4243**	allXL	AA	320	366	BS2G	22.77
**4243**	allXL	CC	370	376	BS2G	17.45
**4243**	allXL	AA	320	357	DBSU	25.52
**4243**	allXL	AA	357	319	DBSU	24.97
**4243**	allXL	DD	372	319	DBSU	28.09
**4243**	allXL	DD	370	357	DBSU	24.8
**4243**	allXL	CC	382	357	DBSU	37.32
**4243**	allXL	AA	372	370	DBSU	6.23

**4243**	allXL	DD	381	370	DBSU	20.41
**4243**	allXL	DD	373	370	DBSU	8.98
**4243**	allXL	DD	381	372	DBSU	14.95
**4243**	allXL	DD	372	382	DBSU	15.72
**4243**	allXL	DD	372	386	DBSU	17.56
**4243**	allXL	DD	373	386	DBSU	20.49
**4243**	allXL	DA	381	386	DBSU	55.59
**4243**	allXL	BD;DB	357	357	DBSU	19.01
**4243**	allXL	CB	373	319	DBSU	38.97
**4243**	allXL	DB	372	357	DBSU	48.14
**4243**	allXL	CA	381	357	DBSU	55.7
**4243**	allXL	DB	373	357	DBSU	46.33
**4243**	allXL	BC	372	370	DBSU	50.07
**4243**	allXL	BC	373	370	DBSU	53.15
**4243**	allXL	DA	382	373	DBSU	61.98
**4244**	allXL	AA	320	357	BS2G	25.7
**4244**	allXL	DD	320	373	BS2G	23.45
**4244**	allXL	DD	373	382	BS2G	17.18
**4244**	allXL	DD	370	386	BS2G	12.9
**4244**	allXL	DD	373	386	BS2G	18.8
**4244**	allXL	AA	319	357	BS2G	26.63
**4244**	allXL	AA	367	373	BS2G	14.16
**4244**	allXL	DD	370	382	BS2G	16.78
**4244**	allXL	CC	376	382	BS2G	14.54
**4244**	allXL	CC	357	373	BS2G	18.13
**4244**	allXL	AA	320	366	BS2G	18.85
**4244**	allXL	BB	370	376	BS2G	15.52
**4244**	allXL	AA	320	357	DBSU	25.7
**4244**	allXL	AA	357	319	DBSU	26.63
**4244**	allXL	DD	372	319	DBSU	20.5
**4244**	allXL	CC	370	357	DBSU	18.91
**4244**	allXL	AA	382	357	DBSU	29.59
**4244**	allXL	AA	372	370	DBSU	6.17
**4244**	allXL	DD	381	370	DBSU	18.06

**4244**	allXL	AA	373	370	DBSU	9.17
**4244**	allXL	DD	381	372	DBSU	18.02
**4244**	allXL	DD	372	382	DBSU	18.26
**4244**	allXL	DD	372	386	DBSU	17.68
**4244**	allXL	DD	373	386	DBSU	18.8
**4244**	allXL	AC	381	386	DBSU	35.89
**4244**	allXL	BD;DB	357	357	DBSU	18.61
**4244**	allXL	DA	373	319	DBSU	24.98
**4244**	allXL	CA	372	357	DBSU	33.84
**4244**	allXL	CA	381	357	DBSU	34.3
**4244**	allXL	CA	373	357	DBSU	30.63
**4244**	allXL	DA	372	370	DBSU	42.71
**4244**	allXL	DA	373	370	DBSU	44.07
**4244**	allXL	CA	382	373	DBSU	36.47
**4245**	allXL	CC	320	357	BS2G	25.98
**4245**	allXL	BB	320	373	BS2G	17.89
**4245**	allXL	BB	373	382	BS2G	19.91
**4245**	allXL	DD	370	386	BS2G	34.01
**4245**	allXL	BB	373	386	BS2G	30.36
**4245**	allXL	AA	319	357	BS2G	25.08
**4245**	allXL	AA	367	373	BS2G	13.31
**4245**	allXL	DD	370	382	BS2G	22.85
**4245**	allXL	CC	376	382	BS2G	13.6
**4245**	allXL	BB	357	373	BS2G	21.59
**4245**	allXL	BB	320	366	BS2G	23.05
**4245**	allXL	DD	370	376	BS2G	17.15
**4245**	allXL	CC	320	357	DBSU	25.98
**4245**	allXL	AA	357	319	DBSU	25.08
**4245**	allXL	BB	372	319	DBSU	21.35
**4245**	allXL	BB	370	357	DBSU	23.35
**4245**	allXL	DD	382	357	DBSU	24.78
**4245**	allXL	AA	372	370	DBSU	6.23
**4245**	allXL	DD	381	370	DBSU	21.43
**4245**	allXL	AA	373	370	DBSU	9.04

**4245**	allXL	DD	381	372	DBSU	21.43
**4245**	allXL	BB	372	382	DBSU	22.44
**4245**	allXL	BB	372	386	DBSU	32.18
**4245**	allXL	BB	373	386	DBSU	30.36
**4245**	allXL	BD	381	386	DBSU	21.44
**4245**	allXL	BD;DB	357	357	DBSU	19.98
**4245**	allXL	CB	373	319	DBSU	12.74
**4245**	allXL	BD	372	357	DBSU	37.5
**4245**	allXL	DB	381	357	DBSU	35.22
**4245**	allXL	BD	373	357	DBSU	35.62
**4245**	allXL	CB	372	370	DBSU	24.47
**4245**	allXL	CB	373	370	DBSU	22.32
**4245**	allXL	CB	382	373	DBSU	22.9
**4246**	allXL	CC	320	357	BS2G	23.53
**4246**	allXL	BB	320	373	BS2G	19.67
**4246**	allXL	AA	373	382	BS2G	18.52
**4246**	allXL	DD	370	386	BS2G	22.42
**4246**	allXL	BB	373	386	BS2G	21.09
**4246**	allXL	CC	319	357	BS2G	26.37
**4246**	allXL	BB	367	373	BS2G	13.59
**4246**	allXL	DD	370	382	BS2G	19.55
**4246**	allXL	BB	376	382	BS2G	13.57
**4246**	allXL	BB	357	373	BS2G	15.94
**4246**	allXL	DD	320	366	BS2G	16.01
**4246**	allXL	DD	370	376	BS2G	15.96
**4246**	allXL	CC	320	357	DBSU	23.53
**4246**	allXL	CC	357	319	DBSU	26.37
**4246**	allXL	DD	372	319	DBSU	18.59
**4246**	allXL	BB	370	357	DBSU	24.76
**4246**	allXL	BB	382	357	DBSU	15.57
**4246**	allXL	AA	372	370	DBSU	6.22
**4246**	allXL	DD	381	370	DBSU	17.48
**4246**	allXL	AA	373	370	DBSU	8.91
**4246**	allXL	DD	381	372	DBSU	18.61

**4246**	allXL	AA	372	382	DBSU	19.8
**4246**	allXL	BB	372	386	DBSU	22.14
**4246**	allXL	BB	373	386	DBSU	21.09
**4246**	allXL	DA	381	386	DBSU	36.17
**4246**	allXL	BD;DB	357	357	DBSU	18.12
**4246**	allXL	CB	373	319	DBSU	13.41
**4246**	allXL	BD	372	357	DBSU	34.39
**4246**	allXL	BD	381	357	DBSU	13.48
**4246**	allXL	BD	373	357	DBSU	30.85
**4246**	allXL	CB	372	370	DBSU	37.05
**4246**	allXL	CB	373	370	DBSU	34.5
**4246**	allXL	AD	382	373	DBSU	32.45
**4247**	allXL	AA	320	357	BS2G	24.02
**4247**	allXL	BB	320	373	BS2G	24.95
**4247**	allXL	CC	373	382	BS2G	16.84
**4247**	allXL	DD	370	386	BS2G	17.58
**4247**	allXL	DD	373	386	BS2G	21.77
**4247**	allXL	CC	319	357	BS2G	24.98
**4247**	allXL	AA	367	373	BS2G	11.87
**4247**	allXL	CC	370	382	BS2G	18.54
**4247**	allXL	AA	376	382	BS2G	13.94
**4247**	allXL	BB	357	373	BS2G	26.73
**4247**	allXL	AA	320	366	BS2G	13.07
**4247**	allXL	CC	370	376	BS2G	14.86
**4247**	allXL	AA	320	357	DBSU	24.02
**4247**	allXL	CC	357	319	DBSU	24.98
**4247**	allXL	BB	372	319	DBSU	22.72
**4247**	allXL	BB;DD	370	357	DBSU	28.21
**4247**	allXL	BB	382	357	DBSU	17.49
**4247**	allXL	CC	372	370	DBSU	6.66
**4247**	allXL	DD	381	370	DBSU	20.06
**4247**	allXL	AA;CC	373	370	DBSU	9.11
**4247**	allXL	CC	381	372	DBSU	20.76
**4247**	allXL	CC	372	382	DBSU	19.24

**4247**	allXL	DD	372	386	DBSU	21.63
**4247**	allXL	DD	373	386	DBSU	21.77
**4247**	allXL	CB	381	386	DBSU	30.94
**4247**	allXL	BD;DB	357	357	DBSU	18.68
**4247**	allXL	CB	373	319	DBSU	13.65
**4247**	allXL	CB	372	357	DBSU	43.01
**4247**	allXL	CB	381	357	DBSU	29.53
**4247**	allXL	CB	373	357	DBSU	39.16
**4247**	allXL	CB	372	370	DBSU	24.22
**4247**	allXL	CB	373	370	DBSU	21.19
**4247**	allXL	BC	382	373	DBSU	30.75
**4248**	allXL	AA	320	357	BS2G	21.91
**4248**	allXL	DD	320	373	BS2G	21.26
**4248**	allXL	DD	373	382	BS2G	17.93
**4248**	allXL	DD	370	386	BS2G	15.03
**4248**	allXL	DD	373	386	BS2G	20.76
**4248**	allXL	AA	319	357	BS2G	19.33
**4248**	allXL	AA	367	373	BS2G	12.34
**4248**	allXL	DD	370	382	BS2G	13.08
**4248**	allXL	CC	376	382	BS2G	13.75
**4248**	allXL	DD	357	373	BS2G	22.81
**4248**	allXL	BB	320	366	BS2G	19.98
**4248**	allXL	CC	370	376	BS2G	14.5
**4248**	allXL	AA	320	357	DBSU	21.91
**4248**	allXL	AA	357	319	DBSU	19.33
**4248**	allXL	AA	372	319	DBSU	26.51
**4248**	allXL	DD	370	357	DBSU	24.29
**4248**	allXL	DD	382	357	DBSU	25.14
**4248**	allXL	CC	372	370	DBSU	6.71
**4248**	allXL	DD	381	370	DBSU	12.58
**4248**	allXL	AA	373	370	DBSU	9.01
**4248**	allXL	DD	381	372	DBSU	16.85
**4248**	allXL	DD	372	382	DBSU	18.56
**4248**	allXL	DD	372	386	DBSU	20.4

**4248**	allXL	DD	373	386	DBSU	20.76
**4248**	allXL	BD	381	386	DBSU	65.66
**4248**	allXL	BD;DB	357	357	DBSU	18.66
**4248**	allXL	AD	373	319	DBSU	37.79
**4248**	allXL	DB	372	357	DBSU	41.94
**4248**	allXL	DB	381	357	DBSU	43.23
**4248**	allXL	DB	373	357	DBSU	40.81
**4248**	allXL	CB	372	370	DBSU	48.1
**4248**	allXL	CB	373	370	DBSU	46.01
**4248**	allXL	DA	382	373	DBSU	46.81
**4249**	allXL	CC	320	357	BS2G	25.82
**4249**	allXL	AA	320	373	BS2G	10.54
**4249**	allXL	BB	373	382	BS2G	23.03
**4249**	allXL	BB	370	386	BS2G	31.8
**4249**	allXL	AA	373	386	BS2G	30.05
**4249**	allXL	CC	319	357	BS2G	25.21
**4249**	allXL	BB	367	373	BS2G	14.03
**4249**	allXL	BB	370	382	BS2G	20.77
**4249**	allXL	CC	376	382	BS2G	15.13
**4249**	allXL	DD	357	373	BS2G	27.63
**4249**	allXL	AA	320	366	BS2G	20.26
**4249**	allXL	BB	370	376	BS2G	14.29
**4249**	allXL	CC	320	357	DBSU	25.82
**4249**	allXL	CC	357	319	DBSU	25.21
**4249**	allXL	AA	372	319	DBSU	14.15
**4249**	allXL	DD	370	357	DBSU	25.72
**4249**	allXL	DD	382	357	DBSU	24.9
**4249**	allXL	AA	372	370	DBSU	6.02
**4249**	allXL	BB	381	370	DBSU	20.35
**4249**	allXL	CC	373	370	DBSU	9.25
**4249**	allXL	BB	381	372	DBSU	23.48
**4249**	allXL	BB	372	382	DBSU	24.69
**4249**	allXL	AA	372	386	DBSU	31.19
**4249**	allXL	AA	373	386	DBSU	30.05

**4249**	allXL	AD	381	386	DBSU	41.41
**4249**	allXL	BD;DB	357	357	DBSU	18.65
**4249**	allXL	AD	373	319	DBSU	32.5
**4249**	allXL	DB	372	357	DBSU	35.77
**4249**	allXL	DB	381	357	DBSU	28.31
**4249**	allXL	DB	373	357	DBSU	32.82
**4249**	allXL	AD	372	370	DBSU	40.62
**4249**	allXL	AD	373	370	DBSU	37.65
**4249**	allXL	DA	382	373	DBSU	27.97
**4250**	allXL	AA	320	357	BS2G	25.2
**4250**	allXL	BB	320	373	BS2G	20.95
**4250**	allXL	CC	373	382	BS2G	18.36
**4250**	allXL	CC	370	386	BS2G	23.64
**4250**	allXL	CC	373	386	BS2G	23.86
**4250**	allXL	AA	319	357	BS2G	27.74
**4250**	allXL	AA	367	373	BS2G	13.0
**4250**	allXL	CC	370	382	BS2G	15.5
**4250**	allXL	CC	376	382	BS2G	15.01
**4250**	allXL	BB	357	373	BS2G	25.58
**4250**	allXL	BB	320	366	BS2G	19.81
**4250**	allXL	CC	370	376	BS2G	11.81
**4250**	allXL	AA	320	357	DBSU	25.2
**4250**	allXL	AA	357	319	DBSU	27.74
**4250**	allXL	BB	372	319	DBSU	23.06
**4250**	allXL	BB	370	357	DBSU	26.69
**4250**	allXL	CC	382	357	DBSU	38.55
**4250**	allXL	AA	372	370	DBSU	6.39
**4250**	allXL	CC	381	370	DBSU	12.37
**4250**	allXL	AA	373	370	DBSU	8.84
**4250**	allXL	CC	381	372	DBSU	13.3
**4250**	allXL	CC	372	382	DBSU	14.99
**4250**	allXL	CC	372	386	DBSU	20.69
**4250**	allXL	CC	373	386	DBSU	23.86
**4250**	allXL	BC	381	386	DBSU	52.05

**4250**	allXL	BD;DB	357	357	DBSU	17.02
**4250**	allXL	CB	373	319	DBSU	25.89
**4250**	allXL	BD	372	357	DBSU	34.57
**4250**	allXL	CB	381	357	DBSU	33.75
**4250**	allXL	BD	373	357	DBSU	35.47
**4250**	allXL	CB	372	370	DBSU	41.65
**4250**	allXL	CB	373	370	DBSU	44.83
**4250**	allXL	CB	382	373	DBSU	31.13
**4251**	allXL	AA	320	357	BS2G	21.82
**4251**	allXL	AA	320	373	BS2G	21.01
**4251**	allXL	BB	373	382	BS2G	20.35
**4251**	allXL	DD	370	386	BS2G	23.21
**4251**	allXL	BB	373	386	BS2G	17.39
**4251**	allXL	AA	319	357	BS2G	20.17
**4251**	allXL	AA	367	373	BS2G	12.6
**4251**	allXL	DD	370	382	BS2G	18.3
**4251**	allXL	BB	376	382	BS2G	13.86
**4251**	allXL	DD	357	373	BS2G	32.52
**4251**	allXL	AA	320	366	BS2G	20.22
**4251**	allXL	DD	370	376	BS2G	16.49
**4251**	allXL	AA	320	357	DBSU	21.82
**4251**	allXL	AA	357	319	DBSU	20.17
**4251**	allXL	AA	372	319	DBSU	20.58
**4251**	allXL	DD	370	357	DBSU	27.19
**4251**	allXL	DD	382	357	DBSU	32.68
**4251**	allXL	AA	372	370	DBSU	6.18
**4251**	allXL	DD	381	370	DBSU	18.29
**4251**	allXL	AA	373	370	DBSU	8.98
**4251**	allXL	DD	381	372	DBSU	20.44
**4251**	allXL	DD	372	382	DBSU	21.68
**4251**	allXL	BB	372	386	DBSU	18.25
**4251**	allXL	BB	373	386	DBSU	17.39
**4251**	allXL	DB	381	386	DBSU	68.86
**4251**	allXL	BD;DB	357	357	DBSU	17.96

**4251**	allXL	DA	373	319	DBSU	28.0
**4251**	allXL	DA	372	357	DBSU	41.32
**4251**	allXL	DA	381	357	DBSU	40.88
**4251**	allXL	DA	373	357	DBSU	37.74
**4251**	allXL	DA	372	370	DBSU	24.54
**4251**	allXL	DA	373	370	DBSU	22.11
**4251**	allXL	DA	382	373	DBSU	42.8
**4252**	allXL	CC	320	357	BS2G	24.48
**4252**	allXL	BB	320	373	BS2G	35.27
**4252**	allXL	DD	373	382	BS2G	16.98
**4252**	allXL	DD	370	386	BS2G	20.99
**4252**	allXL	AA	373	386	BS2G	20.83
**4252**	allXL	CC	319	357	BS2G	28.14
**4252**	allXL	BB	367	373	BS2G	11.35
**4252**	allXL	DD	370	382	BS2G	16.68
**4252**	allXL	BB	376	382	BS2G	13.76
**4252**	allXL	BB	357	373	BS2G	35.29
**4252**	allXL	AA	320	366	BS2G	27.04
**4252**	allXL	CC	370	376	BS2G	15.36
**4252**	allXL	CC	320	357	DBSU	24.48
**4252**	allXL	CC	357	319	DBSU	28.14
**4252**	allXL	BB	372	319	DBSU	34.78
**4252**	allXL	DD	370	357	DBSU	31.38
**4252**	allXL	BB	382	357	DBSU	32.66
**4252**	allXL	AA	372	370	DBSU	6.39
**4252**	allXL	DD	381	370	DBSU	16.35
**4252**	allXL	AA	373	370	DBSU	9.12
**4252**	allXL	DD	381	372	DBSU	15.28
**4252**	allXL	DD	372	382	DBSU	16.89
**4252**	allXL	DD	372	386	DBSU	22.64
**4252**	allXL	AA	373	386	DBSU	20.83
**4252**	allXL	AD	381	386	DBSU	49.88
**4252**	allXL	BD;DB	357	357	DBSU	17.69
**4252**	allXL	AD	373	319	DBSU	36.81

**4252**	allXL	CB	372	357	DBSU	49.8
**4252**	allXL	BD	381	357	DBSU	44.98
**4252**	allXL	BD	373	357	DBSU	50.61
**4252**	allXL	CB	372	370	DBSU	33.92
**4252**	allXL	CB	373	370	DBSU	35.28
**4252**	allXL	BC	382	373	DBSU	42.86
**4253**	allXL	AA	320	357	BS2G	23.46
**4253**	allXL	BB	320	373	BS2G	21.11
**4253**	allXL	DD	373	382	BS2G	18.75
**4253**	allXL	DD	370	386	BS2G	24.45
**4253**	allXL	AA	373	386	BS2G	28.69
**4253**	allXL	CC	319	357	BS2G	24.99
**4253**	allXL	AA	367	373	BS2G	11.62
**4253**	allXL	DD	370	382	BS2G	13.84
**4253**	allXL	CC	376	382	BS2G	14.81
**4253**	allXL	DD	357	373	BS2G	25.66
**4253**	allXL	BB	320	366	BS2G	22.75
**4253**	allXL	BB	370	376	BS2G	14.16
**4253**	allXL	AA	320	357	DBSU	23.46
**4253**	allXL	CC	357	319	DBSU	24.99
**4253**	allXL	BB	372	319	DBSU	20.68
**4253**	allXL	DD	370	357	DBSU	27.69
**4253**	allXL	DD	382	357	DBSU	24.29
**4253**	allXL	AA	372	370	DBSU	6.32
**4253**	allXL	DD	381	370	DBSU	12.87
**4253**	allXL	BB	373	370	DBSU	9.11
**4253**	allXL	DD	381	372	DBSU	17.48
**4253**	allXL	DD	372	382	DBSU	18.76
**4253**	allXL	AA	372	386	DBSU	27.54
**4253**	allXL	AA	373	386	DBSU	28.69
**4253**	allXL	DB	381	386	DBSU	38.73
**4253**	allXL	BD;DB	357	357	DBSU	17.88
**4253**	allXL	DA	373	319	DBSU	21.68
**4253**	allXL	DA	372	357	DBSU	35.33

**4253**	allXL	DB	381	357	DBSU	29.99
**4253**	allXL	DA	373	357	DBSU	33.4
**4253**	allXL	BC	372	370	DBSU	23.36
**4253**	allXL	BC	373	370	DBSU	26.82
**4253**	allXL	AD	382	373	DBSU	35.07
**4254**	allXL	AA	320	357	BS2G	23.19
**4254**	allXL	DD	320	373	BS2G	27.38
**4254**	allXL	BB	373	382	BS2G	17.45
**4254**	allXL	CC	370	386	BS2G	22.72
**4254**	allXL	BB	373	386	BS2G	23.64
**4254**	allXL	AA	319	357	BS2G	23.28
**4254**	allXL	BB	367	373	BS2G	10.91
**4254**	allXL	CC	370	382	BS2G	20.11
**4254**	allXL	CC	376	382	BS2G	13.39
**4254**	allXL	BB	357	373	BS2G	27.39
**4254**	allXL	CC	320	366	BS2G	23.31
**4254**	allXL	CC	370	376	BS2G	14.86
**4254**	allXL	AA	320	357	DBSU	23.19
**4254**	allXL	AA	357	319	DBSU	23.28
**4254**	allXL	DD	372	319	DBSU	29.73
**4254**	allXL	DD	370	357	DBSU	31.95
**4254**	allXL	BB	382	357	DBSU	32.81
**4254**	allXL	AA	372	370	DBSU	6.38
**4254**	allXL	DD	381	370	DBSU	22.21
**4254**	allXL	CC	373	370	DBSU	8.92
**4254**	allXL	DD	381	372	DBSU	17.99
**4254**	allXL	DD	372	382	DBSU	18.37
**4254**	allXL	DD	372	386	DBSU	22.61
**4254**	allXL	BB	373	386	DBSU	23.64
**4254**	allXL	DA	381	386	DBSU	50.2
**4254**	allXL	AC;CA	357	357	DBSU	19.52
**4254**	allXL	BC	373	319	DBSU	32.83
**4254**	allXL	BD	372	357	DBSU	42.1
**4254**	allXL	BD	381	357	DBSU	32.59

**4254**	allXL	BD	373	357	DBSU	38.8
**4254**	allXL	DA	372	370	DBSU	45.91
**4254**	allXL	DA	373	370	DBSU	42.71
**4254**	allXL	DA	382	373	DBSU	45.16
**4255**	allXL	CC	320	357	BS2G	24.46
**4255**	allXL	CC	320	373	BS2G	12.48
**4255**	allXL	CC	373	382	BS2G	16.97
**4255**	allXL	DD	370	386	BS2G	20.0
**4255**	allXL	DD	373	386	BS2G	23.13
**4255**	allXL	AA	319	357	BS2G	25.61
**4255**	allXL	BB	367	373	BS2G	13.44
**4255**	allXL	DD	370	382	BS2G	17.8
**4255**	allXL	CC	376	382	BS2G	14.66
**4255**	allXL	BB	357	373	BS2G	14.27
**4255**	allXL	CC	320	366	BS2G	15.5
**4255**	allXL	CC	370	376	BS2G	14.83
**4255**	allXL	CC	320	357	DBSU	24.46
**4255**	allXL	AA	357	319	DBSU	25.61
**4255**	allXL	CC	372	319	DBSU	11.06
**4255**	allXL	BB	370	357	DBSU	20.0
**4255**	allXL	BB	382	357	DBSU	21.0
**4255**	allXL	AA	372	370	DBSU	6.22
**4255**	allXL	DD	381	370	DBSU	17.43
**4255**	allXL	CC	373	370	DBSU	9.18
**4255**	allXL	DD	381	372	DBSU	16.62
**4255**	allXL	DD	372	382	DBSU	17.66
**4255**	allXL	DD	372	386	DBSU	20.56
**4255**	allXL	DD	373	386	DBSU	23.13
**4255**	allXL	DB	381	386	DBSU	33.08
**4255**	allXL	AC;CA	357	357	DBSU	20.04
**4255**	allXL	AD	373	319	DBSU	26.16
**4255**	allXL	AD	372	357	DBSU	35.1
**4255**	allXL	BD	381	357	DBSU	25.51
**4255**	allXL	BD	373	357	DBSU	33.96

**4255**	allXL	AD	372	370	DBSU	11.21
**4255**	allXL	AD	373	370	DBSU	13.44
**4255**	allXL	DA	382	373	DBSU	21.38
**4256**	allXL	AA	320	357	BS2G	24.69
**4256**	allXL	AA	320	373	BS2G	20.42
**4256**	allXL	DD	373	382	BS2G	18.02
**4256**	allXL	DD	370	386	BS2G	20.21
**4256**	allXL	DD	373	386	BS2G	24.97
**4256**	allXL	AA	319	357	BS2G	27.41
**4256**	allXL	AA	367	373	BS2G	12.53
**4256**	allXL	DD	370	382	BS2G	17.41
**4256**	allXL	CC	376	382	BS2G	13.51
**4256**	allXL	AA	357	373	BS2G	32.88
**4256**	allXL	DD	320	366	BS2G	20.87
**4256**	allXL	CC	370	376	BS2G	16.27
**4256**	allXL	AA	320	357	DBSU	24.69
**4256**	allXL	AA	357	319	DBSU	27.41
**4256**	allXL	AA	372	319	DBSU	25.55
**4256**	allXL	DD	370	357	DBSU	31.27
**4256**	allXL	AA	382	357	DBSU	19.05
**4256**	allXL	AA	372	370	DBSU	6.37
**4256**	allXL	DD	381	370	DBSU	16.94
**4256**	allXL	CC	373	370	DBSU	8.74
**4256**	allXL	DD	381	372	DBSU	17.11
**4256**	allXL	DD	372	382	DBSU	18.95
**4256**	allXL	DD	372	386	DBSU	24.86
**4256**	allXL	DD	373	386	DBSU	24.97
**4256**	allXL	CB	381	386	DBSU	18.23
**4256**	allXL	BD;DB	357	357	DBSU	18.17
**4256**	allXL	AD	373	319	DBSU	25.71
**4256**	allXL	CB	372	357	DBSU	24.29
**4256**	allXL	AD	381	357	DBSU	28.92
**4256**	allXL	CB	373	357	DBSU	27.74
**4256**	allXL	BC	372	370	DBSU	28.47

**4256**	allXL	CB	373	370	DBSU	29.2
**4256**	allXL	DA	382	373	DBSU	21.55
**4257**	allXL	CC	320	357	BS2G	24.75
**4257**	allXL	DD	320	373	BS2G	23.82
**4257**	allXL	DD	373	382	BS2G	17.55
**4257**	allXL	DD	370	386	BS2G	25.96
**4257**	allXL	AA	373	386	BS2G	23.46
**4257**	allXL	CC	319	357	BS2G	24.0
**4257**	allXL	BB	367	373	BS2G	14.23
**4257**	allXL	DD	370	382	BS2G	20.18
**4257**	allXL	BB	376	382	BS2G	14.25
**4257**	allXL	DD	357	373	BS2G	24.51
**4257**	allXL	AA	320	366	BS2G	21.38
**4257**	allXL	DD	370	376	BS2G	13.76
**4257**	allXL	CC	320	357	DBSU	24.75
**4257**	allXL	CC	357	319	DBSU	24.0
**4257**	allXL	DD	372	319	DBSU	24.67
**4257**	allXL	DD	370	357	DBSU	21.65
**4257**	allXL	CC	382	357	DBSU	16.14
**4257**	allXL	AA	372	370	DBSU	5.91
**4257**	allXL	DD	381	370	DBSU	18.47
**4257**	allXL	AA	373	370	DBSU	9.0
**4257**	allXL	DD	381	372	DBSU	18.36
**4257**	allXL	DD	372	382	DBSU	19.17
**4257**	allXL	DD	372	386	DBSU	24.18
**4257**	allXL	AA	373	386	DBSU	23.46
**4257**	allXL	AD	381	386	DBSU	46.92
**4257**	allXL	BD;DB	357	357	DBSU	18.12
**4257**	allXL	AD	373	319	DBSU	23.19
**4257**	allXL	AD	372	357	DBSU	25.04
**4257**	allXL	DB	381	357	DBSU	27.45
**4257**	allXL	AD	373	357	DBSU	27.3
**4257**	allXL	AD	372	370	DBSU	22.44
**4257**	allXL	AD	373	370	DBSU	21.53

**4257**	allXL	DA	382	373	DBSU	37.42
**4258**	allXL	AA	320	357	BS2G	23.57
**4258**	allXL	BB	320	373	BS2G	15.86
**4258**	allXL	BB	373	382	BS2G	21.24
**4258**	allXL	AA	370	386	BS2G	31.4
**4258**	allXL	AA	373	386	BS2G	30.1
**4258**	allXL	AA	319	357	BS2G	26.58
**4258**	allXL	BB	367	373	BS2G	12.3
**4258**	allXL	AA	370	382	BS2G	25.99
**4258**	allXL	BB	376	382	BS2G	14.31
**4258**	allXL	DD	357	373	BS2G	14.96
**4258**	allXL	BB	320	366	BS2G	19.23
**4258**	allXL	DD	370	376	BS2G	17.06
**4258**	allXL	AA	320	357	DBSU	23.57
**4258**	allXL	AA	357	319	DBSU	26.58
**4258**	allXL	BB	372	319	DBSU	17.43
**4258**	allXL	DD	370	357	DBSU	19.82
**4258**	allXL	BB	382	357	DBSU	27.82
**4258**	allXL	AA	372	370	DBSU	5.98
**4258**	allXL	DD	381	370	DBSU	24.99
**4258**	allXL	DD	373	370	DBSU	9.13
**4258**	allXL	AA	381	372	DBSU	23.24
**4258**	allXL	AA	372	382	DBSU	21.93
**4258**	allXL	AA	372	386	DBSU	29.21
**4258**	allXL	AA	373	386	DBSU	30.1
**4258**	allXL	BD	381	386	DBSU	30.47
**4258**	allXL	BD;DB	357	357	DBSU	18.08
**4258**	allXL	AD	373	319	DBSU	25.17
**4258**	allXL	DB	372	357	DBSU	18.89
**4258**	allXL	DB	381	357	DBSU	34.47
**4258**	allXL	DB	373	357	DBSU	18.52
**4258**	allXL	CB	372	370	DBSU	31.34
**4258**	allXL	CB	373	370	DBSU	33.94
**4258**	allXL	BD	382	373	DBSU	29.79

**4259**	allXL	AA	320	357	BS2G	26.89
**4259**	allXL	BB	320	373	BS2G	16.05
**4259**	allXL	DD	373	382	BS2G	19.89
**4259**	allXL	AA	370	386	BS2G	27.06
**4259**	allXL	AA	373	386	BS2G	28.43
**4259**	allXL	AA	319	357	BS2G	27.43
**4259**	allXL	AA	367	373	BS2G	11.9
**4259**	allXL	AA	370	382	BS2G	23.31
**4259**	allXL	CC	376	382	BS2G	15.3
**4259**	allXL	BB	357	373	BS2G	17.38
**4259**	allXL	DD	320	366	BS2G	24.38
**4259**	allXL	CC	370	376	BS2G	16.99
**4259**	allXL	AA	320	357	DBSU	26.89
**4259**	allXL	AA	357	319	DBSU	27.43
**4259**	allXL	BB	372	319	DBSU	16.84
**4259**	allXL	BB	370	357	DBSU	25.92
**4259**	allXL	BB	382	357	DBSU	21.01
**4259**	allXL	AA	372	370	DBSU	6.39
**4259**	allXL	BB	381	370	DBSU	23.29
**4259**	allXL	AA	373	370	DBSU	8.93
**4259**	allXL	BB	381	372	DBSU	20.8
**4259**	allXL	CC	372	382	DBSU	21.85
**4259**	allXL	AA	372	386	DBSU	28.57
**4259**	allXL	AA	373	386	DBSU	28.43
**4259**	allXL	DB	381	386	DBSU	62.82
**4259**	allXL	BD;DB	357	357	DBSU	18.74
**4259**	allXL	CB	373	319	DBSU	37.15
**4259**	allXL	BD	372	357	DBSU	37.61
**4259**	allXL	BD	381	357	DBSU	26.37
**4259**	allXL	BD	373	357	DBSU	34.3
**4259**	allXL	BC	372	370	DBSU	38.93
**4259**	allXL	BC	373	370	DBSU	38.31
**4259**	allXL	BD	382	373	DBSU	53.17
**4260**	allXL	CC	320	357	BS2G	23.87

**4260**	allXL	AA	320	373	BS2G	15.12
**4260**	allXL	AA	373	382	BS2G	19.41
**4260**	allXL	CC	370	386	BS2G	17.82
**4260**	allXL	CC	373	386	BS2G	23.17
**4260**	allXL	CC	319	357	BS2G	27.05
**4260**	allXL	AA	367	373	BS2G	10.48
**4260**	allXL	CC	370	382	BS2G	20.58
**4260**	allXL	CC	376	382	BS2G	14.86
**4260**	allXL	DD	357	373	BS2G	28.03
**4260**	allXL	CC	320	366	BS2G	16.95
**4260**	allXL	CC	370	376	BS2G	16.16
**4260**	allXL	CC	320	357	DBSU	23.87
**4260**	allXL	CC	357	319	DBSU	27.05
**4260**	allXL	AA	372	319	DBSU	14.03
**4260**	allXL	DD	370	357	DBSU	24.86
**4260**	allXL	BB	382	357	DBSU	19.42
**4260**	allXL	CC	372	370	DBSU	6.89
**4260**	allXL	AA	381	370	DBSU	19.64
**4260**	allXL	AA	373	370	DBSU	8.63
**4260**	allXL	AA	381	372	DBSU	21.01
**4260**	allXL	CC	372	382	DBSU	21.95
**4260**	allXL	CC	372	386	DBSU	22.47
**4260**	allXL	CC	373	386	DBSU	23.17
**4260**	allXL	DB	381	386	DBSU	36.6
**4260**	allXL	BD;DB	357	357	DBSU	18.5
**4260**	allXL	BC	373	319	DBSU	32.88
**4260**	allXL	BC	372	357	DBSU	41.72
**4260**	allXL	BD	381	357	DBSU	37.15
**4260**	allXL	DB	373	357	DBSU	41.34
**4260**	allXL	BC	372	370	DBSU	25.28
**4260**	allXL	BC	373	370	DBSU	27.0
**4260**	allXL	CB	382	373	DBSU	44.89
**4261**	allXL	AA	320	357	BS2G	24.24
**4261**	allXL	DD	320	373	BS2G	15.6

**4261**	allXL	CC	373	382	BS2G	18.27
**4261**	allXL	DD	370	386	BS2G	18.68
**4261**	allXL	CC	373	386	BS2G	24.06
**4261**	allXL	AA	319	357	BS2G	23.64
**4261**	allXL	BB	367	373	BS2G	12.58
**4261**	allXL	DD	370	382	BS2G	13.36
**4261**	allXL	CC	376	382	BS2G	13.48
**4261**	allXL	CC	357	373	BS2G	21.55
**4261**	allXL	DD	320	366	BS2G	15.9
**4261**	allXL	CC	370	376	BS2G	14.57
**4261**	allXL	AA	320	357	DBSU	24.24
**4261**	allXL	AA	357	319	DBSU	23.64
**4261**	allXL	DD	372	319	DBSU	11.94
**4261**	allXL	CC	370	357	DBSU	20.02
**4261**	allXL	BB	382	357	DBSU	26.73
**4261**	allXL	AA	372	370	DBSU	5.95
**4261**	allXL	DD	381	370	DBSU	11.54
**4261**	allXL	AA	373	370	DBSU	9.31
**4261**	allXL	DD	381	372	DBSU	16.41
**4261**	allXL	DD	372	382	DBSU	18.99
**4261**	allXL	CC	372	386	DBSU	23.79
**4261**	allXL	CC	373	386	DBSU	24.06
**4261**	allXL	DA	381	386	DBSU	31.38
**4261**	allXL	BD;DB	357	357	DBSU	18.62
**4261**	allXL	DA	373	319	DBSU	26.01
**4261**	allXL	DA	372	357	DBSU	27.57
**4261**	allXL	DA	381	357	DBSU	32.95
**4261**	allXL	DA	373	357	DBSU	24.69
**4261**	allXL	DA	372	370	DBSU	27.39
**4261**	allXL	DA	373	370	DBSU	29.69
**4261**	allXL	AD	382	373	DBSU	38.13
**4262**	allXL	AA	320	357	BS2G	23.84
**4262**	allXL	DD	320	373	BS2G	37.86
**4262**	allXL	CC	373	382	BS2G	17.19

**4262**	allXL	DD	370	386	BS2G	28.67
**4262**	allXL	CC	373	386	BS2G	27.39
**4262**	allXL	AA	319	357	BS2G	26.73
**4262**	allXL	AA	367	373	BS2G	11.62
**4262**	allXL	DD	370	382	BS2G	23.57
**4262**	allXL	CC	376	382	BS2G	14.49
**4262**	allXL	AA	357	373	BS2G	34.06
**4262**	allXL	CC	320	366	BS2G	26.57
**4262**	allXL	CC	370	376	BS2G	16.76
**4262**	allXL	AA	320	357	DBSU	23.84
**4262**	allXL	AA	357	319	DBSU	26.73
**4262**	allXL	CC	372	319	DBSU	34.24
**4262**	allXL	DD	370	357	DBSU	27.76
**4262**	allXL	DD	382	357	DBSU	45.27
**4262**	allXL	AA	372	370	DBSU	6.22
**4262**	allXL	DD	381	370	DBSU	24.06
**4262**	allXL	AA	373	370	DBSU	9.2
**4262**	allXL	CC	381	372	DBSU	21.3
**4262**	allXL	AA	372	382	DBSU	20.56
**4262**	allXL	AA	372	386	DBSU	26.97
**4262**	allXL	CC	373	386	DBSU	27.39
**4262**	allXL	BC	381	386	DBSU	54.72
**4262**	allXL	BD;DB	357	357	DBSU	18.59
**4262**	allXL	AD	373	319	DBSU	34.6
**4262**	allXL	AC	372	357	DBSU	49.05
**4262**	allXL	AC	381	357	DBSU	53.92
**4262**	allXL	AC	373	357	DBSU	46.0
**4262**	allXL	BC	372	370	DBSU	40.1
**4262**	allXL	BC	373	370	DBSU	40.62
**4262**	allXL	CB	382	373	DBSU	39.7
**4263**	allXL	BB	320	357	BS2G	25.46
**4263**	allXL	DD	320	373	BS2G	23.35
**4263**	allXL	BB	373	382	BS2G	21.26
**4263**	allXL	CC	370	386	BS2G	19.5

**4263**	allXL	CC	373	386	BS2G	26.09
**4263**	allXL	BB	319	357	BS2G	28.5
**4263**	allXL	AA	367	373	BS2G	11.75
**4263**	allXL	CC	370	382	BS2G	19.93
**4263**	allXL	BB	376	382	BS2G	14.04
**4263**	allXL	AA	357	373	BS2G	30.02
**4263**	allXL	CC	320	366	BS2G	24.59
**4263**	allXL	CC	370	376	BS2G	15.11
**4263**	allXL	BB	320	357	DBSU	25.46
**4263**	allXL	BB	357	319	DBSU	28.5
**4263**	allXL	DD	372	319	DBSU	20.66
**4263**	allXL	DD	370	357	DBSU	25.6
**4263**	allXL	BB	382	357	DBSU	21.06
**4263**	allXL	AA	372	370	DBSU	6.57
**4263**	allXL	CC	381	370	DBSU	21.97
**4263**	allXL	DD	373	370	DBSU	8.93
**4263**	allXL	DD	381	372	DBSU	22.29
**4263**	allXL	CC	372	382	DBSU	21.73
**4263**	allXL	CC	372	386	DBSU	24.92
**4263**	allXL	CC	373	386	DBSU	26.09
**4263**	allXL	CB	381	386	DBSU	34.53
**4263**	allXL	BD;DB	357	357	DBSU	19.15
**4263**	allXL	AD	373	319	DBSU	35.36
**4263**	allXL	CB	372	357	DBSU	33.07
**4263**	allXL	BD	381	357	DBSU	35.41
**4263**	allXL	CB	373	357	DBSU	35.18
**4263**	allXL	AD	372	370	DBSU	42.55
**4263**	allXL	AD	373	370	DBSU	39.79
**4263**	allXL	BC	382	373	DBSU	17.61
**4264**	allXL	AA	320	357	BS2G	14.94
**4264**	allXL	BB	320	373	BS2G	34.71
**4264**	allXL	CC	373	382	BS2G	21.1
**4264**	allXL	DD	370	386	BS2G	28.51
**4264**	allXL	DD	373	386	BS2G	25.68

**4264**	allXL	AA	319	357	BS2G	11.82
**4264**	allXL	BB	367	373	BS2G	13.25
**4264**	allXL	BB	370	382	BS2G	23.8
**4264**	allXL	CC	376	382	BS2G	14.14
**4264**	allXL	BB	357	373	BS2G	36.2
**4264**	allXL	AA	320	366	BS2G	24.2
**4264**	allXL	CC	370	376	BS2G	16.12
**4264**	allXL	AA	320	357	DBSU	14.94
**4264**	allXL	AA	357	319	DBSU	11.82
**4264**	allXL	DD	372	319	DBSU	33.44
**4264**	allXL	DD	370	357	DBSU	28.2
**4264**	allXL	DD	382	357	DBSU	54.33
**4264**	allXL	AA	372	370	DBSU	6.09
**4264**	allXL	BB	381	370	DBSU	23.59
**4264**	allXL	AA	373	370	DBSU	9.07
**4264**	allXL	DD	381	372	DBSU	23.13
**4264**	allXL	CC	372	382	DBSU	22.6
**4264**	allXL	DD	372	386	DBSU	27.94
**4264**	allXL	DD	373	386	DBSU	25.68
**4264**	allXL	BC	381	386	DBSU	54.39
**4264**	allXL	BD;DB	357	357	DBSU	19.04
**4264**	allXL	AD	373	319	DBSU	28.12
**4264**	allXL	BD	372	357	DBSU	50.84
**4264**	allXL	AC	381	357	DBSU	68.35
**4264**	allXL	AC	373	357	DBSU	53.48
**4264**	allXL	BC	372	370	DBSU	40.62
**4264**	allXL	BC	373	370	DBSU	40.67
**4264**	allXL	BC	382	373	DBSU	52.61
**4265**	allXL	CC	320	357	BS2G	23.85
**4265**	allXL	DD	320	373	BS2G	18.07
**4265**	allXL	DD	373	382	BS2G	18.08
**4265**	allXL	DD	370	386	BS2G	17.82
**4265**	allXL	AA	373	386	BS2G	24.59
**4265**	allXL	CC	319	357	BS2G	24.77

**4265**	allXL	AA	367	373	BS2G	11.6
**4265**	allXL	DD	370	382	BS2G	11.9
**4265**	allXL	AA	376	382	BS2G	14.13
**4265**	allXL	DD	357	373	BS2G	25.4
**4265**	allXL	BB	320	366	BS2G	23.6
**4265**	allXL	DD	370	376	BS2G	14.42
**4265**	allXL	CC	320	357	DBSU	23.85
**4265**	allXL	CC	357	319	DBSU	24.77
**4265**	allXL	BB	372	319	DBSU	19.87
**4265**	allXL	BB	370	357	DBSU	27.8
**4265**	allXL	DD	382	357	DBSU	32.07
**4265**	allXL	AA	372	370	DBSU	6.35
**4265**	allXL	DD	381	370	DBSU	13.3
**4265**	allXL	AA;DD	373	370	DBSU	9.02
**4265**	allXL	DD	381	372	DBSU	18.74
**4265**	allXL	DD	372	382	DBSU	18.37
**4265**	allXL	CC	372	386	DBSU	23.92
**4265**	allXL	AA	373	386	DBSU	24.59
**4265**	allXL	CB	381	386	DBSU	27.34
**4265**	allXL	BD;DB	357	357	DBSU	19.28
**4265**	allXL	CB	373	319	DBSU	24.65
**4265**	allXL	BD	372	357	DBSU	42.02
**4265**	allXL	DB	381	357	DBSU	44.92
**4265**	allXL	DA	373	357	DBSU	43.11
**4265**	allXL	DA	372	370	DBSU	38.2
**4265**	allXL	DA	373	370	DBSU	36.86
**4265**	allXL	CB	382	373	DBSU	28.98
**4266**	allXL	CC	320	357	BS2G	23.1
**4266**	allXL	AA	320	373	BS2G	18.93
**4266**	allXL	CC	373	382	BS2G	17.58
**4266**	allXL	CC	370	386	BS2G	18.26
**4266**	allXL	CC	373	386	BS2G	23.28
**4266**	allXL	CC	319	357	BS2G	23.35
**4266**	allXL	AA	367	373	BS2G	13.78

**4266**	allXL	CC	370	382	BS2G	17.6
**4266**	allXL	BB	376	382	BS2G	14.26
**4266**	allXL	DD	357	373	BS2G	31.59
**4266**	allXL	AA	320	366	BS2G	15.75
**4266**	allXL	CC	370	376	BS2G	15.31
**4266**	allXL	CC	320	357	DBSU	23.1
**4266**	allXL	CC	357	319	DBSU	23.35
**4266**	allXL	AA	372	319	DBSU	17.37
**4266**	allXL	DD	370	357	DBSU	24.46
**4266**	allXL	CC	382	357	DBSU	35.14
**4266**	allXL	AA	372	370	DBSU	6.26
**4266**	allXL	CC	381	370	DBSU	20.69
**4266**	allXL	AA	373	370	DBSU	9.09
**4266**	allXL	CC	381	372	DBSU	21.76
**4266**	allXL	CC	372	382	DBSU	19.39
**4266**	allXL	CC	372	386	DBSU	23.21
**4266**	allXL	CC	373	386	DBSU	23.28
**4266**	allXL	DA	381	386	DBSU	27.97
**4266**	allXL	BD;DB	357	357	DBSU	17.82
**4266**	allXL	AD	373	319	DBSU	31.56
**4266**	allXL	AD	372	357	DBSU	42.64
**4266**	allXL	AD	381	357	DBSU	45.82
**4266**	allXL	AD	373	357	DBSU	45.24
**4266**	allXL	AD	372	370	DBSU	40.68
**4266**	allXL	AD	373	370	DBSU	41.46
**4266**	allXL	AD	382	373	DBSU	30.68
**4267**	allXL	CC	320	357	BS2G	25.42
**4267**	allXL	DD	320	373	BS2G	24.24
**4267**	allXL	BB	373	382	BS2G	16.15
**4267**	allXL	BB	370	386	BS2G	23.87
**4267**	allXL	BB	373	386	BS2G	26.19
**4267**	allXL	AA	319	357	BS2G	26.73
**4267**	allXL	DD	367	373	BS2G	13.63
**4267**	allXL	BB	370	382	BS2G	17.08

**4267**	allXL	BB	376	382	BS2G	12.85
**4267**	allXL	DD	357	373	BS2G	20.4
**4267**	allXL	AA	320	366	BS2G	19.95
**4267**	allXL	BB	370	376	BS2G	15.28
**4267**	allXL	CC	320	357	DBSU	25.42
**4267**	allXL	AA	357	319	DBSU	26.73
**4267**	allXL	DD	372	319	DBSU	28.86
**4267**	allXL	DD	370	357	DBSU	23.19
**4267**	allXL	BB	382	357	DBSU	31.78
**4267**	allXL	AA	372	370	DBSU	6.38
**4267**	allXL	BB	381	370	DBSU	17.78
**4267**	allXL	DD	373	370	DBSU	8.94
**4267**	allXL	BB	381	372	DBSU	19.53
**4267**	allXL	BB	372	382	DBSU	18.68
**4267**	allXL	CC	372	386	DBSU	25.64
**4267**	allXL	BB	373	386	DBSU	26.19
**4267**	allXL	CB	381	386	DBSU	51.59
**4267**	allXL	BD;DB	357	357	DBSU	18.91
**4267**	allXL	CB	373	319	DBSU	26.57
**4267**	allXL	DB	372	357	DBSU	40.15
**4267**	allXL	DB	381	357	DBSU	37.54
**4267**	allXL	DB	373	357	DBSU	36.81
**4267**	allXL	CB	372	370	DBSU	43.74
**4267**	allXL	CB	373	370	DBSU	41.75
**4267**	allXL	BC	382	373	DBSU	46.16
**4268**	allXL	AA	320	357	BS2G	24.62
**4268**	allXL	DD	320	373	BS2G	15.34
**4268**	allXL	BB	373	382	BS2G	18.94
**4268**	allXL	DD	370	386	BS2G	32.71
**4268**	allXL	BB	373	386	BS2G	29.24
**4268**	allXL	AA	319	357	BS2G	27.33
**4268**	allXL	BB	367	373	BS2G	13.24
**4268**	allXL	DD	370	382	BS2G	23.94
**4268**	allXL	CC	376	382	BS2G	14.64

**4268**	allXL	BB	357	373	BS2G	18.86
**4268**	allXL	DD	320	366	BS2G	22.17
**4268**	allXL	CC	370	376	BS2G	16.63
**4268**	allXL	AA	320	357	DBSU	24.62
**4268**	allXL	AA	357	319	DBSU	27.33
**4268**	allXL	DD	372	319	DBSU	16.46
**4268**	allXL	DD	370	357	DBSU	18.91
**4268**	allXL	BB	382	357	DBSU	21.82
**4268**	allXL	AA	372	370	DBSU	6.25
**4268**	allXL	DD	381	370	DBSU	21.76
**4268**	allXL	AA	373	370	DBSU	8.76
**4268**	allXL	DD	381	372	DBSU	20.73
**4268**	allXL	BB	372	382	DBSU	20.82
**4268**	allXL	BB	372	386	DBSU	30.05
**4268**	allXL	BB	373	386	DBSU	29.24
**4268**	allXL	BC	381	386	DBSU	17.21
**4268**	allXL	BD;DB	357	357	DBSU	18.0
**4268**	allXL	CB	373	319	DBSU	24.2
**4268**	allXL	BD	372	357	DBSU	37.59
**4268**	allXL	BD	381	357	DBSU	36.65
**4268**	allXL	BD	373	357	DBSU	34.21
**4268**	allXL	CB	372	370	DBSU	24.47
**4268**	allXL	CB	373	370	DBSU	20.88
**4268**	allXL	CB	382	373	DBSU	19.72
**4269**	allXL	CC	320	357	BS2G	25.39
**4269**	allXL	DD	320	373	BS2G	12.6
**4269**	allXL	CC	373	382	BS2G	20.14
**4269**	allXL	DD	370	386	BS2G	22.4
**4269**	allXL	CC	373	386	BS2G	27.26
**4269**	allXL	AA	319	357	BS2G	26.64
**4269**	allXL	AA	367	373	BS2G	11.74
**4269**	allXL	DD	370	382	BS2G	16.17
**4269**	allXL	CC	376	382	BS2G	14.79
**4269**	allXL	BB	357	373	BS2G	22.65

**4269**	allXL	AA	320	366	BS2G	19.23
**4269**	allXL	DD	370	376	BS2G	15.43
**4269**	allXL	CC	320	357	DBSU	25.39
**4269**	allXL	AA	357	319	DBSU	26.64
**4269**	allXL	DD	372	319	DBSU	15.0
**4269**	allXL	DD	370	357	DBSU	27.96
**4269**	allXL	DD	382	357	DBSU	32.6
**4269**	allXL	CC	372	370	DBSU	6.49
**4269**	allXL	DD	381	370	DBSU	16.15
**4269**	allXL	CC	373	370	DBSU	9.17
**4269**	allXL	DD	381	372	DBSU	20.25
**4269**	allXL	CC	372	382	DBSU	21.09
**4269**	allXL	CC	372	386	DBSU	26.41
**4269**	allXL	CC	373	386	DBSU	27.26
**4269**	allXL	BC	381	386	DBSU	56.82
**4269**	allXL	BD;DB	357	357	DBSU	17.71
**4269**	allXL	CB	373	319	DBSU	35.36
**4269**	allXL	BD	372	357	DBSU	35.7
**4269**	allXL	BD	381	357	DBSU	43.75
**4269**	allXL	BD	373	357	DBSU	34.68
**4269**	allXL	DA	372	370	DBSU	47.26
**4269**	allXL	DA	373	370	DBSU	43.6
**4269**	allXL	DA	382	373	DBSU	56.82
**4270**	allXL	CC	320	357	BS2G	23.92
**4270**	allXL	AA	320	373	BS2G	28.33
**4270**	allXL	DD	373	382	BS2G	17.72
**4270**	allXL	CC	370	386	BS2G	23.45
**4270**	allXL	AA	373	386	BS2G	25.15
**4270**	allXL	AA	319	357	BS2G	23.32
**4270**	allXL	DD	367	373	BS2G	13.47
**4270**	allXL	CC	370	382	BS2G	19.78
**4270**	allXL	BB	376	382	BS2G	12.83
**4270**	allXL	DD	357	373	BS2G	26.39
**4270**	allXL	AA	320	366	BS2G	17.77

**4270**	allXL	CC	370	376	BS2G	16.05
**4270**	allXL	CC	320	357	DBSU	23.92
**4270**	allXL	AA	357	319	DBSU	23.32
**4270**	allXL	AA	372	319	DBSU	25.08
**4270**	allXL	DD	370	357	DBSU	28.21
**4270**	allXL	BB	382	357	DBSU	20.74
**4270**	allXL	AA	372	370	DBSU	6.21
**4270**	allXL	DD	381	370	DBSU	19.98
**4270**	allXL	DD	373	370	DBSU	8.99
**4270**	allXL	DD	381	372	DBSU	13.75
**4270**	allXL	DD	372	382	DBSU	15.85
**4270**	allXL	DD	372	386	DBSU	22.42
**4270**	allXL	AA	373	386	DBSU	25.15
**4270**	allXL	CB	381	386	DBSU	43.6
**4270**	allXL	BD;DB	357	357	DBSU	18.68
**4270**	allXL	CB	373	319	DBSU	14.65
**4270**	allXL	CB	372	357	DBSU	38.06
**4270**	allXL	BD	381	357	DBSU	36.58
**4270**	allXL	CB	373	357	DBSU	36.52
**4270**	allXL	CB	372	370	DBSU	35.84
**4270**	allXL	CB	373	370	DBSU	32.32
**4270**	allXL	BC	382	373	DBSU	33.5
**4271**	allXL	CC	320	357	BS2G	24.77
**4271**	allXL	AA	320	373	BS2G	23.62
**4271**	allXL	CC	373	382	BS2G	20.78
**4271**	allXL	DD	370	386	BS2G	16.0
**4271**	allXL	DD	373	386	BS2G	21.94
**4271**	allXL	AA	319	357	BS2G	25.52
**4271**	allXL	AA	367	373	BS2G	13.92
**4271**	allXL	CC	370	382	BS2G	16.05
**4271**	allXL	CC	376	382	BS2G	15.12
**4271**	allXL	BB	357	373	BS2G	28.65
**4271**	allXL	CC	320	366	BS2G	24.13
**4271**	allXL	CC	370	376	BS2G	13.65

**4271**	allXL	CC	320	357	DBSU	24.77
**4271**	allXL	AA	357	319	DBSU	25.52
**4271**	allXL	CC	372	319	DBSU	20.84
**4271**	allXL	DD	370	357	DBSU	27.0
**4271**	allXL	DD	382	357	DBSU	31.24
**4271**	allXL	AA	372	370	DBSU	6.09
**4271**	allXL	DD	381	370	DBSU	16.64
**4271**	allXL	AA	373	370	DBSU	9.14
**4271**	allXL	DD	381	372	DBSU	19.47
**4271**	allXL	CC	372	382	DBSU	20.43
**4271**	allXL	DD	372	386	DBSU	20.21
**4271**	allXL	DD	373	386	DBSU	21.94
**4271**	allXL	BC	381	386	DBSU	44.72
**4271**	allXL	BD;DB	357	357	DBSU	17.6
**4271**	allXL	BC	373	319	DBSU	21.27
**4271**	allXL	BD	372	357	DBSU	34.57
**4271**	allXL	BC	381	357	DBSU	29.68
**4271**	allXL	BC	373	357	DBSU	34.71
**4271**	allXL	CB	372	370	DBSU	20.92
**4271**	allXL	CB	373	370	DBSU	23.33
**4271**	allXL	BC	382	373	DBSU	31.58
**4272**	allXL	AA	320	357	BS2G	25.17
**4272**	allXL	DD	320	373	BS2G	19.39
**4272**	allXL	BB	373	382	BS2G	19.29
**4272**	allXL	DD	370	386	BS2G	32.28
**4272**	allXL	DD	373	386	BS2G	28.39
**4272**	allXL	AA	319	357	BS2G	24.03
**4272**	allXL	AA	367	373	BS2G	11.04
**4272**	allXL	DD	370	382	BS2G	23.26
**4272**	allXL	CC	376	382	BS2G	14.07
**4272**	allXL	AA	357	373	BS2G	29.3
**4272**	allXL	DD	320	366	BS2G	23.47
**4272**	allXL	CC	370	376	BS2G	15.17
**4272**	allXL	AA	320	357	DBSU	25.17

**4272**	allXL	AA	357	319	DBSU	24.03
**4272**	allXL	DD	372	319	DBSU	16.74
**4272**	allXL	BB	370	357	DBSU	20.84
**4272**	allXL	AA	382	357	DBSU	28.07
**4272**	allXL	AA	372	370	DBSU	6.49
**4272**	allXL	DD	381	370	DBSU	22.85
**4272**	allXL	AA	373	370	DBSU	8.92
**4272**	allXL	BB	381	372	DBSU	21.3
**4272**	allXL	BB	372	382	DBSU	20.75
**4272**	allXL	DD	372	386	DBSU	29.73
**4272**	allXL	DD	373	386	DBSU	28.39
**4272**	allXL	AD	381	386	DBSU	41.62
**4272**	allXL	BD;DB	357	357	DBSU	18.94
**4272**	allXL	DA	373	319	DBSU	19.74
**4272**	allXL	DA	372	357	DBSU	22.74
**4272**	allXL	AC	381	357	DBSU	25.55
**4272**	allXL	DA	373	357	DBSU	22.09
**4272**	allXL	DA	372	370	DBSU	36.61
**4272**	allXL	DA	373	370	DBSU	34.21
**4272**	allXL	DA	382	373	DBSU	33.94
**4273**	allXL	AA	320	357	BS2G	26.89
**4273**	allXL	DD	320	373	BS2G	20.9
**4273**	allXL	BB	373	382	BS2G	18.43
**4273**	allXL	AA	370	386	BS2G	26.58
**4273**	allXL	DD	373	386	BS2G	25.22
**4273**	allXL	AA	319	357	BS2G	28.69
**4273**	allXL	AA	367	373	BS2G	12.86
**4273**	allXL	DD	370	382	BS2G	23.82
**4273**	allXL	BB	376	382	BS2G	12.97
**4273**	allXL	BB	357	373	BS2G	19.62
**4273**	allXL	BB	320	366	BS2G	15.08
**4273**	allXL	CC	370	376	BS2G	15.19
**4273**	allXL	AA	320	357	DBSU	26.89
**4273**	allXL	AA	357	319	DBSU	28.69

**4273**	allXL	DD	372	319	DBSU	17.1
**4273**	allXL	BB	370	357	DBSU	24.45
**4273**	allXL	BB	382	357	DBSU	26.61
**4273**	allXL	AA	372	370	DBSU	6.26
**4273**	allXL	DD	381	370	DBSU	22.3
**4273**	allXL	AA	373	370	DBSU	9.05
**4273**	allXL	DD	381	372	DBSU	16.24
**4273**	allXL	DD	372	382	DBSU	18.16
**4273**	allXL	DD	372	386	DBSU	24.16
**4273**	allXL	DD	373	386	DBSU	25.22
**4273**	allXL	AD	381	386	DBSU	31.9
**4273**	allXL	BD;DB	357	357	DBSU	19.24
**4273**	allXL	CB	373	319	DBSU	13.15
**4273**	allXL	CB	372	357	DBSU	30.66
**4273**	allXL	BD	381	357	DBSU	27.53
**4273**	allXL	BD	373	357	DBSU	31.58
**4273**	allXL	CB	372	370	DBSU	26.31
**4273**	allXL	CB	373	370	DBSU	25.96
**4273**	allXL	DA	382	373	DBSU	41.46
**4274**	allXL	AA	320	357	BS2G	22.31
**4274**	allXL	BB	320	373	BS2G	27.91
**4274**	allXL	AA	373	382	BS2G	17.31
**4274**	allXL	CC	370	386	BS2G	23.92
**4274**	allXL	AA	373	386	BS2G	19.61
**4274**	allXL	AA	319	357	BS2G	21.05
**4274**	allXL	AA	367	373	BS2G	11.69
**4274**	allXL	CC	370	382	BS2G	22.23
**4274**	allXL	AA	376	382	BS2G	13.47
**4274**	allXL	BB	357	373	BS2G	28.44
**4274**	allXL	BB	320	366	BS2G	18.57
**4274**	allXL	CC	370	376	BS2G	14.37
**4274**	allXL	AA	320	357	DBSU	22.31
**4274**	allXL	AA	357	319	DBSU	21.05
**4274**	allXL	CC	372	319	DBSU	22.87

**4274**	allXL	DD	370	357	DBSU	25.37
**4274**	allXL	AA	382	357	DBSU	33.81
**4274**	allXL	AA	372	370	DBSU	6.56
**4274**	allXL	CC	381	370	DBSU	21.88
**4274**	allXL	CC	373	370	DBSU	9.19
**4274**	allXL	AA	381	372	DBSU	21.97
**4274**	allXL	AA	372	382	DBSU	20.57
**4274**	allXL	AA	372	386	DBSU	21.43
**4274**	allXL	AA	373	386	DBSU	19.61
**4274**	allXL	BC	381	386	DBSU	42.67
**4274**	allXL	BD;DB	357	357	DBSU	18.7
**4274**	allXL	DA	373	319	DBSU	36.51
**4274**	allXL	DB	372	357	DBSU	42.62
**4274**	allXL	CB	381	357	DBSU	44.31
**4274**	allXL	BD	373	357	DBSU	45.49
**4274**	allXL	BC	372	370	DBSU	43.46
**4274**	allXL	CB	373	370	DBSU	46.35
**4274**	allXL	CB	382	373	DBSU	32.26
**4275**	allXL	AA	320	357	BS2G	18.1
**4275**	allXL	CC	320	373	BS2G	26.49
**4275**	allXL	DD	373	382	BS2G	15.04
**4275**	allXL	DD	370	386	BS2G	12.07
**4275**	allXL	DD	373	386	BS2G	17.46
**4275**	allXL	AA	319	357	BS2G	15.56
**4275**	allXL	AA	367	373	BS2G	13.02
**4275**	allXL	DD	370	382	BS2G	11.79
**4275**	allXL	DD	376	382	BS2G	14.76
**4275**	allXL	CC	357	373	BS2G	26.51
**4275**	allXL	AA	320	366	BS2G	25.39
**4275**	allXL	DD	370	376	BS2G	16.41
**4275**	allXL	AA	320	357	DBSU	18.1
**4275**	allXL	AA	357	319	DBSU	15.56
**4275**	allXL	CC	372	319	DBSU	26.43
**4275**	allXL	DD	370	357	DBSU	25.53

**4275**	allXL	DD	382	357	DBSU	21.42
**4275**	allXL	AA	372	370	DBSU	6.44
**4275**	allXL	DD	381	370	DBSU	14.88
**4275**	allXL	AA	373	370	DBSU	9.43
**4275**	allXL	DD	381	372	DBSU	17.35
**4275**	allXL	DD	372	382	DBSU	15.14
**4275**	allXL	DD	372	386	DBSU	15.12
**4275**	allXL	DD	373	386	DBSU	17.46
**4275**	allXL	AD	381	386	DBSU	52.72
**4275**	allXL	BD;DB	357	357	DBSU	18.56
**4275**	allXL	AD	373	319	DBSU	20.68
**4275**	allXL	BD	372	357	DBSU	37.73
**4275**	allXL	DB	381	357	DBSU	40.4
**4275**	allXL	BD	373	357	DBSU	38.93
**4275**	allXL	DA	372	370	DBSU	43.98
**4275**	allXL	DA	373	370	DBSU	46.25
**4275**	allXL	DA	382	373	DBSU	43.04
**4276**	allXL	AA	320	357	BS2G	16.83
**4276**	allXL	BB	320	373	BS2G	16.85
**4276**	allXL	DD	373	382	BS2G	17.15
**4276**	allXL	CC	370	386	BS2G	24.13
**4276**	allXL	CC	373	386	BS2G	16.82
**4276**	allXL	AA	319	357	BS2G	14.05
**4276**	allXL	AA	367	373	BS2G	10.43
**4276**	allXL	DD	370	382	BS2G	21.04
**4276**	allXL	CC	376	382	BS2G	13.48
**4276**	allXL	BB	357	373	BS2G	18.68
**4276**	allXL	BB	320	366	BS2G	24.91
**4276**	allXL	CC	370	376	BS2G	16.77
**4276**	allXL	AA	320	357	DBSU	16.83
**4276**	allXL	AA	357	319	DBSU	14.05
**4276**	allXL	BB	372	319	DBSU	15.67
**4276**	allXL	AA	370	357	DBSU	23.55
**4276**	allXL	BB	382	357	DBSU	8.14

**4276**	allXL	AA	372	370	DBSU	6.6
**4276**	allXL	DD	381	370	DBSU	21.26
**4276**	allXL	AA	373	370	DBSU	8.78
**4276**	allXL	DD	381	372	DBSU	15.02
**4276**	allXL	DD	372	382	DBSU	15.21
**4276**	allXL	CC	372	386	DBSU	18.91
**4276**	allXL	CC	373	386	DBSU	16.82
**4276**	allXL	BC	381	386	DBSU	41.99
**4276**	allXL	BD;DB	357	357	DBSU	19.2
**4276**	allXL	CB	373	319	DBSU	27.23
**4276**	allXL	AC	372	357	DBSU	17.45
**4276**	allXL	BD	381	357	DBSU	21.11
**4276**	allXL	AC	373	357	DBSU	20.82
**4276**	allXL	CB	372	370	DBSU	15.64
**4276**	allXL	CB	373	370	DBSU	19.14
**4276**	allXL	CB	382	373	DBSU	37.15
**4277**	allXL	AA	320	357	BS2G	23.84
**4277**	allXL	BB	320	373	BS2G	15.38
**4277**	allXL	DD	373	382	BS2G	17.83
**4277**	allXL	DD	370	386	BS2G	14.63
**4277**	allXL	DD	373	386	BS2G	15.85
**4277**	allXL	AA	319	357	BS2G	24.44
**4277**	allXL	BB	367	373	BS2G	14.69
**4277**	allXL	CC	370	382	BS2G	17.37
**4277**	allXL	BB	376	382	BS2G	13.8
**4277**	allXL	BB	357	373	BS2G	29.47
**4277**	allXL	BB	320	366	BS2G	27.57
**4277**	allXL	CC	370	376	BS2G	12.45
**4277**	allXL	AA	320	357	DBSU	23.84
**4277**	allXL	AA	357	319	DBSU	24.44
**4277**	allXL	BB	372	319	DBSU	20.6
**4277**	allXL	DD	370	357	DBSU	26.59
**4277**	allXL	BB	382	357	DBSU	34.66
**4277**	allXL	AA	372	370	DBSU	6.21

**4277**	allXL	CC	381	370	DBSU	17.86
**4277**	allXL	AA	373	370	DBSU	9.4
**4277**	allXL	DD	381	372	DBSU	19.59
**4277**	allXL	DD	372	382	DBSU	19.38
**4277**	allXL	DD	372	386	DBSU	15.03
**4277**	allXL	DD	373	386	DBSU	15.85
**4277**	allXL	CB	381	386	DBSU	28.15
**4277**	allXL	BD;DB	357	357	DBSU	18.01
**4277**	allXL	DA	373	319	DBSU	12.85
**4277**	allXL	DA	372	357	DBSU	30.58
**4277**	allXL	DA	381	357	DBSU	43.46
**4277**	allXL	DA	373	357	DBSU	31.14
**4277**	allXL	CB	372	370	DBSU	15.0
**4277**	allXL	CB	373	370	DBSU	11.42
**4277**	allXL	BC	382	373	DBSU	25.23
**4278**	allXL	CC	320	357	BS2G	25.23
**4278**	allXL	AA	320	373	BS2G	26.0
**4278**	allXL	AA	373	382	BS2G	14.45
**4278**	allXL	AA	370	386	BS2G	15.98
**4278**	allXL	AA	373	386	BS2G	16.09
**4278**	allXL	AA	319	357	BS2G	25.66
**4278**	allXL	DD	367	373	BS2G	14.32
**4278**	allXL	AA	370	382	BS2G	19.6
**4278**	allXL	AA	376	382	BS2G	13.64
**4278**	allXL	BB	357	373	BS2G	24.13
**4278**	allXL	AA	320	366	BS2G	19.08
**4278**	allXL	CC	370	376	BS2G	16.45
**4278**	allXL	CC	320	357	DBSU	25.23
**4278**	allXL	AA	357	319	DBSU	25.66
**4278**	allXL	AA	372	319	DBSU	24.85
**4278**	allXL	BB	370	357	DBSU	24.16
**4278**	allXL	BB	382	357	DBSU	36.94
**4278**	allXL	AA	372	370	DBSU	5.97
**4278**	allXL	AA	381	370	DBSU	20.91

**4278**	allXL	DD	373	370	DBSU	8.81
**4278**	allXL	AA	381	372	DBSU	18.19
**4278**	allXL	AA	372	382	DBSU	17.16
**4278**	allXL	AA	372	386	DBSU	16.93
**4278**	allXL	AA	373	386	DBSU	16.09
**4278**	allXL	DB	381	386	DBSU	63.58
**4278**	allXL	AC;CA	357	357	DBSU	20.02
**4278**	allXL	AD	373	319	DBSU	34.08
**4278**	allXL	BD	372	357	DBSU	34.74
**4278**	allXL	BD	381	357	DBSU	43.4
**4278**	allXL	BD	373	357	DBSU	35.4
**4278**	allXL	BD	372	370	DBSU	49.17
**4278**	allXL	BD	373	370	DBSU	47.7
**4278**	allXL	BD	382	373	DBSU	47.74
**4279**	allXL	AA	320	357	BS2G	25.84
**4279**	allXL	DD	320	373	BS2G	17.47
**4279**	allXL	DD	373	382	BS2G	15.57
**4279**	allXL	DD	370	386	BS2G	17.87
**4279**	allXL	DD	373	386	BS2G	25.92
**4279**	allXL	AA	319	357	BS2G	24.37
**4279**	allXL	BB	367	373	BS2G	12.57
**4279**	allXL	DD	370	382	BS2G	9.74
**4279**	allXL	BB	376	382	BS2G	12.77
**4279**	allXL	BB	357	373	BS2G	24.9
**4279**	allXL	CC	320	366	BS2G	25.6
**4279**	allXL	DD	370	376	BS2G	15.45
**4279**	allXL	AA	320	357	DBSU	25.84
**4279**	allXL	AA	357	319	DBSU	24.37
**4279**	allXL	DD	372	319	DBSU	17.39
**4279**	allXL	CC	370	357	DBSU	22.22
**4279**	allXL	DD	382	357	DBSU	28.01
**4279**	allXL	AA	372	370	DBSU	6.22
**4279**	allXL	DD	381	370	DBSU	10.01
**4279**	allXL	AA	373	370	DBSU	9.09

**4279**	allXL	DD	381	372	DBSU	12.94
**4279**	allXL	DD	372	382	DBSU	14.05
**4279**	allXL	DD	372	386	DBSU	23.78
**4279**	allXL	DD	373	386	DBSU	25.92
**4279**	allXL	DA	381	386	DBSU	25.36
**4279**	allXL	AC;CA	357	357	DBSU	19.55
**4279**	allXL	AD	373	319	DBSU	40.49
**4279**	allXL	CA	372	357	DBSU	39.1
**4279**	allXL	DA	381	357	DBSU	34.47
**4279**	allXL	CA	373	357	DBSU	37.76
**4279**	allXL	AD	372	370	DBSU	50.79
**4279**	allXL	AD	373	370	DBSU	48.43
**4279**	allXL	AD	382	373	DBSU	38.37
**4280**	allXL	AA	320	357	BS2G	24.34
**4280**	allXL	DD	320	373	BS2G	29.1
**4280**	allXL	AA	373	382	BS2G	17.81
**4280**	allXL	AA	370	386	BS2G	19.89
**4280**	allXL	AA	373	386	BS2G	24.58
**4280**	allXL	AA	319	357	BS2G	24.2
**4280**	allXL	AA	367	373	BS2G	13.73
**4280**	allXL	DD	370	382	BS2G	16.8
**4280**	allXL	CC	376	382	BS2G	14.04
**4280**	allXL	DD	357	373	BS2G	17.52
**4280**	allXL	CC	320	366	BS2G	25.97
**4280**	allXL	DD	370	376	BS2G	16.67
**4280**	allXL	AA	320	357	DBSU	24.34
**4280**	allXL	AA	357	319	DBSU	24.2
**4280**	allXL	CC	372	319	DBSU	24.78
**4280**	allXL	DD	370	357	DBSU	23.61
**4280**	allXL	DD	382	357	DBSU	31.31
**4280**	allXL	AA	372	370	DBSU	6.43
**4280**	allXL	DD	381	370	DBSU	15.23
**4280**	allXL	AA	373	370	DBSU	9.06
**4280**	allXL	DD	381	372	DBSU	17.18

**4280**	allXL	AA	372	382	DBSU	16.48
**4280**	allXL	AA	372	386	DBSU	22.04
**4280**	allXL	AA	373	386	DBSU	24.58
**4280**	allXL	DB	381	386	DBSU	65.63
**4280**	allXL	BD;DB	357	357	DBSU	16.93
**4280**	allXL	DA	373	319	DBSU	31.7
**4280**	allXL	DB	372	357	DBSU	31.74
**4280**	allXL	BD	381	357	DBSU	36.63
**4280**	allXL	DB	373	357	DBSU	28.59
**4280**	allXL	CB	372	370	DBSU	30.67
**4280**	allXL	CB	373	370	DBSU	31.03
**4280**	allXL	BD	382	373	DBSU	48.82
**4281**	allXL	CC	320	357	BS2G	25.21
**4281**	allXL	DD	320	373	BS2G	16.64
**4281**	allXL	BB	373	382	BS2G	18.31
**4281**	allXL	DD	370	386	BS2G	19.58
**4281**	allXL	BB	373	386	BS2G	26.13
**4281**	allXL	AA	319	357	BS2G	25.53
**4281**	allXL	BB	367	373	BS2G	14.91
**4281**	allXL	DD	370	382	BS2G	13.4
**4281**	allXL	BB	376	382	BS2G	14.15
**4281**	allXL	DD	357	373	BS2G	34.49
**4281**	allXL	AA	320	366	BS2G	21.35
**4281**	allXL	DD	370	376	BS2G	15.34
**4281**	allXL	CC	320	357	DBSU	25.21
**4281**	allXL	AA	357	319	DBSU	25.53
**4281**	allXL	DD	372	319	DBSU	20.65
**4281**	allXL	DD	370	357	DBSU	28.39
**4281**	allXL	DD	382	357	DBSU	24.07
**4281**	allXL	AA	372	370	DBSU	6.31
**4281**	allXL	DD	381	370	DBSU	11.61
**4281**	allXL	AA	373	370	DBSU	9.14
**4281**	allXL	DD	381	372	DBSU	15.99
**4281**	allXL	DD	372	382	DBSU	18.84

**4281**	allXL	DD	372	386	DBSU	26.65
**4281**	allXL	BB	373	386	DBSU	26.13
**4281**	allXL	DA	381	386	DBSU	47.01
**4281**	allXL	BD;DB	357	357	DBSU	18.32
**4281**	allXL	AD	373	319	DBSU	41.07
**4281**	allXL	DA	372	357	DBSU	43.66
**4281**	allXL	DA	381	357	DBSU	30.12
**4281**	allXL	DA	373	357	DBSU	43.2
**4281**	allXL	DA	372	370	DBSU	26.44
**4281**	allXL	DA	373	370	DBSU	27.98
**4281**	allXL	BC	382	373	DBSU	38.86
**4282**	allXL	CC	320	357	BS2G	23.66
**4282**	allXL	DD	320	373	BS2G	17.21
**4282**	allXL	AA	373	382	BS2G	18.64
**4282**	allXL	DD	370	386	BS2G	16.48
**4282**	allXL	DD	373	386	BS2G	20.44
**4282**	allXL	AA	319	357	BS2G	24.02
**4282**	allXL	AA	367	373	BS2G	12.25
**4282**	allXL	DD	370	382	BS2G	21.14
**4282**	allXL	CC	376	382	BS2G	15.07
**4282**	allXL	BB	357	373	BS2G	23.46
**4282**	allXL	CC	320	366	BS2G	25.52
**4282**	allXL	CC	370	376	BS2G	16.5
**4282**	allXL	CC	320	357	DBSU	23.66
**4282**	allXL	AA	357	319	DBSU	24.02
**4282**	allXL	DD	372	319	DBSU	17.26
**4282**	allXL	DD	370	357	DBSU	25.57
**4282**	allXL	AA	382	357	DBSU	23.38
**4282**	allXL	AA	372	370	DBSU	6.39
**4282**	allXL	AA	381	370	DBSU	20.72
**4282**	allXL	AA	373	370	DBSU	9.0
**4282**	allXL	AA	381	372	DBSU	19.17
**4282**	allXL	AA	372	382	DBSU	21.43
**4282**	allXL	DD	372	386	DBSU	20.43

**4282**	allXL	DD	373	386	DBSU	20.44
**4282**	allXL	AD	381	386	DBSU	27.47
**4282**	allXL	BD;DB	357	357	DBSU	18.91
**4282**	allXL	CB	373	319	DBSU	27.83
**4282**	allXL	BD	372	357	DBSU	42.56
**4282**	allXL	CB	381	357	DBSU	32.49
**4282**	allXL	DA	373	357	DBSU	40.87
**4282**	allXL	CB	372	370	DBSU	31.76
**4282**	allXL	CB	373	370	DBSU	28.82
**4282**	allXL	CB	382	373	DBSU	12.72
**4283**	allXL	CC	320	357	BS2G	20.54
**4283**	allXL	AA	320	373	BS2G	28.99
**4283**	allXL	BB	373	382	BS2G	19.5
**4283**	allXL	AA	370	386	BS2G	25.13
**4283**	allXL	AA	373	386	BS2G	25.47
**4283**	allXL	CC	319	357	BS2G	22.3
**4283**	allXL	AA	367	373	BS2G	14.65
**4283**	allXL	DD	370	382	BS2G	21.28
**4283**	allXL	CC	376	382	BS2G	14.95
**4283**	allXL	CC	357	373	BS2G	23.48
**4283**	allXL	BB	320	366	BS2G	24.36
**4283**	allXL	CC	370	376	BS2G	14.42
**4283**	allXL	CC	320	357	DBSU	20.54
**4283**	allXL	CC	357	319	DBSU	22.3
**4283**	allXL	BB	372	319	DBSU	31.3
**4283**	allXL	BB	370	357	DBSU	24.51
**4283**	allXL	BB	382	357	DBSU	43.19
**4283**	allXL	AA	372	370	DBSU	6.14
**4283**	allXL	DD	381	370	DBSU	19.73
**4283**	allXL	CC	373	370	DBSU	9.06
**4283**	allXL	DD	381	372	DBSU	20.01
**4283**	allXL	AA	372	382	DBSU	20.13
**4283**	allXL	AA	372	386	DBSU	24.48
**4283**	allXL	AA	373	386	DBSU	25.47

**4283**	allXL	BD	381	386	DBSU	30.51
**4283**	allXL	BD;DB	357	357	DBSU	17.84
**4283**	allXL	DA	373	319	DBSU	27.7
**4283**	allXL	CA	372	357	DBSU	22.05
**4283**	allXL	CA	381	357	DBSU	30.09
**4283**	allXL	CA	373	357	DBSU	18.66
**4283**	allXL	DA	372	370	DBSU	27.13
**4283**	allXL	DA	373	370	DBSU	28.51
**4283**	allXL	AD	382	373	DBSU	34.88
**4284**	allXL	AA	320	357	BS2G	22.83
**4284**	allXL	DD	320	373	BS2G	25.76
**4284**	allXL	DD	373	382	BS2G	19.58
**4284**	allXL	DD	370	386	BS2G	15.67
**4284**	allXL	DD	373	386	BS2G	19.37
**4284**	allXL	AA	319	357	BS2G	20.38
**4284**	allXL	AA;BB	367	373	BS2G	13.66
**4284**	allXL	DD	370	382	BS2G	17.68
**4284**	allXL	BB	376	382	BS2G	15.01
**4284**	allXL	DD	357	373	BS2G	25.55
**4284**	allXL	CC	320	366	BS2G	21.37
**4284**	allXL	CC	370	376	BS2G	14.1
**4284**	allXL	AA	320	357	DBSU	22.83
**4284**	allXL	AA	357	319	DBSU	20.38
**4284**	allXL	DD	372	319	DBSU	25.09
**4284**	allXL	DD	370	357	DBSU	19.71
**4284**	allXL	DD	382	357	DBSU	36.29
**4284**	allXL	AA	372	370	DBSU	6.44
**4284**	allXL	DD	381	370	DBSU	17.75
**4284**	allXL	BB	373	370	DBSU	9.17
**4284**	allXL	DD	381	372	DBSU	19.09
**4284**	allXL	DD	372	382	DBSU	20.09
**4284**	allXL	DD	372	386	DBSU	18.38
**4284**	allXL	DD	373	386	DBSU	19.37
**4284**	allXL	DA	381	386	DBSU	47.39

**4284**	allXL	BD;DB	357	357	DBSU	18.26
**4284**	allXL	AD	373	319	DBSU	22.31
**4284**	allXL	DA	372	357	DBSU	33.97
**4284**	allXL	DA	381	357	DBSU	46.27
**4284**	allXL	DA	373	357	DBSU	31.87
**4284**	allXL	DA	372	370	DBSU	37.37
**4284**	allXL	DA	373	370	DBSU	37.01
**4284**	allXL	AD	382	373	DBSU	42.84
**4285**	allXL	AA	320	357	BS2G	23.2
**4285**	allXL	DD	320	373	BS2G	18.6
**4285**	allXL	AA	373	382	BS2G	19.07
**4285**	allXL	AA	370	386	BS2G	21.85
**4285**	allXL	BB	373	386	BS2G	21.67
**4285**	allXL	CC	319	357	BS2G	24.75
**4285**	allXL	AA	367	373	BS2G	12.5
**4285**	allXL	AA	370	382	BS2G	21.16
**4285**	allXL	CC	376	382	BS2G	14.47
**4285**	allXL	BB	357	373	BS2G	28.21
**4285**	allXL	AA	320	366	BS2G	24.66
**4285**	allXL	CC	370	376	BS2G	16.49
**4285**	allXL	AA	320	357	DBSU	23.2
**4285**	allXL	CC	357	319	DBSU	24.75
**4285**	allXL	DD	372	319	DBSU	15.47
**4285**	allXL	DD	370	357	DBSU	22.35
**4285**	allXL	AA	382	357	DBSU	28.82
**4285**	allXL	AA	372	370	DBSU	6.46
**4285**	allXL	AA	381	370	DBSU	23.96
**4285**	allXL	AA	373	370	DBSU	9.21
**4285**	allXL	BB	381	372	DBSU	17.83
**4285**	allXL	AA	372	382	DBSU	19.14
**4285**	allXL	BB	372	386	DBSU	20.64
**4285**	allXL	BB	373	386	DBSU	21.67
**4285**	allXL	BC	381	386	DBSU	45.69
**4285**	allXL	BD;DB	357	357	DBSU	18.57

**4285**	allXL	CB	373	319	DBSU	29.35
**4285**	allXL	DB	372	357	DBSU	43.85
**4285**	allXL	BD	381	357	DBSU	43.71
**4285**	allXL	BD	373	357	DBSU	45.8
**4285**	allXL	DA	372	370	DBSU	34.43
**4285**	allXL	DA	373	370	DBSU	33.45
**4285**	allXL	CB	382	373	DBSU	33.01
**4286**	allXL	CC	320	357	BS2G	25.56
**4286**	allXL	CC	320	373	BS2G	16.91
**4286**	allXL	DD	373	382	BS2G	14.32
**4286**	allXL	DD	370	386	BS2G	16.38
**4286**	allXL	BB	373	386	BS2G	22.35
**4286**	allXL	AA	319	357	BS2G	26.48
**4286**	allXL	DD	367	373	BS2G	13.23
**4286**	allXL	DD	370	382	BS2G	8.77
**4286**	allXL	CC	376	382	BS2G	14.71
**4286**	allXL	DD	357	373	BS2G	27.43
**4286**	allXL	CC	320	366	BS2G	20.73
**4286**	allXL	DD	370	376	BS2G	13.86
**4286**	allXL	CC	320	357	DBSU	25.56
**4286**	allXL	AA	357	319	DBSU	26.48
**4286**	allXL	CC	372	319	DBSU	10.95
**4286**	allXL	BB	370	357	DBSU	29.15
**4286**	allXL	BB	382	357	DBSU	37.67
**4286**	allXL	AA	372	370	DBSU	5.94
**4286**	allXL	DD	381	370	DBSU	8.13
**4286**	allXL	AA	373	370	DBSU	9.2
**4286**	allXL	DD	381	372	DBSU	12.91
**4286**	allXL	DD	372	382	DBSU	12.95
**4286**	allXL	DD	372	386	DBSU	19.9
**4286**	allXL	BB	373	386	DBSU	22.35
**4286**	allXL	AD	381	386	DBSU	50.76
**4286**	allXL	BD;DB	357	357	DBSU	19.02
**4286**	allXL	DA	373	319	DBSU	33.36

**4286**	allXL	BD	372	357	DBSU	39.86
**4286**	allXL	BD	381	357	DBSU	43.62
**4286**	allXL	BD	373	357	DBSU	41.98
**4286**	allXL	DA	372	370	DBSU	44.68
**4286**	allXL	DA	373	370	DBSU	46.66
**4286**	allXL	BD	382	373	DBSU	50.26
**4287**	allXL	CC	320	357	BS2G	24.34
**4287**	allXL	BB	320	373	BS2G	25.04
**4287**	allXL	AA	373	382	BS2G	22.31
**4287**	allXL	AA	370	386	BS2G	18.9
**4287**	allXL	AA	373	386	BS2G	23.94
**4287**	allXL	AA	319	357	BS2G	26.17
**4287**	allXL	AA	367	373	BS2G	12.72
**4287**	allXL	AA	370	382	BS2G	21.35
**4287**	allXL	CC	376	382	BS2G	14.99
**4287**	allXL	BB	357	373	BS2G	28.45
**4287**	allXL	BB	320	366	BS2G	20.05
**4287**	allXL	CC	370	376	BS2G	15.25
**4287**	allXL	CC	320	357	DBSU	24.34
**4287**	allXL	AA	357	319	DBSU	26.17
**4287**	allXL	BB	372	319	DBSU	23.49
**4287**	allXL	BB	370	357	DBSU	29.34
**4287**	allXL	AA	382	357	DBSU	44.57
**4287**	allXL	AA	372	370	DBSU	6.26
**4287**	allXL	DD	381	370	DBSU	22.11
**4287**	allXL	AA	373	370	DBSU	9.23
**4287**	allXL	DD	381	372	DBSU	20.12
**4287**	allXL	AA	372	382	DBSU	20.75
**4287**	allXL	AA	372	386	DBSU	20.87
**4287**	allXL	AA	373	386	DBSU	23.94
**4287**	allXL	DA	381	386	DBSU	57.91
**4287**	allXL	BD;DB	357	357	DBSU	18.66
**4287**	allXL	AD	373	319	DBSU	35.28
**4287**	allXL	BD	372	357	DBSU	43.55

**4287**	allXL	CB	381	357	DBSU	41.93
**4287**	allXL	BD	373	357	DBSU	45.12
**4287**	allXL	CB	372	370	DBSU	52.49
**4287**	allXL	CB	373	370	DBSU	49.65
**4287**	allXL	CB	382	373	DBSU	37.45
**4288**	allXL	AA	320	357	BS2G	26.07
**4288**	allXL	CC	320	373	BS2G	22.66
**4288**	allXL	CC	373	382	BS2G	17.25
**4288**	allXL	CC	370	386	BS2G	23.39
**4288**	allXL	DD	373	386	BS2G	23.07
**4288**	allXL	AA	319	357	BS2G	25.76
**4288**	allXL	BB	367	373	BS2G	11.64
**4288**	allXL	CC	370	382	BS2G	17.74
**4288**	allXL	BB	376	382	BS2G	15.29
**4288**	allXL	CC	357	373	BS2G	30.4
**4288**	allXL	BB	320	366	BS2G	24.83
**4288**	allXL	BB	370	376	BS2G	14.92
**4288**	allXL	AA	320	357	DBSU	26.07
**4288**	allXL	AA	357	319	DBSU	25.76
**4288**	allXL	CC	372	319	DBSU	25.65
**4288**	allXL	DD	370	357	DBSU	25.15
**4288**	allXL	CC	382	357	DBSU	19.81
**4288**	allXL	AA	372	370	DBSU	6.2
**4288**	allXL	CC	381	370	DBSU	18.75
**4288**	allXL	CC	373	370	DBSU	9.07
**4288**	allXL	DD	381	372	DBSU	17.8
**4288**	allXL	DD	372	382	DBSU	16.91
**4288**	allXL	DD	372	386	DBSU	21.32
**4288**	allXL	DD	373	386	DBSU	23.07
**4288**	allXL	BC	381	386	DBSU	29.39
**4288**	allXL	BD;DB	357	357	DBSU	18.99
**4288**	allXL	AD	373	319	DBSU	36.31
**4288**	allXL	DB	372	357	DBSU	50.08
**4288**	allXL	CB	381	357	DBSU	35.47

**4288**	allXL	CB	373	357	DBSU	49.39
**4288**	allXL	BC	372	370	DBSU	45.88
**4288**	allXL	BC	373	370	DBSU	43.94
**4288**	allXL	BC	382	373	DBSU	34.04
**4289**	allXL	CC	320	357	BS2G	22.81
**4289**	allXL	DD	320	373	BS2G	33.33
**4289**	allXL	CC	373	382	BS2G	17.71
**4289**	allXL	AA	370	386	BS2G	29.84
**4289**	allXL	AA	373	386	BS2G	25.67
**4289**	allXL	CC	319	357	BS2G	21.31
**4289**	allXL	AA	367	373	BS2G	11.88
**4289**	allXL	CC	370	382	BS2G	22.99
**4289**	allXL	BB	376	382	BS2G	14.24
**4289**	allXL	BB	357	373	BS2G	28.98
**4289**	allXL	BB	320	366	BS2G	23.31
**4289**	allXL	CC	370	376	BS2G	16.75
**4289**	allXL	CC	320	357	DBSU	22.81
**4289**	allXL	CC	357	319	DBSU	21.31
**4289**	allXL	CC	372	319	DBSU	28.74
**4289**	allXL	BB	370	357	DBSU	30.21
**4289**	allXL	AA	382	357	DBSU	27.88
**4289**	allXL	AA	372	370	DBSU	6.37
**4289**	allXL	DD	381	370	DBSU	23.43
**4289**	allXL	AA	373	370	DBSU	8.98
**4289**	allXL	DD	381	372	DBSU	18.76
**4289**	allXL	BB	372	382	DBSU	20.75
**4289**	allXL	AA	372	386	DBSU	26.0
**4289**	allXL	AA	373	386	DBSU	25.67
**4289**	allXL	DA	381	386	DBSU	72.34
**4289**	allXL	BD;DB	357	357	DBSU	18.1
**4289**	allXL	AD	373	319	DBSU	21.7
**4289**	allXL	BD	372	357	DBSU	44.95
**4289**	allXL	AC	381	357	DBSU	24.81
**4289**	allXL	AC	373	357	DBSU	41.94

**4289**	allXL	DA	372	370	DBSU	40.22
**4289**	allXL	DA	373	370	DBSU	41.13
**4289**	allXL	AD	382	373	DBSU	59.26
**4290**	allXL	AA	320	357	BS2G	23.09
**4290**	allXL	DD	320	373	BS2G	30.82
**4290**	allXL	DD	373	382	BS2G	14.67
**4290**	allXL	DD	370	386	BS2G	16.64
**4290**	allXL	DD	373	386	BS2G	20.93
**4290**	allXL	CC	319	357	BS2G	24.85
**4290**	allXL	AA	367	373	BS2G	12.22
**4290**	allXL	DD	370	382	BS2G	13.85
**4290**	allXL	BB	376	382	BS2G	12.83
**4290**	allXL	DD	357	373	BS2G	34.67
**4290**	allXL	DD	320	366	BS2G	28.38
**4290**	allXL	DD	370	376	BS2G	16.18
**4290**	allXL	AA	320	357	DBSU	23.09
**4290**	allXL	CC	357	319	DBSU	24.85
**4290**	allXL	DD	372	319	DBSU	26.98
**4290**	allXL	DD	370	357	DBSU	28.69
**4290**	allXL	DD	382	357	DBSU	39.73
**4290**	allXL	AA	372	370	DBSU	6.35
**4290**	allXL	DD	381	370	DBSU	16.02
**4290**	allXL	AA	373	370	DBSU	8.95
**4290**	allXL	DD	381	372	DBSU	13.78
**4290**	allXL	DD	372	382	DBSU	12.37
**4290**	allXL	DD	372	386	DBSU	17.35
**4290**	allXL	DD	373	386	DBSU	20.93
**4290**	allXL	CB	381	386	DBSU	32.82
**4290**	allXL	BD;DB	357	357	DBSU	18.1
**4290**	allXL	AD	373	319	DBSU	22.15
**4290**	allXL	DA	372	357	DBSU	50.11
**4290**	allXL	CB	381	357	DBSU	39.87
**4290**	allXL	CB	373	357	DBSU	49.96
**4290**	allXL	AD	372	370	DBSU	38.2

**4290**	allXL	AD	373	370	DBSU	40.28
**4290**	allXL	BC	382	373	DBSU	33.99
**4291**	allXL	AA	320	357	BS2G	24.46
**4291**	allXL	BB	320	373	BS2G	27.75
**4291**	allXL	AA	373	382	BS2G	19.44
**4291**	allXL	CC	370	386	BS2G	27.41
**4291**	allXL	AA	373	386	BS2G	26.59
**4291**	allXL	AA	319	357	BS2G	22.54
**4291**	allXL	AA	367	373	BS2G	14.84
**4291**	allXL	DD	370	382	BS2G	24.37
**4291**	allXL	AA	376	382	BS2G	14.08
**4291**	allXL	BB	357	373	BS2G	25.32
**4291**	allXL	BB	320	366	BS2G	17.92
**4291**	allXL	DD	370	376	BS2G	15.71
**4291**	allXL	AA	320	357	DBSU	24.46
**4291**	allXL	AA	357	319	DBSU	22.54
**4291**	allXL	CC	372	319	DBSU	26.53
**4291**	allXL	DD	370	357	DBSU	26.49
**4291**	allXL	BB	382	357	DBSU	25.65
**4291**	allXL	AA	372	370	DBSU	6.29
**4291**	allXL	DD	381	370	DBSU	23.24
**4291**	allXL	CC	373	370	DBSU	9.22
**4291**	allXL	CC	381	372	DBSU	21.41
**4291**	allXL	AA	372	382	DBSU	20.4
**4291**	allXL	AA	372	386	DBSU	26.19
**4291**	allXL	AA	373	386	DBSU	26.59
**4291**	allXL	DB	381	386	DBSU	68.43
**4291**	allXL	BD;DB	357	357	DBSU	18.37
**4291**	allXL	AD	373	319	DBSU	43.01
**4291**	allXL	BD	372	357	DBSU	40.87
**4291**	allXL	BD	381	357	DBSU	33.06
**4291**	allXL	BD	373	357	DBSU	40.39
**4291**	allXL	BD	372	370	DBSU	65.38
**4291**	allXL	BD	373	370	DBSU	64.21

**4291**	allXL	BD	382	373	DBSU	53.18
**4292**	allXL	AA	320	357	BS2G	23.54
**4292**	allXL	DD	320	373	BS2G	21.98
**4292**	allXL	AA	373	382	BS2G	17.01
**4292**	allXL	AA	370	386	BS2G	21.23
**4292**	allXL	AA	373	386	BS2G	24.34
**4292**	allXL	AA	319	357	BS2G	23.25
**4292**	allXL	AA	367	373	BS2G	14.37
**4292**	allXL	DD	370	382	BS2G	17.71
**4292**	allXL	AA	376	382	BS2G	14.6
**4292**	allXL	BB	357	373	BS2G	24.0
**4292**	allXL	DD	320	366	BS2G	20.42
**4292**	allXL	CC	370	376	BS2G	13.05
**4292**	allXL	AA	320	357	DBSU	23.54
**4292**	allXL	AA	357	319	DBSU	23.25
**4292**	allXL	DD	372	319	DBSU	20.14
**4292**	allXL	DD	370	357	DBSU	21.59
**4292**	allXL	BB	382	357	DBSU	19.78
**4292**	allXL	AA	372	370	DBSU	6.17
**4292**	allXL	DD	381	370	DBSU	15.76
**4292**	allXL	AA	373	370	DBSU	9.07
**4292**	allXL	DD	381	372	DBSU	17.42
**4292**	allXL	AA	372	382	DBSU	16.57
**4292**	allXL	AA	372	386	DBSU	22.1
**4292**	allXL	AA	373	386	DBSU	24.34
**4292**	allXL	DB	381	386	DBSU	53.65
**4292**	allXL	BD;DB	357	357	DBSU	19.35
**4292**	allXL	DA	373	319	DBSU	22.18
**4292**	allXL	DA	372	357	DBSU	22.04
**4292**	allXL	BD	381	357	DBSU	30.01
**4292**	allXL	DA	373	357	DBSU	19.08
**4292**	allXL	DA	372	370	DBSU	44.8
**4292**	allXL	DA	373	370	DBSU	41.7
**4292**	allXL	DA	382	373	DBSU	45.45

**4293**	allXL	DD	320	357	BS2G	26.29
**4293**	allXL	BB	320	373	BS2G	24.65
**4293**	allXL	DD	373	382	BS2G	16.41
**4293**	allXL	DD	370	386	BS2G	16.14
**4293**	allXL	DD	373	386	BS2G	18.81
**4293**	allXL	DD	319	357	BS2G	25.53
**4293**	allXL	BB	367	373	BS2G	13.48
**4293**	allXL	DD	370	382	BS2G	17.6
**4293**	allXL	CC	376	382	BS2G	12.44
**4293**	allXL	BB	357	373	BS2G	19.09
**4293**	allXL	BB	320	366	BS2G	20.74
**4293**	allXL	CC	370	376	BS2G	16.68
**4293**	allXL	DD	320	357	DBSU	26.29
**4293**	allXL	DD	357	319	DBSU	25.53
**4293**	allXL	BB	372	319	DBSU	27.57
**4293**	allXL	BB	370	357	DBSU	23.37
**4293**	allXL	BB	382	357	DBSU	30.44
**4293**	allXL	AA	372	370	DBSU	5.96
**4293**	allXL	DD	381	370	DBSU	17.85
**4293**	allXL	AA	373	370	DBSU	9.31
**4293**	allXL	DD	381	372	DBSU	17.4
**4293**	allXL	DD	372	382	DBSU	18.71
**4293**	allXL	DD	372	386	DBSU	20.2
**4293**	allXL	DD	373	386	DBSU	18.81
**4293**	allXL	DA	381	386	DBSU	32.41
**4293**	allXL	BD;DB	357	357	DBSU	18.21
**4293**	allXL	AD	373	319	DBSU	17.19
**4293**	allXL	AD	372	357	DBSU	38.22
**4293**	allXL	BD	381	357	DBSU	36.32
**4293**	allXL	BD	373	357	DBSU	35.67
**4293**	allXL	AD	372	370	DBSU	33.46
**4293**	allXL	AD	373	370	DBSU	31.47
**4293**	allXL	DA	382	373	DBSU	22.31
**4294**	allXL	CC	320	357	BS2G	24.77

**4294**	allXL	BB	320	373	BS2G	22.9
**4294**	allXL	BB	373	382	BS2G	17.88
**4294**	allXL	DD	370	386	BS2G	20.86
**4294**	allXL	DD	373	386	BS2G	27.15
**4294**	allXL	CC	319	357	BS2G	25.35
**4294**	allXL	AA	367	373	BS2G	12.61
**4294**	allXL	DD	370	382	BS2G	17.51
**4294**	allXL	BB	376	382	BS2G	14.75
**4294**	allXL	CC	357	373	BS2G	20.87
**4294**	allXL	BB	320	366	BS2G	22.26
**4294**	allXL	CC	370	376	BS2G	16.1
**4294**	allXL	CC	320	357	DBSU	24.77
**4294**	allXL	CC	357	319	DBSU	25.35
**4294**	allXL	BB	372	319	DBSU	21.45
**4294**	allXL	CC	370	357	DBSU	23.67
**4294**	allXL	BB	382	357	DBSU	28.79
**4294**	allXL	AA	372	370	DBSU	6.35
**4294**	allXL	DD	381	370	DBSU	18.8
**4294**	allXL	AA	373	370	DBSU	9.05
**4294**	allXL	DD	381	372	DBSU	20.09
**4294**	allXL	DD	372	382	DBSU	20.09
**4294**	allXL	DD	372	386	DBSU	25.98
**4294**	allXL	DD	373	386	DBSU	27.15
**4294**	allXL	CA	381	386	DBSU	55.56
**4294**	allXL	BD;DB	357	357	DBSU	19.01
**4294**	allXL	DA	373	319	DBSU	35.82
**4294**	allXL	CA	372	357	DBSU	37.02
**4294**	allXL	CA	381	357	DBSU	27.79
**4294**	allXL	CA	373	357	DBSU	34.38
**4294**	allXL	DA	372	370	DBSU	39.46
**4294**	allXL	DA	373	370	DBSU	36.17
**4294**	allXL	DA	382	373	DBSU	38.41
**4295**	allXL	BB	320	357	BS2G	26.85
**4295**	allXL	CC	320	373	BS2G	12.96

**4295**	allXL	DD	373	382	BS2G	20.28
**4295**	allXL	DD	370	386	BS2G	32.44
**4295**	allXL	DD	373	386	BS2G	29.95
**4295**	allXL	AA	319	357	BS2G	27.23
**4295**	allXL	BB	367	373	BS2G	11.77
**4295**	allXL	DD	370	382	BS2G	21.23
**4295**	allXL	CC	376	382	BS2G	14.76
**4295**	allXL	DD	357	373	BS2G	26.33
**4295**	allXL	BB	320	366	BS2G	23.55
**4295**	allXL	CC	370	376	BS2G	15.36
**4295**	allXL	BB	320	357	DBSU	26.85
**4295**	allXL	AA	357	319	DBSU	27.23
**4295**	allXL	CC	372	319	DBSU	15.23
**4295**	allXL	DD	370	357	DBSU	20.71
**4295**	allXL	BB	382	357	DBSU	26.44
**4295**	allXL	AA	372	370	DBSU	6.42
**4295**	allXL	DD	381	370	DBSU	19.68
**4295**	allXL	AA	373	370	DBSU	9.26
**4295**	allXL	DD	381	372	DBSU	18.65
**4295**	allXL	DD	372	382	DBSU	20.29
**4295**	allXL	DD	372	386	DBSU	30.46
**4295**	allXL	DD	373	386	DBSU	29.95
**4295**	allXL	CB	381	386	DBSU	62.7
**4295**	allXL	BD;DB	357	357	DBSU	19.06
**4295**	allXL	DA	373	319	DBSU	32.77
**4295**	allXL	DA	372	357	DBSU	38.08
**4295**	allXL	BD	381	357	DBSU	41.08
**4295**	allXL	DA	373	357	DBSU	36.0
**4295**	allXL	DA	372	370	DBSU	43.46
**4295**	allXL	DA	373	370	DBSU	39.78
**4295**	allXL	CB	382	373	DBSU	40.95
**4296**	allXL	AA	320	357	BS2G	23.14
**4296**	allXL	CC	320	373	BS2G	22.77
**4296**	allXL	DD	373	382	BS2G	18.57

**4296**	allXL	DD	370	386	BS2G	17.35
**4296**	allXL	DD	373	386	BS2G	20.39
**4296**	allXL	AA	319	357	BS2G	21.09
**4296**	allXL	AA	367	373	BS2G	12.44
**4296**	allXL	DD	370	382	BS2G	21.29
**4296**	allXL	CC	376	382	BS2G	14.11
**4296**	allXL	DD	357	373	BS2G	27.67
**4296**	allXL	DD	320	366	BS2G	16.87
**4296**	allXL	CC	370	376	BS2G	11.59
**4296**	allXL	AA	320	357	DBSU	23.14
**4296**	allXL	AA	357	319	DBSU	21.09
**4296**	allXL	CC	372	319	DBSU	21.94
**4296**	allXL	DD	370	357	DBSU	23.43
**4296**	allXL	BB	382	357	DBSU	19.48
**4296**	allXL	AA	372	370	DBSU	6.51
**4296**	allXL	DD	381	370	DBSU	21.22
**4296**	allXL	AA	373	370	DBSU	9.29
**4296**	allXL	DD	381	372	DBSU	17.55
**4296**	allXL	DD	372	382	DBSU	18.42
**4296**	allXL	DD	372	386	DBSU	18.82
**4296**	allXL	DD	373	386	DBSU	20.39
**4296**	allXL	DB	381	386	DBSU	51.35
**4296**	allXL	BD;DB	357	357	DBSU	18.73
**4296**	allXL	DA	373	319	DBSU	23.8
**4296**	allXL	DA	372	357	DBSU	29.4
**4296**	allXL	BD	381	357	DBSU	32.19
**4296**	allXL	DA	373	357	DBSU	31.65
**4296**	allXL	AD	372	370	DBSU	45.07
**4296**	allXL	AD	373	370	DBSU	43.9
**4296**	allXL	DA	382	373	DBSU	38.78
**4297**	allXL	CC	320	357	BS2G	20.97
**4297**	allXL	DD	320	373	BS2G	21.08
**4297**	allXL	DD	373	382	BS2G	17.87
**4297**	allXL	DD	370	386	BS2G	20.52

**4297**	allXL	DD	373	386	BS2G	25.55
**4297**	allXL	CC	319	357	BS2G	23.25
**4297**	allXL	AA	367	373	BS2G	13.44
**4297**	allXL	DD	370	382	BS2G	17.4
**4297**	allXL	BB	376	382	BS2G	15.3
**4297**	allXL	BB	357	373	BS2G	19.8
**4297**	allXL	AA	320	366	BS2G	25.26
**4297**	allXL	CC	370	376	BS2G	16.03
**4297**	allXL	CC	320	357	DBSU	20.97
**4297**	allXL	CC	357	319	DBSU	23.25
**4297**	allXL	DD	372	319	DBSU	22.77
**4297**	allXL	DD	370	357	DBSU	24.45
**4297**	allXL	BB	382	357	DBSU	18.4
**4297**	allXL	AA	372	370	DBSU	6.28
**4297**	allXL	DD	381	370	DBSU	18.38
**4297**	allXL	AA	373	370	DBSU	9.24
**4297**	allXL	DD	381	372	DBSU	16.49
**4297**	allXL	DD	372	382	DBSU	16.59
**4297**	allXL	DD	372	386	DBSU	23.83
**4297**	allXL	DD	373	386	DBSU	25.55
**4297**	allXL	BD	381	386	DBSU	63.15
**4297**	allXL	BD;DB	357	357	DBSU	19.5
**4297**	allXL	BC	373	319	DBSU	30.83
**4297**	allXL	BD	372	357	DBSU	35.24
**4297**	allXL	BD	381	357	DBSU	37.06
**4297**	allXL	BD	373	357	DBSU	35.11
**4297**	allXL	BD	372	370	DBSU	57.09
**4297**	allXL	BD	373	370	DBSU	57.23
**4297**	allXL	AC	382	373	DBSU	60.04
**4298**	allXL	CC	320	357	BS2G	24.12
**4298**	allXL	BB	320	373	BS2G	14.03
**4298**	allXL	BB	373	382	BS2G	17.74
**4298**	allXL	DD	370	386	BS2G	22.03
**4298**	allXL	CC	373	386	BS2G	25.18

**4298**	allXL	CC	319	357	BS2G	23.72
**4298**	allXL	AA	367	373	BS2G	13.93
**4298**	allXL	AA	370	382	BS2G	19.55
**4298**	allXL	BB	376	382	BS2G	14.38
**4298**	allXL	DD	357	373	BS2G	30.03
**4298**	allXL	BB	320	366	BS2G	18.08
**4298**	allXL	BB	370	376	BS2G	16.81
**4298**	allXL	CC	320	357	DBSU	24.12
**4298**	allXL	CC	357	319	DBSU	23.72
**4298**	allXL	BB	372	319	DBSU	14.82
**4298**	allXL	DD	370	357	DBSU	22.89
**4298**	allXL	BB	382	357	DBSU	25.88
**4298**	allXL	AA	372	370	DBSU	6.19
**4298**	allXL	DD	381	370	DBSU	19.77
**4298**	allXL	DD	373	370	DBSU	9.01
**4298**	allXL	AA	381	372	DBSU	18.31
**4298**	allXL	AA	372	382	DBSU	17.06
**4298**	allXL	CC	372	386	DBSU	23.74
**4298**	allXL	CC	373	386	DBSU	25.18
**4298**	allXL	BC	381	386	DBSU	50.24
**4298**	allXL	BD;DB	357	357	DBSU	18.32
**4298**	allXL	AD	373	319	DBSU	28.1
**4298**	allXL	DB	372	357	DBSU	47.5
**4298**	allXL	BD	381	357	DBSU	39.51
**4298**	allXL	DB	373	357	DBSU	47.51
**4298**	allXL	DA	372	370	DBSU	37.12
**4298**	allXL	AD	373	370	DBSU	38.1
**4298**	allXL	CB	382	373	DBSU	45.41
**4299**	allXL	CC	320	357	BS2G	22.99
**4299**	allXL	BB	320	373	BS2G	24.36
**4299**	allXL	DD	373	382	BS2G	18.15
**4299**	allXL	DD	370	386	BS2G	22.37
**4299**	allXL	DD	373	386	BS2G	22.7
**4299**	allXL	CC	319	357	BS2G	20.85

**4299**	allXL	BB	367	373	BS2G	11.9
**4299**	allXL	DD	370	382	BS2G	16.33
**4299**	allXL	BB	376	382	BS2G	13.85
**4299**	allXL	CC	357	373	BS2G	24.43
**4299**	allXL	BB	320	366	BS2G	24.39
**4299**	allXL	DD	370	376	BS2G	16.89
**4299**	allXL	CC	320	357	DBSU	22.99
**4299**	allXL	CC	357	319	DBSU	20.85
**4299**	allXL	BB	372	319	DBSU	20.45
**4299**	allXL	CC	370	357	DBSU	23.67
**4299**	allXL	BB	382	357	DBSU	29.32
**4299**	allXL	AA	372	370	DBSU	6.16
**4299**	allXL	DD	381	370	DBSU	17.31
**4299**	allXL	AA	373	370	DBSU	9.24
**4299**	allXL	DD	381	372	DBSU	18.84
**4299**	allXL	DD	372	382	DBSU	18.56
**4299**	allXL	DD	372	386	DBSU	22.92
**4299**	allXL	DD	373	386	DBSU	22.7
**4299**	allXL	CA	381	386	DBSU	58.0
**4299**	allXL	BD;DB	357	357	DBSU	17.89
**4299**	allXL	AD	373	319	DBSU	27.27
**4299**	allXL	CA	372	357	DBSU	39.16
**4299**	allXL	BD	381	357	DBSU	49.73
**4299**	allXL	CA	373	357	DBSU	40.49
**4299**	allXL	DA	372	370	DBSU	34.64
**4299**	allXL	DA	373	370	DBSU	35.51
**4299**	allXL	DA	382	373	DBSU	60.11
**4300**	allXL	CC	320	357	BS2G	26.93
**4300**	allXL	BB	320	373	BS2G	25.14
**4300**	allXL	DD	373	382	BS2G	19.73
**4300**	allXL	DD	370	386	BS2G	25.48
**4300**	allXL	DD	373	386	BS2G	26.78
**4300**	allXL	AA	319	357	BS2G	28.8
**4300**	allXL	AA	367	373	BS2G	11.4

**4300**	allXL	DD	370	382	BS2G	22.19
**4300**	allXL	CC	376	382	BS2G	13.78
**4300**	allXL	AA	357	373	BS2G	27.3
**4300**	allXL	AA	320	366	BS2G	23.8
**4300**	allXL	CC	370	376	BS2G	16.76
**4300**	allXL	CC	320	357	DBSU	26.93
**4300**	allXL	AA	357	319	DBSU	28.8
**4300**	allXL	BB	372	319	DBSU	26.22
**4300**	allXL	DD	370	357	DBSU	24.48
**4300**	allXL	AA	382	357	DBSU	35.57
**4300**	allXL	AA	372	370	DBSU	6.6
**4300**	allXL	AA	381	370	DBSU	20.1
**4300**	allXL	CC	373	370	DBSU	9.28
**4300**	allXL	DD	381	372	DBSU	17.36
**4300**	allXL	DD	372	382	DBSU	18.73
**4300**	allXL	DD	372	386	DBSU	24.71
**4300**	allXL	DD	373	386	DBSU	26.78
**4300**	allXL	BC	381	386	DBSU	31.68
**4300**	allXL	BD;DB	357	357	DBSU	19.24
**4300**	allXL	CB	373	319	DBSU	12.28
**4300**	allXL	DA	372	357	DBSU	27.31
**4300**	allXL	DA	381	357	DBSU	38.29
**4300**	allXL	DA	373	357	DBSU	28.03
**4300**	allXL	DA	372	370	DBSU	25.79
**4300**	allXL	DA	373	370	DBSU	22.15
**4300**	allXL	CB	382	373	DBSU	29.26
**4301**	allXL	CC	320	357	BS2G	27.33
**4301**	allXL	BB	320	373	BS2G	25.32
**4301**	allXL	CC	373	382	BS2G	19.6
**4301**	allXL	BB	370	386	BS2G	29.88
**4301**	allXL	CC	373	386	BS2G	27.28
**4301**	allXL	CC	319	357	BS2G	27.28
**4301**	allXL	AA	367	373	BS2G	12.66
**4301**	allXL	BB	370	382	BS2G	20.57

**4301**	allXL	CC	376	382	BS2G	12.63
**4301**	allXL	CC	357	373	BS2G	29.89
**4301**	allXL	BB	320	366	BS2G	27.62
**4301**	allXL	BB	370	376	BS2G	16.31
**4301**	allXL	CC	320	357	DBSU	27.33
**4301**	allXL	CC	357	319	DBSU	27.28
**4301**	allXL	BB	372	319	DBSU	22.81
**4301**	allXL	CC	370	357	DBSU	22.07
**4301**	allXL	CC	382	357	DBSU	41.44
**4301**	allXL	AA	372	370	DBSU	6.11
**4301**	allXL	BB	381	370	DBSU	22.13
**4301**	allXL	AA	373	370	DBSU	8.89
**4301**	allXL	AA	381	372	DBSU	21.24
**4301**	allXL	AA	372	382	DBSU	21.05
**4301**	allXL	CC	372	386	DBSU	27.4
**4301**	allXL	CC	373	386	DBSU	27.28
**4301**	allXL	CB	381	386	DBSU	39.0
**4301**	allXL	BD;DB	357	357	DBSU	18.35
**4301**	allXL	AD	373	319	DBSU	16.55
**4301**	allXL	BC	372	357	DBSU	31.35
**4301**	allXL	BC	381	357	DBSU	42.26
**4301**	allXL	BC	373	357	DBSU	29.97
**4301**	allXL	DA	372	370	DBSU	38.82
**4301**	allXL	DA	373	370	DBSU	36.24
**4301**	allXL	CB	382	373	DBSU	41.73
**4302**	allXL	CC	320	357	BS2G	26.97
**4302**	allXL	BB	320	373	BS2G	23.57
**4302**	allXL	CC	373	382	BS2G	17.79
**4302**	allXL	CC	370	386	BS2G	18.91
**4302**	allXL	CC	373	386	BS2G	13.54
**4302**	allXL	CC	319	357	BS2G	29.68
**4302**	allXL	BB	367	373	BS2G	12.89
**4302**	allXL	AA	370	382	BS2G	24.33
**4302**	allXL	CC	376	382	BS2G	14.09

**4302**	allXL	BB	357	373	BS2G	24.64
**4302**	allXL	CC	320	366	BS2G	19.21
**4302**	allXL	CC	370	376	BS2G	17.14
**4302**	allXL	CC	320	357	DBSU	26.97
**4302**	allXL	CC	357	319	DBSU	29.68
**4302**	allXL	BB	372	319	DBSU	26.69
**4302**	allXL	BB	370	357	DBSU	32.24
**4302**	allXL	BB	382	357	DBSU	31.65
**4302**	allXL	AA	372	370	DBSU	6.12
**4302**	allXL	BB	381	370	DBSU	25.17
**4302**	allXL	AA	373	370	DBSU	9.09
**4302**	allXL	BB	381	372	DBSU	18.41
**4302**	allXL	CC	372	382	DBSU	19.99
**4302**	allXL	CC	372	386	DBSU	13.19
**4302**	allXL	CC	373	386	DBSU	13.54
**4302**	allXL	AD	381	386	DBSU	43.74
**4302**	allXL	BD;DB	357	357	DBSU	18.1
**4302**	allXL	AD	373	319	DBSU	23.06
**4302**	allXL	BD	372	357	DBSU	43.35
**4302**	allXL	BD	381	357	DBSU	45.79
**4302**	allXL	BD	373	357	DBSU	41.29
**4302**	allXL	DA	372	370	DBSU	26.13
**4302**	allXL	DA	373	370	DBSU	29.79
**4302**	allXL	DA	382	373	DBSU	33.95
**4303**	allXL	CC	320	357	BS2G	22.75
**4303**	allXL	BB	320	373	BS2G	14.92
**4303**	allXL	BB	373	382	BS2G	18.86
**4303**	allXL	BB	370	386	BS2G	21.98
**4303**	allXL	BB	373	386	BS2G	24.37
**4303**	allXL	CC	319	357	BS2G	19.96
**4303**	allXL	AA	367	373	BS2G	12.0
**4303**	allXL	DD	370	382	BS2G	19.06
**4303**	allXL	BB	376	382	BS2G	14.7
**4303**	allXL	BB	357	373	BS2G	28.47

**4303**	allXL	CC	320	366	BS2G	22.07
**4303**	allXL	DD	370	376	BS2G	15.95
**4303**	allXL	CC	320	357	DBSU	22.75
**4303**	allXL	CC	357	319	DBSU	19.96
**4303**	allXL	BB	372	319	DBSU	15.95
**4303**	allXL	DD	370	357	DBSU	21.98
**4303**	allXL	DD	382	357	DBSU	27.8
**4303**	allXL	AA;CC	372	370	DBSU	6.49
**4303**	allXL	DD	381	370	DBSU	15.88
**4303**	allXL	AA	373	370	DBSU	8.96
**4303**	allXL	BB	381	372	DBSU	18.61
**4303**	allXL	BB	372	382	DBSU	21.05
**4303**	allXL	BB	372	386	DBSU	24.79
**4303**	allXL	BB	373	386	DBSU	24.37
**4303**	allXL	BC	381	386	DBSU	40.19
**4303**	allXL	BD;DB	357	357	DBSU	19.27
**4303**	allXL	CB	373	319	DBSU	24.07
**4303**	allXL	BC	372	357	DBSU	33.51
**4303**	allXL	BD	381	357	DBSU	43.67
**4303**	allXL	BC	373	357	DBSU	36.01
**4303**	allXL	BC	372	370	DBSU	13.76
**4303**	allXL	BC	373	370	DBSU	16.43
**4303**	allXL	BC	382	373	DBSU	32.37
**4304**	allXL	CC	320	357	BS2G	12.64
**4304**	allXL	BB	320	373	BS2G	20.61
**4304**	allXL	CC	373	382	BS2G	18.41
**4304**	allXL	DD	370	386	BS2G	18.31
**4304**	allXL	CC	373	386	BS2G	21.31
**4304**	allXL	CC	319	357	BS2G	12.5
**4304**	allXL	AA	367	373	BS2G	13.26
**4304**	allXL	DD	370	382	BS2G	20.15
**4304**	allXL	CC	376	382	BS2G	14.61
**4304**	allXL	DD	357	373	BS2G	14.42
**4304**	allXL	AA	320	366	BS2G	23.43

**4304**	allXL	CC	370	376	BS2G	16.54
**4304**	allXL	CC	320	357	DBSU	12.64
**4304**	allXL	CC	357	319	DBSU	12.5
**4304**	allXL	AA	372	319	DBSU	19.18
**4304**	allXL	DD	370	357	DBSU	12.59
**4304**	allXL	DD	382	357	DBSU	16.48
**4304**	allXL	AA	372	370	DBSU	6.26
**4304**	allXL	DD	381	370	DBSU	20.17
**4304**	allXL	AA	373	370	DBSU	9.05
**4304**	allXL	DD	381	372	DBSU	18.24
**4304**	allXL	CC	372	382	DBSU	18.23
**4304**	allXL	CC	372	386	DBSU	18.8
**4304**	allXL	CC	373	386	DBSU	21.31
**4304**	allXL	DB	381	386	DBSU	37.09
**4304**	allXL	BD;DB	357	357	DBSU	19.83
**4304**	allXL	CB	373	319	DBSU	10.5
**4304**	allXL	DB	372	357	DBSU	15.79
**4304**	allXL	DB	381	357	DBSU	21.97
**4304**	allXL	DB	373	357	DBSU	12.12
**4304**	allXL	CB	372	370	DBSU	28.96
**4304**	allXL	CB	373	370	DBSU	27.92
**4304**	allXL	CB	382	373	DBSU	20.59
**4305**	allXL	AA	320	357	BS2G	25.44
**4305**	allXL	CC	320	373	BS2G	22.65
**4305**	allXL	DD	373	382	BS2G	17.23
**4305**	allXL	DD	370	386	BS2G	18.84
**4305**	allXL	DD	373	386	BS2G	18.58
**4305**	allXL	AA	319	357	BS2G	26.55
**4305**	allXL	BB	367	373	BS2G	13.11
**4305**	allXL	CC	370	382	BS2G	21.0
**4305**	allXL	BB	376	382	BS2G	14.76
**4305**	allXL	BB	357	373	BS2G	35.03
**4305**	allXL	BB	320	366	BS2G	21.23
**4305**	allXL	CC	370	376	BS2G	13.64

**4305**	allXL	AA	320	357	DBSU	25.44
**4305**	allXL	AA	357	319	DBSU	26.55
**4305**	allXL	CC	372	319	DBSU	16.68
**4305**	allXL	BB	370	357	DBSU	31.22
**4305**	allXL	BB	382	357	DBSU	23.12
**4305**	allXL	AA	372	370	DBSU	6.19
**4305**	allXL	CC	381	370	DBSU	19.71
**4305**	allXL	AA	373	370	DBSU	9.12
**4305**	allXL	DD	381	372	DBSU	18.14
**4305**	allXL	DD	372	382	DBSU	17.01
**4305**	allXL	DD	372	386	DBSU	17.92
**4305**	allXL	DD	373	386	DBSU	18.58
**4305**	allXL	AD	381	386	DBSU	17.15
**4305**	allXL	BD;DB	357	357	DBSU	19.21
**4305**	allXL	AD	373	319	DBSU	21.6
**4305**	allXL	BC	372	357	DBSU	44.43
**4305**	allXL	AD	381	357	DBSU	42.73
**4305**	allXL	BC	373	357	DBSU	47.64
**4305**	allXL	BC	372	370	DBSU	22.53
**4305**	allXL	BC	373	370	DBSU	25.15
**4305**	allXL	AD	382	373	DBSU	23.02
**4306**	allXL	AA	320	357	BS2G	24.42
**4306**	allXL	CC	320	373	BS2G	20.62
**4306**	allXL	DD	373	382	BS2G	19.39
**4306**	allXL	CC	370	386	BS2G	17.91
**4306**	allXL	CC	373	386	BS2G	24.75
**4306**	allXL	AA	319	357	BS2G	23.35
**4306**	allXL	AA	367	373	BS2G	12.84
**4306**	allXL	CC	370	382	BS2G	16.38
**4306**	allXL	CC	376	382	BS2G	14.5
**4306**	allXL	BB	357	373	BS2G	31.7
**4306**	allXL	CC	320	366	BS2G	20.7
**4306**	allXL	CC	370	376	BS2G	11.57
**4306**	allXL	AA	320	357	DBSU	24.42

**4306**	allXL	AA	357	319	DBSU	23.35
**4306**	allXL	CC	372	319	DBSU	18.21
**4306**	allXL	DD	370	357	DBSU	27.09
**4306**	allXL	BB	382	357	DBSU	17.59
**4306**	allXL	AA	372	370	DBSU	6.31
**4306**	allXL	CC	381	370	DBSU	17.85
**4306**	allXL	AA	373	370	DBSU	9.02
**4306**	allXL	DD	381	372	DBSU	18.85
**4306**	allXL	DD	372	382	DBSU	19.83
**4306**	allXL	CC	372	386	DBSU	23.86
**4306**	allXL	CC	373	386	DBSU	24.75
**4306**	allXL	DA	381	386	DBSU	42.78
**4306**	allXL	BD;DB	357	357	DBSU	18.72
**4306**	allXL	AD	373	319	DBSU	23.31
**4306**	allXL	DA	372	357	DBSU	41.41
**4306**	allXL	BD	381	357	DBSU	39.67
**4306**	allXL	DA	373	357	DBSU	44.65
**4306**	allXL	DA	372	370	DBSU	25.79
**4306**	allXL	DA	373	370	DBSU	26.5
**4306**	allXL	AD	382	373	DBSU	32.42
**4307**	allXL	CC	320	357	BS2G	20.27
**4307**	allXL	DD	320	373	BS2G	27.34
**4307**	allXL	BB	373	382	BS2G	14.73
**4307**	allXL	DD	370	386	BS2G	23.22
**4307**	allXL	AA	373	386	BS2G	23.35
**4307**	allXL	CC	319	357	BS2G	22.54
**4307**	allXL	AA	367	373	BS2G	12.88
**4307**	allXL	BB	370	382	BS2G	20.73
**4307**	allXL	BB	376	382	BS2G	12.89
**4307**	allXL	AA	357	373	BS2G	29.53
**4307**	allXL	DD	320	366	BS2G	29.34
**4307**	allXL	CC	370	376	BS2G	16.49
**4307**	allXL	CC	320	357	DBSU	20.27
**4307**	allXL	CC	357	319	DBSU	22.54

**4307**	allXL	DD	372	319	DBSU	27.2
**4307**	allXL	DD	370	357	DBSU	24.49
**4307**	allXL	AA	382	357	DBSU	20.86
**4307**	allXL	AA	372	370	DBSU	6.49
**4307**	allXL	DD	381	370	DBSU	20.34
**4307**	allXL	AA	373	370	DBSU	9.21
**4307**	allXL	BB	381	372	DBSU	15.38
**4307**	allXL	BB	372	382	DBSU	13.75
**4307**	allXL	BB	372	386	DBSU	22.14
**4307**	allXL	AA	373	386	DBSU	23.35
**4307**	allXL	AD	381	386	DBSU	46.0
**4307**	allXL	BD;DB	357	357	DBSU	18.09
**4307**	allXL	AD	373	319	DBSU	35.48
**4307**	allXL	CA	372	357	DBSU	27.6
**4307**	allXL	AC	381	357	DBSU	20.41
**4307**	allXL	CA	373	357	DBSU	30.75
**4307**	allXL	CA	372	370	DBSU	56.42
**4307**	allXL	AC	373	370	DBSU	53.31
**4307**	allXL	AC	382	373	DBSU	40.54
**4308**	allXL	AA	320	357	BS2G	21.04
**4308**	allXL	BB	320	373	BS2G	28.02
**4308**	allXL	CC	373	382	BS2G	20.1
**4308**	allXL	DD	370	386	BS2G	18.93
**4308**	allXL	DD	373	386	BS2G	22.52
**4308**	allXL	AA	319	357	BS2G	19.61
**4308**	allXL	BB	367	373	BS2G	12.33
**4308**	allXL	DD	370	382	BS2G	23.5
**4308**	allXL	CC	376	382	BS2G	14.15
**4308**	allXL	BB	357	373	BS2G	19.23
**4308**	allXL	DD	320	366	BS2G	26.21
**4308**	allXL	DD	370	376	BS2G	17.1
**4308**	allXL	AA	320	357	DBSU	21.04
**4308**	allXL	AA	357	319	DBSU	19.61
**4308**	allXL	BB	372	319	DBSU	30.22

**4308**	allXL	DD	370	357	DBSU	27.16
**4308**	allXL	BB	382	357	DBSU	8.25
**4308**	allXL	AA	372	370	DBSU	5.94
**4308**	allXL	DD	381	370	DBSU	22.51
**4308**	allXL	CC	373	370	DBSU	9.05
**4308**	allXL	DD	381	372	DBSU	22.84
**4308**	allXL	CC	372	382	DBSU	23.9
**4308**	allXL	DD	372	386	DBSU	23.43
**4308**	allXL	DD	373	386	DBSU	22.52
**4308**	allXL	BD	381	386	DBSU	44.09
**4308**	allXL	BD;DB	357	357	DBSU	19.47
**4308**	allXL	BC	373	319	DBSU	24.23
**4308**	allXL	BD	372	357	DBSU	38.61
**4308**	allXL	BD	381	357	DBSU	15.33
**4308**	allXL	BD	373	357	DBSU	35.28
**4308**	allXL	BC	372	370	DBSU	52.6
**4308**	allXL	BC	373	370	DBSU	53.41
**4308**	allXL	BD	382	373	DBSU	52.53
**4309**	allXL	AA	320	357	BS2G	27.18
**4309**	allXL	CC	320	373	BS2G	16.56
**4309**	allXL	DD	373	382	BS2G	18.33
**4309**	allXL	DD	370	386	BS2G	18.06
**4309**	allXL	DD	373	386	BS2G	24.37
**4309**	allXL	CC	319	357	BS2G	29.27
**4309**	allXL	BB	367	373	BS2G	11.77
**4309**	allXL	DD	370	382	BS2G	16.86
**4309**	allXL	BB	376	382	BS2G	14.37
**4309**	allXL	BB	357	373	BS2G	21.59
**4309**	allXL	AA	320	366	BS2G	20.08
**4309**	allXL	CC	370	376	BS2G	15.36
**4309**	allXL	AA	320	357	DBSU	27.18
**4309**	allXL	CC	357	319	DBSU	29.27
**4309**	allXL	CC	372	319	DBSU	12.89
**4309**	allXL	DD	370	357	DBSU	26.98

**4309**	allXL	CC	382	357	DBSU	18.43
**4309**	allXL	AA	372	370	DBSU	6.34
**4309**	allXL	DD	381	370	DBSU	16.46
**4309**	allXL	AA	373	370	DBSU	9.32
**4309**	allXL	DD	381	372	DBSU	16.94
**4309**	allXL	DD	372	382	DBSU	18.86
**4309**	allXL	DD	372	386	DBSU	23.46
**4309**	allXL	DD	373	386	DBSU	24.37
**4309**	allXL	CB	381	386	DBSU	58.72
**4309**	allXL	BD;DB	357	357	DBSU	19.01
**4309**	allXL	BC	373	319	DBSU	32.78
**4309**	allXL	CB	372	357	DBSU	38.21
**4309**	allXL	CA	381	357	DBSU	35.31
**4309**	allXL	CB	373	357	DBSU	34.83
**4309**	allXL	CB	372	370	DBSU	24.6
**4309**	allXL	CB	373	370	DBSU	21.44
**4309**	allXL	BC	382	373	DBSU	30.92
**4310**	allXL	CC	320	357	BS2G	27.63
**4310**	allXL	BB	320	373	BS2G	16.17
**4310**	allXL	DD	373	382	BS2G	19.36
**4310**	allXL	DD	370	386	BS2G	18.47
**4310**	allXL	DD	373	386	BS2G	24.62
**4310**	allXL	DD	319	357	BS2G	28.82
**4310**	allXL	AA	367	373	BS2G	12.79
**4310**	allXL	DD	370	382	BS2G	16.36
**4310**	allXL	BB	376	382	BS2G	15.05
**4310**	allXL	DD	357	373	BS2G	33.26
**4310**	allXL	AA	320	366	BS2G	17.34
**4310**	allXL	DD	370	376	BS2G	16.43
**4310**	allXL	CC	320	357	DBSU	27.63
**4310**	allXL	DD	357	319	DBSU	28.82
**4310**	allXL	BB	372	319	DBSU	15.8
**4310**	allXL	AA	370	357	DBSU	29.24
**4310**	allXL	DD	382	357	DBSU	28.55

**4310**	allXL	AA	372	370	DBSU	6.37
**4310**	allXL	DD	381	370	DBSU	15.12
**4310**	allXL	DD	373	370	DBSU	9.04
**4310**	allXL	DD	381	372	DBSU	18.66
**4310**	allXL	DD	372	382	DBSU	20.95
**4310**	allXL	DD	372	386	DBSU	25.11
**4310**	allXL	DD	373	386	DBSU	24.62
**4310**	allXL	DA	381	386	DBSU	45.16
**4310**	allXL	BD;DB	357	357	DBSU	18.21
**4310**	allXL	CB	373	319	DBSU	31.81
**4310**	allXL	CB	372	357	DBSU	40.84
**4310**	allXL	DB	381	357	DBSU	48.86
**4310**	allXL	CB	373	357	DBSU	40.39
**4310**	allXL	BC	372	370	DBSU	34.52
**4310**	allXL	BC	373	370	DBSU	35.67
**4310**	allXL	BC	382	373	DBSU	50.45
**4311**	allXL	AA	320	357	BS2G	24.4
**4311**	allXL	CC	320	373	BS2G	12.13
**4311**	allXL	AA	373	382	BS2G	18.9
**4311**	allXL	AA	370	386	BS2G	27.4
**4311**	allXL	AA	373	386	BS2G	26.36
**4311**	allXL	AA	319	357	BS2G	24.16
**4311**	allXL	AA	367	373	BS2G	12.68
**4311**	allXL	AA	370	382	BS2G	23.5
**4311**	allXL	AA	376	382	BS2G	14.78
**4311**	allXL	BB	357	373	BS2G	21.73
**4311**	allXL	CC	320	366	BS2G	17.07
**4311**	allXL	CC	370	376	BS2G	14.65
**4311**	allXL	AA	320	357	DBSU	24.4
**4311**	allXL	AA	357	319	DBSU	24.16
**4311**	allXL	CC	372	319	DBSU	10.88
**4311**	allXL	BB	370	357	DBSU	25.16
**4311**	allXL	BB	382	357	DBSU	17.82
**4311**	allXL	AA	372	370	DBSU	6.28

**4311**	allXL	AA	381	370	DBSU	25.75
**4311**	allXL	DD	373	370	DBSU	8.93
**4311**	allXL	AA	381	372	DBSU	22.95
**4311**	allXL	AA	372	382	DBSU	21.51
**4311**	allXL	AA	372	386	DBSU	28.08
**4311**	allXL	AA	373	386	DBSU	26.36
**4311**	allXL	DB	381	386	DBSU	31.21
**4311**	allXL	BD;DB	357	357	DBSU	18.82
**4311**	allXL	AD	373	319	DBSU	26.56
**4311**	allXL	BD	372	357	DBSU	43.01
**4311**	allXL	BD	381	357	DBSU	27.7
**4311**	allXL	BD	373	357	DBSU	40.31
**4311**	allXL	CB	372	370	DBSU	34.56
**4311**	allXL	CB	373	370	DBSU	36.86
**4311**	allXL	BD	382	373	DBSU	42.97
**4312**	allXL	AA	320	357	BS2G	20.36
**4312**	allXL	CC	320	373	BS2G	14.52
**4312**	allXL	DD	373	382	BS2G	14.76
**4312**	allXL	DD	370	386	BS2G	18.18
**4312**	allXL	CC	373	386	BS2G	23.68
**4312**	allXL	AA	319	357	BS2G	18.82
**4312**	allXL	BB	367	373	BS2G	12.19
**4312**	allXL	DD	370	382	BS2G	11.5
**4312**	allXL	CC	376	382	BS2G	13.65
**4312**	allXL	BB	357	373	BS2G	17.72
**4312**	allXL	CC	320	366	BS2G	22.04
**4312**	allXL	BB	370	376	BS2G	16.3
**4312**	allXL	AA	320	357	DBSU	20.36
**4312**	allXL	AA	357	319	DBSU	18.82
**4312**	allXL	CC	372	319	DBSU	10.29
**4312**	allXL	BB	370	357	DBSU	24.39
**4312**	allXL	AA	382	357	DBSU	32.12
**4312**	allXL	AA	372	370	DBSU	6.2
**4312**	allXL	DD	381	370	DBSU	12.13

**4312**	allXL	AA	373	370	DBSU	9.0
**4312**	allXL	DD	381	372	DBSU	14.3
**4312**	allXL	DD	372	382	DBSU	14.97
**4312**	allXL	CC	372	386	DBSU	22.83
**4312**	allXL	CC	373	386	DBSU	23.68
**4312**	allXL	CB	381	386	DBSU	50.67
**4312**	allXL	BD;DB	357	357	DBSU	17.37
**4312**	allXL	AD	373	319	DBSU	25.4
**4312**	allXL	BD	372	357	DBSU	32.33
**4312**	allXL	BD	381	357	DBSU	44.89
**4312**	allXL	BD	373	357	DBSU	32.9
**4312**	allXL	DA	372	370	DBSU	35.45
**4312**	allXL	AD	373	370	DBSU	37.0
**4312**	allXL	DA	382	373	DBSU	40.77
**4313**	allXL	AA	320	357	BS2G	26.39
**4313**	allXL	DD	320	373	BS2G	15.23
**4313**	allXL	DD	373	382	BS2G	18.1
**4313**	allXL	DD	370	386	BS2G	25.05
**4313**	allXL	DD	373	386	BS2G	22.17
**4313**	allXL	AA	319	357	BS2G	25.93
**4313**	allXL	BB	367	373	BS2G	13.35
**4313**	allXL	DD	370	382	BS2G	24.69
**4313**	allXL	BB	376	382	BS2G	14.52
**4313**	allXL	BB	357	373	BS2G	19.81
**4313**	allXL	BB	320	366	BS2G	23.84
**4313**	allXL	CC	370	376	BS2G	17.29
**4313**	allXL	AA	320	357	DBSU	26.39
**4313**	allXL	AA	357	319	DBSU	25.93
**4313**	allXL	DD	372	319	DBSU	17.3
**4313**	allXL	CC	370	357	DBSU	23.27
**4313**	allXL	BB	382	357	DBSU	23.25
**4313**	allXL	AA	372	370	DBSU	6.2
**4313**	allXL	DD	381	370	DBSU	26.0
**4313**	allXL	CC	373	370	DBSU	9.12

**4313**	allXL	DD	381	372	DBSU	19.07
**4313**	allXL	DD	372	382	DBSU	18.16
**4313**	allXL	DD	372	386	DBSU	20.09
**4313**	allXL	DD	373	386	DBSU	22.17
**4313**	allXL	DA	381	386	DBSU	30.28
**4313**	allXL	BD;DB	357	357	DBSU	18.39
**4313**	allXL	DA	373	319	DBSU	37.3
**4313**	allXL	CA	372	357	DBSU	26.78
**4313**	allXL	BD	381	357	DBSU	23.95
**4313**	allXL	CA	373	357	DBSU	26.94
**4313**	allXL	DA	372	370	DBSU	28.82
**4313**	allXL	DA	373	370	DBSU	28.26
**4313**	allXL	DA	382	373	DBSU	22.44
**4314**	allXL	AA	320	357	BS2G	17.82
**4314**	allXL	AA	320	373	BS2G	21.29
**4314**	allXL	AA	373	382	BS2G	12.37
**4314**	allXL	AA	370	386	BS2G	16.41
**4314**	allXL	AA	373	386	BS2G	16.04
**4314**	allXL	AA	319	357	BS2G	14.97
**4314**	allXL	AA	367	373	BS2G	9.36
**4314**	allXL	AA	370	382	BS2G	11.91
**4314**	allXL	AA	376	382	BS2G	12.03
**4314**	allXL	BB	357	373	BS2G	20.91
**4314**	allXL	AA	320	366	BS2G	19.13
**4314**	allXL	CC	370	376	BS2G	14.27
**4314**	allXL	AA	320	357	DBSU	17.82
**4314**	allXL	AA	357	319	DBSU	14.97
**4314**	allXL	AA	372	319	DBSU	21.73
**4314**	allXL	BB	370	357	DBSU	20.76
**4314**	allXL	AA	382	357	DBSU	27.98
**4314**	allXL	CC	372	370	DBSU	6.6
**4314**	allXL	AA	381	370	DBSU	13.81
**4314**	allXL	AA	373	370	DBSU	8.67
**4314**	allXL	AA	381	372	DBSU	17.2

**4314**	allXL	AA	372	382	DBSU	14.57
**4314**	allXL	AA	372	386	DBSU	17.35
**4314**	allXL	AA	373	386	DBSU	16.04
**4314**	allXL	DA	381	386	DBSU	22.52
**4314**	allXL	BD;DB	357	357	DBSU	19.16
**4314**	allXL	AD	373	319	DBSU	13.91
**4314**	allXL	AD	372	357	DBSU	30.04
**4314**	allXL	AC	381	357	DBSU	40.23
**4314**	allXL	AD	373	357	DBSU	31.74
**4314**	allXL	AD	372	370	DBSU	26.63
**4314**	allXL	AD	373	370	DBSU	29.81
**4314**	allXL	DA	382	373	DBSU	31.97
**4315**	allXL	AA	320	357	BS2G	24.52
**4315**	allXL	BB	320	373	BS2G	25.33
**4315**	allXL	DD	373	382	BS2G	15.95
**4315**	allXL	CC	370	386	BS2G	17.57
**4315**	allXL	DD	373	386	BS2G	17.72
**4315**	allXL	AA	319	357	BS2G	23.44
**4315**	allXL	AA	367	373	BS2G	12.3
**4315**	allXL	DD	370	382	BS2G	15.52
**4315**	allXL	CC	376	382	BS2G	13.8
**4315**	allXL	DD	357	373	BS2G	19.58
**4315**	allXL	CC	320	366	BS2G	18.15
**4315**	allXL	CC	370	376	BS2G	15.06
**4315**	allXL	AA	320	357	DBSU	24.52
**4315**	allXL	AA	357	319	DBSU	23.44
**4315**	allXL	DD	372	319	DBSU	26.95
**4315**	allXL	DD	370	357	DBSU	25.28
**4315**	allXL	BB	382	357	DBSU	27.97
**4315**	allXL	AA	372	370	DBSU	6.29
**4315**	allXL	DD	381	370	DBSU	16.83
**4315**	allXL	AA	373	370	DBSU	9.1
**4315**	allXL	DD	381	372	DBSU	17.71
**4315**	allXL	DD	372	382	DBSU	16.14

**4315**	allXL	DD	372	386	DBSU	16.67
**4315**	allXL	DD	373	386	DBSU	17.72
**4315**	allXL	CB	381	386	DBSU	26.22
**4315**	allXL	BD;DB	357	357	DBSU	18.54
**4315**	allXL	AD	373	319	DBSU	33.49
**4315**	allXL	DB	372	357	DBSU	35.56
**4315**	allXL	CB	381	357	DBSU	36.32
**4315**	allXL	DB	373	357	DBSU	32.73
**4315**	allXL	CB	372	370	DBSU	29.01
**4315**	allXL	CB	373	370	DBSU	30.49
**4315**	allXL	CB	382	373	DBSU	30.92
**4316**	allXL	AA	320	357	BS2G	24.52
**4316**	allXL	BB	320	373	BS2G	27.73
**4316**	allXL	CC	373	382	BS2G	19.33
**4316**	allXL	CC	370	386	BS2G	20.96
**4316**	allXL	DD	373	386	BS2G	18.65
**4316**	allXL	AA	319	357	BS2G	25.14
**4316**	allXL	AA	367	373	BS2G	11.06
**4316**	allXL	CC	370	382	BS2G	22.63
**4316**	allXL	CC	376	382	BS2G	13.45
**4316**	allXL	BB	357	373	BS2G	31.59
**4316**	allXL	CC	320	366	BS2G	24.45
**4316**	allXL	CC	370	376	BS2G	17.08
**4316**	allXL	AA	320	357	DBSU	24.52
**4316**	allXL	AA	357	319	DBSU	25.14
**4316**	allXL	BB	372	319	DBSU	26.61
**4316**	allXL	BB	370	357	DBSU	27.88
**4316**	allXL	AA	382	357	DBSU	21.57
**4316**	allXL	AA	372	370	DBSU	6.69
**4316**	allXL	CC	381	370	DBSU	25.13
**4316**	allXL	DD	373	370	DBSU	9.02
**4316**	allXL	DD	381	372	DBSU	19.24
**4316**	allXL	DD	372	382	DBSU	19.55
**4316**	allXL	DD	372	386	DBSU	16.22

**4316**	allXL	DD	373	386	DBSU	18.65
**4316**	allXL	BC	381	386	DBSU	41.54
**4316**	allXL	BD;DB	357	357	DBSU	19.44
**4316**	allXL	AD	373	319	DBSU	24.28
**4316**	allXL	BC	372	357	DBSU	49.09
**4316**	allXL	AC	381	357	DBSU	44.15
**4316**	allXL	DA	373	357	DBSU	47.37
**4316**	allXL	CB	372	370	DBSU	31.86
**4316**	allXL	BC	373	370	DBSU	34.43
**4316**	allXL	AD	382	373	DBSU	30.06
**4317**	allXL	AA	320	357	BS2G	27.0
**4317**	allXL	CC	320	373	BS2G	14.73
**4317**	allXL	CC	373	382	BS2G	17.33
**4317**	allXL	CC	370	386	BS2G	33.86
**4317**	allXL	CC	373	386	BS2G	24.54
**4317**	allXL	AA	319	357	BS2G	28.97
**4317**	allXL	BB	367	373	BS2G	14.56
**4317**	allXL	DD	370	382	BS2G	23.17
**4317**	allXL	CC	376	382	BS2G	14.91
**4317**	allXL	BB	357	373	BS2G	21.97
**4317**	allXL	BB	320	366	BS2G	16.89
**4317**	allXL	CC	370	376	BS2G	16.49
**4317**	allXL	AA	320	357	DBSU	27.0
**4317**	allXL	AA	357	319	DBSU	28.97
**4317**	allXL	CC	372	319	DBSU	14.94
**4317**	allXL	CC	370	357	DBSU	19.58
**4317**	allXL	CC	382	357	DBSU	37.14
**4317**	allXL	AA	372	370	DBSU	6.22
**4317**	allXL	DD	381	370	DBSU	21.51
**4317**	allXL	AA	373	370	DBSU	8.92
**4317**	allXL	CC	381	372	DBSU	20.06
**4317**	allXL	CC	372	382	DBSU	20.62
**4317**	allXL	CC	372	386	DBSU	27.7
**4317**	allXL	CC	373	386	DBSU	24.54

**4317**	allXL	AC	381	386	DBSU	41.57
**4317**	allXL	BD;DB	357	357	DBSU	18.29
**4317**	allXL	AD	373	319	DBSU	18.56
**4317**	allXL	BD	372	357	DBSU	33.55
**4317**	allXL	BD	381	357	DBSU	39.03
**4317**	allXL	BD	373	357	DBSU	32.17
**4317**	allXL	DA	372	370	DBSU	46.06
**4317**	allXL	DA	373	370	DBSU	43.92
**4317**	allXL	CA	382	373	DBSU	48.14
**4318**	allXL	AA	320	357	BS2G	22.53
**4318**	allXL	CC	320	373	BS2G	14.78
**4318**	allXL	DD	373	382	BS2G	17.31
**4318**	allXL	CC	370	386	BS2G	18.39
**4318**	allXL	AA	373	386	BS2G	21.3
**4318**	allXL	AA	319	357	BS2G	21.35
**4318**	allXL	BB	367	373	BS2G	13.91
**4318**	allXL	CC	370	382	BS2G	16.78
**4318**	allXL	CC	376	382	BS2G	14.09
**4318**	allXL	CC	357	373	BS2G	16.0
**4318**	allXL	CC	320	366	BS2G	21.34
**4318**	allXL	CC	370	376	BS2G	15.62
**4318**	allXL	AA	320	357	DBSU	22.53
**4318**	allXL	AA	357	319	DBSU	21.35
**4318**	allXL	CC	372	319	DBSU	16.53
**4318**	allXL	CC	370	357	DBSU	15.67
**4318**	allXL	CC	382	357	DBSU	27.05
**4318**	allXL	AA	372	370	DBSU	5.77
**4318**	allXL	CC	381	370	DBSU	19.7
**4318**	allXL	AA	373	370	DBSU	8.98
**4318**	allXL	DD	381	372	DBSU	16.68
**4318**	allXL	DD	372	382	DBSU	16.76
**4318**	allXL	CC	372	386	DBSU	23.49
**4318**	allXL	AA	373	386	DBSU	21.3
**4318**	allXL	CB	381	386	DBSU	41.02

**4318**	allXL	BD;DB	357	357	DBSU	18.71
**4318**	allXL	BC	373	319	DBSU	22.14
**4318**	allXL	CA	372	357	DBSU	30.4
**4318**	allXL	CA	381	357	DBSU	47.45
**4318**	allXL	CA	373	357	DBSU	30.08
**4318**	allXL	BC	372	370	DBSU	29.89
**4318**	allXL	BC	373	370	DBSU	32.81
**4318**	allXL	CB	382	373	DBSU	38.33
**4319**	allXL	AA	320	357	BS2G	22.39
**4319**	allXL	BB	320	373	BS2G	23.57
**4319**	allXL	BB	373	382	BS2G	19.23
**4319**	allXL	CC	370	386	BS2G	29.78
**4319**	allXL	BB	373	386	BS2G	30.3
**4319**	allXL	AA	319	357	BS2G	24.34
**4319**	allXL	BB	367	373	BS2G	11.21
**4319**	allXL	BB	370	382	BS2G	22.84
**4319**	allXL	CC	376	382	BS2G	13.87
**4319**	allXL	BB	357	373	BS2G	26.02
**4319**	allXL	CC	320	366	BS2G	25.26
**4319**	allXL	CC	370	376	BS2G	15.53
**4319**	allXL	AA	320	357	DBSU	22.39
**4319**	allXL	AA	357	319	DBSU	24.34
**4319**	allXL	CC	372	319	DBSU	22.19
**4319**	allXL	BB	370	357	DBSU	30.08
**4319**	allXL	BB	382	357	DBSU	28.0
**4319**	allXL	AA	372	370	DBSU	6.06
**4319**	allXL	BB	381	370	DBSU	23.94
**4319**	allXL	AA	373	370	DBSU	9.25
**4319**	allXL	BB	381	372	DBSU	21.41
**4319**	allXL	BB	372	382	DBSU	20.72
**4319**	allXL	BB	372	386	DBSU	31.21
**4319**	allXL	BB	373	386	DBSU	30.3
**4319**	allXL	DB	381	386	DBSU	72.08
**4319**	allXL	BD;DB	357	357	DBSU	19.11

**4319**	allXL	AD	373	319	DBSU	32.65
**4319**	allXL	BD	372	357	DBSU	45.49
**4319**	allXL	BD	381	357	DBSU	33.97
**4319**	allXL	BD	373	357	DBSU	41.72
**4319**	allXL	CB	372	370	DBSU	45.57
**4319**	allXL	CB	373	370	DBSU	47.47
**4319**	allXL	BD	382	373	DBSU	60.94
**4320**	allXL	AA	320	357	BS2G	22.41
**4320**	allXL	CC	320	373	BS2G	25.94
**4320**	allXL	DD	373	382	BS2G	17.45
**4320**	allXL	BB	370	386	BS2G	24.71
**4320**	allXL	BB	373	386	BS2G	19.92
**4320**	allXL	AA	319	357	BS2G	19.73
**4320**	allXL	AA	367	373	BS2G	12.78
**4320**	allXL	DD	370	382	BS2G	15.95
**4320**	allXL	CC	376	382	BS2G	13.76
**4320**	allXL	BB	357	373	BS2G	25.33
**4320**	allXL	BB	320	366	BS2G	16.42
**4320**	allXL	CC	370	376	BS2G	16.26
**4320**	allXL	AA	320	357	DBSU	22.41
**4320**	allXL	AA	357	319	DBSU	19.73
**4320**	allXL	BB	372	319	DBSU	25.09
**4320**	allXL	DD	370	357	DBSU	26.45
**4320**	allXL	DD	382	357	DBSU	25.31
**4320**	allXL	AA	372	370	DBSU	6.19
**4320**	allXL	DD	381	370	DBSU	14.92
**4320**	allXL	CC	373	370	DBSU	8.92
**4320**	allXL	DD	381	372	DBSU	16.9
**4320**	allXL	DD	372	382	DBSU	18.38
**4320**	allXL	BB	372	386	DBSU	23.42
**4320**	allXL	BB	373	386	DBSU	19.92
**4320**	allXL	BC	381	386	DBSU	30.12
**4320**	allXL	BD;DB	357	357	DBSU	18.52
**4320**	allXL	AD	373	319	DBSU	15.66

**4320**	allXL	DA	372	357	DBSU	31.04
**4320**	allXL	DB	381	357	DBSU	33.31
**4320**	allXL	DA	373	357	DBSU	32.07
**4320**	allXL	CB	372	370	DBSU	17.65
**4320**	allXL	CB	373	370	DBSU	17.68
**4320**	allXL	CB	382	373	DBSU	29.08
**4321**	allXL	AA	320	357	BS2G	24.82
**4321**	allXL	AA	320	373	BS2G	14.44
**4321**	allXL	BB	373	382	BS2G	18.52
**4321**	allXL	BB	370	386	BS2G	17.78
**4321**	allXL	CC	373	386	BS2G	22.3
**4321**	allXL	AA	319	357	BS2G	23.32
**4321**	allXL	AA	367	373	BS2G	11.86
**4321**	allXL	BB	370	382	BS2G	18.62
**4321**	allXL	CC	376	382	BS2G	13.53
**4321**	allXL	BB	357	373	BS2G	29.46
**4321**	allXL	AA	320	366	BS2G	14.4
**4321**	allXL	CC	370	376	BS2G	14.57
**4321**	allXL	AA	320	357	DBSU	24.82
**4321**	allXL	AA	357	319	DBSU	23.32
**4321**	allXL	AA	372	319	DBSU	14.56
**4321**	allXL	DD	370	357	DBSU	24.55
**4321**	allXL	AA	382	357	DBSU	31.36
**4321**	allXL	AA	372	370	DBSU	6.42
**4321**	allXL	BB	381	370	DBSU	17.22
**4321**	allXL	AA	373	370	DBSU	8.99
**4321**	allXL	BB	381	372	DBSU	18.59
**4321**	allXL	DD	372	382	DBSU	19.94
**4321**	allXL	BB	372	386	DBSU	23.57
**4321**	allXL	CC	373	386	DBSU	22.3
**4321**	allXL	DA	381	386	DBSU	32.29
**4321**	allXL	BD;DB	357	357	DBSU	19.19
**4321**	allXL	DA	373	319	DBSU	31.53
**4321**	allXL	DA	372	357	DBSU	39.48

**4321**	allXL	AC	381	357	DBSU	45.19
**4321**	allXL	DA	373	357	DBSU	39.41
**4321**	allXL	DA	372	370	DBSU	20.15
**4321**	allXL	DA	373	370	DBSU	21.18
**4321**	allXL	AD	382	373	DBSU	29.32
**4322**	allXL	CC	320	357	BS2G	22.64
**4322**	allXL	BB	320	373	BS2G	22.37
**4322**	allXL	AA	373	382	BS2G	16.75
**4322**	allXL	DD	370	386	BS2G	23.72
**4322**	allXL	DD	373	386	BS2G	23.53
**4322**	allXL	AA	319	357	BS2G	23.34
**4322**	allXL	DD	367	373	BS2G	12.71
**4322**	allXL	DD	370	382	BS2G	21.65
**4322**	allXL	AA	376	382	BS2G	13.77
**4322**	allXL	DD	357	373	BS2G	26.35
**4322**	allXL	CC	320	366	BS2G	20.13
**4322**	allXL	CC	370	376	BS2G	14.88
**4322**	allXL	CC	320	357	DBSU	22.64
**4322**	allXL	AA	357	319	DBSU	23.34
**4322**	allXL	CC	372	319	DBSU	21.25
**4322**	allXL	DD	370	357	DBSU	28.93
**4322**	allXL	CC	382	357	DBSU	35.08
**4322**	allXL	AA	372	370	DBSU	6.55
**4322**	allXL	DD	381	370	DBSU	20.57
**4322**	allXL	DD	373	370	DBSU	8.75
**4322**	allXL	DD	381	372	DBSU	16.41
**4322**	allXL	DD	372	382	DBSU	17.85
**4322**	allXL	DD	372	386	DBSU	22.0
**4322**	allXL	DD	373	386	DBSU	23.53
**4322**	allXL	AC	381	386	DBSU	71.05
**4322**	allXL	BD;DB	357	357	DBSU	18.9
**4322**	allXL	AD	373	319	DBSU	32.89
**4322**	allXL	BC	372	357	DBSU	43.54
**4322**	allXL	DB	381	357	DBSU	48.69

**4322**	allXL	DB	373	357	DBSU	40.83
**4322**	allXL	BC	372	370	DBSU	33.91
**4322**	allXL	BC	373	370	DBSU	35.15
**4322**	allXL	CB	382	373	DBSU	54.33
**4323**	allXL	CC	320	357	BS2G	24.17
**4323**	allXL	BB	320	373	BS2G	28.19
**4323**	allXL	BB	373	382	BS2G	17.07
**4323**	allXL	CC	370	386	BS2G	27.86
**4323**	allXL	BB	373	386	BS2G	25.5
**4323**	allXL	CC	319	357	BS2G	26.66
**4323**	allXL	BB	367	373	BS2G	13.88
**4323**	allXL	CC	370	382	BS2G	20.26
**4323**	allXL	BB	376	382	BS2G	13.33
**4323**	allXL	DD	357	373	BS2G	28.13
**4323**	allXL	CC	320	366	BS2G	22.81
**4323**	allXL	CC	370	376	BS2G	13.27
**4323**	allXL	CC	320	357	DBSU	24.17
**4323**	allXL	CC	357	319	DBSU	26.66
**4323**	allXL	DD	372	319	DBSU	28.95
**4323**	allXL	DD	370	357	DBSU	30.86
**4323**	allXL	BB	382	357	DBSU	28.79
**4323**	allXL	AA	372	370	DBSU	6.22
**4323**	allXL	CC	381	370	DBSU	20.87
**4323**	allXL	CC	373	370	DBSU	8.83
**4323**	allXL	BB	381	372	DBSU	18.92
**4323**	allXL	BB	372	382	DBSU	19.96
**4323**	allXL	BB	372	386	DBSU	27.2
**4323**	allXL	BB	373	386	DBSU	25.5
**4323**	allXL	BC	381	386	DBSU	35.66
**4323**	allXL	BD;DB	357	357	DBSU	18.36
**4323**	allXL	AD	373	319	DBSU	33.72
**4323**	allXL	DB	372	357	DBSU	46.94
**4323**	allXL	BD	381	357	DBSU	46.19
**4323**	allXL	DB	373	357	DBSU	44.99

**4323**	allXL	DA	372	370	DBSU	49.12
**4323**	allXL	DA	373	370	DBSU	47.67
**4323**	allXL	CB	382	373	DBSU	47.13
**4324**	allXL	CC	320	357	BS2G	23.53
**4324**	allXL	DD	320	373	BS2G	26.03
**4324**	allXL	AA	373	382	BS2G	20.14
**4324**	allXL	CC	370	386	BS2G	27.31
**4324**	allXL	CC	373	386	BS2G	27.56
**4324**	allXL	CC	319	357	BS2G	22.96
**4324**	allXL	BB	367	373	BS2G	12.71
**4324**	allXL	CC	370	382	BS2G	23.78
**4324**	allXL	CC	376	382	BS2G	14.09
**4324**	allXL	DD	357	373	BS2G	30.04
**4324**	allXL	BB	320	366	BS2G	21.52
**4324**	allXL	CC	370	376	BS2G	16.41
**4324**	allXL	CC	320	357	DBSU	23.53
**4324**	allXL	CC	357	319	DBSU	22.96
**4324**	allXL	DD	372	319	DBSU	26.65
**4324**	allXL	DD	370	357	DBSU	22.94
**4324**	allXL	BB	382	357	DBSU	21.57
**4324**	allXL	AA	372	370	DBSU	6.44
**4324**	allXL	DD	381	370	DBSU	22.85
**4324**	allXL	AA	373	370	DBSU	9.25
**4324**	allXL	DD	381	372	DBSU	19.57
**4324**	allXL	CC	372	382	DBSU	21.78
**4324**	allXL	DD	372	386	DBSU	26.28
**4324**	allXL	CC	373	386	DBSU	27.56
**4324**	allXL	BC	381	386	DBSU	41.21
**4324**	allXL	BD;DB	357	357	DBSU	19.25
**4324**	allXL	AD	373	319	DBSU	26.14
**4324**	allXL	CB	372	357	DBSU	32.17
**4324**	allXL	BD	381	357	DBSU	35.42
**4324**	allXL	CB	373	357	DBSU	33.64
**4324**	allXL	CB	372	370	DBSU	29.51

**4324**	allXL	CB	373	370	DBSU	29.69
**4324**	allXL	CB	382	373	DBSU	20.29
**4325**	allXL	CC	320	357	BS2G	26.11
**4325**	allXL	CC	320	373	BS2G	15.02
**4325**	allXL	AA	373	382	BS2G	15.92
**4325**	allXL	AA	370	386	BS2G	14.88
**4325**	allXL	AA	373	386	BS2G	20.76
**4325**	allXL	AA	319	357	BS2G	29.1
**4325**	allXL	BB	367	373	BS2G	14.22
**4325**	allXL	AA	370	382	BS2G	16.03
**4325**	allXL	CC	376	382	BS2G	13.21
**4325**	allXL	BB	357	373	BS2G	15.42
**4325**	allXL	CC	320	366	BS2G	20.38
**4325**	allXL	CC	370	376	BS2G	16.61
**4325**	allXL	CC	320	357	DBSU	26.11
**4325**	allXL	AA	357	319	DBSU	29.1
**4325**	allXL	CC	372	319	DBSU	16.93
**4325**	allXL	DD	370	357	DBSU	16.92
**4325**	allXL	BB	382	357	DBSU	17.24
**4325**	allXL	AA	372	370	DBSU	5.94
**4325**	allXL	AA	381	370	DBSU	19.03
**4325**	allXL	AA	373	370	DBSU	9.2
**4325**	allXL	AA	381	372	DBSU	16.27
**4325**	allXL	AA	372	382	DBSU	13.93
**4325**	allXL	AA	372	386	DBSU	17.27
**4325**	allXL	AA	373	386	DBSU	20.76
**4325**	allXL	BC	381	386	DBSU	34.35
**4325**	allXL	BD;DB	357	357	DBSU	18.39
**4325**	allXL	BC	373	319	DBSU	35.23
**4325**	allXL	BD	372	357	DBSU	32.71
**4325**	allXL	BD	381	357	DBSU	31.9
**4325**	allXL	BD	373	357	DBSU	31.13
**4325**	allXL	CB	372	370	DBSU	16.52
**4325**	allXL	CB	373	370	DBSU	17.08

**4325**	allXL	CB	382	373	DBSU	29.44
**4326**	allXL	AA	320	357	BS2G	23.98
**4326**	allXL	BB	320	373	BS2G	34.27
**4326**	allXL	BB	373	382	BS2G	15.92
**4326**	allXL	DD	370	386	BS2G	20.62
**4326**	allXL	BB	373	386	BS2G	23.41
**4326**	allXL	AA	319	357	BS2G	25.6
**4326**	allXL	BB	367	373	BS2G	13.04
**4326**	allXL	DD	370	382	BS2G	15.04
**4326**	allXL	BB	376	382	BS2G	13.66
**4326**	allXL	DD	357	373	BS2G	29.37
**4326**	allXL	CC	320	366	BS2G	28.25
**4326**	allXL	DD	370	376	BS2G	14.64
**4326**	allXL	AA	320	357	DBSU	23.98
**4326**	allXL	AA	357	319	DBSU	25.6
**4326**	allXL	BB	372	319	DBSU	29.6
**4326**	allXL	DD	370	357	DBSU	26.38
**4326**	allXL	DD	382	357	DBSU	16.34
**4326**	allXL	AA	372	370	DBSU	6.06
**4326**	allXL	DD	381	370	DBSU	13.34
**4326**	allXL	AA	373	370	DBSU	9.29
**4326**	allXL	DD	381	372	DBSU	18.45
**4326**	allXL	BB	372	382	DBSU	17.49
**4326**	allXL	BB	372	386	DBSU	23.35
**4326**	allXL	BB	373	386	DBSU	23.41
**4326**	allXL	DB	381	386	DBSU	75.49
**4326**	allXL	BD;DB	357	357	DBSU	18.37
**4326**	allXL	DA	373	319	DBSU	19.13
**4326**	allXL	DA	372	357	DBSU	33.15
**4326**	allXL	DB	381	357	DBSU	23.19
**4326**	allXL	DA	373	357	DBSU	31.8
**4326**	allXL	DA	372	370	DBSU	52.33
**4326**	allXL	DA	373	370	DBSU	53.14
**4326**	allXL	DB	382	373	DBSU	55.3

**4327**	allXL	CC	320	357	BS2G	25.05
**4327**	allXL	AA	320	373	BS2G	21.31
**4327**	allXL	DD	373	382	BS2G	19.54
**4327**	allXL	CC	370	386	BS2G	31.53
**4327**	allXL	DD	373	386	BS2G	28.73
**4327**	allXL	CC	319	357	BS2G	27.17
**4327**	allXL	AA	367	373	BS2G	14.57
**4327**	allXL	DD	370	382	BS2G	24.67
**4327**	allXL	CC	376	382	BS2G	14.67
**4327**	allXL	CC	357	373	BS2G	15.33
**4327**	allXL	AA	320	366	BS2G	21.24
**4327**	allXL	CC	370	376	BS2G	15.08
**4327**	allXL	CC	320	357	DBSU	25.05
**4327**	allXL	CC	357	319	DBSU	27.17
**4327**	allXL	AA	372	319	DBSU	21.27
**4327**	allXL	CC	370	357	DBSU	20.25
**4327**	allXL	CC	382	357	DBSU	27.25
**4327**	allXL	AA	372	370	DBSU	6.16
**4327**	allXL	DD	381	370	DBSU	24.02
**4327**	allXL	DD	373	370	DBSU	9.14
**4327**	allXL	DD	381	372	DBSU	19.9
**4327**	allXL	DD	372	382	DBSU	20.59
**4327**	allXL	DD	372	386	DBSU	28.68
**4327**	allXL	DD	373	386	DBSU	28.73
**4327**	allXL	BC	381	386	DBSU	45.99
**4327**	allXL	BD;DB	357	357	DBSU	18.63
**4327**	allXL	AD	373	319	DBSU	34.23
**4327**	allXL	CA	372	357	DBSU	27.32
**4327**	allXL	CB	381	357	DBSU	35.79
**4327**	allXL	CA	373	357	DBSU	24.97
**4327**	allXL	AD	372	370	DBSU	35.87
**4327**	allXL	AD	373	370	DBSU	37.28
**4327**	allXL	AD	382	373	DBSU	47.04
**4328**	allXL	AA	320	357	BS2G	24.28

**4328**	allXL	DD	320	373	BS2G	20.15
**4328**	allXL	DD	373	382	BS2G	18.04
**4328**	allXL	CC	370	386	BS2G	26.36
**4328**	allXL	AA	373	386	BS2G	22.61
**4328**	allXL	AA	319	357	BS2G	23.62
**4328**	allXL	AA	367	373	BS2G	13.49
**4328**	allXL	DD	370	382	BS2G	18.16
**4328**	allXL	CC	376	382	BS2G	14.07
**4328**	allXL	DD	357	373	BS2G	23.59
**4328**	allXL	CC	320	366	BS2G	18.85
**4328**	allXL	CC	370	376	BS2G	14.67
**4328**	allXL	AA	320	357	DBSU	24.28
**4328**	allXL	AA	357	319	DBSU	23.62
**4328**	allXL	CC	372	319	DBSU	17.18
**4328**	allXL	DD	370	357	DBSU	20.64
**4328**	allXL	DD	382	357	DBSU	19.1
**4328**	allXL	AA	372	370	DBSU	6.18
**4328**	allXL	DD	381	370	DBSU	16.98
**4328**	allXL	AA	373	370	DBSU	9.03
**4328**	allXL	DD	381	372	DBSU	17.13
**4328**	allXL	DD	372	382	DBSU	19.44
**4328**	allXL	AA	372	386	DBSU	25.83
**4328**	allXL	AA	373	386	DBSU	22.61
**4328**	allXL	CB	381	386	DBSU	41.62
**4328**	allXL	BD;DB	357	357	DBSU	19.39
**4328**	allXL	CB	373	319	DBSU	34.7
**4328**	allXL	CB	372	357	DBSU	35.7
**4328**	allXL	DB	381	357	DBSU	35.38
**4328**	allXL	CB	373	357	DBSU	36.46
**4328**	allXL	BC	372	370	DBSU	22.67
**4328**	allXL	CB	373	370	DBSU	21.53
**4328**	allXL	CB	382	373	DBSU	21.2
**4329**	allXL	AA	320	357	BS2G	24.14
**4329**	allXL	CC	320	373	BS2G	21.47

**4329**	allXL	BB	373	382	BS2G	17.09
**4329**	allXL	AA	370	386	BS2G	23.06
**4329**	allXL	AA	373	386	BS2G	23.59
**4329**	allXL	AA	319	357	BS2G	26.16
**4329**	allXL	BB	367	373	BS2G	12.15
**4329**	allXL	CC	370	382	BS2G	19.6
**4329**	allXL	BB	376	382	BS2G	13.38
**4329**	allXL	BB	357	373	BS2G	28.98
**4329**	allXL	AA	320	366	BS2G	25.64
**4329**	allXL	CC	370	376	BS2G	15.79
**4329**	allXL	AA	320	357	DBSU	24.14
**4329**	allXL	AA	357	319	DBSU	26.16
**4329**	allXL	CC	372	319	DBSU	24.08
**4329**	allXL	DD	370	357	DBSU	26.11
**4329**	allXL	BB	382	357	DBSU	38.49
**4329**	allXL	AA	372	370	DBSU	6.05
**4329**	allXL	CC	381	370	DBSU	19.79
**4329**	allXL	AA	373	370	DBSU	8.97
**4329**	allXL	CC	381	372	DBSU	20.24
**4329**	allXL	BB	372	382	DBSU	18.92
**4329**	allXL	AA	372	386	DBSU	24.07
**4329**	allXL	AA	373	386	DBSU	23.59
**4329**	allXL	DA	381	386	DBSU	45.7
**4329**	allXL	BD;DB	357	357	DBSU	19.67
**4329**	allXL	AD	373	319	DBSU	23.34
**4329**	allXL	AD	372	357	DBSU	39.15
**4329**	allXL	BD	381	357	DBSU	52.37
**4329**	allXL	AD	373	357	DBSU	42.4
**4329**	allXL	AD	372	370	DBSU	31.54
**4329**	allXL	AD	373	370	DBSU	34.52
**4329**	allXL	AD	382	373	DBSU	47.55
**4330**	allXL	AA	320	357	BS2G	19.04
**4330**	allXL	DD	320	373	BS2G	15.23
**4330**	allXL	CC	373	382	BS2G	20.29

**4330**	allXL	AA	370	386	BS2G	29.36
**4330**	allXL	CC	373	386	BS2G	23.3
**4330**	allXL	AA	319	357	BS2G	16.95
**4330**	allXL	AA	367	373	BS2G	12.87
**4330**	allXL	BB	370	382	BS2G	25.58
**4330**	allXL	CC	376	382	BS2G	14.15
**4330**	allXL	AA	357	373	BS2G	22.86
**4330**	allXL	BB	320	366	BS2G	13.43
**4330**	allXL	CC	370	376	BS2G	16.53
**4330**	allXL	AA	320	357	DBSU	19.04
**4330**	allXL	AA	357	319	DBSU	16.95
**4330**	allXL	DD	372	319	DBSU	10.84
**4330**	allXL	BB	370	357	DBSU	21.03
**4330**	allXL	AA	382	357	DBSU	35.86
**4330**	allXL	AA	372	370	DBSU	6.24
**4330**	allXL	DD	381	370	DBSU	26.2
**4330**	allXL	DD	373	370	DBSU	9.02
**4330**	allXL	DD	381	372	DBSU	22.3
**4330**	allXL	CC	372	382	DBSU	22.24
**4330**	allXL	CC	372	386	DBSU	24.34
**4330**	allXL	CC	373	386	DBSU	23.3
**4330**	allXL	DA	381	386	DBSU	47.86
**4330**	allXL	BD;DB	357	357	DBSU	18.76
**4330**	allXL	AD	373	319	DBSU	21.11
**4330**	allXL	AC	372	357	DBSU	34.58
**4330**	allXL	AC	381	357	DBSU	31.62
**4330**	allXL	AC	373	357	DBSU	30.86
**4330**	allXL	DA	372	370	DBSU	20.45
**4330**	allXL	DA	373	370	DBSU	20.14
**4330**	allXL	DA	382	373	DBSU	36.3
**4331**	allXL	CC	320	357	BS2G	23.36
**4331**	allXL	BB	320	373	BS2G	16.03
**4331**	allXL	AA	373	382	BS2G	16.86
**4331**	allXL	AA	370	386	BS2G	18.37

**4331**	allXL	AA	373	386	BS2G	23.63
**4331**	allXL	AA	319	357	BS2G	21.87
**4331**	allXL	AA	367	373	BS2G	12.0
**4331**	allXL	AA	370	382	BS2G	15.67
**4331**	allXL	BB	376	382	BS2G	14.04
**4331**	allXL	AA	357	373	BS2G	24.12
**4331**	allXL	BB	320	366	BS2G	15.83
**4331**	allXL	CC	370	376	BS2G	15.11
**4331**	allXL	CC	320	357	DBSU	23.36
**4331**	allXL	AA	357	319	DBSU	21.87
**4331**	allXL	BB	372	319	DBSU	13.23
**4331**	allXL	BB	370	357	DBSU	23.65
**4331**	allXL	AA	382	357	DBSU	28.92
**4331**	allXL	AA	372	370	DBSU	6.56
**4331**	allXL	AA	381	370	DBSU	18.55
**4331**	allXL	DD	373	370	DBSU	9.22
**4331**	allXL	AA	381	372	DBSU	17.55
**4331**	allXL	AA	372	382	DBSU	15.19
**4331**	allXL	AA	372	386	DBSU	20.49
**4331**	allXL	AA	373	386	DBSU	23.63
**4331**	allXL	DA	381	386	DBSU	45.86
**4331**	allXL	BD;DB	357	357	DBSU	19.28
**4331**	allXL	AD	373	319	DBSU	26.15
**4331**	allXL	AC	372	357	DBSU	36.66
**4331**	allXL	AD	381	357	DBSU	41.11
**4331**	allXL	AC	373	357	DBSU	33.52
**4331**	allXL	BC	372	370	DBSU	55.49
**4331**	allXL	DA	373	370	DBSU	57.88
**4331**	allXL	AD	382	373	DBSU	43.73
**4332**	allXL	BB	320	357	BS2G	25.6
**4332**	allXL	CC	320	373	BS2G	17.96
**4332**	allXL	CC	373	382	BS2G	18.65
**4332**	allXL	CC	370	386	BS2G	17.7
**4332**	allXL	CC	373	386	BS2G	23.58

**4332**	allXL	AA	319	357	BS2G	27.13
**4332**	allXL	AA	367	373	BS2G	12.72
**4332**	allXL	CC	370	382	BS2G	17.45
**4332**	allXL	CC	376	382	BS2G	12.61
**4332**	allXL	AA	357	373	BS2G	24.06
**4332**	allXL	BB	320	366	BS2G	21.07
**4332**	allXL	CC	370	376	BS2G	15.1
**4332**	allXL	BB	320	357	DBSU	25.6
**4332**	allXL	AA	357	319	DBSU	27.13
**4332**	allXL	CC	372	319	DBSU	15.82
**4332**	allXL	AA	370	357	DBSU	24.15
**4332**	allXL	AA	382	357	DBSU	44.05
**4332**	allXL	AA	372	370	DBSU	6.37
**4332**	allXL	DD	381	370	DBSU	17.96
**4332**	allXL	AA	373	370	DBSU	9.07
**4332**	allXL	DD	381	372	DBSU	18.71
**4332**	allXL	CC	372	382	DBSU	18.89
**4332**	allXL	CC	372	386	DBSU	21.59
**4332**	allXL	CC	373	386	DBSU	23.58
**4332**	allXL	CB	381	386	DBSU	32.58
**4332**	allXL	BD;DB	357	357	DBSU	19.23
**4332**	allXL	DA	373	319	DBSU	26.29
**4332**	allXL	AC	372	357	DBSU	30.6
**4332**	allXL	DA	381	357	DBSU	33.75
**4332**	allXL	AC	373	357	DBSU	31.82
**4332**	allXL	CB	372	370	DBSU	36.95
**4332**	allXL	CB	373	370	DBSU	37.78
**4332**	allXL	CB	382	373	DBSU	35.64
**4333**	allXL	AA	320	357	BS2G	26.39
**4333**	allXL	DD	320	373	BS2G	21.08
**4333**	allXL	BB	373	382	BS2G	17.57
**4333**	allXL	AA	370	386	BS2G	27.39
**4333**	allXL	AA	373	386	BS2G	21.57
**4333**	allXL	AA	319	357	BS2G	25.39

**4333**	allXL	AA	367	373	BS2G	12.59
**4333**	allXL	BB	370	382	BS2G	21.81
**4333**	allXL	CC	376	382	BS2G	14.67
**4333**	allXL	CC	357	373	BS2G	26.88
**4333**	allXL	DD	320	366	BS2G	24.78
**4333**	allXL	CC	370	376	BS2G	14.75
**4333**	allXL	AA	320	357	DBSU	26.39
**4333**	allXL	AA	357	319	DBSU	25.39
**4333**	allXL	BB	372	319	DBSU	17.19
**4333**	allXL	BB	370	357	DBSU	19.97
**4333**	allXL	BB	382	357	DBSU	34.76
**4333**	allXL	AA	372	370	DBSU	6.36
**4333**	allXL	BB	381	370	DBSU	22.7
**4333**	allXL	AA	373	370	DBSU	9.18
**4333**	allXL	DD	381	372	DBSU	19.99
**4333**	allXL	DD	372	382	DBSU	20.51
**4333**	allXL	AA	372	386	DBSU	24.09
**4333**	allXL	AA	373	386	DBSU	21.57
**4333**	allXL	CA	381	386	DBSU	50.99
**4333**	allXL	BD;DB	357	357	DBSU	18.41
**4333**	allXL	AD	373	319	DBSU	21.53
**4333**	allXL	CA	372	357	DBSU	37.41
**4333**	allXL	CA	381	357	DBSU	44.85
**4333**	allXL	CA	373	357	DBSU	36.13
**4333**	allXL	DA	372	370	DBSU	35.95
**4333**	allXL	DA	373	370	DBSU	33.31
**4333**	allXL	AC	382	373	DBSU	41.46
**4334**	allXL	CC	320	357	BS2G	25.23
**4334**	allXL	BB	320	373	BS2G	19.22
**4334**	allXL	BB	373	382	BS2G	18.74
**4334**	allXL	DD	370	386	BS2G	20.74
**4334**	allXL	DD	373	386	BS2G	21.35
**4334**	allXL	AA	319	357	BS2G	26.8
**4334**	allXL	AA	367	373	BS2G	12.74

**4334**	allXL	BB	370	382	BS2G	23.61
**4334**	allXL	BB	376	382	BS2G	13.51
**4334**	allXL	CC	357	373	BS2G	29.72
**4334**	allXL	BB	320	366	BS2G	26.64
**4334**	allXL	CC	370	376	BS2G	15.79
**4334**	allXL	CC	320	357	DBSU	25.23
**4334**	allXL	AA	357	319	DBSU	26.8
**4334**	allXL	BB	372	319	DBSU	22.49
**4334**	allXL	BB	370	357	DBSU	26.62
**4334**	allXL	CC	382	357	DBSU	28.16
**4334**	allXL	AA	372	370	DBSU	6.34
**4334**	allXL	DD	381	370	DBSU	24.49
**4334**	allXL	CC	373	370	DBSU	9.15
**4334**	allXL	DD	381	372	DBSU	20.92
**4334**	allXL	BB	372	382	DBSU	20.48
**4334**	allXL	DD	372	386	DBSU	19.92
**4334**	allXL	DD	373	386	DBSU	21.35
**4334**	allXL	CB	381	386	DBSU	39.13
**4334**	allXL	BD;DB	357	357	DBSU	18.59
**4334**	allXL	AD	373	319	DBSU	25.11
**4334**	allXL	CA	372	357	DBSU	44.87
**4334**	allXL	CA	381	357	DBSU	30.72
**4334**	allXL	CA	373	357	DBSU	41.57
**4334**	allXL	DA	372	370	DBSU	34.05
**4334**	allXL	DA	373	370	DBSU	33.81
**4334**	allXL	BC	382	373	DBSU	51.91
**4335**	allXL	AA	320	357	BS2G	24.24
**4335**	allXL	DD	320	373	BS2G	14.78
**4335**	allXL	BB	373	382	BS2G	17.42
**4335**	allXL	BB	370	386	BS2G	25.43
**4335**	allXL	AA	373	386	BS2G	24.7
**4335**	allXL	AA	319	357	BS2G	27.81
**4335**	allXL	BB	367	373	BS2G	13.27
**4335**	allXL	BB	370	382	BS2G	19.83

**4335**	allXL	BB	376	382	BS2G	13.43
**4335**	allXL	BB	357	373	BS2G	32.72
**4335**	allXL	DD	320	366	BS2G	12.86
**4335**	allXL	CC	370	376	BS2G	16.89
**4335**	allXL	AA	320	357	DBSU	24.24
**4335**	allXL	AA	357	319	DBSU	27.81
**4335**	allXL	CC	372	319	DBSU	14.29
**4335**	allXL	DD	370	357	DBSU	28.26
**4335**	allXL	BB	382	357	DBSU	28.69
**4335**	allXL	AA	372	370	DBSU	6.31
**4335**	allXL	BB	381	370	DBSU	22.28
**4335**	allXL	AA	373	370	DBSU	9.08
**4335**	allXL	CC	381	372	DBSU	20.77
**4335**	allXL	CC	372	382	DBSU	19.37
**4335**	allXL	CC	372	386	DBSU	24.28
**4335**	allXL	AA	373	386	DBSU	24.7
**4335**	allXL	BC	381	386	DBSU	29.99
**4335**	allXL	BD;DB	357	357	DBSU	18.56
**4335**	allXL	AD	373	319	DBSU	39.45
**4335**	allXL	CB	372	357	DBSU	43.47
**4335**	allXL	BD	381	357	DBSU	47.0
**4335**	allXL	CB	373	357	DBSU	45.42
**4335**	allXL	CB	372	370	DBSU	35.11
**4335**	allXL	CB	373	370	DBSU	36.34
**4335**	allXL	CB	382	373	DBSU	29.74
**4336**	allXL	CC	320	357	BS2G	21.25
**4336**	allXL	DD	320	373	BS2G	20.15
**4336**	allXL	CC	373	382	BS2G	15.98
**4336**	allXL	CC	370	386	BS2G	12.73
**4336**	allXL	CC	373	386	BS2G	16.48
**4336**	allXL	AA	319	357	BS2G	21.85
**4336**	allXL	AA	367	373	BS2G	14.26
**4336**	allXL	CC	370	382	BS2G	17.39
**4336**	allXL	AA	376	382	BS2G	13.27

**4336**	allXL	BB	357	373	BS2G	16.44
**4336**	allXL	BB	320	366	BS2G	22.73
**4336**	allXL	CC	370	376	BS2G	16.85
**4336**	allXL	CC	320	357	DBSU	21.25
**4336**	allXL	AA	357	319	DBSU	21.85
**4336**	allXL	DD	372	319	DBSU	22.51
**4336**	allXL	DD	370	357	DBSU	19.42
**4336**	allXL	BB	382	357	DBSU	24.52
**4336**	allXL	AA	372	370	DBSU	6.35
**4336**	allXL	CC	381	370	DBSU	18.45
**4336**	allXL	DD	373	370	DBSU	8.83
**4336**	allXL	DD	381	372	DBSU	13.93
**4336**	allXL	DD	372	382	DBSU	15.35
**4336**	allXL	CC	372	386	DBSU	14.27
**4336**	allXL	CC	373	386	DBSU	16.48
**4336**	allXL	DA	381	386	DBSU	33.54
**4336**	allXL	BD;DB	357	357	DBSU	19.92
**4336**	allXL	CB	373	319	DBSU	22.49
**4336**	allXL	BD	372	357	DBSU	27.63
**4336**	allXL	BD	381	357	DBSU	13.22
**4336**	allXL	BD	373	357	DBSU	25.73
**4336**	allXL	AD	372	370	DBSU	39.52
**4336**	allXL	AD	373	370	DBSU	40.13
**4336**	allXL	BD	382	373	DBSU	23.76
**4337**	allXL	AA	320	357	BS2G	22.94
**4337**	allXL	BB	320	373	BS2G	22.35
**4337**	allXL	BB	373	382	BS2G	17.63
**4337**	allXL	DD	370	386	BS2G	24.79
**4337**	allXL	DD	373	386	BS2G	26.67
**4337**	allXL	AA	319	357	BS2G	23.16
**4337**	allXL	BB	367	373	BS2G	11.31
**4337**	allXL	DD	370	382	BS2G	20.89
**4337**	allXL	BB	376	382	BS2G	13.72
**4337**	allXL	BB	357	373	BS2G	28.2

**4337**	allXL	CC	320	366	BS2G	23.19
**4337**	allXL	CC	370	376	BS2G	17.0
**4337**	allXL	AA	320	357	DBSU	22.94
**4337**	allXL	AA	357	319	DBSU	23.16
**4337**	allXL	BB	372	319	DBSU	23.4
**4337**	allXL	BB	370	357	DBSU	29.94
**4337**	allXL	BB	382	357	DBSU	26.76
**4337**	allXL	AA	372	370	DBSU	6.23
**4337**	allXL	DD	381	370	DBSU	21.25
**4337**	allXL	CC	373	370	DBSU	9.04
**4337**	allXL	BB	381	372	DBSU	20.08
**4337**	allXL	BB	372	382	DBSU	20.33
**4337**	allXL	DD	372	386	DBSU	26.09
**4337**	allXL	DD	373	386	DBSU	26.67
**4337**	allXL	DA	381	386	DBSU	60.38
**4337**	allXL	AC;CA	357	357	DBSU	18.29
**4337**	allXL	CB	373	319	DBSU	13.73
**4337**	allXL	CB	372	357	DBSU	37.28
**4337**	allXL	BD	381	357	DBSU	40.52
**4337**	allXL	DA	373	357	DBSU	38.72
**4337**	allXL	BC	372	370	DBSU	17.94
**4337**	allXL	BC	373	370	DBSU	18.28
**4337**	allXL	BC	382	373	DBSU	37.33
**4338**	allXL	AA	320	357	BS2G	24.51
**4338**	allXL	BB	320	373	BS2G	20.79
**4338**	allXL	DD	373	382	BS2G	19.46
**4338**	allXL	DD	370	386	BS2G	12.73
**4338**	allXL	DD	373	386	BS2G	20.3
**4338**	allXL	AA	319	357	BS2G	24.16
**4338**	allXL	BB	367	373	BS2G	12.92
**4338**	allXL	DD	370	382	BS2G	15.43
**4338**	allXL	BB	376	382	BS2G	13.68
**4338**	allXL	DD	357	373	BS2G	30.42
**4338**	allXL	BB	320	366	BS2G	25.48

**4338**	allXL	CC	370	376	BS2G	15.2
**4338**	allXL	AA	320	357	DBSU	24.51
**4338**	allXL	AA	357	319	DBSU	24.16
**4338**	allXL	BB	372	319	DBSU	24.89
**4338**	allXL	DD	370	357	DBSU	28.29
**4338**	allXL	DD	382	357	DBSU	35.98
**4338**	allXL	AA	372	370	DBSU	6.17
**4338**	allXL	DD	381	370	DBSU	16.33
**4338**	allXL	AA	373	370	DBSU	8.8
**4338**	allXL	DD	381	372	DBSU	18.84
**4338**	allXL	DD	372	382	DBSU	19.21
**4338**	allXL	DD	372	386	DBSU	18.0
**4338**	allXL	DD	373	386	DBSU	20.3
**4338**	allXL	AD	381	386	DBSU	58.44
**4338**	allXL	BD;DB	357	357	DBSU	17.78
**4338**	allXL	AD	373	319	DBSU	21.73
**4338**	allXL	CB	372	357	DBSU	40.26
**4338**	allXL	DB	381	357	DBSU	45.56
**4338**	allXL	CB	373	357	DBSU	40.37
**4338**	allXL	BC	372	370	DBSU	43.45
**4338**	allXL	BC	373	370	DBSU	40.84
**4338**	allXL	BC	382	373	DBSU	42.3
**4339**	allXL	AA	320	357	BS2G	22.77
**4339**	allXL	BB	320	373	BS2G	12.97
**4339**	allXL	CC	373	382	BS2G	19.22
**4339**	allXL	DD	370	386	BS2G	21.14
**4339**	allXL	DD	373	386	BS2G	24.54
**4339**	allXL	AA	319	357	BS2G	21.06
**4339**	allXL	AA	367	373	BS2G	11.3
**4339**	allXL	DD	370	382	BS2G	20.42
**4339**	allXL	CC	376	382	BS2G	14.6
**4339**	allXL	BB	357	373	BS2G	24.09
**4339**	allXL	DD	320	366	BS2G	25.94
**4339**	allXL	CC	370	376	BS2G	17.43

**4339**	allXL	AA	320	357	DBSU	22.77
**4339**	allXL	AA	357	319	DBSU	21.06
**4339**	allXL	BB	372	319	DBSU	15.8
**4339**	allXL	BB	370	357	DBSU	28.73
**4339**	allXL	AA	382	357	DBSU	24.49
**4339**	allXL	AA	372	370	DBSU	6.34
**4339**	allXL	DD	381	370	DBSU	19.83
**4339**	allXL	AA	373	370	DBSU	8.98
**4339**	allXL	DD	381	372	DBSU	19.69
**4339**	allXL	DD	372	382	DBSU	21.53
**4339**	allXL	DD	372	386	DBSU	25.41
**4339**	allXL	DD	373	386	DBSU	24.54
**4339**	allXL	BC	381	386	DBSU	38.08
**4339**	allXL	BD;DB	357	357	DBSU	18.95
**4339**	allXL	CB	373	319	DBSU	28.97
**4339**	allXL	BD	372	357	DBSU	41.58
**4339**	allXL	AC	381	357	DBSU	28.02
**4339**	allXL	BD	373	357	DBSU	42.46
**4339**	allXL	CB	372	370	DBSU	32.08
**4339**	allXL	CB	373	370	DBSU	29.58
**4339**	allXL	CB	382	373	DBSU	32.06
**4340**	allXL	CC	320	357	BS2G	23.67
**4340**	allXL	BB	320	373	BS2G	16.15
**4340**	allXL	AA	373	382	BS2G	17.64
**4340**	allXL	AA	370	386	BS2G	19.47
**4340**	allXL	AA	373	386	BS2G	22.59
**4340**	allXL	CC	319	357	BS2G	25.31
**4340**	allXL	AA	367	373	BS2G	12.68
**4340**	allXL	AA	370	382	BS2G	19.89
**4340**	allXL	CC	376	382	BS2G	13.79
**4340**	allXL	BB	357	373	BS2G	21.58
**4340**	allXL	BB	320	366	BS2G	20.82
**4340**	allXL	CC	370	376	BS2G	16.25
**4340**	allXL	CC	320	357	DBSU	23.67

**4340**	allXL	CC	357	319	DBSU	25.31
**4340**	allXL	BB	372	319	DBSU	20.19
**4340**	allXL	BB	370	357	DBSU	27.14
**4340**	allXL	BB	382	357	DBSU	37.87
**4340**	allXL	AA	372	370	DBSU	6.83
**4340**	allXL	AA	381	370	DBSU	20.0
**4340**	allXL	DD	373	370	DBSU	8.97
**4340**	allXL	DD	381	372	DBSU	17.68
**4340**	allXL	DD	372	382	DBSU	19.58
**4340**	allXL	DD	372	386	DBSU	22.24
**4340**	allXL	AA	373	386	DBSU	22.59
**4340**	allXL	BC	381	386	DBSU	32.93
**4340**	allXL	BD;DB	357	357	DBSU	19.28
**4340**	allXL	AD	373	319	DBSU	26.33
**4340**	allXL	BD	372	357	DBSU	42.24
**4340**	allXL	CB	381	357	DBSU	41.17
**4340**	allXL	BD	373	357	DBSU	39.75
**4340**	allXL	AD	372	370	DBSU	35.83
**4340**	allXL	CB	373	370	DBSU	38.36
**4340**	allXL	CB	382	373	DBSU	30.56
**4341**	allXL	CC	320	357	BS2G	22.38
**4341**	allXL	DD	320	373	BS2G	28.68
**4341**	allXL	CC	373	382	BS2G	21.06
**4341**	allXL	DD	370	386	BS2G	19.84
**4341**	allXL	DD	373	386	BS2G	27.7
**4341**	allXL	CC	319	357	BS2G	23.68
**4341**	allXL	BB	367	373	BS2G	12.99
**4341**	allXL	DD	370	382	BS2G	17.35
**4341**	allXL	CC	376	382	BS2G	14.79
**4341**	allXL	CC	357	373	BS2G	25.75
**4341**	allXL	AA	320	366	BS2G	17.98
**4341**	allXL	CC	370	376	BS2G	11.93
**4341**	allXL	CC	320	357	DBSU	22.38
**4341**	allXL	CC	357	319	DBSU	23.68

**4341**	allXL	DD	372	319	DBSU	29.5
**4341**	allXL	CC	370	357	DBSU	25.83
**4341**	allXL	DD	382	357	DBSU	19.14
**4341**	allXL	AA	372	370	DBSU	6.19
**4341**	allXL	DD	381	370	DBSU	16.53
**4341**	allXL	AA	373	370	DBSU	9.02
**4341**	allXL	DD	381	372	DBSU	20.62
**4341**	allXL	CC	372	382	DBSU	21.62
**4341**	allXL	DD	372	386	DBSU	26.91
**4341**	allXL	DD	373	386	DBSU	27.7
**4341**	allXL	AD	381	386	DBSU	41.4
**4341**	allXL	BD;DB	357	357	DBSU	18.37
**4341**	allXL	AD	373	319	DBSU	23.6
**4341**	allXL	CA	372	357	DBSU	39.31
**4341**	allXL	DB	381	357	DBSU	35.03
**4341**	allXL	DA	373	357	DBSU	36.89
**4341**	allXL	DA	372	370	DBSU	18.54
**4341**	allXL	DA	373	370	DBSU	20.14
**4341**	allXL	AD	382	373	DBSU	38.21
**4342**	allXL	CC	320	357	BS2G	25.7
**4342**	allXL	BB	320	373	BS2G	14.66
**4342**	allXL	CC	373	382	BS2G	18.87
**4342**	allXL	CC	370	386	BS2G	27.87
**4342**	allXL	CC	373	386	BS2G	21.97
**4342**	allXL	CC	319	357	BS2G	27.39
**4342**	allXL	AA	367	373	BS2G	11.64
**4342**	allXL	CC	370	382	BS2G	25.73
**4342**	allXL	CC	376	382	BS2G	13.32
**4342**	allXL	DD	357	373	BS2G	34.58
**4342**	allXL	BB	320	366	BS2G	18.19
**4342**	allXL	CC	370	376	BS2G	16.14
**4342**	allXL	CC	320	357	DBSU	25.7
**4342**	allXL	CC	357	319	DBSU	27.39
**4342**	allXL	BB	372	319	DBSU	13.67

**4342**	allXL	DD	370	357	DBSU	31.22
**4342**	allXL	DD	382	357	DBSU	28.99
**4342**	allXL	AA	372	370	DBSU	6.49
**4342**	allXL	DD	381	370	DBSU	24.24
**4342**	allXL	AA	373	370	DBSU	9.08
**4342**	allXL	DD	381	372	DBSU	23.43
**4342**	allXL	CC	372	382	DBSU	22.58
**4342**	allXL	CC	372	386	DBSU	24.83
**4342**	allXL	CC	373	386	DBSU	21.97
**4342**	allXL	CB	381	386	DBSU	29.89
**4342**	allXL	BD;DB	357	357	DBSU	18.23
**4342**	allXL	BC	373	319	DBSU	34.52
**4342**	allXL	BC	372	357	DBSU	38.28
**4342**	allXL	DB	381	357	DBSU	43.17
**4342**	allXL	BC	373	357	DBSU	38.52
**4342**	allXL	BC	372	370	DBSU	20.28
**4342**	allXL	BC	373	370	DBSU	20.19
**4342**	allXL	CB	382	373	DBSU	35.45
**4343**	allXL	AA	320	357	BS2G	24.43
**4343**	allXL	CC	320	373	BS2G	17.91
**4343**	allXL	BB	373	382	BS2G	18.05
**4343**	allXL	BB	370	386	BS2G	22.59
**4343**	allXL	AA	373	386	BS2G	24.23
**4343**	allXL	AA	319	357	BS2G	23.33
**4343**	allXL	AA	367	373	BS2G	13.13
**4343**	allXL	BB	370	382	BS2G	18.9
**4343**	allXL	BB	376	382	BS2G	14.56
**4343**	allXL	DD	357	373	BS2G	19.45
**4343**	allXL	AA	320	366	BS2G	15.52
**4343**	allXL	DD	370	376	BS2G	16.39
**4343**	allXL	AA	320	357	DBSU	24.43
**4343**	allXL	AA	357	319	DBSU	23.33
**4343**	allXL	CC	372	319	DBSU	16.4
**4343**	allXL	CC	370	357	DBSU	24.79

**4343**	allXL	DD	382	357	DBSU	38.24
**4343**	allXL	AA	372	370	DBSU	6.12
**4343**	allXL	BB	381	370	DBSU	19.15
**4343**	allXL	AA	373	370	DBSU	9.1
**4343**	allXL	BB	381	372	DBSU	19.24
**4343**	allXL	BB	372	382	DBSU	19.83
**4343**	allXL	AA	372	386	DBSU	24.62
**4343**	allXL	AA	373	386	DBSU	24.23
**4343**	allXL	DA	381	386	DBSU	47.67
**4343**	allXL	BD;DB	357	357	DBSU	17.89
**4343**	allXL	BC	373	319	DBSU	33.08
**4343**	allXL	DB	372	357	DBSU	24.36
**4343**	allXL	AD	381	357	DBSU	30.2
**4343**	allXL	DB	373	357	DBSU	22.96
**4343**	allXL	BC	372	370	DBSU	24.06
**4343**	allXL	BC	373	370	DBSU	21.73
**4343**	allXL	AD	382	373	DBSU	34.28
**4344**	allXL	AA	320	357	BS2G	22.71
**4344**	allXL	DD	320	373	BS2G	25.35
**4344**	allXL	DD	373	382	BS2G	19.79
**4344**	allXL	DD	370	386	BS2G	10.63
**4344**	allXL	DD	373	386	BS2G	17.62
**4344**	allXL	AA	319	357	BS2G	22.68
**4344**	allXL	BB	367	373	BS2G	12.13
**4344**	allXL	DD	370	382	BS2G	17.85
**4344**	allXL	BB	376	382	BS2G	14.97
**4344**	allXL	BB	357	373	BS2G	26.74
**4344**	allXL	AA	320	366	BS2G	27.95
**4344**	allXL	CC	370	376	BS2G	14.08
**4344**	allXL	AA	320	357	DBSU	22.71
**4344**	allXL	AA	357	319	DBSU	22.68
**4344**	allXL	DD	372	319	DBSU	24.86
**4344**	allXL	DD	370	357	DBSU	24.02
**4344**	allXL	CC	382	357	DBSU	17.96

**4344**	allXL	AA	372	370	DBSU	6.39
**4344**	allXL	DD	381	370	DBSU	18.41
**4344**	allXL	AA	373	370	DBSU	9.11
**4344**	allXL	DD	381	372	DBSU	20.68
**4344**	allXL	DD	372	382	DBSU	21.35
**4344**	allXL	DD	372	386	DBSU	17.18
**4344**	allXL	DD	373	386	DBSU	17.62
**4344**	allXL	BC	381	386	DBSU	34.64
**4344**	allXL	BD;DB	357	357	DBSU	17.73
**4344**	allXL	BC	373	319	DBSU	16.65
**4344**	allXL	DA	372	357	DBSU	35.28
**4344**	allXL	CB	381	357	DBSU	32.33
**4344**	allXL	BC	373	357	DBSU	36.27
**4344**	allXL	CB	372	370	DBSU	19.1
**4344**	allXL	CB	373	370	DBSU	15.27
**4344**	allXL	CB	382	373	DBSU	18.71
**4345**	allXL	AA	320	357	BS2G	24.83
**4345**	allXL	BB	320	373	BS2G	18.55
**4345**	allXL	DD	373	382	BS2G	17.45
**4345**	allXL	DD	370	386	BS2G	19.76
**4345**	allXL	DD	373	386	BS2G	21.3
**4345**	allXL	AA	319	357	BS2G	27.95
**4345**	allXL	BB	367	373	BS2G	11.6
**4345**	allXL	CC	370	382	BS2G	18.0
**4345**	allXL	CC	376	382	BS2G	14.45
**4345**	allXL	BB	357	373	BS2G	29.85
**4345**	allXL	AA	320	366	BS2G	17.2
**4345**	allXL	CC	370	376	BS2G	15.37
**4345**	allXL	AA	320	357	DBSU	24.83
**4345**	allXL	AA	357	319	DBSU	27.95
**4345**	allXL	BB	372	319	DBSU	16.66
**4345**	allXL	DD	370	357	DBSU	25.98
**4345**	allXL	BB	382	357	DBSU	20.08
**4345**	allXL	AA	372	370	DBSU	6.21

**4345**	allXL	CC	381	370	DBSU	19.68
**4345**	allXL	CC	373	370	DBSU	9.03
**4345**	allXL	DD	381	372	DBSU	14.58
**4345**	allXL	DD	372	382	DBSU	15.05
**4345**	allXL	DD	372	386	DBSU	18.02
**4345**	allXL	DD	373	386	DBSU	21.3
**4345**	allXL	AD	381	386	DBSU	27.01
**4345**	allXL	BD;DB	357	357	DBSU	18.9
**4345**	allXL	DA	373	319	DBSU	23.17
**4345**	allXL	DA	372	357	DBSU	43.64
**4345**	allXL	CA	381	357	DBSU	34.87
**4345**	allXL	DA	373	357	DBSU	40.18
**4345**	allXL	DA	372	370	DBSU	26.19
**4345**	allXL	DA	373	370	DBSU	23.94
**4345**	allXL	DA	382	373	DBSU	19.99
**4346**	allXL	AA	320	357	BS2G	23.56
**4346**	allXL	CC	320	373	BS2G	9.52
**4346**	allXL	BB	373	382	BS2G	18.27
**4346**	allXL	CC	370	386	BS2G	22.61
**4346**	allXL	BB	373	386	BS2G	22.17
**4346**	allXL	AA	319	357	BS2G	22.57
**4346**	allXL	BB	367	373	BS2G	13.93
**4346**	allXL	CC	370	382	BS2G	21.74
**4346**	allXL	CC	376	382	BS2G	14.18
**4346**	allXL	DD	357	373	BS2G	32.93
**4346**	allXL	CC	320	366	BS2G	19.29
**4346**	allXL	CC	370	376	BS2G	17.04
**4346**	allXL	AA	320	357	DBSU	23.56
**4346**	allXL	AA	357	319	DBSU	22.57
**4346**	allXL	CC	372	319	DBSU	11.69
**4346**	allXL	DD	370	357	DBSU	27.31
**4346**	allXL	BB	382	357	DBSU	39.37
**4346**	allXL	AA	372	370	DBSU	6.03
**4346**	allXL	CC	381	370	DBSU	24.19

**4346**	allXL	DD	373	370	DBSU	9.09
**4346**	allXL	BB	381	372	DBSU	18.87
**4346**	allXL	BB	372	382	DBSU	20.47
**4346**	allXL	BB	372	386	DBSU	23.06
**4346**	allXL	BB	373	386	DBSU	22.17
**4346**	allXL	DA	381	386	DBSU	58.33
**4346**	allXL	BD;DB	357	357	DBSU	19.36
**4346**	allXL	DA	373	319	DBSU	27.28
**4346**	allXL	DA	372	357	DBSU	43.07
**4346**	allXL	DA	381	357	DBSU	35.64
**4346**	allXL	DA	373	357	DBSU	40.66
**4346**	allXL	CB	372	370	DBSU	43.82
**4346**	allXL	DA	373	370	DBSU	43.26
**4346**	allXL	DA	382	373	DBSU	39.78
**4347**	allXL	AA	320	357	BS2G	23.08
**4347**	allXL	BB	320	373	BS2G	14.48
**4347**	allXL	CC	373	382	BS2G	17.62
**4347**	allXL	CC	370	386	BS2G	25.33
**4347**	allXL	CC	373	386	BS2G	22.33
**4347**	allXL	CC	319	357	BS2G	25.14
**4347**	allXL	BB	367	373	BS2G	14.2
**4347**	allXL	AA	370	382	BS2G	21.94
**4347**	allXL	CC	376	382	BS2G	14.7
**4347**	allXL	BB	357	373	BS2G	18.36
**4347**	allXL	DD	320	366	BS2G	19.63
**4347**	allXL	DD	370	376	BS2G	16.5
**4347**	allXL	AA	320	357	DBSU	23.08
**4347**	allXL	CC	357	319	DBSU	25.14
**4347**	allXL	BB	372	319	DBSU	17.8
**4347**	allXL	AA	370	357	DBSU	23.13
**4347**	allXL	BB	382	357	DBSU	32.58
**4347**	allXL	AA	372	370	DBSU	6.15
**4347**	allXL	DD	381	370	DBSU	23.72
**4347**	allXL	CC	373	370	DBSU	9.18

**4347**	allXL	AA	381	372	DBSU	21.28
**4347**	allXL	AA	372	382	DBSU	19.68
**4347**	allXL	CC	372	386	DBSU	24.22
**4347**	allXL	CC	373	386	DBSU	22.33
**4347**	allXL	CB	381	386	DBSU	53.85
**4347**	allXL	BD;DB	357	357	DBSU	18.67
**4347**	allXL	AD	373	319	DBSU	29.3
**4347**	allXL	AC	372	357	DBSU	23.5
**4347**	allXL	BD	381	357	DBSU	42.09
**4347**	allXL	AC	373	357	DBSU	23.23
**4347**	allXL	CB	372	370	DBSU	44.51
**4347**	allXL	CB	373	370	DBSU	47.7
**4347**	allXL	BC	382	373	DBSU	41.58
**4348**	allXL	AA	320	357	BS2G	22.96
**4348**	allXL	BB	320	373	BS2G	16.88
**4348**	allXL	CC	373	382	BS2G	21.06
**4348**	allXL	DD	370	386	BS2G	29.29
**4348**	allXL	AA	373	386	BS2G	28.26
**4348**	allXL	AA	319	357	BS2G	21.99
**4348**	allXL	AA	367	373	BS2G	12.83
**4348**	allXL	CC	370	382	BS2G	21.52
**4348**	allXL	CC	376	382	BS2G	14.93
**4348**	allXL	BB	357	373	BS2G	19.68
**4348**	allXL	DD	320	366	BS2G	18.83
**4348**	allXL	CC	370	376	BS2G	13.77
**4348**	allXL	AA	320	357	DBSU	22.96
**4348**	allXL	AA	357	319	DBSU	21.99
**4348**	allXL	BB	372	319	DBSU	23.48
**4348**	allXL	CC	370	357	DBSU	21.04
**4348**	allXL	BB	382	357	DBSU	16.24
**4348**	allXL	AA	372	370	DBSU	6.31
**4348**	allXL	CC	381	370	DBSU	22.1
**4348**	allXL	CC	373	370	DBSU	9.13
**4348**	allXL	CC	381	372	DBSU	22.67

**4348**	allXL	CC	372	382	DBSU	23.14
**4348**	allXL	AA	372	386	DBSU	27.36
**4348**	allXL	AA	373	386	DBSU	28.26
**4348**	allXL	DB	381	386	DBSU	16.89
**4348**	allXL	BD;DB	357	357	DBSU	16.96
**4348**	allXL	AD	373	319	DBSU	23.83
**4348**	allXL	AC	372	357	DBSU	28.98
**4348**	allXL	DB	381	357	DBSU	27.06
**4348**	allXL	AC	373	357	DBSU	30.87
**4348**	allXL	CA	372	370	DBSU	14.39
**4348**	allXL	CA	373	370	DBSU	13.51
**4348**	allXL	AC	382	373	DBSU	34.88
**4349**	allXL	AA	320	357	BS2G	11.35
**4349**	allXL	CC	320	373	BS2G	29.56
**4349**	allXL	BB	373	382	BS2G	19.14
**4349**	allXL	DD	370	386	BS2G	24.52
**4349**	allXL	AA	373	386	BS2G	22.45
**4349**	allXL	AA	319	357	BS2G	14.46
**4349**	allXL	AA	367	373	BS2G	11.24
**4349**	allXL	DD	370	382	BS2G	21.53
**4349**	allXL	BB	376	382	BS2G	14.73
**4349**	allXL	AA	357	373	BS2G	28.41
**4349**	allXL	CC	320	366	BS2G	26.52
**4349**	allXL	CC	370	376	BS2G	16.16
**4349**	allXL	AA	320	357	DBSU	11.35
**4349**	allXL	AA	357	319	DBSU	14.46
**4349**	allXL	DD	372	319	DBSU	26.11
**4349**	allXL	DD	370	357	DBSU	26.54
**4349**	allXL	DD	382	357	DBSU	28.07
**4349**	allXL	CC	372	370	DBSU	6.65
**4349**	allXL	BB	381	370	DBSU	21.09
**4349**	allXL	AA	373	370	DBSU	9.03
**4349**	allXL	BB	381	372	DBSU	20.3
**4349**	allXL	BB	372	382	DBSU	21.85

**4349**	allXL	AA	372	386	DBSU	25.73
**4349**	allXL	AA	373	386	DBSU	22.45
**4349**	allXL	BC	381	386	DBSU	36.55
**4349**	allXL	BD;DB	357	357	DBSU	18.24
**4349**	allXL	DA	373	319	DBSU	23.53
**4349**	allXL	DA	372	357	DBSU	33.55
**4349**	allXL	DB	381	357	DBSU	31.72
**4349**	allXL	DA	373	357	DBSU	32.24
**4349**	allXL	CB	372	370	DBSU	22.14
**4349**	allXL	CB	373	370	DBSU	23.88
**4349**	allXL	BC	382	373	DBSU	27.05
**4350**	allXL	AA	320	357	BS2G	26.37
**4350**	allXL	CC	320	373	BS2G	15.22
**4350**	allXL	AA	373	382	BS2G	17.65
**4350**	allXL	DD	370	386	BS2G	21.74
**4350**	allXL	AA	373	386	BS2G	23.78
**4350**	allXL	AA	319	357	BS2G	26.22
**4350**	allXL	BB	367	373	BS2G	12.21
**4350**	allXL	DD	370	382	BS2G	16.24
**4350**	allXL	CC	376	382	BS2G	13.9
**4350**	allXL	BB	357	373	BS2G	28.03
**4350**	allXL	AA	320	366	BS2G	22.31
**4350**	allXL	CC	370	376	BS2G	14.75
**4350**	allXL	AA	320	357	DBSU	26.37
**4350**	allXL	AA	357	319	DBSU	26.22
**4350**	allXL	CC	372	319	DBSU	16.35
**4350**	allXL	DD	370	357	DBSU	23.61
**4350**	allXL	BB	382	357	DBSU	30.49
**4350**	allXL	AA	372	370	DBSU	6.47
**4350**	allXL	DD	381	370	DBSU	14.96
**4350**	allXL	AA	373	370	DBSU	8.04
**4350**	allXL	DD	381	372	DBSU	16.4
**4350**	allXL	AA	372	382	DBSU	18.08
**4350**	allXL	AA	372	386	DBSU	22.33

**4350**	allXL	AA	373	386	DBSU	23.78
**4350**	allXL	BD	381	386	DBSU	51.32
**4350**	allXL	BD;DB	357	357	DBSU	18.16
**4350**	allXL	DA	373	319	DBSU	27.35
**4350**	allXL	DA	372	357	DBSU	39.54
**4350**	allXL	BD	381	357	DBSU	39.78
**4350**	allXL	DA	373	357	DBSU	40.42
**4350**	allXL	DA	372	370	DBSU	27.04
**4350**	allXL	DA	373	370	DBSU	24.55
**4350**	allXL	DA	382	373	DBSU	42.39
**4351**	allXL	CC	320	357	BS2G	22.46
**4351**	allXL	DD	320	373	BS2G	17.07
**4351**	allXL	CC	373	382	BS2G	19.42
**4351**	allXL	DD	370	386	BS2G	30.38
**4351**	allXL	CC	373	386	BS2G	29.11
**4351**	allXL	CC	319	357	BS2G	21.39
**4351**	allXL	BB	367	373	BS2G	10.16
**4351**	allXL	DD	370	382	BS2G	22.98
**4351**	allXL	CC	376	382	BS2G	14.84
**4351**	allXL	DD	357	373	BS2G	27.24
**4351**	allXL	AA	320	366	BS2G	22.62
**4351**	allXL	BB	370	376	BS2G	15.22
**4351**	allXL	CC	320	357	DBSU	22.46
**4351**	allXL	CC	357	319	DBSU	21.39
**4351**	allXL	DD	372	319	DBSU	24.05
**4351**	allXL	DD	370	357	DBSU	26.3
**4351**	allXL	DD	382	357	DBSU	17.73
**4351**	allXL	AA	372	370	DBSU	6.34
**4351**	allXL	DD	381	370	DBSU	19.92
**4351**	allXL	BB	373	370	DBSU	9.21
**4351**	allXL	CC	381	372	DBSU	21.63
**4351**	allXL	CC	372	382	DBSU	21.5
**4351**	allXL	CC	372	386	DBSU	30.99
**4351**	allXL	CC	373	386	DBSU	29.11

**4351**	allXL	BC	381	386	DBSU	33.55
**4351**	allXL	BD;DB	357	357	DBSU	18.67
**4351**	allXL	CB	373	319	DBSU	29.76
**4351**	allXL	DB	372	357	DBSU	46.0
**4351**	allXL	DA	381	357	DBSU	33.24
**4351**	allXL	DB	373	357	DBSU	44.92
**4351**	allXL	BC	372	370	DBSU	43.3
**4351**	allXL	BC	373	370	DBSU	42.89
**4351**	allXL	CB	382	373	DBSU	25.38
**4352**	allXL	CC	320	357	BS2G	23.26
**4352**	allXL	DD	320	373	BS2G	14.11
**4352**	allXL	BB	373	382	BS2G	18.96
**4352**	allXL	DD	370	386	BS2G	19.64
**4352**	allXL	DD	373	386	BS2G	22.51
**4352**	allXL	CC	319	357	BS2G	21.15
**4352**	allXL	BB	367	373	BS2G	12.74
**4352**	allXL	AA	370	382	BS2G	20.85
**4352**	allXL	BB	376	382	BS2G	13.44
**4352**	allXL	BB	357	373	BS2G	20.37
**4352**	allXL	DD	320	366	BS2G	20.31
**4352**	allXL	CC	370	376	BS2G	17.05
**4352**	allXL	CC	320	357	DBSU	23.26
**4352**	allXL	CC	357	319	DBSU	21.15
**4352**	allXL	DD	372	319	DBSU	12.26
**4352**	allXL	DD	370	357	DBSU	18.61
**4352**	allXL	BB	382	357	DBSU	26.58
**4352**	allXL	AA	372	370	DBSU	6.15
**4352**	allXL	DD	381	370	DBSU	21.58
**4352**	allXL	AA	373	370	DBSU	9.17
**4352**	allXL	DD	381	372	DBSU	18.32
**4352**	allXL	AA	372	382	DBSU	18.06
**4352**	allXL	DD	372	386	DBSU	20.86
**4352**	allXL	DD	373	386	DBSU	22.51
**4352**	allXL	AD	381	386	DBSU	22.42

**4352**	allXL	BD;DB	357	357	DBSU	17.42
**4352**	allXL	AD	373	319	DBSU	15.51
**4352**	allXL	CA	372	357	DBSU	35.87
**4352**	allXL	BD	381	357	DBSU	32.62
**4352**	allXL	CA	373	357	DBSU	34.83
**4352**	allXL	DA	372	370	DBSU	21.07
**4352**	allXL	DA	373	370	DBSU	24.71
**4352**	allXL	AD	382	373	DBSU	25.28
**4353**	allXL	CC	320	357	BS2G	26.03
**4353**	allXL	CC	320	373	BS2G	14.49
**4353**	allXL	BB	373	382	BS2G	19.66
**4353**	allXL	DD	370	386	BS2G	29.57
**4353**	allXL	CC	373	386	BS2G	25.12
**4353**	allXL	BB	319	357	BS2G	25.82
**4353**	allXL	AA	367	373	BS2G	12.38
**4353**	allXL	BB	370	382	BS2G	25.05
**4353**	allXL	CC	376	382	BS2G	13.65
**4353**	allXL	CC	357	373	BS2G	32.63
**4353**	allXL	AA	320	366	BS2G	19.5
**4353**	allXL	CC	370	376	BS2G	15.67
**4353**	allXL	CC	320	357	DBSU	26.03
**4353**	allXL	BB	357	319	DBSU	25.82
**4353**	allXL	CC	372	319	DBSU	12.24
**4353**	allXL	DD	370	357	DBSU	24.45
**4353**	allXL	CC	382	357	DBSU	39.48
**4353**	allXL	AA	372	370	DBSU	6.3
**4353**	allXL	BB	381	370	DBSU	23.82
**4353**	allXL	AA	373	370	DBSU	9.01
**4353**	allXL	AA	381	372	DBSU	20.71
**4353**	allXL	AA	372	382	DBSU	21.29
**4353**	allXL	CC	372	386	DBSU	28.69
**4353**	allXL	CC	373	386	DBSU	25.12
**4353**	allXL	BC	381	386	DBSU	46.41
**4353**	allXL	AC;CA	357	357	DBSU	19.49

**4353**	allXL	AD	373	319	DBSU	40.7
**4353**	allXL	DB	372	357	DBSU	48.94
**4353**	allXL	CB	381	357	DBSU	32.88
**4353**	allXL	CB	373	357	DBSU	49.42
**4353**	allXL	CB	372	370	DBSU	45.79
**4353**	allXL	CB	373	370	DBSU	42.28
**4353**	allXL	CB	382	373	DBSU	38.25
**4354**	allXL	AA	320	357	BS2G	26.62
**4354**	allXL	BB	320	373	BS2G	19.11
**4354**	allXL	DD	373	382	BS2G	20.75
**4354**	allXL	DD	370	386	BS2G	20.5
**4354**	allXL	DD	373	386	BS2G	24.76
**4354**	allXL	DD	319	357	BS2G	27.8
**4354**	allXL	AA	367	373	BS2G	12.55
**4354**	allXL	DD	370	382	BS2G	20.93
**4354**	allXL	CC	376	382	BS2G	15.13
**4354**	allXL	BB	357	373	BS2G	25.49
**4354**	allXL	AA	320	366	BS2G	22.45
**4354**	allXL	CC	370	376	BS2G	16.17
**4354**	allXL	AA	320	357	DBSU	26.62
**4354**	allXL	DD	357	319	DBSU	27.8
**4354**	allXL	DD	372	319	DBSU	21.34
**4354**	allXL	BB	370	357	DBSU	23.63
**4354**	allXL	BB	382	357	DBSU	31.58
**4354**	allXL	CC	372	370	DBSU	6.33
**4354**	allXL	DD	381	370	DBSU	21.24
**4354**	allXL	AA	373	370	DBSU	9.1
**4354**	allXL	DD	381	372	DBSU	20.22
**4354**	allXL	DD	372	382	DBSU	21.14
**4354**	allXL	DD	372	386	DBSU	24.5
**4354**	allXL	DD	373	386	DBSU	24.76
**4354**	allXL	AD	381	386	DBSU	60.9
**4354**	allXL	BD;DB	357	357	DBSU	16.7
**4354**	allXL	CA	373	319	DBSU	17.82

**4354**	allXL	CA	372	357	DBSU	24.07
**4354**	allXL	AC;AD	381	357	DBSU	45.83
**4354**	allXL	CA	373	357	DBSU	27.39
**4354**	allXL	DA	372	370	DBSU	22.49
**4354**	allXL	DA	373	370	DBSU	23.68
**4354**	allXL	AD	382	373	DBSU	42.13
**4355**	allXL	AA	320	357	BS2G	24.94
**4355**	allXL	CC	320	373	BS2G	21.65
**4355**	allXL	BB	373	382	BS2G	19.03
**4355**	allXL	BB	370	386	BS2G	17.47
**4355**	allXL	BB	373	386	BS2G	20.36
**4355**	allXL	AA	319	357	BS2G	27.03
**4355**	allXL	AA	367	373	BS2G	12.9
**4355**	allXL	DD	370	382	BS2G	18.74
**4355**	allXL	BB	376	382	BS2G	13.26
**4355**	allXL	BB	357	373	BS2G	20.28
**4355**	allXL	CC	320	366	BS2G	25.3
**4355**	allXL	CC	370	376	BS2G	14.25
**4355**	allXL	AA	320	357	DBSU	24.94
**4355**	allXL	AA	357	319	DBSU	27.03
**4355**	allXL	CC	372	319	DBSU	19.3
**4355**	allXL	CC	370	357	DBSU	23.82
**4355**	allXL	BB	382	357	DBSU	26.74
**4355**	allXL	AA	372	370	DBSU	6.43
**4355**	allXL	DD	381	370	DBSU	19.61
**4355**	allXL	AA	373	370	DBSU	8.62
**4355**	allXL	DD	381	372	DBSU	21.06
**4355**	allXL	DD	372	382	DBSU	21.47
**4355**	allXL	BB	372	386	DBSU	21.17
**4355**	allXL	BB	373	386	DBSU	20.36
**4355**	allXL	AD	381	386	DBSU	66.26
**4355**	allXL	BD;DB	357	357	DBSU	17.15
**4355**	allXL	AD	373	319	DBSU	26.53
**4355**	allXL	BD	372	357	DBSU	34.54

**4355**	allXL	BD	381	357	DBSU	41.88
**4355**	allXL	BD	373	357	DBSU	33.14
**4355**	allXL	AD	372	370	DBSU	55.11
**4355**	allXL	CA	373	370	DBSU	54.85
**4355**	allXL	DB	382	373	DBSU	55.87
**4356**	allXL	CC	320	357	BS2G	26.26
**4356**	allXL	BB	320	373	BS2G	27.79
**4356**	allXL	CC	373	382	BS2G	17.78
**4356**	allXL	DD	370	386	BS2G	27.92
**4356**	allXL	CC	373	386	BS2G	27.15
**4356**	allXL	AA	319	357	BS2G	27.09
**4356**	allXL	AA	367	373	BS2G	12.72
**4356**	allXL	DD	370	382	BS2G	21.72
**4356**	allXL	BB	376	382	BS2G	15.28
**4356**	allXL	CC	357	373	BS2G	23.17
**4356**	allXL	DD	320	366	BS2G	18.81
**4356**	allXL	BB	370	376	BS2G	17.2
**4356**	allXL	CC	320	357	DBSU	26.26
**4356**	allXL	AA	357	319	DBSU	27.09
**4356**	allXL	DD	372	319	DBSU	26.32
**4356**	allXL	CC	370	357	DBSU	23.33
**4356**	allXL	CC	382	357	DBSU	20.02
**4356**	allXL	AA	372	370	DBSU	6.27
**4356**	allXL	DD	381	370	DBSU	22.02
**4356**	allXL	CC	373	370	DBSU	8.95
**4356**	allXL	CC	381	372	DBSU	20.62
**4356**	allXL	CC	372	382	DBSU	19.35
**4356**	allXL	CC	372	386	DBSU	27.55
**4356**	allXL	CC	373	386	DBSU	27.15
**4356**	allXL	CB	381	386	DBSU	58.39
**4356**	allXL	BD;DB	357	357	DBSU	18.96
**4356**	allXL	CB	373	319	DBSU	21.36
**4356**	allXL	CA	372	357	DBSU	32.32
**4356**	allXL	CA	381	357	DBSU	18.31

**4356**	allXL	CA	373	357	DBSU	30.31
**4356**	allXL	BC	372	370	DBSU	31.5
**4356**	allXL	BC	373	370	DBSU	31.57
**4356**	allXL	BC	382	373	DBSU	37.22
**4357**	allXL	AA	320	357	BS2G	22.98
**4357**	allXL	DD	320	373	BS2G	24.78
**4357**	allXL	DD	373	382	BS2G	15.78
**4357**	allXL	DD	370	386	BS2G	12.41
**4357**	allXL	DD	373	386	BS2G	19.57
**4357**	allXL	AA	319	357	BS2G	22.12
**4357**	allXL	BB	367	373	BS2G	12.69
**4357**	allXL	DD	370	382	BS2G	14.33
**4357**	allXL	CC	376	382	BS2G	14.83
**4357**	allXL	DD	357	373	BS2G	22.07
**4357**	allXL	BB	320	366	BS2G	23.92
**4357**	allXL	CC	370	376	BS2G	14.22
**4357**	allXL	AA	320	357	DBSU	22.98
**4357**	allXL	AA	357	319	DBSU	22.12
**4357**	allXL	DD	372	319	DBSU	24.79
**4357**	allXL	DD	370	357	DBSU	19.64
**4357**	allXL	DD	382	357	DBSU	23.41
**4357**	allXL	AA	372	370	DBSU	6.19
**4357**	allXL	DD	381	370	DBSU	14.74
**4357**	allXL	CC	373	370	DBSU	9.23
**4357**	allXL	DD	381	372	DBSU	15.23
**4357**	allXL	DD	372	382	DBSU	16.2
**4357**	allXL	DD	372	386	DBSU	18.81
**4357**	allXL	DD	373	386	DBSU	19.57
**4357**	allXL	AD	381	386	DBSU	28.5
**4357**	allXL	BD;DB	357	357	DBSU	19.18
**4357**	allXL	CB	373	319	DBSU	22.29
**4357**	allXL	CB	372	357	DBSU	38.55
**4357**	allXL	DB	381	357	DBSU	36.0
**4357**	allXL	DB	373	357	DBSU	37.0

**4357**	allXL	CB	372	370	DBSU	35.47
**4357**	allXL	CB	373	370	DBSU	34.02
**4357**	allXL	DA	382	373	DBSU	34.07
**4358**	allXL	AA	320	357	BS2G	25.1
**4358**	allXL	BB	320	373	BS2G	23.99
**4358**	allXL	BB	373	382	BS2G	19.43
**4358**	allXL	DD	370	386	BS2G	24.18
**4358**	allXL	CC	373	386	BS2G	21.19
**4358**	allXL	AA	319	357	BS2G	25.28
**4358**	allXL	AA	367	373	BS2G	9.67
**4358**	allXL	AA	370	382	BS2G	17.76
**4358**	allXL	CC	376	382	BS2G	13.96
**4358**	allXL	AA	357	373	BS2G	30.58
**4358**	allXL	BB	320	366	BS2G	24.15
**4358**	allXL	AA	370	376	BS2G	14.24
**4358**	allXL	AA	320	357	DBSU	25.1
**4358**	allXL	AA	357	319	DBSU	25.28
**4358**	allXL	BB	372	319	DBSU	23.26
**4358**	allXL	BB	370	357	DBSU	25.19
**4358**	allXL	BB	382	357	DBSU	40.43
**4358**	allXL	CC	372	370	DBSU	6.5
**4358**	allXL	AA	381	370	DBSU	14.66
**4358**	allXL	AA	373	370	DBSU	8.21
**4358**	allXL	AA	381	372	DBSU	18.56
**4358**	allXL	BB	372	382	DBSU	22.07
**4358**	allXL	CC	372	386	DBSU	23.04
**4358**	allXL	CC	373	386	DBSU	21.19
**4358**	allXL	AD	381	386	DBSU	32.15
**4358**	allXL	BD;DB	357	357	DBSU	17.3
**4358**	allXL	CB	373	319	DBSU	8.58
**4358**	allXL	CB	372	357	DBSU	37.43
**4358**	allXL	CB	381	357	DBSU	38.24
**4358**	allXL	CB	373	357	DBSU	34.12
**4358**	allXL	CB	372	370	DBSU	18.28

**4358**	allXL	CB	373	370	DBSU	15.15
**4358**	allXL	CB	382	373	DBSU	25.65
**4359**	allXL	AA	320	357	BS2G	26.26
**4359**	allXL	BB	320	373	BS2G	20.27
**4359**	allXL	DD	373	382	BS2G	17.63
**4359**	allXL	CC	370	386	BS2G	20.09
**4359**	allXL	DD	373	386	BS2G	20.84
**4359**	allXL	AA	319	357	BS2G	27.21
**4359**	allXL	AA	367	373	BS2G	12.21
**4359**	allXL	CC	370	382	BS2G	15.41
**4359**	allXL	CC	376	382	BS2G	13.74
**4359**	allXL	AA	357	373	BS2G	32.46
**4359**	allXL	AA	320	366	BS2G	20.56
**4359**	allXL	CC	370	376	BS2G	14.45
**4359**	allXL	AA	320	357	DBSU	26.26
**4359**	allXL	AA	357	319	DBSU	27.21
**4359**	allXL	BB	372	319	DBSU	21.35
**4359**	allXL	DD	370	357	DBSU	27.58
**4359**	allXL	CC	382	357	DBSU	18.24
**4359**	allXL	AA	372	370	DBSU	6.42
**4359**	allXL	CC	381	370	DBSU	18.26
**4359**	allXL	AA	373	370	DBSU	9.16
**4359**	allXL	DD	381	372	DBSU	17.81
**4359**	allXL	DD	372	382	DBSU	16.66
**4359**	allXL	DD	372	386	DBSU	17.89
**4359**	allXL	DD	373	386	DBSU	20.84
**4359**	allXL	BC	381	386	DBSU	48.03
**4359**	allXL	BD;DB	357	357	DBSU	18.93
**4359**	allXL	AD	373	319	DBSU	35.59
**4359**	allXL	DA	372	357	DBSU	45.76
**4359**	allXL	CA	381	357	DBSU	34.79
**4359**	allXL	CB	373	357	DBSU	47.28
**4359**	allXL	DA	372	370	DBSU	29.14
**4359**	allXL	DA	373	370	DBSU	31.58

**4359**	allXL	DA	382	373	DBSU	31.62
**4360**	allXL	AA	320	357	BS2G	22.88
**4360**	allXL	BB	320	373	BS2G	21.87
**4360**	allXL	BB	373	382	BS2G	18.77
**4360**	allXL	BB	370	386	BS2G	26.29
**4360**	allXL	BB	373	386	BS2G	25.91
**4360**	allXL	AA	319	357	BS2G	21.8
**4360**	allXL	AA	367	373	BS2G	13.28
**4360**	allXL	BB	370	382	BS2G	23.4
**4360**	allXL	BB	376	382	BS2G	12.88
**4360**	allXL	BB	357	373	BS2G	26.46
**4360**	allXL	DD	320	366	BS2G	24.24
**4360**	allXL	DD	370	376	BS2G	14.06
**4360**	allXL	AA	320	357	DBSU	22.88
**4360**	allXL	AA	357	319	DBSU	21.8
**4360**	allXL	BB	372	319	DBSU	27.46
**4360**	allXL	BB	370	357	DBSU	30.87
**4360**	allXL	BB	382	357	DBSU	41.1
**4360**	allXL	AA	372	370	DBSU	6.16
**4360**	allXL	DD	381	370	DBSU	23.01
**4360**	allXL	CC	373	370	DBSU	9.07
**4360**	allXL	CC	381	372	DBSU	22.14
**4360**	allXL	CC	372	382	DBSU	20.51
**4360**	allXL	CC	372	386	DBSU	25.72
**4360**	allXL	BB	373	386	DBSU	25.91
**4360**	allXL	BC	381	386	DBSU	66.96
**4360**	allXL	BD;DB	357	357	DBSU	18.92
**4360**	allXL	AD	373	319	DBSU	33.47
**4360**	allXL	BD	372	357	DBSU	44.15
**4360**	allXL	BD	381	357	DBSU	59.01
**4360**	allXL	CA	373	357	DBSU	44.76
**4360**	allXL	BC	372	370	DBSU	48.88
**4360**	allXL	BC	373	370	DBSU	47.35
**4360**	allXL	BC	382	373	DBSU	60.48

**4361**	allXL	AA	320	357	BS2G	20.19
**4361**	allXL	DD	320	373	BS2G	23.44
**4361**	allXL	CC	373	382	BS2G	19.02
**4361**	allXL	CC	370	386	BS2G	21.38
**4361**	allXL	CC	373	386	BS2G	23.31
**4361**	allXL	AA	319	357	BS2G	17.73
**4361**	allXL	AA	367	373	BS2G	12.13
**4361**	allXL	CC	370	382	BS2G	22.16
**4361**	allXL	CC	376	382	BS2G	12.61
**4361**	allXL	DD	357	373	BS2G	24.24
**4361**	allXL	BB	320	366	BS2G	18.31
**4361**	allXL	DD	370	376	BS2G	14.7
**4361**	allXL	AA	320	357	DBSU	20.19
**4361**	allXL	AA	357	319	DBSU	17.73
**4361**	allXL	BB	372	319	DBSU	24.74
**4361**	allXL	DD	370	357	DBSU	28.37
**4361**	allXL	DD	382	357	DBSU	44.87
**4361**	allXL	AA	372	370	DBSU	6.28
**4361**	allXL	DD	381	370	DBSU	22.53
**4361**	allXL	AA	373	370	DBSU	9.17
**4361**	allXL	DD	381	372	DBSU	21.11
**4361**	allXL	CC	372	382	DBSU	20.27
**4361**	allXL	CC	372	386	DBSU	22.41
**4361**	allXL	CC	373	386	DBSU	23.31
**4361**	allXL	AD	381	386	DBSU	51.33
**4361**	allXL	BD;DB	357	357	DBSU	18.77
**4361**	allXL	AD	373	319	DBSU	23.32
**4361**	allXL	DB	372	357	DBSU	42.86
**4361**	allXL	CA	381	357	DBSU	59.15
**4361**	allXL	DB	373	357	DBSU	42.98
**4361**	allXL	DA	372	370	DBSU	40.39
**4361**	allXL	DA	373	370	DBSU	36.8
**4361**	allXL	DA	382	373	DBSU	41.72
**4362**	allXL	AA	320	357	BS2G	23.85

**4362**	allXL	CC	320	373	BS2G	17.86
**4362**	allXL	AA	373	382	BS2G	17.64
**4362**	allXL	CC	370	386	BS2G	23.78
**4362**	allXL	AA	373	386	BS2G	23.44
**4362**	allXL	AA	319	357	BS2G	22.8
**4362**	allXL	BB	367	373	BS2G	12.9
**4362**	allXL	CC	370	382	BS2G	17.55
**4362**	allXL	AA	376	382	BS2G	13.27
**4362**	allXL	BB	357	373	BS2G	28.25
**4362**	allXL	BB	320	366	BS2G	22.96
**4362**	allXL	CC	370	376	BS2G	12.69
**4362**	allXL	AA	320	357	DBSU	23.85
**4362**	allXL	AA	357	319	DBSU	22.8
**4362**	allXL	CC	372	319	DBSU	16.49
**4362**	allXL	CC	370	357	DBSU	27.15
**4362**	allXL	CC	382	357	DBSU	27.02
**4362**	allXL	AA	372	370	DBSU	6.19
**4362**	allXL	CC	381	370	DBSU	17.02
**4362**	allXL	AA	373	370	DBSU	9.1
**4362**	allXL	DD	381	372	DBSU	15.03
**4362**	allXL	DD	372	382	DBSU	17.32
**4362**	allXL	DD	372	386	DBSU	24.91
**4362**	allXL	AA	373	386	DBSU	23.44
**4362**	allXL	CB	381	386	DBSU	44.46
**4362**	allXL	BD;DB	357	357	DBSU	19.12
**4362**	allXL	DA	373	319	DBSU	29.78
**4362**	allXL	DA	372	357	DBSU	45.68
**4362**	allXL	AC	381	357	DBSU	39.41
**4362**	allXL	DA	373	357	DBSU	41.95
**4362**	allXL	CB	372	370	DBSU	45.28
**4362**	allXL	CB	373	370	DBSU	42.73
**4362**	allXL	CB	382	373	DBSU	38.11
**4363**	allXL	CC	320	357	BS2G	22.97
**4363**	allXL	AA	320	373	BS2G	30.22

**4363**	allXL	CC	373	382	BS2G	19.41
**4363**	allXL	CC	370	386	BS2G	22.6
**4363**	allXL	CC	373	386	BS2G	24.88
**4363**	allXL	CC	319	357	BS2G	22.34
**4363**	allXL	AA	367	373	BS2G	11.57
**4363**	allXL	CC	370	382	BS2G	20.35
**4363**	allXL	CC	376	382	BS2G	13.88
**4363**	allXL	BB	357	373	BS2G	29.88
**4363**	allXL	DD	320	366	BS2G	25.8
**4363**	allXL	CC	370	376	BS2G	15.6
**4363**	allXL	CC	320	357	DBSU	22.97
**4363**	allXL	CC	357	319	DBSU	22.34
**4363**	allXL	AA	372	319	DBSU	30.12
**4363**	allXL	DD	370	357	DBSU	32.23
**4363**	allXL	AA	382	357	DBSU	30.42
**4363**	allXL	AA	372	370	DBSU	6.57
**4363**	allXL	CC	381	370	DBSU	22.78
**4363**	allXL	DD	373	370	DBSU	8.89
**4363**	allXL	CC	381	372	DBSU	21.8
**4363**	allXL	CC	372	382	DBSU	19.5
**4363**	allXL	CC	372	386	DBSU	22.98
**4363**	allXL	CC	373	386	DBSU	24.88
**4363**	allXL	DA	381	386	DBSU	60.05
**4363**	allXL	BD;DB	357	357	DBSU	18.5
**4363**	allXL	AD	373	319	DBSU	23.68
**4363**	allXL	BD	372	357	DBSU	45.59
**4363**	allXL	AC	381	357	DBSU	38.59
**4363**	allXL	BD	373	357	DBSU	45.98
**4363**	allXL	DA	372	370	DBSU	41.33
**4363**	allXL	DA	373	370	DBSU	43.27
**4363**	allXL	AD	382	373	DBSU	55.52
**4364**	allXL	AA	320	357	BS2G	21.03
**4364**	allXL	DD	320	373	BS2G	18.43
**4364**	allXL	DD	373	382	BS2G	13.98

**4364**	allXL	DD	370	386	BS2G	16.32
**4364**	allXL	DD	373	386	BS2G	19.33
**4364**	allXL	AA	319	357	BS2G	24.61
**4364**	allXL	AA	367	373	BS2G	14.0
**4364**	allXL	DD	370	382	BS2G	11.75
**4364**	allXL	CC	376	382	BS2G	13.55
**4364**	allXL	DD	357	373	BS2G	16.12
**4364**	allXL	BB	320	366	BS2G	23.73
**4364**	allXL	CC	370	376	BS2G	14.89
**4364**	allXL	AA	320	357	DBSU	21.03
**4364**	allXL	AA	357	319	DBSU	24.61
**4364**	allXL	DD	372	319	DBSU	20.45
**4364**	allXL	DD	370	357	DBSU	21.74
**4364**	allXL	BB	382	357	DBSU	19.43
**4364**	allXL	AA	372	370	DBSU	6.25
**4364**	allXL	DD	381	370	DBSU	13.73
**4364**	allXL	AA	373	370	DBSU	9.04
**4364**	allXL	DD	381	372	DBSU	15.73
**4364**	allXL	DD	372	382	DBSU	13.83
**4364**	allXL	DD	372	386	DBSU	17.27
**4364**	allXL	DD	373	386	DBSU	19.33
**4364**	allXL	DB	381	386	DBSU	20.08
**4364**	allXL	BD;DB	357	357	DBSU	17.62
**4364**	allXL	AD	373	319	DBSU	22.7
**4364**	allXL	DB	372	357	DBSU	35.06
**4364**	allXL	BD	381	357	DBSU	24.63
**4364**	allXL	DB	373	357	DBSU	32.16
**4364**	allXL	CB	372	370	DBSU	43.81
**4364**	allXL	DA	373	370	DBSU	42.9
**4364**	allXL	BD	382	373	DBSU	28.11
**4365**	allXL	CC	320	357	BS2G	24.17
**4365**	allXL	BB	320	373	BS2G	18.66
**4365**	allXL	CC	373	382	BS2G	19.7
**4365**	allXL	CC	370	386	BS2G	21.79

**4365**	allXL	CC	373	386	BS2G	29.38
**4365**	allXL	AA	319	357	BS2G	25.14
**4365**	allXL	AA	367	373	BS2G	14.22
**4365**	allXL	CC	370	382	BS2G	15.03
**4365**	allXL	CC	376	382	BS2G	14.83
**4365**	allXL	BB	357	373	BS2G	25.36
**4365**	allXL	AA	320	366	BS2G	21.01
**4365**	allXL	CC	370	376	BS2G	11.94
**4365**	allXL	CC	320	357	DBSU	24.17
**4365**	allXL	AA	357	319	DBSU	25.14
**4365**	allXL	BB	372	319	DBSU	22.2
**4365**	allXL	BB	370	357	DBSU	27.26
**4365**	allXL	BB	382	357	DBSU	15.41
**4365**	allXL	AA	372	370	DBSU	6.14
**4365**	allXL	CC	381	370	DBSU	16.89
**4365**	allXL	CC	373	370	DBSU	8.98
**4365**	allXL	CC	381	372	DBSU	20.96
**4365**	allXL	CC	372	382	DBSU	20.01
**4365**	allXL	CC	372	386	DBSU	28.28
**4365**	allXL	CC	373	386	DBSU	29.38
**4365**	allXL	DB	381	386	DBSU	25.12
**4365**	allXL	BD;DB	357	357	DBSU	18.51
**4365**	allXL	AD	373	319	DBSU	24.76
**4365**	allXL	DB	372	357	DBSU	44.69
**4365**	allXL	BD	381	357	DBSU	32.27
**4365**	allXL	DB	373	357	DBSU	43.28
**4365**	allXL	AD	372	370	DBSU	43.03
**4365**	allXL	AD	373	370	DBSU	39.31
**4365**	allXL	AD	382	373	DBSU	36.92
**4366**	allXL	CC	320	357	BS2G	20.5
**4366**	allXL	BB	320	373	BS2G	28.06
**4366**	allXL	CC	373	382	BS2G	17.06
**4366**	allXL	CC	370	386	BS2G	22.61
**4366**	allXL	CC	373	386	BS2G	18.74

**4366**	allXL	CC	319	357	BS2G	22.0
**4366**	allXL	AA	367	373	BS2G	12.39
**4366**	allXL	CC	370	382	BS2G	22.94
**4366**	allXL	CC	376	382	BS2G	13.36
**4366**	allXL	DD	357	373	BS2G	30.23
**4366**	allXL	AA	320	366	BS2G	27.41
**4366**	allXL	DD	370	376	BS2G	17.54
**4366**	allXL	CC	320	357	DBSU	20.5
**4366**	allXL	CC	357	319	DBSU	22.0
**4366**	allXL	BB	372	319	DBSU	28.7
**4366**	allXL	DD	370	357	DBSU	24.33
**4366**	allXL	BB	382	357	DBSU	32.44
**4366**	allXL	AA	372	370	DBSU	6.34
**4366**	allXL	DD	381	370	DBSU	22.5
**4366**	allXL	AA	373	370	DBSU	9.02
**4366**	allXL	CC	381	372	DBSU	20.47
**4366**	allXL	CC	372	382	DBSU	17.75
**4366**	allXL	CC	372	386	DBSU	17.86
**4366**	allXL	CC	373	386	DBSU	18.74
**4366**	allXL	BC	381	386	DBSU	55.97
**4366**	allXL	BD;DB	357	357	DBSU	18.33
**4366**	allXL	DA	373	319	DBSU	24.0
**4366**	allXL	DB	372	357	DBSU	38.55
**4366**	allXL	BC	381	357	DBSU	35.92
**4366**	allXL	DA	373	357	DBSU	40.98
**4366**	allXL	DA	372	370	DBSU	48.36
**4366**	allXL	DA	373	370	DBSU	44.98
**4366**	allXL	DA	382	373	DBSU	42.7
**4367**	allXL	CC	320	357	BS2G	23.88
**4367**	allXL	DD	320	373	BS2G	23.15
**4367**	allXL	AA	373	382	BS2G	19.38
**4367**	allXL	DD	370	386	BS2G	23.89
**4367**	allXL	DD	373	386	BS2G	23.11
**4367**	allXL	AA	319	357	BS2G	25.42

**4367**	allXL	BB	367	373	BS2G	12.19
**4367**	allXL	DD	370	382	BS2G	24.99
**4367**	allXL	CC	376	382	BS2G	14.29
**4367**	allXL	BB	357	373	BS2G	24.93
**4367**	allXL	CC	320	366	BS2G	24.9
**4367**	allXL	CC	370	376	BS2G	16.94
**4367**	allXL	CC	320	357	DBSU	23.88
**4367**	allXL	AA	357	319	DBSU	25.42
**4367**	allXL	DD	372	319	DBSU	19.87
**4367**	allXL	DD	370	357	DBSU	22.29
**4367**	allXL	BB	382	357	DBSU	17.06
**4367**	allXL	CC	372	370	DBSU	6.96
**4367**	allXL	DD	381	370	DBSU	25.25
**4367**	allXL	AA	373	370	DBSU	9.19
**4367**	allXL	DD	381	372	DBSU	18.64
**4367**	allXL	DD	372	382	DBSU	19.02
**4367**	allXL	DD	372	386	DBSU	20.82
**4367**	allXL	DD	373	386	DBSU	23.11
**4367**	allXL	AD	381	386	DBSU	59.71
**4367**	allXL	BD;DB	357	357	DBSU	18.84
**4367**	allXL	CB	373	319	DBSU	35.85
**4367**	allXL	CB	372	357	DBSU	45.33
**4367**	allXL	BD	381	357	DBSU	28.24
**4367**	allXL	BD	373	357	DBSU	42.86
**4367**	allXL	CB	372	370	DBSU	28.03
**4367**	allXL	CB	373	370	DBSU	27.57
**4367**	allXL	DA	382	373	DBSU	49.7
**4368**	allXL	AA	320	357	BS2G	24.7
**4368**	allXL	CC	320	373	BS2G	28.68
**4368**	allXL	CC	373	382	BS2G	17.97
**4368**	allXL	CC	370	386	BS2G	19.88
**4368**	allXL	CC	373	386	BS2G	21.79
**4368**	allXL	AA	319	357	BS2G	27.48
**4368**	allXL	AA	367	373	BS2G	11.84

**4368**	allXL	CC	370	382	BS2G	21.03
**4368**	allXL	CC	376	382	BS2G	13.45
**4368**	allXL	BB	357	373	BS2G	20.5
**4368**	allXL	AA	320	366	BS2G	20.01
**4368**	allXL	CC	370	376	BS2G	15.1
**4368**	allXL	AA	320	357	DBSU	24.7
**4368**	allXL	AA	357	319	DBSU	27.48
**4368**	allXL	CC	372	319	DBSU	25.8
**4368**	allXL	BB	370	357	DBSU	27.03
**4368**	allXL	BB	382	357	DBSU	26.06
**4368**	allXL	AA	372	370	DBSU	6.45
**4368**	allXL	CC	381	370	DBSU	23.37
**4368**	allXL	AA	373	370	DBSU	8.69
**4368**	allXL	DD	381	372	DBSU	19.7
**4368**	allXL	DD	372	382	DBSU	20.63
**4368**	allXL	DD	372	386	DBSU	21.73
**4368**	allXL	CC	373	386	DBSU	21.79
**4368**	allXL	DA	381	386	DBSU	23.62
**4368**	allXL	BD;DB	357	357	DBSU	18.95
**4368**	allXL	AD	373	319	DBSU	29.95
**4368**	allXL	BD	372	357	DBSU	23.42
**4368**	allXL	BD	381	357	DBSU	15.36
**4368**	allXL	BD	373	357	DBSU	20.19
**4368**	allXL	AD	372	370	DBSU	37.33
**4368**	allXL	AD	373	370	DBSU	34.27
**4368**	allXL	AD	382	373	DBSU	20.41
**4369**	allXL	CC	320	357	BS2G	23.28
**4369**	allXL	BB	320	373	BS2G	21.97
**4369**	allXL	AA	373	382	BS2G	19.88
**4369**	allXL	CC	370	386	BS2G	23.61
**4369**	allXL	BB	373	386	BS2G	27.7
**4369**	allXL	CC	319	357	BS2G	25.07
**4369**	allXL	AA	367	373	BS2G	11.45
**4369**	allXL	CC	370	382	BS2G	17.28

**4369**	allXL	CC	376	382	BS2G	14.04
**4369**	allXL	BB	357	373	BS2G	20.48
**4369**	allXL	DD	320	366	BS2G	17.02
**4369**	allXL	CC	370	376	BS2G	11.76
**4369**	allXL	CC	320	357	DBSU	23.28
**4369**	allXL	CC	357	319	DBSU	25.07
**4369**	allXL	BB	372	319	DBSU	21.03
**4369**	allXL	BB	370	357	DBSU	16.19
**4369**	allXL	BB	382	357	DBSU	37.94
**4369**	allXL	AA	372	370	DBSU	6.61
**4369**	allXL	CC	381	370	DBSU	18.08
**4369**	allXL	AA	373	370	DBSU	8.61
**4369**	allXL	DD	381	372	DBSU	20.33
**4369**	allXL	CC	372	382	DBSU	20.65
**4369**	allXL	CC	372	386	DBSU	27.25
**4369**	allXL	BB	373	386	DBSU	27.7
**4369**	allXL	DB	381	386	DBSU	63.73
**4369**	allXL	BD;DB	357	357	DBSU	18.24
**4369**	allXL	CB	373	319	DBSU	12.81
**4369**	allXL	BD	372	357	DBSU	30.65
**4369**	allXL	BD	381	357	DBSU	40.76
**4369**	allXL	BD	373	357	DBSU	31.35
**4369**	allXL	CB	372	370	DBSU	35.29
**4369**	allXL	CB	373	370	DBSU	33.14
**4369**	allXL	CB	382	373	DBSU	51.12
**4370**	allXL	BB	320	357	BS2G	24.72
**4370**	allXL	DD	320	373	BS2G	31.72
**4370**	allXL	AA	373	382	BS2G	19.82
**4370**	allXL	CC	370	386	BS2G	21.57
**4370**	allXL	AA	373	386	BS2G	25.49
**4370**	allXL	AA	319	357	BS2G	26.05
**4370**	allXL	AA	367	373	BS2G	12.3
**4370**	allXL	CC	370	382	BS2G	18.55
**4370**	allXL	AA	376	382	BS2G	14.46

**4370**	allXL	CC	357	373	BS2G	22.89
**4370**	allXL	AA	320	366	BS2G	26.54
**4370**	allXL	CC	370	376	BS2G	13.65
**4370**	allXL	BB	320	357	DBSU	24.72
**4370**	allXL	AA	357	319	DBSU	26.05
**4370**	allXL	DD	372	319	DBSU	32.59
**4370**	allXL	CC	370	357	DBSU	25.49
**4370**	allXL	BB	382	357	DBSU	18.64
**4370**	allXL	AA	372	370	DBSU	6.44
**4370**	allXL	CC	381	370	DBSU	18.9
**4370**	allXL	CC	373	370	DBSU	8.97
**4370**	allXL	DD	381	372	DBSU	19.9
**4370**	allXL	CC	372	382	DBSU	21.46
**4370**	allXL	AA	372	386	DBSU	25.74
**4370**	allXL	AA	373	386	DBSU	25.49
**4370**	allXL	AD	381	386	DBSU	54.59
**4370**	allXL	BD;DB	357	357	DBSU	19.24
**4370**	allXL	AD	373	319	DBSU	36.27
**4370**	allXL	CA	372	357	DBSU	40.3
**4370**	allXL	BD	381	357	DBSU	36.78
**4370**	allXL	CA	373	357	DBSU	38.18
**4370**	allXL	AD	372	370	DBSU	53.56
**4370**	allXL	AD	373	370	DBSU	50.24
**4370**	allXL	AD	382	373	DBSU	39.78
**4371**	allXL	AA	320	357	BS2G	22.46
**4371**	allXL	BB	320	373	BS2G	19.99
**4371**	allXL	DD	373	382	BS2G	17.46
**4371**	allXL	DD	370	386	BS2G	21.21
**4371**	allXL	BB	373	386	BS2G	23.53
**4371**	allXL	AA	319	357	BS2G	24.96
**4371**	allXL	AA	367	373	BS2G	12.14
**4371**	allXL	DD	370	382	BS2G	13.51
**4371**	allXL	BB	376	382	BS2G	14.29
**4371**	allXL	AA	357	373	BS2G	21.0

**4371**	allXL	AA	320	366	BS2G	27.86
**4371**	allXL	CC	370	376	BS2G	15.09
**4371**	allXL	AA	320	357	DBSU	22.46
**4371**	allXL	AA	357	319	DBSU	24.96
**4371**	allXL	BB	372	319	DBSU	24.78
**4371**	allXL	AA	370	357	DBSU	21.51
**4371**	allXL	DD	382	357	DBSU	12.75
**4371**	allXL	AA	372	370	DBSU	6.37
**4371**	allXL	DD	381	370	DBSU	12.44
**4371**	allXL	AA	373	370	DBSU	9.07
**4371**	allXL	DD	381	372	DBSU	16.22
**4371**	allXL	DD	372	382	DBSU	17.85
**4371**	allXL	BB	372	386	DBSU	22.71
**4371**	allXL	BB	373	386	DBSU	23.53
**4371**	allXL	DB	381	386	DBSU	36.72
**4371**	allXL	BD;DB	357	357	DBSU	18.48
**4371**	allXL	AD	373	319	DBSU	38.89
**4371**	allXL	DB	372	357	DBSU	30.32
**4371**	allXL	DB	381	357	DBSU	17.88
**4371**	allXL	DB	373	357	DBSU	29.55
**4371**	allXL	BC	372	370	DBSU	41.86
**4371**	allXL	BC	373	370	DBSU	38.74
**4371**	allXL	BD	382	373	DBSU	37.94
**4372**	allXL	AA	320	357	BS2G	24.74
**4372**	allXL	CC	320	373	BS2G	23.99
**4372**	allXL	CC	373	382	BS2G	22.48
**4372**	allXL	CC	370	386	BS2G	27.41
**4372**	allXL	DD	373	386	BS2G	31.15
**4372**	allXL	AA	319	357	BS2G	23.29
**4372**	allXL	AA	367	373	BS2G	13.16
**4372**	allXL	CC	370	382	BS2G	18.74
**4372**	allXL	CC	376	382	BS2G	14.53
**4372**	allXL	CC	357	373	BS2G	22.07
**4372**	allXL	CC	320	366	BS2G	20.49

**4372**	allXL	CC	370	376	BS2G	10.94
**4372**	allXL	AA	320	357	DBSU	24.74
**4372**	allXL	AA	357	319	DBSU	23.29
**4372**	allXL	CC	372	319	DBSU	20.73
**4372**	allXL	CC	370	357	DBSU	21.25
**4372**	allXL	CC	382	357	DBSU	37.7
**4372**	allXL	AA	372	370	DBSU	6.33
**4372**	allXL	CC	381	370	DBSU	19.53
**4372**	allXL	CC	373	370	DBSU	9.0
**4372**	allXL	AA	381	372	DBSU	20.7
**4372**	allXL	CC	372	382	DBSU	21.91
**4372**	allXL	CC	372	386	DBSU	29.95
**4372**	allXL	DD	373	386	DBSU	31.15
**4372**	allXL	BC	381	386	DBSU	61.65
**4372**	allXL	BD;DB	357	357	DBSU	19.27
**4372**	allXL	AD	373	319	DBSU	25.31
**4372**	allXL	CA	372	357	DBSU	39.36
**4372**	allXL	CA	381	357	DBSU	57.11
**4372**	allXL	CA	373	357	DBSU	39.02
**4372**	allXL	DA	372	370	DBSU	45.61
**4372**	allXL	AD	373	370	DBSU	45.58
**4372**	allXL	AD	382	373	DBSU	47.26
**4373**	allXL	AA	320	357	BS2G	24.44
**4373**	allXL	CC	320	373	BS2G	28.09
**4373**	allXL	CC	373	382	BS2G	18.91
**4373**	allXL	DD	370	386	BS2G	18.4
**4373**	allXL	DD	373	386	BS2G	22.94
**4373**	allXL	AA	319	357	BS2G	25.61
**4373**	allXL	DD	367	373	BS2G	14.95
**4373**	allXL	DD	370	382	BS2G	17.65
**4373**	allXL	CC	376	382	BS2G	13.42
**4373**	allXL	DD	357	373	BS2G	21.14
**4373**	allXL	AA	320	366	BS2G	14.4
**4373**	allXL	DD	370	376	BS2G	14.59

**4373**	allXL	AA	320	357	DBSU	24.44
**4373**	allXL	AA	357	319	DBSU	25.61
**4373**	allXL	AA	372	319	DBSU	29.7
**4373**	allXL	DD	370	357	DBSU	24.37
**4373**	allXL	CC	382	357	DBSU	35.71
**4373**	allXL	AA	372	370	DBSU	5.99
**4373**	allXL	DD	381	370	DBSU	17.1
**4373**	allXL	AA	373	370	DBSU	9.15
**4373**	allXL	DD	381	372	DBSU	20.75
**4373**	allXL	DD	372	382	DBSU	22.03
**4373**	allXL	DD	372	386	DBSU	21.76
**4373**	allXL	DD	373	386	DBSU	22.94
**4373**	allXL	AD	381	386	DBSU	64.67
**4373**	allXL	BD;DB	357	357	DBSU	18.71
**4373**	allXL	CB	373	319	DBSU	31.0
**4373**	allXL	DB	372	357	DBSU	26.79
**4373**	allXL	DB	381	357	DBSU	36.92
**4373**	allXL	DB	373	357	DBSU	23.58
**4373**	allXL	DB	372	370	DBSU	37.42
**4373**	allXL	DB	373	370	DBSU	33.99
**4373**	allXL	DB	382	373	DBSU	43.19
**4374**	allXL	CC	320	357	BS2G	24.18
**4374**	allXL	CC	320	373	BS2G	20.34
**4374**	allXL	BB	373	382	BS2G	16.86
**4374**	allXL	BB	370	386	BS2G	21.76
**4374**	allXL	BB	373	386	BS2G	22.39
**4374**	allXL	CC	319	357	BS2G	26.18
**4374**	allXL	AA	367	373	BS2G	12.24
**4374**	allXL	BB	370	382	BS2G	21.31
**4374**	allXL	CC	376	382	BS2G	14.22
**4374**	allXL	CC	357	373	BS2G	20.12
**4374**	allXL	AA	320	366	BS2G	23.15
**4374**	allXL	CC	370	376	BS2G	13.89
**4374**	allXL	CC	320	357	DBSU	24.18

**4374**	allXL	CC	357	319	DBSU	26.18
**4374**	allXL	CC	372	319	DBSU	22.54
**4374**	allXL	BB	370	357	DBSU	16.72
**4374**	allXL	AA	382	357	DBSU	19.03
**4374**	allXL	CC	372	370	DBSU	6.7
**4374**	allXL	BB	381	370	DBSU	19.75
**4374**	allXL	CC	373	370	DBSU	9.01
**4374**	allXL	BB	381	372	DBSU	16.85
**4374**	allXL	BB	372	382	DBSU	18.45
**4374**	allXL	BB	372	386	DBSU	22.84
**4374**	allXL	BB	373	386	DBSU	22.39
**4374**	allXL	DB	381	386	DBSU	44.37
**4374**	allXL	BD;DB	357	357	DBSU	18.64
**4374**	allXL	AD	373	319	DBSU	18.69
**4374**	allXL	BD	372	357	DBSU	31.34
**4374**	allXL	AC	381	357	DBSU	25.73
**4374**	allXL	BD	373	357	DBSU	33.57
**4374**	allXL	BD	372	370	DBSU	45.25
**4374**	allXL	BD	373	370	DBSU	44.91
**4374**	allXL	AC	382	373	DBSU	31.52
**4375**	allXL	AA	320	357	BS2G	20.75
**4375**	allXL	DD	320	373	BS2G	15.03
**4375**	allXL	CC	373	382	BS2G	17.85
**4375**	allXL	CC	370	386	BS2G	18.33
**4375**	allXL	DD	373	386	BS2G	23.25
**4375**	allXL	AA	319	357	BS2G	18.89
**4375**	allXL	AA	367	373	BS2G	13.69
**4375**	allXL	CC	370	382	BS2G	15.78
**4375**	allXL	CC	376	382	BS2G	14.24
**4375**	allXL	BB	357	373	BS2G	23.72
**4375**	allXL	BB	320	366	BS2G	18.73
**4375**	allXL	CC	370	376	BS2G	13.83
**4375**	allXL	AA	320	357	DBSU	20.75
**4375**	allXL	AA	357	319	DBSU	18.89

**4375**	allXL	DD	372	319	DBSU	15.0
**4375**	allXL	CC	370	357	DBSU	23.31
**4375**	allXL	BB	382	357	DBSU	26.05
**4375**	allXL	AA	372	370	DBSU	6.43
**4375**	allXL	CC	381	370	DBSU	18.19
**4375**	allXL	AA	373	370	DBSU	9.42
**4375**	allXL	CC	381	372	DBSU	19.66
**4375**	allXL	CC	372	382	DBSU	18.47
**4375**	allXL	DD	372	386	DBSU	23.43
**4375**	allXL	DD	373	386	DBSU	23.25
**4375**	allXL	DA	381	386	DBSU	43.42
**4375**	allXL	BD;DB	357	357	DBSU	18.97
**4375**	allXL	AD	373	319	DBSU	28.16
**4375**	allXL	BD	372	357	DBSU	38.17
**4375**	allXL	BD	381	357	DBSU	25.5
**4375**	allXL	BD	373	357	DBSU	36.3
**4375**	allXL	DA	372	370	DBSU	40.65
**4375**	allXL	DA	373	370	DBSU	37.47
**4375**	allXL	AD	382	373	DBSU	27.96
**4376**	allXL	AA	320	357	BS2G	24.08
**4376**	allXL	DD	320	373	BS2G	18.21
**4376**	allXL	BB	373	382	BS2G	17.27
**4376**	allXL	BB	370	386	BS2G	23.52
**4376**	allXL	BB	373	386	BS2G	22.37
**4376**	allXL	AA	319	357	BS2G	23.44
**4376**	allXL	AA	367	373	BS2G	12.51
**4376**	allXL	BB	370	382	BS2G	23.45
**4376**	allXL	AA	376	382	BS2G	14.42
**4376**	allXL	BB	357	373	BS2G	32.58
**4376**	allXL	DD	320	366	BS2G	15.47
**4376**	allXL	CC	370	376	BS2G	15.37
**4376**	allXL	AA	320	357	DBSU	24.08
**4376**	allXL	AA	357	319	DBSU	23.44
**4376**	allXL	DD	372	319	DBSU	17.38

**4376**	allXL	BB	370	357	DBSU	27.91
**4376**	allXL	AA	382	357	DBSU	41.95
**4376**	allXL	AA	372	370	DBSU	6.51
**4376**	allXL	BB	381	370	DBSU	21.64
**4376**	allXL	AA	373	370	DBSU	8.87
**4376**	allXL	BB	381	372	DBSU	17.93
**4376**	allXL	DD	372	382	DBSU	19.37
**4376**	allXL	BB	372	386	DBSU	23.51
**4376**	allXL	BB	373	386	DBSU	22.37
**4376**	allXL	DA	381	386	DBSU	33.13
**4376**	allXL	BD;DB	357	357	DBSU	18.83
**4376**	allXL	AD	373	319	DBSU	29.99
**4376**	allXL	BD	372	357	DBSU	44.33
**4376**	allXL	AC	381	357	DBSU	48.83
**4376**	allXL	BD	373	357	DBSU	47.84
**4376**	allXL	DA	372	370	DBSU	35.11
**4376**	allXL	DA	373	370	DBSU	33.47
**4376**	allXL	DA	382	373	DBSU	41.21
**4377**	allXL	CC	320	357	BS2G	22.57
**4377**	allXL	DD	320	373	BS2G	18.4
**4377**	allXL	CC	373	382	BS2G	16.99
**4377**	allXL	DD	370	386	BS2G	23.26
**4377**	allXL	CC	373	386	BS2G	27.99
**4377**	allXL	AA	319	357	BS2G	22.64
**4377**	allXL	AA	367	373	BS2G	13.08
**4377**	allXL	CC	370	382	BS2G	15.33
**4377**	allXL	BB	376	382	BS2G	15.08
**4377**	allXL	BB	357	373	BS2G	30.43
**4377**	allXL	BB	320	366	BS2G	24.95
**4377**	allXL	CC	370	376	BS2G	13.54
**4377**	allXL	CC	320	357	DBSU	22.57
**4377**	allXL	AA	357	319	DBSU	22.64
**4377**	allXL	BB	372	319	DBSU	19.53
**4377**	allXL	DD	370	357	DBSU	26.95

**4377**	allXL	BB	382	357	DBSU	21.83
**4377**	allXL	AA	372	370	DBSU	6.14
**4377**	allXL	CC	381	370	DBSU	14.45
**4377**	allXL	CC	373	370	DBSU	9.09
**4377**	allXL	CC	381	372	DBSU	15.94
**4377**	allXL	CC	372	382	DBSU	18.17
**4377**	allXL	CC	372	386	DBSU	27.9
**4377**	allXL	CC	373	386	DBSU	27.99
**4377**	allXL	CB	381	386	DBSU	55.47
**4377**	allXL	BD;DB	357	357	DBSU	19.46
**4377**	allXL	CB	373	319	DBSU	24.96
**4377**	allXL	BC	372	357	DBSU	40.22
**4377**	allXL	BD	381	357	DBSU	43.31
**4377**	allXL	BC	373	357	DBSU	43.75
**4377**	allXL	CB	372	370	DBSU	18.86
**4377**	allXL	CB	373	370	DBSU	15.87
**4377**	allXL	CB	382	373	DBSU	31.28
**4378**	allXL	CC	320	357	BS2G	24.72
**4378**	allXL	BB	320	373	BS2G	13.68
**4378**	allXL	AA	373	382	BS2G	18.01
**4378**	allXL	AA	370	386	BS2G	22.53
**4378**	allXL	AA	373	386	BS2G	20.47
**4378**	allXL	BB	319	357	BS2G	27.08
**4378**	allXL	AA	367	373	BS2G	11.96
**4378**	allXL	BB	370	382	BS2G	21.96
**4378**	allXL	BB	376	382	BS2G	13.92
**4378**	allXL	BB	357	373	BS2G	17.67
**4378**	allXL	AA	320	366	BS2G	15.46
**4378**	allXL	CC	370	376	BS2G	15.86
**4378**	allXL	CC	320	357	DBSU	24.72
**4378**	allXL	BB	357	319	DBSU	27.08
**4378**	allXL	BB	372	319	DBSU	14.72
**4378**	allXL	BB	370	357	DBSU	18.45
**4378**	allXL	BB	382	357	DBSU	30.01

**4378**	allXL	AA	372	370	DBSU	6.53
**4378**	allXL	DD	381	370	DBSU	20.23
**4378**	allXL	AA	373	370	DBSU	9.21
**4378**	allXL	AA	381	372	DBSU	17.67
**4378**	allXL	AA	372	382	DBSU	19.9
**4378**	allXL	AA	372	386	DBSU	20.18
**4378**	allXL	AA	373	386	DBSU	20.47
**4378**	allXL	CB	381	386	DBSU	24.01
**4378**	allXL	BD;DB	357	357	DBSU	18.25
**4378**	allXL	CB	373	319	DBSU	15.78
**4378**	allXL	CB	372	357	DBSU	33.32
**4378**	allXL	BD	381	357	DBSU	43.37
**4378**	allXL	CB	373	357	DBSU	34.53
**4378**	allXL	CB	372	370	DBSU	15.36
**4378**	allXL	CB	373	370	DBSU	16.19
**4378**	allXL	BC	382	373	DBSU	25.24
**4379**	allXL	AA	320	357	BS2G	24.58
**4379**	allXL	BB	320	373	BS2G	27.27
**4379**	allXL	DD	373	382	BS2G	22.63
**4379**	allXL	CC	370	386	BS2G	23.43
**4379**	allXL	DD	373	386	BS2G	23.64
**4379**	allXL	AA	319	357	BS2G	23.16
**4379**	allXL	DD	367	373	BS2G	12.88
**4379**	allXL	CC	370	382	BS2G	25.71
**4379**	allXL	CC	376	382	BS2G	15.08
**4379**	allXL	DD	357	373	BS2G	30.25
**4379**	allXL	BB	320	366	BS2G	21.74
**4379**	allXL	CC	370	376	BS2G	16.23
**4379**	allXL	AA	320	357	DBSU	24.58
**4379**	allXL	AA	357	319	DBSU	23.16
**4379**	allXL	BB	372	319	DBSU	28.81
**4379**	allXL	DD	370	357	DBSU	30.15
**4379**	allXL	BB	382	357	DBSU	29.05
**4379**	allXL	AA	372	370	DBSU	6.18

**4379**	allXL	AA	381	370	DBSU	25.95
**4379**	allXL	DD	373	370	DBSU	8.82
**4379**	allXL	AA	381	372	DBSU	22.38
**4379**	allXL	AA	372	382	DBSU	22.82
**4379**	allXL	DD	372	386	DBSU	24.39
**4379**	allXL	DD	373	386	DBSU	23.64
**4379**	allXL	DB	381	386	DBSU	47.57
**4379**	allXL	BD;DB	357	357	DBSU	17.96
**4379**	allXL	CB	373	319	DBSU	15.93
**4379**	allXL	CB	372	357	DBSU	36.6
**4379**	allXL	BD	381	357	DBSU	40.24
**4379**	allXL	CB	373	357	DBSU	40.01
**4379**	allXL	CB	372	370	DBSU	23.45
**4379**	allXL	CB	373	370	DBSU	23.76
**4379**	allXL	BC	382	373	DBSU	36.66
**4380**	allXL	AA	320	357	BS2G	25.21
**4380**	allXL	DD	320	373	BS2G	19.6
**4380**	allXL	DD	373	382	BS2G	16.9
**4380**	allXL	DD	370	386	BS2G	26.86
**4380**	allXL	DD	373	386	BS2G	25.65
**4380**	allXL	AA	319	357	BS2G	28.02
**4380**	allXL	BB	367	373	BS2G	12.86
**4380**	allXL	DD	370	382	BS2G	17.06
**4380**	allXL	CC	376	382	BS2G	15.29
**4380**	allXL	BB	357	373	BS2G	24.92
**4380**	allXL	AA	320	366	BS2G	17.95
**4380**	allXL	CC	370	376	BS2G	15.21
**4380**	allXL	AA	320	357	DBSU	25.21
**4380**	allXL	AA	357	319	DBSU	28.02
**4380**	allXL	DD	372	319	DBSU	18.41
**4380**	allXL	BB	370	357	DBSU	25.38
**4380**	allXL	BB	382	357	DBSU	20.71
**4380**	allXL	AA	372	370	DBSU	6.29
**4380**	allXL	DD	381	370	DBSU	16.54

**4380**	allXL	AA	373	370	DBSU	9.0
**4380**	allXL	DD	381	372	DBSU	16.52
**4380**	allXL	DD	372	382	DBSU	16.64
**4380**	allXL	DD	372	386	DBSU	24.97
**4380**	allXL	DD	373	386	DBSU	25.65
**4380**	allXL	AD	381	386	DBSU	43.17
**4380**	allXL	BD;DB	357	357	DBSU	17.4
**4380**	allXL	DA	373	319	DBSU	29.26
**4380**	allXL	CB	372	357	DBSU	32.69
**4380**	allXL	BD	381	357	DBSU	33.83
**4380**	allXL	CB	373	357	DBSU	34.51
**4380**	allXL	CB	372	370	DBSU	11.78
**4380**	allXL	CB	373	370	DBSU	11.73
**4380**	allXL	CB	382	373	DBSU	30.03
**4381**	allXL	AA	320	357	BS2G	16.53
**4381**	allXL	DD	320	373	BS2G	23.96
**4381**	allXL	DD	373	382	BS2G	17.26
**4381**	allXL	DD	370	386	BS2G	17.36
**4381**	allXL	DD	373	386	BS2G	21.49
**4381**	allXL	AA	319	357	BS2G	13.43
**4381**	allXL	AA	367	373	BS2G	12.78
**4381**	allXL	DD	370	382	BS2G	17.55
**4381**	allXL	AA	376	382	BS2G	14.73
**4381**	allXL	BB	357	373	BS2G	21.34
**4381**	allXL	CC	320	366	BS2G	25.48
**4381**	allXL	AA	370	376	BS2G	16.44
**4381**	allXL	AA	320	357	DBSU	16.53
**4381**	allXL	AA	357	319	DBSU	13.43
**4381**	allXL	DD	372	319	DBSU	28.24
**4381**	allXL	DD	370	357	DBSU	23.63
**4381**	allXL	BB	382	357	DBSU	35.83
**4381**	allXL	AA	372	370	DBSU	6.39
**4381**	allXL	DD	381	370	DBSU	17.68
**4381**	allXL	DD	373	370	DBSU	8.95

**4381**	allXL	DD	381	372	DBSU	14.91
**4381**	allXL	DD	372	382	DBSU	16.09
**4381**	allXL	DD	372	386	DBSU	18.49
**4381**	allXL	DD	373	386	DBSU	21.49
**4381**	allXL	AD	381	386	DBSU	42.87
**4381**	allXL	BD;DB	357	357	DBSU	18.85
**4381**	allXL	AD	373	319	DBSU	23.78
**4381**	allXL	BD	372	357	DBSU	31.83
**4381**	allXL	BD	381	357	DBSU	33.92
**4381**	allXL	BD	373	357	DBSU	30.11
**4381**	allXL	BC	372	370	DBSU	41.49
**4381**	allXL	BC	373	370	DBSU	43.68
**4381**	allXL	DA	382	373	DBSU	40.95
**4382**	allXL	AA	320	357	BS2G	21.38
**4382**	allXL	CC	320	373	BS2G	24.98
**4382**	allXL	AA	373	382	BS2G	20.14
**4382**	allXL	AA	370	386	BS2G	23.95
**4382**	allXL	DD	373	386	BS2G	22.83
**4382**	allXL	AA	319	357	BS2G	19.67
**4382**	allXL	AA	367	373	BS2G	11.97
**4382**	allXL	AA	370	382	BS2G	23.78
**4382**	allXL	BB	376	382	BS2G	14.06
**4382**	allXL	AA	357	373	BS2G	32.76
**4382**	allXL	BB	320	366	BS2G	23.32
**4382**	allXL	CC	370	376	BS2G	16.87
**4382**	allXL	AA	320	357	DBSU	21.38
**4382**	allXL	AA	357	319	DBSU	19.67
**4382**	allXL	CC	372	319	DBSU	22.2
**4382**	allXL	CC	370	357	DBSU	31.94
**4382**	allXL	AA	382	357	DBSU	46.42
**4382**	allXL	AA	372	370	DBSU	6.37
**4382**	allXL	AA	381	370	DBSU	26.22
**4382**	allXL	AA	373	370	DBSU	9.12
**4382**	allXL	DD	381	372	DBSU	20.87

**4382**	allXL	AA	372	382	DBSU	20.61
**4382**	allXL	DD	372	386	DBSU	21.15
**4382**	allXL	DD	373	386	DBSU	22.83
**4382**	allXL	BC	381	386	DBSU	43.42
**4382**	allXL	BD;DB	357	357	DBSU	17.62
**4382**	allXL	CB	373	319	DBSU	34.32
**4382**	allXL	CB	372	357	DBSU	46.95
**4382**	allXL	AC	381	357	DBSU	61.34
**4382**	allXL	CB	373	357	DBSU	49.49
**4382**	allXL	CB	372	370	DBSU	36.77
**4382**	allXL	CB	373	370	DBSU	38.58
**4382**	allXL	CB	382	373	DBSU	34.95
**4383**	allXL	AA	320	357	BS2G	24.76
**4383**	allXL	AA	320	373	BS2G	23.54
**4383**	allXL	DD	373	382	BS2G	18.03
**4383**	allXL	CC	370	386	BS2G	18.14
**4383**	allXL	CC	373	386	BS2G	23.1
**4383**	allXL	AA	319	357	BS2G	23.41
**4383**	allXL	AA	367	373	BS2G	11.98
**4383**	allXL	DD	370	382	BS2G	14.85
**4383**	allXL	CC	376	382	BS2G	13.93
**4383**	allXL	BB	357	373	BS2G	26.34
**4383**	allXL	AA	320	366	BS2G	12.97
**4383**	allXL	DD	370	376	BS2G	16.29
**4383**	allXL	AA	320	357	DBSU	24.76
**4383**	allXL	AA	357	319	DBSU	23.41
**4383**	allXL	DD	372	319	DBSU	22.72
**4383**	allXL	BB	370	357	DBSU	28.07
**4383**	allXL	DD	382	357	DBSU	32.75
**4383**	allXL	AA	372	370	DBSU	6.26
**4383**	allXL	DD	381	370	DBSU	16.31
**4383**	allXL	AA	373	370	DBSU	9.06
**4383**	allXL	DD	381	372	DBSU	19.36
**4383**	allXL	CC	372	382	DBSU	18.49

**4383**	allXL	CC	372	386	DBSU	21.12
**4383**	allXL	CC	373	386	DBSU	23.1
**4383**	allXL	BD	381	386	DBSU	38.52
**4383**	allXL	BD;DB	357	357	DBSU	17.33
**4383**	allXL	AD	373	319	DBSU	28.16
**4383**	allXL	BD	372	357	DBSU	33.02
**4383**	allXL	DB	381	357	DBSU	41.77
**4383**	allXL	BD	373	357	DBSU	34.49
**4383**	allXL	DA	372	370	DBSU	31.34
**4383**	allXL	DA	373	370	DBSU	29.48
**4383**	allXL	AD	382	373	DBSU	31.81
**4384**	allXL	BB	320	357	BS2G	23.89
**4384**	allXL	DD	320	373	BS2G	14.95
**4384**	allXL	AA	373	382	BS2G	16.09
**4384**	allXL	AA	370	386	BS2G	13.67
**4384**	allXL	AA	373	386	BS2G	16.48
**4384**	allXL	AA	319	357	BS2G	26.57
**4384**	allXL	AA	367	373	BS2G	12.38
**4384**	allXL	AA	370	382	BS2G	18.32
**4384**	allXL	AA	376	382	BS2G	13.31
**4384**	allXL	DD	357	373	BS2G	21.81
**4384**	allXL	BB	320	366	BS2G	22.69
**4384**	allXL	CC	370	376	BS2G	16.57
**4384**	allXL	BB	320	357	DBSU	23.89
**4384**	allXL	AA	357	319	DBSU	26.57
**4384**	allXL	DD	372	319	DBSU	14.17
**4384**	allXL	DD	370	357	DBSU	24.54
**4384**	allXL	BB	382	357	DBSU	29.76
**4384**	allXL	AA	372	370	DBSU	6.25
**4384**	allXL	DD	381	370	DBSU	20.13
**4384**	allXL	CC	373	370	DBSU	9.13
**4384**	allXL	AA	381	372	DBSU	19.37
**4384**	allXL	AA	372	382	DBSU	16.41
**4384**	allXL	AA	372	386	DBSU	15.67

**4384**	allXL	AA	373	386	DBSU	16.48
**4384**	allXL	BC	381	386	DBSU	44.82
**4384**	allXL	BD;DB	357	357	DBSU	19.62
**4384**	allXL	AD	373	319	DBSU	27.03
**4384**	allXL	DB	372	357	DBSU	40.13
**4384**	allXL	CA	381	357	DBSU	41.39
**4384**	allXL	CA	373	357	DBSU	40.01
**4384**	allXL	DA	372	370	DBSU	30.27
**4384**	allXL	DA	373	370	DBSU	31.55
**4384**	allXL	AD	382	373	DBSU	47.21
**4385**	allXL	DD	320	357	BS2G	24.91
**4385**	allXL	AA	320	373	BS2G	17.39
**4385**	allXL	CC	373	382	BS2G	17.23
**4385**	allXL	CC	370	386	BS2G	18.94
**4385**	allXL	CC	373	386	BS2G	21.3
**4385**	allXL	DD	319	357	BS2G	23.75
**4385**	allXL	AA	367	373	BS2G	10.4
**4385**	allXL	CC	370	382	BS2G	18.16
**4385**	allXL	BB	376	382	BS2G	13.65
**4385**	allXL	BB	357	373	BS2G	21.24
**4385**	allXL	BB	320	366	BS2G	14.42
**4385**	allXL	DD	370	376	BS2G	13.57
**4385**	allXL	DD	320	357	DBSU	24.91
**4385**	allXL	DD	357	319	DBSU	23.75
**4385**	allXL	AA	372	319	DBSU	13.38
**4385**	allXL	BB	370	357	DBSU	19.17
**4385**	allXL	CC	382	357	DBSU	25.33
**4385**	allXL	AA	372	370	DBSU	6.39
**4385**	allXL	CC	381	370	DBSU	19.96
**4385**	allXL	AA	373	370	DBSU	9.05
**4385**	allXL	CC	381	372	DBSU	19.64
**4385**	allXL	CC	372	382	DBSU	19.18
**4385**	allXL	CC	372	386	DBSU	21.45
**4385**	allXL	CC	373	386	DBSU	21.3

**4385**	allXL	DB	381	386	DBSU	49.32
**4385**	allXL	BD;DB	357	357	DBSU	17.88
**4385**	allXL	AD	373	319	DBSU	25.7
**4385**	allXL	BD	372	357	DBSU	29.14
**4385**	allXL	AD	381	357	DBSU	38.82
**4385**	allXL	BD	373	357	DBSU	28.48
**4385**	allXL	DA	372	370	DBSU	38.31
**4385**	allXL	DA	373	370	DBSU	36.32
**4385**	allXL	AD	382	373	DBSU	39.89
**4386**	allXL	AA	320	357	BS2G	23.52
**4386**	allXL	BB	320	373	BS2G	20.54
**4386**	allXL	DD	373	382	BS2G	17.59
**4386**	allXL	DD	370	386	BS2G	18.68
**4386**	allXL	DD	373	386	BS2G	23.93
**4386**	allXL	AA	319	357	BS2G	25.53
**4386**	allXL	AA	367	373	BS2G	13.38
**4386**	allXL	DD	370	382	BS2G	16.35
**4386**	allXL	BB	376	382	BS2G	13.53
**4386**	allXL	CC	357	373	BS2G	18.91
**4386**	allXL	BB	320	366	BS2G	29.09
**4386**	allXL	CC	370	376	BS2G	15.46
**4386**	allXL	AA	320	357	DBSU	23.52
**4386**	allXL	AA	357	319	DBSU	25.53
**4386**	allXL	BB	372	319	DBSU	24.11
**4386**	allXL	DD	370	357	DBSU	19.68
**4386**	allXL	DD	382	357	DBSU	22.35
**4386**	allXL	AA	372	370	DBSU	6.22
**4386**	allXL	DD	381	370	DBSU	16.28
**4386**	allXL	CC	373	370	DBSU	9.11
**4386**	allXL	DD	381	372	DBSU	14.86
**4386**	allXL	DD	372	382	DBSU	16.26
**4386**	allXL	DD	372	386	DBSU	20.97
**4386**	allXL	DD	373	386	DBSU	23.93
**4386**	allXL	CB	381	386	DBSU	34.15

**4386**	allXL	BD;DB	357	357	DBSU	18.43
**4386**	allXL	AD	373	319	DBSU	38.12
**4386**	allXL	CA	372	357	DBSU	26.29
**4386**	allXL	CA	381	357	DBSU	30.91
**4386**	allXL	CA	373	357	DBSU	24.02
**4386**	allXL	CA	372	370	DBSU	52.5
**4386**	allXL	CB	373	370	DBSU	50.81
**4386**	allXL	CB	382	373	DBSU	43.98
**4387**	allXL	CC	320	357	BS2G	23.01
**4387**	allXL	DD	320	373	BS2G	25.88
**4387**	allXL	AA	373	382	BS2G	17.47
**4387**	allXL	DD	370	386	BS2G	20.41
**4387**	allXL	AA	373	386	BS2G	21.49
**4387**	allXL	CC	319	357	BS2G	24.3
**4387**	allXL	AA	367	373	BS2G	13.0
**4387**	allXL	DD	370	382	BS2G	16.02
**4387**	allXL	BB	376	382	BS2G	14.49
**4387**	allXL	CC	357	373	BS2G	29.73
**4387**	allXL	DD	320	366	BS2G	23.32
**4387**	allXL	CC	370	376	BS2G	13.14
**4387**	allXL	CC	320	357	DBSU	23.01
**4387**	allXL	CC	357	319	DBSU	24.3
**4387**	allXL	DD	372	319	DBSU	25.68
**4387**	allXL	CC	370	357	DBSU	30.94
**4387**	allXL	CC	382	357	DBSU	17.07
**4387**	allXL	AA	372	370	DBSU	6.35
**4387**	allXL	DD	381	370	DBSU	14.83
**4387**	allXL	CC	373	370	DBSU	9.11
**4387**	allXL	AA	381	372	DBSU	19.22
**4387**	allXL	AA	372	382	DBSU	20.83
**4387**	allXL	AA	372	386	DBSU	24.06
**4387**	allXL	AA	373	386	DBSU	21.49
**4387**	allXL	AD	381	386	DBSU	44.85
**4387**	allXL	BD;DB	357	357	DBSU	18.31

**4387**	allXL	AD	373	319	DBSU	25.89
**4387**	allXL	DA	372	357	DBSU	47.54
**4387**	allXL	CA	381	357	DBSU	30.82
**4387**	allXL	DA	373	357	DBSU	43.82
**4387**	allXL	DA	372	370	DBSU	31.48
**4387**	allXL	DA	373	370	DBSU	29.08
**4387**	allXL	AD	382	373	DBSU	23.04
**4388**	allXL	AA	320	357	BS2G	26.16
**4388**	allXL	BB	320	373	BS2G	15.67
**4388**	allXL	CC	373	382	BS2G	13.47
**4388**	allXL	CC	370	386	BS2G	19.97
**4388**	allXL	CC	373	386	BS2G	17.66
**4388**	allXL	AA	319	357	BS2G	25.46
**4388**	allXL	AA	367	373	BS2G	14.27
**4388**	allXL	CC	370	382	BS2G	17.89
**4388**	allXL	BB	376	382	BS2G	14.12
**4388**	allXL	BB	357	373	BS2G	21.22
**4388**	allXL	AA	320	366	BS2G	19.85
**4388**	allXL	CC	370	376	BS2G	16.08
**4388**	allXL	AA	320	357	DBSU	26.16
**4388**	allXL	AA	357	319	DBSU	25.46
**4388**	allXL	AA	372	319	DBSU	17.19
**4388**	allXL	AA	370	357	DBSU	27.87
**4388**	allXL	BB	382	357	DBSU	34.9
**4388**	allXL	AA	372	370	DBSU	6.49
**4388**	allXL	CC	381	370	DBSU	20.65
**4388**	allXL	CC	373	370	DBSU	9.07
**4388**	allXL	CC	381	372	DBSU	18.67
**4388**	allXL	CC	372	382	DBSU	16.13
**4388**	allXL	CC	372	386	DBSU	18.08
**4388**	allXL	CC	373	386	DBSU	17.66
**4388**	allXL	BC	381	386	DBSU	61.57
**4388**	allXL	BD;DB	357	357	DBSU	18.32
**4388**	allXL	AD	373	319	DBSU	40.96

**4388**	allXL	BD	372	357	DBSU	39.34
**4388**	allXL	BD	381	357	DBSU	47.08
**4388**	allXL	BD	373	357	DBSU	39.01
**4388**	allXL	DA	372	370	DBSU	46.73
**4388**	allXL	DA	373	370	DBSU	50.2
**4388**	allXL	CB	382	373	DBSU	43.33
**4389**	allXL	AA	320	357	BS2G	24.84
**4389**	allXL	CC	320	373	BS2G	22.09
**4389**	allXL	BB	373	382	BS2G	17.68
**4389**	allXL	BB	370	386	BS2G	25.2
**4389**	allXL	BB	373	386	BS2G	24.23
**4389**	allXL	AA	319	357	BS2G	24.82
**4389**	allXL	AA	367	373	BS2G	11.92
**4389**	allXL	BB	370	382	BS2G	22.9
**4389**	allXL	BB	376	382	BS2G	12.92
**4389**	allXL	AA	357	373	BS2G	22.1
**4389**	allXL	BB	320	366	BS2G	22.6
**4389**	allXL	CC	370	376	BS2G	16.97
**4389**	allXL	AA	320	357	DBSU	24.84
**4389**	allXL	AA	357	319	DBSU	24.82
**4389**	allXL	CC	372	319	DBSU	16.11
**4389**	allXL	AA;DD	370	357	DBSU	29.41
**4389**	allXL	AA	382	357	DBSU	13.07
**4389**	allXL	CC	372	370	DBSU	6.61
**4389**	allXL	DD	381	370	DBSU	23.56
**4389**	allXL	AA	373	370	DBSU	9.2
**4389**	allXL	DD	381	372	DBSU	16.55
**4389**	allXL	DD	372	382	DBSU	19.14
**4389**	allXL	DD	372	386	DBSU	24.32
**4389**	allXL	BB	373	386	DBSU	24.23
**4389**	allXL	AD	381	386	DBSU	40.8
**4389**	allXL	BD;DB	357	357	DBSU	18.98
**4389**	allXL	BC	373	319	DBSU	30.15
**4389**	allXL	BC	372	357	DBSU	38.28

**4389**	allXL	AC	381	357	DBSU	24.79
**4389**	allXL	AC	373	357	DBSU	35.6
**4389**	allXL	BC	372	370	DBSU	28.19
**4389**	allXL	BC	373	370	DBSU	29.68
**4389**	allXL	BC	382	373	DBSU	40.64
**4390**	allXL	CC	320	357	BS2G	24.76
**4390**	allXL	BB	320	373	BS2G	10.86
**4390**	allXL	DD	373	382	BS2G	19.01
**4390**	allXL	DD	370	386	BS2G	23.6
**4390**	allXL	DD	373	386	BS2G	24.47
**4390**	allXL	AA	319	357	BS2G	24.6
**4390**	allXL	AA	367	373	BS2G	13.46
**4390**	allXL	BB	370	382	BS2G	22.36
**4390**	allXL	BB	376	382	BS2G	14.66
**4390**	allXL	BB	357	373	BS2G	22.65
**4390**	allXL	BB	320	366	BS2G	27.27
**4390**	allXL	CC	370	376	BS2G	15.4
**4390**	allXL	CC	320	357	DBSU	24.76
**4390**	allXL	AA	357	319	DBSU	24.6
**4390**	allXL	BB	372	319	DBSU	17.82
**4390**	allXL	DD	370	357	DBSU	22.78
**4390**	allXL	BB	382	357	DBSU	32.26
**4390**	allXL	AA	372	370	DBSU	6.17
**4390**	allXL	BB	381	370	DBSU	22.58
**4390**	allXL	AA	373	370	DBSU	9.15
**4390**	allXL	DD	381	372	DBSU	21.41
**4390**	allXL	DD	372	382	DBSU	21.42
**4390**	allXL	DD	372	386	DBSU	25.4
**4390**	allXL	DD	373	386	DBSU	24.47
**4390**	allXL	CB	381	386	DBSU	38.13
**4390**	allXL	BD;DB	357	357	DBSU	18.78
**4390**	allXL	AD	373	319	DBSU	27.99
**4390**	allXL	BD	372	357	DBSU	40.37
**4390**	allXL	BC	381	357	DBSU	32.5

**4390**	allXL	CA	373	357	DBSU	37.53
**4390**	allXL	AD	372	370	DBSU	53.15
**4390**	allXL	AD	373	370	DBSU	50.22
**4390**	allXL	AD	382	373	DBSU	41.96
**4391**	allXL	AA	320	357	BS2G	27.04
**4391**	allXL	BB	320	373	BS2G	19.85
**4391**	allXL	DD	373	382	BS2G	20.68
**4391**	allXL	DD	370	386	BS2G	15.8
**4391**	allXL	DD	373	386	BS2G	24.63
**4391**	allXL	AA	319	357	BS2G	28.63
**4391**	allXL	BB	367	373	BS2G	13.31
**4391**	allXL	DD	370	382	BS2G	15.57
**4391**	allXL	CC	376	382	BS2G	15.4
**4391**	allXL	BB	357	373	BS2G	15.12
**4391**	allXL	CC	320	366	BS2G	25.77
**4391**	allXL	CC	370	376	BS2G	13.89
**4391**	allXL	AA	320	357	DBSU	27.04
**4391**	allXL	AA	357	319	DBSU	28.63
**4391**	allXL	BB	372	319	DBSU	22.37
**4391**	allXL	BB	370	357	DBSU	14.11
**4391**	allXL	BB	382	357	DBSU	18.19
**4391**	allXL	AA	372	370	DBSU	6.17
**4391**	allXL	DD	381	370	DBSU	14.83
**4391**	allXL	AA	373	370	DBSU	9.0
**4391**	allXL	DD	381	372	DBSU	17.99
**4391**	allXL	DD	372	382	DBSU	20.0
**4391**	allXL	DD	372	386	DBSU	22.71
**4391**	allXL	DD	373	386	DBSU	24.63
**4391**	allXL	BD	381	386	DBSU	42.28
**4391**	allXL	BD;DB	357	357	DBSU	15.74
**4391**	allXL	CB	373	319	DBSU	21.41
**4391**	allXL	CB	372	357	DBSU	29.61
**4391**	allXL	BD	381	357	DBSU	26.31
**4391**	allXL	CB	373	357	DBSU	27.57

**4391**	allXL	CB	372	370	DBSU	18.15
**4391**	allXL	CB	373	370	DBSU	16.49
**4391**	allXL	CB	382	373	DBSU	37.53
**4392**	allXL	AA	320	357	BS2G	23.72
**4392**	allXL	DD	320	373	BS2G	12.49
**4392**	allXL	DD	373	382	BS2G	16.92
**4392**	allXL	DD	370	386	BS2G	14.82
**4392**	allXL	DD	373	386	BS2G	23.69
**4392**	allXL	AA	319	357	BS2G	22.03
**4392**	allXL	AA	367	373	BS2G	12.85
**4392**	allXL	DD	370	382	BS2G	12.05
**4392**	allXL	CC	376	382	BS2G	15.26
**4392**	allXL	CC	357	373	BS2G	17.59
**4392**	allXL	DD	320	366	BS2G	23.93
**4392**	allXL	CC	370	376	BS2G	14.88
**4392**	allXL	AA	320	357	DBSU	23.72
**4392**	allXL	AA	357	319	DBSU	22.03
**4392**	allXL	DD	372	319	DBSU	18.03
**4392**	allXL	CC	370	357	DBSU	19.14
**4392**	allXL	CC	382	357	DBSU	21.83
**4392**	allXL	AA	372	370	DBSU	6.22
**4392**	allXL	DD	381	370	DBSU	12.08
**4392**	allXL	CC	373	370	DBSU	8.97
**4392**	allXL	DD	381	372	DBSU	14.84
**4392**	allXL	DD	372	382	DBSU	16.15
**4392**	allXL	DD	372	386	DBSU	21.39
**4392**	allXL	DD	373	386	DBSU	23.69
**4392**	allXL	DB	381	386	DBSU	34.74
**4392**	allXL	BD;DB	357	357	DBSU	17.94
**4392**	allXL	AD	373	319	DBSU	41.6
**4392**	allXL	BD	372	357	DBSU	32.76
**4392**	allXL	CA	381	357	DBSU	19.72
**4392**	allXL	CA	373	357	DBSU	29.6
**4392**	allXL	CA	372	370	DBSU	44.03

**4392**	allXL	CA	373	370	DBSU	40.62
**4392**	allXL	CA	382	373	DBSU	34.43
**4393**	allXL	CC	320	357	BS2G	25.88
**4393**	allXL	BB	320	373	BS2G	13.61
**4393**	allXL	AA	373	382	BS2G	14.21
**4393**	allXL	AA	370	386	BS2G	11.44
**4393**	allXL	AA	373	386	BS2G	16.79
**4393**	allXL	AA	319	357	BS2G	26.12
**4393**	allXL	AA	367	373	BS2G	12.05
**4393**	allXL	AA	370	382	BS2G	15.55
**4393**	allXL	AA	376	382	BS2G	13.45
**4393**	allXL	CC	357	373	BS2G	23.63
**4393**	allXL	AA	320	366	BS2G	11.29
**4393**	allXL	CC	370	376	BS2G	15.65
**4393**	allXL	CC	320	357	DBSU	25.88
**4393**	allXL	AA	357	319	DBSU	26.12
**4393**	allXL	AA	372	319	DBSU	16.37
**4393**	allXL	CC	370	357	DBSU	23.61
**4393**	allXL	CC	382	357	DBSU	10.57
**4393**	allXL	AA	372	370	DBSU	6.51
**4393**	allXL	AA	381	370	DBSU	19.03
**4393**	allXL	AA	373	370	DBSU	9.1
**4393**	allXL	AA	381	372	DBSU	14.96
**4393**	allXL	AA	372	382	DBSU	12.07
**4393**	allXL	AA	372	386	DBSU	13.6
**4393**	allXL	AA	373	386	DBSU	16.79
**4393**	allXL	DB	381	386	DBSU	40.9
**4393**	allXL	BD;DB	357	357	DBSU	19.58
**4393**	allXL	AD	373	319	DBSU	42.95
**4393**	allXL	DB	372	357	DBSU	27.91
**4393**	allXL	CA	381	357	DBSU	16.15
**4393**	allXL	DB	373	357	DBSU	26.04
**4393**	allXL	DB	372	370	DBSU	53.27
**4393**	allXL	DB	373	370	DBSU	50.38

**4393**	allXL	CA	382	373	DBSU	46.49
**4394**	allXL	CC	320	357	BS2G	27.25
**4394**	allXL	DD	320	373	BS2G	15.95
**4394**	allXL	BB	373	382	BS2G	19.75
**4394**	allXL	DD	370	386	BS2G	28.68
**4394**	allXL	BB	373	386	BS2G	26.8
**4394**	allXL	BB	319	357	BS2G	29.75
**4394**	allXL	AA	367	373	BS2G	13.22
**4394**	allXL	DD	370	382	BS2G	24.4
**4394**	allXL	BB	376	382	BS2G	14.11
**4394**	allXL	BB	357	373	BS2G	35.89
**4394**	allXL	DD	320	366	BS2G	17.29
**4394**	allXL	CC	370	376	BS2G	16.79
**4394**	allXL	CC	320	357	DBSU	27.25
**4394**	allXL	BB	357	319	DBSU	29.75
**4394**	allXL	DD	372	319	DBSU	13.92
**4394**	allXL	DD	370	357	DBSU	27.27
**4394**	allXL	BB	382	357	DBSU	42.04
**4394**	allXL	AA;DD	372	370	DBSU	6.5
**4394**	allXL	DD	381	370	DBSU	24.26
**4394**	allXL	AA	373	370	DBSU	9.61
**4394**	allXL	DD	381	372	DBSU	22.88
**4394**	allXL	BB	372	382	DBSU	21.56
**4394**	allXL	BB	372	386	DBSU	27.04
**4394**	allXL	BB	373	386	DBSU	26.8
**4394**	allXL	DA	381	386	DBSU	52.66
**4394**	allXL	BD;DB	357	357	DBSU	18.38
**4394**	allXL	AD	373	319	DBSU	26.65
**4394**	allXL	DA	372	357	DBSU	34.16
**4394**	allXL	BC	381	357	DBSU	48.01
**4394**	allXL	DA	373	357	DBSU	35.39
**4394**	allXL	DA	372	370	DBSU	15.45
**4394**	allXL	DA	373	370	DBSU	12.16
**4394**	allXL	DA	382	373	DBSU	30.72

**4395**	allXL	AA	320	357	BS2G	23.37
**4395**	allXL	AA	320	373	BS2G	20.61
**4395**	allXL	CC	373	382	BS2G	16.24
**4395**	allXL	CC	370	386	BS2G	24.3
**4395**	allXL	CC	373	386	BS2G	18.58
**4395**	allXL	AA	319	357	BS2G	22.4
**4395**	allXL	AA	367	373	BS2G	11.89
**4395**	allXL	DD	370	382	BS2G	21.66
**4395**	allXL	CC	376	382	BS2G	14.05
**4395**	allXL	BB	357	373	BS2G	33.17
**4395**	allXL	AA	320	366	BS2G	9.05
**4395**	allXL	CC	370	376	BS2G	16.77
**4395**	allXL	AA	320	357	DBSU	23.37
**4395**	allXL	AA	357	319	DBSU	22.4
**4395**	allXL	AA	372	319	DBSU	22.09
**4395**	allXL	BB	370	357	DBSU	25.47
**4395**	allXL	AA	382	357	DBSU	37.9
**4395**	allXL	AA	372	370	DBSU	6.39
**4395**	allXL	DD	381	370	DBSU	22.64
**4395**	allXL	AA	373	370	DBSU	9.22
**4395**	allXL	DD	381	372	DBSU	17.12
**4395**	allXL	DD	372	382	DBSU	16.79
**4395**	allXL	CC	372	386	DBSU	18.35
**4395**	allXL	CC	373	386	DBSU	18.58
**4395**	allXL	AD	381	386	DBSU	65.8
**4395**	allXL	BD;DB	357	357	DBSU	18.34
**4395**	allXL	AD	373	319	DBSU	39.83
**4395**	allXL	BD	372	357	DBSU	43.79
**4395**	allXL	AC	381	357	DBSU	53.17
**4395**	allXL	BD	373	357	DBSU	44.31
**4395**	allXL	DA	372	370	DBSU	44.86
**4395**	allXL	DA	373	370	DBSU	45.49
**4395**	allXL	DA	382	373	DBSU	60.34
**4396**	allXL	AA	320	357	BS2G	24.18

**4396**	allXL	BB	320	373	BS2G	21.26
**4396**	allXL	BB	373	382	BS2G	18.78
**4396**	allXL	DD	370	386	BS2G	19.63
**4396**	allXL	AA	373	386	BS2G	23.53
**4396**	allXL	AA	319	357	BS2G	22.6
**4396**	allXL	BB	367	373	BS2G	13.77
**4396**	allXL	DD	370	382	BS2G	17.73
**4396**	allXL	AA	376	382	BS2G	14.63
**4396**	allXL	DD	357	373	BS2G	24.49
**4396**	allXL	AA	320	366	BS2G	17.63
**4396**	allXL	BB	370	376	BS2G	16.67
**4396**	allXL	AA	320	357	DBSU	24.18
**4396**	allXL	AA	357	319	DBSU	22.6
**4396**	allXL	BB	372	319	DBSU	23.28
**4396**	allXL	DD	370	357	DBSU	19.28
**4396**	allXL	BB	382	357	DBSU	22.47
**4396**	allXL	AA	372	370	DBSU	6.04
**4396**	allXL	DD	381	370	DBSU	17.29
**4396**	allXL	AA	373	370	DBSU	9.17
**4396**	allXL	DD	381	372	DBSU	18.95
**4396**	allXL	DD	372	382	DBSU	20.52
**4396**	allXL	DD	372	386	DBSU	25.03
**4396**	allXL	AA	373	386	DBSU	23.53
**4396**	allXL	DB	381	386	DBSU	44.83
**4396**	allXL	BD;DB	357	357	DBSU	19.27
**4396**	allXL	CB	373	319	DBSU	31.1
**4396**	allXL	CA	372	357	DBSU	31.08
**4396**	allXL	CA	381	357	DBSU	23.11
**4396**	allXL	CA	373	357	DBSU	28.56
**4396**	allXL	CB	372	370	DBSU	43.55
**4396**	allXL	BC	373	370	DBSU	44.26
**4396**	allXL	BD	382	373	DBSU	44.11
**4397**	allXL	AA	320	357	BS2G	25.66
**4397**	allXL	BB	320	373	BS2G	18.4

**4397**	allXL	AA	373	382	BS2G	15.52
**4397**	allXL	AA	370	386	BS2G	15.59
**4397**	allXL	AA	373	386	BS2G	14.68
**4397**	allXL	AA	319	357	BS2G	24.27
**4397**	allXL	AA	367	373	BS2G	11.74
**4397**	allXL	AA	370	382	BS2G	19.67
**4397**	allXL	BB	376	382	BS2G	14.2
**4397**	allXL	BB	357	373	BS2G	13.8
**4397**	allXL	BB	320	366	BS2G	24.42
**4397**	allXL	CC	370	376	BS2G	17.24
**4397**	allXL	AA	320	357	DBSU	25.66
**4397**	allXL	AA	357	319	DBSU	24.27
**4397**	allXL	BB	372	319	DBSU	18.56
**4397**	allXL	BB	370	357	DBSU	15.2
**4397**	allXL	BB	382	357	DBSU	22.75
**4397**	allXL	AA	372	370	DBSU	6.49
**4397**	allXL	AA	381	370	DBSU	19.66
**4397**	allXL	AA	373	370	DBSU	9.04
**4397**	allXL	DD	381	372	DBSU	16.17
**4397**	allXL	DD	372	382	DBSU	17.11
**4397**	allXL	AA	372	386	DBSU	15.67
**4397**	allXL	AA	373	386	DBSU	14.68
**4397**	allXL	BC	381	386	DBSU	35.95
**4397**	allXL	BD;DB	357	357	DBSU	18.7
**4397**	allXL	CB	373	319	DBSU	17.92
**4397**	allXL	CB	372	357	DBSU	23.98
**4397**	allXL	BD	381	357	DBSU	29.12
**4397**	allXL	CB	373	357	DBSU	24.7
**4397**	allXL	CB	372	370	DBSU	9.6
**4397**	allXL	CB	373	370	DBSU	11.65
**4397**	allXL	CB	382	373	DBSU	23.28
**4398**	allXL	AA	320	357	BS2G	27.16
**4398**	allXL	BB	320	373	BS2G	31.81
**4398**	allXL	CC	373	382	BS2G	21.35

**4398**	allXL	BB	370	386	BS2G	26.89
**4398**	allXL	BB	373	386	BS2G	23.27
**4398**	allXL	AA	319	357	BS2G	28.6
**4398**	allXL	AA	367	373	BS2G	13.19
**4398**	allXL	CC	370	382	BS2G	26.22
**4398**	allXL	BB	376	382	BS2G	13.84
**4398**	allXL	BB	357	373	BS2G	26.12
**4398**	allXL	AA	320	366	BS2G	20.89
**4398**	allXL	DD	370	376	BS2G	14.69
**4398**	allXL	AA	320	357	DBSU	27.16
**4398**	allXL	AA	357	319	DBSU	28.6
**4398**	allXL	BB	372	319	DBSU	29.93
**4398**	allXL	BB	370	357	DBSU	27.67
**4398**	allXL	BB	382	357	DBSU	36.16
**4398**	allXL	AA	372	370	DBSU	6.37
**4398**	allXL	DD	381	370	DBSU	24.06
**4398**	allXL	AA	373	370	DBSU	9.21
**4398**	allXL	CC	381	372	DBSU	23.9
**4398**	allXL	CC	372	382	DBSU	22.3
**4398**	allXL	BB	372	386	DBSU	24.99
**4398**	allXL	BB	373	386	DBSU	23.27
**4398**	allXL	BC	381	386	DBSU	52.6
**4398**	allXL	BD;DB	357	357	DBSU	18.14
**4398**	allXL	CB	373	319	DBSU	22.67
**4398**	allXL	CB	372	357	DBSU	36.48
**4398**	allXL	BD	381	357	DBSU	39.92
**4398**	allXL	CB	373	357	DBSU	37.96
**4398**	allXL	CB	372	370	DBSU	27.3
**4398**	allXL	CB	373	370	DBSU	28.71
**4398**	allXL	CB	382	373	DBSU	39.0
**4399**	allXL	AA	320	357	BS2G	25.82
**4399**	allXL	DD	320	373	BS2G	11.92
**4399**	allXL	BB	373	382	BS2G	18.33
**4399**	allXL	CC	370	386	BS2G	16.51

**4399**	allXL	AA	373	386	BS2G	14.92
**4399**	allXL	AA	319	357	BS2G	25.03
**4399**	allXL	AA	367	373	BS2G	11.04
**4399**	allXL	CC	370	382	BS2G	12.74
**4399**	allXL	CC	376	382	BS2G	14.16
**4399**	allXL	CC	357	373	BS2G	28.8
**4399**	allXL	DD	320	366	BS2G	11.77
**4399**	allXL	CC	370	376	BS2G	13.83
**4399**	allXL	AA	320	357	DBSU	25.82
**4399**	allXL	AA	357	319	DBSU	25.03
**4399**	allXL	DD	372	319	DBSU	14.92
**4399**	allXL	DD	370	357	DBSU	22.52
**4399**	allXL	BB	382	357	DBSU	28.37
**4399**	allXL	AA	372	370	DBSU	6.51
**4399**	allXL	CC	381	370	DBSU	15.73
**4399**	allXL	AA	373	370	DBSU	9.06
**4399**	allXL	CC	381	372	DBSU	19.89
**4399**	allXL	CC	372	382	DBSU	17.84
**4399**	allXL	AA	372	386	DBSU	16.68
**4399**	allXL	AA	373	386	DBSU	14.92
**4399**	allXL	DA	381	386	DBSU	24.66
**4399**	allXL	BD;DB	357	357	DBSU	20.01
**4399**	allXL	AD	373	319	DBSU	28.33
**4399**	allXL	DB	372	357	DBSU	43.38
**4399**	allXL	DA	381	357	DBSU	42.55
**4399**	allXL	DB	373	357	DBSU	44.67
**4399**	allXL	AD	372	370	DBSU	40.53
**4399**	allXL	AD	373	370	DBSU	39.86
**4399**	allXL	DA	382	373	DBSU	23.08
**4400**	allXL	AA	320	357	BS2G	25.04
**4400**	allXL	BB	320	373	BS2G	13.0
**4400**	allXL	CC	373	382	BS2G	17.2
**4400**	allXL	DD	370	386	BS2G	24.15
**4400**	allXL	CC	373	386	BS2G	22.3

**4400**	allXL	AA	319	357	BS2G	23.56
**4400**	allXL	AA	367	373	BS2G	12.28
**4400**	allXL	BB	370	382	BS2G	22.76
**4400**	allXL	CC	376	382	BS2G	13.66
**4400**	allXL	BB	357	373	BS2G	19.28
**4400**	allXL	AA	320	366	BS2G	20.15
**4400**	allXL	CC	370	376	BS2G	16.53
**4400**	allXL	AA	320	357	DBSU	25.04
**4400**	allXL	AA	357	319	DBSU	23.56
**4400**	allXL	BB	372	319	DBSU	17.41
**4400**	allXL	DD	370	357	DBSU	20.96
**4400**	allXL	BB	382	357	DBSU	32.37
**4400**	allXL	AA	372	370	DBSU	6.26
**4400**	allXL	BB	381	370	DBSU	23.79
**4400**	allXL	AA	373	370	DBSU	9.01
**4400**	allXL	DD	381	372	DBSU	21.71
**4400**	allXL	CC	372	382	DBSU	20.52
**4400**	allXL	CC	372	386	DBSU	23.95
**4400**	allXL	CC	373	386	DBSU	22.3
**4400**	allXL	BD	381	386	DBSU	56.44
**4400**	allXL	BD;DB	357	357	DBSU	18.84
**4400**	allXL	AD	373	319	DBSU	42.69
**4400**	allXL	DB	372	357	DBSU	26.6
**4400**	allXL	BC	381	357	DBSU	41.8
**4400**	allXL	DB	373	357	DBSU	28.96
**4400**	allXL	BD	372	370	DBSU	43.74
**4400**	allXL	BD	373	370	DBSU	41.22
**4400**	allXL	BD	382	373	DBSU	41.85
**4401**	allXL	CC	320	357	BS2G	26.79
**4401**	allXL	DD	320	373	BS2G	13.87
**4401**	allXL	CC	373	382	BS2G	16.76
**4401**	allXL	BB	370	386	BS2G	26.92
**4401**	allXL	CC	373	386	BS2G	24.16
**4401**	allXL	AA	319	357	BS2G	28.78

**4401**	allXL	AA	367	373	BS2G	14.13
**4401**	allXL	BB	370	382	BS2G	23.11
**4401**	allXL	CC	376	382	BS2G	13.22
**4401**	allXL	CC	357	373	BS2G	28.34
**4401**	allXL	BB	320	366	BS2G	20.9
**4401**	allXL	CC	370	376	BS2G	16.41
**4401**	allXL	CC	320	357	DBSU	26.79
**4401**	allXL	AA	357	319	DBSU	28.78
**4401**	allXL	DD	372	319	DBSU	13.73
**4401**	allXL	DD	370	357	DBSU	22.98
**4401**	allXL	CC	382	357	DBSU	36.39
**4401**	allXL	AA	372	370	DBSU	6.16
**4401**	allXL	BB	381	370	DBSU	21.85
**4401**	allXL	AA	373	370	DBSU	9.15
**4401**	allXL	BB	381	372	DBSU	19.48
**4401**	allXL	CC	372	382	DBSU	18.67
**4401**	allXL	CC	372	386	DBSU	25.97
**4401**	allXL	CC	373	386	DBSU	24.16
**4401**	allXL	AC	381	386	DBSU	76.09
**4401**	allXL	BD;DB	357	357	DBSU	17.89
**4401**	allXL	AD	373	319	DBSU	41.33
**4401**	allXL	BC	372	357	DBSU	39.85
**4401**	allXL	CA	381	357	DBSU	44.91
**4401**	allXL	CA	373	357	DBSU	40.34
**4401**	allXL	DA	372	370	DBSU	33.77
**4401**	allXL	DA	373	370	DBSU	36.72
**4401**	allXL	DA	382	373	DBSU	57.83
**4402**	allXL	CC	320	357	BS2G	26.18
**4402**	allXL	DD	320	373	BS2G	13.9
**4402**	allXL	AA	373	382	BS2G	17.6
**4402**	allXL	DD	370	386	BS2G	20.41
**4402**	allXL	AA	373	386	BS2G	24.1
**4402**	allXL	AA	319	357	BS2G	27.71
**4402**	allXL	AA	367	373	BS2G	10.73

**4402**	allXL	DD	370	382	BS2G	18.46
**4402**	allXL	AA	376	382	BS2G	14.4
**4402**	allXL	AA	357	373	BS2G	23.26
**4402**	allXL	AA	320	366	BS2G	23.82
**4402**	allXL	CC	370	376	BS2G	16.17
**4402**	allXL	CC	320	357	DBSU	26.18
**4402**	allXL	AA	357	319	DBSU	27.71
**4402**	allXL	DD	372	319	DBSU	18.94
**4402**	allXL	DD	370	357	DBSU	21.56
**4402**	allXL	BB	382	357	DBSU	22.19
**4402**	allXL	CC	372	370	DBSU	6.61
**4402**	allXL	DD	381	370	DBSU	19.85
**4402**	allXL	AA	373	370	DBSU	9.07
**4402**	allXL	DD	381	372	DBSU	17.68
**4402**	allXL	DD	372	382	DBSU	17.46
**4402**	allXL	DD	372	386	DBSU	23.37
**4402**	allXL	AA	373	386	DBSU	24.1
**4402**	allXL	DA	381	386	DBSU	35.04
**4402**	allXL	BD;DB	357	357	DBSU	18.4
**4402**	allXL	AD	373	319	DBSU	16.62
**4402**	allXL	DA	372	357	DBSU	30.99
**4402**	allXL	BD	381	357	DBSU	39.75
**4402**	allXL	DA	373	357	DBSU	31.16
**4402**	allXL	DA	372	370	DBSU	12.29
**4402**	allXL	DA	373	370	DBSU	9.53
**4402**	allXL	DA	382	373	DBSU	30.43
**4403**	allXL	CC	320	357	BS2G	25.08
**4403**	allXL	BB	320	373	BS2G	21.83
**4403**	allXL	BB	373	382	BS2G	17.56
**4403**	allXL	BB	370	386	BS2G	24.44
**4403**	allXL	BB	373	386	BS2G	21.53
**4403**	allXL	AA	319	357	BS2G	24.6
**4403**	allXL	AA	367	373	BS2G	11.9
**4403**	allXL	DD	370	382	BS2G	19.33

**4403**	allXL	BB	376	382	BS2G	12.96
**4403**	allXL	BB	357	373	BS2G	31.47
**4403**	allXL	BB	320	366	BS2G	17.24
**4403**	allXL	CC	370	376	BS2G	13.83
**4403**	allXL	CC	320	357	DBSU	25.08
**4403**	allXL	AA	357	319	DBSU	24.6
**4403**	allXL	BB	372	319	DBSU	24.53
**4403**	allXL	DD	370	357	DBSU	31.06
**4403**	allXL	AA	382	357	DBSU	14.26
**4403**	allXL	AA	372	370	DBSU	6.48
**4403**	allXL	DD	381	370	DBSU	20.01
**4403**	allXL	CC	373	370	DBSU	9.14
**4403**	allXL	CC	381	372	DBSU	22.14
**4403**	allXL	BB	372	382	DBSU	20.76
**4403**	allXL	BB	372	386	DBSU	23.26
**4403**	allXL	BB	373	386	DBSU	21.53
**4403**	allXL	DB	381	386	DBSU	21.99
**4403**	allXL	BD;DB	357	357	DBSU	17.47
**4403**	allXL	AD	373	319	DBSU	18.5
**4403**	allXL	DB	372	357	DBSU	46.18
**4403**	allXL	DB	381	357	DBSU	28.21
**4403**	allXL	DB	373	357	DBSU	43.74
**4403**	allXL	AD	372	370	DBSU	57.64
**4403**	allXL	AD	373	370	DBSU	55.14
**4403**	allXL	BD	382	373	DBSU	39.85
**4404**	allXL	AA	320	357	BS2G	25.84
**4404**	allXL	DD	320	373	BS2G	23.47
**4404**	allXL	BB	373	382	BS2G	19.08
**4404**	allXL	BB	370	386	BS2G	22.02
**4404**	allXL	AA	373	386	BS2G	23.36
**4404**	allXL	AA	319	357	BS2G	25.77
**4404**	allXL	AA	367	373	BS2G	13.22
**4404**	allXL	BB	370	382	BS2G	20.65
**4404**	allXL	BB	376	382	BS2G	14.14

**4404**	allXL	CC	357	373	BS2G	31.72
**4404**	allXL	AA	320	366	BS2G	21.82
**4404**	allXL	CC	370	376	BS2G	15.06
**4404**	allXL	AA	320	357	DBSU	25.84
**4404**	allXL	AA	357	319	DBSU	25.77
**4404**	allXL	BB	372	319	DBSU	23.37
**4404**	allXL	DD	370	357	DBSU	29.78
**4404**	allXL	CC	382	357	DBSU	40.84
**4404**	allXL	AA	372	370	DBSU	6.36
**4404**	allXL	BB	381	370	DBSU	23.43
**4404**	allXL	DD	373	370	DBSU	9.03
**4404**	allXL	DD	381	372	DBSU	19.58
**4404**	allXL	DD	372	382	DBSU	20.74
**4404**	allXL	DD	372	386	DBSU	23.49
**4404**	allXL	AA	373	386	DBSU	23.36
**4404**	allXL	DA	381	386	DBSU	69.06
**4404**	allXL	BD;DB	357	357	DBSU	17.51
**4404**	allXL	AD	373	319	DBSU	38.82
**4404**	allXL	BD	372	357	DBSU	43.97
**4404**	allXL	CA	381	357	DBSU	55.55
**4404**	allXL	BD	373	357	DBSU	46.86
**4404**	allXL	DA	372	370	DBSU	47.26
**4404**	allXL	DA	373	370	DBSU	44.18
**4404**	allXL	DA	382	373	DBSU	59.96
**4405**	allXL	AA	320	357	BS2G	23.75
**4405**	allXL	BB	320	373	BS2G	16.64
**4405**	allXL	DD	373	382	BS2G	19.54
**4405**	allXL	DD	370	386	BS2G	26.15
**4405**	allXL	AA	373	386	BS2G	27.03
**4405**	allXL	AA	319	357	BS2G	23.19
**4405**	allXL	AA	367	373	BS2G	13.7
**4405**	allXL	DD	370	382	BS2G	20.42
**4405**	allXL	CC	376	382	BS2G	13.55
**4405**	allXL	BB	357	373	BS2G	30.77

**4405**	allXL	BB	320	366	BS2G	14.41
**4405**	allXL	CC	370	376	BS2G	15.6
**4405**	allXL	AA	320	357	DBSU	23.75
**4405**	allXL	AA	357	319	DBSU	23.19
**4405**	allXL	BB	372	319	DBSU	15.04
**4405**	allXL	DD	370	357	DBSU	29.58
**4405**	allXL	BA	382	357	DBSU	35.26
**4405**	allXL	AA	372	370	DBSU	6.25
**4405**	allXL	DD	381	370	DBSU	18.81
**4405**	allXL	AA	373	370	DBSU	9.1
**4405**	allXL	DD	381	372	DBSU	17.79
**4405**	allXL	DD	372	382	DBSU	20.08
**4405**	allXL	DD	372	386	DBSU	28.4
**4405**	allXL	AA	373	386	DBSU	27.03
**4405**	allXL	BC	381	386	DBSU	44.93
**4405**	allXL	BD;DB	357	357	DBSU	18.96
**4405**	allXL	DA	373	319	DBSU	34.37
**4405**	allXL	BC	372	357	DBSU	28.47
**4405**	allXL	BC	381	357	DBSU	32.16
**4405**	allXL	BC	373	357	DBSU	25.21
**4405**	allXL	BC	372	370	DBSU	39.61
**4405**	allXL	BC	373	370	DBSU	39.11
**4405**	allXL	CB	382	373	DBSU	48.26
**4406**	allXL	CC	320	357	BS2G	23.13
**4406**	allXL	DD	320	373	BS2G	15.91
**4406**	allXL	CC	373	382	BS2G	19.06
**4406**	allXL	BB	370	386	BS2G	23.5
**4406**	allXL	BB	373	386	BS2G	24.96
**4406**	allXL	AA	319	357	BS2G	20.83
**4406**	allXL	BB	367	373	BS2G	12.17
**4406**	allXL	DD	370	382	BS2G	25.68
**4406**	allXL	CC	376	382	BS2G	15.16
**4406**	allXL	BB	357	373	BS2G	19.35
**4406**	allXL	AA	320	366	BS2G	22.38

**4406**	allXL	CC	370	376	BS2G	17.38
**4406**	allXL	CC	320	357	DBSU	23.13
**4406**	allXL	AA	357	319	DBSU	20.83
**4406**	allXL	DD	372	319	DBSU	16.5
**4406**	allXL	DD	370	357	DBSU	20.32
**4406**	allXL	BB	382	357	DBSU	28.0
**4406**	allXL	AA	372	370	DBSU	6.01
**4406**	allXL	DD	381	370	DBSU	23.91
**4406**	allXL	AA	373	370	DBSU	9.26
**4406**	allXL	DD	381	372	DBSU	22.05
**4406**	allXL	CC	372	382	DBSU	22.61
**4406**	allXL	BB	372	386	DBSU	26.53
**4406**	allXL	BB	373	386	DBSU	24.96
**4406**	allXL	AD	381	386	DBSU	47.55
**4406**	allXL	BD;DB	357	357	DBSU	19.52
**4406**	allXL	AD	373	319	DBSU	29.26
**4406**	allXL	BD	372	357	DBSU	33.72
**4406**	allXL	BD	381	357	DBSU	41.1
**4406**	allXL	BD	373	357	DBSU	32.02
**4406**	allXL	DA	372	370	DBSU	27.96
**4406**	allXL	DA	373	370	DBSU	28.86
**4406**	allXL	DA	382	373	DBSU	33.82
**4407**	allXL	CC	320	357	BS2G	25.8
**4407**	allXL	CC	320	373	BS2G	18.8
**4407**	allXL	BB	373	382	BS2G	15.76
**4407**	allXL	DD	370	386	BS2G	20.93
**4407**	allXL	DD	373	386	BS2G	22.44
**4407**	allXL	CC	319	357	BS2G	27.51
**4407**	allXL	AA	367	373	BS2G	10.67
**4407**	allXL	AA	370	382	BS2G	18.83
**4407**	allXL	BB	376	382	BS2G	13.32
**4407**	allXL	BB	357	373	BS2G	28.09
**4407**	allXL	AA	320	366	BS2G	15.86
**4407**	allXL	AA	370	376	BS2G	15.57

**4407**	allXL	CC	320	357	DBSU	25.8
**4407**	allXL	CC	357	319	DBSU	27.51
**4407**	allXL	CC	372	319	DBSU	16.69
**4407**	allXL	BB	370	357	DBSU	27.93
**4407**	allXL	BB	382	357	DBSU	24.19
**4407**	allXL	CC	372	370	DBSU	6.84
**4407**	allXL	AA	381	370	DBSU	20.23
**4407**	allXL	AA	373	370	DBSU	8.68
**4407**	allXL	BB	381	372	DBSU	18.31
**4407**	allXL	BB	372	382	DBSU	15.96
**4407**	allXL	DD	372	386	DBSU	21.47
**4407**	allXL	DD	373	386	DBSU	22.44
**4407**	allXL	BC	381	386	DBSU	51.92
**4407**	allXL	AC;CA	357	357	DBSU	19.09
**4407**	allXL	AD	373	319	DBSU	30.33
**4407**	allXL	BD	372	357	DBSU	38.22
**4407**	allXL	BD	381	357	DBSU	30.56
**4407**	allXL	BD	373	357	DBSU	40.3
**4407**	allXL	AD	372	370	DBSU	49.64
**4407**	allXL	AD	373	370	DBSU	51.77
**4407**	allXL	CB	382	373	DBSU	56.93
**4408**	allXL	AA	320	357	BS2G	24.7
**4408**	allXL	CC	320	373	BS2G	13.96
**4408**	allXL	AA	373	382	BS2G	18.69
**4408**	allXL	AA	370	386	BS2G	15.8
**4408**	allXL	AA	373	386	BS2G	18.58
**4408**	allXL	AA	319	357	BS2G	23.7
**4408**	allXL	AA	367	373	BS2G	14.51
**4408**	allXL	DD	370	382	BS2G	20.58
**4408**	allXL	CC	376	382	BS2G	13.45
**4408**	allXL	DD	357	373	BS2G	28.13
**4408**	allXL	DD	320	366	BS2G	18.1
**4408**	allXL	CC	370	376	BS2G	15.62
**4408**	allXL	AA	320	357	DBSU	24.7

**4408**	allXL	AA	357	319	DBSU	23.7
**4408**	allXL	CC	372	319	DBSU	8.75
**4408**	allXL	DD	370	357	DBSU	22.49
**4408**	allXL	DD	382	357	DBSU	36.85
**4408**	allXL	AA	372	370	DBSU	6.27
**4408**	allXL	DD	381	370	DBSU	17.98
**4408**	allXL	AA	373	370	DBSU	9.19
**4408**	allXL	AA	381	372	DBSU	18.69
**4408**	allXL	AA	372	382	DBSU	17.38
**4408**	allXL	AA	372	386	DBSU	15.38
**4408**	allXL	AA	373	386	DBSU	18.58
**4408**	allXL	AD	381	386	DBSU	36.4
**4408**	allXL	BD;DB	357	357	DBSU	18.78
**4408**	allXL	AD	373	319	DBSU	24.03
**4408**	allXL	DA	372	357	DBSU	28.7
**4408**	allXL	DA	381	357	DBSU	40.76
**4408**	allXL	DA	373	357	DBSU	26.61
**4408**	allXL	DA	372	370	DBSU	31.58
**4408**	allXL	DA	373	370	DBSU	30.27
**4408**	allXL	DA	382	373	DBSU	24.42
**4409**	allXL	AA	320	357	BS2G	24.93
**4409**	allXL	BB	320	373	BS2G	27.73
**4409**	allXL	DD	373	382	BS2G	15.02
**4409**	allXL	DD	370	386	BS2G	16.83
**4409**	allXL	DD	373	386	BS2G	23.93
**4409**	allXL	AA	319	357	BS2G	24.74
**4409**	allXL	AA	367	373	BS2G	11.24
**4409**	allXL	DD	370	382	BS2G	12.39
**4409**	allXL	BB	376	382	BS2G	13.5
**4409**	allXL	AA	357	373	BS2G	22.21
**4409**	allXL	AA	320	366	BS2G	27.1
**4409**	allXL	CC	370	376	BS2G	15.5
**4409**	allXL	AA	320	357	DBSU	24.93
**4409**	allXL	AA	357	319	DBSU	24.74

**4409**	allXL	CC	372	319	DBSU	29.4
**4409**	allXL	AA	370	357	DBSU	29.39
**4409**	allXL	BB	382	357	DBSU	20.56
**4409**	allXL	AA	372	370	DBSU	6.2
**4409**	allXL	DD	381	370	DBSU	12.86
**4409**	allXL	AA	373	370	DBSU	9.21
**4409**	allXL	DD	381	372	DBSU	14.23
**4409**	allXL	DD	372	382	DBSU	14.94
**4409**	allXL	DD	372	386	DBSU	22.36
**4409**	allXL	DD	373	386	DBSU	23.93
**4409**	allXL	DB	381	386	DBSU	54.26
**4409**	allXL	BD;DB	357	357	DBSU	19.35
**4409**	allXL	DA	373	319	DBSU	33.95
**4409**	allXL	CB	372	357	DBSU	38.6
**4409**	allXL	BD	381	357	DBSU	35.61
**4409**	allXL	AC	373	357	DBSU	36.09
**4409**	allXL	CB	372	370	DBSU	30.71
**4409**	allXL	CB	373	370	DBSU	32.21
**4409**	allXL	CB	382	373	DBSU	41.1
**4410**	allXL	AA	320	357	BS2G	24.7
**4410**	allXL	BB	320	373	BS2G	13.85
**4410**	allXL	CC	373	382	BS2G	16.84
**4410**	allXL	CC	370	386	BS2G	20.93
**4410**	allXL	AA	373	386	BS2G	26.48
**4410**	allXL	AA	319	357	BS2G	24.78
**4410**	allXL	AA	367	373	BS2G	12.39
**4410**	allXL	CC	370	382	BS2G	14.65
**4410**	allXL	CC	376	382	BS2G	14.32
**4410**	allXL	BB	357	373	BS2G	22.53
**4410**	allXL	DD	320	366	BS2G	16.46
**4410**	allXL	CC	370	376	BS2G	14.78
**4410**	allXL	AA	320	357	DBSU	24.7
**4410**	allXL	AA	357	319	DBSU	24.78
**4410**	allXL	BB	372	319	DBSU	16.15

**4410**	allXL	BB	370	357	DBSU	27.8
**4410**	allXL	BB	382	357	DBSU	39.43
**4410**	allXL	AA	372	370	DBSU	6.21
**4410**	allXL	CC	381	370	DBSU	17.78
**4410**	allXL	AA	373	370	DBSU	9.07
**4410**	allXL	CC	381	372	DBSU	19.92
**4410**	allXL	CC	372	382	DBSU	17.74
**4410**	allXL	AA	372	386	DBSU	24.11
**4410**	allXL	AA	373	386	DBSU	26.48
**4410**	allXL	AD	381	386	DBSU	43.48
**4410**	allXL	BD;DB	357	357	DBSU	20.21
**4410**	allXL	DA	373	319	DBSU	17.32
**4410**	allXL	DA	372	357	DBSU	35.22
**4410**	allXL	BD	381	357	DBSU	47.69
**4410**	allXL	DA	373	357	DBSU	34.79
**4410**	allXL	DA	372	370	DBSU	12.99
**4410**	allXL	DA	373	370	DBSU	16.66
**4410**	allXL	DA	382	373	DBSU	25.21
**4411**	allXL	AA	320	357	BS2G	23.76
**4411**	allXL	BB	320	373	BS2G	21.8
**4411**	allXL	CC	373	382	BS2G	16.84
**4411**	allXL	DD	370	386	BS2G	28.4
**4411**	allXL	CC	373	386	BS2G	24.65
**4411**	allXL	AA	319	357	BS2G	23.53
**4411**	allXL	AA	367	373	BS2G	14.44
**4411**	allXL	DD	370	382	BS2G	23.21
**4411**	allXL	BB	376	382	BS2G	14.42
**4411**	allXL	BB	357	373	BS2G	28.28
**4411**	allXL	AA	320	366	BS2G	23.9
**4411**	allXL	DD	370	376	BS2G	16.57
**4411**	allXL	AA	320	357	DBSU	23.76
**4411**	allXL	AA	357	319	DBSU	23.53
**4411**	allXL	DD	372	319	DBSU	22.19
**4411**	allXL	DD	370	357	DBSU	24.99

**4411**	allXL	BB	382	357	DBSU	26.88
**4411**	allXL	AA	372	370	DBSU	5.88
**4411**	allXL	DD	381	370	DBSU	20.94
**4411**	allXL	AA	373	370	DBSU	9.35
**4411**	allXL	DD	381	372	DBSU	21.84
**4411**	allXL	CC	372	382	DBSU	20.12
**4411**	allXL	CC	372	386	DBSU	26.68
**4411**	allXL	CC	373	386	DBSU	24.65
**4411**	allXL	BD	381	386	DBSU	37.59
**4411**	allXL	BD;DB	357	357	DBSU	19.01
**4411**	allXL	DA	373	319	DBSU	17.97
**4411**	allXL	DA	372	357	DBSU	25.45
**4411**	allXL	DA	381	357	DBSU	41.6
**4411**	allXL	DA	373	357	DBSU	24.77
**4411**	allXL	DA	372	370	DBSU	23.22
**4411**	allXL	DA	373	370	DBSU	21.23
**4411**	allXL	CB	382	373	DBSU	42.05
**4412**	allXL	BB	320	357	BS2G	26.6
**4412**	allXL	DD	320	373	BS2G	12.43
**4412**	allXL	AA	373	382	BS2G	18.25
**4412**	allXL	AA	370	386	BS2G	17.19
**4412**	allXL	AA	373	386	BS2G	20.92
**4412**	allXL	DD	319	357	BS2G	28.94
**4412**	allXL	AA	367	373	BS2G	13.42
**4412**	allXL	AA	370	382	BS2G	20.17
**4412**	allXL	AA	376	382	BS2G	13.44
**4412**	allXL	BB	357	373	BS2G	27.74
**4412**	allXL	AA	320	366	BS2G	20.93
**4412**	allXL	BB	370	376	BS2G	16.37
**4412**	allXL	BB	320	357	DBSU	26.6
**4412**	allXL	DD	357	319	DBSU	28.94
**4412**	allXL	DD	372	319	DBSU	12.44
**4412**	allXL	DD	370	357	DBSU	26.12
**4412**	allXL	BB	382	357	DBSU	38.92

**4412**	allXL	AA	372	370	DBSU	6.15
**4412**	allXL	AA	381	370	DBSU	23.37
**4412**	allXL	AA	373	370	DBSU	9.1
**4412**	allXL	AA	381	372	DBSU	20.8
**4412**	allXL	AA	372	382	DBSU	18.04
**4412**	allXL	AA	372	386	DBSU	18.89
**4412**	allXL	AA	373	386	DBSU	20.92
**4412**	allXL	BD	381	386	DBSU	55.62
**4412**	allXL	BD;DB	357	357	DBSU	18.06
**4412**	allXL	AD	373	319	DBSU	29.59
**4412**	allXL	BD	372	357	DBSU	29.03
**4412**	allXL	BD	381	357	DBSU	30.68
**4412**	allXL	BD	373	357	DBSU	26.89
**4412**	allXL	DA	372	370	DBSU	34.67
**4412**	allXL	AD	373	370	DBSU	31.8
**4412**	allXL	AD	382	373	DBSU	39.48
**4413**	allXL	AA	320	357	BS2G	16.98
**4413**	allXL	DD	320	373	BS2G	29.29
**4413**	allXL	DD	373	382	BS2G	18.3
**4413**	allXL	BB	370	386	BS2G	18.1
**4413**	allXL	BB	373	386	BS2G	16.42
**4413**	allXL	AA	319	357	BS2G	20.54
**4413**	allXL	AA	367	373	BS2G	13.19
**4413**	allXL	DD	370	382	BS2G	23.23
**4413**	allXL	BB	376	382	BS2G	13.36
**4413**	allXL	BB	357	373	BS2G	29.76
**4413**	allXL	DD	320	366	BS2G	24.37
**4413**	allXL	AA	370	376	BS2G	16.53
**4413**	allXL	AA	320	357	DBSU	16.98
**4413**	allXL	AA	357	319	DBSU	20.54
**4413**	allXL	DD	372	319	DBSU	26.95
**4413**	allXL	DD	370	357	DBSU	29.67
**4413**	allXL	BB	382	357	DBSU	33.57
**4413**	allXL	AA	372	370	DBSU	6.14

**4413**	allXL	DD	381	370	DBSU	21.16
**4413**	allXL	AA	373	370	DBSU	9.23
**4413**	allXL	DD	381	372	DBSU	14.45
**4413**	allXL	DD	372	382	DBSU	17.01
**4413**	allXL	BB	372	386	DBSU	18.17
**4413**	allXL	BB	373	386	DBSU	16.42
**4413**	allXL	CB	381	386	DBSU	34.56
**4413**	allXL	BD;DB	357	357	DBSU	19.37
**4413**	allXL	CB	373	319	DBSU	36.47
**4413**	allXL	AC	372	357	DBSU	39.47
**4413**	allXL	AC	381	357	DBSU	49.95
**4413**	allXL	AC	373	357	DBSU	36.42
**4413**	allXL	CB	372	370	DBSU	46.91
**4413**	allXL	CB	373	370	DBSU	46.85
**4413**	allXL	BC	382	373	DBSU	38.25
**4414**	allXL	CC	320	357	BS2G	26.44
**4414**	allXL	AA	320	373	BS2G	19.44
**4414**	allXL	BB	373	382	BS2G	15.91
**4414**	allXL	CC	370	386	BS2G	20.08
**4414**	allXL	DD	373	386	BS2G	25.0
**4414**	allXL	AA	319	357	BS2G	26.82
**4414**	allXL	BB	367	373	BS2G	13.74
**4414**	allXL	BB	370	382	BS2G	19.89
**4414**	allXL	CC	376	382	BS2G	14.51
**4414**	allXL	CC	357	373	BS2G	34.22
**4414**	allXL	AA	320	366	BS2G	19.28
**4414**	allXL	CC	370	376	BS2G	16.49
**4414**	allXL	CC	320	357	DBSU	26.44
**4414**	allXL	AA	357	319	DBSU	26.82
**4414**	allXL	AA	372	319	DBSU	24.79
**4414**	allXL	DD	370	357	DBSU	26.45
**4414**	allXL	BB	382	357	DBSU	34.98
**4414**	allXL	AA	372	370	DBSU	6.33
**4414**	allXL	BB	381	370	DBSU	19.77

**4414**	allXL	AA	373	370	DBSU	9.23
**4414**	allXL	BB	381	372	DBSU	18.04
**4414**	allXL	BB	372	382	DBSU	17.98
**4414**	allXL	CC	372	386	DBSU	24.23
**4414**	allXL	DD	373	386	DBSU	25.0
**4414**	allXL	BC	381	386	DBSU	46.02
**4414**	allXL	BD;DB	357	357	DBSU	18.02
**4414**	allXL	BC	373	319	DBSU	19.79
**4414**	allXL	BC	372	357	DBSU	30.08
**4414**	allXL	BC	381	357	DBSU	37.77
**4414**	allXL	BC	373	357	DBSU	29.64
**4414**	allXL	BC	372	370	DBSU	38.64
**4414**	allXL	BC	373	370	DBSU	35.87
**4414**	allXL	BC	382	373	DBSU	51.36
**4415**	allXL	AA	320	357	BS2G	24.66
**4415**	allXL	BB	320	373	BS2G	12.21
**4415**	allXL	DD	373	382	BS2G	16.53
**4415**	allXL	DD	370	386	BS2G	21.68
**4415**	allXL	DD	373	386	BS2G	26.97
**4415**	allXL	AA	319	357	BS2G	22.91
**4415**	allXL	BB	367	373	BS2G	14.74
**4415**	allXL	DD	370	382	BS2G	12.78
**4415**	allXL	CC	376	382	BS2G	14.74
**4415**	allXL	BB	357	373	BS2G	25.13
**4415**	allXL	BB	320	366	BS2G	15.49
**4415**	allXL	DD	370	376	BS2G	16.0
**4415**	allXL	AA	320	357	DBSU	24.66
**4415**	allXL	AA	357	319	DBSU	22.91
**4415**	allXL	AA	372	319	DBSU	16.37
**4415**	allXL	AA	370	357	DBSU	26.35
**4415**	allXL	BB	382	357	DBSU	42.83
**4415**	allXL	AA	372	370	DBSU	6.18
**4415**	allXL	DD	381	370	DBSU	11.98
**4415**	allXL	AA	373	370	DBSU	9.22

**4415**	allXL	DD	381	372	DBSU	13.95
**4415**	allXL	DD	372	382	DBSU	15.12
**4415**	allXL	DD	372	386	DBSU	24.81
**4415**	allXL	DD	373	386	DBSU	26.97
**4415**	allXL	AD	381	386	DBSU	40.63
**4415**	allXL	BD;DB	357	357	DBSU	20.07
**4415**	allXL	AD	373	319	DBSU	27.38
**4415**	allXL	AD	372	357	DBSU	37.77
**4415**	allXL	BC	381	357	DBSU	46.06
**4415**	allXL	AD	373	357	DBSU	41.13
**4415**	allXL	AD	372	370	DBSU	31.05
**4415**	allXL	AD	373	370	DBSU	31.91
**4415**	allXL	AD	382	373	DBSU	39.08
**4416**	allXL	AA	320	357	BS2G	24.15
**4416**	allXL	DD	320	373	BS2G	19.05
**4416**	allXL	AA	373	382	BS2G	19.55
**4416**	allXL	DD	370	386	BS2G	32.54
**4416**	allXL	BB	373	386	BS2G	29.22
**4416**	allXL	AA	319	357	BS2G	22.34
**4416**	allXL	BB	367	373	BS2G	11.76
**4416**	allXL	DD	370	382	BS2G	24.14
**4416**	allXL	BB	376	382	BS2G	14.65
**4416**	allXL	DD	357	373	BS2G	22.95
**4416**	allXL	AA	320	366	BS2G	15.14
**4416**	allXL	CC	370	376	BS2G	15.68
**4416**	allXL	AA	320	357	DBSU	24.15
**4416**	allXL	AA	357	319	DBSU	22.34
**4416**	allXL	DD	372	319	DBSU	21.74
**4416**	allXL	DD	370	357	DBSU	16.34
**4416**	allXL	BB	382	357	DBSU	29.62
**4416**	allXL	AA	372	370	DBSU	6.3
**4416**	allXL	DD	381	370	DBSU	21.98
**4416**	allXL	CC	373	370	DBSU	9.05
**4416**	allXL	DD	381	372	DBSU	20.17

**4416**	allXL	AA	372	382	DBSU	22.98
**4416**	allXL	BB	372	386	DBSU	31.22
**4416**	allXL	BB	373	386	DBSU	29.22
**4416**	allXL	CB	381	386	DBSU	21.96
**4416**	allXL	BD;DB	357	357	DBSU	18.4
**4416**	allXL	CB	373	319	DBSU	20.1
**4416**	allXL	CB	372	357	DBSU	28.69
**4416**	allXL	CB	381	357	DBSU	42.94
**4416**	allXL	CB	373	357	DBSU	31.04
**4416**	allXL	CB	372	370	DBSU	8.84
**4416**	allXL	CB	373	370	DBSU	10.07
**4416**	allXL	BC	382	373	DBSU	25.47
**4417**	allXL	CC	320	357	BS2G	22.95
**4417**	allXL	BB	320	373	BS2G	22.45
**4417**	allXL	BB	373	382	BS2G	14.34
**4417**	allXL	BB	370	386	BS2G	14.13
**4417**	allXL	BB	373	386	BS2G	22.13
**4417**	allXL	CC	319	357	BS2G	23.97
**4417**	allXL	AA	367	373	BS2G	11.39
**4417**	allXL	BB	370	382	BS2G	9.48
**4417**	allXL	BB	376	382	BS2G	13.83
**4417**	allXL	BB	357	373	BS2G	9.41
**4417**	allXL	DD	320	366	BS2G	28.74
**4417**	allXL	CC	370	376	BS2G	12.98
**4417**	allXL	CC	320	357	DBSU	22.95
**4417**	allXL	CC	357	319	DBSU	23.97
**4417**	allXL	BB	372	319	DBSU	29.66
**4417**	allXL	BB	370	357	DBSU	15.99
**4417**	allXL	BB	382	357	DBSU	22.83
**4417**	allXL	AA	372	370	DBSU	6.36
**4417**	allXL	BB	381	370	DBSU	11.31
**4417**	allXL	AA	373	370	DBSU	8.99
**4417**	allXL	BB	381	372	DBSU	15.14
**4417**	allXL	BB	372	382	DBSU	14.79

**4417**	allXL	BB	372	386	DBSU	20.75
**4417**	allXL	BB	373	386	DBSU	22.13
**4417**	allXL	DB	381	386	DBSU	41.25
**4417**	allXL	BD;DB	357	357	DBSU	18.41
**4417**	allXL	CA	373	319	DBSU	20.19
**4417**	allXL	CA	372	357	DBSU	12.67
**4417**	allXL	BD	381	357	DBSU	30.52
**4417**	allXL	CA	373	357	DBSU	15.55
**4417**	allXL	AD	372	370	DBSU	41.85
**4417**	allXL	AD	373	370	DBSU	41.29
**4417**	allXL	AD	382	373	DBSU	40.59
**4418**	allXL	AA	320	357	BS2G	22.64
**4418**	allXL	CC	320	373	BS2G	25.74
**4418**	allXL	BB	373	382	BS2G	18.76
**4418**	allXL	DD	370	386	BS2G	22.97
**4418**	allXL	AA	373	386	BS2G	23.32
**4418**	allXL	AA	319	357	BS2G	22.45
**4418**	allXL	AA	367	373	BS2G	12.52
**4418**	allXL	BB	370	382	BS2G	20.09
**4418**	allXL	BB	376	382	BS2G	14.48
**4418**	allXL	AA	357	373	BS2G	27.67
**4418**	allXL	BB	320	366	BS2G	23.39
**4418**	allXL	BB	370	376	BS2G	16.14
**4418**	allXL	AA	320	357	DBSU	22.64
**4418**	allXL	AA	357	319	DBSU	22.45
**4418**	allXL	CC	372	319	DBSU	23.39
**4418**	allXL	DD	370	357	DBSU	26.19
**4418**	allXL	AA	382	357	DBSU	24.36
**4418**	allXL	AA	372	370	DBSU	6.29
**4418**	allXL	BB	381	370	DBSU	22.09
**4418**	allXL	AA	373	370	DBSU	8.98
**4418**	allXL	DD	381	372	DBSU	19.19
**4418**	allXL	DD	372	382	DBSU	20.65
**4418**	allXL	DD	372	386	DBSU	23.23

**4418**	allXL	AA	373	386	DBSU	23.32
**4418**	allXL	AD	381	386	DBSU	57.49
**4418**	allXL	BD;DB	357	357	DBSU	18.88
**4418**	allXL	DA	373	319	DBSU	28.37
**4418**	allXL	DA	372	357	DBSU	39.69
**4418**	allXL	AC	381	357	DBSU	45.49
**4418**	allXL	DA	373	357	DBSU	40.69
**4418**	allXL	AD	372	370	DBSU	40.49
**4418**	allXL	AD	373	370	DBSU	38.21
**4418**	allXL	AD	382	373	DBSU	38.16
**4419**	allXL	AA	320	357	BS2G	24.93
**4419**	allXL	AA	320	373	BS2G	23.1
**4419**	allXL	CC	373	382	BS2G	16.61
**4419**	allXL	CC	370	386	BS2G	23.64
**4419**	allXL	CC	373	386	BS2G	25.02
**4419**	allXL	AA	319	357	BS2G	23.53
**4419**	allXL	AA	367	373	BS2G	13.16
**4419**	allXL	CC	370	382	BS2G	19.06
**4419**	allXL	CC	376	382	BS2G	14.53
**4419**	allXL	BB	357	373	BS2G	23.9
**4419**	allXL	AA	320	366	BS2G	16.72
**4419**	allXL	CC	370	376	BS2G	14.78
**4419**	allXL	AA	320	357	DBSU	24.93
**4419**	allXL	AA	357	319	DBSU	23.53
**4419**	allXL	AA	372	319	DBSU	22.63
**4419**	allXL	BB	370	357	DBSU	23.23
**4419**	allXL	DD	382	357	DBSU	15.93
**4419**	allXL	AA	372	370	DBSU	6.23
**4419**	allXL	DD	381	370	DBSU	20.49
**4419**	allXL	AA	373	370	DBSU	9.04
**4419**	allXL	CC	381	372	DBSU	19.63
**4419**	allXL	CC	372	382	DBSU	18.99
**4419**	allXL	CC	372	386	DBSU	25.81
**4419**	allXL	CC	373	386	DBSU	25.02

**4419**	allXL	DA	381	386	DBSU	33.08
**4419**	allXL	BD;DB	357	357	DBSU	19.06
**4419**	allXL	AD	373	319	DBSU	35.08
**4419**	allXL	BD	372	357	DBSU	29.4
**4419**	allXL	DB	381	357	DBSU	26.99
**4419**	allXL	BD	373	357	DBSU	31.9
**4419**	allXL	DA	372	370	DBSU	28.84
**4419**	allXL	DA	373	370	DBSU	31.16
**4419**	allXL	AD	382	373	DBSU	30.14
**4420**	allXL	AA	320	357	BS2G	25.61
**4420**	allXL	AA	320	373	BS2G	19.73
**4420**	allXL	DD	373	382	BS2G	13.6
**4420**	allXL	DD	370	386	BS2G	13.34
**4420**	allXL	DD	373	386	BS2G	21.88
**4420**	allXL	AA	319	357	BS2G	24.06
**4420**	allXL	AA	367	373	BS2G	13.28
**4420**	allXL	DD	370	382	BS2G	5.16
**4420**	allXL	BB	376	382	BS2G	13.44
**4420**	allXL	AA	357	373	BS2G	35.79
**4420**	allXL	DD	320	366	BS2G	18.31
**4420**	allXL	DD	370	376	BS2G	14.62
**4420**	allXL	AA	320	357	DBSU	25.61
**4420**	allXL	AA	357	319	DBSU	24.06
**4420**	allXL	AA	372	319	DBSU	20.1
**4420**	allXL	BB	370	357	DBSU	28.7
**4420**	allXL	DD	382	357	DBSU	30.35
**4420**	allXL	AA	372	370	DBSU	6.37
**4420**	allXL	DD	381	370	DBSU	7.49
**4420**	allXL	AA	373	370	DBSU	9.13
**4420**	allXL	DD	381	372	DBSU	13.31
**4420**	allXL	DD	372	382	DBSU	12.03
**4420**	allXL	DD	372	386	DBSU	18.91
**4420**	allXL	DD	373	386	DBSU	21.88
**4420**	allXL	CB	381	386	DBSU	41.64

**4420**	allXL	BD;DB	357	357	DBSU	18.18
**4420**	allXL	CB	373	319	DBSU	25.36
**4420**	allXL	AD	372	357	DBSU	45.22
**4420**	allXL	DB	381	357	DBSU	46.89
**4420**	allXL	DA	373	357	DBSU	43.97
**4420**	allXL	DA	372	370	DBSU	19.34
**4420**	allXL	DA	373	370	DBSU	16.37
**4420**	allXL	DA	382	373	DBSU	32.07
**4421**	allXL	AA	320	357	BS2G	28.09
**4421**	allXL	CC	320	373	BS2G	7.76
**4421**	allXL	AA	373	382	BS2G	18.17
**4421**	allXL	AA	370	386	BS2G	23.37
**4421**	allXL	DD	373	386	BS2G	24.03
**4421**	allXL	AA	319	357	BS2G	29.51
**4421**	allXL	AA	367	373	BS2G	13.92
**4421**	allXL	AA	370	382	BS2G	22.03
**4421**	allXL	CC	376	382	BS2G	14.13
**4421**	allXL	AA	357	373	BS2G	11.94
**4421**	allXL	CC	320	366	BS2G	20.98
**4421**	allXL	DD	370	376	BS2G	17.71
**4421**	allXL	AA	320	357	DBSU	28.09
**4421**	allXL	AA	357	319	DBSU	29.51
**4421**	allXL	CC	372	319	DBSU	9.17
**4421**	allXL	AA	370	357	DBSU	14.0
**4421**	allXL	AA	382	357	DBSU	29.28
**4421**	allXL	AA	372	370	DBSU	6.05
**4421**	allXL	AA	381	370	DBSU	24.4
**4421**	allXL	AA	373	370	DBSU	9.11
**4421**	allXL	BB	381	372	DBSU	20.83
**4421**	allXL	AA	372	382	DBSU	19.23
**4421**	allXL	DD	372	386	DBSU	22.08
**4421**	allXL	DD	373	386	DBSU	24.03
**4421**	allXL	DA	381	386	DBSU	32.08
**4421**	allXL	BD;DB	357	357	DBSU	20.05

**4421**	allXL	DA	373	319	DBSU	32.11
**4421**	allXL	AC	372	357	DBSU	21.45
**4421**	allXL	DA	381	357	DBSU	21.83
**4421**	allXL	AC	373	357	DBSU	20.5
**4421**	allXL	AD	372	370	DBSU	38.5
**4421**	allXL	AD	373	370	DBSU	36.52
**4421**	allXL	DA	382	373	DBSU	23.33
**4422**	allXL	AA	320	357	BS2G	20.34
**4422**	allXL	CC	320	373	BS2G	23.94
**4422**	allXL	AA	373	382	BS2G	16.86
**4422**	allXL	DD	370	386	BS2G	25.18
**4422**	allXL	AA	373	386	BS2G	23.35
**4422**	allXL	AA	319	357	BS2G	22.14
**4422**	allXL	AA	367	373	BS2G	12.67
**4422**	allXL	DD	370	382	BS2G	22.75
**4422**	allXL	CC	376	382	BS2G	14.34
**4422**	allXL	BB	357	373	BS2G	13.65
**4422**	allXL	AA	320	366	BS2G	22.63
**4422**	allXL	CC	370	376	BS2G	14.42
**4422**	allXL	AA	320	357	DBSU	20.34
**4422**	allXL	AA	357	319	DBSU	22.14
**4422**	allXL	CC	372	319	DBSU	19.73
**4422**	allXL	BB	370	357	DBSU	17.45
**4422**	allXL	BB	382	357	DBSU	35.13
**4422**	allXL	AA	372	370	DBSU	6.68
**4422**	allXL	DD	381	370	DBSU	21.17
**4422**	allXL	AA	373	370	DBSU	9.14
**4422**	allXL	DD	381	372	DBSU	18.16
**4422**	allXL	AA	372	382	DBSU	20.22
**4422**	allXL	DD	372	386	DBSU	24.91
**4422**	allXL	AA	373	386	DBSU	23.35
**4422**	allXL	DB	381	386	DBSU	49.77
**4422**	allXL	BD;DB	357	357	DBSU	19.04
**4422**	allXL	CB	373	319	DBSU	32.95

**4422**	allXL	BD	372	357	DBSU	18.5
**4422**	allXL	BD	381	357	DBSU	40.84
**4422**	allXL	BD	373	357	DBSU	21.73
**4422**	allXL	BD	372	370	DBSU	39.2
**4422**	allXL	DB	373	370	DBSU	40.34
**4422**	allXL	DB	382	373	DBSU	49.68
**4423**	allXL	CC	320	357	BS2G	23.5
**4423**	allXL	CC	320	373	BS2G	28.16
**4423**	allXL	AA	373	382	BS2G	17.09
**4423**	allXL	DD	370	386	BS2G	24.26
**4423**	allXL	DD	373	386	BS2G	24.39
**4423**	allXL	CC	319	357	BS2G	22.67
**4423**	allXL	AA	367	373	BS2G	11.96
**4423**	allXL	DD	370	382	BS2G	21.39
**4423**	allXL	CC	376	382	BS2G	13.51
**4423**	allXL	BB	357	373	BS2G	29.71
**4423**	allXL	CC	320	366	BS2G	18.69
**4423**	allXL	BB	370	376	BS2G	16.7
**4423**	allXL	CC	320	357	DBSU	23.5
**4423**	allXL	CC	357	319	DBSU	22.67
**4423**	allXL	CC	372	319	DBSU	26.72
**4423**	allXL	BB	370	357	DBSU	29.34
**4423**	allXL	AA	382	357	DBSU	48.56
**4423**	allXL	AA	372	370	DBSU	6.36
**4423**	allXL	DD	381	370	DBSU	20.29
**4423**	allXL	AA	373	370	DBSU	8.95
**4423**	allXL	DD	381	372	DBSU	17.58
**4423**	allXL	AA	372	382	DBSU	17.43
**4423**	allXL	AA	372	386	DBSU	23.8
**4423**	allXL	DD	373	386	DBSU	24.39
**4423**	allXL	CB	381	386	DBSU	47.85
**4423**	allXL	BD;DB	357	357	DBSU	18.47
**4423**	allXL	DA	373	319	DBSU	26.58
**4423**	allXL	BD	372	357	DBSU	29.51

**4423**	allXL	CB	381	357	DBSU	45.34
**4423**	allXL	BD	373	357	DBSU	31.74
**4423**	allXL	CB	372	370	DBSU	30.68
**4423**	allXL	CB	373	370	DBSU	30.47
**4423**	allXL	CB	382	373	DBSU	35.24
**4424**	allXL	CC	320	357	BS2G	20.48
**4424**	allXL	AA	320	373	BS2G	16.22
**4424**	allXL	BB	373	382	BS2G	20.82
**4424**	allXL	CC	370	386	BS2G	28.81
**4424**	allXL	DD	373	386	BS2G	30.23
**4424**	allXL	CC	319	357	BS2G	20.83
**4424**	allXL	BB	367	373	BS2G	12.63
**4424**	allXL	DD	370	382	BS2G	22.34
**4424**	allXL	CC	376	382	BS2G	14.89
**4424**	allXL	BB	357	373	BS2G	21.36
**4424**	allXL	BB	320	366	BS2G	24.14
**4424**	allXL	CC	370	376	BS2G	15.7
**4424**	allXL	CC	320	357	DBSU	20.48
**4424**	allXL	CC	357	319	DBSU	20.83
**4424**	allXL	AA	372	319	DBSU	17.26
**4424**	allXL	AA	370	357	DBSU	27.22
**4424**	allXL	BB	382	357	DBSU	18.37
**4424**	allXL	AA	372	370	DBSU	6.2
**4424**	allXL	DD	381	370	DBSU	20.08
**4424**	allXL	AA	373	370	DBSU	8.94
**4424**	allXL	DD	381	372	DBSU	18.16
**4424**	allXL	DD	372	382	DBSU	21.17
**4424**	allXL	DD	372	386	DBSU	29.64
**4424**	allXL	DD	373	386	DBSU	30.23
**4424**	allXL	AD	381	386	DBSU	34.64
**4424**	allXL	BD;DB	357	357	DBSU	18.86
**4424**	allXL	CB	373	319	DBSU	22.87
**4424**	allXL	CB	372	357	DBSU	35.99
**4424**	allXL	BD	381	357	DBSU	32.21

**4424**	allXL	BD	373	357	DBSU	36.41
**4424**	allXL	AD	372	370	DBSU	38.73
**4424**	allXL	DA	373	370	DBSU	40.87
**4424**	allXL	DA	382	373	DBSU	27.33
**4425**	allXL	CC	320	357	BS2G	19.89
**4425**	allXL	DD	320	373	BS2G	18.98
**4425**	allXL	AA	373	382	BS2G	19.9
**4425**	allXL	AA	370	386	BS2G	22.89
**4425**	allXL	AA	373	386	BS2G	24.16
**4425**	allXL	AA	319	357	BS2G	18.97
**4425**	allXL	AA	367	373	BS2G	10.83
**4425**	allXL	DD	370	382	BS2G	20.48
**4425**	allXL	CC	376	382	BS2G	15.64
**4425**	allXL	DD	357	373	BS2G	25.68
**4425**	allXL	AA	320	366	BS2G	18.15
**4425**	allXL	CC	370	376	BS2G	13.87
**4425**	allXL	CC	320	357	DBSU	19.89
**4425**	allXL	AA	357	319	DBSU	18.97
**4425**	allXL	DD	372	319	DBSU	16.4
**4425**	allXL	DD	370	357	DBSU	28.35
**4425**	allXL	DD	382	357	DBSU	34.66
**4425**	allXL	AA	372	370	DBSU	6.56
**4425**	allXL	DD	381	370	DBSU	17.79
**4425**	allXL	AA	373	370	DBSU	8.84
**4425**	allXL	AA	381	372	DBSU	18.03
**4425**	allXL	AA	372	382	DBSU	20.18
**4425**	allXL	AA	372	386	DBSU	22.65
**4425**	allXL	AA	373	386	DBSU	24.16
**4425**	allXL	CB	381	386	DBSU	14.89
**4425**	allXL	BD;DB	357	357	DBSU	19.02
**4425**	allXL	BC	373	319	DBSU	22.6
**4425**	allXL	DA	372	357	DBSU	34.04
**4425**	allXL	BC	381	357	DBSU	36.33
**4425**	allXL	DA	373	357	DBSU	31.95

**4425**	allXL	DA	372	370	DBSU	27.0
**4425**	allXL	DA	373	370	DBSU	29.28
**4425**	allXL	BC	382	373	DBSU	21.41
**4426**	allXL	BB	320	357	BS2G	26.03
**4426**	allXL	BB	320	373	BS2G	18.24
**4426**	allXL	AA	373	382	BS2G	17.64
**4426**	allXL	BB	370	386	BS2G	18.3
**4426**	allXL	BB	373	386	BS2G	20.7
**4426**	allXL	AA	319	357	BS2G	26.23
**4426**	allXL	BB	367	373	BS2G	10.99
**4426**	allXL	DD	370	382	BS2G	16.15
**4426**	allXL	BB	376	382	BS2G	12.71
**4426**	allXL	BB	357	373	BS2G	29.06
**4426**	allXL	BB	320	366	BS2G	18.68
**4426**	allXL	DD	370	376	BS2G	14.6
**4426**	allXL	BB	320	357	DBSU	26.03
**4426**	allXL	AA	357	319	DBSU	26.23
**4426**	allXL	CC	372	319	DBSU	17.58
**4426**	allXL	BB	370	357	DBSU	26.15
**4426**	allXL	BB	382	357	DBSU	33.75
**4426**	allXL	AA	372	370	DBSU	6.39
**4426**	allXL	DD	381	370	DBSU	14.6
**4426**	allXL	AA	373	370	DBSU	9.07
**4426**	allXL	DD	381	372	DBSU	15.99
**4426**	allXL	DD	372	382	DBSU	18.02
**4426**	allXL	BB	372	386	DBSU	22.34
**4426**	allXL	BB	373	386	DBSU	20.7
**4426**	allXL	DA	381	386	DBSU	27.95
**4426**	allXL	BD;DB	357	357	DBSU	18.06
**4426**	allXL	AD	373	319	DBSU	26.58
**4426**	allXL	BD	372	357	DBSU	42.6
**4426**	allXL	BD	381	357	DBSU	49.72
**4426**	allXL	BD	373	357	DBSU	43.9
**4426**	allXL	DA	372	370	DBSU	46.87

**4426**	allXL	DA	373	370	DBSU	43.64
**4426**	allXL	AD	382	373	DBSU	24.47
**4427**	allXL	BB	320	357	BS2G	25.1
**4427**	allXL	BB	320	373	BS2G	19.63
**4427**	allXL	DD	373	382	BS2G	18.94
**4427**	allXL	AA	370	386	BS2G	20.44
**4427**	allXL	AA	373	386	BS2G	25.29
**4427**	allXL	AA	319	357	BS2G	26.75
**4427**	allXL	AA	367	373	BS2G	11.41
**4427**	allXL	DD	370	382	BS2G	16.63
**4427**	allXL	BB	376	382	BS2G	13.29
**4427**	allXL	DD	357	373	BS2G	26.37
**4427**	allXL	BB	320	366	BS2G	16.33
**4427**	allXL	AA	370	376	BS2G	14.57
**4427**	allXL	BB	320	357	DBSU	25.1
**4427**	allXL	AA	357	319	DBSU	26.75
**4427**	allXL	BB	372	319	DBSU	19.66
**4427**	allXL	DD	370	357	DBSU	28.46
**4427**	allXL	DD	382	357	DBSU	23.77
**4427**	allXL	AA	372	370	DBSU	6.56
**4427**	allXL	DD	381	370	DBSU	15.71
**4427**	allXL	AA	373	370	DBSU	9.11
**4427**	allXL	DD	381	372	DBSU	17.84
**4427**	allXL	AA	372	382	DBSU	18.79
**4427**	allXL	AA	372	386	DBSU	22.76
**4427**	allXL	AA	373	386	DBSU	25.29
**4427**	allXL	AD	381	386	DBSU	55.09
**4427**	allXL	BD;DB	357	357	DBSU	18.78
**4427**	allXL	DA	373	319	DBSU	24.96
**4427**	allXL	DB	372	357	DBSU	36.84
**4427**	allXL	DB	381	357	DBSU	30.94
**4427**	allXL	DB	373	357	DBSU	34.11
**4427**	allXL	DA	372	370	DBSU	48.29
**4427**	allXL	CB	373	370	DBSU	49.49

**4427**	allXL	AD	382	373	DBSU	50.08
**4428**	allXL	AA	320	357	BS2G	24.2
**4428**	allXL	BB	320	373	BS2G	19.84
**4428**	allXL	DD	373	382	BS2G	16.11
**4428**	allXL	CC	370	386	BS2G	19.97
**4428**	allXL	DD	373	386	BS2G	22.76
**4428**	allXL	AA	319	357	BS2G	25.9
**4428**	allXL	AA	367	373	BS2G	12.0
**4428**	allXL	DD	370	382	BS2G	13.99
**4428**	allXL	BB	376	382	BS2G	14.68
**4428**	allXL	DD	357	373	BS2G	28.0
**4428**	allXL	AA	320	366	BS2G	19.38
**4428**	allXL	DD	370	376	BS2G	13.81
**4428**	allXL	AA	320	357	DBSU	24.2
**4428**	allXL	AA	357	319	DBSU	25.9
**4428**	allXL	BB	372	319	DBSU	19.25
**4428**	allXL	DD	370	357	DBSU	26.45
**4428**	allXL	AA	382	357	DBSU	27.01
**4428**	allXL	AA	372	370	DBSU	6.51
**4428**	allXL	DD	381	370	DBSU	12.33
**4428**	allXL	AA	373	370	DBSU	9.2
**4428**	allXL	DD	381	372	DBSU	12.39
**4428**	allXL	DD	372	382	DBSU	14.2
**4428**	allXL	DD	372	386	DBSU	19.67
**4428**	allXL	DD	373	386	DBSU	22.76
**4428**	allXL	BC	381	386	DBSU	50.0
**4428**	allXL	BD;DB	357	357	DBSU	18.43
**4428**	allXL	AD	373	319	DBSU	35.89
**4428**	allXL	BC	372	357	DBSU	42.51
**4428**	allXL	AC	381	357	DBSU	42.75
**4428**	allXL	DB	373	357	DBSU	42.97
**4428**	allXL	BC	372	370	DBSU	37.17
**4428**	allXL	BC	373	370	DBSU	40.55
**4428**	allXL	CB	382	373	DBSU	40.64

**4429**	allXL	CC	320	357	BS2G	26.33
**4429**	allXL	AA	320	373	BS2G	25.23
**4429**	allXL	AA	373	382	BS2G	19.25
**4429**	allXL	CC	370	386	BS2G	21.95
**4429**	allXL	AA	373	386	BS2G	24.32
**4429**	allXL	CC	319	357	BS2G	29.05
**4429**	allXL	BB	367	373	BS2G	10.28
**4429**	allXL	CC	370	382	BS2G	20.69
**4429**	allXL	AA;CC	376	382	BS2G	14.31
**4429**	allXL	BB	357	373	BS2G	12.95
**4429**	allXL	AA	320	366	BS2G	24.12
**4429**	allXL	CC	370	376	BS2G	14.67
**4429**	allXL	CC	320	357	DBSU	26.33
**4429**	allXL	CC	357	319	DBSU	29.05
**4429**	allXL	BB	372	319	DBSU	25.0
**4429**	allXL	BB	370	357	DBSU	22.78
**4429**	allXL	BB	382	357	DBSU	24.19
**4429**	allXL	AA	372	370	DBSU	6.17
**4429**	allXL	CC	381	370	DBSU	21.79
**4429**	allXL	AA	373	370	DBSU	9.06
**4429**	allXL	DD	381	372	DBSU	18.88
**4429**	allXL	AA	372	382	DBSU	21.12
**4429**	allXL	AA	372	386	DBSU	24.99
**4429**	allXL	AA	373	386	DBSU	24.32
**4429**	allXL	BC	381	386	DBSU	50.32
**4429**	allXL	BD;DB	357	357	DBSU	17.84
**4429**	allXL	BC	373	319	DBSU	36.2
**4429**	allXL	BD	372	357	DBSU	29.45
**4429**	allXL	BD	381	357	DBSU	32.76
**4429**	allXL	BD	373	357	DBSU	26.7
**4429**	allXL	CB	372	370	DBSU	46.58
**4429**	allXL	CB	373	370	DBSU	45.71
**4429**	allXL	CB	382	373	DBSU	44.08
**4430**	allXL	AA	320	357	BS2G	21.09

**4430**	allXL	BB	320	373	BS2G	25.3
**4430**	allXL	CC	373	382	BS2G	18.51
**4430**	allXL	CC	370	386	BS2G	19.67
**4430**	allXL	BB	373	386	BS2G	20.27
**4430**	allXL	AA	319	357	BS2G	18.89
**4430**	allXL	AA	367	373	BS2G	12.52
**4430**	allXL	CC	370	382	BS2G	18.16
**4430**	allXL	CC	376	382	BS2G	14.72
**4430**	allXL	DD	357	373	BS2G	29.46
**4430**	allXL	AA	320	366	BS2G	20.7
**4430**	allXL	CC	370	376	BS2G	14.23
**4430**	allXL	AA	320	357	DBSU	21.09
**4430**	allXL	AA	357	319	DBSU	18.89
**4430**	allXL	DD	372	319	DBSU	25.77
**4430**	allXL	DD	370	357	DBSU	22.25
**4430**	allXL	DD	382	357	DBSU	32.67
**4430**	allXL	AA	372	370	DBSU	6.47
**4430**	allXL	DD	381	370	DBSU	16.18
**4430**	allXL	AA	373	370	DBSU	9.3
**4430**	allXL	DD	381	372	DBSU	17.87
**4430**	allXL	CC	372	382	DBSU	19.6
**4430**	allXL	BB	372	386	DBSU	23.51
**4430**	allXL	BB	373	386	DBSU	20.27
**4430**	allXL	DB	381	386	DBSU	53.14
**4430**	allXL	BD;DB	357	357	DBSU	18.73
**4430**	allXL	DA	373	319	DBSU	22.08
**4430**	allXL	DA	372	357	DBSU	31.25
**4430**	allXL	DB	381	357	DBSU	39.8
**4430**	allXL	DA	373	357	DBSU	29.9
**4430**	allXL	DA	372	370	DBSU	40.65
**4430**	allXL	DA	373	370	DBSU	39.91
**4430**	allXL	AD	382	373	DBSU	50.77
**4431**	allXL	CC	320	357	BS2G	22.58
**4431**	allXL	BB	320	373	BS2G	20.41

**4431**	allXL	CC	373	382	BS2G	20.04
**4431**	allXL	CC	370	386	BS2G	23.26
**4431**	allXL	CC	373	386	BS2G	28.27
**4431**	allXL	CC	319	357	BS2G	23.13
**4431**	allXL	BB	367	373	BS2G	11.08
**4431**	allXL	CC	370	382	BS2G	17.72
**4431**	allXL	BB;CC	376	382	BS2G	14.85
**4431**	allXL	DD	357	373	BS2G	20.99
**4431**	allXL	BB	320	366	BS2G	23.58
**4431**	allXL	CC	370	376	BS2G	14.72
**4431**	allXL	CC	320	357	DBSU	22.58
**4431**	allXL	CC	357	319	DBSU	23.13
**4431**	allXL	BB	372	319	DBSU	19.21
**4431**	allXL	DD	370	357	DBSU	21.56
**4431**	allXL	CC	382	357	DBSU	18.07
**4431**	allXL	AA	372	370	DBSU	5.87
**4431**	allXL	CC	381	370	DBSU	19.23
**4431**	allXL	DD	373	370	DBSU	9.08
**4431**	allXL	CC	381	372	DBSU	21.12
**4431**	allXL	CC	372	382	DBSU	19.83
**4431**	allXL	CC	372	386	DBSU	26.49
**4431**	allXL	CC	373	386	DBSU	28.27
**4431**	allXL	BD	381	386	DBSU	25.36
**4431**	allXL	BD;DB	357	357	DBSU	19.66
**4431**	allXL	DA	373	319	DBSU	33.1
**4431**	allXL	DB	372	357	DBSU	36.12
**4431**	allXL	DB	381	357	DBSU	16.22
**4431**	allXL	DB	373	357	DBSU	32.66
**4431**	allXL	DA	372	370	DBSU	38.75
**4431**	allXL	DA	373	370	DBSU	38.21
**4431**	allXL	DB	382	373	DBSU	32.76
**4432**	allXL	AA	320	357	BS2G	24.82
**4432**	allXL	CC	320	373	BS2G	7.99
**4432**	allXL	DD	373	382	BS2G	16.94

**4432**	allXL	BB	370	386	BS2G	22.61
**4432**	allXL	BB	373	386	BS2G	20.07
**4432**	allXL	AA	319	357	BS2G	25.9
**4432**	allXL	AA	367	373	BS2G	13.78
**4432**	allXL	DD	370	382	BS2G	19.05
**4432**	allXL	CC	376	382	BS2G	13.32
**4432**	allXL	DD	357	373	BS2G	28.24
**4432**	allXL	DD	320	366	BS2G	22.39
**4432**	allXL	DD	370	376	BS2G	13.4
**4432**	allXL	AA	320	357	DBSU	24.82
**4432**	allXL	AA	357	319	DBSU	25.9
**4432**	allXL	CC	372	319	DBSU	10.64
**4432**	allXL	BB	370	357	DBSU	23.38
**4432**	allXL	CC	382	357	DBSU	41.5
**4432**	allXL	AA	372	370	DBSU	6.17
**4432**	allXL	DD	381	370	DBSU	17.63
**4432**	allXL	CC;DD	373	370	DBSU	9.16
**4432**	allXL	DD	381	372	DBSU	17.8
**4432**	allXL	DD	372	382	DBSU	17.8
**4432**	allXL	BB	372	386	DBSU	21.67
**4432**	allXL	BB	373	386	DBSU	20.07
**4432**	allXL	AD	381	386	DBSU	51.89
**4432**	allXL	BD;DB	357	357	DBSU	18.45
**4432**	allXL	DA	373	319	DBSU	31.59
**4432**	allXL	CB	372	357	DBSU	41.31
**4432**	allXL	DA	381	357	DBSU	50.52
**4432**	allXL	DA	373	357	DBSU	41.31
**4432**	allXL	DA	372	370	DBSU	38.67
**4432**	allXL	DA	373	370	DBSU	38.57
**4432**	allXL	DA	382	373	DBSU	36.46
**4433**	allXL	AA	320	357	BS2G	26.31
**4433**	allXL	BB	320	373	BS2G	22.46
**4433**	allXL	DD	373	382	BS2G	21.15
**4433**	allXL	DD	370	386	BS2G	28.67

**4433**	allXL	AA	373	386	BS2G	24.76
**4433**	allXL	AA	319	357	BS2G	25.64
**4433**	allXL	BB	367	373	BS2G	13.27
**4433**	allXL	DD	370	382	BS2G	18.67
**4433**	allXL	CC	376	382	BS2G	14.72
**4433**	allXL	BB	357	373	BS2G	15.56
**4433**	allXL	AA	320	366	BS2G	21.77
**4433**	allXL	CC	370	376	BS2G	14.32
**4433**	allXL	AA	320	357	DBSU	26.31
**4433**	allXL	AA	357	319	DBSU	25.64
**4433**	allXL	AA	372	319	DBSU	22.49
**4433**	allXL	CC	370	357	DBSU	15.84
**4433**	allXL	BB	382	357	DBSU	19.76
**4433**	allXL	AA	372	370	DBSU	5.99
**4433**	allXL	DD	381	370	DBSU	15.54
**4433**	allXL	AA	373	370	DBSU	9.27
**4433**	allXL	DD	381	372	DBSU	17.91
**4433**	allXL	DD	372	382	DBSU	21.34
**4433**	allXL	AA	372	386	DBSU	26.75
**4433**	allXL	AA	373	386	DBSU	24.76
**4433**	allXL	DA	381	386	DBSU	57.26
**4433**	allXL	BD;DB	357	357	DBSU	18.55
**4433**	allXL	AD	373	319	DBSU	30.17
**4433**	allXL	CA	372	357	DBSU	19.95
**4433**	allXL	BD	381	357	DBSU	30.73
**4433**	allXL	CA	373	357	DBSU	18.72
**4433**	allXL	DA	372	370	DBSU	39.69
**4433**	allXL	DA	373	370	DBSU	41.17
**4433**	allXL	AD	382	373	DBSU	43.34
**4434**	allXL	AA	320	357	BS2G	26.42
**4434**	allXL	CC	320	373	BS2G	17.11
**4434**	allXL	DD	373	382	BS2G	18.25
**4434**	allXL	CC	370	386	BS2G	17.87
**4434**	allXL	CC	373	386	BS2G	26.11

**4434**	allXL	AA	319	357	BS2G	28.37
**4434**	allXL	AA	367	373	BS2G	11.33
**4434**	allXL	DD	370	382	BS2G	16.55
**4434**	allXL	CC	376	382	BS2G	13.75
**4434**	allXL	AA	357	373	BS2G	28.56
**4434**	allXL	CC	320	366	BS2G	11.76
**4434**	allXL	CC	370	376	BS2G	13.17
**4434**	allXL	AA	320	357	DBSU	26.42
**4434**	allXL	AA	357	319	DBSU	28.37
**4434**	allXL	CC	372	319	DBSU	15.63
**4434**	allXL	CC	370	357	DBSU	28.53
**4434**	allXL	AA	382	357	DBSU	32.99
**4434**	allXL	AA	372	370	DBSU	6.3
**4434**	allXL	DD	381	370	DBSU	17.04
**4434**	allXL	AA	373	370	DBSU	8.54
**4434**	allXL	DD	381	372	DBSU	17.9
**4434**	allXL	DD	372	382	DBSU	18.2
**4434**	allXL	CC	372	386	DBSU	24.41
**4434**	allXL	CC	373	386	DBSU	26.11
**4434**	allXL	AC	381	386	DBSU	63.23
**4434**	allXL	BD;DB	357	357	DBSU	18.94
**4434**	allXL	DA	373	319	DBSU	29.18
**4434**	allXL	CA	372	357	DBSU	46.2
**4434**	allXL	AC	381	357	DBSU	42.31
**4434**	allXL	CA	373	357	DBSU	46.73
**4434**	allXL	DA	372	370	DBSU	44.24
**4434**	allXL	DA	373	370	DBSU	41.24
**4434**	allXL	AC	382	373	DBSU	46.66
**4435**	allXL	CC	320	357	BS2G	24.39
**4435**	allXL	DD	320	373	BS2G	26.59
**4435**	allXL	BB	373	382	BS2G	18.13
**4435**	allXL	DD	370	386	BS2G	30.21
**4435**	allXL	BB	373	386	BS2G	29.05
**4435**	allXL	AA	319	357	BS2G	25.74

**4435**	allXL	AA	367	373	BS2G	14.38
**4435**	allXL	DD	370	382	BS2G	21.36
**4435**	allXL	BB	376	382	BS2G	13.23
**4435**	allXL	DD	357	373	BS2G	22.59
**4435**	allXL	DD	320	366	BS2G	23.34
**4435**	allXL	DD	370	376	BS2G	14.91
**4435**	allXL	CC	320	357	DBSU	24.39
**4435**	allXL	AA	357	319	DBSU	25.74
**4435**	allXL	DD	372	319	DBSU	23.38
**4435**	allXL	CC	370	357	DBSU	25.04
**4435**	allXL	DD	382	357	DBSU	32.64
**4435**	allXL	AA	372	370	DBSU	6.06
**4435**	allXL	DD	381	370	DBSU	18.1
**4435**	allXL	CC	373	370	DBSU	9.2
**4435**	allXL	DD	381	372	DBSU	20.09
**4435**	allXL	BB	372	382	DBSU	20.45
**4435**	allXL	BB	372	386	DBSU	30.64
**4435**	allXL	BB	373	386	DBSU	29.05
**4435**	allXL	CB	381	386	DBSU	71.58
**4435**	allXL	BD;DB	357	357	DBSU	18.95
**4435**	allXL	AD	373	319	DBSU	17.66
**4435**	allXL	DB	372	357	DBSU	39.79
**4435**	allXL	DB	381	357	DBSU	38.57
**4435**	allXL	DB	373	357	DBSU	36.46
**4435**	allXL	DA	372	370	DBSU	36.21
**4435**	allXL	AD	373	370	DBSU	33.17
**4435**	allXL	AD	382	373	DBSU	49.89
**4436**	allXL	AA	320	357	BS2G	25.55
**4436**	allXL	CC	320	373	BS2G	14.42
**4436**	allXL	DD	373	382	BS2G	19.62
**4436**	allXL	DD	370	386	BS2G	20.25
**4436**	allXL	DD	373	386	BS2G	25.01
**4436**	allXL	AA	319	357	BS2G	24.57
**4436**	allXL	AA	367	373	BS2G	11.79

**4436**	allXL	DD	370	382	BS2G	19.53
**4436**	allXL	BB	376	382	BS2G	15.48
**4436**	allXL	AA	357	373	BS2G	21.5
**4436**	allXL	BB	320	366	BS2G	20.23
**4436**	allXL	BB	370	376	BS2G	14.38
**4436**	allXL	AA	320	357	DBSU	25.55
**4436**	allXL	AA	357	319	DBSU	24.57
**4436**	allXL	CC	372	319	DBSU	15.05
**4436**	allXL	DD	370	357	DBSU	24.51
**4436**	allXL	AA	382	357	DBSU	34.28
**4436**	allXL	AA	372	370	DBSU	6.21
**4436**	allXL	DD	381	370	DBSU	21.68
**4436**	allXL	AA	373	370	DBSU	8.95
**4436**	allXL	AA	381	372	DBSU	21.48
**4436**	allXL	DD	372	382	DBSU	20.74
**4436**	allXL	DD	372	386	DBSU	24.39
**4436**	allXL	DD	373	386	DBSU	25.01
**4436**	allXL	BC	381	386	DBSU	47.12
**4436**	allXL	BD;DB	357	357	DBSU	17.68
**4436**	allXL	BC	373	319	DBSU	26.61
**4436**	allXL	CB	372	357	DBSU	35.2
**4436**	allXL	AC	381	357	DBSU	30.38
**4436**	allXL	AC	373	357	DBSU	33.68
**4436**	allXL	BC	372	370	DBSU	13.04
**4436**	allXL	BC	373	370	DBSU	11.07
**4436**	allXL	BC	382	373	DBSU	26.11
**4437**	allXL	CC	320	357	BS2G	23.02
**4437**	allXL	DD	320	373	BS2G	26.32
**4437**	allXL	CC	373	382	BS2G	19.1
**4437**	allXL	DD	370	386	BS2G	15.48
**4437**	allXL	DD	373	386	BS2G	22.2
**4437**	allXL	CC	319	357	BS2G	24.18
**4437**	allXL	AA	367	373	BS2G	12.32
**4437**	allXL	DD	370	382	BS2G	18.07

**4437**	allXL	CC	376	382	BS2G	13.29
**4437**	allXL	BB	357	373	BS2G	34.35
**4437**	allXL	CC	320	366	BS2G	21.71
**4437**	allXL	CC	370	376	BS2G	14.29
**4437**	allXL	CC	320	357	DBSU	23.02
**4437**	allXL	CC	357	319	DBSU	24.18
**4437**	allXL	DD	372	319	DBSU	26.82
**4437**	allXL	DD	370	357	DBSU	29.32
**4437**	allXL	DD	382	357	DBSU	40.7
**4437**	allXL	AA	372	370	DBSU	6.48
**4437**	allXL	DD	381	370	DBSU	19.73
**4437**	allXL	AA	373	370	DBSU	9.15
**4437**	allXL	DD	381	372	DBSU	21.47
**4437**	allXL	CC	372	382	DBSU	20.98
**4437**	allXL	DD	372	386	DBSU	21.53
**4437**	allXL	DD	373	386	DBSU	22.2
**4437**	allXL	DA	381	386	DBSU	24.82
**4437**	allXL	BD;DB	357	357	DBSU	18.81
**4437**	allXL	CB	373	319	DBSU	18.26
**4437**	allXL	CB	372	357	DBSU	35.9
**4437**	allXL	CB	381	357	DBSU	50.81
**4437**	allXL	CB	373	357	DBSU	36.51
**4437**	allXL	CB	372	370	DBSU	27.55
**4437**	allXL	CB	373	370	DBSU	28.64
**4437**	allXL	AD	382	373	DBSU	38.37
**4438**	allXL	AA	320	357	BS2G	27.45
**4438**	allXL	AA	320	373	BS2G	27.93
**4438**	allXL	CC	373	382	BS2G	18.65
**4438**	allXL	DD	370	386	BS2G	18.43
**4438**	allXL	CC	373	386	BS2G	25.18
**4438**	allXL	AA	319	357	BS2G	27.39
**4438**	allXL	AA	367	373	BS2G	13.92
**4438**	allXL	DD	370	382	BS2G	17.92
**4438**	allXL	CC	376	382	BS2G	13.42

**4438**	allXL	BB	357	373	BS2G	28.59
**4438**	allXL	AA	320	366	BS2G	19.58
**4438**	allXL	DD	370	376	BS2G	16.58
**4438**	allXL	AA	320	357	DBSU	27.45
**4438**	allXL	AA	357	319	DBSU	27.39
**4438**	allXL	AA	372	319	DBSU	24.05
**4438**	allXL	DD	370	357	DBSU	27.4
**4438**	allXL	DD	382	357	DBSU	18.6
**4438**	allXL	AA	372	370	DBSU	6.39
**4438**	allXL	DD	381	370	DBSU	16.5
**4438**	allXL	AA	373	370	DBSU	9.29
**4438**	allXL	DD	381	372	DBSU	19.0
**4438**	allXL	DD	372	382	DBSU	21.56
**4438**	allXL	DD	372	386	DBSU	24.97
**4438**	allXL	CC	373	386	DBSU	25.18
**4438**	allXL	BD	381	386	DBSU	27.6
**4438**	allXL	BD;DB	357	357	DBSU	18.28
**4438**	allXL	CB	373	319	DBSU	22.6
**4438**	allXL	BD	372	357	DBSU	26.99
**4438**	allXL	DB	381	357	DBSU	27.0
**4438**	allXL	BD	373	357	DBSU	29.16
**4438**	allXL	BD	372	370	DBSU	38.03
**4438**	allXL	BD	373	370	DBSU	37.25
**4438**	allXL	DB	382	373	DBSU	25.65
**4439**	allXL	AA	320	357	BS2G	25.27
**4439**	allXL	BB	320	373	BS2G	19.45
**4439**	allXL	DD	373	382	BS2G	17.09
**4439**	allXL	DD	370	386	BS2G	19.86
**4439**	allXL	DD	373	386	BS2G	23.16
**4439**	allXL	AA	319	357	BS2G	24.13
**4439**	allXL	AA	367	373	BS2G	10.74
**4439**	allXL	DD	370	382	BS2G	15.77
**4439**	allXL	CC	376	382	BS2G	13.49
**4439**	allXL	BB	357	373	BS2G	20.2

**4439**	allXL	CC	320	366	BS2G	21.67
**4439**	allXL	CC	370	376	BS2G	16.46
**4439**	allXL	AA	320	357	DBSU	25.27
**4439**	allXL	AA	357	319	DBSU	24.13
**4439**	allXL	BB	372	319	DBSU	26.42
**4439**	allXL	BB	370	357	DBSU	26.56
**4439**	allXL	AA	382	357	DBSU	19.45
**4439**	allXL	AA	372	370	DBSU	6.45
**4439**	allXL	DD	381	370	DBSU	15.59
**4439**	allXL	AA	373	370	DBSU	9.02
**4439**	allXL	DD	381	372	DBSU	15.83
**4439**	allXL	DD	372	382	DBSU	17.52
**4439**	allXL	DD	372	386	DBSU	23.51
**4439**	allXL	DD	373	386	DBSU	23.16
**4439**	allXL	AD;DA	381	386	DBSU	49.34
**4439**	allXL	BD;DB	357	357	DBSU	18.27
**4439**	allXL	CB	373	319	DBSU	33.41
**4439**	allXL	CB	372	357	DBSU	22.42
**4439**	allXL	CB	381	357	DBSU	34.34
**4439**	allXL	CB	373	357	DBSU	20.12
**4439**	allXL	CB	372	370	DBSU	34.48
**4439**	allXL	CB	373	370	DBSU	35.05
**4439**	allXL	CB	382	373	DBSU	45.37
**4440**	allXL	CC	320	357	BS2G	23.61
**4440**	allXL	BB	320	373	BS2G	23.8
**4440**	allXL	DD	373	382	BS2G	16.43
**4440**	allXL	DD	370	386	BS2G	9.92
**4440**	allXL	DD	373	386	BS2G	19.38
**4440**	allXL	AA	319	357	BS2G	23.7
**4440**	allXL	AA	367	373	BS2G	13.94
**4440**	allXL	DD	370	382	BS2G	10.98
**4440**	allXL	CC	376	382	BS2G	15.71
**4440**	allXL	DD	357	373	BS2G	31.72
**4440**	allXL	CC	320	366	BS2G	20.65

**4440**	allXL	BB	370	376	BS2G	14.31
**4440**	allXL	CC	320	357	DBSU	23.61
**4440**	allXL	AA	357	319	DBSU	23.7
**4440**	allXL	DD	372	319	DBSU	25.37
**4440**	allXL	BB	370	357	DBSU	28.33
**4440**	allXL	DD	382	357	DBSU	20.71
**4440**	allXL	AA	372	370	DBSU	6.16
**4440**	allXL	DD	381	370	DBSU	13.81
**4440**	allXL	AA	373	370	DBSU	9.0
**4440**	allXL	DD	381	372	DBSU	17.63
**4440**	allXL	DD	372	382	DBSU	15.98
**4440**	allXL	DD	372	386	DBSU	16.93
**4440**	allXL	DD	373	386	DBSU	19.38
**4440**	allXL	DB	381	386	DBSU	45.02
**4440**	allXL	BD;DB	357	357	DBSU	19.04
**4440**	allXL	DA	373	319	DBSU	22.31
**4440**	allXL	DA	372	357	DBSU	37.16
**4440**	allXL	DB	381	357	DBSU	32.3
**4440**	allXL	DA	373	357	DBSU	33.66
**4440**	allXL	DA	372	370	DBSU	25.57
**4440**	allXL	DA	373	370	DBSU	25.5
**4440**	allXL	DA	382	373	DBSU	39.69
**4441**	allXL	CC	320	357	BS2G	22.36
**4441**	allXL	DD	320	373	BS2G	18.07
**4441**	allXL	CC	373	382	BS2G	19.33
**4441**	allXL	BB	370	386	BS2G	24.66
**4441**	allXL	BB	373	386	BS2G	16.89
**4441**	allXL	AA	319	357	BS2G	25.13
**4441**	allXL	BB	367	373	BS2G	12.91
**4441**	allXL	DD	370	382	BS2G	25.27
**4441**	allXL	CC	376	382	BS2G	13.8
**4441**	allXL	AA	357	373	BS2G	23.84
**4441**	allXL	CC	320	366	BS2G	14.73
**4441**	allXL	CC	370	376	BS2G	17.05

**4441**	allXL	CC	320	357	DBSU	22.36
**4441**	allXL	AA	357	319	DBSU	25.13
**4441**	allXL	DD	372	319	DBSU	19.11
**4441**	allXL	AA	370	357	DBSU	26.67
**4441**	allXL	BB	382	357	DBSU	18.43
**4441**	allXL	AA	372	370	DBSU	6.2
**4441**	allXL	DD	381	370	DBSU	24.24
**4441**	allXL	AA	373	370	DBSU	9.0
**4441**	allXL	DD	381	372	DBSU	20.63
**4441**	allXL	CC	372	382	DBSU	21.4
**4441**	allXL	BB	372	386	DBSU	17.53
**4441**	allXL	BB	373	386	DBSU	16.89
**4441**	allXL	DA	381	386	DBSU	50.0
**4441**	allXL	BD;DB	357	357	DBSU	18.14
**4441**	allXL	AD	373	319	DBSU	24.83
**4441**	allXL	AC	372	357	DBSU	30.38
**4441**	allXL	BD	381	357	DBSU	17.65
**4441**	allXL	AC	373	357	DBSU	30.3
**4441**	allXL	DA	372	370	DBSU	48.57
**4441**	allXL	DA	373	370	DBSU	45.06
**4441**	allXL	AD	382	373	DBSU	31.8
**4442**	allXL	AA	320	357	BS2G	24.79
**4442**	allXL	DD	320	373	BS2G	25.16
**4442**	allXL	BB	373	382	BS2G	18.57
**4442**	allXL	BB	370	386	BS2G	25.05
**4442**	allXL	BB	373	386	BS2G	25.75
**4442**	allXL	AA	319	357	BS2G	25.36
**4442**	allXL	AA	367	373	BS2G	14.92
**4442**	allXL	BB	370	382	BS2G	20.71
**4442**	allXL	BB	376	382	BS2G	14.61
**4442**	allXL	BB	357	373	BS2G	38.71
**4442**	allXL	CC	320	366	BS2G	21.7
**4442**	allXL	CC	370	376	BS2G	16.06
**4442**	allXL	AA	320	357	DBSU	24.79

**4442**	allXL	AA	357	319	DBSU	25.36
**4442**	allXL	DD	372	319	DBSU	18.67
**4442**	allXL	DD	370	357	DBSU	28.93
**4442**	allXL	DD	382	357	DBSU	44.72
**4442**	allXL	AA	372	370	DBSU	6.19
**4442**	allXL	BB	381	370	DBSU	21.48
**4442**	allXL	AA	373	370	DBSU	9.18
**4442**	allXL	BB	381	372	DBSU	20.8
**4442**	allXL	BB	372	382	DBSU	21.13
**4442**	allXL	BB	372	386	DBSU	27.71
**4442**	allXL	BB	373	386	DBSU	25.75
**4442**	allXL	DA	381	386	DBSU	56.3
**4442**	allXL	BD;DB	357	357	DBSU	17.84
**4442**	allXL	DA	373	319	DBSU	19.76
**4442**	allXL	DA	372	357	DBSU	30.79
**4442**	allXL	DA	381	357	DBSU	36.6
**4442**	allXL	DA	373	357	DBSU	32.13
**4442**	allXL	DA	372	370	DBSU	12.99
**4442**	allXL	DA	373	370	DBSU	11.1
**4442**	allXL	AD	382	373	DBSU	32.43
**4443**	allXL	AA	320	357	BS2G	22.79
**4443**	allXL	BB	320	373	BS2G	12.99
**4443**	allXL	BB	373	382	BS2G	18.41
**4443**	allXL	DD	370	386	BS2G	17.58
**4443**	allXL	DD	373	386	BS2G	21.62
**4443**	allXL	AA	319	357	BS2G	23.7
**4443**	allXL	AA	367	373	BS2G	11.46
**4443**	allXL	CC	370	382	BS2G	20.39
**4443**	allXL	BB	376	382	BS2G	12.87
**4443**	allXL	BB	357	373	BS2G	25.8
**4443**	allXL	AA	320	366	BS2G	24.62
**4443**	allXL	CC	370	376	BS2G	14.73
**4443**	allXL	AA	320	357	DBSU	22.79
**4443**	allXL	AA	357	319	DBSU	23.7

**4443**	allXL	BB	372	319	DBSU	17.74
**4443**	allXL	BB	370	357	DBSU	22.27
**4443**	allXL	BB	382	357	DBSU	22.7
**4443**	allXL	AA	372	370	DBSU	6.33
**4443**	allXL	DD	381	370	DBSU	22.34
**4443**	allXL	AA	373	370	DBSU	9.06
**4443**	allXL	DD	381	372	DBSU	22.74
**4443**	allXL	AA;BB	372	382	DBSU	21.91
**4443**	allXL	DD	372	386	DBSU	22.46
**4443**	allXL	DD	373	386	DBSU	21.62
**4443**	allXL	BC	381	386	DBSU	62.51
**4443**	allXL	BD;DB	357	357	DBSU	18.56
**4443**	allXL	AD	373	319	DBSU	30.52
**4443**	allXL	BC	372	357	DBSU	37.16
**4443**	allXL	AC	381	357	DBSU	40.82
**4443**	allXL	BC	373	357	DBSU	39.38
**4443**	allXL	BC	372	370	DBSU	38.25
**4443**	allXL	DA	373	370	DBSU	39.86
**4443**	allXL	DA	382	373	DBSU	46.76
**4444**	allXL	AA;CC	320	357	BS2G	25.33
**4444**	allXL	DD	320	373	BS2G	19.96
**4444**	allXL	AA	373	382	BS2G	14.33
**4444**	allXL	AA	370	386	BS2G	16.43
**4444**	allXL	AA	373	386	BS2G	19.39
**4444**	allXL	CC	319	357	BS2G	27.56
**4444**	allXL	AA	367	373	BS2G	14.74
**4444**	allXL	AA	370	382	BS2G	17.74
**4444**	allXL	AA	376	382	BS2G	12.97
**4444**	allXL	DD	357	373	BS2G	30.21
**4444**	allXL	AA	320	366	BS2G	25.98
**4444**	allXL	BB	370	376	BS2G	14.9
**4444**	allXL	AA;CC	320	357	DBSU	25.33
**4444**	allXL	CC	357	319	DBSU	27.56
**4444**	allXL	DD	372	319	DBSU	20.24

**4444**	allXL	DD	370	357	DBSU	28.69
**4444**	allXL	AA	382	357	DBSU	44.2
**4444**	allXL	CC	372	370	DBSU	6.74
**4444**	allXL	AA	381	370	DBSU	19.54
**4444**	allXL	AA	373	370	DBSU	9.31
**4444**	allXL	DD	381	372	DBSU	16.77
**4444**	allXL	AA	372	382	DBSU	17.17
**4444**	allXL	AA	372	386	DBSU	20.18
**4444**	allXL	AA	373	386	DBSU	19.39
**4444**	allXL	DA	381	386	DBSU	41.6
**4444**	allXL	BD;DB	357	357	DBSU	18.27
**4444**	allXL	DA	373	319	DBSU	23.72
**4444**	allXL	DA	372	357	DBSU	36.98
**4444**	allXL	DA	381	357	DBSU	36.98
**4444**	allXL	DA	373	357	DBSU	33.28
**4444**	allXL	DA	372	370	DBSU	42.25
**4444**	allXL	DA	373	370	DBSU	39.87
**4444**	allXL	DA	382	373	DBSU	35.63
**4445**	allXL	AA	320	357	BS2G	21.92
**4445**	allXL	CC	320	373	BS2G	30.39
**4445**	allXL	DD	373	382	BS2G	17.29
**4445**	allXL	DD	370	386	BS2G	23.46
**4445**	allXL	CC	373	386	BS2G	25.63
**4445**	allXL	AA	319	357	BS2G	22.23
**4445**	allXL	AA	367	373	BS2G	13.93
**4445**	allXL	DD	370	382	BS2G	16.45
**4445**	allXL	CC	376	382	BS2G	13.82
**4445**	allXL	BB	357	373	BS2G	24.41
**4445**	allXL	CC	320	366	BS2G	28.14
**4445**	allXL	CC	370	376	BS2G	16.92
**4445**	allXL	AA	320	357	DBSU	21.92
**4445**	allXL	AA	357	319	DBSU	22.23
**4445**	allXL	CC	372	319	DBSU	30.43
**4445**	allXL	CC	370	357	DBSU	27.25

**4445**	allXL	BB	382	357	DBSU	21.43
**4445**	allXL	AA	372	370	DBSU	6.42
**4445**	allXL	DD	381	370	DBSU	15.9
**4445**	allXL	CC	373	370	DBSU	9.13
**4445**	allXL	DD	381	372	DBSU	17.84
**4445**	allXL	DD	372	382	DBSU	18.95
**4445**	allXL	DD	372	386	DBSU	27.36
**4445**	allXL	CC	373	386	DBSU	25.63
**4445**	allXL	AC	381	386	DBSU	38.46
**4445**	allXL	BD;DB	357	357	DBSU	18.72
**4445**	allXL	AD	373	319	DBSU	28.85
**4445**	allXL	BD	372	357	DBSU	37.17
**4445**	allXL	BD	381	357	DBSU	31.2
**4445**	allXL	BD	373	357	DBSU	36.27
**4445**	allXL	BD	372	370	DBSU	60.26
**4445**	allXL	BD	373	370	DBSU	57.71
**4445**	allXL	CA	382	373	DBSU	44.95
**4446**	allXL	AA	320	357	BS2G	25.33
**4446**	allXL	AA	320	373	BS2G	21.09
**4446**	allXL	AA	373	382	BS2G	18.38
**4446**	allXL	BB	370	386	BS2G	24.64
**4446**	allXL	AA	373	386	BS2G	22.05
**4446**	allXL	CC	319	357	BS2G	23.48
**4446**	allXL	DD	367	373	BS2G	13.68
**4446**	allXL	BB	370	382	BS2G	19.42
**4446**	allXL	AA	376	382	BS2G	13.22
**4446**	allXL	DD	357	373	BS2G	19.99
**4446**	allXL	BB	320	366	BS2G	22.83
**4446**	allXL	CC	370	376	BS2G	15.5
**4446**	allXL	AA	320	357	DBSU	25.33
**4446**	allXL	CC	357	319	DBSU	23.48
**4446**	allXL	AA	372	319	DBSU	22.38
**4446**	allXL	DD	370	357	DBSU	25.44
**4446**	allXL	DD	382	357	DBSU	22.28

**4446**	allXL	AA	372	370	DBSU	6.2
**4446**	allXL	CC	381	370	DBSU	18.42
**4446**	allXL	AA	373	370	DBSU	9.13
**4446**	allXL	CC	381	372	DBSU	18.88
**4446**	allXL	CC	372	382	DBSU	19.11
**4446**	allXL	AA	372	386	DBSU	24.58
**4446**	allXL	AA	373	386	DBSU	22.05
**4446**	allXL	BC	381	386	DBSU	37.41
**4446**	allXL	BD;DB	357	357	DBSU	19.16
**4446**	allXL	AD	373	319	DBSU	33.25
**4446**	allXL	DB	372	357	DBSU	38.39
**4446**	allXL	DB	381	357	DBSU	27.74
**4446**	allXL	DB	373	357	DBSU	35.13
**4446**	allXL	CB	372	370	DBSU	34.37
**4446**	allXL	CB	373	370	DBSU	35.44
**4446**	allXL	CB	382	373	DBSU	38.12
**4447**	allXL	AA	320	357	BS2G	14.95
**4447**	allXL	CC	320	373	BS2G	23.4
**4447**	allXL	BB	373	382	BS2G	20.12
**4447**	allXL	DD	370	386	BS2G	19.29
**4447**	allXL	DD	373	386	BS2G	26.39
**4447**	allXL	AA	319	357	BS2G	16.67
**4447**	allXL	AA	367	373	BS2G	13.03
**4447**	allXL	DD	370	382	BS2G	19.07
**4447**	allXL	BB	376	382	BS2G	13.82
**4447**	allXL	DD	357	373	BS2G	19.53
**4447**	allXL	AA	320	366	BS2G	26.96
**4447**	allXL	DD	370	376	BS2G	15.24
**4447**	allXL	AA	320	357	DBSU	14.95
**4447**	allXL	AA	357	319	DBSU	16.67
**4447**	allXL	CC	372	319	DBSU	27.35
**4447**	allXL	DD	370	357	DBSU	24.33
**4447**	allXL	CC	382	357	DBSU	37.44
**4447**	allXL	AA	372	370	DBSU	6.19

**4447**	allXL	DD	381	370	DBSU	17.16
**4447**	allXL	AA	373	370	DBSU	9.11
**4447**	allXL	DD	381	372	DBSU	20.49
**4447**	allXL	AA	372	382	DBSU	22.09
**4447**	allXL	DD	372	386	DBSU	25.64
**4447**	allXL	DD	373	386	DBSU	26.39
**4447**	allXL	BC	381	386	DBSU	42.64
**4447**	allXL	BD;DB	357	357	DBSU	19.87
**4447**	allXL	AD	373	319	DBSU	21.53
**4447**	allXL	DB	372	357	DBSU	21.35
**4447**	allXL	DB	381	357	DBSU	37.14
**4447**	allXL	DB	373	357	DBSU	20.11
**4447**	allXL	CA	372	370	DBSU	49.76
**4447**	allXL	BD	373	370	DBSU	49.24
**4447**	allXL	CB	382	373	DBSU	40.49
**4448**	allXL	CC	320	357	BS2G	24.14
**4448**	allXL	AA	320	373	BS2G	28.76
**4448**	allXL	DD	373	382	BS2G	19.5
**4448**	allXL	DD	370	386	BS2G	24.13
**4448**	allXL	DD	373	386	BS2G	23.06
**4448**	allXL	CC	319	357	BS2G	27.45
**4448**	allXL	BB	367	373	BS2G	12.67
**4448**	allXL	DD	370	382	BS2G	24.69
**4448**	allXL	CC	376	382	BS2G	14.87
**4448**	allXL	CC	357	373	BS2G	34.5
**4448**	allXL	BB	320	366	BS2G	20.0
**4448**	allXL	CC	370	376	BS2G	17.79
**4448**	allXL	CC	320	357	DBSU	24.14
**4448**	allXL	CC	357	319	DBSU	27.45
**4448**	allXL	BB	372	319	DBSU	23.49
**4448**	allXL	CC	370	357	DBSU	26.54
**4448**	allXL	BB	382	357	DBSU	47.15
**4448**	allXL	AA	372	370	DBSU	6.11
**4448**	allXL	DD	381	370	DBSU	26.03

**4448**	allXL	AA	373	370	DBSU	9.33
**4448**	allXL	DD	381	372	DBSU	19.05
**4448**	allXL	DD	372	382	DBSU	18.21
**4448**	allXL	DD	372	386	DBSU	20.09
**4448**	allXL	DD	373	386	DBSU	23.06
**4448**	allXL	DA	381	386	DBSU	65.67
**4448**	allXL	BD;DB	357	357	DBSU	18.79
**4448**	allXL	AD	373	319	DBSU	32.16
**4448**	allXL	BC	372	357	DBSU	43.16
**4448**	allXL	CA	381	357	DBSU	55.4
**4448**	allXL	BC	373	357	DBSU	44.99
**4448**	allXL	DA	372	370	DBSU	50.45
**4448**	allXL	DA	373	370	DBSU	48.07
**4448**	allXL	AD	382	373	DBSU	56.75
**4449**	allXL	CC	320	357	BS2G	22.79
**4449**	allXL	AA	320	373	BS2G	13.48
**4449**	allXL	AA	373	382	BS2G	18.57
**4449**	allXL	CC	370	386	BS2G	21.81
**4449**	allXL	CC	373	386	BS2G	25.65
**4449**	allXL	CC	319	357	BS2G	22.41
**4449**	allXL	AA	367	373	BS2G	11.59
**4449**	allXL	AA	370	382	BS2G	21.51
**4449**	allXL	CC	376	382	BS2G	13.07
**4449**	allXL	AA	357	373	BS2G	25.5
**4449**	allXL	AA	320	366	BS2G	17.78
**4449**	allXL	CC	370	376	BS2G	16.09
**4449**	allXL	CC	320	357	DBSU	22.79
**4449**	allXL	CC	357	319	DBSU	22.41
**4449**	allXL	AA	372	319	DBSU	13.67
**4449**	allXL	BB	370	357	DBSU	18.63
**4449**	allXL	AA	382	357	DBSU	22.87
**4449**	allXL	AA	372	370	DBSU	6.58
**4449**	allXL	AA	381	370	DBSU	20.42
**4449**	allXL	AA	373	370	DBSU	8.85

**4449**	allXL	AA	381	372	DBSU	19.93
**4449**	allXL	AA	372	382	DBSU	21.31
**4449**	allXL	CC	372	386	DBSU	24.45
**4449**	allXL	CC	373	386	DBSU	25.65
**4449**	allXL	BC	381	386	DBSU	39.45
**4449**	allXL	BD;DB	357	357	DBSU	19.7
**4449**	allXL	AD	373	319	DBSU	34.54
**4449**	allXL	AD	372	357	DBSU	37.33
**4449**	allXL	AC	381	357	DBSU	37.67
**4449**	allXL	AD	373	357	DBSU	36.5
**4449**	allXL	DA	372	370	DBSU	33.65
**4449**	allXL	DA	373	370	DBSU	30.71
**4449**	allXL	AD	382	373	DBSU	35.34
**4450**	allXL	AA	320	357	BS2G	26.95
**4450**	allXL	AA	320	373	BS2G	13.45
**4450**	allXL	AA	373	382	BS2G	17.59
**4450**	allXL	DD	370	386	BS2G	15.36
**4450**	allXL	DD	373	386	BS2G	23.59
**4450**	allXL	AA	319	357	BS2G	26.39
**4450**	allXL	AA	367	373	BS2G	10.42
**4450**	allXL	DD	370	382	BS2G	15.84
**4450**	allXL	AA	376	382	BS2G	14.44
**4450**	allXL	BB	357	373	BS2G	14.26
**4450**	allXL	DD	320	366	BS2G	15.95
**4450**	allXL	DD	370	376	BS2G	15.29
**4450**	allXL	AA	320	357	DBSU	26.95
**4450**	allXL	AA	357	319	DBSU	26.39
**4450**	allXL	AA	372	319	DBSU	11.07
**4450**	allXL	CC	370	357	DBSU	22.8
**4450**	allXL	BB	382	357	DBSU	20.69
**4450**	allXL	CC	372	370	DBSU	6.57
**4450**	allXL	DD	381	370	DBSU	15.52
**4450**	allXL	AA	373	370	DBSU	9.02
**4450**	allXL	DD	381	372	DBSU	19.26

**4450**	allXL	CC	372	382	DBSU	20.68
**4450**	allXL	DD	372	386	DBSU	21.9
**4450**	allXL	DD	373	386	DBSU	23.59
**4450**	allXL	AD	381	386	DBSU	49.66
**4450**	allXL	BD;DB	357	357	DBSU	18.01
**4450**	allXL	AD	373	319	DBSU	30.01
**4450**	allXL	CA	372	357	DBSU	27.22
**4450**	allXL	BD	381	357	DBSU	25.79
**4450**	allXL	CA	373	357	DBSU	24.07
**4450**	allXL	DA	372	370	DBSU	30.75
**4450**	allXL	DA	373	370	DBSU	30.4
**4450**	allXL	AD	382	373	DBSU	35.58
**4451**	allXL	CC	320	357	BS2G	25.21
**4451**	allXL	DD	320	373	BS2G	20.95
**4451**	allXL	DD	373	382	BS2G	18.29
**4451**	allXL	CC	370	386	BS2G	26.16
**4451**	allXL	BB	373	386	BS2G	21.53
**4451**	allXL	AA	319	357	BS2G	26.25
**4451**	allXL	BB	367	373	BS2G	12.22
**4451**	allXL	DD	370	382	BS2G	20.63
**4451**	allXL	CC	376	382	BS2G	12.66
**4451**	allXL	BB	357	373	BS2G	27.03
**4451**	allXL	AA	320	366	BS2G	23.81
**4451**	allXL	CC	370	376	BS2G	16.95
**4451**	allXL	CC	320	357	DBSU	25.21
**4451**	allXL	AA	357	319	DBSU	26.25
**4451**	allXL	DD	372	319	DBSU	20.93
**4451**	allXL	DD	370	357	DBSU	29.15
**4451**	allXL	BB	382	357	DBSU	23.79
**4451**	allXL	AA	372	370	DBSU	6.14
**4451**	allXL	DD	381	370	DBSU	19.75
**4451**	allXL	AA	373	370	DBSU	9.19
**4451**	allXL	DD	381	372	DBSU	16.24
**4451**	allXL	DD	372	382	DBSU	17.14

**4451**	allXL	CC	372	386	DBSU	22.68
**4451**	allXL	BB	373	386	DBSU	21.53
**4451**	allXL	DA	381	386	DBSU	51.54
**4451**	allXL	BD;DB	357	357	DBSU	18.24
**4451**	allXL	DA	373	319	DBSU	23.51
**4451**	allXL	DA	372	357	DBSU	35.96
**4451**	allXL	BD	381	357	DBSU	35.49
**4451**	allXL	DA	373	357	DBSU	32.86
**4451**	allXL	DA	372	370	DBSU	29.44
**4451**	allXL	DA	373	370	DBSU	26.96
**4451**	allXL	DA	382	373	DBSU	32.32
**4452**	allXL	CC	320	357	BS2G	26.89
**4452**	allXL	DD	320	373	BS2G	16.69
**4452**	allXL	BB	373	382	BS2G	14.76
**4452**	allXL	BB	370	386	BS2G	23.82
**4452**	allXL	BB	373	386	BS2G	20.62
**4452**	allXL	AA	319	357	BS2G	28.12
**4452**	allXL	AA	367	373	BS2G	13.13
**4452**	allXL	BB	370	382	BS2G	22.64
**4452**	allXL	CC	376	382	BS2G	14.03
**4452**	allXL	CC	357	373	BS2G	20.55
**4452**	allXL	DD	320	366	BS2G	24.72
**4452**	allXL	CC	370	376	BS2G	14.46
**4452**	allXL	CC	320	357	DBSU	26.89
**4452**	allXL	AA	357	319	DBSU	28.12
**4452**	allXL	DD	372	319	DBSU	16.12
**4452**	allXL	CC	370	357	DBSU	24.79
**4452**	allXL	BB	382	357	DBSU	29.86
**4452**	allXL	AA	372	370	DBSU	6.35
**4452**	allXL	BB	381	370	DBSU	21.43
**4452**	allXL	AA	373	370	DBSU	8.96
**4452**	allXL	BB	381	372	DBSU	14.3
**4452**	allXL	BB	372	382	DBSU	15.44
**4452**	allXL	BB	372	386	DBSU	18.9

**4452**	allXL	BB	373	386	DBSU	20.62
**4452**	allXL	DA	381	386	DBSU	57.52
**4452**	allXL	BD;DB	357	357	DBSU	19.12
**4452**	allXL	BC	373	319	DBSU	39.01
**4452**	allXL	AC	372	357	DBSU	36.92
**4452**	allXL	AC	381	357	DBSU	32.08
**4452**	allXL	AC	373	357	DBSU	36.05
**4452**	allXL	BC	372	370	DBSU	46.02
**4452**	allXL	BC	373	370	DBSU	43.68
**4452**	allXL	AC	382	373	DBSU	43.47
**4453**	allXL	AA	320	357	BS2G	20.72
**4453**	allXL	BB	320	373	BS2G	27.52
**4453**	allXL	DD	373	382	BS2G	18.49
**4453**	allXL	DD	370	386	BS2G	17.99
**4453**	allXL	BB	373	386	BS2G	13.79
**4453**	allXL	AA	319	357	BS2G	18.23
**4453**	allXL	AA	367	373	BS2G	12.15
**4453**	allXL	DD	370	382	BS2G	19.93
**4453**	allXL	CC	376	382	BS2G	13.63
**4453**	allXL	DD	357	373	BS2G	26.7
**4453**	allXL	DD	320	366	BS2G	19.96
**4453**	allXL	CC	370	376	BS2G	16.92
**4453**	allXL	AA	320	357	DBSU	20.72
**4453**	allXL	AA	357	319	DBSU	18.23
**4453**	allXL	DD	372	319	DBSU	29.5
**4453**	allXL	DD	370	357	DBSU	28.04
**4453**	allXL	BB	382	357	DBSU	34.3
**4453**	allXL	AA	372	370	DBSU	6.75
**4453**	allXL	DD	381	370	DBSU	19.4
**4453**	allXL	AA	373	370	DBSU	9.2
**4453**	allXL	DD	381	372	DBSU	17.52
**4453**	allXL	DD	372	382	DBSU	18.62
**4453**	allXL	BB	372	386	DBSU	13.5
**4453**	allXL	BB	373	386	DBSU	13.79

**4453**	allXL	DB	381	386	DBSU	52.91
**4453**	allXL	BD;DB	357	357	DBSU	18.41
**4453**	allXL	AD	373	319	DBSU	22.03
**4453**	allXL	DB	372	357	DBSU	42.92
**4453**	allXL	BD	381	357	DBSU	46.09
**4453**	allXL	DB	373	357	DBSU	39.99
**4453**	allXL	DA	372	370	DBSU	42.81
**4453**	allXL	DA	373	370	DBSU	45.17
**4453**	allXL	BD	382	373	DBSU	47.17
**4454**	allXL	AA	320	357	BS2G	25.49
**4454**	allXL	BB	320	373	BS2G	27.65
**4454**	allXL	DD	373	382	BS2G	15.27
**4454**	allXL	DD	370	386	BS2G	18.83
**4454**	allXL	DD	373	386	BS2G	20.95
**4454**	allXL	AA	319	357	BS2G	24.55
**4454**	allXL	BB	367	373	BS2G	14.16
**4454**	allXL	DD	370	382	BS2G	18.72
**4454**	allXL	BB	376	382	BS2G	13.54
**4454**	allXL	BB	357	373	BS2G	12.32
**4454**	allXL	CC	320	366	BS2G	16.98
**4454**	allXL	AA	370	376	BS2G	16.99
**4454**	allXL	AA	320	357	DBSU	25.49
**4454**	allXL	AA	357	319	DBSU	24.55
**4454**	allXL	BB	372	319	DBSU	27.7
**4454**	allXL	BB	370	357	DBSU	19.44
**4454**	allXL	BB	382	357	DBSU	12.5
**4454**	allXL	AA	372	370	DBSU	6.18
**4454**	allXL	DD	381	370	DBSU	21.19
**4454**	allXL	AA	373	370	DBSU	9.11
**4454**	allXL	DD	381	372	DBSU	16.36
**4454**	allXL	DD	372	382	DBSU	14.92
**4454**	allXL	DD	372	386	DBSU	18.82
**4454**	allXL	DD	373	386	DBSU	20.95
**4454**	allXL	BD	381	386	DBSU	52.31

**4454**	allXL	BD;DB	357	357	DBSU	18.27
**4454**	allXL	DA	373	319	DBSU	18.51
**4454**	allXL	AC	372	357	DBSU	27.11
**4454**	allXL	BD	381	357	DBSU	25.95
**4454**	allXL	BD	373	357	DBSU	24.42
**4454**	allXL	BC	372	370	DBSU	39.05
**4454**	allXL	BD	373	370	DBSU	39.59
**4454**	allXL	BD	382	373	DBSU	49.23
**4455**	allXL	AA	320	357	BS2G	23.01
**4455**	allXL	BB	320	373	BS2G	25.74
**4455**	allXL	AA	373	382	BS2G	16.41
**4455**	allXL	AA	370	386	BS2G	18.17
**4455**	allXL	AA	373	386	BS2G	20.63
**4455**	allXL	AA	319	357	BS2G	20.99
**4455**	allXL	BB	367	373	BS2G	12.33
**4455**	allXL	AA	370	382	BS2G	18.81
**4455**	allXL	CC	376	382	BS2G	14.19
**4455**	allXL	CC	357	373	BS2G	28.61
**4455**	allXL	CC	320	366	BS2G	21.59
**4455**	allXL	BB	370	376	BS2G	17.45
**4455**	allXL	AA	320	357	DBSU	23.01
**4455**	allXL	AA	357	319	DBSU	20.99
**4455**	allXL	BB	372	319	DBSU	22.47
**4455**	allXL	CC	370	357	DBSU	26.44
**4455**	allXL	BB	382	357	DBSU	24.05
**4455**	allXL	AA	372	370	DBSU	5.98
**4455**	allXL	AA	381	370	DBSU	21.64
**4455**	allXL	AA	373	370	DBSU	8.97
**4455**	allXL	AA	381	372	DBSU	17.96
**4455**	allXL	AA	372	382	DBSU	15.81
**4455**	allXL	AA	372	386	DBSU	18.92
**4455**	allXL	AA	373	386	DBSU	20.63
**4455**	allXL	DB	381	386	DBSU	52.21
**4455**	allXL	BD;DB	357	357	DBSU	19.32

**4455**	allXL	AD	373	319	DBSU	32.72
**4455**	allXL	CA	372	357	DBSU	46.16
**4455**	allXL	BD	381	357	DBSU	37.3
**4455**	allXL	CA	373	357	DBSU	47.5
**4455**	allXL	AD	372	370	DBSU	58.23
**4455**	allXL	AD	373	370	DBSU	59.54
**4455**	allXL	BD	382	373	DBSU	59.43
**4456**	allXL	AA	320	357	BS2G	20.33
**4456**	allXL	CC	320	373	BS2G	14.09
**4456**	allXL	AA	373	382	BS2G	20.58
**4456**	allXL	DD	370	386	BS2G	23.15
**4456**	allXL	DD	373	386	BS2G	26.28
**4456**	allXL	AA	319	357	BS2G	18.24
**4456**	allXL	AA	367	373	BS2G	12.48
**4456**	allXL	DD	370	382	BS2G	20.79
**4456**	allXL	AA	376	382	BS2G	13.86
**4456**	allXL	BB	357	373	BS2G	23.55
**4456**	allXL	BB	320	366	BS2G	9.63
**4456**	allXL	BB	370	376	BS2G	16.1
**4456**	allXL	AA	320	357	DBSU	20.33
**4456**	allXL	AA	357	319	DBSU	18.24
**4456**	allXL	CC	372	319	DBSU	11.9
**4456**	allXL	BB	370	357	DBSU	23.89
**4456**	allXL	DD	382	357	DBSU	31.6
**4456**	allXL	AA	372	370	DBSU	6.54
**4456**	allXL	DD	381	370	DBSU	21.06
**4456**	allXL	DD	373	370	DBSU	9.29
**4456**	allXL	DD	381	372	DBSU	22.19
**4456**	allXL	AA	372	382	DBSU	21.16
**4456**	allXL	AA	372	386	DBSU	25.83
**4456**	allXL	DD	373	386	DBSU	26.28
**4456**	allXL	BD	381	386	DBSU	40.78
**4456**	allXL	BD;DB	357	357	DBSU	18.04
**4456**	allXL	CB	373	319	DBSU	34.96

**4456**	allXL	BD	372	357	DBSU	33.03
**4456**	allXL	CB	381	357	DBSU	25.6
**4456**	allXL	CB	373	357	DBSU	34.39
**4456**	allXL	BD	372	370	DBSU	38.66
**4456**	allXL	BD	373	370	DBSU	37.21
**4456**	allXL	DB	382	373	DBSU	39.74
**4457**	allXL	AA	320	357	BS2G	25.35
**4457**	allXL	DD	320	373	BS2G	29.86
**4457**	allXL	BB	373	382	BS2G	18.08
**4457**	allXL	BB	370	386	BS2G	25.72
**4457**	allXL	AA	373	386	BS2G	24.26
**4457**	allXL	AA	319	357	BS2G	28.41
**4457**	allXL	BB	367	373	BS2G	13.56
**4457**	allXL	BB	370	382	BS2G	17.51
**4457**	allXL	BB	376	382	BS2G	14.42
**4457**	allXL	BB	357	373	BS2G	33.93
**4457**	allXL	BB	320	366	BS2G	17.2
**4457**	allXL	CC	370	376	BS2G	16.42
**4457**	allXL	AA	320	357	DBSU	25.35
**4457**	allXL	AA	357	319	DBSU	28.41
**4457**	allXL	BB	372	319	DBSU	27.58
**4457**	allXL	DD	370	357	DBSU	28.51
**4457**	allXL	BB	382	357	DBSU	38.35
**4457**	allXL	AA	372	370	DBSU	6.0
**4457**	allXL	BB	381	370	DBSU	19.41
**4457**	allXL	DD	373	370	DBSU	8.51
**4457**	allXL	BB	381	372	DBSU	20.69
**4457**	allXL	BB	372	382	DBSU	19.85
**4457**	allXL	AA	372	386	DBSU	25.08
**4457**	allXL	AA	373	386	DBSU	24.26
**4457**	allXL	AD	381	386	DBSU	41.88
**4457**	allXL	BD;DB	357	357	DBSU	18.3
**4457**	allXL	AD	373	319	DBSU	21.1
**4457**	allXL	AD	372	357	DBSU	48.76

**4457**	allXL	BD	381	357	DBSU	57.02
**4457**	allXL	AD	373	357	DBSU	50.01
**4457**	allXL	AD	372	370	DBSU	36.15
**4457**	allXL	AD	373	370	DBSU	36.64
**4457**	allXL	DA	382	373	DBSU	45.53
**4458**	allXL	AA	320	357	BS2G	23.41
**4458**	allXL	BB	320	373	BS2G	9.32
**4458**	allXL	DD	373	382	BS2G	17.66
**4458**	allXL	DD	370	386	BS2G	23.97
**4458**	allXL	DD	373	386	BS2G	22.11
**4458**	allXL	AA	319	357	BS2G	21.75
**4458**	allXL	AA	367	373	BS2G	13.93
**4458**	allXL	DD	370	382	BS2G	22.49
**4458**	allXL	BB	376	382	BS2G	14.62
**4458**	allXL	BB	357	373	BS2G	26.84
**4458**	allXL	BB	320	366	BS2G	19.34
**4458**	allXL	CC	370	376	BS2G	15.25
**4458**	allXL	AA	320	357	DBSU	23.41
**4458**	allXL	AA	357	319	DBSU	21.75
**4458**	allXL	BB	372	319	DBSU	12.8
**4458**	allXL	DD	370	357	DBSU	28.01
**4458**	allXL	BB	382	357	DBSU	33.64
**4458**	allXL	AA	372	370	DBSU	6.25
**4458**	allXL	DD	381	370	DBSU	24.03
**4458**	allXL	AA	373	370	DBSU	9.24
**4458**	allXL	DD	381	372	DBSU	18.05
**4458**	allXL	DD	372	382	DBSU	17.03
**4458**	allXL	DD	372	386	DBSU	20.09
**4458**	allXL	DD	373	386	DBSU	22.11
**4458**	allXL	BC	381	386	DBSU	28.68
**4458**	allXL	BD;DB	357	357	DBSU	18.53
**4458**	allXL	DA	373	319	DBSU	15.76
**4458**	allXL	DA	372	357	DBSU	35.1
**4458**	allXL	DA	381	357	DBSU	33.5

**4458**	allXL	DA	373	357	DBSU	32.45
**4458**	allXL	CB	372	370	DBSU	37.98
**4458**	allXL	CB	373	370	DBSU	35.49
**4458**	allXL	BC	382	373	DBSU	15.14
**4459**	allXL	CC	320	357	BS2G	23.41
**4459**	allXL	BB	320	373	BS2G	18.68
**4459**	allXL	DD	373	382	BS2G	20.0
**4459**	allXL	DD	370	386	BS2G	19.1
**4459**	allXL	DD	373	386	BS2G	24.59
**4459**	allXL	CC	319	357	BS2G	22.33
**4459**	allXL	AA	367	373	BS2G	10.16
**4459**	allXL	DD	370	382	BS2G	16.4
**4459**	allXL	CC	376	382	BS2G	14.61
**4459**	allXL	BB	357	373	BS2G	19.48
**4459**	allXL	BB	320	366	BS2G	21.36
**4459**	allXL	DD	370	376	BS2G	15.02
**4459**	allXL	CC	320	357	DBSU	23.41
**4459**	allXL	CC	357	319	DBSU	22.33
**4459**	allXL	BB	372	319	DBSU	20.39
**4459**	allXL	CC	370	357	DBSU	20.4
**4459**	allXL	BB	382	357	DBSU	31.01
**4459**	allXL	CC	372	370	DBSU	6.53
**4459**	allXL	DD	381	370	DBSU	16.14
**4459**	allXL	DD	373	370	DBSU	9.08
**4459**	allXL	DD	381	372	DBSU	19.3
**4459**	allXL	DD	372	382	DBSU	20.56
**4459**	allXL	DD	372	386	DBSU	23.43
**4459**	allXL	DD	373	386	DBSU	24.59
**4459**	allXL	CB	381	386	DBSU	22.75
**4459**	allXL	BD;DB	357	357	DBSU	18.93
**4459**	allXL	AD	373	319	DBSU	14.14
**4459**	allXL	DB	372	357	DBSU	14.5
**4459**	allXL	CA	381	357	DBSU	26.94
**4459**	allXL	DB	373	357	DBSU	13.59

**4459**	allXL	DB	372	370	DBSU	27.84
**4459**	allXL	BD	373	370	DBSU	25.09
**4459**	allXL	BD	382	373	DBSU	33.75
**4460**	allXL	AA	320	357	BS2G	24.28
**4460**	allXL	BB	320	373	BS2G	15.25
**4460**	allXL	BB	373	382	BS2G	17.16
**4460**	allXL	BB	370	386	BS2G	24.35
**4460**	allXL	BB	373	386	BS2G	24.89
**4460**	allXL	AA	319	357	BS2G	23.63
**4460**	allXL	BB	367	373	BS2G	14.07
**4460**	allXL	BB	370	382	BS2G	20.78
**4460**	allXL	BB	376	382	BS2G	14.23
**4460**	allXL	BB	357	373	BS2G	16.72
**4460**	allXL	BB	320	366	BS2G	19.35
**4460**	allXL	CC	370	376	BS2G	16.43
**4460**	allXL	AA	320	357	DBSU	24.28
**4460**	allXL	AA	357	319	DBSU	23.63
**4460**	allXL	BB	372	319	DBSU	17.11
**4460**	allXL	BB	370	357	DBSU	21.27
**4460**	allXL	BB	382	357	DBSU	22.66
**4460**	allXL	AA	372	370	DBSU	6.19
**4460**	allXL	BB	381	370	DBSU	21.24
**4460**	allXL	AA	373	370	DBSU	9.36
**4460**	allXL	BB	381	372	DBSU	19.68
**4460**	allXL	BB	372	382	DBSU	20.33
**4460**	allXL	BB	372	386	DBSU	26.82
**4460**	allXL	BB	373	386	DBSU	24.89
**4460**	allXL	BD	381	386	DBSU	41.06
**4460**	allXL	BD;DB	357	357	DBSU	18.41
**4460**	allXL	CB	373	319	DBSU	33.44
**4460**	allXL	BD	372	357	DBSU	35.64
**4460**	allXL	BD	381	357	DBSU	31.04
**4460**	allXL	BD	373	357	DBSU	32.78
**4460**	allXL	CB	372	370	DBSU	41.27

**4460**	allXL	CB	373	370	DBSU	37.89
**4460**	allXL	CB	382	373	DBSU	38.55
**4461**	allXL	AA	320	357	BS2G	25.15
**4461**	allXL	BB	320	373	BS2G	27.55
**4461**	allXL	DD	373	382	BS2G	17.06
**4461**	allXL	DD	370	386	BS2G	20.25
**4461**	allXL	DD	373	386	BS2G	21.96
**4461**	allXL	AA	319	357	BS2G	24.32
**4461**	allXL	AA	367	373	BS2G	12.17
**4461**	allXL	CC	370	382	BS2G	16.19
**4461**	allXL	AA	376	382	BS2G	14.76
**4461**	allXL	AA	357	373	BS2G	31.82
**4461**	allXL	AA	320	366	BS2G	24.92
**4461**	allXL	CC	370	376	BS2G	11.68
**4461**	allXL	AA	320	357	DBSU	25.15
**4461**	allXL	AA	357	319	DBSU	24.32
**4461**	allXL	BB	372	319	DBSU	23.55
**4461**	allXL	DD	370	357	DBSU	26.14
**4461**	allXL	CC	382	357	DBSU	32.73
**4461**	allXL	AA	372	370	DBSU	6.34
**4461**	allXL	CC	381	370	DBSU	15.33
**4461**	allXL	AA	373	370	DBSU	8.91
**4461**	allXL	DD	381	372	DBSU	15.96
**4461**	allXL	AA	372	382	DBSU	17.56
**4461**	allXL	DD	372	386	DBSU	21.43
**4461**	allXL	DD	373	386	DBSU	21.96
**4461**	allXL	BD	381	386	DBSU	68.28
**4461**	allXL	BD;DB	357	357	DBSU	17.96
**4461**	allXL	DA	373	319	DBSU	20.7
**4461**	allXL	DA	372	357	DBSU	38.89
**4461**	allXL	DA	381	357	DBSU	31.82
**4461**	allXL	DA	373	357	DBSU	35.28
**4461**	allXL	CB	372	370	DBSU	26.63
**4461**	allXL	CB	373	370	DBSU	25.88

**4461**	allXL	CB	382	373	DBSU	45.48
**4462**	allXL	CC	320	357	BS2G	22.65
**4462**	allXL	DD	320	373	BS2G	33.37
**4462**	allXL	BB	373	382	BS2G	17.65
**4462**	allXL	DD	370	386	BS2G	14.99
**4462**	allXL	DD	373	386	BS2G	21.59
**4462**	allXL	CC	319	357	BS2G	23.49
**4462**	allXL	AA	367	373	BS2G	10.63
**4462**	allXL	DD	370	382	BS2G	17.95
**4462**	allXL	BB	376	382	BS2G	12.53
**4462**	allXL	BB	357	373	BS2G	28.12
**4462**	allXL	CC	320	366	BS2G	25.06
**4462**	allXL	CC	370	376	BS2G	15.84
**4462**	allXL	CC	320	357	DBSU	22.65
**4462**	allXL	CC	357	319	DBSU	23.49
**4462**	allXL	DD	372	319	DBSU	32.7
**4462**	allXL	DD	370	357	DBSU	25.9
**4462**	allXL	BB	382	357	DBSU	27.87
**4462**	allXL	CC	372	370	DBSU	6.44
**4462**	allXL	DD	381	370	DBSU	17.7
**4462**	allXL	AA	373	370	DBSU	9.11
**4462**	allXL	DD	381	372	DBSU	18.57
**4462**	allXL	DD	372	382	DBSU	20.06
**4462**	allXL	DD	372	386	DBSU	20.84
**4462**	allXL	DD	373	386	DBSU	21.59
**4462**	allXL	BD	381	386	DBSU	48.67
**4462**	allXL	BD;DB	357	357	DBSU	18.67
**4462**	allXL	AD	373	319	DBSU	25.96
**4462**	allXL	BD	372	357	DBSU	46.9
**4462**	allXL	BD	381	357	DBSU	36.61
**4462**	allXL	BD	373	357	DBSU	43.48
**4462**	allXL	DA	372	370	DBSU	39.57
**4462**	allXL	DA	373	370	DBSU	41.0
**4462**	allXL	AD	382	373	DBSU	63.28

**4463**	allXL	CC	320	357	BS2G	24.36
**4463**	allXL	BB	320	373	BS2G	11.44
**4463**	allXL	BB	373	382	BS2G	17.99
**4463**	allXL	DD	370	386	BS2G	14.17
**4463**	allXL	DD	373	386	BS2G	18.8
**4463**	allXL	CC	319	357	BS2G	25.32
**4463**	allXL	AA	367	373	BS2G	13.47
**4463**	allXL	DD	370	382	BS2G	19.53
**4463**	allXL	BB	376	382	BS2G	13.34
**4463**	allXL	BB	357	373	BS2G	19.33
**4463**	allXL	CC	320	366	BS2G	16.87
**4463**	allXL	CC	370	376	BS2G	15.49
**4463**	allXL	CC	320	357	DBSU	24.36
**4463**	allXL	CC	357	319	DBSU	25.32
**4463**	allXL	BB	372	319	DBSU	13.91
**4463**	allXL	BB	370	357	DBSU	24.61
**4463**	allXL	BB	382	357	DBSU	28.6
**4463**	allXL	AA	372	370	DBSU	6.19
**4463**	allXL	DD	381	370	DBSU	19.75
**4463**	allXL	AA	373	370	DBSU	9.18
**4463**	allXL	DD	381	372	DBSU	19.58
**4463**	allXL	BB	372	382	DBSU	19.29
**4463**	allXL	DD	372	386	DBSU	19.31
**4463**	allXL	DD	373	386	DBSU	18.8
**4463**	allXL	AD	381	386	DBSU	51.22
**4463**	allXL	BD;DB	357	357	DBSU	17.79
**4463**	allXL	AD	373	319	DBSU	20.42
**4463**	allXL	BD	372	357	DBSU	38.16
**4463**	allXL	BD	381	357	DBSU	40.04
**4463**	allXL	BD	373	357	DBSU	34.95
**4463**	allXL	CB	372	370	DBSU	38.49
**4463**	allXL	CB	373	370	DBSU	39.47
**4463**	allXL	DA	382	373	DBSU	47.46
**4464**	allXL	AA	320	357	BS2G	22.59

**4464**	allXL	BB	320	373	BS2G	26.43
**4464**	allXL	DD	373	382	BS2G	15.64
**4464**	allXL	DD	370	386	BS2G	23.76
**4464**	allXL	DD	373	386	BS2G	21.66
**4464**	allXL	AA	319	357	BS2G	19.78
**4464**	allXL	AA	367	373	BS2G	11.0
**4464**	allXL	DD	370	382	BS2G	19.74
**4464**	allXL	CC	376	382	BS2G	14.05
**4464**	allXL	BB	357	373	BS2G	25.02
**4464**	allXL	BB	320	366	BS2G	16.82
**4464**	allXL	CC	370	376	BS2G	15.85
**4464**	allXL	AA	320	357	DBSU	22.59
**4464**	allXL	AA	357	319	DBSU	19.78
**4464**	allXL	BB	372	319	DBSU	27.66
**4464**	allXL	BB	370	357	DBSU	23.11
**4464**	allXL	BB	382	357	DBSU	32.46
**4464**	allXL	CC	372	370	DBSU	6.48
**4464**	allXL	DD	381	370	DBSU	21.49
**4464**	allXL	AA	373	370	DBSU	9.05
**4464**	allXL	DD	381	372	DBSU	16.8
**4464**	allXL	DD	372	382	DBSU	15.34
**4464**	allXL	DD	372	386	DBSU	19.45
**4464**	allXL	DD	373	386	DBSU	21.66
**4464**	allXL	DB	381	386	DBSU	28.22
**4464**	allXL	BD;DB	357	357	DBSU	18.15
**4464**	allXL	DA	373	319	DBSU	22.55
**4464**	allXL	BD	372	357	DBSU	37.03
**4464**	allXL	DA	381	357	DBSU	31.78
**4464**	allXL	BD	373	357	DBSU	35.66
**4464**	allXL	BD	372	370	DBSU	41.9
**4464**	allXL	BD	373	370	DBSU	39.2
**4464**	allXL	BD	382	373	DBSU	25.23
**4465**	allXL	AA	320	357	BS2G	20.86
**4465**	allXL	BB	320	373	BS2G	18.38

**4465**	allXL	CC	373	382	BS2G	20.2
**4465**	allXL	CC	370	386	BS2G	24.04
**4465**	allXL	AA	373	386	BS2G	27.32
**4465**	allXL	AA	319	357	BS2G	21.76
**4465**	allXL	AA	367	373	BS2G	13.35
**4465**	allXL	CC	370	382	BS2G	19.28
**4465**	allXL	CC	376	382	BS2G	14.24
**4465**	allXL	BB	357	373	BS2G	27.04
**4465**	allXL	BB	320	366	BS2G	27.24
**4465**	allXL	CC	370	376	BS2G	13.99
**4465**	allXL	AA	320	357	DBSU	20.86
**4465**	allXL	AA	357	319	DBSU	21.76
**4465**	allXL	BB	372	319	DBSU	18.47
**4465**	allXL	BB	370	357	DBSU	25.61
**4465**	allXL	BB	382	357	DBSU	33.73
**4465**	allXL	AA	372	370	DBSU	6.26
**4465**	allXL	DD	381	370	DBSU	19.63
**4465**	allXL	AA	373	370	DBSU	9.04
**4465**	allXL	DD	381	372	DBSU	20.05
**4465**	allXL	CC	372	382	DBSU	21.68
**4465**	allXL	AA	372	386	DBSU	27.12
**4465**	allXL	AA	373	386	DBSU	27.32
**4465**	allXL	BC	381	386	DBSU	23.13
**4465**	allXL	BD;DB	357	357	DBSU	18.26
**4465**	allXL	AD	373	319	DBSU	19.64
**4465**	allXL	BD	372	357	DBSU	43.79
**4465**	allXL	DB	381	357	DBSU	47.84
**4465**	allXL	BD	373	357	DBSU	44.62
**4465**	allXL	CB	372	370	DBSU	31.76
**4465**	allXL	CB	373	370	DBSU	34.34
**4465**	allXL	CB	382	373	DBSU	36.58
**4466**	allXL	CC	320	357	BS2G	24.27
**4466**	allXL	BB	320	373	BS2G	34.27
**4466**	allXL	BB	373	382	BS2G	18.03

**4466**	allXL	BB	370	386	BS2G	28.48
**4466**	allXL	CC	373	386	BS2G	27.49
**4466**	allXL	AA	319	357	BS2G	24.19
**4466**	allXL	AA	367	373	BS2G	12.51
**4466**	allXL	BB	370	382	BS2G	21.3
**4466**	allXL	BB	376	382	BS2G	12.75
**4466**	allXL	AA	357	373	BS2G	31.58
**4466**	allXL	BB	320	366	BS2G	31.49
**4466**	allXL	CC	370	376	BS2G	16.66
**4466**	allXL	CC	320	357	DBSU	24.27
**4466**	allXL	AA	357	319	DBSU	24.19
**4466**	allXL	DD	372	319	DBSU	35.78
**4466**	allXL	DD	370	357	DBSU	23.12
**4466**	allXL	BB	382	357	DBSU	37.32
**4466**	allXL	AA	372	370	DBSU	6.21
**4466**	allXL	BB	381	370	DBSU	24.12
**4466**	allXL	AA	373	370	DBSU	9.13
**4466**	allXL	BB	381	372	DBSU	22.96
**4466**	allXL	BB	372	382	DBSU	20.99
**4466**	allXL	CC	372	386	DBSU	28.03
**4466**	allXL	CC	373	386	DBSU	27.49
**4466**	allXL	BC	381	386	DBSU	46.63
**4466**	allXL	BD;DB	357	357	DBSU	17.27
**4466**	allXL	DA	373	319	DBSU	29.87
**4466**	allXL	DB	372	357	DBSU	39.43
**4466**	allXL	AC	381	357	DBSU	42.25
**4466**	allXL	AC	373	357	DBSU	42.81
**4466**	allXL	CB	372	370	DBSU	36.92
**4466**	allXL	BC	373	370	DBSU	37.96
**4466**	allXL	BC	382	373	DBSU	40.87
**4467**	allXL	AA	320	357	BS2G	24.65
**4467**	allXL	AA	320	373	BS2G	25.81
**4467**	allXL	CC	373	382	BS2G	21.86
**4467**	allXL	DD	370	386	BS2G	17.27

**4467**	allXL	AA	373	386	BS2G	20.46
**4467**	allXL	AA	319	357	BS2G	26.2
**4467**	allXL	BB	367	373	BS2G	12.44
**4467**	allXL	DD	370	382	BS2G	22.82
**4467**	allXL	CC	376	382	BS2G	13.63
**4467**	allXL	BB	357	373	BS2G	29.08
**4467**	allXL	BB	320	366	BS2G	23.83
**4467**	allXL	CC	370	376	BS2G	15.64
**4467**	allXL	AA	320	357	DBSU	24.65
**4467**	allXL	AA	357	319	DBSU	26.2
**4467**	allXL	AA	372	319	DBSU	30.57
**4467**	allXL	BB	370	357	DBSU	25.66
**4467**	allXL	BB	382	357	DBSU	31.95
**4467**	allXL	AA	372	370	DBSU	6.11
**4467**	allXL	DD	381	370	DBSU	22.43
**4467**	allXL	AA	373	370	DBSU	9.12
**4467**	allXL	DD	381	372	DBSU	23.3
**4467**	allXL	CC	372	382	DBSU	24.08
**4467**	allXL	DD	372	386	DBSU	21.83
**4467**	allXL	AA	373	386	DBSU	20.46
**4467**	allXL	BD	381	386	DBSU	24.62
**4467**	allXL	BD;DB	357	357	DBSU	19.24
**4467**	allXL	AD	373	319	DBSU	34.82
**4467**	allXL	DB	372	357	DBSU	41.07
**4467**	allXL	BD	381	357	DBSU	41.66
**4467**	allXL	BD	373	357	DBSU	42.5
**4467**	allXL	BD	372	370	DBSU	42.02
**4467**	allXL	BD	373	370	DBSU	39.23
**4467**	allXL	BD	382	373	DBSU	32.1
**4468**	allXL	AA	320	357	BS2G	27.0
**4468**	allXL	BB	320	373	BS2G	16.61
**4468**	allXL	BB	373	382	BS2G	15.47
**4468**	allXL	BB	370	386	BS2G	15.61
**4468**	allXL	BB	373	386	BS2G	22.7

**4468**	allXL	AA	319	357	BS2G	28.04
**4468**	allXL	AA	367	373	BS2G	11.01
**4468**	allXL	BB	370	382	BS2G	13.7
**4468**	allXL	AA	376	382	BS2G	14.29
**4468**	allXL	BB	357	373	BS2G	25.43
**4468**	allXL	AA	320	366	BS2G	16.52
**4468**	allXL	BB	370	376	BS2G	14.88
**4468**	allXL	AA	320	357	DBSU	27.0
**4468**	allXL	AA	357	319	DBSU	28.04
**4468**	allXL	BB	372	319	DBSU	19.88
**4468**	allXL	BB	370	357	DBSU	22.65
**4468**	allXL	AA	382	357	DBSU	25.64
**4468**	allXL	AA	372	370	DBSU	6.53
**4468**	allXL	BB	381	370	DBSU	12.37
**4468**	allXL	AA	373	370	DBSU	9.17
**4468**	allXL	BB	381	372	DBSU	14.94
**4468**	allXL	BB	372	382	DBSU	16.05
**4468**	allXL	BB	372	386	DBSU	21.44
**4468**	allXL	BB	373	386	DBSU	22.7
**4468**	allXL	AD	381	386	DBSU	62.13
**4468**	allXL	BD;DB	357	357	DBSU	18.85
**4468**	allXL	AD	373	319	DBSU	31.05
**4468**	allXL	BD	372	357	DBSU	35.5
**4468**	allXL	AC	381	357	DBSU	41.24
**4468**	allXL	BD	373	357	DBSU	39.0
**4468**	allXL	AD	372	370	DBSU	45.12
**4468**	allXL	AD	373	370	DBSU	45.74
**4468**	allXL	DA	382	373	DBSU	51.42
**4469**	allXL	AA	320	357	BS2G	24.77
**4469**	allXL	DD	320	373	BS2G	27.79
**4469**	allXL	DD	373	382	BS2G	18.81
**4469**	allXL	CC	370	386	BS2G	21.3
**4469**	allXL	BB	373	386	BS2G	25.89
**4469**	allXL	AA	319	357	BS2G	26.06

**4469**	allXL	BB	367	373	BS2G	13.0
**4469**	allXL	CC	370	382	BS2G	15.6
**4469**	allXL	CC	376	382	BS2G	14.42
**4469**	allXL	BB	357	373	BS2G	26.54
**4469**	allXL	BB	320	366	BS2G	25.87
**4469**	allXL	CC	370	376	BS2G	12.13
**4469**	allXL	AA	320	357	DBSU	24.77
**4469**	allXL	AA	357	319	DBSU	26.06
**4469**	allXL	DD	372	319	DBSU	31.59
**4469**	allXL	DD	370	357	DBSU	25.56
**4469**	allXL	BB	382	357	DBSU	30.17
**4469**	allXL	AA	372	370	DBSU	6.06
**4469**	allXL	CC	381	370	DBSU	15.6
**4469**	allXL	AA	373	370	DBSU	9.22
**4469**	allXL	DD	381	372	DBSU	15.14
**4469**	allXL	DD	372	382	DBSU	17.62
**4469**	allXL	CC	372	386	DBSU	25.85
**4469**	allXL	BB	373	386	DBSU	25.89
**4469**	allXL	DB	381	386	DBSU	45.51
**4469**	allXL	BD;DB	357	357	DBSU	17.94
**4469**	allXL	DA	373	319	DBSU	23.15
**4469**	allXL	DA	372	357	DBSU	32.65
**4469**	allXL	BD	381	357	DBSU	29.66
**4469**	allXL	DA	373	357	DBSU	32.47
**4469**	allXL	DA	372	370	DBSU	40.0
**4469**	allXL	DA	373	370	DBSU	36.51
**4469**	allXL	BD	382	373	DBSU	54.22
**4470**	allXL	AA	320	357	BS2G	26.79
**4470**	allXL	CC	320	373	BS2G	15.83
**4470**	allXL	AA	373	382	BS2G	15.41
**4470**	allXL	AA	370	386	BS2G	28.42
**4470**	allXL	AA	373	386	BS2G	25.37
**4470**	allXL	AA	319	357	BS2G	27.45
**4470**	allXL	BB	367	373	BS2G	11.62

**4470**	allXL	AA	370	382	BS2G	21.49
**4470**	allXL	CC	376	382	BS2G	14.02
**4470**	allXL	BB	357	373	BS2G	21.57
**4470**	allXL	AA	320	366	BS2G	11.58
**4470**	allXL	CC	370	376	BS2G	13.36
**4470**	allXL	AA	320	357	DBSU	26.79
**4470**	allXL	AA	357	319	DBSU	27.45
**4470**	allXL	CC	372	319	DBSU	13.53
**4470**	allXL	BB	370	357	DBSU	21.82
**4470**	allXL	BB	382	357	DBSU	24.25
**4470**	allXL	AA	372	370	DBSU	6.1
**4470**	allXL	CC	381	370	DBSU	20.39
**4470**	allXL	AA	373	370	DBSU	9.19
**4470**	allXL	AA	381	372	DBSU	19.4
**4470**	allXL	AA	372	382	DBSU	18.58
**4470**	allXL	AA	372	386	DBSU	27.72
**4470**	allXL	AA	373	386	DBSU	25.37
**4470**	allXL	AD	381	386	DBSU	57.19
**4470**	allXL	BD;DB	357	357	DBSU	18.5
**4470**	allXL	AD	373	319	DBSU	26.13
**4470**	allXL	AD	372	357	DBSU	32.35
**4470**	allXL	AD	381	357	DBSU	19.51
**4470**	allXL	AD	373	357	DBSU	29.36
**4470**	allXL	AD	372	370	DBSU	30.87
**4470**	allXL	AD	373	370	DBSU	28.27
**4470**	allXL	AD	382	373	DBSU	36.37
**4471**	allXL	CC	320	357	BS2G	20.0
**4471**	allXL	BB	320	373	BS2G	23.22
**4471**	allXL	DD	373	382	BS2G	16.65
**4471**	allXL	DD	370	386	BS2G	19.82
**4471**	allXL	CC	373	386	BS2G	25.3
**4471**	allXL	CC	319	357	BS2G	22.52
**4471**	allXL	AA	367	373	BS2G	14.69
**4471**	allXL	DD	370	382	BS2G	12.59

**4471**	allXL	BB	376	382	BS2G	12.84
**4471**	allXL	BB	357	373	BS2G	23.99
**4471**	allXL	BB	320	366	BS2G	15.54
**4471**	allXL	DD	370	376	BS2G	16.14
**4471**	allXL	CC	320	357	DBSU	20.0
**4471**	allXL	CC	357	319	DBSU	22.52
**4471**	allXL	BB	372	319	DBSU	21.75
**4471**	allXL	BB	370	357	DBSU	27.99
**4471**	allXL	DD	382	357	DBSU	29.33
**4471**	allXL	AA	372	370	DBSU	6.07
**4471**	allXL	DD	381	370	DBSU	13.13
**4471**	allXL	AA	373	370	DBSU	9.17
**4471**	allXL	DD	381	372	DBSU	16.62
**4471**	allXL	DD	372	382	DBSU	17.38
**4471**	allXL	DD	372	386	DBSU	26.15
**4471**	allXL	CC	373	386	DBSU	25.3
**4471**	allXL	BD	381	386	DBSU	36.8
**4471**	allXL	BD;DB	357	357	DBSU	19.15
**4471**	allXL	CB	373	319	DBSU	22.68
**4471**	allXL	BD	372	357	DBSU	36.69
**4471**	allXL	BD	381	357	DBSU	30.76
**4471**	allXL	BD	373	357	DBSU	34.66
**4471**	allXL	CB	372	370	DBSU	43.66
**4471**	allXL	CB	373	370	DBSU	42.29
**4471**	allXL	CB	382	373	DBSU	47.58
**4472**	allXL	CC	320	357	BS2G	23.45
**4472**	allXL	BB	320	373	BS2G	15.03
**4472**	allXL	CC	373	382	BS2G	19.73
**4472**	allXL	CC	370	386	BS2G	21.12
**4472**	allXL	BB	373	386	BS2G	26.11
**4472**	allXL	CC	319	357	BS2G	26.52
**4472**	allXL	DD	367	373	BS2G	14.95
**4472**	allXL	CC	370	382	BS2G	17.17
**4472**	allXL	CC	376	382	BS2G	14.05

**4472**	allXL	CC	357	373	BS2G	19.14
**4472**	allXL	BB	320	366	BS2G	17.79
**4472**	allXL	CC	370	376	BS2G	13.89
**4472**	allXL	CC	320	357	DBSU	23.45
**4472**	allXL	CC	357	319	DBSU	26.52
**4472**	allXL	BB	372	319	DBSU	17.03
**4472**	allXL	CC	370	357	DBSU	23.75
**4472**	allXL	CC	382	357	DBSU	32.54
**4472**	allXL	AA	372	370	DBSU	6.06
**4472**	allXL	CC	381	370	DBSU	18.59
**4472**	allXL	CC	373	370	DBSU	9.07
**4472**	allXL	CC	381	372	DBSU	21.32
**4472**	allXL	CC	372	382	DBSU	20.34
**4472**	allXL	CC	372	386	DBSU	26.86
**4472**	allXL	BB	373	386	DBSU	26.11
**4472**	allXL	DB	381	386	DBSU	45.89
**4472**	allXL	BD;DB	357	357	DBSU	18.88
**4472**	allXL	DA	373	319	DBSU	19.71
**4472**	allXL	CA	372	357	DBSU	34.75
**4472**	allXL	CA	381	357	DBSU	34.35
**4472**	allXL	CA	373	357	DBSU	31.06
**4472**	allXL	DA	372	370	DBSU	41.01
**4472**	allXL	DA	373	370	DBSU	37.4
**4472**	allXL	DA	382	373	DBSU	45.64
**4473**	allXL	AA	320	357	BS2G	19.2
**4473**	allXL	DD	320	373	BS2G	24.09
**4473**	allXL	BB	373	382	BS2G	16.46
**4473**	allXL	DD	370	386	BS2G	23.1
**4473**	allXL	BB	373	386	BS2G	26.07
**4473**	allXL	AA	319	357	BS2G	17.68
**4473**	allXL	BB	367	373	BS2G	12.62
**4473**	allXL	DD	370	382	BS2G	21.95
**4473**	allXL	BB	376	382	BS2G	13.69
**4473**	allXL	CC	357	373	BS2G	28.57

**4473**	allXL	AA	320	366	BS2G	28.31
**4473**	allXL	CC	370	376	BS2G	15.45
**4473**	allXL	AA	320	357	DBSU	19.2
**4473**	allXL	AA	357	319	DBSU	17.68
**4473**	allXL	DD	372	319	DBSU	20.24
**4473**	allXL	DD	370	357	DBSU	22.65
**4473**	allXL	DD	382	357	DBSU	22.87
**4473**	allXL	CC	372	370	DBSU	6.61
**4473**	allXL	DD	381	370	DBSU	21.76
**4473**	allXL	CC	373	370	DBSU	9.23
**4473**	allXL	BB	381	372	DBSU	20.13
**4473**	allXL	BB	372	382	DBSU	18.48
**4473**	allXL	BB	372	386	DBSU	26.75
**4473**	allXL	BB	373	386	DBSU	26.07
**4473**	allXL	BD	381	386	DBSU	52.72
**4473**	allXL	BD;DB	357	357	DBSU	20.27
**4473**	allXL	AD	373	319	DBSU	34.2
**4473**	allXL	CA	372	357	DBSU	42.36
**4473**	allXL	DB	381	357	DBSU	42.64
**4473**	allXL	CA	373	357	DBSU	43.51
**4473**	allXL	DA	372	370	DBSU	41.97
**4473**	allXL	DA	373	370	DBSU	42.92
**4473**	allXL	AD	382	373	DBSU	61.0
**4474**	allXL	CC	320	357	BS2G	22.69
**4474**	allXL	BB	320	373	BS2G	22.62
**4474**	allXL	BB	373	382	BS2G	16.96
**4474**	allXL	AA	370	386	BS2G	31.06
**4474**	allXL	BB	373	386	BS2G	27.22
**4474**	allXL	CC	319	357	BS2G	24.36
**4474**	allXL	BB	367	373	BS2G	12.48
**4474**	allXL	BB	370	382	BS2G	23.26
**4474**	allXL	BB	376	382	BS2G	12.55
**4474**	allXL	DD	357	373	BS2G	30.17
**4474**	allXL	AA	320	366	BS2G	13.71

**4474**	allXL	CC	370	376	BS2G	15.24
**4474**	allXL	CC	320	357	DBSU	22.69
**4474**	allXL	CC	357	319	DBSU	24.36
**4474**	allXL	AA	372	319	DBSU	21.02
**4474**	allXL	BB	370	357	DBSU	30.25
**4474**	allXL	AA	382	357	DBSU	31.16
**4474**	allXL	AA	372	370	DBSU	6.33
**4474**	allXL	AA	381	370	DBSU	22.12
**4474**	allXL	AA	373	370	DBSU	8.91
**4474**	allXL	CC	381	372	DBSU	19.69
**4474**	allXL	BB	372	382	DBSU	19.79
**4474**	allXL	BB	372	386	DBSU	29.51
**4474**	allXL	BB	373	386	DBSU	27.22
**4474**	allXL	BD	381	386	DBSU	38.37
**4474**	allXL	BD;DB	357	357	DBSU	18.84
**4474**	allXL	CB	373	319	DBSU	33.74
**4474**	allXL	DB	372	357	DBSU	39.61
**4474**	allXL	DB	381	357	DBSU	37.41
**4474**	allXL	DB	373	357	DBSU	39.18
**4474**	allXL	CB	372	370	DBSU	37.0
**4474**	allXL	CB	373	370	DBSU	37.36
**4474**	allXL	DB	382	373	DBSU	49.71
**4475**	allXL	AA	320	357	BS2G	26.04
**4475**	allXL	BB	320	373	BS2G	11.56
**4475**	allXL	AA	373	382	BS2G	19.76
**4475**	allXL	AA	370	386	BS2G	17.31
**4475**	allXL	AA	373	386	BS2G	20.34
**4475**	allXL	AA	319	357	BS2G	26.47
**4475**	allXL	DD	367	373	BS2G	14.71
**4475**	allXL	CC	370	382	BS2G	19.82
**4475**	allXL	CC	376	382	BS2G	14.32
**4475**	allXL	BB	357	373	BS2G	26.45
**4475**	allXL	BB	320	366	BS2G	19.79
**4475**	allXL	CC	370	376	BS2G	13.87

**4475**	allXL	AA	320	357	DBSU	26.04
**4475**	allXL	AA	357	319	DBSU	26.47
**4475**	allXL	BB	372	319	DBSU	15.6
**4475**	allXL	DD	370	357	DBSU	27.17
**4475**	allXL	CC	382	357	DBSU	36.51
**4475**	allXL	AA	372	370	DBSU	6.55
**4475**	allXL	CC	381	370	DBSU	21.76
**4475**	allXL	AA	373	370	DBSU	7.81
**4475**	allXL	DD	381	372	DBSU	18.94
**4475**	allXL	DD	372	382	DBSU	19.81
**4475**	allXL	AA	372	386	DBSU	21.5
**4475**	allXL	AA	373	386	DBSU	20.34
**4475**	allXL	DA	381	386	DBSU	36.52
**4475**	allXL	BD;DB	357	357	DBSU	18.88
**4475**	allXL	AD	373	319	DBSU	15.26
**4475**	allXL	BD	372	357	DBSU	42.29
**4475**	allXL	AD	381	357	DBSU	42.79
**4475**	allXL	BD	373	357	DBSU	42.87
**4475**	allXL	AD	372	370	DBSU	37.74
**4475**	allXL	AD	373	370	DBSU	35.7
**4475**	allXL	AD	382	373	DBSU	21.58
**4476**	allXL	AA	320	357	BS2G	26.53
**4476**	allXL	CC	320	373	BS2G	16.88
**4476**	allXL	DD	373	382	BS2G	17.69
**4476**	allXL	CC	370	386	BS2G	18.35
**4476**	allXL	CC	373	386	BS2G	23.4
**4476**	allXL	AA	319	357	BS2G	26.42
**4476**	allXL	AA	367	373	BS2G	12.36
**4476**	allXL	CC	370	382	BS2G	16.03
**4476**	allXL	CC	376	382	BS2G	13.29
**4476**	allXL	BB	357	373	BS2G	23.51
**4476**	allXL	BB	320	366	BS2G	18.07
**4476**	allXL	CC	370	376	BS2G	13.69
**4476**	allXL	AA	320	357	DBSU	26.53

**4476**	allXL	AA	357	319	DBSU	26.42
**4476**	allXL	CC	372	319	DBSU	13.34
**4476**	allXL	BB	370	357	DBSU	25.44
**4476**	allXL	AA	382	357	DBSU	29.51
**4476**	allXL	AA	372	370	DBSU	6.56
**4476**	allXL	CC	381	370	DBSU	18.62
**4476**	allXL	AA	373	370	DBSU	9.2
**4476**	allXL	DD	381	372	DBSU	17.93
**4476**	allXL	CC	372	382	DBSU	18.54
**4476**	allXL	CC	372	386	DBSU	23.19
**4476**	allXL	CC	373	386	DBSU	23.4
**4476**	allXL	DA	381	386	DBSU	43.25
**4476**	allXL	BD;DB	357	357	DBSU	18.21
**4476**	allXL	DA	373	319	DBSU	20.34
**4476**	allXL	BD	372	357	DBSU	30.67
**4476**	allXL	DA	381	357	DBSU	45.77
**4476**	allXL	BD	373	357	DBSU	31.2
**4476**	allXL	DA	372	370	DBSU	27.56
**4476**	allXL	DA	373	370	DBSU	24.04
**4476**	allXL	DA	382	373	DBSU	20.37
**4477**	allXL	AA	320	357	BS2G	23.63
**4477**	allXL	BB	320	373	BS2G	14.06
**4477**	allXL	BB	373	382	BS2G	16.74
**4477**	allXL	AA	370	386	BS2G	17.76
**4477**	allXL	AA	373	386	BS2G	21.48
**4477**	allXL	AA	319	357	BS2G	21.48
**4477**	allXL	BB	367	373	BS2G	10.67
**4477**	allXL	BB	370	382	BS2G	13.4
**4477**	allXL	BB	376	382	BS2G	13.97
**4477**	allXL	BB	357	373	BS2G	22.18
**4477**	allXL	DD	320	366	BS2G	24.16
**4477**	allXL	BB	370	376	BS2G	14.87
**4477**	allXL	AA	320	357	DBSU	23.63
**4477**	allXL	AA	357	319	DBSU	21.48

**4477**	allXL	BB	372	319	DBSU	17.31
**4477**	allXL	DD	370	357	DBSU	26.32
**4477**	allXL	BB	382	357	DBSU	24.61
**4477**	allXL	CC	372	370	DBSU	6.6
**4477**	allXL	BB	381	370	DBSU	15.43
**4477**	allXL	CC	373	370	DBSU	9.31
**4477**	allXL	BB	381	372	DBSU	18.62
**4477**	allXL	BB	372	382	DBSU	18.02
**4477**	allXL	AA	372	386	DBSU	20.3
**4477**	allXL	AA	373	386	DBSU	21.48
**4477**	allXL	BD	381	386	DBSU	71.47
**4477**	allXL	BD;DB	357	357	DBSU	17.66
**4477**	allXL	AD	373	319	DBSU	31.15
**4477**	allXL	CA	372	357	DBSU	31.89
**4477**	allXL	BD	381	357	DBSU	36.33
**4477**	allXL	CA	373	357	DBSU	32.57
**4477**	allXL	AD	372	370	DBSU	47.45
**4477**	allXL	AD	373	370	DBSU	50.51
**4477**	allXL	BC	382	373	DBSU	63.85
**4478**	allXL	CC	320	357	BS2G	24.7
**4478**	allXL	AA	320	373	BS2G	20.62
**4478**	allXL	AA	373	382	BS2G	19.0
**4478**	allXL	AA	370	386	BS2G	25.8
**4478**	allXL	AA	373	386	BS2G	22.65
**4478**	allXL	CC	319	357	BS2G	23.98
**4478**	allXL	BB	367	373	BS2G	13.1
**4478**	allXL	CC	370	382	BS2G	25.2
**4478**	allXL	AA	376	382	BS2G	13.88
**4478**	allXL	BB	357	373	BS2G	24.57
**4478**	allXL	AA	320	366	BS2G	19.87
**4478**	allXL	CC	370	376	BS2G	13.88
**4478**	allXL	CC	320	357	DBSU	24.7
**4478**	allXL	CC	357	319	DBSU	23.98
**4478**	allXL	CC	372	319	DBSU	22.12

**4478**	allXL	DD	370	357	DBSU	26.6
**4478**	allXL	BB	382	357	DBSU	17.23
**4478**	allXL	AA	372	370	DBSU	6.25
**4478**	allXL	CC	381	370	DBSU	23.75
**4478**	allXL	DD	373	370	DBSU	9.08
**4478**	allXL	DD	381	372	DBSU	17.6
**4478**	allXL	AA	372	382	DBSU	20.03
**4478**	allXL	AA	372	386	DBSU	21.6
**4478**	allXL	AA	373	386	DBSU	22.65
**4478**	allXL	DA	381	386	DBSU	48.6
**4478**	allXL	BD;DB	357	357	DBSU	18.83
**4478**	allXL	AD	373	319	DBSU	32.92
**4478**	allXL	BD	372	357	DBSU	37.73
**4478**	allXL	BD	381	357	DBSU	29.08
**4478**	allXL	BD	373	357	DBSU	36.63
**4478**	allXL	AD	372	370	DBSU	36.94
**4478**	allXL	DA	373	370	DBSU	38.27
**4478**	allXL	AD	382	373	DBSU	45.42
**4479**	allXL	CC	320	357	BS2G	21.84
**4479**	allXL	BB	320	373	BS2G	22.39
**4479**	allXL	BB	373	382	BS2G	18.15
**4479**	allXL	BB	370	386	BS2G	18.71
**4479**	allXL	AA	373	386	BS2G	16.08
**4479**	allXL	CC	319	357	BS2G	25.15
**4479**	allXL	AA	367	373	BS2G	13.84
**4479**	allXL	DD	370	382	BS2G	18.7
**4479**	allXL	BB	376	382	BS2G	12.84
**4479**	allXL	BB	357	373	BS2G	18.04
**4479**	allXL	AA	320	366	BS2G	28.5
**4479**	allXL	CC	370	376	BS2G	16.28
**4479**	allXL	CC	320	357	DBSU	21.84
**4479**	allXL	CC	357	319	DBSU	25.15
**4479**	allXL	BB	372	319	DBSU	25.32
**4479**	allXL	BB	370	357	DBSU	23.81

**4479**	allXL	BB	382	357	DBSU	27.48
**4479**	allXL	AA	372	370	DBSU	6.26
**4479**	allXL	DD	381	370	DBSU	20.5
**4479**	allXL	AA	373	370	DBSU	9.3
**4479**	allXL	DD	381	372	DBSU	20.11
**4479**	allXL	DD	372	382	DBSU	19.53
**4479**	allXL	AA	372	386	DBSU	18.4
**4479**	allXL	AA	373	386	DBSU	16.08
**4479**	allXL	BD	381	386	DBSU	35.76
**4479**	allXL	BD;DB	357	357	DBSU	18.69
**4479**	allXL	AD	373	319	DBSU	18.33
**4479**	allXL	BD	372	357	DBSU	30.97
**4479**	allXL	BD	381	357	DBSU	29.33
**4479**	allXL	BD	373	357	DBSU	30.65
**4479**	allXL	DA	372	370	DBSU	41.35
**4479**	allXL	BD	373	370	DBSU	40.94
**4479**	allXL	BD	382	373	DBSU	28.56
**4480**	allXL	BB	320	357	BS2G	25.11
**4480**	allXL	BB	320	373	BS2G	25.97
**4480**	allXL	AA	373	382	BS2G	19.04
**4480**	allXL	CC	370	386	BS2G	27.08
**4480**	allXL	AA	373	386	BS2G	27.48
**4480**	allXL	CC	319	357	BS2G	26.64
**4480**	allXL	AA	367	373	BS2G	10.96
**4480**	allXL	DD	370	382	BS2G	20.86
**4480**	allXL	BB	376	382	BS2G	13.96
**4480**	allXL	BB	357	373	BS2G	14.25
**4480**	allXL	AA	320	366	BS2G	21.89
**4480**	allXL	CC	370	376	BS2G	14.89
**4480**	allXL	BB	320	357	DBSU	25.11
**4480**	allXL	CC	357	319	DBSU	26.64
**4480**	allXL	CC	372	319	DBSU	21.65
**4480**	allXL	BB	370	357	DBSU	21.02
**4480**	allXL	DD	382	357	DBSU	23.37

**4480**	allXL	AA	372	370	DBSU	6.52
**4480**	allXL	DD	381	370	DBSU	17.96
**4480**	allXL	AA	373	370	DBSU	8.77
**4480**	allXL	DD	381	372	DBSU	21.45
**4480**	allXL	AA	372	382	DBSU	22.35
**4480**	allXL	AA	372	386	DBSU	30.15
**4480**	allXL	AA	373	386	DBSU	27.48
**4480**	allXL	DB	381	386	DBSU	15.53
**4480**	allXL	BD;DB	357	357	DBSU	18.82
**4480**	allXL	DA	373	319	DBSU	30.64
**4480**	allXL	BD	372	357	DBSU	26.8
**4480**	allXL	BD	381	357	DBSU	28.98
**4480**	allXL	BD	373	357	DBSU	27.71
**4480**	allXL	DA	372	370	DBSU	43.77
**4480**	allXL	BD	373	370	DBSU	45.03
**4480**	allXL	DB	382	373	DBSU	27.15
**4481**	allXL	CC	320	357	BS2G	24.3
**4481**	allXL	AA	320	373	BS2G	27.37
**4481**	allXL	AA	373	382	BS2G	18.62
**4481**	allXL	CC	370	386	BS2G	14.45
**4481**	allXL	BB	373	386	BS2G	19.98
**4481**	allXL	CC	319	357	BS2G	26.69
**4481**	allXL	DD	367	373	BS2G	13.87
**4481**	allXL	CC	370	382	BS2G	18.11
**4481**	allXL	CC	376	382	BS2G	13.8
**4481**	allXL	BB	357	373	BS2G	27.48
**4481**	allXL	AA	320	366	BS2G	21.27
**4481**	allXL	CC	370	376	BS2G	15.66
**4481**	allXL	CC	320	357	DBSU	24.3
**4481**	allXL	CC	357	319	DBSU	26.69
**4481**	allXL	AA	372	319	DBSU	27.65
**4481**	allXL	BB	370	357	DBSU	30.0
**4481**	allXL	BB	382	357	DBSU	34.53
**4481**	allXL	AA	372	370	DBSU	6.08

**4481**	allXL	DD	381	370	DBSU	15.75
**4481**	allXL	AA	373	370	DBSU	9.04
**4481**	allXL	DD	381	372	DBSU	18.06
**4481**	allXL	CC	372	382	DBSU	19.1
**4481**	allXL	CC	372	386	DBSU	18.93
**4481**	allXL	BB	373	386	DBSU	19.98
**4481**	allXL	AD	381	386	DBSU	21.85
**4481**	allXL	BD;DB	357	357	DBSU	18.76
**4481**	allXL	BC	373	319	DBSU	21.83
**4481**	allXL	BC	372	357	DBSU	36.91
**4481**	allXL	BD	381	357	DBSU	38.09
**4481**	allXL	BC	373	357	DBSU	36.03
**4481**	allXL	AD	372	370	DBSU	38.93
**4481**	allXL	AD	373	370	DBSU	41.83
**4481**	allXL	DA	382	373	DBSU	36.57
**4482**	allXL	AA	320	357	BS2G	21.93
**4482**	allXL	CC	320	373	BS2G	18.66
**4482**	allXL	BB	373	382	BS2G	18.38
**4482**	allXL	CC	370	386	BS2G	30.36
**4482**	allXL	BB	373	386	BS2G	27.79
**4482**	allXL	AA	319	357	BS2G	20.0
**4482**	allXL	AA	367	373	BS2G	13.85
**4482**	allXL	BB	370	382	BS2G	21.56
**4482**	allXL	CC	376	382	BS2G	13.84
**4482**	allXL	AA	357	373	BS2G	30.3
**4482**	allXL	CC	320	366	BS2G	14.63
**4482**	allXL	BB	370	376	BS2G	16.14
**4482**	allXL	AA	320	357	DBSU	21.93
**4482**	allXL	AA	357	319	DBSU	20.0
**4482**	allXL	CC	372	319	DBSU	17.89
**4482**	allXL	CC	370	357	DBSU	26.13
**4482**	allXL	DD	382	357	DBSU	41.81
**4482**	allXL	AA	372	370	DBSU	6.41
**4482**	allXL	BB	381	370	DBSU	21.74

**4482**	allXL	AA	373	370	DBSU	8.87
**4482**	allXL	BB	381	372	DBSU	20.6
**4482**	allXL	BB	372	382	DBSU	20.22
**4482**	allXL	DD	372	386	DBSU	28.81
**4482**	allXL	BB	373	386	DBSU	27.79
**4482**	allXL	CB	381	386	DBSU	68.76
**4482**	allXL	AC;CA	357	357	DBSU	14.47
**4482**	allXL	CB	373	319	DBSU	34.45
**4482**	allXL	CB	372	357	DBSU	36.5
**4482**	allXL	CB	381	357	DBSU	47.78
**4482**	allXL	CB	373	357	DBSU	33.76
**4482**	allXL	CB	372	370	DBSU	41.95
**4482**	allXL	CB	373	370	DBSU	40.27
**4482**	allXL	CB	382	373	DBSU	57.62
**4483**	allXL	CC	320	357	BS2G	25.74
**4483**	allXL	BB	320	373	BS2G	27.71
**4483**	allXL	CC	373	382	BS2G	17.71
**4483**	allXL	DD	370	386	BS2G	13.38
**4483**	allXL	CC	373	386	BS2G	20.44
**4483**	allXL	AA	319	357	BS2G	25.91
**4483**	allXL	BB	367	373	BS2G	14.06
**4483**	allXL	DD	370	382	BS2G	15.9
**4483**	allXL	CC	376	382	BS2G	14.06
**4483**	allXL	CC	357	373	BS2G	23.67
**4483**	allXL	BB	320	366	BS2G	28.06
**4483**	allXL	CC	370	376	BS2G	16.69
**4483**	allXL	CC	320	357	DBSU	25.74
**4483**	allXL	AA	357	319	DBSU	25.91
**4483**	allXL	BB	372	319	DBSU	30.69
**4483**	allXL	CC	370	357	DBSU	22.17
**4483**	allXL	BB	382	357	DBSU	24.62
**4483**	allXL	AA	372	370	DBSU	6.34
**4483**	allXL	DD	381	370	DBSU	17.18
**4483**	allXL	AA	373	370	DBSU	9.11

**4483**	allXL	DD	381	372	DBSU	17.05
**4483**	allXL	DD	372	382	DBSU	16.87
**4483**	allXL	DD	372	386	DBSU	18.29
**4483**	allXL	CC	373	386	DBSU	20.44
**4483**	allXL	DB	381	386	DBSU	35.75
**4483**	allXL	BD;DB	357	357	DBSU	19.48
**4483**	allXL	DA	373	319	DBSU	23.78
**4483**	allXL	CA	372	357	DBSU	34.46
**4483**	allXL	BD	381	357	DBSU	38.65
**4483**	allXL	CA	373	357	DBSU	33.01
**4483**	allXL	CA	372	370	DBSU	54.1
**4483**	allXL	CA	373	370	DBSU	50.68
**4483**	allXL	BD	382	373	DBSU	39.57
**4484**	allXL	CC	320	357	BS2G	22.35
**4484**	allXL	DD	320	373	BS2G	15.74
**4484**	allXL	AA	373	382	BS2G	16.06
**4484**	allXL	AA	370	386	BS2G	17.27
**4484**	allXL	AA	373	386	BS2G	23.04
**4484**	allXL	CC	319	357	BS2G	21.58
**4484**	allXL	AA	367	373	BS2G	10.35
**4484**	allXL	AA	370	382	BS2G	10.31
**4484**	allXL	BB	376	382	BS2G	14.07
**4484**	allXL	BB	357	373	BS2G	26.32
**4484**	allXL	DD	320	366	BS2G	28.09
**4484**	allXL	CC	370	376	BS2G	13.33
**4484**	allXL	CC	320	357	DBSU	22.35
**4484**	allXL	CC	357	319	DBSU	21.58
**4484**	allXL	DD	372	319	DBSU	20.54
**4484**	allXL	BB	370	357	DBSU	22.75
**4484**	allXL	AA	382	357	DBSU	41.89
**4484**	allXL	AA	372	370	DBSU	6.44
**4484**	allXL	AA	381	370	DBSU	12.87
**4484**	allXL	AA	373	370	DBSU	8.96
**4484**	allXL	AA	381	372	DBSU	15.41

**4484**	allXL	AA	372	382	DBSU	13.82
**4484**	allXL	AA	372	386	DBSU	19.71
**4484**	allXL	AA	373	386	DBSU	23.04
**4484**	allXL	AD	381	386	DBSU	43.67
**4484**	allXL	BD;DB	357	357	DBSU	17.08
**4484**	allXL	AD	373	319	DBSU	26.39
**4484**	allXL	BD	372	357	DBSU	40.76
**4484**	allXL	DA	381	357	DBSU	50.3
**4484**	allXL	BD	373	357	DBSU	42.87
**4484**	allXL	BC	372	370	DBSU	31.18
**4484**	allXL	BC	373	370	DBSU	33.44
**4484**	allXL	DA	382	373	DBSU	41.2
**4485**	allXL	CC	320	357	BS2G	21.88
**4485**	allXL	DD	320	373	BS2G	30.55
**4485**	allXL	CC	373	382	BS2G	16.09
**4485**	allXL	AA	370	386	BS2G	27.31
**4485**	allXL	CC	373	386	BS2G	22.33
**4485**	allXL	CC	319	357	BS2G	21.71
**4485**	allXL	BB	367	373	BS2G	10.32
**4485**	allXL	AA	370	382	BS2G	24.42
**4485**	allXL	CC	376	382	BS2G	12.99
**4485**	allXL	BB	357	373	BS2G	23.33
**4485**	allXL	CC	320	366	BS2G	22.85
**4485**	allXL	DD	370	376	BS2G	15.88
**4485**	allXL	CC	320	357	DBSU	21.88
**4485**	allXL	CC	357	319	DBSU	21.71
**4485**	allXL	BB	372	319	DBSU	32.0
**4485**	allXL	DD	370	357	DBSU	18.58
**4485**	allXL	BB	382	357	DBSU	13.64
**4485**	allXL	AA	372	370	DBSU	6.08
**4485**	allXL	AA	381	370	DBSU	26.34
**4485**	allXL	AA	373	370	DBSU	9.08
**4485**	allXL	CC	381	372	DBSU	20.79
**4485**	allXL	CC	372	382	DBSU	18.85

**4485**	allXL	CC	372	386	DBSU	24.97
**4485**	allXL	CC	373	386	DBSU	22.33
**4485**	allXL	DA	381	386	DBSU	66.41
**4485**	allXL	BD;DB	357	357	DBSU	19.41
**4485**	allXL	AD	373	319	DBSU	23.05
**4485**	allXL	DB	372	357	DBSU	41.74
**4485**	allXL	BD	381	357	DBSU	22.26
**4485**	allXL	BD	373	357	DBSU	39.26
**4485**	allXL	AD	372	370	DBSU	46.09
**4485**	allXL	DA	373	370	DBSU	47.41
**4485**	allXL	BD	382	373	DBSU	49.56
**4486**	allXL	AA	320	357	BS2G	25.63
**4486**	allXL	AA	320	373	BS2G	18.22
**4486**	allXL	AA	373	382	BS2G	18.76
**4486**	allXL	DD	370	386	BS2G	19.68
**4486**	allXL	DD	373	386	BS2G	23.86
**4486**	allXL	AA	319	357	BS2G	27.08
**4486**	allXL	BB	367	373	BS2G	13.03
**4486**	allXL	DD	370	382	BS2G	21.08
**4486**	allXL	CC	376	382	BS2G	14.3
**4486**	allXL	BB	357	373	BS2G	30.24
**4486**	allXL	AA	320	366	BS2G	26.13
**4486**	allXL	CC	370	376	BS2G	16.35
**4486**	allXL	AA	320	357	DBSU	25.63
**4486**	allXL	AA	357	319	DBSU	27.08
**4486**	allXL	BB	372	319	DBSU	20.03
**4486**	allXL	BB	370	357	DBSU	26.32
**4486**	allXL	DD	382	357	DBSU	19.91
**4486**	allXL	AA	372	370	DBSU	6.2
**4486**	allXL	DD	381	370	DBSU	20.14
**4486**	allXL	AA	373	370	DBSU	9.06
**4486**	allXL	DD	381	372	DBSU	21.7
**4486**	allXL	CC	372	382	DBSU	21.73
**4486**	allXL	DD	372	386	DBSU	24.66

**4486**	allXL	DD	373	386	DBSU	23.86
**4486**	allXL	DB	381	386	DBSU	48.56
**4486**	allXL	BD;DB	357	357	DBSU	18.9
**4486**	allXL	AD	373	319	DBSU	21.72
**4486**	allXL	BC	372	357	DBSU	30.88
**4486**	allXL	DB	381	357	DBSU	39.52
**4486**	allXL	BC	373	357	DBSU	33.68
**4486**	allXL	AD	372	370	DBSU	19.34
**4486**	allXL	AD	373	370	DBSU	22.55
**4486**	allXL	DA	382	373	DBSU	40.49
**4487**	allXL	CC	320	357	BS2G	21.05
**4487**	allXL	CC	320	373	BS2G	36.48
**4487**	allXL	CC	373	382	BS2G	18.04
**4487**	allXL	CC	370	386	BS2G	17.22
**4487**	allXL	AA	373	386	BS2G	24.06
**4487**	allXL	CC	319	357	BS2G	19.7
**4487**	allXL	AA	367	373	BS2G	15.15
**4487**	allXL	CC	370	382	BS2G	12.97
**4487**	allXL	CC	376	382	BS2G	15.15
**4487**	allXL	BB	357	373	BS2G	31.23
**4487**	allXL	BB	320	366	BS2G	27.28
**4487**	allXL	CC	370	376	BS2G	14.69
**4487**	allXL	CC	320	357	DBSU	21.05
**4487**	allXL	CC	357	319	DBSU	19.7
**4487**	allXL	CC	372	319	DBSU	29.67
**4487**	allXL	BB	370	357	DBSU	21.56
**4487**	allXL	BB	382	357	DBSU	36.05
**4487**	allXL	AA	372	370	DBSU	5.94
**4487**	allXL	CC	381	370	DBSU	15.15
**4487**	allXL	AA	373	370	DBSU	9.19
**4487**	allXL	DD	381	372	DBSU	15.49
**4487**	allXL	CC	372	382	DBSU	15.97
**4487**	allXL	CC	372	386	DBSU	22.65
**4487**	allXL	AA	373	386	DBSU	24.06

**4487**	allXL	DB	381	386	DBSU	53.56
**4487**	allXL	BD;DB	357	357	DBSU	18.47
**4487**	allXL	AD	373	319	DBSU	36.68
**4487**	allXL	BD	372	357	DBSU	43.77
**4487**	allXL	BD	381	357	DBSU	41.9
**4487**	allXL	BD	373	357	DBSU	45.48
**4487**	allXL	AD	372	370	DBSU	51.02
**4487**	allXL	AD	373	370	DBSU	52.64
**4487**	allXL	AD	382	373	DBSU	62.07
**4488**	allXL	AA	320	357	BS2G	26.02
**4488**	allXL	DD	320	373	BS2G	18.05
**4488**	allXL	DD	373	382	BS2G	17.32
**4488**	allXL	DD	370	386	BS2G	23.21
**4488**	allXL	DD	373	386	BS2G	21.42
**4488**	allXL	CC	319	357	BS2G	27.16
**4488**	allXL	BB	367	373	BS2G	14.05
**4488**	allXL	CC	370	382	BS2G	22.58
**4488**	allXL	CC	376	382	BS2G	14.53
**4488**	allXL	BB	357	373	BS2G	18.78
**4488**	allXL	AA	320	366	BS2G	15.8
**4488**	allXL	CC	370	376	BS2G	14.11
**4488**	allXL	AA	320	357	DBSU	26.02
**4488**	allXL	CC	357	319	DBSU	27.16
**4488**	allXL	DD	372	319	DBSU	19.24
**4488**	allXL	BB	370	357	DBSU	23.42
**4488**	allXL	BB	382	357	DBSU	33.97
**4488**	allXL	AA	372	370	DBSU	6.11
**4488**	allXL	CC	381	370	DBSU	23.75
**4488**	allXL	CC	373	370	DBSU	9.04
**4488**	allXL	DD	381	372	DBSU	17.21
**4488**	allXL	DD	372	382	DBSU	16.01
**4488**	allXL	DD	372	386	DBSU	18.57
**4488**	allXL	DD	373	386	DBSU	21.42
**4488**	allXL	CB	381	386	DBSU	55.31

**4488**	allXL	BD;DB	357	357	DBSU	18.91
**4488**	allXL	AD	373	319	DBSU	41.0
**4488**	allXL	BD	372	357	DBSU	35.12
**4488**	allXL	BD	381	357	DBSU	33.39
**4488**	allXL	BD	373	357	DBSU	31.78
**4488**	allXL	DA	372	370	DBSU	44.71
**4488**	allXL	DA	373	370	DBSU	46.67
**4488**	allXL	CB	382	373	DBSU	57.03
**4489**	allXL	CC	320	357	BS2G	26.39
**4489**	allXL	BB	320	373	BS2G	11.06
**4489**	allXL	DD	373	382	BS2G	19.27
**4489**	allXL	CC	370	386	BS2G	21.35
**4489**	allXL	DD	373	386	BS2G	25.69
**4489**	allXL	AA	319	357	BS2G	25.48
**4489**	allXL	AA	367	373	BS2G	14.05
**4489**	allXL	CC	370	382	BS2G	16.4
**4489**	allXL	CC	376	382	BS2G	14.64
**4489**	allXL	BB	357	373	BS2G	25.0
**4489**	allXL	AA	320	366	BS2G	21.93
**4489**	allXL	CC	370	376	BS2G	12.71
**4489**	allXL	CC	320	357	DBSU	26.39
**4489**	allXL	AA	357	319	DBSU	25.48
**4489**	allXL	AA	372	319	DBSU	16.57
**4489**	allXL	DD	370	357	DBSU	25.83
**4489**	allXL	CC	382	357	DBSU	33.39
**4489**	allXL	AA	372	370	DBSU	6.34
**4489**	allXL	CC	381	370	DBSU	17.87
**4489**	allXL	DD	373	370	DBSU	8.97
**4489**	allXL	DD	381	372	DBSU	16.89
**4489**	allXL	DD	372	382	DBSU	18.71
**4489**	allXL	DD	372	386	DBSU	23.68
**4489**	allXL	DD	373	386	DBSU	25.69
**4489**	allXL	DB	381	386	DBSU	27.99
**4489**	allXL	BD;DB	357	357	DBSU	18.34

**4489**	allXL	DB	373	319	DBSU	29.26
**4489**	allXL	DB	372	357	DBSU	26.26
**4489**	allXL	DB	381	357	DBSU	34.36
**4489**	allXL	DB	373	357	DBSU	23.12
**4489**	allXL	DB	372	370	DBSU	25.16
**4489**	allXL	DB	373	370	DBSU	22.51
**4489**	allXL	DB	382	373	DBSU	21.38
**4490**	allXL	AA	320	357	BS2G	18.95
**4490**	allXL	BB	320	373	BS2G	23.72
**4490**	allXL	BB	373	382	BS2G	17.71
**4490**	allXL	AA	370	386	BS2G	26.87
**4490**	allXL	BB	373	386	BS2G	25.88
**4490**	allXL	AA	319	357	BS2G	16.12
**4490**	allXL	AA	367	373	BS2G	10.9
**4490**	allXL	AA	370	382	BS2G	21.48
**4490**	allXL	BB	376	382	BS2G	14.44
**4490**	allXL	AA	357	373	BS2G	26.8
**4490**	allXL	CC	320	366	BS2G	25.59
**4490**	allXL	AA	370	376	BS2G	16.81
**4490**	allXL	AA	320	357	DBSU	18.95
**4490**	allXL	AA	357	319	DBSU	16.12
**4490**	allXL	CC	372	319	DBSU	24.15
**4490**	allXL	BB	370	357	DBSU	26.7
**4490**	allXL	AA	382	357	DBSU	31.97
**4490**	allXL	AA	372	370	DBSU	6.49
**4490**	allXL	BB	381	370	DBSU	22.83
**4490**	allXL	AA	373	370	DBSU	8.95
**4490**	allXL	DD	381	372	DBSU	18.84
**4490**	allXL	BB	372	382	DBSU	20.17
**4490**	allXL	DD	372	386	DBSU	26.96
**4490**	allXL	BB	373	386	DBSU	25.88
**4490**	allXL	CB	381	386	DBSU	54.45
**4490**	allXL	BD;DB	357	357	DBSU	18.52
**4490**	allXL	AD	373	319	DBSU	24.86

**4490**	allXL	BC	372	357	DBSU	29.14
**4490**	allXL	AC	381	357	DBSU	44.88
**4490**	allXL	BC	373	357	DBSU	31.05
**4490**	allXL	BC	372	370	DBSU	16.16
**4490**	allXL	BC	373	370	DBSU	17.21
**4490**	allXL	BC	382	373	DBSU	32.1
**4491**	allXL	AA	320	357	BS2G	24.58
**4491**	allXL	DD	320	373	BS2G	19.76
**4491**	allXL	CC	373	382	BS2G	18.97
**4491**	allXL	AA	370	386	BS2G	19.8
**4491**	allXL	AA	373	386	BS2G	19.5
**4491**	allXL	AA	319	357	BS2G	23.07
**4491**	allXL	AA;BB	367	373	BS2G	15.02
**4491**	allXL	BB	370	382	BS2G	20.68
**4491**	allXL	CC	376	382	BS2G	13.38
**4491**	allXL	BB	357	373	BS2G	21.54
**4491**	allXL	AA	320	366	BS2G	20.2
**4491**	allXL	BB	370	376	BS2G	16.36
**4491**	allXL	AA	320	357	DBSU	24.58
**4491**	allXL	AA	357	319	DBSU	23.07
**4491**	allXL	DD	372	319	DBSU	20.45
**4491**	allXL	DD	370	357	DBSU	25.51
**4491**	allXL	BB	382	357	DBSU	33.85
**4491**	allXL	AA	372	370	DBSU	5.89
**4491**	allXL	DD	381	370	DBSU	20.63
**4491**	allXL	AA	373	370	DBSU	9.15
**4491**	allXL	AA	381	372	DBSU	20.09
**4491**	allXL	AA	372	382	DBSU	19.7
**4491**	allXL	AA	372	386	DBSU	18.69
**4491**	allXL	AA	373	386	DBSU	19.5
**4491**	allXL	DA	381	386	DBSU	43.61
**4491**	allXL	BD;DB	357	357	DBSU	18.63
**4491**	allXL	AD	373	319	DBSU	40.86
**4491**	allXL	BD	372	357	DBSU	35.0

**4491**	allXL	BD	381	357	DBSU	47.93
**4491**	allXL	BD	373	357	DBSU	34.53
**4491**	allXL	DA	372	370	DBSU	44.98
**4491**	allXL	DA	373	370	DBSU	43.45
**4491**	allXL	DA	382	373	DBSU	43.23
**4492**	allXL	AA	320	357	BS2G	24.47
**4492**	allXL	CC	320	373	BS2G	16.06
**4492**	allXL	DD	373	382	BS2G	17.71
**4492**	allXL	DD	370	386	BS2G	21.56
**4492**	allXL	DD	373	386	BS2G	23.33
**4492**	allXL	AA	319	357	BS2G	27.32
**4492**	allXL	AA	367	373	BS2G	10.82
**4492**	allXL	DD	370	382	BS2G	18.77
**4492**	allXL	BB	376	382	BS2G	14.95
**4492**	allXL	AA	357	373	BS2G	12.91
**4492**	allXL	BB	320	366	BS2G	27.7
**4492**	allXL	AA	370	376	BS2G	13.96
**4492**	allXL	AA	320	357	DBSU	24.47
**4492**	allXL	AA	357	319	DBSU	27.32
**4492**	allXL	CC	372	319	DBSU	17.78
**4492**	allXL	AA	370	357	DBSU	17.13
**4492**	allXL	DD	382	357	DBSU	34.63
**4492**	allXL	CC	372	370	DBSU	6.39
**4492**	allXL	DD	381	370	DBSU	18.6
**4492**	allXL	AA	373	370	DBSU	8.89
**4492**	allXL	DD	381	372	DBSU	17.05
**4492**	allXL	DD	372	382	DBSU	18.04
**4492**	allXL	DD	372	386	DBSU	22.14
**4492**	allXL	DD	373	386	DBSU	23.33
**4492**	allXL	AD	381	386	DBSU	42.07
**4492**	allXL	BD;DB	357	357	DBSU	18.01
**4492**	allXL	DA	373	319	DBSU	24.79
**4492**	allXL	AC	372	357	DBSU	18.88
**4492**	allXL	DA	381	357	DBSU	28.03

**4492**	allXL	AC	373	357	DBSU	19.4
**4492**	allXL	AC	372	370	DBSU	41.6
**4492**	allXL	AC	373	370	DBSU	42.01
**4492**	allXL	DA	382	373	DBSU	36.53
**4493**	allXL	CC	320	357	BS2G	24.43
**4493**	allXL	AA	320	373	BS2G	32.73
**4493**	allXL	DD	373	382	BS2G	19.36
**4493**	allXL	AA	370	386	BS2G	24.91
**4493**	allXL	AA	373	386	BS2G	27.15
**4493**	allXL	AA	319	357	BS2G	24.96
**4493**	allXL	BB	367	373	BS2G	10.94
**4493**	allXL	DD	370	382	BS2G	21.8
**4493**	allXL	CC	376	382	BS2G	15.17
**4493**	allXL	BB	357	373	BS2G	27.32
**4493**	allXL	DD	320	366	BS2G	27.86
**4493**	allXL	DD	370	376	BS2G	15.78
**4493**	allXL	CC	320	357	DBSU	24.43
**4493**	allXL	AA	357	319	DBSU	24.96
**4493**	allXL	AA	372	319	DBSU	35.94
**4493**	allXL	DD	370	357	DBSU	31.21
**4493**	allXL	BB	382	357	DBSU	18.96
**4493**	allXL	AA	372	370	DBSU	6.37
**4493**	allXL	AA	381	370	DBSU	20.4
**4493**	allXL	DD	373	370	DBSU	9.06
**4493**	allXL	AA	381	372	DBSU	16.29
**4493**	allXL	AA	372	382	DBSU	18.53
**4493**	allXL	AA	372	386	DBSU	24.4
**4493**	allXL	AA	373	386	DBSU	27.15
**4493**	allXL	DB	381	386	DBSU	57.22
**4493**	allXL	BD;DB	357	357	DBSU	18.39
**4493**	allXL	AD	373	319	DBSU	31.06
**4493**	allXL	BD	372	357	DBSU	47.05
**4493**	allXL	BD	381	357	DBSU	28.39
**4493**	allXL	BD	373	357	DBSU	44.15

**4493**	allXL	CB	372	370	DBSU	32.03
**4493**	allXL	CB	373	370	DBSU	31.22
**4493**	allXL	CB	382	373	DBSU	51.35
**4494**	allXL	CC	320	357	BS2G	26.3
**4494**	allXL	AA	320	373	BS2G	17.11
**4494**	allXL	DD	373	382	BS2G	21.01
**4494**	allXL	DD	370	386	BS2G	23.33
**4494**	allXL	DD	373	386	BS2G	25.51
**4494**	allXL	CC	319	357	BS2G	27.05
**4494**	allXL	AA	367	373	BS2G	12.77
**4494**	allXL	DD	370	382	BS2G	20.86
**4494**	allXL	CC	376	382	BS2G	15.02
**4494**	allXL	BB	357	373	BS2G	30.42
**4494**	allXL	AA	320	366	BS2G	15.55
**4494**	allXL	BB	370	376	BS2G	14.92
**4494**	allXL	CC	320	357	DBSU	26.3
**4494**	allXL	CC	357	319	DBSU	27.05
**4494**	allXL	AA	372	319	DBSU	13.66
**4494**	allXL	BB	370	357	DBSU	24.17
**4494**	allXL	BB	382	357	DBSU	25.98
**4494**	allXL	AA	372	370	DBSU	6.4
**4494**	allXL	DD	381	370	DBSU	19.43
**4494**	allXL	AA	373	370	DBSU	9.11
**4494**	allXL	DD	381	372	DBSU	19.09
**4494**	allXL	DD	372	382	DBSU	21.63
**4494**	allXL	DD	372	386	DBSU	26.55
**4494**	allXL	DD	373	386	DBSU	25.51
**4494**	allXL	CB	381	386	DBSU	55.69
**4494**	allXL	BD;DB	357	357	DBSU	19.04
**4494**	allXL	CB	373	319	DBSU	21.19
**4494**	allXL	CB	372	357	DBSU	44.1
**4494**	allXL	BD	381	357	DBSU	44.58
**4494**	allXL	CB	373	357	DBSU	41.09
**4494**	allXL	CB	372	370	DBSU	21.75

**4494**	allXL	CB	373	370	DBSU	18.34
**4494**	allXL	CB	382	373	DBSU	28.88
**4495**	allXL	AA	320	357	BS2G	26.84
**4495**	allXL	BB	320	373	BS2G	21.4
**4495**	allXL	AA	373	382	BS2G	17.81
**4495**	allXL	AA	370	386	BS2G	28.16
**4495**	allXL	AA	373	386	BS2G	24.76
**4495**	allXL	AA	319	357	BS2G	26.62
**4495**	allXL	AA	367	373	BS2G	12.87
**4495**	allXL	CC	370	382	BS2G	22.3
**4495**	allXL	AA	376	382	BS2G	13.19
**4495**	allXL	AA	357	373	BS2G	36.71
**4495**	allXL	BB	320	366	BS2G	14.39
**4495**	allXL	CC	370	376	BS2G	13.08
**4495**	allXL	AA	320	357	DBSU	26.84
**4495**	allXL	AA	357	319	DBSU	26.62
**4495**	allXL	BB	372	319	DBSU	18.27
**4495**	allXL	BB	370	357	DBSU	32.8
**4495**	allXL	AA	382	357	DBSU	24.43
**4495**	allXL	AA	372	370	DBSU	6.44
**4495**	allXL	CC	381	370	DBSU	19.95
**4495**	allXL	DD	373	370	DBSU	8.98
**4495**	allXL	CC	381	372	DBSU	22.38
**4495**	allXL	AA	372	382	DBSU	21.01
**4495**	allXL	AA	372	386	DBSU	27.51
**4495**	allXL	AA	373	386	DBSU	24.76
**4495**	allXL	BC	381	386	DBSU	40.77
**4495**	allXL	BD;DB	357	357	DBSU	19.12
**4495**	allXL	CB	373	319	DBSU	20.77
**4495**	allXL	CB	372	357	DBSU	30.35
**4495**	allXL	AC	381	357	DBSU	43.98
**4495**	allXL	CB	373	357	DBSU	31.0
**4495**	allXL	CB	372	370	DBSU	16.93
**4495**	allXL	CB	373	370	DBSU	19.83

**4495**	allXL	CB	382	373	DBSU	23.19
**4496**	allXL	CC	320	357	BS2G	23.27
**4496**	allXL	DD	320	373	BS2G	27.97
**4496**	allXL	DD	373	382	BS2G	18.65
**4496**	allXL	CC	370	386	BS2G	26.18
**4496**	allXL	AA	373	386	BS2G	22.6
**4496**	allXL	AA	319	357	BS2G	26.64
**4496**	allXL	AA	367	373	BS2G	13.28
**4496**	allXL	DD	370	382	BS2G	24.21
**4496**	allXL	CC	376	382	BS2G	12.53
**4496**	allXL	AA	357	373	BS2G	34.14
**4496**	allXL	CC	320	366	BS2G	20.93
**4496**	allXL	CC	370	376	BS2G	16.54
**4496**	allXL	CC	320	357	DBSU	23.27
**4496**	allXL	AA	357	319	DBSU	26.64
**4496**	allXL	DD	372	319	DBSU	24.51
**4496**	allXL	CC	370	357	DBSU	28.42
**4496**	allXL	CC	382	357	DBSU	31.87
**4496**	allXL	AA	372	370	DBSU	6.23
**4496**	allXL	DD	381	370	DBSU	24.76
**4496**	allXL	CC	373	370	DBSU	9.31
**4496**	allXL	DD	381	372	DBSU	19.61
**4496**	allXL	DD	372	382	DBSU	19.18
**4496**	allXL	DD	372	386	DBSU	23.29
**4496**	allXL	AA	373	386	DBSU	22.6
**4496**	allXL	AD	381	386	DBSU	50.84
**4496**	allXL	BD;DB	357	357	DBSU	19.16
**4496**	allXL	CB	373	319	DBSU	14.48
**4496**	allXL	CB	372	357	DBSU	40.71
**4496**	allXL	AC	381	357	DBSU	40.81
**4496**	allXL	AD	373	357	DBSU	44.1
**4496**	allXL	DA	372	370	DBSU	29.51
**4496**	allXL	DA	373	370	DBSU	32.15
**4496**	allXL	DA	382	373	DBSU	31.62

**4497**	allXL	AA	320	357	BS2G	21.68
**4497**	allXL	CC	320	373	BS2G	23.86
**4497**	allXL	DD	373	382	BS2G	18.36
**4497**	allXL	CC	370	386	BS2G	18.1
**4497**	allXL	CC	373	386	BS2G	25.47
**4497**	allXL	AA	319	357	BS2G	19.66
**4497**	allXL	BB	367	373	BS2G	13.89
**4497**	allXL	CC	370	382	BS2G	15.1
**4497**	allXL	CC	376	382	BS2G	15.07
**4497**	allXL	BB	357	373	BS2G	23.91
**4497**	allXL	AA	320	366	BS2G	10.9
**4497**	allXL	CC	370	376	BS2G	14.64
**4497**	allXL	AA	320	357	DBSU	21.68
**4497**	allXL	AA	357	319	DBSU	19.66
**4497**	allXL	CC	372	319	DBSU	19.42
**4497**	allXL	DD	370	357	DBSU	24.43
**4497**	allXL	BA	382	357	DBSU	39.98
**4497**	allXL	AA	372	370	DBSU	6.1
**4497**	allXL	CC	381	370	DBSU	15.85
**4497**	allXL	AA	373	370	DBSU	9.11
**4497**	allXL	DD	381	372	DBSU	18.44
**4497**	allXL	CC	372	382	DBSU	18.1
**4497**	allXL	CC	372	386	DBSU	22.79
**4497**	allXL	CC	373	386	DBSU	25.47
**4497**	allXL	BC	381	386	DBSU	57.92
**4497**	allXL	BD;DB	357	357	DBSU	17.83
**4497**	allXL	BC	373	319	DBSU	29.2
**4497**	allXL	BD	372	357	DBSU	32.06
**4497**	allXL	BC	381	357	DBSU	30.72
**4497**	allXL	BD	373	357	DBSU	30.39
**4497**	allXL	BC	372	370	DBSU	42.15
**4497**	allXL	BC	373	370	DBSU	41.61
**4497**	allXL	BC	382	373	DBSU	46.98
**4498**	allXL	AA	320	357	BS2G	26.33

**4498**	allXL	BB	320	373	BS2G	23.39
**4498**	allXL	CC	373	382	BS2G	18.04
**4498**	allXL	BB	370	386	BS2G	33.07
**4498**	allXL	CC	373	386	BS2G	24.33
**4498**	allXL	AA	319	357	BS2G	27.17
**4498**	allXL	AA	367	373	BS2G	11.76
**4498**	allXL	CC	370	382	BS2G	25.5
**4498**	allXL	BB	376	382	BS2G	14.65
**4498**	allXL	AA	357	373	BS2G	28.26
**4498**	allXL	BB	320	366	BS2G	20.14
**4498**	allXL	CC	370	376	BS2G	14.21
**4498**	allXL	AA	320	357	DBSU	26.33
**4498**	allXL	AA	357	319	DBSU	27.17
**4498**	allXL	BB	372	319	DBSU	20.42
**4498**	allXL	DD	370	357	DBSU	28.63
**4498**	allXL	CC	382	357	DBSU	35.34
**4498**	allXL	AA	372	370	DBSU	6.38
**4498**	allXL	CC	381	370	DBSU	26.09
**4498**	allXL	AA	373	370	DBSU	9.05
**4498**	allXL	CC	381	372	DBSU	22.89
**4498**	allXL	CC	372	382	DBSU	21.87
**4498**	allXL	CC	372	386	DBSU	27.67
**4498**	allXL	CC	373	386	DBSU	24.33
**4498**	allXL	AD	381	386	DBSU	61.58
**4498**	allXL	BD;DB	357	357	DBSU	18.5
**4498**	allXL	CB	373	319	DBSU	20.93
**4498**	allXL	CB	372	357	DBSU	40.41
**4498**	allXL	AC	381	357	DBSU	46.8
**4498**	allXL	CB	373	357	DBSU	43.46
**4498**	allXL	CB	372	370	DBSU	17.52
**4498**	allXL	CB	373	370	DBSU	21.01
**4498**	allXL	BC	382	373	DBSU	42.96
**4499**	allXL	AA	320	357	BS2G	24.58
**4499**	allXL	BB	320	373	BS2G	14.56

**4499**	allXL	AA	373	382	BS2G	18.17
**4499**	allXL	CC	370	386	BS2G	25.05
**4499**	allXL	DD	373	386	BS2G	25.23
**4499**	allXL	AA	319	357	BS2G	25.25
**4499**	allXL	BB	367	373	BS2G	14.57
**4499**	allXL	AA	370	382	BS2G	20.51
**4499**	allXL	CC	376	382	BS2G	14.58
**4499**	allXL	BB	357	373	BS2G	25.85
**4499**	allXL	AA	320	366	BS2G	18.2
**4499**	allXL	CC	370	376	BS2G	14.37
**4499**	allXL	AA	320	357	DBSU	24.58
**4499**	allXL	AA	357	319	DBSU	25.25
**4499**	allXL	CC	372	319	DBSU	14.58
**4499**	allXL	BB	370	357	DBSU	25.96
**4499**	allXL	BB	382	357	DBSU	35.65
**4499**	allXL	AA	372	370	DBSU	6.21
**4499**	allXL	CC	381	370	DBSU	21.88
**4499**	allXL	AA	373	370	DBSU	9.03
**4499**	allXL	AA	381	372	DBSU	20.01
**4499**	allXL	AA	372	382	DBSU	18.85
**4499**	allXL	DD	372	386	DBSU	26.71
**4499**	allXL	DD	373	386	DBSU	25.23
**4499**	allXL	AD	381	386	DBSU	35.48
**4499**	allXL	BD;DB	357	357	DBSU	18.27
**4499**	allXL	AD	373	319	DBSU	25.21
**4499**	allXL	AD	372	357	DBSU	30.16
**4499**	allXL	BC	381	357	DBSU	32.03
**4499**	allXL	AD	373	357	DBSU	33.41
**4499**	allXL	AD	372	370	DBSU	10.81
**4499**	allXL	AD	373	370	DBSU	10.69
**4499**	allXL	DA	382	373	DBSU	32.8
**4500**	allXL	AA	320	357	BS2G	10.29
**4500**	allXL	DD	320	373	BS2G	19.18
**4500**	allXL	CC	373	382	BS2G	18.99

**4500**	allXL	CC	370	386	BS2G	25.19
**4500**	allXL	CC	373	386	BS2G	25.98
**4500**	allXL	AA	319	357	BS2G	8.67
**4500**	allXL	AA	367	373	BS2G	12.81
**4500**	allXL	CC	370	382	BS2G	22.86
**4500**	allXL	CC	376	382	BS2G	13.14
**4500**	allXL	AA	357	373	BS2G	29.5
**4500**	allXL	DD	320	366	BS2G	16.96
**4500**	allXL	CC	370	376	BS2G	15.98
**4500**	allXL	AA	320	357	DBSU	10.29
**4500**	allXL	AA	357	319	DBSU	8.67
**4500**	allXL	DD	372	319	DBSU	19.26
**4500**	allXL	DD	370	357	DBSU	27.09
**4500**	allXL	CC	382	357	DBSU	49.08
**4500**	allXL	AA	372	370	DBSU	6.17
**4500**	allXL	CC	381	370	DBSU	25.36
**4500**	allXL	DD	373	370	DBSU	8.88
**4500**	allXL	DD	381	372	DBSU	21.94
**4500**	allXL	CC	372	382	DBSU	21.44
**4500**	allXL	CC	372	386	DBSU	26.9
**4500**	allXL	CC	373	386	DBSU	25.98
**4500**	allXL	AD	381	386	DBSU	31.44
**4500**	allXL	BD;DB	357	357	DBSU	18.98
**4500**	allXL	AD	373	319	DBSU	24.16
**4500**	allXL	AC	372	357	DBSU	37.58
**4500**	allXL	CB	381	357	DBSU	47.08
**4500**	allXL	AC	373	357	DBSU	37.14
**4500**	allXL	CB	372	370	DBSU	37.63
**4500**	allXL	CB	373	370	DBSU	35.64
**4500**	allXL	CB	382	373	DBSU	29.14
**4501**	allXL	AA	320	357	BS2G	25.77
**4501**	allXL	CC	320	373	BS2G	17.46
**4501**	allXL	AA	373	382	BS2G	18.34
**4501**	allXL	DD	370	386	BS2G	23.1

**4501**	allXL	CC	373	386	BS2G	26.78
**4501**	allXL	AA	319	357	BS2G	26.2
**4501**	allXL	AA	367	373	BS2G	12.1
**4501**	allXL	DD	370	382	BS2G	20.57
**4501**	allXL	BB	376	382	BS2G	14.11
**4501**	allXL	BB	357	373	BS2G	24.29
**4501**	allXL	DD	320	366	BS2G	19.15
**4501**	allXL	DD	370	376	BS2G	13.86
**4501**	allXL	AA	320	357	DBSU	25.77
**4501**	allXL	AA	357	319	DBSU	26.2
**4501**	allXL	CC	372	319	DBSU	20.66
**4501**	allXL	CC	370	357	DBSU	26.35
**4501**	allXL	AA	382	357	DBSU	16.35
**4501**	allXL	CC	372	370	DBSU	6.67
**4501**	allXL	DD	381	370	DBSU	17.66
**4501**	allXL	AA	373	370	DBSU	8.98
**4501**	allXL	DD	381	372	DBSU	20.15
**4501**	allXL	AA	372	382	DBSU	20.95
**4501**	allXL	CC	372	386	DBSU	27.36
**4501**	allXL	CC	373	386	DBSU	26.78
**4501**	allXL	BC	381	386	DBSU	46.63
**4501**	allXL	BD;DB	357	357	DBSU	18.03
**4501**	allXL	AD	373	319	DBSU	14.62
**4501**	allXL	CB	372	357	DBSU	33.78
**4501**	allXL	AC	381	357	DBSU	26.24
**4501**	allXL	AD	373	357	DBSU	35.2
**4501**	allXL	CB	372	370	DBSU	30.5
**4501**	allXL	AD	373	370	DBSU	33.05
**4501**	allXL	CB	382	373	DBSU	37.63
**4502**	allXL	CC	320	357	BS2G	24.64
**4502**	allXL	DD	320	373	BS2G	24.47
**4502**	allXL	DD	373	382	BS2G	17.67
**4502**	allXL	CC	370	386	BS2G	28.2
**4502**	allXL	DD	373	386	BS2G	28.25

**4502**	allXL	AA	319	357	BS2G	23.88
**4502**	allXL	AA	367	373	BS2G	13.6
**4502**	allXL	DD	370	382	BS2G	20.97
**4502**	allXL	BB	376	382	BS2G	13.92
**4502**	allXL	DD	357	373	BS2G	20.07
**4502**	allXL	BB	320	366	BS2G	22.86
**4502**	allXL	CC	370	376	BS2G	14.06
**4502**	allXL	CC	320	357	DBSU	24.64
**4502**	allXL	AA	357	319	DBSU	23.88
**4502**	allXL	DD	372	319	DBSU	27.81
**4502**	allXL	DD	370	357	DBSU	19.31
**4502**	allXL	DD	382	357	DBSU	11.35
**4502**	allXL	AA	372	370	DBSU	6.23
**4502**	allXL	DD	381	370	DBSU	19.6
**4502**	allXL	AA;DD	373	370	DBSU	9.06
**4502**	allXL	DD	381	372	DBSU	19.24
**4502**	allXL	DD	372	382	DBSU	20.77
**4502**	allXL	AA	372	386	DBSU	28.94
**4502**	allXL	DD	373	386	DBSU	28.25
**4502**	allXL	DB	381	386	DBSU	29.53
**4502**	allXL	BD;DB	357	357	DBSU	18.22
**4502**	allXL	DA	373	319	DBSU	31.72
**4502**	allXL	DB	372	357	DBSU	36.72
**4502**	allXL	DB	381	357	DBSU	28.38
**4502**	allXL	DB	373	357	DBSU	35.45
**4502**	allXL	BD	372	370	DBSU	52.91
**4502**	allXL	BD	373	370	DBSU	51.63
**4502**	allXL	BD	382	373	DBSU	42.05
**4503**	allXL	CC	320	357	BS2G	24.2
**4503**	allXL	DD	320	373	BS2G	12.44
**4503**	allXL	AA	373	382	BS2G	20.14
**4503**	allXL	AA	370	386	BS2G	29.2
**4503**	allXL	AA	373	386	BS2G	23.77
**4503**	allXL	CC	319	357	BS2G	24.78

**4503**	allXL	AA	367	373	BS2G	12.57
**4503**	allXL	BB	370	382	BS2G	24.21
**4503**	allXL	BB	376	382	BS2G	15.0
**4503**	allXL	DD	357	373	BS2G	28.45
**4503**	allXL	CC	320	366	BS2G	16.89
**4503**	allXL	BB	370	376	BS2G	17.23
**4503**	allXL	CC	320	357	DBSU	24.2
**4503**	allXL	CC	357	319	DBSU	24.78
**4503**	allXL	DD	372	319	DBSU	16.71
**4503**	allXL	DD	370	357	DBSU	23.59
**4503**	allXL	AA	382	357	DBSU	39.72
**4503**	allXL	AA	372	370	DBSU	6.31
**4503**	allXL	BB	381	370	DBSU	24.06
**4503**	allXL	DD	373	370	DBSU	9.24
**4503**	allXL	DD	381	372	DBSU	21.17
**4503**	allXL	DD	372	382	DBSU	20.97
**4503**	allXL	DD	372	386	DBSU	24.48
**4503**	allXL	AA	373	386	DBSU	23.77
**4503**	allXL	BC	381	386	DBSU	16.44
**4503**	allXL	BD;DB	357	357	DBSU	17.39
**4503**	allXL	AD	373	319	DBSU	35.93
**4503**	allXL	CB	372	357	DBSU	30.6
**4503**	allXL	CB	381	357	DBSU	41.24
**4503**	allXL	CB	373	357	DBSU	30.92
**4503**	allXL	BC	372	370	DBSU	22.87
**4503**	allXL	BC	373	370	DBSU	25.28
**4503**	allXL	CB	382	373	DBSU	23.0
**4504**	allXL	AA	320	357	BS2G	26.1
**4504**	allXL	CC	320	373	BS2G	26.42
**4504**	allXL	CC	373	382	BS2G	20.7
**4504**	allXL	CC	370	386	BS2G	31.51
**4504**	allXL	AA	373	386	BS2G	26.69
**4504**	allXL	AA	319	357	BS2G	26.01
**4504**	allXL	AA	367	373	BS2G	10.32

**4504**	allXL	CC	370	382	BS2G	23.99
**4504**	allXL	CC	376	382	BS2G	15.1
**4504**	allXL	BB	357	373	BS2G	30.27
**4504**	allXL	AA	320	366	BS2G	18.58
**4504**	allXL	CC	370	376	BS2G	13.54
**4504**	allXL	AA	320	357	DBSU	26.1
**4504**	allXL	AA	357	319	DBSU	26.01
**4504**	allXL	CC	372	319	DBSU	28.63
**4504**	allXL	DD	370	357	DBSU	30.93
**4504**	allXL	CC	382	357	DBSU	27.83
**4504**	allXL	AA	372	370	DBSU	6.47
**4504**	allXL	CC	381	370	DBSU	25.13
**4504**	allXL	DD	373	370	DBSU	8.75
**4504**	allXL	BB	381	372	DBSU	20.27
**4504**	allXL	AA	372	382	DBSU	22.54
**4504**	allXL	AA	372	386	DBSU	26.59
**4504**	allXL	AA	373	386	DBSU	26.69
**4504**	allXL	BC	381	386	DBSU	38.0
**4504**	allXL	BD;DB	357	357	DBSU	18.52
**4504**	allXL	AD	373	319	DBSU	21.56
**4504**	allXL	BD	372	357	DBSU	45.84
**4504**	allXL	CB	381	357	DBSU	41.3
**4504**	allXL	DB	373	357	DBSU	45.64
**4504**	allXL	DA	372	370	DBSU	35.0
**4504**	allXL	DA	373	370	DBSU	38.08
**4504**	allXL	CB	382	373	DBSU	35.1
**4505**	allXL	CC	320	357	BS2G	23.55
**4505**	allXL	CC	320	373	BS2G	9.06
**4505**	allXL	DD	373	382	BS2G	21.99
**4505**	allXL	AA	370	386	BS2G	29.76
**4505**	allXL	AA	373	386	BS2G	29.04
**4505**	allXL	AA	319	357	BS2G	25.39
**4505**	allXL	AA	367	373	BS2G	13.67
**4505**	allXL	DD	370	382	BS2G	21.26

**4505**	allXL	BB	376	382	BS2G	14.99
**4505**	allXL	BB	357	373	BS2G	26.57
**4505**	allXL	CC	320	366	BS2G	19.05
**4505**	allXL	DD	370	376	BS2G	16.45
**4505**	allXL	CC	320	357	DBSU	23.55
**4505**	allXL	AA	357	319	DBSU	25.39
**4505**	allXL	CC	372	319	DBSU	12.32
**4505**	allXL	DD	370	357	DBSU	26.03
**4505**	allXL	DD	382	357	DBSU	14.17
**4505**	allXL	AA	372	370	DBSU	6.05
**4505**	allXL	DD	381	370	DBSU	19.34
**4505**	allXL	CC	373	370	DBSU	9.16
**4505**	allXL	DD	381	372	DBSU	21.6
**4505**	allXL	AA	372	382	DBSU	22.9
**4505**	allXL	AA	372	386	DBSU	27.69
**4505**	allXL	AA	373	386	DBSU	29.04
**4505**	allXL	BD	381	386	DBSU	29.27
**4505**	allXL	BD;DB	357	357	DBSU	16.54
**4505**	allXL	AD	373	319	DBSU	32.07
**4505**	allXL	BD	372	357	DBSU	28.69
**4505**	allXL	DB	381	357	DBSU	32.82
**4505**	allXL	BD	373	357	DBSU	28.63
**4505**	allXL	CB	372	370	DBSU	20.15
**4505**	allXL	CB	373	370	DBSU	21.42
**4505**	allXL	CB	382	373	DBSU	26.91
**4506**	allXL	AA	320	357	BS2G	22.48
**4506**	allXL	BB	320	373	BS2G	16.9
**4506**	allXL	CC	373	382	BS2G	17.95
**4506**	allXL	DD	370	386	BS2G	22.04
**4506**	allXL	DD	373	386	BS2G	21.71
**4506**	allXL	AA	319	357	BS2G	21.71
**4506**	allXL	AA	367	373	BS2G	10.26
**4506**	allXL	CC	370	382	BS2G	19.65
**4506**	allXL	CC	376	382	BS2G	14.81

**4506**	allXL	CC	357	373	BS2G	14.24
**4506**	allXL	AA	320	366	BS2G	28.85
**4506**	allXL	CC	370	376	BS2G	15.51
**4506**	allXL	AA	320	357	DBSU	22.48
**4506**	allXL	AA	357	319	DBSU	21.71
**4506**	allXL	BB	372	319	DBSU	20.55
**4506**	allXL	CC	370	357	DBSU	17.5
**4506**	allXL	BB	382	357	DBSU	7.07
**4506**	allXL	CC	372	370	DBSU	6.7
**4506**	allXL	CC	381	370	DBSU	20.36
**4506**	allXL	AA	373	370	DBSU	8.64
**4506**	allXL	CC	381	372	DBSU	20.02
**4506**	allXL	CC	372	382	DBSU	20.49
**4506**	allXL	DD	372	386	DBSU	20.97
**4506**	allXL	DD	373	386	DBSU	21.71
**4506**	allXL	CA	381	386	DBSU	40.09
**4506**	allXL	BD;DB	357	357	DBSU	19.06
**4506**	allXL	AD	373	319	DBSU	32.59
**4506**	allXL	CA	372	357	DBSU	22.41
**4506**	allXL	BD	381	357	DBSU	21.96
**4506**	allXL	CA	373	357	DBSU	20.19
**4506**	allXL	CB	372	370	DBSU	50.18
**4506**	allXL	CB	373	370	DBSU	50.6
**4506**	allXL	AC	382	373	DBSU	34.44
**4507**	allXL	AA	320	357	BS2G	25.67
**4507**	allXL	BB	320	373	BS2G	25.0
**4507**	allXL	AA	373	382	BS2G	17.63
**4507**	allXL	DD	370	386	BS2G	18.84
**4507**	allXL	AA	373	386	BS2G	19.9
**4507**	allXL	AA	319	357	BS2G	24.81
**4507**	allXL	AA	367	373	BS2G	12.87
**4507**	allXL	DD	370	382	BS2G	20.55
**4507**	allXL	AA	376	382	BS2G	13.28
**4507**	allXL	BB	357	373	BS2G	28.96

**4507**	allXL	BB	320	366	BS2G	19.43
**4507**	allXL	CC	370	376	BS2G	15.95
**4507**	allXL	AA	320	357	DBSU	25.67
**4507**	allXL	AA	357	319	DBSU	24.81
**4507**	allXL	CC	372	319	DBSU	25.38
**4507**	allXL	DD	370	357	DBSU	28.86
**4507**	allXL	AA	382	357	DBSU	23.23
**4507**	allXL	AA	372	370	DBSU	6.4
**4507**	allXL	DD	381	370	DBSU	20.19
**4507**	allXL	AA	373	370	DBSU	9.04
**4507**	allXL	DD	381	372	DBSU	21.2
**4507**	allXL	AA	372	382	DBSU	19.33
**4507**	allXL	AA	372	386	DBSU	19.37
**4507**	allXL	AA	373	386	DBSU	19.9
**4507**	allXL	AD	381	386	DBSU	49.36
**4507**	allXL	BD;DB	357	357	DBSU	18.49
**4507**	allXL	AD	373	319	DBSU	28.73
**4507**	allXL	BD	372	357	DBSU	40.86
**4507**	allXL	AD	381	357	DBSU	38.28
**4507**	allXL	BD	373	357	DBSU	42.43
**4507**	allXL	AD	372	370	DBSU	50.2
**4507**	allXL	AD	373	370	DBSU	47.18
**4507**	allXL	AD	382	373	DBSU	52.75
**4508**	allXL	AA	320	357	BS2G	18.27
**4508**	allXL	BB	320	373	BS2G	21.7
**4508**	allXL	BB	373	382	BS2G	16.11
**4508**	allXL	BB	370	386	BS2G	30.24
**4508**	allXL	BB	373	386	BS2G	26.13
**4508**	allXL	AA	319	357	BS2G	14.93
**4508**	allXL	AA	367	373	BS2G	14.12
**4508**	allXL	BB	370	382	BS2G	21.63
**4508**	allXL	BB	376	382	BS2G	13.73
**4508**	allXL	BB	357	373	BS2G	28.87
**4508**	allXL	DD	320	366	BS2G	26.54

**4508**	allXL	CC	370	376	BS2G	15.99
**4508**	allXL	AA	320	357	DBSU	18.27
**4508**	allXL	AA	357	319	DBSU	14.93
**4508**	allXL	CC	372	319	DBSU	22.71
**4508**	allXL	CC	370	357	DBSU	30.56
**4508**	allXL	BB	382	357	DBSU	13.22
**4508**	allXL	AA	372	370	DBSU	6.0
**4508**	allXL	DD	381	370	DBSU	22.46
**4508**	allXL	DD	373	370	DBSU	9.19
**4508**	allXL	DD	381	372	DBSU	16.04
**4508**	allXL	BB	372	382	DBSU	17.39
**4508**	allXL	BB	372	386	DBSU	26.18
**4508**	allXL	BB	373	386	DBSU	26.13
**4508**	allXL	CA	381	386	DBSU	47.75
**4508**	allXL	BD;DB	357	357	DBSU	18.33
**4508**	allXL	DA	373	319	DBSU	38.66
**4508**	allXL	BD	372	357	DBSU	48.44
**4508**	allXL	BD	381	357	DBSU	29.88
**4508**	allXL	BD	373	357	DBSU	46.83
**4508**	allXL	BC	372	370	DBSU	36.16
**4508**	allXL	BC	373	370	DBSU	33.17
**4508**	allXL	BC	382	373	DBSU	45.29
**4509**	allXL	CC	320	357	BS2G	26.95
**4509**	allXL	DD	320	373	BS2G	30.45
**4509**	allXL	DD	373	382	BS2G	20.01
**4509**	allXL	BB	370	386	BS2G	25.4
**4509**	allXL	DD	373	386	BS2G	28.89
**4509**	allXL	AA	319	357	BS2G	26.97
**4509**	allXL	AA	367	373	BS2G	14.04
**4509**	allXL	BB	370	382	BS2G	18.11
**4509**	allXL	BB	376	382	BS2G	14.55
**4509**	allXL	DD	357	373	BS2G	24.77
**4509**	allXL	BB	320	366	BS2G	16.82
**4509**	allXL	BB	370	376	BS2G	14.41

**4509**	allXL	CC	320	357	DBSU	26.95
**4509**	allXL	AA	357	319	DBSU	26.97
**4509**	allXL	AA	372	319	DBSU	30.76
**4509**	allXL	DD	370	357	DBSU	20.57
**4509**	allXL	BB	382	357	DBSU	40.7
**4509**	allXL	AA	372	370	DBSU	6.38
**4509**	allXL	BB	381	370	DBSU	20.21
**4509**	allXL	AA	373	370	DBSU	8.79
**4509**	allXL	DD	381	372	DBSU	17.13
**4509**	allXL	DD	372	382	DBSU	19.45
**4509**	allXL	DD	372	386	DBSU	27.95
**4509**	allXL	DD	373	386	DBSU	28.89
**4509**	allXL	CB	381	386	DBSU	16.6
**4509**	allXL	BD;DB	357	357	DBSU	19.16
**4509**	allXL	CB	373	319	DBSU	6.59
**4509**	allXL	CB	372	357	DBSU	37.48
**4509**	allXL	CB	381	357	DBSU	51.84
**4509**	allXL	CB	373	357	DBSU	38.56
**4509**	allXL	CB	372	370	DBSU	32.53
**4509**	allXL	CB	373	370	DBSU	31.61
**4509**	allXL	BC	382	373	DBSU	27.96
**4510**	allXL	AA	320	357	BS2G	24.24
**4510**	allXL	DD	320	373	BS2G	27.79
**4510**	allXL	BB	373	382	BS2G	16.77
**4510**	allXL	BB	370	386	BS2G	22.03
**4510**	allXL	BB	373	386	BS2G	26.72
**4510**	allXL	AA	319	357	BS2G	25.0
**4510**	allXL	AA	367	373	BS2G	12.19
**4510**	allXL	BB	370	382	BS2G	15.83
**4510**	allXL	BB	376	382	BS2G	13.75
**4510**	allXL	BB	357	373	BS2G	18.94
**4510**	allXL	DD	320	366	BS2G	26.89
**4510**	allXL	CC	370	376	BS2G	15.44
**4510**	allXL	AA	320	357	DBSU	24.24

**4510**	allXL	AA	357	319	DBSU	25.0
**4510**	allXL	DD	372	319	DBSU	29.56
**4510**	allXL	BB	370	357	DBSU	22.85
**4510**	allXL	BB	382	357	DBSU	34.43
**4510**	allXL	CC	372	370	DBSU	6.65
**4510**	allXL	BB	381	370	DBSU	18.76
**4510**	allXL	AA	373	370	DBSU	9.22
**4510**	allXL	BB	381	372	DBSU	19.84
**4510**	allXL	BB	372	382	DBSU	18.04
**4510**	allXL	BB	372	386	DBSU	26.98
**4510**	allXL	BB	373	386	DBSU	26.72
**4510**	allXL	BD	381	386	DBSU	46.41
**4510**	allXL	BD;DB	357	357	DBSU	18.92
**4510**	allXL	CB	373	319	DBSU	9.95
**4510**	allXL	BD	372	357	DBSU	27.78
**4510**	allXL	CB	381	357	DBSU	34.95
**4510**	allXL	BD	373	357	DBSU	26.6
**4510**	allXL	CB	372	370	DBSU	46.44
**4510**	allXL	CB	373	370	DBSU	43.16
**4510**	allXL	CB	382	373	DBSU	39.25
**4511**	allXL	CC	320	357	BS2G	25.01
**4511**	allXL	DD	320	373	BS2G	24.12
**4511**	allXL	CC	373	382	BS2G	17.45
**4511**	allXL	DD	370	386	BS2G	16.58
**4511**	allXL	CC	373	386	BS2G	21.19
**4511**	allXL	AA	319	357	BS2G	26.94
**4511**	allXL	BB	367	373	BS2G	12.73
**4511**	allXL	DD	370	382	BS2G	19.35
**4511**	allXL	CC	376	382	BS2G	14.13
**4511**	allXL	BB	357	373	BS2G	22.08
**4511**	allXL	BB	320	366	BS2G	17.91
**4511**	allXL	BB	370	376	BS2G	15.88
**4511**	allXL	CC	320	357	DBSU	25.01
**4511**	allXL	AA	357	319	DBSU	26.94

**4511**	allXL	DD	372	319	DBSU	20.08
**4511**	allXL	DD	370	357	DBSU	27.83
**4511**	allXL	BB	382	357	DBSU	26.87
**4511**	allXL	AA	372	370	DBSU	6.45
**4511**	allXL	DD	381	370	DBSU	18.99
**4511**	allXL	AA	373	370	DBSU	9.27
**4511**	allXL	DD	381	372	DBSU	17.39
**4511**	allXL	CC	372	382	DBSU	17.46
**4511**	allXL	DD	372	386	DBSU	18.73
**4511**	allXL	CC	373	386	DBSU	21.19
**4511**	allXL	DA	381	386	DBSU	46.09
**4511**	allXL	BD;DB	357	357	DBSU	19.31
**4511**	allXL	CB	373	319	DBSU	22.32
**4511**	allXL	BD	372	357	DBSU	42.35
**4511**	allXL	BD	381	357	DBSU	33.07
**4511**	allXL	BD	373	357	DBSU	38.98
**4511**	allXL	DA	372	370	DBSU	31.42
**4511**	allXL	DA	373	370	DBSU	33.15
**4511**	allXL	DA	382	373	DBSU	38.2
**4512**	allXL	AA	320	357	BS2G	25.62
**4512**	allXL	DD	320	373	BS2G	22.82
**4512**	allXL	DD	373	382	BS2G	16.16
**4512**	allXL	DD	370	386	BS2G	20.79
**4512**	allXL	DD	373	386	BS2G	18.01
**4512**	allXL	AA	319	357	BS2G	27.02
**4512**	allXL	AA	367	373	BS2G	13.84
**4512**	allXL	DD	370	382	BS2G	18.61
**4512**	allXL	CC	376	382	BS2G	15.29
**4512**	allXL	DD	357	373	BS2G	33.2
**4512**	allXL	BB	320	366	BS2G	24.26
**4512**	allXL	DD	370	376	BS2G	15.74
**4512**	allXL	AA	320	357	DBSU	25.62
**4512**	allXL	AA	357	319	DBSU	27.02
**4512**	allXL	DD	372	319	DBSU	16.62

**4512**	allXL	DD	370	357	DBSU	24.91
**4512**	allXL	DD	382	357	DBSU	42.65
**4512**	allXL	AA	372	370	DBSU	6.15
**4512**	allXL	DD	381	370	DBSU	18.34
**4512**	allXL	BB	373	370	DBSU	8.83
**4512**	allXL	DD	381	372	DBSU	16.64
**4512**	allXL	DD	372	382	DBSU	17.14
**4512**	allXL	DD	372	386	DBSU	17.93
**4512**	allXL	DD	373	386	DBSU	18.01
**4512**	allXL	AD	381	386	DBSU	30.21
**4512**	allXL	BD;DB	357	357	DBSU	17.88
**4512**	allXL	CB	373	319	DBSU	26.63
**4512**	allXL	DA	372	357	DBSU	46.96
**4512**	allXL	AD	381	357	DBSU	47.87
**4512**	allXL	CB	373	357	DBSU	46.71
**4512**	allXL	CB	372	370	DBSU	33.19
**4512**	allXL	CB	373	370	DBSU	30.48
**4512**	allXL	AD	382	373	DBSU	34.95
**4513**	allXL	AA	320	357	BS2G	23.19
**4513**	allXL	DD	320	373	BS2G	27.1
**4513**	allXL	DD	373	382	BS2G	17.81
**4513**	allXL	DD	370	386	BS2G	26.72
**4513**	allXL	BB	373	386	BS2G	24.79
**4513**	allXL	AA	319	357	BS2G	21.11
**4513**	allXL	BB	367	373	BS2G	13.72
**4513**	allXL	DD	370	382	BS2G	18.92
**4513**	allXL	BB	376	382	BS2G	14.79
**4513**	allXL	BB	357	373	BS2G	22.03
**4513**	allXL	CC	320	366	BS2G	21.09
**4513**	allXL	CC	370	376	BS2G	16.39
**4513**	allXL	AA	320	357	DBSU	23.19
**4513**	allXL	AA	357	319	DBSU	21.11
**4513**	allXL	CC	372	319	DBSU	24.55
**4513**	allXL	BB	370	357	DBSU	26.16

**4513**	allXL	DD	382	357	DBSU	24.28
**4513**	allXL	AA	372	370	DBSU	6.28
**4513**	allXL	DD	381	370	DBSU	17.87
**4513**	allXL	DD	373	370	DBSU	9.19
**4513**	allXL	DD	381	372	DBSU	13.62
**4513**	allXL	DD	372	382	DBSU	15.72
**4513**	allXL	DD	372	386	DBSU	24.83
**4513**	allXL	BB	373	386	DBSU	24.79
**4513**	allXL	BD	381	386	DBSU	57.39
**4513**	allXL	BD;DB	357	357	DBSU	18.84
**4513**	allXL	DA	373	319	DBSU	35.49
**4513**	allXL	BD	372	357	DBSU	25.01
**4513**	allXL	DA	381	357	DBSU	37.94
**4513**	allXL	BD	373	357	DBSU	28.43
**4513**	allXL	DA	372	370	DBSU	44.1
**4513**	allXL	DA	373	370	DBSU	40.96
**4513**	allXL	DB	382	373	DBSU	51.28
**4514**	allXL	AA	320	357	BS2G	20.96
**4514**	allXL	DD	320	373	BS2G	16.93
**4514**	allXL	CC	373	382	BS2G	19.45
**4514**	allXL	CC	370	386	BS2G	31.26
**4514**	allXL	CC	373	386	BS2G	23.52
**4514**	allXL	AA	319	357	BS2G	20.74
**4514**	allXL	BB	367	373	BS2G	11.4
**4514**	allXL	DD	370	382	BS2G	25.81
**4514**	allXL	CC	376	382	BS2G	13.28
**4514**	allXL	BB	357	373	BS2G	28.68
**4514**	allXL	BB	320	366	BS2G	19.74
**4514**	allXL	BB	370	376	BS2G	16.08
**4514**	allXL	AA	320	357	DBSU	20.96
**4514**	allXL	AA	357	319	DBSU	20.74
**4514**	allXL	DD	372	319	DBSU	16.87
**4514**	allXL	BB	370	357	DBSU	23.72
**4514**	allXL	BB	382	357	DBSU	38.03

**4514**	allXL	AA	372	370	DBSU	6.3
**4514**	allXL	DD	381	370	DBSU	23.97
**4514**	allXL	AA	373	370	DBSU	9.44
**4514**	allXL	DD	381	372	DBSU	21.07
**4514**	allXL	CC	372	382	DBSU	23.29
**4514**	allXL	CC	372	386	DBSU	26.73
**4514**	allXL	CC	373	386	DBSU	23.52
**4514**	allXL	AD	381	386	DBSU	68.03
**4514**	allXL	BD;DB	357	357	DBSU	18.24
**4514**	allXL	AD	373	319	DBSU	23.3
**4514**	allXL	DA	372	357	DBSU	39.33
**4514**	allXL	DA	381	357	DBSU	46.67
**4514**	allXL	DA	373	357	DBSU	40.22
**4514**	allXL	DA	372	370	DBSU	26.49
**4514**	allXL	DA	373	370	DBSU	25.73
**4514**	allXL	DA	382	373	DBSU	47.42
**4515**	allXL	AA	320	357	BS2G	20.5
**4515**	allXL	DD	320	373	BS2G	9.57
**4515**	allXL	CC	373	382	BS2G	17.91
**4515**	allXL	BB	370	386	BS2G	23.09
**4515**	allXL	BB	373	386	BS2G	26.59
**4515**	allXL	AA	319	357	BS2G	18.12
**4515**	allXL	AA	367	373	BS2G	12.84
**4515**	allXL	BB	370	382	BS2G	18.21
**4515**	allXL	BB	376	382	BS2G	14.44
**4515**	allXL	DD	357	373	BS2G	23.28
**4515**	allXL	AA	320	366	BS2G	21.24
**4515**	allXL	BB	370	376	BS2G	15.24
**4515**	allXL	AA	320	357	DBSU	20.5
**4515**	allXL	AA	357	319	DBSU	18.12
**4515**	allXL	DD	372	319	DBSU	11.64
**4515**	allXL	DD	370	357	DBSU	19.39
**4515**	allXL	DD	382	357	DBSU	31.98
**4515**	allXL	AA	372	370	DBSU	6.29

**4515**	allXL	DD	381	370	DBSU	19.84
**4515**	allXL	BB	373	370	DBSU	9.3
**4515**	allXL	DD	381	372	DBSU	21.01
**4515**	allXL	BB	372	382	DBSU	20.86
**4515**	allXL	BB	372	386	DBSU	27.44
**4515**	allXL	BB	373	386	DBSU	26.59
**4515**	allXL	BC	381	386	DBSU	64.11
**4515**	allXL	BD;DB	357	357	DBSU	18.25
**4515**	allXL	AD	373	319	DBSU	35.84
**4515**	allXL	DA	372	357	DBSU	35.39
**4515**	allXL	BC	381	357	DBSU	32.34
**4515**	allXL	DA	373	357	DBSU	38.59
**4515**	allXL	DA	372	370	DBSU	34.81
**4515**	allXL	DA	373	370	DBSU	35.83
**4515**	allXL	DA	382	373	DBSU	42.49
**4516**	allXL	AA	320	357	BS2G	24.46
**4516**	allXL	DD	320	373	BS2G	32.78
**4516**	allXL	AA	373	382	BS2G	18.13
**4516**	allXL	DD	370	386	BS2G	19.94
**4516**	allXL	DD	373	386	BS2G	18.92
**4516**	allXL	AA	319	357	BS2G	23.39
**4516**	allXL	AA	367	373	BS2G	12.9
**4516**	allXL	DD	370	382	BS2G	24.87
**4516**	allXL	CC	376	382	BS2G	14.03
**4516**	allXL	BB	357	373	BS2G	24.81
**4516**	allXL	AA	320	366	BS2G	23.55
**4516**	allXL	CC	370	376	BS2G	16.19
**4516**	allXL	AA	320	357	DBSU	24.46
**4516**	allXL	AA	357	319	DBSU	23.39
**4516**	allXL	DD	372	319	DBSU	33.05
**4516**	allXL	BB	370	357	DBSU	29.36
**4516**	allXL	AA	382	357	DBSU	26.13
**4516**	allXL	AA	372	370	DBSU	6.23
**4516**	allXL	DD	381	370	DBSU	24.06

**4516**	allXL	DD	373	370	DBSU	8.83
**4516**	allXL	DD	381	372	DBSU	17.39
**4516**	allXL	DD	372	382	DBSU	18.6
**4516**	allXL	DD	372	386	DBSU	15.89
**4516**	allXL	DD	373	386	DBSU	18.92
**4516**	allXL	AD	381	386	DBSU	40.99
**4516**	allXL	BD;DB	357	357	DBSU	18.77
**4516**	allXL	CB	373	319	DBSU	29.06
**4516**	allXL	BD	372	357	DBSU	37.61
**4516**	allXL	AC	381	357	DBSU	33.21
**4516**	allXL	BD	373	357	DBSU	36.47
**4516**	allXL	DA	372	370	DBSU	45.92
**4516**	allXL	DA	373	370	DBSU	47.39
**4516**	allXL	DA	382	373	DBSU	31.39
**4517**	allXL	AA	320	357	BS2G	23.56
**4517**	allXL	BB	320	373	BS2G	21.47
**4517**	allXL	DD	373	382	BS2G	20.26
**4517**	allXL	DD	370	386	BS2G	27.19
**4517**	allXL	BB	373	386	BS2G	28.23
**4517**	allXL	AA	319	357	BS2G	22.49
**4517**	allXL	AA	367	373	BS2G	11.26
**4517**	allXL	DD	370	382	BS2G	20.04
**4517**	allXL	BB	376	382	BS2G	14.86
**4517**	allXL	CC	357	373	BS2G	32.07
**4517**	allXL	BB	320	366	BS2G	17.94
**4517**	allXL	CC	370	376	BS2G	16.53
**4517**	allXL	AA	320	357	DBSU	23.56
**4517**	allXL	AA	357	319	DBSU	22.49
**4517**	allXL	BB	372	319	DBSU	22.02
**4517**	allXL	DD	370	357	DBSU	25.7
**4517**	allXL	DD	382	357	DBSU	37.75
**4517**	allXL	AA	372	370	DBSU	6.36
**4517**	allXL	DD	381	370	DBSU	18.15
**4517**	allXL	AA	373	370	DBSU	9.1

**4517**	allXL	DD	381	372	DBSU	17.03
**4517**	allXL	DD	372	382	DBSU	19.85
**4517**	allXL	DD	372	386	DBSU	28.34
**4517**	allXL	BB	373	386	DBSU	28.23
**4517**	allXL	BD	381	386	DBSU	50.19
**4517**	allXL	BD;DB	357	357	DBSU	19.07
**4517**	allXL	DA	373	319	DBSU	26.83
**4517**	allXL	DA	372	357	DBSU	39.95
**4517**	allXL	DA	381	357	DBSU	44.2
**4517**	allXL	DA	373	357	DBSU	40.5
**4517**	allXL	DA	372	370	DBSU	34.41
**4517**	allXL	DA	373	370	DBSU	32.52
**4517**	allXL	AC	382	373	DBSU	51.93
**4518**	allXL	CC	320	357	BS2G	21.09
**4518**	allXL	BB	320	373	BS2G	18.66
**4518**	allXL	BB	373	382	BS2G	17.63
**4518**	allXL	DD	370	386	BS2G	14.65
**4518**	allXL	DD	373	386	BS2G	21.52
**4518**	allXL	CC	319	357	BS2G	20.85
**4518**	allXL	BB	367	373	BS2G	14.53
**4518**	allXL	BB	370	382	BS2G	17.26
**4518**	allXL	BB	376	382	BS2G	13.98
**4518**	allXL	DD	357	373	BS2G	32.2
**4518**	allXL	BB	320	366	BS2G	24.56
**4518**	allXL	BB	370	376	BS2G	16.35
**4518**	allXL	CC	320	357	DBSU	21.09
**4518**	allXL	CC	357	319	DBSU	20.85
**4518**	allXL	BB	372	319	DBSU	17.23
**4518**	allXL	DD	370	357	DBSU	22.67
**4518**	allXL	DD	382	357	DBSU	32.44
**4518**	allXL	AA	372	370	DBSU	6.4
**4518**	allXL	DD	381	370	DBSU	19.36
**4518**	allXL	AA	373	370	DBSU	9.31
**4518**	allXL	DD	381	372	DBSU	20.88

**4518**	allXL	BB	372	382	DBSU	19.44
**4518**	allXL	DD	372	386	DBSU	20.52
**4518**	allXL	DD	373	386	DBSU	21.52
**4518**	allXL	BC	381	386	DBSU	74.74
**4518**	allXL	BD;DB	357	357	DBSU	18.88
**4518**	allXL	BC	373	319	DBSU	30.81
**4518**	allXL	BC	372	357	DBSU	35.7
**4518**	allXL	CA	381	357	DBSU	50.97
**4518**	allXL	BC	373	357	DBSU	35.45
**4518**	allXL	BC	372	370	DBSU	51.08
**4518**	allXL	BC	373	370	DBSU	48.43
**4518**	allXL	BC	382	373	DBSU	58.14
**4519**	allXL	BB	320	357	BS2G	24.91
**4519**	allXL	AA	320	373	BS2G	35.77
**4519**	allXL	AA	373	382	BS2G	18.59
**4519**	allXL	AA	370	386	BS2G	23.85
**4519**	allXL	AA	373	386	BS2G	20.65
**4519**	allXL	AA	319	357	BS2G	25.37
**4519**	allXL	DD	367	373	BS2G	14.15
**4519**	allXL	BB	370	382	BS2G	25.64
**4519**	allXL	AA	376	382	BS2G	14.62
**4519**	allXL	DD	357	373	BS2G	38.74
**4519**	allXL	AA	320	366	BS2G	20.55
**4519**	allXL	CC	370	376	BS2G	14.84
**4519**	allXL	BB	320	357	DBSU	24.91
**4519**	allXL	AA	357	319	DBSU	25.37
**4519**	allXL	AA	372	319	DBSU	32.33
**4519**	allXL	CC	370	357	DBSU	32.07
**4519**	allXL	DD	382	357	DBSU	36.54
**4519**	allXL	AA	372	370	DBSU	6.41
**4519**	allXL	DD	381	370	DBSU	24.11
**4519**	allXL	AA	373	370	DBSU	9.12
**4519**	allXL	AA	381	372	DBSU	21.03
**4519**	allXL	AA	372	382	DBSU	21.93

**4519**	allXL	AA	372	386	DBSU	22.82
**4519**	allXL	AA	373	386	DBSU	20.65
**4519**	allXL	DA	381	386	DBSU	54.26
**4519**	allXL	BD;DB	357	357	DBSU	18.88
**4519**	allXL	CB	373	319	DBSU	6.26
**4519**	allXL	CB	372	357	DBSU	30.22
**4519**	allXL	CB	381	357	DBSU	45.27
**4519**	allXL	CB	373	357	DBSU	30.38
**4519**	allXL	AD	372	370	DBSU	47.8
**4519**	allXL	CB	373	370	DBSU	47.2
**4519**	allXL	CB	382	373	DBSU	54.59
**4520**	allXL	AA	320	357	BS2G	24.55
**4520**	allXL	CC	320	373	BS2G	35.48
**4520**	allXL	DD	373	382	BS2G	18.26
**4520**	allXL	AA	370	386	BS2G	16.27
**4520**	allXL	AA	373	386	BS2G	23.42
**4520**	allXL	AA	319	357	BS2G	23.56
**4520**	allXL	AA	367	373	BS2G	10.31
**4520**	allXL	AA	370	382	BS2G	16.49
**4520**	allXL	CC	376	382	BS2G	13.84
**4520**	allXL	BB	357	373	BS2G	29.49
**4520**	allXL	AA	320	366	BS2G	22.67
**4520**	allXL	AA	370	376	BS2G	15.83
**4520**	allXL	AA	320	357	DBSU	24.55
**4520**	allXL	AA	357	319	DBSU	23.56
**4520**	allXL	BB	372	319	DBSU	33.03
**4520**	allXL	DD	370	357	DBSU	30.37
**4520**	allXL	AA	382	357	DBSU	33.46
**4520**	allXL	AA	372	370	DBSU	6.55
**4520**	allXL	AA	381	370	DBSU	16.45
**4520**	allXL	AA	373	370	DBSU	8.8
**4520**	allXL	DD	381	372	DBSU	17.71
**4520**	allXL	DD	372	382	DBSU	18.54
**4520**	allXL	AA	372	386	DBSU	22.31

**4520**	allXL	AA	373	386	DBSU	23.42
**4520**	allXL	BC	381	386	DBSU	57.46
**4520**	allXL	BD;DB	357	357	DBSU	17.76
**4520**	allXL	CB	373	319	DBSU	32.11
**4520**	allXL	BD	372	357	DBSU	43.27
**4520**	allXL	AC	381	357	DBSU	49.61
**4520**	allXL	BD	373	357	DBSU	45.37
**4520**	allXL	CB	372	370	DBSU	23.67
**4520**	allXL	CB	373	370	DBSU	22.84
**4520**	allXL	AD	382	373	DBSU	43.51
**4521**	allXL	AA	320	357	BS2G	24.29
**4521**	allXL	BB	320	373	BS2G	20.99
**4521**	allXL	CC	373	382	BS2G	20.34
**4521**	allXL	CC	370	386	BS2G	33.79
**4521**	allXL	CC	373	386	BS2G	24.92
**4521**	allXL	AA	319	357	BS2G	25.89
**4521**	allXL	BB	367	373	BS2G	9.88
**4521**	allXL	CC	370	382	BS2G	28.37
**4521**	allXL	CC	376	382	BS2G	13.4
**4521**	allXL	BB	357	373	BS2G	22.64
**4521**	allXL	BB	320	366	BS2G	25.74
**4521**	allXL	CC	370	376	BS2G	15.85
**4521**	allXL	AA	320	357	DBSU	24.29
**4521**	allXL	AA	357	319	DBSU	25.89
**4521**	allXL	BB	372	319	DBSU	23.53
**4521**	allXL	DD	370	357	DBSU	27.71
**4521**	allXL	BB	382	357	DBSU	23.08
**4521**	allXL	AA	372	370	DBSU	6.24
**4521**	allXL	DD	381	370	DBSU	26.95
**4521**	allXL	DD	373	370	DBSU	8.76
**4521**	allXL	DD	381	372	DBSU	20.0
**4521**	allXL	CC	372	382	DBSU	23.26
**4521**	allXL	CC	372	386	DBSU	27.28
**4521**	allXL	CC	373	386	DBSU	24.92

**4521**	allXL	DB	381	386	DBSU	35.83
**4521**	allXL	BD;DB	357	357	DBSU	19.25
**4521**	allXL	AD	373	319	DBSU	21.17
**4521**	allXL	DB	372	357	DBSU	36.16
**4521**	allXL	AC	381	357	DBSU	20.81
**4521**	allXL	DB	373	357	DBSU	35.56
**4521**	allXL	CB	372	370	DBSU	28.13
**4521**	allXL	CB	373	370	DBSU	28.72
**4521**	allXL	BD	382	373	DBSU	44.37
**4522**	allXL	AA	320	357	BS2G	24.66
**4522**	allXL	BB	320	373	BS2G	25.15
**4522**	allXL	BB	373	382	BS2G	19.13
**4522**	allXL	DD	370	386	BS2G	20.01
**4522**	allXL	DD	373	386	BS2G	22.39
**4522**	allXL	AA	319	357	BS2G	23.31
**4522**	allXL	AA	367	373	BS2G	13.5
**4522**	allXL	DD	370	382	BS2G	24.16
**4522**	allXL	BB	376	382	BS2G	13.16
**4522**	allXL	BB	357	373	BS2G	30.05
**4522**	allXL	CC	320	366	BS2G	20.93
**4522**	allXL	CC	370	376	BS2G	16.14
**4522**	allXL	AA	320	357	DBSU	24.66
**4522**	allXL	AA	357	319	DBSU	23.31
**4522**	allXL	CC	372	319	DBSU	24.58
**4522**	allXL	DD	370	357	DBSU	23.7
**4522**	allXL	BB	382	357	DBSU	28.01
**4522**	allXL	AA	372	370	DBSU	6.17
**4522**	allXL	CC	381	370	DBSU	23.06
**4522**	allXL	AA	373	370	DBSU	9.16
**4522**	allXL	CC	381	372	DBSU	20.21
**4522**	allXL	BB	372	382	DBSU	22.37
**4522**	allXL	DD	372	386	DBSU	23.5
**4522**	allXL	DD	373	386	DBSU	22.39
**4522**	allXL	BC	381	386	DBSU	19.47

**4522**	allXL	BD;DB	357	357	DBSU	18.63
**4522**	allXL	CB	373	319	DBSU	20.76
**4522**	allXL	CB	372	357	DBSU	29.17
**4522**	allXL	CB	381	357	DBSU	39.77
**4522**	allXL	CB	373	357	DBSU	27.24
**4522**	allXL	CB	372	370	DBSU	19.3
**4522**	allXL	CB	373	370	DBSU	15.87
**4522**	allXL	CB	382	373	DBSU	15.13
**4523**	allXL	AA	320	357	BS2G	26.16
**4523**	allXL	CC	320	373	BS2G	9.46
**4523**	allXL	DD	373	382	BS2G	18.71
**4523**	allXL	DD	370	386	BS2G	23.37
**4523**	allXL	AA	373	386	BS2G	26.18
**4523**	allXL	AA	319	357	BS2G	27.41
**4523**	allXL	AA	367	373	BS2G	11.81
**4523**	allXL	DD	370	382	BS2G	16.1
**4523**	allXL	CC	376	382	BS2G	14.05
**4523**	allXL	DD	357	373	BS2G	20.87
**4523**	allXL	CC	320	366	BS2G	18.85
**4523**	allXL	CC	370	376	BS2G	13.82
**4523**	allXL	AA	320	357	DBSU	26.16
**4523**	allXL	AA	357	319	DBSU	27.41
**4523**	allXL	CC	372	319	DBSU	13.45
**4523**	allXL	DD	370	357	DBSU	22.79
**4523**	allXL	AA	382	357	DBSU	17.82
**4523**	allXL	CC	372	370	DBSU	6.65
**4523**	allXL	DD	381	370	DBSU	14.18
**4523**	allXL	CC	373	370	DBSU	9.09
**4523**	allXL	DD	381	372	DBSU	17.65
**4523**	allXL	DD	372	382	DBSU	19.81
**4523**	allXL	DD	372	386	DBSU	27.88
**4523**	allXL	AA	373	386	DBSU	26.18
**4523**	allXL	DB	381	386	DBSU	22.63
**4523**	allXL	BD;DB	357	357	DBSU	18.0

**4523**	allXL	DA	373	319	DBSU	32.88
**4523**	allXL	DA	372	357	DBSU	33.38
**4523**	allXL	DB	381	357	DBSU	30.01
**4523**	allXL	DA	373	357	DBSU	31.98
**4523**	allXL	BC	372	370	DBSU	37.97
**4523**	allXL	BC	373	370	DBSU	40.13
**4523**	allXL	BD	382	373	DBSU	44.65
**4524**	allXL	AA	320	357	BS2G	22.48
**4524**	allXL	DD	320	373	BS2G	12.1
**4524**	allXL	DD	373	382	BS2G	19.59
**4524**	allXL	DD	370	386	BS2G	27.38
**4524**	allXL	AA	373	386	BS2G	28.27
**4524**	allXL	AA	319	357	BS2G	23.95
**4524**	allXL	AA	367	373	BS2G	12.61
**4524**	allXL	DD	370	382	BS2G	20.45
**4524**	allXL	BB	376	382	BS2G	14.7
**4524**	allXL	DD	357	373	BS2G	28.15
**4524**	allXL	CC	320	366	BS2G	17.54
**4524**	allXL	CC	370	376	BS2G	17.08
**4524**	allXL	AA	320	357	DBSU	22.48
**4524**	allXL	AA	357	319	DBSU	23.95
**4524**	allXL	DD	372	319	DBSU	11.78
**4524**	allXL	DD	370	357	DBSU	21.75
**4524**	allXL	BB	382	357	DBSU	28.94
**4524**	allXL	AA	372	370	DBSU	6.26
**4524**	allXL	DD	381	370	DBSU	20.8
**4524**	allXL	AA	373	370	DBSU	9.16
**4524**	allXL	DD	381	372	DBSU	20.21
**4524**	allXL	DD	372	382	DBSU	21.02
**4524**	allXL	AA	372	386	DBSU	27.25
**4524**	allXL	AA	373	386	DBSU	28.27
**4524**	allXL	DA	381	386	DBSU	47.68
**4524**	allXL	BD;DB	357	357	DBSU	18.84
**4524**	allXL	BC	373	319	DBSU	38.31

**4524**	allXL	CB	372	357	DBSU	41.71
**4524**	allXL	BD	381	357	DBSU	50.0
**4524**	allXL	CB	373	357	DBSU	44.95
**4524**	allXL	BC	372	370	DBSU	21.96
**4524**	allXL	BC	373	370	DBSU	25.49
**4524**	allXL	CB	382	373	DBSU	30.83
**4525**	allXL	AA	320	357	BS2G	23.03
**4525**	allXL	BB	320	373	BS2G	13.69
**4525**	allXL	DD	373	382	BS2G	17.51
**4525**	allXL	CC	370	386	BS2G	17.88
**4525**	allXL	AA	373	386	BS2G	25.31
**4525**	allXL	CC	319	357	BS2G	23.5
**4525**	allXL	AA	367	373	BS2G	12.69
**4525**	allXL	CC	370	382	BS2G	15.81
**4525**	allXL	BB;CC	376	382	BS2G	14.31
**4525**	allXL	CC	357	373	BS2G	30.96
**4525**	allXL	BB	320	366	BS2G	22.36
**4525**	allXL	CC	370	376	BS2G	11.36
**4525**	allXL	AA	320	357	DBSU	23.03
**4525**	allXL	CC	357	319	DBSU	23.5
**4525**	allXL	BB	372	319	DBSU	11.23
**4525**	allXL	CC	370	357	DBSU	30.12
**4525**	allXL	CC	382	357	DBSU	27.95
**4525**	allXL	AA	372	370	DBSU	6.27
**4525**	allXL	CC	381	370	DBSU	16.36
**4525**	allXL	CC	373	370	DBSU	9.12
**4525**	allXL	DD	381	372	DBSU	16.79
**4525**	allXL	DD	372	382	DBSU	18.09
**4525**	allXL	AA	372	386	DBSU	23.65
**4525**	allXL	AA	373	386	DBSU	25.31
**4525**	allXL	DA	381	386	DBSU	32.35
**4525**	allXL	BD;DB	357	357	DBSU	17.7
**4525**	allXL	AD	373	319	DBSU	19.77
**4525**	allXL	AD	372	357	DBSU	41.95

**4525**	allXL	BC	381	357	DBSU	31.8
**4525**	allXL	BC	373	357	DBSU	42.05
**4525**	allXL	AD	372	370	DBSU	37.49
**4525**	allXL	AD	373	370	DBSU	39.07
**4525**	allXL	AD	382	373	DBSU	32.55
**4526**	allXL	AA	320	357	BS2G	23.41
**4526**	allXL	BB	320	373	BS2G	25.14
**4526**	allXL	BB	373	382	BS2G	16.59
**4526**	allXL	DD	370	386	BS2G	21.61
**4526**	allXL	DD	373	386	BS2G	24.33
**4526**	allXL	AA	319	357	BS2G	22.49
**4526**	allXL	BB	367	373	BS2G	15.3
**4526**	allXL	BB	370	382	BS2G	16.91
**4526**	allXL	BB	376	382	BS2G	12.95
**4526**	allXL	CC	357	373	BS2G	30.89
**4526**	allXL	BB	320	366	BS2G	23.21
**4526**	allXL	CC	370	376	BS2G	15.86
**4526**	allXL	AA	320	357	DBSU	23.41
**4526**	allXL	AA	357	319	DBSU	22.49
**4526**	allXL	DD	372	319	DBSU	22.31
**4526**	allXL	CC	370	357	DBSU	26.69
**4526**	allXL	BB	382	357	DBSU	26.32
**4526**	allXL	AA	372	370	DBSU	5.97
**4526**	allXL	BB	381	370	DBSU	18.11
**4526**	allXL	AA	373	370	DBSU	9.27
**4526**	allXL	AA	381	372	DBSU	17.77
**4526**	allXL	BB	372	382	DBSU	18.51
**4526**	allXL	DD	372	386	DBSU	25.49
**4526**	allXL	DD	373	386	DBSU	24.33
**4526**	allXL	AD	381	386	DBSU	51.18
**4526**	allXL	BD;DB	357	357	DBSU	18.54
**4526**	allXL	DA	373	319	DBSU	14.13
**4526**	allXL	DA	372	357	DBSU	29.29
**4526**	allXL	BD	381	357	DBSU	45.17

**4526**	allXL	DA	373	357	DBSU	31.0
**4526**	allXL	DA	372	370	DBSU	30.48
**4526**	allXL	DA	373	370	DBSU	30.11
**4526**	allXL	DA	382	373	DBSU	33.46
**4527**	allXL	AA	320	357	BS2G	20.34
**4527**	allXL	CC	320	373	BS2G	16.69
**4527**	allXL	CC	373	382	BS2G	21.02
**4527**	allXL	BB	370	386	BS2G	25.74
**4527**	allXL	DD	373	386	BS2G	28.27
**4527**	allXL	AA	319	357	BS2G	17.84
**4527**	allXL	AA	367	373	BS2G	12.38
**4527**	allXL	CC	370	382	BS2G	18.0
**4527**	allXL	CC	376	382	BS2G	14.8
**4527**	allXL	CC	357	373	BS2G	24.69
**4527**	allXL	AA	320	366	BS2G	11.1
**4527**	allXL	CC	370	376	BS2G	12.68
**4527**	allXL	AA	320	357	DBSU	20.34
**4527**	allXL	AA	357	319	DBSU	17.84
**4527**	allXL	CC	372	319	DBSU	15.61
**4527**	allXL	CC	370	357	DBSU	25.29
**4527**	allXL	CC	382	357	DBSU	23.31
**4527**	allXL	AA	372	370	DBSU	6.38
**4527**	allXL	CC	381	370	DBSU	18.6
**4527**	allXL	AA	373	370	DBSU	9.14
**4527**	allXL	CC	381	372	DBSU	21.38
**4527**	allXL	CC	372	382	DBSU	21.73
**4527**	allXL	DD	372	386	DBSU	30.28
**4527**	allXL	DD	373	386	DBSU	28.27
**4527**	allXL	DA	381	386	DBSU	32.57
**4527**	allXL	BD;DB	357	357	DBSU	18.44
**4527**	allXL	AD	373	319	DBSU	28.08
**4527**	allXL	AD	372	357	DBSU	40.16
**4527**	allXL	CA	381	357	DBSU	33.62
**4527**	allXL	AD	373	357	DBSU	39.66

**4527**	allXL	AD	372	370	DBSU	24.58
**4527**	allXL	DA	373	370	DBSU	24.3
**4527**	allXL	AD	382	373	DBSU	23.88
**4528**	allXL	CC	320	357	BS2G	25.46
**4528**	allXL	CC	320	373	BS2G	21.23
**4528**	allXL	DD	373	382	BS2G	19.78
**4528**	allXL	DD	370	386	BS2G	24.78
**4528**	allXL	AA	373	386	BS2G	20.39
**4528**	allXL	AA	319	357	BS2G	26.17
**4528**	allXL	BB	367	373	BS2G	13.4
**4528**	allXL	DD	370	382	BS2G	20.6
**4528**	allXL	CC	376	382	BS2G	14.8
**4528**	allXL	BB	357	373	BS2G	31.24
**4528**	allXL	AA	320	366	BS2G	22.66
**4528**	allXL	DD	370	376	BS2G	16.4
**4528**	allXL	CC	320	357	DBSU	25.46
**4528**	allXL	AA	357	319	DBSU	26.17
**4528**	allXL	CC	372	319	DBSU	17.37
**4528**	allXL	DD	370	357	DBSU	27.33
**4528**	allXL	BB	382	357	DBSU	26.63
**4528**	allXL	AA	372	370	DBSU	6.05
**4528**	allXL	DD	381	370	DBSU	20.71
**4528**	allXL	AA	373	370	DBSU	9.12
**4528**	allXL	DD	381	372	DBSU	19.91
**4528**	allXL	DD	372	382	DBSU	20.2
**4528**	allXL	AA	372	386	DBSU	23.28
**4528**	allXL	AA	373	386	DBSU	20.39
**4528**	allXL	BD	381	386	DBSU	46.85
**4528**	allXL	BD;DB	357	357	DBSU	18.38
**4528**	allXL	AD	373	319	DBSU	32.41
**4528**	allXL	CB	372	357	DBSU	43.81
**4528**	allXL	BD	381	357	DBSU	38.11
**4528**	allXL	CB	373	357	DBSU	45.6
**4528**	allXL	CB	372	370	DBSU	45.55

**4528**	allXL	CB	373	370	DBSU	45.6
**4528**	allXL	CB	382	373	DBSU	39.22
**4529**	allXL	AA	320	357	BS2G	22.03
**4529**	allXL	DD	320	373	BS2G	22.5
**4529**	allXL	BB	373	382	BS2G	18.26
**4529**	allXL	BB	370	386	BS2G	23.64
**4529**	allXL	BB	373	386	BS2G	29.06
**4529**	allXL	AA	319	357	BS2G	23.98
**4529**	allXL	AA	367	373	BS2G	15.46
**4529**	allXL	BB	370	382	BS2G	15.76
**4529**	allXL	BB	376	382	BS2G	14.2
**4529**	allXL	AA	357	373	BS2G	31.09
**4529**	allXL	DD	320	366	BS2G	22.24
**4529**	allXL	BB	370	376	BS2G	15.12
**4529**	allXL	AA	320	357	DBSU	22.03
**4529**	allXL	AA	357	319	DBSU	23.98
**4529**	allXL	DD	372	319	DBSU	22.33
**4529**	allXL	DD	370	357	DBSU	26.05
**4529**	allXL	BB	382	357	DBSU	32.32
**4529**	allXL	AA	372	370	DBSU	6.02
**4529**	allXL	BB	381	370	DBSU	17.61
**4529**	allXL	AA	373	370	DBSU	9.18
**4529**	allXL	BB	381	372	DBSU	20.52
**4529**	allXL	BB	372	382	DBSU	19.74
**4529**	allXL	AA	372	386	DBSU	28.75
**4529**	allXL	BB	373	386	DBSU	29.06
**4529**	allXL	BC	381	386	DBSU	45.58
**4529**	allXL	BD;DB	357	357	DBSU	18.16
**4529**	allXL	CB	373	319	DBSU	6.42
**4529**	allXL	CB	372	357	DBSU	30.53
**4529**	allXL	AD	381	357	DBSU	36.19
**4529**	allXL	CB	373	357	DBSU	33.05
**4529**	allXL	BC	372	370	DBSU	21.47
**4529**	allXL	BC	373	370	DBSU	18.98

**4529**	allXL	BC	382	373	DBSU	29.69
**4530**	allXL	AA	320	357	BS2G	23.18
**4530**	allXL	DD	320	373	BS2G	19.54
**4530**	allXL	DD	373	382	BS2G	17.17
**4530**	allXL	DD	370	386	BS2G	21.02
**4530**	allXL	AA	373	386	BS2G	24.25
**4530**	allXL	CC	319	357	BS2G	23.4
**4530**	allXL	BB	367	373	BS2G	13.21
**4530**	allXL	DD	370	382	BS2G	13.29
**4530**	allXL	CC	376	382	BS2G	14.63
**4530**	allXL	BB	357	373	BS2G	26.35
**4530**	allXL	DD	320	366	BS2G	12.09
**4530**	allXL	CC	370	376	BS2G	14.89
**4530**	allXL	AA	320	357	DBSU	23.18
**4530**	allXL	CC	357	319	DBSU	23.4
**4530**	allXL	DD	372	319	DBSU	17.06
**4530**	allXL	BB	370	357	DBSU	26.5
**4530**	allXL	DD	382	357	DBSU	26.57
**4530**	allXL	AA	372	370	DBSU	6.03
**4530**	allXL	DD	381	370	DBSU	13.4
**4530**	allXL	AA	373	370	DBSU	9.06
**4530**	allXL	DD	381	372	DBSU	16.69
**4530**	allXL	CC	372	382	DBSU	17.9
**4530**	allXL	AA	372	386	DBSU	21.91
**4530**	allXL	AA	373	386	DBSU	24.25
**4530**	allXL	DA	381	386	DBSU	62.61
**4530**	allXL	BD;DB	357	357	DBSU	18.16
**4530**	allXL	AD	373	319	DBSU	17.62
**4530**	allXL	BD	372	357	DBSU	39.48
**4530**	allXL	DB	381	357	DBSU	46.07
**4530**	allXL	BD	373	357	DBSU	40.88
**4530**	allXL	DA	372	370	DBSU	35.26
**4530**	allXL	AD	373	370	DBSU	31.84
**4530**	allXL	AD	382	373	DBSU	40.61

**4531**	allXL	AA	320	357	BS2G	24.62
**4531**	allXL	BB	320	373	BS2G	20.43
**4531**	allXL	AA	373	382	BS2G	20.71
**4531**	allXL	AA	370	386	BS2G	26.91
**4531**	allXL	AA	373	386	BS2G	23.96
**4531**	allXL	AA	319	357	BS2G	27.71
**4531**	allXL	AA	367	373	BS2G	13.83
**4531**	allXL	DD	370	382	BS2G	22.66
**4531**	allXL	CC	376	382	BS2G	14.8
**4531**	allXL	DD	357	373	BS2G	24.66
**4531**	allXL	DD	320	366	BS2G	20.9
**4531**	allXL	DD	370	376	BS2G	14.78
**4531**	allXL	AA	320	357	DBSU	24.62
**4531**	allXL	AA	357	319	DBSU	27.71
**4531**	allXL	BB	372	319	DBSU	22.16
**4531**	allXL	DD	370	357	DBSU	21.8
**4531**	allXL	BB	382	357	DBSU	17.87
**4531**	allXL	AA	372	370	DBSU	6.12
**4531**	allXL	DD	381	370	DBSU	21.72
**4531**	allXL	AA	373	370	DBSU	9.15
**4531**	allXL	DD	381	372	DBSU	21.62
**4531**	allXL	AA	372	382	DBSU	21.14
**4531**	allXL	AA	372	386	DBSU	22.98
**4531**	allXL	AA	373	386	DBSU	23.96
**4531**	allXL	DB	381	386	DBSU	52.79
**4531**	allXL	BD;DB	357	357	DBSU	17.6
**4531**	allXL	AD	373	319	DBSU	26.43
**4531**	allXL	DB	372	357	DBSU	39.94
**4531**	allXL	BD	381	357	DBSU	30.82
**4531**	allXL	DB	373	357	DBSU	37.84
**4531**	allXL	AD	372	370	DBSU	44.64
**4531**	allXL	AD	373	370	DBSU	45.15
**4531**	allXL	DA	382	373	DBSU	47.44
**4532**	allXL	CC	320	357	BS2G	20.55

**4532**	allXL	AA	320	373	BS2G	24.47
**4532**	allXL	AA	373	382	BS2G	16.58
**4532**	allXL	AA	370	386	BS2G	15.54
**4532**	allXL	AA	373	386	BS2G	20.58
**4532**	allXL	CC	319	357	BS2G	19.85
**4532**	allXL	DD	367	373	BS2G	12.26
**4532**	allXL	AA	370	382	BS2G	16.43
**4532**	allXL	CC	376	382	BS2G	13.49
**4532**	allXL	BB	357	373	BS2G	27.7
**4532**	allXL	AA	320	366	BS2G	24.51
**4532**	allXL	DD	370	376	BS2G	14.05
**4532**	allXL	CC	320	357	DBSU	20.55
**4532**	allXL	CC	357	319	DBSU	19.85
**4532**	allXL	AA	372	319	DBSU	25.81
**4532**	allXL	BB	370	357	DBSU	28.92
**4532**	allXL	BB	382	357	DBSU	32.4
**4532**	allXL	AA	372	370	DBSU	6.36
**4532**	allXL	AA	381	370	DBSU	18.73
**4532**	allXL	AA	373	370	DBSU	9.03
**4532**	allXL	AA	381	372	DBSU	19.16
**4532**	allXL	AA	372	382	DBSU	16.99
**4532**	allXL	AA	372	386	DBSU	18.75
**4532**	allXL	AA	373	386	DBSU	20.58
**4532**	allXL	BD	381	386	DBSU	50.44
**4532**	allXL	BD;DB	357	357	DBSU	18.2
**4532**	allXL	AD	373	319	DBSU	26.55
**4532**	allXL	BD	372	357	DBSU	45.94
**4532**	allXL	DB	381	357	DBSU	40.77
**4532**	allXL	DB	373	357	DBSU	44.0
**4532**	allXL	AD	372	370	DBSU	42.04
**4532**	allXL	AD	373	370	DBSU	44.86
**4532**	allXL	DB	382	373	DBSU	52.93
**4533**	allXL	CC	320	357	BS2G	24.77
**4533**	allXL	CC	320	373	BS2G	20.92

**4533**	allXL	DD	373	382	BS2G	18.65
**4533**	allXL	DD	370	386	BS2G	29.57
**4533**	allXL	DD	373	386	BS2G	26.44
**4533**	allXL	AA	319	357	BS2G	26.3
**4533**	allXL	AA	367	373	BS2G	10.93
**4533**	allXL	DD	370	382	BS2G	24.49
**4533**	allXL	BB	376	382	BS2G	14.81
**4533**	allXL	BB	357	373	BS2G	24.83
**4533**	allXL	DD	320	366	BS2G	22.95
**4533**	allXL	CC	370	376	BS2G	16.52
**4533**	allXL	CC	320	357	DBSU	24.77
**4533**	allXL	AA	357	319	DBSU	26.3
**4533**	allXL	CC	372	319	DBSU	21.93
**4533**	allXL	CC	370	357	DBSU	25.72
**4533**	allXL	BB	382	357	DBSU	21.52
**4533**	allXL	AA	372	370	DBSU	6.4
**4533**	allXL	DD	381	370	DBSU	24.83
**4533**	allXL	DD	373	370	DBSU	8.91
**4533**	allXL	DD	381	372	DBSU	18.68
**4533**	allXL	DD	372	382	DBSU	18.98
**4533**	allXL	DD	372	386	DBSU	25.2
**4533**	allXL	DD	373	386	DBSU	26.44
**4533**	allXL	AD	381	386	DBSU	53.06
**4533**	allXL	BD;DB	357	357	DBSU	17.98
**4533**	allXL	AD	373	319	DBSU	37.37
**4533**	allXL	BD	372	357	DBSU	40.05
**4533**	allXL	BD	381	357	DBSU	25.65
**4533**	allXL	BD	373	357	DBSU	38.35
**4533**	allXL	AD	372	370	DBSU	49.45
**4533**	allXL	AD	373	370	DBSU	47.92
**4533**	allXL	AD	382	373	DBSU	42.75
**4534**	allXL	AA	320	357	BS2G	25.89
**4534**	allXL	DD	320	373	BS2G	20.51
**4534**	allXL	DD	373	382	BS2G	19.77

**4534**	allXL	CC	370	386	BS2G	25.3
**4534**	allXL	AA	373	386	BS2G	25.34
**4534**	allXL	AA	319	357	BS2G	25.59
**4534**	allXL	AA	367	373	BS2G	13.73
**4534**	allXL	CC	370	382	BS2G	22.32
**4534**	allXL	CC	376	382	BS2G	14.11
**4534**	allXL	CC	357	373	BS2G	20.34
**4534**	allXL	DD	320	366	BS2G	20.46
**4534**	allXL	CC	370	376	BS2G	16.61
**4534**	allXL	AA	320	357	DBSU	25.89
**4534**	allXL	AA	357	319	DBSU	25.59
**4534**	allXL	DD	372	319	DBSU	17.21
**4534**	allXL	CC	370	357	DBSU	19.56
**4534**	allXL	BB	382	357	DBSU	19.32
**4534**	allXL	AA	372	370	DBSU	5.97
**4534**	allXL	CC	381	370	DBSU	22.59
**4534**	allXL	AA	373	370	DBSU	8.94
**4534**	allXL	DD	381	372	DBSU	17.81
**4534**	allXL	DD	372	382	DBSU	19.71
**4534**	allXL	CC	372	386	DBSU	26.89
**4534**	allXL	AA	373	386	DBSU	25.34
**4534**	allXL	CA	381	386	DBSU	27.93
**4534**	allXL	BD;DB	357	357	DBSU	18.03
**4534**	allXL	AD	373	319	DBSU	24.69
**4534**	allXL	CA	372	357	DBSU	32.27
**4534**	allXL	CA	381	357	DBSU	22.83
**4534**	allXL	CA	373	357	DBSU	29.05
**4534**	allXL	DA	372	370	DBSU	21.48
**4534**	allXL	DA	373	370	DBSU	22.46
**4534**	allXL	DA	382	373	DBSU	16.12
**4535**	allXL	AA	320	357	BS2G	24.82
**4535**	allXL	CC	320	373	BS2G	20.83
**4535**	allXL	AA	373	382	BS2G	20.4
**4535**	allXL	AA	370	386	BS2G	20.53

**4535**	allXL	AA	373	386	BS2G	24.04
**4535**	allXL	AA	319	357	BS2G	22.89
**4535**	allXL	AA	367	373	BS2G	13.02
**4535**	allXL	DD	370	382	BS2G	18.33
**4535**	allXL	AA	376	382	BS2G	15.76
**4535**	allXL	DD	357	373	BS2G	33.78
**4535**	allXL	CC	320	366	BS2G	25.89
**4535**	allXL	CC	370	376	BS2G	14.57
**4535**	allXL	AA	320	357	DBSU	24.82
**4535**	allXL	AA	357	319	DBSU	22.89
**4535**	allXL	CC	372	319	DBSU	17.89
**4535**	allXL	BB	370	357	DBSU	28.16
**4535**	allXL	AA	382	357	DBSU	46.72
**4535**	allXL	AA	372	370	DBSU	6.32
**4535**	allXL	DD	381	370	DBSU	18.0
**4535**	allXL	CC	373	370	DBSU	9.0
**4535**	allXL	DD	381	372	DBSU	20.34
**4535**	allXL	AA	372	382	DBSU	20.0
**4535**	allXL	AA	372	386	DBSU	21.9
**4535**	allXL	AA	373	386	DBSU	24.04
**4535**	allXL	BC	381	386	DBSU	52.44
**4535**	allXL	BD;DB	357	357	DBSU	19.36
**4535**	allXL	BC	373	319	DBSU	35.93
**4535**	allXL	BC	372	357	DBSU	35.94
**4535**	allXL	AC	381	357	DBSU	51.1
**4535**	allXL	BC	373	357	DBSU	37.26
**4535**	allXL	BC	372	370	DBSU	11.27
**4535**	allXL	BC	373	370	DBSU	14.74
**4535**	allXL	CB	382	373	DBSU	30.67
**4536**	allXL	AA	320	357	BS2G	24.54
**4536**	allXL	DD	320	373	BS2G	38.91
**4536**	allXL	DD	373	382	BS2G	17.73
**4536**	allXL	DD	370	386	BS2G	16.47
**4536**	allXL	DD	373	386	BS2G	19.28

**4536**	allXL	AA	319	357	BS2G	24.58
**4536**	allXL	AA	367	373	BS2G	12.78
**4536**	allXL	DD	370	382	BS2G	20.68
**4536**	allXL	BB	376	382	BS2G	13.59
**4536**	allXL	BB	357	373	BS2G	36.2
**4536**	allXL	AA	320	366	BS2G	25.91
**4536**	allXL	CC	370	376	BS2G	14.45
**4536**	allXL	AA	320	357	DBSU	24.54
**4536**	allXL	AA	357	319	DBSU	24.58
**4536**	allXL	DD	372	319	DBSU	38.12
**4536**	allXL	DD	370	357	DBSU	27.92
**4536**	allXL	DD	382	357	DBSU	45.43
**4536**	allXL	AA	372	370	DBSU	6.26
**4536**	allXL	BB	381	370	DBSU	21.25
**4536**	allXL	AA	373	370	DBSU	9.12
**4536**	allXL	BB	381	372	DBSU	20.87
**4536**	allXL	DD	372	382	DBSU	20.63
**4536**	allXL	DD	372	386	DBSU	20.35
**4536**	allXL	DD	373	386	DBSU	19.28
**4536**	allXL	DA	381	386	DBSU	58.16
**4536**	allXL	BD;DB	357	357	DBSU	18.43
**4536**	allXL	CB	373	319	DBSU	11.61
**4536**	allXL	CB	372	357	DBSU	36.94
**4536**	allXL	AD	381	357	DBSU	61.49
**4536**	allXL	CB	373	357	DBSU	39.24
**4536**	allXL	BC	372	370	DBSU	51.89
**4536**	allXL	BC	373	370	DBSU	50.17
**4536**	allXL	BC	382	373	DBSU	44.53
**4537**	allXL	AA	320	357	BS2G	21.33
**4537**	allXL	AA	320	373	BS2G	38.57
**4537**	allXL	AA	373	382	BS2G	21.44
**4537**	allXL	CC	370	386	BS2G	17.47
**4537**	allXL	AA	373	386	BS2G	21.77
**4537**	allXL	AA	319	357	BS2G	23.96

**4537**	allXL	AA	367	373	BS2G	12.61
**4537**	allXL	CC	370	382	BS2G	20.46
**4537**	allXL	CC	376	382	BS2G	14.72
**4537**	allXL	BB	357	373	BS2G	34.15
**4537**	allXL	AA	320	366	BS2G	25.72
**4537**	allXL	CC	370	376	BS2G	12.37
**4537**	allXL	AA	320	357	DBSU	21.33
**4537**	allXL	AA	357	319	DBSU	23.96
**4537**	allXL	BB	372	319	DBSU	38.57
**4537**	allXL	AA	370	357	DBSU	32.4
**4537**	allXL	AA	382	357	DBSU	42.03
**4537**	allXL	AA	372	370	DBSU	6.57
**4537**	allXL	DD	381	370	DBSU	21.57
**4537**	allXL	CC	373	370	DBSU	8.96
**4537**	allXL	DD	381	372	DBSU	19.03
**4537**	allXL	DD	372	382	DBSU	21.45
**4537**	allXL	CC	372	386	DBSU	22.72
**4537**	allXL	AA	373	386	DBSU	21.77
**4537**	allXL	BC	381	386	DBSU	69.72
**4537**	allXL	BD;DB	357	357	DBSU	18.42
**4537**	allXL	AD	373	319	DBSU	25.49
**4537**	allXL	AC	372	357	DBSU	40.59
**4537**	allXL	DB	381	357	DBSU	57.11
**4537**	allXL	AC	373	357	DBSU	42.45
**4537**	allXL	BC	372	370	DBSU	59.13
**4537**	allXL	BC	373	370	DBSU	60.89
**4537**	allXL	AD	382	373	DBSU	63.09
**4538**	allXL	AA	320	357	BS2G	24.34
**4538**	allXL	DD	320	373	BS2G	31.44
**4538**	allXL	DD	373	382	BS2G	17.7
**4538**	allXL	DD	370	386	BS2G	22.66
**4538**	allXL	BB	373	386	BS2G	27.14
**4538**	allXL	AA	319	357	BS2G	26.79
**4538**	allXL	BB	367	373	BS2G	12.05

**4538**	allXL	DD	370	382	BS2G	12.09
**4538**	allXL	BB	376	382	BS2G	12.67
**4538**	allXL	BB	357	373	BS2G	27.75
**4538**	allXL	AA	320	366	BS2G	25.36
**4538**	allXL	DD	370	376	BS2G	14.54
**4538**	allXL	AA	320	357	DBSU	24.34
**4538**	allXL	AA	357	319	DBSU	26.79
**4538**	allXL	DD	372	319	DBSU	34.1
**4538**	allXL	DD	370	357	DBSU	28.52
**4538**	allXL	DD	382	357	DBSU	29.07
**4538**	allXL	AA	372	370	DBSU	6.07
**4538**	allXL	DD	381	370	DBSU	9.45
**4538**	allXL	AA	373	370	DBSU	9.02
**4538**	allXL	DD	381	372	DBSU	14.07
**4538**	allXL	DD	372	382	DBSU	16.84
**4538**	allXL	DD	372	386	DBSU	28.1
**4538**	allXL	BB	373	386	DBSU	27.14
**4538**	allXL	BD	381	386	DBSU	66.46
**4538**	allXL	BD;DB	357	357	DBSU	18.65
**4538**	allXL	DA	373	319	DBSU	30.98
**4538**	allXL	BD	372	357	DBSU	41.78
**4538**	allXL	DB	381	357	DBSU	40.07
**4538**	allXL	BD	373	357	DBSU	40.18
**4538**	allXL	DA	372	370	DBSU	43.67
**4538**	allXL	DA	373	370	DBSU	40.55
**4538**	allXL	DA	382	373	DBSU	49.88
**4539**	allXL	AA	320	357	BS2G	25.6
**4539**	allXL	BB	320	373	BS2G	19.12
**4539**	allXL	DD	373	382	BS2G	16.14
**4539**	allXL	DD	370	386	BS2G	8.61
**4539**	allXL	DD	373	386	BS2G	16.28
**4539**	allXL	AA	319	357	BS2G	24.82
**4539**	allXL	AA	367	373	BS2G	12.97
**4539**	allXL	DD	370	382	BS2G	13.96

**4539**	allXL	AA	376	382	BS2G	13.69
**4539**	allXL	DD	357	373	BS2G	27.96
**4539**	allXL	DD	320	366	BS2G	23.47
**4539**	allXL	BB	370	376	BS2G	16.03
**4539**	allXL	AA	320	357	DBSU	25.6
**4539**	allXL	AA	357	319	DBSU	24.82
**4539**	allXL	BB	372	319	DBSU	21.05
**4539**	allXL	DD	370	357	DBSU	24.04
**4539**	allXL	DD	382	357	DBSU	16.9
**4539**	allXL	AA	372	370	DBSU	6.29
**4539**	allXL	DD	381	370	DBSU	15.65
**4539**	allXL	AA	373	370	DBSU	9.28
**4539**	allXL	DD	381	372	DBSU	15.69
**4539**	allXL	DD	372	382	DBSU	15.67
**4539**	allXL	DD	372	386	DBSU	14.9
**4539**	allXL	DD	373	386	DBSU	16.28
**4539**	allXL	AD	381	386	DBSU	56.74
**4539**	allXL	BD;DB	357	357	DBSU	18.7
**4539**	allXL	AD	373	319	DBSU	31.45
**4539**	allXL	CA	372	357	DBSU	30.94
**4539**	allXL	AC	381	357	DBSU	33.98
**4539**	allXL	CA	373	357	DBSU	28.65
**4539**	allXL	BC	372	370	DBSU	47.34
**4539**	allXL	BC	373	370	DBSU	44.19
**4539**	allXL	BC	382	373	DBSU	44.72
**4540**	allXL	CC	320	357	BS2G	21.4
**4540**	allXL	DD	320	373	BS2G	18.11
**4540**	allXL	BB	373	382	BS2G	16.09
**4540**	allXL	CC	370	386	BS2G	19.93
**4540**	allXL	BB	373	386	BS2G	23.1
**4540**	allXL	CC	319	357	BS2G	20.41
**4540**	allXL	AA	367	373	BS2G	14.01
**4540**	allXL	CC	370	382	BS2G	20.58
**4540**	allXL	BB	376	382	BS2G	13.38

**4540**	allXL	DD	357	373	BS2G	24.66
**4540**	allXL	DD	320	366	BS2G	26.41
**4540**	allXL	CC	370	376	BS2G	15.08
**4540**	allXL	CC	320	357	DBSU	21.4
**4540**	allXL	CC	357	319	DBSU	20.41
**4540**	allXL	DD	372	319	DBSU	17.27
**4540**	allXL	DD	370	357	DBSU	22.13
**4540**	allXL	BB	382	357	DBSU	19.99
**4540**	allXL	AA	372	370	DBSU	6.03
**4540**	allXL	CC	381	370	DBSU	21.22
**4540**	allXL	AA	373	370	DBSU	9.3
**4540**	allXL	BB	381	372	DBSU	18.36
**4540**	allXL	BB	372	382	DBSU	16.31
**4540**	allXL	BB	372	386	DBSU	21.43
**4540**	allXL	BB	373	386	DBSU	23.1
**4540**	allXL	BC	381	386	DBSU	26.89
**4540**	allXL	BD;DB	357	357	DBSU	18.38
**4540**	allXL	CB	373	319	DBSU	19.37
**4540**	allXL	CA	372	357	DBSU	34.92
**4540**	allXL	DB	381	357	DBSU	33.64
**4540**	allXL	BC	373	357	DBSU	31.46
**4540**	allXL	BC	372	370	DBSU	36.47
**4540**	allXL	BC	373	370	DBSU	33.75
**4540**	allXL	BC	382	373	DBSU	28.43
**4541**	allXL	CC	320	357	BS2G	25.87
**4541**	allXL	CC	320	373	BS2G	17.63
**4541**	allXL	CC	373	382	BS2G	20.03
**4541**	allXL	AA	370	386	BS2G	29.88
**4541**	allXL	AA	373	386	BS2G	26.4
**4541**	allXL	AA	319	357	BS2G	24.82
**4541**	allXL	AA	367	373	BS2G	12.36
**4541**	allXL	CC	370	382	BS2G	18.37
**4541**	allXL	CC	376	382	BS2G	15.82
**4541**	allXL	BB	357	373	BS2G	26.25

**4541**	allXL	BB	320	366	BS2G	21.66
**4541**	allXL	CC	370	376	BS2G	12.65
**4541**	allXL	CC	320	357	DBSU	25.87
**4541**	allXL	AA	357	319	DBSU	24.82
**4541**	allXL	CC	372	319	DBSU	17.65
**4541**	allXL	BB	370	357	DBSU	24.88
**4541**	allXL	CC	382	357	DBSU	16.59
**4541**	allXL	AA	372	370	DBSU	6.15
**4541**	allXL	CC	381	370	DBSU	16.91
**4541**	allXL	AA	373	370	DBSU	9.1
**4541**	allXL	CC	381	372	DBSU	18.83
**4541**	allXL	CC	372	382	DBSU	21.28
**4541**	allXL	AA	372	386	DBSU	26.78
**4541**	allXL	AA	373	386	DBSU	26.4
**4541**	allXL	BC	381	386	DBSU	41.12
**4541**	allXL	BD;DB	357	357	DBSU	17.86
**4541**	allXL	AD	373	319	DBSU	20.12
**4541**	allXL	BD	372	357	DBSU	34.59
**4541**	allXL	CB	381	357	DBSU	29.87
**4541**	allXL	CB	373	357	DBSU	33.76
**4541**	allXL	DA	372	370	DBSU	37.59
**4541**	allXL	DA	373	370	DBSU	40.14
**4541**	allXL	CB	382	373	DBSU	39.69
**4542**	allXL	AA	320	357	BS2G	25.07
**4542**	allXL	AA	320	373	BS2G	30.68
**4542**	allXL	AA	373	382	BS2G	18.92
**4542**	allXL	AA	370	386	BS2G	23.52
**4542**	allXL	AA	373	386	BS2G	21.11
**4542**	allXL	AA	319	357	BS2G	23.53
**4542**	allXL	AA	367	373	BS2G	13.51
**4542**	allXL	CC	370	382	BS2G	21.15
**4542**	allXL	BB	376	382	BS2G	13.94
**4542**	allXL	BB	357	373	BS2G	9.66
**4542**	allXL	AA	320	366	BS2G	17.59

**4542**	allXL	CC	370	376	BS2G	14.85
**4542**	allXL	AA	320	357	DBSU	25.07
**4542**	allXL	AA	357	319	DBSU	23.53
**4542**	allXL	AA	372	319	DBSU	29.78
**4542**	allXL	BB	370	357	DBSU	17.96
**4542**	allXL	BB	382	357	DBSU	20.78
**4542**	allXL	AA	372	370	DBSU	6.21
**4542**	allXL	CC	381	370	DBSU	23.39
**4542**	allXL	AA;CC	373	370	DBSU	9.18
**4542**	allXL	DD	381	372	DBSU	21.87
**4542**	allXL	AA	372	382	DBSU	22.0
**4542**	allXL	AA	372	386	DBSU	22.77
**4542**	allXL	AA	373	386	DBSU	21.11
**4542**	allXL	CB	381	386	DBSU	60.89
**4542**	allXL	BD;DB	357	357	DBSU	19.12
**4542**	allXL	CB	373	319	DBSU	13.36
**4542**	allXL	BD	372	357	DBSU	13.16
**4542**	allXL	BD	381	357	DBSU	18.47
**4542**	allXL	BD	373	357	DBSU	11.03
**4542**	allXL	DB	372	370	DBSU	41.96
**4542**	allXL	BD	373	370	DBSU	41.65
**4542**	allXL	BD	382	373	DBSU	47.15
**4543**	allXL	AA	320	357	BS2G	26.27
**4543**	allXL	CC	320	373	BS2G	26.43
**4543**	allXL	DD	373	382	BS2G	18.44
**4543**	allXL	DD	370	386	BS2G	21.05
**4543**	allXL	BB	373	386	BS2G	25.78
**4543**	allXL	AA	319	357	BS2G	27.62
**4543**	allXL	AA	367	373	BS2G	13.35
**4543**	allXL	DD	370	382	BS2G	12.16
**4543**	allXL	BB	376	382	BS2G	14.14
**4543**	allXL	AA	357	373	BS2G	31.42
**4543**	allXL	CC	320	366	BS2G	24.65
**4543**	allXL	CC	370	376	BS2G	14.07

**4543**	allXL	AA	320	357	DBSU	26.27
**4543**	allXL	AA	357	319	DBSU	27.62
**4543**	allXL	CC	372	319	DBSU	24.71
**4543**	allXL	CC	370	357	DBSU	30.81
**4543**	allXL	DD	382	357	DBSU	32.96
**4543**	allXL	AA	372	370	DBSU	6.19
**4543**	allXL	DD	381	370	DBSU	13.15
**4543**	allXL	AA	373	370	DBSU	9.09
**4543**	allXL	DD	381	372	DBSU	18.34
**4543**	allXL	DD	372	382	DBSU	17.97
**4543**	allXL	AA	372	386	DBSU	24.12
**4543**	allXL	BB	373	386	DBSU	25.78
**4543**	allXL	DA	381	386	DBSU	55.91
**4543**	allXL	BD;DB	357	357	DBSU	18.75
**4543**	allXL	CB	373	319	DBSU	32.76
**4543**	allXL	CB	372	357	DBSU	34.06
**4543**	allXL	CB	381	357	DBSU	38.94
**4543**	allXL	CB	373	357	DBSU	34.82
**4543**	allXL	CB	372	370	DBSU	30.27
**4543**	allXL	CB	373	370	DBSU	27.23
**4543**	allXL	CB	382	373	DBSU	42.93
**4544**	allXL	AA	320	357	BS2G	23.11
**4544**	allXL	BB	320	373	BS2G	20.08
**4544**	allXL	DD	373	382	BS2G	21.88
**4544**	allXL	DD	370	386	BS2G	27.13
**4544**	allXL	DD	373	386	BS2G	26.65
**4544**	allXL	AA	319	357	BS2G	20.65
**4544**	allXL	AA	367	373	BS2G	13.52
**4544**	allXL	DD	370	382	BS2G	26.49
**4544**	allXL	CC	376	382	BS2G	13.35
**4544**	allXL	AA	357	373	BS2G	25.37
**4544**	allXL	BB	320	366	BS2G	27.69
**4544**	allXL	CC	370	376	BS2G	14.66
**4544**	allXL	AA	320	357	DBSU	23.11

**4544**	allXL	AA	357	319	DBSU	20.65
**4544**	allXL	BB	372	319	DBSU	22.07
**4544**	allXL	AA	370	357	DBSU	27.26
**4544**	allXL	AA	382	357	DBSU	18.82
**4544**	allXL	AA	372	370	DBSU	6.28
**4544**	allXL	DD	381	370	DBSU	25.83
**4544**	allXL	AA	373	370	DBSU	9.2
**4544**	allXL	DD	381	372	DBSU	22.23
**4544**	allXL	DD	372	382	DBSU	23.74
**4544**	allXL	DD	372	386	DBSU	27.11
**4544**	allXL	DD	373	386	DBSU	26.65
**4544**	allXL	CA	381	386	DBSU	57.03
**4544**	allXL	BD;DB	357	357	DBSU	18.54
**4544**	allXL	CB	373	319	DBSU	29.35
**4544**	allXL	AC	372	357	DBSU	43.51
**4544**	allXL	AC	381	357	DBSU	38.95
**4544**	allXL	AC	373	357	DBSU	44.39
**4544**	allXL	BC	372	370	DBSU	43.31
**4544**	allXL	BC	373	370	DBSU	42.84
**4544**	allXL	BC	382	373	DBSU	41.83
**4545**	allXL	CC	320	357	BS2G	23.68
**4545**	allXL	CC	320	373	BS2G	31.26
**4545**	allXL	DD	373	382	BS2G	19.94
**4545**	allXL	DD	370	386	BS2G	25.92
**4545**	allXL	BB	373	386	BS2G	26.76
**4545**	allXL	CC	319	357	BS2G	26.39
**4545**	allXL	BB	367	373	BS2G	12.18
**4545**	allXL	DD	370	382	BS2G	20.91
**4545**	allXL	BB	376	382	BS2G	13.85
**4545**	allXL	BB	357	373	BS2G	29.18
**4545**	allXL	CC	320	366	BS2G	21.75
**4545**	allXL	CC	370	376	BS2G	15.73
**4545**	allXL	CC	320	357	DBSU	23.68
**4545**	allXL	CC	357	319	DBSU	26.39

**4545**	allXL	CC	372	319	DBSU	30.5
**4545**	allXL	BB	370	357	DBSU	27.87
**4545**	allXL	BB	382	357	DBSU	29.6
**4545**	allXL	AA	372	370	DBSU	6.04
**4545**	allXL	DD	381	370	DBSU	18.52
**4545**	allXL	AA	373	370	DBSU	9.04
**4545**	allXL	DD	381	372	DBSU	18.58
**4545**	allXL	DD	372	382	DBSU	21.4
**4545**	allXL	DD	372	386	DBSU	28.69
**4545**	allXL	BB	373	386	DBSU	26.76
**4545**	allXL	BC	381	386	DBSU	38.48
**4545**	allXL	BD;DB	357	357	DBSU	18.61
**4545**	allXL	DA	373	319	DBSU	33.8
**4545**	allXL	AC	372	357	DBSU	44.73
**4545**	allXL	BD	381	357	DBSU	42.73
**4545**	allXL	BD	373	357	DBSU	46.7
**4545**	allXL	CB	372	370	DBSU	39.81
**4545**	allXL	CB	373	370	DBSU	40.43
**4545**	allXL	CB	382	373	DBSU	29.03
**4546**	allXL	CC	320	357	BS2G	24.97
**4546**	allXL	BB	320	373	BS2G	14.44
**4546**	allXL	CC	373	382	BS2G	16.72
**4546**	allXL	DD	370	386	BS2G	18.04
**4546**	allXL	DD	373	386	BS2G	20.0
**4546**	allXL	CC	319	357	BS2G	26.06
**4546**	allXL	AA	367	373	BS2G	11.82
**4546**	allXL	AA	370	382	BS2G	19.64
**4546**	allXL	CC	376	382	BS2G	14.51
**4546**	allXL	AA	357	373	BS2G	19.57
**4546**	allXL	BB	320	366	BS2G	18.82
**4546**	allXL	AA	370	376	BS2G	15.76
**4546**	allXL	CC	320	357	DBSU	24.97
**4546**	allXL	CC	357	319	DBSU	26.06
**4546**	allXL	BB	372	319	DBSU	15.64

**4546**	allXL	AA	370	357	DBSU	24.11
**4546**	allXL	BB	382	357	DBSU	28.37
**4546**	allXL	AA	372	370	DBSU	6.35
**4546**	allXL	AA	381	370	DBSU	20.7
**4546**	allXL	AA	373	370	DBSU	9.05
**4546**	allXL	DD	381	372	DBSU	15.62
**4546**	allXL	DD	372	382	DBSU	15.6
**4546**	allXL	DD	372	386	DBSU	16.47
**4546**	allXL	DD	373	386	DBSU	20.0
**4546**	allXL	DB	381	386	DBSU	44.73
**4546**	allXL	BD;DB	357	357	DBSU	18.04
**4546**	allXL	DA	373	319	DBSU	35.67
**4546**	allXL	AC	372	357	DBSU	35.62
**4546**	allXL	BD	381	357	DBSU	32.93
**4546**	allXL	AC	373	357	DBSU	34.34
**4546**	allXL	AD	372	370	DBSU	50.85
**4546**	allXL	AD	373	370	DBSU	47.77
**4546**	allXL	BD	382	373	DBSU	47.88
**4547**	allXL	AA	320	357	BS2G	18.44
**4547**	allXL	DD	320	373	BS2G	21.61
**4547**	allXL	DD	373	382	BS2G	17.52
**4547**	allXL	DD	370	386	BS2G	20.34
**4547**	allXL	AA	373	386	BS2G	25.92
**4547**	allXL	AA	319	357	BS2G	19.44
**4547**	allXL	AA	367	373	BS2G	11.47
**4547**	allXL	DD	370	382	BS2G	12.86
**4547**	allXL	CC	376	382	BS2G	15.69
**4547**	allXL	BB	357	373	BS2G	24.35
**4547**	allXL	DD	320	366	BS2G	15.26
**4547**	allXL	DD	370	376	BS2G	16.05
**4547**	allXL	AA	320	357	DBSU	18.44
**4547**	allXL	AA	357	319	DBSU	19.44
**4547**	allXL	DD	372	319	DBSU	22.57
**4547**	allXL	BB	370	357	DBSU	21.12

**4547**	allXL	DD	382	357	DBSU	26.6
**4547**	allXL	AA	372	370	DBSU	6.44
**4547**	allXL	DD	381	370	DBSU	13.82
**4547**	allXL	AA	373	370	DBSU	9.36
**4547**	allXL	DD	381	372	DBSU	17.12
**4547**	allXL	DD	372	382	DBSU	17.67
**4547**	allXL	DD	372	386	DBSU	26.93
**4547**	allXL	AA	373	386	DBSU	25.92
**4547**	allXL	BD	381	386	DBSU	26.47
**4547**	allXL	BD;DB	357	357	DBSU	18.24
**4547**	allXL	AD	373	319	DBSU	21.77
**4547**	allXL	BD	372	357	DBSU	32.94
**4547**	allXL	BD	381	357	DBSU	23.85
**4547**	allXL	BD	373	357	DBSU	32.42
**4547**	allXL	DA	372	370	DBSU	36.03
**4547**	allXL	DA	373	370	DBSU	33.69
**4547**	allXL	DA	382	373	DBSU	40.2
**4548**	allXL	AA	320	357	BS2G	26.18
**4548**	allXL	CC	320	373	BS2G	26.15
**4548**	allXL	AA	373	382	BS2G	20.26
**4548**	allXL	DD	370	386	BS2G	28.79
**4548**	allXL	DD	373	386	BS2G	23.64
**4548**	allXL	AA	319	357	BS2G	28.26
**4548**	allXL	AA	367	373	BS2G	10.08
**4548**	allXL	AA	370	382	BS2G	22.99
**4548**	allXL	BB	376	382	BS2G	15.38
**4548**	allXL	BB	357	373	BS2G	27.63
**4548**	allXL	CC	320	366	BS2G	29.03
**4548**	allXL	CC	370	376	BS2G	16.38
**4548**	allXL	AA	320	357	DBSU	26.18
**4548**	allXL	AA	357	319	DBSU	28.26
**4548**	allXL	DD	372	319	DBSU	23.6
**4548**	allXL	DD	370	357	DBSU	23.72
**4548**	allXL	BB	382	357	DBSU	23.44

**4548**	allXL	CC	372	370	DBSU	6.57
**4548**	allXL	AA	381	370	DBSU	21.94
**4548**	allXL	DD	373	370	DBSU	8.65
**4548**	allXL	AA	381	372	DBSU	21.61
**4548**	allXL	BB	372	382	DBSU	22.16
**4548**	allXL	DD	372	386	DBSU	23.67
**4548**	allXL	DD	373	386	DBSU	23.64
**4548**	allXL	AD	381	386	DBSU	31.99
**4548**	allXL	BD;DB	357	357	DBSU	18.39
**4548**	allXL	DA	373	319	DBSU	19.05
**4548**	allXL	DA	372	357	DBSU	35.67
**4548**	allXL	BD	381	357	DBSU	33.36
**4548**	allXL	DA	373	357	DBSU	39.21
**4548**	allXL	CB	372	370	DBSU	46.86
**4548**	allXL	AD	373	370	DBSU	48.89
**4548**	allXL	DA	382	373	DBSU	44.19
**4549**	allXL	AA	320	357	BS2G	21.48
**4549**	allXL	DD	320	373	BS2G	22.39
**4549**	allXL	DD	373	382	BS2G	19.06
**4549**	allXL	DD	370	386	BS2G	27.67
**4549**	allXL	DD	373	386	BS2G	23.2
**4549**	allXL	AA	319	357	BS2G	19.41
**4549**	allXL	DD	367	373	BS2G	14.51
**4549**	allXL	DD	370	382	BS2G	25.23
**4549**	allXL	CC	376	382	BS2G	14.51
**4549**	allXL	DD	357	373	BS2G	28.47
**4549**	allXL	CC	320	366	BS2G	15.77
**4549**	allXL	CC	370	376	BS2G	15.54
**4549**	allXL	AA	320	357	DBSU	21.48
**4549**	allXL	AA	357	319	DBSU	19.41
**4549**	allXL	CC	372	319	DBSU	18.09
**4549**	allXL	DD	370	357	DBSU	28.07
**4549**	allXL	DD	382	357	DBSU	46.13
**4549**	allXL	AA	372	370	DBSU	6.13

**4549**	allXL	AA	381	370	DBSU	25.18
**4549**	allXL	DD	373	370	DBSU	8.5
**4549**	allXL	DD	381	372	DBSU	19.23
**4549**	allXL	DD	372	382	DBSU	19.19
**4549**	allXL	DD	372	386	DBSU	21.44
**4549**	allXL	DD	373	386	DBSU	23.2
**4549**	allXL	AD	381	386	DBSU	54.06
**4549**	allXL	BD;DB	357	357	DBSU	19.79
**4549**	allXL	AD	373	319	DBSU	32.96
**4549**	allXL	BD	372	357	DBSU	49.37
**4549**	allXL	DA	381	357	DBSU	61.23
**4549**	allXL	DB	373	357	DBSU	48.14
**4549**	allXL	AD	372	370	DBSU	49.3
**4549**	allXL	AD	373	370	DBSU	45.66
**4549**	allXL	DA	382	373	DBSU	40.62
**4550**	allXL	AA	320	357	BS2G	20.47
**4550**	allXL	DD	320	373	BS2G	18.21
**4550**	allXL	CC	373	382	BS2G	18.92
**4550**	allXL	AA	370	386	BS2G	25.91
**4550**	allXL	AA	373	386	BS2G	23.77
**4550**	allXL	AA	319	357	BS2G	18.72
**4550**	allXL	AA	367	373	BS2G	14.32
**4550**	allXL	CC	370	382	BS2G	24.6
**4550**	allXL	CC	376	382	BS2G	12.9
**4550**	allXL	BB	357	373	BS2G	22.46
**4550**	allXL	BB	320	366	BS2G	11.21
**4550**	allXL	CC	370	376	BS2G	16.22
**4550**	allXL	AA	320	357	DBSU	20.47
**4550**	allXL	AA	357	319	DBSU	18.72
**4550**	allXL	DD	372	319	DBSU	20.7
**4550**	allXL	BB	370	357	DBSU	24.26
**4550**	allXL	BB	382	357	DBSU	27.39
**4550**	allXL	AA	372	370	DBSU	6.08
**4550**	allXL	AA	381	370	DBSU	23.61

**4550**	allXL	AA	373	370	DBSU	9.25
**4550**	allXL	AA	381	372	DBSU	20.4
**4550**	allXL	CC	372	382	DBSU	20.14
**4550**	allXL	DD	372	386	DBSU	25.21
**4550**	allXL	AA	373	386	DBSU	23.77
**4550**	allXL	CB	381	386	DBSU	30.8
**4550**	allXL	BD;DB	357	357	DBSU	19.52
**4550**	allXL	CA	373	319	DBSU	30.73
**4550**	allXL	CA	372	357	DBSU	23.95
**4550**	allXL	CA	381	357	DBSU	13.44
**4550**	allXL	CA	373	357	DBSU	21.45
**4550**	allXL	DA	372	370	DBSU	50.97
**4550**	allXL	DA	373	370	DBSU	49.64
**4550**	allXL	CA	382	373	DBSU	40.88
**4551**	allXL	CC	320	357	BS2G	23.69
**4551**	allXL	BB	320	373	BS2G	18.84
**4551**	allXL	DD	373	382	BS2G	16.94
**4551**	allXL	CC	370	386	BS2G	20.2
**4551**	allXL	DD	373	386	BS2G	18.95
**4551**	allXL	AA	319	357	BS2G	25.68
**4551**	allXL	AA	367	373	BS2G	12.63
**4551**	allXL	CC	370	382	BS2G	17.67
**4551**	allXL	DD	376	382	BS2G	13.38
**4551**	allXL	BB	357	373	BS2G	20.33
**4551**	allXL	BB	320	366	BS2G	12.68
**4551**	allXL	CC	370	376	BS2G	14.76
**4551**	allXL	CC	320	357	DBSU	23.69
**4551**	allXL	AA	357	319	DBSU	25.68
**4551**	allXL	BB	372	319	DBSU	20.07
**4551**	allXL	DD	370	357	DBSU	21.31
**4551**	allXL	AA	382	357	DBSU	34.09
**4551**	allXL	AA	372	370	DBSU	6.36
**4551**	allXL	CC	381	370	DBSU	19.76
**4551**	allXL	CC	373	370	DBSU	9.04

**4551**	allXL	DD	381	372	DBSU	18.43
**4551**	allXL	DD	372	382	DBSU	17.43
**4551**	allXL	DD	372	386	DBSU	19.62
**4551**	allXL	DD	373	386	DBSU	18.95
**4551**	allXL	BC	381	386	DBSU	47.69
**4551**	allXL	BD;DB	357	357	DBSU	18.35
**4551**	allXL	AD	373	319	DBSU	13.61
**4551**	allXL	BD	372	357	DBSU	30.79
**4551**	allXL	CB	381	357	DBSU	34.03
**4551**	allXL	BD	373	357	DBSU	29.82
**4551**	allXL	AD	372	370	DBSU	43.95
**4551**	allXL	AD	373	370	DBSU	44.53
**4551**	allXL	CB	382	373	DBSU	36.46
**4552**	allXL	AA	320	357	BS2G	25.45
**4552**	allXL	DD	320	373	BS2G	15.99
**4552**	allXL	CC	373	382	BS2G	16.47
**4552**	allXL	DD	370	386	BS2G	16.64
**4552**	allXL	BB	373	386	BS2G	20.91
**4552**	allXL	AA	319	357	BS2G	27.35
**4552**	allXL	BB	367	373	BS2G	13.68
**4552**	allXL	DD	370	382	BS2G	14.98
**4552**	allXL	CC	376	382	BS2G	14.42
**4552**	allXL	DD	357	373	BS2G	21.27
**4552**	allXL	BB	320	366	BS2G	21.58
**4552**	allXL	CC	370	376	BS2G	15.07
**4552**	allXL	AA	320	357	DBSU	25.45
**4552**	allXL	AA	357	319	DBSU	27.35
**4552**	allXL	DD	372	319	DBSU	14.64
**4552**	allXL	DD	370	357	DBSU	26.69
**4552**	allXL	BB	382	357	DBSU	22.42
**4552**	allXL	AA	372	370	DBSU	6.15
**4552**	allXL	DD	381	370	DBSU	13.83
**4552**	allXL	BB	373	370	DBSU	9.03
**4552**	allXL	DD	381	372	DBSU	17.69

**4552**	allXL	CC	372	382	DBSU	19.38
**4552**	allXL	CC	372	386	DBSU	22.46
**4552**	allXL	BB	373	386	DBSU	20.91
**4552**	allXL	DA	381	386	DBSU	48.62
**4552**	allXL	BD;DB	357	357	DBSU	19.9
**4552**	allXL	AD	373	319	DBSU	32.0
**4552**	allXL	DA	372	357	DBSU	36.38
**4552**	allXL	BD	381	357	DBSU	28.88
**4552**	allXL	DA	373	357	DBSU	33.97
**4552**	allXL	DA	372	370	DBSU	30.0
**4552**	allXL	DA	373	370	DBSU	31.64
**4552**	allXL	AD	382	373	DBSU	41.9
**4553**	allXL	AA	320	357	BS2G	25.59
**4553**	allXL	BB	320	373	BS2G	17.06
**4553**	allXL	BB	373	382	BS2G	17.87
**4553**	allXL	CC	370	386	BS2G	25.14
**4553**	allXL	CC	373	386	BS2G	22.89
**4553**	allXL	DD	319	357	BS2G	25.79
**4553**	allXL	DD	367	373	BS2G	14.64
**4553**	allXL	DD	370	382	BS2G	22.71
**4553**	allXL	CC	376	382	BS2G	14.11
**4553**	allXL	BB	357	373	BS2G	15.64
**4553**	allXL	AA	320	366	BS2G	22.86
**4553**	allXL	CC	370	376	BS2G	16.44
**4553**	allXL	AA	320	357	DBSU	25.59
**4553**	allXL	DD	357	319	DBSU	25.79
**4553**	allXL	CC	372	319	DBSU	20.78
**4553**	allXL	BB	370	357	DBSU	18.87
**4553**	allXL	BB	382	357	DBSU	31.94
**4553**	allXL	AA	372	370	DBSU	6.24
**4553**	allXL	DD	381	370	DBSU	22.99
**4553**	allXL	DD	373	370	DBSU	8.94
**4553**	allXL	BB	381	372	DBSU	19.5
**4553**	allXL	BB	372	382	DBSU	17.85

**4553**	allXL	CC	372	386	DBSU	21.98
**4553**	allXL	CC	373	386	DBSU	22.89
**4553**	allXL	DB	381	386	DBSU	50.93
**4553**	allXL	BD;DB	357	357	DBSU	18.33
**4553**	allXL	AD	373	319	DBSU	26.11
**4553**	allXL	DB	372	357	DBSU	31.18
**4553**	allXL	DB	381	357	DBSU	26.81
**4553**	allXL	DB	373	357	DBSU	29.28
**4553**	allXL	BD	372	370	DBSU	42.52
**4553**	allXL	BD	373	370	DBSU	41.0
**4553**	allXL	DB	382	373	DBSU	35.45
**4554**	allXL	AA	320	357	BS2G	24.36
**4554**	allXL	CC	320	373	BS2G	16.31
**4554**	allXL	BB	373	382	BS2G	17.4
**4554**	allXL	AA	370	386	BS2G	20.27
**4554**	allXL	AA	373	386	BS2G	23.38
**4554**	allXL	AA	319	357	BS2G	24.71
**4554**	allXL	AA	367	373	BS2G	13.18
**4554**	allXL	AA	370	382	BS2G	20.57
**4554**	allXL	BB	376	382	BS2G	12.63
**4554**	allXL	AA	357	373	BS2G	31.53
**4554**	allXL	BB	320	366	BS2G	20.04
**4554**	allXL	CC	370	376	BS2G	16.04
**4554**	allXL	AA	320	357	DBSU	24.36
**4554**	allXL	AA	357	319	DBSU	24.71
**4554**	allXL	CC	372	319	DBSU	13.16
**4554**	allXL	DD	370	357	DBSU	26.96
**4554**	allXL	BB	382	357	DBSU	37.6
**4554**	allXL	AA	372	370	DBSU	6.26
**4554**	allXL	AA	381	370	DBSU	22.24
**4554**	allXL	AA	373	370	DBSU	9.03
**4554**	allXL	AA	381	372	DBSU	20.86
**4554**	allXL	AA	372	382	DBSU	20.09
**4554**	allXL	AA	372	386	DBSU	23.09

**4554**	allXL	AA	373	386	DBSU	23.38
**4554**	allXL	DA	381	386	DBSU	51.37
**4554**	allXL	BD;DB	357	357	DBSU	19.5
**4554**	allXL	AD	373	319	DBSU	33.78
**4554**	allXL	AC	372	357	DBSU	45.45
**4554**	allXL	BD	381	357	DBSU	48.64
**4554**	allXL	AC	373	357	DBSU	43.77
**4554**	allXL	DA	372	370	DBSU	53.8
**4554**	allXL	AD	373	370	DBSU	51.8
**4554**	allXL	AD	382	373	DBSU	38.81
**4555**	allXL	CC	320	357	BS2G	23.92
**4555**	allXL	DD	320	373	BS2G	12.43
**4555**	allXL	DD	373	382	BS2G	17.94
**4555**	allXL	AA	370	386	BS2G	22.73
**4555**	allXL	AA	373	386	BS2G	26.66
**4555**	allXL	CC	319	357	BS2G	25.35
**4555**	allXL	BB	367	373	BS2G	12.05
**4555**	allXL	DD	370	382	BS2G	19.17
**4555**	allXL	CC	376	382	BS2G	14.85
**4555**	allXL	BB	357	373	BS2G	24.44
**4555**	allXL	DD	320	366	BS2G	14.62
**4555**	allXL	CC	370	376	BS2G	15.31
**4555**	allXL	CC	320	357	DBSU	23.92
**4555**	allXL	CC	357	319	DBSU	25.35
**4555**	allXL	DD	372	319	DBSU	12.16
**4555**	allXL	DD	370	357	DBSU	29.18
**4555**	allXL	BB	382	357	DBSU	33.69
**4555**	allXL	AA	372	370	DBSU	6.2
**4555**	allXL	DD	381	370	DBSU	19.07
**4555**	allXL	AA	373	370	DBSU	9.32
**4555**	allXL	DD	381	372	DBSU	18.71
**4555**	allXL	AA	372	382	DBSU	19.05
**4555**	allXL	AA	372	386	DBSU	23.66
**4555**	allXL	AA	373	386	DBSU	26.66

**4555**	allXL	DA	381	386	DBSU	36.36
**4555**	allXL	BD;DB	357	357	DBSU	18.55
**4555**	allXL	CB	373	319	DBSU	25.2
**4555**	allXL	CB	372	357	DBSU	39.78
**4555**	allXL	DA	381	357	DBSU	35.75
**4555**	allXL	CB	373	357	DBSU	39.04
**4555**	allXL	CB	372	370	DBSU	28.13
**4555**	allXL	CB	373	370	DBSU	24.81
**4555**	allXL	BC	382	373	DBSU	11.83
**4556**	allXL	AA	320	357	BS2G	21.18
**4556**	allXL	CC	320	373	BS2G	23.45
**4556**	allXL	BB	373	382	BS2G	19.31
**4556**	allXL	CC	370	386	BS2G	20.6
**4556**	allXL	CC	373	386	BS2G	26.29
**4556**	allXL	AA	319	357	BS2G	18.46
**4556**	allXL	AA	367	373	BS2G	13.89
**4556**	allXL	CC	370	382	BS2G	16.41
**4556**	allXL	CC	376	382	BS2G	12.87
**4556**	allXL	DD	357	373	BS2G	35.18
**4556**	allXL	AA	320	366	BS2G	22.82
**4556**	allXL	CC	370	376	BS2G	13.03
**4556**	allXL	AA	320	357	DBSU	21.18
**4556**	allXL	AA	357	319	DBSU	18.46
**4556**	allXL	CC	372	319	DBSU	27.31
**4556**	allXL	DD	370	357	DBSU	27.07
**4556**	allXL	BB	382	357	DBSU	30.03
**4556**	allXL	AA	372	370	DBSU	6.08
**4556**	allXL	CC	381	370	DBSU	18.36
**4556**	allXL	AA	373	370	DBSU	8.91
**4556**	allXL	CC	381	372	DBSU	21.08
**4556**	allXL	CC	372	382	DBSU	19.54
**4556**	allXL	CC	372	386	DBSU	24.43
**4556**	allXL	CC	373	386	DBSU	26.29
**4556**	allXL	AD	381	386	DBSU	30.43

**4556**	allXL	BD;DB	357	357	DBSU	17.57
**4556**	allXL	AD	373	319	DBSU	27.06
**4556**	allXL	BC	372	357	DBSU	45.74
**4556**	allXL	BD	381	357	DBSU	43.5
**4556**	allXL	BC	373	357	DBSU	49.11
**4556**	allXL	BC	372	370	DBSU	20.97
**4556**	allXL	BC	373	370	DBSU	24.37
**4556**	allXL	DA	382	373	DBSU	31.73
**4557**	allXL	AA	320	357	BS2G	23.51
**4557**	allXL	BB	320	373	BS2G	24.88
**4557**	allXL	AA	373	382	BS2G	16.89
**4557**	allXL	CC	370	386	BS2G	19.61
**4557**	allXL	AA	373	386	BS2G	20.51
**4557**	allXL	AA	319	357	BS2G	26.81
**4557**	allXL	AA	367	373	BS2G	11.06
**4557**	allXL	BB	370	382	BS2G	17.13
**4557**	allXL	AA	376	382	BS2G	14.13
**4557**	allXL	BB	357	373	BS2G	16.83
**4557**	allXL	AA	320	366	BS2G	20.11
**4557**	allXL	BB	370	376	BS2G	13.93
**4557**	allXL	AA	320	357	DBSU	23.51
**4557**	allXL	AA	357	319	DBSU	26.81
**4557**	allXL	BB	372	319	DBSU	29.67
**4557**	allXL	BB	370	357	DBSU	23.34
**4557**	allXL	AA	382	357	DBSU	23.4
**4557**	allXL	AA	372	370	DBSU	6.6
**4557**	allXL	BB	381	370	DBSU	19.02
**4557**	allXL	AA	373	370	DBSU	9.04
**4557**	allXL	DD	381	372	DBSU	18.56
**4557**	allXL	CC	372	382	DBSU	20.05
**4557**	allXL	CC	372	386	DBSU	20.24
**4557**	allXL	AA	373	386	DBSU	20.51
**4557**	allXL	BD	381	386	DBSU	42.97
**4557**	allXL	BD;DB	357	357	DBSU	19.79

**4557**	allXL	AD	373	319	DBSU	32.43
**4557**	allXL	BD	372	357	DBSU	32.62
**4557**	allXL	AC	381	357	DBSU	37.68
**4557**	allXL	BD	373	357	DBSU	32.84
**4557**	allXL	BD	372	370	DBSU	46.8
**4557**	allXL	BD	373	370	DBSU	45.16
**4557**	allXL	DB	382	373	DBSU	44.85
**4558**	allXL	CC	320	357	BS2G	22.97
**4558**	allXL	BB	320	373	BS2G	23.12
**4558**	allXL	AA	373	382	BS2G	18.25
**4558**	allXL	AA	370	386	BS2G	20.17
**4558**	allXL	AA	373	386	BS2G	23.84
**4558**	allXL	CC	319	357	BS2G	25.25
**4558**	allXL	BB	367	373	BS2G	12.86
**4558**	allXL	AA	370	382	BS2G	19.09
**4558**	allXL	CC	376	382	BS2G	13.5
**4558**	allXL	BB	357	373	BS2G	17.39
**4558**	allXL	CC	320	366	BS2G	23.7
**4558**	allXL	CC	370	376	BS2G	16.77
**4558**	allXL	CC	320	357	DBSU	22.97
**4558**	allXL	CC	357	319	DBSU	25.25
**4558**	allXL	BB	372	319	DBSU	28.79
**4558**	allXL	AA	370	357	DBSU	23.4
**4558**	allXL	BB	382	357	DBSU	24.83
**4558**	allXL	AA	372	370	DBSU	6.2
**4558**	allXL	BB	381	370	DBSU	21.39
**4558**	allXL	DD	373	370	DBSU	8.81
**4558**	allXL	BB	381	372	DBSU	18.55
**4558**	allXL	AA	372	382	DBSU	17.24
**4558**	allXL	AA	372	386	DBSU	21.1
**4558**	allXL	AA	373	386	DBSU	23.84
**4558**	allXL	BC	381	386	DBSU	51.65
**4558**	allXL	BD;DB	357	357	DBSU	18.45
**4558**	allXL	CB	373	319	DBSU	19.37

**4558**	allXL	AC	372	357	DBSU	24.0
**4558**	allXL	AC	381	357	DBSU	29.57
**4558**	allXL	AC	373	357	DBSU	20.52
**4558**	allXL	BC	372	370	DBSU	34.18
**4558**	allXL	BC	373	370	DBSU	34.13
**4558**	allXL	CB	382	373	DBSU	46.12
**4559**	allXL	AA	320	357	BS2G	24.33
**4559**	allXL	BB	320	373	BS2G	24.5
**4559**	allXL	DD	373	382	BS2G	18.44
**4559**	allXL	DD	370	386	BS2G	22.63
**4559**	allXL	CC	373	386	BS2G	25.28
**4559**	allXL	AA	319	357	BS2G	27.44
**4559**	allXL	AA	367	373	BS2G	13.75
**4559**	allXL	DD	370	382	BS2G	16.2
**4559**	allXL	CC	376	382	BS2G	13.31
**4559**	allXL	CC	357	373	BS2G	22.48
**4559**	allXL	BB	320	366	BS2G	20.84
**4559**	allXL	BB	370	376	BS2G	13.66
**4559**	allXL	AA	320	357	DBSU	24.33
**4559**	allXL	AA	357	319	DBSU	27.44
**4559**	allXL	BB	372	319	DBSU	22.85
**4559**	allXL	CC	370	357	DBSU	27.07
**4559**	allXL	DD	382	357	DBSU	22.1
**4559**	allXL	AA	372	370	DBSU	6.08
**4559**	allXL	DD	381	370	DBSU	15.28
**4559**	allXL	AA	373	370	DBSU	8.97
**4559**	allXL	DD	381	372	DBSU	17.83
**4559**	allXL	DD	372	382	DBSU	19.75
**4559**	allXL	AA	372	386	DBSU	25.71
**4559**	allXL	CC	373	386	DBSU	25.28
**4559**	allXL	DA	381	386	DBSU	41.42
**4559**	allXL	BD;DB	357	357	DBSU	19.65
**4559**	allXL	AD	373	319	DBSU	22.39
**4559**	allXL	CA	372	357	DBSU	37.4

**4559**	allXL	CA	381	357	DBSU	22.5
**4559**	allXL	CA	373	357	DBSU	33.72
**4559**	allXL	DA	372	370	DBSU	22.58
**4559**	allXL	DA	373	370	DBSU	19.58
**4559**	allXL	DA	382	373	DBSU	33.32
**4560**	allXL	CC	320	357	BS2G	23.12
**4560**	allXL	DD	320	373	BS2G	15.1
**4560**	allXL	BB	373	382	BS2G	15.2
**4560**	allXL	BB	370	386	BS2G	24.56
**4560**	allXL	BB	373	386	BS2G	23.05
**4560**	allXL	CC	319	357	BS2G	25.81
**4560**	allXL	AA	367	373	BS2G	13.79
**4560**	allXL	BB	370	382	BS2G	21.22
**4560**	allXL	BB	376	382	BS2G	14.26
**4560**	allXL	BB	357	373	BS2G	29.81
**4560**	allXL	CC	320	366	BS2G	18.73
**4560**	allXL	CC	370	376	BS2G	13.76
**4560**	allXL	CC	320	357	DBSU	23.12
**4560**	allXL	CC	357	319	DBSU	25.81
**4560**	allXL	DD	372	319	DBSU	16.65
**4560**	allXL	CC	370	357	DBSU	28.85
**4560**	allXL	BB	382	357	DBSU	27.74
**4560**	allXL	AA	372	370	DBSU	6.21
**4560**	allXL	BB	381	370	DBSU	20.55
**4560**	allXL	AA	373	370	DBSU	9.15
**4560**	allXL	BB	381	372	DBSU	13.87
**4560**	allXL	BB	372	382	DBSU	14.74
**4560**	allXL	BB	372	386	DBSU	20.79
**4560**	allXL	BB	373	386	DBSU	23.05
**4560**	allXL	CB	381	386	DBSU	26.28
**4560**	allXL	BD;DB	357	357	DBSU	18.03
**4560**	allXL	CB	373	319	DBSU	9.85
**4560**	allXL	CB	372	357	DBSU	32.8
**4560**	allXL	BD	381	357	DBSU	40.2

**4560**	allXL	CB	373	357	DBSU	29.92
**4560**	allXL	DA	372	370	DBSU	28.17
**4560**	allXL	DA	373	370	DBSU	25.25
**4560**	allXL	BC	382	373	DBSU	22.11
**4561**	allXL	AA	320	357	BS2G	26.0
**4561**	allXL	CC	320	373	BS2G	15.72
**4561**	allXL	DD	373	382	BS2G	19.19
**4561**	allXL	CC	370	386	BS2G	25.03
**4561**	allXL	AA	373	386	BS2G	26.52
**4561**	allXL	AA	319	357	BS2G	24.59
**4561**	allXL	AA	367	373	BS2G	13.84
**4561**	allXL	DD	370	382	BS2G	21.15
**4561**	allXL	CC	376	382	BS2G	15.1
**4561**	allXL	DD	357	373	BS2G	25.17
**4561**	allXL	AA	320	366	BS2G	13.93
**4561**	allXL	CC	370	376	BS2G	14.49
**4561**	allXL	AA	320	357	DBSU	26.0
**4561**	allXL	AA	357	319	DBSU	24.59
**4561**	allXL	DD	372	319	DBSU	9.96
**4561**	allXL	DD	370	357	DBSU	18.13
**4561**	allXL	CC	382	357	DBSU	16.24
**4561**	allXL	AA	372	370	DBSU	6.19
**4561**	allXL	DD	381	370	DBSU	19.52
**4561**	allXL	AA	373	370	DBSU	9.03
**4561**	allXL	DD	381	372	DBSU	18.93
**4561**	allXL	DD	372	382	DBSU	21.64
**4561**	allXL	AA	372	386	DBSU	26.98
**4561**	allXL	AA	373	386	DBSU	26.52
**4561**	allXL	DA	381	386	DBSU	51.26
**4561**	allXL	BD;DB	357	357	DBSU	19.32
**4561**	allXL	BC	373	319	DBSU	35.4
**4561**	allXL	DB	372	357	DBSU	41.91
**4561**	allXL	CA	381	357	DBSU	34.94
**4561**	allXL	DB	373	357	DBSU	42.42

**4561**	allXL	CB	372	370	DBSU	26.39
**4561**	allXL	CB	373	370	DBSU	26.23
**4561**	allXL	AD	382	373	DBSU	39.14
**4562**	allXL	AA	320	357	BS2G	26.98
**4562**	allXL	AA	320	373	BS2G	16.83
**4562**	allXL	CC	373	382	BS2G	19.53
**4562**	allXL	CC	370	386	BS2G	29.96
**4562**	allXL	DD	373	386	BS2G	24.2
**4562**	allXL	AA	319	357	BS2G	27.37
**4562**	allXL	AA	367	373	BS2G	13.76
**4562**	allXL	BB	370	382	BS2G	24.18
**4562**	allXL	CC	376	382	BS2G	14.84
**4562**	allXL	BB	357	373	BS2G	12.82
**4562**	allXL	DD	320	366	BS2G	21.29
**4562**	allXL	CC	370	376	BS2G	15.27
**4562**	allXL	AA	320	357	DBSU	26.98
**4562**	allXL	AA	357	319	DBSU	27.37
**4562**	allXL	CC	372	319	DBSU	17.92
**4562**	allXL	BB	370	357	DBSU	15.07
**4562**	allXL	BB	382	357	DBSU	24.7
**4562**	allXL	CC	372	370	DBSU	6.53
**4562**	allXL	BB	381	370	DBSU	25.85
**4562**	allXL	AA	373	370	DBSU	9.18
**4562**	allXL	DD	381	372	DBSU	23.11
**4562**	allXL	CC	372	382	DBSU	22.85
**4562**	allXL	DD	372	386	DBSU	25.61
**4562**	allXL	DD	373	386	DBSU	24.2
**4562**	allXL	DA	381	386	DBSU	39.75
**4562**	allXL	BD;DB	357	357	DBSU	18.74
**4562**	allXL	AD	373	319	DBSU	39.08
**4562**	allXL	BD	372	357	DBSU	29.52
**4562**	allXL	BD	381	357	DBSU	25.94
**4562**	allXL	BD	373	357	DBSU	27.68
**4562**	allXL	DA	372	370	DBSU	43.47

**4562**	allXL	DA	373	370	DBSU	44.52
**4562**	allXL	AD	382	373	DBSU	41.29
**4563**	allXL	AA	320	357	BS2G	19.87
**4563**	allXL	CC	320	373	BS2G	20.18
**4563**	allXL	CC	373	382	BS2G	15.14
**4563**	allXL	BB	370	386	BS2G	24.53
**4563**	allXL	CC	373	386	BS2G	24.56
**4563**	allXL	AA	319	357	BS2G	17.1
**4563**	allXL	AA	367	373	BS2G	11.26
**4563**	allXL	CC	370	382	BS2G	21.29
**4563**	allXL	CC	376	382	BS2G	14.53
**4563**	allXL	BB	357	373	BS2G	21.79
**4563**	allXL	CC	320	366	BS2G	16.96
**4563**	allXL	BB	370	376	BS2G	15.94
**4563**	allXL	AA	320	357	DBSU	19.87
**4563**	allXL	AA	357	319	DBSU	17.1
**4563**	allXL	CC	372	319	DBSU	15.82
**4563**	allXL	CC	370	357	DBSU	24.05
**4563**	allXL	BB	382	357	DBSU	31.95
**4563**	allXL	AA	372	370	DBSU	6.74
**4563**	allXL	CC	381	370	DBSU	23.67
**4563**	allXL	AA	373	370	DBSU	8.95
**4563**	allXL	DD	381	372	DBSU	18.93
**4563**	allXL	CC	372	382	DBSU	18.07
**4563**	allXL	CC	372	386	DBSU	26.0
**4563**	allXL	CC	373	386	DBSU	24.56
**4563**	allXL	AD	381	386	DBSU	33.92
**4563**	allXL	BD;DB	357	357	DBSU	18.9
**4563**	allXL	AD	373	319	DBSU	27.88
**4563**	allXL	BD	372	357	DBSU	37.92
**4563**	allXL	BD	381	357	DBSU	38.14
**4563**	allXL	BD	373	357	DBSU	35.93
**4563**	allXL	DA	372	370	DBSU	26.43
**4563**	allXL	DA	373	370	DBSU	27.82

**4563**	allXL	DA	382	373	DBSU	23.4
**4564**	allXL	CC	320	357	BS2G	26.34
**4564**	allXL	BB	320	373	BS2G	19.56
**4564**	allXL	CC	373	382	BS2G	15.92
**4564**	allXL	CC	370	386	BS2G	29.78
**4564**	allXL	CC	373	386	BS2G	23.81
**4564**	allXL	AA	319	357	BS2G	27.5
**4564**	allXL	AA	367	373	BS2G	13.89
**4564**	allXL	CC	370	382	BS2G	23.12
**4564**	allXL	BB	376	382	BS2G	14.24
**4564**	allXL	BB	357	373	BS2G	27.76
**4564**	allXL	DD	320	366	BS2G	23.84
**4564**	allXL	CC	370	376	BS2G	14.85
**4564**	allXL	CC	320	357	DBSU	26.34
**4564**	allXL	AA	357	319	DBSU	27.5
**4564**	allXL	DD	372	319	DBSU	23.17
**4564**	allXL	BB	370	357	DBSU	24.37
**4564**	allXL	CC	382	357	DBSU	32.89
**4564**	allXL	AA	372	370	DBSU	6.2
**4564**	allXL	CC	381	370	DBSU	24.62
**4564**	allXL	CC	373	370	DBSU	9.07
**4564**	allXL	DD	381	372	DBSU	19.77
**4564**	allXL	CC	372	382	DBSU	19.48
**4564**	allXL	DD	372	386	DBSU	25.03
**4564**	allXL	CC	373	386	DBSU	23.81
**4564**	allXL	BC	381	386	DBSU	35.04
**4564**	allXL	BD;DB	357	357	DBSU	18.73
**4564**	allXL	CB	373	319	DBSU	12.47
**4564**	allXL	DA	372	357	DBSU	18.65
**4564**	allXL	DA	381	357	DBSU	15.69
**4564**	allXL	DA	373	357	DBSU	19.53
**4564**	allXL	CB	372	370	DBSU	16.66
**4564**	allXL	BC	373	370	DBSU	14.94
**4564**	allXL	BC	382	373	DBSU	26.39

**4565**	allXL	AA	320	357	BS2G	26.57
**4565**	allXL	DD	320	373	BS2G	21.27
**4565**	allXL	BB	373	382	BS2G	19.07
**4565**	allXL	DD	370	386	BS2G	25.74
**4565**	allXL	DD	373	386	BS2G	23.05
**4565**	allXL	AA	319	357	BS2G	26.88
**4565**	allXL	BB	367	373	BS2G	10.75
**4565**	allXL	BB	370	382	BS2G	18.02
**4565**	allXL	BB	376	382	BS2G	14.42
**4565**	allXL	BB	357	373	BS2G	29.9
**4565**	allXL	DD	320	366	BS2G	24.17
**4565**	allXL	BB	370	376	BS2G	15.69
**4565**	allXL	AA	320	357	DBSU	26.57
**4565**	allXL	AA	357	319	DBSU	26.88
**4565**	allXL	DD	372	319	DBSU	17.44
**4565**	allXL	DD	370	357	DBSU	26.87
**4565**	allXL	BB	382	357	DBSU	31.41
**4565**	allXL	AA	372	370	DBSU	6.4
**4565**	allXL	BB	381	370	DBSU	20.22
**4565**	allXL	AA	373	370	DBSU	9.03
**4565**	allXL	DD	381	372	DBSU	19.0
**4565**	allXL	DD	372	382	DBSU	19.6
**4565**	allXL	DD	372	386	DBSU	20.79
**4565**	allXL	DD	373	386	DBSU	23.05
**4565**	allXL	DA	381	386	DBSU	29.56
**4565**	allXL	BD;DB	357	357	DBSU	18.92
**4565**	allXL	CB	373	319	DBSU	22.47
**4565**	allXL	CB	372	357	DBSU	27.83
**4565**	allXL	CB	381	357	DBSU	45.95
**4565**	allXL	CB	373	357	DBSU	25.99
**4565**	allXL	CB	372	370	DBSU	27.54
**4565**	allXL	CB	373	370	DBSU	24.24
**4565**	allXL	CB	382	373	DBSU	20.31
**4566**	allXL	CC	320	357	BS2G	26.65

**4566**	allXL	CC	320	373	BS2G	12.5
**4566**	allXL	DD	373	382	BS2G	12.78
**4566**	allXL	DD	370	386	BS2G	14.42
**4566**	allXL	DD	373	386	BS2G	19.28
**4566**	allXL	AA	319	357	BS2G	26.91
**4566**	allXL	AA	367	373	BS2G	12.91
**4566**	allXL	DD	370	382	BS2G	12.69
**4566**	allXL	AA;BB	376	382	BS2G	14.84
**4566**	allXL	CC	357	373	BS2G	18.13
**4566**	allXL	AA	320	366	BS2G	22.34
**4566**	allXL	CC	370	376	BS2G	15.53
**4566**	allXL	CC	320	357	DBSU	26.65
**4566**	allXL	AA	357	319	DBSU	26.91
**4566**	allXL	CC	372	319	DBSU	18.08
**4566**	allXL	CC	370	357	DBSU	19.42
**4566**	allXL	BB	382	357	DBSU	19.98
**4566**	allXL	AA	372	370	DBSU	6.28
**4566**	allXL	DD	381	370	DBSU	15.09
**4566**	allXL	DD	373	370	DBSU	9.26
**4566**	allXL	DD	381	372	DBSU	14.26
**4566**	allXL	DD	372	382	DBSU	12.7
**4566**	allXL	DD	372	386	DBSU	16.95
**4566**	allXL	DD	373	386	DBSU	19.28
**4566**	allXL	BC	381	386	DBSU	46.09
**4566**	allXL	BD;DB	357	357	DBSU	17.82
**4566**	allXL	AD	373	319	DBSU	33.91
**4566**	allXL	CA	372	357	DBSU	34.83
**4566**	allXL	BD	381	357	DBSU	25.75
**4566**	allXL	CA	373	357	DBSU	35.39
**4566**	allXL	DA	372	370	DBSU	40.31
**4566**	allXL	DA	373	370	DBSU	42.53
**4566**	allXL	DA	382	373	DBSU	40.7
**4567**	allXL	CC	320	357	BS2G	23.48
**4567**	allXL	CC	320	373	BS2G	15.1

**4567**	allXL	AA	373	382	BS2G	17.79
**4567**	allXL	CC	370	386	BS2G	21.98
**4567**	allXL	AA	373	386	BS2G	25.09
**4567**	allXL	CC	319	357	BS2G	23.85
**4567**	allXL	BB	367	373	BS2G	14.14
**4567**	allXL	CC	370	382	BS2G	20.31
**4567**	allXL	AA	376	382	BS2G	12.96
**4567**	allXL	CC	357	373	BS2G	30.68
**4567**	allXL	CC	320	366	BS2G	21.84
**4567**	allXL	CC	370	376	BS2G	17.47
**4567**	allXL	CC	320	357	DBSU	23.48
**4567**	allXL	CC	357	319	DBSU	23.85
**4567**	allXL	CC	372	319	DBSU	15.77
**4567**	allXL	CC	370	357	DBSU	23.0
**4567**	allXL	CD	382	357	DBSU	33.58
**4567**	allXL	AA	372	370	DBSU	6.12
**4567**	allXL	CC	381	370	DBSU	23.16
**4567**	allXL	DD	373	370	DBSU	9.19
**4567**	allXL	CC	381	372	DBSU	19.91
**4567**	allXL	CC	372	382	DBSU	17.74
**4567**	allXL	CC	372	386	DBSU	23.32
**4567**	allXL	AA	373	386	DBSU	25.09
**4567**	allXL	BC	381	386	DBSU	40.37
**4567**	allXL	BD;DB	357	357	DBSU	17.71
**4567**	allXL	AD	373	319	DBSU	26.82
**4567**	allXL	CB	372	357	DBSU	37.72
**4567**	allXL	CB	381	357	DBSU	20.24
**4567**	allXL	CB	373	357	DBSU	38.06
**4567**	allXL	BC	372	370	DBSU	35.6
**4567**	allXL	CB	373	370	DBSU	36.97
**4567**	allXL	CB	382	373	DBSU	25.14
**4568**	allXL	CC	320	357	BS2G	16.02
**4568**	allXL	DD	320	373	BS2G	18.4
**4568**	allXL	BB	373	382	BS2G	19.52

**4568**	allXL	DD	370	386	BS2G	20.87
**4568**	allXL	DD	373	386	BS2G	24.71
**4568**	allXL	CC	319	357	BS2G	15.67
**4568**	allXL	AA	367	373	BS2G	10.51
**4568**	allXL	BB	370	382	BS2G	19.62
**4568**	allXL	CC	376	382	BS2G	14.12
**4568**	allXL	BB	357	373	BS2G	25.38
**4568**	allXL	AA	320	366	BS2G	24.96
**4568**	allXL	BB	370	376	BS2G	16.01
**4568**	allXL	CC	320	357	DBSU	16.02
**4568**	allXL	CC	357	319	DBSU	15.67
**4568**	allXL	DD	372	319	DBSU	19.89
**4568**	allXL	DD	370	357	DBSU	22.41
**4568**	allXL	AA	382	357	DBSU	22.23
**4568**	allXL	AA	372	370	DBSU	6.49
**4568**	allXL	DD	381	370	DBSU	21.4
**4568**	allXL	AA	373	370	DBSU	8.85
**4568**	allXL	DD	381	372	DBSU	19.42
**4568**	allXL	DD	372	382	DBSU	20.68
**4568**	allXL	DD	372	386	DBSU	22.32
**4568**	allXL	DD	373	386	DBSU	24.71
**4568**	allXL	DA	381	386	DBSU	50.17
**4568**	allXL	BD;DB	357	357	DBSU	19.02
**4568**	allXL	CB	373	319	DBSU	10.49
**4568**	allXL	DA	372	357	DBSU	27.93
**4568**	allXL	BD	381	357	DBSU	33.76
**4568**	allXL	DA	373	357	DBSU	29.92
**4568**	allXL	DA	372	370	DBSU	25.4
**4568**	allXL	DA	373	370	DBSU	23.03
**4568**	allXL	DA	382	373	DBSU	37.53
**4569**	allXL	AA	320	357	BS2G	25.84
**4569**	allXL	BB	320	373	BS2G	22.47
**4569**	allXL	AA	373	382	BS2G	17.24
**4569**	allXL	AA	370	386	BS2G	16.81

**4569**	allXL	AA	373	386	BS2G	16.6
**4569**	allXL	AA	319	357	BS2G	26.56
**4569**	allXL	BB	367	373	BS2G	14.12
**4569**	allXL	BB	370	382	BS2G	19.98
**4569**	allXL	AA	376	382	BS2G	14.13
**4569**	allXL	BB	357	373	BS2G	26.54
**4569**	allXL	CC	320	366	BS2G	16.44
**4569**	allXL	CC	370	376	BS2G	17.11
**4569**	allXL	AA	320	357	DBSU	25.84
**4569**	allXL	AA	357	319	DBSU	26.56
**4569**	allXL	AA	372	319	DBSU	25.37
**4569**	allXL	DD	370	357	DBSU	28.65
**4569**	allXL	BB	382	357	DBSU	43.45
**4569**	allXL	AA	372	370	DBSU	6.04
**4569**	allXL	BB	381	370	DBSU	20.76
**4569**	allXL	AA	373	370	DBSU	8.97
**4569**	allXL	DD	381	372	DBSU	19.08
**4569**	allXL	AA	372	382	DBSU	19.49
**4569**	allXL	AA	372	386	DBSU	17.87
**4569**	allXL	AA	373	386	DBSU	16.6
**4569**	allXL	CB	381	386	DBSU	39.27
**4569**	allXL	BD;DB	357	357	DBSU	19.73
**4569**	allXL	AD	373	319	DBSU	25.74
**4569**	allXL	AD	372	357	DBSU	36.64
**4569**	allXL	AD	381	357	DBSU	56.25
**4569**	allXL	AD	373	357	DBSU	39.5
**4569**	allXL	AD	372	370	DBSU	20.63
**4569**	allXL	AD	373	370	DBSU	21.68
**4569**	allXL	AD	382	373	DBSU	37.77
**4570**	allXL	AA	320	357	BS2G	24.55
**4570**	allXL	CC	320	373	BS2G	22.03
**4570**	allXL	DD	373	382	BS2G	18.91
**4570**	allXL	DD	370	386	BS2G	22.54
**4570**	allXL	DD	373	386	BS2G	26.08

**4570**	allXL	CC	319	357	BS2G	23.77
**4570**	allXL	AA	367	373	BS2G	13.83
**4570**	allXL	DD	370	382	BS2G	12.95
**4570**	allXL	CC	376	382	BS2G	15.32
**4570**	allXL	BB	357	373	BS2G	24.44
**4570**	allXL	CC	320	366	BS2G	20.19
**4570**	allXL	DD	370	376	BS2G	14.0
**4570**	allXL	AA	320	357	DBSU	24.55
**4570**	allXL	CC	357	319	DBSU	23.77
**4570**	allXL	CC	372	319	DBSU	19.52
**4570**	allXL	BB	370	357	DBSU	26.61
**4570**	allXL	DD	382	357	DBSU	35.78
**4570**	allXL	AA	372	370	DBSU	6.38
**4570**	allXL	DD	381	370	DBSU	12.02
**4570**	allXL	AA	373	370	DBSU	9.09
**4570**	allXL	DD	381	372	DBSU	17.29
**4570**	allXL	DD	372	382	DBSU	18.36
**4570**	allXL	AA	372	386	DBSU	25.29
**4570**	allXL	DD	373	386	DBSU	26.08
**4570**	allXL	CB	381	386	DBSU	58.35
**4570**	allXL	BD;DB	357	357	DBSU	18.7
**4570**	allXL	DA	373	319	DBSU	22.89
**4570**	allXL	BD	372	357	DBSU	35.61
**4570**	allXL	BC	381	357	DBSU	24.92
**4570**	allXL	BD	373	357	DBSU	36.24
**4570**	allXL	BC	372	370	DBSU	42.29
**4570**	allXL	BC	373	370	DBSU	38.99
**4570**	allXL	BC	382	373	DBSU	33.27
**4571**	allXL	CC	320	357	BS2G	23.11
**4571**	allXL	DD	320	373	BS2G	12.64
**4571**	allXL	AA	373	382	BS2G	20.64
**4571**	allXL	DD	370	386	BS2G	20.06
**4571**	allXL	AA	373	386	BS2G	24.62
**4571**	allXL	CC	319	357	BS2G	22.08

**4571**	allXL	AA	367	373	BS2G	14.71
**4571**	allXL	DD	370	382	BS2G	22.08
**4571**	allXL	AA	376	382	BS2G	14.64
**4571**	allXL	DD	357	373	BS2G	24.35
**4571**	allXL	BB	320	366	BS2G	27.38
**4571**	allXL	BB	370	376	BS2G	16.43
**4571**	allXL	CC	320	357	DBSU	23.11
**4571**	allXL	CC	357	319	DBSU	22.08
**4571**	allXL	DD	372	319	DBSU	17.5
**4571**	allXL	DD	370	357	DBSU	22.14
**4571**	allXL	DD	382	357	DBSU	36.95
**4571**	allXL	AA	372	370	DBSU	6.12
**4571**	allXL	DD	381	370	DBSU	21.21
**4571**	allXL	AA	373	370	DBSU	9.15
**4571**	allXL	DD	381	372	DBSU	20.68
**4571**	allXL	CC	372	382	DBSU	21.82
**4571**	allXL	DD	372	386	DBSU	23.9
**4571**	allXL	AA	373	386	DBSU	24.62
**4571**	allXL	BC	381	386	DBSU	15.56
**4571**	allXL	BD;DB	357	357	DBSU	18.1
**4571**	allXL	BC	373	319	DBSU	30.5
**4571**	allXL	BC	372	357	DBSU	39.99
**4571**	allXL	BC	381	357	DBSU	48.88
**4571**	allXL	BC	373	357	DBSU	39.56
**4571**	allXL	DA	372	370	DBSU	30.69
**4571**	allXL	BC	373	370	DBSU	28.74
**4571**	allXL	BC	382	373	DBSU	18.41
**4572**	allXL	CC	320	357	BS2G	19.93
**4572**	allXL	DD	320	373	BS2G	21.02
**4572**	allXL	DD	373	382	BS2G	18.43
**4572**	allXL	BB	370	386	BS2G	28.18
**4572**	allXL	BB	373	386	BS2G	22.74
**4572**	allXL	CC	319	357	BS2G	20.33
**4572**	allXL	AA	367	373	BS2G	14.0

**4572**	allXL	DD	370	382	BS2G	25.18
**4572**	allXL	AA	376	382	BS2G	13.57
**4572**	allXL	BB	357	373	BS2G	26.87
**4572**	allXL	CC	320	366	BS2G	24.26
**4572**	allXL	DD	370	376	BS2G	16.46
**4572**	allXL	CC	320	357	DBSU	19.93
**4572**	allXL	CC	357	319	DBSU	20.33
**4572**	allXL	DD	372	319	DBSU	24.81
**4572**	allXL	CC	370	357	DBSU	26.08
**4572**	allXL	BB	382	357	DBSU	26.32
**4572**	allXL	AA	372	370	DBSU	6.8
**4572**	allXL	DD	381	370	DBSU	23.48
**4572**	allXL	DD	373	370	DBSU	9.2
**4572**	allXL	DD	381	372	DBSU	18.52
**4572**	allXL	DD	372	382	DBSU	19.42
**4572**	allXL	BB	372	386	DBSU	25.09
**4572**	allXL	BB	373	386	DBSU	22.74
**4572**	allXL	DA	381	386	DBSU	62.08
**4572**	allXL	BD;DB	357	357	DBSU	18.94
**4572**	allXL	CB	373	319	DBSU	12.12
**4572**	allXL	CA	372	357	DBSU	35.96
**4572**	allXL	BD	381	357	DBSU	43.0
**4572**	allXL	CA	373	357	DBSU	39.15
**4572**	allXL	CB	372	370	DBSU	35.43
**4572**	allXL	CB	373	370	DBSU	35.83
**4572**	allXL	BC	382	373	DBSU	46.28
**4573**	allXL	CC	320	357	BS2G	24.07
**4573**	allXL	AA	320	373	BS2G	24.32
**4573**	allXL	BB	373	382	BS2G	20.08
**4573**	allXL	DD	370	386	BS2G	25.5
**4573**	allXL	BB	373	386	BS2G	29.62
**4573**	allXL	CC	319	357	BS2G	25.44
**4573**	allXL	BB	367	373	BS2G	13.18
**4573**	allXL	DD	370	382	BS2G	18.03

**4573**	allXL	CC	376	382	BS2G	14.75
**4573**	allXL	DD	357	373	BS2G	17.06
**4573**	allXL	CC	320	366	BS2G	18.8
**4573**	allXL	DD	370	376	BS2G	15.95
**4573**	allXL	CC	320	357	DBSU	24.07
**4573**	allXL	CC	357	319	DBSU	25.44
**4573**	allXL	AA	372	319	DBSU	26.15
**4573**	allXL	DD	370	357	DBSU	22.99
**4573**	allXL	DD	382	357	DBSU	19.29
**4573**	allXL	AA	372	370	DBSU	6.11
**4573**	allXL	DD	381	370	DBSU	17.66
**4573**	allXL	AA	373	370	DBSU	8.97
**4573**	allXL	BB	381	372	DBSU	20.41
**4573**	allXL	BB	372	382	DBSU	20.79
**4573**	allXL	BB	372	386	DBSU	30.33
**4573**	allXL	BB	373	386	DBSU	29.62
**4573**	allXL	DB	381	386	DBSU	34.17
**4573**	allXL	BD;DB	357	357	DBSU	19.84
**4573**	allXL	AD	373	319	DBSU	25.11
**4573**	allXL	CB	372	357	DBSU	17.26
**4573**	allXL	DB	381	357	DBSU	30.01
**4573**	allXL	CB	373	357	DBSU	15.67
**4573**	allXL	AD	372	370	DBSU	30.13
**4573**	allXL	DA	373	370	DBSU	31.39
**4573**	allXL	BC	382	373	DBSU	28.06
**4574**	allXL	AA	320	357	BS2G	24.26
**4574**	allXL	CC	320	373	BS2G	18.42
**4574**	allXL	CC	373	382	BS2G	16.98
**4574**	allXL	AA	370	386	BS2G	17.32
**4574**	allXL	AA	373	386	BS2G	17.55
**4574**	allXL	AA	319	357	BS2G	23.68
**4574**	allXL	AA	367	373	BS2G	13.97
**4574**	allXL	BB	370	382	BS2G	20.68
**4574**	allXL	BB	376	382	BS2G	13.87

**4574**	allXL	BB	357	373	BS2G	23.79
**4574**	allXL	DD	320	366	BS2G	17.12
**4574**	allXL	CC	370	376	BS2G	17.29
**4574**	allXL	AA	320	357	DBSU	24.26
**4574**	allXL	AA	357	319	DBSU	23.68
**4574**	allXL	CC	372	319	DBSU	19.21
**4574**	allXL	BB	370	357	DBSU	26.51
**4574**	allXL	BB	382	357	DBSU	33.23
**4574**	allXL	AA	372	370	DBSU	6.18
**4574**	allXL	BB	381	370	DBSU	23.03
**4574**	allXL	CC	373	370	DBSU	9.03
**4574**	allXL	CC	381	372	DBSU	20.87
**4574**	allXL	CC	372	382	DBSU	20.2
**4574**	allXL	AA	372	386	DBSU	17.72
**4574**	allXL	AA	373	386	DBSU	17.55
**4574**	allXL	DA	381	386	DBSU	56.97
**4574**	allXL	BD;DB	357	357	DBSU	18.63
**4574**	allXL	AD	373	319	DBSU	13.76
**4574**	allXL	CB	372	357	DBSU	33.61
**4574**	allXL	BD	381	357	DBSU	41.7
**4574**	allXL	CB	373	357	DBSU	34.52
**4574**	allXL	CB	372	370	DBSU	37.47
**4574**	allXL	AD	373	370	DBSU	38.18
**4574**	allXL	DA	382	373	DBSU	47.45
**4575**	allXL	CC	320	357	BS2G	22.51
**4575**	allXL	DD	320	373	BS2G	15.22
**4575**	allXL	DD	373	382	BS2G	18.31
**4575**	allXL	AA	370	386	BS2G	26.37
**4575**	allXL	AA	373	386	BS2G	26.83
**4575**	allXL	CC	319	357	BS2G	22.29
**4575**	allXL	BB	367	373	BS2G	13.78
**4575**	allXL	AA	370	382	BS2G	23.73
**4575**	allXL	CC	376	382	BS2G	14.35
**4575**	allXL	BB	357	373	BS2G	20.41

**4575**	allXL	AA	320	366	BS2G	21.72
**4575**	allXL	CC	370	376	BS2G	14.99
**4575**	allXL	CC	320	357	DBSU	22.51
**4575**	allXL	CC	357	319	DBSU	22.29
**4575**	allXL	DD	372	319	DBSU	20.43
**4575**	allXL	DD	370	357	DBSU	23.1
**4575**	allXL	BB	382	357	DBSU	20.79
**4575**	allXL	AA	372	370	DBSU	6.28
**4575**	allXL	DD	381	370	DBSU	25.25
**4575**	allXL	DD	373	370	DBSU	9.02
**4575**	allXL	DD	381	372	DBSU	17.96
**4575**	allXL	DD	372	382	DBSU	17.58
**4575**	allXL	AA	372	386	DBSU	24.88
**4575**	allXL	AA	373	386	DBSU	26.83
**4575**	allXL	AD	381	386	DBSU	58.55
**4575**	allXL	BD;DB	357	357	DBSU	18.05
**4575**	allXL	AD	373	319	DBSU	27.31
**4575**	allXL	BD	372	357	DBSU	39.57
**4575**	allXL	BD	381	357	DBSU	28.36
**4575**	allXL	AD	373	357	DBSU	37.08
**4575**	allXL	AD	372	370	DBSU	28.71
**4575**	allXL	AD	373	370	DBSU	27.05
**4575**	allXL	AD	382	373	DBSU	41.22
**4576**	allXL	AA	320	357	BS2G	16.99
**4576**	allXL	BB	320	373	BS2G	23.47
**4576**	allXL	CC	373	382	BS2G	19.8
**4576**	allXL	BB	370	386	BS2G	18.48
**4576**	allXL	BB	373	386	BS2G	10.85
**4576**	allXL	AA	319	357	BS2G	15.34
**4576**	allXL	DD	367	373	BS2G	14.18
**4576**	allXL	CC	370	382	BS2G	20.86
**4576**	allXL	CC	376	382	BS2G	14.11
**4576**	allXL	CC	357	373	BS2G	34.33
**4576**	allXL	AA	320	366	BS2G	24.4

**4576**	allXL	CC	370	376	BS2G	13.72
**4576**	allXL	AA	320	357	DBSU	16.99
**4576**	allXL	AA	357	319	DBSU	15.34
**4576**	allXL	BB	372	319	DBSU	19.44
**4576**	allXL	CC	370	357	DBSU	28.08
**4576**	allXL	CC	382	357	DBSU	37.28
**4576**	allXL	AA	372	370	DBSU	6.22
**4576**	allXL	CC	381	370	DBSU	20.98
**4576**	allXL	AA	373	370	DBSU	8.94
**4576**	allXL	DD	381	372	DBSU	21.99
**4576**	allXL	CC	372	382	DBSU	21.57
**4576**	allXL	BB	372	386	DBSU	12.69
**4576**	allXL	BB	373	386	DBSU	10.85
**4576**	allXL	CB	381	386	DBSU	22.8
**4576**	allXL	BD;DB	357	357	DBSU	18.97
**4576**	allXL	CB	373	319	DBSU	29.34
**4576**	allXL	BC	372	357	DBSU	44.71
**4576**	allXL	CB	381	357	DBSU	40.39
**4576**	allXL	BC	373	357	DBSU	45.28
**4576**	allXL	BC	372	370	DBSU	21.95
**4576**	allXL	BC	373	370	DBSU	20.95
**4576**	allXL	BC	382	373	DBSU	14.37
**4577**	allXL	CC	320	357	BS2G	22.3
**4577**	allXL	BB	320	373	BS2G	10.96
**4577**	allXL	DD	373	382	BS2G	18.91
**4577**	allXL	DD	370	386	BS2G	22.92
**4577**	allXL	DD	373	386	BS2G	26.68
**4577**	allXL	AA	319	357	BS2G	21.62
**4577**	allXL	AA	367	373	BS2G	12.2
**4577**	allXL	DD	370	382	BS2G	16.18
**4577**	allXL	CC	376	382	BS2G	14.7
**4577**	allXL	BB	357	373	BS2G	23.22
**4577**	allXL	CC	320	366	BS2G	17.39
**4577**	allXL	CC	370	376	BS2G	14.39

**4577**	allXL	CC	320	357	DBSU	22.3
**4577**	allXL	AA	357	319	DBSU	21.62
**4577**	allXL	DD	372	319	DBSU	14.81
**4577**	allXL	DD	370	357	DBSU	26.12
**4577**	allXL	BB	382	357	DBSU	35.32
**4577**	allXL	AA	372	370	DBSU	6.38
**4577**	allXL	DD	381	370	DBSU	13.74
**4577**	allXL	AA	373	370	DBSU	8.75
**4577**	allXL	DD	381	372	DBSU	15.76
**4577**	allXL	DD	372	382	DBSU	18.72
**4577**	allXL	DD	372	386	DBSU	25.69
**4577**	allXL	DD	373	386	DBSU	26.68
**4577**	allXL	DA	381	386	DBSU	56.94
**4577**	allXL	BD;DB	357	357	DBSU	18.16
**4577**	allXL	AD	373	319	DBSU	33.39
**4577**	allXL	BD	372	357	DBSU	40.21
**4577**	allXL	BC	381	357	DBSU	39.3
**4577**	allXL	BD	373	357	DBSU	39.41
**4577**	allXL	DA	372	370	DBSU	29.93
**4577**	allXL	DA	373	370	DBSU	26.37
**4577**	allXL	AD	382	373	DBSU	43.55
**4578**	allXL	AA	320	357	BS2G	25.9
**4578**	allXL	BB	320	373	BS2G	28.58
**4578**	allXL	DD	373	382	BS2G	18.0
**4578**	allXL	DD	370	386	BS2G	18.69
**4578**	allXL	DD	373	386	BS2G	18.64
**4578**	allXL	AA	319	357	BS2G	27.31
**4578**	allXL	DD	367	373	BS2G	14.96
**4578**	allXL	DD	370	382	BS2G	23.24
**4578**	allXL	BB	376	382	BS2G	14.46
**4578**	allXL	BB	357	373	BS2G	18.85
**4578**	allXL	AA	320	366	BS2G	24.4
**4578**	allXL	CC	370	376	BS2G	17.09
**4578**	allXL	AA	320	357	DBSU	25.9

**4578**	allXL	AA	357	319	DBSU	27.31
**4578**	allXL	BB	372	319	DBSU	29.05
**4578**	allXL	BB	370	357	DBSU	24.07
**4578**	allXL	BB	382	357	DBSU	22.19
**4578**	allXL	AA	372	370	DBSU	5.89
**4578**	allXL	DD	381	370	DBSU	23.8
**4578**	allXL	AA	373	370	DBSU	9.26
**4578**	allXL	DD	381	372	DBSU	17.9
**4578**	allXL	DD	372	382	DBSU	18.1
**4578**	allXL	DD	372	386	DBSU	17.03
**4578**	allXL	DD	373	386	DBSU	18.64
**4578**	allXL	BD	381	386	DBSU	60.38
**4578**	allXL	BD;DB	357	357	DBSU	17.19
**4578**	allXL	BC	373	319	DBSU	29.01
**4578**	allXL	BD	372	357	DBSU	30.35
**4578**	allXL	BD	381	357	DBSU	21.75
**4578**	allXL	BD	373	357	DBSU	29.11
**4578**	allXL	CB	372	370	DBSU	41.87
**4578**	allXL	CB	373	370	DBSU	43.77
**4578**	allXL	BD	382	373	DBSU	55.84
**4579**	allXL	CC	320	357	BS2G	24.69
**4579**	allXL	AA	320	373	BS2G	8.63
**4579**	allXL	AA	373	382	BS2G	19.27
**4579**	allXL	AA	370	386	BS2G	19.58
**4579**	allXL	AA	373	386	BS2G	22.4
**4579**	allXL	CC	319	357	BS2G	25.3
**4579**	allXL	BB	367	373	BS2G	11.26
**4579**	allXL	AA	370	382	BS2G	20.64
**4579**	allXL	CC	376	382	BS2G	14.67
**4579**	allXL	BB	357	373	BS2G	24.09
**4579**	allXL	AA	320	366	BS2G	15.05
**4579**	allXL	CC	370	376	BS2G	16.18
**4579**	allXL	CC	320	357	DBSU	24.69
**4579**	allXL	CC	357	319	DBSU	25.3

**4579**	allXL	AA	372	319	DBSU	13.91
**4579**	allXL	CC	370	357	DBSU	25.18
**4579**	allXL	BB	382	357	DBSU	29.12
**4579**	allXL	AA	372	370	DBSU	6.13
**4579**	allXL	AA	381	370	DBSU	21.9
**4579**	allXL	DD	373	370	DBSU	8.42
**4579**	allXL	AA	381	372	DBSU	21.49
**4579**	allXL	AA	372	382	DBSU	20.84
**4579**	allXL	AA	372	386	DBSU	22.74
**4579**	allXL	AA	373	386	DBSU	22.4
**4579**	allXL	CA	381	386	DBSU	43.29
**4579**	allXL	BD;DB	357	357	DBSU	18.66
**4579**	allXL	AD	373	319	DBSU	34.08
**4579**	allXL	AD	372	357	DBSU	32.35
**4579**	allXL	CA	381	357	DBSU	22.61
**4579**	allXL	AD	373	357	DBSU	35.82
**4579**	allXL	AD	372	370	DBSU	39.48
**4579**	allXL	AD	373	370	DBSU	42.5
**4579**	allXL	AD	382	373	DBSU	50.35
**4580**	allXL	AA	320	357	BS2G	22.02
**4580**	allXL	BB	320	373	BS2G	16.44
**4580**	allXL	DD	373	382	BS2G	18.73
**4580**	allXL	DD	370	386	BS2G	22.41
**4580**	allXL	DD	373	386	BS2G	28.01
**4580**	allXL	AA	319	357	BS2G	19.88
**4580**	allXL	AA	367	373	BS2G	13.41
**4580**	allXL	DD	370	382	BS2G	16.05
**4580**	allXL	CC	376	382	BS2G	14.26
**4580**	allXL	BB	357	373	BS2G	21.43
**4580**	allXL	BB	320	366	BS2G	21.34
**4580**	allXL	CC	370	376	BS2G	14.94
**4580**	allXL	AA	320	357	DBSU	22.02
**4580**	allXL	AA	357	319	DBSU	19.88
**4580**	allXL	BB	372	319	DBSU	20.13

**4580**	allXL	BB	370	357	DBSU	25.64
**4580**	allXL	DD	382	357	DBSU	27.99
**4580**	allXL	AA	372	370	DBSU	6.08
**4580**	allXL	DD	381	370	DBSU	14.93
**4580**	allXL	AA	373	370	DBSU	9.08
**4580**	allXL	DD	381	372	DBSU	17.69
**4580**	allXL	DD	372	382	DBSU	20.02
**4580**	allXL	DD	372	386	DBSU	28.45
**4580**	allXL	DD	373	386	DBSU	28.01
**4580**	allXL	BD	381	386	DBSU	33.23
**4580**	allXL	BD;DB	357	357	DBSU	18.66
**4580**	allXL	AD	373	319	DBSU	35.76
**4580**	allXL	BD	372	357	DBSU	32.43
**4580**	allXL	BC	381	357	DBSU	38.64
**4580**	allXL	BD	373	357	DBSU	33.13
**4580**	allXL	BD	372	370	DBSU	56.01
**4580**	allXL	BD	373	370	DBSU	56.32
**4580**	allXL	DB	382	373	DBSU	47.84
**4581**	allXL	AA	320	357	BS2G	24.37
**4581**	allXL	BB	320	373	BS2G	12.5
**4581**	allXL	DD	373	382	BS2G	18.65
**4581**	allXL	DD	370	386	BS2G	24.46
**4581**	allXL	DD	373	386	BS2G	22.43
**4581**	allXL	AA	319	357	BS2G	25.4
**4581**	allXL	AA	367	373	BS2G	12.17
**4581**	allXL	BB	370	382	BS2G	23.53
**4581**	allXL	CC	376	382	BS2G	14.46
**4581**	allXL	CC	357	373	BS2G	21.56
**4581**	allXL	AA	320	366	BS2G	17.17
**4581**	allXL	BB	370	376	BS2G	16.56
**4581**	allXL	AA	320	357	DBSU	24.37
**4581**	allXL	AA	357	319	DBSU	25.4
**4581**	allXL	BB	372	319	DBSU	17.85
**4581**	allXL	CC	370	357	DBSU	24.82

**4581**	allXL	BB	382	357	DBSU	21.25
**4581**	allXL	AA	372	370	DBSU	6.37
**4581**	allXL	BB	381	370	DBSU	24.34
**4581**	allXL	AA	373	370	DBSU	9.13
**4581**	allXL	DD	381	372	DBSU	18.34
**4581**	allXL	DD	372	382	DBSU	17.53
**4581**	allXL	DD	372	386	DBSU	19.68
**4581**	allXL	DD	373	386	DBSU	22.43
**4581**	allXL	AD	381	386	DBSU	59.5
**4581**	allXL	BD;DB	357	357	DBSU	18.72
**4581**	allXL	AD	373	319	DBSU	29.79
**4581**	allXL	CA	372	357	DBSU	28.97
**4581**	allXL	CA	381	357	DBSU	35.69
**4581**	allXL	CA	373	357	DBSU	25.81
**4581**	allXL	AD	372	370	DBSU	39.8
**4581**	allXL	AD	373	370	DBSU	36.82
**4581**	allXL	AD	382	373	DBSU	47.24
**4582**	allXL	AA	320	357	BS2G	25.28
**4582**	allXL	CC	320	373	BS2G	21.64
**4582**	allXL	CC	373	382	BS2G	18.83
**4582**	allXL	BB	370	386	BS2G	23.64
**4582**	allXL	CC	373	386	BS2G	23.64
**4582**	allXL	AA	319	357	BS2G	24.4
**4582**	allXL	BB	367	373	BS2G	12.54
**4582**	allXL	CC	370	382	BS2G	22.27
**4582**	allXL	CC	376	382	BS2G	13.5
**4582**	allXL	CC	357	373	BS2G	31.14
**4582**	allXL	BB	320	366	BS2G	23.5
**4582**	allXL	CC	370	376	BS2G	15.21
**4582**	allXL	AA	320	357	DBSU	25.28
**4582**	allXL	AA	357	319	DBSU	24.4
**4582**	allXL	CC	372	319	DBSU	21.56
**4582**	allXL	CC	370	357	DBSU	28.92
**4582**	allXL	BB	382	357	DBSU	20.27

**4582**	allXL	AA	372	370	DBSU	6.24
**4582**	allXL	BB	381	370	DBSU	20.85
**4582**	allXL	DD	373	370	DBSU	8.8
**4582**	allXL	BB	381	372	DBSU	20.58
**4582**	allXL	AA	372	382	DBSU	21.64
**4582**	allXL	CC	372	386	DBSU	25.48
**4582**	allXL	CC	373	386	DBSU	23.64
**4582**	allXL	BC	381	386	DBSU	31.25
**4582**	allXL	BD;DB	357	357	DBSU	18.12
**4582**	allXL	AD	373	319	DBSU	24.44
**4582**	allXL	BC	372	357	DBSU	40.85
**4582**	allXL	CB	381	357	DBSU	26.59
**4582**	allXL	BC	373	357	DBSU	38.12
**4582**	allXL	BC	372	370	DBSU	22.45
**4582**	allXL	BC	373	370	DBSU	21.07
**4582**	allXL	CB	382	373	DBSU	10.5
**4583**	allXL	AA	320	357	BS2G	21.48
**4583**	allXL	BB	320	373	BS2G	15.53
**4583**	allXL	CC	373	382	BS2G	15.55
**4583**	allXL	CC	370	386	BS2G	20.26
**4583**	allXL	CC	373	386	BS2G	21.49
**4583**	allXL	AA	319	357	BS2G	18.58
**4583**	allXL	AA	367	373	BS2G	12.69
**4583**	allXL	CC	370	382	BS2G	19.3
**4583**	allXL	CC	376	382	BS2G	14.41
**4583**	allXL	DD	357	373	BS2G	21.76
**4583**	allXL	CC	320	366	BS2G	23.01
**4583**	allXL	CC	370	376	BS2G	15.84
**4583**	allXL	AA	320	357	DBSU	21.48
**4583**	allXL	AA	357	319	DBSU	18.58
**4583**	allXL	CC	372	319	DBSU	16.24
**4583**	allXL	DD	370	357	DBSU	22.06
**4583**	allXL	BB	382	357	DBSU	23.45
**4583**	allXL	AA	372	370	DBSU	6.27

**4583**	allXL	CC	381	370	DBSU	21.87
**4583**	allXL	DD	373	370	DBSU	9.0
**4583**	allXL	DD	381	372	DBSU	19.14
**4583**	allXL	CC	372	382	DBSU	18.04
**4583**	allXL	DD	372	386	DBSU	20.72
**4583**	allXL	CC	373	386	DBSU	21.49
**4583**	allXL	BC	381	386	DBSU	46.71
**4583**	allXL	BD;DB	357	357	DBSU	19.16
**4583**	allXL	AD	373	319	DBSU	23.91
**4583**	allXL	AC	372	357	DBSU	42.09
**4583**	allXL	BD	381	357	DBSU	32.67
**4583**	allXL	AC	373	357	DBSU	40.46
**4583**	allXL	CB	372	370	DBSU	40.71
**4583**	allXL	CB	373	370	DBSU	40.71
**4583**	allXL	CB	382	373	DBSU	36.49
**4584**	allXL	DD	320	357	BS2G	23.11
**4584**	allXL	DD	320	373	BS2G	14.09
**4584**	allXL	CC	373	382	BS2G	18.16
**4584**	allXL	CC	370	386	BS2G	22.66
**4584**	allXL	CC	373	386	BS2G	18.11
**4584**	allXL	AA	319	357	BS2G	25.47
**4584**	allXL	AA	367	373	BS2G	12.48
**4584**	allXL	BB	370	382	BS2G	22.43
**4584**	allXL	BB	376	382	BS2G	13.96
**4584**	allXL	BB	357	373	BS2G	29.89
**4584**	allXL	DD	320	366	BS2G	19.67
**4584**	allXL	CC	370	376	BS2G	16.43
**4584**	allXL	DD	320	357	DBSU	23.11
**4584**	allXL	AA	357	319	DBSU	25.47
**4584**	allXL	DD	372	319	DBSU	11.88
**4584**	allXL	DD	370	357	DBSU	29.09
**4584**	allXL	BB	382	357	DBSU	44.0
**4584**	allXL	AA	372	370	DBSU	6.4
**4584**	allXL	DD	381	370	DBSU	23.58

**4584**	allXL	AA	373	370	DBSU	9.28
**4584**	allXL	DD	381	372	DBSU	19.44
**4584**	allXL	CC	372	382	DBSU	19.08
**4584**	allXL	CC	372	386	DBSU	17.39
**4584**	allXL	CC	373	386	DBSU	18.11
**4584**	allXL	BC	381	386	DBSU	34.2
**4584**	allXL	BD;DB	357	357	DBSU	18.37
**4584**	allXL	CB	373	319	DBSU	31.86
**4584**	allXL	BD	372	357	DBSU	44.9
**4584**	allXL	DA	381	357	DBSU	54.01
**4584**	allXL	DA	373	357	DBSU	46.05
**4584**	allXL	DA	372	370	DBSU	32.1
**4584**	allXL	DA	373	370	DBSU	28.68
**4584**	allXL	DA	382	373	DBSU	33.23
**4585**	allXL	CC	320	357	BS2G	24.73
**4585**	allXL	DD	320	373	BS2G	15.43
**4585**	allXL	DD	373	382	BS2G	17.81
**4585**	allXL	AA	370	386	BS2G	15.35
**4585**	allXL	CC	373	386	BS2G	21.48
**4585**	allXL	CC	319	357	BS2G	24.86
**4585**	allXL	AA	367	373	BS2G	11.93
**4585**	allXL	AA	370	382	BS2G	15.94
**4585**	allXL	BB	376	382	BS2G	14.07
**4585**	allXL	DD	357	373	BS2G	18.44
**4585**	allXL	DD	320	366	BS2G	23.36
**4585**	allXL	DD	370	376	BS2G	15.11
**4585**	allXL	CC	320	357	DBSU	24.73
**4585**	allXL	CC	357	319	DBSU	24.86
**4585**	allXL	DD	372	319	DBSU	16.48
**4585**	allXL	DD	370	357	DBSU	19.63
**4585**	allXL	DD	382	357	DBSU	25.96
**4585**	allXL	AA	372	370	DBSU	6.33
**4585**	allXL	DD	381	370	DBSU	16.85
**4585**	allXL	AA	373	370	DBSU	9.16

**4585**	allXL	DD	381	372	DBSU	17.48
**4585**	allXL	DD	372	382	DBSU	16.68
**4585**	allXL	CC	372	386	DBSU	19.59
**4585**	allXL	CC	373	386	DBSU	21.48
**4585**	allXL	CB	381	386	DBSU	28.65
**4585**	allXL	BD;DB	357	357	DBSU	18.77
**4585**	allXL	CB	373	319	DBSU	26.97
**4585**	allXL	DB	372	357	DBSU	38.63
**4585**	allXL	DB	381	357	DBSU	41.77
**4585**	allXL	DB	373	357	DBSU	35.82
**4585**	allXL	BC	372	370	DBSU	33.57
**4585**	allXL	BC	373	370	DBSU	30.48
**4585**	allXL	CB	382	373	DBSU	19.35
**4586**	allXL	CC	320	357	BS2G	23.5
**4586**	allXL	DD	320	373	BS2G	27.16
**4586**	allXL	CC	373	382	BS2G	16.55
**4586**	allXL	DD	370	386	BS2G	20.23
**4586**	allXL	AA	373	386	BS2G	21.08
**4586**	allXL	DD	319	357	BS2G	24.12
**4586**	allXL	AA	367	373	BS2G	13.13
**4586**	allXL	DD	370	382	BS2G	16.78
**4586**	allXL	CC	376	382	BS2G	14.8
**4586**	allXL	BB	357	373	BS2G	34.02
**4586**	allXL	DD	320	366	BS2G	21.25
**4586**	allXL	CC	370	376	BS2G	15.04
**4586**	allXL	CC	320	357	DBSU	23.5
**4586**	allXL	DD	357	319	DBSU	24.12
**4586**	allXL	DD	372	319	DBSU	20.63
**4586**	allXL	DD	370	357	DBSU	26.91
**4586**	allXL	DD	382	357	DBSU	26.5
**4586**	allXL	AA	372	370	DBSU	6.2
**4586**	allXL	DD	381	370	DBSU	15.49
**4586**	allXL	AA	373	370	DBSU	9.08
**4586**	allXL	DD	381	372	DBSU	17.84

**4586**	allXL	CC	372	382	DBSU	19.25
**4586**	allXL	AA	372	386	DBSU	22.14
**4586**	allXL	AA	373	386	DBSU	21.08
**4586**	allXL	DA	381	386	DBSU	44.31
**4586**	allXL	BD;DB	357	357	DBSU	18.15
**4586**	allXL	CB	373	319	DBSU	27.04
**4586**	allXL	CA	372	357	DBSU	41.17
**4586**	allXL	CA	381	357	DBSU	31.08
**4586**	allXL	CA	373	357	DBSU	39.25
**4586**	allXL	AD	372	370	DBSU	47.24
**4586**	allXL	AD	373	370	DBSU	44.1
**4586**	allXL	AD	382	373	DBSU	25.41
**4587**	allXL	CC	320	357	BS2G	23.72
**4587**	allXL	AA	320	373	BS2G	41.76
**4587**	allXL	BB	373	382	BS2G	18.82
**4587**	allXL	BB	370	386	BS2G	21.86
**4587**	allXL	BB	373	386	BS2G	23.04
**4587**	allXL	CC	319	357	BS2G	24.29
**4587**	allXL	AA	367	373	BS2G	11.9
**4587**	allXL	CC	370	382	BS2G	20.13
**4587**	allXL	BB	376	382	BS2G	13.78
**4587**	allXL	DD	357	373	BS2G	33.4
**4587**	allXL	AA	320	366	BS2G	26.67
**4587**	allXL	CC	370	376	BS2G	15.26
**4587**	allXL	CC	320	357	DBSU	23.72
**4587**	allXL	CC	357	319	DBSU	24.29
**4587**	allXL	AA	372	319	DBSU	40.27
**4587**	allXL	DD	370	357	DBSU	25.91
**4587**	allXL	BB	382	357	DBSU	39.31
**4587**	allXL	AA	372	370	DBSU	6.31
**4587**	allXL	CC	381	370	DBSU	17.84
**4587**	allXL	AA	373	370	DBSU	9.01
**4587**	allXL	DD	381	372	DBSU	17.84
**4587**	allXL	CC	372	382	DBSU	19.66

**4587**	allXL	DD	372	386	DBSU	22.42
**4587**	allXL	BB	373	386	DBSU	23.04
**4587**	allXL	DB	381	386	DBSU	73.62
**4587**	allXL	BD;DB	357	357	DBSU	18.83
**4587**	allXL	CB	373	319	DBSU	20.54
**4587**	allXL	DB	372	357	DBSU	46.6
**4587**	allXL	BD	381	357	DBSU	55.09
**4587**	allXL	CB	373	357	DBSU	47.31
**4587**	allXL	CB	372	370	DBSU	54.29
**4587**	allXL	CB	373	370	DBSU	51.83
**4587**	allXL	BC	382	373	DBSU	64.21
**4588**	allXL	AA	320	357	BS2G	14.28
**4588**	allXL	BB	320	373	BS2G	32.71
**4588**	allXL	AA	373	382	BS2G	18.28
**4588**	allXL	BB	370	386	BS2G	24.99
**4588**	allXL	BB	373	386	BS2G	23.04
**4588**	allXL	AA	319	357	BS2G	17.42
**4588**	allXL	BB	367	373	BS2G	12.35
**4588**	allXL	AA	370	382	BS2G	22.9
**4588**	allXL	AA	376	382	BS2G	14.5
**4588**	allXL	BB	357	373	BS2G	28.48
**4588**	allXL	BB	320	366	BS2G	18.63
**4588**	allXL	CC	370	376	BS2G	17.8
**4588**	allXL	AA	320	357	DBSU	14.28
**4588**	allXL	AA	357	319	DBSU	17.42
**4588**	allXL	AA	372	319	DBSU	32.09
**4588**	allXL	DD	370	357	DBSU	25.74
**4588**	allXL	BB	382	357	DBSU	19.68
**4588**	allXL	CC	372	370	DBSU	6.92
**4588**	allXL	AA	381	370	DBSU	25.3
**4588**	allXL	AA	373	370	DBSU	9.05
**4588**	allXL	DD	381	372	DBSU	19.5
**4588**	allXL	DD	372	382	DBSU	20.29
**4588**	allXL	DD	372	386	DBSU	21.09

**4588**	allXL	BB	373	386	DBSU	23.04
**4588**	allXL	BD	381	386	DBSU	66.05
**4588**	allXL	BD;DB	357	357	DBSU	19.5
**4588**	allXL	AD	373	319	DBSU	40.51
**4588**	allXL	CA	372	357	DBSU	44.3
**4588**	allXL	BD	381	357	DBSU	30.14
**4588**	allXL	CA	373	357	DBSU	43.94
**4588**	allXL	BD	372	370	DBSU	69.59
**4588**	allXL	BD	373	370	DBSU	66.22
**4588**	allXL	BD	382	373	DBSU	48.44
**4589**	allXL	AA	320	357	BS2G	22.75
**4589**	allXL	BB	320	373	BS2G	19.63
**4589**	allXL	BB	373	382	BS2G	16.4
**4589**	allXL	CC	370	386	BS2G	21.82
**4589**	allXL	CC	373	386	BS2G	25.15
**4589**	allXL	AA	319	357	BS2G	22.24
**4589**	allXL	BB	367	373	BS2G	13.8
**4589**	allXL	BB	370	382	BS2G	18.45
**4589**	allXL	BB	376	382	BS2G	13.47
**4589**	allXL	BB	357	373	BS2G	23.26
**4589**	allXL	AA	320	366	BS2G	21.92
**4589**	allXL	CC	370	376	BS2G	16.35
**4589**	allXL	AA	320	357	DBSU	22.75
**4589**	allXL	AA	357	319	DBSU	22.24
**4589**	allXL	BB	372	319	DBSU	25.07
**4589**	allXL	DD	370	357	DBSU	29.62
**4589**	allXL	BB	382	357	DBSU	28.18
**4589**	allXL	AA	372	370	DBSU	6.16
**4589**	allXL	BB	381	370	DBSU	21.34
**4589**	allXL	AA	373	370	DBSU	8.89
**4589**	allXL	DD	381	372	DBSU	20.02
**4589**	allXL	BB	372	382	DBSU	18.89
**4589**	allXL	CC	372	386	DBSU	24.43
**4589**	allXL	CC	373	386	DBSU	25.15

**4589**	allXL	BC	381	386	DBSU	34.15
**4589**	allXL	BD;DB	357	357	DBSU	18.65
**4589**	allXL	CB	373	319	DBSU	13.91
**4589**	allXL	CB	372	357	DBSU	30.47
**4589**	allXL	BC	381	357	DBSU	27.67
**4589**	allXL	CB	373	357	DBSU	28.67
**4589**	allXL	CB	372	370	DBSU	19.71
**4589**	allXL	CB	373	370	DBSU	16.12
**4589**	allXL	BC	382	373	DBSU	10.81
**4590**	allXL	AA	320	357	BS2G	25.11
**4590**	allXL	BB	320	373	BS2G	21.93
**4590**	allXL	DD	373	382	BS2G	19.0
**4590**	allXL	CC	370	386	BS2G	23.63
**4590**	allXL	DD	373	386	BS2G	27.11
**4590**	allXL	AA	319	357	BS2G	24.24
**4590**	allXL	AA	367	373	BS2G	12.62
**4590**	allXL	DD	370	382	BS2G	19.58
**4590**	allXL	CC	376	382	BS2G	14.11
**4590**	allXL	BB	357	373	BS2G	29.58
**4590**	allXL	BB	320	366	BS2G	22.21
**4590**	allXL	CC	370	376	BS2G	15.98
**4590**	allXL	AA	320	357	DBSU	25.11
**4590**	allXL	AA	357	319	DBSU	24.24
**4590**	allXL	BB	372	319	DBSU	24.07
**4590**	allXL	DD	370	357	DBSU	26.46
**4590**	allXL	CC	382	357	DBSU	19.93
**4590**	allXL	AA	372	370	DBSU	6.25
**4590**	allXL	DD	381	370	DBSU	20.75
**4590**	allXL	AA	373	370	DBSU	9.14
**4590**	allXL	DD	381	372	DBSU	18.09
**4590**	allXL	DD	372	382	DBSU	18.26
**4590**	allXL	DD	372	386	DBSU	25.63
**4590**	allXL	DD	373	386	DBSU	27.11
**4590**	allXL	BD	381	386	DBSU	55.02

**4590**	allXL	BD;DB	357	357	DBSU	18.8
**4590**	allXL	AD	373	319	DBSU	21.17
**4590**	allXL	AC	372	357	DBSU	38.37
**4590**	allXL	CA	381	357	DBSU	31.35
**4590**	allXL	AC	373	357	DBSU	38.07
**4590**	allXL	AD	372	370	DBSU	50.18
**4590**	allXL	AD	373	370	DBSU	46.84
**4590**	allXL	CA	382	373	DBSU	41.46
**4591**	allXL	AA	320	357	BS2G	22.87
**4591**	allXL	DD	320	373	BS2G	18.45
**4591**	allXL	CC	373	382	BS2G	17.98
**4591**	allXL	DD	370	386	BS2G	14.67
**4591**	allXL	DD	373	386	BS2G	18.92
**4591**	allXL	AA	319	357	BS2G	20.97
**4591**	allXL	AA	367	373	BS2G	13.82
**4591**	allXL	DD	370	382	BS2G	18.71
**4591**	allXL	CC	376	382	BS2G	14.22
**4591**	allXL	AA	357	373	BS2G	32.77
**4591**	allXL	BB	320	366	BS2G	20.91
**4591**	allXL	DD	370	376	BS2G	15.95
**4591**	allXL	AA	320	357	DBSU	22.87
**4591**	allXL	AA	357	319	DBSU	20.97
**4591**	allXL	BB	372	319	DBSU	19.91
**4591**	allXL	DD	370	357	DBSU	31.0
**4591**	allXL	DD	382	357	DBSU	28.0
**4591**	allXL	AA	372	370	DBSU	6.17
**4591**	allXL	DD	381	370	DBSU	19.42
**4591**	allXL	AA	373	370	DBSU	9.16
**4591**	allXL	DD	381	372	DBSU	20.21
**4591**	allXL	CC	372	382	DBSU	18.51
**4591**	allXL	DD	372	386	DBSU	18.33
**4591**	allXL	DD	373	386	DBSU	18.92
**4591**	allXL	DA	381	386	DBSU	41.47
**4591**	allXL	BD;DB	357	357	DBSU	18.53

**4591**	allXL	AD	373	319	DBSU	43.48
**4591**	allXL	BC	372	357	DBSU	48.24
**4591**	allXL	DA	381	357	DBSU	38.23
**4591**	allXL	AC	373	357	DBSU	48.12
**4591**	allXL	BC	372	370	DBSU	44.64
**4591**	allXL	BC	373	370	DBSU	46.0
**4591**	allXL	BC	382	373	DBSU	44.81
**4592**	allXL	CC	320	357	BS2G	25.98
**4592**	allXL	DD	320	373	BS2G	28.69
**4592**	allXL	BB	373	382	BS2G	19.79
**4592**	allXL	DD	370	386	BS2G	25.74
**4592**	allXL	AA	373	386	BS2G	27.55
**4592**	allXL	AA	319	357	BS2G	28.89
**4592**	allXL	AA	367	373	BS2G	14.08
**4592**	allXL	DD	370	382	BS2G	22.87
**4592**	allXL	CC	376	382	BS2G	13.88
**4592**	allXL	BB	357	373	BS2G	25.52
**4592**	allXL	AA	320	366	BS2G	24.21
**4592**	allXL	CC	370	376	BS2G	16.09
**4592**	allXL	CC	320	357	DBSU	25.98
**4592**	allXL	AA	357	319	DBSU	28.89
**4592**	allXL	DD	372	319	DBSU	27.73
**4592**	allXL	DD	370	357	DBSU	27.21
**4592**	allXL	BB	382	357	DBSU	33.48
**4592**	allXL	AA	372	370	DBSU	6.11
**4592**	allXL	DD	381	370	DBSU	23.49
**4592**	allXL	AA	373	370	DBSU	9.16
**4592**	allXL	DD	381	372	DBSU	22.22
**4592**	allXL	BB	372	382	DBSU	22.08
**4592**	allXL	DD	372	386	DBSU	28.47
**4592**	allXL	AA	373	386	DBSU	27.55
**4592**	allXL	DB	381	386	DBSU	56.75
**4592**	allXL	AC;CA	357	357	DBSU	19.68
**4592**	allXL	AD	373	319	DBSU	33.81

**4592**	allXL	BD	372	357	DBSU	35.38
**4592**	allXL	BD	381	357	DBSU	32.09
**4592**	allXL	BD	373	357	DBSU	35.82
**4592**	allXL	DA	372	370	DBSU	41.78
**4592**	allXL	DA	373	370	DBSU	45.39
**4592**	allXL	BD	382	373	DBSU	55.31
**4593**	allXL	CC	320	357	BS2G	22.83
**4593**	allXL	BB	320	373	BS2G	22.22
**4593**	allXL	DD	373	382	BS2G	16.99
**4593**	allXL	DD	370	386	BS2G	18.52
**4593**	allXL	DD	373	386	BS2G	19.51
**4593**	allXL	CC	319	357	BS2G	24.17
**4593**	allXL	AA	367	373	BS2G	12.23
**4593**	allXL	BB	370	382	BS2G	20.81
**4593**	allXL	CC	376	382	BS2G	14.45
**4593**	allXL	BB	357	373	BS2G	25.87
**4593**	allXL	CC	320	366	BS2G	17.28
**4593**	allXL	CC	370	376	BS2G	14.18
**4593**	allXL	CC	320	357	DBSU	22.83
**4593**	allXL	CC	357	319	DBSU	24.17
**4593**	allXL	BB	372	319	DBSU	23.08
**4593**	allXL	BB	370	357	DBSU	25.36
**4593**	allXL	BB	382	357	DBSU	13.98
**4593**	allXL	DD	372	370	DBSU	6.97
**4593**	allXL	DD	381	370	DBSU	20.82
**4593**	allXL	DD	373	370	DBSU	9.03
**4593**	allXL	DD	381	372	DBSU	18.67
**4593**	allXL	DD	372	382	DBSU	20.37
**4593**	allXL	DD	372	386	DBSU	21.41
**4593**	allXL	DD	373	386	DBSU	19.51
**4593**	allXL	CB	381	386	DBSU	45.44
**4593**	allXL	BD;DB	357	357	DBSU	17.62
**4593**	allXL	CB	373	319	DBSU	15.21
**4593**	allXL	CB	372	357	DBSU	26.36

**4593**	allXL	CA	381	357	DBSU	27.15
**4593**	allXL	CB	373	357	DBSU	28.11
**4593**	allXL	CB	372	370	DBSU	24.34
**4593**	allXL	CB	373	370	DBSU	25.43
**4593**	allXL	BC	382	373	DBSU	30.53
**4594**	allXL	CC	320	357	BS2G	25.06
**4594**	allXL	CC	320	373	BS2G	16.07
**4594**	allXL	CC	373	382	BS2G	19.11
**4594**	allXL	DD	370	386	BS2G	18.08
**4594**	allXL	CC	373	386	BS2G	18.59
**4594**	allXL	CC	319	357	BS2G	25.85
**4594**	allXL	AA	367	373	BS2G	14.46
**4594**	allXL	DD	370	382	BS2G	22.08
**4594**	allXL	CC	376	382	BS2G	13.46
**4594**	allXL	CC	357	373	BS2G	14.69
**4594**	allXL	CC	320	366	BS2G	17.57
**4594**	allXL	CC	370	376	BS2G	17.44
**4594**	allXL	CC	320	357	DBSU	25.06
**4594**	allXL	CC	357	319	DBSU	25.85
**4594**	allXL	CC	372	319	DBSU	15.48
**4594**	allXL	CC	370	357	DBSU	18.02
**4594**	allXL	CC	382	357	DBSU	31.02
**4594**	allXL	AA	372	370	DBSU	6.32
**4594**	allXL	DD	381	370	DBSU	21.45
**4594**	allXL	AA	373	370	DBSU	9.15
**4594**	allXL	DD	381	372	DBSU	20.72
**4594**	allXL	CC	372	382	DBSU	20.46
**4594**	allXL	CC	372	386	DBSU	17.59
**4594**	allXL	CC	373	386	DBSU	18.59
**4594**	allXL	CA	381	386	DBSU	36.37
**4594**	allXL	BD;DB	357	357	DBSU	18.5
**4594**	allXL	AD	373	319	DBSU	20.77
**4594**	allXL	CA	372	357	DBSU	34.1
**4594**	allXL	CA	381	357	DBSU	34.65

**4594**	allXL	CA	373	357	DBSU	30.47
**4594**	allXL	CA	372	370	DBSU	52.23
**4594**	allXL	CA	373	370	DBSU	48.86
**4594**	allXL	CA	382	373	DBSU	32.17
**4595**	allXL	AA	320	357	BS2G	22.46
**4595**	allXL	CC	320	373	BS2G	15.05
**4595**	allXL	CC	373	382	BS2G	19.88
**4595**	allXL	CC	370	386	BS2G	19.34
**4595**	allXL	DD	373	386	BS2G	24.97
**4595**	allXL	AA	319	357	BS2G	21.98
**4595**	allXL	BB	367	373	BS2G	15.19
**4595**	allXL	CC	370	382	BS2G	16.77
**4595**	allXL	CC	376	382	BS2G	14.52
**4595**	allXL	BB	357	373	BS2G	28.15
**4595**	allXL	DD	320	366	BS2G	17.19
**4595**	allXL	CC	370	376	BS2G	14.62
**4595**	allXL	AA	320	357	DBSU	22.46
**4595**	allXL	AA	357	319	DBSU	21.98
**4595**	allXL	DD	372	319	DBSU	10.23
**4595**	allXL	DD	370	357	DBSU	28.58
**4595**	allXL	CC	382	357	DBSU	37.52
**4595**	allXL	AA	372	370	DBSU	6.14
**4595**	allXL	CC	381	370	DBSU	19.02
**4595**	allXL	AA	373	370	DBSU	8.91
**4595**	allXL	CC	381	372	DBSU	21.28
**4595**	allXL	CC	372	382	DBSU	20.05
**4595**	allXL	DD	372	386	DBSU	24.23
**4595**	allXL	DD	373	386	DBSU	24.97
**4595**	allXL	CA	381	386	DBSU	62.59
**4595**	allXL	BD;DB	357	357	DBSU	18.18
**4595**	allXL	DA	373	319	DBSU	32.79
**4595**	allXL	AC	372	357	DBSU	39.19
**4595**	allXL	AC	381	357	DBSU	52.25
**4595**	allXL	AC	373	357	DBSU	36.93

**4595**	allXL	DA	372	370	DBSU	41.6
**4595**	allXL	DA	373	370	DBSU	41.39
**4595**	allXL	AC	382	373	DBSU	47.35
**4596**	allXL	AA	320	357	BS2G	22.52
**4596**	allXL	DD	320	373	BS2G	18.87
**4596**	allXL	BB	373	382	BS2G	14.83
**4596**	allXL	CC	370	386	BS2G	20.84
**4596**	allXL	CC	373	386	BS2G	16.65
**4596**	allXL	AA	319	357	BS2G	21.11
**4596**	allXL	AA	367	373	BS2G	13.06
**4596**	allXL	BB	370	382	BS2G	18.5
**4596**	allXL	BB	376	382	BS2G	14.1
**4596**	allXL	BB	357	373	BS2G	28.27
**4596**	allXL	DD	320	366	BS2G	17.08
**4596**	allXL	CC	370	376	BS2G	17.77
**4596**	allXL	AA	320	357	DBSU	22.52
**4596**	allXL	AA	357	319	DBSU	21.11
**4596**	allXL	DD	372	319	DBSU	12.87
**4596**	allXL	DD	370	357	DBSU	28.23
**4596**	allXL	BB	382	357	DBSU	17.49
**4596**	allXL	AA	372	370	DBSU	6.09
**4596**	allXL	BB	381	370	DBSU	20.82
**4596**	allXL	AA	373	370	DBSU	9.09
**4596**	allXL	CC	381	372	DBSU	17.66
**4596**	allXL	BB	372	382	DBSU	16.39
**4596**	allXL	CC	372	386	DBSU	16.17
**4596**	allXL	CC	373	386	DBSU	16.65
**4596**	allXL	AD	381	386	DBSU	33.51
**4596**	allXL	BD;DB	357	357	DBSU	18.15
**4596**	allXL	AD	373	319	DBSU	17.24
**4596**	allXL	CA	372	357	DBSU	40.1
**4596**	allXL	BD	381	357	DBSU	31.96
**4596**	allXL	CA	373	357	DBSU	41.85
**4596**	allXL	AD	372	370	DBSU	25.34

**4596**	allXL	AD	373	370	DBSU	23.85
**4596**	allXL	DA	382	373	DBSU	33.55
**4597**	allXL	AA	320	357	BS2G	17.77
**4597**	allXL	AA	320	373	BS2G	36.2
**4597**	allXL	BB	373	382	BS2G	17.76
**4597**	allXL	DD	370	386	BS2G	18.42
**4597**	allXL	DD	373	386	BS2G	20.53
**4597**	allXL	AA	319	357	BS2G	16.19
**4597**	allXL	AA	367	373	BS2G	13.32
**4597**	allXL	DD	370	382	BS2G	20.23
**4597**	allXL	BB	376	382	BS2G	13.26
**4597**	allXL	DD	357	373	BS2G	28.29
**4597**	allXL	CC	320	366	BS2G	26.52
**4597**	allXL	CC	370	376	BS2G	15.41
**4597**	allXL	AA	320	357	DBSU	17.77
**4597**	allXL	AA	357	319	DBSU	16.19
**4597**	allXL	CC	372	319	DBSU	37.64
**4597**	allXL	DD	370	357	DBSU	28.53
**4597**	allXL	BB	382	357	DBSU	33.34
**4597**	allXL	AA	372	370	DBSU	6.25
**4597**	allXL	DD	381	370	DBSU	19.88
**4597**	allXL	CC	373	370	DBSU	9.08
**4597**	allXL	DD	381	372	DBSU	21.39
**4597**	allXL	BB	372	382	DBSU	19.69
**4597**	allXL	DD	372	386	DBSU	20.14
**4597**	allXL	DD	373	386	DBSU	20.53
**4597**	allXL	CB	381	386	DBSU	70.78
**4597**	allXL	BD;DB	357	357	DBSU	19.74
**4597**	allXL	CB	373	319	DBSU	20.26
**4597**	allXL	CB	372	357	DBSU	38.96
**4597**	allXL	BD	381	357	DBSU	42.43
**4597**	allXL	CB	373	357	DBSU	40.55
**4597**	allXL	CB	372	370	DBSU	49.45
**4597**	allXL	CB	373	370	DBSU	47.99

**4597**	allXL	BC	382	373	DBSU	59.69
**4598**	allXL	AA	320	357	BS2G	25.5
**4598**	allXL	CC	320	373	BS2G	21.9
**4598**	allXL	DD	373	382	BS2G	17.45
**4598**	allXL	DD	370	386	BS2G	23.38
**4598**	allXL	DD	373	386	BS2G	22.57
**4598**	allXL	AA	319	357	BS2G	24.47
**4598**	allXL	AA	367	373	BS2G	12.13
**4598**	allXL	AA	370	382	BS2G	18.73
**4598**	allXL	CC	376	382	BS2G	13.51
**4598**	allXL	AA	357	373	BS2G	19.68
**4598**	allXL	CC	320	366	BS2G	21.69
**4598**	allXL	AA	370	376	BS2G	15.19
**4598**	allXL	AA	320	357	DBSU	25.5
**4598**	allXL	AA	357	319	DBSU	24.47
**4598**	allXL	CC	372	319	DBSU	22.12
**4598**	allXL	AA	370	357	DBSU	20.43
**4598**	allXL	BB	382	357	DBSU	31.71
**4598**	allXL	AA	372	370	DBSU	6.32
**4598**	allXL	AA	381	370	DBSU	17.98
**4598**	allXL	DD	373	370	DBSU	9.21
**4598**	allXL	DD	381	372	DBSU	17.71
**4598**	allXL	DD	372	382	DBSU	17.46
**4598**	allXL	DD	372	386	DBSU	20.95
**4598**	allXL	DD	373	386	DBSU	22.57
**4598**	allXL	DB	381	386	DBSU	71.01
**4598**	allXL	BD;DB	357	357	DBSU	17.3
**4598**	allXL	AD	373	319	DBSU	41.88
**4598**	allXL	AC	372	357	DBSU	31.56
**4598**	allXL	BD	381	357	DBSU	45.04
**4598**	allXL	AC	373	357	DBSU	31.2
**4598**	allXL	BC	372	370	DBSU	55.44
**4598**	allXL	AC	373	370	DBSU	57.97
**4598**	allXL	BD	382	373	DBSU	67.8

**4599**	allXL	CC	320	357	BS2G	24.29
**4599**	allXL	BB	320	373	BS2G	21.53
**4599**	allXL	AA	373	382	BS2G	20.31
**4599**	allXL	CC	370	386	BS2G	21.86
**4599**	allXL	CC	373	386	BS2G	25.44
**4599**	allXL	AA	319	357	BS2G	26.68
**4599**	allXL	BB	367	373	BS2G	13.71
**4599**	allXL	CC	370	382	BS2G	22.14
**4599**	allXL	CC	376	382	BS2G	14.38
**4599**	allXL	BB	357	373	BS2G	30.1
**4599**	allXL	AA	320	366	BS2G	23.64
**4599**	allXL	CC	370	376	BS2G	15.69
**4599**	allXL	CC	320	357	DBSU	24.29
**4599**	allXL	AA	357	319	DBSU	26.68
**4599**	allXL	DD	372	319	DBSU	27.63
**4599**	allXL	DD	370	357	DBSU	29.15
**4599**	allXL	BB	382	357	DBSU	13.94
**4599**	allXL	AA	372	370	DBSU	6.0
**4599**	allXL	CC	381	370	DBSU	22.77
**4599**	allXL	AA	373	370	DBSU	8.9
**4599**	allXL	DD	381	372	DBSU	20.83
**4599**	allXL	DD	372	382	DBSU	21.87
**4599**	allXL	CC	372	386	DBSU	25.6
**4599**	allXL	CC	373	386	DBSU	25.44
**4599**	allXL	AD	381	386	DBSU	24.95
**4599**	allXL	BD;DB	357	357	DBSU	19.07
**4599**	allXL	DA	373	319	DBSU	14.05
**4599**	allXL	DA	372	357	DBSU	33.19
**4599**	allXL	BD	381	357	DBSU	32.28
**4599**	allXL	DA	373	357	DBSU	32.9
**4599**	allXL	AD	372	370	DBSU	27.26
**4599**	allXL	AD	373	370	DBSU	26.63
**4599**	allXL	DA	382	373	DBSU	30.2
**4600**	allXL	AA	320	357	BS2G	24.74

**4600**	allXL	DD	320	373	BS2G	27.44
**4600**	allXL	AA	373	382	BS2G	18.18
**4600**	allXL	DD	370	386	BS2G	17.21
**4600**	allXL	DD	373	386	BS2G	24.47
**4600**	allXL	AA	319	357	BS2G	23.37
**4600**	allXL	AA	367	373	BS2G	12.39
**4600**	allXL	AA	370	382	BS2G	16.45
**4600**	allXL	BB	376	382	BS2G	14.32
**4600**	allXL	BB	357	373	BS2G	19.21
**4600**	allXL	BB	320	366	BS2G	25.91
**4600**	allXL	AA	370	376	BS2G	16.38
**4600**	allXL	AA	320	357	DBSU	24.74
**4600**	allXL	AA	357	319	DBSU	23.37
**4600**	allXL	DD	372	319	DBSU	30.59
**4600**	allXL	BB	370	357	DBSU	19.86
**4600**	allXL	DD	382	357	DBSU	23.22
**4600**	allXL	AA	372	370	DBSU	6.56
**4600**	allXL	DD	381	370	DBSU	17.96
**4600**	allXL	AA	373	370	DBSU	9.24
**4600**	allXL	AA	381	372	DBSU	17.94
**4600**	allXL	AA	372	382	DBSU	16.88
**4600**	allXL	DD	372	386	DBSU	23.85
**4600**	allXL	DD	373	386	DBSU	24.47
**4600**	allXL	BD	381	386	DBSU	24.4
**4600**	allXL	BD;DB	357	357	DBSU	18.36
**4600**	allXL	CB	373	319	DBSU	23.68
**4600**	allXL	BD	372	357	DBSU	29.83
**4600**	allXL	BD	381	357	DBSU	18.5
**4600**	allXL	BD	373	357	DBSU	26.22
**4600**	allXL	AD	372	370	DBSU	52.28
**4600**	allXL	AD	373	370	DBSU	49.84
**4600**	allXL	DA	382	373	DBSU	34.65
**4601**	allXL	AA	320	357	BS2G	25.13
**4601**	allXL	AA	320	373	BS2G	16.1

**4601**	allXL	AA	373	382	BS2G	18.45
**4601**	allXL	AA	370	386	BS2G	26.66
**4601**	allXL	BB	373	386	BS2G	23.96
**4601**	allXL	BB	319	357	BS2G	26.7
**4601**	allXL	BB	367	373	BS2G	10.56
**4601**	allXL	AA	370	382	BS2G	20.41
**4601**	allXL	CC	376	382	BS2G	14.8
**4601**	allXL	CC	357	373	BS2G	28.01
**4601**	allXL	DD	320	366	BS2G	17.15
**4601**	allXL	BB	370	376	BS2G	15.95
**4601**	allXL	AA	320	357	DBSU	25.13
**4601**	allXL	BB	357	319	DBSU	26.7
**4601**	allXL	AA	372	319	DBSU	15.23
**4601**	allXL	DD	370	357	DBSU	23.8
**4601**	allXL	BB	382	357	DBSU	20.33
**4601**	allXL	AA	372	370	DBSU	6.27
**4601**	allXL	DD	381	370	DBSU	21.08
**4601**	allXL	AA	373	370	DBSU	8.84
**4601**	allXL	DD	381	372	DBSU	18.9
**4601**	allXL	AA	372	382	DBSU	17.45
**4601**	allXL	AA	372	386	DBSU	24.99
**4601**	allXL	BB	373	386	DBSU	23.96
**4601**	allXL	AD	381	386	DBSU	46.08
**4601**	allXL	BD;DB	357	357	DBSU	18.64
**4601**	allXL	AD	373	319	DBSU	23.78
**4601**	allXL	AD	372	357	DBSU	30.84
**4601**	allXL	AD	381	357	DBSU	25.94
**4601**	allXL	AD	373	357	DBSU	33.5
**4601**	allXL	AD	372	370	DBSU	30.97
**4601**	allXL	AD	373	370	DBSU	31.47
**4601**	allXL	AD	382	373	DBSU	37.4
**4602**	allXL	AA	320	357	BS2G	22.37
**4602**	allXL	BB	320	373	BS2G	19.31
**4602**	allXL	DD	373	382	BS2G	19.92

**4602**	allXL	DD	370	386	BS2G	25.55
**4602**	allXL	DD	373	386	BS2G	24.22
**4602**	allXL	AA	319	357	BS2G	20.77
**4602**	allXL	AA;DD	367	373	BS2G	13.69
**4602**	allXL	CC	370	382	BS2G	24.51
**4602**	allXL	CC	376	382	BS2G	14.32
**4602**	allXL	BB	357	373	BS2G	23.0
**4602**	allXL	BB	320	366	BS2G	21.21
**4602**	allXL	CC	370	376	BS2G	14.79
**4602**	allXL	AA	320	357	DBSU	22.37
**4602**	allXL	AA	357	319	DBSU	20.77
**4602**	allXL	BB	372	319	DBSU	21.5
**4602**	allXL	BB	370	357	DBSU	23.56
**4602**	allXL	BB	382	357	DBSU	26.89
**4602**	allXL	AA	372	370	DBSU	6.32
**4602**	allXL	DD	381	370	DBSU	24.52
**4602**	allXL	AA	373	370	DBSU	8.97
**4602**	allXL	DD	381	372	DBSU	19.02
**4602**	allXL	DD	372	382	DBSU	20.25
**4602**	allXL	DD	372	386	DBSU	23.03
**4602**	allXL	DD	373	386	DBSU	24.22
**4602**	allXL	DB	381	386	DBSU	47.2
**4602**	allXL	BD;DB	357	357	DBSU	18.13
**4602**	allXL	AD	373	319	DBSU	16.23
**4602**	allXL	AD	372	357	DBSU	38.07
**4602**	allXL	BD	381	357	DBSU	27.05
**4602**	allXL	BD	373	357	DBSU	37.87
**4602**	allXL	AD	372	370	DBSU	25.84
**4602**	allXL	DA	373	370	DBSU	28.83
**4602**	allXL	BD	382	373	DBSU	38.25
**4603**	allXL	BB	320	357	BS2G	25.19
**4603**	allXL	AA	320	373	BS2G	19.4
**4603**	allXL	CC	373	382	BS2G	13.98
**4603**	allXL	CC	370	386	BS2G	18.28

**4603**	allXL	CC	373	386	BS2G	16.38
**4603**	allXL	AA	319	357	BS2G	25.83
**4603**	allXL	AA	367	373	BS2G	13.32
**4603**	allXL	CC	370	382	BS2G	21.07
**4603**	allXL	CC	376	382	BS2G	13.39
**4603**	allXL	DD	357	373	BS2G	28.57
**4603**	allXL	BB	320	366	BS2G	18.46
**4603**	allXL	BB	370	376	BS2G	17.59
**4603**	allXL	BB	320	357	DBSU	25.19
**4603**	allXL	AA	357	319	DBSU	25.83
**4603**	allXL	BB	372	319	DBSU	20.58
**4603**	allXL	DD	370	357	DBSU	23.24
**4603**	allXL	BB	382	357	DBSU	31.2
**4603**	allXL	AA	372	370	DBSU	6.29
**4603**	allXL	CC	381	370	DBSU	23.98
**4603**	allXL	AA	373	370	DBSU	8.94
**4603**	allXL	CC	381	372	DBSU	17.17
**4603**	allXL	CC	372	382	DBSU	14.49
**4603**	allXL	CC	372	386	DBSU	14.46
**4603**	allXL	CC	373	386	DBSU	16.38
**4603**	allXL	BC	381	386	DBSU	38.4
**4603**	allXL	BD;DB	357	357	DBSU	19.55
**4603**	allXL	CB	373	319	DBSU	23.97
**4603**	allXL	AD	372	357	DBSU	39.4
**4603**	allXL	BD	381	357	DBSU	50.04
**4603**	allXL	AD	373	357	DBSU	42.49
**4603**	allXL	CB	372	370	DBSU	28.25
**4603**	allXL	CB	373	370	DBSU	30.94
**4603**	allXL	CB	382	373	DBSU	39.71
**4604**	allXL	CC	320	357	BS2G	23.99
**4604**	allXL	BB	320	373	BS2G	30.6
**4604**	allXL	CC	373	382	BS2G	18.07
**4604**	allXL	BB	370	386	BS2G	22.82
**4604**	allXL	AA	373	386	BS2G	22.62

**4604**	allXL	CC	319	357	BS2G	23.48
**4604**	allXL	AA	367	373	BS2G	13.74
**4604**	allXL	CC	370	382	BS2G	18.51
**4604**	allXL	CC	376	382	BS2G	14.52
**4604**	allXL	AA	357	373	BS2G	16.41
**4604**	allXL	BB	320	366	BS2G	17.81
**4604**	allXL	BB	370	376	BS2G	15.16
**4604**	allXL	CC	320	357	DBSU	23.99
**4604**	allXL	CC	357	319	DBSU	23.48
**4604**	allXL	BB	372	319	DBSU	28.11
**4604**	allXL	AA	370	357	DBSU	21.79
**4604**	allXL	AA	382	357	DBSU	33.78
**4604**	allXL	AA	372	370	DBSU	6.13
**4604**	allXL	DD	381	370	DBSU	17.14
**4604**	allXL	AA	373	370	DBSU	9.16
**4604**	allXL	DD	381	372	DBSU	17.96
**4604**	allXL	CC	372	382	DBSU	19.61
**4604**	allXL	AA	372	386	DBSU	22.61
**4604**	allXL	AA	373	386	DBSU	22.62
**4604**	allXL	AC	381	386	DBSU	59.8
**4604**	allXL	BD;DB	357	357	DBSU	18.9
**4604**	allXL	AC	373	319	DBSU	36.41
**4604**	allXL	AC	372	357	DBSU	23.75
**4604**	allXL	AC	381	357	DBSU	26.15
**4604**	allXL	AC	373	357	DBSU	21.6
**4604**	allXL	AC	372	370	DBSU	46.67
**4604**	allXL	AC	373	370	DBSU	46.55
**4604**	allXL	AC	382	373	DBSU	49.42
**4605**	allXL	BB	320	357	BS2G	26.76
**4605**	allXL	AA	320	373	BS2G	25.16
**4605**	allXL	CC	373	382	BS2G	17.57
**4605**	allXL	BB	370	386	BS2G	27.14
**4605**	allXL	AA	373	386	BS2G	22.94
**4605**	allXL	AA	319	357	BS2G	27.84

**4605**	allXL	BB	367	373	BS2G	13.35
**4605**	allXL	CC	370	382	BS2G	24.02
**4605**	allXL	CC	376	382	BS2G	14.86
**4605**	allXL	BB	357	373	BS2G	21.94
**4605**	allXL	AA	320	366	BS2G	21.51
**4605**	allXL	CC	370	376	BS2G	17.3
**4605**	allXL	BB	320	357	DBSU	26.76
**4605**	allXL	AA	357	319	DBSU	27.84
**4605**	allXL	AA	372	319	DBSU	25.02
**4605**	allXL	DD	370	357	DBSU	27.17
**4605**	allXL	BB	382	357	DBSU	27.93
**4605**	allXL	AA	372	370	DBSU	6.21
**4605**	allXL	BB	381	370	DBSU	22.39
**4605**	allXL	DD	373	370	DBSU	9.18
**4605**	allXL	DD	381	372	DBSU	18.98
**4605**	allXL	CC	372	382	DBSU	17.96
**4605**	allXL	CC	372	386	DBSU	22.97
**4605**	allXL	AA	373	386	DBSU	22.94
**4605**	allXL	BC	381	386	DBSU	18.06
**4605**	allXL	BD;DB	357	357	DBSU	18.82
**4605**	allXL	CB	373	319	DBSU	22.38
**4605**	allXL	BD	372	357	DBSU	37.0
**4605**	allXL	BD	381	357	DBSU	42.22
**4605**	allXL	BD	373	357	DBSU	34.43
**4605**	allXL	AD	372	370	DBSU	34.42
**4605**	allXL	AD	373	370	DBSU	36.78
**4605**	allXL	BC	382	373	DBSU	17.89
**4606**	allXL	AA	320	357	BS2G	23.09
**4606**	allXL	CC	320	373	BS2G	26.07
**4606**	allXL	BB	373	382	BS2G	16.37
**4606**	allXL	BB	370	386	BS2G	24.67
**4606**	allXL	DD	373	386	BS2G	23.64
**4606**	allXL	AA	319	357	BS2G	21.39
**4606**	allXL	AA	367	373	BS2G	13.42

**4606**	allXL	BB	370	382	BS2G	16.83
**4606**	allXL	BB	376	382	BS2G	13.46
**4606**	allXL	DD	357	373	BS2G	35.11
**4606**	allXL	AA	320	366	BS2G	20.87
**4606**	allXL	CC	370	376	BS2G	15.55
**4606**	allXL	AA	320	357	DBSU	23.09
**4606**	allXL	AA	357	319	DBSU	21.39
**4606**	allXL	CC	372	319	DBSU	24.02
**4606**	allXL	BB	370	357	DBSU	28.81
**4606**	allXL	BB	382	357	DBSU	37.06
**4606**	allXL	AA	372	370	DBSU	6.12
**4606**	allXL	BB	381	370	DBSU	19.3
**4606**	allXL	CC	373	370	DBSU	9.09
**4606**	allXL	BB	381	372	DBSU	19.23
**4606**	allXL	BB	372	382	DBSU	17.69
**4606**	allXL	DD	372	386	DBSU	24.63
**4606**	allXL	DD	373	386	DBSU	23.64
**4606**	allXL	CB	381	386	DBSU	82.66
**4606**	allXL	BD;DB	357	357	DBSU	19.71
**4606**	allXL	AD	373	319	DBSU	38.51
**4606**	allXL	CB	372	357	DBSU	51.57
**4606**	allXL	BD	381	357	DBSU	59.55
**4606**	allXL	DB	373	357	DBSU	53.86
**4606**	allXL	BC	372	370	DBSU	34.88
**4606**	allXL	BC	373	370	DBSU	37.62
**4606**	allXL	BC	382	373	DBSU	56.45
**4607**	allXL	CC	320	357	BS2G	24.43
**4607**	allXL	DD	320	373	BS2G	16.25
**4607**	allXL	BB	373	382	BS2G	17.79
**4607**	allXL	CC	370	386	BS2G	22.5
**4607**	allXL	AA	373	386	BS2G	26.16
**4607**	allXL	CC	319	357	BS2G	24.51
**4607**	allXL	BB	367	373	BS2G	14.97
**4607**	allXL	CC	370	382	BS2G	14.17

**4607**	allXL	BB	376	382	BS2G	14.01
**4607**	allXL	CC	357	373	BS2G	23.41
**4607**	allXL	DD	320	366	BS2G	14.22
**4607**	allXL	CC	370	376	BS2G	12.5
**4607**	allXL	CC	320	357	DBSU	24.43
**4607**	allXL	CC	357	319	DBSU	24.51
**4607**	allXL	DD	372	319	DBSU	14.91
**4607**	allXL	CC	370	357	DBSU	26.03
**4607**	allXL	CC	382	357	DBSU	39.45
**4607**	allXL	AA	372	370	DBSU	5.96
**4607**	allXL	CC	381	370	DBSU	16.46
**4607**	allXL	CC	373	370	DBSU	9.26
**4607**	allXL	CC	381	372	DBSU	19.38
**4607**	allXL	CC	372	382	DBSU	18.17
**4607**	allXL	AA	372	386	DBSU	26.67
**4607**	allXL	AA	373	386	DBSU	26.16
**4607**	allXL	BC	381	386	DBSU	34.01
**4607**	allXL	BD;DB	357	357	DBSU	19.87
**4607**	allXL	CB	373	319	DBSU	19.45
**4607**	allXL	CA	372	357	DBSU	31.63
**4607**	allXL	CB	381	357	DBSU	42.37
**4607**	allXL	CA	373	357	DBSU	30.92
**4607**	allXL	DA	372	370	DBSU	25.77
**4607**	allXL	DA	373	370	DBSU	24.4
**4607**	allXL	CB	382	373	DBSU	30.51
**4608**	allXL	AA	320	357	BS2G	24.71
**4608**	allXL	CC	320	373	BS2G	14.65
**4608**	allXL	DD	373	382	BS2G	18.93
**4608**	allXL	DD	370	386	BS2G	22.52
**4608**	allXL	DD	373	386	BS2G	27.24
**4608**	allXL	AA	319	357	BS2G	25.57
**4608**	allXL	AA	367	373	BS2G	11.63
**4608**	allXL	DD	370	382	BS2G	16.38
**4608**	allXL	BB	376	382	BS2G	14.27

**4608**	allXL	DD	357	373	BS2G	24.93
**4608**	allXL	AA	320	366	BS2G	29.41
**4608**	allXL	DD	370	376	BS2G	14.38
**4608**	allXL	AA	320	357	DBSU	24.71
**4608**	allXL	AA	357	319	DBSU	25.57
**4608**	allXL	CC	372	319	DBSU	16.91
**4608**	allXL	DD	370	357	DBSU	21.48
**4608**	allXL	DD	382	357	DBSU	25.31
**4608**	allXL	AA	372	370	DBSU	6.38
**4608**	allXL	DD	381	370	DBSU	13.83
**4608**	allXL	CC	373	370	DBSU	9.29
**4608**	allXL	DD	381	372	DBSU	16.48
**4608**	allXL	DD	372	382	DBSU	19.34
**4608**	allXL	DD	372	386	DBSU	26.34
**4608**	allXL	DD	373	386	DBSU	27.24
**4608**	allXL	AD	381	386	DBSU	48.86
**4608**	allXL	BD;DB	357	357	DBSU	19.23
**4608**	allXL	AD	373	319	DBSU	26.19
**4608**	allXL	CB	372	357	DBSU	39.2
**4608**	allXL	DB	381	357	DBSU	40.88
**4608**	allXL	AC	373	357	DBSU	39.41
**4608**	allXL	DA	372	370	DBSU	50.6
**4608**	allXL	DA	373	370	DBSU	49.6
**4608**	allXL	DA	382	373	DBSU	39.27
**4609**	allXL	AA	320	357	BS2G	24.41
**4609**	allXL	CC	320	373	BS2G	9.99
**4609**	allXL	DD	373	382	BS2G	16.47
**4609**	allXL	CC	370	386	BS2G	26.16
**4609**	allXL	AA	373	386	BS2G	23.05
**4609**	allXL	AA	319	357	BS2G	23.86
**4609**	allXL	AA	367	373	BS2G	11.82
**4609**	allXL	CC	370	382	BS2G	20.5
**4609**	allXL	AA	376	382	BS2G	13.53
**4609**	allXL	CC	357	373	BS2G	22.32

**4609**	allXL	AA	320	366	BS2G	14.54
**4609**	allXL	CC	370	376	BS2G	13.62
**4609**	allXL	AA	320	357	DBSU	24.41
**4609**	allXL	AA	357	319	DBSU	23.86
**4609**	allXL	CC	372	319	DBSU	13.07
**4609**	allXL	CC	370	357	DBSU	24.15
**4609**	allXL	CC	382	357	DBSU	17.14
**4609**	allXL	AA	372	370	DBSU	6.25
**4609**	allXL	DD	381	370	DBSU	21.36
**4609**	allXL	DD	373	370	DBSU	8.82
**4609**	allXL	DD	381	372	DBSU	14.58
**4609**	allXL	DD	372	382	DBSU	14.8
**4609**	allXL	DD	372	386	DBSU	21.69
**4609**	allXL	AA	373	386	DBSU	23.05
**4609**	allXL	CA	381	386	DBSU	60.44
**4609**	allXL	BD;DB	357	357	DBSU	17.75
**4609**	allXL	AD	373	319	DBSU	23.77
**4609**	allXL	BD	372	357	DBSU	38.88
**4609**	allXL	CA	381	357	DBSU	34.53
**4609**	allXL	CA	373	357	DBSU	40.69
**4609**	allXL	BD	372	370	DBSU	57.28
**4609**	allXL	BD	373	370	DBSU	58.4
**4609**	allXL	AC	382	373	DBSU	48.78
**4610**	allXL	CC	320	357	BS2G	26.41
**4610**	allXL	DD	320	373	BS2G	22.66
**4610**	allXL	BB	373	382	BS2G	18.61
**4610**	allXL	AA	370	386	BS2G	19.28
**4610**	allXL	AA	373	386	BS2G	23.48
**4610**	allXL	AA	319	357	BS2G	25.43
**4610**	allXL	BB	367	373	BS2G	10.43
**4610**	allXL	AA	370	382	BS2G	18.19
**4610**	allXL	BB	376	382	BS2G	14.02
**4610**	allXL	BB	357	373	BS2G	20.49
**4610**	allXL	CC	320	366	BS2G	16.64

**4610**	allXL	AA	370	376	BS2G	14.68
**4610**	allXL	CC	320	357	DBSU	26.41
**4610**	allXL	AA	357	319	DBSU	25.43
**4610**	allXL	DD	372	319	DBSU	20.25
**4610**	allXL	CC	370	357	DBSU	23.58
**4610**	allXL	BB	382	357	DBSU	27.72
**4610**	allXL	CC	372	370	DBSU	6.62
**4610**	allXL	AA	381	370	DBSU	19.86
**4610**	allXL	AA	373	370	DBSU	7.54
**4610**	allXL	BB	381	372	DBSU	20.61
**4610**	allXL	BB	372	382	DBSU	19.95
**4610**	allXL	AA	372	386	DBSU	25.03
**4610**	allXL	AA	373	386	DBSU	23.48
**4610**	allXL	DA	381	386	DBSU	43.1
**4610**	allXL	BD;DB	357	357	DBSU	18.38
**4610**	allXL	AD	373	319	DBSU	7.97
**4610**	allXL	AD	372	357	DBSU	38.2
**4610**	allXL	BD	381	357	DBSU	35.33
**4610**	allXL	AD	373	357	DBSU	35.17
**4610**	allXL	AD	372	370	DBSU	21.49
**4610**	allXL	AD	373	370	DBSU	19.61
**4610**	allXL	AD	382	373	DBSU	23.68
**4611**	allXL	CC	320	357	BS2G	21.73
**4611**	allXL	DD	320	373	BS2G	18.77
**4611**	allXL	DD	373	382	BS2G	19.46
**4611**	allXL	DD	370	386	BS2G	14.17
**4611**	allXL	DD	373	386	BS2G	22.67
**4611**	allXL	CC	319	357	BS2G	23.74
**4611**	allXL	BB	367	373	BS2G	13.54
**4611**	allXL	DD	370	382	BS2G	13.16
**4611**	allXL	BB	376	382	BS2G	14.59
**4611**	allXL	DD	357	373	BS2G	33.68
**4611**	allXL	DD	320	366	BS2G	22.49
**4611**	allXL	DD	370	376	BS2G	14.82

**4611**	allXL	CC	320	357	DBSU	21.73
**4611**	allXL	CC	357	319	DBSU	23.74
**4611**	allXL	DD	372	319	DBSU	20.38
**4611**	allXL	DD	370	357	DBSU	29.41
**4611**	allXL	DD	382	357	DBSU	32.16
**4611**	allXL	AA	372	370	DBSU	6.21
**4611**	allXL	DD	381	370	DBSU	13.39
**4611**	allXL	AA	373	370	DBSU	9.07
**4611**	allXL	DD	381	372	DBSU	18.38
**4611**	allXL	DD	372	382	DBSU	19.28
**4611**	allXL	DD	372	386	DBSU	20.52
**4611**	allXL	DD	373	386	DBSU	22.67
**4611**	allXL	DA	381	386	DBSU	67.84
**4611**	allXL	BD;DB	357	357	DBSU	18.58
**4611**	allXL	AD	373	319	DBSU	27.55
**4611**	allXL	CA	372	357	DBSU	46.94
**4611**	allXL	DB	381	357	DBSU	50.3
**4611**	allXL	DA	373	357	DBSU	45.87
**4611**	allXL	DA	372	370	DBSU	27.02
**4611**	allXL	DA	373	370	DBSU	24.46
**4611**	allXL	AD	382	373	DBSU	48.98
**4612**	allXL	CC	320	357	BS2G	26.07
**4612**	allXL	BB	320	373	BS2G	18.17
**4612**	allXL	CC	373	382	BS2G	21.12
**4612**	allXL	CC	370	386	BS2G	19.14
**4612**	allXL	AA	373	386	BS2G	27.54
**4612**	allXL	AA	319	357	BS2G	26.13
**4612**	allXL	AA	367	373	BS2G	14.12
**4612**	allXL	CC	370	382	BS2G	15.95
**4612**	allXL	BB	376	382	BS2G	13.91
**4612**	allXL	DD	357	373	BS2G	33.06
**4612**	allXL	DD	320	366	BS2G	16.15
**4612**	allXL	CC	370	376	BS2G	11.45
**4612**	allXL	CC	320	357	DBSU	26.07

**4612**	allXL	AA	357	319	DBSU	26.13
**4612**	allXL	BB	372	319	DBSU	18.77
**4612**	allXL	DD	370	357	DBSU	28.1
**4612**	allXL	CC	382	357	DBSU	34.75
**4612**	allXL	AA	372	370	DBSU	6.32
**4612**	allXL	CC	381	370	DBSU	17.69
**4612**	allXL	AA	373	370	DBSU	9.06
**4612**	allXL	DD	381	372	DBSU	20.19
**4612**	allXL	CC	372	382	DBSU	20.12
**4612**	allXL	CC	372	386	DBSU	25.15
**4612**	allXL	AA	373	386	DBSU	27.54
**4612**	allXL	DA	381	386	DBSU	43.8
**4612**	allXL	BD;DB	357	357	DBSU	19.24
**4612**	allXL	DA	373	319	DBSU	46.28
**4612**	allXL	BC	372	357	DBSU	46.95
**4612**	allXL	CB	381	357	DBSU	47.27
**4612**	allXL	BC	373	357	DBSU	49.72
**4612**	allXL	BC	372	370	DBSU	45.35
**4612**	allXL	BC	373	370	DBSU	48.36
**4612**	allXL	CB	382	373	DBSU	41.59
**4613**	allXL	AA	320	357	BS2G	21.68
**4613**	allXL	DD	320	373	BS2G	21.45
**4613**	allXL	AA	373	382	BS2G	20.49
**4613**	allXL	BB	370	386	BS2G	25.79
**4613**	allXL	AA	373	386	BS2G	29.02
**4613**	allXL	AA	319	357	BS2G	24.94
**4613**	allXL	DD	367	373	BS2G	14.55
**4613**	allXL	AA	370	382	BS2G	22.51
**4613**	allXL	CC	376	382	BS2G	14.54
**4613**	allXL	DD	357	373	BS2G	20.63
**4613**	allXL	BB	320	366	BS2G	19.89
**4613**	allXL	AA	370	376	BS2G	15.6
**4613**	allXL	AA	320	357	DBSU	21.68
**4613**	allXL	AA	357	319	DBSU	24.94

**4613**	allXL	BB	372	319	DBSU	25.28
**4613**	allXL	DD	370	357	DBSU	21.12
**4613**	allXL	DD	382	357	DBSU	31.05
**4613**	allXL	CC	372	370	DBSU	6.42
**4613**	allXL	BB	381	370	DBSU	20.35
**4613**	allXL	AA	373	370	DBSU	7.4
**4613**	allXL	DD	381	372	DBSU	19.02
**4613**	allXL	DD	372	382	DBSU	21.97
**4613**	allXL	BB	372	386	DBSU	29.95
**4613**	allXL	AA	373	386	DBSU	29.02
**4613**	allXL	CB	381	386	DBSU	21.8
**4613**	allXL	BD;DB	357	357	DBSU	18.18
**4613**	allXL	CB	373	319	DBSU	24.04
**4613**	allXL	BD	372	357	DBSU	34.18
**4613**	allXL	DB	381	357	DBSU	38.99
**4613**	allXL	BD	373	357	DBSU	35.35
**4613**	allXL	CB	372	370	DBSU	39.23
**4613**	allXL	CB	373	370	DBSU	39.18
**4613**	allXL	BC	382	373	DBSU	44.37
**4614**	allXL	AA	320	357	BS2G	25.07
**4614**	allXL	DD	320	373	BS2G	23.81
**4614**	allXL	AA	373	382	BS2G	19.03
**4614**	allXL	AA	370	386	BS2G	21.35
**4614**	allXL	AA	373	386	BS2G	22.55
**4614**	allXL	AA	319	357	BS2G	27.0
**4614**	allXL	AA	367	373	BS2G	14.94
**4614**	allXL	BB	370	382	BS2G	22.12
**4614**	allXL	AA	376	382	BS2G	15.39
**4614**	allXL	BB	357	373	BS2G	30.15
**4614**	allXL	CC	320	366	BS2G	24.02
**4614**	allXL	CC	370	376	BS2G	16.03
**4614**	allXL	AA	320	357	DBSU	25.07
**4614**	allXL	AA	357	319	DBSU	27.0
**4614**	allXL	DD	372	319	DBSU	22.62

**4614**	allXL	DD	370	357	DBSU	28.19
**4614**	allXL	BB	382	357	DBSU	50.53
**4614**	allXL	AA	372	370	DBSU	5.98
**4614**	allXL	BB	381	370	DBSU	23.28
**4614**	allXL	AA	373	370	DBSU	9.22
**4614**	allXL	AA	381	372	DBSU	20.87
**4614**	allXL	AA	372	382	DBSU	19.03
**4614**	allXL	AA	372	386	DBSU	20.7
**4614**	allXL	AA	373	386	DBSU	22.55
**4614**	allXL	DA	381	386	DBSU	29.58
**4614**	allXL	BD;DB	357	357	DBSU	19.01
**4614**	allXL	AD	373	319	DBSU	32.54
**4614**	allXL	BD	372	357	DBSU	40.98
**4614**	allXL	BD	381	357	DBSU	63.98
**4614**	allXL	BD	373	357	DBSU	43.72
**4614**	allXL	DA	372	370	DBSU	39.13
**4614**	allXL	DA	373	370	DBSU	41.47
**4614**	allXL	AD	382	373	DBSU	40.16
**4615**	allXL	CC	320	357	BS2G	22.31
**4615**	allXL	CC	320	373	BS2G	15.93
**4615**	allXL	CC	373	382	BS2G	20.2
**4615**	allXL	CC	370	386	BS2G	20.81
**4615**	allXL	DD	373	386	BS2G	20.8
**4615**	allXL	CC	319	357	BS2G	21.0
**4615**	allXL	AA	367	373	BS2G	13.17
**4615**	allXL	CC	370	382	BS2G	16.26
**4615**	allXL	CC	376	382	BS2G	14.69
**4615**	allXL	DD	357	373	BS2G	30.98
**4615**	allXL	CC	320	366	BS2G	18.18
**4615**	allXL	CC	370	376	BS2G	12.71
**4615**	allXL	CC	320	357	DBSU	22.31
**4615**	allXL	CC	357	319	DBSU	21.0
**4615**	allXL	CC	372	319	DBSU	16.18
**4615**	allXL	DD	370	357	DBSU	25.82

**4615**	allXL	BB	382	357	DBSU	28.06
**4615**	allXL	CC	372	370	DBSU	6.52
**4615**	allXL	CC	381	370	DBSU	17.14
**4615**	allXL	CC	373	370	DBSU	9.08
**4615**	allXL	DD	381	372	DBSU	19.24
**4615**	allXL	CC	372	382	DBSU	20.18
**4615**	allXL	DD	372	386	DBSU	20.2
**4615**	allXL	DD	373	386	DBSU	20.8
**4615**	allXL	BC	381	386	DBSU	28.66
**4615**	allXL	BD;DB	357	357	DBSU	19.19
**4615**	allXL	DA	373	319	DBSU	36.62
**4615**	allXL	DB	372	357	DBSU	34.51
**4615**	allXL	CB	381	357	DBSU	26.57
**4615**	allXL	DB	373	357	DBSU	34.73
**4615**	allXL	CB	372	370	DBSU	38.43
**4615**	allXL	CB	373	370	DBSU	37.1
**4615**	allXL	CB	382	373	DBSU	27.15
**4616**	allXL	CC	320	357	BS2G	24.54
**4616**	allXL	BB	320	373	BS2G	14.93
**4616**	allXL	DD	373	382	BS2G	16.66
**4616**	allXL	AA	370	386	BS2G	23.25
**4616**	allXL	AA	373	386	BS2G	21.04
**4616**	allXL	AA	319	357	BS2G	25.67
**4616**	allXL	AA	367	373	BS2G	13.2
**4616**	allXL	DD	370	382	BS2G	22.21
**4616**	allXL	BB	376	382	BS2G	14.05
**4616**	allXL	CC	357	373	BS2G	17.11
**4616**	allXL	CC	320	366	BS2G	16.96
**4616**	allXL	CC	370	376	BS2G	15.15
**4616**	allXL	CC	320	357	DBSU	24.54
**4616**	allXL	AA	357	319	DBSU	25.67
**4616**	allXL	BB	372	319	DBSU	17.94
**4616**	allXL	CC	370	357	DBSU	18.66
**4616**	allXL	CC	382	357	DBSU	20.41

**4616**	allXL	AA	372	370	DBSU	6.23
**4616**	allXL	CC	381	370	DBSU	20.25
**4616**	allXL	DD	373	370	DBSU	8.94
**4616**	allXL	DD	381	372	DBSU	16.55
**4616**	allXL	DD	372	382	DBSU	15.7
**4616**	allXL	DD	372	386	DBSU	21.13
**4616**	allXL	AA	373	386	DBSU	21.04
**4616**	allXL	BC	381	386	DBSU	42.41
**4616**	allXL	BD;DB	357	357	DBSU	18.9
**4616**	allXL	AD	373	319	DBSU	35.18
**4616**	allXL	CA	372	357	DBSU	34.4
**4616**	allXL	CA	381	357	DBSU	36.1
**4616**	allXL	CA	373	357	DBSU	31.41
**4616**	allXL	AD	372	370	DBSU	47.05
**4616**	allXL	AD	373	370	DBSU	46.55
**4616**	allXL	CB	382	373	DBSU	39.5
**4617**	allXL	CC	320	357	BS2G	24.14
**4617**	allXL	BB	320	373	BS2G	23.67
**4617**	allXL	AA	373	382	BS2G	16.11
**4617**	allXL	AA	370	386	BS2G	17.55
**4617**	allXL	AA	373	386	BS2G	20.87
**4617**	allXL	AA	319	357	BS2G	27.62
**4617**	allXL	BB	367	373	BS2G	10.11
**4617**	allXL	AA	370	382	BS2G	15.01
**4617**	allXL	BB	376	382	BS2G	14.95
**4617**	allXL	CC	357	373	BS2G	26.54
**4617**	allXL	BB	320	366	BS2G	25.78
**4617**	allXL	CC	370	376	BS2G	14.69
**4617**	allXL	CC	320	357	DBSU	24.14
**4617**	allXL	AA	357	319	DBSU	27.62
**4617**	allXL	BB	372	319	DBSU	25.44
**4617**	allXL	BB	370	357	DBSU	28.79
**4617**	allXL	AA	382	357	DBSU	22.41
**4617**	allXL	AA	372	370	DBSU	6.86

**4617**	allXL	AA	381	370	DBSU	13.05
**4617**	allXL	AA	373	370	DBSU	9.13
**4617**	allXL	AA	381	372	DBSU	15.3
**4617**	allXL	AA	372	382	DBSU	17.4
**4617**	allXL	AA	372	386	DBSU	20.49
**4617**	allXL	AA	373	386	DBSU	20.87
**4617**	allXL	DA	381	386	DBSU	27.53
**4617**	allXL	BD;DB	357	357	DBSU	18.83
**4617**	allXL	AD	373	319	DBSU	36.59
**4617**	allXL	CA	372	357	DBSU	45.65
**4617**	allXL	CB	381	357	DBSU	40.25
**4617**	allXL	CA	373	357	DBSU	42.21
**4617**	allXL	DA	372	370	DBSU	38.19
**4617**	allXL	DA	373	370	DBSU	35.28
**4617**	allXL	CB	382	373	DBSU	29.8
**4618**	allXL	BB	320	357	BS2G	23.11
**4618**	allXL	BB	320	373	BS2G	33.56
**4618**	allXL	AA	373	382	BS2G	19.54
**4618**	allXL	DD	370	386	BS2G	21.49
**4618**	allXL	CC	373	386	BS2G	25.14
**4618**	allXL	BB	319	357	BS2G	23.84
**4618**	allXL	AA	367	373	BS2G	13.03
**4618**	allXL	DD	370	382	BS2G	20.62
**4618**	allXL	BB	376	382	BS2G	14.41
**4618**	allXL	BB	357	373	BS2G	28.12
**4618**	allXL	BB	320	366	BS2G	17.99
**4618**	allXL	DD	370	376	BS2G	15.38
**4618**	allXL	BB	320	357	DBSU	23.11
**4618**	allXL	BB	357	319	DBSU	23.84
**4618**	allXL	DD	372	319	DBSU	31.81
**4618**	allXL	BB	370	357	DBSU	26.48
**4618**	allXL	AA	382	357	DBSU	33.34
**4618**	allXL	AA	372	370	DBSU	6.37
**4618**	allXL	DD	381	370	DBSU	18.53

**4618**	allXL	DD	373	370	DBSU	9.11
**4618**	allXL	DD	381	372	DBSU	20.54
**4618**	allXL	AA	372	382	DBSU	21.77
**4618**	allXL	AA	372	386	DBSU	25.92
**4618**	allXL	CC	373	386	DBSU	25.14
**4618**	allXL	CB	381	386	DBSU	49.85
**4618**	allXL	BD;DB	357	357	DBSU	17.44
**4618**	allXL	CB	373	319	DBSU	17.61
**4618**	allXL	BD	372	357	DBSU	31.88
**4618**	allXL	BD	381	357	DBSU	43.34
**4618**	allXL	BD	373	357	DBSU	33.46
**4618**	allXL	CB	372	370	DBSU	36.74
**4618**	allXL	CB	373	370	DBSU	38.55
**4618**	allXL	BC	382	373	DBSU	48.26
**4619**	allXL	AA	320	357	BS2G	16.62
**4619**	allXL	DD	320	373	BS2G	18.36
**4619**	allXL	BB	373	382	BS2G	16.39
**4619**	allXL	AA	370	386	BS2G	27.97
**4619**	allXL	BB	373	386	BS2G	23.78
**4619**	allXL	AA	319	357	BS2G	13.92
**4619**	allXL	AA	367	373	BS2G	12.81
**4619**	allXL	DD	370	382	BS2G	21.25
**4619**	allXL	BB	376	382	BS2G	14.23
**4619**	allXL	BB	357	373	BS2G	22.82
**4619**	allXL	CC	320	366	BS2G	16.69
**4619**	allXL	CC	370	376	BS2G	14.63
**4619**	allXL	AA	320	357	DBSU	16.62
**4619**	allXL	AA	357	319	DBSU	13.92
**4619**	allXL	DD	372	319	DBSU	16.21
**4619**	allXL	DD	370	357	DBSU	22.62
**4619**	allXL	BB	382	357	DBSU	18.05
**4619**	allXL	AA	372	370	DBSU	6.27
**4619**	allXL	DD	381	370	DBSU	19.77
**4619**	allXL	AA	373	370	DBSU	9.09

**4619**	allXL	BB	381	372	DBSU	17.91
**4619**	allXL	BB	372	382	DBSU	18.27
**4619**	allXL	BB	372	386	DBSU	24.26
**4619**	allXL	BB	373	386	DBSU	23.78
**4619**	allXL	BD	381	386	DBSU	51.05
**4619**	allXL	BD;DB	357	357	DBSU	19.09
**4619**	allXL	BD	373	319	DBSU	37.84
**4619**	allXL	BD	372	357	DBSU	18.02
**4619**	allXL	AC	381	357	DBSU	15.77
**4619**	allXL	BD	373	357	DBSU	17.93
**4619**	allXL	BD	372	370	DBSU	32.25
**4619**	allXL	BD	373	370	DBSU	31.79
**4619**	allXL	AC	382	373	DBSU	45.58
**4620**	allXL	DD	320	357	BS2G	25.74
**4620**	allXL	DD	320	373	BS2G	30.34
**4620**	allXL	BB	373	382	BS2G	16.59
**4620**	allXL	AA	370	386	BS2G	21.57
**4620**	allXL	AA	373	386	BS2G	21.88
**4620**	allXL	AA	319	357	BS2G	27.8
**4620**	allXL	AA	367	373	BS2G	12.96
**4620**	allXL	BB	370	382	BS2G	15.89
**4620**	allXL	BB	376	382	BS2G	13.43
**4620**	allXL	DD	357	373	BS2G	32.11
**4620**	allXL	AA	320	366	BS2G	25.25
**4620**	allXL	CC	370	376	BS2G	15.18
**4620**	allXL	DD	320	357	DBSU	25.74
**4620**	allXL	AA	357	319	DBSU	27.8
**4620**	allXL	DD	372	319	DBSU	32.4
**4620**	allXL	DD	370	357	DBSU	26.66
**4620**	allXL	BB	382	357	DBSU	33.01
**4620**	allXL	AA	372	370	DBSU	6.2
**4620**	allXL	BB	381	370	DBSU	18.75
**4620**	allXL	AA	373	370	DBSU	9.18
**4620**	allXL	DD	381	372	DBSU	16.86

**4620**	allXL	DD	372	382	DBSU	17.6
**4620**	allXL	AA	372	386	DBSU	19.92
**4620**	allXL	AA	373	386	DBSU	21.88
**4620**	allXL	CB	381	386	DBSU	33.16
**4620**	allXL	BD;DB	357	357	DBSU	18.26
**4620**	allXL	CB	373	319	DBSU	19.84
**4620**	allXL	CB	372	357	DBSU	36.35
**4620**	allXL	CB	381	357	DBSU	49.62
**4620**	allXL	CB	373	357	DBSU	39.88
**4620**	allXL	CB	372	370	DBSU	26.88
**4620**	allXL	CB	373	370	DBSU	27.48
**4620**	allXL	BC	382	373	DBSU	35.38
**4621**	allXL	AA	320	357	BS2G	26.19
**4621**	allXL	CC	320	373	BS2G	25.02
**4621**	allXL	CC	373	382	BS2G	20.01
**4621**	allXL	AA	370	386	BS2G	27.39
**4621**	allXL	AA	373	386	BS2G	26.92
**4621**	allXL	AA	319	357	BS2G	26.14
**4621**	allXL	AA	367	373	BS2G	10.42
**4621**	allXL	BB	370	382	BS2G	22.75
**4621**	allXL	CC	376	382	BS2G	15.32
**4621**	allXL	DD	357	373	BS2G	31.07
**4621**	allXL	DD	320	366	BS2G	23.75
**4621**	allXL	CC	370	376	BS2G	15.23
**4621**	allXL	AA	320	357	DBSU	26.19
**4621**	allXL	AA	357	319	DBSU	26.14
**4621**	allXL	CC	372	319	DBSU	20.5
**4621**	allXL	CC	370	357	DBSU	25.3
**4621**	allXL	BB	382	357	DBSU	22.82
**4621**	allXL	AA	372	370	DBSU	6.61
**4621**	allXL	BB	381	370	DBSU	24.25
**4621**	allXL	DD	373	370	DBSU	8.75
**4621**	allXL	BB	381	372	DBSU	24.48
**4621**	allXL	CC	372	382	DBSU	23.73

**4621**	allXL	AA	372	386	DBSU	27.23
**4621**	allXL	AA	373	386	DBSU	26.92
**4621**	allXL	BC	381	386	DBSU	16.33
**4621**	allXL	BD;DB	357	357	DBSU	17.89
**4621**	allXL	CB	373	319	DBSU	24.52
**4621**	allXL	CB	372	357	DBSU	31.82
**4621**	allXL	CB	381	357	DBSU	28.47
**4621**	allXL	CB	373	357	DBSU	29.95
**4621**	allXL	CB	372	370	DBSU	22.48
**4621**	allXL	CB	373	370	DBSU	21.4
**4621**	allXL	CB	382	373	DBSU	22.97
**4622**	allXL	CC	320	357	BS2G	24.82
**4622**	allXL	CC	320	373	BS2G	20.91
**4622**	allXL	BB	373	382	BS2G	19.2
**4622**	allXL	CC	370	386	BS2G	22.26
**4622**	allXL	BB	373	386	BS2G	22.46
**4622**	allXL	AA	319	357	BS2G	28.34
**4622**	allXL	BB	367	373	BS2G	13.63
**4622**	allXL	CC	370	382	BS2G	16.7
**4622**	allXL	BB	376	382	BS2G	13.15
**4622**	allXL	DD	357	373	BS2G	25.82
**4622**	allXL	CC	320	366	BS2G	15.78
**4622**	allXL	CC	370	376	BS2G	12.17
**4622**	allXL	CC	320	357	DBSU	24.82
**4622**	allXL	AA	357	319	DBSU	28.34
**4622**	allXL	CC	372	319	DBSU	16.04
**4622**	allXL	DD	370	357	DBSU	19.99
**4622**	allXL	CC	382	357	DBSU	35.46
**4622**	allXL	AA	372	370	DBSU	6.44
**4622**	allXL	CC	381	370	DBSU	16.97
**4622**	allXL	AA	373	370	DBSU	9.4
**4622**	allXL	CC	381	372	DBSU	21.06
**4622**	allXL	CC	372	382	DBSU	20.52
**4622**	allXL	BB	372	386	DBSU	22.4

**4622**	allXL	BB	373	386	DBSU	22.46
**4622**	allXL	AD	381	386	DBSU	27.46
**4622**	allXL	BD;DB	357	357	DBSU	19.05
**4622**	allXL	DA	373	319	DBSU	23.31
**4622**	allXL	DA	372	357	DBSU	28.94
**4622**	allXL	CB	381	357	DBSU	32.05
**4622**	allXL	DA	373	357	DBSU	27.26
**4622**	allXL	DA	372	370	DBSU	43.0
**4622**	allXL	DA	373	370	DBSU	39.14
**4622**	allXL	DA	382	373	DBSU	23.2
**4623**	allXL	AA	320	357	BS2G	22.66
**4623**	allXL	CC	320	373	BS2G	21.71
**4623**	allXL	CC	373	382	BS2G	18.62
**4623**	allXL	CC	370	386	BS2G	23.11
**4623**	allXL	DD	373	386	BS2G	21.8
**4623**	allXL	AA	319	357	BS2G	21.58
**4623**	allXL	AA	367	373	BS2G	12.36
**4623**	allXL	CC	370	382	BS2G	20.01
**4623**	allXL	BB	376	382	BS2G	13.69
**4623**	allXL	CC	357	373	BS2G	24.58
**4623**	allXL	DD	320	366	BS2G	16.95
**4623**	allXL	CC	370	376	BS2G	15.05
**4623**	allXL	AA	320	357	DBSU	22.66
**4623**	allXL	AA	357	319	DBSU	21.58
**4623**	allXL	CC	372	319	DBSU	19.82
**4623**	allXL	CC	370	357	DBSU	23.6
**4623**	allXL	CC	382	357	DBSU	18.05
**4623**	allXL	AA	372	370	DBSU	6.24
**4623**	allXL	CC	381	370	DBSU	22.48
**4623**	allXL	AA	373	370	DBSU	9.09
**4623**	allXL	AA	381	372	DBSU	20.06
**4623**	allXL	CC	372	382	DBSU	20.92
**4623**	allXL	DD	372	386	DBSU	23.58
**4623**	allXL	DD	373	386	DBSU	21.8

**4623**	allXL	BD	381	386	DBSU	35.81
**4623**	allXL	BD;DB	357	357	DBSU	19.36
**4623**	allXL	AD	373	319	DBSU	26.58
**4623**	allXL	CA	372	357	DBSU	42.3
**4623**	allXL	CA	381	357	DBSU	27.31
**4623**	allXL	CA	373	357	DBSU	40.08
**4623**	allXL	AD	372	370	DBSU	46.73
**4623**	allXL	AD	373	370	DBSU	43.61
**4623**	allXL	AD	382	373	DBSU	43.83
**4624**	allXL	AA	320	357	BS2G	25.02
**4624**	allXL	BB	320	373	BS2G	28.73
**4624**	allXL	AA	373	382	BS2G	19.5
**4624**	allXL	DD	370	386	BS2G	22.33
**4624**	allXL	BB	373	386	BS2G	26.38
**4624**	allXL	AA	319	357	BS2G	25.64
**4624**	allXL	BB	367	373	BS2G	14.16
**4624**	allXL	DD	370	382	BS2G	21.99
**4624**	allXL	AA	376	382	BS2G	13.55
**4624**	allXL	BB	357	373	BS2G	17.03
**4624**	allXL	BB	320	366	BS2G	16.09
**4624**	allXL	CC	370	376	BS2G	16.0
**4624**	allXL	AA	320	357	DBSU	25.02
**4624**	allXL	AA	357	319	DBSU	25.64
**4624**	allXL	DD	372	319	DBSU	27.39
**4624**	allXL	BB	370	357	DBSU	22.42
**4624**	allXL	BB	382	357	DBSU	16.44
**4624**	allXL	AA	372	370	DBSU	6.2
**4624**	allXL	DD	381	370	DBSU	21.06
**4624**	allXL	AA	373	370	DBSU	9.13
**4624**	allXL	BB	381	372	DBSU	22.03
**4624**	allXL	AA	372	382	DBSU	22.73
**4624**	allXL	DD	372	386	DBSU	27.25
**4624**	allXL	BB	373	386	DBSU	26.38
**4624**	allXL	BD	381	386	DBSU	36.81

**4624**	allXL	BD;DB	357	357	DBSU	18.7
**4624**	allXL	CB	373	319	DBSU	24.44
**4624**	allXL	BD	372	357	DBSU	35.06
**4624**	allXL	BD	381	357	DBSU	18.5
**4624**	allXL	BD	373	357	DBSU	31.83
**4624**	allXL	DA	372	370	DBSU	26.64
**4624**	allXL	DA	373	370	DBSU	29.2
**4624**	allXL	BD	382	373	DBSU	44.99
**4625**	allXL	BB	320	357	BS2G	26.23
**4625**	allXL	DD	320	373	BS2G	12.97
**4625**	allXL	DD	373	382	BS2G	16.66
**4625**	allXL	BB	370	386	BS2G	26.15
**4625**	allXL	DD	373	386	BS2G	22.05
**4625**	allXL	AA	319	357	BS2G	27.46
**4625**	allXL	AA	367	373	BS2G	13.81
**4625**	allXL	DD	370	382	BS2G	21.83
**4625**	allXL	BB	376	382	BS2G	14.27
**4625**	allXL	CC	357	373	BS2G	15.15
**4625**	allXL	BB	320	366	BS2G	17.78
**4625**	allXL	CC	370	376	BS2G	16.16
**4625**	allXL	BB	320	357	DBSU	26.23
**4625**	allXL	AA	357	319	DBSU	27.46
**4625**	allXL	DD	372	319	DBSU	15.52
**4625**	allXL	CC	370	357	DBSU	19.96
**4625**	allXL	CC	382	357	DBSU	16.9
**4625**	allXL	AA	372	370	DBSU	6.33
**4625**	allXL	DD	381	370	DBSU	21.96
**4625**	allXL	AA	373	370	DBSU	9.19
**4625**	allXL	DD	381	372	DBSU	16.9
**4625**	allXL	DD	372	382	DBSU	16.28
**4625**	allXL	DD	372	386	DBSU	21.24
**4625**	allXL	DD	373	386	DBSU	22.05
**4625**	allXL	BC	381	386	DBSU	49.1
**4625**	allXL	BD;DB	357	357	DBSU	19.18

**4625**	allXL	AD	373	319	DBSU	19.55
**4625**	allXL	CA	372	357	DBSU	32.89
**4625**	allXL	CA	381	357	DBSU	23.71
**4625**	allXL	CA	373	357	DBSU	30.02
**4625**	allXL	DA	372	370	DBSU	23.18
**4625**	allXL	DA	373	370	DBSU	20.59
**4625**	allXL	DA	382	373	DBSU	36.51
**4626**	allXL	AA	320	357	BS2G	25.29
**4626**	allXL	DD	320	373	BS2G	15.55
**4626**	allXL	CC	373	382	BS2G	18.25
**4626**	allXL	DD	370	386	BS2G	28.86
**4626**	allXL	BB	373	386	BS2G	22.63
**4626**	allXL	AA	319	357	BS2G	24.3
**4626**	allXL	BB	367	373	BS2G	14.0
**4626**	allXL	DD	370	382	BS2G	23.89
**4626**	allXL	CC	376	382	BS2G	14.4
**4626**	allXL	BB	357	373	BS2G	28.4
**4626**	allXL	CC	320	366	BS2G	21.53
**4626**	allXL	CC	370	376	BS2G	15.73
**4626**	allXL	AA	320	357	DBSU	25.29
**4626**	allXL	AA	357	319	DBSU	24.3
**4626**	allXL	DD	372	319	DBSU	14.9
**4626**	allXL	DD	370	357	DBSU	25.51
**4626**	allXL	BB	382	357	DBSU	26.9
**4626**	allXL	AA	372	370	DBSU	6.03
**4626**	allXL	DD	381	370	DBSU	23.84
**4626**	allXL	DD	373	370	DBSU	9.05
**4626**	allXL	DD	381	372	DBSU	19.68
**4626**	allXL	DD	372	382	DBSU	19.8
**4626**	allXL	BB	372	386	DBSU	23.13
**4626**	allXL	BB	373	386	DBSU	22.63
**4626**	allXL	AD	381	386	DBSU	50.22
**4626**	allXL	BD;DB	357	357	DBSU	18.63
**4626**	allXL	AD	373	319	DBSU	16.2

**4626**	allXL	DA	372	357	DBSU	39.02
**4626**	allXL	CB	381	357	DBSU	41.83
**4626**	allXL	DA	373	357	DBSU	35.88
**4626**	allXL	DA	372	370	DBSU	28.6
**4626**	allXL	DA	373	370	DBSU	25.03
**4626**	allXL	DA	382	373	DBSU	35.35
**4627**	allXL	CC	320	357	BS2G	21.9
**4627**	allXL	CC	320	373	BS2G	13.16
**4627**	allXL	DD	373	382	BS2G	18.03
**4627**	allXL	DD	370	386	BS2G	25.18
**4627**	allXL	DD	373	386	BS2G	22.98
**4627**	allXL	CC	319	357	BS2G	24.33
**4627**	allXL	AA	367	373	BS2G	13.74
**4627**	allXL	CC	370	382	BS2G	22.28
**4627**	allXL	BB	376	382	BS2G	13.79
**4627**	allXL	CC	357	373	BS2G	15.91
**4627**	allXL	CC	320	366	BS2G	22.45
**4627**	allXL	CC	370	376	BS2G	13.47
**4627**	allXL	CC	320	357	DBSU	21.9
**4627**	allXL	CC	357	319	DBSU	24.33
**4627**	allXL	CC	372	319	DBSU	11.03
**4627**	allXL	CC	370	357	DBSU	17.78
**4627**	allXL	CC	382	357	DBSU	34.47
**4627**	allXL	AA	372	370	DBSU	6.27
**4627**	allXL	CC	381	370	DBSU	23.71
**4627**	allXL	DD	373	370	DBSU	8.93
**4627**	allXL	DD	381	372	DBSU	18.61
**4627**	allXL	DD	372	382	DBSU	18.33
**4627**	allXL	DD	372	386	DBSU	23.63
**4627**	allXL	DD	373	386	DBSU	22.98
**4627**	allXL	CA	381	386	DBSU	73.04
**4627**	allXL	BD;DB	357	357	DBSU	19.94
**4627**	allXL	AD	373	319	DBSU	25.59
**4627**	allXL	CA	372	357	DBSU	35.04

**4627**	allXL	DA	381	357	DBSU	43.89
**4627**	allXL	CA	373	357	DBSU	35.03
**4627**	allXL	DA	372	370	DBSU	32.7
**4627**	allXL	DA	373	370	DBSU	36.07
**4627**	allXL	DA	382	373	DBSU	50.41
**4628**	allXL	AA	320	357	BS2G	23.92
**4628**	allXL	DD	320	373	BS2G	27.32
**4628**	allXL	CC	373	382	BS2G	15.94
**4628**	allXL	CC	370	386	BS2G	18.9
**4628**	allXL	CC	373	386	BS2G	23.98
**4628**	allXL	AA	319	357	BS2G	22.44
**4628**	allXL	AA	367	373	BS2G	14.42
**4628**	allXL	CC	370	382	BS2G	14.02
**4628**	allXL	BB	376	382	BS2G	14.74
**4628**	allXL	BB	357	373	BS2G	28.02
**4628**	allXL	CC	320	366	BS2G	22.26
**4628**	allXL	CC	370	376	BS2G	14.61
**4628**	allXL	AA	320	357	DBSU	23.92
**4628**	allXL	AA	357	319	DBSU	22.44
**4628**	allXL	DD	372	319	DBSU	29.99
**4628**	allXL	BB	370	357	DBSU	22.3
**4628**	allXL	BB	382	357	DBSU	21.34
**4628**	allXL	AA	372	370	DBSU	6.16
**4628**	allXL	CC	381	370	DBSU	16.91
**4628**	allXL	DD	373	370	DBSU	9.1
**4628**	allXL	CC	381	372	DBSU	18.52
**4628**	allXL	CC	372	382	DBSU	16.41
**4628**	allXL	CC	372	386	DBSU	23.05
**4628**	allXL	CC	373	386	DBSU	23.98
**4628**	allXL	AD	381	386	DBSU	58.47
**4628**	allXL	BD;DB	357	357	DBSU	19.1
**4628**	allXL	AD	373	319	DBSU	30.91
**4628**	allXL	BD	372	357	DBSU	25.48
**4628**	allXL	BD	381	357	DBSU	30.51

**4628**	allXL	BD	373	357	DBSU	26.42
**4628**	allXL	DB	372	370	DBSU	40.19
**4628**	allXL	DB	373	370	DBSU	40.32
**4628**	allXL	BD	382	373	DBSU	51.6
**4629**	allXL	CC	320	357	BS2G	25.7
**4629**	allXL	DD	320	373	BS2G	24.22
**4629**	allXL	CC	373	382	BS2G	20.33
**4629**	allXL	CC	370	386	BS2G	29.69
**4629**	allXL	CC	373	386	BS2G	21.32
**4629**	allXL	CC	319	357	BS2G	28.72
**4629**	allXL	BB	367	373	BS2G	13.21
**4629**	allXL	DD	370	382	BS2G	21.08
**4629**	allXL	BB	376	382	BS2G	14.52
**4629**	allXL	DD	357	373	BS2G	29.95
**4629**	allXL	DD	320	366	BS2G	23.83
**4629**	allXL	CC	370	376	BS2G	16.4
**4629**	allXL	CC	320	357	DBSU	25.7
**4629**	allXL	CC	357	319	DBSU	28.72
**4629**	allXL	CC	372	319	DBSU	24.58
**4629**	allXL	DD	370	357	DBSU	22.46
**4629**	allXL	DD	382	357	DBSU	39.49
**4629**	allXL	AA	372	370	DBSU	6.15
**4629**	allXL	DD	381	370	DBSU	20.84
**4629**	allXL	AA	373	370	DBSU	9.19
**4629**	allXL	DD	381	372	DBSU	21.22
**4629**	allXL	DD	372	382	DBSU	21.93
**4629**	allXL	CC	372	386	DBSU	23.39
**4629**	allXL	CC	373	386	DBSU	21.32
**4629**	allXL	BC	381	386	DBSU	46.74
**4629**	allXL	BD;DB	357	357	DBSU	18.75
**4629**	allXL	AD	373	319	DBSU	22.96
**4629**	allXL	DB	372	357	DBSU	40.99
**4629**	allXL	CB	381	357	DBSU	30.44
**4629**	allXL	DB	373	357	DBSU	43.6

**4629**	allXL	DA	372	370	DBSU	39.95
**4629**	allXL	DA	373	370	DBSU	38.82
**4629**	allXL	CB	382	373	DBSU	30.64
**4630**	allXL	AA	320	357	BS2G	23.5
**4630**	allXL	DD	320	373	BS2G	28.73
**4630**	allXL	CC	373	382	BS2G	21.76
**4630**	allXL	CC	370	386	BS2G	26.71
**4630**	allXL	CC	373	386	BS2G	26.55
**4630**	allXL	CC	319	357	BS2G	24.48
**4630**	allXL	AA	367	373	BS2G	14.03
**4630**	allXL	CC	370	382	BS2G	26.48
**4630**	allXL	CC	376	382	BS2G	14.41
**4630**	allXL	CC	357	373	BS2G	31.47
**4630**	allXL	CC	320	366	BS2G	25.37
**4630**	allXL	CC	370	376	BS2G	17.04
**4630**	allXL	AA	320	357	DBSU	23.5
**4630**	allXL	CC	357	319	DBSU	24.48
**4630**	allXL	BB	372	319	DBSU	26.52
**4630**	allXL	CC	370	357	DBSU	28.22
**4630**	allXL	CC	382	357	DBSU	25.47
**4630**	allXL	AA	372	370	DBSU	6.17
**4630**	allXL	CC	381	370	DBSU	27.44
**4630**	allXL	AA	373	370	DBSU	9.0
**4630**	allXL	DD	381	372	DBSU	22.79
**4630**	allXL	CC	372	382	DBSU	23.08
**4630**	allXL	CC	372	386	DBSU	25.95
**4630**	allXL	CC	373	386	DBSU	26.55
**4630**	allXL	BC	381	386	DBSU	63.6
**4630**	allXL	BD;DB	357	357	DBSU	18.95
**4630**	allXL	AD	373	319	DBSU	15.92
**4630**	allXL	AD	372	357	DBSU	42.9
**4630**	allXL	CA	381	357	DBSU	34.43
**4630**	allXL	AD	373	357	DBSU	42.71
**4630**	allXL	DA	372	370	DBSU	31.35

**4630**	allXL	DA	373	370	DBSU	29.3
**4630**	allXL	AD	382	373	DBSU	49.49
**4631**	allXL	CC	320	357	BS2G	26.33
**4631**	allXL	BB	320	373	BS2G	29.73
**4631**	allXL	CC	373	382	BS2G	18.75
**4631**	allXL	CC	370	386	BS2G	25.83
**4631**	allXL	DD	373	386	BS2G	26.55
**4631**	allXL	DD	319	357	BS2G	26.98
**4631**	allXL	BB	367	373	BS2G	12.0
**4631**	allXL	CC	370	382	BS2G	16.87
**4631**	allXL	CC	376	382	BS2G	15.37
**4631**	allXL	BB	357	373	BS2G	26.33
**4631**	allXL	AA	320	366	BS2G	24.92
**4631**	allXL	CC	370	376	BS2G	14.72
**4631**	allXL	CC	320	357	DBSU	26.33
**4631**	allXL	DD	357	319	DBSU	26.98
**4631**	allXL	DD	372	319	DBSU	25.71
**4631**	allXL	DD	370	357	DBSU	31.3
**4631**	allXL	BB	382	357	DBSU	22.21
**4631**	allXL	AA	372	370	DBSU	6.03
**4631**	allXL	CC	381	370	DBSU	17.32
**4631**	allXL	AA	373	370	DBSU	9.03
**4631**	allXL	DD	381	372	DBSU	18.6
**4631**	allXL	CC	372	382	DBSU	19.41
**4631**	allXL	DD	372	386	DBSU	26.21
**4631**	allXL	DD	373	386	DBSU	26.55
**4631**	allXL	DA	381	386	DBSU	36.55
**4631**	allXL	BD;DB	357	357	DBSU	17.23
**4631**	allXL	BC	373	319	DBSU	31.72
**4631**	allXL	BC	372	357	DBSU	36.1
**4631**	allXL	BD	381	357	DBSU	24.82
**4631**	allXL	BC	373	357	DBSU	36.33
**4631**	allXL	CB	372	370	DBSU	16.91
**4631**	allXL	CB	373	370	DBSU	18.0

**4631**	allXL	AD	382	373	DBSU	35.98
**4632**	allXL	AA	320	357	BS2G	25.4
**4632**	allXL	BB	320	373	BS2G	22.45
**4632**	allXL	AA	373	382	BS2G	20.19
**4632**	allXL	DD	370	386	BS2G	21.81
**4632**	allXL	AA	373	386	BS2G	23.73
**4632**	allXL	AA	319	357	BS2G	24.6
**4632**	allXL	AA	367	373	BS2G	11.06
**4632**	allXL	DD	370	382	BS2G	21.48
**4632**	allXL	CC	376	382	BS2G	13.48
**4632**	allXL	BB	357	373	BS2G	19.48
**4632**	allXL	BB	320	366	BS2G	29.5
**4632**	allXL	CC	370	376	BS2G	16.63
**4632**	allXL	AA	320	357	DBSU	25.4
**4632**	allXL	AA	357	319	DBSU	24.6
**4632**	allXL	AA	372	319	DBSU	28.58
**4632**	allXL	DD	370	357	DBSU	26.18
**4632**	allXL	AA	382	357	DBSU	16.57
**4632**	allXL	AA	372	370	DBSU	6.56
**4632**	allXL	DD	381	370	DBSU	19.85
**4632**	allXL	AA	373	370	DBSU	9.03
**4632**	allXL	DD	381	372	DBSU	19.37
**4632**	allXL	DD	372	382	DBSU	22.03
**4632**	allXL	DD	372	386	DBSU	25.3
**4632**	allXL	AA	373	386	DBSU	23.73
**4632**	allXL	DB	381	386	DBSU	30.6
**4632**	allXL	BD;DB	357	357	DBSU	18.34
**4632**	allXL	CB	373	319	DBSU	19.21
**4632**	allXL	CB	372	357	DBSU	30.63
**4632**	allXL	BD	381	357	DBSU	25.51
**4632**	allXL	CB	373	357	DBSU	32.03
**4632**	allXL	CB	372	370	DBSU	31.61
**4632**	allXL	CB	373	370	DBSU	30.28
**4632**	allXL	BD	382	373	DBSU	27.83

**4633**	allXL	CC	320	357	BS2G	25.45
**4633**	allXL	CC	320	373	BS2G	33.08
**4633**	allXL	AA	373	382	BS2G	18.63
**4633**	allXL	AA	370	386	BS2G	23.99
**4633**	allXL	AA	373	386	BS2G	26.15
**4633**	allXL	AA	319	357	BS2G	27.11
**4633**	allXL	AA	367	373	BS2G	12.08
**4633**	allXL	AA	370	382	BS2G	20.65
**4633**	allXL	CC	376	382	BS2G	14.51
**4633**	allXL	DD	357	373	BS2G	29.5
**4633**	allXL	AA	320	366	BS2G	26.52
**4633**	allXL	CC	370	376	BS2G	14.04
**4633**	allXL	CC	320	357	DBSU	25.45
**4633**	allXL	AA	357	319	DBSU	27.11
**4633**	allXL	CC	372	319	DBSU	32.47
**4633**	allXL	DD	370	357	DBSU	28.25
**4633**	allXL	BB	382	357	DBSU	42.84
**4633**	allXL	AA	372	370	DBSU	6.37
**4633**	allXL	DD	381	370	DBSU	20.64
**4633**	allXL	AA	373	370	DBSU	9.02
**4633**	allXL	AA	381	372	DBSU	18.6
**4633**	allXL	AA	372	382	DBSU	16.81
**4633**	allXL	AA	372	386	DBSU	23.18
**4633**	allXL	AA	373	386	DBSU	26.15
**4633**	allXL	DB	381	386	DBSU	66.16
**4633**	allXL	BD;DB	357	357	DBSU	18.79
**4633**	allXL	AD	373	319	DBSU	16.29
**4633**	allXL	DB	372	357	DBSU	28.71
**4633**	allXL	DB	381	357	DBSU	46.78
**4633**	allXL	DB	373	357	DBSU	32.23
**4633**	allXL	DB	372	370	DBSU	50.22
**4633**	allXL	DB	373	370	DBSU	53.62
**4633**	allXL	BC	382	373	DBSU	56.76
**4634**	allXL	CC	320	357	BS2G	24.88

**4634**	allXL	BB	320	373	BS2G	27.53
**4634**	allXL	DD	373	382	BS2G	16.33
**4634**	allXL	DD	370	386	BS2G	19.18
**4634**	allXL	DD	373	386	BS2G	25.24
**4634**	allXL	CC	319	357	BS2G	27.28
**4634**	allXL	AA	367	373	BS2G	11.74
**4634**	allXL	DD	370	382	BS2G	11.0
**4634**	allXL	CC	376	382	BS2G	15.3
**4634**	allXL	BB	357	373	BS2G	27.1
**4634**	allXL	AA	320	366	BS2G	23.42
**4634**	allXL	DD	370	376	BS2G	13.4
**4634**	allXL	CC	320	357	DBSU	24.88
**4634**	allXL	CC	357	319	DBSU	27.28
**4634**	allXL	DD	372	319	DBSU	27.8
**4634**	allXL	DD	370	357	DBSU	30.69
**4634**	allXL	BB	382	357	DBSU	34.46
**4634**	allXL	AA	372	370	DBSU	6.47
**4634**	allXL	DD	381	370	DBSU	8.23
**4634**	allXL	AA	373	370	DBSU	9.22
**4634**	allXL	DD	381	372	DBSU	12.58
**4634**	allXL	DD	372	382	DBSU	15.11
**4634**	allXL	DD	372	386	DBSU	22.75
**4634**	allXL	DD	373	386	DBSU	25.24
**4634**	allXL	AD	381	386	DBSU	68.72
**4634**	allXL	BD;DB	357	357	DBSU	18.16
**4634**	allXL	AD	373	319	DBSU	25.01
**4634**	allXL	BD	372	357	DBSU	43.15
**4634**	allXL	BC	381	357	DBSU	36.66
**4634**	allXL	BD	373	357	DBSU	43.75
**4634**	allXL	DA	372	370	DBSU	29.27
**4634**	allXL	DA	373	370	DBSU	28.77
**4634**	allXL	DA	382	373	DBSU	49.74
**4635**	allXL	AA	320	357	BS2G	22.54
**4635**	allXL	AA	320	373	BS2G	26.59

**4635**	allXL	CC	373	382	BS2G	14.24
**4635**	allXL	CC	370	386	BS2G	19.43
**4635**	allXL	CC	373	386	BS2G	16.82
**4635**	allXL	AA	319	357	BS2G	21.1
**4635**	allXL	BB	367	373	BS2G	14.6
**4635**	allXL	CC	370	382	BS2G	20.61
**4635**	allXL	AA	376	382	BS2G	13.73
**4635**	allXL	AA	357	373	BS2G	32.47
**4635**	allXL	CC	320	366	BS2G	27.37
**4635**	allXL	BB	370	376	BS2G	16.38
**4635**	allXL	AA	320	357	DBSU	22.54
**4635**	allXL	AA	357	319	DBSU	21.1
**4635**	allXL	CC	372	319	DBSU	28.87
**4635**	allXL	CC	370	357	DBSU	27.59
**4635**	allXL	CD	382	357	DBSU	35.75
**4635**	allXL	AA	372	370	DBSU	6.07
**4635**	allXL	DD	381	370	DBSU	20.6
**4635**	allXL	AA	373	370	DBSU	9.16
**4635**	allXL	DD	381	372	DBSU	16.71
**4635**	allXL	CC	372	382	DBSU	16.91
**4635**	allXL	CC	372	386	DBSU	17.2
**4635**	allXL	CC	373	386	DBSU	16.82
**4635**	allXL	AD	381	386	DBSU	35.2
**4635**	allXL	BD;DB	357	357	DBSU	18.05
**4635**	allXL	AD	373	319	DBSU	14.49
**4635**	allXL	CB	372	357	DBSU	22.26
**4635**	allXL	CB	381	357	DBSU	17.02
**4635**	allXL	CB	373	357	DBSU	18.63
**4635**	allXL	AD	372	370	DBSU	26.32
**4635**	allXL	AD	373	370	DBSU	23.1
**4635**	allXL	CB	382	373	DBSU	20.77
**4636**	allXL	AA	320	357	BS2G	24.95
**4636**	allXL	BB	320	373	BS2G	24.65
**4636**	allXL	AA	373	382	BS2G	16.01

**4636**	allXL	BB	370	386	BS2G	18.01
**4636**	allXL	BB	373	386	BS2G	23.31
**4636**	allXL	AA	319	357	BS2G	27.88
**4636**	allXL	AA	367	373	BS2G	13.74
**4636**	allXL	AA	370	382	BS2G	16.61
**4636**	allXL	BB	376	382	BS2G	13.84
**4636**	allXL	BB	357	373	BS2G	30.12
**4636**	allXL	AA	320	366	BS2G	20.54
**4636**	allXL	CC	370	376	BS2G	15.93
**4636**	allXL	AA	320	357	DBSU	24.95
**4636**	allXL	AA	357	319	DBSU	27.88
**4636**	allXL	CC	372	319	DBSU	25.65
**4636**	allXL	DD	370	357	DBSU	24.0
**4636**	allXL	DD	382	357	DBSU	36.22
**4636**	allXL	AA	372	370	DBSU	6.19
**4636**	allXL	AA	381	370	DBSU	18.31
**4636**	allXL	CC	373	370	DBSU	9.15
**4636**	allXL	AA	381	372	DBSU	17.5
**4636**	allXL	AA	372	382	DBSU	16.7
**4636**	allXL	BB	372	386	DBSU	23.4
**4636**	allXL	BB	373	386	DBSU	23.31
**4636**	allXL	CB	381	386	DBSU	24.25
**4636**	allXL	BD;DB	357	357	DBSU	17.62
**4636**	allXL	CB	373	319	DBSU	24.83
**4636**	allXL	CB	372	357	DBSU	35.71
**4636**	allXL	CB	381	357	DBSU	32.89
**4636**	allXL	CB	373	357	DBSU	36.33
**4636**	allXL	CB	372	370	DBSU	26.1
**4636**	allXL	CB	373	370	DBSU	23.26
**4636**	allXL	CB	382	373	DBSU	10.26
**4637**	allXL	CC	320	357	BS2G	22.9
**4637**	allXL	AA	320	373	BS2G	31.24
**4637**	allXL	BB	373	382	BS2G	20.4
**4637**	allXL	AA	370	386	BS2G	27.66

**4637**	allXL	AA	373	386	BS2G	23.47
**4637**	allXL	CC	319	357	BS2G	24.91
**4637**	allXL	AA	367	373	BS2G	11.82
**4637**	allXL	CC	370	382	BS2G	25.25
**4637**	allXL	BB	376	382	BS2G	13.52
**4637**	allXL	BB	357	373	BS2G	25.72
**4637**	allXL	AA	320	366	BS2G	19.28
**4637**	allXL	CC	370	376	BS2G	15.22
**4637**	allXL	CC	320	357	DBSU	22.9
**4637**	allXL	CC	357	319	DBSU	24.91
**4637**	allXL	AA	372	319	DBSU	32.59
**4637**	allXL	BB	370	357	DBSU	27.41
**4637**	allXL	AA	382	357	DBSU	28.06
**4637**	allXL	CC	372	370	DBSU	6.53
**4637**	allXL	CC	381	370	DBSU	26.9
**4637**	allXL	AA	373	370	DBSU	9.23
**4637**	allXL	DD	381	372	DBSU	20.64
**4637**	allXL	DD	372	382	DBSU	23.21
**4637**	allXL	AA	372	386	DBSU	24.07
**4637**	allXL	AA	373	386	DBSU	23.47
**4637**	allXL	DA	381	386	DBSU	64.02
**4637**	allXL	BD;DB	357	357	DBSU	19.31
**4637**	allXL	BC	373	319	DBSU	27.15
**4637**	allXL	BD	372	357	DBSU	31.13
**4637**	allXL	BD	381	357	DBSU	34.89
**4637**	allXL	BD	373	357	DBSU	31.14
**4637**	allXL	DA	372	370	DBSU	48.87
**4637**	allXL	DA	373	370	DBSU	46.45
**4637**	allXL	DA	382	373	DBSU	59.0
**4638**	allXL	AA	320	357	BS2G	25.68
**4638**	allXL	CC	320	373	BS2G	22.36
**4638**	allXL	DD	373	382	BS2G	14.06
**4638**	allXL	DD	370	386	BS2G	12.11
**4638**	allXL	DD	373	386	BS2G	14.45

**4638**	allXL	AA	319	357	BS2G	27.95
**4638**	allXL	AA	367	373	BS2G	13.18
**4638**	allXL	DD	370	382	BS2G	7.72
**4638**	allXL	BB	376	382	BS2G	13.72
**4638**	allXL	AA	357	373	BS2G	22.07
**4638**	allXL	CC	320	366	BS2G	22.13
**4638**	allXL	CC	370	376	BS2G	15.89
**4638**	allXL	AA	320	357	DBSU	25.68
**4638**	allXL	AA	357	319	DBSU	27.95
**4638**	allXL	CC	372	319	DBSU	19.23
**4638**	allXL	DD	370	357	DBSU	26.34
**4638**	allXL	DD	382	357	DBSU	23.5
**4638**	allXL	AA	372	370	DBSU	6.19
**4638**	allXL	DD	381	370	DBSU	10.45
**4638**	allXL	AA	373	370	DBSU	9.16
**4638**	allXL	DD	381	372	DBSU	14.42
**4638**	allXL	DD	372	382	DBSU	12.98
**4638**	allXL	DD	372	386	DBSU	13.35
**4638**	allXL	DD	373	386	DBSU	14.45
**4638**	allXL	AD	381	386	DBSU	48.72
**4638**	allXL	BD;DB	357	357	DBSU	18.67
**4638**	allXL	DA	373	319	DBSU	14.83
**4638**	allXL	DA	372	357	DBSU	24.41
**4638**	allXL	AC	381	357	DBSU	26.76
**4638**	allXL	AC	373	357	DBSU	21.49
**4638**	allXL	CB	372	370	DBSU	39.44
**4638**	allXL	CB	373	370	DBSU	43.09
**4638**	allXL	AD	382	373	DBSU	52.38
**4639**	allXL	AA	320	357	BS2G	23.95
**4639**	allXL	CC	320	373	BS2G	15.3
**4639**	allXL	CC	373	382	BS2G	16.04
**4639**	allXL	AA	370	386	BS2G	22.23
**4639**	allXL	CC	373	386	BS2G	24.8
**4639**	allXL	AA	319	357	BS2G	22.8

**4639**	allXL	AA;DD	367	373	BS2G	14.02
**4639**	allXL	DD	370	382	BS2G	21.75
**4639**	allXL	BB	376	382	BS2G	14.25
**4639**	allXL	CC	357	373	BS2G	18.47
**4639**	allXL	DD	320	366	BS2G	27.17
**4639**	allXL	DD	370	376	BS2G	14.82
**4639**	allXL	AA	320	357	DBSU	23.95
**4639**	allXL	AA	357	319	DBSU	22.8
**4639**	allXL	CC	372	319	DBSU	12.48
**4639**	allXL	CC	370	357	DBSU	21.99
**4639**	allXL	CC	382	357	DBSU	17.04
**4639**	allXL	AA	372	370	DBSU	6.09
**4639**	allXL	DD	381	370	DBSU	20.5
**4639**	allXL	AA	373	370	DBSU	9.17
**4639**	allXL	CC	381	372	DBSU	19.91
**4639**	allXL	CC	372	382	DBSU	18.89
**4639**	allXL	AA	372	386	DBSU	22.58
**4639**	allXL	CC	373	386	DBSU	24.8
**4639**	allXL	DB	381	386	DBSU	55.0
**4639**	allXL	BD;DB	357	357	DBSU	19.78
**4639**	allXL	AD	373	319	DBSU	29.48
**4639**	allXL	CA	372	357	DBSU	38.53
**4639**	allXL	CA	381	357	DBSU	30.35
**4639**	allXL	CA	373	357	DBSU	37.14
**4639**	allXL	AD	372	370	DBSU	40.66
**4639**	allXL	AD	373	370	DBSU	37.98
**4639**	allXL	DA	382	373	DBSU	57.63
**4640**	allXL	CC	320	357	BS2G	25.52
**4640**	allXL	BB	320	373	BS2G	13.25
**4640**	allXL	BB	373	382	BS2G	17.49
**4640**	allXL	BB	370	386	BS2G	24.05
**4640**	allXL	BB	373	386	BS2G	22.41
**4640**	allXL	AA	319	357	BS2G	24.88
**4640**	allXL	AA	367	373	BS2G	13.28

**4640**	allXL	BB	370	382	BS2G	22.36
**4640**	allXL	BB	376	382	BS2G	14.38
**4640**	allXL	BB	357	373	BS2G	18.96
**4640**	allXL	CC	320	366	BS2G	17.59
**4640**	allXL	DD	370	376	BS2G	16.44
**4640**	allXL	CC	320	357	DBSU	25.52
**4640**	allXL	AA	357	319	DBSU	24.88
**4640**	allXL	DD	372	319	DBSU	13.25
**4640**	allXL	BB	370	357	DBSU	26.86
**4640**	allXL	BB	382	357	DBSU	13.83
**4640**	allXL	AA	372	370	DBSU	6.24
**4640**	allXL	DD	381	370	DBSU	20.79
**4640**	allXL	AA	373	370	DBSU	9.08
**4640**	allXL	BB	381	372	DBSU	17.59
**4640**	allXL	BB	372	382	DBSU	18.81
**4640**	allXL	BB	372	386	DBSU	21.65
**4640**	allXL	BB	373	386	DBSU	22.41
**4640**	allXL	DA	381	386	DBSU	49.18
**4640**	allXL	BD;DB	357	357	DBSU	18.28
**4640**	allXL	CB	373	319	DBSU	16.07
**4640**	allXL	CB	372	357	DBSU	31.66
**4640**	allXL	BD	381	357	DBSU	25.02
**4640**	allXL	CB	373	357	DBSU	34.45
**4640**	allXL	DA	372	370	DBSU	20.57
**4640**	allXL	DA	373	370	DBSU	17.56
**4640**	allXL	CB	382	373	DBSU	26.9
**4641**	allXL	CC	320	357	BS2G	24.92
**4641**	allXL	BB	320	373	BS2G	19.41
**4641**	allXL	DD	373	382	BS2G	18.59
**4641**	allXL	BB	370	386	BS2G	27.93
**4641**	allXL	BB	373	386	BS2G	25.49
**4641**	allXL	AA	319	357	BS2G	25.23
**4641**	allXL	AA	367	373	BS2G	12.24
**4641**	allXL	DD	370	382	BS2G	24.79

**4641**	allXL	BB	376	382	BS2G	14.32
**4641**	allXL	AA	357	373	BS2G	26.38
**4641**	allXL	DD	320	366	BS2G	19.15
**4641**	allXL	CC	370	376	BS2G	17.01
**4641**	allXL	CC	320	357	DBSU	24.92
**4641**	allXL	AA	357	319	DBSU	25.23
**4641**	allXL	BB	372	319	DBSU	19.46
**4641**	allXL	BB	370	357	DBSU	28.96
**4641**	allXL	AA	382	357	DBSU	37.84
**4641**	allXL	AA	372	370	DBSU	6.25
**4641**	allXL	DD	381	370	DBSU	24.83
**4641**	allXL	AA	373	370	DBSU	9.08
**4641**	allXL	DD	381	372	DBSU	17.98
**4641**	allXL	DD	372	382	DBSU	18.52
**4641**	allXL	DD	372	386	DBSU	24.71
**4641**	allXL	BB	373	386	DBSU	25.49
**4641**	allXL	CB	381	386	DBSU	28.83
**4641**	allXL	BD;DB	357	357	DBSU	18.09
**4641**	allXL	CB	373	319	DBSU	26.41
**4641**	allXL	BC	372	357	DBSU	42.39
**4641**	allXL	AC	381	357	DBSU	49.95
**4641**	allXL	AC	373	357	DBSU	43.91
**4641**	allXL	CB	372	370	DBSU	28.37
**4641**	allXL	BC	373	370	DBSU	26.7
**4641**	allXL	BC	382	373	DBSU	12.05
**4642**	allXL	CC	320	357	BS2G	23.14
**4642**	allXL	CC	320	373	BS2G	26.81
**4642**	allXL	BB	373	382	BS2G	18.3
**4642**	allXL	BB	370	386	BS2G	23.22
**4642**	allXL	AA	373	386	BS2G	25.88
**4642**	allXL	AA	319	357	BS2G	26.2
**4642**	allXL	AA	367	373	BS2G	12.84
**4642**	allXL	BB	370	382	BS2G	15.91
**4642**	allXL	BB	376	382	BS2G	13.79

**4642**	allXL	DD	357	373	BS2G	17.87
**4642**	allXL	BB	320	366	BS2G	19.22
**4642**	allXL	CC	370	376	BS2G	14.23
**4642**	allXL	CC	320	357	DBSU	23.14
**4642**	allXL	AA	357	319	DBSU	26.2
**4642**	allXL	CC	372	319	DBSU	21.67
**4642**	allXL	DD	370	357	DBSU	11.82
**4642**	allXL	BB	382	357	DBSU	17.6
**4642**	allXL	AA	372	370	DBSU	6.29
**4642**	allXL	BB	381	370	DBSU	18.73
**4642**	allXL	AA	373	370	DBSU	9.34
**4642**	allXL	CC	381	372	DBSU	21.21
**4642**	allXL	BB	372	382	DBSU	19.76
**4642**	allXL	AA	372	386	DBSU	26.63
**4642**	allXL	AA	373	386	DBSU	25.88
**4642**	allXL	BD	381	386	DBSU	13.55
**4642**	allXL	BD;DB	357	357	DBSU	18.67
**4642**	allXL	AD	373	319	DBSU	30.72
**4642**	allXL	DB	372	357	DBSU	29.97
**4642**	allXL	BD	381	357	DBSU	18.64
**4642**	allXL	DB	373	357	DBSU	29.44
**4642**	allXL	DB	372	370	DBSU	44.73
**4642**	allXL	DB	373	370	DBSU	43.21
**4642**	allXL	BD	382	373	DBSU	28.81
**4643**	allXL	AA	320	357	BS2G	21.68
**4643**	allXL	CC	320	373	BS2G	31.03
**4643**	allXL	DD	373	382	BS2G	17.44
**4643**	allXL	DD	370	386	BS2G	24.56
**4643**	allXL	DD	373	386	BS2G	22.62
**4643**	allXL	AA	319	357	BS2G	20.22
**4643**	allXL	BB	367	373	BS2G	13.83
**4643**	allXL	BB	370	382	BS2G	22.07
**4643**	allXL	BB	376	382	BS2G	14.02
**4643**	allXL	BB	357	373	BS2G	17.71

**4643**	allXL	AA	320	366	BS2G	26.19
**4643**	allXL	CC	370	376	BS2G	16.62
**4643**	allXL	AA	320	357	DBSU	21.68
**4643**	allXL	AA	357	319	DBSU	20.22
**4643**	allXL	CC	372	319	DBSU	27.67
**4643**	allXL	BB	370	357	DBSU	26.92
**4643**	allXL	BB	382	357	DBSU	31.12
**4643**	allXL	AA	372	370	DBSU	6.07
**4643**	allXL	DD	381	370	DBSU	22.75
**4643**	allXL	DD	373	370	DBSU	8.97
**4643**	allXL	DD	381	372	DBSU	15.74
**4643**	allXL	DD	372	382	DBSU	16.68
**4643**	allXL	DD	372	386	DBSU	20.3
**4643**	allXL	DD	373	386	DBSU	22.62
**4643**	allXL	AD	381	386	DBSU	62.49
**4643**	allXL	BD;DB	357	357	DBSU	18.91
**4643**	allXL	CB	373	319	DBSU	41.56
**4643**	allXL	BD	372	357	DBSU	23.89
**4643**	allXL	BD	381	357	DBSU	29.46
**4643**	allXL	BD	373	357	DBSU	22.76
**4643**	allXL	BD	372	370	DBSU	51.94
**4643**	allXL	BD	373	370	DBSU	50.39
**4643**	allXL	BD	382	373	DBSU	54.23
**4644**	allXL	AA	320	357	BS2G	26.91
**4644**	allXL	CC	320	373	BS2G	29.75
**4644**	allXL	AA	373	382	BS2G	16.7
**4644**	allXL	AA	370	386	BS2G	14.84
**4644**	allXL	AA	373	386	BS2G	20.66
**4644**	allXL	AA	319	357	BS2G	27.54
**4644**	allXL	AA	367	373	BS2G	12.5
**4644**	allXL	AA	370	382	BS2G	16.73
**4644**	allXL	CC	376	382	BS2G	14.12
**4644**	allXL	AA	357	373	BS2G	20.82
**4644**	allXL	DD	320	366	BS2G	27.4

**4644**	allXL	CC	370	376	BS2G	15.54
**4644**	allXL	AA	320	357	DBSU	26.91
**4644**	allXL	AA	357	319	DBSU	27.54
**4644**	allXL	CC	372	319	DBSU	31.45
**4644**	allXL	DD	370	357	DBSU	25.06
**4644**	allXL	DD	382	357	DBSU	34.09
**4644**	allXL	AA	372	370	DBSU	6.32
**4644**	allXL	AA	381	370	DBSU	20.11
**4644**	allXL	BB	373	370	DBSU	9.18
**4644**	allXL	AA	381	372	DBSU	19.11
**4644**	allXL	AA	372	382	DBSU	16.44
**4644**	allXL	AA	372	386	DBSU	18.33
**4644**	allXL	AA	373	386	DBSU	20.66
**4644**	allXL	BC	381	386	DBSU	33.56
**4644**	allXL	BD;DB	357	357	DBSU	18.51
**4644**	allXL	DA	373	319	DBSU	15.36
**4644**	allXL	DA	372	357	DBSU	30.53
**4644**	allXL	DA	381	357	DBSU	41.72
**4644**	allXL	DA	373	357	DBSU	28.34
**4644**	allXL	CB	372	370	DBSU	14.05
**4644**	allXL	CB	373	370	DBSU	15.28
**4644**	allXL	BC	382	373	DBSU	27.04
**4645**	allXL	BB	320	357	BS2G	25.33
**4645**	allXL	DD	320	373	BS2G	20.88
**4645**	allXL	DD	373	382	BS2G	15.92
**4645**	allXL	DD	370	386	BS2G	10.26
**4645**	allXL	DD	373	386	BS2G	16.46
**4645**	allXL	BB	319	357	BS2G	26.11
**4645**	allXL	AA	367	373	BS2G	13.06
**4645**	allXL	DD	370	382	BS2G	15.29
**4645**	allXL	BB	376	382	BS2G	14.27
**4645**	allXL	DD	357	373	BS2G	30.4
**4645**	allXL	AA	320	366	BS2G	21.82
**4645**	allXL	CC	370	376	BS2G	13.94

**4645**	allXL	BB	320	357	DBSU	25.33
**4645**	allXL	BB	357	319	DBSU	26.11
**4645**	allXL	DD	372	319	DBSU	23.62
**4645**	allXL	DD	370	357	DBSU	27.8
**4645**	allXL	BB	382	357	DBSU	31.13
**4645**	allXL	AA	372	370	DBSU	6.38
**4645**	allXL	DD	381	370	DBSU	17.91
**4645**	allXL	DD	373	370	DBSU	8.98
**4645**	allXL	DD	381	372	DBSU	16.85
**4645**	allXL	DD	372	382	DBSU	15.66
**4645**	allXL	DD	372	386	DBSU	14.41
**4645**	allXL	DD	373	386	DBSU	16.46
**4645**	allXL	AD	381	386	DBSU	41.49
**4645**	allXL	BD;DB	357	357	DBSU	18.54
**4645**	allXL	AD	373	319	DBSU	36.64
**4645**	allXL	DA	372	357	DBSU	48.62
**4645**	allXL	DA	381	357	DBSU	36.1
**4645**	allXL	DA	373	357	DBSU	48.01
**4645**	allXL	DA	372	370	DBSU	30.55
**4645**	allXL	DA	373	370	DBSU	32.27
**4645**	allXL	DA	382	373	DBSU	26.51
**4646**	allXL	AA	320	357	BS2G	26.11
**4646**	allXL	AA	320	373	BS2G	25.42
**4646**	allXL	DD	373	382	BS2G	16.71
**4646**	allXL	CC	370	386	BS2G	21.04
**4646**	allXL	DD	373	386	BS2G	23.08
**4646**	allXL	AA	319	357	BS2G	27.65
**4646**	allXL	BB	367	373	BS2G	13.05
**4646**	allXL	CC	370	382	BS2G	16.51
**4646**	allXL	AA	376	382	BS2G	13.74
**4646**	allXL	BB	357	373	BS2G	16.4
**4646**	allXL	AA	320	366	BS2G	12.63
**4646**	allXL	CC	370	376	BS2G	12.62
**4646**	allXL	AA	320	357	DBSU	26.11

**4646**	allXL	AA	357	319	DBSU	27.65
**4646**	allXL	AA	372	319	DBSU	25.84
**4646**	allXL	BB	370	357	DBSU	23.77
**4646**	allXL	CC	382	357	DBSU	33.02
**4646**	allXL	AA	372	370	DBSU	6.12
**4646**	allXL	DD	381	370	DBSU	17.34
**4646**	allXL	AA	373	370	DBSU	9.02
**4646**	allXL	DD	381	372	DBSU	15.79
**4646**	allXL	DD	372	382	DBSU	17.32
**4646**	allXL	DD	372	386	DBSU	21.86
**4646**	allXL	DD	373	386	DBSU	23.08
**4646**	allXL	DA	381	386	DBSU	34.3
**4646**	allXL	BD;DB	357	357	DBSU	18.79
**4646**	allXL	DA	373	319	DBSU	19.83
**4646**	allXL	BD	372	357	DBSU	22.28
**4646**	allXL	BD	381	357	DBSU	23.42
**4646**	allXL	BD	373	357	DBSU	19.92
**4646**	allXL	AD	372	370	DBSU	12.12
**4646**	allXL	AD	373	370	DBSU	15.41
**4646**	allXL	DA	382	373	DBSU	20.91
**4647**	allXL	AA	320	357	BS2G	25.51
**4647**	allXL	BB	320	373	BS2G	20.4
**4647**	allXL	CC	373	382	BS2G	17.62
**4647**	allXL	DD	370	386	BS2G	21.85
**4647**	allXL	CC	373	386	BS2G	20.43
**4647**	allXL	AA	319	357	BS2G	24.84
**4647**	allXL	AA	367	373	BS2G	14.36
**4647**	allXL	BB	370	382	BS2G	20.8
**4647**	allXL	CC	376	382	BS2G	12.56
**4647**	allXL	BB	357	373	BS2G	22.82
**4647**	allXL	BB	320	366	BS2G	27.44
**4647**	allXL	DD	370	376	BS2G	16.42
**4647**	allXL	AA	320	357	DBSU	25.51
**4647**	allXL	AA	357	319	DBSU	24.84

**4647**	allXL	BB	372	319	DBSU	19.9
**4647**	allXL	BB	370	357	DBSU	25.68
**4647**	allXL	DD	382	357	DBSU	27.38
**4647**	allXL	AA	372	370	DBSU	6.15
**4647**	allXL	BB	381	370	DBSU	20.78
**4647**	allXL	AA	373	370	DBSU	8.85
**4647**	allXL	BB	381	372	DBSU	18.28
**4647**	allXL	BB	372	382	DBSU	19.61
**4647**	allXL	CC	372	386	DBSU	20.9
**4647**	allXL	CC	373	386	DBSU	20.43
**4647**	allXL	BD	381	386	DBSU	34.25
**4647**	allXL	BD;DB	357	357	DBSU	18.96
**4647**	allXL	DA	373	319	DBSU	32.14
**4647**	allXL	BD	372	357	DBSU	36.22
**4647**	allXL	BD	381	357	DBSU	38.8
**4647**	allXL	BD	373	357	DBSU	36.73
**4647**	allXL	BD	372	370	DBSU	44.43
**4647**	allXL	BD	373	370	DBSU	42.35
**4647**	allXL	DB	382	373	DBSU	46.16
**4648**	allXL	AA	320	357	BS2G	21.89
**4648**	allXL	CC	320	373	BS2G	13.86
**4648**	allXL	BB	373	382	BS2G	20.26
**4648**	allXL	CC	370	386	BS2G	27.17
**4648**	allXL	AA	373	386	BS2G	29.83
**4648**	allXL	AA	319	357	BS2G	20.06
**4648**	allXL	AA	367	373	BS2G	10.37
**4648**	allXL	CC	370	382	BS2G	20.71
**4648**	allXL	BB	376	382	BS2G	14.06
**4648**	allXL	CC	357	373	BS2G	30.58
**4648**	allXL	AA	320	366	BS2G	18.21
**4648**	allXL	CC	370	376	BS2G	14.24
**4648**	allXL	AA	320	357	DBSU	21.89
**4648**	allXL	AA	357	319	DBSU	20.06
**4648**	allXL	CC	372	319	DBSU	17.12

**4648**	allXL	CC	370	357	DBSU	28.88
**4648**	allXL	AA	382	357	DBSU	24.28
**4648**	allXL	AA	372	370	DBSU	6.57
**4648**	allXL	CC	381	370	DBSU	21.25
**4648**	allXL	AA	373	370	DBSU	9.03
**4648**	allXL	DD	381	372	DBSU	20.12
**4648**	allXL	DD	372	382	DBSU	21.61
**4648**	allXL	DD	372	386	DBSU	30.66
**4648**	allXL	AA	373	386	DBSU	29.83
**4648**	allXL	DA	381	386	DBSU	61.0
**4648**	allXL	BD;DB	357	357	DBSU	18.36
**4648**	allXL	AD	373	319	DBSU	37.18
**4648**	allXL	CB	372	357	DBSU	32.23
**4648**	allXL	AC	381	357	DBSU	41.24
**4648**	allXL	CB	373	357	DBSU	35.31
**4648**	allXL	AD	372	370	DBSU	43.03
**4648**	allXL	AD	373	370	DBSU	41.18
**4648**	allXL	AD	382	373	DBSU	45.74
**4649**	allXL	BB	320	357	BS2G	26.03
**4649**	allXL	BB	320	373	BS2G	27.13
**4649**	allXL	CC	373	382	BS2G	19.87
**4649**	allXL	AA	370	386	BS2G	21.98
**4649**	allXL	AA	373	386	BS2G	22.92
**4649**	allXL	AA	319	357	BS2G	28.48
**4649**	allXL	BB	367	373	BS2G	12.69
**4649**	allXL	AA	370	382	BS2G	25.14
**4649**	allXL	CC	376	382	BS2G	13.75
**4649**	allXL	BB	357	373	BS2G	32.61
**4649**	allXL	AA	320	366	BS2G	26.14
**4649**	allXL	CC	370	376	BS2G	16.27
**4649**	allXL	BB	320	357	DBSU	26.03
**4649**	allXL	AA	357	319	DBSU	28.48
**4649**	allXL	BB	372	319	DBSU	26.08
**4649**	allXL	DD	370	357	DBSU	29.85

**4649**	allXL	BB	382	357	DBSU	20.32
**4649**	allXL	AA	372	370	DBSU	6.25
**4649**	allXL	AA	381	370	DBSU	23.2
**4649**	allXL	AA	373	370	DBSU	9.2
**4649**	allXL	AA	381	372	DBSU	20.7
**4649**	allXL	CC	372	382	DBSU	21.91
**4649**	allXL	AA	372	386	DBSU	23.27
**4649**	allXL	AA	373	386	DBSU	22.92
**4649**	allXL	DA	381	386	DBSU	49.3
**4649**	allXL	BD;DB	357	357	DBSU	17.71
**4649**	allXL	AD	373	319	DBSU	25.08
**4649**	allXL	CB	372	357	DBSU	45.55
**4649**	allXL	BD	381	357	DBSU	34.87
**4649**	allXL	CB	373	357	DBSU	47.9
**4649**	allXL	CB	372	370	DBSU	16.81
**4649**	allXL	CB	373	370	DBSU	17.51
**4649**	allXL	DA	382	373	DBSU	33.21
**4650**	allXL	AA	320	357	BS2G	22.05
**4650**	allXL	BB	320	373	BS2G	15.72
**4650**	allXL	CC	373	382	BS2G	18.19
**4650**	allXL	CC	370	386	BS2G	21.66
**4650**	allXL	CC	373	386	BS2G	19.73
**4650**	allXL	AA	319	357	BS2G	20.7
**4650**	allXL	BB	367	373	BS2G	13.65
**4650**	allXL	BB	370	382	BS2G	22.29
**4650**	allXL	CC	376	382	BS2G	13.71
**4650**	allXL	BB	357	373	BS2G	29.37
**4650**	allXL	AA	320	366	BS2G	20.03
**4650**	allXL	CC	370	376	BS2G	16.43
**4650**	allXL	AA	320	357	DBSU	22.05
**4650**	allXL	AA	357	319	DBSU	20.7
**4650**	allXL	BB	372	319	DBSU	18.22
**4650**	allXL	BB	370	357	DBSU	25.13
**4650**	allXL	BB	382	357	DBSU	38.26

**4650**	allXL	AA	372	370	DBSU	6.03
**4650**	allXL	BB	381	370	DBSU	22.41
**4650**	allXL	AA	373	370	DBSU	8.99
**4650**	allXL	CC	381	372	DBSU	20.69
**4650**	allXL	CC	372	382	DBSU	18.78
**4650**	allXL	CC	372	386	DBSU	17.9
**4650**	allXL	CC	373	386	DBSU	19.73
**4650**	allXL	DA	381	386	DBSU	36.41
**4650**	allXL	BD;DB	357	357	DBSU	18.53
**4650**	allXL	DA	373	319	DBSU	32.54
**4650**	allXL	DA	372	357	DBSU	43.87
**4650**	allXL	BC	381	357	DBSU	52.22
**4650**	allXL	DA	373	357	DBSU	43.86
**4650**	allXL	DA	372	370	DBSU	28.22
**4650**	allXL	DA	373	370	DBSU	26.35
**4650**	allXL	DA	382	373	DBSU	34.22
**4651**	allXL	AA	320	357	BS2G	24.35
**4651**	allXL	BB	320	373	BS2G	9.65
**4651**	allXL	DD	373	382	BS2G	15.57
**4651**	allXL	DD	370	386	BS2G	15.42
**4651**	allXL	DD	373	386	BS2G	23.32
**4651**	allXL	AA	319	357	BS2G	22.69
**4651**	allXL	AA	367	373	BS2G	13.15
**4651**	allXL	DD	370	382	BS2G	7.76
**4651**	allXL	CC	376	382	BS2G	14.29
**4651**	allXL	DD	357	373	BS2G	21.54
**4651**	allXL	BB	320	366	BS2G	17.06
**4651**	allXL	DD	370	376	BS2G	13.03
**4651**	allXL	AA	320	357	DBSU	24.35
**4651**	allXL	AA	357	319	DBSU	22.69
**4651**	allXL	CC	372	319	DBSU	12.75
**4651**	allXL	BB	370	357	DBSU	19.92
**4651**	allXL	CC	382	357	DBSU	18.01
**4651**	allXL	AA	372	370	DBSU	6.32

**4651**	allXL	DD	381	370	DBSU	6.65
**4651**	allXL	CC	373	370	DBSU	9.04
**4651**	allXL	DD	381	372	DBSU	13.43
**4651**	allXL	DD	372	382	DBSU	14.59
**4651**	allXL	DD	372	386	DBSU	20.95
**4651**	allXL	DD	373	386	DBSU	23.32
**4651**	allXL	DB	381	386	DBSU	34.88
**4651**	allXL	BD;DB	357	357	DBSU	18.41
**4651**	allXL	AD	373	319	DBSU	28.79
**4651**	allXL	DA	372	357	DBSU	29.0
**4651**	allXL	AC	381	357	DBSU	22.99
**4651**	allXL	DA	373	357	DBSU	29.27
**4651**	allXL	DA	372	370	DBSU	36.09
**4651**	allXL	DA	373	370	DBSU	38.66
**4651**	allXL	BD	382	373	DBSU	43.69
**4652**	allXL	CC	320	357	BS2G	24.72
**4652**	allXL	AA	320	373	BS2G	21.14
**4652**	allXL	CC	373	382	BS2G	17.34
**4652**	allXL	CC	370	386	BS2G	27.42
**4652**	allXL	CC	373	386	BS2G	21.52
**4652**	allXL	AA	319	357	BS2G	26.21
**4652**	allXL	AA	367	373	BS2G	9.95
**4652**	allXL	CC	370	382	BS2G	23.48
**4652**	allXL	CC	376	382	BS2G	13.29
**4652**	allXL	BB	357	373	BS2G	15.36
**4652**	allXL	AA	320	366	BS2G	10.78
**4652**	allXL	BB	370	376	BS2G	14.89
**4652**	allXL	CC	320	357	DBSU	24.72
**4652**	allXL	AA	357	319	DBSU	26.21
**4652**	allXL	AA	372	319	DBSU	21.52
**4652**	allXL	BB	370	357	DBSU	24.2
**4652**	allXL	BB	382	357	DBSU	17.37
**4652**	allXL	AA	372	370	DBSU	6.67
**4652**	allXL	DD	381	370	DBSU	24.78

**4652**	allXL	AA	373	370	DBSU	9.06
**4652**	allXL	CC	381	372	DBSU	20.9
**4652**	allXL	CC	372	382	DBSU	18.53
**4652**	allXL	CC	372	386	DBSU	21.77
**4652**	allXL	CC	373	386	DBSU	21.52
**4652**	allXL	DA	381	386	DBSU	43.95
**4652**	allXL	BD;DB	357	357	DBSU	18.37
**4652**	allXL	BC	373	319	DBSU	29.66
**4652**	allXL	BD	372	357	DBSU	28.39
**4652**	allXL	BD	381	357	DBSU	25.28
**4652**	allXL	BD	373	357	DBSU	25.27
**4652**	allXL	DA	372	370	DBSU	25.79
**4652**	allXL	DA	373	370	DBSU	23.93
**4652**	allXL	DA	382	373	DBSU	22.8
**4653**	allXL	AA	320	357	BS2G	27.52
**4653**	allXL	BB	320	373	BS2G	12.96
**4653**	allXL	DD	373	382	BS2G	19.54
**4653**	allXL	DD	370	386	BS2G	20.6
**4653**	allXL	DD	373	386	BS2G	24.94
**4653**	allXL	AA	319	357	BS2G	27.52
**4653**	allXL	BB	367	373	BS2G	12.91
**4653**	allXL	DD	370	382	BS2G	19.91
**4653**	allXL	CC	376	382	BS2G	13.04
**4653**	allXL	BB	357	373	BS2G	22.29
**4653**	allXL	BB	320	366	BS2G	26.33
**4653**	allXL	BB	370	376	BS2G	16.15
**4653**	allXL	AA	320	357	DBSU	27.52
**4653**	allXL	AA	357	319	DBSU	27.52
**4653**	allXL	BB	372	319	DBSU	18.38
**4653**	allXL	BB	370	357	DBSU	21.88
**4653**	allXL	BB	382	357	DBSU	41.52
**4653**	allXL	AA	372	370	DBSU	6.18
**4653**	allXL	DD	381	370	DBSU	19.3
**4653**	allXL	AA	373	370	DBSU	9.18

**4653**	allXL	DD	381	372	DBSU	18.26
**4653**	allXL	DD	372	382	DBSU	19.92
**4653**	allXL	DD	372	386	DBSU	23.93
**4653**	allXL	DD	373	386	DBSU	24.94
**4653**	allXL	BC	381	386	DBSU	26.98
**4653**	allXL	BD;DB	357	357	DBSU	18.6
**4653**	allXL	CB	373	319	DBSU	16.38
**4653**	allXL	BD	372	357	DBSU	37.02
**4653**	allXL	BC	381	357	DBSU	58.78
**4653**	allXL	BD	373	357	DBSU	40.26
**4653**	allXL	CB	372	370	DBSU	33.12
**4653**	allXL	BC	373	370	DBSU	29.8
**4653**	allXL	BC	382	373	DBSU	23.95
**4654**	allXL	CC	320	357	BS2G	26.04
**4654**	allXL	CC	320	373	BS2G	24.25
**4654**	allXL	CC	373	382	BS2G	17.69
**4654**	allXL	DD	370	386	BS2G	26.13
**4654**	allXL	CC	373	386	BS2G	24.05
**4654**	allXL	AA	319	357	BS2G	27.98
**4654**	allXL	AA	367	373	BS2G	11.2
**4654**	allXL	BB	370	382	BS2G	21.43
**4654**	allXL	CC	376	382	BS2G	14.44
**4654**	allXL	BB	357	373	BS2G	20.76
**4654**	allXL	CC	320	366	BS2G	21.95
**4654**	allXL	AA	370	376	BS2G	16.49
**4654**	allXL	CC	320	357	DBSU	26.04
**4654**	allXL	AA	357	319	DBSU	27.98
**4654**	allXL	CC	372	319	DBSU	19.72
**4654**	allXL	AA	370	357	DBSU	27.37
**4654**	allXL	AA	382	357	DBSU	29.63
**4654**	allXL	AA	372	370	DBSU	6.44
**4654**	allXL	BB	381	370	DBSU	19.78
**4654**	allXL	AA	373	370	DBSU	9.08
**4654**	allXL	DD	381	372	DBSU	16.29

**4654**	allXL	CC	372	382	DBSU	18.96
**4654**	allXL	CC	372	386	DBSU	23.8
**4654**	allXL	CC	373	386	DBSU	24.05
**4654**	allXL	BD	381	386	DBSU	61.87
**4654**	allXL	BD;DB	357	357	DBSU	19.09
**4654**	allXL	AD	373	319	DBSU	23.97
**4654**	allXL	BD	372	357	DBSU	34.48
**4654**	allXL	BD	381	357	DBSU	36.6
**4654**	allXL	BD	373	357	DBSU	31.96
**4654**	allXL	AD	372	370	DBSU	55.02
**4654**	allXL	AD	373	370	DBSU	52.39
**4654**	allXL	AD	382	373	DBSU	54.67
**4655**	allXL	AA	320	357	BS2G	24.77
**4655**	allXL	BB	320	373	BS2G	13.56
**4655**	allXL	DD	373	382	BS2G	16.33
**4655**	allXL	CC	370	386	BS2G	20.56
**4655**	allXL	DD	373	386	BS2G	23.99
**4655**	allXL	AA	319	357	BS2G	24.13
**4655**	allXL	BB	367	373	BS2G	13.24
**4655**	allXL	CC	370	382	BS2G	17.41
**4655**	allXL	CC	376	382	BS2G	14.22
**4655**	allXL	BB	357	373	BS2G	26.49
**4655**	allXL	DD	320	366	BS2G	22.48
**4655**	allXL	CC	370	376	BS2G	14.62
**4655**	allXL	AA	320	357	DBSU	24.77
**4655**	allXL	AA	357	319	DBSU	24.13
**4655**	allXL	BB	372	319	DBSU	13.24
**4655**	allXL	BB	370	357	DBSU	25.29
**4655**	allXL	BB	382	357	DBSU	20.59
**4655**	allXL	AA	372	370	DBSU	6.18
**4655**	allXL	DD	381	370	DBSU	18.69
**4655**	allXL	BB	373	370	DBSU	9.11
**4655**	allXL	DD	381	372	DBSU	17.15
**4655**	allXL	DD	372	382	DBSU	16.34

**4655**	allXL	DD	372	386	DBSU	23.79
**4655**	allXL	DD	373	386	DBSU	23.99
**4655**	allXL	DB	381	386	DBSU	61.44
**4655**	allXL	BD;DB	357	357	DBSU	16.66
**4655**	allXL	AD	373	319	DBSU	36.78
**4655**	allXL	BD	372	357	DBSU	45.03
**4655**	allXL	BD	381	357	DBSU	28.35
**4655**	allXL	BD	373	357	DBSU	42.78
**4655**	allXL	AD	372	370	DBSU	37.99
**4655**	allXL	AD	373	370	DBSU	40.64
**4655**	allXL	CB	382	373	DBSU	49.76
**4656**	allXL	CC	320	357	BS2G	23.98
**4656**	allXL	BB	320	373	BS2G	13.37
**4656**	allXL	DD	373	382	BS2G	18.37
**4656**	allXL	DD	370	386	BS2G	16.0
**4656**	allXL	AA	373	386	BS2G	19.61
**4656**	allXL	CC	319	357	BS2G	25.69
**4656**	allXL	DD	367	373	BS2G	13.28
**4656**	allXL	DD	370	382	BS2G	16.73
**4656**	allXL	AA;CC	376	382	BS2G	15.02
**4656**	allXL	DD	357	373	BS2G	25.4
**4656**	allXL	BB	320	366	BS2G	20.37
**4656**	allXL	DD	370	376	BS2G	16.26
**4656**	allXL	CC	320	357	DBSU	23.98
**4656**	allXL	CC	357	319	DBSU	25.69
**4656**	allXL	BB	372	319	DBSU	15.43
**4656**	allXL	DD	370	357	DBSU	21.51
**4656**	allXL	DD	382	357	DBSU	34.07
**4656**	allXL	AA	372	370	DBSU	6.08
**4656**	allXL	DD	381	370	DBSU	18.3
**4656**	allXL	AA	373	370	DBSU	9.0
**4656**	allXL	DD	381	372	DBSU	17.96
**4656**	allXL	DD	372	382	DBSU	17.37
**4656**	allXL	AA	372	386	DBSU	20.62

**4656**	allXL	AA	373	386	DBSU	19.61
**4656**	allXL	BD	381	386	DBSU	35.37
**4656**	allXL	BD;DB	357	357	DBSU	18.29
**4656**	allXL	DA	373	319	DBSU	38.16
**4656**	allXL	BD	372	357	DBSU	39.88
**4656**	allXL	BC	381	357	DBSU	39.66
**4656**	allXL	DB	373	357	DBSU	41.59
**4656**	allXL	BD	372	370	DBSU	47.98
**4656**	allXL	BD	373	370	DBSU	48.83
**4656**	allXL	AD	382	373	DBSU	45.2
**4657**	allXL	AA	320	357	BS2G	26.5
**4657**	allXL	AA	320	373	BS2G	20.58
**4657**	allXL	DD	373	382	BS2G	17.1
**4657**	allXL	DD	370	386	BS2G	18.97
**4657**	allXL	CC	373	386	BS2G	19.06
**4657**	allXL	AA	319	357	BS2G	26.75
**4657**	allXL	DD	367	373	BS2G	13.96
**4657**	allXL	DD	370	382	BS2G	19.09
**4657**	allXL	CC	376	382	BS2G	14.09
**4657**	allXL	BB	357	373	BS2G	21.65
**4657**	allXL	AA	320	366	BS2G	14.09
**4657**	allXL	CC	370	376	BS2G	17.35
**4657**	allXL	AA	320	357	DBSU	26.5
**4657**	allXL	AA	357	319	DBSU	26.75
**4657**	allXL	AA	372	319	DBSU	19.44
**4657**	allXL	DD	370	357	DBSU	26.02
**4657**	allXL	BB	382	357	DBSU	30.17
**4657**	allXL	AA	372	370	DBSU	6.27
**4657**	allXL	DD	381	370	DBSU	18.68
**4657**	allXL	DD	373	370	DBSU	9.21
**4657**	allXL	DD	381	372	DBSU	14.67
**4657**	allXL	DD	372	382	DBSU	16.01
**4657**	allXL	CC	372	386	DBSU	17.51
**4657**	allXL	CC	373	386	DBSU	19.06

**4657**	allXL	AD	381	386	DBSU	49.38
**4657**	allXL	BD;DB	357	357	DBSU	20.35
**4657**	allXL	AD	373	319	DBSU	42.44
**4657**	allXL	DB	372	357	DBSU	40.85
**4657**	allXL	BD	381	357	DBSU	35.54
**4657**	allXL	BD	373	357	DBSU	40.25
**4657**	allXL	CB	372	370	DBSU	51.77
**4657**	allXL	CB	373	370	DBSU	52.65
**4657**	allXL	AD	382	373	DBSU	46.22
**4658**	allXL	AA	320	357	BS2G	26.07
**4658**	allXL	BB	320	373	BS2G	12.83
**4658**	allXL	AA	373	382	BS2G	17.54
**4658**	allXL	AA	370	386	BS2G	24.68
**4658**	allXL	CC	373	386	BS2G	24.13
**4658**	allXL	AA	319	357	BS2G	26.79
**4658**	allXL	AA	367	373	BS2G	11.32
**4658**	allXL	AA	370	382	BS2G	20.59
**4658**	allXL	CC	376	382	BS2G	13.08
**4658**	allXL	BB	357	373	BS2G	25.35
**4658**	allXL	BB	320	366	BS2G	22.43
**4658**	allXL	CC	370	376	BS2G	16.69
**4658**	allXL	AA	320	357	DBSU	26.07
**4658**	allXL	AA	357	319	DBSU	26.79
**4658**	allXL	DD	372	319	DBSU	16.26
**4658**	allXL	DD	370	357	DBSU	24.93
**4658**	allXL	AA	382	357	DBSU	23.35
**4658**	allXL	AA	372	370	DBSU	6.49
**4658**	allXL	AA	381	370	DBSU	21.75
**4658**	allXL	AA	373	370	DBSU	9.05
**4658**	allXL	AA	381	372	DBSU	21.48
**4658**	allXL	AA	372	382	DBSU	20.28
**4658**	allXL	AA	372	386	DBSU	26.07
**4658**	allXL	CC	373	386	DBSU	24.13
**4658**	allXL	AD	381	386	DBSU	41.25

**4658**	allXL	BD;DB	357	357	DBSU	19.01
**4658**	allXL	AD	373	319	DBSU	21.23
**4658**	allXL	DA	372	357	DBSU	35.72
**4658**	allXL	AC	381	357	DBSU	33.3
**4658**	allXL	DA	373	357	DBSU	35.35
**4658**	allXL	DA	372	370	DBSU	26.85
**4658**	allXL	DA	373	370	DBSU	24.25
**4658**	allXL	AD	382	373	DBSU	24.87
**4659**	allXL	CC	320	357	BS2G	24.94
**4659**	allXL	CC	320	373	BS2G	20.09
**4659**	allXL	BB	373	382	BS2G	16.46
**4659**	allXL	BB	370	386	BS2G	18.27
**4659**	allXL	DD	373	386	BS2G	24.59
**4659**	allXL	AA	319	357	BS2G	28.18
**4659**	allXL	AA	367	373	BS2G	14.45
**4659**	allXL	BB	370	382	BS2G	11.23
**4659**	allXL	BB	376	382	BS2G	14.59
**4659**	allXL	CC	357	373	BS2G	22.54
**4659**	allXL	AA	320	366	BS2G	27.88
**4659**	allXL	BB	370	376	BS2G	14.23
**4659**	allXL	CC	320	357	DBSU	24.94
**4659**	allXL	AA	357	319	DBSU	28.18
**4659**	allXL	CC	372	319	DBSU	23.42
**4659**	allXL	DD	370	357	DBSU	20.81
**4659**	allXL	CC	382	357	DBSU	9.75
**4659**	allXL	AA	372	370	DBSU	6.44
**4659**	allXL	BB	381	370	DBSU	12.54
**4659**	allXL	BB	373	370	DBSU	9.31
**4659**	allXL	BB	381	372	DBSU	17.16
**4659**	allXL	BB	372	382	DBSU	16.98
**4659**	allXL	DD	372	386	DBSU	23.31
**4659**	allXL	DD	373	386	DBSU	24.59
**4659**	allXL	AC	381	386	DBSU	33.41
**4659**	allXL	BD;DB	357	357	DBSU	18.4

**4659**	allXL	CB	373	319	DBSU	29.97
**4659**	allXL	DB	372	357	DBSU	21.84
**4659**	allXL	DB	381	357	DBSU	24.1
**4659**	allXL	DB	373	357	DBSU	19.06
**4659**	allXL	CB	372	370	DBSU	46.56
**4659**	allXL	DB	373	370	DBSU	45.69
**4659**	allXL	DB	382	373	DBSU	32.54
**4660**	allXL	AA	320	357	BS2G	23.51
**4660**	allXL	BB	320	373	BS2G	13.44
**4660**	allXL	DD	373	382	BS2G	15.96
**4660**	allXL	DD	370	386	BS2G	18.04
**4660**	allXL	DD	373	386	BS2G	18.38
**4660**	allXL	AA	319	357	BS2G	24.02
**4660**	allXL	AA	367	373	BS2G	13.71
**4660**	allXL	DD	370	382	BS2G	19.35
**4660**	allXL	BB	376	382	BS2G	15.37
**4660**	allXL	BB	357	373	BS2G	17.47
**4660**	allXL	DD	320	366	BS2G	23.2
**4660**	allXL	CC	370	376	BS2G	12.46
**4660**	allXL	AA	320	357	DBSU	23.51
**4660**	allXL	AA	357	319	DBSU	24.02
**4660**	allXL	CC	372	319	DBSU	16.21
**4660**	allXL	BB	370	357	DBSU	16.59
**4660**	allXL	DD	382	357	DBSU	25.55
**4660**	allXL	AA	372	370	DBSU	6.4
**4660**	allXL	DD	381	370	DBSU	19.36
**4660**	allXL	BB	373	370	DBSU	9.12
**4660**	allXL	DD	381	372	DBSU	14.24
**4660**	allXL	DD	372	382	DBSU	14.28
**4660**	allXL	DD	372	386	DBSU	15.08
**4660**	allXL	DD	373	386	DBSU	18.38
**4660**	allXL	CB	381	386	DBSU	40.3
**4660**	allXL	BD;DB	357	357	DBSU	18.49
**4660**	allXL	CB	373	319	DBSU	29.9

**4660**	allXL	CB	372	357	DBSU	25.42
**4660**	allXL	DB	381	357	DBSU	35.99
**4660**	allXL	CB	373	357	DBSU	28.74
**4660**	allXL	BC	372	370	DBSU	28.85
**4660**	allXL	BC	373	370	DBSU	29.87
**4660**	allXL	CB	382	373	DBSU	36.69
**4661**	allXL	BB	320	357	BS2G	25.76
**4661**	allXL	BB	320	373	BS2G	19.27
**4661**	allXL	CC	373	382	BS2G	18.19
**4661**	allXL	DD	370	386	BS2G	23.86
**4661**	allXL	CC	373	386	BS2G	24.26
**4661**	allXL	AA	319	357	BS2G	25.46
**4661**	allXL	AA	367	373	BS2G	14.0
**4661**	allXL	DD	370	382	BS2G	14.22
**4661**	allXL	CC	376	382	BS2G	12.65
**4661**	allXL	DD	357	373	BS2G	31.89
**4661**	allXL	BB	320	366	BS2G	13.48
**4661**	allXL	DD	370	376	BS2G	14.42
**4661**	allXL	BB	320	357	DBSU	25.76
**4661**	allXL	AA	357	319	DBSU	25.46
**4661**	allXL	BB	372	319	DBSU	19.2
**4661**	allXL	DD	370	357	DBSU	30.85
**4661**	allXL	DD	382	357	DBSU	43.02
**4661**	allXL	AA	372	370	DBSU	5.89
**4661**	allXL	DD	381	370	DBSU	13.36
**4661**	allXL	CC	373	370	DBSU	9.04
**4661**	allXL	DD	381	372	DBSU	17.72
**4661**	allXL	DD	372	382	DBSU	18.42
**4661**	allXL	CC	372	386	DBSU	25.94
**4661**	allXL	CC	373	386	DBSU	24.26
**4661**	allXL	CB	381	386	DBSU	71.55
**4661**	allXL	BD;DB	357	357	DBSU	17.76
**4661**	allXL	AD	373	319	DBSU	34.64
**4661**	allXL	DB	372	357	DBSU	45.37

**4661**	allXL	DB	381	357	DBSU	53.16
**4661**	allXL	DB	373	357	DBSU	44.58
**4661**	allXL	DA	372	370	DBSU	32.91
**4661**	allXL	DA	373	370	DBSU	34.53
**4661**	allXL	DA	382	373	DBSU	54.33
**4662**	allXL	CC	320	357	BS2G	23.44
**4662**	allXL	AA	320	373	BS2G	15.78
**4662**	allXL	DD	373	382	BS2G	18.65
**4662**	allXL	DD	370	386	BS2G	22.97
**4662**	allXL	DD	373	386	BS2G	26.74
**4662**	allXL	CC	319	357	BS2G	24.74
**4662**	allXL	BB	367	373	BS2G	11.02
**4662**	allXL	DD	370	382	BS2G	17.94
**4662**	allXL	CC	376	382	BS2G	14.97
**4662**	allXL	AA	357	373	BS2G	24.74
**4662**	allXL	DD	320	366	BS2G	26.68
**4662**	allXL	CC	370	376	BS2G	15.5
**4662**	allXL	CC	320	357	DBSU	23.44
**4662**	allXL	CC	357	319	DBSU	24.74
**4662**	allXL	AA	372	319	DBSU	21.05
**4662**	allXL	CC	370	357	DBSU	26.76
**4662**	allXL	AA	382	357	DBSU	18.81
**4662**	allXL	AA	372	370	DBSU	6.14
**4662**	allXL	DD	381	370	DBSU	16.97
**4662**	allXL	AA	373	370	DBSU	9.2
**4662**	allXL	DD	381	372	DBSU	18.44
**4662**	allXL	DD	372	382	DBSU	20.71
**4662**	allXL	DD	372	386	DBSU	27.92
**4662**	allXL	DD	373	386	DBSU	26.74
**4662**	allXL	BD	381	386	DBSU	41.45
**4662**	allXL	BD;DB	357	357	DBSU	19.0
**4662**	allXL	AD	373	319	DBSU	24.75
**4662**	allXL	BC	372	357	DBSU	31.08
**4662**	allXL	DB	381	357	DBSU	38.76

**4662**	allXL	BC	373	357	DBSU	32.52
**4662**	allXL	DA	372	370	DBSU	23.69
**4662**	allXL	DA	373	370	DBSU	20.98
**4662**	allXL	DA	382	373	DBSU	27.06
**4663**	allXL	AA	320	357	BS2G	23.87
**4663**	allXL	DD	320	373	BS2G	20.24
**4663**	allXL	DD	373	382	BS2G	19.09
**4663**	allXL	CC	370	386	BS2G	26.26
**4663**	allXL	DD	373	386	BS2G	24.48
**4663**	allXL	AA	319	357	BS2G	22.5
**4663**	allXL	AA	367	373	BS2G	13.49
**4663**	allXL	CC	370	382	BS2G	21.28
**4663**	allXL	CC	376	382	BS2G	14.3
**4663**	allXL	BB	357	373	BS2G	35.65
**4663**	allXL	DD	320	366	BS2G	17.92
**4663**	allXL	CC	370	376	BS2G	14.39
**4663**	allXL	AA	320	357	DBSU	23.87
**4663**	allXL	AA	357	319	DBSU	22.5
**4663**	allXL	DD	372	319	DBSU	14.59
**4663**	allXL	DD	370	357	DBSU	31.47
**4663**	allXL	CC	382	357	DBSU	43.71
**4663**	allXL	AA	372	370	DBSU	6.21
**4663**	allXL	BB	381	370	DBSU	22.99
**4663**	allXL	AA	373	370	DBSU	8.96
**4663**	allXL	DD	381	372	DBSU	21.36
**4663**	allXL	DD	372	382	DBSU	20.43
**4663**	allXL	DD	372	386	DBSU	24.56
**4663**	allXL	DD	373	386	DBSU	24.48
**4663**	allXL	AD	381	386	DBSU	32.03
**4663**	allXL	BD;DB	357	357	DBSU	18.71
**4663**	allXL	AD	373	319	DBSU	38.28
**4663**	allXL	DA	372	357	DBSU	50.82
**4663**	allXL	CB	381	357	DBSU	45.49
**4663**	allXL	DA	373	357	DBSU	51.82

**4663**	allXL	BC	372	370	DBSU	40.56
**4663**	allXL	BC	373	370	DBSU	38.15
**4663**	allXL	CB	382	373	DBSU	30.57
**4664**	allXL	AA	320	357	BS2G	24.72
**4664**	allXL	BB	320	373	BS2G	19.2
**4664**	allXL	AA	373	382	BS2G	17.04
**4664**	allXL	AA	370	386	BS2G	16.39
**4664**	allXL	AA	373	386	BS2G	21.07
**4664**	allXL	AA	319	357	BS2G	24.3
**4664**	allXL	AA	367	373	BS2G	14.76
**4664**	allXL	AA	370	382	BS2G	16.04
**4664**	allXL	BB	376	382	BS2G	13.91
**4664**	allXL	CC	357	373	BS2G	17.93
**4664**	allXL	BB	320	366	BS2G	21.4
**4664**	allXL	DD	370	376	BS2G	16.5
**4664**	allXL	AA	320	357	DBSU	24.72
**4664**	allXL	AA	357	319	DBSU	24.3
**4664**	allXL	BB	372	319	DBSU	19.45
**4664**	allXL	CC	370	357	DBSU	17.51
**4664**	allXL	DD	382	357	DBSU	21.25
**4664**	allXL	AA	372	370	DBSU	6.18
**4664**	allXL	AA	381	370	DBSU	19.13
**4664**	allXL	AA	373	370	DBSU	9.19
**4664**	allXL	AA	381	372	DBSU	18.57
**4664**	allXL	AA	372	382	DBSU	16.41
**4664**	allXL	AA	372	386	DBSU	20.07
**4664**	allXL	AA	373	386	DBSU	21.07
**4664**	allXL	BD	381	386	DBSU	45.74
**4664**	allXL	BD;DB	357	357	DBSU	18.99
**4664**	allXL	AD	373	319	DBSU	25.22
**4664**	allXL	CA	372	357	DBSU	25.52
**4664**	allXL	CA	381	357	DBSU	24.6
**4664**	allXL	CA	373	357	DBSU	26.17
**4664**	allXL	DA	372	370	DBSU	39.12

**4664**	allXL	AD	373	370	DBSU	39.19
**4664**	allXL	CA	382	373	DBSU	40.66
**4665**	allXL	CC	320	357	BS2G	25.62
**4665**	allXL	BB	320	373	BS2G	16.07
**4665**	allXL	BB	373	382	BS2G	18.96
**4665**	allXL	DD	370	386	BS2G	26.44
**4665**	allXL	AA	373	386	BS2G	25.93
**4665**	allXL	AA	319	357	BS2G	29.07
**4665**	allXL	AA	367	373	BS2G	9.38
**4665**	allXL	DD	370	382	BS2G	18.25
**4665**	allXL	BB	376	382	BS2G	13.41
**4665**	allXL	CC	357	373	BS2G	16.68
**4665**	allXL	AA	320	366	BS2G	12.59
**4665**	allXL	DD	370	376	BS2G	15.76
**4665**	allXL	CC	320	357	DBSU	25.62
**4665**	allXL	AA	357	319	DBSU	29.07
**4665**	allXL	BB	372	319	DBSU	19.38
**4665**	allXL	CC	370	357	DBSU	12.4
**4665**	allXL	CC	382	357	DBSU	21.64
**4665**	allXL	AA	372	370	DBSU	6.59
**4665**	allXL	DD	381	370	DBSU	17.64
**4665**	allXL	AA	373	370	DBSU	9.13
**4665**	allXL	DD	381	372	DBSU	20.69
**4665**	allXL	AA	372	382	DBSU	20.0
**4665**	allXL	AA	372	386	DBSU	23.77
**4665**	allXL	AA	373	386	DBSU	25.93
**4665**	allXL	DB	381	386	DBSU	51.06
**4665**	allXL	BD;DB	357	357	DBSU	19.25
**4665**	allXL	AD	373	319	DBSU	30.88
**4665**	allXL	CA	372	357	DBSU	21.23
**4665**	allXL	CA	381	357	DBSU	17.2
**4665**	allXL	CA	373	357	DBSU	17.92
**4665**	allXL	DA	372	370	DBSU	32.41
**4665**	allXL	DA	373	370	DBSU	30.76

**4665**	allXL	CA	382	373	DBSU	38.96
**4666**	allXL	CC	320	357	BS2G	24.64
**4666**	allXL	DD	320	373	BS2G	17.48
**4666**	allXL	BB	373	382	BS2G	18.94
**4666**	allXL	CC	370	386	BS2G	19.29
**4666**	allXL	AA	373	386	BS2G	24.27
**4666**	allXL	CC	319	357	BS2G	26.44
**4666**	allXL	AA	367	373	BS2G	12.18
**4666**	allXL	DD	370	382	BS2G	18.47
**4666**	allXL	BB	376	382	BS2G	14.56
**4666**	allXL	AA	357	373	BS2G	29.09
**4666**	allXL	DD	320	366	BS2G	20.97
**4666**	allXL	CC	370	376	BS2G	15.27
**4666**	allXL	CC	320	357	DBSU	24.64
**4666**	allXL	CC	357	319	DBSU	26.44
**4666**	allXL	DD	372	319	DBSU	15.73
**4666**	allXL	DD	370	357	DBSU	25.05
**4666**	allXL	CC	382	357	DBSU	23.35
**4666**	allXL	AA	372	370	DBSU	6.35
**4666**	allXL	DD	381	370	DBSU	18.06
**4666**	allXL	AA	373	370	DBSU	9.04
**4666**	allXL	DD	381	372	DBSU	19.22
**4666**	allXL	BB	372	382	DBSU	19.79
**4666**	allXL	CC	372	386	DBSU	23.08
**4666**	allXL	AA	373	386	DBSU	24.27
**4666**	allXL	BC	381	386	DBSU	57.6
**4666**	allXL	BD;DB	357	357	DBSU	18.19
**4666**	allXL	AD	373	319	DBSU	32.86
**4666**	allXL	BD	372	357	DBSU	46.85
**4666**	allXL	CA	381	357	DBSU	33.22
**4666**	allXL	BC	373	357	DBSU	45.9
**4666**	allXL	DA	372	370	DBSU	30.5
**4666**	allXL	DA	373	370	DBSU	30.7
**4666**	allXL	AD	382	373	DBSU	47.23

**4667**	allXL	CC	320	357	BS2G	24.33
**4667**	allXL	BB	320	373	BS2G	29.92
**4667**	allXL	BB	373	382	BS2G	20.81
**4667**	allXL	DD	370	386	BS2G	22.84
**4667**	allXL	CC	373	386	BS2G	28.54
**4667**	allXL	AA	319	357	BS2G	24.41
**4667**	allXL	AA	367	373	BS2G	11.97
**4667**	allXL	DD	370	382	BS2G	18.42
**4667**	allXL	CC	376	382	BS2G	14.59
**4667**	allXL	DD	357	373	BS2G	28.23
**4667**	allXL	BB	320	366	BS2G	17.27
**4667**	allXL	DD	370	376	BS2G	14.63
**4667**	allXL	CC	320	357	DBSU	24.33
**4667**	allXL	AA	357	319	DBSU	24.41
**4667**	allXL	BB	372	319	DBSU	28.96
**4667**	allXL	DD	370	357	DBSU	28.01
**4667**	allXL	BB	382	357	DBSU	21.61
**4667**	allXL	AA	372	370	DBSU	6.13
**4667**	allXL	DD	381	370	DBSU	15.66
**4667**	allXL	AA	373	370	DBSU	9.2
**4667**	allXL	DD	381	372	DBSU	19.79
**4667**	allXL	AA	372	382	DBSU	22.52
**4667**	allXL	CC	372	386	DBSU	29.29
**4667**	allXL	CC	373	386	DBSU	28.54
**4667**	allXL	DB	381	386	DBSU	40.98
**4667**	allXL	BD;DB	357	357	DBSU	19.47
**4667**	allXL	DA	373	319	DBSU	24.29
**4667**	allXL	DA	372	357	DBSU	38.73
**4667**	allXL	BD	381	357	DBSU	40.11
**4667**	allXL	DB	373	357	DBSU	37.31
**4667**	allXL	DA	372	370	DBSU	43.2
**4667**	allXL	DA	373	370	DBSU	45.9
**4667**	allXL	BD	382	373	DBSU	52.24
**4668**	allXL	CC	320	357	BS2G	23.82

**4668**	allXL	DD	320	373	BS2G	15.8
**4668**	allXL	AA	373	382	BS2G	17.48
**4668**	allXL	DD	370	386	BS2G	21.76
**4668**	allXL	AA	373	386	BS2G	21.47
**4668**	allXL	CC	319	357	BS2G	25.88
**4668**	allXL	AA	367	373	BS2G	12.06
**4668**	allXL	DD	370	382	BS2G	19.2
**4668**	allXL	AA	376	382	BS2G	14.44
**4668**	allXL	DD	357	373	BS2G	27.12
**4668**	allXL	BB	320	366	BS2G	25.29
**4668**	allXL	DD	370	376	BS2G	13.86
**4668**	allXL	CC	320	357	DBSU	23.82
**4668**	allXL	CC	357	319	DBSU	25.88
**4668**	allXL	DD	372	319	DBSU	19.41
**4668**	allXL	DD	370	357	DBSU	25.28
**4668**	allXL	BB	382	357	DBSU	25.7
**4668**	allXL	AA	372	370	DBSU	6.51
**4668**	allXL	DD	381	370	DBSU	17.64
**4668**	allXL	AA	373	370	DBSU	8.93
**4668**	allXL	AA	381	372	DBSU	19.23
**4668**	allXL	AA	372	382	DBSU	16.8
**4668**	allXL	AA	372	386	DBSU	20.29
**4668**	allXL	AA	373	386	DBSU	21.47
**4668**	allXL	DB	381	386	DBSU	36.2
**4668**	allXL	BD;DB	357	357	DBSU	17.88
**4668**	allXL	CB	373	319	DBSU	6.35
**4668**	allXL	CB	372	357	DBSU	32.06
**4668**	allXL	DA	381	357	DBSU	27.46
**4668**	allXL	CB	373	357	DBSU	31.71
**4668**	allXL	CB	372	370	DBSU	39.86
**4668**	allXL	CB	373	370	DBSU	38.57
**4668**	allXL	BC	382	373	DBSU	34.26
**4669**	allXL	BB	320	357	BS2G	25.22
**4669**	allXL	BB	320	373	BS2G	19.76

**4669**	allXL	BB	373	382	BS2G	19.94
**4669**	allXL	CC	370	386	BS2G	32.26
**4669**	allXL	CC	373	386	BS2G	29.33
**4669**	allXL	AA	319	357	BS2G	24.45
**4669**	allXL	BB	367	373	BS2G	12.97
**4669**	allXL	CC	370	382	BS2G	27.57
**4669**	allXL	CC	376	382	BS2G	14.34
**4669**	allXL	CC	357	373	BS2G	18.99
**4669**	allXL	AA	320	366	BS2G	18.53
**4669**	allXL	CC	370	376	BS2G	16.82
**4669**	allXL	BB	320	357	DBSU	25.22
**4669**	allXL	AA	357	319	DBSU	24.45
**4669**	allXL	BB	372	319	DBSU	17.43
**4669**	allXL	CC	370	357	DBSU	25.83
**4669**	allXL	CC	382	357	DBSU	33.88
**4669**	allXL	AA	372	370	DBSU	6.09
**4669**	allXL	DD	381	370	DBSU	27.15
**4669**	allXL	AA	373	370	DBSU	9.11
**4669**	allXL	DD	381	372	DBSU	22.36
**4669**	allXL	BB	372	382	DBSU	21.2
**4669**	allXL	CC	372	386	DBSU	29.6
**4669**	allXL	CC	373	386	DBSU	29.33
**4669**	allXL	DA	381	386	DBSU	59.92
**4669**	allXL	BD;DB	357	357	DBSU	18.14
**4669**	allXL	CB	373	319	DBSU	29.65
**4669**	allXL	CA	372	357	DBSU	34.41
**4669**	allXL	CA	381	357	DBSU	46.4
**4669**	allXL	CA	373	357	DBSU	32.33
**4669**	allXL	CB	372	370	DBSU	26.51
**4669**	allXL	BC	373	370	DBSU	29.14
**4669**	allXL	CB	382	373	DBSU	51.1
**4670**	allXL	CC	320	357	BS2G	22.54
**4670**	allXL	BB	320	373	BS2G	15.16
**4670**	allXL	AA	373	382	BS2G	19.86

**4670**	allXL	AA	370	386	BS2G	21.38
**4670**	allXL	AA	373	386	BS2G	27.15
**4670**	allXL	CC	319	357	BS2G	24.84
**4670**	allXL	DD	367	373	BS2G	12.73
**4670**	allXL	AA	370	382	BS2G	18.43
**4670**	allXL	CC	376	382	BS2G	14.03
**4670**	allXL	CC	357	373	BS2G	30.44
**4670**	allXL	BB	320	366	BS2G	17.9
**4670**	allXL	BB	370	376	BS2G	16.13
**4670**	allXL	CC	320	357	DBSU	22.54
**4670**	allXL	CC	357	319	DBSU	24.84
**4670**	allXL	BB	372	319	DBSU	15.69
**4670**	allXL	CC	370	357	DBSU	31.5
**4670**	allXL	DD	382	357	DBSU	27.89
**4670**	allXL	AA	372	370	DBSU	6.45
**4670**	allXL	AA	381	370	DBSU	19.29
**4670**	allXL	DD	373	370	DBSU	9.18
**4670**	allXL	AA	381	372	DBSU	18.58
**4670**	allXL	AA	372	382	DBSU	18.13
**4670**	allXL	AA	372	386	DBSU	24.32
**4670**	allXL	AA	373	386	DBSU	27.15
**4670**	allXL	BC	381	386	DBSU	56.89
**4670**	allXL	BD;DB	357	357	DBSU	19.58
**4670**	allXL	CB	373	319	DBSU	26.44
**4670**	allXL	CB	372	357	DBSU	39.95
**4670**	allXL	DB	381	357	DBSU	43.83
**4670**	allXL	CB	373	357	DBSU	38.24
**4670**	allXL	CB	372	370	DBSU	28.2
**4670**	allXL	CB	373	370	DBSU	29.75
**4670**	allXL	CB	382	373	DBSU	44.81
**4671**	allXL	BB	320	357	BS2G	25.82
**4671**	allXL	DD	320	373	BS2G	22.78
**4671**	allXL	BB	373	382	BS2G	17.58
**4671**	allXL	DD	370	386	BS2G	22.82

**4671**	allXL	DD	373	386	BS2G	25.32
**4671**	allXL	AA	319	357	BS2G	27.61
**4671**	allXL	AA	367	373	BS2G	12.56
**4671**	allXL	DD	370	382	BS2G	18.08
**4671**	allXL	BB	376	382	BS2G	13.89
**4671**	allXL	DD	357	373	BS2G	29.98
**4671**	allXL	AA	320	366	BS2G	28.52
**4671**	allXL	CC	370	376	BS2G	15.82
**4671**	allXL	BB	320	357	DBSU	25.82
**4671**	allXL	AA	357	319	DBSU	27.61
**4671**	allXL	DD	372	319	DBSU	24.72
**4671**	allXL	DD	370	357	DBSU	25.26
**4671**	allXL	DD	382	357	DBSU	35.27
**4671**	allXL	AA	372	370	DBSU	6.4
**4671**	allXL	DD	381	370	DBSU	16.82
**4671**	allXL	AA	373	370	DBSU	8.74
**4671**	allXL	DD	381	372	DBSU	12.86
**4671**	allXL	DD	372	382	DBSU	15.3
**4671**	allXL	DD	372	386	DBSU	22.36
**4671**	allXL	DD	373	386	DBSU	25.32
**4671**	allXL	AD	381	386	DBSU	41.14
**4671**	allXL	BD;DB	357	357	DBSU	16.96
**4671**	allXL	AD	373	319	DBSU	30.3
**4671**	allXL	DB	372	357	DBSU	45.5
**4671**	allXL	DA	381	357	DBSU	47.14
**4671**	allXL	DB	373	357	DBSU	46.87
**4671**	allXL	DA	372	370	DBSU	31.25
**4671**	allXL	DA	373	370	DBSU	33.78
**4671**	allXL	DA	382	373	DBSU	23.27
**4672**	allXL	AA	320	357	BS2G	21.18
**4672**	allXL	BB	320	373	BS2G	20.6
**4672**	allXL	CC	373	382	BS2G	17.83
**4672**	allXL	CC	370	386	BS2G	25.17
**4672**	allXL	CC	373	386	BS2G	21.87

**4672**	allXL	AA	319	357	BS2G	20.29
**4672**	allXL	AA	367	373	BS2G	12.96
**4672**	allXL	DD	370	382	BS2G	23.99
**4672**	allXL	CC	376	382	BS2G	13.33
**4672**	allXL	AA	357	373	BS2G	27.78
**4672**	allXL	BB	320	366	BS2G	20.58
**4672**	allXL	DD	370	376	BS2G	14.86
**4672**	allXL	AA	320	357	DBSU	21.18
**4672**	allXL	AA	357	319	DBSU	20.29
**4672**	allXL	BB	372	319	DBSU	17.11
**4672**	allXL	AA	370	357	DBSU	27.07
**4672**	allXL	BB	382	357	DBSU	19.06
**4672**	allXL	AA	372	370	DBSU	6.41
**4672**	allXL	DD	381	370	DBSU	20.26
**4672**	allXL	AA	373	370	DBSU	8.82
**4672**	allXL	DD	381	372	DBSU	19.99
**4672**	allXL	CC	372	382	DBSU	18.73
**4672**	allXL	CC	372	386	DBSU	20.45
**4672**	allXL	CC	373	386	DBSU	21.87
**4672**	allXL	AD	381	386	DBSU	21.92
**4672**	allXL	BD;DB	357	357	DBSU	19.05
**4672**	allXL	AD	373	319	DBSU	39.97
**4672**	allXL	AC	372	357	DBSU	37.95
**4672**	allXL	BD	381	357	DBSU	37.47
**4672**	allXL	AC	373	357	DBSU	39.39
**4672**	allXL	CB	372	370	DBSU	50.85
**4672**	allXL	CB	373	370	DBSU	51.68
**4672**	allXL	DA	382	373	DBSU	49.35
**4673**	allXL	CC	320	357	BS2G	23.54
**4673**	allXL	DD	320	373	BS2G	31.64
**4673**	allXL	DD	373	382	BS2G	19.18
**4673**	allXL	DD	370	386	BS2G	20.58
**4673**	allXL	DD	373	386	BS2G	25.29
**4673**	allXL	CC	319	357	BS2G	25.99

**4673**	allXL	AA	367	373	BS2G	13.87
**4673**	allXL	DD	370	382	BS2G	18.89
**4673**	allXL	CC	376	382	BS2G	14.2
**4673**	allXL	AA	357	373	BS2G	27.18
**4673**	allXL	DD	320	366	BS2G	21.02
**4673**	allXL	CC	370	376	BS2G	15.46
**4673**	allXL	CC	320	357	DBSU	23.54
**4673**	allXL	CC	357	319	DBSU	25.99
**4673**	allXL	DD	372	319	DBSU	31.39
**4673**	allXL	DD	370	357	DBSU	28.84
**4673**	allXL	CC	382	357	DBSU	28.22
**4673**	allXL	AA	372	370	DBSU	6.18
**4673**	allXL	DD	381	370	DBSU	19.22
**4673**	allXL	AA	373	370	DBSU	9.04
**4673**	allXL	DD	381	372	DBSU	20.36
**4673**	allXL	DD	372	382	DBSU	21.15
**4673**	allXL	DD	372	386	DBSU	25.77
**4673**	allXL	DD	373	386	DBSU	25.29
**4673**	allXL	CB	381	386	DBSU	49.01
**4673**	allXL	BD;DB	357	357	DBSU	19.08
**4673**	allXL	CB	373	319	DBSU	27.08
**4673**	allXL	CA	372	357	DBSU	34.88
**4673**	allXL	CB	381	357	DBSU	29.09
**4673**	allXL	CA	373	357	DBSU	32.54
**4673**	allXL	DA	372	370	DBSU	58.77
**4673**	allXL	CB	373	370	DBSU	57.34
**4673**	allXL	CB	382	373	DBSU	50.57
**4674**	allXL	CC	320	357	BS2G	24.26
**4674**	allXL	CC	320	373	BS2G	22.01
**4674**	allXL	DD	373	382	BS2G	12.14
**4674**	allXL	DD	370	386	BS2G	23.51
**4674**	allXL	DD	373	386	BS2G	21.09
**4674**	allXL	AA	319	357	BS2G	25.11
**4674**	allXL	AA	367	373	BS2G	12.1

**4674**	allXL	DD	370	382	BS2G	12.78
**4674**	allXL	DD	376	382	BS2G	13.67
**4674**	allXL	BB	357	373	BS2G	24.43
**4674**	allXL	BB	320	366	BS2G	13.86
**4674**	allXL	DD	370	376	BS2G	15.63
**4674**	allXL	CC	320	357	DBSU	24.26
**4674**	allXL	AA	357	319	DBSU	25.11
**4674**	allXL	CC	372	319	DBSU	21.25
**4674**	allXL	BB	370	357	DBSU	16.73
**4674**	allXL	BB	382	357	DBSU	37.18
**4674**	allXL	AA	372	370	DBSU	6.49
**4674**	allXL	DD	381	370	DBSU	11.38
**4674**	allXL	AA	373	370	DBSU	8.7
**4674**	allXL	DD	381	372	DBSU	12.17
**4674**	allXL	DD	372	382	DBSU	11.98
**4674**	allXL	DD	372	386	DBSU	21.05
**4674**	allXL	DD	373	386	DBSU	21.09
**4674**	allXL	DB	381	386	DBSU	52.87
**4674**	allXL	AC;CA	357	357	DBSU	19.7
**4674**	allXL	AD	373	319	DBSU	20.81
**4674**	allXL	CB	372	357	DBSU	28.34
**4674**	allXL	BD	381	357	DBSU	39.95
**4674**	allXL	CB	373	357	DBSU	29.68
**4674**	allXL	DA	372	370	DBSU	36.25
**4674**	allXL	DA	373	370	DBSU	39.51
**4674**	allXL	BD	382	373	DBSU	49.72
**4675**	allXL	CC	320	357	BS2G	25.65
**4675**	allXL	CC	320	373	BS2G	18.32
**4675**	allXL	AA	373	382	BS2G	18.53
**4675**	allXL	DD	370	386	BS2G	15.61
**4675**	allXL	DD	373	386	BS2G	20.94
**4675**	allXL	CC	319	357	BS2G	29.12
**4675**	allXL	AA	367	373	BS2G	11.58
**4675**	allXL	DD	370	382	BS2G	18.28

**4675**	allXL	AA	376	382	BS2G	14.27
**4675**	allXL	BB	357	373	BS2G	30.26
**4675**	allXL	AA	320	366	BS2G	10.14
**4675**	allXL	DD	370	376	BS2G	15.71
**4675**	allXL	CC	320	357	DBSU	25.65
**4675**	allXL	CC	357	319	DBSU	29.12
**4675**	allXL	CC	372	319	DBSU	15.42
**4675**	allXL	DD	370	357	DBSU	30.56
**4675**	allXL	AA	382	357	DBSU	28.42
**4675**	allXL	CC	372	370	DBSU	6.76
**4675**	allXL	DD	381	370	DBSU	18.16
**4675**	allXL	AA	373	370	DBSU	8.98
**4675**	allXL	DD	381	372	DBSU	19.16
**4675**	allXL	DD	372	382	DBSU	20.29
**4675**	allXL	DD	372	386	DBSU	18.81
**4675**	allXL	DD	373	386	DBSU	20.94
**4675**	allXL	BD	381	386	DBSU	63.67
**4675**	allXL	BD;DB	357	357	DBSU	18.54
**4675**	allXL	AD	373	319	DBSU	37.89
**4675**	allXL	CB	372	357	DBSU	39.28
**4675**	allXL	BD	381	357	DBSU	48.8
**4675**	allXL	BD	373	357	DBSU	39.04
**4675**	allXL	CB	372	370	DBSU	34.41
**4675**	allXL	CB	373	370	DBSU	35.44
**4675**	allXL	CB	382	373	DBSU	52.11
**4676**	allXL	CC	320	357	BS2G	25.46
**4676**	allXL	DD	320	373	BS2G	17.65
**4676**	allXL	DD	373	382	BS2G	16.7
**4676**	allXL	DD	370	386	BS2G	18.36
**4676**	allXL	DD	373	386	BS2G	19.61
**4676**	allXL	AA	319	357	BS2G	24.57
**4676**	allXL	AA	367	373	BS2G	11.56
**4676**	allXL	DD	370	382	BS2G	21.03
**4676**	allXL	AA	376	382	BS2G	14.17

**4676**	allXL	BB	357	373	BS2G	28.78
**4676**	allXL	CC	320	366	BS2G	22.09
**4676**	allXL	CC	370	376	BS2G	16.08
**4676**	allXL	CC	320	357	DBSU	25.46
**4676**	allXL	AA	357	319	DBSU	24.57
**4676**	allXL	DD	372	319	DBSU	16.68
**4676**	allXL	BB	370	357	DBSU	25.59
**4676**	allXL	BB	382	357	DBSU	25.58
**4676**	allXL	CC	372	370	DBSU	6.61
**4676**	allXL	DD	381	370	DBSU	21.32
**4676**	allXL	CC	373	370	DBSU	9.25
**4676**	allXL	DD	381	372	DBSU	14.4
**4676**	allXL	DD	372	382	DBSU	14.85
**4676**	allXL	DD	372	386	DBSU	16.09
**4676**	allXL	DD	373	386	DBSU	19.61
**4676**	allXL	BC	381	386	DBSU	47.25
**4676**	allXL	BD;DB	357	357	DBSU	18.42
**4676**	allXL	AD	373	319	DBSU	22.23
**4676**	allXL	DA	372	357	DBSU	41.87
**4676**	allXL	AC	381	357	DBSU	40.76
**4676**	allXL	DA	373	357	DBSU	41.39
**4676**	allXL	DA	372	370	DBSU	24.44
**4676**	allXL	DA	373	370	DBSU	21.95
**4676**	allXL	DA	382	373	DBSU	36.11
**4677**	allXL	AA	320	357	BS2G	19.37
**4677**	allXL	BB	320	373	BS2G	15.84
**4677**	allXL	BB	373	382	BS2G	16.23
**4677**	allXL	CC	370	386	BS2G	22.88
**4677**	allXL	DD	373	386	BS2G	24.89
**4677**	allXL	AA	319	357	BS2G	18.64
**4677**	allXL	AA	367	373	BS2G	13.89
**4677**	allXL	BB	370	382	BS2G	19.46
**4677**	allXL	BB	376	382	BS2G	13.23
**4677**	allXL	BB	357	373	BS2G	26.58

**4677**	allXL	BB	320	366	BS2G	18.63
**4677**	allXL	CC	370	376	BS2G	15.82
**4677**	allXL	AA	320	357	DBSU	19.37
**4677**	allXL	AA	357	319	DBSU	18.64
**4677**	allXL	BB	372	319	DBSU	18.05
**4677**	allXL	DD	370	357	DBSU	23.37
**4677**	allXL	BB	382	357	DBSU	19.84
**4677**	allXL	AA	372	370	DBSU	6.14
**4677**	allXL	BB	381	370	DBSU	21.52
**4677**	allXL	DD	373	370	DBSU	9.0
**4677**	allXL	DD	381	372	DBSU	16.86
**4677**	allXL	BB	372	382	DBSU	16.89
**4677**	allXL	DD	372	386	DBSU	23.87
**4677**	allXL	DD	373	386	DBSU	24.89
**4677**	allXL	DB	381	386	DBSU	47.29
**4677**	allXL	BD;DB	357	357	DBSU	18.49
**4677**	allXL	DA	373	319	DBSU	21.58
**4677**	allXL	DA	372	357	DBSU	27.69
**4677**	allXL	BD	381	357	DBSU	25.89
**4677**	allXL	DA	373	357	DBSU	25.48
**4677**	allXL	BD	372	370	DBSU	41.83
**4677**	allXL	BD	373	370	DBSU	40.27
**4677**	allXL	BD	382	373	DBSU	36.2
**4678**	allXL	CC	320	357	BS2G	25.52
**4678**	allXL	DD	320	373	BS2G	19.61
**4678**	allXL	CC	373	382	BS2G	19.04
**4678**	allXL	CC	370	386	BS2G	23.44
**4678**	allXL	CC	373	386	BS2G	26.92
**4678**	allXL	AA	319	357	BS2G	24.09
**4678**	allXL	AA	367	373	BS2G	12.62
**4678**	allXL	CC	370	382	BS2G	20.2
**4678**	allXL	CC	376	382	BS2G	14.7
**4678**	allXL	BB	357	373	BS2G	23.74
**4678**	allXL	BB	320	366	BS2G	19.95

**4678**	allXL	CC	370	376	BS2G	14.31
**4678**	allXL	CC	320	357	DBSU	25.52
**4678**	allXL	AA	357	319	DBSU	24.09
**4678**	allXL	DD	372	319	DBSU	16.63
**4678**	allXL	BB	370	357	DBSU	22.95
**4678**	allXL	CC	382	357	DBSU	38.23
**4678**	allXL	AA	372	370	DBSU	6.26
**4678**	allXL	CC	381	370	DBSU	22.2
**4678**	allXL	CC	373	370	DBSU	9.06
**4678**	allXL	BB	381	372	DBSU	21.48
**4678**	allXL	CC	372	382	DBSU	21.46
**4678**	allXL	CC	372	386	DBSU	27.75
**4678**	allXL	CC	373	386	DBSU	26.92
**4678**	allXL	BC	381	386	DBSU	45.38
**4678**	allXL	BD;DB	357	357	DBSU	18.43
**4678**	allXL	CB	373	319	DBSU	18.74
**4678**	allXL	BD	372	357	DBSU	35.24
**4678**	allXL	BD	381	357	DBSU	46.54
**4678**	allXL	BD	373	357	DBSU	33.75
**4678**	allXL	CB	372	370	DBSU	29.99
**4678**	allXL	CB	373	370	DBSU	30.66
**4678**	allXL	CB	382	373	DBSU	45.7
**4679**	allXL	CC	320	357	BS2G	24.4
**4679**	allXL	BB	320	373	BS2G	31.24
**4679**	allXL	CC	373	382	BS2G	18.56
**4679**	allXL	DD	370	386	BS2G	16.58
**4679**	allXL	DD	373	386	BS2G	20.61
**4679**	allXL	AA	319	357	BS2G	25.36
**4679**	allXL	AA	367	373	BS2G	13.21
**4679**	allXL	CC	370	382	BS2G	15.92
**4679**	allXL	BB	376	382	BS2G	13.82
**4679**	allXL	BB	357	373	BS2G	30.25
**4679**	allXL	AA	320	366	BS2G	25.9
**4679**	allXL	CC	370	376	BS2G	14.52

**4679**	allXL	CC	320	357	DBSU	24.4
**4679**	allXL	AA	357	319	DBSU	25.36
**4679**	allXL	CC	372	319	DBSU	31.38
**4679**	allXL	DD	370	357	DBSU	23.86
**4679**	allXL	DD	382	357	DBSU	30.64
**4679**	allXL	AA	372	370	DBSU	6.23
**4679**	allXL	CC	381	370	DBSU	17.37
**4679**	allXL	AA	373	370	DBSU	9.08
**4679**	allXL	DD	381	372	DBSU	19.78
**4679**	allXL	CC	372	382	DBSU	19.56
**4679**	allXL	DD	372	386	DBSU	19.93
**4679**	allXL	DD	373	386	DBSU	20.61
**4679**	allXL	CB	381	386	DBSU	38.47
**4679**	allXL	BD;DB	357	357	DBSU	18.64
**4679**	allXL	DA	373	319	DBSU	27.63
**4679**	allXL	DB	372	357	DBSU	28.9
**4679**	allXL	DB	381	357	DBSU	32.34
**4679**	allXL	DB	373	357	DBSU	29.09
**4679**	allXL	CB	372	370	DBSU	42.98
**4679**	allXL	CB	373	370	DBSU	44.61
**4679**	allXL	BC	382	373	DBSU	29.72
**4680**	allXL	AA	320	357	BS2G	24.16
**4680**	allXL	BB	320	373	BS2G	10.69
**4680**	allXL	DD	373	382	BS2G	17.29
**4680**	allXL	CC	370	386	BS2G	18.8
**4680**	allXL	CC	373	386	BS2G	21.94
**4680**	allXL	AA	319	357	BS2G	22.33
**4680**	allXL	BB	367	373	BS2G	13.64
**4680**	allXL	DD	370	382	BS2G	18.41
**4680**	allXL	CC	376	382	BS2G	14.1
**4680**	allXL	BB	357	373	BS2G	25.47
**4680**	allXL	AA	320	366	BS2G	18.73
**4680**	allXL	CC	370	376	BS2G	15.83
**4680**	allXL	AA	320	357	DBSU	24.16

**4680**	allXL	AA	357	319	DBSU	22.33
**4680**	allXL	BB	372	319	DBSU	14.78
**4680**	allXL	DD	370	357	DBSU	28.72
**4680**	allXL	DD	382	357	DBSU	38.27
**4680**	allXL	AA	372	370	DBSU	6.08
**4680**	allXL	DD	381	370	DBSU	20.23
**4680**	allXL	AA	373	370	DBSU	9.15
**4680**	allXL	DD	381	372	DBSU	17.24
**4680**	allXL	DD	372	382	DBSU	16.04
**4680**	allXL	DD	372	386	DBSU	19.75
**4680**	allXL	CC	373	386	DBSU	21.94
**4680**	allXL	BC	381	386	DBSU	39.84
**4680**	allXL	BD;DB	357	357	DBSU	18.46
**4680**	allXL	CB	373	319	DBSU	28.25
**4680**	allXL	DB	372	357	DBSU	42.44
**4680**	allXL	BC	381	357	DBSU	31.24
**4680**	allXL	BD	373	357	DBSU	42.18
**4680**	allXL	AD	372	370	DBSU	33.78
**4680**	allXL	AD	373	370	DBSU	30.3
**4680**	allXL	AD	382	373	DBSU	22.99
**4681**	allXL	AA	320	357	BS2G	20.23
**4681**	allXL	BB	320	373	BS2G	16.08
**4681**	allXL	AA	373	382	BS2G	20.31
**4681**	allXL	AA	370	386	BS2G	21.43
**4681**	allXL	AA	373	386	BS2G	24.43
**4681**	allXL	AA	319	357	BS2G	17.46
**4681**	allXL	AA	367	373	BS2G	10.21
**4681**	allXL	AA	370	382	BS2G	21.88
**4681**	allXL	CC	376	382	BS2G	14.3
**4681**	allXL	DD	357	373	BS2G	28.81
**4681**	allXL	DD	320	366	BS2G	13.61
**4681**	allXL	AA	370	376	BS2G	15.75
**4681**	allXL	AA	320	357	DBSU	20.23
**4681**	allXL	AA	357	319	DBSU	17.46

**4681**	allXL	BB	372	319	DBSU	11.89
**4681**	allXL	DD	370	357	DBSU	19.25
**4681**	allXL	AA	382	357	DBSU	29.04
**4681**	allXL	AA	372	370	DBSU	6.63
**4681**	allXL	AA	381	370	DBSU	23.26
**4681**	allXL	AA	373	370	DBSU	9.0
**4681**	allXL	DD	381	372	DBSU	21.21
**4681**	allXL	AA	372	382	DBSU	22.74
**4681**	allXL	AA	372	386	DBSU	25.25
**4681**	allXL	AA	373	386	DBSU	24.43
**4681**	allXL	BC	381	386	DBSU	48.06
**4681**	allXL	BD;DB	357	357	DBSU	19.42
**4681**	allXL	AD	373	319	DBSU	26.38
**4681**	allXL	DB	372	357	DBSU	40.37
**4681**	allXL	AC	381	357	DBSU	42.66
**4681**	allXL	DB	373	357	DBSU	43.99
**4681**	allXL	BC	372	370	DBSU	38.99
**4681**	allXL	BC	373	370	DBSU	42.12
**4681**	allXL	CB	382	373	DBSU	32.81
**4682**	allXL	AA	320	357	BS2G	25.91
**4682**	allXL	DD	320	373	BS2G	26.57
**4682**	allXL	DD	373	382	BS2G	18.41
**4682**	allXL	CC	370	386	BS2G	25.94
**4682**	allXL	DD	373	386	BS2G	28.48
**4682**	allXL	AA	319	357	BS2G	26.14
**4682**	allXL	AA	367	373	BS2G	12.31
**4682**	allXL	DD	370	382	BS2G	21.02
**4682**	allXL	BB	376	382	BS2G	14.72
**4682**	allXL	BB	357	373	BS2G	16.45
**4682**	allXL	DD	320	366	BS2G	23.2
**4682**	allXL	CC	370	376	BS2G	15.01
**4682**	allXL	AA	320	357	DBSU	25.91
**4682**	allXL	AA	357	319	DBSU	26.14
**4682**	allXL	DD	372	319	DBSU	26.63

**4682**	allXL	BB	370	357	DBSU	16.79
**4682**	allXL	BB	382	357	DBSU	36.26
**4682**	allXL	AA	372	370	DBSU	6.45
**4682**	allXL	DD	381	370	DBSU	18.21
**4682**	allXL	AA	373	370	DBSU	8.98
**4682**	allXL	DD	381	372	DBSU	15.77
**4682**	allXL	DD	372	382	DBSU	18.3
**4682**	allXL	CC	372	386	DBSU	26.42
**4682**	allXL	DD	373	386	DBSU	28.48
**4682**	allXL	DA	381	386	DBSU	46.72
**4682**	allXL	BD;DB	357	357	DBSU	20.04
**4682**	allXL	CB	373	319	DBSU	21.65
**4682**	allXL	BD	372	357	DBSU	22.98
**4682**	allXL	BD	381	357	DBSU	33.43
**4682**	allXL	BD	373	357	DBSU	21.27
**4682**	allXL	AD	372	370	DBSU	39.74
**4682**	allXL	AD	373	370	DBSU	36.19
**4682**	allXL	AD	382	373	DBSU	27.97
**4683**	allXL	AA	320	357	BS2G	26.41
**4683**	allXL	BB	320	373	BS2G	31.08
**4683**	allXL	DD	373	382	BS2G	18.47
**4683**	allXL	DD	370	386	BS2G	24.26
**4683**	allXL	DD	373	386	BS2G	22.39
**4683**	allXL	AA	319	357	BS2G	28.78
**4683**	allXL	BB	367	373	BS2G	11.92
**4683**	allXL	CC	370	382	BS2G	23.53
**4683**	allXL	BB	376	382	BS2G	14.02
**4683**	allXL	BB	357	373	BS2G	23.63
**4683**	allXL	BB	320	366	BS2G	21.83
**4683**	allXL	BB	370	376	BS2G	15.75
**4683**	allXL	AA	320	357	DBSU	26.41
**4683**	allXL	AA	357	319	DBSU	28.78
**4683**	allXL	BB	372	319	DBSU	32.19
**4683**	allXL	BB	370	357	DBSU	15.73

**4683**	allXL	BB	382	357	DBSU	29.52
**4683**	allXL	AA	372	370	DBSU	6.16
**4683**	allXL	BB	381	370	DBSU	24.87
**4683**	allXL	DD	373	370	DBSU	9.05
**4683**	allXL	DD	381	372	DBSU	18.92
**4683**	allXL	DD	372	382	DBSU	17.92
**4683**	allXL	DD	372	386	DBSU	20.19
**4683**	allXL	DD	373	386	DBSU	22.39
**4683**	allXL	AD	381	386	DBSU	50.7
**4683**	allXL	BD;DB	357	357	DBSU	18.13
**4683**	allXL	DA	373	319	DBSU	41.21
**4683**	allXL	BD	372	357	DBSU	30.93
**4683**	allXL	BD	381	357	DBSU	34.55
**4683**	allXL	BD	373	357	DBSU	32.68
**4683**	allXL	DB	372	370	DBSU	48.64
**4683**	allXL	DB	373	370	DBSU	46.26
**4683**	allXL	DA	382	373	DBSU	54.08
**4684**	allXL	AA	320	357	BS2G	26.35
**4684**	allXL	AA	320	373	BS2G	13.14
**4684**	allXL	DD	373	382	BS2G	19.44
**4684**	allXL	DD	370	386	BS2G	24.88
**4684**	allXL	DD	373	386	BS2G	24.48
**4684**	allXL	AA	319	357	BS2G	24.85
**4684**	allXL	BB	367	373	BS2G	11.32
**4684**	allXL	AA	370	382	BS2G	23.35
**4684**	allXL	CC	376	382	BS2G	13.93
**4684**	allXL	BB	357	373	BS2G	26.14
**4684**	allXL	AA	320	366	BS2G	19.1
**4684**	allXL	CC	370	376	BS2G	16.29
**4684**	allXL	AA	320	357	DBSU	26.35
**4684**	allXL	AA	357	319	DBSU	24.85
**4684**	allXL	CC	372	319	DBSU	13.62
**4684**	allXL	DD	370	357	DBSU	27.55
**4684**	allXL	BB	382	357	DBSU	16.36

**4684**	allXL	AA	372	370	DBSU	6.49
**4684**	allXL	DD	381	370	DBSU	25.28
**4684**	allXL	AA	373	370	DBSU	9.29
**4684**	allXL	DD	381	372	DBSU	22.4
**4684**	allXL	CC	372	382	DBSU	21.79
**4684**	allXL	DD	372	386	DBSU	25.86
**4684**	allXL	DD	373	386	DBSU	24.48
**4684**	allXL	AD	381	386	DBSU	50.98
**4684**	allXL	BD;DB	357	357	DBSU	19.06
**4684**	allXL	AD	373	319	DBSU	34.47
**4684**	allXL	CB	372	357	DBSU	44.77
**4684**	allXL	BD	381	357	DBSU	30.84
**4684**	allXL	BD	373	357	DBSU	44.86
**4684**	allXL	CB	372	370	DBSU	34.57
**4684**	allXL	AD	373	370	DBSU	34.71
**4684**	allXL	AD	382	373	DBSU	37.04
**4685**	allXL	AA	320	357	BS2G	23.0
**4685**	allXL	DD	320	373	BS2G	26.01
**4685**	allXL	CC	373	382	BS2G	18.56
**4685**	allXL	CC	370	386	BS2G	18.75
**4685**	allXL	CC	373	386	BS2G	23.91
**4685**	allXL	AA	319	357	BS2G	22.55
**4685**	allXL	AA	367	373	BS2G	12.84
**4685**	allXL	CC	370	382	BS2G	16.8
**4685**	allXL	BB	376	382	BS2G	14.87
**4685**	allXL	AA	357	373	BS2G	27.97
**4685**	allXL	DD	320	366	BS2G	20.58
**4685**	allXL	CC	370	376	BS2G	15.97
**4685**	allXL	AA	320	357	DBSU	23.0
**4685**	allXL	AA	357	319	DBSU	22.55
**4685**	allXL	DD	372	319	DBSU	24.35
**4685**	allXL	DD	370	357	DBSU	31.21
**4685**	allXL	AA	382	357	DBSU	39.66
**4685**	allXL	AA	372	370	DBSU	6.3

**4685**	allXL	CC	381	370	DBSU	18.2
**4685**	allXL	AA	373	370	DBSU	9.24
**4685**	allXL	CC	381	372	DBSU	18.69
**4685**	allXL	CC	372	382	DBSU	17.2
**4685**	allXL	CC	372	386	DBSU	20.86
**4685**	allXL	CC	373	386	DBSU	23.91
**4685**	allXL	BC	381	386	DBSU	69.55
**4685**	allXL	BD;DB	357	357	DBSU	18.25
**4685**	allXL	CB	373	319	DBSU	29.8
**4685**	allXL	BD	372	357	DBSU	43.12
**4685**	allXL	AC	381	357	DBSU	44.97
**4685**	allXL	BD	373	357	DBSU	42.83
**4685**	allXL	DA	372	370	DBSU	39.37
**4685**	allXL	DA	373	370	DBSU	37.85
**4685**	allXL	CB	382	373	DBSU	52.76
**4686**	allXL	AA	320	357	BS2G	25.81
**4686**	allXL	BB	320	373	BS2G	16.29
**4686**	allXL	DD	373	382	BS2G	15.26
**4686**	allXL	DD	370	386	BS2G	15.91
**4686**	allXL	DD	373	386	BS2G	22.5
**4686**	allXL	AA	319	357	BS2G	25.45
**4686**	allXL	AA	367	373	BS2G	12.1
**4686**	allXL	DD	370	382	BS2G	12.56
**4686**	allXL	BB	376	382	BS2G	14.68
**4686**	allXL	BB	357	373	BS2G	33.33
**4686**	allXL	DD	320	366	BS2G	15.55
**4686**	allXL	CC	370	376	BS2G	16.69
**4686**	allXL	AA	320	357	DBSU	25.81
**4686**	allXL	AA	357	319	DBSU	25.45
**4686**	allXL	DD	372	319	DBSU	15.53
**4686**	allXL	BB	370	357	DBSU	29.36
**4686**	allXL	AA	382	357	DBSU	29.14
**4686**	allXL	AA	372	370	DBSU	6.36
**4686**	allXL	DD	381	370	DBSU	12.89

**4686**	allXL	AA	373	370	DBSU	8.96
**4686**	allXL	DD	381	372	DBSU	13.91
**4686**	allXL	DD	372	382	DBSU	15.43
**4686**	allXL	DD	372	386	DBSU	22.29
**4686**	allXL	DD	373	386	DBSU	22.5
**4686**	allXL	BC	381	386	DBSU	42.19
**4686**	allXL	BD;DB	357	357	DBSU	19.34
**4686**	allXL	AD	373	319	DBSU	21.95
**4686**	allXL	CB	372	357	DBSU	42.35
**4686**	allXL	AC	381	357	DBSU	41.79
**4686**	allXL	DA	373	357	DBSU	40.27
**4686**	allXL	DA	372	370	DBSU	14.66
**4686**	allXL	DA	373	370	DBSU	12.0
**4686**	allXL	CB	382	373	DBSU	26.59
**4687**	allXL	AA	320	357	BS2G	25.87
**4687**	allXL	CC	320	373	BS2G	11.46
**4687**	allXL	BB	373	382	BS2G	18.54
**4687**	allXL	BB	370	386	BS2G	20.98
**4687**	allXL	BB	373	386	BS2G	26.22
**4687**	allXL	AA	319	357	BS2G	27.93
**4687**	allXL	AA	367	373	BS2G	13.64
**4687**	allXL	BB	370	382	BS2G	17.86
**4687**	allXL	CC	376	382	BS2G	15.18
**4687**	allXL	CC	357	373	BS2G	22.83
**4687**	allXL	AA	320	366	BS2G	21.53
**4687**	allXL	CC	370	376	BS2G	16.4
**4687**	allXL	AA	320	357	DBSU	25.87
**4687**	allXL	AA	357	319	DBSU	27.93
**4687**	allXL	CC	372	319	DBSU	11.83
**4687**	allXL	CC	370	357	DBSU	23.17
**4687**	allXL	CC	382	357	DBSU	17.82
**4687**	allXL	AA	372	370	DBSU	6.14
**4687**	allXL	BB	381	370	DBSU	16.73
**4687**	allXL	CC	373	370	DBSU	9.02

**4687**	allXL	BB	381	372	DBSU	18.16
**4687**	allXL	BB	372	382	DBSU	20.11
**4687**	allXL	BB	372	386	DBSU	26.22
**4687**	allXL	BB	373	386	DBSU	26.22
**4687**	allXL	AD	381	386	DBSU	26.09
**4687**	allXL	BD;DB	357	357	DBSU	18.37
**4687**	allXL	AD	373	319	DBSU	17.87
**4687**	allXL	CA	372	357	DBSU	44.85
**4687**	allXL	CA	381	357	DBSU	26.66
**4687**	allXL	CA	373	357	DBSU	42.08
**4687**	allXL	AD	372	370	DBSU	34.94
**4687**	allXL	AD	373	370	DBSU	31.62
**4687**	allXL	AD	382	373	DBSU	23.24
**4688**	allXL	CC	320	357	BS2G	24.51
**4688**	allXL	DD	320	373	BS2G	9.61
**4688**	allXL	DD	373	382	BS2G	17.17
**4688**	allXL	AA	370	386	BS2G	17.8
**4688**	allXL	AA	373	386	BS2G	20.99
**4688**	allXL	AA	319	357	BS2G	27.31
**4688**	allXL	AA	367	373	BS2G	9.98
**4688**	allXL	AA	370	382	BS2G	18.95
**4688**	allXL	CC	376	382	BS2G	13.91
**4688**	allXL	BB	357	373	BS2G	18.68
**4688**	allXL	DD	320	366	BS2G	12.25
**4688**	allXL	AA	370	376	BS2G	16.44
**4688**	allXL	CC	320	357	DBSU	24.51
**4688**	allXL	AA	357	319	DBSU	27.31
**4688**	allXL	DD	372	319	DBSU	12.43
**4688**	allXL	BB	370	357	DBSU	26.28
**4688**	allXL	BB	382	357	DBSU	27.98
**4688**	allXL	AA	372	370	DBSU	6.63
**4688**	allXL	AA	381	370	DBSU	20.36
**4688**	allXL	AA	373	370	DBSU	8.82
**4688**	allXL	DD	381	372	DBSU	16.76

4688	allXL	DD	372	382	DBSU	16.77
4688	allXL	AA	372	386	DBSU	18.66
4688	allXL	AA	373	386	DBSU	20.99
4688	allXL	AD	381	386	DBSU	55.12
4688	allXL	BD;DB	357	357	DBSU	18.91
4688	allXL	AD	373	319	DBSU	39.29
4688	allXL	BD	372	357	DBSU	36.62
4688	allXL	BD	381	357	DBSU	27.52
4688	allXL	BD	373	357	DBSU	33.76
4688	allXL	AD	372	370	DBSU	51.56
4688	allXL	AD	373	370	DBSU	48.5
4688	allXL	AD	382	373	DBSU	48.23
4689	allXL	CC	320	357	BS2G	22.19
4689	allXL	DD	320	373	BS2G	25.51
4689	allXL	BB	373	382	BS2G	17.45
4689	allXL	CC	370	386	BS2G	20.8
4689	allXL	BB	373	386	BS2G	25.07
4689	allXL	CC	319	357	BS2G	25.36
4689	allXL	BB	367	373	BS2G	14.09
4689	allXL	DD	370	382	BS2G	17.01
4689	allXL	CC	376	382	BS2G	14.34
4689	allXL	DD	357	373	BS2G	29.74
4689	allXL	CC	320	366	BS2G	27.98
4689	allXL	DD	370	376	BS2G	15.88
4689	allXL	CC	320	357	DBSU	22.19
4689	allXL	CC	357	319	DBSU	25.36
4689	allXL	DD	372	319	DBSU	25.24
4689	allXL	DD	370	357	DBSU	21.43
4689	allXL	DD	382	357	DBSU	33.47
4689	allXL	AA	372	370	DBSU	5.79
4689	allXL	DD	381	370	DBSU	17.43
4689	allXL	AA	373	370	DBSU	9.11
4689	allXL	BB	381	372	DBSU	18.37
4689	allXL	BB	372	382	DBSU	19.97

**4689**	allXL	CC	372	386	DBSU	24.88
**4689**	allXL	BB	373	386	DBSU	25.07
**4689**	allXL	BC	381	386	DBSU	48.86
**4689**	allXL	BD;DB	357	357	DBSU	18.34
**4689**	allXL	AD	373	319	DBSU	36.99
**4689**	allXL	DB	372	357	DBSU	44.65
**4689**	allXL	DB	381	357	DBSU	52.82
**4689**	allXL	DB	373	357	DBSU	47.97
**4689**	allXL	AD	372	370	DBSU	47.82
**4689**	allXL	AD	373	370	DBSU	48.36
**4689**	allXL	DA	382	373	DBSU	40.07
**4690**	allXL	CC	320	357	BS2G	22.99
**4690**	allXL	DD	320	373	BS2G	24.86
**4690**	allXL	DD	373	382	BS2G	21.74
**4690**	allXL	AA	370	386	BS2G	27.43
**4690**	allXL	AA	373	386	BS2G	24.15
**4690**	allXL	CC	319	357	BS2G	22.63
**4690**	allXL	BB	367	373	BS2G	12.07
**4690**	allXL	DD	370	382	BS2G	26.87
**4690**	allXL	CC	376	382	BS2G	14.82
**4690**	allXL	CC	357	373	BS2G	19.94
**4690**	allXL	AA	320	366	BS2G	19.42
**4690**	allXL	BB	370	376	BS2G	15.99
**4690**	allXL	CC	320	357	DBSU	22.99
**4690**	allXL	CC	357	319	DBSU	22.63
**4690**	allXL	DD	372	319	DBSU	22.47
**4690**	allXL	CC	370	357	DBSU	18.56
**4690**	allXL	AA	382	357	DBSU	36.58
**4690**	allXL	AA	372	370	DBSU	6.29
**4690**	allXL	DD	381	370	DBSU	23.72
**4690**	allXL	AA	373	370	DBSU	9.12
**4690**	allXL	DD	381	372	DBSU	19.97
**4690**	allXL	DD	372	382	DBSU	23.12
**4690**	allXL	AA	372	386	DBSU	24.96

**4690**	allXL	AA	373	386	DBSU	24.15
**4690**	allXL	AC	381	386	DBSU	46.38
**4690**	allXL	BD;DB	357	357	DBSU	19.94
**4690**	allXL	DA	373	319	DBSU	23.87
**4690**	allXL	CA	372	357	DBSU	21.8
**4690**	allXL	CA	381	357	DBSU	40.87
**4690**	allXL	CA	373	357	DBSU	21.69
**4690**	allXL	DA	372	370	DBSU	31.38
**4690**	allXL	DA	373	370	DBSU	27.72
**4690**	allXL	AD	382	373	DBSU	41.95
**4691**	allXL	AA	320	357	BS2G	25.77
**4691**	allXL	BB	320	373	BS2G	7.77
**4691**	allXL	AA	373	382	BS2G	15.3
**4691**	allXL	AA	370	386	BS2G	9.47
**4691**	allXL	AA	373	386	BS2G	12.76
**4691**	allXL	AA	319	357	BS2G	24.23
**4691**	allXL	AA	367	373	BS2G	12.55
**4691**	allXL	AA	370	382	BS2G	18.21
**4691**	allXL	AA	376	382	BS2G	14.15
**4691**	allXL	BB	357	373	BS2G	28.02
**4691**	allXL	BB	320	366	BS2G	21.49
**4691**	allXL	BB	370	376	BS2G	15.72
**4691**	allXL	AA	320	357	DBSU	25.77
**4691**	allXL	AA	357	319	DBSU	24.23
**4691**	allXL	BB	372	319	DBSU	11.73
**4691**	allXL	BB	370	357	DBSU	26.37
**4691**	allXL	BB	382	357	DBSU	30.65
**4691**	allXL	AA	372	370	DBSU	6.42
**4691**	allXL	DD	381	370	DBSU	17.93
**4691**	allXL	AA	373	370	DBSU	9.13
**4691**	allXL	DD	381	372	DBSU	13.93
**4691**	allXL	AA	372	382	DBSU	14.28
**4691**	allXL	AA	372	386	DBSU	9.27
**4691**	allXL	AA	373	386	DBSU	12.76

**4691**	allXL	BC	381	386	DBSU	30.14
**4691**	allXL	BD;DB	357	357	DBSU	18.25
**4691**	allXL	AD	373	319	DBSU	23.18
**4691**	allXL	BD	372	357	DBSU	44.21
**4691**	allXL	BC	381	357	DBSU	32.57
**4691**	allXL	BD	373	357	DBSU	44.72
**4691**	allXL	CB	372	370	DBSU	30.03
**4691**	allXL	CB	373	370	DBSU	30.26
**4691**	allXL	CB	382	373	DBSU	32.98
**4692**	allXL	CC	320	357	BS2G	26.0
**4692**	allXL	CC	320	373	BS2G	11.68
**4692**	allXL	CC	373	382	BS2G	20.31
**4692**	allXL	CC	370	386	BS2G	22.21
**4692**	allXL	BB	373	386	BS2G	27.68
**4692**	allXL	AA	319	357	BS2G	27.11
**4692**	allXL	AA	367	373	BS2G	9.79
**4692**	allXL	CC	370	382	BS2G	16.09
**4692**	allXL	BB;CC	376	382	BS2G	14.54
**4692**	allXL	BB	357	373	BS2G	23.94
**4692**	allXL	BB	320	366	BS2G	14.03
**4692**	allXL	CC	370	376	BS2G	12.58
**4692**	allXL	CC	320	357	DBSU	26.0
**4692**	allXL	AA	357	319	DBSU	27.11
**4692**	allXL	CC	372	319	DBSU	8.66
**4692**	allXL	BB	370	357	DBSU	21.2
**4692**	allXL	CC	382	357	DBSU	32.23
**4692**	allXL	AA	372	370	DBSU	6.39
**4692**	allXL	CC	381	370	DBSU	18.24
**4692**	allXL	AA	373	370	DBSU	8.88
**4692**	allXL	BB	381	372	DBSU	21.32
**4692**	allXL	CC	372	382	DBSU	20.21
**4692**	allXL	CC	372	386	DBSU	27.79
**4692**	allXL	BB	373	386	DBSU	27.68
**4692**	allXL	DB	381	386	DBSU	60.44

**4692**	allXL	BD;DB	357	357	DBSU	19.11
**4692**	allXL	AD	373	319	DBSU	31.02
**4692**	allXL	BD	372	357	DBSU	31.06
**4692**	allXL	BD	381	357	DBSU	34.62
**4692**	allXL	BD	373	357	DBSU	29.86
**4692**	allXL	DA	372	370	DBSU	27.9
**4692**	allXL	DA	373	370	DBSU	28.25
**4692**	allXL	DA	382	373	DBSU	54.02
**4693**	allXL	CC	320	357	BS2G	24.24
**4693**	allXL	BB	320	373	BS2G	13.99
**4693**	allXL	DD	373	382	BS2G	17.4
**4693**	allXL	DD	370	386	BS2G	15.93
**4693**	allXL	DD	373	386	BS2G	21.23
**4693**	allXL	AA	319	357	BS2G	23.87
**4693**	allXL	AA	367	373	BS2G	11.79
**4693**	allXL	DD	370	382	BS2G	17.51
**4693**	allXL	BB	376	382	BS2G	13.93
**4693**	allXL	AA	357	373	BS2G	29.72
**4693**	allXL	DD	320	366	BS2G	21.73
**4693**	allXL	CC	370	376	BS2G	16.02
**4693**	allXL	CC	320	357	DBSU	24.24
**4693**	allXL	AA	357	319	DBSU	23.87
**4693**	allXL	DD	372	319	DBSU	17.26
**4693**	allXL	BB	370	357	DBSU	26.32
**4693**	allXL	CC	382	357	DBSU	20.01
**4693**	allXL	AA	372	370	DBSU	6.38
**4693**	allXL	DD	381	370	DBSU	18.24
**4693**	allXL	AA	373	370	DBSU	8.87
**4693**	allXL	DD	381	372	DBSU	16.15
**4693**	allXL	DD	372	382	DBSU	16.95
**4693**	allXL	DD	372	386	DBSU	19.06
**4693**	allXL	DD	373	386	DBSU	21.23
**4693**	allXL	DA	381	386	DBSU	33.68
**4693**	allXL	BD;DB	357	357	DBSU	19.01

**4693**	allXL	AD	373	319	DBSU	19.55
**4693**	allXL	AC	372	357	DBSU	45.92
**4693**	allXL	CA	381	357	DBSU	34.62
**4693**	allXL	AC	373	357	DBSU	43.02
**4693**	allXL	DA	372	370	DBSU	32.68
**4693**	allXL	DA	373	370	DBSU	29.24
**4693**	allXL	AD	382	373	DBSU	19.58
**4694**	allXL	AA	320	357	BS2G	24.32
**4694**	allXL	CC	320	373	BS2G	22.95
**4694**	allXL	DD	373	382	BS2G	16.08
**4694**	allXL	DD	370	386	BS2G	22.43
**4694**	allXL	DD	373	386	BS2G	23.32
**4694**	allXL	CC	319	357	BS2G	25.64
**4694**	allXL	BB	367	373	BS2G	11.12
**4694**	allXL	DD	370	382	BS2G	19.53
**4694**	allXL	BB	376	382	BS2G	15.07
**4694**	allXL	BB	357	373	BS2G	18.87
**4694**	allXL	CC	320	366	BS2G	19.89
**4694**	allXL	CC	370	376	BS2G	16.46
**4694**	allXL	AA	320	357	DBSU	24.32
**4694**	allXL	CC	357	319	DBSU	25.64
**4694**	allXL	CC	372	319	DBSU	21.65
**4694**	allXL	CC	370	357	DBSU	22.49
**4694**	allXL	BB	382	357	DBSU	21.35
**4694**	allXL	AA	372	370	DBSU	6.26
**4694**	allXL	DD	381	370	DBSU	21.01
**4694**	allXL	AA	373	370	DBSU	9.02
**4694**	allXL	DD	381	372	DBSU	16.8
**4694**	allXL	DD	372	382	DBSU	15.9
**4694**	allXL	DD	372	386	DBSU	21.26
**4694**	allXL	DD	373	386	DBSU	23.32
**4694**	allXL	DA	381	386	DBSU	54.07
**4694**	allXL	BD;DB	357	357	DBSU	19.19
**4694**	allXL	DA	373	319	DBSU	24.08

**4694**	allXL	BD	372	357	DBSU	39.43
**4694**	allXL	BD	381	357	DBSU	28.28
**4694**	allXL	BD	373	357	DBSU	36.08
**4694**	allXL	DA	372	370	DBSU	39.05
**4694**	allXL	DA	373	370	DBSU	37.52
**4694**	allXL	AD	382	373	DBSU	47.8
**4695**	allXL	AA	320	357	BS2G	26.17
**4695**	allXL	CC	320	373	BS2G	25.44
**4695**	allXL	AA	373	382	BS2G	14.7
**4695**	allXL	AA	370	386	BS2G	20.42
**4695**	allXL	AA	373	386	BS2G	19.86
**4695**	allXL	AA	319	357	BS2G	28.99
**4695**	allXL	AA	367	373	BS2G	12.87
**4695**	allXL	AA	370	382	BS2G	20.29
**4695**	allXL	AA	376	382	BS2G	13.38
**4695**	allXL	BB	357	373	BS2G	32.27
**4695**	allXL	BB	320	366	BS2G	17.74
**4695**	allXL	CC	370	376	BS2G	16.89
**4695**	allXL	AA	320	357	DBSU	26.17
**4695**	allXL	AA	357	319	DBSU	28.99
**4695**	allXL	CC	372	319	DBSU	23.13
**4695**	allXL	DD	370	357	DBSU	29.77
**4695**	allXL	AA	382	357	DBSU	32.29
**4695**	allXL	AA	372	370	DBSU	6.34
**4695**	allXL	AA	381	370	DBSU	22.94
**4695**	allXL	AA	373	370	DBSU	9.06
**4695**	allXL	AA	381	372	DBSU	20.32
**4695**	allXL	AA	372	382	DBSU	17.9
**4695**	allXL	AA	372	386	DBSU	21.35
**4695**	allXL	AA	373	386	DBSU	19.86
**4695**	allXL	DB	381	386	DBSU	81.53
**4695**	allXL	BD;DB	357	357	DBSU	18.87
**4695**	allXL	AD	373	319	DBSU	39.87
**4695**	allXL	BD	372	357	DBSU	47.98

**4695**	allXL	AC	381	357	DBSU	51.05
**4695**	allXL	BD	373	357	DBSU	49.25
**4695**	allXL	DA	372	370	DBSU	54.27
**4695**	allXL	DA	373	370	DBSU	56.96
**4695**	allXL	AD	382	373	DBSU	76.7
**4696**	allXL	AA	320	357	BS2G	26.36
**4696**	allXL	AA	320	373	BS2G	23.51
**4696**	allXL	AA	373	382	BS2G	18.19
**4696**	allXL	DD	370	386	BS2G	27.32
**4696**	allXL	CC	373	386	BS2G	23.79
**4696**	allXL	CC	319	357	BS2G	27.06
**4696**	allXL	AA	367	373	BS2G	11.51
**4696**	allXL	DD	370	382	BS2G	19.85
**4696**	allXL	BB	376	382	BS2G	14.81
**4696**	allXL	BB	357	373	BS2G	28.96
**4696**	allXL	BB	320	366	BS2G	15.64
**4696**	allXL	DD	370	376	BS2G	16.32
**4696**	allXL	AA	320	357	DBSU	26.36
**4696**	allXL	CC	357	319	DBSU	27.06
**4696**	allXL	AA	372	319	DBSU	26.73
**4696**	allXL	DD	370	357	DBSU	26.59
**4696**	allXL	DD	382	357	DBSU	34.92
**4696**	allXL	CC	372	370	DBSU	6.6
**4696**	allXL	DD	381	370	DBSU	20.78
**4696**	allXL	AA	373	370	DBSU	9.23
**4696**	allXL	AA	381	372	DBSU	20.78
**4696**	allXL	AA	372	382	DBSU	19.53
**4696**	allXL	CC	372	386	DBSU	23.22
**4696**	allXL	CC	373	386	DBSU	23.79
**4696**	allXL	DA	381	386	DBSU	31.81
**4696**	allXL	BD;DB	357	357	DBSU	19.05
**4696**	allXL	CB	373	319	DBSU	14.9
**4696**	allXL	CB	372	357	DBSU	44.05
**4696**	allXL	AD	381	357	DBSU	45.02

**4696**	allXL	CA	373	357	DBSU	44.23
**4696**	allXL	CB	372	370	DBSU	30.73
**4696**	allXL	CB	373	370	DBSU	32.3
**4696**	allXL	CB	382	373	DBSU	43.85
**4697**	allXL	AA	320	357	BS2G	22.33
**4697**	allXL	BB	320	373	BS2G	17.19
**4697**	allXL	DD	373	382	BS2G	17.88
**4697**	allXL	DD	370	386	BS2G	24.62
**4697**	allXL	DD	373	386	BS2G	25.07
**4697**	allXL	AA	319	357	BS2G	24.86
**4697**	allXL	AA	367	373	BS2G	11.64
**4697**	allXL	DD	370	382	BS2G	19.66
**4697**	allXL	CC	376	382	BS2G	13.92
**4697**	allXL	AA	357	373	BS2G	23.41
**4697**	allXL	BB	320	366	BS2G	21.31
**4697**	allXL	AA	370	376	BS2G	15.15
**4697**	allXL	AA	320	357	DBSU	22.33
**4697**	allXL	AA	357	319	DBSU	24.86
**4697**	allXL	BB	372	319	DBSU	19.26
**4697**	allXL	BB	370	357	DBSU	28.01
**4697**	allXL	BB	382	357	DBSU	27.2
**4697**	allXL	CC	372	370	DBSU	6.52
**4697**	allXL	DD	381	370	DBSU	20.63
**4697**	allXL	AA	373	370	DBSU	8.72
**4697**	allXL	DD	381	372	DBSU	19.41
**4697**	allXL	DD	372	382	DBSU	19.7
**4697**	allXL	DD	372	386	DBSU	26.98
**4697**	allXL	DD	373	386	DBSU	25.07
**4697**	allXL	DB	381	386	DBSU	46.4
**4697**	allXL	BD;DB	357	357	DBSU	20.0
**4697**	allXL	AD	373	319	DBSU	27.11
**4697**	allXL	CB	372	357	DBSU	32.19
**4697**	allXL	BD	381	357	DBSU	41.43
**4697**	allXL	CB	373	357	DBSU	33.46

**4697**	allXL	CB	372	370	DBSU	24.15
**4697**	allXL	CB	373	370	DBSU	25.12
**4697**	allXL	BC	382	373	DBSU	40.72
**4698**	allXL	AA	320	357	BS2G	24.88
**4698**	allXL	AA	320	373	BS2G	23.72
**4698**	allXL	CC	373	382	BS2G	19.01
**4698**	allXL	CC	370	386	BS2G	26.88
**4698**	allXL	CC	373	386	BS2G	27.44
**4698**	allXL	AA	319	357	BS2G	25.29
**4698**	allXL	AA	367	373	BS2G	13.74
**4698**	allXL	CC	370	382	BS2G	19.3
**4698**	allXL	BB	376	382	BS2G	13.84
**4698**	allXL	BB	357	373	BS2G	23.9
**4698**	allXL	AA	320	366	BS2G	10.33
**4698**	allXL	CC	370	376	BS2G	12.39
**4698**	allXL	AA	320	357	DBSU	24.88
**4698**	allXL	AA	357	319	DBSU	25.29
**4698**	allXL	AA	372	319	DBSU	24.23
**4698**	allXL	DD	370	357	DBSU	25.86
**4698**	allXL	BB	382	357	DBSU	26.53
**4698**	allXL	AA	372	370	DBSU	6.15
**4698**	allXL	CC	381	370	DBSU	20.08
**4698**	allXL	AA	373	370	DBSU	9.12
**4698**	allXL	CC	381	372	DBSU	21.74
**4698**	allXL	CC	372	382	DBSU	20.56
**4698**	allXL	CC	372	386	DBSU	28.36
**4698**	allXL	CC	373	386	DBSU	27.44
**4698**	allXL	BD	381	386	DBSU	22.54
**4698**	allXL	BD;DB	357	357	DBSU	19.51
**4698**	allXL	AD	373	319	DBSU	33.22
**4698**	allXL	BD	372	357	DBSU	43.71
**4698**	allXL	DB	381	357	DBSU	27.96
**4698**	allXL	BD	373	357	DBSU	41.82
**4698**	allXL	CB	372	370	DBSU	54.81

**4698**	allXL	CB	373	370	DBSU	52.51
**4698**	allXL	DB	382	373	DBSU	31.32
**4699**	allXL	CC	320	357	BS2G	25.41
**4699**	allXL	CC	320	373	BS2G	21.89
**4699**	allXL	CC	373	382	BS2G	18.91
**4699**	allXL	AA	370	386	BS2G	24.66
**4699**	allXL	BB	373	386	BS2G	24.21
**4699**	allXL	AA	319	357	BS2G	26.23
**4699**	allXL	AA	367	373	BS2G	11.73
**4699**	allXL	DD	370	382	BS2G	22.29
**4699**	allXL	CC	376	382	BS2G	15.0
**4699**	allXL	DD	357	373	BS2G	24.48
**4699**	allXL	CC	320	366	BS2G	19.56
**4699**	allXL	CC	370	376	BS2G	16.48
**4699**	allXL	CC	320	357	DBSU	25.41
**4699**	allXL	AA	357	319	DBSU	26.23
**4699**	allXL	CC	372	319	DBSU	19.77
**4699**	allXL	DD	370	357	DBSU	24.99
**4699**	allXL	DD	382	357	DBSU	11.6
**4699**	allXL	AA	372	370	DBSU	6.42
**4699**	allXL	DD	381	370	DBSU	19.67
**4699**	allXL	AA	373	370	DBSU	9.26
**4699**	allXL	AA	381	372	DBSU	17.6
**4699**	allXL	AA	372	382	DBSU	19.38
**4699**	allXL	AA	372	386	DBSU	22.06
**4699**	allXL	BB	373	386	DBSU	24.21
**4699**	allXL	BD	381	386	DBSU	63.22
**4699**	allXL	BD;DB	357	357	DBSU	17.21
**4699**	allXL	DA	373	319	DBSU	21.78
**4699**	allXL	DB	372	357	DBSU	39.45
**4699**	allXL	DB	381	357	DBSU	18.88
**4699**	allXL	DB	373	357	DBSU	36.73
**4699**	allXL	DA	372	370	DBSU	37.12
**4699**	allXL	DA	373	370	DBSU	35.97

**4699**	allXL	DB	382	373	DBSU	53.04
**4700**	allXL	AA	320	357	BS2G	24.13
**4700**	allXL	BB	320	373	BS2G	29.03
**4700**	allXL	DD	373	382	BS2G	19.48
**4700**	allXL	DD	370	386	BS2G	19.59
**4700**	allXL	DD	373	386	BS2G	24.0
**4700**	allXL	AA	319	357	BS2G	22.95
**4700**	allXL	AA	367	373	BS2G	12.84
**4700**	allXL	DD	370	382	BS2G	15.39
**4700**	allXL	BB	376	382	BS2G	15.15
**4700**	allXL	CC	357	373	BS2G	28.54
**4700**	allXL	CC	320	366	BS2G	24.66
**4700**	allXL	DD	370	376	BS2G	15.82
**4700**	allXL	AA	320	357	DBSU	24.13
**4700**	allXL	AA	357	319	DBSU	22.95
**4700**	allXL	DD	372	319	DBSU	29.25
**4700**	allXL	DD	370	357	DBSU	27.64
**4700**	allXL	BB	382	357	DBSU	19.81
**4700**	allXL	AA	372	370	DBSU	6.47
**4700**	allXL	DD	381	370	DBSU	15.97
**4700**	allXL	AA	373	370	DBSU	9.11
**4700**	allXL	DD	381	372	DBSU	19.74
**4700**	allXL	DD	372	382	DBSU	20.15
**4700**	allXL	DD	372	386	DBSU	23.52
**4700**	allXL	DD	373	386	DBSU	24.0
**4700**	allXL	DB	381	386	DBSU	30.03
**4700**	allXL	BD;DB	357	357	DBSU	18.86
**4700**	allXL	DA	373	319	DBSU	24.08
**4700**	allXL	DA	372	357	DBSU	40.8
**4700**	allXL	CA	381	357	DBSU	26.55
**4700**	allXL	DA	373	357	DBSU	37.8
**4700**	allXL	CB	372	370	DBSU	49.21
**4700**	allXL	AD	373	370	DBSU	50.84
**4700**	allXL	AD	382	373	DBSU	48.08

**4701**	allXL	AA	320	357	BS2G	19.87
**4701**	allXL	CC	320	373	BS2G	20.89
**4701**	allXL	CC	373	382	BS2G	18.29
**4701**	allXL	BB	370	386	BS2G	25.97
**4701**	allXL	CC	373	386	BS2G	27.3
**4701**	allXL	AA	319	357	BS2G	17.01
**4701**	allXL	BB	367	373	BS2G	13.36
**4701**	allXL	BB	370	382	BS2G	22.32
**4701**	allXL	CC	376	382	BS2G	14.8
**4701**	allXL	BB	357	373	BS2G	16.52
**4701**	allXL	CC	320	366	BS2G	18.87
**4701**	allXL	CC	370	376	BS2G	16.03
**4701**	allXL	AA	320	357	DBSU	19.87
**4701**	allXL	AA	357	319	DBSU	17.01
**4701**	allXL	CC	372	319	DBSU	17.93
**4701**	allXL	AA	370	357	DBSU	21.05
**4701**	allXL	BB	382	357	DBSU	26.78
**4701**	allXL	AA	372	370	DBSU	6.21
**4701**	allXL	CC	381	370	DBSU	22.59
**4701**	allXL	DD	373	370	DBSU	8.88
**4701**	allXL	DD	381	372	DBSU	19.47
**4701**	allXL	DD	372	382	DBSU	20.42
**4701**	allXL	CC	372	386	DBSU	28.52
**4701**	allXL	CC	373	386	DBSU	27.3
**4701**	allXL	DA	381	386	DBSU	41.29
**4701**	allXL	BD;DB	357	357	DBSU	18.36
**4701**	allXL	AD	373	319	DBSU	22.77
**4701**	allXL	AC	372	357	DBSU	25.9
**4701**	allXL	BD	381	357	DBSU	26.87
**4701**	allXL	AC	373	357	DBSU	29.06
**4701**	allXL	AC	372	370	DBSU	32.77
**4701**	allXL	AC	373	370	DBSU	34.19
**4701**	allXL	BD	382	373	DBSU	40.17
**4702**	allXL	AA	320	357	BS2G	25.29

**4702**	allXL	DD	320	373	BS2G	14.04
**4702**	allXL	CC	373	382	BS2G	17.34
**4702**	allXL	AA	370	386	BS2G	21.48
**4702**	allXL	AA	373	386	BS2G	20.99
**4702**	allXL	AA	319	357	BS2G	28.18
**4702**	allXL	AA	367	373	BS2G	12.52
**4702**	allXL	AA	370	382	BS2G	21.53
**4702**	allXL	AA	376	382	BS2G	13.48
**4702**	allXL	BB	357	373	BS2G	29.77
**4702**	allXL	DD	320	366	BS2G	21.84
**4702**	allXL	BB	370	376	BS2G	14.77
**4702**	allXL	AA	320	357	DBSU	25.29
**4702**	allXL	AA	357	319	DBSU	28.18
**4702**	allXL	DD	372	319	DBSU	10.65
**4702**	allXL	DD	370	357	DBSU	23.43
**4702**	allXL	CD	382	357	DBSU	30.14
**4702**	allXL	AA	372	370	DBSU	6.54
**4702**	allXL	AA	381	370	DBSU	24.04
**4702**	allXL	AA	373	370	DBSU	8.74
**4702**	allXL	AA	381	372	DBSU	20.44
**4702**	allXL	AA	372	382	DBSU	17.89
**4702**	allXL	AA	372	386	DBSU	19.07
**4702**	allXL	AA	373	386	DBSU	20.99
**4702**	allXL	DA	381	386	DBSU	36.61
**4702**	allXL	BD;DB	357	357	DBSU	18.44
**4702**	allXL	CB	373	319	DBSU	23.13
**4702**	allXL	CB	372	357	DBSU	29.62
**4702**	allXL	CB	381	357	DBSU	20.73
**4702**	allXL	CB	373	357	DBSU	26.69
**4702**	allXL	DA	372	370	DBSU	19.65
**4702**	allXL	DA	373	370	DBSU	19.68
**4702**	allXL	BC	382	373	DBSU	35.57
**4703**	allXL	AA	320	357	BS2G	20.19
**4703**	allXL	BB	320	373	BS2G	24.17

**4703**	allXL	DD	373	382	BS2G	16.46
**4703**	allXL	DD	370	386	BS2G	22.01
**4703**	allXL	DD	373	386	BS2G	20.65
**4703**	allXL	AA	319	357	BS2G	17.55
**4703**	allXL	BB	367	373	BS2G	11.11
**4703**	allXL	DD	370	382	BS2G	22.32
**4703**	allXL	CC	376	382	BS2G	13.97
**4703**	allXL	BB	357	373	BS2G	24.81
**4703**	allXL	CC	320	366	BS2G	26.56
**4703**	allXL	CC	370	376	BS2G	16.73
**4703**	allXL	AA	320	357	DBSU	20.19
**4703**	allXL	AA	357	319	DBSU	17.55
**4703**	allXL	BB	372	319	DBSU	26.73
**4703**	allXL	DD	370	357	DBSU	27.82
**4703**	allXL	BB	382	357	DBSU	16.65
**4703**	allXL	AA	372	370	DBSU	6.48
**4703**	allXL	DD	381	370	DBSU	24.19
**4703**	allXL	CC	373	370	DBSU	9.15
**4703**	allXL	DD	381	372	DBSU	17.22
**4703**	allXL	DD	372	382	DBSU	15.57
**4703**	allXL	DD	372	386	DBSU	17.79
**4703**	allXL	DD	373	386	DBSU	20.65
**4703**	allXL	BC	381	386	DBSU	57.15
**4703**	allXL	BD;DB	357	357	DBSU	18.75
**4703**	allXL	BC	373	319	DBSU	28.52
**4703**	allXL	BC	372	357	DBSU	40.43
**4703**	allXL	BD	381	357	DBSU	33.22
**4703**	allXL	BC	373	357	DBSU	41.04
**4703**	allXL	BC	372	370	DBSU	17.61
**4703**	allXL	BC	373	370	DBSU	20.47
**4703**	allXL	CB	382	373	DBSU	43.54
**4704**	allXL	AA	320	357	BS2G	24.01
**4704**	allXL	AA	320	373	BS2G	29.68
**4704**	allXL	BB	373	382	BS2G	20.43

**4704**	allXL	DD	370	386	BS2G	25.05
**4704**	allXL	BB	373	386	BS2G	25.7
**4704**	allXL	AA	319	357	BS2G	23.49
**4704**	allXL	AA	367	373	BS2G	12.96
**4704**	allXL	DD	370	382	BS2G	19.23
**4704**	allXL	BB	376	382	BS2G	13.28
**4704**	allXL	AA	357	373	BS2G	34.77
**4704**	allXL	AA	320	366	BS2G	19.72
**4704**	allXL	DD	370	376	BS2G	15.78
**4704**	allXL	AA	320	357	DBSU	24.01
**4704**	allXL	AA	357	319	DBSU	23.49
**4704**	allXL	AA	372	319	DBSU	25.64
**4704**	allXL	DD	370	357	DBSU	26.94
**4704**	allXL	DD	382	357	DBSU	33.99
**4704**	allXL	AA	372	370	DBSU	6.43
**4704**	allXL	DD	381	370	DBSU	19.15
**4704**	allXL	CC	373	370	DBSU	9.18
**4704**	allXL	DD	381	372	DBSU	21.68
**4704**	allXL	DD	372	382	DBSU	22.59
**4704**	allXL	DD	372	386	DBSU	27.75
**4704**	allXL	BB	373	386	DBSU	25.7
**4704**	allXL	BC	381	386	DBSU	38.91
**4704**	allXL	BD;DB	357	357	DBSU	18.79
**4704**	allXL	CB	373	319	DBSU	18.14
**4704**	allXL	CB	372	357	DBSU	42.32
**4704**	allXL	DB	381	357	DBSU	50.55
**4704**	allXL	CB	373	357	DBSU	43.3
**4704**	allXL	CB	372	370	DBSU	21.92
**4704**	allXL	CB	373	370	DBSU	20.34
**4704**	allXL	CB	382	373	DBSU	33.4
**4705**	allXL	AA	320	357	BS2G	23.27
**4705**	allXL	BB	320	373	BS2G	30.76
**4705**	allXL	DD	373	382	BS2G	20.88
**4705**	allXL	DD	370	386	BS2G	31.19

**4705**	allXL	DD	373	386	BS2G	27.97
**4705**	allXL	AA	319	357	BS2G	21.47
**4705**	allXL	AA	367	373	BS2G	12.67
**4705**	allXL	CC	370	382	BS2G	21.33
**4705**	allXL	BB	376	382	BS2G	14.52
**4705**	allXL	AA	357	373	BS2G	31.14
**4705**	allXL	BB	320	366	BS2G	19.28
**4705**	allXL	CC	370	376	BS2G	14.03
**4705**	allXL	AA	320	357	DBSU	23.27
**4705**	allXL	AA	357	319	DBSU	21.47
**4705**	allXL	BB	372	319	DBSU	27.44
**4705**	allXL	DD	370	357	DBSU	29.41
**4705**	allXL	DD	382	357	DBSU	45.97
**4705**	allXL	AA	372	370	DBSU	6.27
**4705**	allXL	CC	381	370	DBSU	20.44
**4705**	allXL	AA	373	370	DBSU	9.06
**4705**	allXL	DD	381	372	DBSU	19.72
**4705**	allXL	DD	372	382	DBSU	21.1
**4705**	allXL	DD	372	386	DBSU	27.57
**4705**	allXL	DD	373	386	DBSU	27.97
**4705**	allXL	BC	381	386	DBSU	62.43
**4705**	allXL	BD;DB	357	357	DBSU	18.01
**4705**	allXL	CB	373	319	DBSU	15.93
**4705**	allXL	CB	372	357	DBSU	39.52
**4705**	allXL	AC	381	357	DBSU	50.19
**4705**	allXL	CB	373	357	DBSU	36.8
**4705**	allXL	CB	372	370	DBSU	38.09
**4705**	allXL	CB	373	370	DBSU	36.87
**4705**	allXL	CB	382	373	DBSU	41.81
**4706**	allXL	BB	320	357	BS2G	25.35
**4706**	allXL	CC	320	373	BS2G	15.02
**4706**	allXL	DD	373	382	BS2G	18.67
**4706**	allXL	DD	370	386	BS2G	22.2
**4706**	allXL	AA	373	386	BS2G	22.43

**4706**	allXL	BB	319	357	BS2G	26.94
**4706**	allXL	AA	367	373	BS2G	12.96
**4706**	allXL	DD	370	382	BS2G	16.8
**4706**	allXL	CC	376	382	BS2G	15.12
**4706**	allXL	DD	357	373	BS2G	21.71
**4706**	allXL	BB	320	366	BS2G	25.32
**4706**	allXL	DD	370	376	BS2G	16.15
**4706**	allXL	BB	320	357	DBSU	25.35
**4706**	allXL	BB	357	319	DBSU	26.94
**4706**	allXL	CC	372	319	DBSU	21.17
**4706**	allXL	DD	370	357	DBSU	16.49
**4706**	allXL	BB	382	357	DBSU	22.89
**4706**	allXL	AA	372	370	DBSU	6.16
**4706**	allXL	DD	381	370	DBSU	14.68
**4706**	allXL	CC	373	370	DBSU	9.3
**4706**	allXL	DD	381	372	DBSU	16.39
**4706**	allXL	DD	372	382	DBSU	19.05
**4706**	allXL	AA	372	386	DBSU	20.94
**4706**	allXL	AA	373	386	DBSU	22.43
**4706**	allXL	DA	381	386	DBSU	32.41
**4706**	allXL	AC;CA	357	357	DBSU	19.73
**4706**	allXL	AD	373	319	DBSU	21.21
**4706**	allXL	DB	372	357	DBSU	36.11
**4706**	allXL	BD	381	357	DBSU	30.49
**4706**	allXL	DB	373	357	DBSU	38.69
**4706**	allXL	DA	372	370	DBSU	34.18
**4706**	allXL	DA	373	370	DBSU	32.59
**4706**	allXL	DA	382	373	DBSU	36.5
**4707**	allXL	AA	320	357	BS2G	26.56
**4707**	allXL	CC	320	373	BS2G	32.04
**4707**	allXL	AA	373	382	BS2G	18.19
**4707**	allXL	AA	370	386	BS2G	20.7
**4707**	allXL	AA	373	386	BS2G	25.58
**4707**	allXL	AA	319	357	BS2G	25.33

**4707**	allXL	AA	367	373	BS2G	13.86
**4707**	allXL	AA	370	382	BS2G	17.9
**4707**	allXL	CC	376	382	BS2G	13.02
**4707**	allXL	BB	357	373	BS2G	25.11
**4707**	allXL	DD	320	366	BS2G	23.12
**4707**	allXL	BB	370	376	BS2G	15.42
**4707**	allXL	AA	320	357	DBSU	26.56
**4707**	allXL	AA	357	319	DBSU	25.33
**4707**	allXL	CC	372	319	DBSU	29.0
**4707**	allXL	BB	370	357	DBSU	28.32
**4707**	allXL	BB	382	357	DBSU	13.63
**4707**	allXL	AA	372	370	DBSU	6.05
**4707**	allXL	DD	381	370	DBSU	16.51
**4707**	allXL	BB	373	370	DBSU	9.32
**4707**	allXL	DD	381	372	DBSU	17.7
**4707**	allXL	AA	372	382	DBSU	16.57
**4707**	allXL	AA	372	386	DBSU	22.82
**4707**	allXL	AA	373	386	DBSU	25.58
**4707**	allXL	CB	381	386	DBSU	53.61
**4707**	allXL	BD;DB	357	357	DBSU	19.76
**4707**	allXL	DA	373	319	DBSU	27.72
**4707**	allXL	AC	372	357	DBSU	29.59
**4707**	allXL	BD	381	357	DBSU	35.1
**4707**	allXL	AC	373	357	DBSU	26.32
**4707**	allXL	CB	372	370	DBSU	25.04
**4707**	allXL	CB	373	370	DBSU	25.99
**4707**	allXL	BC	382	373	DBSU	43.84
**4708**	allXL	CC	320	357	BS2G	25.54
**4708**	allXL	AA	320	373	BS2G	28.08
**4708**	allXL	BB	373	382	BS2G	17.44
**4708**	allXL	CC	370	386	BS2G	17.6
**4708**	allXL	CC	373	386	BS2G	25.73
**4708**	allXL	AA	319	357	BS2G	27.51
**4708**	allXL	AA	367	373	BS2G	13.03

**4708**	allXL	CC	370	382	BS2G	15.37
**4708**	allXL	BB	376	382	BS2G	14.21
**4708**	allXL	DD	357	373	BS2G	27.95
**4708**	allXL	CC	320	366	BS2G	20.84
**4708**	allXL	CC	370	376	BS2G	13.54
**4708**	allXL	CC	320	357	DBSU	25.54
**4708**	allXL	AA	357	319	DBSU	27.51
**4708**	allXL	CC	372	319	DBSU	25.3
**4708**	allXL	DD	370	357	DBSU	27.01
**4708**	allXL	BB	382	357	DBSU	20.18
**4708**	allXL	AA	372	370	DBSU	6.24
**4708**	allXL	CC	381	370	DBSU	18.13
**4708**	allXL	AA	373	370	DBSU	9.12
**4708**	allXL	DD	381	372	DBSU	16.09
**4708**	allXL	DD	372	382	DBSU	18.46
**4708**	allXL	CC	372	386	DBSU	23.17
**4708**	allXL	CC	373	386	DBSU	25.73
**4708**	allXL	AD	381	386	DBSU	31.16
**4708**	allXL	BD;DB	357	357	DBSU	18.61
**4708**	allXL	AD	373	319	DBSU	19.11
**4708**	allXL	DA	372	357	DBSU	32.27
**4708**	allXL	BD	381	357	DBSU	34.83
**4708**	allXL	DA	373	357	DBSU	31.65
**4708**	allXL	AD	372	370	DBSU	30.21
**4708**	allXL	AD	373	370	DBSU	27.28
**4708**	allXL	AD	382	373	DBSU	26.96
**4709**	allXL	BB	320	357	BS2G	25.6
**4709**	allXL	AA	320	373	BS2G	18.38
**4709**	allXL	BB	373	382	BS2G	18.31
**4709**	allXL	DD	370	386	BS2G	17.83
**4709**	allXL	CC	373	386	BS2G	21.02
**4709**	allXL	BB	319	357	BS2G	29.03
**4709**	allXL	AA	367	373	BS2G	12.6
**4709**	allXL	DD	370	382	BS2G	13.43

**4709**	allXL	CC	376	382	BS2G	13.38
**4709**	allXL	CC	357	373	BS2G	32.2
**4709**	allXL	AA	320	366	BS2G	20.33
**4709**	allXL	DD	370	376	BS2G	14.87
**4709**	allXL	BB	320	357	DBSU	25.6
**4709**	allXL	BB	357	319	DBSU	29.03
**4709**	allXL	AA	372	319	DBSU	18.42
**4709**	allXL	AA	370	357	DBSU	29.23
**4709**	allXL	DD	382	357	DBSU	29.84
**4709**	allXL	AA	372	370	DBSU	6.54
**4709**	allXL	DD	381	370	DBSU	13.62
**4709**	allXL	CC	373	370	DBSU	8.88
**4709**	allXL	DD	381	372	DBSU	18.41
**4709**	allXL	BB	372	382	DBSU	19.21
**4709**	allXL	CC	372	386	DBSU	24.0
**4709**	allXL	CC	373	386	DBSU	21.02
**4709**	allXL	AD	381	386	DBSU	39.77
**4709**	allXL	BD;DB	357	357	DBSU	18.33
**4709**	allXL	CB	373	319	DBSU	21.7
**4709**	allXL	AD	372	357	DBSU	41.99
**4709**	allXL	CB	381	357	DBSU	39.55
**4709**	allXL	CB	373	357	DBSU	43.88
**4709**	allXL	AD	372	370	DBSU	25.98
**4709**	allXL	DA	373	370	DBSU	26.02
**4709**	allXL	AD	382	373	DBSU	17.08
**4710**	allXL	CC	320	357	BS2G	24.0
**4710**	allXL	BB	320	373	BS2G	17.99
**4710**	allXL	DD	373	382	BS2G	16.55
**4710**	allXL	DD	370	386	BS2G	29.16
**4710**	allXL	DD	373	386	BS2G	24.25
**4710**	allXL	CC	319	357	BS2G	23.13
**4710**	allXL	BB	367	373	BS2G	14.22
**4710**	allXL	DD	370	382	BS2G	20.74
**4710**	allXL	BB	376	382	BS2G	13.41

**4710**	allXL	BB	357	373	BS2G	23.25
**4710**	allXL	AA	320	366	BS2G	16.68
**4710**	allXL	CC	370	376	BS2G	15.9
**4710**	allXL	CC	320	357	DBSU	24.0
**4710**	allXL	CC	357	319	DBSU	23.13
**4710**	allXL	BB	372	319	DBSU	20.35
**4710**	allXL	BB	370	357	DBSU	30.24
**4710**	allXL	BB	382	357	DBSU	35.07
**4710**	allXL	AA	372	370	DBSU	6.04
**4710**	allXL	DD	381	370	DBSU	19.34
**4710**	allXL	CC	373	370	DBSU	9.26
**4710**	allXL	DD	381	372	DBSU	15.68
**4710**	allXL	DD	372	382	DBSU	16.4
**4710**	allXL	DD	372	386	DBSU	23.73
**4710**	allXL	DD	373	386	DBSU	24.25
**4710**	allXL	CB	381	386	DBSU	24.93
**4710**	allXL	BD;DB	357	357	DBSU	19.72
**4710**	allXL	CB	373	319	DBSU	33.77
**4710**	allXL	BC	372	357	DBSU	35.3
**4710**	allXL	BD	381	357	DBSU	52.45
**4710**	allXL	BC	373	357	DBSU	35.31
**4710**	allXL	CB	372	370	DBSU	21.49
**4710**	allXL	CB	373	370	DBSU	22.49
**4710**	allXL	BC	382	373	DBSU	32.06
**4711**	allXL	CC	320	357	BS2G	18.65
**4711**	allXL	CC	320	373	BS2G	20.48
**4711**	allXL	DD	373	382	BS2G	21.04
**4711**	allXL	BB	370	386	BS2G	18.05
**4711**	allXL	BB	373	386	BS2G	16.75
**4711**	allXL	CC	319	357	BS2G	15.33
**4711**	allXL	AA	367	373	BS2G	14.56
**4711**	allXL	CC	370	382	BS2G	24.43
**4711**	allXL	CC	376	382	BS2G	14.32
**4711**	allXL	BB	357	373	BS2G	28.93

**4711**	allXL	AA	320	366	BS2G	15.57
**4711**	allXL	CC	370	376	BS2G	15.73
**4711**	allXL	CC	320	357	DBSU	18.65
**4711**	allXL	CC	357	319	DBSU	15.33
**4711**	allXL	CC	372	319	DBSU	21.5
**4711**	allXL	DD	370	357	DBSU	29.41
**4711**	allXL	BB	382	357	DBSU	39.27
**4711**	allXL	AA	372	370	DBSU	6.28
**4711**	allXL	DD	381	370	DBSU	25.25
**4711**	allXL	AA	373	370	DBSU	9.07
**4711**	allXL	DD	381	372	DBSU	20.73
**4711**	allXL	DD	372	382	DBSU	20.71
**4711**	allXL	BB	372	386	DBSU	17.88
**4711**	allXL	BB	373	386	DBSU	16.75
**4711**	allXL	DA	381	386	DBSU	41.71
**4711**	allXL	BD;DB	357	357	DBSU	18.87
**4711**	allXL	AD	373	319	DBSU	28.84
**4711**	allXL	CB	372	357	DBSU	34.75
**4711**	allXL	BD	381	357	DBSU	43.91
**4711**	allXL	CB	373	357	DBSU	34.72
**4711**	allXL	AD	372	370	DBSU	32.23
**4711**	allXL	AD	373	370	DBSU	33.15
**4711**	allXL	AD	382	373	DBSU	34.64
**4712**	allXL	AA	320	357	BS2G	23.2
**4712**	allXL	BB	320	373	BS2G	26.58
**4712**	allXL	CC	373	382	BS2G	19.06
**4712**	allXL	DD	370	386	BS2G	15.67
**4712**	allXL	DD	373	386	BS2G	17.11
**4712**	allXL	AA	319	357	BS2G	23.2
**4712**	allXL	AA	367	373	BS2G	11.45
**4712**	allXL	CC	370	382	BS2G	23.29
**4712**	allXL	CC	376	382	BS2G	14.25
**4712**	allXL	CC	357	373	BS2G	27.99
**4712**	allXL	BB	320	366	BS2G	27.44

**4712**	allXL	CC	370	376	BS2G	14.82
**4712**	allXL	AA	320	357	DBSU	23.2
**4712**	allXL	AA	357	319	DBSU	23.2
**4712**	allXL	BB	372	319	DBSU	25.67
**4712**	allXL	CC	370	357	DBSU	20.44
**4712**	allXL	BB	382	357	DBSU	34.68
**4712**	allXL	AA	372	370	DBSU	6.44
**4712**	allXL	DD	381	370	DBSU	25.08
**4712**	allXL	AA	373	370	DBSU	8.94
**4712**	allXL	DD	381	372	DBSU	22.44
**4712**	allXL	CC	372	382	DBSU	22.33
**4712**	allXL	DD	372	386	DBSU	16.72
**4712**	allXL	DD	373	386	DBSU	17.11
**4712**	allXL	BD	381	386	DBSU	69.93
**4712**	allXL	BD;DB	357	357	DBSU	19.34
**4712**	allXL	AD	373	319	DBSU	16.23
**4712**	allXL	CA	372	357	DBSU	33.13
**4712**	allXL	BD	381	357	DBSU	47.21
**4712**	allXL	CA	373	357	DBSU	36.64
**4712**	allXL	CA	372	370	DBSU	49.82
**4712**	allXL	CA	373	370	DBSU	51.52
**4712**	allXL	BD	382	373	DBSU	61.31
**4713**	allXL	AA	320	357	BS2G	24.14
**4713**	allXL	CC	320	373	BS2G	14.92
**4713**	allXL	BB	373	382	BS2G	18.14
**4713**	allXL	DD	370	386	BS2G	26.66
**4713**	allXL	AA	373	386	BS2G	23.66
**4713**	allXL	AA	319	357	BS2G	27.33
**4713**	allXL	AA	367	373	BS2G	12.35
**4713**	allXL	DD	370	382	BS2G	17.02
**4713**	allXL	BB	376	382	BS2G	14.16
**4713**	allXL	AA	357	373	BS2G	26.49
**4713**	allXL	BB	320	366	BS2G	18.26
**4713**	allXL	BB	370	376	BS2G	16.1

**4713**	allXL	AA	320	357	DBSU	24.14
**4713**	allXL	AA	357	319	DBSU	27.33
**4713**	allXL	CC	372	319	DBSU	12.78
**4713**	allXL	CC	370	357	DBSU	30.91
**4713**	allXL	BB	382	357	DBSU	30.02
**4713**	allXL	AA	372	370	DBSU	6.42
**4713**	allXL	DD	381	370	DBSU	15.97
**4713**	allXL	AA	373	370	DBSU	9.04
**4713**	allXL	DD	381	372	DBSU	18.22
**4713**	allXL	DD	372	382	DBSU	19.67
**4713**	allXL	AA	372	386	DBSU	23.12
**4713**	allXL	AA	373	386	DBSU	23.66
**4713**	allXL	BC	381	386	DBSU	48.02
**4713**	allXL	BD;DB	357	357	DBSU	19.49
**4713**	allXL	BC	373	319	DBSU	33.45
**4713**	allXL	AC	372	357	DBSU	41.73
**4713**	allXL	AC	381	357	DBSU	37.01
**4713**	allXL	AC	373	357	DBSU	38.96
**4713**	allXL	BC	372	370	DBSU	33.35
**4713**	allXL	BC	373	370	DBSU	37.11
**4713**	allXL	CB	382	373	DBSU	33.48
**4714**	allXL	CC	320	357	BS2G	25.19
**4714**	allXL	BB	320	373	BS2G	24.84
**4714**	allXL	BB	373	382	BS2G	18.09
**4714**	allXL	DD	370	386	BS2G	19.02
**4714**	allXL	DD	373	386	BS2G	20.7
**4714**	allXL	CC	319	357	BS2G	28.0
**4714**	allXL	DD	367	373	BS2G	12.53
**4714**	allXL	DD	370	382	BS2G	22.23
**4714**	allXL	BB	376	382	BS2G	12.4
**4714**	allXL	BB	357	373	BS2G	21.21
**4714**	allXL	AA	320	366	BS2G	20.2
**4714**	allXL	BB	370	376	BS2G	16.76
**4714**	allXL	CC	320	357	DBSU	25.19

**4714**	allXL	CC	357	319	DBSU	28.0
**4714**	allXL	BB	372	319	DBSU	27.73
**4714**	allXL	BB	370	357	DBSU	29.01
**4714**	allXL	BB	382	357	DBSU	22.34
**4714**	allXL	AA	372	370	DBSU	6.26
**4714**	allXL	DD	381	370	DBSU	22.66
**4714**	allXL	AA	373	370	DBSU	8.86
**4714**	allXL	DD	381	372	DBSU	16.07
**4714**	allXL	DD	372	382	DBSU	16.42
**4714**	allXL	DD	372	386	DBSU	17.02
**4714**	allXL	DD	373	386	DBSU	20.7
**4714**	allXL	DA	381	386	DBSU	55.44
**4714**	allXL	BD;DB	357	357	DBSU	18.7
**4714**	allXL	AD	373	319	DBSU	39.04
**4714**	allXL	BD	372	357	DBSU	39.65
**4714**	allXL	BD	381	357	DBSU	30.66
**4714**	allXL	BD	373	357	DBSU	37.74
**4714**	allXL	AD	372	370	DBSU	57.08
**4714**	allXL	BC	373	370	DBSU	56.05
**4714**	allXL	AD	382	373	DBSU	55.01
**4715**	allXL	AA	320	357	BS2G	27.9
**4715**	allXL	DD	320	373	BS2G	24.13
**4715**	allXL	CC	373	382	BS2G	15.79
**4715**	allXL	CC	370	386	BS2G	20.26
**4715**	allXL	CC	373	386	BS2G	15.57
**4715**	allXL	AA	319	357	BS2G	29.18
**4715**	allXL	AA	367	373	BS2G	13.24
**4715**	allXL	DD	370	382	BS2G	20.83
**4715**	allXL	CC	376	382	BS2G	14.25
**4715**	allXL	BB	357	373	BS2G	31.66
**4715**	allXL	DD	320	366	BS2G	17.97
**4715**	allXL	CC	370	376	BS2G	15.76
**4715**	allXL	AA	320	357	DBSU	27.9
**4715**	allXL	AA	357	319	DBSU	29.18

**4715**	allXL	DD	372	319	DBSU	24.95
**4715**	allXL	BB	370	357	DBSU	24.34
**4715**	allXL	BB	382	357	DBSU	27.65
**4715**	allXL	AA	372	370	DBSU	6.26
**4715**	allXL	DD	381	370	DBSU	17.98
**4715**	allXL	AA	373	370	DBSU	8.94
**4715**	allXL	DD	381	372	DBSU	18.56
**4715**	allXL	CC	372	382	DBSU	19.22
**4715**	allXL	CC	372	386	DBSU	16.74
**4715**	allXL	CC	373	386	DBSU	15.57
**4715**	allXL	DA	381	386	DBSU	26.88
**4715**	allXL	BD;DB	357	357	DBSU	17.82
**4715**	allXL	AD	373	319	DBSU	19.55
**4715**	allXL	BD	372	357	DBSU	46.6
**4715**	allXL	BD	381	357	DBSU	40.25
**4715**	allXL	AD	373	357	DBSU	47.19
**4715**	allXL	DA	372	370	DBSU	33.73
**4715**	allXL	DA	373	370	DBSU	33.34
**4715**	allXL	AD	382	373	DBSU	23.11
**4716**	allXL	AA	320	357	BS2G	26.11
**4716**	allXL	CC	320	373	BS2G	22.01
**4716**	allXL	CC	373	382	BS2G	17.65
**4716**	allXL	DD	370	386	BS2G	26.48
**4716**	allXL	CC	373	386	BS2G	25.32
**4716**	allXL	AA	319	357	BS2G	28.53
**4716**	allXL	AA	367	373	BS2G	13.55
**4716**	allXL	BB	370	382	BS2G	20.49
**4716**	allXL	BB	376	382	BS2G	13.74
**4716**	allXL	CC	357	373	BS2G	20.8
**4716**	allXL	AA	320	366	BS2G	20.59
**4716**	allXL	CC	370	376	BS2G	16.38
**4716**	allXL	AA	320	357	DBSU	26.11
**4716**	allXL	AA	357	319	DBSU	28.53
**4716**	allXL	CC	372	319	DBSU	19.2

**4716**	allXL	CC	370	357	DBSU	21.29
**4716**	allXL	CC	382	357	DBSU	24.97
**4716**	allXL	AA	372	370	DBSU	6.21
**4716**	allXL	BB	381	370	DBSU	21.85
**4716**	allXL	CC	373	370	DBSU	8.95
**4716**	allXL	DD	381	372	DBSU	17.43
**4716**	allXL	DD	372	382	DBSU	17.43
**4716**	allXL	DD	372	386	DBSU	25.39
**4716**	allXL	CC	373	386	DBSU	25.32
**4716**	allXL	CA	381	386	DBSU	46.43
**4716**	allXL	BD;DB	357	357	DBSU	18.76
**4716**	allXL	DA	373	319	DBSU	26.52
**4716**	allXL	CA	372	357	DBSU	39.1
**4716**	allXL	CA	381	357	DBSU	34.49
**4716**	allXL	CA	373	357	DBSU	37.29
**4716**	allXL	DA	372	370	DBSU	36.92
**4716**	allXL	DA	373	370	DBSU	36.36
**4716**	allXL	DA	382	373	DBSU	29.2
**4717**	allXL	AA	320	357	BS2G	26.01
**4717**	allXL	BB	320	373	BS2G	23.72
**4717**	allXL	DD	373	382	BS2G	19.39
**4717**	allXL	DD	370	386	BS2G	14.13
**4717**	allXL	DD	373	386	BS2G	22.78
**4717**	allXL	AA	319	357	BS2G	26.56
**4717**	allXL	AA;BB	367	373	BS2G	13.17
**4717**	allXL	DD	370	382	BS2G	14.27
**4717**	allXL	BB	376	382	BS2G	15.54
**4717**	allXL	DD	357	373	BS2G	17.41
**4717**	allXL	AA	320	366	BS2G	21.64
**4717**	allXL	DD	370	376	BS2G	15.14
**4717**	allXL	AA	320	357	DBSU	26.01
**4717**	allXL	AA	357	319	DBSU	26.56
**4717**	allXL	BB	372	319	DBSU	21.05
**4717**	allXL	DD	370	357	DBSU	21.38

**4717**	allXL	BB	382	357	DBSU	17.51
**4717**	allXL	AA	372	370	DBSU	6.23
**4717**	allXL	DD	381	370	DBSU	14.2
**4717**	allXL	AA	373	370	DBSU	9.16
**4717**	allXL	DD	381	372	DBSU	18.89
**4717**	allXL	DD	372	382	DBSU	19.85
**4717**	allXL	DD	372	386	DBSU	21.25
**4717**	allXL	DD	373	386	DBSU	22.78
**4717**	allXL	BC	381	386	DBSU	38.52
**4717**	allXL	BD;DB	357	357	DBSU	18.6
**4717**	allXL	CB	373	319	DBSU	15.87
**4717**	allXL	CB	372	357	DBSU	29.92
**4717**	allXL	BD	381	357	DBSU	29.17
**4717**	allXL	DB	373	357	DBSU	31.83
**4717**	allXL	CB	372	370	DBSU	11.22
**4717**	allXL	CB	373	370	DBSU	11.22
**4717**	allXL	CB	382	373	DBSU	17.03
**4718**	allXL	AA	320	357	BS2G	26.55
**4718**	allXL	CC	320	373	BS2G	22.59
**4718**	allXL	BB	373	382	BS2G	18.52
**4718**	allXL	DD	370	386	BS2G	30.93
**4718**	allXL	AA	373	386	BS2G	24.84
**4718**	allXL	AA	319	357	BS2G	25.31
**4718**	allXL	AA	367	373	BS2G	13.83
**4718**	allXL	DD	370	382	BS2G	21.22
**4718**	allXL	CC	376	382	BS2G	13.92
**4718**	allXL	DD	357	373	BS2G	18.54
**4718**	allXL	CC	320	366	BS2G	25.56
**4718**	allXL	CC	370	376	BS2G	15.56
**4718**	allXL	AA	320	357	DBSU	26.55
**4718**	allXL	AA	357	319	DBSU	25.31
**4718**	allXL	CC	372	319	DBSU	18.29
**4718**	allXL	DD	370	357	DBSU	15.96
**4718**	allXL	DD	382	357	DBSU	33.28

**4718**	allXL	AA	372	370	DBSU	6.12
**4718**	allXL	DD	381	370	DBSU	19.41
**4718**	allXL	AA	373	370	DBSU	9.11
**4718**	allXL	BB	381	372	DBSU	18.74
**4718**	allXL	BB	372	382	DBSU	19.29
**4718**	allXL	BB	372	386	DBSU	26.9
**4718**	allXL	AA	373	386	DBSU	24.84
**4718**	allXL	BC	381	386	DBSU	16.96
**4718**	allXL	BD;DB	357	357	DBSU	18.99
**4718**	allXL	BC	373	319	DBSU	19.6
**4718**	allXL	DB	372	357	DBSU	24.11
**4718**	allXL	DB	381	357	DBSU	42.78
**4718**	allXL	DB	373	357	DBSU	26.6
**4718**	allXL	CB	372	370	DBSU	20.15
**4718**	allXL	BC	373	370	DBSU	16.77
**4718**	allXL	BC	382	373	DBSU	21.35
**4719**	allXL	AA	320	357	BS2G	21.48
**4719**	allXL	BB	320	373	BS2G	21.97
**4719**	allXL	AA	373	382	BS2G	16.81
**4719**	allXL	AA	370	386	BS2G	15.66
**4719**	allXL	AA	373	386	BS2G	21.22
**4719**	allXL	AA	319	357	BS2G	19.48
**4719**	allXL	AA	367	373	BS2G	13.12
**4719**	allXL	AA	370	382	BS2G	17.13
**4719**	allXL	CC	376	382	BS2G	13.51
**4719**	allXL	AA	357	373	BS2G	17.47
**4719**	allXL	DD	320	366	BS2G	23.06
**4719**	allXL	CC	370	376	BS2G	15.18
**4719**	allXL	AA	320	357	DBSU	21.48
**4719**	allXL	AA	357	319	DBSU	19.48
**4719**	allXL	DD	372	319	DBSU	21.4
**4719**	allXL	AA	370	357	DBSU	25.15
**4719**	allXL	AA	382	357	DBSU	32.63
**4719**	allXL	AA	372	370	DBSU	6.13

**4719**	allXL	BB	381	370	DBSU	16.48
**4719**	allXL	DD	373	370	DBSU	8.94
**4719**	allXL	DD	381	372	DBSU	16.04
**4719**	allXL	AA	372	382	DBSU	15.34
**4719**	allXL	AA	372	386	DBSU	18.03
**4719**	allXL	AA	373	386	DBSU	21.22
**4719**	allXL	CB	381	386	DBSU	23.08
**4719**	allXL	BD;DB	357	357	DBSU	18.96
**4719**	allXL	DA	373	319	DBSU	23.65
**4719**	allXL	DA	372	357	DBSU	28.0
**4719**	allXL	DA	381	357	DBSU	19.06
**4719**	allXL	DA	373	357	DBSU	25.39
**4719**	allXL	CB	372	370	DBSU	33.52
**4719**	allXL	CB	373	370	DBSU	30.61
**4719**	allXL	DA	382	373	DBSU	25.06
**4720**	allXL	AA	320	357	BS2G	23.93
**4720**	allXL	CC	320	373	BS2G	20.46
**4720**	allXL	DD	373	382	BS2G	16.92
**4720**	allXL	DD	370	386	BS2G	19.1
**4720**	allXL	DD	373	386	BS2G	26.28
**4720**	allXL	AA	319	357	BS2G	24.41
**4720**	allXL	AA	367	373	BS2G	11.0
**4720**	allXL	DD	370	382	BS2G	13.22
**4720**	allXL	AA	376	382	BS2G	13.57
**4720**	allXL	CC	357	373	BS2G	19.08
**4720**	allXL	AA	320	366	BS2G	22.49
**4720**	allXL	CC	370	376	BS2G	16.19
**4720**	allXL	AA	320	357	DBSU	23.93
**4720**	allXL	AA	357	319	DBSU	24.41
**4720**	allXL	CC	372	319	DBSU	19.17
**4720**	allXL	BB	370	357	DBSU	25.44
**4720**	allXL	CC	382	357	DBSU	21.27
**4720**	allXL	AA	372	370	DBSU	6.65
**4720**	allXL	DD	381	370	DBSU	13.97

**4720**	allXL	AA	373	370	DBSU	9.1
**4720**	allXL	DD	381	372	DBSU	16.74
**4720**	allXL	DD	372	382	DBSU	17.57
**4720**	allXL	DD	372	386	DBSU	25.72
**4720**	allXL	DD	373	386	DBSU	26.28
**4720**	allXL	AC	381	386	DBSU	58.35
**4720**	allXL	BD;DB	357	357	DBSU	19.24
**4720**	allXL	CB	373	319	DBSU	39.35
**4720**	allXL	CA	372	357	DBSU	41.08
**4720**	allXL	CA	381	357	DBSU	29.89
**4720**	allXL	CA	373	357	DBSU	38.38
**4720**	allXL	DB	372	370	DBSU	55.44
**4720**	allXL	DB	373	370	DBSU	54.18
**4720**	allXL	BD	382	373	DBSU	52.75
**4721**	allXL	CC	320	357	BS2G	19.61
**4721**	allXL	CC	320	373	BS2G	23.49
**4721**	allXL	CC	373	382	BS2G	17.33
**4721**	allXL	DD	370	386	BS2G	22.63
**4721**	allXL	CC	373	386	BS2G	17.47
**4721**	allXL	CC	319	357	BS2G	19.43
**4721**	allXL	DD	367	373	BS2G	13.93
**4721**	allXL	DD	370	382	BS2G	24.04
**4721**	allXL	CC	376	382	BS2G	12.39
**4721**	allXL	BB	357	373	BS2G	28.36
**4721**	allXL	CC	320	366	BS2G	11.29
**4721**	allXL	CC	370	376	BS2G	16.75
**4721**	allXL	CC	320	357	DBSU	19.61
**4721**	allXL	CC	357	319	DBSU	19.43
**4721**	allXL	CC	372	319	DBSU	22.86
**4721**	allXL	DD	370	357	DBSU	30.35
**4721**	allXL	DD	382	357	DBSU	33.61
**4721**	allXL	AA	372	370	DBSU	5.89
**4721**	allXL	DD	381	370	DBSU	23.97
**4721**	allXL	AA	373	370	DBSU	9.1

**4721**	allXL	AA	381	372	DBSU	20.88
**4721**	allXL	CC	372	382	DBSU	21.16
**4721**	allXL	CC	372	386	DBSU	20.37
**4721**	allXL	CC	373	386	DBSU	17.47
**4721**	allXL	DB	381	386	DBSU	34.8
**4721**	allXL	BD;DB	357	357	DBSU	19.3
**4721**	allXL	DA	373	319	DBSU	18.7
**4721**	allXL	CB	372	357	DBSU	33.59
**4721**	allXL	DB	381	357	DBSU	36.05
**4721**	allXL	CB	373	357	DBSU	34.28
**4721**	allXL	BC	372	370	DBSU	36.39
**4721**	allXL	CB	373	370	DBSU	37.93
**4721**	allXL	AD	382	373	DBSU	48.61
**4722**	allXL	AA	320	357	BS2G	23.57
**4722**	allXL	BB	320	373	BS2G	13.28
**4722**	allXL	DD	373	382	BS2G	18.99
**4722**	allXL	AA	370	386	BS2G	17.71
**4722**	allXL	AA	373	386	BS2G	17.21
**4722**	allXL	AA	319	357	BS2G	22.49
**4722**	allXL	DD	367	373	BS2G	13.48
**4722**	allXL	DD	370	382	BS2G	24.15
**4722**	allXL	CC	376	382	BS2G	13.53
**4722**	allXL	BB	357	373	BS2G	22.49
**4722**	allXL	BB	320	366	BS2G	17.06
**4722**	allXL	CC	370	376	BS2G	16.45
**4722**	allXL	AA	320	357	DBSU	23.57
**4722**	allXL	AA	357	319	DBSU	22.49
**4722**	allXL	BB	372	319	DBSU	13.41
**4722**	allXL	BB	370	357	DBSU	22.52
**4722**	allXL	BB	382	357	DBSU	31.16
**4722**	allXL	AA	372	370	DBSU	6.19
**4722**	allXL	DD	381	370	DBSU	21.39
**4722**	allXL	AA	373	370	DBSU	9.08
**4722**	allXL	DD	381	372	DBSU	16.83

**4722**	allXL	DD	372	382	DBSU	19.38
**4722**	allXL	AA	372	386	DBSU	15.12
**4722**	allXL	AA	373	386	DBSU	17.21
**4722**	allXL	DB	381	386	DBSU	55.7
**4722**	allXL	BD;DB	357	357	DBSU	18.83
**4722**	allXL	CB	373	319	DBSU	20.87
**4722**	allXL	BD	372	357	DBSU	36.88
**4722**	allXL	BD	381	357	DBSU	28.97
**4722**	allXL	AC	373	357	DBSU	34.48
**4722**	allXL	BC	372	370	DBSU	31.65
**4722**	allXL	CB	373	370	DBSU	33.9
**4722**	allXL	CB	382	373	DBSU	43.48
**4723**	allXL	CC	320	357	BS2G	24.34
**4723**	allXL	BB	320	373	BS2G	28.63
**4723**	allXL	DD	373	382	BS2G	18.35
**4723**	allXL	DD	370	386	BS2G	15.83
**4723**	allXL	DD	373	386	BS2G	19.76
**4723**	allXL	AA	319	357	BS2G	24.71
**4723**	allXL	BB	367	373	BS2G	11.49
**4723**	allXL	DD	370	382	BS2G	15.63
**4723**	allXL	CC	376	382	BS2G	14.8
**4723**	allXL	CC	357	373	BS2G	22.94
**4723**	allXL	CC	320	366	BS2G	17.5
**4723**	allXL	CC	370	376	BS2G	15.49
**4723**	allXL	CC	320	357	DBSU	24.34
**4723**	allXL	AA	357	319	DBSU	24.71
**4723**	allXL	BB	372	319	DBSU	28.98
**4723**	allXL	CC	370	357	DBSU	21.64
**4723**	allXL	BB	382	357	DBSU	30.59
**4723**	allXL	AA	372	370	DBSU	6.24
**4723**	allXL	DD	381	370	DBSU	16.58
**4723**	allXL	AA	373	370	DBSU	9.34
**4723**	allXL	DD	381	372	DBSU	18.97
**4723**	allXL	DD	372	382	DBSU	19.24

**4723**	allXL	DD	372	386	DBSU	19.38
**4723**	allXL	DD	373	386	DBSU	19.76
**4723**	allXL	AD	381	386	DBSU	48.86
**4723**	allXL	BD;DB	357	357	DBSU	19.22
**4723**	allXL	DA	373	319	DBSU	25.19
**4723**	allXL	CA	372	357	DBSU	35.79
**4723**	allXL	CA	381	357	DBSU	39.66
**4723**	allXL	CA	373	357	DBSU	33.0
**4723**	allXL	AD	372	370	DBSU	45.27
**4723**	allXL	AD	373	370	DBSU	43.46
**4723**	allXL	DA	382	373	DBSU	38.69
**4724**	allXL	AA	320	357	BS2G	23.45
**4724**	allXL	CC	320	373	BS2G	20.31
**4724**	allXL	AA	373	382	BS2G	21.46
**4724**	allXL	AA	370	386	BS2G	25.62
**4724**	allXL	AA	373	386	BS2G	20.06
**4724**	allXL	AA	319	357	BS2G	23.71
**4724**	allXL	BB	367	373	BS2G	12.35
**4724**	allXL	CC	370	382	BS2G	23.3
**4724**	allXL	BB	376	382	BS2G	13.52
**4724**	allXL	BB	357	373	BS2G	15.68
**4724**	allXL	CC	320	366	BS2G	26.14
**4724**	allXL	CC	370	376	BS2G	14.21
**4724**	allXL	AA	320	357	DBSU	23.45
**4724**	allXL	AA	357	319	DBSU	23.71
**4724**	allXL	CC	372	319	DBSU	23.29
**4724**	allXL	CC	370	357	DBSU	19.42
**4724**	allXL	CC	382	357	DBSU	17.4
**4724**	allXL	AA	372	370	DBSU	6.42
**4724**	allXL	CC	381	370	DBSU	25.08
**4724**	allXL	AA	373	370	DBSU	9.24
**4724**	allXL	DD	381	372	DBSU	23.61
**4724**	allXL	CC	372	382	DBSU	22.82
**4724**	allXL	AA	372	386	DBSU	23.31

**4724**	allXL	AA	373	386	DBSU	20.06
**4724**	allXL	DB	381	386	DBSU	35.08
**4724**	allXL	BD;DB	357	357	DBSU	18.82
**4724**	allXL	DA	373	319	DBSU	17.56
**4724**	allXL	DA	372	357	DBSU	30.05
**4724**	allXL	BD	381	357	DBSU	19.57
**4724**	allXL	DA	373	357	DBSU	28.58
**4724**	allXL	DA	372	370	DBSU	33.95
**4724**	allXL	DA	373	370	DBSU	35.61
**4724**	allXL	BD	382	373	DBSU	45.03
**4725**	allXL	CC	320	357	BS2G	21.81
**4725**	allXL	BB	320	373	BS2G	18.43
**4725**	allXL	AA	373	382	BS2G	20.7
**4725**	allXL	CC	370	386	BS2G	26.76
**4725**	allXL	CC	373	386	BS2G	18.16
**4725**	allXL	CC	319	357	BS2G	20.29
**4725**	allXL	AA	367	373	BS2G	12.17
**4725**	allXL	BB	370	382	BS2G	24.29
**4725**	allXL	AA	376	382	BS2G	14.49
**4725**	allXL	BB	357	373	BS2G	20.29
**4725**	allXL	BB	320	366	BS2G	15.25
**4725**	allXL	BB	370	376	BS2G	16.62
**4725**	allXL	CC	320	357	DBSU	21.81
**4725**	allXL	CC	357	319	DBSU	20.29
**4725**	allXL	DD	372	319	DBSU	20.84
**4725**	allXL	BB	370	357	DBSU	20.1
**4725**	allXL	AA	382	357	DBSU	25.79
**4725**	allXL	CC	372	370	DBSU	6.55
**4725**	allXL	BB	381	370	DBSU	23.53
**4725**	allXL	CC	373	370	DBSU	9.12
**4725**	allXL	BB	381	372	DBSU	22.27
**4725**	allXL	AA	372	382	DBSU	24.09
**4725**	allXL	CC	372	386	DBSU	20.92
**4725**	allXL	CC	373	386	DBSU	18.16

**4725**	allXL	BC	381	386	DBSU	44.36
**4725**	allXL	BD;DB	357	357	DBSU	19.12
**4725**	allXL	AD	373	319	DBSU	37.32
**4725**	allXL	BD	372	357	DBSU	27.94
**4725**	allXL	AC	381	357	DBSU	26.43
**4725**	allXL	BD	373	357	DBSU	29.61
**4725**	allXL	DA	372	370	DBSU	46.78
**4725**	allXL	DA	373	370	DBSU	45.17
**4725**	allXL	BC	382	373	DBSU	47.21
**4726**	allXL	AA	320	357	BS2G	24.77
**4726**	allXL	DD	320	373	BS2G	17.83
**4726**	allXL	BB	373	382	BS2G	18.18
**4726**	allXL	DD	370	386	BS2G	23.92
**4726**	allXL	AA	373	386	BS2G	24.39
**4726**	allXL	AA	319	357	BS2G	23.66
**4726**	allXL	AA	367	373	BS2G	13.64
**4726**	allXL	DD	370	382	BS2G	18.6
**4726**	allXL	BB	376	382	BS2G	13.68
**4726**	allXL	CC	357	373	BS2G	25.81
**4726**	allXL	AA	320	366	BS2G	21.11
**4726**	allXL	BB	370	376	BS2G	16.75
**4726**	allXL	AA	320	357	DBSU	24.77
**4726**	allXL	AA	357	319	DBSU	23.66
**4726**	allXL	DD	372	319	DBSU	11.88
**4726**	allXL	CC	370	357	DBSU	22.64
**4726**	allXL	BB	382	357	DBSU	25.92
**4726**	allXL	AA	372	370	DBSU	6.22
**4726**	allXL	DD	381	370	DBSU	16.81
**4726**	allXL	AA	373	370	DBSU	9.13
**4726**	allXL	DD	381	372	DBSU	18.06
**4726**	allXL	BB	372	382	DBSU	19.53
**4726**	allXL	AA	372	386	DBSU	25.29
**4726**	allXL	AA	373	386	DBSU	24.39
**4726**	allXL	BC	381	386	DBSU	29.75

**4726**	allXL	BD;DB	357	357	DBSU	19.16
**4726**	allXL	AD	373	319	DBSU	30.64
**4726**	allXL	BC	372	357	DBSU	34.14
**4726**	allXL	DA	381	357	DBSU	41.48
**4726**	allXL	BC	373	357	DBSU	37.72
**4726**	allXL	DA	372	370	DBSU	22.34
**4726**	allXL	DA	373	370	DBSU	20.52
**4726**	allXL	DA	382	373	DBSU	29.94
**4727**	allXL	CC	320	357	BS2G	24.65
**4727**	allXL	AA	320	373	BS2G	22.86
**4727**	allXL	BB	373	382	BS2G	15.96
**4727**	allXL	AA	370	386	BS2G	13.17
**4727**	allXL	AA	373	386	BS2G	14.22
**4727**	allXL	AA	319	357	BS2G	25.54
**4727**	allXL	AA	367	373	BS2G	11.44
**4727**	allXL	BB	370	382	BS2G	20.53
**4727**	allXL	BB	376	382	BS2G	14.5
**4727**	allXL	AA	357	373	BS2G	33.47
**4727**	allXL	AA	320	366	BS2G	17.96
**4727**	allXL	CC	370	376	BS2G	15.31
**4727**	allXL	CC	320	357	DBSU	24.65
**4727**	allXL	AA	357	319	DBSU	25.54
**4727**	allXL	CC	372	319	DBSU	23.47
**4727**	allXL	DD	370	357	DBSU	28.49
**4727**	allXL	BB	382	357	DBSU	28.32
**4727**	allXL	AA	372	370	DBSU	6.58
**4727**	allXL	BB	381	370	DBSU	22.97
**4727**	allXL	AA	373	370	DBSU	8.75
**4727**	allXL	DD	381	372	DBSU	18.53
**4727**	allXL	BB	372	382	DBSU	16.84
**4727**	allXL	AA	372	386	DBSU	14.42
**4727**	allXL	AA	373	386	DBSU	14.22
**4727**	allXL	DA	381	386	DBSU	43.51
**4727**	allXL	BD;DB	357	357	DBSU	18.68

**4727**	allXL	AD	373	319	DBSU	28.36
**4727**	allXL	AD	372	357	DBSU	45.95
**4727**	allXL	AD	381	357	DBSU	34.61
**4727**	allXL	AD	373	357	DBSU	42.65
**4727**	allXL	AD	372	370	DBSU	29.77
**4727**	allXL	AD	373	370	DBSU	28.33
**4727**	allXL	AD	382	373	DBSU	37.52
**4728**	allXL	CC	320	357	BS2G	27.33
**4728**	allXL	BB	320	373	BS2G	32.31
**4728**	allXL	DD	373	382	BS2G	14.84
**4728**	allXL	CC	370	386	BS2G	19.5
**4728**	allXL	CC	373	386	BS2G	23.59
**4728**	allXL	CC	319	357	BS2G	29.22
**4728**	allXL	AA	367	373	BS2G	10.16
**4728**	allXL	DD	370	382	BS2G	15.75
**4728**	allXL	CC	376	382	BS2G	12.53
**4728**	allXL	CC	357	373	BS2G	23.5
**4728**	allXL	AA	320	366	BS2G	23.05
**4728**	allXL	CC	370	376	BS2G	15.0
**4728**	allXL	CC	320	357	DBSU	27.33
**4728**	allXL	CC	357	319	DBSU	29.22
**4728**	allXL	BB	372	319	DBSU	33.8
**4728**	allXL	CC	370	357	DBSU	23.93
**4728**	allXL	BB	382	357	DBSU	27.62
**4728**	allXL	CC	372	370	DBSU	6.66
**4728**	allXL	DD	381	370	DBSU	16.31
**4728**	allXL	AA	373	370	DBSU	9.13
**4728**	allXL	DD	381	372	DBSU	11.37
**4728**	allXL	DD	372	382	DBSU	12.24
**4728**	allXL	DD	372	386	DBSU	21.59
**4728**	allXL	CC	373	386	DBSU	23.59
**4728**	allXL	BC	381	386	DBSU	46.04
**4728**	allXL	BD;DB	357	357	DBSU	19.33
**4728**	allXL	CB	373	319	DBSU	32.22

**4728**	allXL	CA	372	357	DBSU	25.93
**4728**	allXL	CA	381	357	DBSU	38.5
**4728**	allXL	CA	373	357	DBSU	24.5
**4728**	allXL	BC;CB	372	370	DBSU	49.58
**4728**	allXL	CB	373	370	DBSU	49.67
**4728**	allXL	CB	382	373	DBSU	52.46
**4729**	allXL	CC	320	357	BS2G	25.74
**4729**	allXL	DD	320	373	BS2G	23.23
**4729**	allXL	CC	373	382	BS2G	17.82
**4729**	allXL	CC	370	386	BS2G	27.31
**4729**	allXL	CC	373	386	BS2G	25.71
**4729**	allXL	CC	319	357	BS2G	28.22
**4729**	allXL	BB	367	373	BS2G	11.6
**4729**	allXL	CC	370	382	BS2G	22.12
**4729**	allXL	CC	376	382	BS2G	14.15
**4729**	allXL	BB	357	373	BS2G	19.14
**4729**	allXL	AA	320	366	BS2G	29.24
**4729**	allXL	BB	370	376	BS2G	16.35
**4729**	allXL	CC	320	357	DBSU	25.74
**4729**	allXL	CC	357	319	DBSU	28.22
**4729**	allXL	BB	372	319	DBSU	28.04
**4729**	allXL	DD	370	357	DBSU	24.77
**4729**	allXL	BB	382	357	DBSU	16.44
**4729**	allXL	AA	372	370	DBSU	6.14
**4729**	allXL	DD	381	370	DBSU	21.23
**4729**	allXL	DD	373	370	DBSU	8.91
**4729**	allXL	DD	381	372	DBSU	16.45
**4729**	allXL	CC	372	382	DBSU	17.35
**4729**	allXL	CC	372	386	DBSU	23.93
**4729**	allXL	CC	373	386	DBSU	25.71
**4729**	allXL	AD	381	386	DBSU	35.16
**4729**	allXL	BD;DB	357	357	DBSU	19.74
**4729**	allXL	CB	373	319	DBSU	16.31
**4729**	allXL	BD	372	357	DBSU	39.49

**4729**	allXL	BD	381	357	DBSU	24.9
**4729**	allXL	BD	373	357	DBSU	35.85
**4729**	allXL	BC	372	370	DBSU	32.96
**4729**	allXL	CB	373	370	DBSU	32.53
**4729**	allXL	DA	382	373	DBSU	31.53
**4730**	allXL	AA	320	357	BS2G	24.58
**4730**	allXL	CC	320	373	BS2G	16.27
**4730**	allXL	BB	373	382	BS2G	14.6
**4730**	allXL	DD	370	386	BS2G	21.13
**4730**	allXL	BB	373	386	BS2G	22.19
**4730**	allXL	AA	319	357	BS2G	22.82
**4730**	allXL	BB	367	373	BS2G	14.48
**4730**	allXL	DD	370	382	BS2G	17.09
**4730**	allXL	BB	376	382	BS2G	14.83
**4730**	allXL	BB	357	373	BS2G	30.43
**4730**	allXL	DD	320	366	BS2G	25.5
**4730**	allXL	CC	370	376	BS2G	14.52
**4730**	allXL	AA	320	357	DBSU	24.58
**4730**	allXL	AA	357	319	DBSU	22.82
**4730**	allXL	CC	372	319	DBSU	15.0
**4730**	allXL	DD	370	357	DBSU	26.66
**4730**	allXL	BB	382	357	DBSU	26.84
**4730**	allXL	AA	372	370	DBSU	6.13
**4730**	allXL	DD	381	370	DBSU	17.97
**4730**	allXL	AA	373	370	DBSU	9.3
**4730**	allXL	BB	381	372	DBSU	16.67
**4730**	allXL	BB	372	382	DBSU	14.7
**4730**	allXL	BB	372	386	DBSU	20.34
**4730**	allXL	BB	373	386	DBSU	22.19
**4730**	allXL	BC	381	386	DBSU	36.6
**4730**	allXL	BD;DB	357	357	DBSU	19.09
**4730**	allXL	BC	373	319	DBSU	37.1
**4730**	allXL	AC	372	357	DBSU	44.55
**4730**	allXL	CB	381	357	DBSU	45.85

**4730**	allXL	BC	373	357	DBSU	44.3
**4730**	allXL	BC	372	370	DBSU	34.52
**4730**	allXL	BC	373	370	DBSU	31.81
**4730**	allXL	CB	382	373	DBSU	26.85
**4731**	allXL	CC	320	357	BS2G	23.57
**4731**	allXL	BB	320	373	BS2G	24.17
**4731**	allXL	CC	373	382	BS2G	18.32
**4731**	allXL	DD	370	386	BS2G	18.46
**4731**	allXL	AA	373	386	BS2G	20.95
**4731**	allXL	CC	319	357	BS2G	25.03
**4731**	allXL	AA	367	373	BS2G	13.7
**4731**	allXL	DD	370	382	BS2G	20.42
**4731**	allXL	CC	376	382	BS2G	13.39
**4731**	allXL	BB	357	373	BS2G	16.78
**4731**	allXL	AA	320	366	BS2G	20.29
**4731**	allXL	CC	370	376	BS2G	15.85
**4731**	allXL	CC	320	357	DBSU	23.57
**4731**	allXL	CC	357	319	DBSU	25.03
**4731**	allXL	BB	372	319	DBSU	23.63
**4731**	allXL	BB	370	357	DBSU	22.95
**4731**	allXL	BB	382	357	DBSU	23.7
**4731**	allXL	AA	372	370	DBSU	6.2
**4731**	allXL	DD	381	370	DBSU	19.9
**4731**	allXL	AA	373	370	DBSU	9.29
**4731**	allXL	DD	381	372	DBSU	19.16
**4731**	allXL	DD	372	382	DBSU	20.33
**4731**	allXL	DD	372	386	DBSU	20.21
**4731**	allXL	AA	373	386	DBSU	20.95
**4731**	allXL	BD	381	386	DBSU	40.84
**4731**	allXL	BD;DB	357	357	DBSU	18.72
**4731**	allXL	DA	373	319	DBSU	28.01
**4731**	allXL	BD	372	357	DBSU	36.06
**4731**	allXL	BD	381	357	DBSU	28.72
**4731**	allXL	BD	373	357	DBSU	33.55

**4731**	allXL	DA	372	370	DBSU	39.51
**4731**	allXL	DA	373	370	DBSU	39.96
**4731**	allXL	DB	382	373	DBSU	45.6
**4732**	allXL	AA	320	357	BS2G	26.1
**4732**	allXL	CC	320	373	BS2G	24.62
**4732**	allXL	BB	373	382	BS2G	18.69
**4732**	allXL	CC	370	386	BS2G	25.36
**4732**	allXL	BB	373	386	BS2G	27.6
**4732**	allXL	AA	319	357	BS2G	27.53
**4732**	allXL	AA	367	373	BS2G	12.89
**4732**	allXL	CC	370	382	BS2G	17.96
**4732**	allXL	BB	376	382	BS2G	13.68
**4732**	allXL	BB	357	373	BS2G	32.42
**4732**	allXL	CC	320	366	BS2G	25.15
**4732**	allXL	CC	370	376	BS2G	11.52
**4732**	allXL	AA	320	357	DBSU	26.1
**4732**	allXL	AA	357	319	DBSU	27.53
**4732**	allXL	DD	372	319	DBSU	20.41
**4732**	allXL	DD	370	357	DBSU	27.68
**4732**	allXL	BB	382	357	DBSU	26.88
**4732**	allXL	AA	372	370	DBSU	6.25
**4732**	allXL	CC	381	370	DBSU	17.92
**4732**	allXL	AA	373	370	DBSU	9.17
**4732**	allXL	BB	381	372	DBSU	20.93
**4732**	allXL	BB	372	382	DBSU	20.08
**4732**	allXL	BB	372	386	DBSU	27.91
**4732**	allXL	BB	373	386	DBSU	27.6
**4732**	allXL	DA	381	386	DBSU	53.66
**4732**	allXL	BD;DB	357	357	DBSU	18.9
**4732**	allXL	DA	373	319	DBSU	28.43
**4732**	allXL	DA	372	357	DBSU	35.51
**4732**	allXL	BD	381	357	DBSU	39.34
**4732**	allXL	DA	373	357	DBSU	36.13
**4732**	allXL	DA	372	370	DBSU	28.72

**4732**	allXL	DA	373	370	DBSU	26.7
**4732**	allXL	DA	382	373	DBSU	34.31
**4733**	allXL	AA	320	357	BS2G	25.76
**4733**	allXL	CC	320	373	BS2G	20.25
**4733**	allXL	DD	373	382	BS2G	16.58
**4733**	allXL	DD	370	386	BS2G	22.13
**4733**	allXL	DD	373	386	BS2G	25.19
**4733**	allXL	AA	319	357	BS2G	27.98
**4733**	allXL	AA	367	373	BS2G	12.49
**4733**	allXL	DD	370	382	BS2G	16.27
**4733**	allXL	BB	376	382	BS2G	12.74
**4733**	allXL	BB	357	373	BS2G	29.83
**4733**	allXL	BB	320	366	BS2G	22.55
**4733**	allXL	CC	370	376	BS2G	13.92
**4733**	allXL	AA	320	357	DBSU	25.76
**4733**	allXL	AA	357	319	DBSU	27.98
**4733**	allXL	CC	372	319	DBSU	19.32
**4733**	allXL	DD	370	357	DBSU	30.22
**4733**	allXL	BB	382	357	DBSU	18.21
**4733**	allXL	AA	372	370	DBSU	6.26
**4733**	allXL	DD	381	370	DBSU	16.88
**4733**	allXL	DD	373	370	DBSU	8.93
**4733**	allXL	DD	381	372	DBSU	16.56
**4733**	allXL	DD	372	382	DBSU	16.31
**4733**	allXL	DD	372	386	DBSU	23.52
**4733**	allXL	DD	373	386	DBSU	25.19
**4733**	allXL	BC	381	386	DBSU	54.41
**4733**	allXL	AC;CA	357	357	DBSU	19.95
**4733**	allXL	DA	373	319	DBSU	29.77
**4733**	allXL	CB	372	357	DBSU	40.52
**4733**	allXL	BD	381	357	DBSU	34.29
**4733**	allXL	CB	373	357	DBSU	42.44
**4733**	allXL	CB	372	370	DBSU	29.04
**4733**	allXL	CB	373	370	DBSU	29.42

**4733**	allXL	CB	382	373	DBSU	37.72
**4734**	allXL	CC	320	357	BS2G	23.89
**4734**	allXL	DD	320	373	BS2G	15.64
**4734**	allXL	BB	373	382	BS2G	17.11
**4734**	allXL	BB	370	386	BS2G	22.64
**4734**	allXL	BB	373	386	BS2G	27.17
**4734**	allXL	CC	319	357	BS2G	22.82
**4734**	allXL	AA	367	373	BS2G	13.6
**4734**	allXL	BB	370	382	BS2G	15.82
**4734**	allXL	BB	376	382	BS2G	14.33
**4734**	allXL	DD	357	373	BS2G	35.23
**4734**	allXL	DD	320	366	BS2G	19.64
**4734**	allXL	CC	370	376	BS2G	15.63
**4734**	allXL	CC	320	357	DBSU	23.89
**4734**	allXL	CC	357	319	DBSU	22.82
**4734**	allXL	DD	372	319	DBSU	12.77
**4734**	allXL	DD	370	357	DBSU	26.53
**4734**	allXL	CC	382	357	DBSU	21.87
**4734**	allXL	AA	372	370	DBSU	6.2
**4734**	allXL	BB	381	370	DBSU	17.16
**4734**	allXL	AA	373	370	DBSU	9.13
**4734**	allXL	BB	381	372	DBSU	18.94
**4734**	allXL	BB	372	382	DBSU	18.71
**4734**	allXL	BB	372	386	DBSU	27.94
**4734**	allXL	BB	373	386	DBSU	27.17
**4734**	allXL	CB	381	386	DBSU	56.21
**4734**	allXL	BD;DB	357	357	DBSU	18.94
**4734**	allXL	AD	373	319	DBSU	35.95
**4734**	allXL	DA	372	357	DBSU	48.41
**4734**	allXL	CA	381	357	DBSU	42.08
**4734**	allXL	DA	373	357	DBSU	51.97
**4734**	allXL	DA	372	370	DBSU	36.56
**4734**	allXL	DA	373	370	DBSU	38.16
**4734**	allXL	BC	382	373	DBSU	57.61

**4735**	allXL	AA	320	357	BS2G	21.37
**4735**	allXL	BB	320	373	BS2G	13.7
**4735**	allXL	AA	373	382	BS2G	20.35
**4735**	allXL	AA	370	386	BS2G	20.94
**4735**	allXL	AA	373	386	BS2G	24.65
**4735**	allXL	CC	319	357	BS2G	24.27
**4735**	allXL	AA	367	373	BS2G	13.56
**4735**	allXL	AA	370	382	BS2G	21.06
**4735**	allXL	CC	376	382	BS2G	14.85
**4735**	allXL	CC	357	373	BS2G	18.86
**4735**	allXL	BB	320	366	BS2G	18.9
**4735**	allXL	BB	370	376	BS2G	15.77
**4735**	allXL	AA	320	357	DBSU	21.37
**4735**	allXL	CC	357	319	DBSU	24.27
**4735**	allXL	BB	372	319	DBSU	14.87
**4735**	allXL	CC	370	357	DBSU	17.57
**4735**	allXL	CC	382	357	DBSU	16.69
**4735**	allXL	AA	372	370	DBSU	6.39
**4735**	allXL	DD	381	370	DBSU	21.98
**4735**	allXL	AA	373	370	DBSU	8.93
**4735**	allXL	AA	381	372	DBSU	19.54
**4735**	allXL	AA	372	382	DBSU	19.02
**4735**	allXL	AA	372	386	DBSU	21.74
**4735**	allXL	AA	373	386	DBSU	24.65
**4735**	allXL	DA	381	386	DBSU	38.11
**4735**	allXL	BD;DB	357	357	DBSU	18.26
**4735**	allXL	DA	373	319	DBSU	20.06
**4735**	allXL	CA	372	357	DBSU	25.8
**4735**	allXL	CA	381	357	DBSU	35.46
**4735**	allXL	CA	373	357	DBSU	27.69
**4735**	allXL	BC	372	370	DBSU	34.15
**4735**	allXL	DA	373	370	DBSU	34.46
**4735**	allXL	BC	382	373	DBSU	32.19
**4736**	allXL	CC	320	357	BS2G	23.3

**4736**	allXL	BB	320	373	BS2G	19.69
**4736**	allXL	DD	373	382	BS2G	17.81
**4736**	allXL	DD	370	386	BS2G	20.23
**4736**	allXL	DD	373	386	BS2G	26.03
**4736**	allXL	CC	319	357	BS2G	23.06
**4736**	allXL	AA	367	373	BS2G	13.3
**4736**	allXL	DD	370	382	BS2G	16.51
**4736**	allXL	BB	376	382	BS2G	15.07
**4736**	allXL	BB	357	373	BS2G	25.07
**4736**	allXL	AA	320	366	BS2G	16.84
**4736**	allXL	DD	370	376	BS2G	17.27
**4736**	allXL	CC	320	357	DBSU	23.3
**4736**	allXL	CC	357	319	DBSU	23.06
**4736**	allXL	BB	372	319	DBSU	15.88
**4736**	allXL	BB	370	357	DBSU	16.3
**4736**	allXL	BB	382	357	DBSU	41.09
**4736**	allXL	AA	372	370	DBSU	6.24
**4736**	allXL	DD	381	370	DBSU	17.7
**4736**	allXL	AA	373	370	DBSU	8.83
**4736**	allXL	DD	381	372	DBSU	16.15
**4736**	allXL	DD	372	382	DBSU	16.28
**4736**	allXL	DD	372	386	DBSU	23.49
**4736**	allXL	DD	373	386	DBSU	26.03
**4736**	allXL	CB	381	386	DBSU	63.38
**4736**	allXL	BD;DB	357	357	DBSU	17.83
**4736**	allXL	BC	373	319	DBSU	28.09
**4736**	allXL	BC	372	357	DBSU	27.9
**4736**	allXL	BC	381	357	DBSU	29.44
**4736**	allXL	BC	373	357	DBSU	25.01
**4736**	allXL	DA	372	370	DBSU	39.56
**4736**	allXL	DA	373	370	DBSU	39.45
**4736**	allXL	BC	382	373	DBSU	44.47
**4737**	allXL	CC	320	357	BS2G	24.9
**4737**	allXL	AA	320	373	BS2G	18.49

**4737**	allXL	DD	373	382	BS2G	19.14
**4737**	allXL	DD	370	386	BS2G	25.06
**4737**	allXL	CC	373	386	BS2G	23.0
**4737**	allXL	CC	319	357	BS2G	26.28
**4737**	allXL	BB	367	373	BS2G	14.2
**4737**	allXL	DD	370	382	BS2G	20.52
**4737**	allXL	CC	376	382	BS2G	13.13
**4737**	allXL	BB	357	373	BS2G	27.2
**4737**	allXL	CC	320	366	BS2G	22.17
**4737**	allXL	DD	370	376	BS2G	14.96
**4737**	allXL	CC	320	357	DBSU	24.9
**4737**	allXL	CC	357	319	DBSU	26.28
**4737**	allXL	AA	372	319	DBSU	21.22
**4737**	allXL	AA	370	357	DBSU	28.53
**4737**	allXL	BB	382	357	DBSU	17.74
**4737**	allXL	AA	372	370	DBSU	6.27
**4737**	allXL	DD	381	370	DBSU	18.44
**4737**	allXL	AA	373	370	DBSU	9.01
**4737**	allXL	DD	381	372	DBSU	16.23
**4737**	allXL	DD	372	382	DBSU	18.65
**4737**	allXL	CC	372	386	DBSU	24.34
**4737**	allXL	CC	373	386	DBSU	23.0
**4737**	allXL	BC	381	386	DBSU	42.98
**4737**	allXL	BD;DB	357	357	DBSU	18.95
**4737**	allXL	AD	373	319	DBSU	21.15
**4737**	allXL	AD	372	357	DBSU	35.94
**4737**	allXL	BD	381	357	DBSU	38.54
**4737**	allXL	AD	373	357	DBSU	39.26
**4737**	allXL	CB	372	370	DBSU	19.44
**4737**	allXL	CB	373	370	DBSU	17.14
**4737**	allXL	CB	382	373	DBSU	35.63
**4738**	allXL	CC	320	357	BS2G	22.85
**4738**	allXL	BB	320	373	BS2G	24.19
**4738**	allXL	DD	373	382	BS2G	19.29

**4738**	allXL	BB	370	386	BS2G	28.09
**4738**	allXL	DD	373	386	BS2G	28.34
**4738**	allXL	CC	319	357	BS2G	22.88
**4738**	allXL	AA	367	373	BS2G	13.9
**4738**	allXL	BB	370	382	BS2G	23.6
**4738**	allXL	BB	376	382	BS2G	14.92
**4738**	allXL	CC	357	373	BS2G	28.12
**4738**	allXL	BB	320	366	BS2G	16.82
**4738**	allXL	CC	370	376	BS2G	14.31
**4738**	allXL	CC	320	357	DBSU	22.85
**4738**	allXL	CC	357	319	DBSU	22.88
**4738**	allXL	BB	372	319	DBSU	24.36
**4738**	allXL	BB	370	357	DBSU	27.19
**4738**	allXL	CC	382	357	DBSU	22.34
**4738**	allXL	AA	372	370	DBSU	6.18
**4738**	allXL	CC	381	370	DBSU	21.07
**4738**	allXL	DD	373	370	DBSU	9.03
**4738**	allXL	DD	381	372	DBSU	15.92
**4738**	allXL	DD	372	382	DBSU	18.82
**4738**	allXL	DD	372	386	DBSU	27.01
**4738**	allXL	DD	373	386	DBSU	28.34
**4738**	allXL	AD	381	386	DBSU	56.4
**4738**	allXL	BD;DB	357	357	DBSU	19.24
**4738**	allXL	DA	373	319	DBSU	29.67
**4738**	allXL	DA	372	357	DBSU	43.51
**4738**	allXL	CA	381	357	DBSU	33.25
**4738**	allXL	CA	373	357	DBSU	45.5
**4738**	allXL	DA	372	370	DBSU	34.36
**4738**	allXL	DA	373	370	DBSU	34.25
**4738**	allXL	DA	382	373	DBSU	38.15
**4739**	allXL	BB	320	357	BS2G	23.48
**4739**	allXL	DD	320	373	BS2G	20.86
**4739**	allXL	CC	373	382	BS2G	13.42
**4739**	allXL	CC	370	386	BS2G	15.01

**4739**	allXL	CC	373	386	BS2G	20.75
**4739**	allXL	BB	319	357	BS2G	25.87
**4739**	allXL	AA	367	373	BS2G	11.63
**4739**	allXL	CC	370	382	BS2G	14.28
**4739**	allXL	CC	376	382	BS2G	13.62
**4739**	allXL	CC	357	373	BS2G	28.63
**4739**	allXL	BB	320	366	BS2G	13.87
**4739**	allXL	BB	370	376	BS2G	14.9
**4739**	allXL	BB	320	357	DBSU	23.48
**4739**	allXL	BB	357	319	DBSU	25.87
**4739**	allXL	DD	372	319	DBSU	20.65
**4739**	allXL	DD	370	357	DBSU	22.14
**4739**	allXL	CC	382	357	DBSU	32.15
**4739**	allXL	CC	372	370	DBSU	6.38
**4739**	allXL	CC	381	370	DBSU	17.4
**4739**	allXL	AA	373	370	DBSU	8.88
**4739**	allXL	CC	381	372	DBSU	13.16
**4739**	allXL	CC	372	382	DBSU	11.07
**4739**	allXL	CC	372	386	DBSU	16.98
**4739**	allXL	CC	373	386	DBSU	20.75
**4739**	allXL	DA	381	386	DBSU	46.2
**4739**	allXL	BD;DB	357	357	DBSU	19.16
**4739**	allXL	DA	373	319	DBSU	29.82
**4739**	allXL	DA	372	357	DBSU	34.5
**4739**	allXL	DA	381	357	DBSU	44.69
**4739**	allXL	DA	373	357	DBSU	34.75
**4739**	allXL	DA	372	370	DBSU	23.2
**4739**	allXL	DA	373	370	DBSU	20.31
**4739**	allXL	DA	382	373	DBSU	26.96
**4740**	allXL	CC	320	357	BS2G	25.21
**4740**	allXL	CC	320	373	BS2G	15.94
**4740**	allXL	CC	373	382	BS2G	17.15
**4740**	allXL	DD	370	386	BS2G	20.91
**4740**	allXL	DD	373	386	BS2G	21.85

**4740**	allXL	AA	319	357	BS2G	24.43
**4740**	allXL	AA	367	373	BS2G	13.53
**4740**	allXL	DD	370	382	BS2G	17.82
**4740**	allXL	CC	376	382	BS2G	14.15
**4740**	allXL	DD	357	373	BS2G	18.23
**4740**	allXL	AA	320	366	BS2G	13.04
**4740**	allXL	DD	370	376	BS2G	15.48
**4740**	allXL	CC	320	357	DBSU	25.21
**4740**	allXL	AA	357	319	DBSU	24.43
**4740**	allXL	CC	372	319	DBSU	11.36
**4740**	allXL	DD	370	357	DBSU	21.33
**4740**	allXL	DD	382	357	DBSU	29.63
**4740**	allXL	AA	372	370	DBSU	6.07
**4740**	allXL	DD	381	370	DBSU	18.08
**4740**	allXL	AA	373	370	DBSU	9.12
**4740**	allXL	DD	381	372	DBSU	19.81
**4740**	allXL	DD	372	382	DBSU	20.17
**4740**	allXL	DD	372	386	DBSU	20.98
**4740**	allXL	DD	373	386	DBSU	21.85
**4740**	allXL	BC	381	386	DBSU	39.45
**4740**	allXL	BD;DB	357	357	DBSU	19.06
**4740**	allXL	DB	373	319	DBSU	34.33
**4740**	allXL	DB	372	357	DBSU	22.65
**4740**	allXL	DB	381	357	DBSU	27.44
**4740**	allXL	DB	373	357	DBSU	19.14
**4740**	allXL	CB	372	370	DBSU	30.06
**4740**	allXL	CB	373	370	DBSU	28.16
**4740**	allXL	CB	382	373	DBSU	27.82
**4741**	allXL	CC	320	357	BS2G	25.9
**4741**	allXL	DD	320	373	BS2G	11.98
**4741**	allXL	CC	373	382	BS2G	18.0
**4741**	allXL	DD	370	386	BS2G	18.4
**4741**	allXL	BB	373	386	BS2G	20.6
**4741**	allXL	AA	319	357	BS2G	27.52

**4741**	allXL	DD	367	373	BS2G	15.08
**4741**	allXL	DD	370	382	BS2G	18.76
**4741**	allXL	BB	376	382	BS2G	13.4
**4741**	allXL	DD	357	373	BS2G	32.75
**4741**	allXL	DD	320	366	BS2G	18.76
**4741**	allXL	DD	370	376	BS2G	16.18
**4741**	allXL	CC	320	357	DBSU	25.9
**4741**	allXL	AA	357	319	DBSU	27.52
**4741**	allXL	DD	372	319	DBSU	14.64
**4741**	allXL	DD	370	357	DBSU	30.46
**4741**	allXL	BB	382	357	DBSU	43.84
**4741**	allXL	AA	372	370	DBSU	5.94
**4741**	allXL	DD	381	370	DBSU	18.92
**4741**	allXL	AA	373	370	DBSU	8.95
**4741**	allXL	DD	381	372	DBSU	20.66
**4741**	allXL	CC	372	382	DBSU	19.55
**4741**	allXL	BB	372	386	DBSU	21.47
**4741**	allXL	BB	373	386	DBSU	20.6
**4741**	allXL	DA	381	386	DBSU	35.6
**4741**	allXL	BD;DB	357	357	DBSU	18.51
**4741**	allXL	AD	373	319	DBSU	31.62
**4741**	allXL	AD	372	357	DBSU	45.5
**4741**	allXL	DA	381	357	DBSU	57.96
**4741**	allXL	DA	373	357	DBSU	48.71
**4741**	allXL	DA	372	370	DBSU	37.1
**4741**	allXL	DA	373	370	DBSU	34.43
**4741**	allXL	DA	382	373	DBSU	35.95
**4742**	allXL	CC	320	357	BS2G	25.0
**4742**	allXL	BB	320	373	BS2G	14.77
**4742**	allXL	DD	373	382	BS2G	17.76
**4742**	allXL	CC	370	386	BS2G	24.31
**4742**	allXL	DD	373	386	BS2G	23.84
**4742**	allXL	CC	319	357	BS2G	23.7
**4742**	allXL	AA	367	373	BS2G	13.91

**4742**	allXL	CC	370	382	BS2G	18.83
**4742**	allXL	AA	376	382	BS2G	14.77
**4742**	allXL	DD	357	373	BS2G	25.99
**4742**	allXL	AA	320	366	BS2G	20.37
**4742**	allXL	CC	370	376	BS2G	14.48
**4742**	allXL	CC	320	357	DBSU	25.0
**4742**	allXL	CC	357	319	DBSU	23.7
**4742**	allXL	BB	372	319	DBSU	15.85
**4742**	allXL	DD	370	357	DBSU	21.42
**4742**	allXL	BB	382	357	DBSU	19.41
**4742**	allXL	AA	372	370	DBSU	6.37
**4742**	allXL	CC	381	370	DBSU	20.55
**4742**	allXL	DD	373	370	DBSU	9.09
**4742**	allXL	DD	381	372	DBSU	14.88
**4742**	allXL	DD	372	382	DBSU	16.43
**4742**	allXL	DD	372	386	DBSU	20.88
**4742**	allXL	DD	373	386	DBSU	23.84
**4742**	allXL	AD	381	386	DBSU	57.83
**4742**	allXL	BD;DB	357	357	DBSU	18.57
**4742**	allXL	BC	373	319	DBSU	32.51
**4742**	allXL	BC	372	357	DBSU	35.0
**4742**	allXL	BD	381	357	DBSU	40.07
**4742**	allXL	BC	373	357	DBSU	37.75
**4742**	allXL	BC	372	370	DBSU	38.9
**4742**	allXL	BC	373	370	DBSU	41.59
**4742**	allXL	CB	382	373	DBSU	47.24
**4743**	allXL	AA	320	357	BS2G	22.67
**4743**	allXL	BB	320	373	BS2G	19.5
**4743**	allXL	CC	373	382	BS2G	19.86
**4743**	allXL	CC	370	386	BS2G	18.06
**4743**	allXL	CC	373	386	BS2G	18.2
**4743**	allXL	AA	319	357	BS2G	20.16
**4743**	allXL	BB	367	373	BS2G	13.55
**4743**	allXL	DD	370	382	BS2G	23.58

**4743**	allXL	CC	376	382	BS2G	13.36
**4743**	allXL	BB	357	373	BS2G	10.97
**4743**	allXL	BB	320	366	BS2G	22.95
**4743**	allXL	DD	370	376	BS2G	16.46
**4743**	allXL	AA	320	357	DBSU	22.67
**4743**	allXL	AA	357	319	DBSU	20.16
**4743**	allXL	BB	372	319	DBSU	18.4
**4743**	allXL	BB	370	357	DBSU	16.95
**4743**	allXL	BB	382	357	DBSU	18.96
**4743**	allXL	AA	372	370	DBSU	6.24
**4743**	allXL	DD	381	370	DBSU	21.48
**4743**	allXL	AA	373	370	DBSU	9.36
**4743**	allXL	DD	381	372	DBSU	17.82
**4743**	allXL	DD	372	382	DBSU	20.42
**4743**	allXL	CC	372	386	DBSU	17.76
**4743**	allXL	CC	373	386	DBSU	18.2
**4743**	allXL	DB	381	386	DBSU	36.93
**4743**	allXL	BD;DB	357	357	DBSU	18.73
**4743**	allXL	CB	373	319	DBSU	24.57
**4743**	allXL	BD	372	357	DBSU	30.25
**4743**	allXL	BD	381	357	DBSU	23.28
**4743**	allXL	BD	373	357	DBSU	28.69
**4743**	allXL	CB	372	370	DBSU	26.53
**4743**	allXL	CB	373	370	DBSU	29.39
**4743**	allXL	CB	382	373	DBSU	43.56
**4744**	allXL	AA	320	357	BS2G	26.36
**4744**	allXL	BB	320	373	BS2G	17.04
**4744**	allXL	AA	373	382	BS2G	19.17
**4744**	allXL	AA	370	386	BS2G	23.26
**4744**	allXL	AA	373	386	BS2G	24.94
**4744**	allXL	AA	319	357	BS2G	25.6
**4744**	allXL	AA	367	373	BS2G	13.12
**4744**	allXL	AA	370	382	BS2G	22.1
**4744**	allXL	CC	376	382	BS2G	14.02

**4744**	allXL	BB	357	373	BS2G	18.77
**4744**	allXL	CC	320	366	BS2G	17.45
**4744**	allXL	CC	370	376	BS2G	17.58
**4744**	allXL	AA	320	357	DBSU	26.36
**4744**	allXL	AA	357	319	DBSU	25.6
**4744**	allXL	CC	372	319	DBSU	16.45
**4744**	allXL	CC	370	357	DBSU	23.03
**4744**	allXL	BB	382	357	DBSU	28.84
**4744**	allXL	AA	372	370	DBSU	6.2
**4744**	allXL	AA	381	370	DBSU	24.51
**4744**	allXL	AA	373	370	DBSU	9.19
**4744**	allXL	AA	381	372	DBSU	20.29
**4744**	allXL	AA	372	382	DBSU	18.57
**4744**	allXL	AA	372	386	DBSU	22.79
**4744**	allXL	AA	373	386	DBSU	24.94
**4744**	allXL	AC	381	386	DBSU	53.24
**4744**	allXL	BD;DB	357	357	DBSU	17.99
**4744**	allXL	DA	373	319	DBSU	22.08
**4744**	allXL	BD	372	357	DBSU	37.36
**4744**	allXL	BD	381	357	DBSU	35.92
**4744**	allXL	BD	373	357	DBSU	36.12
**4744**	allXL	DA	372	370	DBSU	41.57
**4744**	allXL	DA	373	370	DBSU	42.69
**4744**	allXL	CA	382	373	DBSU	48.05
**4745**	allXL	AA	320	357	BS2G	23.62
**4745**	allXL	CC	320	373	BS2G	27.61
**4745**	allXL	DD	373	382	BS2G	21.48
**4745**	allXL	DD	370	386	BS2G	20.78
**4745**	allXL	DD	373	386	BS2G	26.97
**4745**	allXL	AA	319	357	BS2G	25.52
**4745**	allXL	DD	367	373	BS2G	14.46
**4745**	allXL	DD	370	382	BS2G	19.5
**4745**	allXL	CC	376	382	BS2G	14.94
**4745**	allXL	AA	357	373	BS2G	31.08

**4745**	allXL	CC	320	366	BS2G	23.12
**4745**	allXL	CC	370	376	BS2G	16.2
**4745**	allXL	AA	320	357	DBSU	23.62
**4745**	allXL	AA	357	319	DBSU	25.52
**4745**	allXL	CC	372	319	DBSU	29.33
**4745**	allXL	CC	370	357	DBSU	25.46
**4745**	allXL	CC	382	357	DBSU	20.08
**4745**	allXL	AA	372	370	DBSU	6.06
**4745**	allXL	DD	381	370	DBSU	19.34
**4745**	allXL	AA	373	370	DBSU	9.24
**4745**	allXL	DD	381	372	DBSU	21.51
**4745**	allXL	CC	372	382	DBSU	22.35
**4745**	allXL	DD	372	386	DBSU	26.86
**4745**	allXL	DD	373	386	DBSU	26.97
**4745**	allXL	AC	381	386	DBSU	54.39
**4745**	allXL	BD;DB	357	357	DBSU	17.99
**4745**	allXL	DA	373	319	DBSU	40.5
**4745**	allXL	DB	372	357	DBSU	36.91
**4745**	allXL	CA	381	357	DBSU	25.85
**4745**	allXL	DB	373	357	DBSU	37.35
**4745**	allXL	DB	372	370	DBSU	48.16
**4745**	allXL	DB	373	370	DBSU	49.71
**4745**	allXL	CA	382	373	DBSU	42.93
**4746**	allXL	AA	320	357	BS2G	12.61
**4746**	allXL	BB	320	373	BS2G	15.15
**4746**	allXL	DD	373	382	BS2G	19.03
**4746**	allXL	CC	370	386	BS2G	26.27
**4746**	allXL	DD	373	386	BS2G	25.58
**4746**	allXL	AA	319	357	BS2G	9.29
**4746**	allXL	DD	367	373	BS2G	13.76
**4746**	allXL	CC	370	382	BS2G	21.88
**4746**	allXL	CC	376	382	BS2G	14.68
**4746**	allXL	BB	357	373	BS2G	20.36
**4746**	allXL	AA	320	366	BS2G	24.56

**4746**	allXL	CC	370	376	BS2G	16.21
**4746**	allXL	AA	320	357	DBSU	12.61
**4746**	allXL	AA	357	319	DBSU	9.29
**4746**	allXL	BB	372	319	DBSU	21.66
**4746**	allXL	CC	370	357	DBSU	22.59
**4746**	allXL	CC	382	357	DBSU	17.66
**4746**	allXL	AA	372	370	DBSU	6.0
**4746**	allXL	DD	381	370	DBSU	23.79
**4746**	allXL	DD	373	370	DBSU	8.91
**4746**	allXL	DD	381	372	DBSU	19.08
**4746**	allXL	DD	372	382	DBSU	19.22
**4746**	allXL	DD	372	386	DBSU	24.31
**4746**	allXL	DD	373	386	DBSU	25.58
**4746**	allXL	DA	381	386	DBSU	39.15
**4746**	allXL	BD;DB	357	357	DBSU	18.6
**4746**	allXL	CA	373	319	DBSU	26.39
**4746**	allXL	CA	372	357	DBSU	23.85
**4746**	allXL	CA	381	357	DBSU	27.63
**4746**	allXL	CA	373	357	DBSU	21.96
**4746**	allXL	BC	372	370	DBSU	36.48
**4746**	allXL	BC	373	370	DBSU	33.54
**4746**	allXL	BC	382	373	DBSU	26.69
**4747**	allXL	CC	320	357	BS2G	23.45
**4747**	allXL	BB	320	373	BS2G	17.39
**4747**	allXL	DD	373	382	BS2G	17.41
**4747**	allXL	CC	370	386	BS2G	19.45
**4747**	allXL	CC	373	386	BS2G	18.79
**4747**	allXL	AA	319	357	BS2G	23.39
**4747**	allXL	AA	367	373	BS2G	14.07
**4747**	allXL	DD	370	382	BS2G	14.16
**4747**	allXL	AA	376	382	BS2G	14.08
**4747**	allXL	BB	357	373	BS2G	25.0
**4747**	allXL	CC	320	366	BS2G	16.77
**4747**	allXL	DD	370	376	BS2G	15.31

**4747**	allXL	CC	320	357	DBSU	23.45
**4747**	allXL	AA	357	319	DBSU	23.39
**4747**	allXL	CC	372	319	DBSU	13.66
**4747**	allXL	BB	370	357	DBSU	23.84
**4747**	allXL	BB	382	357	DBSU	27.06
**4747**	allXL	AA	372	370	DBSU	6.33
**4747**	allXL	DD	381	370	DBSU	14.49
**4747**	allXL	CC	373	370	DBSU	9.41
**4747**	allXL	DD	381	372	DBSU	16.46
**4747**	allXL	DD	372	382	DBSU	16.36
**4747**	allXL	CC	372	386	DBSU	20.3
**4747**	allXL	CC	373	386	DBSU	18.79
**4747**	allXL	BC	381	386	DBSU	53.04
**4747**	allXL	BD;DB	357	357	DBSU	18.18
**4747**	allXL	DA	373	319	DBSU	24.62
**4747**	allXL	DA	372	357	DBSU	34.67
**4747**	allXL	BD	381	357	DBSU	43.7
**4747**	allXL	DA	373	357	DBSU	33.72
**4747**	allXL	DA	372	370	DBSU	34.06
**4747**	allXL	DA	373	370	DBSU	36.6
**4747**	allXL	DA	382	373	DBSU	47.94
**4748**	allXL	AA	320	357	BS2G	23.21
**4748**	allXL	DD	320	373	BS2G	30.9
**4748**	allXL	DD	373	382	BS2G	15.24
**4748**	allXL	DD	370	386	BS2G	20.29
**4748**	allXL	BB	373	386	BS2G	25.26
**4748**	allXL	AA	319	357	BS2G	26.15
**4748**	allXL	AA	367	373	BS2G	11.32
**4748**	allXL	DD	370	382	BS2G	13.15
**4748**	allXL	AA	376	382	BS2G	14.3
**4748**	allXL	AA	357	373	BS2G	27.68
**4748**	allXL	AA	320	366	BS2G	24.38
**4748**	allXL	CC	370	376	BS2G	15.76
**4748**	allXL	AA	320	357	DBSU	23.21

**4748**	allXL	AA	357	319	DBSU	26.15
**4748**	allXL	DD	372	319	DBSU	32.36
**4748**	allXL	DD	370	357	DBSU	31.97
**4748**	allXL	AA	382	357	DBSU	21.49
**4748**	allXL	AA	372	370	DBSU	6.5
**4748**	allXL	DD	381	370	DBSU	12.61
**4748**	allXL	DD	373	370	DBSU	9.03
**4748**	allXL	DD	381	372	DBSU	13.28
**4748**	allXL	DD	372	382	DBSU	14.46
**4748**	allXL	DD	372	386	DBSU	24.2
**4748**	allXL	BB	373	386	DBSU	25.26
**4748**	allXL	BC	381	386	DBSU	50.48
**4748**	allXL	BD;DB	357	357	DBSU	16.65
**4748**	allXL	AD	373	319	DBSU	34.92
**4748**	allXL	AC	372	357	DBSU	46.45
**4748**	allXL	AC	381	357	DBSU	25.46
**4748**	allXL	AC	373	357	DBSU	42.78
**4748**	allXL	BC	372	370	DBSU	55.8
**4748**	allXL	DA	373	370	DBSU	54.42
**4748**	allXL	CB	382	373	DBSU	44.08
**4749**	allXL	AA	320	357	BS2G	19.63
**4749**	allXL	CC	320	373	BS2G	16.56
**4749**	allXL	BB	373	382	BS2G	17.39
**4749**	allXL	CC	370	386	BS2G	22.28
**4749**	allXL	DD	373	386	BS2G	23.83
**4749**	allXL	AA	319	357	BS2G	22.61
**4749**	allXL	AA	367	373	BS2G	13.81
**4749**	allXL	CC	370	382	BS2G	15.32
**4749**	allXL	BB	376	382	BS2G	13.3
**4749**	allXL	DD	357	373	BS2G	25.54
**4749**	allXL	AA	320	366	BS2G	21.51
**4749**	allXL	CC	370	376	BS2G	13.68
**4749**	allXL	AA	320	357	DBSU	19.63
**4749**	allXL	AA	357	319	DBSU	22.61

**4749**	allXL	CC	372	319	DBSU	14.33
**4749**	allXL	DD	370	357	DBSU	22.45
**4749**	allXL	CC	382	357	DBSU	33.93
**4749**	allXL	AA	372	370	DBSU	6.26
**4749**	allXL	CC	381	370	DBSU	17.48
**4749**	allXL	AA	373	370	DBSU	9.31
**4749**	allXL	DD	381	372	DBSU	17.29
**4749**	allXL	BB	372	382	DBSU	18.4
**4749**	allXL	DD	372	386	DBSU	21.72
**4749**	allXL	DD	373	386	DBSU	23.83
**4749**	allXL	DB	381	386	DBSU	32.09
**4749**	allXL	BD;DB	357	357	DBSU	18.78
**4749**	allXL	CB	373	319	DBSU	30.85
**4749**	allXL	CB	372	357	DBSU	30.99
**4749**	allXL	BD	381	357	DBSU	47.72
**4749**	allXL	CB	373	357	DBSU	32.98
**4749**	allXL	CB	372	370	DBSU	26.54
**4749**	allXL	CB	373	370	DBSU	24.5
**4749**	allXL	BD	382	373	DBSU	45.93
**4750**	allXL	CC	320	357	BS2G	22.75
**4750**	allXL	AA	320	373	BS2G	22.36
**4750**	allXL	AA	373	382	BS2G	19.33
**4750**	allXL	AA	370	386	BS2G	23.97
**4750**	allXL	BB	373	386	BS2G	23.9
**4750**	allXL	CC	319	357	BS2G	26.2
**4750**	allXL	AA	367	373	BS2G	14.72
**4750**	allXL	AA	370	382	BS2G	22.92
**4750**	allXL	AA	376	382	BS2G	14.96
**4750**	allXL	DD	357	373	BS2G	34.21
**4750**	allXL	AA	320	366	BS2G	17.95
**4750**	allXL	DD	370	376	BS2G	16.11
**4750**	allXL	CC	320	357	DBSU	22.75
**4750**	allXL	CC	357	319	DBSU	26.2
**4750**	allXL	AA	372	319	DBSU	20.18

**4750**	allXL	DD	370	357	DBSU	30.77
**4750**	allXL	CC	382	357	DBSU	35.82
**4750**	allXL	AA	372	370	DBSU	6.2
**4750**	allXL	DD	381	370	DBSU	22.2
**4750**	allXL	AA	373	370	DBSU	9.08
**4750**	allXL	DD	381	372	DBSU	19.28
**4750**	allXL	AA	372	382	DBSU	20.23
**4750**	allXL	AA	372	386	DBSU	24.36
**4750**	allXL	BB	373	386	DBSU	23.9
**4750**	allXL	DA	381	386	DBSU	36.21
**4750**	allXL	BD;DB	357	357	DBSU	18.75
**4750**	allXL	CB	373	319	DBSU	22.85
**4750**	allXL	CB	372	357	DBSU	41.75
**4750**	allXL	CB	381	357	DBSU	41.95
**4750**	allXL	CB	373	357	DBSU	39.39
**4750**	allXL	CB	372	370	DBSU	35.59
**4750**	allXL	CB	373	370	DBSU	32.05
**4750**	allXL	AD	382	373	DBSU	40.43
**4751**	allXL	AA	320	357	BS2G	22.44
**4751**	allXL	AA	320	373	BS2G	27.93
**4751**	allXL	CC	373	382	BS2G	15.91
**4751**	allXL	AA	370	386	BS2G	27.35
**4751**	allXL	AA	373	386	BS2G	26.08
**4751**	allXL	AA	319	357	BS2G	24.95
**4751**	allXL	AA	367	373	BS2G	12.62
**4751**	allXL	CC	370	382	BS2G	20.34
**4751**	allXL	AA	376	382	BS2G	14.14
**4751**	allXL	BB	357	373	BS2G	30.22
**4751**	allXL	CC	320	366	BS2G	17.89
**4751**	allXL	BB	370	376	BS2G	14.95
**4751**	allXL	AA	320	357	DBSU	22.44
**4751**	allXL	AA	357	319	DBSU	24.95
**4751**	allXL	BB	372	319	DBSU	31.23
**4751**	allXL	BB	370	357	DBSU	30.36

**4751**	allXL	CC	382	357	DBSU	34.81
**4751**	allXL	AA	372	370	DBSU	6.46
**4751**	allXL	CC	381	370	DBSU	21.1
**4751**	allXL	AA	373	370	DBSU	8.82
**4751**	allXL	CC	381	372	DBSU	18.24
**4751**	allXL	CC	372	382	DBSU	18.7
**4751**	allXL	AA	372	386	DBSU	26.05
**4751**	allXL	AA	373	386	DBSU	26.08
**4751**	allXL	AD	381	386	DBSU	44.41
**4751**	allXL	BD;DB	357	357	DBSU	18.08
**4751**	allXL	CB	373	319	DBSU	27.52
**4751**	allXL	BD	372	357	DBSU	46.5
**4751**	allXL	AD	381	357	DBSU	47.45
**4751**	allXL	BD	373	357	DBSU	47.35
**4751**	allXL	CB	372	370	DBSU	27.7
**4751**	allXL	CB	373	370	DBSU	30.78
**4751**	allXL	AD	382	373	DBSU	32.82
**4752**	allXL	AA	320	357	BS2G	22.59
**4752**	allXL	DD	320	373	BS2G	15.48
**4752**	allXL	AA	373	382	BS2G	16.8
**4752**	allXL	BB	370	386	BS2G	14.93
**4752**	allXL	BB	373	386	BS2G	20.63
**4752**	allXL	AA	319	357	BS2G	22.21
**4752**	allXL	DD	367	373	BS2G	13.97
**4752**	allXL	AA	370	382	BS2G	19.22
**4752**	allXL	CC	376	382	BS2G	13.13
**4752**	allXL	BB	357	373	BS2G	30.17
**4752**	allXL	DD	320	366	BS2G	25.54
**4752**	allXL	BB	370	376	BS2G	16.73
**4752**	allXL	AA	320	357	DBSU	22.59
**4752**	allXL	AA	357	319	DBSU	22.21
**4752**	allXL	DD	372	319	DBSU	20.35
**4752**	allXL	BB	370	357	DBSU	27.53
**4752**	allXL	DD	382	357	DBSU	34.62

**4752**	allXL	AA	372	370	DBSU	6.06
**4752**	allXL	BB	381	370	DBSU	17.25
**4752**	allXL	AA	373	370	DBSU	8.64
**4752**	allXL	DD	381	372	DBSU	15.71
**4752**	allXL	AA	372	382	DBSU	18.34
**4752**	allXL	BB	372	386	DBSU	19.75
**4752**	allXL	BB	373	386	DBSU	20.63
**4752**	allXL	BC	381	386	DBSU	33.79
**4752**	allXL	BD;DB	357	357	DBSU	17.81
**4752**	allXL	CB	373	319	DBSU	16.26
**4752**	allXL	CB	372	357	DBSU	36.1
**4752**	allXL	CB	381	357	DBSU	46.17
**4752**	allXL	CB	373	357	DBSU	38.76
**4752**	allXL	CB	372	370	DBSU	13.83
**4752**	allXL	CB	373	370	DBSU	17.11
**4752**	allXL	BC	382	373	DBSU	20.9
**4753**	allXL	AA	320	357	BS2G	25.46
**4753**	allXL	DD	320	373	BS2G	24.85
**4753**	allXL	BB	373	382	BS2G	20.64
**4753**	allXL	DD	370	386	BS2G	24.86
**4753**	allXL	AA	373	386	BS2G	29.53
**4753**	allXL	AA	319	357	BS2G	26.43
**4753**	allXL	AA	367	373	BS2G	11.83
**4753**	allXL	DD	370	382	BS2G	20.31
**4753**	allXL	CC	376	382	BS2G	14.24
**4753**	allXL	DD	357	373	BS2G	35.75
**4753**	allXL	DD	320	366	BS2G	16.14
**4753**	allXL	DD	370	376	BS2G	15.22
**4753**	allXL	AA	320	357	DBSU	25.46
**4753**	allXL	AA	357	319	DBSU	26.43
**4753**	allXL	DD	372	319	DBSU	20.82
**4753**	allXL	DD	370	357	DBSU	29.98
**4753**	allXL	CC	382	357	DBSU	24.9
**4753**	allXL	AA	372	370	DBSU	6.42

**4753**	allXL	DD	381	370	DBSU	17.68
**4753**	allXL	AA	373	370	DBSU	9.17
**4753**	allXL	DD	381	372	DBSU	20.91
**4753**	allXL	BB	372	382	DBSU	22.93
**4753**	allXL	AA	372	386	DBSU	30.06
**4753**	allXL	AA	373	386	DBSU	29.53
**4753**	allXL	CB	381	386	DBSU	23.97
**4753**	allXL	BD;DB	357	357	DBSU	18.29
**4753**	allXL	AD	373	319	DBSU	18.61
**4753**	allXL	AD	372	357	DBSU	43.24
**4753**	allXL	BC	381	357	DBSU	36.68
**4753**	allXL	DA	373	357	DBSU	41.65
**4753**	allXL	DA	372	370	DBSU	15.67
**4753**	allXL	DA	373	370	DBSU	16.75
**4753**	allXL	BC	382	373	DBSU	20.64
**4754**	allXL	AA	320	357	BS2G	26.39
**4754**	allXL	CC	320	373	BS2G	21.67
**4754**	allXL	CC	373	382	BS2G	14.16
**4754**	allXL	CC	370	386	BS2G	20.4
**4754**	allXL	CC	373	386	BS2G	24.45
**4754**	allXL	AA	319	357	BS2G	26.18
**4754**	allXL	BB	367	373	BS2G	12.19
**4754**	allXL	CC	370	382	BS2G	10.98
**4754**	allXL	AA	376	382	BS2G	14.47
**4754**	allXL	BB	357	373	BS2G	28.28
**4754**	allXL	CC	320	366	BS2G	23.32
**4754**	allXL	CC	370	376	BS2G	11.26
**4754**	allXL	AA	320	357	DBSU	26.39
**4754**	allXL	AA	357	319	DBSU	26.18
**4754**	allXL	CC	372	319	DBSU	20.6
**4754**	allXL	BB	370	357	DBSU	20.96
**4754**	allXL	CC	382	357	DBSU	30.31
**4754**	allXL	AA	372	370	DBSU	6.68
**4754**	allXL	CC	381	370	DBSU	13.04

**4754**	allXL	BB	373	370	DBSU	9.21
**4754**	allXL	CC	381	372	DBSU	15.64
**4754**	allXL	CC	372	382	DBSU	13.82
**4754**	allXL	CC	372	386	DBSU	22.98
**4754**	allXL	CC	373	386	DBSU	24.45
**4754**	allXL	BC	381	386	DBSU	27.46
**4754**	allXL	BD;DB	357	357	DBSU	17.93
**4754**	allXL	AD	373	319	DBSU	24.33
**4754**	allXL	BC	372	357	DBSU	38.31
**4754**	allXL	CA	381	357	DBSU	45.0
**4754**	allXL	BC	373	357	DBSU	41.47
**4754**	allXL	BC	372	370	DBSU	28.57
**4754**	allXL	BC	373	370	DBSU	31.42
**4754**	allXL	CB	382	373	DBSU	29.76
**4755**	allXL	CC	320	357	BS2G	22.83
**4755**	allXL	DD	320	373	BS2G	17.12
**4755**	allXL	AA	373	382	BS2G	17.83
**4755**	allXL	AA	370	386	BS2G	22.41
**4755**	allXL	AA	373	386	BS2G	20.73
**4755**	allXL	CC	319	357	BS2G	26.0
**4755**	allXL	AA	367	373	BS2G	12.67
**4755**	allXL	DD	370	382	BS2G	22.05
**4755**	allXL	BB	376	382	BS2G	12.73
**4755**	allXL	CC	357	373	BS2G	28.44
**4755**	allXL	AA	320	366	BS2G	17.5
**4755**	allXL	CC	370	376	BS2G	15.56
**4755**	allXL	CC	320	357	DBSU	22.83
**4755**	allXL	CC	357	319	DBSU	26.0
**4755**	allXL	DD	372	319	DBSU	18.62
**4755**	allXL	CC	370	357	DBSU	27.06
**4755**	allXL	CC	382	357	DBSU	19.24
**4755**	allXL	DD	372	370	DBSU	6.7
**4755**	allXL	DD	381	370	DBSU	22.0
**4755**	allXL	AA	373	370	DBSU	9.21

**4755**	allXL	DD	381	372	DBSU	19.24
**4755**	allXL	BB	372	382	DBSU	19.96
**4755**	allXL	AA	372	386	DBSU	23.19
**4755**	allXL	AA	373	386	DBSU	20.73
**4755**	allXL	AD	381	386	DBSU	61.35
**4755**	allXL	BD;DB	357	357	DBSU	18.89
**4755**	allXL	AD	373	319	DBSU	31.79
**4755**	allXL	CA	372	357	DBSU	36.5
**4755**	allXL	CA	381	357	DBSU	35.78
**4755**	allXL	CA	373	357	DBSU	36.89
**4755**	allXL	DA	372	370	DBSU	30.68
**4755**	allXL	DA	373	370	DBSU	32.94
**4755**	allXL	DA	382	373	DBSU	44.02
**4756**	allXL	AA	320	357	BS2G	21.36
**4756**	allXL	CC	320	373	BS2G	14.7
**4756**	allXL	DD	373	382	BS2G	18.93
**4756**	allXL	CC	370	386	BS2G	24.31
**4756**	allXL	BB	373	386	BS2G	25.92
**4756**	allXL	AA	319	357	BS2G	20.28
**4756**	allXL	AA	367	373	BS2G	14.71
**4756**	allXL	CC	370	382	BS2G	18.22
**4756**	allXL	BB	376	382	BS2G	13.0
**4756**	allXL	BB	357	373	BS2G	29.54
**4756**	allXL	AA	320	366	BS2G	18.59
**4756**	allXL	CC	370	376	BS2G	13.59
**4756**	allXL	AA	320	357	DBSU	21.36
**4756**	allXL	AA	357	319	DBSU	20.28
**4756**	allXL	CC	372	319	DBSU	12.98
**4756**	allXL	CC	370	357	DBSU	26.88
**4756**	allXL	CC	382	357	DBSU	37.17
**4756**	allXL	AA	372	370	DBSU	6.2
**4756**	allXL	DD	381	370	DBSU	17.9
**4756**	allXL	AA	373	370	DBSU	8.99
**4756**	allXL	DD	381	372	DBSU	18.87

**4756**	allXL	DD	372	382	DBSU	19.62
**4756**	allXL	CC	372	386	DBSU	28.42
**4756**	allXL	BB	373	386	DBSU	25.92
**4756**	allXL	BC	381	386	DBSU	35.14
**4756**	allXL	BD;DB	357	357	DBSU	17.88
**4756**	allXL	BC	373	319	DBSU	12.86
**4756**	allXL	BC	372	357	DBSU	34.2
**4756**	allXL	BC	381	357	DBSU	37.66
**4756**	allXL	BC	373	357	DBSU	32.43
**4756**	allXL	BC	372	370	DBSU	25.08
**4756**	allXL	BC	373	370	DBSU	24.14
**4756**	allXL	CB	382	373	DBSU	19.34
**4757**	allXL	CC	320	357	BS2G	26.06
**4757**	allXL	AA	320	373	BS2G	19.83
**4757**	allXL	AA	373	382	BS2G	20.11
**4757**	allXL	AA	370	386	BS2G	30.8
**4757**	allXL	AA	373	386	BS2G	28.9
**4757**	allXL	AA	319	357	BS2G	27.45
**4757**	allXL	BB	367	373	BS2G	12.62
**4757**	allXL	AA	370	382	BS2G	25.18
**4757**	allXL	CC	376	382	BS2G	15.17
**4757**	allXL	AA	357	373	BS2G	25.92
**4757**	allXL	BB	320	366	BS2G	26.49
**4757**	allXL	CC	370	376	BS2G	15.4
**4757**	allXL	CC	320	357	DBSU	26.06
**4757**	allXL	AA	357	319	DBSU	27.45
**4757**	allXL	BB	372	319	DBSU	22.96
**4757**	allXL	AA	370	357	DBSU	28.67
**4757**	allXL	BB	382	357	DBSU	25.59
**4757**	allXL	AA	372	370	DBSU	6.16
**4757**	allXL	CC	381	370	DBSU	26.47
**4757**	allXL	CC	373	370	DBSU	9.07
**4757**	allXL	AA	381	372	DBSU	22.39
**4757**	allXL	AA	372	382	DBSU	21.29

**4757**	allXL	AA	372	386	DBSU	28.98
**4757**	allXL	AA	373	386	DBSU	28.9
**4757**	allXL	DA	381	386	DBSU	24.43
**4757**	allXL	BD;DB	357	357	DBSU	17.97
**4757**	allXL	BC	373	319	DBSU	28.57
**4757**	allXL	BC	372	357	DBSU	38.16
**4757**	allXL	BD	381	357	DBSU	33.87
**4757**	allXL	BC	373	357	DBSU	36.74
**4757**	allXL	BC	372	370	DBSU	32.43
**4757**	allXL	BC	373	370	DBSU	32.88
**4757**	allXL	AD	382	373	DBSU	32.96
**4758**	allXL	CC	320	357	BS2G	24.92
**4758**	allXL	DD	320	373	BS2G	21.4
**4758**	allXL	DD	373	382	BS2G	17.7
**4758**	allXL	DD	370	386	BS2G	22.44
**4758**	allXL	CC	373	386	BS2G	25.6
**4758**	allXL	AA	319	357	BS2G	23.67
**4758**	allXL	BB	367	373	BS2G	15.03
**4758**	allXL	DD	370	382	BS2G	16.73
**4758**	allXL	CC	376	382	BS2G	14.44
**4758**	allXL	DD	357	373	BS2G	35.5
**4758**	allXL	DD	320	366	BS2G	26.43
**4758**	allXL	CC	370	376	BS2G	15.5
**4758**	allXL	CC	320	357	DBSU	24.92
**4758**	allXL	AA	357	319	DBSU	23.67
**4758**	allXL	DD	372	319	DBSU	21.75
**4758**	allXL	DD	370	357	DBSU	28.71
**4758**	allXL	CC	382	357	DBSU	27.79
**4758**	allXL	AA	372	370	DBSU	5.98
**4758**	allXL	DD	381	370	DBSU	17.27
**4758**	allXL	AA	373	370	DBSU	9.14
**4758**	allXL	DD	381	372	DBSU	16.24
**4758**	allXL	DD	372	382	DBSU	17.28
**4758**	allXL	DD	372	386	DBSU	25.48

**4758**	allXL	CC	373	386	DBSU	25.6
**4758**	allXL	DA	381	386	DBSU	47.35
**4758**	allXL	BD;DB	357	357	DBSU	18.85
**4758**	allXL	CB	373	319	DBSU	22.76
**4758**	allXL	DA	372	357	DBSU	40.5
**4758**	allXL	CB	381	357	DBSU	30.47
**4758**	allXL	DA	373	357	DBSU	40.29
**4758**	allXL	DA	372	370	DBSU	33.04
**4758**	allXL	DA	373	370	DBSU	30.66
**4758**	allXL	AD	382	373	DBSU	44.86
**4759**	allXL	CC	320	357	BS2G	23.4
**4759**	allXL	DD	320	373	BS2G	25.84
**4759**	allXL	BB	373	382	BS2G	15.82
**4759**	allXL	DD	370	386	BS2G	26.49
**4759**	allXL	DD	373	386	BS2G	23.91
**4759**	allXL	CC	319	357	BS2G	25.97
**4759**	allXL	BB	367	373	BS2G	11.99
**4759**	allXL	BB	370	382	BS2G	23.59
**4759**	allXL	BB	376	382	BS2G	13.17
**4759**	allXL	BB	357	373	BS2G	24.07
**4759**	allXL	DD	320	366	BS2G	26.51
**4759**	allXL	CC	370	376	BS2G	15.89
**4759**	allXL	CC	320	357	DBSU	23.4
**4759**	allXL	CC	357	319	DBSU	25.97
**4759**	allXL	DD	372	319	DBSU	20.13
**4759**	allXL	DD	370	357	DBSU	28.82
**4759**	allXL	BB	382	357	DBSU	19.77
**4759**	allXL	AA	372	370	DBSU	6.37
**4759**	allXL	DD	381	370	DBSU	24.15
**4759**	allXL	CC	373	370	DBSU	9.25
**4759**	allXL	BB	381	372	DBSU	18.46
**4759**	allXL	BB	372	382	DBSU	16.66
**4759**	allXL	DD	372	386	DBSU	23.48
**4759**	allXL	DD	373	386	DBSU	23.91

**4759**	allXL	AD	381	386	DBSU	54.18
**4759**	allXL	BD;DB	357	357	DBSU	20.07
**4759**	allXL	CB	373	319	DBSU	28.76
**4759**	allXL	DA	372	357	DBSU	43.67
**4759**	allXL	BD	381	357	DBSU	33.83
**4759**	allXL	BD	373	357	DBSU	43.54
**4759**	allXL	DA	372	370	DBSU	26.32
**4759**	allXL	DA	373	370	DBSU	28.23
**4759**	allXL	DA	382	373	DBSU	41.15
**4760**	allXL	CC	320	357	BS2G	24.02
**4760**	allXL	BB	320	373	BS2G	33.14
**4760**	allXL	CC	373	382	BS2G	18.42
**4760**	allXL	DD	370	386	BS2G	30.11
**4760**	allXL	CC	373	386	BS2G	24.89
**4760**	allXL	CC	319	357	BS2G	22.8
**4760**	allXL	AA	367	373	BS2G	12.84
**4760**	allXL	BB	370	382	BS2G	22.55
**4760**	allXL	CC	376	382	BS2G	14.7
**4760**	allXL	DD	357	373	BS2G	31.83
**4760**	allXL	BB	320	366	BS2G	19.8
**4760**	allXL	BB	370	376	BS2G	16.67
**4760**	allXL	CC	320	357	DBSU	24.02
**4760**	allXL	CC	357	319	DBSU	22.8
**4760**	allXL	BB	372	319	DBSU	29.78
**4760**	allXL	DD	370	357	DBSU	24.31
**4760**	allXL	DD	382	357	DBSU	35.0
**4760**	allXL	AA	372	370	DBSU	6.18
**4760**	allXL	BB	381	370	DBSU	24.08
**4760**	allXL	AA	373	370	DBSU	9.07
**4760**	allXL	CC	381	372	DBSU	21.54
**4760**	allXL	CC	372	382	DBSU	20.83
**4760**	allXL	CC	372	386	DBSU	25.83
**4760**	allXL	CC	373	386	DBSU	24.89
**4760**	allXL	DB	381	386	DBSU	50.57

**4760**	allXL	BD;DB	357	357	DBSU	19.24
**4760**	allXL	AD	373	319	DBSU	29.51
**4760**	allXL	DB	372	357	DBSU	46.55
**4760**	allXL	DB	381	357	DBSU	40.82
**4760**	allXL	DB	373	357	DBSU	46.89
**4760**	allXL	CB	372	370	DBSU	49.17
**4760**	allXL	CB	373	370	DBSU	52.42
**4760**	allXL	CB	382	373	DBSU	65.85
**4761**	allXL	CC	320	357	BS2G	25.02
**4761**	allXL	BB	320	373	BS2G	17.88
**4761**	allXL	AA	373	382	BS2G	20.13
**4761**	allXL	AA	370	386	BS2G	30.81
**4761**	allXL	AA	373	386	BS2G	28.32
**4761**	allXL	CC	319	357	BS2G	25.35
**4761**	allXL	BB	367	373	BS2G	12.95
**4761**	allXL	AA	370	382	BS2G	24.77
**4761**	allXL	CC	376	382	BS2G	15.08
**4761**	allXL	BB	357	373	BS2G	21.16
**4761**	allXL	DD	320	366	BS2G	24.28
**4761**	allXL	CC	370	376	BS2G	16.44
**4761**	allXL	CC	320	357	DBSU	25.02
**4761**	allXL	CC	357	319	DBSU	25.35
**4761**	allXL	BB	372	319	DBSU	22.84
**4761**	allXL	CC	370	357	DBSU	23.47
**4761**	allXL	BB	382	357	DBSU	25.63
**4761**	allXL	AA	372	370	DBSU	6.18
**4761**	allXL	DD	381	370	DBSU	24.49
**4761**	allXL	AA	373	370	DBSU	8.55
**4761**	allXL	DD	381	372	DBSU	19.92
**4761**	allXL	AA	372	382	DBSU	21.55
**4761**	allXL	AA	372	386	DBSU	28.34
**4761**	allXL	AA	373	386	DBSU	28.32
**4761**	allXL	AD	381	386	DBSU	59.64
**4761**	allXL	BD;DB	357	357	DBSU	17.63

**4761**	allXL	AD	373	319	DBSU	32.26
**4761**	allXL	CA	372	357	DBSU	32.61
**4761**	allXL	BD	381	357	DBSU	36.35
**4761**	allXL	CA	373	357	DBSU	32.36
**4761**	allXL	CA	372	370	DBSU	47.4
**4761**	allXL	CA	373	370	DBSU	47.37
**4761**	allXL	AC	382	373	DBSU	48.4
**4762**	allXL	BB	320	357	BS2G	25.08
**4762**	allXL	DD	320	373	BS2G	16.77
**4762**	allXL	DD	373	382	BS2G	17.15
**4762**	allXL	DD	370	386	BS2G	18.69
**4762**	allXL	DD	373	386	BS2G	20.0
**4762**	allXL	AA	319	357	BS2G	26.42
**4762**	allXL	AA	367	373	BS2G	13.82
**4762**	allXL	DD	370	382	BS2G	18.37
**4762**	allXL	BB	376	382	BS2G	14.86
**4762**	allXL	DD	357	373	BS2G	22.05
**4762**	allXL	BB	320	366	BS2G	21.5
**4762**	allXL	CC	370	376	BS2G	16.16
**4762**	allXL	BB	320	357	DBSU	25.08
**4762**	allXL	AA	357	319	DBSU	26.42
**4762**	allXL	DD	372	319	DBSU	21.8
**4762**	allXL	DD	370	357	DBSU	17.06
**4762**	allXL	BB	382	357	DBSU	26.09
**4762**	allXL	AA	372	370	DBSU	6.11
**4762**	allXL	DD	381	370	DBSU	17.77
**4762**	allXL	CC	373	370	DBSU	9.15
**4762**	allXL	DD	381	372	DBSU	16.47
**4762**	allXL	DD	372	382	DBSU	17.42
**4762**	allXL	DD	372	386	DBSU	18.45
**4762**	allXL	DD	373	386	DBSU	20.0
**4762**	allXL	DB	381	386	DBSU	48.4
**4762**	allXL	BD;DB	357	357	DBSU	18.76
**4762**	allXL	CB	373	319	DBSU	18.41

**4762**	allXL	DB	372	357	DBSU	35.7
**4762**	allXL	BD	381	357	DBSU	35.14
**4762**	allXL	DB	373	357	DBSU	37.57
**4762**	allXL	CB	372	370	DBSU	49.2
**4762**	allXL	CB	373	370	DBSU	47.21
**4762**	allXL	BD	382	373	DBSU	46.29
**4763**	allXL	AA	320	357	BS2G	26.15
**4763**	allXL	BB	320	373	BS2G	17.52
**4763**	allXL	BB	373	382	BS2G	14.52
**4763**	allXL	BB	370	386	BS2G	26.63
**4763**	allXL	BB	373	386	BS2G	24.67
**4763**	allXL	AA	319	357	BS2G	26.67
**4763**	allXL	AA	367	373	BS2G	12.99
**4763**	allXL	BB	370	382	BS2G	20.04
**4763**	allXL	BB	376	382	BS2G	13.56
**4763**	allXL	BB	357	373	BS2G	27.22
**4763**	allXL	DD	320	366	BS2G	24.24
**4763**	allXL	CC	370	376	BS2G	17.06
**4763**	allXL	AA	320	357	DBSU	26.15
**4763**	allXL	AA	357	319	DBSU	26.67
**4763**	allXL	DD	372	319	DBSU	20.13
**4763**	allXL	DD	370	357	DBSU	23.17
**4763**	allXL	BB	382	357	DBSU	29.08
**4763**	allXL	AA	372	370	DBSU	6.18
**4763**	allXL	BB	381	370	DBSU	21.09
**4763**	allXL	AA	373	370	DBSU	8.96
**4763**	allXL	BB	381	372	DBSU	16.92
**4763**	allXL	BB	372	382	DBSU	15.78
**4763**	allXL	BB	372	386	DBSU	24.72
**4763**	allXL	BB	373	386	DBSU	24.67
**4763**	allXL	DB	381	386	DBSU	58.23
**4763**	allXL	BD;DB	357	357	DBSU	18.93
**4763**	allXL	AD	373	319	DBSU	31.96
**4763**	allXL	DB	372	357	DBSU	40.23

**4763**	allXL	AC	381	357	DBSU	40.46
**4763**	allXL	DB	373	357	DBSU	40.67
**4763**	allXL	DA	372	370	DBSU	48.32
**4763**	allXL	AD	373	370	DBSU	50.6
**4763**	allXL	AD	382	373	DBSU	54.12
**4764**	allXL	AA	320	357	BS2G	23.46
**4764**	allXL	BB	320	373	BS2G	7.84
**4764**	allXL	BB	373	382	BS2G	18.46
**4764**	allXL	DD	370	386	BS2G	23.68
**4764**	allXL	DD	373	386	BS2G	24.31
**4764**	allXL	AA	319	357	BS2G	21.6
**4764**	allXL	AA	367	373	BS2G	13.48
**4764**	allXL	BB	370	382	BS2G	18.56
**4764**	allXL	BB	376	382	BS2G	13.97
**4764**	allXL	BB	357	373	BS2G	23.14
**4764**	allXL	BB	320	366	BS2G	19.84
**4764**	allXL	BB	370	376	BS2G	16.26
**4764**	allXL	AA	320	357	DBSU	23.46
**4764**	allXL	AA	357	319	DBSU	21.6
**4764**	allXL	BB	372	319	DBSU	11.67
**4764**	allXL	BB	370	357	DBSU	21.81
**4764**	allXL	DD	382	357	DBSU	30.57
**4764**	allXL	AA	372	370	DBSU	6.28
**4764**	allXL	BB	381	370	DBSU	21.23
**4764**	allXL	DD	373	370	DBSU	8.87
**4764**	allXL	BB	381	372	DBSU	22.01
**4764**	allXL	BB	372	382	DBSU	20.45
**4764**	allXL	DD	372	386	DBSU	26.48
**4764**	allXL	DD	373	386	DBSU	24.31
**4764**	allXL	AD	381	386	DBSU	62.73
**4764**	allXL	BD;DB	357	357	DBSU	18.44
**4764**	allXL	AD	373	319	DBSU	27.99
**4764**	allXL	BD	372	357	DBSU	36.11
**4764**	allXL	DB	381	357	DBSU	44.39

**4764**	allXL	BD	373	357	DBSU	37.07
**4764**	allXL	AD	372	370	DBSU	47.79
**4764**	allXL	AD	373	370	DBSU	51.37
**4764**	allXL	DA	382	373	DBSU	61.52
**4765**	allXL	AA	320	357	BS2G	21.36
**4765**	allXL	DD	320	373	BS2G	15.56
**4765**	allXL	CC	373	382	BS2G	20.78
**4765**	allXL	DD	370	386	BS2G	29.93
**4765**	allXL	DD	373	386	BS2G	28.71
**4765**	allXL	AA	319	357	BS2G	18.81
**4765**	allXL	AA	367	373	BS2G	14.57
**4765**	allXL	DD	370	382	BS2G	25.28
**4765**	allXL	AA	376	382	BS2G	15.13
**4765**	allXL	DD	357	373	BS2G	32.95
**4765**	allXL	BB	320	366	BS2G	13.39
**4765**	allXL	CC	370	376	BS2G	16.68
**4765**	allXL	AA	320	357	DBSU	21.36
**4765**	allXL	AA	357	319	DBSU	18.81
**4765**	allXL	DD	372	319	DBSU	15.11
**4765**	allXL	DD	370	357	DBSU	25.06
**4765**	allXL	DD	382	357	DBSU	39.81
**4765**	allXL	AA	372	370	DBSU	6.13
**4765**	allXL	DD	381	370	DBSU	22.66
**4765**	allXL	DD	373	370	DBSU	9.11
**4765**	allXL	CC	381	372	DBSU	19.77
**4765**	allXL	CC	372	382	DBSU	21.73
**4765**	allXL	DD	372	386	DBSU	31.5
**4765**	allXL	DD	373	386	DBSU	28.71
**4765**	allXL	AD	381	386	DBSU	30.5
**4765**	allXL	BD;DB	357	357	DBSU	19.28
**4765**	allXL	AD	373	319	DBSU	19.51
**4765**	allXL	AC	372	357	DBSU	37.0
**4765**	allXL	AD	381	357	DBSU	47.46
**4765**	allXL	AC	373	357	DBSU	39.35

**4765**	allXL	AD	372	370	DBSU	31.64
**4765**	allXL	AD	373	370	DBSU	28.27
**4765**	allXL	AD	382	373	DBSU	19.12
**4766**	allXL	BB	320	357	BS2G	25.62
**4766**	allXL	CC	320	373	BS2G	28.0
**4766**	allXL	BB	373	382	BS2G	19.65
**4766**	allXL	CC	370	386	BS2G	20.92
**4766**	allXL	BB	373	386	BS2G	22.03
**4766**	allXL	BB	319	357	BS2G	27.03
**4766**	allXL	AA	367	373	BS2G	12.25
**4766**	allXL	CC	370	382	BS2G	18.47
**4766**	allXL	BB	376	382	BS2G	14.27
**4766**	allXL	CC	357	373	BS2G	21.63
**4766**	allXL	CC	320	366	BS2G	24.18
**4766**	allXL	CC	370	376	BS2G	14.35
**4766**	allXL	BB	320	357	DBSU	25.62
**4766**	allXL	BB	357	319	DBSU	27.03
**4766**	allXL	BB	372	319	DBSU	27.52
**4766**	allXL	CC	370	357	DBSU	23.79
**4766**	allXL	CC	382	357	DBSU	13.49
**4766**	allXL	AA	372	370	DBSU	6.38
**4766**	allXL	CC	381	370	DBSU	17.65
**4766**	allXL	AA	373	370	DBSU	9.18
**4766**	allXL	CC	381	372	DBSU	20.61
**4766**	allXL	BB	372	382	DBSU	21.1
**4766**	allXL	BB	372	386	DBSU	21.65
**4766**	allXL	BB	373	386	DBSU	22.03
**4766**	allXL	CA	381	386	DBSU	40.12
**4766**	allXL	BD;DB	357	357	DBSU	18.53
**4766**	allXL	AD	373	319	DBSU	28.69
**4766**	allXL	CA	372	357	DBSU	35.09
**4766**	allXL	CA	381	357	DBSU	29.13
**4766**	allXL	CA	373	357	DBSU	32.05
**4766**	allXL	CA	372	370	DBSU	58.3

**4766**	allXL	CA	373	370	DBSU	54.85
**4766**	allXL	AC	382	373	DBSU	31.28
**4767**	allXL	AA	320	357	BS2G	25.88
**4767**	allXL	DD	320	373	BS2G	22.63
**4767**	allXL	BB	373	382	BS2G	19.52
**4767**	allXL	DD	370	386	BS2G	20.35
**4767**	allXL	DD	373	386	BS2G	21.82
**4767**	allXL	AA	319	357	BS2G	25.93
**4767**	allXL	AA	367	373	BS2G	11.37
**4767**	allXL	CC	370	382	BS2G	20.56
**4767**	allXL	BB	376	382	BS2G	13.16
**4767**	allXL	DD	357	373	BS2G	26.82
**4767**	allXL	DD	320	366	BS2G	13.88
**4767**	allXL	CC	370	376	BS2G	13.02
**4767**	allXL	AA	320	357	DBSU	25.88
**4767**	allXL	AA	357	319	DBSU	25.93
**4767**	allXL	CC	372	319	DBSU	22.85
**4767**	allXL	DD	370	357	DBSU	21.8
**4767**	allXL	DD	382	357	DBSU	38.71
**4767**	allXL	CC	372	370	DBSU	6.63
**4767**	allXL	DD	381	370	DBSU	22.12
**4767**	allXL	CC	373	370	DBSU	9.2
**4767**	allXL	DD	381	372	DBSU	20.97
**4767**	allXL	DD	372	382	DBSU	21.5
**4767**	allXL	DD	372	386	DBSU	21.24
**4767**	allXL	DD	373	386	DBSU	21.82
**4767**	allXL	CB	381	386	DBSU	41.18
**4767**	allXL	BD;DB	357	357	DBSU	18.72
**4767**	allXL	CB	373	319	DBSU	24.47
**4767**	allXL	CB	372	357	DBSU	21.23
**4767**	allXL	CB	381	357	DBSU	26.44
**4767**	allXL	CB	373	357	DBSU	19.51
**4767**	allXL	CB	372	370	DBSU	32.92
**4767**	allXL	CB	373	370	DBSU	30.83

**4767**	allXL	CB	382	373	DBSU	23.01
**4768**	allXL	AA	320	357	BS2G	26.0
**4768**	allXL	BB	320	373	BS2G	17.78
**4768**	allXL	DD	373	382	BS2G	16.03
**4768**	allXL	DD	370	386	BS2G	25.41
**4768**	allXL	DD	373	386	BS2G	26.62
**4768**	allXL	AA	319	357	BS2G	25.19
**4768**	allXL	AA	367	373	BS2G	12.75
**4768**	allXL	DD	370	382	BS2G	17.44
**4768**	allXL	BB	376	382	BS2G	14.14
**4768**	allXL	BB	357	373	BS2G	22.32
**4768**	allXL	BB	320	366	BS2G	20.67
**4768**	allXL	CC	370	376	BS2G	11.78
**4768**	allXL	AA	320	357	DBSU	26.0
**4768**	allXL	AA	357	319	DBSU	25.19
**4768**	allXL	BB	372	319	DBSU	21.54
**4768**	allXL	BB	370	357	DBSU	25.92
**4768**	allXL	AA	382	357	DBSU	40.33
**4768**	allXL	AA	372	370	DBSU	6.45
**4768**	allXL	CC	381	370	DBSU	15.7
**4768**	allXL	AA	373	370	DBSU	9.02
**4768**	allXL	DD	381	372	DBSU	14.93
**4768**	allXL	DD	372	382	DBSU	15.13
**4768**	allXL	DD	372	386	DBSU	24.7
**4768**	allXL	DD	373	386	DBSU	26.62
**4768**	allXL	BC	381	386	DBSU	41.1
**4768**	allXL	BD;DB	357	357	DBSU	18.64
**4768**	allXL	DA	373	319	DBSU	16.89
**4768**	allXL	DA	372	357	DBSU	37.89
**4768**	allXL	DA	381	357	DBSU	42.22
**4768**	allXL	DA	373	357	DBSU	34.23
**4768**	allXL	DA	372	370	DBSU	27.15
**4768**	allXL	DA	373	370	DBSU	25.06
**4768**	allXL	DA	382	373	DBSU	36.24

**4769**	allXL	AA	320	357	BS2G	23.31
**4769**	allXL	BB	320	373	BS2G	25.56
**4769**	allXL	DD	373	382	BS2G	13.41
**4769**	allXL	DD	370	386	BS2G	20.91
**4769**	allXL	DD	373	386	BS2G	19.02
**4769**	allXL	AA	319	357	BS2G	21.5
**4769**	allXL	AA	367	373	BS2G	13.42
**4769**	allXL	DD	370	382	BS2G	18.68
**4769**	allXL	CC	376	382	BS2G	14.27
**4769**	allXL	BB	357	373	BS2G	24.58
**4769**	allXL	CC	320	366	BS2G	21.61
**4769**	allXL	CC	370	376	BS2G	15.97
**4769**	allXL	AA	320	357	DBSU	23.31
**4769**	allXL	AA	357	319	DBSU	21.5
**4769**	allXL	CC	372	319	DBSU	25.67
**4769**	allXL	DD	370	357	DBSU	24.59
**4769**	allXL	BB	382	357	DBSU	33.85
**4769**	allXL	AA	372	370	DBSU	6.12
**4769**	allXL	DD	381	370	DBSU	20.86
**4769**	allXL	DD	373	370	DBSU	8.69
**4769**	allXL	DD	381	372	DBSU	14.95
**4769**	allXL	DD	372	382	DBSU	12.67
**4769**	allXL	DD	372	386	DBSU	16.06
**4769**	allXL	DD	373	386	DBSU	19.02
**4769**	allXL	DA	381	386	DBSU	45.46
**4769**	allXL	BD;DB	357	357	DBSU	18.72
**4769**	allXL	DA	373	319	DBSU	30.71
**4769**	allXL	DA	372	357	DBSU	40.5
**4769**	allXL	DA	381	357	DBSU	36.33
**4769**	allXL	DA	373	357	DBSU	42.78
**4769**	allXL	DA	372	370	DBSU	37.06
**4769**	allXL	DA	373	370	DBSU	37.37
**4769**	allXL	DA	382	373	DBSU	32.75
**4770**	allXL	AA	320	357	BS2G	24.51

**4770**	allXL	DD	320	373	BS2G	20.82
**4770**	allXL	AA	373	382	BS2G	16.52
**4770**	allXL	DD	370	386	BS2G	18.26
**4770**	allXL	AA	373	386	BS2G	24.05
**4770**	allXL	AA	319	357	BS2G	22.72
**4770**	allXL	AA	367	373	BS2G	13.88
**4770**	allXL	DD	370	382	BS2G	14.19
**4770**	allXL	AA	376	382	BS2G	14.58
**4770**	allXL	DD	357	373	BS2G	27.41
**4770**	allXL	CC	320	366	BS2G	23.39
**4770**	allXL	DD	370	376	BS2G	16.08
**4770**	allXL	AA	320	357	DBSU	24.51
**4770**	allXL	AA	357	319	DBSU	22.72
**4770**	allXL	CC	372	319	DBSU	20.37
**4770**	allXL	DD	370	357	DBSU	26.77
**4770**	allXL	DD	382	357	DBSU	21.57
**4770**	allXL	AA	372	370	DBSU	6.3
**4770**	allXL	DD	381	370	DBSU	15.54
**4770**	allXL	AA	373	370	DBSU	9.21
**4770**	allXL	DD	381	372	DBSU	19.08
**4770**	allXL	DD	372	382	DBSU	18.86
**4770**	allXL	DD	372	386	DBSU	24.43
**4770**	allXL	AA	373	386	DBSU	24.05
**4770**	allXL	AD	381	386	DBSU	57.46
**4770**	allXL	BD;DB	357	357	DBSU	19.24
**4770**	allXL	DA	373	319	DBSU	25.68
**4770**	allXL	DA	372	357	DBSU	37.44
**4770**	allXL	DB	381	357	DBSU	29.94
**4770**	allXL	DA	373	357	DBSU	34.19
**4770**	allXL	CB	372	370	DBSU	42.27
**4770**	allXL	CB	373	370	DBSU	42.61
**4770**	allXL	AD	382	373	DBSU	38.17
**4771**	allXL	CC	320	357	BS2G	20.01
**4771**	allXL	DD	320	373	BS2G	23.09

**4771**	allXL	BB	373	382	BS2G	15.97
**4771**	allXL	BB	370	386	BS2G	20.29
**4771**	allXL	BB	373	386	BS2G	15.56
**4771**	allXL	CC	319	357	BS2G	20.42
**4771**	allXL	AA	367	373	BS2G	12.8
**4771**	allXL	AA	370	382	BS2G	22.03
**4771**	allXL	BB	376	382	BS2G	12.45
**4771**	allXL	BB	357	373	BS2G	29.76
**4771**	allXL	BB	320	366	BS2G	19.75
**4771**	allXL	CC	370	376	BS2G	16.41
**4771**	allXL	CC	320	357	DBSU	20.01
**4771**	allXL	CC	357	319	DBSU	20.42
**4771**	allXL	BB	372	319	DBSU	23.35
**4771**	allXL	DD	370	357	DBSU	23.7
**4771**	allXL	BB	382	357	DBSU	32.41
**4771**	allXL	AA	372	370	DBSU	6.19
**4771**	allXL	DD	381	370	DBSU	22.89
**4771**	allXL	AA	373	370	DBSU	9.07
**4771**	allXL	DD	381	372	DBSU	21.01
**4771**	allXL	BB	372	382	DBSU	18.0
**4771**	allXL	BB	372	386	DBSU	15.56
**4771**	allXL	BB	373	386	DBSU	15.56
**4771**	allXL	DA	381	386	DBSU	52.03
**4771**	allXL	BD;DB	357	357	DBSU	20.04
**4771**	allXL	AD	373	319	DBSU	34.72
**4771**	allXL	BD	372	357	DBSU	40.47
**4771**	allXL	BD	381	357	DBSU	42.33
**4771**	allXL	BD	373	357	DBSU	42.05
**4771**	allXL	DA	372	370	DBSU	52.63
**4771**	allXL	DA	373	370	DBSU	51.57
**4771**	allXL	AD	382	373	DBSU	42.32
**4772**	allXL	CC	320	357	BS2G	23.01
**4772**	allXL	BB	320	373	BS2G	22.3
**4772**	allXL	DD	373	382	BS2G	18.89

**4772**	allXL	AA	370	386	BS2G	29.13
**4772**	allXL	AA	373	386	BS2G	28.97
**4772**	allXL	CC	319	357	BS2G	22.18
**4772**	allXL	AA	367	373	BS2G	13.11
**4772**	allXL	DD	370	382	BS2G	21.3
**4772**	allXL	CC	376	382	BS2G	15.1
**4772**	allXL	DD	357	373	BS2G	22.81
**4772**	allXL	AA	320	366	BS2G	24.98
**4772**	allXL	CC	370	376	BS2G	14.9
**4772**	allXL	CC	320	357	DBSU	23.01
**4772**	allXL	CC	357	319	DBSU	22.18
**4772**	allXL	BB	372	319	DBSU	25.75
**4772**	allXL	DD	370	357	DBSU	19.42
**4772**	allXL	CC	382	357	DBSU	19.47
**4772**	allXL	AA	372	370	DBSU	6.13
**4772**	allXL	DD	381	370	DBSU	19.88
**4772**	allXL	AA	373	370	DBSU	9.17
**4772**	allXL	DD	381	372	DBSU	15.49
**4772**	allXL	DD	372	382	DBSU	18.0
**4772**	allXL	AA	372	386	DBSU	27.94
**4772**	allXL	AA	373	386	DBSU	28.97
**4772**	allXL	CB	381	386	DBSU	47.01
**4772**	allXL	BD;DB	357	357	DBSU	19.03
**4772**	allXL	CB	373	319	DBSU	25.62
**4772**	allXL	DB	372	357	DBSU	24.8
**4772**	allXL	CA	381	357	DBSU	34.17
**4772**	allXL	DB	373	357	DBSU	23.23
**4772**	allXL	DB	372	370	DBSU	40.12
**4772**	allXL	DB	373	370	DBSU	38.1
**4772**	allXL	BD	382	373	DBSU	40.7
**4773**	allXL	CC	320	357	BS2G	23.92
**4773**	allXL	AA	320	373	BS2G	9.61
**4773**	allXL	BB	373	382	BS2G	17.78
**4773**	allXL	AA	370	386	BS2G	26.6

**4773**	allXL	DD	373	386	BS2G	22.48
**4773**	allXL	AA	319	357	BS2G	27.13
**4773**	allXL	AA	367	373	BS2G	13.47
**4773**	allXL	BB	370	382	BS2G	23.59
**4773**	allXL	CC	376	382	BS2G	14.1
**4773**	allXL	BB	357	373	BS2G	18.38
**4773**	allXL	AA	320	366	BS2G	18.78
**4773**	allXL	CC	370	376	BS2G	15.63
**4773**	allXL	CC	320	357	DBSU	23.92
**4773**	allXL	AA	357	319	DBSU	27.13
**4773**	allXL	AA	372	319	DBSU	13.87
**4773**	allXL	BB	370	357	DBSU	26.86
**4773**	allXL	BB	382	357	DBSU	12.84
**4773**	allXL	AA	372	370	DBSU	6.32
**4773**	allXL	BB	381	370	DBSU	24.34
**4773**	allXL	CC	373	370	DBSU	9.21
**4773**	allXL	BB	381	372	DBSU	20.08
**4773**	allXL	BB	372	382	DBSU	18.97
**4773**	allXL	DD	372	386	DBSU	23.74
**4773**	allXL	DD	373	386	DBSU	22.48
**4773**	allXL	BC	381	386	DBSU	40.7
**4773**	allXL	BD;DB	357	357	DBSU	19.35
**4773**	allXL	DA	373	319	DBSU	28.65
**4773**	allXL	CB	372	357	DBSU	36.51
**4773**	allXL	BD	381	357	DBSU	21.63
**4773**	allXL	BD	373	357	DBSU	34.88
**4773**	allXL	DA	372	370	DBSU	17.02
**4773**	allXL	DA	373	370	DBSU	16.36
**4773**	allXL	CB	382	373	DBSU	27.8
**4774**	allXL	CC	320	357	BS2G	22.17
**4774**	allXL	BB	320	373	BS2G	27.9
**4774**	allXL	DD	373	382	BS2G	18.22
**4774**	allXL	DD	370	386	BS2G	26.0
**4774**	allXL	DD	373	386	BS2G	23.26

**4774**	allXL	CC	319	357	BS2G	20.51
**4774**	allXL	BB	367	373	BS2G	13.77
**4774**	allXL	DD	370	382	BS2G	22.62
**4774**	allXL	AA	376	382	BS2G	15.4
**4774**	allXL	BB	357	373	BS2G	31.39
**4774**	allXL	CC	320	366	BS2G	21.08
**4774**	allXL	BB	370	376	BS2G	16.31
**4774**	allXL	CC	320	357	DBSU	22.17
**4774**	allXL	CC	357	319	DBSU	20.51
**4774**	allXL	BB	372	319	DBSU	32.01
**4774**	allXL	DD	370	357	DBSU	32.69
**4774**	allXL	BB	382	357	DBSU	22.98
**4774**	allXL	AA	372	370	DBSU	6.28
**4774**	allXL	BB	381	370	DBSU	22.69
**4774**	allXL	AA	373	370	DBSU	8.93
**4774**	allXL	BB	381	372	DBSU	19.85
**4774**	allXL	DD	372	382	DBSU	20.97
**4774**	allXL	AA	372	386	DBSU	24.48
**4774**	allXL	DD	373	386	DBSU	23.26
**4774**	allXL	AD	381	386	DBSU	22.76
**4774**	allXL	BD;DB	357	357	DBSU	19.68
**4774**	allXL	AD	373	319	DBSU	16.95
**4774**	allXL	CB	372	357	DBSU	48.07
**4774**	allXL	BD	381	357	DBSU	37.42
**4774**	allXL	AD	373	357	DBSU	48.18
**4774**	allXL	CB	372	370	DBSU	36.82
**4774**	allXL	CB	373	370	DBSU	38.03
**4774**	allXL	DA	382	373	DBSU	37.51
**4775**	allXL	CC	320	357	BS2G	21.94
**4775**	allXL	BB	320	373	BS2G	25.44
**4775**	allXL	AA	373	382	BS2G	19.66
**4775**	allXL	DD	370	386	BS2G	23.21
**4775**	allXL	DD	373	386	BS2G	23.07
**4775**	allXL	CC	319	357	BS2G	21.82

**4775**	allXL	AA	367	373	BS2G	13.64
**4775**	allXL	BB	370	382	BS2G	22.24
**4775**	allXL	CC	376	382	BS2G	14.09
**4775**	allXL	DD	357	373	BS2G	33.22
**4775**	allXL	DD	320	366	BS2G	18.88
**4775**	allXL	CC	370	376	BS2G	15.11
**4775**	allXL	CC	320	357	DBSU	21.94
**4775**	allXL	CC	357	319	DBSU	21.82
**4775**	allXL	BB	372	319	DBSU	27.51
**4775**	allXL	DD	370	357	DBSU	31.98
**4775**	allXL	DD	382	357	DBSU	23.34
**4775**	allXL	AA	372	370	DBSU	6.15
**4775**	allXL	BB	381	370	DBSU	22.62
**4775**	allXL	DD	373	370	DBSU	9.13
**4775**	allXL	DD	381	372	DBSU	18.51
**4775**	allXL	DD	372	382	DBSU	19.39
**4775**	allXL	DD	372	386	DBSU	21.64
**4775**	allXL	DD	373	386	DBSU	23.07
**4775**	allXL	BC	381	386	DBSU	33.76
**4775**	allXL	BD;DB	357	357	DBSU	19.76
**4775**	allXL	CB	373	319	DBSU	24.69
**4775**	allXL	CB	372	357	DBSU	34.02
**4775**	allXL	CB	381	357	DBSU	39.99
**4775**	allXL	CB	373	357	DBSU	37.56
**4775**	allXL	CB	372	370	DBSU	14.1
**4775**	allXL	CB	373	370	DBSU	14.84
**4775**	allXL	CB	382	373	DBSU	16.79
**4776**	allXL	BB	320	357	BS2G	25.31
**4776**	allXL	DD	320	373	BS2G	22.91
**4776**	allXL	DD	373	382	BS2G	17.26
**4776**	allXL	DD	370	386	BS2G	15.57
**4776**	allXL	DD	373	386	BS2G	24.38
**4776**	allXL	AA	319	357	BS2G	28.24
**4776**	allXL	AA	367	373	BS2G	10.76

**4776**	allXL	DD	370	382	BS2G	11.86
**4776**	allXL	BB	376	382	BS2G	14.44
**4776**	allXL	DD	357	373	BS2G	30.88
**4776**	allXL	BB	320	366	BS2G	21.51
**4776**	allXL	CC	370	376	BS2G	14.58
**4776**	allXL	BB	320	357	DBSU	25.31
**4776**	allXL	AA	357	319	DBSU	28.24
**4776**	allXL	CC	372	319	DBSU	28.57
**4776**	allXL	CC	370	357	DBSU	30.02
**4776**	allXL	DD	382	357	DBSU	25.25
**4776**	allXL	AA	372	370	DBSU	6.42
**4776**	allXL	DD	381	370	DBSU	11.99
**4776**	allXL	AA	373	370	DBSU	8.65
**4776**	allXL	DD	381	372	DBSU	16.18
**4776**	allXL	DD	372	382	DBSU	17.3
**4776**	allXL	DD	372	386	DBSU	22.82
**4776**	allXL	DD	373	386	DBSU	24.38
**4776**	allXL	DA	381	386	DBSU	59.73
**4776**	allXL	BD;DB	357	357	DBSU	18.73
**4776**	allXL	CB	373	319	DBSU	24.88
**4776**	allXL	CB	372	357	DBSU	40.26
**4776**	allXL	DB	381	357	DBSU	39.19
**4776**	allXL	CB	373	357	DBSU	39.59
**4776**	allXL	DA	372	370	DBSU	30.75
**4776**	allXL	DA	373	370	DBSU	27.93
**4776**	allXL	DA	382	373	DBSU	37.58
**4777**	allXL	AA	320	357	BS2G	17.05
**4777**	allXL	CC	320	373	BS2G	22.04
**4777**	allXL	DD	373	382	BS2G	19.46
**4777**	allXL	BB	370	386	BS2G	24.28
**4777**	allXL	DD	373	386	BS2G	24.49
**4777**	allXL	AA	319	357	BS2G	13.93
**4777**	allXL	DD	367	373	BS2G	12.3
**4777**	allXL	BB	370	382	BS2G	18.29

**4777**	allXL	CC	376	382	BS2G	13.89
**4777**	allXL	DD	357	373	BS2G	20.03
**4777**	allXL	AA	320	366	BS2G	22.35
**4777**	allXL	BB	370	376	BS2G	14.66
**4777**	allXL	AA	320	357	DBSU	17.05
**4777**	allXL	AA	357	319	DBSU	13.93
**4777**	allXL	CC	372	319	DBSU	22.24
**4777**	allXL	DD	370	357	DBSU	25.62
**4777**	allXL	DD	382	357	DBSU	33.97
**4777**	allXL	AA	372	370	DBSU	6.41
**4777**	allXL	BB	381	370	DBSU	19.56
**4777**	allXL	DD	373	370	DBSU	8.75
**4777**	allXL	DD	381	372	DBSU	19.25
**4777**	allXL	DD	372	382	DBSU	19.15
**4777**	allXL	DD	372	386	DBSU	22.8
**4777**	allXL	DD	373	386	DBSU	24.49
**4777**	allXL	DA	381	386	DBSU	29.74
**4777**	allXL	BD;DB	357	357	DBSU	18.88
**4777**	allXL	AD	373	319	DBSU	33.44
**4777**	allXL	CB	372	357	DBSU	37.85
**4777**	allXL	DA	381	357	DBSU	42.39
**4777**	allXL	DB	373	357	DBSU	36.42
**4777**	allXL	BC	372	370	DBSU	25.81
**4777**	allXL	BC	373	370	DBSU	26.68
**4777**	allXL	CB	382	373	DBSU	20.37
**4778**	allXL	AA	320	357	BS2G	23.33
**4778**	allXL	AA	320	373	BS2G	25.37
**4778**	allXL	BB	373	382	BS2G	18.29
**4778**	allXL	CC	370	386	BS2G	20.67
**4778**	allXL	CC	373	386	BS2G	24.16
**4778**	allXL	CC	319	357	BS2G	23.75
**4778**	allXL	AA	367	373	BS2G	13.39
**4778**	allXL	BB	370	382	BS2G	19.95
**4778**	allXL	CC	376	382	BS2G	13.68

**4778**	allXL	BB	357	373	BS2G	22.42
**4778**	allXL	AA	320	366	BS2G	20.66
**4778**	allXL	CC	370	376	BS2G	15.3
**4778**	allXL	AA	320	357	DBSU	23.33
**4778**	allXL	CC	357	319	DBSU	23.75
**4778**	allXL	CC	372	319	DBSU	26.97
**4778**	allXL	DD	370	357	DBSU	25.19
**4778**	allXL	CC	382	357	DBSU	19.48
**4778**	allXL	AA	372	370	DBSU	6.33
**4778**	allXL	BB	381	370	DBSU	22.59
**4778**	allXL	AA	373	370	DBSU	8.88
**4778**	allXL	DD	381	372	DBSU	17.13
**4778**	allXL	DD	372	382	DBSU	19.11
**4778**	allXL	CC	372	386	DBSU	22.56
**4778**	allXL	CC	373	386	DBSU	24.16
**4778**	allXL	BD	381	386	DBSU	47.44
**4778**	allXL	BD;DB	357	357	DBSU	18.99
**4778**	allXL	CB	373	319	DBSU	20.45
**4778**	allXL	CB	372	357	DBSU	33.18
**4778**	allXL	CA	381	357	DBSU	32.31
**4778**	allXL	CB	373	357	DBSU	30.68
**4778**	allXL	CB	372	370	DBSU	42.48
**4778**	allXL	CB	373	370	DBSU	39.78
**4778**	allXL	DB	382	373	DBSU	50.89
**4779**	allXL	AA	320	357	BS2G	26.9
**4779**	allXL	DD	320	373	BS2G	23.27
**4779**	allXL	BB	373	382	BS2G	16.87
**4779**	allXL	DD	370	386	BS2G	22.98
**4779**	allXL	DD	373	386	BS2G	22.24
**4779**	allXL	AA	319	357	BS2G	27.76
**4779**	allXL	AA	367	373	BS2G	11.96
**4779**	allXL	DD	370	382	BS2G	16.24
**4779**	allXL	BB	376	382	BS2G	12.54
**4779**	allXL	BB	357	373	BS2G	27.52

**4779**	allXL	DD	320	366	BS2G	18.92
**4779**	allXL	DD	370	376	BS2G	14.37
**4779**	allXL	AA	320	357	DBSU	26.9
**4779**	allXL	AA	357	319	DBSU	27.76
**4779**	allXL	DD	372	319	DBSU	17.87
**4779**	allXL	DD	370	357	DBSU	25.96
**4779**	allXL	DD	382	357	DBSU	32.0
**4779**	allXL	AA	372	370	DBSU	6.34
**4779**	allXL	DD	381	370	DBSU	14.32
**4779**	allXL	AA	373	370	DBSU	9.09
**4779**	allXL	DD	381	372	DBSU	16.47
**4779**	allXL	DD	372	382	DBSU	18.68
**4779**	allXL	DD	372	386	DBSU	22.35
**4779**	allXL	DD	373	386	DBSU	22.24
**4779**	allXL	CB	381	386	DBSU	32.0
**4779**	allXL	BD;DB	357	357	DBSU	18.7
**4779**	allXL	AD	373	319	DBSU	29.92
**4779**	allXL	CB	372	357	DBSU	41.0
**4779**	allXL	CB	381	357	DBSU	48.41
**4779**	allXL	CB	373	357	DBSU	42.95
**4779**	allXL	CB	372	370	DBSU	37.06
**4779**	allXL	CB	373	370	DBSU	36.46
**4779**	allXL	CB	382	373	DBSU	37.63
**4780**	allXL	CC	320	357	BS2G	22.19
**4780**	allXL	AA	320	373	BS2G	16.18
**4780**	allXL	DD	373	382	BS2G	12.15
**4780**	allXL	DD	370	386	BS2G	20.96
**4780**	allXL	DD	373	386	BS2G	20.8
**4780**	allXL	CC	319	357	BS2G	24.46
**4780**	allXL	AA	367	373	BS2G	13.26
**4780**	allXL	DD	370	382	BS2G	14.41
**4780**	allXL	DD	376	382	BS2G	14.72
**4780**	allXL	BB	357	373	BS2G	23.44
**4780**	allXL	BB	320	366	BS2G	16.53

**4780**	allXL	BB	370	376	BS2G	16.48
**4780**	allXL	CC	320	357	DBSU	22.19
**4780**	allXL	CC	357	319	DBSU	24.46
**4780**	allXL	DD	372	319	DBSU	17.97
**4780**	allXL	BB	370	357	DBSU	25.92
**4780**	allXL	BB	382	357	DBSU	30.75
**4780**	allXL	AA	372	370	DBSU	6.29
**4780**	allXL	DD	381	370	DBSU	15.68
**4780**	allXL	AA;DD	373	370	DBSU	9.03
**4780**	allXL	DD	381	372	DBSU	11.38
**4780**	allXL	DD	372	382	DBSU	10.14
**4780**	allXL	DD	372	386	DBSU	17.61
**4780**	allXL	DD	373	386	DBSU	20.8
**4780**	allXL	CB	381	386	DBSU	42.44
**4780**	allXL	BD;DB	357	357	DBSU	18.84
**4780**	allXL	CB	373	319	DBSU	13.65
**4780**	allXL	CB	372	357	DBSU	31.38
**4780**	allXL	DA	381	357	DBSU	34.56
**4780**	allXL	DA	373	357	DBSU	30.61
**4780**	allXL	DA	372	370	DBSU	22.18
**4780**	allXL	DA	373	370	DBSU	21.68
**4780**	allXL	BC	382	373	DBSU	22.21
**4781**	allXL	BB	320	357	BS2G	25.41
**4781**	allXL	AA	320	373	BS2G	10.81
**4781**	allXL	AA	373	382	BS2G	18.63
**4781**	allXL	AA	370	386	BS2G	29.53
**4781**	allXL	AA	373	386	BS2G	26.24
**4781**	allXL	BB	319	357	BS2G	26.19
**4781**	allXL	AA	367	373	BS2G	13.91
**4781**	allXL	DD	370	382	BS2G	22.32
**4781**	allXL	AA	376	382	BS2G	14.56
**4781**	allXL	BB	357	373	BS2G	12.43
**4781**	allXL	CC	320	366	BS2G	12.78
**4781**	allXL	CC	370	376	BS2G	15.25

**4781**	allXL	BB	320	357	DBSU	25.41
**4781**	allXL	BB	357	319	DBSU	26.19
**4781**	allXL	AA	372	319	DBSU	17.88
**4781**	allXL	BB	370	357	DBSU	18.44
**4781**	allXL	BB	382	357	DBSU	19.66
**4781**	allXL	AA	372	370	DBSU	6.14
**4781**	allXL	DD	381	370	DBSU	19.64
**4781**	allXL	AA	373	370	DBSU	9.14
**4781**	allXL	DD	381	372	DBSU	19.89
**4781**	allXL	AA	372	382	DBSU	21.83
**4781**	allXL	AA	372	386	DBSU	28.79
**4781**	allXL	AA	373	386	DBSU	26.24
**4781**	allXL	BD	381	386	DBSU	36.95
**4781**	allXL	BD;DB	357	357	DBSU	18.59
**4781**	allXL	AD	373	319	DBSU	30.85
**4781**	allXL	BD	372	357	DBSU	17.63
**4781**	allXL	BD	381	357	DBSU	9.71
**4781**	allXL	BD	373	357	DBSU	15.43
**4781**	allXL	BD	372	370	DBSU	30.73
**4781**	allXL	BD	373	370	DBSU	28.03
**4781**	allXL	BD	382	373	DBSU	34.9
**4782**	allXL	BB	320	357	BS2G	25.52
**4782**	allXL	DD	320	373	BS2G	30.78
**4782**	allXL	DD	373	382	BS2G	15.36
**4782**	allXL	BB	370	386	BS2G	25.32
**4782**	allXL	DD	373	386	BS2G	25.26
**4782**	allXL	AA	319	357	BS2G	28.23
**4782**	allXL	AA	367	373	BS2G	12.41
**4782**	allXL	BB	370	382	BS2G	18.54
**4782**	allXL	BB	376	382	BS2G	12.76
**4782**	allXL	BB	357	373	BS2G	27.93
**4782**	allXL	CC	320	366	BS2G	28.1
**4782**	allXL	CC	370	376	BS2G	15.95
**4782**	allXL	BB	320	357	DBSU	25.52

**4782**	allXL	AA	357	319	DBSU	28.23
**4782**	allXL	DD	372	319	DBSU	30.05
**4782**	allXL	DD	370	357	DBSU	26.18
**4782**	allXL	BB	382	357	DBSU	20.42
**4782**	allXL	AA	372	370	DBSU	6.26
**4782**	allXL	DD	381	370	DBSU	19.68
**4782**	allXL	AA	373	370	DBSU	9.0
**4782**	allXL	DD	381	372	DBSU	14.25
**4782**	allXL	DD	372	382	DBSU	14.23
**4782**	allXL	DD	372	386	DBSU	23.0
**4782**	allXL	DD	373	386	DBSU	25.26
**4782**	allXL	CB	381	386	DBSU	56.94
**4782**	allXL	BD;DB	357	357	DBSU	18.92
**4782**	allXL	CB	373	319	DBSU	25.66
**4782**	allXL	BD	372	357	DBSU	41.88
**4782**	allXL	BD	381	357	DBSU	25.57
**4782**	allXL	BD	373	357	DBSU	38.78
**4782**	allXL	BC	372	370	DBSU	50.71
**4782**	allXL	BC	373	370	DBSU	51.39
**4782**	allXL	DA	382	373	DBSU	51.29
**4783**	allXL	BB	320	357	BS2G	24.0
**4783**	allXL	BB	320	373	BS2G	19.7
**4783**	allXL	DD	373	382	BS2G	19.62
**4783**	allXL	DD	370	386	BS2G	16.1
**4783**	allXL	DD	373	386	BS2G	21.4
**4783**	allXL	AA	319	357	BS2G	23.61
**4783**	allXL	AA	367	373	BS2G	12.0
**4783**	allXL	DD	370	382	BS2G	19.42
**4783**	allXL	CC	376	382	BS2G	14.76
**4783**	allXL	BB	357	373	BS2G	25.96
**4783**	allXL	BB	320	366	BS2G	12.56
**4783**	allXL	CC	370	376	BS2G	14.22
**4783**	allXL	BB	320	357	DBSU	24.0
**4783**	allXL	AA	357	319	DBSU	23.61

**4783**	allXL	BB	372	319	DBSU	19.45
**4783**	allXL	DD	370	357	DBSU	18.35
**4783**	allXL	BB	382	357	DBSU	16.24
**4783**	allXL	AA	372	370	DBSU	6.46
**4783**	allXL	DD	381	370	DBSU	20.16
**4783**	allXL	AA	373	370	DBSU	9.03
**4783**	allXL	DD	381	372	DBSU	20.18
**4783**	allXL	DD	372	382	DBSU	20.7
**4783**	allXL	DD	372	386	DBSU	20.36
**4783**	allXL	DD	373	386	DBSU	21.4
**4783**	allXL	BD	381	386	DBSU	47.78
**4783**	allXL	BD;DB	357	357	DBSU	18.74
**4783**	allXL	AD	373	319	DBSU	28.7
**4783**	allXL	DB	372	357	DBSU	38.39
**4783**	allXL	BD	381	357	DBSU	25.03
**4783**	allXL	DB	373	357	DBSU	41.9
**4783**	allXL	BC	372	370	DBSU	59.21
**4783**	allXL	AD	373	370	DBSU	57.08
**4783**	allXL	BD	382	373	DBSU	50.07
**4784**	allXL	CC	320	357	BS2G	24.37
**4784**	allXL	DD	320	373	BS2G	18.05
**4784**	allXL	CC	373	382	BS2G	16.99
**4784**	allXL	DD	370	386	BS2G	17.53
**4784**	allXL	DD	373	386	BS2G	25.04
**4784**	allXL	AA	319	357	BS2G	26.69
**4784**	allXL	AA	367	373	BS2G	12.28
**4784**	allXL	DD	370	382	BS2G	16.55
**4784**	allXL	CC	376	382	BS2G	14.8
**4784**	allXL	DD	357	373	BS2G	28.83
**4784**	allXL	AA	320	366	BS2G	12.72
**4784**	allXL	BB	370	376	BS2G	15.2
**4784**	allXL	CC	320	357	DBSU	24.37
**4784**	allXL	AA	357	319	DBSU	26.69
**4784**	allXL	AA	372	319	DBSU	17.36

**4784**	allXL	AA	370	357	DBSU	25.56
**4784**	allXL	DD	382	357	DBSU	40.8
**4784**	allXL	AA	372	370	DBSU	6.34
**4784**	allXL	DD	381	370	DBSU	16.13
**4784**	allXL	AA	373	370	DBSU	8.85
**4784**	allXL	DD	381	372	DBSU	18.27
**4784**	allXL	CC	372	382	DBSU	19.59
**4784**	allXL	DD	372	386	DBSU	23.79
**4784**	allXL	DD	373	386	DBSU	25.04
**4784**	allXL	AD	381	386	DBSU	30.42
**4784**	allXL	BD;DB	357	357	DBSU	18.48
**4784**	allXL	BC	373	319	DBSU	28.92
**4784**	allXL	BC	372	357	DBSU	30.28
**4784**	allXL	DA	381	357	DBSU	29.33
**4784**	allXL	BC	373	357	DBSU	29.58
**4784**	allXL	AD	372	370	DBSU	37.46
**4784**	allXL	AD	373	370	DBSU	35.72
**4784**	allXL	DA	382	373	DBSU	19.78
**4785**	allXL	BB	320	357	BS2G	26.21
**4785**	allXL	BB	320	373	BS2G	19.01
**4785**	allXL	DD	373	382	BS2G	18.63
**4785**	allXL	DD	370	386	BS2G	16.99
**4785**	allXL	DD	373	386	BS2G	24.96
**4785**	allXL	AA	319	357	BS2G	27.62
**4785**	allXL	BB	367	373	BS2G	13.27
**4785**	allXL	DD	370	382	BS2G	13.78
**4785**	allXL	BB	376	382	BS2G	14.09
**4785**	allXL	CC	357	373	BS2G	19.54
**4785**	allXL	BB	320	366	BS2G	15.04
**4785**	allXL	DD	370	376	BS2G	15.56
**4785**	allXL	BB	320	357	DBSU	26.21
**4785**	allXL	AA	357	319	DBSU	27.62
**4785**	allXL	BB	372	319	DBSU	17.02
**4785**	allXL	CC	370	357	DBSU	21.97

**4785**	allXL	DD	382	357	DBSU	28.0
**4785**	allXL	AA	372	370	DBSU	6.21
**4785**	allXL	DD	381	370	DBSU	12.61
**4785**	allXL	AA	373	370	DBSU	9.17
**4785**	allXL	DD	381	372	DBSU	16.84
**4785**	allXL	DD	372	382	DBSU	19.21
**4785**	allXL	DD	372	386	DBSU	24.02
**4785**	allXL	DD	373	386	DBSU	24.96
**4785**	allXL	DB	381	386	DBSU	57.42
**4785**	allXL	BD;DB	357	357	DBSU	18.21
**4785**	allXL	AD	373	319	DBSU	27.4
**4785**	allXL	CA	372	357	DBSU	31.02
**4785**	allXL	CA	381	357	DBSU	32.67
**4785**	allXL	CA	373	357	DBSU	28.7
**4785**	allXL	AD	372	370	DBSU	46.97
**4785**	allXL	DA	373	370	DBSU	44.8
**4785**	allXL	DA	382	373	DBSU	36.79
**4786**	allXL	CC	320	357	BS2G	26.72
**4786**	allXL	DD	320	373	BS2G	13.87
**4786**	allXL	BB	373	382	BS2G	15.32
**4786**	allXL	DD	370	386	BS2G	27.21
**4786**	allXL	BB	373	386	BS2G	25.6
**4786**	allXL	AA	319	357	BS2G	27.08
**4786**	allXL	AA	367	373	BS2G	14.0
**4786**	allXL	DD	370	382	BS2G	17.03
**4786**	allXL	CC	376	382	BS2G	13.37
**4786**	allXL	DD	357	373	BS2G	25.76
**4786**	allXL	AA	320	366	BS2G	20.01
**4786**	allXL	DD	370	376	BS2G	14.54
**4786**	allXL	CC	320	357	DBSU	26.72
**4786**	allXL	AA	357	319	DBSU	27.08
**4786**	allXL	DD	372	319	DBSU	13.67
**4786**	allXL	DD	370	357	DBSU	27.51
**4786**	allXL	DD	382	357	DBSU	35.66

**4786**	allXL	AA	372	370	DBSU	6.11
**4786**	allXL	DD	381	370	DBSU	14.64
**4786**	allXL	AA	373	370	DBSU	9.11
**4786**	allXL	BB	381	372	DBSU	18.11
**4786**	allXL	BB	372	382	DBSU	16.28
**4786**	allXL	BB	372	386	DBSU	25.19
**4786**	allXL	BB	373	386	DBSU	25.6
**4786**	allXL	BC	381	386	DBSU	39.91
**4786**	allXL	BD;DB	357	357	DBSU	19.38
**4786**	allXL	CB	373	319	DBSU	31.35
**4786**	allXL	CB	372	357	DBSU	41.85
**4786**	allXL	CB	381	357	DBSU	41.16
**4786**	allXL	DA	373	357	DBSU	38.48
**4786**	allXL	DA	372	370	DBSU	30.46
**4786**	allXL	DA	373	370	DBSU	28.44
**4786**	allXL	CB	382	373	DBSU	30.87
**4787**	allXL	CC	320	357	BS2G	27.36
**4787**	allXL	BB	320	373	BS2G	25.92
**4787**	allXL	CC	373	382	BS2G	16.11
**4787**	allXL	CC	370	386	BS2G	23.49
**4787**	allXL	CC	373	386	BS2G	21.5
**4787**	allXL	AA	319	357	BS2G	29.06
**4787**	allXL	BB	367	373	BS2G	12.77
**4787**	allXL	BB	370	382	BS2G	18.73
**4787**	allXL	CC	376	382	BS2G	14.27
**4787**	allXL	BB	357	373	BS2G	28.23
**4787**	allXL	DD	320	366	BS2G	18.7
**4787**	allXL	BB	370	376	BS2G	15.21
**4787**	allXL	CC	320	357	DBSU	27.36
**4787**	allXL	AA	357	319	DBSU	29.06
**4787**	allXL	BB	372	319	DBSU	27.9
**4787**	allXL	DD	370	357	DBSU	28.39
**4787**	allXL	BB	382	357	DBSU	30.74
**4787**	allXL	AA	372	370	DBSU	6.1

**4787**	allXL	BB	381	370	DBSU	20.59
**4787**	allXL	AA	373	370	DBSU	9.14
**4787**	allXL	DD	381	372	DBSU	18.84
**4787**	allXL	AA	372	382	DBSU	19.03
**4787**	allXL	CC	372	386	DBSU	22.71
**4787**	allXL	CC	373	386	DBSU	21.5
**4787**	allXL	BC	381	386	DBSU	24.79
**4787**	allXL	BD;DB	357	357	DBSU	18.62
**4787**	allXL	AD	373	319	DBSU	11.29
**4787**	allXL	AC	372	357	DBSU	37.39
**4787**	allXL	BD	381	357	DBSU	44.43
**4787**	allXL	AD	373	357	DBSU	34.56
**4787**	allXL	AD	372	370	DBSU	38.8
**4787**	allXL	AD	373	370	DBSU	35.63
**4787**	allXL	CB	382	373	DBSU	26.83
**4788**	allXL	AA	320	357	BS2G	25.04
**4788**	allXL	DD	320	373	BS2G	19.23
**4788**	allXL	DD	373	382	BS2G	20.12
**4788**	allXL	DD	370	386	BS2G	24.0
**4788**	allXL	CC	373	386	BS2G	21.91
**4788**	allXL	AA	319	357	BS2G	23.66
**4788**	allXL	AA	367	373	BS2G	13.36
**4788**	allXL	DD	370	382	BS2G	16.21
**4788**	allXL	CC	376	382	BS2G	12.43
**4788**	allXL	DD	357	373	BS2G	33.46
**4788**	allXL	AA	320	366	BS2G	19.09
**4788**	allXL	DD	370	376	BS2G	15.44
**4788**	allXL	AA	320	357	DBSU	25.04
**4788**	allXL	AA	357	319	DBSU	23.66
**4788**	allXL	DD	372	319	DBSU	21.99
**4788**	allXL	DD	370	357	DBSU	27.9
**4788**	allXL	DD	382	357	DBSU	41.47
**4788**	allXL	AA	372	370	DBSU	6.4
**4788**	allXL	DD	381	370	DBSU	14.36

**4788**	allXL	CC	373	370	DBSU	9.34
**4788**	allXL	DD	381	372	DBSU	18.06
**4788**	allXL	DD	372	382	DBSU	20.66
**4788**	allXL	CC	372	386	DBSU	25.12
**4788**	allXL	CC	373	386	DBSU	21.91
**4788**	allXL	BC	381	386	DBSU	37.4
**4788**	allXL	BD;DB	357	357	DBSU	19.13
**4788**	allXL	AD	373	319	DBSU	23.76
**4788**	allXL	BD	372	357	DBSU	48.26
**4788**	allXL	AD	381	357	DBSU	59.07
**4788**	allXL	AD	373	357	DBSU	50.78
**4788**	allXL	AD	372	370	DBSU	45.98
**4788**	allXL	AD	373	370	DBSU	43.49
**4788**	allXL	AD	382	373	DBSU	35.17
**4789**	allXL	BB	320	357	BS2G	25.38
**4789**	allXL	BB	320	373	BS2G	24.04
**4789**	allXL	AA	373	382	BS2G	17.52
**4789**	allXL	DD	370	386	BS2G	20.96
**4789**	allXL	AA	373	386	BS2G	23.75
**4789**	allXL	AA	319	357	BS2G	24.82
**4789**	allXL	AA	367	373	BS2G	11.07
**4789**	allXL	DD	370	382	BS2G	16.33
**4789**	allXL	BB	376	382	BS2G	14.03
**4789**	allXL	DD	357	373	BS2G	28.38
**4789**	allXL	BB	320	366	BS2G	18.09
**4789**	allXL	CC	370	376	BS2G	16.06
**4789**	allXL	BB	320	357	DBSU	25.38
**4789**	allXL	AA	357	319	DBSU	24.82
**4789**	allXL	BB	372	319	DBSU	20.87
**4789**	allXL	BB	370	357	DBSU	27.69
**4789**	allXL	AA	382	357	DBSU	20.83
**4789**	allXL	CC	372	370	DBSU	6.58
**4789**	allXL	DD	381	370	DBSU	16.89
**4789**	allXL	CC	373	370	DBSU	9.3

**4789**	allXL	DD	381	372	DBSU	19.42
**4789**	allXL	DD	372	382	DBSU	19.77
**4789**	allXL	DD	372	386	DBSU	24.36
**4789**	allXL	AA	373	386	DBSU	23.75
**4789**	allXL	CB	381	386	DBSU	24.65
**4789**	allXL	BD;DB	357	357	DBSU	19.36
**4789**	allXL	DA	373	319	DBSU	21.11
**4789**	allXL	CB	372	357	DBSU	32.18
**4789**	allXL	CB	381	357	DBSU	32.57
**4789**	allXL	DA	373	357	DBSU	30.84
**4789**	allXL	CB	372	370	DBSU	24.67
**4789**	allXL	CB	373	370	DBSU	23.61
**4789**	allXL	CB	382	373	DBSU	21.63
**4790**	allXL	CC	320	357	BS2G	26.26
**4790**	allXL	DD	320	373	BS2G	20.43
**4790**	allXL	DD	373	382	BS2G	17.89
**4790**	allXL	CC	370	386	BS2G	17.86
**4790**	allXL	DD	373	386	BS2G	24.81
**4790**	allXL	AA	319	357	BS2G	27.74
**4790**	allXL	BB	367	373	BS2G	12.62
**4790**	allXL	CC	370	382	BS2G	14.85
**4790**	allXL	CC	376	382	BS2G	13.77
**4790**	allXL	DD	357	373	BS2G	29.1
**4790**	allXL	AA	320	366	BS2G	13.82
**4790**	allXL	CC	370	376	BS2G	13.39
**4790**	allXL	CC	320	357	DBSU	26.26
**4790**	allXL	AA	357	319	DBSU	27.74
**4790**	allXL	CC	372	319	DBSU	16.67
**4790**	allXL	DD	370	357	DBSU	28.12
**4790**	allXL	CC	382	357	DBSU	33.0
**4790**	allXL	AA	372	370	DBSU	6.04
**4790**	allXL	CC	381	370	DBSU	17.75
**4790**	allXL	AA	373	370	DBSU	9.13
**4790**	allXL	DD	381	372	DBSU	17.61

**4790**	allXL	DD	372	382	DBSU	17.19
**4790**	allXL	CC	372	386	DBSU	23.8
**4790**	allXL	DD	373	386	DBSU	24.81
**4790**	allXL	BC	381	386	DBSU	52.36
**4790**	allXL	BD;DB	357	357	DBSU	18.7
**4790**	allXL	AD	373	319	DBSU	28.92
**4790**	allXL	AD	372	357	DBSU	40.71
**4790**	allXL	CB	381	357	DBSU	24.2
**4790**	allXL	CB	373	357	DBSU	43.83
**4790**	allXL	AD	372	370	DBSU	29.12
**4790**	allXL	DA	373	370	DBSU	29.96
**4790**	allXL	CB	382	373	DBSU	34.49
**4791**	allXL	AA	320	357	BS2G	22.52
**4791**	allXL	DD	320	373	BS2G	25.18
**4791**	allXL	DD	373	382	BS2G	17.49
**4791**	allXL	DD	370	386	BS2G	22.82
**4791**	allXL	DD	373	386	BS2G	23.98
**4791**	allXL	CC	319	357	BS2G	22.9
**4791**	allXL	BB	367	373	BS2G	12.93
**4791**	allXL	DD	370	382	BS2G	20.99
**4791**	allXL	CC	376	382	BS2G	14.64
**4791**	allXL	BB	357	373	BS2G	37.13
**4791**	allXL	BB	320	366	BS2G	20.21
**4791**	allXL	CC	370	376	BS2G	16.86
**4791**	allXL	AA	320	357	DBSU	22.52
**4791**	allXL	CC	357	319	DBSU	22.9
**4791**	allXL	BB	372	319	DBSU	22.23
**4791**	allXL	BB	370	357	DBSU	31.79
**4791**	allXL	DD	382	357	DBSU	32.69
**4791**	allXL	AA	372	370	DBSU	6.14
**4791**	allXL	DD	381	370	DBSU	20.07
**4791**	allXL	AA	373	370	DBSU	9.18
**4791**	allXL	DD	381	372	DBSU	15.23
**4791**	allXL	DD	372	382	DBSU	17.09

**4791**	allXL	DD	372	386	DBSU	21.96
**4791**	allXL	DD	373	386	DBSU	23.98
**4791**	allXL	DA	381	386	DBSU	53.02
**4791**	allXL	BD;DB	357	357	DBSU	18.11
**4791**	allXL	AD	373	319	DBSU	24.66
**4791**	allXL	AD	372	357	DBSU	48.76
**4791**	allXL	DB	381	357	DBSU	51.65
**4791**	allXL	AD	373	357	DBSU	50.79
**4791**	allXL	DA	372	370	DBSU	30.05
**4791**	allXL	DA	373	370	DBSU	26.5
**4791**	allXL	AD	382	373	DBSU	34.4
**4792**	allXL	CC	320	357	BS2G	22.88
**4792**	allXL	BB	320	373	BS2G	21.73
**4792**	allXL	CC	373	382	BS2G	17.2
**4792**	allXL	CC	370	386	BS2G	21.82
**4792**	allXL	AA	373	386	BS2G	24.29
**4792**	allXL	AA	319	357	BS2G	24.66
**4792**	allXL	AA	367	373	BS2G	14.53
**4792**	allXL	CC	370	382	BS2G	18.63
**4792**	allXL	BB	376	382	BS2G	13.18
**4792**	allXL	BB	357	373	BS2G	29.19
**4792**	allXL	CC	320	366	BS2G	24.46
**4792**	allXL	CC	370	376	BS2G	15.98
**4792**	allXL	CC	320	357	DBSU	22.88
**4792**	allXL	AA	357	319	DBSU	24.66
**4792**	allXL	CC	372	319	DBSU	22.56
**4792**	allXL	BB	370	357	DBSU	27.99
**4792**	allXL	BB	382	357	DBSU	28.51
**4792**	allXL	AA	372	370	DBSU	6.21
**4792**	allXL	CC	381	370	DBSU	21.15
**4792**	allXL	AA	373	370	DBSU	9.09
**4792**	allXL	DD	381	372	DBSU	17.92
**4792**	allXL	CC	372	382	DBSU	19.24
**4792**	allXL	AA	372	386	DBSU	23.24

**4792**	allXL	AA	373	386	DBSU	24.29
**4792**	allXL	AD	381	386	DBSU	39.9
**4792**	allXL	BD;DB	357	357	DBSU	19.34
**4792**	allXL	AD	373	319	DBSU	20.11
**4792**	allXL	AD	372	357	DBSU	44.28
**4792**	allXL	BD	381	357	DBSU	46.22
**4792**	allXL	DA	373	357	DBSU	45.19
**4792**	allXL	DA	372	370	DBSU	23.2
**4792**	allXL	DA	373	370	DBSU	25.16
**4792**	allXL	AD	382	373	DBSU	35.1
**4793**	allXL	AA	320	357	BS2G	25.51
**4793**	allXL	BB	320	373	BS2G	24.73
**4793**	allXL	AA	373	382	BS2G	17.36
**4793**	allXL	AA	370	386	BS2G	27.39
**4793**	allXL	AA	373	386	BS2G	25.4
**4793**	allXL	AA	319	357	BS2G	24.2
**4793**	allXL	AA	367	373	BS2G	11.44
**4793**	allXL	AA	370	382	BS2G	22.92
**4793**	allXL	CC	376	382	BS2G	14.21
**4793**	allXL	DD	357	373	BS2G	19.81
**4793**	allXL	BB	320	366	BS2G	21.65
**4793**	allXL	DD	370	376	BS2G	16.34
**4793**	allXL	AA	320	357	DBSU	25.51
**4793**	allXL	AA	357	319	DBSU	24.2
**4793**	allXL	BB	372	319	DBSU	28.89
**4793**	allXL	DD	370	357	DBSU	12.62
**4793**	allXL	BB	382	357	DBSU	22.64
**4793**	allXL	AA	372	370	DBSU	6.74
**4793**	allXL	AA	381	370	DBSU	25.34
**4793**	allXL	AA	373	370	DBSU	9.12
**4793**	allXL	AA	381	372	DBSU	21.77
**4793**	allXL	AA	372	382	DBSU	20.19
**4793**	allXL	AA	372	386	DBSU	27.22
**4793**	allXL	AA	373	386	DBSU	25.4

**4793**	allXL	AC	381	386	DBSU	35.78
**4793**	allXL	BD;DB	357	357	DBSU	18.78
**4793**	allXL	AD	373	319	DBSU	22.74
**4793**	allXL	CA	372	357	DBSU	25.0
**4793**	allXL	BD	381	357	DBSU	30.36
**4793**	allXL	CA	373	357	DBSU	24.83
**4793**	allXL	CA	372	370	DBSU	43.34
**4793**	allXL	CA	373	370	DBSU	40.08
**4793**	allXL	CA	382	373	DBSU	34.47
**4794**	allXL	CC	320	357	BS2G	25.36
**4794**	allXL	DD	320	373	BS2G	19.22
**4794**	allXL	AA	373	382	BS2G	18.94
**4794**	allXL	AA	370	386	BS2G	21.32
**4794**	allXL	DD	373	386	BS2G	20.94
**4794**	allXL	CC	319	357	BS2G	26.6
**4794**	allXL	AA	367	373	BS2G	11.82
**4794**	allXL	AA	370	382	BS2G	19.09
**4794**	allXL	AA	376	382	BS2G	14.99
**4794**	allXL	BB	357	373	BS2G	24.58
**4794**	allXL	BB	320	366	BS2G	19.07
**4794**	allXL	CC	370	376	BS2G	15.71
**4794**	allXL	CC	320	357	DBSU	25.36
**4794**	allXL	CC	357	319	DBSU	26.6
**4794**	allXL	BB	372	319	DBSU	20.29
**4794**	allXL	CC	370	357	DBSU	19.92
**4794**	allXL	BB	382	357	DBSU	35.56
**4794**	allXL	AA	372	370	DBSU	6.4
**4794**	allXL	AA	381	370	DBSU	21.65
**4794**	allXL	AA	373	370	DBSU	9.21
**4794**	allXL	DD	381	372	DBSU	19.33
**4794**	allXL	AA	372	382	DBSU	18.43
**4794**	allXL	DD	372	386	DBSU	18.3
**4794**	allXL	DD	373	386	DBSU	20.94
**4794**	allXL	DA	381	386	DBSU	29.17

**4794**	allXL	BD;DB	357	357	DBSU	19.4
**4794**	allXL	DA	373	319	DBSU	24.2
**4794**	allXL	CA	372	357	DBSU	24.7
**4794**	allXL	DA	381	357	DBSU	27.26
**4794**	allXL	CA	373	357	DBSU	22.97
**4794**	allXL	DA	372	370	DBSU	41.46
**4794**	allXL	DA	373	370	DBSU	41.21
**4794**	allXL	DA	382	373	DBSU	26.28
**4795**	allXL	CC	320	357	BS2G	24.58
**4795**	allXL	AA	320	373	BS2G	20.33
**4795**	allXL	BB	373	382	BS2G	15.19
**4795**	allXL	AA	370	386	BS2G	24.45
**4795**	allXL	AA	373	386	BS2G	24.44
**4795**	allXL	CC	319	357	BS2G	25.75
**4795**	allXL	BB	367	373	BS2G	11.3
**4795**	allXL	BB	370	382	BS2G	18.52
**4795**	allXL	BB	376	382	BS2G	13.36
**4795**	allXL	DD	357	373	BS2G	32.44
**4795**	allXL	AA	320	366	BS2G	14.63
**4795**	allXL	CC	370	376	BS2G	16.33
**4795**	allXL	CC	320	357	DBSU	24.58
**4795**	allXL	CC	357	319	DBSU	25.75
**4795**	allXL	AA	372	319	DBSU	20.74
**4795**	allXL	DD	370	357	DBSU	28.17
**4795**	allXL	BB	382	357	DBSU	30.39
**4795**	allXL	AA	372	370	DBSU	6.2
**4795**	allXL	CC	381	370	DBSU	18.61
**4795**	allXL	AA	373	370	DBSU	8.89
**4795**	allXL	CC	381	372	DBSU	17.65
**4795**	allXL	BB	372	382	DBSU	17.1
**4795**	allXL	BB	372	386	DBSU	26.11
**4795**	allXL	AA	373	386	DBSU	24.44
**4795**	allXL	AD	381	386	DBSU	28.83
**4795**	allXL	BD;DB	357	357	DBSU	18.32

**4795**	allXL	AD	373	319	DBSU	27.15
**4795**	allXL	AD	372	357	DBSU	41.19
**4795**	allXL	BD	381	357	DBSU	39.34
**4795**	allXL	AD	373	357	DBSU	42.74
**4795**	allXL	CB	372	370	DBSU	32.36
**4795**	allXL	CB	373	370	DBSU	32.81
**4795**	allXL	AD	382	373	DBSU	34.66
**4796**	allXL	AA	320	357	BS2G	27.28
**4796**	allXL	CC	320	373	BS2G	21.85
**4796**	allXL	CC	373	382	BS2G	17.36
**4796**	allXL	DD	370	386	BS2G	21.68
**4796**	allXL	CC	373	386	BS2G	25.13
**4796**	allXL	AA	319	357	BS2G	28.07
**4796**	allXL	AA	367	373	BS2G	12.07
**4796**	allXL	CC	370	382	BS2G	19.06
**4796**	allXL	CC	376	382	BS2G	13.35
**4796**	allXL	CC	357	373	BS2G	23.34
**4796**	allXL	AA	320	366	BS2G	16.16
**4796**	allXL	CC	370	376	BS2G	15.21
**4796**	allXL	AA	320	357	DBSU	27.28
**4796**	allXL	AA	357	319	DBSU	28.07
**4796**	allXL	CC	372	319	DBSU	23.03
**4796**	allXL	CC	370	357	DBSU	21.18
**4796**	allXL	AA	382	357	DBSU	20.84
**4796**	allXL	AA	372	370	DBSU	6.32
**4796**	allXL	DD	381	370	DBSU	20.35
**4796**	allXL	AA	373	370	DBSU	8.99
**4796**	allXL	CC	381	372	DBSU	21.46
**4796**	allXL	CC	372	382	DBSU	19.51
**4796**	allXL	CC	372	386	DBSU	25.53
**4796**	allXL	CC	373	386	DBSU	25.13
**4796**	allXL	CA	381	386	DBSU	54.5
**4796**	allXL	BD;DB	357	357	DBSU	18.9
**4796**	allXL	AD	373	319	DBSU	29.24

**4796**	allXL	CA	372	357	DBSU	37.87
**4796**	allXL	AC	381	357	DBSU	36.04
**4796**	allXL	CA	373	357	DBSU	39.84
**4796**	allXL	AD	372	370	DBSU	50.85
**4796**	allXL	AD	373	370	DBSU	48.39
**4796**	allXL	DA	382	373	DBSU	44.02
**4797**	allXL	CC	320	357	BS2G	23.07
**4797**	allXL	DD	320	373	BS2G	20.82
**4797**	allXL	CC	373	382	BS2G	16.31
**4797**	allXL	CC	370	386	BS2G	25.33
**4797**	allXL	CC	373	386	BS2G	24.24
**4797**	allXL	CC	319	357	BS2G	24.83
**4797**	allXL	BB	367	373	BS2G	12.3
**4797**	allXL	CC	370	382	BS2G	20.68
**4797**	allXL	CC	376	382	BS2G	14.94
**4797**	allXL	BB	357	373	BS2G	19.89
**4797**	allXL	AA	320	366	BS2G	32.08
**4797**	allXL	CC	370	376	BS2G	15.41
**4797**	allXL	CC	320	357	DBSU	23.07
**4797**	allXL	CC	357	319	DBSU	24.83
**4797**	allXL	DD	372	319	DBSU	24.43
**4797**	allXL	DD	370	357	DBSU	23.85
**4797**	allXL	CC	382	357	DBSU	29.18
**4797**	allXL	AA	372	370	DBSU	6.04
**4797**	allXL	CC	381	370	DBSU	22.18
**4797**	allXL	CC	373	370	DBSU	9.06
**4797**	allXL	CC	381	372	DBSU	19.59
**4797**	allXL	CC	372	382	DBSU	19.21
**4797**	allXL	CC	372	386	DBSU	25.57
**4797**	allXL	CC	373	386	DBSU	24.24
**4797**	allXL	BD	381	386	DBSU	51.88
**4797**	allXL	BD;DB	357	357	DBSU	19.19
**4797**	allXL	AD	373	319	DBSU	30.83
**4797**	allXL	BD	372	357	DBSU	19.9

**4797**	allXL	BD	381	357	DBSU	40.5
**4797**	allXL	BD	373	357	DBSU	20.94
**4797**	allXL	BD	372	370	DBSU	34.66
**4797**	allXL	DB	373	370	DBSU	33.02
**4797**	allXL	DB	382	373	DBSU	36.41
**4798**	allXL	AA	320	357	BS2G	27.06
**4798**	allXL	AA	320	373	BS2G	26.47
**4798**	allXL	DD	373	382	BS2G	19.76
**4798**	allXL	CC	370	386	BS2G	21.44
**4798**	allXL	CC	373	386	BS2G	26.0
**4798**	allXL	AA	319	357	BS2G	28.16
**4798**	allXL	BB	367	373	BS2G	12.3
**4798**	allXL	BB	370	382	BS2G	20.0
**4798**	allXL	CC	376	382	BS2G	14.25
**4798**	allXL	BB	357	373	BS2G	33.32
**4798**	allXL	AA	320	366	BS2G	15.02
**4798**	allXL	CC	370	376	BS2G	14.62
**4798**	allXL	AA	320	357	DBSU	27.06
**4798**	allXL	AA	357	319	DBSU	28.16
**4798**	allXL	AA	372	319	DBSU	28.44
**4798**	allXL	DD	370	357	DBSU	31.28
**4798**	allXL	BB	382	357	DBSU	44.16
**4798**	allXL	AA	372	370	DBSU	6.19
**4798**	allXL	BB	381	370	DBSU	21.24
**4798**	allXL	AA	373	370	DBSU	9.08
**4798**	allXL	DD	381	372	DBSU	21.72
**4798**	allXL	DD	372	382	DBSU	22.12
**4798**	allXL	CC	372	386	DBSU	25.84
**4798**	allXL	CC	373	386	DBSU	26.0
**4798**	allXL	AD	381	386	DBSU	44.79
**4798**	allXL	BD;DB	357	357	DBSU	16.28
**4798**	allXL	CB	373	319	DBSU	19.1
**4798**	allXL	CB	372	357	DBSU	44.07
**4798**	allXL	AD	381	357	DBSU	47.86

**4798**	allXL	CB	373	357	DBSU	44.55
**4798**	allXL	AD	372	370	DBSU	39.53
**4798**	allXL	AD	373	370	DBSU	36.42
**4798**	allXL	AD	382	373	DBSU	35.82
**4799**	allXL	CC	320	357	BS2G	21.78
**4799**	allXL	AA	320	373	BS2G	32.09
**4799**	allXL	BB	373	382	BS2G	21.9
**4799**	allXL	AA	370	386	BS2G	35.76
**4799**	allXL	CC	373	386	BS2G	31.22
**4799**	allXL	CC	319	357	BS2G	22.64
**4799**	allXL	BB	367	373	BS2G	12.62
**4799**	allXL	BB	370	382	BS2G	26.51
**4799**	allXL	CC	376	382	BS2G	13.89
**4799**	allXL	BB	357	373	BS2G	28.45
**4799**	allXL	CC	320	366	BS2G	27.21
**4799**	allXL	CC	370	376	BS2G	14.33
**4799**	allXL	CC	320	357	DBSU	21.78
**4799**	allXL	CC	357	319	DBSU	22.64
**4799**	allXL	CC	372	319	DBSU	33.89
**4799**	allXL	BB	370	357	DBSU	28.78
**4799**	allXL	BB	382	357	DBSU	16.09
**4799**	allXL	AA	372	370	DBSU	5.99
**4799**	allXL	BB	381	370	DBSU	27.09
**4799**	allXL	DD	373	370	DBSU	9.22
**4799**	allXL	AA	381	372	DBSU	23.23
**4799**	allXL	AA	372	382	DBSU	22.47
**4799**	allXL	AA	372	386	DBSU	32.56
**4799**	allXL	CC	373	386	DBSU	31.22
**4799**	allXL	AD	381	386	DBSU	32.08
**4799**	allXL	BD;DB	357	357	DBSU	18.48
**4799**	allXL	CB	373	319	DBSU	15.58
**4799**	allXL	CB	372	357	DBSU	34.63
**4799**	allXL	BD	381	357	DBSU	29.48
**4799**	allXL	CB	373	357	DBSU	36.36

**4799**	allXL	CB	372	370	DBSU	26.23
**4799**	allXL	CB	373	370	DBSU	26.92
**4799**	allXL	AD	382	373	DBSU	34.33
**4800**	allXL	AA	320	357	BS2G	24.96
**4800**	allXL	AA	320	373	BS2G	20.16
**4800**	allXL	CC	373	382	BS2G	17.71
**4800**	allXL	AA	370	386	BS2G	21.71
**4800**	allXL	CC	373	386	BS2G	17.89
**4800**	allXL	AA	319	357	BS2G	23.34
**4800**	allXL	AA	367	373	BS2G	12.7
**4800**	allXL	AA	370	382	BS2G	22.43
**4800**	allXL	CC	376	382	BS2G	13.67
**4800**	allXL	BB	357	373	BS2G	23.42
**4800**	allXL	AA	320	366	BS2G	21.68
**4800**	allXL	DD	370	376	BS2G	15.88
**4800**	allXL	AA	320	357	DBSU	24.96
**4800**	allXL	AA	357	319	DBSU	23.34
**4800**	allXL	AA	372	319	DBSU	20.37
**4800**	allXL	BB	370	357	DBSU	25.63
**4800**	allXL	AA	382	357	DBSU	23.57
**4800**	allXL	AA	372	370	DBSU	6.36
**4800**	allXL	AA	381	370	DBSU	24.04
**4800**	allXL	DD	373	370	DBSU	8.82
**4800**	allXL	AA	381	372	DBSU	22.09
**4800**	allXL	AA	372	382	DBSU	21.37
**4800**	allXL	CC	372	386	DBSU	20.51
**4800**	allXL	CC	373	386	DBSU	17.89
**4800**	allXL	DA	381	386	DBSU	46.16
**4800**	allXL	BD;DB	357	357	DBSU	17.9
**4800**	allXL	CB	373	319	DBSU	18.49
**4800**	allXL	BD	372	357	DBSU	39.93
**4800**	allXL	BD	381	357	DBSU	32.04
**4800**	allXL	BD	373	357	DBSU	36.85
**4800**	allXL	DA	372	370	DBSU	26.31

**4800**	allXL	DA	373	370	DBSU	25.24
**4800**	allXL	DA	382	373	DBSU	34.66
**4801**	allXL	AA	320	357	BS2G	23.37
**4801**	allXL	CC	320	373	BS2G	8.78
**4801**	allXL	DD	373	382	BS2G	19.16
**4801**	allXL	DD	370	386	BS2G	18.16
**4801**	allXL	AA	373	386	BS2G	26.15
**4801**	allXL	AA	319	357	BS2G	22.55
**4801**	allXL	AA	367	373	BS2G	11.42
**4801**	allXL	DD	370	382	BS2G	13.92
**4801**	allXL	CC	376	382	BS2G	14.04
**4801**	allXL	BB	357	373	BS2G	22.01
**4801**	allXL	AA	320	366	BS2G	20.01
**4801**	allXL	DD	370	376	BS2G	15.3
**4801**	allXL	AA	320	357	DBSU	23.37
**4801**	allXL	AA	357	319	DBSU	22.55
**4801**	allXL	CC	372	319	DBSU	11.37
**4801**	allXL	BB	370	357	DBSU	17.7
**4801**	allXL	AA	382	357	DBSU	27.05
**4801**	allXL	AA	372	370	DBSU	6.69
**4801**	allXL	DD	381	370	DBSU	13.28
**4801**	allXL	AA	373	370	DBSU	9.5
**4801**	allXL	DD	381	372	DBSU	17.44
**4801**	allXL	DD	372	382	DBSU	19.23
**4801**	allXL	DD	372	386	DBSU	24.67
**4801**	allXL	AA	373	386	DBSU	26.15
**4801**	allXL	DA	381	386	DBSU	43.94
**4801**	allXL	BD;DB	357	357	DBSU	18.27
**4801**	allXL	AD	373	319	DBSU	28.26
**4801**	allXL	BD	372	357	DBSU	38.37
**4801**	allXL	BD	381	357	DBSU	34.99
**4801**	allXL	BD	373	357	DBSU	36.68
**4801**	allXL	DA	372	370	DBSU	23.03
**4801**	allXL	DA	373	370	DBSU	19.35

**4801**	allXL	DA	382	373	DBSU	33.63
**4802**	allXL	CC	320	357	BS2G	23.29
**4802**	allXL	AA	320	373	BS2G	25.82
**4802**	allXL	BB	373	382	BS2G	18.61
**4802**	allXL	DD	370	386	BS2G	20.34
**4802**	allXL	DD	373	386	BS2G	26.14
**4802**	allXL	CC	319	357	BS2G	21.83
**4802**	allXL	DD	367	373	BS2G	14.34
**4802**	allXL	BB	370	382	BS2G	16.73
**4802**	allXL	CC	376	382	BS2G	13.36
**4802**	allXL	CC	357	373	BS2G	27.77
**4802**	allXL	AA	320	366	BS2G	21.67
**4802**	allXL	BB	370	376	BS2G	16.18
**4802**	allXL	CC	320	357	DBSU	23.29
**4802**	allXL	CC	357	319	DBSU	21.83
**4802**	allXL	AA	372	319	DBSU	22.49
**4802**	allXL	CC	370	357	DBSU	29.85
**4802**	allXL	DD	382	357	DBSU	29.55
**4802**	allXL	AA	372	370	DBSU	5.95
**4802**	allXL	BB	381	370	DBSU	17.11
**4802**	allXL	AA	373	370	DBSU	9.1
**4802**	allXL	BB	381	372	DBSU	18.66
**4802**	allXL	BB	372	382	DBSU	19.76
**4802**	allXL	DD	372	386	DBSU	26.15
**4802**	allXL	DD	373	386	DBSU	26.14
**4802**	allXL	DA	381	386	DBSU	32.12
**4802**	allXL	BD;DB	357	357	DBSU	18.23
**4802**	allXL	DA	373	319	DBSU	35.14
**4802**	allXL	CA	372	357	DBSU	39.95
**4802**	allXL	CA	381	357	DBSU	42.09
**4802**	allXL	CA	373	357	DBSU	39.67
**4802**	allXL	DA	372	370	DBSU	28.3
**4802**	allXL	DA	373	370	DBSU	30.63
**4802**	allXL	AD	382	373	DBSU	23.68

**4803**	allXL	CC	320	357	BS2G	25.93
**4803**	allXL	DD	320	373	BS2G	33.31
**4803**	allXL	DD	373	382	BS2G	22.23
**4803**	allXL	CC	370	386	BS2G	24.93
**4803**	allXL	DD	373	386	BS2G	24.13
**4803**	allXL	AA	319	357	BS2G	27.3
**4803**	allXL	AA	367	373	BS2G	14.38
**4803**	allXL	AA	370	382	BS2G	25.78
**4803**	allXL	CC	376	382	BS2G	14.05
**4803**	allXL	DD	357	373	BS2G	6.22
**4803**	allXL	DD	320	366	BS2G	25.92
**4803**	allXL	CC	370	376	BS2G	16.36
**4803**	allXL	CC	320	357	DBSU	25.93
**4803**	allXL	AA	357	319	DBSU	27.3
**4803**	allXL	AA	372	319	DBSU	33.04
**4803**	allXL	DD	370	357	DBSU	6.76
**4803**	allXL	DD	382	357	DBSU	20.34
**4803**	allXL	AA	372	370	DBSU	6.26
**4803**	allXL	DD	381	370	DBSU	25.44
**4803**	allXL	AA;CC	373	370	DBSU	9.08
**4803**	allXL	DD	381	372	DBSU	23.29
**4803**	allXL	AA	372	382	DBSU	24.27
**4803**	allXL	DD	372	386	DBSU	25.29
**4803**	allXL	DD	373	386	DBSU	24.13
**4803**	allXL	DA	381	386	DBSU	62.29
**4803**	allXL	BD;DB	357	357	DBSU	18.17
**4803**	allXL	BC	373	319	DBSU	39.0
**4803**	allXL	DB	372	357	DBSU	13.21
**4803**	allXL	DB	381	357	DBSU	27.96
**4803**	allXL	DB	373	357	DBSU	12.02
**4803**	allXL	DB	372	370	DBSU	41.96
**4803**	allXL	DB	373	370	DBSU	42.63
**4803**	allXL	DA	382	373	DBSU	66.19
**4804**	allXL	CC	320	357	BS2G	26.12

**4804**	allXL	BB	320	373	BS2G	11.89
**4804**	allXL	BB	373	382	BS2G	16.28
**4804**	allXL	BB	370	386	BS2G	17.32
**4804**	allXL	BB	373	386	BS2G	21.0
**4804**	allXL	AA	319	357	BS2G	27.12
**4804**	allXL	AA	367	373	BS2G	12.94
**4804**	allXL	BB	370	382	BS2G	15.86
**4804**	allXL	BB	376	382	BS2G	15.4
**4804**	allXL	DD	357	373	BS2G	28.9
**4804**	allXL	BB	320	366	BS2G	19.56
**4804**	allXL	CC	370	376	BS2G	14.19
**4804**	allXL	CC	320	357	DBSU	26.12
**4804**	allXL	AA	357	319	DBSU	27.12
**4804**	allXL	BB	372	319	DBSU	10.87
**4804**	allXL	DD	370	357	DBSU	25.38
**4804**	allXL	CC	382	357	DBSU	34.4
**4804**	allXL	AA	372	370	DBSU	6.4
**4804**	allXL	BB	381	370	DBSU	13.35
**4804**	allXL	AA	373	370	DBSU	9.12
**4804**	allXL	BB	381	372	DBSU	14.77
**4804**	allXL	BB	372	382	DBSU	17.89
**4804**	allXL	BB	372	386	DBSU	20.66
**4804**	allXL	BB	373	386	DBSU	21.0
**4804**	allXL	BC	381	386	DBSU	40.28
**4804**	allXL	BD;DB	357	357	DBSU	19.57
**4804**	allXL	AD	373	319	DBSU	36.75
**4804**	allXL	BC	372	357	DBSU	34.52
**4804**	allXL	BC	381	357	DBSU	42.07
**4804**	allXL	BC	373	357	DBSU	33.69
**4804**	allXL	BC	372	370	DBSU	31.78
**4804**	allXL	BC	373	370	DBSU	31.28
**4804**	allXL	BC	382	373	DBSU	37.47
**4805**	allXL	CC	320	357	BS2G	23.79
**4805**	allXL	BB	320	373	BS2G	22.39

**4805**	allXL	DD	373	382	BS2G	19.14
**4805**	allXL	DD	370	386	BS2G	26.15
**4805**	allXL	AA	373	386	BS2G	28.28
**4805**	allXL	CC	319	357	BS2G	26.82
**4805**	allXL	BB	367	373	BS2G	12.5
**4805**	allXL	DD	370	382	BS2G	17.83
**4805**	allXL	BB	376	382	BS2G	13.28
**4805**	allXL	BB	357	373	BS2G	23.89
**4805**	allXL	BB	320	366	BS2G	22.11
**4805**	allXL	CC	370	376	BS2G	15.42
**4805**	allXL	CC	320	357	DBSU	23.79
**4805**	allXL	CC	357	319	DBSU	26.82
**4805**	allXL	BB	372	319	DBSU	23.44
**4805**	allXL	DD	370	357	DBSU	26.22
**4805**	allXL	BB	382	357	DBSU	32.06
**4805**	allXL	AA	372	370	DBSU	6.09
**4805**	allXL	DD	381	370	DBSU	17.62
**4805**	allXL	AA	373	370	DBSU	9.15
**4805**	allXL	DD	381	372	DBSU	16.8
**4805**	allXL	DD	372	382	DBSU	18.34
**4805**	allXL	AA	372	386	DBSU	27.38
**4805**	allXL	AA	373	386	DBSU	28.28
**4805**	allXL	BD	381	386	DBSU	49.6
**4805**	allXL	BD;DB	357	357	DBSU	19.05
**4805**	allXL	DA	373	319	DBSU	22.85
**4805**	allXL	CB	372	357	DBSU	34.48
**4805**	allXL	DB	381	357	DBSU	44.7
**4805**	allXL	CB	373	357	DBSU	36.77
**4805**	allXL	CB	372	370	DBSU	11.63
**4805**	allXL	CB	373	370	DBSU	12.32
**4805**	allXL	CB	382	373	DBSU	36.49
**4806**	allXL	AA	320	357	BS2G	24.75
**4806**	allXL	DD	320	373	BS2G	16.6
**4806**	allXL	AA	373	382	BS2G	18.77

**4806**	allXL	DD	370	386	BS2G	14.7
**4806**	allXL	AA	373	386	BS2G	17.89
**4806**	allXL	AA	319	357	BS2G	23.07
**4806**	allXL	BB	367	373	BS2G	13.09
**4806**	allXL	DD	370	382	BS2G	14.34
**4806**	allXL	CC	376	382	BS2G	12.47
**4806**	allXL	BB	357	373	BS2G	14.59
**4806**	allXL	DD	320	366	BS2G	15.86
**4806**	allXL	DD	370	376	BS2G	15.74
**4806**	allXL	AA	320	357	DBSU	24.75
**4806**	allXL	AA	357	319	DBSU	23.07
**4806**	allXL	DD	372	319	DBSU	15.58
**4806**	allXL	BB	370	357	DBSU	23.1
**4806**	allXL	BB	382	357	DBSU	20.52
**4806**	allXL	AA	372	370	DBSU	6.4
**4806**	allXL	DD	381	370	DBSU	15.02
**4806**	allXL	CC	373	370	DBSU	9.19
**4806**	allXL	DD	381	372	DBSU	19.26
**4806**	allXL	DD	372	382	DBSU	19.83
**4806**	allXL	AA	372	386	DBSU	18.73
**4806**	allXL	AA	373	386	DBSU	17.89
**4806**	allXL	DB	381	386	DBSU	45.92
**4806**	allXL	BD;DB	357	357	DBSU	18.71
**4806**	allXL	CB	373	319	DBSU	25.12
**4806**	allXL	DA	372	357	DBSU	31.53
**4806**	allXL	BD	381	357	DBSU	28.59
**4806**	allXL	DA	373	357	DBSU	27.81
**4806**	allXL	DA	372	370	DBSU	26.96
**4806**	allXL	DA	373	370	DBSU	26.37
**4806**	allXL	DA	382	373	DBSU	44.29
**4807**	allXL	AA	320	357	BS2G	20.09
**4807**	allXL	DD	320	373	BS2G	13.19
**4807**	allXL	CC	373	382	BS2G	19.29
**4807**	allXL	CC	370	386	BS2G	35.74

**4807**	allXL	CC	373	386	BS2G	26.33
**4807**	allXL	AA	319	357	BS2G	22.02
**4807**	allXL	AA	367	373	BS2G	14.52
**4807**	allXL	CC	370	382	BS2G	29.07
**4807**	allXL	CC	376	382	BS2G	13.4
**4807**	allXL	BB	357	373	BS2G	20.36
**4807**	allXL	AA	320	366	BS2G	19.48
**4807**	allXL	CC	370	376	BS2G	17.53
**4807**	allXL	AA	320	357	DBSU	20.09
**4807**	allXL	AA	357	319	DBSU	22.02
**4807**	allXL	DD	372	319	DBSU	14.51
**4807**	allXL	DD	370	357	DBSU	19.85
**4807**	allXL	BB	382	357	DBSU	29.82
**4807**	allXL	AA	372	370	DBSU	6.06
**4807**	allXL	DD	381	370	DBSU	28.82
**4807**	allXL	AA	373	370	DBSU	9.27
**4807**	allXL	CC	381	372	DBSU	24.6
**4807**	allXL	CC	372	382	DBSU	22.89
**4807**	allXL	CC	372	386	DBSU	29.51
**4807**	allXL	CC	373	386	DBSU	26.33
**4807**	allXL	DA	381	386	DBSU	48.07
**4807**	allXL	BD;DB	357	357	DBSU	18.64
**4807**	allXL	DA	373	319	DBSU	22.33
**4807**	allXL	DA	372	357	DBSU	28.22
**4807**	allXL	BD	381	357	DBSU	36.17
**4807**	allXL	DA	373	357	DBSU	27.68
**4807**	allXL	DA	372	370	DBSU	30.79
**4807**	allXL	DA	373	370	DBSU	27.14
**4807**	allXL	DA	382	373	DBSU	23.83
**4808**	allXL	AA	320	357	BS2G	24.17
**4808**	allXL	AA	320	373	BS2G	28.85
**4808**	allXL	BB	373	382	BS2G	15.97
**4808**	allXL	CC	370	386	BS2G	24.48
**4808**	allXL	CC	373	386	BS2G	20.34

**4808**	allXL	AA	319	357	BS2G	24.09
**4808**	allXL	AA	367	373	BS2G	12.69
**4808**	allXL	BB	370	382	BS2G	20.26
**4808**	allXL	BB	376	382	BS2G	13.17
**4808**	allXL	DD	357	373	BS2G	38.04
**4808**	allXL	AA	320	366	BS2G	24.73
**4808**	allXL	DD	370	376	BS2G	16.32
**4808**	allXL	AA	320	357	DBSU	24.17
**4808**	allXL	AA	357	319	DBSU	24.09
**4808**	allXL	AA	372	319	DBSU	30.9
**4808**	allXL	DD	370	357	DBSU	30.08
**4808**	allXL	DD	382	357	DBSU	34.88
**4808**	allXL	CC	372	370	DBSU	6.34
**4808**	allXL	AA	381	370	DBSU	17.85
**4808**	allXL	AA	373	370	DBSU	9.02
**4808**	allXL	AA	381	372	DBSU	17.82
**4808**	allXL	CC	372	382	DBSU	17.71
**4808**	allXL	CC	372	386	DBSU	19.9
**4808**	allXL	CC	373	386	DBSU	20.34
**4808**	allXL	CB	381	386	DBSU	41.1
**4808**	allXL	BD;DB	357	357	DBSU	18.97
**4808**	allXL	CB	373	319	DBSU	16.6
**4808**	allXL	CB	372	357	DBSU	42.98
**4808**	allXL	CB	381	357	DBSU	38.2
**4808**	allXL	CB	373	357	DBSU	42.28
**4808**	allXL	AD	372	370	DBSU	36.87
**4808**	allXL	AD	373	370	DBSU	33.66
**4808**	allXL	DA	382	373	DBSU	30.83
**4809**	allXL	CC	320	357	BS2G	25.18
**4809**	allXL	DD	320	373	BS2G	12.58
**4809**	allXL	BB	373	382	BS2G	20.47
**4809**	allXL	CC	370	386	BS2G	26.84
**4809**	allXL	BB	373	386	BS2G	25.92
**4809**	allXL	AA	319	357	BS2G	26.79

**4809**	allXL	AA	367	373	BS2G	12.32
**4809**	allXL	CC	370	382	BS2G	23.02
**4809**	allXL	CC	376	382	BS2G	14.6
**4809**	allXL	DD	357	373	BS2G	29.57
**4809**	allXL	BB	320	366	BS2G	24.84
**4809**	allXL	CC	370	376	BS2G	16.46
**4809**	allXL	CC	320	357	DBSU	25.18
**4809**	allXL	AA	357	319	DBSU	26.79
**4809**	allXL	DD	372	319	DBSU	12.91
**4809**	allXL	DD	370	357	DBSU	22.17
**4809**	allXL	BB	382	357	DBSU	36.87
**4809**	allXL	AA	372	370	DBSU	6.34
**4809**	allXL	DD	381	370	DBSU	24.03
**4809**	allXL	AA	373	370	DBSU	9.23
**4809**	allXL	DD	381	372	DBSU	20.88
**4809**	allXL	CC	372	382	DBSU	21.98
**4809**	allXL	BB	372	386	DBSU	26.1
**4809**	allXL	BB	373	386	DBSU	25.92
**4809**	allXL	BC	381	386	DBSU	29.43
**4809**	allXL	BD;DB	357	357	DBSU	18.76
**4809**	allXL	AD	373	319	DBSU	18.28
**4809**	allXL	DB	372	357	DBSU	43.39
**4809**	allXL	AC	381	357	DBSU	55.4
**4809**	allXL	BC	373	357	DBSU	45.2
**4809**	allXL	CB	372	370	DBSU	26.57
**4809**	allXL	BC	373	370	DBSU	25.16
**4809**	allXL	CB	382	373	DBSU	30.59
**4810**	allXL	CC	320	357	BS2G	24.86
**4810**	allXL	DD	320	373	BS2G	23.05
**4810**	allXL	BB	373	382	BS2G	17.23
**4810**	allXL	DD	370	386	BS2G	21.47
**4810**	allXL	DD	373	386	BS2G	25.93
**4810**	allXL	AA	319	357	BS2G	25.39
**4810**	allXL	AA	367	373	BS2G	13.78

**4810**	allXL	DD	370	382	BS2G	17.01
**4810**	allXL	BB	376	382	BS2G	13.39
**4810**	allXL	DD	357	373	BS2G	33.57
**4810**	allXL	AA	320	366	BS2G	18.11
**4810**	allXL	DD	370	376	BS2G	16.83
**4810**	allXL	CC	320	357	DBSU	24.86
**4810**	allXL	AA	357	319	DBSU	25.39
**4810**	allXL	DD	372	319	DBSU	17.99
**4810**	allXL	DD	370	357	DBSU	25.32
**4810**	allXL	DD	382	357	DBSU	37.96
**4810**	allXL	AA	372	370	DBSU	6.12
**4810**	allXL	DD	381	370	DBSU	17.68
**4810**	allXL	AA;CC	373	370	DBSU	9.38
**4810**	allXL	CC	381	372	DBSU	18.84
**4810**	allXL	DD	372	382	DBSU	19.54
**4810**	allXL	DD	372	386	DBSU	25.51
**4810**	allXL	DD	373	386	DBSU	25.93
**4810**	allXL	CB	381	386	DBSU	29.02
**4810**	allXL	BD;DB	357	357	DBSU	18.62
**4810**	allXL	CB	373	319	DBSU	20.27
**4810**	allXL	CB	372	357	DBSU	40.89
**4810**	allXL	CB	381	357	DBSU	49.87
**4810**	allXL	CB	373	357	DBSU	41.43
**4810**	allXL	CB	372	370	DBSU	28.46
**4810**	allXL	CB	373	370	DBSU	25.6
**4810**	allXL	CB	382	373	DBSU	33.7
**4811**	allXL	CC	320	357	BS2G	24.37
**4811**	allXL	CC	320	373	BS2G	21.44
**4811**	allXL	CC	373	382	BS2G	20.1
**4811**	allXL	DD	370	386	BS2G	20.67
**4811**	allXL	DD	373	386	BS2G	22.07
**4811**	allXL	AA	319	357	BS2G	24.81
**4811**	allXL	AA	367	373	BS2G	12.07
**4811**	allXL	CC	370	382	BS2G	17.38

**4811**	allXL	BB	376	382	BS2G	14.54
**4811**	allXL	AA	357	373	BS2G	34.03
**4811**	allXL	DD	320	366	BS2G	20.52
**4811**	allXL	CC	370	376	BS2G	15.31
**4811**	allXL	CC	320	357	DBSU	24.37
**4811**	allXL	AA	357	319	DBSU	24.81
**4811**	allXL	CC	372	319	DBSU	17.69
**4811**	allXL	CC	370	357	DBSU	31.97
**4811**	allXL	CC	382	357	DBSU	39.41
**4811**	allXL	AA	372	370	DBSU	6.46
**4811**	allXL	CC	381	370	DBSU	19.79
**4811**	allXL	DD	373	370	DBSU	8.9
**4811**	allXL	CC	381	372	DBSU	20.79
**4811**	allXL	CC	372	382	DBSU	19.33
**4811**	allXL	DD	372	386	DBSU	21.6
**4811**	allXL	DD	373	386	DBSU	22.07
**4811**	allXL	CB	381	386	DBSU	19.03
**4811**	allXL	BD;DB	357	357	DBSU	19.06
**4811**	allXL	DA	373	319	DBSU	25.36
**4811**	allXL	BC	372	357	DBSU	43.81
**4811**	allXL	BC	381	357	DBSU	47.85
**4811**	allXL	DA	373	357	DBSU	43.76
**4811**	allXL	CB	372	370	DBSU	19.84
**4811**	allXL	CB	373	370	DBSU	19.05
**4811**	allXL	BC	382	373	DBSU	15.73
**4812**	allXL	AA	320	357	BS2G	24.46
**4812**	allXL	DD	320	373	BS2G	7.86
**4812**	allXL	DD	373	382	BS2G	21.5
**4812**	allXL	DD	370	386	BS2G	20.24
**4812**	allXL	DD	373	386	BS2G	25.05
**4812**	allXL	AA	319	357	BS2G	27.26
**4812**	allXL	AA	367	373	BS2G	12.48
**4812**	allXL	DD	370	382	BS2G	19.29
**4812**	allXL	CC	376	382	BS2G	14.7

**4812**	allXL	DD	357	373	BS2G	32.39
**4812**	allXL	DD	320	366	BS2G	17.32
**4812**	allXL	CC	370	376	BS2G	14.79
**4812**	allXL	AA	320	357	DBSU	24.46
**4812**	allXL	AA	357	319	DBSU	27.26
**4812**	allXL	DD	372	319	DBSU	13.24
**4812**	allXL	DD	370	357	DBSU	24.09
**4812**	allXL	DD	382	357	DBSU	37.22
**4812**	allXL	AA	372	370	DBSU	6.17
**4812**	allXL	DD	381	370	DBSU	19.34
**4812**	allXL	CC	373	370	DBSU	9.04
**4812**	allXL	AA	381	372	DBSU	19.06
**4812**	allXL	AA	372	382	DBSU	21.51
**4812**	allXL	AA	372	386	DBSU	23.65
**4812**	allXL	DD	373	386	DBSU	25.05
**4812**	allXL	DA	381	386	DBSU	35.0
**4812**	allXL	BD;DB	357	357	DBSU	17.7
**4812**	allXL	AD	373	319	DBSU	27.49
**4812**	allXL	DB	372	357	DBSU	43.63
**4812**	allXL	BC	381	357	DBSU	30.51
**4812**	allXL	DA	373	357	DBSU	43.75
**4812**	allXL	DA	372	370	DBSU	39.57
**4812**	allXL	DA	373	370	DBSU	36.03
**4812**	allXL	AD	382	373	DBSU	32.45
**4813**	allXL	AA	320	357	BS2G	25.66
**4813**	allXL	BB	320	373	BS2G	27.82
**4813**	allXL	AA	373	382	BS2G	20.75
**4813**	allXL	CC	370	386	BS2G	26.92
**4813**	allXL	BB	373	386	BS2G	23.72
**4813**	allXL	AA	319	357	BS2G	24.95
**4813**	allXL	AA	367	373	BS2G	13.77
**4813**	allXL	CC	370	382	BS2G	23.02
**4813**	allXL	CC	376	382	BS2G	13.54
**4813**	allXL	BB	357	373	BS2G	32.03

**4813**	allXL	AA	320	366	BS2G	19.25
**4813**	allXL	CC	370	376	BS2G	14.32
**4813**	allXL	AA	320	357	DBSU	25.66
**4813**	allXL	AA	357	319	DBSU	24.95
**4813**	allXL	CC	372	319	DBSU	23.83
**4813**	allXL	BB	370	357	DBSU	28.54
**4813**	allXL	CC	382	357	DBSU	47.94
**4813**	allXL	AA	372	370	DBSU	6.24
**4813**	allXL	CC	381	370	DBSU	25.15
**4813**	allXL	AA	373	370	DBSU	8.8
**4813**	allXL	DD	381	372	DBSU	20.21
**4813**	allXL	AA	372	382	DBSU	20.57
**4813**	allXL	BB	372	386	DBSU	24.61
**4813**	allXL	BB	373	386	DBSU	23.72
**4813**	allXL	AD	381	386	DBSU	35.56
**4813**	allXL	BD;DB	357	357	DBSU	20.04
**4813**	allXL	DA	373	319	DBSU	26.42
**4813**	allXL	DA	372	357	DBSU	45.03
**4813**	allXL	DA	381	357	DBSU	54.08
**4813**	allXL	DA	373	357	DBSU	48.52
**4813**	allXL	DA	372	370	DBSU	41.82
**4813**	allXL	AD	373	370	DBSU	43.19
**4813**	allXL	DA	382	373	DBSU	28.34
**4814**	allXL	BB	320	357	BS2G	24.67
**4814**	allXL	BB	320	373	BS2G	18.67
**4814**	allXL	DD	373	382	BS2G	18.25
**4814**	allXL	DD	370	386	BS2G	18.13
**4814**	allXL	DD	373	386	BS2G	22.13
**4814**	allXL	AA	319	357	BS2G	25.58
**4814**	allXL	AA	367	373	BS2G	13.5
**4814**	allXL	DD	370	382	BS2G	19.15
**4814**	allXL	BB	376	382	BS2G	14.44
**4814**	allXL	BB	357	373	BS2G	28.78
**4814**	allXL	AA	320	366	BS2G	20.1

**4814**	allXL	CC	370	376	BS2G	16.65
**4814**	allXL	BB	320	357	DBSU	24.67
**4814**	allXL	AA	357	319	DBSU	25.58
**4814**	allXL	BB	372	319	DBSU	18.81
**4814**	allXL	BB	370	357	DBSU	30.25
**4814**	allXL	DD	382	357	DBSU	37.95
**4814**	allXL	AA	372	370	DBSU	6.4
**4814**	allXL	DD	381	370	DBSU	19.47
**4814**	allXL	CC	373	370	DBSU	9.19
**4814**	allXL	DD	381	372	DBSU	20.21
**4814**	allXL	DD	372	382	DBSU	20.43
**4814**	allXL	DD	372	386	DBSU	22.39
**4814**	allXL	DD	373	386	DBSU	22.13
**4814**	allXL	DA	381	386	DBSU	53.03
**4814**	allXL	BD;DB	357	357	DBSU	19.8
**4814**	allXL	AD	373	319	DBSU	27.34
**4814**	allXL	CB	372	357	DBSU	31.05
**4814**	allXL	CB	381	357	DBSU	44.41
**4814**	allXL	CB	373	357	DBSU	30.94
**4814**	allXL	CB	372	370	DBSU	15.08
**4814**	allXL	CB	373	370	DBSU	18.27
**4814**	allXL	DA	382	373	DBSU	30.86
**4815**	allXL	CC	320	357	BS2G	25.64
**4815**	allXL	AA	320	373	BS2G	25.76
**4815**	allXL	AA	373	382	BS2G	11.27
**4815**	allXL	AA	370	386	BS2G	15.86
**4815**	allXL	AA	373	386	BS2G	16.22
**4815**	allXL	AA	319	357	BS2G	26.1
**4815**	allXL	AA	367	373	BS2G	11.44
**4815**	allXL	AA	370	382	BS2G	15.83
**4815**	allXL	AA	376	382	BS2G	11.72
**4815**	allXL	AA	357	373	BS2G	23.98
**4815**	allXL	DD	320	366	BS2G	22.91
**4815**	allXL	CC	370	376	BS2G	16.15

**4815**	allXL	CC	320	357	DBSU	25.64
**4815**	allXL	AA	357	319	DBSU	26.1
**4815**	allXL	CC	372	319	DBSU	24.13
**4815**	allXL	BB	370	357	DBSU	29.88
**4815**	allXL	AA	382	357	DBSU	30.82
**4815**	allXL	CC	372	370	DBSU	6.55
**4815**	allXL	AA	381	370	DBSU	19.34
**4815**	allXL	AA	373	370	DBSU	9.18
**4815**	allXL	AA	381	372	DBSU	17.48
**4815**	allXL	AA	372	382	DBSU	14.07
**4815**	allXL	AA	372	386	DBSU	16.34
**4815**	allXL	AA	373	386	DBSU	16.22
**4815**	allXL	CB	381	386	DBSU	38.41
**4815**	allXL	BD;DB	357	357	DBSU	18.46
**4815**	allXL	DA	373	319	DBSU	32.16
**4815**	allXL	CB	372	357	DBSU	44.02
**4815**	allXL	CB	381	357	DBSU	45.89
**4815**	allXL	DA	373	357	DBSU	42.29
**4815**	allXL	CB	372	370	DBSU	35.39
**4815**	allXL	DA	373	370	DBSU	33.76
**4815**	allXL	AD	382	373	DBSU	28.78
**4816**	allXL	AA	320	357	BS2G	15.49
**4816**	allXL	DD	320	373	BS2G	37.42
**4816**	allXL	BB	373	382	BS2G	20.57
**4816**	allXL	DD	370	386	BS2G	22.93
**4816**	allXL	CC	373	386	BS2G	24.22
**4816**	allXL	AA	319	357	BS2G	14.81
**4816**	allXL	AA	367	373	BS2G	13.76
**4816**	allXL	DD	370	382	BS2G	22.75
**4816**	allXL	CC	376	382	BS2G	13.43
**4816**	allXL	DD	357	373	BS2G	26.12
**4816**	allXL	CC	320	366	BS2G	25.39
**4816**	allXL	CC	370	376	BS2G	17.4
**4816**	allXL	AA	320	357	DBSU	15.49

**4816**	allXL	AA	357	319	DBSU	14.81
**4816**	allXL	CC	372	319	DBSU	34.38
**4816**	allXL	DD	370	357	DBSU	24.9
**4816**	allXL	DD	382	357	DBSU	32.41
**4816**	allXL	AA	372	370	DBSU	6.29
**4816**	allXL	DD	381	370	DBSU	21.74
**4816**	allXL	DD	373	370	DBSU	8.94
**4816**	allXL	AA	381	372	DBSU	20.88
**4816**	allXL	BB	372	382	DBSU	23.08
**4816**	allXL	CC	372	386	DBSU	26.22
**4816**	allXL	CC	373	386	DBSU	24.22
**4816**	allXL	BC	381	386	DBSU	42.87
**4816**	allXL	BD;DB	357	357	DBSU	17.3
**4816**	allXL	CB	373	319	DBSU	21.82
**4816**	allXL	DB	372	357	DBSU	38.06
**4816**	allXL	DB	381	357	DBSU	32.43
**4816**	allXL	DB	373	357	DBSU	35.05
**4816**	allXL	CB	372	370	DBSU	29.71
**4816**	allXL	CB	373	370	DBSU	26.57
**4816**	allXL	CB	382	373	DBSU	23.11
**4817**	allXL	AA	320	357	BS2G	23.75
**4817**	allXL	BB	320	373	BS2G	21.72
**4817**	allXL	DD	373	382	BS2G	19.07
**4817**	allXL	BB	370	386	BS2G	25.01
**4817**	allXL	BB	373	386	BS2G	25.07
**4817**	allXL	AA	319	357	BS2G	22.38
**4817**	allXL	AA	367	373	BS2G	12.94
**4817**	allXL	DD	370	382	BS2G	20.86
**4817**	allXL	BB	376	382	BS2G	14.53
**4817**	allXL	DD	357	373	BS2G	27.28
**4817**	allXL	AA	320	366	BS2G	17.27
**4817**	allXL	CC	370	376	BS2G	16.9
**4817**	allXL	AA	320	357	DBSU	23.75
**4817**	allXL	AA	357	319	DBSU	22.38

**4817**	allXL	BB	372	319	DBSU	22.13
**4817**	allXL	BB	370	357	DBSU	26.78
**4817**	allXL	DD	382	357	DBSU	44.07
**4817**	allXL	CC	372	370	DBSU	6.55
**4817**	allXL	DD	381	370	DBSU	18.89
**4817**	allXL	AA	373	370	DBSU	9.18
**4817**	allXL	DD	381	372	DBSU	17.39
**4817**	allXL	DD	372	382	DBSU	19.71
**4817**	allXL	AA	372	386	DBSU	26.88
**4817**	allXL	BB	373	386	DBSU	25.07
**4817**	allXL	DB	381	386	DBSU	69.93
**4817**	allXL	BD;DB	357	357	DBSU	18.61
**4817**	allXL	AD	373	319	DBSU	37.08
**4817**	allXL	DB	372	357	DBSU	42.59
**4817**	allXL	DB	381	357	DBSU	55.7
**4817**	allXL	DB	373	357	DBSU	41.23
**4817**	allXL	AD	372	370	DBSU	50.44
**4817**	allXL	AD	373	370	DBSU	53.72
**4817**	allXL	BD	382	373	DBSU	62.28
**4818**	allXL	CC	320	357	BS2G	23.45
**4818**	allXL	BB	320	373	BS2G	23.71
**4818**	allXL	AA	373	382	BS2G	16.7
**4818**	allXL	DD	370	386	BS2G	23.11
**4818**	allXL	AA	373	386	BS2G	19.64
**4818**	allXL	AA	319	357	BS2G	22.99
**4818**	allXL	AA	367	373	BS2G	12.67
**4818**	allXL	DD	370	382	BS2G	15.24
**4818**	allXL	AA	376	382	BS2G	12.39
**4818**	allXL	BB	357	373	BS2G	11.26
**4818**	allXL	AA	320	366	BS2G	26.3
**4818**	allXL	DD	370	376	BS2G	14.4
**4818**	allXL	CC	320	357	DBSU	23.45
**4818**	allXL	AA	357	319	DBSU	22.99
**4818**	allXL	DD	372	319	DBSU	27.78

**4818**	allXL	BB	370	357	DBSU	14.21
**4818**	allXL	BB	382	357	DBSU	6.78
**4818**	allXL	AA	372	370	DBSU	6.59
**4818**	allXL	DD	381	370	DBSU	13.28
**4818**	allXL	AA	373	370	DBSU	9.28
**4818**	allXL	DD	381	372	DBSU	15.21
**4818**	allXL	DD	372	382	DBSU	16.54
**4818**	allXL	AA	372	386	DBSU	22.07
**4818**	allXL	AA	373	386	DBSU	19.64
**4818**	allXL	DA	381	386	DBSU	45.41
**4818**	allXL	BD;DB	357	357	DBSU	17.96
**4818**	allXL	AD	373	319	DBSU	21.31
**4818**	allXL	BD	372	357	DBSU	24.76
**4818**	allXL	BD	381	357	DBSU	19.5
**4818**	allXL	BD	373	357	DBSU	24.31
**4818**	allXL	DA	372	370	DBSU	38.35
**4818**	allXL	DA	373	370	DBSU	36.92
**4818**	allXL	DA	382	373	DBSU	32.41
**4819**	allXL	AA	320	357	BS2G	23.68
**4819**	allXL	BB	320	373	BS2G	22.8
**4819**	allXL	BB	373	382	BS2G	18.95
**4819**	allXL	BB	370	386	BS2G	28.47
**4819**	allXL	BB	373	386	BS2G	29.72
**4819**	allXL	AA	319	357	BS2G	23.05
**4819**	allXL	BB	367	373	BS2G	12.92
**4819**	allXL	BB	370	382	BS2G	20.72
**4819**	allXL	BB	376	382	BS2G	13.88
**4819**	allXL	BB	357	373	BS2G	27.51
**4819**	allXL	BB	320	366	BS2G	18.02
**4819**	allXL	DD	370	376	BS2G	16.81
**4819**	allXL	AA	320	357	DBSU	23.68
**4819**	allXL	AA	357	319	DBSU	23.05
**4819**	allXL	DD	372	319	DBSU	20.23
**4819**	allXL	DD	370	357	DBSU	29.82

**4819**	allXL	BB	382	357	DBSU	27.97
**4819**	allXL	AA	372	370	DBSU	6.29
**4819**	allXL	DD	381	370	DBSU	21.85
**4819**	allXL	CC	373	370	DBSU	9.09
**4819**	allXL	DD	381	372	DBSU	21.59
**4819**	allXL	BB	372	382	DBSU	21.32
**4819**	allXL	BB	372	386	DBSU	31.36
**4819**	allXL	BB	373	386	DBSU	29.72
**4819**	allXL	CB	381	386	DBSU	48.12
**4819**	allXL	BD;DB	357	357	DBSU	18.18
**4819**	allXL	CB	373	319	DBSU	27.11
**4819**	allXL	DA	372	357	DBSU	41.9
**4819**	allXL	CA	381	357	DBSU	37.43
**4819**	allXL	DA	373	357	DBSU	42.86
**4819**	allXL	CB	372	370	DBSU	39.03
**4819**	allXL	DA	373	370	DBSU	40.01
**4819**	allXL	BC	382	373	DBSU	49.44
**4820**	allXL	AA	320	357	BS2G	25.24
**4820**	allXL	BB	320	373	BS2G	12.19
**4820**	allXL	DD	373	382	BS2G	17.66
**4820**	allXL	DD	370	386	BS2G	21.1
**4820**	allXL	AA	373	386	BS2G	24.35
**4820**	allXL	AA	319	357	BS2G	27.35
**4820**	allXL	AA	367	373	BS2G	10.94
**4820**	allXL	DD	370	382	BS2G	11.24
**4820**	allXL	CC	376	382	BS2G	14.74
**4820**	allXL	DD	357	373	BS2G	24.47
**4820**	allXL	AA	320	366	BS2G	20.9
**4820**	allXL	DD	370	376	BS2G	13.43
**4820**	allXL	AA	320	357	DBSU	25.24
**4820**	allXL	AA	357	319	DBSU	27.35
**4820**	allXL	BB	372	319	DBSU	12.41
**4820**	allXL	CC	370	357	DBSU	21.2
**4820**	allXL	AA	382	357	DBSU	17.14

**4820**	allXL	AA	372	370	DBSU	6.42
**4820**	allXL	DD	381	370	DBSU	9.1
**4820**	allXL	AA	373	370	DBSU	8.96
**4820**	allXL	DD	381	372	DBSU	15.36
**4820**	allXL	DD	372	382	DBSU	17.32
**4820**	allXL	AA	372	386	DBSU	27.2
**4820**	allXL	AA	373	386	DBSU	24.35
**4820**	allXL	AD	381	386	DBSU	49.44
**4820**	allXL	BD;DB	357	357	DBSU	19.22
**4820**	allXL	DA	373	319	DBSU	25.96
**4820**	allXL	DA	372	357	DBSU	27.7
**4820**	allXL	AC	381	357	DBSU	34.26
**4820**	allXL	DA	373	357	DBSU	27.45
**4820**	allXL	DA	372	370	DBSU	28.16
**4820**	allXL	DA	373	370	DBSU	31.62
**4820**	allXL	AD	382	373	DBSU	32.81
**4821**	allXL	AA	320	357	BS2G	19.73
**4821**	allXL	CC	320	373	BS2G	20.6
**4821**	allXL	AA	373	382	BS2G	19.62
**4821**	allXL	BB	370	386	BS2G	26.68
**4821**	allXL	DD	373	386	BS2G	25.94
**4821**	allXL	AA	319	357	BS2G	23.33
**4821**	allXL	AA	367	373	BS2G	11.12
**4821**	allXL	AA	370	382	BS2G	23.28
**4821**	allXL	CC	376	382	BS2G	14.77
**4821**	allXL	BB	357	373	BS2G	24.95
**4821**	allXL	AA	320	366	BS2G	13.76
**4821**	allXL	DD	370	376	BS2G	12.93
**4821**	allXL	AA	320	357	DBSU	19.73
**4821**	allXL	AA	357	319	DBSU	23.33
**4821**	allXL	CC	372	319	DBSU	16.3
**4821**	allXL	BB	370	357	DBSU	26.88
**4821**	allXL	BB	382	357	DBSU	20.87
**4821**	allXL	CC	372	370	DBSU	6.58

**4821**	allXL	DD	381	370	DBSU	21.47
**4821**	allXL	AA	373	370	DBSU	9.02
**4821**	allXL	BB	381	372	DBSU	21.31
**4821**	allXL	CC	372	382	DBSU	21.8
**4821**	allXL	CC	372	386	DBSU	26.78
**4821**	allXL	DD	373	386	DBSU	25.94
**4821**	allXL	BC	381	386	DBSU	13.26
**4821**	allXL	BD;DB	357	357	DBSU	18.13
**4821**	allXL	BC	373	319	DBSU	25.53
**4821**	allXL	BC	372	357	DBSU	28.85
**4821**	allXL	BD	381	357	DBSU	22.83
**4821**	allXL	BD	373	357	DBSU	28.02
**4821**	allXL	CB	372	370	DBSU	15.25
**4821**	allXL	CB	373	370	DBSU	17.76
**4821**	allXL	CB	382	373	DBSU	23.72
**4822**	allXL	AA	320	357	BS2G	24.07
**4822**	allXL	CC	320	373	BS2G	33.86
**4822**	allXL	BB	373	382	BS2G	22.43
**4822**	allXL	DD	370	386	BS2G	22.46
**4822**	allXL	DD	373	386	BS2G	23.7
**4822**	allXL	AA	319	357	BS2G	26.02
**4822**	allXL	AA	367	373	BS2G	13.07
**4822**	allXL	BB	370	382	BS2G	21.09
**4822**	allXL	CC	376	382	BS2G	15.46
**4822**	allXL	DD	357	373	BS2G	33.57
**4822**	allXL	DD	320	366	BS2G	26.06
**4822**	allXL	BB	370	376	BS2G	14.52
**4822**	allXL	AA	320	357	DBSU	24.07
**4822**	allXL	AA	357	319	DBSU	26.02
**4822**	allXL	CC	372	319	DBSU	39.15
**4822**	allXL	DD	370	357	DBSU	28.13
**4822**	allXL	CC	382	357	DBSU	37.8
**4822**	allXL	AA	372	370	DBSU	6.23
**4822**	allXL	BB	381	370	DBSU	21.68

**4822**	allXL	AA	373	370	DBSU	9.07
**4822**	allXL	AA	381	372	DBSU	23.05
**4822**	allXL	CC	372	382	DBSU	24.05
**4822**	allXL	DD	372	386	DBSU	23.8
**4822**	allXL	DD	373	386	DBSU	23.7
**4822**	allXL	CB	381	386	DBSU	68.38
**4822**	allXL	BD;DB	357	357	DBSU	18.19
**4822**	allXL	AD	373	319	DBSU	34.31
**4822**	allXL	BD	372	357	DBSU	48.07
**4822**	allXL	AC	381	357	DBSU	53.74
**4822**	allXL	DB	373	357	DBSU	48.2
**4822**	allXL	BC	372	370	DBSU	43.09
**4822**	allXL	BC	373	370	DBSU	41.6
**4822**	allXL	CB	382	373	DBSU	50.18
**4823**	allXL	CC	320	357	BS2G	25.77
**4823**	allXL	CC	320	373	BS2G	16.53
**4823**	allXL	BB	373	382	BS2G	18.73
**4823**	allXL	DD	370	386	BS2G	14.42
**4823**	allXL	DD	373	386	BS2G	21.43
**4823**	allXL	CC	319	357	BS2G	27.62
**4823**	allXL	DD	367	373	BS2G	13.22
**4823**	allXL	DD	370	382	BS2G	17.43
**4823**	allXL	BB	376	382	BS2G	13.06
**4823**	allXL	DD	357	373	BS2G	18.01
**4823**	allXL	AA	320	366	BS2G	22.53
**4823**	allXL	DD	370	376	BS2G	16.66
**4823**	allXL	CC	320	357	DBSU	25.77
**4823**	allXL	CC	357	319	DBSU	27.62
**4823**	allXL	CC	372	319	DBSU	16.1
**4823**	allXL	DD	370	357	DBSU	24.63
**4823**	allXL	DD	382	357	DBSU	31.73
**4823**	allXL	AA	372	370	DBSU	5.95
**4823**	allXL	DD	381	370	DBSU	17.33
**4823**	allXL	AA	373	370	DBSU	8.98

**4823**	allXL	DD	381	372	DBSU	19.75
**4823**	allXL	DD	372	382	DBSU	21.18
**4823**	allXL	DD	372	386	DBSU	20.93
**4823**	allXL	DD	373	386	DBSU	21.43
**4823**	allXL	BC	381	386	DBSU	55.19
**4823**	allXL	BD;DB	357	357	DBSU	17.81
**4823**	allXL	AD	373	319	DBSU	21.05
**4823**	allXL	DB	372	357	DBSU	32.31
**4823**	allXL	CB	381	357	DBSU	32.02
**4823**	allXL	DB	373	357	DBSU	29.65
**4823**	allXL	CB	372	370	DBSU	27.35
**4823**	allXL	CB	373	370	DBSU	28.99
**4823**	allXL	CB	382	373	DBSU	37.68
**4824**	allXL	AA	320	357	BS2G	25.84
**4824**	allXL	DD	320	373	BS2G	32.68
**4824**	allXL	DD	373	382	BS2G	14.13
**4824**	allXL	DD	370	386	BS2G	18.42
**4824**	allXL	DD	373	386	BS2G	19.51
**4824**	allXL	AA	319	357	BS2G	26.56
**4824**	allXL	AA	367	373	BS2G	12.49
**4824**	allXL	DD	370	382	BS2G	16.95
**4824**	allXL	CC	376	382	BS2G	13.39
**4824**	allXL	CC	357	373	BS2G	33.2
**4824**	allXL	AA	320	366	BS2G	27.12
**4824**	allXL	CC	370	376	BS2G	12.79
**4824**	allXL	AA	320	357	DBSU	25.84
**4824**	allXL	AA	357	319	DBSU	26.56
**4824**	allXL	BB	372	319	DBSU	34.09
**4824**	allXL	BB	370	357	DBSU	30.02
**4824**	allXL	CC	382	357	DBSU	15.78
**4824**	allXL	AA	372	370	DBSU	6.49
**4824**	allXL	CC	381	370	DBSU	18.98
**4824**	allXL	CC	373	370	DBSU	9.19
**4824**	allXL	DD	381	372	DBSU	14.37

**4824**	allXL	DD	372	382	DBSU	12.01
**4824**	allXL	DD	372	386	DBSU	16.74
**4824**	allXL	DD	373	386	DBSU	19.51
**4824**	allXL	AD	381	386	DBSU	38.44
**4824**	allXL	BD;DB	357	357	DBSU	18.26
**4824**	allXL	CB	373	319	DBSU	14.23
**4824**	allXL	CB	372	357	DBSU	34.73
**4824**	allXL	CA	381	357	DBSU	25.62
**4824**	allXL	CB	373	357	DBSU	35.44
**4824**	allXL	CB	372	370	DBSU	37.72
**4824**	allXL	CB	373	370	DBSU	40.88
**4824**	allXL	AD	382	373	DBSU	23.13
**4825**	allXL	AA	320	357	BS2G	25.29
**4825**	allXL	DD	320	373	BS2G	15.59
**4825**	allXL	AA	373	382	BS2G	16.15
**4825**	allXL	DD	370	386	BS2G	15.14
**4825**	allXL	DD	373	386	BS2G	22.01
**4825**	allXL	AA	319	357	BS2G	27.5
**4825**	allXL	AA	367	373	BS2G	12.34
**4825**	allXL	DD	370	382	BS2G	15.69
**4825**	allXL	BB	376	382	BS2G	13.88
**4825**	allXL	DD	357	373	BS2G	34.17
**4825**	allXL	DD	320	366	BS2G	16.07
**4825**	allXL	CC	370	376	BS2G	14.88
**4825**	allXL	AA	320	357	DBSU	25.29
**4825**	allXL	AA	357	319	DBSU	27.5
**4825**	allXL	DD	372	319	DBSU	10.04
**4825**	allXL	DD	370	357	DBSU	29.45
**4825**	allXL	DD	382	357	DBSU	33.86
**4825**	allXL	AA	372	370	DBSU	6.79
**4825**	allXL	DD	381	370	DBSU	15.16
**4825**	allXL	AA	373	370	DBSU	9.09
**4825**	allXL	DD	381	372	DBSU	19.17
**4825**	allXL	AA	372	382	DBSU	17.41

**4825**	allXL	DD	372	386	DBSU	21.23
**4825**	allXL	DD	373	386	DBSU	22.01
**4825**	allXL	DA	381	386	DBSU	50.58
**4825**	allXL	BD;DB	357	357	DBSU	18.76
**4825**	allXL	AD	373	319	DBSU	22.15
**4825**	allXL	DA	372	357	DBSU	34.4
**4825**	allXL	DA	381	357	DBSU	38.99
**4825**	allXL	DA	373	357	DBSU	31.31
**4825**	allXL	DA	372	370	DBSU	15.87
**4825**	allXL	DA	373	370	DBSU	15.06
**4825**	allXL	AD	382	373	DBSU	34.63
**4826**	allXL	CC	320	357	BS2G	24.78
**4826**	allXL	BB	320	373	BS2G	20.72
**4826**	allXL	DD	373	382	BS2G	16.22
**4826**	allXL	DD	370	386	BS2G	15.5
**4826**	allXL	DD	373	386	BS2G	16.54
**4826**	allXL	CC	319	357	BS2G	25.46
**4826**	allXL	AA	367	373	BS2G	15.29
**4826**	allXL	DD	370	382	BS2G	20.25
**4826**	allXL	BB	376	382	BS2G	13.1
**4826**	allXL	DD	357	373	BS2G	24.87
**4826**	allXL	BB	320	366	BS2G	14.63
**4826**	allXL	CC	370	376	BS2G	16.6
**4826**	allXL	CC	320	357	DBSU	24.78
**4826**	allXL	CC	357	319	DBSU	25.46
**4826**	allXL	BB	372	319	DBSU	20.68
**4826**	allXL	DD	370	357	DBSU	19.2
**4826**	allXL	CC	382	357	DBSU	25.97
**4826**	allXL	AA	372	370	DBSU	6.06
**4826**	allXL	BB	381	370	DBSU	22.64
**4826**	allXL	AA	373	370	DBSU	9.04
**4826**	allXL	DD	381	372	DBSU	19.29
**4826**	allXL	DD	372	382	DBSU	17.79
**4826**	allXL	DD	372	386	DBSU	16.67

**4826**	allXL	DD	373	386	DBSU	16.54
**4826**	allXL	AD	381	386	DBSU	48.37
**4826**	allXL	BD;DB	357	357	DBSU	18.45
**4826**	allXL	AD	373	319	DBSU	20.2
**4826**	allXL	DA	372	357	DBSU	33.53
**4826**	allXL	DA	381	357	DBSU	26.88
**4826**	allXL	DA	373	357	DBSU	31.51
**4826**	allXL	DA	372	370	DBSU	28.1
**4826**	allXL	DA	373	370	DBSU	24.69
**4826**	allXL	DA	382	373	DBSU	28.67
**4827**	allXL	AA	320	357	BS2G	24.21
**4827**	allXL	DD	320	373	BS2G	17.96
**4827**	allXL	BB	373	382	BS2G	15.23
**4827**	allXL	BB	370	386	BS2G	21.54
**4827**	allXL	BB	373	386	BS2G	22.53
**4827**	allXL	AA	319	357	BS2G	27.0
**4827**	allXL	BB	367	373	BS2G	13.27
**4827**	allXL	BB	370	382	BS2G	19.68
**4827**	allXL	BB	376	382	BS2G	12.67
**4827**	allXL	CC	357	373	BS2G	25.49
**4827**	allXL	CC	320	366	BS2G	25.18
**4827**	allXL	CC	370	376	BS2G	14.93
**4827**	allXL	AA	320	357	DBSU	24.21
**4827**	allXL	AA	357	319	DBSU	27.0
**4827**	allXL	DD	372	319	DBSU	19.1
**4827**	allXL	DD	370	357	DBSU	22.47
**4827**	allXL	CC	382	357	DBSU	9.41
**4827**	allXL	AA	372	370	DBSU	5.96
**4827**	allXL	BB	381	370	DBSU	22.75
**4827**	allXL	CC	373	370	DBSU	9.1
**4827**	allXL	BB	381	372	DBSU	20.43
**4827**	allXL	BB	372	382	DBSU	18.34
**4827**	allXL	BB	372	386	DBSU	24.16
**4827**	allXL	BB	373	386	DBSU	22.53

**4827**	allXL	BC	381	386	DBSU	21.83
**4827**	allXL	BD;DB	357	357	DBSU	18.56
**4827**	allXL	AD	373	319	DBSU	31.06
**4827**	allXL	AC	372	357	DBSU	41.27
**4827**	allXL	CA	381	357	DBSU	24.57
**4827**	allXL	CA	373	357	DBSU	43.65
**4827**	allXL	DA	372	370	DBSU	41.88
**4827**	allXL	DA	373	370	DBSU	41.4
**4827**	allXL	BC	382	373	DBSU	34.14
**4828**	allXL	CC	320	357	BS2G	22.75
**4828**	allXL	AA	320	373	BS2G	17.06
**4828**	allXL	DD	373	382	BS2G	16.32
**4828**	allXL	AA	370	386	BS2G	17.36
**4828**	allXL	AA	373	386	BS2G	17.95
**4828**	allXL	CC	319	357	BS2G	22.57
**4828**	allXL	AA	367	373	BS2G	12.26
**4828**	allXL	DD	370	382	BS2G	21.61
**4828**	allXL	AA	376	382	BS2G	14.96
**4828**	allXL	DD	357	373	BS2G	27.76
**4828**	allXL	BB	320	366	BS2G	23.39
**4828**	allXL	CC	370	376	BS2G	14.52
**4828**	allXL	CC	320	357	DBSU	22.75
**4828**	allXL	CC	357	319	DBSU	22.57
**4828**	allXL	AA	372	319	DBSU	19.55
**4828**	allXL	BB	370	357	DBSU	23.42
**4828**	allXL	DD	382	357	DBSU	43.43
**4828**	allXL	AA	372	370	DBSU	6.41
**4828**	allXL	CC	381	370	DBSU	19.23
**4828**	allXL	AA	373	370	DBSU	9.03
**4828**	allXL	DD	381	372	DBSU	14.13
**4828**	allXL	DD	372	382	DBSU	15.01
**4828**	allXL	AA	372	386	DBSU	15.32
**4828**	allXL	AA	373	386	DBSU	17.95
**4828**	allXL	AD	381	386	DBSU	74.94

**4828**	allXL	BD;DB	357	357	DBSU	18.31
**4828**	allXL	AD	373	319	DBSU	42.3
**4828**	allXL	DB	372	357	DBSU	43.55
**4828**	allXL	DB	381	357	DBSU	54.84
**4828**	allXL	DB	373	357	DBSU	41.79
**4828**	allXL	BC	372	370	DBSU	60.34
**4828**	allXL	BC	373	370	DBSU	63.54
**4828**	allXL	CB	382	373	DBSU	62.32
**4829**	allXL	CC	320	357	BS2G	27.69
**4829**	allXL	BB	320	373	BS2G	18.69
**4829**	allXL	AA	373	382	BS2G	20.16
**4829**	allXL	DD	370	386	BS2G	20.6
**4829**	allXL	AA	373	386	BS2G	20.08
**4829**	allXL	AA	319	357	BS2G	26.47
**4829**	allXL	AA	367	373	BS2G	13.27
**4829**	allXL	BB	370	382	BS2G	24.28
**4829**	allXL	AA	376	382	BS2G	14.25
**4829**	allXL	AA	357	373	BS2G	27.3
**4829**	allXL	BB	320	366	BS2G	19.41
**4829**	allXL	BB	370	376	BS2G	16.09
**4829**	allXL	CC	320	357	DBSU	27.69
**4829**	allXL	AA	357	319	DBSU	26.47
**4829**	allXL	BB	372	319	DBSU	19.54
**4829**	allXL	BB	370	357	DBSU	26.36
**4829**	allXL	AA	382	357	DBSU	31.1
**4829**	allXL	AA	372	370	DBSU	6.24
**4829**	allXL	DD	381	370	DBSU	23.66
**4829**	allXL	CC	373	370	DBSU	8.88
**4829**	allXL	CC	381	372	DBSU	22.85
**4829**	allXL	AA	372	382	DBSU	23.56
**4829**	allXL	AA	372	386	DBSU	22.18
**4829**	allXL	AA	373	386	DBSU	20.08
**4829**	allXL	CB	381	386	DBSU	30.17
**4829**	allXL	BD;DB	357	357	DBSU	19.27

**4829**	allXL	CB	373	319	DBSU	23.64
**4829**	allXL	AC	372	357	DBSU	49.03
**4829**	allXL	CB	381	357	DBSU	46.37
**4829**	allXL	AC	373	357	DBSU	46.7
**4829**	allXL	CB	372	370	DBSU	32.24
**4829**	allXL	CB	373	370	DBSU	30.93
**4829**	allXL	CB	382	373	DBSU	29.26
**4830**	allXL	DD	320	357	BS2G	26.49
**4830**	allXL	CC	320	373	BS2G	10.26
**4830**	allXL	DD	373	382	BS2G	18.12
**4830**	allXL	AA	370	386	BS2G	17.72
**4830**	allXL	AA	373	386	BS2G	21.18
**4830**	allXL	AA	319	357	BS2G	28.12
**4830**	allXL	AA	367	373	BS2G	13.19
**4830**	allXL	AA	370	382	BS2G	19.67
**4830**	allXL	AA	376	382	BS2G	15.05
**4830**	allXL	DD	357	373	BS2G	27.78
**4830**	allXL	AA	320	366	BS2G	24.97
**4830**	allXL	CC	370	376	BS2G	14.74
**4830**	allXL	DD	320	357	DBSU	26.49
**4830**	allXL	AA	357	319	DBSU	28.12
**4830**	allXL	CC	372	319	DBSU	9.41
**4830**	allXL	BB	370	357	DBSU	25.42
**4830**	allXL	CC	382	357	DBSU	19.65
**4830**	allXL	AA	372	370	DBSU	6.23
**4830**	allXL	CC	381	370	DBSU	20.01
**4830**	allXL	DD	373	370	DBSU	8.93
**4830**	allXL	AA	381	372	DBSU	17.84
**4830**	allXL	AA	372	382	DBSU	18.04
**4830**	allXL	AA	372	386	DBSU	19.71
**4830**	allXL	AA	373	386	DBSU	21.18
**4830**	allXL	DA	381	386	DBSU	22.92
**4830**	allXL	BD;DB	357	357	DBSU	18.79
**4830**	allXL	AD	373	319	DBSU	28.49

**4830**	allXL	BD	372	357	DBSU	33.22
**4830**	allXL	CA	381	357	DBSU	36.92
**4830**	allXL	BD	373	357	DBSU	36.81
**4830**	allXL	AD	372	370	DBSU	20.01
**4830**	allXL	AD	373	370	DBSU	22.5
**4830**	allXL	AD	382	373	DBSU	19.75
**4831**	allXL	AA	320	357	BS2G	23.73
**4831**	allXL	BB	320	373	BS2G	31.04
**4831**	allXL	AA	373	382	BS2G	20.37
**4831**	allXL	AA	370	386	BS2G	26.05
**4831**	allXL	AA	373	386	BS2G	21.63
**4831**	allXL	AA	319	357	BS2G	21.39
**4831**	allXL	DD	367	373	BS2G	13.17
**4831**	allXL	BB	370	382	BS2G	25.58
**4831**	allXL	BB	376	382	BS2G	15.06
**4831**	allXL	DD	357	373	BS2G	28.41
**4831**	allXL	BB	320	366	BS2G	16.88
**4831**	allXL	CC	370	376	BS2G	15.99
**4831**	allXL	AA	320	357	DBSU	23.73
**4831**	allXL	AA	357	319	DBSU	21.39
**4831**	allXL	BB	372	319	DBSU	33.08
**4831**	allXL	CC	370	357	DBSU	26.78
**4831**	allXL	BB	382	357	DBSU	32.71
**4831**	allXL	AA	372	370	DBSU	6.2
**4831**	allXL	CC	381	370	DBSU	25.64
**4831**	allXL	DD	373	370	DBSU	8.98
**4831**	allXL	DD	381	372	DBSU	21.75
**4831**	allXL	CC	372	382	DBSU	23.59
**4831**	allXL	AA	372	386	DBSU	24.55
**4831**	allXL	AA	373	386	DBSU	21.63
**4831**	allXL	DB	381	386	DBSU	39.58
**4831**	allXL	BD;DB	357	357	DBSU	19.44
**4831**	allXL	DA	373	319	DBSU	38.0
**4831**	allXL	DB	372	357	DBSU	44.06

**4831**	allXL	BD	381	357	DBSU	38.78
**4831**	allXL	DB	373	357	DBSU	43.52
**4831**	allXL	AD	372	370	DBSU	53.79
**4831**	allXL	AD	373	370	DBSU	56.73
**4831**	allXL	BD	382	373	DBSU	45.47
**4832**	allXL	AA	320	357	BS2G	20.78
**4832**	allXL	DD	320	373	BS2G	13.63
**4832**	allXL	CC	373	382	BS2G	14.84
**4832**	allXL	CC	370	386	BS2G	21.65
**4832**	allXL	CC	373	386	BS2G	20.74
**4832**	allXL	AA	319	357	BS2G	20.27
**4832**	allXL	AA	367	373	BS2G	8.67
**4832**	allXL	CC	370	382	BS2G	19.4
**4832**	allXL	CC	376	382	BS2G	13.35
**4832**	allXL	BB	357	373	BS2G	15.23
**4832**	allXL	DD	320	366	BS2G	23.87
**4832**	allXL	AA	370	376	BS2G	14.53
**4832**	allXL	AA	320	357	DBSU	20.78
**4832**	allXL	AA	357	319	DBSU	20.27
**4832**	allXL	DD	372	319	DBSU	10.6
**4832**	allXL	DD	370	357	DBSU	22.22
**4832**	allXL	BB	382	357	DBSU	7.18
**4832**	allXL	CC	372	370	DBSU	6.72
**4832**	allXL	AA	381	370	DBSU	21.99
**4832**	allXL	AA	373	370	DBSU	8.42
**4832**	allXL	BB	381	372	DBSU	19.51
**4832**	allXL	CC	372	382	DBSU	16.94
**4832**	allXL	CC	372	386	DBSU	20.82
**4832**	allXL	CC	373	386	DBSU	20.74
**4832**	allXL	CB	381	386	DBSU	49.18
**4832**	allXL	BD;DB	357	357	DBSU	18.4
**4832**	allXL	CB	373	319	DBSU	16.48
**4832**	allXL	BD	372	357	DBSU	26.56
**4832**	allXL	BD	381	357	DBSU	21.12

**4832**	allXL	BD	373	357	DBSU	24.36
**4832**	allXL	BD	372	370	DBSU	38.94
**4832**	allXL	BD	373	370	DBSU	38.31
**4832**	allXL	AD	382	373	DBSU	34.76
**4833**	allXL	AA	320	357	BS2G	25.13
**4833**	allXL	CC	320	373	BS2G	18.48
**4833**	allXL	BB	373	382	BS2G	20.05
**4833**	allXL	DD	370	386	BS2G	30.07
**4833**	allXL	CC	373	386	BS2G	25.5
**4833**	allXL	AA	319	357	BS2G	25.76
**4833**	allXL	AA	367	373	BS2G	12.11
**4833**	allXL	BB	370	382	BS2G	27.53
**4833**	allXL	BB	376	382	BS2G	16.03
**4833**	allXL	BB	357	373	BS2G	28.87
**4833**	allXL	AA	320	366	BS2G	17.71
**4833**	allXL	CC	370	376	BS2G	15.91
**4833**	allXL	AA	320	357	DBSU	25.13
**4833**	allXL	AA	357	319	DBSU	25.76
**4833**	allXL	CC	372	319	DBSU	12.96
**4833**	allXL	DD	370	357	DBSU	28.23
**4833**	allXL	BB	382	357	DBSU	22.47
**4833**	allXL	AA	372	370	DBSU	6.22
**4833**	allXL	BB	381	370	DBSU	25.54
**4833**	allXL	AA	373	370	DBSU	8.88
**4833**	allXL	BB	381	372	DBSU	18.75
**4833**	allXL	BB	372	382	DBSU	20.77
**4833**	allXL	BB	372	386	DBSU	26.76
**4833**	allXL	CC	373	386	DBSU	25.5
**4833**	allXL	BD	381	386	DBSU	25.85
**4833**	allXL	BD;DB	357	357	DBSU	19.01
**4833**	allXL	CB	373	319	DBSU	30.36
**4833**	allXL	CB	372	357	DBSU	35.02
**4833**	allXL	CB	381	357	DBSU	23.55
**4833**	allXL	CB	373	357	DBSU	32.53

**4833**	allXL	DA	372	370	DBSU	26.31
**4833**	allXL	DA	373	370	DBSU	26.93
**4833**	allXL	CB	382	373	DBSU	28.74
**4834**	allXL	AA	320	357	BS2G	24.16
**4834**	allXL	BB	320	373	BS2G	19.23
**4834**	allXL	BB	373	382	BS2G	17.46
**4834**	allXL	CC	370	386	BS2G	26.5
**4834**	allXL	CC	373	386	BS2G	21.44
**4834**	allXL	AA	319	357	BS2G	26.9
**4834**	allXL	AA	367	373	BS2G	12.07
**4834**	allXL	BB	370	382	BS2G	20.22
**4834**	allXL	BB	376	382	BS2G	13.25
**4834**	allXL	BB;DD	357	373	BS2G	28.69
**4834**	allXL	BB	320	366	BS2G	19.39
**4834**	allXL	CC	370	376	BS2G	15.42
**4834**	allXL	AA	320	357	DBSU	24.16
**4834**	allXL	AA	357	319	DBSU	26.9
**4834**	allXL	BB	372	319	DBSU	21.49
**4834**	allXL	DD	370	357	DBSU	26.55
**4834**	allXL	DD	382	357	DBSU	27.56
**4834**	allXL	CC	372	370	DBSU	6.53
**4834**	allXL	BB	381	370	DBSU	22.1
**4834**	allXL	AA	373	370	DBSU	9.2
**4834**	allXL	BB	381	372	DBSU	21.87
**4834**	allXL	BB	372	382	DBSU	20.57
**4834**	allXL	CC	372	386	DBSU	21.71
**4834**	allXL	CC	373	386	DBSU	21.44
**4834**	allXL	BD	381	386	DBSU	25.08
**4834**	allXL	BD;DB	357	357	DBSU	17.54
**4834**	allXL	DA	373	319	DBSU	21.41
**4834**	allXL	BD	372	357	DBSU	40.99
**4834**	allXL	DB	381	357	DBSU	26.91
**4834**	allXL	DA	373	357	DBSU	38.69
**4834**	allXL	DA	372	370	DBSU	28.47

**4834**	allXL	DA	373	370	DBSU	28.75
**4834**	allXL	DB	382	373	DBSU	25.86
**4835**	allXL	CC	320	357	BS2G	24.06
**4835**	allXL	BB	320	373	BS2G	22.95
**4835**	allXL	AA	373	382	BS2G	17.95
**4835**	allXL	CC	370	386	BS2G	24.88
**4835**	allXL	AA	373	386	BS2G	23.96
**4835**	allXL	CC	319	357	BS2G	27.24
**4835**	allXL	BB	367	373	BS2G	12.63
**4835**	allXL	CC	370	382	BS2G	19.93
**4835**	allXL	CC	376	382	BS2G	13.43
**4835**	allXL	BB	357	373	BS2G	24.59
**4835**	allXL	DD	320	366	BS2G	29.61
**4835**	allXL	CC	370	376	BS2G	15.57
**4835**	allXL	CC	320	357	DBSU	24.06
**4835**	allXL	CC	357	319	DBSU	27.24
**4835**	allXL	DD	372	319	DBSU	23.03
**4835**	allXL	BB	370	357	DBSU	24.51
**4835**	allXL	BB	382	357	DBSU	22.16
**4835**	allXL	AA	372	370	DBSU	6.03
**4835**	allXL	DD	381	370	DBSU	19.26
**4835**	allXL	CC	373	370	DBSU	9.13
**4835**	allXL	DD	381	372	DBSU	20.97
**4835**	allXL	CC	372	382	DBSU	21.14
**4835**	allXL	AA	372	386	DBSU	26.65
**4835**	allXL	AA	373	386	DBSU	23.96
**4835**	allXL	BD	381	386	DBSU	53.71
**4835**	allXL	BD;DB	357	357	DBSU	18.31
**4835**	allXL	CB	373	319	DBSU	29.44
**4835**	allXL	CA	372	357	DBSU	27.91
**4835**	allXL	BD	381	357	DBSU	37.36
**4835**	allXL	CA	373	357	DBSU	27.84
**4835**	allXL	DA	372	370	DBSU	22.29
**4835**	allXL	DA	373	370	DBSU	24.9

**4835**	allXL	CB	382	373	DBSU	41.0
**4836**	allXL	CC	320	357	BS2G	25.81
**4836**	allXL	BB	320	373	BS2G	19.69
**4836**	allXL	BB	373	382	BS2G	15.81
**4836**	allXL	BB	370	386	BS2G	23.13
**4836**	allXL	DD	373	386	BS2G	23.88
**4836**	allXL	AA	319	357	BS2G	27.56
**4836**	allXL	BB	367	373	BS2G	12.26
**4836**	allXL	BB	370	382	BS2G	15.18
**4836**	allXL	BB	376	382	BS2G	13.65
**4836**	allXL	CC	357	373	BS2G	23.7
**4836**	allXL	DD	320	366	BS2G	18.69
**4836**	allXL	CC	370	376	BS2G	12.5
**4836**	allXL	CC	320	357	DBSU	25.81
**4836**	allXL	AA	357	319	DBSU	27.56
**4836**	allXL	BB	372	319	DBSU	20.09
**4836**	allXL	DD	370	357	DBSU	21.68
**4836**	allXL	CC	382	357	DBSU	24.51
**4836**	allXL	AA	372	370	DBSU	6.16
**4836**	allXL	BB	381	370	DBSU	17.0
**4836**	allXL	DD	373	370	DBSU	9.07
**4836**	allXL	DD	381	372	DBSU	16.13
**4836**	allXL	BB	372	382	DBSU	16.25
**4836**	allXL	DD	372	386	DBSU	22.77
**4836**	allXL	DD	373	386	DBSU	23.88
**4836**	allXL	BC	381	386	DBSU	41.21
**4836**	allXL	BD;DB	357	357	DBSU	19.06
**4836**	allXL	CB	373	319	DBSU	23.49
**4836**	allXL	CB	372	357	DBSU	33.6
**4836**	allXL	CA	381	357	DBSU	29.74
**4836**	allXL	CB	373	357	DBSU	30.46
**4836**	allXL	CB	372	370	DBSU	15.77
**4836**	allXL	CB	373	370	DBSU	16.98
**4836**	allXL	CB	382	373	DBSU	28.96

**4837**	allXL	AA	320	357	BS2G	26.16
**4837**	allXL	DD	320	373	BS2G	15.32
**4837**	allXL	CC	373	382	BS2G	17.05
**4837**	allXL	BB	370	386	BS2G	27.63
**4837**	allXL	BB	373	386	BS2G	26.63
**4837**	allXL	AA	319	357	BS2G	25.94
**4837**	allXL	AA	367	373	BS2G	13.05
**4837**	allXL	BB	370	382	BS2G	21.99
**4837**	allXL	BB	376	382	BS2G	12.76
**4837**	allXL	DD	357	373	BS2G	24.48
**4837**	allXL	DD	320	366	BS2G	16.36
**4837**	allXL	DD	370	376	BS2G	15.92
**4837**	allXL	AA	320	357	DBSU	26.16
**4837**	allXL	AA	357	319	DBSU	25.94
**4837**	allXL	DD	372	319	DBSU	14.68
**4837**	allXL	DD	370	357	DBSU	17.43
**4837**	allXL	DD	382	357	DBSU	17.1
**4837**	allXL	AA	372	370	DBSU	6.4
**4837**	allXL	DD	381	370	DBSU	21.59
**4837**	allXL	CC	373	370	DBSU	9.03
**4837**	allXL	CC	381	372	DBSU	19.93
**4837**	allXL	CC	372	382	DBSU	20.12
**4837**	allXL	BB	372	386	DBSU	29.01
**4837**	allXL	BB	373	386	DBSU	26.63
**4837**	allXL	DB	381	386	DBSU	36.08
**4837**	allXL	BD;DB	357	357	DBSU	18.4
**4837**	allXL	CB	373	319	DBSU	13.71
**4837**	allXL	DB	372	357	DBSU	39.28
**4837**	allXL	DB	381	357	DBSU	33.99
**4837**	allXL	CB	373	357	DBSU	40.62
**4837**	allXL	DA	372	370	DBSU	35.09
**4837**	allXL	DA	373	370	DBSU	36.79
**4837**	allXL	DB	382	373	DBSU	48.2
**4838**	allXL	AA	320	357	BS2G	23.68

**4838**	allXL	DD	320	373	BS2G	13.84
**4838**	allXL	BB	373	382	BS2G	18.09
**4838**	allXL	DD	370	386	BS2G	19.84
**4838**	allXL	AA	373	386	BS2G	24.85
**4838**	allXL	AA	319	357	BS2G	21.64
**4838**	allXL	AA	367	373	BS2G	11.62
**4838**	allXL	DD	370	382	BS2G	18.57
**4838**	allXL	CC	376	382	BS2G	13.69
**4838**	allXL	AA	357	373	BS2G	25.04
**4838**	allXL	BB	320	366	BS2G	22.48
**4838**	allXL	CC	370	376	BS2G	14.52
**4838**	allXL	AA	320	357	DBSU	23.68
**4838**	allXL	AA	357	319	DBSU	21.64
**4838**	allXL	DD	372	319	DBSU	11.92
**4838**	allXL	DD	370	357	DBSU	25.94
**4838**	allXL	AA	382	357	DBSU	22.15
**4838**	allXL	AA	372	370	DBSU	6.49
**4838**	allXL	DD	381	370	DBSU	20.38
**4838**	allXL	CC	373	370	DBSU	9.09
**4838**	allXL	DD	381	372	DBSU	19.9
**4838**	allXL	DD	372	382	DBSU	19.34
**4838**	allXL	DD	372	386	DBSU	23.99
**4838**	allXL	AA	373	386	DBSU	24.85
**4838**	allXL	DA	381	386	DBSU	37.64
**4838**	allXL	BD;DB	357	357	DBSU	18.81
**4838**	allXL	AD	373	319	DBSU	38.94
**4838**	allXL	CA	372	357	DBSU	37.25
**4838**	allXL	AC	381	357	DBSU	33.96
**4838**	allXL	CA	373	357	DBSU	35.58
**4838**	allXL	DA	372	370	DBSU	42.0
**4838**	allXL	DA	373	370	DBSU	42.43
**4838**	allXL	AD	382	373	DBSU	29.83
**4839**	allXL	BB	320	357	BS2G	21.85
**4839**	allXL	CC	320	373	BS2G	27.65

**4839**	allXL	AA	373	382	BS2G	16.86
**4839**	allXL	AA	370	386	BS2G	26.65
**4839**	allXL	AA	373	386	BS2G	22.06
**4839**	allXL	BB	319	357	BS2G	20.75
**4839**	allXL	AA	367	373	BS2G	10.55
**4839**	allXL	DD	370	382	BS2G	21.46
**4839**	allXL	AA	376	382	BS2G	13.58
**4839**	allXL	DD	357	373	BS2G	23.96
**4839**	allXL	AA	320	366	BS2G	22.13
**4839**	allXL	CC	370	376	BS2G	16.78
**4839**	allXL	BB	320	357	DBSU	21.85
**4839**	allXL	BB	357	319	DBSU	20.75
**4839**	allXL	CC	372	319	DBSU	25.79
**4839**	allXL	DD	370	357	DBSU	27.51
**4839**	allXL	AA	382	357	DBSU	25.07
**4839**	allXL	CC	372	370	DBSU	6.56
**4839**	allXL	DD	381	370	DBSU	21.21
**4839**	allXL	DD	373	370	DBSU	8.89
**4839**	allXL	DD	381	372	DBSU	17.19
**4839**	allXL	DD	372	382	DBSU	17.06
**4839**	allXL	DD	372	386	DBSU	23.06
**4839**	allXL	AA	373	386	DBSU	22.06
**4839**	allXL	BD	381	386	DBSU	65.77
**4839**	allXL	BD;DB	357	357	DBSU	19.8
**4839**	allXL	AD	373	319	DBSU	37.0
**4839**	allXL	DB	372	357	DBSU	42.37
**4839**	allXL	AC	381	357	DBSU	37.7
**4839**	allXL	DB	373	357	DBSU	42.58
**4839**	allXL	CB	372	370	DBSU	33.2
**4839**	allXL	CB	373	370	DBSU	33.31
**4839**	allXL	BC	382	373	DBSU	59.11
**4840**	allXL	AA	320	357	BS2G	26.14
**4840**	allXL	AA	320	373	BS2G	19.49
**4840**	allXL	CC	373	382	BS2G	20.76

**4840**	allXL	BB	370	386	BS2G	20.06
**4840**	allXL	BB	373	386	BS2G	24.21
**4840**	allXL	AA	319	357	BS2G	24.94
**4840**	allXL	DD	367	373	BS2G	13.82
**4840**	allXL	DD	370	382	BS2G	20.23
**4840**	allXL	CC	376	382	BS2G	14.38
**4840**	allXL	CC	357	373	BS2G	25.45
**4840**	allXL	AA	320	366	BS2G	21.2
**4840**	allXL	BB	370	376	BS2G	15.88
**4840**	allXL	AA	320	357	DBSU	26.14
**4840**	allXL	AA	357	319	DBSU	24.94
**4840**	allXL	AA	372	319	DBSU	19.3
**4840**	allXL	CC	370	357	DBSU	22.35
**4840**	allXL	DD	382	357	DBSU	23.65
**4840**	allXL	AA	372	370	DBSU	6.07
**4840**	allXL	DD	381	370	DBSU	18.04
**4840**	allXL	AA	373	370	DBSU	9.21
**4840**	allXL	DD	381	372	DBSU	18.74
**4840**	allXL	CC	372	382	DBSU	21.08
**4840**	allXL	BB	372	386	DBSU	24.89
**4840**	allXL	BB	373	386	DBSU	24.21
**4840**	allXL	DA	381	386	DBSU	56.63
**4840**	allXL	BD;DB	357	357	DBSU	19.11
**4840**	allXL	AD	373	319	DBSU	36.02
**4840**	allXL	CA	372	357	DBSU	38.34
**4840**	allXL	DB	381	357	DBSU	41.72
**4840**	allXL	CA	373	357	DBSU	40.01
**4840**	allXL	DA	372	370	DBSU	51.27
**4840**	allXL	DA	373	370	DBSU	48.78
**4840**	allXL	DA	382	373	DBSU	48.03
**4841**	allXL	AA	320	357	BS2G	24.19
**4841**	allXL	BB	320	373	BS2G	22.38
**4841**	allXL	DD	373	382	BS2G	18.11
**4841**	allXL	DD	370	386	BS2G	12.13

**4841**	allXL	DD	373	386	BS2G	19.99
**4841**	allXL	AA	319	357	BS2G	24.01
**4841**	allXL	AA	367	373	BS2G	13.21
**4841**	allXL	DD	370	382	BS2G	15.52
**4841**	allXL	BB	376	382	BS2G	14.26
**4841**	allXL	BB	357	373	BS2G	31.3
**4841**	allXL	BB	320	366	BS2G	23.22
**4841**	allXL	BB	370	376	BS2G	16.42
**4841**	allXL	AA	320	357	DBSU	24.19
**4841**	allXL	AA	357	319	DBSU	24.01
**4841**	allXL	BB	372	319	DBSU	24.73
**4841**	allXL	DD	370	357	DBSU	31.93
**4841**	allXL	BB	382	357	DBSU	21.48
**4841**	allXL	AA	372	370	DBSU	6.26
**4841**	allXL	DD	381	370	DBSU	15.62
**4841**	allXL	AA	373	370	DBSU	9.14
**4841**	allXL	DD	381	372	DBSU	15.41
**4841**	allXL	DD	372	382	DBSU	17.0
**4841**	allXL	DD	372	386	DBSU	18.1
**4841**	allXL	DD	373	386	DBSU	19.99
**4841**	allXL	CB	381	386	DBSU	20.64
**4841**	allXL	BD;DB	357	357	DBSU	18.28
**4841**	allXL	CB	373	319	DBSU	23.58
**4841**	allXL	CB	372	357	DBSU	26.1
**4841**	allXL	BD	381	357	DBSU	34.09
**4841**	allXL	CB	373	357	DBSU	27.63
**4841**	allXL	CB	372	370	DBSU	26.23
**4841**	allXL	CB	373	370	DBSU	23.88
**4841**	allXL	BC	382	373	DBSU	27.99
**4842**	allXL	CC	320	357	BS2G	21.51
**4842**	allXL	AA	320	373	BS2G	31.88
**4842**	allXL	BB	373	382	BS2G	18.56
**4842**	allXL	BB	370	386	BS2G	28.49
**4842**	allXL	CC	373	386	BS2G	24.94

**4842**	allXL	CC	319	357	BS2G	20.54
**4842**	allXL	BB	367	373	BS2G	10.63
**4842**	allXL	BB	370	382	BS2G	23.66
**4842**	allXL	CC	376	382	BS2G	12.91
**4842**	allXL	DD	357	373	BS2G	19.8
**4842**	allXL	AA	320	366	BS2G	23.37
**4842**	allXL	CC	370	376	BS2G	16.84
**4842**	allXL	CC	320	357	DBSU	21.51
**4842**	allXL	CC	357	319	DBSU	20.54
**4842**	allXL	AA	372	319	DBSU	28.68
**4842**	allXL	DD	370	357	DBSU	17.36
**4842**	allXL	DD	382	357	DBSU	39.3
**4842**	allXL	AA	372	370	DBSU	6.04
**4842**	allXL	DD	381	370	DBSU	22.74
**4842**	allXL	CC	373	370	DBSU	8.98
**4842**	allXL	DD	381	372	DBSU	16.32
**4842**	allXL	DD	372	382	DBSU	18.62
**4842**	allXL	CC	372	386	DBSU	24.27
**4842**	allXL	CC	373	386	DBSU	24.94
**4842**	allXL	CB	381	386	DBSU	62.7
**4842**	allXL	BD;DB	357	357	DBSU	18.12
**4842**	allXL	BC	373	319	DBSU	31.24
**4842**	allXL	DB	372	357	DBSU	33.02
**4842**	allXL	DB	381	357	DBSU	43.91
**4842**	allXL	DB	373	357	DBSU	29.97
**4842**	allXL	BD	372	370	DBSU	45.27
**4842**	allXL	DB	373	370	DBSU	45.38
**4842**	allXL	BC	382	373	DBSU	50.51
**4843**	allXL	CC	320	357	BS2G	26.04
**4843**	allXL	CC	320	373	BS2G	14.94
**4843**	allXL	DD	373	382	BS2G	17.6
**4843**	allXL	CC	370	386	BS2G	25.96
**4843**	allXL	CC	373	386	BS2G	27.32
**4843**	allXL	AA	319	357	BS2G	27.61

**4843**	allXL	AA	367	373	BS2G	14.32
**4843**	allXL	CC	370	382	BS2G	21.46
**4843**	allXL	CC	376	382	BS2G	12.55
**4843**	allXL	DD	357	373	BS2G	27.56
**4843**	allXL	AA	320	366	BS2G	23.71
**4843**	allXL	CC	370	376	BS2G	14.86
**4843**	allXL	CC	320	357	DBSU	26.04
**4843**	allXL	AA	357	319	DBSU	27.61
**4843**	allXL	CC	372	319	DBSU	12.42
**4843**	allXL	DD	370	357	DBSU	27.71
**4843**	allXL	BB	382	357	DBSU	18.17
**4843**	allXL	AA	372	370	DBSU	6.13
**4843**	allXL	DD	381	370	DBSU	21.22
**4843**	allXL	AA	373	370	DBSU	9.13
**4843**	allXL	DD	381	372	DBSU	14.56
**4843**	allXL	DD	372	382	DBSU	16.25
**4843**	allXL	DD	372	386	DBSU	27.29
**4843**	allXL	CC	373	386	DBSU	27.32
**4843**	allXL	AD	381	386	DBSU	43.76
**4843**	allXL	BD;DB	357	357	DBSU	18.75
**4843**	allXL	DA	373	319	DBSU	25.86
**4843**	allXL	DA	372	357	DBSU	40.28
**4843**	allXL	BD	381	357	DBSU	35.98
**4843**	allXL	DA	373	357	DBSU	36.89
**4843**	allXL	DA	372	370	DBSU	29.11
**4843**	allXL	DA	373	370	DBSU	29.21
**4843**	allXL	DA	382	373	DBSU	27.08
**4844**	allXL	AA	320	357	BS2G	25.19
**4844**	allXL	BB	320	373	BS2G	15.74
**4844**	allXL	DD	373	382	BS2G	20.04
**4844**	allXL	DD	370	386	BS2G	20.9
**4844**	allXL	DD	373	386	BS2G	25.41
**4844**	allXL	AA	319	357	BS2G	23.76
**4844**	allXL	AA	367	373	BS2G	12.72

**4844**	allXL	CC	370	382	BS2G	17.44
**4844**	allXL	CC	376	382	BS2G	15.13
**4844**	allXL	BB	357	373	BS2G	28.57
**4844**	allXL	DD	320	366	BS2G	20.98
**4844**	allXL	CC	370	376	BS2G	12.93
**4844**	allXL	AA	320	357	DBSU	25.19
**4844**	allXL	AA	357	319	DBSU	23.76
**4844**	allXL	BB	372	319	DBSU	18.66
**4844**	allXL	BB	370	357	DBSU	28.06
**4844**	allXL	BB	382	357	DBSU	10.46
**4844**	allXL	DD	372	370	DBSU	6.49
**4844**	allXL	CC	381	370	DBSU	17.33
**4844**	allXL	AA	373	370	DBSU	9.39
**4844**	allXL	DD	381	372	DBSU	19.38
**4844**	allXL	CC	372	382	DBSU	19.55
**4844**	allXL	DD	372	386	DBSU	24.68
**4844**	allXL	DD	373	386	DBSU	25.41
**4844**	allXL	AC	381	386	DBSU	42.49
**4844**	allXL	BD;DB	357	357	DBSU	17.79
**4844**	allXL	DA	373	319	DBSU	25.45
**4844**	allXL	DA	372	357	DBSU	34.23
**4844**	allXL	BD	381	357	DBSU	25.97
**4844**	allXL	DA	373	357	DBSU	34.91
**4844**	allXL	DA	372	370	DBSU	36.75
**4844**	allXL	DA	373	370	DBSU	34.09
**4844**	allXL	CB	382	373	DBSU	53.94
**4845**	allXL	AA	320	357	BS2G	24.58
**4845**	allXL	BB	320	373	BS2G	26.14
**4845**	allXL	DD	373	382	BS2G	16.59
**4845**	allXL	DD	370	386	BS2G	16.52
**4845**	allXL	DD	373	386	BS2G	17.76
**4845**	allXL	AA	319	357	BS2G	23.29
**4845**	allXL	BB	367	373	BS2G	12.25
**4845**	allXL	DD	370	382	BS2G	21.04

**4845**	allXL	CC	376	382	BS2G	14.65
**4845**	allXL	DD	357	373	BS2G	25.77
**4845**	allXL	BB	320	366	BS2G	26.38
**4845**	allXL	CC	370	376	BS2G	16.92
**4845**	allXL	AA	320	357	DBSU	24.58
**4845**	allXL	AA	357	319	DBSU	23.29
**4845**	allXL	BB	372	319	DBSU	24.26
**4845**	allXL	DD	370	357	DBSU	27.37
**4845**	allXL	DD	382	357	DBSU	35.15
**4845**	allXL	AA	372	370	DBSU	6.23
**4845**	allXL	DD	381	370	DBSU	21.47
**4845**	allXL	AA	373	370	DBSU	9.03
**4845**	allXL	DD	381	372	DBSU	14.59
**4845**	allXL	DD	372	382	DBSU	14.77
**4845**	allXL	DD	372	386	DBSU	14.17
**4845**	allXL	DD	373	386	DBSU	17.76
**4845**	allXL	CB	381	386	DBSU	41.38
**4845**	allXL	BD;DB	357	357	DBSU	19.21
**4845**	allXL	CB	373	319	DBSU	32.05
**4845**	allXL	BC	372	357	DBSU	37.13
**4845**	allXL	DB	381	357	DBSU	48.39
**4845**	allXL	BC	373	357	DBSU	40.57
**4845**	allXL	BC	372	370	DBSU	12.23
**4845**	allXL	BC	373	370	DBSU	15.21
**4845**	allXL	CB	382	373	DBSU	19.51
**4846**	allXL	AA	320	357	BS2G	21.77
**4846**	allXL	BB	320	373	BS2G	17.0
**4846**	allXL	CC	373	382	BS2G	18.29
**4846**	allXL	CC	370	386	BS2G	22.34
**4846**	allXL	AA	373	386	BS2G	18.48
**4846**	allXL	AA	319	357	BS2G	20.22
**4846**	allXL	AA	367	373	BS2G	12.96
**4846**	allXL	DD	370	382	BS2G	18.88
**4846**	allXL	CC	376	382	BS2G	14.22

**4846**	allXL	DD	357	373	BS2G	25.39
**4846**	allXL	AA	320	366	BS2G	20.98
**4846**	allXL	CC	370	376	BS2G	15.96
**4846**	allXL	AA	320	357	DBSU	21.77
**4846**	allXL	AA	357	319	DBSU	20.22
**4846**	allXL	BB	372	319	DBSU	21.24
**4846**	allXL	CC	370	357	DBSU	27.13
**4846**	allXL	CC	382	357	DBSU	33.71
**4846**	allXL	AA	372	370	DBSU	6.22
**4846**	allXL	DD	381	370	DBSU	19.55
**4846**	allXL	AA	373	370	DBSU	9.08
**4846**	allXL	DD	381	372	DBSU	20.09
**4846**	allXL	DD	372	382	DBSU	20.1
**4846**	allXL	AA	372	386	DBSU	20.7
**4846**	allXL	AA	373	386	DBSU	18.48
**4846**	allXL	DB	381	386	DBSU	47.46
**4846**	allXL	BD;DB	357	357	DBSU	18.33
**4846**	allXL	DA	373	319	DBSU	31.89
**4846**	allXL	DB	372	357	DBSU	36.33
**4846**	allXL	DB	381	357	DBSU	45.6
**4846**	allXL	DB	373	357	DBSU	33.9
**4846**	allXL	DA	372	370	DBSU	49.91
**4846**	allXL	DA	373	370	DBSU	52.77
**4846**	allXL	BD	382	373	DBSU	48.22
**4847**	allXL	CC	320	357	BS2G	26.07
**4847**	allXL	BB	320	373	BS2G	14.49
**4847**	allXL	AA	373	382	BS2G	19.03
**4847**	allXL	CC	370	386	BS2G	28.44
**4847**	allXL	AA	373	386	BS2G	27.1
**4847**	allXL	AA	319	357	BS2G	28.48
**4847**	allXL	AA	367	373	BS2G	12.74
**4847**	allXL	CC	370	382	BS2G	21.64
**4847**	allXL	CC	376	382	BS2G	14.8
**4847**	allXL	CC	357	373	BS2G	26.78

**4847**	allXL	BB	320	366	BS2G	13.23
**4847**	allXL	CC	370	376	BS2G	14.29
**4847**	allXL	CC	320	357	DBSU	26.07
**4847**	allXL	AA	357	319	DBSU	28.48
**4847**	allXL	BB	372	319	DBSU	15.61
**4847**	allXL	CC	370	357	DBSU	27.62
**4847**	allXL	CC	382	357	DBSU	27.12
**4847**	allXL	AA	372	370	DBSU	6.58
**4847**	allXL	CC	381	370	DBSU	22.53
**4847**	allXL	CC	373	370	DBSU	9.14
**4847**	allXL	DD	381	372	DBSU	19.49
**4847**	allXL	AA	372	382	DBSU	22.21
**4847**	allXL	AA	372	386	DBSU	29.36
**4847**	allXL	AA	373	386	DBSU	27.1
**4847**	allXL	CB	381	386	DBSU	34.92
**4847**	allXL	BD;DB	357	357	DBSU	19.04
**4847**	allXL	DA	373	319	DBSU	32.85
**4847**	allXL	AC	372	357	DBSU	34.74
**4847**	allXL	CA	381	357	DBSU	41.4
**4847**	allXL	AC	373	357	DBSU	35.46
**4847**	allXL	AC;DA	372	370	DBSU	54.4
**4847**	allXL	CB;DA	373	370	DBSU	52.31
**4847**	allXL	CB	382	373	DBSU	39.75
**4848**	allXL	AA	320	357	BS2G	24.21
**4848**	allXL	CC	320	373	BS2G	17.13
**4848**	allXL	DD	373	382	BS2G	14.04
**4848**	allXL	DD	370	386	BS2G	17.04
**4848**	allXL	DD	373	386	BS2G	21.35
**4848**	allXL	AA	319	357	BS2G	22.29
**4848**	allXL	BB	367	373	BS2G	11.32
**4848**	allXL	DD	370	382	BS2G	14.13
**4848**	allXL	CC	376	382	BS2G	14.64
**4848**	allXL	DD	357	373	BS2G	23.51
**4848**	allXL	DD	320	366	BS2G	24.45

**4848**	allXL	CC	370	376	BS2G	12.89
**4848**	allXL	AA	320	357	DBSU	24.21
**4848**	allXL	AA	357	319	DBSU	22.29
**4848**	allXL	BB	372	319	DBSU	20.81
**4848**	allXL	BB	370	357	DBSU	23.39
**4848**	allXL	CC	382	357	DBSU	30.4
**4848**	allXL	AA	372	370	DBSU	6.22
**4848**	allXL	DD	381	370	DBSU	16.7
**4848**	allXL	CC	373	370	DBSU	8.93
**4848**	allXL	DD	381	372	DBSU	15.23
**4848**	allXL	DD	372	382	DBSU	13.39
**4848**	allXL	DD	372	386	DBSU	18.64
**4848**	allXL	DD	373	386	DBSU	21.35
**4848**	allXL	CB	381	386	DBSU	59.94
**4848**	allXL	BD;DB	357	357	DBSU	18.32
**4848**	allXL	BC	373	319	DBSU	30.82
**4848**	allXL	DB	372	357	DBSU	31.01
**4848**	allXL	DB	381	357	DBSU	39.65
**4848**	allXL	DB	373	357	DBSU	29.74
**4848**	allXL	CB	372	370	DBSU	44.39
**4848**	allXL	CB	373	370	DBSU	42.83
**4848**	allXL	CB	382	373	DBSU	41.06
**4849**	allXL	AA	320	357	BS2G	23.86
**4849**	allXL	CC	320	373	BS2G	19.27
**4849**	allXL	CC	373	382	BS2G	15.14
**4849**	allXL	CC	370	386	BS2G	13.85
**4849**	allXL	CC	373	386	BS2G	17.43
**4849**	allXL	AA	319	357	BS2G	21.62
**4849**	allXL	BB	367	373	BS2G	12.2
**4849**	allXL	CC	370	382	BS2G	12.7
**4849**	allXL	CC	376	382	BS2G	14.53
**4849**	allXL	BB	357	373	BS2G	24.98
**4849**	allXL	CC	320	366	BS2G	22.96
**4849**	allXL	CC	370	376	BS2G	11.62

**4849**	allXL	AA	320	357	DBSU	23.86
**4849**	allXL	AA	357	319	DBSU	21.62
**4849**	allXL	CC	372	319	DBSU	17.95
**4849**	allXL	DD	370	357	DBSU	28.71
**4849**	allXL	BB	382	357	DBSU	24.4
**4849**	allXL	AA	372	370	DBSU	6.32
**4849**	allXL	CC	381	370	DBSU	13.75
**4849**	allXL	AA	373	370	DBSU	8.86
**4849**	allXL	DD	381	372	DBSU	16.14
**4849**	allXL	CC	372	382	DBSU	15.44
**4849**	allXL	CC	372	386	DBSU	15.61
**4849**	allXL	CC	373	386	DBSU	17.43
**4849**	allXL	BC	381	386	DBSU	52.82
**4849**	allXL	BD;DB	357	357	DBSU	18.42
**4849**	allXL	DA	373	319	DBSU	27.03
**4849**	allXL	DB	372	357	DBSU	39.11
**4849**	allXL	BD	381	357	DBSU	32.79
**4849**	allXL	DB	373	357	DBSU	36.09
**4849**	allXL	BC	372	370	DBSU	39.95
**4849**	allXL	BC	373	370	DBSU	37.84
**4849**	allXL	CB	382	373	DBSU	28.0
**4850**	allXL	AA	320	357	BS2G	26.67
**4850**	allXL	BB	320	373	BS2G	18.66
**4850**	allXL	BB	373	382	BS2G	19.79
**4850**	allXL	DD	370	386	BS2G	23.03
**4850**	allXL	CC	373	386	BS2G	25.9
**4850**	allXL	AA	319	357	BS2G	26.31
**4850**	allXL	DD	367	373	BS2G	13.76
**4850**	allXL	DD	370	382	BS2G	20.12
**4850**	allXL	CC	376	382	BS2G	13.86
**4850**	allXL	BB	357	373	BS2G	27.41
**4850**	allXL	CC	320	366	BS2G	16.76
**4850**	allXL	CC	370	376	BS2G	13.53
**4850**	allXL	AA	320	357	DBSU	26.67

**4850**	allXL	AA	357	319	DBSU	26.31
**4850**	allXL	CC	372	319	DBSU	15.06
**4850**	allXL	DD	370	357	DBSU	28.35
**4850**	allXL	DD	382	357	DBSU	19.64
**4850**	allXL	AA	372	370	DBSU	5.9
**4850**	allXL	DD	381	370	DBSU	20.65
**4850**	allXL	AA	373	370	DBSU	8.95
**4850**	allXL	BB	381	372	DBSU	22.43
**4850**	allXL	BB	372	382	DBSU	22.58
**4850**	allXL	DD	372	386	DBSU	28.56
**4850**	allXL	CC	373	386	DBSU	25.9
**4850**	allXL	DB	381	386	DBSU	19.7
**4850**	allXL	BD;DB	357	357	DBSU	17.71
**4850**	allXL	DA	373	319	DBSU	26.07
**4850**	allXL	BD	372	357	DBSU	44.21
**4850**	allXL	DB	381	357	DBSU	25.63
**4850**	allXL	DB	373	357	DBSU	41.28
**4850**	allXL	CB	372	370	DBSU	35.85
**4850**	allXL	CB	373	370	DBSU	39.37
**4850**	allXL	BD	382	373	DBSU	47.48
**4851**	allXL	CC	320	357	BS2G	22.55
**4851**	allXL	BB	320	373	BS2G	24.61
**4851**	allXL	CC	373	382	BS2G	19.0
**4851**	allXL	DD	370	386	BS2G	26.35
**4851**	allXL	CC	373	386	BS2G	25.17
**4851**	allXL	CC	319	357	BS2G	21.05
**4851**	allXL	AA	367	373	BS2G	13.45
**4851**	allXL	DD	370	382	BS2G	24.7
**4851**	allXL	CC	376	382	BS2G	14.45
**4851**	allXL	DD	357	373	BS2G	22.57
**4851**	allXL	DD	320	366	BS2G	21.84
**4851**	allXL	CC	370	376	BS2G	16.12
**4851**	allXL	CC	320	357	DBSU	22.55
**4851**	allXL	CC	357	319	DBSU	21.05

**4851**	allXL	DD	372	319	DBSU	24.15
**4851**	allXL	DD	370	357	DBSU	18.84
**4851**	allXL	DD	382	357	DBSU	28.09
**4851**	allXL	AA	372	370	DBSU	6.25
**4851**	allXL	AA	381	370	DBSU	23.07
**4851**	allXL	AA	373	370	DBSU	9.29
**4851**	allXL	AA	381	372	DBSU	20.8
**4851**	allXL	CC	372	382	DBSU	22.56
**4851**	allXL	CC	372	386	DBSU	27.74
**4851**	allXL	CC	373	386	DBSU	25.17
**4851**	allXL	AD	381	386	DBSU	56.3
**4851**	allXL	BD;DB	357	357	DBSU	18.68
**4851**	allXL	AD	373	319	DBSU	31.36
**4851**	allXL	AC	372	357	DBSU	39.31
**4851**	allXL	DB	381	357	DBSU	40.55
**4851**	allXL	AC	373	357	DBSU	38.0
**4851**	allXL	AD	372	370	DBSU	51.71
**4851**	allXL	AD	373	370	DBSU	48.12
**4851**	allXL	AD	382	373	DBSU	50.09
**4852**	allXL	CC	320	357	BS2G	24.86
**4852**	allXL	AA	320	373	BS2G	15.69
**4852**	allXL	AA	373	382	BS2G	21.15
**4852**	allXL	AA	370	386	BS2G	33.71
**4852**	allXL	AA	373	386	BS2G	31.04
**4852**	allXL	CC	319	357	BS2G	26.43
**4852**	allXL	AA	367	373	BS2G	12.85
**4852**	allXL	BB	370	382	BS2G	25.45
**4852**	allXL	BB	376	382	BS2G	14.89
**4852**	allXL	BB	357	373	BS2G	26.52
**4852**	allXL	AA	320	366	BS2G	17.79
**4852**	allXL	CC	370	376	BS2G	16.35
**4852**	allXL	CC	320	357	DBSU	24.86
**4852**	allXL	CC	357	319	DBSU	26.43
**4852**	allXL	AA	372	319	DBSU	15.5

**4852**	allXL	DD	370	357	DBSU	28.39
**4852**	allXL	BB	382	357	DBSU	43.43
**4852**	allXL	CC	372	370	DBSU	6.64
**4852**	allXL	BB	381	370	DBSU	24.77
**4852**	allXL	AA	373	370	DBSU	9.26
**4852**	allXL	BB	381	372	DBSU	23.76
**4852**	allXL	AA	372	382	DBSU	24.44
**4852**	allXL	AA	372	386	DBSU	33.81
**4852**	allXL	AA	373	386	DBSU	31.04
**4852**	allXL	DA	381	386	DBSU	29.42
**4852**	allXL	BD;DB	357	357	DBSU	19.88
**4852**	allXL	CB	373	319	DBSU	18.7
**4852**	allXL	AD	372	357	DBSU	41.06
**4852**	allXL	AD	381	357	DBSU	49.52
**4852**	allXL	AD	373	357	DBSU	40.48
**4852**	allXL	AD	372	370	DBSU	31.4
**4852**	allXL	AD	373	370	DBSU	28.42
**4852**	allXL	AD	382	373	DBSU	29.76
**4853**	allXL	AA	320	357	BS2G	24.56
**4853**	allXL	CC	320	373	BS2G	23.19
**4853**	allXL	DD	373	382	BS2G	18.07
**4853**	allXL	DD	370	386	BS2G	23.25
**4853**	allXL	DD	373	386	BS2G	26.12
**4853**	allXL	AA	319	357	BS2G	23.47
**4853**	allXL	DD	367	373	BS2G	13.83
**4853**	allXL	DD	370	382	BS2G	19.2
**4853**	allXL	BB	376	382	BS2G	14.84
**4853**	allXL	CC	357	373	BS2G	26.93
**4853**	allXL	AA	320	366	BS2G	20.42
**4853**	allXL	CC	370	376	BS2G	14.06
**4853**	allXL	AA	320	357	DBSU	24.56
**4853**	allXL	AA	357	319	DBSU	23.47
**4853**	allXL	CC	372	319	DBSU	21.76
**4853**	allXL	CC	370	357	DBSU	25.55

**4853**	allXL	BB	382	357	DBSU	30.16
**4853**	allXL	AA	372	370	DBSU	6.02
**4853**	allXL	DD	381	370	DBSU	20.79
**4853**	allXL	AA	373	370	DBSU	9.12
**4853**	allXL	DD	381	372	DBSU	21.13
**4853**	allXL	DD	372	382	DBSU	20.83
**4853**	allXL	DD	372	386	DBSU	27.74
**4853**	allXL	DD	373	386	DBSU	26.12
**4853**	allXL	DB	381	386	DBSU	50.99
**4853**	allXL	BD;DB	357	357	DBSU	18.96
**4853**	allXL	AD	373	319	DBSU	21.59
**4853**	allXL	CA	372	357	DBSU	44.01
**4853**	allXL	DB	381	357	DBSU	43.85
**4853**	allXL	AD	373	357	DBSU	42.71
**4853**	allXL	AD	372	370	DBSU	42.71
**4853**	allXL	AD	373	370	DBSU	42.02
**4853**	allXL	AD	382	373	DBSU	51.66
**4854**	allXL	AA	320	357	BS2G	25.25
**4854**	allXL	AA	320	373	BS2G	17.31
**4854**	allXL	AA	373	382	BS2G	20.69
**4854**	allXL	CC	370	386	BS2G	21.43
**4854**	allXL	AA	373	386	BS2G	23.38
**4854**	allXL	AA	319	357	BS2G	23.43
**4854**	allXL	AA	367	373	BS2G	12.98
**4854**	allXL	CC	370	382	BS2G	21.12
**4854**	allXL	CC	376	382	BS2G	14.56
**4854**	allXL	BB	357	373	BS2G	30.49
**4854**	allXL	AA	320	366	BS2G	17.58
**4854**	allXL	CC	370	376	BS2G	13.27
**4854**	allXL	AA	320	357	DBSU	25.25
**4854**	allXL	AA	357	319	DBSU	23.43
**4854**	allXL	AA	372	319	DBSU	16.85
**4854**	allXL	CC	370	357	DBSU	28.57
**4854**	allXL	AA	382	357	DBSU	42.19

**4854**	allXL	AA	372	370	DBSU	6.34
**4854**	allXL	CC	381	370	DBSU	22.75
**4854**	allXL	DD	373	370	DBSU	8.49
**4854**	allXL	AA	381	372	DBSU	22.09
**4854**	allXL	AA	372	382	DBSU	22.4
**4854**	allXL	AA	372	386	DBSU	23.29
**4854**	allXL	AA	373	386	DBSU	23.38
**4854**	allXL	DA	381	386	DBSU	38.69
**4854**	allXL	BD;DB	357	357	DBSU	18.62
**4854**	allXL	AD	373	319	DBSU	42.01
**4854**	allXL	CA	372	357	DBSU	43.29
**4854**	allXL	CA	381	357	DBSU	53.02
**4854**	allXL	CA	373	357	DBSU	42.86
**4854**	allXL	DA	372	370	DBSU	44.03
**4854**	allXL	DA	373	370	DBSU	41.67
**4854**	allXL	DA	382	373	DBSU	51.23
**4855**	allXL	CC	320	357	BS2G	24.69
**4855**	allXL	CC	320	373	BS2G	18.92
**4855**	allXL	DD	373	382	BS2G	18.94
**4855**	allXL	CC	370	386	BS2G	22.1
**4855**	allXL	DD	373	386	BS2G	28.05
**4855**	allXL	CC	319	357	BS2G	25.11
**4855**	allXL	BB	367	373	BS2G	13.38
**4855**	allXL	CC	370	382	BS2G	19.49
**4855**	allXL	CC	376	382	BS2G	14.63
**4855**	allXL	CC	357	373	BS2G	25.32
**4855**	allXL	AA	320	366	BS2G	25.69
**4855**	allXL	CC	370	376	BS2G	13.48
**4855**	allXL	CC	320	357	DBSU	24.69
**4855**	allXL	CC	357	319	DBSU	25.11
**4855**	allXL	DD	372	319	DBSU	19.59
**4855**	allXL	CC	370	357	DBSU	24.18
**4855**	allXL	DD	382	357	DBSU	36.15
**4855**	allXL	AA	372	370	DBSU	6.4

**4855**	allXL	CC	381	370	DBSU	21.8
**4855**	allXL	AA	373	370	DBSU	9.29
**4855**	allXL	DD	381	372	DBSU	17.99
**4855**	allXL	DD	372	382	DBSU	18.3
**4855**	allXL	CC	372	386	DBSU	26.84
**4855**	allXL	DD	373	386	DBSU	28.05
**4855**	allXL	BC	381	386	DBSU	44.06
**4855**	allXL	BD;DB	357	357	DBSU	17.91
**4855**	allXL	DA	373	319	DBSU	28.68
**4855**	allXL	CB	372	357	DBSU	25.96
**4855**	allXL	CB	381	357	DBSU	38.81
**4855**	allXL	CB	373	357	DBSU	28.01
**4855**	allXL	CB	372	370	DBSU	35.3
**4855**	allXL	CB	373	370	DBSU	36.77
**4855**	allXL	CB	382	373	DBSU	31.6
**4856**	allXL	CC	320	357	BS2G	24.22
**4856**	allXL	DD	320	373	BS2G	22.14
**4856**	allXL	DD	373	382	BS2G	17.18
**4856**	allXL	BB	370	386	BS2G	20.68
**4856**	allXL	BB	373	386	BS2G	25.52
**4856**	allXL	CC	319	357	BS2G	25.74
**4856**	allXL	AA	367	373	BS2G	12.36
**4856**	allXL	BB	370	382	BS2G	18.12
**4856**	allXL	CC	376	382	BS2G	14.72
**4856**	allXL	BB	357	373	BS2G	23.9
**4856**	allXL	AA	320	366	BS2G	18.29
**4856**	allXL	BB;DD	370	376	BS2G	15.65
**4856**	allXL	CC	320	357	DBSU	24.22
**4856**	allXL	CC	357	319	DBSU	25.74
**4856**	allXL	DD	372	319	DBSU	21.98
**4856**	allXL	BB	370	357	DBSU	19.56
**4856**	allXL	DD	382	357	DBSU	25.03
**4856**	allXL	AA	372	370	DBSU	6.27
**4856**	allXL	BB	381	370	DBSU	17.97

**4856**	allXL	AA	373	370	DBSU	9.24
**4856**	allXL	DD	381	372	DBSU	15.8
**4856**	allXL	DD	372	382	DBSU	16.42
**4856**	allXL	BB	372	386	DBSU	25.78
**4856**	allXL	BB	373	386	DBSU	25.52
**4856**	allXL	DB	381	386	DBSU	23.61
**4856**	allXL	BD;DB	357	357	DBSU	17.94
**4856**	allXL	CB	373	319	DBSU	27.61
**4856**	allXL	CB	372	357	DBSU	20.02
**4856**	allXL	CB	381	357	DBSU	12.1
**4856**	allXL	CB	373	357	DBSU	19.35
**4856**	allXL	CB	372	370	DBSU	38.81
**4856**	allXL	CB	373	370	DBSU	38.6
**4856**	allXL	CB	382	373	DBSU	27.19
**4857**	allXL	AA	320	357	BS2G	23.97
**4857**	allXL	BB	320	373	BS2G	19.54
**4857**	allXL	DD	373	382	BS2G	18.47
**4857**	allXL	DD	370	386	BS2G	15.02
**4857**	allXL	DD	373	386	BS2G	19.09
**4857**	allXL	AA	319	357	BS2G	22.55
**4857**	allXL	AA	367	373	BS2G	13.49
**4857**	allXL	DD	370	382	BS2G	19.51
**4857**	allXL	CC	376	382	BS2G	14.75
**4857**	allXL	BB	357	373	BS2G	24.09
**4857**	allXL	BB	320	366	BS2G	25.91
**4857**	allXL	BB	370	376	BS2G	16.68
**4857**	allXL	AA	320	357	DBSU	23.97
**4857**	allXL	AA	357	319	DBSU	22.55
**4857**	allXL	BB	372	319	DBSU	20.89
**4857**	allXL	DD	370	357	DBSU	23.48
**4857**	allXL	DD	382	357	DBSU	34.0
**4857**	allXL	AA	372	370	DBSU	6.32
**4857**	allXL	DD	381	370	DBSU	19.58
**4857**	allXL	DD	373	370	DBSU	9.11

**4857**	allXL	DD	381	372	DBSU	17.71
**4857**	allXL	DD	372	382	DBSU	18.91
**4857**	allXL	DD	372	386	DBSU	17.53
**4857**	allXL	DD	373	386	DBSU	19.09
**4857**	allXL	BC	381	386	DBSU	57.91
**4857**	allXL	BD;DB	357	357	DBSU	18.43
**4857**	allXL	DA	373	319	DBSU	24.99
**4857**	allXL	DA	372	357	DBSU	33.63
**4857**	allXL	AC	381	357	DBSU	33.66
**4857**	allXL	DA	373	357	DBSU	30.63
**4857**	allXL	BC	372	370	DBSU	37.25
**4857**	allXL	DA	373	370	DBSU	39.77
**4857**	allXL	DA	382	373	DBSU	46.45
**4858**	allXL	AA	320	357	BS2G	26.85
**4858**	allXL	BB	320	373	BS2G	26.41
**4858**	allXL	BB	373	382	BS2G	14.08
**4858**	allXL	BB	370	386	BS2G	21.07
**4858**	allXL	BB	373	386	BS2G	23.75
**4858**	allXL	AA	319	357	BS2G	26.52
**4858**	allXL	DD	367	373	BS2G	14.73
**4858**	allXL	BB	370	382	BS2G	14.71
**4858**	allXL	BB	376	382	BS2G	12.76
**4858**	allXL	DD	357	373	BS2G	34.46
**4858**	allXL	BB	320	366	BS2G	26.87
**4858**	allXL	CC	370	376	BS2G	13.85
**4858**	allXL	AA	320	357	DBSU	26.85
**4858**	allXL	AA	357	319	DBSU	26.52
**4858**	allXL	CC	372	319	DBSU	24.84
**4858**	allXL	BB	370	357	DBSU	28.01
**4858**	allXL	BB	382	357	DBSU	38.98
**4858**	allXL	AA	372	370	DBSU	6.17
**4858**	allXL	BB	381	370	DBSU	17.64
**4858**	allXL	DD	373	370	DBSU	9.13
**4858**	allXL	DD	381	372	DBSU	17.31

**4858**	allXL	BB	372	382	DBSU	16.09
**4858**	allXL	BB	372	386	DBSU	24.36
**4858**	allXL	BB	373	386	DBSU	23.75
**4858**	allXL	CB	381	386	DBSU	49.44
**4858**	allXL	BD;DB	357	357	DBSU	18.36
**4858**	allXL	DA	373	319	DBSU	23.36
**4858**	allXL	CB	372	357	DBSU	37.82
**4858**	allXL	DA	381	357	DBSU	32.91
**4858**	allXL	DA	373	357	DBSU	35.74
**4858**	allXL	BC	372	370	DBSU	26.79
**4858**	allXL	BC	373	370	DBSU	29.4
**4858**	allXL	CB	382	373	DBSU	36.59
**4859**	allXL	AA	320	357	BS2G	25.22
**4859**	allXL	BB	320	373	BS2G	22.76
**4859**	allXL	AA	373	382	BS2G	21.82
**4859**	allXL	CC	370	386	BS2G	33.89
**4859**	allXL	AA	373	386	BS2G	31.43
**4859**	allXL	AA	319	357	BS2G	23.85
**4859**	allXL	AA	367	373	BS2G	11.02
**4859**	allXL	CC	370	382	BS2G	24.87
**4859**	allXL	CC	376	382	BS2G	15.15
**4859**	allXL	AA	357	373	BS2G	25.08
**4859**	allXL	DD	320	366	BS2G	17.4
**4859**	allXL	CC	370	376	BS2G	15.23
**4859**	allXL	AA	320	357	DBSU	25.22
**4859**	allXL	AA	357	319	DBSU	23.85
**4859**	allXL	DD	372	319	DBSU	16.79
**4859**	allXL	CC	370	357	DBSU	24.8
**4859**	allXL	CC	382	357	DBSU	27.67
**4859**	allXL	AA	372	370	DBSU	6.68
**4859**	allXL	CC	381	370	DBSU	25.14
**4859**	allXL	AA	373	370	DBSU	8.89
**4859**	allXL	BB	381	372	DBSU	22.56
**4859**	allXL	AA	372	382	DBSU	22.48

**4859**	allXL	AA	372	386	DBSU	30.96
**4859**	allXL	AA	373	386	DBSU	31.43
**4859**	allXL	CB	381	386	DBSU	44.55
**4859**	allXL	BD;DB	357	357	DBSU	20.63
**4859**	allXL	CB	373	319	DBSU	16.08
**4859**	allXL	CB	372	357	DBSU	28.91
**4859**	allXL	CA	381	357	DBSU	35.81
**4859**	allXL	CB	373	357	DBSU	26.08
**4859**	allXL	DA	372	370	DBSU	23.57
**4859**	allXL	DA	373	370	DBSU	24.73
**4859**	allXL	DA	382	373	DBSU	32.41
**4860**	allXL	AA	320	357	BS2G	26.13
**4860**	allXL	BB	320	373	BS2G	18.25
**4860**	allXL	BB	373	382	BS2G	18.45
**4860**	allXL	DD	370	386	BS2G	26.94
**4860**	allXL	AA	373	386	BS2G	24.25
**4860**	allXL	AA	319	357	BS2G	24.81
**4860**	allXL	AA	367	373	BS2G	12.3
**4860**	allXL	BB	370	382	BS2G	23.01
**4860**	allXL	BB	376	382	BS2G	14.59
**4860**	allXL	DD	357	373	BS2G	31.3
**4860**	allXL	AA	320	366	BS2G	15.04
**4860**	allXL	DD	370	376	BS2G	16.95
**4860**	allXL	AA	320	357	DBSU	26.13
**4860**	allXL	AA	357	319	DBSU	24.81
**4860**	allXL	BB	372	319	DBSU	19.62
**4860**	allXL	DD	370	357	DBSU	23.7
**4860**	allXL	AA	382	357	DBSU	28.65
**4860**	allXL	AA	372	370	DBSU	6.37
**4860**	allXL	DD	381	370	DBSU	20.99
**4860**	allXL	AA	373	370	DBSU	9.11
**4860**	allXL	BB	381	372	DBSU	21.67
**4860**	allXL	BB	372	382	DBSU	20.66
**4860**	allXL	AA	372	386	DBSU	26.94

**4860**	allXL	AA	373	386	DBSU	24.25
**4860**	allXL	BC	381	386	DBSU	46.0
**4860**	allXL	BD;DB	357	357	DBSU	19.26
**4860**	allXL	AD	373	319	DBSU	16.56
**4860**	allXL	BC	372	357	DBSU	27.31
**4860**	allXL	DB	381	357	DBSU	44.77
**4860**	allXL	BC	373	357	DBSU	28.0
**4860**	allXL	BC	372	370	DBSU	12.86
**4860**	allXL	BC	373	370	DBSU	12.64
**4860**	allXL	BC	382	373	DBSU	26.44
**4861**	allXL	AA	320	357	BS2G	21.83
**4861**	allXL	CC	320	373	BS2G	22.09
**4861**	allXL	BB	373	382	BS2G	20.22
**4861**	allXL	CC	370	386	BS2G	24.44
**4861**	allXL	BB	373	386	BS2G	29.79
**4861**	allXL	AA	319	357	BS2G	20.71
**4861**	allXL	BB	367	373	BS2G	13.95
**4861**	allXL	CC;DD	370	382	BS2G	19.69
**4861**	allXL	BB	376	382	BS2G	14.44
**4861**	allXL	BB	357	373	BS2G	26.34
**4861**	allXL	BB	320	366	BS2G	23.48
**4861**	allXL	CC	370	376	BS2G	15.01
**4861**	allXL	AA	320	357	DBSU	21.83
**4861**	allXL	AA	357	319	DBSU	20.71
**4861**	allXL	CC	372	319	DBSU	21.43
**4861**	allXL	DD	370	357	DBSU	25.73
**4861**	allXL	BB	382	357	DBSU	21.36
**4861**	allXL	AA	372	370	DBSU	6.01
**4861**	allXL	DD	381	370	DBSU	17.5
**4861**	allXL	CC	373	370	DBSU	9.33
**4861**	allXL	DD	381	372	DBSU	19.33
**4861**	allXL	AA	372	382	DBSU	21.21
**4861**	allXL	CC	372	386	DBSU	28.6
**4861**	allXL	BB	373	386	DBSU	29.79

**4861**	allXL	BC	381	386	DBSU	47.0
**4861**	allXL	BD;DB	357	357	DBSU	18.05
**4861**	allXL	CB	373	319	DBSU	35.74
**4861**	allXL	CB	372	357	DBSU	44.89
**4861**	allXL	BD	381	357	DBSU	34.24
**4861**	allXL	BD	373	357	DBSU	43.18
**4861**	allXL	CB	372	370	DBSU	40.95
**4861**	allXL	CB	373	370	DBSU	44.37
**4861**	allXL	CB	382	373	DBSU	49.19
**4862**	allXL	CC	320	357	BS2G	22.39
**4862**	allXL	DD	320	373	BS2G	18.71
**4862**	allXL	DD	373	382	BS2G	19.71
**4862**	allXL	CC	370	386	BS2G	25.89
**4862**	allXL	AA	373	386	BS2G	22.99
**4862**	allXL	CC	319	357	BS2G	25.63
**4862**	allXL	AA	367	373	BS2G	12.31
**4862**	allXL	DD	370	382	BS2G	23.3
**4862**	allXL	CC	376	382	BS2G	14.81
**4862**	allXL	BB	357	373	BS2G	19.4
**4862**	allXL	AA	320	366	BS2G	22.23
**4862**	allXL	DD	370	376	BS2G	16.06
**4862**	allXL	CC	320	357	DBSU	22.39
**4862**	allXL	CC	357	319	DBSU	25.63
**4862**	allXL	DD	372	319	DBSU	22.4
**4862**	allXL	BB	370	357	DBSU	23.94
**4862**	allXL	BB	382	357	DBSU	31.19
**4862**	allXL	AA	372	370	DBSU	6.59
**4862**	allXL	DD	381	370	DBSU	21.86
**4862**	allXL	AA	373	370	DBSU	9.29
**4862**	allXL	DD	381	372	DBSU	20.77
**4862**	allXL	DD	372	382	DBSU	21.44
**4862**	allXL	AA	372	386	DBSU	25.05
**4862**	allXL	AA	373	386	DBSU	22.99
**4862**	allXL	AD	381	386	DBSU	37.49

**4862**	allXL	BD;DB	357	357	DBSU	19.02
**4862**	allXL	DA	373	319	DBSU	30.32
**4862**	allXL	BD	372	357	DBSU	32.08
**4862**	allXL	BD	381	357	DBSU	31.47
**4862**	allXL	BD	373	357	DBSU	32.35
**4862**	allXL	DA	372	370	DBSU	35.39
**4862**	allXL	DA	373	370	DBSU	35.66
**4862**	allXL	DA	382	373	DBSU	21.16
**4863**	allXL	CC	320	357	BS2G	25.41
**4863**	allXL	DD	320	373	BS2G	10.33
**4863**	allXL	DD	373	382	BS2G	16.58
**4863**	allXL	CC	370	386	BS2G	20.86
**4863**	allXL	CC	373	386	BS2G	26.16
**4863**	allXL	CC	319	357	BS2G	24.57
**4863**	allXL	AA	367	373	BS2G	12.19
**4863**	allXL	DD	370	382	BS2G	19.84
**4863**	allXL	CC	376	382	BS2G	13.64
**4863**	allXL	BB	357	373	BS2G	25.53
**4863**	allXL	AA	320	366	BS2G	12.15
**4863**	allXL	CC	370	376	BS2G	15.21
**4863**	allXL	CC	320	357	DBSU	25.41
**4863**	allXL	CC	357	319	DBSU	24.57
**4863**	allXL	DD	372	319	DBSU	8.75
**4863**	allXL	DD	370	357	DBSU	25.55
**4863**	allXL	AA	382	357	DBSU	27.18
**4863**	allXL	AA	372	370	DBSU	6.56
**4863**	allXL	DD	381	370	DBSU	19.46
**4863**	allXL	DD	373	370	DBSU	9.03
**4863**	allXL	DD	381	372	DBSU	14.41
**4863**	allXL	DD	372	382	DBSU	14.83
**4863**	allXL	DD	372	386	DBSU	23.96
**4863**	allXL	CC	373	386	DBSU	26.16
**4863**	allXL	DA	381	386	DBSU	32.07
**4863**	allXL	BD;DB	357	357	DBSU	19.14

**4863**	allXL	BC	373	319	DBSU	34.31
**4863**	allXL	CB	372	357	DBSU	40.53
**4863**	allXL	BD	381	357	DBSU	38.05
**4863**	allXL	CB	373	357	DBSU	40.34
**4863**	allXL	CB	372	370	DBSU	23.96
**4863**	allXL	CB	373	370	DBSU	20.91
**4863**	allXL	BC	382	373	DBSU	21.4
**4864**	allXL	AA	320	357	BS2G	18.34
**4864**	allXL	DD	320	373	BS2G	23.93
**4864**	allXL	CC	373	382	BS2G	18.88
**4864**	allXL	AA	370	386	BS2G	20.83
**4864**	allXL	AA	373	386	BS2G	19.48
**4864**	allXL	AA	319	357	BS2G	21.49
**4864**	allXL	BB	367	373	BS2G	11.31
**4864**	allXL	DD	370	382	BS2G	21.59
**4864**	allXL	CC	376	382	BS2G	12.39
**4864**	allXL	BB	357	373	BS2G	26.06
**4864**	allXL	CC	320	366	BS2G	17.25
**4864**	allXL	BB	370	376	BS2G	16.54
**4864**	allXL	AA	320	357	DBSU	18.34
**4864**	allXL	AA	357	319	DBSU	21.49
**4864**	allXL	CC	372	319	DBSU	23.94
**4864**	allXL	BB	370	357	DBSU	25.54
**4864**	allXL	BB	382	357	DBSU	21.15
**4864**	allXL	AA	372	370	DBSU	6.06
**4864**	allXL	DD	381	370	DBSU	21.61
**4864**	allXL	AA	373	370	DBSU	9.21
**4864**	allXL	BB	381	372	DBSU	19.69
**4864**	allXL	CC	372	382	DBSU	20.68
**4864**	allXL	AA	372	386	DBSU	19.8
**4864**	allXL	AA	373	386	DBSU	19.48
**4864**	allXL	DA	381	386	DBSU	27.39
**4864**	allXL	BD;DB	357	357	DBSU	18.67
**4864**	allXL	AD	373	319	DBSU	32.35

**4864**	allXL	BD	372	357	DBSU	35.26
**4864**	allXL	BD	381	357	DBSU	29.7
**4864**	allXL	BD	373	357	DBSU	35.51
**4864**	allXL	DA	372	370	DBSU	28.37
**4864**	allXL	DA	373	370	DBSU	26.88
**4864**	allXL	DA	382	373	DBSU	17.57
**4865**	allXL	AA	320	357	BS2G	24.66
**4865**	allXL	DD	320	373	BS2G	14.7
**4865**	allXL	CC	373	382	BS2G	15.18
**4865**	allXL	DD	370	386	BS2G	14.97
**4865**	allXL	CC	373	386	BS2G	18.56
**4865**	allXL	AA	319	357	BS2G	26.34
**4865**	allXL	AA	367	373	BS2G	13.52
**4865**	allXL	DD	370	382	BS2G	17.57
**4865**	allXL	BB	376	382	BS2G	12.3
**4865**	allXL	DD	357	373	BS2G	20.48
**4865**	allXL	BB	320	366	BS2G	20.31
**4865**	allXL	DD	370	376	BS2G	15.99
**4865**	allXL	AA	320	357	DBSU	24.66
**4865**	allXL	AA	357	319	DBSU	26.34
**4865**	allXL	DD	372	319	DBSU	10.97
**4865**	allXL	DD	370	357	DBSU	26.01
**4865**	allXL	DD	382	357	DBSU	23.22
**4865**	allXL	AA	372	370	DBSU	6.3
**4865**	allXL	DD	381	370	DBSU	17.54
**4865**	allXL	CC	373	370	DBSU	9.01
**4865**	allXL	CC	381	372	DBSU	20.44
**4865**	allXL	CC	372	382	DBSU	18.32
**4865**	allXL	CC	372	386	DBSU	20.7
**4865**	allXL	CC	373	386	DBSU	18.56
**4865**	allXL	BC	381	386	DBSU	62.96
**4865**	allXL	BD;DB	357	357	DBSU	18.74
**4865**	allXL	AD	373	319	DBSU	21.48
**4865**	allXL	DB	372	357	DBSU	40.64

**4865**	allXL	DB	381	357	DBSU	37.39
**4865**	allXL	DB	373	357	DBSU	38.04
**4865**	allXL	DA	372	370	DBSU	23.07
**4865**	allXL	DA	373	370	DBSU	23.73
**4865**	allXL	AD	382	373	DBSU	47.45
**4866**	allXL	BB	320	357	BS2G	26.31
**4866**	allXL	CC	320	373	BS2G	34.85
**4866**	allXL	DD	373	382	BS2G	17.33
**4866**	allXL	BB	370	386	BS2G	19.65
**4866**	allXL	BB	373	386	BS2G	18.47
**4866**	allXL	AA	319	357	BS2G	27.67
**4866**	allXL	AA	367	373	BS2G	12.55
**4866**	allXL	BB	370	382	BS2G	19.72
**4866**	allXL	BB	376	382	BS2G	13.01
**4866**	allXL	AA	357	373	BS2G	29.72
**4866**	allXL	BB	320	366	BS2G	20.24
**4866**	allXL	CC	370	376	BS2G	17.23
**4866**	allXL	BB	320	357	DBSU	26.31
**4866**	allXL	AA	357	319	DBSU	27.67
**4866**	allXL	CC	372	319	DBSU	34.95
**4866**	allXL	DD	370	357	DBSU	28.96
**4866**	allXL	AA	382	357	DBSU	32.03
**4866**	allXL	AA	372	370	DBSU	6.25
**4866**	allXL	BB	381	370	DBSU	22.88
**4866**	allXL	AA	373	370	DBSU	9.03
**4866**	allXL	DD	381	372	DBSU	17.1
**4866**	allXL	DD	372	382	DBSU	18.68
**4866**	allXL	BB	372	386	DBSU	20.55
**4866**	allXL	BB	373	386	DBSU	18.47
**4866**	allXL	AC	381	386	DBSU	66.78
**4866**	allXL	BD;DB	357	357	DBSU	20.03
**4866**	allXL	CB	373	319	DBSU	26.37
**4866**	allXL	CB	372	357	DBSU	40.28
**4866**	allXL	AC	381	357	DBSU	32.0

**4866**	allXL	AC	373	357	DBSU	41.19
**4866**	allXL	CB	372	370	DBSU	37.58
**4866**	allXL	CB	373	370	DBSU	41.28
**4866**	allXL	BC	382	373	DBSU	57.69
**4867**	allXL	AA	320	357	BS2G	23.53
**4867**	allXL	BB	320	373	BS2G	9.0
**4867**	allXL	BB	373	382	BS2G	17.38
**4867**	allXL	DD	370	386	BS2G	15.52
**4867**	allXL	DD	373	386	BS2G	22.16
**4867**	allXL	AA	319	357	BS2G	22.16
**4867**	allXL	AA	367	373	BS2G	12.56
**4867**	allXL	DD	370	382	BS2G	15.98
**4867**	allXL	CC	376	382	BS2G	13.83
**4867**	allXL	AA	357	373	BS2G	21.04
**4867**	allXL	DD	320	366	BS2G	18.9
**4867**	allXL	DD	370	376	BS2G	15.75
**4867**	allXL	AA	320	357	DBSU	23.53
**4867**	allXL	AA	357	319	DBSU	22.16
**4867**	allXL	BB	372	319	DBSU	13.08
**4867**	allXL	AA	370	357	DBSU	24.05
**4867**	allXL	BB	382	357	DBSU	24.19
**4867**	allXL	AA	372	370	DBSU	6.34
**4867**	allXL	DD	381	370	DBSU	16.27
**4867**	allXL	AA	373	370	DBSU	9.12
**4867**	allXL	DD	381	372	DBSU	19.27
**4867**	allXL	BB	372	382	DBSU	19.83
**4867**	allXL	DD	372	386	DBSU	20.34
**4867**	allXL	DD	373	386	DBSU	22.16
**4867**	allXL	DB	381	386	DBSU	55.95
**4867**	allXL	BD;DB	357	357	DBSU	18.45
**4867**	allXL	CB	373	319	DBSU	23.62
**4867**	allXL	AC	372	357	DBSU	21.44
**4867**	allXL	BD	381	357	DBSU	33.08
**4867**	allXL	AC	373	357	DBSU	20.72

**4867**	allXL	CB	372	370	DBSU	15.61
**4867**	allXL	CB	373	370	DBSU	13.2
**4867**	allXL	CB	382	373	DBSU	37.55
**4868**	allXL	CC	320	357	BS2G	23.57
**4868**	allXL	DD	320	373	BS2G	22.31
**4868**	allXL	DD	373	382	BS2G	17.67
**4868**	allXL	DD	370	386	BS2G	11.68
**4868**	allXL	DD	373	386	BS2G	15.79
**4868**	allXL	CC	319	357	BS2G	27.11
**4868**	allXL	AA	367	373	BS2G	10.93
**4868**	allXL	DD	370	382	BS2G	18.29
**4868**	allXL	CC	376	382	BS2G	14.75
**4868**	allXL	BB	357	373	BS2G	35.48
**4868**	allXL	DD	320	366	BS2G	18.8
**4868**	allXL	BB	370	376	BS2G	16.47
**4868**	allXL	CC	320	357	DBSU	23.57
**4868**	allXL	CC	357	319	DBSU	27.11
**4868**	allXL	DD	372	319	DBSU	18.32
**4868**	allXL	DD	370	357	DBSU	26.32
**4868**	allXL	BB	382	357	DBSU	29.99
**4868**	allXL	CC	372	370	DBSU	6.53
**4868**	allXL	DD	381	370	DBSU	19.76
**4868**	allXL	AA	373	370	DBSU	9.18
**4868**	allXL	DD	381	372	DBSU	18.02
**4868**	allXL	DD	372	382	DBSU	17.85
**4868**	allXL	DD	372	386	DBSU	15.3
**4868**	allXL	DD	373	386	DBSU	15.79
**4868**	allXL	AD	381	386	DBSU	54.41
**4868**	allXL	BD;DB	357	357	DBSU	18.75
**4868**	allXL	AD	373	319	DBSU	28.45
**4868**	allXL	DB	372	357	DBSU	51.29
**4868**	allXL	BD	381	357	DBSU	48.45
**4868**	allXL	DB	373	357	DBSU	53.68
**4868**	allXL	AD	372	370	DBSU	42.87

**4868**	allXL	AD	373	370	DBSU	40.67
**4868**	allXL	DA	382	373	DBSU	39.42
**4869**	allXL	AA	320	357	BS2G	19.81
**4869**	allXL	DD	320	373	BS2G	19.94
**4869**	allXL	CC	373	382	BS2G	17.87
**4869**	allXL	CC	370	386	BS2G	24.99
**4869**	allXL	CC	373	386	BS2G	23.09
**4869**	allXL	AA	319	357	BS2G	17.46
**4869**	allXL	AA	367	373	BS2G	11.13
**4869**	allXL	BB	370	382	BS2G	20.74
**4869**	allXL	CC	376	382	BS2G	13.0
**4869**	allXL	CC	357	373	BS2G	27.54
**4869**	allXL	BB	320	366	BS2G	19.4
**4869**	allXL	BB	370	376	BS2G	16.31
**4869**	allXL	AA	320	357	DBSU	19.81
**4869**	allXL	AA	357	319	DBSU	17.46
**4869**	allXL	DD	372	319	DBSU	16.77
**4869**	allXL	CC	370	357	DBSU	22.29
**4869**	allXL	AA	382	357	DBSU	25.83
**4869**	allXL	AA	372	370	DBSU	6.7
**4869**	allXL	BB	381	370	DBSU	20.11
**4869**	allXL	AA	373	370	DBSU	9.3
**4869**	allXL	DD	381	372	DBSU	18.12
**4869**	allXL	DD	372	382	DBSU	19.31
**4869**	allXL	CC	372	386	DBSU	24.81
**4869**	allXL	CC	373	386	DBSU	23.09
**4869**	allXL	CA	381	386	DBSU	50.27
**4869**	allXL	BD;DB	357	357	DBSU	18.97
**4869**	allXL	AD	373	319	DBSU	28.78
**4869**	allXL	CA	372	357	DBSU	39.65
**4869**	allXL	AC	381	357	DBSU	36.61
**4869**	allXL	CA	373	357	DBSU	40.43
**4869**	allXL	AD	372	370	DBSU	42.37
**4869**	allXL	AD	373	370	DBSU	44.92

**4869**	allXL	DA	382	373	DBSU	40.53
**4870**	allXL	BB	320	357	BS2G	27.37
**4870**	allXL	BB	320	373	BS2G	14.35
**4870**	allXL	CC	373	382	BS2G	16.24
**4870**	allXL	BB	370	386	BS2G	25.53
**4870**	allXL	CC	373	386	BS2G	21.48
**4870**	allXL	AA	319	357	BS2G	28.44
**4870**	allXL	BB	367	373	BS2G	12.28
**4870**	allXL	BB	370	382	BS2G	17.17
**4870**	allXL	CC	376	382	BS2G	14.2
**4870**	allXL	BB	357	373	BS2G	23.34
**4870**	allXL	CC	320	366	BS2G	26.28
**4870**	allXL	BB	370	376	BS2G	17.28
**4870**	allXL	BB	320	357	DBSU	27.37
**4870**	allXL	AA	357	319	DBSU	28.44
**4870**	allXL	BB	372	319	DBSU	19.31
**4870**	allXL	BB	370	357	DBSU	29.9
**4870**	allXL	BB	382	357	DBSU	17.72
**4870**	allXL	AA	372	370	DBSU	6.51
**4870**	allXL	BB	381	370	DBSU	19.57
**4870**	allXL	CC	373	370	DBSU	9.09
**4870**	allXL	BB	381	372	DBSU	19.98
**4870**	allXL	CC	372	382	DBSU	18.09
**4870**	allXL	CC	372	386	DBSU	21.6
**4870**	allXL	CC	373	386	DBSU	21.48
**4870**	allXL	BC	381	386	DBSU	41.87
**4870**	allXL	BD;DB	357	357	DBSU	18.71
**4870**	allXL	CB	373	319	DBSU	14.29
**4870**	allXL	CB	372	357	DBSU	36.01
**4870**	allXL	BD	381	357	DBSU	29.17
**4870**	allXL	CB	373	357	DBSU	33.04
**4870**	allXL	CB	372	370	DBSU	11.81
**4870**	allXL	CB	373	370	DBSU	10.74
**4870**	allXL	CB	382	373	DBSU	21.04

**4871**	allXL	CC	320	357	BS2G	26.06
**4871**	allXL	AA	320	373	BS2G	20.17
**4871**	allXL	BB	373	382	BS2G	19.3
**4871**	allXL	DD	370	386	BS2G	17.74
**4871**	allXL	DD	373	386	BS2G	25.58
**4871**	allXL	CC	319	357	BS2G	29.35
**4871**	allXL	AA	367	373	BS2G	12.47
**4871**	allXL	DD	370	382	BS2G	17.39
**4871**	allXL	BB	376	382	BS2G	13.75
**4871**	allXL	BB	357	373	BS2G	21.8
**4871**	allXL	AA	320	366	BS2G	9.96
**4871**	allXL	DD	370	376	BS2G	14.46
**4871**	allXL	CC	320	357	DBSU	26.06
**4871**	allXL	CC	357	319	DBSU	29.35
**4871**	allXL	AA	372	319	DBSU	20.18
**4871**	allXL	BB	370	357	DBSU	27.27
**4871**	allXL	BB	382	357	DBSU	16.31
**4871**	allXL	AA	372	370	DBSU	6.3
**4871**	allXL	DD	381	370	DBSU	15.27
**4871**	allXL	AA	373	370	DBSU	8.95
**4871**	allXL	DD	381	372	DBSU	18.9
**4871**	allXL	BB	372	382	DBSU	20.48
**4871**	allXL	DD	372	386	DBSU	24.16
**4871**	allXL	DD	373	386	DBSU	25.58
**4871**	allXL	DB	381	386	DBSU	45.76
**4871**	allXL	BD;DB	357	357	DBSU	17.91
**4871**	allXL	AD	373	319	DBSU	40.83
**4871**	allXL	BD	372	357	DBSU	39.53
**4871**	allXL	BD	381	357	DBSU	22.59
**4871**	allXL	BD	373	357	DBSU	38.3
**4871**	allXL	DA	372	370	DBSU	45.52
**4871**	allXL	DA	373	370	DBSU	41.76
**4871**	allXL	AD	382	373	DBSU	42.59
**4872**	allXL	BB	320	357	BS2G	26.93

**4872**	allXL	CC	320	373	BS2G	13.71
**4872**	allXL	BB	373	382	BS2G	18.02
**4872**	allXL	CC	370	386	BS2G	15.45
**4872**	allXL	CC	373	386	BS2G	24.08
**4872**	allXL	AA	319	357	BS2G	27.09
**4872**	allXL	AA	367	373	BS2G	13.57
**4872**	allXL	CC	370	382	BS2G	14.17
**4872**	allXL	BB	376	382	BS2G	14.28
**4872**	allXL	DD	357	373	BS2G	18.71
**4872**	allXL	AA	320	366	BS2G	24.24
**4872**	allXL	CC	370	376	BS2G	13.17
**4872**	allXL	BB	320	357	DBSU	26.93
**4872**	allXL	AA	357	319	DBSU	27.09
**4872**	allXL	CC	372	319	DBSU	12.79
**4872**	allXL	DD	370	357	DBSU	23.14
**4872**	allXL	DD	382	357	DBSU	28.03
**4872**	allXL	AA	372	370	DBSU	6.18
**4872**	allXL	CC	381	370	DBSU	15.17
**4872**	allXL	AA	373	370	DBSU	8.99
**4872**	allXL	CC	381	372	DBSU	18.66
**4872**	allXL	CC	372	382	DBSU	18.59
**4872**	allXL	CC	372	386	DBSU	22.08
**4872**	allXL	CC	373	386	DBSU	24.08
**4872**	allXL	DB	381	386	DBSU	39.66
**4872**	allXL	BD;DB	357	357	DBSU	19.56
**4872**	allXL	AD	373	319	DBSU	19.64
**4872**	allXL	DB	372	357	DBSU	30.92
**4872**	allXL	DB	381	357	DBSU	17.52
**4872**	allXL	DB	373	357	DBSU	27.38
**4872**	allXL	DB	372	370	DBSU	37.32
**4872**	allXL	DB	373	370	DBSU	35.67
**4872**	allXL	DB	382	373	DBSU	17.78
**4873**	allXL	AA	320	357	BS2G	26.45
**4873**	allXL	AA	320	373	BS2G	15.13

**4873**	allXL	DD	373	382	BS2G	20.03
**4873**	allXL	DD	370	386	BS2G	26.26
**4873**	allXL	DD	373	386	BS2G	24.66
**4873**	allXL	AA	319	357	BS2G	25.66
**4873**	allXL	DD	367	373	BS2G	13.75
**4873**	allXL	BB	370	382	BS2G	20.63
**4873**	allXL	CC	376	382	BS2G	13.93
**4873**	allXL	CC	357	373	BS2G	30.76
**4873**	allXL	AA	320	366	BS2G	19.54
**4873**	allXL	BB	370	376	BS2G	16.03
**4873**	allXL	AA	320	357	DBSU	26.45
**4873**	allXL	AA	357	319	DBSU	25.66
**4873**	allXL	AA	372	319	DBSU	18.1
**4873**	allXL	CC	370	357	DBSU	28.14
**4873**	allXL	CC	382	357	DBSU	30.73
**4873**	allXL	AA	372	370	DBSU	6.26
**4873**	allXL	BB	381	370	DBSU	22.19
**4873**	allXL	DD	373	370	DBSU	8.99
**4873**	allXL	DD	381	372	DBSU	17.69
**4873**	allXL	DD	372	382	DBSU	18.73
**4873**	allXL	DD	372	386	DBSU	21.83
**4873**	allXL	DD	373	386	DBSU	24.66
**4873**	allXL	AC	381	386	DBSU	49.22
**4873**	allXL	BD;DB	357	357	DBSU	18.72
**4873**	allXL	AD	373	319	DBSU	32.8
**4873**	allXL	AD	372	357	DBSU	38.49
**4873**	allXL	CA	381	357	DBSU	41.11
**4873**	allXL	AD	373	357	DBSU	41.95
**4873**	allXL	DA	372	370	DBSU	39.94
**4873**	allXL	DA	373	370	DBSU	36.72
**4873**	allXL	AD	382	373	DBSU	54.96
**4874**	allXL	AA	320	357	BS2G	22.68
**4874**	allXL	CC	320	373	BS2G	25.51
**4874**	allXL	AA	373	382	BS2G	19.08

**4874**	allXL	AA	370	386	BS2G	25.36
**4874**	allXL	CC	373	386	BS2G	28.88
**4874**	allXL	AA	319	357	BS2G	22.07
**4874**	allXL	AA	367	373	BS2G	11.38
**4874**	allXL	AA	370	382	BS2G	18.57
**4874**	allXL	CC	376	382	BS2G	13.55
**4874**	allXL	CC	357	373	BS2G	32.45
**4874**	allXL	DD	320	366	BS2G	23.2
**4874**	allXL	CC	370	376	BS2G	15.28
**4874**	allXL	AA	320	357	DBSU	22.68
**4874**	allXL	AA	357	319	DBSU	22.07
**4874**	allXL	DD	372	319	DBSU	25.24
**4874**	allXL	DD	370	357	DBSU	30.16
**4874**	allXL	CC	382	357	DBSU	36.26
**4874**	allXL	AA	372	370	DBSU	6.39
**4874**	allXL	AA	381	370	DBSU	19.71
**4874**	allXL	AA	373	370	DBSU	8.83
**4874**	allXL	AA	381	372	DBSU	19.95
**4874**	allXL	CC	372	382	DBSU	19.57
**4874**	allXL	CC	372	386	DBSU	27.7
**4874**	allXL	CC	373	386	DBSU	28.88
**4874**	allXL	DA	381	386	DBSU	68.58
**4874**	allXL	BD;DB	357	357	DBSU	18.47
**4874**	allXL	AD	373	319	DBSU	20.93
**4874**	allXL	CB	372	357	DBSU	42.2
**4874**	allXL	BD	381	357	DBSU	51.3
**4874**	allXL	CB	373	357	DBSU	40.57
**4874**	allXL	CB	372	370	DBSU	27.62
**4874**	allXL	CB	373	370	DBSU	27.23
**4874**	allXL	DA	382	373	DBSU	44.25
**4875**	allXL	AA	320	357	BS2G	25.52
**4875**	allXL	BB	320	373	BS2G	26.95
**4875**	allXL	AA	373	382	BS2G	18.42
**4875**	allXL	CC	370	386	BS2G	17.69

**4875**	allXL	CC	373	386	BS2G	23.74
**4875**	allXL	AA	319	357	BS2G	26.91
**4875**	allXL	BB	367	373	BS2G	14.61
**4875**	allXL	CC	370	382	BS2G	18.99
**4875**	allXL	CC	376	382	BS2G	14.05
**4875**	allXL	DD	357	373	BS2G	27.54
**4875**	allXL	BB	320	366	BS2G	27.52
**4875**	allXL	CC	370	376	BS2G	17.13
**4875**	allXL	AA	320	357	DBSU	25.52
**4875**	allXL	AA	357	319	DBSU	26.91
**4875**	allXL	CC	372	319	DBSU	26.12
**4875**	allXL	DD	370	357	DBSU	26.03
**4875**	allXL	DD	382	357	DBSU	30.92
**4875**	allXL	AA	372	370	DBSU	6.11
**4875**	allXL	CC	381	370	DBSU	21.7
**4875**	allXL	AA	373	370	DBSU	9.16
**4875**	allXL	CC	381	372	DBSU	21.47
**4875**	allXL	CC	372	382	DBSU	19.68
**4875**	allXL	CC	372	386	DBSU	22.28
**4875**	allXL	CC	373	386	DBSU	23.74
**4875**	allXL	CB	381	386	DBSU	39.32
**4875**	allXL	BD;DB	357	357	DBSU	19.0
**4875**	allXL	DA	373	319	DBSU	21.21
**4875**	allXL	DB	372	357	DBSU	37.9
**4875**	allXL	DB	381	357	DBSU	27.78
**4875**	allXL	DB	373	357	DBSU	35.99
**4875**	allXL	CB	372	370	DBSU	13.59
**4875**	allXL	CB	373	370	DBSU	11.54
**4875**	allXL	CB	382	373	DBSU	22.57
**4876**	allXL	AA	320	357	BS2G	23.59
**4876**	allXL	BB	320	373	BS2G	15.47
**4876**	allXL	BB	373	382	BS2G	18.55
**4876**	allXL	AA	370	386	BS2G	24.02
**4876**	allXL	BB	373	386	BS2G	24.3

**4876**	allXL	AA	319	357	BS2G	21.77
**4876**	allXL	AA	367	373	BS2G	13.96
**4876**	allXL	BB	370	382	BS2G	23.39
**4876**	allXL	BB	376	382	BS2G	14.18
**4876**	allXL	BB	357	373	BS2G	21.09
**4876**	allXL	BB	320	366	BS2G	22.32
**4876**	allXL	CC	370	376	BS2G	16.37
**4876**	allXL	AA	320	357	DBSU	23.59
**4876**	allXL	AA	357	319	DBSU	21.77
**4876**	allXL	BB	372	319	DBSU	17.86
**4876**	allXL	BB	370	357	DBSU	25.9
**4876**	allXL	BB	382	357	DBSU	37.32
**4876**	allXL	AA	372	370	DBSU	5.91
**4876**	allXL	BB	381	370	DBSU	23.35
**4876**	allXL	CC	373	370	DBSU	9.02
**4876**	allXL	BB	381	372	DBSU	18.18
**4876**	allXL	BB	372	382	DBSU	19.27
**4876**	allXL	AA	372	386	DBSU	23.0
**4876**	allXL	BB	373	386	DBSU	24.3
**4876**	allXL	BC	381	386	DBSU	47.64
**4876**	allXL	BD;DB	357	357	DBSU	18.31
**4876**	allXL	AD	373	319	DBSU	32.41
**4876**	allXL	CA	372	357	DBSU	31.27
**4876**	allXL	CA	381	357	DBSU	40.12
**4876**	allXL	CA	373	357	DBSU	30.79
**4876**	allXL	DA	372	370	DBSU	40.24
**4876**	allXL	DA	373	370	DBSU	41.61
**4876**	allXL	CB	382	373	DBSU	43.88
**4877**	allXL	AA	320	357	BS2G	24.98
**4877**	allXL	BB	320	373	BS2G	21.99
**4877**	allXL	CC	373	382	BS2G	17.45
**4877**	allXL	CC	370	386	BS2G	17.33
**4877**	allXL	CC	373	386	BS2G	25.56
**4877**	allXL	AA	319	357	BS2G	23.95

**4877**	allXL	AA	367	373	BS2G	14.18
**4877**	allXL	CC	370	382	BS2G	12.7
**4877**	allXL	CC	376	382	BS2G	13.78
**4877**	allXL	DD	357	373	BS2G	35.31
**4877**	allXL	BB	320	366	BS2G	24.21
**4877**	allXL	CC	370	376	BS2G	13.63
**4877**	allXL	AA	320	357	DBSU	24.98
**4877**	allXL	AA	357	319	DBSU	23.95
**4877**	allXL	BB	372	319	DBSU	22.6
**4877**	allXL	BB;DD	370	357	DBSU	26.73
**4877**	allXL	CC	382	357	DBSU	43.53
**4877**	allXL	AA	372	370	DBSU	6.26
**4877**	allXL	CC	381	370	DBSU	15.37
**4877**	allXL	CC	373	370	DBSU	9.01
**4877**	allXL	AA	381	372	DBSU	17.46
**4877**	allXL	CC	372	382	DBSU	16.53
**4877**	allXL	CC	372	386	DBSU	23.0
**4877**	allXL	CC	373	386	DBSU	25.56
**4877**	allXL	AD	381	386	DBSU	17.34
**4877**	allXL	BD;DB	357	357	DBSU	18.41
**4877**	allXL	AD	373	319	DBSU	30.91
**4877**	allXL	BD	372	357	DBSU	50.47
**4877**	allXL	CB	381	357	DBSU	45.2
**4877**	allXL	AD	373	357	DBSU	52.95
**4877**	allXL	BC	372	370	DBSU	39.54
**4877**	allXL	AD	373	370	DBSU	39.17
**4877**	allXL	CB	382	373	DBSU	32.24
**4878**	allXL	AA	320	357	BS2G	22.52
**4878**	allXL	DD	320	373	BS2G	19.96
**4878**	allXL	CC	373	382	BS2G	16.29
**4878**	allXL	CC	370	386	BS2G	19.43
**4878**	allXL	CC	373	386	BS2G	22.34
**4878**	allXL	AA	319	357	BS2G	23.86
**4878**	allXL	AA	367	373	BS2G	10.46

**4878**	allXL	CC	370	382	BS2G	18.97
**4878**	allXL	CC	376	382	BS2G	13.69
**4878**	allXL	CC	357	373	BS2G	26.31
**4878**	allXL	BB	320	366	BS2G	26.44
**4878**	allXL	CC	370	376	BS2G	15.31
**4878**	allXL	AA	320	357	DBSU	22.52
**4878**	allXL	AA	357	319	DBSU	23.86
**4878**	allXL	DD	372	319	DBSU	17.76
**4878**	allXL	CC	370	357	DBSU	24.99
**4878**	allXL	CC	382	357	DBSU	35.63
**4878**	allXL	CC	372	370	DBSU	6.46
**4878**	allXL	CC	381	370	DBSU	22.08
**4878**	allXL	AA	373	370	DBSU	8.81
**4878**	allXL	CC	381	372	DBSU	21.23
**4878**	allXL	CC	372	382	DBSU	18.9
**4878**	allXL	CC	372	386	DBSU	23.0
**4878**	allXL	CC	373	386	DBSU	22.34
**4878**	allXL	DA	381	386	DBSU	21.92
**4878**	allXL	BD;DB	357	357	DBSU	18.2
**4878**	allXL	AD	373	319	DBSU	21.8
**4878**	allXL	CA	372	357	DBSU	21.35
**4878**	allXL	CA	381	357	DBSU	24.92
**4878**	allXL	CA	373	357	DBSU	19.55
**4878**	allXL	DA	372	370	DBSU	19.39
**4878**	allXL	DA	373	370	DBSU	16.74
**4878**	allXL	AD	382	373	DBSU	31.37
**4879**	allXL	CC	320	357	BS2G	26.29
**4879**	allXL	BB	320	373	BS2G	10.97
**4879**	allXL	DD	373	382	BS2G	17.39
**4879**	allXL	DD	370	386	BS2G	15.01
**4879**	allXL	DD	373	386	BS2G	22.04
**4879**	allXL	CC	319	357	BS2G	26.61
**4879**	allXL	BB	367	373	BS2G	12.16
**4879**	allXL	DD	370	382	BS2G	15.98

**4879**	allXL	CC	376	382	BS2G	14.09
**4879**	allXL	DD	357	373	BS2G	25.48
**4879**	allXL	BB	320	366	BS2G	19.54
**4879**	allXL	DD	370	376	BS2G	17.15
**4879**	allXL	CC	320	357	DBSU	26.29
**4879**	allXL	CC	357	319	DBSU	26.61
**4879**	allXL	BB	372	319	DBSU	11.16
**4879**	allXL	BB	370	357	DBSU	27.14
**4879**	allXL	DD	382	357	DBSU	32.07
**4879**	allXL	AA	372	370	DBSU	6.13
**4879**	allXL	DD	381	370	DBSU	17.39
**4879**	allXL	AA	373	370	DBSU	9.12
**4879**	allXL	DD	381	372	DBSU	18.57
**4879**	allXL	DD	372	382	DBSU	18.54
**4879**	allXL	DD	372	386	DBSU	21.33
**4879**	allXL	DD	373	386	DBSU	22.04
**4879**	allXL	CB	381	386	DBSU	45.15
**4879**	allXL	BD;DB	357	357	DBSU	18.73
**4879**	allXL	DA	373	319	DBSU	33.52
**4879**	allXL	DB	372	357	DBSU	32.18
**4879**	allXL	DB	381	357	DBSU	43.71
**4879**	allXL	DB	373	357	DBSU	32.08
**4879**	allXL	BC	372	370	DBSU	20.72
**4879**	allXL	CB	373	370	DBSU	23.53
**4879**	allXL	BC	382	373	DBSU	33.66
**4880**	allXL	AA	320	357	BS2G	8.89
**4880**	allXL	DD	320	373	BS2G	25.42
**4880**	allXL	BB	373	382	BS2G	17.84
**4880**	allXL	DD	370	386	BS2G	20.52
**4880**	allXL	BB	373	386	BS2G	22.71
**4880**	allXL	AA	319	357	BS2G	6.38
**4880**	allXL	AA	367	373	BS2G	12.86
**4880**	allXL	DD	370	382	BS2G	20.51
**4880**	allXL	BB	376	382	BS2G	12.77

**4880**	allXL	BB	357	373	BS2G	30.31
**4880**	allXL	AA	320	366	BS2G	27.89
**4880**	allXL	CC	370	376	BS2G	14.63
**4880**	allXL	AA	320	357	DBSU	8.89
**4880**	allXL	AA	357	319	DBSU	6.38
**4880**	allXL	CC	372	319	DBSU	28.35
**4880**	allXL	DD	370	357	DBSU	30.53
**4880**	allXL	BB	382	357	DBSU	29.28
**4880**	allXL	AA	372	370	DBSU	6.24
**4880**	allXL	DD	381	370	DBSU	20.01
**4880**	allXL	DD	373	370	DBSU	9.03
**4880**	allXL	DD	381	372	DBSU	18.79
**4880**	allXL	DD	372	382	DBSU	20.35
**4880**	allXL	BB	372	386	DBSU	23.86
**4880**	allXL	BB	373	386	DBSU	22.71
**4880**	allXL	BC	381	386	DBSU	27.62
**4880**	allXL	BD;DB	357	357	DBSU	19.23
**4880**	allXL	CB	373	319	DBSU	28.35
**4880**	allXL	CB	372	357	DBSU	30.98
**4880**	allXL	CB	381	357	DBSU	34.62
**4880**	allXL	CB	373	357	DBSU	33.71
**4880**	allXL	CB	372	370	DBSU	25.45
**4880**	allXL	CB	373	370	DBSU	24.4
**4880**	allXL	CB	382	373	DBSU	18.37
**4881**	allXL	AA	320	357	BS2G	20.77
**4881**	allXL	BB	320	373	BS2G	20.96
**4881**	allXL	CC	373	382	BS2G	18.53
**4881**	allXL	DD	370	386	BS2G	25.66
**4881**	allXL	CC	373	386	BS2G	25.43
**4881**	allXL	AA	319	357	BS2G	20.62
**4881**	allXL	AA	367	373	BS2G	12.27
**4881**	allXL	DD	370	382	BS2G	19.06
**4881**	allXL	CC	376	382	BS2G	13.03
**4881**	allXL	BB	357	373	BS2G	32.34

**4881**	allXL	BB	320	366	BS2G	20.13
**4881**	allXL	CC	370	376	BS2G	16.4
**4881**	allXL	AA	320	357	DBSU	20.77
**4881**	allXL	AA	357	319	DBSU	20.62
**4881**	allXL	BB	372	319	DBSU	18.98
**4881**	allXL	BB	370	357	DBSU	31.11
**4881**	allXL	BB	382	357	DBSU	22.4
**4881**	allXL	AA	372	370	DBSU	6.43
**4881**	allXL	DD	381	370	DBSU	17.0
**4881**	allXL	AA	373	370	DBSU	9.19
**4881**	allXL	DD	381	372	DBSU	18.95
**4881**	allXL	DD	372	382	DBSU	21.91
**4881**	allXL	AA	372	386	DBSU	27.61
**4881**	allXL	CC	373	386	DBSU	25.43
**4881**	allXL	BD	381	386	DBSU	43.64
**4881**	allXL	BD;DB	357	357	DBSU	18.43
**4881**	allXL	AD	373	319	DBSU	29.08
**4881**	allXL	AC	372	357	DBSU	47.4
**4881**	allXL	CB	381	357	DBSU	37.43
**4881**	allXL	AC	373	357	DBSU	47.03
**4881**	allXL	CB	372	370	DBSU	35.7
**4881**	allXL	CB	373	370	DBSU	32.91
**4881**	allXL	CB	382	373	DBSU	27.59
**4882**	allXL	AA	320	357	BS2G	24.91
**4882**	allXL	DD	320	373	BS2G	31.91
**4882**	allXL	CC	373	382	BS2G	22.24
**4882**	allXL	BB	370	386	BS2G	32.66
**4882**	allXL	BB	373	386	BS2G	29.69
**4882**	allXL	AA	319	357	BS2G	24.71
**4882**	allXL	AA	367	373	BS2G	10.96
**4882**	allXL	DD	370	382	BS2G	26.02
**4882**	allXL	CC	376	382	BS2G	14.24
**4882**	allXL	AA	357	373	BS2G	26.84
**4882**	allXL	AA	320	366	BS2G	28.65

**4882**	allXL	CC	370	376	BS2G	16.14
**4882**	allXL	AA	320	357	DBSU	24.91
**4882**	allXL	AA	357	319	DBSU	24.71
**4882**	allXL	CC	372	319	DBSU	31.56
**4882**	allXL	DD	370	357	DBSU	31.56
**4882**	allXL	AA	382	357	DBSU	31.85
**4882**	allXL	AA	372	370	DBSU	6.31
**4882**	allXL	DD	381	370	DBSU	22.89
**4882**	allXL	AA	373	370	DBSU	8.96
**4882**	allXL	DD	381	372	DBSU	21.06
**4882**	allXL	DD	372	382	DBSU	24.57
**4882**	allXL	BB	372	386	DBSU	32.25
**4882**	allXL	BB	373	386	DBSU	29.69
**4882**	allXL	AC	381	386	DBSU	89.33
**4882**	allXL	BD;DB	357	357	DBSU	19.16
**4882**	allXL	AD	373	319	DBSU	30.55
**4882**	allXL	BD	372	357	DBSU	41.76
**4882**	allXL	AC	381	357	DBSU	30.53
**4882**	allXL	AC	373	357	DBSU	39.34
**4882**	allXL	DA	372	370	DBSU	57.41
**4882**	allXL	DA	373	370	DBSU	54.49
**4882**	allXL	AC	382	373	DBSU	63.62
**4883**	allXL	AA	320	357	BS2G	27.1
**4883**	allXL	BB	320	373	BS2G	9.82
**4883**	allXL	BB	373	382	BS2G	17.15
**4883**	allXL	BB	370	386	BS2G	24.16
**4883**	allXL	DD	373	386	BS2G	26.36
**4883**	allXL	AA	319	357	BS2G	28.68
**4883**	allXL	AA	367	373	BS2G	12.44
**4883**	allXL	BB	370	382	BS2G	16.18
**4883**	allXL	BB	376	382	BS2G	14.35
**4883**	allXL	BB	357	373	BS2G	24.43
**4883**	allXL	BB	320	366	BS2G	19.39
**4883**	allXL	CC	370	376	BS2G	15.5

**4883**	allXL	AA	320	357	DBSU	27.1
**4883**	allXL	AA	357	319	DBSU	28.68
**4883**	allXL	BB	372	319	DBSU	15.7
**4883**	allXL	DD	370	357	DBSU	25.51
**4883**	allXL	BB	382	357	DBSU	40.78
**4883**	allXL	AA	372	370	DBSU	6.36
**4883**	allXL	BB	381	370	DBSU	18.53
**4883**	allXL	AA	373	370	DBSU	9.17
**4883**	allXL	BB	381	372	DBSU	19.57
**4883**	allXL	BB	372	382	DBSU	18.44
**4883**	allXL	DD	372	386	DBSU	26.15
**4883**	allXL	DD	373	386	DBSU	26.36
**4883**	allXL	CB	381	386	DBSU	33.0
**4883**	allXL	AC;CA	357	357	DBSU	19.3
**4883**	allXL	DA	373	319	DBSU	25.22
**4883**	allXL	CB	372	357	DBSU	32.1
**4883**	allXL	BC	381	357	DBSU	44.3
**4883**	allXL	DB	373	357	DBSU	32.1
**4883**	allXL	CB	372	370	DBSU	22.36
**4883**	allXL	CB	373	370	DBSU	23.74
**4883**	allXL	BC	382	373	DBSU	31.25
**4884**	allXL	CC	320	357	BS2G	25.39
**4884**	allXL	DD	320	373	BS2G	25.48
**4884**	allXL	DD	373	382	BS2G	19.59
**4884**	allXL	CC	370	386	BS2G	21.87
**4884**	allXL	DD	373	386	BS2G	23.23
**4884**	allXL	AA	319	357	BS2G	25.99
**4884**	allXL	AA	367	373	BS2G	12.86
**4884**	allXL	CC	370	382	BS2G	21.36
**4884**	allXL	CC	376	382	BS2G	14.52
**4884**	allXL	AA	357	373	BS2G	33.36
**4884**	allXL	DD	320	366	BS2G	20.07
**4884**	allXL	CC	370	376	BS2G	13.22
**4884**	allXL	CC	320	357	DBSU	25.39

**4884**	allXL	AA	357	319	DBSU	25.99
**4884**	allXL	DD	372	319	DBSU	28.75
**4884**	allXL	DD	370	357	DBSU	30.61
**4884**	allXL	CC	382	357	DBSU	25.95
**4884**	allXL	AA	372	370	DBSU	5.95
**4884**	allXL	CC	381	370	DBSU	22.91
**4884**	allXL	AA	373	370	DBSU	9.04
**4884**	allXL	DD	381	372	DBSU	19.93
**4884**	allXL	DD	372	382	DBSU	19.67
**4884**	allXL	DD	372	386	DBSU	22.3
**4884**	allXL	DD	373	386	DBSU	23.23
**4884**	allXL	CB	381	386	DBSU	63.12
**4884**	allXL	BD;DB	357	357	DBSU	18.38
**4884**	allXL	CB	373	319	DBSU	31.72
**4884**	allXL	AC	372	357	DBSU	47.66
**4884**	allXL	CA	381	357	DBSU	37.4
**4884**	allXL	AC	373	357	DBSU	46.12
**4884**	allXL	BC	372	370	DBSU	40.34
**4884**	allXL	BC	373	370	DBSU	38.03
**4884**	allXL	CB	382	373	DBSU	43.52
**4885**	allXL	AA	320	357	BS2G	23.44
**4885**	allXL	CC	320	373	BS2G	14.4
**4885**	allXL	BB	373	382	BS2G	17.44
**4885**	allXL	CC	370	386	BS2G	23.32
**4885**	allXL	BB	373	386	BS2G	19.63
**4885**	allXL	AA	319	357	BS2G	21.03
**4885**	allXL	AA	367	373	BS2G	13.52
**4885**	allXL	CC	370	382	BS2G	19.42
**4885**	allXL	BB	376	382	BS2G	12.41
**4885**	allXL	BB	357	373	BS2G	26.4
**4885**	allXL	AA	320	366	BS2G	18.56
**4885**	allXL	CC	370	376	BS2G	15.66
**4885**	allXL	AA	320	357	DBSU	23.44
**4885**	allXL	AA	357	319	DBSU	21.03

**4885**	allXL	CC	372	319	DBSU	17.15
**4885**	allXL	DD	370	357	DBSU	25.81
**4885**	allXL	CC	382	357	DBSU	11.34
**4885**	allXL	AA	372	370	DBSU	6.19
**4885**	allXL	CC	381	370	DBSU	20.18
**4885**	allXL	AA	373	370	DBSU	9.09
**4885**	allXL	CC	381	372	DBSU	21.86
**4885**	allXL	BB	372	382	DBSU	20.03
**4885**	allXL	BB	372	386	DBSU	21.07
**4885**	allXL	BB	373	386	DBSU	19.63
**4885**	allXL	BC	381	386	DBSU	48.45
**4885**	allXL	BD;DB	357	357	DBSU	18.45
**4885**	allXL	DA	373	319	DBSU	24.23
**4885**	allXL	DB	372	357	DBSU	39.85
**4885**	allXL	CA	381	357	DBSU	28.81
**4885**	allXL	DA	373	357	DBSU	41.02
**4885**	allXL	DA	372	370	DBSU	40.7
**4885**	allXL	DA	373	370	DBSU	38.99
**4885**	allXL	DA	382	373	DBSU	38.54
**4886**	allXL	AA	320	357	BS2G	18.28
**4886**	allXL	CC	320	373	BS2G	24.13
**4886**	allXL	DD	373	382	BS2G	17.41
**4886**	allXL	DD	370	386	BS2G	19.34
**4886**	allXL	DD	373	386	BS2G	23.56
**4886**	allXL	AA	319	357	BS2G	21.15
**4886**	allXL	AA	367	373	BS2G	13.48
**4886**	allXL	DD	370	382	BS2G	16.29
**4886**	allXL	CC	376	382	BS2G	14.15
**4886**	allXL	BB	357	373	BS2G	35.79
**4886**	allXL	BB	320	366	BS2G	22.23
**4886**	allXL	CC	370	376	BS2G	15.98
**4886**	allXL	AA	320	357	DBSU	18.28
**4886**	allXL	AA	357	319	DBSU	21.15
**4886**	allXL	CC	372	319	DBSU	22.46

**4886**	allXL	DD	370	357	DBSU	28.84
**4886**	allXL	BB	382	357	DBSU	40.17
**4886**	allXL	AA	372	370	DBSU	6.27
**4886**	allXL	DD	381	370	DBSU	18.3
**4886**	allXL	AA	373	370	DBSU	8.75
**4886**	allXL	DD	381	372	DBSU	17.29
**4886**	allXL	DD	372	382	DBSU	16.63
**4886**	allXL	DD	372	386	DBSU	22.67
**4886**	allXL	DD	373	386	DBSU	23.56
**4886**	allXL	CB	381	386	DBSU	29.96
**4886**	allXL	BD;DB	357	357	DBSU	18.66
**4886**	allXL	AD	373	319	DBSU	38.51
**4886**	allXL	CB	372	357	DBSU	42.34
**4886**	allXL	BD	381	357	DBSU	58.47
**4886**	allXL	CB	373	357	DBSU	46.04
**4886**	allXL	CB	372	370	DBSU	43.73
**4886**	allXL	BC	373	370	DBSU	45.05
**4886**	allXL	BC	382	373	DBSU	33.69
**4887**	allXL	CC	320	357	BS2G	24.09
**4887**	allXL	CC	320	373	BS2G	18.65
**4887**	allXL	BB	373	382	BS2G	18.97
**4887**	allXL	CC	370	386	BS2G	26.31
**4887**	allXL	BB	373	386	BS2G	25.51
**4887**	allXL	AA	319	357	BS2G	22.56
**4887**	allXL	AA	367	373	BS2G	11.71
**4887**	allXL	CC	370	382	BS2G	22.22
**4887**	allXL	CC	376	382	BS2G	13.37
**4887**	allXL	CC	357	373	BS2G	23.64
**4887**	allXL	AA	320	366	BS2G	16.48
**4887**	allXL	CC	370	376	BS2G	16.45
**4887**	allXL	CC	320	357	DBSU	24.09
**4887**	allXL	AA	357	319	DBSU	22.56
**4887**	allXL	AA	372	319	DBSU	19.97
**4887**	allXL	AA	370	357	DBSU	22.98

**4887**	allXL	CC	382	357	DBSU	16.11
**4887**	allXL	AA	372	370	DBSU	6.59
**4887**	allXL	DD	381	370	DBSU	22.84
**4887**	allXL	AA	373	370	DBSU	8.64
**4887**	allXL	BB	381	372	DBSU	20.52
**4887**	allXL	AA	372	382	DBSU	22.19
**4887**	allXL	BB	372	386	DBSU	28.03
**4887**	allXL	BB	373	386	DBSU	25.51
**4887**	allXL	CB	381	386	DBSU	59.09
**4887**	allXL	BD;DB	357	357	DBSU	18.59
**4887**	allXL	CB	373	319	DBSU	34.1
**4887**	allXL	AC	372	357	DBSU	44.26
**4887**	allXL	CA	381	357	DBSU	35.26
**4887**	allXL	CA	373	357	DBSU	43.48
**4887**	allXL	CB	372	370	DBSU	51.69
**4887**	allXL	CB	373	370	DBSU	49.44
**4887**	allXL	BC	382	373	DBSU	58.98
**4888**	allXL	CC	320	357	BS2G	23.89
**4888**	allXL	BB	320	373	BS2G	16.41
**4888**	allXL	DD	373	382	BS2G	14.88
**4888**	allXL	CC	370	386	BS2G	20.97
**4888**	allXL	DD	373	386	BS2G	20.73
**4888**	allXL	CC	319	357	BS2G	24.71
**4888**	allXL	AA	367	373	BS2G	12.4
**4888**	allXL	CC	370	382	BS2G	19.57
**4888**	allXL	BB	376	382	BS2G	14.18
**4888**	allXL	BB	357	373	BS2G	16.25
**4888**	allXL	CC	320	366	BS2G	25.72
**4888**	allXL	CC	370	376	BS2G	14.09
**4888**	allXL	CC	320	357	DBSU	23.89
**4888**	allXL	CC	357	319	DBSU	24.71
**4888**	allXL	BB	372	319	DBSU	16.37
**4888**	allXL	BB	370	357	DBSU	15.74
**4888**	allXL	BB	382	357	DBSU	13.02

**4888**	allXL	AA	372	370	DBSU	6.33
**4888**	allXL	CC	381	370	DBSU	21.96
**4888**	allXL	AA	373	370	DBSU	9.17
**4888**	allXL	DD	381	372	DBSU	17.84
**4888**	allXL	DD	372	382	DBSU	16.78
**4888**	allXL	DD	372	386	DBSU	21.33
**4888**	allXL	DD	373	386	DBSU	20.73
**4888**	allXL	AD	381	386	DBSU	54.9
**4888**	allXL	BD;DB	357	357	DBSU	18.84
**4888**	allXL	DA	373	319	DBSU	24.09
**4888**	allXL	BD	372	357	DBSU	35.33
**4888**	allXL	BD	381	357	DBSU	29.47
**4888**	allXL	BD	373	357	DBSU	34.05
**4888**	allXL	DB	372	370	DBSU	48.86
**4888**	allXL	DB	373	370	DBSU	46.38
**4888**	allXL	BD	382	373	DBSU	47.57
**4889**	allXL	AA	320	357	BS2G	23.14
**4889**	allXL	CC	320	373	BS2G	14.13
**4889**	allXL	BB	373	382	BS2G	20.78
**4889**	allXL	BB	370	386	BS2G	22.26
**4889**	allXL	BB	373	386	BS2G	22.54
**4889**	allXL	AA	319	357	BS2G	26.07
**4889**	allXL	AA	367	373	BS2G	11.52
**4889**	allXL	CC	370	382	BS2G	22.96
**4889**	allXL	BB	376	382	BS2G	13.29
**4889**	allXL	AA	357	373	BS2G	30.73
**4889**	allXL	BB	320	366	BS2G	21.93
**4889**	allXL	CC	370	376	BS2G	13.96
**4889**	allXL	AA	320	357	DBSU	23.14
**4889**	allXL	AA	357	319	DBSU	26.07
**4889**	allXL	CC	372	319	DBSU	12.58
**4889**	allXL	DD	370	357	DBSU	23.05
**4889**	allXL	BB	382	357	DBSU	35.62
**4889**	allXL	AA	372	370	DBSU	6.39

**4889**	allXL	AA	381	370	DBSU	21.69
**4889**	allXL	AA	373	370	DBSU	8.85
**4889**	allXL	AA	381	372	DBSU	20.2
**4889**	allXL	CC	372	382	DBSU	23.42
**4889**	allXL	BB	372	386	DBSU	24.68
**4889**	allXL	BB	373	386	DBSU	22.54
**4889**	allXL	CB	381	386	DBSU	45.21
**4889**	allXL	BD;DB	357	357	DBSU	18.84
**4889**	allXL	AD	373	319	DBSU	30.38
**4889**	allXL	AC	372	357	DBSU	40.03
**4889**	allXL	DB	381	357	DBSU	50.97
**4889**	allXL	AC	373	357	DBSU	39.69
**4889**	allXL	BC	372	370	DBSU	23.41
**4889**	allXL	BC	373	370	DBSU	25.57
**4889**	allXL	CB	382	373	DBSU	28.3
**4890**	allXL	CC	320	357	BS2G	24.43
**4890**	allXL	AA	320	373	BS2G	31.33
**4890**	allXL	DD	373	382	BS2G	19.98
**4890**	allXL	CC	370	386	BS2G	23.81
**4890**	allXL	CC	373	386	BS2G	26.68
**4890**	allXL	CC	319	357	BS2G	24.39
**4890**	allXL	AA	367	373	BS2G	13.89
**4890**	allXL	DD	370	382	BS2G	16.99
**4890**	allXL	CC	376	382	BS2G	13.81
**4890**	allXL	BB	357	373	BS2G	28.77
**4890**	allXL	AA	320	366	BS2G	14.73
**4890**	allXL	CC	370	376	BS2G	14.92
**4890**	allXL	CC	320	357	DBSU	24.43
**4890**	allXL	CC	357	319	DBSU	24.39
**4890**	allXL	AA	372	319	DBSU	33.69
**4890**	allXL	AA	370	357	DBSU	27.73
**4890**	allXL	CC	382	357	DBSU	25.08
**4890**	allXL	AA	372	370	DBSU	6.13
**4890**	allXL	DD	381	370	DBSU	16.83

**4890**	allXL	AA	373	370	DBSU	9.18
**4890**	allXL	DD	381	372	DBSU	20.11
**4890**	allXL	CC	372	382	DBSU	20.05
**4890**	allXL	CC	372	386	DBSU	24.86
**4890**	allXL	CC	373	386	DBSU	26.68
**4890**	allXL	DA	381	386	DBSU	62.82
**4890**	allXL	BD;DB	357	357	DBSU	18.82
**4890**	allXL	AD	373	319	DBSU	8.96
**4890**	allXL	AD	372	357	DBSU	30.75
**4890**	allXL	DB	381	357	DBSU	34.77
**4890**	allXL	AD	373	357	DBSU	34.19
**4890**	allXL	DA	372	370	DBSU	25.19
**4890**	allXL	DA	373	370	DBSU	24.61
**4890**	allXL	AD	382	373	DBSU	41.67
**4891**	allXL	AA	320	357	BS2G	22.54
**4891**	allXL	DD	320	373	BS2G	17.3
**4891**	allXL	BB	373	382	BS2G	17.16
**4891**	allXL	BB	370	386	BS2G	19.58
**4891**	allXL	BB	373	386	BS2G	22.47
**4891**	allXL	AA	319	357	BS2G	20.89
**4891**	allXL	AA	367	373	BS2G	11.63
**4891**	allXL	CC	370	382	BS2G	15.37
**4891**	allXL	BB	376	382	BS2G	14.46
**4891**	allXL	BB	357	373	BS2G	16.99
**4891**	allXL	DD	320	366	BS2G	23.81
**4891**	allXL	CC	370	376	BS2G	12.47
**4891**	allXL	AA	320	357	DBSU	22.54
**4891**	allXL	AA	357	319	DBSU	20.89
**4891**	allXL	DD	372	319	DBSU	14.51
**4891**	allXL	DD	370	357	DBSU	22.27
**4891**	allXL	BB	382	357	DBSU	26.92
**4891**	allXL	AA	372	370	DBSU	6.38
**4891**	allXL	CC	381	370	DBSU	15.53
**4891**	allXL	AA	373	370	DBSU	8.89

**4891**	allXL	BB	381	372	DBSU	17.65
**4891**	allXL	CC	372	382	DBSU	19.5
**4891**	allXL	BB	372	386	DBSU	23.29
**4891**	allXL	BB	373	386	DBSU	22.47
**4891**	allXL	CB	381	386	DBSU	32.84
**4891**	allXL	AC;CA	357	357	DBSU	19.64
**4891**	allXL	CB	373	319	DBSU	19.45
**4891**	allXL	CB	372	357	DBSU	19.15
**4891**	allXL	AC	381	357	DBSU	29.53
**4891**	allXL	CB	373	357	DBSU	20.81
**4891**	allXL	CB	372	370	DBSU	22.6
**4891**	allXL	CB	373	370	DBSU	20.99
**4891**	allXL	BC	382	373	DBSU	24.44
**4892**	allXL	CC	320	357	BS2G	24.6
**4892**	allXL	BB	320	373	BS2G	25.08
**4892**	allXL	CC	373	382	BS2G	19.3
**4892**	allXL	DD	370	386	BS2G	15.65
**4892**	allXL	DD	373	386	BS2G	21.1
**4892**	allXL	AA	319	357	BS2G	24.75
**4892**	allXL	BB	367	373	BS2G	12.87
**4892**	allXL	DD	370	382	BS2G	18.09
**4892**	allXL	CC	376	382	BS2G	14.04
**4892**	allXL	DD	357	373	BS2G	26.0
**4892**	allXL	DD	320	366	BS2G	24.3
**4892**	allXL	BB	370	376	BS2G	15.53
**4892**	allXL	CC	320	357	DBSU	24.6
**4892**	allXL	AA	357	319	DBSU	24.75
**4892**	allXL	BB	372	319	DBSU	21.74
**4892**	allXL	DD	370	357	DBSU	26.4
**4892**	allXL	DD	382	357	DBSU	29.74
**4892**	allXL	AA	372	370	DBSU	6.32
**4892**	allXL	DD	381	370	DBSU	18.61
**4892**	allXL	CC	373	370	DBSU	9.18
**4892**	allXL	DD	381	372	DBSU	19.28

**4892**	allXL	DD	372	382	DBSU	19.9
**4892**	allXL	DD	372	386	DBSU	19.51
**4892**	allXL	DD	373	386	DBSU	21.1
**4892**	allXL	BC	381	386	DBSU	44.76
**4892**	allXL	BD;DB	357	357	DBSU	18.2
**4892**	allXL	BC	373	319	DBSU	26.28
**4892**	allXL	DB	372	357	DBSU	36.76
**4892**	allXL	DA	381	357	DBSU	35.82
**4892**	allXL	BC	373	357	DBSU	35.53
**4892**	allXL	BC	372	370	DBSU	22.35
**4892**	allXL	BC	373	370	DBSU	22.08
**4892**	allXL	BC	382	373	DBSU	41.25
**4893**	allXL	AA	320	357	BS2G	26.02
**4893**	allXL	AA	320	373	BS2G	24.05
**4893**	allXL	AA	373	382	BS2G	19.88
**4893**	allXL	DD	370	386	BS2G	22.36
**4893**	allXL	AA	373	386	BS2G	24.98
**4893**	allXL	AA	319	357	BS2G	24.93
**4893**	allXL	AA	367	373	BS2G	11.6
**4893**	allXL	BB	370	382	BS2G	19.52
**4893**	allXL	CC	376	382	BS2G	13.56
**4893**	allXL	DD	357	373	BS2G	31.17
**4893**	allXL	AA	320	366	BS2G	11.25
**4893**	allXL	BB	370	376	BS2G	14.84
**4893**	allXL	AA	320	357	DBSU	26.02
**4893**	allXL	AA	357	319	DBSU	24.93
**4893**	allXL	AA	372	319	DBSU	22.27
**4893**	allXL	DD	370	357	DBSU	22.72
**4893**	allXL	DD	382	357	DBSU	30.78
**4893**	allXL	CC	372	370	DBSU	6.54
**4893**	allXL	BB	381	370	DBSU	21.4
**4893**	allXL	BB	373	370	DBSU	9.34
**4893**	allXL	DD	381	372	DBSU	21.46
**4893**	allXL	CC	372	382	DBSU	21.94

**4893**	allXL	DD	372	386	DBSU	25.0
**4893**	allXL	AA	373	386	DBSU	24.98
**4893**	allXL	BD	381	386	DBSU	65.34
**4893**	allXL	BD;DB	357	357	DBSU	18.16
**4893**	allXL	AD	373	319	DBSU	26.85
**4893**	allXL	AD	372	357	DBSU	43.2
**4893**	allXL	DB	381	357	DBSU	50.32
**4893**	allXL	AD	373	357	DBSU	46.4
**4893**	allXL	DA	372	370	DBSU	19.15
**4893**	allXL	DA	373	370	DBSU	20.47
**4893**	allXL	AD	382	373	DBSU	45.87
**4894**	allXL	AA	320	357	BS2G	25.79
**4894**	allXL	DD	320	373	BS2G	24.01
**4894**	allXL	DD	373	382	BS2G	19.44
**4894**	allXL	DD	370	386	BS2G	25.74
**4894**	allXL	DD	373	386	BS2G	29.28
**4894**	allXL	AA	319	357	BS2G	28.21
**4894**	allXL	AA	367	373	BS2G	12.18
**4894**	allXL	DD	370	382	BS2G	17.99
**4894**	allXL	BB	376	382	BS2G	15.09
**4894**	allXL	AA	357	373	BS2G	24.15
**4894**	allXL	CC	320	366	BS2G	18.56
**4894**	allXL	CC	370	376	BS2G	14.95
**4894**	allXL	AA	320	357	DBSU	25.79
**4894**	allXL	AA	357	319	DBSU	28.21
**4894**	allXL	DD	372	319	DBSU	22.76
**4894**	allXL	DD	370	357	DBSU	24.3
**4894**	allXL	AA	382	357	DBSU	31.7
**4894**	allXL	AA	372	370	DBSU	6.11
**4894**	allXL	DD	381	370	DBSU	18.81
**4894**	allXL	AA	373	370	DBSU	9.14
**4894**	allXL	DD	381	372	DBSU	19.59
**4894**	allXL	DD	372	382	DBSU	19.81
**4894**	allXL	DD	372	386	DBSU	28.75

**4894**	allXL	DD	373	386	DBSU	29.28
**4894**	allXL	BC	381	386	DBSU	61.27
**4894**	allXL	BD;DB	357	357	DBSU	19.57
**4894**	allXL	DA	373	319	DBSU	17.67
**4894**	allXL	DA	372	357	DBSU	25.57
**4894**	allXL	AC	381	357	DBSU	31.5
**4894**	allXL	DA	373	357	DBSU	24.75
**4894**	allXL	DA	372	370	DBSU	37.19
**4894**	allXL	DA	373	370	DBSU	33.63
**4894**	allXL	CB	382	373	DBSU	41.36
**4895**	allXL	AA	320	357	BS2G	12.3
**4895**	allXL	CC	320	373	BS2G	16.0
**4895**	allXL	DD	373	382	BS2G	16.5
**4895**	allXL	DD	370	386	BS2G	24.94
**4895**	allXL	AA	373	386	BS2G	22.21
**4895**	allXL	AA	319	357	BS2G	8.78
**4895**	allXL	BB	367	373	BS2G	14.11
**4895**	allXL	DD	370	382	BS2G	16.29
**4895**	allXL	CC	376	382	BS2G	13.54
**4895**	allXL	DD	357	373	BS2G	27.89
**4895**	allXL	CC	320	366	BS2G	26.65
**4895**	allXL	DD	370	376	BS2G	14.99
**4895**	allXL	AA	320	357	DBSU	12.3
**4895**	allXL	AA	357	319	DBSU	8.78
**4895**	allXL	CC	372	319	DBSU	19.99
**4895**	allXL	BB	370	357	DBSU	24.64
**4895**	allXL	BB	382	357	DBSU	18.6
**4895**	allXL	AA	372	370	DBSU	6.11
**4895**	allXL	DD	381	370	DBSU	15.5
**4895**	allXL	DD	373	370	DBSU	9.21
**4895**	allXL	DD	381	372	DBSU	16.31
**4895**	allXL	DD	372	382	DBSU	16.16
**4895**	allXL	DD	372	386	DBSU	22.11
**4895**	allXL	AA	373	386	DBSU	22.21

**4895**	allXL	BC	381	386	DBSU	43.73
**4895**	allXL	BD;DB	357	357	DBSU	19.46
**4895**	allXL	AD	373	319	DBSU	28.43
**4895**	allXL	CB	372	357	DBSU	28.48
**4895**	allXL	BD	381	357	DBSU	31.62
**4895**	allXL	CB	373	357	DBSU	29.28
**4895**	allXL	CB	372	370	DBSU	31.24
**4895**	allXL	CB	373	370	DBSU	34.35
**4895**	allXL	BC	382	373	DBSU	44.57
**4896**	allXL	AA	320	357	BS2G	21.74
**4896**	allXL	BB	320	373	BS2G	21.51
**4896**	allXL	BB	373	382	BS2G	19.31
**4896**	allXL	DD	370	386	BS2G	21.45
**4896**	allXL	DD	373	386	BS2G	24.47
**4896**	allXL	AA	319	357	BS2G	19.38
**4896**	allXL	AA	367	373	BS2G	11.34
**4896**	allXL	DD	370	382	BS2G	22.18
**4896**	allXL	BB	376	382	BS2G	14.77
**4896**	allXL	DD	357	373	BS2G	29.5
**4896**	allXL	BB	320	366	BS2G	19.07
**4896**	allXL	CC	370	376	BS2G	14.83
**4896**	allXL	AA	320	357	DBSU	21.74
**4896**	allXL	AA	357	319	DBSU	19.38
**4896**	allXL	BB	372	319	DBSU	24.79
**4896**	allXL	DD	370	357	DBSU	21.5
**4896**	allXL	DD	382	357	DBSU	25.96
**4896**	allXL	CC	372	370	DBSU	6.51
**4896**	allXL	BB	381	370	DBSU	21.16
**4896**	allXL	AA	373	370	DBSU	9.23
**4896**	allXL	BB	381	372	DBSU	20.02
**4896**	allXL	DD	372	382	DBSU	20.08
**4896**	allXL	DD	372	386	DBSU	22.97
**4896**	allXL	DD	373	386	DBSU	24.47
**4896**	allXL	BC	381	386	DBSU	20.18

**4896**	allXL	BD;DB	357	357	DBSU	19.45
**4896**	allXL	CB	373	319	DBSU	30.71
**4896**	allXL	CB	372	357	DBSU	36.87
**4896**	allXL	DB	381	357	DBSU	35.33
**4896**	allXL	CB	373	357	DBSU	36.65
**4896**	allXL	BC	372	370	DBSU	41.37
**4896**	allXL	BC	373	370	DBSU	38.72
**4896**	allXL	CB	382	373	DBSU	17.8
**4897**	allXL	CC	320	357	BS2G	25.94
**4897**	allXL	BB	320	373	BS2G	14.43
**4897**	allXL	DD	373	382	BS2G	19.78
**4897**	allXL	DD	370	386	BS2G	22.06
**4897**	allXL	DD	373	386	BS2G	20.14
**4897**	allXL	AA	319	357	BS2G	26.1
**4897**	allXL	DD	367	373	BS2G	14.51
**4897**	allXL	DD	370	382	BS2G	21.65
**4897**	allXL	CC	376	382	BS2G	14.27
**4897**	allXL	BB	357	373	BS2G	29.75
**4897**	allXL	BB	320	366	BS2G	21.37
**4897**	allXL	CC	370	376	BS2G	14.54
**4897**	allXL	CC	320	357	DBSU	25.94
**4897**	allXL	AA	357	319	DBSU	26.1
**4897**	allXL	BB	372	319	DBSU	17.31
**4897**	allXL	BB	370	357	DBSU	26.63
**4897**	allXL	CC	382	357	DBSU	22.42
**4897**	allXL	AA	372	370	DBSU	6.11
**4897**	allXL	DD	381	370	DBSU	22.01
**4897**	allXL	AA	373	370	DBSU	9.13
**4897**	allXL	DD	381	372	DBSU	20.39
**4897**	allXL	DD	372	382	DBSU	20.46
**4897**	allXL	DD	372	386	DBSU	19.21
**4897**	allXL	DD	373	386	DBSU	20.14
**4897**	allXL	DA	381	386	DBSU	38.89
**4897**	allXL	BD;DB	357	357	DBSU	19.03

4897	allXL	DA	373	319	DBSU	16.9
4897	allXL	DA	372	357	DBSU	33.96
4897	allXL	CA	381	357	DBSU	41.85
4897	allXL	DA	373	357	DBSU	37.03
4897	allXL	AD	372	370	DBSU	27.71
4897	allXL	AD	373	370	DBSU	27.33
4897	allXL	BD	382	373	DBSU	36.04
4898	allXL	CC	320	357	BS2G	23.5
4898	allXL	AA	320	373	BS2G	21.61
4898	allXL	BB	373	382	BS2G	18.16
4898	allXL	BB	370	386	BS2G	27.99
4898	allXL	AA	373	386	BS2G	23.6
4898	allXL	AA	319	357	BS2G	26.3
4898	allXL	AA;BB	367	373	BS2G	11.55
4898	allXL	BB	370	382	BS2G	18.17
4898	allXL	AA	376	382	BS2G	13.2
4898	allXL	BB	357	373	BS2G	26.49
4898	allXL	AA	320	366	BS2G	22.56
4898	allXL	CC	370	376	BS2G	14.27
4898	allXL	CC	320	357	DBSU	23.5
4898	allXL	AA	357	319	DBSU	26.3
4898	allXL	AA	372	319	DBSU	20.44
4898	allXL	BB	370	357	DBSU	25.58
4898	allXL	CC	382	357	DBSU	26.66
4898	allXL	DD	372	370	DBSU	6.93
4898	allXL	BB	381	370	DBSU	18.96
4898	allXL	AA	373	370	DBSU	8.58
4898	allXL	BB	381	372	DBSU	18.95
4898	allXL	BB	372	382	DBSU	18.47
4898	allXL	AA	372	386	DBSU	26.19
4898	allXL	AA	373	386	DBSU	23.6
4898	allXL	BC	381	386	DBSU	55.28
4898	allXL	BD;DB	357	357	DBSU	18.81
4898	allXL	BC	373	319	DBSU	31.41

**4898**	allXL	BD	372	357	DBSU	39.41
**4898**	allXL	DB	381	357	DBSU	37.43
**4898**	allXL	BC	373	357	DBSU	39.01
**4898**	allXL	BC	372	370	DBSU	37.14
**4898**	allXL	BC	373	370	DBSU	35.83
**4898**	allXL	CB	382	373	DBSU	45.35
**4899**	allXL	CC	320	357	BS2G	25.48
**4899**	allXL	BB	320	373	BS2G	21.39
**4899**	allXL	DD	373	382	BS2G	20.04
**4899**	allXL	AA	370	386	BS2G	34.32
**4899**	allXL	AA	373	386	BS2G	29.21
**4899**	allXL	AA	319	357	BS2G	26.05
**4899**	allXL	AA	367	373	BS2G	11.41
**4899**	allXL	DD	370	382	BS2G	26.36
**4899**	allXL	CC	376	382	BS2G	15.33
**4899**	allXL	DD	357	373	BS2G	25.52
**4899**	allXL	AA	320	366	BS2G	21.23
**4899**	allXL	CC	370	376	BS2G	16.37
**4899**	allXL	CC	320	357	DBSU	25.48
**4899**	allXL	AA	357	319	DBSU	26.05
**4899**	allXL	BB	372	319	DBSU	20.19
**4899**	allXL	BB	370	357	DBSU	25.74
**4899**	allXL	AA	382	357	DBSU	34.69
**4899**	allXL	AA	372	370	DBSU	6.46
**4899**	allXL	DD	381	370	DBSU	25.49
**4899**	allXL	DD	373	370	DBSU	9.09
**4899**	allXL	DD	381	372	DBSU	18.74
**4899**	allXL	DD	372	382	DBSU	19.55
**4899**	allXL	DD	372	386	DBSU	28.41
**4899**	allXL	AA	373	386	DBSU	29.21
**4899**	allXL	CB	381	386	DBSU	74.52
**4899**	allXL	BD;DB	357	357	DBSU	18.39
**4899**	allXL	CB	373	319	DBSU	24.27
**4899**	allXL	BC	372	357	DBSU	38.95

**4899**	allXL	CA	381	357	DBSU	38.26
**4899**	allXL	DB	373	357	DBSU	38.64
**4899**	allXL	BC	372	370	DBSU	23.03
**4899**	allXL	BC	373	370	DBSU	24.9
**4899**	allXL	BC	382	373	DBSU	50.22
**4900**	allXL	CC	320	357	BS2G	23.29
**4900**	allXL	DD	320	373	BS2G	17.49
**4900**	allXL	CC	373	382	BS2G	16.17
**4900**	allXL	AA	370	386	BS2G	22.64
**4900**	allXL	AA	373	386	BS2G	22.56
**4900**	allXL	CC	319	357	BS2G	24.92
**4900**	allXL	BB	367	373	BS2G	10.4
**4900**	allXL	DD	370	382	BS2G	22.3
**4900**	allXL	BB	376	382	BS2G	14.43
**4900**	allXL	DD	357	373	BS2G	34.82
**4900**	allXL	CC	320	366	BS2G	18.3
**4900**	allXL	DD	370	376	BS2G	17.06
**4900**	allXL	CC	320	357	DBSU	23.29
**4900**	allXL	CC	357	319	DBSU	24.92
**4900**	allXL	DD	372	319	DBSU	13.94
**4900**	allXL	DD	370	357	DBSU	25.66
**4900**	allXL	BB	382	357	DBSU	27.8
**4900**	allXL	AA	372	370	DBSU	6.1
**4900**	allXL	AA	381	370	DBSU	21.44
**4900**	allXL	AA	373	370	DBSU	9.03
**4900**	allXL	CC	381	372	DBSU	17.38
**4900**	allXL	CC	372	382	DBSU	17.62
**4900**	allXL	AA	372	386	DBSU	24.04
**4900**	allXL	AA	373	386	DBSU	22.56
**4900**	allXL	DA	381	386	DBSU	48.81
**4900**	allXL	BD;DB	357	357	DBSU	18.59
**4900**	allXL	AD	373	319	DBSU	35.34
**4900**	allXL	DA	372	357	DBSU	46.04
**4900**	allXL	BD	381	357	DBSU	46.83

**4900**	allXL	DA	373	357	DBSU	49.66
**4900**	allXL	CB	372	370	DBSU	26.88
**4900**	allXL	CB	373	370	DBSU	27.43
**4900**	allXL	AD	382	373	DBSU	27.35
**4901**	allXL	DD	320	357	BS2G	25.98
**4901**	allXL	BB	320	373	BS2G	16.93
**4901**	allXL	DD	373	382	BS2G	17.87
**4901**	allXL	DD	370	386	BS2G	15.05
**4901**	allXL	DD	373	386	BS2G	21.28
**4901**	allXL	AA	319	357	BS2G	26.09
**4901**	allXL	DD	367	373	BS2G	13.78
**4901**	allXL	DD	370	382	BS2G	16.89
**4901**	allXL	CC	376	382	BS2G	13.92
**4901**	allXL	DD	357	373	BS2G	28.11
**4901**	allXL	BB	320	366	BS2G	18.83
**4901**	allXL	CC	370	376	BS2G	15.25
**4901**	allXL	DD	320	357	DBSU	25.98
**4901**	allXL	AA	357	319	DBSU	26.09
**4901**	allXL	BB	372	319	DBSU	18.84
**4901**	allXL	BB	370	357	DBSU	21.08
**4901**	allXL	DD	382	357	DBSU	31.98
**4901**	allXL	AA	372	370	DBSU	6.06
**4901**	allXL	DD	381	370	DBSU	15.69
**4901**	allXL	AA	373	370	DBSU	9.02
**4901**	allXL	DD	381	372	DBSU	17.48
**4901**	allXL	AA;DD	372	382	DBSU	19.95
**4901**	allXL	DD	372	386	DBSU	21.3
**4901**	allXL	DD	373	386	DBSU	21.28
**4901**	allXL	BC	381	386	DBSU	44.24
**4901**	allXL	BD;DB	357	357	DBSU	19.99
**4901**	allXL	AD	373	319	DBSU	16.95
**4901**	allXL	DB	372	357	DBSU	32.13
**4901**	allXL	DB	381	357	DBSU	41.96
**4901**	allXL	DB	373	357	DBSU	32.73

**4901**	allXL	BC	372	370	DBSU	29.13
**4901**	allXL	BC	373	370	DBSU	31.57
**4901**	allXL	CB	382	373	DBSU	35.52
**4902**	allXL	AA	320	357	BS2G	24.57
**4902**	allXL	AA	320	373	BS2G	26.95
**4902**	allXL	CC	373	382	BS2G	19.55
**4902**	allXL	CC	370	386	BS2G	32.59
**4902**	allXL	CC	373	386	BS2G	22.71
**4902**	allXL	AA	319	357	BS2G	23.53
**4902**	allXL	AA	367	373	BS2G	11.75
**4902**	allXL	BB	370	382	BS2G	22.81
**4902**	allXL	BB	376	382	BS2G	14.81
**4902**	allXL	BB	357	373	BS2G	29.73
**4902**	allXL	AA	320	366	BS2G	22.35
**4902**	allXL	CC	370	376	BS2G	16.23
**4902**	allXL	AA	320	357	DBSU	24.57
**4902**	allXL	AA	357	319	DBSU	23.53
**4902**	allXL	AA	372	319	DBSU	31.07
**4902**	allXL	DD	370	357	DBSU	31.83
**4902**	allXL	BB	382	357	DBSU	27.09
**4902**	allXL	AA	372	370	DBSU	6.47
**4902**	allXL	DD	381	370	DBSU	22.61
**4902**	allXL	AA	373	370	DBSU	8.96
**4902**	allXL	DD	381	372	DBSU	16.53
**4902**	allXL	DD	372	382	DBSU	18.75
**4902**	allXL	CC	372	386	DBSU	25.92
**4902**	allXL	CC	373	386	DBSU	22.71
**4902**	allXL	AD	381	386	DBSU	42.23
**4902**	allXL	BD;DB	357	357	DBSU	20.13
**4902**	allXL	AD	373	319	DBSU	27.73
**4902**	allXL	BD	372	357	DBSU	43.68
**4902**	allXL	BD	381	357	DBSU	31.34
**4902**	allXL	BD	373	357	DBSU	41.76
**4902**	allXL	AD	372	370	DBSU	18.55

**4902**	allXL	DA	373	370	DBSU	21.03
**4902**	allXL	DA	382	373	DBSU	26.3
**4903**	allXL	CC	320	357	BS2G	22.77
**4903**	allXL	CC	320	373	BS2G	15.84
**4903**	allXL	CC	373	382	BS2G	19.52
**4903**	allXL	CC	370	386	BS2G	22.35
**4903**	allXL	DD	373	386	BS2G	25.08
**4903**	allXL	CC	319	357	BS2G	22.39
**4903**	allXL	AA	367	373	BS2G	12.97
**4903**	allXL	CC	370	382	BS2G	19.29
**4903**	allXL	CC	376	382	BS2G	14.49
**4903**	allXL	BB	357	373	BS2G	28.34
**4903**	allXL	CC	320	366	BS2G	18.62
**4903**	allXL	CC	370	376	BS2G	14.14
**4903**	allXL	CC	320	357	DBSU	22.77
**4903**	allXL	CC	357	319	DBSU	22.39
**4903**	allXL	CC	372	319	DBSU	13.86
**4903**	allXL	DD	370	357	DBSU	28.29
**4903**	allXL	DD	382	357	DBSU	37.37
**4903**	allXL	AA	372	370	DBSU	6.25
**4903**	allXL	CC	381	370	DBSU	20.04
**4903**	allXL	AA	373	370	DBSU	9.08
**4903**	allXL	DD	381	372	DBSU	19.23
**4903**	allXL	DD	372	382	DBSU	20.56
**4903**	allXL	DD	372	386	DBSU	22.47
**4903**	allXL	DD	373	386	DBSU	25.08
**4903**	allXL	AD	381	386	DBSU	69.88
**4903**	allXL	BD;DB	357	357	DBSU	18.8
**4903**	allXL	DA	373	319	DBSU	26.7
**4903**	allXL	BD	372	357	DBSU	39.2
**4903**	allXL	CB	381	357	DBSU	47.57
**4903**	allXL	BD	373	357	DBSU	39.53
**4903**	allXL	DA	372	370	DBSU	23.25
**4903**	allXL	DA	373	370	DBSU	21.93

**4903**	allXL	CB	382	373	DBSU	47.65
**4904**	allXL	AA	320	357	BS2G	24.39
**4904**	allXL	CC	320	373	BS2G	25.59
**4904**	allXL	BB	373	382	BS2G	21.31
**4904**	allXL	CC	370	386	BS2G	20.81
**4904**	allXL	DD	373	386	BS2G	25.91
**4904**	allXL	AA	319	357	BS2G	23.12
**4904**	allXL	AA	367	373	BS2G	13.59
**4904**	allXL	CC	370	382	BS2G	21.32
**4904**	allXL	CC	376	382	BS2G	15.25
**4904**	allXL	CC	357	373	BS2G	13.0
**4904**	allXL	CC	320	366	BS2G	15.14
**4904**	allXL	CC	370	376	BS2G	13.69
**4904**	allXL	AA	320	357	DBSU	24.39
**4904**	allXL	AA	357	319	DBSU	23.12
**4904**	allXL	CC	372	319	DBSU	25.66
**4904**	allXL	CC	370	357	DBSU	14.31
**4904**	allXL	CC	382	357	DBSU	20.18
**4904**	allXL	AA	372	370	DBSU	6.49
**4904**	allXL	CC	381	370	DBSU	22.07
**4904**	allXL	CC	373	370	DBSU	9.15
**4904**	allXL	DD	381	372	DBSU	19.66
**4904**	allXL	DD	372	382	DBSU	21.89
**4904**	allXL	DD	372	386	DBSU	25.2
**4904**	allXL	DD	373	386	DBSU	25.91
**4904**	allXL	BC	381	386	DBSU	59.8
**4904**	allXL	BD;DB	357	357	DBSU	18.29
**4904**	allXL	BC	373	319	DBSU	21.9
**4904**	allXL	CA	372	357	DBSU	25.95
**4904**	allXL	CA	381	357	DBSU	35.53
**4904**	allXL	CA	373	357	DBSU	23.62
**4904**	allXL	DA	372	370	DBSU	28.9
**4904**	allXL	DA	373	370	DBSU	26.76
**4904**	allXL	DA	382	373	DBSU	44.96

**4905**	allXL	CC	320	357	BS2G	22.66
**4905**	allXL	CC	320	373	BS2G	16.77
**4905**	allXL	BB	373	382	BS2G	17.17
**4905**	allXL	CC	370	386	BS2G	27.17
**4905**	allXL	BB	373	386	BS2G	27.69
**4905**	allXL	CC	319	357	BS2G	25.68
**4905**	allXL	AA	367	373	BS2G	13.73
**4905**	allXL	CC	370	382	BS2G	20.36
**4905**	allXL	BB	376	382	BS2G	13.98
**4905**	allXL	CC	357	373	BS2G	28.8
**4905**	allXL	CC	320	366	BS2G	22.26
**4905**	allXL	CC	370	376	BS2G	14.71
**4905**	allXL	CC	320	357	DBSU	22.66
**4905**	allXL	CC	357	319	DBSU	25.68
**4905**	allXL	CC	372	319	DBSU	14.93
**4905**	allXL	DD	370	357	DBSU	26.81
**4905**	allXL	CC	382	357	DBSU	17.79
**4905**	allXL	AA	372	370	DBSU	6.05
**4905**	allXL	CC	381	370	DBSU	22.22
**4905**	allXL	DD	373	370	DBSU	9.07
**4905**	allXL	DD	381	372	DBSU	18.6
**4905**	allXL	BB	372	382	DBSU	19.71
**4905**	allXL	BB;DD	372	386	DBSU	29.43
**4905**	allXL	BB	373	386	DBSU	27.69
**4905**	allXL	AC	381	386	DBSU	74.05
**4905**	allXL	BD;DB	357	357	DBSU	18.63
**4905**	allXL	AD	373	319	DBSU	20.35
**4905**	allXL	DA	372	357	DBSU	40.99
**4905**	allXL	CA	381	357	DBSU	37.49
**4905**	allXL	DA	373	357	DBSU	37.35
**4905**	allXL	DA	372	370	DBSU	38.85
**4905**	allXL	DA	373	370	DBSU	35.77
**4905**	allXL	AD	382	373	DBSU	56.67
**4906**	allXL	CC	320	357	BS2G	23.77

**4906**	allXL	BB	320	373	BS2G	20.84
**4906**	allXL	CC	373	382	BS2G	19.23
**4906**	allXL	CC	370	386	BS2G	24.42
**4906**	allXL	CC	373	386	BS2G	24.31
**4906**	allXL	CC	319	357	BS2G	26.75
**4906**	allXL	AA	367	373	BS2G	11.47
**4906**	allXL	AA	370	382	BS2G	19.57
**4906**	allXL	CC	376	382	BS2G	14.99
**4906**	allXL	BB	357	373	BS2G	20.56
**4906**	allXL	BB	320	366	BS2G	21.26
**4906**	allXL	AA	370	376	BS2G	15.51
**4906**	allXL	CC	320	357	DBSU	23.77
**4906**	allXL	CC	357	319	DBSU	26.75
**4906**	allXL	BB	372	319	DBSU	19.22
**4906**	allXL	BB	370	357	DBSU	22.36
**4906**	allXL	BB	382	357	DBSU	30.75
**4906**	allXL	AA	372	370	DBSU	6.44
**4906**	allXL	AA	381	370	DBSU	21.29
**4906**	allXL	AA	373	370	DBSU	8.92
**4906**	allXL	CC	381	372	DBSU	20.54
**4906**	allXL	CC	372	382	DBSU	19.57
**4906**	allXL	CC	372	386	DBSU	22.61
**4906**	allXL	CC	373	386	DBSU	24.31
**4906**	allXL	DA	381	386	DBSU	53.17
**4906**	allXL	BD;DB	357	357	DBSU	19.21
**4906**	allXL	AD	373	319	DBSU	18.38
**4906**	allXL	BD	372	357	DBSU	38.74
**4906**	allXL	BD	381	357	DBSU	41.26
**4906**	allXL	BD	373	357	DBSU	38.37
**4906**	allXL	DA	372	370	DBSU	20.26
**4906**	allXL	DA	373	370	DBSU	19.08
**4906**	allXL	AD	382	373	DBSU	34.32
**4907**	allXL	AA	320	357	BS2G	25.76
**4907**	allXL	BB	320	373	BS2G	10.74

**4907**	allXL	BB	373	382	BS2G	20.97
**4907**	allXL	DD	370	386	BS2G	26.59
**4907**	allXL	DD	373	386	BS2G	30.78
**4907**	allXL	AA	319	357	BS2G	24.66
**4907**	allXL	AA	367	373	BS2G	14.23
**4907**	allXL	DD	370	382	BS2G	17.35
**4907**	allXL	BB	376	382	BS2G	14.77
**4907**	allXL	BB	357	373	BS2G	33.37
**4907**	allXL	CC	320	366	BS2G	18.43
**4907**	allXL	DD	370	376	BS2G	14.3
**4907**	allXL	AA	320	357	DBSU	25.76
**4907**	allXL	AA	357	319	DBSU	24.66
**4907**	allXL	BB	372	319	DBSU	11.44
**4907**	allXL	BB	370	357	DBSU	28.31
**4907**	allXL	BA	382	357	DBSU	34.31
**4907**	allXL	AA	372	370	DBSU	6.35
**4907**	allXL	DD	381	370	DBSU	16.53
**4907**	allXL	AA	373	370	DBSU	8.92
**4907**	allXL	DD	381	372	DBSU	20.47
**4907**	allXL	DD	372	382	DBSU	21.57
**4907**	allXL	DD	372	386	DBSU	30.63
**4907**	allXL	DD	373	386	DBSU	30.78
**4907**	allXL	BC	381	386	DBSU	61.55
**4907**	allXL	BD;DB	357	357	DBSU	19.5
**4907**	allXL	DA	373	319	DBSU	21.94
**4907**	allXL	BC	372	357	DBSU	32.7
**4907**	allXL	BC	381	357	DBSU	30.12
**4907**	allXL	BC	373	357	DBSU	32.3
**4907**	allXL	CB	372	370	DBSU	23.72
**4907**	allXL	CB	373	370	DBSU	26.29
**4907**	allXL	BC	382	373	DBSU	46.33
**4908**	allXL	AA	320	357	BS2G	25.48
**4908**	allXL	CC	320	373	BS2G	23.05
**4908**	allXL	AA	373	382	BS2G	20.16

**4908**	allXL	AA	370	386	BS2G	17.95
**4908**	allXL	AA	373	386	BS2G	18.1
**4908**	allXL	AA	319	357	BS2G	26.13
**4908**	allXL	AA	367	373	BS2G	12.5
**4908**	allXL	CC	370	382	BS2G	21.3
**4908**	allXL	CC	376	382	BS2G	14.66
**4908**	allXL	BB	357	373	BS2G	20.09
**4908**	allXL	DD	320	366	BS2G	17.11
**4908**	allXL	BB	370	376	BS2G	15.41
**4908**	allXL	AA	320	357	DBSU	25.48
**4908**	allXL	AA	357	319	DBSU	26.13
**4908**	allXL	CC	372	319	DBSU	19.35
**4908**	allXL	CC	370	357	DBSU	26.15
**4908**	allXL	DD	382	357	DBSU	17.11
**4908**	allXL	AA	372	370	DBSU	6.31
**4908**	allXL	DD	381	370	DBSU	21.2
**4908**	allXL	AA	373	370	DBSU	9.14
**4908**	allXL	CC	381	372	DBSU	22.5
**4908**	allXL	CC	372	382	DBSU	20.72
**4908**	allXL	AA	372	386	DBSU	17.69
**4908**	allXL	AA	373	386	DBSU	18.1
**4908**	allXL	BD	381	386	DBSU	58.18
**4908**	allXL	BD;DB	357	357	DBSU	18.07
**4908**	allXL	AD	373	319	DBSU	36.64
**4908**	allXL	BD	372	357	DBSU	36.13
**4908**	allXL	DB	381	357	DBSU	30.4
**4908**	allXL	BD	373	357	DBSU	33.9
**4908**	allXL	DA	372	370	DBSU	54.57
**4908**	allXL	DA	373	370	DBSU	55.09
**4908**	allXL	DB	382	373	DBSU	39.96
**4909**	allXL	AA	320	357	BS2G	23.68
**4909**	allXL	BB	320	373	BS2G	32.72
**4909**	allXL	BB	373	382	BS2G	18.61
**4909**	allXL	CC	370	386	BS2G	27.97

**4909**	allXL	BB	373	386	BS2G	23.48
**4909**	allXL	AA	319	357	BS2G	22.34
**4909**	allXL	DD	367	373	BS2G	13.54
**4909**	allXL	CC	370	382	BS2G	25.02
**4909**	allXL	BB	376	382	BS2G	13.76
**4909**	allXL	DD	357	373	BS2G	25.53
**4909**	allXL	AA	320	366	BS2G	29.92
**4909**	allXL	CC	370	376	BS2G	15.83
**4909**	allXL	AA	320	357	DBSU	23.68
**4909**	allXL	AA	357	319	DBSU	22.34
**4909**	allXL	BB	372	319	DBSU	34.38
**4909**	allXL	DD	370	357	DBSU	21.88
**4909**	allXL	BB	382	357	DBSU	32.13
**4909**	allXL	AA	372	370	DBSU	6.18
**4909**	allXL	DD	381	370	DBSU	23.61
**4909**	allXL	DD	373	370	DBSU	8.92
**4909**	allXL	DD	381	372	DBSU	19.4
**4909**	allXL	BB	372	382	DBSU	20.16
**4909**	allXL	BB	372	386	DBSU	23.24
**4909**	allXL	BB	373	386	DBSU	23.48
**4909**	allXL	BC	381	386	DBSU	42.79
**4909**	allXL	BD;DB	357	357	DBSU	18.25
**4909**	allXL	AD	373	319	DBSU	20.89
**4909**	allXL	DB	372	357	DBSU	37.57
**4909**	allXL	BC	381	357	DBSU	31.49
**4909**	allXL	DB	373	357	DBSU	35.15
**4909**	allXL	AD	372	370	DBSU	50.3
**4909**	allXL	AD	373	370	DBSU	51.72
**4909**	allXL	CB	382	373	DBSU	50.28
**4910**	allXL	CC	320	357	BS2G	27.15
**4910**	allXL	BB	320	373	BS2G	18.47
**4910**	allXL	DD	373	382	BS2G	19.18
**4910**	allXL	DD	370	386	BS2G	12.8
**4910**	allXL	DD	373	386	BS2G	19.6

**4910**	allXL	CC	319	357	BS2G	29.04
**4910**	allXL	AA	367	373	BS2G	14.57
**4910**	allXL	DD	370	382	BS2G	17.64
**4910**	allXL	CC	376	382	BS2G	15.04
**4910**	allXL	CC	357	373	BS2G	22.6
**4910**	allXL	CC	320	366	BS2G	16.74
**4910**	allXL	CC	370	376	BS2G	16.54
**4910**	allXL	CC	320	357	DBSU	27.15
**4910**	allXL	CC	357	319	DBSU	29.04
**4910**	allXL	BB	372	319	DBSU	24.27
**4910**	allXL	CC	370	357	DBSU	20.21
**4910**	allXL	CC	382	357	DBSU	31.09
**4910**	allXL	AA	372	370	DBSU	6.18
**4910**	allXL	DD	381	370	DBSU	18.27
**4910**	allXL	AA	373	370	DBSU	8.7
**4910**	allXL	DD	381	372	DBSU	18.05
**4910**	allXL	DD	372	382	DBSU	18.91
**4910**	allXL	DD	372	386	DBSU	17.88
**4910**	allXL	DD	373	386	DBSU	19.6
**4910**	allXL	AD	381	386	DBSU	54.2
**4910**	allXL	BD;DB	357	357	DBSU	18.79
**4910**	allXL	AD	373	319	DBSU	23.91
**4910**	allXL	CA	372	357	DBSU	32.54
**4910**	allXL	CA	381	357	DBSU	29.2
**4910**	allXL	CA	373	357	DBSU	29.42
**4910**	allXL	AD	372	370	DBSU	44.98
**4910**	allXL	AD	373	370	DBSU	45.72
**4910**	allXL	DA	382	373	DBSU	38.72
**4911**	allXL	AA	320	357	BS2G	24.34
**4911**	allXL	DD	320	373	BS2G	20.38
**4911**	allXL	CC	373	382	BS2G	16.82
**4911**	allXL	AA	370	386	BS2G	22.57
**4911**	allXL	AA	373	386	BS2G	20.82
**4911**	allXL	AA	319	357	BS2G	23.64

**4911**	allXL	AA	367	373	BS2G	13.8
**4911**	allXL	AA	370	382	BS2G	22.7
**4911**	allXL	BB	376	382	BS2G	13.05
**4911**	allXL	AA	357	373	BS2G	34.78
**4911**	allXL	BB	320	366	BS2G	22.79
**4911**	allXL	DD	370	376	BS2G	16.16
**4911**	allXL	AA	320	357	DBSU	24.34
**4911**	allXL	AA	357	319	DBSU	23.64
**4911**	allXL	DD	372	319	DBSU	16.89
**4911**	allXL	DD	370	357	DBSU	27.06
**4911**	allXL	DD	382	357	DBSU	45.66
**4911**	allXL	AA	372	370	DBSU	6.1
**4911**	allXL	DD	381	370	DBSU	20.62
**4911**	allXL	AA	373	370	DBSU	9.15
**4911**	allXL	CC	381	372	DBSU	18.75
**4911**	allXL	CC	372	382	DBSU	17.38
**4911**	allXL	AA	372	386	DBSU	20.53
**4911**	allXL	AA	373	386	DBSU	20.82
**4911**	allXL	CB	381	386	DBSU	21.4
**4911**	allXL	BD;DB	357	357	DBSU	17.82
**4911**	allXL	AD	373	319	DBSU	16.18
**4911**	allXL	AD	372	357	DBSU	39.7
**4911**	allXL	AD	381	357	DBSU	42.05
**4911**	allXL	AD	373	357	DBSU	39.82
**4911**	allXL	CB	372	370	DBSU	21.43
**4911**	allXL	CB	373	370	DBSU	17.71
**4911**	allXL	CB	382	373	DBSU	24.09
**4912**	allXL	AA	320	357	BS2G	25.11
**4912**	allXL	CC	320	373	BS2G	11.51
**4912**	allXL	BB	373	382	BS2G	17.46
**4912**	allXL	DD	370	386	BS2G	14.76
**4912**	allXL	DD	373	386	BS2G	18.65
**4912**	allXL	AA	319	357	BS2G	26.09
**4912**	allXL	AA	367	373	BS2G	12.42

**4912**	allXL	BB	370	382	BS2G	16.24
**4912**	allXL	CC	376	382	BS2G	13.78
**4912**	allXL	AA	357	373	BS2G	31.2
**4912**	allXL	CC	320	366	BS2G	22.19
**4912**	allXL	CC	370	376	BS2G	15.19
**4912**	allXL	AA	320	357	DBSU	25.11
**4912**	allXL	AA	357	319	DBSU	26.09
**4912**	allXL	CC	372	319	DBSU	10.1
**4912**	allXL	DD	370	357	DBSU	29.46
**4912**	allXL	CC	382	357	DBSU	24.29
**4912**	allXL	AA	372	370	DBSU	6.61
**4912**	allXL	BB	381	370	DBSU	18.1
**4912**	allXL	AA	373	370	DBSU	9.25
**4912**	allXL	DD	381	372	DBSU	16.05
**4912**	allXL	DD	372	382	DBSU	16.23
**4912**	allXL	DD	372	386	DBSU	15.07
**4912**	allXL	DD	373	386	DBSU	18.65
**4912**	allXL	BC	381	386	DBSU	33.13
**4912**	allXL	BD;DB	357	357	DBSU	18.46
**4912**	allXL	BC	373	319	DBSU	33.99
**4912**	allXL	BC	372	357	DBSU	40.05
**4912**	allXL	CB	381	357	DBSU	28.34
**4912**	allXL	BC	373	357	DBSU	41.1
**4912**	allXL	BC	372	370	DBSU	24.5
**4912**	allXL	CB	373	370	DBSU	22.19
**4912**	allXL	CB	382	373	DBSU	17.37
**4913**	allXL	CC	320	357	BS2G	25.51
**4913**	allXL	AA	320	373	BS2G	7.21
**4913**	allXL	CC	373	382	BS2G	14.59
**4913**	allXL	AA	370	386	BS2G	23.25
**4913**	allXL	CC	373	386	BS2G	21.98
**4913**	allXL	CC	319	357	BS2G	26.65
**4913**	allXL	BB	367	373	BS2G	12.86
**4913**	allXL	AA	370	382	BS2G	21.09

**4913**	allXL	CC	376	382	BS2G	13.97
**4913**	allXL	CC	357	373	BS2G	18.87
**4913**	allXL	AA	320	366	BS2G	21.11
**4913**	allXL	DD	370	376	BS2G	16.08
**4913**	allXL	CC	320	357	DBSU	25.51
**4913**	allXL	CC	357	319	DBSU	26.65
**4913**	allXL	AA	372	319	DBSU	9.2
**4913**	allXL	CC	370	357	DBSU	15.15
**4913**	allXL	DD	382	357	DBSU	22.28
**4913**	allXL	CC	372	370	DBSU	6.71
**4913**	allXL	DD	381	370	DBSU	19.05
**4913**	allXL	AA	373	370	DBSU	8.0
**4913**	allXL	DD	381	372	DBSU	19.7
**4913**	allXL	CC	372	382	DBSU	17.72
**4913**	allXL	CC	372	386	DBSU	23.53
**4913**	allXL	CC	373	386	DBSU	21.98
**4913**	allXL	AD	381	386	DBSU	22.69
**4913**	allXL	BD;DB	357	357	DBSU	19.25
**4913**	allXL	AD	373	319	DBSU	29.8
**4913**	allXL	CA	372	357	DBSU	24.42
**4913**	allXL	AD	381	357	DBSU	29.08
**4913**	allXL	CA	373	357	DBSU	26.59
**4913**	allXL	DA	372	370	DBSU	44.64
**4913**	allXL	DA	373	370	DBSU	41.73
**4913**	allXL	AD	382	373	DBSU	30.11
**4914**	allXL	BB	320	357	BS2G	25.7
**4914**	allXL	CC	320	373	BS2G	21.3
**4914**	allXL	AA	373	382	BS2G	20.43
**4914**	allXL	DD	370	386	BS2G	20.64
**4914**	allXL	AA	373	386	BS2G	22.61
**4914**	allXL	BB	319	357	BS2G	26.82
**4914**	allXL	DD	367	373	BS2G	13.36
**4914**	allXL	DD	370	382	BS2G	19.84
**4914**	allXL	CC	376	382	BS2G	14.88

**4914**	allXL	CC	357	373	BS2G	24.05
**4914**	allXL	BB	320	366	BS2G	14.66
**4914**	allXL	CC	370	376	BS2G	15.7
**4914**	allXL	BB	320	357	DBSU	25.7
**4914**	allXL	BB	357	319	DBSU	26.82
**4914**	allXL	CC	372	319	DBSU	16.11
**4914**	allXL	CC	370	357	DBSU	24.28
**4914**	allXL	DD	382	357	DBSU	30.71
**4914**	allXL	AA	372	370	DBSU	6.17
**4914**	allXL	DD	381	370	DBSU	19.34
**4914**	allXL	CC	373	370	DBSU	9.09
**4914**	allXL	DD	381	372	DBSU	20.35
**4914**	allXL	DD	372	382	DBSU	21.67
**4914**	allXL	AA	372	386	DBSU	25.53
**4914**	allXL	AA	373	386	DBSU	22.61
**4914**	allXL	BD	381	386	DBSU	40.49
**4914**	allXL	BD;DB	357	357	DBSU	18.93
**4914**	allXL	DA	373	319	DBSU	35.84
**4914**	allXL	CA	372	357	DBSU	39.34
**4914**	allXL	DB	381	357	DBSU	43.96
**4914**	allXL	CA	373	357	DBSU	39.51
**4914**	allXL	DA	372	370	DBSU	28.92
**4914**	allXL	DA	373	370	DBSU	32.39
**4914**	allXL	AD	382	373	DBSU	33.97
**4915**	allXL	CC	320	357	BS2G	25.89
**4915**	allXL	AA	320	373	BS2G	20.44
**4915**	allXL	AA	373	382	BS2G	16.84
**4915**	allXL	AA	370	386	BS2G	24.46
**4915**	allXL	AA	373	386	BS2G	24.13
**4915**	allXL	AA	319	357	BS2G	26.09
**4915**	allXL	BB	367	373	BS2G	10.14
**4915**	allXL	AA	370	382	BS2G	21.53
**4915**	allXL	BB	376	382	BS2G	13.65
**4915**	allXL	CC	357	373	BS2G	26.66

**4915**	allXL	AA	320	366	BS2G	17.6
**4915**	allXL	CC	370	376	BS2G	17.18
**4915**	allXL	CC	320	357	DBSU	25.89
**4915**	allXL	AA	357	319	DBSU	26.09
**4915**	allXL	AA	372	319	DBSU	15.83
**4915**	allXL	CC	370	357	DBSU	24.05
**4915**	allXL	BB	382	357	DBSU	34.09
**4915**	allXL	AA	372	370	DBSU	6.21
**4915**	allXL	DD	381	370	DBSU	21.03
**4915**	allXL	AA;CC	373	370	DBSU	9.1
**4915**	allXL	DD	381	372	DBSU	16.73
**4915**	allXL	AA	372	382	DBSU	16.49
**4915**	allXL	AA	372	386	DBSU	22.05
**4915**	allXL	AA	373	386	DBSU	24.13
**4915**	allXL	CB	381	386	DBSU	44.99
**4915**	allXL	BD;DB	357	357	DBSU	18.25
**4915**	allXL	AD	373	319	DBSU	40.0
**4915**	allXL	CA	372	357	DBSU	45.41
**4915**	allXL	BC	381	357	DBSU	49.82
**4915**	allXL	CA	373	357	DBSU	44.44
**4915**	allXL	DA	372	370	DBSU	41.19
**4915**	allXL	DA	373	370	DBSU	44.61
**4915**	allXL	BC	382	373	DBSU	53.9
**4916**	allXL	AA	320	357	BS2G	25.48
**4916**	allXL	DD	320	373	BS2G	19.6
**4916**	allXL	DD	373	382	BS2G	19.67
**4916**	allXL	CC	370	386	BS2G	23.69
**4916**	allXL	CC	373	386	BS2G	26.23
**4916**	allXL	AA	319	357	BS2G	27.48
**4916**	allXL	AA	367	373	BS2G	14.42
**4916**	allXL	DD	370	382	BS2G	22.58
**4916**	allXL	CC	376	382	BS2G	14.31
**4916**	allXL	CC	357	373	BS2G	15.35
**4916**	allXL	AA	320	366	BS2G	22.95

**4916**	allXL	CC	370	376	BS2G	15.16
**4916**	allXL	AA	320	357	DBSU	25.48
**4916**	allXL	AA	357	319	DBSU	27.48
**4916**	allXL	DD	372	319	DBSU	20.67
**4916**	allXL	CC	370	357	DBSU	12.91
**4916**	allXL	CC	382	357	DBSU	20.89
**4916**	allXL	AA	372	370	DBSU	6.35
**4916**	allXL	DD	381	370	DBSU	22.81
**4916**	allXL	AA	373	370	DBSU	9.3
**4916**	allXL	DD	381	372	DBSU	21.68
**4916**	allXL	DD	372	382	DBSU	22.7
**4916**	allXL	CC	372	386	DBSU	25.31
**4916**	allXL	CC	373	386	DBSU	26.23
**4916**	allXL	CA	381	386	DBSU	43.77
**4916**	allXL	BD;DB	357	357	DBSU	19.61
**4916**	allXL	BC	373	319	DBSU	18.35
**4916**	allXL	CA	372	357	DBSU	18.38
**4916**	allXL	CA	381	357	DBSU	20.88
**4916**	allXL	CA	373	357	DBSU	14.68
**4916**	allXL	DA	372	370	DBSU	26.11
**4916**	allXL	DA	373	370	DBSU	22.73
**4916**	allXL	DA	382	373	DBSU	29.93
**4917**	allXL	BB	320	357	BS2G	26.02
**4917**	allXL	DD	320	373	BS2G	14.82
**4917**	allXL	CC	373	382	BS2G	18.14
**4917**	allXL	CC	370	386	BS2G	27.97
**4917**	allXL	CC	373	386	BS2G	20.49
**4917**	allXL	AA	319	357	BS2G	25.46
**4917**	allXL	AA	367	373	BS2G	13.06
**4917**	allXL	DD	370	382	BS2G	21.19
**4917**	allXL	CC	376	382	BS2G	14.81
**4917**	allXL	BB	357	373	BS2G	30.45
**4917**	allXL	DD	320	366	BS2G	24.39
**4917**	allXL	DD	370	376	BS2G	15.19

**4917**	allXL	BB	320	357	DBSU	26.02
**4917**	allXL	AA	357	319	DBSU	25.46
**4917**	allXL	DD	372	319	DBSU	11.44
**4917**	allXL	DD	370	357	DBSU	24.52
**4917**	allXL	BB	382	357	DBSU	13.16
**4917**	allXL	AA	372	370	DBSU	6.23
**4917**	allXL	DD	381	370	DBSU	18.78
**4917**	allXL	AA	373	370	DBSU	9.09
**4917**	allXL	CC	381	372	DBSU	21.33
**4917**	allXL	CC	372	382	DBSU	20.73
**4917**	allXL	CC	372	386	DBSU	21.54
**4917**	allXL	CC	373	386	DBSU	20.49
**4917**	allXL	BC	381	386	DBSU	39.86
**4917**	allXL	BD;DB	357	357	DBSU	18.98
**4917**	allXL	CB	373	319	DBSU	15.0
**4917**	allXL	DA	372	357	DBSU	33.16
**4917**	allXL	BD	381	357	DBSU	26.93
**4917**	allXL	DA	373	357	DBSU	36.05
**4917**	allXL	DA	372	370	DBSU	31.06
**4917**	allXL	DA	373	370	DBSU	30.14
**4917**	allXL	BC	382	373	DBSU	45.46
**4918**	allXL	AA	320	357	BS2G	25.31
**4918**	allXL	BB	320	373	BS2G	22.77
**4918**	allXL	BB	373	382	BS2G	15.72
**4918**	allXL	AA	370	386	BS2G	20.99
**4918**	allXL	AA	373	386	BS2G	21.91
**4918**	allXL	AA	319	357	BS2G	26.87
**4918**	allXL	AA	367	373	BS2G	11.5
**4918**	allXL	CC	370	382	BS2G	20.94
**4918**	allXL	BB	376	382	BS2G	13.5
**4918**	allXL	AA	357	373	BS2G	29.93
**4918**	allXL	CC	320	366	BS2G	17.69
**4918**	allXL	CC	370	376	BS2G	16.68
**4918**	allXL	AA	320	357	DBSU	25.31

**4918**	allXL	AA	357	319	DBSU	26.87
**4918**	allXL	BB	372	319	DBSU	22.64
**4918**	allXL	DD	370	357	DBSU	30.13
**4918**	allXL	AA	382	357	DBSU	19.54
**4918**	allXL	AA	372	370	DBSU	6.42
**4918**	allXL	CC	381	370	DBSU	22.94
**4918**	allXL	AA	373	370	DBSU	9.08
**4918**	allXL	BB	381	372	DBSU	19.29
**4918**	allXL	BB	372	382	DBSU	18.14
**4918**	allXL	AA	372	386	DBSU	23.07
**4918**	allXL	AA	373	386	DBSU	21.91
**4918**	allXL	DA	381	386	DBSU	25.87
**4918**	allXL	BD;DB	357	357	DBSU	18.82
**4918**	allXL	CB	373	319	DBSU	26.99
**4918**	allXL	CB	372	357	DBSU	41.77
**4918**	allXL	AC	381	357	DBSU	36.76
**4918**	allXL	CB	373	357	DBSU	44.81
**4918**	allXL	CB	372	370	DBSU	12.4
**4918**	allXL	CB	373	370	DBSU	14.82
**4918**	allXL	CB	382	373	DBSU	34.04
**4919**	allXL	AA	320	357	BS2G	25.42
**4919**	allXL	DD	320	373	BS2G	16.58
**4919**	allXL	DD	373	382	BS2G	21.2
**4919**	allXL	DD	370	386	BS2G	27.35
**4919**	allXL	DD	373	386	BS2G	30.83
**4919**	allXL	CC	319	357	BS2G	26.83
**4919**	allXL	DD	367	373	BS2G	13.38
**4919**	allXL	DD	370	382	BS2G	19.9
**4919**	allXL	CC	376	382	BS2G	14.27
**4919**	allXL	DD	357	373	BS2G	19.92
**4919**	allXL	CC	320	366	BS2G	27.13
**4919**	allXL	DD	370	376	BS2G	16.55
**4919**	allXL	AA	320	357	DBSU	25.42
**4919**	allXL	CC	357	319	DBSU	26.83

**4919**	allXL	DD	372	319	DBSU	20.14
**4919**	allXL	DD	370	357	DBSU	23.24
**4919**	allXL	DD	382	357	DBSU	13.16
**4919**	allXL	AA	372	370	DBSU	6.07
**4919**	allXL	DD	381	370	DBSU	19.8
**4919**	allXL	AA	373	370	DBSU	9.09
**4919**	allXL	DD	381	372	DBSU	21.61
**4919**	allXL	DD	372	382	DBSU	22.93
**4919**	allXL	CC	372	386	DBSU	31.45
**4919**	allXL	DD	373	386	DBSU	30.83
**4919**	allXL	DB	381	386	DBSU	49.49
**4919**	allXL	BD;DB	357	357	DBSU	18.74
**4919**	allXL	AD	373	319	DBSU	11.46
**4919**	allXL	BD	372	357	DBSU	28.75
**4919**	allXL	DB	381	357	DBSU	27.52
**4919**	allXL	BD	373	357	DBSU	26.89
**4919**	allXL	DA	372	370	DBSU	35.81
**4919**	allXL	DA	373	370	DBSU	35.21
**4919**	allXL	AD	382	373	DBSU	35.59
**4920**	allXL	CC	320	357	BS2G	24.39
**4920**	allXL	BB	320	373	BS2G	20.37
**4920**	allXL	AA	373	382	BS2G	16.99
**4920**	allXL	AA	370	386	BS2G	23.08
**4920**	allXL	AA	373	386	BS2G	19.61
**4920**	allXL	AA	319	357	BS2G	24.23
**4920**	allXL	AA	367	373	BS2G	15.01
**4920**	allXL	CC	370	382	BS2G	22.46
**4920**	allXL	BB	376	382	BS2G	13.97
**4920**	allXL	DD	357	373	BS2G	30.8
**4920**	allXL	BB	320	366	BS2G	21.86
**4920**	allXL	CC	370	376	BS2G	15.85
**4920**	allXL	CC	320	357	DBSU	24.39
**4920**	allXL	AA	357	319	DBSU	24.23
**4920**	allXL	BB	372	319	DBSU	19.08

**4920**	allXL	DD	370	357	DBSU	22.49
**4920**	allXL	AA	382	357	DBSU	39.19
**4920**	allXL	AA	372	370	DBSU	6.08
**4920**	allXL	CC	381	370	DBSU	22.86
**4920**	allXL	AA	373	370	DBSU	9.28
**4920**	allXL	BB	381	372	DBSU	19.83
**4920**	allXL	BB	372	382	DBSU	19.08
**4920**	allXL	AA	372	386	DBSU	21.57
**4920**	allXL	AA	373	386	DBSU	19.61
**4920**	allXL	BC	381	386	DBSU	28.32
**4920**	allXL	BD;DB	357	357	DBSU	18.97
**4920**	allXL	CB	373	319	DBSU	27.49
**4920**	allXL	BC	372	357	DBSU	28.51
**4920**	allXL	BC	381	357	DBSU	40.98
**4920**	allXL	BC	373	357	DBSU	31.8
**4920**	allXL	BC	372	370	DBSU	19.65
**4920**	allXL	BC	373	370	DBSU	17.89
**4920**	allXL	CB	382	373	DBSU	28.24
**4921**	allXL	BB	320	357	BS2G	25.46
**4921**	allXL	CC	320	373	BS2G	17.3
**4921**	allXL	AA	373	382	BS2G	14.18
**4921**	allXL	AA	370	386	BS2G	8.82
**4921**	allXL	DD	373	386	BS2G	15.4
**4921**	allXL	AA	319	357	BS2G	27.58
**4921**	allXL	BB	367	373	BS2G	11.45
**4921**	allXL	AA	370	382	BS2G	14.26
**4921**	allXL	AA	376	382	BS2G	13.67
**4921**	allXL	BB	357	373	BS2G	18.07
**4921**	allXL	AA	320	366	BS2G	20.95
**4921**	allXL	CC	370	376	BS2G	14.72
**4921**	allXL	BB	320	357	DBSU	25.46
**4921**	allXL	AA	357	319	DBSU	27.58
**4921**	allXL	CC	372	319	DBSU	20.84
**4921**	allXL	CC	370	357	DBSU	24.88

**4921**	allXL	BB	382	357	DBSU	33.46
**4921**	allXL	AA	372	370	DBSU	6.24
**4921**	allXL	AA	381	370	DBSU	17.73
**4921**	allXL	AA	373	370	DBSU	9.2
**4921**	allXL	DD	381	372	DBSU	14.68
**4921**	allXL	AA	372	382	DBSU	13.33
**4921**	allXL	AA	372	386	DBSU	12.78
**4921**	allXL	DD	373	386	DBSU	15.4
**4921**	allXL	CB	381	386	DBSU	26.08
**4921**	allXL	BD;DB	357	357	DBSU	17.83
**4921**	allXL	CB	373	319	DBSU	29.87
**4921**	allXL	BD	372	357	DBSU	19.62
**4921**	allXL	CB	381	357	DBSU	38.43
**4921**	allXL	BD	373	357	DBSU	22.34
**4921**	allXL	CB	372	370	DBSU	41.89
**4921**	allXL	CB	373	370	DBSU	38.42
**4921**	allXL	BC	382	373	DBSU	29.15
**4922**	allXL	AA	320	357	BS2G	26.23
**4922**	allXL	CC	320	373	BS2G	23.93
**4922**	allXL	AA	373	382	BS2G	19.99
**4922**	allXL	AA	370	386	BS2G	23.17
**4922**	allXL	AA	373	386	BS2G	26.43
**4922**	allXL	AA	319	357	BS2G	25.72
**4922**	allXL	AA	367	373	BS2G	10.51
**4922**	allXL	AA	370	382	BS2G	20.61
**4922**	allXL	BB	376	382	BS2G	13.76
**4922**	allXL	BB	357	373	BS2G	20.64
**4922**	allXL	CC	320	366	BS2G	17.25
**4922**	allXL	CC	370	376	BS2G	14.8
**4922**	allXL	AA	320	357	DBSU	26.23
**4922**	allXL	AA	357	319	DBSU	25.72
**4922**	allXL	CC	372	319	DBSU	20.19
**4922**	allXL	DD	370	357	DBSU	24.2
**4922**	allXL	CC	382	357	DBSU	35.35

**4922**	allXL	AA	372	370	DBSU	6.33
**4922**	allXL	AA	381	370	DBSU	22.72
**4922**	allXL	AA	373	370	DBSU	8.97
**4922**	allXL	AA	381	372	DBSU	20.46
**4922**	allXL	AA	372	382	DBSU	19.07
**4922**	allXL	AA	372	386	DBSU	23.99
**4922**	allXL	AA	373	386	DBSU	26.43
**4922**	allXL	DA	381	386	DBSU	50.77
**4922**	allXL	BD;DB	357	357	DBSU	18.85
**4922**	allXL	AD	373	319	DBSU	17.98
**4922**	allXL	BD	372	357	DBSU	36.47
**4922**	allXL	AD	381	357	DBSU	38.74
**4922**	allXL	BD	373	357	DBSU	37.41
**4922**	allXL	DA	372	370	DBSU	29.17
**4922**	allXL	DA	373	370	DBSU	31.11
**4922**	allXL	AD	382	373	DBSU	42.64
**4923**	allXL	CC	320	357	BS2G	22.99
**4923**	allXL	DD	320	373	BS2G	35.5
**4923**	allXL	DD	373	382	BS2G	17.59
**4923**	allXL	DD	370	386	BS2G	22.73
**4923**	allXL	DD	373	386	BS2G	17.75
**4923**	allXL	AA	319	357	BS2G	21.22
**4923**	allXL	AA	367	373	BS2G	13.4
**4923**	allXL	DD	370	382	BS2G	25.76
**4923**	allXL	CC	376	382	BS2G	14.26
**4923**	allXL	DD	357	373	BS2G	36.78
**4923**	allXL	DD	320	366	BS2G	23.53
**4923**	allXL	BB	370	376	BS2G	16.7
**4923**	allXL	CC	320	357	DBSU	22.99
**4923**	allXL	AA	357	319	DBSU	21.22
**4923**	allXL	DD	372	319	DBSU	30.52
**4923**	allXL	DD	370	357	DBSU	28.4
**4923**	allXL	AA	382	357	DBSU	43.82
**4923**	allXL	AA	372	370	DBSU	6.46

**4923**	allXL	BB	381	370	DBSU	25.57
**4923**	allXL	AA	373	370	DBSU	9.13
**4923**	allXL	DD	381	372	DBSU	21.66
**4923**	allXL	DD	372	382	DBSU	20.76
**4923**	allXL	DD	372	386	DBSU	19.27
**4923**	allXL	DD	373	386	DBSU	17.75
**4923**	allXL	BC	381	386	DBSU	53.96
**4923**	allXL	BD;DB	357	357	DBSU	18.28
**4923**	allXL	CB	373	319	DBSU	23.33
**4923**	allXL	CB	372	357	DBSU	47.42
**4923**	allXL	AC	381	357	DBSU	46.47
**4923**	allXL	AC	373	357	DBSU	47.88
**4923**	allXL	CB	372	370	DBSU	53.25
**4923**	allXL	CB	373	370	DBSU	54.77
**4923**	allXL	CB	382	373	DBSU	43.69
**4924**	allXL	AA	320	357	BS2G	25.94
**4924**	allXL	AA	320	373	BS2G	27.94
**4924**	allXL	BB	373	382	BS2G	22.11
**4924**	allXL	DD	370	386	BS2G	29.71
**4924**	allXL	DD	373	386	BS2G	29.15
**4924**	allXL	AA	319	357	BS2G	25.32
**4924**	allXL	BB	367	373	BS2G	11.8
**4924**	allXL	BB	370	382	BS2G	25.27
**4924**	allXL	CC	376	382	BS2G	15.15
**4924**	allXL	BB	357	373	BS2G	18.89
**4924**	allXL	CC	320	366	BS2G	26.04
**4924**	allXL	BB	370	376	BS2G	16.63
**4924**	allXL	AA	320	357	DBSU	25.94
**4924**	allXL	AA	357	319	DBSU	25.32
**4924**	allXL	DD	372	319	DBSU	26.76
**4924**	allXL	DD	370	357	DBSU	21.16
**4924**	allXL	BB	382	357	DBSU	32.29
**4924**	allXL	AA	372	370	DBSU	6.0
**4924**	allXL	BB	381	370	DBSU	24.53

**4924**	allXL	DD	373	370	DBSU	8.92
**4924**	allXL	BB	381	372	DBSU	23.67
**4924**	allXL	CC	372	382	DBSU	24.61
**4924**	allXL	CC	372	386	DBSU	30.15
**4924**	allXL	DD	373	386	DBSU	29.15
**4924**	allXL	BD	381	386	DBSU	47.85
**4924**	allXL	BD;DB	357	357	DBSU	19.05
**4924**	allXL	AD	373	319	DBSU	17.67
**4924**	allXL	BD	372	357	DBSU	22.27
**4924**	allXL	BD	381	357	DBSU	31.93
**4924**	allXL	BD	373	357	DBSU	21.18
**4924**	allXL	AD	372	370	DBSU	28.36
**4924**	allXL	AD	373	370	DBSU	28.97
**4924**	allXL	BD	382	373	DBSU	48.08
**4925**	allXL	AA	320	357	BS2G	17.11
**4925**	allXL	CC	320	373	BS2G	31.75
**4925**	allXL	DD	373	382	BS2G	17.23
**4925**	allXL	AA	370	386	BS2G	23.41
**4925**	allXL	AA	373	386	BS2G	24.81
**4925**	allXL	AA	319	357	BS2G	14.09
**4925**	allXL	AA	367	373	BS2G	13.03
**4925**	allXL	DD	370	382	BS2G	19.23
**4925**	allXL	BB	376	382	BS2G	14.38
**4925**	allXL	BB	357	373	BS2G	31.75
**4925**	allXL	BB	320	366	BS2G	23.96
**4925**	allXL	CC	370	376	BS2G	14.26
**4925**	allXL	AA	320	357	DBSU	17.11
**4925**	allXL	AA	357	319	DBSU	14.09
**4925**	allXL	DD	372	319	DBSU	30.8
**4925**	allXL	DD	370	357	DBSU	28.76
**4925**	allXL	BB	382	357	DBSU	40.01
**4925**	allXL	AA	372	370	DBSU	6.22
**4925**	allXL	DD	381	370	DBSU	18.67
**4925**	allXL	AA	373	370	DBSU	9.0

**4925**	allXL	DD	381	372	DBSU	13.0
**4925**	allXL	DD	372	382	DBSU	14.57
**4925**	allXL	DD	372	386	DBSU	24.38
**4925**	allXL	AA	373	386	DBSU	24.81
**4925**	allXL	AD	381	386	DBSU	67.78
**4925**	allXL	BD;DB	357	357	DBSU	18.68
**4925**	allXL	CB	373	319	DBSU	31.24
**4925**	allXL	CB	372	357	DBSU	33.29
**4925**	allXL	BD	381	357	DBSU	49.73
**4925**	allXL	CB	373	357	DBSU	34.27
**4925**	allXL	CB	372	370	DBSU	42.47
**4925**	allXL	CB	373	370	DBSU	40.92
**4925**	allXL	CB	382	373	DBSU	52.71
**4926**	allXL	AA	320	357	BS2G	13.51
**4926**	allXL	DD	320	373	BS2G	25.76
**4926**	allXL	AA	373	382	BS2G	16.96
**4926**	allXL	AA	370	386	BS2G	21.2
**4926**	allXL	AA	373	386	BS2G	19.61
**4926**	allXL	AA	319	357	BS2G	11.08
**4926**	allXL	AA	367	373	BS2G	12.73
**4926**	allXL	CC	370	382	BS2G	20.91
**4926**	allXL	AA	376	382	BS2G	14.32
**4926**	allXL	BB	357	373	BS2G	13.71
**4926**	allXL	AA	320	366	BS2G	18.89
**4926**	allXL	CC	370	376	BS2G	15.87
**4926**	allXL	AA	320	357	DBSU	13.51
**4926**	allXL	AA	357	319	DBSU	11.08
**4926**	allXL	DD	372	319	DBSU	23.9
**4926**	allXL	BB	370	357	DBSU	21.95
**4926**	allXL	BB	382	357	DBSU	27.13
**4926**	allXL	AA	372	370	DBSU	6.29
**4926**	allXL	DD	381	370	DBSU	22.57
**4926**	allXL	DD	373	370	DBSU	8.99
**4926**	allXL	DD	381	372	DBSU	15.32

**4926**	allXL	DD	372	382	DBSU	16.9
**4926**	allXL	AA	372	386	DBSU	21.58
**4926**	allXL	AA	373	386	DBSU	19.61
**4926**	allXL	AD	381	386	DBSU	49.54
**4926**	allXL	BD;DB	357	357	DBSU	18.57
**4926**	allXL	AD	373	319	DBSU	39.6
**4926**	allXL	BD	372	357	DBSU	29.73
**4926**	allXL	BD	381	357	DBSU	27.01
**4926**	allXL	BD	373	357	DBSU	26.47
**4926**	allXL	BD	372	370	DBSU	55.46
**4926**	allXL	BD	373	370	DBSU	52.15
**4926**	allXL	AD	382	373	DBSU	51.76
**4927**	allXL	AA	320	357	BS2G	25.38
**4927**	allXL	DD	320	373	BS2G	19.44
**4927**	allXL	CC	373	382	BS2G	17.45
**4927**	allXL	BB	370	386	BS2G	23.73
**4927**	allXL	BB	373	386	BS2G	25.52
**4927**	allXL	AA	319	357	BS2G	24.39
**4927**	allXL	BB	367	373	BS2G	14.37
**4927**	allXL	CC	370	382	BS2G	18.83
**4927**	allXL	CC	376	382	BS2G	13.76
**4927**	allXL	CC	357	373	BS2G	21.48
**4927**	allXL	DD	320	366	BS2G	21.89
**4927**	allXL	CC	370	376	BS2G	14.25
**4927**	allXL	AA	320	357	DBSU	25.38
**4927**	allXL	AA	357	319	DBSU	24.39
**4927**	allXL	DD	372	319	DBSU	17.56
**4927**	allXL	CC	370	357	DBSU	19.62
**4927**	allXL	CC	382	357	DBSU	13.9
**4927**	allXL	AA	372	370	DBSU	6.09
**4927**	allXL	BB	381	370	DBSU	18.43
**4927**	allXL	CC	373	370	DBSU	8.85
**4927**	allXL	BB	381	372	DBSU	19.67
**4927**	allXL	CC	372	382	DBSU	20.15

**4927**	allXL	BB	372	386	DBSU	26.78
**4927**	allXL	BB	373	386	DBSU	25.52
**4927**	allXL	AD	381	386	DBSU	39.84
**4927**	allXL	BD;DB	357	357	DBSU	19.21
**4927**	allXL	AD	373	319	DBSU	23.72
**4927**	allXL	CA	372	357	DBSU	33.44
**4927**	allXL	CA	381	357	DBSU	27.67
**4927**	allXL	CA	373	357	DBSU	31.65
**4927**	allXL	DA	372	370	DBSU	14.58
**4927**	allXL	DA	373	370	DBSU	12.05
**4927**	allXL	DA	382	373	DBSU	30.93
**4928**	allXL	AA	320	357	BS2G	24.42
**4928**	allXL	BB	320	373	BS2G	20.56
**4928**	allXL	DD	373	382	BS2G	15.37
**4928**	allXL	AA	370	386	BS2G	20.47
**4928**	allXL	AA	373	386	BS2G	24.01
**4928**	allXL	AA	319	357	BS2G	23.57
**4928**	allXL	AA	367	373	BS2G	11.85
**4928**	allXL	DD	370	382	BS2G	18.25
**4928**	allXL	CC	376	382	BS2G	14.89
**4928**	allXL	AA	357	373	BS2G	30.12
**4928**	allXL	BB	320	366	BS2G	22.49
**4928**	allXL	CC	370	376	BS2G	13.59
**4928**	allXL	AA	320	357	DBSU	24.42
**4928**	allXL	AA	357	319	DBSU	23.57
**4928**	allXL	BB	372	319	DBSU	23.61
**4928**	allXL	DD	370	357	DBSU	30.72
**4928**	allXL	BB	382	357	DBSU	35.86
**4928**	allXL	AA	372	370	DBSU	6.36
**4928**	allXL	DD	381	370	DBSU	19.51
**4928**	allXL	AA	373	370	DBSU	8.93
**4928**	allXL	DD	381	372	DBSU	16.09
**4928**	allXL	DD	372	382	DBSU	15.09
**4928**	allXL	DD	372	386	DBSU	22.19

**4928**	allXL	AA	373	386	DBSU	24.01
**4928**	allXL	CB	381	386	DBSU	32.0
**4928**	allXL	BD;DB	357	357	DBSU	18.61
**4928**	allXL	CB	373	319	DBSU	24.88
**4928**	allXL	CB	372	357	DBSU	45.77
**4928**	allXL	BC	381	357	DBSU	44.62
**4928**	allXL	CB	373	357	DBSU	46.34
**4928**	allXL	CB	372	370	DBSU	18.11
**4928**	allXL	CB	373	370	DBSU	17.93
**4928**	allXL	BC	382	373	DBSU	29.94
**4929**	allXL	CC	320	357	BS2G	26.95
**4929**	allXL	DD	320	373	BS2G	25.31
**4929**	allXL	DD	373	382	BS2G	16.91
**4929**	allXL	DD	370	386	BS2G	22.39
**4929**	allXL	DD	373	386	BS2G	23.98
**4929**	allXL	AA	319	357	BS2G	28.86
**4929**	allXL	BB	367	373	BS2G	11.61
**4929**	allXL	DD	370	382	BS2G	19.23
**4929**	allXL	CC	376	382	BS2G	15.14
**4929**	allXL	BB	357	373	BS2G	25.21
**4929**	allXL	BB	320	366	BS2G	14.53
**4929**	allXL	CC	370	376	BS2G	16.3
**4929**	allXL	CC	320	357	DBSU	26.95
**4929**	allXL	AA	357	319	DBSU	28.86
**4929**	allXL	DD	372	319	DBSU	20.72
**4929**	allXL	BB	370	357	DBSU	28.02
**4929**	allXL	BB	382	357	DBSU	14.37
**4929**	allXL	AA	372	370	DBSU	6.25
**4929**	allXL	DD	381	370	DBSU	20.93
**4929**	allXL	AA	373	370	DBSU	9.08
**4929**	allXL	DD	381	372	DBSU	20.31
**4929**	allXL	DD	372	382	DBSU	19.12
**4929**	allXL	DD	372	386	DBSU	24.66
**4929**	allXL	DD	373	386	DBSU	23.98

**4929**	allXL	BD	381	386	DBSU	53.78
**4929**	allXL	AC;CA	357	357	DBSU	19.98
**4929**	allXL	DA	373	319	DBSU	22.16
**4929**	allXL	DA	372	357	DBSU	31.69
**4929**	allXL	BD	381	357	DBSU	30.54
**4929**	allXL	DA	373	357	DBSU	34.81
**4929**	allXL	DA	372	370	DBSU	16.6
**4929**	allXL	DA	373	370	DBSU	15.31
**4929**	allXL	DA	382	373	DBSU	38.71
**4930**	allXL	CC	320	357	BS2G	25.95
**4930**	allXL	AA	320	373	BS2G	23.27
**4930**	allXL	BB	373	382	BS2G	18.49
**4930**	allXL	BB	370	386	BS2G	27.8
**4930**	allXL	CC	373	386	BS2G	24.32
**4930**	allXL	AA	319	357	BS2G	26.7
**4930**	allXL	AA	367	373	BS2G	11.08
**4930**	allXL	BB	370	382	BS2G	18.92
**4930**	allXL	BB	376	382	BS2G	14.43
**4930**	allXL	BB	357	373	BS2G	29.8
**4930**	allXL	AA	320	366	BS2G	21.12
**4930**	allXL	BB	370	376	BS2G	16.61
**4930**	allXL	CC	320	357	DBSU	25.95
**4930**	allXL	AA	357	319	DBSU	26.7
**4930**	allXL	CC	372	319	DBSU	21.39
**4930**	allXL	BB	370	357	DBSU	26.79
**4930**	allXL	BB	382	357	DBSU	22.51
**4930**	allXL	AA	372	370	DBSU	6.78
**4930**	allXL	BB	381	370	DBSU	20.51
**4930**	allXL	AA	373	370	DBSU	9.08
**4930**	allXL	DD	381	372	DBSU	20.69
**4930**	allXL	BB	372	382	DBSU	20.43
**4930**	allXL	CC	372	386	DBSU	26.1
**4930**	allXL	CC	373	386	DBSU	24.32
**4930**	allXL	BC	381	386	DBSU	42.55

**4930**	allXL	BD;DB	357	357	DBSU	18.06
**4930**	allXL	AD	373	319	DBSU	26.42
**4930**	allXL	AD	372	357	DBSU	39.19
**4930**	allXL	BD	381	357	DBSU	42.51
**4930**	allXL	AD	373	357	DBSU	41.82
**4930**	allXL	CB	372	370	DBSU	27.65
**4930**	allXL	CB	373	370	DBSU	24.01
**4930**	allXL	CB	382	373	DBSU	30.15
**4931**	allXL	AA	320	357	BS2G	20.03
**4931**	allXL	BB	320	373	BS2G	17.36
**4931**	allXL	DD	373	382	BS2G	18.09
**4931**	allXL	DD	370	386	BS2G	23.73
**4931**	allXL	DD	373	386	BS2G	26.38
**4931**	allXL	AA	319	357	BS2G	19.58
**4931**	allXL	AA	367	373	BS2G	13.26
**4931**	allXL	DD	370	382	BS2G	14.72
**4931**	allXL	BB	376	382	BS2G	14.64
**4931**	allXL	AA	357	373	BS2G	29.69
**4931**	allXL	DD	320	366	BS2G	20.14
**4931**	allXL	CC	370	376	BS2G	13.79
**4931**	allXL	AA	320	357	DBSU	20.03
**4931**	allXL	AA	357	319	DBSU	19.58
**4931**	allXL	BB	372	319	DBSU	19.43
**4931**	allXL	DD	370	357	DBSU	29.16
**4931**	allXL	BB	382	357	DBSU	29.19
**4931**	allXL	AA	372	370	DBSU	6.17
**4931**	allXL	DD	381	370	DBSU	14.89
**4931**	allXL	AA	373	370	DBSU	9.15
**4931**	allXL	DD	381	372	DBSU	18.42
**4931**	allXL	DD	372	382	DBSU	18.39
**4931**	allXL	DD	372	386	DBSU	26.07
**4931**	allXL	DD	373	386	DBSU	26.38
**4931**	allXL	BC	381	386	DBSU	48.75
**4931**	allXL	BD;DB	357	357	DBSU	19.03

**4931**	allXL	CB	373	319	DBSU	21.19
**4931**	allXL	AC	372	357	DBSU	32.5
**4931**	allXL	BD	381	357	DBSU	45.59
**4931**	allXL	AC	373	357	DBSU	32.19
**4931**	allXL	CB	372	370	DBSU	17.28
**4931**	allXL	CB	373	370	DBSU	18.71
**4931**	allXL	BC	382	373	DBSU	33.59
**4932**	allXL	AA	320	357	BS2G	26.23
**4932**	allXL	AA	320	373	BS2G	29.05
**4932**	allXL	AA	373	382	BS2G	20.78
**4932**	allXL	DD	370	386	BS2G	29.58
**4932**	allXL	AA	373	386	BS2G	29.53
**4932**	allXL	AA	319	357	BS2G	26.53
**4932**	allXL	BB	367	373	BS2G	14.22
**4932**	allXL	CC	370	382	BS2G	24.03
**4932**	allXL	CC	376	382	BS2G	13.57
**4932**	allXL	CC	357	373	BS2G	30.41
**4932**	allXL	AA	320	366	BS2G	19.13
**4932**	allXL	CC	370	376	BS2G	14.34
**4932**	allXL	AA	320	357	DBSU	26.23
**4932**	allXL	AA	357	319	DBSU	26.53
**4932**	allXL	AA	372	319	DBSU	31.43
**4932**	allXL	BB	370	357	DBSU	29.87
**4932**	allXL	BB	382	357	DBSU	33.45
**4932**	allXL	AA	372	370	DBSU	6.4
**4932**	allXL	CC	381	370	DBSU	22.35
**4932**	allXL	AA	373	370	DBSU	9.11
**4932**	allXL	DD	381	372	DBSU	22.27
**4932**	allXL	AA	372	382	DBSU	22.48
**4932**	allXL	AA	372	386	DBSU	30.02
**4932**	allXL	AA	373	386	DBSU	29.53
**4932**	allXL	BD	381	386	DBSU	51.67
**4932**	allXL	BD;DB	357	357	DBSU	18.5
**4932**	allXL	AD	373	319	DBSU	32.07

**4932**	allXL	DA	372	357	DBSU	47.68
**4932**	allXL	BD	381	357	DBSU	46.27
**4932**	allXL	CA	373	357	DBSU	47.91
**4932**	allXL	DA	372	370	DBSU	16.0
**4932**	allXL	DA	373	370	DBSU	18.24
**4932**	allXL	AD	382	373	DBSU	34.85
**4933**	allXL	AA	320	357	BS2G	22.33
**4933**	allXL	DD	320	373	BS2G	30.26
**4933**	allXL	DD	373	382	BS2G	15.79
**4933**	allXL	DD	370	386	BS2G	20.15
**4933**	allXL	DD	373	386	BS2G	19.76
**4933**	allXL	AA	319	357	BS2G	25.39
**4933**	allXL	AA	367	373	BS2G	13.34
**4933**	allXL	DD	370	382	BS2G	19.44
**4933**	allXL	AA	376	382	BS2G	13.78
**4933**	allXL	DD	357	373	BS2G	17.43
**4933**	allXL	CC	320	366	BS2G	14.56
**4933**	allXL	CC	370	376	BS2G	15.26
**4933**	allXL	AA	320	357	DBSU	22.33
**4933**	allXL	AA	357	319	DBSU	25.39
**4933**	allXL	CC	372	319	DBSU	31.28
**4933**	allXL	CC	370	357	DBSU	20.43
**4933**	allXL	CC	382	357	DBSU	21.44
**4933**	allXL	AA	372	370	DBSU	6.24
**4933**	allXL	DD	381	370	DBSU	20.65
**4933**	allXL	DD	373	370	DBSU	8.74
**4933**	allXL	DD	381	372	DBSU	14.84
**4933**	allXL	DD	372	382	DBSU	14.13
**4933**	allXL	DD	372	386	DBSU	16.38
**4933**	allXL	DD	373	386	DBSU	19.76
**4933**	allXL	DB	381	386	DBSU	32.61
**4933**	allXL	BD;DB	357	357	DBSU	18.51
**4933**	allXL	AD	373	319	DBSU	38.04
**4933**	allXL	CA	372	357	DBSU	34.45

**4933**	allXL	CA	381	357	DBSU	33.68
**4933**	allXL	CA	373	357	DBSU	32.28
**4933**	allXL	CA	372	370	DBSU	40.69
**4933**	allXL	CA	373	370	DBSU	40.29
**4933**	allXL	AC	382	373	DBSU	41.23
**4934**	allXL	AA	320	357	BS2G	23.1
**4934**	allXL	CC	320	373	BS2G	20.15
**4934**	allXL	DD	373	382	BS2G	15.4
**4934**	allXL	CC	370	386	BS2G	12.47
**4934**	allXL	CC	373	386	BS2G	18.26
**4934**	allXL	AA	319	357	BS2G	22.5
**4934**	allXL	AA	367	373	BS2G	12.42
**4934**	allXL	CC	370	382	BS2G	16.79
**4934**	allXL	CC	376	382	BS2G	13.75
**4934**	allXL	DD	357	373	BS2G	27.99
**4934**	allXL	CC	320	366	BS2G	22.92
**4934**	allXL	BB	370	376	BS2G	15.69
**4934**	allXL	AA	320	357	DBSU	23.1
**4934**	allXL	AA	357	319	DBSU	22.5
**4934**	allXL	CC	372	319	DBSU	24.04
**4934**	allXL	DD	370	357	DBSU	26.02
**4934**	allXL	DD	382	357	DBSU	26.39
**4934**	allXL	AA	372	370	DBSU	6.33
**4934**	allXL	CC	381	370	DBSU	19.08
**4934**	allXL	DD	373	370	DBSU	9.1
**4934**	allXL	DD	381	372	DBSU	14.45
**4934**	allXL	DD	372	382	DBSU	14.7
**4934**	allXL	CC	372	386	DBSU	15.29
**4934**	allXL	CC	373	386	DBSU	18.26
**4934**	allXL	BC	381	386	DBSU	39.69
**4934**	allXL	BD;DB	357	357	DBSU	18.08
**4934**	allXL	AD	373	319	DBSU	31.43
**4934**	allXL	DB	372	357	DBSU	34.35
**4934**	allXL	DB	381	357	DBSU	30.37

**4934**	allXL	DB	373	357	DBSU	32.04
**4934**	allXL	CB	372	370	DBSU	12.52
**4934**	allXL	CB	373	370	DBSU	13.1
**4934**	allXL	BC	382	373	DBSU	34.27
**4935**	allXL	CC	320	357	BS2G	23.33
**4935**	allXL	BB	320	373	BS2G	26.54
**4935**	allXL	AA	373	382	BS2G	20.94
**4935**	allXL	AA	370	386	BS2G	31.03
**4935**	allXL	AA	373	386	BS2G	29.19
**4935**	allXL	AA	319	357	BS2G	26.72
**4935**	allXL	BB	367	373	BS2G	14.25
**4935**	allXL	AA	370	382	BS2G	25.92
**4935**	allXL	CC	376	382	BS2G	15.12
**4935**	allXL	CC	357	373	BS2G	16.42
**4935**	allXL	BB	320	366	BS2G	27.76
**4935**	allXL	CC	370	376	BS2G	15.5
**4935**	allXL	CC	320	357	DBSU	23.33
**4935**	allXL	AA	357	319	DBSU	26.72
**4935**	allXL	AA	372	319	DBSU	27.52
**4935**	allXL	CC	370	357	DBSU	13.79
**4935**	allXL	CC	382	357	DBSU	38.69
**4935**	allXL	AA	372	370	DBSU	6.24
**4935**	allXL	DD	381	370	DBSU	26.05
**4935**	allXL	AA	373	370	DBSU	9.21
**4935**	allXL	AA	381	372	DBSU	22.36
**4935**	allXL	AA	372	382	DBSU	21.11
**4935**	allXL	AA	372	386	DBSU	28.07
**4935**	allXL	AA	373	386	DBSU	29.19
**4935**	allXL	AC	381	386	DBSU	48.29
**4935**	allXL	BD;DB	357	357	DBSU	18.41
**4935**	allXL	DA	373	319	DBSU	42.38
**4935**	allXL	CA	372	357	DBSU	17.46
**4935**	allXL	CA	381	357	DBSU	30.69
**4935**	allXL	CA	373	357	DBSU	16.68

**4935**	allXL	CA	372	370	DBSU	44.99
**4935**	allXL	CA	373	370	DBSU	41.88
**4935**	allXL	CA	382	373	DBSU	38.49
**4936**	allXL	AA	320	357	BS2G	21.71
**4936**	allXL	BB	320	373	BS2G	15.51
**4936**	allXL	DD	373	382	BS2G	20.26
**4936**	allXL	DD	370	386	BS2G	22.62
**4936**	allXL	DD	373	386	BS2G	23.43
**4936**	allXL	AA	319	357	BS2G	20.81
**4936**	allXL	BB	367	373	BS2G	13.21
**4936**	allXL	DD	370	382	BS2G	21.06
**4936**	allXL	CC	376	382	BS2G	14.36
**4936**	allXL	CC	357	373	BS2G	18.12
**4936**	allXL	CC	320	366	BS2G	18.19
**4936**	allXL	CC	370	376	BS2G	15.22
**4936**	allXL	AA	320	357	DBSU	21.71
**4936**	allXL	AA	357	319	DBSU	20.81
**4936**	allXL	BB	372	319	DBSU	19.43
**4936**	allXL	CC	370	357	DBSU	18.89
**4936**	allXL	DD	382	357	DBSU	26.99
**4936**	allXL	AA	372	370	DBSU	6.18
**4936**	allXL	DD	381	370	DBSU	19.71
**4936**	allXL	AA	373	370	DBSU	9.08
**4936**	allXL	DD	381	372	DBSU	20.55
**4936**	allXL	DD	372	382	DBSU	22.1
**4936**	allXL	DD	372	386	DBSU	23.82
**4936**	allXL	DD	373	386	DBSU	23.43
**4936**	allXL	AD	381	386	DBSU	11.84
**4936**	allXL	BD;DB	357	357	DBSU	18.71
**4936**	allXL	AD	373	319	DBSU	23.67
**4936**	allXL	CA	372	357	DBSU	32.43
**4936**	allXL	BD	381	357	DBSU	36.73
**4936**	allXL	CA	373	357	DBSU	31.8
**4936**	allXL	CA	372	370	DBSU	44.92

**4936**	allXL	CA	373	370	DBSU	46.22
**4936**	allXL	AD	382	373	DBSU	26.43
**4937**	allXL	AA	320	357	BS2G	22.49
**4937**	allXL	AA	320	373	BS2G	17.11
**4937**	allXL	AA	373	382	BS2G	18.08
**4937**	allXL	AA	370	386	BS2G	22.18
**4937**	allXL	DD	373	386	BS2G	25.43
**4937**	allXL	AA	319	357	BS2G	25.48
**4937**	allXL	AA	367	373	BS2G	13.85
**4937**	allXL	AA	370	382	BS2G	14.78
**4937**	allXL	CC	376	382	BS2G	13.91
**4937**	allXL	AA	357	373	BS2G	31.61
**4937**	allXL	AA	320	366	BS2G	21.23
**4937**	allXL	CC	370	376	BS2G	14.92
**4937**	allXL	AA	320	357	DBSU	22.49
**4937**	allXL	AA	357	319	DBSU	25.48
**4937**	allXL	AA	372	319	DBSU	18.99
**4937**	allXL	AA	370	357	DBSU	30.53
**4937**	allXL	AA	382	357	DBSU	36.97
**4937**	allXL	AA	372	370	DBSU	6.38
**4937**	allXL	AA	381	370	DBSU	16.84
**4937**	allXL	DD	373	370	DBSU	9.0
**4937**	allXL	AA	381	372	DBSU	18.11
**4937**	allXL	AA	372	382	DBSU	16.96
**4937**	allXL	DD	372	386	DBSU	24.6
**4937**	allXL	DD	373	386	DBSU	25.43
**4937**	allXL	CB	381	386	DBSU	31.17
**4937**	allXL	BD;DB	357	357	DBSU	19.4
**4937**	allXL	CB	373	319	DBSU	19.94
**4937**	allXL	CB	372	357	DBSU	31.58
**4937**	allXL	CB	381	357	DBSU	38.14
**4937**	allXL	CB	373	357	DBSU	32.32
**4937**	allXL	CB	372	370	DBSU	14.66
**4937**	allXL	CB	373	370	DBSU	14.08

**4937**	allXL	BC	382	373	DBSU	25.08
**4938**	allXL	BB	320	357	BS2G	26.93
**4938**	allXL	CC	320	373	BS2G	14.59
**4938**	allXL	DD	373	382	BS2G	19.04
**4938**	allXL	CC	370	386	BS2G	27.15
**4938**	allXL	CC	373	386	BS2G	28.13
**4938**	allXL	AA	319	357	BS2G	28.05
**4938**	allXL	AA	367	373	BS2G	13.27
**4938**	allXL	CC	370	382	BS2G	18.87
**4938**	allXL	CC	376	382	BS2G	14.65
**4938**	allXL	CC	357	373	BS2G	25.26
**4938**	allXL	AA	320	366	BS2G	14.15
**4938**	allXL	CC	370	376	BS2G	14.78
**4938**	allXL	BB	320	357	DBSU	26.93
**4938**	allXL	AA	357	319	DBSU	28.05
**4938**	allXL	CC	372	319	DBSU	15.63
**4938**	allXL	CC	370	357	DBSU	19.36
**4938**	allXL	CC	382	357	DBSU	23.23
**4938**	allXL	AA	372	370	DBSU	6.19
**4938**	allXL	CC	381	370	DBSU	19.63
**4938**	allXL	DD	373	370	DBSU	8.88
**4938**	allXL	DD	381	372	DBSU	16.99
**4938**	allXL	DD	372	382	DBSU	18.73
**4938**	allXL	CC	372	386	DBSU	27.12
**4938**	allXL	CC	373	386	DBSU	28.13
**4938**	allXL	AD	381	386	DBSU	48.25
**4938**	allXL	AC;CA	357	357	DBSU	18.84
**4938**	allXL	AD	373	319	DBSU	40.0
**4938**	allXL	CA	372	357	DBSU	36.67
**4938**	allXL	BD	381	357	DBSU	42.39
**4938**	allXL	CA	373	357	DBSU	40.25
**4938**	allXL	AD	372	370	DBSU	40.03
**4938**	allXL	AD	373	370	DBSU	39.59
**4938**	allXL	AD	382	373	DBSU	43.29

**4939**	allXL	AA	320	357	BS2G	22.13
**4939**	allXL	DD	320	373	BS2G	19.5
**4939**	allXL	DD	373	382	BS2G	17.04
**4939**	allXL	AA	370	386	BS2G	23.11
**4939**	allXL	AA	373	386	BS2G	26.45
**4939**	allXL	AA	319	357	BS2G	24.03
**4939**	allXL	AA	367	373	BS2G	10.01
**4939**	allXL	AA	370	382	BS2G	19.21
**4939**	allXL	CC	376	382	BS2G	13.01
**4939**	allXL	DD	357	373	BS2G	23.12
**4939**	allXL	BB	320	366	BS2G	13.56
**4939**	allXL	CC	370	376	BS2G	16.2
**4939**	allXL	AA	320	357	DBSU	22.13
**4939**	allXL	AA	357	319	DBSU	24.03
**4939**	allXL	BB	372	319	DBSU	20.85
**4939**	allXL	DD	370	357	DBSU	22.89
**4939**	allXL	AA	382	357	DBSU	35.37
**4939**	allXL	AA	372	370	DBSU	6.49
**4939**	allXL	DD	381	370	DBSU	18.62
**4939**	allXL	AA	373	370	DBSU	8.83
**4939**	allXL	DD	381	372	DBSU	14.66
**4939**	allXL	DD	372	382	DBSU	16.37
**4939**	allXL	DD	372	386	DBSU	26.19
**4939**	allXL	AA	373	386	DBSU	26.45
**4939**	allXL	DA	381	386	DBSU	29.63
**4939**	allXL	BD;DB	357	357	DBSU	19.38
**4939**	allXL	CB	373	319	DBSU	24.48
**4939**	allXL	CB	372	357	DBSU	39.31
**4939**	allXL	AD	381	357	DBSU	40.54
**4939**	allXL	DB	373	357	DBSU	38.83
**4939**	allXL	CB	372	370	DBSU	25.37
**4939**	allXL	CB	373	370	DBSU	25.44
**4939**	allXL	CB	382	373	DBSU	19.41
**4940**	allXL	CC	320	357	BS2G	24.02

**4940**	allXL	AA	320	373	BS2G	21.81
**4940**	allXL	BB	373	382	BS2G	19.76
**4940**	allXL	DD	370	386	BS2G	23.18
**4940**	allXL	DD	373	386	BS2G	25.53
**4940**	allXL	AA	319	357	BS2G	25.35
**4940**	allXL	BB	367	373	BS2G	8.79
**4940**	allXL	BB	370	382	BS2G	21.3
**4940**	allXL	BB	376	382	BS2G	14.31
**4940**	allXL	BB	357	373	BS2G	30.99
**4940**	allXL	AA	320	366	BS2G	10.88
**4940**	allXL	DD	370	376	BS2G	16.78
**4940**	allXL	CC	320	357	DBSU	24.02
**4940**	allXL	AA	357	319	DBSU	25.35
**4940**	allXL	AA	372	319	DBSU	23.11
**4940**	allXL	DD	370	357	DBSU	24.95
**4940**	allXL	BB	382	357	DBSU	37.3
**4940**	allXL	AA	372	370	DBSU	6.3
**4940**	allXL	BB	381	370	DBSU	22.48
**4940**	allXL	AA	373	370	DBSU	9.11
**4940**	allXL	DD	381	372	DBSU	20.45
**4940**	allXL	DD	372	382	DBSU	19.99
**4940**	allXL	DD	372	386	DBSU	23.58
**4940**	allXL	DD	373	386	DBSU	25.53
**4940**	allXL	AD	381	386	DBSU	30.9
**4940**	allXL	BD;DB	357	357	DBSU	18.2
**4940**	allXL	AD	373	319	DBSU	28.24
**4940**	allXL	AD	372	357	DBSU	43.69
**4940**	allXL	AD	381	357	DBSU	27.32
**4940**	allXL	AD	373	357	DBSU	41.14
**4940**	allXL	AD	372	370	DBSU	32.91
**4940**	allXL	AD	373	370	DBSU	30.69
**4940**	allXL	AD	382	373	DBSU	16.47
**4941**	allXL	CC	320	357	BS2G	23.08
**4941**	allXL	BB	320	373	BS2G	27.83

**4941**	allXL	BB	373	382	BS2G	18.51
**4941**	allXL	DD	370	386	BS2G	17.67
**4941**	allXL	BB	373	386	BS2G	24.53
**4941**	allXL	CC	319	357	BS2G	22.1
**4941**	allXL	AA	367	373	BS2G	11.39
**4941**	allXL	DD	370	382	BS2G	16.47
**4941**	allXL	BB	376	382	BS2G	14.37
**4941**	allXL	AA	357	373	BS2G	25.28
**4941**	allXL	AA	320	366	BS2G	27.29
**4941**	allXL	BB	370	376	BS2G	15.42
**4941**	allXL	CC	320	357	DBSU	23.08
**4941**	allXL	CC	357	319	DBSU	22.1
**4941**	allXL	DD	372	319	DBSU	29.05
**4941**	allXL	DD	370	357	DBSU	21.57
**4941**	allXL	DD	382	357	DBSU	28.46
**4941**	allXL	AA	372	370	DBSU	6.35
**4941**	allXL	DD	381	370	DBSU	16.93
**4941**	allXL	AA	373	370	DBSU	9.01
**4941**	allXL	DD	381	372	DBSU	18.12
**4941**	allXL	DD	372	382	DBSU	18.98
**4941**	allXL	DD	372	386	DBSU	22.92
**4941**	allXL	BB	373	386	DBSU	24.53
**4941**	allXL	AD	381	386	DBSU	59.32
**4941**	allXL	BD;DB	357	357	DBSU	19.77
**4941**	allXL	AD	373	319	DBSU	34.02
**4941**	allXL	DB	372	357	DBSU	41.44
**4941**	allXL	AC	381	357	DBSU	34.34
**4941**	allXL	AC	373	357	DBSU	41.46
**4941**	allXL	BC	372	370	DBSU	58.78
**4941**	allXL	AD	373	370	DBSU	59.43
**4941**	allXL	AD	382	373	DBSU	54.43
**4942**	allXL	AA	320	357	BS2G	14.43
**4942**	allXL	CC	320	373	BS2G	20.75
**4942**	allXL	BB	373	382	BS2G	15.05

**4942**	allXL	CC	370	386	BS2G	28.25
**4942**	allXL	CC	373	386	BS2G	19.86
**4942**	allXL	AA	319	357	BS2G	15.87
**4942**	allXL	AA	367	373	BS2G	14.31
**4942**	allXL	BB	370	382	BS2G	19.86
**4942**	allXL	BB	376	382	BS2G	12.93
**4942**	allXL	CC	357	373	BS2G	24.64
**4942**	allXL	CC	320	366	BS2G	19.67
**4942**	allXL	CC	370	376	BS2G	16.06
**4942**	allXL	AA	320	357	DBSU	14.43
**4942**	allXL	AA	357	319	DBSU	15.87
**4942**	allXL	CC	372	319	DBSU	17.82
**4942**	allXL	DD	370	357	DBSU	24.58
**4942**	allXL	CC	382	357	DBSU	25.47
**4942**	allXL	AA	372	370	DBSU	5.98
**4942**	allXL	BB	381	370	DBSU	20.4
**4942**	allXL	AA	373	370	DBSU	9.26
**4942**	allXL	BB	381	372	DBSU	18.36
**4942**	allXL	BB	372	382	DBSU	17.03
**4942**	allXL	CC	372	386	DBSU	22.63
**4942**	allXL	CC	373	386	DBSU	19.86
**4942**	allXL	AC	381	386	DBSU	57.61
**4942**	allXL	BD;DB	357	357	DBSU	17.89
**4942**	allXL	AD	373	319	DBSU	24.68
**4942**	allXL	BC	372	357	DBSU	38.24
**4942**	allXL	CA	381	357	DBSU	33.1
**4942**	allXL	BC	373	357	DBSU	37.86
**4942**	allXL	BC	372	370	DBSU	33.79
**4942**	allXL	BC	373	370	DBSU	32.51
**4942**	allXL	CA	382	373	DBSU	48.67
**4943**	allXL	AA	320	357	BS2G	25.29
**4943**	allXL	CC	320	373	BS2G	22.04
**4943**	allXL	CC	373	382	BS2G	18.96
**4943**	allXL	AA	370	386	BS2G	27.6

**4943**	allXL	CC	373	386	BS2G	21.74
**4943**	allXL	AA	319	357	BS2G	24.61
**4943**	allXL	BB	367	373	BS2G	10.74
**4943**	allXL	BB	370	382	BS2G	22.89
**4943**	allXL	AA	376	382	BS2G	13.31
**4943**	allXL	BB	357	373	BS2G	30.19
**4943**	allXL	DD	320	366	BS2G	22.82
**4943**	allXL	BB	370	376	BS2G	12.74
**4943**	allXL	AA	320	357	DBSU	25.29
**4943**	allXL	AA	357	319	DBSU	24.61
**4943**	allXL	DD	372	319	DBSU	20.4
**4943**	allXL	CC	370	357	DBSU	25.22
**4943**	allXL	AA	382	357	DBSU	25.09
**4943**	allXL	AA	372	370	DBSU	6.36
**4943**	allXL	BB	381	370	DBSU	19.91
**4943**	allXL	BB	373	370	DBSU	9.04
**4943**	allXL	DD	381	372	DBSU	17.96
**4943**	allXL	DD	372	382	DBSU	20.83
**4943**	allXL	CC	372	386	DBSU	24.25
**4943**	allXL	CC	373	386	DBSU	21.74
**4943**	allXL	DA	381	386	DBSU	42.95
**4943**	allXL	BD;DB	357	357	DBSU	20.21
**4943**	allXL	DA	373	319	DBSU	21.56
**4943**	allXL	CB	372	357	DBSU	29.64
**4943**	allXL	CB	381	357	DBSU	26.9
**4943**	allXL	CB	373	357	DBSU	26.95
**4943**	allXL	DA	372	370	DBSU	32.58
**4943**	allXL	DA	373	370	DBSU	32.01
**4943**	allXL	CB	382	373	DBSU	25.82
**4944**	allXL	AA	320	357	BS2G	18.36
**4944**	allXL	BB	320	373	BS2G	38.33
**4944**	allXL	DD	373	382	BS2G	17.55
**4944**	allXL	CC	370	386	BS2G	20.09
**4944**	allXL	CC	373	386	BS2G	24.54

**4944**	allXL	AA	319	357	BS2G	21.1
**4944**	allXL	BB	367	373	BS2G	14.85
**4944**	allXL	CC	370	382	BS2G	17.02
**4944**	allXL	CC	376	382	BS2G	14.11
**4944**	allXL	AA	357	373	BS2G	27.41
**4944**	allXL	CC	320	366	BS2G	29.4
**4944**	allXL	CC	370	376	BS2G	13.45
**4944**	allXL	AA	320	357	DBSU	18.36
**4944**	allXL	AA	357	319	DBSU	21.1
**4944**	allXL	BB	372	319	DBSU	38.93
**4944**	allXL	AA	370	357	DBSU	27.75
**4944**	allXL	CC	382	357	DBSU	23.91
**4944**	allXL	AA	372	370	DBSU	5.88
**4944**	allXL	CC	381	370	DBSU	19.07
**4944**	allXL	AA	373	370	DBSU	9.21
**4944**	allXL	DD	381	372	DBSU	19.35
**4944**	allXL	CC	372	382	DBSU	19.89
**4944**	allXL	CC	372	386	DBSU	24.55
**4944**	allXL	CC	373	386	DBSU	24.54
**4944**	allXL	BC	381	386	DBSU	37.75
**4944**	allXL	BD;DB	357	357	DBSU	19.58
**4944**	allXL	AD	373	319	DBSU	31.93
**4944**	allXL	AC	372	357	DBSU	29.63
**4944**	allXL	CA	381	357	DBSU	42.74
**4944**	allXL	AC	373	357	DBSU	27.13
**4944**	allXL	BC	372	370	DBSU	53.17
**4944**	allXL	BC	373	370	DBSU	50.15
**4944**	allXL	CA	382	373	DBSU	45.88
**4945**	allXL	CC	320	357	BS2G	25.43
**4945**	allXL	AA	320	373	BS2G	16.91
**4945**	allXL	BB	373	382	BS2G	16.18
**4945**	allXL	BB	370	386	BS2G	23.53
**4945**	allXL	CC	373	386	BS2G	23.9
**4945**	allXL	AA	319	357	BS2G	25.07

**4945**	allXL	AA	367	373	BS2G	12.74
**4945**	allXL	BB	370	382	BS2G	16.95
**4945**	allXL	BB	376	382	BS2G	12.63
**4945**	allXL	AA	357	373	BS2G	24.25
**4945**	allXL	AA	320	366	BS2G	22.74
**4945**	allXL	CC	370	376	BS2G	13.33
**4945**	allXL	CC	320	357	DBSU	25.43
**4945**	allXL	AA	357	319	DBSU	25.07
**4945**	allXL	AA	372	319	DBSU	12.28
**4945**	allXL	BB	370	357	DBSU	22.14
**4945**	allXL	AA	382	357	DBSU	16.15
**4945**	allXL	AA	372	370	DBSU	6.23
**4945**	allXL	BB	381	370	DBSU	18.79
**4945**	allXL	AA	373	370	DBSU	9.08
**4945**	allXL	BB	381	372	DBSU	19.48
**4945**	allXL	BB	372	382	DBSU	18.09
**4945**	allXL	CC	372	386	DBSU	22.92
**4945**	allXL	CC	373	386	DBSU	23.9
**4945**	allXL	BC	381	386	DBSU	31.27
**4945**	allXL	BD;DB	357	357	DBSU	18.56
**4945**	allXL	BC	373	319	DBSU	17.64
**4945**	allXL	BC	372	357	DBSU	26.14
**4945**	allXL	AC	381	357	DBSU	34.81
**4945**	allXL	BC	373	357	DBSU	27.79
**4945**	allXL	CB	372	370	DBSU	8.57
**4945**	allXL	CB	373	370	DBSU	10.21
**4945**	allXL	BC	382	373	DBSU	14.98
**4946**	allXL	CC	320	357	BS2G	26.51
**4946**	allXL	BB	320	373	BS2G	15.01
**4946**	allXL	CC	373	382	BS2G	18.57
**4946**	allXL	AA	370	386	BS2G	26.89
**4946**	allXL	DD	373	386	BS2G	23.09
**4946**	allXL	AA	319	357	BS2G	28.15
**4946**	allXL	AA	367	373	BS2G	11.07

**4946**	allXL	DD	370	382	BS2G	23.95
**4946**	allXL	AA	376	382	BS2G	14.25
**4946**	allXL	BB	357	373	BS2G	22.24
**4946**	allXL	BB	320	366	BS2G	24.57
**4946**	allXL	DD	370	376	BS2G	14.26
**4946**	allXL	CC	320	357	DBSU	26.51
**4946**	allXL	AA	357	319	DBSU	28.15
**4946**	allXL	BB	372	319	DBSU	18.24
**4946**	allXL	BB	370	357	DBSU	26.08
**4946**	allXL	DD	382	357	DBSU	19.9
**4946**	allXL	AA	372	370	DBSU	6.28
**4946**	allXL	DD	381	370	DBSU	20.71
**4946**	allXL	DD	373	370	DBSU	9.1
**4946**	allXL	DD	381	372	DBSU	19.04
**4946**	allXL	CC	372	382	DBSU	21.25
**4946**	allXL	DD	372	386	DBSU	24.0
**4946**	allXL	DD	373	386	DBSU	23.09
**4946**	allXL	CB	381	386	DBSU	52.04
**4946**	allXL	BD;DB	357	357	DBSU	18.97
**4946**	allXL	CB	373	319	DBSU	28.85
**4946**	allXL	CB	372	357	DBSU	34.76
**4946**	allXL	CB	381	357	DBSU	26.37
**4946**	allXL	CB	373	357	DBSU	34.72
**4946**	allXL	CB	372	370	DBSU	12.77
**4946**	allXL	CB	373	370	DBSU	15.17
**4946**	allXL	CB	382	373	DBSU	20.83
**4947**	allXL	AA	320	357	BS2G	24.99
**4947**	allXL	CC	320	373	BS2G	18.91
**4947**	allXL	BB	373	382	BS2G	16.8
**4947**	allXL	DD	370	386	BS2G	17.91
**4947**	allXL	CC	373	386	BS2G	22.92
**4947**	allXL	AA	319	357	BS2G	24.49
**4947**	allXL	DD	367	373	BS2G	12.95
**4947**	allXL	DD	370	382	BS2G	18.79

**4947**	allXL	CC	376	382	BS2G	12.79
**4947**	allXL	AA	357	373	BS2G	17.97
**4947**	allXL	AA	320	366	BS2G	16.37
**4947**	allXL	DD	370	376	BS2G	15.9
**4947**	allXL	AA	320	357	DBSU	24.99
**4947**	allXL	AA	357	319	DBSU	24.49
**4947**	allXL	CC	372	319	DBSU	24.15
**4947**	allXL	AA	370	357	DBSU	20.88
**4947**	allXL	AA	382	357	DBSU	10.43
**4947**	allXL	AA	372	370	DBSU	6.11
**4947**	allXL	DD	381	370	DBSU	18.57
**4947**	allXL	AA	373	370	DBSU	9.23
**4947**	allXL	BB	381	372	DBSU	19.65
**4947**	allXL	BB	372	382	DBSU	19.0
**4947**	allXL	DD	372	386	DBSU	24.25
**4947**	allXL	CC	373	386	DBSU	22.92
**4947**	allXL	AD	381	386	DBSU	45.04
**4947**	allXL	BD;DB	357	357	DBSU	18.95
**4947**	allXL	AD	373	319	DBSU	19.1
**4947**	allXL	CB	372	357	DBSU	24.42
**4947**	allXL	AC	381	357	DBSU	11.28
**4947**	allXL	CB	373	357	DBSU	27.6
**4947**	allXL	AC	372	370	DBSU	37.39
**4947**	allXL	DA	373	370	DBSU	38.87
**4947**	allXL	DA	382	373	DBSU	26.86
**4948**	allXL	CC	320	357	BS2G	23.52
**4948**	allXL	DD	320	373	BS2G	24.74
**4948**	allXL	CC	373	382	BS2G	19.98
**4948**	allXL	DD	370	386	BS2G	25.64
**4948**	allXL	DD	373	386	BS2G	30.16
**4948**	allXL	CC	319	357	BS2G	26.7
**4948**	allXL	BB	367	373	BS2G	10.22
**4948**	allXL	DD	370	382	BS2G	17.98
**4948**	allXL	CC	376	382	BS2G	15.27

**4948**	allXL	AA	357	373	BS2G	24.44
**4948**	allXL	AA	320	366	BS2G	24.77
**4948**	allXL	DD	370	376	BS2G	12.7
**4948**	allXL	CC	320	357	DBSU	23.52
**4948**	allXL	CC	357	319	DBSU	26.7
**4948**	allXL	DD	372	319	DBSU	27.46
**4948**	allXL	CC	370	357	DBSU	24.14
**4948**	allXL	DD	382	357	DBSU	14.31
**4948**	allXL	AA;BB	372	370	DBSU	6.44
**4948**	allXL	DD	381	370	DBSU	16.7
**4948**	allXL	DD	373	370	DBSU	9.19
**4948**	allXL	DD	381	372	DBSU	19.79
**4948**	allXL	DD	372	382	DBSU	21.28
**4948**	allXL	DD	372	386	DBSU	30.09
**4948**	allXL	DD	373	386	DBSU	30.16
**4948**	allXL	BD	381	386	DBSU	44.41
**4948**	allXL	BD;DB	357	357	DBSU	18.61
**4948**	allXL	AD	373	319	DBSU	23.83
**4948**	allXL	DA	372	357	DBSU	38.07
**4948**	allXL	DB	381	357	DBSU	31.26
**4948**	allXL	DA	373	357	DBSU	35.37
**4948**	allXL	DA	372	370	DBSU	30.53
**4948**	allXL	DA	373	370	DBSU	27.64
**4948**	allXL	DA	382	373	DBSU	45.54
**4949**	allXL	AA	320	357	BS2G	24.26
**4949**	allXL	DD	320	373	BS2G	15.44
**4949**	allXL	DD	373	382	BS2G	13.73
**4949**	allXL	DD	370	386	BS2G	16.85
**4949**	allXL	BB	373	386	BS2G	24.2
**4949**	allXL	AA	319	357	BS2G	25.77
**4949**	allXL	BB	367	373	BS2G	12.14
**4949**	allXL	DD	370	382	BS2G	7.97
**4949**	allXL	BB	376	382	BS2G	13.0
**4949**	allXL	DD	357	373	BS2G	20.72

**4949**	allXL	DD	320	366	BS2G	28.01
**4949**	allXL	AA	370	376	BS2G	13.4
**4949**	allXL	AA	320	357	DBSU	24.26
**4949**	allXL	AA	357	319	DBSU	25.77
**4949**	allXL	DD	372	319	DBSU	16.92
**4949**	allXL	DD	370	357	DBSU	23.13
**4949**	allXL	DD	382	357	DBSU	28.42
**4949**	allXL	AA	372	370	DBSU	6.41
**4949**	allXL	DD	381	370	DBSU	7.03
**4949**	allXL	AA	373	370	DBSU	9.04
**4949**	allXL	DD	381	372	DBSU	11.95
**4949**	allXL	DD	372	382	DBSU	12.7
**4949**	allXL	DD	372	386	DBSU	22.58
**4949**	allXL	BB	373	386	DBSU	24.2
**4949**	allXL	CB	381	386	DBSU	34.84
**4949**	allXL	BD;DB	357	357	DBSU	17.44
**4949**	allXL	AD	373	319	DBSU	32.12
**4949**	allXL	CB	372	357	DBSU	33.5
**4949**	allXL	DB	381	357	DBSU	39.98
**4949**	allXL	AC	373	357	DBSU	32.65
**4949**	allXL	CB	372	370	DBSU	18.61
**4949**	allXL	CB	373	370	DBSU	15.77
**4949**	allXL	CB	382	373	DBSU	28.46
**4950**	allXL	CC	320	357	BS2G	24.14
**4950**	allXL	DD	320	373	BS2G	18.72
**4950**	allXL	BB	373	382	BS2G	14.13
**4950**	allXL	CC	370	386	BS2G	24.14
**4950**	allXL	BB	373	386	BS2G	23.01
**4950**	allXL	AA	319	357	BS2G	24.68
**4950**	allXL	AA	367	373	BS2G	13.73
**4950**	allXL	DD	370	382	BS2G	19.11
**4950**	allXL	BB	376	382	BS2G	12.47
**4950**	allXL	CC	357	373	BS2G	21.55
**4950**	allXL	BB	320	366	BS2G	23.86

**4950**	allXL	DD	370	376	BS2G	13.55
**4950**	allXL	CC	320	357	DBSU	24.14
**4950**	allXL	AA	357	319	DBSU	24.68
**4950**	allXL	DD	372	319	DBSU	15.79
**4950**	allXL	CC	370	357	DBSU	21.6
**4950**	allXL	DD	382	357	DBSU	14.51
**4950**	allXL	AA	372	370	DBSU	6.03
**4950**	allXL	DD	381	370	DBSU	18.2
**4950**	allXL	AA	373	370	DBSU	9.18
**4950**	allXL	BB	381	372	DBSU	17.06
**4950**	allXL	BB	372	382	DBSU	14.35
**4950**	allXL	BB	372	386	DBSU	21.48
**4950**	allXL	BB	373	386	DBSU	23.01
**4950**	allXL	BD	381	386	DBSU	34.97
**4950**	allXL	BD;DB	357	357	DBSU	18.86
**4950**	allXL	DA	373	319	DBSU	30.8
**4950**	allXL	CA	372	357	DBSU	29.32
**4950**	allXL	CA	381	357	DBSU	20.66
**4950**	allXL	CA	373	357	DBSU	28.35
**4950**	allXL	DA	372	370	DBSU	32.32
**4950**	allXL	DA	373	370	DBSU	34.56
**4950**	allXL	CA	382	373	DBSU	32.56
**4951**	allXL	AA	320	357	BS2G	25.76
**4951**	allXL	CC	320	373	BS2G	13.66
**4951**	allXL	DD	373	382	BS2G	20.81
**4951**	allXL	DD	370	386	BS2G	23.75
**4951**	allXL	DD	373	386	BS2G	26.29
**4951**	allXL	AA	319	357	BS2G	26.05
**4951**	allXL	BB	367	373	BS2G	11.88
**4951**	allXL	CC	370	382	BS2G	20.47
**4951**	allXL	BB	376	382	BS2G	14.74
**4951**	allXL	CC	357	373	BS2G	17.43
**4951**	allXL	AA	320	366	BS2G	22.23
**4951**	allXL	CC	370	376	BS2G	13.5

**4951**	allXL	AA	320	357	DBSU	25.76
**4951**	allXL	AA	357	319	DBSU	26.05
**4951**	allXL	CC	372	319	DBSU	16.18
**4951**	allXL	CC	370	357	DBSU	20.97
**4951**	allXL	BB	382	357	DBSU	21.37
**4951**	allXL	AA	372	370	DBSU	6.13
**4951**	allXL	CC	381	370	DBSU	19.86
**4951**	allXL	AA	373	370	DBSU	9.05
**4951**	allXL	DD	381	372	DBSU	17.19
**4951**	allXL	DD	372	382	DBSU	18.86
**4951**	allXL	DD	372	386	DBSU	23.03
**4951**	allXL	DD	373	386	DBSU	26.29
**4951**	allXL	AD	381	386	DBSU	36.9
**4951**	allXL	BD;DB	357	357	DBSU	18.83
**4951**	allXL	AD	373	319	DBSU	35.74
**4951**	allXL	CA	372	357	DBSU	37.65
**4951**	allXL	BD	381	357	DBSU	41.84
**4951**	allXL	CA	373	357	DBSU	36.54
**4951**	allXL	BC	372	370	DBSU	30.09
**4951**	allXL	BC	373	370	DBSU	33.5
**4951**	allXL	DA	382	373	DBSU	35.38
**4952**	allXL	BB	320	357	BS2G	23.39
**4952**	allXL	BB	320	373	BS2G	28.43
**4952**	allXL	CC	373	382	BS2G	14.17
**4952**	allXL	CC	370	386	BS2G	23.09
**4952**	allXL	CC	373	386	BS2G	21.66
**4952**	allXL	AA	319	357	BS2G	23.54
**4952**	allXL	BB	367	373	BS2G	12.09
**4952**	allXL	CC	370	382	BS2G	20.85
**4952**	allXL	CC	376	382	BS2G	14.04
**4952**	allXL	BB	357	373	BS2G	29.83
**4952**	allXL	AA	320	366	BS2G	21.25
**4952**	allXL	BB	370	376	BS2G	15.21
**4952**	allXL	BB	320	357	DBSU	23.39

**4952**	allXL	AA	357	319	DBSU	23.54
**4952**	allXL	BB	372	319	DBSU	26.83
**4952**	allXL	DD	370	357	DBSU	28.81
**4952**	allXL	CC	382	357	DBSU	28.75
**4952**	allXL	AA	372	370	DBSU	6.34
**4952**	allXL	DD	381	370	DBSU	19.75
**4952**	allXL	AA	373	370	DBSU	8.76
**4952**	allXL	DD	381	372	DBSU	19.12
**4952**	allXL	CC	372	382	DBSU	17.28
**4952**	allXL	CC	372	386	DBSU	22.97
**4952**	allXL	CC	373	386	DBSU	21.66
**4952**	allXL	DA	381	386	DBSU	55.61
**4952**	allXL	BD;DB	357	357	DBSU	18.75
**4952**	allXL	AD	373	319	DBSU	28.86
**4952**	allXL	BD	372	357	DBSU	43.89
**4952**	allXL	CA	381	357	DBSU	40.17
**4952**	allXL	DA	373	357	DBSU	45.96
**4952**	allXL	DA	372	370	DBSU	26.95
**4952**	allXL	DA	373	370	DBSU	25.6
**4952**	allXL	AD	382	373	DBSU	44.31
**4953**	allXL	AA	320	357	BS2G	25.38
**4953**	allXL	BB	320	373	BS2G	23.24
**4953**	allXL	AA	373	382	BS2G	18.85
**4953**	allXL	AA	370	386	BS2G	22.69
**4953**	allXL	BB	373	386	BS2G	22.02
**4953**	allXL	AA	319	357	BS2G	26.25
**4953**	allXL	BB	367	373	BS2G	13.13
**4953**	allXL	AA	370	382	BS2G	17.8
**4953**	allXL	CC	376	382	BS2G	13.95
**4953**	allXL	BB	357	373	BS2G	23.89
**4953**	allXL	CC	320	366	BS2G	31.83
**4953**	allXL	AA	370	376	BS2G	16.6
**4953**	allXL	AA	320	357	DBSU	25.38
**4953**	allXL	AA	357	319	DBSU	26.25

**4953**	allXL	BB	372	319	DBSU	26.15
**4953**	allXL	BB	370	357	DBSU	23.0
**4953**	allXL	BB	382	357	DBSU	27.89
**4953**	allXL	AA	372	370	DBSU	6.12
**4953**	allXL	AA	381	370	DBSU	19.14
**4953**	allXL	AA	373	370	DBSU	9.15
**4953**	allXL	AA	381	372	DBSU	16.58
**4953**	allXL	AA	372	382	DBSU	16.36
**4953**	allXL	BB	372	386	DBSU	24.11
**4953**	allXL	BB	373	386	DBSU	22.02
**4953**	allXL	BC	381	386	DBSU	45.1
**4953**	allXL	BD;DB	357	357	DBSU	18.51
**4953**	allXL	CB	373	319	DBSU	20.81
**4953**	allXL	DB	372	357	DBSU	33.86
**4953**	allXL	DB	381	357	DBSU	41.12
**4953**	allXL	DB	373	357	DBSU	34.93
**4953**	allXL	CB	372	370	DBSU	16.08
**4953**	allXL	CB	373	370	DBSU	15.17
**4953**	allXL	CB	382	373	DBSU	29.44
**4954**	allXL	AA	320	357	BS2G	24.78
**4954**	allXL	BB	320	373	BS2G	18.5
**4954**	allXL	AA	373	382	BS2G	17.72
**4954**	allXL	AA	370	386	BS2G	24.66
**4954**	allXL	AA	373	386	BS2G	19.22
**4954**	allXL	AA	319	357	BS2G	24.82
**4954**	allXL	AA	367	373	BS2G	14.54
**4954**	allXL	DD	370	382	BS2G	23.98
**4954**	allXL	AA	376	382	BS2G	13.75
**4954**	allXL	CC	357	373	BS2G	27.13
**4954**	allXL	BB	320	366	BS2G	21.29
**4954**	allXL	CC	370	376	BS2G	16.57
**4954**	allXL	AA	320	357	DBSU	24.78
**4954**	allXL	AA	357	319	DBSU	24.82
**4954**	allXL	BB	372	319	DBSU	16.15

**4954**	allXL	DD	370	357	DBSU	24.19
**4954**	allXL	CC	382	357	DBSU	19.91
**4954**	allXL	AA	372	370	DBSU	6.17
**4954**	allXL	DD	381	370	DBSU	22.74
**4954**	allXL	AA	373	370	DBSU	9.27
**4954**	allXL	DD	381	372	DBSU	21.04
**4954**	allXL	AA	372	382	DBSU	21.44
**4954**	allXL	AA	372	386	DBSU	22.25
**4954**	allXL	AA	373	386	DBSU	19.22
**4954**	allXL	CA	381	386	DBSU	16.64
**4954**	allXL	BD;DB	357	357	DBSU	18.88
**4954**	allXL	AD	373	319	DBSU	19.78
**4954**	allXL	AC	372	357	DBSU	32.36
**4954**	allXL	AC	381	357	DBSU	15.48
**4954**	allXL	AC	373	357	DBSU	28.94
**4954**	allXL	DA	372	370	DBSU	25.1
**4954**	allXL	DA	373	370	DBSU	26.72
**4954**	allXL	AC	382	373	DBSU	26.48
**4955**	allXL	AA	320	357	BS2G	23.81
**4955**	allXL	BB	320	373	BS2G	22.48
**4955**	allXL	DD	373	382	BS2G	18.83
**4955**	allXL	AA	370	386	BS2G	24.26
**4955**	allXL	AA	373	386	BS2G	20.27
**4955**	allXL	AA	319	357	BS2G	25.56
**4955**	allXL	BB	367	373	BS2G	14.59
**4955**	allXL	BB	370	382	BS2G	26.41
**4955**	allXL	BB	376	382	BS2G	15.11
**4955**	allXL	BB	357	373	BS2G	29.16
**4955**	allXL	AA	320	366	BS2G	18.09
**4955**	allXL	BB	370	376	BS2G	17.61
**4955**	allXL	AA	320	357	DBSU	23.81
**4955**	allXL	AA	357	319	DBSU	25.56
**4955**	allXL	CC	372	319	DBSU	21.69
**4955**	allXL	BB	370	357	DBSU	23.48

**4955**	allXL	BB	382	357	DBSU	27.55
**4955**	allXL	AA	372	370	DBSU	6.4
**4955**	allXL	DD	381	370	DBSU	25.04
**4955**	allXL	AA	373	370	DBSU	9.12
**4955**	allXL	DD	381	372	DBSU	19.74
**4955**	allXL	DD	372	382	DBSU	21.62
**4955**	allXL	AA	372	386	DBSU	19.61
**4955**	allXL	AA	373	386	DBSU	20.27
**4955**	allXL	AD	381	386	DBSU	24.7
**4955**	allXL	BD;DB	357	357	DBSU	17.44
**4955**	allXL	AD	373	319	DBSU	24.25
**4955**	allXL	AD	372	357	DBSU	38.23
**4955**	allXL	AD	381	357	DBSU	35.79
**4955**	allXL	AD	373	357	DBSU	40.19
**4955**	allXL	AD	372	370	DBSU	13.98
**4955**	allXL	AD	373	370	DBSU	16.72
**4955**	allXL	DA	382	373	DBSU	19.78
**4956**	allXL	AA	320	357	BS2G	25.62
**4956**	allXL	BB	320	373	BS2G	13.2
**4956**	allXL	AA	373	382	BS2G	16.85
**4956**	allXL	CC	370	386	BS2G	22.3
**4956**	allXL	AA	373	386	BS2G	21.89
**4956**	allXL	BB	319	357	BS2G	24.04
**4956**	allXL	BB	367	373	BS2G	12.71
**4956**	allXL	CC	370	382	BS2G	16.99
**4956**	allXL	AA	376	382	BS2G	14.3
**4956**	allXL	BB	357	373	BS2G	20.31
**4956**	allXL	AA	320	366	BS2G	25.31
**4956**	allXL	CC	370	376	BS2G	13.43
**4956**	allXL	AA	320	357	DBSU	25.62
**4956**	allXL	BB	357	319	DBSU	24.04
**4956**	allXL	BB	372	319	DBSU	12.15
**4956**	allXL	BB	370	357	DBSU	23.22
**4956**	allXL	BB	382	357	DBSU	10.29

**4956**	allXL	AA	372	370	DBSU	6.23
**4956**	allXL	CC	381	370	DBSU	18.54
**4956**	allXL	AA	373	370	DBSU	9.12
**4956**	allXL	DD	381	372	DBSU	21.52
**4956**	allXL	AA	372	382	DBSU	20.26
**4956**	allXL	AA	372	386	DBSU	24.05
**4956**	allXL	AA	373	386	DBSU	21.89
**4956**	allXL	DB	381	386	DBSU	48.38
**4956**	allXL	BD;DB	357	357	DBSU	18.34
**4956**	allXL	AD	373	319	DBSU	22.91
**4956**	allXL	BD	372	357	DBSU	41.11
**4956**	allXL	BD	381	357	DBSU	21.11
**4956**	allXL	BD	373	357	DBSU	38.35
**4956**	allXL	AD	372	370	DBSU	27.72
**4956**	allXL	AD	373	370	DBSU	25.89
**4956**	allXL	AD	382	373	DBSU	33.36
**4957**	allXL	CC	320	357	BS2G	25.39
**4957**	allXL	DD	320	373	BS2G	22.87
**4957**	allXL	CC	373	382	BS2G	18.63
**4957**	allXL	DD	370	386	BS2G	23.65
**4957**	allXL	CC	373	386	BS2G	23.78
**4957**	allXL	CC	319	357	BS2G	26.13
**4957**	allXL	AA	367	373	BS2G	13.31
**4957**	allXL	DD	370	382	BS2G	21.44
**4957**	allXL	BB	376	382	BS2G	13.28
**4957**	allXL	BB	357	373	BS2G	31.65
**4957**	allXL	DD	320	366	BS2G	19.48
**4957**	allXL	CC	370	376	BS2G	17.03
**4957**	allXL	CC	320	357	DBSU	25.39
**4957**	allXL	CC	357	319	DBSU	26.13
**4957**	allXL	BB	372	319	DBSU	25.0
**4957**	allXL	BB	370	357	DBSU	23.41
**4957**	allXL	BB	382	357	DBSU	41.77
**4957**	allXL	AA	372	370	DBSU	6.15

**4957**	allXL	DD	381	370	DBSU	20.42
**4957**	allXL	AA	373	370	DBSU	9.18
**4957**	allXL	DD	381	372	DBSU	18.82
**4957**	allXL	CC	372	382	DBSU	19.01
**4957**	allXL	DD	372	386	DBSU	22.83
**4957**	allXL	CC	373	386	DBSU	23.78
**4957**	allXL	DA	381	386	DBSU	39.26
**4957**	allXL	BD;DB	357	357	DBSU	18.16
**4957**	allXL	CB	373	319	DBSU	24.94
**4957**	allXL	BD	372	357	DBSU	40.86
**4957**	allXL	BD	381	357	DBSU	48.35
**4957**	allXL	BD	373	357	DBSU	41.96
**4957**	allXL	CB	372	370	DBSU	47.86
**4957**	allXL	CB	373	370	DBSU	49.8
**4957**	allXL	DA	382	373	DBSU	47.96
**4958**	allXL	AA	320	357	BS2G	24.01
**4958**	allXL	CC	320	373	BS2G	23.34
**4958**	allXL	CC	373	382	BS2G	19.17
**4958**	allXL	CC	370	386	BS2G	16.8
**4958**	allXL	CC	373	386	BS2G	20.64
**4958**	allXL	AA	319	357	BS2G	25.04
**4958**	allXL	DD	367	373	BS2G	14.81
**4958**	allXL	AA	370	382	BS2G	20.89
**4958**	allXL	AA	376	382	BS2G	13.89
**4958**	allXL	BB	357	373	BS2G	28.29
**4958**	allXL	DD	320	366	BS2G	20.65
**4958**	allXL	AA	370	376	BS2G	16.22
**4958**	allXL	AA	320	357	DBSU	24.01
**4958**	allXL	AA	357	319	DBSU	25.04
**4958**	allXL	CC	372	319	DBSU	22.43
**4958**	allXL	CC	370	357	DBSU	27.31
**4958**	allXL	CC	382	357	DBSU	38.84
**4958**	allXL	AA	372	370	DBSU	6.25
**4958**	allXL	CC	381	370	DBSU	22.79

**4958**	allXL	AA	373	370	DBSU	9.05
**4958**	allXL	CC	381	372	DBSU	19.5
**4958**	allXL	CC	372	382	DBSU	18.31
**4958**	allXL	CC	372	386	DBSU	17.61
**4958**	allXL	CC	373	386	DBSU	20.64
**4958**	allXL	AD	381	386	DBSU	53.66
**4958**	allXL	BD;DB	357	357	DBSU	17.85
**4958**	allXL	DA	373	319	DBSU	25.67
**4958**	allXL	BD	372	357	DBSU	37.06
**4958**	allXL	DA	381	357	DBSU	36.47
**4958**	allXL	BD	373	357	DBSU	38.1
**4958**	allXL	BD	372	370	DBSU	48.0
**4958**	allXL	BD	373	370	DBSU	46.72
**4958**	allXL	DA	382	373	DBSU	31.28
**4959**	allXL	BB	320	357	BS2G	24.58
**4959**	allXL	AA	320	373	BS2G	22.7
**4959**	allXL	CC	373	382	BS2G	15.09
**4959**	allXL	CC	370	386	BS2G	22.44
**4959**	allXL	CC	373	386	BS2G	17.55
**4959**	allXL	CC	319	357	BS2G	26.7
**4959**	allXL	BB	367	373	BS2G	12.41
**4959**	allXL	BB	370	382	BS2G	21.03
**4959**	allXL	CC	376	382	BS2G	13.46
**4959**	allXL	BB	357	373	BS2G	27.4
**4959**	allXL	CC	320	366	BS2G	29.44
**4959**	allXL	BB	370	376	BS2G	16.03
**4959**	allXL	BB	320	357	DBSU	24.58
**4959**	allXL	CC	357	319	DBSU	26.7
**4959**	allXL	AA	372	319	DBSU	29.96
**4959**	allXL	AA	370	357	DBSU	32.32
**4959**	allXL	AA	382	357	DBSU	31.66
**4959**	allXL	AA	372	370	DBSU	6.25
**4959**	allXL	BB	381	370	DBSU	22.07
**4959**	allXL	AA	373	370	DBSU	9.13

4959	allXL	CC	381	372	DBSU	19.15
4959	allXL	CC	372	382	DBSU	16.23
4959	allXL	CC	372	386	DBSU	18.04
4959	allXL	CC	373	386	DBSU	17.55
4959	allXL	BC	381	386	DBSU	32.55
4959	allXL	BD;DB	357	357	DBSU	18.0
4959	allXL	AD	373	319	DBSU	18.8
4959	allXL	AD	372	357	DBSU	29.06
4959	allXL	CB	381	357	DBSU	30.82
4959	allXL	AD	373	357	DBSU	30.75
4959	allXL	AD	372	370	DBSU	27.05
4959	allXL	AD	373	370	DBSU	28.62
4959	allXL	CB	382	373	DBSU	14.1
4960	allXL	CC	320	357	BS2G	25.45
4960	allXL	DD	320	373	BS2G	18.32
4960	allXL	DD	373	382	BS2G	17.06
4960	allXL	DD	370	386	BS2G	18.04
4960	allXL	DD	373	386	BS2G	25.7
4960	allXL	AA	319	357	BS2G	24.75
4960	allXL	BB	367	373	BS2G	13.19
4960	allXL	DD	370	382	BS2G	10.57
4960	allXL	BB	376	382	BS2G	15.1
4960	allXL	BB	357	373	BS2G	24.31
4960	allXL	DD	320	366	BS2G	23.69
4960	allXL	CC	370	376	BS2G	14.58
4960	allXL	CC	320	357	DBSU	25.45
4960	allXL	AA	357	319	DBSU	24.75
4960	allXL	DD	372	319	DBSU	19.44
4960	allXL	DD	370	357	DBSU	29.78
4960	allXL	BB	382	357	DBSU	23.71
4960	allXL	AA	372	370	DBSU	6.17
4960	allXL	DD	381	370	DBSU	10.08
4960	allXL	AA	373	370	DBSU	9.07
4960	allXL	DD	381	372	DBSU	15.01

**4960**	allXL	DD	372	382	DBSU	16.85
**4960**	allXL	DD	372	386	DBSU	24.95
**4960**	allXL	DD	373	386	DBSU	25.7
**4960**	allXL	BD	381	386	DBSU	53.76
**4960**	allXL	BD;DB	357	357	DBSU	18.19
**4960**	allXL	BC	373	319	DBSU	25.05
**4960**	allXL	BC	372	357	DBSU	37.83
**4960**	allXL	BD	381	357	DBSU	27.7
**4960**	allXL	BC	373	357	DBSU	35.58
**4960**	allXL	DA	372	370	DBSU	20.11
**4960**	allXL	DA	373	370	DBSU	18.56
**4960**	allXL	DA	382	373	DBSU	36.47
**4961**	allXL	AA	320	357	BS2G	25.16
**4961**	allXL	CC	320	373	BS2G	15.8
**4961**	allXL	AA	373	382	BS2G	15.08
**4961**	allXL	AA	370	386	BS2G	20.98
**4961**	allXL	AA	373	386	BS2G	21.47
**4961**	allXL	AA	319	357	BS2G	26.38
**4961**	allXL	AA	367	373	BS2G	14.29
**4961**	allXL	AA;CC	370	382	BS2G	19.62
**4961**	allXL	AA	376	382	BS2G	13.71
**4961**	allXL	CC	357	373	BS2G	23.06
**4961**	allXL	CC	320	366	BS2G	20.03
**4961**	allXL	CC	370	376	BS2G	15.33
**4961**	allXL	AA	320	357	DBSU	25.16
**4961**	allXL	AA	357	319	DBSU	26.38
**4961**	allXL	CC	372	319	DBSU	14.31
**4961**	allXL	CC	370	357	DBSU	16.92
**4961**	allXL	CC	382	357	DBSU	26.62
**4961**	allXL	AA	372	370	DBSU	6.48
**4961**	allXL	CC	381	370	DBSU	20.57
**4961**	allXL	DD	373	370	DBSU	9.0
**4961**	allXL	AA	381	372	DBSU	19.01
**4961**	allXL	AA	372	382	DBSU	17.04

**4961**	allXL	AA	372	386	DBSU	21.42
**4961**	allXL	AA	373	386	DBSU	21.47
**4961**	allXL	AD	381	386	DBSU	27.94
**4961**	allXL	BD;DB	357	357	DBSU	19.4
**4961**	allXL	DA	373	319	DBSU	18.47
**4961**	allXL	DA	372	357	DBSU	28.9
**4961**	allXL	DA	381	357	DBSU	13.85
**4961**	allXL	DA	373	357	DBSU	26.16
**4961**	allXL	DA	372	370	DBSU	40.11
**4961**	allXL	DA	373	370	DBSU	40.57
**4961**	allXL	DA	382	373	DBSU	24.41
**4962**	allXL	CC	320	357	BS2G	22.05
**4962**	allXL	BB	320	373	BS2G	29.19
**4962**	allXL	BB	373	382	BS2G	18.8
**4962**	allXL	AA	370	386	BS2G	20.74
**4962**	allXL	AA	373	386	BS2G	19.52
**4962**	allXL	CC	319	357	BS2G	24.95
**4962**	allXL	DD	367	373	BS2G	14.15
**4962**	allXL	AA	370	382	BS2G	25.0
**4962**	allXL	CC	376	382	BS2G	13.88
**4962**	allXL	CC	357	373	BS2G	29.94
**4962**	allXL	BB	320	366	BS2G	25.47
**4962**	allXL	CC	370	376	BS2G	17.59
**4962**	allXL	CC	320	357	DBSU	22.05
**4962**	allXL	CC	357	319	DBSU	24.95
**4962**	allXL	BB	372	319	DBSU	29.54
**4962**	allXL	BB	370	357	DBSU	22.96
**4962**	allXL	BB	382	357	DBSU	35.22
**4962**	allXL	AA	372	370	DBSU	6.15
**4962**	allXL	BB	381	370	DBSU	23.75
**4962**	allXL	DD	373	370	DBSU	9.07
**4962**	allXL	DD	381	372	DBSU	17.55
**4962**	allXL	DD	372	382	DBSU	19.04
**4962**	allXL	AA	372	386	DBSU	20.32

**4962**	allXL	AA	373	386	DBSU	19.52
**4962**	allXL	AD	381	386	DBSU	45.25
**4962**	allXL	BD;DB	357	357	DBSU	19.24
**4962**	allXL	AD	373	319	DBSU	25.3
**4962**	allXL	CA	372	357	DBSU	34.71
**4962**	allXL	BD	381	357	DBSU	39.29
**4962**	allXL	CA	373	357	DBSU	33.3
**4962**	allXL	AD	372	370	DBSU	36.32
**4962**	allXL	AD	373	370	DBSU	38.34
**4962**	allXL	DA	382	373	DBSU	45.39
**4963**	allXL	AA	320	357	BS2G	26.63
**4963**	allXL	CC	320	373	BS2G	23.05
**4963**	allXL	BB	373	382	BS2G	17.41
**4963**	allXL	BB	370	386	BS2G	23.35
**4963**	allXL	BB	373	386	BS2G	14.92
**4963**	allXL	AA	319	357	BS2G	25.66
**4963**	allXL	AA	367	373	BS2G	13.05
**4963**	allXL	DD	370	382	BS2G	21.33
**4963**	allXL	BB	376	382	BS2G	13.6
**4963**	allXL	CC	357	373	BS2G	30.65
**4963**	allXL	CC	320	366	BS2G	18.26
**4963**	allXL	CC	370	376	BS2G	15.73
**4963**	allXL	AA	320	357	DBSU	26.63
**4963**	allXL	AA	357	319	DBSU	25.66
**4963**	allXL	CC	372	319	DBSU	22.08
**4963**	allXL	DD	370	357	DBSU	26.24
**4963**	allXL	BB	382	357	DBSU	22.81
**4963**	allXL	AA	372	370	DBSU	6.27
**4963**	allXL	DD	381	370	DBSU	21.27
**4963**	allXL	AA	373	370	DBSU	8.89
**4963**	allXL	BB	381	372	DBSU	22.16
**4963**	allXL	BB	372	382	DBSU	19.83
**4963**	allXL	BB	372	386	DBSU	15.89
**4963**	allXL	BB	373	386	DBSU	14.92

**4963**	allXL	DA	381	386	DBSU	33.84
**4963**	allXL	BD;DB	357	357	DBSU	18.05
**4963**	allXL	AD	373	319	DBSU	27.49
**4963**	allXL	AD	372	357	DBSU	31.83
**4963**	allXL	BD	381	357	DBSU	36.85
**4963**	allXL	AD	373	357	DBSU	35.46
**4963**	allXL	AD	372	370	DBSU	25.61
**4963**	allXL	DA	373	370	DBSU	28.03
**4963**	allXL	DA	382	373	DBSU	20.27
**4964**	allXL	AA	320	357	BS2G	23.77
**4964**	allXL	CC	320	373	BS2G	22.19
**4964**	allXL	CC	373	382	BS2G	19.41
**4964**	allXL	DD	370	386	BS2G	21.29
**4964**	allXL	AA	373	386	BS2G	23.61
**4964**	allXL	AA	319	357	BS2G	26.11
**4964**	allXL	BB	367	373	BS2G	13.08
**4964**	allXL	DD	370	382	BS2G	17.16
**4964**	allXL	CC	376	382	BS2G	14.95
**4964**	allXL	BB	357	373	BS2G	14.21
**4964**	allXL	AA	320	366	BS2G	15.88
**4964**	allXL	DD	370	376	BS2G	16.26
**4964**	allXL	AA	320	357	DBSU	23.77
**4964**	allXL	AA	357	319	DBSU	26.11
**4964**	allXL	CC	372	319	DBSU	22.65
**4964**	allXL	BB	370	357	DBSU	15.91
**4964**	allXL	BB	382	357	DBSU	17.36
**4964**	allXL	AA	372	370	DBSU	5.94
**4964**	allXL	DD	381	370	DBSU	14.97
**4964**	allXL	AA	373	370	DBSU	9.16
**4964**	allXL	DD	381	372	DBSU	18.07
**4964**	allXL	CC	372	382	DBSU	19.95
**4964**	allXL	AA	372	386	DBSU	24.56
**4964**	allXL	AA	373	386	DBSU	23.61
**4964**	allXL	BD	381	386	DBSU	32.19

**4964**	allXL	BD;DB	357	357	DBSU	18.68
**4964**	allXL	BC	373	319	DBSU	37.93
**4964**	allXL	BD	372	357	DBSU	24.55
**4964**	allXL	BD	381	357	DBSU	13.88
**4964**	allXL	BD	373	357	DBSU	23.22
**4964**	allXL	BD	372	370	DBSU	35.97
**4964**	allXL	BD	373	370	DBSU	35.84
**4964**	allXL	BD	382	373	DBSU	40.92
**4965**	allXL	AA	320	357	BS2G	24.03
**4965**	allXL	BB	320	373	BS2G	15.22
**4965**	allXL	DD	373	382	BS2G	18.36
**4965**	allXL	DD	370	386	BS2G	23.14
**4965**	allXL	DD	373	386	BS2G	21.12
**4965**	allXL	AA	319	357	BS2G	22.48
**4965**	allXL	AA	367	373	BS2G	11.36
**4965**	allXL	DD	370	382	BS2G	23.6
**4965**	allXL	BB	376	382	BS2G	13.7
**4965**	allXL	BB	357	373	BS2G	21.64
**4965**	allXL	DD	320	366	BS2G	18.92
**4965**	allXL	CC	370	376	BS2G	16.82
**4965**	allXL	AA	320	357	DBSU	24.03
**4965**	allXL	AA	357	319	DBSU	22.48
**4965**	allXL	BB	372	319	DBSU	14.64
**4965**	allXL	BB	370	357	DBSU	25.5
**4965**	allXL	BB	382	357	DBSU	22.48
**4965**	allXL	CC	372	370	DBSU	6.93
**4965**	allXL	DD	381	370	DBSU	22.32
**4965**	allXL	AA	373	370	DBSU	9.38
**4965**	allXL	DD	381	372	DBSU	15.63
**4965**	allXL	DD	372	382	DBSU	17.25
**4965**	allXL	DD	372	386	DBSU	18.34
**4965**	allXL	DD	373	386	DBSU	21.12
**4965**	allXL	DA	381	386	DBSU	46.69
**4965**	allXL	BD;DB	357	357	DBSU	18.99

**4965**	allXL	AD	373	319	DBSU	26.82
**4965**	allXL	BC	372	357	DBSU	42.29
**4965**	allXL	BD	381	357	DBSU	37.86
**4965**	allXL	BD	373	357	DBSU	40.11
**4965**	allXL	BC	372	370	DBSU	39.51
**4965**	allXL	BC	373	370	DBSU	42.87
**4965**	allXL	DA	382	373	DBSU	36.03
**4966**	allXL	AA	320	357	BS2G	24.46
**4966**	allXL	DD	320	373	BS2G	17.19
**4966**	allXL	DD	373	382	BS2G	19.24
**4966**	allXL	DD	370	386	BS2G	17.57
**4966**	allXL	DD	373	386	BS2G	19.2
**4966**	allXL	AA	319	357	BS2G	24.23
**4966**	allXL	AA	367	373	BS2G	13.69
**4966**	allXL	DD	370	382	BS2G	21.14
**4966**	allXL	CC	376	382	BS2G	14.53
**4966**	allXL	BB	357	373	BS2G	28.43
**4966**	allXL	BB	320	366	BS2G	18.08
**4966**	allXL	CC	370	376	BS2G	15.66
**4966**	allXL	AA	320	357	DBSU	24.46
**4966**	allXL	AA	357	319	DBSU	24.23
**4966**	allXL	DD	372	319	DBSU	20.56
**4966**	allXL	DD	370	357	DBSU	27.72
**4966**	allXL	BB	382	357	DBSU	16.85
**4966**	allXL	AA	372	370	DBSU	6.11
**4966**	allXL	DD	381	370	DBSU	21.5
**4966**	allXL	AA	373	370	DBSU	9.31
**4966**	allXL	DD	381	372	DBSU	18.0
**4966**	allXL	DD	372	382	DBSU	18.47
**4966**	allXL	DD	372	386	DBSU	16.37
**4966**	allXL	DD	373	386	DBSU	19.2
**4966**	allXL	BC	381	386	DBSU	63.26
**4966**	allXL	BD;DB	357	357	DBSU	19.01
**4966**	allXL	CB	373	319	DBSU	32.69

**4966**	allXL	CB	372	357	DBSU	37.91
**4966**	allXL	BD	381	357	DBSU	34.38
**4966**	allXL	CB	373	357	DBSU	40.37
**4966**	allXL	CB	372	370	DBSU	33.19
**4966**	allXL	CB	373	370	DBSU	36.96
**4966**	allXL	CB	382	373	DBSU	48.1
**4967**	allXL	AA	320	357	BS2G	24.86
**4967**	allXL	CC	320	373	BS2G	13.23
**4967**	allXL	DD	373	382	BS2G	20.15
**4967**	allXL	DD	370	386	BS2G	26.21
**4967**	allXL	BB	373	386	BS2G	21.7
**4967**	allXL	AA	319	357	BS2G	27.8
**4967**	allXL	BB	367	373	BS2G	11.82
**4967**	allXL	DD	370	382	BS2G	26.33
**4967**	allXL	BB	376	382	BS2G	13.66
**4967**	allXL	CC	357	373	BS2G	13.92
**4967**	allXL	CC	320	366	BS2G	23.91
**4967**	allXL	CC	370	376	BS2G	16.64
**4967**	allXL	AA	320	357	DBSU	24.86
**4967**	allXL	AA	357	319	DBSU	27.8
**4967**	allXL	CC	372	319	DBSU	12.1
**4967**	allXL	DD	370	357	DBSU	17.2
**4967**	allXL	CC	382	357	DBSU	27.51
**4967**	allXL	AA	372	370	DBSU	6.24
**4967**	allXL	AA	381	370	DBSU	26.37
**4967**	allXL	AA	373	370	DBSU	9.03
**4967**	allXL	DD	381	372	DBSU	19.52
**4967**	allXL	DD	372	382	DBSU	19.67
**4967**	allXL	DD	372	386	DBSU	21.83
**4967**	allXL	BB	373	386	DBSU	21.7
**4967**	allXL	DB	381	386	DBSU	37.6
**4967**	allXL	BD;DB	357	357	DBSU	17.47
**4967**	allXL	DA	373	319	DBSU	35.16
**4967**	allXL	DB	372	357	DBSU	26.43

**4967**	allXL	DB	381	357	DBSU	40.85
**4967**	allXL	DB	373	357	DBSU	24.79
**4967**	allXL	BD	372	370	DBSU	26.71
**4967**	allXL	BD	373	370	DBSU	27.85
**4967**	allXL	DB	382	373	DBSU	35.68
**4968**	allXL	AA	320	357	BS2G	25.53
**4968**	allXL	BB	320	373	BS2G	19.05
**4968**	allXL	DD	373	382	BS2G	16.24
**4968**	allXL	DD	370	386	BS2G	24.16
**4968**	allXL	CC	373	386	BS2G	22.91
**4968**	allXL	AA	319	357	BS2G	25.66
**4968**	allXL	AA	367	373	BS2G	13.25
**4968**	allXL	DD	370	382	BS2G	20.87
**4968**	allXL	CC	376	382	BS2G	14.48
**4968**	allXL	BB	357	373	BS2G	24.25
**4968**	allXL	BB	320	366	BS2G	24.04
**4968**	allXL	CC	370	376	BS2G	17.67
**4968**	allXL	AA	320	357	DBSU	25.53
**4968**	allXL	AA	357	319	DBSU	25.66
**4968**	allXL	BB	372	319	DBSU	20.81
**4968**	allXL	CC	370	357	DBSU	24.23
**4968**	allXL	BB	382	357	DBSU	28.18
**4968**	allXL	AA	372	370	DBSU	6.21
**4968**	allXL	DD	381	370	DBSU	22.13
**4968**	allXL	AA	373	370	DBSU	9.09
**4968**	allXL	DD	381	372	DBSU	15.64
**4968**	allXL	DD	372	382	DBSU	14.74
**4968**	allXL	DD	372	386	DBSU	20.0
**4968**	allXL	CC	373	386	DBSU	22.91
**4968**	allXL	AC	381	386	DBSU	29.92
**4968**	allXL	BD;DB	357	357	DBSU	18.99
**4968**	allXL	AD	373	319	DBSU	37.03
**4968**	allXL	CA	372	357	DBSU	39.95
**4968**	allXL	CA	381	357	DBSU	38.86

**4968**	allXL	CA	373	357	DBSU	41.82
**4968**	allXL	CB	372	370	DBSU	57.68
**4968**	allXL	AC	373	370	DBSU	56.66
**4968**	allXL	CA	382	373	DBSU	36.22
**4969**	allXL	AA	320	357	BS2G	24.83
**4969**	allXL	BB	320	373	BS2G	8.8
**4969**	allXL	DD	373	382	BS2G	17.87
**4969**	allXL	CC	370	386	BS2G	20.71
**4969**	allXL	CC	373	386	BS2G	27.61
**4969**	allXL	AA	319	357	BS2G	25.83
**4969**	allXL	AA	367	373	BS2G	13.49
**4969**	allXL	CC	370	382	BS2G	15.86
**4969**	allXL	CC	376	382	BS2G	15.21
**4969**	allXL	BB	357	373	BS2G	26.25
**4969**	allXL	BB	320	366	BS2G	23.95
**4969**	allXL	CC	370	376	BS2G	13.57
**4969**	allXL	AA	320	357	DBSU	24.83
**4969**	allXL	AA	357	319	DBSU	25.83
**4969**	allXL	BB	372	319	DBSU	7.95
**4969**	allXL	BB	370	357	DBSU	22.63
**4969**	allXL	BB	382	357	DBSU	28.98
**4969**	allXL	AA	372	370	DBSU	6.36
**4969**	allXL	CC	381	370	DBSU	17.79
**4969**	allXL	AA	373	370	DBSU	9.01
**4969**	allXL	DD	381	372	DBSU	17.62
**4969**	allXL	CC	372	382	DBSU	19.15
**4969**	allXL	CC	372	386	DBSU	26.24
**4969**	allXL	CC	373	386	DBSU	27.61
**4969**	allXL	BC	381	386	DBSU	64.13
**4969**	allXL	BD;DB	357	357	DBSU	18.75
**4969**	allXL	AD	373	319	DBSU	21.21
**4969**	allXL	BC	372	357	DBSU	37.86
**4969**	allXL	BD	381	357	DBSU	39.82
**4969**	allXL	BC	373	357	DBSU	39.93

**4969**	allXL	BC	372	370	DBSU	42.62
**4969**	allXL	BC	373	370	DBSU	43.99
**4969**	allXL	CB	382	373	DBSU	55.89
**4970**	allXL	DD	320	357	BS2G	25.51
**4970**	allXL	BB	320	373	BS2G	18.11
**4970**	allXL	DD	373	382	BS2G	18.7
**4970**	allXL	DD	370	386	BS2G	20.52
**4970**	allXL	DD	373	386	BS2G	25.05
**4970**	allXL	AA	319	357	BS2G	24.66
**4970**	allXL	BB	367	373	BS2G	14.34
**4970**	allXL	DD	370	382	BS2G	18.91
**4970**	allXL	CC	376	382	BS2G	14.85
**4970**	allXL	DD	357	373	BS2G	23.67
**4970**	allXL	BB	320	366	BS2G	29.67
**4970**	allXL	CC	370	376	BS2G	16.28
**4970**	allXL	DD	320	357	DBSU	25.51
**4970**	allXL	AA	357	319	DBSU	24.66
**4970**	allXL	CC	372	319	DBSU	17.28
**4970**	allXL	DD	370	357	DBSU	21.79
**4970**	allXL	BB	382	357	DBSU	14.96
**4970**	allXL	AA	372	370	DBSU	6.13
**4970**	allXL	DD	381	370	DBSU	18.34
**4970**	allXL	CC	373	370	DBSU	9.2
**4970**	allXL	DD	381	372	DBSU	17.13
**4970**	allXL	DD	372	382	DBSU	18.98
**4970**	allXL	DD	372	386	DBSU	23.84
**4970**	allXL	DD	373	386	DBSU	25.05
**4970**	allXL	DB	381	386	DBSU	32.12
**4970**	allXL	BD;DB	357	357	DBSU	19.52
**4970**	allXL	BC	373	319	DBSU	32.36
**4970**	allXL	BC	372	357	DBSU	32.71
**4970**	allXL	BD	381	357	DBSU	34.59
**4970**	allXL	DB	373	357	DBSU	33.17
**4970**	allXL	CB	372	370	DBSU	15.86

**4970**	allXL	CB	373	370	DBSU	19.46
**4970**	allXL	BD	382	373	DBSU	38.88
**4971**	allXL	CC	320	357	BS2G	26.39
**4971**	allXL	DD	320	373	BS2G	14.2
**4971**	allXL	DD	373	382	BS2G	18.0
**4971**	allXL	DD	370	386	BS2G	26.55
**4971**	allXL	DD	373	386	BS2G	24.61
**4971**	allXL	CC	319	357	BS2G	27.84
**4971**	allXL	BB	367	373	BS2G	11.92
**4971**	allXL	CC	370	382	BS2G	21.78
**4971**	allXL	CC	376	382	BS2G	14.06
**4971**	allXL	BB	357	373	BS2G	21.06
**4971**	allXL	DD	320	366	BS2G	26.78
**4971**	allXL	CC	370	376	BS2G	12.76
**4971**	allXL	CC	320	357	DBSU	26.39
**4971**	allXL	CC	357	319	DBSU	27.84
**4971**	allXL	DD	372	319	DBSU	17.34
**4971**	allXL	DD	370	357	DBSU	23.41
**4971**	allXL	AA	382	357	DBSU	15.97
**4971**	allXL	AA	372	370	DBSU	6.22
**4971**	allXL	CC	381	370	DBSU	20.8
**4971**	allXL	DD	373	370	DBSU	8.73
**4971**	allXL	DD	381	372	DBSU	16.41
**4971**	allXL	DD	372	382	DBSU	16.05
**4971**	allXL	DD	372	386	DBSU	22.04
**4971**	allXL	DD	373	386	DBSU	24.61
**4971**	allXL	CA	381	386	DBSU	48.42
**4971**	allXL	BD;DB	357	357	DBSU	18.7
**4971**	allXL	AD	373	319	DBSU	33.88
**4971**	allXL	AC	372	357	DBSU	35.07
**4971**	allXL	AC	381	357	DBSU	11.78
**4971**	allXL	AC	373	357	DBSU	31.81
**4971**	allXL	DA	372	370	DBSU	41.0
**4971**	allXL	DA	373	370	DBSU	40.81

**4971**	allXL	AC	382	373	DBSU	28.46
**4972**	allXL	AA	320	357	BS2G	22.64
**4972**	allXL	BB	320	373	BS2G	27.61
**4972**	allXL	CC	373	382	BS2G	21.02
**4972**	allXL	CC	370	386	BS2G	25.36
**4972**	allXL	CC	373	386	BS2G	23.34
**4972**	allXL	AA	319	357	BS2G	21.67
**4972**	allXL	AA	367	373	BS2G	13.25
**4972**	allXL	CC	370	382	BS2G	22.3
**4972**	allXL	CC	376	382	BS2G	14.64
**4972**	allXL	BB	357	373	BS2G	33.69
**4972**	allXL	BB	320	366	BS2G	21.78
**4972**	allXL	CC	370	376	BS2G	13.51
**4972**	allXL	AA	320	357	DBSU	22.64
**4972**	allXL	AA	357	319	DBSU	21.67
**4972**	allXL	BB	372	319	DBSU	26.91
**4972**	allXL	DD	370	357	DBSU	25.84
**4972**	allXL	AA	382	357	DBSU	42.01
**4972**	allXL	AA	372	370	DBSU	6.25
**4972**	allXL	CC	381	370	DBSU	23.02
**4972**	allXL	AA	373	370	DBSU	9.01
**4972**	allXL	AA	381	372	DBSU	23.46
**4972**	allXL	CC	372	382	DBSU	23.25
**4972**	allXL	CC	372	386	DBSU	25.44
**4972**	allXL	CC	373	386	DBSU	23.34
**4972**	allXL	CB	381	386	DBSU	43.21
**4972**	allXL	BD;DB	357	357	DBSU	19.09
**4972**	allXL	DA	373	319	DBSU	15.43
**4972**	allXL	DA	372	357	DBSU	21.45
**4972**	allXL	DA	381	357	DBSU	37.47
**4972**	allXL	DA	373	357	DBSU	20.33
**4972**	allXL	BC	372	370	DBSU	29.22
**4972**	allXL	CB	373	370	DBSU	31.94
**4972**	allXL	DA	382	373	DBSU	37.39

**4973**	allXL	AA	320	357	BS2G	25.96
**4973**	allXL	BB	320	373	BS2G	14.14
**4973**	allXL	DD	373	382	BS2G	17.9
**4973**	allXL	DD	370	386	BS2G	15.28
**4973**	allXL	DD	373	386	BS2G	20.28
**4973**	allXL	AA	319	357	BS2G	27.99
**4973**	allXL	DD	367	373	BS2G	13.96
**4973**	allXL	DD	370	382	BS2G	18.42
**4973**	allXL	CC	376	382	BS2G	14.33
**4973**	allXL	CC	357	373	BS2G	16.77
**4973**	allXL	CC	320	366	BS2G	13.97
**4973**	allXL	BB	370	376	BS2G	16.26
**4973**	allXL	AA	320	357	DBSU	25.96
**4973**	allXL	AA	357	319	DBSU	27.99
**4973**	allXL	BB	372	319	DBSU	16.43
**4973**	allXL	CC	370	357	DBSU	16.67
**4973**	allXL	BB	382	357	DBSU	25.01
**4973**	allXL	AA	372	370	DBSU	6.12
**4973**	allXL	DD	381	370	DBSU	18.68
**4973**	allXL	DD	373	370	DBSU	9.18
**4973**	allXL	DD	381	372	DBSU	18.04
**4973**	allXL	DD	372	382	DBSU	19.14
**4973**	allXL	DD	372	386	DBSU	19.67
**4973**	allXL	DD	373	386	DBSU	20.28
**4973**	allXL	CA	381	386	DBSU	55.21
**4973**	allXL	BD;DB	357	357	DBSU	19.57
**4973**	allXL	AD	373	319	DBSU	28.66
**4973**	allXL	CA	372	357	DBSU	30.48
**4973**	allXL	CA	381	357	DBSU	26.59
**4973**	allXL	CA	373	357	DBSU	30.79
**4973**	allXL	AD	372	370	DBSU	37.39
**4973**	allXL	AD	373	370	DBSU	36.34
**4973**	allXL	DA	382	373	DBSU	53.76
**4974**	allXL	CC	320	357	BS2G	23.56

**4974**	allXL	AA	320	373	BS2G	20.1
**4974**	allXL	DD	373	382	BS2G	19.05
**4974**	allXL	DD	370	386	BS2G	11.78
**4974**	allXL	DD	373	386	BS2G	15.1
**4974**	allXL	AA	319	357	BS2G	25.81
**4974**	allXL	AA	367	373	BS2G	11.6
**4974**	allXL	DD	370	382	BS2G	19.98
**4974**	allXL	CC	376	382	BS2G	15.45
**4974**	allXL	AA	357	373	BS2G	33.29
**4974**	allXL	AA	320	366	BS2G	22.58
**4974**	allXL	AA	370	376	BS2G	16.62
**4974**	allXL	CC	320	357	DBSU	23.56
**4974**	allXL	AA	357	319	DBSU	25.81
**4974**	allXL	AA	372	319	DBSU	21.35
**4974**	allXL	DD	370	357	DBSU	27.16
**4974**	allXL	AA	382	357	DBSU	22.15
**4974**	allXL	AA	372	370	DBSU	6.32
**4974**	allXL	DD	381	370	DBSU	20.07
**4974**	allXL	AA	373	370	DBSU	8.95
**4974**	allXL	BB	381	372	DBSU	19.17
**4974**	allXL	CC	372	382	DBSU	19.86
**4974**	allXL	DD	372	386	DBSU	14.63
**4974**	allXL	DD	373	386	DBSU	15.1
**4974**	allXL	AD	381	386	DBSU	48.16
**4974**	allXL	BD;DB	357	357	DBSU	18.25
**4974**	allXL	DA	373	319	DBSU	22.04
**4974**	allXL	DA	372	357	DBSU	32.31
**4974**	allXL	BD	381	357	DBSU	40.01
**4974**	allXL	DA	373	357	DBSU	31.1
**4974**	allXL	DA	372	370	DBSU	13.3
**4974**	allXL	DA	373	370	DBSU	12.04
**4974**	allXL	AD	382	373	DBSU	32.7
**4975**	allXL	CC	320	357	BS2G	25.13
**4975**	allXL	DD	320	373	BS2G	22.22

**4975**	allXL	DD	373	382	BS2G	17.61
**4975**	allXL	DD	370	386	BS2G	21.55
**4975**	allXL	DD	373	386	BS2G	22.87
**4975**	allXL	CC	319	357	BS2G	24.53
**4975**	allXL	AA	367	373	BS2G	12.03
**4975**	allXL	DD	370	382	BS2G	21.39
**4975**	allXL	BB	376	382	BS2G	13.61
**4975**	allXL	DD	357	373	BS2G	27.13
**4975**	allXL	DD	320	366	BS2G	26.44
**4975**	allXL	CC	370	376	BS2G	16.57
**4975**	allXL	CC	320	357	DBSU	25.13
**4975**	allXL	CC	357	319	DBSU	24.53
**4975**	allXL	DD	372	319	DBSU	18.41
**4975**	allXL	BB	370	357	DBSU	20.79
**4975**	allXL	DD	382	357	DBSU	44.25
**4975**	allXL	AA	372	370	DBSU	6.39
**4975**	allXL	DD	381	370	DBSU	20.24
**4975**	allXL	DD	373	370	DBSU	9.24
**4975**	allXL	DD	381	372	DBSU	13.62
**4975**	allXL	DD	372	382	DBSU	15.45
**4975**	allXL	DD	372	386	DBSU	19.36
**4975**	allXL	DD	373	386	DBSU	22.87
**4975**	allXL	AD	381	386	DBSU	44.32
**4975**	allXL	BD;DB	357	357	DBSU	17.7
**4975**	allXL	CB	373	319	DBSU	13.01
**4975**	allXL	BD	372	357	DBSU	28.76
**4975**	allXL	BD	381	357	DBSU	50.71
**4975**	allXL	BD	373	357	DBSU	31.71
**4975**	allXL	DA	372	370	DBSU	29.62
**4975**	allXL	DA	373	370	DBSU	32.91
**4975**	allXL	DA	382	373	DBSU	37.31
**4976**	allXL	AA	320	357	BS2G	25.12
**4976**	allXL	CC	320	373	BS2G	35.44
**4976**	allXL	DD	373	382	BS2G	17.7

**4976**	allXL	BB	370	386	BS2G	18.74
**4976**	allXL	DD	373	386	BS2G	25.54
**4976**	allXL	AA	319	357	BS2G	24.57
**4976**	allXL	DD	367	373	BS2G	14.25
**4976**	allXL	DD	370	382	BS2G	15.64
**4976**	allXL	CC	376	382	BS2G	14.77
**4976**	allXL	AA	357	373	BS2G	30.18
**4976**	allXL	DD	320	366	BS2G	24.39
**4976**	allXL	BB	370	376	BS2G	13.32
**4976**	allXL	AA	320	357	DBSU	25.12
**4976**	allXL	AA	357	319	DBSU	24.57
**4976**	allXL	CC	372	319	DBSU	34.63
**4976**	allXL	DD	370	357	DBSU	24.17
**4976**	allXL	DD	382	357	DBSU	28.82
**4976**	allXL	AA	372	370	DBSU	6.89
**4976**	allXL	BB	381	370	DBSU	17.11
**4976**	allXL	AA	373	370	DBSU	7.79
**4976**	allXL	DD	381	372	DBSU	18.6
**4976**	allXL	DD	372	382	DBSU	18.27
**4976**	allXL	BB	372	386	DBSU	24.19
**4976**	allXL	DD	373	386	DBSU	25.54
**4976**	allXL	AD	381	386	DBSU	54.66
**4976**	allXL	BD;DB	357	357	DBSU	18.54
**4976**	allXL	AD	373	319	DBSU	21.54
**4976**	allXL	AC	372	357	DBSU	38.21
**4976**	allXL	DB	381	357	DBSU	49.14
**4976**	allXL	AC	373	357	DBSU	40.55
**4976**	allXL	BC	372	370	DBSU	28.81
**4976**	allXL	BC	373	370	DBSU	27.98
**4976**	allXL	CB	382	373	DBSU	35.84
**4977**	allXL	CC	320	357	BS2G	22.22
**4977**	allXL	BB	320	373	BS2G	12.86
**4977**	allXL	BB	373	382	BS2G	14.91
**4977**	allXL	AA	370	386	BS2G	23.46

**4977**	allXL	BB	373	386	BS2G	24.34
**4977**	allXL	AA	319	357	BS2G	21.96
**4977**	allXL	AA	367	373	BS2G	11.4
**4977**	allXL	AA	370	382	BS2G	18.51
**4977**	allXL	BB	376	382	BS2G	12.23
**4977**	allXL	DD	357	373	BS2G	24.94
**4977**	allXL	BB	320	366	BS2G	19.67
**4977**	allXL	CC	370	376	BS2G	15.88
**4977**	allXL	CC	320	357	DBSU	22.22
**4977**	allXL	AA	357	319	DBSU	21.96
**4977**	allXL	BB	372	319	DBSU	14.57
**4977**	allXL	DD	370	357	DBSU	25.71
**4977**	allXL	DD	382	357	DBSU	18.26
**4977**	allXL	CC	372	370	DBSU	6.79
**4977**	allXL	AA	381	370	DBSU	20.9
**4977**	allXL	CC	373	370	DBSU	8.9
**4977**	allXL	AA	381	372	DBSU	18.89
**4977**	allXL	AA	372	382	DBSU	17.24
**4977**	allXL	AA	372	386	DBSU	24.27
**4977**	allXL	BB	373	386	DBSU	24.34
**4977**	allXL	BD	381	386	DBSU	27.53
**4977**	allXL	BD;DB	357	357	DBSU	18.61
**4977**	allXL	DA	373	319	DBSU	25.82
**4977**	allXL	CB	372	357	DBSU	25.08
**4977**	allXL	DB	381	357	DBSU	22.82
**4977**	allXL	CB	373	357	DBSU	26.91
**4977**	allXL	CB	372	370	DBSU	19.56
**4977**	allXL	CB	373	370	DBSU	20.03
**4977**	allXL	BC	382	373	DBSU	30.49
**4978**	allXL	AA	320	357	BS2G	24.01
**4978**	allXL	BB	320	373	BS2G	29.39
**4978**	allXL	CC	373	382	BS2G	15.71
**4978**	allXL	CC	370	386	BS2G	32.24
**4978**	allXL	CC	373	386	BS2G	24.9

**4978**	allXL	AA	319	357	BS2G	23.89
**4978**	allXL	AA	367	373	BS2G	13.9
**4978**	allXL	CC	370	382	BS2G	25.02
**4978**	allXL	CC	376	382	BS2G	14.26
**4978**	allXL	BB	357	373	BS2G	23.75
**4978**	allXL	AA	320	366	BS2G	19.46
**4978**	allXL	BB	370	376	BS2G	17.13
**4978**	allXL	AA	320	357	DBSU	24.01
**4978**	allXL	AA	357	319	DBSU	23.89
**4978**	allXL	CC	372	319	DBSU	29.69
**4978**	allXL	BB	370	357	DBSU	17.09
**4978**	allXL	BB	382	357	DBSU	35.67
**4978**	allXL	AA	372	370	DBSU	6.19
**4978**	allXL	CC	381	370	DBSU	27.02
**4978**	allXL	AA	373	370	DBSU	9.15
**4978**	allXL	CC	381	372	DBSU	20.52
**4978**	allXL	CC	372	382	DBSU	18.88
**4978**	allXL	CC	372	386	DBSU	27.8
**4978**	allXL	CC	373	386	DBSU	24.9
**4978**	allXL	DB	381	386	DBSU	42.37
**4978**	allXL	BD;DB	357	357	DBSU	16.86
**4978**	allXL	AD	373	319	DBSU	31.97
**4978**	allXL	BD	372	357	DBSU	28.06
**4978**	allXL	DB	381	357	DBSU	33.15
**4978**	allXL	BD	373	357	DBSU	29.72
**4978**	allXL	DB	372	370	DBSU	20.13
**4978**	allXL	DB	373	370	DBSU	18.77
**4978**	allXL	DB	382	373	DBSU	22.31
**4979**	allXL	AA	320	357	BS2G	24.99
**4979**	allXL	DD	320	373	BS2G	25.12
**4979**	allXL	CC	373	382	BS2G	20.36
**4979**	allXL	CC	370	386	BS2G	31.73
**4979**	allXL	CC	373	386	BS2G	27.39
**4979**	allXL	CC	319	357	BS2G	26.87

**4979**	allXL	AA	367	373	BS2G	12.87
**4979**	allXL	DD	370	382	BS2G	25.12
**4979**	allXL	CC	376	382	BS2G	13.58
**4979**	allXL	BB	357	373	BS2G	17.68
**4979**	allXL	CC	320	366	BS2G	20.92
**4979**	allXL	CC	370	376	BS2G	16.87
**4979**	allXL	AA	320	357	DBSU	24.99
**4979**	allXL	CC	357	319	DBSU	26.87
**4979**	allXL	DD	372	319	DBSU	25.75
**4979**	allXL	DD	370	357	DBSU	18.8
**4979**	allXL	BB	382	357	DBSU	8.23
**4979**	allXL	AA	372	370	DBSU	6.06
**4979**	allXL	AA	381	370	DBSU	24.86
**4979**	allXL	AA	373	370	DBSU	9.05
**4979**	allXL	DD	381	372	DBSU	20.99
**4979**	allXL	CC	372	382	DBSU	21.54
**4979**	allXL	CC	372	386	DBSU	27.27
**4979**	allXL	CC	373	386	DBSU	27.39
**4979**	allXL	AD	381	386	DBSU	28.36
**4979**	allXL	BD;DB	357	357	DBSU	19.34
**4979**	allXL	CB	373	319	DBSU	18.18
**4979**	allXL	CB	372	357	DBSU	28.54
**4979**	allXL	BD	381	357	DBSU	18.47
**4979**	allXL	CB	373	357	DBSU	27.58
**4979**	allXL	CB	372	370	DBSU	8.96
**4979**	allXL	CB	373	370	DBSU	8.97
**4979**	allXL	BC	382	373	DBSU	32.45
**4980**	allXL	CC	320	357	BS2G	24.93
**4980**	allXL	BB	320	373	BS2G	13.06
**4980**	allXL	DD	373	382	BS2G	18.1
**4980**	allXL	BB	370	386	BS2G	25.74
**4980**	allXL	AA	373	386	BS2G	27.04
**4980**	allXL	AA	319	357	BS2G	23.76
**4980**	allXL	DD	367	373	BS2G	13.81

**4980**	allXL	BB	370	382	BS2G	17.55
**4980**	allXL	AA	376	382	BS2G	15.18
**4980**	allXL	AA	357	373	BS2G	18.57
**4980**	allXL	DD	320	366	BS2G	24.42
**4980**	allXL	BB	370	376	BS2G	14.98
**4980**	allXL	CC	320	357	DBSU	24.93
**4980**	allXL	AA	357	319	DBSU	23.76
**4980**	allXL	BB	372	319	DBSU	18.61
**4980**	allXL	BB	370	357	DBSU	14.7
**4980**	allXL	BB	382	357	DBSU	18.29
**4980**	allXL	AA	372	370	DBSU	6.14
**4980**	allXL	BB	381	370	DBSU	19.52
**4980**	allXL	DD	373	370	DBSU	8.89
**4980**	allXL	DD	381	372	DBSU	14.58
**4980**	allXL	DD	372	382	DBSU	16.66
**4980**	allXL	DD	372	386	DBSU	26.4
**4980**	allXL	AA	373	386	DBSU	27.04
**4980**	allXL	BC	381	386	DBSU	39.08
**4980**	allXL	BD;DB	357	357	DBSU	18.18
**4980**	allXL	CB	373	319	DBSU	12.47
**4980**	allXL	AC	372	357	DBSU	23.04
**4980**	allXL	BD	381	357	DBSU	25.08
**4980**	allXL	AC	373	357	DBSU	19.79
**4980**	allXL	BC	372	370	DBSU	36.74
**4980**	allXL	BC	373	370	DBSU	34.91
**4980**	allXL	CB	382	373	DBSU	33.26
**4981**	allXL	AA	320	357	BS2G	21.96
**4981**	allXL	AA	320	373	BS2G	22.57
**4981**	allXL	BB	373	382	BS2G	14.68
**4981**	allXL	BB	370	386	BS2G	23.3
**4981**	allXL	BB	373	386	BS2G	23.35
**4981**	allXL	AA	319	357	BS2G	23.84
**4981**	allXL	AA	367	373	BS2G	13.22
**4981**	allXL	BB	370	382	BS2G	18.56

**4981**	allXL	BB	376	382	BS2G	12.13
**4981**	allXL	AA	357	373	BS2G	34.74
**4981**	allXL	BB	320	366	BS2G	22.13
**4981**	allXL	CC	370	376	BS2G	16.47
**4981**	allXL	AA	320	357	DBSU	21.96
**4981**	allXL	AA	357	319	DBSU	23.84
**4981**	allXL	BB	372	319	DBSU	23.05
**4981**	allXL	AA	370	357	DBSU	31.89
**4981**	allXL	BB	382	357	DBSU	26.64
**4981**	allXL	AA	372	370	DBSU	6.32
**4981**	allXL	BB	381	370	DBSU	19.06
**4981**	allXL	AA	373	370	DBSU	9.01
**4981**	allXL	BB	381	372	DBSU	17.35
**4981**	allXL	BB	372	382	DBSU	16.2
**4981**	allXL	BB	372	386	DBSU	23.05
**4981**	allXL	BB	373	386	DBSU	23.35
**4981**	allXL	BC	381	386	DBSU	57.48
**4981**	allXL	BD;DB	357	357	DBSU	18.17
**4981**	allXL	CB	373	319	DBSU	34.03
**4981**	allXL	CA	372	357	DBSU	48.35
**4981**	allXL	BD	381	357	DBSU	41.72
**4981**	allXL	CA	373	357	DBSU	49.59
**4981**	allXL	BC	372	370	DBSU	40.24
**4981**	allXL	BC	373	370	DBSU	37.62
**4981**	allXL	DA	382	373	DBSU	48.32
**4982**	allXL	CC	320	357	BS2G	27.14
**4982**	allXL	DD	320	373	BS2G	13.6
**4982**	allXL	AA	373	382	BS2G	20.02
**4982**	allXL	AA	370	386	BS2G	24.79
**4982**	allXL	AA	373	386	BS2G	18.22
**4982**	allXL	AA	319	357	BS2G	28.76
**4982**	allXL	AA	367	373	BS2G	15.06
**4982**	allXL	DD	370	382	BS2G	25.49
**4982**	allXL	CC	376	382	BS2G	14.17

**4982**	allXL	DD	357	373	BS2G	30.69
**4982**	allXL	DD	320	366	BS2G	25.84
**4982**	allXL	CC	370	376	BS2G	16.63
**4982**	allXL	CC	320	357	DBSU	27.14
**4982**	allXL	AA	357	319	DBSU	28.76
**4982**	allXL	DD	372	319	DBSU	13.84
**4982**	allXL	DD	370	357	DBSU	21.73
**4982**	allXL	DD	382	357	DBSU	38.68
**4982**	allXL	AA	372	370	DBSU	6.04
**4982**	allXL	DD	381	370	DBSU	23.22
**4982**	allXL	AA	373	370	DBSU	9.13
**4982**	allXL	DD	381	372	DBSU	20.28
**4982**	allXL	DD	372	382	DBSU	23.3
**4982**	allXL	AA	372	386	DBSU	21.29
**4982**	allXL	AA	373	386	DBSU	18.22
**4982**	allXL	DA	381	386	DBSU	52.41
**4982**	allXL	BD;DB	357	357	DBSU	17.92
**4982**	allXL	DA	373	319	DBSU	27.42
**4982**	allXL	DA	372	357	DBSU	31.19
**4982**	allXL	DA	381	357	DBSU	49.34
**4982**	allXL	DA	373	357	DBSU	31.45
**4982**	allXL	DA	372	370	DBSU	25.27
**4982**	allXL	DA	373	370	DBSU	23.65
**4982**	allXL	DA	382	373	DBSU	38.65
**4983**	allXL	AA	320	357	BS2G	21.49
**4983**	allXL	BB	320	373	BS2G	28.59
**4983**	allXL	AA	373	382	BS2G	19.0
**4983**	allXL	AA	370	386	BS2G	21.95
**4983**	allXL	AA	373	386	BS2G	23.74
**4983**	allXL	AA	319	357	BS2G	18.47
**4983**	allXL	BB	367	373	BS2G	13.42
**4983**	allXL	BB	370	382	BS2G	19.46
**4983**	allXL	AA	376	382	BS2G	14.76
**4983**	allXL	DD	357	373	BS2G	21.47

**4983**	allXL	AA	320	366	BS2G	19.79
**4983**	allXL	BB	370	376	BS2G	15.56
**4983**	allXL	AA	320	357	DBSU	21.49
**4983**	allXL	AA	357	319	DBSU	18.47
**4983**	allXL	BB	372	319	DBSU	28.39
**4983**	allXL	DD	370	357	DBSU	22.44
**4983**	allXL	DD	382	357	DBSU	33.84
**4983**	allXL	AA	372	370	DBSU	6.17
**4983**	allXL	BB	381	370	DBSU	20.23
**4983**	allXL	AA	373	370	DBSU	9.18
**4983**	allXL	AA	381	372	DBSU	21.01
**4983**	allXL	AA	372	382	DBSU	18.76
**4983**	allXL	AA	372	386	DBSU	21.64
**4983**	allXL	AA	373	386	DBSU	23.74
**4983**	allXL	BC	381	386	DBSU	57.94
**4983**	allXL	BD;DB	357	357	DBSU	17.83
**4983**	allXL	AD	373	319	DBSU	36.86
**4983**	allXL	DB	372	357	DBSU	37.5
**4983**	allXL	DB	381	357	DBSU	45.64
**4983**	allXL	DB	373	357	DBSU	35.95
**4983**	allXL	BC	372	370	DBSU	50.95
**4983**	allXL	BC	373	370	DBSU	51.16
**4983**	allXL	CB	382	373	DBSU	54.88
**4984**	allXL	AA	320	357	BS2G	20.1
**4984**	allXL	CC	320	373	BS2G	23.73
**4984**	allXL	DD	373	382	BS2G	19.68
**4984**	allXL	DD	370	386	BS2G	25.11
**4984**	allXL	DD	373	386	BS2G	25.33
**4984**	allXL	AA	319	357	BS2G	17.69
**4984**	allXL	BB	367	373	BS2G	12.73
**4984**	allXL	CC	370	382	BS2G	20.06
**4984**	allXL	CC	376	382	BS2G	14.87
**4984**	allXL	BB	357	373	BS2G	11.4
**4984**	allXL	BB	320	366	BS2G	20.09

**4984**	allXL	CC	370	376	BS2G	14.26
**4984**	allXL	AA	320	357	DBSU	20.1
**4984**	allXL	AA	357	319	DBSU	17.69
**4984**	allXL	CC	372	319	DBSU	19.36
**4984**	allXL	BB	370	357	DBSU	18.1
**4984**	allXL	BB	382	357	DBSU	17.45
**4984**	allXL	AA	372	370	DBSU	6.31
**4984**	allXL	CC	381	370	DBSU	22.39
**4984**	allXL	AA	373	370	DBSU	8.8
**4984**	allXL	AA	381	372	DBSU	20.67
**4984**	allXL	DD	372	382	DBSU	21.83
**4984**	allXL	DD	372	386	DBSU	26.25
**4984**	allXL	DD	373	386	DBSU	25.33
**4984**	allXL	DB	381	386	DBSU	37.74
**4984**	allXL	BD;DB	357	357	DBSU	18.23
**4984**	allXL	AD	373	319	DBSU	29.4
**4984**	allXL	BD	372	357	DBSU	31.77
**4984**	allXL	BD	381	357	DBSU	15.57
**4984**	allXL	BD	373	357	DBSU	28.04
**4984**	allXL	BD	372	370	DBSU	52.02
**4984**	allXL	BD	373	370	DBSU	49.35
**4984**	allXL	BD	382	373	DBSU	32.04
**4985**	allXL	AA	320	357	BS2G	25.83
**4985**	allXL	BB	320	373	BS2G	22.84
**4985**	allXL	DD	373	382	BS2G	17.98
**4985**	allXL	DD	370	386	BS2G	19.29
**4985**	allXL	DD	373	386	BS2G	24.28
**4985**	allXL	AA	319	357	BS2G	26.1
**4985**	allXL	BB	367	373	BS2G	13.37
**4985**	allXL	DD	370	382	BS2G	14.27
**4985**	allXL	BB	376	382	BS2G	13.09
**4985**	allXL	BB	357	373	BS2G	13.82
**4985**	allXL	BB	320	366	BS2G	24.04
**4985**	allXL	DD	370	376	BS2G	15.73

**4985**	allXL	AA	320	357	DBSU	25.83
**4985**	allXL	AA	357	319	DBSU	26.1
**4985**	allXL	BB	372	319	DBSU	21.64
**4985**	allXL	BB	370	357	DBSU	21.03
**4985**	allXL	BB	382	357	DBSU	9.89
**4985**	allXL	AA	372	370	DBSU	6.28
**4985**	allXL	DD	381	370	DBSU	15.37
**4985**	allXL	AA	373	370	DBSU	8.87
**4985**	allXL	DD	381	372	DBSU	19.12
**4985**	allXL	DD	372	382	DBSU	18.81
**4985**	allXL	DD	372	386	DBSU	23.86
**4985**	allXL	DD	373	386	DBSU	24.28
**4985**	allXL	DB	381	386	DBSU	48.66
**4985**	allXL	BD;DB	357	357	DBSU	18.99
**4985**	allXL	CB	373	319	DBSU	23.67
**4985**	allXL	BD	372	357	DBSU	29.11
**4985**	allXL	BD	381	357	DBSU	12.11
**4985**	allXL	BD	373	357	DBSU	25.46
**4985**	allXL	CB	372	370	DBSU	21.07
**4985**	allXL	CB	373	370	DBSU	23.8
**4985**	allXL	BD	382	373	DBSU	40.54
**4986**	allXL	CC	320	357	BS2G	26.55
**4986**	allXL	AA	320	373	BS2G	19.38
**4986**	allXL	AA	373	382	BS2G	16.36
**4986**	allXL	AA	370	386	BS2G	19.82
**4986**	allXL	AA	373	386	BS2G	19.87
**4986**	allXL	AA	319	357	BS2G	27.69
**4986**	allXL	BB	367	373	BS2G	11.57
**4986**	allXL	AA	370	382	BS2G	21.4
**4986**	allXL	BB	376	382	BS2G	12.44
**4986**	allXL	BB	357	373	BS2G	22.12
**4986**	allXL	AA	320	366	BS2G	19.97
**4986**	allXL	BB	370	376	BS2G	15.0
**4986**	allXL	CC	320	357	DBSU	26.55

**4986**	allXL	AA	357	319	DBSU	27.69
**4986**	allXL	CC	372	319	DBSU	16.62
**4986**	allXL	DD	370	357	DBSU	24.11
**4986**	allXL	BB	382	357	DBSU	31.27
**4986**	allXL	AA	372	370	DBSU	6.5
**4986**	allXL	DD	381	370	DBSU	20.07
**4986**	allXL	AA	373	370	DBSU	9.26
**4986**	allXL	DD	381	372	DBSU	19.05
**4986**	allXL	AA	372	382	DBSU	19.45
**4986**	allXL	AA	372	386	DBSU	21.23
**4986**	allXL	AA	373	386	DBSU	19.87
**4986**	allXL	DA	381	386	DBSU	48.95
**4986**	allXL	BD;DB	357	357	DBSU	17.45
**4986**	allXL	DA	373	319	DBSU	24.99
**4986**	allXL	DA	372	357	DBSU	39.13
**4986**	allXL	DA	381	357	DBSU	31.75
**4986**	allXL	DA	373	357	DBSU	35.42
**4986**	allXL	DA	372	370	DBSU	28.61
**4986**	allXL	DA	373	370	DBSU	27.19
**4986**	allXL	DA	382	373	DBSU	33.74
**4987**	allXL	CC	320	357	BS2G	25.78
**4987**	allXL	CC	320	373	BS2G	19.79
**4987**	allXL	DD	373	382	BS2G	16.74
**4987**	allXL	CC	370	386	BS2G	22.44
**4987**	allXL	DD	373	386	BS2G	21.83
**4987**	allXL	AA	319	357	BS2G	26.28
**4987**	allXL	AA	367	373	BS2G	11.38
**4987**	allXL	BB	370	382	BS2G	21.85
**4987**	allXL	CC	376	382	BS2G	14.04
**4987**	allXL	CC	357	373	BS2G	21.81
**4987**	allXL	DD	320	366	BS2G	22.1
**4987**	allXL	BB	370	376	BS2G	14.5
**4987**	allXL	CC	320	357	DBSU	25.78
**4987**	allXL	AA	357	319	DBSU	26.28

**4987**	allXL	DD	372	319	DBSU	19.48
**4987**	allXL	CC	370	357	DBSU	22.42
**4987**	allXL	BB	382	357	DBSU	25.89
**4987**	allXL	CC	372	370	DBSU	6.43
**4987**	allXL	BB	381	370	DBSU	21.27
**4987**	allXL	AA	373	370	DBSU	9.01
**4987**	allXL	DD	381	372	DBSU	19.08
**4987**	allXL	DD	372	382	DBSU	17.96
**4987**	allXL	DD	372	386	DBSU	21.74
**4987**	allXL	DD	373	386	DBSU	21.83
**4987**	allXL	BC	381	386	DBSU	38.3
**4987**	allXL	BD;DB	357	357	DBSU	17.77
**4987**	allXL	CB	373	319	DBSU	27.21
**4987**	allXL	CB	372	357	DBSU	34.32
**4987**	allXL	CB	381	357	DBSU	31.11
**4987**	allXL	CB	373	357	DBSU	30.7
**4987**	allXL	AD	372	370	DBSU	26.91
**4987**	allXL	AD	373	370	DBSU	29.26
**4987**	allXL	CB	382	373	DBSU	27.46
**4988**	allXL	DD	320	357	BS2G	26.13
**4988**	allXL	BB	320	373	BS2G	11.38
**4988**	allXL	BB	373	382	BS2G	19.21
**4988**	allXL	DD	370	386	BS2G	21.3
**4988**	allXL	DD	373	386	BS2G	24.93
**4988**	allXL	DD	319	357	BS2G	26.35
**4988**	allXL	AA	367	373	BS2G	11.75
**4988**	allXL	DD	370	382	BS2G	20.93
**4988**	allXL	BB	376	382	BS2G	12.64
**4988**	allXL	CC	357	373	BS2G	18.37
**4988**	allXL	CC	320	366	BS2G	18.65
**4988**	allXL	DD	370	376	BS2G	15.87
**4988**	allXL	DD	320	357	DBSU	26.13
**4988**	allXL	DD	357	319	DBSU	26.35
**4988**	allXL	BB	372	319	DBSU	17.37

**4988**	allXL	CC	370	357	DBSU	14.72
**4988**	allXL	BB	382	357	DBSU	33.26
**4988**	allXL	AA	372	370	DBSU	6.77
**4988**	allXL	DD	381	370	DBSU	20.02
**4988**	allXL	DD	373	370	DBSU	8.83
**4988**	allXL	DD	381	372	DBSU	20.3
**4988**	allXL	DD	372	382	DBSU	21.9
**4988**	allXL	DD	372	386	DBSU	23.73
**4988**	allXL	DD	373	386	DBSU	24.93
**4988**	allXL	AC	381	386	DBSU	47.72
**4988**	allXL	BD;DB	357	357	DBSU	17.18
**4988**	allXL	DA	373	319	DBSU	35.88
**4988**	allXL	CA	372	357	DBSU	22.09
**4988**	allXL	CA	381	357	DBSU	21.38
**4988**	allXL	CA	373	357	DBSU	18.83
**4988**	allXL	CA	372	370	DBSU	48.11
**4988**	allXL	CA	373	370	DBSU	44.31
**4988**	allXL	CA	382	373	DBSU	34.53
**4989**	allXL	AA	320	357	BS2G	24.4
**4989**	allXL	BB	320	373	BS2G	41.02
**4989**	allXL	DD	373	382	BS2G	17.75
**4989**	allXL	DD	370	386	BS2G	25.3
**4989**	allXL	DD	373	386	BS2G	25.68
**4989**	allXL	AA	319	357	BS2G	24.0
**4989**	allXL	AA	367	373	BS2G	14.09
**4989**	allXL	DD	370	382	BS2G	21.43
**4989**	allXL	DD	376	382	BS2G	15.18
**4989**	allXL	BB	357	373	BS2G	23.7
**4989**	allXL	CC	320	366	BS2G	25.99
**4989**	allXL	CC	370	376	BS2G	16.4
**4989**	allXL	AA	320	357	DBSU	24.4
**4989**	allXL	AA	357	319	DBSU	24.0
**4989**	allXL	DD	372	319	DBSU	37.89
**4989**	allXL	BB	370	357	DBSU	24.75

**4989**	allXL	DD	382	357	DBSU	39.09
**4989**	allXL	AA	372	370	DBSU	6.2
**4989**	allXL	BB	381	370	DBSU	20.85
**4989**	allXL	AA	373	370	DBSU	9.04
**4989**	allXL	DD	381	372	DBSU	18.48
**4989**	allXL	DD	372	382	DBSU	17.69
**4989**	allXL	DD	372	386	DBSU	24.49
**4989**	allXL	DD	373	386	DBSU	25.68
**4989**	allXL	BD	381	386	DBSU	65.69
**4989**	allXL	BD;DB	357	357	DBSU	19.26
**4989**	allXL	CB	373	319	DBSU	15.01
**4989**	allXL	BD	372	357	DBSU	27.29
**4989**	allXL	BD	381	357	DBSU	44.85
**4989**	allXL	BD	373	357	DBSU	30.49
**4989**	allXL	DA	372	370	DBSU	43.95
**4989**	allXL	DA	373	370	DBSU	47.77
**4989**	allXL	DA	382	373	DBSU	56.39
**4990**	allXL	CC	320	357	BS2G	25.4
**4990**	allXL	AA	320	373	BS2G	20.05
**4990**	allXL	AA	373	382	BS2G	20.15
**4990**	allXL	DD	370	386	BS2G	20.57
**4990**	allXL	DD	373	386	BS2G	23.97
**4990**	allXL	CC	319	357	BS2G	25.98
**4990**	allXL	AA	367	373	BS2G	14.59
**4990**	allXL	DD	370	382	BS2G	21.4
**4990**	allXL	CC	376	382	BS2G	14.57
**4990**	allXL	CC	357	373	BS2G	28.66
**4990**	allXL	AA	320	366	BS2G	23.19
**4990**	allXL	CC	370	376	BS2G	14.62
**4990**	allXL	CC	320	357	DBSU	25.4
**4990**	allXL	CC	357	319	DBSU	25.98
**4990**	allXL	AA	372	319	DBSU	23.08
**4990**	allXL	DD	370	357	DBSU	27.29
**4990**	allXL	CC	382	357	DBSU	33.61

**4990**	allXL	AA	372	370	DBSU	6.14
**4990**	allXL	DD	381	370	DBSU	21.8
**4990**	allXL	AA	373	370	DBSU	9.27
**4990**	allXL	DD	381	372	DBSU	21.48
**4990**	allXL	AA	372	382	DBSU	20.59
**4990**	allXL	DD	372	386	DBSU	23.56
**4990**	allXL	DD	373	386	DBSU	23.97
**4990**	allXL	CB	381	386	DBSU	28.55
**4990**	allXL	BD;DB	357	357	DBSU	17.9
**4990**	allXL	BC	373	319	DBSU	33.48
**4990**	allXL	CA	372	357	DBSU	46.06
**4990**	allXL	CB	381	357	DBSU	42.64
**4990**	allXL	CA	373	357	DBSU	47.31
**4990**	allXL	DA	372	370	DBSU	35.88
**4990**	allXL	DA	373	370	DBSU	37.2
**4990**	allXL	CB	382	373	DBSU	38.53
**4991**	allXL	CC	320	357	BS2G	25.0
**4991**	allXL	DD	320	373	BS2G	26.83
**4991**	allXL	CC	373	382	BS2G	17.13
**4991**	allXL	BB	370	386	BS2G	24.07
**4991**	allXL	BB	373	386	BS2G	25.11
**4991**	allXL	AA	319	357	BS2G	27.36
**4991**	allXL	AA	367	373	BS2G	13.25
**4991**	allXL	DD	370	382	BS2G	16.96
**4991**	allXL	CC	376	382	BS2G	14.72
**4991**	allXL	BB	357	373	BS2G	19.45
**4991**	allXL	AA	320	366	BS2G	24.59
**4991**	allXL	DD	370	376	BS2G	15.13
**4991**	allXL	CC	320	357	DBSU	25.0
**4991**	allXL	AA	357	319	DBSU	27.36
**4991**	allXL	DD	372	319	DBSU	30.35
**4991**	allXL	BB	370	357	DBSU	23.77
**4991**	allXL	BB	382	357	DBSU	27.19
**4991**	allXL	AA	372	370	DBSU	6.27

**4991**	allXL	DD	381	370	DBSU	15.47
**4991**	allXL	AA	373	370	DBSU	9.11
**4991**	allXL	DD	381	372	DBSU	18.27
**4991**	allXL	DD	372	382	DBSU	19.26
**4991**	allXL	BB	372	386	DBSU	26.76
**4991**	allXL	BB	373	386	DBSU	25.11
**4991**	allXL	DA	381	386	DBSU	44.69
**4991**	allXL	BD;DB	357	357	DBSU	18.32
**4991**	allXL	AD	373	319	DBSU	25.65
**4991**	allXL	BD	372	357	DBSU	26.94
**4991**	allXL	BD	381	357	DBSU	25.47
**4991**	allXL	BD	373	357	DBSU	24.53
**4991**	allXL	DA	372	370	DBSU	38.88
**4991**	allXL	DA	373	370	DBSU	35.6
**4991**	allXL	DA	382	373	DBSU	38.35
**4992**	allXL	AA	320	357	BS2G	19.66
**4992**	allXL	CC	320	373	BS2G	15.7
**4992**	allXL	AA	373	382	BS2G	19.88
**4992**	allXL	AA	370	386	BS2G	30.69
**4992**	allXL	AA	373	386	BS2G	27.36
**4992**	allXL	AA	319	357	BS2G	17.08
**4992**	allXL	AA	367	373	BS2G	11.83
**4992**	allXL	AA	370	382	BS2G	26.24
**4992**	allXL	CC	376	382	BS2G	14.15
**4992**	allXL	CC	357	373	BS2G	23.28
**4992**	allXL	BB	320	366	BS2G	27.77
**4992**	allXL	BB	370	376	BS2G	16.03
**4992**	allXL	AA	320	357	DBSU	19.66
**4992**	allXL	AA	357	319	DBSU	17.08
**4992**	allXL	CC	372	319	DBSU	21.56
**4992**	allXL	CC	370	357	DBSU	22.07
**4992**	allXL	AA	382	357	DBSU	19.67
**4992**	allXL	CC	372	370	DBSU	6.67
**4992**	allXL	BB	381	370	DBSU	22.92

**4992**	allXL	DD	373	370	DBSU	9.04
**4992**	allXL	DD	381	372	DBSU	21.07
**4992**	allXL	DD	372	382	DBSU	22.38
**4992**	allXL	DD	372	386	DBSU	26.78
**4992**	allXL	AA	373	386	DBSU	27.36
**4992**	allXL	AD	381	386	DBSU	47.6
**4992**	allXL	BD;DB	357	357	DBSU	18.16
**4992**	allXL	AD	373	319	DBSU	28.21
**4992**	allXL	DB	372	357	DBSU	30.74
**4992**	allXL	AC	381	357	DBSU	38.74
**4992**	allXL	DB	373	357	DBSU	28.48
**4992**	allXL	DA	372	370	DBSU	41.01
**4992**	allXL	CB	373	370	DBSU	43.36
**4992**	allXL	CB	382	373	DBSU	41.99
**4993**	allXL	CC	320	357	BS2G	25.27
**4993**	allXL	DD	320	373	BS2G	18.67
**4993**	allXL	DD	373	382	BS2G	18.21
**4993**	allXL	DD	370	386	BS2G	18.53
**4993**	allXL	DD	373	386	BS2G	26.95
**4993**	allXL	CC	319	357	BS2G	25.67
**4993**	allXL	AA	367	373	BS2G	13.2
**4993**	allXL	DD	370	382	BS2G	12.73
**4993**	allXL	BB	376	382	BS2G	14.56
**4993**	allXL	BB	357	373	BS2G	17.65
**4993**	allXL	AA	320	366	BS2G	14.86
**4993**	allXL	DD	370	376	BS2G	15.36
**4993**	allXL	CC	320	357	DBSU	25.27
**4993**	allXL	CC	357	319	DBSU	25.67
**4993**	allXL	DD	372	319	DBSU	18.61
**4993**	allXL	BB	370	357	DBSU	24.77
**4993**	allXL	BB	382	357	DBSU	21.13
**4993**	allXL	AA	372	370	DBSU	6.23
**4993**	allXL	DD	381	370	DBSU	11.66
**4993**	allXL	AA	373	370	DBSU	9.0

**4993**	allXL	DD	381	372	DBSU	16.12
**4993**	allXL	DD	372	382	DBSU	18.18
**4993**	allXL	DD	372	386	DBSU	25.58
**4993**	allXL	DD	373	386	DBSU	26.95
**4993**	allXL	DB	381	386	DBSU	30.46
**4993**	allXL	BD;DB	357	357	DBSU	19.04
**4993**	allXL	CB	373	319	DBSU	19.1
**4993**	allXL	BD	372	357	DBSU	14.69
**4993**	allXL	BD	381	357	DBSU	26.69
**4993**	allXL	BD	373	357	DBSU	16.28
**4993**	allXL	AD	372	370	DBSU	25.76
**4993**	allXL	AD	373	370	DBSU	25.11
**4993**	allXL	AD	382	373	DBSU	28.59
**4994**	allXL	BB	320	357	BS2G	25.42
**4994**	allXL	BB	320	373	BS2G	22.92
**4994**	allXL	DD	373	382	BS2G	18.06
**4994**	allXL	AA	370	386	BS2G	22.69
**4994**	allXL	AA	373	386	BS2G	23.39
**4994**	allXL	AA	319	357	BS2G	24.99
**4994**	allXL	AA	367	373	BS2G	13.94
**4994**	allXL	DD	370	382	BS2G	19.55
**4994**	allXL	CC	376	382	BS2G	13.32
**4994**	allXL	DD	357	373	BS2G	21.4
**4994**	allXL	DD	320	366	BS2G	23.66
**4994**	allXL	CC	370	376	BS2G	16.09
**4994**	allXL	BB	320	357	DBSU	25.42
**4994**	allXL	AA	357	319	DBSU	24.99
**4994**	allXL	BB	372	319	DBSU	24.27
**4994**	allXL	DD	370	357	DBSU	20.05
**4994**	allXL	DD	382	357	DBSU	24.72
**4994**	allXL	AA	372	370	DBSU	6.11
**4994**	allXL	DD	381	370	DBSU	18.31
**4994**	allXL	AA	373	370	DBSU	9.04
**4994**	allXL	DD	381	372	DBSU	16.61

**4994**	allXL	AA	372	382	DBSU	18.04
**4994**	allXL	AA	372	386	DBSU	20.92
**4994**	allXL	AA	373	386	DBSU	23.39
**4994**	allXL	DB	381	386	DBSU	29.87
**4994**	allXL	BD;DB	357	357	DBSU	19.16
**4994**	allXL	AD	373	319	DBSU	25.81
**4994**	allXL	DB	372	357	DBSU	31.54
**4994**	allXL	DB	381	357	DBSU	24.95
**4994**	allXL	DB	373	357	DBSU	27.94
**4994**	allXL	AD	372	370	DBSU	41.79
**4994**	allXL	DA	373	370	DBSU	43.28
**4994**	allXL	DB	382	373	DBSU	43.36
**4995**	allXL	DD	320	357	BS2G	25.08
**4995**	allXL	BB	320	373	BS2G	11.33
**4995**	allXL	BB	373	382	BS2G	19.98
**4995**	allXL	AA;BB	370	386	BS2G	33.35
**4995**	allXL	CC	373	386	BS2G	26.09
**4995**	allXL	AA	319	357	BS2G	25.7
**4995**	allXL	BB	367	373	BS2G	14.03
**4995**	allXL	BB	370	382	BS2G	27.4
**4995**	allXL	BB	376	382	BS2G	14.93
**4995**	allXL	BB	357	373	BS2G	21.68
**4995**	allXL	AA	320	366	BS2G	16.43
**4995**	allXL	AA	370	376	BS2G	16.22
**4995**	allXL	DD	320	357	DBSU	25.08
**4995**	allXL	AA	357	319	DBSU	25.7
**4995**	allXL	BB	372	319	DBSU	12.2
**4995**	allXL	BB	370	357	DBSU	26.24
**4995**	allXL	BB	382	357	DBSU	35.27
**4995**	allXL	AA	372	370	DBSU	5.91
**4995**	allXL	BB	381	370	DBSU	26.51
**4995**	allXL	CC	373	370	DBSU	9.03
**4995**	allXL	BB	381	372	DBSU	19.75
**4995**	allXL	BB	372	382	DBSU	21.13

**4995**	allXL	BB	372	386	DBSU	27.48
**4995**	allXL	CC	373	386	DBSU	26.09
**4995**	allXL	BD	381	386	DBSU	59.4
**4995**	allXL	BD;DB	357	357	DBSU	18.4
**4995**	allXL	AD	373	319	DBSU	37.3
**4995**	allXL	BD	372	357	DBSU	40.94
**4995**	allXL	BD	381	357	DBSU	47.09
**4995**	allXL	BD	373	357	DBSU	39.18
**4995**	allXL	CB	372	370	DBSU	29.89
**4995**	allXL	CB	373	370	DBSU	30.22
**4995**	allXL	BC	382	373	DBSU	53.23
**4996**	allXL	AA	320	357	BS2G	25.13
**4996**	allXL	BB	320	373	BS2G	23.61
**4996**	allXL	CC	373	382	BS2G	22.89
**4996**	allXL	CC	370	386	BS2G	31.29
**4996**	allXL	CC	373	386	BS2G	33.13
**4996**	allXL	AA	319	357	BS2G	24.01
**4996**	allXL	AA	367	373	BS2G	13.95
**4996**	allXL	CC	370	382	BS2G	23.88
**4996**	allXL	CC	376	382	BS2G	14.22
**4996**	allXL	BB	357	373	BS2G	22.13
**4996**	allXL	CC	320	366	BS2G	27.6
**4996**	allXL	CC	370	376	BS2G	15.62
**4996**	allXL	AA	320	357	DBSU	25.13
**4996**	allXL	AA	357	319	DBSU	24.01
**4996**	allXL	BB	372	319	DBSU	26.98
**4996**	allXL	DD	370	357	DBSU	25.03
**4996**	allXL	CC	382	357	DBSU	27.82
**4996**	allXL	AA	372	370	DBSU	6.07
**4996**	allXL	AA	381	370	DBSU	24.81
**4996**	allXL	AA	373	370	DBSU	9.18
**4996**	allXL	AA	381	372	DBSU	21.11
**4996**	allXL	DD	372	382	DBSU	23.91
**4996**	allXL	AA	372	386	DBSU	32.94

**4996**	allXL	CC	373	386	DBSU	33.13
**4996**	allXL	CB	381	386	DBSU	49.69
**4996**	allXL	BD;DB	357	357	DBSU	18.23
**4996**	allXL	CB	373	319	DBSU	14.62
**4996**	allXL	CB	372	357	DBSU	34.49
**4996**	allXL	BD	381	357	DBSU	29.5
**4996**	allXL	CB	373	357	DBSU	32.09
**4996**	allXL	DA	372	370	DBSU	43.73
**4996**	allXL	CB	373	370	DBSU	44.08
**4996**	allXL	CB	382	373	DBSU	38.89
**4997**	allXL	AA	320	357	BS2G	26.0
**4997**	allXL	BB	320	373	BS2G	37.71
**4997**	allXL	BB	373	382	BS2G	18.67
**4997**	allXL	DD	370	386	BS2G	21.46
**4997**	allXL	DD	373	386	BS2G	23.65
**4997**	allXL	CC	319	357	BS2G	26.77
**4997**	allXL	AA	367	373	BS2G	12.11
**4997**	allXL	BB	370	382	BS2G	19.7
**4997**	allXL	BB	376	382	BS2G	13.84
**4997**	allXL	BB	357	373	BS2G	31.58
**4997**	allXL	CC	320	366	BS2G	31.74
**4997**	allXL	CC	370	376	BS2G	12.91
**4997**	allXL	AA	320	357	DBSU	26.0
**4997**	allXL	CC	357	319	DBSU	26.77
**4997**	allXL	BB	372	319	DBSU	37.21
**4997**	allXL	DD	370	357	DBSU	27.73
**4997**	allXL	BB	382	357	DBSU	35.69
**4997**	allXL	AA	372	370	DBSU	6.42
**4997**	allXL	BB	381	370	DBSU	17.02
**4997**	allXL	AA	373	370	DBSU	9.01
**4997**	allXL	BB	381	372	DBSU	18.54
**4997**	allXL	BB	372	382	DBSU	20.57
**4997**	allXL	DD	372	386	DBSU	24.54
**4997**	allXL	DD	373	386	DBSU	23.65

**4997**	allXL	CB	381	386	DBSU	35.34
**4997**	allXL	BD;DB	357	357	DBSU	17.86
**4997**	allXL	CB	373	319	DBSU	24.14
**4997**	allXL	BD	372	357	DBSU	42.32
**4997**	allXL	CB	381	357	DBSU	40.4
**4997**	allXL	BD	373	357	DBSU	43.86
**4997**	allXL	CB	372	370	DBSU	34.18
**4997**	allXL	CB	373	370	DBSU	34.21
**4997**	allXL	CB	382	373	DBSU	42.53
**4998**	allXL	AA	320	357	BS2G	22.5
**4998**	allXL	DD	320	373	BS2G	10.04
**4998**	allXL	BB	373	382	BS2G	17.3
**4998**	allXL	DD	370	386	BS2G	26.07
**4998**	allXL	DD	373	386	BS2G	25.44
**4998**	allXL	AA	319	357	BS2G	21.87
**4998**	allXL	AA	367	373	BS2G	11.66
**4998**	allXL	DD	370	382	BS2G	25.66
**4998**	allXL	CC	376	382	BS2G	13.68
**4998**	allXL	AA	357	373	BS2G	26.29
**4998**	allXL	DD	320	366	BS2G	18.66
**4998**	allXL	CC	370	376	BS2G	16.83
**4998**	allXL	AA	320	357	DBSU	22.5
**4998**	allXL	AA	357	319	DBSU	21.87
**4998**	allXL	DD	372	319	DBSU	13.0
**4998**	allXL	DD	370	357	DBSU	22.38
**4998**	allXL	AA	382	357	DBSU	25.2
**4998**	allXL	AA	372	370	DBSU	6.58
**4998**	allXL	DD	381	370	DBSU	24.84
**4998**	allXL	AA	373	370	DBSU	9.21
**4998**	allXL	DD	381	372	DBSU	20.13
**4998**	allXL	BB	372	382	DBSU	18.67
**4998**	allXL	DD	372	386	DBSU	24.93
**4998**	allXL	DD	373	386	DBSU	25.44
**4998**	allXL	BC	381	386	DBSU	53.37

**4998**	allXL	BD;DB	357	357	DBSU	18.05
**4998**	allXL	CB	373	319	DBSU	26.22
**4998**	allXL	AC	372	357	DBSU	33.87
**4998**	allXL	AC	381	357	DBSU	31.11
**4998**	allXL	AC	373	357	DBSU	31.9
**4998**	allXL	DA	372	370	DBSU	51.59
**4998**	allXL	DA	373	370	DBSU	51.22
**4998**	allXL	BC	382	373	DBSU	64.03
**4999**	allXL	AA	320	357	BS2G	21.48
**4999**	allXL	DD	320	373	BS2G	23.47
**4999**	allXL	CC	373	382	BS2G	16.84
**4999**	allXL	CC	370	386	BS2G	26.03
**4999**	allXL	CC	373	386	BS2G	19.52
**4999**	allXL	AA	319	357	BS2G	20.04
**4999**	allXL	BB	367	373	BS2G	11.86
**4999**	allXL	AA	370	382	BS2G	23.37
**4999**	allXL	CC	376	382	BS2G	13.8
**4999**	allXL	DD	357	373	BS2G	23.66
**4999**	allXL	BB	320	366	BS2G	25.77
**4999**	allXL	CC	370	376	BS2G	16.02
**4999**	allXL	AA	320	357	DBSU	21.48
**4999**	allXL	AA	357	319	DBSU	20.04
**4999**	allXL	DD	372	319	DBSU	22.02
**4999**	allXL	DD	370	357	DBSU	22.95
**4999**	allXL	AA	382	357	DBSU	18.8
**4999**	allXL	AA	372	370	DBSU	6.14
**4999**	allXL	DD	381	370	DBSU	24.33
**4999**	allXL	AA	373	370	DBSU	9.05
**4999**	allXL	DD	381	372	DBSU	19.28
**4999**	allXL	CC	372	382	DBSU	19.98
**4999**	allXL	CC	372	386	DBSU	21.01
**4999**	allXL	CC	373	386	DBSU	19.52
**4999**	allXL	DA	381	386	DBSU	69.72
**4999**	allXL	BD;DB	357	357	DBSU	18.54

**4999**	allXL	CB	373	319	DBSU	28.4
**4999**	allXL	DB	372	357	DBSU	42.78
**4999**	allXL	AC	381	357	DBSU	32.49
**4999**	allXL	AC	373	357	DBSU	40.82
**4999**	allXL	DA	372	370	DBSU	42.23
**4999**	allXL	DA	373	370	DBSU	45.45
**4999**	allXL	CB	382	373	DBSU	57.18
**5000**	allXL	CC	320	357	BS2G	26.61
**5000**	allXL	BB	320	373	BS2G	12.32
**5000**	allXL	BB	373	382	BS2G	19.13
**5000**	allXL	DD	370	386	BS2G	22.79
**5000**	allXL	DD	373	386	BS2G	19.24
**5000**	allXL	AA	319	357	BS2G	28.6
**5000**	allXL	AA	367	373	BS2G	13.13
**5000**	allXL	BB	370	382	BS2G	25.76
**5000**	allXL	BB	376	382	BS2G	13.41
**5000**	allXL	BB	357	373	BS2G	25.67
**5000**	allXL	BB	320	366	BS2G	24.27
**5000**	allXL	CC	370	376	BS2G	17.06
**5000**	allXL	CC	320	357	DBSU	26.61
**5000**	allXL	AA	357	319	DBSU	28.6
**5000**	allXL	BB	372	319	DBSU	18.2
**5000**	allXL	BB	370	357	DBSU	24.24
**5000**	allXL	BB	382	357	DBSU	39.95
**5000**	allXL	AA	372	370	DBSU	6.29
**5000**	allXL	DD	381	370	DBSU	26.75
**5000**	allXL	CC	373	370	DBSU	9.45
**5000**	allXL	DD	381	372	DBSU	19.84
**5000**	allXL	DD	372	382	DBSU	20.99
**5000**	allXL	DD	372	386	DBSU	18.23
**5000**	allXL	DD	373	386	DBSU	19.24
**5000**	allXL	AD	381	386	DBSU	66.78
**5000**	allXL	BD;DB	357	357	DBSU	18.82
**5000**	allXL	DA	373	319	DBSU	37.0

**5000**	allXL	BD	372	357	DBSU	37.92
**5000**	allXL	BD	381	357	DBSU	47.91
**5000**	allXL	BD	373	357	DBSU	40.45
**5000**	allXL	DA	372	370	DBSU	40.88
**5000**	allXL	DA	373	370	DBSU	39.45
**5000**	allXL	DA	382	373	DBSU	43.8
**5001**	allXL	AA	320	357	BS2G	23.62
**5001**	allXL	DD	320	373	BS2G	30.06
**5001**	allXL	BB	373	382	BS2G	19.41
**5001**	allXL	DD	370	386	BS2G	21.2
**5001**	allXL	DD	373	386	BS2G	25.86
**5001**	allXL	AA	319	357	BS2G	22.46
**5001**	allXL	AA	367	373	BS2G	13.92
**5001**	allXL	DD	370	382	BS2G	19.51
**5001**	allXL	BB	376	382	BS2G	14.12
**5001**	allXL	AA	357	373	BS2G	25.01
**5001**	allXL	DD	320	366	BS2G	31.41
**5001**	allXL	CC	370	376	BS2G	15.82
**5001**	allXL	AA	320	357	DBSU	23.62
**5001**	allXL	AA	357	319	DBSU	22.46
**5001**	allXL	DD	372	319	DBSU	29.85
**5001**	allXL	AA	370	357	DBSU	24.78
**5001**	allXL	AA	382	357	DBSU	20.26
**5001**	allXL	AA	372	370	DBSU	6.38
**5001**	allXL	DD	381	370	DBSU	19.48
**5001**	allXL	AA	373	370	DBSU	9.29
**5001**	allXL	DD	381	372	DBSU	19.86
**5001**	allXL	DD	372	382	DBSU	21.15
**5001**	allXL	DD	372	386	DBSU	25.98
**5001**	allXL	DD	373	386	DBSU	25.86
**5001**	allXL	CA	381	386	DBSU	42.93
**5001**	allXL	BD;DB	357	357	DBSU	18.48
**5001**	allXL	DA	373	319	DBSU	27.24
**5001**	allXL	AC	372	357	DBSU	27.04

**5001**	allXL	AC	381	357	DBSU	27.75
**5001**	allXL	AC	373	357	DBSU	29.6
**5001**	allXL	AC	372	370	DBSU	47.28
**5001**	allXL	AC	373	370	DBSU	47.81
**5001**	allXL	AC	382	373	DBSU	34.53
**5002**	allXL	AA	320	357	BS2G	24.68
**5002**	allXL	BB	320	373	BS2G	14.06
**5002**	allXL	AA	373	382	BS2G	19.1
**5002**	allXL	CC	370	386	BS2G	27.08
**5002**	allXL	CC	373	386	BS2G	22.34
**5002**	allXL	AA	319	357	BS2G	27.68
**5002**	allXL	AA	367	373	BS2G	13.87
**5002**	allXL	AA	370	382	BS2G	21.86
**5002**	allXL	CC	376	382	BS2G	14.13
**5002**	allXL	BB	357	373	BS2G	23.78
**5002**	allXL	BB	320	366	BS2G	28.59
**5002**	allXL	CC	370	376	BS2G	15.7
**5002**	allXL	AA	320	357	DBSU	24.68
**5002**	allXL	AA	357	319	DBSU	27.68
**5002**	allXL	BB	372	319	DBSU	17.72
**5002**	allXL	BB	370	357	DBSU	25.56
**5002**	allXL	CC	382	357	DBSU	34.66
**5002**	allXL	AA	372	370	DBSU	6.42
**5002**	allXL	DD	381	370	DBSU	23.33
**5002**	allXL	AA	373	370	DBSU	8.59
**5002**	allXL	DD	381	372	DBSU	20.71
**5002**	allXL	AA	372	382	DBSU	20.81
**5002**	allXL	CC	372	386	DBSU	25.35
**5002**	allXL	CC	373	386	DBSU	22.34
**5002**	allXL	BC	381	386	DBSU	50.8
**5002**	allXL	BD;DB	357	357	DBSU	17.38
**5002**	allXL	AD	373	319	DBSU	32.87
**5002**	allXL	BC	372	357	DBSU	31.88
**5002**	allXL	BC	381	357	DBSU	35.62

**5002**	allXL	BC	373	357	DBSU	30.7
**5002**	allXL	BC	372	370	DBSU	27.26
**5002**	allXL	CB	373	370	DBSU	27.86
**5002**	allXL	CB	382	373	DBSU	34.22
**5003**	allXL	CC	320	357	BS2G	24.83
**5003**	allXL	AA	320	373	BS2G	28.97
**5003**	allXL	BB	373	382	BS2G	20.74
**5003**	allXL	DD	370	386	BS2G	24.75
**5003**	allXL	DD	373	386	BS2G	26.07
**5003**	allXL	CC	319	357	BS2G	26.99
**5003**	allXL	AA	367	373	BS2G	11.75
**5003**	allXL	DD	370	382	BS2G	21.51
**5003**	allXL	BB	376	382	BS2G	14.42
**5003**	allXL	BB	357	373	BS2G	32.71
**5003**	allXL	AA	320	366	BS2G	20.89
**5003**	allXL	DD	370	376	BS2G	14.58
**5003**	allXL	CC	320	357	DBSU	24.83
**5003**	allXL	CC	357	319	DBSU	26.99
**5003**	allXL	BB	372	319	DBSU	24.04
**5003**	allXL	DD	370	357	DBSU	26.92
**5003**	allXL	AA	382	357	DBSU	20.51
**5003**	allXL	AA	372	370	DBSU	6.37
**5003**	allXL	DD	381	370	DBSU	19.71
**5003**	allXL	CC	373	370	DBSU	9.07
**5003**	allXL	DD	381	372	DBSU	20.48
**5003**	allXL	DD	372	382	DBSU	21.99
**5003**	allXL	DD	372	386	DBSU	25.13
**5003**	allXL	DD	373	386	DBSU	26.07
**5003**	allXL	DB	381	386	DBSU	44.91
**5003**	allXL	BD;DB	357	357	DBSU	18.67
**5003**	allXL	CB	373	319	DBSU	15.55
**5003**	allXL	CA	372	357	DBSU	38.62
**5003**	allXL	BD	381	357	DBSU	37.54
**5003**	allXL	CB	373	357	DBSU	36.68

**5003**	allXL	DA	372	370	DBSU	21.79
**5003**	allXL	DA	373	370	DBSU	21.12
**5003**	allXL	CB	382	373	DBSU	34.85
**5004**	allXL	AA	320	357	BS2G	16.27
**5004**	allXL	DD	320	373	BS2G	18.11
**5004**	allXL	DD	373	382	BS2G	19.9
**5004**	allXL	DD	370	386	BS2G	15.62
**5004**	allXL	DD	373	386	BS2G	21.86
**5004**	allXL	AA	319	357	BS2G	15.48
**5004**	allXL	AA	367	373	BS2G	13.79
**5004**	allXL	DD	370	382	BS2G	18.21
**5004**	allXL	CC	376	382	BS2G	14.83
**5004**	allXL	BB	357	373	BS2G	24.67
**5004**	allXL	DD	320	366	BS2G	19.38
**5004**	allXL	CC	370	376	BS2G	15.03
**5004**	allXL	AA	320	357	DBSU	16.27
**5004**	allXL	AA	357	319	DBSU	15.48
**5004**	allXL	DD	372	319	DBSU	16.56
**5004**	allXL	BB	370	357	DBSU	25.26
**5004**	allXL	BB	382	357	DBSU	18.62
**5004**	allXL	AA	372	370	DBSU	6.18
**5004**	allXL	DD	381	370	DBSU	18.07
**5004**	allXL	AA	373	370	DBSU	9.17
**5004**	allXL	DD	381	372	DBSU	17.9
**5004**	allXL	DD	372	382	DBSU	18.82
**5004**	allXL	DD	372	386	DBSU	18.92
**5004**	allXL	DD	373	386	DBSU	21.86
**5004**	allXL	DB	381	386	DBSU	44.34
**5004**	allXL	BD;DB	357	357	DBSU	18.13
**5004**	allXL	DA	373	319	DBSU	32.48
**5004**	allXL	DA	372	357	DBSU	37.48
**5004**	allXL	BD	381	357	DBSU	28.17
**5004**	allXL	AC	373	357	DBSU	37.94
**5004**	allXL	DA	372	370	DBSU	43.78

**5004**	allXL	DA	373	370	DBSU	42.06
**5004**	allXL	AD	382	373	DBSU	42.95
**5005**	allXL	CC	320	357	BS2G	23.94
**5005**	allXL	AA	320	373	BS2G	11.37
**5005**	allXL	BB	373	382	BS2G	17.65
**5005**	allXL	DD	370	386	BS2G	15.91
**5005**	allXL	CC	373	386	BS2G	22.28
**5005**	allXL	CC	319	357	BS2G	23.41
**5005**	allXL	BB	367	373	BS2G	13.54
**5005**	allXL	DD	370	382	BS2G	18.79
**5005**	allXL	BB	376	382	BS2G	13.5
**5005**	allXL	BB	357	373	BS2G	22.31
**5005**	allXL	AA	320	366	BS2G	20.25
**5005**	allXL	CC	370	376	BS2G	14.95
**5005**	allXL	CC	320	357	DBSU	23.94
**5005**	allXL	CC	357	319	DBSU	23.41
**5005**	allXL	AA	372	319	DBSU	7.2
**5005**	allXL	AA	370	357	DBSU	22.29
**5005**	allXL	BB	382	357	DBSU	19.41
**5005**	allXL	AA	372	370	DBSU	6.46
**5005**	allXL	DD	381	370	DBSU	18.47
**5005**	allXL	AA	373	370	DBSU	9.33
**5005**	allXL	DD	381	372	DBSU	19.12
**5005**	allXL	BB	372	382	DBSU	19.54
**5005**	allXL	DD	372	386	DBSU	20.68
**5005**	allXL	CC	373	386	DBSU	22.28
**5005**	allXL	BD	381	386	DBSU	45.35
**5005**	allXL	BD;DB	357	357	DBSU	19.46
**5005**	allXL	DA	373	319	DBSU	18.45
**5005**	allXL	DA	372	357	DBSU	33.94
**5005**	allXL	BD	381	357	DBSU	35.0
**5005**	allXL	DA	373	357	DBSU	34.35
**5005**	allXL	DA	372	370	DBSU	22.12
**5005**	allXL	DA	373	370	DBSU	20.21

**5005**	allXL	DA	382	373	DBSU	32.6
**5006**	allXL	CC	320	357	BS2G	22.01
**5006**	allXL	BB	320	373	BS2G	16.84
**5006**	allXL	DD	373	382	BS2G	17.53
**5006**	allXL	CC	370	386	BS2G	24.4
**5006**	allXL	DD	373	386	BS2G	28.04
**5006**	allXL	CC	319	357	BS2G	22.38
**5006**	allXL	AA	367	373	BS2G	11.49
**5006**	allXL	CC	370	382	BS2G	19.04
**5006**	allXL	CC	376	382	BS2G	14.11
**5006**	allXL	BB	357	373	BS2G	19.94
**5006**	allXL	CC	320	366	BS2G	22.05
**5006**	allXL	CC	370	376	BS2G	14.49
**5006**	allXL	CC	320	357	DBSU	22.01
**5006**	allXL	CC	357	319	DBSU	22.38
**5006**	allXL	BB	372	319	DBSU	18.74
**5006**	allXL	BB	370	357	DBSU	26.22
**5006**	allXL	BB	382	357	DBSU	12.13
**5006**	allXL	CC	372	370	DBSU	6.78
**5006**	allXL	CC	381	370	DBSU	21.59
**5006**	allXL	AA	373	370	DBSU	9.03
**5006**	allXL	DD	381	372	DBSU	15.71
**5006**	allXL	DD	372	382	DBSU	17.26
**5006**	allXL	DD	372	386	DBSU	27.26
**5006**	allXL	DD	373	386	DBSU	28.04
**5006**	allXL	CB	381	386	DBSU	45.54
**5006**	allXL	BD;DB	357	357	DBSU	17.87
**5006**	allXL	AD	373	319	DBSU	21.51
**5006**	allXL	BD	372	357	DBSU	40.72
**5006**	allXL	BD	381	357	DBSU	24.06
**5006**	allXL	BD	373	357	DBSU	37.77
**5006**	allXL	CB	372	370	DBSU	30.44
**5006**	allXL	CB	373	370	DBSU	27.22
**5006**	allXL	CB	382	373	DBSU	22.42

**5007**	allXL	CC	320	357	BS2G	23.36
**5007**	allXL	CC	320	373	BS2G	27.22
**5007**	allXL	CC	373	382	BS2G	17.81
**5007**	allXL	DD	370	386	BS2G	25.2
**5007**	allXL	CC	373	386	BS2G	24.54
**5007**	allXL	AA	319	357	BS2G	25.1
**5007**	allXL	AA	367	373	BS2G	13.71
**5007**	allXL	AA	370	382	BS2G	20.87
**5007**	allXL	CC	376	382	BS2G	15.02
**5007**	allXL	AA	357	373	BS2G	38.36
**5007**	allXL	BB	320	366	BS2G	18.97
**5007**	allXL	BB	370	376	BS2G	15.59
**5007**	allXL	CC	320	357	DBSU	23.36
**5007**	allXL	AA	357	319	DBSU	25.1
**5007**	allXL	CC	372	319	DBSU	24.59
**5007**	allXL	BB	370	357	DBSU	32.14
**5007**	allXL	DD	382	357	DBSU	41.56
**5007**	allXL	AA	372	370	DBSU	6.15
**5007**	allXL	AA	381	370	DBSU	22.74
**5007**	allXL	AA	373	370	DBSU	9.11
**5007**	allXL	DD	381	372	DBSU	17.7
**5007**	allXL	DD	372	382	DBSU	17.07
**5007**	allXL	DD	372	386	DBSU	22.04
**5007**	allXL	CC	373	386	DBSU	24.54
**5007**	allXL	BC	381	386	DBSU	57.17
**5007**	allXL	BD;DB	357	357	DBSU	18.8
**5007**	allXL	DA	373	319	DBSU	24.02
**5007**	allXL	DA	372	357	DBSU	44.25
**5007**	allXL	DA	381	357	DBSU	43.44
**5007**	allXL	DA	373	357	DBSU	46.7
**5007**	allXL	AD	372	370	DBSU	28.35
**5007**	allXL	AD	373	370	DBSU	30.65
**5007**	allXL	DA	382	373	DBSU	46.47
**5008**	allXL	AA	320	357	BS2G	26.74

**5008**	allXL	DD	320	373	BS2G	23.78
**5008**	allXL	AA	373	382	BS2G	20.4
**5008**	allXL	AA	370	386	BS2G	24.52
**5008**	allXL	AA	373	386	BS2G	25.66
**5008**	allXL	AA	319	357	BS2G	26.62
**5008**	allXL	AA	367	373	BS2G	13.14
**5008**	allXL	DD	370	382	BS2G	21.91
**5008**	allXL	CC	376	382	BS2G	14.95
**5008**	allXL	CC	357	373	BS2G	33.31
**5008**	allXL	AA	320	366	BS2G	15.58
**5008**	allXL	CC	370	376	BS2G	14.97
**5008**	allXL	AA	320	357	DBSU	26.74
**5008**	allXL	AA	357	319	DBSU	26.62
**5008**	allXL	AA	372	319	DBSU	21.5
**5008**	allXL	CC	370	357	DBSU	30.31
**5008**	allXL	AA	382	357	DBSU	31.13
**5008**	allXL	AA	372	370	DBSU	6.36
**5008**	allXL	DD	381	370	DBSU	19.39
**5008**	allXL	CC	373	370	DBSU	9.01
**5008**	allXL	DD	381	372	DBSU	19.53
**5008**	allXL	AA	372	382	DBSU	22.53
**5008**	allXL	AA	372	386	DBSU	26.19
**5008**	allXL	AA	373	386	DBSU	25.66
**5008**	allXL	DA	381	386	DBSU	36.66
**5008**	allXL	BD;DB	357	357	DBSU	18.51
**5008**	allXL	DA	373	319	DBSU	39.95
**5008**	allXL	CA	372	357	DBSU	51.47
**5008**	allXL	DA	381	357	DBSU	50.73
**5008**	allXL	DA	373	357	DBSU	50.7
**5008**	allXL	AD	372	370	DBSU	33.3
**5008**	allXL	DA	373	370	DBSU	31.32
**5008**	allXL	DA	382	373	DBSU	21.16
**5009**	allXL	AA	320	357	BS2G	23.46
**5009**	allXL	BB	320	373	BS2G	16.51

**5009**	allXL	AA	373	382	BS2G	13.0
**5009**	allXL	DD	370	386	BS2G	20.54
**5009**	allXL	AA	373	386	BS2G	22.73
**5009**	allXL	AA	319	357	BS2G	21.65
**5009**	allXL	BB	367	373	BS2G	14.22
**5009**	allXL	DD	370	382	BS2G	15.06
**5009**	allXL	BB	376	382	BS2G	14.05
**5009**	allXL	BB	357	373	BS2G	27.92
**5009**	allXL	BB	320	366	BS2G	23.13
**5009**	allXL	DD	370	376	BS2G	15.99
**5009**	allXL	AA	320	357	DBSU	23.46
**5009**	allXL	AA	357	319	DBSU	21.65
**5009**	allXL	BB	372	319	DBSU	14.84
**5009**	allXL	DD	370	357	DBSU	22.17
**5009**	allXL	DD	382	357	DBSU	24.22
**5009**	allXL	AA	372	370	DBSU	6.13
**5009**	allXL	DD	381	370	DBSU	13.67
**5009**	allXL	AA	373	370	DBSU	9.03
**5009**	allXL	AA	381	372	DBSU	16.53
**5009**	allXL	AA	372	382	DBSU	15.23
**5009**	allXL	AA	372	386	DBSU	24.78
**5009**	allXL	AA	373	386	DBSU	22.73
**5009**	allXL	DA	381	386	DBSU	46.99
**5009**	allXL	BD;DB	357	357	DBSU	19.9
**5009**	allXL	DA	373	319	DBSU	20.68
**5009**	allXL	DA	372	357	DBSU	35.22
**5009**	allXL	DA	381	357	DBSU	43.38
**5009**	allXL	DA	373	357	DBSU	33.37
**5009**	allXL	BC	372	370	DBSU	25.14
**5009**	allXL	BC	373	370	DBSU	27.26
**5009**	allXL	DA	382	373	DBSU	40.48
**5010**	allXL	AA	320	357	BS2G	26.01
**5010**	allXL	DD	320	373	BS2G	18.41
**5010**	allXL	CC	373	382	BS2G	18.08

**5010**	allXL	DD	370	386	BS2G	25.41
**5010**	allXL	CC	373	386	BS2G	24.58
**5010**	allXL	AA	319	357	BS2G	25.91
**5010**	allXL	AA	367	373	BS2G	13.69
**5010**	allXL	DD	370	382	BS2G	22.87
**5010**	allXL	BB	376	382	BS2G	12.54
**5010**	allXL	BB	357	373	BS2G	14.03
**5010**	allXL	DD	320	366	BS2G	22.63
**5010**	allXL	AA	370	376	BS2G	16.94
**5010**	allXL	AA	320	357	DBSU	26.01
**5010**	allXL	AA	357	319	DBSU	25.91
**5010**	allXL	DD	372	319	DBSU	19.17
**5010**	allXL	BB	370	357	DBSU	21.66
**5010**	allXL	BB	382	357	DBSU	24.0
**5010**	allXL	AA	372	370	DBSU	6.36
**5010**	allXL	DD	381	370	DBSU	23.02
**5010**	allXL	AA	373	370	DBSU	8.89
**5010**	allXL	CC	381	372	DBSU	20.64
**5010**	allXL	CC	372	382	DBSU	18.39
**5010**	allXL	CC	372	386	DBSU	23.97
**5010**	allXL	CC	373	386	DBSU	24.58
**5010**	allXL	BC	381	386	DBSU	33.0
**5010**	allXL	BD;DB	357	357	DBSU	18.56
**5010**	allXL	CB	373	319	DBSU	22.33
**5010**	allXL	BD	372	357	DBSU	25.28
**5010**	allXL	BD	381	357	DBSU	29.3
**5010**	allXL	BD	373	357	DBSU	23.56
**5010**	allXL	DA	372	370	DBSU	23.7
**5010**	allXL	DA	373	370	DBSU	26.9
**5010**	allXL	CB	382	373	DBSU	40.74
**5011**	allXL	AA	320	357	BS2G	25.41
**5011**	allXL	DD	320	373	BS2G	20.12
**5011**	allXL	BB	373	382	BS2G	14.88
**5011**	allXL	BB	370	386	BS2G	19.8

**5011**	allXL	BB	373	386	BS2G	22.22
**5011**	allXL	AA	319	357	BS2G	24.89
**5011**	allXL	AA	367	373	BS2G	11.54
**5011**	allXL	BB	370	382	BS2G	17.23
**5011**	allXL	BB	376	382	BS2G	13.46
**5011**	allXL	DD	357	373	BS2G	25.56
**5011**	allXL	AA	320	366	BS2G	18.36
**5011**	allXL	DD	370	376	BS2G	15.49
**5011**	allXL	AA	320	357	DBSU	25.41
**5011**	allXL	AA	357	319	DBSU	24.89
**5011**	allXL	DD	372	319	DBSU	22.31
**5011**	allXL	DD	370	357	DBSU	15.8
**5011**	allXL	DD	382	357	DBSU	24.91
**5011**	allXL	CC	372	370	DBSU	6.56
**5011**	allXL	DD	381	370	DBSU	17.99
**5011**	allXL	AA	373	370	DBSU	8.59
**5011**	allXL	BB	381	372	DBSU	15.53
**5011**	allXL	BB	372	382	DBSU	15.61
**5011**	allXL	BB	372	386	DBSU	20.82
**5011**	allXL	BB	373	386	DBSU	22.22
**5011**	allXL	BC	381	386	DBSU	31.73
**5011**	allXL	BD;DB	357	357	DBSU	19.06
**5011**	allXL	AD	373	319	DBSU	18.53
**5011**	allXL	CB	372	357	DBSU	35.33
**5011**	allXL	DB	381	357	DBSU	44.45
**5011**	allXL	CB	373	357	DBSU	34.72
**5011**	allXL	CB	372	370	DBSU	18.36
**5011**	allXL	CB	373	370	DBSU	15.2
**5011**	allXL	BC	382	373	DBSU	23.59
**5012**	allXL	AA	320	357	BS2G	22.38
**5012**	allXL	CC	320	373	BS2G	16.37
**5012**	allXL	BB	373	382	BS2G	17.43
**5012**	allXL	CC	370	386	BS2G	23.3
**5012**	allXL	CC	373	386	BS2G	25.46

**5012**	allXL	AA	319	357	BS2G	22.77
**5012**	allXL	BB	367	373	BS2G	13.38
**5012**	allXL	CC	370	382	BS2G	21.69
**5012**	allXL	BB	376	382	BS2G	12.01
**5012**	allXL	CC	357	373	BS2G	24.55
**5012**	allXL	CC	320	366	BS2G	25.46
**5012**	allXL	CC	370	376	BS2G	13.55
**5012**	allXL	AA	320	357	DBSU	22.38
**5012**	allXL	AA	357	319	DBSU	22.77
**5012**	allXL	CC	372	319	DBSU	14.56
**5012**	allXL	CC	370	357	DBSU	24.99
**5012**	allXL	CC	382	357	DBSU	29.15
**5012**	allXL	AA	372	370	DBSU	6.09
**5012**	allXL	DD	381	370	DBSU	18.83
**5012**	allXL	AA	373	370	DBSU	9.06
**5012**	allXL	BB	381	372	DBSU	20.61
**5012**	allXL	BB	372	382	DBSU	18.5
**5012**	allXL	BB	372	386	DBSU	25.98
**5012**	allXL	CC	373	386	DBSU	25.46
**5012**	allXL	BC	381	386	DBSU	41.08
**5012**	allXL	BD;DB	357	357	DBSU	18.65
**5012**	allXL	AD	373	319	DBSU	25.65
**5012**	allXL	DA	372	357	DBSU	34.22
**5012**	allXL	DA	381	357	DBSU	28.95
**5012**	allXL	DA	373	357	DBSU	30.99
**5012**	allXL	CB	372	370	DBSU	23.04
**5012**	allXL	CB	373	370	DBSU	20.67
**5012**	allXL	CB	382	373	DBSU	24.09
**5013**	allXL	CC	320	357	BS2G	21.73
**5013**	allXL	DD	320	373	BS2G	18.46
**5013**	allXL	CC	373	382	BS2G	17.85
**5013**	allXL	AA	370	386	BS2G	28.15
**5013**	allXL	AA	373	386	BS2G	24.22
**5013**	allXL	CC	319	357	BS2G	24.92

**5013**	allXL	AA	367	373	BS2G	14.08
**5013**	allXL	AA	370	382	BS2G	25.97
**5013**	allXL	CC	376	382	BS2G	14.11
**5013**	allXL	BB	357	373	BS2G	34.3
**5013**	allXL	DD	320	366	BS2G	14.7
**5013**	allXL	CC	370	376	BS2G	17.51
**5013**	allXL	CC	320	357	DBSU	21.73
**5013**	allXL	CC	357	319	DBSU	24.92
**5013**	allXL	DD	372	319	DBSU	13.74
**5013**	allXL	DD	370	357	DBSU	28.96
**5013**	allXL	BB	382	357	DBSU	34.98
**5013**	allXL	AA	372	370	DBSU	6.37
**5013**	allXL	CC	381	370	DBSU	27.97
**5013**	allXL	DD	373	370	DBSU	8.87
**5013**	allXL	DD	381	372	DBSU	21.15
**5013**	allXL	CC	372	382	DBSU	19.86
**5013**	allXL	AA	372	386	DBSU	24.08
**5013**	allXL	AA	373	386	DBSU	24.22
**5013**	allXL	DA	381	386	DBSU	53.83
**5013**	allXL	BD;DB	357	357	DBSU	19.0
**5013**	allXL	AD	373	319	DBSU	20.17
**5013**	allXL	AC	372	357	DBSU	43.03
**5013**	allXL	DA	381	357	DBSU	46.85
**5013**	allXL	AC	373	357	DBSU	41.79
**5013**	allXL	DA	372	370	DBSU	23.25
**5013**	allXL	DA	373	370	DBSU	26.59
**5013**	allXL	DA	382	373	DBSU	34.43
**5014**	allXL	CC	320	357	BS2G	23.96
**5014**	allXL	DD	320	373	BS2G	28.07
**5014**	allXL	DD	373	382	BS2G	20.57
**5014**	allXL	BB	370	386	BS2G	21.33
**5014**	allXL	BB	373	386	BS2G	24.01
**5014**	allXL	AA	319	357	BS2G	22.65
**5014**	allXL	BB	367	373	BS2G	10.63

**5014**	allXL	DD	370	382	BS2G	21.76
**5014**	allXL	BB	376	382	BS2G	14.51
**5014**	allXL	BB	357	373	BS2G	27.6
**5014**	allXL	DD	320	366	BS2G	23.72
**5014**	allXL	CC	370	376	BS2G	15.02
**5014**	allXL	CC	320	357	DBSU	23.96
**5014**	allXL	AA	357	319	DBSU	22.65
**5014**	allXL	DD	372	319	DBSU	27.29
**5014**	allXL	DD	370	357	DBSU	26.0
**5014**	allXL	AA	382	357	DBSU	38.09
**5014**	allXL	AA	372	370	DBSU	6.13
**5014**	allXL	DD	381	370	DBSU	20.06
**5014**	allXL	AA	373	370	DBSU	9.23
**5014**	allXL	DD	381	372	DBSU	19.51
**5014**	allXL	DD	372	382	DBSU	22.17
**5014**	allXL	BB	372	386	DBSU	24.56
**5014**	allXL	BB	373	386	DBSU	24.01
**5014**	allXL	DA	381	386	DBSU	63.29
**5014**	allXL	BD;DB	357	357	DBSU	18.8
**5014**	allXL	CB	373	319	DBSU	19.59
**5014**	allXL	CB	372	357	DBSU	34.22
**5014**	allXL	AC	381	357	DBSU	39.08
**5014**	allXL	CB	373	357	DBSU	36.96
**5014**	allXL	BC	372	370	DBSU	35.25
**5014**	allXL	CB	373	370	DBSU	37.58
**5014**	allXL	BC	382	373	DBSU	55.5
**5015**	allXL	AA	320	357	BS2G	19.92
**5015**	allXL	AA	320	373	BS2G	13.95
**5015**	allXL	CC	373	382	BS2G	17.08
**5015**	allXL	CC	370	386	BS2G	21.19
**5015**	allXL	AA	373	386	BS2G	23.91
**5015**	allXL	AA	319	357	BS2G	17.54
**5015**	allXL	DD	367	373	BS2G	14.57
**5015**	allXL	CC	370	382	BS2G	14.52

**5015**	allXL	BB	376	382	BS2G	15.38
**5015**	allXL	AA	357	373	BS2G	31.03
**5015**	allXL	AA	320	366	BS2G	18.92
**5015**	allXL	CC	370	376	BS2G	14.93
**5015**	allXL	AA	320	357	DBSU	19.92
**5015**	allXL	AA	357	319	DBSU	17.54
**5015**	allXL	AA	372	319	DBSU	12.81
**5015**	allXL	BB	370	357	DBSU	27.3
**5015**	allXL	AA	382	357	DBSU	27.89
**5015**	allXL	CC	372	370	DBSU	6.58
**5015**	allXL	CC	381	370	DBSU	17.26
**5015**	allXL	AA	373	370	DBSU	8.6
**5015**	allXL	CC	381	372	DBSU	19.49
**5015**	allXL	CC	372	382	DBSU	17.8
**5015**	allXL	AA	372	386	DBSU	22.18
**5015**	allXL	AA	373	386	DBSU	23.91
**5015**	allXL	BC	381	386	DBSU	27.05
**5015**	allXL	BD;DB	357	357	DBSU	18.56
**5015**	allXL	BC	373	319	DBSU	30.5
**5015**	allXL	BC	372	357	DBSU	33.69
**5015**	allXL	BC	381	357	DBSU	32.69
**5015**	allXL	BC	373	357	DBSU	32.67
**5015**	allXL	BC	372	370	DBSU	42.81
**5015**	allXL	BC	373	370	DBSU	40.96
**5015**	allXL	CB	382	373	DBSU	34.12
**5016**	allXL	AA	320	357	BS2G	25.31
**5016**	allXL	DD	320	373	BS2G	23.79
**5016**	allXL	AA	373	382	BS2G	17.35
**5016**	allXL	AA	370	386	BS2G	20.67
**5016**	allXL	AA	373	386	BS2G	22.48
**5016**	allXL	CC	319	357	BS2G	27.43
**5016**	allXL	AA	367	373	BS2G	12.19
**5016**	allXL	AA	370	382	BS2G	20.32
**5016**	allXL	AA	376	382	BS2G	13.38

**5016**	allXL	CC	357	373	BS2G	23.81
**5016**	allXL	AA	320	366	BS2G	23.94
**5016**	allXL	CC	370	376	BS2G	16.35
**5016**	allXL	AA	320	357	DBSU	25.31
**5016**	allXL	CC	357	319	DBSU	27.43
**5016**	allXL	DD	372	319	DBSU	21.59
**5016**	allXL	CC	370	357	DBSU	19.67
**5016**	allXL	BB	382	357	DBSU	16.6
**5016**	allXL	AA	372	370	DBSU	6.37
**5016**	allXL	AA	381	370	DBSU	23.05
**5016**	allXL	AA	373	370	DBSU	9.05
**5016**	allXL	AA	381	372	DBSU	19.49
**5016**	allXL	AA	372	382	DBSU	17.14
**5016**	allXL	AA	372	386	DBSU	20.29
**5016**	allXL	AA	373	386	DBSU	22.48
**5016**	allXL	DA	381	386	DBSU	57.97
**5016**	allXL	BD;DB	357	357	DBSU	19.92
**5016**	allXL	AD	373	319	DBSU	37.55
**5016**	allXL	CA	372	357	DBSU	28.66
**5016**	allXL	BD	381	357	DBSU	36.76
**5016**	allXL	CA	373	357	DBSU	30.68
**5016**	allXL	DA	372	370	DBSU	42.2
**5016**	allXL	DA	373	370	DBSU	44.4
**5016**	allXL	AD	382	373	DBSU	57.87
**5017**	allXL	AA	320	357	BS2G	16.89
**5017**	allXL	DD	320	373	BS2G	28.72
**5017**	allXL	BB	373	382	BS2G	17.01
**5017**	allXL	DD	370	386	BS2G	15.99
**5017**	allXL	DD	373	386	BS2G	22.09
**5017**	allXL	AA	319	357	BS2G	13.58
**5017**	allXL	AA	367	373	BS2G	11.62
**5017**	allXL	AA	370	382	BS2G	17.64
**5017**	allXL	BB	376	382	BS2G	13.76
**5017**	allXL	AA	357	373	BS2G	31.44

**5017**	allXL	DD	320	366	BS2G	24.32
**5017**	allXL	CC	370	376	BS2G	14.29
**5017**	allXL	AA	320	357	DBSU	16.89
**5017**	allXL	AA	357	319	DBSU	13.58
**5017**	allXL	AA	372	319	DBSU	29.76
**5017**	allXL	BB	370	357	DBSU	30.39
**5017**	allXL	DD	382	357	DBSU	27.85
**5017**	allXL	AA	372	370	DBSU	6.26
**5017**	allXL	DD	381	370	DBSU	18.74
**5017**	allXL	AA	373	370	DBSU	8.81
**5017**	allXL	BB	381	372	DBSU	18.21
**5017**	allXL	AA	372	382	DBSU	19.02
**5017**	allXL	DD	372	386	DBSU	20.48
**5017**	allXL	DD	373	386	DBSU	22.09
**5017**	allXL	BC	381	386	DBSU	49.92
**5017**	allXL	BD;DB	357	357	DBSU	17.99
**5017**	allXL	AD	373	319	DBSU	29.45
**5017**	allXL	BD	372	357	DBSU	45.51
**5017**	allXL	BC	381	357	DBSU	42.8
**5017**	allXL	AC	373	357	DBSU	47.5
**5017**	allXL	CB	372	370	DBSU	37.69
**5017**	allXL	CB	373	370	DBSU	39.9
**5017**	allXL	DA	382	373	DBSU	40.04
**5018**	allXL	CC	320	357	BS2G	22.62
**5018**	allXL	BB	320	373	BS2G	24.71
**5018**	allXL	DD	373	382	BS2G	18.53
**5018**	allXL	DD	370	386	BS2G	20.83
**5018**	allXL	DD	373	386	BS2G	22.07
**5018**	allXL	AA	319	357	BS2G	23.3
**5018**	allXL	BB	367	373	BS2G	13.58
**5018**	allXL	DD	370	382	BS2G	21.79
**5018**	allXL	CC	376	382	BS2G	14.12
**5018**	allXL	AA	357	373	BS2G	31.92
**5018**	allXL	AA	320	366	BS2G	21.98

**5018**	allXL	CC	370	376	BS2G	14.71
**5018**	allXL	CC	320	357	DBSU	22.62
**5018**	allXL	AA	357	319	DBSU	23.3
**5018**	allXL	BB	372	319	DBSU	25.43
**5018**	allXL	DD	370	357	DBSU	30.75
**5018**	allXL	BB	382	357	DBSU	26.58
**5018**	allXL	AA	372	370	DBSU	6.21
**5018**	allXL	DD	381	370	DBSU	21.42
**5018**	allXL	CC	373	370	DBSU	9.02
**5018**	allXL	DD	381	372	DBSU	18.18
**5018**	allXL	DD	372	382	DBSU	19.19
**5018**	allXL	DD	372	386	DBSU	20.68
**5018**	allXL	DD	373	386	DBSU	22.07
**5018**	allXL	CB	381	386	DBSU	26.49
**5018**	allXL	BD;DB	357	357	DBSU	18.88
**5018**	allXL	AD	373	319	DBSU	16.79
**5018**	allXL	CB	372	357	DBSU	38.91
**5018**	allXL	CB	381	357	DBSU	42.91
**5018**	allXL	CB	373	357	DBSU	40.79
**5018**	allXL	CB	372	370	DBSU	12.12
**5018**	allXL	CB	373	370	DBSU	11.54
**5018**	allXL	CB	382	373	DBSU	24.42
**5019**	allXL	CC	320	357	BS2G	25.71
**5019**	allXL	CC	320	373	BS2G	25.0
**5019**	allXL	CC	373	382	BS2G	17.88
**5019**	allXL	CC	370	386	BS2G	20.12
**5019**	allXL	DD	373	386	BS2G	22.94
**5019**	allXL	AA	319	357	BS2G	27.28
**5019**	allXL	AA	367	373	BS2G	10.2
**5019**	allXL	CC	370	382	BS2G	13.75
**5019**	allXL	CC	376	382	BS2G	15.0
**5019**	allXL	DD	357	373	BS2G	22.68
**5019**	allXL	CC	320	366	BS2G	16.8
**5019**	allXL	CC	370	376	BS2G	12.27

**5019**	allXL	CC	320	357	DBSU	25.71
**5019**	allXL	AA	357	319	DBSU	27.28
**5019**	allXL	CC	372	319	DBSU	21.45
**5019**	allXL	DD	370	357	DBSU	26.77
**5019**	allXL	BB	382	357	DBSU	14.36
**5019**	allXL	CC	372	370	DBSU	6.6
**5019**	allXL	CC	381	370	DBSU	15.98
**5019**	allXL	AA	373	370	DBSU	8.98
**5019**	allXL	DD	381	372	DBSU	18.45
**5019**	allXL	CC	372	382	DBSU	18.05
**5019**	allXL	DD	372	386	DBSU	23.29
**5019**	allXL	DD	373	386	DBSU	22.94
**5019**	allXL	DA	381	386	DBSU	34.55
**5019**	allXL	BD;DB	357	357	DBSU	18.79
**5019**	allXL	AD	373	319	DBSU	16.25
**5019**	allXL	AC	372	357	DBSU	35.21
**5019**	allXL	BD	381	357	DBSU	27.56
**5019**	allXL	AC	373	357	DBSU	35.54
**5019**	allXL	DA	372	370	DBSU	28.6
**5019**	allXL	DA	373	370	DBSU	26.28
**5019**	allXL	DA	382	373	DBSU	24.37
**5020**	allXL	BB	320	357	BS2G	27.39
**5020**	allXL	DD	320	373	BS2G	12.95
**5020**	allXL	AA	373	382	BS2G	19.55
**5020**	allXL	DD	370	386	BS2G	19.98
**5020**	allXL	AA	373	386	BS2G	23.99
**5020**	allXL	AA	319	357	BS2G	29.96
**5020**	allXL	AA	367	373	BS2G	15.22
**5020**	allXL	DD	370	382	BS2G	20.9
**5020**	allXL	AA	376	382	BS2G	13.61
**5020**	allXL	CC	357	373	BS2G	21.29
**5020**	allXL	AA	320	366	BS2G	17.81
**5020**	allXL	CC	370	376	BS2G	16.22
**5020**	allXL	BB	320	357	DBSU	27.39

**5020**	allXL	AA	357	319	DBSU	29.96
**5020**	allXL	DD	372	319	DBSU	11.0
**5020**	allXL	CC	370	357	DBSU	17.13
**5020**	allXL	CC	382	357	DBSU	18.49
**5020**	allXL	AA	372	370	DBSU	6.16
**5020**	allXL	DD	381	370	DBSU	20.53
**5020**	allXL	AA	373	370	DBSU	9.22
**5020**	allXL	CC	381	372	DBSU	21.53
**5020**	allXL	CC	372	382	DBSU	21.76
**5020**	allXL	CC	372	386	DBSU	22.55
**5020**	allXL	AA	373	386	DBSU	23.99
**5020**	allXL	CA	381	386	DBSU	52.4
**5020**	allXL	BD;DB	357	357	DBSU	19.1
**5020**	allXL	AD	373	319	DBSU	45.06
**5020**	allXL	CA	372	357	DBSU	32.28
**5020**	allXL	CA	381	357	DBSU	14.49
**5020**	allXL	CA	373	357	DBSU	31.25
**5020**	allXL	AD	372	370	DBSU	43.27
**5020**	allXL	AD	373	370	DBSU	43.62
**5020**	allXL	AD	382	373	DBSU	41.67
**5021**	allXL	AA	320	357	BS2G	24.25
**5021**	allXL	DD	320	373	BS2G	20.44
**5021**	allXL	BB	373	382	BS2G	19.67
**5021**	allXL	CC	370	386	BS2G	27.06
**5021**	allXL	AA	373	386	BS2G	25.68
**5021**	allXL	AA	319	357	BS2G	23.09
**5021**	allXL	AA	367	373	BS2G	13.02
**5021**	allXL	CC	370	382	BS2G	18.29
**5021**	allXL	BB	376	382	BS2G	14.48
**5021**	allXL	BB	357	373	BS2G	21.97
**5021**	allXL	AA	320	366	BS2G	21.15
**5021**	allXL	CC	370	376	BS2G	12.31
**5021**	allXL	AA	320	357	DBSU	24.25
**5021**	allXL	AA	357	319	DBSU	23.09

**5021**	allXL	DD	372	319	DBSU	21.22
**5021**	allXL	DD	370	357	DBSU	26.75
**5021**	allXL	BB	382	357	DBSU	36.67
**5021**	allXL	AA	372	370	DBSU	6.29
**5021**	allXL	CC	381	370	DBSU	19.15
**5021**	allXL	AA	373	370	DBSU	9.09
**5021**	allXL	BB	381	372	DBSU	21.45
**5021**	allXL	BB	372	382	DBSU	21.66
**5021**	allXL	AA	372	386	DBSU	24.66
**5021**	allXL	AA	373	386	DBSU	25.68
**5021**	allXL	BC	381	386	DBSU	34.7
**5021**	allXL	BD;DB	357	357	DBSU	18.48
**5021**	allXL	CB	373	319	DBSU	30.06
**5021**	allXL	BD	372	357	DBSU	40.71
**5021**	allXL	BD	381	357	DBSU	52.83
**5021**	allXL	BD	373	357	DBSU	39.48
**5021**	allXL	CB	372	370	DBSU	31.07
**5021**	allXL	CB	373	370	DBSU	34.14
**5021**	allXL	AD	382	373	DBSU	35.71
**5022**	allXL	CC	320	357	BS2G	26.38
**5022**	allXL	BB	320	373	BS2G	23.83
**5022**	allXL	CC	373	382	BS2G	13.57
**5022**	allXL	CC	370	386	BS2G	21.19
**5022**	allXL	CC	373	386	BS2G	20.32
**5022**	allXL	CC	319	357	BS2G	26.53
**5022**	allXL	BB	367	373	BS2G	14.73
**5022**	allXL	DD	370	382	BS2G	18.87
**5022**	allXL	CC	376	382	BS2G	13.54
**5022**	allXL	BB	357	373	BS2G	37.11
**5022**	allXL	BB	320	366	BS2G	25.33
**5022**	allXL	CC	370	376	BS2G	16.21
**5022**	allXL	CC	320	357	DBSU	26.38
**5022**	allXL	CC	357	319	DBSU	26.53
**5022**	allXL	BB	372	319	DBSU	23.14

**5022**	allXL	BB	370	357	DBSU	30.77
**5022**	allXL	DD	382	357	DBSU	38.98
**5022**	allXL	AA	372	370	DBSU	5.93
**5022**	allXL	DD	381	370	DBSU	17.6
**5022**	allXL	AA	373	370	DBSU	9.34
**5022**	allXL	DD	381	372	DBSU	17.85
**5022**	allXL	CC	372	382	DBSU	16.53
**5022**	allXL	CC	372	386	DBSU	21.07
**5022**	allXL	CC	373	386	DBSU	20.32
**5022**	allXL	CB	381	386	DBSU	20.61
**5022**	allXL	BD;DB	357	357	DBSU	18.51
**5022**	allXL	DA	373	319	DBSU	15.54
**5022**	allXL	DA	372	357	DBSU	35.88
**5022**	allXL	DB	381	357	DBSU	48.66
**5022**	allXL	DA	373	357	DBSU	38.7
**5022**	allXL	AD	372	370	DBSU	49.37
**5022**	allXL	AD	373	370	DBSU	47.75
**5022**	allXL	BC	382	373	DBSU	32.12
**5023**	allXL	CC	320	357	BS2G	25.37
**5023**	allXL	DD	320	373	BS2G	16.32
**5023**	allXL	CC	373	382	BS2G	14.76
**5023**	allXL	DD	370	386	BS2G	21.72
**5023**	allXL	DD	373	386	BS2G	20.22
**5023**	allXL	CC	319	357	BS2G	26.41
**5023**	allXL	AA	367	373	BS2G	11.57
**5023**	allXL	CC	370	382	BS2G	19.37
**5023**	allXL	CC	376	382	BS2G	14.49
**5023**	allXL	DD	357	373	BS2G	27.69
**5023**	allXL	AA	320	366	BS2G	18.55
**5023**	allXL	CC	370	376	BS2G	16.98
**5023**	allXL	CC	320	357	DBSU	25.37
**5023**	allXL	CC	357	319	DBSU	26.41
**5023**	allXL	DD	372	319	DBSU	21.15
**5023**	allXL	DD	370	357	DBSU	23.59

**5023**	allXL	AA	382	357	DBSU	40.51
**5023**	allXL	AA	372	370	DBSU	6.33
**5023**	allXL	CC	381	370	DBSU	21.99
**5023**	allXL	AA	373	370	DBSU	9.0
**5023**	allXL	DD	381	372	DBSU	16.31
**5023**	allXL	DD	372	382	DBSU	14.94
**5023**	allXL	DD	372	386	DBSU	17.27
**5023**	allXL	DD	373	386	DBSU	20.22
**5023**	allXL	DA	381	386	DBSU	22.06
**5023**	allXL	BD;DB	357	357	DBSU	19.18
**5023**	allXL	DA	373	319	DBSU	32.59
**5023**	allXL	DA	372	357	DBSU	36.84
**5023**	allXL	DA	381	357	DBSU	39.48
**5023**	allXL	DA	373	357	DBSU	34.35
**5023**	allXL	DA	372	370	DBSU	26.38
**5023**	allXL	DA	373	370	DBSU	26.98
**5023**	allXL	DA	382	373	DBSU	21.28
**5024**	allXL	AA	320	357	BS2G	19.92
**5024**	allXL	BB	320	373	BS2G	29.37
**5024**	allXL	CC	373	382	BS2G	16.14
**5024**	allXL	CC	370	386	BS2G	16.86
**5024**	allXL	CC	373	386	BS2G	14.32
**5024**	allXL	AA	319	357	BS2G	23.4
**5024**	allXL	BB	367	373	BS2G	10.23
**5024**	allXL	DD	370	382	BS2G	21.52
**5024**	allXL	BB	376	382	BS2G	12.46
**5024**	allXL	AA	357	373	BS2G	27.09
**5024**	allXL	BB	320	366	BS2G	26.98
**5024**	allXL	AA	370	376	BS2G	16.49
**5024**	allXL	AA	320	357	DBSU	19.92
**5024**	allXL	AA	357	319	DBSU	23.4
**5024**	allXL	BB	372	319	DBSU	30.98
**5024**	allXL	AA	370	357	DBSU	23.46
**5024**	allXL	BB	382	357	DBSU	22.87

**5024**	allXL	AA	372	370	DBSU	6.42
**5024**	allXL	DD	381	370	DBSU	21.61
**5024**	allXL	AA	373	370	DBSU	8.54
**5024**	allXL	DD	381	372	DBSU	16.8
**5024**	allXL	CC	372	382	DBSU	17.21
**5024**	allXL	CC	372	386	DBSU	12.6
**5024**	allXL	CC	373	386	DBSU	14.32
**5024**	allXL	BC	381	386	DBSU	46.5
**5024**	allXL	BD;DB	357	357	DBSU	18.42
**5024**	allXL	CB	373	319	DBSU	21.52
**5024**	allXL	AC	372	357	DBSU	25.42
**5024**	allXL	AC	381	357	DBSU	35.79
**5024**	allXL	AC	373	357	DBSU	28.1
**5024**	allXL	BC	372	370	DBSU	38.85
**5024**	allXL	BC	373	370	DBSU	36.3
**5024**	allXL	BC	382	373	DBSU	35.56
**5025**	allXL	CC	320	357	BS2G	23.23
**5025**	allXL	CC	320	373	BS2G	16.54
**5025**	allXL	DD	373	382	BS2G	20.37
**5025**	allXL	AA	370	386	BS2G	21.05
**5025**	allXL	AA	373	386	BS2G	19.75
**5025**	allXL	CC	319	357	BS2G	26.11
**5025**	allXL	BB	367	373	BS2G	13.48
**5025**	allXL	DD	370	382	BS2G	23.14
**5025**	allXL	CC	376	382	BS2G	14.87
**5025**	allXL	BB	357	373	BS2G	15.17
**5025**	allXL	BB	320	366	BS2G	27.03
**5025**	allXL	CC	370	376	BS2G	14.64
**5025**	allXL	CC	320	357	DBSU	23.23
**5025**	allXL	CC	357	319	DBSU	26.11
**5025**	allXL	BB	372	319	DBSU	16.9
**5025**	allXL	BB	370	357	DBSU	19.0
**5025**	allXL	BB	382	357	DBSU	18.99
**5025**	allXL	AA	372	370	DBSU	6.38

**5025**	allXL	DD	381	370	DBSU	22.62
**5025**	allXL	DD	373	370	DBSU	9.1
**5025**	allXL	DD	381	372	DBSU	19.78
**5025**	allXL	DD	372	382	DBSU	21.23
**5025**	allXL	AA	372	386	DBSU	18.24
**5025**	allXL	AA	373	386	DBSU	19.75
**5025**	allXL	DB	381	386	DBSU	53.11
**5025**	allXL	BD;DB	357	357	DBSU	18.19
**5025**	allXL	CB	373	319	DBSU	36.69
**5025**	allXL	BD	372	357	DBSU	35.75
**5025**	allXL	BD	381	357	DBSU	20.62
**5025**	allXL	BD	373	357	DBSU	32.54
**5025**	allXL	CB	372	370	DBSU	35.15
**5025**	allXL	CB	373	370	DBSU	36.96
**5025**	allXL	BD	382	373	DBSU	46.97
**5026**	allXL	AA	320	357	BS2G	25.17
**5026**	allXL	BB	320	373	BS2G	34.83
**5026**	allXL	CC	373	382	BS2G	19.82
**5026**	allXL	DD	370	386	BS2G	24.36
**5026**	allXL	DD	373	386	BS2G	24.73
**5026**	allXL	AA	319	357	BS2G	24.76
**5026**	allXL	DD	367	373	BS2G	14.3
**5026**	allXL	DD	370	382	BS2G	23.12
**5026**	allXL	BB;CC	376	382	BS2G	15.37
**5026**	allXL	BB	357	373	BS2G	24.9
**5026**	allXL	BB	320	366	BS2G	26.05
**5026**	allXL	BB	370	376	BS2G	17.5
**5026**	allXL	AA	320	357	DBSU	25.17
**5026**	allXL	AA	357	319	DBSU	24.76
**5026**	allXL	BB	372	319	DBSU	38.41
**5026**	allXL	BB	370	357	DBSU	30.55
**5026**	allXL	BB	382	357	DBSU	16.48
**5026**	allXL	AA	372	370	DBSU	6.1
**5026**	allXL	DD	381	370	DBSU	22.99

**5026**	allXL	DD	373	370	DBSU	8.91
**5026**	allXL	DD	381	372	DBSU	20.13
**5026**	allXL	CC	372	382	DBSU	19.95
**5026**	allXL	DD	372	386	DBSU	23.98
**5026**	allXL	DD	373	386	DBSU	24.73
**5026**	allXL	AD	381	386	DBSU	28.29
**5026**	allXL	BD;DB	357	357	DBSU	19.46
**5026**	allXL	AD	373	319	DBSU	16.32
**5026**	allXL	AD	372	357	DBSU	38.07
**5026**	allXL	BD	381	357	DBSU	34.03
**5026**	allXL	AD	373	357	DBSU	38.9
**5026**	allXL	AD	372	370	DBSU	37.31
**5026**	allXL	AD	373	370	DBSU	35.0
**5026**	allXL	AD	382	373	DBSU	39.63
**5027**	allXL	AA	320	357	BS2G	23.77
**5027**	allXL	BB	320	373	BS2G	12.75
**5027**	allXL	CC	373	382	BS2G	17.5
**5027**	allXL	CC	370	386	BS2G	25.19
**5027**	allXL	CC	373	386	BS2G	21.13
**5027**	allXL	AA	319	357	BS2G	23.05
**5027**	allXL	BB	367	373	BS2G	14.14
**5027**	allXL	CC	370	382	BS2G	24.52
**5027**	allXL	BB	376	382	BS2G	14.1
**5027**	allXL	BB	357	373	BS2G	20.0
**5027**	allXL	BB	320	366	BS2G	19.04
**5027**	allXL	CC	370	376	BS2G	17.29
**5027**	allXL	AA	320	357	DBSU	23.77
**5027**	allXL	AA	357	319	DBSU	23.05
**5027**	allXL	BB	372	319	DBSU	15.78
**5027**	allXL	DD	370	357	DBSU	25.96
**5027**	allXL	BB	382	357	DBSU	34.49
**5027**	allXL	AA	372	370	DBSU	6.23
**5027**	allXL	CC	381	370	DBSU	26.81
**5027**	allXL	AA	373	370	DBSU	8.85

**5027**	allXL	CC	381	372	DBSU	20.41
**5027**	allXL	CC	372	382	DBSU	18.19
**5027**	allXL	CC	372	386	DBSU	20.11
**5027**	allXL	CC	373	386	DBSU	21.13
**5027**	allXL	BD	381	386	DBSU	32.21
**5027**	allXL	BD;DB	357	357	DBSU	17.97
**5027**	allXL	AD	373	319	DBSU	23.48
**5027**	allXL	DB	372	357	DBSU	37.22
**5027**	allXL	BD	381	357	DBSU	40.1
**5027**	allXL	BD	373	357	DBSU	35.89
**5027**	allXL	AD	372	370	DBSU	50.88
**5027**	allXL	BD	373	370	DBSU	49.05
**5027**	allXL	BD	382	373	DBSU	37.27
**5028**	allXL	CC	320	357	BS2G	24.16
**5028**	allXL	DD	320	373	BS2G	13.35
**5028**	allXL	BB	373	382	BS2G	19.31
**5028**	allXL	BB	370	386	BS2G	24.73
**5028**	allXL	BB	373	386	BS2G	21.77
**5028**	allXL	AA	319	357	BS2G	24.78
**5028**	allXL	AA	367	373	BS2G	13.54
**5028**	allXL	DD	370	382	BS2G	20.65
**5028**	allXL	BB	376	382	BS2G	13.49
**5028**	allXL	DD	357	373	BS2G	29.16
**5028**	allXL	AA	320	366	BS2G	27.21
**5028**	allXL	CC	370	376	BS2G	15.39
**5028**	allXL	CC	320	357	DBSU	24.16
**5028**	allXL	AA	357	319	DBSU	24.78
**5028**	allXL	DD	372	319	DBSU	12.5
**5028**	allXL	DD	370	357	DBSU	21.93
**5028**	allXL	DD	382	357	DBSU	37.92
**5028**	allXL	AA	372	370	DBSU	6.22
**5028**	allXL	DD	381	370	DBSU	18.21
**5028**	allXL	BB	373	370	DBSU	8.94
**5028**	allXL	DD	381	372	DBSU	18.51

**5028**	allXL	BB	372	382	DBSU	20.76
**5028**	allXL	BB	372	386	DBSU	22.43
**5028**	allXL	BB	373	386	DBSU	21.77
**5028**	allXL	AD	381	386	DBSU	41.09
**5028**	allXL	BD;DB	357	357	DBSU	17.12
**5028**	allXL	CB	373	319	DBSU	28.35
**5028**	allXL	DA	372	357	DBSU	39.0
**5028**	allXL	DA	381	357	DBSU	36.07
**5028**	allXL	DA	373	357	DBSU	38.67
**5028**	allXL	DA	372	370	DBSU	41.64
**5028**	allXL	DA	373	370	DBSU	38.54
**5028**	allXL	DA	382	373	DBSU	29.63
**5029**	allXL	AA	320	357	BS2G	24.33
**5029**	allXL	BB	320	373	BS2G	13.92
**5029**	allXL	DD	373	382	BS2G	17.87
**5029**	allXL	CC	370	386	BS2G	20.15
**5029**	allXL	AA	373	386	BS2G	22.65
**5029**	allXL	AA	319	357	BS2G	24.37
**5029**	allXL	AA	367	373	BS2G	13.97
**5029**	allXL	CC	370	382	BS2G	20.78
**5029**	allXL	CC	376	382	BS2G	14.1
**5029**	allXL	BB	357	373	BS2G	22.23
**5029**	allXL	BB	320	366	BS2G	25.01
**5029**	allXL	CC	370	376	BS2G	15.67
**5029**	allXL	AA	320	357	DBSU	24.33
**5029**	allXL	AA	357	319	DBSU	24.37
**5029**	allXL	BB	372	319	DBSU	17.24
**5029**	allXL	BB	370	357	DBSU	25.15
**5029**	allXL	BB	382	357	DBSU	27.12
**5029**	allXL	AA	372	370	DBSU	6.07
**5029**	allXL	CC	381	370	DBSU	22.05
**5029**	allXL	AA	373	370	DBSU	9.08
**5029**	allXL	DD	381	372	DBSU	21.25
**5029**	allXL	DD	372	382	DBSU	20.95

**5029**	allXL	CC	372	386	DBSU	23.93
**5029**	allXL	AA	373	386	DBSU	22.65
**5029**	allXL	BC	381	386	DBSU	39.15
**5029**	allXL	BD;DB	357	357	DBSU	18.9
**5029**	allXL	AD	373	319	DBSU	18.65
**5029**	allXL	AD	372	357	DBSU	41.05
**5029**	allXL	BD	381	357	DBSU	33.79
**5029**	allXL	BD	373	357	DBSU	39.73
**5029**	allXL	CB	372	370	DBSU	40.81
**5029**	allXL	CB	373	370	DBSU	41.43
**5029**	allXL	CB	382	373	DBSU	31.05
**5030**	allXL	AA	320	357	BS2G	24.99
**5030**	allXL	BB	320	373	BS2G	31.76
**5030**	allXL	CC	373	382	BS2G	18.79
**5030**	allXL	CC	370	386	BS2G	28.15
**5030**	allXL	CC	373	386	BS2G	25.03
**5030**	allXL	AA	319	357	BS2G	25.93
**5030**	allXL	BB	367	373	BS2G	12.76
**5030**	allXL	CC	370	382	BS2G	25.58
**5030**	allXL	CC	376	382	BS2G	13.14
**5030**	allXL	BB	357	373	BS2G	18.13
**5030**	allXL	DD	320	366	BS2G	26.09
**5030**	allXL	CC	370	376	BS2G	16.79
**5030**	allXL	AA	320	357	DBSU	24.99
**5030**	allXL	AA	357	319	DBSU	25.93
**5030**	allXL	DD	372	319	DBSU	30.48
**5030**	allXL	BB	370	357	DBSU	20.33
**5030**	allXL	BB	382	357	DBSU	25.54
**5030**	allXL	AA	372	370	DBSU	6.57
**5030**	allXL	BB	381	370	DBSU	25.29
**5030**	allXL	AA	373	370	DBSU	8.5
**5030**	allXL	DD	381	372	DBSU	20.2
**5030**	allXL	DD	372	382	DBSU	21.73
**5030**	allXL	CC	372	386	DBSU	27.48

**5030**	allXL	CC	373	386	DBSU	25.03
**5030**	allXL	CB	381	386	DBSU	59.71
**5030**	allXL	BD;DB	357	357	DBSU	18.34
**5030**	allXL	AD	373	319	DBSU	17.06
**5030**	allXL	BD	372	357	DBSU	20.74
**5030**	allXL	CA	381	357	DBSU	36.11
**5030**	allXL	BD	373	357	DBSU	23.88
**5030**	allXL	DA	372	370	DBSU	37.78
**5030**	allXL	DA	373	370	DBSU	41.36
**5030**	allXL	BC	382	373	DBSU	42.95
**5031**	allXL	AA	320	357	BS2G	19.66
**5031**	allXL	CC	320	373	BS2G	13.01
**5031**	allXL	DD	373	382	BS2G	18.17
**5031**	allXL	DD	370	386	BS2G	18.29
**5031**	allXL	AA	373	386	BS2G	21.12
**5031**	allXL	AA	319	357	BS2G	16.81
**5031**	allXL	AA	367	373	BS2G	14.45
**5031**	allXL	DD	370	382	BS2G	13.6
**5031**	allXL	CC	376	382	BS2G	13.71
**5031**	allXL	CC	357	373	BS2G	26.01
**5031**	allXL	AA	320	366	BS2G	25.3
**5031**	allXL	CC	370	376	BS2G	15.31
**5031**	allXL	AA	320	357	DBSU	19.66
**5031**	allXL	AA	357	319	DBSU	16.81
**5031**	allXL	CC	372	319	DBSU	18.14
**5031**	allXL	CC	370	357	DBSU	26.05
**5031**	allXL	CC	382	357	DBSU	28.11
**5031**	allXL	AA	372	370	DBSU	6.07
**5031**	allXL	DD	381	370	DBSU	12.45
**5031**	allXL	CC	373	370	DBSU	9.15
**5031**	allXL	DD	381	372	DBSU	16.28
**5031**	allXL	DD	372	382	DBSU	18.64
**5031**	allXL	AA	372	386	DBSU	19.42
**5031**	allXL	AA	373	386	DBSU	21.12

**5031**	allXL	BC	381	386	DBSU	35.27
**5031**	allXL	BD;DB	357	357	DBSU	18.08
**5031**	allXL	BC	373	319	DBSU	35.17
**5031**	allXL	CB	372	357	DBSU	42.96
**5031**	allXL	BC	381	357	DBSU	31.63
**5031**	allXL	BC	373	357	DBSU	44.02
**5031**	allXL	BC	372	370	DBSU	28.1
**5031**	allXL	BC	373	370	DBSU	25.0
**5031**	allXL	BC	382	373	DBSU	26.88
**5032**	allXL	AA	320	357	BS2G	18.06
**5032**	allXL	DD	320	373	BS2G	19.38
**5032**	allXL	CC	373	382	BS2G	17.58
**5032**	allXL	DD	370	386	BS2G	26.17
**5032**	allXL	CC	373	386	BS2G	25.04
**5032**	allXL	AA	319	357	BS2G	15.39
**5032**	allXL	AA	367	373	BS2G	13.87
**5032**	allXL	DD	370	382	BS2G	21.09
**5032**	allXL	CC	376	382	BS2G	14.77
**5032**	allXL	CC	357	373	BS2G	31.51
**5032**	allXL	DD	320	366	BS2G	20.7
**5032**	allXL	CC	370	376	BS2G	16.71
**5032**	allXL	AA	320	357	DBSU	18.06
**5032**	allXL	AA	357	319	DBSU	15.39
**5032**	allXL	CC	372	319	DBSU	18.82
**5032**	allXL	BB	370	357	DBSU	27.68
**5032**	allXL	BB	382	357	DBSU	42.1
**5032**	allXL	AA	372	370	DBSU	6.21
**5032**	allXL	DD	381	370	DBSU	19.34
**5032**	allXL	AA	373	370	DBSU	8.92
**5032**	allXL	DD	381	372	DBSU	13.83
**5032**	allXL	DD	372	382	DBSU	16.34
**5032**	allXL	DD	372	386	DBSU	24.39
**5032**	allXL	CC	373	386	DBSU	25.04
**5032**	allXL	DA	381	386	DBSU	44.87

**5032**	allXL	BD;DB	357	357	DBSU	18.47
**5032**	allXL	CB	373	319	DBSU	25.42
**5032**	allXL	CB	372	357	DBSU	32.24
**5032**	allXL	CB	381	357	DBSU	49.22
**5032**	allXL	CB	373	357	DBSU	34.65
**5032**	allXL	CB	372	370	DBSU	19.69
**5032**	allXL	CB	373	370	DBSU	19.57
**5032**	allXL	CB	382	373	DBSU	29.79
**5033**	allXL	CC	320	357	BS2G	24.96
**5033**	allXL	AA	320	373	BS2G	26.74
**5033**	allXL	BB	373	382	BS2G	17.72
**5033**	allXL	DD	370	386	BS2G	29.8
**5033**	allXL	BB	373	386	BS2G	27.12
**5033**	allXL	CC	319	357	BS2G	25.18
**5033**	allXL	BB	367	373	BS2G	13.34
**5033**	allXL	BB	370	382	BS2G	23.73
**5033**	allXL	BB	376	382	BS2G	14.2
**5033**	allXL	BB	357	373	BS2G	21.05
**5033**	allXL	AA	320	366	BS2G	22.45
**5033**	allXL	CC	370	376	BS2G	17.01
**5033**	allXL	CC	320	357	DBSU	24.96
**5033**	allXL	CC	357	319	DBSU	25.18
**5033**	allXL	AA	372	319	DBSU	22.92
**5033**	allXL	BB	370	357	DBSU	28.28
**5033**	allXL	BB	382	357	DBSU	19.85
**5033**	allXL	AA	372	370	DBSU	6.07
**5033**	allXL	BB	381	370	DBSU	23.25
**5033**	allXL	DD	373	370	DBSU	9.1
**5033**	allXL	BB	381	372	DBSU	20.2
**5033**	allXL	BB	372	382	DBSU	20.36
**5033**	allXL	DD	372	386	DBSU	26.89
**5033**	allXL	BB	373	386	DBSU	27.12
**5033**	allXL	DA	381	386	DBSU	54.66
**5033**	allXL	BD;DB	357	357	DBSU	18.55

**5033**	allXL	BC	373	319	DBSU	27.74
**5033**	allXL	BD	372	357	DBSU	34.66
**5033**	allXL	BD	381	357	DBSU	34.03
**5033**	allXL	BD	373	357	DBSU	33.18
**5033**	allXL	CB	372	370	DBSU	30.49
**5033**	allXL	CB	373	370	DBSU	34.29
**5033**	allXL	AD	382	373	DBSU	56.68
**5034**	allXL	CC	320	357	BS2G	26.14
**5034**	allXL	DD	320	373	BS2G	19.01
**5034**	allXL	BB	373	382	BS2G	20.16
**5034**	allXL	DD	370	386	BS2G	22.92
**5034**	allXL	CC	373	386	BS2G	24.08
**5034**	allXL	BB	319	357	BS2G	27.42
**5034**	allXL	BB	367	373	BS2G	13.16
**5034**	allXL	DD	370	382	BS2G	21.27
**5034**	allXL	CC	376	382	BS2G	13.61
**5034**	allXL	DD	357	373	BS2G	29.89
**5034**	allXL	BB	320	366	BS2G	16.07
**5034**	allXL	DD	370	376	BS2G	15.23
**5034**	allXL	CC	320	357	DBSU	26.14
**5034**	allXL	BB	357	319	DBSU	27.42
**5034**	allXL	DD	372	319	DBSU	22.64
**5034**	allXL	CC	370	357	DBSU	30.82
**5034**	allXL	BB	382	357	DBSU	17.68
**5034**	allXL	AA	372	370	DBSU	6.2
**5034**	allXL	DD	381	370	DBSU	20.27
**5034**	allXL	AA	373	370	DBSU	9.02
**5034**	allXL	BB	381	372	DBSU	20.47
**5034**	allXL	AA	372	382	DBSU	20.38
**5034**	allXL	DD	372	386	DBSU	24.95
**5034**	allXL	CC	373	386	DBSU	24.08
**5034**	allXL	AD	381	386	DBSU	42.54
**5034**	allXL	BD;DB	357	357	DBSU	19.17
**5034**	allXL	AD	373	319	DBSU	17.99

**5034**	allXL	DB	372	357	DBSU	48.21
**5034**	allXL	BD	381	357	DBSU	38.65
**5034**	allXL	DA	373	357	DBSU	46.95
**5034**	allXL	DA	372	370	DBSU	34.78
**5034**	allXL	DA	373	370	DBSU	31.21
**5034**	allXL	DA	382	373	DBSU	36.51
**5035**	allXL	CC	320	357	BS2G	25.33
**5035**	allXL	BB	320	373	BS2G	35.32
**5035**	allXL	AA	373	382	BS2G	18.18
**5035**	allXL	DD	370	386	BS2G	19.49
**5035**	allXL	DD	373	386	BS2G	21.62
**5035**	allXL	AA	319	357	BS2G	25.06
**5035**	allXL	AA	367	373	BS2G	12.35
**5035**	allXL	CC	370	382	BS2G	17.83
**5035**	allXL	CC	376	382	BS2G	14.36
**5035**	allXL	CC	357	373	BS2G	19.81
**5035**	allXL	BB	320	366	BS2G	27.04
**5035**	allXL	CC	370	376	BS2G	14.57
**5035**	allXL	CC	320	357	DBSU	25.33
**5035**	allXL	AA	357	319	DBSU	25.06
**5035**	allXL	BB	372	319	DBSU	35.28
**5035**	allXL	CC	370	357	DBSU	20.32
**5035**	allXL	BB	382	357	DBSU	21.09
**5035**	allXL	AA	372	370	DBSU	6.48
**5035**	allXL	CC	381	370	DBSU	18.63
**5035**	allXL	AA	373	370	DBSU	8.96
**5035**	allXL	DD	381	372	DBSU	16.75
**5035**	allXL	DD	372	382	DBSU	18.57
**5035**	allXL	DD	372	386	DBSU	18.59
**5035**	allXL	DD	373	386	DBSU	21.62
**5035**	allXL	CA	381	386	DBSU	38.93
**5035**	allXL	BD;DB	357	357	DBSU	17.87
**5035**	allXL	BC	373	319	DBSU	34.37
**5035**	allXL	CA	372	357	DBSU	18.6

**5035**	allXL	BD	381	357	DBSU	29.36
**5035**	allXL	CA	373	357	DBSU	17.81
**5035**	allXL	CA	372	370	DBSU	44.18
**5035**	allXL	CA	373	370	DBSU	44.96
**5035**	allXL	AC	382	373	DBSU	32.81
**5036**	allXL	AA	320	357	BS2G	25.65
**5036**	allXL	CC	320	373	BS2G	29.47
**5036**	allXL	CC	373	382	BS2G	13.93
**5036**	allXL	CC	370	386	BS2G	24.07
**5036**	allXL	CC	373	386	BS2G	20.95
**5036**	allXL	AA	319	357	BS2G	24.54
**5036**	allXL	DD	367	373	BS2G	12.9
**5036**	allXL	DD	370	382	BS2G	21.64
**5036**	allXL	CC	376	382	BS2G	13.64
**5036**	allXL	BB	357	373	BS2G	20.53
**5036**	allXL	DD	320	366	BS2G	20.74
**5036**	allXL	BB	370	376	BS2G	16.28
**5036**	allXL	AA	320	357	DBSU	25.65
**5036**	allXL	AA	357	319	DBSU	24.54
**5036**	allXL	CC	372	319	DBSU	24.45
**5036**	allXL	BB	370	357	DBSU	21.87
**5036**	allXL	DD	382	357	DBSU	33.52
**5036**	allXL	AA	372	370	DBSU	6.4
**5036**	allXL	DD	381	370	DBSU	18.47
**5036**	allXL	DD	373	370	DBSU	9.28
**5036**	allXL	CC	381	372	DBSU	17.78
**5036**	allXL	CC	372	382	DBSU	15.67
**5036**	allXL	CC	372	386	DBSU	20.81
**5036**	allXL	CC	373	386	DBSU	20.95
**5036**	allXL	BD	381	386	DBSU	42.82
**5036**	allXL	BD;DB	357	357	DBSU	19.38
**5036**	allXL	AD	373	319	DBSU	35.35
**5036**	allXL	BD	372	357	DBSU	12.91
**5036**	allXL	BD	381	357	DBSU	30.69

**5036**	allXL	BD	373	357	DBSU	12.72
**5036**	allXL	DA	372	370	DBSU	36.43
**5036**	allXL	DA	373	370	DBSU	35.63
**5036**	allXL	DB	382	373	DBSU	38.44
**5037**	allXL	AA	320	357	BS2G	24.77
**5037**	allXL	DD	320	373	BS2G	13.45
**5037**	allXL	DD	373	382	BS2G	16.71
**5037**	allXL	DD	370	386	BS2G	9.66
**5037**	allXL	DD	373	386	BS2G	18.71
**5037**	allXL	AA	319	357	BS2G	24.05
**5037**	allXL	BB	367	373	BS2G	10.37
**5037**	allXL	DD	370	382	BS2G	11.75
**5037**	allXL	BB	376	382	BS2G	14.46
**5037**	allXL	BB	357	373	BS2G	20.82
**5037**	allXL	BB	320	366	BS2G	23.76
**5037**	allXL	CC	370	376	BS2G	16.11
**5037**	allXL	AA	320	357	DBSU	24.77
**5037**	allXL	AA	357	319	DBSU	24.05
**5037**	allXL	DD	372	319	DBSU	19.86
**5037**	allXL	DD	370	357	DBSU	28.52
**5037**	allXL	BB	382	357	DBSU	37.08
**5037**	allXL	AA	372	370	DBSU	6.31
**5037**	allXL	DD	381	370	DBSU	12.69
**5037**	allXL	CC	373	370	DBSU	9.01
**5037**	allXL	DD	381	372	DBSU	16.44
**5037**	allXL	DD	372	382	DBSU	16.75
**5037**	allXL	DD	372	386	DBSU	16.51
**5037**	allXL	DD	373	386	DBSU	18.71
**5037**	allXL	CB	381	386	DBSU	25.91
**5037**	allXL	BD;DB	357	357	DBSU	18.78
**5037**	allXL	CB	373	319	DBSU	33.87
**5037**	allXL	BD	372	357	DBSU	38.9
**5037**	allXL	BD	381	357	DBSU	50.06
**5037**	allXL	BD	373	357	DBSU	37.66

**5037**	allXL	CB	372	370	DBSU	28.3
**5037**	allXL	CB	373	370	DBSU	26.17
**5037**	allXL	CB	382	373	DBSU	35.42
**5038**	allXL	AA	320	357	BS2G	13.53
**5038**	allXL	BB	320	373	BS2G	18.16
**5038**	allXL	BB	373	382	BS2G	17.83
**5038**	allXL	DD	370	386	BS2G	27.93
**5038**	allXL	BB	373	386	BS2G	27.78
**5038**	allXL	AA	319	357	BS2G	14.1
**5038**	allXL	AA	367	373	BS2G	14.39
**5038**	allXL	DD	370	382	BS2G	25.19
**5038**	allXL	BB	376	382	BS2G	13.28
**5038**	allXL	DD	357	373	BS2G	18.5
**5038**	allXL	BB	320	366	BS2G	23.87
**5038**	allXL	CC	370	376	BS2G	16.61
**5038**	allXL	AA	320	357	DBSU	13.53
**5038**	allXL	AA	357	319	DBSU	14.1
**5038**	allXL	BB	372	319	DBSU	19.54
**5038**	allXL	DD	370	357	DBSU	22.67
**5038**	allXL	DD	382	357	DBSU	27.4
**5038**	allXL	AA	372	370	DBSU	6.31
**5038**	allXL	DD	381	370	DBSU	23.05
**5038**	allXL	AA	373	370	DBSU	9.09
**5038**	allXL	DD	381	372	DBSU	20.5
**5038**	allXL	BB	372	382	DBSU	20.56
**5038**	allXL	DD	372	386	DBSU	27.22
**5038**	allXL	BB	373	386	DBSU	27.78
**5038**	allXL	CB	381	386	DBSU	59.61
**5038**	allXL	BD;DB	357	357	DBSU	18.57
**5038**	allXL	AD	373	319	DBSU	24.26
**5038**	allXL	BC	372	357	DBSU	23.01
**5038**	allXL	DB	381	357	DBSU	27.86
**5038**	allXL	BC	373	357	DBSU	22.99
**5038**	allXL	BC	372	370	DBSU	32.92

**5038**	allXL	BC	373	370	DBSU	32.73
**5038**	allXL	BC	382	373	DBSU	38.35
**5039**	allXL	AA	320	357	BS2G	22.51
**5039**	allXL	CC	320	373	BS2G	32.63
**5039**	allXL	AA	373	382	BS2G	18.37
**5039**	allXL	DD	370	386	BS2G	25.31
**5039**	allXL	DD	373	386	BS2G	18.07
**5039**	allXL	AA	319	357	BS2G	21.26
**5039**	allXL	AA	367	373	BS2G	12.34
**5039**	allXL	AA	370	382	BS2G	21.74
**5039**	allXL	AA	376	382	BS2G	14.5
**5039**	allXL	CC	357	373	BS2G	27.11
**5039**	allXL	CC	320	366	BS2G	15.85
**5039**	allXL	DD	370	376	BS2G	13.23
**5039**	allXL	AA	320	357	DBSU	22.51
**5039**	allXL	AA	357	319	DBSU	21.26
**5039**	allXL	CC	372	319	DBSU	30.94
**5039**	allXL	CC	370	357	DBSU	24.85
**5039**	allXL	DD	382	357	DBSU	37.09
**5039**	allXL	AA	372	370	DBSU	6.25
**5039**	allXL	DD	381	370	DBSU	21.17
**5039**	allXL	AA	373	370	DBSU	8.98
**5039**	allXL	AA	381	372	DBSU	18.57
**5039**	allXL	AA	372	382	DBSU	17.19
**5039**	allXL	DD	372	386	DBSU	20.89
**5039**	allXL	DD	373	386	DBSU	18.07
**5039**	allXL	CA	381	386	DBSU	57.86
**5039**	allXL	BD;DB	357	357	DBSU	20.07
**5039**	allXL	AD	373	319	DBSU	19.19
**5039**	allXL	AC	372	357	DBSU	36.8
**5039**	allXL	AD	381	357	DBSU	48.27
**5039**	allXL	AC	373	357	DBSU	39.66
**5039**	allXL	CA	372	370	DBSU	47.94
**5039**	allXL	AD	373	370	DBSU	48.54

**5039**	allXL	DA	382	373	DBSU	46.88
**5040**	allXL	CC	320	357	BS2G	26.93
**5040**	allXL	BB	320	373	BS2G	23.47
**5040**	allXL	BB	373	382	BS2G	17.68
**5040**	allXL	BB	370	386	BS2G	19.88
**5040**	allXL	BB	373	386	BS2G	20.48
**5040**	allXL	AA	319	357	BS2G	27.47
**5040**	allXL	AA	367	373	BS2G	10.49
**5040**	allXL	DD	370	382	BS2G	17.55
**5040**	allXL	CC	376	382	BS2G	14.56
**5040**	allXL	DD	357	373	BS2G	21.76
**5040**	allXL	CC	320	366	BS2G	25.21
**5040**	allXL	DD	370	376	BS2G	17.04
**5040**	allXL	CC	320	357	DBSU	26.93
**5040**	allXL	AA	357	319	DBSU	27.47
**5040**	allXL	BB	372	319	DBSU	28.9
**5040**	allXL	BB	370	357	DBSU	18.92
**5040**	allXL	DD	382	357	DBSU	24.84
**5040**	allXL	CC	372	370	DBSU	6.64
**5040**	allXL	DD	381	370	DBSU	19.26
**5040**	allXL	AA	373	370	DBSU	9.26
**5040**	allXL	BB	381	372	DBSU	15.03
**5040**	allXL	BB	372	382	DBSU	17.01
**5040**	allXL	BB	372	386	DBSU	17.67
**5040**	allXL	BB	373	386	DBSU	20.48
**5040**	allXL	BC	381	386	DBSU	37.9
**5040**	allXL	BD;DB	357	357	DBSU	18.98
**5040**	allXL	AD	373	319	DBSU	20.65
**5040**	allXL	DB	372	357	DBSU	34.32
**5040**	allXL	DB	381	357	DBSU	38.66
**5040**	allXL	DB	373	357	DBSU	31.75
**5040**	allXL	DB	372	370	DBSU	39.2
**5040**	allXL	DB	373	370	DBSU	35.56
**5040**	allXL	CB	382	373	DBSU	44.55

**5041**	allXL	AA	320	357	BS2G	25.53
**5041**	allXL	DD	320	373	BS2G	24.96
**5041**	allXL	CC	373	382	BS2G	15.17
**5041**	allXL	DD	370	386	BS2G	17.5
**5041**	allXL	DD	373	386	BS2G	19.55
**5041**	allXL	AA	319	357	BS2G	27.41
**5041**	allXL	AA	367	373	BS2G	13.64
**5041**	allXL	DD	370	382	BS2G	19.44
**5041**	allXL	BB	376	382	BS2G	14.27
**5041**	allXL	AA	357	373	BS2G	31.08
**5041**	allXL	DD	320	366	BS2G	21.67
**5041**	allXL	DD	370	376	BS2G	16.33
**5041**	allXL	AA	320	357	DBSU	25.53
**5041**	allXL	AA	357	319	DBSU	27.41
**5041**	allXL	DD	372	319	DBSU	22.54
**5041**	allXL	DD	370	357	DBSU	29.82
**5041**	allXL	CC	382	357	DBSU	28.69
**5041**	allXL	CC	372	370	DBSU	6.29
**5041**	allXL	DD	381	370	DBSU	19.79
**5041**	allXL	CC	373	370	DBSU	9.15
**5041**	allXL	CC	381	372	DBSU	17.94
**5041**	allXL	CC	372	382	DBSU	16.52
**5041**	allXL	DD	372	386	DBSU	17.23
**5041**	allXL	DD	373	386	DBSU	19.55
**5041**	allXL	CB	381	386	DBSU	37.86
**5041**	allXL	BD;DB	357	357	DBSU	18.43
**5041**	allXL	CB	373	319	DBSU	17.02
**5041**	allXL	CB	372	357	DBSU	40.31
**5041**	allXL	CB	381	357	DBSU	27.12
**5041**	allXL	CB	373	357	DBSU	38.79
**5041**	allXL	DA	372	370	DBSU	42.37
**5041**	allXL	DA	373	370	DBSU	40.67
**5041**	allXL	AD	382	373	DBSU	46.06
**5042**	allXL	CC	320	357	BS2G	23.47

**5042**	allXL	DD	320	373	BS2G	19.31
**5042**	allXL	AA	373	382	BS2G	17.07
**5042**	allXL	AA	370	386	BS2G	25.55
**5042**	allXL	AA	373	386	BS2G	23.25
**5042**	allXL	AA	319	357	BS2G	24.88
**5042**	allXL	AA	367	373	BS2G	10.67
**5042**	allXL	BB	370	382	BS2G	21.4
**5042**	allXL	BB	376	382	BS2G	14.28
**5042**	allXL	CC	357	373	BS2G	28.44
**5042**	allXL	CC	320	366	BS2G	13.37
**5042**	allXL	CC	370	376	BS2G	14.26
**5042**	allXL	CC	320	357	DBSU	23.47
**5042**	allXL	AA	357	319	DBSU	24.88
**5042**	allXL	DD	372	319	DBSU	17.35
**5042**	allXL	CC	370	357	DBSU	23.41
**5042**	allXL	BB	382	357	DBSU	17.36
**5042**	allXL	AA	372	370	DBSU	6.51
**5042**	allXL	DD	381	370	DBSU	20.22
**5042**	allXL	AA	373	370	DBSU	9.07
**5042**	allXL	DD	381	372	DBSU	19.73
**5042**	allXL	AA	372	382	DBSU	20.39
**5042**	allXL	AA	372	386	DBSU	25.35
**5042**	allXL	AA	373	386	DBSU	23.25
**5042**	allXL	CA	381	386	DBSU	49.27
**5042**	allXL	BD;DB	357	357	DBSU	18.45
**5042**	allXL	AD	373	319	DBSU	34.75
**5042**	allXL	CA	372	357	DBSU	41.17
**5042**	allXL	BD	381	357	DBSU	31.97
**5042**	allXL	CA	373	357	DBSU	39.85
**5042**	allXL	AD	372	370	DBSU	41.59
**5042**	allXL	AD	373	370	DBSU	40.61
**5042**	allXL	AC	382	373	DBSU	38.22
**5043**	allXL	AA	320	357	BS2G	24.97
**5043**	allXL	BB	320	373	BS2G	19.88

**5043**	allXL	DD	373	382	BS2G	19.08
**5043**	allXL	CC	370	386	BS2G	16.68
**5043**	allXL	CC	373	386	BS2G	22.71
**5043**	allXL	AA	319	357	BS2G	24.37
**5043**	allXL	AA	367	373	BS2G	12.62
**5043**	allXL	CC	370	382	BS2G	19.12
**5043**	allXL	BB	376	382	BS2G	13.8
**5043**	allXL	BB	357	373	BS2G	17.33
**5043**	allXL	CC	320	366	BS2G	26.87
**5043**	allXL	CC	370	376	BS2G	16.0
**5043**	allXL	AA	320	357	DBSU	24.97
**5043**	allXL	AA	357	319	DBSU	24.37
**5043**	allXL	BB	372	319	DBSU	26.42
**5043**	allXL	BB	370	357	DBSU	25.18
**5043**	allXL	BB	382	357	DBSU	19.1
**5043**	allXL	AA	372	370	DBSU	6.27
**5043**	allXL	CC	381	370	DBSU	21.86
**5043**	allXL	AA	373	370	DBSU	9.1
**5043**	allXL	DD	381	372	DBSU	21.26
**5043**	allXL	CC	372	382	DBSU	20.26
**5043**	allXL	CC	372	386	DBSU	20.34
**5043**	allXL	CC	373	386	DBSU	22.71
**5043**	allXL	BC	381	386	DBSU	67.38
**5043**	allXL	BD;DB	357	357	DBSU	18.17
**5043**	allXL	AD	373	319	DBSU	16.53
**5043**	allXL	BD	372	357	DBSU	36.33
**5043**	allXL	BD	381	357	DBSU	31.47
**5043**	allXL	BD	373	357	DBSU	35.24
**5043**	allXL	CB	372	370	DBSU	27.32
**5043**	allXL	CB	373	370	DBSU	25.82
**5043**	allXL	BD	382	373	DBSU	48.65
**5044**	allXL	AA	320	357	BS2G	26.62
**5044**	allXL	DD	320	373	BS2G	19.19
**5044**	allXL	CC	373	382	BS2G	17.25

**5044**	allXL	CC	370	386	BS2G	15.03
**5044**	allXL	CC	373	386	BS2G	13.44
**5044**	allXL	AA	319	357	BS2G	25.79
**5044**	allXL	BB	367	373	BS2G	12.89
**5044**	allXL	DD	370	382	BS2G	19.83
**5044**	allXL	CC	376	382	BS2G	12.99
**5044**	allXL	BB	357	373	BS2G	30.58
**5044**	allXL	DD	320	366	BS2G	23.47
**5044**	allXL	CC	370	376	BS2G	14.28
**5044**	allXL	AA	320	357	DBSU	26.62
**5044**	allXL	AA	357	319	DBSU	25.79
**5044**	allXL	DD	372	319	DBSU	16.8
**5044**	allXL	DD	370	357	DBSU	24.19
**5044**	allXL	BB	382	357	DBSU	19.39
**5044**	allXL	AA	372	370	DBSU	6.07
**5044**	allXL	DD	381	370	DBSU	17.31
**5044**	allXL	CC	373	370	DBSU	8.88
**5044**	allXL	DD	381	372	DBSU	19.23
**5044**	allXL	BB	372	382	DBSU	18.99
**5044**	allXL	CC	372	386	DBSU	14.85
**5044**	allXL	CC	373	386	DBSU	13.44
**5044**	allXL	BC	381	386	DBSU	48.83
**5044**	allXL	BD;DB	357	357	DBSU	18.53
**5044**	allXL	DA	373	319	DBSU	31.68
**5044**	allXL	DA	372	357	DBSU	42.08
**5044**	allXL	BD	381	357	DBSU	35.63
**5044**	allXL	BC	373	357	DBSU	44.73
**5044**	allXL	CB	372	370	DBSU	37.3
**5044**	allXL	DA	373	370	DBSU	38.25
**5044**	allXL	CB	382	373	DBSU	44.9
**5045**	allXL	CC	320	357	BS2G	21.4
**5045**	allXL	DD	320	373	BS2G	14.68
**5045**	allXL	CC	373	382	BS2G	15.35
**5045**	allXL	BB	370	386	BS2G	26.91

**5045**	allXL	CC	373	386	BS2G	21.69
**5045**	allXL	CC	319	357	BS2G	20.11
**5045**	allXL	BB	367	373	BS2G	13.4
**5045**	allXL	BB	370	382	BS2G	20.21
**5045**	allXL	BB	376	382	BS2G	13.77
**5045**	allXL	CC	357	373	BS2G	27.33
**5045**	allXL	DD	320	366	BS2G	21.88
**5045**	allXL	DD	370	376	BS2G	16.68
**5045**	allXL	CC	320	357	DBSU	21.4
**5045**	allXL	CC	357	319	DBSU	20.11
**5045**	allXL	DD	372	319	DBSU	13.55
**5045**	allXL	DD	370	357	DBSU	23.16
**5045**	allXL	CC	382	357	DBSU	35.28
**5045**	allXL	AA	372	370	DBSU	6.25
**5045**	allXL	BB	381	370	DBSU	22.44
**5045**	allXL	AA	373	370	DBSU	8.85
**5045**	allXL	CC	381	372	DBSU	19.33
**5045**	allXL	CC	372	382	DBSU	18.05
**5045**	allXL	CC	372	386	DBSU	22.98
**5045**	allXL	CC	373	386	DBSU	21.69
**5045**	allXL	DA	381	386	DBSU	40.42
**5045**	allXL	BD;DB	357	357	DBSU	18.37
**5045**	allXL	AD	373	319	DBSU	14.02
**5045**	allXL	CA	372	357	DBSU	31.11
**5045**	allXL	CA	381	357	DBSU	32.45
**5045**	allXL	CA	373	357	DBSU	32.88
**5045**	allXL	DA	372	370	DBSU	21.99
**5045**	allXL	DA	373	370	DBSU	25.37
**5045**	allXL	AD	382	373	DBSU	33.83
**5046**	allXL	AA	320	357	BS2G	24.81
**5046**	allXL	BB	320	373	BS2G	30.49
**5046**	allXL	CC	373	382	BS2G	21.81
**5046**	allXL	BB	370	386	BS2G	34.67
**5046**	allXL	DD	373	386	BS2G	29.56

**5046**	allXL	AA	319	357	BS2G	23.63
**5046**	allXL	AA	367	373	BS2G	13.59
**5046**	allXL	BB	370	382	BS2G	26.18
**5046**	allXL	CC	376	382	BS2G	14.4
**5046**	allXL	BB	357	373	BS2G	38.06
**5046**	allXL	AA	320	366	BS2G	25.16
**5046**	allXL	DD	370	376	BS2G	14.57
**5046**	allXL	AA	320	357	DBSU	24.81
**5046**	allXL	AA	357	319	DBSU	23.63
**5046**	allXL	BB	372	319	DBSU	29.12
**5046**	allXL	DD	370	357	DBSU	30.05
**5046**	allXL	DD	382	357	DBSU	50.6
**5046**	allXL	AA	372	370	DBSU	6.21
**5046**	allXL	DD	381	370	DBSU	25.05
**5046**	allXL	AA	373	370	DBSU	9.18
**5046**	allXL	DD	381	372	DBSU	23.81
**5046**	allXL	DD	372	382	DBSU	25.44
**5046**	allXL	DD	372	386	DBSU	33.11
**5046**	allXL	DD	373	386	DBSU	29.56
**5046**	allXL	DA	381	386	DBSU	37.24
**5046**	allXL	BD;DB	357	357	DBSU	20.04
**5046**	allXL	AD	373	319	DBSU	29.53
**5046**	allXL	DA	372	357	DBSU	50.26
**5046**	allXL	DA	381	357	DBSU	56.06
**5046**	allXL	DA	373	357	DBSU	52.59
**5046**	allXL	DA	372	370	DBSU	27.26
**5046**	allXL	DA	373	370	DBSU	26.85
**5046**	allXL	DA	382	373	DBSU	26.07
**5047**	allXL	CC	320	357	BS2G	22.31
**5047**	allXL	AA	320	373	BS2G	15.69
**5047**	allXL	AA	373	382	BS2G	21.82
**5047**	allXL	DD	370	386	BS2G	32.95
**5047**	allXL	DD	373	386	BS2G	29.88
**5047**	allXL	CC	319	357	BS2G	24.17

**5047**	allXL	BB	367	373	BS2G	14.13
**5047**	allXL	AA	370	382	BS2G	26.03
**5047**	allXL	BB	376	382	BS2G	15.46
**5047**	allXL	AA	357	373	BS2G	34.79
**5047**	allXL	AA	320	366	BS2G	14.04
**5047**	allXL	AA	370	376	BS2G	16.38
**5047**	allXL	CC	320	357	DBSU	22.31
**5047**	allXL	CC	357	319	DBSU	24.17
**5047**	allXL	AA	372	319	DBSU	17.79
**5047**	allXL	AA	370	357	DBSU	29.16
**5047**	allXL	AA	382	357	DBSU	40.98
**5047**	allXL	CC	372	370	DBSU	6.45
**5047**	allXL	DD	381	370	DBSU	25.69
**5047**	allXL	AA	373	370	DBSU	7.46
**5047**	allXL	DD	381	372	DBSU	19.04
**5047**	allXL	DD	372	382	DBSU	22.22
**5047**	allXL	DD	372	386	DBSU	28.43
**5047**	allXL	DD	373	386	DBSU	29.88
**5047**	allXL	CB	381	386	DBSU	43.38
**5047**	allXL	BD;DB	357	357	DBSU	18.42
**5047**	allXL	CB	373	319	DBSU	32.56
**5047**	allXL	AD	372	357	DBSU	48.94
**5047**	allXL	AC	381	357	DBSU	63.42
**5047**	allXL	AD	373	357	DBSU	51.12
**5047**	allXL	CB	372	370	DBSU	40.46
**5047**	allXL	CB	373	370	DBSU	38.25
**5047**	allXL	BC	382	373	DBSU	43.29
**5048**	allXL	AA	320	357	BS2G	27.3
**5048**	allXL	BB	320	373	BS2G	26.44
**5048**	allXL	DD	373	382	BS2G	22.54
**5048**	allXL	BB	370	386	BS2G	30.92
**5048**	allXL	BB	373	386	BS2G	31.77
**5048**	allXL	AA	319	357	BS2G	28.17
**5048**	allXL	AA	367	373	BS2G	14.82

**5048**	allXL	BB	370	382	BS2G	24.12
**5048**	allXL	CC	376	382	BS2G	15.24
**5048**	allXL	BB	357	373	BS2G	31.93
**5048**	allXL	BB	320	366	BS2G	21.24
**5048**	allXL	CC	370	376	BS2G	14.99
**5048**	allXL	AA	320	357	DBSU	27.3
**5048**	allXL	AA	357	319	DBSU	28.17
**5048**	allXL	DD	372	319	DBSU	24.28
**5048**	allXL	DD	370	357	DBSU	25.74
**5048**	allXL	DD	382	357	DBSU	48.45
**5048**	allXL	AA	372	370	DBSU	6.03
**5048**	allXL	DD	381	370	DBSU	23.47
**5048**	allXL	AA	373	370	DBSU	9.27
**5048**	allXL	DD	381	372	DBSU	21.19
**5048**	allXL	DD	372	382	DBSU	22.97
**5048**	allXL	DD	372	386	DBSU	33.18
**5048**	allXL	BB	373	386	DBSU	31.77
**5048**	allXL	BC	381	386	DBSU	21.19
**5048**	allXL	BD;DB	357	357	DBSU	18.45
**5048**	allXL	AD	373	319	DBSU	14.9
**5048**	allXL	DA	372	357	DBSU	40.65
**5048**	allXL	CB	381	357	DBSU	50.79
**5048**	allXL	DA	373	357	DBSU	42.69
**5048**	allXL	DA	372	370	DBSU	33.56
**5048**	allXL	DA	373	370	DBSU	32.07
**5048**	allXL	CB	382	373	DBSU	32.45
**5049**	allXL	CC	320	357	BS2G	25.73
**5049**	allXL	CC	320	373	BS2G	14.99
**5049**	allXL	CC	373	382	BS2G	18.17
**5049**	allXL	DD	370	386	BS2G	13.72
**5049**	allXL	DD	373	386	BS2G	17.02
**5049**	allXL	AA	319	357	BS2G	25.48
**5049**	allXL	AA	367	373	BS2G	11.25
**5049**	allXL	DD	370	382	BS2G	16.18

**5049**	allXL	CC	376	382	BS2G	13.16
**5049**	allXL	BB	357	373	BS2G	20.05
**5049**	allXL	CC	320	366	BS2G	17.94
**5049**	allXL	DD	370	376	BS2G	14.94
**5049**	allXL	CC	320	357	DBSU	25.73
**5049**	allXL	AA	357	319	DBSU	25.48
**5049**	allXL	CC	372	319	DBSU	12.1
**5049**	allXL	BB	370	357	DBSU	27.55
**5049**	allXL	DD	382	357	DBSU	18.37
**5049**	allXL	CC	372	370	DBSU	6.46
**5049**	allXL	DD	381	370	DBSU	15.83
**5049**	allXL	AA	373	370	DBSU	9.05
**5049**	allXL	DD	381	372	DBSU	18.49
**5049**	allXL	DD	372	382	DBSU	19.04
**5049**	allXL	DD	372	386	DBSU	15.9
**5049**	allXL	DD	373	386	DBSU	17.02
**5049**	allXL	AC	381	386	DBSU	44.46
**5049**	allXL	BD;DB	357	357	DBSU	19.19
**5049**	allXL	AD	373	319	DBSU	34.88
**5049**	allXL	BD	372	357	DBSU	39.8
**5049**	allXL	DB	381	357	DBSU	37.98
**5049**	allXL	BD	373	357	DBSU	39.21
**5049**	allXL	CA	372	370	DBSU	58.8
**5049**	allXL	CA	373	370	DBSU	55.37
**5049**	allXL	DA	382	373	DBSU	51.27
**5050**	allXL	AA	320	357	BS2G	22.83
**5050**	allXL	BB	320	373	BS2G	16.74
**5050**	allXL	CC	373	382	BS2G	20.51
**5050**	allXL	CC	370	386	BS2G	28.98
**5050**	allXL	AA	373	386	BS2G	25.29
**5050**	allXL	AA	319	357	BS2G	23.61
**5050**	allXL	BB	367	373	BS2G	13.21
**5050**	allXL	CC	370	382	BS2G	22.85
**5050**	allXL	CC	376	382	BS2G	14.45

**5050**	allXL	CC	357	373	BS2G	30.68
**5050**	allXL	CC	320	366	BS2G	12.47
**5050**	allXL	CC	370	376	BS2G	14.89
**5050**	allXL	AA	320	357	DBSU	22.83
**5050**	allXL	AA	357	319	DBSU	23.61
**5050**	allXL	BB	372	319	DBSU	18.48
**5050**	allXL	CC	370	357	DBSU	27.66
**5050**	allXL	CC	382	357	DBSU	27.92
**5050**	allXL	AA	372	370	DBSU	6.26
**5050**	allXL	DD	381	370	DBSU	20.77
**5050**	allXL	AA	373	370	DBSU	9.32
**5050**	allXL	DD	381	372	DBSU	19.16
**5050**	allXL	DD	372	382	DBSU	22.32
**5050**	allXL	AA	372	386	DBSU	27.02
**5050**	allXL	AA	373	386	DBSU	25.29
**5050**	allXL	BC	381	386	DBSU	49.21
**5050**	allXL	BD;DB	357	357	DBSU	19.38
**5050**	allXL	AD	373	319	DBSU	31.26
**5050**	allXL	BC	372	357	DBSU	39.02
**5050**	allXL	BD	381	357	DBSU	50.89
**5050**	allXL	BC	373	357	DBSU	40.63
**5050**	allXL	DA	372	370	DBSU	32.54
**5050**	allXL	DA	373	370	DBSU	30.03
**5050**	allXL	CB	382	373	DBSU	32.1
**5051**	allXL	AA	320	357	BS2G	22.88
**5051**	allXL	CC	320	373	BS2G	17.39
**5051**	allXL	BB	373	382	BS2G	17.48
**5051**	allXL	DD	370	386	BS2G	19.43
**5051**	allXL	AA	373	386	BS2G	23.47
**5051**	allXL	AA	319	357	BS2G	21.91
**5051**	allXL	AA	367	373	BS2G	14.18
**5051**	allXL	DD	370	382	BS2G	18.94
**5051**	allXL	BB	376	382	BS2G	14.53
**5051**	allXL	BB	357	373	BS2G	26.79

**5051**	allXL	CC	320	366	BS2G	10.06
**5051**	allXL	CC	370	376	BS2G	13.4
**5051**	allXL	AA	320	357	DBSU	22.88
**5051**	allXL	AA	357	319	DBSU	21.91
**5051**	allXL	CC	372	319	DBSU	11.91
**5051**	allXL	DD	370	357	DBSU	26.55
**5051**	allXL	BB	382	357	DBSU	15.44
**5051**	allXL	AA	372	370	DBSU	6.16
**5051**	allXL	DD	381	370	DBSU	18.04
**5051**	allXL	AA	373	370	DBSU	9.26
**5051**	allXL	DD	381	372	DBSU	18.58
**5051**	allXL	BB	372	382	DBSU	18.49
**5051**	allXL	AA	372	386	DBSU	24.52
**5051**	allXL	AA	373	386	DBSU	23.47
**5051**	allXL	CA	381	386	DBSU	53.02
**5051**	allXL	BD;DB	357	357	DBSU	18.18
**5051**	allXL	AD	373	319	DBSU	16.95
**5051**	allXL	AD	372	357	DBSU	37.82
**5051**	allXL	BD	381	357	DBSU	30.36
**5051**	allXL	AD	373	357	DBSU	39.93
**5051**	allXL	DA	372	370	DBSU	26.15
**5051**	allXL	DA	373	370	DBSU	27.34
**5051**	allXL	AC	382	373	DBSU	35.25
**5052**	allXL	CC	320	357	BS2G	22.41
**5052**	allXL	CC	320	373	BS2G	31.67
**5052**	allXL	BB	373	382	BS2G	15.0
**5052**	allXL	CC	370	386	BS2G	18.25
**5052**	allXL	BB	373	386	BS2G	22.98
**5052**	allXL	CC	319	357	BS2G	20.89
**5052**	allXL	BB	367	373	BS2G	13.76
**5052**	allXL	CC	370	382	BS2G	13.63
**5052**	allXL	BB	376	382	BS2G	13.14
**5052**	allXL	BB	357	373	BS2G	31.19
**5052**	allXL	CC	320	366	BS2G	23.05

**5052**	allXL	CC	370	376	BS2G	13.71
**5052**	allXL	CC	320	357	DBSU	22.41
**5052**	allXL	CC	357	319	DBSU	20.89
**5052**	allXL	CC	372	319	DBSU	29.28
**5052**	allXL	DD	370	357	DBSU	27.78
**5052**	allXL	CC	382	357	DBSU	24.1
**5052**	allXL	AA	372	370	DBSU	5.97
**5052**	allXL	DD	381	370	DBSU	14.63
**5052**	allXL	AA	373	370	DBSU	9.02
**5052**	allXL	DD	381	372	DBSU	14.41
**5052**	allXL	BB	372	382	DBSU	15.75
**5052**	allXL	BB	372	386	DBSU	21.93
**5052**	allXL	BB	373	386	DBSU	22.98
**5052**	allXL	CB	381	386	DBSU	39.92
**5052**	allXL	BD;DB	357	357	DBSU	17.91
**5052**	allXL	CB	373	319	DBSU	21.54
**5052**	allXL	DB	372	357	DBSU	35.15
**5052**	allXL	CB	381	357	DBSU	28.33
**5052**	allXL	CB	373	357	DBSU	34.7
**5052**	allXL	BC	372	370	DBSU	46.58
**5052**	allXL	BC	373	370	DBSU	43.68
**5052**	allXL	BC	382	373	DBSU	27.76
**5053**	allXL	AA	320	357	BS2G	26.76
**5053**	allXL	BB	320	373	BS2G	25.72
**5053**	allXL	BB	373	382	BS2G	14.66
**5053**	allXL	CC	370	386	BS2G	18.44
**5053**	allXL	AA	373	386	BS2G	17.3
**5053**	allXL	AA	319	357	BS2G	27.85
**5053**	allXL	AA	367	373	BS2G	10.82
**5053**	allXL	BB	370	382	BS2G	17.87
**5053**	allXL	BB	376	382	BS2G	12.35
**5053**	allXL	BB	357	373	BS2G	12.64
**5053**	allXL	AA	320	366	BS2G	28.73
**5053**	allXL	DD	370	376	BS2G	15.91

**5053**	allXL	AA	320	357	DBSU	26.76
**5053**	allXL	AA	357	319	DBSU	27.85
**5053**	allXL	BB	372	319	DBSU	32.03
**5053**	allXL	BB	370	357	DBSU	20.81
**5053**	allXL	BB	382	357	DBSU	16.44
**5053**	allXL	AA	372	370	DBSU	6.71
**5053**	allXL	DD	381	370	DBSU	19.62
**5053**	allXL	AA	373	370	DBSU	8.81
**5053**	allXL	BB	381	372	DBSU	18.56
**5053**	allXL	BB	372	382	DBSU	17.01
**5053**	allXL	AA	372	386	DBSU	18.25
**5053**	allXL	AA	373	386	DBSU	17.3
**5053**	allXL	DB	381	386	DBSU	17.32
**5053**	allXL	BD;DB	357	357	DBSU	18.76
**5053**	allXL	CB	373	319	DBSU	32.47
**5053**	allXL	BD	372	357	DBSU	31.05
**5053**	allXL	BD	381	357	DBSU	19.38
**5053**	allXL	BD	373	357	DBSU	29.47
**5053**	allXL	BC	372	370	DBSU	44.9
**5053**	allXL	BC	373	370	DBSU	44.52
**5053**	allXL	DB	382	373	DBSU	34.97
**5054**	allXL	CC	320	357	BS2G	25.34
**5054**	allXL	DD	320	373	BS2G	19.62
**5054**	allXL	BB	373	382	BS2G	17.87
**5054**	allXL	DD	370	386	BS2G	27.9
**5054**	allXL	BB	373	386	BS2G	28.76
**5054**	allXL	AA	319	357	BS2G	27.94
**5054**	allXL	BB	367	373	BS2G	13.43
**5054**	allXL	DD	370	382	BS2G	20.12
**5054**	allXL	BB	376	382	BS2G	13.74
**5054**	allXL	CC	357	373	BS2G	25.94
**5054**	allXL	CC	320	366	BS2G	23.01
**5054**	allXL	CC	370	376	BS2G	16.64
**5054**	allXL	CC	320	357	DBSU	25.34

**5054**	allXL	AA	357	319	DBSU	27.94
**5054**	allXL	DD	372	319	DBSU	19.76
**5054**	allXL	BB	370	357	DBSU	24.95
**5054**	allXL	CC	382	357	DBSU	26.64
**5054**	allXL	AA	372	370	DBSU	6.42
**5054**	allXL	DD	381	370	DBSU	20.06
**5054**	allXL	CC	373	370	DBSU	9.2
**5054**	allXL	BB	381	372	DBSU	19.82
**5054**	allXL	BB	372	382	DBSU	21.46
**5054**	allXL	DD	372	386	DBSU	30.59
**5054**	allXL	BB	373	386	DBSU	28.76
**5054**	allXL	DA	381	386	DBSU	69.86
**5054**	allXL	BD;DB	357	357	DBSU	18.79
**5054**	allXL	CB	373	319	DBSU	19.89
**5054**	allXL	CA	372	357	DBSU	40.34
**5054**	allXL	CA	381	357	DBSU	33.63
**5054**	allXL	CA	373	357	DBSU	40.68
**5054**	allXL	DA	372	370	DBSU	26.85
**5054**	allXL	DA	373	370	DBSU	27.74
**5054**	allXL	BC	382	373	DBSU	47.46
**5055**	allXL	AA	320	357	BS2G	24.82
**5055**	allXL	BB	320	373	BS2G	18.03
**5055**	allXL	DD	373	382	BS2G	19.04
**5055**	allXL	DD	370	386	BS2G	26.57
**5055**	allXL	DD	373	386	BS2G	21.81
**5055**	allXL	AA	319	357	BS2G	24.94
**5055**	allXL	BB	367	373	BS2G	11.87
**5055**	allXL	CC	370	382	BS2G	25.12
**5055**	allXL	BB	376	382	BS2G	13.47
**5055**	allXL	CC	357	373	BS2G	16.6
**5055**	allXL	BB	320	366	BS2G	16.25
**5055**	allXL	CC	370	376	BS2G	13.89
**5055**	allXL	AA	320	357	DBSU	24.82
**5055**	allXL	AA	357	319	DBSU	24.94

**5055**	allXL	BB	372	319	DBSU	21.47
**5055**	allXL	CC	370	357	DBSU	18.77
**5055**	allXL	BB	382	357	DBSU	26.87
**5055**	allXL	AA	372	370	DBSU	6.34
**5055**	allXL	DD	381	370	DBSU	24.66
**5055**	allXL	DD	373	370	DBSU	8.95
**5055**	allXL	DD	381	372	DBSU	17.28
**5055**	allXL	DD	372	382	DBSU	19.26
**5055**	allXL	DD	372	386	DBSU	20.12
**5055**	allXL	DD	373	386	DBSU	21.81
**5055**	allXL	BC	381	386	DBSU	71.53
**5055**	allXL	BD;DB	357	357	DBSU	18.79
**5055**	allXL	AD	373	319	DBSU	33.07
**5055**	allXL	CA	372	357	DBSU	26.37
**5055**	allXL	CA	381	357	DBSU	32.28
**5055**	allXL	CA	373	357	DBSU	23.61
**5055**	allXL	DA	372	370	DBSU	46.45
**5055**	allXL	DA	373	370	DBSU	43.84
**5055**	allXL	DA	382	373	DBSU	64.27
**5056**	allXL	CC	320	357	BS2G	25.49
**5056**	allXL	CC	320	373	BS2G	19.9
**5056**	allXL	DD	373	382	BS2G	14.81
**5056**	allXL	DD	370	386	BS2G	22.81
**5056**	allXL	CC	373	386	BS2G	21.41
**5056**	allXL	AA	319	357	BS2G	28.51
**5056**	allXL	AA	367	373	BS2G	12.83
**5056**	allXL	DD	370	382	BS2G	18.39
**5056**	allXL	CC	376	382	BS2G	13.48
**5056**	allXL	DD	357	373	BS2G	26.38
**5056**	allXL	BB	320	366	BS2G	20.63
**5056**	allXL	CC	370	376	BS2G	16.74
**5056**	allXL	CC	320	357	DBSU	25.49
**5056**	allXL	AA	357	319	DBSU	28.51
**5056**	allXL	DD	372	319	DBSU	21.05

**5056**	allXL	DD	370	357	DBSU	26.77
**5056**	allXL	AA	382	357	DBSU	25.77
**5056**	allXL	AA	372	370	DBSU	6.29
**5056**	allXL	DD	381	370	DBSU	18.1
**5056**	allXL	CC	373	370	DBSU	8.79
**5056**	allXL	DD	381	372	DBSU	13.37
**5056**	allXL	DD	372	382	DBSU	13.71
**5056**	allXL	DD	372	386	DBSU	20.39
**5056**	allXL	CC	373	386	DBSU	21.41
**5056**	allXL	DA	381	386	DBSU	25.2
**5056**	allXL	AC;CA	357	357	DBSU	19.48
**5056**	allXL	DA	373	319	DBSU	25.48
**5056**	allXL	DA	372	357	DBSU	32.5
**5056**	allXL	DA	381	357	DBSU	25.29
**5056**	allXL	DA	373	357	DBSU	29.58
**5056**	allXL	DA	372	370	DBSU	20.72
**5056**	allXL	DA	373	370	DBSU	22.08
**5056**	allXL	DA	382	373	DBSU	15.99
**5057**	allXL	AA	320	357	BS2G	21.24
**5057**	allXL	BB	320	373	BS2G	20.74
**5057**	allXL	AA	373	382	BS2G	19.62
**5057**	allXL	AA	370	386	BS2G	24.39
**5057**	allXL	BB	373	386	BS2G	22.09
**5057**	allXL	AA	319	357	BS2G	18.46
**5057**	allXL	AA	367	373	BS2G	13.71
**5057**	allXL	AA	370	382	BS2G	22.93
**5057**	allXL	CC	376	382	BS2G	14.27
**5057**	allXL	AA	357	373	BS2G	30.39
**5057**	allXL	CC	320	366	BS2G	19.53
**5057**	allXL	CC	370	376	BS2G	15.93
**5057**	allXL	AA	320	357	DBSU	21.24
**5057**	allXL	AA	357	319	DBSU	18.46
**5057**	allXL	BB	372	319	DBSU	22.86
**5057**	allXL	DD	370	357	DBSU	21.75

**5057**	allXL	BB	382	357	DBSU	36.65
**5057**	allXL	AA	372	370	DBSU	6.27
**5057**	allXL	AA	381	370	DBSU	22.85
**5057**	allXL	AA	373	370	DBSU	9.06
**5057**	allXL	AA	381	372	DBSU	20.96
**5057**	allXL	AA	372	382	DBSU	21.7
**5057**	allXL	BB	372	386	DBSU	23.95
**5057**	allXL	BB	373	386	DBSU	22.09
**5057**	allXL	BC	381	386	DBSU	8.99
**5057**	allXL	BD;DB	357	357	DBSU	18.16
**5057**	allXL	CB	373	319	DBSU	17.53
**5057**	allXL	CB	372	357	DBSU	19.56
**5057**	allXL	CB	381	357	DBSU	30.12
**5057**	allXL	CB	373	357	DBSU	18.85
**5057**	allXL	CB	372	370	DBSU	29.95
**5057**	allXL	CB	373	370	DBSU	27.16
**5057**	allXL	CB	382	373	DBSU	11.99
**5058**	allXL	AA	320	357	BS2G	24.48
**5058**	allXL	DD	320	373	BS2G	24.01
**5058**	allXL	DD	373	382	BS2G	18.44
**5058**	allXL	DD	370	386	BS2G	12.87
**5058**	allXL	DD	373	386	BS2G	20.67
**5058**	allXL	CC	319	357	BS2G	26.35
**5058**	allXL	AA	367	373	BS2G	10.62
**5058**	allXL	DD	370	382	BS2G	10.41
**5058**	allXL	CC	376	382	BS2G	14.9
**5058**	allXL	BB	357	373	BS2G	27.28
**5058**	allXL	AA	320	366	BS2G	24.12
**5058**	allXL	DD	370	376	BS2G	13.7
**5058**	allXL	AA	320	357	DBSU	24.48
**5058**	allXL	CC	357	319	DBSU	26.35
**5058**	allXL	DD	372	319	DBSU	23.57
**5058**	allXL	BB	370	357	DBSU	24.01
**5058**	allXL	DD	382	357	DBSU	34.87

**5058**	allXL	AA	372	370	DBSU	6.62
**5058**	allXL	DD	381	370	DBSU	10.31
**5058**	allXL	DD	373	370	DBSU	9.1
**5058**	allXL	DD	381	372	DBSU	16.88
**5058**	allXL	DD	372	382	DBSU	17.64
**5058**	allXL	DD	372	386	DBSU	18.47
**5058**	allXL	DD	373	386	DBSU	20.67
**5058**	allXL	DB	381	386	DBSU	30.19
**5058**	allXL	BD;DB	357	357	DBSU	18.05
**5058**	allXL	CB	373	319	DBSU	15.57
**5058**	allXL	BD	372	357	DBSU	33.92
**5058**	allXL	BD	381	357	DBSU	39.86
**5058**	allXL	BD	373	357	DBSU	33.98
**5058**	allXL	DA	372	370	DBSU	31.32
**5058**	allXL	DA	373	370	DBSU	27.54
**5058**	allXL	DA	382	373	DBSU	46.13
**5059**	allXL	AA	320	357	BS2G	24.54
**5059**	allXL	DD	320	373	BS2G	18.65
**5059**	allXL	DD	373	382	BS2G	17.7
**5059**	allXL	AA	370	386	BS2G	30.79
**5059**	allXL	DD	373	386	BS2G	27.92
**5059**	allXL	AA	319	357	BS2G	23.44
**5059**	allXL	BB	367	373	BS2G	12.81
**5059**	allXL	DD	370	382	BS2G	22.76
**5059**	allXL	CC	376	382	BS2G	13.39
**5059**	allXL	DD	357	373	BS2G	18.23
**5059**	allXL	AA	320	366	BS2G	16.7
**5059**	allXL	CC	370	376	BS2G	17.11
**5059**	allXL	AA	320	357	DBSU	24.54
**5059**	allXL	AA	357	319	DBSU	23.44
**5059**	allXL	DD	372	319	DBSU	15.74
**5059**	allXL	DD	370	357	DBSU	17.73
**5059**	allXL	AA	382	357	DBSU	24.29
**5059**	allXL	AA	372	370	DBSU	6.37

**5059**	allXL	DD	381	370	DBSU	21.51
**5059**	allXL	AA	373	370	DBSU	9.06
**5059**	allXL	DD	381	372	DBSU	15.31
**5059**	allXL	DD	372	382	DBSU	17.26
**5059**	allXL	DD	372	386	DBSU	26.28
**5059**	allXL	DD	373	386	DBSU	27.92
**5059**	allXL	BC	381	386	DBSU	33.15
**5059**	allXL	BD;DB	357	357	DBSU	17.92
**5059**	allXL	BC	373	319	DBSU	28.29
**5059**	allXL	DB	372	357	DBSU	37.58
**5059**	allXL	BD	381	357	DBSU	40.59
**5059**	allXL	DB	373	357	DBSU	35.18
**5059**	allXL	BC	372	370	DBSU	22.65
**5059**	allXL	BC	373	370	DBSU	23.11
**5059**	allXL	CB	382	373	DBSU	28.19
**5060**	allXL	AA	320	357	BS2G	23.67
**5060**	allXL	BB	320	373	BS2G	15.69
**5060**	allXL	CC	373	382	BS2G	19.36
**5060**	allXL	AA	370	386	BS2G	22.49
**5060**	allXL	AA	373	386	BS2G	21.47
**5060**	allXL	AA	319	357	BS2G	21.81
**5060**	allXL	BB	367	373	BS2G	14.82
**5060**	allXL	DD	370	382	BS2G	21.31
**5060**	allXL	CC	376	382	BS2G	13.37
**5060**	allXL	BB	357	373	BS2G	14.31
**5060**	allXL	BB	320	366	BS2G	18.72
**5060**	allXL	CC	370	376	BS2G	15.83
**5060**	allXL	AA	320	357	DBSU	23.67
**5060**	allXL	AA	357	319	DBSU	21.81
**5060**	allXL	CC	372	319	DBSU	16.59
**5060**	allXL	BB	370	357	DBSU	20.92
**5060**	allXL	BB	382	357	DBSU	27.38
**5060**	allXL	AA	372	370	DBSU	6.24
**5060**	allXL	DD	381	370	DBSU	19.66

**5060**	allXL	AA	373	370	DBSU	9.14
**5060**	allXL	DD	381	372	DBSU	18.6
**5060**	allXL	DD	372	382	DBSU	20.58
**5060**	allXL	AA	372	386	DBSU	20.49
**5060**	allXL	AA	373	386	DBSU	21.47
**5060**	allXL	AD	381	386	DBSU	39.68
**5060**	allXL	BD;DB	357	357	DBSU	18.31
**5060**	allXL	AD	373	319	DBSU	16.05
**5060**	allXL	BD	372	357	DBSU	32.41
**5060**	allXL	BD	381	357	DBSU	32.15
**5060**	allXL	BD	373	357	DBSU	30.37
**5060**	allXL	AD	372	370	DBSU	42.38
**5060**	allXL	AD	373	370	DBSU	41.56
**5060**	allXL	DA	382	373	DBSU	30.71
**5061**	allXL	AA	320	357	BS2G	21.3
**5061**	allXL	BB	320	373	BS2G	23.11
**5061**	allXL	DD	373	382	BS2G	18.46
**5061**	allXL	DD	370	386	BS2G	28.09
**5061**	allXL	DD	373	386	BS2G	26.8
**5061**	allXL	AA	319	357	BS2G	19.42
**5061**	allXL	DD	367	373	BS2G	13.66
**5061**	allXL	DD	370	382	BS2G	21.88
**5061**	allXL	BB	376	382	BS2G	14.67
**5061**	allXL	DD	357	373	BS2G	32.65
**5061**	allXL	DD	320	366	BS2G	21.14
**5061**	allXL	BB	370	376	BS2G	15.17
**5061**	allXL	AA	320	357	DBSU	21.3
**5061**	allXL	AA	357	319	DBSU	19.42
**5061**	allXL	BB	372	319	DBSU	18.56
**5061**	allXL	DD	370	357	DBSU	29.3
**5061**	allXL	BB	382	357	DBSU	32.55
**5061**	allXL	AA	372	370	DBSU	5.96
**5061**	allXL	DD	381	370	DBSU	20.74
**5061**	allXL	DD	373	370	DBSU	8.77

**5061**	allXL	DD	381	372	DBSU	18.32
**5061**	allXL	DD	372	382	DBSU	19.42
**5061**	allXL	DD	372	386	DBSU	26.44
**5061**	allXL	DD	373	386	DBSU	26.8
**5061**	allXL	AD	381	386	DBSU	43.8
**5061**	allXL	BD;DB	357	357	DBSU	18.55
**5061**	allXL	AD	373	319	DBSU	24.19
**5061**	allXL	BC	372	357	DBSU	36.44
**5061**	allXL	BC	381	357	DBSU	43.93
**5061**	allXL	BC	373	357	DBSU	38.53
**5061**	allXL	BC	372	370	DBSU	13.47
**5061**	allXL	BC	373	370	DBSU	11.52
**5061**	allXL	BC	382	373	DBSU	28.29
**5062**	allXL	BB	320	357	BS2G	24.99
**5062**	allXL	BB	320	373	BS2G	22.0
**5062**	allXL	CC	373	382	BS2G	16.92
**5062**	allXL	CC	370	386	BS2G	20.1
**5062**	allXL	AA	373	386	BS2G	22.65
**5062**	allXL	AA	319	357	BS2G	27.83
**5062**	allXL	AA	367	373	BS2G	14.18
**5062**	allXL	CC	370	382	BS2G	16.86
**5062**	allXL	AA	376	382	BS2G	14.77
**5062**	allXL	DD	357	373	BS2G	31.23
**5062**	allXL	AA	320	366	BS2G	30.27
**5062**	allXL	CC	370	376	BS2G	15.35
**5062**	allXL	BB	320	357	DBSU	24.99
**5062**	allXL	AA	357	319	DBSU	27.83
**5062**	allXL	BB	372	319	DBSU	25.21
**5062**	allXL	DD	370	357	DBSU	27.66
**5062**	allXL	DD	382	357	DBSU	23.05
**5062**	allXL	AA	372	370	DBSU	6.14
**5062**	allXL	CC	381	370	DBSU	19.63
**5062**	allXL	AA	373	370	DBSU	9.04
**5062**	allXL	CC	381	372	DBSU	20.59

**5062**	allXL	AA	372	382	DBSU	18.46
**5062**	allXL	AA	372	386	DBSU	20.03
**5062**	allXL	AA	373	386	DBSU	22.65
**5062**	allXL	CB	381	386	DBSU	41.72
**5062**	allXL	BD;DB	357	357	DBSU	18.73
**5062**	allXL	CB	373	319	DBSU	21.82
**5062**	allXL	CB	372	357	DBSU	38.63
**5062**	allXL	DB	381	357	DBSU	41.35
**5062**	allXL	BC	373	357	DBSU	40.91
**5062**	allXL	BC	372	370	DBSU	20.44
**5062**	allXL	BC	373	370	DBSU	18.56
**5062**	allXL	BC	382	373	DBSU	24.47
**5063**	allXL	CC	320	357	BS2G	26.93
**5063**	allXL	BB	320	373	BS2G	22.56
**5063**	allXL	DD	373	382	BS2G	17.83
**5063**	allXL	DD	370	386	BS2G	21.82
**5063**	allXL	DD	373	386	BS2G	24.63
**5063**	allXL	AA	319	357	BS2G	27.73
**5063**	allXL	BB	367	373	BS2G	13.17
**5063**	allXL	DD	370	382	BS2G	19.14
**5063**	allXL	CC	376	382	BS2G	14.46
**5063**	allXL	BB	357	373	BS2G	28.02
**5063**	allXL	BB	320	366	BS2G	22.46
**5063**	allXL	BB	370	376	BS2G	15.22
**5063**	allXL	CC	320	357	DBSU	26.93
**5063**	allXL	AA	357	319	DBSU	27.73
**5063**	allXL	BB	372	319	DBSU	24.22
**5063**	allXL	BB	370	357	DBSU	27.03
**5063**	allXL	BB	382	357	DBSU	36.2
**5063**	allXL	AA	372	370	DBSU	6.12
**5063**	allXL	DD	381	370	DBSU	19.28
**5063**	allXL	AA	373	370	DBSU	9.24
**5063**	allXL	DD	381	372	DBSU	18.7
**5063**	allXL	DD	372	382	DBSU	18.86

**5063**	allXL	DD	372	386	DBSU	23.93
**5063**	allXL	DD	373	386	DBSU	24.63
**5063**	allXL	CB	381	386	DBSU	57.93
**5063**	allXL	BD;DB	357	357	DBSU	17.85
**5063**	allXL	CB	373	319	DBSU	29.9
**5063**	allXL	CB	372	357	DBSU	46.42
**5063**	allXL	CB	381	357	DBSU	46.49
**5063**	allXL	CB	373	357	DBSU	43.01
**5063**	allXL	CB	372	370	DBSU	27.9
**5063**	allXL	CB	373	370	DBSU	24.13
**5063**	allXL	CB	382	373	DBSU	31.78
**5064**	allXL	AA	320	357	BS2G	26.98
**5064**	allXL	BB	320	373	BS2G	21.44
**5064**	allXL	DD	373	382	BS2G	20.05
**5064**	allXL	DD	370	386	BS2G	24.66
**5064**	allXL	AA	373	386	BS2G	22.17
**5064**	allXL	AA	319	357	BS2G	28.55
**5064**	allXL	AA	367	373	BS2G	13.89
**5064**	allXL	BB	370	382	BS2G	19.21
**5064**	allXL	CC	376	382	BS2G	14.01
**5064**	allXL	DD	357	373	BS2G	33.14
**5064**	allXL	CC	320	366	BS2G	15.58
**5064**	allXL	BB	370	376	BS2G	14.7
**5064**	allXL	AA	320	357	DBSU	26.98
**5064**	allXL	AA	357	319	DBSU	28.55
**5064**	allXL	BB	372	319	DBSU	19.67
**5064**	allXL	DD	370	357	DBSU	24.87
**5064**	allXL	CC	382	357	DBSU	36.14
**5064**	allXL	AA	372	370	DBSU	6.08
**5064**	allXL	DD	381	370	DBSU	18.53
**5064**	allXL	AA	373	370	DBSU	9.12
**5064**	allXL	DD	381	372	DBSU	18.69
**5064**	allXL	DD	372	382	DBSU	20.96
**5064**	allXL	AA	372	386	DBSU	24.52

**5064**	allXL	AA	373	386	DBSU	22.17
**5064**	allXL	DA	381	386	DBSU	28.07
**5064**	allXL	BD;DB	357	357	DBSU	18.77
**5064**	allXL	AD	373	319	DBSU	29.54
**5064**	allXL	DB	372	357	DBSU	47.27
**5064**	allXL	AD	381	357	DBSU	48.38
**5064**	allXL	BC	373	357	DBSU	49.95
**5064**	allXL	DA	372	370	DBSU	44.08
**5064**	allXL	AD	373	370	DBSU	41.58
**5064**	allXL	AD	382	373	DBSU	20.28
**5065**	allXL	CC	320	357	BS2G	24.72
**5065**	allXL	DD	320	373	BS2G	18.49
**5065**	allXL	BB	373	382	BS2G	18.18
**5065**	allXL	BB	370	386	BS2G	20.2
**5065**	allXL	AA	373	386	BS2G	20.78
**5065**	allXL	CC	319	357	BS2G	24.63
**5065**	allXL	AA	367	373	BS2G	13.57
**5065**	allXL	BB	370	382	BS2G	16.87
**5065**	allXL	BB	376	382	BS2G	13.44
**5065**	allXL	DD	357	373	BS2G	30.8
**5065**	allXL	CC	320	366	BS2G	16.88
**5065**	allXL	BB	370	376	BS2G	15.36
**5065**	allXL	CC	320	357	DBSU	24.72
**5065**	allXL	CC	357	319	DBSU	24.63
**5065**	allXL	DD	372	319	DBSU	18.71
**5065**	allXL	DD	370	357	DBSU	25.69
**5065**	allXL	AA	382	357	DBSU	40.87
**5065**	allXL	AA	372	370	DBSU	6.16
**5065**	allXL	BB	381	370	DBSU	19.04
**5065**	allXL	AA	373	370	DBSU	9.1
**5065**	allXL	DD	381	372	DBSU	19.06
**5065**	allXL	DD	372	382	DBSU	18.66
**5065**	allXL	AA	372	386	DBSU	22.32
**5065**	allXL	AA	373	386	DBSU	20.78

**5065**	allXL	CA	381	386	DBSU	45.65
**5065**	allXL	BD;DB	357	357	DBSU	17.77
**5065**	allXL	AD	373	319	DBSU	25.33
**5065**	allXL	CA	372	357	DBSU	46.48
**5065**	allXL	AC	381	357	DBSU	51.86
**5065**	allXL	DB	373	357	DBSU	46.53
**5065**	allXL	DA	372	370	DBSU	36.51
**5065**	allXL	DA	373	370	DBSU	39.27
**5065**	allXL	AD	382	373	DBSU	43.8
**5066**	allXL	AA	320	357	BS2G	21.21
**5066**	allXL	BB	320	373	BS2G	20.39
**5066**	allXL	DD	373	382	BS2G	18.86
**5066**	allXL	AA	370	386	BS2G	16.8
**5066**	allXL	AA	373	386	BS2G	22.77
**5066**	allXL	AA	319	357	BS2G	23.63
**5066**	allXL	AA	367	373	BS2G	10.59
**5066**	allXL	AA	370	382	BS2G	18.45
**5066**	allXL	CC	376	382	BS2G	14.06
**5066**	allXL	BB	357	373	BS2G	28.07
**5066**	allXL	AA	320	366	BS2G	18.59
**5066**	allXL	CC	370	376	BS2G	12.87
**5066**	allXL	AA	320	357	DBSU	21.21
**5066**	allXL	AA	357	319	DBSU	23.63
**5066**	allXL	BB	372	319	DBSU	23.57
**5066**	allXL	BB	370	357	DBSU	23.38
**5066**	allXL	BB	382	357	DBSU	18.92
**5066**	allXL	AA	372	370	DBSU	6.51
**5066**	allXL	AA	381	370	DBSU	18.22
**5066**	allXL	AA	373	370	DBSU	8.94
**5066**	allXL	DD	381	372	DBSU	19.14
**5066**	allXL	DD	372	382	DBSU	19.79
**5066**	allXL	AA	372	386	DBSU	21.77
**5066**	allXL	AA	373	386	DBSU	22.77
**5066**	allXL	DB	381	386	DBSU	41.24

**5066**	allXL	BD;DB	357	357	DBSU	17.82
**5066**	allXL	CB	373	319	DBSU	26.06
**5066**	allXL	CB	372	357	DBSU	35.93
**5066**	allXL	BD	381	357	DBSU	33.1
**5066**	allXL	CB	373	357	DBSU	36.53
**5066**	allXL	CB	372	370	DBSU	26.75
**5066**	allXL	CB	373	370	DBSU	24.96
**5066**	allXL	CB	382	373	DBSU	39.76
**5067**	allXL	CC	320	357	BS2G	24.51
**5067**	allXL	BB	320	373	BS2G	14.03
**5067**	allXL	DD	373	382	BS2G	17.32
**5067**	allXL	AA	370	386	BS2G	21.0
**5067**	allXL	AA	373	386	BS2G	22.08
**5067**	allXL	AA	319	357	BS2G	24.92
**5067**	allXL	AA	367	373	BS2G	14.6
**5067**	allXL	AA	370	382	BS2G	22.77
**5067**	allXL	DD	376	382	BS2G	14.19
**5067**	allXL	BB	357	373	BS2G	25.93
**5067**	allXL	CC	320	366	BS2G	26.49
**5067**	allXL	CC	370	376	BS2G	16.92
**5067**	allXL	CC	320	357	DBSU	24.51
**5067**	allXL	AA	357	319	DBSU	24.92
**5067**	allXL	BB	372	319	DBSU	19.94
**5067**	allXL	BB	370	357	DBSU	27.93
**5067**	allXL	BB	382	357	DBSU	27.79
**5067**	allXL	AA	372	370	DBSU	6.11
**5067**	allXL	DD	381	370	DBSU	23.82
**5067**	allXL	AA	373	370	DBSU	8.87
**5067**	allXL	DD	381	372	DBSU	17.19
**5067**	allXL	DD	372	382	DBSU	17.61
**5067**	allXL	AA	372	386	DBSU	21.37
**5067**	allXL	AA	373	386	DBSU	22.08
**5067**	allXL	DB	381	386	DBSU	45.42
**5067**	allXL	BD;DB	357	357	DBSU	17.25

**5067**	allXL	AD	373	319	DBSU	13.15
**5067**	allXL	AD	372	357	DBSU	34.54
**5067**	allXL	BD	381	357	DBSU	38.52
**5067**	allXL	AD	373	357	DBSU	38.02
**5067**	allXL	AD	372	370	DBSU	49.43
**5067**	allXL	AD	373	370	DBSU	52.21
**5067**	allXL	BD	382	373	DBSU	58.65
**5068**	allXL	AA	320	357	BS2G	15.83
**5068**	allXL	CC	320	373	BS2G	25.47
**5068**	allXL	BB	373	382	BS2G	17.4
**5068**	allXL	BB	370	386	BS2G	25.97
**5068**	allXL	BB	373	386	BS2G	23.79
**5068**	allXL	AA	319	357	BS2G	12.65
**5068**	allXL	AA	367	373	BS2G	12.92
**5068**	allXL	DD	370	382	BS2G	21.12
**5068**	allXL	CC	376	382	BS2G	14.43
**5068**	allXL	DD	357	373	BS2G	24.58
**5068**	allXL	AA	320	366	BS2G	24.42
**5068**	allXL	CC	370	376	BS2G	13.12
**5068**	allXL	AA	320	357	DBSU	15.83
**5068**	allXL	AA	357	319	DBSU	12.65
**5068**	allXL	CC	372	319	DBSU	26.11
**5068**	allXL	DD	370	357	DBSU	26.2
**5068**	allXL	BB	382	357	DBSU	30.67
**5068**	allXL	AA	372	370	DBSU	6.87
**5068**	allXL	DD	381	370	DBSU	20.21
**5068**	allXL	AA	373	370	DBSU	9.05
**5068**	allXL	BB	381	372	DBSU	17.15
**5068**	allXL	BB	372	382	DBSU	18.95
**5068**	allXL	BB	372	386	DBSU	23.75
**5068**	allXL	BB	373	386	DBSU	23.79
**5068**	allXL	DB	381	386	DBSU	51.7
**5068**	allXL	BD;DB	357	357	DBSU	18.58
**5068**	allXL	DA	373	319	DBSU	27.22

**5068**	allXL	DA	372	357	DBSU	32.72
**5068**	allXL	DB	381	357	DBSU	37.57
**5068**	allXL	DA	373	357	DBSU	31.27
**5068**	allXL	DA	372	370	DBSU	39.52
**5068**	allXL	DA	373	370	DBSU	41.96
**5068**	allXL	CB	382	373	DBSU	37.25
**5069**	allXL	AA	320	357	BS2G	22.87
**5069**	allXL	DD	320	373	BS2G	26.66
**5069**	allXL	BB	373	382	BS2G	19.26
**5069**	allXL	DD	370	386	BS2G	18.63
**5069**	allXL	DD	373	386	BS2G	21.75
**5069**	allXL	AA	319	357	BS2G	20.56
**5069**	allXL	AA	367	373	BS2G	11.99
**5069**	allXL	DD	370	382	BS2G	19.99
**5069**	allXL	BB	376	382	BS2G	13.23
**5069**	allXL	BB	357	373	BS2G	25.91
**5069**	allXL	DD	320	366	BS2G	20.48
**5069**	allXL	CC	370	376	BS2G	15.04
**5069**	allXL	AA	320	357	DBSU	22.87
**5069**	allXL	AA	357	319	DBSU	20.56
**5069**	allXL	CC	372	319	DBSU	25.46
**5069**	allXL	BB	370	357	DBSU	23.93
**5069**	allXL	BB	382	357	DBSU	37.51
**5069**	allXL	AA	372	370	DBSU	6.31
**5069**	allXL	DD	381	370	DBSU	19.84
**5069**	allXL	AA	373	370	DBSU	8.86
**5069**	allXL	DD	381	372	DBSU	18.04
**5069**	allXL	DD	372	382	DBSU	19.25
**5069**	allXL	DD	372	386	DBSU	19.1
**5069**	allXL	DD	373	386	DBSU	21.75
**5069**	allXL	AD	381	386	DBSU	44.14
**5069**	allXL	BD;DB	357	357	DBSU	18.35
**5069**	allXL	AD	373	319	DBSU	31.02
**5069**	allXL	BD	372	357	DBSU	32.77

**5069**	allXL	BD	381	357	DBSU	39.13
**5069**	allXL	BD	373	357	DBSU	30.88
**5069**	allXL	BD	372	370	DBSU	57.64
**5069**	allXL	BD	373	370	DBSU	55.54
**5069**	allXL	DA	382	373	DBSU	45.91
**5070**	allXL	AA	320	357	BS2G	23.05
**5070**	allXL	BB	320	373	BS2G	14.2
**5070**	allXL	BB	373	382	BS2G	16.68
**5070**	allXL	DD	370	386	BS2G	18.21
**5070**	allXL	DD	373	386	BS2G	22.27
**5070**	allXL	AA	319	357	BS2G	22.64
**5070**	allXL	DD	367	373	BS2G	13.81
**5070**	allXL	BB	370	382	BS2G	14.95
**5070**	allXL	BB	376	382	BS2G	13.73
**5070**	allXL	CC	357	373	BS2G	29.14
**5070**	allXL	BB	320	366	BS2G	25.74
**5070**	allXL	CC	370	376	BS2G	15.23
**5070**	allXL	AA	320	357	DBSU	23.05
**5070**	allXL	AA	357	319	DBSU	22.64
**5070**	allXL	BB	372	319	DBSU	15.24
**5070**	allXL	CC	370	357	DBSU	23.17
**5070**	allXL	BB	382	357	DBSU	34.73
**5070**	allXL	AA	372	370	DBSU	6.29
**5070**	allXL	BB	381	370	DBSU	18.24
**5070**	allXL	AA	373	370	DBSU	9.05
**5070**	allXL	CC	381	372	DBSU	19.22
**5070**	allXL	BB	372	382	DBSU	18.24
**5070**	allXL	DD	372	386	DBSU	21.99
**5070**	allXL	DD	373	386	DBSU	22.27
**5070**	allXL	BC	381	386	DBSU	44.71
**5070**	allXL	BD;DB	357	357	DBSU	18.61
**5070**	allXL	DA	373	319	DBSU	30.99
**5070**	allXL	CA	372	357	DBSU	38.56
**5070**	allXL	BC	381	357	DBSU	34.26

**5070**	allXL	CA	373	357	DBSU	41.42
**5070**	allXL	DA	372	370	DBSU	44.1
**5070**	allXL	DA	373	370	DBSU	43.52
**5070**	allXL	BC	382	373	DBSU	54.61
**5071**	allXL	AA	320	357	BS2G	22.08
**5071**	allXL	CC	320	373	BS2G	6.69
**5071**	allXL	DD	373	382	BS2G	17.03
**5071**	allXL	DD	370	386	BS2G	15.81
**5071**	allXL	BB	373	386	BS2G	22.11
**5071**	allXL	AA	319	357	BS2G	19.6
**5071**	allXL	AA	367	373	BS2G	12.36
**5071**	allXL	DD	370	382	BS2G	14.2
**5071**	allXL	CC	376	382	BS2G	13.4
**5071**	allXL	BB	357	373	BS2G	27.97
**5071**	allXL	CC	320	366	BS2G	21.98
**5071**	allXL	CC	370	376	BS2G	15.66
**5071**	allXL	AA	320	357	DBSU	22.08
**5071**	allXL	AA	357	319	DBSU	19.6
**5071**	allXL	CC	372	319	DBSU	6.39
**5071**	allXL	CC	370	357	DBSU	25.17
**5071**	allXL	BB	382	357	DBSU	31.2
**5071**	allXL	AA	372	370	DBSU	6.49
**5071**	allXL	DD	381	370	DBSU	16.6
**5071**	allXL	AA	373	370	DBSU	9.23
**5071**	allXL	DD	381	372	DBSU	18.87
**5071**	allXL	DD	372	382	DBSU	17.49
**5071**	allXL	DD	372	386	DBSU	20.83
**5071**	allXL	BB	373	386	DBSU	22.11
**5071**	allXL	BC	381	386	DBSU	42.85
**5071**	allXL	BD;DB	357	357	DBSU	19.24
**5071**	allXL	DA	373	319	DBSU	19.73
**5071**	allXL	BC	372	357	DBSU	36.92
**5071**	allXL	CB	381	357	DBSU	31.46
**5071**	allXL	DA	373	357	DBSU	36.56

**5071**	allXL	CB	372	370	DBSU	26.99
**5071**	allXL	CB	373	370	DBSU	27.33
**5071**	allXL	CB	382	373	DBSU	34.22
**5072**	allXL	AA	320	357	BS2G	23.45
**5072**	allXL	CC	320	373	BS2G	22.04
**5072**	allXL	CC	373	382	BS2G	19.78
**5072**	allXL	CC	370	386	BS2G	20.29
**5072**	allXL	AA	373	386	BS2G	23.92
**5072**	allXL	AA	319	357	BS2G	24.15
**5072**	allXL	DD	367	373	BS2G	13.21
**5072**	allXL	CC	370	382	BS2G	17.14
**5072**	allXL	AA	376	382	BS2G	14.63
**5072**	allXL	CC	357	373	BS2G	16.95
**5072**	allXL	CC	320	366	BS2G	9.98
**5072**	allXL	CC	370	376	BS2G	14.17
**5072**	allXL	AA	320	357	DBSU	23.45
**5072**	allXL	AA	357	319	DBSU	24.15
**5072**	allXL	CC	372	319	DBSU	20.37
**5072**	allXL	CC	370	357	DBSU	16.89
**5072**	allXL	CC	382	357	DBSU	9.4
**5072**	allXL	AA	372	370	DBSU	6.13
**5072**	allXL	CC	381	370	DBSU	18.76
**5072**	allXL	AA	373	370	DBSU	9.11
**5072**	allXL	CC	381	372	DBSU	21.18
**5072**	allXL	CC	372	382	DBSU	20.6
**5072**	allXL	CC	372	386	DBSU	24.92
**5072**	allXL	AA	373	386	DBSU	23.92
**5072**	allXL	DB	381	386	DBSU	29.52
**5072**	allXL	BD;DB	357	357	DBSU	18.44
**5072**	allXL	DA	373	319	DBSU	31.15
**5072**	allXL	CA	372	357	DBSU	26.69
**5072**	allXL	DB	381	357	DBSU	23.07
**5072**	allXL	CA	373	357	DBSU	23.38
**5072**	allXL	DA	372	370	DBSU	37.92

**5072**	allXL	DA	373	370	DBSU	40.48
**5072**	allXL	BD	382	373	DBSU	40.61
**5073**	allXL	AA	320	357	BS2G	25.39
**5073**	allXL	DD	320	373	BS2G	21.27
**5073**	allXL	DD	373	382	BS2G	16.9
**5073**	allXL	CC	370	386	BS2G	24.34
**5073**	allXL	BB	373	386	BS2G	23.6
**5073**	allXL	AA	319	357	BS2G	26.34
**5073**	allXL	BB	367	373	BS2G	10.22
**5073**	allXL	DD	370	382	BS2G	15.64
**5073**	allXL	CC	376	382	BS2G	13.37
**5073**	allXL	CC	357	373	BS2G	33.92
**5073**	allXL	AA	320	366	BS2G	17.28
**5073**	allXL	DD	370	376	BS2G	15.57
**5073**	allXL	AA	320	357	DBSU	25.39
**5073**	allXL	AA	357	319	DBSU	26.34
**5073**	allXL	DD	372	319	DBSU	18.79
**5073**	allXL	BB	370	357	DBSU	30.7
**5073**	allXL	DD	382	357	DBSU	18.64
**5073**	allXL	AA	372	370	DBSU	6.34
**5073**	allXL	DD	381	370	DBSU	14.93
**5073**	allXL	AA	373	370	DBSU	9.13
**5073**	allXL	DD	381	372	DBSU	14.94
**5073**	allXL	DD	372	382	DBSU	16.59
**5073**	allXL	BB	372	386	DBSU	23.79
**5073**	allXL	BB	373	386	DBSU	23.6
**5073**	allXL	DA	381	386	DBSU	43.22
**5073**	allXL	BD;DB	357	357	DBSU	19.19
**5073**	allXL	AD	373	319	DBSU	28.64
**5073**	allXL	BC	372	357	DBSU	45.25
**5073**	allXL	DB	381	357	DBSU	39.34
**5073**	allXL	BC	373	357	DBSU	46.02
**5073**	allXL	DA	372	370	DBSU	20.13
**5073**	allXL	DA	373	370	DBSU	16.39

**5073**	allXL	DA	382	373	DBSU	33.21
**5074**	allXL	AA	320	357	BS2G	22.86
**5074**	allXL	CC	320	373	BS2G	16.11
**5074**	allXL	AA	373	382	BS2G	18.12
**5074**	allXL	DD	370	386	BS2G	21.09
**5074**	allXL	DD	373	386	BS2G	23.33
**5074**	allXL	AA	319	357	BS2G	20.8
**5074**	allXL	AA	367	373	BS2G	13.69
**5074**	allXL	DD	370	382	BS2G	15.37
**5074**	allXL	CC	376	382	BS2G	13.82
**5074**	allXL	BB	357	373	BS2G	28.31
**5074**	allXL	BB	320	366	BS2G	19.57
**5074**	allXL	CC	370	376	BS2G	13.33
**5074**	allXL	AA	320	357	DBSU	22.86
**5074**	allXL	AA	357	319	DBSU	20.8
**5074**	allXL	CC	372	319	DBSU	16.45
**5074**	allXL	DD	370	357	DBSU	22.36
**5074**	allXL	CC	382	357	DBSU	18.82
**5074**	allXL	AA	372	370	DBSU	6.37
**5074**	allXL	DD	381	370	DBSU	16.34
**5074**	allXL	AA	373	370	DBSU	9.34
**5074**	allXL	DD	381	372	DBSU	18.01
**5074**	allXL	DD	372	382	DBSU	17.94
**5074**	allXL	DD	372	386	DBSU	22.58
**5074**	allXL	DD	373	386	DBSU	23.33
**5074**	allXL	BD	381	386	DBSU	45.63
**5074**	allXL	BD;DB	357	357	DBSU	18.11
**5074**	allXL	AD	373	319	DBSU	20.39
**5074**	allXL	DB	372	357	DBSU	34.1
**5074**	allXL	CA	381	357	DBSU	31.17
**5074**	allXL	BD	373	357	DBSU	35.49
**5074**	allXL	BD	372	370	DBSU	32.28
**5074**	allXL	BD	373	370	DBSU	31.37
**5074**	allXL	DB	382	373	DBSU	41.76

**5075**	allXL	CC	320	357	BS2G	23.58
**5075**	allXL	BB	320	373	BS2G	20.23
**5075**	allXL	DD	373	382	BS2G	18.33
**5075**	allXL	DD	370	386	BS2G	22.9
**5075**	allXL	DD	373	386	BS2G	23.72
**5075**	allXL	AA	319	357	BS2G	23.39
**5075**	allXL	AA	367	373	BS2G	10.03
**5075**	allXL	DD	370	382	BS2G	22.0
**5075**	allXL	CC	376	382	BS2G	14.29
**5075**	allXL	BB	357	373	BS2G	28.13
**5075**	allXL	DD	320	366	BS2G	25.67
**5075**	allXL	CC	370	376	BS2G	16.52
**5075**	allXL	CC	320	357	DBSU	23.58
**5075**	allXL	AA	357	319	DBSU	23.39
**5075**	allXL	BB	372	319	DBSU	22.21
**5075**	allXL	DD	370	357	DBSU	31.14
**5075**	allXL	BB	382	357	DBSU	18.11
**5075**	allXL	CC	372	370	DBSU	7.0
**5075**	allXL	DD	381	370	DBSU	23.93
**5075**	allXL	AA	373	370	DBSU	8.87
**5075**	allXL	BB	381	372	DBSU	19.7
**5075**	allXL	BB	372	382	DBSU	19.23
**5075**	allXL	CC	372	386	DBSU	24.33
**5075**	allXL	DD	373	386	DBSU	23.72
**5075**	allXL	BC	381	386	DBSU	51.78
**5075**	allXL	BD;DB	357	357	DBSU	17.33
**5075**	allXL	CB	373	319	DBSU	16.62
**5075**	allXL	CB	372	357	DBSU	39.82
**5075**	allXL	BD	381	357	DBSU	30.92
**5075**	allXL	CB	373	357	DBSU	41.15
**5075**	allXL	CB	372	370	DBSU	26.21
**5075**	allXL	CB	373	370	DBSU	27.0
**5075**	allXL	DA	382	373	DBSU	28.08
**5076**	allXL	CC	320	357	BS2G	21.46

**5076**	allXL	BB	320	373	BS2G	21.6
**5076**	allXL	DD	373	382	BS2G	15.37
**5076**	allXL	AA	370	386	BS2G	25.43
**5076**	allXL	DD	373	386	BS2G	22.27
**5076**	allXL	CC	319	357	BS2G	20.42
**5076**	allXL	AA	367	373	BS2G	13.52
**5076**	allXL	DD	370	382	BS2G	21.12
**5076**	allXL	DD	376	382	BS2G	14.2
**5076**	allXL	BB	357	373	BS2G	30.24
**5076**	allXL	DD	320	366	BS2G	22.5
**5076**	allXL	CC	370	376	BS2G	16.17
**5076**	allXL	CC	320	357	DBSU	21.46
**5076**	allXL	CC	357	319	DBSU	20.42
**5076**	allXL	BB	372	319	DBSU	20.23
**5076**	allXL	BB	370	357	DBSU	24.4
**5076**	allXL	BB	382	357	DBSU	39.83
**5076**	allXL	CC	372	370	DBSU	6.77
**5076**	allXL	DD	381	370	DBSU	20.71
**5076**	allXL	DD	373	370	DBSU	9.06
**5076**	allXL	DD	381	372	DBSU	14.48
**5076**	allXL	DD	372	382	DBSU	14.51
**5076**	allXL	DD	372	386	DBSU	20.08
**5076**	allXL	DD	373	386	DBSU	22.27
**5076**	allXL	AD	381	386	DBSU	26.4
**5076**	allXL	BD;DB	357	357	DBSU	18.85
**5076**	allXL	DA	373	319	DBSU	25.05
**5076**	allXL	CA	372	357	DBSU	30.67
**5076**	allXL	DA	381	357	DBSU	37.71
**5076**	allXL	CA	373	357	DBSU	31.51
**5076**	allXL	DA	372	370	DBSU	42.22
**5076**	allXL	DA	373	370	DBSU	39.15
**5076**	allXL	AD	382	373	DBSU	27.87
**5077**	allXL	CC	320	357	BS2G	25.37
**5077**	allXL	DD	320	373	BS2G	27.76

**5077**	allXL	DD	373	382	BS2G	17.25
**5077**	allXL	DD	370	386	BS2G	15.33
**5077**	allXL	DD	373	386	BS2G	21.54
**5077**	allXL	AA	319	357	BS2G	28.78
**5077**	allXL	DD	367	373	BS2G	13.42
**5077**	allXL	DD	370	382	BS2G	16.79
**5077**	allXL	BB	376	382	BS2G	13.69
**5077**	allXL	DD	357	373	BS2G	34.9
**5077**	allXL	BB	320	366	BS2G	25.0
**5077**	allXL	DD	370	376	BS2G	15.93
**5077**	allXL	CC	320	357	DBSU	25.37
**5077**	allXL	AA	357	319	DBSU	28.78
**5077**	allXL	BB	372	319	DBSU	27.72
**5077**	allXL	BB	370	357	DBSU	31.36
**5077**	allXL	DD	382	357	DBSU	36.4
**5077**	allXL	AA	372	370	DBSU	5.96
**5077**	allXL	DD	381	370	DBSU	18.67
**5077**	allXL	AA	373	370	DBSU	9.26
**5077**	allXL	DD	381	372	DBSU	17.12
**5077**	allXL	DD	372	382	DBSU	15.97
**5077**	allXL	DD	372	386	DBSU	19.04
**5077**	allXL	DD	373	386	DBSU	21.54
**5077**	allXL	BC	381	386	DBSU	11.61
**5077**	allXL	AC;CA	357	357	DBSU	19.44
**5077**	allXL	AD	373	319	DBSU	23.93
**5077**	allXL	DA	372	357	DBSU	45.35
**5077**	allXL	CB	381	357	DBSU	45.1
**5077**	allXL	DA	373	357	DBSU	44.82
**5077**	allXL	CB	372	370	DBSU	27.74
**5077**	allXL	CB	373	370	DBSU	26.21
**5077**	allXL	CB	382	373	DBSU	15.5
**5078**	allXL	AA	320	357	BS2G	24.59
**5078**	allXL	DD	320	373	BS2G	8.81
**5078**	allXL	CC	373	382	BS2G	18.37

**5078**	allXL	BB	370	386	BS2G	23.86
**5078**	allXL	CC	373	386	BS2G	23.48
**5078**	allXL	AA	319	357	BS2G	24.22
**5078**	allXL	BB	367	373	BS2G	10.51
**5078**	allXL	AA	370	382	BS2G	22.1
**5078**	allXL	CC	376	382	BS2G	14.34
**5078**	allXL	BB	357	373	BS2G	21.36
**5078**	allXL	DD	320	366	BS2G	19.68
**5078**	allXL	BB	370	376	BS2G	16.51
**5078**	allXL	AA	320	357	DBSU	24.59
**5078**	allXL	AA	357	319	DBSU	24.22
**5078**	allXL	DD	372	319	DBSU	11.39
**5078**	allXL	DD	370	357	DBSU	24.83
**5078**	allXL	BB	382	357	DBSU	27.89
**5078**	allXL	AA	372	370	DBSU	6.22
**5078**	allXL	AA	381	370	DBSU	23.87
**5078**	allXL	DD	373	370	DBSU	8.98
**5078**	allXL	DD	381	372	DBSU	18.88
**5078**	allXL	AA	372	382	DBSU	20.22
**5078**	allXL	CC	372	386	DBSU	25.23
**5078**	allXL	CC	373	386	DBSU	23.48
**5078**	allXL	DA	381	386	DBSU	36.0
**5078**	allXL	BD;DB	357	357	DBSU	18.64
**5078**	allXL	CB	373	319	DBSU	28.46
**5078**	allXL	BD	372	357	DBSU	33.12
**5078**	allXL	BD	381	357	DBSU	36.07
**5078**	allXL	BD	373	357	DBSU	30.59
**5078**	allXL	DA	372	370	DBSU	42.7
**5078**	allXL	DA	373	370	DBSU	44.52
**5078**	allXL	AD	382	373	DBSU	44.25
**5079**	allXL	CC	320	357	BS2G	24.11
**5079**	allXL	BB	320	373	BS2G	19.48
**5079**	allXL	BB	373	382	BS2G	14.39
**5079**	allXL	BB	370	386	BS2G	21.76

**5079**	allXL	BB	373	386	BS2G	23.44
**5079**	allXL	AA	319	357	BS2G	26.18
**5079**	allXL	BB	367	373	BS2G	14.5
**5079**	allXL	BB	370	382	BS2G	17.73
**5079**	allXL	BB	376	382	BS2G	13.9
**5079**	allXL	DD	357	373	BS2G	31.67
**5079**	allXL	AA	320	366	BS2G	19.13
**5079**	allXL	BB	370	376	BS2G	16.36
**5079**	allXL	CC	320	357	DBSU	24.11
**5079**	allXL	AA	357	319	DBSU	26.18
**5079**	allXL	BB	372	319	DBSU	21.88
**5079**	allXL	BB	370	357	DBSU	26.45
**5079**	allXL	BB	382	357	DBSU	28.01
**5079**	allXL	AA	372	370	DBSU	6.15
**5079**	allXL	BB	381	370	DBSU	18.88
**5079**	allXL	DD	373	370	DBSU	9.38
**5079**	allXL	BB	381	372	DBSU	16.74
**5079**	allXL	BB	372	382	DBSU	15.33
**5079**	allXL	BB	372	386	DBSU	22.8
**5079**	allXL	BB	373	386	DBSU	23.44
**5079**	allXL	AD	381	386	DBSU	44.72
**5079**	allXL	BD;DB	357	357	DBSU	18.96
**5079**	allXL	AD	373	319	DBSU	35.04
**5079**	allXL	AD	372	357	DBSU	48.52
**5079**	allXL	BD	381	357	DBSU	44.93
**5079**	allXL	DB	373	357	DBSU	50.1
**5079**	allXL	AD	372	370	DBSU	37.02
**5079**	allXL	AD	373	370	DBSU	38.16
**5079**	allXL	DA	382	373	DBSU	40.53
**5080**	allXL	AA	320	357	BS2G	25.18
**5080**	allXL	DD	320	373	BS2G	21.34
**5080**	allXL	AA	373	382	BS2G	15.99
**5080**	allXL	AA	370	386	BS2G	13.65
**5080**	allXL	AA	373	386	BS2G	19.04

**5080**	allXL	AA	319	357	BS2G	24.38
**5080**	allXL	AA	367	373	BS2G	12.5
**5080**	allXL	AA	370	382	BS2G	16.59
**5080**	allXL	AA	376	382	BS2G	13.92
**5080**	allXL	DD	357	373	BS2G	34.41
**5080**	allXL	CC	320	366	BS2G	21.04
**5080**	allXL	BB	370	376	BS2G	15.29
**5080**	allXL	AA	320	357	DBSU	25.18
**5080**	allXL	AA	357	319	DBSU	24.38
**5080**	allXL	DD	372	319	DBSU	21.21
**5080**	allXL	DD	370	357	DBSU	28.73
**5080**	allXL	BB	382	357	DBSU	34.01
**5080**	allXL	AA	372	370	DBSU	6.24
**5080**	allXL	AA	381	370	DBSU	20.0
**5080**	allXL	AA	373	370	DBSU	9.05
**5080**	allXL	AA	381	372	DBSU	17.33
**5080**	allXL	AA	372	382	DBSU	14.34
**5080**	allXL	AA	372	386	DBSU	15.61
**5080**	allXL	AA	373	386	DBSU	19.04
**5080**	allXL	DA	381	386	DBSU	12.5
**5080**	allXL	BD;DB	357	357	DBSU	18.91
**5080**	allXL	DA	373	319	DBSU	24.77
**5080**	allXL	DA	372	357	DBSU	36.3
**5080**	allXL	DA	381	357	DBSU	44.41
**5080**	allXL	DA	373	357	DBSU	33.7
**5080**	allXL	DA	372	370	DBSU	22.53
**5080**	allXL	DA	373	370	DBSU	19.67
**5080**	allXL	DA	382	373	DBSU	25.93
**5081**	allXL	CC	320	357	BS2G	26.47
**5081**	allXL	DD	320	373	BS2G	16.74
**5081**	allXL	BB	373	382	BS2G	15.89
**5081**	allXL	DD	370	386	BS2G	21.25
**5081**	allXL	AA	373	386	BS2G	23.04
**5081**	allXL	CC	319	357	BS2G	28.01

**5081**	allXL	AA	367	373	BS2G	15.11
**5081**	allXL	BB	370	382	BS2G	18.74
**5081**	allXL	BB	376	382	BS2G	13.13
**5081**	allXL	BB	357	373	BS2G	33.01
**5081**	allXL	BB	320	366	BS2G	19.28
**5081**	allXL	CC	370	376	BS2G	15.04
**5081**	allXL	CC	320	357	DBSU	26.47
**5081**	allXL	CC	357	319	DBSU	28.01
**5081**	allXL	DD	372	319	DBSU	12.3
**5081**	allXL	BB	370	357	DBSU	24.67
**5081**	allXL	BB	382	357	DBSU	33.38
**5081**	allXL	AA	372	370	DBSU	5.94
**5081**	allXL	BB	381	370	DBSU	21.9
**5081**	allXL	CC	373	370	DBSU	9.19
**5081**	allXL	BB	381	372	DBSU	19.96
**5081**	allXL	BB	372	382	DBSU	17.8
**5081**	allXL	DD	372	386	DBSU	23.33
**5081**	allXL	AA	373	386	DBSU	23.04
**5081**	allXL	AD	381	386	DBSU	40.09
**5081**	allXL	BD;DB	357	357	DBSU	19.34
**5081**	allXL	AD	373	319	DBSU	34.82
**5081**	allXL	DA	372	357	DBSU	46.73
**5081**	allXL	BD	381	357	DBSU	49.68
**5081**	allXL	BD	373	357	DBSU	49.37
**5081**	allXL	DA	372	370	DBSU	36.76
**5081**	allXL	DA	373	370	DBSU	37.11
**5081**	allXL	AD	382	373	DBSU	32.09
**5082**	allXL	AA	320	357	BS2G	26.11
**5082**	allXL	BB	320	373	BS2G	19.8
**5082**	allXL	CC	373	382	BS2G	21.35
**5082**	allXL	AA	370	386	BS2G	31.31
**5082**	allXL	BB	373	386	BS2G	25.45
**5082**	allXL	AA	319	357	BS2G	28.13
**5082**	allXL	AA	367	373	BS2G	10.86

**5082**	allXL	DD	370	382	BS2G	28.41
**5082**	allXL	CC	376	382	BS2G	14.4
**5082**	allXL	BB	357	373	BS2G	28.9
**5082**	allXL	BB	320	366	BS2G	16.88
**5082**	allXL	BB	370	376	BS2G	16.81
**5082**	allXL	AA	320	357	DBSU	26.11
**5082**	allXL	AA	357	319	DBSU	28.13
**5082**	allXL	BB	372	319	DBSU	19.54
**5082**	allXL	BB	370	357	DBSU	21.01
**5082**	allXL	BB	382	357	DBSU	48.39
**5082**	allXL	AA	372	370	DBSU	6.5
**5082**	allXL	DD	381	370	DBSU	26.59
**5082**	allXL	BB	373	370	DBSU	8.95
**5082**	allXL	DD	381	372	DBSU	20.15
**5082**	allXL	DD	372	382	DBSU	21.75
**5082**	allXL	AA	372	386	DBSU	25.86
**5082**	allXL	BB	373	386	DBSU	25.45
**5082**	allXL	AD	381	386	DBSU	33.07
**5082**	allXL	BD;DB	357	357	DBSU	18.03
**5082**	allXL	DA	373	319	DBSU	19.71
**5082**	allXL	DA	372	357	DBSU	35.25
**5082**	allXL	DA	381	357	DBSU	36.41
**5082**	allXL	DA	373	357	DBSU	31.59
**5082**	allXL	DA	372	370	DBSU	23.05
**5082**	allXL	DA	373	370	DBSU	21.55
**5082**	allXL	DA	382	373	DBSU	26.54
**5083**	allXL	AA	320	357	BS2G	26.84
**5083**	allXL	CC	320	373	BS2G	29.9
**5083**	allXL	DD	373	382	BS2G	19.07
**5083**	allXL	DD	370	386	BS2G	27.23
**5083**	allXL	DD	373	386	BS2G	24.83
**5083**	allXL	CC	319	357	BS2G	28.45
**5083**	allXL	AA	367	373	BS2G	12.84
**5083**	allXL	CC	370	382	BS2G	20.67

**5083**	allXL	CC	376	382	BS2G	15.5
**5083**	allXL	BB	357	373	BS2G	16.65
**5083**	allXL	CC	320	366	BS2G	27.13
**5083**	allXL	CC	370	376	BS2G	13.39
**5083**	allXL	AA	320	357	DBSU	26.84
**5083**	allXL	CC	357	319	DBSU	28.45
**5083**	allXL	CC	372	319	DBSU	25.78
**5083**	allXL	BB	370	357	DBSU	24.18
**5083**	allXL	BB	382	357	DBSU	15.32
**5083**	allXL	AA	372	370	DBSU	6.24
**5083**	allXL	CC	381	370	DBSU	21.62
**5083**	allXL	DD	373	370	DBSU	8.98
**5083**	allXL	DD	381	372	DBSU	16.83
**5083**	allXL	DD	372	382	DBSU	18.85
**5083**	allXL	DD	372	386	DBSU	23.56
**5083**	allXL	DD	373	386	DBSU	24.83
**5083**	allXL	AD	381	386	DBSU	55.61
**5083**	allXL	BD;DB	357	357	DBSU	18.9
**5083**	allXL	AD	373	319	DBSU	33.25
**5083**	allXL	BD	372	357	DBSU	30.46
**5083**	allXL	BD	381	357	DBSU	17.13
**5083**	allXL	BD	373	357	DBSU	27.84
**5083**	allXL	DA	372	370	DBSU	40.74
**5083**	allXL	DA	373	370	DBSU	44.05
**5083**	allXL	BD	382	373	DBSU	47.77
**5084**	allXL	AA	320	357	BS2G	24.25
**5084**	allXL	DD	320	373	BS2G	22.36
**5084**	allXL	DD	373	382	BS2G	17.68
**5084**	allXL	BB	370	386	BS2G	28.22
**5084**	allXL	DD	373	386	BS2G	26.99
**5084**	allXL	AA	319	357	BS2G	24.56
**5084**	allXL	AA	367	373	BS2G	13.81
**5084**	allXL	DD	370	382	BS2G	21.81
**5084**	allXL	CC	376	382	BS2G	14.0

**5084**	allXL	DD	357	373	BS2G	25.42
**5084**	allXL	DD	320	366	BS2G	21.32
**5084**	allXL	BB	370	376	BS2G	16.42
**5084**	allXL	AA	320	357	DBSU	24.25
**5084**	allXL	AA	357	319	DBSU	24.56
**5084**	allXL	DD	372	319	DBSU	22.29
**5084**	allXL	DD	370	357	DBSU	23.44
**5084**	allXL	DD	382	357	DBSU	37.8
**5084**	allXL	AA	372	370	DBSU	6.03
**5084**	allXL	DD	381	370	DBSU	20.79
**5084**	allXL	AA	373	370	DBSU	9.08
**5084**	allXL	DD	381	372	DBSU	17.19
**5084**	allXL	DD	372	382	DBSU	17.76
**5084**	allXL	DD	372	386	DBSU	25.58
**5084**	allXL	DD	373	386	DBSU	26.99
**5084**	allXL	DA	381	386	DBSU	46.53
**5084**	allXL	BD;DB	357	357	DBSU	18.13
**5084**	allXL	CB	373	319	DBSU	9.74
**5084**	allXL	CB	372	357	DBSU	36.22
**5084**	allXL	DB	381	357	DBSU	39.67
**5084**	allXL	DB	373	357	DBSU	35.3
**5084**	allXL	CB	372	370	DBSU	44.85
**5084**	allXL	CB	373	370	DBSU	45.63
**5084**	allXL	DA	382	373	DBSU	51.22
**5085**	allXL	AA	320	357	BS2G	24.27
**5085**	allXL	BB	320	373	BS2G	21.88
**5085**	allXL	DD	373	382	BS2G	17.27
**5085**	allXL	DD	370	386	BS2G	11.69
**5085**	allXL	DD	373	386	BS2G	15.04
**5085**	allXL	AA	319	357	BS2G	25.7
**5085**	allXL	AA	367	373	BS2G	12.03
**5085**	allXL	DD	370	382	BS2G	18.19
**5085**	allXL	CC	376	382	BS2G	14.62
**5085**	allXL	AA	357	373	BS2G	29.69

**5085**	allXL	CC	320	366	BS2G	26.76
**5085**	allXL	DD	370	376	BS2G	17.01
**5085**	allXL	AA	320	357	DBSU	24.27
**5085**	allXL	AA	357	319	DBSU	25.7
**5085**	allXL	BB	372	319	DBSU	23.95
**5085**	allXL	DD	370	357	DBSU	25.44
**5085**	allXL	DD	382	357	DBSU	26.13
**5085**	allXL	AA	372	370	DBSU	6.33
**5085**	allXL	DD	381	370	DBSU	18.24
**5085**	allXL	AA	373	370	DBSU	8.98
**5085**	allXL	DD	381	372	DBSU	18.57
**5085**	allXL	DD	372	382	DBSU	18.91
**5085**	allXL	DD	372	386	DBSU	14.18
**5085**	allXL	DD	373	386	DBSU	15.04
**5085**	allXL	DA	381	386	DBSU	46.97
**5085**	allXL	BD;DB	357	357	DBSU	19.1
**5085**	allXL	DA	373	319	DBSU	14.21
**5085**	allXL	DA	372	357	DBSU	31.6
**5085**	allXL	DA	381	357	DBSU	38.71
**5085**	allXL	DA	373	357	DBSU	29.51
**5085**	allXL	DA	372	370	DBSU	33.61
**5085**	allXL	DA	373	370	DBSU	30.19
**5085**	allXL	DA	382	373	DBSU	28.55
**5086**	allXL	CC	320	357	BS2G	26.67
**5086**	allXL	DD	320	373	BS2G	20.42
**5086**	allXL	CC	373	382	BS2G	20.7
**5086**	allXL	DD	370	386	BS2G	21.74
**5086**	allXL	CC	373	386	BS2G	27.75
**5086**	allXL	AA	319	357	BS2G	28.15
**5086**	allXL	AA	367	373	BS2G	10.38
**5086**	allXL	CC	370	382	BS2G	19.32
**5086**	allXL	CC	376	382	BS2G	13.91
**5086**	allXL	BB	357	373	BS2G	14.82
**5086**	allXL	CC	320	366	BS2G	16.4

**5086**	allXL	CC	370	376	BS2G	13.81
**5086**	allXL	CC	320	357	DBSU	26.67
**5086**	allXL	AA	357	319	DBSU	28.15
**5086**	allXL	CC	372	319	DBSU	22.08
**5086**	allXL	BB	370	357	DBSU	19.43
**5086**	allXL	AA	382	357	DBSU	19.84
**5086**	allXL	CC	372	370	DBSU	6.59
**5086**	allXL	DD	381	370	DBSU	19.51
**5086**	allXL	AA	373	370	DBSU	9.04
**5086**	allXL	DD	381	372	DBSU	20.13
**5086**	allXL	DD	372	382	DBSU	21.12
**5086**	allXL	DD	372	386	DBSU	26.64
**5086**	allXL	CC	373	386	DBSU	27.75
**5086**	allXL	BD	381	386	DBSU	21.13
**5086**	allXL	BD;DB	357	357	DBSU	18.65
**5086**	allXL	DA	373	319	DBSU	25.03
**5086**	allXL	BD	372	357	DBSU	17.33
**5086**	allXL	BD	381	357	DBSU	12.9
**5086**	allXL	BD	373	357	DBSU	14.0
**5086**	allXL	BD	372	370	DBSU	31.94
**5086**	allXL	BD	373	370	DBSU	29.96
**5086**	allXL	BD	382	373	DBSU	37.5
**5087**	allXL	AA	320	357	BS2G	22.23
**5087**	allXL	CC	320	373	BS2G	21.3
**5087**	allXL	CC	373	382	BS2G	19.37
**5087**	allXL	AA	370	386	BS2G	29.84
**5087**	allXL	CC	373	386	BS2G	25.14
**5087**	allXL	AA	319	357	BS2G	21.73
**5087**	allXL	AA	367	373	BS2G	12.56
**5087**	allXL	DD	370	382	BS2G	24.89
**5087**	allXL	CC	376	382	BS2G	13.6
**5087**	allXL	BB	357	373	BS2G	24.14
**5087**	allXL	DD	320	366	BS2G	21.71
**5087**	allXL	CC	370	376	BS2G	16.6

**5087**	allXL	AA	320	357	DBSU	22.23
**5087**	allXL	AA	357	319	DBSU	21.73
**5087**	allXL	CC	372	319	DBSU	17.91
**5087**	allXL	DD	370	357	DBSU	20.17
**5087**	allXL	BB	382	357	DBSU	40.53
**5087**	allXL	AA	372	370	DBSU	6.22
**5087**	allXL	DD	381	370	DBSU	25.28
**5087**	allXL	AA	373	370	DBSU	9.14
**5087**	allXL	DD	381	372	DBSU	20.26
**5087**	allXL	DD	372	382	DBSU	20.63
**5087**	allXL	AA	372	386	DBSU	28.13
**5087**	allXL	CC	373	386	DBSU	25.14
**5087**	allXL	DB	381	386	DBSU	65.97
**5087**	allXL	BD;DB	357	357	DBSU	18.87
**5087**	allXL	AD	373	319	DBSU	36.05
**5087**	allXL	BD	372	357	DBSU	28.78
**5087**	allXL	BD	381	357	DBSU	32.19
**5087**	allXL	BD	373	357	DBSU	30.09
**5087**	allXL	BD	372	370	DBSU	47.3
**5087**	allXL	BD	373	370	DBSU	48.64
**5087**	allXL	BD	382	373	DBSU	51.44
**5088**	allXL	CC	320	357	BS2G	22.04
**5088**	allXL	BB	320	373	BS2G	25.82
**5088**	allXL	BB	373	382	BS2G	14.54
**5088**	allXL	BB	370	386	BS2G	24.62
**5088**	allXL	CC	373	386	BS2G	20.97
**5088**	allXL	CC	319	357	BS2G	21.22
**5088**	allXL	BB	367	373	BS2G	12.55
**5088**	allXL	BB	370	382	BS2G	19.57
**5088**	allXL	BB	376	382	BS2G	12.65
**5088**	allXL	BB	357	373	BS2G	25.65
**5088**	allXL	AA	320	366	BS2G	19.09
**5088**	allXL	CC	370	376	BS2G	16.98
**5088**	allXL	CC	320	357	DBSU	22.04

**5088**	allXL	CC	357	319	DBSU	21.22
**5088**	allXL	CC	372	319	DBSU	24.43
**5088**	allXL	BB	370	357	DBSU	31.22
**5088**	allXL	BB	382	357	DBSU	19.83
**5088**	allXL	AA	372	370	DBSU	6.48
**5088**	allXL	BB	381	370	DBSU	22.09
**5088**	allXL	AA	373	370	DBSU	9.31
**5088**	allXL	BB	381	372	DBSU	16.48
**5088**	allXL	BB	372	382	DBSU	14.69
**5088**	allXL	CC	372	386	DBSU	21.03
**5088**	allXL	CC	373	386	DBSU	20.97
**5088**	allXL	BC	381	386	DBSU	38.16
**5088**	allXL	BD;DB	357	357	DBSU	17.99
**5088**	allXL	CB	373	319	DBSU	22.16
**5088**	allXL	CB	372	357	DBSU	38.92
**5088**	allXL	BD	381	357	DBSU	32.06
**5088**	allXL	CB	373	357	DBSU	41.46
**5088**	allXL	BC	372	370	DBSU	41.82
**5088**	allXL	BC	373	370	DBSU	38.56
**5088**	allXL	CB	382	373	DBSU	25.89
**5089**	allXL	AA	320	357	BS2G	24.34
**5089**	allXL	CC	320	373	BS2G	14.64
**5089**	allXL	CC	373	382	BS2G	17.99
**5089**	allXL	AA	370	386	BS2G	23.56
**5089**	allXL	AA	373	386	BS2G	21.3
**5089**	allXL	AA	319	357	BS2G	24.05
**5089**	allXL	AA	367	373	BS2G	12.67
**5089**	allXL	CC	370	382	BS2G	22.43
**5089**	allXL	AA	376	382	BS2G	13.3
**5089**	allXL	CC	357	373	BS2G	28.1
**5089**	allXL	CC	320	366	BS2G	23.18
**5089**	allXL	CC	370	376	BS2G	17.06
**5089**	allXL	AA	320	357	DBSU	24.34
**5089**	allXL	AA	357	319	DBSU	24.05

**5089**	allXL	CC	372	319	DBSU	14.09
**5089**	allXL	DD	370	357	DBSU	25.29
**5089**	allXL	CC	382	357	DBSU	37.71
**5089**	allXL	AA	372	370	DBSU	6.5
**5089**	allXL	CC	381	370	DBSU	23.22
**5089**	allXL	AA	373	370	DBSU	8.85
**5089**	allXL	CC	381	372	DBSU	19.67
**5089**	allXL	CC	372	382	DBSU	18.36
**5089**	allXL	AA	372	386	DBSU	19.94
**5089**	allXL	AA	373	386	DBSU	21.3
**5089**	allXL	DB	381	386	DBSU	47.27
**5089**	allXL	BD;DB	357	357	DBSU	19.03
**5089**	allXL	AD	373	319	DBSU	26.42
**5089**	allXL	BD	372	357	DBSU	30.47
**5089**	allXL	BD	381	357	DBSU	41.45
**5089**	allXL	BD	373	357	DBSU	33.56
**5089**	allXL	CB	372	370	DBSU	34.99
**5089**	allXL	CB	373	370	DBSU	34.7
**5089**	allXL	BD	382	373	DBSU	51.26
**5090**	allXL	CC	320	357	BS2G	25.44
**5090**	allXL	BB	320	373	BS2G	28.53
**5090**	allXL	DD	373	382	BS2G	18.09
**5090**	allXL	AA	370	386	BS2G	21.52
**5090**	allXL	AA	373	386	BS2G	23.76
**5090**	allXL	AA	319	357	BS2G	24.98
**5090**	allXL	AA	367	373	BS2G	12.24
**5090**	allXL	BB	370	382	BS2G	22.4
**5090**	allXL	CC	376	382	BS2G	14.25
**5090**	allXL	BB	357	373	BS2G	28.6
**5090**	allXL	BB	320	366	BS2G	23.22
**5090**	allXL	CC	370	376	BS2G	16.74
**5090**	allXL	CC	320	357	DBSU	25.44
**5090**	allXL	AA	357	319	DBSU	24.98
**5090**	allXL	BB	372	319	DBSU	26.98

**5090**	allXL	DD	370	357	DBSU	27.24
**5090**	allXL	BB	382	357	DBSU	30.77
**5090**	allXL	AA	372	370	DBSU	6.31
**5090**	allXL	AA	381	370	DBSU	23.19
**5090**	allXL	DD	373	370	DBSU	8.88
**5090**	allXL	DD	381	372	DBSU	19.65
**5090**	allXL	DD	372	382	DBSU	18.89
**5090**	allXL	DD	372	386	DBSU	23.28
**5090**	allXL	AA	373	386	DBSU	23.76
**5090**	allXL	BC	381	386	DBSU	14.81
**5090**	allXL	BD;DB	357	357	DBSU	18.66
**5090**	allXL	AD	373	319	DBSU	12.65
**5090**	allXL	AD	372	357	DBSU	32.35
**5090**	allXL	AC	381	357	DBSU	35.79
**5090**	allXL	AD	373	357	DBSU	35.12
**5090**	allXL	CB	372	370	DBSU	33.83
**5090**	allXL	CB	373	370	DBSU	32.38
**5090**	allXL	BC	382	373	DBSU	30.02
**5091**	allXL	AA	320	357	BS2G	24.42
**5091**	allXL	AA	320	373	BS2G	15.37
**5091**	allXL	DD	373	382	BS2G	18.12
**5091**	allXL	AA	370	386	BS2G	22.23
**5091**	allXL	AA	373	386	BS2G	22.76
**5091**	allXL	AA	319	357	BS2G	23.13
**5091**	allXL	DD	367	373	BS2G	13.55
**5091**	allXL	DD	370	382	BS2G	21.6
**5091**	allXL	BB	376	382	BS2G	14.23
**5091**	allXL	BB	357	373	BS2G	22.47
**5091**	allXL	DD	320	366	BS2G	19.25
**5091**	allXL	CC	370	376	BS2G	15.41
**5091**	allXL	AA	320	357	DBSU	24.42
**5091**	allXL	AA	357	319	DBSU	23.13
**5091**	allXL	CC	372	319	DBSU	16.83
**5091**	allXL	BB	370	357	DBSU	24.23

**5091**	allXL	DD	382	357	DBSU	17.68
**5091**	allXL	AA	372	370	DBSU	6.04
**5091**	allXL	DD	381	370	DBSU	22.88
**5091**	allXL	DD	373	370	DBSU	9.02
**5091**	allXL	DD	381	372	DBSU	19.21
**5091**	allXL	DD	372	382	DBSU	19.15
**5091**	allXL	AA	372	386	DBSU	21.11
**5091**	allXL	AA	373	386	DBSU	22.76
**5091**	allXL	DA	381	386	DBSU	21.07
**5091**	allXL	BD;DB	357	357	DBSU	19.52
**5091**	allXL	AD	373	319	DBSU	35.12
**5091**	allXL	BD	372	357	DBSU	37.78
**5091**	allXL	BD	381	357	DBSU	29.82
**5091**	allXL	BD	373	357	DBSU	39.07
**5091**	allXL	DA	372	370	DBSU	24.42
**5091**	allXL	DA	373	370	DBSU	25.5
**5091**	allXL	AD	382	373	DBSU	24.06
**5092**	allXL	AA	320	357	BS2G	25.07
**5092**	allXL	BB	320	373	BS2G	23.02
**5092**	allXL	BB	373	382	BS2G	18.96
**5092**	allXL	DD	370	386	BS2G	22.76
**5092**	allXL	DD	373	386	BS2G	24.6
**5092**	allXL	AA	319	357	BS2G	23.93
**5092**	allXL	BB	367	373	BS2G	12.49
**5092**	allXL	DD	370	382	BS2G	20.28
**5092**	allXL	BB	376	382	BS2G	14.12
**5092**	allXL	BB	357	373	BS2G	24.76
**5092**	allXL	AA	320	366	BS2G	16.98
**5092**	allXL	CC	370	376	BS2G	17.18
**5092**	allXL	AA	320	357	DBSU	25.07
**5092**	allXL	AA	357	319	DBSU	23.93
**5092**	allXL	BB	372	319	DBSU	24.5
**5092**	allXL	CC	370	357	DBSU	28.58
**5092**	allXL	BB	382	357	DBSU	29.79

**5092**	allXL	AA	372	370	DBSU	6.41
**5092**	allXL	DD	381	370	DBSU	21.62
**5092**	allXL	CC	373	370	DBSU	9.16
**5092**	allXL	DD	381	372	DBSU	22.28
**5092**	allXL	BB	372	382	DBSU	20.93
**5092**	allXL	DD	372	386	DBSU	25.16
**5092**	allXL	DD	373	386	DBSU	24.6
**5092**	allXL	BC	381	386	DBSU	47.82
**5092**	allXL	BD;DB	357	357	DBSU	17.86
**5092**	allXL	CB	373	319	DBSU	25.0
**5092**	allXL	CB	372	357	DBSU	36.64
**5092**	allXL	CB	381	357	DBSU	23.75
**5092**	allXL	CB	373	357	DBSU	32.85
**5092**	allXL	CB	372	370	DBSU	30.33
**5092**	allXL	CB	373	370	DBSU	28.04
**5092**	allXL	CB	382	373	DBSU	24.51
**5093**	allXL	CC	320	357	BS2G	24.4
**5093**	allXL	BB	320	373	BS2G	23.83
**5093**	allXL	DD	373	382	BS2G	18.62
**5093**	allXL	CC	370	386	BS2G	16.07
**5093**	allXL	CC	373	386	BS2G	17.9
**5093**	allXL	CC	319	357	BS2G	24.92
**5093**	allXL	AA	367	373	BS2G	13.78
**5093**	allXL	CC	370	382	BS2G	22.61
**5093**	allXL	CC	376	382	BS2G	12.66
**5093**	allXL	CC	357	373	BS2G	32.59
**5093**	allXL	AA	320	366	BS2G	21.34
**5093**	allXL	CC	370	376	BS2G	16.47
**5093**	allXL	CC	320	357	DBSU	24.4
**5093**	allXL	CC	357	319	DBSU	24.92
**5093**	allXL	BB	372	319	DBSU	22.59
**5093**	allXL	CC	370	357	DBSU	29.33
**5093**	allXL	AA	382	357	DBSU	44.89
**5093**	allXL	AA	372	370	DBSU	6.2

**5093**	allXL	DD	381	370	DBSU	23.28
**5093**	allXL	AA	373	370	DBSU	9.16
**5093**	allXL	DD	381	372	DBSU	16.83
**5093**	allXL	DD	372	382	DBSU	19.29
**5093**	allXL	CC	372	386	DBSU	16.21
**5093**	allXL	CC	373	386	DBSU	17.9
**5093**	allXL	BC	381	386	DBSU	59.04
**5093**	allXL	BD;DB	357	357	DBSU	19.34
**5093**	allXL	BC	373	319	DBSU	29.14
**5093**	allXL	BC	372	357	DBSU	37.19
**5093**	allXL	DB	381	357	DBSU	55.94
**5093**	allXL	BC	373	357	DBSU	38.94
**5093**	allXL	AD	372	370	DBSU	36.26
**5093**	allXL	DA	373	370	DBSU	38.63
**5093**	allXL	DA	382	373	DBSU	58.6
**5094**	allXL	AA	320	357	BS2G	25.15
**5094**	allXL	BB	320	373	BS2G	16.15
**5094**	allXL	AA	373	382	BS2G	18.11
**5094**	allXL	AA	370	386	BS2G	24.69
**5094**	allXL	CC	373	386	BS2G	21.69
**5094**	allXL	AA	319	357	BS2G	25.84
**5094**	allXL	AA	367	373	BS2G	13.34
**5094**	allXL	DD	370	382	BS2G	17.9
**5094**	allXL	CC	376	382	BS2G	14.23
**5094**	allXL	DD	357	373	BS2G	22.14
**5094**	allXL	DD	320	366	BS2G	17.22
**5094**	allXL	DD	370	376	BS2G	14.2
**5094**	allXL	AA	320	357	DBSU	25.15
**5094**	allXL	AA	357	319	DBSU	25.84
**5094**	allXL	BB	372	319	DBSU	20.5
**5094**	allXL	DD	370	357	DBSU	23.93
**5094**	allXL	DD	382	357	DBSU	33.57
**5094**	allXL	AA	372	370	DBSU	6.53
**5094**	allXL	DD	381	370	DBSU	14.78

**5094**	allXL	DD	373	370	DBSU	9.26
**5094**	allXL	DD	381	372	DBSU	19.95
**5094**	allXL	AA	372	382	DBSU	21.34
**5094**	allXL	AA	372	386	DBSU	23.89
**5094**	allXL	CC	373	386	DBSU	21.69
**5094**	allXL	BC	381	386	DBSU	30.75
**5094**	allXL	BD;DB	357	357	DBSU	19.06
**5094**	allXL	CB	373	319	DBSU	16.56
**5094**	allXL	DA	372	357	DBSU	27.35
**5094**	allXL	CB	381	357	DBSU	41.02
**5094**	allXL	DA	373	357	DBSU	28.01
**5094**	allXL	BC	372	370	DBSU	28.58
**5094**	allXL	BC	373	370	DBSU	29.32
**5094**	allXL	CB	382	373	DBSU	16.9
**5095**	allXL	BB	320	357	BS2G	26.41
**5095**	allXL	BB	320	373	BS2G	14.42
**5095**	allXL	AA	373	382	BS2G	18.93
**5095**	allXL	AA	370	386	BS2G	29.17
**5095**	allXL	DD	373	386	BS2G	27.02
**5095**	allXL	AA	319	357	BS2G	26.76
**5095**	allXL	AA	367	373	BS2G	12.92
**5095**	allXL	AA	370	382	BS2G	21.88
**5095**	allXL	CC	376	382	BS2G	13.62
**5095**	allXL	BB	357	373	BS2G	21.03
**5095**	allXL	CC	320	366	BS2G	24.7
**5095**	allXL	CC	370	376	BS2G	17.23
**5095**	allXL	BB	320	357	DBSU	26.41
**5095**	allXL	AA	357	319	DBSU	26.76
**5095**	allXL	CC	372	319	DBSU	14.3
**5095**	allXL	BB	370	357	DBSU	28.84
**5095**	allXL	BB	382	357	DBSU	16.83
**5095**	allXL	AA	372	370	DBSU	6.18
**5095**	allXL	AA	381	370	DBSU	23.34
**5095**	allXL	DD	373	370	DBSU	9.04

**5095**	allXL	DD	381	372	DBSU	20.03
**5095**	allXL	AA	372	382	DBSU	21.08
**5095**	allXL	DD	372	386	DBSU	25.9
**5095**	allXL	DD	373	386	DBSU	27.02
**5095**	allXL	BC	381	386	DBSU	35.25
**5095**	allXL	BD;DB	357	357	DBSU	19.03
**5095**	allXL	AD	373	319	DBSU	21.55
**5095**	allXL	CB	372	357	DBSU	35.26
**5095**	allXL	BD	381	357	DBSU	30.72
**5095**	allXL	CB	373	357	DBSU	36.85
**5095**	allXL	CB	372	370	DBSU	18.27
**5095**	allXL	CB	373	370	DBSU	21.13
**5095**	allXL	CB	382	373	DBSU	29.98
**5096**	allXL	AA	320	357	BS2G	25.45
**5096**	allXL	BB	320	373	BS2G	20.01
**5096**	allXL	BB	373	382	BS2G	19.33
**5096**	allXL	CC	370	386	BS2G	22.16
**5096**	allXL	BB	373	386	BS2G	20.41
**5096**	allXL	AA	319	357	BS2G	24.65
**5096**	allXL	AA	367	373	BS2G	13.44
**5096**	allXL	CC	370	382	BS2G	20.4
**5096**	allXL	CC	376	382	BS2G	13.01
**5096**	allXL	CC	357	373	BS2G	8.92
**5096**	allXL	BB	320	366	BS2G	23.17
**5096**	allXL	CC	370	376	BS2G	14.29
**5096**	allXL	AA	320	357	DBSU	25.45
**5096**	allXL	AA	357	319	DBSU	24.65
**5096**	allXL	BB	372	319	DBSU	21.45
**5096**	allXL	CC	370	357	DBSU	16.03
**5096**	allXL	CC	382	357	DBSU	26.55
**5096**	allXL	AA	372	370	DBSU	6.32
**5096**	allXL	CC	381	370	DBSU	23.19
**5096**	allXL	AA	373	370	DBSU	9.02
**5096**	allXL	BB	381	372	DBSU	22.85

**5096**	allXL	BB	372	382	DBSU	22.15
**5096**	allXL	BB	372	386	DBSU	21.37
**5096**	allXL	BB	373	386	DBSU	20.41
**5096**	allXL	CB	381	386	DBSU	77.82
**5096**	allXL	BD;DB	357	357	DBSU	18.82
**5096**	allXL	CB	373	319	DBSU	40.05
**5096**	allXL	CA	372	357	DBSU	25.5
**5096**	allXL	CA	381	357	DBSU	39.71
**5096**	allXL	CA	373	357	DBSU	24.0
**5096**	allXL	CA	372	370	DBSU	58.69
**5096**	allXL	CB	373	370	DBSU	56.57
**5096**	allXL	CB	382	373	DBSU	60.03
**5097**	allXL	AA	320	357	BS2G	17.06
**5097**	allXL	DD	320	373	BS2G	12.5
**5097**	allXL	CC	373	382	BS2G	18.25
**5097**	allXL	CC	370	386	BS2G	20.97
**5097**	allXL	CC	373	386	BS2G	23.98
**5097**	allXL	AA	319	357	BS2G	14.27
**5097**	allXL	AA	367	373	BS2G	13.25
**5097**	allXL	CC	370	382	BS2G	20.34
**5097**	allXL	CC	376	382	BS2G	14.59
**5097**	allXL	DD	357	373	BS2G	25.93
**5097**	allXL	CC	320	366	BS2G	15.43
**5097**	allXL	CC	370	376	BS2G	15.04
**5097**	allXL	AA	320	357	DBSU	17.06
**5097**	allXL	AA	357	319	DBSU	14.27
**5097**	allXL	CC	372	319	DBSU	13.39
**5097**	allXL	DD	370	357	DBSU	28.02
**5097**	allXL	CC	382	357	DBSU	43.72
**5097**	allXL	AA	372	370	DBSU	6.28
**5097**	allXL	DD	381	370	DBSU	19.09
**5097**	allXL	AA	373	370	DBSU	9.3
**5097**	allXL	DD	381	372	DBSU	17.99
**5097**	allXL	CC	372	382	DBSU	20.57

**5097**	allXL	CC	372	386	DBSU	24.4
**5097**	allXL	CC	373	386	DBSU	23.98
**5097**	allXL	BC	381	386	DBSU	21.89
**5097**	allXL	BD;DB	357	357	DBSU	18.85
**5097**	allXL	AD	373	319	DBSU	28.37
**5097**	allXL	BD	372	357	DBSU	42.37
**5097**	allXL	AC	381	357	DBSU	44.92
**5097**	allXL	DB	373	357	DBSU	43.26
**5097**	allXL	CB	372	370	DBSU	34.05
**5097**	allXL	CB	373	370	DBSU	31.38
**5097**	allXL	CB	382	373	DBSU	22.23
**5098**	allXL	AA	320	357	BS2G	24.25
**5098**	allXL	DD	320	373	BS2G	17.74
**5098**	allXL	DD	373	382	BS2G	19.71
**5098**	allXL	BB	370	386	BS2G	27.11
**5098**	allXL	BB	373	386	BS2G	28.09
**5098**	allXL	AA	319	357	BS2G	26.61
**5098**	allXL	BB	367	373	BS2G	12.6
**5098**	allXL	BB	370	382	BS2G	22.75
**5098**	allXL	CC	376	382	BS2G	14.63
**5098**	allXL	CC	357	373	BS2G	23.26
**5098**	allXL	DD	320	366	BS2G	22.29
**5098**	allXL	CC	370	376	BS2G	15.67
**5098**	allXL	AA	320	357	DBSU	24.25
**5098**	allXL	AA	357	319	DBSU	26.61
**5098**	allXL	DD	372	319	DBSU	14.77
**5098**	allXL	CC	370	357	DBSU	18.16
**5098**	allXL	AA	382	357	DBSU	19.83
**5098**	allXL	AA	372	370	DBSU	6.55
**5098**	allXL	BB	381	370	DBSU	22.37
**5098**	allXL	BB	373	370	DBSU	9.1
**5098**	allXL	DD	381	372	DBSU	21.83
**5098**	allXL	BB	372	382	DBSU	22.94
**5098**	allXL	BB	372	386	DBSU	30.2

**5098**	allXL	BB	373	386	DBSU	28.09
**5098**	allXL	DA	381	386	DBSU	54.6
**5098**	allXL	BD;DB	357	357	DBSU	18.67
**5098**	allXL	AD	373	319	DBSU	24.11
**5098**	allXL	CA	372	357	DBSU	16.02
**5098**	allXL	AC	381	357	DBSU	30.97
**5098**	allXL	CA	373	357	DBSU	18.53
**5098**	allXL	AD	372	370	DBSU	37.57
**5098**	allXL	AD	373	370	DBSU	34.74
**5098**	allXL	AD	382	373	DBSU	35.52
**5099**	allXL	CC	320	357	BS2G	23.12
**5099**	allXL	BB	320	373	BS2G	21.72
**5099**	allXL	BB	373	382	BS2G	17.87
**5099**	allXL	BB	370	386	BS2G	24.39
**5099**	allXL	BB	373	386	BS2G	26.54
**5099**	allXL	CC	319	357	BS2G	25.14
**5099**	allXL	AA	367	373	BS2G	15.2
**5099**	allXL	BB	370	382	BS2G	17.3
**5099**	allXL	BB	376	382	BS2G	14.07
**5099**	allXL	CC	357	373	BS2G	24.92
**5099**	allXL	AA	320	366	BS2G	21.0
**5099**	allXL	BB	370	376	BS2G	15.22
**5099**	allXL	CC	320	357	DBSU	23.12
**5099**	allXL	CC	357	319	DBSU	25.14
**5099**	allXL	DD	372	319	DBSU	19.21
**5099**	allXL	DD	370	357	DBSU	23.06
**5099**	allXL	BB	382	357	DBSU	23.65
**5099**	allXL	AA	372	370	DBSU	5.97
**5099**	allXL	BB	381	370	DBSU	18.98
**5099**	allXL	AA	373	370	DBSU	9.19
**5099**	allXL	BB	381	372	DBSU	20.11
**5099**	allXL	BB	372	382	DBSU	19.0
**5099**	allXL	BB	372	386	DBSU	26.68
**5099**	allXL	BB	373	386	DBSU	26.54

**5099**	allXL	DA	381	386	DBSU	48.64
**5099**	allXL	BD;DB	357	357	DBSU	19.07
**5099**	allXL	BC	373	319	DBSU	23.58
**5099**	allXL	BC	372	357	DBSU	35.82
**5099**	allXL	BD	381	357	DBSU	45.7
**5099**	allXL	BC	373	357	DBSU	36.99
**5099**	allXL	DA	372	370	DBSU	26.38
**5099**	allXL	DA	373	370	DBSU	24.77
**5099**	allXL	AD	382	373	DBSU	41.11
**5100**	allXL	CC	320	357	BS2G	27.9
**5100**	allXL	BB	320	373	BS2G	17.0
**5100**	allXL	CC	373	382	BS2G	19.62
**5100**	allXL	DD	370	386	BS2G	28.12
**5100**	allXL	DD	373	386	BS2G	22.37
**5100**	allXL	AA	319	357	BS2G	28.24
**5100**	allXL	AA	367	373	BS2G	10.82
**5100**	allXL	CC	370	382	BS2G	23.72
**5100**	allXL	CC	376	382	BS2G	15.54
**5100**	allXL	BB	357	373	BS2G	22.91
**5100**	allXL	AA	320	366	BS2G	14.61
**5100**	allXL	DD	370	376	BS2G	13.57
**5100**	allXL	CC	320	357	DBSU	27.9
**5100**	allXL	AA	357	319	DBSU	28.24
**5100**	allXL	BB	372	319	DBSU	21.97
**5100**	allXL	CC	370	357	DBSU	25.54
**5100**	allXL	AA	382	357	DBSU	27.59
**5100**	allXL	CC	372	370	DBSU	6.44
**5100**	allXL	DD	381	370	DBSU	21.58
**5100**	allXL	AA	373	370	DBSU	8.92
**5100**	allXL	CC	381	372	DBSU	18.84
**5100**	allXL	CC	372	382	DBSU	19.37
**5100**	allXL	DD	372	386	DBSU	24.23
**5100**	allXL	DD	373	386	DBSU	22.37
**5100**	allXL	BC	381	386	DBSU	28.94

**5100**	allXL	BD;DB	357	357	DBSU	18.08
**5100**	allXL	CB	373	319	DBSU	22.99
**5100**	allXL	CB	372	357	DBSU	35.06
**5100**	allXL	DA	381	357	DBSU	34.37
**5100**	allXL	CB	373	357	DBSU	31.3
**5100**	allXL	CB	372	370	DBSU	34.45
**5100**	allXL	CB	373	370	DBSU	32.0
**5100**	allXL	DA	382	373	DBSU	20.53
**5101**	allXL	CC	320	357	BS2G	26.28
**5101**	allXL	BB	320	373	BS2G	12.09
**5101**	allXL	BB	373	382	BS2G	18.72
**5101**	allXL	DD	370	386	BS2G	27.86
**5101**	allXL	CC	373	386	BS2G	25.54
[truncated: 436,908 more chars]
