# Supplementary material for: Structural assessment of the full-length wild-type tumor suppressor protein p53 by mass spectrometry-guided computational modeling
Source: Sci Rep. 2023 May 25;13:8497. doi: 10.1038/s41598-023-35437-5 (PMC10213020; doi:10.1038/s41598-023-35437-5)
Supplement: Supplementary file 3 — Supplementary Table 2. [file 41598_2023_35437_MOESM3_ESM.pdf]

TableS2

ModelID	Residue	Chain	SASA	Score
**2**	305	A	80.53	1
**2**	319	A	80.3867	1
**2**	320	A	80.2222	-1
**2**	321	A	79.9269	0
**2**	351	A	3.1862	1
**2**	357	A	69.2814	1
**2**	370	A	80.3122	1
**2**	372	A	80.2167	0
**2**	373	A	80.2276	1
**2**	381	A	80.2533	1
**2**	382	A	80.1101	1
**2**	386	A	79.9858	1
**2**	305	B	80.2573	1
**2**	319	B	80.1742	1
**2**	320	B	80.1482	-1
**2**	321	B	79.6751	0
**2**	351	B	7.6785	1
**2**	357	B	79.9628	1
**2**	370	B	73.1427	1
**2**	372	B	80.3076	0
**2**	373	B	76.0438	1
**2**	381	B	78.0461	1
**2**	382	B	69.0635	1
**2**	386	B	64.8531	1
**2**	305	C	80.9306	1
**2**	319	C	80.4329	1
**2**	320	C	80.0773	-1
**2**	321	C	79.9572	0
**2**	351	C	3.0542	1
**2**	357	C	66.3374	1
**2**	370	C	80.188	1
**2**	372	C	79.8524	0

**2**	373	C	80.2864	1
**2**	381	C	80.2689	1
**2**	382	C	72.8918	1
**2**	386	C	42.7719	0
**2**	305	D	80.3503	1
**2**	319	D	80.0805	1
**2**	320	D	80.1253	-1
**2**	321	D	79.8214	0
**2**	351	D	6.1861	1
**2**	357	D	79.8683	1
**2**	370	D	80.3027	1
**2**	372	D	80.3302	0
**2**	373	D	80.1025	1
**2**	381	D	80.2351	1
**2**	382	D	80.2289	1
**2**	386	D	80.1322	1
**3986**	305	A	53.6689	1
**3986**	319	A	36.408	0
**3986**	320	A	72.8706	-1
**3986**	321	A	79.5305	0
**3986**	351	A	4.031	1
**3986**	357	A	74.699	1
**3986**	370	A	46.8306	0
**3986**	372	A	48.7914	1
**3986**	373	A	73.7609	1
**3986**	381	A	79.647	1
**3986**	382	A	51.3513	1
**3986**	386	A	79.2379	1
**3986**	305	B	69.6173	1
**3986**	319	B	78.0104	1
**3986**	320	B	79.5686	-1
**3986**	321	B	71.7909	0
**3986**	351	B	14.3158	1
**3986**	357	B	73.7446	1

**3986**	370	B	48.1616	0
**3986**	372	B	79.5724	0
**3986**	373	B	44.1565	0
**3986**	381	B	62.9992	1
**3986**	382	B	46.1576	0
**3986**	386	B	47.6264	0
**3986**	305	C	51.7882	1
**3986**	319	C	72.669	1
**3986**	320	C	79.5253	-1
**3986**	321	C	79.5225	0
**3986**	351	C	9.7606	1
**3986**	357	C	73.5408	1
**3986**	370	C	79.5689	1
**3986**	372	C	73.3352	0
**3986**	373	C	74.0089	1
**3986**	381	C	68.4153	1
**3986**	382	C	65.7623	1
**3986**	386	C	45.1215	0
**3986**	305	D	67.5277	1
**3986**	319	D	51.8238	1
**3986**	320	D	61.1651	-1
**3986**	321	D	68.7311	0
**3986**	351	D	11.7307	1
**3986**	357	D	57.7217	1
**3986**	370	D	79.4748	1
**3986**	372	D	79.5083	0
**3986**	373	D	77.8666	1
**3986**	381	D	57.5775	1
**3986**	382	D	64.3222	1
**3986**	386	D	67.1291	1
**3987**	305	A	79.5763	1
**3987**	319	A	79.1552	1
**3987**	320	A	64.7438	-1
**3987**	321	A	77.1063	0

**3987**	351	A	1.6211	1
**3987**	357	A	51.2676	1
**3987**	370	A	79.6217	1
**3987**	372	A	62.0721	0
**3987**	373	A	27.5542	0
**3987**	381	A	72.7018	1
**3987**	382	A	42.0325	0
**3987**	386	A	69.3127	1
**3987**	305	B	79.5337	1
**3987**	319	B	64.7862	1
**3987**	320	B	79.5494	-1
**3987**	321	B	79.5802	0
**3987**	351	B	14.8397	1
**3987**	357	B	70.7002	1
**3987**	370	B	79.6322	1
**3987**	372	B	64.8166	0
**3987**	373	B	40.3361	0
**3987**	381	B	44.3638	0
**3987**	382	B	56.5136	1
**3987**	386	B	75.2395	1
**3987**	305	C	79.7205	1
**3987**	319	C	71.5079	1
**3987**	320	C	79.4111	-1
**3987**	321	C	39.8267	1
**3987**	351	C	2.9683	1
**3987**	357	C	69.0389	1
**3987**	370	C	74.3324	1
**3987**	372	C	73.7326	0
**3987**	373	C	79.4259	1
**3987**	381	C	79.5602	1
**3987**	382	C	72.2072	1
**3987**	386	C	55.4837	1
**3987**	305	D	79.5605	1
**3987**	319	D	72.9407	1

**3987**	320	D	79.4054	-1
**3987**	321	D	63.5256	0
**3987**	351	D	7.4542	1
**3987**	357	D	77.4377	1
**3987**	370	D	68.553	1
**3987**	372	D	79.4701	0
**3987**	373	D	1.6182	-1
**3987**	381	D	73.327	1
**3987**	382	D	73.1553	1
**3987**	386	D	79.7066	1
**3988**	305	A	73.5222	1
**3988**	319	A	60.9779	1
**3988**	320	A	79.5835	-1
**3988**	321	A	36.0456	1
**3988**	351	A	19.3253	1
**3988**	357	A	54.9947	1
**3988**	370	A	79.6585	1
**3988**	372	A	79.5086	0
**3988**	373	A	69.2827	1
**3988**	381	A	79.6483	1
**3988**	382	A	76.5871	1
**3988**	386	A	73.5412	1
**3988**	305	B	74.699	1
**3988**	319	B	54.2632	1
**3988**	320	B	61.178	-1
**3988**	321	B	44.5053	1
**3988**	351	B	8.3962	1
**3988**	357	B	69.4863	1
**3988**	370	B	30.2123	0
**3988**	372	B	64.3808	0
**3988**	373	B	79.4851	1
**3988**	381	B	71.6233	1
**3988**	382	B	23.6004	0
**3988**	386	B	79.5624	1

**3988**	305	C	79.4404	1
**3988**	319	C	77.8392	1
**3988**	320	C	73.2177	-1
**3988**	321	C	31.2621	1
**3988**	351	C	14.5182	1
**3988**	357	C	79.5574	1
**3988**	370	C	73.1665	1
**3988**	372	C	77.5361	0
**3988**	373	C	54.8937	1
**3988**	381	C	30.5805	0
**3988**	382	C	55.9425	1
**3988**	386	C	43.4395	0
**3988**	305	D	62.4893	1
**3988**	319	D	76.3992	1
**3988**	320	D	79.5341	-1
**3988**	321	D	65.6637	0
**3988**	351	D	3.2175	1
**3988**	357	D	70.1241	1
**3988**	370	D	73.2364	1
**3988**	372	D	73.9691	0
**3988**	373	D	66.7803	1
**3988**	381	D	79.6117	1
**3988**	382	D	38.0522	0
**3988**	386	D	79.5215	1
**3989**	305	A	70.8991	1
**3989**	319	A	58.4499	1
**3989**	320	A	67.7306	-1
**3989**	321	A	72.9379	0
**3989**	351	A	32.9524	-1
**3989**	357	A	79.6993	1
**3989**	370	A	72.0854	1
**3989**	372	A	52.9084	0
**3989**	373	A	75.0872	1
**3989**	381	A	76.8018	1

**3989**	382	A	58.3013	1
**3989**	386	A	60.2684	1
**3989**	305	B	79.5419	1
**3989**	319	B	52.8538	1
**3989**	320	B	72.1787	-1
**3989**	321	B	79.527	0
**3989**	351	B	16.3521	1
**3989**	357	B	71.6515	1
**3989**	370	B	76.4096	1
**3989**	372	B	45.1763	1
**3989**	373	B	33.8199	0
**3989**	381	B	43.0476	0
**3989**	382	B	43.5808	0
**3989**	386	B	37.3609	0
**3989**	305	C	40.1023	0
**3989**	319	C	78.7282	1
**3989**	320	C	76.0904	-1
**3989**	321	C	64.1906	0
**3989**	351	C	20.0803	-1
**3989**	357	C	79.7448	1
**3989**	370	C	51.1787	1
**3989**	372	C	79.5025	0
**3989**	373	C	72.8659	1
**3989**	381	C	79.6017	1
**3989**	382	C	67.0095	1
**3989**	386	C	60.3768	1
**3989**	305	D	71.872	1
**3989**	319	D	79.6589	1
**3989**	320	D	79.5212	-1
**3989**	321	D	79.4335	0
**3989**	351	D	22.1706	-1
**3989**	357	D	57.6991	1
**3989**	370	D	77.4275	1
**3989**	372	D	74.7835	0

**3989**	373	D	79.5358	1
**3989**	381	D	58.0611	1
**3989**	382	D	61.1973	1
**3989**	386	D	72.4317	1
**3990**	305	A	76.548	1
**3990**	319	A	29.9401	0
**3990**	320	A	71.0707	-1
**3990**	321	A	56.5827	0
**3990**	351	A	3.6866	1
**3990**	357	A	79.5647	1
**3990**	370	A	74.7446	1
**3990**	372	A	72.924	0
**3990**	373	A	79.6506	1
**3990**	381	A	48.5979	0
**3990**	382	A	45.27	0
**3990**	386	A	45.4895	0
**3990**	305	B	71.2697	1
**3990**	319	B	79.557	1
**3990**	320	B	71.8191	-1
**3990**	321	B	78.6191	0
**3990**	351	B	0.3424	1
**3990**	357	B	79.4997	1
**3990**	370	B	45.8994	0
**3990**	372	B	64.3424	0
**3990**	373	B	32.1056	0
**3990**	381	B	65.3294	1
**3990**	382	B	76.7344	1
**3990**	386	B	58.2421	1
**3990**	305	C	79.5916	1
**3990**	319	C	79.5877	1
**3990**	320	C	73.6768	-1
**3990**	321	C	54.6644	0
**3990**	351	C	2.8255	1
**3990**	357	C	65.0425	1

**3990**	370	C	74.8749	1
**3990**	372	C	75.8472	0
**3990**	373	C	79.6122	1
**3990**	381	C	49.0795	0
**3990**	382	C	43.2708	0
**3990**	386	C	28.272	0
**3990**	305	D	63.7538	1
**3990**	319	D	67.5404	1
**3990**	320	D	62.873	-1
**3990**	321	D	54.307	0
**3990**	351	D	19.5918	1
**3990**	357	D	74.3425	1
**3990**	370	D	61.4765	1
**3990**	372	D	35.3896	1
**3990**	373	D	66.1351	1
**3990**	381	D	63.0824	1
**3990**	382	D	79.5715	1
**3990**	386	D	79.6576	1
**3991**	305	A	79.4718	1
**3991**	319	A	61.8719	1
**3991**	320	A	72.5897	-1
**3991**	321	A	79.5315	0
**3991**	351	A	5.4552	1
**3991**	357	A	67.3366	1
**3991**	370	A	58.3265	1
**3991**	372	A	64.2521	0
**3991**	373	A	47.0449	0
**3991**	381	A	79.6018	1
**3991**	382	A	38.8117	0
**3991**	386	A	78.0027	1
**3991**	305	B	73.8078	1
**3991**	319	B	35.0525	0
**3991**	320	B	79.5592	-1
**3991**	321	B	73.5249	0

**3991**	351	B	14.8286	1
**3991**	357	B	37.1744	0
**3991**	370	B	72.7723	1
**3991**	372	B	79.5793	0
**3991**	373	B	43.9545	0
**3991**	381	B	62.8258	1
**3991**	382	B	58.299	1
**3991**	386	B	55.7545	1
**3991**	305	C	79.5429	1
**3991**	319	C	68.5785	1
**3991**	320	C	79.5696	-1
**3991**	321	C	57.3281	0
**3991**	351	C	6.8968	1
**3991**	357	C	59.0468	1
**3991**	370	C	32.645	0
**3991**	372	C	73.8735	0
**3991**	373	C	72.5942	1
**3991**	381	C	53.8423	1
**3991**	382	C	73.2752	1
**3991**	386	C	13.9424	-1
**3991**	305	D	46.1051	0
**3991**	319	D	40.2632	0
**3991**	320	D	53.6518	-1
**3991**	321	D	79.7193	0
**3991**	351	D	10.1654	1
**3991**	357	D	79.5654	1
**3991**	370	D	59.0241	1
**3991**	372	D	72.3283	0
**3991**	373	D	15.3043	-1
**3991**	381	D	57.3788	1
**3991**	382	D	79.558	1
**3991**	386	D	79.5413	1
**3992**	305	A	61.147	1
**3992**	319	A	62.9262	1

**3992**	320	A	79.5301	-1
**3992**	321	A	73.1219	0
**3992**	351	A	4.0049	1
**3992**	357	A	66.986	1
**3992**	370	A	47.306	0
**3992**	372	A	79.5764	0
**3992**	373	A	46.9283	0
**3992**	381	A	37.7948	0
**3992**	382	A	56.8673	1
**3992**	386	A	79.5635	1
**3992**	305	B	69.6039	1
**3992**	319	B	69.12	1
**3992**	320	B	54.946	-1
**3992**	321	B	73.4436	0
**3992**	351	B	11.7372	1
**3992**	357	B	70.0618	1
**3992**	370	B	78.95	1
**3992**	372	B	66.8993	0
**3992**	373	B	63.4221	1
**3992**	381	B	68.0323	1
**3992**	382	B	70.0679	1
**3992**	386	B	50.384	1
**3992**	305	C	79.5797	1
**3992**	319	C	79.6041	1
**3992**	320	C	58.2319	-1
**3992**	321	C	70.4267	0
**3992**	351	C	3.0834	1
**3992**	357	C	79.5324	1
**3992**	370	C	79.509	1
**3992**	372	C	62.8457	0
**3992**	373	C	72.3923	1
**3992**	381	C	53.8705	1
**3992**	382	C	73.0894	1
**3992**	386	C	50.4206	1

**3992**	305	D	45.5318	0
**3992**	319	D	71.7372	1
**3992**	320	D	55.8467	-1
**3992**	321	D	79.5317	0
**3992**	351	D	10.2086	1
**3992**	357	D	63.6028	1
**3992**	370	D	79.4483	1
**3992**	372	D	79.5452	0
**3992**	373	D	79.5644	1
**3992**	381	D	74.4073	1
**3992**	382	D	79.5598	1
**3992**	386	D	73.4266	1
**3993**	305	A	75.0906	1
**3993**	319	A	67.8306	1
**3993**	320	A	73.9715	-1
**3993**	321	A	54.7273	0
**3993**	351	A	3.0951	1
**3993**	357	A	67.3086	1
**3993**	370	A	22.5867	0
**3993**	372	A	55.7684	0
**3993**	373	A	79.6107	1
**3993**	381	A	64.7877	1
**3993**	382	A	74.4741	1
**3993**	386	A	79.5828	1
**3993**	305	B	60.0409	1
**3993**	319	B	79.563	1
**3993**	320	B	71.9005	-1
**3993**	321	B	47.6257	1
**3993**	351	B	12.264	1
**3993**	357	B	79.576	1
**3993**	370	B	67.7682	1
**3993**	372	B	44.1045	1
**3993**	373	B	21.9124	0
**3993**	381	B	33.3296	0

**3993**	382	B	76.6556	1
**3993**	386	B	56.8	1
**3993**	305	C	54.9316	1
**3993**	319	C	79.6132	1
**3993**	320	C	78.0781	-1
**3993**	321	C	79.4769	0
**3993**	351	C	14.3062	1
**3993**	357	C	57.9812	1
**3993**	370	C	79.5134	1
**3993**	372	C	79.5965	0
**3993**	373	C	71.2641	1
**3993**	381	C	79.2718	1
**3993**	382	C	37.1766	0
**3993**	386	C	27.3264	0
**3993**	305	D	79.5973	1
**3993**	319	D	79.51	1
**3993**	320	D	64.411	-1
**3993**	321	D	79.5379	0
**3993**	351	D	1.9935	1
**3993**	357	D	68.5792	1
**3993**	370	D	21.2335	0
**3993**	372	D	79.5266	0
**3993**	373	D	68.1273	1
**3993**	381	D	63.5749	1
**3993**	382	D	75.981	1
**3993**	386	D	51.9801	1
**3994**	305	A	52.2021	1
**3994**	319	A	78.0147	1
**3994**	320	A	79.5378	-1
**3994**	321	A	78.4244	0
**3994**	351	A	11.6578	1
**3994**	357	A	79.5642	1
**3994**	370	A	79.5939	1
**3994**	372	A	28.5578	1

**3994**	373	A	37.9131	0
**3994**	381	A	79.5622	1
**3994**	382	A	62.7899	1
**3994**	386	A	72.5393	1
**3994**	305	B	50.6061	1
**3994**	319	B	79.5055	1
**3994**	320	B	55.4733	-1
**3994**	321	B	75.4583	0
**3994**	351	B	13.9719	1
**3994**	357	B	67.2759	1
**3994**	370	B	67.5911	1
**3994**	372	B	71.7076	0
**3994**	373	B	51.5681	1
**3994**	381	B	72.2801	1
**3994**	382	B	69.5382	1
**3994**	386	B	79.4999	1
**3994**	305	C	71.1508	1
**3994**	319	C	79.6301	1
**3994**	320	C	73.382	-1
**3994**	321	C	79.6425	0
**3994**	351	C	5.9417	1
**3994**	357	C	77.6718	1
**3994**	370	C	79.6159	1
**3994**	372	C	79.4521	0
**3994**	373	C	73.0161	1
**3994**	381	C	70.8536	1
**3994**	382	C	73.3604	1
**3994**	386	C	56.2863	1
**3994**	305	D	66.2622	1
**3994**	319	D	63.9547	1
**3994**	320	D	79.5715	-1
**3994**	321	D	72.8958	0
**3994**	351	D	15.061	1
**3994**	357	D	79.5356	1

**3994**	370	D	79.5574	1
**3994**	372	D	79.4944	0
**3994**	373	D	62.5629	1
**3994**	381	D	71.5638	1
**3994**	382	D	79.5301	1
**3994**	386	D	71.03	1
**3995**	305	A	63.8421	1
**3995**	319	A	67.3723	1
**3995**	320	A	57.2356	-1
**3995**	321	A	79.5261	0
**3995**	351	A	0.4517	1
**3995**	357	A	69.5506	1
**3995**	370	A	79.5158	1
**3995**	372	A	79.5045	0
**3995**	373	A	72.3696	1
**3995**	381	A	70.1981	1
**3995**	382	A	61.9844	1
**3995**	386	A	73.8577	1
**3995**	305	B	67.0245	1
**3995**	319	B	75.3105	1
**3995**	320	B	70.0332	-1
**3995**	321	B	79.5655	0
**3995**	351	B	7.4108	1
**3995**	357	B	45.8985	0
**3995**	370	B	24.6177	0
**3995**	372	B	75.5349	0
**3995**	373	B	74.1618	1
**3995**	381	B	51.9936	1
**3995**	382	B	28.7466	0
**3995**	386	B	79.5804	1
**3995**	305	C	72.875	1
**3995**	319	C	79.3549	1
**3995**	320	C	31.2078	-1
**3995**	321	C	64.7985	0

**3995**	351	C	0.0022	1
**3995**	357	C	79.5092	1
**3995**	370	C	78.293	1
**3995**	372	C	79.5705	0
**3995**	373	C	79.6755	1
**3995**	381	C	56.5218	1
**3995**	382	C	54.0382	1
**3995**	386	C	53.0888	1
**3995**	305	D	73.1856	1
**3995**	319	D	48.2963	0
**3995**	320	D	65.3252	-1
**3995**	321	D	79.6722	0
**3995**	351	D	15.3961	1
**3995**	357	D	72.055	1
**3995**	370	D	53.6239	1
**3995**	372	D	46.5489	1
**3995**	373	D	79.6138	1
**3995**	381	D	76.0926	1
**3995**	382	D	60.3334	1
**3995**	386	D	74.8329	1
**3996**	305	A	58.724	1
**3996**	319	A	53.6326	1
**3996**	320	A	77.5688	-1
**3996**	321	A	68.1138	0
**3996**	351	A	0.8989	1
**3996**	357	A	79.5917	1
**3996**	370	A	74.4877	1
**3996**	372	A	33.6926	1
**3996**	373	A	69.4146	1
**3996**	381	A	49.3373	0
**3996**	382	A	55.3239	1
**3996**	386	A	47.9151	0
**3996**	305	B	62.7475	1
**3996**	319	B	58.521	1

**3996**	320	B	79.5191	-1
**3996**	321	B	79.5345	0
**3996**	351	B	14.6927	1
**3996**	357	B	70.54	1
**3996**	370	B	69.2131	1
**3996**	372	B	74.0795	0
**3996**	373	B	53.1882	1
**3996**	381	B	46.3927	0
**3996**	382	B	79.7121	1
**3996**	386	B	79.6577	1
**3996**	305	C	79.5638	1
**3996**	319	C	19.1078	-1
**3996**	320	C	79.5614	-1
**3996**	321	C	79.5133	0
**3996**	351	C	2.7205	1
**3996**	357	C	72.1054	1
**3996**	370	C	76.342	1
**3996**	372	C	50.21	0
**3996**	373	C	79.5481	1
**3996**	381	C	43.2577	0
**3996**	382	C	21.2936	0
**3996**	386	C	47.5736	0
**3996**	305	D	37.3619	0
**3996**	319	D	79.5208	1
**3996**	320	D	73.7884	-1
**3996**	321	D	64.9269	0
**3996**	351	D	14.5159	1
**3996**	357	D	43.1013	0
**3996**	370	D	79.5731	1
**3996**	372	D	79.1683	0
**3996**	373	D	57.2049	1
**3996**	381	D	79.569	1
**3996**	382	D	79.4203	1
**3996**	386	D	79.3816	1

**3997**	305	A	69.5116	1
**3997**	319	A	62.2184	1
**3997**	320	A	69.0354	-1
**3997**	321	A	50.0133	0
**3997**	351	A	4.8749	1
**3997**	357	A	55.5255	1
**3997**	370	A	78.7671	1
**3997**	372	A	64.4689	0
**3997**	373	A	50.9401	1
**3997**	381	A	79.3986	1
**3997**	382	A	52.4906	1
**3997**	386	A	79.4507	1
**3997**	305	B	34.3372	0
**3997**	319	B	56.4385	1
**3997**	320	B	33.1076	-1
**3997**	321	B	72.8956	0
**3997**	351	B	0.0	1
**3997**	357	B	47.8883	0
**3997**	370	B	61.831	1
**3997**	372	B	68.3145	0
**3997**	373	B	26.8073	0
**3997**	381	B	79.4565	1
**3997**	382	B	74.3308	1
**3997**	386	B	50.3232	1
**3997**	305	C	48.2616	0
**3997**	319	C	79.5077	1
**3997**	320	C	79.4646	-1
**3997**	321	C	68.2294	0
**3997**	351	C	20.3506	-1
**3997**	357	C	60.922	1
**3997**	370	C	62.4811	1
**3997**	372	C	74.0889	0
**3997**	373	C	71.2623	1
**3997**	381	C	79.6163	1

**3997**	382	C	73.5619	1
**3997**	386	C	40.5195	0
**3997**	305	D	49.8734	0
**3997**	319	D	79.5904	1
**3997**	320	D	79.2686	-1
**3997**	321	D	79.5434	0
**3997**	351	D	15.5856	1
**3997**	357	D	79.5965	1
**3997**	370	D	41.53	0
**3997**	372	D	78.7346	0
**3997**	373	D	79.5726	1
**3997**	381	D	67.639	1
**3997**	382	D	69.967	1
**3997**	386	D	79.703	1
**3998**	305	A	37.2772	0
**3998**	319	A	62.562	1
**3998**	320	A	64.1092	-1
**3998**	321	A	29.6084	1
**3998**	351	A	11.2407	1
**3998**	357	A	69.7769	1
**3998**	370	A	79.4689	1
**3998**	372	A	65.2107	0
**3998**	373	A	65.1118	1
**3998**	381	A	72.3684	1
**3998**	382	A	77.7245	1
**3998**	386	A	70.8886	1
**3998**	305	B	79.6755	1
**3998**	319	B	44.1349	0
**3998**	320	B	79.3506	-1
**3998**	321	B	60.5032	0
**3998**	351	B	8.5289	1
**3998**	357	B	34.6172	0
**3998**	370	B	79.5018	1
**3998**	372	B	37.011	1

**3998**	373	B	72.0492	1
**3998**	381	B	25.1339	0
**3998**	382	B	15.3291	-1
**3998**	386	B	79.677	1
**3998**	305	C	77.7998	1
**3998**	319	C	56.2173	1
**3998**	320	C	69.6909	-1
**3998**	321	C	79.4458	0
**3998**	351	C	5.9699	1
**3998**	357	C	75.7553	1
**3998**	370	C	67.6139	1
**3998**	372	C	79.6089	0
**3998**	373	C	70.138	1
**3998**	381	C	72.0912	1
**3998**	382	C	79.5123	1
**3998**	386	C	41.6849	0
**3998**	305	D	73.9494	1
**3998**	319	D	36.8813	0
**3998**	320	D	59.1551	-1
**3998**	321	D	40.959	1
**3998**	351	D	14.5518	1
**3998**	357	D	70.7195	1
**3998**	370	D	72.4842	1
**3998**	372	D	63.7744	0
**3998**	373	D	31.5277	0
**3998**	381	D	58.4205	1
**3998**	382	D	79.5139	1
**3998**	386	D	79.5741	1
**3999**	305	A	79.6126	1
**3999**	319	A	74.1017	1
**3999**	320	A	77.4939	-1
**3999**	321	A	47.8851	1
**3999**	351	A	6.9997	1
**3999**	357	A	71.1127	1

**3999**	370	A	73.9073	1
**3999**	372	A	79.5331	0
**3999**	373	A	79.6069	1
**3999**	381	A	38.4216	0
**3999**	382	A	59.2621	1
**3999**	386	A	58.9084	1
**3999**	305	B	52.4833	1
**3999**	319	B	62.8177	1
**3999**	320	B	79.5048	-1
**3999**	321	B	79.3103	0
**3999**	351	B	6.4333	1
**3999**	357	B	74.8503	1
**3999**	370	B	79.5078	1
**3999**	372	B	43.0185	1
**3999**	373	B	27.1682	0
**3999**	381	B	71.4315	1
**3999**	382	B	58.6384	1
**3999**	386	B	79.4113	1
**3999**	305	C	61.5448	1
**3999**	319	C	60.6275	1
**3999**	320	C	79.4959	-1
**3999**	321	C	52.5585	0
**3999**	351	C	7.2929	1
**3999**	357	C	79.564	1
**3999**	370	C	76.1183	1
**3999**	372	C	70.9487	0
**3999**	373	C	75.5748	1
**3999**	381	C	79.4638	1
**3999**	382	C	59.84	1
**3999**	386	C	43.2206	0
**3999**	305	D	54.5289	1
**3999**	319	D	79.5791	1
**3999**	320	D	43.4437	-1
**3999**	321	D	73.973	0

**3999**	351	D	18.8779	1
**3999**	357	D	52.9535	1
**3999**	370	D	77.3502	1
**3999**	372	D	70.8706	0
**3999**	373	D	41.3797	0
**3999**	381	D	47.4746	0
**3999**	382	D	60.7027	1
**3999**	386	D	76.7636	1
**4000**	305	A	79.5761	1
**4000**	319	A	46.8568	0
**4000**	320	A	79.557	-1
**4000**	321	A	69.5697	0
**4000**	351	A	11.8066	1
**4000**	357	A	70.6255	1
**4000**	370	A	78.934	1
**4000**	372	A	71.1491	0
**4000**	373	A	71.866	1
**4000**	381	A	71.8575	1
**4000**	382	A	70.518	1
**4000**	386	A	79.2949	1
**4000**	305	B	79.5552	1
**4000**	319	B	67.044	1
**4000**	320	B	79.5564	-1
**4000**	321	B	18.6478	0
**4000**	351	B	14.5292	1
**4000**	357	B	55.6321	1
**4000**	370	B	79.5728	1
**4000**	372	B	79.5524	0
**4000**	373	B	72.0773	1
**4000**	381	B	79.5403	1
**4000**	382	B	40.9782	0
**4000**	386	B	79.6425	1
**4000**	305	C	79.524	1
**4000**	319	C	53.8655	1

**4000**	320	C	71.0572	-1
**4000**	321	C	41.4834	1
**4000**	351	C	0.2987	1
**4000**	357	C	79.6064	1
**4000**	370	C	70.9059	1
**4000**	372	C	74.4281	0
**4000**	373	C	79.5269	1
**4000**	381	C	79.5502	1
**4000**	382	C	70.9117	1
**4000**	386	C	39.7836	0
**4000**	305	D	79.5228	1
**4000**	319	D	61.4527	1
**4000**	320	D	79.5647	-1
**4000**	321	D	61.0989	0
**4000**	351	D	5.2886	1
**4000**	357	D	51.2531	1
**4000**	370	D	73.3084	1
**4000**	372	D	71.8945	0
**4000**	373	D	79.5605	1
**4000**	381	D	75.6036	1
**4000**	382	D	79.5434	1
**4000**	386	D	18.5368	-1
**4001**	305	A	64.1415	1
**4001**	319	A	76.0587	1
**4001**	320	A	42.1668	-1
**4001**	321	A	22.3829	1
**4001**	351	A	0.6099	1
**4001**	357	A	72.3457	1
**4001**	370	A	45.6362	0
**4001**	372	A	79.5706	0
**4001**	373	A	62.8246	1
**4001**	381	A	78.9752	1
**4001**	382	A	59.8022	1
**4001**	386	A	79.5891	1

**4001**	305	B	62.5948	1
**4001**	319	B	38.3384	0
**4001**	320	B	57.5391	-1
**4001**	321	B	79.3129	0
**4001**	351	B	13.656	1
**4001**	357	B	79.5531	1
**4001**	370	B	79.5227	1
**4001**	372	B	74.4348	0
**4001**	373	B	55.0491	1
**4001**	381	B	79.5488	1
**4001**	382	B	54.8511	1
**4001**	386	B	43.7681	0
**4001**	305	C	62.8971	1
**4001**	319	C	55.0151	1
**4001**	320	C	49.4317	-1
**4001**	321	C	26.1706	1
**4001**	351	C	25.6166	-1
**4001**	357	C	79.5758	1
**4001**	370	C	79.5751	1
**4001**	372	C	58.3521	0
**4001**	373	C	79.5801	1
**4001**	381	C	79.5706	1
**4001**	382	C	79.603	1
**4001**	386	C	34.539	0
**4001**	305	D	28.8039	0
**4001**	319	D	59.614	1
**4001**	320	D	79.541	-1
**4001**	321	D	57.6287	0
**4001**	351	D	10.874	1
**4001**	357	D	72.476	1
**4001**	370	D	76.8797	1
**4001**	372	D	53.5412	0
**4001**	373	D	70.6687	1
**4001**	381	D	74.2763	1

**4001**	382	D	45.1329	0
**4001**	386	D	49.2013	0
**4002**	305	A	79.5872	1
**4002**	319	A	79.5732	1
**4002**	320	A	79.5458	-1
**4002**	321	A	73.5298	0
**4002**	351	A	1.4142	1
**4002**	357	A	72.1961	1
**4002**	370	A	79.5472	1
**4002**	372	A	73.1119	0
**4002**	373	A	79.4637	1
**4002**	381	A	78.3822	1
**4002**	382	A	79.5631	1
**4002**	386	A	68.194	1
**4002**	305	B	77.7695	1
**4002**	319	B	79.5861	1
**4002**	320	B	69.9131	-1
**4002**	321	B	79.6106	0
**4002**	351	B	14.7041	1
**4002**	357	B	66.769	1
**4002**	370	B	79.5683	1
**4002**	372	B	79.4827	0
**4002**	373	B	50.2848	1
**4002**	381	B	72.1427	1
**4002**	382	B	42.7633	0
**4002**	386	B	79.6014	1
**4002**	305	C	79.551	1
**4002**	319	C	78.5469	1
**4002**	320	C	79.5753	-1
**4002**	321	C	73.7483	0
**4002**	351	C	8.0692	1
**4002**	357	C	49.1174	0
**4002**	370	C	65.609	1
**4002**	372	C	73.4229	0

**4002**	373	C	79.567	1
**4002**	381	C	73.0435	1
**4002**	382	C	72.3705	1
**4002**	386	C	58.5634	1
**4002**	305	D	39.6611	0
**4002**	319	D	74.0222	1
**4002**	320	D	73.7201	-1
**4002**	321	D	79.6125	0
**4002**	351	D	11.2266	1
**4002**	357	D	63.1308	1
**4002**	370	D	79.5456	1
**4002**	372	D	72.5333	0
**4002**	373	D	66.7701	1
**4002**	381	D	74.2193	1
**4002**	382	D	68.2978	1
**4002**	386	D	79.5541	1
**4003**	305	A	73.312	1
**4003**	319	A	55.772	1
**4003**	320	A	73.6084	-1
**4003**	321	A	79.5843	0
**4003**	351	A	6.8924	1
**4003**	357	A	65.6755	1
**4003**	370	A	45.66	0
**4003**	372	A	79.5043	0
**4003**	373	A	79.5576	1
**4003**	381	A	41.8834	0
**4003**	382	A	79.5587	1
**4003**	386	A	79.5528	1
**4003**	305	B	79.5267	1
**4003**	319	B	79.5625	1
**4003**	320	B	28.5313	-1
**4003**	321	B	72.9999	0
**4003**	351	B	8.5339	1
**4003**	357	B	73.1998	1

**4003**	370	B	78.2025	1
**4003**	372	B	50.6807	0
**4003**	373	B	73.1701	1
**4003**	381	B	79.4675	1
**4003**	382	B	52.7808	1
**4003**	386	B	48.1615	0
**4003**	305	C	73.0279	1
**4003**	319	C	72.7728	1
**4003**	320	C	72.8255	-1
**4003**	321	C	70.8794	0
**4003**	351	C	0.6298	1
**4003**	357	C	57.9446	1
**4003**	370	C	79.5844	1
**4003**	372	C	53.3824	0
**4003**	373	C	73.6984	1
**4003**	381	C	46.1492	0
**4003**	382	C	78.6662	1
**4003**	386	C	20.6223	0
**4003**	305	D	74.5397	1
**4003**	319	D	79.5231	1
**4003**	320	D	58.1055	-1
**4003**	321	D	61.969	0
**4003**	351	D	11.9422	1
**4003**	357	D	71.3354	1
**4003**	370	D	77.8484	1
**4003**	372	D	66.8279	0
**4003**	373	D	45.9887	0
**4003**	381	D	69.6357	1
**4003**	382	D	48.4138	0
**4003**	386	D	13.8656	-1
**4004**	305	A	79.5163	1
**4004**	319	A	70.4617	1
**4004**	320	A	39.0283	-1
**4004**	321	A	79.4678	0

**4004**	351	A	7.9751	1
**4004**	357	A	78.6218	1
**4004**	370	A	79.4759	1
**4004**	372	A	59.4672	0
**4004**	373	A	74.7561	1
**4004**	381	A	56.5579	1
**4004**	382	A	68.0511	1
**4004**	386	A	33.42	0
**4004**	305	B	79.5703	1
**4004**	319	B	79.5294	1
**4004**	320	B	65.4769	-1
**4004**	321	B	73.9445	0
**4004**	351	B	21.7626	-1
**4004**	357	B	79.5683	1
**4004**	370	B	34.1167	0
**4004**	372	B	79.5788	0
**4004**	373	B	13.5214	-1
**4004**	381	B	73.0692	1
**4004**	382	B	53.4133	1
**4004**	386	B	79.3363	1
**4004**	305	C	57.6004	1
**4004**	319	C	74.0174	1
**4004**	320	C	79.541	-1
**4004**	321	C	62.2038	0
**4004**	351	C	4.9557	1
**4004**	357	C	79.3387	1
**4004**	370	C	67.5314	1
**4004**	372	C	79.4323	0
**4004**	373	C	69.7147	1
**4004**	381	C	72.0423	1
**4004**	382	C	54.6115	1
**4004**	386	C	67.4441	1
**4004**	305	D	79.4697	1
**4004**	319	D	79.5077	1

**4004**	320	D	79.4484	-1
**4004**	321	D	79.6268	0
**4004**	351	D	12.6759	1
**4004**	357	D	50.4984	1
**4004**	370	D	74.216	1
**4004**	372	D	71.7982	0
**4004**	373	D	73.7974	1
**4004**	381	D	79.592	1
**4004**	382	D	73.141	1
**4004**	386	D	64.8922	1
**4005**	305	A	75.7022	1
**4005**	319	A	60.4876	1
**4005**	320	A	72.3556	-1
**4005**	321	A	56.8067	0
**4005**	351	A	8.0712	1
**4005**	357	A	65.6049	1
**4005**	370	A	59.0352	1
**4005**	372	A	50.4294	0
**4005**	373	A	79.5911	1
**4005**	381	A	76.1207	1
**4005**	382	A	67.093	1
**4005**	386	A	79.4871	1
**4005**	305	B	76.2627	1
**4005**	319	B	79.635	1
**4005**	320	B	70.5542	-1
**4005**	321	B	78.7712	0
**4005**	351	B	10.3331	1
**4005**	357	B	79.5735	1
**4005**	370	B	35.8335	0
**4005**	372	B	60.9519	0
**4005**	373	B	57.6134	1
**4005**	381	B	59.8072	1
**4005**	382	B	49.9328	0
**4005**	386	B	69.7264	1

**4005**	305	C	68.7023	1
**4005**	319	C	79.5564	1
**4005**	320	C	49.5649	-1
**4005**	321	C	79.4912	0
**4005**	351	C	2.8513	1
**4005**	357	C	79.4443	1
**4005**	370	C	69.7964	1
**4005**	372	C	79.6445	0
**4005**	373	C	75.7162	1
**4005**	381	C	64.9283	1
**4005**	382	C	27.8236	0
**4005**	386	C	44.3948	0
**4005**	305	D	79.6177	1
**4005**	319	D	21.85	0
**4005**	320	D	79.5522	-1
**4005**	321	D	72.778	0
**4005**	351	D	16.2126	1
**4005**	357	D	73.2424	1
**4005**	370	D	74.6865	1
**4005**	372	D	78.9952	0
**4005**	373	D	79.684	1
**4005**	381	D	25.252	0
**4005**	382	D	71.7707	1
**4005**	386	D	70.8575	1
**4006**	305	A	3.8586	-1
**4006**	319	A	46.7673	0
**4006**	320	A	51.9032	-1
**4006**	321	A	79.5674	0
**4006**	351	A	7.9212	1
**4006**	357	A	79.5464	1
**4006**	370	A	54.2618	1
**4006**	372	A	79.5342	0
**4006**	373	A	72.4472	1
**4006**	381	A	53.6944	1

**4006**	382	A	79.592	1
**4006**	386	A	79.6026	1
**4006**	305	B	79.478	1
**4006**	319	B	72.918	1
**4006**	320	B	68.7708	-1
**4006**	321	B	79.5348	0
**4006**	351	B	14.908	1
**4006**	357	B	64.2671	1
**4006**	370	B	71.3666	1
**4006**	372	B	79.524	0
**4006**	373	B	63.7297	1
**4006**	381	B	79.5456	1
**4006**	382	B	79.6144	1
**4006**	386	B	79.4993	1
**4006**	305	C	38.6685	0
**4006**	319	C	77.1119	1
**4006**	320	C	79.6044	-1
**4006**	321	C	79.6675	0
**4006**	351	C	7.715	1
**4006**	357	C	72.8122	1
**4006**	370	C	70.7977	1
**4006**	372	C	79.6081	0
**4006**	373	C	79.4273	1
**4006**	381	C	60.0494	1
**4006**	382	C	50.8413	1
**4006**	386	C	46.9522	0
**4006**	305	D	79.5355	1
**4006**	319	D	79.6049	1
**4006**	320	D	79.5136	-1
**4006**	321	D	51.1936	0
**4006**	351	D	10.5515	1
**4006**	357	D	73.8257	1
**4006**	370	D	79.5771	1
**4006**	372	D	72.723	0

**4006**	373	D	71.0204	1
**4006**	381	D	9.8453	-1
**4006**	382	D	57.879	1
**4006**	386	D	43.9538	0
**4007**	305	A	73.0532	1
**4007**	319	A	54.9013	1
**4007**	320	A	63.57	-1
**4007**	321	A	71.3319	0
**4007**	351	A	8.3747	1
**4007**	357	A	75.4069	1
**4007**	370	A	79.5459	1
**4007**	372	A	79.5864	0
**4007**	373	A	78.0077	1
**4007**	381	A	35.3231	0
**4007**	382	A	71.818	1
**4007**	386	A	72.5597	1
**4007**	305	B	72.1112	1
**4007**	319	B	15.8659	-1
**4007**	320	B	79.5531	-1
**4007**	321	B	79.6205	0
**4007**	351	B	15.9364	1
**4007**	357	B	72.0063	1
**4007**	370	B	79.5814	1
**4007**	372	B	79.5435	0
**4007**	373	B	79.5451	1
**4007**	381	B	79.4981	1
**4007**	382	B	29.2302	0
**4007**	386	B	40.8173	0
**4007**	305	C	57.0678	1
**4007**	319	C	51.6885	1
**4007**	320	C	63.724	-1
**4007**	321	C	60.9493	0
**4007**	351	C	6.4187	1
**4007**	357	C	72.625	1

**4007**	370	C	79.5715	1
**4007**	372	C	79.6231	0
**4007**	373	C	79.4333	1
**4007**	381	C	77.5644	1
**4007**	382	C	63.4421	1
**4007**	386	C	36.7942	0
**4007**	305	D	59.5344	1
**4007**	319	D	40.0935	0
**4007**	320	D	79.5607	-1
**4007**	321	D	59.6489	0
**4007**	351	D	9.3648	1
**4007**	357	D	56.8943	1
**4007**	370	D	72.3263	1
**4007**	372	D	42.833	1
**4007**	373	D	73.0103	1
**4007**	381	D	79.5645	1
**4007**	382	D	62.704	1
**4007**	386	D	79.527	1
**4008**	305	A	72.4404	1
**4008**	319	A	41.1424	0
**4008**	320	A	45.3561	-1
**4008**	321	A	43.2301	1
**4008**	351	A	2.7484	1
**4008**	357	A	75.1633	1
**4008**	370	A	77.5479	1
**4008**	372	A	24.682	1
**4008**	373	A	60.9818	1
**4008**	381	A	79.4827	1
**4008**	382	A	76.2782	1
**4008**	386	A	74.0754	1
**4008**	305	B	68.2787	1
**4008**	319	B	70.5454	1
**4008**	320	B	62.7608	-1
**4008**	321	B	48.7024	1

**4008**	351	B	9.1328	1
**4008**	357	B	72.4141	1
**4008**	370	B	58.3296	1
**4008**	372	B	68.9963	0
**4008**	373	B	54.7424	1
**4008**	381	B	79.4854	1
**4008**	382	B	53.5825	1
**4008**	386	B	65.0751	1
**4008**	305	C	48.3131	0
**4008**	319	C	68.5161	1
**4008**	320	C	56.2099	-1
**4008**	321	C	69.6185	0
**4008**	351	C	3.8498	1
**4008**	357	C	59.9677	1
**4008**	370	C	33.9267	0
**4008**	372	C	79.6104	0
**4008**	373	C	79.6048	1
**4008**	381	C	63.6633	1
**4008**	382	C	47.0013	0
**4008**	386	C	42.4257	0
**4008**	305	D	72.4795	1
**4008**	319	D	79.5283	1
**4008**	320	D	67.7197	-1
**4008**	321	D	79.6336	0
**4008**	351	D	16.015	1
**4008**	357	D	63.416	1
**4008**	370	D	47.1827	0
**4008**	372	D	79.5759	0
**4008**	373	D	64.4567	1
**4008**	381	D	79.6187	1
**4008**	382	D	75.7316	1
**4008**	386	D	79.5388	1
**4009**	305	A	79.5706	1
**4009**	319	A	50.3368	1

**4009**	320	A	47.3162	-1
**4009**	321	A	71.4923	0
**4009**	351	A	14.4299	1
**4009**	357	A	76.892	1
**4009**	370	A	60.7243	1
**4009**	372	A	78.3019	0
**4009**	373	A	73.1543	1
**4009**	381	A	79.5855	1
**4009**	382	A	65.4956	1
**4009**	386	A	67.3631	1
**4009**	305	B	52.8427	1
**4009**	319	B	71.3227	1
**4009**	320	B	51.5945	-1
**4009**	321	B	55.7605	0
**4009**	351	B	10.2463	1
**4009**	357	B	79.5735	1
**4009**	370	B	59.1572	1
**4009**	372	B	79.6098	0
**4009**	373	B	79.5121	1
**4009**	381	B	67.6749	1
**4009**	382	B	42.5448	0
**4009**	386	B	63.0276	1
**4009**	305	C	62.63	1
**4009**	319	C	73.9109	1
**4009**	320	C	63.4647	-1
**4009**	321	C	78.3846	0
**4009**	351	C	7.5799	1
**4009**	357	C	79.5193	1
**4009**	370	C	73.0796	1
**4009**	372	C	79.5759	0
**4009**	373	C	74.2813	1
**4009**	381	C	64.7498	1
**4009**	382	C	73.8123	1
**4009**	386	C	34.8383	0

**4009**	305	D	76.6948	1
**4009**	319	D	79.593	1
**4009**	320	D	35.7012	-1
**4009**	321	D	79.5089	0
**4009**	351	D	11.9012	1
**4009**	357	D	72.9408	1
**4009**	370	D	74.5119	1
**4009**	372	D	79.5253	0
**4009**	373	D	79.464	1
**4009**	381	D	78.8943	1
**4009**	382	D	67.4401	1
**4009**	386	D	79.5459	1
**4010**	305	A	48.3328	0
**4010**	319	A	57.8326	1
**4010**	320	A	49.8929	-1
**4010**	321	A	72.7174	0
**4010**	351	A	7.5429	1
**4010**	357	A	79.4411	1
**4010**	370	A	78.8573	1
**4010**	372	A	62.333	0
**4010**	373	A	79.6445	1
**4010**	381	A	59.3127	1
**4010**	382	A	79.3503	1
**4010**	386	A	78.398	1
**4010**	305	B	72.2429	1
**4010**	319	B	79.4988	1
**4010**	320	B	74.0861	-1
**4010**	321	B	67.9143	0
**4010**	351	B	20.6282	-1
**4010**	357	B	79.4569	1
**4010**	370	B	51.136	1
**4010**	372	B	79.5526	0
**4010**	373	B	37.7218	0
**4010**	381	B	4.3323	-1

**4010**	382	B	79.4659	1
**4010**	386	B	47.3779	0
**4010**	305	C	34.6186	0
**4010**	319	C	72.7996	1
**4010**	320	C	60.4961	-1
**4010**	321	C	75.8497	0
**4010**	351	C	2.4533	1
**4010**	357	C	51.7319	1
**4010**	370	C	63.2475	1
**4010**	372	C	44.8424	1
**4010**	373	C	63.9863	1
**4010**	381	C	57.0631	1
**4010**	382	C	31.0365	0
**4010**	386	C	55.9694	1
**4010**	305	D	79.5639	1
**4010**	319	D	22.7598	0
**4010**	320	D	69.5342	-1
**4010**	321	D	79.6186	0
**4010**	351	D	3.1864	1
**4010**	357	D	43.2273	0
**4010**	370	D	74.7016	1
**4010**	372	D	76.066	0
**4010**	373	D	69.1984	1
**4010**	381	D	79.5608	1
**4010**	382	D	79.6228	1
**4010**	386	D	66.0571	1
**4011**	305	A	79.5716	1
**4011**	319	A	50.2243	1
**4011**	320	A	79.5236	-1
**4011**	321	A	42.104	1
**4011**	351	A	13.1871	1
**4011**	357	A	74.8127	1
**4011**	370	A	38.7281	0
**4011**	372	A	79.5808	0

**4011**	373	A	79.5885	1
**4011**	381	A	79.5482	1
**4011**	382	A	65.8341	1
**4011**	386	A	79.5282	1
**4011**	305	B	73.9919	1
**4011**	319	B	40.3126	0
**4011**	320	B	74.5823	-1
**4011**	321	B	73.2947	0
**4011**	351	B	15.6704	1
**4011**	357	B	79.3986	1
**4011**	370	B	52.536	1
**4011**	372	B	42.705	1
**4011**	373	B	56.8468	1
**4011**	381	B	52.0127	1
**4011**	382	B	43.3695	0
**4011**	386	B	79.5929	1
**4011**	305	C	37.8378	0
**4011**	319	C	73.4148	1
**4011**	320	C	31.0148	-1
**4011**	321	C	79.5488	0
**4011**	351	C	2.0109	1
**4011**	357	C	34.2084	0
**4011**	370	C	78.3339	1
**4011**	372	C	65.0388	0
**4011**	373	C	66.1756	1
**4011**	381	C	49.9298	0
**4011**	382	C	51.6948	1
**4011**	386	C	45.0541	0
**4011**	305	D	79.5127	1
**4011**	319	D	74.8783	1
**4011**	320	D	20.1372	-1
**4011**	321	D	73.9665	0
**4011**	351	D	12.1058	1
**4011**	357	D	79.6871	1

**4011**	370	D	78.391	1
**4011**	372	D	79.0809	0
**4011**	373	D	73.3595	1
**4011**	381	D	79.6032	1
**4011**	382	D	71.8237	1
**4011**	386	D	39.0116	0
**4012**	305	A	68.6626	1
**4012**	319	A	35.0087	0
**4012**	320	A	73.5652	-1
**4012**	321	A	79.4768	0
**4012**	351	A	3.3794	1
**4012**	357	A	79.6125	1
**4012**	370	A	79.4431	1
**4012**	372	A	79.6217	0
**4012**	373	A	79.6151	1
**4012**	381	A	74.5386	1
**4012**	382	A	79.5535	1
**4012**	386	A	59.3809	1
**4012**	305	B	79.7577	1
**4012**	319	B	79.6423	1
**4012**	320	B	66.15	-1
**4012**	321	B	66.7309	0
**4012**	351	B	22.5844	-1
**4012**	357	B	78.3374	1
**4012**	370	B	73.2078	1
**4012**	372	B	61.44	0
**4012**	373	B	51.9277	1
**4012**	381	B	73.0765	1
**4012**	382	B	79.5331	1
**4012**	386	B	58.4477	1
**4012**	305	C	66.5936	1
**4012**	319	C	39.9492	0
**4012**	320	C	53.6644	-1
**4012**	321	C	58.4065	0

**4012**	351	C	10.5622	1
**4012**	357	C	79.6072	1
**4012**	370	C	53.5783	1
**4012**	372	C	79.3419	0
**4012**	373	C	79.5551	1
**4012**	381	C	77.6878	1
**4012**	382	C	70.8865	1
**4012**	386	C	65.4341	1
**4012**	305	D	58.6014	1
**4012**	319	D	74.8591	1
**4012**	320	D	30.638	-1
**4012**	321	D	79.5746	0
**4012**	351	D	33.6597	-1
**4012**	357	D	35.4457	0
**4012**	370	D	49.3559	0
**4012**	372	D	79.5489	0
**4012**	373	D	79.3417	1
**4012**	381	D	79.7751	1
**4012**	382	D	79.6869	1
**4012**	386	D	79.5453	1
**4013**	305	A	79.5334	1
**4013**	319	A	46.8393	0
**4013**	320	A	78.9264	-1
**4013**	321	A	76.5828	0
**4013**	351	A	12.8225	1
**4013**	357	A	57.7582	1
**4013**	370	A	37.072	0
**4013**	372	A	32.4573	1
**4013**	373	A	73.8456	1
**4013**	381	A	71.9956	1
**4013**	382	A	42.5287	0
**4013**	386	A	79.5891	1
**4013**	305	B	51.7428	1
**4013**	319	B	68.5676	1

**4013**	320	B	74.8801	-1
**4013**	321	B	71.037	0
**4013**	351	B	10.0718	1
**4013**	357	B	45.0639	0
**4013**	370	B	79.4669	1
**4013**	372	B	55.9617	0
**4013**	373	B	78.7084	1
**4013**	381	B	79.4548	1
**4013**	382	B	40.9301	0
**4013**	386	B	75.3119	1
**4013**	305	C	55.6064	1
**4013**	319	C	48.3083	0
**4013**	320	C	67.1406	-1
**4013**	321	C	78.7107	0
**4013**	351	C	8.6499	1
**4013**	357	C	27.4777	0
**4013**	370	C	79.5944	1
**4013**	372	C	46.9105	1
**4013**	373	C	69.1627	1
**4013**	381	C	78.0963	1
**4013**	382	C	74.3544	1
**4013**	386	C	46.4105	0
**4013**	305	D	36.3504	0
**4013**	319	D	50.0017	1
**4013**	320	D	77.7048	-1
**4013**	321	D	50.7497	0
**4013**	351	D	7.6505	1
**4013**	357	D	73.8743	1
**4013**	370	D	45.1319	0
**4013**	372	D	70.3309	0
**4013**	373	D	49.2192	0
**4013**	381	D	51.2993	1
**4013**	382	D	30.6597	0
**4013**	386	D	52.8571	1

**4014**	305	A	51.382	1
**4014**	319	A	66.0775	1
**4014**	320	A	73.9199	-1
**4014**	321	A	71.9742	0
**4014**	351	A	5.5309	1
**4014**	357	A	58.7113	1
**4014**	370	A	79.2235	1
**4014**	372	A	44.5917	1
**4014**	373	A	47.0723	0
**4014**	381	A	79.492	1
**4014**	382	A	79.5083	1
**4014**	386	A	43.7454	0
**4014**	305	B	69.8012	1
**4014**	319	B	61.757	1
**4014**	320	B	71.3471	-1
**4014**	321	B	79.4925	0
**4014**	351	B	12.1812	1
**4014**	357	B	79.5092	1
**4014**	370	B	56.8719	1
**4014**	372	B	71.8878	0
**4014**	373	B	70.9267	1
**4014**	381	B	69.2391	1
**4014**	382	B	50.9947	1
**4014**	386	B	79.5379	1
**4014**	305	C	78.9367	1
**4014**	319	C	72.8333	1
**4014**	320	C	70.5841	-1
**4014**	321	C	79.5814	0
**4014**	351	C	4.9475	1
**4014**	357	C	72.7114	1
**4014**	370	C	79.6303	1
**4014**	372	C	69.1183	0
**4014**	373	C	79.5652	1
**4014**	381	C	79.5122	1

**4014**	382	C	62.6265	1
**4014**	386	C	68.5843	1
**4014**	305	D	63.3681	1
**4014**	319	D	63.8925	1
**4014**	320	D	79.6217	-1
**4014**	321	D	79.6398	0
**4014**	351	D	8.2678	1
**4014**	357	D	79.5841	1
**4014**	370	D	79.599	1
**4014**	372	D	43.4735	1
**4014**	373	D	42.6235	0
**4014**	381	D	74.0199	1
**4014**	382	D	59.8978	1
**4014**	386	D	68.727	1
**4015**	305	A	56.8848	1
**4015**	319	A	66.3503	1
**4015**	320	A	79.5285	-1
**4015**	321	A	69.4551	0
**4015**	351	A	6.3305	1
**4015**	357	A	73.9188	1
**4015**	370	A	75.8707	1
**4015**	372	A	68.5071	0
**4015**	373	A	49.6859	0
**4015**	381	A	73.8964	1
**4015**	382	A	74.4909	1
**4015**	386	A	58.7859	1
**4015**	305	B	78.0388	1
**4015**	319	B	75.443	1
**4015**	320	B	63.4359	-1
**4015**	321	B	74.6496	0
**4015**	351	B	29.8733	-1
**4015**	357	B	57.9945	1
**4015**	370	B	79.8118	1
**4015**	372	B	22.1217	1

**4015**	373	B	75.5262	1
**4015**	381	B	52.8334	1
**4015**	382	B	41.2121	0
**4015**	386	B	59.8708	1
**4015**	305	C	78.2452	1
**4015**	319	C	68.1684	1
**4015**	320	C	78.1929	-1
**4015**	321	C	68.5524	0
**4015**	351	C	10.8685	1
**4015**	357	C	53.692	1
**4015**	370	C	72.2877	1
**4015**	372	C	79.5318	0
**4015**	373	C	79.5814	1
**4015**	381	C	61.4271	1
**4015**	382	C	16.506	-1
**4015**	386	C	69.8326	1
**4015**	305	D	79.5263	1
**4015**	319	D	57.336	1
**4015**	320	D	61.8893	-1
**4015**	321	D	58.5358	0
**4015**	351	D	10.2456	1
**4015**	357	D	29.4553	0
**4015**	370	D	36.7979	0
**4015**	372	D	21.6731	1
**4015**	373	D	55.1166	1
**4015**	381	D	44.5828	0
**4015**	382	D	68.5079	1
**4015**	386	D	79.5668	1
**4016**	305	A	79.5868	1
**4016**	319	A	56.1131	1
**4016**	320	A	73.3395	-1
**4016**	321	A	76.945	0
**4016**	351	A	11.6489	1
**4016**	357	A	79.55	1

**4016**	370	A	50.6268	1
**4016**	372	A	79.572	0
**4016**	373	A	79.5582	1
**4016**	381	A	78.3797	1
**4016**	382	A	38.2991	0
**4016**	386	A	62.7781	1
**4016**	305	B	73.2786	1
**4016**	319	B	51.5264	1
**4016**	320	B	79.6216	-1
**4016**	321	B	79.4905	0
**4016**	351	B	13.6703	1
**4016**	357	B	75.1205	1
**4016**	370	B	79.5099	1
**4016**	372	B	74.9977	0
**4016**	373	B	72.6488	1
**4016**	381	B	71.2246	1
**4016**	382	B	53.5646	1
**4016**	386	B	79.5264	1
**4016**	305	C	72.0525	1
**4016**	319	C	56.9807	1
**4016**	320	C	63.6727	-1
**4016**	321	C	50.1687	0
**4016**	351	C	5.6046	1
**4016**	357	C	79.5766	1
**4016**	370	C	79.4957	1
**4016**	372	C	51.3423	0
**4016**	373	C	79.6214	1
**4016**	381	C	72.6377	1
**4016**	382	C	69.3032	1
**4016**	386	C	50.8436	1
**4016**	305	D	24.0422	0
**4016**	319	D	79.6119	1
**4016**	320	D	54.9535	-1
**4016**	321	D	79.5842	0

**4016**	351	D	10.4016	1
**4016**	357	D	67.1378	1
**4016**	370	D	79.3483	1
**4016**	372	D	72.008	0
**4016**	373	D	48.7041	0
**4016**	381	D	76.3084	1
**4016**	382	D	79.5175	1
**4016**	386	D	65.529	1
**4017**	305	A	65.1022	1
**4017**	319	A	71.8309	1
**4017**	320	A	54.7249	-1
**4017**	321	A	79.3113	0
**4017**	351	A	6.0869	1
**4017**	357	A	74.7408	1
**4017**	370	A	42.0228	0
**4017**	372	A	46.6433	1
**4017**	373	A	71.803	1
**4017**	381	A	41.9877	0
**4017**	382	A	74.5359	1
**4017**	386	A	79.5673	1
**4017**	305	B	29.3831	0
**4017**	319	B	79.7351	1
**4017**	320	B	63.6438	-1
**4017**	321	B	45.6141	1
**4017**	351	B	7.115	1
**4017**	357	B	79.5551	1
**4017**	370	B	45.2347	0
**4017**	372	B	58.7421	0
**4017**	373	B	62.7922	1
**4017**	381	B	73.12	1
**4017**	382	B	55.4602	1
**4017**	386	B	59.7188	1
**4017**	305	C	54.1106	1
**4017**	319	C	79.5459	1

**4017**	320	C	79.281	-1
**4017**	321	C	47.79	1
**4017**	351	C	15.2645	1
**4017**	357	C	54.2711	1
**4017**	370	C	70.5073	1
**4017**	372	C	78.8833	0
**4017**	373	C	79.5691	1
**4017**	381	C	79.4999	1
**4017**	382	C	32.9169	0
**4017**	386	C	15.2436	-1
**4017**	305	D	57.6717	1
**4017**	319	D	75.9937	1
**4017**	320	D	79.5838	-1
**4017**	321	D	79.5804	0
**4017**	351	D	10.9375	1
**4017**	357	D	31.398	0
**4017**	370	D	37.2347	0
**4017**	372	D	60.3277	0
**4017**	373	D	79.5688	1
**4017**	381	D	63.4051	1
**4017**	382	D	79.5741	1
**4017**	386	D	66.1877	1
**4018**	305	A	21.1821	0
**4018**	319	A	72.5218	1
**4018**	320	A	79.567	-1
**4018**	321	A	64.3918	0
**4018**	351	A	6.0428	1
**4018**	357	A	79.5498	1
**4018**	370	A	73.4317	1
**4018**	372	A	79.4854	0
**4018**	373	A	79.6018	1
**4018**	381	A	72.4649	1
**4018**	382	A	55.3915	1
**4018**	386	A	50.7192	1

**4018**	305	B	73.9275	1
**4018**	319	B	72.76	1
**4018**	320	B	26.7016	-1
**4018**	321	B	72.6015	0
**4018**	351	B	13.1991	1
**4018**	357	B	79.5913	1
**4018**	370	B	79.5592	1
**4018**	372	B	79.5699	0
**4018**	373	B	62.6369	1
**4018**	381	B	72.9689	1
**4018**	382	B	53.2294	1
**4018**	386	B	40.9276	0
**4018**	305	C	72.9647	1
**4018**	319	C	72.5428	1
**4018**	320	C	50.7505	-1
**4018**	321	C	72.83	0
**4018**	351	C	3.5639	1
**4018**	357	C	27.8103	0
**4018**	370	C	66.6448	1
**4018**	372	C	79.5793	0
**4018**	373	C	45.7239	0
**4018**	381	C	71.4106	1
**4018**	382	C	66.3744	1
**4018**	386	C	47.5957	0
**4018**	305	D	41.2655	0
**4018**	319	D	58.152	1
**4018**	320	D	48.308	-1
**4018**	321	D	38.5942	1
**4018**	351	D	13.623	1
**4018**	357	D	73.2022	1
**4018**	370	D	40.5047	0
**4018**	372	D	73.0331	0
**4018**	373	D	71.2024	1
**4018**	381	D	62.7318	1

**4018**	382	D	79.5733	1
**4018**	386	D	78.2824	1
**4019**	305	A	48.5206	0
**4019**	319	A	55.3406	1
**4019**	320	A	73.5985	-1
**4019**	321	A	67.8694	0
**4019**	351	A	12.3682	1
**4019**	357	A	61.1394	1
**4019**	370	A	73.7126	1
**4019**	372	A	72.6488	0
**4019**	373	A	79.5893	1
**4019**	381	A	79.4742	1
**4019**	382	A	79.5447	1
**4019**	386	A	70.2812	1
**4019**	305	B	79.4737	1
**4019**	319	B	43.7035	0
**4019**	320	B	55.5385	-1
**4019**	321	B	70.6807	0
**4019**	351	B	20.4074	-1
**4019**	357	B	79.5593	1
**4019**	370	B	50.6631	1
**4019**	372	B	64.9031	0
**4019**	373	B	74.6257	1
**4019**	381	B	72.7512	1
**4019**	382	B	16.3162	-1
**4019**	386	B	73.7891	1
**4019**	305	C	54.1265	1
**4019**	319	C	45.4976	0
**4019**	320	C	76.8194	-1
**4019**	321	C	72.8694	0
**4019**	351	C	0.7596	1
**4019**	357	C	65.281	1
**4019**	370	C	79.4626	1
**4019**	372	C	62.0549	0

**4019**	373	C	79.5435	1
**4019**	381	C	79.5788	1
**4019**	382	C	79.5618	1
**4019**	386	C	57.3078	1
**4019**	305	D	28.9229	0
**4019**	319	D	79.5809	1
**4019**	320	D	79.4674	-1
**4019**	321	D	74.1689	0
**4019**	351	D	6.9687	1
**4019**	357	D	73.1198	1
**4019**	370	D	79.4164	1
**4019**	372	D	73.0786	0
**4019**	373	D	79.551	1
**4019**	381	D	79.5301	1
**4019**	382	D	62.2461	1
**4019**	386	D	79.5293	1
**4020**	305	A	64.9469	1
**4020**	319	A	55.2549	1
**4020**	320	A	79.5398	-1
**4020**	321	A	51.308	0
**4020**	351	A	9.6697	1
**4020**	357	A	64.9311	1
**4020**	370	A	72.2322	1
**4020**	372	A	77.2777	0
**4020**	373	A	79.5508	1
**4020**	381	A	74.5349	1
**4020**	382	A	73.702	1
**4020**	386	A	72.1878	1
**4020**	305	B	60.0035	1
**4020**	319	B	79.5738	1
**4020**	320	B	36.1581	-1
**4020**	321	B	73.0541	0
**4020**	351	B	7.8336	1
**4020**	357	B	79.4395	1

**4020**	370	B	64.978	1
**4020**	372	B	79.5585	0
**4020**	373	B	79.5297	1
**4020**	381	B	61.1502	1
**4020**	382	B	74.7078	1
**4020**	386	B	69.6327	1
**4020**	305	C	79.4711	1
**4020**	319	C	48.5433	0
**4020**	320	C	79.5326	-1
**4020**	321	C	72.8555	0
**4020**	351	C	3.5101	1
**4020**	357	C	73.0269	1
**4020**	370	C	65.6697	1
**4020**	372	C	79.5039	0
**4020**	373	C	53.393	1
**4020**	381	C	79.5834	1
**4020**	382	C	40.7049	0
**4020**	386	C	58.9715	1
**4020**	305	D	79.6643	1
**4020**	319	D	79.5368	1
**4020**	320	D	78.4167	-1
**4020**	321	D	79.5399	0
**4020**	351	D	12.5843	1
**4020**	357	D	74.4562	1
**4020**	370	D	71.8138	1
**4020**	372	D	79.5282	0
**4020**	373	D	79.5506	1
**4020**	381	D	79.5407	1
**4020**	382	D	79.5826	1
**4020**	386	D	78.7061	1
**4021**	305	A	60.7004	1
**4021**	319	A	55.204	1
**4021**	320	A	79.6725	-1
**4021**	321	A	77.6373	0

**4021**	351	A	13.1526	1
**4021**	357	A	69.9209	1
**4021**	370	A	73.7046	1
**4021**	372	A	67.4819	0
**4021**	373	A	46.1523	0
**4021**	381	A	51.5875	1
**4021**	382	A	69.3747	1
**4021**	386	A	79.5361	1
**4021**	305	B	76.659	1
**4021**	319	B	69.5034	1
**4021**	320	B	23.4904	-1
**4021**	321	B	67.7166	0
**4021**	351	B	22.9281	-1
**4021**	357	B	50.7898	1
**4021**	370	B	54.2044	1
**4021**	372	B	78.9024	0
**4021**	373	B	66.7055	1
**4021**	381	B	43.9412	0
**4021**	382	B	24.3377	0
**4021**	386	B	79.7759	1
**4021**	305	C	51.9771	1
**4021**	319	C	79.554	1
**4021**	320	C	77.3893	-1
**4021**	321	C	79.6122	0
**4021**	351	C	4.6362	1
**4021**	357	C	61.9871	1
**4021**	370	C	69.1866	1
**4021**	372	C	79.6019	0
**4021**	373	C	74.3214	1
**4021**	381	C	71.1961	1
**4021**	382	C	79.576	1
**4021**	386	C	52.1435	1
**4021**	305	D	43.4723	0
**4021**	319	D	69.1879	1

**4021**	320	D	37.2188	-1
**4021**	321	D	64.1356	0
**4021**	351	D	14.6373	1
**4021**	357	D	66.9813	1
**4021**	370	D	50.3844	1
**4021**	372	D	79.676	0
**4021**	373	D	59.7003	1
**4021**	381	D	79.7938	1
**4021**	382	D	79.7036	1
**4021**	386	D	79.511	1
**4022**	305	A	48.7452	0
**4022**	319	A	79.5555	1
**4022**	320	A	79.5668	-1
**4022**	321	A	43.4879	1
**4022**	351	A	8.2537	1
**4022**	357	A	79.5339	1
**4022**	370	A	74.5993	1
**4022**	372	A	63.2222	0
**4022**	373	A	79.5382	1
**4022**	381	A	70.7239	1
**4022**	382	A	69.3363	1
**4022**	386	A	55.828	1
**4022**	305	B	74.4625	1
**4022**	319	B	58.6805	1
**4022**	320	B	73.9926	-1
**4022**	321	B	63.5223	0
**4022**	351	B	11.743	1
**4022**	357	B	75.7223	1
**4022**	370	B	79.5684	1
**4022**	372	B	42.9148	1
**4022**	373	B	79.2983	1
**4022**	381	B	36.0959	0
**4022**	382	B	79.6267	1
**4022**	386	B	62.9301	1

**4022**	305	C	63.8568	1
**4022**	319	C	79.5697	1
**4022**	320	C	79.4477	-1
**4022**	321	C	62.9301	0
**4022**	351	C	3.2548	1
**4022**	357	C	74.4698	1
**4022**	370	C	76.82	1
**4022**	372	C	79.5712	0
**4022**	373	C	41.7888	0
**4022**	381	C	52.538	1
**4022**	382	C	32.4611	0
**4022**	386	C	62.7386	1
**4022**	305	D	47.684	0
**4022**	319	D	62.3718	1
**4022**	320	D	79.5912	-1
**4022**	321	D	31.9279	1
**4022**	351	D	12.7428	1
**4022**	357	D	44.3492	0
**4022**	370	D	62.2451	1
**4022**	372	D	79.5557	0
**4022**	373	D	78.1871	1
**4022**	381	D	67.39	1
**4022**	382	D	79.5443	1
**4022**	386	D	79.5827	1
**4023**	305	A	59.293	1
**4023**	319	A	61.1351	1
**4023**	320	A	79.5994	-1
**4023**	321	A	40.7529	1
**4023**	351	A	11.6573	1
**4023**	357	A	65.5256	1
**4023**	370	A	79.5263	1
**4023**	372	A	59.0446	0
**4023**	373	A	59.5018	1
**4023**	381	A	75.1311	1

**4023**	382	A	64.3505	1
**4023**	386	A	79.5154	1
**4023**	305	B	71.7798	1
**4023**	319	B	79.6156	1
**4023**	320	B	79.4364	-1
**4023**	321	B	58.276	0
**4023**	351	B	12.9436	1
**4023**	357	B	79.6539	1
**4023**	370	B	29.268	0
**4023**	372	B	71.8972	0
**4023**	373	B	79.0594	1
**4023**	381	B	70.3824	1
**4023**	382	B	79.3094	1
**4023**	386	B	79.5883	1
**4023**	305	C	79.6036	1
**4023**	319	C	68.2152	1
**4023**	320	C	67.7171	-1
**4023**	321	C	72.9659	0
**4023**	351	C	15.9904	1
**4023**	357	C	79.5584	1
**4023**	370	C	79.5235	1
**4023**	372	C	40.1566	1
**4023**	373	C	57.9032	1
**4023**	381	C	59.2856	1
**4023**	382	C	66.7724	1
**4023**	386	C	55.0609	1
**4023**	305	D	50.3397	1
**4023**	319	D	75.9349	1
**4023**	320	D	67.1691	-1
**4023**	321	D	79.63	0
**4023**	351	D	24.0685	-1
**4023**	357	D	79.6044	1
**4023**	370	D	73.4716	1
**4023**	372	D	79.5115	0

**4023**	373	D	37.1656	0
**4023**	381	D	60.2663	1
**4023**	382	D	47.6161	0
**4023**	386	D	79.6248	1
**4024**	305	A	52.4468	1
**4024**	319	A	76.3315	1
**4024**	320	A	75.4193	-1
**4024**	321	A	60.3421	0
**4024**	351	A	12.1755	1
**4024**	357	A	70.6696	1
**4024**	370	A	79.6149	1
**4024**	372	A	55.8337	0
**4024**	373	A	79.6787	1
**4024**	381	A	55.8977	1
**4024**	382	A	60.8569	1
**4024**	386	A	52.3156	1
**4024**	305	B	69.4982	1
**4024**	319	B	71.7673	1
**4024**	320	B	78.8468	-1
**4024**	321	B	74.4741	0
**4024**	351	B	14.1242	1
**4024**	357	B	63.166	1
**4024**	370	B	43.3039	0
**4024**	372	B	69.5093	0
**4024**	373	B	59.1557	1
**4024**	381	B	69.8741	1
**4024**	382	B	40.4432	0
**4024**	386	B	60.5575	1
**4024**	305	C	69.5598	1
**4024**	319	C	53.6008	1
**4024**	320	C	79.6308	-1
**4024**	321	C	79.4501	0
**4024**	351	C	0.0	1
**4024**	357	C	79.2854	1

**4024**	370	C	73.188	1
**4024**	372	C	57.378	0
**4024**	373	C	61.5941	1
**4024**	381	C	72.8864	1
**4024**	382	C	70.2688	1
**4024**	386	C	21.0473	0
**4024**	305	D	79.809	1
**4024**	319	D	48.7523	0
**4024**	320	D	75.8088	-1
**4024**	321	D	68.6751	0
**4024**	351	D	18.5489	1
**4024**	357	D	73.8275	1
**4024**	370	D	70.8295	1
**4024**	372	D	58.2543	0
**4024**	373	D	52.0337	1
**4024**	381	D	79.5781	1
**4024**	382	D	74.1708	1
**4024**	386	D	79.4318	1
**4025**	305	A	79.5819	1
**4025**	319	A	60.1612	1
**4025**	320	A	79.6708	-1
**4025**	321	A	53.9813	0
**4025**	351	A	3.5412	1
**4025**	357	A	49.4508	0
**4025**	370	A	34.8726	0
**4025**	372	A	73.4483	0
**4025**	373	A	79.3541	1
**4025**	381	A	47.7659	0
**4025**	382	A	68.4845	1
**4025**	386	A	73.8247	1
**4025**	305	B	79.611	1
**4025**	319	B	76.7251	1
**4025**	320	B	79.2107	-1
**4025**	321	B	69.7164	0

**4025**	351	B	16.5852	1
**4025**	357	B	66.7056	1
**4025**	370	B	38.9804	0
**4025**	372	B	68.5454	0
**4025**	373	B	64.8234	1
**4025**	381	B	40.4952	0
**4025**	382	B	62.2263	1
**4025**	386	B	70.481	1
**4025**	305	C	73.9351	1
**4025**	319	C	50.6701	1
**4025**	320	C	71.9959	-1
**4025**	321	C	51.9851	0
**4025**	351	C	0.1054	1
**4025**	357	C	76.311	1
**4025**	370	C	54.1387	1
**4025**	372	C	71.846	0
**4025**	373	C	79.4813	1
**4025**	381	C	79.652	1
**4025**	382	C	26.6399	0
**4025**	386	C	37.0183	0
**4025**	305	D	72.0428	1
**4025**	319	D	79.1668	1
**4025**	320	D	67.3357	-1
**4025**	321	D	79.3236	0
**4025**	351	D	18.5307	1
**4025**	357	D	79.6654	1
**4025**	370	D	31.1627	0
**4025**	372	D	79.6782	0
**4025**	373	D	79.3152	1
**4025**	381	D	66.1829	1
**4025**	382	D	40.6361	0
**4025**	386	D	60.9899	1
**4026**	305	A	60.9716	1
**4026**	319	A	70.2751	1

**4026**	320	A	79.555	-1
**4026**	321	A	78.8536	0
**4026**	351	A	4.4684	1
**4026**	357	A	67.3487	1
**4026**	370	A	49.2246	0
**4026**	372	A	66.4466	0
**4026**	373	A	68.721	1
**4026**	381	A	79.3736	1
**4026**	382	A	71.558	1
**4026**	386	A	53.3652	1
**4026**	305	B	74.0062	1
**4026**	319	B	79.563	1
**4026**	320	B	79.0583	-1
**4026**	321	B	43.093	1
**4026**	351	B	17.8194	1
**4026**	357	B	31.9897	0
**4026**	370	B	64.7704	1
**4026**	372	B	75.5974	0
**4026**	373	B	77.6119	1
**4026**	381	B	49.4037	0
**4026**	382	B	61.4657	1
**4026**	386	B	79.366	1
**4026**	305	C	70.3479	1
**4026**	319	C	79.5611	1
**4026**	320	C	52.8061	-1
**4026**	321	C	79.5335	0
**4026**	351	C	0.9559	1
**4026**	357	C	67.8492	1
**4026**	370	C	79.605	1
**4026**	372	C	43.5602	1
**4026**	373	C	45.0861	0
**4026**	381	C	67.7699	1
**4026**	382	C	54.3346	1
**4026**	386	C	61.0553	1

**4026**	305	D	30.5128	0
**4026**	319	D	68.4438	1
**4026**	320	D	68.125	-1
**4026**	321	D	63.2813	0
**4026**	351	D	10.5687	1
**4026**	357	D	72.7728	1
**4026**	370	D	18.8999	-1
**4026**	372	D	73.9828	0
**4026**	373	D	50.6687	1
**4026**	381	D	38.2947	0
**4026**	382	D	75.7386	1
**4026**	386	D	79.6122	1
**4027**	305	A	66.7898	1
**4027**	319	A	72.4838	1
**4027**	320	A	79.5697	-1
**4027**	321	A	71.7611	0
**4027**	351	A	10.5111	1
**4027**	357	A	73.7277	1
**4027**	370	A	78.1652	1
**4027**	372	A	78.4049	0
**4027**	373	A	77.4982	1
**4027**	381	A	71.7336	1
**4027**	382	A	56.2619	1
**4027**	386	A	71.9119	1
**4027**	305	B	44.7809	0
**4027**	319	B	45.8087	0
**4027**	320	B	54.0291	-1
**4027**	321	B	73.4106	0
**4027**	351	B	19.5297	1
**4027**	357	B	79.6102	1
**4027**	370	B	79.5446	1
**4027**	372	B	63.4786	0
**4027**	373	B	62.3821	1
**4027**	381	B	69.1031	1

**4027**	382	B	79.57	1
**4027**	386	B	79.4778	1
**4027**	305	C	42.1967	0
**4027**	319	C	72.8723	1
**4027**	320	C	68.2026	-1
**4027**	321	C	38.8352	1
**4027**	351	C	4.876	1
**4027**	357	C	79.5554	1
**4027**	370	C	79.5562	1
**4027**	372	C	79.5319	0
**4027**	373	C	73.8483	1
**4027**	381	C	39.7323	0
**4027**	382	C	39.2945	0
**4027**	386	C	18.6743	-1
**4027**	305	D	57.0739	1
**4027**	319	D	79.5469	1
**4027**	320	D	79.5615	-1
**4027**	321	D	79.5427	0
**4027**	351	D	4.4398	1
**4027**	357	D	71.5005	1
**4027**	370	D	43.9422	0
**4027**	372	D	79.5397	0
**4027**	373	D	48.8824	0
**4027**	381	D	76.7953	1
**4027**	382	D	79.5774	1
**4027**	386	D	78.3607	1
**4028**	305	A	42.6413	0
**4028**	319	A	73.5617	1
**4028**	320	A	73.961	-1
**4028**	321	A	76.8269	0
**4028**	351	A	14.3635	1
**4028**	357	A	68.7368	1
**4028**	370	A	57.4671	1
**4028**	372	A	50.0553	0

**4028**	373	A	33.6888	0
**4028**	381	A	79.6119	1
**4028**	382	A	76.4321	1
**4028**	386	A	78.9996	1
**4028**	305	B	79.51	1
**4028**	319	B	78.424	1
**4028**	320	B	71.3021	-1
**4028**	321	B	79.5511	0
**4028**	351	B	40.1959	-1
**4028**	357	B	66.6861	1
**4028**	370	B	63.117	1
**4028**	372	B	79.5638	0
**4028**	373	B	66.0148	1
**4028**	381	B	79.5545	1
**4028**	382	B	79.5791	1
**4028**	386	B	78.5639	1
**4028**	305	C	72.1132	1
**4028**	319	C	69.1959	1
**4028**	320	C	38.6749	-1
**4028**	321	C	37.6055	1
**4028**	351	C	6.8897	1
**4028**	357	C	79.6021	1
**4028**	370	C	33.7984	0
**4028**	372	C	79.532	0
**4028**	373	C	70.6963	1
**4028**	381	C	79.5407	1
**4028**	382	C	32.5072	0
**4028**	386	C	37.3673	0
**4028**	305	D	60.3975	1
**4028**	319	D	73.4384	1
**4028**	320	D	79.6193	-1
**4028**	321	D	58.4133	0
**4028**	351	D	10.7153	1
**4028**	357	D	67.8684	1

**4028**	370	D	68.5336	1
**4028**	372	D	73.2199	0
**4028**	373	D	71.4071	1
**4028**	381	D	66.4936	1
**4028**	382	D	59.5746	1
**4028**	386	D	78.543	1
**4029**	305	A	55.3109	1
**4029**	319	A	41.9838	0
**4029**	320	A	79.4722	-1
**4029**	321	A	44.9249	1
**4029**	351	A	8.2659	1
**4029**	357	A	72.3597	1
**4029**	370	A	79.4826	1
**4029**	372	A	23.9031	1
**4029**	373	A	77.9483	1
**4029**	381	A	76.4341	1
**4029**	382	A	67.3212	1
**4029**	386	A	77.3044	1
**4029**	305	B	79.5898	1
**4029**	319	B	79.6667	1
**4029**	320	B	49.1138	-1
**4029**	321	B	79.4982	0
**4029**	351	B	18.188	1
**4029**	357	B	36.9464	0
**4029**	370	B	78.7092	1
**4029**	372	B	58.7	0
**4029**	373	B	76.6644	1
**4029**	381	B	60.7162	1
**4029**	382	B	64.5693	1
**4029**	386	B	55.4126	1
**4029**	305	C	49.8737	0
**4029**	319	C	75.5854	1
**4029**	320	C	48.5163	-1
**4029**	321	C	69.3491	0

**4029**	351	C	24.273	-1
**4029**	357	C	79.4774	1
**4029**	370	C	79.6435	1
**4029**	372	C	63.6902	0
**4029**	373	C	53.2976	1
**4029**	381	C	79.3453	1
**4029**	382	C	47.2597	0
**4029**	386	C	51.7052	1
**4029**	305	D	23.3481	0
**4029**	319	D	74.0974	1
**4029**	320	D	68.8324	-1
**4029**	321	D	77.6837	0
**4029**	351	D	28.127	-1
**4029**	357	D	79.5377	1
**4029**	370	D	41.238	0
**4029**	372	D	79.4639	0
**4029**	373	D	77.7924	1
**4029**	381	D	77.6088	1
**4029**	382	D	79.4548	1
**4029**	386	D	65.7349	1
**4030**	305	A	65.675	1
**4030**	319	A	35.4657	0
**4030**	320	A	79.6115	-1
**4030**	321	A	75.3578	0
**4030**	351	A	1.5046	1
**4030**	357	A	19.67	-1
**4030**	370	A	71.6816	1
**4030**	372	A	63.7375	0
**4030**	373	A	79.6125	1
**4030**	381	A	46.9098	0
**4030**	382	A	79.4417	1
**4030**	386	A	47.4707	0
**4030**	305	B	56.2148	1
**4030**	319	B	72.5658	1

**4030**	320	B	25.2875	-1
**4030**	321	B	75.2221	0
**4030**	351	B	10.0144	1
**4030**	357	B	77.8624	1
**4030**	370	B	54.219	1
**4030**	372	B	74.2221	0
**4030**	373	B	75.7951	1
**4030**	381	B	79.6508	1
**4030**	382	B	37.1122	0
**4030**	386	B	79.4753	1
**4030**	305	C	72.4079	1
**4030**	319	C	77.852	1
**4030**	320	C	59.0326	-1
**4030**	321	C	49.6987	1
**4030**	351	C	0.0174	1
**4030**	357	C	79.5775	1
**4030**	370	C	72.7497	1
**4030**	372	C	79.6428	0
**4030**	373	C	79.3593	1
**4030**	381	C	50.9302	1
**4030**	382	C	54.5531	1
**4030**	386	C	23.2873	0
**4030**	305	D	65.6252	1
**4030**	319	D	65.9127	1
**4030**	320	D	25.0802	-1
**4030**	321	D	65.8283	0
**4030**	351	D	11.7873	1
**4030**	357	D	79.5164	1
**4030**	370	D	72.5166	1
**4030**	372	D	79.4627	0
**4030**	373	D	74.1788	1
**4030**	381	D	79.5017	1
**4030**	382	D	79.6311	1
**4030**	386	D	70.1338	1

**4031**	305	A	74.8856	1
**4031**	319	A	66.3948	1
**4031**	320	A	79.5354	-1
**4031**	321	A	56.9098	0
**4031**	351	A	2.0027	1
**4031**	357	A	79.4993	1
**4031**	370	A	64.6363	1
**4031**	372	A	79.5497	0
**4031**	373	A	79.5456	1
**4031**	381	A	35.9528	0
**4031**	382	A	64.319	1
**4031**	386	A	63.2252	1
**4031**	305	B	79.3461	1
**4031**	319	B	79.5319	1
**4031**	320	B	70.9875	-1
**4031**	321	B	79.4984	0
**4031**	351	B	14.9718	1
**4031**	357	B	76.3658	1
**4031**	370	B	52.586	1
**4031**	372	B	73.4678	0
**4031**	373	B	30.4473	0
**4031**	381	B	51.4779	1
**4031**	382	B	31.892	0
**4031**	386	B	72.4444	1
**4031**	305	C	73.1356	1
**4031**	319	C	79.6819	1
**4031**	320	C	78.9653	-1
**4031**	321	C	52.8456	0
**4031**	351	C	0.0	1
**4031**	357	C	72.0156	1
**4031**	370	C	42.1009	0
**4031**	372	C	79.4983	0
**4031**	373	C	32.1627	0
**4031**	381	C	64.5107	1

**4031**	382	C	70.6234	1
**4031**	386	C	37.1414	0
**4031**	305	D	66.8601	1
**4031**	319	D	62.9712	1
**4031**	320	D	79.5952	-1
**4031**	321	D	35.6411	1
**4031**	351	D	0.0011	1
**4031**	357	D	65.7402	1
**4031**	370	D	79.6815	1
**4031**	372	D	44.6252	1
**4031**	373	D	44.9664	0
**4031**	381	D	68.5436	1
**4031**	382	D	79.6963	1
**4031**	386	D	75.1586	1
**4032**	305	A	77.9272	1
**4032**	319	A	52.0849	1
**4032**	320	A	79.5347	-1
**4032**	321	A	55.8452	0
**4032**	351	A	8.0756	1
**4032**	357	A	79.5642	1
**4032**	370	A	79.6125	1
**4032**	372	A	79.569	0
**4032**	373	A	27.069	0
**4032**	381	A	62.1392	1
**4032**	382	A	73.6876	1
**4032**	386	A	44.9942	0
**4032**	305	B	43.3491	0
**4032**	319	B	72.1899	1
**4032**	320	B	68.1514	-1
**4032**	321	B	72.5016	0
**4032**	351	B	15.6718	1
**4032**	357	B	66.4555	1
**4032**	370	B	72.3331	1
**4032**	372	B	79.6014	0

**4032**	373	B	79.5545	1
**4032**	381	B	38.0149	0
**4032**	382	B	14.1665	-1
**4032**	386	B	56.0434	1
**4032**	305	C	73.3228	1
**4032**	319	C	79.565	1
**4032**	320	C	66.5085	-1
**4032**	321	C	73.1154	0
**4032**	351	C	4.5094	1
**4032**	357	C	76.9398	1
**4032**	370	C	79.5344	1
**4032**	372	C	79.541	0
**4032**	373	C	14.7899	-1
**4032**	381	C	77.1978	1
**4032**	382	C	79.5604	1
**4032**	386	C	41.3342	0
**4032**	305	D	79.4949	1
**4032**	319	D	48.4095	0
**4032**	320	D	36.8369	-1
**4032**	321	D	79.6052	0
**4032**	351	D	12.5046	1
**4032**	357	D	79.4255	1
**4032**	370	D	77.7344	1
**4032**	372	D	79.5417	0
**4032**	373	D	79.5209	1
**4032**	381	D	79.5505	1
**4032**	382	D	73.8932	1
**4032**	386	D	78.9178	1
**4033**	305	A	72.5937	1
**4033**	319	A	28.7225	0
**4033**	320	A	71.8257	-1
**4033**	321	A	71.5745	0
**4033**	351	A	2.3912	1
**4033**	357	A	74.0687	1

**4033**	370	A	72.9597	1
**4033**	372	A	74.6727	0
**4033**	373	A	71.4402	1
**4033**	381	A	79.5472	1
**4033**	382	A	63.0615	1
**4033**	386	A	79.5696	1
**4033**	305	B	65.8385	1
**4033**	319	B	73.893	1
**4033**	320	B	61.3119	-1
**4033**	321	B	60.0726	0
**4033**	351	B	15.12	1
**4033**	357	B	72.0967	1
**4033**	370	B	58.9699	1
**4033**	372	B	55.1576	0
**4033**	373	B	11.0299	-1
**4033**	381	B	73.9785	1
**4033**	382	B	79.6177	1
**4033**	386	B	40.2339	0
**4033**	305	C	79.5671	1
**4033**	319	C	79.51	1
**4033**	320	C	79.5322	-1
**4033**	321	C	46.9717	1
**4033**	351	C	0.0464	1
**4033**	357	C	79.5535	1
**4033**	370	C	47.1092	0
**4033**	372	C	79.3671	0
**4033**	373	C	79.1765	1
**4033**	381	C	79.1095	1
**4033**	382	C	54.1273	1
**4033**	386	C	37.805	0
**4033**	305	D	72.6769	1
**4033**	319	D	53.902	1
**4033**	320	D	35.3668	-1
**4033**	321	D	70.6567	0

**4033**	351	D	1.7411	1
**4033**	357	D	79.605	1
**4033**	370	D	52.2475	1
**4033**	372	D	79.5703	0
**4033**	373	D	42.6117	0
**4033**	381	D	61.6525	1
**4033**	382	D	68.643	1
**4033**	386	D	78.7517	1
**4034**	305	A	79.5293	1
**4034**	319	A	72.5888	1
**4034**	320	A	39.7041	-1
**4034**	321	A	72.5903	0
**4034**	351	A	14.5044	1
**4034**	357	A	63.0871	1
**4034**	370	A	71.6186	1
**4034**	372	A	58.1322	0
**4034**	373	A	65.5381	1
**4034**	381	A	59.167	1
**4034**	382	A	72.8669	1
**4034**	386	A	56.7422	1
**4034**	305	B	75.3455	1
**4034**	319	B	79.5357	1
**4034**	320	B	77.3011	-1
**4034**	321	B	60.7133	0
**4034**	351	B	19.4635	1
**4034**	357	B	72.1033	1
**4034**	370	B	55.949	1
**4034**	372	B	79.5777	0
**4034**	373	B	68.1857	1
**4034**	381	B	40.8197	0
**4034**	382	B	31.4665	0
**4034**	386	B	25.2829	0
**4034**	305	C	57.2501	1
**4034**	319	C	65.3919	1

**4034**	320	C	75.6504	-1
**4034**	321	C	72.3392	0
**4034**	351	C	6.2641	1
**4034**	357	C	52.5707	1
**4034**	370	C	79.5676	1
**4034**	372	C	73.6571	0
**4034**	373	C	63.5812	1
**4034**	381	C	79.5794	1
**4034**	382	C	60.1698	1
**4034**	386	C	59.8771	1
**4034**	305	D	45.527	0
**4034**	319	D	62.4847	1
**4034**	320	D	35.7747	-1
**4034**	321	D	79.6082	0
**4034**	351	D	17.3368	1
**4034**	357	D	79.5633	1
**4034**	370	D	79.5392	1
**4034**	372	D	30.6421	1
**4034**	373	D	79.6037	1
**4034**	381	D	60.5877	1
**4034**	382	D	79.5938	1
**4034**	386	D	33.928	0
**4035**	305	A	56.4417	1
**4035**	319	A	79.5767	1
**4035**	320	A	79.5636	-1
**4035**	321	A	79.5251	0
**4035**	351	A	9.5356	1
**4035**	357	A	79.5706	1
**4035**	370	A	67.9978	1
**4035**	372	A	60.6746	0
**4035**	373	A	58.4216	1
**4035**	381	A	47.9094	0
**4035**	382	A	23.4579	0
**4035**	386	A	77.9121	1

**4035**	305	B	73.7959	1
**4035**	319	B	72.2351	1
**4035**	320	B	47.3571	-1
**4035**	321	B	55.1005	0
**4035**	351	B	11.6538	1
**4035**	357	B	79.5437	1
**4035**	370	B	79.5754	1
**4035**	372	B	79.5417	0
**4035**	373	B	79.6089	1
**4035**	381	B	51.5195	1
**4035**	382	B	74.3455	1
**4035**	386	B	38.9341	0
**4035**	305	C	48.3225	0
**4035**	319	C	57.2748	1
**4035**	320	C	60.7836	-1
**4035**	321	C	51.3494	0
**4035**	351	C	4.6221	1
**4035**	357	C	79.5578	1
**4035**	370	C	79.567	1
**4035**	372	C	79.5812	0
**4035**	373	C	72.8011	1
**4035**	381	C	78.8753	1
**4035**	382	C	62.6336	1
**4035**	386	C	52.469	1
**4035**	305	D	58.8972	1
**4035**	319	D	77.1385	1
**4035**	320	D	72.1229	-1
**4035**	321	D	79.5459	0
**4035**	351	D	2.4329	1
**4035**	357	D	42.0575	0
**4035**	370	D	70.9362	1
**4035**	372	D	79.3387	0
**4035**	373	D	70.7504	1
**4035**	381	D	74.9143	1

**4035**	382	D	72.7328	1
**4035**	386	D	78.2408	1
**4036**	305	A	74.4601	1
**4036**	319	A	66.5792	1
**4036**	320	A	79.4296	-1
**4036**	321	A	63.0938	0
**4036**	351	A	11.4139	1
**4036**	357	A	72.1033	1
**4036**	370	A	62.2526	1
**4036**	372	A	72.2741	0
**4036**	373	A	65.0725	1
**4036**	381	A	74.3334	1
**4036**	382	A	61.4138	1
**4036**	386	A	34.2259	0
**4036**	305	B	73.3926	1
**4036**	319	B	72.3439	1
**4036**	320	B	61.2903	-1
**4036**	321	B	70.7976	0
**4036**	351	B	9.7484	1
**4036**	357	B	69.2166	1
**4036**	370	B	64.0881	1
**4036**	372	B	78.1714	0
**4036**	373	B	79.5399	1
**4036**	381	B	79.314	1
**4036**	382	B	41.7876	0
**4036**	386	B	79.6108	1
**4036**	305	C	49.998	0
**4036**	319	C	71.3104	1
**4036**	320	C	67.5442	-1
**4036**	321	C	56.0654	0
**4036**	351	C	2.229	1
**4036**	357	C	71.1193	1
**4036**	370	C	51.6994	1
**4036**	372	C	79.482	0

**4036**	373	C	48.1437	0
**4036**	381	C	76.1011	1
**4036**	382	C	47.2561	0
**4036**	386	C	64.6443	1
**4036**	305	D	67.8478	1
**4036**	319	D	73.3812	1
**4036**	320	D	57.9664	-1
**4036**	321	D	79.6407	0
**4036**	351	D	5.2762	1
**4036**	357	D	53.3098	1
**4036**	370	D	76.2877	1
**4036**	372	D	77.4458	0
**4036**	373	D	57.0094	1
**4036**	381	D	75.8994	1
**4036**	382	D	76.1074	1
**4036**	386	D	79.5225	1
**4037**	305	A	73.6098	1
**4037**	319	A	68.7722	1
**4037**	320	A	47.59	-1
**4037**	321	A	31.6016	1
**4037**	351	A	6.4901	1
**4037**	357	A	79.4946	1
**4037**	370	A	79.6102	1
**4037**	372	A	79.5797	0
**4037**	373	A	19.1002	-1
**4037**	381	A	79.5578	1
**4037**	382	A	79.6432	1
**4037**	386	A	62.7743	1
**4037**	305	B	27.6977	0
**4037**	319	B	36.3522	0
**4037**	320	B	50.751	-1
**4037**	321	B	50.6708	0
**4037**	351	B	20.2692	-1
**4037**	357	B	29.868	0

**4037**	370	B	50.1958	1
**4037**	372	B	73.7413	0
**4037**	373	B	73.6216	1
**4037**	381	B	51.0247	1
**4037**	382	B	67.8541	1
**4037**	386	B	59.352	1
**4037**	305	C	69.8453	1
**4037**	319	C	79.5562	1
**4037**	320	C	79.6016	-1
**4037**	321	C	76.3674	0
**4037**	351	C	4.116	1
**4037**	357	C	66.8602	1
**4037**	370	C	79.4974	1
**4037**	372	C	74.0181	0
**4037**	373	C	79.5911	1
**4037**	381	C	41.6824	0
**4037**	382	C	72.2717	1
**4037**	386	C	63.8647	1
**4037**	305	D	35.8365	0
**4037**	319	D	44.8837	0
**4037**	320	D	79.5756	-1
**4037**	321	D	71.1789	0
**4037**	351	D	8.3539	1
**4037**	357	D	73.6857	1
**4037**	370	D	78.5003	1
**4037**	372	D	49.3346	1
**4037**	373	D	79.571	1
**4037**	381	D	51.9027	1
**4037**	382	D	51.7271	1
**4037**	386	D	57.0908	1
**4038**	305	A	73.6759	1
**4038**	319	A	79.5324	1
**4038**	320	A	53.7669	-1
**4038**	321	A	74.4078	0

**4038**	351	A	13.5357	1
**4038**	357	A	63.9682	1
**4038**	370	A	79.6313	1
**4038**	372	A	56.6536	0
**4038**	373	A	38.9475	0
**4038**	381	A	45.0334	0
**4038**	382	A	23.7706	0
**4038**	386	A	79.6734	1
**4038**	305	B	79.4697	1
**4038**	319	B	75.338	1
**4038**	320	B	71.3769	-1
**4038**	321	B	61.0495	0
**4038**	351	B	13.3272	1
**4038**	357	B	79.5246	1
**4038**	370	B	51.7078	1
**4038**	372	B	79.6057	0
**4038**	373	B	8.6766	-1
**4038**	381	B	68.521	1
**4038**	382	B	74.013	1
**4038**	386	B	71.1711	1
**4038**	305	C	32.9092	0
**4038**	319	C	48.3699	0
**4038**	320	C	79.5219	-1
**4038**	321	C	34.641	1
**4038**	351	C	6.7513	1
**4038**	357	C	73.0326	1
**4038**	370	C	79.5029	1
**4038**	372	C	79.487	0
**4038**	373	C	79.4797	1
**4038**	381	C	76.6993	1
**4038**	382	C	79.5411	1
**4038**	386	C	53.489	1
**4038**	305	D	44.1241	0
**4038**	319	D	55.5477	1

**4038**	320	D	41.6072	-1
**4038**	321	D	77.8125	0
**4038**	351	D	18.2605	1
**4038**	357	D	79.0671	1
**4038**	370	D	66.7722	1
**4038**	372	D	77.9369	0
**4038**	373	D	71.2596	1
**4038**	381	D	74.1624	1
**4038**	382	D	79.4578	1
**4038**	386	D	18.2581	-1
**4039**	305	A	55.4635	1
**4039**	319	A	79.4896	1
**4039**	320	A	79.528	-1
**4039**	321	A	41.4286	1
**4039**	351	A	1.6876	1
**4039**	357	A	77.6214	1
**4039**	370	A	79.6319	1
**4039**	372	A	79.6048	0
**4039**	373	A	43.7799	0
**4039**	381	A	35.1105	0
**4039**	382	A	79.609	1
**4039**	386	A	63.2759	1
**4039**	305	B	79.5588	1
**4039**	319	B	20.723	0
**4039**	320	B	22.6808	-1
**4039**	321	B	75.3335	0
**4039**	351	B	19.0325	1
**4039**	357	B	54.923	1
**4039**	370	B	40.6963	0
**4039**	372	B	72.8373	0
**4039**	373	B	79.4758	1
**4039**	381	B	79.5918	1
**4039**	382	B	38.9426	0
**4039**	386	B	47.5726	0

**4039**	305	C	72.1238	1
**4039**	319	C	75.3618	1
**4039**	320	C	49.7435	-1
**4039**	321	C	42.3497	1
**4039**	351	C	0.5982	1
**4039**	357	C	52.0606	1
**4039**	370	C	77.1753	1
**4039**	372	C	79.4561	0
**4039**	373	C	40.3804	0
**4039**	381	C	36.7546	0
**4039**	382	C	67.1511	1
**4039**	386	C	27.0222	0
**4039**	305	D	79.5412	1
**4039**	319	D	79.558	1
**4039**	320	D	71.199	-1
**4039**	321	D	73.986	0
**4039**	351	D	12.5267	1
**4039**	357	D	45.1188	0
**4039**	370	D	72.8186	1
**4039**	372	D	79.5354	0
**4039**	373	D	79.5661	1
**4039**	381	D	77.2415	1
**4039**	382	D	79.6178	1
**4039**	386	D	79.5623	1
**4040**	305	A	79.6401	1
**4040**	319	A	71.6668	1
**4040**	320	A	79.4751	-1
**4040**	321	A	62.6905	0
**4040**	351	A	1.8675	1
**4040**	357	A	79.4998	1
**4040**	370	A	61.1363	1
**4040**	372	A	48.5972	1
**4040**	373	A	55.3975	1
**4040**	381	A	63.0346	1

**4040**	382	A	36.2712	0
**4040**	386	A	79.509	1
**4040**	305	B	65.4062	1
**4040**	319	B	47.6249	0
**4040**	320	B	49.4451	-1
**4040**	321	B	73.2671	0
**4040**	351	B	12.3306	1
**4040**	357	B	59.1717	1
**4040**	370	B	50.2935	1
**4040**	372	B	73.2288	0
**4040**	373	B	66.8914	1
**4040**	381	B	69.7039	1
**4040**	382	B	22.7192	0
**4040**	386	B	34.665	0
**4040**	305	C	63.9228	1
**4040**	319	C	70.5622	1
**4040**	320	C	79.4771	-1
**4040**	321	C	47.3799	1
**4040**	351	C	5.3523	1
**4040**	357	C	74.4653	1
**4040**	370	C	37.6594	0
**4040**	372	C	78.9177	0
**4040**	373	C	79.4734	1
**4040**	381	C	79.4735	1
**4040**	382	C	7.831	-1
**4040**	386	C	55.0729	1
**4040**	305	D	60.1663	1
**4040**	319	D	66.6348	1
**4040**	320	D	79.5793	-1
**4040**	321	D	59.9493	0
**4040**	351	D	10.9124	1
**4040**	357	D	69.2643	1
**4040**	370	D	79.6278	1
**4040**	372	D	70.6823	0

**4040**	373	D	79.4719	1
**4040**	381	D	79.5117	1
**4040**	382	D	77.0671	1
**4040**	386	D	72.5692	1
**4041**	305	A	67.4808	1
**4041**	319	A	50.3764	1
**4041**	320	A	73.806	-1
**4041**	321	A	58.6077	0
**4041**	351	A	0.0165	1
**4041**	357	A	53.1886	1
**4041**	370	A	79.4299	1
**4041**	372	A	32.182	1
**4041**	373	A	63.6956	1
**4041**	381	A	33.9722	0
**4041**	382	A	76.6948	1
**4041**	386	A	76.3597	1
**4041**	305	B	79.5829	1
**4041**	319	B	79.4799	1
**4041**	320	B	79.5742	-1
**4041**	321	B	64.6956	0
**4041**	351	B	12.3695	1
**4041**	357	B	61.995	1
**4041**	370	B	60.3374	1
**4041**	372	B	72.9527	0
**4041**	373	B	56.8227	1
**4041**	381	B	77.697	1
**4041**	382	B	77.4795	1
**4041**	386	B	73.8593	1
**4041**	305	C	67.8354	1
**4041**	319	C	66.9545	1
**4041**	320	C	46.6313	-1
**4041**	321	C	49.2428	1
**4041**	351	C	2.3793	1
**4041**	357	C	79.6184	1

**4041**	370	C	79.6362	1
**4041**	372	C	79.5661	0
**4041**	373	C	78.5072	1
**4041**	381	C	72.7204	1
**4041**	382	C	37.6486	0
**4041**	386	C	53.552	1
**4041**	305	D	60.5691	1
**4041**	319	D	79.5073	1
**4041**	320	D	57.2985	-1
**4041**	321	D	60.7404	0
**4041**	351	D	2.3133	1
**4041**	357	D	38.9299	0
**4041**	370	D	42.4828	0
**4041**	372	D	67.949	0
**4041**	373	D	79.5018	1
**4041**	381	D	79.4594	1
**4041**	382	D	72.2068	1
**4041**	386	D	47.2069	0
**4042**	305	A	71.3152	1
**4042**	319	A	47.8679	0
**4042**	320	A	79.6019	-1
**4042**	321	A	29.1563	1
**4042**	351	A	15.0864	1
**4042**	357	A	60.3985	1
**4042**	370	A	73.9777	1
**4042**	372	A	58.0319	0
**4042**	373	A	29.379	0
**4042**	381	A	78.0733	1
**4042**	382	A	72.7483	1
**4042**	386	A	79.5596	1
**4042**	305	B	79.3141	1
**4042**	319	B	72.3547	1
**4042**	320	B	73.4861	-1
**4042**	321	B	67.9199	0

**4042**	351	B	14.8892	1
**4042**	357	B	76.6678	1
**4042**	370	B	58.0018	1
**4042**	372	B	52.2355	0
**4042**	373	B	52.5674	1
**4042**	381	B	54.7615	1
**4042**	382	B	66.1505	1
**4042**	386	B	71.3113	1
**4042**	305	C	79.5291	1
**4042**	319	C	79.5601	1
**4042**	320	C	39.1859	-1
**4042**	321	C	62.2973	0
**4042**	351	C	7.4539	1
**4042**	357	C	74.8454	1
**4042**	370	C	66.6734	1
**4042**	372	C	79.5074	0
**4042**	373	C	73.1266	1
**4042**	381	C	72.6662	1
**4042**	382	C	51.7553	1
**4042**	386	C	45.2077	0
**4042**	305	D	29.8309	0
**4042**	319	D	79.5841	1
**4042**	320	D	35.4582	-1
**4042**	321	D	79.4904	0
**4042**	351	D	14.4307	1
**4042**	357	D	35.9616	0
**4042**	370	D	67.7062	1
**4042**	372	D	79.5336	0
**4042**	373	D	40.9366	0
**4042**	381	D	75.9965	1
**4042**	382	D	79.4896	1
**4042**	386	D	77.175	1
**4043**	305	A	57.1023	1
**4043**	319	A	63.3143	1

**4043**	320	A	79.5144	-1
**4043**	321	A	71.2773	0
**4043**	351	A	15.3923	1
**4043**	357	A	79.5761	1
**4043**	370	A	79.5992	1
**4043**	372	A	67.382	0
**4043**	373	A	54.9369	1
**4043**	381	A	79.6078	1
**4043**	382	A	47.7269	0
**4043**	386	A	38.4339	0
**4043**	305	B	58.111	1
**4043**	319	B	79.5832	1
**4043**	320	B	67.0331	-1
**4043**	321	B	73.5102	0
**4043**	351	B	7.9511	1
**4043**	357	B	79.7484	1
**4043**	370	B	79.5172	1
**4043**	372	B	79.4466	0
**4043**	373	B	70.8727	1
**4043**	381	B	69.1873	1
**4043**	382	B	43.5599	0
**4043**	386	B	60.024	1
**4043**	305	C	48.786	0
**4043**	319	C	76.2251	1
**4043**	320	C	79.5993	-1
**4043**	321	C	68.4395	0
**4043**	351	C	1.1847	1
**4043**	357	C	25.505	0
**4043**	370	C	44.348	0
**4043**	372	C	78.5964	0
**4043**	373	C	47.24	0
**4043**	381	C	61.5952	1
**4043**	382	C	72.4034	1
**4043**	386	C	63.2661	1

**4043**	305	D	60.0543	1
**4043**	319	D	62.0706	1
**4043**	320	D	75.5447	-1
**4043**	321	D	79.5944	0
**4043**	351	D	18.9657	1
**4043**	357	D	79.5233	1
**4043**	370	D	68.0274	1
**4043**	372	D	69.2957	0
**4043**	373	D	46.2332	0
**4043**	381	D	79.4689	1
**4043**	382	D	72.8424	1
**4043**	386	D	50.0072	1
**4044**	305	A	72.7623	1
**4044**	319	A	31.6102	0
**4044**	320	A	75.7556	-1
**4044**	321	A	79.5247	0
**4044**	351	A	6.2245	1
**4044**	357	A	79.706	1
**4044**	370	A	72.8127	1
**4044**	372	A	76.0118	0
**4044**	373	A	65.9203	1
**4044**	381	A	68.765	1
**4044**	382	A	59.0367	1
**4044**	386	A	60.4325	1
**4044**	305	B	79.4646	1
**4044**	319	B	71.7397	1
**4044**	320	B	54.019	-1
**4044**	321	B	73.6584	0
**4044**	351	B	10.3869	1
**4044**	357	B	71.9932	1
**4044**	370	B	79.4074	1
**4044**	372	B	58.2926	0
**4044**	373	B	78.5324	1
**4044**	381	B	79.5781	1

**4044**	382	B	33.699	0
**4044**	386	B	61.3597	1
**4044**	305	C	79.5759	1
**4044**	319	C	73.7984	1
**4044**	320	C	69.5407	-1
**4044**	321	C	35.7816	1
**4044**	351	C	12.2608	1
**4044**	357	C	76.9553	1
**4044**	370	C	75.8095	1
**4044**	372	C	36.8383	1
**4044**	373	C	79.4892	1
**4044**	381	C	72.2223	1
**4044**	382	C	54.6373	1
**4044**	386	C	42.398	0
**4044**	305	D	79.624	1
**4044**	319	D	79.6644	1
**4044**	320	D	76.5531	-1
**4044**	321	D	72.3042	0
**4044**	351	D	10.4798	1
**4044**	357	D	27.1741	0
**4044**	370	D	51.4763	1
**4044**	372	D	78.7983	0
**4044**	373	D	79.3969	1
**4044**	381	D	79.7442	1
**4044**	382	D	79.6079	1
**4044**	386	D	16.9001	-1
**4045**	305	A	61.8805	1
**4045**	319	A	60.8785	1
**4045**	320	A	73.3288	-1
**4045**	321	A	31.8275	1
**4045**	351	A	17.0306	1
**4045**	357	A	43.2859	0
**4045**	370	A	79.4991	1
**4045**	372	A	76.7723	0

**4045**	373	A	79.4513	1
**4045**	381	A	74.6087	1
**4045**	382	A	71.3018	1
**4045**	386	A	77.5583	1
**4045**	305	B	64.4245	1
**4045**	319	B	72.8308	1
**4045**	320	B	34.7103	-1
**4045**	321	B	62.7453	0
**4045**	351	B	12.2707	1
**4045**	357	B	36.7169	0
**4045**	370	B	79.513	1
**4045**	372	B	71.07	0
**4045**	373	B	56.852	1
**4045**	381	B	73.8454	1
**4045**	382	B	71.0755	1
**4045**	386	B	71.714	1
**4045**	305	C	27.4669	0
**4045**	319	C	79.5088	1
**4045**	320	C	43.8675	-1
**4045**	321	C	75.1674	0
**4045**	351	C	11.7858	1
**4045**	357	C	64.4092	1
**4045**	370	C	60.5965	1
**4045**	372	C	73.0523	0
**4045**	373	C	75.9892	1
**4045**	381	C	40.5846	0
**4045**	382	C	63.7092	1
**4045**	386	C	39.0502	0
**4045**	305	D	79.6245	1
**4045**	319	D	76.3289	1
**4045**	320	D	73.5935	-1
**4045**	321	D	73.5313	0
**4045**	351	D	13.9216	1
**4045**	357	D	65.1323	1

**4045**	370	D	79.5264	1
**4045**	372	D	54.5055	0
**4045**	373	D	47.2367	0
**4045**	381	D	67.7265	1
**4045**	382	D	70.7137	1
**4045**	386	D	75.9003	1
**4046**	305	A	65.2121	1
**4046**	319	A	67.4195	1
**4046**	320	A	72.9998	-1
**4046**	321	A	74.0274	0
**4046**	351	A	4.3201	1
**4046**	357	A	57.0233	1
**4046**	370	A	51.3923	1
**4046**	372	A	18.4921	0
**4046**	373	A	79.6225	1
**4046**	381	A	52.5176	1
**4046**	382	A	74.2822	1
**4046**	386	A	72.27	1
**4046**	305	B	79.4831	1
**4046**	319	B	43.297	0
**4046**	320	B	72.7477	-1
**4046**	321	B	79.5909	0
**4046**	351	B	16.0132	1
**4046**	357	B	39.5914	0
**4046**	370	B	68.3446	1
**4046**	372	B	66.3708	0
**4046**	373	B	78.4485	1
**4046**	381	B	48.8415	0
**4046**	382	B	55.1104	1
**4046**	386	B	79.6052	1
**4046**	305	C	45.5784	0
**4046**	319	C	77.0805	1
**4046**	320	C	68.7298	-1
**4046**	321	C	77.0821	0

**4046**	351	C	0.9309	1
**4046**	357	C	77.485	1
**4046**	370	C	79.5805	1
**4046**	372	C	72.3282	0
**4046**	373	C	52.586	1
**4046**	381	C	45.9893	0
**4046**	382	C	57.0606	1
**4046**	386	C	50.2444	1
**4046**	305	D	79.5693	1
**4046**	319	D	79.6476	1
**4046**	320	D	79.5839	-1
**4046**	321	D	70.3569	0
**4046**	351	D	2.1303	1
**4046**	357	D	79.6612	1
**4046**	370	D	79.647	1
**4046**	372	D	79.5149	0
**4046**	373	D	65.1769	1
**4046**	381	D	72.7877	1
**4046**	382	D	72.174	1
**4046**	386	D	69.7415	1
**4047**	305	A	79.406	1
**4047**	319	A	79.4208	1
**4047**	320	A	79.5516	-1
**4047**	321	A	64.5662	0
**4047**	351	A	14.8522	1
**4047**	357	A	60.9526	1
**4047**	370	A	79.5613	1
**4047**	372	A	69.1272	0
**4047**	373	A	74.5724	1
**4047**	381	A	79.5228	1
**4047**	382	A	75.9555	1
**4047**	386	A	50.4117	1
**4047**	305	B	71.1679	1
**4047**	319	B	79.483	1

**4047**	320	B	79.512	-1
**4047**	321	B	71.9706	0
**4047**	351	B	12.205	1
**4047**	357	B	79.5612	1
**4047**	370	B	46.8534	0
**4047**	372	B	79.0243	0
**4047**	373	B	31.6139	0
**4047**	381	B	79.5487	1
**4047**	382	B	79.3973	1
**4047**	386	B	62.3682	1
**4047**	305	C	79.5028	1
**4047**	319	C	79.5409	1
**4047**	320	C	48.9005	-1
**4047**	321	C	53.7505	0
**4047**	351	C	41.5731	-1
**4047**	357	C	79.4722	1
**4047**	370	C	69.3799	1
**4047**	372	C	77.266	0
**4047**	373	C	65.6932	1
**4047**	381	C	60.3356	1
**4047**	382	C	63.1427	1
**4047**	386	C	44.1727	0
**4047**	305	D	30.9066	0
**4047**	319	D	79.5278	1
**4047**	320	D	58.6274	-1
**4047**	321	D	73.0525	0
**4047**	351	D	18.4404	1
**4047**	357	D	44.3545	0
**4047**	370	D	75.8998	1
**4047**	372	D	69.7642	0
**4047**	373	D	72.6852	1
**4047**	381	D	65.1657	1
**4047**	382	D	74.0255	1
**4047**	386	D	79.4881	1

**4048**	305	A	59.2678	1
**4048**	319	A	73.1443	1
**4048**	320	A	65.7393	-1
**4048**	321	A	71.9881	0
**4048**	351	A	3.4566	1
**4048**	357	A	53.2984	1
**4048**	370	A	34.1489	0
**4048**	372	A	79.5778	0
**4048**	373	A	74.4625	1
**4048**	381	A	36.8825	0
**4048**	382	A	43.5274	0
**4048**	386	A	70.6365	1
**4048**	305	B	68.6945	1
**4048**	319	B	59.6612	1
**4048**	320	B	72.9691	-1
**4048**	321	B	77.0499	0
**4048**	351	B	16.9494	1
**4048**	357	B	79.5897	1
**4048**	370	B	79.6754	1
**4048**	372	B	64.431	0
**4048**	373	B	54.532	1
**4048**	381	B	79.6375	1
**4048**	382	B	43.7368	0
**4048**	386	B	51.001	1
**4048**	305	C	79.4875	1
**4048**	319	C	74.2669	1
**4048**	320	C	57.0777	-1
**4048**	321	C	79.5637	0
**4048**	351	C	7.1415	1
**4048**	357	C	67.8395	1
**4048**	370	C	79.5739	1
**4048**	372	C	79.5304	0
**4048**	373	C	65.182	1
**4048**	381	C	72.3652	1

**4048**	382	C	79.5336	1
**4048**	386	C	57.7678	1
**4048**	305	D	75.2102	1
**4048**	319	D	57.0776	1
**4048**	320	D	73.4401	-1
**4048**	321	D	52.4444	0
**4048**	351	D	8.9842	1
**4048**	357	D	63.6568	1
**4048**	370	D	59.2162	1
**4048**	372	D	36.3314	1
**4048**	373	D	66.7616	1
**4048**	381	D	71.9417	1
**4048**	382	D	72.8643	1
**4048**	386	D	79.4868	1
**4049**	305	A	73.2178	1
**4049**	319	A	61.4539	1
**4049**	320	A	79.4985	-1
**4049**	321	A	73.1665	0
**4049**	351	A	2.7316	1
**4049**	357	A	79.5297	1
**4049**	370	A	72.1287	1
**4049**	372	A	57.0643	0
**4049**	373	A	79.5976	1
**4049**	381	A	62.4903	1
**4049**	382	A	79.5272	1
**4049**	386	A	79.6617	1
**4049**	305	B	79.5388	1
**4049**	319	B	75.4277	1
**4049**	320	B	77.2576	-1
**4049**	321	B	51.5863	0
**4049**	351	B	1.4211	1
**4049**	357	B	52.7153	1
**4049**	370	B	79.5737	1
**4049**	372	B	70.9552	0

**4049**	373	B	79.6742	1
**4049**	381	B	69.9022	1
**4049**	382	B	79.4109	1
**4049**	386	B	79.122	1
**4049**	305	C	50.8799	1
**4049**	319	C	70.6031	1
**4049**	320	C	79.5572	-1
**4049**	321	C	31.7732	1
**4049**	351	C	9.8104	1
**4049**	357	C	56.462	1
**4049**	370	C	79.5825	1
**4049**	372	C	51.6262	0
**4049**	373	C	62.4989	1
**4049**	381	C	35.4713	0
**4049**	382	C	56.4806	1
**4049**	386	C	32.0168	0
**4049**	305	D	49.0616	0
**4049**	319	D	78.5453	1
**4049**	320	D	52.9755	-1
**4049**	321	D	31.73	1
**4049**	351	D	7.5547	1
**4049**	357	D	79.6261	1
**4049**	370	D	67.8187	1
**4049**	372	D	79.5354	0
**4049**	373	D	73.3262	1
**4049**	381	D	71.203	1
**4049**	382	D	60.1916	1
**4049**	386	D	33.6468	0
**4050**	305	A	79.5694	1
**4050**	319	A	61.5797	1
**4050**	320	A	59.4418	-1
**4050**	321	A	70.2294	0
**4050**	351	A	5.1964	1
**4050**	357	A	79.5827	1

**4050**	370	A	79.5638	1
**4050**	372	A	44.9742	1
**4050**	373	A	53.7412	1
**4050**	381	A	60.1268	1
**4050**	382	A	75.3709	1
**4050**	386	A	57.0333	1
**4050**	305	B	79.5333	1
**4050**	319	B	79.5499	1
**4050**	320	B	72.8484	-1
**4050**	321	B	79.5568	0
**4050**	351	B	10.1827	1
**4050**	357	B	73.0	1
**4050**	370	B	79.5827	1
**4050**	372	B	74.9966	0
**4050**	373	B	67.6324	1
**4050**	381	B	53.7157	1
**4050**	382	B	78.6748	1
**4050**	386	B	49.5828	0
**4050**	305	C	73.735	1
**4050**	319	C	79.5769	1
**4050**	320	C	73.0486	-1
**4050**	321	C	73.171	0
**4050**	351	C	2.5328	1
**4050**	357	C	67.388	1
**4050**	370	C	70.5536	1
**4050**	372	C	72.47	0
**4050**	373	C	74.1078	1
**4050**	381	C	68.2068	1
**4050**	382	C	79.5877	1
**4050**	386	C	43.7175	0
**4050**	305	D	30.6238	0
**4050**	319	D	76.3537	1
**4050**	320	D	79.5315	-1
**4050**	321	D	71.6843	0

**4050**	351	D	15.5855	1
**4050**	357	D	34.369	0
**4050**	370	D	77.2315	1
**4050**	372	D	62.6801	0
**4050**	373	D	79.5631	1
**4050**	381	D	58.384	1
**4050**	382	D	53.4421	1
**4050**	386	D	70.0254	1
**4051**	305	A	79.2726	1
**4051**	319	A	65.8497	1
**4051**	320	A	71.9276	-1
**4051**	321	A	66.5204	0
**4051**	351	A	9.6127	1
**4051**	357	A	34.0948	0
**4051**	370	A	79.544	1
**4051**	372	A	45.0351	1
**4051**	373	A	79.5693	1
**4051**	381	A	79.542	1
**4051**	382	A	64.9045	1
**4051**	386	A	79.2418	1
**4051**	305	B	67.8469	1
**4051**	319	B	72.7591	1
**4051**	320	B	35.3075	-1
**4051**	321	B	69.5724	0
**4051**	351	B	15.925	1
**4051**	357	B	79.5647	1
**4051**	370	B	61.6574	1
**4051**	372	B	65.2872	0
**4051**	373	B	66.9085	1
**4051**	381	B	60.2948	1
**4051**	382	B	36.526	0
**4051**	386	B	79.5065	1
**4051**	305	C	56.2453	1
**4051**	319	C	74.5028	1

**4051**	320	C	79.4618	-1
**4051**	321	C	54.2571	0
**4051**	351	C	3.1065	1
**4051**	357	C	62.1597	1
**4051**	370	C	71.8553	1
**4051**	372	C	66.2383	0
**4051**	373	C	79.6429	1
**4051**	381	C	53.8849	1
**4051**	382	C	14.693	-1
**4051**	386	C	35.3988	0
**4051**	305	D	79.4889	1
**4051**	319	D	67.4819	1
**4051**	320	D	76.5415	-1
**4051**	321	D	48.5462	1
**4051**	351	D	16.2894	1
**4051**	357	D	72.6897	1
**4051**	370	D	42.7892	0
**4051**	372	D	73.7015	0
**4051**	373	D	79.5395	1
**4051**	381	D	45.7176	0
**4051**	382	D	45.738	0
**4051**	386	D	79.6129	1
**4052**	305	A	72.2958	1
**4052**	319	A	65.2982	1
**4052**	320	A	60.9985	-1
**4052**	321	A	42.8858	1
**4052**	351	A	17.5497	1
**4052**	357	A	68.1862	1
**4052**	370	A	53.496	1
**4052**	372	A	50.8901	0
**4052**	373	A	79.5309	1
**4052**	381	A	68.234	1
**4052**	382	A	65.2907	1
**4052**	386	A	39.0755	0

**4052**	305	B	79.5793	1
**4052**	319	B	74.7625	1
**4052**	320	B	78.4641	-1
**4052**	321	B	72.9198	0
**4052**	351	B	17.9311	1
**4052**	357	B	79.7465	1
**4052**	370	B	79.4459	1
**4052**	372	B	79.5104	0
**4052**	373	B	74.1443	1
**4052**	381	B	70.5236	1
**4052**	382	B	41.6774	0
**4052**	386	B	35.7705	0
**4052**	305	C	63.7044	1
**4052**	319	C	79.4697	1
**4052**	320	C	65.395	-1
**4052**	321	C	71.1503	0
**4052**	351	C	8.3197	1
**4052**	357	C	62.5409	1
**4052**	370	C	76.3277	1
**4052**	372	C	72.7729	0
**4052**	373	C	43.9925	0
**4052**	381	C	76.4679	1
**4052**	382	C	35.3499	0
**4052**	386	C	52.5473	1
**4052**	305	D	78.801	1
**4052**	319	D	79.3657	1
**4052**	320	D	71.1257	-1
**4052**	321	D	79.5909	0
**4052**	351	D	4.4934	1
**4052**	357	D	77.9803	1
**4052**	370	D	64.283	1
**4052**	372	D	64.8368	0
**4052**	373	D	47.4524	0
**4052**	381	D	55.6124	1

**4052**	382	D	79.5894	1
**4052**	386	D	79.6479	1
**4053**	305	A	73.4246	1
**4053**	319	A	55.346	1
**4053**	320	A	73.5571	-1
**4053**	321	A	66.9514	0
**4053**	351	A	1.204	1
**4053**	357	A	36.6907	0
**4053**	370	A	79.5597	1
**4053**	372	A	34.35	1
**4053**	373	A	65.684	1
**4053**	381	A	53.2442	1
**4053**	382	A	46.7093	0
**4053**	386	A	76.6364	1
**4053**	305	B	70.5742	1
**4053**	319	B	71.396	1
**4053**	320	B	38.6779	-1
**4053**	321	B	79.5652	0
**4053**	351	B	8.0284	1
**4053**	357	B	23.3278	0
**4053**	370	B	72.5571	1
**4053**	372	B	53.0898	0
**4053**	373	B	70.8653	1
**4053**	381	B	69.4897	1
**4053**	382	B	59.4844	1
**4053**	386	B	44.1285	0
**4053**	305	C	24.3569	0
**4053**	319	C	79.3084	1
**4053**	320	C	64.1418	-1
**4053**	321	C	76.8073	0
**4053**	351	C	9.6803	1
**4053**	357	C	79.3563	1
**4053**	370	C	2.6283	-1
**4053**	372	C	79.5808	0

**4053**	373	C	42.2018	0
**4053**	381	C	75.6208	1
**4053**	382	C	79.6089	1
**4053**	386	C	53.1651	1
**4053**	305	D	79.5702	1
**4053**	319	D	76.6216	1
**4053**	320	D	68.9424	-1
**4053**	321	D	63.1798	0
**4053**	351	D	12.9536	1
**4053**	357	D	77.6783	1
**4053**	370	D	79.5562	1
**4053**	372	D	79.5577	0
**4053**	373	D	79.5735	1
**4053**	381	D	79.5658	1
**4053**	382	D	63.5999	1
**4053**	386	D	79.5578	1
**4054**	305	A	79.5478	1
**4054**	319	A	54.8744	1
**4054**	320	A	79.5693	-1
**4054**	321	A	44.3788	1
**4054**	351	A	7.4109	1
**4054**	357	A	67.9528	1
**4054**	370	A	68.6267	1
**4054**	372	A	23.953	1
**4054**	373	A	38.6766	0
**4054**	381	A	79.5652	1
**4054**	382	A	72.6484	1
**4054**	386	A	71.8108	1
**4054**	305	B	57.6013	1
**4054**	319	B	70.4502	1
**4054**	320	B	69.1554	-1
**4054**	321	B	74.444	0
**4054**	351	B	15.8667	1
**4054**	357	B	62.5018	1

**4054**	370	B	79.5649	1
**4054**	372	B	46.1996	1
**4054**	373	B	39.8151	0
**4054**	381	B	77.5625	1
**4054**	382	B	44.9242	0
**4054**	386	B	79.5757	1
**4054**	305	C	72.6229	1
**4054**	319	C	76.0211	1
**4054**	320	C	44.2947	-1
**4054**	321	C	63.2979	0
**4054**	351	C	3.6419	1
**4054**	357	C	79.5447	1
**4054**	370	C	73.6542	1
**4054**	372	C	66.5694	0
**4054**	373	C	76.435	1
**4054**	381	C	70.7638	1
**4054**	382	C	79.5144	1
**4054**	386	C	53.2005	1
**4054**	305	D	18.6885	-1
**4054**	319	D	79.5487	1
**4054**	320	D	76.1553	-1
**4054**	321	D	79.5768	0
**4054**	351	D	10.7842	1
**4054**	357	D	79.5638	1
**4054**	370	D	72.2246	1
**4054**	372	D	68.2142	0
**4054**	373	D	73.2033	1
**4054**	381	D	38.0568	0
**4054**	382	D	73.9366	1
**4054**	386	D	79.5675	1
**4055**	305	A	79.5447	1
**4055**	319	A	63.6407	1
**4055**	320	A	79.4445	-1
**4055**	321	A	70.5954	0

**4055**	351	A	14.7776	1
**4055**	357	A	79.6147	1
**4055**	370	A	45.2698	0
**4055**	372	A	76.9449	0
**4055**	373	A	79.5963	1
**4055**	381	A	61.531	1
**4055**	382	A	72.8679	1
**4055**	386	A	57.9886	1
**4055**	305	B	69.9328	1
**4055**	319	B	78.6476	1
**4055**	320	B	47.0599	-1
**4055**	321	B	79.503	0
**4055**	351	B	12.7897	1
**4055**	357	B	62.3916	1
**4055**	370	B	57.1385	1
**4055**	372	B	67.9795	0
**4055**	373	B	69.4205	1
**4055**	381	B	79.4633	1
**4055**	382	B	49.7815	0
**4055**	386	B	73.9101	1
**4055**	305	C	68.1335	1
**4055**	319	C	57.8868	1
**4055**	320	C	79.6324	-1
**4055**	321	C	73.9027	0
**4055**	351	C	9.7826	1
**4055**	357	C	71.6456	1
**4055**	370	C	79.4438	1
**4055**	372	C	79.5455	0
**4055**	373	C	79.4576	1
**4055**	381	C	72.737	1
**4055**	382	C	35.0569	0
**4055**	386	C	52.2614	1
**4055**	305	D	70.3967	1
**4055**	319	D	72.5853	1

**4055**	320	D	79.6065	-1
**4055**	321	D	79.4073	0
**4055**	351	D	46.8621	-1
**4055**	357	D	79.6005	1
**4055**	370	D	36.0843	0
**4055**	372	D	79.5399	0
**4055**	373	D	55.0182	1
**4055**	381	D	79.5533	1
**4055**	382	D	76.7202	1
**4055**	386	D	77.0612	1
**4056**	305	A	68.7457	1
**4056**	319	A	37.047	0
**4056**	320	A	70.8728	-1
**4056**	321	A	79.539	0
**4056**	351	A	14.9407	1
**4056**	357	A	77.5189	1
**4056**	370	A	63.6832	1
**4056**	372	A	79.6405	0
**4056**	373	A	79.5787	1
**4056**	381	A	79.5278	1
**4056**	382	A	70.8435	1
**4056**	386	A	70.3484	1
**4056**	305	B	69.8899	1
**4056**	319	B	75.5307	1
**4056**	320	B	79.5446	-1
**4056**	321	B	79.5296	0
**4056**	351	B	14.8495	1
**4056**	357	B	79.5183	1
**4056**	370	B	69.768	1
**4056**	372	B	74.181	0
**4056**	373	B	65.423	1
**4056**	381	B	78.1248	1
**4056**	382	B	58.8257	1
**4056**	386	B	79.4846	1

**4056**	305	C	59.083	1
**4056**	319	C	65.2016	1
**4056**	320	C	64.3095	-1
**4056**	321	C	79.5092	0
**4056**	351	C	769	1
**4056**	357	C	59.7245	1
**4056**	370	C	79.53	1
**4056**	372	C	63.5619	0
**4056**	373	C	75.6093	1
**4056**	381	C	77.7827	1
**4056**	382	C	29.5927	0
**4056**	386	C	61.7266	1
**4056**	305	D	64.1969	1
**4056**	319	D	79.5959	1
**4056**	320	D	79.5633	-1
**4056**	321	D	69.7918	0
**4056**	351	D	6.5573	1
**4056**	357	D	79.5368	1
**4056**	370	D	73.8803	1
**4056**	372	D	63.2056	0
**4056**	373	D	68.1789	1
**4056**	381	D	60.9021	1
**4056**	382	D	33.9506	0
**4056**	386	D	79.5481	1
**4057**	305	A	59.5399	1
**4057**	319	A	59.3854	1
**4057**	320	A	52.7417	-1
**4057**	321	A	79.484	0
**4057**	351	A	0.0341	1
**4057**	357	A	79.5574	1
**4057**	370	A	57.9814	1
**4057**	372	A	73.868	0
**4057**	373	A	73.1378	1
**4057**	381	A	58.1907	1

**4057**	382	A	79.5924	1
**4057**	386	A	79.4473	1
**4057**	305	B	79.5804	1
**4057**	319	B	72.9431	1
**4057**	320	B	55.6591	-1
**4057**	321	B	79.5399	0
**4057**	351	B	9.6932	1
**4057**	357	B	79.542	1
**4057**	370	B	79.6147	1
**4057**	372	B	34.9495	1
**4057**	373	B	59.1052	1
**4057**	381	B	75.3267	1
**4057**	382	B	55.5125	1
**4057**	386	B	79.5593	1
**4057**	305	C	34.353	0
**4057**	319	C	72.8239	1
**4057**	320	C	71.8484	-1
**4057**	321	C	79.5559	0
**4057**	351	C	2.4685	1
**4057**	357	C	51.6943	1
**4057**	370	C	79.5636	1
**4057**	372	C	79.5452	0
**4057**	373	C	72.9584	1
**4057**	381	C	66.4562	1
**4057**	382	C	78.5508	1
**4057**	386	C	57.4693	1
**4057**	305	D	74.8655	1
**4057**	319	D	70.9133	1
**4057**	320	D	74.9488	-1
**4057**	321	D	77.0358	0
**4057**	351	D	20.5631	-1
**4057**	357	D	42.6543	0
**4057**	370	D	79.4357	1
**4057**	372	D	67.9991	0

**4057**	373	D	79.6034	1
**4057**	381	D	66.9675	1
**4057**	382	D	79.5316	1
**4057**	386	D	32.8702	0
**4058**	305	A	60.1284	1
**4058**	319	A	66.209	1
**4058**	320	A	79.2795	-1
**4058**	321	A	75.3895	0
**4058**	351	A	5.5837	1
**4058**	357	A	66.687	1
**4058**	370	A	79.6338	1
**4058**	372	A	79.5782	0
**4058**	373	A	47.8174	0
**4058**	381	A	79.5624	1
**4058**	382	A	72.4537	1
**4058**	386	A	73.3016	1
**4058**	305	B	62.1252	1
**4058**	319	B	72.0497	1
**4058**	320	B	57.4557	-1
**4058**	321	B	70.0222	0
**4058**	351	B	8.0708	1
**4058**	357	B	50.8872	1
**4058**	370	B	57.8443	1
**4058**	372	B	79.6425	0
**4058**	373	B	59.0324	1
**4058**	381	B	74.2647	1
**4058**	382	B	61.0816	1
**4058**	386	B	79.4768	1
**4058**	305	C	59.5698	1
**4058**	319	C	59.4005	1
**4058**	320	C	69.4093	-1
**4058**	321	C	36.3319	1
**4058**	351	C	10.7387	1
**4058**	357	C	60.7615	1

**4058**	370	C	60.9748	1
**4058**	372	C	79.5574	0
**4058**	373	C	48.8435	0
**4058**	381	C	78.7273	1
**4058**	382	C	48.2394	0
**4058**	386	C	60.0448	1
**4058**	305	D	68.79	1
**4058**	319	D	32.9713	0
**4058**	320	D	79.5309	-1
**4058**	321	D	43.482	1
**4058**	351	D	19.1649	1
**4058**	357	D	45.9232	0
**4058**	370	D	69.8436	1
**4058**	372	D	79.3475	0
**4058**	373	D	72.0556	1
**4058**	381	D	62.276	1
**4058**	382	D	79.4725	1
**4058**	386	D	25.6078	0
**4059**	305	A	79.3027	1
**4059**	319	A	61.0026	1
**4059**	320	A	70.4353	-1
**4059**	321	A	44.1243	1
**4059**	351	A	4.8159	1
**4059**	357	A	79.6556	1
**4059**	370	A	79.9539	1
**4059**	372	A	69.7407	0
**4059**	373	A	72.6788	1
**4059**	381	A	65.5857	1
**4059**	382	A	73.22	1
**4059**	386	A	76.7277	1
**4059**	305	B	79.6096	1
**4059**	319	B	63.8282	1
**4059**	320	B	35.845	-1
**4059**	321	B	79.4114	0

**4059**	351	B	10.5662	1
**4059**	357	B	79.3874	1
**4059**	370	B	31.2419	0
**4059**	372	B	79.5292	0
**4059**	373	B	64.5256	1
**4059**	381	B	67.3712	1
**4059**	382	B	47.2431	0
**4059**	386	B	75.0499	1
**4059**	305	C	59.6308	1
**4059**	319	C	61.8037	1
**4059**	320	C	77.6679	-1
**4059**	321	C	21.6881	1
**4059**	351	C	9.1671	1
**4059**	357	C	64.3087	1
**4059**	370	C	79.4116	1
**4059**	372	C	71.9223	0
**4059**	373	C	65.9914	1
**4059**	381	C	79.3626	1
**4059**	382	C	64.8927	1
**4059**	386	C	46.7697	0
**4059**	305	D	58.4822	1
**4059**	319	D	79.5484	1
**4059**	320	D	62.7138	-1
**4059**	321	D	49.2902	1
**4059**	351	D	13.6921	1
**4059**	357	D	63.3222	1
**4059**	370	D	79.264	1
**4059**	372	D	79.6719	0
**4059**	373	D	79.6406	1
**4059**	381	D	76.1034	1
**4059**	382	D	79.5248	1
**4059**	386	D	70.8823	1
**4060**	305	A	54.7802	1
**4060**	319	A	43.3817	0

**4060**	320	A	73.2162	-1
**4060**	321	A	70.7035	0
**4060**	351	A	1.017	1
**4060**	357	A	52.5965	1
**4060**	370	A	79.5538	1
**4060**	372	A	79.5484	0
**4060**	373	A	57.4023	1
**4060**	381	A	53.9745	1
**4060**	382	A	79.3304	1
**4060**	386	A	50.8924	1
**4060**	305	B	79.5657	1
**4060**	319	B	74.6491	1
**4060**	320	B	73.6898	-1
**4060**	321	B	79.5892	0
**4060**	351	B	11.7431	1
**4060**	357	B	79.5548	1
**4060**	370	B	79.2374	1
**4060**	372	B	79.5594	0
**4060**	373	B	79.5513	1
**4060**	381	B	79.5879	1
**4060**	382	B	41.5406	0
**4060**	386	B	77.9888	1
**4060**	305	C	62.3863	1
**4060**	319	C	76.6039	1
**4060**	320	C	74.1215	-1
**4060**	321	C	68.6266	0
**4060**	351	C	11.1205	1
**4060**	357	C	69.3113	1
**4060**	370	C	72.6963	1
**4060**	372	C	79.5358	0
**4060**	373	C	79.5469	1
**4060**	381	C	74.0644	1
**4060**	382	C	57.9072	1
**4060**	386	C	22.5659	0

**4060**	305	D	79.5266	1
**4060**	319	D	44.6343	0
**4060**	320	D	69.4691	-1
**4060**	321	D	59.7554	0
**4060**	351	D	15.2718	1
**4060**	357	D	71.8337	1
**4060**	370	D	72.7265	1
**4060**	372	D	79.5662	0
**4060**	373	D	76.7194	1
**4060**	381	D	68.1495	1
**4060**	382	D	79.5611	1
**4060**	386	D	43.4686	0
**4061**	305	A	65.1375	1
**4061**	319	A	78.5083	1
**4061**	320	A	79.5784	-1
**4061**	321	A	68.7855	0
**4061**	351	A	7.8778	1
**4061**	357	A	54.4727	1
**4061**	370	A	53.1006	1
**4061**	372	A	59.7893	0
**4061**	373	A	71.1545	1
**4061**	381	A	58.3927	1
**4061**	382	A	73.069	1
**4061**	386	A	35.3615	0
**4061**	305	B	59.858	1
**4061**	319	B	71.6622	1
**4061**	320	B	79.5182	-1
**4061**	321	B	79.5024	0
**4061**	351	B	17.7319	1
**4061**	357	B	56.8293	1
**4061**	370	B	72.6643	1
**4061**	372	B	79.5753	0
**4061**	373	B	79.5033	1
**4061**	381	B	54.896	1

**4061**	382	B	70.8371	1
**4061**	386	B	64.9405	1
**4061**	305	C	35.1568	0
**4061**	319	C	79.5361	1
**4061**	320	C	79.5923	-1
**4061**	321	C	79.2468	0
**4061**	351	C	7.6686	1
**4061**	357	C	53.6765	1
**4061**	370	C	79.537	1
**4061**	372	C	79.5608	0
**4061**	373	C	48.9316	0
**4061**	381	C	73.4322	1
**4061**	382	C	78.5511	1
**4061**	386	C	54.7345	1
**4061**	305	D	75.696	1
**4061**	319	D	45.6371	0
**4061**	320	D	79.7183	-1
**4061**	321	D	71.4527	0
**4061**	351	D	13.7658	1
**4061**	357	D	74.9057	1
**4061**	370	D	79.5726	1
**4061**	372	D	24.2304	1
**4061**	373	D	62.4557	1
**4061**	381	D	62.1359	1
**4061**	382	D	67.8412	1
**4061**	386	D	79.5392	1
**4062**	305	A	75.8911	1
**4062**	319	A	26.0472	0
**4062**	320	A	79.4568	-1
**4062**	321	A	61.867	0
**4062**	351	A	11.0474	1
**4062**	357	A	79.6949	1
**4062**	370	A	78.3288	1
**4062**	372	A	79.5504	0

**4062**	373	A	55.7866	1
**4062**	381	A	46.9698	0
**4062**	382	A	32.1302	0
**4062**	386	A	56.3308	1
**4062**	305	B	75.6591	1
**4062**	319	B	50.0881	1
**4062**	320	B	72.8313	-1
**4062**	321	B	57.2328	0
**4062**	351	B	43.806	-1
**4062**	357	B	67.669	1
**4062**	370	B	79.5041	1
**4062**	372	B	73.5321	0
**4062**	373	B	45.0137	0
**4062**	381	B	56.0317	1
**4062**	382	B	30.1144	0
**4062**	386	B	53.5157	1
**4062**	305	C	57.9795	1
**4062**	319	C	60.2996	1
**4062**	320	C	79.585	-1
**4062**	321	C	79.606	0
**4062**	351	C	7.4947	1
**4062**	357	C	79.4821	1
**4062**	370	C	79.5655	1
**4062**	372	C	78.5775	0
**4062**	373	C	43.1408	0
**4062**	381	C	57.3259	1
**4062**	382	C	70.5517	1
**4062**	386	C	63.0847	1
**4062**	305	D	60.8855	1
**4062**	319	D	74.9607	1
**4062**	320	D	41.2587	-1
**4062**	321	D	79.6287	0
**4062**	351	D	12.3997	1
**4062**	357	D	79.5631	1

**4062**	370	D	48.048	0
**4062**	372	D	36.4601	1
**4062**	373	D	79.619	1
**4062**	381	D	61.2275	1
**4062**	382	D	79.7406	1
**4062**	386	D	79.3675	1
**4063**	305	A	54.6263	1
**4063**	319	A	63.3118	1
**4063**	320	A	65.373	-1
**4063**	321	A	78.9092	0
**4063**	351	A	4.9757	1
**4063**	357	A	77.179	1
**4063**	370	A	59.5776	1
**4063**	372	A	71.8807	0
**4063**	373	A	62.7759	1
**4063**	381	A	65.8407	1
**4063**	382	A	70.9639	1
**4063**	386	A	72.6844	1
**4063**	305	B	64.5117	1
**4063**	319	B	73.9916	1
**4063**	320	B	72.3718	-1
**4063**	321	B	67.8041	0
**4063**	351	B	9.2361	1
**4063**	357	B	28.7878	0
**4063**	370	B	58.418	1
**4063**	372	B	77.7748	0
**4063**	373	B	79.5741	1
**4063**	381	B	65.6381	1
**4063**	382	B	69.9089	1
**4063**	386	B	46.7758	0
**4063**	305	C	79.1298	1
**4063**	319	C	79.6056	1
**4063**	320	C	68.9754	-1
**4063**	321	C	79.5515	0

**4063**	351	C	1.7118	1
**4063**	357	C	79.5154	1
**4063**	370	C	70.1888	1
**4063**	372	C	79.5851	0
**4063**	373	C	64.9277	1
**4063**	381	C	79.5383	1
**4063**	382	C	79.5484	1
**4063**	386	C	61.163	1
**4063**	305	D	60.5914	1
**4063**	319	D	79.5495	1
**4063**	320	D	79.6159	-1
**4063**	321	D	79.5496	0
**4063**	351	D	17.0596	1
**4063**	357	D	46.0439	0
**4063**	370	D	34.24	0
**4063**	372	D	79.581	0
**4063**	373	D	69.7022	1
**4063**	381	D	50.9208	1
**4063**	382	D	59.7171	1
**4063**	386	D	23.8297	0
**4064**	305	A	76.5236	1
**4064**	319	A	61.0921	1
**4064**	320	A	55.3474	-1
**4064**	321	A	70.8163	0
**4064**	351	A	8.2636	1
**4064**	357	A	79.115	1
**4064**	370	A	77.4262	1
**4064**	372	A	48.0266	1
**4064**	373	A	78.2897	1
**4064**	381	A	51.1478	1
**4064**	382	A	79.5709	1
**4064**	386	A	41.2295	0
**4064**	305	B	78.302	1
**4064**	319	B	61.8379	1

**4064**	320	B	79.5888	-1
**4064**	321	B	69.4446	0
**4064**	351	B	15.7077	1
**4064**	357	B	78.8135	1
**4064**	370	B	69.4489	1
**4064**	372	B	70.608	0
**4064**	373	B	69.4459	1
**4064**	381	B	66.1841	1
**4064**	382	B	37.3765	0
**4064**	386	B	79.5413	1
**4064**	305	C	78.4664	1
**4064**	319	C	79.5358	1
**4064**	320	C	79.636	-1
**4064**	321	C	25.0672	1
**4064**	351	C	1.2205	1
**4064**	357	C	75.7268	1
**4064**	370	C	34.5805	0
**4064**	372	C	79.5108	0
**4064**	373	C	75.6493	1
**4064**	381	C	72.1932	1
**4064**	382	C	50.2953	1
**4064**	386	C	60.3473	1
**4064**	305	D	79.5086	1
**4064**	319	D	68.5791	1
**4064**	320	D	72.1381	-1
**4064**	321	D	73.7114	0
**4064**	351	D	14.7887	1
**4064**	357	D	72.9757	1
**4064**	370	D	74.1032	1
**4064**	372	D	79.5041	0
**4064**	373	D	74.1278	1
**4064**	381	D	71.3561	1
**4064**	382	D	39.3008	0
**4064**	386	D	47.6009	0

**4065**	305	A	37.1853	0
**4065**	319	A	42.9946	0
**4065**	320	A	78.7176	-1
**4065**	321	A	73.0777	0
**4065**	351	A	19.3724	1
**4065**	357	A	78.8189	1
**4065**	370	A	69.9852	1
**4065**	372	A	72.8563	0
**4065**	373	A	79.5856	1
**4065**	381	A	74.0987	1
**4065**	382	A	68.9149	1
**4065**	386	A	78.4278	1
**4065**	305	B	57.6793	1
**4065**	319	B	79.6381	1
**4065**	320	B	25.3883	-1
**4065**	321	B	38.8332	1
**4065**	351	B	4.0404	1
**4065**	357	B	78.1478	1
**4065**	370	B	64.5337	1
**4065**	372	B	42.0047	1
**4065**	373	B	54.2801	1
**4065**	381	B	63.0691	1
**4065**	382	B	33.4126	0
**4065**	386	B	79.5609	1
**4065**	305	C	63.9586	1
**4065**	319	C	62.8406	1
**4065**	320	C	79.2596	-1
**4065**	321	C	56.2381	0
**4065**	351	C	5.1578	1
**4065**	357	C	58.2293	1
**4065**	370	C	79.5698	1
**4065**	372	C	79.5299	0
**4065**	373	C	73.4935	1
**4065**	381	C	79.5612	1

**4065**	382	C	77.6082	1
**4065**	386	C	44.3402	0
**4065**	305	D	70.4969	1
**4065**	319	D	79.4584	1
**4065**	320	D	79.5989	-1
**4065**	321	D	79.4974	0
**4065**	351	D	14.8529	1
**4065**	357	D	79.4732	1
**4065**	370	D	79.6146	1
**4065**	372	D	73.3922	0
**4065**	373	D	50.9006	1
**4065**	381	D	28.5987	0
**4065**	382	D	79.4958	1
**4065**	386	D	40.569	0
**4066**	305	A	49.7978	0
**4066**	319	A	73.6203	1
**4066**	320	A	79.5222	-1
**4066**	321	A	34.237	1
**4066**	351	A	12.0318	1
**4066**	357	A	65.8943	1
**4066**	370	A	79.4383	1
**4066**	372	A	60.2932	0
**4066**	373	A	79.6675	1
**4066**	381	A	79.5907	1
**4066**	382	A	72.6392	1
**4066**	386	A	71.349	1
**4066**	305	B	71.8039	1
**4066**	319	B	73.3125	1
**4066**	320	B	67.5096	-1
**4066**	321	B	69.0859	0
**4066**	351	B	2.9093	1
**4066**	357	B	48.9022	0
**4066**	370	B	72.858	1
**4066**	372	B	69.8264	0

**4066**	373	B	79.5709	1
**4066**	381	B	67.1633	1
**4066**	382	B	46.4825	0
**4066**	386	B	59.7943	1
**4066**	305	C	79.5191	1
**4066**	319	C	73.468	1
**4066**	320	C	62.0162	-1
**4066**	321	C	71.52	0
**4066**	351	C	14.1404	1
**4066**	357	C	74.4189	1
**4066**	370	C	77.5191	1
**4066**	372	C	67.7493	0
**4066**	373	C	42.5631	0
**4066**	381	C	79.5695	1
**4066**	382	C	62.5629	1
**4066**	386	C	43.7807	0
**4066**	305	D	73.3051	1
**4066**	319	D	75.9931	1
**4066**	320	D	38.6913	-1
**4066**	321	D	70.298	0
**4066**	351	D	0.8288	1
**4066**	357	D	73.8222	1
**4066**	370	D	57.3873	1
**4066**	372	D	69.8713	0
**4066**	373	D	79.6218	1
**4066**	381	D	70.6116	1
**4066**	382	D	79.5149	1
**4066**	386	D	79.5653	1
**4067**	305	A	76.9986	1
**4067**	319	A	73.5612	1
**4067**	320	A	79.4958	-1
**4067**	321	A	67.0543	0
**4067**	351	A	11.6698	1
**4067**	357	A	57.9562	1

**4067**	370	A	71.6233	1
**4067**	372	A	65.6065	0
**4067**	373	A	78.8041	1
**4067**	381	A	73.8858	1
**4067**	382	A	79.6308	1
**4067**	386	A	79.5952	1
**4067**	305	B	75.0214	1
**4067**	319	B	79.6844	1
**4067**	320	B	67.957	-1
**4067**	321	B	79.5906	0
**4067**	351	B	24.3384	-1
**4067**	357	B	79.5842	1
**4067**	370	B	72.1207	1
**4067**	372	B	64.6578	0
**4067**	373	B	23.0186	0
**4067**	381	B	79.689	1
**4067**	382	B	78.6736	1
**4067**	386	B	71.8923	1
**4067**	305	C	53.3752	1
**4067**	319	C	65.8518	1
**4067**	320	C	79.2929	-1
**4067**	321	C	79.5847	0
**4067**	351	C	14.7017	1
**4067**	357	C	79.5752	1
**4067**	370	C	79.6737	1
**4067**	372	C	79.4123	0
**4067**	373	C	72.8154	1
**4067**	381	C	79.7554	1
**4067**	382	C	44.4488	0
**4067**	386	C	60.6494	1
**4067**	305	D	79.4445	1
**4067**	319	D	74.5556	1
**4067**	320	D	48.9544	-1
**4067**	321	D	79.4388	0

**4067**	351	D	8.67	1
**4067**	357	D	79.5409	1
**4067**	370	D	40.3401	0
**4067**	372	D	79.5266	0
**4067**	373	D	58.5858	1
**4067**	381	D	70.0907	1
**4067**	382	D	79.4624	1
**4067**	386	D	79.6656	1
**4068**	305	A	72.484	1
**4068**	319	A	47.2266	0
**4068**	320	A	79.6081	-1
**4068**	321	A	73.4843	0
**4068**	351	A	4.2641	1
**4068**	357	A	51.8969	1
**4068**	370	A	71.7461	1
**4068**	372	A	57.0923	0
**4068**	373	A	74.3821	1
**4068**	381	A	79.7056	1
**4068**	382	A	79.6206	1
**4068**	386	A	72.4432	1
**4068**	305	B	62.7718	1
**4068**	319	B	79.7442	1
**4068**	320	B	26.6083	-1
**4068**	321	B	71.2942	0
**4068**	351	B	9.611	1
**4068**	357	B	51.2474	1
**4068**	370	B	54.5908	1
**4068**	372	B	77.0687	0
**4068**	373	B	79.4121	1
**4068**	381	B	71.138	1
**4068**	382	B	77.842	1
**4068**	386	B	71.8261	1
**4068**	305	C	58.8803	1
**4068**	319	C	79.596	1

**4068**	320	C	73.3967	-1
**4068**	321	C	64.5967	0
**4068**	351	C	10.6184	1
**4068**	357	C	79.5622	1
**4068**	370	C	56.4831	1
**4068**	372	C	74.4524	0
**4068**	373	C	67.142	1
**4068**	381	C	78.5262	1
**4068**	382	C	40.6225	0
**4068**	386	C	15.1791	-1
**4068**	305	D	39.871	0
**4068**	319	D	79.3228	1
**4068**	320	D	79.6013	-1
**4068**	321	D	79.4887	0
**4068**	351	D	8.5263	1
**4068**	357	D	79.5701	1
**4068**	370	D	75.1378	1
**4068**	372	D	79.5163	0
**4068**	373	D	28.359	0
**4068**	381	D	51.7103	1
**4068**	382	D	73.1245	1
**4068**	386	D	30.8581	0
**4069**	305	A	79.5278	1
**4069**	319	A	41.0227	0
**4069**	320	A	57.9583	-1
**4069**	321	A	72.8094	0
**4069**	351	A	0.1621	1
**4069**	357	A	58.7419	1
**4069**	370	A	79.5736	1
**4069**	372	A	58.688	0
**4069**	373	A	68.8707	1
**4069**	381	A	39.7564	0
**4069**	382	A	62.4256	1
**4069**	386	A	63.3182	1

**4069**	305	B	42.0313	0
**4069**	319	B	74.8495	1
**4069**	320	B	79.5938	-1
**4069**	321	B	57.6694	0
**4069**	351	B	6.6375	1
**4069**	357	B	70.2366	1
**4069**	370	B	73.1035	1
**4069**	372	B	79.5223	0
**4069**	373	B	63.7352	1
**4069**	381	B	79.5728	1
**4069**	382	B	58.8474	1
**4069**	386	B	69.2751	1
**4069**	305	C	79.5094	1
**4069**	319	C	76.924	1
**4069**	320	C	64.8702	-1
**4069**	321	C	74.207	0
**4069**	351	C	3.1943	1
**4069**	357	C	79.5684	1
**4069**	370	C	66.0682	1
**4069**	372	C	79.5687	0
**4069**	373	C	59.7622	1
**4069**	381	C	65.5734	1
**4069**	382	C	73.4875	1
**4069**	386	C	40.1536	0
**4069**	305	D	41.9323	0
**4069**	319	D	71.4833	1
**4069**	320	D	58.4239	-1
**4069**	321	D	75.9206	0
**4069**	351	D	16.2435	1
**4069**	357	D	63.1473	1
**4069**	370	D	79.5519	1
**4069**	372	D	79.5643	0
**4069**	373	D	79.5845	1
**4069**	381	D	62.3927	1

**4069**	382	D	78.3549	1
**4069**	386	D	63.6688	1
**4070**	305	A	72.3846	1
**4070**	319	A	76.7568	1
**4070**	320	A	79.5023	-1
**4070**	321	A	26.198	1
**4070**	351	A	9.535	1
**4070**	357	A	47.1962	0
**4070**	370	A	20.3163	0
**4070**	372	A	56.9217	0
**4070**	373	A	37.6715	0
**4070**	381	A	70.9067	1
**4070**	382	A	63.2376	1
**4070**	386	A	79.1557	1
**4070**	305	B	76.8325	1
**4070**	319	B	74.5329	1
**4070**	320	B	60.8018	-1
**4070**	321	B	79.5156	0
**4070**	351	B	10.5289	1
**4070**	357	B	60.6425	1
**4070**	370	B	79.5351	1
**4070**	372	B	69.7404	0
**4070**	373	B	45.5702	0
**4070**	381	B	74.6565	1
**4070**	382	B	21.9592	0
**4070**	386	B	45.7736	0
**4070**	305	C	73.367	1
**4070**	319	C	42.984	0
**4070**	320	C	75.9592	-1
**4070**	321	C	77.214	0
**4070**	351	C	7.844	1
**4070**	357	C	25.2961	0
**4070**	370	C	68.8652	1
**4070**	372	C	68.1084	0

**4070**	373	C	51.5408	1
**4070**	381	C	79.5261	1
**4070**	382	C	79.2998	1
**4070**	386	C	66.0568	1
**4070**	305	D	68.7769	1
**4070**	319	D	72.2904	1
**4070**	320	D	67.6319	-1
**4070**	321	D	59.759	0
**4070**	351	D	21.0395	-1
**4070**	357	D	79.4752	1
**4070**	370	D	61.2573	1
**4070**	372	D	79.5704	0
**4070**	373	D	79.5838	1
**4070**	381	D	65.3414	1
**4070**	382	D	64.5165	1
**4070**	386	D	53.7836	1
**4071**	305	A	69.9498	1
**4071**	319	A	79.1198	1
**4071**	320	A	75.931	-1
**4071**	321	A	79.5362	0
**4071**	351	A	15.5307	1
**4071**	357	A	72.8425	1
**4071**	370	A	79.5447	1
**4071**	372	A	79.5601	0
**4071**	373	A	45.0149	0
**4071**	381	A	79.0767	1
**4071**	382	A	65.524	1
**4071**	386	A	79.5584	1
**4071**	305	B	52.0275	1
**4071**	319	B	79.7038	1
**4071**	320	B	51.4918	-1
**4071**	321	B	79.6159	0
**4071**	351	B	14.7933	1
**4071**	357	B	61.941	1

**4071**	370	B	73.813	1
**4071**	372	B	79.5347	0
**4071**	373	B	49.768	0
**4071**	381	B	76.7861	1
**4071**	382	B	41.4314	0
**4071**	386	B	46.2032	0
**4071**	305	C	77.2631	1
**4071**	319	C	68.4752	1
**4071**	320	C	50.7671	-1
**4071**	321	C	79.5654	0
**4071**	351	C	7.6877	1
**4071**	357	C	74.9774	1
**4071**	370	C	72.4719	1
**4071**	372	C	75.9722	0
**4071**	373	C	79.5734	1
**4071**	381	C	79.077	1
**4071**	382	C	72.8798	1
**4071**	386	C	24.5003	0
**4071**	305	D	60.1279	1
**4071**	319	D	66.9636	1
**4071**	320	D	79.5731	-1
**4071**	321	D	54.9108	0
**4071**	351	D	12.7831	1
**4071**	357	D	79.5626	1
**4071**	370	D	43.9956	0
**4071**	372	D	73.0507	0
**4071**	373	D	78.8933	1
**4071**	381	D	71.6507	1
**4071**	382	D	79.5453	1
**4071**	386	D	79.3824	1
**4072**	305	A	59.2879	1
**4072**	319	A	64.2503	1
**4072**	320	A	79.5314	-1
**4072**	321	A	77.3272	0

**4072**	351	A	3.7207	1
**4072**	357	A	53.8067	1
**4072**	370	A	79.6302	1
**4072**	372	A	79.5292	0
**4072**	373	A	79.649	1
**4072**	381	A	79.5603	1
**4072**	382	A	79.5563	1
**4072**	386	A	72.7326	1
**4072**	305	B	62.6385	1
**4072**	319	B	71.6956	1
**4072**	320	B	66.8117	-1
**4072**	321	B	73.066	0
**4072**	351	B	11.5366	1
**4072**	357	B	74.6931	1
**4072**	370	B	73.0209	1
**4072**	372	B	70.8361	0
**4072**	373	B	47.0007	0
**4072**	381	B	58.0992	1
**4072**	382	B	75.5436	1
**4072**	386	B	79.6169	1
**4072**	305	C	75.8844	1
**4072**	319	C	79.2964	1
**4072**	320	C	61.2116	-1
**4072**	321	C	31.4767	1
**4072**	351	C	6.0164	1
**4072**	357	C	79.5483	1
**4072**	370	C	72.7966	1
**4072**	372	C	79.5565	0
**4072**	373	C	79.5658	1
**4072**	381	C	72.4655	1
**4072**	382	C	72.9518	1
**4072**	386	C	57.5272	1
**4072**	305	D	59.0644	1
**4072**	319	D	76.4189	1

**4072**	320	D	76.5986	-1
**4072**	321	D	71.931	0
**4072**	351	D	11.3828	1
**4072**	357	D	79.5305	1
**4072**	370	D	79.2974	1
**4072**	372	D	78.7327	0
**4072**	373	D	75.5644	1
**4072**	381	D	79.5691	1
**4072**	382	D	79.5821	1
**4072**	386	D	79.5495	1
**4073**	305	A	56.1667	1
**4073**	319	A	58.6549	1
**4073**	320	A	79.601	-1
**4073**	321	A	75.3187	0
**4073**	351	A	15.8177	1
**4073**	357	A	73.2041	1
**4073**	370	A	67.7689	1
**4073**	372	A	67.1169	0
**4073**	373	A	70.0056	1
**4073**	381	A	79.6155	1
**4073**	382	A	67.4764	1
**4073**	386	A	72.6441	1
**4073**	305	B	71.3877	1
**4073**	319	B	79.6207	1
**4073**	320	B	49.582	-1
**4073**	321	B	57.3025	0
**4073**	351	B	9.9028	1
**4073**	357	B	50.5408	1
**4073**	370	B	71.7916	1
**4073**	372	B	79.5288	0
**4073**	373	B	77.5596	1
**4073**	381	B	74.2939	1
**4073**	382	B	77.3797	1
**4073**	386	B	40.3708	0

**4073**	305	C	69.2914	1
**4073**	319	C	38.1046	0
**4073**	320	C	62.0611	-1
**4073**	321	C	79.584	0
**4073**	351	C	11.4102	1
**4073**	357	C	49.4891	0
**4073**	370	C	79.5543	1
**4073**	372	C	74.5896	0
**4073**	373	C	58.5197	1
**4073**	381	C	79.6178	1
**4073**	382	C	79.5268	1
**4073**	386	C	62.2836	1
**4073**	305	D	48.8482	0
**4073**	319	D	63.4196	1
**4073**	320	D	71.8465	-1
**4073**	321	D	65.6864	0
**4073**	351	D	27.7667	-1
**4073**	357	D	60.7522	1
**4073**	370	D	72.99	1
**4073**	372	D	72.8885	0
**4073**	373	D	56.9454	1
**4073**	381	D	33.2839	0
**4073**	382	D	75.1945	1
**4073**	386	D	79.6073	1
**4074**	305	A	75.5857	1
**4074**	319	A	69.5455	1
**4074**	320	A	79.6158	-1
**4074**	321	A	53.6868	0
**4074**	351	A	9.6108	1
**4074**	357	A	69.057	1
**4074**	370	A	71.8119	1
**4074**	372	A	62.1319	0
**4074**	373	A	21.5894	0
**4074**	381	A	79.5075	1

**4074**	382	A	62.18	1
**4074**	386	A	51.6485	1
**4074**	305	B	73.6097	1
**4074**	319	B	64.6253	1
**4074**	320	B	47.5177	-1
**4074**	321	B	73.547	0
**4074**	351	B	8.1927	1
**4074**	357	B	71.6477	1
**4074**	370	B	79.5773	1
**4074**	372	B	74.3348	0
**4074**	373	B	60.3031	1
**4074**	381	B	74.765	1
**4074**	382	B	36.0253	0
**4074**	386	B	69.9135	1
**4074**	305	C	68.9337	1
**4074**	319	C	43.6066	0
**4074**	320	C	61.8412	-1
**4074**	321	C	49.7848	1
**4074**	351	C	8.3685	1
**4074**	357	C	75.0516	1
**4074**	370	C	79.5569	1
**4074**	372	C	72.0816	0
**4074**	373	C	79.4701	1
**4074**	381	C	61.7873	1
**4074**	382	C	79.5749	1
**4074**	386	C	43.909	0
**4074**	305	D	79.6807	1
**4074**	319	D	59.9739	1
**4074**	320	D	79.5791	-1
**4074**	321	D	69.4614	0
**4074**	351	D	13.7181	1
**4074**	357	D	68.3637	1
**4074**	370	D	79.7172	1
**4074**	372	D	79.7067	0

**4074**	373	D	79.6676	1
**4074**	381	D	52.0223	1
**4074**	382	D	68.9684	1
**4074**	386	D	74.0273	1
**4075**	305	A	78.5085	1
**4075**	319	A	68.0333	1
**4075**	320	A	79.5578	-1
**4075**	321	A	79.5651	0
**4075**	351	A	4.4476	1
**4075**	357	A	66.2868	1
**4075**	370	A	79.5092	1
**4075**	372	A	73.7894	0
**4075**	373	A	52.9474	1
**4075**	381	A	73.8642	1
**4075**	382	A	62.3573	1
**4075**	386	A	73.3318	1
**4075**	305	B	79.5677	1
**4075**	319	B	76.7967	1
**4075**	320	B	79.5505	-1
**4075**	321	B	79.5611	0
**4075**	351	B	13.2685	1
**4075**	357	B	57.5001	1
**4075**	370	B	72.3127	1
**4075**	372	B	71.3882	0
**4075**	373	B	49.382	0
**4075**	381	B	79.579	1
**4075**	382	B	73.3294	1
**4075**	386	B	78.2433	1
**4075**	305	C	79.577	1
**4075**	319	C	79.5704	1
**4075**	320	C	78.7017	-1
**4075**	321	C	79.5609	0
**4075**	351	C	244	1
**4075**	357	C	78.7179	1

**4075**	370	C	70.8241	1
**4075**	372	C	64.8512	0
**4075**	373	C	63.1677	1
**4075**	381	C	79.5979	1
**4075**	382	C	69.7428	1
**4075**	386	C	56.7189	1
**4075**	305	D	73.1293	1
**4075**	319	D	75.0939	1
**4075**	320	D	53.1335	-1
**4075**	321	D	74.8471	0
**4075**	351	D	12.9788	1
**4075**	357	D	74.8211	1
**4075**	370	D	49.9521	0
**4075**	372	D	65.2174	0
**4075**	373	D	79.5755	1
**4075**	381	D	64.1148	1
**4075**	382	D	70.779	1
**4075**	386	D	72.6031	1
**4076**	305	A	79.5824	1
**4076**	319	A	73.8493	1
**4076**	320	A	46.3144	-1
**4076**	321	A	69.1484	0
**4076**	351	A	3.5662	1
**4076**	357	A	74.0837	1
**4076**	370	A	79.5949	1
**4076**	372	A	38.2305	1
**4076**	373	A	71.3584	1
**4076**	381	A	54.5718	1
**4076**	382	A	30.0598	0
**4076**	386	A	72.3291	1
**4076**	305	B	71.5492	1
**4076**	319	B	67.3319	1
**4076**	320	B	79.3846	-1
**4076**	321	B	66.8982	0

**4076**	351	B	6.5272	1
**4076**	357	B	79.4469	1
**4076**	370	B	71.9017	1
**4076**	372	B	57.5027	0
**4076**	373	B	66.9513	1
**4076**	381	B	35.8093	0
**4076**	382	B	34.7793	0
**4076**	386	B	45.7675	0
**4076**	305	C	73.1781	1
**4076**	319	C	73.1679	1
**4076**	320	C	79.5356	-1
**4076**	321	C	79.5962	0
**4076**	351	C	5.4699	1
**4076**	357	C	61.6803	1
**4076**	370	C	50.2889	1
**4076**	372	C	79.5079	0
**4076**	373	C	67.6064	1
**4076**	381	C	55.4978	1
**4076**	382	C	74.7207	1
**4076**	386	C	49.6322	0
**4076**	305	D	61.4112	1
**4076**	319	D	50.7614	1
**4076**	320	D	79.5885	-1
**4076**	321	D	72.7906	0
**4076**	351	D	14.4595	1
**4076**	357	D	67.328	1
**4076**	370	D	79.553	1
**4076**	372	D	70.5024	0
**4076**	373	D	79.3759	1
**4076**	381	D	50.7366	1
**4076**	382	D	69.7488	1
**4076**	386	D	79.5809	1
**4077**	305	A	79.6641	1
**4077**	319	A	61.0748	1

**4077**	320	A	78.9115	-1
**4077**	321	A	66.9589	0
**4077**	351	A	2.7823	1
**4077**	357	A	59.0425	1
**4077**	370	A	75.7835	1
**4077**	372	A	49.8169	1
**4077**	373	A	79.6521	1
**4077**	381	A	49.1458	0
**4077**	382	A	76.0367	1
**4077**	386	A	49.7271	0
**4077**	305	B	79.711	1
**4077**	319	B	70.5992	1
**4077**	320	B	51.9097	-1
**4077**	321	B	73.9726	0
**4077**	351	B	13.9329	1
**4077**	357	B	47.4702	0
**4077**	370	B	41.8897	0
**4077**	372	B	79.5057	0
**4077**	373	B	22.2965	0
**4077**	381	B	55.6138	1
**4077**	382	B	79.305	1
**4077**	386	B	69.9006	1
**4077**	305	C	62.492	1
**4077**	319	C	55.5845	1
**4077**	320	C	79.4953	-1
**4077**	321	C	79.4341	0
**4077**	351	C	6.3416	1
**4077**	357	C	50.2016	1
**4077**	370	C	60.1266	1
**4077**	372	C	21.9368	1
**4077**	373	C	74.9018	1
**4077**	381	C	66.7958	1
**4077**	382	C	71.7875	1
**4077**	386	C	59.2381	1

**4077**	305	D	32.3749	0
**4077**	319	D	76.9812	1
**4077**	320	D	54.3292	-1
**4077**	321	D	72.2813	0
**4077**	351	D	17.155	1
**4077**	357	D	56.6029	1
**4077**	370	D	63.9231	1
**4077**	372	D	46.7174	1
**4077**	373	D	61.2284	1
**4077**	381	D	72.0009	1
**4077**	382	D	79.4272	1
**4077**	386	D	43.7298	0
**4078**	305	A	62.121	1
**4078**	319	A	74.1103	1
**4078**	320	A	29.3376	-1
**4078**	321	A	71.9758	0
**4078**	351	A	2.9118	1
**4078**	357	A	69.7926	1
**4078**	370	A	67.5503	1
**4078**	372	A	45.3986	1
**4078**	373	A	44.3227	0
**4078**	381	A	60.7108	1
**4078**	382	A	36.4022	0
**4078**	386	A	73.5095	1
**4078**	305	B	66.6262	1
**4078**	319	B	79.5149	1
**4078**	320	B	69.1382	-1
**4078**	321	B	30.61	1
**4078**	351	B	5.2186	1
**4078**	357	B	58.5067	1
**4078**	370	B	79.5237	1
**4078**	372	B	68.9091	0
**4078**	373	B	39.1455	0
**4078**	381	B	79.4827	1

**4078**	382	B	74.8945	1
**4078**	386	B	79.3724	1
**4078**	305	C	77.5713	1
**4078**	319	C	77.9217	1
**4078**	320	C	74.6109	-1
**4078**	321	C	76.5184	0
**4078**	351	C	5.7322	1
**4078**	357	C	79.4656	1
**4078**	370	C	72.6576	1
**4078**	372	C	45.0448	1
**4078**	373	C	66.7917	1
**4078**	381	C	64.0421	1
**4078**	382	C	69.6145	1
**4078**	386	C	46.0192	0
**4078**	305	D	62.5788	1
**4078**	319	D	76.9839	1
**4078**	320	D	72.8778	-1
**4078**	321	D	59.3062	0
**4078**	351	D	13.906	1
**4078**	357	D	52.9768	1
**4078**	370	D	69.6026	1
**4078**	372	D	75.9221	0
**4078**	373	D	50.3295	1
**4078**	381	D	44.901	0
**4078**	382	D	79.4788	1
**4078**	386	D	61.9443	1
**4079**	305	A	72.9713	1
**4079**	319	A	79.5272	1
**4079**	320	A	79.5757	-1
**4079**	321	A	44.3666	1
**4079**	351	A	11.8947	1
**4079**	357	A	63.7524	1
**4079**	370	A	41.4713	0
**4079**	372	A	8.5291	0

**4079**	373	A	74.4107	1
**4079**	381	A	79.5316	1
**4079**	382	A	61.8271	1
**4079**	386	A	60.9795	1
**4079**	305	B	79.5278	1
**4079**	319	B	67.4039	1
**4079**	320	B	79.5597	-1
**4079**	321	B	53.5851	0
**4079**	351	B	6.3879	1
**4079**	357	B	47.3532	0
**4079**	370	B	79.5255	1
**4079**	372	B	79.5912	0
**4079**	373	B	67.5961	1
**4079**	381	B	78.7141	1
**4079**	382	B	31.754	0
**4079**	386	B	77.9297	1
**4079**	305	C	73.9434	1
**4079**	319	C	53.6013	1
**4079**	320	C	48.7505	-1
**4079**	321	C	70.955	0
**4079**	351	C	5.1945	1
**4079**	357	C	62.7164	1
**4079**	370	C	57.4986	1
**4079**	372	C	79.5116	0
**4079**	373	C	79.5229	1
**4079**	381	C	79.6255	1
**4079**	382	C	70.7275	1
**4079**	386	C	17.0164	-1
**4079**	305	D	4.6319	-1
**4079**	319	D	71.529	1
**4079**	320	D	29.8754	-1
**4079**	321	D	68.8884	0
**4079**	351	D	15.7128	1
**4079**	357	D	73.4832	1

**4079**	370	D	79.4256	1
**4079**	372	D	55.8337	0
**4079**	373	D	52.7795	1
**4079**	381	D	70.9454	1
**4079**	382	D	79.5316	1
**4079**	386	D	76.1782	1
**4080**	305	A	48.8387	0
**4080**	319	A	57.4213	1
**4080**	320	A	79.5274	-1
**4080**	321	A	69.3696	0
**4080**	351	A	7.1531	1
**4080**	357	A	79.5926	1
**4080**	370	A	73.3431	1
**4080**	372	A	79.6341	0
**4080**	373	A	47.4794	0
**4080**	381	A	79.4438	1
**4080**	382	A	79.7397	1
**4080**	386	A	68.6382	1
**4080**	305	B	79.4596	1
**4080**	319	B	79.5336	1
**4080**	320	B	41.9296	-1
**4080**	321	B	79.5719	0
**4080**	351	B	44.8639	-1
**4080**	357	B	34.6228	0
**4080**	370	B	68.8444	1
**4080**	372	B	79.6542	0
**4080**	373	B	41.2942	0
**4080**	381	B	59.525	1
**4080**	382	B	10.0742	-1
**4080**	386	B	79.666	1
**4080**	305	C	79.1259	1
**4080**	319	C	59.3915	1
**4080**	320	C	79.6398	-1
**4080**	321	C	28.8791	1

**4080**	351	C	2.9354	1
**4080**	357	C	79.2342	1
**4080**	370	C	64.2442	1
**4080**	372	C	79.5869	0
**4080**	373	C	73.0802	1
**4080**	381	C	73.6915	1
**4080**	382	C	53.5117	1
**4080**	386	C	30.6816	0
**4080**	305	D	79.544	1
**4080**	319	D	34.7995	0
**4080**	320	D	74.4864	-1
**4080**	321	D	79.6181	0
**4080**	351	D	12.8378	1
**4080**	357	D	57.1096	1
**4080**	370	D	41.7282	0
**4080**	372	D	54.2578	0
**4080**	373	D	63.6848	1
**4080**	381	D	41.4471	0
**4080**	382	D	74.257	1
**4080**	386	D	79.084	1
**4081**	305	A	73.8426	1
**4081**	319	A	76.7242	1
**4081**	320	A	73.1418	-1
**4081**	321	A	36.5818	1
**4081**	351	A	15.3902	1
**4081**	357	A	52.3862	1
**4081**	370	A	72.34	1
**4081**	372	A	50.3615	0
**4081**	373	A	79.5241	1
**4081**	381	A	43.3923	0
**4081**	382	A	72.0686	1
**4081**	386	A	11.6363	-1
**4081**	305	B	79.5608	1
**4081**	319	B	79.5674	1

**4081**	320	B	79.581	-1
**4081**	321	B	68.0384	0
**4081**	351	B	13.8774	1
**4081**	357	B	58.8466	1
**4081**	370	B	50.7485	1
**4081**	372	B	79.5742	0
**4081**	373	B	70.8082	1
**4081**	381	B	77.2296	1
**4081**	382	B	35.5073	0
**4081**	386	B	76.6903	1
**4081**	305	C	75.5432	1
**4081**	319	C	75.9548	1
**4081**	320	C	61.9353	-1
**4081**	321	C	59.3933	0
**4081**	351	C	5.5745	1
**4081**	357	C	66.6627	1
**4081**	370	C	79.549	1
**4081**	372	C	79.5793	0
**4081**	373	C	79.5605	1
**4081**	381	C	79.541	1
**4081**	382	C	47.0995	0
**4081**	386	C	32.1184	0
**4081**	305	D	78.9238	1
**4081**	319	D	69.9509	1
**4081**	320	D	48.9373	-1
**4081**	321	D	79.5259	0
**4081**	351	D	10.3293	1
**4081**	357	D	79.5914	1
**4081**	370	D	62.7661	1
**4081**	372	D	79.5653	0
**4081**	373	D	79.5616	1
**4081**	381	D	79.5702	1
**4081**	382	D	79.178	1
**4081**	386	D	53.0504	1

**4082**	305	A	72.2312	1
**4082**	319	A	57.8845	1
**4082**	320	A	66.6073	-1
**4082**	321	A	79.5807	0
**4082**	351	A	6.014	1
**4082**	357	A	65.5094	1
**4082**	370	A	62.4774	1
**4082**	372	A	72.2351	0
**4082**	373	A	79.5523	1
**4082**	381	A	67.9224	1
**4082**	382	A	14.4622	-1
**4082**	386	A	79.6509	1
**4082**	305	B	79.5621	1
**4082**	319	B	55.6572	1
**4082**	320	B	79.7035	-1
**4082**	321	B	79.6041	0
**4082**	351	B	30.39	-1
**4082**	357	B	79.5912	1
**4082**	370	B	76.4751	1
**4082**	372	B	76.3858	0
**4082**	373	B	46.3326	0
**4082**	381	B	39.627	0
**4082**	382	B	57.4658	1
**4082**	386	B	78.8982	1
**4082**	305	C	79.4984	1
**4082**	319	C	70.8844	1
**4082**	320	C	71.7478	-1
**4082**	321	C	74.5001	0
**4082**	351	C	6.2953	1
**4082**	357	C	45.6298	0
**4082**	370	C	53.3485	1
**4082**	372	C	79.6382	0
**4082**	373	C	58.4598	1
**4082**	381	C	77.1269	1

**4082**	382	C	73.2863	1
**4082**	386	C	60.3505	1
**4082**	305	D	76.3028	1
**4082**	319	D	22.0616	0
**4082**	320	D	74.9268	-1
**4082**	321	D	78.8036	0
**4082**	351	D	16.59	1
**4082**	357	D	55.2911	1
**4082**	370	D	79.349	1
**4082**	372	D	79.5482	0
**4082**	373	D	22.7659	0
**4082**	381	D	76.243	1
**4082**	382	D	59.4052	1
**4082**	386	D	79.7177	1
**4083**	305	A	70.1006	1
**4083**	319	A	51.3748	1
**4083**	320	A	79.558	-1
**4083**	321	A	58.4277	0
**4083**	351	A	18.1472	1
**4083**	357	A	56.2722	1
**4083**	370	A	48.4427	0
**4083**	372	A	79.5126	0
**4083**	373	A	45.6493	0
**4083**	381	A	64.9774	1
**4083**	382	A	56.932	1
**4083**	386	A	68.3025	1
**4083**	305	B	74.6614	1
**4083**	319	B	79.5716	1
**4083**	320	B	31.7316	-1
**4083**	321	B	46.2863	1
**4083**	351	B	5.6615	1
**4083**	357	B	64.3791	1
**4083**	370	B	38.8735	0
**4083**	372	B	13.0144	0

**4083**	373	B	20.8098	0
**4083**	381	B	14.9205	-1
**4083**	382	B	63.7708	1
**4083**	386	B	45.7851	0
**4083**	305	C	64.3272	1
**4083**	319	C	50.6266	1
**4083**	320	C	62.8648	-1
**4083**	321	C	79.4767	0
**4083**	351	C	1.2619	1
**4083**	357	C	63.434	1
**4083**	370	C	72.9209	1
**4083**	372	C	79.5609	0
**4083**	373	C	63.0672	1
**4083**	381	C	60.7646	1
**4083**	382	C	79.1467	1
**4083**	386	C	45.3571	0
**4083**	305	D	71.9353	1
**4083**	319	D	53.2428	1
**4083**	320	D	54.3128	-1
**4083**	321	D	71.1398	0
**4083**	351	D	13.3106	1
**4083**	357	D	49.0748	0
**4083**	370	D	76.9895	1
**4083**	372	D	78.579	0
**4083**	373	D	62.1777	1
**4083**	381	D	79.5607	1
**4083**	382	D	72.6646	1
**4083**	386	D	79.4727	1
**4084**	305	A	79.5837	1
**4084**	319	A	60.4436	1
**4084**	320	A	67.8199	-1
**4084**	321	A	72.78	0
**4084**	351	A	9.3942	1
**4084**	357	A	79.5666	1

**4084**	370	A	61.7118	1
**4084**	372	A	79.5892	0
**4084**	373	A	52.9333	1
**4084**	381	A	64.3678	1
**4084**	382	A	36.355	0
**4084**	386	A	79.5721	1
**4084**	305	B	76.7917	1
**4084**	319	B	65.4729	1
**4084**	320	B	78.9825	-1
**4084**	321	B	79.5105	0
**4084**	351	B	19.09	1
**4084**	357	B	64.1565	1
**4084**	370	B	62.2134	1
**4084**	372	B	70.2985	0
**4084**	373	B	45.0738	0
**4084**	381	B	66.5823	1
**4084**	382	B	79.5543	1
**4084**	386	B	77.9757	1
**4084**	305	C	78.5469	1
**4084**	319	C	79.4823	1
**4084**	320	C	47.1248	-1
**4084**	321	C	36.2492	1
**4084**	351	C	17.1297	1
**4084**	357	C	66.681	1
**4084**	370	C	79.3566	1
**4084**	372	C	79.5479	0
**4084**	373	C	78.7948	1
**4084**	381	C	61.321	1
**4084**	382	C	71.0464	1
**4084**	386	C	42.7095	0
**4084**	305	D	24.887	0
**4084**	319	D	67.5383	1
**4084**	320	D	38.1506	-1
**4084**	321	D	15.5975	0

**4084**	351	D	6.5328	1
**4084**	357	D	78.2795	1
**4084**	370	D	40.3484	0
**4084**	372	D	33.2546	1
**4084**	373	D	79.5243	1
**4084**	381	D	78.9255	1
**4084**	382	D	72.2104	1
**4084**	386	D	53.7053	1
**4085**	305	A	79.5923	1
**4085**	319	A	31.9032	0
**4085**	320	A	79.5177	-1
**4085**	321	A	69.4066	0
**4085**	351	A	41.0668	-1
**4085**	357	A	75.9853	1
**4085**	370	A	72.8059	1
**4085**	372	A	72.9294	0
**4085**	373	A	79.5673	1
**4085**	381	A	76.0274	1
**4085**	382	A	79.6804	1
**4085**	386	A	60.1954	1
**4085**	305	B	79.4728	1
**4085**	319	B	54.758	1
**4085**	320	B	79.6043	-1
**4085**	321	B	55.6548	0
**4085**	351	B	0.9007	1
**4085**	357	B	79.5672	1
**4085**	370	B	79.656	1
**4085**	372	B	67.5521	0
**4085**	373	B	40.1527	0
**4085**	381	B	79.5011	1
**4085**	382	B	50.1167	1
**4085**	386	B	65.2134	1
**4085**	305	C	74.6048	1
**4085**	319	C	63.5519	1

**4085**	320	C	64.4378	-1
**4085**	321	C	57.8397	0
**4085**	351	C	2.0481	1
**4085**	357	C	79.6564	1
**4085**	370	C	79.5029	1
**4085**	372	C	70.1064	0
**4085**	373	C	49.7587	0
**4085**	381	C	76.9754	1
**4085**	382	C	59.8843	1
**4085**	386	C	67.2977	1
**4085**	305	D	75.5783	1
**4085**	319	D	79.4772	1
**4085**	320	D	52.8326	-1
**4085**	321	D	71.4449	0
**4085**	351	D	16.5887	1
**4085**	357	D	49.966	0
**4085**	370	D	42.0761	0
**4085**	372	D	79.5319	0
**4085**	373	D	63.9173	1
**4085**	381	D	66.4532	1
**4085**	382	D	73.8757	1
**4085**	386	D	72.2649	1
**4086**	305	A	72.0049	1
**4086**	319	A	78.9861	1
**4086**	320	A	78.5007	-1
**4086**	321	A	69.8358	0
**4086**	351	A	0.1636	1
**4086**	357	A	48.5306	0
**4086**	370	A	73.3457	1
**4086**	372	A	75.3802	0
**4086**	373	A	79.5972	1
**4086**	381	A	70.9202	1
**4086**	382	A	63.5906	1
**4086**	386	A	79.2941	1

**4086**	305	B	65.8054	1
**4086**	319	B	56.5318	1
**4086**	320	B	79.4569	-1
**4086**	321	B	74.1567	0
**4086**	351	B	14.207	1
**4086**	357	B	79.3235	1
**4086**	370	B	79.7317	1
**4086**	372	B	69.3025	0
**4086**	373	B	62.2919	1
**4086**	381	B	60.4119	1
**4086**	382	B	28.0309	0
**4086**	386	B	74.244	1
**4086**	305	C	61.92	1
**4086**	319	C	48.3029	0
**4086**	320	C	69.8516	-1
**4086**	321	C	67.4707	0
**4086**	351	C	10.9796	1
**4086**	357	C	79.7466	1
**4086**	370	C	73.3705	1
**4086**	372	C	79.504	0
**4086**	373	C	53.2294	1
**4086**	381	C	35.03	0
**4086**	382	C	79.2911	1
**4086**	386	C	11.1796	-1
**4086**	305	D	74.2246	1
**4086**	319	D	79.4683	1
**4086**	320	D	75.5273	-1
**4086**	321	D	70.0173	0
**4086**	351	D	17.8793	1
**4086**	357	D	79.5148	1
**4086**	370	D	40.4577	0
**4086**	372	D	69.0286	0
**4086**	373	D	75.2202	1
**4086**	381	D	79.5651	1

**4086**	382	D	79.5218	1
**4086**	386	D	79.3323	1
**4087**	305	A	79.0802	1
**4087**	319	A	57.9836	1
**4087**	320	A	72.2242	-1
**4087**	321	A	68.3217	0
**4087**	351	A	1.0307	1
**4087**	357	A	60.187	1
**4087**	370	A	56.6856	1
**4087**	372	A	7.6606	0
**4087**	373	A	79.5885	1
**4087**	381	A	70.1017	1
**4087**	382	A	62.3397	1
**4087**	386	A	78.5625	1
**4087**	305	B	39.2585	0
**4087**	319	B	52.8508	1
**4087**	320	B	79.6793	-1
**4087**	321	B	66.8559	0
**4087**	351	B	9.7135	1
**4087**	357	B	62.5971	1
**4087**	370	B	56.8788	1
**4087**	372	B	74.2619	0
**4087**	373	B	52.3108	1
**4087**	381	B	20.0146	0
**4087**	382	B	59.6648	1
**4087**	386	B	79.6246	1
**4087**	305	C	69.6817	1
**4087**	319	C	79.4556	1
**4087**	320	C	63.2265	-1
**4087**	321	C	43.097	1
**4087**	351	C	1.8303	1
**4087**	357	C	61.6445	1
**4087**	370	C	65.2999	1
**4087**	372	C	79.5421	0

**4087**	373	C	53.287	1
**4087**	381	C	79.4458	1
**4087**	382	C	72.9634	1
**4087**	386	C	55.83	1
**4087**	305	D	72.697	1
**4087**	319	D	66.6473	1
**4087**	320	D	56.3735	-1
**4087**	321	D	31.1561	1
**4087**	351	D	15.9117	1
**4087**	357	D	58.8741	1
**4087**	370	D	38.3685	0
**4087**	372	D	68.6485	0
**4087**	373	D	64.9514	1
**4087**	381	D	77.8518	1
**4087**	382	D	71.3528	1
**4087**	386	D	72.4134	1
**4088**	305	A	65.8665	1
**4088**	319	A	52.8217	1
**4088**	320	A	79.5359	-1
**4088**	321	A	73.449	0
**4088**	351	A	13.023	1
**4088**	357	A	79.6439	1
**4088**	370	A	73.7039	1
**4088**	372	A	72.4357	0
**4088**	373	A	69.85	1
**4088**	381	A	79.4598	1
**4088**	382	A	79.4513	1
**4088**	386	A	64.9531	1
**4088**	305	B	73.4024	1
**4088**	319	B	66.7097	1
**4088**	320	B	58.065	-1
**4088**	321	B	59.5073	0
**4088**	351	B	29.92	-1
**4088**	357	B	43.78	0

**4088**	370	B	79.6251	1
**4088**	372	B	69.0145	0
**4088**	373	B	76.9915	1
**4088**	381	B	50.654	1
**4088**	382	B	56.3486	1
**4088**	386	B	65.2971	1
**4088**	305	C	48.7149	0
**4088**	319	C	78.1827	1
**4088**	320	C	57.4388	-1
**4088**	321	C	69.2044	0
**4088**	351	C	8.9593	1
**4088**	357	C	79.4059	1
**4088**	370	C	77.0004	1
**4088**	372	C	73.9847	0
**4088**	373	C	65.3972	1
**4088**	381	C	78.5389	1
**4088**	382	C	74.6513	1
**4088**	386	C	19.3782	-1
**4088**	305	D	56.1198	1
**4088**	319	D	79.5466	1
**4088**	320	D	79.4811	-1
**4088**	321	D	79.5839	0
**4088**	351	D	17.0599	1
**4088**	357	D	49.0059	0
**4088**	370	D	33.6844	0
**4088**	372	D	66.9044	0
**4088**	373	D	62.7065	1
**4088**	381	D	79.6236	1
**4088**	382	D	79.4822	1
**4088**	386	D	79.4408	1
**4089**	305	A	72.6639	1
**4089**	319	A	71.575	1
**4089**	320	A	73.5425	-1
**4089**	321	A	79.6461	0

**4089**	351	A	18.0407	1
**4089**	357	A	79.657	1
**4089**	370	A	79.4976	1
**4089**	372	A	79.6184	0
**4089**	373	A	55.3471	1
**4089**	381	A	79.5845	1
**4089**	382	A	60.908	1
**4089**	386	A	79.4901	1
**4089**	305	B	44.5517	0
**4089**	319	B	79.1852	1
**4089**	320	B	54.9051	-1
**4089**	321	B	59.606	0
**4089**	351	B	8.3438	1
**4089**	357	B	73.426	1
**4089**	370	B	72.8603	1
**4089**	372	B	79.052	0
**4089**	373	B	59.2736	1
**4089**	381	B	79.6461	1
**4089**	382	B	46.4524	0
**4089**	386	B	79.5623	1
**4089**	305	C	74.8425	1
**4089**	319	C	20.2899	0
**4089**	320	C	79.5243	-1
**4089**	321	C	56.1696	0
**4089**	351	C	0.5482	1
**4089**	357	C	36.6115	0
**4089**	370	C	46.3996	0
**4089**	372	C	55.5494	0
**4089**	373	C	30.9091	0
**4089**	381	C	64.5946	1
**4089**	382	C	79.66	1
**4089**	386	C	27.5157	0
**4089**	305	D	59.6505	1
**4089**	319	D	79.6912	1

**4089**	320	D	79.6166	-1
**4089**	321	D	78.5198	0
**4089**	351	D	13.4273	1
**4089**	357	D	35.3104	0
**4089**	370	D	79.6333	1
**4089**	372	D	79.5379	0
**4089**	373	D	32.1809	0
**4089**	381	D	11.1196	-1
**4089**	382	D	79.5625	1
**4089**	386	D	79.4934	1
**4090**	305	A	63.2016	1
**4090**	319	A	75.3575	1
**4090**	320	A	79.6212	-1
**4090**	321	A	77.7773	0
**4090**	351	A	25.4974	-1
**4090**	357	A	76.1916	1
**4090**	370	A	66.2274	1
**4090**	372	A	79.622	0
**4090**	373	A	79.6184	1
**4090**	381	A	70.4607	1
**4090**	382	A	30.572	0
**4090**	386	A	25.0041	0
**4090**	305	B	79.6545	1
**4090**	319	B	66.254	1
**4090**	320	B	79.6168	-1
**4090**	321	B	35.9506	1
**4090**	351	B	13.5955	1
**4090**	357	B	67.1049	1
**4090**	370	B	51.9406	1
**4090**	372	B	66.2203	0
**4090**	373	B	18.1726	-1
**4090**	381	B	52.653	1
**4090**	382	B	26.1801	0
**4090**	386	B	71.7794	1

**4090**	305	C	26.2269	0
**4090**	319	C	79.4796	1
**4090**	320	C	79.6463	-1
**4090**	321	C	79.4838	0
**4090**	351	C	17.2204	1
**4090**	357	C	79.6498	1
**4090**	370	C	30.3789	0
**4090**	372	C	54.8425	0
**4090**	373	C	54.6377	1
**4090**	381	C	75.5085	1
**4090**	382	C	34.7936	0
**4090**	386	C	66.2073	1
**4090**	305	D	23.4122	0
**4090**	319	D	41.0576	0
**4090**	320	D	73.4455	-1
**4090**	321	D	75.6149	0
**4090**	351	D	10.6445	1
**4090**	357	D	26.2599	0
**4090**	370	D	54.6758	1
**4090**	372	D	73.1526	0
**4090**	373	D	79.4812	1
**4090**	381	D	79.6465	1
**4090**	382	D	79.6075	1
**4090**	386	D	79.6476	1
**4091**	305	A	62.4351	1
**4091**	319	A	68.03	1
**4091**	320	A	73.6347	-1
**4091**	321	A	73.8429	0
**4091**	351	A	6.4621	1
**4091**	357	A	49.9976	0
**4091**	370	A	61.7758	1
**4091**	372	A	79.5702	0
**4091**	373	A	79.5483	1
**4091**	381	A	79.1794	1

**4091**	382	A	70.219	1
**4091**	386	A	66.0506	1
**4091**	305	B	62.5217	1
**4091**	319	B	79.5508	1
**4091**	320	B	47.9341	-1
**4091**	321	B	52.5811	0
**4091**	351	B	21.5221	-1
**4091**	357	B	79.5578	1
**4091**	370	B	74.697	1
**4091**	372	B	68.5115	0
**4091**	373	B	79.5253	1
**4091**	381	B	29.951	0
**4091**	382	B	57.4856	1
**4091**	386	B	73.4279	1
**4091**	305	C	67.9748	1
**4091**	319	C	42.3455	0
**4091**	320	C	74.4279	-1
**4091**	321	C	79.535	0
**4091**	351	C	6.9292	1
**4091**	357	C	76.8312	1
**4091**	370	C	79.5691	1
**4091**	372	C	54.827	0
**4091**	373	C	70.154	1
**4091**	381	C	79.3735	1
**4091**	382	C	61.8471	1
**4091**	386	C	61.6253	1
**4091**	305	D	15.2297	-1
**4091**	319	D	72.7463	1
**4091**	320	D	79.5473	-1
**4091**	321	D	71.5035	0
**4091**	351	D	16.9182	1
**4091**	357	D	74.2494	1
**4091**	370	D	73.6162	1
**4091**	372	D	79.5747	0

**4091**	373	D	79.5827	1
**4091**	381	D	78.5817	1
**4091**	382	D	71.1127	1
**4091**	386	D	79.5559	1
**4092**	305	A	77.5355	1
**4092**	319	A	37.7843	0
**4092**	320	A	79.545	-1
**4092**	321	A	79.5538	0
**4092**	351	A	12.1216	1
**4092**	357	A	68.8427	1
**4092**	370	A	79.5673	1
**4092**	372	A	37.4608	1
**4092**	373	A	79.5437	1
**4092**	381	A	61.3654	1
**4092**	382	A	38.0987	0
**4092**	386	A	79.549	1
**4092**	305	B	79.5757	1
**4092**	319	B	79.5732	1
**4092**	320	B	63.3496	-1
**4092**	321	B	79.5583	0
**4092**	351	B	12.8542	1
**4092**	357	B	72.6717	1
**4092**	370	B	79.488	1
**4092**	372	B	79.5543	0
**4092**	373	B	73.3979	1
**4092**	381	B	79.5343	1
**4092**	382	B	45.1824	0
**4092**	386	B	79.6006	1
**4092**	305	C	78.6649	1
**4092**	319	C	66.4301	1
**4092**	320	C	76.9257	-1
**4092**	321	C	79.6206	0
**4092**	351	C	6.109	1
**4092**	357	C	73.3308	1

**4092**	370	C	68.0557	1
**4092**	372	C	73.523	0
**4092**	373	C	55.3451	1
**4092**	381	C	79.5658	1
**4092**	382	C	38.7532	0
**4092**	386	C	36.6868	0
**4092**	305	D	63.3565	1
**4092**	319	D	71.3967	1
**4092**	320	D	79.5606	-1
**4092**	321	D	79.5504	0
**4092**	351	D	13.6354	1
**4092**	357	D	79.5877	1
**4092**	370	D	73.4389	1
**4092**	372	D	78.6367	0
**4092**	373	D	68.6686	1
**4092**	381	D	63.5851	1
**4092**	382	D	61.924	1
**4092**	386	D	79.5347	1
**4093**	305	A	70.7516	1
**4093**	319	A	53.5918	1
**4093**	320	A	50.6311	-1
**4093**	321	A	65.5614	0
**4093**	351	A	7.2335	1
**4093**	357	A	71.8383	1
**4093**	370	A	44.1891	0
**4093**	372	A	66.7381	0
**4093**	373	A	79.4912	1
**4093**	381	A	73.1807	1
**4093**	382	A	53.4551	1
**4093**	386	A	79.6378	1
**4093**	305	B	54.8185	1
**4093**	319	B	79.5442	1
**4093**	320	B	79.5857	-1
**4093**	321	B	79.5545	0

**4093**	351	B	0.0293	1
**4093**	357	B	49.2811	0
**4093**	370	B	79.1071	1
**4093**	372	B	79.6363	0
**4093**	373	B	60.8606	1
**4093**	381	B	71.7492	1
**4093**	382	B	69.9452	1
**4093**	386	B	59.0586	1
**4093**	305	C	79.5029	1
**4093**	319	C	29.9595	0
**4093**	320	C	79.599	-1
**4093**	321	C	79.4628	0
**4093**	351	C	13.0532	1
**4093**	357	C	79.4867	1
**4093**	370	C	79.5237	1
**4093**	372	C	78.0666	0
**4093**	373	C	79.5117	1
**4093**	381	C	79.5174	1
**4093**	382	C	21.8562	0
**4093**	386	C	63.5911	1
**4093**	305	D	79.0013	1
**4093**	319	D	79.527	1
**4093**	320	D	73.6927	-1
**4093**	321	D	45.9474	1
**4093**	351	D	18.3415	1
**4093**	357	D	26.5172	0
**4093**	370	D	79.5303	1
**4093**	372	D	69.2557	0
**4093**	373	D	79.4181	1
**4093**	381	D	79.5493	1
**4093**	382	D	79.4256	1
**4093**	386	D	7.1474	-1
**4094**	305	A	55.1993	1
**4094**	319	A	73.0681	1

**4094**	320	A	75.4467	-1
**4094**	321	A	79.5652	0
**4094**	351	A	11.6866	1
**4094**	357	A	52.731	1
**4094**	370	A	71.619	1
**4094**	372	A	79.5547	0
**4094**	373	A	65.3269	1
**4094**	381	A	54.191	1
**4094**	382	A	48.5789	0
**4094**	386	A	56.6189	1
**4094**	305	B	79.6508	1
**4094**	319	B	67.7531	1
**4094**	320	B	78.3458	-1
**4094**	321	B	79.6226	0
**4094**	351	B	13.3155	1
**4094**	357	B	74.1306	1
**4094**	370	B	79.4179	1
**4094**	372	B	79.5189	0
**4094**	373	B	67.8625	1
**4094**	381	B	63.877	1
**4094**	382	B	64.0687	1
**4094**	386	B	79.5458	1
**4094**	305	C	71.1974	1
**4094**	319	C	43.3647	0
**4094**	320	C	79.4773	-1
**4094**	321	C	79.0225	0
**4094**	351	C	7.8629	1
**4094**	357	C	51.7976	1
**4094**	370	C	72.4497	1
**4094**	372	C	72.1467	0
**4094**	373	C	67.3963	1
**4094**	381	C	48.5051	0
**4094**	382	C	43.561	0
**4094**	386	C	50.3937	1

**4094**	305	D	59.2732	1
**4094**	319	D	72.215	1
**4094**	320	D	72.1775	-1
**4094**	321	D	79.6076	0
**4094**	351	D	2.4224	1
**4094**	357	D	69.0325	1
**4094**	370	D	56.6661	1
**4094**	372	D	68.3691	0
**4094**	373	D	46.6184	0
**4094**	381	D	57.7071	1
**4094**	382	D	71.1406	1
**4094**	386	D	57.3073	1
**4095**	305	A	72.8006	1
**4095**	319	A	58.9105	1
**4095**	320	A	61.8184	-1
**4095**	321	A	59.6343	0
**4095**	351	A	12.5841	1
**4095**	357	A	65.3096	1
**4095**	370	A	49.9123	0
**4095**	372	A	46.576	1
**4095**	373	A	59.1238	1
**4095**	381	A	79.5086	1
**4095**	382	A	56.9619	1
**4095**	386	A	79.5407	1
**4095**	305	B	56.255	1
**4095**	319	B	76.4851	1
**4095**	320	B	67.8126	-1
**4095**	321	B	72.3145	0
**4095**	351	B	20.5327	-1
**4095**	357	B	53.2535	1
**4095**	370	B	79.5918	1
**4095**	372	B	79.5666	0
**4095**	373	B	70.7653	1
**4095**	381	B	75.7488	1

**4095**	382	B	55.1004	1
**4095**	386	B	79.4947	1
**4095**	305	C	79.5758	1
**4095**	319	C	79.5402	1
**4095**	320	C	79.5136	-1
**4095**	321	C	79.5644	0
**4095**	351	C	10.1303	1
**4095**	357	C	69.1251	1
**4095**	370	C	79.0743	1
**4095**	372	C	72.8388	0
**4095**	373	C	71.0919	1
**4095**	381	C	23.5127	0
**4095**	382	C	51.0076	1
**4095**	386	C	76.5733	1
**4095**	305	D	18.222	-1
**4095**	319	D	63.9817	1
**4095**	320	D	79.4848	-1
**4095**	321	D	73.3599	0
**4095**	351	D	12.743	1
**4095**	357	D	52.5707	1
**4095**	370	D	79.568	1
**4095**	372	D	79.5196	0
**4095**	373	D	77.5729	1
**4095**	381	D	70.9694	1
**4095**	382	D	78.6987	1
**4095**	386	D	75.1928	1
**4096**	305	A	75.6567	1
**4096**	319	A	42.0582	0
**4096**	320	A	38.1253	-1
**4096**	321	A	77.0001	0
**4096**	351	A	1.6273	1
**4096**	357	A	40.851	0
**4096**	370	A	40.8338	0
**4096**	372	A	52.2228	0

**4096**	373	A	2.869	-1
**4096**	381	A	69.6635	1
**4096**	382	A	75.7507	1
**4096**	386	A	76.4467	1
**4096**	305	B	79.55	1
**4096**	319	B	45.5648	0
**4096**	320	B	59.3881	-1
**4096**	321	B	54.5297	0
**4096**	351	B	9.0585	1
**4096**	357	B	79.5813	1
**4096**	370	B	48.0289	0
**4096**	372	B	68.1192	0
**4096**	373	B	63.8841	1
**4096**	381	B	79.6124	1
**4096**	382	B	16.6396	-1
**4096**	386	B	79.7333	1
**4096**	305	C	70.4558	1
**4096**	319	C	39.7969	0
**4096**	320	C	77.7808	-1
**4096**	321	C	79.6261	0
**4096**	351	C	15.8088	1
**4096**	357	C	71.1218	1
**4096**	370	C	74.849	1
**4096**	372	C	75.2553	0
**4096**	373	C	52.1911	1
**4096**	381	C	79.5643	1
**4096**	382	C	23.3474	0
**4096**	386	C	34.4695	0
**4096**	305	D	70.7196	1
**4096**	319	D	79.5862	1
**4096**	320	D	68.5433	-1
**4096**	321	D	79.1709	0
**4096**	351	D	9.8456	1
**4096**	357	D	79.4971	1

**4096**	370	D	55.8717	1
**4096**	372	D	79.5383	0
**4096**	373	D	79.5904	1
**4096**	381	D	60.1635	1
**4096**	382	D	73.1228	1
**4096**	386	D	79.6039	1
**4097**	305	A	74.8259	1
**4097**	319	A	68.0808	1
**4097**	320	A	29.7002	-1
**4097**	321	A	67.828	0
**4097**	351	A	0.7916	1
**4097**	357	A	69.6637	1
**4097**	370	A	69.9361	1
**4097**	372	A	70.7162	0
**4097**	373	A	79.5286	1
**4097**	381	A	33.7718	0
**4097**	382	A	59.8624	1
**4097**	386	A	75.093	1
**4097**	305	B	79.5963	1
**4097**	319	B	79.5909	1
**4097**	320	B	28.0327	-1
**4097**	321	B	75.1316	0
**4097**	351	B	9.2698	1
**4097**	357	B	79.5304	1
**4097**	370	B	79.5894	1
**4097**	372	B	79.5647	0
**4097**	373	B	79.1086	1
**4097**	381	B	78.9264	1
**4097**	382	B	70.4908	1
**4097**	386	B	77.3133	1
**4097**	305	C	45.936	0
**4097**	319	C	71.3705	1
**4097**	320	C	79.5652	-1
**4097**	321	C	66.6768	0

**4097**	351	C	0.2724	1
**4097**	357	C	73.1023	1
**4097**	370	C	79.5053	1
**4097**	372	C	73.013	0
**4097**	373	C	74.4199	1
**4097**	381	C	73.6787	1
**4097**	382	C	79.1994	1
**4097**	386	C	21.7057	0
**4097**	305	D	35.1323	0
**4097**	319	D	57.6522	1
**4097**	320	D	73.7378	-1
**4097**	321	D	59.0732	0
**4097**	351	D	12.8856	1
**4097**	357	D	68.1823	1
**4097**	370	D	72.6826	1
**4097**	372	D	79.5581	0
**4097**	373	D	65.4736	1
**4097**	381	D	76.3662	1
**4097**	382	D	79.5928	1
**4097**	386	D	79.5634	1
**4098**	305	A	79.6085	1
**4098**	319	A	59.1438	1
**4098**	320	A	79.4681	-1
**4098**	321	A	71.1748	0
**4098**	351	A	14.9442	1
**4098**	357	A	79.537	1
**4098**	370	A	79.5787	1
**4098**	372	A	79.5169	0
**4098**	373	A	57.426	1
**4098**	381	A	42.0263	0
**4098**	382	A	67.8404	1
**4098**	386	A	79.5643	1
**4098**	305	B	74.8768	1
**4098**	319	B	71.7909	1

**4098**	320	B	69.2501	-1
**4098**	321	B	44.7372	1
**4098**	351	B	11.6857	1
**4098**	357	B	38.3984	0
**4098**	370	B	79.5839	1
**4098**	372	B	68.3108	0
**4098**	373	B	44.5914	0
**4098**	381	B	73.7242	1
**4098**	382	B	60.3166	1
**4098**	386	B	79.578	1
**4098**	305	C	79.5333	1
**4098**	319	C	71.2534	1
**4098**	320	C	70.4224	-1
**4098**	321	C	56.6883	0
**4098**	351	C	0.1084	1
**4098**	357	C	79.4853	1
**4098**	370	C	76.6441	1
**4098**	372	C	29.5595	1
**4098**	373	C	48.5712	0
**4098**	381	C	76.7644	1
**4098**	382	C	60.6783	1
**4098**	386	C	34.5542	0
**4098**	305	D	46.0795	0
**4098**	319	D	67.6385	1
**4098**	320	D	74.1195	-1
**4098**	321	D	36.1735	1
**4098**	351	D	17.0122	1
**4098**	357	D	65.9572	1
**4098**	370	D	57.0326	1
**4098**	372	D	66.2012	0
**4098**	373	D	65.2664	1
**4098**	381	D	70.8492	1
**4098**	382	D	43.4521	0
**4098**	386	D	78.6254	1

**4099**	305	A	74.1224	1
**4099**	319	A	73.1981	1
**4099**	320	A	61.8447	-1
**4099**	321	A	79.4851	0
**4099**	351	A	1.4901	1
**4099**	357	A	67.442	1
**4099**	370	A	77.5934	1
**4099**	372	A	79.5253	0
**4099**	373	A	50.8665	1
**4099**	381	A	79.552	1
**4099**	382	A	78.8482	1
**4099**	386	A	79.5039	1
**4099**	305	B	79.3295	1
**4099**	319	B	79.557	1
**4099**	320	B	29.8059	-1
**4099**	321	B	78.5518	0
**4099**	351	B	22.043	-1
**4099**	357	B	79.5942	1
**4099**	370	B	72.9003	1
**4099**	372	B	68.5039	0
**4099**	373	B	79.3049	1
**4099**	381	B	79.4744	1
**4099**	382	B	58.6608	1
**4099**	386	B	58.8867	1
**4099**	305	C	73.9716	1
**4099**	319	C	48.2129	0
**4099**	320	C	67.8299	-1
**4099**	321	C	79.3775	0
**4099**	351	C	0.5146	1
**4099**	357	C	69.4054	1
**4099**	370	C	49.7923	0
**4099**	372	C	79.6635	0
**4099**	373	C	79.559	1
**4099**	381	C	79.4688	1

**4099**	382	C	78.6303	1
**4099**	386	C	41.1863	0
**4099**	305	D	60.0536	1
**4099**	319	D	60.5354	1
**4099**	320	D	67.2494	-1
**4099**	321	D	75.3727	0
**4099**	351	D	32.6892	-1
**4099**	357	D	30.4442	0
**4099**	370	D	79.5824	1
**4099**	372	D	70.1793	0
**4099**	373	D	50.8715	1
**4099**	381	D	42.3643	0
**4099**	382	D	60.485	1
**4099**	386	D	79.4915	1
**4100**	305	A	52.7611	1
**4100**	319	A	79.4237	1
**4100**	320	A	72.1851	-1
**4100**	321	A	54.6524	0
**4100**	351	A	13.4447	1
**4100**	357	A	32.8173	0
**4100**	370	A	79.579	1
**4100**	372	A	70.5043	0
**4100**	373	A	79.5371	1
**4100**	381	A	57.4181	1
**4100**	382	A	74.6495	1
**4100**	386	A	66.8975	1
**4100**	305	B	79.6072	1
**4100**	319	B	72.527	1
**4100**	320	B	79.6439	-1
**4100**	321	B	71.1783	0
**4100**	351	B	14.5421	1
**4100**	357	B	40.3295	0
**4100**	370	B	79.5529	1
**4100**	372	B	79.5875	0

**4100**	373	B	79.5811	1
**4100**	381	B	36.3188	0
**4100**	382	B	79.5792	1
**4100**	386	B	67.0107	1
**4100**	305	C	66.7722	1
**4100**	319	C	79.5448	1
**4100**	320	C	70.9364	-1
**4100**	321	C	79.4857	0
**4100**	351	C	17.3143	1
**4100**	357	C	76.5416	1
**4100**	370	C	67.3093	1
**4100**	372	C	52.4466	0
**4100**	373	C	64.914	1
**4100**	381	C	74.1634	1
**4100**	382	C	49.6135	0
**4100**	386	C	44.883	0
**4100**	305	D	77.1898	1
**4100**	319	D	77.1951	1
**4100**	320	D	51.9416	-1
**4100**	321	D	64.9112	0
**4100**	351	D	21.1998	-1
**4100**	357	D	63.7831	1
**4100**	370	D	54.7355	1
**4100**	372	D	79.4408	0
**4100**	373	D	64.4608	1
**4100**	381	D	45.696	0
**4100**	382	D	44.0047	0
**4100**	386	D	66.2351	1
**4101**	305	A	65.3492	1
**4101**	319	A	75.4164	1
**4101**	320	A	79.5632	-1
**4101**	321	A	67.2608	0
**4101**	351	A	15.494	1
**4101**	357	A	70.2372	1

**4101**	370	A	78.4132	1
**4101**	372	A	50.3102	0
**4101**	373	A	4.1116	-1
**4101**	381	A	75.172	1
**4101**	382	A	43.2729	0
**4101**	386	A	78.0318	1
**4101**	305	B	79.5624	1
**4101**	319	B	79.5665	1
**4101**	320	B	79.5781	-1
**4101**	321	B	72.0564	0
**4101**	351	B	9.5398	1
**4101**	357	B	66.4755	1
**4101**	370	B	79.3923	1
**4101**	372	B	73.263	0
**4101**	373	B	66.7753	1
**4101**	381	B	49.8267	0
**4101**	382	B	24.0247	0
**4101**	386	B	74.2623	1
**4101**	305	C	46.7877	0
**4101**	319	C	35.4677	0
**4101**	320	C	79.4943	-1
**4101**	321	C	59.1291	0
**4101**	351	C	10.7887	1
**4101**	357	C	63.9588	1
**4101**	370	C	74.0614	1
**4101**	372	C	79.5164	0
**4101**	373	C	70.1843	1
**4101**	381	C	72.4706	1
**4101**	382	C	79.5289	1
**4101**	386	C	46.6942	0
**4101**	305	D	51.5327	1
**4101**	319	D	75.7973	1
**4101**	320	D	49.2874	-1
**4101**	321	D	62.3022	0

**4101**	351	D	7.3223	1
**4101**	357	D	46.8861	0
**4101**	370	D	20.1227	0
**4101**	372	D	79.487	0
**4101**	373	D	48.3396	0
**4101**	381	D	78.0403	1
**4101**	382	D	47.8603	0
**4101**	386	D	63.9809	1
**4102**	305	A	62.8682	1
**4102**	319	A	53.6886	1
**4102**	320	A	79.3973	-1
**4102**	321	A	79.5556	0
**4102**	351	A	9.8765	1
**4102**	357	A	79.5605	1
**4102**	370	A	78.9612	1
**4102**	372	A	52.5786	0
**4102**	373	A	70.0053	1
**4102**	381	A	39.6056	0
**4102**	382	A	71.5205	1
**4102**	386	A	79.5605	1
**4102**	305	B	73.6667	1
**4102**	319	B	55.9027	1
**4102**	320	B	59.4006	-1
**4102**	321	B	53.3433	0
**4102**	351	B	12.3452	1
**4102**	357	B	22.8016	0
**4102**	370	B	79.3956	1
**4102**	372	B	79.5775	0
**4102**	373	B	77.2313	1
**4102**	381	B	79.5574	1
**4102**	382	B	33.7801	0
**4102**	386	B	75.3764	1
**4102**	305	C	41.332	0
**4102**	319	C	77.3117	1

**4102**	320	C	38.638	-1
**4102**	321	C	64.2334	0
**4102**	351	C	9.5447	1
**4102**	357	C	75.2682	1
**4102**	370	C	79.5472	1
**4102**	372	C	46.3379	1
**4102**	373	C	53.0394	1
**4102**	381	C	79.5611	1
**4102**	382	C	65.4834	1
**4102**	386	C	8.5714	-1
**4102**	305	D	16.0548	-1
**4102**	319	D	79.5504	1
**4102**	320	D	72.9142	-1
**4102**	321	D	76.2665	0
**4102**	351	D	12.8359	1
**4102**	357	D	79.5788	1
**4102**	370	D	32.6521	0
**4102**	372	D	79.5517	0
**4102**	373	D	46.8534	0
**4102**	381	D	79.56	1
**4102**	382	D	79.5658	1
**4102**	386	D	78.4267	1
**4103**	305	A	79.5948	1
**4103**	319	A	69.4036	1
**4103**	320	A	79.5181	-1
**4103**	321	A	59.3052	0
**4103**	351	A	4.1999	1
**4103**	357	A	72.8123	1
**4103**	370	A	78.4941	1
**4103**	372	A	78.391	0
**4103**	373	A	29.6364	0
**4103**	381	A	53.2738	1
**4103**	382	A	73.7516	1
**4103**	386	A	74.2025	1

**4103**	305	B	79.5963	1
**4103**	319	B	59.7312	1
**4103**	320	B	16.7369	1
**4103**	321	B	73.3896	0
**4103**	351	B	13.6131	1
**4103**	357	B	79.5512	1
**4103**	370	B	56.6474	1
**4103**	372	B	61.0045	0
**4103**	373	B	70.1597	1
**4103**	381	B	41.2515	0
**4103**	382	B	56.0101	1
**4103**	386	B	58.751	1
**4103**	305	C	54.7116	1
**4103**	319	C	69.5574	1
**4103**	320	C	35.9052	-1
**4103**	321	C	75.0455	0
**4103**	351	C	5.412	1
**4103**	357	C	73.5785	1
**4103**	370	C	61.1229	1
**4103**	372	C	73.5971	0
**4103**	373	C	56.8911	1
**4103**	381	C	79.5925	1
**4103**	382	C	71.719	1
**4103**	386	C	50.8255	1
**4103**	305	D	79.5685	1
**4103**	319	D	79.5958	1
**4103**	320	D	79.533	-1
**4103**	321	D	79.5425	0
**4103**	351	D	0.5493	1
**4103**	357	D	72.4533	1
**4103**	370	D	40.7268	0
**4103**	372	D	53.1606	0
**4103**	373	D	79.6014	1
**4103**	381	D	73.0128	1

**4103**	382	D	79.5054	1
**4103**	386	D	79.5947	1
**4104**	305	A	79.6437	1
**4104**	319	A	79.4697	1
**4104**	320	A	68.251	-1
**4104**	321	A	21.3867	1
**4104**	351	A	41.3761	-1
**4104**	357	A	50.481	1
**4104**	370	A	75.9534	1
**4104**	372	A	68.5781	0
**4104**	373	A	79.4605	1
**4104**	381	A	65.8731	1
**4104**	382	A	58.6999	1
**4104**	386	A	72.5453	1
**4104**	305	B	35.5283	0
**4104**	319	B	55.2785	1
**4104**	320	B	66.8746	-1
**4104**	321	B	69.9424	0
**4104**	351	B	42.9918	-1
**4104**	357	B	79.5367	1
**4104**	370	B	79.2119	1
**4104**	372	B	66.3804	0
**4104**	373	B	68.533	1
**4104**	381	B	79.5181	1
**4104**	382	B	71.8392	1
**4104**	386	B	65.9156	1
**4104**	305	C	49.636	0
**4104**	319	C	74.7588	1
**4104**	320	C	75.0028	-1
**4104**	321	C	63.8994	0
**4104**	351	C	5.2298	1
**4104**	357	C	77.7631	1
**4104**	370	C	77.5026	1
**4104**	372	C	71.6325	0

**4104**	373	C	72.4336	1
**4104**	381	C	54.6297	1
**4104**	382	C	71.7342	1
**4104**	386	C	46.0854	0
**4104**	305	D	68.2124	1
**4104**	319	D	75.7313	1
**4104**	320	D	72.4267	-1
**4104**	321	D	63.6863	0
**4104**	351	D	10.3771	1
**4104**	357	D	79.5456	1
**4104**	370	D	63.4263	1
**4104**	372	D	79.5394	0
**4104**	373	D	79.6082	1
**4104**	381	D	79.495	1
**4104**	382	D	60.9694	1
**4104**	386	D	79.6524	1
**4105**	305	A	73.6488	1
**4105**	319	A	70.3983	1
**4105**	320	A	79.2818	-1
**4105**	321	A	55.3066	0
**4105**	351	A	4.5006	1
**4105**	357	A	44.6209	0
**4105**	370	A	64.1258	1
**4105**	372	A	79.4361	0
**4105**	373	A	48.4217	0
**4105**	381	A	59.1132	1
**4105**	382	A	66.9273	1
**4105**	386	A	72.5569	1
**4105**	305	B	71.0282	1
**4105**	319	B	70.1954	1
**4105**	320	B	77.5943	-1
**4105**	321	B	79.533	0
**4105**	351	B	15.5537	1
**4105**	357	B	65.4011	1

**4105**	370	B	79.533	1
**4105**	372	B	72.3503	0
**4105**	373	B	31.0683	0
**4105**	381	B	79.5283	1
**4105**	382	B	43.2309	0
**4105**	386	B	53.9366	1
**4105**	305	C	51.82	1
**4105**	319	C	64.2159	1
**4105**	320	C	78.8702	-1
**4105**	321	C	63.4892	0
**4105**	351	C	3.2577	1
**4105**	357	C	79.5141	1
**4105**	370	C	79.609	1
**4105**	372	C	79.6452	0
**4105**	373	C	72.874	1
**4105**	381	C	79.5218	1
**4105**	382	C	50.1097	1
**4105**	386	C	62.9471	1
**4105**	305	D	40.5504	0
**4105**	319	D	77.2265	1
**4105**	320	D	79.549	-1
**4105**	321	D	67.6403	0
**4105**	351	D	18.3713	1
**4105**	357	D	73.0308	1
**4105**	370	D	79.5946	1
**4105**	372	D	69.7965	0
**4105**	373	D	79.6161	1
**4105**	381	D	73.7342	1
**4105**	382	D	65.0484	1
**4105**	386	D	42.4183	0
**4106**	305	A	54.9363	1
**4106**	319	A	79.5689	1
**4106**	320	A	50.5651	-1
**4106**	321	A	78.144	0

**4106**	351	A	8.7634	1
**4106**	357	A	67.9358	1
**4106**	370	A	72.8793	1
**4106**	372	A	66.7899	0
**4106**	373	A	79.5121	1
**4106**	381	A	79.5633	1
**4106**	382	A	52.5591	1
**4106**	386	A	77.7411	1
**4106**	305	B	63.2417	1
**4106**	319	B	42.2659	0
**4106**	320	B	56.6227	-1
**4106**	321	B	52.9134	0
**4106**	351	B	12.6355	1
**4106**	357	B	72.9412	1
**4106**	370	B	59.0976	1
**4106**	372	B	70.5187	0
**4106**	373	B	48.7091	0
**4106**	381	B	62.8103	1
**4106**	382	B	63.0234	1
**4106**	386	B	79.4923	1
**4106**	305	C	79.5476	1
**4106**	319	C	71.7137	1
**4106**	320	C	53.1819	-1
**4106**	321	C	79.5754	0
**4106**	351	C	6.0324	1
**4106**	357	C	48.4249	0
**4106**	370	C	69.0948	1
**4106**	372	C	79.5661	0
**4106**	373	C	79.5524	1
**4106**	381	C	79.0545	1
**4106**	382	C	79.5571	1
**4106**	386	C	20.975	0
**4106**	305	D	79.5828	1
**4106**	319	D	56.0501	1

**4106**	320	D	49.9842	-1
**4106**	321	D	77.7756	0
**4106**	351	D	9.4974	1
**4106**	357	D	43.0432	0
**4106**	370	D	75.6627	1
**4106**	372	D	79.5557	0
**4106**	373	D	72.3368	1
**4106**	381	D	22.4262	0
**4106**	382	D	60.9492	1
**4106**	386	D	62.0733	1
**4107**	305	A	60.6388	1
**4107**	319	A	48.9455	0
**4107**	320	A	77.5413	-1
**4107**	321	A	28.7549	1
**4107**	351	A	0.4535	1
**4107**	357	A	75.4989	1
**4107**	370	A	52.5921	1
**4107**	372	A	79.4124	0
**4107**	373	A	40.0161	0
**4107**	381	A	65.9968	1
**4107**	382	A	15.7747	-1
**4107**	386	A	49.2021	0
**4107**	305	B	79.6191	1
**4107**	319	B	73.5503	1
**4107**	320	B	72.2508	-1
**4107**	321	B	65.0179	0
**4107**	351	B	11.1564	1
**4107**	357	B	79.419	1
**4107**	370	B	73.3972	1
**4107**	372	B	79.2324	0
**4107**	373	B	54.0028	1
**4107**	381	B	62.5013	1
**4107**	382	B	79.4782	1
**4107**	386	B	55.4062	1

**4107**	305	C	34.4043	0
**4107**	319	C	79.5392	1
**4107**	320	C	64.3898	-1
**4107**	321	C	79.608	0
**4107**	351	C	9.018	1
**4107**	357	C	50.6974	1
**4107**	370	C	79.0245	1
**4107**	372	C	79.5077	0
**4107**	373	C	79.6192	1
**4107**	381	C	43.7665	0
**4107**	382	C	68.1092	1
**4107**	386	C	34.7529	0
**4107**	305	D	73.6683	1
**4107**	319	D	79.7304	1
**4107**	320	D	74.0741	-1
**4107**	321	D	79.6356	0
**4107**	351	D	14.9153	1
**4107**	357	D	79.6937	1
**4107**	370	D	58.6175	1
**4107**	372	D	79.6004	0
**4107**	373	D	76.7841	1
**4107**	381	D	61.8468	1
**4107**	382	D	75.9279	1
**4107**	386	D	79.7775	1
**4108**	305	A	55.5052	1
**4108**	319	A	41.9906	0
**4108**	320	A	73.4523	-1
**4108**	321	A	79.6093	0
**4108**	351	A	14.8941	1
**4108**	357	A	58.0422	1
**4108**	370	A	79.5448	1
**4108**	372	A	79.495	0
**4108**	373	A	76.1281	1
**4108**	381	A	48.9114	0

**4108**	382	A	65.9164	1
**4108**	386	A	72.8758	1
**4108**	305	B	61.4125	1
**4108**	319	B	79.5757	1
**4108**	320	B	12.8349	1
**4108**	321	B	73.4679	0
**4108**	351	B	19.8463	1
**4108**	357	B	79.599	1
**4108**	370	B	56.2969	1
**4108**	372	B	79.5741	0
**4108**	373	B	79.5573	1
**4108**	381	B	74.3495	1
**4108**	382	B	75.8168	1
**4108**	386	B	79.4365	1
**4108**	305	C	51.7573	1
**4108**	319	C	42.7368	0
**4108**	320	C	44.8639	-1
**4108**	321	C	56.2583	0
**4108**	351	C	1.8178	1
**4108**	357	C	72.5459	1
**4108**	370	C	79.4308	1
**4108**	372	C	74.0293	0
**4108**	373	C	79.5255	1
**4108**	381	C	72.2503	1
**4108**	382	C	73.0386	1
**4108**	386	C	65.4425	1
**4108**	305	D	22.3914	0
**4108**	319	D	64.8847	1
**4108**	320	D	50.1281	-1
**4108**	321	D	79.5429	0
**4108**	351	D	12.3444	1
**4108**	357	D	73.0845	1
**4108**	370	D	79.5871	1
**4108**	372	D	74.7762	0

**4108**	373	D	73.2645	1
**4108**	381	D	59.6275	1
**4108**	382	D	62.9199	1
**4108**	386	D	56.0664	1
**4109**	305	A	70.6019	1
**4109**	319	A	47.0982	0
**4109**	320	A	65.9328	-1
**4109**	321	A	54.2101	0
**4109**	351	A	0.3431	1
**4109**	357	A	79.6122	1
**4109**	370	A	78.9613	1
**4109**	372	A	35.4881	1
**4109**	373	A	57.1441	1
**4109**	381	A	45.6065	0
**4109**	382	A	79.1826	1
**4109**	386	A	72.4869	1
**4109**	305	B	57.0783	1
**4109**	319	B	66.4408	1
**4109**	320	B	79.5172	-1
**4109**	321	B	66.7323	0
**4109**	351	B	12.2089	1
**4109**	357	B	71.2903	1
**4109**	370	B	29.0593	0
**4109**	372	B	43.4044	1
**4109**	373	B	21.8207	0
**4109**	381	B	79.2913	1
**4109**	382	B	56.3032	1
**4109**	386	B	68.711	1
**4109**	305	C	66.0188	1
**4109**	319	C	79.6116	1
**4109**	320	C	79.3889	-1
**4109**	321	C	72.1578	0
**4109**	351	C	4.3458	1
**4109**	357	C	79.6697	1

**4109**	370	C	72.8721	1
**4109**	372	C	79.4796	0
**4109**	373	C	72.0129	1
**4109**	381	C	72.8754	1
**4109**	382	C	54.4947	1
**4109**	386	C	69.7873	1
**4109**	305	D	79.6251	1
**4109**	319	D	71.6181	1
**4109**	320	D	69.4631	-1
**4109**	321	D	41.5038	1
**4109**	351	D	9.3322	1
**4109**	357	D	67.4666	1
**4109**	370	D	72.0815	1
**4109**	372	D	54.8811	0
**4109**	373	D	75.3999	1
**4109**	381	D	45.1113	0
**4109**	382	D	64.2924	1
**4109**	386	D	46.6923	0
**4110**	305	A	74.3466	1
**4110**	319	A	73.9614	1
**4110**	320	A	51.7481	-1
**4110**	321	A	79.4914	0
**4110**	351	A	14.1827	1
**4110**	357	A	75.2338	1
**4110**	370	A	71.8085	1
**4110**	372	A	37.1131	1
**4110**	373	A	43.0887	0
**4110**	381	A	71.8718	1
**4110**	382	A	72.7136	1
**4110**	386	A	79.4021	1
**4110**	305	B	76.4225	1
**4110**	319	B	79.5266	1
**4110**	320	B	73.1897	-1
**4110**	321	B	65.0178	0

**4110**	351	B	8.752	1
**4110**	357	B	77.2503	1
**4110**	370	B	54.5139	1
**4110**	372	B	67.6616	0
**4110**	373	B	76.9376	1
**4110**	381	B	74.8221	1
**4110**	382	B	36.2638	0
**4110**	386	B	79.5921	1
**4110**	305	C	64.2907	1
**4110**	319	C	73.5122	1
**4110**	320	C	79.6361	-1
**4110**	321	C	41.2939	1
**4110**	351	C	8.0324	1
**4110**	357	C	78.6696	1
**4110**	370	C	56.0777	1
**4110**	372	C	71.3722	0
**4110**	373	C	72.264	1
**4110**	381	C	59.7133	1
**4110**	382	C	53.7861	1
**4110**	386	C	48.0856	0
**4110**	305	D	72.9741	1
**4110**	319	D	79.5226	1
**4110**	320	D	67.4646	-1
**4110**	321	D	79.6402	0
**4110**	351	D	6.004	1
**4110**	357	D	26.6505	0
**4110**	370	D	78.8118	1
**4110**	372	D	79.6449	0
**4110**	373	D	56.3704	1
**4110**	381	D	69.9614	1
**4110**	382	D	69.4094	1
**4110**	386	D	79.6172	1
**4111**	305	A	79.5545	1
**4111**	319	A	68.021	1

**4111**	320	A	79.5804	-1
**4111**	321	A	37.6668	1
**4111**	351	A	6.7306	1
**4111**	357	A	79.5594	1
**4111**	370	A	70.5303	1
**4111**	372	A	68.75	0
**4111**	373	A	79.5756	1
**4111**	381	A	79.5382	1
**4111**	382	A	70.5687	1
**4111**	386	A	34.8305	0
**4111**	305	B	73.6143	1
**4111**	319	B	48.628	0
**4111**	320	B	57.0269	-1
**4111**	321	B	59.6493	0
**4111**	351	B	15.6496	1
**4111**	357	B	79.5529	1
**4111**	370	B	57.5282	1
**4111**	372	B	72.3986	0
**4111**	373	B	44.1931	0
**4111**	381	B	74.9352	1
**4111**	382	B	31.3322	0
**4111**	386	B	73.3349	1
**4111**	305	C	44.1388	0
**4111**	319	C	54.1438	1
**4111**	320	C	75.0718	-1
**4111**	321	C	56.868	0
**4111**	351	C	5.4879	1
**4111**	357	C	72.8112	1
**4111**	370	C	73.8226	1
**4111**	372	C	79.551	0
**4111**	373	C	79.4905	1
**4111**	381	C	73.8919	1
**4111**	382	C	39.5927	0
**4111**	386	C	59.2269	1

**4111**	305	D	79.1405	1
**4111**	319	D	70.9492	1
**4111**	320	D	75.0776	-1
**4111**	321	D	79.5396	0
**4111**	351	D	2.1487	1
**4111**	357	D	72.3293	1
**4111**	370	D	79.5799	1
**4111**	372	D	72.0895	0
**4111**	373	D	79.5622	1
**4111**	381	D	24.4514	0
**4111**	382	D	73.3612	1
**4111**	386	D	79.5511	1
**4112**	305	A	79.5908	1
**4112**	319	A	30.4346	0
**4112**	320	A	79.55	-1
**4112**	321	A	62.5164	0
**4112**	351	A	2.0527	1
**4112**	357	A	56.1301	1
**4112**	370	A	79.6191	1
**4112**	372	A	58.4471	0
**4112**	373	A	71.582	1
**4112**	381	A	61.8524	1
**4112**	382	A	29.5125	0
**4112**	386	A	40.8711	0
**4112**	305	B	79.0149	1
**4112**	319	B	60.5421	1
**4112**	320	B	67.3319	-1
**4112**	321	B	72.4101	0
**4112**	351	B	23.7232	-1
**4112**	357	B	77.9582	1
**4112**	370	B	57.557	1
**4112**	372	B	79.5337	0
**4112**	373	B	23.8079	0
**4112**	381	B	78.8517	1

**4112**	382	B	18.7702	-1
**4112**	386	B	63.4654	1
**4112**	305	C	53.3752	1
**4112**	319	C	67.6327	1
**4112**	320	C	79.591	-1
**4112**	321	C	48.0486	1
**4112**	351	C	1.7123	1
**4112**	357	C	79.5228	1
**4112**	370	C	78.5535	1
**4112**	372	C	79.534	0
**4112**	373	C	46.9299	0
**4112**	381	C	77.3214	1
**4112**	382	C	67.4515	1
**4112**	386	C	48.7217	0
**4112**	305	D	53.874	1
**4112**	319	D	75.946	1
**4112**	320	D	72.8536	-1
**4112**	321	D	71.7019	0
**4112**	351	D	12.9611	1
**4112**	357	D	79.6219	1
**4112**	370	D	58.0391	1
**4112**	372	D	79.4908	0
**4112**	373	D	76.031	1
**4112**	381	D	59.4158	1
**4112**	382	D	57.3044	1
**4112**	386	D	75.0544	1
**4113**	305	A	75.9014	1
**4113**	319	A	67.3753	1
**4113**	320	A	54.5096	-1
**4113**	321	A	64.1219	0
**4113**	351	A	5.5581	1
**4113**	357	A	67.3252	1
**4113**	370	A	69.6394	1
**4113**	372	A	79.5506	0

**4113**	373	A	50.8205	1
**4113**	381	A	79.5486	1
**4113**	382	A	79.5181	1
**4113**	386	A	57.0853	1
**4113**	305	B	57.056	1
**4113**	319	B	79.5343	1
**4113**	320	B	55.4465	-1
**4113**	321	B	40.7066	1
**4113**	351	B	19.3278	1
**4113**	357	B	79.5315	1
**4113**	370	B	72.2995	1
**4113**	372	B	18.5845	0
**4113**	373	B	79.5248	1
**4113**	381	B	78.1929	1
**4113**	382	B	18.0316	-1
**4113**	386	B	79.6292	1
**4113**	305	C	72.8507	1
**4113**	319	C	73.8659	1
**4113**	320	C	70.3013	-1
**4113**	321	C	79.5102	0
**4113**	351	C	5.9021	1
**4113**	357	C	74.5137	1
**4113**	370	C	67.8257	1
**4113**	372	C	79.5049	0
**4113**	373	C	74.8504	1
**4113**	381	C	79.5653	1
**4113**	382	C	72.7656	1
**4113**	386	C	40.7813	0
**4113**	305	D	79.6193	1
**4113**	319	D	79.6308	1
**4113**	320	D	73.6815	-1
**4113**	321	D	72.6755	0
**4113**	351	D	14.4527	1
**4113**	357	D	72.6192	1

**4113**	370	D	65.6188	1
**4113**	372	D	72.3121	0
**4113**	373	D	55.9134	1
**4113**	381	D	54.5337	1
**4113**	382	D	78.6696	1
**4113**	386	D	76.3144	1
**4114**	305	A	79.576	1
**4114**	319	A	58.8588	1
**4114**	320	A	72.7135	-1
**4114**	321	A	78.0223	0
**4114**	351	A	7.8517	1
**4114**	357	A	60.2344	1
**4114**	370	A	79.5167	1
**4114**	372	A	57.192	0
**4114**	373	A	17.7838	-1
**4114**	381	A	72.3106	1
**4114**	382	A	42.9886	0
**4114**	386	A	74.4444	1
**4114**	305	B	57.3387	1
**4114**	319	B	79.5465	1
**4114**	320	B	55.454	-1
**4114**	321	B	72.9336	0
**4114**	351	B	18.4395	1
**4114**	357	B	79.5741	1
**4114**	370	B	79.5252	1
**4114**	372	B	57.1592	0
**4114**	373	B	24.764	0
**4114**	381	B	79.576	1
**4114**	382	B	57.118	1
**4114**	386	B	63.0152	1
**4114**	305	C	70.1476	1
**4114**	319	C	38.115	0
**4114**	320	C	71.0614	-1
**4114**	321	C	6.1419	0

**4114**	351	C	7.2408	1
**4114**	357	C	79.5951	1
**4114**	370	C	79.5444	1
**4114**	372	C	51.9844	0
**4114**	373	C	79.5615	1
**4114**	381	C	79.5366	1
**4114**	382	C	72.8419	1
**4114**	386	C	50.0302	1
**4114**	305	D	70.7075	1
**4114**	319	D	41.2251	0
**4114**	320	D	79.6054	-1
**4114**	321	D	70.4712	0
**4114**	351	D	12.4808	1
**4114**	357	D	68.007	1
**4114**	370	D	46.1873	0
**4114**	372	D	49.4119	1
**4114**	373	D	45.7085	0
**4114**	381	D	79.5265	1
**4114**	382	D	78.4594	1
**4114**	386	D	79.5817	1
**4115**	305	A	46.125	0
**4115**	319	A	66.0939	1
**4115**	320	A	79.5361	-1
**4115**	321	A	79.5455	0
**4115**	351	A	3.8151	1
**4115**	357	A	38.7536	0
**4115**	370	A	74.3371	1
**4115**	372	A	56.2726	0
**4115**	373	A	73.6849	1
**4115**	381	A	62.8757	1
**4115**	382	A	67.0737	1
**4115**	386	A	63.3757	1
**4115**	305	B	79.6826	1
**4115**	319	B	50.6239	1

**4115**	320	B	79.4564	-1
**4115**	321	B	79.2952	0
**4115**	351	B	11.648	1
**4115**	357	B	79.5164	1
**4115**	370	B	79.6083	1
**4115**	372	B	75.7395	0
**4115**	373	B	79.2685	1
**4115**	381	B	73.6112	1
**4115**	382	B	79.5667	1
**4115**	386	B	44.8463	0
**4115**	305	C	70.5159	1
**4115**	319	C	53.025	1
**4115**	320	C	78.4212	-1
**4115**	321	C	48.7023	1
**4115**	351	C	7.1389	1
**4115**	357	C	73.5241	1
**4115**	370	C	62.8082	1
**4115**	372	C	62.8715	0
**4115**	373	C	79.5661	1
**4115**	381	C	40.2269	0
**4115**	382	C	79.555	1
**4115**	386	C	56.5553	1
**4115**	305	D	52.7004	1
**4115**	319	D	79.5935	1
**4115**	320	D	79.5518	-1
**4115**	321	D	68.3411	0
**4115**	351	D	16.9655	1
**4115**	357	D	66.6678	1
**4115**	370	D	79.5614	1
**4115**	372	D	52.6201	0
**4115**	373	D	79.4077	1
**4115**	381	D	79.5478	1
**4115**	382	D	79.5254	1
**4115**	386	D	78.4642	1

**4116**	305	A	79.5386	1
**4116**	319	A	19.7222	-1
**4116**	320	A	79.6006	-1
**4116**	321	A	72.8388	0
**4116**	351	A	8.0956	1
**4116**	357	A	67.8872	1
**4116**	370	A	79.594	1
**4116**	372	A	70.1351	0
**4116**	373	A	76.3353	1
**4116**	381	A	66.0936	1
**4116**	382	A	57.2359	1
**4116**	386	A	55.5085	1
**4116**	305	B	53.0929	1
**4116**	319	B	66.2494	1
**4116**	320	B	79.5463	-1
**4116**	321	B	69.6237	0
**4116**	351	B	25.3921	-1
**4116**	357	B	73.515	1
**4116**	370	B	54.692	1
**4116**	372	B	79.5054	0
**4116**	373	B	76.6832	1
**4116**	381	B	62.4716	1
**4116**	382	B	73.7936	1
**4116**	386	B	58.8845	1
**4116**	305	C	70.4495	1
**4116**	319	C	79.5084	1
**4116**	320	C	76.2435	-1
**4116**	321	C	72.9609	0
**4116**	351	C	19.4673	1
**4116**	357	C	78.2524	1
**4116**	370	C	40.8431	0
**4116**	372	C	79.6238	0
**4116**	373	C	79.5555	1
**4116**	381	C	31.9008	0

**4116**	382	C	79.512	1
**4116**	386	C	43.7184	0
**4116**	305	D	41.1674	0
**4116**	319	D	79.6255	1
**4116**	320	D	48.6475	-1
**4116**	321	D	56.4365	0
**4116**	351	D	12.4395	1
**4116**	357	D	71.6492	1
**4116**	370	D	79.6273	1
**4116**	372	D	74.0982	0
**4116**	373	D	77.7552	1
**4116**	381	D	79.4332	1
**4116**	382	D	69.583	1
**4116**	386	D	54.9315	1
**4117**	305	A	60.8955	1
**4117**	319	A	79.5161	1
**4117**	320	A	79.5879	-1
**4117**	321	A	38.8264	1
**4117**	351	A	7.0428	1
**4117**	357	A	79.5265	1
**4117**	370	A	53.9676	1
**4117**	372	A	67.7635	0
**4117**	373	A	55.3972	1
**4117**	381	A	74.4774	1
**4117**	382	A	79.5631	1
**4117**	386	A	75.932	1
**4117**	305	B	79.5659	1
**4117**	319	B	73.7473	1
**4117**	320	B	77.2034	-1
**4117**	321	B	68.2121	0
**4117**	351	B	21.485	-1
**4117**	357	B	79.5523	1
**4117**	370	B	79.6718	1
**4117**	372	B	79.5993	0

**4117**	373	B	45.0405	0
**4117**	381	B	65.9836	1
**4117**	382	B	72.8637	1
**4117**	386	B	73.3127	1
**4117**	305	C	79.5958	1
**4117**	319	C	58.75	1
**4117**	320	C	79.5332	-1
**4117**	321	C	43.0104	1
**4117**	351	C	9.2535	1
**4117**	357	C	25.6374	0
**4117**	370	C	79.5094	1
**4117**	372	C	41.4063	1
**4117**	373	C	46.9356	0
**4117**	381	C	73.7468	1
**4117**	382	C	60.6596	1
**4117**	386	C	44.2497	0
**4117**	305	D	65.4892	1
**4117**	319	D	79.022	1
**4117**	320	D	79.6204	-1
**4117**	321	D	45.1939	1
**4117**	351	D	8.1492	1
**4117**	357	D	72.0332	1
**4117**	370	D	50.8523	1
**4117**	372	D	79.4474	0
**4117**	373	D	74.6539	1
**4117**	381	D	72.4723	1
**4117**	382	D	54.8741	1
**4117**	386	D	73.1762	1
**4118**	305	A	76.6377	1
**4118**	319	A	54.1029	1
**4118**	320	A	71.163	-1
**4118**	321	A	70.1604	0
**4118**	351	A	5.5426	1
**4118**	357	A	34.9049	0

**4118**	370	A	47.2258	0
**4118**	372	A	24.2562	1
**4118**	373	A	69.8577	1
**4118**	381	A	60.8312	1
**4118**	382	A	79.5215	1
**4118**	386	A	79.5785	1
**4118**	305	B	79.5042	1
**4118**	319	B	65.7654	1
**4118**	320	B	67.0554	-1
**4118**	321	B	73.762	0
**4118**	351	B	9.9895	1
**4118**	357	B	79.6014	1
**4118**	370	B	72.1498	1
**4118**	372	B	79.4459	0
**4118**	373	B	79.5101	1
**4118**	381	B	71.5505	1
**4118**	382	B	71.5037	1
**4118**	386	B	66.2267	1
**4118**	305	C	46.1171	0
**4118**	319	C	47.4251	0
**4118**	320	C	71.3771	-1
**4118**	321	C	71.3334	0
**4118**	351	C	5.5242	1
**4118**	357	C	79.5643	1
**4118**	370	C	77.6202	1
**4118**	372	C	58.9461	0
**4118**	373	C	53.9841	1
**4118**	381	C	71.2872	1
**4118**	382	C	45.705	0
**4118**	386	C	31.5837	0
**4118**	305	D	79.5397	1
**4118**	319	D	29.4404	0
**4118**	320	D	72.593	-1
**4118**	321	D	72.8323	0

**4118**	351	D	13.6566	1
**4118**	357	D	61.7195	1
**4118**	370	D	67.694	1
**4118**	372	D	79.5599	0
**4118**	373	D	61.6543	1
**4118**	381	D	30.574	0
**4118**	382	D	79.5738	1
**4118**	386	D	17.8831	-1
**4119**	305	A	74.5716	1
**4119**	319	A	79.4986	1
**4119**	320	A	79.7311	-1
**4119**	321	A	71.658	0
**4119**	351	A	2.088	1
**4119**	357	A	63.5056	1
**4119**	370	A	72.716	1
**4119**	372	A	79.6233	0
**4119**	373	A	71.6901	1
**4119**	381	A	79.6885	1
**4119**	382	A	61.9884	1
**4119**	386	A	70.8349	1
**4119**	305	B	79.741	1
**4119**	319	B	72.4236	1
**4119**	320	B	79.659	-1
**4119**	321	B	66.5733	0
**4119**	351	B	4.1296	1
**4119**	357	B	75.2882	1
**4119**	370	B	76.4898	1
**4119**	372	B	79.5369	0
**4119**	373	B	74.8689	1
**4119**	381	B	23.8795	0
**4119**	382	B	21.6857	0
**4119**	386	B	79.5531	1
**4119**	305	C	71.6012	1
**4119**	319	C	78.3153	1

**4119**	320	C	74.6172	-1
**4119**	321	C	63.9492	0
**4119**	351	C	5.2805	1
**4119**	357	C	71.1247	1
**4119**	370	C	40.9347	0
**4119**	372	C	55.4974	0
**4119**	373	C	66.8313	1
**4119**	381	C	79.5568	1
**4119**	382	C	69.2095	1
**4119**	386	C	25.1899	0
**4119**	305	D	24.4089	0
**4119**	319	D	79.4852	1
**4119**	320	D	79.6439	-1
**4119**	321	D	39.7655	1
**4119**	351	D	3.9477	1
**4119**	357	D	54.9244	1
**4119**	370	D	67.3254	1
**4119**	372	D	79.507	0
**4119**	373	D	79.4149	1
**4119**	381	D	62.3789	1
**4119**	382	D	78.4367	1
**4119**	386	D	55.9124	1
**4120**	305	A	56.8257	1
**4120**	319	A	66.6585	1
**4120**	320	A	78.6983	-1
**4120**	321	A	75.9985	0
**4120**	351	A	0.3587	1
**4120**	357	A	69.3043	1
**4120**	370	A	47.6704	0
**4120**	372	A	79.6012	0
**4120**	373	A	79.4031	1
**4120**	381	A	79.6339	1
**4120**	382	A	67.3188	1
**4120**	386	A	67.7342	1

**4120**	305	B	76.6763	1
**4120**	319	B	21.2067	0
**4120**	320	B	76.2218	-1
**4120**	321	B	35.0696	1
**4120**	351	B	8.5143	1
**4120**	357	B	78.0393	1
**4120**	370	B	78.4164	1
**4120**	372	B	60.4569	0
**4120**	373	B	26.7879	0
**4120**	381	B	79.5977	1
**4120**	382	B	39.388	0
**4120**	386	B	79.541	1
**4120**	305	C	76.8635	1
**4120**	319	C	79.6603	1
**4120**	320	C	79.4795	-1
**4120**	321	C	79.5268	0
**4120**	351	C	2.7132	1
**4120**	357	C	69.6252	1
**4120**	370	C	79.595	1
**4120**	372	C	73.1577	0
**4120**	373	C	73.493	1
**4120**	381	C	79.5692	1
**4120**	382	C	52.4106	1
**4120**	386	C	55.7776	1
**4120**	305	D	62.1205	1
**4120**	319	D	40.2267	0
**4120**	320	D	72.7222	-1
**4120**	321	D	30.219	1
**4120**	351	D	14.6087	1
**4120**	357	D	79.4265	1
**4120**	370	D	77.7029	1
**4120**	372	D	79.5372	0
**4120**	373	D	70.4793	1
**4120**	381	D	74.4754	1

**4120**	382	D	79.5216	1
**4120**	386	D	68.3504	1
**4121**	305	A	79.5424	1
**4121**	319	A	39.1342	0
**4121**	320	A	48.6208	-1
**4121**	321	A	75.3232	0
**4121**	351	A	1.1338	1
**4121**	357	A	71.2184	1
**4121**	370	A	75.6184	1
**4121**	372	A	72.1097	0
**4121**	373	A	79.0168	1
**4121**	381	A	79.4689	1
**4121**	382	A	67.7799	1
**4121**	386	A	46.1524	0
**4121**	305	B	24.1337	0
**4121**	319	B	50.5797	1
**4121**	320	B	56.3357	-1
**4121**	321	B	79.5483	0
**4121**	351	B	13.1604	1
**4121**	357	B	72.0526	1
**4121**	370	B	72.2717	1
**4121**	372	B	70.868	0
**4121**	373	B	44.0916	0
**4121**	381	B	72.1786	1
**4121**	382	B	79.4939	1
**4121**	386	B	63.8943	1
**4121**	305	C	38.954	0
**4121**	319	C	75.1889	1
**4121**	320	C	79.5602	-1
**4121**	321	C	76.0273	0
**4121**	351	C	4.9457	1
**4121**	357	C	63.6611	1
**4121**	370	C	79.5034	1
**4121**	372	C	60.2493	0

**4121**	373	C	79.4587	1
**4121**	381	C	77.2189	1
**4121**	382	C	62.2138	1
**4121**	386	C	41.8952	0
**4121**	305	D	79.5123	1
**4121**	319	D	70.974	1
**4121**	320	D	79.3287	-1
**4121**	321	D	79.3723	0
**4121**	351	D	9.4254	1
**4121**	357	D	75.6273	1
**4121**	370	D	57.6624	1
**4121**	372	D	71.4678	0
**4121**	373	D	79.4914	1
**4121**	381	D	79.6317	1
**4121**	382	D	79.6197	1
**4121**	386	D	56.758	1
**4122**	305	A	27.2919	0
**4122**	319	A	69.0649	1
**4122**	320	A	75.1948	-1
**4122**	321	A	60.9965	0
**4122**	351	A	7.7302	1
**4122**	357	A	73.0915	1
**4122**	370	A	13.6197	-1
**4122**	372	A	23.3058	1
**4122**	373	A	79.5906	1
**4122**	381	A	79.6098	1
**4122**	382	A	50.446	1
**4122**	386	A	72.8781	1
**4122**	305	B	79.619	1
**4122**	319	B	29.3072	0
**4122**	320	B	79.6469	-1
**4122**	321	B	61.1314	0
**4122**	351	B	17.1066	1
**4122**	357	B	79.6041	1

**4122**	370	B	76.9333	1
**4122**	372	B	79.5235	0
**4122**	373	B	71.8071	1
**4122**	381	B	55.5521	1
**4122**	382	B	50.3693	1
**4122**	386	B	71.39	1
**4122**	305	C	73.5526	1
**4122**	319	C	79.6087	1
**4122**	320	C	71.2799	-1
**4122**	321	C	55.4585	0
**4122**	351	C	7.4075	1
**4122**	357	C	79.4563	1
**4122**	370	C	78.7587	1
**4122**	372	C	64.5394	0
**4122**	373	C	73.687	1
**4122**	381	C	46.6562	0
**4122**	382	C	79.6671	1
**4122**	386	C	8.8989	-1
**4122**	305	D	70.2758	1
**4122**	319	D	78.4758	1
**4122**	320	D	79.5655	-1
**4122**	321	D	70.9172	0
**4122**	351	D	9.4676	1
**4122**	357	D	76.1802	1
**4122**	370	D	67.2588	1
**4122**	372	D	79.5373	0
**4122**	373	D	76.2972	1
**4122**	381	D	69.1931	1
**4122**	382	D	64.3001	1
**4122**	386	D	51.8083	1
**4123**	305	A	79.5854	1
**4123**	319	A	67.594	1
**4123**	320	A	71.4535	-1
**4123**	321	A	45.6333	1

**4123**	351	A	5.1632	1
**4123**	357	A	78.9662	1
**4123**	370	A	73.6657	1
**4123**	372	A	79.6271	0
**4123**	373	A	79.5158	1
**4123**	381	A	62.9236	1
**4123**	382	A	55.3315	1
**4123**	386	A	79.6029	1
**4123**	305	B	70.4141	1
**4123**	319	B	78.923	1
**4123**	320	B	79.5757	-1
**4123**	321	B	79.639	0
**4123**	351	B	12.5398	1
**4123**	357	B	79.5232	1
**4123**	370	B	79.5283	1
**4123**	372	B	79.6033	0
**4123**	373	B	75.337	1
**4123**	381	B	79.5209	1
**4123**	382	B	36.5926	0
**4123**	386	B	79.6705	1
**4123**	305	C	75.699	1
**4123**	319	C	79.4916	1
**4123**	320	C	60.2126	-1
**4123**	321	C	78.2844	0
**4123**	351	C	2.5335	1
**4123**	357	C	72.5753	1
**4123**	370	C	76.4785	1
**4123**	372	C	71.894	0
**4123**	373	C	73.5398	1
**4123**	381	C	70.982	1
**4123**	382	C	70.0271	1
**4123**	386	C	38.6319	0
**4123**	305	D	79.1886	1
**4123**	319	D	79.494	1

**4123**	320	D	75.5336	-1
**4123**	321	D	46.5307	1
**4123**	351	D	11.8286	1
**4123**	357	D	55.2558	1
**4123**	370	D	79.4367	1
**4123**	372	D	79.4985	0
**4123**	373	D	79.5733	1
**4123**	381	D	75.9724	1
**4123**	382	D	63.3433	1
**4123**	386	D	79.591	1
**4124**	305	A	75.1864	1
**4124**	319	A	71.5619	1
**4124**	320	A	79.6938	-1
**4124**	321	A	79.375	0
**4124**	351	A	3.8974	1
**4124**	357	A	71.6276	1
**4124**	370	A	29.415	0
**4124**	372	A	66.1725	0
**4124**	373	A	69.6721	1
**4124**	381	A	79.6252	1
**4124**	382	A	65.6759	1
**4124**	386	A	79.514	1
**4124**	305	B	65.9187	1
**4124**	319	B	79.5154	1
**4124**	320	B	74.1735	-1
**4124**	321	B	43.6258	1
**4124**	351	B	14.7627	1
**4124**	357	B	79.4671	1
**4124**	370	B	70.7738	1
**4124**	372	B	79.4979	0
**4124**	373	B	79.6001	1
**4124**	381	B	79.5913	1
**4124**	382	B	48.6459	0
**4124**	386	B	40.7607	0

**4124**	305	C	47.5391	0
**4124**	319	C	76.3133	1
**4124**	320	C	76.8276	-1
**4124**	321	C	35.7843	1
**4124**	351	C	0.9416	1
**4124**	357	C	74.5215	1
**4124**	370	C	49.3834	0
**4124**	372	C	25.1334	1
**4124**	373	C	77.4231	1
**4124**	381	C	62.9955	1
**4124**	382	C	39.4744	0
**4124**	386	C	42.3189	0
**4124**	305	D	78.7783	1
**4124**	319	D	79.4742	1
**4124**	320	D	71.8731	-1
**4124**	321	D	79.6465	0
**4124**	351	D	7.5297	1
**4124**	357	D	73.2518	1
**4124**	370	D	76.2186	1
**4124**	372	D	68.5999	0
**4124**	373	D	52.0588	1
**4124**	381	D	78.5104	1
**4124**	382	D	79.6024	1
**4124**	386	D	68.5304	1
**4125**	305	A	72.1899	1
**4125**	319	A	71.945	1
**4125**	320	A	71.4493	-1
**4125**	321	A	70.9101	0
**4125**	351	A	2.3395	1
**4125**	357	A	62.0606	1
**4125**	370	A	79.574	1
**4125**	372	A	52.4445	0
**4125**	373	A	23.7193	0
**4125**	381	A	73.5019	1

**4125**	382	A	79.5426	1
**4125**	386	A	79.5718	1
**4125**	305	B	79.5361	1
**4125**	319	B	68.9542	1
**4125**	320	B	79.5344	-1
**4125**	321	B	55.9026	0
**4125**	351	B	8.7828	1
**4125**	357	B	79.5443	1
**4125**	370	B	58.8128	1
**4125**	372	B	79.5448	0
**4125**	373	B	79.5589	1
**4125**	381	B	71.8564	1
**4125**	382	B	15.4625	-1
**4125**	386	B	79.6095	1
**4125**	305	C	69.9558	1
**4125**	319	C	75.4427	1
**4125**	320	C	79.4166	-1
**4125**	321	C	60.5993	0
**4125**	351	C	6.3765	1
**4125**	357	C	79.5734	1
**4125**	370	C	78.2432	1
**4125**	372	C	78.2603	0
**4125**	373	C	79.5346	1
**4125**	381	C	70.0306	1
**4125**	382	C	79.5297	1
**4125**	386	C	56.0392	1
**4125**	305	D	72.815	1
**4125**	319	D	79.5333	1
**4125**	320	D	47.9902	-1
**4125**	321	D	79.055	0
**4125**	351	D	13.7801	1
**4125**	357	D	76.3569	1
**4125**	370	D	71.2036	1
**4125**	372	D	70.0467	0

**4125**	373	D	79.5602	1
**4125**	381	D	79.5893	1
**4125**	382	D	68.2115	1
**4125**	386	D	79.558	1
**4126**	305	A	61.0619	1
**4126**	319	A	78.8539	1
**4126**	320	A	79.5389	-1
**4126**	321	A	79.6016	0
**4126**	351	A	0.6265	1
**4126**	357	A	55.2185	1
**4126**	370	A	58.6353	1
**4126**	372	A	76.0373	0
**4126**	373	A	32.1363	0
**4126**	381	A	79.5561	1
**4126**	382	A	78.8401	1
**4126**	386	A	79.5517	1
**4126**	305	B	69.9882	1
**4126**	319	B	76.3908	1
**4126**	320	B	78.6886	-1
**4126**	321	B	79.5278	0
**4126**	351	B	9.992	1
**4126**	357	B	59.5628	1
**4126**	370	B	73.6386	1
**4126**	372	B	79.5338	0
**4126**	373	B	54.6982	1
**4126**	381	B	79.5486	1
**4126**	382	B	33.7638	0
**4126**	386	B	60.2893	1
**4126**	305	C	45.7581	0
**4126**	319	C	75.4032	1
**4126**	320	C	59.0312	-1
**4126**	321	C	68.8743	0
**4126**	351	C	5.8147	1
**4126**	357	C	79.5847	1

**4126**	370	C	55.9192	1
**4126**	372	C	79.5611	0
**4126**	373	C	79.5378	1
**4126**	381	C	71.8047	1
**4126**	382	C	66.4407	1
**4126**	386	C	57.327	1
**4126**	305	D	79.5116	1
**4126**	319	D	76.6027	1
**4126**	320	D	78.657	-1
**4126**	321	D	79.567	0
**4126**	351	D	14.0957	1
**4126**	357	D	58.5073	1
**4126**	370	D	79.587	1
**4126**	372	D	79.5306	0
**4126**	373	D	28.0511	0
**4126**	381	D	63.0571	1
**4126**	382	D	73.9215	1
**4126**	386	D	79.5985	1
**4127**	305	A	44.5498	0
**4127**	319	A	41.5664	0
**4127**	320	A	70.6936	-1
**4127**	321	A	79.3521	0
**4127**	351	A	2.6064	1
**4127**	357	A	76.2755	1
**4127**	370	A	73.3616	1
**4127**	372	A	79.5208	0
**4127**	373	A	42.6782	0
**4127**	381	A	69.4977	1
**4127**	382	A	63.6848	1
**4127**	386	A	66.8937	1
**4127**	305	B	58.0823	1
**4127**	319	B	74.5173	1
**4127**	320	B	79.7097	-1
**4127**	321	B	72.7745	0

**4127**	351	B	2.0026	1
**4127**	357	B	64.5502	1
**4127**	370	B	41.9738	0
**4127**	372	B	70.9541	0
**4127**	373	B	79.4265	1
**4127**	381	B	63.4891	1
**4127**	382	B	77.6846	1
**4127**	386	B	60.6525	1
**4127**	305	C	66.1034	1
**4127**	319	C	79.7756	1
**4127**	320	C	74.9828	-1
**4127**	321	C	64.0624	0
**4127**	351	C	20.0578	-1
**4127**	357	C	79.5503	1
**4127**	370	C	64.2557	1
**4127**	372	C	72.5287	0
**4127**	373	C	55.511	1
**4127**	381	C	70.8055	1
**4127**	382	C	78.3488	1
**4127**	386	C	57.8653	1
**4127**	305	D	79.2666	1
**4127**	319	D	50.3901	1
**4127**	320	D	79.4509	-1
**4127**	321	D	58.1624	0
**4127**	351	D	671	1
**4127**	357	D	68.6733	1
**4127**	370	D	56.8466	1
**4127**	372	D	79.4657	0
**4127**	373	D	79.4431	1
**4127**	381	D	76.9916	1
**4127**	382	D	79.5654	1
**4127**	386	D	79.5674	1
**4128**	305	A	65.7156	1
**4128**	319	A	40.3926	0

**4128**	320	A	79.6435	-1
**4128**	321	A	54.8175	0
**4128**	351	A	8.5909	1
**4128**	357	A	56.1034	1
**4128**	370	A	76.735	1
**4128**	372	A	43.5954	1
**4128**	373	A	31.6428	0
**4128**	381	A	67.7844	1
**4128**	382	A	65.8953	1
**4128**	386	A	79.3575	1
**4128**	305	B	79.5737	1
**4128**	319	B	78.1682	1
**4128**	320	B	51.7802	-1
**4128**	321	B	53.2496	0
**4128**	351	B	19.9939	1
**4128**	357	B	71.3628	1
**4128**	370	B	28.3801	0
**4128**	372	B	79.5596	0
**4128**	373	B	54.3884	1
**4128**	381	B	79.5616	1
**4128**	382	B	71.99	1
**4128**	386	B	79.6439	1
**4128**	305	C	69.1847	1
**4128**	319	C	72.5942	1
**4128**	320	C	50.7539	-1
**4128**	321	C	63.5471	0
**4128**	351	C	17.7671	1
**4128**	357	C	47.0187	0
**4128**	370	C	47.2833	0
**4128**	372	C	79.5729	0
**4128**	373	C	79.6683	1
**4128**	381	C	72.917	1
**4128**	382	C	61.4109	1
**4128**	386	C	54.8936	1

**4128**	305	D	79.5724	1
**4128**	319	D	67.4292	1
**4128**	320	D	61.5699	-1
**4128**	321	D	54.9867	0
**4128**	351	D	14.0256	1
**4128**	357	D	79.5946	1
**4128**	370	D	74.2957	1
**4128**	372	D	79.5314	0
**4128**	373	D	71.1751	1
**4128**	381	D	42.8616	0
**4128**	382	D	76.5687	1
**4128**	386	D	78.8334	1
**4129**	305	A	79.5399	1
**4129**	319	A	44.3609	0
**4129**	320	A	69.2376	-1
**4129**	321	A	77.7295	0
**4129**	351	A	7.0517	1
**4129**	357	A	32.9324	0
**4129**	370	A	74.2004	1
**4129**	372	A	79.6975	0
**4129**	373	A	69.9929	1
**4129**	381	A	79.409	1
**4129**	382	A	65.5919	1
**4129**	386	A	79.5607	1
**4129**	305	B	74.5141	1
**4129**	319	B	52.7306	1
**4129**	320	B	73.6874	-1
**4129**	321	B	69.6022	0
**4129**	351	B	9.938	1
**4129**	357	B	73.0902	1
**4129**	370	B	39.9565	0
**4129**	372	B	79.5765	0
**4129**	373	B	53.642	1
**4129**	381	B	63.5705	1

**4129**	382	B	71.5725	1
**4129**	386	B	67.1929	1
**4129**	305	C	78.7142	1
**4129**	319	C	79.5375	1
**4129**	320	C	79.5389	-1
**4129**	321	C	72.7941	0
**4129**	351	C	3.7955	1
**4129**	357	C	73.2577	1
**4129**	370	C	60.4706	1
**4129**	372	C	73.1253	0
**4129**	373	C	77.0993	1
**4129**	381	C	66.4038	1
**4129**	382	C	57.3297	1
**4129**	386	C	63.6468	1
**4129**	305	D	64.1219	1
**4129**	319	D	79.5043	1
**4129**	320	D	23.551	-1
**4129**	321	D	55.9936	0
**4129**	351	D	38.4957	-1
**4129**	357	D	75.1781	1
**4129**	370	D	48.8254	0
**4129**	372	D	79.5993	0
**4129**	373	D	76.8192	1
**4129**	381	D	69.3398	1
**4129**	382	D	71.1557	1
**4129**	386	D	76.4161	1
**4130**	305	A	79.5415	1
**4130**	319	A	19.6227	-1
**4130**	320	A	79.7305	-1
**4130**	321	A	78.3541	0
**4130**	351	A	4.5941	1
**4130**	357	A	44.8051	0
**4130**	370	A	52.6412	1
**4130**	372	A	58.1522	0

**4130**	373	A	70.1652	1
**4130**	381	A	79.6334	1
**4130**	382	A	64.9843	1
**4130**	386	A	35.9836	0
**4130**	305	B	72.0212	1
**4130**	319	B	72.0531	1
**4130**	320	B	72.9387	-1
**4130**	321	B	79.5647	0
**4130**	351	B	37.2662	-1
**4130**	357	B	79.5844	1
**4130**	370	B	79.4572	1
**4130**	372	B	69.8715	0
**4130**	373	B	79.1588	1
**4130**	381	B	72.3603	1
**4130**	382	B	79.6556	1
**4130**	386	B	79.6015	1
**4130**	305	C	72.8829	1
**4130**	319	C	67.9756	1
**4130**	320	C	58.8805	-1
**4130**	321	C	32.4713	1
**4130**	351	C	0.6375	1
**4130**	357	C	79.5318	1
**4130**	370	C	67.3844	1
**4130**	372	C	72.3808	0
**4130**	373	C	46.5434	0
**4130**	381	C	79.6929	1
**4130**	382	C	53.8023	1
**4130**	386	C	48.3242	0
**4130**	305	D	43.3028	0
**4130**	319	D	79.5695	1
**4130**	320	D	30.749	-1
**4130**	321	D	60.0187	0
**4130**	351	D	16.6382	1
**4130**	357	D	57.2193	1

**4130**	370	D	61.2982	1
**4130**	372	D	73.2135	0
**4130**	373	D	54.1347	1
**4130**	381	D	63.1184	1
**4130**	382	D	73.1409	1
**4130**	386	D	79.52	1
**4131**	305	A	66.9092	1
**4131**	319	A	77.4666	1
**4131**	320	A	67.5244	-1
**4131**	321	A	72.7391	0
**4131**	351	A	0.0	1
**4131**	357	A	76.2275	1
**4131**	370	A	79.5634	1
**4131**	372	A	79.5466	0
**4131**	373	A	69.0576	1
**4131**	381	A	73.0497	1
**4131**	382	A	79.5379	1
**4131**	386	A	54.2376	1
**4131**	305	B	78.0169	1
**4131**	319	B	72.8274	1
**4131**	320	B	73.8067	-1
**4131**	321	B	49.3028	1
**4131**	351	B	16.567	1
**4131**	357	B	79.492	1
**4131**	370	B	6.7566	-1
**4131**	372	B	51.2404	0
**4131**	373	B	42.1609	0
**4131**	381	B	79.5255	1
**4131**	382	B	78.692	1
**4131**	386	B	57.6009	1
**4131**	305	C	64.5971	1
**4131**	319	C	79.4225	1
**4131**	320	C	79.5315	-1
**4131**	321	C	37.7693	1

**4131**	351	C	0.9747	1
**4131**	357	C	62.9458	1
**4131**	370	C	31.8483	0
**4131**	372	C	6.6297	0
**4131**	373	C	79.5587	1
**4131**	381	C	79.5291	1
**4131**	382	C	75.9025	1
**4131**	386	C	50.892	1
**4131**	305	D	56.6535	1
**4131**	319	D	36.2889	0
**4131**	320	D	77.428	-1
**4131**	321	D	45.9224	1
**4131**	351	D	14.0061	1
**4131**	357	D	49.2235	0
**4131**	370	D	64.9381	1
**4131**	372	D	79.5621	0
**4131**	373	D	79.5854	1
**4131**	381	D	73.3681	1
**4131**	382	D	73.2134	1
**4131**	386	D	79.5538	1
**4132**	305	A	72.7492	1
**4132**	319	A	74.0699	1
**4132**	320	A	79.5852	-1
**4132**	321	A	72.4366	0
**4132**	351	A	0.7688	1
**4132**	357	A	79.6713	1
**4132**	370	A	57.2397	1
**4132**	372	A	66.2575	0
**4132**	373	A	32.8155	0
**4132**	381	A	67.7678	1
**4132**	382	A	72.8271	1
**4132**	386	A	32.0474	0
**4132**	305	B	75.6702	1
**4132**	319	B	59.4308	1

**4132**	320	B	64.8809	-1
**4132**	321	B	79.5572	0
**4132**	351	B	7.3703	1
**4132**	357	B	51.5596	1
**4132**	370	B	72.4379	1
**4132**	372	B	70.193	0
**4132**	373	B	50.129	1
**4132**	381	B	32.4	0
**4132**	382	B	79.5024	1
**4132**	386	B	71.5719	1
**4132**	305	C	62.6629	1
**4132**	319	C	57.9534	1
**4132**	320	C	30.5778	-1
**4132**	321	C	47.0708	1
**4132**	351	C	16.3638	1
**4132**	357	C	63.3657	1
**4132**	370	C	74.3797	1
**4132**	372	C	68.0592	0
**4132**	373	C	34.0861	0
**4132**	381	C	79.5345	1
**4132**	382	C	72.0262	1
**4132**	386	C	54.3796	1
**4132**	305	D	72.0254	1
**4132**	319	D	65.8154	1
**4132**	320	D	26.6585	-1
**4132**	321	D	74.1944	0
**4132**	351	D	11.0557	1
**4132**	357	D	77.8737	1
**4132**	370	D	69.5378	1
**4132**	372	D	79.5044	0
**4132**	373	D	73.3837	1
**4132**	381	D	76.5057	1
**4132**	382	D	69.9316	1
**4132**	386	D	79.5123	1

**4133**	305	A	63.3219	1
**4133**	319	A	79.5603	1
**4133**	320	A	64.5217	-1
**4133**	321	A	60.9722	0
**4133**	351	A	2.853	1
**4133**	357	A	72.0925	1
**4133**	370	A	79.5844	1
**4133**	372	A	74.4678	0
**4133**	373	A	37.1094	0
**4133**	381	A	51.9542	1
**4133**	382	A	72.2751	1
**4133**	386	A	79.5654	1
**4133**	305	B	79.5689	1
**4133**	319	B	72.3106	1
**4133**	320	B	33.9429	-1
**4133**	321	B	67.9591	0
**4133**	351	B	16.5685	1
**4133**	357	B	79.5453	1
**4133**	370	B	79.5545	1
**4133**	372	B	53.0901	0
**4133**	373	B	74.0874	1
**4133**	381	B	79.5677	1
**4133**	382	B	40.6773	0
**4133**	386	B	72.9886	1
**4133**	305	C	0.9487	-1
**4133**	319	C	74.0557	1
**4133**	320	C	64.0873	-1
**4133**	321	C	74.493	0
**4133**	351	C	6.5261	1
**4133**	357	C	79.5871	1
**4133**	370	C	51.1355	1
**4133**	372	C	54.1234	0
**4133**	373	C	32.4975	0
**4133**	381	C	79.5792	1

**4133**	382	C	58.8442	1
**4133**	386	C	27.8077	0
**4133**	305	D	50.2757	1
**4133**	319	D	25.3595	0
**4133**	320	D	79.5582	-1
**4133**	321	D	39.7578	1
**4133**	351	D	11.5059	1
**4133**	357	D	72.262	1
**4133**	370	D	79.5766	1
**4133**	372	D	70.9022	0
**4133**	373	D	78.2409	1
**4133**	381	D	79.4514	1
**4133**	382	D	65.4981	1
**4133**	386	D	79.0721	1
**4134**	305	A	54.7342	1
**4134**	319	A	76.7134	1
**4134**	320	A	79.4329	-1
**4134**	321	A	63.8706	0
**4134**	351	A	7.9491	1
**4134**	357	A	33.1392	0
**4134**	370	A	50.4305	1
**4134**	372	A	72.6202	0
**4134**	373	A	75.5652	1
**4134**	381	A	50.4602	1
**4134**	382	A	45.4613	0
**4134**	386	A	59.7208	1
**4134**	305	B	79.5737	1
**4134**	319	B	59.8469	1
**4134**	320	B	47.6353	-1
**4134**	321	B	76.9105	0
**4134**	351	B	13.6114	1
**4134**	357	B	48.4649	0
**4134**	370	B	74.8349	1
**4134**	372	B	79.5182	0

**4134**	373	B	52.8954	1
**4134**	381	B	79.73	1
**4134**	382	B	79.6966	1
**4134**	386	B	78.3841	1
**4134**	305	C	65.7276	1
**4134**	319	C	73.6629	1
**4134**	320	C	75.1094	-1
**4134**	321	C	44.6963	1
**4134**	351	C	10.7722	1
**4134**	357	C	48.2269	0
**4134**	370	C	79.5572	1
**4134**	372	C	79.5707	0
**4134**	373	C	54.7993	1
**4134**	381	C	77.1491	1
**4134**	382	C	44.3661	0
**4134**	386	C	60.1092	1
**4134**	305	D	77.8026	1
**4134**	319	D	74.4778	1
**4134**	320	D	70.0303	-1
**4134**	321	D	57.7329	0
**4134**	351	D	15.4563	1
**4134**	357	D	79.5665	1
**4134**	370	D	75.3801	1
**4134**	372	D	28.2248	1
**4134**	373	D	79.042	1
**4134**	381	D	79.5081	1
**4134**	382	D	79.5097	1
**4134**	386	D	63.9528	1
**4135**	305	A	53.9305	1
**4135**	319	A	72.6286	1
**4135**	320	A	79.4833	-1
**4135**	321	A	79.531	0
**4135**	351	A	9.9197	1
**4135**	357	A	78.208	1

**4135**	370	A	79.0211	1
**4135**	372	A	32.4385	1
**4135**	373	A	32.5855	0
**4135**	381	A	71.1003	1
**4135**	382	A	43.1843	0
**4135**	386	A	79.5219	1
**4135**	305	B	79.5209	1
**4135**	319	B	78.6992	1
**4135**	320	B	73.8334	-1
**4135**	321	B	72.1962	0
**4135**	351	B	11.7372	1
**4135**	357	B	78.8925	1
**4135**	370	B	79.5724	1
**4135**	372	B	77.0808	0
**4135**	373	B	62.8085	1
**4135**	381	B	72.7435	1
**4135**	382	B	79.6962	1
**4135**	386	B	79.4975	1
**4135**	305	C	68.6994	1
**4135**	319	C	77.337	1
**4135**	320	C	73.4713	-1
**4135**	321	C	74.1661	0
**4135**	351	C	3.4109	1
**4135**	357	C	65.4607	1
**4135**	370	C	40.9148	0
**4135**	372	C	71.867	0
**4135**	373	C	73.9778	1
**4135**	381	C	60.4547	1
**4135**	382	C	68.4836	1
**4135**	386	C	39.3811	0
**4135**	305	D	61.7913	1
**4135**	319	D	79.5511	1
**4135**	320	D	79.6225	-1
**4135**	321	D	63.7177	0

**4135**	351	D	13.3485	1
**4135**	357	D	65.6507	1
**4135**	370	D	79.5544	1
**4135**	372	D	79.5248	0
**4135**	373	D	37.393	0
**4135**	381	D	79.5228	1
**4135**	382	D	73.0227	1
**4135**	386	D	58.8361	1
**4136**	305	A	66.8791	1
**4136**	319	A	67.7885	1
**4136**	320	A	56.9477	-1
**4136**	321	A	74.7489	0
**4136**	351	A	7.1614	1
**4136**	357	A	68.99	1
**4136**	370	A	79.5367	1
**4136**	372	A	79.5939	0
**4136**	373	A	68.2873	1
**4136**	381	A	73.1681	1
**4136**	382	A	61.2849	1
**4136**	386	A	53.2214	1
**4136**	305	B	79.5348	1
**4136**	319	B	36.7479	0
**4136**	320	B	72.7075	-1
**4136**	321	B	66.5734	0
**4136**	351	B	12.1483	1
**4136**	357	B	62.1879	1
**4136**	370	B	79.5739	1
**4136**	372	B	58.9498	0
**4136**	373	B	71.8231	1
**4136**	381	B	67.1751	1
**4136**	382	B	42.5873	0
**4136**	386	B	79.5771	1
**4136**	305	C	66.0314	1
**4136**	319	C	72.4373	1

**4136**	320	C	62.8158	-1
**4136**	321	C	66.4662	0
**4136**	351	C	3.6437	1
**4136**	357	C	64.4598	1
**4136**	370	C	51.1194	1
**4136**	372	C	79.5868	0
**4136**	373	C	63.9861	1
**4136**	381	C	79.5211	1
**4136**	382	C	71.2701	1
**4136**	386	C	15.8854	-1
**4136**	305	D	66.5447	1
**4136**	319	D	72.8483	1
**4136**	320	D	52.4959	-1
**4136**	321	D	69.7677	0
**4136**	351	D	19.14	1
**4136**	357	D	59.8759	1
**4136**	370	D	67.2726	1
**4136**	372	D	79.5501	0
**4136**	373	D	69.3851	1
**4136**	381	D	29.5423	0
**4136**	382	D	66.1228	1
**4136**	386	D	79.5794	1
**4137**	305	A	35.3163	0
**4137**	319	A	79.5971	1
**4137**	320	A	79.446	-1
**4137**	321	A	79.5307	0
**4137**	351	A	4.4688	1
**4137**	357	A	79.5624	1
**4137**	370	A	66.8306	1
**4137**	372	A	61.7423	0
**4137**	373	A	58.157	1
**4137**	381	A	47.4117	0
**4137**	382	A	43.4054	0
**4137**	386	A	54.1002	1

**4137**	305	B	72.3032	1
**4137**	319	B	79.3228	1
**4137**	320	B	75.2572	-1
**4137**	321	B	66.9786	0
**4137**	351	B	15.2842	1
**4137**	357	B	53.1712	1
**4137**	370	B	52.8684	1
**4137**	372	B	56.3666	0
**4137**	373	B	46.1458	0
**4137**	381	B	79.536	1
**4137**	382	B	63.3236	1
**4137**	386	B	79.6034	1
**4137**	305	C	63.1635	1
**4137**	319	C	74.6851	1
**4137**	320	C	68.7195	-1
**4137**	321	C	79.6082	0
**4137**	351	C	6.6272	1
**4137**	357	C	63.9632	1
**4137**	370	C	78.231	1
**4137**	372	C	68.2153	0
**4137**	373	C	75.412	1
**4137**	381	C	79.4876	1
**4137**	382	C	51.0965	1
**4137**	386	C	30.5448	0
**4137**	305	D	72.1706	1
**4137**	319	D	63.7341	1
**4137**	320	D	79.5301	-1
**4137**	321	D	63.0224	0
**4137**	351	D	6.1906	1
**4137**	357	D	37.4672	0
**4137**	370	D	75.7695	1
**4137**	372	D	79.4864	0
**4137**	373	D	73.2317	1
**4137**	381	D	74.2137	1

**4137**	382	D	68.1503	1
**4137**	386	D	75.6578	1
**4138**	305	A	43.7172	0
**4138**	319	A	79.4961	1
**4138**	320	A	50.609	-1
**4138**	321	A	78.9633	0
**4138**	351	A	16.5582	1
**4138**	357	A	76.683	1
**4138**	370	A	79.5519	1
**4138**	372	A	78.6104	0
**4138**	373	A	57.9301	1
**4138**	381	A	79.3943	1
**4138**	382	A	72.5573	1
**4138**	386	A	79.5788	1
**4138**	305	B	63.2048	1
**4138**	319	B	79.5995	1
**4138**	320	B	55.0467	-1
**4138**	321	B	79.5626	0
**4138**	351	B	12.5339	1
**4138**	357	B	77.0476	1
**4138**	370	B	51.9466	1
**4138**	372	B	71.3487	0
**4138**	373	B	65.6013	1
**4138**	381	B	79.5829	1
**4138**	382	B	75.7571	1
**4138**	386	B	51.6017	1
**4138**	305	C	79.5363	1
**4138**	319	C	74.5707	1
**4138**	320	C	27.6985	-1
**4138**	321	C	69.4415	0
**4138**	351	C	10.4373	1
**4138**	357	C	49.0751	0
**4138**	370	C	79.4749	1
**4138**	372	C	59.67	0

**4138**	373	C	79.5963	1
**4138**	381	C	79.5772	1
**4138**	382	C	51.2123	1
**4138**	386	C	42.1496	0
**4138**	305	D	73.5456	1
**4138**	319	D	79.6438	1
**4138**	320	D	30.8895	-1
**4138**	321	D	76.8228	0
**4138**	351	D	16.1743	1
**4138**	357	D	79.6491	1
**4138**	370	D	71.6362	1
**4138**	372	D	77.5112	0
**4138**	373	D	78.2939	1
**4138**	381	D	76.472	1
**4138**	382	D	52.3504	1
**4138**	386	D	61.7889	1
**4139**	305	A	66.9254	1
**4139**	319	A	35.3226	0
**4139**	320	A	78.8637	-1
**4139**	321	A	73.6345	0
**4139**	351	A	1.1495	1
**4139**	357	A	67.1894	1
**4139**	370	A	79.5446	1
**4139**	372	A	72.3348	0
**4139**	373	A	27.6586	0
**4139**	381	A	79.5851	1
**4139**	382	A	49.3921	0
**4139**	386	A	73.349	1
**4139**	305	B	67.5448	1
**4139**	319	B	79.5852	1
**4139**	320	B	59.6576	-1
**4139**	321	B	44.5872	1
**4139**	351	B	13.1499	1
**4139**	357	B	65.1507	1

**4139**	370	B	57.3742	1
**4139**	372	B	79.5319	0
**4139**	373	B	76.9635	1
**4139**	381	B	79.5887	1
**4139**	382	B	48.3425	0
**4139**	386	B	55.1923	1
**4139**	305	C	71.706	1
**4139**	319	C	79.5979	1
**4139**	320	C	68.8845	-1
**4139**	321	C	79.6189	0
**4139**	351	C	2.0122	1
**4139**	357	C	14.3148	-1
**4139**	370	C	68.5995	1
**4139**	372	C	79.5822	0
**4139**	373	C	46.4398	0
**4139**	381	C	79.6317	1
**4139**	382	C	73.9328	1
**4139**	386	C	48.2867	0
**4139**	305	D	71.1182	1
**4139**	319	D	79.1675	1
**4139**	320	D	63.0568	-1
**4139**	321	D	34.9852	1
**4139**	351	D	14.4204	1
**4139**	357	D	64.1915	1
**4139**	370	D	79.5955	1
**4139**	372	D	79.5426	0
**4139**	373	D	66.6129	1
**4139**	381	D	63.5884	1
**4139**	382	D	70.0341	1
**4139**	386	D	54.9966	1
**4140**	305	A	79.5483	1
**4140**	319	A	79.4834	1
**4140**	320	A	79.5038	-1
**4140**	321	A	76.8062	0

**4140**	351	A	26.712	-1
**4140**	357	A	79.5577	1
**4140**	370	A	78.0412	1
**4140**	372	A	79.4557	0
**4140**	373	A	76.5407	1
**4140**	381	A	75.7598	1
**4140**	382	A	33.2801	0
**4140**	386	A	77.5767	1
**4140**	305	B	29.1326	0
**4140**	319	B	53.7692	1
**4140**	320	B	70.2346	-1
**4140**	321	B	54.6146	0
**4140**	351	B	18.4752	1
**4140**	357	B	79.5611	1
**4140**	370	B	74.2439	1
**4140**	372	B	79.7224	0
**4140**	373	B	73.8245	1
**4140**	381	B	79.4188	1
**4140**	382	B	79.2172	1
**4140**	386	B	65.8545	1
**4140**	305	C	79.4361	1
**4140**	319	C	31.7242	0
**4140**	320	C	79.6041	-1
**4140**	321	C	60.012	0
**4140**	351	C	19.9212	1
**4140**	357	C	79.6384	1
**4140**	370	C	79.6202	1
**4140**	372	C	79.5191	0
**4140**	373	C	50.6795	1
**4140**	381	C	72.1867	1
**4140**	382	C	63.2811	1
**4140**	386	C	42.8361	0
**4140**	305	D	79.5549	1
**4140**	319	D	38.4223	0

**4140**	320	D	47.6467	-1
**4140**	321	D	49.1281	1
**4140**	351	D	5.9139	1
**4140**	357	D	79.5778	1
**4140**	370	D	59.8402	1
**4140**	372	D	79.5368	0
**4140**	373	D	79.5684	1
**4140**	381	D	72.8728	1
**4140**	382	D	74.0635	1
**4140**	386	D	25.067	0
**4141**	305	A	6.8507	-1
**4141**	319	A	13.281	-1
**4141**	320	A	27.3283	-1
**4141**	321	A	36.7984	1
**4141**	351	A	3.7766	1
**4141**	357	A	51.7267	1
**4141**	370	A	52.8402	1
**4141**	372	A	47.5427	1
**4141**	373	A	60.9309	1
**4141**	381	A	79.5827	1
**4141**	382	A	31.452	0
**4141**	386	A	44.2798	0
**4141**	305	B	62.8171	1
**4141**	319	B	79.5257	1
**4141**	320	B	79.5053	-1
**4141**	321	B	39.6549	1
**4141**	351	B	6.943	1
**4141**	357	B	79.5644	1
**4141**	370	B	64.958	1
**4141**	372	B	60.8949	0
**4141**	373	B	40.9546	0
**4141**	381	B	73.5623	1
**4141**	382	B	54.7769	1
**4141**	386	B	76.2544	1

**4141**	305	C	74.3442	1
**4141**	319	C	58.1897	1
**4141**	320	C	79.54	-1
**4141**	321	C	50.7636	0
**4141**	351	C	2.6433	1
**4141**	357	C	57.7822	1
**4141**	370	C	77.3225	1
**4141**	372	C	79.5398	0
**4141**	373	C	79.5605	1
**4141**	381	C	79.275	1
**4141**	382	C	71.2818	1
**4141**	386	C	8.155	-1
**4141**	305	D	77.4971	1
**4141**	319	D	79.4611	1
**4141**	320	D	79.5538	-1
**4141**	321	D	69.0837	0
**4141**	351	D	7.6976	1
**4141**	357	D	79.5789	1
**4141**	370	D	78.6337	1
**4141**	372	D	73.7639	0
**4141**	373	D	78.8379	1
**4141**	381	D	79.5567	1
**4141**	382	D	72.2038	1
**4141**	386	D	79.5607	1
**4142**	305	A	79.4745	1
**4142**	319	A	79.587	1
**4142**	320	A	79.7028	-1
**4142**	321	A	79.6953	0
**4142**	351	A	7.7814	1
**4142**	357	A	79.5582	1
**4142**	370	A	44.9408	0
**4142**	372	A	79.8053	0
**4142**	373	A	74.7619	1
**4142**	381	A	79.6577	1

**4142**	382	A	65.5599	1
**4142**	386	A	47.2946	0
**4142**	305	B	73.3004	1
**4142**	319	B	79.5666	1
**4142**	320	B	62.2943	-1
**4142**	321	B	77.9458	0
**4142**	351	B	11.9477	1
**4142**	357	B	46.663	0
**4142**	370	B	79.6743	1
**4142**	372	B	72.3877	0
**4142**	373	B	58.0366	1
**4142**	381	B	65.3204	1
**4142**	382	B	38.1971	0
**4142**	386	B	67.0199	1
**4142**	305	C	25.2749	0
**4142**	319	C	25.0918	0
**4142**	320	C	61.8323	-1
**4142**	321	C	79.6306	0
**4142**	351	C	18.3055	1
**4142**	357	C	73.643	1
**4142**	370	C	79.4487	1
**4142**	372	C	73.5441	0
**4142**	373	C	65.1596	1
**4142**	381	C	51.6286	1
**4142**	382	C	72.0835	1
**4142**	386	C	72.4659	1
**4142**	305	D	71.2879	1
**4142**	319	D	60.8618	1
**4142**	320	D	46.4761	-1
**4142**	321	D	48.5873	1
**4142**	351	D	15.1145	1
**4142**	357	D	68.7738	1
**4142**	370	D	40.4232	0
**4142**	372	D	57.3233	0

**4142**	373	D	54.0173	1
**4142**	381	D	25.9948	0
**4142**	382	D	55.8857	1
**4142**	386	D	79.5762	1
**4143**	305	A	79.5074	1
**4143**	319	A	61.6141	1
**4143**	320	A	48.0913	-1
**4143**	321	A	79.5398	0
**4143**	351	A	8.7018	1
**4143**	357	A	64.3358	1
**4143**	370	A	43.0573	0
**4143**	372	A	27.7349	1
**4143**	373	A	79.592	1
**4143**	381	A	64.9255	1
**4143**	382	A	69.9163	1
**4143**	386	A	32.7318	0
**4143**	305	B	79.6185	1
**4143**	319	B	73.444	1
**4143**	320	B	26.3334	-1
**4143**	321	B	79.6002	0
**4143**	351	B	11.802	1
**4143**	357	B	79.5613	1
**4143**	370	B	24.0829	0
**4143**	372	B	73.4497	0
**4143**	373	B	79.6453	1
**4143**	381	B	71.656	1
**4143**	382	B	30.598	0
**4143**	386	B	54.0923	1
**4143**	305	C	37.1016	0
**4143**	319	C	77.9991	1
**4143**	320	C	68.1142	-1
**4143**	321	C	76.9802	0
**4143**	351	C	10.8876	1
**4143**	357	C	46.2352	0

**4143**	370	C	73.7378	1
**4143**	372	C	76.0711	0
**4143**	373	C	73.0755	1
**4143**	381	C	54.0716	1
**4143**	382	C	54.1443	1
**4143**	386	C	30.9793	0
**4143**	305	D	79.4142	1
**4143**	319	D	72.2605	1
**4143**	320	D	78.7558	-1
**4143**	321	D	79.5317	0
**4143**	351	D	14.5334	1
**4143**	357	D	79.5552	1
**4143**	370	D	65.5338	1
**4143**	372	D	69.6379	0
**4143**	373	D	64.3546	1
**4143**	381	D	66.4386	1
**4143**	382	D	66.9458	1
**4143**	386	D	75.3698	1
**4144**	305	A	77.9236	1
**4144**	319	A	69.394	1
**4144**	320	A	79.4907	-1
**4144**	321	A	57.9526	0
**4144**	351	A	7.1432	1
**4144**	357	A	57.4143	1
**4144**	370	A	79.359	1
**4144**	372	A	79.5502	0
**4144**	373	A	79.55	1
**4144**	381	A	64.8432	1
**4144**	382	A	72.2779	1
**4144**	386	A	79.5163	1
**4144**	305	B	79.6036	1
**4144**	319	B	77.4004	1
**4144**	320	B	57.5777	-1
**4144**	321	B	79.0969	0

**4144**	351	B	0.0234	1
**4144**	357	B	67.7041	1
**4144**	370	B	25.4534	0
**4144**	372	B	79.5386	0
**4144**	373	B	74.4774	1
**4144**	381	B	79.4931	1
**4144**	382	B	28.5581	0
**4144**	386	B	72.969	1
**4144**	305	C	74.5298	1
**4144**	319	C	76.6174	1
**4144**	320	C	27.4738	-1
**4144**	321	C	79.6018	0
**4144**	351	C	8.4957	1
**4144**	357	C	61.1505	1
**4144**	370	C	79.4892	1
**4144**	372	C	79.5399	0
**4144**	373	C	74.1226	1
**4144**	381	C	78.8677	1
**4144**	382	C	75.2597	1
**4144**	386	C	52.5722	1
**4144**	305	D	75.1102	1
**4144**	319	D	30.2989	0
**4144**	320	D	63.0063	-1
**4144**	321	D	79.5137	0
**4144**	351	D	8.4729	1
**4144**	357	D	61.8254	1
**4144**	370	D	79.3755	1
**4144**	372	D	79.4383	0
**4144**	373	D	75.1227	1
**4144**	381	D	47.0786	0
**4144**	382	D	34.506	0
**4144**	386	D	79.5704	1
**4145**	305	A	65.9251	1
**4145**	319	A	22.1574	0

**4145**	320	A	59.0266	-1
**4145**	321	A	79.5978	0
**4145**	351	A	2.5559	1
**4145**	357	A	79.3848	1
**4145**	370	A	62.2365	1
**4145**	372	A	62.8316	0
**4145**	373	A	76.1676	1
**4145**	381	A	42.711	0
**4145**	382	A	79.7466	1
**4145**	386	A	72.5538	1
**4145**	305	B	50.2008	1
**4145**	319	B	79.5849	1
**4145**	320	B	70.7309	-1
**4145**	321	B	72.6451	0
**4145**	351	B	1.7249	1
**4145**	357	B	53.3807	1
**4145**	370	B	13.8056	-1
**4145**	372	B	63.7371	0
**4145**	373	B	57.8905	1
**4145**	381	B	53.1787	1
**4145**	382	B	44.0312	0
**4145**	386	B	71.302	1
**4145**	305	C	74.2966	1
**4145**	319	C	79.372	1
**4145**	320	C	72.5122	-1
**4145**	321	C	79.6056	0
**4145**	351	C	3.8961	1
**4145**	357	C	24.2504	0
**4145**	370	C	57.827	1
**4145**	372	C	74.6797	0
**4145**	373	C	48.8027	0
**4145**	381	C	79.7172	1
**4145**	382	C	77.7608	1
**4145**	386	C	51.3565	1

**4145**	305	D	71.7149	1
**4145**	319	D	79.425	1
**4145**	320	D	70.8227	-1
**4145**	321	D	55.7614	0
**4145**	351	D	9.6067	1
**4145**	357	D	46.8375	0
**4145**	370	D	53.6344	1
**4145**	372	D	76.0865	0
**4145**	373	D	73.8616	1
**4145**	381	D	79.6192	1
**4145**	382	D	79.635	1
**4145**	386	D	73.2587	1
**4146**	305	A	79.6022	1
**4146**	319	A	67.9574	1
**4146**	320	A	72.3478	-1
**4146**	321	A	79.5907	0
**4146**	351	A	5.2779	1
**4146**	357	A	50.2887	1
**4146**	370	A	50.6854	1
**4146**	372	A	21.654	1
**4146**	373	A	65.8003	1
**4146**	381	A	71.3657	1
**4146**	382	A	79.3527	1
**4146**	386	A	79.4746	1
**4146**	305	B	72.2051	1
**4146**	319	B	79.5065	1
**4146**	320	B	37.4276	-1
**4146**	321	B	72.361	0
**4146**	351	B	0.0001	1
**4146**	357	B	79.6237	1
**4146**	370	B	79.5136	1
**4146**	372	B	40.3496	1
**4146**	373	B	52.5116	1
**4146**	381	B	79.5107	1

**4146**	382	B	79.5187	1
**4146**	386	B	79.6873	1
**4146**	305	C	67.0054	1
**4146**	319	C	57.3428	1
**4146**	320	C	63.8495	-1
**4146**	321	C	79.6488	0
**4146**	351	C	8.804	1
**4146**	357	C	79.3985	1
**4146**	370	C	78.6578	1
**4146**	372	C	78.5345	0
**4146**	373	C	78.1366	1
**4146**	381	C	32.1577	0
**4146**	382	C	74.99	1
**4146**	386	C	48.9841	0
**4146**	305	D	72.522	1
**4146**	319	D	79.031	1
**4146**	320	D	53.9848	-1
**4146**	321	D	45.1717	1
**4146**	351	D	12.2514	1
**4146**	357	D	63.6724	1
**4146**	370	D	79.6276	1
**4146**	372	D	79.5643	0
**4146**	373	D	79.6026	1
**4146**	381	D	43.3269	0
**4146**	382	D	73.2185	1
**4146**	386	D	48.2093	0
**4147**	305	A	76.3917	1
**4147**	319	A	37.6224	0
**4147**	320	A	66.0348	-1
**4147**	321	A	67.7537	0
**4147**	351	A	2.955	1
**4147**	357	A	78.0414	1
**4147**	370	A	73.9436	1
**4147**	372	A	32.4575	1

**4147**	373	A	76.2087	1
**4147**	381	A	44.4875	0
**4147**	382	A	26.129	0
**4147**	386	A	48.4476	0
**4147**	305	B	70.4617	1
**4147**	319	B	72.2466	1
**4147**	320	B	73.106	-1
**4147**	321	B	79.6054	0
**4147**	351	B	12.4269	1
**4147**	357	B	62.5941	1
**4147**	370	B	39.7921	0
**4147**	372	B	70.2198	0
**4147**	373	B	78.5538	1
**4147**	381	B	79.5403	1
**4147**	382	B	62.3573	1
**4147**	386	B	73.2327	1
**4147**	305	C	78.4293	1
**4147**	319	C	79.495	1
**4147**	320	C	62.234	-1
**4147**	321	C	76.0909	0
**4147**	351	C	0.5195	1
**4147**	357	C	64.8703	1
**4147**	370	C	65.1856	1
**4147**	372	C	71.5218	0
**4147**	373	C	45.1294	0
**4147**	381	C	79.4262	1
**4147**	382	C	29.1631	0
**4147**	386	C	29.1219	0
**4147**	305	D	45.2664	0
**4147**	319	D	60.469	1
**4147**	320	D	50.7346	-1
**4147**	321	D	69.1818	0
**4147**	351	D	1.4001	1
**4147**	357	D	79.5887	1

**4147**	370	D	65.2777	1
**4147**	372	D	79.5439	0
**4147**	373	D	79.5694	1
**4147**	381	D	77.3215	1
**4147**	382	D	77.3649	1
**4147**	386	D	79.5191	1
**4148**	305	A	79.6057	1
**4148**	319	A	79.6304	1
**4148**	320	A	69.1875	-1
**4148**	321	A	79.6106	0
**4148**	351	A	1.8182	1
**4148**	357	A	79.6505	1
**4148**	370	A	79.6166	1
**4148**	372	A	79.4953	0
**4148**	373	A	73.2331	1
**4148**	381	A	41.8298	0
**4148**	382	A	79.5754	1
**4148**	386	A	73.1281	1
**4148**	305	B	71.9244	1
**4148**	319	B	59.8084	1
**4148**	320	B	49.1548	-1
**4148**	321	B	72.0601	0
**4148**	351	B	13.5265	1
**4148**	357	B	75.1554	1
**4148**	370	B	79.5452	1
**4148**	372	B	79.5376	0
**4148**	373	B	68.9995	1
**4148**	381	B	58.4045	1
**4148**	382	B	44.5068	0
**4148**	386	B	79.5967	1
**4148**	305	C	79.5988	1
**4148**	319	C	79.5541	1
**4148**	320	C	72.924	-1
**4148**	321	C	79.4679	0

**4148**	351	C	2.3718	1
**4148**	357	C	25.3948	0
**4148**	370	C	67.4869	1
**4148**	372	C	79.6712	0
**4148**	373	C	79.6312	1
**4148**	381	C	79.5437	1
**4148**	382	C	46.6807	0
**4148**	386	C	61.5619	1
**4148**	305	D	49.7904	0
**4148**	319	D	75.5997	1
**4148**	320	D	79.5613	-1
**4148**	321	D	65.7923	0
**4148**	351	D	18.7759	1
**4148**	357	D	69.3649	1
**4148**	370	D	70.8665	1
**4148**	372	D	79.6255	0
**4148**	373	D	73.6849	1
**4148**	381	D	79.5275	1
**4148**	382	D	73.4894	1
**4148**	386	D	79.5965	1
**4149**	305	A	79.3131	1
**4149**	319	A	64.1986	1
**4149**	320	A	79.5035	-1
**4149**	321	A	73.8397	0
**4149**	351	A	3.7449	1
**4149**	357	A	78.4077	1
**4149**	370	A	40.6592	0
**4149**	372	A	55.2625	0
**4149**	373	A	77.1555	1
**4149**	381	A	61.0757	1
**4149**	382	A	46.1145	0
**4149**	386	A	74.6593	1
**4149**	305	B	72.0498	1
**4149**	319	B	75.1961	1

**4149**	320	B	65.1912	-1
**4149**	321	B	79.5028	0
**4149**	351	B	15.417	1
**4149**	357	B	79.4994	1
**4149**	370	B	51.7635	1
**4149**	372	B	79.6443	0
**4149**	373	B	67.9275	1
**4149**	381	B	79.3297	1
**4149**	382	B	63.8886	1
**4149**	386	B	79.6247	1
**4149**	305	C	79.6851	1
**4149**	319	C	64.0692	1
**4149**	320	C	69.8558	-1
**4149**	321	C	79.6212	0
**4149**	351	C	8.0493	1
**4149**	357	C	61.8422	1
**4149**	370	C	79.4522	1
**4149**	372	C	76.5667	0
**4149**	373	C	79.4372	1
**4149**	381	C	75.6836	1
**4149**	382	C	64.3374	1
**4149**	386	C	51.7772	1
**4149**	305	D	79.8286	1
**4149**	319	D	79.4742	1
**4149**	320	D	63.8629	-1
**4149**	321	D	57.111	0
**4149**	351	D	10.4354	1
**4149**	357	D	79.7358	1
**4149**	370	D	70.8923	1
**4149**	372	D	54.0956	0
**4149**	373	D	79.2944	1
**4149**	381	D	65.1241	1
**4149**	382	D	67.5357	1
**4149**	386	D	52.7514	1

**4150**	305	A	76.4673	1
**4150**	319	A	72.9964	1
**4150**	320	A	69.1802	-1
**4150**	321	A	79.5349	0
**4150**	351	A	0.0	1
**4150**	357	A	31.0747	0
**4150**	370	A	79.5466	1
**4150**	372	A	79.5229	0
**4150**	373	A	61.1317	1
**4150**	381	A	79.5392	1
**4150**	382	A	74.5797	1
**4150**	386	A	58.0124	1
**4150**	305	B	74.7042	1
**4150**	319	B	69.9872	1
**4150**	320	B	79.4431	-1
**4150**	321	B	62.8173	0
**4150**	351	B	15.3115	1
**4150**	357	B	79.621	1
**4150**	370	B	79.5609	1
**4150**	372	B	74.9689	0
**4150**	373	B	34.1636	0
**4150**	381	B	79.554	1
**4150**	382	B	34.2986	0
**4150**	386	B	60.3501	1
**4150**	305	C	79.5453	1
**4150**	319	C	67.1686	1
**4150**	320	C	74.1824	-1
**4150**	321	C	79.5595	0
**4150**	351	C	4.2484	1
**4150**	357	C	63.3181	1
**4150**	370	C	79.5264	1
**4150**	372	C	79.4908	0
**4150**	373	C	79.6345	1
**4150**	381	C	79.609	1

**4150**	382	C	79.5984	1
**4150**	386	C	63.0959	1
**4150**	305	D	25.5744	0
**4150**	319	D	79.4805	1
**4150**	320	D	63.0757	-1
**4150**	321	D	79.505	0
**4150**	351	D	6.3734	1
**4150**	357	D	30.694	0
**4150**	370	D	75.2983	1
**4150**	372	D	79.4417	0
**4150**	373	D	73.8914	1
**4150**	381	D	59.3806	1
**4150**	382	D	57.5952	1
**4150**	386	D	79.5334	1
**4151**	305	A	66.2769	1
**4151**	319	A	28.0199	0
**4151**	320	A	67.4746	-1
**4151**	321	A	71.1995	0
**4151**	351	A	2.3528	1
**4151**	357	A	73.6996	1
**4151**	370	A	57.2625	1
**4151**	372	A	51.7598	0
**4151**	373	A	69.701	1
**4151**	381	A	45.2783	0
**4151**	382	A	71.8663	1
**4151**	386	A	38.831	0
**4151**	305	B	72.9111	1
**4151**	319	B	70.5513	1
**4151**	320	B	23.1118	-1
**4151**	321	B	79.4273	0
**4151**	351	B	13.1358	1
**4151**	357	B	73.4044	1
**4151**	370	B	69.5063	1
**4151**	372	B	65.1287	0

**4151**	373	B	64.3691	1
**4151**	381	B	79.705	1
**4151**	382	B	67.0049	1
**4151**	386	B	55.6959	1
**4151**	305	C	78.4484	1
**4151**	319	C	78.364	1
**4151**	320	C	79.3594	-1
**4151**	321	C	79.5574	0
**4151**	351	C	5.7595	1
**4151**	357	C	76.5198	1
**4151**	370	C	53.7248	1
**4151**	372	C	70.7506	0
**4151**	373	C	32.5441	0
**4151**	381	C	79.546	1
**4151**	382	C	67.7162	1
**4151**	386	C	12.337	-1
**4151**	305	D	54.2493	1
**4151**	319	D	79.7301	1
**4151**	320	D	38.4882	-1
**4151**	321	D	74.9989	0
**4151**	351	D	9.3905	1
**4151**	357	D	47.8295	0
**4151**	370	D	66.6819	1
**4151**	372	D	79.7663	0
**4151**	373	D	36.3667	0
**4151**	381	D	60.9491	1
**4151**	382	D	72.6435	1
**4151**	386	D	79.4419	1
**4152**	305	A	65.9396	1
**4152**	319	A	46.433	0
**4152**	320	A	79.5122	-1
**4152**	321	A	38.6152	1
**4152**	351	A	8.2876	1
**4152**	357	A	63.6557	1

**4152**	370	A	56.7226	1
**4152**	372	A	77.2743	0
**4152**	373	A	79.6007	1
**4152**	381	A	70.9858	1
**4152**	382	A	33.2935	0
**4152**	386	A	67.8069	1
**4152**	305	B	72.7456	1
**4152**	319	B	31.9296	0
**4152**	320	B	79.5694	-1
**4152**	321	B	72.7095	0
**4152**	351	B	9.0858	1
**4152**	357	B	55.1768	1
**4152**	370	B	63.644	1
**4152**	372	B	77.6559	0
**4152**	373	B	74.9583	1
**4152**	381	B	79.5103	1
**4152**	382	B	77.3326	1
**4152**	386	B	53.4152	1
**4152**	305	C	79.532	1
**4152**	319	C	73.3768	1
**4152**	320	C	33.5407	-1
**4152**	321	C	50.3886	0
**4152**	351	C	4.7127	1
**4152**	357	C	74.2578	1
**4152**	370	C	51.7318	1
**4152**	372	C	68.2755	0
**4152**	373	C	79.5949	1
**4152**	381	C	75.1382	1
**4152**	382	C	50.7445	1
**4152**	386	C	42.1261	0
**4152**	305	D	61.6073	1
**4152**	319	D	78.5785	1
**4152**	320	D	79.5397	-1
**4152**	321	D	75.0459	0

**4152**	351	D	7.8581	1
**4152**	357	D	74.3038	1
**4152**	370	D	79.6325	1
**4152**	372	D	79.5117	0
**4152**	373	D	70.0541	1
**4152**	381	D	74.864	1
**4152**	382	D	79.5774	1
**4152**	386	D	77.6242	1
**4153**	305	A	69.8731	1
**4153**	319	A	61.0681	1
**4153**	320	A	72.7793	-1
**4153**	321	A	79.5162	0
**4153**	351	A	11.2483	1
**4153**	357	A	71.99	1
**4153**	370	A	79.4431	1
**4153**	372	A	43.1219	1
**4153**	373	A	50.739	1
**4153**	381	A	47.0848	0
**4153**	382	A	76.5567	1
**4153**	386	A	79.6496	1
**4153**	305	B	58.904	1
**4153**	319	B	49.1097	0
**4153**	320	B	79.4251	-1
**4153**	321	B	79.5098	0
**4153**	351	B	13.9104	1
**4153**	357	B	71.2854	1
**4153**	370	B	68.3559	1
**4153**	372	B	54.4489	0
**4153**	373	B	26.0304	0
**4153**	381	B	71.6576	1
**4153**	382	B	64.8292	1
**4153**	386	B	56.6834	1
**4153**	305	C	79.6955	1
**4153**	319	C	74.4205	1

**4153**	320	C	38.8462	-1
**4153**	321	C	56.3404	0
**4153**	351	C	0.3494	1
**4153**	357	C	66.0281	1
**4153**	370	C	55.2711	1
**4153**	372	C	79.5609	0
**4153**	373	C	71.2111	1
**4153**	381	C	61.2931	1
**4153**	382	C	61.292	1
**4153**	386	C	11.779	-1
**4153**	305	D	77.2784	1
**4153**	319	D	30.6497	0
**4153**	320	D	79.5695	-1
**4153**	321	D	79.5214	0
**4153**	351	D	12.8775	1
**4153**	357	D	70.5284	1
**4153**	370	D	57.7333	1
**4153**	372	D	44.8118	1
**4153**	373	D	79.6915	1
**4153**	381	D	79.5995	1
**4153**	382	D	53.7397	1
**4153**	386	D	28.9252	0
**4154**	305	A	73.03	1
**4154**	319	A	79.5011	1
**4154**	320	A	63.5784	-1
**4154**	321	A	64.9994	0
**4154**	351	A	4.3298	1
**4154**	357	A	56.2078	1
**4154**	370	A	72.1056	1
**4154**	372	A	67.5384	0
**4154**	373	A	79.5799	1
**4154**	381	A	74.4226	1
**4154**	382	A	79.5465	1
**4154**	386	A	71.7642	1

**4154**	305	B	50.9359	1
**4154**	319	B	78.238	1
**4154**	320	B	71.1343	-1
**4154**	321	B	79.4765	0
**4154**	351	B	14.644	1
**4154**	357	B	49.3541	0
**4154**	370	B	43.8966	0
**4154**	372	B	79.631	0
**4154**	373	B	66.9423	1
**4154**	381	B	58.9375	1
**4154**	382	B	24.8054	0
**4154**	386	B	74.0233	1
**4154**	305	C	75.1919	1
**4154**	319	C	73.2897	1
**4154**	320	C	61.4341	-1
**4154**	321	C	79.575	0
**4154**	351	C	34.3515	-1
**4154**	357	C	25.3292	0
**4154**	370	C	79.4296	1
**4154**	372	C	79.4541	0
**4154**	373	C	79.6717	1
**4154**	381	C	42.068	0
**4154**	382	C	79.5438	1
**4154**	386	C	59.7866	1
**4154**	305	D	79.5765	1
**4154**	319	D	74.2818	1
**4154**	320	D	79.5841	-1
**4154**	321	D	69.4225	0
**4154**	351	D	10.8533	1
**4154**	357	D	65.952	1
**4154**	370	D	71.4959	1
**4154**	372	D	79.6209	0
**4154**	373	D	68.7066	1
**4154**	381	D	79.4939	1

**4154**	382	D	79.4398	1
**4154**	386	D	79.5882	1
**4155**	305	A	79.1878	1
**4155**	319	A	47.7531	0
**4155**	320	A	73.601	-1
**4155**	321	A	79.5346	0
**4155**	351	A	0.0	1
**4155**	357	A	79.6434	1
**4155**	370	A	57.3111	1
**4155**	372	A	79.6089	0
**4155**	373	A	38.9247	0
**4155**	381	A	79.536	1
**4155**	382	A	74.342	1
**4155**	386	A	78.7029	1
**4155**	305	B	32.2663	0
**4155**	319	B	44.2739	0
**4155**	320	B	75.3237	-1
**4155**	321	B	70.2714	0
**4155**	351	B	14.1404	1
**4155**	357	B	79.5607	1
**4155**	370	B	79.5372	1
**4155**	372	B	72.0006	0
**4155**	373	B	72.2687	1
**4155**	381	B	79.6046	1
**4155**	382	B	76.8945	1
**4155**	386	B	79.5532	1
**4155**	305	C	74.8669	1
**4155**	319	C	47.8869	0
**4155**	320	C	75.2144	-1
**4155**	321	C	43.014	1
**4155**	351	C	38.3168	-1
**4155**	357	C	60.9303	1
**4155**	370	C	76.6736	1
**4155**	372	C	73.4055	0

**4155**	373	C	74.3892	1
**4155**	381	C	79.565	1
**4155**	382	C	62.9566	1
**4155**	386	C	49.3678	0
**4155**	305	D	41.6919	0
**4155**	319	D	79.5755	1
**4155**	320	D	79.5995	-1
**4155**	321	D	74.4329	0
**4155**	351	D	16.9097	1
**4155**	357	D	55.3978	1
**4155**	370	D	79.5446	1
**4155**	372	D	67.5506	0
**4155**	373	D	73.6361	1
**4155**	381	D	79.5911	1
**4155**	382	D	70.0456	1
**4155**	386	D	79.6131	1
**4156**	305	A	79.5597	1
**4156**	319	A	27.2681	0
**4156**	320	A	74.7505	-1
**4156**	321	A	67.8344	0
**4156**	351	A	5.7587	1
**4156**	357	A	35.35	0
**4156**	370	A	67.1095	1
**4156**	372	A	64.7986	0
**4156**	373	A	13.6942	-1
**4156**	381	A	62.791	1
**4156**	382	A	63.3991	1
**4156**	386	A	79.4627	1
**4156**	305	B	65.1319	1
**4156**	319	B	70.9952	1
**4156**	320	B	37.5544	-1
**4156**	321	B	79.5828	0
**4156**	351	B	6.1706	1
**4156**	357	B	79.5397	1

**4156**	370	B	79.6151	1
**4156**	372	B	62.8735	0
**4156**	373	B	54.3201	1
**4156**	381	B	37.0289	0
**4156**	382	B	74.5678	1
**4156**	386	B	79.4991	1
**4156**	305	C	37.1377	0
**4156**	319	C	78.5698	1
**4156**	320	C	56.1633	-1
**4156**	321	C	62.2046	0
**4156**	351	C	19.1686	1
**4156**	357	C	57.2927	1
**4156**	370	C	73.7505	1
**4156**	372	C	79.5261	0
**4156**	373	C	55.9757	1
**4156**	381	C	79.6554	1
**4156**	382	C	58.8727	1
**4156**	386	C	63.5902	1
**4156**	305	D	64.5315	1
**4156**	319	D	62.9488	1
**4156**	320	D	73.2341	-1
**4156**	321	D	55.6225	0
**4156**	351	D	16.4359	1
**4156**	357	D	48.4273	0
**4156**	370	D	73.6516	1
**4156**	372	D	77.9824	0
**4156**	373	D	75.2007	1
**4156**	381	D	79.4974	1
**4156**	382	D	77.9697	1
**4156**	386	D	69.2717	1
**4157**	305	A	79.4799	1
**4157**	319	A	57.0242	1
**4157**	320	A	45.4132	-1
**4157**	321	A	79.3782	0

**4157**	351	A	10.38	1
**4157**	357	A	79.4996	1
**4157**	370	A	77.8122	1
**4157**	372	A	72.833	0
**4157**	373	A	72.0553	1
**4157**	381	A	75.4034	1
**4157**	382	A	73.3446	1
**4157**	386	A	59.702	1
**4157**	305	B	34.3161	0
**4157**	319	B	79.6429	1
**4157**	320	B	38.5374	-1
**4157**	321	B	74.2345	0
**4157**	351	B	14.4759	1
**4157**	357	B	79.5928	1
**4157**	370	B	79.4901	1
**4157**	372	B	64.9744	0
**4157**	373	B	61.3124	1
**4157**	381	B	73.0225	1
**4157**	382	B	31.079	0
**4157**	386	B	79.5294	1
**4157**	305	C	69.775	1
**4157**	319	C	59.4622	1
**4157**	320	C	57.6357	-1
**4157**	321	C	72.5329	0
**4157**	351	C	6.8618	1
**4157**	357	C	69.8662	1
**4157**	370	C	79.509	1
**4157**	372	C	73.3614	0
**4157**	373	C	79.5106	1
**4157**	381	C	73.6004	1
**4157**	382	C	71.3992	1
**4157**	386	C	40.1578	0
**4157**	305	D	30.1959	0
**4157**	319	D	79.5489	1

**4157**	320	D	79.6194	-1
**4157**	321	D	60.189	0
**4157**	351	D	11.101	1
**4157**	357	D	53.9622	1
**4157**	370	D	68.7547	1
**4157**	372	D	79.6374	0
**4157**	373	D	60.4835	1
**4157**	381	D	46.7523	0
**4157**	382	D	65.0796	1
**4157**	386	D	65.38	1
**4158**	305	A	67.4855	1
**4158**	319	A	75.3419	1
**4158**	320	A	79.6075	-1
**4158**	321	A	79.515	0
**4158**	351	A	2.7624	1
**4158**	357	A	79.5146	1
**4158**	370	A	79.5188	1
**4158**	372	A	72.8603	0
**4158**	373	A	33.3415	0
**4158**	381	A	74.4692	1
**4158**	382	A	76.1525	1
**4158**	386	A	79.5212	1
**4158**	305	B	79.5659	1
**4158**	319	B	79.6759	1
**4158**	320	B	69.565	-1
**4158**	321	B	61.0062	0
**4158**	351	B	16.638	1
**4158**	357	B	79.5888	1
**4158**	370	B	73.9957	1
**4158**	372	B	52.8907	0
**4158**	373	B	77.1842	1
**4158**	381	B	66.0045	1
**4158**	382	B	79.5278	1
**4158**	386	B	79.5347	1

**4158**	305	C	56.4042	1
**4158**	319	C	79.6479	1
**4158**	320	C	79.6382	-1
**4158**	321	C	31.0449	1
**4158**	351	C	10.0288	1
**4158**	357	C	74.2245	1
**4158**	370	C	68.3934	1
**4158**	372	C	53.0714	0
**4158**	373	C	72.9321	1
**4158**	381	C	70.6724	1
**4158**	382	C	77.6485	1
**4158**	386	C	58.1309	1
**4158**	305	D	77.5776	1
**4158**	319	D	55.353	1
**4158**	320	D	70.8861	-1
**4158**	321	D	79.5538	0
**4158**	351	D	16.2732	1
**4158**	357	D	71.0342	1
**4158**	370	D	79.495	1
**4158**	372	D	79.5347	0
**4158**	373	D	60.9377	1
**4158**	381	D	71.5962	1
**4158**	382	D	73.2923	1
**4158**	386	D	73.788	1
**4159**	305	A	75.7419	1
**4159**	319	A	48.4598	0
**4159**	320	A	59.9774	-1
**4159**	321	A	40.0042	1
**4159**	351	A	9.4529	1
**4159**	357	A	20.0885	0
**4159**	370	A	79.6335	1
**4159**	372	A	71.9307	0
**4159**	373	A	20.1345	0
**4159**	381	A	79.5077	1

**4159**	382	A	32.6373	0
**4159**	386	A	79.562	1
**4159**	305	B	58.4128	1
**4159**	319	B	65.3699	1
**4159**	320	B	79.5124	-1
**4159**	321	B	63.804	0
**4159**	351	B	21.9126	-1
**4159**	357	B	79.5287	1
**4159**	370	B	73.3228	1
**4159**	372	B	66.3864	0
**4159**	373	B	74.9888	1
**4159**	381	B	72.6774	1
**4159**	382	B	23.7648	0
**4159**	386	B	49.1825	0
**4159**	305	C	73.2067	1
**4159**	319	C	57.0899	1
**4159**	320	C	35.5725	-1
**4159**	321	C	78.9086	0
**4159**	351	C	8.7028	1
**4159**	357	C	65.6377	1
**4159**	370	C	65.8421	1
**4159**	372	C	73.1896	0
**4159**	373	C	47.0606	0
**4159**	381	C	45.1013	0
**4159**	382	C	59.4665	1
**4159**	386	C	36.1238	0
**4159**	305	D	32.972	0
**4159**	319	D	73.0421	1
**4159**	320	D	41.4969	-1
**4159**	321	D	64.8286	0
**4159**	351	D	12.1698	1
**4159**	357	D	39.3844	0
**4159**	370	D	79.6471	1
**4159**	372	D	79.5732	0

**4159**	373	D	54.9618	1
**4159**	381	D	58.3854	1
**4159**	382	D	79.5879	1
**4159**	386	D	52.6259	1
**4160**	305	A	79.6623	1
**4160**	319	A	61.0397	1
**4160**	320	A	49.6124	-1
**4160**	321	A	56.3256	0
**4160**	351	A	1.0665	1
**4160**	357	A	79.6147	1
**4160**	370	A	67.2529	1
**4160**	372	A	67.2906	0
**4160**	373	A	62.3008	1
**4160**	381	A	79.4496	1
**4160**	382	A	75.7144	1
**4160**	386	A	79.4893	1
**4160**	305	B	72.0658	1
**4160**	319	B	79.5475	1
**4160**	320	B	79.4909	-1
**4160**	321	B	72.7366	0
**4160**	351	B	15.4325	1
**4160**	357	B	64.3382	1
**4160**	370	B	77.8911	1
**4160**	372	B	72.8985	0
**4160**	373	B	60.8272	1
**4160**	381	B	52.5544	1
**4160**	382	B	71.911	1
**4160**	386	B	42.7758	0
**4160**	305	C	72.215	1
**4160**	319	C	79.6162	1
**4160**	320	C	21.137	-1
**4160**	321	C	78.2068	0
**4160**	351	C	15.8127	1
**4160**	357	C	79.6158	1

**4160**	370	C	78.4345	1
**4160**	372	C	69.4317	0
**4160**	373	C	43.0843	0
**4160**	381	C	36.4808	0
**4160**	382	C	56.0395	1
**4160**	386	C	57.2695	1
**4160**	305	D	79.5181	1
**4160**	319	D	79.5145	1
**4160**	320	D	78.3684	-1
**4160**	321	D	79.6928	0
**4160**	351	D	18.5884	1
**4160**	357	D	78.0359	1
**4160**	370	D	50.712	1
**4160**	372	D	65.6602	0
**4160**	373	D	48.0091	0
**4160**	381	D	31.6152	0
**4160**	382	D	59.6103	1
**4160**	386	D	71.1565	1
**4161**	305	A	79.6015	1
**4161**	319	A	79.5002	1
**4161**	320	A	79.556	-1
**4161**	321	A	76.3949	0
**4161**	351	A	9.8835	1
**4161**	357	A	71.6736	1
**4161**	370	A	79.6141	1
**4161**	372	A	68.6442	0
**4161**	373	A	34.4931	0
**4161**	381	A	79.597	1
**4161**	382	A	72.1428	1
**4161**	386	A	74.2122	1
**4161**	305	B	65.1679	1
**4161**	319	B	53.6106	1
**4161**	320	B	62.1381	-1
**4161**	321	B	73.8164	0

**4161**	351	B	22.035	-1
**4161**	357	B	63.8613	1
**4161**	370	B	49.4361	0
**4161**	372	B	72.6438	0
**4161**	373	B	69.6064	1
**4161**	381	B	74.6062	1
**4161**	382	B	27.4972	0
**4161**	386	B	79.4521	1
**4161**	305	C	47.9114	0
**4161**	319	C	65.44	1
**4161**	320	C	30.9805	-1
**4161**	321	C	71.5132	0
**4161**	351	C	0.1641	1
**4161**	357	C	74.587	1
**4161**	370	C	65.5231	1
**4161**	372	C	70.9578	0
**4161**	373	C	65.903	1
**4161**	381	C	79.6011	1
**4161**	382	C	58.5736	1
**4161**	386	C	55.1382	1
**4161**	305	D	44.5573	0
**4161**	319	D	57.4193	1
**4161**	320	D	27.2747	-1
**4161**	321	D	79.5144	0
**4161**	351	D	0.7513	1
**4161**	357	D	64.7705	1
**4161**	370	D	56.262	1
**4161**	372	D	74.2823	0
**4161**	373	D	79.3797	1
**4161**	381	D	67.0977	1
**4161**	382	D	72.5746	1
**4161**	386	D	43.7531	0
**4162**	305	A	45.5354	0
**4162**	319	A	45.3715	0

**4162**	320	A	51.3235	-1
**4162**	321	A	69.0432	0
**4162**	351	A	13.1564	1
**4162**	357	A	79.6104	1
**4162**	370	A	79.5645	1
**4162**	372	A	36.5213	1
**4162**	373	A	74.73	1
**4162**	381	A	76.3734	1
**4162**	382	A	73.9699	1
**4162**	386	A	77.1505	1
**4162**	305	B	73.7838	1
**4162**	319	B	49.9393	0
**4162**	320	B	72.6699	-1
**4162**	321	B	72.2477	0
**4162**	351	B	8.8583	1
**4162**	357	B	79.5316	1
**4162**	370	B	71.7779	1
**4162**	372	B	65.66	0
**4162**	373	B	64.5277	1
**4162**	381	B	79.552	1
**4162**	382	B	48.9557	0
**4162**	386	B	79.5843	1
**4162**	305	C	68.5248	1
**4162**	319	C	55.0927	1
**4162**	320	C	54.4428	-1
**4162**	321	C	79.6056	0
**4162**	351	C	15.4281	1
**4162**	357	C	29.7429	0
**4162**	370	C	70.7835	1
**4162**	372	C	79.5644	0
**4162**	373	C	79.5775	1
**4162**	381	C	79.5665	1
**4162**	382	C	79.5694	1
**4162**	386	C	12.1584	-1

**4162**	305	D	65.174	1
**4162**	319	D	79.1432	1
**4162**	320	D	71.3661	-1
**4162**	321	D	31.2616	1
**4162**	351	D	21.4238	-1
**4162**	357	D	79.6101	1
**4162**	370	D	78.5417	1
**4162**	372	D	79.5637	0
**4162**	373	D	29.4415	0
**4162**	381	D	79.5629	1
**4162**	382	D	71.7263	1
**4162**	386	D	79.5657	1
**4163**	305	A	79.6468	1
**4163**	319	A	72.8459	1
**4163**	320	A	79.5366	-1
**4163**	321	A	79.5195	0
**4163**	351	A	2.2419	1
**4163**	357	A	79.5381	1
**4163**	370	A	79.5953	1
**4163**	372	A	59.6077	0
**4163**	373	A	71.8565	1
**4163**	381	A	79.6912	1
**4163**	382	A	10.9298	-1
**4163**	386	A	33.5107	0
**4163**	305	B	79.5625	1
**4163**	319	B	69.2632	1
**4163**	320	B	72.2675	-1
**4163**	321	B	64.0449	0
**4163**	351	B	18.2424	1
**4163**	357	B	36.3473	0
**4163**	370	B	71.7058	1
**4163**	372	B	79.6101	0
**4163**	373	B	73.6035	1
**4163**	381	B	67.9683	1

**4163**	382	B	44.488	0
**4163**	386	B	39.4895	0
**4163**	305	C	70.6173	1
**4163**	319	C	60.9604	1
**4163**	320	C	71.9609	-1
**4163**	321	C	79.6791	0
**4163**	351	C	8.2957	1
**4163**	357	C	63.6118	1
**4163**	370	C	71.2454	1
**4163**	372	C	72.2756	0
**4163**	373	C	79.5908	1
**4163**	381	C	77.7677	1
**4163**	382	C	79.4904	1
**4163**	386	C	53.9608	1
**4163**	305	D	30.0184	0
**4163**	319	D	57.6417	1
**4163**	320	D	61.0841	-1
**4163**	321	D	40.8192	1
**4163**	351	D	9.6352	1
**4163**	357	D	79.1589	1
**4163**	370	D	50.3167	1
**4163**	372	D	51.0898	0
**4163**	373	D	49.1945	0
**4163**	381	D	73.9969	1
**4163**	382	D	71.3686	1
**4163**	386	D	48.8819	0
**4164**	305	A	70.8742	1
**4164**	319	A	24.6647	0
**4164**	320	A	79.5748	-1
**4164**	321	A	79.5387	0
**4164**	351	A	5.3333	1
**4164**	357	A	79.5399	1
**4164**	370	A	78.4569	1
**4164**	372	A	68.5918	0

**4164**	373	A	71.2182	1
**4164**	381	A	62.2352	1
**4164**	382	A	79.5792	1
**4164**	386	A	78.9874	1
**4164**	305	B	79.5772	1
**4164**	319	B	68.9141	1
**4164**	320	B	73.155	-1
**4164**	321	B	68.2715	0
**4164**	351	B	0.1017	1
**4164**	357	B	61.2477	1
**4164**	370	B	70.4889	1
**4164**	372	B	79.5525	0
**4164**	373	B	65.1401	1
**4164**	381	B	79.5755	1
**4164**	382	B	25.2196	0
**4164**	386	B	46.6895	0
**4164**	305	C	75.5782	1
**4164**	319	C	65.4298	1
**4164**	320	C	77.9994	-1
**4164**	321	C	49.6937	1
**4164**	351	C	2.1063	1
**4164**	357	C	78.6525	1
**4164**	370	C	78.8569	1
**4164**	372	C	79.5646	0
**4164**	373	C	58.1918	1
**4164**	381	C	76.8644	1
**4164**	382	C	52.8196	1
**4164**	386	C	59.6725	1
**4164**	305	D	48.7619	0
**4164**	319	D	71.7947	1
**4164**	320	D	27.5887	-1
**4164**	321	D	79.513	0
**4164**	351	D	10.2699	1
**4164**	357	D	58.5845	1

**4164**	370	D	58.7565	1
**4164**	372	D	79.5647	0
**4164**	373	D	56.2897	1
**4164**	381	D	79.6024	1
**4164**	382	D	71.0536	1
**4164**	386	D	58.6606	1
**4165**	305	A	79.4579	1
**4165**	319	A	79.5873	1
**4165**	320	A	79.489	-1
**4165**	321	A	72.3712	0
**4165**	351	A	18.8099	1
**4165**	357	A	70.275	1
**4165**	370	A	79.6381	1
**4165**	372	A	79.5264	0
**4165**	373	A	76.4794	1
**4165**	381	A	79.5262	1
**4165**	382	A	79.6209	1
**4165**	386	A	64.6053	1
**4165**	305	B	71.6771	1
**4165**	319	B	79.5239	1
**4165**	320	B	79.5693	-1
**4165**	321	B	79.6487	0
**4165**	351	B	19.5174	1
**4165**	357	B	72.7252	1
**4165**	370	B	75.1125	1
**4165**	372	B	73.5906	0
**4165**	373	B	36.3608	0
**4165**	381	B	41.2989	0
**4165**	382	B	67.3908	1
**4165**	386	B	48.1222	0
**4165**	305	C	73.8738	1
**4165**	319	C	62.8483	1
**4165**	320	C	63.49	-1
**4165**	321	C	79.1938	0

**4165**	351	C	6.1571	1
**4165**	357	C	72.6083	1
**4165**	370	C	70.4186	1
**4165**	372	C	79.6169	0
**4165**	373	C	65.2789	1
**4165**	381	C	51.166	1
**4165**	382	C	54.456	1
**4165**	386	C	40.5236	0
**4165**	305	D	75.5102	1
**4165**	319	D	75.7466	1
**4165**	320	D	39.9334	-1
**4165**	321	D	50.5317	0
**4165**	351	D	13.6916	1
**4165**	357	D	32.6046	0
**4165**	370	D	70.4602	1
**4165**	372	D	76.0319	0
**4165**	373	D	62.8652	1
**4165**	381	D	67.8089	1
**4165**	382	D	42.3763	0
**4165**	386	D	49.0561	0
**4166**	305	A	59.4259	1
**4166**	319	A	29.5285	0
**4166**	320	A	59.3139	-1
**4166**	321	A	54.8999	0
**4166**	351	A	23.657	-1
**4166**	357	A	45.9269	0
**4166**	370	A	65.1949	1
**4166**	372	A	79.4768	0
**4166**	373	A	49.0414	0
**4166**	381	A	79.3553	1
**4166**	382	A	79.5795	1
**4166**	386	A	74.7719	1
**4166**	305	B	79.5755	1
**4166**	319	B	79.5929	1

**4166**	320	B	66.2091	-1
**4166**	321	B	79.5603	0
**4166**	351	B	19.1789	1
**4166**	357	B	74.2178	1
**4166**	370	B	62.6119	1
**4166**	372	B	79.573	0
**4166**	373	B	75.1837	1
**4166**	381	B	79.6786	1
**4166**	382	B	24.7123	0
**4166**	386	B	71.4566	1
**4166**	305	C	62.6148	1
**4166**	319	C	79.4546	1
**4166**	320	C	63.6608	-1
**4166**	321	C	55.4774	0
**4166**	351	C	14.635	1
**4166**	357	C	43.1905	0
**4166**	370	C	65.6648	1
**4166**	372	C	73.3198	0
**4166**	373	C	79.4988	1
**4166**	381	C	64.0233	1
**4166**	382	C	60.7837	1
**4166**	386	C	58.7893	1
**4166**	305	D	25.4336	0
**4166**	319	D	68.5639	1
**4166**	320	D	71.7609	-1
**4166**	321	D	66.5245	0
**4166**	351	D	6.446	1
**4166**	357	D	59.0858	1
**4166**	370	D	61.0403	1
**4166**	372	D	77.1536	0
**4166**	373	D	44.2389	0
**4166**	381	D	73.3701	1
**4166**	382	D	63.5184	1
**4166**	386	D	30.2086	0

**4167**	305	A	67.8787	1
**4167**	319	A	79.6914	1
**4167**	320	A	79.6794	-1
**4167**	321	A	72.5133	0
**4167**	351	A	5.0285	1
**4167**	357	A	77.5472	1
**4167**	370	A	79.5692	1
**4167**	372	A	48.797	1
**4167**	373	A	60.6348	1
**4167**	381	A	77.8469	1
**4167**	382	A	60.6218	1
**4167**	386	A	72.5807	1
**4167**	305	B	79.5826	1
**4167**	319	B	38.1467	0
**4167**	320	B	79.516	-1
**4167**	321	B	71.7867	0
**4167**	351	B	15.3941	1
**4167**	357	B	74.4973	1
**4167**	370	B	76.7875	1
**4167**	372	B	67.5662	0
**4167**	373	B	53.5352	1
**4167**	381	B	79.4982	1
**4167**	382	B	44.9044	0
**4167**	386	B	79.552	1
**4167**	305	C	79.5297	1
**4167**	319	C	77.9213	1
**4167**	320	C	73.7614	-1
**4167**	321	C	79.6677	0
**4167**	351	C	0.7147	1
**4167**	357	C	67.9369	1
**4167**	370	C	59.1063	1
**4167**	372	C	54.7024	0
**4167**	373	C	50.8239	1
**4167**	381	C	67.6455	1

**4167**	382	C	73.6649	1
**4167**	386	C	42.3804	0
**4167**	305	D	79.517	1
**4167**	319	D	59.9073	1
**4167**	320	D	74.4999	-1
**4167**	321	D	59.6262	0
**4167**	351	D	8.5647	1
**4167**	357	D	79.5467	1
**4167**	370	D	79.5478	1
**4167**	372	D	79.5507	0
**4167**	373	D	78.7879	1
**4167**	381	D	79.4986	1
**4167**	382	D	59.8592	1
**4167**	386	D	47.0325	0
**4168**	305	A	73.971	1
**4168**	319	A	76.1827	1
**4168**	320	A	69.8562	-1
**4168**	321	A	77.3812	0
**4168**	351	A	14.3283	1
**4168**	357	A	70.9996	1
**4168**	370	A	78.9815	1
**4168**	372	A	58.7161	0
**4168**	373	A	31.4184	0
**4168**	381	A	54.9546	1
**4168**	382	A	20.999	0
**4168**	386	A	69.4399	1
**4168**	305	B	69.6087	1
**4168**	319	B	66.991	1
**4168**	320	B	49.723	-1
**4168**	321	B	70.3047	0
**4168**	351	B	14.6307	1
**4168**	357	B	71.4378	1
**4168**	370	B	78.2549	1
**4168**	372	B	72.8728	0

**4168**	373	B	52.6965	1
**4168**	381	B	79.5666	1
**4168**	382	B	74.866	1
**4168**	386	B	71.0286	1
**4168**	305	C	74.6139	1
**4168**	319	C	72.7029	1
**4168**	320	C	79.5646	-1
**4168**	321	C	79.5681	0
**4168**	351	C	16.0184	1
**4168**	357	C	63.8348	1
**4168**	370	C	79.5671	1
**4168**	372	C	79.5633	0
**4168**	373	C	79.5684	1
**4168**	381	C	44.8102	0
**4168**	382	C	43.312	0
**4168**	386	C	25.1406	0
**4168**	305	D	28.6998	0
**4168**	319	D	79.5405	1
**4168**	320	D	66.9599	-1
**4168**	321	D	79.5338	0
**4168**	351	D	14.036	1
**4168**	357	D	33.895	0
**4168**	370	D	56.577	1
**4168**	372	D	79.5637	0
**4168**	373	D	67.539	1
**4168**	381	D	37.3673	0
**4168**	382	D	79.6061	1
**4168**	386	D	24.812	0
**4169**	305	A	79.4819	1
**4169**	319	A	56.4418	1
**4169**	320	A	79.4823	-1
**4169**	321	A	75.695	0
**4169**	351	A	15.07	1
**4169**	357	A	79.5235	1

**4169**	370	A	79.1883	1
**4169**	372	A	79.5252	0
**4169**	373	A	79.6058	1
**4169**	381	A	79.597	1
**4169**	382	A	60.0468	1
**4169**	386	A	69.0425	1
**4169**	305	B	72.8466	1
**4169**	319	B	79.6224	1
**4169**	320	B	77.0489	-1
**4169**	321	B	63.6583	0
**4169**	351	B	21.9716	-1
**4169**	357	B	79.649	1
**4169**	370	B	53.8299	1
**4169**	372	B	79.5833	0
**4169**	373	B	22.4607	0
**4169**	381	B	75.3608	1
**4169**	382	B	46.2004	0
**4169**	386	B	52.7978	1
**4169**	305	C	79.5606	1
**4169**	319	C	22.493	0
**4169**	320	C	62.0885	-1
**4169**	321	C	70.7377	0
**4169**	351	C	13.305	1
**4169**	357	C	51.6213	1
**4169**	370	C	79.4314	1
**4169**	372	C	79.5992	0
**4169**	373	C	79.6067	1
**4169**	381	C	62.4102	1
**4169**	382	C	74.4873	1
**4169**	386	C	74.0552	1
**4169**	305	D	75.9917	1
**4169**	319	D	58.7212	1
**4169**	320	D	43.3186	-1
**4169**	321	D	79.595	0

**4169**	351	D	8.9222	1
**4169**	357	D	72.6099	1
**4169**	370	D	18.5236	-1
**4169**	372	D	68.5371	0
**4169**	373	D	71.7075	1
**4169**	381	D	32.3211	0
**4169**	382	D	79.5702	1
**4169**	386	D	17.0875	-1
**4170**	305	A	78.3721	1
**4170**	319	A	61.0955	1
**4170**	320	A	73.8768	-1
**4170**	321	A	70.0251	0
**4170**	351	A	173	1
**4170**	357	A	79.534	1
**4170**	370	A	79.572	1
**4170**	372	A	79.5732	0
**4170**	373	A	45.1721	0
**4170**	381	A	42.3078	0
**4170**	382	A	78.0221	1
**4170**	386	A	72.8834	1
**4170**	305	B	62.2896	1
**4170**	319	B	40.0386	0
**4170**	320	B	50.6539	-1
**4170**	321	B	53.6555	0
**4170**	351	B	13.9676	1
**4170**	357	B	30.1534	0
**4170**	370	B	73.1197	1
**4170**	372	B	56.9028	0
**4170**	373	B	69.6692	1
**4170**	381	B	79.5475	1
**4170**	382	B	56.3918	1
**4170**	386	B	66.6496	1
**4170**	305	C	79.1725	1
**4170**	319	C	74.9096	1

**4170**	320	C	59.2322	-1
**4170**	321	C	37.941	1
**4170**	351	C	4.0109	1
**4170**	357	C	62.0755	1
**4170**	370	C	68.8566	1
**4170**	372	C	79.5686	0
**4170**	373	C	14.5744	-1
**4170**	381	C	34.5327	0
**4170**	382	C	40.5978	0
**4170**	386	C	34.8836	0
**4170**	305	D	69.7111	1
**4170**	319	D	65.4119	1
**4170**	320	D	79.5506	-1
**4170**	321	D	66.5908	0
**4170**	351	D	13.166	1
**4170**	357	D	79.6002	1
**4170**	370	D	70.0923	1
**4170**	372	D	51.761	0
**4170**	373	D	39.7816	0
**4170**	381	D	61.3765	1
**4170**	382	D	49.4871	0
**4170**	386	D	79.5418	1
**4171**	305	A	66.2409	1
**4171**	319	A	74.445	1
**4171**	320	A	74.1447	-1
**4171**	321	A	77.6761	0
**4171**	351	A	4.3759	1
**4171**	357	A	73.8589	1
**4171**	370	A	72.6528	1
**4171**	372	A	79.5612	0
**4171**	373	A	79.523	1
**4171**	381	A	20.0533	0
**4171**	382	A	29.5388	0
**4171**	386	A	47.9042	0

**4171**	305	B	79.6049	1
**4171**	319	B	79.6497	1
**4171**	320	B	62.4852	-1
**4171**	321	B	79.6484	0
**4171**	351	B	13.2666	1
**4171**	357	B	34.6721	0
**4171**	370	B	79.501	1
**4171**	372	B	74.4956	0
**4171**	373	B	61.2283	1
**4171**	381	B	72.7485	1
**4171**	382	B	35.3554	0
**4171**	386	B	79.5685	1
**4171**	305	C	73.2276	1
**4171**	319	C	73.3223	1
**4171**	320	C	79.5312	-1
**4171**	321	C	79.5841	0
**4171**	351	C	11.1494	1
**4171**	357	C	73.9309	1
**4171**	370	C	76.2003	1
**4171**	372	C	71.6921	0
**4171**	373	C	72.1315	1
**4171**	381	C	53.6401	1
**4171**	382	C	73.6263	1
**4171**	386	C	28.6919	0
**4171**	305	D	79.5528	1
**4171**	319	D	79.5125	1
**4171**	320	D	59.1519	-1
**4171**	321	D	71.6995	0
**4171**	351	D	9.7966	1
**4171**	357	D	63.7289	1
**4171**	370	D	66.7288	1
**4171**	372	D	79.5485	0
**4171**	373	D	64.6311	1
**4171**	381	D	56.4166	1

**4171**	382	D	75.3534	1
**4171**	386	D	79.5553	1
**4172**	305	A	79.4089	1
**4172**	319	A	24.1081	0
**4172**	320	A	73.0109	-1
**4172**	321	A	70.9671	0
**4172**	351	A	9.2935	1
**4172**	357	A	79.6299	1
**4172**	370	A	79.5694	1
**4172**	372	A	79.5615	0
**4172**	373	A	38.4846	0
**4172**	381	A	61.2271	1
**4172**	382	A	34.7876	0
**4172**	386	A	37.1014	0
**4172**	305	B	38.2193	0
**4172**	319	B	59.1271	1
**4172**	320	B	67.1831	-1
**4172**	321	B	66.4746	0
**4172**	351	B	12.7841	1
**4172**	357	B	73.2413	1
**4172**	370	B	79.5466	1
**4172**	372	B	79.5133	0
**4172**	373	B	70.4966	1
**4172**	381	B	44.634	0
**4172**	382	B	79.561	1
**4172**	386	B	72.7187	1
**4172**	305	C	79.5483	1
**4172**	319	C	73.1826	1
**4172**	320	C	62.359	-1
**4172**	321	C	79.4951	0
**4172**	351	C	8.5336	1
**4172**	357	C	63.4241	1
**4172**	370	C	78.5874	1
**4172**	372	C	57.9327	0

**4172**	373	C	56.1802	1
**4172**	381	C	79.5301	1
**4172**	382	C	68.0915	1
**4172**	386	C	49.0962	0
**4172**	305	D	14.4427	-1
**4172**	319	D	42.625	0
**4172**	320	D	79.5582	-1
**4172**	321	D	79.5428	0
**4172**	351	D	16.4037	1
**4172**	357	D	79.5576	1
**4172**	370	D	76.2059	1
**4172**	372	D	79.5456	0
**4172**	373	D	58.1516	1
**4172**	381	D	77.4758	1
**4172**	382	D	68.5135	1
**4172**	386	D	11.0098	-1
**4173**	305	A	66.8039	1
**4173**	319	A	74.5323	1
**4173**	320	A	72.6671	-1
**4173**	321	A	79.0876	0
**4173**	351	A	12.5376	1
**4173**	357	A	79.5524	1
**4173**	370	A	79.5628	1
**4173**	372	A	72.5431	0
**4173**	373	A	74.7176	1
**4173**	381	A	71.8242	1
**4173**	382	A	65.2073	1
**4173**	386	A	63.1446	1
**4173**	305	B	79.3582	1
**4173**	319	B	48.6597	0
**4173**	320	B	79.5788	-1
**4173**	321	B	79.4719	0
**4173**	351	B	12.284	1
**4173**	357	B	62.3826	1

**4173**	370	B	78.8825	1
**4173**	372	B	79.6429	0
**4173**	373	B	73.7873	1
**4173**	381	B	70.1475	1
**4173**	382	B	35.2385	0
**4173**	386	B	57.9549	1
**4173**	305	C	72.6896	1
**4173**	319	C	68.3452	1
**4173**	320	C	76.7124	-1
**4173**	321	C	49.9986	1
**4173**	351	C	11.6203	1
**4173**	357	C	46.7238	0
**4173**	370	C	42.9486	0
**4173**	372	C	74.1804	0
**4173**	373	C	76.0472	1
**4173**	381	C	67.2425	1
**4173**	382	C	49.8087	0
**4173**	386	C	58.5827	1
**4173**	305	D	65.9495	1
**4173**	319	D	69.3154	1
**4173**	320	D	41.9432	-1
**4173**	321	D	49.8585	1
**4173**	351	D	11.3164	1
**4173**	357	D	72.7319	1
**4173**	370	D	58.0273	1
**4173**	372	D	68.6595	0
**4173**	373	D	79.5613	1
**4173**	381	D	16.8635	-1
**4173**	382	D	26.1006	0
**4173**	386	D	53.5159	1
**4174**	305	A	79.5366	1
**4174**	319	A	79.5631	1
**4174**	320	A	78.881	-1
**4174**	321	A	79.335	0

**4174**	351	A	9.8582	1
**4174**	357	A	50.6195	1
**4174**	370	A	41.1072	0
**4174**	372	A	79.5686	0
**4174**	373	A	24.3589	0
**4174**	381	A	71.2021	1
**4174**	382	A	79.5542	1
**4174**	386	A	73.6878	1
**4174**	305	B	65.2389	1
**4174**	319	B	67.1361	1
**4174**	320	B	71.655	-1
**4174**	321	B	79.5599	0
**4174**	351	B	19.8031	1
**4174**	357	B	64.931	1
**4174**	370	B	72.5396	1
**4174**	372	B	78.6987	0
**4174**	373	B	72.4565	1
**4174**	381	B	79.5647	1
**4174**	382	B	76.1091	1
**4174**	386	B	79.5495	1
**4174**	305	C	56.7981	1
**4174**	319	C	65.5059	1
**4174**	320	C	79.1847	-1
**4174**	321	C	49.313	1
**4174**	351	C	0.0	1
**4174**	357	C	75.3225	1
**4174**	370	C	65.3187	1
**4174**	372	C	79.5616	0
**4174**	373	C	79.5715	1
**4174**	381	C	51.1637	1
**4174**	382	C	79.5647	1
**4174**	386	C	63.5319	1
**4174**	305	D	26.09	0
**4174**	319	D	78.8484	1

**4174**	320	D	79.5411	-1
**4174**	321	D	74.7974	0
**4174**	351	D	1.2849	1
**4174**	357	D	79.5636	1
**4174**	370	D	74.4594	1
**4174**	372	D	79.533	0
**4174**	373	D	64.3374	1
**4174**	381	D	51.6268	1
**4174**	382	D	72.7558	1
**4174**	386	D	78.2771	1
**4175**	305	A	79.5064	1
**4175**	319	A	69.6687	1
**4175**	320	A	45.6872	-1
**4175**	321	A	70.3983	0
**4175**	351	A	6.5392	1
**4175**	357	A	79.5468	1
**4175**	370	A	75.1393	1
**4175**	372	A	79.5625	0
**4175**	373	A	71.2229	1
**4175**	381	A	79.4065	1
**4175**	382	A	69.1506	1
**4175**	386	A	75.6028	1
**4175**	305	B	67.1997	1
**4175**	319	B	74.6654	1
**4175**	320	B	79.3597	-1
**4175**	321	B	74.6983	0
**4175**	351	B	11.9292	1
**4175**	357	B	76.0347	1
**4175**	370	B	79.6007	1
**4175**	372	B	44.0406	1
**4175**	373	B	63.4743	1
**4175**	381	B	79.4485	1
**4175**	382	B	48.1987	0
**4175**	386	B	42.8598	0

**4175**	305	C	42.1067	0
**4175**	319	C	55.1248	1
**4175**	320	C	79.5776	-1
**4175**	321	C	22.9531	1
**4175**	351	C	9.1117	1
**4175**	357	C	79.4909	1
**4175**	370	C	63.1632	1
**4175**	372	C	49.4357	1
**4175**	373	C	79.4801	1
**4175**	381	C	79.6212	1
**4175**	382	C	44.6727	0
**4175**	386	C	35.9574	0
**4175**	305	D	63.9812	1
**4175**	319	D	78.322	1
**4175**	320	D	40.4262	-1
**4175**	321	D	66.1253	0
**4175**	351	D	5.55	1
**4175**	357	D	79.7101	1
**4175**	370	D	74.2853	1
**4175**	372	D	79.6129	0
**4175**	373	D	79.3024	1
**4175**	381	D	56.6962	1
**4175**	382	D	50.9969	1
**4175**	386	D	48.2604	0
**4176**	305	A	50.655	1
**4176**	319	A	70.1816	1
**4176**	320	A	57.2632	-1
**4176**	321	A	39.227	1
**4176**	351	A	0.9466	1
**4176**	357	A	72.7067	1
**4176**	370	A	79.7501	1
**4176**	372	A	73.3318	0
**4176**	373	A	67.2007	1
**4176**	381	A	74.7217	1

**4176**	382	A	45.7076	0
**4176**	386	A	79.6172	1
**4176**	305	B	79.6175	1
**4176**	319	B	66.8444	1
**4176**	320	B	77.9747	-1
**4176**	321	B	73.3374	0
**4176**	351	B	19.5194	1
**4176**	357	B	35.46	0
**4176**	370	B	73.4355	1
**4176**	372	B	62.6921	0
**4176**	373	B	74.4235	1
**4176**	381	B	69.33	1
**4176**	382	B	44.551	0
**4176**	386	B	79.5852	1
**4176**	305	C	52.0843	1
**4176**	319	C	79.2217	1
**4176**	320	C	79.5348	-1
**4176**	321	C	77.5484	0
**4176**	351	C	0.1445	1
**4176**	357	C	58.0201	1
**4176**	370	C	79.6018	1
**4176**	372	C	24.6651	1
**4176**	373	C	53.7237	1
**4176**	381	C	47.5865	0
**4176**	382	C	79.6286	1
**4176**	386	C	35.265	0
**4176**	305	D	79.5667	1
**4176**	319	D	79.6718	1
**4176**	320	D	79.6312	-1
**4176**	321	D	79.3572	0
**4176**	351	D	9.6761	1
**4176**	357	D	32.1068	0
**4176**	370	D	67.7731	1
**4176**	372	D	79.5557	0

**4176**	373	D	73.6383	1
**4176**	381	D	60.2682	1
**4176**	382	D	66.0962	1
**4176**	386	D	79.5799	1
**4177**	305	A	71.612	1
**4177**	319	A	75.6258	1
**4177**	320	A	79.5626	-1
**4177**	321	A	35.6694	1
**4177**	351	A	3.7229	1
**4177**	357	A	74.5338	1
**4177**	370	A	73.3099	1
**4177**	372	A	71.3017	0
**4177**	373	A	79.5811	1
**4177**	381	A	79.5213	1
**4177**	382	A	78.6772	1
**4177**	386	A	37.0401	0
**4177**	305	B	67.7065	1
**4177**	319	B	75.6619	1
**4177**	320	B	77.6653	-1
**4177**	321	B	79.664	0
**4177**	351	B	8.2634	1
**4177**	357	B	30.0895	0
**4177**	370	B	21.2266	0
**4177**	372	B	79.5266	0
**4177**	373	B	64.313	1
**4177**	381	B	68.7779	1
**4177**	382	B	17.9917	-1
**4177**	386	B	73.7222	1
**4177**	305	C	59.6747	1
**4177**	319	C	61.0094	1
**4177**	320	C	72.3607	-1
**4177**	321	C	64.0761	0
**4177**	351	C	3.0723	1
**4177**	357	C	72.7469	1

**4177**	370	C	79.5373	1
**4177**	372	C	59.608	0
**4177**	373	C	72.0444	1
**4177**	381	C	60.3742	1
**4177**	382	C	79.539	1
**4177**	386	C	49.632	0
**4177**	305	D	78.7192	1
**4177**	319	D	63.6333	1
**4177**	320	D	79.5969	-1
**4177**	321	D	42.0663	1
**4177**	351	D	8.6171	1
**4177**	357	D	79.5554	1
**4177**	370	D	72.4324	1
**4177**	372	D	55.2327	0
**4177**	373	D	72.9175	1
**4177**	381	D	52.8381	1
**4177**	382	D	72.9092	1
**4177**	386	D	66.6411	1
**4178**	305	A	69.6517	1
**4178**	319	A	69.2913	1
**4178**	320	A	62.2523	-1
**4178**	321	A	67.0405	0
**4178**	351	A	5.4728	1
**4178**	357	A	59.6107	1
**4178**	370	A	49.2414	0
**4178**	372	A	63.2706	0
**4178**	373	A	78.5512	1
**4178**	381	A	60.8142	1
**4178**	382	A	79.4885	1
**4178**	386	A	76.9288	1
**4178**	305	B	79.4997	1
**4178**	319	B	51.3187	1
**4178**	320	B	56.8522	-1
**4178**	321	B	79.4156	0

**4178**	351	B	9.972	1
**4178**	357	B	79.1429	1
**4178**	370	B	40.2863	0
**4178**	372	B	44.1863	1
**4178**	373	B	42.5825	0
**4178**	381	B	77.4355	1
**4178**	382	B	77.4176	1
**4178**	386	B	79.4751	1
**4178**	305	C	72.5344	1
**4178**	319	C	76.8602	1
**4178**	320	C	79.5883	-1
**4178**	321	C	72.9169	0
**4178**	351	C	9.4473	1
**4178**	357	C	75.4907	1
**4178**	370	C	78.8081	1
**4178**	372	C	25.017	1
**4178**	373	C	54.7091	1
**4178**	381	C	28.7769	0
**4178**	382	C	32.0781	0
**4178**	386	C	46.6401	0
**4178**	305	D	59.114	1
**4178**	319	D	72.4639	1
**4178**	320	D	71.1396	-1
**4178**	321	D	67.7882	0
**4178**	351	D	14.9186	1
**4178**	357	D	79.4877	1
**4178**	370	D	79.341	1
**4178**	372	D	32.7442	1
**4178**	373	D	43.0161	0
**4178**	381	D	40.0329	0
**4178**	382	D	79.5372	1
**4178**	386	D	74.2449	1
**4179**	305	A	69.8241	1
**4179**	319	A	79.6332	1

**4179**	320	A	72.7969	-1
**4179**	321	A	79.4844	0
**4179**	351	A	2.8207	1
**4179**	357	A	43.2301	0
**4179**	370	A	79.6188	1
**4179**	372	A	42.8182	1
**4179**	373	A	71.4016	1
**4179**	381	A	79.5869	1
**4179**	382	A	79.5982	1
**4179**	386	A	79.6067	1
**4179**	305	B	64.7961	1
**4179**	319	B	79.4952	1
**4179**	320	B	64.9902	-1
**4179**	321	B	79.6048	0
**4179**	351	B	4.9315	1
**4179**	357	B	45.4577	0
**4179**	370	B	52.708	1
**4179**	372	B	79.5481	0
**4179**	373	B	50.0619	1
**4179**	381	B	79.5401	1
**4179**	382	B	33.9609	0
**4179**	386	B	65.313	1
**4179**	305	C	58.3078	1
**4179**	319	C	57.7902	1
**4179**	320	C	79.4881	-1
**4179**	321	C	54.6173	0
**4179**	351	C	10.0002	1
**4179**	357	C	63.9887	1
**4179**	370	C	65.1453	1
**4179**	372	C	68.6515	0
**4179**	373	C	79.5059	1
**4179**	381	C	79.4683	1
**4179**	382	C	8.0832	-1
**4179**	386	C	26.8003	0

**4179**	305	D	67.2957	1
**4179**	319	D	79.5361	1
**4179**	320	D	75.2757	-1
**4179**	321	D	25.5426	1
**4179**	351	D	15.297	1
**4179**	357	D	79.5139	1
**4179**	370	D	29.8669	0
**4179**	372	D	65.7176	0
**4179**	373	D	55.3997	1
**4179**	381	D	79.6766	1
**4179**	382	D	67.6717	1
**4179**	386	D	42.3067	0
**4180**	305	A	79.4497	1
**4180**	319	A	64.1541	1
**4180**	320	A	73.3192	-1
**4180**	321	A	70.7936	0
**4180**	351	A	2.1417	1
**4180**	357	A	76.8928	1
**4180**	370	A	37.4089	0
**4180**	372	A	46.96	1
**4180**	373	A	64.6109	1
**4180**	381	A	76.3797	1
**4180**	382	A	23.7289	0
**4180**	386	A	56.4879	1
**4180**	305	B	71.279	1
**4180**	319	B	56.8046	1
**4180**	320	B	57.8934	-1
**4180**	321	B	72.2511	0
**4180**	351	B	19.7339	1
**4180**	357	B	79.5187	1
**4180**	370	B	79.6568	1
**4180**	372	B	34.2542	1
**4180**	373	B	57.4593	1
**4180**	381	B	66.8134	1

**4180**	382	B	60.703	1
**4180**	386	B	65.4824	1
**4180**	305	C	76.6959	1
**4180**	319	C	79.5614	1
**4180**	320	C	72.9741	-1
**4180**	321	C	77.6099	0
**4180**	351	C	11.3189	1
**4180**	357	C	77.2272	1
**4180**	370	C	57.6894	1
**4180**	372	C	79.6071	0
**4180**	373	C	79.5168	1
**4180**	381	C	54.9331	1
**4180**	382	C	79.465	1
**4180**	386	C	41.9901	0
**4180**	305	D	79.552	1
**4180**	319	D	50.4065	1
**4180**	320	D	79.592	-1
**4180**	321	D	79.4255	0
**4180**	351	D	14.8834	1
**4180**	357	D	78.9871	1
**4180**	370	D	64.2154	1
**4180**	372	D	69.4301	0
**4180**	373	D	39.7812	0
**4180**	381	D	54.3546	1
**4180**	382	D	29.7753	0
**4180**	386	D	49.704	0
**4181**	305	A	58.178	1
**4181**	319	A	79.3949	1
**4181**	320	A	79.4297	-1
**4181**	321	A	71.7338	0
**4181**	351	A	21.101	-1
**4181**	357	A	79.6364	1
**4181**	370	A	69.7059	1
**4181**	372	A	67.4712	0

**4181**	373	A	79.7544	1
**4181**	381	A	68.1326	1
**4181**	382	A	66.4358	1
**4181**	386	A	74.5966	1
**4181**	305	B	79.481	1
**4181**	319	B	76.7959	1
**4181**	320	B	63.9103	-1
**4181**	321	B	72.8424	0
**4181**	351	B	17.5066	1
**4181**	357	B	37.4156	0
**4181**	370	B	63.6087	1
**4181**	372	B	73.7574	0
**4181**	373	B	37.2776	0
**4181**	381	B	74.0545	1
**4181**	382	B	79.5897	1
**4181**	386	B	68.3927	1
**4181**	305	C	45.1892	0
**4181**	319	C	79.4946	1
**4181**	320	C	68.0404	-1
**4181**	321	C	79.4533	0
**4181**	351	C	1.0609	1
**4181**	357	C	59.9237	1
**4181**	370	C	79.617	1
**4181**	372	C	79.4867	0
**4181**	373	C	79.4548	1
**4181**	381	C	75.9436	1
**4181**	382	C	77.8398	1
**4181**	386	C	49.9809	0
**4181**	305	D	37.0632	0
**4181**	319	D	77.1438	1
**4181**	320	D	67.5744	-1
**4181**	321	D	68.0029	0
**4181**	351	D	10.9519	1
**4181**	357	D	73.6948	1

**4181**	370	D	79.3358	1
**4181**	372	D	77.1737	0
**4181**	373	D	41.1055	0
**4181**	381	D	29.1361	0
**4181**	382	D	63.0551	1
**4181**	386	D	79.4603	1
**4182**	305	A	72.5052	1
**4182**	319	A	16.4479	-1
**4182**	320	A	79.563	-1
**4182**	321	A	43.8425	1
**4182**	351	A	9.0244	1
**4182**	357	A	79.5629	1
**4182**	370	A	66.5888	1
**4182**	372	A	45.2214	1
**4182**	373	A	59.4311	1
**4182**	381	A	78.8178	1
**4182**	382	A	79.5841	1
**4182**	386	A	72.6888	1
**4182**	305	B	73.7297	1
**4182**	319	B	65.0232	1
**4182**	320	B	78.3536	-1
**4182**	321	B	52.3042	0
**4182**	351	B	8.9825	1
**4182**	357	B	57.0792	1
**4182**	370	B	78.5034	1
**4182**	372	B	65.6891	0
**4182**	373	B	58.7437	1
**4182**	381	B	20.7775	0
**4182**	382	B	74.0042	1
**4182**	386	B	45.6243	0
**4182**	305	C	75.6794	1
**4182**	319	C	62.1546	1
**4182**	320	C	71.6444	-1
**4182**	321	C	62.6666	0

**4182**	351	C	8.017	1
**4182**	357	C	73.7372	1
**4182**	370	C	73.0883	1
**4182**	372	C	79.5355	0
**4182**	373	C	78.994	1
**4182**	381	C	50.9372	1
**4182**	382	C	52.61	1
**4182**	386	C	15.9545	-1
**4182**	305	D	64.0092	1
**4182**	319	D	78.6917	1
**4182**	320	D	68.5475	-1
**4182**	321	D	51.4333	0
**4182**	351	D	0.0	1
**4182**	357	D	68.177	1
**4182**	370	D	36.9525	0
**4182**	372	D	70.3336	0
**4182**	373	D	79.5628	1
**4182**	381	D	73.9126	1
**4182**	382	D	34.4035	0
**4182**	386	D	60.3734	1
**4183**	305	A	42.7397	0
**4183**	319	A	78.2755	1
**4183**	320	A	71.9579	-1
**4183**	321	A	79.4838	0
**4183**	351	A	9.5958	1
**4183**	357	A	73.0395	1
**4183**	370	A	76.244	1
**4183**	372	A	20.109	1
**4183**	373	A	73.8589	1
**4183**	381	A	30.7058	0
**4183**	382	A	70.9465	1
**4183**	386	A	67.211	1
**4183**	305	B	46.1123	0
**4183**	319	B	76.3363	1

**4183**	320	B	56.4757	-1
**4183**	321	B	76.1165	0
**4183**	351	B	14.1546	1
**4183**	357	B	74.0894	1
**4183**	370	B	68.3719	1
**4183**	372	B	79.4615	0
**4183**	373	B	65.214	1
**4183**	381	B	79.5943	1
**4183**	382	B	44.9358	0
**4183**	386	B	71.4624	1
**4183**	305	C	77.075	1
**4183**	319	C	71.4172	1
**4183**	320	C	79.6638	-1
**4183**	321	C	78.9447	0
**4183**	351	C	6.6412	1
**4183**	357	C	72.6773	1
**4183**	370	C	65.6318	1
**4183**	372	C	79.6028	0
**4183**	373	C	51.5587	1
**4183**	381	C	79.5354	1
**4183**	382	C	68.9823	1
**4183**	386	C	18.5484	-1
**4183**	305	D	47.2826	0
**4183**	319	D	55.5072	1
**4183**	320	D	79.523	-1
**4183**	321	D	71.8042	0
**4183**	351	D	5.6386	1
**4183**	357	D	53.1792	1
**4183**	370	D	37.7877	0
**4183**	372	D	71.4626	0
**4183**	373	D	57.7977	1
**4183**	381	D	79.6253	1
**4183**	382	D	26.4874	0
**4183**	386	D	54.3818	1

**4184**	305	A	48.7339	0
**4184**	319	A	69.0245	1
**4184**	320	A	77.5454	-1
**4184**	321	A	62.0519	0
**4184**	351	A	33.8604	-1
**4184**	357	A	73.184	1
**4184**	370	A	79.5727	1
**4184**	372	A	79.5563	0
**4184**	373	A	72.1096	1
**4184**	381	A	79.5633	1
**4184**	382	A	77.661	1
**4184**	386	A	47.778	0
**4184**	305	B	69.2163	1
**4184**	319	B	54.5927	1
**4184**	320	B	79.5627	-1
**4184**	321	B	61.9148	0
**4184**	351	B	19.6701	1
**4184**	357	B	52.9095	1
**4184**	370	B	79.4767	1
**4184**	372	B	78.0779	0
**4184**	373	B	76.1875	1
**4184**	381	B	72.2676	1
**4184**	382	B	60.2629	1
**4184**	386	B	45.3283	0
**4184**	305	C	47.5708	0
**4184**	319	C	63.0589	1
**4184**	320	C	57.0254	-1
**4184**	321	C	62.1363	0
**4184**	351	C	6.9947	1
**4184**	357	C	69.5278	1
**4184**	370	C	69.355	1
**4184**	372	C	52.3017	0
**4184**	373	C	75.7547	1
**4184**	381	C	73.244	1

**4184**	382	C	73.8049	1
**4184**	386	C	58.3473	1
**4184**	305	D	61.5859	1
**4184**	319	D	67.3686	1
**4184**	320	D	56.9665	-1
**4184**	321	D	44.587	1
**4184**	351	D	10.1547	1
**4184**	357	D	59.4739	1
**4184**	370	D	32.8375	0
**4184**	372	D	77.1915	0
**4184**	373	D	43.3461	0
**4184**	381	D	48.6478	0
**4184**	382	D	38.6326	0
**4184**	386	D	79.545	1
**4185**	305	A	68.997	1
**4185**	319	A	75.3732	1
**4185**	320	A	79.5214	-1
**4185**	321	A	32.5051	1
**4185**	351	A	18.5066	1
**4185**	357	A	79.3523	1
**4185**	370	A	69.7094	1
**4185**	372	A	79.5097	0
**4185**	373	A	69.323	1
**4185**	381	A	79.5729	1
**4185**	382	A	24.0058	0
**4185**	386	A	29.2668	0
**4185**	305	B	79.4923	1
**4185**	319	B	32.6495	0
**4185**	320	B	79.5599	-1
**4185**	321	B	39.241	1
**4185**	351	B	9.0387	1
**4185**	357	B	77.7617	1
**4185**	370	B	79.4051	1
**4185**	372	B	73.6831	0

**4185**	373	B	79.5773	1
**4185**	381	B	55.4125	1
**4185**	382	B	38.5765	0
**4185**	386	B	57.4706	1
**4185**	305	C	55.9041	1
**4185**	319	C	47.9746	0
**4185**	320	C	57.3809	-1
**4185**	321	C	65.6353	0
**4185**	351	C	3.2634	1
**4185**	357	C	68.8852	1
**4185**	370	C	47.831	0
**4185**	372	C	79.5349	0
**4185**	373	C	61.4149	1
**4185**	381	C	79.5871	1
**4185**	382	C	46.4687	0
**4185**	386	C	44.8134	0
**4185**	305	D	12.606	-1
**4185**	319	D	78.5071	1
**4185**	320	D	54.1151	-1
**4185**	321	D	79.5817	0
**4185**	351	D	16.4079	1
**4185**	357	D	74.9008	1
**4185**	370	D	73.0067	1
**4185**	372	D	74.4937	0
**4185**	373	D	74.3396	1
**4185**	381	D	78.921	1
**4185**	382	D	74.0106	1
**4185**	386	D	72.9183	1
**4186**	305	A	5.9834	-1
**4186**	319	A	54.2282	1
**4186**	320	A	79.5879	-1
**4186**	321	A	75.4906	0
**4186**	351	A	9.5263	1
**4186**	357	A	60.6901	1

**4186**	370	A	14.6596	-1
**4186**	372	A	76.3741	0
**4186**	373	A	77.2978	1
**4186**	381	A	46.9274	0
**4186**	382	A	74.5488	1
**4186**	386	A	79.6171	1
**4186**	305	B	65.0821	1
**4186**	319	B	62.13	1
**4186**	320	B	60.4151	-1
**4186**	321	B	75.5407	0
**4186**	351	B	9.4887	1
**4186**	357	B	79.534	1
**4186**	370	B	79.5305	1
**4186**	372	B	79.549	0
**4186**	373	B	53.656	1
**4186**	381	B	37.6591	0
**4186**	382	B	54.593	1
**4186**	386	B	75.1624	1
**4186**	305	C	63.1709	1
**4186**	319	C	78.8383	1
**4186**	320	C	32.7904	-1
**4186**	321	C	67.3841	0
**4186**	351	C	1.4402	1
**4186**	357	C	58.8905	1
**4186**	370	C	79.5824	1
**4186**	372	C	79.5915	0
**4186**	373	C	79.5585	1
**4186**	381	C	79.5825	1
**4186**	382	C	67.967	1
**4186**	386	C	60.227	1
**4186**	305	D	43.2143	0
**4186**	319	D	79.5425	1
**4186**	320	D	79.5535	-1
**4186**	321	D	72.864	0

**4186**	351	D	11.5233	1
**4186**	357	D	72.0194	1
**4186**	370	D	77.7582	1
**4186**	372	D	67.5214	0
**4186**	373	D	70.8898	1
**4186**	381	D	68.6065	1
**4186**	382	D	71.6074	1
**4186**	386	D	77.7883	1
**4187**	305	A	71.5478	1
**4187**	319	A	51.6552	1
**4187**	320	A	79.6722	-1
**4187**	321	A	62.9329	0
**4187**	351	A	0.0	1
**4187**	357	A	62.9211	1
**4187**	370	A	79.6899	1
**4187**	372	A	79.6437	0
**4187**	373	A	62.8984	1
**4187**	381	A	79.6568	1
**4187**	382	A	45.616	0
**4187**	386	A	74.0533	1
**4187**	305	B	65.1399	1
**4187**	319	B	60.9391	1
**4187**	320	B	46.436	-1
**4187**	321	B	71.8513	0
**4187**	351	B	16.4589	1
**4187**	357	B	51.0028	1
**4187**	370	B	29.0617	0
**4187**	372	B	79.5439	0
**4187**	373	B	43.7652	0
**4187**	381	B	79.4228	1
**4187**	382	B	24.0806	0
**4187**	386	B	79.6935	1
**4187**	305	C	79.4989	1
**4187**	319	C	39.2249	0

**4187**	320	C	56.487	-1
**4187**	321	C	79.4453	0
**4187**	351	C	39.4303	-1
**4187**	357	C	79.5292	1
**4187**	370	C	52.4078	1
**4187**	372	C	79.4052	0
**4187**	373	C	48.1655	0
**4187**	381	C	70.7159	1
**4187**	382	C	74.196	1
**4187**	386	C	46.6509	0
**4187**	305	D	79.5141	1
**4187**	319	D	66.9086	1
**4187**	320	D	79.5025	-1
**4187**	321	D	79.5838	0
**4187**	351	D	5.9008	1
**4187**	357	D	79.5443	1
**4187**	370	D	79.5675	1
**4187**	372	D	72.4557	0
**4187**	373	D	79.5713	1
**4187**	381	D	66.1406	1
**4187**	382	D	11.6578	-1
**4187**	386	D	78.4966	1
**4188**	305	A	30.1873	0
**4188**	319	A	60.4113	1
**4188**	320	A	79.4788	-1
**4188**	321	A	44.0355	1
**4188**	351	A	0.5775	1
**4188**	357	A	72.7261	1
**4188**	370	A	34.6377	0
**4188**	372	A	67.9288	0
**4188**	373	A	35.4514	0
**4188**	381	A	65.8105	1
**4188**	382	A	41.1574	0
**4188**	386	A	79.5127	1

**4188**	305	B	69.2004	1
**4188**	319	B	63.8967	1
**4188**	320	B	78.5138	-1
**4188**	321	B	50.4301	0
**4188**	351	B	14.7872	1
**4188**	357	B	79.61	1
**4188**	370	B	50.5903	1
**4188**	372	B	46.8455	1
**4188**	373	B	71.7394	1
**4188**	381	B	79.5308	1
**4188**	382	B	73.1111	1
**4188**	386	B	49.6574	0
**4188**	305	C	57.9022	1
**4188**	319	C	79.6457	1
**4188**	320	C	76.7995	-1
**4188**	321	C	28.8584	1
**4188**	351	C	5.2478	1
**4188**	357	C	63.4869	1
**4188**	370	C	56.339	1
**4188**	372	C	79.5272	0
**4188**	373	C	15.444	-1
**4188**	381	C	58.3688	1
**4188**	382	C	73.7111	1
**4188**	386	C	78.2365	1
**4188**	305	D	64.6299	1
**4188**	319	D	79.6241	1
**4188**	320	D	61.3462	-1
**4188**	321	D	50.574	0
**4188**	351	D	10.1994	1
**4188**	357	D	57.8652	1
**4188**	370	D	42.3378	0
**4188**	372	D	72.6511	0
**4188**	373	D	48.0322	0
**4188**	381	D	53.896	1

**4188**	382	D	70.2322	1
**4188**	386	D	68.9785	1
**4189**	305	A	76.2206	1
**4189**	319	A	34.9776	0
**4189**	320	A	79.6157	-1
**4189**	321	A	79.347	0
**4189**	351	A	4.274	1
**4189**	357	A	79.5172	1
**4189**	370	A	76.6872	1
**4189**	372	A	70.196	0
**4189**	373	A	47.9788	0
**4189**	381	A	54.3173	1
**4189**	382	A	74.139	1
**4189**	386	A	55.441	1
**4189**	305	B	79.4872	1
**4189**	319	B	73.6751	1
**4189**	320	B	79.5848	-1
**4189**	321	B	79.5498	0
**4189**	351	B	13.4001	1
**4189**	357	B	50.5391	1
**4189**	370	B	73.8646	1
**4189**	372	B	70.6984	0
**4189**	373	B	36.5405	0
**4189**	381	B	71.5843	1
**4189**	382	B	50.8276	1
**4189**	386	B	79.3218	1
**4189**	305	C	64.0471	1
**4189**	319	C	79.4835	1
**4189**	320	C	39.4487	-1
**4189**	321	C	51.4238	0
**4189**	351	C	1.5438	1
**4189**	357	C	79.6091	1
**4189**	370	C	79.6324	1
**4189**	372	C	28.5528	1

**4189**	373	C	79.6153	1
**4189**	381	C	79.5704	1
**4189**	382	C	75.823	1
**4189**	386	C	22.1588	0
**4189**	305	D	79.6148	1
**4189**	319	D	73.4747	1
**4189**	320	D	79.5911	-1
**4189**	321	D	71.4263	0
**4189**	351	D	13.0558	1
**4189**	357	D	67.6272	1
**4189**	370	D	79.5655	1
**4189**	372	D	78.7604	0
**4189**	373	D	79.6022	1
**4189**	381	D	51.3431	1
**4189**	382	D	79.5998	1
**4189**	386	D	79.5501	1
**4190**	305	A	30.5793	0
**4190**	319	A	66.1052	1
**4190**	320	A	74.3538	-1
**4190**	321	A	72.8596	0
**4190**	351	A	4.9698	1
**4190**	357	A	72.3215	1
**4190**	370	A	76.2019	1
**4190**	372	A	74.8936	0
**4190**	373	A	75.662	1
**4190**	381	A	22.2806	0
**4190**	382	A	71.8109	1
**4190**	386	A	72.673	1
**4190**	305	B	79.6417	1
**4190**	319	B	72.2924	1
**4190**	320	B	38.5163	-1
**4190**	321	B	61.6932	0
**4190**	351	B	14.3131	1
**4190**	357	B	58.9967	1

**4190**	370	B	71.7755	1
**4190**	372	B	79.7121	0
**4190**	373	B	65.1314	1
**4190**	381	B	79.5668	1
**4190**	382	B	51.0054	1
**4190**	386	B	79.3157	1
**4190**	305	C	66.8348	1
**4190**	319	C	74.7566	1
**4190**	320	C	44.7445	-1
**4190**	321	C	79.4708	0
**4190**	351	C	13.3502	1
**4190**	357	C	73.8262	1
**4190**	370	C	76.0094	1
**4190**	372	C	79.5482	0
**4190**	373	C	71.407	1
**4190**	381	C	75.11	1
**4190**	382	C	79.482	1
**4190**	386	C	32.9636	0
**4190**	305	D	37.549	0
**4190**	319	D	71.5006	1
**4190**	320	D	73.6802	-1
**4190**	321	D	71.6784	0
**4190**	351	D	17.801	1
**4190**	357	D	71.3679	1
**4190**	370	D	77.4482	1
**4190**	372	D	74.5482	0
**4190**	373	D	39.7594	0
**4190**	381	D	79.5933	1
**4190**	382	D	66.9363	1
**4190**	386	D	74.3608	1
**4191**	305	A	79.5768	1
**4191**	319	A	54.7853	1
**4191**	320	A	25.7931	-1
**4191**	321	A	62.3473	0

**4191**	351	A	12.7296	1
**4191**	357	A	42.8646	0
**4191**	370	A	34.6043	0
**4191**	372	A	65.9363	0
**4191**	373	A	79.5682	1
**4191**	381	A	67.1699	1
**4191**	382	A	58.6586	1
**4191**	386	A	79.5438	1
**4191**	305	B	63.7121	1
**4191**	319	B	79.5574	1
**4191**	320	B	79.6008	-1
**4191**	321	B	79.5406	0
**4191**	351	B	5.5957	1
**4191**	357	B	79.5633	1
**4191**	370	B	79.3195	1
**4191**	372	B	79.5762	0
**4191**	373	B	65.97	1
**4191**	381	B	24.0878	0
**4191**	382	B	23.6639	0
**4191**	386	B	79.5843	1
**4191**	305	C	48.1787	0
**4191**	319	C	73.034	1
**4191**	320	C	49.0009	-1
**4191**	321	C	78.9935	0
**4191**	351	C	6.8045	1
**4191**	357	C	61.4548	1
**4191**	370	C	77.2638	1
**4191**	372	C	60.9346	0
**4191**	373	C	72.8873	1
**4191**	381	C	79.5423	1
**4191**	382	C	79.5769	1
**4191**	386	C	50.4089	1
**4191**	305	D	34.2379	0
**4191**	319	D	43.3192	0

**4191**	320	D	34.3961	-1
**4191**	321	D	46.0522	1
**4191**	351	D	16.0663	1
**4191**	357	D	36.7712	0
**4191**	370	D	79.5763	1
**4191**	372	D	72.3457	0
**4191**	373	D	46.4877	0
**4191**	381	D	40.1265	0
**4191**	382	D	73.5581	1
**4191**	386	D	79.5776	1
**4192**	305	A	74.9073	1
**4192**	319	A	36.854	0
**4192**	320	A	55.5614	-1
**4192**	321	A	73.0363	0
**4192**	351	A	2.9927	1
**4192**	357	A	62.1796	1
**4192**	370	A	79.6656	1
**4192**	372	A	79.5484	0
**4192**	373	A	62.667	1
**4192**	381	A	79.4964	1
**4192**	382	A	65.8342	1
**4192**	386	A	79.4566	1
**4192**	305	B	64.4103	1
**4192**	319	B	71.7919	1
**4192**	320	B	62.5292	-1
**4192**	321	B	62.0779	0
**4192**	351	B	6.6269	1
**4192**	357	B	71.6669	1
**4192**	370	B	72.1566	1
**4192**	372	B	76.1038	0
**4192**	373	B	59.052	1
**4192**	381	B	73.167	1
**4192**	382	B	78.7898	1
**4192**	386	B	69.8596	1

**4192**	305	C	32.524	0
**4192**	319	C	54.6935	1
**4192**	320	C	71.5054	-1
**4192**	321	C	15.4332	0
**4192**	351	C	4.2491	1
**4192**	357	C	79.5855	1
**4192**	370	C	79.4758	1
**4192**	372	C	73.2558	0
**4192**	373	C	79.4637	1
**4192**	381	C	79.5745	1
**4192**	382	C	50.1475	1
**4192**	386	C	65.8747	1
**4192**	305	D	79.6254	1
**4192**	319	D	79.582	1
**4192**	320	D	60.2986	-1
**4192**	321	D	51.8352	0
**4192**	351	D	15.0196	1
**4192**	357	D	47.9634	0
**4192**	370	D	79.5662	1
**4192**	372	D	79.4022	0
**4192**	373	D	71.3142	1
**4192**	381	D	52.0443	1
**4192**	382	D	52.9679	1
**4192**	386	D	79.5991	1
**4193**	305	A	77.122	1
**4193**	319	A	59.2422	1
**4193**	320	A	71.5835	-1
**4193**	321	A	9.6639	0
**4193**	351	A	8.683	1
**4193**	357	A	60.3355	1
**4193**	370	A	76.3556	1
**4193**	372	A	79.5502	0
**4193**	373	A	79.5826	1
**4193**	381	A	56.5207	1

**4193**	382	A	79.517	1
**4193**	386	A	50.4668	1
**4193**	305	B	73.9036	1
**4193**	319	B	70.2849	1
**4193**	320	B	63.0139	-1
**4193**	321	B	79.5874	0
**4193**	351	B	12.1364	1
**4193**	357	B	67.2652	1
**4193**	370	B	25.7303	0
**4193**	372	B	60.9368	0
**4193**	373	B	55.4969	1
**4193**	381	B	79.5451	1
**4193**	382	B	79.2812	1
**4193**	386	B	49.3519	0
**4193**	305	C	77.7352	1
**4193**	319	C	60.5962	1
**4193**	320	C	53.8016	-1
**4193**	321	C	63.5407	0
**4193**	351	C	1.2407	1
**4193**	357	C	79.569	1
**4193**	370	C	79.607	1
**4193**	372	C	74.5661	0
**4193**	373	C	63.1526	1
**4193**	381	C	66.4402	1
**4193**	382	C	79.5066	1
**4193**	386	C	55.373	1
**4193**	305	D	72.1872	1
**4193**	319	D	78.8568	1
**4193**	320	D	79.4928	-1
**4193**	321	D	79.5065	0
**4193**	351	D	15.7364	1
**4193**	357	D	39.366	0
**4193**	370	D	77.8608	1
**4193**	372	D	79.5232	0

**4193**	373	D	68.7071	1
**4193**	381	D	79.543	1
**4193**	382	D	76.594	1
**4193**	386	D	79.3664	1
**4194**	305	A	54.9908	1
**4194**	319	A	66.0387	1
**4194**	320	A	79.6932	-1
**4194**	321	A	66.762	0
**4194**	351	A	15.575	1
**4194**	357	A	60.8832	1
**4194**	370	A	79.6506	1
**4194**	372	A	74.9113	0
**4194**	373	A	35.4547	0
**4194**	381	A	79.5276	1
**4194**	382	A	79.5434	1
**4194**	386	A	75.5774	1
**4194**	305	B	71.725	1
**4194**	319	B	76.2914	1
**4194**	320	B	73.0732	-1
**4194**	321	B	44.7593	1
**4194**	351	B	9.2076	1
**4194**	357	B	79.5884	1
**4194**	370	B	70.0344	1
**4194**	372	B	68.6167	0
**4194**	373	B	66.2736	1
**4194**	381	B	54.1113	1
**4194**	382	B	49.0609	0
**4194**	386	B	46.4671	0
**4194**	305	C	60.9885	1
**4194**	319	C	66.1121	1
**4194**	320	C	65.7243	-1
**4194**	321	C	39.2512	1
**4194**	351	C	6.7536	1
**4194**	357	C	79.0161	1

**4194**	370	C	17.1255	-1
**4194**	372	C	79.5222	0
**4194**	373	C	75.8941	1
**4194**	381	C	48.3366	0
**4194**	382	C	75.4996	1
**4194**	386	C	28.6742	0
**4194**	305	D	29.9682	0
**4194**	319	D	70.2115	1
**4194**	320	D	79.5012	-1
**4194**	321	D	62.391	0
**4194**	351	D	11.2042	1
**4194**	357	D	49.738	0
**4194**	370	D	57.6562	1
**4194**	372	D	27.2468	1
**4194**	373	D	67.1657	1
**4194**	381	D	79.5134	1
**4194**	382	D	79.4749	1
**4194**	386	D	73.2204	1
**4195**	305	A	62.3398	1
**4195**	319	A	78.1097	1
**4195**	320	A	79.5192	-1
**4195**	321	A	67.9646	0
**4195**	351	A	12.5061	1
**4195**	357	A	79.4975	1
**4195**	370	A	79.6554	1
**4195**	372	A	65.6056	0
**4195**	373	A	52.0429	1
**4195**	381	A	60.7033	1
**4195**	382	A	41.0484	0
**4195**	386	A	70.0549	1
**4195**	305	B	79.5682	1
**4195**	319	B	62.1852	1
**4195**	320	B	79.5655	-1
**4195**	321	B	72.1632	0

**4195**	351	B	15.022	1
**4195**	357	B	61.2256	1
**4195**	370	B	40.5898	0
**4195**	372	B	72.329	0
**4195**	373	B	79.5279	1
**4195**	381	B	39.0995	0
**4195**	382	B	40.6187	0
**4195**	386	B	68.353	1
**4195**	305	C	79.5358	1
**4195**	319	C	75.1606	1
**4195**	320	C	52.2003	-1
**4195**	321	C	60.6128	0
**4195**	351	C	6.262	1
**4195**	357	C	52.3591	1
**4195**	370	C	76.1208	1
**4195**	372	C	79.5608	0
**4195**	373	C	68.5849	1
**4195**	381	C	47.9001	0
**4195**	382	C	27.5036	0
**4195**	386	C	50.3913	1
**4195**	305	D	34.5072	0
**4195**	319	D	60.6884	1
**4195**	320	D	72.3829	-1
**4195**	321	D	51.071	0
**4195**	351	D	4.3374	1
**4195**	357	D	79.6341	1
**4195**	370	D	79.3863	1
**4195**	372	D	46.0845	1
**4195**	373	D	79.6082	1
**4195**	381	D	79.6866	1
**4195**	382	D	68.7196	1
**4195**	386	D	49.7875	0
**4196**	305	A	53.7762	1
**4196**	319	A	63.6714	1

**4196**	320	A	47.2369	-1
**4196**	321	A	50.8179	0
**4196**	351	A	6.2591	1
**4196**	357	A	79.1481	1
**4196**	370	A	79.5576	1
**4196**	372	A	16.1157	0
**4196**	373	A	79.4546	1
**4196**	381	A	79.5732	1
**4196**	382	A	75.7329	1
**4196**	386	A	79.5401	1
**4196**	305	B	64.792	1
**4196**	319	B	73.3756	1
**4196**	320	B	55.4327	-1
**4196**	321	B	79.6007	0
**4196**	351	B	2.5259	1
**4196**	357	B	79.4614	1
**4196**	370	B	79.3399	1
**4196**	372	B	73.4234	0
**4196**	373	B	79.2585	1
**4196**	381	B	68.2137	1
**4196**	382	B	74.687	1
**4196**	386	B	64.7593	1
**4196**	305	C	79.5737	1
**4196**	319	C	79.4983	1
**4196**	320	C	78.8952	-1
**4196**	321	C	65.1151	0
**4196**	351	C	16.8598	1
**4196**	357	C	76.4537	1
**4196**	370	C	63.7983	1
**4196**	372	C	60.3048	0
**4196**	373	C	79.6323	1
**4196**	381	C	65.6139	1
**4196**	382	C	79.5674	1
**4196**	386	C	19.8537	-1

**4196**	305	D	79.3718	1
**4196**	319	D	79.5874	1
**4196**	320	D	79.0859	-1
**4196**	321	D	52.5246	0
**4196**	351	D	17.7585	1
**4196**	357	D	65.2294	1
**4196**	370	D	62.8111	1
**4196**	372	D	74.6415	0
**4196**	373	D	79.4982	1
**4196**	381	D	72.0488	1
**4196**	382	D	72.2751	1
**4196**	386	D	79.5688	1
**4197**	305	A	73.223	1
**4197**	319	A	64.3948	1
**4197**	320	A	79.5407	-1
**4197**	321	A	68.8978	0
**4197**	351	A	6.0693	1
**4197**	357	A	56.5501	1
**4197**	370	A	79.5783	1
**4197**	372	A	21.1229	1
**4197**	373	A	79.5677	1
**4197**	381	A	59.5045	1
**4197**	382	A	78.7685	1
**4197**	386	A	72.7334	1
**4197**	305	B	79.5703	1
**4197**	319	B	70.7171	1
**4197**	320	B	79.5607	-1
**4197**	321	B	79.5695	0
**4197**	351	B	42.8901	-1
**4197**	357	B	58.1492	1
**4197**	370	B	79.565	1
**4197**	372	B	44.8618	1
**4197**	373	B	60.3338	1
**4197**	381	B	79.5913	1

**4197**	382	B	34.388	0
**4197**	386	B	79.5656	1
**4197**	305	C	79.6854	1
**4197**	319	C	60.3589	1
**4197**	320	C	74.604	-1
**4197**	321	C	79.4846	0
**4197**	351	C	12.7226	1
**4197**	357	C	79.5693	1
**4197**	370	C	79.5303	1
**4197**	372	C	79.5457	0
**4197**	373	C	79.6029	1
**4197**	381	C	66.0537	1
**4197**	382	C	73.6183	1
**4197**	386	C	66.7508	1
**4197**	305	D	66.5632	1
**4197**	319	D	79.5165	1
**4197**	320	D	39.8746	-1
**4197**	321	D	73.3983	0
**4197**	351	D	12.6596	1
**4197**	357	D	71.6182	1
**4197**	370	D	72.3825	1
**4197**	372	D	73.3521	0
**4197**	373	D	34.6591	0
**4197**	381	D	65.9267	1
**4197**	382	D	79.5613	1
**4197**	386	D	24.3328	0
**4198**	305	A	77.1671	1
**4198**	319	A	65.0003	1
**4198**	320	A	41.3009	-1
**4198**	321	A	45.9173	1
**4198**	351	A	9.3586	1
**4198**	357	A	79.6362	1
**4198**	370	A	67.3766	1
**4198**	372	A	72.272	0

**4198**	373	A	76.5572	1
**4198**	381	A	79.6407	1
**4198**	382	A	78.2739	1
**4198**	386	A	79.6903	1
**4198**	305	B	59.9547	1
**4198**	319	B	79.672	1
**4198**	320	B	79.6537	-1
**4198**	321	B	79.5361	0
**4198**	351	B	9.6897	1
**4198**	357	B	79.5313	1
**4198**	370	B	79.6656	1
**4198**	372	B	56.2787	0
**4198**	373	B	79.5701	1
**4198**	381	B	79.6511	1
**4198**	382	B	60.3089	1
**4198**	386	B	46.5492	0
**4198**	305	C	58.8935	1
**4198**	319	C	79.5346	1
**4198**	320	C	79.501	-1
**4198**	321	C	39.1325	1
**4198**	351	C	1.9621	1
**4198**	357	C	79.5871	1
**4198**	370	C	76.3675	1
**4198**	372	C	72.9603	0
**4198**	373	C	70.5792	1
**4198**	381	C	70.7148	1
**4198**	382	C	56.8675	1
**4198**	386	C	67.1181	1
**4198**	305	D	74.8114	1
**4198**	319	D	79.5701	1
**4198**	320	D	40.5494	-1
**4198**	321	D	56.2589	0
**4198**	351	D	8.959	1
**4198**	357	D	79.551	1

**4198**	370	D	37.5354	0
**4198**	372	D	70.6233	0
**4198**	373	D	79.4346	1
**4198**	381	D	74.9967	1
**4198**	382	D	79.4822	1
**4198**	386	D	73.9275	1
**4199**	305	A	73.1598	1
**4199**	319	A	36.0177	0
**4199**	320	A	79.5693	-1
**4199**	321	A	65.3264	0
**4199**	351	A	10.6895	1
**4199**	357	A	70.249	1
**4199**	370	A	64.647	1
**4199**	372	A	28.3473	1
**4199**	373	A	79.6112	1
**4199**	381	A	57.4906	1
**4199**	382	A	68.1981	1
**4199**	386	A	79.5468	1
**4199**	305	B	78.7775	1
**4199**	319	B	75.061	1
**4199**	320	B	25.9731	-1
**4199**	321	B	68.0039	0
**4199**	351	B	18.7042	1
**4199**	357	B	79.5553	1
**4199**	370	B	79.5743	1
**4199**	372	B	79.5698	0
**4199**	373	B	77.6976	1
**4199**	381	B	70.9878	1
**4199**	382	B	50.1299	1
**4199**	386	B	79.6208	1
**4199**	305	C	55.6029	1
**4199**	319	C	72.9419	1
**4199**	320	C	74.0358	-1
**4199**	321	C	77.5122	0

**4199**	351	C	11.89	1
**4199**	357	C	35.3398	0
**4199**	370	C	68.7375	1
**4199**	372	C	69.4441	0
**4199**	373	C	71.9161	1
**4199**	381	C	63.5895	1
**4199**	382	C	72.0374	1
**4199**	386	C	43.0734	0
**4199**	305	D	49.2477	0
**4199**	319	D	69.9875	1
**4199**	320	D	41.9389	-1
**4199**	321	D	33.1638	1
**4199**	351	D	34.6899	-1
**4199**	357	D	75.4117	1
**4199**	370	D	55.7657	1
**4199**	372	D	79.5558	0
**4199**	373	D	72.1368	1
**4199**	381	D	45.0518	0
**4199**	382	D	68.6809	1
**4199**	386	D	79.5766	1
**4200**	305	A	59.629	1
**4200**	319	A	59.5488	1
**4200**	320	A	79.5616	-1
**4200**	321	A	79.5761	0
**4200**	351	A	36.2922	-1
**4200**	357	A	62.1879	1
**4200**	370	A	79.5802	1
**4200**	372	A	55.8231	0
**4200**	373	A	73.7273	1
**4200**	381	A	57.9734	1
**4200**	382	A	11.4499	-1
**4200**	386	A	73.4528	1
**4200**	305	B	79.5318	1
**4200**	319	B	42.5772	0

**4200**	320	B	72.9514	-1
**4200**	321	B	79.5572	0
**4200**	351	B	28.1551	-1
**4200**	357	B	65.5184	1
**4200**	370	B	72.428	1
**4200**	372	B	76.4793	0
**4200**	373	B	46.8693	0
**4200**	381	B	63.5409	1
**4200**	382	B	40.766	0
**4200**	386	B	61.6488	1
**4200**	305	C	43.4259	0
**4200**	319	C	70.7823	1
**4200**	320	C	79.603	-1
**4200**	321	C	33.9703	1
**4200**	351	C	12.0212	1
**4200**	357	C	79.4424	1
**4200**	370	C	31.7624	0
**4200**	372	C	49.275	1
**4200**	373	C	79.5271	1
**4200**	381	C	28.2963	0
**4200**	382	C	4.7587	-1
**4200**	386	C	54.6218	1
**4200**	305	D	75.0858	1
**4200**	319	D	22.4482	0
**4200**	320	D	71.748	-1
**4200**	321	D	47.6799	1
**4200**	351	D	20.7557	-1
**4200**	357	D	79.5176	1
**4200**	370	D	76.0381	1
**4200**	372	D	64.9834	0
**4200**	373	D	76.0616	1
**4200**	381	D	79.3698	1
**4200**	382	D	73.0184	1
**4200**	386	D	20.9947	0

**4201**	305	A	58.9041	1
**4201**	319	A	76.9883	1
**4201**	320	A	79.5212	-1
**4201**	321	A	35.6838	1
**4201**	351	A	0.0	1
**4201**	357	A	55.4117	1
**4201**	370	A	79.6046	1
**4201**	372	A	52.9138	0
**4201**	373	A	72.9967	1
**4201**	381	A	79.5572	1
**4201**	382	A	73.2808	1
**4201**	386	A	79.595	1
**4201**	305	B	70.471	1
**4201**	319	B	74.3316	1
**4201**	320	B	35.1154	-1
**4201**	321	B	79.5211	0
**4201**	351	B	5.2483	1
**4201**	357	B	47.5754	0
**4201**	370	B	79.5879	1
**4201**	372	B	41.2309	1
**4201**	373	B	23.0862	0
**4201**	381	B	79.6115	1
**4201**	382	B	33.3651	0
**4201**	386	B	74.0484	1
**4201**	305	C	57.1983	1
**4201**	319	C	79.5424	1
**4201**	320	C	79.481	-1
**4201**	321	C	73.776	0
**4201**	351	C	4.0696	1
**4201**	357	C	75.907	1
**4201**	370	C	75.8734	1
**4201**	372	C	71.591	0
**4201**	373	C	78.7306	1
**4201**	381	C	69.6843	1

**4201**	382	C	31.6902	0
**4201**	386	C	50.4889	1
**4201**	305	D	79.5762	1
**4201**	319	D	62.3358	1
**4201**	320	D	79.6645	-1
**4201**	321	D	72.3077	0
**4201**	351	D	12.9431	1
**4201**	357	D	49.2893	0
**4201**	370	D	78.6294	1
**4201**	372	D	79.6688	0
**4201**	373	D	77.373	1
**4201**	381	D	71.5947	1
**4201**	382	D	78.3294	1
**4201**	386	D	72.634	1
**4202**	305	A	69.7467	1
**4202**	319	A	79.5309	1
**4202**	320	A	76.3606	-1
**4202**	321	A	76.8848	0
**4202**	351	A	9.4591	1
**4202**	357	A	79.5956	1
**4202**	370	A	79.6102	1
**4202**	372	A	79.4473	0
**4202**	373	A	43.3919	0
**4202**	381	A	79.5609	1
**4202**	382	A	66.4015	1
**4202**	386	A	77.3039	1
**4202**	305	B	66.1064	1
**4202**	319	B	73.2366	1
**4202**	320	B	79.6266	-1
**4202**	321	B	79.5171	0
**4202**	351	B	19.7086	1
**4202**	357	B	79.5305	1
**4202**	370	B	27.3675	0
**4202**	372	B	31.1988	1

**4202**	373	B	70.961	1
**4202**	381	B	79.6757	1
**4202**	382	B	47.1669	0
**4202**	386	B	53.5536	1
**4202**	305	C	79.5921	1
**4202**	319	C	79.6089	1
**4202**	320	C	37.1987	-1
**4202**	321	C	48.1847	1
**4202**	351	C	34.8107	-1
**4202**	357	C	54.8028	1
**4202**	370	C	78.5612	1
**4202**	372	C	75.1431	0
**4202**	373	C	43.9616	0
**4202**	381	C	38.4756	0
**4202**	382	C	79.4046	1
**4202**	386	C	4.9203	-1
**4202**	305	D	78.8021	1
**4202**	319	D	79.5064	1
**4202**	320	D	63.3037	-1
**4202**	321	D	79.5712	0
**4202**	351	D	9.1977	1
**4202**	357	D	79.5384	1
**4202**	370	D	61.6468	1
**4202**	372	D	65.9123	0
**4202**	373	D	79.5335	1
**4202**	381	D	63.0046	1
**4202**	382	D	63.9325	1
**4202**	386	D	79.4736	1
**4203**	305	A	74.7373	1
**4203**	319	A	58.9309	1
**4203**	320	A	79.5445	-1
**4203**	321	A	79.6067	0
**4203**	351	A	3.8133	1
**4203**	357	A	72.9282	1

**4203**	370	A	57.9246	1
**4203**	372	A	73.1382	0
**4203**	373	A	63.8722	1
**4203**	381	A	71.3669	1
**4203**	382	A	73.0407	1
**4203**	386	A	27.2645	0
**4203**	305	B	79.4539	1
**4203**	319	B	75.2869	1
**4203**	320	B	50.859	-1
**4203**	321	B	79.6531	0
**4203**	351	B	8.9071	1
**4203**	357	B	68.7053	1
**4203**	370	B	52.0805	1
**4203**	372	B	30.4232	1
**4203**	373	B	70.9887	1
**4203**	381	B	72.2537	1
**4203**	382	B	18.7893	-1
**4203**	386	B	61.4176	1
**4203**	305	C	79.6505	1
**4203**	319	C	45.5513	0
**4203**	320	C	72.4498	-1
**4203**	321	C	72.7952	0
**4203**	351	C	1.9832	1
**4203**	357	C	79.5777	1
**4203**	370	C	77.704	1
**4203**	372	C	76.8492	0
**4203**	373	C	71.1072	1
**4203**	381	C	79.5955	1
**4203**	382	C	79.5264	1
**4203**	386	C	61.697	1
**4203**	305	D	63.205	1
**4203**	319	D	79.4087	1
**4203**	320	D	79.433	-1
**4203**	321	D	72.453	0

**4203**	351	D	18.435	1
**4203**	357	D	20.716	0
**4203**	370	D	79.5891	1
**4203**	372	D	79.2979	0
**4203**	373	D	67.5218	1
**4203**	381	D	52.9499	1
**4203**	382	D	74.8263	1
**4203**	386	D	53.3724	1
**4204**	305	A	42.9035	0
**4204**	319	A	72.466	1
**4204**	320	A	79.5408	-1
**4204**	321	A	79.3737	0
**4204**	351	A	31.6345	-1
**4204**	357	A	26.1786	0
**4204**	370	A	41.4212	0
**4204**	372	A	76.3224	0
**4204**	373	A	36.1531	0
**4204**	381	A	57.3511	1
**4204**	382	A	79.5106	1
**4204**	386	A	79.6156	1
**4204**	305	B	77.4934	1
**4204**	319	B	75.0982	1
**4204**	320	B	62.2976	-1
**4204**	321	B	79.4895	0
**4204**	351	B	0.0206	1
**4204**	357	B	67.6521	1
**4204**	370	B	79.6108	1
**4204**	372	B	79.5617	0
**4204**	373	B	77.0255	1
**4204**	381	B	73.4163	1
**4204**	382	B	30.7673	0
**4204**	386	B	57.7923	1
**4204**	305	C	63.4495	1
**4204**	319	C	79.6438	1

**4204**	320	C	76.9225	-1
**4204**	321	C	72.3629	0
**4204**	351	C	5.2347	1
**4204**	357	C	72.1672	1
**4204**	370	C	55.8302	1
**4204**	372	C	64.6314	0
**4204**	373	C	79.7177	1
**4204**	381	C	79.5579	1
**4204**	382	C	79.6623	1
**4204**	386	C	46.691	0
**4204**	305	D	61.7769	1
**4204**	319	D	35.2738	0
**4204**	320	D	79.5417	-1
**4204**	321	D	79.4925	0
**4204**	351	D	13.107	1
**4204**	357	D	43.6692	0
**4204**	370	D	74.1834	1
**4204**	372	D	57.0859	0
**4204**	373	D	45.7262	0
**4204**	381	D	79.6231	1
**4204**	382	D	59.4768	1
**4204**	386	D	79.6547	1
**4205**	305	A	79.4812	1
**4205**	319	A	49.0558	0
**4205**	320	A	79.51	-1
**4205**	321	A	68.7236	0
**4205**	351	A	32.0746	-1
**4205**	357	A	43.4648	0
**4205**	370	A	36.105	0
**4205**	372	A	54.6182	0
**4205**	373	A	79.5355	1
**4205**	381	A	73.0775	1
**4205**	382	A	54.2766	1
**4205**	386	A	79.623	1

**4205**	305	B	72.0162	1
**4205**	319	B	43.267	0
**4205**	320	B	64.2422	-1
**4205**	321	B	32.8177	1
**4205**	351	B	21.5646	-1
**4205**	357	B	63.3141	1
**4205**	370	B	79.3966	1
**4205**	372	B	79.6464	0
**4205**	373	B	41.8601	0
**4205**	381	B	58.5655	1
**4205**	382	B	70.8094	1
**4205**	386	B	79.596	1
**4205**	305	C	70.7587	1
**4205**	319	C	79.4555	1
**4205**	320	C	36.8158	-1
**4205**	321	C	74.1636	0
**4205**	351	C	0.0	1
**4205**	357	C	51.7228	1
**4205**	370	C	63.8156	1
**4205**	372	C	79.497	0
**4205**	373	C	30.7264	0
**4205**	381	C	23.9515	0
**4205**	382	C	79.5095	1
**4205**	386	C	57.3451	1
**4205**	305	D	56.7133	1
**4205**	319	D	62.177	1
**4205**	320	D	74.5796	-1
**4205**	321	D	79.3846	0
**4205**	351	D	15.8776	1
**4205**	357	D	61.641	1
**4205**	370	D	44.2212	0
**4205**	372	D	75.4852	0
**4205**	373	D	55.5148	1
**4205**	381	D	79.6261	1

**4205**	382	D	79.5864	1
**4205**	386	D	79.5349	1
**4206**	305	A	75.2935	1
**4206**	319	A	79.6165	1
**4206**	320	A	67.5031	-1
**4206**	321	A	68.755	0
**4206**	351	A	7.5073	1
**4206**	357	A	40.836	0
**4206**	370	A	59.8121	1
**4206**	372	A	35.3331	1
**4206**	373	A	79.4996	1
**4206**	381	A	56.3435	1
**4206**	382	A	43.0472	0
**4206**	386	A	34.7102	0
**4206**	305	B	76.2424	1
**4206**	319	B	74.6254	1
**4206**	320	B	28.889	-1
**4206**	321	B	79.4673	0
**4206**	351	B	22.2047	-1
**4206**	357	B	79.5496	1
**4206**	370	B	77.221	1
**4206**	372	B	71.1872	0
**4206**	373	B	57.4099	1
**4206**	381	B	79.619	1
**4206**	382	B	21.0373	0
**4206**	386	B	79.6235	1
**4206**	305	C	70.6443	1
**4206**	319	C	79.6748	1
**4206**	320	C	79.5654	-1
**4206**	321	C	79.5657	0
**4206**	351	C	2.4104	1
**4206**	357	C	72.1556	1
**4206**	370	C	73.8948	1
**4206**	372	C	58.5129	0

**4206**	373	C	74.9384	1
**4206**	381	C	78.0386	1
**4206**	382	C	71.5342	1
**4206**	386	C	34.3975	0
**4206**	305	D	78.9503	1
**4206**	319	D	70.5859	1
**4206**	320	D	74.1844	-1
**4206**	321	D	73.5231	0
**4206**	351	D	4.0141	1
**4206**	357	D	79.5547	1
**4206**	370	D	57.8839	1
**4206**	372	D	68.3194	0
**4206**	373	D	70.7497	1
**4206**	381	D	79.6444	1
**4206**	382	D	47.3095	0
**4206**	386	D	79.5641	1
**4207**	305	A	61.7952	1
**4207**	319	A	56.1241	1
**4207**	320	A	71.2944	-1
**4207**	321	A	69.8946	0
**4207**	351	A	9.1847	1
**4207**	357	A	79.5544	1
**4207**	370	A	79.5688	1
**4207**	372	A	79.5582	0
**4207**	373	A	74.5062	1
**4207**	381	A	72.7883	1
**4207**	382	A	64.213	1
**4207**	386	A	71.5318	1
**4207**	305	B	79.5415	1
**4207**	319	B	79.5433	1
**4207**	320	B	70.832	-1
**4207**	321	B	75.8222	0
**4207**	351	B	18.0861	1
**4207**	357	B	73.466	1

**4207**	370	B	57.3833	1
**4207**	372	B	79.5625	0
**4207**	373	B	79.5198	1
**4207**	381	B	79.5751	1
**4207**	382	B	70.5378	1
**4207**	386	B	65.0782	1
**4207**	305	C	67.9567	1
**4207**	319	C	74.2743	1
**4207**	320	C	66.6515	-1
**4207**	321	C	74.7663	0
**4207**	351	C	6.7894	1
**4207**	357	C	65.8143	1
**4207**	370	C	79.575	1
**4207**	372	C	71.8934	0
**4207**	373	C	78.9342	1
**4207**	381	C	31.688	0
**4207**	382	C	73.7024	1
**4207**	386	C	49.2948	0
**4207**	305	D	51.4867	1
**4207**	319	D	79.546	1
**4207**	320	D	73.1669	-1
**4207**	321	D	79.2848	0
**4207**	351	D	13.2198	1
**4207**	357	D	73.1471	1
**4207**	370	D	44.8484	0
**4207**	372	D	79.5685	0
**4207**	373	D	51.4339	1
**4207**	381	D	79.5848	1
**4207**	382	D	65.2672	1
**4207**	386	D	79.5605	1
**4208**	305	A	73.123	1
**4208**	319	A	52.4895	1
**4208**	320	A	49.4581	-1
**4208**	321	A	62.2194	0

**4208**	351	A	3.0447	1
**4208**	357	A	21.7759	0
**4208**	370	A	79.507	1
**4208**	372	A	79.5705	0
**4208**	373	A	68.7899	1
**4208**	381	A	79.5571	1
**4208**	382	A	13.768	-1
**4208**	386	A	78.8744	1
**4208**	305	B	67.7203	1
**4208**	319	B	66.5238	1
**4208**	320	B	54.0616	-1
**4208**	321	B	70.27	0
**4208**	351	B	0.8257	1
**4208**	357	B	79.5366	1
**4208**	370	B	75.7443	1
**4208**	372	B	79.5917	0
**4208**	373	B	77.4628	1
**4208**	381	B	79.5561	1
**4208**	382	B	67.7209	1
**4208**	386	B	57.2229	1
**4208**	305	C	43.5314	0
**4208**	319	C	38.3162	0
**4208**	320	C	51.1273	-1
**4208**	321	C	29.686	1
**4208**	351	C	3.5976	1
**4208**	357	C	50.2342	1
**4208**	370	C	75.7341	1
**4208**	372	C	73.8337	0
**4208**	373	C	58.8225	1
**4208**	381	C	79.5448	1
**4208**	382	C	46.7273	0
**4208**	386	C	26.8402	0
**4208**	305	D	34.3538	0
**4208**	319	D	79.5117	1

**4208**	320	D	76.0583	-1
**4208**	321	D	63.9784	0
**4208**	351	D	15.843	1
**4208**	357	D	79.5759	1
**4208**	370	D	35.6477	0
**4208**	372	D	79.5475	0
**4208**	373	D	79.1438	1
**4208**	381	D	79.5717	1
**4208**	382	D	72.8089	1
**4208**	386	D	79.5907	1
**4209**	305	A	79.4507	1
**4209**	319	A	78.0123	1
**4209**	320	A	79.7337	-1
**4209**	321	A	76.1825	0
**4209**	351	A	16.7648	1
**4209**	357	A	52.5799	1
**4209**	370	A	78.4422	1
**4209**	372	A	68.6282	0
**4209**	373	A	79.5126	1
**4209**	381	A	74.8251	1
**4209**	382	A	41.5237	0
**4209**	386	A	79.5889	1
**4209**	305	B	79.473	1
**4209**	319	B	67.2445	1
**4209**	320	B	49.1016	-1
**4209**	321	B	46.2249	1
**4209**	351	B	18.4691	1
**4209**	357	B	79.7135	1
**4209**	370	B	74.7368	1
**4209**	372	B	73.8498	0
**4209**	373	B	62.9401	1
**4209**	381	B	76.8182	1
**4209**	382	B	37.6038	0
**4209**	386	B	36.0138	0

**4209**	305	C	29.7196	0
**4209**	319	C	73.2941	1
**4209**	320	C	74.3444	-1
**4209**	321	C	68.5981	0
**4209**	351	C	6.3026	1
**4209**	357	C	69.548	1
**4209**	370	C	57.5981	1
**4209**	372	C	70.8481	0
**4209**	373	C	68.5834	1
**4209**	381	C	79.5411	1
**4209**	382	C	75.0123	1
**4209**	386	C	58.1098	1
**4209**	305	D	74.6914	1
**4209**	319	D	69.8922	1
**4209**	320	D	79.6003	-1
**4209**	321	D	79.5921	0
**4209**	351	D	34.4743	-1
**4209**	357	D	70.2386	1
**4209**	370	D	79.5632	1
**4209**	372	D	72.7852	0
**4209**	373	D	29.2097	0
**4209**	381	D	79.3612	1
**4209**	382	D	72.8043	1
**4209**	386	D	63.12	1
**4210**	305	A	17.9468	-1
**4210**	319	A	71.1966	1
**4210**	320	A	73.7	-1
**4210**	321	A	78.9404	0
**4210**	351	A	1.976	1
**4210**	357	A	79.5467	1
**4210**	370	A	72.9079	1
**4210**	372	A	70.0081	0
**4210**	373	A	71.4531	1
**4210**	381	A	51.2149	1

**4210**	382	A	45.7181	0
**4210**	386	A	43.2718	0
**4210**	305	B	79.5308	1
**4210**	319	B	73.0191	1
**4210**	320	B	61.9358	-1
**4210**	321	B	68.3075	0
**4210**	351	B	11.3544	1
**4210**	357	B	79.6283	1
**4210**	370	B	79.6469	1
**4210**	372	B	71.7853	0
**4210**	373	B	66.3383	1
**4210**	381	B	79.6145	1
**4210**	382	B	72.794	1
**4210**	386	B	79.6198	1
**4210**	305	C	79.4503	1
**4210**	319	C	73.7042	1
**4210**	320	C	74.4102	-1
**4210**	321	C	70.5218	0
**4210**	351	C	13.1802	1
**4210**	357	C	29.2121	0
**4210**	370	C	79.7109	1
**4210**	372	C	79.3798	0
**4210**	373	C	50.7175	1
**4210**	381	C	74.5447	1
**4210**	382	C	31.4558	0
**4210**	386	C	60.371	1
**4210**	305	D	63.5122	1
**4210**	319	D	79.583	1
**4210**	320	D	55.5786	-1
**4210**	321	D	53.6828	0
**4210**	351	D	23.9917	-1
**4210**	357	D	79.4611	1
**4210**	370	D	37.9078	0
**4210**	372	D	72.5472	0

**4210**	373	D	74.1793	1
**4210**	381	D	46.1636	0
**4210**	382	D	76.3728	1
**4210**	386	D	79.6221	1
**4211**	305	A	48.1572	0
**4211**	319	A	71.4034	1
**4211**	320	A	60.2045	-1
**4211**	321	A	70.2048	0
**4211**	351	A	35.6208	-1
**4211**	357	A	77.0804	1
**4211**	370	A	79.6083	1
**4211**	372	A	50.8196	0
**4211**	373	A	79.509	1
**4211**	381	A	79.5108	1
**4211**	382	A	66.5433	1
**4211**	386	A	34.2273	0
**4211**	305	B	57.9813	1
**4211**	319	B	75.2617	1
**4211**	320	B	68.835	-1
**4211**	321	B	58.592	0
**4211**	351	B	20.929	-1
**4211**	357	B	45.8265	0
**4211**	370	B	71.643	1
**4211**	372	B	79.5632	0
**4211**	373	B	73.6651	1
**4211**	381	B	74.3957	1
**4211**	382	B	24.8844	0
**4211**	386	B	79.6559	1
**4211**	305	C	69.3197	1
**4211**	319	C	73.0187	1
**4211**	320	C	69.9015	-1
**4211**	321	C	74.5889	0
**4211**	351	C	2.5017	1
**4211**	357	C	75.6147	1

**4211**	370	C	78.4714	1
**4211**	372	C	79.6136	0
**4211**	373	C	64.0357	1
**4211**	381	C	38.8282	0
**4211**	382	C	79.2718	1
**4211**	386	C	26.3666	0
**4211**	305	D	79.6273	1
**4211**	319	D	66.7699	1
**4211**	320	D	55.2318	-1
**4211**	321	D	69.2966	0
**4211**	351	D	8.7231	1
**4211**	357	D	43.8354	0
**4211**	370	D	67.8509	1
**4211**	372	D	79.529	0
**4211**	373	D	54.5953	1
**4211**	381	D	71.4389	1
**4211**	382	D	63.4942	1
**4211**	386	D	31.2865	0
**4212**	305	A	76.2674	1
**4212**	319	A	39.3878	0
**4212**	320	A	71.1222	-1
**4212**	321	A	53.1954	0
**4212**	351	A	4.4345	1
**4212**	357	A	65.3357	1
**4212**	370	A	79.6337	1
**4212**	372	A	53.0338	0
**4212**	373	A	79.5986	1
**4212**	381	A	41.0335	0
**4212**	382	A	72.512	1
**4212**	386	A	33.8461	0
**4212**	305	B	79.5159	1
**4212**	319	B	49.1039	0
**4212**	320	B	79.1943	-1
**4212**	321	B	79.5528	0

**4212**	351	B	13.6254	1
**4212**	357	B	73.837	1
**4212**	370	B	79.6456	1
**4212**	372	B	73.3076	0
**4212**	373	B	49.503	0
**4212**	381	B	79.5869	1
**4212**	382	B	17.0525	-1
**4212**	386	B	68.7829	1
**4212**	305	C	52.856	1
**4212**	319	C	79.6304	1
**4212**	320	C	62.5244	-1
**4212**	321	C	62.3337	0
**4212**	351	C	6.2616	1
**4212**	357	C	79.54	1
**4212**	370	C	79.5189	1
**4212**	372	C	79.4939	0
**4212**	373	C	75.7451	1
**4212**	381	C	43.253	0
**4212**	382	C	64.2829	1
**4212**	386	C	64.5855	1
**4212**	305	D	79.6325	1
**4212**	319	D	67.3532	1
**4212**	320	D	72.778	-1
**4212**	321	D	71.5654	0
**4212**	351	D	9.715	1
**4212**	357	D	79.5819	1
**4212**	370	D	31.5651	0
**4212**	372	D	77.2547	0
**4212**	373	D	48.1758	0
**4212**	381	D	72.2541	1
**4212**	382	D	70.5985	1
**4212**	386	D	36.1824	0
**4213**	305	A	79.5746	1
**4213**	319	A	62.8998	1

**4213**	320	A	73.3527	-1
**4213**	321	A	69.9384	0
**4213**	351	A	6.8773	1
**4213**	357	A	79.1172	1
**4213**	370	A	79.492	1
**4213**	372	A	11.0007	0
**4213**	373	A	73.6455	1
**4213**	381	A	69.7104	1
**4213**	382	A	69.2864	1
**4213**	386	A	79.6221	1
**4213**	305	B	51.1065	1
**4213**	319	B	72.7583	1
**4213**	320	B	70.8729	-1
**4213**	321	B	66.2442	0
**4213**	351	B	19.7576	1
**4213**	357	B	74.5619	1
**4213**	370	B	64.8073	1
**4213**	372	B	68.2657	0
**4213**	373	B	78.2664	1
**4213**	381	B	79.638	1
**4213**	382	B	54.8443	1
**4213**	386	B	65.9651	1
**4213**	305	C	79.3674	1
**4213**	319	C	74.2945	1
**4213**	320	C	79.4942	-1
**4213**	321	C	70.7067	0
**4213**	351	C	4.0978	1
**4213**	357	C	72.8828	1
**4213**	370	C	78.6301	1
**4213**	372	C	73.5706	0
**4213**	373	C	75.6113	1
**4213**	381	C	79.5443	1
**4213**	382	C	79.2014	1
**4213**	386	C	40.8489	0

**4213**	305	D	68.9298	1
**4213**	319	D	69.436	1
**4213**	320	D	59.1729	-1
**4213**	321	D	79.4949	0
**4213**	351	D	15.3146	1
**4213**	357	D	70.2053	1
**4213**	370	D	73.0201	1
**4213**	372	D	79.3617	0
**4213**	373	D	63.7222	1
**4213**	381	D	74.2089	1
**4213**	382	D	79.6278	1
**4213**	386	D	79.5471	1
**4214**	305	A	79.6579	1
**4214**	319	A	62.2169	1
**4214**	320	A	71.5717	-1
**4214**	321	A	78.9664	0
**4214**	351	A	6.6472	1
**4214**	357	A	30.521	0
**4214**	370	A	56.2732	1
**4214**	372	A	61.494	0
**4214**	373	A	69.97	1
**4214**	381	A	73.5476	1
**4214**	382	A	78.0825	1
**4214**	386	A	48.3599	0
**4214**	305	B	67.2066	1
**4214**	319	B	79.5778	1
**4214**	320	B	59.5829	-1
**4214**	321	B	79.5591	0
**4214**	351	B	8.8892	1
**4214**	357	B	72.1371	1
**4214**	370	B	66.384	1
**4214**	372	B	79.4999	0
**4214**	373	B	45.108	0
**4214**	381	B	56.7807	1

**4214**	382	B	50.9441	1
**4214**	386	B	78.1626	1
**4214**	305	C	79.5538	1
**4214**	319	C	79.0708	1
**4214**	320	C	72.4506	-1
**4214**	321	C	71.6627	0
**4214**	351	C	13.9858	1
**4214**	357	C	51.3313	1
**4214**	370	C	79.6081	1
**4214**	372	C	79.5484	0
**4214**	373	C	41.4411	0
**4214**	381	C	73.4366	1
**4214**	382	C	77.4219	1
**4214**	386	C	61.9463	1
**4214**	305	D	79.5935	1
**4214**	319	D	79.4208	1
**4214**	320	D	79.6827	-1
**4214**	321	D	56.3849	0
**4214**	351	D	29.8682	-1
**4214**	357	D	64.2021	1
**4214**	370	D	62.997	1
**4214**	372	D	79.5765	0
**4214**	373	D	79.5151	1
**4214**	381	D	71.0451	1
**4214**	382	D	71.4049	1
**4214**	386	D	61.4267	1
**4215**	305	A	8.986	-1
**4215**	319	A	58.6361	1
**4215**	320	A	79.509	-1
**4215**	321	A	65.9644	0
**4215**	351	A	9.1553	1
**4215**	357	A	40.0956	0
**4215**	370	A	74.0292	1
**4215**	372	A	62.1337	0

**4215**	373	A	74.3464	1
**4215**	381	A	79.5603	1
**4215**	382	A	79.5586	1
**4215**	386	A	62.5344	1
**4215**	305	B	79.557	1
**4215**	319	B	79.5865	1
**4215**	320	B	31.9546	-1
**4215**	321	B	63.6741	0
**4215**	351	B	9.7355	1
**4215**	357	B	79.5583	1
**4215**	370	B	72.4433	1
**4215**	372	B	79.5583	0
**4215**	373	B	66.3766	1
**4215**	381	B	71.2502	1
**4215**	382	B	52.1333	1
**4215**	386	B	79.544	1
**4215**	305	C	62.2442	1
**4215**	319	C	77.475	1
**4215**	320	C	67.8418	-1
**4215**	321	C	25.1179	1
**4215**	351	C	5.269	1
**4215**	357	C	77.0768	1
**4215**	370	C	53.5496	1
**4215**	372	C	79.5448	0
**4215**	373	C	79.5608	1
**4215**	381	C	58.1749	1
**4215**	382	C	79.5814	1
**4215**	386	C	19.376	-1
**4215**	305	D	25.9785	0
**4215**	319	D	79.5418	1
**4215**	320	D	79.5769	-1
**4215**	321	D	38.6915	1
**4215**	351	D	15.0221	1
**4215**	357	D	79.564	1

**4215**	370	D	77.5269	1
**4215**	372	D	79.2597	0
**4215**	373	D	69.7569	1
**4215**	381	D	63.6488	1
**4215**	382	D	79.5342	1
**4215**	386	D	24.1909	0
**4216**	305	A	79.5627	1
**4216**	319	A	64.6062	1
**4216**	320	A	69.6232	-1
**4216**	321	A	79.6835	0
**4216**	351	A	3.272	1
**4216**	357	A	79.543	1
**4216**	370	A	34.913	0
**4216**	372	A	79.5512	0
**4216**	373	A	79.5783	1
**4216**	381	A	41.3929	0
**4216**	382	A	74.6107	1
**4216**	386	A	79.5137	1
**4216**	305	B	71.8377	1
**4216**	319	B	60.0246	1
**4216**	320	B	79.4122	-1
**4216**	321	B	79.0121	0
**4216**	351	B	9.8542	1
**4216**	357	B	60.3474	1
**4216**	370	B	66.5831	1
**4216**	372	B	62.2695	0
**4216**	373	B	71.0744	1
**4216**	381	B	18.7175	-1
**4216**	382	B	37.4723	0
**4216**	386	B	79.4493	1
**4216**	305	C	69.6354	1
**4216**	319	C	79.5397	1
**4216**	320	C	52.9232	-1
**4216**	321	C	44.1914	1

**4216**	351	C	7.8004	1
**4216**	357	C	64.0037	1
**4216**	370	C	66.9297	1
**4216**	372	C	68.495	0
**4216**	373	C	60.8797	1
**4216**	381	C	65.6878	1
**4216**	382	C	69.6293	1
**4216**	386	C	43.3702	0
**4216**	305	D	57.5926	1
**4216**	319	D	65.805	1
**4216**	320	D	79.544	-1
**4216**	321	D	68.5538	0
**4216**	351	D	12.696	1
**4216**	357	D	79.5211	1
**4216**	370	D	66.7546	1
**4216**	372	D	46.364	1
**4216**	373	D	79.5161	1
**4216**	381	D	38.7223	0
**4216**	382	D	67.1461	1
**4216**	386	D	77.2225	1
**4217**	305	A	79.5263	1
**4217**	319	A	60.5598	1
**4217**	320	A	73.7526	-1
**4217**	321	A	75.4411	0
**4217**	351	A	12.9192	1
**4217**	357	A	79.7167	1
**4217**	370	A	69.1222	1
**4217**	372	A	79.5919	0
**4217**	373	A	79.5199	1
**4217**	381	A	75.3358	1
**4217**	382	A	26.0928	0
**4217**	386	A	42.7133	0
**4217**	305	B	53.6753	1
**4217**	319	B	79.4822	1

**4217**	320	B	79.5228	-1
**4217**	321	B	67.7415	0
**4217**	351	B	10.2993	1
**4217**	357	B	57.584	1
**4217**	370	B	79.7253	1
**4217**	372	B	79.491	0
**4217**	373	B	38.1312	0
**4217**	381	B	77.3247	1
**4217**	382	B	21.0941	0
**4217**	386	B	71.7639	1
**4217**	305	C	56.8188	1
**4217**	319	C	60.2486	1
**4217**	320	C	71.9751	-1
**4217**	321	C	52.7582	0
**4217**	351	C	11.6053	1
**4217**	357	C	79.2752	1
**4217**	370	C	73.0873	1
**4217**	372	C	50.511	0
**4217**	373	C	68.1581	1
**4217**	381	C	79.5278	1
**4217**	382	C	36.7709	0
**4217**	386	C	41.2653	0
**4217**	305	D	68.8667	1
**4217**	319	D	24.7523	0
**4217**	320	D	79.319	-1
**4217**	321	D	71.3206	0
**4217**	351	D	17.173	1
**4217**	357	D	54.358	1
**4217**	370	D	50.7375	1
**4217**	372	D	56.6149	0
**4217**	373	D	79.5375	1
**4217**	381	D	60.0241	1
**4217**	382	D	69.4028	1
**4217**	386	D	49.9536	0

**4218**	305	A	52.0357	1
**4218**	319	A	75.2142	1
**4218**	320	A	66.3272	-1
**4218**	321	A	70.8766	0
**4218**	351	A	10.1073	1
**4218**	357	A	65.5242	1
**4218**	370	A	79.6049	1
**4218**	372	A	68.4638	0
**4218**	373	A	79.5939	1
**4218**	381	A	73.0163	1
**4218**	382	A	67.1342	1
**4218**	386	A	69.532	1
**4218**	305	B	54.9691	1
**4218**	319	B	71.3338	1
**4218**	320	B	66.6908	-1
**4218**	321	B	73.5605	0
**4218**	351	B	8.3395	1
**4218**	357	B	30.2658	0
**4218**	370	B	52.8478	1
**4218**	372	B	72.1466	0
**4218**	373	B	65.4961	1
**4218**	381	B	73.9173	1
**4218**	382	B	36.5018	0
**4218**	386	B	39.2266	0
**4218**	305	C	75.5808	1
**4218**	319	C	75.8243	1
**4218**	320	C	79.5631	-1
**4218**	321	C	62.5037	0
**4218**	351	C	8.0089	1
**4218**	357	C	78.3227	1
**4218**	370	C	78.1727	1
**4218**	372	C	79.5838	0
**4218**	373	C	79.5727	1
**4218**	381	C	66.1591	1

**4218**	382	C	77.5137	1
**4218**	386	C	64.0537	1
**4218**	305	D	69.5602	1
**4218**	319	D	79.5811	1
**4218**	320	D	72.6338	-1
**4218**	321	D	73.4934	0
**4218**	351	D	15.0635	1
**4218**	357	D	79.5433	1
**4218**	370	D	69.4789	1
**4218**	372	D	74.8642	0
**4218**	373	D	78.9373	1
**4218**	381	D	60.5075	1
**4218**	382	D	79.5919	1
**4218**	386	D	45.9297	0
**4219**	305	A	79.497	1
**4219**	319	A	71.4493	1
**4219**	320	A	60.7696	-1
**4219**	321	A	73.1992	0
**4219**	351	A	9.1352	1
**4219**	357	A	65.6982	1
**4219**	370	A	37.2502	0
**4219**	372	A	79.4625	0
**4219**	373	A	72.6272	1
**4219**	381	A	78.5226	1
**4219**	382	A	69.0283	1
**4219**	386	A	79.5034	1
**4219**	305	B	73.9717	1
**4219**	319	B	34.7122	0
**4219**	320	B	68.7466	-1
**4219**	321	B	72.0041	0
**4219**	351	B	7.2567	1
**4219**	357	B	71.7345	1
**4219**	370	B	79.0267	1
**4219**	372	B	74.7718	0

**4219**	373	B	79.5651	1
**4219**	381	B	64.1683	1
**4219**	382	B	28.5807	0
**4219**	386	B	79.3885	1
**4219**	305	C	71.7714	1
**4219**	319	C	79.5485	1
**4219**	320	C	70.7447	-1
**4219**	321	C	79.547	0
**4219**	351	C	9.1135	1
**4219**	357	C	24.9679	0
**4219**	370	C	79.4827	1
**4219**	372	C	26.4412	1
**4219**	373	C	79.5034	1
**4219**	381	C	75.483	1
**4219**	382	C	79.4532	1
**4219**	386	C	37.3033	0
**4219**	305	D	79.5871	1
**4219**	319	D	57.5929	1
**4219**	320	D	60.3616	-1
**4219**	321	D	79.5001	0
**4219**	351	D	8.2582	1
**4219**	357	D	60.1462	1
**4219**	370	D	43.0917	0
**4219**	372	D	75.236	0
**4219**	373	D	78.5744	1
**4219**	381	D	74.5087	1
**4219**	382	D	79.5448	1
**4219**	386	D	79.0526	1
**4220**	305	A	66.7638	1
**4220**	319	A	10.952	-1
**4220**	320	A	71.9569	-1
**4220**	321	A	79.5151	0
**4220**	351	A	4.0457	1
**4220**	357	A	63.9382	1

**4220**	370	A	79.1318	1
**4220**	372	A	79.545	0
**4220**	373	A	68.5207	1
**4220**	381	A	71.2367	1
**4220**	382	A	79.5843	1
**4220**	386	A	54.886	1
**4220**	305	B	79.6361	1
**4220**	319	B	75.3776	1
**4220**	320	B	79.5062	-1
**4220**	321	B	79.6052	0
**4220**	351	B	26.2515	-1
**4220**	357	B	45.8175	0
**4220**	370	B	79.1982	1
**4220**	372	B	65.0037	0
**4220**	373	B	55.99	1
**4220**	381	B	76.7914	1
**4220**	382	B	20.3372	0
**4220**	386	B	53.7176	1
**4220**	305	C	75.6426	1
**4220**	319	C	69.0402	1
**4220**	320	C	72.9643	-1
**4220**	321	C	79.6123	0
**4220**	351	C	12.3851	1
**4220**	357	C	57.0996	1
**4220**	370	C	69.0415	1
**4220**	372	C	74.5074	0
**4220**	373	C	78.3519	1
**4220**	381	C	79.5119	1
**4220**	382	C	79.2826	1
**4220**	386	C	35.1219	0
**4220**	305	D	61.4479	1
**4220**	319	D	47.8593	0
**4220**	320	D	75.8403	-1
**4220**	321	D	72.7164	0

**4220**	351	D	27.7081	-1
**4220**	357	D	79.6641	1
**4220**	370	D	79.3038	1
**4220**	372	D	68.5101	0
**4220**	373	D	17.2099	-1
**4220**	381	D	71.2105	1
**4220**	382	D	77.167	1
**4220**	386	D	47.9859	0
**4221**	305	A	79.5859	1
**4221**	319	A	68.836	1
**4221**	320	A	52.474	-1
**4221**	321	A	61.44	0
**4221**	351	A	6.1416	1
**4221**	357	A	35.1679	0
**4221**	370	A	42.8282	0
**4221**	372	A	21.126	1
**4221**	373	A	76.5152	1
**4221**	381	A	42.2989	0
**4221**	382	A	72.6263	1
**4221**	386	A	65.6937	1
**4221**	305	B	79.6439	1
**4221**	319	B	48.8636	0
**4221**	320	B	79.5559	-1
**4221**	321	B	20.2578	1
**4221**	351	B	13.3259	1
**4221**	357	B	49.6034	0
**4221**	370	B	59.4846	1
**4221**	372	B	73.8594	0
**4221**	373	B	79.6393	1
**4221**	381	B	79.5644	1
**4221**	382	B	78.8384	1
**4221**	386	B	38.2012	0
**4221**	305	C	79.6729	1
**4221**	319	C	76.8527	1

**4221**	320	C	79.5948	-1
**4221**	321	C	79.5137	0
**4221**	351	C	7.5798	1
**4221**	357	C	79.2965	1
**4221**	370	C	70.8282	1
**4221**	372	C	79.5428	0
**4221**	373	C	38.6743	0
**4221**	381	C	74.4614	1
**4221**	382	C	79.5488	1
**4221**	386	C	65.334	1
**4221**	305	D	65.9132	1
**4221**	319	D	79.5435	1
**4221**	320	D	51.3901	-1
**4221**	321	D	68.6304	0
**4221**	351	D	14.0137	1
**4221**	357	D	79.4965	1
**4221**	370	D	49.9722	0
**4221**	372	D	79.5214	0
**4221**	373	D	79.5428	1
**4221**	381	D	58.5249	1
**4221**	382	D	79.5425	1
**4221**	386	D	76.5777	1
**4222**	305	A	64.7436	1
**4222**	319	A	79.5727	1
**4222**	320	A	79.5133	-1
**4222**	321	A	73.7046	0
**4222**	351	A	4.5052	1
**4222**	357	A	79.5863	1
**4222**	370	A	63.907	1
**4222**	372	A	36.191	1
**4222**	373	A	73.064	1
**4222**	381	A	65.3768	1
**4222**	382	A	61.1473	1
**4222**	386	A	63.3647	1

**4222**	305	B	79.5667	1
**4222**	319	B	67.0143	1
**4222**	320	B	75.8924	-1
**4222**	321	B	69.9898	0
**4222**	351	B	6.5008	1
**4222**	357	B	79.568	1
**4222**	370	B	79.5507	1
**4222**	372	B	78.3953	0
**4222**	373	B	51.273	1
**4222**	381	B	56.0903	1
**4222**	382	B	38.9807	0
**4222**	386	B	56.2564	1
**4222**	305	C	55.5807	1
**4222**	319	C	70.074	1
**4222**	320	C	79.5507	-1
**4222**	321	C	57.3135	0
**4222**	351	C	5.7008	1
**4222**	357	C	72.3174	1
**4222**	370	C	79.3832	1
**4222**	372	C	77.6372	0
**4222**	373	C	66.7363	1
**4222**	381	C	79.5512	1
**4222**	382	C	54.1263	1
**4222**	386	C	38.9365	0
**4222**	305	D	51.6327	1
**4222**	319	D	79.6068	1
**4222**	320	D	79.5166	-1
**4222**	321	D	72.8516	0
**4222**	351	D	4.5525	1
**4222**	357	D	53.6207	1
**4222**	370	D	30.4606	0
**4222**	372	D	79.5882	0
**4222**	373	D	51.6794	1
**4222**	381	D	79.5525	1

**4222**	382	D	69.306	1
**4222**	386	D	58.1087	1
**4223**	305	A	65.1838	1
**4223**	319	A	79.5572	1
**4223**	320	A	62.8923	-1
**4223**	321	A	57.0057	0
**4223**	351	A	14.0274	1
**4223**	357	A	62.5673	1
**4223**	370	A	79.4693	1
**4223**	372	A	79.7163	0
**4223**	373	A	68.6184	1
**4223**	381	A	79.5895	1
**4223**	382	A	78.1335	1
**4223**	386	A	61.435	1
**4223**	305	B	79.5817	1
**4223**	319	B	78.77	1
**4223**	320	B	73.222	-1
**4223**	321	B	49.6327	1
**4223**	351	B	10.2961	1
**4223**	357	B	57.4326	1
**4223**	370	B	67.7401	1
**4223**	372	B	66.6011	0
**4223**	373	B	1.4636	-1
**4223**	381	B	58.2369	1
**4223**	382	B	42.4433	0
**4223**	386	B	16.5115	-1
**4223**	305	C	79.456	1
**4223**	319	C	46.0798	0
**4223**	320	C	37.4439	-1
**4223**	321	C	72.107	0
**4223**	351	C	10.0464	1
**4223**	357	C	79.5281	1
**4223**	370	C	67.8095	1
**4223**	372	C	62.5123	0

**4223**	373	C	78.4598	1
**4223**	381	C	79.3794	1
**4223**	382	C	63.5214	1
**4223**	386	C	37.4554	0
**4223**	305	D	68.7466	1
**4223**	319	D	65.9579	1
**4223**	320	D	79.1701	-1
**4223**	321	D	60.5951	0
**4223**	351	D	14.2756	1
**4223**	357	D	62.2054	1
**4223**	370	D	79.5291	1
**4223**	372	D	72.9763	0
**4223**	373	D	68.4967	1
**4223**	381	D	67.4816	1
**4223**	382	D	79.5823	1
**4223**	386	D	79.5867	1
**4224**	305	A	14.4352	-1
**4224**	319	A	73.2919	1
**4224**	320	A	65.1597	-1
**4224**	321	A	67.7915	0
**4224**	351	A	8.4087	1
**4224**	357	A	67.7935	1
**4224**	370	A	48.6487	0
**4224**	372	A	79.56	0
**4224**	373	A	62.1117	1
**4224**	381	A	79.5687	1
**4224**	382	A	79.5366	1
**4224**	386	A	17.4988	-1
**4224**	305	B	72.3224	1
**4224**	319	B	79.576	1
**4224**	320	B	40.0833	-1
**4224**	321	B	53.8345	0
**4224**	351	B	13.1839	1
**4224**	357	B	48.5363	0

**4224**	370	B	79.5686	1
**4224**	372	B	70.0077	0
**4224**	373	B	44.2051	0
**4224**	381	B	46.1128	0
**4224**	382	B	79.664	1
**4224**	386	B	58.9674	1
**4224**	305	C	79.1257	1
**4224**	319	C	66.396	1
**4224**	320	C	78.824	-1
**4224**	321	C	72.1599	0
**4224**	351	C	6.1248	1
**4224**	357	C	45.8814	0
**4224**	370	C	48.8372	0
**4224**	372	C	61.1587	0
**4224**	373	C	63.7752	1
**4224**	381	C	77.7238	1
**4224**	382	C	54.5311	1
**4224**	386	C	32.2379	0
**4224**	305	D	38.3201	0
**4224**	319	D	71.3009	1
**4224**	320	D	79.4988	-1
**4224**	321	D	79.5008	0
**4224**	351	D	19.4742	1
**4224**	357	D	73.4528	1
**4224**	370	D	78.1039	1
**4224**	372	D	79.5695	0
**4224**	373	D	79.5221	1
**4224**	381	D	68.9099	1
**4224**	382	D	72.0735	1
**4224**	386	D	51.1448	1
**4225**	305	A	61.1029	1
**4225**	319	A	72.3214	1
**4225**	320	A	79.5945	-1
**4225**	321	A	74.3532	0

**4225**	351	A	10.0898	1
**4225**	357	A	79.6102	1
**4225**	370	A	49.6229	0
**4225**	372	A	65.8342	0
**4225**	373	A	79.5504	1
**4225**	381	A	79.6128	1
**4225**	382	A	46.8157	0
**4225**	386	A	74.02	1
**4225**	305	B	79.5112	1
**4225**	319	B	79.6175	1
**4225**	320	B	79.4492	-1
**4225**	321	B	32.5856	1
**4225**	351	B	5.6943	1
**4225**	357	B	74.2864	1
**4225**	370	B	73.7416	1
**4225**	372	B	75.8551	0
**4225**	373	B	19.8881	-1
**4225**	381	B	63.7042	1
**4225**	382	B	49.6286	0
**4225**	386	B	65.2998	1
**4225**	305	C	73.122	1
**4225**	319	C	68.155	1
**4225**	320	C	59.8071	-1
**4225**	321	C	55.6823	0
**4225**	351	C	9.2652	1
**4225**	357	C	79.6784	1
**4225**	370	C	73.2093	1
**4225**	372	C	79.6034	0
**4225**	373	C	71.4877	1
**4225**	381	C	79.6294	1
**4225**	382	C	79.6425	1
**4225**	386	C	46.6848	0
**4225**	305	D	73.1749	1
**4225**	319	D	62.5485	1

**4225**	320	D	54.3423	-1
**4225**	321	D	78.6961	0
**4225**	351	D	14.4364	1
**4225**	357	D	79.5957	1
**4225**	370	D	59.8413	1
**4225**	372	D	79.548	0
**4225**	373	D	48.5419	0
**4225**	381	D	46.6218	0
**4225**	382	D	67.5094	1
**4225**	386	D	79.6089	1
**4226**	305	A	53.0286	1
**4226**	319	A	49.1841	0
**4226**	320	A	79.6404	-1
**4226**	321	A	78.0438	0
**4226**	351	A	3.1205	1
**4226**	357	A	59.9217	1
**4226**	370	A	59.6612	1
**4226**	372	A	73.8072	0
**4226**	373	A	68.6886	1
**4226**	381	A	66.0028	1
**4226**	382	A	73.9344	1
**4226**	386	A	72.1754	1
**4226**	305	B	60.3359	1
**4226**	319	B	76.7952	1
**4226**	320	B	57.9498	-1
**4226**	321	B	71.2644	0
**4226**	351	B	16.7862	1
**4226**	357	B	72.8792	1
**4226**	370	B	76.8134	1
**4226**	372	B	73.4007	0
**4226**	373	B	72.2615	1
**4226**	381	B	79.5114	1
**4226**	382	B	54.1441	1
**4226**	386	B	79.5689	1

**4226**	305	C	79.6033	1
**4226**	319	C	79.6032	1
**4226**	320	C	67.6217	-1
**4226**	321	C	79.5629	0
**4226**	351	C	0.0318	1
**4226**	357	C	62.907	1
**4226**	370	C	73.781	1
**4226**	372	C	44.4036	1
**4226**	373	C	54.0537	1
**4226**	381	C	79.1933	1
**4226**	382	C	56.4105	1
**4226**	386	C	63.934	1
**4226**	305	D	77.215	1
**4226**	319	D	79.5907	1
**4226**	320	D	70.1656	-1
**4226**	321	D	79.5705	0
**4226**	351	D	10.1729	1
**4226**	357	D	68.0148	1
**4226**	370	D	27.2593	0
**4226**	372	D	61.3286	0
**4226**	373	D	79.5487	1
**4226**	381	D	39.785	0
**4226**	382	D	57.5982	1
**4226**	386	D	79.5312	1
**4227**	305	A	76.8227	1
**4227**	319	A	67.3277	1
**4227**	320	A	43.2097	-1
**4227**	321	A	79.5783	0
**4227**	351	A	15.5941	1
**4227**	357	A	73.3431	1
**4227**	370	A	76.8375	1
**4227**	372	A	49.4312	1
**4227**	373	A	23.4405	0
**4227**	381	A	79.5971	1

**4227**	382	A	39.3607	0
**4227**	386	A	77.9832	1
**4227**	305	B	66.4385	1
**4227**	319	B	54.704	1
**4227**	320	B	67.2557	-1
**4227**	321	B	24.3399	1
**4227**	351	B	18.7363	1
**4227**	357	B	79.5545	1
**4227**	370	B	79.5669	1
**4227**	372	B	76.4791	0
**4227**	373	B	76.8224	1
**4227**	381	B	57.9828	1
**4227**	382	B	79.6239	1
**4227**	386	B	64.9455	1
**4227**	305	C	35.5781	0
**4227**	319	C	66.3785	1
**4227**	320	C	40.5071	-1
**4227**	321	C	43.6599	1
**4227**	351	C	5.0386	1
**4227**	357	C	58.3443	1
**4227**	370	C	79.6113	1
**4227**	372	C	79.5251	0
**4227**	373	C	57.412	1
**4227**	381	C	41.1207	0
**4227**	382	C	71.202	1
**4227**	386	C	24.9037	0
**4227**	305	D	54.7885	1
**4227**	319	D	79.4726	1
**4227**	320	D	44.6282	-1
**4227**	321	D	73.5292	0
**4227**	351	D	15.2279	1
**4227**	357	D	57.2288	1
**4227**	370	D	79.5239	1
**4227**	372	D	53.2208	0

**4227**	373	D	70.769	1
**4227**	381	D	79.5419	1
**4227**	382	D	79.4849	1
**4227**	386	D	45.8084	0
**4228**	305	A	75.5437	1
**4228**	319	A	18.2949	-1
**4228**	320	A	65.1772	-1
**4228**	321	A	67.0905	0
**4228**	351	A	18.9864	1
**4228**	357	A	58.6508	1
**4228**	370	A	57.1438	1
**4228**	372	A	71.4053	0
**4228**	373	A	72.8769	1
**4228**	381	A	75.2401	1
**4228**	382	A	65.4102	1
**4228**	386	A	71.423	1
**4228**	305	B	79.0432	1
**4228**	319	B	72.8326	1
**4228**	320	B	79.4637	-1
**4228**	321	B	72.8052	0
**4228**	351	B	10.7253	1
**4228**	357	B	50.5626	1
**4228**	370	B	56.0859	1
**4228**	372	B	79.273	0
**4228**	373	B	49.7688	0
**4228**	381	B	79.4986	1
**4228**	382	B	78.1724	1
**4228**	386	B	47.5926	0
**4228**	305	C	63.3885	1
**4228**	319	C	78.2658	1
**4228**	320	C	60.8495	-1
**4228**	321	C	79.653	0
**4228**	351	C	5.3095	1
**4228**	357	C	50.1644	1

**4228**	370	C	65.0656	1
**4228**	372	C	79.5802	0
**4228**	373	C	50.4106	1
**4228**	381	C	79.5611	1
**4228**	382	C	79.4734	1
**4228**	386	C	5.3928	-1
**4228**	305	D	74.1413	1
**4228**	319	D	79.5213	1
**4228**	320	D	60.8149	-1
**4228**	321	D	69.7978	0
**4228**	351	D	21.9048	-1
**4228**	357	D	59.3128	1
**4228**	370	D	79.3477	1
**4228**	372	D	79.5927	0
**4228**	373	D	79.5108	1
**4228**	381	D	42.3209	0
**4228**	382	D	69.9151	1
**4228**	386	D	53.9564	1
**4229**	305	A	79.4339	1
**4229**	319	A	44.5285	0
**4229**	320	A	13.9475	1
**4229**	321	A	56.3301	0
**4229**	351	A	4.3299	1
**4229**	357	A	79.6248	1
**4229**	370	A	60.8285	1
**4229**	372	A	79.4502	0
**4229**	373	A	51.3255	1
**4229**	381	A	53.226	1
**4229**	382	A	70.3295	1
**4229**	386	A	74.1145	1
**4229**	305	B	69.3034	1
**4229**	319	B	73.8998	1
**4229**	320	B	68.4336	-1
**4229**	321	B	58.3756	0

**4229**	351	B	8.9598	1
**4229**	357	B	78.4625	1
**4229**	370	B	79.4929	1
**4229**	372	B	60.3876	0
**4229**	373	B	56.8912	1
**4229**	381	B	73.4288	1
**4229**	382	B	58.5099	1
**4229**	386	B	59.2046	1
**4229**	305	C	60.9508	1
**4229**	319	C	77.8555	1
**4229**	320	C	62.6939	-1
**4229**	321	C	68.3298	0
**4229**	351	C	13.5083	1
**4229**	357	C	77.5779	1
**4229**	370	C	39.9476	0
**4229**	372	C	58.0622	0
**4229**	373	C	74.853	1
**4229**	381	C	64.421	1
**4229**	382	C	72.3942	1
**4229**	386	C	68.4004	1
**4229**	305	D	79.5003	1
**4229**	319	D	46.2369	0
**4229**	320	D	60.4353	-1
**4229**	321	D	79.0166	0
**4229**	351	D	13.0551	1
**4229**	357	D	79.2418	1
**4229**	370	D	79.4284	1
**4229**	372	D	74.1708	0
**4229**	373	D	73.0394	1
**4229**	381	D	43.1883	0
**4229**	382	D	71.0685	1
**4229**	386	D	75.8675	1
**4230**	305	A	61.9854	1
**4230**	319	A	69.4098	1

**4230**	320	A	79.5591	-1
**4230**	321	A	65.7863	0
**4230**	351	A	7.6856	1
**4230**	357	A	66.6501	1
**4230**	370	A	79.5391	1
**4230**	372	A	60.0393	0
**4230**	373	A	32.6951	0
**4230**	381	A	51.3578	1
**4230**	382	A	79.552	1
**4230**	386	A	72.9546	1
**4230**	305	B	79.5503	1
**4230**	319	B	62.5878	1
**4230**	320	B	31.234	-1
**4230**	321	B	79.5753	0
**4230**	351	B	13.5737	1
**4230**	357	B	59.8412	1
**4230**	370	B	79.5774	1
**4230**	372	B	34.6909	1
**4230**	373	B	79.2372	1
**4230**	381	B	67.5964	1
**4230**	382	B	34.6613	0
**4230**	386	B	70.9831	1
**4230**	305	C	77.8951	1
**4230**	319	C	66.362	1
**4230**	320	C	61.3922	-1
**4230**	321	C	57.6635	0
**4230**	351	C	4.1494	1
**4230**	357	C	73.6956	1
**4230**	370	C	53.525	1
**4230**	372	C	79.6085	0
**4230**	373	C	79.5493	1
**4230**	381	C	79.5197	1
**4230**	382	C	79.5854	1
**4230**	386	C	55.3393	1

**4230**	305	D	73.8136	1
**4230**	319	D	79.5721	1
**4230**	320	D	71.7798	-1
**4230**	321	D	78.4001	0
**4230**	351	D	13.2706	1
**4230**	357	D	79.5456	1
**4230**	370	D	47.9321	0
**4230**	372	D	79.5477	0
**4230**	373	D	79.5864	1
**4230**	381	D	72.9932	1
**4230**	382	D	79.5872	1
**4230**	386	D	72.5257	1
**4231**	305	A	66.86	1
**4231**	319	A	79.6473	1
**4231**	320	A	74.3906	-1
**4231**	321	A	33.6947	1
**4231**	351	A	8.0439	1
**4231**	357	A	60.5327	1
**4231**	370	A	35.8915	0
**4231**	372	A	64.4902	0
**4231**	373	A	47.0123	0
**4231**	381	A	30.1593	0
**4231**	382	A	79.4902	1
**4231**	386	A	59.8536	1
**4231**	305	B	52.6393	1
**4231**	319	B	75.0632	1
**4231**	320	B	23.131	-1
**4231**	321	B	73.6344	0
**4231**	351	B	15.2618	1
**4231**	357	B	57.4254	1
**4231**	370	B	63.497	1
**4231**	372	B	61.8724	0
**4231**	373	B	79.5485	1
**4231**	381	B	70.2801	1

**4231**	382	B	36.2735	0
**4231**	386	B	79.6558	1
**4231**	305	C	70.5827	1
**4231**	319	C	57.3218	1
**4231**	320	C	76.5717	-1
**4231**	321	C	79.5083	0
**4231**	351	C	9.684	1
**4231**	357	C	72.9848	1
**4231**	370	C	77.1367	1
**4231**	372	C	74.2363	0
**4231**	373	C	71.2503	1
**4231**	381	C	79.6041	1
**4231**	382	C	76.0068	1
**4231**	386	C	40.6639	0
**4231**	305	D	45.2133	0
**4231**	319	D	71.8027	1
**4231**	320	D	32.8123	-1
**4231**	321	D	56.3217	0
**4231**	351	D	11.1101	1
**4231**	357	D	79.5473	1
**4231**	370	D	56.0355	1
**4231**	372	D	71.5898	0
**4231**	373	D	79.6168	1
**4231**	381	D	44.3651	0
**4231**	382	D	52.8526	1
**4231**	386	D	54.5792	1
**4232**	305	A	58.5609	1
**4232**	319	A	43.4305	0
**4232**	320	A	79.6417	-1
**4232**	321	A	76.7747	0
**4232**	351	A	7.5007	1
**4232**	357	A	69.0773	1
**4232**	370	A	79.6473	1
**4232**	372	A	67.1195	0

**4232**	373	A	35.8672	0
**4232**	381	A	35.735	0
**4232**	382	A	49.3866	0
**4232**	386	A	73.5204	1
**4232**	305	B	79.5105	1
**4232**	319	B	63.3899	1
**4232**	320	B	77.2133	-1
**4232**	321	B	57.0656	0
**4232**	351	B	17.3598	1
**4232**	357	B	72.4644	1
**4232**	370	B	78.7271	1
**4232**	372	B	79.3558	0
**4232**	373	B	44.2892	0
**4232**	381	B	79.6846	1
**4232**	382	B	64.6286	1
**4232**	386	B	72.4942	1
**4232**	305	C	79.5621	1
**4232**	319	C	69.6756	1
**4232**	320	C	46.4688	-1
**4232**	321	C	79.5917	0
**4232**	351	C	0.5475	1
**4232**	357	C	49.6895	0
**4232**	370	C	78.6704	1
**4232**	372	C	67.9992	0
**4232**	373	C	79.5802	1
**4232**	381	C	79.4912	1
**4232**	382	C	50.5237	1
**4232**	386	C	65.7214	1
**4232**	305	D	54.926	1
**4232**	319	D	79.4875	1
**4232**	320	D	72.8423	-1
**4232**	321	D	53.0054	0
**4232**	351	D	5.2774	1
**4232**	357	D	69.8724	1

**4232**	370	D	58.0302	1
**4232**	372	D	79.576	0
**4232**	373	D	73.9106	1
**4232**	381	D	72.7945	1
**4232**	382	D	79.5445	1
**4232**	386	D	58.213	1
**4233**	305	A	79.5817	1
**4233**	319	A	72.9276	1
**4233**	320	A	79.5482	-1
**4233**	321	A	73.45	0
**4233**	351	A	0.7312	1
**4233**	357	A	78.8371	1
**4233**	370	A	79.3143	1
**4233**	372	A	55.3014	0
**4233**	373	A	60.8734	1
**4233**	381	A	79.7	1
**4233**	382	A	51.0662	1
**4233**	386	A	63.6715	1
**4233**	305	B	79.5638	1
**4233**	319	B	68.7327	1
**4233**	320	B	79.6359	-1
**4233**	321	B	49.0991	1
**4233**	351	B	8.7321	1
**4233**	357	B	65.2535	1
**4233**	370	B	72.3648	1
**4233**	372	B	22.8037	1
**4233**	373	B	40.2318	0
**4233**	381	B	79.673	1
**4233**	382	B	62.1225	1
**4233**	386	B	70.7747	1
**4233**	305	C	62.6824	1
**4233**	319	C	67.9958	1
**4233**	320	C	79.5176	-1
**4233**	321	C	78.8885	0

**4233**	351	C	0.0766	1
**4233**	357	C	79.5852	1
**4233**	370	C	71.6399	1
**4233**	372	C	79.6921	0
**4233**	373	C	67.067	1
**4233**	381	C	64.3677	1
**4233**	382	C	50.2371	1
**4233**	386	C	52.6688	1
**4233**	305	D	79.4889	1
**4233**	319	D	79.5068	1
**4233**	320	D	38.5603	-1
**4233**	321	D	79.4459	0
**4233**	351	D	9.2856	1
**4233**	357	D	62.1659	1
**4233**	370	D	73.1634	1
**4233**	372	D	58.6034	0
**4233**	373	D	77.6407	1
**4233**	381	D	73.9241	1
**4233**	382	D	59.0245	1
**4233**	386	D	79.336	1
**4234**	305	A	68.8977	1
**4234**	319	A	64.724	1
**4234**	320	A	56.3655	-1
**4234**	321	A	59.5824	0
**4234**	351	A	6.3744	1
**4234**	357	A	78.7254	1
**4234**	370	A	62.7577	1
**4234**	372	A	67.5736	0
**4234**	373	A	33.724	0
**4234**	381	A	79.5492	1
**4234**	382	A	36.9177	0
**4234**	386	A	35.3883	0
**4234**	305	B	62.909	1
**4234**	319	B	53.4633	1

**4234**	320	B	77.5146	-1
**4234**	321	B	79.5635	0
**4234**	351	B	13.9586	1
**4234**	357	B	78.1819	1
**4234**	370	B	59.4993	1
**4234**	372	B	46.2751	1
**4234**	373	B	62.5119	1
**4234**	381	B	58.335	1
**4234**	382	B	36.8973	0
**4234**	386	B	52.4456	1
**4234**	305	C	76.0668	1
**4234**	319	C	78.9098	1
**4234**	320	C	58.8135	-1
**4234**	321	C	63.8288	0
**4234**	351	C	3.4409	1
**4234**	357	C	79.5116	1
**4234**	370	C	79.5641	1
**4234**	372	C	79.5579	0
**4234**	373	C	79.1807	1
**4234**	381	C	79.5575	1
**4234**	382	C	16.7292	-1
**4234**	386	C	55.1284	1
**4234**	305	D	57.2204	1
**4234**	319	D	40.8151	0
**4234**	320	D	79.5546	-1
**4234**	321	D	79.5825	0
**4234**	351	D	14.7727	1
**4234**	357	D	79.5127	1
**4234**	370	D	46.292	0
**4234**	372	D	79.5442	0
**4234**	373	D	79.549	1
**4234**	381	D	69.2377	1
**4234**	382	D	40.923	0
**4234**	386	D	72.6961	1

**4235**	305	A	72.9166	1
**4235**	319	A	57.955	1
**4235**	320	A	79.5382	-1
**4235**	321	A	52.5155	0
**4235**	351	A	7.8252	1
**4235**	357	A	79.6046	1
**4235**	370	A	79.5452	1
**4235**	372	A	79.7676	0
**4235**	373	A	79.4803	1
**4235**	381	A	79.4808	1
**4235**	382	A	74.254	1
**4235**	386	A	74.1882	1
**4235**	305	B	67.8161	1
**4235**	319	B	74.2838	1
**4235**	320	B	68.8707	-1
**4235**	321	B	70.4234	0
**4235**	351	B	17.1662	1
**4235**	357	B	79.4452	1
**4235**	370	B	79.6328	1
**4235**	372	B	79.5353	0
**4235**	373	B	71.7236	1
**4235**	381	B	79.6584	1
**4235**	382	B	47.2361	0
**4235**	386	B	67.0382	1
**4235**	305	C	72.5197	1
**4235**	319	C	73.1685	1
**4235**	320	C	79.5062	-1
**4235**	321	C	73.1476	0
**4235**	351	C	1.8077	1
**4235**	357	C	79.6157	1
**4235**	370	C	77.4081	1
**4235**	372	C	77.0129	0
**4235**	373	C	60.8352	1
**4235**	381	C	79.5552	1

**4235**	382	C	78.2305	1
**4235**	386	C	53.8105	1
**4235**	305	D	79.6114	1
**4235**	319	D	56.2696	1
**4235**	320	D	66.5251	-1
**4235**	321	D	66.3487	0
**4235**	351	D	9.3303	1
**4235**	357	D	69.0251	1
**4235**	370	D	75.1597	1
**4235**	372	D	79.5873	0
**4235**	373	D	56.9816	1
**4235**	381	D	59.1068	1
**4235**	382	D	79.5374	1
**4235**	386	D	79.4978	1
**4236**	305	A	64.0867	1
**4236**	319	A	37.601	0
**4236**	320	A	79.6304	-1
**4236**	321	A	79.5327	0
**4236**	351	A	7.197	1
**4236**	357	A	63.8741	1
**4236**	370	A	59.9263	1
**4236**	372	A	44.7277	1
**4236**	373	A	37.1061	0
**4236**	381	A	35.3839	0
**4236**	382	A	71.3535	1
**4236**	386	A	79.5155	1
**4236**	305	B	79.2061	1
**4236**	319	B	79.7331	1
**4236**	320	B	78.792	-1
**4236**	321	B	63.597	0
**4236**	351	B	1.498	1
**4236**	357	B	79.6616	1
**4236**	370	B	58.0911	1
**4236**	372	B	61.8227	0

**4236**	373	B	51.3732	1
**4236**	381	B	67.4027	1
**4236**	382	B	46.4081	0
**4236**	386	B	79.4539	1
**4236**	305	C	79.2275	1
**4236**	319	C	79.5834	1
**4236**	320	C	67.2197	-1
**4236**	321	C	59.9369	0
**4236**	351	C	12.6198	1
**4236**	357	C	77.4145	1
**4236**	370	C	64.4946	1
**4236**	372	C	78.5423	0
**4236**	373	C	75.588	1
**4236**	381	C	72.7228	1
**4236**	382	C	75.1755	1
**4236**	386	C	11.7393	-1
**4236**	305	D	13.917	-1
**4236**	319	D	71.857	1
**4236**	320	D	70.3891	-1
**4236**	321	D	67.2669	0
**4236**	351	D	16.0356	1
**4236**	357	D	74.101	1
**4236**	370	D	62.1479	1
**4236**	372	D	79.6325	0
**4236**	373	D	52.5003	1
**4236**	381	D	62.558	1
**4236**	382	D	41.0095	0
**4236**	386	D	79.6596	1
**4237**	305	A	69.7765	1
**4237**	319	A	66.6039	1
**4237**	320	A	79.5421	-1
**4237**	321	A	79.5249	0
**4237**	351	A	4.5507	1
**4237**	357	A	79.5406	1

**4237**	370	A	74.2933	1
**4237**	372	A	62.9708	0
**4237**	373	A	73.3406	1
**4237**	381	A	57.0221	1
**4237**	382	A	72.4955	1
**4237**	386	A	75.7449	1
**4237**	305	B	79.2358	1
**4237**	319	B	65.4346	1
**4237**	320	B	40.2435	-1
**4237**	321	B	74.1398	0
**4237**	351	B	16.0701	1
**4237**	357	B	76.952	1
**4237**	370	B	79.486	1
**4237**	372	B	67.5428	0
**4237**	373	B	59.5051	1
**4237**	381	B	71.3012	1
**4237**	382	B	77.3625	1
**4237**	386	B	65.0383	1
**4237**	305	C	58.3962	1
**4237**	319	C	72.1961	1
**4237**	320	C	57.2183	-1
**4237**	321	C	73.8737	0
**4237**	351	C	31.4129	-1
**4237**	357	C	73.6984	1
**4237**	370	C	53.7839	1
**4237**	372	C	52.1497	0
**4237**	373	C	72.2869	1
**4237**	381	C	57.6147	1
**4237**	382	C	79.5816	1
**4237**	386	C	48.2443	0
**4237**	305	D	71.5729	1
**4237**	319	D	71.2508	1
**4237**	320	D	30.414	-1
**4237**	321	D	73.9271	0

**4237**	351	D	13.792	1
**4237**	357	D	79.5517	1
**4237**	370	D	79.5956	1
**4237**	372	D	79.4973	0
**4237**	373	D	66.4485	1
**4237**	381	D	67.6226	1
**4237**	382	D	79.6135	1
**4237**	386	D	79.5495	1
**4238**	305	A	66.9198	1
**4238**	319	A	48.2376	0
**4238**	320	A	79.2504	-1
**4238**	321	A	79.534	0
**4238**	351	A	8.8222	1
**4238**	357	A	67.6689	1
**4238**	370	A	77.1416	1
**4238**	372	A	63.1264	0
**4238**	373	A	71.6676	1
**4238**	381	A	72.6282	1
**4238**	382	A	78.228	1
**4238**	386	A	65.4771	1
**4238**	305	B	79.5566	1
**4238**	319	B	79.5576	1
**4238**	320	B	79.5546	-1
**4238**	321	B	45.9058	1
**4238**	351	B	0.0208	1
**4238**	357	B	79.5458	1
**4238**	370	B	73.0263	1
**4238**	372	B	79.5148	0
**4238**	373	B	68.227	1
**4238**	381	B	74.5886	1
**4238**	382	B	70.6408	1
**4238**	386	B	79.5436	1
**4238**	305	C	51.2725	1
**4238**	319	C	72.2226	1

**4238**	320	C	65.8823	-1
**4238**	321	C	66.2241	0
**4238**	351	C	11.1351	1
**4238**	357	C	48.2583	0
**4238**	370	C	77.5116	1
**4238**	372	C	79.6498	0
**4238**	373	C	38.5405	0
**4238**	381	C	72.3173	1
**4238**	382	C	79.6029	1
**4238**	386	C	40.5202	0
**4238**	305	D	79.4485	1
**4238**	319	D	77.7244	1
**4238**	320	D	59.8375	-1
**4238**	321	D	12.28	0
**4238**	351	D	8.2308	1
**4238**	357	D	30.5058	0
**4238**	370	D	37.8521	0
**4238**	372	D	79.5226	0
**4238**	373	D	66.0619	1
**4238**	381	D	53.3736	1
**4238**	382	D	70.6126	1
**4238**	386	D	39.3791	0
**4239**	305	A	78.0069	1
**4239**	319	A	52.8926	1
**4239**	320	A	45.7402	-1
**4239**	321	A	63.5523	0
**4239**	351	A	8.4844	1
**4239**	357	A	42.4063	0
**4239**	370	A	69.7586	1
**4239**	372	A	38.5294	1
**4239**	373	A	72.8802	1
**4239**	381	A	21.8451	0
**4239**	382	A	42.0098	0
**4239**	386	A	79.5682	1

**4239**	305	B	72.7646	1
**4239**	319	B	74.0106	1
**4239**	320	B	71.2443	-1
**4239**	321	B	49.7336	1
**4239**	351	B	0.2488	1
**4239**	357	B	54.8749	1
**4239**	370	B	53.251	1
**4239**	372	B	40.1649	1
**4239**	373	B	60.7434	1
**4239**	381	B	79.5293	1
**4239**	382	B	51.6527	1
**4239**	386	B	33.3772	0
**4239**	305	C	79.18	1
**4239**	319	C	79.5241	1
**4239**	320	C	10.8163	1
**4239**	321	C	76.0797	0
**4239**	351	C	11.1405	1
**4239**	357	C	70.1613	1
**4239**	370	C	79.5932	1
**4239**	372	C	72.1948	0
**4239**	373	C	75.9241	1
**4239**	381	C	79.5261	1
**4239**	382	C	79.5449	1
**4239**	386	C	31.7534	0
**4239**	305	D	79.6089	1
**4239**	319	D	36.046	0
**4239**	320	D	78.1683	-1
**4239**	321	D	74.9113	0
**4239**	351	D	11.612	1
**4239**	357	D	69.469	1
**4239**	370	D	79.5147	1
**4239**	372	D	74.3498	0
**4239**	373	D	78.5442	1
**4239**	381	D	78.8552	1

**4239**	382	D	59.715	1
**4239**	386	D	79.5459	1
**4240**	305	A	72.5198	1
**4240**	319	A	67.4543	1
**4240**	320	A	59.4135	-1
**4240**	321	A	42.2474	1
**4240**	351	A	17.3256	1
**4240**	357	A	79.5278	1
**4240**	370	A	70.8668	1
**4240**	372	A	72.3552	0
**4240**	373	A	75.2388	1
**4240**	381	A	26.393	0
**4240**	382	A	79.5823	1
**4240**	386	A	64.2637	1
**4240**	305	B	63.7012	1
**4240**	319	B	71.2171	1
**4240**	320	B	79.6175	-1
**4240**	321	B	79.6276	0
**4240**	351	B	16.95	1
**4240**	357	B	19.0372	-1
**4240**	370	B	59.127	1
**4240**	372	B	79.5199	0
**4240**	373	B	79.4799	1
**4240**	381	B	47.493	0
**4240**	382	B	72.5077	1
**4240**	386	B	79.5268	1
**4240**	305	C	69.8084	1
**4240**	319	C	79.536	1
**4240**	320	C	63.7293	-1
**4240**	321	C	79.5635	0
**4240**	351	C	4.688	1
**4240**	357	C	57.3108	1
**4240**	370	C	79.3236	1
**4240**	372	C	79.5345	0

**4240**	373	C	79.5216	1
**4240**	381	C	79.5634	1
**4240**	382	C	19.6738	-1
**4240**	386	C	23.1371	0
**4240**	305	D	51.1432	1
**4240**	319	D	79.5693	1
**4240**	320	D	79.5246	-1
**4240**	321	D	79.5941	0
**4240**	351	D	17.2247	1
**4240**	357	D	79.5665	1
**4240**	370	D	76.0661	1
**4240**	372	D	79.5651	0
**4240**	373	D	79.5776	1
**4240**	381	D	36.905	0
**4240**	382	D	79.5589	1
**4240**	386	D	79.5157	1
**4241**	305	A	36.4988	0
**4241**	319	A	73.2521	1
**4241**	320	A	69.6075	-1
**4241**	321	A	79.3997	0
**4241**	351	A	1.6843	1
**4241**	357	A	56.1414	1
**4241**	370	A	44.3156	0
**4241**	372	A	68.587	0
**4241**	373	A	61.6901	1
**4241**	381	A	79.4966	1
**4241**	382	A	78.4188	1
**4241**	386	A	79.6117	1
**4241**	305	B	61.8872	1
**4241**	319	B	59.9089	1
**4241**	320	B	72.8789	-1
**4241**	321	B	53.0022	0
**4241**	351	B	17.4499	1
**4241**	357	B	73.1269	1

**4241**	370	B	73.0618	1
**4241**	372	B	77.069	0
**4241**	373	B	49.9807	0
**4241**	381	B	79.5181	1
**4241**	382	B	60.1409	1
**4241**	386	B	46.3688	0
**4241**	305	C	64.8711	1
**4241**	319	C	79.5555	1
**4241**	320	C	79.5548	-1
**4241**	321	C	38.3315	1
**4241**	351	C	10.482	1
**4241**	357	C	30.1856	0
**4241**	370	C	69.7055	1
**4241**	372	C	50.1098	0
**4241**	373	C	65.0668	1
**4241**	381	C	75.4697	1
**4241**	382	C	51.1794	1
**4241**	386	C	67.2608	1
**4241**	305	D	71.4001	1
**4241**	319	D	11.9314	-1
**4241**	320	D	45.0612	-1
**4241**	321	D	79.5698	0
**4241**	351	D	10.8011	1
**4241**	357	D	74.5521	1
**4241**	370	D	73.3506	1
**4241**	372	D	79.6433	0
**4241**	373	D	35.5509	0
**4241**	381	D	60.1098	1
**4241**	382	D	79.0849	1
**4241**	386	D	79.5375	1
**4242**	305	A	64.5381	1
**4242**	319	A	72.4525	1
**4242**	320	A	64.5266	-1
**4242**	321	A	69.1938	0

**4242**	351	A	0.0	1
**4242**	357	A	46.4903	0
**4242**	370	A	78.592	1
**4242**	372	A	79.559	0
**4242**	373	A	79.6228	1
**4242**	381	A	36.58	0
**4242**	382	A	75.9833	1
**4242**	386	A	79.5957	1
**4242**	305	B	71.7292	1
**4242**	319	B	47.0833	0
**4242**	320	B	77.3098	-1
**4242**	321	B	77.4799	0
**4242**	351	B	9.0792	1
**4242**	357	B	65.1652	1
**4242**	370	B	75.3454	1
**4242**	372	B	79.7587	0
**4242**	373	B	20.055	0
**4242**	381	B	73.4294	1
**4242**	382	B	43.4354	0
**4242**	386	B	49.5512	0
**4242**	305	C	74.8062	1
**4242**	319	C	46.2212	0
**4242**	320	C	79.4265	-1
**4242**	321	C	74.3285	0
**4242**	351	C	8.6101	1
**4242**	357	C	55.1864	1
**4242**	370	C	79.5842	1
**4242**	372	C	71.8813	0
**4242**	373	C	44.2778	0
**4242**	381	C	62.8832	1
**4242**	382	C	47.1199	0
**4242**	386	C	43.9402	0
**4242**	305	D	57.811	1
**4242**	319	D	68.4568	1

**4242**	320	D	79.4251	-1
**4242**	321	D	79.7083	0
**4242**	351	D	19.2522	1
**4242**	357	D	57.8053	1
**4242**	370	D	79.5332	1
**4242**	372	D	74.9088	0
**4242**	373	D	79.5953	1
**4242**	381	D	74.4585	1
**4242**	382	D	70.0179	1
**4242**	386	D	71.2631	1
**4243**	305	A	79.5229	1
**4243**	319	A	76.6877	1
**4243**	320	A	72.2366	-1
**4243**	321	A	78.8404	0
**4243**	351	A	12.0775	1
**4243**	357	A	22.3445	0
**4243**	370	A	79.5765	1
**4243**	372	A	79.5574	0
**4243**	373	A	75.2519	1
**4243**	381	A	79.5319	1
**4243**	382	A	69.5409	1
**4243**	386	A	75.4162	1
**4243**	305	B	44.7867	0
**4243**	319	B	64.8541	1
**4243**	320	B	79.6102	-1
**4243**	321	B	78.2471	0
**4243**	351	B	13.8663	1
**4243**	357	B	79.5651	1
**4243**	370	B	78.1199	1
**4243**	372	B	79.5698	0
**4243**	373	B	67.6255	1
**4243**	381	B	75.8116	1
**4243**	382	B	72.0409	1
**4243**	386	B	66.8609	1

**4243**	305	C	68.807	1
**4243**	319	C	79.5943	1
**4243**	320	C	75.3126	-1
**4243**	321	C	79.5542	0
**4243**	351	C	6.6367	1
**4243**	357	C	73.6443	1
**4243**	370	C	52.3897	1
**4243**	372	C	79.5289	0
**4243**	373	C	79.1569	1
**4243**	381	C	79.5682	1
**4243**	382	C	77.7474	1
**4243**	386	C	44.4663	0
**4243**	305	D	73.2936	1
**4243**	319	D	40.8358	0
**4243**	320	D	79.5701	-1
**4243**	321	D	61.7579	0
**4243**	351	D	13.1038	1
**4243**	357	D	70.3559	1
**4243**	370	D	21.3223	0
**4243**	372	D	71.6244	0
**4243**	373	D	79.5735	1
**4243**	381	D	34.1292	0
**4243**	382	D	79.575	1
**4243**	386	D	78.9754	1
**4244**	305	A	59.528	1
**4244**	319	A	61.5671	1
**4244**	320	A	67.8077	-1
**4244**	321	A	59.2329	0
**4244**	351	A	11.2141	1
**4244**	357	A	75.2334	1
**4244**	370	A	63.4418	1
**4244**	372	A	79.5447	0
**4244**	373	A	74.971	1
**4244**	381	A	74.2221	1

**4244**	382	A	67.1721	1
**4244**	386	A	56.9911	1
**4244**	305	B	79.0032	1
**4244**	319	B	79.4635	1
**4244**	320	B	79.6258	-1
**4244**	321	B	74.8063	0
**4244**	351	B	14.3875	1
**4244**	357	B	45.1962	0
**4244**	370	B	69.9048	1
**4244**	372	B	71.7948	0
**4244**	373	B	79.4687	1
**4244**	381	B	74.7183	1
**4244**	382	B	68.2026	1
**4244**	386	B	73.6489	1
**4244**	305	C	51.3189	1
**4244**	319	C	58.9393	1
**4244**	320	C	79.529	-1
**4244**	321	C	63.9021	0
**4244**	351	C	15.3013	1
**4244**	357	C	79.6478	1
**4244**	370	C	79.5519	1
**4244**	372	C	71.5662	0
**4244**	373	C	67.7873	1
**4244**	381	C	76.5328	1
**4244**	382	C	52.7584	1
**4244**	386	C	54.8479	1
**4244**	305	D	74.7778	1
**4244**	319	D	74.5152	1
**4244**	320	D	79.5698	-1
**4244**	321	D	72.7419	0
**4244**	351	D	21.6474	-1
**4244**	357	D	79.5839	1
**4244**	370	D	70.9054	1
**4244**	372	D	79.5793	0

**4244**	373	D	67.2054	1
**4244**	381	D	60.6276	1
**4244**	382	D	79.4623	1
**4244**	386	D	71.0368	1
**4245**	305	A	56.3417	1
**4245**	319	A	79.493	1
**4245**	320	A	52.689	-1
**4245**	321	A	36.5848	1
**4245**	351	A	7.2684	1
**4245**	357	A	65.6151	1
**4245**	370	A	69.43	1
**4245**	372	A	27.6095	1
**4245**	373	A	77.0237	1
**4245**	381	A	61.722	1
**4245**	382	A	70.4821	1
**4245**	386	A	77.6127	1
**4245**	305	B	79.5549	1
**4245**	319	B	18.3482	-1
**4245**	320	B	79.6401	-1
**4245**	321	B	72.3039	0
**4245**	351	B	4.4782	1
**4245**	357	B	39.2596	0
**4245**	370	B	63.4691	1
**4245**	372	B	69.5648	0
**4245**	373	B	76.929	1
**4245**	381	B	66.0869	1
**4245**	382	B	79.6814	1
**4245**	386	B	79.3349	1
**4245**	305	C	76.2504	1
**4245**	319	C	50.0749	1
**4245**	320	C	71.6326	-1
**4245**	321	C	78.8895	0
**4245**	351	C	5.7284	1
**4245**	357	C	75.2535	1

**4245**	370	C	52.3651	1
**4245**	372	C	56.8221	0
**4245**	373	C	78.8049	1
**4245**	381	C	79.3169	1
**4245**	382	C	68.883	1
**4245**	386	C	45.2141	0
**4245**	305	D	55.4733	1
**4245**	319	D	79.006	1
**4245**	320	D	37.4236	-1
**4245**	321	D	49.1682	1
**4245**	351	D	8.9699	1
**4245**	357	D	79.2091	1
**4245**	370	D	50.1352	1
**4245**	372	D	79.5028	0
**4245**	373	D	79.5069	1
**4245**	381	D	62.7091	1
**4245**	382	D	78.284	1
**4245**	386	D	63.5453	1
**4246**	305	A	69.737	1
**4246**	319	A	47.3833	0
**4246**	320	A	79.5401	-1
**4246**	321	A	60.3708	0
**4246**	351	A	4.473	1
**4246**	357	A	79.5741	1
**4246**	370	A	64.4686	1
**4246**	372	A	50.0926	0
**4246**	373	A	73.739	1
**4246**	381	A	73.0301	1
**4246**	382	A	63.1566	1
**4246**	386	A	79.5386	1
**4246**	305	B	79.6404	1
**4246**	319	B	79.3176	1
**4246**	320	B	79.5388	-1
**4246**	321	B	72.0911	0

**4246**	351	B	14.2018	1
**4246**	357	B	79.6472	1
**4246**	370	B	59.436	1
**4246**	372	B	64.2184	0
**4246**	373	B	49.5025	0
**4246**	381	B	53.875	1
**4246**	382	B	24.3524	0
**4246**	386	B	79.5655	1
**4246**	305	C	73.5144	1
**4246**	319	C	76.3636	1
**4246**	320	C	75.7528	-1
**4246**	321	C	54.4	0
**4246**	351	C	26.2048	-1
**4246**	357	C	57.0238	1
**4246**	370	C	79.592	1
**4246**	372	C	79.549	0
**4246**	373	C	79.533	1
**4246**	381	C	66.9632	1
**4246**	382	C	46.1489	0
**4246**	386	C	53.4527	1
**4246**	305	D	49.1424	0
**4246**	319	D	75.9435	1
**4246**	320	D	79.5358	-1
**4246**	321	D	61.1223	0
**4246**	351	D	12.2325	1
**4246**	357	D	57.493	1
**4246**	370	D	79.515	1
**4246**	372	D	74.4131	0
**4246**	373	D	79.476	1
**4246**	381	D	67.2884	1
**4246**	382	D	74.4091	1
**4246**	386	D	79.5504	1
**4247**	305	A	71.1402	1
**4247**	319	A	34.6161	0

**4247**	320	A	79.5836	-1
**4247**	321	A	79.5799	0
**4247**	351	A	7.6112	1
**4247**	357	A	57.257	1
**4247**	370	A	33.4007	0
**4247**	372	A	72.0179	0
**4247**	373	A	69.7076	1
**4247**	381	A	79.5986	1
**4247**	382	A	79.5675	1
**4247**	386	A	79.5294	1
**4247**	305	B	79.5144	1
**4247**	319	B	79.5503	1
**4247**	320	B	75.1977	-1
**4247**	321	B	29.2413	1
**4247**	351	B	10.9885	1
**4247**	357	B	57.6837	1
**4247**	370	B	64.2732	1
**4247**	372	B	73.2625	0
**4247**	373	B	71.7328	1
**4247**	381	B	47.8086	0
**4247**	382	B	69.7957	1
**4247**	386	B	79.5983	1
**4247**	305	C	72.5565	1
**4247**	319	C	79.5286	1
**4247**	320	C	75.8906	-1
**4247**	321	C	79.5946	0
**4247**	351	C	4.0533	1
**4247**	357	C	52.1989	1
**4247**	370	C	51.3823	1
**4247**	372	C	63.7352	0
**4247**	373	C	45.1339	0
**4247**	381	C	72.861	1
**4247**	382	C	70.3838	1
**4247**	386	C	51.7221	1

**4247**	305	D	38.8376	0
**4247**	319	D	65.9059	1
**4247**	320	D	68.0473	-1
**4247**	321	D	62.3926	0
**4247**	351	D	10.2172	1
**4247**	357	D	73.7814	1
**4247**	370	D	60.9068	1
**4247**	372	D	55.4109	0
**4247**	373	D	79.6015	1
**4247**	381	D	72.7354	1
**4247**	382	D	79.5666	1
**4247**	386	D	69.5839	1
**4248**	305	A	51.402	1
**4248**	319	A	24.868	0
**4248**	320	A	79.5335	-1
**4248**	321	A	79.6019	0
**4248**	351	A	8.3932	1
**4248**	357	A	73.9512	1
**4248**	370	A	73.3075	1
**4248**	372	A	74.5211	0
**4248**	373	A	52.7462	1
**4248**	381	A	73.2942	1
**4248**	382	A	79.6311	1
**4248**	386	A	67.0582	1
**4248**	305	B	79.1306	1
**4248**	319	B	30.7562	0
**4248**	320	B	43.4384	-1
**4248**	321	B	75.3962	0
**4248**	351	B	11.554	1
**4248**	357	B	61.2292	1
**4248**	370	B	79.6094	1
**4248**	372	B	72.8281	0
**4248**	373	B	58.8214	1
**4248**	381	B	70.2659	1

**4248**	382	B	78.5359	1
**4248**	386	B	42.0489	0
**4248**	305	C	67.6104	1
**4248**	319	C	69.1618	1
**4248**	320	C	79.5346	-1
**4248**	321	C	62.6763	0
**4248**	351	C	4.6257	1
**4248**	357	C	60.932	1
**4248**	370	C	41.9521	0
**4248**	372	C	79.5227	0
**4248**	373	C	50.4827	1
**4248**	381	C	52.9333	1
**4248**	382	C	69.7359	1
**4248**	386	C	52.9605	1
**4248**	305	D	51.9413	1
**4248**	319	D	79.596	1
**4248**	320	D	79.692	-1
**4248**	321	D	79.554	0
**4248**	351	D	15.1279	1
**4248**	357	D	79.5172	1
**4248**	370	D	62.3637	1
**4248**	372	D	69.7915	0
**4248**	373	D	38.4669	0
**4248**	381	D	41.4091	0
**4248**	382	D	79.3246	1
**4248**	386	D	79.5258	1
**4249**	305	A	73.0083	1
**4249**	319	A	63.6314	1
**4249**	320	A	56.7101	-1
**4249**	321	A	76.0103	0
**4249**	351	A	17.4588	1
**4249**	357	A	66.2149	1
**4249**	370	A	79.6349	1
**4249**	372	A	78.4606	0

**4249**	373	A	50.2392	1
**4249**	381	A	76.7343	1
**4249**	382	A	73.4171	1
**4249**	386	A	56.7278	1
**4249**	305	B	78.8886	1
**4249**	319	B	79.6004	1
**4249**	320	B	39.5041	-1
**4249**	321	B	79.6395	0
**4249**	351	B	3.7449	1
**4249**	357	B	73.1776	1
**4249**	370	B	68.4909	1
**4249**	372	B	79.5374	0
**4249**	373	B	64.6164	1
**4249**	381	B	63.4065	1
**4249**	382	B	77.4889	1
**4249**	386	B	47.3023	0
**4249**	305	C	79.3932	1
**4249**	319	C	32.8953	0
**4249**	320	C	52.1611	-1
**4249**	321	C	79.6756	0
**4249**	351	C	3.9764	1
**4249**	357	C	70.9242	1
**4249**	370	C	59.2811	1
**4249**	372	C	79.4497	0
**4249**	373	C	49.8161	0
**4249**	381	C	73.0584	1
**4249**	382	C	50.5753	1
**4249**	386	C	51.0808	1
**4249**	305	D	79.6142	1
**4249**	319	D	73.1948	1
**4249**	320	D	70.1084	-1
**4249**	321	D	38.4519	1
**4249**	351	D	24.6994	-1
**4249**	357	D	65.9449	1

**4249**	370	D	79.4868	1
**4249**	372	D	79.427	0
**4249**	373	D	60.9633	1
**4249**	381	D	80.6022	1
**4249**	382	D	77.8998	1
**4249**	386	D	79.6378	1
**4250**	305	A	71.767	1
**4250**	319	A	49.2765	0
**4250**	320	A	25.4994	-1
**4250**	321	A	79.194	0
**4250**	351	A	2.4282	1
**4250**	357	A	79.5999	1
**4250**	370	A	79.5795	1
**4250**	372	A	79.6052	0
**4250**	373	A	41.4626	0
**4250**	381	A	73.3931	1
**4250**	382	A	79.6466	1
**4250**	386	A	79.5615	1
**4250**	305	B	71.7386	1
**4250**	319	B	67.2329	1
**4250**	320	B	62.6829	-1
**4250**	321	B	13.5119	0
**4250**	351	B	4.6502	1
**4250**	357	B	70.1735	1
**4250**	370	B	74.474	1
**4250**	372	B	79.487	0
**4250**	373	B	79.4514	1
**4250**	381	B	79.6095	1
**4250**	382	B	52.2864	1
**4250**	386	B	47.9418	0
**4250**	305	C	79.3074	1
**4250**	319	C	69.0927	1
**4250**	320	C	72.1455	-1
**4250**	321	C	79.5233	0

**4250**	351	C	0.1449	1
**4250**	357	C	77.001	1
**4250**	370	C	62.5535	1
**4250**	372	C	29.9405	1
**4250**	373	C	37.3761	0
**4250**	381	C	57.1136	1
**4250**	382	C	38.9097	0
**4250**	386	C	46.6033	0
**4250**	305	D	63.1079	1
**4250**	319	D	72.8306	1
**4250**	320	D	79.5445	-1
**4250**	321	D	75.8237	0
**4250**	351	D	11.1377	1
**4250**	357	D	53.7969	1
**4250**	370	D	71.7987	1
**4250**	372	D	78.4477	0
**4250**	373	D	79.5669	1
**4250**	381	D	69.9305	1
**4250**	382	D	79.5192	1
**4250**	386	D	20.7399	0
**4251**	305	A	67.3689	1
**4251**	319	A	79.5571	1
**4251**	320	A	69.2108	-1
**4251**	321	A	68.5738	0
**4251**	351	A	16.1971	1
**4251**	357	A	62.5444	1
**4251**	370	A	79.3931	1
**4251**	372	A	69.4608	0
**4251**	373	A	64.9428	1
**4251**	381	A	75.1587	1
**4251**	382	A	39.32	0
**4251**	386	A	79.6139	1
**4251**	305	B	79.1658	1
**4251**	319	B	44.0846	0

**4251**	320	B	79.6189	-1
**4251**	321	B	69.653	0
**4251**	351	B	4.9122	1
**4251**	357	B	79.5262	1
**4251**	370	B	49.5583	0
**4251**	372	B	57.5505	0
**4251**	373	B	51.6979	1
**4251**	381	B	46.747	0
**4251**	382	B	41.9092	0
**4251**	386	B	73.1407	1
**4251**	305	C	48.6932	0
**4251**	319	C	78.8829	1
**4251**	320	C	79.4361	-1
**4251**	321	C	77.2941	0
**4251**	351	C	10.5909	1
**4251**	357	C	62.4888	1
**4251**	370	C	55.6002	1
**4251**	372	C	72.2988	0
**4251**	373	C	79.5404	1
**4251**	381	C	65.7937	1
**4251**	382	C	60.4264	1
**4251**	386	C	64.6444	1
**4251**	305	D	47.6621	0
**4251**	319	D	79.665	1
**4251**	320	D	79.4215	-1
**4251**	321	D	64.7324	0
**4251**	351	D	10.6442	1
**4251**	357	D	71.0982	1
**4251**	370	D	79.173	1
**4251**	372	D	49.1705	1
**4251**	373	D	79.4984	1
**4251**	381	D	79.5026	1
**4251**	382	D	79.5229	1
**4251**	386	D	31.2521	0

**4252**	305	A	77.3858	1
**4252**	319	A	47.7572	0
**4252**	320	A	72.8782	-1
**4252**	321	A	57.7061	0
**4252**	351	A	9.4087	1
**4252**	357	A	68.1254	1
**4252**	370	A	79.511	1
**4252**	372	A	55.6256	0
**4252**	373	A	41.6991	0
**4252**	381	A	72.5537	1
**4252**	382	A	66.9195	1
**4252**	386	A	79.6122	1
**4252**	305	B	72.2538	1
**4252**	319	B	79.6116	1
**4252**	320	B	75.4364	-1
**4252**	321	B	42.854	1
**4252**	351	B	0.1828	1
**4252**	357	B	50.9154	1
**4252**	370	B	79.5896	1
**4252**	372	B	69.0809	0
**4252**	373	B	79.3587	1
**4252**	381	B	71.7008	1
**4252**	382	B	73.6678	1
**4252**	386	B	44.33	0
**4252**	305	C	79.6456	1
**4252**	319	C	79.5338	1
**4252**	320	C	49.7645	-1
**4252**	321	C	78.5636	0
**4252**	351	C	11.3154	1
**4252**	357	C	41.2758	0
**4252**	370	C	74.9697	1
**4252**	372	C	71.2037	0
**4252**	373	C	79.6231	1
**4252**	381	C	79.5633	1

**4252**	382	C	60.5968	1
**4252**	386	C	15.1771	-1
**4252**	305	D	79.4833	1
**4252**	319	D	39.3279	0
**4252**	320	D	79.5051	-1
**4252**	321	D	18.6419	0
**4252**	351	D	13.5199	1
**4252**	357	D	79.568	1
**4252**	370	D	79.5551	1
**4252**	372	D	71.1089	0
**4252**	373	D	51.4471	1
**4252**	381	D	67.1186	1
**4252**	382	D	79.5173	1
**4252**	386	D	70.8919	1
**4253**	305	A	79.521	1
**4253**	319	A	79.5528	1
**4253**	320	A	69.8438	-1
**4253**	321	A	79.5695	0
**4253**	351	A	7.9376	1
**4253**	357	A	50.3956	1
**4253**	370	A	31.1136	0
**4253**	372	A	64.1447	0
**4253**	373	A	54.5692	1
**4253**	381	A	74.7351	1
**4253**	382	A	51.4905	1
**4253**	386	A	68.9011	1
**4253**	305	B	72.8107	1
**4253**	319	B	79.5972	1
**4253**	320	B	79.541	-1
**4253**	321	B	54.7612	0
**4253**	351	B	14.0599	1
**4253**	357	B	79.6491	1
**4253**	370	B	79.5909	1
**4253**	372	B	79.484	0

**4253**	373	B	71.9517	1
**4253**	381	B	75.226	1
**4253**	382	B	35.6315	0
**4253**	386	B	51.9178	1
**4253**	305	C	79.6295	1
**4253**	319	C	50.8879	1
**4253**	320	C	73.4211	-1
**4253**	321	C	72.0075	0
**4253**	351	C	3.021	1
**4253**	357	C	61.663	1
**4253**	370	C	65.6738	1
**4253**	372	C	79.5561	0
**4253**	373	C	49.4135	0
**4253**	381	C	48.8421	0
**4253**	382	C	67.2831	1
**4253**	386	C	52.8869	1
**4253**	305	D	44.1634	0
**4253**	319	D	46.6568	0
**4253**	320	D	69.2945	-1
**4253**	321	D	79.5211	0
**4253**	351	D	16.7112	1
**4253**	357	D	63.7315	1
**4253**	370	D	38.4498	0
**4253**	372	D	71.2297	0
**4253**	373	D	78.088	1
**4253**	381	D	59.8481	1
**4253**	382	D	73.6157	1
**4253**	386	D	69.6756	1
**4254**	305	A	76.1184	1
**4254**	319	A	70.9163	1
**4254**	320	A	79.469	-1
**4254**	321	A	77.5759	0
**4254**	351	A	12.7677	1
**4254**	357	A	73.569	1

**4254**	370	A	74.7207	1
**4254**	372	A	71.4741	0
**4254**	373	A	79.5829	1
**4254**	381	A	79.7315	1
**4254**	382	A	75.2434	1
**4254**	386	A	72.8494	1
**4254**	305	B	33.1845	0
**4254**	319	B	76.2349	1
**4254**	320	B	58.3376	-1
**4254**	321	B	79.6624	0
**4254**	351	B	15.42	1
**4254**	357	B	63.3305	1
**4254**	370	B	79.5546	1
**4254**	372	B	72.4344	0
**4254**	373	B	59.438	1
**4254**	381	B	79.3622	1
**4254**	382	B	45.9401	0
**4254**	386	B	79.6029	1
**4254**	305	C	75.8128	1
**4254**	319	C	78.2201	1
**4254**	320	C	79.6197	-1
**4254**	321	C	76.3637	0
**4254**	351	C	9.1871	1
**4254**	357	C	43.7121	0
**4254**	370	C	79.5679	1
**4254**	372	C	63.9215	0
**4254**	373	C	75.0526	1
**4254**	381	C	52.607	1
**4254**	382	C	28.4612	0
**4254**	386	C	30.555	0
**4254**	305	D	63.4893	1
**4254**	319	D	48.8834	0
**4254**	320	D	43.2845	-1
**4254**	321	D	75.8237	0

**4254**	351	D	17.2256	1
**4254**	357	D	78.9777	1
**4254**	370	D	74.0439	1
**4254**	372	D	79.4896	0
**4254**	373	D	66.339	1
**4254**	381	D	68.2075	1
**4254**	382	D	63.313	1
**4254**	386	D	34.9803	0
**4255**	305	A	60.0256	1
**4255**	319	A	77.0186	1
**4255**	320	A	79.5847	-1
**4255**	321	A	79.0618	0
**4255**	351	A	1.994	1
**4255**	357	A	76.319	1
**4255**	370	A	3.5786	-1
**4255**	372	A	16.4999	0
**4255**	373	A	46.7847	0
**4255**	381	A	79.6694	1
**4255**	382	A	79.5926	1
**4255**	386	A	61.4245	1
**4255**	305	B	79.1924	1
**4255**	319	B	79.4615	1
**4255**	320	B	29.4938	-1
**4255**	321	B	61.9339	0
**4255**	351	B	19.7334	1
**4255**	357	B	53.1955	1
**4255**	370	B	70.2243	1
**4255**	372	B	50.4804	0
**4255**	373	B	79.5297	1
**4255**	381	B	79.5553	1
**4255**	382	B	41.5722	0
**4255**	386	B	64.285	1
**4255**	305	C	79.6356	1
**4255**	319	C	57.8177	1

**4255**	320	C	74.2438	-1
**4255**	321	C	67.9813	0
**4255**	351	C	6.7031	1
**4255**	357	C	76.9181	1
**4255**	370	C	25.132	0
**4255**	372	C	79.5186	0
**4255**	373	C	43.5647	0
**4255**	381	C	64.8062	1
**4255**	382	C	66.9803	1
**4255**	386	C	63.9623	1
**4255**	305	D	73.127	1
**4255**	319	D	79.479	1
**4255**	320	D	77.1618	-1
**4255**	321	D	55.0666	0
**4255**	351	D	20.6544	-1
**4255**	357	D	70.909	1
**4255**	370	D	65.3006	1
**4255**	372	D	55.0561	0
**4255**	373	D	65.628	1
**4255**	381	D	77.091	1
**4255**	382	D	69.6455	1
**4255**	386	D	75.8805	1
**4256**	305	A	46.4947	0
**4256**	319	A	62.1855	1
**4256**	320	A	79.6212	-1
**4256**	321	A	62.4186	0
**4256**	351	A	0.8523	1
**4256**	357	A	28.8101	0
**4256**	370	A	79.6753	1
**4256**	372	A	67.0341	0
**4256**	373	A	54.0455	1
**4256**	381	A	30.2172	0
**4256**	382	A	50.1748	1
**4256**	386	A	33.2676	0

**4256**	305	B	79.4991	1
**4256**	319	B	79.6055	1
**4256**	320	B	67.1664	-1
**4256**	321	B	67.9937	0
**4256**	351	B	0.1271	1
**4256**	357	B	68.6151	1
**4256**	370	B	79.5123	1
**4256**	372	B	79.5522	0
**4256**	373	B	79.481	1
**4256**	381	B	75.2001	1
**4256**	382	B	79.7117	1
**4256**	386	B	51.9676	1
**4256**	305	C	79.1231	1
**4256**	319	C	19.4912	-1
**4256**	320	C	56.0859	-1
**4256**	321	C	50.7419	0
**4256**	351	C	5.8984	1
**4256**	357	C	66.4304	1
**4256**	370	C	71.9969	1
**4256**	372	C	79.541	0
**4256**	373	C	70.6051	1
**4256**	381	C	79.5733	1
**4256**	382	C	73.8298	1
**4256**	386	C	41.9844	0
**4256**	305	D	44.5022	0
**4256**	319	D	79.5748	1
**4256**	320	D	53.1459	-1
**4256**	321	D	79.5911	0
**4256**	351	D	38.5768	-1
**4256**	357	D	23.2834	0
**4256**	370	D	79.182	1
**4256**	372	D	79.4419	0
**4256**	373	D	79.6082	1
**4256**	381	D	71.4135	1

**4256**	382	D	63.4717	1
**4256**	386	D	27.9251	0
**4257**	305	A	79.6166	1
**4257**	319	A	78.3229	1
**4257**	320	A	71.434	-1
**4257**	321	A	46.9332	1
**4257**	351	A	0.0	1
**4257**	357	A	79.5949	1
**4257**	370	A	60.7077	1
**4257**	372	A	79.6239	0
**4257**	373	A	71.0347	1
**4257**	381	A	75.2357	1
**4257**	382	A	79.4884	1
**4257**	386	A	53.0758	1
**4257**	305	B	47.2375	0
**4257**	319	B	63.0411	1
**4257**	320	B	65.7947	-1
**4257**	321	B	79.5044	0
**4257**	351	B	14.8911	1
**4257**	357	B	79.5117	1
**4257**	370	B	49.7193	0
**4257**	372	B	79.5766	0
**4257**	373	B	77.4758	1
**4257**	381	B	78.7334	1
**4257**	382	B	46.4116	0
**4257**	386	B	79.4534	1
**4257**	305	C	75.765	1
**4257**	319	C	62.6152	1
**4257**	320	C	79.6281	-1
**4257**	321	C	79.5865	0
**4257**	351	C	28.6581	-1
**4257**	357	C	46.9446	0
**4257**	370	C	78.4475	1
**4257**	372	C	72.0476	0

**4257**	373	C	60.9765	1
**4257**	381	C	65.9304	1
**4257**	382	C	47.9133	0
**4257**	386	C	34.4341	0
**4257**	305	D	51.4266	1
**4257**	319	D	79.5966	1
**4257**	320	D	59.9664	-1
**4257**	321	D	59.4063	0
**4257**	351	D	13.3607	1
**4257**	357	D	79.5636	1
**4257**	370	D	72.7511	1
**4257**	372	D	62.2289	0
**4257**	373	D	79.5432	1
**4257**	381	D	56.7582	1
**4257**	382	D	59.9299	1
**4257**	386	D	74.08	1
**4258**	305	A	52.9771	1
**4258**	319	A	79.7973	1
**4258**	320	A	36.271	-1
**4258**	321	A	39.5199	1
**4258**	351	A	1.2787	1
**4258**	357	A	65.9177	1
**4258**	370	A	73.766	1
**4258**	372	A	72.5179	0
**4258**	373	A	79.6881	1
**4258**	381	A	61.77	1
**4258**	382	A	63.8785	1
**4258**	386	A	72.0419	1
**4258**	305	B	79.6678	1
**4258**	319	B	72.8033	1
**4258**	320	B	51.0028	-1
**4258**	321	B	46.7299	1
**4258**	351	B	13.1271	1
**4258**	357	B	79.7099	1

**4258**	370	B	65.7418	1
**4258**	372	B	79.6305	0
**4258**	373	B	65.8304	1
**4258**	381	B	79.6696	1
**4258**	382	B	78.0285	1
**4258**	386	B	78.7429	1
**4258**	305	C	63.9365	1
**4258**	319	C	53.7299	1
**4258**	320	C	79.5982	-1
**4258**	321	C	65.0767	0
**4258**	351	C	3.8171	1
**4258**	357	C	79.4028	1
**4258**	370	C	79.6532	1
**4258**	372	C	72.9984	0
**4258**	373	C	74.8819	1
**4258**	381	C	76.8523	1
**4258**	382	C	74.2291	1
**4258**	386	C	47.8078	0
**4258**	305	D	35.2374	0
**4258**	319	D	79.8321	1
**4258**	320	D	75.4547	-1
**4258**	321	D	73.4914	0
**4258**	351	D	29.3438	-1
**4258**	357	D	79.5425	1
**4258**	370	D	73.0976	1
**4258**	372	D	36.7143	1
**4258**	373	D	70.4905	1
**4258**	381	D	78.4736	1
**4258**	382	D	70.9153	1
**4258**	386	D	69.4911	1
**4259**	305	A	70.1337	1
**4259**	319	A	45.5991	0
**4259**	320	A	73.7529	-1
**4259**	321	A	79.3327	0

**4259**	351	A	2.2225	1
**4259**	357	A	75.9125	1
**4259**	370	A	79.4101	1
**4259**	372	A	79.55	0
**4259**	373	A	56.0386	1
**4259**	381	A	68.8016	1
**4259**	382	A	62.6029	1
**4259**	386	A	44.6306	0
**4259**	305	B	79.5247	1
**4259**	319	B	39.9703	0
**4259**	320	B	71.668	-1
**4259**	321	B	74.6352	0
**4259**	351	B	9.3886	1
**4259**	357	B	79.5582	1
**4259**	370	B	56.3166	1
**4259**	372	B	45.9816	1
**4259**	373	B	65.6569	1
**4259**	381	B	79.6153	1
**4259**	382	B	72.2255	1
**4259**	386	B	72.1412	1
**4259**	305	C	79.4769	1
**4259**	319	C	74.9879	1
**4259**	320	C	79.6851	-1
**4259**	321	C	69.0724	0
**4259**	351	C	31.6733	-1
**4259**	357	C	77.6375	1
**4259**	370	C	73.6178	1
**4259**	372	C	67.7828	0
**4259**	373	C	79.5927	1
**4259**	381	C	51.4321	1
**4259**	382	C	73.2609	1
**4259**	386	C	24.9489	0
**4259**	305	D	65.2907	1
**4259**	319	D	57.7133	1

**4259**	320	D	66.1559	-1
**4259**	321	D	75.21	0
**4259**	351	D	11.4417	1
**4259**	357	D	62.4264	1
**4259**	370	D	71.7738	1
**4259**	372	D	79.5411	0
**4259**	373	D	69.61	1
**4259**	381	D	79.6952	1
**4259**	382	D	79.511	1
**4259**	386	D	79.5434	1
**4260**	305	A	2.2506	-1
**4260**	319	A	56.1212	1
**4260**	320	A	58.6025	-1
**4260**	321	A	49.2385	1
**4260**	351	A	5.5179	1
**4260**	357	A	67.6342	1
**4260**	370	A	73.0024	1
**4260**	372	A	74.2479	0
**4260**	373	A	72.7057	1
**4260**	381	A	61.0231	1
**4260**	382	A	69.9986	1
**4260**	386	A	74.3859	1
**4260**	305	B	71.2188	1
**4260**	319	B	79.5551	1
**4260**	320	B	68.3496	-1
**4260**	321	B	75.808	0
**4260**	351	B	11.0157	1
**4260**	357	B	57.8656	1
**4260**	370	B	72.5096	1
**4260**	372	B	58.5579	0
**4260**	373	B	79.5832	1
**4260**	381	B	79.4144	1
**4260**	382	B	79.567	1
**4260**	386	B	54.2573	1

**4260**	305	C	71.954	1
**4260**	319	C	69.7568	1
**4260**	320	C	24.3303	-1
**4260**	321	C	55.2585	0
**4260**	351	C	1.2453	1
**4260**	357	C	79.67	1
**4260**	370	C	58.6234	1
**4260**	372	C	72.7039	0
**4260**	373	C	78.428	1
**4260**	381	C	62.66	1
**4260**	382	C	79.6262	1
**4260**	386	C	41.2084	0
**4260**	305	D	65.4004	1
**4260**	319	D	79.5599	1
**4260**	320	D	27.0643	-1
**4260**	321	D	70.4512	0
**4260**	351	D	12.4822	1
**4260**	357	D	65.9719	1
**4260**	370	D	40.8971	0
**4260**	372	D	64.6649	0
**4260**	373	D	54.6021	1
**4260**	381	D	79.5282	1
**4260**	382	D	77.4094	1
**4260**	386	D	73.942	1
**4261**	305	A	74.559	1
**4261**	319	A	72.7694	1
**4261**	320	A	73.2788	-1
**4261**	321	A	70.0353	0
**4261**	351	A	10.7228	1
**4261**	357	A	75.124	1
**4261**	370	A	65.1441	1
**4261**	372	A	74.3842	0
**4261**	373	A	37.0942	0
**4261**	381	A	79.5726	1

**4261**	382	A	61.8097	1
**4261**	386	A	64.416	1
**4261**	305	B	70.0668	1
**4261**	319	B	79.5585	1
**4261**	320	B	79.5743	-1
**4261**	321	B	49.0849	1
**4261**	351	B	12.0687	1
**4261**	357	B	79.5569	1
**4261**	370	B	72.524	1
**4261**	372	B	79.5717	0
**4261**	373	B	65.6996	1
**4261**	381	B	79.5616	1
**4261**	382	B	44.5233	0
**4261**	386	B	77.0413	1
**4261**	305	C	26.0004	0
**4261**	319	C	71.7415	1
**4261**	320	C	57.0324	-1
**4261**	321	C	29.8588	1
**4261**	351	C	7.8677	1
**4261**	357	C	47.453	0
**4261**	370	C	47.688	0
**4261**	372	C	79.5677	0
**4261**	373	C	79.5466	1
**4261**	381	C	67.8802	1
**4261**	382	C	57.6464	1
**4261**	386	C	55.2021	1
**4261**	305	D	79.5772	1
**4261**	319	D	45.5093	0
**4261**	320	D	79.5511	-1
**4261**	321	D	63.5887	0
**4261**	351	D	20.5988	-1
**4261**	357	D	51.4401	1
**4261**	370	D	54.993	1
**4261**	372	D	57.0643	0

**4261**	373	D	65.0747	1
**4261**	381	D	54.5674	1
**4261**	382	D	73.2518	1
**4261**	386	D	35.7342	0
**4262**	305	A	72.6698	1
**4262**	319	A	60.7888	1
**4262**	320	A	72.3262	-1
**4262**	321	A	79.5908	0
**4262**	351	A	4.2534	1
**4262**	357	A	71.4564	1
**4262**	370	A	79.5746	1
**4262**	372	A	69.5604	0
**4262**	373	A	62.4513	1
**4262**	381	A	73.8417	1
**4262**	382	A	73.0614	1
**4262**	386	A	66.6337	1
**4262**	305	B	67.259	1
**4262**	319	B	78.2898	1
**4262**	320	B	69.4608	-1
**4262**	321	B	72.2504	0
**4262**	351	B	16.9835	1
**4262**	357	B	62.0067	1
**4262**	370	B	56.0679	1
**4262**	372	B	73.0475	0
**4262**	373	B	62.9851	1
**4262**	381	B	59.0554	1
**4262**	382	B	70.5088	1
**4262**	386	B	59.9538	1
**4262**	305	C	28.6566	0
**4262**	319	C	41.8219	0
**4262**	320	C	77.7642	-1
**4262**	321	C	79.4362	0
**4262**	351	C	8.0894	1
**4262**	357	C	46.4773	0

**4262**	370	C	79.5107	1
**4262**	372	C	71.8751	0
**4262**	373	C	50.947	1
**4262**	381	C	56.4176	1
**4262**	382	C	61.7818	1
**4262**	386	C	38.2267	0
**4262**	305	D	68.4403	1
**4262**	319	D	58.1963	1
**4262**	320	D	54.5496	-1
**4262**	321	D	69.7139	0
**4262**	351	D	20.9512	-1
**4262**	357	D	55.3259	1
**4262**	370	D	79.5936	1
**4262**	372	D	79.5818	0
**4262**	373	D	72.0787	1
**4262**	381	D	79.5743	1
**4262**	382	D	72.5826	1
**4262**	386	D	40.3344	0
**4263**	305	A	64.4518	1
**4263**	319	A	66.2644	1
**4263**	320	A	29.3776	-1
**4263**	321	A	26.3778	1
**4263**	351	A	7.2342	1
**4263**	357	A	74.2866	1
**4263**	370	A	57.7361	1
**4263**	372	A	79.5259	0
**4263**	373	A	40.0527	0
**4263**	381	A	50.6515	1
**4263**	382	A	19.4858	-1
**4263**	386	A	39.7791	0
**4263**	305	B	79.6149	1
**4263**	319	B	57.2593	1
**4263**	320	B	73.6538	-1
**4263**	321	B	70.8448	0

**4263**	351	B	16.5277	1
**4263**	357	B	78.3185	1
**4263**	370	B	45.8397	0
**4263**	372	B	41.6685	1
**4263**	373	B	38.1251	0
**4263**	381	B	74.3914	1
**4263**	382	B	66.2912	1
**4263**	386	B	34.6151	0
**4263**	305	C	62.6704	1
**4263**	319	C	70.1866	1
**4263**	320	C	74.3827	-1
**4263**	321	C	79.741	0
**4263**	351	C	6.0606	1
**4263**	357	C	74.2355	1
**4263**	370	C	79.55	1
**4263**	372	C	67.8211	0
**4263**	373	C	35.8034	0
**4263**	381	C	79.4981	1
**4263**	382	C	66.918	1
**4263**	386	C	41.3947	0
**4263**	305	D	56.316	1
**4263**	319	D	79.6404	1
**4263**	320	D	79.5177	-1
**4263**	321	D	79.6059	0
**4263**	351	D	5.9455	1
**4263**	357	D	53.4623	1
**4263**	370	D	38.3259	0
**4263**	372	D	69.8601	0
**4263**	373	D	19.6183	-1
**4263**	381	D	66.8176	1
**4263**	382	D	79.7076	1
**4263**	386	D	28.6156	0
**4264**	305	A	71.7757	1
**4264**	319	A	69.0553	1

**4264**	320	A	40.5856	-1
**4264**	321	A	79.5354	0
**4264**	351	A	11.7373	1
**4264**	357	A	61.1279	1
**4264**	370	A	55.1862	1
**4264**	372	A	35.1763	1
**4264**	373	A	79.551	1
**4264**	381	A	39.285	0
**4264**	382	A	69.3861	1
**4264**	386	A	79.6376	1
**4264**	305	B	72.5579	1
**4264**	319	B	79.5503	1
**4264**	320	B	79.6659	-1
**4264**	321	B	69.6529	0
**4264**	351	B	14.4513	1
**4264**	357	B	58.6971	1
**4264**	370	B	77.109	1
**4264**	372	B	72.9999	0
**4264**	373	B	29.9377	0
**4264**	381	B	77.3834	1
**4264**	382	B	36.1841	0
**4264**	386	B	49.8882	0
**4264**	305	C	69.5873	1
**4264**	319	C	54.1559	1
**4264**	320	C	41.6493	-1
**4264**	321	C	79.5994	0
**4264**	351	C	10.636	1
**4264**	357	C	79.5474	1
**4264**	370	C	79.4591	1
**4264**	372	C	79.5205	0
**4264**	373	C	78.754	1
**4264**	381	C	73.0708	1
**4264**	382	C	49.0459	0
**4264**	386	C	38.6077	0

**4264**	305	D	79.6028	1
**4264**	319	D	74.2391	1
**4264**	320	D	37.716	-1
**4264**	321	D	79.6349	0
**4264**	351	D	9.8926	1
**4264**	357	D	77.3458	1
**4264**	370	D	64.0309	1
**4264**	372	D	66.7759	0
**4264**	373	D	79.4376	1
**4264**	381	D	51.7255	1
**4264**	382	D	72.9213	1
**4264**	386	D	54.6244	1
**4265**	305	A	79.63	1
**4265**	319	A	79.5714	1
**4265**	320	A	79.5191	-1
**4265**	321	A	79.5018	0
**4265**	351	A	9.0465	1
**4265**	357	A	79.541	1
**4265**	370	A	77.3419	1
**4265**	372	A	79.5031	0
**4265**	373	A	76.4666	1
**4265**	381	A	69.1627	1
**4265**	382	A	76.8614	1
**4265**	386	A	41.6643	0
**4265**	305	B	79.5984	1
**4265**	319	B	38.8063	0
**4265**	320	B	73.9301	-1
**4265**	321	B	79.6041	0
**4265**	351	B	26.8865	-1
**4265**	357	B	55.5428	1
**4265**	370	B	43.7109	0
**4265**	372	B	60.022	0
**4265**	373	B	67.7748	1
**4265**	381	B	76.2522	1

**4265**	382	B	45.943	0
**4265**	386	B	30.7302	0
**4265**	305	C	57.1261	1
**4265**	319	C	40.9694	0
**4265**	320	C	70.4475	-1
**4265**	321	C	63.3353	0
**4265**	351	C	8.7973	1
**4265**	357	C	43.867	0
**4265**	370	C	57.3941	1
**4265**	372	C	79.6214	0
**4265**	373	C	66.6374	1
**4265**	381	C	49.5232	0
**4265**	382	C	57.4472	1
**4265**	386	C	53.3182	1
**4265**	305	D	79.5475	1
**4265**	319	D	67.5975	1
**4265**	320	D	55.2719	-1
**4265**	321	D	79.6253	0
**4265**	351	D	5.0701	1
**4265**	357	D	33.0558	0
**4265**	370	D	73.312	1
**4265**	372	D	76.9946	0
**4265**	373	D	64.7649	1
**4265**	381	D	73.5604	1
**4265**	382	D	43.0851	0
**4265**	386	D	79.5739	1
**4266**	305	A	45.3304	0
**4266**	319	A	77.0034	1
**4266**	320	A	67.7368	-1
**4266**	321	A	54.2283	0
**4266**	351	A	7.2365	1
**4266**	357	A	73.855	1
**4266**	370	A	40.8765	0
**4266**	372	A	57.5934	0

**4266**	373	A	50.5675	1
**4266**	381	A	79.5522	1
**4266**	382	A	76.258	1
**4266**	386	A	73.3833	1
**4266**	305	B	79.4111	1
**4266**	319	B	79.5903	1
**4266**	320	B	68.0403	-1
**4266**	321	B	79.5051	0
**4266**	351	B	7.5703	1
**4266**	357	B	54.184	1
**4266**	370	B	79.5919	1
**4266**	372	B	70.5867	0
**4266**	373	B	68.1676	1
**4266**	381	B	79.6021	1
**4266**	382	B	29.0339	0
**4266**	386	B	72.0161	1
**4266**	305	C	79.4592	1
**4266**	319	C	79.621	1
**4266**	320	C	71.7867	-1
**4266**	321	C	47.6247	1
**4266**	351	C	9.1906	1
**4266**	357	C	43.875	0
**4266**	370	C	52.2145	1
**4266**	372	C	69.8084	0
**4266**	373	C	74.1976	1
**4266**	381	C	59.7756	1
**4266**	382	C	66.8807	1
**4266**	386	C	64.7221	1
**4266**	305	D	38.8694	0
**4266**	319	D	58.5581	1
**4266**	320	D	73.5347	-1
**4266**	321	D	62.7503	0
**4266**	351	D	9.838	1
**4266**	357	D	66.4945	1

**4266**	370	D	79.5481	1
**4266**	372	D	71.4925	0
**4266**	373	D	45.8375	0
**4266**	381	D	79.4936	1
**4266**	382	D	79.4053	1
**4266**	386	D	79.5868	1
**4267**	305	A	79.4666	1
**4267**	319	A	68.5481	1
**4267**	320	A	79.5369	-1
**4267**	321	A	78.7107	0
**4267**	351	A	13.2779	1
**4267**	357	A	79.5025	1
**4267**	370	A	57.0554	1
**4267**	372	A	40.6875	1
**4267**	373	A	57.6836	1
**4267**	381	A	67.0502	1
**4267**	382	A	65.0435	1
**4267**	386	A	76.649	1
**4267**	305	B	43.1279	0
**4267**	319	B	39.5425	0
**4267**	320	B	72.3105	-1
**4267**	321	B	79.6141	0
**4267**	351	B	11.4551	1
**4267**	357	B	77.3782	1
**4267**	370	B	57.9101	1
**4267**	372	B	70.9211	0
**4267**	373	B	52.8532	1
**4267**	381	B	79.5644	1
**4267**	382	B	72.0634	1
**4267**	386	B	71.7145	1
**4267**	305	C	34.8772	0
**4267**	319	C	72.2437	1
**4267**	320	C	13.5413	1
**4267**	321	C	79.5718	0

**4267**	351	C	8.3453	1
**4267**	357	C	79.5242	1
**4267**	370	C	46.4247	0
**4267**	372	C	79.5907	0
**4267**	373	C	72.4158	1
**4267**	381	C	79.5056	1
**4267**	382	C	50.0909	1
**4267**	386	C	44.2155	0
**4267**	305	D	79.644	1
**4267**	319	D	60.6556	1
**4267**	320	D	79.5371	-1
**4267**	321	D	79.5355	0
**4267**	351	D	2.1045	1
**4267**	357	D	74.2316	1
**4267**	370	D	79.5678	1
**4267**	372	D	79.4989	0
**4267**	373	D	79.4769	1
**4267**	381	D	79.6298	1
**4267**	382	D	58.4841	1
**4267**	386	D	73.7501	1
**4268**	305	A	79.5956	1
**4268**	319	A	65.0928	1
**4268**	320	A	38.2959	-1
**4268**	321	A	39.9983	1
**4268**	351	A	10.5886	1
**4268**	357	A	73.2745	1
**4268**	370	A	39.7144	0
**4268**	372	A	54.015	0
**4268**	373	A	79.532	1
**4268**	381	A	79.5793	1
**4268**	382	A	75.5781	1
**4268**	386	A	72.7554	1
**4268**	305	B	69.7062	1
**4268**	319	B	74.9871	1

**4268**	320	B	60.4815	-1
**4268**	321	B	71.4021	0
**4268**	351	B	11.008	1
**4268**	357	B	79.4835	1
**4268**	370	B	79.6326	1
**4268**	372	B	79.4884	0
**4268**	373	B	13.4538	-1
**4268**	381	B	77.9265	1
**4268**	382	B	40.2503	0
**4268**	386	B	54.7673	1
**4268**	305	C	78.3942	1
**4268**	319	C	74.5324	1
**4268**	320	C	69.1683	-1
**4268**	321	C	79.569	0
**4268**	351	C	8.3009	1
**4268**	357	C	79.5814	1
**4268**	370	C	79.5695	1
**4268**	372	C	79.5477	0
**4268**	373	C	73.2759	1
**4268**	381	C	79.6047	1
**4268**	382	C	79.5609	1
**4268**	386	C	58.8472	1
**4268**	305	D	42.5983	0
**4268**	319	D	58.9338	1
**4268**	320	D	64.126	-1
**4268**	321	D	71.4482	0
**4268**	351	D	19.362	1
**4268**	357	D	73.601	1
**4268**	370	D	79.5754	1
**4268**	372	D	73.5684	0
**4268**	373	D	72.125	1
**4268**	381	D	79.5153	1
**4268**	382	D	79.5952	1
**4268**	386	D	64.3183	1

**4269**	305	A	48.8332	0
**4269**	319	A	71.2707	1
**4269**	320	A	79.5998	-1
**4269**	321	A	40.6848	1
**4269**	351	A	0.0	1
**4269**	357	A	76.3726	1
**4269**	370	A	79.5724	1
**4269**	372	A	73.6206	0
**4269**	373	A	38.7129	0
**4269**	381	A	72.5572	1
**4269**	382	A	68.0525	1
**4269**	386	A	79.5459	1
**4269**	305	B	79.6306	1
**4269**	319	B	79.5994	1
**4269**	320	B	73.037	-1
**4269**	321	B	70.2346	0
**4269**	351	B	17.0265	1
**4269**	357	B	79.5583	1
**4269**	370	B	79.5709	1
**4269**	372	B	79.5608	0
**4269**	373	B	65.0389	1
**4269**	381	B	76.5381	1
**4269**	382	B	41.1773	0
**4269**	386	B	57.1587	1
**4269**	305	C	72.9713	1
**4269**	319	C	78.7756	1
**4269**	320	C	77.2404	-1
**4269**	321	C	76.3044	0
**4269**	351	C	8.4484	1
**4269**	357	C	61.8916	1
**4269**	370	C	71.9471	1
**4269**	372	C	52.8276	0
**4269**	373	C	72.8297	1
**4269**	381	C	73.0241	1

**4269**	382	C	73.856	1
**4269**	386	C	34.636	0
**4269**	305	D	71.582	1
**4269**	319	D	67.8918	1
**4269**	320	D	79.5605	-1
**4269**	321	D	78.0785	0
**4269**	351	D	10.0975	1
**4269**	357	D	55.0819	1
**4269**	370	D	54.7592	1
**4269**	372	D	68.8106	0
**4269**	373	D	57.3225	1
**4269**	381	D	68.5797	1
**4269**	382	D	79.5245	1
**4269**	386	D	46.9049	0
**4270**	305	A	63.5167	1
**4270**	319	A	52.5699	1
**4270**	320	A	47.9558	-1
**4270**	321	A	57.6122	0
**4270**	351	A	2.0692	1
**4270**	357	A	56.2476	1
**4270**	370	A	60.3618	1
**4270**	372	A	30.2233	1
**4270**	373	A	79.5501	1
**4270**	381	A	72.1237	1
**4270**	382	A	50.11	1
**4270**	386	A	75.7444	1
**4270**	305	B	28.6713	0
**4270**	319	B	67.7378	1
**4270**	320	B	46.2206	-1
**4270**	321	B	78.8501	0
**4270**	351	B	19.2587	1
**4270**	357	B	51.9986	1
**4270**	370	B	67.5066	1
**4270**	372	B	79.5713	0

**4270**	373	B	26.8603	0
**4270**	381	B	47.6808	0
**4270**	382	B	20.4585	0
**4270**	386	B	40.3894	0
**4270**	305	C	34.0337	0
**4270**	319	C	79.5772	1
**4270**	320	C	79.589	-1
**4270**	321	C	37.0308	1
**4270**	351	C	3.1256	1
**4270**	357	C	52.0359	1
**4270**	370	C	34.5772	0
**4270**	372	C	54.0769	0
**4270**	373	C	79.5695	1
**4270**	381	C	64.2976	1
**4270**	382	C	78.4246	1
**4270**	386	C	14.9371	-1
**4270**	305	D	56.7767	1
**4270**	319	D	79.5449	1
**4270**	320	D	79.603	-1
**4270**	321	D	62.5075	0
**4270**	351	D	0.0666	1
**4270**	357	D	74.1717	1
**4270**	370	D	79.5838	1
**4270**	372	D	73.7307	0
**4270**	373	D	46.7651	0
**4270**	381	D	79.5198	1
**4270**	382	D	79.5762	1
**4270**	386	D	60.5221	1
**4271**	305	A	78.9506	1
**4271**	319	A	72.2763	1
**4271**	320	A	71.9794	-1
**4271**	321	A	79.4102	0
**4271**	351	A	5.9671	1
**4271**	357	A	72.7537	1

**4271**	370	A	79.6437	1
**4271**	372	A	61.4742	0
**4271**	373	A	67.3958	1
**4271**	381	A	79.5499	1
**4271**	382	A	73.9705	1
**4271**	386	A	50.6779	1
**4271**	305	B	79.5949	1
**4271**	319	B	78.1984	1
**4271**	320	B	78.9023	-1
**4271**	321	B	68.9126	0
**4271**	351	B	7.7649	1
**4271**	357	B	79.7667	1
**4271**	370	B	74.8768	1
**4271**	372	B	69.7637	0
**4271**	373	B	71.607	1
**4271**	381	B	37.5109	0
**4271**	382	B	20.9716	0
**4271**	386	B	69.5154	1
**4271**	305	C	75.1031	1
**4271**	319	C	69.5348	1
**4271**	320	C	30.946	-1
**4271**	321	C	56.082	0
**4271**	351	C	4.5461	1
**4271**	357	C	73.5103	1
**4271**	370	C	74.2701	1
**4271**	372	C	79.5713	0
**4271**	373	C	75.0005	1
**4271**	381	C	60.3334	1
**4271**	382	C	55.9266	1
**4271**	386	C	14.6825	-1
**4271**	305	D	49.8712	0
**4271**	319	D	79.6747	1
**4271**	320	D	73.5581	-1
**4271**	321	D	63.9674	0

**4271**	351	D	4.026	1
**4271**	357	D	49.4339	0
**4271**	370	D	43.9151	0
**4271**	372	D	79.3402	0
**4271**	373	D	79.528	1
**4271**	381	D	68.8634	1
**4271**	382	D	78.233	1
**4271**	386	D	37.2098	0
**4272**	305	A	59.7749	1
**4272**	319	A	77.5687	1
**4272**	320	A	71.8545	-1
**4272**	321	A	76.8967	0
**4272**	351	A	10.3671	1
**4272**	357	A	55.7422	1
**4272**	370	A	79.5058	1
**4272**	372	A	73.7584	0
**4272**	373	A	73.5837	1
**4272**	381	A	61.0748	1
**4272**	382	A	46.1662	0
**4272**	386	A	79.0664	1
**4272**	305	B	64.8751	1
**4272**	319	B	73.145	1
**4272**	320	B	79.0069	-1
**4272**	321	B	77.2301	0
**4272**	351	B	17.185	1
**4272**	357	B	79.5626	1
**4272**	370	B	79.5658	1
**4272**	372	B	71.7449	0
**4272**	373	B	79.548	1
**4272**	381	B	54.1821	1
**4272**	382	B	78.1714	1
**4272**	386	B	79.5537	1
**4272**	305	C	79.6047	1
**4272**	319	C	77.0544	1

**4272**	320	C	79.5409	-1
**4272**	321	C	67.1228	0
**4272**	351	C	13.3398	1
**4272**	357	C	73.4203	1
**4272**	370	C	44.9925	0
**4272**	372	C	78.7369	0
**4272**	373	C	70.3671	1
**4272**	381	C	79.5291	1
**4272**	382	C	73.0401	1
**4272**	386	C	40.1692	0
**4272**	305	D	69.1163	1
**4272**	319	D	79.4881	1
**4272**	320	D	79.546	-1
**4272**	321	D	79.5881	0
**4272**	351	D	9.7372	1
**4272**	357	D	79.5342	1
**4272**	370	D	72.8993	1
**4272**	372	D	57.2905	0
**4272**	373	D	79.0326	1
**4272**	381	D	73.2686	1
**4272**	382	D	55.6671	1
**4272**	386	D	79.5956	1
**4273**	305	A	74.7349	1
**4273**	319	A	51.882	1
**4273**	320	A	79.5875	-1
**4273**	321	A	63.2746	0
**4273**	351	A	2.1871	1
**4273**	357	A	61.8355	1
**4273**	370	A	79.5367	1
**4273**	372	A	66.5228	0
**4273**	373	A	61.536	1
**4273**	381	A	69.7584	1
**4273**	382	A	63.9216	1
**4273**	386	A	79.5665	1

**4273**	305	B	79.5117	1
**4273**	319	B	73.7949	1
**4273**	320	B	79.556	-1
**4273**	321	B	79.0602	0
**4273**	351	B	6.9717	1
**4273**	357	B	79.3878	1
**4273**	370	B	79.5756	1
**4273**	372	B	75.2877	0
**4273**	373	B	22.9286	0
**4273**	381	B	72.6945	1
**4273**	382	B	79.6023	1
**4273**	386	B	69.3549	1
**4273**	305	C	65.1573	1
**4273**	319	C	35.0978	0
**4273**	320	C	44.1567	-1
**4273**	321	C	31.2327	1
**4273**	351	C	8.056	1
**4273**	357	C	79.5884	1
**4273**	370	C	54.8389	1
**4273**	372	C	79.5521	0
**4273**	373	C	51.3469	1
**4273**	381	C	69.1548	1
**4273**	382	C	78.9883	1
**4273**	386	C	53.497	1
**4273**	305	D	45.2909	0
**4273**	319	D	74.0004	1
**4273**	320	D	79.6091	-1
**4273**	321	D	55.8301	0
**4273**	351	D	1.1234	1
**4273**	357	D	79.5599	1
**4273**	370	D	72.6922	1
**4273**	372	D	79.2519	0
**4273**	373	D	79.5483	1
**4273**	381	D	79.5609	1

**4273**	382	D	79.5625	1
**4273**	386	D	78.2388	1
**4274**	305	A	60.9706	1
**4274**	319	A	59.3176	1
**4274**	320	A	79.5178	-1
**4274**	321	A	70.25	0
**4274**	351	A	7.3654	1
**4274**	357	A	66.287	1
**4274**	370	A	79.575	1
**4274**	372	A	46.1312	1
**4274**	373	A	79.6123	1
**4274**	381	A	72.2147	1
**4274**	382	A	72.2074	1
**4274**	386	A	79.3171	1
**4274**	305	B	66.9035	1
**4274**	319	B	79.5642	1
**4274**	320	B	49.2532	-1
**4274**	321	B	78.5737	0
**4274**	351	B	6.4477	1
**4274**	357	B	79.5852	1
**4274**	370	B	73.035	1
**4274**	372	B	71.065	0
**4274**	373	B	49.723	0
**4274**	381	B	79.5446	1
**4274**	382	B	54.2249	1
**4274**	386	B	60.8203	1
**4274**	305	C	79.5925	1
**4274**	319	C	47.106	0
**4274**	320	C	70.4226	-1
**4274**	321	C	77.9616	0
**4274**	351	C	3.5399	1
**4274**	357	C	72.8271	1
**4274**	370	C	65.1245	1
**4274**	372	C	77.1131	0

**4274**	373	C	79.5963	1
**4274**	381	C	11.834	-1
**4274**	382	C	57.6525	1
**4274**	386	C	35.1756	0
**4274**	305	D	74.8711	1
**4274**	319	D	39.6996	0
**4274**	320	D	69.348	-1
**4274**	321	D	66.7788	0
**4274**	351	D	13.4167	1
**4274**	357	D	79.5602	1
**4274**	370	D	79.5174	1
**4274**	372	D	79.6091	0
**4274**	373	D	72.8838	1
**4274**	381	D	33.1282	0
**4274**	382	D	79.601	1
**4274**	386	D	79.5723	1
**4275**	305	A	51.9064	1
**4275**	319	A	79.5584	1
**4275**	320	A	79.483	-1
**4275**	321	A	72.8301	0
**4275**	351	A	8.0536	1
**4275**	357	A	69.2872	1
**4275**	370	A	54.6137	1
**4275**	372	A	37.451	1
**4275**	373	A	79.6113	1
**4275**	381	A	40.4259	0
**4275**	382	A	69.9016	1
**4275**	386	A	69.4169	1
**4275**	305	B	72.1468	1
**4275**	319	B	73.1552	1
**4275**	320	B	58.3464	-1
**4275**	321	B	62.2189	0
**4275**	351	B	13.741	1
**4275**	357	B	45.688	0

**4275**	370	B	49.9324	0
**4275**	372	B	52.2213	0
**4275**	373	B	64.8141	1
**4275**	381	B	72.8885	1
**4275**	382	B	55.4992	1
**4275**	386	B	57.242	1
**4275**	305	C	66.7157	1
**4275**	319	C	72.1964	1
**4275**	320	C	64.5679	-1
**4275**	321	C	44.91	1
**4275**	351	C	4.5353	1
**4275**	357	C	79.5111	1
**4275**	370	C	71.4851	1
**4275**	372	C	79.6404	0
**4275**	373	C	51.8618	1
**4275**	381	C	79.4298	1
**4275**	382	C	50.2418	1
**4275**	386	C	64.185	1
**4275**	305	D	75.622	1
**4275**	319	D	65.8229	1
**4275**	320	D	79.6456	-1
**4275**	321	D	59.6879	0
**4275**	351	D	14.5618	1
**4275**	357	D	79.1792	1
**4275**	370	D	79.6388	1
**4275**	372	D	79.603	0
**4275**	373	D	73.0202	1
**4275**	381	D	69.2877	1
**4275**	382	D	54.9871	1
**4275**	386	D	55.4353	1
**4276**	305	A	71.411	1
**4276**	319	A	68.4974	1
**4276**	320	A	79.5296	-1
**4276**	321	A	79.5626	0

**4276**	351	A	1.6145	1
**4276**	357	A	72.5127	1
**4276**	370	A	68.4271	1
**4276**	372	A	79.5719	0
**4276**	373	A	55.5028	1
**4276**	381	A	67.2146	1
**4276**	382	A	52.2949	1
**4276**	386	A	60.3379	1
**4276**	305	B	70.0624	1
**4276**	319	B	60.7037	1
**4276**	320	B	73.4857	-1
**4276**	321	B	72.3373	0
**4276**	351	B	16.7527	1
**4276**	357	B	24.7664	0
**4276**	370	B	72.317	1
**4276**	372	B	79.5186	0
**4276**	373	B	48.2848	0
**4276**	381	B	72.4693	1
**4276**	382	B	79.5887	1
**4276**	386	B	40.8015	0
**4276**	305	C	55.9602	1
**4276**	319	C	54.2488	1
**4276**	320	C	59.0176	-1
**4276**	321	C	66.9522	0
**4276**	351	C	4.1219	1
**4276**	357	C	72.7934	1
**4276**	370	C	79.5671	1
**4276**	372	C	54.7555	0
**4276**	373	C	79.5352	1
**4276**	381	C	75.5027	1
**4276**	382	C	56.2903	1
**4276**	386	C	32.997	0
**4276**	305	D	27.8249	0
**4276**	319	D	79.4562	1

**4276**	320	D	60.196	-1
**4276**	321	D	79.5946	0
**4276**	351	D	15.0638	1
**4276**	357	D	58.3067	1
**4276**	370	D	78.6945	1
**4276**	372	D	79.5951	0
**4276**	373	D	79.5342	1
**4276**	381	D	75.6351	1
**4276**	382	D	52.9958	1
**4276**	386	D	75.866	1
**4277**	305	A	78.1248	1
**4277**	319	A	73.9801	1
**4277**	320	A	60.4923	-1
**4277**	321	A	64.1502	0
**4277**	351	A	1.2166	1
**4277**	357	A	79.5822	1
**4277**	370	A	73.5084	1
**4277**	372	A	51.894	0
**4277**	373	A	79.5519	1
**4277**	381	A	79.6193	1
**4277**	382	A	73.0586	1
**4277**	386	A	79.5882	1
**4277**	305	B	79.6252	1
**4277**	319	B	66.8971	1
**4277**	320	B	68.6162	-1
**4277**	321	B	70.1436	0
**4277**	351	B	6.456	1
**4277**	357	B	76.7329	1
**4277**	370	B	56.8838	1
**4277**	372	B	71.3144	0
**4277**	373	B	75.9073	1
**4277**	381	B	65.6747	1
**4277**	382	B	32.1715	0
**4277**	386	B	70.1557	1

**4277**	305	C	68.6075	1
**4277**	319	C	66.1192	1
**4277**	320	C	54.854	-1
**4277**	321	C	71.7209	0
**4277**	351	C	12.9292	1
**4277**	357	C	75.1996	1
**4277**	370	C	71.2134	1
**4277**	372	C	79.5308	0
**4277**	373	C	71.7922	1
**4277**	381	C	55.0211	1
**4277**	382	C	66.3982	1
**4277**	386	C	38.8373	0
**4277**	305	D	44.8028	0
**4277**	319	D	73.5231	1
**4277**	320	D	79.5422	-1
**4277**	321	D	79.6436	0
**4277**	351	D	11.1238	1
**4277**	357	D	69.5456	1
**4277**	370	D	32.3034	0
**4277**	372	D	52.3044	0
**4277**	373	D	44.5421	0
**4277**	381	D	56.6221	1
**4277**	382	D	71.8797	1
**4277**	386	D	73.189	1
**4278**	305	A	48.8184	0
**4278**	319	A	9.7903	-1
**4278**	320	A	38.123	-1
**4278**	321	A	77.3021	0
**4278**	351	A	6.8559	1
**4278**	357	A	35.0794	0
**4278**	370	A	79.4397	1
**4278**	372	A	7.405	0
**4278**	373	A	73.5343	1
**4278**	381	A	50.4326	1

**4278**	382	A	62.0711	1
**4278**	386	A	67.5881	1
**4278**	305	B	79.3889	1
**4278**	319	B	49.7227	0
**4278**	320	B	79.6491	-1
**4278**	321	B	69.4879	0
**4278**	351	B	17.1529	1
**4278**	357	B	55.6881	1
**4278**	370	B	51.9436	1
**4278**	372	B	78.8087	0
**4278**	373	B	67.078	1
**4278**	381	B	75.6677	1
**4278**	382	B	74.6345	1
**4278**	386	B	47.0438	0
**4278**	305	C	53.8291	1
**4278**	319	C	46.6357	0
**4278**	320	C	66.8539	-1
**4278**	321	C	73.1147	0
**4278**	351	C	1.672	1
**4278**	357	C	79.5014	1
**4278**	370	C	64.5955	1
**4278**	372	C	61.2539	0
**4278**	373	C	68.3996	1
**4278**	381	C	79.5228	1
**4278**	382	C	19.4793	-1
**4278**	386	C	36.6995	0
**4278**	305	D	30.6778	0
**4278**	319	D	73.0064	1
**4278**	320	D	75.8508	-1
**4278**	321	D	79.554	0
**4278**	351	D	10.4266	1
**4278**	357	D	79.6559	1
**4278**	370	D	56.1029	1
**4278**	372	D	68.3258	0

**4278**	373	D	58.0161	1
**4278**	381	D	38.0994	0
**4278**	382	D	50.1792	1
**4278**	386	D	72.7793	1
**4279**	305	A	62.315	1
**4279**	319	A	70.4497	1
**4279**	320	A	79.5497	-1
**4279**	321	A	65.2849	0
**4279**	351	A	26.5924	-1
**4279**	357	A	77.3925	1
**4279**	370	A	79.6271	1
**4279**	372	A	77.3718	0
**4279**	373	A	70.1494	1
**4279**	381	A	79.4739	1
**4279**	382	A	73.0553	1
**4279**	386	A	79.6948	1
**4279**	305	B	43.2221	0
**4279**	319	B	55.6432	1
**4279**	320	B	79.576	-1
**4279**	321	B	37.3935	1
**4279**	351	B	12.1176	1
**4279**	357	B	79.3275	1
**4279**	370	B	79.543	1
**4279**	372	B	72.0567	0
**4279**	373	B	57.5562	1
**4279**	381	B	49.0926	0
**4279**	382	B	19.5937	-1
**4279**	386	B	71.4451	1
**4279**	305	C	72.6908	1
**4279**	319	C	79.0315	1
**4279**	320	C	76.2101	-1
**4279**	321	C	73.9163	0
**4279**	351	C	9.7822	1
**4279**	357	C	79.5114	1

**4279**	370	C	79.2043	1
**4279**	372	C	74.9458	0
**4279**	373	C	77.6675	1
**4279**	381	C	79.6216	1
**4279**	382	C	74.6613	1
**4279**	386	C	34.8637	0
**4279**	305	D	14.7568	-1
**4279**	319	D	66.4995	1
**4279**	320	D	79.5976	-1
**4279**	321	D	79.4214	0
**4279**	351	D	19.1767	1
**4279**	357	D	59.3073	1
**4279**	370	D	29.567	0
**4279**	372	D	70.7206	0
**4279**	373	D	68.5256	1
**4279**	381	D	32.5365	0
**4279**	382	D	54.8074	1
**4279**	386	D	79.6104	1
**4280**	305	A	73.5475	1
**4280**	319	A	63.0057	1
**4280**	320	A	78.9483	-1
**4280**	321	A	72.1682	0
**4280**	351	A	5.9018	1
**4280**	357	A	79.4933	1
**4280**	370	A	71.9145	1
**4280**	372	A	79.4325	0
**4280**	373	A	79.5314	1
**4280**	381	A	67.3352	1
**4280**	382	A	64.5713	1
**4280**	386	A	79.4983	1
**4280**	305	B	79.6004	1
**4280**	319	B	72.653	1
**4280**	320	B	55.0698	-1
**4280**	321	B	79.5284	0

**4280**	351	B	18.9972	1
**4280**	357	B	73.6298	1
**4280**	370	B	79.6193	1
**4280**	372	B	79.4785	0
**4280**	373	B	68.3225	1
**4280**	381	B	49.5787	0
**4280**	382	B	53.3765	1
**4280**	386	B	31.2968	0
**4280**	305	C	65.1684	1
**4280**	319	C	74.0103	1
**4280**	320	C	52.99	-1
**4280**	321	C	70.3847	0
**4280**	351	C	11.5637	1
**4280**	357	C	73.7728	1
**4280**	370	C	79.1204	1
**4280**	372	C	79.5475	0
**4280**	373	C	79.1845	1
**4280**	381	C	66.6337	1
**4280**	382	C	79.4626	1
**4280**	386	C	74.4652	1
**4280**	305	D	71.0245	1
**4280**	319	D	68.3962	1
**4280**	320	D	55.7174	-1
**4280**	321	D	32.2953	1
**4280**	351	D	18.6653	1
**4280**	357	D	75.5221	1
**4280**	370	D	65.3735	1
**4280**	372	D	75.0979	0
**4280**	373	D	72.3097	1
**4280**	381	D	76.285	1
**4280**	382	D	75.4916	1
**4280**	386	D	75.0526	1
**4281**	305	A	66.8866	1
**4281**	319	A	25.7113	0

**4281**	320	A	79.4644	-1
**4281**	321	A	78.0226	0
**4281**	351	A	4.7787	1
**4281**	357	A	73.9207	1
**4281**	370	A	69.405	1
**4281**	372	A	79.6957	0
**4281**	373	A	79.1064	1
**4281**	381	A	62.8489	1
**4281**	382	A	72.7403	1
**4281**	386	A	79.5213	1
**4281**	305	B	68.7276	1
**4281**	319	B	58.0179	1
**4281**	320	B	72.6421	-1
**4281**	321	B	79.5692	0
**4281**	351	B	28.2039	-1
**4281**	357	B	33.2622	0
**4281**	370	B	79.5781	1
**4281**	372	B	79.4967	0
**4281**	373	B	66.7304	1
**4281**	381	B	37.773	0
**4281**	382	B	78.9814	1
**4281**	386	B	54.7194	1
**4281**	305	C	79.4031	1
**4281**	319	C	79.5718	1
**4281**	320	C	34.375	-1
**4281**	321	C	64.5015	0
**4281**	351	C	4.8439	1
**4281**	357	C	48.5094	0
**4281**	370	C	79.4392	1
**4281**	372	C	79.1767	0
**4281**	373	C	65.0475	1
**4281**	381	C	57.6267	1
**4281**	382	C	40.2308	0
**4281**	386	C	50.4249	1

**4281**	305	D	67.3824	1
**4281**	319	D	79.5833	1
**4281**	320	D	71.9373	-1
**4281**	321	D	36.8347	1
**4281**	351	D	31.8534	-1
**4281**	357	D	67.3707	1
**4281**	370	D	60.3028	1
**4281**	372	D	79.5992	0
**4281**	373	D	79.7274	1
**4281**	381	D	61.4328	1
**4281**	382	D	79.4842	1
**4281**	386	D	19.0175	-1
**4282**	305	A	64.2589	1
**4282**	319	A	74.9788	1
**4282**	320	A	79.5633	-1
**4282**	321	A	75.818	0
**4282**	351	A	6.0238	1
**4282**	357	A	79.561	1
**4282**	370	A	79.5674	1
**4282**	372	A	79.5717	0
**4282**	373	A	73.9769	1
**4282**	381	A	41.3586	0
**4282**	382	A	60.7467	1
**4282**	386	A	41.7208	0
**4282**	305	B	73.0138	1
**4282**	319	B	55.219	1
**4282**	320	B	65.0675	-1
**4282**	321	B	47.0601	1
**4282**	351	B	13.2747	1
**4282**	357	B	73.1773	1
**4282**	370	B	19.1486	-1
**4282**	372	B	79.5908	0
**4282**	373	B	67.26	1
**4282**	381	B	12.4966	-1

**4282**	382	B	47.5379	0
**4282**	386	B	70.7652	1
**4282**	305	C	60.9941	1
**4282**	319	C	74.2138	1
**4282**	320	C	79.4915	-1
**4282**	321	C	70.7715	0
**4282**	351	C	8.8624	1
**4282**	357	C	70.6291	1
**4282**	370	C	79.597	1
**4282**	372	C	52.534	0
**4282**	373	C	79.5429	1
**4282**	381	C	77.114	1
**4282**	382	C	49.7065	0
**4282**	386	C	38.5094	0
**4282**	305	D	57.9494	1
**4282**	319	D	79.5532	1
**4282**	320	D	78.757	-1
**4282**	321	D	75.0156	0
**4282**	351	D	11.716	1
**4282**	357	D	79.5641	1
**4282**	370	D	34.7497	0
**4282**	372	D	62.6405	0
**4282**	373	D	75.6603	1
**4282**	381	D	79.5819	1
**4282**	382	D	75.006	1
**4282**	386	D	72.1581	1
**4283**	305	A	56.4254	1
**4283**	319	A	79.3386	1
**4283**	320	A	65.3464	-1
**4283**	321	A	79.5417	0
**4283**	351	A	5.0483	1
**4283**	357	A	48.5768	0
**4283**	370	A	68.8118	1
**4283**	372	A	37.0868	1

**4283**	373	A	58.5178	1
**4283**	381	A	56.8806	1
**4283**	382	A	39.8416	0
**4283**	386	A	79.5478	1
**4283**	305	B	75.9667	1
**4283**	319	B	56.4913	1
**4283**	320	B	77.3878	-1
**4283**	321	B	65.5767	0
**4283**	351	B	10.766	1
**4283**	357	B	73.0557	1
**4283**	370	B	64.6072	1
**4283**	372	B	68.3851	0
**4283**	373	B	79.2348	1
**4283**	381	B	79.6056	1
**4283**	382	B	73.3555	1
**4283**	386	B	79.4314	1
**4283**	305	C	66.9468	1
**4283**	319	C	72.3528	1
**4283**	320	C	71.0917	-1
**4283**	321	C	62.395	0
**4283**	351	C	15.11	1
**4283**	357	C	79.578	1
**4283**	370	C	69.7298	1
**4283**	372	C	71.9449	0
**4283**	373	C	79.591	1
**4283**	381	C	77.0624	1
**4283**	382	C	77.1658	1
**4283**	386	C	42.8878	0
**4283**	305	D	79.5712	1
**4283**	319	D	79.6151	1
**4283**	320	D	76.2452	-1
**4283**	321	D	79.4709	0
**4283**	351	D	19.5682	1
**4283**	357	D	54.7554	1

**4283**	370	D	51.4886	1
**4283**	372	D	74.1058	0
**4283**	373	D	68.1559	1
**4283**	381	D	70.0704	1
**4283**	382	D	72.5911	1
**4283**	386	D	47.9311	0
**4284**	305	A	71.702	1
**4284**	319	A	79.4577	1
**4284**	320	A	79.5736	-1
**4284**	321	A	71.5836	0
**4284**	351	A	0.9951	1
**4284**	357	A	71.7036	1
**4284**	370	A	72.0352	1
**4284**	372	A	76.9128	0
**4284**	373	A	35.5243	0
**4284**	381	A	72.5264	1
**4284**	382	A	71.5322	1
**4284**	386	A	62.9371	1
**4284**	305	B	72.2441	1
**4284**	319	B	55.3263	1
**4284**	320	B	57.7125	-1
**4284**	321	B	75.4409	0
**4284**	351	B	24.0939	-1
**4284**	357	B	37.3998	0
**4284**	370	B	75.7269	1
**4284**	372	B	79.5635	0
**4284**	373	B	73.1785	1
**4284**	381	B	78.6759	1
**4284**	382	B	26.295	0
**4284**	386	B	79.2838	1
**4284**	305	C	56.4577	1
**4284**	319	C	73.5612	1
**4284**	320	C	47.012	-1
**4284**	321	C	48.1725	1

**4284**	351	C	6.6939	1
**4284**	357	C	29.0443	0
**4284**	370	C	68.1949	1
**4284**	372	C	72.7748	0
**4284**	373	C	53.8801	1
**4284**	381	C	66.1842	1
**4284**	382	C	18.8851	-1
**4284**	386	C	56.8055	1
**4284**	305	D	79.4547	1
**4284**	319	D	72.9543	1
**4284**	320	D	11.6252	1
**4284**	321	D	76.5767	0
**4284**	351	D	12.4322	1
**4284**	357	D	45.4157	0
**4284**	370	D	79.5377	1
**4284**	372	D	41.9645	1
**4284**	373	D	79.5333	1
**4284**	381	D	77.1848	1
**4284**	382	D	79.5594	1
**4284**	386	D	19.9705	-1
**4285**	305	A	71.3456	1
**4285**	319	A	67.2213	1
**4285**	320	A	79.3811	-1
**4285**	321	A	78.9611	0
**4285**	351	A	0.3402	1
**4285**	357	A	70.8668	1
**4285**	370	A	24.9136	0
**4285**	372	A	79.5145	0
**4285**	373	A	45.5195	0
**4285**	381	A	79.6046	1
**4285**	382	A	77.7879	1
**4285**	386	A	47.9718	0
**4285**	305	B	66.318	1
**4285**	319	B	79.7274	1

**4285**	320	B	68.1579	-1
**4285**	321	B	67.149	0
**4285**	351	B	7.3756	1
**4285**	357	B	38.5244	0
**4285**	370	B	57.2582	1
**4285**	372	B	79.5968	0
**4285**	373	B	68.0092	1
**4285**	381	B	77.2015	1
**4285**	382	B	65.9085	1
**4285**	386	B	79.2339	1
**4285**	305	C	72.0458	1
**4285**	319	C	79.5588	1
**4285**	320	C	79.6143	-1
**4285**	321	C	60.4498	0
**4285**	351	C	0.2472	1
**4285**	357	C	78.6355	1
**4285**	370	C	73.6828	1
**4285**	372	C	79.5655	0
**4285**	373	C	79.5728	1
**4285**	381	C	77.4498	1
**4285**	382	C	79.4273	1
**4285**	386	C	45.1867	0
**4285**	305	D	21.5528	0
**4285**	319	D	79.5348	1
**4285**	320	D	79.5916	-1
**4285**	321	D	28.2246	1
**4285**	351	D	12.2767	1
**4285**	357	D	79.5993	1
**4285**	370	D	71.7188	1
**4285**	372	D	66.1009	0
**4285**	373	D	79.5048	1
**4285**	381	D	68.6733	1
**4285**	382	D	79.6108	1
**4285**	386	D	79.6491	1

**4286**	305	A	71.0286	1
**4286**	319	A	69.0482	1
**4286**	320	A	72.8073	-1
**4286**	321	A	72.2108	0
**4286**	351	A	3.5846	1
**4286**	357	A	79.6103	1
**4286**	370	A	49.5322	0
**4286**	372	A	79.5542	0
**4286**	373	A	56.345	1
**4286**	381	A	78.784	1
**4286**	382	A	67.4039	1
**4286**	386	A	53.1984	1
**4286**	305	B	74.4543	1
**4286**	319	B	66.4453	1
**4286**	320	B	61.7534	-1
**4286**	321	B	79.6061	0
**4286**	351	B	17.8633	1
**4286**	357	B	79.5606	1
**4286**	370	B	79.5524	1
**4286**	372	B	44.439	1
**4286**	373	B	79.5843	1
**4286**	381	B	79.4704	1
**4286**	382	B	79.6549	1
**4286**	386	B	66.1438	1
**4286**	305	C	60.5218	1
**4286**	319	C	37.9984	0
**4286**	320	C	79.4675	-1
**4286**	321	C	35.9867	1
**4286**	351	C	3.5338	1
**4286**	357	C	67.0631	1
**4286**	370	C	79.6333	1
**4286**	372	C	44.5703	1
**4286**	373	C	73.3129	1
**4286**	381	C	79.5708	1

**4286**	382	C	41.8553	0
**4286**	386	C	8.1224	-1
**4286**	305	D	71.8443	1
**4286**	319	D	72.1665	1
**4286**	320	D	79.6303	-1
**4286**	321	D	78.6289	0
**4286**	351	D	1.2545	1
**4286**	357	D	51.3708	1
**4286**	370	D	70.3877	1
**4286**	372	D	72.2052	0
**4286**	373	D	79.4121	1
**4286**	381	D	51.8572	1
**4286**	382	D	75.5493	1
**4286**	386	D	79.537	1
**4287**	305	A	60.3577	1
**4287**	319	A	35.8209	0
**4287**	320	A	79.6015	-1
**4287**	321	A	36.3402	1
**4287**	351	A	4.9101	1
**4287**	357	A	79.5874	1
**4287**	370	A	43.7908	0
**4287**	372	A	79.6791	0
**4287**	373	A	64.5137	1
**4287**	381	A	7.0942	-1
**4287**	382	A	65.3656	1
**4287**	386	A	34.4917	0
**4287**	305	B	79.6208	1
**4287**	319	B	79.5918	1
**4287**	320	B	79.5119	-1
**4287**	321	B	79.5476	0
**4287**	351	B	6.6043	1
**4287**	357	B	66.0554	1
**4287**	370	B	58.6394	1
**4287**	372	B	79.5753	0

**4287**	373	B	50.5091	1
**4287**	381	B	58.8651	1
**4287**	382	B	58.1481	1
**4287**	386	B	48.2542	0
**4287**	305	C	72.1587	1
**4287**	319	C	61.2579	1
**4287**	320	C	79.6256	-1
**4287**	321	C	76.8204	0
**4287**	351	C	8.6412	1
**4287**	357	C	67.9371	1
**4287**	370	C	66.4648	1
**4287**	372	C	65.5412	0
**4287**	373	C	72.8746	1
**4287**	381	C	67.7987	1
**4287**	382	C	34.886	0
**4287**	386	C	47.4599	0
**4287**	305	D	79.5263	1
**4287**	319	D	78.9604	1
**4287**	320	D	73.0902	-1
**4287**	321	D	79.4568	0
**4287**	351	D	12.8629	1
**4287**	357	D	73.5807	1
**4287**	370	D	58.9082	1
**4287**	372	D	62.7176	0
**4287**	373	D	79.6261	1
**4287**	381	D	75.6938	1
**4287**	382	D	48.4394	0
**4287**	386	D	79.4042	1
**4288**	305	A	28.9513	0
**4288**	319	A	50.786	1
**4288**	320	A	72.6088	-1
**4288**	321	A	72.8387	0
**4288**	351	A	14.1045	1
**4288**	357	A	53.5009	1

**4288**	370	A	79.5591	1
**4288**	372	A	79.5478	0
**4288**	373	A	45.0045	0
**4288**	381	A	77.6203	1
**4288**	382	A	61.6115	1
**4288**	386	A	79.0006	1
**4288**	305	B	72.9065	1
**4288**	319	B	72.4473	1
**4288**	320	B	73.6462	-1
**4288**	321	B	75.915	0
**4288**	351	B	9.0664	1
**4288**	357	B	44.5192	0
**4288**	370	B	77.3867	1
**4288**	372	B	64.6682	0
**4288**	373	B	63.8995	1
**4288**	381	B	79.5488	1
**4288**	382	B	79.6232	1
**4288**	386	B	58.3776	1
**4288**	305	C	68.9696	1
**4288**	319	C	67.0059	1
**4288**	320	C	47.1568	-1
**4288**	321	C	79.3353	0
**4288**	351	C	3.1711	1
**4288**	357	C	79.4192	1
**4288**	370	C	73.5428	1
**4288**	372	C	72.4121	0
**4288**	373	C	48.8754	0
**4288**	381	C	41.8728	0
**4288**	382	C	62.4767	1
**4288**	386	C	14.8533	-1
**4288**	305	D	48.6267	0
**4288**	319	D	71.3713	1
**4288**	320	D	69.4416	-1
**4288**	321	D	58.3173	0

**4288**	351	D	10.0913	1
**4288**	357	D	72.7256	1
**4288**	370	D	34.0435	0
**4288**	372	D	79.5586	0
**4288**	373	D	78.8328	1
**4288**	381	D	79.5545	1
**4288**	382	D	73.1983	1
**4288**	386	D	78.8567	1
**4289**	305	A	72.9429	1
**4289**	319	A	73.8697	1
**4289**	320	A	67.6083	-1
**4289**	321	A	73.5342	0
**4289**	351	A	5.5738	1
**4289**	357	A	68.4079	1
**4289**	370	A	79.5083	1
**4289**	372	A	79.5544	0
**4289**	373	A	73.3856	1
**4289**	381	A	60.8299	1
**4289**	382	A	62.1494	1
**4289**	386	A	76.0613	1
**4289**	305	B	79.5671	1
**4289**	319	B	72.8052	1
**4289**	320	B	72.3679	-1
**4289**	321	B	55.5457	0
**4289**	351	B	9.8673	1
**4289**	357	B	53.0895	1
**4289**	370	B	72.8669	1
**4289**	372	B	79.5256	0
**4289**	373	B	69.0728	1
**4289**	381	B	79.5203	1
**4289**	382	B	78.0069	1
**4289**	386	B	64.808	1
**4289**	305	C	65.1874	1
**4289**	319	C	63.3985	1

**4289**	320	C	79.5896	-1
**4289**	321	C	65.3321	0
**4289**	351	C	5.3711	1
**4289**	357	C	51.123	1
**4289**	370	C	79.5333	1
**4289**	372	C	43.1774	1
**4289**	373	C	59.0714	1
**4289**	381	C	72.1516	1
**4289**	382	C	77.7673	1
**4289**	386	C	52.762	1
**4289**	305	D	52.4359	1
**4289**	319	D	63.3879	1
**4289**	320	D	74.9526	-1
**4289**	321	D	79.5336	0
**4289**	351	D	10.9902	1
**4289**	357	D	79.3409	1
**4289**	370	D	79.5076	1
**4289**	372	D	79.5549	0
**4289**	373	D	28.9387	0
**4289**	381	D	58.4812	1
**4289**	382	D	39.3829	0
**4289**	386	D	42.3604	0
**4290**	305	A	52.663	1
**4290**	319	A	71.3137	1
**4290**	320	A	62.1714	-1
**4290**	321	A	65.706	0
**4290**	351	A	6.1657	1
**4290**	357	A	66.8415	1
**4290**	370	A	76.1468	1
**4290**	372	A	74.9364	0
**4290**	373	A	57.0235	1
**4290**	381	A	79.6617	1
**4290**	382	A	79.5433	1
**4290**	386	A	79.566	1

**4290**	305	B	79.4803	1
**4290**	319	B	76.2291	1
**4290**	320	B	79.6132	-1
**4290**	321	B	60.707	0
**4290**	351	B	24.0635	-1
**4290**	357	B	50.8796	1
**4290**	370	B	66.499	1
**4290**	372	B	54.5022	0
**4290**	373	B	70.9521	1
**4290**	381	B	30.8329	0
**4290**	382	B	26.9631	0
**4290**	386	B	79.5727	1
**4290**	305	C	79.5285	1
**4290**	319	C	39.4891	0
**4290**	320	C	61.991	-1
**4290**	321	C	65.8573	0
**4290**	351	C	7.3155	1
**4290**	357	C	38.7634	0
**4290**	370	C	72.8151	1
**4290**	372	C	72.9202	0
**4290**	373	C	79.6594	1
**4290**	381	C	41.0292	0
**4290**	382	C	79.4591	1
**4290**	386	C	44.75	0
**4290**	305	D	66.558	1
**4290**	319	D	44.7127	0
**4290**	320	D	78.1655	-1
**4290**	321	D	78.8826	0
**4290**	351	D	14.105	1
**4290**	357	D	79.6347	1
**4290**	370	D	78.7731	1
**4290**	372	D	76.3164	0
**4290**	373	D	77.1264	1
**4290**	381	D	79.5887	1

**4290**	382	D	44.8459	0
**4290**	386	D	59.8017	1
**4291**	305	A	52.8503	1
**4291**	319	A	72.2878	1
**4291**	320	A	76.5428	-1
**4291**	321	A	63.8145	0
**4291**	351	A	2.4832	1
**4291**	357	A	79.6201	1
**4291**	370	A	71.0244	1
**4291**	372	A	60.2823	0
**4291**	373	A	63.8365	1
**4291**	381	A	74.7707	1
**4291**	382	A	79.568	1
**4291**	386	A	50.0154	1
**4291**	305	B	66.1352	1
**4291**	319	B	74.0681	1
**4291**	320	B	62.8505	-1
**4291**	321	B	79.5263	0
**4291**	351	B	22.3205	-1
**4291**	357	B	74.1818	1
**4291**	370	B	69.892	1
**4291**	372	B	79.5718	0
**4291**	373	B	56.8577	1
**4291**	381	B	79.5504	1
**4291**	382	B	42.9944	0
**4291**	386	B	71.3057	1
**4291**	305	C	52.8697	1
**4291**	319	C	45.9347	0
**4291**	320	C	79.4905	-1
**4291**	321	C	79.5288	0
**4291**	351	C	9.5355	1
**4291**	357	C	75.0628	1
**4291**	370	C	28.3012	0
**4291**	372	C	51.9652	0

**4291**	373	C	22.9737	0
**4291**	381	C	68.8614	1
**4291**	382	C	67.1886	1
**4291**	386	C	39.0282	0
**4291**	305	D	62.8118	1
**4291**	319	D	64.9708	1
**4291**	320	D	79.5106	-1
**4291**	321	D	65.7457	0
**4291**	351	D	5.8648	1
**4291**	357	D	47.6834	0
**4291**	370	D	79.6096	1
**4291**	372	D	79.5997	0
**4291**	373	D	67.7276	1
**4291**	381	D	43.9521	0
**4291**	382	D	75.1203	1
**4291**	386	D	51.7216	1
**4292**	305	A	64.9476	1
**4292**	319	A	50.9827	1
**4292**	320	A	53.3414	-1
**4292**	321	A	29.2179	1
**4292**	351	A	12.3481	1
**4292**	357	A	67.7165	1
**4292**	370	A	79.5995	1
**4292**	372	A	73.9406	0
**4292**	373	A	46.5631	0
**4292**	381	A	79.5539	1
**4292**	382	A	62.395	1
**4292**	386	A	76.6349	1
**4292**	305	B	73.0237	1
**4292**	319	B	79.575	1
**4292**	320	B	55.8186	-1
**4292**	321	B	50.6734	0
**4292**	351	B	10.0288	1
**4292**	357	B	78.8999	1

**4292**	370	B	37.5767	0
**4292**	372	B	79.5571	0
**4292**	373	B	65.7823	1
**4292**	381	B	79.5042	1
**4292**	382	B	35.614	0
**4292**	386	B	79.548	1
**4292**	305	C	78.4307	1
**4292**	319	C	76.4801	1
**4292**	320	C	78.1826	-1
**4292**	321	C	79.4652	0
**4292**	351	C	15.0945	1
**4292**	357	C	79.5634	1
**4292**	370	C	65.0284	1
**4292**	372	C	79.5231	0
**4292**	373	C	72.314	1
**4292**	381	C	73.6903	1
**4292**	382	C	65.6841	1
**4292**	386	C	32.6042	0
**4292**	305	D	67.9486	1
**4292**	319	D	55.3685	1
**4292**	320	D	74.1513	-1
**4292**	321	D	79.5494	0
**4292**	351	D	11.0631	1
**4292**	357	D	60.2261	1
**4292**	370	D	42.6943	0
**4292**	372	D	11.6914	0
**4292**	373	D	55.7848	1
**4292**	381	D	43.0158	0
**4292**	382	D	72.9732	1
**4292**	386	D	79.5077	1
**4293**	305	A	76.5795	1
**4293**	319	A	54.6462	1
**4293**	320	A	76.7024	-1
**4293**	321	A	60.6859	0

**4293**	351	A	5.0198	1
**4293**	357	A	79.4538	1
**4293**	370	A	70.0171	1
**4293**	372	A	54.0304	0
**4293**	373	A	24.7323	0
**4293**	381	A	44.7314	0
**4293**	382	A	66.5107	1
**4293**	386	A	79.5492	1
**4293**	305	B	67.5865	1
**4293**	319	B	59.2893	1
**4293**	320	B	73.6949	-1
**4293**	321	B	78.1637	0
**4293**	351	B	14.1879	1
**4293**	357	B	49.4421	0
**4293**	370	B	79.5597	1
**4293**	372	B	78.7923	0
**4293**	373	B	68.2703	1
**4293**	381	B	41.5688	0
**4293**	382	B	16.7242	-1
**4293**	386	B	79.68	1
**4293**	305	C	62.6696	1
**4293**	319	C	78.487	1
**4293**	320	C	43.6947	-1
**4293**	321	C	79.4938	0
**4293**	351	C	3.3655	1
**4293**	357	C	79.5959	1
**4293**	370	C	71.577	1
**4293**	372	C	79.6267	0
**4293**	373	C	72.8875	1
**4293**	381	C	53.2634	1
**4293**	382	C	73.5657	1
**4293**	386	C	48.5144	0
**4293**	305	D	79.5379	1
**4293**	319	D	74.9441	1

**4293**	320	D	75.2326	-1
**4293**	321	D	79.5544	0
**4293**	351	D	13.1329	1
**4293**	357	D	79.7025	1
**4293**	370	D	73.4319	1
**4293**	372	D	71.9244	0
**4293**	373	D	71.3055	1
**4293**	381	D	41.1602	0
**4293**	382	D	74.4591	1
**4293**	386	D	49.3222	0
**4294**	305	A	46.1482	0
**4294**	319	A	66.1867	1
**4294**	320	A	79.4997	-1
**4294**	321	A	31.8575	1
**4294**	351	A	3.9242	1
**4294**	357	A	62.4837	1
**4294**	370	A	79.4634	1
**4294**	372	A	74.3223	0
**4294**	373	A	71.8349	1
**4294**	381	A	79.4488	1
**4294**	382	A	52.1624	1
**4294**	386	A	79.5082	1
**4294**	305	B	52.8032	1
**4294**	319	B	49.1464	0
**4294**	320	B	54.5536	-1
**4294**	321	B	55.2692	0
**4294**	351	B	10.2066	1
**4294**	357	B	45.8274	0
**4294**	370	B	60.5027	1
**4294**	372	B	72.5878	0
**4294**	373	B	46.9701	0
**4294**	381	B	60.4528	1
**4294**	382	B	42.4705	0
**4294**	386	B	79.5752	1

**4294**	305	C	72.8579	1
**4294**	319	C	79.6597	1
**4294**	320	C	79.5276	-1
**4294**	321	C	79.4056	0
**4294**	351	C	0.5943	1
**4294**	357	C	79.563	1
**4294**	370	C	79.5479	1
**4294**	372	C	79.6118	0
**4294**	373	C	79.4497	1
**4294**	381	C	79.4998	1
**4294**	382	C	43.8475	0
**4294**	386	C	54.0276	1
**4294**	305	D	53.4733	1
**4294**	319	D	44.985	0
**4294**	320	D	47.6084	-1
**4294**	321	D	70.3549	0
**4294**	351	D	12.6768	1
**4294**	357	D	50.8102	1
**4294**	370	D	71.5909	1
**4294**	372	D	79.5271	0
**4294**	373	D	42.8085	0
**4294**	381	D	72.942	1
**4294**	382	D	79.5791	1
**4294**	386	D	68.7536	1
**4295**	305	A	66.137	1
**4295**	319	A	75.365	1
**4295**	320	A	79.3868	-1
**4295**	321	A	74.7425	0
**4295**	351	A	0.0	1
**4295**	357	A	79.3383	1
**4295**	370	A	79.6619	1
**4295**	372	A	63.5675	0
**4295**	373	A	49.0997	0
**4295**	381	A	37.9112	0

**4295**	382	A	65.8939	1
**4295**	386	A	78.4569	1
**4295**	305	B	79.8286	1
**4295**	319	B	70.3454	1
**4295**	320	B	59.7153	-1
**4295**	321	B	64.5028	0
**4295**	351	B	20.5733	-1
**4295**	357	B	50.801	1
**4295**	370	B	79.1758	1
**4295**	372	B	75.6384	0
**4295**	373	B	66.9942	1
**4295**	381	B	73.9559	1
**4295**	382	B	32.0042	0
**4295**	386	B	79.4904	1
**4295**	305	C	30.222	0
**4295**	319	C	29.8771	0
**4295**	320	C	56.3968	-1
**4295**	321	C	39.1411	1
**4295**	351	C	1.7781	1
**4295**	357	C	73.1857	1
**4295**	370	C	79.5472	1
**4295**	372	C	75.7395	0
**4295**	373	C	39.3646	0
**4295**	381	C	78.1869	1
**4295**	382	C	71.7116	1
**4295**	386	C	40.7236	0
**4295**	305	D	73.6663	1
**4295**	319	D	55.6733	1
**4295**	320	D	50.9477	-1
**4295**	321	D	73.5861	0
**4295**	351	D	19.5304	1
**4295**	357	D	75.4849	1
**4295**	370	D	64.1593	1
**4295**	372	D	70.5531	0

**4295**	373	D	62.7676	1
**4295**	381	D	79.5657	1
**4295**	382	D	79.5731	1
**4295**	386	D	73.7713	1
**4296**	305	A	78.3836	1
**4296**	319	A	49.1692	0
**4296**	320	A	78.0867	-1
**4296**	321	A	17.2641	0
**4296**	351	A	12.5195	1
**4296**	357	A	79.501	1
**4296**	370	A	76.42	1
**4296**	372	A	62.5963	0
**4296**	373	A	40.153	0
**4296**	381	A	70.6932	1
**4296**	382	A	74.6993	1
**4296**	386	A	72.4671	1
**4296**	305	B	67.7646	1
**4296**	319	B	73.9661	1
**4296**	320	B	69.8737	-1
**4296**	321	B	64.0772	0
**4296**	351	B	10.5511	1
**4296**	357	B	29.8994	0
**4296**	370	B	79.5898	1
**4296**	372	B	78.2074	0
**4296**	373	B	79.5264	1
**4296**	381	B	67.8104	1
**4296**	382	B	50.3295	1
**4296**	386	B	71.4001	1
**4296**	305	C	63.2755	1
**4296**	319	C	72.537	1
**4296**	320	C	14.6378	1
**4296**	321	C	63.0748	0
**4296**	351	C	12.3516	1
**4296**	357	C	79.5509	1

**4296**	370	C	63.1318	1
**4296**	372	C	79.6543	0
**4296**	373	C	55.9287	1
**4296**	381	C	63.6025	1
**4296**	382	C	78.4895	1
**4296**	386	C	63.3059	1
**4296**	305	D	79.5444	1
**4296**	319	D	39.6358	0
**4296**	320	D	78.6496	-1
**4296**	321	D	58.5514	0
**4296**	351	D	8.269	1
**4296**	357	D	47.7113	0
**4296**	370	D	73.0669	1
**4296**	372	D	13.8391	0
**4296**	373	D	79.5226	1
**4296**	381	D	71.7027	1
**4296**	382	D	68.7888	1
**4296**	386	D	75.9516	1
**4297**	305	A	73.3884	1
**4297**	319	A	66.6126	1
**4297**	320	A	59.7705	-1
**4297**	321	A	63.4809	0
**4297**	351	A	10.8943	1
**4297**	357	A	71.9206	1
**4297**	370	A	79.5743	1
**4297**	372	A	42.6385	1
**4297**	373	A	69.7581	1
**4297**	381	A	79.6077	1
**4297**	382	A	79.6217	1
**4297**	386	A	70.9118	1
**4297**	305	B	79.583	1
**4297**	319	B	65.7758	1
**4297**	320	B	58.5161	-1
**4297**	321	B	78.1321	0

**4297**	351	B	12.5768	1
**4297**	357	B	55.0616	1
**4297**	370	B	79.2799	1
**4297**	372	B	69.013	0
**4297**	373	B	39.4427	0
**4297**	381	B	62.8198	1
**4297**	382	B	75.7521	1
**4297**	386	B	68.2498	1
**4297**	305	C	78.8649	1
**4297**	319	C	74.2321	1
**4297**	320	C	79.6562	-1
**4297**	321	C	71.602	0
**4297**	351	C	4.6642	1
**4297**	357	C	56.542	1
**4297**	370	C	79.482	1
**4297**	372	C	79.6118	0
**4297**	373	C	70.8561	1
**4297**	381	C	63.9848	1
**4297**	382	C	48.6395	0
**4297**	386	C	30.2446	0
**4297**	305	D	73.3463	1
**4297**	319	D	78.0383	1
**4297**	320	D	74.9882	-1
**4297**	321	D	79.5506	0
**4297**	351	D	14.7039	1
**4297**	357	D	77.113	1
**4297**	370	D	69.7474	1
**4297**	372	D	70.4294	0
**4297**	373	D	67.1716	1
**4297**	381	D	41.3393	0
**4297**	382	D	66.8166	1
**4297**	386	D	44.8137	0
**4298**	305	A	72.6057	1
**4298**	319	A	69.5899	1

**4298**	320	A	79.4915	-1
**4298**	321	A	79.6893	0
**4298**	351	A	2.9237	1
**4298**	357	A	69.9409	1
**4298**	370	A	56.3744	1
**4298**	372	A	79.6454	0
**4298**	373	A	47.7088	0
**4298**	381	A	74.0153	1
**4298**	382	A	79.5732	1
**4298**	386	A	79.5327	1
**4298**	305	B	52.7341	1
**4298**	319	B	64.0805	1
**4298**	320	B	53.554	-1
**4298**	321	B	36.7417	1
**4298**	351	B	10.151	1
**4298**	357	B	79.6426	1
**4298**	370	B	67.7779	1
**4298**	372	B	79.6442	0
**4298**	373	B	58.1093	1
**4298**	381	B	54.5894	1
**4298**	382	B	76.9924	1
**4298**	386	B	51.8741	1
**4298**	305	C	51.7742	1
**4298**	319	C	51.6683	1
**4298**	320	C	79.4725	-1
**4298**	321	C	79.5314	0
**4298**	351	C	7.0295	1
**4298**	357	C	62.0912	1
**4298**	370	C	72.5984	1
**4298**	372	C	79.6056	0
**4298**	373	C	79.4329	1
**4298**	381	C	79.4584	1
**4298**	382	C	62.6201	1
**4298**	386	C	46.5617	0

**4298**	305	D	79.5495	1
**4298**	319	D	37.5321	0
**4298**	320	D	79.6022	-1
**4298**	321	D	45.4844	1
**4298**	351	D	12.9765	1
**4298**	357	D	79.5187	1
**4298**	370	D	62.8551	1
**4298**	372	D	71.1952	0
**4298**	373	D	79.55	1
**4298**	381	D	72.1238	1
**4298**	382	D	64.1539	1
**4298**	386	D	42.4573	0
**4299**	305	A	76.2716	1
**4299**	319	A	61.791	1
**4299**	320	A	79.7712	-1
**4299**	321	A	75.1925	0
**4299**	351	A	9.8517	1
**4299**	357	A	74.2879	1
**4299**	370	A	63.4458	1
**4299**	372	A	79.6417	0
**4299**	373	A	78.2988	1
**4299**	381	A	79.4975	1
**4299**	382	A	32.7829	0
**4299**	386	A	69.697	1
**4299**	305	B	42.4986	0
**4299**	319	B	75.0199	1
**4299**	320	B	47.8252	-1
**4299**	321	B	44.3489	1
**4299**	351	B	10.1722	1
**4299**	357	B	41.433	0
**4299**	370	B	68.1483	1
**4299**	372	B	54.8607	0
**4299**	373	B	29.0412	0
**4299**	381	B	45.161	0

**4299**	382	B	27.1542	0
**4299**	386	B	79.3434	1
**4299**	305	C	71.1767	1
**4299**	319	C	65.8038	1
**4299**	320	C	79.476	-1
**4299**	321	C	55.1878	0
**4299**	351	C	4.3737	1
**4299**	357	C	79.5844	1
**4299**	370	C	79.3944	1
**4299**	372	C	79.5788	0
**4299**	373	C	62.0007	1
**4299**	381	C	79.6236	1
**4299**	382	C	59.5998	1
**4299**	386	C	67.1047	1
**4299**	305	D	79.0304	1
**4299**	319	D	35.7016	0
**4299**	320	D	79.4236	-1
**4299**	321	D	76.1571	0
**4299**	351	D	14.4369	1
**4299**	357	D	55.827	1
**4299**	370	D	68.8643	1
**4299**	372	D	69.4572	0
**4299**	373	D	43.9206	0
**4299**	381	D	72.2623	1
**4299**	382	D	40.918	0
**4299**	386	D	79.4911	1
**4300**	305	A	79.5728	1
**4300**	319	A	73.3433	1
**4300**	320	A	66.0016	-1
**4300**	321	A	40.2264	1
**4300**	351	A	3.8897	1
**4300**	357	A	55.4098	1
**4300**	370	A	66.4169	1
**4300**	372	A	70.5407	0

**4300**	373	A	63.5258	1
**4300**	381	A	29.8843	0
**4300**	382	A	72.3315	1
**4300**	386	A	78.0071	1
**4300**	305	B	64.2724	1
**4300**	319	B	79.6427	1
**4300**	320	B	73.1411	-1
**4300**	321	B	79.4842	0
**4300**	351	B	9.6624	1
**4300**	357	B	63.3041	1
**4300**	370	B	37.5544	0
**4300**	372	B	64.8885	0
**4300**	373	B	61.1989	1
**4300**	381	B	62.8212	1
**4300**	382	B	79.4575	1
**4300**	386	B	79.5283	1
**4300**	305	C	61.1432	1
**4300**	319	C	74.0933	1
**4300**	320	C	79.5243	-1
**4300**	321	C	79.729	0
**4300**	351	C	0.5562	1
**4300**	357	C	72.5681	1
**4300**	370	C	53.8329	1
**4300**	372	C	45.338	1
**4300**	373	C	42.7102	0
**4300**	381	C	78.7378	1
**4300**	382	C	26.7596	0
**4300**	386	C	51.7548	1
**4300**	305	D	48.5045	0
**4300**	319	D	79.449	1
**4300**	320	D	79.5497	-1
**4300**	321	D	79.5744	0
**4300**	351	D	13.3901	1
**4300**	357	D	65.1259	1

**4300**	370	D	60.3322	1
**4300**	372	D	63.88	0
**4300**	373	D	35.2846	0
**4300**	381	D	67.5096	1
**4300**	382	D	47.3037	0
**4300**	386	D	70.0938	1
**4301**	305	A	75.9366	1
**4301**	319	A	65.566	1
**4301**	320	A	79.483	-1
**4301**	321	A	79.4964	0
**4301**	351	A	0.5514	1
**4301**	357	A	43.9992	0
**4301**	370	A	69.6279	1
**4301**	372	A	79.5413	0
**4301**	373	A	62.8269	1
**4301**	381	A	79.5881	1
**4301**	382	A	75.1509	1
**4301**	386	A	73.0892	1
**4301**	305	B	75.6275	1
**4301**	319	B	77.5656	1
**4301**	320	B	72.7665	-1
**4301**	321	B	75.5678	0
**4301**	351	B	21.3746	-1
**4301**	357	B	79.6537	1
**4301**	370	B	66.239	1
**4301**	372	B	79.695	0
**4301**	373	B	36.9645	0
**4301**	381	B	79.5916	1
**4301**	382	B	44.4411	0
**4301**	386	B	56.5563	1
**4301**	305	C	76.6434	1
**4301**	319	C	79.5934	1
**4301**	320	C	63.3326	-1
**4301**	321	C	79.6157	0

**4301**	351	C	9.1273	1
**4301**	357	C	76.8644	1
**4301**	370	C	78.5853	1
**4301**	372	C	45.2921	1
**4301**	373	C	79.427	1
**4301**	381	C	74.9967	1
**4301**	382	C	64.1684	1
**4301**	386	C	71.9189	1
**4301**	305	D	62.3197	1
**4301**	319	D	71.4024	1
**4301**	320	D	74.9017	-1
**4301**	321	D	79.5456	0
**4301**	351	D	21.556	-1
**4301**	357	D	74.7381	1
**4301**	370	D	73.8018	1
**4301**	372	D	67.0194	0
**4301**	373	D	59.3925	1
**4301**	381	D	39.563	0
**4301**	382	D	79.334	1
**4301**	386	D	43.065	0
**4302**	305	A	60.4385	1
**4302**	319	A	63.1076	1
**4302**	320	A	73.0292	-1
**4302**	321	A	70.5509	0
**4302**	351	A	6.9982	1
**4302**	357	A	79.5562	1
**4302**	370	A	57.4132	1
**4302**	372	A	73.0114	0
**4302**	373	A	78.8477	1
**4302**	381	A	69.2776	1
**4302**	382	A	75.3866	1
**4302**	386	A	68.0377	1
**4302**	305	B	79.4418	1
**4302**	319	B	79.4951	1

**4302**	320	B	79.5984	-1
**4302**	321	B	49.1949	1
**4302**	351	B	9.9913	1
**4302**	357	B	79.584	1
**4302**	370	B	65.0187	1
**4302**	372	B	72.2607	0
**4302**	373	B	61.9261	1
**4302**	381	B	58.9851	1
**4302**	382	B	70.9221	1
**4302**	386	B	72.1445	1
**4302**	305	C	57.4331	1
**4302**	319	C	79.525	1
**4302**	320	C	69.7209	-1
**4302**	321	C	79.522	0
**4302**	351	C	13.4895	1
**4302**	357	C	79.6694	1
**4302**	370	C	52.4656	1
**4302**	372	C	67.7646	0
**4302**	373	C	78.9129	1
**4302**	381	C	79.3724	1
**4302**	382	C	61.0143	1
**4302**	386	C	41.0417	0
**4302**	305	D	48.2035	0
**4302**	319	D	64.298	1
**4302**	320	D	57.0551	-1
**4302**	321	D	32.9523	1
**4302**	351	D	10.5743	1
**4302**	357	D	59.4571	1
**4302**	370	D	78.9546	1
**4302**	372	D	78.4222	0
**4302**	373	D	79.3572	1
**4302**	381	D	68.4588	1
**4302**	382	D	79.5401	1
**4302**	386	D	45.3006	0

**4303**	305	A	56.1269	1
**4303**	319	A	79.5744	1
**4303**	320	A	79.5655	-1
**4303**	321	A	77.4394	0
**4303**	351	A	7.1788	1
**4303**	357	A	63.069	1
**4303**	370	A	78.8054	1
**4303**	372	A	79.5071	0
**4303**	373	A	67.3056	1
**4303**	381	A	69.551	1
**4303**	382	A	67.8598	1
**4303**	386	A	62.1698	1
**4303**	305	B	72.0572	1
**4303**	319	B	54.9781	1
**4303**	320	B	40.1725	-1
**4303**	321	B	60.7354	0
**4303**	351	B	10.7565	1
**4303**	357	B	74.9079	1
**4303**	370	B	72.5626	1
**4303**	372	B	52.4974	0
**4303**	373	B	33.5351	0
**4303**	381	B	21.5001	0
**4303**	382	B	42.4452	0
**4303**	386	B	78.5694	1
**4303**	305	C	71.7048	1
**4303**	319	C	74.3069	1
**4303**	320	C	52.4704	-1
**4303**	321	C	75.2716	0
**4303**	351	C	8.9812	1
**4303**	357	C	65.5954	1
**4303**	370	C	78.8983	1
**4303**	372	C	79.5675	0
**4303**	373	C	73.8438	1
**4303**	381	C	16.9947	-1

**4303**	382	C	75.8357	1
**4303**	386	C	52.6276	1
**4303**	305	D	63.1592	1
**4303**	319	D	73.154	1
**4303**	320	D	79.5545	-1
**4303**	321	D	79.5886	0
**4303**	351	D	11.8307	1
**4303**	357	D	50.1242	1
**4303**	370	D	79.5748	1
**4303**	372	D	75.5037	0
**4303**	373	D	50.3307	1
**4303**	381	D	77.2052	1
**4303**	382	D	79.5444	1
**4303**	386	D	75.7017	1
**4304**	305	A	64.135	1
**4304**	319	A	72.0342	1
**4304**	320	A	66.6825	-1
**4304**	321	A	73.9749	0
**4304**	351	A	11.055	1
**4304**	357	A	56.5984	1
**4304**	370	A	73.5184	1
**4304**	372	A	61.3952	0
**4304**	373	A	39.7104	0
**4304**	381	A	79.8228	1
**4304**	382	A	69.5855	1
**4304**	386	A	70.906	1
**4304**	305	B	79.6977	1
**4304**	319	B	30.5085	0
**4304**	320	B	52.2618	-1
**4304**	321	B	72.0791	0
**4304**	351	B	14.1855	1
**4304**	357	B	79.6472	1
**4304**	370	B	76.0164	1
**4304**	372	B	44.2894	1

**4304**	373	B	71.8077	1
**4304**	381	B	51.9656	1
**4304**	382	B	36.4439	0
**4304**	386	B	69.3104	1
**4304**	305	C	77.7629	1
**4304**	319	C	45.8816	0
**4304**	320	C	54.2342	-1
**4304**	321	C	77.9084	0
**4304**	351	C	10.4969	1
**4304**	357	C	79.4642	1
**4304**	370	C	50.4767	1
**4304**	372	C	56.5382	0
**4304**	373	C	60.5293	1
**4304**	381	C	79.5072	1
**4304**	382	C	79.6063	1
**4304**	386	C	43.3979	0
**4304**	305	D	71.6392	1
**4304**	319	D	64.9356	1
**4304**	320	D	79.4659	-1
**4304**	321	D	63.6643	0
**4304**	351	D	15.0679	1
**4304**	357	D	61.9959	1
**4304**	370	D	63.9993	1
**4304**	372	D	79.4832	0
**4304**	373	D	59.0271	1
**4304**	381	D	74.3278	1
**4304**	382	D	63.8225	1
**4304**	386	D	79.473	1
**4305**	305	A	63.4426	1
**4305**	319	A	78.7426	1
**4305**	320	A	79.4712	-1
**4305**	321	A	58.2913	0
**4305**	351	A	5.5328	1
**4305**	357	A	62.5337	1

**4305**	370	A	79.4424	1
**4305**	372	A	47.603	1
**4305**	373	A	57.7567	1
**4305**	381	A	79.5375	1
**4305**	382	A	79.5031	1
**4305**	386	A	79.0324	1
**4305**	305	B	79.7823	1
**4305**	319	B	45.1493	0
**4305**	320	B	72.1906	-1
**4305**	321	B	77.2823	0
**4305**	351	B	14.2702	1
**4305**	357	B	79.5837	1
**4305**	370	B	78.9756	1
**4305**	372	B	79.5492	0
**4305**	373	B	79.5386	1
**4305**	381	B	34.2117	0
**4305**	382	B	50.3144	1
**4305**	386	B	73.15	1
**4305**	305	C	62.7805	1
**4305**	319	C	73.1484	1
**4305**	320	C	52.5257	-1
**4305**	321	C	74.1302	0
**4305**	351	C	7.4253	1
**4305**	357	C	63.3261	1
**4305**	370	C	57.4511	1
**4305**	372	C	71.1839	0
**4305**	373	C	79.5823	1
**4305**	381	C	58.9657	1
**4305**	382	C	65.8357	1
**4305**	386	C	64.8345	1
**4305**	305	D	76.4186	1
**4305**	319	D	73.7793	1
**4305**	320	D	24.3005	-1
**4305**	321	D	77.8194	0

**4305**	351	D	12.1752	1
**4305**	357	D	79.5781	1
**4305**	370	D	75.4525	1
**4305**	372	D	79.5661	0
**4305**	373	D	43.5756	0
**4305**	381	D	70.3578	1
**4305**	382	D	69.2634	1
**4305**	386	D	73.61	1
**4306**	305	A	75.1778	1
**4306**	319	A	61.925	1
**4306**	320	A	65.182	-1
**4306**	321	A	76.3585	0
**4306**	351	A	8.9673	1
**4306**	357	A	73.3196	1
**4306**	370	A	79.4906	1
**4306**	372	A	79.5653	0
**4306**	373	A	63.8573	1
**4306**	381	A	23.9671	0
**4306**	382	A	70.4434	1
**4306**	386	A	79.5915	1
**4306**	305	B	61.8573	1
**4306**	319	B	71.0964	1
**4306**	320	B	76.2826	-1
**4306**	321	B	65.1029	0
**4306**	351	B	16.7579	1
**4306**	357	B	79.5949	1
**4306**	370	B	79.4833	1
**4306**	372	B	53.2794	0
**4306**	373	B	67.7131	1
**4306**	381	B	52.9446	1
**4306**	382	B	79.5515	1
**4306**	386	B	69.384	1
**4306**	305	C	68.7725	1
**4306**	319	C	79.4738	1

**4306**	320	C	77.6242	-1
**4306**	321	C	67.6713	0
**4306**	351	C	13.5311	1
**4306**	357	C	43.3035	0
**4306**	370	C	79.5388	1
**4306**	372	C	74.8566	0
**4306**	373	C	79.6177	1
**4306**	381	C	79.7193	1
**4306**	382	C	59.1209	1
**4306**	386	C	47.6459	0
**4306**	305	D	65.6165	1
**4306**	319	D	63.2621	1
**4306**	320	D	79.1273	-1
**4306**	321	D	54.6788	0
**4306**	351	D	13.5457	1
**4306**	357	D	72.9771	1
**4306**	370	D	59.3371	1
**4306**	372	D	74.6951	0
**4306**	373	D	79.6381	1
**4306**	381	D	79.6002	1
**4306**	382	D	79.6021	1
**4306**	386	D	79.5996	1
**4307**	305	A	79.5217	1
**4307**	319	A	70.9652	1
**4307**	320	A	72.5276	-1
**4307**	321	A	61.663	0
**4307**	351	A	11.4822	1
**4307**	357	A	79.6079	1
**4307**	370	A	79.5532	1
**4307**	372	A	76.266	0
**4307**	373	A	50.6294	1
**4307**	381	A	75.6567	1
**4307**	382	A	20.4529	0
**4307**	386	A	79.057	1

**4307**	305	B	79.7358	1
**4307**	319	B	79.6221	1
**4307**	320	B	49.7375	-1
**4307**	321	B	40.5438	1
**4307**	351	B	19.9349	1
**4307**	357	B	79.623	1
**4307**	370	B	79.5623	1
**4307**	372	B	47.2743	1
**4307**	373	B	50.2829	1
**4307**	381	B	75.1064	1
**4307**	382	B	53.7292	1
**4307**	386	B	63.8918	1
**4307**	305	C	79.5455	1
**4307**	319	C	40.9551	0
**4307**	320	C	65.9014	-1
**4307**	321	C	70.725	0
**4307**	351	C	8.9109	1
**4307**	357	C	55.254	1
**4307**	370	C	73.0316	1
**4307**	372	C	79.734	0
**4307**	373	C	70.6614	1
**4307**	381	C	79.5094	1
**4307**	382	C	77.2743	1
**4307**	386	C	58.3004	1
**4307**	305	D	68.1539	1
**4307**	319	D	78.3559	1
**4307**	320	D	74.7953	-1
**4307**	321	D	60.1921	0
**4307**	351	D	14.1997	1
**4307**	357	D	56.4079	1
**4307**	370	D	29.0954	0
**4307**	372	D	73.3916	0
**4307**	373	D	79.4348	1
**4307**	381	D	39.1074	0

**4307**	382	D	70.4589	1
**4307**	386	D	53.8639	1
**4308**	305	A	79.6029	1
**4308**	319	A	39.5449	0
**4308**	320	A	79.6908	-1
**4308**	321	A	72.1228	0
**4308**	351	A	4.792	1
**4308**	357	A	73.4402	1
**4308**	370	A	71.8088	1
**4308**	372	A	45.388	1
**4308**	373	A	70.39	1
**4308**	381	A	64.3109	1
**4308**	382	A	74.0384	1
**4308**	386	A	79.1541	1
**4308**	305	B	68.0194	1
**4308**	319	B	72.0735	1
**4308**	320	B	74.4256	-1
**4308**	321	B	79.5101	0
**4308**	351	B	11.756	1
**4308**	357	B	45.7539	0
**4308**	370	B	79.5195	1
**4308**	372	B	67.019	0
**4308**	373	B	46.2971	0
**4308**	381	B	32.0631	0
**4308**	382	B	5.8138	-1
**4308**	386	B	69.7604	1
**4308**	305	C	66.1218	1
**4308**	319	C	61.3259	1
**4308**	320	C	79.5924	-1
**4308**	321	C	74.5101	0
**4308**	351	C	1.8019	1
**4308**	357	C	50.4597	1
**4308**	370	C	56.9612	1
**4308**	372	C	46.9849	1

**4308**	373	C	79.498	1
**4308**	381	C	61.1219	1
**4308**	382	C	79.5662	1
**4308**	386	C	47.5449	0
**4308**	305	D	31.9084	0
**4308**	319	D	73.5947	1
**4308**	320	D	79.5926	-1
**4308**	321	D	79.5599	0
**4308**	351	D	10.1233	1
**4308**	357	D	70.6394	1
**4308**	370	D	77.0729	1
**4308**	372	D	79.5787	0
**4308**	373	D	79.5425	1
**4308**	381	D	68.0068	1
**4308**	382	D	42.3077	0
**4308**	386	D	77.7226	1
**4309**	305	A	40.2306	0
**4309**	319	A	79.7078	1
**4309**	320	A	79.4302	-1
**4309**	321	A	73.5701	0
**4309**	351	A	9.9663	1
**4309**	357	A	79.2123	1
**4309**	370	A	43.2558	0
**4309**	372	A	61.91	0
**4309**	373	A	79.3905	1
**4309**	381	A	76.6861	1
**4309**	382	A	58.0139	1
**4309**	386	A	79.6278	1
**4309**	305	B	56.1233	1
**4309**	319	B	50.4505	1
**4309**	320	B	74.1841	-1
**4309**	321	B	71.7295	0
**4309**	351	B	7.6812	1
**4309**	357	B	73.3777	1

**4309**	370	B	79.5909	1
**4309**	372	B	79.6434	0
**4309**	373	B	71.33	1
**4309**	381	B	79.4967	1
**4309**	382	B	27.9798	0
**4309**	386	B	69.4044	1
**4309**	305	C	68.0393	1
**4309**	319	C	32.8054	0
**4309**	320	C	51.5423	-1
**4309**	321	C	79.7487	0
**4309**	351	C	8.1006	1
**4309**	357	C	58.7759	1
**4309**	370	C	43.6053	0
**4309**	372	C	42.0573	1
**4309**	373	C	57.0099	1
**4309**	381	C	79.6757	1
**4309**	382	C	42.3751	0
**4309**	386	C	37.0919	0
**4309**	305	D	53.2658	1
**4309**	319	D	76.5431	1
**4309**	320	D	69.9806	-1
**4309**	321	D	70.7861	0
**4309**	351	D	46.2299	-1
**4309**	357	D	79.6874	1
**4309**	370	D	73.3801	1
**4309**	372	D	65.2836	0
**4309**	373	D	79.4521	1
**4309**	381	D	57.6528	1
**4309**	382	D	70.8454	1
**4309**	386	D	79.5674	1
**4310**	305	A	66.4236	1
**4310**	319	A	46.0869	0
**4310**	320	A	48.5924	-1
**4310**	321	A	21.8416	1

**4310**	351	A	6.9142	1
**4310**	357	A	79.4767	1
**4310**	370	A	54.1164	1
**4310**	372	A	55.9007	0
**4310**	373	A	79.5582	1
**4310**	381	A	52.2852	1
**4310**	382	A	76.9659	1
**4310**	386	A	71.7708	1
**4310**	305	B	51.7227	1
**4310**	319	B	77.7636	1
**4310**	320	B	75.3981	-1
**4310**	321	B	75.5763	0
**4310**	351	B	9.0911	1
**4310**	357	B	54.8176	1
**4310**	370	B	65.5682	1
**4310**	372	B	78.479	0
**4310**	373	B	38.0303	0
**4310**	381	B	70.0497	1
**4310**	382	B	60.5577	1
**4310**	386	B	79.561	1
**4310**	305	C	68.8377	1
**4310**	319	C	79.1126	1
**4310**	320	C	79.5102	-1
**4310**	321	C	73.3638	0
**4310**	351	C	12.5295	1
**4310**	357	C	79.5039	1
**4310**	370	C	79.638	1
**4310**	372	C	72.6416	0
**4310**	373	C	79.5773	1
**4310**	381	C	79.6283	1
**4310**	382	C	79.5484	1
**4310**	386	C	67.4271	1
**4310**	305	D	60.78	1
**4310**	319	D	51.3992	1

**4310**	320	D	75.8908	-1
**4310**	321	D	77.1377	0
**4310**	351	D	9.0053	1
**4310**	357	D	79.5333	1
**4310**	370	D	59.0543	1
**4310**	372	D	67.7345	0
**4310**	373	D	55.8326	1
**4310**	381	D	24.7526	0
**4310**	382	D	62.3428	1
**4310**	386	D	62.2138	1
**4311**	305	A	79.5099	1
**4311**	319	A	72.7298	1
**4311**	320	A	71.5941	-1
**4311**	321	A	48.9798	1
**4311**	351	A	6.1313	1
**4311**	357	A	73.7089	1
**4311**	370	A	79.581	1
**4311**	372	A	40.587	1
**4311**	373	A	79.5764	1
**4311**	381	A	78.9169	1
**4311**	382	A	79.1183	1
**4311**	386	A	79.5878	1
**4311**	305	B	47.3855	0
**4311**	319	B	79.3529	1
**4311**	320	B	79.6659	-1
**4311**	321	B	35.0305	1
**4311**	351	B	14.1054	1
**4311**	357	B	55.7706	1
**4311**	370	B	71.6371	1
**4311**	372	B	79.5397	0
**4311**	373	B	51.2671	1
**4311**	381	B	79.5756	1
**4311**	382	B	42.0288	0
**4311**	386	B	33.8989	0

**4311**	305	C	21.0016	0
**4311**	319	C	61.7191	1
**4311**	320	C	72.2479	-1
**4311**	321	C	79.5427	0
**4311**	351	C	4.0739	1
**4311**	357	C	68.8187	1
**4311**	370	C	75.6612	1
**4311**	372	C	75.1971	0
**4311**	373	C	79.5487	1
**4311**	381	C	59.9876	1
**4311**	382	C	58.8939	1
**4311**	386	C	20.0058	0
**4311**	305	D	59.3349	1
**4311**	319	D	44.5722	0
**4311**	320	D	73.5815	-1
**4311**	321	D	79.56	0
**4311**	351	D	12.594	1
**4311**	357	D	57.4819	1
**4311**	370	D	72.4117	1
**4311**	372	D	79.5476	0
**4311**	373	D	39.5864	0
**4311**	381	D	79.5657	1
**4311**	382	D	79.5578	1
**4311**	386	D	79.5567	1
**4312**	305	A	78.3941	1
**4312**	319	A	79.5582	1
**4312**	320	A	72.0783	-1
**4312**	321	A	72.4315	0
**4312**	351	A	7.491	1
**4312**	357	A	69.8368	1
**4312**	370	A	73.8207	1
**4312**	372	A	76.73	0
**4312**	373	A	79.5071	1
**4312**	381	A	71.8441	1

**4312**	382	A	57.9106	1
**4312**	386	A	79.495	1
**4312**	305	B	62.7854	1
**4312**	319	B	79.6289	1
**4312**	320	B	73.085	-1
**4312**	321	B	72.2916	0
**4312**	351	B	12.7098	1
**4312**	357	B	79.6875	1
**4312**	370	B	79.5753	1
**4312**	372	B	46.595	1
**4312**	373	B	54.2498	1
**4312**	381	B	40.3033	0
**4312**	382	B	50.6282	1
**4312**	386	B	51.1694	1
**4312**	305	C	79.5148	1
**4312**	319	C	77.1314	1
**4312**	320	C	55.1552	-1
**4312**	321	C	79.5201	0
**4312**	351	C	128	1
**4312**	357	C	79.5541	1
**4312**	370	C	46.2673	0
**4312**	372	C	65.4683	0
**4312**	373	C	79.5173	1
**4312**	381	C	52.1166	1
**4312**	382	C	79.5341	1
**4312**	386	C	38.9981	0
**4312**	305	D	64.3527	1
**4312**	319	D	62.7065	1
**4312**	320	D	40.3273	-1
**4312**	321	D	55.528	0
**4312**	351	D	16.7134	1
**4312**	357	D	79.423	1
**4312**	370	D	71.7402	1
**4312**	372	D	73.1412	0

**4312**	373	D	63.7825	1
**4312**	381	D	54.6581	1
**4312**	382	D	79.5521	1
**4312**	386	D	79.5814	1
**4313**	305	A	54.4991	1
**4313**	319	A	70.7074	1
**4313**	320	A	79.7174	-1
**4313**	321	A	71.3396	0
**4313**	351	A	12.5074	1
**4313**	357	A	79.5719	1
**4313**	370	A	37.7253	0
**4313**	372	A	79.4649	0
**4313**	373	A	71.1474	1
**4313**	381	A	51.0418	1
**4313**	382	A	59.118	1
**4313**	386	A	76.6993	1
**4313**	305	B	79.392	1
**4313**	319	B	78.881	1
**4313**	320	B	79.0206	-1
**4313**	321	B	79.5026	0
**4313**	351	B	10.8845	1
**4313**	357	B	59.6874	1
**4313**	370	B	79.7107	1
**4313**	372	B	61.2283	0
**4313**	373	B	58.2893	1
**4313**	381	B	79.5109	1
**4313**	382	B	79.5485	1
**4313**	386	B	64.7362	1
**4313**	305	C	56.9041	1
**4313**	319	C	78.0794	1
**4313**	320	C	28.8631	-1
**4313**	321	C	50.588	0
**4313**	351	C	19.2987	1
**4313**	357	C	79.524	1

**4313**	370	C	69.9101	1
**4313**	372	C	79.4948	0
**4313**	373	C	79.5496	1
**4313**	381	C	72.4918	1
**4313**	382	C	79.6547	1
**4313**	386	C	43.7125	0
**4313**	305	D	76.8151	1
**4313**	319	D	75.4593	1
**4313**	320	D	62.1485	-1
**4313**	321	D	72.2073	0
**4313**	351	D	9.1641	1
**4313**	357	D	28.4639	0
**4313**	370	D	79.09	1
**4313**	372	D	79.5444	0
**4313**	373	D	47.2923	0
**4313**	381	D	24.3564	0
**4313**	382	D	79.4967	1
**4313**	386	D	79.5469	1
**4314**	305	A	57.0493	1
**4314**	319	A	64.5927	1
**4314**	320	A	79.511	-1
**4314**	321	A	78.0501	0
**4314**	351	A	7.1267	1
**4314**	357	A	76.6862	1
**4314**	370	A	58.6772	1
**4314**	372	A	46.1217	1
**4314**	373	A	25.5964	0
**4314**	381	A	79.5251	1
**4314**	382	A	16.4277	-1
**4314**	386	A	28.0931	0
**4314**	305	B	79.4652	1
**4314**	319	B	72.2313	1
**4314**	320	B	79.5328	-1
**4314**	321	B	79.4831	0

**4314**	351	B	19.6725	1
**4314**	357	B	20.8935	0
**4314**	370	B	59.9925	1
**4314**	372	B	72.9502	0
**4314**	373	B	74.372	1
**4314**	381	B	79.6118	1
**4314**	382	B	24.77	0
**4314**	386	B	79.6289	1
**4314**	305	C	66.9973	1
**4314**	319	C	70.0874	1
**4314**	320	C	79.6371	-1
**4314**	321	C	64.5806	0
**4314**	351	C	6.9211	1
**4314**	357	C	79.5656	1
**4314**	370	C	79.4443	1
**4314**	372	C	73.2552	0
**4314**	373	C	32.7291	0
**4314**	381	C	79.501	1
**4314**	382	C	53.5811	1
**4314**	386	C	61.3525	1
**4314**	305	D	75.4328	1
**4314**	319	D	27.4263	0
**4314**	320	D	58.2304	-1
**4314**	321	D	77.379	0
**4314**	351	D	19.6329	1
**4314**	357	D	78.1789	1
**4314**	370	D	76.8449	1
**4314**	372	D	71.3227	0
**4314**	373	D	79.3518	1
**4314**	381	D	71.0699	1
**4314**	382	D	74.2715	1
**4314**	386	D	77.4297	1
**4315**	305	A	79.4067	1
**4315**	319	A	77.5499	1

**4315**	320	A	62.3147	-1
**4315**	321	A	10.3627	0
**4315**	351	A	10.558	1
**4315**	357	A	79.5633	1
**4315**	370	A	69.5276	1
**4315**	372	A	63.4713	0
**4315**	373	A	79.5597	1
**4315**	381	A	79.5434	1
**4315**	382	A	79.5807	1
**4315**	386	A	72.5725	1
**4315**	305	B	58.1473	1
**4315**	319	B	73.9855	1
**4315**	320	B	24.6204	-1
**4315**	321	B	68.0579	0
**4315**	351	B	14.0574	1
**4315**	357	B	56.7439	1
**4315**	370	B	79.5822	1
**4315**	372	B	79.5666	0
**4315**	373	B	46.6815	0
**4315**	381	B	57.9343	1
**4315**	382	B	70.8841	1
**4315**	386	B	69.3086	1
**4315**	305	C	44.8321	0
**4315**	319	C	60.1384	1
**4315**	320	C	40.5067	-1
**4315**	321	C	79.56	0
**4315**	351	C	4.0853	1
**4315**	357	C	69.8196	1
**4315**	370	C	36.0393	0
**4315**	372	C	79.6287	0
**4315**	373	C	79.5703	1
**4315**	381	C	79.5261	1
**4315**	382	C	50.9216	1
**4315**	386	C	34.4801	0

**4315**	305	D	75.4596	1
**4315**	319	D	67.9702	1
**4315**	320	D	79.2049	-1
**4315**	321	D	53.5249	0
**4315**	351	D	15.4509	1
**4315**	357	D	79.5436	1
**4315**	370	D	53.9218	1
**4315**	372	D	59.9605	0
**4315**	373	D	43.7958	0
**4315**	381	D	54.7327	1
**4315**	382	D	78.851	1
**4315**	386	D	78.8026	1
**4316**	305	A	71.0996	1
**4316**	319	A	79.4039	1
**4316**	320	A	79.5373	-1
**4316**	321	A	75.3816	0
**4316**	351	A	7.7158	1
**4316**	357	A	72.5283	1
**4316**	370	A	67.8719	1
**4316**	372	A	79.427	0
**4316**	373	A	75.3963	1
**4316**	381	A	67.1445	1
**4316**	382	A	52.6017	1
**4316**	386	A	50.2119	1
**4316**	305	B	75.5428	1
**4316**	319	B	66.3851	1
**4316**	320	B	60.6653	-1
**4316**	321	B	79.5677	0
**4316**	351	B	23.1027	-1
**4316**	357	B	61.2539	1
**4316**	370	B	79.5461	1
**4316**	372	B	74.1558	0
**4316**	373	B	79.616	1
**4316**	381	B	68.8928	1

**4316**	382	B	55.2219	1
**4316**	386	B	36.2902	0
**4316**	305	C	56.9326	1
**4316**	319	C	79.5212	1
**4316**	320	C	79.5347	-1
**4316**	321	C	79.6417	0
**4316**	351	C	3.1223	1
**4316**	357	C	59.2268	1
**4316**	370	C	71.9084	1
**4316**	372	C	67.4184	0
**4316**	373	C	60.6254	1
**4316**	381	C	79.5959	1
**4316**	382	C	64.668	1
**4316**	386	C	64.8593	1
**4316**	305	D	76.0709	1
**4316**	319	D	79.5899	1
**4316**	320	D	49.2516	-1
**4316**	321	D	73.8574	0
**4316**	351	D	11.083	1
**4316**	357	D	71.3604	1
**4316**	370	D	71.9046	1
**4316**	372	D	60.9214	0
**4316**	373	D	59.1953	1
**4316**	381	D	75.1817	1
**4316**	382	D	77.7248	1
**4316**	386	D	60.9277	1
**4317**	305	A	56.8408	1
**4317**	319	A	55.0454	1
**4317**	320	A	72.0692	-1
**4317**	321	A	74.5844	0
**4317**	351	A	11.3994	1
**4317**	357	A	79.5091	1
**4317**	370	A	79.6328	1
**4317**	372	A	42.337	1

**4317**	373	A	79.5547	1
**4317**	381	A	60.9628	1
**4317**	382	A	52.3821	1
**4317**	386	A	79.4815	1
**4317**	305	B	73.8483	1
**4317**	319	B	34.2735	0
**4317**	320	B	44.1874	-1
**4317**	321	B	76.3736	0
**4317**	351	B	9.4228	1
**4317**	357	B	32.6202	0
**4317**	370	B	73.4578	1
**4317**	372	B	79.6159	0
**4317**	373	B	56.9864	1
**4317**	381	B	63.3556	1
**4317**	382	B	79.5802	1
**4317**	386	B	72.7848	1
**4317**	305	C	60.6463	1
**4317**	319	C	77.0217	1
**4317**	320	C	79.6006	-1
**4317**	321	C	66.6453	0
**4317**	351	C	6.3842	1
**4317**	357	C	68.068	1
**4317**	370	C	79.5789	1
**4317**	372	C	68.5618	0
**4317**	373	C	79.5516	1
**4317**	381	C	56.2972	1
**4317**	382	C	57.7001	1
**4317**	386	C	65.5942	1
**4317**	305	D	79.5476	1
**4317**	319	D	70.2854	1
**4317**	320	D	79.5461	-1
**4317**	321	D	73.3562	0
**4317**	351	D	46.3373	-1
**4317**	357	D	71.571	1

**4317**	370	D	77.7128	1
**4317**	372	D	78.1251	0
**4317**	373	D	77.3812	1
**4317**	381	D	33.9391	0
**4317**	382	D	79.4795	1
**4317**	386	D	79.4492	1
**4318**	305	A	63.0938	1
**4318**	319	A	76.724	1
**4318**	320	A	75.0233	-1
**4318**	321	A	79.2116	0
**4318**	351	A	9.8694	1
**4318**	357	A	72.3254	1
**4318**	370	A	62.6287	1
**4318**	372	A	73.8682	0
**4318**	373	A	69.3112	1
**4318**	381	A	75.2937	1
**4318**	382	A	77.8223	1
**4318**	386	A	79.4728	1
**4318**	305	B	72.2466	1
**4318**	319	B	72.2556	1
**4318**	320	B	67.1729	-1
**4318**	321	B	59.6746	0
**4318**	351	B	3.5151	1
**4318**	357	B	79.7312	1
**4318**	370	B	79.575	1
**4318**	372	B	79.6796	0
**4318**	373	B	64.7634	1
**4318**	381	B	61.8568	1
**4318**	382	B	47.0403	0
**4318**	386	B	41.0989	0
**4318**	305	C	35.2958	0
**4318**	319	C	51.7586	1
**4318**	320	C	71.1045	-1
**4318**	321	C	63.3499	0

**4318**	351	C	3.5284	1
**4318**	357	C	48.6538	0
**4318**	370	C	41.7129	0
**4318**	372	C	70.1472	0
**4318**	373	C	75.0806	1
**4318**	381	C	75.0255	1
**4318**	382	C	69.2507	1
**4318**	386	C	29.7028	0
**4318**	305	D	79.6229	1
**4318**	319	D	69.8161	1
**4318**	320	D	56.6183	-1
**4318**	321	D	79.5178	0
**4318**	351	D	1.891	1
**4318**	357	D	66.0362	1
**4318**	370	D	78.1439	1
**4318**	372	D	79.6283	0
**4318**	373	D	73.6918	1
**4318**	381	D	79.5408	1
**4318**	382	D	60.0716	1
**4318**	386	D	79.6274	1
**4319**	305	A	36.0443	0
**4319**	319	A	73.2466	1
**4319**	320	A	79.5468	-1
**4319**	321	A	79.53	0
**4319**	351	A	11.0261	1
**4319**	357	A	79.4597	1
**4319**	370	A	55.1877	1
**4319**	372	A	56.0159	0
**4319**	373	A	79.5024	1
**4319**	381	A	79.4622	1
**4319**	382	A	68.8634	1
**4319**	386	A	79.6098	1
**4319**	305	B	66.9118	1
**4319**	319	B	72.4104	1

**4319**	320	B	79.389	-1
**4319**	321	B	79.5627	0
**4319**	351	B	19.9124	1
**4319**	357	B	79.6157	1
**4319**	370	B	39.7134	0
**4319**	372	B	79.703	0
**4319**	373	B	43.3412	0
**4319**	381	B	45.1633	0
**4319**	382	B	39.9745	0
**4319**	386	B	55.5597	1
**4319**	305	C	79.456	1
**4319**	319	C	51.1238	1
**4319**	320	C	67.4601	-1
**4319**	321	C	35.9906	1
**4319**	351	C	1.2174	1
**4319**	357	C	69.1477	1
**4319**	370	C	29.468	0
**4319**	372	C	73.3336	0
**4319**	373	C	70.0808	1
**4319**	381	C	32.3259	0
**4319**	382	C	79.5184	1
**4319**	386	C	46.7356	0
**4319**	305	D	77.9493	1
**4319**	319	D	79.5918	1
**4319**	320	D	79.5459	-1
**4319**	321	D	60.0452	0
**4319**	351	D	12.0796	1
**4319**	357	D	50.6342	1
**4319**	370	D	71.828	1
**4319**	372	D	66.4516	0
**4319**	373	D	74.5845	1
**4319**	381	D	79.5912	1
**4319**	382	D	79.4003	1
**4319**	386	D	79.6042	1

**4320**	305	A	62.9086	1
**4320**	319	A	68.6748	1
**4320**	320	A	79.4716	-1
**4320**	321	A	67.8259	0
**4320**	351	A	9.1384	1
**4320**	357	A	79.5163	1
**4320**	370	A	79.576	1
**4320**	372	A	79.5678	0
**4320**	373	A	72.0488	1
**4320**	381	A	79.538	1
**4320**	382	A	61.1223	1
**4320**	386	A	79.6136	1
**4320**	305	B	54.037	1
**4320**	319	B	79.5461	1
**4320**	320	B	61.7464	-1
**4320**	321	B	64.4397	0
**4320**	351	B	11.5149	1
**4320**	357	B	79.646	1
**4320**	370	B	73.8357	1
**4320**	372	B	68.6273	0
**4320**	373	B	66.6045	1
**4320**	381	B	79.4945	1
**4320**	382	B	62.522	1
**4320**	386	B	79.5571	1
**4320**	305	C	78.7714	1
**4320**	319	C	79.6178	1
**4320**	320	C	75.0629	-1
**4320**	321	C	52.9746	0
**4320**	351	C	7.1434	1
**4320**	357	C	77.5033	1
**4320**	370	C	28.2475	0
**4320**	372	C	79.5975	0
**4320**	373	C	79.4066	1
**4320**	381	C	78.4925	1

**4320**	382	C	79.5391	1
**4320**	386	C	18.9559	-1
**4320**	305	D	56.7516	1
**4320**	319	D	79.6944	1
**4320**	320	D	55.3237	-1
**4320**	321	D	64.087	0
**4320**	351	D	4.7678	1
**4320**	357	D	77.792	1
**4320**	370	D	79.4706	1
**4320**	372	D	78.9054	0
**4320**	373	D	63.2939	1
**4320**	381	D	60.4308	1
**4320**	382	D	72.8718	1
**4320**	386	D	79.4828	1
**4321**	305	A	73.5193	1
**4321**	319	A	66.9361	1
**4321**	320	A	71.2658	-1
**4321**	321	A	69.7565	0
**4321**	351	A	20.6839	-1
**4321**	357	A	70.357	1
**4321**	370	A	60.0063	1
**4321**	372	A	79.5492	0
**4321**	373	A	78.5888	1
**4321**	381	A	79.5976	1
**4321**	382	A	72.8552	1
**4321**	386	A	79.5015	1
**4321**	305	B	79.5715	1
**4321**	319	B	79.5266	1
**4321**	320	B	71.9323	-1
**4321**	321	B	79.5644	0
**4321**	351	B	13.0506	1
**4321**	357	B	79.5396	1
**4321**	370	B	79.5636	1
**4321**	372	B	42.8227	1

**4321**	373	B	71.0107	1
**4321**	381	B	17.2981	-1
**4321**	382	B	73.7077	1
**4321**	386	B	37.6536	0
**4321**	305	C	43.1922	0
**4321**	319	C	79.4932	1
**4321**	320	C	63.9424	-1
**4321**	321	C	27.3931	1
**4321**	351	C	3.6349	1
**4321**	357	C	73.9181	1
**4321**	370	C	79.5789	1
**4321**	372	C	79.0271	0
**4321**	373	C	62.8418	1
**4321**	381	C	79.5725	1
**4321**	382	C	37.9154	0
**4321**	386	C	26.1956	0
**4321**	305	D	21.1335	0
**4321**	319	D	64.3654	1
**4321**	320	D	79.5505	-1
**4321**	321	D	60.5749	0
**4321**	351	D	17.8154	1
**4321**	357	D	79.577	1
**4321**	370	D	61.1269	1
**4321**	372	D	30.7747	1
**4321**	373	D	52.7406	1
**4321**	381	D	63.2795	1
**4321**	382	D	68.0306	1
**4321**	386	D	71.952	1
**4322**	305	A	29.9051	0
**4322**	319	A	25.6646	0
**4322**	320	A	79.5509	-1
**4322**	321	A	70.9455	0
**4322**	351	A	9.9036	1
**4322**	357	A	63.3374	1

**4322**	370	A	72.8965	1
**4322**	372	A	47.7862	1
**4322**	373	A	38.937	0
**4322**	381	A	65.8533	1
**4322**	382	A	74.953	1
**4322**	386	A	64.297	1
**4322**	305	B	63.1607	1
**4322**	319	B	52.48	1
**4322**	320	B	79.3197	-1
**4322**	321	B	79.535	0
**4322**	351	B	9.9817	1
**4322**	357	B	72.4763	1
**4322**	370	B	72.8822	1
**4322**	372	B	73.5617	0
**4322**	373	B	79.5177	1
**4322**	381	B	72.3473	1
**4322**	382	B	78.0667	1
**4322**	386	B	75.4311	1
**4322**	305	C	79.534	1
**4322**	319	C	73.9537	1
**4322**	320	C	73.3687	-1
**4322**	321	C	63.0001	0
**4322**	351	C	7.4854	1
**4322**	357	C	32.8966	0
**4322**	370	C	75.9355	1
**4322**	372	C	59.2976	0
**4322**	373	C	32.1264	0
**4322**	381	C	78.1518	1
**4322**	382	C	38.9616	0
**4322**	386	C	48.9366	0
**4322**	305	D	79.553	1
**4322**	319	D	79.384	1
**4322**	320	D	79.5521	-1
**4322**	321	D	72.3092	0

**4322**	351	D	12.9621	1
**4322**	357	D	70.5653	1
**4322**	370	D	37.1192	0
**4322**	372	D	79.5372	0
**4322**	373	D	61.7018	1
**4322**	381	D	36.3305	0
**4322**	382	D	63.7599	1
**4322**	386	D	73.3652	1
**4323**	305	A	67.9601	1
**4323**	319	A	76.6155	1
**4323**	320	A	59.4595	-1
**4323**	321	A	62.5823	0
**4323**	351	A	6.5171	1
**4323**	357	A	79.5705	1
**4323**	370	A	52.4164	1
**4323**	372	A	69.2227	0
**4323**	373	A	47.271	0
**4323**	381	A	72.1699	1
**4323**	382	A	70.115	1
**4323**	386	A	67.8609	1
**4323**	305	B	38.0668	0
**4323**	319	B	61.5449	1
**4323**	320	B	78.3674	-1
**4323**	321	B	75.3165	0
**4323**	351	B	10.8406	1
**4323**	357	B	79.4492	1
**4323**	370	B	44.1443	0
**4323**	372	B	47.9095	1
**4323**	373	B	60.7154	1
**4323**	381	B	77.386	1
**4323**	382	B	79.5698	1
**4323**	386	B	79.5004	1
**4323**	305	C	77.6534	1
**4323**	319	C	72.4596	1

**4323**	320	C	77.679	-1
**4323**	321	C	79.5433	0
**4323**	351	C	5.3466	1
**4323**	357	C	31.3461	0
**4323**	370	C	79.5294	1
**4323**	372	C	79.5385	0
**4323**	373	C	79.5512	1
**4323**	381	C	70.5873	1
**4323**	382	C	28.2505	0
**4323**	386	C	48.5083	0
**4323**	305	D	61.4086	1
**4323**	319	D	78.8817	1
**4323**	320	D	72.1437	-1
**4323**	321	D	65.6482	0
**4323**	351	D	10.2198	1
**4323**	357	D	69.633	1
**4323**	370	D	42.2886	0
**4323**	372	D	73.6963	0
**4323**	373	D	79.6352	1
**4323**	381	D	77.5878	1
**4323**	382	D	37.2108	0
**4323**	386	D	76.7541	1
**4324**	305	A	26.3391	0
**4324**	319	A	60.625	1
**4324**	320	A	79.5593	-1
**4324**	321	A	55.8256	0
**4324**	351	A	8.2844	1
**4324**	357	A	79.5798	1
**4324**	370	A	31.1093	0
**4324**	372	A	79.5748	0
**4324**	373	A	63.1247	1
**4324**	381	A	73.3996	1
**4324**	382	A	74.5624	1
**4324**	386	A	76.0164	1

**4324**	305	B	62.717	1
**4324**	319	B	72.6507	1
**4324**	320	B	69.7115	-1
**4324**	321	B	79.553	0
**4324**	351	B	7.3537	1
**4324**	357	B	76.1452	1
**4324**	370	B	73.9667	1
**4324**	372	B	64.3375	0
**4324**	373	B	68.9355	1
**4324**	381	B	69.2415	1
**4324**	382	B	79.6027	1
**4324**	386	B	79.5945	1
**4324**	305	C	79.7959	1
**4324**	319	C	55.6983	1
**4324**	320	C	71.6352	-1
**4324**	321	C	36.9123	1
**4324**	351	C	6.6978	1
**4324**	357	C	75.4991	1
**4324**	370	C	70.0818	1
**4324**	372	C	46.7979	1
**4324**	373	C	78.9155	1
**4324**	381	C	73.5211	1
**4324**	382	C	57.6495	1
**4324**	386	C	24.9368	0
**4324**	305	D	38.854	0
**4324**	319	D	79.5363	1
**4324**	320	D	79.5359	-1
**4324**	321	D	79.5742	0
**4324**	351	D	13.538	1
**4324**	357	D	79.57	1
**4324**	370	D	71.3018	1
**4324**	372	D	65.8507	0
**4324**	373	D	76.6047	1
**4324**	381	D	68.3053	1

**4324**	382	D	79.5835	1
**4324**	386	D	79.57	1
**4325**	305	A	69.9198	1
**4325**	319	A	41.9882	0
**4325**	320	A	63.8451	-1
**4325**	321	A	76.9368	0
**4325**	351	A	0.0	1
**4325**	357	A	70.9748	1
**4325**	370	A	79.6093	1
**4325**	372	A	46.521	1
**4325**	373	A	72.3067	1
**4325**	381	A	46.8284	0
**4325**	382	A	51.8744	1
**4325**	386	A	70.9717	1
**4325**	305	B	30.9981	0
**4325**	319	B	73.9226	1
**4325**	320	B	79.5736	-1
**4325**	321	B	73.2767	0
**4325**	351	B	10.5082	1
**4325**	357	B	79.5481	1
**4325**	370	B	79.5652	1
**4325**	372	B	69.4797	0
**4325**	373	B	64.8675	1
**4325**	381	B	49.2466	0
**4325**	382	B	64.7288	1
**4325**	386	B	79.5732	1
**4325**	305	C	27.0626	0
**4325**	319	C	79.5534	1
**4325**	320	C	62.4467	-1
**4325**	321	C	56.1123	0
**4325**	351	C	18.0419	1
**4325**	357	C	46.9022	0
**4325**	370	C	74.8625	1
**4325**	372	C	73.3856	0

**4325**	373	C	65.8888	1
**4325**	381	C	42.8982	0
**4325**	382	C	78.8114	1
**4325**	386	C	61.5129	1
**4325**	305	D	73.5445	1
**4325**	319	D	79.0569	1
**4325**	320	D	79.4951	-1
**4325**	321	D	59.8829	0
**4325**	351	D	12.6895	1
**4325**	357	D	75.0327	1
**4325**	370	D	78.9004	1
**4325**	372	D	73.409	0
**4325**	373	D	56.1045	1
**4325**	381	D	61.674	1
**4325**	382	D	79.5222	1
**4325**	386	D	63.6056	1
**4326**	305	A	65.4006	1
**4326**	319	A	79.5369	1
**4326**	320	A	53.5836	-1
**4326**	321	A	79.4812	0
**4326**	351	A	36.8213	-1
**4326**	357	A	69.4678	1
**4326**	370	A	63.0886	1
**4326**	372	A	70.3671	0
**4326**	373	A	79.6416	1
**4326**	381	A	74.4271	1
**4326**	382	A	78.8408	1
**4326**	386	A	79.3925	1
**4326**	305	B	52.1225	1
**4326**	319	B	74.5727	1
**4326**	320	B	47.3158	-1
**4326**	321	B	60.938	0
**4326**	351	B	16.102	1
**4326**	357	B	79.5366	1

**4326**	370	B	52.0002	1
**4326**	372	B	79.499	0
**4326**	373	B	47.9681	0
**4326**	381	B	74.8527	1
**4326**	382	B	57.5449	1
**4326**	386	B	64.1952	1
**4326**	305	C	64.2009	1
**4326**	319	C	60.4108	1
**4326**	320	C	72.6792	-1
**4326**	321	C	54.1165	0
**4326**	351	C	11.8322	1
**4326**	357	C	42.8913	0
**4326**	370	C	66.5566	1
**4326**	372	C	66.1741	0
**4326**	373	C	77.1733	1
**4326**	381	C	28.3894	0
**4326**	382	C	34.8447	0
**4326**	386	C	54.5327	1
**4326**	305	D	32.5533	0
**4326**	319	D	76.5627	1
**4326**	320	D	52.1057	-1
**4326**	321	D	79.6196	0
**4326**	351	D	20.2397	-1
**4326**	357	D	66.2432	1
**4326**	370	D	53.1306	1
**4326**	372	D	59.3696	0
**4326**	373	D	45.4529	0
**4326**	381	D	34.5901	0
**4326**	382	D	79.538	1
**4326**	386	D	34.7871	0
**4327**	305	A	79.4943	1
**4327**	319	A	44.4378	0
**4327**	320	A	79.5169	-1
**4327**	321	A	74.9204	0

**4327**	351	A	16.0498	1
**4327**	357	A	74.7359	1
**4327**	370	A	74.1087	1
**4327**	372	A	79.5951	0
**4327**	373	A	70.0906	1
**4327**	381	A	32.7433	0
**4327**	382	A	75.497	1
**4327**	386	A	79.6123	1
**4327**	305	B	49.8674	0
**4327**	319	B	71.0364	1
**4327**	320	B	41.6011	-1
**4327**	321	B	69.584	0
**4327**	351	B	9.3331	1
**4327**	357	B	78.6476	1
**4327**	370	B	79.4869	1
**4327**	372	B	79.5884	0
**4327**	373	B	65.5989	1
**4327**	381	B	37.3983	0
**4327**	382	B	72.8073	1
**4327**	386	B	47.6682	0
**4327**	305	C	53.0333	1
**4327**	319	C	72.8741	1
**4327**	320	C	67.8617	-1
**4327**	321	C	72.6677	0
**4327**	351	C	0.5241	1
**4327**	357	C	69.2416	1
**4327**	370	C	72.4346	1
**4327**	372	C	70.7967	0
**4327**	373	C	68.5087	1
**4327**	381	C	63.788	1
**4327**	382	C	19.3926	-1
**4327**	386	C	45.2939	0
**4327**	305	D	36.3104	0
**4327**	319	D	79.6838	1

**4327**	320	D	63.1209	-1
**4327**	321	D	74.6094	0
**4327**	351	D	27.2354	-1
**4327**	357	D	57.0921	1
**4327**	370	D	46.2934	0
**4327**	372	D	72.1826	0
**4327**	373	D	52.4565	1
**4327**	381	D	66.1648	1
**4327**	382	D	34.7775	0
**4327**	386	D	67.5616	1
**4328**	305	A	79.1108	1
**4328**	319	A	61.5557	1
**4328**	320	A	79.5831	-1
**4328**	321	A	73.2571	0
**4328**	351	A	2.6148	1
**4328**	357	A	79.3354	1
**4328**	370	A	79.2891	1
**4328**	372	A	73.5933	0
**4328**	373	A	47.6621	0
**4328**	381	A	79.5677	1
**4328**	382	A	55.2806	1
**4328**	386	A	68.5493	1
**4328**	305	B	74.655	1
**4328**	319	B	37.5026	0
**4328**	320	B	56.0993	-1
**4328**	321	B	77.5641	0
**4328**	351	B	15.8763	1
**4328**	357	B	65.5137	1
**4328**	370	B	75.606	1
**4328**	372	B	50.2865	0
**4328**	373	B	72.9366	1
**4328**	381	B	79.5254	1
**4328**	382	B	53.1494	1
**4328**	386	B	71.4587	1

**4328**	305	C	78.4403	1
**4328**	319	C	74.5416	1
**4328**	320	C	62.8772	-1
**4328**	321	C	60.7995	0
**4328**	351	C	4.0181	1
**4328**	357	C	79.5925	1
**4328**	370	C	39.2445	0
**4328**	372	C	79.5288	0
**4328**	373	C	79.1356	1
**4328**	381	C	72.5112	1
**4328**	382	C	52.0949	1
**4328**	386	C	49.438	0
**4328**	305	D	65.0405	1
**4328**	319	D	30.6223	0
**4328**	320	D	79.5529	-1
**4328**	321	D	77.9931	0
**4328**	351	D	13.1157	1
**4328**	357	D	79.5694	1
**4328**	370	D	46.769	0
**4328**	372	D	79.5669	0
**4328**	373	D	57.156	1
**4328**	381	D	72.943	1
**4328**	382	D	68.721	1
**4328**	386	D	79.4209	1
**4329**	305	A	76.8602	1
**4329**	319	A	65.3439	1
**4329**	320	A	79.5113	-1
**4329**	321	A	75.9029	0
**4329**	351	A	10.5046	1
**4329**	357	A	63.5576	1
**4329**	370	A	66.5399	1
**4329**	372	A	79.4871	0
**4329**	373	A	73.39	1
**4329**	381	A	52.9954	1

**4329**	382	A	65.8312	1
**4329**	386	A	69.1089	1
**4329**	305	B	38.1306	0
**4329**	319	B	79.4822	1
**4329**	320	B	77.129	-1
**4329**	321	B	48.348	1
**4329**	351	B	14.025	1
**4329**	357	B	51.7629	1
**4329**	370	B	79.5226	1
**4329**	372	B	79.7608	0
**4329**	373	B	76.5758	1
**4329**	381	B	79.5535	1
**4329**	382	B	30.4349	0
**4329**	386	B	79.751	1
**4329**	305	C	63.1584	1
**4329**	319	C	79.6346	1
**4329**	320	C	61.8863	-1
**4329**	321	C	79.5041	0
**4329**	351	C	6.7014	1
**4329**	357	C	77.2494	1
**4329**	370	C	43.0199	0
**4329**	372	C	79.6331	0
**4329**	373	C	79.6679	1
**4329**	381	C	75.2572	1
**4329**	382	C	79.6009	1
**4329**	386	C	42.039	0
**4329**	305	D	74.4598	1
**4329**	319	D	61.1926	1
**4329**	320	D	79.5612	-1
**4329**	321	D	48.3167	1
**4329**	351	D	4.4363	1
**4329**	357	D	55.5955	1
**4329**	370	D	72.6294	1
**4329**	372	D	79.5404	0

**4329**	373	D	79.6441	1
**4329**	381	D	34.0055	0
**4329**	382	D	74.2726	1
**4329**	386	D	57.8503	1
**4330**	305	A	79.5688	1
**4330**	319	A	71.5094	1
**4330**	320	A	45.6725	-1
**4330**	321	A	74.1283	0
**4330**	351	A	10.4807	1
**4330**	357	A	59.9943	1
**4330**	370	A	79.5536	1
**4330**	372	A	35.6535	1
**4330**	373	A	73.1977	1
**4330**	381	A	73.5451	1
**4330**	382	A	79.55	1
**4330**	386	A	50.2982	1
**4330**	305	B	72.9502	1
**4330**	319	B	79.5758	1
**4330**	320	B	70.9317	-1
**4330**	321	B	79.5621	0
**4330**	351	B	14.8724	1
**4330**	357	B	79.6058	1
**4330**	370	B	72.474	1
**4330**	372	B	79.5716	0
**4330**	373	B	75.5516	1
**4330**	381	B	79.5669	1
**4330**	382	B	23.3427	0
**4330**	386	B	79.5875	1
**4330**	305	C	55.2476	1
**4330**	319	C	57.0498	1
**4330**	320	C	72.5701	-1
**4330**	321	C	38.7056	1
**4330**	351	C	5.2643	1
**4330**	357	C	79.5635	1

**4330**	370	C	69.6585	1
**4330**	372	C	58.2182	0
**4330**	373	C	79.5855	1
**4330**	381	C	79.5837	1
**4330**	382	C	65.3638	1
**4330**	386	C	13.88	-1
**4330**	305	D	59.396	1
**4330**	319	D	79.5521	1
**4330**	320	D	79.5568	-1
**4330**	321	D	59.2592	0
**4330**	351	D	15.4029	1
**4330**	357	D	29.8281	0
**4330**	370	D	79.0592	1
**4330**	372	D	52.1379	0
**4330**	373	D	28.2272	0
**4330**	381	D	68.6645	1
**4330**	382	D	79.1997	1
**4330**	386	D	36.5897	0
**4331**	305	A	49.7374	0
**4331**	319	A	65.6631	1
**4331**	320	A	22.6429	-1
**4331**	321	A	58.7036	0
**4331**	351	A	4.1968	1
**4331**	357	A	79.5256	1
**4331**	370	A	79.6738	1
**4331**	372	A	79.6054	0
**4331**	373	A	73.1887	1
**4331**	381	A	68.5503	1
**4331**	382	A	63.9407	1
**4331**	386	A	41.5206	0
**4331**	305	B	75.3799	1
**4331**	319	B	44.619	0
**4331**	320	B	38.5379	-1
**4331**	321	B	79.4299	0

**4331**	351	B	10.3126	1
**4331**	357	B	79.5628	1
**4331**	370	B	43.493	0
**4331**	372	B	41.3866	1
**4331**	373	B	75.2196	1
**4331**	381	B	33.1304	0
**4331**	382	B	36.305	0
**4331**	386	B	56.9556	1
**4331**	305	C	79.6464	1
**4331**	319	C	78.9616	1
**4331**	320	C	79.5008	-1
**4331**	321	C	35.1524	1
**4331**	351	C	13.425	1
**4331**	357	C	74.2556	1
**4331**	370	C	69.0724	1
**4331**	372	C	79.5126	0
**4331**	373	C	69.3885	1
**4331**	381	C	52.7215	1
**4331**	382	C	68.4012	1
**4331**	386	C	57.9822	1
**4331**	305	D	59.4033	1
**4331**	319	D	79.6273	1
**4331**	320	D	14.5462	1
**4331**	321	D	39.9961	1
**4331**	351	D	15.7403	1
**4331**	357	D	69.6058	1
**4331**	370	D	79.6547	1
**4331**	372	D	79.6381	0
**4331**	373	D	61.3326	1
**4331**	381	D	73.0621	1
**4331**	382	D	64.738	1
**4331**	386	D	46.9511	0
**4332**	305	A	55.5296	1
**4332**	319	A	66.7925	1

**4332**	320	A	22.7836	-1
**4332**	321	A	77.9813	0
**4332**	351	A	5.9139	1
**4332**	357	A	79.3965	1
**4332**	370	A	79.6442	1
**4332**	372	A	77.3661	0
**4332**	373	A	40.7809	0
**4332**	381	A	79.3638	1
**4332**	382	A	66.2417	1
**4332**	386	A	79.7165	1
**4332**	305	B	50.7425	1
**4332**	319	B	78.8602	1
**4332**	320	B	74.8368	-1
**4332**	321	B	55.9245	0
**4332**	351	B	6.9271	1
**4332**	357	B	73.7716	1
**4332**	370	B	74.5071	1
**4332**	372	B	79.3942	0
**4332**	373	B	72.8286	1
**4332**	381	B	65.7271	1
**4332**	382	B	70.7209	1
**4332**	386	B	77.0954	1
**4332**	305	C	75.6747	1
**4332**	319	C	79.4463	1
**4332**	320	C	79.7151	-1
**4332**	321	C	72.9014	0
**4332**	351	C	4.5093	1
**4332**	357	C	52.6934	1
**4332**	370	C	74.992	1
**4332**	372	C	67.169	0
**4332**	373	C	74.577	1
**4332**	381	C	79.642	1
**4332**	382	C	79.5109	1
**4332**	386	C	48.5946	0

**4332**	305	D	79.5452	1
**4332**	319	D	67.7415	1
**4332**	320	D	79.2825	-1
**4332**	321	D	77.854	0
**4332**	351	D	16.9817	1
**4332**	357	D	79.5397	1
**4332**	370	D	79.5774	1
**4332**	372	D	79.4446	0
**4332**	373	D	79.5358	1
**4332**	381	D	73.7301	1
**4332**	382	D	72.7617	1
**4332**	386	D	56.2075	1
**4333**	305	A	79.5941	1
**4333**	319	A	73.3022	1
**4333**	320	A	79.5628	-1
**4333**	321	A	76.1913	0
**4333**	351	A	2.7862	1
**4333**	357	A	79.5932	1
**4333**	370	A	79.0898	1
**4333**	372	A	32.3754	1
**4333**	373	A	73.483	1
**4333**	381	A	74.804	1
**4333**	382	A	33.4414	0
**4333**	386	A	79.6005	1
**4333**	305	B	79.5836	1
**4333**	319	B	75.8778	1
**4333**	320	B	79.0194	-1
**4333**	321	B	79.5465	0
**4333**	351	B	21.2138	-1
**4333**	357	B	42.4277	0
**4333**	370	B	42.2684	0
**4333**	372	B	78.3475	0
**4333**	373	B	79.559	1
**4333**	381	B	79.6314	1

**4333**	382	B	47.0812	0
**4333**	386	B	29.6649	0
**4333**	305	C	79.428	1
**4333**	319	C	79.6402	1
**4333**	320	C	66.8832	-1
**4333**	321	C	44.9771	1
**4333**	351	C	5.0946	1
**4333**	357	C	72.4218	1
**4333**	370	C	65.5227	1
**4333**	372	C	72.0423	0
**4333**	373	C	47.7601	0
**4333**	381	C	72.7626	1
**4333**	382	C	59.9302	1
**4333**	386	C	64.6339	1
**4333**	305	D	79.5322	1
**4333**	319	D	60.5802	1
**4333**	320	D	35.9674	-1
**4333**	321	D	30.7051	1
**4333**	351	D	6.5115	1
**4333**	357	D	66.83	1
**4333**	370	D	79.5411	1
**4333**	372	D	76.9254	0
**4333**	373	D	79.53	1
**4333**	381	D	74.2505	1
**4333**	382	D	66.2188	1
**4333**	386	D	55.1881	1
**4334**	305	A	39.9307	0
**4334**	319	A	77.8616	1
**4334**	320	A	79.5439	-1
**4334**	321	A	46.0557	1
**4334**	351	A	12.114	1
**4334**	357	A	17.5744	-1
**4334**	370	A	38.8848	0
**4334**	372	A	79.5168	0

**4334**	373	A	57.1452	1
**4334**	381	A	24.4954	0
**4334**	382	A	78.313	1
**4334**	386	A	52.1037	1
**4334**	305	B	79.4805	1
**4334**	319	B	42.8326	0
**4334**	320	B	26.7063	-1
**4334**	321	B	61.9803	0
**4334**	351	B	5.9132	1
**4334**	357	B	60.5354	1
**4334**	370	B	71.1629	1
**4334**	372	B	49.5259	1
**4334**	373	B	67.4489	1
**4334**	381	B	34.4933	0
**4334**	382	B	27.6477	0
**4334**	386	B	79.5225	1
**4334**	305	C	43.3501	0
**4334**	319	C	79.492	1
**4334**	320	C	53.6093	-1
**4334**	321	C	79.637	0
**4334**	351	C	30.1839	-1
**4334**	357	C	62.1275	1
**4334**	370	C	76.561	1
**4334**	372	C	69.4598	0
**4334**	373	C	71.4089	1
**4334**	381	C	76.7272	1
**4334**	382	C	48.1198	0
**4334**	386	C	48.0304	0
**4334**	305	D	64.1274	1
**4334**	319	D	79.5212	1
**4334**	320	D	60.9896	-1
**4334**	321	D	79.7594	0
**4334**	351	D	12.7765	1
**4334**	357	D	79.562	1

**4334**	370	D	52.4591	1
**4334**	372	D	79.529	0
**4334**	373	D	72.36	1
**4334**	381	D	59.9059	1
**4334**	382	D	66.1923	1
**4334**	386	D	75.3724	1
**4335**	305	A	79.6333	1
**4335**	319	A	77.5551	1
**4335**	320	A	79.4669	-1
**4335**	321	A	51.9972	0
**4335**	351	A	0.0408	1
**4335**	357	A	79.6337	1
**4335**	370	A	79.5931	1
**4335**	372	A	50.9356	0
**4335**	373	A	79.5802	1
**4335**	381	A	79.5835	1
**4335**	382	A	60.6961	1
**4335**	386	A	78.7094	1
**4335**	305	B	79.5718	1
**4335**	319	B	75.1063	1
**4335**	320	B	75.0185	-1
**4335**	321	B	79.5402	0
**4335**	351	B	12.2018	1
**4335**	357	B	79.486	1
**4335**	370	B	70.9325	1
**4335**	372	B	54.9074	0
**4335**	373	B	62.7094	1
**4335**	381	B	73.9053	1
**4335**	382	B	8.8889	-1
**4335**	386	B	66.3615	1
**4335**	305	C	79.5798	1
**4335**	319	C	70.0784	1
**4335**	320	C	61.5757	-1
**4335**	321	C	68.5619	0

**4335**	351	C	24.0297	-1
**4335**	357	C	55.6876	1
**4335**	370	C	58.605	1
**4335**	372	C	58.9231	0
**4335**	373	C	71.7796	1
**4335**	381	C	70.6175	1
**4335**	382	C	36.4561	0
**4335**	386	C	59.8942	1
**4335**	305	D	79.5587	1
**4335**	319	D	44.1654	0
**4335**	320	D	68.6493	-1
**4335**	321	D	54.4995	0
**4335**	351	D	7.6362	1
**4335**	357	D	28.3168	0
**4335**	370	D	74.7685	1
**4335**	372	D	79.6351	0
**4335**	373	D	45.3813	0
**4335**	381	D	61.2095	1
**4335**	382	D	26.3127	0
**4335**	386	D	59.5349	1
**4336**	305	A	52.6832	1
**4336**	319	A	61.0534	1
**4336**	320	A	72.8817	-1
**4336**	321	A	71.7513	0
**4336**	351	A	2.3001	1
**4336**	357	A	64.2358	1
**4336**	370	A	79.5196	1
**4336**	372	A	61.1519	0
**4336**	373	A	79.5057	1
**4336**	381	A	52.2242	1
**4336**	382	A	63.4109	1
**4336**	386	A	76.486	1
**4336**	305	B	58.5309	1
**4336**	319	B	43.2077	0

**4336**	320	B	69.9968	-1
**4336**	321	B	37.6342	1
**4336**	351	B	0.2237	1
**4336**	357	B	73.0562	1
**4336**	370	B	59.4799	1
**4336**	372	B	79.5821	0
**4336**	373	B	78.1181	1
**4336**	381	B	39.5674	0
**4336**	382	B	79.1759	1
**4336**	386	B	56.2985	1
**4336**	305	C	72.0723	1
**4336**	319	C	55.0856	1
**4336**	320	C	78.1929	-1
**4336**	321	C	35.1753	1
**4336**	351	C	7.4781	1
**4336**	357	C	61.4428	1
**4336**	370	C	74.1268	1
**4336**	372	C	79.5184	0
**4336**	373	C	61.2108	1
**4336**	381	C	79.6926	1
**4336**	382	C	50.5573	1
**4336**	386	C	36.7581	0
**4336**	305	D	71.8819	1
**4336**	319	D	68.4637	1
**4336**	320	D	79.5772	-1
**4336**	321	D	79.5907	0
**4336**	351	D	38.2845	-1
**4336**	357	D	74.9356	1
**4336**	370	D	79.5557	1
**4336**	372	D	50.1839	0
**4336**	373	D	79.6386	1
**4336**	381	D	75.1677	1
**4336**	382	D	79.5378	1
**4336**	386	D	44.7746	0

**4337**	305	A	55.4589	1
**4337**	319	A	73.0848	1
**4337**	320	A	78.1569	-1
**4337**	321	A	33.5428	1
**4337**	351	A	2.1437	1
**4337**	357	A	51.8525	1
**4337**	370	A	79.5817	1
**4337**	372	A	69.0507	0
**4337**	373	A	79.5995	1
**4337**	381	A	59.1069	1
**4337**	382	A	79.4095	1
**4337**	386	A	61.5012	1
**4337**	305	B	79.5016	1
**4337**	319	B	79.6378	1
**4337**	320	B	73.6308	-1
**4337**	321	B	65.3517	0
**4337**	351	B	16.6188	1
**4337**	357	B	79.462	1
**4337**	370	B	73.9721	1
**4337**	372	B	76.155	0
**4337**	373	B	57.42	1
**4337**	381	B	79.6165	1
**4337**	382	B	37.0048	0
**4337**	386	B	33.8534	0
**4337**	305	C	73.1365	1
**4337**	319	C	73.6283	1
**4337**	320	C	79.5895	-1
**4337**	321	C	50.8881	0
**4337**	351	C	14.3264	1
**4337**	357	C	73.7531	1
**4337**	370	C	76.4669	1
**4337**	372	C	56.7127	0
**4337**	373	C	32.3508	0
**4337**	381	C	79.6659	1

**4337**	382	C	13.2219	-1
**4337**	386	C	54.9012	1
**4337**	305	D	48.097	0
**4337**	319	D	79.5587	1
**4337**	320	D	52.9891	-1
**4337**	321	D	72.5285	0
**4337**	351	D	18.0429	1
**4337**	357	D	79.4998	1
**4337**	370	D	68.5916	1
**4337**	372	D	78.2993	0
**4337**	373	D	79.7184	1
**4337**	381	D	29.7695	0
**4337**	382	D	79.4571	1
**4337**	386	D	70.8682	1
**4338**	305	A	79.3437	1
**4338**	319	A	71.0692	1
**4338**	320	A	48.9142	-1
**4338**	321	A	77.2716	0
**4338**	351	A	2.5147	1
**4338**	357	A	72.8834	1
**4338**	370	A	79.5227	1
**4338**	372	A	74.3714	0
**4338**	373	A	65.5621	1
**4338**	381	A	79.6248	1
**4338**	382	A	70.8735	1
**4338**	386	A	79.6515	1
**4338**	305	B	79.5058	1
**4338**	319	B	67.6045	1
**4338**	320	B	65.008	-1
**4338**	321	B	66.2636	0
**4338**	351	B	8.3629	1
**4338**	357	B	68.3735	1
**4338**	370	B	79.4697	1
**4338**	372	B	72.8505	0

**4338**	373	B	54.7305	1
**4338**	381	B	76.3063	1
**4338**	382	B	74.5362	1
**4338**	386	B	68.4464	1
**4338**	305	C	79.5015	1
**4338**	319	C	79.4667	1
**4338**	320	C	46.8271	-1
**4338**	321	C	74.946	0
**4338**	351	C	8.0014	1
**4338**	357	C	76.0615	1
**4338**	370	C	56.0722	1
**4338**	372	C	72.0391	0
**4338**	373	C	79.5275	1
**4338**	381	C	79.6513	1
**4338**	382	C	75.5857	1
**4338**	386	C	42.355	0
**4338**	305	D	55.5065	1
**4338**	319	D	73.6667	1
**4338**	320	D	79.4609	-1
**4338**	321	D	48.789	1
**4338**	351	D	10.8916	1
**4338**	357	D	56.1443	1
**4338**	370	D	79.6785	1
**4338**	372	D	54.6253	0
**4338**	373	D	62.9776	1
**4338**	381	D	67.4752	1
**4338**	382	D	69.7387	1
**4338**	386	D	48.5115	0
**4339**	305	A	46.3684	0
**4339**	319	A	62.675	1
**4339**	320	A	61.4474	-1
**4339**	321	A	79.5745	0
**4339**	351	A	9.0374	1
**4339**	357	A	79.5067	1

**4339**	370	A	79.5749	1
**4339**	372	A	66.1523	0
**4339**	373	A	73.0307	1
**4339**	381	A	24.3899	0
**4339**	382	A	47.5535	0
**4339**	386	A	54.1122	1
**4339**	305	B	57.3633	1
**4339**	319	B	44.0103	0
**4339**	320	B	34.4808	-1
**4339**	321	B	27.9747	1
**4339**	351	B	12.4065	1
**4339**	357	B	79.5786	1
**4339**	370	B	78.8822	1
**4339**	372	B	79.5603	0
**4339**	373	B	55.0605	1
**4339**	381	B	58.349	1
**4339**	382	B	79.6162	1
**4339**	386	B	55.4568	1
**4339**	305	C	43.2828	0
**4339**	319	C	79.607	1
**4339**	320	C	79.5599	-1
**4339**	321	C	79.5272	0
**4339**	351	C	1.309	1
**4339**	357	C	79.6113	1
**4339**	370	C	55.8847	1
**4339**	372	C	78.6076	0
**4339**	373	C	74.1811	1
**4339**	381	C	54.9775	1
**4339**	382	C	79.5891	1
**4339**	386	C	25.2112	0
**4339**	305	D	75.3869	1
**4339**	319	D	79.5642	1
**4339**	320	D	79.5461	-1
**4339**	321	D	71.5214	0

**4339**	351	D	8.2672	1
**4339**	357	D	31.0535	0
**4339**	370	D	60.0332	1
**4339**	372	D	79.5871	0
**4339**	373	D	74.1621	1
**4339**	381	D	79.5571	1
**4339**	382	D	67.2847	1
**4339**	386	D	73.8847	1
**4340**	305	A	75.2216	1
**4340**	319	A	79.5331	1
**4340**	320	A	79.6	-1
**4340**	321	A	74.9173	0
**4340**	351	A	13.7011	1
**4340**	357	A	72.5434	1
**4340**	370	A	79.6409	1
**4340**	372	A	49.9584	1
**4340**	373	A	79.4869	1
**4340**	381	A	79.4598	1
**4340**	382	A	69.5258	1
**4340**	386	A	75.863	1
**4340**	305	B	79.5241	1
**4340**	319	B	77.8421	1
**4340**	320	B	64.5849	-1
**4340**	321	B	73.9108	0
**4340**	351	B	14.3055	1
**4340**	357	B	63.9786	1
**4340**	370	B	79.5583	1
**4340**	372	B	63.0825	0
**4340**	373	B	77.7727	1
**4340**	381	B	79.5745	1
**4340**	382	B	49.1347	0
**4340**	386	B	59.3061	1
**4340**	305	C	75.675	1
**4340**	319	C	39.4368	0

**4340**	320	C	27.7722	-1
**4340**	321	C	34.4347	1
**4340**	351	C	3.8543	1
**4340**	357	C	63.3547	1
**4340**	370	C	59.2489	1
**4340**	372	C	63.9333	0
**4340**	373	C	56.9792	1
**4340**	381	C	11.2372	-1
**4340**	382	C	65.9624	1
**4340**	386	C	38.8306	0
**4340**	305	D	69.5409	1
**4340**	319	D	79.4167	1
**4340**	320	D	70.1667	-1
**4340**	321	D	79.5628	0
**4340**	351	D	15.6188	1
**4340**	357	D	64.7749	1
**4340**	370	D	38.2519	0
**4340**	372	D	79.577	0
**4340**	373	D	79.5544	1
**4340**	381	D	69.278	1
**4340**	382	D	79.5564	1
**4340**	386	D	34.719	0
**4341**	305	A	79.4305	1
**4341**	319	A	79.526	1
**4341**	320	A	79.5661	-1
**4341**	321	A	79.5238	0
**4341**	351	A	7.0882	1
**4341**	357	A	67.3789	1
**4341**	370	A	79.5945	1
**4341**	372	A	79.5139	0
**4341**	373	A	63.8835	1
**4341**	381	A	79.6891	1
**4341**	382	A	72.9395	1
**4341**	386	A	46.5009	0

**4341**	305	B	55.1893	1
**4341**	319	B	59.4777	1
**4341**	320	B	79.5311	-1
**4341**	321	B	74.5407	0
**4341**	351	B	15.2956	1
**4341**	357	B	64.8778	1
**4341**	370	B	72.6268	1
**4341**	372	B	79.5844	0
**4341**	373	B	29.8119	0
**4341**	381	B	79.514	1
**4341**	382	B	41.0144	0
**4341**	386	B	58.9111	1
**4341**	305	C	74.7695	1
**4341**	319	C	79.7048	1
**4341**	320	C	70.5451	-1
**4341**	321	C	63.5281	0
**4341**	351	C	8.0187	1
**4341**	357	C	79.659	1
**4341**	370	C	79.6201	1
**4341**	372	C	62.8247	0
**4341**	373	C	79.5617	1
**4341**	381	C	58.4554	1
**4341**	382	C	78.2393	1
**4341**	386	C	39.1334	0
**4341**	305	D	69.0819	1
**4341**	319	D	77.5561	1
**4341**	320	D	79.6195	-1
**4341**	321	D	39.3509	1
**4341**	351	D	7.8235	1
**4341**	357	D	53.739	1
**4341**	370	D	72.6314	1
**4341**	372	D	55.431	0
**4341**	373	D	30.3898	0
**4341**	381	D	65.3453	1

**4341**	382	D	37.2865	0
**4341**	386	D	44.8	0
**4342**	305	A	79.8008	1
**4342**	319	A	44.7028	0
**4342**	320	A	79.5482	-1
**4342**	321	A	69.1656	0
**4342**	351	A	24.2098	-1
**4342**	357	A	67.482	1
**4342**	370	A	78.9255	1
**4342**	372	A	79.6035	0
**4342**	373	A	72.1945	1
**4342**	381	A	73.9522	1
**4342**	382	A	74.1326	1
**4342**	386	A	54.4014	1
**4342**	305	B	66.5896	1
**4342**	319	B	79.7595	1
**4342**	320	B	47.91	-1
**4342**	321	B	51.2773	0
**4342**	351	B	9.2364	1
**4342**	357	B	69.5163	1
**4342**	370	B	60.0672	1
**4342**	372	B	70.451	0
**4342**	373	B	57.9457	1
**4342**	381	B	50.1174	1
**4342**	382	B	76.4184	1
**4342**	386	B	54.6459	1
**4342**	305	C	33.9887	0
**4342**	319	C	77.7393	1
**4342**	320	C	70.9182	-1
**4342**	321	C	73.3905	0
**4342**	351	C	3.1843	1
**4342**	357	C	74.6271	1
**4342**	370	C	34.2796	0
**4342**	372	C	67.237	0

**4342**	373	C	73.2239	1
**4342**	381	C	53.8339	1
**4342**	382	C	36.1576	0
**4342**	386	C	6.3757	-1
**4342**	305	D	20.5193	0
**4342**	319	D	79.5467	1
**4342**	320	D	79.5226	-1
**4342**	321	D	79.563	0
**4342**	351	D	25.2093	-1
**4342**	357	D	79.4433	1
**4342**	370	D	79.5251	1
**4342**	372	D	79.652	0
**4342**	373	D	79.6437	1
**4342**	381	D	79.8463	1
**4342**	382	D	78.1542	1
**4342**	386	D	79.54	1
**4343**	305	A	44.7096	0
**4343**	319	A	48.5236	0
**4343**	320	A	46.9592	-1
**4343**	321	A	77.0517	0
**4343**	351	A	4.2767	1
**4343**	357	A	34.2059	0
**4343**	370	A	74.462	1
**4343**	372	A	79.5457	0
**4343**	373	A	71.3904	1
**4343**	381	A	40.2293	0
**4343**	382	A	66.2245	1
**4343**	386	A	73.2676	1
**4343**	305	B	79.4413	1
**4343**	319	B	79.5924	1
**4343**	320	B	79.5214	-1
**4343**	321	B	47.4289	1
**4343**	351	B	11.3325	1
**4343**	357	B	79.497	1

**4343**	370	B	46.8205	0
**4343**	372	B	79.6332	0
**4343**	373	B	59.2044	1
**4343**	381	B	63.3189	1
**4343**	382	B	30.6554	0
**4343**	386	B	73.1291	1
**4343**	305	C	74.1411	1
**4343**	319	C	79.6004	1
**4343**	320	C	59.8754	-1
**4343**	321	C	73.2592	0
**4343**	351	C	7.5612	1
**4343**	357	C	71.7351	1
**4343**	370	C	67.8304	1
**4343**	372	C	79.5528	0
**4343**	373	C	79.5345	1
**4343**	381	C	79.4901	1
**4343**	382	C	52.816	1
**4343**	386	C	70.3289	1
**4343**	305	D	78.5177	1
**4343**	319	D	48.7699	0
**4343**	320	D	79.5625	-1
**4343**	321	D	73.3175	0
**4343**	351	D	8.8972	1
**4343**	357	D	50.0279	1
**4343**	370	D	26.868	0
**4343**	372	D	79.5533	0
**4343**	373	D	79.5925	1
**4343**	381	D	69.5142	1
**4343**	382	D	75.0132	1
**4343**	386	D	77.9445	1
**4344**	305	A	64.1379	1
**4344**	319	A	37.9957	0
**4344**	320	A	50.0898	-1
**4344**	321	A	76.1447	0

**4344**	351	A	9.8923	1
**4344**	357	A	53.6682	1
**4344**	370	A	36.4937	0
**4344**	372	A	28.3142	1
**4344**	373	A	72.3772	1
**4344**	381	A	56.6172	1
**4344**	382	A	71.7051	1
**4344**	386	A	72.7092	1
**4344**	305	B	79.5862	1
**4344**	319	B	78.3396	1
**4344**	320	B	23.5061	-1
**4344**	321	B	79.4085	0
**4344**	351	B	44.5861	-1
**4344**	357	B	69.3974	1
**4344**	370	B	77.0287	1
**4344**	372	B	68.589	0
**4344**	373	B	70.1196	1
**4344**	381	B	62.9519	1
**4344**	382	B	68.7194	1
**4344**	386	B	73.3831	1
**4344**	305	C	70.9359	1
**4344**	319	C	72.7026	1
**4344**	320	C	70.2325	-1
**4344**	321	C	79.4624	0
**4344**	351	C	12.4644	1
**4344**	357	C	60.8471	1
**4344**	370	C	79.6313	1
**4344**	372	C	79.4675	0
**4344**	373	C	71.8872	1
**4344**	381	C	76.8512	1
**4344**	382	C	58.7045	1
**4344**	386	C	46.4542	0
**4344**	305	D	72.642	1
**4344**	319	D	28.5698	0

**4344**	320	D	78.7735	-1
**4344**	321	D	79.4284	0
**4344**	351	D	18.999	1
**4344**	357	D	72.2357	1
**4344**	370	D	63.302	1
**4344**	372	D	79.6473	0
**4344**	373	D	37.9133	0
**4344**	381	D	79.5472	1
**4344**	382	D	65.8247	1
**4344**	386	D	79.3528	1
**4345**	305	A	70.3102	1
**4345**	319	A	15.0469	-1
**4345**	320	A	79.6108	-1
**4345**	321	A	72.3717	0
**4345**	351	A	0.2293	1
**4345**	357	A	78.9381	1
**4345**	370	A	53.861	1
**4345**	372	A	63.8662	0
**4345**	373	A	79.1069	1
**4345**	381	A	51.9363	1
**4345**	382	A	66.6074	1
**4345**	386	A	79.6076	1
**4345**	305	B	36.8726	0
**4345**	319	B	66.1445	1
**4345**	320	B	30.9397	-1
**4345**	321	B	67.9772	0
**4345**	351	B	32.185	-1
**4345**	357	B	64.8837	1
**4345**	370	B	70.5776	1
**4345**	372	B	76.946	0
**4345**	373	B	70.3138	1
**4345**	381	B	37.445	0
**4345**	382	B	43.1074	0
**4345**	386	B	54.0568	1

**4345**	305	C	79.6272	1
**4345**	319	C	79.6992	1
**4345**	320	C	56.2145	-1
**4345**	321	C	79.6075	0
**4345**	351	C	0.0	1
**4345**	357	C	79.5434	1
**4345**	370	C	32.0718	0
**4345**	372	C	59.1918	0
**4345**	373	C	72.9682	1
**4345**	381	C	60.1488	1
**4345**	382	C	75.8555	1
**4345**	386	C	64.4424	1
**4345**	305	D	72.552	1
**4345**	319	D	64.7408	1
**4345**	320	D	79.5885	-1
**4345**	321	D	79.5825	0
**4345**	351	D	34.9836	-1
**4345**	357	D	42.0181	0
**4345**	370	D	79.3218	1
**4345**	372	D	51.93	0
**4345**	373	D	47.9215	0
**4345**	381	D	73.3315	1
**4345**	382	D	59.4248	1
**4345**	386	D	47.2037	0
**4346**	305	A	78.191	1
**4346**	319	A	65.3549	1
**4346**	320	A	35.2071	-1
**4346**	321	A	33.7459	1
**4346**	351	A	14.0086	1
**4346**	357	A	76.9048	1
**4346**	370	A	71.618	1
**4346**	372	A	50.3732	0
**4346**	373	A	66.964	1
**4346**	381	A	79.5525	1

**4346**	382	A	77.9804	1
**4346**	386	A	73.5708	1
**4346**	305	B	71.7793	1
**4346**	319	B	76.7224	1
**4346**	320	B	74.6147	-1
**4346**	321	B	67.0278	0
**4346**	351	B	9.3827	1
**4346**	357	B	72.3223	1
**4346**	370	B	79.2405	1
**4346**	372	B	68.4163	0
**4346**	373	B	72.8101	1
**4346**	381	B	61.8279	1
**4346**	382	B	18.9775	-1
**4346**	386	B	60.5193	1
**4346**	305	C	70.9533	1
**4346**	319	C	78.2837	1
**4346**	320	C	62.8854	-1
**4346**	321	C	68.9668	0
**4346**	351	C	3.6775	1
**4346**	357	C	68.4145	1
**4346**	370	C	45.5675	0
**4346**	372	C	78.378	0
**4346**	373	C	39.7879	0
**4346**	381	C	44.0192	0
**4346**	382	C	67.5168	1
**4346**	386	C	46.2152	0
**4346**	305	D	72.7433	1
**4346**	319	D	76.0271	1
**4346**	320	D	68.0899	-1
**4346**	321	D	63.1886	0
**4346**	351	D	17.9506	1
**4346**	357	D	69.9752	1
**4346**	370	D	72.7032	1
**4346**	372	D	79.5869	0

**4346**	373	D	79.5957	1
**4346**	381	D	54.1116	1
**4346**	382	D	79.5279	1
**4346**	386	D	68.5133	1
**4347**	305	A	69.0824	1
**4347**	319	A	61.2015	1
**4347**	320	A	79.4928	-1
**4347**	321	A	79.2291	0
**4347**	351	A	11.7857	1
**4347**	357	A	74.4575	1
**4347**	370	A	65.6028	1
**4347**	372	A	79.5132	0
**4347**	373	A	76.2814	1
**4347**	381	A	56.2503	1
**4347**	382	A	71.5628	1
**4347**	386	A	79.5082	1
**4347**	305	B	72.4147	1
**4347**	319	B	60.3157	1
**4347**	320	B	40.9446	-1
**4347**	321	B	77.8644	0
**4347**	351	B	13.495	1
**4347**	357	B	79.5506	1
**4347**	370	B	64.6015	1
**4347**	372	B	79.4674	0
**4347**	373	B	28.2265	0
**4347**	381	B	58.1173	1
**4347**	382	B	52.0263	1
**4347**	386	B	43.0525	0
**4347**	305	C	69.9399	1
**4347**	319	C	30.8048	0
**4347**	320	C	66.3763	-1
**4347**	321	C	75.4653	0
**4347**	351	C	6.336	1
**4347**	357	C	73.7084	1

**4347**	370	C	79.519	1
**4347**	372	C	79.6003	0
**4347**	373	C	79.5901	1
**4347**	381	C	71.578	1
**4347**	382	C	79.454	1
**4347**	386	C	49.905	0
**4347**	305	D	75.9422	1
**4347**	319	D	43.638	0
**4347**	320	D	79.4908	-1
**4347**	321	D	67.1217	0
**4347**	351	D	17.7771	1
**4347**	357	D	79.3926	1
**4347**	370	D	79.5468	1
**4347**	372	D	79.5816	0
**4347**	373	D	79.5456	1
**4347**	381	D	79.583	1
**4347**	382	D	79.5459	1
**4347**	386	D	79.5559	1
**4348**	305	A	79.5807	1
**4348**	319	A	79.5266	1
**4348**	320	A	69.9313	-1
**4348**	321	A	64.3826	0
**4348**	351	A	8.0502	1
**4348**	357	A	43.1797	0
**4348**	370	A	59.2634	1
**4348**	372	A	72.356	0
**4348**	373	A	41.3647	0
**4348**	381	A	77.5173	1
**4348**	382	A	76.5845	1
**4348**	386	A	65.801	1
**4348**	305	B	73.4091	1
**4348**	319	B	79.6938	1
**4348**	320	B	37.4143	-1
**4348**	321	B	73.8805	0

**4348**	351	B	13.2958	1
**4348**	357	B	26.7344	0
**4348**	370	B	79.3635	1
**4348**	372	B	79.5538	0
**4348**	373	B	79.5217	1
**4348**	381	B	21.7716	0
**4348**	382	B	26.3929	0
**4348**	386	B	47.6508	0
**4348**	305	C	39.2043	0
**4348**	319	C	79.62	1
**4348**	320	C	79.4572	-1
**4348**	321	C	75.891	0
**4348**	351	C	17.3064	1
**4348**	357	C	55.7795	1
**4348**	370	C	33.2046	0
**4348**	372	C	40.4266	1
**4348**	373	C	11.7154	-1
**4348**	381	C	79.4924	1
**4348**	382	C	52.9196	1
**4348**	386	C	50.4185	1
**4348**	305	D	79.7404	1
**4348**	319	D	63.7384	1
**4348**	320	D	79.4899	-1
**4348**	321	D	79.4214	0
**4348**	351	D	18.7931	1
**4348**	357	D	46.3247	0
**4348**	370	D	69.1119	1
**4348**	372	D	45.3836	1
**4348**	373	D	79.4836	1
**4348**	381	D	45.2984	0
**4348**	382	D	57.7424	1
**4348**	386	D	36.6021	0
**4349**	305	A	74.1323	1
**4349**	319	A	65.3001	1

**4349**	320	A	79.5203	-1
**4349**	321	A	56.9762	0
**4349**	351	A	0.5832	1
**4349**	357	A	39.5475	0
**4349**	370	A	79.4453	1
**4349**	372	A	66.8761	0
**4349**	373	A	47.4141	0
**4349**	381	A	74.8552	1
**4349**	382	A	32.4566	0
**4349**	386	A	79.5589	1
**4349**	305	B	79.5373	1
**4349**	319	B	72.5896	1
**4349**	320	B	13.5383	1
**4349**	321	B	79.5768	0
**4349**	351	B	14.2462	1
**4349**	357	B	69.2342	1
**4349**	370	B	61.472	1
**4349**	372	B	39.0723	1
**4349**	373	B	37.2431	0
**4349**	381	B	13.0607	-1
**4349**	382	B	30.7489	0
**4349**	386	B	58.2709	1
**4349**	305	C	30.4768	0
**4349**	319	C	79.5155	1
**4349**	320	C	79.5196	-1
**4349**	321	C	78.3736	0
**4349**	351	C	4.4582	1
**4349**	357	C	69.3101	1
**4349**	370	C	79.5798	1
**4349**	372	C	64.536	0
**4349**	373	C	79.3073	1
**4349**	381	C	71.9529	1
**4349**	382	C	44.6072	0
**4349**	386	C	11.7237	-1

**4349**	305	D	67.8072	1
**4349**	319	D	68.4395	1
**4349**	320	D	57.9035	-1
**4349**	321	D	51.2842	0
**4349**	351	D	8.2305	1
**4349**	357	D	65.4836	1
**4349**	370	D	25.2199	0
**4349**	372	D	79.4922	0
**4349**	373	D	77.5368	1
**4349**	381	D	74.2428	1
**4349**	382	D	65.279	1
**4349**	386	D	39.528	0
**4350**	305	A	79.5457	1
**4350**	319	A	79.5454	1
**4350**	320	A	65.3303	-1
**4350**	321	A	73.3882	0
**4350**	351	A	5.7959	1
**4350**	357	A	79.5759	1
**4350**	370	A	72.9875	1
**4350**	372	A	29.4515	1
**4350**	373	A	79.5802	1
**4350**	381	A	67.7159	1
**4350**	382	A	67.925	1
**4350**	386	A	78.0194	1
**4350**	305	B	78.6722	1
**4350**	319	B	61.6836	1
**4350**	320	B	29.8062	-1
**4350**	321	B	79.5222	0
**4350**	351	B	15.7636	1
**4350**	357	B	65.8363	1
**4350**	370	B	51.9625	1
**4350**	372	B	63.4123	0
**4350**	373	B	51.993	1
**4350**	381	B	79.5317	1

**4350**	382	B	17.2367	-1
**4350**	386	B	48.1463	0
**4350**	305	C	78.7515	1
**4350**	319	C	64.2563	1
**4350**	320	C	79.5714	-1
**4350**	321	C	79.573	0
**4350**	351	C	5.885	1
**4350**	357	C	72.3121	1
**4350**	370	C	68.9729	1
**4350**	372	C	72.7179	0
**4350**	373	C	74.2075	1
**4350**	381	C	74.4447	1
**4350**	382	C	79.5758	1
**4350**	386	C	40.1169	0
**4350**	305	D	67.5717	1
**4350**	319	D	78.8359	1
**4350**	320	D	79.6159	-1
**4350**	321	D	79.5614	0
**4350**	351	D	14.3023	1
**4350**	357	D	79.5448	1
**4350**	370	D	70.3654	1
**4350**	372	D	48.2625	1
**4350**	373	D	79.3882	1
**4350**	381	D	46.3172	0
**4350**	382	D	79.5304	1
**4350**	386	D	21.6112	0
**4351**	305	A	79.5771	1
**4351**	319	A	79.6596	1
**4351**	320	A	65.3485	-1
**4351**	321	A	72.6419	0
**4351**	351	A	1.0664	1
**4351**	357	A	70.8736	1
**4351**	370	A	56.4104	1
**4351**	372	A	48.1809	1

**4351**	373	A	69.1355	1
**4351**	381	A	60.7959	1
**4351**	382	A	52.8502	1
**4351**	386	A	79.0955	1
**4351**	305	B	68.0052	1
**4351**	319	B	71.3661	1
**4351**	320	B	76.0229	-1
**4351**	321	B	74.1996	0
**4351**	351	B	15.1944	1
**4351**	357	B	79.6105	1
**4351**	370	B	79.6195	1
**4351**	372	B	58.6714	0
**4351**	373	B	41.532	0
**4351**	381	B	73.4518	1
**4351**	382	B	79.5746	1
**4351**	386	B	79.6706	1
**4351**	305	C	79.6574	1
**4351**	319	C	70.8597	1
**4351**	320	C	69.822	-1
**4351**	321	C	77.583	0
**4351**	351	C	1.9461	1
**4351**	357	C	58.3027	1
**4351**	370	C	74.1447	1
**4351**	372	C	71.8567	0
**4351**	373	C	72.7181	1
**4351**	381	C	79.5561	1
**4351**	382	C	47.6272	0
**4351**	386	C	35.7719	0
**4351**	305	D	79.6344	1
**4351**	319	D	40.6092	0
**4351**	320	D	79.4584	-1
**4351**	321	D	68.2575	0
**4351**	351	D	3.3192	1
**4351**	357	D	61.2087	1

**4351**	370	D	63.7618	1
**4351**	372	D	79.5649	0
**4351**	373	D	79.1939	1
**4351**	381	D	79.5455	1
**4351**	382	D	43.2969	0
**4351**	386	D	27.7929	0
**4352**	305	A	40.3542	0
**4352**	319	A	64.8482	1
**4352**	320	A	70.2954	-1
**4352**	321	A	70.3988	0
**4352**	351	A	3.4298	1
**4352**	357	A	26.6542	0
**4352**	370	A	54.303	1
**4352**	372	A	65.3713	0
**4352**	373	A	53.973	1
**4352**	381	A	68.6315	1
**4352**	382	A	77.5333	1
**4352**	386	A	79.5755	1
**4352**	305	B	79.5505	1
**4352**	319	B	79.5677	1
**4352**	320	B	35.4331	-1
**4352**	321	B	79.5644	0
**4352**	351	B	8.6537	1
**4352**	357	B	41.0044	0
**4352**	370	B	79.6189	1
**4352**	372	B	57.9157	0
**4352**	373	B	46.06	0
**4352**	381	B	70.9552	1
**4352**	382	B	35.5734	0
**4352**	386	B	56.202	1
**4352**	305	C	67.7506	1
**4352**	319	C	79.5671	1
**4352**	320	C	79.5145	-1
**4352**	321	C	30.7683	1

**4352**	351	C	7.7132	1
**4352**	357	C	62.0855	1
**4352**	370	C	79.598	1
**4352**	372	C	79.6178	0
**4352**	373	C	72.2031	1
**4352**	381	C	73.9601	1
**4352**	382	C	79.5658	1
**4352**	386	C	51.6375	1
**4352**	305	D	79.5973	1
**4352**	319	D	4.204	-1
**4352**	320	D	59.2803	-1
**4352**	321	D	77.8903	0
**4352**	351	D	16.054	1
**4352**	357	D	79.556	1
**4352**	370	D	79.4964	1
**4352**	372	D	77.7876	0
**4352**	373	D	78.6309	1
**4352**	381	D	37.5753	0
**4352**	382	D	79.6179	1
**4352**	386	D	77.7695	1
**4353**	305	A	75.3776	1
**4353**	319	A	56.3891	1
**4353**	320	A	74.1405	-1
**4353**	321	A	72.3202	0
**4353**	351	A	14.8422	1
**4353**	357	A	50.8297	1
**4353**	370	A	72.6179	1
**4353**	372	A	50.9087	0
**4353**	373	A	61.1659	1
**4353**	381	A	68.2029	1
**4353**	382	A	70.5631	1
**4353**	386	A	57.5288	1
**4353**	305	B	50.0749	1
**4353**	319	B	63.6732	1

**4353**	320	B	74.1717	-1
**4353**	321	B	79.7199	0
**4353**	351	B	14.6603	1
**4353**	357	B	61.0971	1
**4353**	370	B	65.4919	1
**4353**	372	B	79.6568	0
**4353**	373	B	64.7915	1
**4353**	381	B	75.9952	1
**4353**	382	B	73.0978	1
**4353**	386	B	79.4244	1
**4353**	305	C	79.5854	1
**4353**	319	C	65.2571	1
**4353**	320	C	37.8253	-1
**4353**	321	C	36.5987	1
**4353**	351	C	20.3934	-1
**4353**	357	C	79.5157	1
**4353**	370	C	74.5789	1
**4353**	372	C	79.4836	0
**4353**	373	C	63.692	1
**4353**	381	C	74.7504	1
**4353**	382	C	74.4936	1
**4353**	386	C	59.5332	1
**4353**	305	D	66.1804	1
**4353**	319	D	74.9995	1
**4353**	320	D	71.3222	-1
**4353**	321	D	79.5904	0
**4353**	351	D	18.9611	1
**4353**	357	D	71.4235	1
**4353**	370	D	79.1975	1
**4353**	372	D	76.2455	0
**4353**	373	D	79.5276	1
**4353**	381	D	79.1621	1
**4353**	382	D	51.5228	1
**4353**	386	D	79.5818	1

**4354**	305	A	75.2842	1
**4354**	319	A	76.8988	1
**4354**	320	A	62.0105	-1
**4354**	321	A	50.0529	0
**4354**	351	A	9.6266	1
**4354**	357	A	77.083	1
**4354**	370	A	74.4587	1
**4354**	372	A	79.5857	0
**4354**	373	A	79.6095	1
**4354**	381	A	71.3103	1
**4354**	382	A	72.9667	1
**4354**	386	A	49.1768	0
**4354**	305	B	34.6077	0
**4354**	319	B	79.5968	1
**4354**	320	B	65.2116	-1
**4354**	321	B	69.1448	0
**4354**	351	B	11.9129	1
**4354**	357	B	58.076	1
**4354**	370	B	79.4106	1
**4354**	372	B	79.5607	0
**4354**	373	B	79.441	1
**4354**	381	B	64.3529	1
**4354**	382	B	46.9927	0
**4354**	386	B	59.6656	1
**4354**	305	C	71.5825	1
**4354**	319	C	77.7073	1
**4354**	320	C	79.6203	-1
**4354**	321	C	55.253	0
**4354**	351	C	3.8823	1
**4354**	357	C	79.3996	1
**4354**	370	C	78.9732	1
**4354**	372	C	34.1448	1
**4354**	373	C	72.1189	1
**4354**	381	C	51.7185	1

**4354**	382	C	53.3818	1
**4354**	386	C	57.8861	1
**4354**	305	D	37.2557	0
**4354**	319	D	48.8045	0
**4354**	320	D	79.5773	-1
**4354**	321	D	62.5621	0
**4354**	351	D	14.1559	1
**4354**	357	D	68.9659	1
**4354**	370	D	78.3324	1
**4354**	372	D	47.255	1
**4354**	373	D	79.5729	1
**4354**	381	D	50.4479	1
**4354**	382	D	79.5589	1
**4354**	386	D	50.8414	1
**4355**	305	A	71.2795	1
**4355**	319	A	66.5774	1
**4355**	320	A	79.5652	-1
**4355**	321	A	67.0451	0
**4355**	351	A	3.7381	1
**4355**	357	A	44.2775	0
**4355**	370	A	42.3896	0
**4355**	372	A	66.2157	0
**4355**	373	A	76.6286	1
**4355**	381	A	65.5757	1
**4355**	382	A	67.5205	1
**4355**	386	A	79.5336	1
**4355**	305	B	79.5618	1
**4355**	319	B	62.1948	1
**4355**	320	B	13.3193	1
**4355**	321	B	71.4601	0
**4355**	351	B	8.9989	1
**4355**	357	B	73.0552	1
**4355**	370	B	70.2833	1
**4355**	372	B	53.5369	0

**4355**	373	B	76.2983	1
**4355**	381	B	79.4856	1
**4355**	382	B	70.8461	1
**4355**	386	B	51.4326	1
**4355**	305	C	71.937	1
**4355**	319	C	53.5608	1
**4355**	320	C	65.0304	-1
**4355**	321	C	56.4137	0
**4355**	351	C	5.2441	1
**4355**	357	C	54.4484	1
**4355**	370	C	79.5426	1
**4355**	372	C	79.5453	0
**4355**	373	C	79.4682	1
**4355**	381	C	69.7036	1
**4355**	382	C	71.1459	1
**4355**	386	C	42.8662	0
**4355**	305	D	53.7289	1
**4355**	319	D	32.4522	0
**4355**	320	D	79.4976	-1
**4355**	321	D	58.3852	0
**4355**	351	D	12.8082	1
**4355**	357	D	53.4784	1
**4355**	370	D	79.5667	1
**4355**	372	D	64.864	0
**4355**	373	D	60.4088	1
**4355**	381	D	79.5081	1
**4355**	382	D	74.4354	1
**4355**	386	D	45.6474	0
**4356**	305	A	72.3615	1
**4356**	319	A	77.5791	1
**4356**	320	A	74.4589	-1
**4356**	321	A	79.6029	0
**4356**	351	A	10.3946	1
**4356**	357	A	71.888	1

**4356**	370	A	75.7828	1
**4356**	372	A	79.6029	0
**4356**	373	A	69.3688	1
**4356**	381	A	21.7742	0
**4356**	382	A	45.4595	0
**4356**	386	A	75.8451	1
**4356**	305	B	75.6268	1
**4356**	319	B	79.5665	1
**4356**	320	B	72.8663	-1
**4356**	321	B	72.432	0
**4356**	351	B	7.7981	1
**4356**	357	B	79.1739	1
**4356**	370	B	68.5866	1
**4356**	372	B	64.0223	0
**4356**	373	B	52.091	1
**4356**	381	B	15.3805	-1
**4356**	382	B	48.4562	0
**4356**	386	B	53.6499	1
**4356**	305	C	79.4133	1
**4356**	319	C	73.4265	1
**4356**	320	C	33.0213	-1
**4356**	321	C	73.3106	0
**4356**	351	C	9.1735	1
**4356**	357	C	74.1069	1
**4356**	370	C	62.1952	1
**4356**	372	C	47.3016	1
**4356**	373	C	38.1346	0
**4356**	381	C	75.7429	1
**4356**	382	C	79.5354	1
**4356**	386	C	54.8925	1
**4356**	305	D	36.0323	0
**4356**	319	D	79.5887	1
**4356**	320	D	63.7093	-1
**4356**	321	D	64.683	0

**4356**	351	D	12.4188	1
**4356**	357	D	73.5879	1
**4356**	370	D	73.5559	1
**4356**	372	D	79.2806	0
**4356**	373	D	79.6943	1
**4356**	381	D	73.919	1
**4356**	382	D	79.6146	1
**4356**	386	D	61.7903	1
**4357**	305	A	44.2662	0
**4357**	319	A	59.9154	1
**4357**	320	A	56.602	-1
**4357**	321	A	75.3475	0
**4357**	351	A	14.0515	1
**4357**	357	A	79.6208	1
**4357**	370	A	79.5375	1
**4357**	372	A	70.2303	0
**4357**	373	A	79.5838	1
**4357**	381	A	77.3165	1
**4357**	382	A	78.1645	1
**4357**	386	A	73.4334	1
**4357**	305	B	68.6721	1
**4357**	319	B	70.9203	1
**4357**	320	B	79.5819	-1
**4357**	321	B	43.4399	1
**4357**	351	B	7.3739	1
**4357**	357	B	42.0297	0
**4357**	370	B	79.4215	1
**4357**	372	B	45.6324	1
**4357**	373	B	79.524	1
**4357**	381	B	79.5899	1
**4357**	382	B	37.5018	0
**4357**	386	B	61.5258	1
**4357**	305	C	65.45	1
**4357**	319	C	79.4523	1

**4357**	320	C	54.8414	-1
**4357**	321	C	79.603	0
**4357**	351	C	5.7491	1
**4357**	357	C	79.6345	1
**4357**	370	C	70.906	1
**4357**	372	C	79.5023	0
**4357**	373	C	57.1927	1
**4357**	381	C	79.5701	1
**4357**	382	C	79.5731	1
**4357**	386	C	31.0617	0
**4357**	305	D	45.2704	0
**4357**	319	D	79.5054	1
**4357**	320	D	61.1922	-1
**4357**	321	D	0.8569	0
**4357**	351	D	9.0646	1
**4357**	357	D	56.7804	1
**4357**	370	D	54.532	1
**4357**	372	D	72.2521	0
**4357**	373	D	66.8321	1
**4357**	381	D	72.8715	1
**4357**	382	D	61.0692	1
**4357**	386	D	63.4583	1
**4358**	305	A	73.1444	1
**4358**	319	A	66.6593	1
**4358**	320	A	79.533	-1
**4358**	321	A	76.9653	0
**4358**	351	A	0.4428	1
**4358**	357	A	70.4305	1
**4358**	370	A	21.0567	0
**4358**	372	A	74.8474	0
**4358**	373	A	65.529	1
**4358**	381	A	52.2729	1
**4358**	382	A	75.8047	1
**4358**	386	A	61.2972	1

**4358**	305	B	79.5079	1
**4358**	319	B	70.941	1
**4358**	320	B	70.9393	-1
**4358**	321	B	71.1377	0
**4358**	351	B	15.1688	1
**4358**	357	B	73.2354	1
**4358**	370	B	31.2938	0
**4358**	372	B	70.1674	0
**4358**	373	B	26.5556	0
**4358**	381	B	76.7463	1
**4358**	382	B	74.5426	1
**4358**	386	B	72.0396	1
**4358**	305	C	67.4718	1
**4358**	319	C	54.5758	1
**4358**	320	C	58.5296	-1
**4358**	321	C	65.5211	0
**4358**	351	C	8.359	1
**4358**	357	C	79.3532	1
**4358**	370	C	73.3198	1
**4358**	372	C	52.1598	0
**4358**	373	C	78.2384	1
**4358**	381	C	66.2381	1
**4358**	382	C	79.5866	1
**4358**	386	C	67.5125	1
**4358**	305	D	57.8227	1
**4358**	319	D	76.033	1
**4358**	320	D	65.6652	-1
**4358**	321	D	79.5667	0
**4358**	351	D	8.8518	1
**4358**	357	D	74.6612	1
**4358**	370	D	57.4359	1
**4358**	372	D	64.892	0
**4358**	373	D	13.3422	-1
**4358**	381	D	61.7811	1

**4358**	382	D	79.5631	1
**4358**	386	D	73.2074	1
**4359**	305	A	79.5617	1
**4359**	319	A	64.0717	1
**4359**	320	A	59.669	-1
**4359**	321	A	44.4713	1
**4359**	351	A	8.838	1
**4359**	357	A	62.201	1
**4359**	370	A	69.2263	1
**4359**	372	A	66.5633	0
**4359**	373	A	77.6668	1
**4359**	381	A	73.4235	1
**4359**	382	A	77.4416	1
**4359**	386	A	79.5706	1
**4359**	305	B	71.9618	1
**4359**	319	B	61.3257	1
**4359**	320	B	52.0432	-1
**4359**	321	B	74.7728	0
**4359**	351	B	17.3383	1
**4359**	357	B	79.5914	1
**4359**	370	B	72.0556	1
**4359**	372	B	79.5733	0
**4359**	373	B	51.6918	1
**4359**	381	B	54.8308	1
**4359**	382	B	47.3784	0
**4359**	386	B	51.5645	1
**4359**	305	C	67.4721	1
**4359**	319	C	63.2718	1
**4359**	320	C	50.6488	-1
**4359**	321	C	63.4543	0
**4359**	351	C	4.8243	1
**4359**	357	C	79.618	1
**4359**	370	C	66.5573	1
**4359**	372	C	79.5425	0

**4359**	373	C	77.7716	1
**4359**	381	C	79.5454	1
**4359**	382	C	71.0023	1
**4359**	386	C	52.4398	1
**4359**	305	D	60.236	1
**4359**	319	D	79.5487	1
**4359**	320	D	71.4913	-1
**4359**	321	D	72.5631	0
**4359**	351	D	13.4827	1
**4359**	357	D	64.4233	1
**4359**	370	D	79.5511	1
**4359**	372	D	79.5971	0
**4359**	373	D	74.9707	1
**4359**	381	D	43.9953	0
**4359**	382	D	79.5503	1
**4359**	386	D	67.3196	1
**4360**	305	A	63.052	1
**4360**	319	A	47.4803	0
**4360**	320	A	79.6103	-1
**4360**	321	A	79.6051	0
**4360**	351	A	8.214	1
**4360**	357	A	67.0388	1
**4360**	370	A	79.7006	1
**4360**	372	A	79.5702	0
**4360**	373	A	50.0613	1
**4360**	381	A	61.6837	1
**4360**	382	A	49.0705	0
**4360**	386	A	79.5814	1
**4360**	305	B	65.9615	1
**4360**	319	B	35.8358	0
**4360**	320	B	42.9399	-1
**4360**	321	B	67.6911	0
**4360**	351	B	13.0715	1
**4360**	357	B	65.6471	1

**4360**	370	B	71.9791	1
**4360**	372	B	30.7552	1
**4360**	373	B	79.3964	1
**4360**	381	B	79.6226	1
**4360**	382	B	66.2521	1
**4360**	386	B	79.5562	1
**4360**	305	C	79.6809	1
**4360**	319	C	79.5965	1
**4360**	320	C	61.6489	-1
**4360**	321	C	72.97	0
**4360**	351	C	12.3867	1
**4360**	357	C	71.8152	1
**4360**	370	C	65.5031	1
**4360**	372	C	61.1618	0
**4360**	373	C	73.8816	1
**4360**	381	C	79.554	1
**4360**	382	C	30.1443	0
**4360**	386	C	48.5973	0
**4360**	305	D	57.3141	1
**4360**	319	D	79.607	1
**4360**	320	D	79.5367	-1
**4360**	321	D	39.6892	1
**4360**	351	D	12.8306	1
**4360**	357	D	36.5101	0
**4360**	370	D	66.1172	1
**4360**	372	D	79.568	0
**4360**	373	D	76.7082	1
**4360**	381	D	79.5176	1
**4360**	382	D	79.4199	1
**4360**	386	D	61.4898	1
**4361**	305	A	76.8116	1
**4361**	319	A	46.6872	0
**4361**	320	A	49.4417	-1
**4361**	321	A	33.8351	1

**4361**	351	A	36.6599	-1
**4361**	357	A	79.4303	1
**4361**	370	A	66.8467	1
**4361**	372	A	79.2094	0
**4361**	373	A	79.4738	1
**4361**	381	A	79.5985	1
**4361**	382	A	77.801	1
**4361**	386	A	79.4964	1
**4361**	305	B	65.4904	1
**4361**	319	B	69.1723	1
**4361**	320	B	79.5209	-1
**4361**	321	B	75.2717	0
**4361**	351	B	16.554	1
**4361**	357	B	78.8778	1
**4361**	370	B	76.9274	1
**4361**	372	B	72.2862	0
**4361**	373	B	71.4464	1
**4361**	381	B	74.7319	1
**4361**	382	B	46.8081	0
**4361**	386	B	72.3608	1
**4361**	305	C	54.7351	1
**4361**	319	C	71.9448	1
**4361**	320	C	79.5321	-1
**4361**	321	C	62.9873	0
**4361**	351	C	19.0011	1
**4361**	357	C	47.421	0
**4361**	370	C	39.5582	0
**4361**	372	C	66.1112	0
**4361**	373	C	79.5127	1
**4361**	381	C	62.5831	1
**4361**	382	C	37.3259	0
**4361**	386	C	33.8088	0
**4361**	305	D	75.5424	1
**4361**	319	D	75.9532	1

**4361**	320	D	79.5373	-1
**4361**	321	D	66.3215	0
**4361**	351	D	7.6488	1
**4361**	357	D	79.4579	1
**4361**	370	D	65.2258	1
**4361**	372	D	79.5898	0
**4361**	373	D	79.479	1
**4361**	381	D	47.4521	0
**4361**	382	D	74.966	1
**4361**	386	D	23.7295	0
**4362**	305	A	73.9257	1
**4362**	319	A	63.1227	1
**4362**	320	A	73.1328	-1
**4362**	321	A	70.8139	0
**4362**	351	A	0.1189	1
**4362**	357	A	73.0698	1
**4362**	370	A	79.3585	1
**4362**	372	A	77.9002	0
**4362**	373	A	37.978	0
**4362**	381	A	79.5158	1
**4362**	382	A	79.5451	1
**4362**	386	A	73.1691	1
**4362**	305	B	72.1163	1
**4362**	319	B	36.4335	0
**4362**	320	B	10.8636	1
**4362**	321	B	73.4947	0
**4362**	351	B	13.3822	1
**4362**	357	B	54.861	1
**4362**	370	B	79.5406	1
**4362**	372	B	73.1208	0
**4362**	373	B	52.3524	1
**4362**	381	B	45.638	0
**4362**	382	B	32.8672	0
**4362**	386	B	55.1746	1

**4362**	305	C	30.3447	0
**4362**	319	C	61.8131	1
**4362**	320	C	70.3576	-1
**4362**	321	C	79.5843	0
**4362**	351	C	9.9091	1
**4362**	357	C	79.543	1
**4362**	370	C	74.5965	1
**4362**	372	C	63.1518	0
**4362**	373	C	25.8837	0
**4362**	381	C	54.3316	1
**4362**	382	C	79.5495	1
**4362**	386	C	43.2379	0
**4362**	305	D	79.5197	1
**4362**	319	D	75.6068	1
**4362**	320	D	56.1613	-1
**4362**	321	D	74.0478	0
**4362**	351	D	12.8407	1
**4362**	357	D	79.5613	1
**4362**	370	D	78.453	1
**4362**	372	D	79.5538	0
**4362**	373	D	79.5589	1
**4362**	381	D	79.5479	1
**4362**	382	D	79.5592	1
**4362**	386	D	79.5652	1
**4363**	305	A	41.9914	0
**4363**	319	A	34.2959	0
**4363**	320	A	79.4432	-1
**4363**	321	A	76.3026	0
**4363**	351	A	14.5797	1
**4363**	357	A	62.8117	1
**4363**	370	A	79.5415	1
**4363**	372	A	79.4909	0
**4363**	373	A	45.6837	0
**4363**	381	A	76.8682	1

**4363**	382	A	79.5957	1
**4363**	386	A	72.2546	1
**4363**	305	B	44.9424	0
**4363**	319	B	76.8836	1
**4363**	320	B	79.5175	-1
**4363**	321	B	74.6671	0
**4363**	351	B	13.5288	1
**4363**	357	B	52.2341	1
**4363**	370	B	39.7288	0
**4363**	372	B	75.6755	0
**4363**	373	B	65.2829	1
**4363**	381	B	37.0889	0
**4363**	382	B	70.8092	1
**4363**	386	B	37.3361	0
**4363**	305	C	64.549	1
**4363**	319	C	37.1932	0
**4363**	320	C	79.4716	-1
**4363**	321	C	79.1128	0
**4363**	351	C	28.327	-1
**4363**	357	C	23.6416	0
**4363**	370	C	59.2131	1
**4363**	372	C	56.3426	0
**4363**	373	C	79.6049	1
**4363**	381	C	79.5184	1
**4363**	382	C	53.74	1
**4363**	386	C	56.3669	1
**4363**	305	D	73.0464	1
**4363**	319	D	77.2341	1
**4363**	320	D	55.9273	-1
**4363**	321	D	50.4762	0
**4363**	351	D	0.2709	1
**4363**	357	D	40.9228	0
**4363**	370	D	43.8904	0
**4363**	372	D	72.4954	0

**4363**	373	D	48.0766	0
**4363**	381	D	79.4811	1
**4363**	382	D	48.363	0
**4363**	386	D	79.5597	1
**4364**	305	A	68.3901	1
**4364**	319	A	73.1869	1
**4364**	320	A	79.5757	-1
**4364**	321	A	76.7468	0
**4364**	351	A	5.572	1
**4364**	357	A	79.5804	1
**4364**	370	A	44.3636	0
**4364**	372	A	58.5059	0
**4364**	373	A	78.8282	1
**4364**	381	A	62.8123	1
**4364**	382	A	70.868	1
**4364**	386	A	51.8689	1
**4364**	305	B	63.1657	1
**4364**	319	B	72.0535	1
**4364**	320	B	16.0091	1
**4364**	321	B	79.606	0
**4364**	351	B	1.5557	1
**4364**	357	B	36.3815	0
**4364**	370	B	79.5746	1
**4364**	372	B	68.3792	0
**4364**	373	B	50.8943	1
**4364**	381	B	79.5531	1
**4364**	382	B	79.3433	1
**4364**	386	B	48.3791	0
**4364**	305	C	73.1483	1
**4364**	319	C	79.53	1
**4364**	320	C	70.5436	-1
**4364**	321	C	79.5949	0
**4364**	351	C	3.7795	1
**4364**	357	C	73.9422	1

**4364**	370	C	70.4463	1
**4364**	372	C	75.8118	0
**4364**	373	C	72.6094	1
**4364**	381	C	47.9078	0
**4364**	382	C	79.3624	1
**4364**	386	C	55.6589	1
**4364**	305	D	35.1729	0
**4364**	319	D	33.9927	0
**4364**	320	D	56.5307	-1
**4364**	321	D	41.5134	1
**4364**	351	D	14.0939	1
**4364**	357	D	55.4334	1
**4364**	370	D	73.0045	1
**4364**	372	D	12.0893	0
**4364**	373	D	79.5635	1
**4364**	381	D	58.4718	1
**4364**	382	D	79.546	1
**4364**	386	D	77.6772	1
**4365**	305	A	79.6159	1
**4365**	319	A	66.8935	1
**4365**	320	A	79.5077	-1
**4365**	321	A	27.6686	1
**4365**	351	A	3.6981	1
**4365**	357	A	41.3679	0
**4365**	370	A	70.7632	1
**4365**	372	A	55.7178	0
**4365**	373	A	74.085	1
**4365**	381	A	79.6295	1
**4365**	382	A	79.2283	1
**4365**	386	A	79.5024	1
**4365**	305	B	61.5443	1
**4365**	319	B	70.9704	1
**4365**	320	B	79.4008	-1
**4365**	321	B	79.6089	0

**4365**	351	B	8.9899	1
**4365**	357	B	79.4903	1
**4365**	370	B	79.5443	1
**4365**	372	B	73.7368	0
**4365**	373	B	79.4544	1
**4365**	381	B	79.6433	1
**4365**	382	B	55.46	1
**4365**	386	B	29.1078	0
**4365**	305	C	48.7332	0
**4365**	319	C	79.5911	1
**4365**	320	C	56.9768	-1
**4365**	321	C	79.4132	0
**4365**	351	C	34.7509	-1
**4365**	357	C	70.6519	1
**4365**	370	C	68.2735	1
**4365**	372	C	79.3089	0
**4365**	373	C	71.7445	1
**4365**	381	C	79.6207	1
**4365**	382	C	79.6097	1
**4365**	386	C	49.1157	0
**4365**	305	D	79.7537	1
**4365**	319	D	78.517	1
**4365**	320	D	66.884	-1
**4365**	321	D	56.4705	0
**4365**	351	D	12.3348	1
**4365**	357	D	74.3915	1
**4365**	370	D	44.3282	0
**4365**	372	D	46.8593	1
**4365**	373	D	79.4947	1
**4365**	381	D	65.8806	1
**4365**	382	D	60.5211	1
**4365**	386	D	79.472	1
**4366**	305	A	79.3335	1
**4366**	319	A	52.4689	1

**4366**	320	A	49.5336	-1
**4366**	321	A	69.8055	0
**4366**	351	A	10.3813	1
**4366**	357	A	74.0959	1
**4366**	370	A	57.0485	1
**4366**	372	A	73.4479	0
**4366**	373	A	76.8398	1
**4366**	381	A	61.7893	1
**4366**	382	A	71.4147	1
**4366**	386	A	70.4267	1
**4366**	305	B	79.6039	1
**4366**	319	B	66.8594	1
**4366**	320	B	74.872	-1
**4366**	321	B	79.5424	0
**4366**	351	B	5.5077	1
**4366**	357	B	79.5162	1
**4366**	370	B	53.304	1
**4366**	372	B	74.4631	0
**4366**	373	B	46.9369	0
**4366**	381	B	64.4861	1
**4366**	382	B	48.4166	0
**4366**	386	B	70.219	1
**4366**	305	C	79.4866	1
**4366**	319	C	75.5819	1
**4366**	320	C	31.6657	-1
**4366**	321	C	59.3596	0
**4366**	351	C	9.8761	1
**4366**	357	C	63.5855	1
**4366**	370	C	79.5048	1
**4366**	372	C	71.4851	0
**4366**	373	C	75.0797	1
**4366**	381	C	52.1489	1
**4366**	382	C	79.4428	1
**4366**	386	C	48.4028	0

**4366**	305	D	72.3478	1
**4366**	319	D	78.9873	1
**4366**	320	D	32.5186	-1
**4366**	321	D	70.8113	0
**4366**	351	D	17.0315	1
**4366**	357	D	79.5359	1
**4366**	370	D	65.928	1
**4366**	372	D	75.8792	0
**4366**	373	D	41.7457	0
**4366**	381	D	79.6007	1
**4366**	382	D	73.1585	1
**4366**	386	D	53.105	1
**4367**	305	A	79.4159	1
**4367**	319	A	60.0066	1
**4367**	320	A	79.5649	-1
**4367**	321	A	77.9758	0
**4367**	351	A	16.8642	1
**4367**	357	A	64.6936	1
**4367**	370	A	55.651	1
**4367**	372	A	78.7763	0
**4367**	373	A	73.8697	1
**4367**	381	A	70.3184	1
**4367**	382	A	55.5321	1
**4367**	386	A	72.6369	1
**4367**	305	B	79.6892	1
**4367**	319	B	50.6648	1
**4367**	320	B	67.9355	-1
**4367**	321	B	79.6116	0
**4367**	351	B	13.1199	1
**4367**	357	B	68.8245	1
**4367**	370	B	56.2078	1
**4367**	372	B	71.4629	0
**4367**	373	B	71.4269	1
**4367**	381	B	79.5609	1

**4367**	382	B	52.2633	1
**4367**	386	B	50.976	1
**4367**	305	C	39.6023	0
**4367**	319	C	79.611	1
**4367**	320	C	73.1205	-1
**4367**	321	C	73.3948	0
**4367**	351	C	13.7381	1
**4367**	357	C	79.5288	1
**4367**	370	C	48.9352	0
**4367**	372	C	60.3875	0
**4367**	373	C	79.6298	1
**4367**	381	C	79.5518	1
**4367**	382	C	78.6998	1
**4367**	386	C	54.8109	1
**4367**	305	D	75.6788	1
**4367**	319	D	70.3838	1
**4367**	320	D	52.07	-1
**4367**	321	D	79.4951	0
**4367**	351	D	6.5053	1
**4367**	357	D	79.5212	1
**4367**	370	D	79.6199	1
**4367**	372	D	77.1867	0
**4367**	373	D	43.3352	0
**4367**	381	D	67.98	1
**4367**	382	D	79.6038	1
**4367**	386	D	35.6282	0
**4368**	305	A	79.5397	1
**4368**	319	A	62.27	1
**4368**	320	A	66.8191	-1
**4368**	321	A	79.5285	0
**4368**	351	A	4.3051	1
**4368**	357	A	62.7193	1
**4368**	370	A	77.7204	1
**4368**	372	A	61.6876	0

**4368**	373	A	79.4993	1
**4368**	381	A	37.8078	0
**4368**	382	A	66.2969	1
**4368**	386	A	78.5474	1
**4368**	305	B	79.6172	1
**4368**	319	B	43.3749	0
**4368**	320	B	79.5739	-1
**4368**	321	B	52.7132	0
**4368**	351	B	14.9122	1
**4368**	357	B	62.4002	1
**4368**	370	B	79.5528	1
**4368**	372	B	79.5562	0
**4368**	373	B	77.5687	1
**4368**	381	B	58.2739	1
**4368**	382	B	24.263	0
**4368**	386	B	79.6052	1
**4368**	305	C	58.0361	1
**4368**	319	C	79.5734	1
**4368**	320	C	79.5489	-1
**4368**	321	C	79.5787	0
**4368**	351	C	5.5496	1
**4368**	357	C	79.6029	1
**4368**	370	C	73.0607	1
**4368**	372	C	79.5378	0
**4368**	373	C	47.3846	0
**4368**	381	C	51.2553	1
**4368**	382	C	79.5932	1
**4368**	386	C	65.9359	1
**4368**	305	D	68.0529	1
**4368**	319	D	67.702	1
**4368**	320	D	56.9049	-1
**4368**	321	D	70.901	0
**4368**	351	D	14.7832	1
**4368**	357	D	54.7034	1

**4368**	370	D	68.7355	1
**4368**	372	D	78.1075	0
**4368**	373	D	11.548	-1
**4368**	381	D	74.0461	1
**4368**	382	D	79.5177	1
**4368**	386	D	79.5591	1
**4369**	305	A	79.613	1
**4369**	319	A	79.5751	1
**4369**	320	A	42.4548	-1
**4369**	321	A	79.4904	0
**4369**	351	A	0.0	1
**4369**	357	A	68.6294	1
**4369**	370	A	78.156	1
**4369**	372	A	79.0316	0
**4369**	373	A	41.3074	0
**4369**	381	A	79.5671	1
**4369**	382	A	64.1468	1
**4369**	386	A	79.5804	1
**4369**	305	B	52.8586	1
**4369**	319	B	79.5591	1
**4369**	320	B	43.3262	-1
**4369**	321	B	61.1233	0
**4369**	351	B	19.9377	1
**4369**	357	B	74.9321	1
**4369**	370	B	67.9342	1
**4369**	372	B	63.4979	0
**4369**	373	B	74.6865	1
**4369**	381	B	69.2572	1
**4369**	382	B	79.492	1
**4369**	386	B	72.4446	1
**4369**	305	C	76.8323	1
**4369**	319	C	65.3301	1
**4369**	320	C	79.645	-1
**4369**	321	C	63.1022	0

**4369**	351	C	4.0806	1
**4369**	357	C	79.674	1
**4369**	370	C	40.7639	0
**4369**	372	C	68.8483	0
**4369**	373	C	72.5638	1
**4369**	381	C	64.4505	1
**4369**	382	C	76.6391	1
**4369**	386	C	44.5534	0
**4369**	305	D	79.5362	1
**4369**	319	D	77.2739	1
**4369**	320	D	79.604	-1
**4369**	321	D	65.0043	0
**4369**	351	D	5.3888	1
**4369**	357	D	47.0276	0
**4369**	370	D	30.1509	0
**4369**	372	D	79.6377	0
**4369**	373	D	68.097	1
**4369**	381	D	79.6246	1
**4369**	382	D	79.5231	1
**4369**	386	D	74.9788	1
**4370**	305	A	79.243	1
**4370**	319	A	75.611	1
**4370**	320	A	69.7661	-1
**4370**	321	A	46.831	1
**4370**	351	A	11.7173	1
**4370**	357	A	57.4126	1
**4370**	370	A	61.2776	1
**4370**	372	A	73.5693	0
**4370**	373	A	73.831	1
**4370**	381	A	79.5391	1
**4370**	382	A	71.9773	1
**4370**	386	A	79.4462	1
**4370**	305	B	72.5512	1
**4370**	319	B	75.4618	1

**4370**	320	B	47.331	-1
**4370**	321	B	47.2266	1
**4370**	351	B	15.0098	1
**4370**	357	B	79.4705	1
**4370**	370	B	78.4153	1
**4370**	372	B	79.3557	0
**4370**	373	B	65.3322	1
**4370**	381	B	79.5722	1
**4370**	382	B	47.1638	0
**4370**	386	B	61.5997	1
**4370**	305	C	74.0582	1
**4370**	319	C	58.6294	1
**4370**	320	C	62.0512	-1
**4370**	321	C	54.9057	0
**4370**	351	C	9.0199	1
**4370**	357	C	45.8374	0
**4370**	370	C	64.0662	1
**4370**	372	C	79.4107	0
**4370**	373	C	75.3852	1
**4370**	381	C	34.0872	0
**4370**	382	C	65.9518	1
**4370**	386	C	50.6204	1
**4370**	305	D	79.5617	1
**4370**	319	D	72.985	1
**4370**	320	D	45.4486	-1
**4370**	321	D	71.4535	0
**4370**	351	D	18.9112	1
**4370**	357	D	79.5801	1
**4370**	370	D	77.1751	1
**4370**	372	D	79.5174	0
**4370**	373	D	79.5046	1
**4370**	381	D	61.7645	1
**4370**	382	D	73.3044	1
**4370**	386	D	79.4992	1

**4371**	305	A	79.5719	1
**4371**	319	A	79.5273	1
**4371**	320	A	79.602	-1
**4371**	321	A	79.5834	0
**4371**	351	A	11.1796	1
**4371**	357	A	79.5874	1
**4371**	370	A	77.6038	1
**4371**	372	A	79.548	0
**4371**	373	A	63.4917	1
**4371**	381	A	52.3341	1
**4371**	382	A	71.5748	1
**4371**	386	A	39.4335	0
**4371**	305	B	50.1322	1
**4371**	319	B	79.5329	1
**4371**	320	B	79.5134	-1
**4371**	321	B	79.5622	0
**4371**	351	B	15.4298	1
**4371**	357	B	79.5789	1
**4371**	370	B	60.3803	1
**4371**	372	B	68.0643	0
**4371**	373	B	53.468	1
**4371**	381	B	65.2169	1
**4371**	382	B	50.3264	1
**4371**	386	B	65.0223	1
**4371**	305	C	75.0297	1
**4371**	319	C	65.6353	1
**4371**	320	C	37.9866	-1
**4371**	321	C	62.5094	0
**4371**	351	C	11.4001	1
**4371**	357	C	73.3983	1
**4371**	370	C	54.6592	1
**4371**	372	C	66.9298	0
**4371**	373	C	79.6005	1
**4371**	381	C	60.7745	1

**4371**	382	C	72.1131	1
**4371**	386	C	51.0129	1
**4371**	305	D	59.3764	1
**4371**	319	D	67.8155	1
**4371**	320	D	79.5959	-1
**4371**	321	D	40.352	1
**4371**	351	D	12.1275	1
**4371**	357	D	70.5925	1
**4371**	370	D	54.5207	1
**4371**	372	D	59.4478	0
**4371**	373	D	72.2667	1
**4371**	381	D	67.833	1
**4371**	382	D	79.5847	1
**4371**	386	D	28.2036	0
**4372**	305	A	70.6543	1
**4372**	319	A	57.6008	1
**4372**	320	A	79.5539	-1
**4372**	321	A	63.0712	0
**4372**	351	A	6.449	1
**4372**	357	A	69.4178	1
**4372**	370	A	48.4911	0
**4372**	372	A	33.455	1
**4372**	373	A	72.9569	1
**4372**	381	A	68.6323	1
**4372**	382	A	56.682	1
**4372**	386	A	70.988	1
**4372**	305	B	77.1428	1
**4372**	319	B	54.5963	1
**4372**	320	B	44.4514	-1
**4372**	321	B	79.5711	0
**4372**	351	B	33.3147	-1
**4372**	357	B	72.8784	1
**4372**	370	B	75.662	1
**4372**	372	B	79.6338	0

**4372**	373	B	57.2495	1
**4372**	381	B	79.7423	1
**4372**	382	B	66.63	1
**4372**	386	B	79.4539	1
**4372**	305	C	78.075	1
**4372**	319	C	63.2925	1
**4372**	320	C	52.5681	-1
**4372**	321	C	67.3792	0
**4372**	351	C	19.2091	1
**4372**	357	C	71.8535	1
**4372**	370	C	71.843	1
**4372**	372	C	71.2526	0
**4372**	373	C	79.5375	1
**4372**	381	C	69.9258	1
**4372**	382	C	74.3297	1
**4372**	386	C	19.6567	-1
**4372**	305	D	34.4282	0
**4372**	319	D	77.7017	1
**4372**	320	D	79.5444	-1
**4372**	321	D	79.5164	0
**4372**	351	D	18.5462	1
**4372**	357	D	73.3004	1
**4372**	370	D	79.6796	1
**4372**	372	D	75.5473	0
**4372**	373	D	77.0216	1
**4372**	381	D	74.4056	1
**4372**	382	D	61.7279	1
**4372**	386	D	79.5258	1
**4373**	305	A	70.0752	1
**4373**	319	A	58.4587	1
**4373**	320	A	79.5935	-1
**4373**	321	A	75.7312	0
**4373**	351	A	13.43	1
**4373**	357	A	72.8641	1

**4373**	370	A	41.4284	0
**4373**	372	A	72.3821	0
**4373**	373	A	18.0136	-1
**4373**	381	A	62.8302	1
**4373**	382	A	64.4778	1
**4373**	386	A	70.8696	1
**4373**	305	B	79.5416	1
**4373**	319	B	79.5105	1
**4373**	320	B	77.9509	-1
**4373**	321	B	59.9807	0
**4373**	351	B	0.9388	1
**4373**	357	B	40.191	0
**4373**	370	B	74.6053	1
**4373**	372	B	63.2208	0
**4373**	373	B	79.5186	1
**4373**	381	B	72.7752	1
**4373**	382	B	63.8085	1
**4373**	386	B	46.6869	0
**4373**	305	C	74.5962	1
**4373**	319	C	78.6892	1
**4373**	320	C	74.3828	-1
**4373**	321	C	34.2478	1
**4373**	351	C	4.3262	1
**4373**	357	C	71.0637	1
**4373**	370	C	79.5409	1
**4373**	372	C	75.0348	0
**4373**	373	C	79.5529	1
**4373**	381	C	73.4388	1
**4373**	382	C	79.6327	1
**4373**	386	C	14.5311	-1
**4373**	305	D	50.4951	1
**4373**	319	D	76.7854	1
**4373**	320	D	60.4253	-1
**4373**	321	D	72.8016	0

**4373**	351	D	0.1463	1
**4373**	357	D	79.5943	1
**4373**	370	D	65.6588	1
**4373**	372	D	60.7408	0
**4373**	373	D	22.6025	0
**4373**	381	D	79.5363	1
**4373**	382	D	71.7496	1
**4373**	386	D	74.5144	1
**4374**	305	A	72.2815	1
**4374**	319	A	61.4917	1
**4374**	320	A	79.5435	-1
**4374**	321	A	70.2766	0
**4374**	351	A	9.9544	1
**4374**	357	A	69.5717	1
**4374**	370	A	58.5538	1
**4374**	372	A	57.584	0
**4374**	373	A	78.6686	1
**4374**	381	A	73.0244	1
**4374**	382	A	79.4312	1
**4374**	386	A	58.7047	1
**4374**	305	B	79.5831	1
**4374**	319	B	73.7062	1
**4374**	320	B	65.9027	-1
**4374**	321	B	79.6332	0
**4374**	351	B	13.3074	1
**4374**	357	B	69.2863	1
**4374**	370	B	38.8523	0
**4374**	372	B	68.2496	0
**4374**	373	B	78.016	1
**4374**	381	B	79.5426	1
**4374**	382	B	41.2397	0
**4374**	386	B	79.5906	1
**4374**	305	C	70.1637	1
**4374**	319	C	78.5627	1

**4374**	320	C	62.4867	-1
**4374**	321	C	58.4991	0
**4374**	351	C	4.7374	1
**4374**	357	C	79.5896	1
**4374**	370	C	59.3885	1
**4374**	372	C	59.7988	0
**4374**	373	C	75.2587	1
**4374**	381	C	72.3049	1
**4374**	382	C	28.4923	0
**4374**	386	C	60.6395	1
**4374**	305	D	57.5273	1
**4374**	319	D	66.1724	1
**4374**	320	D	70.66	-1
**4374**	321	D	71.5876	0
**4374**	351	D	2.8944	1
**4374**	357	D	79.4931	1
**4374**	370	D	76.7118	1
**4374**	372	D	59.424	0
**4374**	373	D	71.8338	1
**4374**	381	D	79.6002	1
**4374**	382	D	79.6265	1
**4374**	386	D	57.0329	1
**4375**	305	A	73.2255	1
**4375**	319	A	79.5289	1
**4375**	320	A	72.4047	-1
**4375**	321	A	79.3885	0
**4375**	351	A	10.1081	1
**4375**	357	A	44.4456	0
**4375**	370	A	79.1949	1
**4375**	372	A	73.3139	0
**4375**	373	A	74.8924	1
**4375**	381	A	65.2932	1
**4375**	382	A	42.4291	0
**4375**	386	A	64.4818	1

**4375**	305	B	76.6509	1
**4375**	319	B	41.389	0
**4375**	320	B	78.7199	-1
**4375**	321	B	53.2954	0
**4375**	351	B	0.1548	1
**4375**	357	B	79.5555	1
**4375**	370	B	58.121	1
**4375**	372	B	79.5018	0
**4375**	373	B	75.4179	1
**4375**	381	B	79.6181	1
**4375**	382	B	79.6194	1
**4375**	386	B	79.556	1
**4375**	305	C	75.8904	1
**4375**	319	C	73.6611	1
**4375**	320	C	79.5515	-1
**4375**	321	C	79.5382	0
**4375**	351	C	6.7524	1
**4375**	357	C	79.541	1
**4375**	370	C	79.5921	1
**4375**	372	C	78.2041	0
**4375**	373	C	77.0828	1
**4375**	381	C	72.038	1
**4375**	382	C	28.2581	0
**4375**	386	C	54.1725	1
**4375**	305	D	58.3698	1
**4375**	319	D	64.8145	1
**4375**	320	D	55.4081	-1
**4375**	321	D	29.2341	1
**4375**	351	D	17.5214	1
**4375**	357	D	79.5275	1
**4375**	370	D	79.5371	1
**4375**	372	D	73.013	0
**4375**	373	D	48.3875	0
**4375**	381	D	26.4462	0

**4375**	382	D	45.9622	0
**4375**	386	D	75.5933	1
**4376**	305	A	64.6447	1
**4376**	319	A	75.0938	1
**4376**	320	A	79.5101	-1
**4376**	321	A	76.8431	0
**4376**	351	A	0.9852	1
**4376**	357	A	79.5705	1
**4376**	370	A	44.2203	0
**4376**	372	A	59.7634	0
**4376**	373	A	79.2602	1
**4376**	381	A	61.2796	1
**4376**	382	A	74.6265	1
**4376**	386	A	70.4273	1
**4376**	305	B	69.9818	1
**4376**	319	B	72.7012	1
**4376**	320	B	69.589	-1
**4376**	321	B	79.5027	0
**4376**	351	B	7.1683	1
**4376**	357	B	59.9667	1
**4376**	370	B	79.6097	1
**4376**	372	B	46.7741	1
**4376**	373	B	57.7788	1
**4376**	381	B	74.3196	1
**4376**	382	B	33.6343	0
**4376**	386	B	40.381	0
**4376**	305	C	66.1203	1
**4376**	319	C	79.519	1
**4376**	320	C	75.8997	-1
**4376**	321	C	74.3082	0
**4376**	351	C	5.9172	1
**4376**	357	C	73.8391	1
**4376**	370	C	42.5566	0
**4376**	372	C	79.4769	0

**4376**	373	C	79.6233	1
**4376**	381	C	66.4959	1
**4376**	382	C	76.6607	1
**4376**	386	C	20.0389	0
**4376**	305	D	79.5983	1
**4376**	319	D	62.8473	1
**4376**	320	D	79.5883	-1
**4376**	321	D	45.541	1
**4376**	351	D	18.2347	1
**4376**	357	D	75.2099	1
**4376**	370	D	49.1052	0
**4376**	372	D	79.5878	0
**4376**	373	D	71.0891	1
**4376**	381	D	60.9561	1
**4376**	382	D	79.5742	1
**4376**	386	D	74.0414	1
**4377**	305	A	67.2631	1
**4377**	319	A	79.6309	1
**4377**	320	A	79.5551	-1
**4377**	321	A	64.6611	0
**4377**	351	A	2.7954	1
**4377**	357	A	32.9147	0
**4377**	370	A	79.5678	1
**4377**	372	A	79.5574	0
**4377**	373	A	79.6191	1
**4377**	381	A	76.9414	1
**4377**	382	A	79.6157	1
**4377**	386	A	58.8417	1
**4377**	305	B	72.1144	1
**4377**	319	B	79.5632	1
**4377**	320	B	69.2578	-1
**4377**	321	B	72.3472	0
**4377**	351	B	5.8877	1
**4377**	357	B	71.8514	1

**4377**	370	B	49.3897	0
**4377**	372	B	79.6888	0
**4377**	373	B	75.5798	1
**4377**	381	B	79.6127	1
**4377**	382	B	32.6931	0
**4377**	386	B	61.1135	1
**4377**	305	C	75.6784	1
**4377**	319	C	79.5404	1
**4377**	320	C	67.7905	-1
**4377**	321	C	54.3153	0
**4377**	351	C	8.279	1
**4377**	357	C	44.2399	0
**4377**	370	C	32.0582	0
**4377**	372	C	68.1375	0
**4377**	373	C	23.0971	0
**4377**	381	C	56.1899	1
**4377**	382	C	56.2541	1
**4377**	386	C	39.2863	0
**4377**	305	D	79.6083	1
**4377**	319	D	79.5444	1
**4377**	320	D	78.0016	-1
**4377**	321	D	79.6005	0
**4377**	351	D	18.4727	1
**4377**	357	D	79.4904	1
**4377**	370	D	75.697	1
**4377**	372	D	73.7297	0
**4377**	373	D	71.8542	1
**4377**	381	D	50.0639	1
**4377**	382	D	64.1363	1
**4377**	386	D	51.3511	1
**4378**	305	A	72.7721	1
**4378**	319	A	79.5811	1
**4378**	320	A	69.3554	-1
**4378**	321	A	72.9951	0

**4378**	351	A	4.8681	1
**4378**	357	A	79.5499	1
**4378**	370	A	79.5736	1
**4378**	372	A	33.9018	1
**4378**	373	A	79.565	1
**4378**	381	A	79.5457	1
**4378**	382	A	70.7822	1
**4378**	386	A	72.9807	1
**4378**	305	B	61.0792	1
**4378**	319	B	6.7217	-1
**4378**	320	B	47.2904	-1
**4378**	321	B	51.387	0
**4378**	351	B	13.5987	1
**4378**	357	B	47.1899	0
**4378**	370	B	57.1452	1
**4378**	372	B	79.5745	0
**4378**	373	B	29.254	0
**4378**	381	B	73.8913	1
**4378**	382	B	20.8328	0
**4378**	386	B	77.5083	1
**4378**	305	C	75.2703	1
**4378**	319	C	39.1153	0
**4378**	320	C	28.5804	-1
**4378**	321	C	72.9766	0
**4378**	351	C	42.5292	-1
**4378**	357	C	72.1556	1
**4378**	370	C	66.8439	1
**4378**	372	C	67.1337	0
**4378**	373	C	71.9542	1
**4378**	381	C	79.5461	1
**4378**	382	C	27.078	0
**4378**	386	C	4.6917	-1
**4378**	305	D	72.7694	1
**4378**	319	D	73.3229	1

**4378**	320	D	48.6557	-1
**4378**	321	D	60.1827	0
**4378**	351	D	2.5011	1
**4378**	357	D	79.502	1
**4378**	370	D	57.6808	1
**4378**	372	D	72.8567	0
**4378**	373	D	71.6891	1
**4378**	381	D	79.5493	1
**4378**	382	D	73.0488	1
**4378**	386	D	79.5281	1
**4379**	305	A	78.3596	1
**4379**	319	A	51.0253	1
**4379**	320	A	79.5442	-1
**4379**	321	A	79.5167	0
**4379**	351	A	1.7923	1
**4379**	357	A	54.7929	1
**4379**	370	A	79.5222	1
**4379**	372	A	72.2794	0
**4379**	373	A	79.622	1
**4379**	381	A	73.7111	1
**4379**	382	A	76.1635	1
**4379**	386	A	73.6173	1
**4379**	305	B	79.4885	1
**4379**	319	B	61.3314	1
**4379**	320	B	73.1601	-1
**4379**	321	B	72.5764	0
**4379**	351	B	12.6109	1
**4379**	357	B	72.2876	1
**4379**	370	B	72.4724	1
**4379**	372	B	73.2912	0
**4379**	373	B	61.2516	1
**4379**	381	B	79.4622	1
**4379**	382	B	66.8638	1
**4379**	386	B	79.5281	1

**4379**	305	C	76.5221	1
**4379**	319	C	79.5971	1
**4379**	320	C	45.6473	-1
**4379**	321	C	74.5012	0
**4379**	351	C	1.0801	1
**4379**	357	C	38.7854	0
**4379**	370	C	75.9355	1
**4379**	372	C	79.612	0
**4379**	373	C	62.2811	1
**4379**	381	C	79.5171	1
**4379**	382	C	47.5246	0
**4379**	386	C	35.1537	0
**4379**	305	D	79.6355	1
**4379**	319	D	49.3348	0
**4379**	320	D	79.5497	-1
**4379**	321	D	58.9367	0
**4379**	351	D	7.1914	1
**4379**	357	D	79.5951	1
**4379**	370	D	57.2888	1
**4379**	372	D	69.9262	0
**4379**	373	D	79.4778	1
**4379**	381	D	66.0629	1
**4379**	382	D	68.5345	1
**4379**	386	D	45.3102	0
**4380**	305	A	49.9662	0
**4380**	319	A	77.9916	1
**4380**	320	A	73.3145	-1
**4380**	321	A	65.9253	0
**4380**	351	A	8.8846	1
**4380**	357	A	72.4401	1
**4380**	370	A	79.6197	1
**4380**	372	A	79.4614	0
**4380**	373	A	79.6376	1
**4380**	381	A	79.5611	1

**4380**	382	A	41.3491	0
**4380**	386	A	72.2356	1
**4380**	305	B	61.4789	1
**4380**	319	B	52.4796	1
**4380**	320	B	68.0284	-1
**4380**	321	B	69.4602	0
**4380**	351	B	6.7727	1
**4380**	357	B	46.5854	0
**4380**	370	B	72.0337	1
**4380**	372	B	79.7203	0
**4380**	373	B	68.0582	1
**4380**	381	B	59.6134	1
**4380**	382	B	38.1811	0
**4380**	386	B	79.7419	1
**4380**	305	C	79.5445	1
**4380**	319	C	62.1024	1
**4380**	320	C	79.5769	-1
**4380**	321	C	58.0676	0
**4380**	351	C	6.7826	1
**4380**	357	C	48.1827	0
**4380**	370	C	67.7728	1
**4380**	372	C	60.8851	0
**4380**	373	C	79.5928	1
**4380**	381	C	46.1221	0
**4380**	382	C	41.1162	0
**4380**	386	C	55.9527	1
**4380**	305	D	78.5816	1
**4380**	319	D	79.388	1
**4380**	320	D	75.7172	-1
**4380**	321	D	73.2408	0
**4380**	351	D	0.9494	1
**4380**	357	D	72.7953	1
**4380**	370	D	79.5305	1
**4380**	372	D	79.505	0

**4380**	373	D	70.2522	1
**4380**	381	D	69.6805	1
**4380**	382	D	79.6081	1
**4380**	386	D	40.2064	0
**4381**	305	A	57.8646	1
**4381**	319	A	69.6866	1
**4381**	320	A	79.5153	-1
**4381**	321	A	34.2809	1
**4381**	351	A	4.2353	1
**4381**	357	A	70.1537	1
**4381**	370	A	68.6507	1
**4381**	372	A	59.0068	0
**4381**	373	A	14.9582	-1
**4381**	381	A	76.4222	1
**4381**	382	A	19.4093	-1
**4381**	386	A	79.5823	1
**4381**	305	B	79.5584	1
**4381**	319	B	79.2605	1
**4381**	320	B	79.485	-1
**4381**	321	B	78.9403	0
**4381**	351	B	8.7477	1
**4381**	357	B	74.2984	1
**4381**	370	B	68.4409	1
**4381**	372	B	79.5668	0
**4381**	373	B	26.4367	0
**4381**	381	B	79.3631	1
**4381**	382	B	56.1358	1
**4381**	386	B	59.0377	1
**4381**	305	C	78.5916	1
**4381**	319	C	65.6255	1
**4381**	320	C	47.0805	-1
**4381**	321	C	29.5694	1
**4381**	351	C	1.6774	1
**4381**	357	C	78.5786	1

**4381**	370	C	76.3431	1
**4381**	372	C	79.3187	0
**4381**	373	C	62.8406	1
**4381**	381	C	79.4292	1
**4381**	382	C	78.299	1
**4381**	386	C	32.0127	0
**4381**	305	D	37.5175	0
**4381**	319	D	59.7459	1
**4381**	320	D	74.1462	-1
**4381**	321	D	76.1233	0
**4381**	351	D	21.4728	-1
**4381**	357	D	56.5366	1
**4381**	370	D	66.3008	1
**4381**	372	D	73.1222	0
**4381**	373	D	70.5239	1
**4381**	381	D	76.0809	1
**4381**	382	D	63.8625	1
**4381**	386	D	70.8634	1
**4382**	305	A	79.3747	1
**4382**	319	A	62.2269	1
**4382**	320	A	65.4991	-1
**4382**	321	A	75.2047	0
**4382**	351	A	4.5617	1
**4382**	357	A	78.3995	1
**4382**	370	A	54.9237	1
**4382**	372	A	79.5282	0
**4382**	373	A	22.6153	0
**4382**	381	A	60.7998	1
**4382**	382	A	73.4482	1
**4382**	386	A	40.9432	0
**4382**	305	B	79.0356	1
**4382**	319	B	79.5617	1
**4382**	320	B	65.0502	-1
**4382**	321	B	79.4742	0

**4382**	351	B	20.7584	-1
**4382**	357	B	73.553	1
**4382**	370	B	72.8048	1
**4382**	372	B	41.2968	1
**4382**	373	B	71.2683	1
**4382**	381	B	79.5554	1
**4382**	382	B	43.7244	0
**4382**	386	B	58.6451	1
**4382**	305	C	47.7805	0
**4382**	319	C	60.243	1
**4382**	320	C	55.8555	-1
**4382**	321	C	72.3136	0
**4382**	351	C	11.8711	1
**4382**	357	C	79.5443	1
**4382**	370	C	68.9168	1
**4382**	372	C	79.5254	0
**4382**	373	C	72.5464	1
**4382**	381	C	79.5741	1
**4382**	382	C	56.1388	1
**4382**	386	C	53.7085	1
**4382**	305	D	10.7155	-1
**4382**	319	D	29.6704	0
**4382**	320	D	77.8313	-1
**4382**	321	D	56.6736	0
**4382**	351	D	13.3645	1
**4382**	357	D	79.5831	1
**4382**	370	D	44.1638	0
**4382**	372	D	73.7948	0
**4382**	373	D	74.7106	1
**4382**	381	D	55.3196	1
**4382**	382	D	79.4597	1
**4382**	386	D	67.5571	1
**4383**	305	A	73.0457	1
**4383**	319	A	20.8557	0

**4383**	320	A	72.8081	-1
**4383**	321	A	79.5027	0
**4383**	351	A	6.4757	1
**4383**	357	A	44.5957	0
**4383**	370	A	78.5706	1
**4383**	372	A	42.0703	1
**4383**	373	A	72.0466	1
**4383**	381	A	66.477	1
**4383**	382	A	52.9028	1
**4383**	386	A	61.9211	1
**4383**	305	B	79.5808	1
**4383**	319	B	43.2548	0
**4383**	320	B	79.6304	-1
**4383**	321	B	54.9832	0
**4383**	351	B	13.9749	1
**4383**	357	B	70.7526	1
**4383**	370	B	79.5834	1
**4383**	372	B	61.0089	0
**4383**	373	B	47.746	0
**4383**	381	B	48.3262	0
**4383**	382	B	43.1444	0
**4383**	386	B	76.7886	1
**4383**	305	C	66.5562	1
**4383**	319	C	72.9178	1
**4383**	320	C	73.1568	-1
**4383**	321	C	79.5501	0
**4383**	351	C	0.5888	1
**4383**	357	C	69.8051	1
**4383**	370	C	76.4032	1
**4383**	372	C	71.979	0
**4383**	373	C	79.1829	1
**4383**	381	C	79.548	1
**4383**	382	C	26.2585	0
**4383**	386	C	47.4201	0

**4383**	305	D	60.7567	1
**4383**	319	D	73.9377	1
**4383**	320	D	64.4809	-1
**4383**	321	D	60.5095	0
**4383**	351	D	17.2929	1
**4383**	357	D	40.7535	0
**4383**	370	D	69.3355	1
**4383**	372	D	73.3305	0
**4383**	373	D	79.4999	1
**4383**	381	D	62.8114	1
**4383**	382	D	47.6344	0
**4383**	386	D	57.3849	1
**4384**	305	A	62.0034	1
**4384**	319	A	63.4208	1
**4384**	320	A	79.4842	-1
**4384**	321	A	74.2169	0
**4384**	351	A	8.9081	1
**4384**	357	A	44.4539	0
**4384**	370	A	79.7142	1
**4384**	372	A	62.6588	0
**4384**	373	A	78.8271	1
**4384**	381	A	50.2386	1
**4384**	382	A	37.6509	0
**4384**	386	A	35.7955	0
**4384**	305	B	70.681	1
**4384**	319	B	64.6029	1
**4384**	320	B	79.6474	-1
**4384**	321	B	76.587	0
**4384**	351	B	16.2582	1
**4384**	357	B	53.7559	1
**4384**	370	B	79.6315	1
**4384**	372	B	79.5248	0
**4384**	373	B	68.1659	1
**4384**	381	B	66.4982	1

**4384**	382	B	26.2506	0
**4384**	386	B	66.9466	1
**4384**	305	C	79.5109	1
**4384**	319	C	69.6508	1
**4384**	320	C	79.042	-1
**4384**	321	C	55.2624	0
**4384**	351	C	13.8968	1
**4384**	357	C	79.5191	1
**4384**	370	C	53.072	1
**4384**	372	C	66.7146	0
**4384**	373	C	79.6103	1
**4384**	381	C	79.5254	1
**4384**	382	C	44.4182	0
**4384**	386	C	20.0429	0
**4384**	305	D	64.5401	1
**4384**	319	D	72.4151	1
**4384**	320	D	70.2376	-1
**4384**	321	D	61.4287	0
**4384**	351	D	7.7717	1
**4384**	357	D	78.2703	1
**4384**	370	D	33.2176	0
**4384**	372	D	64.6589	0
**4384**	373	D	70.6923	1
**4384**	381	D	24.2267	0
**4384**	382	D	56.7358	1
**4384**	386	D	41.3569	0
**4385**	305	A	44.1583	0
**4385**	319	A	51.7736	1
**4385**	320	A	75.8166	-1
**4385**	321	A	25.3538	1
**4385**	351	A	9.6438	1
**4385**	357	A	69.5753	1
**4385**	370	A	71.2872	1
**4385**	372	A	37.0726	1

**4385**	373	A	56.2486	1
**4385**	381	A	73.3747	1
**4385**	382	A	77.918	1
**4385**	386	A	53.3243	1
**4385**	305	B	70.4861	1
**4385**	319	B	73.6755	1
**4385**	320	B	79.5673	-1
**4385**	321	B	59.8341	0
**4385**	351	B	16.8004	1
**4385**	357	B	65.4826	1
**4385**	370	B	68.8473	1
**4385**	372	B	79.6114	0
**4385**	373	B	67.7176	1
**4385**	381	B	55.1103	1
**4385**	382	B	35.6294	0
**4385**	386	B	70.8228	1
**4385**	305	C	79.3615	1
**4385**	319	C	78.8171	1
**4385**	320	C	50.7858	-1
**4385**	321	C	49.1665	1
**4385**	351	C	0.5969	1
**4385**	357	C	79.5092	1
**4385**	370	C	79.4986	1
**4385**	372	C	77.3146	0
**4385**	373	C	78.6714	1
**4385**	381	C	56.7311	1
**4385**	382	C	79.5757	1
**4385**	386	C	32.7244	0
**4385**	305	D	52.7456	1
**4385**	319	D	62.5927	1
**4385**	320	D	28.515	-1
**4385**	321	D	79.2231	0
**4385**	351	D	11.2551	1
**4385**	357	D	67.8611	1

**4385**	370	D	79.5303	1
**4385**	372	D	74.3728	0
**4385**	373	D	71.6245	1
**4385**	381	D	58.6846	1
**4385**	382	D	53.8803	1
**4385**	386	D	79.5421	1
**4386**	305	A	58.4932	1
**4386**	319	A	68.8279	1
**4386**	320	A	72.5425	-1
**4386**	321	A	77.45	0
**4386**	351	A	3.6401	1
**4386**	357	A	79.6816	1
**4386**	370	A	59.4434	1
**4386**	372	A	73.6177	0
**4386**	373	A	72.099	1
**4386**	381	A	71.7309	1
**4386**	382	A	78.8558	1
**4386**	386	A	34.4583	0
**4386**	305	B	68.4204	1
**4386**	319	B	67.5523	1
**4386**	320	B	60.0036	-1
**4386**	321	B	75.242	0
**4386**	351	B	38.2135	-1
**4386**	357	B	79.6393	1
**4386**	370	B	73.0158	1
**4386**	372	B	61.8972	0
**4386**	373	B	14.4633	-1
**4386**	381	B	79.5674	1
**4386**	382	B	53.4771	1
**4386**	386	B	77.822	1
**4386**	305	C	79.6539	1
**4386**	319	C	67.4005	1
**4386**	320	C	79.5637	-1
**4386**	321	C	72.4121	0

**4386**	351	C	47.2125	-1
**4386**	357	C	58.7992	1
**4386**	370	C	70.8835	1
**4386**	372	C	69.206	0
**4386**	373	C	79.5486	1
**4386**	381	C	79.5558	1
**4386**	382	C	63.7843	1
**4386**	386	C	57.0449	1
**4386**	305	D	59.4626	1
**4386**	319	D	73.7385	1
**4386**	320	D	79.5582	-1
**4386**	321	D	79.5629	0
**4386**	351	D	2.1441	1
**4386**	357	D	51.5507	1
**4386**	370	D	79.5787	1
**4386**	372	D	77.1023	0
**4386**	373	D	38.2321	0
**4386**	381	D	60.6571	1
**4386**	382	D	73.4897	1
**4386**	386	D	79.5492	1
**4387**	305	A	67.3125	1
**4387**	319	A	79.6679	1
**4387**	320	A	66.2424	-1
**4387**	321	A	72.8391	0
**4387**	351	A	10.3339	1
**4387**	357	A	79.6196	1
**4387**	370	A	79.3587	1
**4387**	372	A	61.7281	0
**4387**	373	A	55.4609	1
**4387**	381	A	68.2426	1
**4387**	382	A	11.2093	-1
**4387**	386	A	67.0466	1
**4387**	305	B	70.1901	1
**4387**	319	B	74.7593	1

**4387**	320	B	67.4568	-1
**4387**	321	B	79.6511	0
**4387**	351	B	6.7782	1
**4387**	357	B	79.6295	1
**4387**	370	B	79.5883	1
**4387**	372	B	79.6392	0
**4387**	373	B	38.2061	0
**4387**	381	B	79.6387	1
**4387**	382	B	35.9297	0
**4387**	386	B	71.1268	1
**4387**	305	C	73.8377	1
**4387**	319	C	67.7668	1
**4387**	320	C	30.5304	-1
**4387**	321	C	64.6339	0
**4387**	351	C	5.531	1
**4387**	357	C	58.1596	1
**4387**	370	C	60.0266	1
**4387**	372	C	61.9504	0
**4387**	373	C	79.2337	1
**4387**	381	C	78.9231	1
**4387**	382	C	74.7365	1
**4387**	386	C	59.876	1
**4387**	305	D	64.5518	1
**4387**	319	D	48.4857	0
**4387**	320	D	73.2256	-1
**4387**	321	D	79.5718	0
**4387**	351	D	39.6259	-1
**4387**	357	D	57.2864	1
**4387**	370	D	50.943	1
**4387**	372	D	64.7059	0
**4387**	373	D	57.3012	1
**4387**	381	D	73.6573	1
**4387**	382	D	67.1642	1
**4387**	386	D	47.1097	0

**4388**	305	A	54.4048	1
**4388**	319	A	53.1775	1
**4388**	320	A	55.1276	-1
**4388**	321	A	74.2024	0
**4388**	351	A	5.2911	1
**4388**	357	A	49.252	0
**4388**	370	A	73.9565	1
**4388**	372	A	52.0461	0
**4388**	373	A	58.3501	1
**4388**	381	A	79.5511	1
**4388**	382	A	79.5735	1
**4388**	386	A	50.7805	1
**4388**	305	B	79.5607	1
**4388**	319	B	58.8729	1
**4388**	320	B	54.5495	-1
**4388**	321	B	18.9482	0
**4388**	351	B	18.7577	1
**4388**	357	B	44.6705	0
**4388**	370	B	79.5188	1
**4388**	372	B	71.5503	0
**4388**	373	B	51.9669	1
**4388**	381	B	74.4838	1
**4388**	382	B	73.5145	1
**4388**	386	B	71.3302	1
**4388**	305	C	62.2115	1
**4388**	319	C	44.5594	0
**4388**	320	C	79.5551	-1
**4388**	321	C	79.5331	0
**4388**	351	C	0.0	1
**4388**	357	C	78.6531	1
**4388**	370	C	79.5194	1
**4388**	372	C	79.5012	0
**4388**	373	C	54.131	1
**4388**	381	C	74.5787	1

**4388**	382	C	79.5878	1
**4388**	386	C	42.1173	0
**4388**	305	D	70.1107	1
**4388**	319	D	28.5471	0
**4388**	320	D	51.7316	-1
**4388**	321	D	61.3857	0
**4388**	351	D	6.0694	1
**4388**	357	D	79.6489	1
**4388**	370	D	79.6195	1
**4388**	372	D	70.3363	0
**4388**	373	D	79.5821	1
**4388**	381	D	65.0195	1
**4388**	382	D	68.6417	1
**4388**	386	D	72.5719	1
**4389**	305	A	72.5425	1
**4389**	319	A	58.476	1
**4389**	320	A	79.5774	-1
**4389**	321	A	63.8155	0
**4389**	351	A	8.3829	1
**4389**	357	A	32.3442	0
**4389**	370	A	79.5214	1
**4389**	372	A	72.8577	0
**4389**	373	A	79.5773	1
**4389**	381	A	74.5605	1
**4389**	382	A	72.3324	1
**4389**	386	A	79.5902	1
**4389**	305	B	67.3158	1
**4389**	319	B	58.4607	1
**4389**	320	B	63.7582	-1
**4389**	321	B	67.8925	0
**4389**	351	B	13.879	1
**4389**	357	B	71.5319	1
**4389**	370	B	79.4525	1
**4389**	372	B	71.3148	0

**4389**	373	B	79.5221	1
**4389**	381	B	70.3021	1
**4389**	382	B	27.2146	0
**4389**	386	B	65.3523	1
**4389**	305	C	56.519	1
**4389**	319	C	79.4189	1
**4389**	320	C	76.7832	-1
**4389**	321	C	76.0072	0
**4389**	351	C	9.8633	1
**4389**	357	C	73.1661	1
**4389**	370	C	73.6987	1
**4389**	372	C	72.7872	0
**4389**	373	C	79.5689	1
**4389**	381	C	44.6797	0
**4389**	382	C	45.9803	0
**4389**	386	C	55.4669	1
**4389**	305	D	44.8367	0
**4389**	319	D	67.6971	1
**4389**	320	D	79.5384	-1
**4389**	321	D	58.1957	0
**4389**	351	D	8.1393	1
**4389**	357	D	79.6115	1
**4389**	370	D	74.7046	1
**4389**	372	D	73.382	0
**4389**	373	D	79.5475	1
**4389**	381	D	68.3146	1
**4389**	382	D	63.601	1
**4389**	386	D	25.2285	0
**4390**	305	A	33.2717	0
**4390**	319	A	67.5401	1
**4390**	320	A	69.3707	-1
**4390**	321	A	61.3634	0
**4390**	351	A	5.9874	1
**4390**	357	A	71.7452	1

**4390**	370	A	62.223	1
**4390**	372	A	41.9059	1
**4390**	373	A	72.7027	1
**4390**	381	A	77.3684	1
**4390**	382	A	78.36	1
**4390**	386	A	65.7698	1
**4390**	305	B	73.0608	1
**4390**	319	B	12.1926	-1
**4390**	320	B	59.541	-1
**4390**	321	B	79.5953	0
**4390**	351	B	17.3686	1
**4390**	357	B	36.835	0
**4390**	370	B	79.617	1
**4390**	372	B	73.6603	0
**4390**	373	B	27.5606	0
**4390**	381	B	44.0895	0
**4390**	382	B	49.555	0
**4390**	386	B	61.1935	1
**4390**	305	C	54.2321	1
**4390**	319	C	55.3236	1
**4390**	320	C	58.7552	-1
**4390**	321	C	75.1886	0
**4390**	351	C	3.569	1
**4390**	357	C	72.2014	1
**4390**	370	C	44.7538	0
**4390**	372	C	73.0433	0
**4390**	373	C	51.9036	1
**4390**	381	C	78.5421	1
**4390**	382	C	79.5699	1
**4390**	386	C	38.15	0
**4390**	305	D	74.5412	1
**4390**	319	D	73.1856	1
**4390**	320	D	79.5259	-1
**4390**	321	D	78.0461	0

**4390**	351	D	11.5695	1
**4390**	357	D	79.5493	1
**4390**	370	D	79.3904	1
**4390**	372	D	58.4666	0
**4390**	373	D	72.9681	1
**4390**	381	D	79.4955	1
**4390**	382	D	79.5112	1
**4390**	386	D	21.1931	0
**4391**	305	A	77.8123	1
**4391**	319	A	74.1414	1
**4391**	320	A	71.3116	-1
**4391**	321	A	69.9569	0
**4391**	351	A	16.0246	1
**4391**	357	A	66.2811	1
**4391**	370	A	73.1731	1
**4391**	372	A	49.8919	1
**4391**	373	A	79.5334	1
**4391**	381	A	79.6404	1
**4391**	382	A	72.5023	1
**4391**	386	A	75.8047	1
**4391**	305	B	48.5148	0
**4391**	319	B	57.2719	1
**4391**	320	B	63.8299	-1
**4391**	321	B	72.4916	0
**4391**	351	B	13.1244	1
**4391**	357	B	42.4224	0
**4391**	370	B	79.5396	1
**4391**	372	B	64.5938	0
**4391**	373	B	68.3815	1
**4391**	381	B	73.6756	1
**4391**	382	B	38.0475	0
**4391**	386	B	64.6857	1
**4391**	305	C	70.8916	1
**4391**	319	C	79.5351	1

**4391**	320	C	58.9928	-1
**4391**	321	C	70.0584	0
**4391**	351	C	7.8469	1
**4391**	357	C	59.452	1
**4391**	370	C	43.1994	0
**4391**	372	C	79.5309	0
**4391**	373	C	79.4775	1
**4391**	381	C	64.2357	1
**4391**	382	C	5.7293	-1
**4391**	386	C	61.236	1
**4391**	305	D	79.5006	1
**4391**	319	D	69.7736	1
**4391**	320	D	69.8522	-1
**4391**	321	D	44.6025	1
**4391**	351	D	18.8419	1
**4391**	357	D	41.8263	0
**4391**	370	D	62.0724	1
**4391**	372	D	79.5735	0
**4391**	373	D	79.6064	1
**4391**	381	D	46.2714	0
**4391**	382	D	79.5158	1
**4391**	386	D	79.5463	1
**4392**	305	A	77.6367	1
**4392**	319	A	40.2585	0
**4392**	320	A	79.5289	-1
**4392**	321	A	42.7187	1
**4392**	351	A	8.3234	1
**4392**	357	A	65.8984	1
**4392**	370	A	50.3055	1
**4392**	372	A	77.4546	0
**4392**	373	A	42.9336	0
**4392**	381	A	79.5356	1
**4392**	382	A	59.6246	1
**4392**	386	A	58.9349	1

**4392**	305	B	49.6952	0
**4392**	319	B	73.6177	1
**4392**	320	B	61.6999	-1
**4392**	321	B	71.6262	0
**4392**	351	B	20.3645	-1
**4392**	357	B	79.5336	1
**4392**	370	B	79.5088	1
**4392**	372	B	79.4848	0
**4392**	373	B	74.8347	1
**4392**	381	B	76.5503	1
**4392**	382	B	68.5956	1
**4392**	386	B	72.9879	1
**4392**	305	C	79.4736	1
**4392**	319	C	76.8129	1
**4392**	320	C	35.245	-1
**4392**	321	C	79.5574	0
**4392**	351	C	9.8915	1
**4392**	357	C	79.2399	1
**4392**	370	C	79.5208	1
**4392**	372	C	79.6128	0
**4392**	373	C	73.9892	1
**4392**	381	C	79.5395	1
**4392**	382	C	79.5645	1
**4392**	386	C	37.1482	0
**4392**	305	D	42.2245	0
**4392**	319	D	79.5175	1
**4392**	320	D	79.53	-1
**4392**	321	D	79.5783	0
**4392**	351	D	0.0	1
**4392**	357	D	65.5803	1
**4392**	370	D	75.8729	1
**4392**	372	D	56.3837	0
**4392**	373	D	79.5001	1
**4392**	381	D	44.6076	0

**4392**	382	D	79.5148	1
**4392**	386	D	79.5668	1
**4393**	305	A	61.1761	1
**4393**	319	A	45.5419	0
**4393**	320	A	71.9046	-1
**4393**	321	A	68.5084	0
**4393**	351	A	11.2183	1
**4393**	357	A	79.5534	1
**4393**	370	A	79.5526	1
**4393**	372	A	55.3934	0
**4393**	373	A	35.7881	0
**4393**	381	A	76.807	1
**4393**	382	A	54.1382	1
**4393**	386	A	79.5663	1
**4393**	305	B	66.8401	1
**4393**	319	B	79.6565	1
**4393**	320	B	79.6529	-1
**4393**	321	B	76.4268	0
**4393**	351	B	1.9124	1
**4393**	357	B	59.7563	1
**4393**	370	B	67.3495	1
**4393**	372	B	71.9362	0
**4393**	373	B	59.7549	1
**4393**	381	B	60.2935	1
**4393**	382	B	74.7325	1
**4393**	386	B	78.5026	1
**4393**	305	C	53.6854	1
**4393**	319	C	58.544	1
**4393**	320	C	55.7823	-1
**4393**	321	C	79.6492	0
**4393**	351	C	4.3258	1
**4393**	357	C	26.6059	0
**4393**	370	C	77.3129	1
**4393**	372	C	79.6416	0

**4393**	373	C	75.8743	1
**4393**	381	C	69.2071	1
**4393**	382	C	41.6221	0
**4393**	386	C	54.0719	1
**4393**	305	D	35.0079	0
**4393**	319	D	79.6244	1
**4393**	320	D	79.6039	-1
**4393**	321	D	69.5335	0
**4393**	351	D	14.5113	1
**4393**	357	D	73.78	1
**4393**	370	D	64.1948	1
**4393**	372	D	73.3605	0
**4393**	373	D	66.5521	1
**4393**	381	D	79.6778	1
**4393**	382	D	79.65	1
**4393**	386	D	79.5037	1
**4394**	305	A	35.1166	0
**4394**	319	A	46.7381	0
**4394**	320	A	73.4218	-1
**4394**	321	A	24.8399	1
**4394**	351	A	8.1174	1
**4394**	357	A	59.3092	1
**4394**	370	A	46.44	0
**4394**	372	A	70.9951	0
**4394**	373	A	71.281	1
**4394**	381	A	65.6855	1
**4394**	382	A	79.5505	1
**4394**	386	A	30.856	0
**4394**	305	B	79.5488	1
**4394**	319	B	63.1855	1
**4394**	320	B	65.5325	-1
**4394**	321	B	73.3804	0
**4394**	351	B	15.192	1
**4394**	357	B	78.9535	1

**4394**	370	B	79.5831	1
**4394**	372	B	67.0785	0
**4394**	373	B	48.3434	0
**4394**	381	B	79.5696	1
**4394**	382	B	33.4919	0
**4394**	386	B	65.7398	1
**4394**	305	C	76.1735	1
**4394**	319	C	69.2134	1
**4394**	320	C	56.1392	-1
**4394**	321	C	79.5984	0
**4394**	351	C	35.322	-1
**4394**	357	C	79.6383	1
**4394**	370	C	63.7944	1
**4394**	372	C	79.6327	0
**4394**	373	C	52.1006	1
**4394**	381	C	79.3651	1
**4394**	382	C	78.834	1
**4394**	386	C	57.1314	1
**4394**	305	D	63.7808	1
**4394**	319	D	79.4859	1
**4394**	320	D	35.9676	-1
**4394**	321	D	36.7829	1
**4394**	351	D	16.376	1
**4394**	357	D	74.0358	1
**4394**	370	D	61.4959	1
**4394**	372	D	30.4788	1
**4394**	373	D	78.7811	1
**4394**	381	D	72.6061	1
**4394**	382	D	46.4894	0
**4394**	386	D	73.1625	1
**4395**	305	A	55.7765	1
**4395**	319	A	57.3272	1
**4395**	320	A	43.5874	-1
**4395**	321	A	60.2984	0

**4395**	351	A	11.0296	1
**4395**	357	A	77.6004	1
**4395**	370	A	79.5068	1
**4395**	372	A	62.699	0
**4395**	373	A	40.9806	0
**4395**	381	A	71.5072	1
**4395**	382	A	47.8638	0
**4395**	386	A	42.6309	0
**4395**	305	B	79.4806	1
**4395**	319	B	73.9951	1
**4395**	320	B	75.0252	-1
**4395**	321	B	41.6272	1
**4395**	351	B	19.9048	1
**4395**	357	B	13.5949	-1
**4395**	370	B	79.4949	1
**4395**	372	B	51.2702	0
**4395**	373	B	71.5597	1
**4395**	381	B	74.8607	1
**4395**	382	B	59.9139	1
**4395**	386	B	57.466	1
**4395**	305	C	42.416	0
**4395**	319	C	41.5634	0
**4395**	320	C	63.5965	-1
**4395**	321	C	61.8482	0
**4395**	351	C	4.0068	1
**4395**	357	C	79.558	1
**4395**	370	C	68.7071	1
**4395**	372	C	79.6463	0
**4395**	373	C	72.6459	1
**4395**	381	C	65.8484	1
**4395**	382	C	36.6152	0
**4395**	386	C	66.1305	1
**4395**	305	D	38.2914	0
**4395**	319	D	79.5109	1

**4395**	320	D	79.6376	-1
**4395**	321	D	52.6736	0
**4395**	351	D	1.1562	1
**4395**	357	D	48.4192	0
**4395**	370	D	71.7789	1
**4395**	372	D	59.1671	0
**4395**	373	D	77.4431	1
**4395**	381	D	69.1849	1
**4395**	382	D	79.4689	1
**4395**	386	D	68.3687	1
**4396**	305	A	70.4048	1
**4396**	319	A	62.7938	1
**4396**	320	A	56.6653	-1
**4396**	321	A	66.3795	0
**4396**	351	A	14.1506	1
**4396**	357	A	49.1359	0
**4396**	370	A	36.0914	0
**4396**	372	A	73.0222	0
**4396**	373	A	79.5721	1
**4396**	381	A	77.7605	1
**4396**	382	A	79.5846	1
**4396**	386	A	48.6254	0
**4396**	305	B	79.5342	1
**4396**	319	B	68.0251	1
**4396**	320	B	73.1326	-1
**4396**	321	B	54.4817	0
**4396**	351	B	15.6472	1
**4396**	357	B	32.8265	0
**4396**	370	B	55.8845	1
**4396**	372	B	79.5341	0
**4396**	373	B	30.5605	0
**4396**	381	B	55.6448	1
**4396**	382	B	37.6405	0
**4396**	386	B	9.5037	-1

**4396**	305	C	48.2778	0
**4396**	319	C	75.7264	1
**4396**	320	C	51.2093	-1
**4396**	321	C	75.7956	0
**4396**	351	C	0.9702	1
**4396**	357	C	72.7113	1
**4396**	370	C	79.5756	1
**4396**	372	C	79.5453	0
**4396**	373	C	79.5659	1
**4396**	381	C	79.5413	1
**4396**	382	C	65.6229	1
**4396**	386	C	27.3633	0
**4396**	305	D	49.574	0
**4396**	319	D	62.1452	1
**4396**	320	D	72.1407	-1
**4396**	321	D	73.5877	0
**4396**	351	D	14.745	1
**4396**	357	D	33.3396	0
**4396**	370	D	79.5288	1
**4396**	372	D	49.3892	1
**4396**	373	D	77.4255	1
**4396**	381	D	70.7991	1
**4396**	382	D	72.9005	1
**4396**	386	D	79.5668	1
**4397**	305	A	79.1658	1
**4397**	319	A	74.2652	1
**4397**	320	A	72.8273	-1
**4397**	321	A	42.5277	1
**4397**	351	A	10.0475	1
**4397**	357	A	73.1364	1
**4397**	370	A	45.2424	0
**4397**	372	A	65.3179	0
**4397**	373	A	78.9532	1
**4397**	381	A	50.921	1

**4397**	382	A	31.8466	0
**4397**	386	A	79.5464	1
**4397**	305	B	79.0682	1
**4397**	319	B	45.1073	0
**4397**	320	B	49.567	-1
**4397**	321	B	48.7546	1
**4397**	351	B	11.8783	1
**4397**	357	B	54.2905	1
**4397**	370	B	54.8174	1
**4397**	372	B	45.968	1
**4397**	373	B	59.7389	1
**4397**	381	B	79.5547	1
**4397**	382	B	30.4992	0
**4397**	386	B	43.976	0
**4397**	305	C	63.8179	1
**4397**	319	C	60.3461	1
**4397**	320	C	55.0435	-1
**4397**	321	C	63.8245	0
**4397**	351	C	1.5137	1
**4397**	357	C	72.7935	1
**4397**	370	C	34.2007	0
**4397**	372	C	35.3282	1
**4397**	373	C	21.4606	0
**4397**	381	C	30.4522	0
**4397**	382	C	39.8749	0
**4397**	386	C	25.8645	0
**4397**	305	D	66.2904	1
**4397**	319	D	79.5294	1
**4397**	320	D	50.767	-1
**4397**	321	D	50.5494	0
**4397**	351	D	13.8059	1
**4397**	357	D	52.1575	1
**4397**	370	D	71.0323	1
**4397**	372	D	72.8773	0

**4397**	373	D	78.7779	1
**4397**	381	D	78.9817	1
**4397**	382	D	79.5707	1
**4397**	386	D	35.5009	0
**4398**	305	A	77.3172	1
**4398**	319	A	69.3933	1
**4398**	320	A	79.6223	-1
**4398**	321	A	72.7492	0
**4398**	351	A	8.3912	1
**4398**	357	A	42.8477	0
**4398**	370	A	79.5887	1
**4398**	372	A	79.5668	0
**4398**	373	A	79.5583	1
**4398**	381	A	63.284	1
**4398**	382	A	59.655	1
**4398**	386	A	72.5205	1
**4398**	305	B	70.3112	1
**4398**	319	B	56.3815	1
**4398**	320	B	79.6128	-1
**4398**	321	B	42.9222	1
**4398**	351	B	31.5558	-1
**4398**	357	B	79.6343	1
**4398**	370	B	72.6067	1
**4398**	372	B	57.6683	0
**4398**	373	B	72.6435	1
**4398**	381	B	79.5647	1
**4398**	382	B	36.2933	0
**4398**	386	B	69.4526	1
**4398**	305	C	60.1814	1
**4398**	319	C	78.1621	1
**4398**	320	C	72.7814	-1
**4398**	321	C	78.9364	0
**4398**	351	C	32.0753	-1
**4398**	357	C	73.6971	1

**4398**	370	C	31.7954	0
**4398**	372	C	59.797	0
**4398**	373	C	32.9034	0
**4398**	381	C	73.9544	1
**4398**	382	C	79.5324	1
**4398**	386	C	42.0256	0
**4398**	305	D	72.911	1
**4398**	319	D	71.1719	1
**4398**	320	D	31.8022	-1
**4398**	321	D	67.8329	0
**4398**	351	D	7.6676	1
**4398**	357	D	68.4848	1
**4398**	370	D	72.6377	1
**4398**	372	D	79.5615	0
**4398**	373	D	62.2554	1
**4398**	381	D	61.9901	1
**4398**	382	D	73.4916	1
**4398**	386	D	66.3259	1
**4399**	305	A	52.3126	1
**4399**	319	A	71.6955	1
**4399**	320	A	56.3164	-1
**4399**	321	A	76.9242	0
**4399**	351	A	10.5297	1
**4399**	357	A	75.2548	1
**4399**	370	A	79.3158	1
**4399**	372	A	79.434	0
**4399**	373	A	79.6422	1
**4399**	381	A	55.5755	1
**4399**	382	A	58.5274	1
**4399**	386	A	72.6262	1
**4399**	305	B	75.4748	1
**4399**	319	B	58.298	1
**4399**	320	B	29.7427	-1
**4399**	321	B	40.8382	1

**4399**	351	B	13.6634	1
**4399**	357	B	72.971	1
**4399**	370	B	64.9878	1
**4399**	372	B	79.5497	0
**4399**	373	B	79.5914	1
**4399**	381	B	74.4617	1
**4399**	382	B	43.2718	0
**4399**	386	B	79.3497	1
**4399**	305	C	72.4627	1
**4399**	319	C	71.116	1
**4399**	320	C	79.7065	-1
**4399**	321	C	77.3278	0
**4399**	351	C	34.961	-1
**4399**	357	C	78.903	1
**4399**	370	C	79.4733	1
**4399**	372	C	70.5858	0
**4399**	373	C	79.5931	1
**4399**	381	C	72.1062	1
**4399**	382	C	33.5827	0
**4399**	386	C	60.0666	1
**4399**	305	D	59.4666	1
**4399**	319	D	50.3135	1
**4399**	320	D	73.239	-1
**4399**	321	D	68.9036	0
**4399**	351	D	22.6183	-1
**4399**	357	D	79.4635	1
**4399**	370	D	55.2241	1
**4399**	372	D	59.3032	0
**4399**	373	D	79.5889	1
**4399**	381	D	39.5964	0
**4399**	382	D	58.7536	1
**4399**	386	D	79.4371	1
**4400**	305	A	79.5591	1
**4400**	319	A	57.7407	1

**4400**	320	A	79.5671	-1
**4400**	321	A	23.9876	1
**4400**	351	A	10.8154	1
**4400**	357	A	72.9954	1
**4400**	370	A	47.3806	0
**4400**	372	A	62.9639	0
**4400**	373	A	63.1523	1
**4400**	381	A	71.8067	1
**4400**	382	A	79.5917	1
**4400**	386	A	76.453	1
**4400**	305	B	79.5455	1
**4400**	319	B	67.087	1
**4400**	320	B	19.5231	1
**4400**	321	B	71.1378	0
**4400**	351	B	16.7256	1
**4400**	357	B	79.5742	1
**4400**	370	B	79.5489	1
**4400**	372	B	79.5502	0
**4400**	373	B	48.3163	0
**4400**	381	B	23.1173	0
**4400**	382	B	21.7452	0
**4400**	386	B	47.8976	0
**4400**	305	C	73.4045	1
**4400**	319	C	65.5637	1
**4400**	320	C	79.5593	-1
**4400**	321	C	50.3631	0
**4400**	351	C	8.9656	1
**4400**	357	C	59.578	1
**4400**	370	C	74.2979	1
**4400**	372	C	76.5976	0
**4400**	373	C	79.529	1
**4400**	381	C	72.3944	1
**4400**	382	C	43.6781	0
**4400**	386	C	5.9668	-1

**4400**	305	D	41.5433	0
**4400**	319	D	79.5554	1
**4400**	320	D	72.7401	-1
**4400**	321	D	59.0749	0
**4400**	351	D	13.9292	1
**4400**	357	D	73.4345	1
**4400**	370	D	79.5628	1
**4400**	372	D	72.4571	0
**4400**	373	D	74.7559	1
**4400**	381	D	71.454	1
**4400**	382	D	79.5636	1
**4400**	386	D	73.893	1
**4401**	305	A	73.9764	1
**4401**	319	A	79.6861	1
**4401**	320	A	71.2283	-1
**4401**	321	A	66.199	0
**4401**	351	A	10.9483	1
**4401**	357	A	79.5432	1
**4401**	370	A	77.8936	1
**4401**	372	A	48.3973	1
**4401**	373	A	73.2446	1
**4401**	381	A	74.2686	1
**4401**	382	A	63.7893	1
**4401**	386	A	75.4901	1
**4401**	305	B	59.375	1
**4401**	319	B	79.4912	1
**4401**	320	B	53.4441	-1
**4401**	321	B	76.9159	0
**4401**	351	B	5.9959	1
**4401**	357	B	79.6928	1
**4401**	370	B	79.5895	1
**4401**	372	B	79.464	0
**4401**	373	B	33.9414	0
**4401**	381	B	20.3536	0

**4401**	382	B	76.1893	1
**4401**	386	B	73.2248	1
**4401**	305	C	73.7584	1
**4401**	319	C	55.4777	1
**4401**	320	C	64.4656	-1
**4401**	321	C	54.8567	0
**4401**	351	C	0.2302	1
**4401**	357	C	74.4406	1
**4401**	370	C	79.5621	1
**4401**	372	C	63.9978	0
**4401**	373	C	65.7885	1
**4401**	381	C	58.1203	1
**4401**	382	C	73.4395	1
**4401**	386	C	48.3915	0
**4401**	305	D	79.5853	1
**4401**	319	D	79.5491	1
**4401**	320	D	79.5364	-1
**4401**	321	D	48.6155	1
**4401**	351	D	14.7268	1
**4401**	357	D	70.0658	1
**4401**	370	D	74.1263	1
**4401**	372	D	61.7034	0
**4401**	373	D	79.5495	1
**4401**	381	D	79.5113	1
**4401**	382	D	75.3484	1
**4401**	386	D	72.0544	1
**4402**	305	A	76.2355	1
**4402**	319	A	77.1442	1
**4402**	320	A	56.4845	-1
**4402**	321	A	79.5762	0
**4402**	351	A	2.1532	1
**4402**	357	A	79.5491	1
**4402**	370	A	6.3681	-1
**4402**	372	A	68.3366	0

**4402**	373	A	42.3895	0
**4402**	381	A	73.5565	1
**4402**	382	A	64.6856	1
**4402**	386	A	79.5935	1
**4402**	305	B	77.0717	1
**4402**	319	B	66.8131	1
**4402**	320	B	68.4187	-1
**4402**	321	B	79.5719	0
**4402**	351	B	14.7825	1
**4402**	357	B	73.4549	1
**4402**	370	B	74.0706	1
**4402**	372	B	79.5501	0
**4402**	373	B	70.5181	1
**4402**	381	B	79.5412	1
**4402**	382	B	42.0551	0
**4402**	386	B	72.9002	1
**4402**	305	C	60.5149	1
**4402**	319	C	44.7497	0
**4402**	320	C	64.5425	-1
**4402**	321	C	79.5422	0
**4402**	351	C	35.6918	-1
**4402**	357	C	79.5534	1
**4402**	370	C	78.6336	1
**4402**	372	C	79.5439	0
**4402**	373	C	79.5522	1
**4402**	381	C	79.3667	1
**4402**	382	C	74.0102	1
**4402**	386	C	42.1858	0
**4402**	305	D	44.9186	0
**4402**	319	D	33.4329	0
**4402**	320	D	68.5021	-1
**4402**	321	D	37.1851	1
**4402**	351	D	16.223	1
**4402**	357	D	79.5641	1

**4402**	370	D	79.5817	1
**4402**	372	D	79.555	0
**4402**	373	D	74.6881	1
**4402**	381	D	79.5554	1
**4402**	382	D	58.9458	1
**4402**	386	D	45.8452	0
**4403**	305	A	57.2345	1
**4403**	319	A	65.9201	1
**4403**	320	A	53.6804	-1
**4403**	321	A	36.5833	1
**4403**	351	A	5.3708	1
**4403**	357	A	79.6337	1
**4403**	370	A	54.9303	1
**4403**	372	A	49.8258	1
**4403**	373	A	79.488	1
**4403**	381	A	35.1229	0
**4403**	382	A	68.0682	1
**4403**	386	A	72.9175	1
**4403**	305	B	79.5633	1
**4403**	319	B	70.8998	1
**4403**	320	B	79.1232	-1
**4403**	321	B	79.6052	0
**4403**	351	B	13.1743	1
**4403**	357	B	57.1758	1
**4403**	370	B	62.2499	1
**4403**	372	B	79.5776	0
**4403**	373	B	42.444	0
**4403**	381	B	45.6242	0
**4403**	382	B	71.3788	1
**4403**	386	B	34.0573	0
**4403**	305	C	79.6086	1
**4403**	319	C	61.2006	1
**4403**	320	C	72.5725	-1
**4403**	321	C	71.4333	0

**4403**	351	C	36.9098	-1
**4403**	357	C	79.4879	1
**4403**	370	C	79.4933	1
**4403**	372	C	79.2304	0
**4403**	373	C	47.1002	0
**4403**	381	C	71.7383	1
**4403**	382	C	79.5185	1
**4403**	386	C	64.5498	1
**4403**	305	D	71.2721	1
**4403**	319	D	76.4382	1
**4403**	320	D	79.5986	-1
**4403**	321	D	76.2092	0
**4403**	351	D	16.1007	1
**4403**	357	D	68.0021	1
**4403**	370	D	69.882	1
**4403**	372	D	79.4587	0
**4403**	373	D	78.8758	1
**4403**	381	D	79.6007	1
**4403**	382	D	77.2185	1
**4403**	386	D	56.5951	1
**4404**	305	A	79.4513	1
**4404**	319	A	32.7142	0
**4404**	320	A	60.7195	-1
**4404**	321	A	66.5379	0
**4404**	351	A	9.0661	1
**4404**	357	A	79.5013	1
**4404**	370	A	72.5346	1
**4404**	372	A	36.4632	1
**4404**	373	A	28.2247	0
**4404**	381	A	49.366	0
**4404**	382	A	77.8529	1
**4404**	386	A	75.8391	1
**4404**	305	B	50.0796	1
**4404**	319	B	79.6931	1

**4404**	320	B	79.6528	-1
**4404**	321	B	69.3602	0
**4404**	351	B	22.1736	-1
**4404**	357	B	77.6978	1
**4404**	370	B	27.5431	0
**4404**	372	B	73.5603	0
**4404**	373	B	60.7308	1
**4404**	381	B	66.9757	1
**4404**	382	B	74.58	1
**4404**	386	B	79.6769	1
**4404**	305	C	72.2641	1
**4404**	319	C	30.4226	0
**4404**	320	C	72.3276	-1
**4404**	321	C	79.5011	0
**4404**	351	C	3.5762	1
**4404**	357	C	69.6904	1
**4404**	370	C	69.0834	1
**4404**	372	C	79.4898	0
**4404**	373	C	79.6478	1
**4404**	381	C	48.9597	0
**4404**	382	C	76.9414	1
**4404**	386	C	27.5215	0
**4404**	305	D	69.9003	1
**4404**	319	D	79.5812	1
**4404**	320	D	79.4885	-1
**4404**	321	D	79.6099	0
**4404**	351	D	8.8519	1
**4404**	357	D	69.4231	1
**4404**	370	D	62.2959	1
**4404**	372	D	79.518	0
**4404**	373	D	38.6457	0
**4404**	381	D	79.6004	1
**4404**	382	D	79.5173	1
**4404**	386	D	79.6373	1

**4405**	305	A	72.851	1
**4405**	319	A	47.6138	0
**4405**	320	A	75.9783	-1
**4405**	321	A	72.879	0
**4405**	351	A	4.4004	1
**4405**	357	A	71.8781	1
**4405**	370	A	77.6805	1
**4405**	372	A	72.1569	0
**4405**	373	A	48.1417	0
**4405**	381	A	79.5031	1
**4405**	382	A	79.5556	1
**4405**	386	A	53.6215	1
**4405**	305	B	45.2474	0
**4405**	319	B	79.5752	1
**4405**	320	B	72.8203	-1
**4405**	321	B	36.2741	1
**4405**	351	B	14.6697	1
**4405**	357	B	79.528	1
**4405**	370	B	79.5394	1
**4405**	372	B	79.5621	0
**4405**	373	B	63.934	1
**4405**	381	B	45.2304	0
**4405**	382	B	39.0911	0
**4405**	386	B	72.6162	1
**4405**	305	C	64.8703	1
**4405**	319	C	71.6397	1
**4405**	320	C	75.6011	-1
**4405**	321	C	76.9957	0
**4405**	351	C	1.3749	1
**4405**	357	C	51.6155	1
**4405**	370	C	79.5359	1
**4405**	372	C	79.568	0
**4405**	373	C	53.5182	1
**4405**	381	C	71.2433	1

**4405**	382	C	74.9099	1
**4405**	386	C	38.3668	0
**4405**	305	D	68.6891	1
**4405**	319	D	73.8097	1
**4405**	320	D	49.6487	-1
**4405**	321	D	79.5889	0
**4405**	351	D	13.4311	1
**4405**	357	D	51.8819	1
**4405**	370	D	61.2301	1
**4405**	372	D	79.5262	0
**4405**	373	D	72.0362	1
**4405**	381	D	68.2589	1
**4405**	382	D	79.5312	1
**4405**	386	D	79.5764	1
**4406**	305	A	76.4209	1
**4406**	319	A	61.7856	1
**4406**	320	A	65.0056	-1
**4406**	321	A	69.5676	0
**4406**	351	A	28.704	-1
**4406**	357	A	49.6804	0
**4406**	370	A	35.7479	0
**4406**	372	A	79.6098	0
**4406**	373	A	79.5827	1
**4406**	381	A	64.5109	1
**4406**	382	A	79.6065	1
**4406**	386	A	73.6499	1
**4406**	305	B	72.9495	1
**4406**	319	B	72.6025	1
**4406**	320	B	72.3346	-1
**4406**	321	B	70.6849	0
**4406**	351	B	19.8724	1
**4406**	357	B	74.2305	1
**4406**	370	B	79.4682	1
**4406**	372	B	67.3504	0

**4406**	373	B	57.2225	1
**4406**	381	B	67.9613	1
**4406**	382	B	60.8523	1
**4406**	386	B	79.6819	1
**4406**	305	C	78.7244	1
**4406**	319	C	79.6012	1
**4406**	320	C	76.1989	-1
**4406**	321	C	75.3813	0
**4406**	351	C	3.7778	1
**4406**	357	C	79.5414	1
**4406**	370	C	75.8714	1
**4406**	372	C	74.3986	0
**4406**	373	C	77.3501	1
**4406**	381	C	79.4937	1
**4406**	382	C	50.7442	1
**4406**	386	C	40.3631	0
**4406**	305	D	69.1778	1
**4406**	319	D	42.3924	0
**4406**	320	D	53.657	-1
**4406**	321	D	52.3406	0
**4406**	351	D	15.2203	1
**4406**	357	D	50.603	1
**4406**	370	D	70.0152	1
**4406**	372	D	61.5004	0
**4406**	373	D	67.7997	1
**4406**	381	D	79.5936	1
**4406**	382	D	64.9542	1
**4406**	386	D	79.5362	1
**4407**	305	A	75.2469	1
**4407**	319	A	43.5024	0
**4407**	320	A	67.0334	-1
**4407**	321	A	79.5769	0
**4407**	351	A	10.1615	1
**4407**	357	A	79.5306	1

**4407**	370	A	79.5873	1
**4407**	372	A	73.9726	0
**4407**	373	A	37.9281	0
**4407**	381	A	68.7114	1
**4407**	382	A	63.8514	1
**4407**	386	A	54.1427	1
**4407**	305	B	59.7707	1
**4407**	319	B	43.0635	0
**4407**	320	B	79.4867	-1
**4407**	321	B	76.7271	0
**4407**	351	B	15.9382	1
**4407**	357	B	79.6051	1
**4407**	370	B	45.3861	0
**4407**	372	B	68.9554	0
**4407**	373	B	79.065	1
**4407**	381	B	79.6057	1
**4407**	382	B	75.535	1
**4407**	386	B	73.0344	1
**4407**	305	C	69.9113	1
**4407**	319	C	73.8065	1
**4407**	320	C	77.7681	-1
**4407**	321	C	79.5497	0
**4407**	351	C	0.9365	1
**4407**	357	C	79.5195	1
**4407**	370	C	79.6125	1
**4407**	372	C	58.8188	0
**4407**	373	C	46.7743	0
**4407**	381	C	77.192	1
**4407**	382	C	65.7615	1
**4407**	386	C	48.0364	0
**4407**	305	D	79.5852	1
**4407**	319	D	55.6186	1
**4407**	320	D	34.3305	-1
**4407**	321	D	79.6322	0

**4407**	351	D	14.3179	1
**4407**	357	D	79.4956	1
**4407**	370	D	36.1098	0
**4407**	372	D	64.1861	0
**4407**	373	D	79.233	1
**4407**	381	D	71.5305	1
**4407**	382	D	74.7813	1
**4407**	386	D	44.9436	0
**4408**	305	A	79.5641	1
**4408**	319	A	72.3944	1
**4408**	320	A	70.0762	-1
**4408**	321	A	74.5994	0
**4408**	351	A	1.7321	1
**4408**	357	A	79.5402	1
**4408**	370	A	52.2839	1
**4408**	372	A	75.8829	0
**4408**	373	A	79.5154	1
**4408**	381	A	79.6068	1
**4408**	382	A	48.7717	0
**4408**	386	A	68.8883	1
**4408**	305	B	77.7303	1
**4408**	319	B	49.4786	0
**4408**	320	B	71.6376	-1
**4408**	321	B	75.1841	0
**4408**	351	B	15.7159	1
**4408**	357	B	68.8386	1
**4408**	370	B	45.7603	0
**4408**	372	B	67.0637	0
**4408**	373	B	71.8432	1
**4408**	381	B	58.9529	1
**4408**	382	B	77.8372	1
**4408**	386	B	62.2945	1
**4408**	305	C	62.1951	1
**4408**	319	C	59.1189	1

**4408**	320	C	64.2414	-1
**4408**	321	C	24.1345	1
**4408**	351	C	5.6704	1
**4408**	357	C	78.7705	1
**4408**	370	C	63.2929	1
**4408**	372	C	37.8115	1
**4408**	373	C	68.0236	1
**4408**	381	C	36.8013	0
**4408**	382	C	79.5162	1
**4408**	386	C	44.2577	0
**4408**	305	D	73.6299	1
**4408**	319	D	47.3382	0
**4408**	320	D	76.3929	-1
**4408**	321	D	53.2903	0
**4408**	351	D	13.8906	1
**4408**	357	D	79.542	1
**4408**	370	D	60.2993	1
**4408**	372	D	71.8141	0
**4408**	373	D	42.4391	0
**4408**	381	D	52.597	1
**4408**	382	D	73.5771	1
**4408**	386	D	78.8837	1
**4409**	305	A	79.5622	1
**4409**	319	A	36.3622	0
**4409**	320	A	67.1568	-1
**4409**	321	A	79.5617	0
**4409**	351	A	6.5324	1
**4409**	357	A	79.6131	1
**4409**	370	A	68.6243	1
**4409**	372	A	71.9819	0
**4409**	373	A	68.0142	1
**4409**	381	A	79.4666	1
**4409**	382	A	74.9079	1
**4409**	386	A	63.4659	1

**4409**	305	B	79.5636	1
**4409**	319	B	53.2352	1
**4409**	320	B	74.8924	-1
**4409**	321	B	79.5574	0
**4409**	351	B	13.5474	1
**4409**	357	B	55.2332	1
**4409**	370	B	78.5488	1
**4409**	372	B	79.2806	0
**4409**	373	B	69.644	1
**4409**	381	B	36.742	0
**4409**	382	B	67.7287	1
**4409**	386	B	71.0268	1
**4409**	305	C	60.2328	1
**4409**	319	C	75.7639	1
**4409**	320	C	74.8677	-1
**4409**	321	C	72.612	0
**4409**	351	C	2.9674	1
**4409**	357	C	72.8373	1
**4409**	370	C	61.3036	1
**4409**	372	C	79.5002	0
**4409**	373	C	79.6087	1
**4409**	381	C	79.5194	1
**4409**	382	C	79.5509	1
**4409**	386	C	41.7071	0
**4409**	305	D	63.5034	1
**4409**	319	D	72.4842	1
**4409**	320	D	79.5985	-1
**4409**	321	D	70.134	0
**4409**	351	D	20.0237	-1
**4409**	357	D	72.764	1
**4409**	370	D	29.914	0
**4409**	372	D	72.6133	0
**4409**	373	D	73.167	1
**4409**	381	D	30.426	0

**4409**	382	D	73.7736	1
**4409**	386	D	32.3954	0
**4410**	305	A	62.1949	1
**4410**	319	A	77.5373	1
**4410**	320	A	55.9169	-1
**4410**	321	A	78.401	0
**4410**	351	A	9.0931	1
**4410**	357	A	79.5183	1
**4410**	370	A	55.3297	1
**4410**	372	A	57.1353	0
**4410**	373	A	13.0484	-1
**4410**	381	A	60.2769	1
**4410**	382	A	79.2721	1
**4410**	386	A	69.7863	1
**4410**	305	B	45.3624	0
**4410**	319	B	74.1071	1
**4410**	320	B	63.6714	-1
**4410**	321	B	38.1974	1
**4410**	351	B	3.3717	1
**4410**	357	B	70.1856	1
**4410**	370	B	31.9549	0
**4410**	372	B	79.5003	0
**4410**	373	B	79.5316	1
**4410**	381	B	75.4997	1
**4410**	382	B	63.4468	1
**4410**	386	B	79.326	1
**4410**	305	C	72.8753	1
**4410**	319	C	79.4959	1
**4410**	320	C	61.9838	-1
**4410**	321	C	79.5167	0
**4410**	351	C	5.1937	1
**4410**	357	C	74.0192	1
**4410**	370	C	31.1592	0
**4410**	372	C	57.1457	0

**4410**	373	C	78.0885	1
**4410**	381	C	79.5548	1
**4410**	382	C	26.7649	0
**4410**	386	C	32.9372	0
**4410**	305	D	69.2709	1
**4410**	319	D	28.8326	0
**4410**	320	D	79.5263	-1
**4410**	321	D	79.6049	0
**4410**	351	D	4.5518	1
**4410**	357	D	71.3517	1
**4410**	370	D	79.5899	1
**4410**	372	D	75.1633	0
**4410**	373	D	19.5281	-1
**4410**	381	D	66.199	1
**4410**	382	D	71.8795	1
**4410**	386	D	73.9422	1
**4411**	305	A	44.5126	0
**4411**	319	A	70.4369	1
**4411**	320	A	79.5694	-1
**4411**	321	A	62.1924	0
**4411**	351	A	34.638	-1
**4411**	357	A	53.9769	1
**4411**	370	A	74.2825	1
**4411**	372	A	66.5006	0
**4411**	373	A	63.5048	1
**4411**	381	A	51.6678	1
**4411**	382	A	79.3262	1
**4411**	386	A	79.5656	1
**4411**	305	B	71.579	1
**4411**	319	B	79.5284	1
**4411**	320	B	79.5816	-1
**4411**	321	B	73.29	0
**4411**	351	B	15.596	1
**4411**	357	B	79.6073	1

**4411**	370	B	57.9249	1
**4411**	372	B	79.6149	0
**4411**	373	B	73.7779	1
**4411**	381	B	36.9237	0
**4411**	382	B	52.5719	1
**4411**	386	B	79.5338	1
**4411**	305	C	79.3922	1
**4411**	319	C	55.1708	1
**4411**	320	C	58.7956	-1
**4411**	321	C	67.7382	0
**4411**	351	C	11.3737	1
**4411**	357	C	79.4835	1
**4411**	370	C	72.6806	1
**4411**	372	C	79.5974	0
**4411**	373	C	70.7427	1
**4411**	381	C	67.4514	1
**4411**	382	C	68.9508	1
**4411**	386	C	49.2446	0
**4411**	305	D	69.6919	1
**4411**	319	D	53.9525	1
**4411**	320	D	79.672	-1
**4411**	321	D	79.5821	0
**4411**	351	D	9.6784	1
**4411**	357	D	51.7212	1
**4411**	370	D	79.5177	1
**4411**	372	D	79.5052	0
**4411**	373	D	60.8501	1
**4411**	381	D	41.1425	0
**4411**	382	D	27.0304	0
**4411**	386	D	73.4423	1
**4412**	305	A	57.2104	1
**4412**	319	A	68.8338	1
**4412**	320	A	79.4962	-1
**4412**	321	A	47.9542	1

**4412**	351	A	10.8915	1
**4412**	357	A	50.9004	1
**4412**	370	A	73.718	1
**4412**	372	A	47.9308	1
**4412**	373	A	33.9683	0
**4412**	381	A	53.5699	1
**4412**	382	A	63.6206	1
**4412**	386	A	79.4247	1
**4412**	305	B	64.3395	1
**4412**	319	B	79.5629	1
**4412**	320	B	48.9851	-1
**4412**	321	B	79.5847	0
**4412**	351	B	14.0815	1
**4412**	357	B	76.7691	1
**4412**	370	B	59.8754	1
**4412**	372	B	73.0058	0
**4412**	373	B	79.4514	1
**4412**	381	B	79.5396	1
**4412**	382	B	31.8253	0
**4412**	386	B	70.3039	1
**4412**	305	C	79.5305	1
**4412**	319	C	72.7204	1
**4412**	320	C	79.5591	-1
**4412**	321	C	73.1891	0
**4412**	351	C	4.5726	1
**4412**	357	C	77.1319	1
**4412**	370	C	34.6817	0
**4412**	372	C	61.9123	0
**4412**	373	C	79.5641	1
**4412**	381	C	79.6075	1
**4412**	382	C	6.811	-1
**4412**	386	C	58.2467	1
**4412**	305	D	71.819	1
**4412**	319	D	78.4978	1

**4412**	320	D	79.5937	-1
**4412**	321	D	55.6699	0
**4412**	351	D	12.071	1
**4412**	357	D	66.4228	1
**4412**	370	D	67.4763	1
**4412**	372	D	79.5776	0
**4412**	373	D	71.9384	1
**4412**	381	D	73.5617	1
**4412**	382	D	79.5759	1
**4412**	386	D	32.1744	0
**4413**	305	A	62.8645	1
**4413**	319	A	70.61	1
**4413**	320	A	78.5385	-1
**4413**	321	A	75.5137	0
**4413**	351	A	29.6157	-1
**4413**	357	A	73.279	1
**4413**	370	A	61.4109	1
**4413**	372	A	50.0126	0
**4413**	373	A	54.7876	1
**4413**	381	A	73.3272	1
**4413**	382	A	75.233	1
**4413**	386	A	72.7423	1
**4413**	305	B	43.2893	0
**4413**	319	B	65.3396	1
**4413**	320	B	71.8674	-1
**4413**	321	B	70.0164	0
**4413**	351	B	12.0764	1
**4413**	357	B	73.6596	1
**4413**	370	B	79.692	1
**4413**	372	B	68.4556	0
**4413**	373	B	37.7683	0
**4413**	381	B	72.3363	1
**4413**	382	B	45.7195	0
**4413**	386	B	71.0232	1

**4413**	305	C	78.3079	1
**4413**	319	C	79.4633	1
**4413**	320	C	79.5857	-1
**4413**	321	C	70.5634	0
**4413**	351	C	3.7009	1
**4413**	357	C	79.2394	1
**4413**	370	C	19.8642	-1
**4413**	372	C	75.3503	0
**4413**	373	C	79.5144	1
**4413**	381	C	79.6152	1
**4413**	382	C	70.8423	1
**4413**	386	C	50.1721	1
**4413**	305	D	21.2517	0
**4413**	319	D	79.5546	1
**4413**	320	D	29.777	-1
**4413**	321	D	79.596	0
**4413**	351	D	13.9924	1
**4413**	357	D	75.4932	1
**4413**	370	D	79.4372	1
**4413**	372	D	71.1214	0
**4413**	373	D	78.3575	1
**4413**	381	D	79.6551	1
**4413**	382	D	47.5658	0
**4413**	386	D	79.5984	1
**4414**	305	A	74.7042	1
**4414**	319	A	52.7803	1
**4414**	320	A	79.503	-1
**4414**	321	A	73.552	0
**4414**	351	A	3.156	1
**4414**	357	A	79.4971	1
**4414**	370	A	68.5759	1
**4414**	372	A	73.3623	0
**4414**	373	A	79.4999	1
**4414**	381	A	59.9588	1

**4414**	382	A	73.4689	1
**4414**	386	A	79.5292	1
**4414**	305	B	70.6952	1
**4414**	319	B	79.5748	1
**4414**	320	B	74.5358	-1
**4414**	321	B	68.5814	0
**4414**	351	B	43.1258	-1
**4414**	357	B	79.5149	1
**4414**	370	B	62.09	1
**4414**	372	B	79.6016	0
**4414**	373	B	79.5552	1
**4414**	381	B	47.0764	0
**4414**	382	B	79.5544	1
**4414**	386	B	66.7689	1
**4414**	305	C	78.9598	1
**4414**	319	C	48.3753	0
**4414**	320	C	79.5889	-1
**4414**	321	C	79.5209	0
**4414**	351	C	12.1085	1
**4414**	357	C	79.5287	1
**4414**	370	C	57.6251	1
**4414**	372	C	76.3957	0
**4414**	373	C	78.7397	1
**4414**	381	C	43.1517	0
**4414**	382	C	51.6934	1
**4414**	386	C	62.8381	1
**4414**	305	D	78.761	1
**4414**	319	D	62.6453	1
**4414**	320	D	69.6806	-1
**4414**	321	D	79.6371	0
**4414**	351	D	18.2381	1
**4414**	357	D	37.0969	0
**4414**	370	D	71.5226	1
**4414**	372	D	79.4655	0

**4414**	373	D	70.9781	1
**4414**	381	D	58.6387	1
**4414**	382	D	79.6662	1
**4414**	386	D	39.4983	0
**4415**	305	A	43.4122	0
**4415**	319	A	60.564	1
**4415**	320	A	76.4979	-1
**4415**	321	A	68.4269	0
**4415**	351	A	6.7225	1
**4415**	357	A	73.8845	1
**4415**	370	A	78.4367	1
**4415**	372	A	47.9088	1
**4415**	373	A	70.0553	1
**4415**	381	A	76.5954	1
**4415**	382	A	72.605	1
**4415**	386	A	79.5943	1
**4415**	305	B	69.3595	1
**4415**	319	B	73.1217	1
**4415**	320	B	79.4038	-1
**4415**	321	B	52.7645	0
**4415**	351	B	16.3022	1
**4415**	357	B	68.9167	1
**4415**	370	B	46.5614	0
**4415**	372	B	79.5279	0
**4415**	373	B	77.3326	1
**4415**	381	B	65.6754	1
**4415**	382	B	24.2521	0
**4415**	386	B	70.9435	1
**4415**	305	C	62.2867	1
**4415**	319	C	79.672	1
**4415**	320	C	71.8289	-1
**4415**	321	C	79.5579	0
**4415**	351	C	12.7248	1
**4415**	357	C	79.385	1

**4415**	370	C	61.8624	1
**4415**	372	C	73.347	0
**4415**	373	C	73.7283	1
**4415**	381	C	79.6084	1
**4415**	382	C	78.2622	1
**4415**	386	C	61.1284	1
**4415**	305	D	76.2766	1
**4415**	319	D	45.1521	0
**4415**	320	D	79.5249	-1
**4415**	321	D	45.3413	1
**4415**	351	D	7.6594	1
**4415**	357	D	76.112	1
**4415**	370	D	63.9723	1
**4415**	372	D	65.3609	0
**4415**	373	D	79.7773	1
**4415**	381	D	23.5634	0
**4415**	382	D	68.3184	1
**4415**	386	D	79.6105	1
**4416**	305	A	79.4303	1
**4416**	319	A	72.4803	1
**4416**	320	A	68.9973	-1
**4416**	321	A	64.2038	0
**4416**	351	A	21.4499	-1
**4416**	357	A	79.6161	1
**4416**	370	A	74.3673	1
**4416**	372	A	74.0505	0
**4416**	373	A	75.9643	1
**4416**	381	A	72.4217	1
**4416**	382	A	78.8367	1
**4416**	386	A	58.7745	1
**4416**	305	B	74.8461	1
**4416**	319	B	57.855	1
**4416**	320	B	79.5492	-1
**4416**	321	B	79.5122	0

**4416**	351	B	15.7763	1
**4416**	357	B	62.5595	1
**4416**	370	B	18.0632	-1
**4416**	372	B	76.4355	0
**4416**	373	B	63.6317	1
**4416**	381	B	77.9761	1
**4416**	382	B	37.893	0
**4416**	386	B	59.4253	1
**4416**	305	C	73.805	1
**4416**	319	C	65.3106	1
**4416**	320	C	79.5761	-1
**4416**	321	C	79.5611	0
**4416**	351	C	10.1384	1
**4416**	357	C	58.4775	1
**4416**	370	C	64.15	1
**4416**	372	C	69.2764	0
**4416**	373	C	65.0976	1
**4416**	381	C	53.5287	1
**4416**	382	C	52.0847	1
**4416**	386	C	68.7742	1
**4416**	305	D	56.7523	1
**4416**	319	D	48.0312	0
**4416**	320	D	72.6032	-1
**4416**	321	D	74.7374	0
**4416**	351	D	14.2325	1
**4416**	357	D	45.5634	0
**4416**	370	D	62.9524	1
**4416**	372	D	62.9884	0
**4416**	373	D	74.2412	1
**4416**	381	D	79.495	1
**4416**	382	D	63.8041	1
**4416**	386	D	23.7848	0
**4417**	305	A	72.9891	1
**4417**	319	A	62.1774	1

**4417**	320	A	54.493	-1
**4417**	321	A	65.9298	0
**4417**	351	A	3.268	1
**4417**	357	A	74.5581	1
**4417**	370	A	68.7548	1
**4417**	372	A	79.5541	0
**4417**	373	A	79.52	1
**4417**	381	A	72.7525	1
**4417**	382	A	68.4473	1
**4417**	386	A	79.5726	1
**4417**	305	B	79.5612	1
**4417**	319	B	49.8514	0
**4417**	320	B	74.9765	-1
**4417**	321	B	79.5749	0
**4417**	351	B	0.2497	1
**4417**	357	B	76.4994	1
**4417**	370	B	53.9397	1
**4417**	372	B	23.2817	1
**4417**	373	B	50.4451	1
**4417**	381	B	64.7099	1
**4417**	382	B	57.9204	1
**4417**	386	B	79.3236	1
**4417**	305	C	60.3997	1
**4417**	319	C	76.5525	1
**4417**	320	C	24.7188	-1
**4417**	321	C	61.1479	0
**4417**	351	C	5.0542	1
**4417**	357	C	62.9566	1
**4417**	370	C	79.5835	1
**4417**	372	C	66.4466	0
**4417**	373	C	79.5291	1
**4417**	381	C	71.4747	1
**4417**	382	C	79.586	1
**4417**	386	C	66.4415	1

**4417**	305	D	78.1682	1
**4417**	319	D	79.4289	1
**4417**	320	D	72.0754	-1
**4417**	321	D	79.5141	0
**4417**	351	D	12.7898	1
**4417**	357	D	72.9457	1
**4417**	370	D	79.5648	1
**4417**	372	D	62.9888	0
**4417**	373	D	56.4119	1
**4417**	381	D	68.4259	1
**4417**	382	D	70.5002	1
**4417**	386	D	72.6644	1
**4418**	305	A	41.9419	0
**4418**	319	A	73.7356	1
**4418**	320	A	68.5815	-1
**4418**	321	A	64.6851	0
**4418**	351	A	11.2686	1
**4418**	357	A	79.4287	1
**4418**	370	A	56.9828	1
**4418**	372	A	39.8099	1
**4418**	373	A	79.5688	1
**4418**	381	A	79.5504	1
**4418**	382	A	78.5444	1
**4418**	386	A	77.754	1
**4418**	305	B	32.8523	0
**4418**	319	B	79.5455	1
**4418**	320	B	79.5404	-1
**4418**	321	B	79.5175	0
**4418**	351	B	9.9444	1
**4418**	357	B	40.626	0
**4418**	370	B	79.4457	1
**4418**	372	B	72.3934	0
**4418**	373	B	79.5829	1
**4418**	381	B	79.5447	1

**4418**	382	B	70.4589	1
**4418**	386	B	76.8	1
**4418**	305	C	79.3386	1
**4418**	319	C	60.9523	1
**4418**	320	C	79.4956	-1
**4418**	321	C	65.2523	0
**4418**	351	C	5.6923	1
**4418**	357	C	79.2303	1
**4418**	370	C	42.3998	0
**4418**	372	C	71.9913	0
**4418**	373	C	40.74	0
**4418**	381	C	59.1007	1
**4418**	382	C	79.1627	1
**4418**	386	C	50.3423	1
**4418**	305	D	78.086	1
**4418**	319	D	79.5812	1
**4418**	320	D	74.0489	-1
**4418**	321	D	79.5282	0
**4418**	351	D	13.1162	1
**4418**	357	D	79.5468	1
**4418**	370	D	43.9771	0
**4418**	372	D	63.19	0
**4418**	373	D	73.8604	1
**4418**	381	D	79.5712	1
**4418**	382	D	79.5587	1
**4418**	386	D	26.2272	0
**4419**	305	A	70.5589	1
**4419**	319	A	62.0356	1
**4419**	320	A	79.5353	-1
**4419**	321	A	53.7407	0
**4419**	351	A	7.4563	1
**4419**	357	A	79.5606	1
**4419**	370	A	79.4113	1
**4419**	372	A	49.2337	1

**4419**	373	A	72.7238	1
**4419**	381	A	79.591	1
**4419**	382	A	64.5447	1
**4419**	386	A	79.5317	1
**4419**	305	B	10.684	-1
**4419**	319	B	28.0674	0
**4419**	320	B	52.5818	-1
**4419**	321	B	60.8842	0
**4419**	351	B	16.1307	1
**4419**	357	B	70.8461	1
**4419**	370	B	79.5792	1
**4419**	372	B	79.5615	0
**4419**	373	B	79.5026	1
**4419**	381	B	45.2142	0
**4419**	382	B	36.2699	0
**4419**	386	B	75.3672	1
**4419**	305	C	76.7504	1
**4419**	319	C	61.4421	1
**4419**	320	C	60.9725	-1
**4419**	321	C	79.5655	0
**4419**	351	C	5.8858	1
**4419**	357	C	68.6072	1
**4419**	370	C	60.3881	1
**4419**	372	C	60.2661	0
**4419**	373	C	51.6882	1
**4419**	381	C	55.1358	1
**4419**	382	C	79.515	1
**4419**	386	C	17.452	-1
**4419**	305	D	79.5524	1
**4419**	319	D	79.5792	1
**4419**	320	D	79.5876	-1
**4419**	321	D	67.2165	0
**4419**	351	D	12.1329	1
**4419**	357	D	79.549	1

**4419**	370	D	79.4988	1
**4419**	372	D	72.8964	0
**4419**	373	D	79.5531	1
**4419**	381	D	40.6791	0
**4419**	382	D	79.5372	1
**4419**	386	D	79.549	1
**4420**	305	A	79.5201	1
**4420**	319	A	42.4235	0
**4420**	320	A	79.6867	-1
**4420**	321	A	79.5588	0
**4420**	351	A	3.0528	1
**4420**	357	A	54.2456	1
**4420**	370	A	51.8399	1
**4420**	372	A	68.7929	0
**4420**	373	A	79.4467	1
**4420**	381	A	79.6095	1
**4420**	382	A	70.7902	1
**4420**	386	A	78.099	1
**4420**	305	B	75.3046	1
**4420**	319	B	79.6024	1
**4420**	320	B	69.1381	-1
**4420**	321	B	79.4675	0
**4420**	351	B	11.8671	1
**4420**	357	B	79.6779	1
**4420**	370	B	74.6435	1
**4420**	372	B	58.6502	0
**4420**	373	B	66.2086	1
**4420**	381	B	79.538	1
**4420**	382	B	42.8878	0
**4420**	386	B	49.6681	0
**4420**	305	C	36.7873	0
**4420**	319	C	50.602	1
**4420**	320	C	64.6166	-1
**4420**	321	C	58.207	0

**4420**	351	C	3.2176	1
**4420**	357	C	47.1455	0
**4420**	370	C	77.8659	1
**4420**	372	C	79.361	0
**4420**	373	C	64.1087	1
**4420**	381	C	74.7176	1
**4420**	382	C	72.9775	1
**4420**	386	C	48.0726	0
**4420**	305	D	52.9008	1
**4420**	319	D	45.1582	0
**4420**	320	D	64.5997	-1
**4420**	321	D	79.7224	0
**4420**	351	D	17.0206	1
**4420**	357	D	79.5945	1
**4420**	370	D	8.3651	-1
**4420**	372	D	79.5202	0
**4420**	373	D	76.0973	1
**4420**	381	D	79.6953	1
**4420**	382	D	77.3838	1
**4420**	386	D	72.3594	1
**4421**	305	A	79.774	1
**4421**	319	A	69.1793	1
**4421**	320	A	70.8076	-1
**4421**	321	A	37.5508	1
**4421**	351	A	5.3328	1
**4421**	357	A	65.9856	1
**4421**	370	A	79.5575	1
**4421**	372	A	40.7193	1
**4421**	373	A	76.5046	1
**4421**	381	A	56.6214	1
**4421**	382	A	69.1522	1
**4421**	386	A	24.1553	0
**4421**	305	B	79.4483	1
**4421**	319	B	79.5481	1

**4421**	320	B	71.6873	-1
**4421**	321	B	79.6922	0
**4421**	351	B	7.1901	1
**4421**	357	B	44.5527	0
**4421**	370	B	79.5141	1
**4421**	372	B	71.7716	0
**4421**	373	B	79.6016	1
**4421**	381	B	79.5733	1
**4421**	382	B	34.761	0
**4421**	386	B	58.5197	1
**4421**	305	C	50.1939	1
**4421**	319	C	79.5833	1
**4421**	320	C	63.5448	-1
**4421**	321	C	33.8398	1
**4421**	351	C	0.7329	1
**4421**	357	C	65.9781	1
**4421**	370	C	68.8961	1
**4421**	372	C	33.4039	1
**4421**	373	C	73.5211	1
**4421**	381	C	32.7829	0
**4421**	382	C	72.1406	1
**4421**	386	C	42.4574	0
**4421**	305	D	69.1077	1
**4421**	319	D	64.898	1
**4421**	320	D	79.6552	-1
**4421**	321	D	63.1205	0
**4421**	351	D	6.0804	1
**4421**	357	D	63.4518	1
**4421**	370	D	69.41	1
**4421**	372	D	79.5949	0
**4421**	373	D	19.8278	-1
**4421**	381	D	73.1479	1
**4421**	382	D	48.9371	0
**4421**	386	D	64.1214	1

**4422**	305	A	73.7742	1
**4422**	319	A	63.7157	1
**4422**	320	A	79.5717	-1
**4422**	321	A	41.6829	1
**4422**	351	A	2.742	1
**4422**	357	A	48.6924	0
**4422**	370	A	79.5648	1
**4422**	372	A	79.555	0
**4422**	373	A	69.9834	1
**4422**	381	A	65.1477	1
**4422**	382	A	62.9694	1
**4422**	386	A	73.6502	1
**4422**	305	B	58.0057	1
**4422**	319	B	79.5067	1
**4422**	320	B	70.8666	-1
**4422**	321	B	79.5485	0
**4422**	351	B	22.4575	-1
**4422**	357	B	43.2684	0
**4422**	370	B	79.4746	1
**4422**	372	B	38.4458	1
**4422**	373	B	56.2251	1
**4422**	381	B	77.1786	1
**4422**	382	B	62.3871	1
**4422**	386	B	79.6076	1
**4422**	305	C	77.64	1
**4422**	319	C	79.526	1
**4422**	320	C	74.3027	-1
**4422**	321	C	64.8004	0
**4422**	351	C	9.5019	1
**4422**	357	C	53.974	1
**4422**	370	C	79.8013	1
**4422**	372	C	75.0091	0
**4422**	373	C	75.0833	1
**4422**	381	C	70.8881	1

**4422**	382	C	75.4372	1
**4422**	386	C	70.1522	1
**4422**	305	D	68.5098	1
**4422**	319	D	61.3747	1
**4422**	320	D	71.3102	-1
**4422**	321	D	12.7853	0
**4422**	351	D	8.782	1
**4422**	357	D	73.5695	1
**4422**	370	D	48.1821	0
**4422**	372	D	72.4957	0
**4422**	373	D	69.6397	1
**4422**	381	D	79.4911	1
**4422**	382	D	79.7367	1
**4422**	386	D	79.5571	1
**4423**	305	A	39.9269	0
**4423**	319	A	55.0019	1
**4423**	320	A	72.62	-1
**4423**	321	A	66.9429	0
**4423**	351	A	5.9228	1
**4423**	357	A	79.6876	1
**4423**	370	A	49.5032	0
**4423**	372	A	72.2734	0
**4423**	373	A	79.5575	1
**4423**	381	A	70.5556	1
**4423**	382	A	74.611	1
**4423**	386	A	79.1062	1
**4423**	305	B	61.3206	1
**4423**	319	B	77.3572	1
**4423**	320	B	72.5327	-1
**4423**	321	B	79.6113	0
**4423**	351	B	14.5342	1
**4423**	357	B	64.255	1
**4423**	370	B	72.8712	1
**4423**	372	B	65.0633	0

**4423**	373	B	70.2255	1
**4423**	381	B	62.4987	1
**4423**	382	B	51.8242	1
**4423**	386	B	70.127	1
**4423**	305	C	79.5266	1
**4423**	319	C	75.554	1
**4423**	320	C	71.9708	-1
**4423**	321	C	61.5574	0
**4423**	351	C	0.9868	1
**4423**	357	C	53.1535	1
**4423**	370	C	79.6701	1
**4423**	372	C	76.2589	0
**4423**	373	C	53.9061	1
**4423**	381	C	78.9164	1
**4423**	382	C	78.0201	1
**4423**	386	C	56.2167	1
**4423**	305	D	75.8552	1
**4423**	319	D	76.0979	1
**4423**	320	D	75.6467	-1
**4423**	321	D	63.1881	0
**4423**	351	D	1.0306	1
**4423**	357	D	63.5887	1
**4423**	370	D	67.6766	1
**4423**	372	D	79.6007	0
**4423**	373	D	79.5704	1
**4423**	381	D	75.027	1
**4423**	382	D	68.6729	1
**4423**	386	D	75.2599	1
**4424**	305	A	64.3196	1
**4424**	319	A	73.4169	1
**4424**	320	A	75.0435	-1
**4424**	321	A	71.0264	0
**4424**	351	A	16.6897	1
**4424**	357	A	69.3057	1

**4424**	370	A	79.5649	1
**4424**	372	A	20.2595	1
**4424**	373	A	79.5922	1
**4424**	381	A	53.3532	1
**4424**	382	A	39.5108	0
**4424**	386	A	79.5603	1
**4424**	305	B	60.8794	1
**4424**	319	B	79.4802	1
**4424**	320	B	74.791	-1
**4424**	321	B	66.3222	0
**4424**	351	B	17.8653	1
**4424**	357	B	70.6175	1
**4424**	370	B	59.9508	1
**4424**	372	B	79.4999	0
**4424**	373	B	69.3265	1
**4424**	381	B	31.7986	0
**4424**	382	B	25.9257	0
**4424**	386	B	50.0039	1
**4424**	305	C	53.7393	1
**4424**	319	C	17.675	-1
**4424**	320	C	64.5173	-1
**4424**	321	C	79.5494	0
**4424**	351	C	9.8023	1
**4424**	357	C	65.68	1
**4424**	370	C	73.7626	1
**4424**	372	C	79.4935	0
**4424**	373	C	59.7248	1
**4424**	381	C	12.8546	-1
**4424**	382	C	79.5934	1
**4424**	386	C	33.9809	0
**4424**	305	D	79.6435	1
**4424**	319	D	72.364	1
**4424**	320	D	79.5881	-1
**4424**	321	D	61.6311	0

**4424**	351	D	14.3022	1
**4424**	357	D	79.5577	1
**4424**	370	D	35.9889	0
**4424**	372	D	79.5652	0
**4424**	373	D	72.687	1
**4424**	381	D	79.4927	1
**4424**	382	D	79.5561	1
**4424**	386	D	78.65	1
**4425**	305	A	79.515	1
**4425**	319	A	69.7243	1
**4425**	320	A	54.1803	-1
**4425**	321	A	51.0771	0
**4425**	351	A	7.9616	1
**4425**	357	A	71.247	1
**4425**	370	A	65.3017	1
**4425**	372	A	79.2529	0
**4425**	373	A	70.4816	1
**4425**	381	A	68.1447	1
**4425**	382	A	79.6008	1
**4425**	386	A	70.6517	1
**4425**	305	B	79.6512	1
**4425**	319	B	79.5175	1
**4425**	320	B	73.6771	-1
**4425**	321	B	75.5487	0
**4425**	351	B	7.1923	1
**4425**	357	B	73.292	1
**4425**	370	B	71.865	1
**4425**	372	B	53.9668	0
**4425**	373	B	78.6924	1
**4425**	381	B	70.3365	1
**4425**	382	B	58.3958	1
**4425**	386	B	47.9711	0
**4425**	305	C	78.8123	1
**4425**	319	C	61.4976	1

**4425**	320	C	22.269	-1
**4425**	321	C	37.1147	1
**4425**	351	C	13.2446	1
**4425**	357	C	54.7683	1
**4425**	370	C	70.2438	1
**4425**	372	C	79.5901	0
**4425**	373	C	79.6559	1
**4425**	381	C	22.1335	0
**4425**	382	C	49.7305	0
**4425**	386	C	12.8251	-1
**4425**	305	D	64.3097	1
**4425**	319	D	79.4834	1
**4425**	320	D	50.4243	-1
**4425**	321	D	70.7677	0
**4425**	351	D	8.7355	1
**4425**	357	D	79.2074	1
**4425**	370	D	79.5963	1
**4425**	372	D	79.475	0
**4425**	373	D	77.4261	1
**4425**	381	D	44.8681	0
**4425**	382	D	69.3932	1
**4425**	386	D	79.6055	1
**4426**	305	A	43.54	0
**4426**	319	A	79.5576	1
**4426**	320	A	79.6181	-1
**4426**	321	A	71.7966	0
**4426**	351	A	10.7302	1
**4426**	357	A	64.9007	1
**4426**	370	A	79.4559	1
**4426**	372	A	76.497	0
**4426**	373	A	79.73	1
**4426**	381	A	76.2231	1
**4426**	382	A	72.8153	1
**4426**	386	A	72.4806	1

**4426**	305	B	75.7437	1
**4426**	319	B	79.5866	1
**4426**	320	B	79.7014	-1
**4426**	321	B	72.6102	0
**4426**	351	B	16.5888	1
**4426**	357	B	66.1592	1
**4426**	370	B	71.7445	1
**4426**	372	B	65.9202	0
**4426**	373	B	48.2844	0
**4426**	381	B	79.4512	1
**4426**	382	B	38.7904	0
**4426**	386	B	38.8436	0
**4426**	305	C	61.7786	1
**4426**	319	C	79.4456	1
**4426**	320	C	74.45	-1
**4426**	321	C	79.4895	0
**4426**	351	C	2.842	1
**4426**	357	C	66.8847	1
**4426**	370	C	79.6177	1
**4426**	372	C	79.5177	0
**4426**	373	C	78.9191	1
**4426**	381	C	44.5003	0
**4426**	382	C	56.2774	1
**4426**	386	C	55.0846	1
**4426**	305	D	79.6909	1
**4426**	319	D	79.5515	1
**4426**	320	D	51.3382	-1
**4426**	321	D	62.6795	0
**4426**	351	D	19.3982	1
**4426**	357	D	58.9858	1
**4426**	370	D	71.9349	1
**4426**	372	D	69.3045	0
**4426**	373	D	68.2104	1
**4426**	381	D	76.0823	1

**4426**	382	D	79.7159	1
**4426**	386	D	79.5171	1
**4427**	305	A	48.7849	0
**4427**	319	A	34.3296	0
**4427**	320	A	52.0719	-1
**4427**	321	A	72.9735	0
**4427**	351	A	0.2634	1
**4427**	357	A	79.3147	1
**4427**	370	A	74.7973	1
**4427**	372	A	73.3329	0
**4427**	373	A	77.5813	1
**4427**	381	A	79.6115	1
**4427**	382	A	75.2188	1
**4427**	386	A	79.598	1
**4427**	305	B	69.4021	1
**4427**	319	B	79.4079	1
**4427**	320	B	68.1322	-1
**4427**	321	B	79.6003	0
**4427**	351	B	18.3181	1
**4427**	357	B	79.5903	1
**4427**	370	B	38.7174	0
**4427**	372	B	73.8082	0
**4427**	373	B	79.5554	1
**4427**	381	B	79.5706	1
**4427**	382	B	52.216	1
**4427**	386	B	59.4296	1
**4427**	305	C	79.6415	1
**4427**	319	C	73.8942	1
**4427**	320	C	77.9768	-1
**4427**	321	C	69.7502	0
**4427**	351	C	0.5271	1
**4427**	357	C	54.9142	1
**4427**	370	C	70.6698	1
**4427**	372	C	64.3503	0

**4427**	373	C	79.5815	1
**4427**	381	C	79.5837	1
**4427**	382	C	53.0589	1
**4427**	386	C	47.3317	0
**4427**	305	D	67.4396	1
**4427**	319	D	39.4973	0
**4427**	320	D	68.651	-1
**4427**	321	D	49.755	1
**4427**	351	D	10.6963	1
**4427**	357	D	48.869	0
**4427**	370	D	79.6043	1
**4427**	372	D	69.6599	0
**4427**	373	D	41.6452	0
**4427**	381	D	79.5076	1
**4427**	382	D	73.0855	1
**4427**	386	D	79.6094	1
**4428**	305	A	32.0718	0
**4428**	319	A	32.9025	0
**4428**	320	A	79.4726	-1
**4428**	321	A	74.0255	0
**4428**	351	A	26.4493	-1
**4428**	357	A	65.2414	1
**4428**	370	A	79.6483	1
**4428**	372	A	73.8944	0
**4428**	373	A	79.5478	1
**4428**	381	A	79.6092	1
**4428**	382	A	56.5213	1
**4428**	386	A	21.3644	0
**4428**	305	B	78.6261	1
**4428**	319	B	79.5324	1
**4428**	320	B	47.6529	-1
**4428**	321	B	79.5901	0
**4428**	351	B	17.3291	1
**4428**	357	B	79.5986	1

**4428**	370	B	74.4084	1
**4428**	372	B	74.1198	0
**4428**	373	B	38.0524	0
**4428**	381	B	71.5717	1
**4428**	382	B	53.7665	1
**4428**	386	B	54.8488	1
**4428**	305	C	53.5536	1
**4428**	319	C	51.4018	1
**4428**	320	C	64.6829	-1
**4428**	321	C	79.5962	0
**4428**	351	C	16.8808	1
**4428**	357	C	79.6633	1
**4428**	370	C	73.2077	1
**4428**	372	C	79.5487	0
**4428**	373	C	79.6073	1
**4428**	381	C	79.5843	1
**4428**	382	C	55.9691	1
**4428**	386	C	32.8562	0
**4428**	305	D	79.5643	1
**4428**	319	D	79.507	1
**4428**	320	D	79.4299	-1
**4428**	321	D	71.2263	0
**4428**	351	D	24.9891	-1
**4428**	357	D	69.96	1
**4428**	370	D	73.3452	1
**4428**	372	D	51.2488	0
**4428**	373	D	79.6651	1
**4428**	381	D	76.8636	1
**4428**	382	D	53.7197	1
**4428**	386	D	79.642	1
**4429**	305	A	79.5683	1
**4429**	319	A	74.2814	1
**4429**	320	A	78.3972	-1
**4429**	321	A	79.5141	0

**4429**	351	A	7.9437	1
**4429**	357	A	79.5639	1
**4429**	370	A	75.6082	1
**4429**	372	A	74.3402	0
**4429**	373	A	79.5368	1
**4429**	381	A	73.307	1
**4429**	382	A	58.958	1
**4429**	386	A	72.9054	1
**4429**	305	B	71.3813	1
**4429**	319	B	67.9719	1
**4429**	320	B	57.9897	-1
**4429**	321	B	71.0539	0
**4429**	351	B	16.7545	1
**4429**	357	B	45.4476	0
**4429**	370	B	43.9712	0
**4429**	372	B	79.6071	0
**4429**	373	B	77.6861	1
**4429**	381	B	66.9175	1
**4429**	382	B	75.2998	1
**4429**	386	B	57.7663	1
**4429**	305	C	76.9455	1
**4429**	319	C	73.4401	1
**4429**	320	C	78.2412	-1
**4429**	321	C	79.526	0
**4429**	351	C	7.0815	1
**4429**	357	C	75.4781	1
**4429**	370	C	75.6914	1
**4429**	372	C	79.5225	0
**4429**	373	C	67.3215	1
**4429**	381	C	73.275	1
**4429**	382	C	72.4408	1
**4429**	386	C	47.9303	0
**4429**	305	D	69.24	1
**4429**	319	D	69.0285	1

**4429**	320	D	79.5531	-1
**4429**	321	D	75.191	0
**4429**	351	D	8.5381	1
**4429**	357	D	56.2836	1
**4429**	370	D	79.5445	1
**4429**	372	D	73.2567	0
**4429**	373	D	53.489	1
**4429**	381	D	72.4364	1
**4429**	382	D	79.6248	1
**4429**	386	D	39.278	0
**4430**	305	A	79.5581	1
**4430**	319	A	53.1512	1
**4430**	320	A	79.5326	-1
**4430**	321	A	75.2172	0
**4430**	351	A	10.4175	1
**4430**	357	A	72.9418	1
**4430**	370	A	68.7005	1
**4430**	372	A	72.4944	0
**4430**	373	A	76.2665	1
**4430**	381	A	50.2858	1
**4430**	382	A	79.5122	1
**4430**	386	A	79.566	1
**4430**	305	B	60.5433	1
**4430**	319	B	79.5575	1
**4430**	320	B	79.6024	-1
**4430**	321	B	38.8272	1
**4430**	351	B	17.1183	1
**4430**	357	B	79.5371	1
**4430**	370	B	79.4274	1
**4430**	372	B	79.5742	0
**4430**	373	B	37.7488	0
**4430**	381	B	79.535	1
**4430**	382	B	44.95	0
**4430**	386	B	43.6878	0

**4430**	305	C	45.2433	0
**4430**	319	C	36.4793	0
**4430**	320	C	65.5999	-1
**4430**	321	C	79.5348	0
**4430**	351	C	3.776	1
**4430**	357	C	79.569	1
**4430**	370	C	74.9676	1
**4430**	372	C	79.5723	0
**4430**	373	C	33.327	0
**4430**	381	C	79.5529	1
**4430**	382	C	75.6667	1
**4430**	386	C	55.2266	1
**4430**	305	D	79.5783	1
**4430**	319	D	33.6527	0
**4430**	320	D	79.5866	-1
**4430**	321	D	79.5703	0
**4430**	351	D	5.7197	1
**4430**	357	D	59.9924	1
**4430**	370	D	49.2316	0
**4430**	372	D	72.3991	0
**4430**	373	D	72.55	1
**4430**	381	D	69.9402	1
**4430**	382	D	79.5718	1
**4430**	386	D	48.0128	0
**4431**	305	A	62.3236	1
**4431**	319	A	64.149	1
**4431**	320	A	31.249	-1
**4431**	321	A	62.2325	0
**4431**	351	A	6.1837	1
**4431**	357	A	70.9461	1
**4431**	370	A	79.5484	1
**4431**	372	A	79.5519	0
**4431**	373	A	35.1867	0
**4431**	381	A	62.8993	1

**4431**	382	A	21.5407	0
**4431**	386	A	31.8626	0
**4431**	305	B	42.4244	0
**4431**	319	B	79.5764	1
**4431**	320	B	61.1549	-1
**4431**	321	B	69.0981	0
**4431**	351	B	6.8476	1
**4431**	357	B	50.3035	1
**4431**	370	B	78.853	1
**4431**	372	B	72.5987	0
**4431**	373	B	79.4259	1
**4431**	381	B	76.4123	1
**4431**	382	B	79.5628	1
**4431**	386	B	64.6499	1
**4431**	305	C	50.9176	1
**4431**	319	C	79.542	1
**4431**	320	C	66.4107	-1
**4431**	321	C	72.4879	0
**4431**	351	C	2.8503	1
**4431**	357	C	48.4896	0
**4431**	370	C	76.6007	1
**4431**	372	C	42.8953	1
**4431**	373	C	59.4908	1
**4431**	381	C	45.7119	0
**4431**	382	C	74.6802	1
**4431**	386	C	28.9511	0
**4431**	305	D	68.9491	1
**4431**	319	D	60.6963	1
**4431**	320	D	68.1751	-1
**4431**	321	D	79.5936	0
**4431**	351	D	25.9186	-1
**4431**	357	D	51.8393	1
**4431**	370	D	46.3874	0
**4431**	372	D	65.209	0

**4431**	373	D	67.2313	1
**4431**	381	D	79.6092	1
**4431**	382	D	69.1807	1
**4431**	386	D	79.5731	1
**4432**	305	A	79.5706	1
**4432**	319	A	25.6478	0
**4432**	320	A	68.7102	-1
**4432**	321	A	44.5324	1
**4432**	351	A	4.2462	1
**4432**	357	A	39.2192	0
**4432**	370	A	65.6188	1
**4432**	372	A	70.2518	0
**4432**	373	A	79.3524	1
**4432**	381	A	79.5626	1
**4432**	382	A	74.1033	1
**4432**	386	A	79.5847	1
**4432**	305	B	67.3633	1
**4432**	319	B	58.6436	1
**4432**	320	B	75.7099	-1
**4432**	321	B	63.6465	0
**4432**	351	B	4.223	1
**4432**	357	B	79.5241	1
**4432**	370	B	74.2612	1
**4432**	372	B	60.074	0
**4432**	373	B	64.0308	1
**4432**	381	B	69.4061	1
**4432**	382	B	68.7015	1
**4432**	386	B	57.7898	1
**4432**	305	C	66.8391	1
**4432**	319	C	75.1804	1
**4432**	320	C	63.6323	-1
**4432**	321	C	70.5546	0
**4432**	351	C	4.0973	1
**4432**	357	C	79.5389	1

**4432**	370	C	42.2416	0
**4432**	372	C	52.1112	0
**4432**	373	C	40.5583	0
**4432**	381	C	69.4321	1
**4432**	382	C	79.501	1
**4432**	386	C	57.5687	1
**4432**	305	D	50.9681	1
**4432**	319	D	70.7231	1
**4432**	320	D	72.6984	-1
**4432**	321	D	79.5388	0
**4432**	351	D	11.9431	1
**4432**	357	D	53.9043	1
**4432**	370	D	74.8422	1
**4432**	372	D	73.941	0
**4432**	373	D	70.8902	1
**4432**	381	D	79.5425	1
**4432**	382	D	72.25	1
**4432**	386	D	21.3098	0
**4433**	305	A	21.8926	0
**4433**	319	A	73.3615	1
**4433**	320	A	72.47	-1
**4433**	321	A	55.8748	0
**4433**	351	A	1.3151	1
**4433**	357	A	37.4827	0
**4433**	370	A	35.6898	0
**4433**	372	A	21.5896	1
**4433**	373	A	74.6678	1
**4433**	381	A	73.5896	1
**4433**	382	A	79.4927	1
**4433**	386	A	75.3399	1
**4433**	305	B	49.6779	0
**4433**	319	B	63.2419	1
**4433**	320	B	37.1063	-1
**4433**	321	B	71.5443	0

**4433**	351	B	16.738	1
**4433**	357	B	56.2474	1
**4433**	370	B	73.6644	1
**4433**	372	B	67.6813	0
**4433**	373	B	67.6511	1
**4433**	381	B	51.6611	1
**4433**	382	B	64.2119	1
**4433**	386	B	77.9844	1
**4433**	305	C	50.769	1
**4433**	319	C	72.3625	1
**4433**	320	C	79.5297	-1
**4433**	321	C	70.4097	0
**4433**	351	C	7.17	1
**4433**	357	C	75.1967	1
**4433**	370	C	77.0382	1
**4433**	372	C	79.512	0
**4433**	373	C	79.6924	1
**4433**	381	C	45.8546	0
**4433**	382	C	79.3576	1
**4433**	386	C	53.4186	1
**4433**	305	D	71.9633	1
**4433**	319	D	69.8735	1
**4433**	320	D	77.1846	-1
**4433**	321	D	45.6401	1
**4433**	351	D	19.1299	1
**4433**	357	D	79.5584	1
**4433**	370	D	56.4406	1
**4433**	372	D	74.3011	0
**4433**	373	D	56.0665	1
**4433**	381	D	47.6383	0
**4433**	382	D	45.6578	0
**4433**	386	D	63.7342	1
**4434**	305	A	59.1529	1
**4434**	319	A	79.5489	1

**4434**	320	A	79.5565	-1
**4434**	321	A	67.6531	0
**4434**	351	A	6.0005	1
**4434**	357	A	68.609	1
**4434**	370	A	37.2142	0
**4434**	372	A	50.6562	0
**4434**	373	A	79.4773	1
**4434**	381	A	57.5868	1
**4434**	382	A	69.9187	1
**4434**	386	A	56.2623	1
**4434**	305	B	73.1861	1
**4434**	319	B	73.3461	1
**4434**	320	B	72.0663	-1
**4434**	321	B	79.2659	0
**4434**	351	B	11.025	1
**4434**	357	B	79.5546	1
**4434**	370	B	63.0832	1
**4434**	372	B	79.5792	0
**4434**	373	B	65.1375	1
**4434**	381	B	79.5455	1
**4434**	382	B	36.7207	0
**4434**	386	B	36.6568	0
**4434**	305	C	59.6482	1
**4434**	319	C	79.5456	1
**4434**	320	C	79.567	-1
**4434**	321	C	73.4411	0
**4434**	351	C	1.9257	1
**4434**	357	C	79.5337	1
**4434**	370	C	77.0598	1
**4434**	372	C	79.3898	0
**4434**	373	C	78.8247	1
**4434**	381	C	79.5532	1
**4434**	382	C	70.9202	1
**4434**	386	C	36.4044	0

**4434**	305	D	29.9493	0
**4434**	319	D	71.5379	1
**4434**	320	D	79.6061	-1
**4434**	321	D	56.2424	0
**4434**	351	D	19.7386	1
**4434**	357	D	61.6558	1
**4434**	370	D	69.5255	1
**4434**	372	D	73.763	0
**4434**	373	D	79.5837	1
**4434**	381	D	59.2303	1
**4434**	382	D	79.529	1
**4434**	386	D	79.5169	1
**4435**	305	A	74.163	1
**4435**	319	A	67.1714	1
**4435**	320	A	79.5491	-1
**4435**	321	A	75.1703	0
**4435**	351	A	6.0231	1
**4435**	357	A	65.2744	1
**4435**	370	A	77.7645	1
**4435**	372	A	69.8581	0
**4435**	373	A	66.6584	1
**4435**	381	A	79.5774	1
**4435**	382	A	79.5498	1
**4435**	386	A	79.6356	1
**4435**	305	B	71.2297	1
**4435**	319	B	72.4878	1
**4435**	320	B	79.5926	-1
**4435**	321	B	73.8031	0
**4435**	351	B	9.7371	1
**4435**	357	B	68.31	1
**4435**	370	B	48.9494	0
**4435**	372	B	71.0531	0
**4435**	373	B	70.9602	1
**4435**	381	B	79.4946	1

**4435**	382	B	38.6794	0
**4435**	386	B	79.4573	1
**4435**	305	C	66.1428	1
**4435**	319	C	79.593	1
**4435**	320	C	79.5702	-1
**4435**	321	C	76.411	0
**4435**	351	C	11.6392	1
**4435**	357	C	61.2779	1
**4435**	370	C	72.7238	1
**4435**	372	C	72.5351	0
**4435**	373	C	58.3443	1
**4435**	381	C	79.6627	1
**4435**	382	C	56.5719	1
**4435**	386	C	55.3752	1
**4435**	305	D	41.3954	0
**4435**	319	D	71.1903	1
**4435**	320	D	74.0237	-1
**4435**	321	D	78.9553	0
**4435**	351	D	17.1188	1
**4435**	357	D	52.7728	1
**4435**	370	D	52.1802	1
**4435**	372	D	79.5369	0
**4435**	373	D	78.9448	1
**4435**	381	D	79.5381	1
**4435**	382	D	79.1076	1
**4435**	386	D	73.1411	1
**4436**	305	A	71.3928	1
**4436**	319	A	78.9163	1
**4436**	320	A	79.6572	-1
**4436**	321	A	79.5648	0
**4436**	351	A	10.877	1
**4436**	357	A	73.7557	1
**4436**	370	A	79.5862	1
**4436**	372	A	73.1216	0

**4436**	373	A	79.5821	1
**4436**	381	A	79.5669	1
**4436**	382	A	70.0199	1
**4436**	386	A	79.6836	1
**4436**	305	B	70.4305	1
**4436**	319	B	79.5532	1
**4436**	320	B	39.9888	-1
**4436**	321	B	63.9958	0
**4436**	351	B	15.4375	1
**4436**	357	B	68.5521	1
**4436**	370	B	51.6006	1
**4436**	372	B	66.2476	0
**4436**	373	B	79.507	1
**4436**	381	B	79.5765	1
**4436**	382	B	60.8032	1
**4436**	386	B	62.8195	1
**4436**	305	C	58.1992	1
**4436**	319	C	63.4144	1
**4436**	320	C	55.0417	-1
**4436**	321	C	32.554	1
**4436**	351	C	9.692	1
**4436**	357	C	51.548	1
**4436**	370	C	35.9893	0
**4436**	372	C	79.5287	0
**4436**	373	C	75.8115	1
**4436**	381	C	64.1446	1
**4436**	382	C	36.7095	0
**4436**	386	C	31.0255	0
**4436**	305	D	59.1522	1
**4436**	319	D	50.4146	1
**4436**	320	D	69.8372	-1
**4436**	321	D	79.6268	0
**4436**	351	D	20.7237	-1
**4436**	357	D	79.6114	1

**4436**	370	D	18.6364	-1
**4436**	372	D	79.5396	0
**4436**	373	D	70.6281	1
**4436**	381	D	55.3556	1
**4436**	382	D	79.5936	1
**4436**	386	D	57.9027	1
**4437**	305	A	73.2218	1
**4437**	319	A	79.5938	1
**4437**	320	A	66.8592	-1
**4437**	321	A	69.7148	0
**4437**	351	A	5.982	1
**4437**	357	A	73.4445	1
**4437**	370	A	61.141	1
**4437**	372	A	59.0439	0
**4437**	373	A	32.1615	0
**4437**	381	A	64.9294	1
**4437**	382	A	79.5287	1
**4437**	386	A	79.5958	1
**4437**	305	B	54.3589	1
**4437**	319	B	66.6342	1
**4437**	320	B	79.5038	-1
**4437**	321	B	71.7511	0
**4437**	351	B	9.0247	1
**4437**	357	B	42.1819	0
**4437**	370	B	37.9201	0
**4437**	372	B	62.4913	0
**4437**	373	B	69.6795	1
**4437**	381	B	55.1807	1
**4437**	382	B	35.3987	0
**4437**	386	B	66.9391	1
**4437**	305	C	79.6023	1
**4437**	319	C	71.341	1
**4437**	320	C	72.0368	-1
**4437**	321	C	40.7084	1

**4437**	351	C	12.6075	1
**4437**	357	C	79.6022	1
**4437**	370	C	44.2061	0
**4437**	372	C	73.8908	0
**4437**	373	C	44.626	0
**4437**	381	C	71.3231	1
**4437**	382	C	64.2251	1
**4437**	386	C	50.855	1
**4437**	305	D	62.2518	1
**4437**	319	D	79.5832	1
**4437**	320	D	79.6193	-1
**4437**	321	D	79.5887	0
**4437**	351	D	2.1753	1
**4437**	357	D	79.5649	1
**4437**	370	D	33.2431	0
**4437**	372	D	71.4222	0
**4437**	373	D	71.0792	1
**4437**	381	D	79.5219	1
**4437**	382	D	68.3088	1
**4437**	386	D	54.8802	1
**4438**	305	A	17.7489	-1
**4438**	319	A	65.3787	1
**4438**	320	A	71.5503	-1
**4438**	321	A	73.5237	0
**4438**	351	A	8.9459	1
**4438**	357	A	77.9387	1
**4438**	370	A	52.8921	1
**4438**	372	A	74.0662	0
**4438**	373	A	71.5044	1
**4438**	381	A	39.3097	0
**4438**	382	A	50.0754	1
**4438**	386	A	79.5432	1
**4438**	305	B	79.5941	1
**4438**	319	B	68.8807	1

**4438**	320	B	65.4734	-1
**4438**	321	B	79.5773	0
**4438**	351	B	13.9455	1
**4438**	357	B	79.5694	1
**4438**	370	B	79.6036	1
**4438**	372	B	79.5852	0
**4438**	373	B	65.2075	1
**4438**	381	B	50.9947	1
**4438**	382	B	79.6024	1
**4438**	386	B	79.6374	1
**4438**	305	C	44.3417	0
**4438**	319	C	79.5594	1
**4438**	320	C	79.5522	-1
**4438**	321	C	72.9834	0
**4438**	351	C	1.0557	1
**4438**	357	C	79.0099	1
**4438**	370	C	79.6255	1
**4438**	372	C	79.5898	0
**4438**	373	C	79.5843	1
**4438**	381	C	79.5737	1
**4438**	382	C	74.433	1
**4438**	386	C	58.7351	1
**4438**	305	D	70.2862	1
**4438**	319	D	79.558	1
**4438**	320	D	38.2885	-1
**4438**	321	D	65.8288	0
**4438**	351	D	13.6196	1
**4438**	357	D	79.5472	1
**4438**	370	D	75.7845	1
**4438**	372	D	61.6901	0
**4438**	373	D	79.5192	1
**4438**	381	D	76.9243	1
**4438**	382	D	79.5664	1
**4438**	386	D	79.5683	1

**4439**	305	A	79.5517	1
**4439**	319	A	53.4148	1
**4439**	320	A	79.5096	-1
**4439**	321	A	64.0264	0
**4439**	351	A	15.4839	1
**4439**	357	A	79.5441	1
**4439**	370	A	75.22	1
**4439**	372	A	78.4649	0
**4439**	373	A	79.5438	1
**4439**	381	A	70.4727	1
**4439**	382	A	23.895	0
**4439**	386	A	70.1274	1
**4439**	305	B	68.4988	1
**4439**	319	B	65.1952	1
**4439**	320	B	79.6601	-1
**4439**	321	B	68.4883	0
**4439**	351	B	21.4098	-1
**4439**	357	B	79.4546	1
**4439**	370	B	58.4137	1
**4439**	372	B	55.8467	0
**4439**	373	B	40.7912	0
**4439**	381	B	54.8233	1
**4439**	382	B	75.5268	1
**4439**	386	B	73.7885	1
**4439**	305	C	75.3321	1
**4439**	319	C	79.5986	1
**4439**	320	C	76.134	-1
**4439**	321	C	65.7115	0
**4439**	351	C	0.0363	1
**4439**	357	C	79.5426	1
**4439**	370	C	78.2926	1
**4439**	372	C	79.6009	0
**4439**	373	C	79.5757	1
**4439**	381	C	64.5552	1

**4439**	382	C	73.3081	1
**4439**	386	C	58.2541	1
**4439**	305	D	33.0595	0
**4439**	319	D	78.5016	1
**4439**	320	D	73.9917	-1
**4439**	321	D	65.4141	0
**4439**	351	D	17.1659	1
**4439**	357	D	79.5054	1
**4439**	370	D	79.438	1
**4439**	372	D	71.4239	0
**4439**	373	D	79.5484	1
**4439**	381	D	79.4743	1
**4439**	382	D	79.4853	1
**4439**	386	D	72.3915	1
**4440**	305	A	79.6374	1
**4440**	319	A	67.0494	1
**4440**	320	A	79.7208	-1
**4440**	321	A	78.9094	0
**4440**	351	A	8.3422	1
**4440**	357	A	79.577	1
**4440**	370	A	79.5026	1
**4440**	372	A	79.5929	0
**4440**	373	A	79.6351	1
**4440**	381	A	79.5603	1
**4440**	382	A	77.9178	1
**4440**	386	A	78.4892	1
**4440**	305	B	79.5855	1
**4440**	319	B	76.1187	1
**4440**	320	B	69.248	-1
**4440**	321	B	69.6664	0
**4440**	351	B	30.038	-1
**4440**	357	B	41.4845	0
**4440**	370	B	55.454	1
**4440**	372	B	73.5901	0

**4440**	373	B	79.5035	1
**4440**	381	B	61.5367	1
**4440**	382	B	66.4526	1
**4440**	386	B	77.7402	1
**4440**	305	C	20.7002	0
**4440**	319	C	79.5067	1
**4440**	320	C	66.2973	-1
**4440**	321	C	61.695	0
**4440**	351	C	31.0238	-1
**4440**	357	C	46.9727	0
**4440**	370	C	79.4687	1
**4440**	372	C	79.6219	0
**4440**	373	C	37.9122	0
**4440**	381	C	79.5774	1
**4440**	382	C	72.0374	1
**4440**	386	C	37.7803	0
**4440**	305	D	15.5112	-1
**4440**	319	D	74.2141	1
**4440**	320	D	79.6185	-1
**4440**	321	D	66.1142	0
**4440**	351	D	13.4032	1
**4440**	357	D	69.59	1
**4440**	370	D	21.524	0
**4440**	372	D	65.1054	0
**4440**	373	D	78.4108	1
**4440**	381	D	63.642	1
**4440**	382	D	6.996	-1
**4440**	386	D	72.4932	1
**4441**	305	A	45.2675	0
**4441**	319	A	78.1175	1
**4441**	320	A	46.8278	-1
**4441**	321	A	73.3012	0
**4441**	351	A	12.7449	1
**4441**	357	A	56.7171	1

**4441**	370	A	37.3379	0
**4441**	372	A	33.1178	1
**4441**	373	A	73.0545	1
**4441**	381	A	49.5226	0
**4441**	382	A	79.5477	1
**4441**	386	A	79.4969	1
**4441**	305	B	79.4274	1
**4441**	319	B	75.646	1
**4441**	320	B	78.482	-1
**4441**	321	B	58.9168	0
**4441**	351	B	18.9151	1
**4441**	357	B	77.4275	1
**4441**	370	B	79.548	1
**4441**	372	B	79.5313	0
**4441**	373	B	67.6979	1
**4441**	381	B	64.8122	1
**4441**	382	B	12.0002	-1
**4441**	386	B	66.9676	1
**4441**	305	C	79.6553	1
**4441**	319	C	44.9808	0
**4441**	320	C	77.896	-1
**4441**	321	C	35.4851	1
**4441**	351	C	33.9665	-1
**4441**	357	C	40.0151	0
**4441**	370	C	62.725	1
**4441**	372	C	72.3284	0
**4441**	373	C	44.1741	0
**4441**	381	C	67.0268	1
**4441**	382	C	77.8036	1
**4441**	386	C	51.7587	1
**4441**	305	D	33.6827	0
**4441**	319	D	5.1169	-1
**4441**	320	D	46.0992	-1
**4441**	321	D	79.6558	0

**4441**	351	D	12.3251	1
**4441**	357	D	41.5751	0
**4441**	370	D	79.5195	1
**4441**	372	D	33.7112	1
**4441**	373	D	74.8577	1
**4441**	381	D	61.8222	1
**4441**	382	D	68.7831	1
**4441**	386	D	63.006	1
**4442**	305	A	66.9374	1
**4442**	319	A	79.576	1
**4442**	320	A	72.3429	-1
**4442**	321	A	70.4322	0
**4442**	351	A	10.9429	1
**4442**	357	A	69.9292	1
**4442**	370	A	76.367	1
**4442**	372	A	18.5895	0
**4442**	373	A	79.5673	1
**4442**	381	A	79.631	1
**4442**	382	A	72.5543	1
**4442**	386	A	79.227	1
**4442**	305	B	31.2511	0
**4442**	319	B	64.6824	1
**4442**	320	B	42.2609	-1
**4442**	321	B	57.6116	0
**4442**	351	B	8.7394	1
**4442**	357	B	79.5505	1
**4442**	370	B	73.2183	1
**4442**	372	B	73.8659	0
**4442**	373	B	77.8379	1
**4442**	381	B	69.8884	1
**4442**	382	B	42.9566	0
**4442**	386	B	32.173	0
**4442**	305	C	79.0434	1
**4442**	319	C	79.5675	1

**4442**	320	C	54.4339	-1
**4442**	321	C	71.8305	0
**4442**	351	C	5.5049	1
**4442**	357	C	79.6779	1
**4442**	370	C	62.561	1
**4442**	372	C	31.1406	1
**4442**	373	C	37.3432	0
**4442**	381	C	70.8509	1
**4442**	382	C	54.4437	1
**4442**	386	C	28.1615	0
**4442**	305	D	79.4258	1
**4442**	319	D	65.6399	1
**4442**	320	D	54.9992	-1
**4442**	321	D	79.5747	0
**4442**	351	D	1.0012	1
**4442**	357	D	79.5728	1
**4442**	370	D	70.7588	1
**4442**	372	D	72.1274	0
**4442**	373	D	49.1404	0
**4442**	381	D	51.9899	1
**4442**	382	D	73.5436	1
**4442**	386	D	56.7828	1
**4443**	305	A	76.9293	1
**4443**	319	A	66.2629	1
**4443**	320	A	79.5531	-1
**4443**	321	A	65.9028	0
**4443**	351	A	8.4835	1
**4443**	357	A	79.5739	1
**4443**	370	A	79.5625	1
**4443**	372	A	71.9445	0
**4443**	373	A	49.3774	0
**4443**	381	A	79.5576	1
**4443**	382	A	58.545	1
**4443**	386	A	79.575	1

**4443**	305	B	69.3745	1
**4443**	319	B	66.1458	1
**4443**	320	B	39.8228	-1
**4443**	321	B	68.1987	0
**4443**	351	B	16.8474	1
**4443**	357	B	71.9658	1
**4443**	370	B	79.253	1
**4443**	372	B	79.5555	0
**4443**	373	B	50.1787	1
**4443**	381	B	71.533	1
**4443**	382	B	27.6231	0
**4443**	386	B	63.0957	1
**4443**	305	C	77.6302	1
**4443**	319	C	79.165	1
**4443**	320	C	74.519	-1
**4443**	321	C	79.5829	0
**4443**	351	C	11.4807	1
**4443**	357	C	53.3148	1
**4443**	370	C	79.5788	1
**4443**	372	C	79.5912	0
**4443**	373	C	79.5467	1
**4443**	381	C	51.1847	1
**4443**	382	C	79.3501	1
**4443**	386	C	47.6542	0
**4443**	305	D	63.9215	1
**4443**	319	D	73.9957	1
**4443**	320	D	72.873	-1
**4443**	321	D	79.6039	0
**4443**	351	D	13.3979	1
**4443**	357	D	74.283	1
**4443**	370	D	66.144	1
**4443**	372	D	79.5072	0
**4443**	373	D	79.5396	1
**4443**	381	D	67.0718	1

**4443**	382	D	79.5399	1
**4443**	386	D	70.9545	1
**4444**	305	A	66.5002	1
**4444**	319	A	73.1849	1
**4444**	320	A	59.42	-1
**4444**	321	A	42.403	1
**4444**	351	A	4.7598	1
**4444**	357	A	73.3813	1
**4444**	370	A	79.5378	1
**4444**	372	A	63.4329	0
**4444**	373	A	60.0098	1
**4444**	381	A	78.9237	1
**4444**	382	A	60.8911	1
**4444**	386	A	79.6114	1
**4444**	305	B	79.5328	1
**4444**	319	B	73.1902	1
**4444**	320	B	79.5227	-1
**4444**	321	B	75.1977	0
**4444**	351	B	11.9436	1
**4444**	357	B	75.7334	1
**4444**	370	B	53.8996	1
**4444**	372	B	79.6861	0
**4444**	373	B	45.9293	0
**4444**	381	B	79.6304	1
**4444**	382	B	22.4758	0
**4444**	386	B	68.1136	1
**4444**	305	C	79.5386	1
**4444**	319	C	62.265	1
**4444**	320	C	70.116	-1
**4444**	321	C	73.589	0
**4444**	351	C	7.0722	1
**4444**	357	C	61.808	1
**4444**	370	C	79.5618	1
**4444**	372	C	73.1112	0

**4444**	373	C	44.9717	0
**4444**	381	C	49.83	0
**4444**	382	C	54.958	1
**4444**	386	C	25.7317	0
**4444**	305	D	79.6417	1
**4444**	319	D	74.55	1
**4444**	320	D	73.9304	-1
**4444**	321	D	49.3536	1
**4444**	351	D	0.0	1
**4444**	357	D	72.4288	1
**4444**	370	D	54.7013	1
**4444**	372	D	79.0188	0
**4444**	373	D	57.5482	1
**4444**	381	D	40.191	0
**4444**	382	D	35.5618	0
**4444**	386	D	61.4755	1
**4445**	305	A	79.2112	1
**4445**	319	A	72.4381	1
**4445**	320	A	79.5438	-1
**4445**	321	A	79.5755	0
**4445**	351	A	6.0434	1
**4445**	357	A	60.053	1
**4445**	370	A	79.5397	1
**4445**	372	A	64.0551	0
**4445**	373	A	45.5628	0
**4445**	381	A	50.0791	1
**4445**	382	A	73.0811	1
**4445**	386	A	57.363	1
**4445**	305	B	79.5814	1
**4445**	319	B	75.8	1
**4445**	320	B	73.0469	-1
**4445**	321	B	79.5896	0
**4445**	351	B	8.3552	1
**4445**	357	B	79.579	1

**4445**	370	B	63.7637	1
**4445**	372	B	79.5561	0
**4445**	373	B	62.8992	1
**4445**	381	B	38.1717	0
**4445**	382	B	48.6863	0
**4445**	386	B	57.9944	1
**4445**	305	C	78.803	1
**4445**	319	C	45.2853	0
**4445**	320	C	71.6504	-1
**4445**	321	C	79.5756	0
**4445**	351	C	6.7082	1
**4445**	357	C	79.407	1
**4445**	370	C	67.3111	1
**4445**	372	C	79.5691	0
**4445**	373	C	40.8304	0
**4445**	381	C	73.3823	1
**4445**	382	C	46.5395	0
**4445**	386	C	57.1154	1
**4445**	305	D	64.3371	1
**4445**	319	D	55.2371	1
**4445**	320	D	79.5417	-1
**4445**	321	D	79.6104	0
**4445**	351	D	9.0053	1
**4445**	357	D	79.5673	1
**4445**	370	D	77.6143	1
**4445**	372	D	79.584	0
**4445**	373	D	79.5338	1
**4445**	381	D	71.92	1
**4445**	382	D	70.618	1
**4445**	386	D	64.4863	1
**4446**	305	A	74.0414	1
**4446**	319	A	74.5452	1
**4446**	320	A	79.5221	-1
**4446**	321	A	79.598	0

**4446**	351	A	4.9649	1
**4446**	357	A	76.4209	1
**4446**	370	A	74.1077	1
**4446**	372	A	79.5889	0
**4446**	373	A	72.1268	1
**4446**	381	A	48.3961	0
**4446**	382	A	68.609	1
**4446**	386	A	73.6648	1
**4446**	305	B	79.5412	1
**4446**	319	B	79.5496	1
**4446**	320	B	71.3952	-1
**4446**	321	B	63.6873	0
**4446**	351	B	18.2089	1
**4446**	357	B	79.5611	1
**4446**	370	B	78.6884	1
**4446**	372	B	79.5602	0
**4446**	373	B	64.0993	1
**4446**	381	B	79.6153	1
**4446**	382	B	41.5514	0
**4446**	386	B	66.5777	1
**4446**	305	C	47.4202	0
**4446**	319	C	79.1501	1
**4446**	320	C	79.5525	-1
**4446**	321	C	61.9809	0
**4446**	351	C	2.2275	1
**4446**	357	C	71.138	1
**4446**	370	C	79.5694	1
**4446**	372	C	69.9977	0
**4446**	373	C	70.4851	1
**4446**	381	C	70.3502	1
**4446**	382	C	72.4784	1
**4446**	386	C	48.8977	0
**4446**	305	D	37.5201	0
**4446**	319	D	79.5573	1

**4446**	320	D	73.5257	-1
**4446**	321	D	79.4995	0
**4446**	351	D	5.1103	1
**4446**	357	D	73.5121	1
**4446**	370	D	45.4591	0
**4446**	372	D	71.6509	0
**4446**	373	D	66.2941	1
**4446**	381	D	79.5457	1
**4446**	382	D	79.5099	1
**4446**	386	D	79.5481	1
**4447**	305	A	63.4249	1
**4447**	319	A	79.5864	1
**4447**	320	A	78.7362	-1
**4447**	321	A	68.8285	0
**4447**	351	A	1.6358	1
**4447**	357	A	71.6031	1
**4447**	370	A	32.7698	0
**4447**	372	A	52.6068	0
**4447**	373	A	42.776	0
**4447**	381	A	74.8039	1
**4447**	382	A	76.1474	1
**4447**	386	A	23.0915	0
**4447**	305	B	71.4006	1
**4447**	319	B	47.3335	0
**4447**	320	B	73.5198	-1
**4447**	321	B	72.3454	0
**4447**	351	B	10.707	1
**4447**	357	B	48.6625	0
**4447**	370	B	79.565	1
**4447**	372	B	51.3535	0
**4447**	373	B	55.6056	1
**4447**	381	B	64.4813	1
**4447**	382	B	79.5145	1
**4447**	386	B	79.6437	1

**4447**	305	C	79.5356	1
**4447**	319	C	58.8445	1
**4447**	320	C	44.1835	-1
**4447**	321	C	67.3586	0
**4447**	351	C	8.8474	1
**4447**	357	C	62.3883	1
**4447**	370	C	79.6464	1
**4447**	372	C	79.4685	0
**4447**	373	C	79.4276	1
**4447**	381	C	79.5077	1
**4447**	382	C	47.3048	0
**4447**	386	C	38.1295	0
**4447**	305	D	14.4785	-1
**4447**	319	D	79.684	1
**4447**	320	D	43.2553	-1
**4447**	321	D	65.6255	0
**4447**	351	D	5.751	1
**4447**	357	D	74.0733	1
**4447**	370	D	79.4507	1
**4447**	372	D	73.757	0
**4447**	373	D	79.4328	1
**4447**	381	D	66.9407	1
**4447**	382	D	76.2628	1
**4447**	386	D	61.8792	1
**4448**	305	A	71.4256	1
**4448**	319	A	70.4102	1
**4448**	320	A	79.4929	-1
**4448**	321	A	76.7452	0
**4448**	351	A	9.4896	1
**4448**	357	A	79.5038	1
**4448**	370	A	73.7021	1
**4448**	372	A	79.4477	0
**4448**	373	A	79.5923	1
**4448**	381	A	65.1528	1

**4448**	382	A	66.7909	1
**4448**	386	A	79.4934	1
**4448**	305	B	40.1825	0
**4448**	319	B	35.2978	0
**4448**	320	B	79.2351	-1
**4448**	321	B	72.2979	0
**4448**	351	B	6.8863	1
**4448**	357	B	79.5605	1
**4448**	370	B	68.075	1
**4448**	372	B	73.1303	0
**4448**	373	B	76.7777	1
**4448**	381	B	66.8087	1
**4448**	382	B	55.8388	1
**4448**	386	B	79.5165	1
**4448**	305	C	79.8703	1
**4448**	319	C	62.747	1
**4448**	320	C	73.2886	-1
**4448**	321	C	68.9949	0
**4448**	351	C	9.4415	1
**4448**	357	C	63.6172	1
**4448**	370	C	79.513	1
**4448**	372	C	79.5812	0
**4448**	373	C	79.6088	1
**4448**	381	C	75.4249	1
**4448**	382	C	73.3135	1
**4448**	386	C	62.3501	1
**4448**	305	D	33.4755	0
**4448**	319	D	79.5763	1
**4448**	320	D	59.9001	-1
**4448**	321	D	79.0806	0
**4448**	351	D	39.1532	-1
**4448**	357	D	74.2052	1
**4448**	370	D	79.5203	1
**4448**	372	D	59.7644	0

**4448**	373	D	79.6294	1
**4448**	381	D	68.1281	1
**4448**	382	D	50.4315	1
**4448**	386	D	79.5251	1
**4449**	305	A	79.4741	1
**4449**	319	A	65.8505	1
**4449**	320	A	79.575	-1
**4449**	321	A	57.3066	0
**4449**	351	A	7.6279	1
**4449**	357	A	72.9829	1
**4449**	370	A	56.0492	1
**4449**	372	A	62.05	0
**4449**	373	A	20.0469	0
**4449**	381	A	40.5326	0
**4449**	382	A	23.1211	0
**4449**	386	A	76.3957	1
**4449**	305	B	79.6943	1
**4449**	319	B	69.6548	1
**4449**	320	B	49.9691	-1
**4449**	321	B	67.4595	0
**4449**	351	B	26.7178	-1
**4449**	357	B	46.1929	0
**4449**	370	B	51.7941	1
**4449**	372	B	41.4411	1
**4449**	373	B	69.0972	1
**4449**	381	B	74.6684	1
**4449**	382	B	34.6927	0
**4449**	386	B	66.1104	1
**4449**	305	C	78.3828	1
**4449**	319	C	79.1506	1
**4449**	320	C	79.5882	-1
**4449**	321	C	64.3015	0
**4449**	351	C	4.9165	1
**4449**	357	C	79.5506	1

**4449**	370	C	79.5773	1
**4449**	372	C	79.4601	0
**4449**	373	C	79.6231	1
**4449**	381	C	37.9608	0
**4449**	382	C	64.4534	1
**4449**	386	C	56.4952	1
**4449**	305	D	25.224	0
**4449**	319	D	69.4839	1
**4449**	320	D	70.6284	-1
**4449**	321	D	61.5317	0
**4449**	351	D	9.6691	1
**4449**	357	D	79.4158	1
**4449**	370	D	74.1471	1
**4449**	372	D	79.5436	0
**4449**	373	D	78.7705	1
**4449**	381	D	35.8109	0
**4449**	382	D	48.6127	0
**4449**	386	D	72.8883	1
**4450**	305	A	78.0458	1
**4450**	319	A	72.537	1
**4450**	320	A	79.5819	-1
**4450**	321	A	40.7906	1
**4450**	351	A	6.3683	1
**4450**	357	A	79.5677	1
**4450**	370	A	69.0682	1
**4450**	372	A	64.221	0
**4450**	373	A	16.8959	-1
**4450**	381	A	68.9198	1
**4450**	382	A	49.7323	0
**4450**	386	A	79.5448	1
**4450**	305	B	57.9432	1
**4450**	319	B	61.3041	1
**4450**	320	B	30.8666	-1
**4450**	321	B	61.5904	0

**4450**	351	B	16.6287	1
**4450**	357	B	77.4829	1
**4450**	370	B	45.2913	0
**4450**	372	B	79.5364	0
**4450**	373	B	48.9083	0
**4450**	381	B	61.903	1
**4450**	382	B	28.15	0
**4450**	386	B	79.5599	1
**4450**	305	C	43.3806	0
**4450**	319	C	49.7709	0
**4450**	320	C	29.6131	-1
**4450**	321	C	30.1883	1
**4450**	351	C	1.8101	1
**4450**	357	C	79.5508	1
**4450**	370	C	73.076	1
**4450**	372	C	79.5808	0
**4450**	373	C	79.5636	1
**4450**	381	C	79.5554	1
**4450**	382	C	79.5816	1
**4450**	386	C	51.9052	1
**4450**	305	D	77.9049	1
**4450**	319	D	72.0598	1
**4450**	320	D	79.5877	-1
**4450**	321	D	60.286	0
**4450**	351	D	10.3004	1
**4450**	357	D	69.3883	1
**4450**	370	D	48.1852	0
**4450**	372	D	79.0798	0
**4450**	373	D	79.5715	1
**4450**	381	D	77.8873	1
**4450**	382	D	79.5671	1
**4450**	386	D	79.5639	1
**4451**	305	A	79.077	1
**4451**	319	A	76.61	1

**4451**	320	A	79.4963	-1
**4451**	321	A	77.1947	0
**4451**	351	A	8.8896	1
**4451**	357	A	74.2619	1
**4451**	370	A	62.0891	1
**4451**	372	A	79.4496	0
**4451**	373	A	79.3993	1
**4451**	381	A	77.376	1
**4451**	382	A	66.1805	1
**4451**	386	A	44.5007	0
**4451**	305	B	74.1586	1
**4451**	319	B	79.5095	1
**4451**	320	B	77.592	-1
**4451**	321	B	59.6532	0
**4451**	351	B	7.7186	1
**4451**	357	B	72.9941	1
**4451**	370	B	67.4388	1
**4451**	372	B	38.8605	1
**4451**	373	B	65.0506	1
**4451**	381	B	61.9939	1
**4451**	382	B	53.6063	1
**4451**	386	B	48.7495	0
**4451**	305	C	64.1368	1
**4451**	319	C	50.6153	1
**4451**	320	C	67.6013	-1
**4451**	321	C	35.6227	1
**4451**	351	C	7.6728	1
**4451**	357	C	79.1799	1
**4451**	370	C	79.4788	1
**4451**	372	C	71.0048	0
**4451**	373	C	74.6995	1
**4451**	381	C	77.6506	1
**4451**	382	C	79.7072	1
**4451**	386	C	41.7922	0

**4451**	305	D	63.2633	1
**4451**	319	D	79.6112	1
**4451**	320	D	72.0843	-1
**4451**	321	D	79.7231	0
**4451**	351	D	6.0352	1
**4451**	357	D	64.5952	1
**4451**	370	D	68.144	1
**4451**	372	D	79.5846	0
**4451**	373	D	71.117	1
**4451**	381	D	73.1164	1
**4451**	382	D	75.1486	1
**4451**	386	D	77.9856	1
**4452**	305	A	79.4916	1
**4452**	319	A	56.1112	1
**4452**	320	A	78.7521	-1
**4452**	321	A	79.7208	0
**4452**	351	A	3.8728	1
**4452**	357	A	76.713	1
**4452**	370	A	79.5834	1
**4452**	372	A	74.6791	0
**4452**	373	A	70.3693	1
**4452**	381	A	66.7363	1
**4452**	382	A	59.5349	1
**4452**	386	A	70.488	1
**4452**	305	B	79.4988	1
**4452**	319	B	79.4281	1
**4452**	320	B	31.3263	-1
**4452**	321	B	65.8227	0
**4452**	351	B	16.6024	1
**4452**	357	B	79.6718	1
**4452**	370	B	56.7718	1
**4452**	372	B	72.9344	0
**4452**	373	B	76.4899	1
**4452**	381	B	45.6154	0

**4452**	382	B	73.7106	1
**4452**	386	B	79.5232	1
**4452**	305	C	73.6082	1
**4452**	319	C	29.9312	0
**4452**	320	C	62.6964	-1
**4452**	321	C	79.7558	0
**4452**	351	C	9.6085	1
**4452**	357	C	79.5368	1
**4452**	370	C	45.0375	0
**4452**	372	C	68.5809	0
**4452**	373	C	64.5866	1
**4452**	381	C	67.9668	1
**4452**	382	C	39.35	0
**4452**	386	C	64.1685	1
**4452**	305	D	72.4347	1
**4452**	319	D	70.8825	1
**4452**	320	D	74.6359	-1
**4452**	321	D	59.1065	0
**4452**	351	D	11.0232	1
**4452**	357	D	79.655	1
**4452**	370	D	79.5013	1
**4452**	372	D	44.7648	1
**4452**	373	D	50.9703	1
**4452**	381	D	68.6583	1
**4452**	382	D	75.2568	1
**4452**	386	D	59.9897	1
**4453**	305	A	21.9381	0
**4453**	319	A	47.0256	0
**4453**	320	A	79.5196	-1
**4453**	321	A	55.9483	0
**4453**	351	A	0.0345	1
**4453**	357	A	65.1204	1
**4453**	370	A	66.1166	1
**4453**	372	A	60.2297	0

**4453**	373	A	79.5672	1
**4453**	381	A	79.5428	1
**4453**	382	A	79.5383	1
**4453**	386	A	72.458	1
**4453**	305	B	79.5605	1
**4453**	319	B	79.3622	1
**4453**	320	B	74.1444	-1
**4453**	321	B	61.5395	0
**4453**	351	B	11.9382	1
**4453**	357	B	77.7712	1
**4453**	370	B	67.0814	1
**4453**	372	B	64.0997	0
**4453**	373	B	26.3686	0
**4453**	381	B	79.5649	1
**4453**	382	B	33.2427	0
**4453**	386	B	39.9199	0
**4453**	305	C	48.549	0
**4453**	319	C	60.7874	1
**4453**	320	C	79.5204	-1
**4453**	321	C	21.9234	1
**4453**	351	C	5.4315	1
**4453**	357	C	67.3027	1
**4453**	370	C	69.4603	1
**4453**	372	C	70.8914	0
**4453**	373	C	53.7375	1
**4453**	381	C	79.1381	1
**4453**	382	C	54.8483	1
**4453**	386	C	48.1559	0
**4453**	305	D	59.6301	1
**4453**	319	D	79.5741	1
**4453**	320	D	79.5747	-1
**4453**	321	D	75.7039	0
**4453**	351	D	9.0824	1
**4453**	357	D	49.4763	0

**4453**	370	D	79.1587	1
**4453**	372	D	79.5622	0
**4453**	373	D	71.9773	1
**4453**	381	D	44.465	0
**4453**	382	D	79.5621	1
**4453**	386	D	77.9587	1
**4454**	305	A	63.9216	1
**4454**	319	A	61.0278	1
**4454**	320	A	72.6585	-1
**4454**	321	A	75.438	0
**4454**	351	A	9.9788	1
**4454**	357	A	79.7062	1
**4454**	370	A	73.0569	1
**4454**	372	A	46.394	1
**4454**	373	A	75.6892	1
**4454**	381	A	79.581	1
**4454**	382	A	69.3264	1
**4454**	386	A	68.5921	1
**4454**	305	B	53.8861	1
**4454**	319	B	55.9231	1
**4454**	320	B	52.5925	-1
**4454**	321	B	79.0506	0
**4454**	351	B	13.2561	1
**4454**	357	B	66.9736	1
**4454**	370	B	65.7184	1
**4454**	372	B	22.7842	1
**4454**	373	B	73.761	1
**4454**	381	B	79.5139	1
**4454**	382	B	27.8036	0
**4454**	386	B	46.6404	0
**4454**	305	C	76.6573	1
**4454**	319	C	22.1783	0
**4454**	320	C	71.6285	-1
**4454**	321	C	67.279	0

**4454**	351	C	9.8377	1
**4454**	357	C	70.9269	1
**4454**	370	C	79.7326	1
**4454**	372	C	69.9457	0
**4454**	373	C	78.1989	1
**4454**	381	C	78.3663	1
**4454**	382	C	58.9706	1
**4454**	386	C	55.7801	1
**4454**	305	D	31.7476	0
**4454**	319	D	47.1201	0
**4454**	320	D	78.1958	-1
**4454**	321	D	73.064	0
**4454**	351	D	20.1925	-1
**4454**	357	D	36.0379	0
**4454**	370	D	47.9858	0
**4454**	372	D	63.9615	0
**4454**	373	D	68.9557	1
**4454**	381	D	72.1421	1
**4454**	382	D	79.748	1
**4454**	386	D	79.5043	1
**4455**	305	A	12.1683	-1
**4455**	319	A	79.4753	1
**4455**	320	A	79.6106	-1
**4455**	321	A	76.3005	0
**4455**	351	A	7.2963	1
**4455**	357	A	65.6067	1
**4455**	370	A	79.5199	1
**4455**	372	A	72.6808	0
**4455**	373	A	59.9467	1
**4455**	381	A	74.4023	1
**4455**	382	A	77.5643	1
**4455**	386	A	71.9157	1
**4455**	305	B	52.2245	1
**4455**	319	B	79.5128	1

**4455**	320	B	79.3465	-1
**4455**	321	B	67.4209	0
**4455**	351	B	11.372	1
**4455**	357	B	61.9748	1
**4455**	370	B	79.5226	1
**4455**	372	B	79.5644	0
**4455**	373	B	39.9947	0
**4455**	381	B	69.3233	1
**4455**	382	B	79.5533	1
**4455**	386	B	42.4642	0
**4455**	305	C	28.4635	0
**4455**	319	C	66.4873	1
**4455**	320	C	79.5503	-1
**4455**	321	C	56.3386	0
**4455**	351	C	2.1506	1
**4455**	357	C	79.504	1
**4455**	370	C	69.9354	1
**4455**	372	C	66.7303	0
**4455**	373	C	57.8205	1
**4455**	381	C	79.6629	1
**4455**	382	C	35.6222	0
**4455**	386	C	38.8928	0
**4455**	305	D	79.6417	1
**4455**	319	D	72.9099	1
**4455**	320	D	35.5472	-1
**4455**	321	D	78.7027	0
**4455**	351	D	11.4882	1
**4455**	357	D	72.475	1
**4455**	370	D	60.6594	1
**4455**	372	D	79.478	0
**4455**	373	D	52.9498	1
**4455**	381	D	26.9896	0
**4455**	382	D	79.5406	1
**4455**	386	D	79.486	1

**4456**	305	A	79.5018	1
**4456**	319	A	45.8278	0
**4456**	320	A	62.5536	-1
**4456**	321	A	59.5617	0
**4456**	351	A	3.8129	1
**4456**	357	A	57.4703	1
**4456**	370	A	72.0385	1
**4456**	372	A	56.7554	0
**4456**	373	A	74.2363	1
**4456**	381	A	79.593	1
**4456**	382	A	73.1749	1
**4456**	386	A	79.3784	1
**4456**	305	B	79.6606	1
**4456**	319	B	79.5042	1
**4456**	320	B	57.3532	-1
**4456**	321	B	79.5559	0
**4456**	351	B	27.5849	-1
**4456**	357	B	63.2753	1
**4456**	370	B	56.7781	1
**4456**	372	B	79.5421	0
**4456**	373	B	57.7635	1
**4456**	381	B	73.5687	1
**4456**	382	B	24.2646	0
**4456**	386	B	48.7728	0
**4456**	305	C	22.6367	0
**4456**	319	C	20.7731	0
**4456**	320	C	72.4173	-1
**4456**	321	C	16.4294	0
**4456**	351	C	8.6433	1
**4456**	357	C	40.2148	0
**4456**	370	C	28.8641	0
**4456**	372	C	22.112	1
**4456**	373	C	79.5728	1
**4456**	381	C	77.5193	1

**4456**	382	C	39.1032	0
**4456**	386	C	56.328	1
**4456**	305	D	15.2467	-1
**4456**	319	D	79.5566	1
**4456**	320	D	79.4375	-1
**4456**	321	D	79.5635	0
**4456**	351	D	24.0251	-1
**4456**	357	D	56.8559	1
**4456**	370	D	67.5635	1
**4456**	372	D	72.0356	0
**4456**	373	D	74.2177	1
**4456**	381	D	79.6084	1
**4456**	382	D	79.5831	1
**4456**	386	D	79.5532	1
**4457**	305	A	79.4829	1
**4457**	319	A	76.2391	1
**4457**	320	A	79.5851	-1
**4457**	321	A	58.0944	0
**4457**	351	A	2.9233	1
**4457**	357	A	73.0347	1
**4457**	370	A	59.0714	1
**4457**	372	A	79.0295	0
**4457**	373	A	66.5277	1
**4457**	381	A	79.018	1
**4457**	382	A	66.7119	1
**4457**	386	A	79.4561	1
**4457**	305	B	79.4634	1
**4457**	319	B	78.2519	1
**4457**	320	B	12.8565	1
**4457**	321	B	65.2137	0
**4457**	351	B	18.9873	1
**4457**	357	B	75.0351	1
**4457**	370	B	79.5672	1
**4457**	372	B	72.0	0

**4457**	373	B	72.382	1
**4457**	381	B	62.0762	1
**4457**	382	B	56.6873	1
**4457**	386	B	46.8384	0
**4457**	305	C	46.7019	0
**4457**	319	C	60.6223	1
**4457**	320	C	79.6059	-1
**4457**	321	C	68.8687	0
**4457**	351	C	37.7228	-1
**4457**	357	C	69.4681	1
**4457**	370	C	74.7685	1
**4457**	372	C	79.5628	0
**4457**	373	C	51.583	1
**4457**	381	C	70.1863	1
**4457**	382	C	69.3825	1
**4457**	386	C	41.9082	0
**4457**	305	D	79.6073	1
**4457**	319	D	66.4148	1
**4457**	320	D	79.5044	-1
**4457**	321	D	50.6576	0
**4457**	351	D	16.7629	1
**4457**	357	D	79.584	1
**4457**	370	D	79.4302	1
**4457**	372	D	79.6257	0
**4457**	373	D	50.9887	1
**4457**	381	D	48.4081	0
**4457**	382	D	73.9212	1
**4457**	386	D	72.3894	1
**4458**	305	A	72.4669	1
**4458**	319	A	60.1877	1
**4458**	320	A	53.1789	-1
**4458**	321	A	78.1823	0
**4458**	351	A	11.3292	1
**4458**	357	A	79.6045	1

**4458**	370	A	79.3754	1
**4458**	372	A	32.8276	1
**4458**	373	A	79.5883	1
**4458**	381	A	79.5558	1
**4458**	382	A	78.3441	1
**4458**	386	A	26.6757	0
**4458**	305	B	79.5407	1
**4458**	319	B	70.6502	1
**4458**	320	B	9.9641	1
**4458**	321	B	79.5616	0
**4458**	351	B	15.5002	1
**4458**	357	B	60.5372	1
**4458**	370	B	31.9595	0
**4458**	372	B	78.4834	0
**4458**	373	B	55.718	1
**4458**	381	B	79.5993	1
**4458**	382	B	47.6184	0
**4458**	386	B	56.9129	1
**4458**	305	C	80.1086	1
**4458**	319	C	73.6239	1
**4458**	320	C	62.4793	-1
**4458**	321	C	41.0858	1
**4458**	351	C	4.6946	1
**4458**	357	C	73.1756	1
**4458**	370	C	45.2797	0
**4458**	372	C	79.5904	0
**4458**	373	C	57.9402	1
**4458**	381	C	54.7892	1
**4458**	382	C	64.191	1
**4458**	386	C	47.7161	0
**4458**	305	D	76.6654	1
**4458**	319	D	77.3243	1
**4458**	320	D	79.0784	-1
**4458**	321	D	64.9337	0

**4458**	351	D	14.8641	1
**4458**	357	D	75.1214	1
**4458**	370	D	40.3781	0
**4458**	372	D	66.8613	0
**4458**	373	D	48.5643	0
**4458**	381	D	73.1354	1
**4458**	382	D	73.7552	1
**4458**	386	D	79.6029	1
**4459**	305	A	79.4316	1
**4459**	319	A	72.6083	1
**4459**	320	A	79.6127	-1
**4459**	321	A	79.4765	0
**4459**	351	A	10.2502	1
**4459**	357	A	48.5051	0
**4459**	370	A	70.8997	1
**4459**	372	A	56.9079	0
**4459**	373	A	68.2415	1
**4459**	381	A	79.7422	1
**4459**	382	A	71.3308	1
**4459**	386	A	77.3658	1
**4459**	305	B	79.4989	1
**4459**	319	B	79.1737	1
**4459**	320	B	69.0833	-1
**4459**	321	B	72.3378	0
**4459**	351	B	15.6967	1
**4459**	357	B	70.2697	1
**4459**	370	B	43.5229	0
**4459**	372	B	79.5313	0
**4459**	373	B	77.6566	1
**4459**	381	B	72.5557	1
**4459**	382	B	63.9057	1
**4459**	386	B	76.9565	1
**4459**	305	C	79.5527	1
**4459**	319	C	73.8374	1

**4459**	320	C	77.8031	-1
**4459**	321	C	68.9472	0
**4459**	351	C	5.5654	1
**4459**	357	C	29.0205	0
**4459**	370	C	55.9546	1
**4459**	372	C	75.1339	0
**4459**	373	C	17.4581	-1
**4459**	381	C	40.129	0
**4459**	382	C	65.4042	1
**4459**	386	C	37.9624	0
**4459**	305	D	79.691	1
**4459**	319	D	21.6201	0
**4459**	320	D	68.5126	-1
**4459**	321	D	66.4456	0
**4459**	351	D	20.3193	-1
**4459**	357	D	53.1855	1
**4459**	370	D	78.5652	1
**4459**	372	D	79.6167	0
**4459**	373	D	79.5777	1
**4459**	381	D	49.0259	0
**4459**	382	D	73.7152	1
**4459**	386	D	75.0404	1
**4460**	305	A	79.6081	1
**4460**	319	A	79.5763	1
**4460**	320	A	79.5564	-1
**4460**	321	A	73.7666	0
**4460**	351	A	0.0338	1
**4460**	357	A	66.5517	1
**4460**	370	A	72.942	1
**4460**	372	A	79.5357	0
**4460**	373	A	51.808	1
**4460**	381	A	77.1711	1
**4460**	382	A	66.7285	1
**4460**	386	A	64.5616	1

**4460**	305	B	79.565	1
**4460**	319	B	79.6146	1
**4460**	320	B	40.454	-1
**4460**	321	B	75.8121	0
**4460**	351	B	16.9115	1
**4460**	357	B	79.5893	1
**4460**	370	B	29.0198	0
**4460**	372	B	71.3287	0
**4460**	373	B	44.9304	0
**4460**	381	B	77.9215	1
**4460**	382	B	16.1114	-1
**4460**	386	B	79.4925	1
**4460**	305	C	58.6351	1
**4460**	319	C	76.4795	1
**4460**	320	C	71.6923	-1
**4460**	321	C	57.1975	0
**4460**	351	C	3.3337	1
**4460**	357	C	72.8572	1
**4460**	370	C	75.5294	1
**4460**	372	C	78.7857	0
**4460**	373	C	58.5448	1
**4460**	381	C	79.6053	1
**4460**	382	C	62.3209	1
**4460**	386	C	50.454	1
**4460**	305	D	78.2399	1
**4460**	319	D	44.4789	0
**4460**	320	D	79.6403	-1
**4460**	321	D	60.7153	0
**4460**	351	D	10.8731	1
**4460**	357	D	79.5292	1
**4460**	370	D	79.1912	1
**4460**	372	D	79.6166	0
**4460**	373	D	79.0105	1
**4460**	381	D	79.5437	1

**4460**	382	D	43.0951	0
**4460**	386	D	79.5403	1
**4461**	305	A	59.222	1
**4461**	319	A	50.2229	1
**4461**	320	A	50.4974	-1
**4461**	321	A	66.5269	0
**4461**	351	A	16.03	1
**4461**	357	A	79.4746	1
**4461**	370	A	79.6648	1
**4461**	372	A	51.647	0
**4461**	373	A	54.5442	1
**4461**	381	A	71.949	1
**4461**	382	A	79.5399	1
**4461**	386	A	73.0512	1
**4461**	305	B	64.4503	1
**4461**	319	B	75.8801	1
**4461**	320	B	69.4826	-1
**4461**	321	B	43.1732	1
**4461**	351	B	13.3576	1
**4461**	357	B	79.6973	1
**4461**	370	B	79.5862	1
**4461**	372	B	57.5057	0
**4461**	373	B	79.2437	1
**4461**	381	B	79.6436	1
**4461**	382	B	33.338	0
**4461**	386	B	53.2481	1
**4461**	305	C	34.6339	0
**4461**	319	C	72.051	1
**4461**	320	C	62.2032	-1
**4461**	321	C	66.8962	0
**4461**	351	C	0.3322	1
**4461**	357	C	79.5149	1
**4461**	370	C	79.5293	1
**4461**	372	C	64.1878	0

**4461**	373	C	79.4857	1
**4461**	381	C	70.2146	1
**4461**	382	C	62.8405	1
**4461**	386	C	53.6117	1
**4461**	305	D	79.592	1
**4461**	319	D	79.5869	1
**4461**	320	D	70.8032	-1
**4461**	321	D	78.9616	0
**4461**	351	D	18.9278	1
**4461**	357	D	48.013	0
**4461**	370	D	77.2776	1
**4461**	372	D	52.5173	0
**4461**	373	D	79.2957	1
**4461**	381	D	50.7204	1
**4461**	382	D	49.513	0
**4461**	386	D	61.7365	1
**4462**	305	A	30.8756	0
**4462**	319	A	75.098	1
**4462**	320	A	79.5887	-1
**4462**	321	A	68.5331	0
**4462**	351	A	3.2052	1
**4462**	357	A	75.5998	1
**4462**	370	A	44.4244	0
**4462**	372	A	48.3808	1
**4462**	373	A	79.773	1
**4462**	381	A	60.294	1
**4462**	382	A	73.8834	1
**4462**	386	A	67.1848	1
**4462**	305	B	57.1656	1
**4462**	319	B	45.0257	0
**4462**	320	B	22.6287	-1
**4462**	321	B	65.5772	0
**4462**	351	B	7.8634	1
**4462**	357	B	75.1261	1

**4462**	370	B	79.5937	1
**4462**	372	B	79.3601	0
**4462**	373	B	57.2364	1
**4462**	381	B	74.6543	1
**4462**	382	B	24.0219	0
**4462**	386	B	64.1928	1
**4462**	305	C	74.7563	1
**4462**	319	C	79.6639	1
**4462**	320	C	45.5261	-1
**4462**	321	C	79.1233	0
**4462**	351	C	32.4084	-1
**4462**	357	C	71.6164	1
**4462**	370	C	66.5503	1
**4462**	372	C	70.5479	0
**4462**	373	C	67.5571	1
**4462**	381	C	71.1213	1
**4462**	382	C	79.6103	1
**4462**	386	C	22.137	0
**4462**	305	D	77.4161	1
**4462**	319	D	71.7782	1
**4462**	320	D	58.1139	-1
**4462**	321	D	79.5753	0
**4462**	351	D	10.909	1
**4462**	357	D	29.9143	0
**4462**	370	D	70.9605	1
**4462**	372	D	73.1039	0
**4462**	373	D	73.3846	1
**4462**	381	D	76.7621	1
**4462**	382	D	46.9922	0
**4462**	386	D	77.9634	1
**4463**	305	A	27.5841	0
**4463**	319	A	73.4433	1
**4463**	320	A	69.3444	-1
**4463**	321	A	64.3249	0

**4463**	351	A	0.8123	1
**4463**	357	A	79.6025	1
**4463**	370	A	62.2344	1
**4463**	372	A	69.8494	0
**4463**	373	A	79.5916	1
**4463**	381	A	64.7798	1
**4463**	382	A	50.1249	1
**4463**	386	A	45.5924	0
**4463**	305	B	79.4189	1
**4463**	319	B	67.5486	1
**4463**	320	B	53.5954	-1
**4463**	321	B	24.5548	1
**4463**	351	B	7.0275	1
**4463**	357	B	79.512	1
**4463**	370	B	79.5761	1
**4463**	372	B	41.7326	1
**4463**	373	B	32.6269	0
**4463**	381	B	71.6259	1
**4463**	382	B	32.0691	0
**4463**	386	B	24.141	0
**4463**	305	C	73.3534	1
**4463**	319	C	57.9871	1
**4463**	320	C	64.0582	-1
**4463**	321	C	79.5219	0
**4463**	351	C	0.7559	1
**4463**	357	C	73.1865	1
**4463**	370	C	60.3668	1
**4463**	372	C	30.7543	1
**4463**	373	C	64.0642	1
**4463**	381	C	71.6021	1
**4463**	382	C	32.1828	0
**4463**	386	C	47.5545	0
**4463**	305	D	56.8944	1
**4463**	319	D	75.6083	1

**4463**	320	D	58.376	-1
**4463**	321	D	70.2592	0
**4463**	351	D	7.3463	1
**4463**	357	D	72.8719	1
**4463**	370	D	79.2413	1
**4463**	372	D	71.3003	0
**4463**	373	D	71.7924	1
**4463**	381	D	79.5558	1
**4463**	382	D	61.2346	1
**4463**	386	D	25.2712	0
**4464**	305	A	71.5333	1
**4464**	319	A	62.7461	1
**4464**	320	A	44.6005	-1
**4464**	321	A	66.1698	0
**4464**	351	A	10.6204	1
**4464**	357	A	73.0802	1
**4464**	370	A	79.5922	1
**4464**	372	A	62.9675	0
**4464**	373	A	70.5322	1
**4464**	381	A	63.2399	1
**4464**	382	A	28.4596	0
**4464**	386	A	73.6429	1
**4464**	305	B	73.8935	1
**4464**	319	B	73.0612	1
**4464**	320	B	71.2138	-1
**4464**	321	B	72.8349	0
**4464**	351	B	17.0394	1
**4464**	357	B	56.8593	1
**4464**	370	B	79.3957	1
**4464**	372	B	79.5578	0
**4464**	373	B	79.3228	1
**4464**	381	B	79.5723	1
**4464**	382	B	46.3683	0
**4464**	386	B	76.4735	1

**4464**	305	C	68.8635	1
**4464**	319	C	79.5917	1
**4464**	320	C	22.792	-1
**4464**	321	C	72.7823	0
**4464**	351	C	7.3509	1
**4464**	357	C	54.1637	1
**4464**	370	C	32.1681	0
**4464**	372	C	72.8461	0
**4464**	373	C	50.6031	1
**4464**	381	C	79.5688	1
**4464**	382	C	53.129	1
**4464**	386	C	53.4726	1
**4464**	305	D	79.4877	1
**4464**	319	D	75.8307	1
**4464**	320	D	79.7717	-1
**4464**	321	D	78.4636	0
**4464**	351	D	10.1723	1
**4464**	357	D	74.8807	1
**4464**	370	D	75.2423	1
**4464**	372	D	61.2597	0
**4464**	373	D	57.1929	1
**4464**	381	D	79.2083	1
**4464**	382	D	76.7067	1
**4464**	386	D	79.5421	1
**4465**	305	A	45.6724	0
**4465**	319	A	52.6045	1
**4465**	320	A	72.5882	-1
**4465**	321	A	73.1757	0
**4465**	351	A	8.8443	1
**4465**	357	A	79.5689	1
**4465**	370	A	32.6112	0
**4465**	372	A	79.5714	0
**4465**	373	A	75.3706	1
**4465**	381	A	79.5595	1

**4465**	382	A	79.571	1
**4465**	386	A	45.8278	0
**4465**	305	B	69.6074	1
**4465**	319	B	78.0564	1
**4465**	320	B	63.9443	-1
**4465**	321	B	51.7484	0
**4465**	351	B	16.6383	1
**4465**	357	B	68.9237	1
**4465**	370	B	54.6377	1
**4465**	372	B	78.8937	0
**4465**	373	B	76.2615	1
**4465**	381	B	54.8819	1
**4465**	382	B	12.7814	-1
**4465**	386	B	69.2962	1
**4465**	305	C	62.6739	1
**4465**	319	C	71.1616	1
**4465**	320	C	59.9016	-1
**4465**	321	C	63.3109	0
**4465**	351	C	5.1629	1
**4465**	357	C	59.0539	1
**4465**	370	C	67.1168	1
**4465**	372	C	72.4783	0
**4465**	373	C	79.5828	1
**4465**	381	C	43.3491	0
**4465**	382	C	77.0878	1
**4465**	386	C	52.0873	1
**4465**	305	D	59.4663	1
**4465**	319	D	74.2644	1
**4465**	320	D	61.5742	-1
**4465**	321	D	79.271	0
**4465**	351	D	12.652	1
**4465**	357	D	64.5399	1
**4465**	370	D	79.5289	1
**4465**	372	D	73.0887	0

**4465**	373	D	79.5728	1
**4465**	381	D	36.448	0
**4465**	382	D	72.3679	1
**4465**	386	D	18.5216	-1
**4466**	305	A	64.9702	1
**4466**	319	A	38.2416	0
**4466**	320	A	58.9425	-1
**4466**	321	A	71.9133	0
**4466**	351	A	11.6084	1
**4466**	357	A	69.4783	1
**4466**	370	A	79.6364	1
**4466**	372	A	79.4553	0
**4466**	373	A	67.57	1
**4466**	381	A	39.4059	0
**4466**	382	A	79.4755	1
**4466**	386	A	79.5119	1
**4466**	305	B	74.0666	1
**4466**	319	B	77.114	1
**4466**	320	B	51.2152	-1
**4466**	321	B	62.2421	0
**4466**	351	B	13.8667	1
**4466**	357	B	64.1572	1
**4466**	370	B	78.6521	1
**4466**	372	B	79.3936	0
**4466**	373	B	79.6592	1
**4466**	381	B	79.6658	1
**4466**	382	B	73.1914	1
**4466**	386	B	57.7555	1
**4466**	305	C	79.5014	1
**4466**	319	C	79.6357	1
**4466**	320	C	32.9055	-1
**4466**	321	C	31.15	1
**4466**	351	C	10.1843	1
**4466**	357	C	76.6181	1

**4466**	370	C	68.2038	1
**4466**	372	C	73.2653	0
**4466**	373	C	75.9475	1
**4466**	381	C	78.0327	1
**4466**	382	C	78.5045	1
**4466**	386	C	67.5129	1
**4466**	305	D	79.6618	1
**4466**	319	D	79.5517	1
**4466**	320	D	75.2259	-1
**4466**	321	D	79.4366	0
**4466**	351	D	6.8772	1
**4466**	357	D	71.8706	1
**4466**	370	D	40.0802	0
**4466**	372	D	55.8176	0
**4466**	373	D	71.3409	1
**4466**	381	D	79.4952	1
**4466**	382	D	77.7494	1
**4466**	386	D	60.7839	1
**4467**	305	A	79.5988	1
**4467**	319	A	78.7266	1
**4467**	320	A	78.9006	-1
**4467**	321	A	45.315	1
**4467**	351	A	7.6652	1
**4467**	357	A	68.557	1
**4467**	370	A	73.3182	1
**4467**	372	A	78.182	0
**4467**	373	A	66.3692	1
**4467**	381	A	65.6502	1
**4467**	382	A	78.2162	1
**4467**	386	A	64.8433	1
**4467**	305	B	79.5121	1
**4467**	319	B	38.4502	0
**4467**	320	B	73.4093	-1
**4467**	321	B	66.556	0

**4467**	351	B	15.6447	1
**4467**	357	B	25.6203	0
**4467**	370	B	79.5474	1
**4467**	372	B	79.5442	0
**4467**	373	B	55.1837	1
**4467**	381	B	67.6197	1
**4467**	382	B	23.8281	0
**4467**	386	B	79.6111	1
**4467**	305	C	63.0615	1
**4467**	319	C	79.5519	1
**4467**	320	C	72.6831	-1
**4467**	321	C	74.0385	0
**4467**	351	C	6.2967	1
**4467**	357	C	56.2397	1
**4467**	370	C	79.5965	1
**4467**	372	C	78.6659	0
**4467**	373	C	79.5035	1
**4467**	381	C	75.9567	1
**4467**	382	C	77.0027	1
**4467**	386	C	49.6357	0
**4467**	305	D	79.5841	1
**4467**	319	D	78.5505	1
**4467**	320	D	60.8828	-1
**4467**	321	D	59.9037	0
**4467**	351	D	22.2121	-1
**4467**	357	D	43.2203	0
**4467**	370	D	79.6367	1
**4467**	372	D	55.1048	0
**4467**	373	D	79.6402	1
**4467**	381	D	78.341	1
**4467**	382	D	70.5218	1
**4467**	386	D	75.3921	1
**4468**	305	A	25.3395	0
**4468**	319	A	79.5578	1

**4468**	320	A	79.5717	-1
**4468**	321	A	64.4536	0
**4468**	351	A	4.1524	1
**4468**	357	A	40.9199	0
**4468**	370	A	74.8397	1
**4468**	372	A	79.5958	0
**4468**	373	A	17.1807	-1
**4468**	381	A	59.3334	1
**4468**	382	A	66.7874	1
**4468**	386	A	77.4284	1
**4468**	305	B	73.3798	1
**4468**	319	B	71.8905	1
**4468**	320	B	56.8208	-1
**4468**	321	B	34.8158	1
**4468**	351	B	3.8134	1
**4468**	357	B	79.5441	1
**4468**	370	B	58.2522	1
**4468**	372	B	79.0566	0
**4468**	373	B	78.6396	1
**4468**	381	B	59.0688	1
**4468**	382	B	79.6256	1
**4468**	386	B	79.6213	1
**4468**	305	C	52.4838	1
**4468**	319	C	67.4354	1
**4468**	320	C	74.072	-1
**4468**	321	C	71.9532	0
**4468**	351	C	2.2236	1
**4468**	357	C	48.4444	0
**4468**	370	C	69.6934	1
**4468**	372	C	79.6021	0
**4468**	373	C	79.6265	1
**4468**	381	C	79.2852	1
**4468**	382	C	47.2676	0
**4468**	386	C	23.3319	0

**4468**	305	D	72.0355	1
**4468**	319	D	28.4299	0
**4468**	320	D	72.4385	-1
**4468**	321	D	79.5632	0
**4468**	351	D	14.645	1
**4468**	357	D	73.7579	1
**4468**	370	D	38.8831	0
**4468**	372	D	79.5797	0
**4468**	373	D	78.3485	1
**4468**	381	D	79.568	1
**4468**	382	D	79.5511	1
**4468**	386	D	79.552	1
**4469**	305	A	63.6166	1
**4469**	319	A	68.0804	1
**4469**	320	A	79.7165	-1
**4469**	321	A	62.3598	0
**4469**	351	A	9.1604	1
**4469**	357	A	60.1387	1
**4469**	370	A	41.4029	0
**4469**	372	A	72.261	0
**4469**	373	A	79.5131	1
**4469**	381	A	72.9996	1
**4469**	382	A	79.5681	1
**4469**	386	A	67.3021	1
**4469**	305	B	79.5041	1
**4469**	319	B	56.2271	1
**4469**	320	B	77.0366	-1
**4469**	321	B	75.0743	0
**4469**	351	B	8.1644	1
**4469**	357	B	66.2408	1
**4469**	370	B	74.4658	1
**4469**	372	B	79.6084	0
**4469**	373	B	73.8505	1
**4469**	381	B	79.5401	1

**4469**	382	B	46.3652	0
**4469**	386	B	79.6038	1
**4469**	305	C	40.3433	0
**4469**	319	C	54.3645	1
**4469**	320	C	79.4739	-1
**4469**	321	C	53.2025	0
**4469**	351	C	16.6271	1
**4469**	357	C	50.1814	1
**4469**	370	C	43.1047	0
**4469**	372	C	73.3377	0
**4469**	373	C	61.0416	1
**4469**	381	C	51.0845	1
**4469**	382	C	73.7694	1
**4469**	386	C	24.0006	0
**4469**	305	D	58.4015	1
**4469**	319	D	70.5245	1
**4469**	320	D	79.6593	-1
**4469**	321	D	44.5787	1
**4469**	351	D	8.6045	1
**4469**	357	D	77.9368	1
**4469**	370	D	77.5049	1
**4469**	372	D	79.5484	0
**4469**	373	D	79.4866	1
**4469**	381	D	48.2525	0
**4469**	382	D	69.6452	1
**4469**	386	D	14.4649	-1
**4470**	305	A	73.2004	1
**4470**	319	A	66.9169	1
**4470**	320	A	27.9258	-1
**4470**	321	A	75.4515	0
**4470**	351	A	16.3874	1
**4470**	357	A	71.4094	1
**4470**	370	A	79.7166	1
**4470**	372	A	50.0362	0

**4470**	373	A	79.4232	1
**4470**	381	A	79.3834	1
**4470**	382	A	77.956	1
**4470**	386	A	79.6015	1
**4470**	305	B	79.7009	1
**4470**	319	B	63.3472	1
**4470**	320	B	75.2878	-1
**4470**	321	B	67.0915	0
**4470**	351	B	18.32	1
**4470**	357	B	57.5765	1
**4470**	370	B	60.5752	1
**4470**	372	B	77.6188	0
**4470**	373	B	53.9567	1
**4470**	381	B	53.1395	1
**4470**	382	B	34.2661	0
**4470**	386	B	42.4704	0
**4470**	305	C	72.1591	1
**4470**	319	C	52.9019	1
**4470**	320	C	64.9975	-1
**4470**	321	C	57.4982	0
**4470**	351	C	6.9805	1
**4470**	357	C	72.1756	1
**4470**	370	C	41.1355	0
**4470**	372	C	79.4795	0
**4470**	373	C	79.5059	1
**4470**	381	C	72.0533	1
**4470**	382	C	79.6871	1
**4470**	386	C	61.6358	1
**4470**	305	D	71.9201	1
**4470**	319	D	78.789	1
**4470**	320	D	60.0216	-1
**4470**	321	D	73.8707	0
**4470**	351	D	18.594	1
**4470**	357	D	79.5886	1

**4470**	370	D	73.7223	1
**4470**	372	D	79.5437	0
**4470**	373	D	73.6993	1
**4470**	381	D	47.7927	0
**4470**	382	D	63.7921	1
**4470**	386	D	79.5012	1
**4471**	305	A	55.4926	1
**4471**	319	A	42.0209	0
**4471**	320	A	49.3766	-1
**4471**	321	A	28.7058	1
**4471**	351	A	3.8632	1
**4471**	357	A	79.6869	1
**4471**	370	A	74.977	1
**4471**	372	A	79.4351	0
**4471**	373	A	56.1467	1
**4471**	381	A	67.6547	1
**4471**	382	A	51.0795	1
**4471**	386	A	79.5929	1
**4471**	305	B	79.6404	1
**4471**	319	B	79.4951	1
**4471**	320	B	79.4944	-1
**4471**	321	B	79.1277	0
**4471**	351	B	48.6213	-1
**4471**	357	B	41.5549	0
**4471**	370	B	79.4471	1
**4471**	372	B	74.5963	0
**4471**	373	B	79.5781	1
**4471**	381	B	60.3479	1
**4471**	382	B	58.3695	1
**4471**	386	B	70.498	1
**4471**	305	C	74.9384	1
**4471**	319	C	72.3414	1
**4471**	320	C	32.6365	-1
**4471**	321	C	73.4974	0

**4471**	351	C	0.1512	1
**4471**	357	C	78.1557	1
**4471**	370	C	76.0399	1
**4471**	372	C	78.0264	0
**4471**	373	C	64.6762	1
**4471**	381	C	79.7171	1
**4471**	382	C	62.548	1
**4471**	386	C	39.5531	0
**4471**	305	D	79.6719	1
**4471**	319	D	50.3225	1
**4471**	320	D	79.5532	-1
**4471**	321	D	66.5428	0
**4471**	351	D	17.0935	1
**4471**	357	D	39.8386	0
**4471**	370	D	50.4802	1
**4471**	372	D	79.7516	0
**4471**	373	D	71.3193	1
**4471**	381	D	76.7473	1
**4471**	382	D	67.4489	1
**4471**	386	D	56.5188	1
**4472**	305	A	64.2996	1
**4472**	319	A	58.0302	1
**4472**	320	A	27.8822	-1
**4472**	321	A	71.1938	0
**4472**	351	A	2.5898	1
**4472**	357	A	74.302	1
**4472**	370	A	57.9148	1
**4472**	372	A	46.1801	1
**4472**	373	A	61.805	1
**4472**	381	A	77.6953	1
**4472**	382	A	79.5865	1
**4472**	386	A	71.0835	1
**4472**	305	B	72.5529	1
**4472**	319	B	32.0902	0

**4472**	320	B	61.5068	-1
**4472**	321	B	52.7891	0
**4472**	351	B	14.2967	1
**4472**	357	B	56.6107	1
**4472**	370	B	26.6823	0
**4472**	372	B	32.6939	1
**4472**	373	B	64.5209	1
**4472**	381	B	69.3078	1
**4472**	382	B	79.6109	1
**4472**	386	B	69.449	1
**4472**	305	C	78.7948	1
**4472**	319	C	79.5475	1
**4472**	320	C	75.8508	-1
**4472**	321	C	58.0922	0
**4472**	351	C	4.1926	1
**4472**	357	C	18.6394	-1
**4472**	370	C	79.5025	1
**4472**	372	C	79.5591	0
**4472**	373	C	79.5801	1
**4472**	381	C	79.4993	1
**4472**	382	C	79.5583	1
**4472**	386	C	46.6164	0
**4472**	305	D	71.9412	1
**4472**	319	D	46.2836	0
**4472**	320	D	67.7214	-1
**4472**	321	D	73.7562	0
**4472**	351	D	14.4865	1
**4472**	357	D	74.9511	1
**4472**	370	D	42.4181	0
**4472**	372	D	75.9156	0
**4472**	373	D	78.2914	1
**4472**	381	D	58.2656	1
**4472**	382	D	79.5984	1
**4472**	386	D	79.6592	1

**4473**	305	A	79.7077	1
**4473**	319	A	34.8474	0
**4473**	320	A	79.6656	-1
**4473**	321	A	75.8976	0
**4473**	351	A	4.6341	1
**4473**	357	A	79.7403	1
**4473**	370	A	23.5948	0
**4473**	372	A	65.9635	0
**4473**	373	A	79.537	1
**4473**	381	A	54.9244	1
**4473**	382	A	78.2278	1
**4473**	386	A	79.6739	1
**4473**	305	B	62.1948	1
**4473**	319	B	70.0796	1
**4473**	320	B	71.6185	-1
**4473**	321	B	54.2045	0
**4473**	351	B	7.0181	1
**4473**	357	B	71.0247	1
**4473**	370	B	79.6672	1
**4473**	372	B	74.6984	0
**4473**	373	B	76.6315	1
**4473**	381	B	46.5135	0
**4473**	382	B	53.6797	1
**4473**	386	B	79.3612	1
**4473**	305	C	74.9098	1
**4473**	319	C	73.6501	1
**4473**	320	C	68.2639	-1
**4473**	321	C	72.3441	0
**4473**	351	C	2.6623	1
**4473**	357	C	68.8021	1
**4473**	370	C	67.6995	1
**4473**	372	C	72.5778	0
**4473**	373	C	73.2195	1
**4473**	381	C	73.4829	1

**4473**	382	C	79.4419	1
**4473**	386	C	24.9185	0
**4473**	305	D	79.7355	1
**4473**	319	D	72.0799	1
**4473**	320	D	79.4703	-1
**4473**	321	D	72.0981	0
**4473**	351	D	0.8175	1
**4473**	357	D	67.7398	1
**4473**	370	D	79.3781	1
**4473**	372	D	61.3159	0
**4473**	373	D	61.8052	1
**4473**	381	D	79.5333	1
**4473**	382	D	58.1351	1
**4473**	386	D	74.7131	1
**4474**	305	A	71.0057	1
**4474**	319	A	77.3478	1
**4474**	320	A	79.4853	-1
**4474**	321	A	54.0036	0
**4474**	351	A	3.023	1
**4474**	357	A	46.5217	0
**4474**	370	A	72.7604	1
**4474**	372	A	63.3445	0
**4474**	373	A	71.5353	1
**4474**	381	A	42.0492	0
**4474**	382	A	79.0079	1
**4474**	386	A	79.5751	1
**4474**	305	B	49.6649	0
**4474**	319	B	78.0305	1
**4474**	320	B	35.9275	-1
**4474**	321	B	38.6274	1
**4474**	351	B	9.376	1
**4474**	357	B	79.5661	1
**4474**	370	B	79.6134	1
**4474**	372	B	79.6669	0

**4474**	373	B	79.6647	1
**4474**	381	B	79.4612	1
**4474**	382	B	55.0995	1
**4474**	386	B	79.5628	1
**4474**	305	C	68.1942	1
**4474**	319	C	79.5747	1
**4474**	320	C	60.9288	-1
**4474**	321	C	77.7461	0
**4474**	351	C	8.0358	1
**4474**	357	C	63.7229	1
**4474**	370	C	37.5728	0
**4474**	372	C	62.8254	0
**4474**	373	C	79.5396	1
**4474**	381	C	79.571	1
**4474**	382	C	79.4397	1
**4474**	386	C	27.621	0
**4474**	305	D	79.5515	1
**4474**	319	D	63.0658	1
**4474**	320	D	70.3835	-1
**4474**	321	D	78.1627	0
**4474**	351	D	18.9127	1
**4474**	357	D	72.6935	1
**4474**	370	D	47.9087	0
**4474**	372	D	72.5993	0
**4474**	373	D	43.9478	0
**4474**	381	D	79.6239	1
**4474**	382	D	79.546	1
**4474**	386	D	23.967	0
**4475**	305	A	47.5667	0
**4475**	319	A	20.7462	0
**4475**	320	A	78.8369	-1
**4475**	321	A	72.5109	0
**4475**	351	A	9.2657	1
**4475**	357	A	79.5457	1

**4475**	370	A	79.5128	1
**4475**	372	A	79.5732	0
**4475**	373	A	68.7097	1
**4475**	381	A	50.0841	1
**4475**	382	A	71.7009	1
**4475**	386	A	59.7326	1
**4475**	305	B	79.5047	1
**4475**	319	B	73.1711	1
**4475**	320	B	76.8876	-1
**4475**	321	B	36.2874	1
**4475**	351	B	13.4941	1
**4475**	357	B	70.981	1
**4475**	370	B	56.7736	1
**4475**	372	B	79.5495	0
**4475**	373	B	54.5102	1
**4475**	381	B	55.0437	1
**4475**	382	B	0.0	-1
**4475**	386	B	65.7513	1
**4475**	305	C	79.5474	1
**4475**	319	C	78.9331	1
**4475**	320	C	57.5123	-1
**4475**	321	C	73.3747	0
**4475**	351	C	1.5786	1
**4475**	357	C	43.4428	0
**4475**	370	C	72.602	1
**4475**	372	C	61.4895	0
**4475**	373	C	79.5465	1
**4475**	381	C	79.6268	1
**4475**	382	C	79.5361	1
**4475**	386	C	42.3667	0
**4475**	305	D	56.1758	1
**4475**	319	D	75.4402	1
**4475**	320	D	31.0673	-1
**4475**	321	D	73.8523	0

**4475**	351	D	11.4802	1
**4475**	357	D	62.1648	1
**4475**	370	D	78.9938	1
**4475**	372	D	73.0823	0
**4475**	373	D	34.0595	0
**4475**	381	D	71.8991	1
**4475**	382	D	72.3865	1
**4475**	386	D	79.5709	1
**4476**	305	A	62.8736	1
**4476**	319	A	73.707	1
**4476**	320	A	79.5365	-1
**4476**	321	A	75.6038	0
**4476**	351	A	19.7826	1
**4476**	357	A	67.299	1
**4476**	370	A	79.7304	1
**4476**	372	A	79.5109	0
**4476**	373	A	52.1863	1
**4476**	381	A	79.5318	1
**4476**	382	A	70.0142	1
**4476**	386	A	79.6885	1
**4476**	305	B	69.5306	1
**4476**	319	B	79.5798	1
**4476**	320	B	65.2826	-1
**4476**	321	B	79.4506	0
**4476**	351	B	0.5842	1
**4476**	357	B	28.921	0
**4476**	370	B	51.4474	1
**4476**	372	B	79.4915	0
**4476**	373	B	52.302	1
**4476**	381	B	53.8857	1
**4476**	382	B	66.5127	1
**4476**	386	B	79.5052	1
**4476**	305	C	35.7334	0
**4476**	319	C	79.4441	1

**4476**	320	C	69.7871	-1
**4476**	321	C	62.6396	0
**4476**	351	C	2.8907	1
**4476**	357	C	64.9247	1
**4476**	370	C	78.7872	1
**4476**	372	C	78.5123	0
**4476**	373	C	65.9269	1
**4476**	381	C	36.5248	0
**4476**	382	C	30.9296	0
**4476**	386	C	21.6064	0
**4476**	305	D	19.5512	-1
**4476**	319	D	73.1309	1
**4476**	320	D	48.6638	-1
**4476**	321	D	75.741	0
**4476**	351	D	3.7941	1
**4476**	357	D	77.1409	1
**4476**	370	D	63.8383	1
**4476**	372	D	79.6281	0
**4476**	373	D	33.7655	0
**4476**	381	D	79.6329	1
**4476**	382	D	67.1205	1
**4476**	386	D	73.6576	1
**4477**	305	A	68.2764	1
**4477**	319	A	72.5718	1
**4477**	320	A	79.5542	-1
**4477**	321	A	27.145	1
**4477**	351	A	7.4169	1
**4477**	357	A	73.1202	1
**4477**	370	A	53.6103	1
**4477**	372	A	60.3279	0
**4477**	373	A	67.0157	1
**4477**	381	A	71.5477	1
**4477**	382	A	71.2578	1
**4477**	386	A	73.9532	1

**4477**	305	B	35.3726	0
**4477**	319	B	68.798	1
**4477**	320	B	59.5348	-1
**4477**	321	B	71.2148	0
**4477**	351	B	19.0616	1
**4477**	357	B	56.702	1
**4477**	370	B	55.986	1
**4477**	372	B	73.0469	0
**4477**	373	B	40.2804	0
**4477**	381	B	73.8416	1
**4477**	382	B	34.3319	0
**4477**	386	B	70.4223	1
**4477**	305	C	58.9493	1
**4477**	319	C	56.9139	1
**4477**	320	C	67.7488	-1
**4477**	321	C	79.5095	0
**4477**	351	C	4.1118	1
**4477**	357	C	24.8667	0
**4477**	370	C	58.0641	1
**4477**	372	C	79.5515	0
**4477**	373	C	60.7893	1
**4477**	381	C	79.5005	1
**4477**	382	C	32.5953	0
**4477**	386	C	10.5872	-1
**4477**	305	D	78.3831	1
**4477**	319	D	72.5256	1
**4477**	320	D	59.1948	-1
**4477**	321	D	79.5801	0
**4477**	351	D	10.6215	1
**4477**	357	D	56.3706	1
**4477**	370	D	70.5464	1
**4477**	372	D	79.4392	0
**4477**	373	D	55.0782	1
**4477**	381	D	47.7707	0

**4477**	382	D	79.5564	1
**4477**	386	D	70.0778	1
**4478**	305	A	60.4764	1
**4478**	319	A	74.1259	1
**4478**	320	A	65.631	-1
**4478**	321	A	40.7001	1
**4478**	351	A	35.9896	-1
**4478**	357	A	55.1702	1
**4478**	370	A	55.5643	1
**4478**	372	A	67.4896	0
**4478**	373	A	65.2097	1
**4478**	381	A	64.8454	1
**4478**	382	A	58.9086	1
**4478**	386	A	79.4732	1
**4478**	305	B	79.6323	1
**4478**	319	B	56.8426	1
**4478**	320	B	38.1182	-1
**4478**	321	B	71.2161	0
**4478**	351	B	11.001	1
**4478**	357	B	79.5664	1
**4478**	370	B	70.9726	1
**4478**	372	B	23.5166	1
**4478**	373	B	78.4532	1
**4478**	381	B	79.6179	1
**4478**	382	B	74.3192	1
**4478**	386	B	46.362	0
**4478**	305	C	69.7777	1
**4478**	319	C	64.3749	1
**4478**	320	C	79.4918	-1
**4478**	321	C	74.2245	0
**4478**	351	C	12.9266	1
**4478**	357	C	49.7904	0
**4478**	370	C	75.6358	1
**4478**	372	C	55.7523	0

**4478**	373	C	70.2927	1
**4478**	381	C	74.2483	1
**4478**	382	C	79.5066	1
**4478**	386	C	60.5646	1
**4478**	305	D	72.9368	1
**4478**	319	D	79.6331	1
**4478**	320	D	79.486	-1
**4478**	321	D	79.5694	0
**4478**	351	D	7.1153	1
**4478**	357	D	49.7715	0
**4478**	370	D	72.761	1
**4478**	372	D	74.0625	0
**4478**	373	D	75.486	1
**4478**	381	D	77.5354	1
**4478**	382	D	79.5029	1
**4478**	386	D	34.5019	0
**4479**	305	A	50.5864	1
**4479**	319	A	71.1332	1
**4479**	320	A	79.6323	-1
**4479**	321	A	74.4978	0
**4479**	351	A	13.2392	1
**4479**	357	A	69.8706	1
**4479**	370	A	65.1912	1
**4479**	372	A	20.1586	1
**4479**	373	A	79.59	1
**4479**	381	A	36.2373	0
**4479**	382	A	24.1328	0
**4479**	386	A	66.0042	1
**4479**	305	B	73.3323	1
**4479**	319	B	79.5554	1
**4479**	320	B	78.06	-1
**4479**	321	B	77.1465	0
**4479**	351	B	3.9247	1
**4479**	357	B	59.589	1

**4479**	370	B	72.4789	1
**4479**	372	B	46.0937	1
**4479**	373	B	50.4947	1
**4479**	381	B	58.7584	1
**4479**	382	B	71.2519	1
**4479**	386	B	79.6035	1
**4479**	305	C	69.9857	1
**4479**	319	C	79.6924	1
**4479**	320	C	43.3526	-1
**4479**	321	C	73.9814	0
**4479**	351	C	10.7524	1
**4479**	357	C	62.2659	1
**4479**	370	C	63.7775	1
**4479**	372	C	69.2661	0
**4479**	373	C	79.5435	1
**4479**	381	C	67.771	1
**4479**	382	C	45.4285	0
**4479**	386	C	65.0851	1
**4479**	305	D	71.2846	1
**4479**	319	D	50.8788	1
**4479**	320	D	74.8792	-1
**4479**	321	D	66.7475	0
**4479**	351	D	16.2927	1
**4479**	357	D	30.7189	0
**4479**	370	D	70.4943	1
**4479**	372	D	77.0557	0
**4479**	373	D	79.5306	1
**4479**	381	D	79.4683	1
**4479**	382	D	61.724	1
**4479**	386	D	78.9037	1
**4480**	305	A	79.5303	1
**4480**	319	A	79.5181	1
**4480**	320	A	50.4399	-1
**4480**	321	A	74.941	0

**4480**	351	A	10.7062	1
**4480**	357	A	49.4652	0
**4480**	370	A	79.4989	1
**4480**	372	A	79.572	0
**4480**	373	A	55.7727	1
**4480**	381	A	79.6176	1
**4480**	382	A	79.4988	1
**4480**	386	A	79.6264	1
**4480**	305	B	79.5289	1
**4480**	319	B	72.5492	1
**4480**	320	B	79.6955	-1
**4480**	321	B	72.9535	0
**4480**	351	B	3.7936	1
**4480**	357	B	79.4917	1
**4480**	370	B	63.0257	1
**4480**	372	B	48.9761	1
**4480**	373	B	76.5506	1
**4480**	381	B	75.2539	1
**4480**	382	B	75.5341	1
**4480**	386	B	79.5447	1
**4480**	305	C	79.5762	1
**4480**	319	C	78.6806	1
**4480**	320	C	79.5888	-1
**4480**	321	C	79.5006	0
**4480**	351	C	4.5391	1
**4480**	357	C	62.6946	1
**4480**	370	C	79.5689	1
**4480**	372	C	73.457	0
**4480**	373	C	63.0138	1
**4480**	381	C	59.9973	1
**4480**	382	C	72.7988	1
**4480**	386	C	36.4367	0
**4480**	305	D	75.8332	1
**4480**	319	D	36.1715	0

**4480**	320	D	79.5732	-1
**4480**	321	D	43.3662	1
**4480**	351	D	7.4824	1
**4480**	357	D	72.8198	1
**4480**	370	D	58.8608	1
**4480**	372	D	79.1393	0
**4480**	373	D	68.1536	1
**4480**	381	D	79.2247	1
**4480**	382	D	79.4668	1
**4480**	386	D	79.7022	1
**4481**	305	A	79.6224	1
**4481**	319	A	52.4492	1
**4481**	320	A	72.1015	-1
**4481**	321	A	78.1681	0
**4481**	351	A	18.1876	1
**4481**	357	A	67.9856	1
**4481**	370	A	37.5557	0
**4481**	372	A	50.8712	0
**4481**	373	A	42.992	0
**4481**	381	A	66.7774	1
**4481**	382	A	40.9134	0
**4481**	386	A	43.5559	0
**4481**	305	B	69.4705	1
**4481**	319	B	79.6031	1
**4481**	320	B	79.6094	-1
**4481**	321	B	79.609	0
**4481**	351	B	8.2697	1
**4481**	357	B	58.5805	1
**4481**	370	B	65.4527	1
**4481**	372	B	78.9568	0
**4481**	373	B	57.2344	1
**4481**	381	B	74.8418	1
**4481**	382	B	50.7422	1
**4481**	386	B	79.5614	1

**4481**	305	C	58.3577	1
**4481**	319	C	10.1211	-1
**4481**	320	C	67.3198	-1
**4481**	321	C	43.4014	1
**4481**	351	C	13.1655	1
**4481**	357	C	79.5845	1
**4481**	370	C	79.591	1
**4481**	372	C	79.5419	0
**4481**	373	C	65.4422	1
**4481**	381	C	48.9602	0
**4481**	382	C	79.6429	1
**4481**	386	C	33.7799	0
**4481**	305	D	79.4884	1
**4481**	319	D	61.2477	1
**4481**	320	D	60.9026	-1
**4481**	321	D	64.5274	0
**4481**	351	D	14.6963	1
**4481**	357	D	79.2348	1
**4481**	370	D	72.5756	1
**4481**	372	D	56.6916	0
**4481**	373	D	78.7734	1
**4481**	381	D	72.4706	1
**4481**	382	D	70.9045	1
**4481**	386	D	49.8345	0
**4482**	305	A	42.5193	0
**4482**	319	A	78.7926	1
**4482**	320	A	79.4846	-1
**4482**	321	A	48.1613	1
**4482**	351	A	2.0611	1
**4482**	357	A	79.5465	1
**4482**	370	A	72.5675	1
**4482**	372	A	79.5047	0
**4482**	373	A	67.9383	1
**4482**	381	A	78.7943	1

**4482**	382	A	74.104	1
**4482**	386	A	47.6636	0
**4482**	305	B	79.4989	1
**4482**	319	B	72.1849	1
**4482**	320	B	75.4619	-1
**4482**	321	B	74.6872	0
**4482**	351	B	14.3523	1
**4482**	357	B	79.5657	1
**4482**	370	B	79.5419	1
**4482**	372	B	73.1095	0
**4482**	373	B	79.5865	1
**4482**	381	B	72.6593	1
**4482**	382	B	35.1271	0
**4482**	386	B	53.1087	1
**4482**	305	C	53.4458	1
**4482**	319	C	72.8029	1
**4482**	320	C	79.5863	-1
**4482**	321	C	32.1854	1
**4482**	351	C	8.0757	1
**4482**	357	C	77.122	1
**4482**	370	C	79.536	1
**4482**	372	C	74.9302	0
**4482**	373	C	73.2156	1
**4482**	381	C	79.4979	1
**4482**	382	C	64.6797	1
**4482**	386	C	59.5708	1
**4482**	305	D	46.5173	0
**4482**	319	D	79.5448	1
**4482**	320	D	51.9196	-1
**4482**	321	D	79.6813	0
**4482**	351	D	22.9187	-1
**4482**	357	D	79.5029	1
**4482**	370	D	73.6657	1
**4482**	372	D	63.4003	0

**4482**	373	D	31.4746	0
**4482**	381	D	62.7414	1
**4482**	382	D	79.5848	1
**4482**	386	D	35.8267	0
**4483**	305	A	79.587	1
**4483**	319	A	54.3938	1
**4483**	320	A	50.0795	-1
**4483**	321	A	79.5618	0
**4483**	351	A	1.1455	1
**4483**	357	A	79.5819	1
**4483**	370	A	78.6717	1
**4483**	372	A	79.511	0
**4483**	373	A	42.068	0
**4483**	381	A	70.6227	1
**4483**	382	A	17.5668	-1
**4483**	386	A	50.874	1
**4483**	305	B	79.1738	1
**4483**	319	B	74.8447	1
**4483**	320	B	72.5572	-1
**4483**	321	B	59.2461	0
**4483**	351	B	11.4633	1
**4483**	357	B	79.1049	1
**4483**	370	B	71.1045	1
**4483**	372	B	57.0951	0
**4483**	373	B	74.2594	1
**4483**	381	B	44.5387	0
**4483**	382	B	77.0681	1
**4483**	386	B	71.4406	1
**4483**	305	C	57.3703	1
**4483**	319	C	69.8517	1
**4483**	320	C	58.5362	-1
**4483**	321	C	51.0655	0
**4483**	351	C	4.307	1
**4483**	357	C	79.586	1

**4483**	370	C	77.6059	1
**4483**	372	C	77.9644	0
**4483**	373	C	79.5874	1
**4483**	381	C	79.6621	1
**4483**	382	C	74.3996	1
**4483**	386	C	61.0849	1
**4483**	305	D	32.9796	0
**4483**	319	D	60.0912	1
**4483**	320	D	29.0137	-1
**4483**	321	D	51.1758	0
**4483**	351	D	15.7554	1
**4483**	357	D	71.7723	1
**4483**	370	D	78.6351	1
**4483**	372	D	79.4827	0
**4483**	373	D	36.3806	0
**4483**	381	D	72.8256	1
**4483**	382	D	79.5641	1
**4483**	386	D	79.6132	1
**4484**	305	A	79.481	1
**4484**	319	A	37.3022	0
**4484**	320	A	70.9756	-1
**4484**	321	A	30.9564	1
**4484**	351	A	11.6172	1
**4484**	357	A	79.5583	1
**4484**	370	A	78.5175	1
**4484**	372	A	46.797	1
**4484**	373	A	48.3812	0
**4484**	381	A	73.5624	1
**4484**	382	A	58.4391	1
**4484**	386	A	28.8576	0
**4484**	305	B	59.1099	1
**4484**	319	B	69.6273	1
**4484**	320	B	79.5597	-1
**4484**	321	B	64.4446	0

**4484**	351	B	16.3556	1
**4484**	357	B	72.7001	1
**4484**	370	B	72.7676	1
**4484**	372	B	66.1248	0
**4484**	373	B	38.7938	0
**4484**	381	B	75.3385	1
**4484**	382	B	49.2472	0
**4484**	386	B	79.4048	1
**4484**	305	C	74.2642	1
**4484**	319	C	76.1106	1
**4484**	320	C	79.5762	-1
**4484**	321	C	71.6777	0
**4484**	351	C	14.1139	1
**4484**	357	C	45.1931	0
**4484**	370	C	79.6394	1
**4484**	372	C	79.6506	0
**4484**	373	C	54.1426	1
**4484**	381	C	79.5996	1
**4484**	382	C	48.0232	0
**4484**	386	C	57.848	1
**4484**	305	D	71.9207	1
**4484**	319	D	79.6014	1
**4484**	320	D	79.5566	-1
**4484**	321	D	79.6241	0
**4484**	351	D	20.0658	-1
**4484**	357	D	79.6456	1
**4484**	370	D	79.6602	1
**4484**	372	D	37.3853	1
**4484**	373	D	52.7511	1
**4484**	381	D	56.6845	1
**4484**	382	D	79.6049	1
**4484**	386	D	78.5182	1
**4485**	305	A	67.287	1
**4485**	319	A	60.3348	1

**4485**	320	A	76.6965	-1
**4485**	321	A	78.7816	0
**4485**	351	A	5.1252	1
**4485**	357	A	72.8326	1
**4485**	370	A	48.956	0
**4485**	372	A	71.8717	0
**4485**	373	A	49.2028	0
**4485**	381	A	79.626	1
**4485**	382	A	79.6572	1
**4485**	386	A	73.8129	1
**4485**	305	B	74.1164	1
**4485**	319	B	79.5874	1
**4485**	320	B	79.2605	-1
**4485**	321	B	57.1411	0
**4485**	351	B	11.1974	1
**4485**	357	B	42.8732	0
**4485**	370	B	76.4062	1
**4485**	372	B	71.0034	0
**4485**	373	B	47.4181	0
**4485**	381	B	34.4017	0
**4485**	382	B	39.0745	0
**4485**	386	B	79.5027	1
**4485**	305	C	59.8621	1
**4485**	319	C	60.7561	1
**4485**	320	C	79.5687	-1
**4485**	321	C	33.0215	1
**4485**	351	C	9.3204	1
**4485**	357	C	78.0722	1
**4485**	370	C	68.1995	1
**4485**	372	C	43.5422	1
**4485**	373	C	72.171	1
**4485**	381	C	79.492	1
**4485**	382	C	18.1358	-1
**4485**	386	C	22.3078	0

**4485**	305	D	64.2676	1
**4485**	319	D	71.3521	1
**4485**	320	D	79.5237	-1
**4485**	321	D	79.6663	0
**4485**	351	D	0.5466	1
**4485**	357	D	79.647	1
**4485**	370	D	75.823	1
**4485**	372	D	73.5696	0
**4485**	373	D	45.8323	0
**4485**	381	D	67.9373	1
**4485**	382	D	70.9765	1
**4485**	386	D	78.6092	1
**4486**	305	A	72.8626	1
**4486**	319	A	30.4401	0
**4486**	320	A	70.6789	-1
**4486**	321	A	72.9439	0
**4486**	351	A	11.3702	1
**4486**	357	A	79.5555	1
**4486**	370	A	51.9124	1
**4486**	372	A	73.6094	0
**4486**	373	A	67.9138	1
**4486**	381	A	64.5458	1
**4486**	382	A	72.0104	1
**4486**	386	A	69.5311	1
**4486**	305	B	79.2791	1
**4486**	319	B	35.6221	0
**4486**	320	B	47.6417	-1
**4486**	321	B	57.555	0
**4486**	351	B	10.9848	1
**4486**	357	B	48.1303	0
**4486**	370	B	66.1634	1
**4486**	372	B	59.552	0
**4486**	373	B	65.3608	1
**4486**	381	B	59.722	1

**4486**	382	B	77.9038	1
**4486**	386	B	62.3784	1
**4486**	305	C	56.2603	1
**4486**	319	C	35.9181	0
**4486**	320	C	61.7716	-1
**4486**	321	C	73.4757	0
**4486**	351	C	4.0542	1
**4486**	357	C	44.1515	0
**4486**	370	C	79.5154	1
**4486**	372	C	71.9246	0
**4486**	373	C	73.1534	1
**4486**	381	C	56.1641	1
**4486**	382	C	44.1254	0
**4486**	386	C	51.2992	1
**4486**	305	D	50.4573	1
**4486**	319	D	33.4616	0
**4486**	320	D	79.5718	-1
**4486**	321	D	66.4341	0
**4486**	351	D	4.8071	1
**4486**	357	D	52.2122	1
**4486**	370	D	71.3535	1
**4486**	372	D	79.5433	0
**4486**	373	D	54.6973	1
**4486**	381	D	56.2557	1
**4486**	382	D	37.4583	0
**4486**	386	D	69.4116	1
**4487**	305	A	69.212	1
**4487**	319	A	61.918	1
**4487**	320	A	73.3892	-1
**4487**	321	A	79.4108	0
**4487**	351	A	11.1504	1
**4487**	357	A	79.5762	1
**4487**	370	A	77.1716	1
**4487**	372	A	45.8341	1

**4487**	373	A	64.7543	1
**4487**	381	A	79.4952	1
**4487**	382	A	51.9577	1
**4487**	386	A	74.7685	1
**4487**	305	B	66.1813	1
**4487**	319	B	79.4303	1
**4487**	320	B	30.7482	-1
**4487**	321	B	78.3724	0
**4487**	351	B	13.2894	1
**4487**	357	B	73.7611	1
**4487**	370	B	79.6062	1
**4487**	372	B	61.4585	0
**4487**	373	B	28.4534	0
**4487**	381	B	47.4578	0
**4487**	382	B	62.5417	1
**4487**	386	B	70.9746	1
**4487**	305	C	55.0057	1
**4487**	319	C	65.309	1
**4487**	320	C	62.1816	-1
**4487**	321	C	65.2355	0
**4487**	351	C	8.8052	1
**4487**	357	C	49.2725	0
**4487**	370	C	56.2841	1
**4487**	372	C	49.377	1
**4487**	373	C	61.2577	1
**4487**	381	C	62.064	1
**4487**	382	C	78.9932	1
**4487**	386	C	37.7815	0
**4487**	305	D	49.2163	0
**4487**	319	D	73.1659	1
**4487**	320	D	32.3009	-1
**4487**	321	D	74.2846	0
**4487**	351	D	6.3949	1
**4487**	357	D	79.263	1

**4487**	370	D	79.5681	1
**4487**	372	D	75.5367	0
**4487**	373	D	79.5273	1
**4487**	381	D	53.6401	1
**4487**	382	D	72.3343	1
**4487**	386	D	76.5809	1
**4488**	305	A	76.9449	1
**4488**	319	A	44.1131	0
**4488**	320	A	69.3382	-1
**4488**	321	A	38.0764	1
**4488**	351	A	9.4292	1
**4488**	357	A	72.5605	1
**4488**	370	A	79.5708	1
**4488**	372	A	63.7715	0
**4488**	373	A	77.4494	1
**4488**	381	A	72.855	1
**4488**	382	A	74.268	1
**4488**	386	A	78.8332	1
**4488**	305	B	61.2417	1
**4488**	319	B	73.9898	1
**4488**	320	B	23.6039	-1
**4488**	321	B	35.1916	1
**4488**	351	B	14.1159	1
**4488**	357	B	79.5378	1
**4488**	370	B	72.4128	1
**4488**	372	B	74.5463	0
**4488**	373	B	79.5571	1
**4488**	381	B	78.1195	1
**4488**	382	B	79.5885	1
**4488**	386	B	44.2066	0
**4488**	305	C	67.7569	1
**4488**	319	C	30.1745	0
**4488**	320	C	42.9248	-1
**4488**	321	C	64.6496	0

**4488**	351	C	3.9484	1
**4488**	357	C	65.9154	1
**4488**	370	C	67.8531	1
**4488**	372	C	73.1215	0
**4488**	373	C	52.0068	1
**4488**	381	C	79.5513	1
**4488**	382	C	79.5437	1
**4488**	386	C	37.2718	0
**4488**	305	D	72.5558	1
**4488**	319	D	79.4958	1
**4488**	320	D	79.5647	-1
**4488**	321	D	79.5912	0
**4488**	351	D	13.1903	1
**4488**	357	D	72.5487	1
**4488**	370	D	74.8682	1
**4488**	372	D	79.5326	0
**4488**	373	D	78.7787	1
**4488**	381	D	64.2914	1
**4488**	382	D	78.3117	1
**4488**	386	D	27.3904	0
**4489**	305	A	79.5756	1
**4489**	319	A	50.1123	1
**4489**	320	A	63.3231	-1
**4489**	321	A	46.6512	1
**4489**	351	A	21.9814	-1
**4489**	357	A	59.4791	1
**4489**	370	A	78.4105	1
**4489**	372	A	79.4616	0
**4489**	373	A	47.0476	0
**4489**	381	A	73.9652	1
**4489**	382	A	77.2355	1
**4489**	386	A	35.1322	0
**4489**	305	B	37.6025	0
**4489**	319	B	79.5295	1

**4489**	320	B	17.8935	1
**4489**	321	B	74.4061	0
**4489**	351	B	16.297	1
**4489**	357	B	79.1372	1
**4489**	370	B	74.38	1
**4489**	372	B	38.253	1
**4489**	373	B	41.1598	0
**4489**	381	B	44.6612	0
**4489**	382	B	23.0122	0
**4489**	386	B	46.4913	0
**4489**	305	C	79.565	1
**4489**	319	C	52.1404	1
**4489**	320	C	75.8971	-1
**4489**	321	C	50.6354	0
**4489**	351	C	8.6663	1
**4489**	357	C	55.4377	1
**4489**	370	C	79.5344	1
**4489**	372	C	72.1876	0
**4489**	373	C	79.5095	1
**4489**	381	C	72.9545	1
**4489**	382	C	79.5522	1
**4489**	386	C	42.4268	0
**4489**	305	D	20.0838	0
**4489**	319	D	72.7803	1
**4489**	320	D	25.6027	-1
**4489**	321	D	75.9462	0
**4489**	351	D	14.6466	1
**4489**	357	D	42.7486	0
**4489**	370	D	79.5646	1
**4489**	372	D	45.4343	1
**4489**	373	D	76.4342	1
**4489**	381	D	70.2605	1
**4489**	382	D	79.5183	1
**4489**	386	D	73.2016	1

**4490**	305	A	59.1108	1
**4490**	319	A	37.7895	0
**4490**	320	A	79.4697	-1
**4490**	321	A	72.7055	0
**4490**	351	A	5.9872	1
**4490**	357	A	33.8422	0
**4490**	370	A	76.567	1
**4490**	372	A	58.3409	0
**4490**	373	A	79.2537	1
**4490**	381	A	71.752	1
**4490**	382	A	71.2873	1
**4490**	386	A	79.507	1
**4490**	305	B	79.4841	1
**4490**	319	B	76.0766	1
**4490**	320	B	69.6681	-1
**4490**	321	B	70.601	0
**4490**	351	B	17.2364	1
**4490**	357	B	75.2779	1
**4490**	370	B	75.8367	1
**4490**	372	B	55.2157	0
**4490**	373	B	67.0361	1
**4490**	381	B	55.8294	1
**4490**	382	B	48.6042	0
**4490**	386	B	36.7497	0
**4490**	305	C	78.171	1
**4490**	319	C	78.2144	1
**4490**	320	C	79.6473	-1
**4490**	321	C	75.5071	0
**4490**	351	C	5.8119	1
**4490**	357	C	73.5888	1
**4490**	370	C	79.6013	1
**4490**	372	C	72.1235	0
**4490**	373	C	79.576	1
**4490**	381	C	79.5704	1

**4490**	382	C	63.4208	1
**4490**	386	C	10.4019	-1
**4490**	305	D	79.578	1
**4490**	319	D	79.5062	1
**4490**	320	D	69.5615	-1
**4490**	321	D	79.5193	0
**4490**	351	D	8.8333	1
**4490**	357	D	44.9454	0
**4490**	370	D	40.0853	0
**4490**	372	D	79.599	0
**4490**	373	D	62.0965	1
**4490**	381	D	70.062	1
**4490**	382	D	72.6362	1
**4490**	386	D	79.5779	1
**4491**	305	A	65.4164	1
**4491**	319	A	71.1185	1
**4491**	320	A	79.6837	-1
**4491**	321	A	40.9739	1
**4491**	351	A	12.7643	1
**4491**	357	A	73.8924	1
**4491**	370	A	68.8676	1
**4491**	372	A	77.662	0
**4491**	373	A	79.5744	1
**4491**	381	A	59.5553	1
**4491**	382	A	47.6581	0
**4491**	386	A	73.1307	1
**4491**	305	B	63.0148	1
**4491**	319	B	66.7941	1
**4491**	320	B	50.3895	-1
**4491**	321	B	79.481	0
**4491**	351	B	12.0999	1
**4491**	357	B	37.3368	0
**4491**	370	B	79.2515	1
**4491**	372	B	77.2952	0

**4491**	373	B	51.9867	1
**4491**	381	B	79.616	1
**4491**	382	B	50.5972	1
**4491**	386	B	79.5095	1
**4491**	305	C	74.4017	1
**4491**	319	C	49.9794	0
**4491**	320	C	74.1988	-1
**4491**	321	C	69.4994	0
**4491**	351	C	14.0216	1
**4491**	357	C	79.6549	1
**4491**	370	C	75.5842	1
**4491**	372	C	72.2859	0
**4491**	373	C	70.7294	1
**4491**	381	C	54.8277	1
**4491**	382	C	68.927	1
**4491**	386	C	49.4887	0
**4491**	305	D	79.4994	1
**4491**	319	D	67.8248	1
**4491**	320	D	79.6081	-1
**4491**	321	D	67.5791	0
**4491**	351	D	19.4901	1
**4491**	357	D	75.3066	1
**4491**	370	D	21.9925	0
**4491**	372	D	79.4704	0
**4491**	373	D	69.6074	1
**4491**	381	D	58.5407	1
**4491**	382	D	79.4694	1
**4491**	386	D	79.6272	1
**4492**	305	A	71.3381	1
**4492**	319	A	49.299	0
**4492**	320	A	58.305	-1
**4492**	321	A	78.8822	0
**4492**	351	A	14.2775	1
**4492**	357	A	73.5423	1

**4492**	370	A	72.321	1
**4492**	372	A	70.4763	0
**4492**	373	A	65.2772	1
**4492**	381	A	71.7194	1
**4492**	382	A	60.5541	1
**4492**	386	A	79.4995	1
**4492**	305	B	79.5412	1
**4492**	319	B	35.5966	0
**4492**	320	B	79.6423	-1
**4492**	321	B	72.9772	0
**4492**	351	B	13.535	1
**4492**	357	B	47.4626	0
**4492**	370	B	45.7899	0
**4492**	372	B	79.5538	0
**4492**	373	B	37.7768	0
**4492**	381	B	62.8761	1
**4492**	382	B	36.772	0
**4492**	386	B	85.2034	1
**4492**	305	C	73.1945	1
**4492**	319	C	79.5232	1
**4492**	320	C	79.5838	-1
**4492**	321	C	79.648	0
**4492**	351	C	6.7011	1
**4492**	357	C	79.4392	1
**4492**	370	C	79.4683	1
**4492**	372	C	69.867	0
**4492**	373	C	79.5774	1
**4492**	381	C	79.5691	1
**4492**	382	C	75.6769	1
**4492**	386	C	33.545	0
**4492**	305	D	35.1777	0
**4492**	319	D	41.9374	0
**4492**	320	D	52.886	-1
**4492**	321	D	79.5267	0

**4492**	351	D	7.3299	1
**4492**	357	D	73.3854	1
**4492**	370	D	62.5516	1
**4492**	372	D	63.5388	0
**4492**	373	D	33.3374	0
**4492**	381	D	57.6356	1
**4492**	382	D	77.5121	1
**4492**	386	D	70.7372	1
**4493**	305	A	72.0861	1
**4493**	319	A	61.2038	1
**4493**	320	A	79.3221	-1
**4493**	321	A	56.2323	0
**4493**	351	A	11.1598	1
**4493**	357	A	72.6821	1
**4493**	370	A	58.9808	1
**4493**	372	A	77.5448	0
**4493**	373	A	76.2186	1
**4493**	381	A	73.7678	1
**4493**	382	A	57.9659	1
**4493**	386	A	68.5262	1
**4493**	305	B	79.6749	1
**4493**	319	B	79.4885	1
**4493**	320	B	79.4952	-1
**4493**	321	B	79.5769	0
**4493**	351	B	21.1397	-1
**4493**	357	B	75.6598	1
**4493**	370	B	79.5477	1
**4493**	372	B	79.5096	0
**4493**	373	B	35.4712	0
**4493**	381	B	67.8596	1
**4493**	382	B	62.331	1
**4493**	386	B	79.5945	1
**4493**	305	C	79.4247	1
**4493**	319	C	74.6402	1

**4493**	320	C	79.5257	-1
**4493**	321	C	64.2632	0
**4493**	351	C	1.88	1
**4493**	357	C	69.8323	1
**4493**	370	C	68.4786	1
**4493**	372	C	71.5963	0
**4493**	373	C	79.5224	1
**4493**	381	C	73.1376	1
**4493**	382	C	59.5322	1
**4493**	386	C	61.4646	1
**4493**	305	D	42.0397	0
**4493**	319	D	79.6676	1
**4493**	320	D	72.7732	-1
**4493**	321	D	60.3589	0
**4493**	351	D	19.6984	1
**4493**	357	D	79.6283	1
**4493**	370	D	44.9371	0
**4493**	372	D	42.0979	1
**4493**	373	D	73.7574	1
**4493**	381	D	25.631	0
**4493**	382	D	79.6226	1
**4493**	386	D	79.4508	1
**4494**	305	A	40.2499	0
**4494**	319	A	68.4137	1
**4494**	320	A	73.1206	-1
**4494**	321	A	44.0675	1
**4494**	351	A	0.0	1
**4494**	357	A	79.5681	1
**4494**	370	A	79.6149	1
**4494**	372	A	62.8754	0
**4494**	373	A	40.0246	0
**4494**	381	A	74.7837	1
**4494**	382	A	47.0935	0
**4494**	386	A	43.381	0

**4494**	305	B	79.4065	1
**4494**	319	B	71.7196	1
**4494**	320	B	68.1595	-1
**4494**	321	B	56.3076	0
**4494**	351	B	15.8396	1
**4494**	357	B	73.1198	1
**4494**	370	B	79.4846	1
**4494**	372	B	79.5966	0
**4494**	373	B	79.5312	1
**4494**	381	B	37.456	0
**4494**	382	B	42.2354	0
**4494**	386	B	69.8337	1
**4494**	305	C	79.306	1
**4494**	319	C	68.0335	1
**4494**	320	C	79.6272	-1
**4494**	321	C	68.1992	0
**4494**	351	C	0.0	1
**4494**	357	C	70.4136	1
**4494**	370	C	74.2328	1
**4494**	372	C	60.3355	0
**4494**	373	C	57.1109	1
**4494**	381	C	79.536	1
**4494**	382	C	72.0256	1
**4494**	386	C	73.7692	1
**4494**	305	D	46.3506	0
**4494**	319	D	65.798	1
**4494**	320	D	79.5729	-1
**4494**	321	D	34.5912	1
**4494**	351	D	11.3128	1
**4494**	357	D	51.0446	1
**4494**	370	D	47.195	0
**4494**	372	D	53.6242	0
**4494**	373	D	36.1105	0
**4494**	381	D	29.9819	0

**4494**	382	D	46.8075	0
**4494**	386	D	70.9907	1
**4495**	305	A	33.8657	0
**4495**	319	A	37.5376	0
**4495**	320	A	72.3752	-1
**4495**	321	A	79.4267	0
**4495**	351	A	38.5741	-1
**4495**	357	A	78.9253	1
**4495**	370	A	60.6272	1
**4495**	372	A	79.4248	0
**4495**	373	A	29.897	0
**4495**	381	A	79.5336	1
**4495**	382	A	70.6491	1
**4495**	386	A	36.5711	0
**4495**	305	B	71.2329	1
**4495**	319	B	79.0749	1
**4495**	320	B	58.4837	-1
**4495**	321	B	48.3335	1
**4495**	351	B	35.3645	-1
**4495**	357	B	79.542	1
**4495**	370	B	57.9282	1
**4495**	372	B	79.5334	0
**4495**	373	B	77.6361	1
**4495**	381	B	79.5557	1
**4495**	382	B	49.4353	0
**4495**	386	B	79.6534	1
**4495**	305	C	78.0748	1
**4495**	319	C	51.1127	1
**4495**	320	C	38.7031	-1
**4495**	321	C	79.634	0
**4495**	351	C	0.7975	1
**4495**	357	C	74.019	1
**4495**	370	C	60.5716	1
**4495**	372	C	76.4367	0

**4495**	373	C	74.7047	1
**4495**	381	C	79.5937	1
**4495**	382	C	72.5352	1
**4495**	386	C	36.2072	0
**4495**	305	D	78.6055	1
**4495**	319	D	49.1719	0
**4495**	320	D	79.6385	-1
**4495**	321	D	70.0288	0
**4495**	351	D	9.8524	1
**4495**	357	D	54.3036	1
**4495**	370	D	42.8534	0
**4495**	372	D	79.6156	0
**4495**	373	D	57.0055	1
**4495**	381	D	79.5231	1
**4495**	382	D	79.6061	1
**4495**	386	D	71.0809	1
**4496**	305	A	43.2669	0
**4496**	319	A	75.7593	1
**4496**	320	A	77.6797	-1
**4496**	321	A	72.5716	0
**4496**	351	A	5.235	1
**4496**	357	A	56.8046	1
**4496**	370	A	79.6483	1
**4496**	372	A	61.3785	0
**4496**	373	A	79.5718	1
**4496**	381	A	79.5495	1
**4496**	382	A	60.37	1
**4496**	386	A	79.6371	1
**4496**	305	B	74.0572	1
**4496**	319	B	21.463	0
**4496**	320	B	58.7716	-1
**4496**	321	B	68.0403	0
**4496**	351	B	48.4143	-1
**4496**	357	B	74.1437	1

**4496**	370	B	79.5892	1
**4496**	372	B	79.4998	0
**4496**	373	B	66.5524	1
**4496**	381	B	72.3722	1
**4496**	382	B	73.037	1
**4496**	386	B	67.8046	1
**4496**	305	C	63.3325	1
**4496**	319	C	79.4459	1
**4496**	320	C	72.4757	-1
**4496**	321	C	71.2088	0
**4496**	351	C	8.1851	1
**4496**	357	C	23.7004	0
**4496**	370	C	27.703	0
**4496**	372	C	69.4259	0
**4496**	373	C	60.4781	1
**4496**	381	C	70.013	1
**4496**	382	C	46.7823	0
**4496**	386	C	48.5695	0
**4496**	305	D	41.9653	0
**4496**	319	D	73.0231	1
**4496**	320	D	64.5372	-1
**4496**	321	D	70.0686	0
**4496**	351	D	14.503	1
**4496**	357	D	50.5721	1
**4496**	370	D	79.5197	1
**4496**	372	D	79.4988	0
**4496**	373	D	79.6655	1
**4496**	381	D	79.5453	1
**4496**	382	D	79.5641	1
**4496**	386	D	74.504	1
**4497**	305	A	57.4726	1
**4497**	319	A	59.4682	1
**4497**	320	A	79.485	-1
**4497**	321	A	27.3784	1

**4497**	351	A	8.0975	1
**4497**	357	A	79.6422	1
**4497**	370	A	36.441	0
**4497**	372	A	79.6149	0
**4497**	373	A	79.6081	1
**4497**	381	A	69.5291	1
**4497**	382	A	55.4261	1
**4497**	386	A	77.9373	1
**4497**	305	B	73.9136	1
**4497**	319	B	79.6336	1
**4497**	320	B	79.5658	-1
**4497**	321	B	63.6539	0
**4497**	351	B	0.1437	1
**4497**	357	B	74.8534	1
**4497**	370	B	79.504	1
**4497**	372	B	75.7943	0
**4497**	373	B	60.7068	1
**4497**	381	B	52.5637	1
**4497**	382	B	36.905	0
**4497**	386	B	30.8307	0
**4497**	305	C	79.6203	1
**4497**	319	C	75.6257	1
**4497**	320	C	26.007	-1
**4497**	321	C	79.6844	0
**4497**	351	C	6.9525	1
**4497**	357	C	57.5825	1
**4497**	370	C	62.3863	1
**4497**	372	C	79.4142	0
**4497**	373	C	79.5919	1
**4497**	381	C	46.3862	0
**4497**	382	C	63.6872	1
**4497**	386	C	43.9443	0
**4497**	305	D	55.5972	1
**4497**	319	D	79.4242	1

**4497**	320	D	79.4873	-1
**4497**	321	D	73.6986	0
**4497**	351	D	37.5111	-1
**4497**	357	D	67.2093	1
**4497**	370	D	47.5854	0
**4497**	372	D	79.4997	0
**4497**	373	D	79.6322	1
**4497**	381	D	74.6451	1
**4497**	382	D	79.5011	1
**4497**	386	D	75.0339	1
**4498**	305	A	71.9109	1
**4498**	319	A	40.0398	0
**4498**	320	A	79.5832	-1
**4498**	321	A	77.5038	0
**4498**	351	A	9.5509	1
**4498**	357	A	79.5483	1
**4498**	370	A	50.7684	1
**4498**	372	A	59.5753	0
**4498**	373	A	38.3713	0
**4498**	381	A	33.2909	0
**4498**	382	A	79.62	1
**4498**	386	A	74.5375	1
**4498**	305	B	55.8376	1
**4498**	319	B	75.7573	1
**4498**	320	B	73.8309	-1
**4498**	321	B	72.1515	0
**4498**	351	B	1.4656	1
**4498**	357	B	60.3268	1
**4498**	370	B	71.6967	1
**4498**	372	B	57.2528	0
**4498**	373	B	44.9818	0
**4498**	381	B	72.5433	1
**4498**	382	B	34.6115	0
**4498**	386	B	39.4492	0

**4498**	305	C	73.1526	1
**4498**	319	C	48.844	0
**4498**	320	C	74.1891	-1
**4498**	321	C	79.5722	0
**4498**	351	C	6.5562	1
**4498**	357	C	79.507	1
**4498**	370	C	59.3862	1
**4498**	372	C	70.5163	0
**4498**	373	C	56.3131	1
**4498**	381	C	29.9318	0
**4498**	382	C	79.6001	1
**4498**	386	C	42.564	0
**4498**	305	D	63.5692	1
**4498**	319	D	73.6694	1
**4498**	320	D	73.6829	-1
**4498**	321	D	78.4747	0
**4498**	351	D	9.2866	1
**4498**	357	D	69.6201	1
**4498**	370	D	70.8506	1
**4498**	372	D	79.5153	0
**4498**	373	D	19.4173	-1
**4498**	381	D	54.7427	1
**4498**	382	D	79.5807	1
**4498**	386	D	61.1257	1
**4499**	305	A	75.5296	1
**4499**	319	A	73.0476	1
**4499**	320	A	76.8913	-1
**4499**	321	A	58.7999	0
**4499**	351	A	6.3592	1
**4499**	357	A	47.3575	0
**4499**	370	A	78.0472	1
**4499**	372	A	55.2153	0
**4499**	373	A	79.5344	1
**4499**	381	A	62.0716	1

**4499**	382	A	53.5572	1
**4499**	386	A	79.5225	1
**4499**	305	B	79.5516	1
**4499**	319	B	56.9861	1
**4499**	320	B	27.7297	-1
**4499**	321	B	79.2806	0
**4499**	351	B	13.0304	1
**4499**	357	B	43.2099	0
**4499**	370	B	44.5868	0
**4499**	372	B	79.5312	0
**4499**	373	B	79.4909	1
**4499**	381	B	79.5475	1
**4499**	382	B	37.1229	0
**4499**	386	B	75.6525	1
**4499**	305	C	49.8475	0
**4499**	319	C	67.4917	1
**4499**	320	C	79.3534	-1
**4499**	321	C	79.6191	0
**4499**	351	C	15.0475	1
**4499**	357	C	73.657	1
**4499**	370	C	45.6454	0
**4499**	372	C	79.5641	0
**4499**	373	C	69.4513	1
**4499**	381	C	62.3747	1
**4499**	382	C	43.6254	0
**4499**	386	C	31.9824	0
**4499**	305	D	78.4297	1
**4499**	319	D	61.0918	1
**4499**	320	D	79.5018	-1
**4499**	321	D	79.5762	0
**4499**	351	D	13.7407	1
**4499**	357	D	66.1586	1
**4499**	370	D	21.1745	0
**4499**	372	D	70.9151	0

**4499**	373	D	79.5447	1
**4499**	381	D	61.0033	1
**4499**	382	D	79.5843	1
**4499**	386	D	33.683	0
**4500**	305	A	79.5441	1
**4500**	319	A	44.1476	0
**4500**	320	A	31.8715	-1
**4500**	321	A	79.1475	0
**4500**	351	A	4.2942	1
**4500**	357	A	73.845	1
**4500**	370	A	62.3983	1
**4500**	372	A	62.0112	0
**4500**	373	A	61.6939	1
**4500**	381	A	79.5499	1
**4500**	382	A	46.6758	0
**4500**	386	A	44.423	0
**4500**	305	B	46.0172	0
**4500**	319	B	79.556	1
**4500**	320	B	68.6398	-1
**4500**	321	B	79.5544	0
**4500**	351	B	17.9342	1
**4500**	357	B	25.6466	0
**4500**	370	B	79.6035	1
**4500**	372	B	79.4956	0
**4500**	373	B	79.5197	1
**4500**	381	B	79.6633	1
**4500**	382	B	78.0287	1
**4500**	386	B	62.9038	1
**4500**	305	C	50.9082	1
**4500**	319	C	50.9709	1
**4500**	320	C	72.6851	-1
**4500**	321	C	43.4162	1
**4500**	351	C	2.5702	1
**4500**	357	C	79.4728	1

**4500**	370	C	73.7106	1
**4500**	372	C	69.8859	0
**4500**	373	C	79.5294	1
**4500**	381	C	23.2376	0
**4500**	382	C	79.5353	1
**4500**	386	C	24.968	0
**4500**	305	D	33.0736	0
**4500**	319	D	59.329	1
**4500**	320	D	47.5485	-1
**4500**	321	D	40.2347	1
**4500**	351	D	13.951	1
**4500**	357	D	73.41	1
**4500**	370	D	53.9471	1
**4500**	372	D	79.7357	0
**4500**	373	D	79.454	1
**4500**	381	D	79.528	1
**4500**	382	D	67.9905	1
**4500**	386	D	48.8768	0
**4501**	305	A	11.5568	-1
**4501**	319	A	74.4748	1
**4501**	320	A	75.5871	-1
**4501**	321	A	52.9585	0
**4501**	351	A	997	1
**4501**	357	A	79.6171	1
**4501**	370	A	79.5558	1
**4501**	372	A	69.7221	0
**4501**	373	A	79.5416	1
**4501**	381	A	58.7711	1
**4501**	382	A	16.5737	-1
**4501**	386	A	72.0423	1
**4501**	305	B	79.5317	1
**4501**	319	B	79.5822	1
**4501**	320	B	68.6325	-1
**4501**	321	B	79.5627	0

**4501**	351	B	14.3724	1
**4501**	357	B	79.6971	1
**4501**	370	B	78.4767	1
**4501**	372	B	62.3697	0
**4501**	373	B	68.7868	1
**4501**	381	B	68.515	1
**4501**	382	B	51.7985	1
**4501**	386	B	79.61	1
**4501**	305	C	79.68	1
**4501**	319	C	79.608	1
**4501**	320	C	79.5438	-1
**4501**	321	C	47.2266	1
**4501**	351	C	10.6551	1
**4501**	357	C	75.6069	1
**4501**	370	C	38.4034	0
**4501**	372	C	79.1014	0
**4501**	373	C	42.6967	0
**4501**	381	C	44.7622	0
**4501**	382	C	44.3476	0
**4501**	386	C	15.8846	-1
**4501**	305	D	79.4953	1
**4501**	319	D	73.404	1
**4501**	320	D	73.3961	-1
**4501**	321	D	67.2059	0
**4501**	351	D	22.4362	-1
**4501**	357	D	78.7271	1
**4501**	370	D	54.2826	1
**4501**	372	D	48.9327	1
**4501**	373	D	72.4432	1
**4501**	381	D	79.4969	1
**4501**	382	D	60.3457	1
**4501**	386	D	72.8473	1
**4502**	305	A	30.4641	0
**4502**	319	A	57.3795	1

**4502**	320	A	41.1218	-1
**4502**	321	A	30.8904	1
**4502**	351	A	3.0392	1
**4502**	357	A	79.4264	1
**4502**	370	A	72.9102	1
**4502**	372	A	77.5616	0
**4502**	373	A	72.4115	1
**4502**	381	A	16.4347	-1
**4502**	382	A	49.8304	0
**4502**	386	A	49.0813	0
**4502**	305	B	79.6188	1
**4502**	319	B	71.9299	1
**4502**	320	B	74.0241	-1
**4502**	321	B	69.1835	0
**4502**	351	B	16.4844	1
**4502**	357	B	70.845	1
**4502**	370	B	68.4358	1
**4502**	372	B	58.1166	0
**4502**	373	B	71.6746	1
**4502**	381	B	41.0091	0
**4502**	382	B	18.8806	-1
**4502**	386	B	79.6011	1
**4502**	305	C	75.3354	1
**4502**	319	C	79.4937	1
**4502**	320	C	66.7144	-1
**4502**	321	C	79.5853	0
**4502**	351	C	3.4885	1
**4502**	357	C	47.6344	0
**4502**	370	C	72.9994	1
**4502**	372	C	79.5617	0
**4502**	373	C	79.6341	1
**4502**	381	C	67.697	1
**4502**	382	C	79.5026	1
**4502**	386	C	52.1442	1

**4502**	305	D	51.6808	1
**4502**	319	D	62.8523	1
**4502**	320	D	59.7376	-1
**4502**	321	D	69.5091	0
**4502**	351	D	11.5878	1
**4502**	357	D	79.5564	1
**4502**	370	D	23.2872	0
**4502**	372	D	79.5167	0
**4502**	373	D	45.3473	0
**4502**	381	D	56.3399	1
**4502**	382	D	79.5954	1
**4502**	386	D	79.4387	1
**4503**	305	A	49.1582	0
**4503**	319	A	79.424	1
**4503**	320	A	73.2501	-1
**4503**	321	A	79.6481	0
**4503**	351	A	6.835	1
**4503**	357	A	74.3613	1
**4503**	370	A	72.1284	1
**4503**	372	A	55.5521	0
**4503**	373	A	42.7841	0
**4503**	381	A	26.3454	0
**4503**	382	A	69.6727	1
**4503**	386	A	33.4983	0
**4503**	305	B	62.2221	1
**4503**	319	B	67.9299	1
**4503**	320	B	79.5965	-1
**4503**	321	B	79.4618	0
**4503**	351	B	19.5839	1
**4503**	357	B	77.0619	1
**4503**	370	B	49.2449	0
**4503**	372	B	60.6213	0
**4503**	373	B	78.9565	1
**4503**	381	B	70.389	1

**4503**	382	B	53.708	1
**4503**	386	B	45.1621	0
**4503**	305	C	30.468	0
**4503**	319	C	17.8754	-1
**4503**	320	C	46.8798	-1
**4503**	321	C	48.731	1
**4503**	351	C	4.5609	1
**4503**	357	C	70.4566	1
**4503**	370	C	72.9234	1
**4503**	372	C	73.0205	0
**4503**	373	C	72.2495	1
**4503**	381	C	79.4536	1
**4503**	382	C	79.0086	1
**4503**	386	C	37.4798	0
**4503**	305	D	79.4983	1
**4503**	319	D	72.0905	1
**4503**	320	D	79.2424	-1
**4503**	321	D	63.8574	0
**4503**	351	D	15.5101	1
**4503**	357	D	38.2314	0
**4503**	370	D	65.4446	1
**4503**	372	D	66.3469	0
**4503**	373	D	79.494	1
**4503**	381	D	79.6162	1
**4503**	382	D	79.522	1
**4503**	386	D	49.4142	0
**4504**	305	A	68.2303	1
**4504**	319	A	68.016	1
**4504**	320	A	72.5167	-1
**4504**	321	A	72.1306	0
**4504**	351	A	5.7681	1
**4504**	357	A	65.2079	1
**4504**	370	A	47.8032	0
**4504**	372	A	61.8774	0

**4504**	373	A	32.4396	0
**4504**	381	A	75.3914	1
**4504**	382	A	78.8742	1
**4504**	386	A	79.5851	1
**4504**	305	B	76.0647	1
**4504**	319	B	79.6537	1
**4504**	320	B	70.6799	-1
**4504**	321	B	79.5605	0
**4504**	351	B	10.9829	1
**4504**	357	B	79.5463	1
**4504**	370	B	64.3218	1
**4504**	372	B	79.5726	0
**4504**	373	B	41.3335	0
**4504**	381	B	79.4819	1
**4504**	382	B	44.5276	0
**4504**	386	B	58.1419	1
**4504**	305	C	72.6001	1
**4504**	319	C	32.2887	0
**4504**	320	C	71.0869	-1
**4504**	321	C	55.6931	0
**4504**	351	C	2.0168	1
**4504**	357	C	71.1453	1
**4504**	370	C	79.595	1
**4504**	372	C	57.3349	0
**4504**	373	C	78.8382	1
**4504**	381	C	50.4664	1
**4504**	382	C	76.3095	1
**4504**	386	C	30.9787	0
**4504**	305	D	76.8755	1
**4504**	319	D	63.4092	1
**4504**	320	D	79.5652	-1
**4504**	321	D	79.5305	0
**4504**	351	D	13.1811	1
**4504**	357	D	79.4233	1

**4504**	370	D	41.5313	0
**4504**	372	D	55.288	0
**4504**	373	D	79.2738	1
**4504**	381	D	79.5362	1
**4504**	382	D	73.7018	1
**4504**	386	D	79.5686	1
**4505**	305	A	51.1877	1
**4505**	319	A	55.3956	1
**4505**	320	A	44.3436	-1
**4505**	321	A	73.4738	0
**4505**	351	A	11.3792	1
**4505**	357	A	71.45	1
**4505**	370	A	72.744	1
**4505**	372	A	74.7761	0
**4505**	373	A	73.3077	1
**4505**	381	A	77.4695	1
**4505**	382	A	79.587	1
**4505**	386	A	70.6024	1
**4505**	305	B	79.5843	1
**4505**	319	B	63.0102	1
**4505**	320	B	79.4695	-1
**4505**	321	B	53.6157	0
**4505**	351	B	9.7826	1
**4505**	357	B	64.7731	1
**4505**	370	B	60.0779	1
**4505**	372	B	79.5623	0
**4505**	373	B	72.328	1
**4505**	381	B	77.2032	1
**4505**	382	B	70.7063	1
**4505**	386	B	68.6587	1
**4505**	305	C	23.1355	0
**4505**	319	C	79.6098	1
**4505**	320	C	58.6609	-1
**4505**	321	C	28.8849	1

**4505**	351	C	7.1746	1
**4505**	357	C	72.2403	1
**4505**	370	C	35.3689	0
**4505**	372	C	79.2523	0
**4505**	373	C	76.1672	1
**4505**	381	C	76.087	1
**4505**	382	C	33.9886	0
**4505**	386	C	56.5291	1
**4505**	305	D	79.4582	1
**4505**	319	D	36.2803	0
**4505**	320	D	72.9345	-1
**4505**	321	D	57.9101	0
**4505**	351	D	5.6482	1
**4505**	357	D	49.9492	0
**4505**	370	D	74.8443	1
**4505**	372	D	79.6311	0
**4505**	373	D	70.1258	1
**4505**	381	D	79.6351	1
**4505**	382	D	46.544	0
**4505**	386	D	17.3929	-1
**4506**	305	A	71.4504	1
**4506**	319	A	58.01	1
**4506**	320	A	44.1121	-1
**4506**	321	A	44.9376	1
**4506**	351	A	12.906	1
**4506**	357	A	74.1557	1
**4506**	370	A	72.1362	1
**4506**	372	A	71.7691	0
**4506**	373	A	42.1555	0
**4506**	381	A	79.4156	1
**4506**	382	A	35.6518	0
**4506**	386	A	79.2632	1
**4506**	305	B	62.7529	1
**4506**	319	B	79.5743	1

**4506**	320	B	73.1672	-1
**4506**	321	B	58.7518	0
**4506**	351	B	15.3616	1
**4506**	357	B	46.9996	0
**4506**	370	B	79.588	1
**4506**	372	B	42.203	1
**4506**	373	B	19.3932	-1
**4506**	381	B	55.1514	1
**4506**	382	B	44.6536	0
**4506**	386	B	43.4973	0
**4506**	305	C	75.6285	1
**4506**	319	C	31.8795	0
**4506**	320	C	71.5763	-1
**4506**	321	C	67.7939	0
**4506**	351	C	4.6461	1
**4506**	357	C	45.5389	0
**4506**	370	C	79.5143	1
**4506**	372	C	76.1504	0
**4506**	373	C	76.6673	1
**4506**	381	C	77.4562	1
**4506**	382	C	79.4172	1
**4506**	386	C	60.4857	1
**4506**	305	D	47.3903	0
**4506**	319	D	60.2941	1
**4506**	320	D	78.7076	-1
**4506**	321	D	79.5354	0
**4506**	351	D	13.2952	1
**4506**	357	D	79.6009	1
**4506**	370	D	60.2018	1
**4506**	372	D	71.4717	0
**4506**	373	D	65.6176	1
**4506**	381	D	33.3413	0
**4506**	382	D	70.1047	1
**4506**	386	D	73.2443	1

**4507**	305	A	78.8945	1
**4507**	319	A	33.0444	0
**4507**	320	A	49.5444	-1
**4507**	321	A	59.3034	0
**4507**	351	A	1.3823	1
**4507**	357	A	74.1813	1
**4507**	370	A	78.4955	1
**4507**	372	A	42.7909	1
**4507**	373	A	79.5571	1
**4507**	381	A	48.1431	0
**4507**	382	A	39.4611	0
**4507**	386	A	37.5207	0
**4507**	305	B	62.2081	1
**4507**	319	B	79.4511	1
**4507**	320	B	66.296	-1
**4507**	321	B	66.2913	0
**4507**	351	B	16.6428	1
**4507**	357	B	79.6013	1
**4507**	370	B	79.5632	1
**4507**	372	B	75.6885	0
**4507**	373	B	79.5085	1
**4507**	381	B	79.5567	1
**4507**	382	B	43.8663	0
**4507**	386	B	79.5392	1
**4507**	305	C	43.2995	0
**4507**	319	C	68.5263	1
**4507**	320	C	70.9074	-1
**4507**	321	C	79.5089	0
**4507**	351	C	3.9456	1
**4507**	357	C	76.556	1
**4507**	370	C	57.9091	1
**4507**	372	C	41.1667	1
**4507**	373	C	67.152	1
**4507**	381	C	75.2279	1

**4507**	382	C	71.2781	1
**4507**	386	C	47.9006	0
**4507**	305	D	79.5873	1
**4507**	319	D	79.5618	1
**4507**	320	D	79.5721	-1
**4507**	321	D	79.5875	0
**4507**	351	D	12.4484	1
**4507**	357	D	79.5791	1
**4507**	370	D	73.0976	1
**4507**	372	D	74.1214	0
**4507**	373	D	65.1765	1
**4507**	381	D	18.5785	-1
**4507**	382	D	58.8863	1
**4507**	386	D	48.3363	0
**4508**	305	A	68.7685	1
**4508**	319	A	29.1167	0
**4508**	320	A	79.522	-1
**4508**	321	A	33.077	1
**4508**	351	A	12.4907	1
**4508**	357	A	64.4026	1
**4508**	370	A	59.1327	1
**4508**	372	A	69.9009	0
**4508**	373	A	74.8898	1
**4508**	381	A	71.8921	1
**4508**	382	A	79.5648	1
**4508**	386	A	55.382	1
**4508**	305	B	65.81	1
**4508**	319	B	79.6323	1
**4508**	320	B	79.5361	-1
**4508**	321	B	77.7697	0
**4508**	351	B	16.1433	1
**4508**	357	B	43.6946	0
**4508**	370	B	79.5998	1
**4508**	372	B	79.5393	0

**4508**	373	B	77.3898	1
**4508**	381	B	42.5872	0
**4508**	382	B	47.3749	0
**4508**	386	B	66.3569	1
**4508**	305	C	76.3521	1
**4508**	319	C	73.7411	1
**4508**	320	C	79.5489	-1
**4508**	321	C	78.0735	0
**4508**	351	C	1.9456	1
**4508**	357	C	54.0118	1
**4508**	370	C	43.345	0
**4508**	372	C	79.5944	0
**4508**	373	C	79.5335	1
**4508**	381	C	44.2156	0
**4508**	382	C	77.4691	1
**4508**	386	C	48.0755	0
**4508**	305	D	79.5809	1
**4508**	319	D	79.575	1
**4508**	320	D	79.503	-1
**4508**	321	D	43.8956	1
**4508**	351	D	13.6444	1
**4508**	357	D	79.5846	1
**4508**	370	D	37.789	0
**4508**	372	D	79.5372	0
**4508**	373	D	71.0108	1
**4508**	381	D	74.4833	1
**4508**	382	D	72.7555	1
**4508**	386	D	34.1369	0
**4509**	305	A	77.9477	1
**4509**	319	A	79.3825	1
**4509**	320	A	52.9385	-1
**4509**	321	A	71.2898	0
**4509**	351	A	7.5256	1
**4509**	357	A	78.9181	1

**4509**	370	A	79.5101	1
**4509**	372	A	75.7097	0
**4509**	373	A	79.7066	1
**4509**	381	A	79.5782	1
**4509**	382	A	67.7514	1
**4509**	386	A	64.9987	1
**4509**	305	B	77.6642	1
**4509**	319	B	44.9501	0
**4509**	320	B	68.7793	-1
**4509**	321	B	79.4765	0
**4509**	351	B	27.2929	-1
**4509**	357	B	49.7421	0
**4509**	370	B	79.6256	1
**4509**	372	B	77.4531	0
**4509**	373	B	56.7676	1
**4509**	381	B	69.3305	1
**4509**	382	B	33.0089	0
**4509**	386	B	59.6049	1
**4509**	305	C	79.4433	1
**4509**	319	C	69.1823	1
**4509**	320	C	61.0728	-1
**4509**	321	C	51.1992	0
**4509**	351	C	12.6276	1
**4509**	357	C	74.2074	1
**4509**	370	C	72.2142	1
**4509**	372	C	66.5229	0
**4509**	373	C	31.1352	0
**4509**	381	C	61.6758	1
**4509**	382	C	61.8915	1
**4509**	386	C	51.0834	1
**4509**	305	D	61.7143	1
**4509**	319	D	78.2049	1
**4509**	320	D	79.6122	-1
**4509**	321	D	79.4628	0

**4509**	351	D	6.9163	1
**4509**	357	D	79.4566	1
**4509**	370	D	76.9973	1
**4509**	372	D	55.7393	0
**4509**	373	D	76.1558	1
**4509**	381	D	69.1245	1
**4509**	382	D	72.0418	1
**4509**	386	D	79.5455	1
**4510**	305	A	54.3576	1
**4510**	319	A	73.5849	1
**4510**	320	A	79.5425	-1
**4510**	321	A	79.4863	0
**4510**	351	A	9.3896	1
**4510**	357	A	43.5349	0
**4510**	370	A	79.6192	1
**4510**	372	A	65.3492	0
**4510**	373	A	78.3521	1
**4510**	381	A	79.4732	1
**4510**	382	A	63.6393	1
**4510**	386	A	74.9208	1
**4510**	305	B	64.9528	1
**4510**	319	B	31.2842	0
**4510**	320	B	12.6552	1
**4510**	321	B	67.834	0
**4510**	351	B	12.8834	1
**4510**	357	B	34.1228	0
**4510**	370	B	64.4775	1
**4510**	372	B	79.4865	0
**4510**	373	B	79.65	1
**4510**	381	B	58.5085	1
**4510**	382	B	41.1761	0
**4510**	386	B	75.4201	1
**4510**	305	C	36.831	0
**4510**	319	C	59.045	1

**4510**	320	C	70.078	-1
**4510**	321	C	45.4779	1
**4510**	351	C	9.2706	1
**4510**	357	C	79.5514	1
**4510**	370	C	58.2954	1
**4510**	372	C	65.8447	0
**4510**	373	C	79.4122	1
**4510**	381	C	79.5158	1
**4510**	382	C	23.6539	0
**4510**	386	C	61.6081	1
**4510**	305	D	75.5921	1
**4510**	319	D	64.7775	1
**4510**	320	D	30.3166	-1
**4510**	321	D	79.5756	0
**4510**	351	D	5.169	1
**4510**	357	D	49.2134	0
**4510**	370	D	69.9531	1
**4510**	372	D	72.6406	0
**4510**	373	D	51.2274	1
**4510**	381	D	79.6104	1
**4510**	382	D	79.5265	1
**4510**	386	D	79.4057	1
**4511**	305	A	37.1412	0
**4511**	319	A	79.6976	1
**4511**	320	A	71.1782	-1
**4511**	321	A	79.5069	0
**4511**	351	A	5.663	1
**4511**	357	A	79.6571	1
**4511**	370	A	79.5813	1
**4511**	372	A	40.8481	1
**4511**	373	A	64.8783	1
**4511**	381	A	79.5438	1
**4511**	382	A	79.6209	1
**4511**	386	A	74.3518	1

**4511**	305	B	45.6392	0
**4511**	319	B	79.5818	1
**4511**	320	B	67.3334	-1
**4511**	321	B	72.3919	0
**4511**	351	B	10.9004	1
**4511**	357	B	79.5196	1
**4511**	370	B	56.5629	1
**4511**	372	B	68.2167	0
**4511**	373	B	42.706	0
**4511**	381	B	77.5704	1
**4511**	382	B	62.319	1
**4511**	386	B	79.5767	1
**4511**	305	C	70.6976	1
**4511**	319	C	68.4577	1
**4511**	320	C	79.5837	-1
**4511**	321	C	56.5035	0
**4511**	351	C	2.7848	1
**4511**	357	C	59.2752	1
**4511**	370	C	70.3229	1
**4511**	372	C	73.3163	0
**4511**	373	C	47.0551	0
**4511**	381	C	79.5249	1
**4511**	382	C	60.4862	1
**4511**	386	C	67.6004	1
**4511**	305	D	72.3064	1
**4511**	319	D	72.5046	1
**4511**	320	D	73.2185	-1
**4511**	321	D	72.471	0
**4511**	351	D	10.7975	1
**4511**	357	D	60.7611	1
**4511**	370	D	72.9612	1
**4511**	372	D	79.507	0
**4511**	373	D	71.8392	1
**4511**	381	D	79.5734	1

**4511**	382	D	79.5337	1
**4511**	386	D	73.0462	1
**4512**	305	A	79.535	1
**4512**	319	A	78.2931	1
**4512**	320	A	53.0026	-1
**4512**	321	A	75.3565	0
**4512**	351	A	4.3639	1
**4512**	357	A	79.4943	1
**4512**	370	A	67.1568	1
**4512**	372	A	55.5588	0
**4512**	373	A	72.0934	1
**4512**	381	A	60.1646	1
**4512**	382	A	46.4492	0
**4512**	386	A	79.512	1
**4512**	305	B	69.885	1
**4512**	319	B	79.6379	1
**4512**	320	B	79.489	-1
**4512**	321	B	76.2973	0
**4512**	351	B	12.9606	1
**4512**	357	B	72.4473	1
**4512**	370	B	45.7706	0
**4512**	372	B	79.5863	0
**4512**	373	B	59.0402	1
**4512**	381	B	71.4859	1
**4512**	382	B	57.4211	1
**4512**	386	B	56.6934	1
**4512**	305	C	47.0269	0
**4512**	319	C	79.5227	1
**4512**	320	C	66.8294	-1
**4512**	321	C	78.0839	0
**4512**	351	C	9.7656	1
**4512**	357	C	77.0085	1
**4512**	370	C	79.6783	1
**4512**	372	C	66.8171	0

**4512**	373	C	51.8906	1
**4512**	381	C	66.114	1
**4512**	382	C	78.072	1
**4512**	386	C	38.3148	0
**4512**	305	D	72.5133	1
**4512**	319	D	79.5976	1
**4512**	320	D	60.2178	-1
**4512**	321	D	73.5395	0
**4512**	351	D	6.5066	1
**4512**	357	D	60.3488	1
**4512**	370	D	79.5818	1
**4512**	372	D	79.6101	0
**4512**	373	D	79.602	1
**4512**	381	D	55.0321	1
**4512**	382	D	71.4515	1
**4512**	386	D	56.2865	1
**4513**	305	A	54.1677	1
**4513**	319	A	38.3052	0
**4513**	320	A	76.6575	-1
**4513**	321	A	58.1082	0
**4513**	351	A	10.1837	1
**4513**	357	A	50.3986	1
**4513**	370	A	77.7246	1
**4513**	372	A	79.4527	0
**4513**	373	A	79.591	1
**4513**	381	A	79.6054	1
**4513**	382	A	47.3511	0
**4513**	386	A	42.7392	0
**4513**	305	B	78.4479	1
**4513**	319	B	74.0693	1
**4513**	320	B	75.2988	-1
**4513**	321	B	46.8856	1
**4513**	351	B	0.2819	1
**4513**	357	B	67.9576	1

**4513**	370	B	79.6895	1
**4513**	372	B	61.2063	0
**4513**	373	B	79.6199	1
**4513**	381	B	61.7291	1
**4513**	382	B	75.8253	1
**4513**	386	B	79.4847	1
**4513**	305	C	62.2821	1
**4513**	319	C	76.0205	1
**4513**	320	C	79.606	-1
**4513**	321	C	79.4765	0
**4513**	351	C	2.0114	1
**4513**	357	C	70.3261	1
**4513**	370	C	40.5388	0
**4513**	372	C	66.2137	0
**4513**	373	C	78.8225	1
**4513**	381	C	48.726	0
**4513**	382	C	30.4673	0
**4513**	386	C	71.1287	1
**4513**	305	D	79.3409	1
**4513**	319	D	67.9831	1
**4513**	320	D	71.9586	-1
**4513**	321	D	79.6047	0
**4513**	351	D	10.2257	1
**4513**	357	D	64.4021	1
**4513**	370	D	76.6528	1
**4513**	372	D	74.9515	0
**4513**	373	D	79.6572	1
**4513**	381	D	78.9539	1
**4513**	382	D	79.4569	1
**4513**	386	D	79.5871	1
**4514**	305	A	59.7092	1
**4514**	319	A	70.7436	1
**4514**	320	A	72.6408	-1
**4514**	321	A	71.1391	0

**4514**	351	A	13.5916	1
**4514**	357	A	69.4555	1
**4514**	370	A	43.2068	0
**4514**	372	A	45.81	1
**4514**	373	A	62.3105	1
**4514**	381	A	79.8071	1
**4514**	382	A	71.6615	1
**4514**	386	A	74.4636	1
**4514**	305	B	58.4817	1
**4514**	319	B	79.6226	1
**4514**	320	B	34.6207	-1
**4514**	321	B	61.8454	0
**4514**	351	B	16.0653	1
**4514**	357	B	79.5255	1
**4514**	370	B	79.5781	1
**4514**	372	B	69.1077	0
**4514**	373	B	54.264	1
**4514**	381	B	78.801	1
**4514**	382	B	38.8439	0
**4514**	386	B	58.1015	1
**4514**	305	C	79.602	1
**4514**	319	C	79.5902	1
**4514**	320	C	75.9282	-1
**4514**	321	C	69.4374	0
**4514**	351	C	15.6496	1
**4514**	357	C	21.7857	0
**4514**	370	C	56.8806	1
**4514**	372	C	79.677	0
**4514**	373	C	62.339	1
**4514**	381	C	79.5823	1
**4514**	382	C	73.5329	1
**4514**	386	C	42.17	0
**4514**	305	D	72.4685	1
**4514**	319	D	79.4597	1

**4514**	320	D	61.7608	-1
**4514**	321	D	57.066	0
**4514**	351	D	39.3631	-1
**4514**	357	D	79.4991	1
**4514**	370	D	61.74	1
**4514**	372	D	63.3082	0
**4514**	373	D	76.2305	1
**4514**	381	D	75.8694	1
**4514**	382	D	74.961	1
**4514**	386	D	69.06	1
**4515**	305	A	67.933	1
**4515**	319	A	35.4976	0
**4515**	320	A	79.6279	-1
**4515**	321	A	60.9065	0
**4515**	351	A	10.7778	1
**4515**	357	A	58.4389	1
**4515**	370	A	73.3076	1
**4515**	372	A	52.1316	0
**4515**	373	A	79.6183	1
**4515**	381	A	79.5072	1
**4515**	382	A	71.7896	1
**4515**	386	A	59.632	1
**4515**	305	B	79.5267	1
**4515**	319	B	27.8505	0
**4515**	320	B	79.5728	-1
**4515**	321	B	79.4433	0
**4515**	351	B	21.2649	-1
**4515**	357	B	63.0257	1
**4515**	370	B	79.6581	1
**4515**	372	B	79.5558	0
**4515**	373	B	47.9668	0
**4515**	381	B	40.8041	0
**4515**	382	B	44.172	0
**4515**	386	B	66.5824	1

**4515**	305	C	79.5513	1
**4515**	319	C	66.2668	1
**4515**	320	C	71.784	-1
**4515**	321	C	75.5047	0
**4515**	351	C	3.8955	1
**4515**	357	C	79.5191	1
**4515**	370	C	74.8263	1
**4515**	372	C	66.9538	0
**4515**	373	C	63.5416	1
**4515**	381	C	42.4338	0
**4515**	382	C	34.0603	0
**4515**	386	C	64.9651	1
**4515**	305	D	79.6211	1
**4515**	319	D	63.6617	1
**4515**	320	D	36.2831	-1
**4515**	321	D	47.5606	1
**4515**	351	D	19.8003	1
**4515**	357	D	79.7311	1
**4515**	370	D	74.8229	1
**4515**	372	D	74.093	0
**4515**	373	D	63.5415	1
**4515**	381	D	79.5909	1
**4515**	382	D	79.5961	1
**4515**	386	D	79.5517	1
**4516**	305	A	76.4253	1
**4516**	319	A	77.8179	1
**4516**	320	A	79.4855	-1
**4516**	321	A	13.7862	0
**4516**	351	A	7.6049	1
**4516**	357	A	46.564	0
**4516**	370	A	79.5735	1
**4516**	372	A	50.2581	0
**4516**	373	A	79.526	1
**4516**	381	A	69.8551	1

**4516**	382	A	73.1191	1
**4516**	386	A	79.5857	1
**4516**	305	B	75.2117	1
**4516**	319	B	73.3288	1
**4516**	320	B	69.3742	-1
**4516**	321	B	67.593	0
**4516**	351	B	14.2715	1
**4516**	357	B	79.54	1
**4516**	370	B	73.9148	1
**4516**	372	B	79.4956	0
**4516**	373	B	53.8027	1
**4516**	381	B	73.425	1
**4516**	382	B	43.7686	0
**4516**	386	B	56.923	1
**4516**	305	C	75.2799	1
**4516**	319	C	79.5897	1
**4516**	320	C	74.3908	-1
**4516**	321	C	79.6549	0
**4516**	351	C	16.2276	1
**4516**	357	C	74.1543	1
**4516**	370	C	76.6417	1
**4516**	372	C	44.6539	1
**4516**	373	C	79.5608	1
**4516**	381	C	74.4249	1
**4516**	382	C	66.4851	1
**4516**	386	C	34.5735	0
**4516**	305	D	62.5575	1
**4516**	319	D	67.0224	1
**4516**	320	D	79.594	-1
**4516**	321	D	79.6086	0
**4516**	351	D	12.9346	1
**4516**	357	D	79.4828	1
**4516**	370	D	78.1681	1
**4516**	372	D	73.2728	0

**4516**	373	D	73.7894	1
**4516**	381	D	79.5193	1
**4516**	382	D	79.5359	1
**4516**	386	D	79.5507	1
**4517**	305	A	50.8956	1
**4517**	319	A	17.444	-1
**4517**	320	A	58.4274	-1
**4517**	321	A	79.6301	0
**4517**	351	A	8.559	1
**4517**	357	A	61.8226	1
**4517**	370	A	79.5576	1
**4517**	372	A	72.5527	0
**4517**	373	A	52.0563	1
**4517**	381	A	75.5754	1
**4517**	382	A	47.232	0
**4517**	386	A	72.3834	1
**4517**	305	B	59.6486	1
**4517**	319	B	47.368	0
**4517**	320	B	79.512	-1
**4517**	321	B	79.5731	0
**4517**	351	B	3.7948	1
**4517**	357	B	72.7612	1
**4517**	370	B	79.5616	1
**4517**	372	B	79.5691	0
**4517**	373	B	79.2345	1
**4517**	381	B	69.0013	1
**4517**	382	B	79.6495	1
**4517**	386	B	57.1318	1
**4517**	305	C	74.9378	1
**4517**	319	C	79.6297	1
**4517**	320	C	79.5199	-1
**4517**	321	C	68.0996	0
**4517**	351	C	11.1098	1
**4517**	357	C	73.4672	1

**4517**	370	C	79.5561	1
**4517**	372	C	50.858	0
**4517**	373	C	40.5494	0
**4517**	381	C	50.4481	1
**4517**	382	C	54.5388	1
**4517**	386	C	38.3604	0
**4517**	305	D	52.7888	1
**4517**	319	D	56.6208	1
**4517**	320	D	79.5223	-1
**4517**	321	D	34.222	1
**4517**	351	D	14.15	1
**4517**	357	D	77.5018	1
**4517**	370	D	23.695	0
**4517**	372	D	66.3449	0
**4517**	373	D	79.5264	1
**4517**	381	D	79.5279	1
**4517**	382	D	61.3973	1
**4517**	386	D	54.7462	1
**4518**	305	A	78.8845	1
**4518**	319	A	72.224	1
**4518**	320	A	39.1882	-1
**4518**	321	A	37.7949	1
**4518**	351	A	15.915	1
**4518**	357	A	55.4646	1
**4518**	370	A	79.5853	1
**4518**	372	A	73.4905	0
**4518**	373	A	69.7366	1
**4518**	381	A	66.2841	1
**4518**	382	A	69.4118	1
**4518**	386	A	76.7631	1
**4518**	305	B	79.5918	1
**4518**	319	B	79.534	1
**4518**	320	B	72.2178	-1
**4518**	321	B	70.3873	0

**4518**	351	B	9.015	1
**4518**	357	B	79.4239	1
**4518**	370	B	79.621	1
**4518**	372	B	43.9226	1
**4518**	373	B	46.1049	0
**4518**	381	B	71.3833	1
**4518**	382	B	15.7049	-1
**4518**	386	B	56.9975	1
**4518**	305	C	63.5593	1
**4518**	319	C	67.8183	1
**4518**	320	C	75.1845	-1
**4518**	321	C	64.7208	0
**4518**	351	C	40.8611	-1
**4518**	357	C	43.3424	0
**4518**	370	C	64.4418	1
**4518**	372	C	71.6651	0
**4518**	373	C	71.8969	1
**4518**	381	C	71.8423	1
**4518**	382	C	68.263	1
**4518**	386	C	44.4412	0
**4518**	305	D	62.7809	1
**4518**	319	D	62.7779	1
**4518**	320	D	51.6061	-1
**4518**	321	D	79.5789	0
**4518**	351	D	3.999	1
**4518**	357	D	79.4291	1
**4518**	370	D	69.0244	1
**4518**	372	D	75.2421	0
**4518**	373	D	79.5553	1
**4518**	381	D	79.6754	1
**4518**	382	D	74.3891	1
**4518**	386	D	49.5421	0
**4519**	305	A	71.9679	1
**4519**	319	A	64.0981	1

**4519**	320	A	75.0105	-1
**4519**	321	A	62.2254	0
**4519**	351	A	25.4935	-1
**4519**	357	A	73.552	1
**4519**	370	A	79.6011	1
**4519**	372	A	49.519	1
**4519**	373	A	76.6452	1
**4519**	381	A	73.2964	1
**4519**	382	A	79.63	1
**4519**	386	A	72.5036	1
**4519**	305	B	79.5147	1
**4519**	319	B	14.5423	-1
**4519**	320	B	43.2841	-1
**4519**	321	B	78.7438	0
**4519**	351	B	13.1807	1
**4519**	357	B	50.6914	1
**4519**	370	B	71.591	1
**4519**	372	B	76.3535	0
**4519**	373	B	60.7361	1
**4519**	381	B	43.9648	0
**4519**	382	B	44.4983	0
**4519**	386	B	79.5719	1
**4519**	305	C	67.1902	1
**4519**	319	C	79.5173	1
**4519**	320	C	71.1674	-1
**4519**	321	C	72.9456	0
**4519**	351	C	5.3338	1
**4519**	357	C	74.7334	1
**4519**	370	C	61.8765	1
**4519**	372	C	47.2642	1
**4519**	373	C	78.5801	1
**4519**	381	C	43.6785	0
**4519**	382	C	55.5394	1
**4519**	386	C	67.967	1

**4519**	305	D	67.1173	1
**4519**	319	D	72.4136	1
**4519**	320	D	79.5795	-1
**4519**	321	D	77.9779	0
**4519**	351	D	10.0437	1
**4519**	357	D	78.1376	1
**4519**	370	D	73.0607	1
**4519**	372	D	79.562	0
**4519**	373	D	79.5932	1
**4519**	381	D	69.9303	1
**4519**	382	D	49.096	0
**4519**	386	D	74.821	1
**4520**	305	A	52.6644	1
**4520**	319	A	59.4558	1
**4520**	320	A	73.7386	-1
**4520**	321	A	79.4934	0
**4520**	351	A	17.8017	1
**4520**	357	A	68.2072	1
**4520**	370	A	41.8059	0
**4520**	372	A	79.6535	0
**4520**	373	A	49.0184	0
**4520**	381	A	79.5951	1
**4520**	382	A	79.5252	1
**4520**	386	A	79.5316	1
**4520**	305	B	79.5794	1
**4520**	319	B	23.605	0
**4520**	320	B	53.5299	-1
**4520**	321	B	58.2329	0
**4520**	351	B	12.7076	1
**4520**	357	B	79.6567	1
**4520**	370	B	79.6249	1
**4520**	372	B	77.9137	0
**4520**	373	B	43.817	0
**4520**	381	B	69.9334	1

**4520**	382	B	67.2813	1
**4520**	386	B	47.2363	0
**4520**	305	C	70.547	1
**4520**	319	C	63.8391	1
**4520**	320	C	57.9391	-1
**4520**	321	C	67.9337	0
**4520**	351	C	42.7245	-1
**4520**	357	C	65.3118	1
**4520**	370	C	79.5641	1
**4520**	372	C	58.1626	0
**4520**	373	C	58.9201	1
**4520**	381	C	74.33	1
**4520**	382	C	75.5358	1
**4520**	386	C	34.0557	0
**4520**	305	D	63.2106	1
**4520**	319	D	73.8204	1
**4520**	320	D	78.2644	-1
**4520**	321	D	79.4951	0
**4520**	351	D	9.3601	1
**4520**	357	D	65.2639	1
**4520**	370	D	74.5938	1
**4520**	372	D	79.6231	0
**4520**	373	D	71.8528	1
**4520**	381	D	36.1874	0
**4520**	382	D	79.5355	1
**4520**	386	D	51.8213	1
**4521**	305	A	56.2854	1
**4521**	319	A	38.413	0
**4521**	320	A	79.4754	-1
**4521**	321	A	54.6012	0
**4521**	351	A	4.556	1
**4521**	357	A	65.9583	1
**4521**	370	A	74.7785	1
**4521**	372	A	48.5388	1

**4521**	373	A	79.5321	1
**4521**	381	A	67.1439	1
**4521**	382	A	63.2534	1
**4521**	386	A	65.0322	1
**4521**	305	B	69.4222	1
**4521**	319	B	72.6721	1
**4521**	320	B	79.5173	-1
**4521**	321	B	71.3415	0
**4521**	351	B	15.4414	1
**4521**	357	B	72.9476	1
**4521**	370	B	79.5675	1
**4521**	372	B	49.0162	1
**4521**	373	B	49.4122	0
**4521**	381	B	52.6205	1
**4521**	382	B	69.3285	1
**4521**	386	B	57.5233	1
**4521**	305	C	68.5935	1
**4521**	319	C	75.2563	1
**4521**	320	C	72.27	-1
**4521**	321	C	44.2481	1
**4521**	351	C	7.9296	1
**4521**	357	C	53.7535	1
**4521**	370	C	71.5349	1
**4521**	372	C	74.7561	0
**4521**	373	C	73.4313	1
**4521**	381	C	72.8985	1
**4521**	382	C	79.4579	1
**4521**	386	C	29.0901	0
**4521**	305	D	79.6005	1
**4521**	319	D	42.1836	0
**4521**	320	D	66.969	-1
**4521**	321	D	72.4384	0
**4521**	351	D	9.4136	1
**4521**	357	D	79.6327	1

**4521**	370	D	72.7558	1
**4521**	372	D	79.5978	0
**4521**	373	D	73.6901	1
**4521**	381	D	79.5271	1
**4521**	382	D	79.5388	1
**4521**	386	D	79.5914	1
**4522**	305	A	75.4936	1
**4522**	319	A	51.0775	1
**4522**	320	A	72.4132	-1
**4522**	321	A	42.9112	1
**4522**	351	A	7.8463	1
**4522**	357	A	72.2224	1
**4522**	370	A	79.5542	1
**4522**	372	A	79.561	0
**4522**	373	A	65.3962	1
**4522**	381	A	50.3227	1
**4522**	382	A	72.4884	1
**4522**	386	A	39.3507	0
**4522**	305	B	32.8105	0
**4522**	319	B	20.4096	0
**4522**	320	B	31.0554	-1
**4522**	321	B	79.5636	0
**4522**	351	B	16.5368	1
**4522**	357	B	79.5572	1
**4522**	370	B	40.503	0
**4522**	372	B	67.3312	0
**4522**	373	B	72.8465	1
**4522**	381	B	41.8093	0
**4522**	382	B	68.4368	1
**4522**	386	B	55.5895	1
**4522**	305	C	75.5311	1
**4522**	319	C	67.715	1
**4522**	320	C	67.7721	-1
**4522**	321	C	66.4135	0

**4522**	351	C	2.6946	1
**4522**	357	C	45.6161	0
**4522**	370	C	60.5256	1
**4522**	372	C	58.4558	0
**4522**	373	C	72.9027	1
**4522**	381	C	79.4688	1
**4522**	382	C	35.8585	0
**4522**	386	C	63.1649	1
**4522**	305	D	79.5584	1
**4522**	319	D	78.6663	1
**4522**	320	D	79.5635	-1
**4522**	321	D	70.1952	0
**4522**	351	D	0.7969	1
**4522**	357	D	30.1155	0
**4522**	370	D	79.1774	1
**4522**	372	D	76.3841	0
**4522**	373	D	79.1808	1
**4522**	381	D	53.606	1
**4522**	382	D	79.2615	1
**4522**	386	D	79.2066	1
**4523**	305	A	53.9476	1
**4523**	319	A	47.9568	0
**4523**	320	A	79.5531	-1
**4523**	321	A	72.5673	0
**4523**	351	A	7.3904	1
**4523**	357	A	65.6112	1
**4523**	370	A	79.6019	1
**4523**	372	A	60.1278	0
**4523**	373	A	71.2093	1
**4523**	381	A	74.9271	1
**4523**	382	A	26.6916	0
**4523**	386	A	79.5706	1
**4523**	305	B	71.9543	1
**4523**	319	B	79.547	1

**4523**	320	B	74.5756	-1
**4523**	321	B	44.9009	1
**4523**	351	B	11.5494	1
**4523**	357	B	79.5137	1
**4523**	370	B	72.3945	1
**4523**	372	B	79.5735	0
**4523**	373	B	51.5649	1
**4523**	381	B	75.2694	1
**4523**	382	B	77.3746	1
**4523**	386	B	66.5529	1
**4523**	305	C	70.7456	1
**4523**	319	C	36.9223	0
**4523**	320	C	33.7343	-1
**4523**	321	C	69.7346	0
**4523**	351	C	9.5607	1
**4523**	357	C	71.9181	1
**4523**	370	C	70.8778	1
**4523**	372	C	79.5704	0
**4523**	373	C	64.4674	1
**4523**	381	C	79.5701	1
**4523**	382	C	79.0791	1
**4523**	386	C	31.3411	0
**4523**	305	D	72.3252	1
**4523**	319	D	76.8929	1
**4523**	320	D	71.6009	-1
**4523**	321	D	72.547	0
**4523**	351	D	7.7209	1
**4523**	357	D	56.755	1
**4523**	370	D	59.6991	1
**4523**	372	D	79.5946	0
**4523**	373	D	51.334	1
**4523**	381	D	36.4588	0
**4523**	382	D	54.5124	1
**4523**	386	D	43.3164	0

**4524**	305	A	79.5733	1
**4524**	319	A	57.4629	1
**4524**	320	A	73.4618	-1
**4524**	321	A	78.552	0
**4524**	351	A	6.5036	1
**4524**	357	A	48.4181	0
**4524**	370	A	67.809	1
**4524**	372	A	79.5646	0
**4524**	373	A	52.8476	1
**4524**	381	A	79.5366	1
**4524**	382	A	64.1926	1
**4524**	386	A	77.9121	1
**4524**	305	B	62.0296	1
**4524**	319	B	32.0733	0
**4524**	320	B	78.734	-1
**4524**	321	B	79.5331	0
**4524**	351	B	12.1329	1
**4524**	357	B	61.283	1
**4524**	370	B	79.5424	1
**4524**	372	B	62.4628	0
**4524**	373	B	49.0301	0
**4524**	381	B	46.3883	0
**4524**	382	B	32.6284	0
**4524**	386	B	32.898	0
**4524**	305	C	75.2341	1
**4524**	319	C	62.3339	1
**4524**	320	C	74.6841	-1
**4524**	321	C	76.7841	0
**4524**	351	C	0.4445	1
**4524**	357	C	69.171	1
**4524**	370	C	73.4151	1
**4524**	372	C	50.8537	0
**4524**	373	C	79.6211	1
**4524**	381	C	79.5673	1

**4524**	382	C	28.2953	0
**4524**	386	C	18.7486	-1
**4524**	305	D	46.7289	0
**4524**	319	D	72.5965	1
**4524**	320	D	79.5827	-1
**4524**	321	D	37.6335	1
**4524**	351	D	13.6582	1
**4524**	357	D	59.599	1
**4524**	370	D	46.9148	0
**4524**	372	D	59.5881	0
**4524**	373	D	30.4086	0
**4524**	381	D	72.8069	1
**4524**	382	D	66.5203	1
**4524**	386	D	41.2762	0
**4525**	305	A	66.4346	1
**4525**	319	A	55.1723	1
**4525**	320	A	45.8069	-1
**4525**	321	A	77.8018	0
**4525**	351	A	0.0	1
**4525**	357	A	79.5859	1
**4525**	370	A	74.649	1
**4525**	372	A	70.9119	0
**4525**	373	A	79.5905	1
**4525**	381	A	18.1373	-1
**4525**	382	A	71.5321	1
**4525**	386	A	23.347	0
**4525**	305	B	78.5106	1
**4525**	319	B	15.4582	-1
**4525**	320	B	67.2395	-1
**4525**	321	B	48.4741	1
**4525**	351	B	16.6114	1
**4525**	357	B	40.7055	0
**4525**	370	B	79.5825	1
**4525**	372	B	49.1803	1

**4525**	373	B	42.0887	0
**4525**	381	B	79.4716	1
**4525**	382	B	44.1307	0
**4525**	386	B	69.8176	1
**4525**	305	C	20.0099	0
**4525**	319	C	59.9621	1
**4525**	320	C	49.8416	-1
**4525**	321	C	79.2025	0
**4525**	351	C	7.795	1
**4525**	357	C	67.3375	1
**4525**	370	C	44.0016	0
**4525**	372	C	36.5613	1
**4525**	373	C	57.9632	1
**4525**	381	C	61.1462	1
**4525**	382	C	79.4858	1
**4525**	386	C	40.3861	0
**4525**	305	D	79.6312	1
**4525**	319	D	69.1734	1
**4525**	320	D	79.3783	-1
**4525**	321	D	68.0668	0
**4525**	351	D	10.5878	1
**4525**	357	D	79.4669	1
**4525**	370	D	79.5455	1
**4525**	372	D	50.2199	0
**4525**	373	D	43.9708	0
**4525**	381	D	79.7231	1
**4525**	382	D	74.93	1
**4525**	386	D	51.29	1
**4526**	305	A	79.5343	1
**4526**	319	A	63.8909	1
**4526**	320	A	72.7576	-1
**4526**	321	A	66.3556	0
**4526**	351	A	7.2604	1
**4526**	357	A	68.2946	1

**4526**	370	A	73.5707	1
**4526**	372	A	49.1254	1
**4526**	373	A	40.2	0
**4526**	381	A	59.4844	1
**4526**	382	A	27.5075	0
**4526**	386	A	79.579	1
**4526**	305	B	60.9032	1
**4526**	319	B	75.3799	1
**4526**	320	B	73.5577	-1
**4526**	321	B	79.5226	0
**4526**	351	B	18.1834	1
**4526**	357	B	50.2649	1
**4526**	370	B	52.132	1
**4526**	372	B	38.2612	1
**4526**	373	B	54.0346	1
**4526**	381	B	72.7623	1
**4526**	382	B	68.9134	1
**4526**	386	B	39.2045	0
**4526**	305	C	79.6752	1
**4526**	319	C	51.5986	1
**4526**	320	C	34.2013	-1
**4526**	321	C	72.9512	0
**4526**	351	C	0.0005	1
**4526**	357	C	70.3513	1
**4526**	370	C	30.2956	0
**4526**	372	C	71.9041	0
**4526**	373	C	79.5837	1
**4526**	381	C	55.9841	1
**4526**	382	C	58.698	1
**4526**	386	C	35.6557	0
**4526**	305	D	72.895	1
**4526**	319	D	58.8549	1
**4526**	320	D	79.5565	-1
**4526**	321	D	79.574	0

**4526**	351	D	9.4926	1
**4526**	357	D	77.2968	1
**4526**	370	D	79.548	1
**4526**	372	D	79.5356	0
**4526**	373	D	79.2802	1
**4526**	381	D	79.3777	1
**4526**	382	D	71.3981	1
**4526**	386	D	49.366	0
**4527**	305	A	61.5172	1
**4527**	319	A	49.3576	0
**4527**	320	A	46.5569	-1
**4527**	321	A	48.915	1
**4527**	351	A	11.7921	1
**4527**	357	A	68.2581	1
**4527**	370	A	69.7145	1
**4527**	372	A	71.9252	0
**4527**	373	A	67.0195	1
**4527**	381	A	68.6281	1
**4527**	382	A	78.5442	1
**4527**	386	A	79.5932	1
**4527**	305	B	74.9961	1
**4527**	319	B	79.5091	1
**4527**	320	B	33.1787	-1
**4527**	321	B	68.838	0
**4527**	351	B	4.2502	1
**4527**	357	B	67.5108	1
**4527**	370	B	79.6058	1
**4527**	372	B	72.6595	0
**4527**	373	B	60.233	1
**4527**	381	B	60.3268	1
**4527**	382	B	53.5688	1
**4527**	386	B	36.7648	0
**4527**	305	C	75.8926	1
**4527**	319	C	77.621	1

**4527**	320	C	79.5653	-1
**4527**	321	C	79.5404	0
**4527**	351	C	9.0453	1
**4527**	357	C	36.1017	0
**4527**	370	C	71.0711	1
**4527**	372	C	79.6188	0
**4527**	373	C	55.0574	1
**4527**	381	C	79.5947	1
**4527**	382	C	66.0557	1
**4527**	386	C	18.3744	-1
**4527**	305	D	69.8951	1
**4527**	319	D	42.7063	0
**4527**	320	D	70.203	-1
**4527**	321	D	63.4672	0
**4527**	351	D	5.0946	1
**4527**	357	D	68.2739	1
**4527**	370	D	79.6281	1
**4527**	372	D	50.5698	0
**4527**	373	D	78.6635	1
**4527**	381	D	31.5708	0
**4527**	382	D	79.505	1
**4527**	386	D	62.68	1
**4528**	305	A	47.1226	0
**4528**	319	A	70.8039	1
**4528**	320	A	79.5194	-1
**4528**	321	A	67.514	0
**4528**	351	A	4.5742	1
**4528**	357	A	79.602	1
**4528**	370	A	79.5882	1
**4528**	372	A	72.4648	0
**4528**	373	A	64.1734	1
**4528**	381	A	58.0339	1
**4528**	382	A	79.571	1
**4528**	386	A	77.2722	1

**4528**	305	B	79.7607	1
**4528**	319	B	79.1793	1
**4528**	320	B	72.15	-1
**4528**	321	B	55.0586	0
**4528**	351	B	16.5066	1
**4528**	357	B	63.7501	1
**4528**	370	B	70.0623	1
**4528**	372	B	79.548	0
**4528**	373	B	71.7488	1
**4528**	381	B	79.5671	1
**4528**	382	B	46.647	0
**4528**	386	B	53.214	1
**4528**	305	C	79.5768	1
**4528**	319	C	71.7226	1
**4528**	320	C	79.5428	-1
**4528**	321	C	79.5727	0
**4528**	351	C	3.1561	1
**4528**	357	C	32.8266	0
**4528**	370	C	73.9407	1
**4528**	372	C	57.5198	0
**4528**	373	C	79.5731	1
**4528**	381	C	72.7446	1
**4528**	382	C	78.7927	1
**4528**	386	C	51.2162	1
**4528**	305	D	73.1261	1
**4528**	319	D	76.4873	1
**4528**	320	D	79.5657	-1
**4528**	321	D	71.8811	0
**4528**	351	D	17.3153	1
**4528**	357	D	79.5538	1
**4528**	370	D	72.6488	1
**4528**	372	D	79.5504	0
**4528**	373	D	72.9216	1
**4528**	381	D	79.5267	1

**4528**	382	D	79.5338	1
**4528**	386	D	72.5588	1
**4529**	305	A	79.5475	1
**4529**	319	A	73.7889	1
**4529**	320	A	79.6101	-1
**4529**	321	A	53.2282	0
**4529**	351	A	14.1014	1
**4529**	357	A	72.861	1
**4529**	370	A	79.5288	1
**4529**	372	A	69.4705	0
**4529**	373	A	14.4802	-1
**4529**	381	A	79.4152	1
**4529**	382	A	79.5207	1
**4529**	386	A	57.7071	1
**4529**	305	B	79.6209	1
**4529**	319	B	47.765	0
**4529**	320	B	79.6443	-1
**4529**	321	B	74.1735	0
**4529**	351	B	27.9453	-1
**4529**	357	B	79.5928	1
**4529**	370	B	25.0003	0
**4529**	372	B	75.1437	0
**4529**	373	B	78.7915	1
**4529**	381	B	79.5329	1
**4529**	382	B	78.5749	1
**4529**	386	B	62.5011	1
**4529**	305	C	38.8323	0
**4529**	319	C	62.4181	1
**4529**	320	C	79.5984	-1
**4529**	321	C	54.3307	0
**4529**	351	C	16.4084	1
**4529**	357	C	74.0017	1
**4529**	370	C	43.9829	0
**4529**	372	C	51.0941	0

**4529**	373	C	63.1972	1
**4529**	381	C	58.436	1
**4529**	382	C	70.6484	1
**4529**	386	C	38.1385	0
**4529**	305	D	74.5507	1
**4529**	319	D	60.7984	1
**4529**	320	D	67.108	-1
**4529**	321	D	28.3777	1
**4529**	351	D	19.0888	1
**4529**	357	D	79.3906	1
**4529**	370	D	77.6771	1
**4529**	372	D	79.5178	0
**4529**	373	D	79.5417	1
**4529**	381	D	79.6061	1
**4529**	382	D	73.2419	1
**4529**	386	D	75.6645	1
**4530**	305	A	77.1202	1
**4530**	319	A	70.3161	1
**4530**	320	A	79.6167	-1
**4530**	321	A	79.5733	0
**4530**	351	A	6.9547	1
**4530**	357	A	79.3471	1
**4530**	370	A	78.2428	1
**4530**	372	A	64.9565	0
**4530**	373	A	74.3241	1
**4530**	381	A	43.8608	0
**4530**	382	A	70.5018	1
**4530**	386	A	72.6103	1
**4530**	305	B	5.8562	-1
**4530**	319	B	33.9095	0
**4530**	320	B	70.5502	-1
**4530**	321	B	76.3862	0
**4530**	351	B	9.2391	1
**4530**	357	B	72.8817	1

**4530**	370	B	59.4111	1
**4530**	372	B	74.7229	0
**4530**	373	B	53.3532	1
**4530**	381	B	59.7316	1
**4530**	382	B	66.1494	1
**4530**	386	B	79.5183	1
**4530**	305	C	78.6	1
**4530**	319	C	68.6577	1
**4530**	320	C	79.5391	-1
**4530**	321	C	77.1262	0
**4530**	351	C	10.075	1
**4530**	357	C	79.5739	1
**4530**	370	C	79.507	1
**4530**	372	C	74.3467	0
**4530**	373	C	72.4594	1
**4530**	381	C	79.6542	1
**4530**	382	C	63.6187	1
**4530**	386	C	39.4781	0
**4530**	305	D	75.0769	1
**4530**	319	D	73.8079	1
**4530**	320	D	78.8975	-1
**4530**	321	D	46.5391	1
**4530**	351	D	8.4313	1
**4530**	357	D	79.523	1
**4530**	370	D	59.4214	1
**4530**	372	D	71.4728	0
**4530**	373	D	39.6495	0
**4530**	381	D	47.3835	0
**4530**	382	D	79.5402	1
**4530**	386	D	69.6607	1
**4531**	305	A	57.4693	1
**4531**	319	A	77.8126	1
**4531**	320	A	60.6869	-1
**4531**	321	A	79.6936	0

**4531**	351	A	19.1561	1
**4531**	357	A	47.6287	0
**4531**	370	A	66.809	1
**4531**	372	A	45.0852	1
**4531**	373	A	29.1488	0
**4531**	381	A	79.4944	1
**4531**	382	A	25.3374	0
**4531**	386	A	79.0564	1
**4531**	305	B	56.5828	1
**4531**	319	B	14.2561	-1
**4531**	320	B	65.4173	-1
**4531**	321	B	68.8531	0
**4531**	351	B	18.9188	1
**4531**	357	B	79.6481	1
**4531**	370	B	79.5663	1
**4531**	372	B	58.6663	0
**4531**	373	B	75.4751	1
**4531**	381	B	79.5825	1
**4531**	382	B	50.672	1
**4531**	386	B	73.3065	1
**4531**	305	C	62.97	1
**4531**	319	C	53.2946	1
**4531**	320	C	27.6477	-1
**4531**	321	C	29.0868	1
**4531**	351	C	1.9954	1
**4531**	357	C	78.2167	1
**4531**	370	C	72.6957	1
**4531**	372	C	65.7101	0
**4531**	373	C	79.5684	1
**4531**	381	C	69.4029	1
**4531**	382	C	78.6727	1
**4531**	386	C	42.2564	0
**4531**	305	D	79.7769	1
**4531**	319	D	37.1351	0

**4531**	320	D	48.6239	-1
**4531**	321	D	33.4431	1
**4531**	351	D	16.0435	1
**4531**	357	D	44.5034	0
**4531**	370	D	51.3711	1
**4531**	372	D	79.4557	0
**4531**	373	D	79.5911	1
**4531**	381	D	73.1416	1
**4531**	382	D	79.5351	1
**4531**	386	D	79.4857	1
**4532**	305	A	62.0248	1
**4532**	319	A	52.086	1
**4532**	320	A	79.7032	-1
**4532**	321	A	37.9371	1
**4532**	351	A	11.3413	1
**4532**	357	A	58.3654	1
**4532**	370	A	54.4647	1
**4532**	372	A	42.1713	1
**4532**	373	A	79.5373	1
**4532**	381	A	79.5566	1
**4532**	382	A	79.4768	1
**4532**	386	A	54.2956	1
**4532**	305	B	79.5863	1
**4532**	319	B	76.868	1
**4532**	320	B	79.5483	-1
**4532**	321	B	60.8433	0
**4532**	351	B	12.8034	1
**4532**	357	B	79.5417	1
**4532**	370	B	50.2682	1
**4532**	372	B	45.2258	1
**4532**	373	B	79.524	1
**4532**	381	B	50.8261	1
**4532**	382	B	66.2091	1
**4532**	386	B	52.7426	1

**4532**	305	C	70.4222	1
**4532**	319	C	73.1902	1
**4532**	320	C	79.5509	-1
**4532**	321	C	40.2546	1
**4532**	351	C	21.587	-1
**4532**	357	C	72.4346	1
**4532**	370	C	76.0398	1
**4532**	372	C	79.6635	0
**4532**	373	C	76.5209	1
**4532**	381	C	76.5814	1
**4532**	382	C	65.6243	1
**4532**	386	C	43.8606	0
**4532**	305	D	61.2996	1
**4532**	319	D	79.2752	1
**4532**	320	D	74.7724	-1
**4532**	321	D	79.5365	0
**4532**	351	D	13.4049	1
**4532**	357	D	79.512	1
**4532**	370	D	75.4793	1
**4532**	372	D	67.9118	0
**4532**	373	D	70.7029	1
**4532**	381	D	78.0698	1
**4532**	382	D	79.5813	1
**4532**	386	D	79.5978	1
**4533**	305	A	44.7245	0
**4533**	319	A	70.6063	1
**4533**	320	A	79.6163	-1
**4533**	321	A	78.5657	0
**4533**	351	A	40.3058	-1
**4533**	357	A	73.1425	1
**4533**	370	A	70.1941	1
**4533**	372	A	79.6101	0
**4533**	373	A	53.864	1
**4533**	381	A	79.5335	1

**4533**	382	A	52.2005	1
**4533**	386	A	57.1964	1
**4533**	305	B	71.9163	1
**4533**	319	B	47.5235	0
**4533**	320	B	72.9516	-1
**4533**	321	B	79.6264	0
**4533**	351	B	17.9784	1
**4533**	357	B	55.5081	1
**4533**	370	B	59.6873	1
**4533**	372	B	79.587	0
**4533**	373	B	79.5588	1
**4533**	381	B	79.6839	1
**4533**	382	B	33.3909	0
**4533**	386	B	74.5117	1
**4533**	305	C	69.081	1
**4533**	319	C	69.9896	1
**4533**	320	C	69.3828	-1
**4533**	321	C	75.831	0
**4533**	351	C	8.2014	1
**4533**	357	C	79.5439	1
**4533**	370	C	64.726	1
**4533**	372	C	67.5044	0
**4533**	373	C	73.4372	1
**4533**	381	C	72.4364	1
**4533**	382	C	60.7653	1
**4533**	386	C	53.931	1
**4533**	305	D	59.0311	1
**4533**	319	D	79.5493	1
**4533**	320	D	79.5737	-1
**4533**	321	D	67.0679	0
**4533**	351	D	14.0093	1
**4533**	357	D	57.8452	1
**4533**	370	D	72.5875	1
**4533**	372	D	79.4843	0

**4533**	373	D	59.6568	1
**4533**	381	D	76.37	1
**4533**	382	D	71.1568	1
**4533**	386	D	68.6078	1
**4534**	305	A	19.6266	-1
**4534**	319	A	61.7576	1
**4534**	320	A	79.5466	-1
**4534**	321	A	60.6362	0
**4534**	351	A	8.2941	1
**4534**	357	A	74.4554	1
**4534**	370	A	79.4739	1
**4534**	372	A	72.3716	0
**4534**	373	A	77.5143	1
**4534**	381	A	79.6024	1
**4534**	382	A	79.494	1
**4534**	386	A	55.0251	1
**4534**	305	B	79.5455	1
**4534**	319	B	59.8213	1
**4534**	320	B	58.9255	-1
**4534**	321	B	53.7841	0
**4534**	351	B	4.4969	1
**4534**	357	B	74.6406	1
**4534**	370	B	72.9371	1
**4534**	372	B	71.9704	0
**4534**	373	B	64.6227	1
**4534**	381	B	64.4126	1
**4534**	382	B	56.8063	1
**4534**	386	B	45.7446	0
**4534**	305	C	32.8734	0
**4534**	319	C	43.4502	0
**4534**	320	C	70.0608	-1
**4534**	321	C	61.4423	0
**4534**	351	C	15.9203	1
**4534**	357	C	54.9554	1

**4534**	370	C	58.7947	1
**4534**	372	C	79.5692	0
**4534**	373	C	79.6187	1
**4534**	381	C	79.6134	1
**4534**	382	C	70.6267	1
**4534**	386	C	49.7808	0
**4534**	305	D	79.5501	1
**4534**	319	D	79.5583	1
**4534**	320	D	68.9884	-1
**4534**	321	D	41.6344	1
**4534**	351	D	14.7863	1
**4534**	357	D	72.4267	1
**4534**	370	D	73.4534	1
**4534**	372	D	60.3861	0
**4534**	373	D	79.5991	1
**4534**	381	D	36.3935	0
**4534**	382	D	53.5495	1
**4534**	386	D	79.5556	1
**4535**	305	A	47.0948	0
**4535**	319	A	71.273	1
**4535**	320	A	79.4668	-1
**4535**	321	A	79.5001	0
**4535**	351	A	5.2391	1
**4535**	357	A	70.2865	1
**4535**	370	A	61.0222	1
**4535**	372	A	79.5728	0
**4535**	373	A	38.3916	0
**4535**	381	A	79.5911	1
**4535**	382	A	62.8798	1
**4535**	386	A	79.5269	1
**4535**	305	B	67.8339	1
**4535**	319	B	79.6147	1
**4535**	320	B	79.5689	-1
**4535**	321	B	72.8117	0

**4535**	351	B	42.6518	-1
**4535**	357	B	70.1979	1
**4535**	370	B	76.9267	1
**4535**	372	B	79.5144	0
**4535**	373	B	61.339	1
**4535**	381	B	73.1541	1
**4535**	382	B	78.9657	1
**4535**	386	B	73.9351	1
**4535**	305	C	79.5192	1
**4535**	319	C	22.2874	0
**4535**	320	C	74.06	-1
**4535**	321	C	54.6705	0
**4535**	351	C	1.7026	1
**4535**	357	C	61.5187	1
**4535**	370	C	79.6755	1
**4535**	372	C	63.0525	0
**4535**	373	C	70.2332	1
**4535**	381	C	36.5755	0
**4535**	382	C	60.498	1
**4535**	386	C	31.5086	0
**4535**	305	D	62.7309	1
**4535**	319	D	57.2188	1
**4535**	320	D	72.4077	-1
**4535**	321	D	72.5226	0
**4535**	351	D	16.8132	1
**4535**	357	D	65.4145	1
**4535**	370	D	79.5531	1
**4535**	372	D	70.5007	0
**4535**	373	D	79.6454	1
**4535**	381	D	64.6084	1
**4535**	382	D	72.4628	1
**4535**	386	D	79.6596	1
**4536**	305	A	72.53	1
**4536**	319	A	79.5801	1

**4536**	320	A	79.561	-1
**4536**	321	A	65.0392	0
**4536**	351	A	15.3699	1
**4536**	357	A	63.4776	1
**4536**	370	A	49.5298	0
**4536**	372	A	43.1051	1
**4536**	373	A	33.7371	0
**4536**	381	A	79.5552	1
**4536**	382	A	71.965	1
**4536**	386	A	79.4513	1
**4536**	305	B	73.8697	1
**4536**	319	B	77.2702	1
**4536**	320	B	71.6517	-1
**4536**	321	B	37.8907	1
**4536**	351	B	21.0794	-1
**4536**	357	B	79.5462	1
**4536**	370	B	55.1127	1
**4536**	372	B	46.4165	1
**4536**	373	B	77.19	1
**4536**	381	B	39.8456	0
**4536**	382	B	60.1363	1
**4536**	386	B	79.6128	1
**4536**	305	C	78.4411	1
**4536**	319	C	32.356	0
**4536**	320	C	79.5589	-1
**4536**	321	C	61.7241	0
**4536**	351	C	12.1336	1
**4536**	357	C	45.786	0
**4536**	370	C	50.6395	1
**4536**	372	C	13.0996	0
**4536**	373	C	73.0875	1
**4536**	381	C	55.8355	1
**4536**	382	C	55.7108	1
**4536**	386	C	38.7878	0

**4536**	305	D	79.3304	1
**4536**	319	D	71.7426	1
**4536**	320	D	72.0975	-1
**4536**	321	D	60.1715	0
**4536**	351	D	13.1919	1
**4536**	357	D	79.4642	1
**4536**	370	D	69.4128	1
**4536**	372	D	64.9789	0
**4536**	373	D	30.0372	0
**4536**	381	D	79.5662	1
**4536**	382	D	79.5479	1
**4536**	386	D	79.5503	1
**4537**	305	A	68.1823	1
**4537**	319	A	78.152	1
**4537**	320	A	70.9548	-1
**4537**	321	A	77.2171	0
**4537**	351	A	6.6367	1
**4537**	357	A	42.9355	0
**4537**	370	A	65.9252	1
**4537**	372	A	73.7221	0
**4537**	373	A	39.1054	0
**4537**	381	A	79.5639	1
**4537**	382	A	50.0525	1
**4537**	386	A	61.2403	1
**4537**	305	B	75.3371	1
**4537**	319	B	50.5775	1
**4537**	320	B	52.4024	-1
**4537**	321	B	79.5505	0
**4537**	351	B	14.6685	1
**4537**	357	B	76.0266	1
**4537**	370	B	79.1504	1
**4537**	372	B	71.848	0
**4537**	373	B	28.267	0
**4537**	381	B	79.5816	1

**4537**	382	B	38.1217	0
**4537**	386	B	55.9094	1
**4537**	305	C	51.6471	1
**4537**	319	C	75.2216	1
**4537**	320	C	45.4278	-1
**4537**	321	C	79.543	0
**4537**	351	C	4.1299	1
**4537**	357	C	79.535	1
**4537**	370	C	78.252	1
**4537**	372	C	75.1096	0
**4537**	373	C	49.4194	0
**4537**	381	C	71.7696	1
**4537**	382	C	66.6644	1
**4537**	386	C	47.338	0
**4537**	305	D	30.5726	0
**4537**	319	D	79.5937	1
**4537**	320	D	52.9228	-1
**4537**	321	D	79.5428	0
**4537**	351	D	6.0119	1
**4537**	357	D	79.5645	1
**4537**	370	D	72.6377	1
**4537**	372	D	79.3562	0
**4537**	373	D	66.3271	1
**4537**	381	D	79.5171	1
**4537**	382	D	79.5106	1
**4537**	386	D	57.0424	1
**4538**	305	A	69.804	1
**4538**	319	A	78.6753	1
**4538**	320	A	79.5394	-1
**4538**	321	A	75.8552	0
**4538**	351	A	15.2713	1
**4538**	357	A	79.4714	1
**4538**	370	A	70.7468	1
**4538**	372	A	79.5208	0

**4538**	373	A	79.4932	1
**4538**	381	A	39.6414	0
**4538**	382	A	22.2297	0
**4538**	386	A	44.9766	0
**4538**	305	B	79.6474	1
**4538**	319	B	75.3565	1
**4538**	320	B	52.5442	-1
**4538**	321	B	79.5668	0
**4538**	351	B	13.0724	1
**4538**	357	B	71.9213	1
**4538**	370	B	79.578	1
**4538**	372	B	75.372	0
**4538**	373	B	66.0586	1
**4538**	381	B	56.6793	1
**4538**	382	B	60.3066	1
**4538**	386	B	35.6303	0
**4538**	305	C	69.6391	1
**4538**	319	C	71.3149	1
**4538**	320	C	74.0883	-1
**4538**	321	C	79.5255	0
**4538**	351	C	3.5101	1
**4538**	357	C	79.263	1
**4538**	370	C	60.3947	1
**4538**	372	C	56.4107	0
**4538**	373	C	60.7371	1
**4538**	381	C	61.905	1
**4538**	382	C	79.5481	1
**4538**	386	C	40.2211	0
**4538**	305	D	62.0714	1
**4538**	319	D	69.0871	1
**4538**	320	D	69.0292	-1
**4538**	321	D	38.409	1
**4538**	351	D	4.4486	1
**4538**	357	D	25.5022	0

**4538**	370	D	45.7203	0
**4538**	372	D	79.4489	0
**4538**	373	D	79.5246	1
**4538**	381	D	49.5484	0
**4538**	382	D	72.0717	1
**4538**	386	D	79.4922	1
**4539**	305	A	72.3626	1
**4539**	319	A	79.6337	1
**4539**	320	A	69.8906	-1
**4539**	321	A	66.2945	0
**4539**	351	A	9.742	1
**4539**	357	A	53.8347	1
**4539**	370	A	51.2975	1
**4539**	372	A	79.5829	0
**4539**	373	A	79.5791	1
**4539**	381	A	48.249	0
**4539**	382	A	45.4578	0
**4539**	386	A	78.597	1
**4539**	305	B	64.2176	1
**4539**	319	B	77.0131	1
**4539**	320	B	76.1677	-1
**4539**	321	B	64.5091	0
**4539**	351	B	11.6921	1
**4539**	357	B	79.5587	1
**4539**	370	B	76.5777	1
**4539**	372	B	79.6196	0
**4539**	373	B	68.9679	1
**4539**	381	B	65.3576	1
**4539**	382	B	18.642	-1
**4539**	386	B	79.598	1
**4539**	305	C	54.9182	1
**4539**	319	C	73.3791	1
**4539**	320	C	79.5971	-1
**4539**	321	C	71.4516	0

**4539**	351	C	5.3696	1
**4539**	357	C	35.0662	0
**4539**	370	C	75.3015	1
**4539**	372	C	78.8651	0
**4539**	373	C	63.7955	1
**4539**	381	C	79.6862	1
**4539**	382	C	77.5605	1
**4539**	386	C	18.7928	-1
**4539**	305	D	78.37	1
**4539**	319	D	79.5497	1
**4539**	320	D	70.5466	-1
**4539**	321	D	51.6753	0
**4539**	351	D	14.5174	1
**4539**	357	D	46.1443	0
**4539**	370	D	49.9114	0
**4539**	372	D	79.5182	0
**4539**	373	D	74.7107	1
**4539**	381	D	76.0919	1
**4539**	382	D	79.6922	1
**4539**	386	D	33.9468	0
**4540**	305	A	54.4035	1
**4540**	319	A	64.2985	1
**4540**	320	A	42.8586	-1
**4540**	321	A	50.7069	0
**4540**	351	A	11.8908	1
**4540**	357	A	69.9328	1
**4540**	370	A	79.4475	1
**4540**	372	A	57.8622	0
**4540**	373	A	79.4604	1
**4540**	381	A	48.0664	0
**4540**	382	A	54.1162	1
**4540**	386	A	69.778	1
**4540**	305	B	79.4899	1
**4540**	319	B	72.7372	1

**4540**	320	B	74.9053	-1
**4540**	321	B	65.7113	0
**4540**	351	B	2.9857	1
**4540**	357	B	79.6483	1
**4540**	370	B	71.7165	1
**4540**	372	B	79.565	0
**4540**	373	B	56.8105	1
**4540**	381	B	79.5286	1
**4540**	382	B	39.2916	0
**4540**	386	B	45.163	0
**4540**	305	C	64.6098	1
**4540**	319	C	71.4257	1
**4540**	320	C	79.6729	-1
**4540**	321	C	47.8411	1
**4540**	351	C	6.004	1
**4540**	357	C	74.6776	1
**4540**	370	C	72.9203	1
**4540**	372	C	77.1766	0
**4540**	373	C	42.0747	0
**4540**	381	C	73.583	1
**4540**	382	C	79.6428	1
**4540**	386	C	42.1043	0
**4540**	305	D	49.7104	0
**4540**	319	D	79.5621	1
**4540**	320	D	79.5944	-1
**4540**	321	D	79.619	0
**4540**	351	D	11.0167	1
**4540**	357	D	73.5525	1
**4540**	370	D	75.5749	1
**4540**	372	D	33.9509	1
**4540**	373	D	45.3246	0
**4540**	381	D	79.5723	1
**4540**	382	D	45.0352	0
**4540**	386	D	73.4495	1

**4541**	305	A	72.7563	1
**4541**	319	A	26.3032	0
**4541**	320	A	79.5449	-1
**4541**	321	A	42.5622	1
**4541**	351	A	8.7035	1
**4541**	357	A	71.9662	1
**4541**	370	A	79.6167	1
**4541**	372	A	70.3738	0
**4541**	373	A	73.1802	1
**4541**	381	A	78.2006	1
**4541**	382	A	51.9553	1
**4541**	386	A	79.5611	1
**4541**	305	B	57.3255	1
**4541**	319	B	66.2783	1
**4541**	320	B	34.8	-1
**4541**	321	B	76.3397	0
**4541**	351	B	15.8241	1
**4541**	357	B	59.577	1
**4541**	370	B	66.0103	1
**4541**	372	B	79.6249	0
**4541**	373	B	77.9478	1
**4541**	381	B	62.9897	1
**4541**	382	B	46.6553	0
**4541**	386	B	79.6185	1
**4541**	305	C	79.5449	1
**4541**	319	C	69.898	1
**4541**	320	C	72.7597	-1
**4541**	321	C	23.492	1
**4541**	351	C	8.6815	1
**4541**	357	C	42.6781	0
**4541**	370	C	35.9751	0
**4541**	372	C	61.0498	0
**4541**	373	C	40.5497	0
**4541**	381	C	45.6547	0

**4541**	382	C	79.3728	1
**4541**	386	C	21.1137	0
**4541**	305	D	79.5915	1
**4541**	319	D	79.5965	1
**4541**	320	D	57.8991	-1
**4541**	321	D	73.5846	0
**4541**	351	D	19.2357	1
**4541**	357	D	56.1694	1
**4541**	370	D	79.5712	1
**4541**	372	D	79.5613	0
**4541**	373	D	73.092	1
**4541**	381	D	79.5344	1
**4541**	382	D	76.6548	1
**4541**	386	D	15.5471	-1
**4542**	305	A	43.6669	0
**4542**	319	A	41.856	0
**4542**	320	A	79.5735	-1
**4542**	321	A	79.2643	0
**4542**	351	A	8.2193	1
**4542**	357	A	72.9436	1
**4542**	370	A	49.3973	0
**4542**	372	A	61.7218	0
**4542**	373	A	78.0918	1
**4542**	381	A	58.7389	1
**4542**	382	A	78.6121	1
**4542**	386	A	75.7814	1
**4542**	305	B	79.5619	1
**4542**	319	B	62.6905	1
**4542**	320	B	79.5515	-1
**4542**	321	B	73.9157	0
**4542**	351	B	13.6846	1
**4542**	357	B	42.091	0
**4542**	370	B	73.0298	1
**4542**	372	B	60.969	0

**4542**	373	B	78.9637	1
**4542**	381	B	73.9135	1
**4542**	382	B	79.1516	1
**4542**	386	B	41.758	0
**4542**	305	C	59.8486	1
**4542**	319	C	49.4153	0
**4542**	320	C	74.2162	-1
**4542**	321	C	42.5511	1
**4542**	351	C	7.9468	1
**4542**	357	C	55.7671	1
**4542**	370	C	61.0166	1
**4542**	372	C	69.5574	0
**4542**	373	C	34.8923	0
**4542**	381	C	79.578	1
**4542**	382	C	78.9743	1
**4542**	386	C	53.2175	1
**4542**	305	D	79.5597	1
**4542**	319	D	79.5503	1
**4542**	320	D	72.9088	-1
**4542**	321	D	60.6462	0
**4542**	351	D	7.1308	1
**4542**	357	D	79.4875	1
**4542**	370	D	77.8193	1
**4542**	372	D	79.5644	0
**4542**	373	D	63.7316	1
**4542**	381	D	79.4614	1
**4542**	382	D	79.5695	1
**4542**	386	D	79.5736	1
**4543**	305	A	60.5633	1
**4543**	319	A	65.798	1
**4543**	320	A	79.5374	-1
**4543**	321	A	64.8889	0
**4543**	351	A	7.9927	1
**4543**	357	A	79.3703	1

**4543**	370	A	79.6639	1
**4543**	372	A	67.8592	0
**4543**	373	A	71.2297	1
**4543**	381	A	74.7546	1
**4543**	382	A	43.27	0
**4543**	386	A	57.9025	1
**4543**	305	B	59.8276	1
**4543**	319	B	21.5117	0
**4543**	320	B	45.8917	-1
**4543**	321	B	79.7318	0
**4543**	351	B	12.4732	1
**4543**	357	B	59.5794	1
**4543**	370	B	74.8079	1
**4543**	372	B	46.1101	1
**4543**	373	B	77.87	1
**4543**	381	B	79.6499	1
**4543**	382	B	31.934	0
**4543**	386	B	47.8578	0
**4543**	305	C	57.1935	1
**4543**	319	C	79.5703	1
**4543**	320	C	79.4706	-1
**4543**	321	C	79.6403	0
**4543**	351	C	6.6176	1
**4543**	357	C	73.6213	1
**4543**	370	C	62.6581	1
**4543**	372	C	54.5167	0
**4543**	373	C	46.8106	0
**4543**	381	C	79.0041	1
**4543**	382	C	79.5211	1
**4543**	386	C	24.5022	0
**4543**	305	D	79.6353	1
**4543**	319	D	79.6079	1
**4543**	320	D	78.9125	-1
**4543**	321	D	74.1594	0

**4543**	351	D	16.6602	1
**4543**	357	D	44.6463	0
**4543**	370	D	40.8032	0
**4543**	372	D	58.9803	0
**4543**	373	D	32.4995	0
**4543**	381	D	74.5845	1
**4543**	382	D	50.1774	1
**4543**	386	D	79.2196	1
**4544**	305	A	66.5434	1
**4544**	319	A	60.2693	1
**4544**	320	A	79.5691	-1
**4544**	321	A	66.177	0
**4544**	351	A	6.659	1
**4544**	357	A	78.456	1
**4544**	370	A	60.4225	1
**4544**	372	A	37.641	1
**4544**	373	A	79.5826	1
**4544**	381	A	57.4792	1
**4544**	382	A	71.5118	1
**4544**	386	A	76.3807	1
**4544**	305	B	73.7006	1
**4544**	319	B	67.6807	1
**4544**	320	B	78.2166	-1
**4544**	321	B	70.6674	0
**4544**	351	B	9.2667	1
**4544**	357	B	74.9611	1
**4544**	370	B	63.426	1
**4544**	372	B	64.6364	0
**4544**	373	B	74.5557	1
**4544**	381	B	79.6917	1
**4544**	382	B	29.4347	0
**4544**	386	B	56.8867	1
**4544**	305	C	58.7093	1
**4544**	319	C	68.7262	1

**4544**	320	C	67.1437	-1
**4544**	321	C	79.4868	0
**4544**	351	C	12.2529	1
**4544**	357	C	74.5342	1
**4544**	370	C	55.6154	1
**4544**	372	C	62.5819	0
**4544**	373	C	79.5259	1
**4544**	381	C	50.7436	1
**4544**	382	C	79.482	1
**4544**	386	C	55.2819	1
**4544**	305	D	45.7219	0
**4544**	319	D	46.4048	0
**4544**	320	D	47.5176	-1
**4544**	321	D	73.3006	0
**4544**	351	D	28.5167	-1
**4544**	357	D	56.8752	1
**4544**	370	D	79.6348	1
**4544**	372	D	55.3607	0
**4544**	373	D	57.0639	1
**4544**	381	D	77.4736	1
**4544**	382	D	44.2693	0
**4544**	386	D	56.9192	1
**4545**	305	A	24.0956	0
**4545**	319	A	77.0191	1
**4545**	320	A	40.6482	-1
**4545**	321	A	77.7182	0
**4545**	351	A	5.7691	1
**4545**	357	A	79.5043	1
**4545**	370	A	73.2791	1
**4545**	372	A	79.5359	0
**4545**	373	A	64.1693	1
**4545**	381	A	79.6308	1
**4545**	382	A	58.3747	1
**4545**	386	A	69.5988	1

**4545**	305	B	72.4793	1
**4545**	319	B	67.6902	1
**4545**	320	B	52.3928	-1
**4545**	321	B	72.5199	0
**4545**	351	B	10.3053	1
**4545**	357	B	73.3188	1
**4545**	370	B	79.4576	1
**4545**	372	B	73.5626	0
**4545**	373	B	79.3153	1
**4545**	381	B	65.6821	1
**4545**	382	B	53.7531	1
**4545**	386	B	79.5593	1
**4545**	305	C	76.0397	1
**4545**	319	C	79.3751	1
**4545**	320	C	51.9287	-1
**4545**	321	C	79.5399	0
**4545**	351	C	7.9147	1
**4545**	357	C	77.4378	1
**4545**	370	C	79.4872	1
**4545**	372	C	79.5733	0
**4545**	373	C	72.8632	1
**4545**	381	C	79.5097	1
**4545**	382	C	77.6092	1
**4545**	386	C	35.8573	0
**4545**	305	D	75.8938	1
**4545**	319	D	79.6124	1
**4545**	320	D	69.7421	-1
**4545**	321	D	79.5884	0
**4545**	351	D	13.412	1
**4545**	357	D	22.8152	0
**4545**	370	D	71.5833	1
**4545**	372	D	73.1185	0
**4545**	373	D	57.9666	1
**4545**	381	D	79.5471	1

**4545**	382	D	79.6552	1
**4545**	386	D	45.5871	0
**4546**	305	A	70.2773	1
**4546**	319	A	47.0875	0
**4546**	320	A	79.5743	-1
**4546**	321	A	79.5677	0
**4546**	351	A	12.2767	1
**4546**	357	A	79.3761	1
**4546**	370	A	71.182	1
**4546**	372	A	79.5291	0
**4546**	373	A	79.5814	1
**4546**	381	A	79.5631	1
**4546**	382	A	68.0981	1
**4546**	386	A	77.7059	1
**4546**	305	B	79.582	1
**4546**	319	B	72.5269	1
**4546**	320	B	14.5317	1
**4546**	321	B	79.5306	0
**4546**	351	B	13.9579	1
**4546**	357	B	79.5524	1
**4546**	370	B	59.2411	1
**4546**	372	B	69.5532	0
**4546**	373	B	33.4606	0
**4546**	381	B	29.6294	0
**4546**	382	B	43.1085	0
**4546**	386	B	57.8139	1
**4546**	305	C	79.7515	1
**4546**	319	C	74.295	1
**4546**	320	C	70.4991	-1
**4546**	321	C	72.6191	0
**4546**	351	C	15.0423	1
**4546**	357	C	79.4831	1
**4546**	370	C	74.8499	1
**4546**	372	C	79.5512	0

**4546**	373	C	79.5803	1
**4546**	381	C	72.3625	1
**4546**	382	C	45.0262	0
**4546**	386	C	28.1012	0
**4546**	305	D	79.5612	1
**4546**	319	D	79.5339	1
**4546**	320	D	52.6869	-1
**4546**	321	D	73.4367	0
**4546**	351	D	13.8033	1
**4546**	357	D	72.7538	1
**4546**	370	D	73.0634	1
**4546**	372	D	79.5787	0
**4546**	373	D	69.687	1
**4546**	381	D	79.5718	1
**4546**	382	D	79.4299	1
**4546**	386	D	48.851	0
**4547**	305	A	72.9247	1
**4547**	319	A	68.9264	1
**4547**	320	A	73.1645	-1
**4547**	321	A	64.2521	0
**4547**	351	A	8.3595	1
**4547**	357	A	66.4548	1
**4547**	370	A	79.6028	1
**4547**	372	A	79.432	0
**4547**	373	A	43.8727	0
**4547**	381	A	60.1125	1
**4547**	382	A	52.58	1
**4547**	386	A	79.5337	1
**4547**	305	B	79.5499	1
**4547**	319	B	62.4069	1
**4547**	320	B	44.0148	-1
**4547**	321	B	74.7797	0
**4547**	351	B	14.4395	1
**4547**	357	B	73.6992	1

**4547**	370	B	45.06	0
**4547**	372	B	74.7163	0
**4547**	373	B	41.8963	0
**4547**	381	B	55.6756	1
**4547**	382	B	55.6241	1
**4547**	386	B	37.0242	0
**4547**	305	C	70.313	1
**4547**	319	C	72.0538	1
**4547**	320	C	57.675	-1
**4547**	321	C	79.5398	0
**4547**	351	C	1.26	1
**4547**	357	C	79.5572	1
**4547**	370	C	71.4551	1
**4547**	372	C	28.2463	1
**4547**	373	C	35.8015	0
**4547**	381	C	42.2155	0
**4547**	382	C	55.8972	1
**4547**	386	C	24.5945	0
**4547**	305	D	34.2245	0
**4547**	319	D	79.5468	1
**4547**	320	D	75.8442	-1
**4547**	321	D	67.7656	0
**4547**	351	D	13.6233	1
**4547**	357	D	79.5892	1
**4547**	370	D	79.3839	1
**4547**	372	D	79.5805	0
**4547**	373	D	60.6638	1
**4547**	381	D	79.5947	1
**4547**	382	D	16.7237	-1
**4547**	386	D	79.6046	1
**4548**	305	A	79.6481	1
**4548**	319	A	54.4965	1
**4548**	320	A	79.6295	-1
**4548**	321	A	62.6316	0

**4548**	351	A	4.5626	1
**4548**	357	A	70.6663	1
**4548**	370	A	79.6096	1
**4548**	372	A	52.4063	0
**4548**	373	A	54.0382	1
**4548**	381	A	26.4416	0
**4548**	382	A	71.606	1
**4548**	386	A	73.6742	1
**4548**	305	B	67.3148	1
**4548**	319	B	62.5179	1
**4548**	320	B	71.2927	-1
**4548**	321	B	62.3737	0
**4548**	351	B	12.5595	1
**4548**	357	B	63.4896	1
**4548**	370	B	79.4582	1
**4548**	372	B	69.0607	0
**4548**	373	B	58.6957	1
**4548**	381	B	47.2046	0
**4548**	382	B	77.0411	1
**4548**	386	B	45.6338	0
**4548**	305	C	50.2311	1
**4548**	319	C	79.5936	1
**4548**	320	C	49.1199	-1
**4548**	321	C	74.8832	0
**4548**	351	C	16.2508	1
**4548**	357	C	75.4551	1
**4548**	370	C	37.4957	0
**4548**	372	C	72.4334	0
**4548**	373	C	66.6705	1
**4548**	381	C	79.5094	1
**4548**	382	C	78.1489	1
**4548**	386	C	29.7589	0
**4548**	305	D	66.2157	1
**4548**	319	D	59.292	1

**4548**	320	D	79.4774	-1
**4548**	321	D	79.5389	0
**4548**	351	D	13.7399	1
**4548**	357	D	79.5295	1
**4548**	370	D	73.0248	1
**4548**	372	D	79.6415	0
**4548**	373	D	65.8059	1
**4548**	381	D	35.4808	0
**4548**	382	D	30.1018	0
**4548**	386	D	79.5539	1
**4549**	305	A	65.9191	1
**4549**	319	A	70.5914	1
**4549**	320	A	79.6164	-1
**4549**	321	A	78.2203	0
**4549**	351	A	12.5514	1
**4549**	357	A	71.5493	1
**4549**	370	A	79.5054	1
**4549**	372	A	78.9286	0
**4549**	373	A	79.3273	1
**4549**	381	A	79.8196	1
**4549**	382	A	73.9329	1
**4549**	386	A	79.5825	1
**4549**	305	B	78.9516	1
**4549**	319	B	72.1712	1
**4549**	320	B	64.6379	-1
**4549**	321	B	61.3353	0
**4549**	351	B	22.8031	-1
**4549**	357	B	75.4921	1
**4549**	370	B	79.6447	1
**4549**	372	B	70.2628	0
**4549**	373	B	43.7479	0
**4549**	381	B	73.8095	1
**4549**	382	B	21.1393	0
**4549**	386	B	47.0243	0

**4549**	305	C	71.0713	1
**4549**	319	C	48.051	0
**4549**	320	C	69.5671	-1
**4549**	321	C	71.7441	0
**4549**	351	C	10.6326	1
**4549**	357	C	79.6083	1
**4549**	370	C	79.5346	1
**4549**	372	C	48.4697	1
**4549**	373	C	63.4994	1
**4549**	381	C	59.0884	1
**4549**	382	C	55.0225	1
**4549**	386	C	21.1728	0
**4549**	305	D	79.3572	1
**4549**	319	D	64.627	1
**4549**	320	D	63.8289	-1
**4549**	321	D	69.0416	0
**4549**	351	D	16.2418	1
**4549**	357	D	74.4106	1
**4549**	370	D	79.7036	1
**4549**	372	D	79.555	0
**4549**	373	D	69.975	1
**4549**	381	D	51.5029	1
**4549**	382	D	79.6576	1
**4549**	386	D	75.7926	1
**4550**	305	A	66.7785	1
**4550**	319	A	53.2291	1
**4550**	320	A	79.514	-1
**4550**	321	A	74.0497	0
**4550**	351	A	3.9792	1
**4550**	357	A	66.6325	1
**4550**	370	A	79.4764	1
**4550**	372	A	79.4633	0
**4550**	373	A	79.5407	1
**4550**	381	A	44.6603	0

**4550**	382	A	75.3612	1
**4550**	386	A	69.6251	1
**4550**	305	B	55.504	1
**4550**	319	B	72.7874	1
**4550**	320	B	53.7785	-1
**4550**	321	B	45.0069	1
**4550**	351	B	12.763	1
**4550**	357	B	79.6603	1
**4550**	370	B	65.8243	1
**4550**	372	B	71.5967	0
**4550**	373	B	77.2872	1
**4550**	381	B	79.5785	1
**4550**	382	B	73.585	1
**4550**	386	B	43.5071	0
**4550**	305	C	76.3424	1
**4550**	319	C	79.5263	1
**4550**	320	C	79.5934	-1
**4550**	321	C	73.0183	0
**4550**	351	C	1.778	1
**4550**	357	C	79.6316	1
**4550**	370	C	73.2352	1
**4550**	372	C	79.603	0
**4550**	373	C	74.1858	1
**4550**	381	C	52.6512	1
**4550**	382	C	79.5977	1
**4550**	386	C	39.7128	0
**4550**	305	D	58.2497	1
**4550**	319	D	41.93	0
**4550**	320	D	58.2285	-1
**4550**	321	D	67.3345	0
**4550**	351	D	17.8734	1
**4550**	357	D	60.3621	1
**4550**	370	D	69.1926	1
**4550**	372	D	79.5923	0

**4550**	373	D	79.5439	1
**4550**	381	D	68.9506	1
**4550**	382	D	74.699	1
**4550**	386	D	79.4727	1
**4551**	305	A	73.9552	1
**4551**	319	A	68.6531	1
**4551**	320	A	63.8061	-1
**4551**	321	A	75.1315	0
**4551**	351	A	11.8625	1
**4551**	357	A	79.534	1
**4551**	370	A	79.6071	1
**4551**	372	A	65.9685	0
**4551**	373	A	44.6715	0
**4551**	381	A	71.3548	1
**4551**	382	A	72.2823	1
**4551**	386	A	54.4926	1
**4551**	305	B	75.028	1
**4551**	319	B	71.1574	1
**4551**	320	B	27.2839	-1
**4551**	321	B	54.8809	0
**4551**	351	B	17.9585	1
**4551**	357	B	79.5583	1
**4551**	370	B	39.0078	0
**4551**	372	B	79.6417	0
**4551**	373	B	78.6936	1
**4551**	381	B	75.5764	1
**4551**	382	B	49.7719	0
**4551**	386	B	44.2799	0
**4551**	305	C	79.5566	1
**4551**	319	C	79.6158	1
**4551**	320	C	59.7714	-1
**4551**	321	C	79.5003	0
**4551**	351	C	9.0003	1
**4551**	357	C	79.6131	1

**4551**	370	C	68.2447	1
**4551**	372	C	79.6044	0
**4551**	373	C	73.016	1
**4551**	381	C	71.1915	1
**4551**	382	C	64.3179	1
**4551**	386	C	19.0336	-1
**4551**	305	D	79.5352	1
**4551**	319	D	45.0471	0
**4551**	320	D	79.5847	-1
**4551**	321	D	65.0895	0
**4551**	351	D	45.4258	-1
**4551**	357	D	79.5889	1
**4551**	370	D	74.9759	1
**4551**	372	D	79.4622	0
**4551**	373	D	73.0458	1
**4551**	381	D	79.6109	1
**4551**	382	D	57.3248	1
**4551**	386	D	58.6506	1
**4552**	305	A	73.1127	1
**4552**	319	A	62.78	1
**4552**	320	A	75.2568	-1
**4552**	321	A	79.5942	0
**4552**	351	A	7.1584	1
**4552**	357	A	70.4693	1
**4552**	370	A	54.9437	1
**4552**	372	A	76.5956	0
**4552**	373	A	50.2056	1
**4552**	381	A	74.4352	1
**4552**	382	A	45.9367	0
**4552**	386	A	76.8349	1
**4552**	305	B	70.6442	1
**4552**	319	B	79.5961	1
**4552**	320	B	76.4599	-1
**4552**	321	B	48.8809	1

**4552**	351	B	25.1821	-1
**4552**	357	B	74.3375	1
**4552**	370	B	79.5179	1
**4552**	372	B	79.5826	0
**4552**	373	B	74.5424	1
**4552**	381	B	60.7088	1
**4552**	382	B	20.055	0
**4552**	386	B	54.466	1
**4552**	305	C	74.4404	1
**4552**	319	C	48.8934	0
**4552**	320	C	55.0594	-1
**4552**	321	C	71.7198	0
**4552**	351	C	0.1831	1
**4552**	357	C	65.7547	1
**4552**	370	C	68.0135	1
**4552**	372	C	79.5494	0
**4552**	373	C	53.8762	1
**4552**	381	C	51.4631	1
**4552**	382	C	79.5914	1
**4552**	386	C	43.709	0
**4552**	305	D	45.6692	0
**4552**	319	D	74.3712	1
**4552**	320	D	76.8032	-1
**4552**	321	D	44.198	1
**4552**	351	D	15.2064	1
**4552**	357	D	79.5924	1
**4552**	370	D	79.0863	1
**4552**	372	D	35.8302	1
**4552**	373	D	54.1492	1
**4552**	381	D	72.384	1
**4552**	382	D	79.5422	1
**4552**	386	D	78.8111	1
**4553**	305	A	76.8612	1
**4553**	319	A	75.8584	1

**4553**	320	A	69.3869	-1
**4553**	321	A	71.8864	0
**4553**	351	A	3.3039	1
**4553**	357	A	79.5311	1
**4553**	370	A	72.0249	1
**4553**	372	A	75.1792	0
**4553**	373	A	77.0005	1
**4553**	381	A	79.5296	1
**4553**	382	A	69.574	1
**4553**	386	A	36.0068	0
**4553**	305	B	79.5516	1
**4553**	319	B	79.5491	1
**4553**	320	B	75.3955	-1
**4553**	321	B	79.5426	0
**4553**	351	B	13.879	1
**4553**	357	B	46.9829	0
**4553**	370	B	79.5082	1
**4553**	372	B	36.9295	1
**4553**	373	B	79.6332	1
**4553**	381	B	79.6149	1
**4553**	382	B	37.7655	0
**4553**	386	B	79.6226	1
**4553**	305	C	78.8582	1
**4553**	319	C	79.5652	1
**4553**	320	C	75.0537	-1
**4553**	321	C	65.4096	0
**4553**	351	C	6.7916	1
**4553**	357	C	79.6002	1
**4553**	370	C	51.7927	1
**4553**	372	C	79.5444	0
**4553**	373	C	73.4113	1
**4553**	381	C	67.9235	1
**4553**	382	C	72.7717	1
**4553**	386	C	66.5618	1

**4553**	305	D	79.5413	1
**4553**	319	D	79.4697	1
**4553**	320	D	79.5909	-1
**4553**	321	D	35.4034	1
**4553**	351	D	14.2275	1
**4553**	357	D	73.4654	1
**4553**	370	D	54.3142	1
**4553**	372	D	79.1845	0
**4553**	373	D	74.4033	1
**4553**	381	D	79.3831	1
**4553**	382	D	73.3279	1
**4553**	386	D	72.1216	1
**4554**	305	A	52.105	1
**4554**	319	A	40.1775	0
**4554**	320	A	79.5917	-1
**4554**	321	A	47.0774	1
**4554**	351	A	4.3454	1
**4554**	357	A	72.5324	1
**4554**	370	A	72.3361	1
**4554**	372	A	74.5476	0
**4554**	373	A	25.7144	0
**4554**	381	A	79.5074	1
**4554**	382	A	49.5609	0
**4554**	386	A	77.6025	1
**4554**	305	B	77.8174	1
**4554**	319	B	68.0235	1
**4554**	320	B	58.6521	-1
**4554**	321	B	79.6355	0
**4554**	351	B	12.417	1
**4554**	357	B	72.0889	1
**4554**	370	B	78.396	1
**4554**	372	B	79.5783	0
**4554**	373	B	56.1222	1
**4554**	381	B	79.5243	1

**4554**	382	B	69.6974	1
**4554**	386	B	70.6435	1
**4554**	305	C	79.1206	1
**4554**	319	C	49.7331	0
**4554**	320	C	70.5317	-1
**4554**	321	C	50.3331	0
**4554**	351	C	2.5809	1
**4554**	357	C	66.9423	1
**4554**	370	C	62.4615	1
**4554**	372	C	58.6133	0
**4554**	373	C	79.548	1
**4554**	381	C	73.658	1
**4554**	382	C	73.5648	1
**4554**	386	C	39.8663	0
**4554**	305	D	79.553	1
**4554**	319	D	63.7806	1
**4554**	320	D	79.5579	-1
**4554**	321	D	79.5833	0
**4554**	351	D	14.1363	1
**4554**	357	D	77.2708	1
**4554**	370	D	59.3351	1
**4554**	372	D	79.5543	0
**4554**	373	D	69.5661	1
**4554**	381	D	39.1127	0
**4554**	382	D	58.0455	1
**4554**	386	D	79.583	1
**4555**	305	A	32.9808	0
**4555**	319	A	58.061	1
**4555**	320	A	79.5508	-1
**4555**	321	A	60.609	0
**4555**	351	A	7.6581	1
**4555**	357	A	52.0699	1
**4555**	370	A	29.9508	0
**4555**	372	A	66.3696	0

**4555**	373	A	37.4991	0
**4555**	381	A	77.9012	1
**4555**	382	A	70.2821	1
**4555**	386	A	78.4828	1
**4555**	305	B	78.1679	1
**4555**	319	B	44.238	0
**4555**	320	B	63.3303	-1
**4555**	321	B	66.8862	0
**4555**	351	B	12.3547	1
**4555**	357	B	79.5882	1
**4555**	370	B	79.2301	1
**4555**	372	B	79.574	0
**4555**	373	B	70.2141	1
**4555**	381	B	72.2657	1
**4555**	382	B	14.033	-1
**4555**	386	B	54.5605	1
**4555**	305	C	52.5547	1
**4555**	319	C	79.5198	1
**4555**	320	C	79.577	-1
**4555**	321	C	61.4579	0
**4555**	351	C	11.8328	1
**4555**	357	C	79.3814	1
**4555**	370	C	56.113	1
**4555**	372	C	40.7353	1
**4555**	373	C	79.5332	1
**4555**	381	C	67.6649	1
**4555**	382	C	79.5521	1
**4555**	386	C	26.4651	0
**4555**	305	D	64.85	1
**4555**	319	D	79.5894	1
**4555**	320	D	59.7622	-1
**4555**	321	D	35.6567	1
**4555**	351	D	11.1782	1
**4555**	357	D	72.2223	1

**4555**	370	D	79.5659	1
**4555**	372	D	61.1635	0
**4555**	373	D	35.6333	0
**4555**	381	D	79.5441	1
**4555**	382	D	72.8222	1
**4555**	386	D	79.5757	1
**4556**	305	A	69.306	1
**4556**	319	A	66.457	1
**4556**	320	A	79.5598	-1
**4556**	321	A	34.2045	1
**4556**	351	A	18.2252	1
**4556**	357	A	60.1842	1
**4556**	370	A	79.6445	1
**4556**	372	A	75.0908	0
**4556**	373	A	79.6283	1
**4556**	381	A	56.0669	1
**4556**	382	A	79.5251	1
**4556**	386	A	43.0494	0
**4556**	305	B	72.5037	1
**4556**	319	B	74.3204	1
**4556**	320	B	73.4044	-1
**4556**	321	B	60.7474	0
**4556**	351	B	7.6416	1
**4556**	357	B	58.4906	1
**4556**	370	B	44.8685	0
**4556**	372	B	79.5726	0
**4556**	373	B	52.073	1
**4556**	381	B	66.2329	1
**4556**	382	B	76.3844	1
**4556**	386	B	71.4847	1
**4556**	305	C	73.2471	1
**4556**	319	C	79.3222	1
**4556**	320	C	73.8451	-1
**4556**	321	C	74.0916	0

**4556**	351	C	5.1575	1
**4556**	357	C	59.7739	1
**4556**	370	C	66.1339	1
**4556**	372	C	73.5973	0
**4556**	373	C	66.5381	1
**4556**	381	C	79.5306	1
**4556**	382	C	79.4639	1
**4556**	386	C	34.931	0
**4556**	305	D	65.8853	1
**4556**	319	D	70.7765	1
**4556**	320	D	79.5901	-1
**4556**	321	D	73.6467	0
**4556**	351	D	12.9603	1
**4556**	357	D	66.132	1
**4556**	370	D	64.1595	1
**4556**	372	D	33.741	1
**4556**	373	D	51.4426	1
**4556**	381	D	54.7112	1
**4556**	382	D	79.6565	1
**4556**	386	D	57.0209	1
**4557**	305	A	66.5295	1
**4557**	319	A	71.7549	1
**4557**	320	A	79.4909	-1
**4557**	321	A	46.3792	1
**4557**	351	A	9.826	1
**4557**	357	A	74.737	1
**4557**	370	A	60.8318	1
**4557**	372	A	79.5699	0
**4557**	373	A	58.4642	1
**4557**	381	A	57.8646	1
**4557**	382	A	67.4284	1
**4557**	386	A	79.4847	1
**4557**	305	B	79.591	1
**4557**	319	B	79.5072	1

**4557**	320	B	70.3089	-1
**4557**	321	B	79.5881	0
**4557**	351	B	13.5221	1
**4557**	357	B	72.6019	1
**4557**	370	B	75.9872	1
**4557**	372	B	79.5046	0
**4557**	373	B	19.5742	-1
**4557**	381	B	73.935	1
**4557**	382	B	42.8503	0
**4557**	386	B	79.4547	1
**4557**	305	C	70.281	1
**4557**	319	C	79.6278	1
**4557**	320	C	67.7843	-1
**4557**	321	C	44.7048	1
**4557**	351	C	0.3253	1
**4557**	357	C	78.5709	1
**4557**	370	C	79.1759	1
**4557**	372	C	67.3771	0
**4557**	373	C	79.5682	1
**4557**	381	C	79.6028	1
**4557**	382	C	75.1247	1
**4557**	386	C	17.5282	-1
**4557**	305	D	79.3237	1
**4557**	319	D	53.4523	1
**4557**	320	D	74.3081	-1
**4557**	321	D	50.6255	0
**4557**	351	D	14.6536	1
**4557**	357	D	79.5632	1
**4557**	370	D	49.2001	0
**4557**	372	D	79.4257	0
**4557**	373	D	50.4117	1
**4557**	381	D	70.3255	1
**4557**	382	D	79.6152	1
**4557**	386	D	66.4107	1

**4558**	305	A	75.6317	1
**4558**	319	A	55.9081	1
**4558**	320	A	74.0341	-1
**4558**	321	A	55.2672	0
**4558**	351	A	4.1489	1
**4558**	357	A	79.5763	1
**4558**	370	A	40.1026	0
**4558**	372	A	79.546	0
**4558**	373	A	45.2101	0
**4558**	381	A	77.5475	1
**4558**	382	A	79.5879	1
**4558**	386	A	47.0307	0
**4558**	305	B	66.1788	1
**4558**	319	B	68.4184	1
**4558**	320	B	79.5276	-1
**4558**	321	B	79.5766	0
**4558**	351	B	10.8299	1
**4558**	357	B	72.5704	1
**4558**	370	B	79.5638	1
**4558**	372	B	33.8133	1
**4558**	373	B	33.6471	0
**4558**	381	B	38.2419	0
**4558**	382	B	79.4842	1
**4558**	386	B	57.9166	1
**4558**	305	C	71.5794	1
**4558**	319	C	73.2334	1
**4558**	320	C	53.8722	-1
**4558**	321	C	65.714	0
**4558**	351	C	7.2386	1
**4558**	357	C	53.7921	1
**4558**	370	C	37.0713	0
**4558**	372	C	71.8183	0
**4558**	373	C	79.5623	1
**4558**	381	C	57.4346	1

**4558**	382	C	70.2379	1
**4558**	386	C	18.7037	-1
**4558**	305	D	34.7118	0
**4558**	319	D	77.1045	1
**4558**	320	D	41.9212	-1
**4558**	321	D	71.6438	0
**4558**	351	D	8.9956	1
**4558**	357	D	73.0236	1
**4558**	370	D	64.8251	1
**4558**	372	D	55.0914	0
**4558**	373	D	79.5211	1
**4558**	381	D	36.5555	0
**4558**	382	D	79.5265	1
**4558**	386	D	71.8275	1
**4559**	305	A	69.4498	1
**4559**	319	A	79.6029	1
**4559**	320	A	77.3526	-1
**4559**	321	A	56.635	0
**4559**	351	A	2.2699	1
**4559**	357	A	79.5046	1
**4559**	370	A	72.2442	1
**4559**	372	A	63.5997	0
**4559**	373	A	79.4989	1
**4559**	381	A	79.6249	1
**4559**	382	A	39.6597	0
**4559**	386	A	64.9259	1
**4559**	305	B	73.8139	1
**4559**	319	B	69.691	1
**4559**	320	B	76.7007	-1
**4559**	321	B	74.5032	0
**4559**	351	B	0.0	1
**4559**	357	B	66.2476	1
**4559**	370	B	79.4555	1
**4559**	372	B	66.6762	0

**4559**	373	B	68.6282	1
**4559**	381	B	39.989	0
**4559**	382	B	78.4926	1
**4559**	386	B	79.347	1
**4559**	305	C	71.1176	1
**4559**	319	C	79.5386	1
**4559**	320	C	58.2931	-1
**4559**	321	C	71.0042	0
**4559**	351	C	2.4756	1
**4559**	357	C	55.2842	1
**4559**	370	C	70.3613	1
**4559**	372	C	71.1742	0
**4559**	373	C	79.5026	1
**4559**	381	C	79.6923	1
**4559**	382	C	22.4077	0
**4559**	386	C	65.2756	1
**4559**	305	D	72.5835	1
**4559**	319	D	73.6882	1
**4559**	320	D	23.1201	-1
**4559**	321	D	79.4115	0
**4559**	351	D	13.7945	1
**4559**	357	D	50.7272	1
**4559**	370	D	79.1349	1
**4559**	372	D	78.9975	0
**4559**	373	D	73.1808	1
**4559**	381	D	75.7318	1
**4559**	382	D	74.5777	1
**4559**	386	D	71.9206	1
**4560**	305	A	79.5803	1
**4560**	319	A	45.5129	0
**4560**	320	A	75.1464	-1
**4560**	321	A	37.8118	1
**4560**	351	A	4.3188	1
**4560**	357	A	60.0719	1

**4560**	370	A	63.0596	1
**4560**	372	A	55.6676	0
**4560**	373	A	70.4606	1
**4560**	381	A	74.0745	1
**4560**	382	A	52.6155	1
**4560**	386	A	79.5308	1
**4560**	305	B	79.6011	1
**4560**	319	B	29.1271	0
**4560**	320	B	79.4993	-1
**4560**	321	B	65.6146	0
**4560**	351	B	0.0671	1
**4560**	357	B	79.5223	1
**4560**	370	B	58.901	1
**4560**	372	B	69.5933	0
**4560**	373	B	44.4205	0
**4560**	381	B	59.7235	1
**4560**	382	B	56.412	1
**4560**	386	B	72.5315	1
**4560**	305	C	59.9034	1
**4560**	319	C	58.2666	1
**4560**	320	C	61.3368	-1
**4560**	321	C	71.0841	0
**4560**	351	C	18.2843	1
**4560**	357	C	79.5216	1
**4560**	370	C	73.4202	1
**4560**	372	C	79.6607	0
**4560**	373	C	54.4028	1
**4560**	381	C	72.6237	1
**4560**	382	C	79.5138	1
**4560**	386	C	59.4943	1
**4560**	305	D	54.1042	1
**4560**	319	D	77.6559	1
**4560**	320	D	79.553	-1
**4560**	321	D	79.6015	0

**4560**	351	D	15.7692	1
**4560**	357	D	79.4501	1
**4560**	370	D	79.5774	1
**4560**	372	D	73.8878	0
**4560**	373	D	35.4334	0
**4560**	381	D	79.5353	1
**4560**	382	D	58.0425	1
**4560**	386	D	79.656	1
**4561**	305	A	64.0602	1
**4561**	319	A	31.7248	0
**4561**	320	A	79.5632	-1
**4561**	321	A	57.2304	0
**4561**	351	A	7.5184	1
**4561**	357	A	30.8835	0
**4561**	370	A	78.8722	1
**4561**	372	A	79.5909	0
**4561**	373	A	72.2521	1
**4561**	381	A	79.594	1
**4561**	382	A	68.7937	1
**4561**	386	A	79.5757	1
**4561**	305	B	79.5153	1
**4561**	319	B	70.7495	1
**4561**	320	B	30.9464	-1
**4561**	321	B	79.3047	0
**4561**	351	B	10.0874	1
**4561**	357	B	49.6483	0
**4561**	370	B	50.6724	1
**4561**	372	B	74.2216	0
**4561**	373	B	40.256	0
**4561**	381	B	42.7706	0
**4561**	382	B	67.4003	1
**4561**	386	B	46.8326	0
**4561**	305	C	79.543	1
**4561**	319	C	39.6419	0

**4561**	320	C	30.842	-1
**4561**	321	C	79.55	0
**4561**	351	C	4.1815	1
**4561**	357	C	34.1565	0
**4561**	370	C	29.0477	0
**4561**	372	C	69.3346	0
**4561**	373	C	79.5817	1
**4561**	381	C	79.5694	1
**4561**	382	C	55.1408	1
**4561**	386	C	46.2292	0
**4561**	305	D	79.4144	1
**4561**	319	D	10.1053	-1
**4561**	320	D	64.2435	-1
**4561**	321	D	46.1142	1
**4561**	351	D	23.5511	-1
**4561**	357	D	79.071	1
**4561**	370	D	76.0878	1
**4561**	372	D	74.5726	0
**4561**	373	D	76.1313	1
**4561**	381	D	79.6001	1
**4561**	382	D	77.0538	1
**4561**	386	D	79.5676	1
**4562**	305	A	63.8027	1
**4562**	319	A	79.5422	1
**4562**	320	A	40.3485	-1
**4562**	321	A	40.1534	1
**4562**	351	A	2.2735	1
**4562**	357	A	79.5676	1
**4562**	370	A	64.8774	1
**4562**	372	A	68.1835	0
**4562**	373	A	40.5723	0
**4562**	381	A	79.5644	1
**4562**	382	A	58.5561	1
**4562**	386	A	55.9021	1

**4562**	305	B	44.8376	0
**4562**	319	B	59.4478	1
**4562**	320	B	79.5977	-1
**4562**	321	B	55.2308	0
**4562**	351	B	10.75	1
**4562**	357	B	79.5593	1
**4562**	370	B	79.5517	1
**4562**	372	B	67.109	0
**4562**	373	B	51.022	1
**4562**	381	B	65.9122	1
**4562**	382	B	13.9679	-1
**4562**	386	B	79.5425	1
**4562**	305	C	78.5908	1
**4562**	319	C	45.0646	0
**4562**	320	C	79.5987	-1
**4562**	321	C	79.5499	0
**4562**	351	C	4.6287	1
**4562**	357	C	26.4789	0
**4562**	370	C	70.0663	1
**4562**	372	C	69.1999	0
**4562**	373	C	73.037	1
**4562**	381	C	79.5759	1
**4562**	382	C	24.3698	0
**4562**	386	C	41.1163	0
**4562**	305	D	73.756	1
**4562**	319	D	74.835	1
**4562**	320	D	48.2741	-1
**4562**	321	D	70.9488	0
**4562**	351	D	0.6056	1
**4562**	357	D	62.9301	1
**4562**	370	D	72.913	1
**4562**	372	D	79.5396	0
**4562**	373	D	79.5072	1
**4562**	381	D	42.0554	0

**4562**	382	D	78.3799	1
**4562**	386	D	79.2762	1
**4563**	305	A	61.0625	1
**4563**	319	A	75.9656	1
**4563**	320	A	79.5625	-1
**4563**	321	A	77.6833	0
**4563**	351	A	11.324	1
**4563**	357	A	79.2322	1
**4563**	370	A	53.9843	1
**4563**	372	A	76.7519	0
**4563**	373	A	79.3571	1
**4563**	381	A	72.4043	1
**4563**	382	A	79.5324	1
**4563**	386	A	79.5576	1
**4563**	305	B	51.9845	1
**4563**	319	B	47.685	0
**4563**	320	B	73.4236	-1
**4563**	321	B	73.2803	0
**4563**	351	B	12.6123	1
**4563**	357	B	45.0176	0
**4563**	370	B	77.6301	1
**4563**	372	B	79.556	0
**4563**	373	B	78.2863	1
**4563**	381	B	79.5835	1
**4563**	382	B	41.3063	0
**4563**	386	B	34.6676	0
**4563**	305	C	42.8057	0
**4563**	319	C	45.0358	0
**4563**	320	C	79.5717	-1
**4563**	321	C	30.9619	1
**4563**	351	C	7.0239	1
**4563**	357	C	64.4071	1
**4563**	370	C	47.7265	0
**4563**	372	C	79.5761	0

**4563**	373	C	62.2388	1
**4563**	381	C	55.8818	1
**4563**	382	C	65.1666	1
**4563**	386	C	53.568	1
**4563**	305	D	77.4172	1
**4563**	319	D	74.4929	1
**4563**	320	D	48.434	-1
**4563**	321	D	79.6435	0
**4563**	351	D	14.2359	1
**4563**	357	D	73.9212	1
**4563**	370	D	69.2826	1
**4563**	372	D	79.5291	0
**4563**	373	D	78.9376	1
**4563**	381	D	68.2956	1
**4563**	382	D	59.6398	1
**4563**	386	D	18.8478	-1
**4564**	305	A	79.5437	1
**4564**	319	A	7.9387	-1
**4564**	320	A	78.4172	-1
**4564**	321	A	50.5393	0
**4564**	351	A	11.9135	1
**4564**	357	A	69.2919	1
**4564**	370	A	79.6697	1
**4564**	372	A	79.5635	0
**4564**	373	A	55.57	1
**4564**	381	A	69.1538	1
**4564**	382	A	36.1631	0
**4564**	386	A	79.2376	1
**4564**	305	B	72.5871	1
**4564**	319	B	76.5462	1
**4564**	320	B	78.0872	-1
**4564**	321	B	49.9058	1
**4564**	351	B	16.3734	1
**4564**	357	B	64.9734	1

**4564**	370	B	75.7581	1
**4564**	372	B	79.5779	0
**4564**	373	B	79.4583	1
**4564**	381	B	79.4592	1
**4564**	382	B	18.3542	-1
**4564**	386	B	50.8351	1
**4564**	305	C	64.6774	1
**4564**	319	C	52.0185	1
**4564**	320	C	67.3944	-1
**4564**	321	C	69.8989	0
**4564**	351	C	9.5276	1
**4564**	357	C	75.1738	1
**4564**	370	C	56.9332	1
**4564**	372	C	79.5688	0
**4564**	373	C	56.8197	1
**4564**	381	C	26.7811	0
**4564**	382	C	64.2881	1
**4564**	386	C	48.5615	0
**4564**	305	D	73.3178	1
**4564**	319	D	77.3737	1
**4564**	320	D	79.5193	-1
**4564**	321	D	61.779	0
**4564**	351	D	28.5096	-1
**4564**	357	D	79.0446	1
**4564**	370	D	50.0365	1
**4564**	372	D	79.5327	0
**4564**	373	D	33.5497	0
**4564**	381	D	59.0629	1
**4564**	382	D	79.4501	1
**4564**	386	D	53.3446	1
**4565**	305	A	60.9511	1
**4565**	319	A	66.6239	1
**4565**	320	A	79.5394	-1
**4565**	321	A	67.1642	0

**4565**	351	A	7.6021	1
**4565**	357	A	72.048	1
**4565**	370	A	79.6457	1
**4565**	372	A	79.5702	0
**4565**	373	A	72.3579	1
**4565**	381	A	79.5679	1
**4565**	382	A	73.8233	1
**4565**	386	A	79.5524	1
**4565**	305	B	54.7809	1
**4565**	319	B	72.212	1
**4565**	320	B	63.2445	-1
**4565**	321	B	70.7899	0
**4565**	351	B	12.4162	1
**4565**	357	B	79.5833	1
**4565**	370	B	79.2717	1
**4565**	372	B	79.631	0
**4565**	373	B	71.3585	1
**4565**	381	B	65.4335	1
**4565**	382	B	51.0193	1
**4565**	386	B	70.4484	1
**4565**	305	C	68.0906	1
**4565**	319	C	50.7254	1
**4565**	320	C	59.1244	-1
**4565**	321	C	60.1545	0
**4565**	351	C	7.5562	1
**4565**	357	C	71.8191	1
**4565**	370	C	77.2889	1
**4565**	372	C	58.2581	0
**4565**	373	C	74.6238	1
**4565**	381	C	65.1427	1
**4565**	382	C	35.4313	0
**4565**	386	C	25.3522	0
**4565**	305	D	74.2336	1
**4565**	319	D	60.2017	1

**4565**	320	D	74.2267	-1
**4565**	321	D	65.2769	0
**4565**	351	D	5.7064	1
**4565**	357	D	66.7088	1
**4565**	370	D	76.0472	1
**4565**	372	D	79.5777	0
**4565**	373	D	74.3853	1
**4565**	381	D	56.4113	1
**4565**	382	D	79.5211	1
**4565**	386	D	61.0373	1
**4566**	305	A	61.8668	1
**4566**	319	A	16.8468	-1
**4566**	320	A	73.9932	-1
**4566**	321	A	75.0595	0
**4566**	351	A	8.4345	1
**4566**	357	A	75.1741	1
**4566**	370	A	79.5469	1
**4566**	372	A	79.5377	0
**4566**	373	A	68.4515	1
**4566**	381	A	71.9349	1
**4566**	382	A	55.9462	1
**4566**	386	A	75.7708	1
**4566**	305	B	54.7392	1
**4566**	319	B	79.6503	1
**4566**	320	B	59.4903	-1
**4566**	321	B	69.4358	0
**4566**	351	B	13.2974	1
**4566**	357	B	79.5182	1
**4566**	370	B	79.5389	1
**4566**	372	B	79.5713	0
**4566**	373	B	41.0857	0
**4566**	381	B	56.7453	1
**4566**	382	B	79.5874	1
**4566**	386	B	79.5861	1

**4566**	305	C	76.554	1
**4566**	319	C	30.8294	0
**4566**	320	C	79.6128	-1
**4566**	321	C	29.8409	1
**4566**	351	C	3.1691	1
**4566**	357	C	79.5598	1
**4566**	370	C	74.9821	1
**4566**	372	C	79.5488	0
**4566**	373	C	75.4262	1
**4566**	381	C	50.9311	1
**4566**	382	C	18.6778	-1
**4566**	386	C	30.1486	0
**4566**	305	D	72.6629	1
**4566**	319	D	78.096	1
**4566**	320	D	79.5758	-1
**4566**	321	D	9.3194	0
**4566**	351	D	10.5555	1
**4566**	357	D	79.5901	1
**4566**	370	D	72.2987	1
**4566**	372	D	67.6977	0
**4566**	373	D	68.1044	1
**4566**	381	D	79.6177	1
**4566**	382	D	25.2224	0
**4566**	386	D	40.8916	0
**4567**	305	A	72.2728	1
**4567**	319	A	68.3018	1
**4567**	320	A	79.4875	-1
**4567**	321	A	73.4911	0
**4567**	351	A	11.6177	1
**4567**	357	A	33.7524	0
**4567**	370	A	72.0519	1
**4567**	372	A	70.6343	0
**4567**	373	A	63.6617	1
**4567**	381	A	79.527	1

**4567**	382	A	77.9141	1
**4567**	386	A	75.5936	1
**4567**	305	B	79.4943	1
**4567**	319	B	67.4782	1
**4567**	320	B	79.5349	-1
**4567**	321	B	70.2066	0
**4567**	351	B	15.9622	1
**4567**	357	B	58.1329	1
**4567**	370	B	32.9573	0
**4567**	372	B	75.1285	0
**4567**	373	B	42.4761	0
**4567**	381	B	71.5624	1
**4567**	382	B	22.5261	0
**4567**	386	B	79.5293	1
**4567**	305	C	76.1646	1
**4567**	319	C	71.3827	1
**4567**	320	C	67.6863	-1
**4567**	321	C	38.5814	1
**4567**	351	C	6.7162	1
**4567**	357	C	59.395	1
**4567**	370	C	45.7168	0
**4567**	372	C	45.0295	1
**4567**	373	C	79.4958	1
**4567**	381	C	70.0709	1
**4567**	382	C	78.124	1
**4567**	386	C	14.6364	-1
**4567**	305	D	71.9726	1
**4567**	319	D	39.4554	0
**4567**	320	D	79.559	-1
**4567**	321	D	67.0069	0
**4567**	351	D	6.6717	1
**4567**	357	D	70.257	1
**4567**	370	D	79.4923	1
**4567**	372	D	74.9185	0

**4567**	373	D	59.5831	1
**4567**	381	D	70.0443	1
**4567**	382	D	70.8814	1
**4567**	386	D	79.495	1
**4568**	305	A	16.5524	-1
**4568**	319	A	71.4858	1
**4568**	320	A	71.441	-1
**4568**	321	A	77.4561	0
**4568**	351	A	8.4936	1
**4568**	357	A	56.4193	1
**4568**	370	A	79.6019	1
**4568**	372	A	50.0773	0
**4568**	373	A	63.5117	1
**4568**	381	A	73.5687	1
**4568**	382	A	79.6259	1
**4568**	386	A	39.8531	0
**4568**	305	B	79.5729	1
**4568**	319	B	54.7364	1
**4568**	320	B	67.0505	-1
**4568**	321	B	46.5437	1
**4568**	351	B	15.3653	1
**4568**	357	B	66.8806	1
**4568**	370	B	79.055	1
**4568**	372	B	71.2865	0
**4568**	373	B	43.0758	0
**4568**	381	B	43.3366	0
**4568**	382	B	41.5696	0
**4568**	386	B	79.5664	1
**4568**	305	C	78.9293	1
**4568**	319	C	60.0916	1
**4568**	320	C	79.5972	-1
**4568**	321	C	46.6475	1
**4568**	351	C	5.2958	1
**4568**	357	C	36.1454	0

**4568**	370	C	75.9196	1
**4568**	372	C	72.648	0
**4568**	373	C	50.1501	1
**4568**	381	C	77.6459	1
**4568**	382	C	61.9685	1
**4568**	386	C	41.1723	0
**4568**	305	D	79.3484	1
**4568**	319	D	74.8797	1
**4568**	320	D	4.1595	1
**4568**	321	D	69.7113	0
**4568**	351	D	9.3437	1
**4568**	357	D	79.5465	1
**4568**	370	D	79.5604	1
**4568**	372	D	79.5527	0
**4568**	373	D	77.295	1
**4568**	381	D	46.082	0
**4568**	382	D	73.4337	1
**4568**	386	D	21.3747	0
**4569**	305	A	30.92	0
**4569**	319	A	77.0062	1
**4569**	320	A	79.5854	-1
**4569**	321	A	75.0853	0
**4569**	351	A	0.8792	1
**4569**	357	A	79.51	1
**4569**	370	A	71.6517	1
**4569**	372	A	79.5499	0
**4569**	373	A	73.786	1
**4569**	381	A	79.4743	1
**4569**	382	A	60.8333	1
**4569**	386	A	78.7052	1
**4569**	305	B	79.5758	1
**4569**	319	B	73.791	1
**4569**	320	B	79.5693	-1
**4569**	321	B	76.9049	0

**4569**	351	B	13.1843	1
**4569**	357	B	68.6198	1
**4569**	370	B	50.0529	1
**4569**	372	B	79.4952	0
**4569**	373	B	68.0609	1
**4569**	381	B	76.1122	1
**4569**	382	B	54.1414	1
**4569**	386	B	74.8427	1
**4569**	305	C	78.5985	1
**4569**	319	C	79.5842	1
**4569**	320	C	51.6428	-1
**4569**	321	C	77.8486	0
**4569**	351	C	1.9463	1
**4569**	357	C	78.4593	1
**4569**	370	C	71.409	1
**4569**	372	C	75.9043	0
**4569**	373	C	35.2774	0
**4569**	381	C	59.7337	1
**4569**	382	C	79.5655	1
**4569**	386	C	62.7125	1
**4569**	305	D	49.0578	0
**4569**	319	D	75.4867	1
**4569**	320	D	68.7456	-1
**4569**	321	D	66.8506	0
**4569**	351	D	8.8682	1
**4569**	357	D	72.3147	1
**4569**	370	D	78.9836	1
**4569**	372	D	72.4123	0
**4569**	373	D	57.6896	1
**4569**	381	D	79.555	1
**4569**	382	D	57.1006	1
**4569**	386	D	76.7627	1
**4570**	305	A	77.6004	1
**4570**	319	A	77.8926	1

**4570**	320	A	71.4382	-1
**4570**	321	A	39.165	1
**4570**	351	A	3.7467	1
**4570**	357	A	79.5764	1
**4570**	370	A	41.7024	0
**4570**	372	A	79.5918	0
**4570**	373	A	79.5673	1
**4570**	381	A	65.1401	1
**4570**	382	A	79.2692	1
**4570**	386	A	79.5226	1
**4570**	305	B	59.8701	1
**4570**	319	B	79.5621	1
**4570**	320	B	54.9082	-1
**4570**	321	B	79.5607	0
**4570**	351	B	16.7507	1
**4570**	357	B	79.634	1
**4570**	370	B	72.8486	1
**4570**	372	B	79.4889	0
**4570**	373	B	67.9949	1
**4570**	381	B	79.5351	1
**4570**	382	B	79.2974	1
**4570**	386	B	58.3608	1
**4570**	305	C	78.4224	1
**4570**	319	C	58.1597	1
**4570**	320	C	79.4441	-1
**4570**	321	C	79.57	0
**4570**	351	C	11.0166	1
**4570**	357	C	79.5236	1
**4570**	370	C	66.563	1
**4570**	372	C	79.541	0
**4570**	373	C	79.5923	1
**4570**	381	C	59.7245	1
**4570**	382	C	71.5273	1
**4570**	386	C	4.9594	-1

**4570**	305	D	74.0547	1
**4570**	319	D	68.9637	1
**4570**	320	D	79.5786	-1
**4570**	321	D	72.5863	0
**4570**	351	D	12.9262	1
**4570**	357	D	55.4644	1
**4570**	370	D	49.0087	0
**4570**	372	D	79.5394	0
**4570**	373	D	79.6032	1
**4570**	381	D	74.071	1
**4570**	382	D	73.6135	1
**4570**	386	D	79.5621	1
**4571**	305	A	73.583	1
**4571**	319	A	79.4808	1
**4571**	320	A	57.929	-1
**4571**	321	A	79.5047	0
**4571**	351	A	21.9087	-1
**4571**	357	A	79.5826	1
**4571**	370	A	65.622	1
**4571**	372	A	79.6137	0
**4571**	373	A	78.6398	1
**4571**	381	A	72.8372	1
**4571**	382	A	79.4743	1
**4571**	386	A	79.5777	1
**4571**	305	B	75.7756	1
**4571**	319	B	35.7678	0
**4571**	320	B	64.9787	-1
**4571**	321	B	79.4767	0
**4571**	351	B	24.2601	-1
**4571**	357	B	79.6985	1
**4571**	370	B	74.2684	1
**4571**	372	B	79.582	0
**4571**	373	B	70.7151	1
**4571**	381	B	79.6118	1

**4571**	382	B	69.9086	1
**4571**	386	B	76.6163	1
**4571**	305	C	79.3802	1
**4571**	319	C	62.9492	1
**4571**	320	C	76.7816	-1
**4571**	321	C	75.9424	0
**4571**	351	C	12.2441	1
**4571**	357	C	55.8213	1
**4571**	370	C	64.7911	1
**4571**	372	C	66.1394	0
**4571**	373	C	61.2637	1
**4571**	381	C	64.8335	1
**4571**	382	C	79.4791	1
**4571**	386	C	24.3201	0
**4571**	305	D	65.8553	1
**4571**	319	D	79.6061	1
**4571**	320	D	79.7857	-1
**4571**	321	D	70.031	0
**4571**	351	D	22.0561	-1
**4571**	357	D	78.9609	1
**4571**	370	D	79.7402	1
**4571**	372	D	76.5084	0
**4571**	373	D	61.6888	1
**4571**	381	D	31.7935	0
**4571**	382	D	75.1669	1
**4571**	386	D	79.5063	1
**4572**	305	A	79.4965	1
**4572**	319	A	54.0137	1
**4572**	320	A	74.4856	-1
**4572**	321	A	36.5104	1
**4572**	351	A	3.994	1
**4572**	357	A	72.8909	1
**4572**	370	A	76.2117	1
**4572**	372	A	77.2266	0

**4572**	373	A	50.3667	1
**4572**	381	A	79.5193	1
**4572**	382	A	74.6025	1
**4572**	386	A	28.7827	0
**4572**	305	B	71.8893	1
**4572**	319	B	50.1329	1
**4572**	320	B	79.5179	-1
**4572**	321	B	68.8913	0
**4572**	351	B	11.6753	1
**4572**	357	B	73.1797	1
**4572**	370	B	79.5745	1
**4572**	372	B	79.4753	0
**4572**	373	B	45.866	0
**4572**	381	B	79.5501	1
**4572**	382	B	45.0627	0
**4572**	386	B	54.6809	1
**4572**	305	C	48.8417	0
**4572**	319	C	75.3897	1
**4572**	320	C	79.5386	-1
**4572**	321	C	77.4856	0
**4572**	351	C	5.5066	1
**4572**	357	C	79.5563	1
**4572**	370	C	74.1806	1
**4572**	372	C	70.4861	0
**4572**	373	C	79.5839	1
**4572**	381	C	38.8809	0
**4572**	382	C	66.9731	1
**4572**	386	C	65.0788	1
**4572**	305	D	37.2422	0
**4572**	319	D	54.2384	1
**4572**	320	D	76.2142	-1
**4572**	321	D	67.4642	0
**4572**	351	D	16.3064	1
**4572**	357	D	79.5425	1

**4572**	370	D	72.0821	1
**4572**	372	D	79.5596	0
**4572**	373	D	79.4925	1
**4572**	381	D	79.499	1
**4572**	382	D	79.5696	1
**4572**	386	D	79.5698	1
**4573**	305	A	49.7186	0
**4573**	319	A	52.7254	1
**4573**	320	A	74.8836	-1
**4573**	321	A	79.6226	0
**4573**	351	A	5.0231	1
**4573**	357	A	72.0158	1
**4573**	370	A	63.1087	1
**4573**	372	A	79.6056	0
**4573**	373	A	74.1981	1
**4573**	381	A	79.5002	1
**4573**	382	A	74.8383	1
**4573**	386	A	64.3736	1
**4573**	305	B	68.5326	1
**4573**	319	B	75.4716	1
**4573**	320	B	79.5978	-1
**4573**	321	B	77.1275	0
**4573**	351	B	13.5316	1
**4573**	357	B	50.6234	1
**4573**	370	B	79.5073	1
**4573**	372	B	79.5187	0
**4573**	373	B	79.5685	1
**4573**	381	B	75.8293	1
**4573**	382	B	19.0934	-1
**4573**	386	B	68.6601	1
**4573**	305	C	79.5119	1
**4573**	319	C	72.4197	1
**4573**	320	C	76.1223	-1
**4573**	321	C	68.3683	0

**4573**	351	C	2.4679	1
**4573**	357	C	79.6248	1
**4573**	370	C	37.8534	0
**4573**	372	C	55.9245	0
**4573**	373	C	23.598	0
**4573**	381	C	79.5644	1
**4573**	382	C	79.5715	1
**4573**	386	C	51.2575	1
**4573**	305	D	79.5279	1
**4573**	319	D	79.6024	1
**4573**	320	D	79.5184	-1
**4573**	321	D	79.5858	0
**4573**	351	D	13.0017	1
**4573**	357	D	54.7661	1
**4573**	370	D	50.2268	1
**4573**	372	D	79.5785	0
**4573**	373	D	79.6909	1
**4573**	381	D	79.4646	1
**4573**	382	D	72.7222	1
**4573**	386	D	25.1392	0
**4574**	305	A	77.4539	1
**4574**	319	A	79.5889	1
**4574**	320	A	79.2195	-1
**4574**	321	A	79.592	0
**4574**	351	A	4.0456	1
**4574**	357	A	45.6059	0
**4574**	370	A	69.1616	1
**4574**	372	A	57.3817	0
**4574**	373	A	34.6866	0
**4574**	381	A	72.232	1
**4574**	382	A	74.7892	1
**4574**	386	A	79.5519	1
**4574**	305	B	79.5444	1
**4574**	319	B	72.5949	1

**4574**	320	B	61.9008	-1
**4574**	321	B	58.5164	0
**4574**	351	B	14.295	1
**4574**	357	B	55.1332	1
**4574**	370	B	79.6006	1
**4574**	372	B	79.5482	0
**4574**	373	B	72.7816	1
**4574**	381	B	79.5656	1
**4574**	382	B	71.4084	1
**4574**	386	B	63.969	1
**4574**	305	C	63.7533	1
**4574**	319	C	79.4863	1
**4574**	320	C	71.5068	-1
**4574**	321	C	78.6687	0
**4574**	351	C	11.7299	1
**4574**	357	C	79.5724	1
**4574**	370	C	70.2051	1
**4574**	372	C	78.7307	0
**4574**	373	C	24.893	0
**4574**	381	C	75.3059	1
**4574**	382	C	79.5623	1
**4574**	386	C	59.1956	1
**4574**	305	D	76.3731	1
**4574**	319	D	79.5808	1
**4574**	320	D	65.9537	-1
**4574**	321	D	71.8972	0
**4574**	351	D	10.0826	1
**4574**	357	D	63.2116	1
**4574**	370	D	63.9736	1
**4574**	372	D	79.5429	0
**4574**	373	D	62.6609	1
**4574**	381	D	78.7029	1
**4574**	382	D	79.5479	1
**4574**	386	D	66.6037	1

**4575**	305	A	73.5541	1
**4575**	319	A	75.673	1
**4575**	320	A	46.1805	-1
**4575**	321	A	59.0114	0
**4575**	351	A	13.7687	1
**4575**	357	A	64.7811	1
**4575**	370	A	72.4253	1
**4575**	372	A	48.2065	1
**4575**	373	A	70.007	1
**4575**	381	A	68.1799	1
**4575**	382	A	73.1954	1
**4575**	386	A	67.7759	1
**4575**	305	B	73.6049	1
**4575**	319	B	73.0667	1
**4575**	320	B	79.5737	-1
**4575**	321	B	79.6098	0
**4575**	351	B	15.0818	1
**4575**	357	B	67.942	1
**4575**	370	B	69.6795	1
**4575**	372	B	60.4286	0
**4575**	373	B	48.313	0
**4575**	381	B	79.5726	1
**4575**	382	B	79.5859	1
**4575**	386	B	46.7689	0
**4575**	305	C	55.7522	1
**4575**	319	C	79.5147	1
**4575**	320	C	59.4079	-1
**4575**	321	C	74.5541	0
**4575**	351	C	4.478	1
**4575**	357	C	78.3562	1
**4575**	370	C	58.3608	1
**4575**	372	C	60.4836	0
**4575**	373	C	79.6229	1
**4575**	381	C	79.5896	1

**4575**	382	C	47.2853	0
**4575**	386	C	34.2599	0
**4575**	305	D	52.1026	1
**4575**	319	D	79.5688	1
**4575**	320	D	65.804	-1
**4575**	321	D	46.9488	1
**4575**	351	D	15.2131	1
**4575**	357	D	77.6526	1
**4575**	370	D	51.2982	1
**4575**	372	D	67.4477	0
**4575**	373	D	79.5946	1
**4575**	381	D	79.6042	1
**4575**	382	D	42.5761	0
**4575**	386	D	75.5622	1
**4576**	305	A	57.126	1
**4576**	319	A	73.1143	1
**4576**	320	A	74.2032	-1
**4576**	321	A	79.5479	0
**4576**	351	A	0.1216	1
**4576**	357	A	79.4876	1
**4576**	370	A	59.9836	1
**4576**	372	A	34.8087	1
**4576**	373	A	22.2378	0
**4576**	381	A	78.9189	1
**4576**	382	A	30.758	0
**4576**	386	A	72.2205	1
**4576**	305	B	79.6211	1
**4576**	319	B	30.4028	0
**4576**	320	B	69.8022	-1
**4576**	321	B	79.5541	0
**4576**	351	B	10.7456	1
**4576**	357	B	79.708	1
**4576**	370	B	69.1967	1
**4576**	372	B	66.1742	0

**4576**	373	B	57.8635	1
**4576**	381	B	73.0844	1
**4576**	382	B	51.4331	1
**4576**	386	B	53.4566	1
**4576**	305	C	53.352	1
**4576**	319	C	75.7876	1
**4576**	320	C	65.112	-1
**4576**	321	C	48.3042	1
**4576**	351	C	2.5657	1
**4576**	357	C	54.4921	1
**4576**	370	C	78.8366	1
**4576**	372	C	79.389	0
**4576**	373	C	67.8025	1
**4576**	381	C	44.4692	0
**4576**	382	C	42.3795	0
**4576**	386	C	48.6399	0
**4576**	305	D	79.6177	1
**4576**	319	D	75.7015	1
**4576**	320	D	52.0529	-1
**4576**	321	D	17.2169	0
**4576**	351	D	14.9425	1
**4576**	357	D	72.9437	1
**4576**	370	D	79.6709	1
**4576**	372	D	75.494	0
**4576**	373	D	36.2616	0
**4576**	381	D	79.5791	1
**4576**	382	D	79.63	1
**4576**	386	D	79.6693	1
**4577**	305	A	23.158	0
**4577**	319	A	22.052	0
**4577**	320	A	44.4187	-1
**4577**	321	A	78.7532	0
**4577**	351	A	11.269	1
**4577**	357	A	79.6695	1

**4577**	370	A	79.5096	1
**4577**	372	A	73.4407	0
**4577**	373	A	39.5891	0
**4577**	381	A	79.5605	1
**4577**	382	A	59.2922	1
**4577**	386	A	79.3525	1
**4577**	305	B	79.6197	1
**4577**	319	B	59.0949	1
**4577**	320	B	44.8634	-1
**4577**	321	B	70.4774	0
**4577**	351	B	13.9401	1
**4577**	357	B	79.5824	1
**4577**	370	B	79.427	1
**4577**	372	B	78.8851	0
**4577**	373	B	53.1927	1
**4577**	381	B	29.098	0
**4577**	382	B	49.205	0
**4577**	386	B	79.5642	1
**4577**	305	C	51.001	1
**4577**	319	C	79.7259	1
**4577**	320	C	61.8667	-1
**4577**	321	C	79.6667	0
**4577**	351	C	1.5582	1
**4577**	357	C	79.4514	1
**4577**	370	C	69.6001	1
**4577**	372	C	79.5224	0
**4577**	373	C	79.5554	1
**4577**	381	C	79.6507	1
**4577**	382	C	60.1493	1
**4577**	386	C	38.0509	0
**4577**	305	D	77.3821	1
**4577**	319	D	70.961	1
**4577**	320	D	68.2307	-1
**4577**	321	D	79.5424	0

**4577**	351	D	11.3782	1
**4577**	357	D	79.5228	1
**4577**	370	D	79.5376	1
**4577**	372	D	50.2172	0
**4577**	373	D	79.3616	1
**4577**	381	D	45.9468	0
**4577**	382	D	73.6868	1
**4577**	386	D	39.565	0
**4578**	305	A	64.2712	1
**4578**	319	A	71.5126	1
**4578**	320	A	74.1071	-1
**4578**	321	A	21.2482	1
**4578**	351	A	2.2397	1
**4578**	357	A	78.8828	1
**4578**	370	A	67.7235	1
**4578**	372	A	69.2545	0
**4578**	373	A	79.5237	1
**4578**	381	A	71.9665	1
**4578**	382	A	18.6309	-1
**4578**	386	A	79.5906	1
**4578**	305	B	79.0071	1
**4578**	319	B	59.1199	1
**4578**	320	B	52.2601	-1
**4578**	321	B	79.6483	0
**4578**	351	B	3.2476	1
**4578**	357	B	59.3895	1
**4578**	370	B	27.5077	0
**4578**	372	B	79.563	0
**4578**	373	B	72.3907	1
**4578**	381	B	79.5871	1
**4578**	382	B	64.1642	1
**4578**	386	B	74.1686	1
**4578**	305	C	57.5413	1
**4578**	319	C	76.3208	1

**4578**	320	C	73.5236	-1
**4578**	321	C	70.2023	0
**4578**	351	C	32.8682	-1
**4578**	357	C	78.9931	1
**4578**	370	C	33.1335	0
**4578**	372	C	75.3165	0
**4578**	373	C	75.3928	1
**4578**	381	C	59.1016	1
**4578**	382	C	79.5959	1
**4578**	386	C	43.0287	0
**4578**	305	D	77.5404	1
**4578**	319	D	79.5616	1
**4578**	320	D	79.5741	-1
**4578**	321	D	72.8562	0
**4578**	351	D	7.7988	1
**4578**	357	D	32.874	0
**4578**	370	D	78.3792	1
**4578**	372	D	66.9634	0
**4578**	373	D	79.5336	1
**4578**	381	D	79.6629	1
**4578**	382	D	73.8634	1
**4578**	386	D	79.481	1
**4579**	305	A	70.8598	1
**4579**	319	A	71.1933	1
**4579**	320	A	79.4679	-1
**4579**	321	A	31.4211	1
**4579**	351	A	5.6802	1
**4579**	357	A	79.5377	1
**4579**	370	A	58.4808	1
**4579**	372	A	65.7173	0
**4579**	373	A	68.5596	1
**4579**	381	A	79.5653	1
**4579**	382	A	39.3789	0
**4579**	386	A	52.4942	1

**4579**	305	B	79.5055	1
**4579**	319	B	59.7456	1
**4579**	320	B	71.8507	-1
**4579**	321	B	79.585	0
**4579**	351	B	9.2668	1
**4579**	357	B	64.7071	1
**4579**	370	B	43.4458	0
**4579**	372	B	48.7238	1
**4579**	373	B	56.403	1
**4579**	381	B	79.4972	1
**4579**	382	B	36.8813	0
**4579**	386	B	48.1741	0
**4579**	305	C	77.366	1
**4579**	319	C	79.576	1
**4579**	320	C	79.5691	-1
**4579**	321	C	76.416	0
**4579**	351	C	5.2609	1
**4579**	357	C	57.7719	1
**4579**	370	C	79.4929	1
**4579**	372	C	77.4681	0
**4579**	373	C	56.5636	1
**4579**	381	C	79.1786	1
**4579**	382	C	68.6183	1
**4579**	386	C	50.4006	1
**4579**	305	D	72.942	1
**4579**	319	D	68.231	1
**4579**	320	D	59.4186	-1
**4579**	321	D	46.0928	1
**4579**	351	D	10.3524	1
**4579**	357	D	68.949	1
**4579**	370	D	46.6339	0
**4579**	372	D	79.5799	0
**4579**	373	D	39.715	0
**4579**	381	D	63.2214	1

**4579**	382	D	72.7967	1
**4579**	386	D	79.4361	1
**4580**	305	A	42.9412	0
**4580**	319	A	17.2002	-1
**4580**	320	A	79.5932	-1
**4580**	321	A	79.5345	0
**4580**	351	A	3.1778	1
**4580**	357	A	59.0102	1
**4580**	370	A	48.967	0
**4580**	372	A	77.6408	0
**4580**	373	A	79.6205	1
**4580**	381	A	79.4734	1
**4580**	382	A	79.3295	1
**4580**	386	A	79.6464	1
**4580**	305	B	64.0212	1
**4580**	319	B	74.7373	1
**4580**	320	B	73.5101	-1
**4580**	321	B	79.5982	0
**4580**	351	B	12.8418	1
**4580**	357	B	57.6047	1
**4580**	370	B	74.9209	1
**4580**	372	B	79.5965	0
**4580**	373	B	37.7265	0
**4580**	381	B	69.6574	1
**4580**	382	B	37.5304	0
**4580**	386	B	79.6019	1
**4580**	305	C	50.9002	1
**4580**	319	C	79.5642	1
**4580**	320	C	67.2051	-1
**4580**	321	C	79.577	0
**4580**	351	C	15.5785	1
**4580**	357	C	72.2951	1
**4580**	370	C	79.6201	1
**4580**	372	C	69.5691	0

**4580**	373	C	79.4911	1
**4580**	381	C	77.5345	1
**4580**	382	C	25.3942	0
**4580**	386	C	28.4438	0
**4580**	305	D	63.8361	1
**4580**	319	D	79.5777	1
**4580**	320	D	79.5401	-1
**4580**	321	D	63.2615	0
**4580**	351	D	6.8967	1
**4580**	357	D	72.6181	1
**4580**	370	D	67.5684	1
**4580**	372	D	79.6079	0
**4580**	373	D	79.5549	1
**4580**	381	D	68.634	1
**4580**	382	D	72.4912	1
**4580**	386	D	55.0678	1
**4581**	305	A	72.5178	1
**4581**	319	A	76.5626	1
**4581**	320	A	79.566	-1
**4581**	321	A	64.2933	0
**4581**	351	A	5.4394	1
**4581**	357	A	59.0609	1
**4581**	370	A	31.411	0
**4581**	372	A	79.3611	0
**4581**	373	A	76.3425	1
**4581**	381	A	71.703	1
**4581**	382	A	41.1735	0
**4581**	386	A	79.5759	1
**4581**	305	B	59.0131	1
**4581**	319	B	33.2914	0
**4581**	320	B	25.3565	-1
**4581**	321	B	49.274	1
**4581**	351	B	15.0302	1
**4581**	357	B	73.9187	1

**4581**	370	B	36.7486	0
**4581**	372	B	79.5735	0
**4581**	373	B	72.7025	1
**4581**	381	B	73.1222	1
**4581**	382	B	79.4474	1
**4581**	386	B	76.5409	1
**4581**	305	C	79.5682	1
**4581**	319	C	79.5399	1
**4581**	320	C	79.5849	-1
**4581**	321	C	77.4167	0
**4581**	351	C	7.8794	1
**4581**	357	C	60.0714	1
**4581**	370	C	79.5526	1
**4581**	372	C	79.5629	0
**4581**	373	C	62.818	1
**4581**	381	C	79.5638	1
**4581**	382	C	79.5544	1
**4581**	386	C	49.1611	0
**4581**	305	D	73.8565	1
**4581**	319	D	79.5119	1
**4581**	320	D	79.55	-1
**4581**	321	D	74.52	0
**4581**	351	D	13.1419	1
**4581**	357	D	73.3634	1
**4581**	370	D	59.0774	1
**4581**	372	D	79.555	0
**4581**	373	D	53.9911	1
**4581**	381	D	79.5889	1
**4581**	382	D	27.0225	0
**4581**	386	D	79.543	1
**4582**	305	A	28.0232	0
**4582**	319	A	51.7277	1
**4582**	320	A	79.5653	-1
**4582**	321	A	72.8628	0

**4582**	351	A	7.7491	1
**4582**	357	A	67.2567	1
**4582**	370	A	79.5697	1
**4582**	372	A	40.3159	1
**4582**	373	A	49.944	0
**4582**	381	A	72.8635	1
**4582**	382	A	62.2202	1
**4582**	386	A	53.2714	1
**4582**	305	B	79.5233	1
**4582**	319	B	64.959	1
**4582**	320	B	41.6131	-1
**4582**	321	B	55.8152	0
**4582**	351	B	11.1793	1
**4582**	357	B	65.2983	1
**4582**	370	B	79.5356	1
**4582**	372	B	36.4864	1
**4582**	373	B	72.7304	1
**4582**	381	B	77.1569	1
**4582**	382	B	79.2396	1
**4582**	386	B	79.5402	1
**4582**	305	C	77.3438	1
**4582**	319	C	79.501	1
**4582**	320	C	38.6551	-1
**4582**	321	C	79.483	0
**4582**	351	C	3.0942	1
**4582**	357	C	72.9534	1
**4582**	370	C	75.5926	1
**4582**	372	C	73.1642	0
**4582**	373	C	53.1288	1
**4582**	381	C	79.5596	1
**4582**	382	C	79.5471	1
**4582**	386	C	32.1173	0
**4582**	305	D	79.5155	1
**4582**	319	D	79.5513	1

**4582**	320	D	62.34	-1
**4582**	321	D	72.3609	0
**4582**	351	D	10.4409	1
**4582**	357	D	79.5634	1
**4582**	370	D	73.2873	1
**4582**	372	D	79.5528	0
**4582**	373	D	79.5514	1
**4582**	381	D	79.5418	1
**4582**	382	D	79.6045	1
**4582**	386	D	26.0709	0
**4583**	305	A	79.5552	1
**4583**	319	A	67.4034	1
**4583**	320	A	65.2056	-1
**4583**	321	A	79.0756	0
**4583**	351	A	6.5034	1
**4583**	357	A	77.7988	1
**4583**	370	A	79.3145	1
**4583**	372	A	65.7385	0
**4583**	373	A	79.5714	1
**4583**	381	A	67.472	1
**4583**	382	A	74.0173	1
**4583**	386	A	78.1635	1
**4583**	305	B	39.9242	0
**4583**	319	B	53.9869	1
**4583**	320	B	45.5942	-1
**4583**	321	B	35.1813	1
**4583**	351	B	9.4162	1
**4583**	357	B	56.3918	1
**4583**	370	B	76.09	1
**4583**	372	B	79.5614	0
**4583**	373	B	58.4299	1
**4583**	381	B	72.6529	1
**4583**	382	B	16.1769	-1
**4583**	386	B	48.8002	0

**4583**	305	C	79.5701	1
**4583**	319	C	78.0859	1
**4583**	320	C	79.5464	-1
**4583**	321	C	79.5606	0
**4583**	351	C	4.6166	1
**4583**	357	C	70.9137	1
**4583**	370	C	64.8055	1
**4583**	372	C	62.6808	0
**4583**	373	C	51.3329	1
**4583**	381	C	72.3643	1
**4583**	382	C	40.9743	0
**4583**	386	C	31.9306	0
**4583**	305	D	41.1898	0
**4583**	319	D	74.2137	1
**4583**	320	D	71.7413	-1
**4583**	321	D	70.6953	0
**4583**	351	D	10.336	1
**4583**	357	D	73.336	1
**4583**	370	D	71.2228	1
**4583**	372	D	69.4299	0
**4583**	373	D	17.0705	-1
**4583**	381	D	30.073	0
**4583**	382	D	79.565	1
**4583**	386	D	26.8353	0
**4584**	305	A	61.4485	1
**4584**	319	A	39.7521	0
**4584**	320	A	74.4308	-1
**4584**	321	A	68.1355	0
**4584**	351	A	9.6242	1
**4584**	357	A	53.0879	1
**4584**	370	A	79.5654	1
**4584**	372	A	72.8575	0
**4584**	373	A	66.6651	1
**4584**	381	A	79.5626	1

**4584**	382	A	72.1018	1
**4584**	386	A	61.9842	1
**4584**	305	B	79.557	1
**4584**	319	B	61.3938	1
**4584**	320	B	73.7009	-1
**4584**	321	B	23.1986	1
**4584**	351	B	17.4535	1
**4584**	357	B	56.7398	1
**4584**	370	B	65.2366	1
**4584**	372	B	75.5691	0
**4584**	373	B	79.4208	1
**4584**	381	B	79.535	1
**4584**	382	B	76.9679	1
**4584**	386	B	71.7788	1
**4584**	305	C	78.5354	1
**4584**	319	C	38.2141	0
**4584**	320	C	53.2102	-1
**4584**	321	C	63.5682	0
**4584**	351	C	8.4715	1
**4584**	357	C	63.3385	1
**4584**	370	C	79.5497	1
**4584**	372	C	79.5641	0
**4584**	373	C	79.5757	1
**4584**	381	C	44.2513	0
**4584**	382	C	55.6912	1
**4584**	386	C	30.7855	0
**4584**	305	D	79.6965	1
**4584**	319	D	63.5042	1
**4584**	320	D	61.3094	-1
**4584**	321	D	75.6782	0
**4584**	351	D	14.0128	1
**4584**	357	D	79.5709	1
**4584**	370	D	64.8137	1
**4584**	372	D	79.571	0

**4584**	373	D	31.6631	0
**4584**	381	D	33.1099	0
**4584**	382	D	77.4705	1
**4584**	386	D	65.3035	1
**4585**	305	A	79.4796	1
**4585**	319	A	79.555	1
**4585**	320	A	57.9668	-1
**4585**	321	A	77.9817	0
**4585**	351	A	4.514	1
**4585**	357	A	72.2835	1
**4585**	370	A	79.3965	1
**4585**	372	A	79.6296	0
**4585**	373	A	28.2736	0
**4585**	381	A	79.6722	1
**4585**	382	A	65.4964	1
**4585**	386	A	39.1969	0
**4585**	305	B	63.1816	1
**4585**	319	B	36.609	0
**4585**	320	B	51.9409	-1
**4585**	321	B	72.8371	0
**4585**	351	B	16.1983	1
**4585**	357	B	65.7108	1
**4585**	370	B	79.5318	1
**4585**	372	B	64.007	0
**4585**	373	B	68.4481	1
**4585**	381	B	56.7894	1
**4585**	382	B	61.5328	1
**4585**	386	B	68.1728	1
**4585**	305	C	73.8444	1
**4585**	319	C	79.6619	1
**4585**	320	C	37.8685	-1
**4585**	321	C	68.3488	0
**4585**	351	C	6.6691	1
**4585**	357	C	76.4554	1

**4585**	370	C	79.5853	1
**4585**	372	C	79.4852	0
**4585**	373	C	79.622	1
**4585**	381	C	66.0756	1
**4585**	382	C	28.7383	0
**4585**	386	C	37.6406	0
**4585**	305	D	59.8301	1
**4585**	319	D	73.2828	1
**4585**	320	D	79.4491	-1
**4585**	321	D	38.972	1
**4585**	351	D	2.7998	1
**4585**	357	D	78.0945	1
**4585**	370	D	66.3462	1
**4585**	372	D	68.5906	0
**4585**	373	D	63.5459	1
**4585**	381	D	56.1395	1
**4585**	382	D	66.6325	1
**4585**	386	D	79.516	1
**4586**	305	A	58.5896	1
**4586**	319	A	73.1133	1
**4586**	320	A	79.6208	-1
**4586**	321	A	61.8243	0
**4586**	351	A	9.212	1
**4586**	357	A	51.8358	1
**4586**	370	A	79.6031	1
**4586**	372	A	69.0612	0
**4586**	373	A	27.5184	0
**4586**	381	A	79.6275	1
**4586**	382	A	20.6943	0
**4586**	386	A	42.9747	0
**4586**	305	B	75.1478	1
**4586**	319	B	68.5118	1
**4586**	320	B	24.8703	-1
**4586**	321	B	72.2494	0

**4586**	351	B	40.3249	-1
**4586**	357	B	70.4413	1
**4586**	370	B	79.5041	1
**4586**	372	B	25.707	1
**4586**	373	B	69.5442	1
**4586**	381	B	79.647	1
**4586**	382	B	40.1564	0
**4586**	386	B	78.667	1
**4586**	305	C	79.5369	1
**4586**	319	C	79.45	1
**4586**	320	C	26.3229	-1
**4586**	321	C	68.1062	0
**4586**	351	C	16.4532	1
**4586**	357	C	62.8206	1
**4586**	370	C	60.9437	1
**4586**	372	C	57.5041	0
**4586**	373	C	76.6924	1
**4586**	381	C	70.5088	1
**4586**	382	C	72.0379	1
**4586**	386	C	52.5625	1
**4586**	305	D	70.7707	1
**4586**	319	D	65.3329	1
**4586**	320	D	79.4009	-1
**4586**	321	D	55.1647	0
**4586**	351	D	10.9938	1
**4586**	357	D	69.1956	1
**4586**	370	D	43.7837	0
**4586**	372	D	42.2682	1
**4586**	373	D	58.8189	1
**4586**	381	D	79.0041	1
**4586**	382	D	64.6079	1
**4586**	386	D	68.3343	1
**4587**	305	A	79.5813	1
**4587**	319	A	67.4466	1

**4587**	320	A	79.5632	-1
**4587**	321	A	68.611	0
**4587**	351	A	6.7575	1
**4587**	357	A	53.6951	1
**4587**	370	A	45.0865	0
**4587**	372	A	79.5676	0
**4587**	373	A	79.4683	1
**4587**	381	A	78.9398	1
**4587**	382	A	53.8774	1
**4587**	386	A	75.1113	1
**4587**	305	B	62.8559	1
**4587**	319	B	54.4193	1
**4587**	320	B	39.9406	-1
**4587**	321	B	72.4418	0
**4587**	351	B	7.6984	1
**4587**	357	B	79.5219	1
**4587**	370	B	70.6779	1
**4587**	372	B	70.5618	0
**4587**	373	B	46.3335	0
**4587**	381	B	64.7856	1
**4587**	382	B	36.0774	0
**4587**	386	B	70.6379	1
**4587**	305	C	58.3946	1
**4587**	319	C	74.1364	1
**4587**	320	C	76.6598	-1
**4587**	321	C	75.7083	0
**4587**	351	C	2.2248	1
**4587**	357	C	79.5953	1
**4587**	370	C	73.7767	1
**4587**	372	C	77.5402	0
**4587**	373	C	33.8354	0
**4587**	381	C	60.4395	1
**4587**	382	C	51.6671	1
**4587**	386	C	65.94	1

**4587**	305	D	57.4795	1
**4587**	319	D	55.4848	1
**4587**	320	D	79.5934	-1
**4587**	321	D	64.16	0
**4587**	351	D	11.4382	1
**4587**	357	D	67.1939	1
**4587**	370	D	73.3284	1
**4587**	372	D	79.5844	0
**4587**	373	D	79.5797	1
**4587**	381	D	41.4915	0
**4587**	382	D	79.6123	1
**4587**	386	D	79.5363	1
**4588**	305	A	52.7175	1
**4588**	319	A	46.2226	0
**4588**	320	A	72.4764	-1
**4588**	321	A	78.9996	0
**4588**	351	A	35.2388	-1
**4588**	357	A	72.9629	1
**4588**	370	A	78.5437	1
**4588**	372	A	79.6334	0
**4588**	373	A	66.1874	1
**4588**	381	A	50.2702	1
**4588**	382	A	64.6293	1
**4588**	386	A	74.579	1
**4588**	305	B	79.3642	1
**4588**	319	B	79.4535	1
**4588**	320	B	79.5405	-1
**4588**	321	B	72.6287	0
**4588**	351	B	15.6325	1
**4588**	357	B	76.4923	1
**4588**	370	B	66.7317	1
**4588**	372	B	77.4155	0
**4588**	373	B	59.3929	1
**4588**	381	B	72.1621	1

**4588**	382	B	79.5263	1
**4588**	386	B	48.325	0
**4588**	305	C	60.0508	1
**4588**	319	C	79.558	1
**4588**	320	C	55.4847	-1
**4588**	321	C	57.0155	0
**4588**	351	C	12.1954	1
**4588**	357	C	79.4798	1
**4588**	370	C	44.685	0
**4588**	372	C	70.7183	0
**4588**	373	C	79.5732	1
**4588**	381	C	65.4007	1
**4588**	382	C	51.8209	1
**4588**	386	C	73.5572	1
**4588**	305	D	64.8497	1
**4588**	319	D	79.5749	1
**4588**	320	D	73.5547	-1
**4588**	321	D	52.993	0
**4588**	351	D	13.4076	1
**4588**	357	D	38.2739	0
**4588**	370	D	79.6159	1
**4588**	372	D	73.3455	0
**4588**	373	D	68.951	1
**4588**	381	D	51.2026	1
**4588**	382	D	79.654	1
**4588**	386	D	59.282	1
**4589**	305	A	66.9952	1
**4589**	319	A	68.0306	1
**4589**	320	A	79.4931	-1
**4589**	321	A	63.0804	0
**4589**	351	A	11.4522	1
**4589**	357	A	44.6085	0
**4589**	370	A	73.2273	1
**4589**	372	A	79.658	0

**4589**	373	A	59.1474	1
**4589**	381	A	63.8056	1
**4589**	382	A	17.889	-1
**4589**	386	A	62.2904	1
**4589**	305	B	78.3507	1
**4589**	319	B	56.583	1
**4589**	320	B	52.6281	-1
**4589**	321	B	18.4163	0
**4589**	351	B	15.3005	1
**4589**	357	B	76.1757	1
**4589**	370	B	79.2663	1
**4589**	372	B	79.6189	0
**4589**	373	B	55.4296	1
**4589**	381	B	68.4601	1
**4589**	382	B	61.5321	1
**4589**	386	B	39.3789	0
**4589**	305	C	46.8059	0
**4589**	319	C	72.0385	1
**4589**	320	C	60.1456	-1
**4589**	321	C	79.579	0
**4589**	351	C	8.042	1
**4589**	357	C	61.7823	1
**4589**	370	C	79.5971	1
**4589**	372	C	55.7035	0
**4589**	373	C	46.3182	0
**4589**	381	C	39.3322	0
**4589**	382	C	79.5787	1
**4589**	386	C	53.6883	1
**4589**	305	D	79.5515	1
**4589**	319	D	72.4041	1
**4589**	320	D	78.2103	-1
**4589**	321	D	64.9763	0
**4589**	351	D	17.7557	1
**4589**	357	D	79.468	1

**4589**	370	D	79.5104	1
**4589**	372	D	48.7014	1
**4589**	373	D	73.4078	1
**4589**	381	D	64.0183	1
**4589**	382	D	74.9538	1
**4589**	386	D	63.439	1
**4590**	305	A	78.4211	1
**4590**	319	A	79.5735	1
**4590**	320	A	16.4505	1
**4590**	321	A	35.7563	1
**4590**	351	A	0.0	1
**4590**	357	A	79.5669	1
**4590**	370	A	79.6004	1
**4590**	372	A	78.3533	0
**4590**	373	A	79.5167	1
**4590**	381	A	70.6835	1
**4590**	382	A	79.5652	1
**4590**	386	A	55.9764	1
**4590**	305	B	73.4891	1
**4590**	319	B	79.4371	1
**4590**	320	B	69.7388	-1
**4590**	321	B	71.4308	0
**4590**	351	B	14.2079	1
**4590**	357	B	64.7046	1
**4590**	370	B	79.5648	1
**4590**	372	B	79.5635	0
**4590**	373	B	71.2852	1
**4590**	381	B	72.4981	1
**4590**	382	B	50.3408	1
**4590**	386	B	59.1552	1
**4590**	305	C	72.2914	1
**4590**	319	C	79.5207	1
**4590**	320	C	79.5525	-1
**4590**	321	C	76.6994	0

**4590**	351	C	5.1508	1
**4590**	357	C	69.0346	1
**4590**	370	C	79.5775	1
**4590**	372	C	79.5724	0
**4590**	373	C	79.5397	1
**4590**	381	C	79.5429	1
**4590**	382	C	35.3178	0
**4590**	386	C	32.4857	0
**4590**	305	D	64.0214	1
**4590**	319	D	79.6034	1
**4590**	320	D	59.8694	-1
**4590**	321	D	77.28	0
**4590**	351	D	14.0343	1
**4590**	357	D	73.0217	1
**4590**	370	D	74.6905	1
**4590**	372	D	79.5701	0
**4590**	373	D	79.5664	1
**4590**	381	D	30.4782	0
**4590**	382	D	79.5661	1
**4590**	386	D	71.4356	1
**4591**	305	A	69.3612	1
**4591**	319	A	76.079	1
**4591**	320	A	79.6051	-1
**4591**	321	A	49.639	1
**4591**	351	A	0.94	1
**4591**	357	A	79.5842	1
**4591**	370	A	60.4743	1
**4591**	372	A	74.6307	0
**4591**	373	A	79.5781	1
**4591**	381	A	79.5706	1
**4591**	382	A	62.7957	1
**4591**	386	A	52.8747	1
**4591**	305	B	71.3285	1
**4591**	319	B	79.3748	1

**4591**	320	B	70.9045	-1
**4591**	321	B	67.1239	0
**4591**	351	B	17.5809	1
**4591**	357	B	67.3322	1
**4591**	370	B	47.1261	0
**4591**	372	B	74.1197	0
**4591**	373	B	63.6862	1
**4591**	381	B	64.1804	1
**4591**	382	B	69.0688	1
**4591**	386	B	58.5956	1
**4591**	305	C	67.5117	1
**4591**	319	C	58.9963	1
**4591**	320	C	79.4781	-1
**4591**	321	C	72.9126	0
**4591**	351	C	3.814	1
**4591**	357	C	79.5099	1
**4591**	370	C	66.8859	1
**4591**	372	C	79.5955	0
**4591**	373	C	45.4625	0
**4591**	381	C	68.0304	1
**4591**	382	C	73.1873	1
**4591**	386	C	37.9494	0
**4591**	305	D	42.1238	0
**4591**	319	D	79.5681	1
**4591**	320	D	79.5376	-1
**4591**	321	D	74.1382	0
**4591**	351	D	11.613	1
**4591**	357	D	79.5653	1
**4591**	370	D	64.2726	1
**4591**	372	D	68.8289	0
**4591**	373	D	63.4417	1
**4591**	381	D	75.5173	1
**4591**	382	D	79.5263	1
**4591**	386	D	72.1274	1

**4592**	305	A	73.1265	1
**4592**	319	A	73.864	1
**4592**	320	A	79.566	-1
**4592**	321	A	32.4411	1
**4592**	351	A	12.3683	1
**4592**	357	A	73.1501	1
**4592**	370	A	45.7539	0
**4592**	372	A	30.5193	1
**4592**	373	A	12.5338	-1
**4592**	381	A	77.7407	1
**4592**	382	A	49.8911	0
**4592**	386	A	79.5789	1
**4592**	305	B	63.3353	1
**4592**	319	B	79.6165	1
**4592**	320	B	73.3821	-1
**4592**	321	B	62.7456	0
**4592**	351	B	15.5147	1
**4592**	357	B	79.5824	1
**4592**	370	B	72.5275	1
**4592**	372	B	47.137	1
**4592**	373	B	49.6196	0
**4592**	381	B	72.4437	1
**4592**	382	B	36.9382	0
**4592**	386	B	79.6186	1
**4592**	305	C	79.5668	1
**4592**	319	C	79.5268	1
**4592**	320	C	79.5138	-1
**4592**	321	C	79.031	0
**4592**	351	C	3.2675	1
**4592**	357	C	73.6019	1
**4592**	370	C	52.0107	1
**4592**	372	C	72.1769	0
**4592**	373	C	79.6523	1
**4592**	381	C	64.4579	1

**4592**	382	C	79.5146	1
**4592**	386	C	7.8929	-1
**4592**	305	D	79.5865	1
**4592**	319	D	79.5228	1
**4592**	320	D	78.3844	-1
**4592**	321	D	74.1384	0
**4592**	351	D	14.0829	1
**4592**	357	D	39.417	0
**4592**	370	D	47.4856	0
**4592**	372	D	73.1691	0
**4592**	373	D	79.5205	1
**4592**	381	D	79.5381	1
**4592**	382	D	52.3642	1
**4592**	386	D	79.673	1
**4593**	305	A	73.5476	1
**4593**	319	A	60.3136	1
**4593**	320	A	72.6986	-1
**4593**	321	A	44.2743	1
**4593**	351	A	4.9905	1
**4593**	357	A	75.8673	1
**4593**	370	A	79.6075	1
**4593**	372	A	73.7533	0
**4593**	373	A	37.5722	0
**4593**	381	A	79.6254	1
**4593**	382	A	79.5945	1
**4593**	386	A	79.2897	1
**4593**	305	B	57.2682	1
**4593**	319	B	76.482	1
**4593**	320	B	79.5653	-1
**4593**	321	B	46.1429	1
**4593**	351	B	2.5364	1
**4593**	357	B	59.9602	1
**4593**	370	B	74.4228	1
**4593**	372	B	54.5568	0

**4593**	373	B	50.2268	1
**4593**	381	B	79.5055	1
**4593**	382	B	26.9301	0
**4593**	386	B	53.3166	1
**4593**	305	C	31.1026	0
**4593**	319	C	79.6114	1
**4593**	320	C	48.6985	-1
**4593**	321	C	54.142	0
**4593**	351	C	10.8073	1
**4593**	357	C	22.6703	0
**4593**	370	C	73.1383	1
**4593**	372	C	46.2727	1
**4593**	373	C	79.6222	1
**4593**	381	C	39.1281	0
**4593**	382	C	79.4398	1
**4593**	386	C	54.9517	1
**4593**	305	D	66.336	1
**4593**	319	D	56.8407	1
**4593**	320	D	62.2884	-1
**4593**	321	D	72.7251	0
**4593**	351	D	3.1712	1
**4593**	357	D	59.3426	1
**4593**	370	D	62.8908	1
**4593**	372	D	72.6059	0
**4593**	373	D	71.8956	1
**4593**	381	D	79.0366	1
**4593**	382	D	60.4862	1
**4593**	386	D	58.757	1
**4594**	305	A	79.6418	1
**4594**	319	A	59.6193	1
**4594**	320	A	71.5048	-1
**4594**	321	A	51.1384	0
**4594**	351	A	2.4346	1
**4594**	357	A	79.5507	1

**4594**	370	A	37.572	0
**4594**	372	A	53.9599	0
**4594**	373	A	58.6403	1
**4594**	381	A	71.0043	1
**4594**	382	A	39.0836	0
**4594**	386	A	66.4507	1
**4594**	305	B	60.3872	1
**4594**	319	B	65.1756	1
**4594**	320	B	77.0568	-1
**4594**	321	B	39.9184	1
**4594**	351	B	23.5242	-1
**4594**	357	B	72.8037	1
**4594**	370	B	72.3104	1
**4594**	372	B	48.4876	1
**4594**	373	B	79.468	1
**4594**	381	B	50.1343	1
**4594**	382	B	26.4484	0
**4594**	386	B	79.4769	1
**4594**	305	C	72.8844	1
**4594**	319	C	49.1514	0
**4594**	320	C	79.5754	-1
**4594**	321	C	71.6815	0
**4594**	351	C	17.5015	1
**4594**	357	C	79.5824	1
**4594**	370	C	61.629	1
**4594**	372	C	17.8169	0
**4594**	373	C	79.549	1
**4594**	381	C	51.6576	1
**4594**	382	C	60.6584	1
**4594**	386	C	48.8654	0
**4594**	305	D	79.6438	1
**4594**	319	D	46.6466	0
**4594**	320	D	69.907	-1
**4594**	321	D	79.5571	0

**4594**	351	D	8.8377	1
**4594**	357	D	65.5521	1
**4594**	370	D	50.67	1
**4594**	372	D	73.574	0
**4594**	373	D	78.8913	1
**4594**	381	D	79.6484	1
**4594**	382	D	21.1867	0
**4594**	386	D	62.6713	1
**4595**	305	A	44.1931	0
**4595**	319	A	21.752	0
**4595**	320	A	53.0231	-1
**4595**	321	A	72.2224	0
**4595**	351	A	0.4984	1
**4595**	357	A	53.9963	1
**4595**	370	A	75.6024	1
**4595**	372	A	44.0543	1
**4595**	373	A	57.4297	1
**4595**	381	A	71.3842	1
**4595**	382	A	79.5529	1
**4595**	386	A	33.869	0
**4595**	305	B	69.7212	1
**4595**	319	B	56.5187	1
**4595**	320	B	74.2717	-1
**4595**	321	B	79.5464	0
**4595**	351	B	8.3464	1
**4595**	357	B	79.5753	1
**4595**	370	B	39.7992	0
**4595**	372	B	79.5613	0
**4595**	373	B	70.7879	1
**4595**	381	B	72.1013	1
**4595**	382	B	21.3182	0
**4595**	386	B	60.1455	1
**4595**	305	C	79.0223	1
**4595**	319	C	77.8554	1

**4595**	320	C	79.5845	-1
**4595**	321	C	79.6015	0
**4595**	351	C	1.6745	1
**4595**	357	C	58.6466	1
**4595**	370	C	69.9277	1
**4595**	372	C	79.5833	0
**4595**	373	C	66.701	1
**4595**	381	C	79.5754	1
**4595**	382	C	54.2389	1
**4595**	386	C	60.8937	1
**4595**	305	D	78.8862	1
**4595**	319	D	78.8857	1
**4595**	320	D	59.071	-1
**4595**	321	D	53.4273	0
**4595**	351	D	2.6536	1
**4595**	357	D	79.5611	1
**4595**	370	D	79.6089	1
**4595**	372	D	66.568	0
**4595**	373	D	50.849	1
**4595**	381	D	76.7109	1
**4595**	382	D	72.1524	1
**4595**	386	D	79.5251	1
**4596**	305	A	53.5861	1
**4596**	319	A	72.1909	1
**4596**	320	A	79.4894	-1
**4596**	321	A	45.9703	1
**4596**	351	A	15.6966	1
**4596**	357	A	79.6283	1
**4596**	370	A	65.2321	1
**4596**	372	A	60.4117	0
**4596**	373	A	42.4243	0
**4596**	381	A	63.4147	1
**4596**	382	A	64.7589	1
**4596**	386	A	51.4257	1

**4596**	305	B	77.6569	1
**4596**	319	B	79.5798	1
**4596**	320	B	69.892	-1
**4596**	321	B	69.8373	0
**4596**	351	B	17.7313	1
**4596**	357	B	79.4815	1
**4596**	370	B	79.5637	1
**4596**	372	B	79.4989	0
**4596**	373	B	75.6358	1
**4596**	381	B	79.526	1
**4596**	382	B	74.8602	1
**4596**	386	B	75.0241	1
**4596**	305	C	62.1656	1
**4596**	319	C	79.6607	1
**4596**	320	C	79.6477	-1
**4596**	321	C	79.5321	0
**4596**	351	C	5.3494	1
**4596**	357	C	70.9922	1
**4596**	370	C	79.5513	1
**4596**	372	C	79.391	0
**4596**	373	C	79.6837	1
**4596**	381	C	70.7092	1
**4596**	382	C	79.2483	1
**4596**	386	C	52.9826	1
**4596**	305	D	40.5779	0
**4596**	319	D	66.9196	1
**4596**	320	D	55.2182	-1
**4596**	321	D	12.29	0
**4596**	351	D	12.4717	1
**4596**	357	D	79.566	1
**4596**	370	D	48.1886	0
**4596**	372	D	77.4524	0
**4596**	373	D	39.2673	0
**4596**	381	D	76.9921	1

**4596**	382	D	69.8757	1
**4596**	386	D	79.5652	1
**4597**	305	A	49.1153	0
**4597**	319	A	37.1318	0
**4597**	320	A	79.6266	-1
**4597**	321	A	78.8467	0
**4597**	351	A	11.256	1
**4597**	357	A	66.3436	1
**4597**	370	A	71.5402	1
**4597**	372	A	79.5185	0
**4597**	373	A	77.6531	1
**4597**	381	A	48.2535	0
**4597**	382	A	71.5168	1
**4597**	386	A	29.4298	0
**4597**	305	B	58.0021	1
**4597**	319	B	72.6541	1
**4597**	320	B	67.2183	-1
**4597**	321	B	79.6574	0
**4597**	351	B	12.8403	1
**4597**	357	B	79.6063	1
**4597**	370	B	71.5083	1
**4597**	372	B	79.6722	0
**4597**	373	B	65.7319	1
**4597**	381	B	79.6728	1
**4597**	382	B	39.3595	0
**4597**	386	B	77.0407	1
**4597**	305	C	79.5945	1
**4597**	319	C	70.1771	1
**4597**	320	C	50.8241	-1
**4597**	321	C	61.0973	0
**4597**	351	C	10.7031	1
**4597**	357	C	79.661	1
**4597**	370	C	79.6114	1
**4597**	372	C	69.5208	0

**4597**	373	C	42.5168	0
**4597**	381	C	79.5452	1
**4597**	382	C	69.0642	1
**4597**	386	C	36.4255	0
**4597**	305	D	79.5817	1
**4597**	319	D	52.7566	1
**4597**	320	D	79.5106	-1
**4597**	321	D	65.6373	0
**4597**	351	D	12.0943	1
**4597**	357	D	35.4305	0
**4597**	370	D	79.4197	1
**4597**	372	D	47.0754	1
**4597**	373	D	71.4994	1
**4597**	381	D	77.8989	1
**4597**	382	D	63.3083	1
**4597**	386	D	74.1377	1
**4598**	305	A	70.6922	1
**4598**	319	A	75.4494	1
**4598**	320	A	65.8563	-1
**4598**	321	A	73.6664	0
**4598**	351	A	0.8812	1
**4598**	357	A	74.0709	1
**4598**	370	A	73.916	1
**4598**	372	A	73.4038	0
**4598**	373	A	65.2643	1
**4598**	381	A	73.5187	1
**4598**	382	A	76.5469	1
**4598**	386	A	53.3086	1
**4598**	305	B	79.5492	1
**4598**	319	B	77.7945	1
**4598**	320	B	78.3268	-1
**4598**	321	B	79.5246	0
**4598**	351	B	44.6641	-1
**4598**	357	B	71.2372	1

**4598**	370	B	45.4391	0
**4598**	372	B	71.027	0
**4598**	373	B	56.4057	1
**4598**	381	B	79.6578	1
**4598**	382	B	37.667	0
**4598**	386	B	79.6143	1
**4598**	305	C	41.1103	0
**4598**	319	C	53.417	1
**4598**	320	C	79.3548	-1
**4598**	321	C	72.1998	0
**4598**	351	C	10.6303	1
**4598**	357	C	72.7571	1
**4598**	370	C	69.8309	1
**4598**	372	C	79.3922	0
**4598**	373	C	79.4961	1
**4598**	381	C	79.4673	1
**4598**	382	C	69.2463	1
**4598**	386	C	48.2089	0
**4598**	305	D	57.2908	1
**4598**	319	D	58.3517	1
**4598**	320	D	43.6135	-1
**4598**	321	D	74.1311	0
**4598**	351	D	22.5305	-1
**4598**	357	D	79.446	1
**4598**	370	D	40.165	0
**4598**	372	D	79.5784	0
**4598**	373	D	53.7904	1
**4598**	381	D	63.3023	1
**4598**	382	D	78.5522	1
**4598**	386	D	79.5227	1
**4599**	305	A	63.7189	1
**4599**	319	A	60.2083	1
**4599**	320	A	79.5943	-1
**4599**	321	A	79.5683	0

**4599**	351	A	2.2868	1
**4599**	357	A	68.1737	1
**4599**	370	A	79.6001	1
**4599**	372	A	79.5522	0
**4599**	373	A	79.6013	1
**4599**	381	A	73.4503	1
**4599**	382	A	76.8496	1
**4599**	386	A	79.2974	1
**4599**	305	B	79.6203	1
**4599**	319	B	78.6809	1
**4599**	320	B	16.8582	1
**4599**	321	B	71.6217	0
**4599**	351	B	21.8045	-1
**4599**	357	B	58.0881	1
**4599**	370	B	60.2051	1
**4599**	372	B	73.2605	0
**4599**	373	B	43.6116	0
**4599**	381	B	30.2979	0
**4599**	382	B	38.5856	0
**4599**	386	B	63.0268	1
**4599**	305	C	79.4113	1
**4599**	319	C	73.9347	1
**4599**	320	C	36.7675	-1
**4599**	321	C	63.2735	0
**4599**	351	C	9.4983	1
**4599**	357	C	72.9771	1
**4599**	370	C	73.0788	1
**4599**	372	C	79.506	0
**4599**	373	C	59.6876	1
**4599**	381	C	79.5707	1
**4599**	382	C	79.4735	1
**4599**	386	C	42.9014	0
**4599**	305	D	72.5145	1
**4599**	319	D	72.7548	1

**4599**	320	D	58.2736	-1
**4599**	321	D	59.2325	0
**4599**	351	D	19.5263	1
**4599**	357	D	44.837	0
**4599**	370	D	79.5583	1
**4599**	372	D	35.6127	1
**4599**	373	D	54.5894	1
**4599**	381	D	57.6517	1
**4599**	382	D	79.5509	1
**4599**	386	D	79.556	1
**4600**	305	A	51.1759	1
**4600**	319	A	73.2114	1
**4600**	320	A	79.5736	-1
**4600**	321	A	72.6881	0
**4600**	351	A	8.5656	1
**4600**	357	A	79.4563	1
**4600**	370	A	79.5602	1
**4600**	372	A	79.5542	0
**4600**	373	A	79.5521	1
**4600**	381	A	79.5821	1
**4600**	382	A	69.968	1
**4600**	386	A	55.4055	1
**4600**	305	B	72.7518	1
**4600**	319	B	65.5165	1
**4600**	320	B	73.6105	-1
**4600**	321	B	55.5033	0
**4600**	351	B	14.6026	1
**4600**	357	B	79.5624	1
**4600**	370	B	47.7847	0
**4600**	372	B	72.8564	0
**4600**	373	B	63.1568	1
**4600**	381	B	79.5584	1
**4600**	382	B	48.7476	0
**4600**	386	B	79.4035	1

**4600**	305	C	61.5946	1
**4600**	319	C	72.0764	1
**4600**	320	C	60.1665	-1
**4600**	321	C	45.8429	1
**4600**	351	C	5.1856	1
**4600**	357	C	50.0585	1
**4600**	370	C	57.4477	1
**4600**	372	C	65.301	0
**4600**	373	C	54.8567	1
**4600**	381	C	72.2899	1
**4600**	382	C	79.4848	1
**4600**	386	C	34.2857	0
**4600**	305	D	29.1566	0
**4600**	319	D	56.8463	1
**4600**	320	D	73.3952	-1
**4600**	321	D	42.5318	1
**4600**	351	D	13.3086	1
**4600**	357	D	76.9849	1
**4600**	370	D	71.0509	1
**4600**	372	D	78.8308	0
**4600**	373	D	79.5767	1
**4600**	381	D	71.8972	1
**4600**	382	D	79.5395	1
**4600**	386	D	77.7058	1
**4601**	305	A	79.5212	1
**4601**	319	A	72.5054	1
**4601**	320	A	79.5586	-1
**4601**	321	A	55.3854	0
**4601**	351	A	4.5362	1
**4601**	357	A	66.652	1
**4601**	370	A	47.0444	0
**4601**	372	A	77.969	0
**4601**	373	A	79.4245	1
**4601**	381	A	79.2855	1

**4601**	382	A	79.5246	1
**4601**	386	A	48.6514	0
**4601**	305	B	79.5851	1
**4601**	319	B	49.176	0
**4601**	320	B	73.9694	-1
**4601**	321	B	79.5421	0
**4601**	351	B	10.1528	1
**4601**	357	B	79.55	1
**4601**	370	B	79.5861	1
**4601**	372	B	79.5003	0
**4601**	373	B	66.046	1
**4601**	381	B	62.859	1
**4601**	382	B	37.1872	0
**4601**	386	B	79.3148	1
**4601**	305	C	64.7808	1
**4601**	319	C	75.8001	1
**4601**	320	C	40.3423	-1
**4601**	321	C	71.5623	0
**4601**	351	C	4.2537	1
**4601**	357	C	79.5135	1
**4601**	370	C	62.4392	1
**4601**	372	C	79.4983	0
**4601**	373	C	62.2844	1
**4601**	381	C	31.281	0
**4601**	382	C	65.5455	1
**4601**	386	C	37.0789	0
**4601**	305	D	47.7976	0
**4601**	319	D	80.0299	1
**4601**	320	D	79.4141	-1
**4601**	321	D	31.52	1
**4601**	351	D	14.0353	1
**4601**	357	D	53.5873	1
**4601**	370	D	74.708	1
**4601**	372	D	67.3814	0

**4601**	373	D	79.5697	1
**4601**	381	D	79.4745	1
**4601**	382	D	72.3263	1
**4601**	386	D	74.9358	1
**4602**	305	A	20.8982	0
**4602**	319	A	51.54	1
**4602**	320	A	79.5137	-1
**4602**	321	A	59.5563	0
**4602**	351	A	2.288	1
**4602**	357	A	79.5446	1
**4602**	370	A	63.4709	1
**4602**	372	A	68.0838	0
**4602**	373	A	77.896	1
**4602**	381	A	79.5687	1
**4602**	382	A	70.8249	1
**4602**	386	A	79.6307	1
**4602**	305	B	79.5696	1
**4602**	319	B	79.6059	1
**4602**	320	B	38.8676	-1
**4602**	321	B	73.3939	0
**4602**	351	B	11.6511	1
**4602**	357	B	79.5587	1
**4602**	370	B	49.0493	0
**4602**	372	B	44.0668	1
**4602**	373	B	78.6399	1
**4602**	381	B	72.9073	1
**4602**	382	B	27.4105	0
**4602**	386	B	15.1365	-1
**4602**	305	C	73.3879	1
**4602**	319	C	51.3181	1
**4602**	320	C	79.5561	-1
**4602**	321	C	26.8997	1
**4602**	351	C	6.712	1
**4602**	357	C	79.5468	1

**4602**	370	C	79.5276	1
**4602**	372	C	72.983	0
**4602**	373	C	79.2691	1
**4602**	381	C	79.6133	1
**4602**	382	C	12.4138	-1
**4602**	386	C	37.8947	0
**4602**	305	D	69.2983	1
**4602**	319	D	54.7069	1
**4602**	320	D	45.4614	-1
**4602**	321	D	29.5099	1
**4602**	351	D	14.3117	1
**4602**	357	D	79.5566	1
**4602**	370	D	73.5566	1
**4602**	372	D	71.277	0
**4602**	373	D	71.738	1
**4602**	381	D	79.6068	1
**4602**	382	D	51.5506	1
**4602**	386	D	72.4064	1
**4603**	305	A	76.4221	1
**4603**	319	A	14.1772	-1
**4603**	320	A	74.8208	-1
**4603**	321	A	43.5725	1
**4603**	351	A	15.8144	1
**4603**	357	A	79.5914	1
**4603**	370	A	79.6032	1
**4603**	372	A	48.5318	1
**4603**	373	A	73.126	1
**4603**	381	A	71.9651	1
**4603**	382	A	40.9305	0
**4603**	386	A	78.8171	1
**4603**	305	B	71.6748	1
**4603**	319	B	79.5682	1
**4603**	320	B	79.3093	-1
**4603**	321	B	54.7846	0

**4603**	351	B	10.2441	1
**4603**	357	B	79.5452	1
**4603**	370	B	33.2123	0
**4603**	372	B	60.9478	0
**4603**	373	B	39.1019	0
**4603**	381	B	73.7582	1
**4603**	382	B	30.7978	0
**4603**	386	B	65.7278	1
**4603**	305	C	78.5231	1
**4603**	319	C	79.5644	1
**4603**	320	C	45.2528	-1
**4603**	321	C	66.9581	0
**4603**	351	C	8.1148	1
**4603**	357	C	74.5903	1
**4603**	370	C	79.5526	1
**4603**	372	C	58.6172	0
**4603**	373	C	57.3772	1
**4603**	381	C	71.967	1
**4603**	382	C	61.9211	1
**4603**	386	C	18.9036	-1
**4603**	305	D	17.9884	-1
**4603**	319	D	74.0905	1
**4603**	320	D	36.1923	-1
**4603**	321	D	76.6797	0
**4603**	351	D	0.0	1
**4603**	357	D	39.7089	0
**4603**	370	D	79.5484	1
**4603**	372	D	67.7122	0
**4603**	373	D	73.78	1
**4603**	381	D	79.5869	1
**4603**	382	D	57.2785	1
**4603**	386	D	79.5742	1
**4604**	305	A	79.0045	1
**4604**	319	A	68.5154	1

**4604**	320	A	54.4178	-1
**4604**	321	A	29.3832	1
**4604**	351	A	10.6423	1
**4604**	357	A	68.5092	1
**4604**	370	A	47.2279	0
**4604**	372	A	74.3566	0
**4604**	373	A	65.8271	1
**4604**	381	A	62.673	1
**4604**	382	A	58.8497	1
**4604**	386	A	50.9576	1
**4604**	305	B	77.5255	1
**4604**	319	B	71.888	1
**4604**	320	B	79.5589	-1
**4604**	321	B	79.5783	0
**4604**	351	B	15.1427	1
**4604**	357	B	79.5597	1
**4604**	370	B	79.5371	1
**4604**	372	B	79.5346	0
**4604**	373	B	79.5272	1
**4604**	381	B	73.529	1
**4604**	382	B	27.3367	0
**4604**	386	B	78.6219	1
**4604**	305	C	39.445	0
**4604**	319	C	70.412	1
**4604**	320	C	60.7846	-1
**4604**	321	C	74.3719	0
**4604**	351	C	4.1848	1
**4604**	357	C	79.5425	1
**4604**	370	C	79.5244	1
**4604**	372	C	79.5525	0
**4604**	373	C	72.5339	1
**4604**	381	C	38.2826	0
**4604**	382	C	55.0744	1
**4604**	386	C	27.5117	0

**4604**	305	D	43.9002	0
**4604**	319	D	72.4672	1
**4604**	320	D	47.9434	-1
**4604**	321	D	79.5901	0
**4604**	351	D	15.0594	1
**4604**	357	D	72.8424	1
**4604**	370	D	79.5518	1
**4604**	372	D	79.5672	0
**4604**	373	D	53.0916	1
**4604**	381	D	79.5481	1
**4604**	382	D	41.9045	0
**4604**	386	D	79.5317	1
**4605**	305	A	27.6685	0
**4605**	319	A	75.6601	1
**4605**	320	A	67.5267	-1
**4605**	321	A	31.8867	1
**4605**	351	A	18.6481	1
**4605**	357	A	79.6083	1
**4605**	370	A	72.6541	1
**4605**	372	A	70.9065	0
**4605**	373	A	79.6009	1
**4605**	381	A	63.0143	1
**4605**	382	A	47.6051	0
**4605**	386	A	19.8354	-1
**4605**	305	B	79.5591	1
**4605**	319	B	39.4604	0
**4605**	320	B	22.2447	-1
**4605**	321	B	70.3366	0
**4605**	351	B	11.3055	1
**4605**	357	B	79.513	1
**4605**	370	B	79.6008	1
**4605**	372	B	67.5813	0
**4605**	373	B	61.1432	1
**4605**	381	B	62.4383	1

**4605**	382	B	37.3439	0
**4605**	386	B	79.5035	1
**4605**	305	C	79.4951	1
**4605**	319	C	55.3701	1
**4605**	320	C	63.0918	-1
**4605**	321	C	38.6447	1
**4605**	351	C	1.1444	1
**4605**	357	C	75.2035	1
**4605**	370	C	60.3896	1
**4605**	372	C	79.5312	0
**4605**	373	C	48.756	0
**4605**	381	C	73.6284	1
**4605**	382	C	77.4562	1
**4605**	386	C	16.8566	-1
**4605**	305	D	73.5585	1
**4605**	319	D	65.1803	1
**4605**	320	D	58.891	-1
**4605**	321	D	58.1854	0
**4605**	351	D	48.8304	-1
**4605**	357	D	62.9624	1
**4605**	370	D	67.7469	1
**4605**	372	D	79.6902	0
**4605**	373	D	32.4956	0
**4605**	381	D	79.5581	1
**4605**	382	D	68.4587	1
**4605**	386	D	79.5669	1
**4606**	305	A	33.4691	0
**4606**	319	A	58.8386	1
**4606**	320	A	73.5844	-1
**4606**	321	A	47.2628	1
**4606**	351	A	10.3549	1
**4606**	357	A	74.1329	1
**4606**	370	A	79.6216	1
**4606**	372	A	76.2449	0

**4606**	373	A	78.035	1
**4606**	381	A	74.0014	1
**4606**	382	A	79.6292	1
**4606**	386	A	79.5635	1
**4606**	305	B	79.6004	1
**4606**	319	B	79.0475	1
**4606**	320	B	73.5809	-1
**4606**	321	B	79.6428	0
**4606**	351	B	15.2044	1
**4606**	357	B	16.7206	-1
**4606**	370	B	79.6455	1
**4606**	372	B	79.5108	0
**4606**	373	B	78.8013	1
**4606**	381	B	71.7086	1
**4606**	382	B	41.6368	0
**4606**	386	B	65.0731	1
**4606**	305	C	79.5917	1
**4606**	319	C	65.0712	1
**4606**	320	C	79.4826	-1
**4606**	321	C	67.8669	0
**4606**	351	C	18.6416	1
**4606**	357	C	67.3979	1
**4606**	370	C	75.9461	1
**4606**	372	C	73.9288	0
**4606**	373	C	79.6111	1
**4606**	381	C	78.0043	1
**4606**	382	C	61.6655	1
**4606**	386	C	16.4585	-1
**4606**	305	D	44.4449	0
**4606**	319	D	74.4452	1
**4606**	320	D	77.5658	-1
**4606**	321	D	13.0274	0
**4606**	351	D	21.2375	-1
**4606**	357	D	74.5477	1

**4606**	370	D	79.552	1
**4606**	372	D	79.5937	0
**4606**	373	D	79.5554	1
**4606**	381	D	51.2831	1
**4606**	382	D	79.6193	1
**4606**	386	D	77.7917	1
**4607**	305	A	65.7327	1
**4607**	319	A	20.6693	0
**4607**	320	A	72.4284	-1
**4607**	321	A	76.2459	0
**4607**	351	A	12.8737	1
**4607**	357	A	72.4263	1
**4607**	370	A	40.4538	0
**4607**	372	A	56.5156	0
**4607**	373	A	61.4159	1
**4607**	381	A	79.4487	1
**4607**	382	A	66.6378	1
**4607**	386	A	55.2892	1
**4607**	305	B	79.6043	1
**4607**	319	B	79.5768	1
**4607**	320	B	79.6201	-1
**4607**	321	B	78.3706	0
**4607**	351	B	17.2751	1
**4607**	357	B	59.1592	1
**4607**	370	B	57.7752	1
**4607**	372	B	79.561	0
**4607**	373	B	78.7316	1
**4607**	381	B	42.0699	0
**4607**	382	B	54.283	1
**4607**	386	B	79.5297	1
**4607**	305	C	79.4677	1
**4607**	319	C	37.2484	0
**4607**	320	C	69.9267	-1
**4607**	321	C	53.468	0

**4607**	351	C	1.9301	1
**4607**	357	C	79.6012	1
**4607**	370	C	72.5457	1
**4607**	372	C	79.5044	0
**4607**	373	C	53.2834	1
**4607**	381	C	64.3609	1
**4607**	382	C	37.9491	0
**4607**	386	C	37.0176	0
**4607**	305	D	50.1571	1
**4607**	319	D	73.4471	1
**4607**	320	D	79.4891	-1
**4607**	321	D	50.3697	0
**4607**	351	D	15.3947	1
**4607**	357	D	74.8079	1
**4607**	370	D	58.8971	1
**4607**	372	D	64.1033	0
**4607**	373	D	79.58	1
**4607**	381	D	70.5361	1
**4607**	382	D	34.991	0
**4607**	386	D	55.4978	1
**4608**	305	A	58.1578	1
**4608**	319	A	77.7173	1
**4608**	320	A	75.5221	-1
**4608**	321	A	66.6245	0
**4608**	351	A	11.1152	1
**4608**	357	A	49.8223	0
**4608**	370	A	76.9246	1
**4608**	372	A	75.3657	0
**4608**	373	A	73.3285	1
**4608**	381	A	57.3846	1
**4608**	382	A	63.7485	1
**4608**	386	A	71.8749	1
**4608**	305	B	66.747	1
**4608**	319	B	79.5865	1

**4608**	320	B	48.6868	-1
**4608**	321	B	47.9935	1
**4608**	351	B	5.8058	1
**4608**	357	B	79.4182	1
**4608**	370	B	60.499	1
**4608**	372	B	79.6038	0
**4608**	373	B	48.7026	0
**4608**	381	B	58.1024	1
**4608**	382	B	36.6317	0
**4608**	386	B	79.6177	1
**4608**	305	C	51.9367	1
**4608**	319	C	68.2332	1
**4608**	320	C	71.5133	-1
**4608**	321	C	44.686	1
**4608**	351	C	1.3154	1
**4608**	357	C	76.1379	1
**4608**	370	C	79.5354	1
**4608**	372	C	63.1566	0
**4608**	373	C	63.0488	1
**4608**	381	C	74.4783	1
**4608**	382	C	79.5166	1
**4608**	386	C	41.5253	0
**4608**	305	D	40.2165	0
**4608**	319	D	74.4157	1
**4608**	320	D	79.5495	-1
**4608**	321	D	67.8288	0
**4608**	351	D	19.8985	1
**4608**	357	D	46.9371	0
**4608**	370	D	43.3821	0
**4608**	372	D	72.1589	0
**4608**	373	D	79.5659	1
**4608**	381	D	72.6264	1
**4608**	382	D	79.4064	1
**4608**	386	D	77.8979	1

**4609**	305	A	66.2037	1
**4609**	319	A	70.9178	1
**4609**	320	A	79.5038	-1
**4609**	321	A	78.6541	0
**4609**	351	A	1.2359	1
**4609**	357	A	49.3011	0
**4609**	370	A	74.0272	1
**4609**	372	A	52.9891	0
**4609**	373	A	39.6376	0
**4609**	381	A	70.0542	1
**4609**	382	A	59.1061	1
**4609**	386	A	71.1748	1
**4609**	305	B	72.5683	1
**4609**	319	B	40.6474	0
**4609**	320	B	74.923	-1
**4609**	321	B	71.4297	0
**4609**	351	B	12.7924	1
**4609**	357	B	74.5359	1
**4609**	370	B	79.7022	1
**4609**	372	B	79.4174	0
**4609**	373	B	72.4389	1
**4609**	381	B	79.5133	1
**4609**	382	B	54.5443	1
**4609**	386	B	52.4826	1
**4609**	305	C	71.7578	1
**4609**	319	C	72.7963	1
**4609**	320	C	64.5105	-1
**4609**	321	C	56.1553	0
**4609**	351	C	3.6162	1
**4609**	357	C	79.5676	1
**4609**	370	C	60.145	1
**4609**	372	C	79.6295	0
**4609**	373	C	57.4317	1
**4609**	381	C	69.6749	1

**4609**	382	C	51.5474	1
**4609**	386	C	18.3881	-1
**4609**	305	D	79.5433	1
**4609**	319	D	75.4207	1
**4609**	320	D	31.9116	-1
**4609**	321	D	68.7878	0
**4609**	351	D	16.1541	1
**4609**	357	D	74.9423	1
**4609**	370	D	74.8348	1
**4609**	372	D	79.4001	0
**4609**	373	D	74.7158	1
**4609**	381	D	62.2406	1
**4609**	382	D	76.5991	1
**4609**	386	D	67.5757	1
**4610**	305	A	66.0558	1
**4610**	319	A	79.5109	1
**4610**	320	A	62.2815	-1
**4610**	321	A	72.8122	0
**4610**	351	A	9.2718	1
**4610**	357	A	38.4955	0
**4610**	370	A	32.1504	0
**4610**	372	A	54.9546	0
**4610**	373	A	30.6486	0
**4610**	381	A	45.4728	0
**4610**	382	A	74.8285	1
**4610**	386	A	79.5492	1
**4610**	305	B	79.6744	1
**4610**	319	B	53.0029	1
**4610**	320	B	38.0017	-1
**4610**	321	B	75.6342	0
**4610**	351	B	20.906	-1
**4610**	357	B	52.4818	1
**4610**	370	B	79.5547	1
**4610**	372	B	70.6781	0

**4610**	373	B	69.2518	1
**4610**	381	B	63.1188	1
**4610**	382	B	57.3876	1
**4610**	386	B	78.0529	1
**4610**	305	C	79.5594	1
**4610**	319	C	79.4923	1
**4610**	320	C	55.8903	-1
**4610**	321	C	79.5401	0
**4610**	351	C	4.3482	1
**4610**	357	C	56.6728	1
**4610**	370	C	72.0446	1
**4610**	372	C	68.4824	0
**4610**	373	C	79.5182	1
**4610**	381	C	50.1993	1
**4610**	382	C	79.5458	1
**4610**	386	C	45.4972	0
**4610**	305	D	78.966	1
**4610**	319	D	30.1228	0
**4610**	320	D	36.9985	-1
**4610**	321	D	79.5826	0
**4610**	351	D	16.4542	1
**4610**	357	D	79.5207	1
**4610**	370	D	72.8295	1
**4610**	372	D	79.5653	0
**4610**	373	D	62.8673	1
**4610**	381	D	79.5656	1
**4610**	382	D	74.7458	1
**4610**	386	D	79.5631	1
**4611**	305	A	79.3507	1
**4611**	319	A	78.7319	1
**4611**	320	A	72.3918	-1
**4611**	321	A	66.9302	0
**4611**	351	A	10.6857	1
**4611**	357	A	75.2022	1

**4611**	370	A	63.0081	1
**4611**	372	A	74.4322	0
**4611**	373	A	79.551	1
**4611**	381	A	70.7415	1
**4611**	382	A	79.5517	1
**4611**	386	A	79.5728	1
**4611**	305	B	58.623	1
**4611**	319	B	65.6321	1
**4611**	320	B	78.9972	-1
**4611**	321	B	72.4844	0
**4611**	351	B	11.6928	1
**4611**	357	B	73.5684	1
**4611**	370	B	45.1958	0
**4611**	372	B	33.8968	1
**4611**	373	B	66.5611	1
**4611**	381	B	79.0838	1
**4611**	382	B	54.4599	1
**4611**	386	B	79.5633	1
**4611**	305	C	68.8073	1
**4611**	319	C	55.6461	1
**4611**	320	C	46.5136	-1
**4611**	321	C	71.1755	0
**4611**	351	C	4.166	1
**4611**	357	C	79.5485	1
**4611**	370	C	40.5634	0
**4611**	372	C	69.11	0
**4611**	373	C	73.8049	1
**4611**	381	C	71.1796	1
**4611**	382	C	72.8479	1
**4611**	386	C	36.5396	0
**4611**	305	D	79.5572	1
**4611**	319	D	64.7428	1
**4611**	320	D	78.5175	-1
**4611**	321	D	61.3973	0

**4611**	351	D	14.4564	1
**4611**	357	D	50.3095	1
**4611**	370	D	79.6087	1
**4611**	372	D	79.582	0
**4611**	373	D	79.554	1
**4611**	381	D	36.8819	0
**4611**	382	D	61.8907	1
**4611**	386	D	55.5215	1
**4612**	305	A	69.7921	1
**4612**	319	A	58.5792	1
**4612**	320	A	74.7376	-1
**4612**	321	A	79.4152	0
**4612**	351	A	1.9796	1
**4612**	357	A	15.618	-1
**4612**	370	A	79.5746	1
**4612**	372	A	75.6899	0
**4612**	373	A	66.1572	1
**4612**	381	A	74.5206	1
**4612**	382	A	56.8082	1
**4612**	386	A	79.6124	1
**4612**	305	B	68.3329	1
**4612**	319	B	79.6335	1
**4612**	320	B	42.9485	-1
**4612**	321	B	57.6877	0
**4612**	351	B	17.4846	1
**4612**	357	B	79.4751	1
**4612**	370	B	58.6184	1
**4612**	372	B	71.7526	0
**4612**	373	B	79.6276	1
**4612**	381	B	79.6652	1
**4612**	382	B	45.1747	0
**4612**	386	B	48.9987	0
**4612**	305	C	56.1329	1
**4612**	319	C	27.1044	0

**4612**	320	C	74.1244	-1
**4612**	321	C	45.3091	1
**4612**	351	C	37.9622	-1
**4612**	357	C	78.6398	1
**4612**	370	C	58.4847	1
**4612**	372	C	79.6301	0
**4612**	373	C	79.5365	1
**4612**	381	C	61.3972	1
**4612**	382	C	10.6553	-1
**4612**	386	C	30.1319	0
**4612**	305	D	35.5457	0
**4612**	319	D	65.9489	1
**4612**	320	D	75.984	-1
**4612**	321	D	79.5149	0
**4612**	351	D	11.3133	1
**4612**	357	D	79.5435	1
**4612**	370	D	31.5099	0
**4612**	372	D	79.5766	0
**4612**	373	D	75.8785	1
**4612**	381	D	79.5819	1
**4612**	382	D	75.2774	1
**4612**	386	D	79.5969	1
**4613**	305	A	71.2967	1
**4613**	319	A	74.5657	1
**4613**	320	A	73.1553	-1
**4613**	321	A	57.8816	0
**4613**	351	A	1.351	1
**4613**	357	A	79.677	1
**4613**	370	A	79.5176	1
**4613**	372	A	77.0302	0
**4613**	373	A	79.4671	1
**4613**	381	A	57.7347	1
**4613**	382	A	55.6193	1
**4613**	386	A	68.6955	1

**4613**	305	B	79.4664	1
**4613**	319	B	79.5338	1
**4613**	320	B	63.4807	-1
**4613**	321	B	78.084	0
**4613**	351	B	7.7792	1
**4613**	357	B	79.58	1
**4613**	370	B	40.1592	0
**4613**	372	B	42.4116	1
**4613**	373	B	77.7162	1
**4613**	381	B	79.5191	1
**4613**	382	B	27.824	0
**4613**	386	B	40.4708	0
**4613**	305	C	61.5526	1
**4613**	319	C	72.8298	1
**4613**	320	C	79.529	-1
**4613**	321	C	72.7426	0
**4613**	351	C	12.9821	1
**4613**	357	C	62.8753	1
**4613**	370	C	41.4702	0
**4613**	372	C	78.8223	0
**4613**	373	C	73.6909	1
**4613**	381	C	47.4306	0
**4613**	382	C	71.0622	1
**4613**	386	C	61.7244	1
**4613**	305	D	63.0542	1
**4613**	319	D	79.4767	1
**4613**	320	D	74.0054	-1
**4613**	321	D	70.884	0
**4613**	351	D	8.1908	1
**4613**	357	D	79.5756	1
**4613**	370	D	74.1293	1
**4613**	372	D	72.7745	0
**4613**	373	D	79.4341	1
**4613**	381	D	72.2932	1

**4613**	382	D	73.9862	1
**4613**	386	D	49.8657	0
**4614**	305	A	79.1838	1
**4614**	319	A	73.5003	1
**4614**	320	A	79.4158	-1
**4614**	321	A	79.5845	0
**4614**	351	A	11.3376	1
**4614**	357	A	76.2979	1
**4614**	370	A	78.2617	1
**4614**	372	A	78.4833	0
**4614**	373	A	79.1576	1
**4614**	381	A	69.5384	1
**4614**	382	A	74.118	1
**4614**	386	A	68.0258	1
**4614**	305	B	69.6839	1
**4614**	319	B	79.7216	1
**4614**	320	B	69.6078	-1
**4614**	321	B	69.6432	0
**4614**	351	B	20.3384	-1
**4614**	357	B	79.5909	1
**4614**	370	B	39.344	0
**4614**	372	B	74.05	0
**4614**	373	B	38.328	0
**4614**	381	B	56.9158	1
**4614**	382	B	79.5316	1
**4614**	386	B	63.6426	1
**4614**	305	C	70.3388	1
**4614**	319	C	71.0283	1
**4614**	320	C	60.8855	-1
**4614**	321	C	47.1838	1
**4614**	351	C	2.4802	1
**4614**	357	C	26.359	0
**4614**	370	C	64.7647	1
**4614**	372	C	79.7069	0

**4614**	373	C	79.2949	1
**4614**	381	C	79.4774	1
**4614**	382	C	69.4808	1
**4614**	386	C	69.5426	1
**4614**	305	D	61.5257	1
**4614**	319	D	77.4989	1
**4614**	320	D	73.8059	-1
**4614**	321	D	65.7699	0
**4614**	351	D	14.183	1
**4614**	357	D	75.4483	1
**4614**	370	D	79.56	1
**4614**	372	D	79.4481	0
**4614**	373	D	62.7684	1
**4614**	381	D	62.9438	1
**4614**	382	D	71.6554	1
**4614**	386	D	64.3394	1
**4615**	305	A	75.4727	1
**4615**	319	A	62.557	1
**4615**	320	A	76.2811	-1
**4615**	321	A	64.9586	0
**4615**	351	A	0.0	1
**4615**	357	A	65.232	1
**4615**	370	A	53.4663	1
**4615**	372	A	79.5996	0
**4615**	373	A	77.9919	1
**4615**	381	A	69.8765	1
**4615**	382	A	77.3825	1
**4615**	386	A	78.8534	1
**4615**	305	B	56.3749	1
**4615**	319	B	61.7249	1
**4615**	320	B	66.5042	-1
**4615**	321	B	37.4592	1
**4615**	351	B	14.1843	1
**4615**	357	B	79.6194	1

**4615**	370	B	46.7794	0
**4615**	372	B	79.5316	0
**4615**	373	B	48.1155	0
**4615**	381	B	72.7252	1
**4615**	382	B	40.473	0
**4615**	386	B	60.3893	1
**4615**	305	C	53.903	1
**4615**	319	C	77.1156	1
**4615**	320	C	70.8093	-1
**4615**	321	C	61.3795	0
**4615**	351	C	14.1424	1
**4615**	357	C	72.871	1
**4615**	370	C	78.4681	1
**4615**	372	C	58.3411	0
**4615**	373	C	73.4972	1
**4615**	381	C	51.9322	1
**4615**	382	C	79.6719	1
**4615**	386	C	50.5888	1
**4615**	305	D	73.3334	1
**4615**	319	D	77.1548	1
**4615**	320	D	79.5924	-1
**4615**	321	D	79.5721	0
**4615**	351	D	16.4236	1
**4615**	357	D	72.5863	1
**4615**	370	D	79.5425	1
**4615**	372	D	77.514	0
**4615**	373	D	79.5587	1
**4615**	381	D	24.0503	0
**4615**	382	D	44.3707	0
**4615**	386	D	79.5676	1
**4616**	305	A	55.1777	1
**4616**	319	A	52.8163	1
**4616**	320	A	79.5488	-1
**4616**	321	A	79.554	0

**4616**	351	A	6.4109	1
**4616**	357	A	43.0233	0
**4616**	370	A	73.5086	1
**4616**	372	A	75.1704	0
**4616**	373	A	79.549	1
**4616**	381	A	79.5763	1
**4616**	382	A	71.4295	1
**4616**	386	A	71.6019	1
**4616**	305	B	57.8774	1
**4616**	319	B	72.4996	1
**4616**	320	B	27.7531	-1
**4616**	321	B	70.6752	0
**4616**	351	B	14.0599	1
**4616**	357	B	46.7731	0
**4616**	370	B	63.3878	1
**4616**	372	B	77.0221	0
**4616**	373	B	53.8936	1
**4616**	381	B	24.5128	0
**4616**	382	B	78.9645	1
**4616**	386	B	61.3995	1
**4616**	305	C	63.173	1
**4616**	319	C	28.5646	0
**4616**	320	C	58.8053	-1
**4616**	321	C	72.3476	0
**4616**	351	C	3.0082	1
**4616**	357	C	79.5652	1
**4616**	370	C	79.1962	1
**4616**	372	C	79.5711	0
**4616**	373	C	63.7577	1
**4616**	381	C	16.4297	-1
**4616**	382	C	76.1148	1
**4616**	386	C	10.1807	-1
**4616**	305	D	79.5587	1
**4616**	319	D	79.5452	1

**4616**	320	D	72.4444	-1
**4616**	321	D	52.8755	0
**4616**	351	D	14.7135	1
**4616**	357	D	68.539	1
**4616**	370	D	79.5475	1
**4616**	372	D	69.9206	0
**4616**	373	D	79.5329	1
**4616**	381	D	60.0092	1
**4616**	382	D	62.0103	1
**4616**	386	D	34.1511	0
**4617**	305	A	79.0504	1
**4617**	319	A	76.2113	1
**4617**	320	A	67.4175	-1
**4617**	321	A	23.0226	1
**4617**	351	A	26.6626	-1
**4617**	357	A	79.528	1
**4617**	370	A	46.8494	0
**4617**	372	A	79.6636	0
**4617**	373	A	49.5739	0
**4617**	381	A	47.0611	0
**4617**	382	A	79.5605	1
**4617**	386	A	71.6758	1
**4617**	305	B	61.0	1
**4617**	319	B	79.633	1
**4617**	320	B	79.5351	-1
**4617**	321	B	79.6665	0
**4617**	351	B	3.4377	1
**4617**	357	B	79.5375	1
**4617**	370	B	62.5568	1
**4617**	372	B	79.6178	0
**4617**	373	B	30.9332	0
**4617**	381	B	56.1045	1
**4617**	382	B	79.2101	1
**4617**	386	B	36.9302	0

**4617**	305	C	76.5855	1
**4617**	319	C	71.6681	1
**4617**	320	C	73.313	-1
**4617**	321	C	72.8023	0
**4617**	351	C	7.8327	1
**4617**	357	C	46.4017	0
**4617**	370	C	56.0292	1
**4617**	372	C	79.4913	0
**4617**	373	C	72.783	1
**4617**	381	C	79.1801	1
**4617**	382	C	63.578	1
**4617**	386	C	31.4543	0
**4617**	305	D	71.4908	1
**4617**	319	D	72.6883	1
**4617**	320	D	75.9612	-1
**4617**	321	D	79.2962	0
**4617**	351	D	19.4239	1
**4617**	357	D	79.4288	1
**4617**	370	D	66.7944	1
**4617**	372	D	79.5177	0
**4617**	373	D	79.4168	1
**4617**	381	D	63.3044	1
**4617**	382	D	73.2243	1
**4617**	386	D	70.2362	1
**4618**	305	A	69.5548	1
**4618**	319	A	74.093	1
**4618**	320	A	79.5636	-1
**4618**	321	A	67.436	0
**4618**	351	A	1.8431	1
**4618**	357	A	78.5077	1
**4618**	370	A	72.7406	1
**4618**	372	A	79.5663	0
**4618**	373	A	74.0163	1
**4618**	381	A	66.8374	1

**4618**	382	A	72.8879	1
**4618**	386	A	75.7038	1
**4618**	305	B	32.6646	0
**4618**	319	B	79.1826	1
**4618**	320	B	72.3669	-1
**4618**	321	B	46.6154	1
**4618**	351	B	11.4959	1
**4618**	357	B	67.7621	1
**4618**	370	B	79.5357	1
**4618**	372	B	79.5233	0
**4618**	373	B	20.1255	0
**4618**	381	B	71.4279	1
**4618**	382	B	76.1215	1
**4618**	386	B	79.5534	1
**4618**	305	C	76.8852	1
**4618**	319	C	29.751	0
**4618**	320	C	79.5595	-1
**4618**	321	C	77.7695	0
**4618**	351	C	3.7296	1
**4618**	357	C	73.1658	1
**4618**	370	C	75.5072	1
**4618**	372	C	60.4554	0
**4618**	373	C	35.9263	0
**4618**	381	C	69.4792	1
**4618**	382	C	79.6509	1
**4618**	386	C	41.0353	0
**4618**	305	D	77.3484	1
**4618**	319	D	70.6695	1
**4618**	320	D	68.9654	-1
**4618**	321	D	79.5103	0
**4618**	351	D	1.8463	1
**4618**	357	D	52.0428	1
**4618**	370	D	79.6289	1
**4618**	372	D	70.1002	0

**4618**	373	D	79.5078	1
**4618**	381	D	79.5375	1
**4618**	382	D	79.5715	1
**4618**	386	D	62.4422	1
**4619**	305	A	79.2848	1
**4619**	319	A	35.2449	0
**4619**	320	A	79.5492	-1
**4619**	321	A	76.6993	0
**4619**	351	A	10.8455	1
**4619**	357	A	67.6676	1
**4619**	370	A	45.4077	0
**4619**	372	A	72.1628	0
**4619**	373	A	67.6266	1
**4619**	381	A	50.2999	1
**4619**	382	A	72.5443	1
**4619**	386	A	66.9431	1
**4619**	305	B	64.0636	1
**4619**	319	B	71.2095	1
**4619**	320	B	79.5098	-1
**4619**	321	B	79.5601	0
**4619**	351	B	15.9311	1
**4619**	357	B	79.5908	1
**4619**	370	B	55.5647	1
**4619**	372	B	73.1865	0
**4619**	373	B	65.9841	1
**4619**	381	B	79.5598	1
**4619**	382	B	75.5728	1
**4619**	386	B	47.1392	0
**4619**	305	C	56.1842	1
**4619**	319	C	37.3529	0
**4619**	320	C	50.3757	-1
**4619**	321	C	59.2695	0
**4619**	351	C	14.5102	1
**4619**	357	C	79.5654	1

**4619**	370	C	74.6555	1
**4619**	372	C	73.6473	0
**4619**	373	C	79.5688	1
**4619**	381	C	79.2987	1
**4619**	382	C	79.5367	1
**4619**	386	C	61.0719	1
**4619**	305	D	48.9078	0
**4619**	319	D	79.5552	1
**4619**	320	D	79.5502	-1
**4619**	321	D	64.6333	0
**4619**	351	D	8.3444	1
**4619**	357	D	72.4988	1
**4619**	370	D	79.5873	1
**4619**	372	D	79.5642	0
**4619**	373	D	79.5472	1
**4619**	381	D	79.3916	1
**4619**	382	D	78.5942	1
**4619**	386	D	78.4034	1
**4620**	305	A	78.5008	1
**4620**	319	A	79.5299	1
**4620**	320	A	79.5349	-1
**4620**	321	A	72.8102	0
**4620**	351	A	16.5977	1
**4620**	357	A	62.351	1
**4620**	370	A	20.1715	0
**4620**	372	A	78.4107	0
**4620**	373	A	79.5958	1
**4620**	381	A	41.3241	0
**4620**	382	A	62.3847	1
**4620**	386	A	62.5517	1
**4620**	305	B	79.5576	1
**4620**	319	B	55.006	1
**4620**	320	B	79.5735	-1
**4620**	321	B	79.5504	0

**4620**	351	B	13.1813	1
**4620**	357	B	49.1031	0
**4620**	370	B	53.2609	1
**4620**	372	B	79.5684	0
**4620**	373	B	57.7384	1
**4620**	381	B	46.5203	0
**4620**	382	B	57.3737	1
**4620**	386	B	52.5581	1
**4620**	305	C	79.577	1
**4620**	319	C	79.5603	1
**4620**	320	C	43.5307	-1
**4620**	321	C	79.5736	0
**4620**	351	C	6.3607	1
**4620**	357	C	79.5758	1
**4620**	370	C	79.5794	1
**4620**	372	C	47.9673	1
**4620**	373	C	79.5302	1
**4620**	381	C	42.0609	0
**4620**	382	C	79.5625	1
**4620**	386	C	37.346	0
**4620**	305	D	70.7015	1
**4620**	319	D	57.0081	1
**4620**	320	D	70.8221	-1
**4620**	321	D	71.7652	0
**4620**	351	D	14.6444	1
**4620**	357	D	32.9506	0
**4620**	370	D	72.48	1
**4620**	372	D	79.5619	0
**4620**	373	D	50.1951	1
**4620**	381	D	76.674	1
**4620**	382	D	79.5489	1
**4620**	386	D	78.5274	1
**4621**	305	A	78.6946	1
**4621**	319	A	72.1552	1

**4621**	320	A	79.4855	-1
**4621**	321	A	71.8952	0
**4621**	351	A	0.0	1
**4621**	357	A	52.0178	1
**4621**	370	A	79.2581	1
**4621**	372	A	72.5559	0
**4621**	373	A	30.9697	0
**4621**	381	A	68.7749	1
**4621**	382	A	6.7862	-1
**4621**	386	A	65.6227	1
**4621**	305	B	63.9543	1
**4621**	319	B	76.9699	1
**4621**	320	B	60.6845	-1
**4621**	321	B	34.1702	1
**4621**	351	B	26.4467	-1
**4621**	357	B	12.0474	-1
**4621**	370	B	69.9266	1
**4621**	372	B	79.6484	0
**4621**	373	B	68.661	1
**4621**	381	B	6.2091	-1
**4621**	382	B	44.2703	0
**4621**	386	B	47.59	0
**4621**	305	C	79.5663	1
**4621**	319	C	79.5098	1
**4621**	320	C	71.7461	-1
**4621**	321	C	73.1711	0
**4621**	351	C	3.6003	1
**4621**	357	C	45.883	0
**4621**	370	C	79.6561	1
**4621**	372	C	79.6072	0
**4621**	373	C	79.4817	1
**4621**	381	C	3.7687	-1
**4621**	382	C	42.3982	0
**4621**	386	C	32.2784	0

**4621**	305	D	17.978	-1
**4621**	319	D	54.825	1
**4621**	320	D	73.6589	-1
**4621**	321	D	73.5857	0
**4621**	351	D	10.664	1
**4621**	357	D	79.5735	1
**4621**	370	D	74.9342	1
**4621**	372	D	79.6754	0
**4621**	373	D	78.6411	1
**4621**	381	D	66.2705	1
**4621**	382	D	63.3781	1
**4621**	386	D	79.5625	1
**4622**	305	A	79.4713	1
**4622**	319	A	14.0679	-1
**4622**	320	A	73.3481	-1
**4622**	321	A	50.08	0
**4622**	351	A	16.711	1
**4622**	357	A	79.3155	1
**4622**	370	A	73.104	1
**4622**	372	A	74.6499	0
**4622**	373	A	58.0379	1
**4622**	381	A	71.6274	1
**4622**	382	A	79.5995	1
**4622**	386	A	79.5467	1
**4622**	305	B	72.8969	1
**4622**	319	B	39.517	0
**4622**	320	B	79.5282	-1
**4622**	321	B	79.5113	0
**4622**	351	B	12.5906	1
**4622**	357	B	67.2444	1
**4622**	370	B	49.7894	0
**4622**	372	B	79.5278	0
**4622**	373	B	44.1917	0
**4622**	381	B	54.8675	1

**4622**	382	B	16.6503	-1
**4622**	386	B	59.0918	1
**4622**	305	C	62.9943	1
**4622**	319	C	79.5561	1
**4622**	320	C	64.9295	-1
**4622**	321	C	55.1786	0
**4622**	351	C	8.236	1
**4622**	357	C	29.9418	0
**4622**	370	C	66.2507	1
**4622**	372	C	79.5339	0
**4622**	373	C	79.5365	1
**4622**	381	C	64.3168	1
**4622**	382	C	62.4583	1
**4622**	386	C	34.0872	0
**4622**	305	D	17.3733	-1
**4622**	319	D	73.7456	1
**4622**	320	D	72.1346	-1
**4622**	321	D	76.8916	0
**4622**	351	D	11.2738	1
**4622**	357	D	73.2855	1
**4622**	370	D	79.5057	1
**4622**	372	D	56.8793	0
**4622**	373	D	68.4718	1
**4622**	381	D	59.8746	1
**4622**	382	D	79.5234	1
**4622**	386	D	79.6369	1
**4623**	305	A	75.7991	1
**4623**	319	A	69.714	1
**4623**	320	A	69.2165	-1
**4623**	321	A	76.8651	0
**4623**	351	A	6.3476	1
**4623**	357	A	62.3531	1
**4623**	370	A	79.5388	1
**4623**	372	A	79.5693	0

**4623**	373	A	74.4868	1
**4623**	381	A	71.819	1
**4623**	382	A	79.559	1
**4623**	386	A	73.7884	1
**4623**	305	B	66.6991	1
**4623**	319	B	69.3914	1
**4623**	320	B	66.4208	-1
**4623**	321	B	79.7043	0
**4623**	351	B	12.721	1
**4623**	357	B	72.9389	1
**4623**	370	B	79.5591	1
**4623**	372	B	73.8762	0
**4623**	373	B	79.4278	1
**4623**	381	B	54.9279	1
**4623**	382	B	33.7219	0
**4623**	386	B	57.1688	1
**4623**	305	C	79.5965	1
**4623**	319	C	57.0655	1
**4623**	320	C	71.1377	-1
**4623**	321	C	53.6771	0
**4623**	351	C	1.519	1
**4623**	357	C	76.7707	1
**4623**	370	C	79.4093	1
**4623**	372	C	73.5578	0
**4623**	373	C	57.3077	1
**4623**	381	C	58.3713	1
**4623**	382	C	59.1607	1
**4623**	386	C	44.0452	0
**4623**	305	D	38.5808	0
**4623**	319	D	78.7152	1
**4623**	320	D	49.7173	-1
**4623**	321	D	58.3575	0
**4623**	351	D	17.8397	1
**4623**	357	D	79.6392	1

**4623**	370	D	41.7295	0
**4623**	372	D	69.4569	0
**4623**	373	D	79.4949	1
**4623**	381	D	76.6566	1
**4623**	382	D	79.6115	1
**4623**	386	D	79.5824	1
**4624**	305	A	71.503	1
**4624**	319	A	62.3162	1
**4624**	320	A	79.6558	-1
**4624**	321	A	32.022	1
**4624**	351	A	10.0519	1
**4624**	357	A	79.5788	1
**4624**	370	A	53.4475	1
**4624**	372	A	79.7351	0
**4624**	373	A	37.7866	0
**4624**	381	A	73.3644	1
**4624**	382	A	66.4399	1
**4624**	386	A	48.6754	0
**4624**	305	B	63.7295	1
**4624**	319	B	79.551	1
**4624**	320	B	54.4937	-1
**4624**	321	B	79.6144	0
**4624**	351	B	18.6327	1
**4624**	357	B	74.3573	1
**4624**	370	B	34.2257	0
**4624**	372	B	69.8169	0
**4624**	373	B	50.1616	1
**4624**	381	B	74.8191	1
**4624**	382	B	45.4154	0
**4624**	386	B	79.4202	1
**4624**	305	C	58.9654	1
**4624**	319	C	74.1642	1
**4624**	320	C	78.5009	-1
**4624**	321	C	79.6882	0

**4624**	351	C	2.5633	1
**4624**	357	C	64.5182	1
**4624**	370	C	57.7518	1
**4624**	372	C	66.8398	0
**4624**	373	C	47.1758	0
**4624**	381	C	75.5455	1
**4624**	382	C	20.071	0
**4624**	386	C	51.6501	1
**4624**	305	D	44.0107	0
**4624**	319	D	79.5723	1
**4624**	320	D	79.6165	-1
**4624**	321	D	45.4589	1
**4624**	351	D	7.4807	1
**4624**	357	D	73.1097	1
**4624**	370	D	79.6028	1
**4624**	372	D	79.6625	0
**4624**	373	D	71.5741	1
**4624**	381	D	79.55	1
**4624**	382	D	76.9084	1
**4624**	386	D	65.478	1
**4625**	305	A	79.5615	1
**4625**	319	A	53.1497	1
**4625**	320	A	68.3315	-1
**4625**	321	A	43.5625	1
**4625**	351	A	0.0	1
**4625**	357	A	79.5495	1
**4625**	370	A	76.2487	1
**4625**	372	A	54.7472	0
**4625**	373	A	79.5561	1
**4625**	381	A	39.3389	0
**4625**	382	A	74.1822	1
**4625**	386	A	53.1856	1
**4625**	305	B	74.989	1
**4625**	319	B	79.5092	1

**4625**	320	B	29.0869	-1
**4625**	321	B	60.6643	0
**4625**	351	B	8.5851	1
**4625**	357	B	74.8027	1
**4625**	370	B	19.2549	-1
**4625**	372	B	67.0616	0
**4625**	373	B	58.5174	1
**4625**	381	B	79.5508	1
**4625**	382	B	50.8909	1
**4625**	386	B	79.6141	1
**4625**	305	C	79.5612	1
**4625**	319	C	43.0932	0
**4625**	320	C	79.4732	-1
**4625**	321	C	79.5763	0
**4625**	351	C	0.1585	1
**4625**	357	C	79.5394	1
**4625**	370	C	76.4257	1
**4625**	372	C	78.638	0
**4625**	373	C	46.6715	0
**4625**	381	C	59.8369	1
**4625**	382	C	55.4033	1
**4625**	386	C	63.2246	1
**4625**	305	D	79.5856	1
**4625**	319	D	52.877	1
**4625**	320	D	79.5738	-1
**4625**	321	D	57.3548	0
**4625**	351	D	15.028	1
**4625**	357	D	74.9746	1
**4625**	370	D	50.6695	1
**4625**	372	D	63.6233	0
**4625**	373	D	45.9421	0
**4625**	381	D	79.5698	1
**4625**	382	D	72.6828	1
**4625**	386	D	79.5244	1

**4626**	305	A	79.4899	1
**4626**	319	A	78.909	1
**4626**	320	A	63.5963	-1
**4626**	321	A	22.5555	1
**4626**	351	A	6.4806	1
**4626**	357	A	74.847	1
**4626**	370	A	61.0095	1
**4626**	372	A	68.6507	0
**4626**	373	A	79.5326	1
**4626**	381	A	72.7817	1
**4626**	382	A	29.8036	0
**4626**	386	A	72.4822	1
**4626**	305	B	71.6533	1
**4626**	319	B	74.2257	1
**4626**	320	B	26.3221	-1
**4626**	321	B	79.5111	0
**4626**	351	B	17.4697	1
**4626**	357	B	79.5506	1
**4626**	370	B	30.8606	0
**4626**	372	B	79.4507	0
**4626**	373	B	79.5711	1
**4626**	381	B	77.9107	1
**4626**	382	B	55.0514	1
**4626**	386	B	64.5312	1
**4626**	305	C	49.0864	0
**4626**	319	C	79.544	1
**4626**	320	C	67.0372	-1
**4626**	321	C	79.578	0
**4626**	351	C	36.1715	-1
**4626**	357	C	79.5245	1
**4626**	370	C	50.6293	1
**4626**	372	C	79.5482	0
**4626**	373	C	79.5068	1
**4626**	381	C	78.7832	1

**4626**	382	C	54.3857	1
**4626**	386	C	64.6318	1
**4626**	305	D	56.508	1
**4626**	319	D	71.0768	1
**4626**	320	D	70.4121	-1
**4626**	321	D	57.3514	0
**4626**	351	D	13.4325	1
**4626**	357	D	79.4928	1
**4626**	370	D	65.2965	1
**4626**	372	D	79.6463	0
**4626**	373	D	79.5893	1
**4626**	381	D	72.5619	1
**4626**	382	D	73.0226	1
**4626**	386	D	67.541	1
**4627**	305	A	79.6075	1
**4627**	319	A	78.4437	1
**4627**	320	A	79.5472	-1
**4627**	321	A	77.1622	0
**4627**	351	A	0.0	1
**4627**	357	A	54.4563	1
**4627**	370	A	78.416	1
**4627**	372	A	58.1136	0
**4627**	373	A	79.3968	1
**4627**	381	A	77.8422	1
**4627**	382	A	62.6458	1
**4627**	386	A	39.2031	0
**4627**	305	B	79.4643	1
**4627**	319	B	32.4506	0
**4627**	320	B	65.0532	-1
**4627**	321	B	62.5795	0
**4627**	351	B	7.4001	1
**4627**	357	B	75.9752	1
**4627**	370	B	53.0362	1
**4627**	372	B	68.1398	0

**4627**	373	B	72.2949	1
**4627**	381	B	78.808	1
**4627**	382	B	72.3882	1
**4627**	386	B	72.7185	1
**4627**	305	C	79.64	1
**4627**	319	C	77.1131	1
**4627**	320	C	67.8504	-1
**4627**	321	C	72.4293	0
**4627**	351	C	21.7206	-1
**4627**	357	C	79.4385	1
**4627**	370	C	79.4956	1
**4627**	372	C	71.4144	0
**4627**	373	C	77.3297	1
**4627**	381	C	79.6953	1
**4627**	382	C	31.3137	0
**4627**	386	C	30.5284	0
**4627**	305	D	74.9459	1
**4627**	319	D	79.5598	1
**4627**	320	D	79.526	-1
**4627**	321	D	79.6959	0
**4627**	351	D	6.4589	1
**4627**	357	D	79.7033	1
**4627**	370	D	68.1709	1
**4627**	372	D	75.5773	0
**4627**	373	D	48.208	0
**4627**	381	D	79.6341	1
**4627**	382	D	78.9831	1
**4627**	386	D	79.5386	1
**4628**	305	A	52.3197	1
**4628**	319	A	55.7311	1
**4628**	320	A	79.5362	-1
**4628**	321	A	77.8035	0
**4628**	351	A	8.7635	1
**4628**	357	A	44.5362	0

**4628**	370	A	45.3863	0
**4628**	372	A	65.848	0
**4628**	373	A	36.4906	0
**4628**	381	A	77.9121	1
**4628**	382	A	67.0054	1
**4628**	386	A	79.5506	1
**4628**	305	B	78.4157	1
**4628**	319	B	75.913	1
**4628**	320	B	79.7232	-1
**4628**	321	B	79.5439	0
**4628**	351	B	12.5835	1
**4628**	357	B	75.7106	1
**4628**	370	B	78.431	1
**4628**	372	B	79.5763	0
**4628**	373	B	69.6991	1
**4628**	381	B	38.4998	0
**4628**	382	B	36.447	0
**4628**	386	B	47.4805	0
**4628**	305	C	47.4459	0
**4628**	319	C	79.5742	1
**4628**	320	C	79.5767	-1
**4628**	321	C	72.5587	0
**4628**	351	C	5.1826	1
**4628**	357	C	74.5001	1
**4628**	370	C	75.757	1
**4628**	372	C	79.5065	0
**4628**	373	C	79.5785	1
**4628**	381	C	63.4832	1
**4628**	382	C	79.5078	1
**4628**	386	C	46.9836	0
**4628**	305	D	79.5633	1
**4628**	319	D	73.4343	1
**4628**	320	D	74.5604	-1
**4628**	321	D	79.5442	0

**4628**	351	D	13.5744	1
**4628**	357	D	64.5442	1
**4628**	370	D	29.403	0
**4628**	372	D	73.4287	0
**4628**	373	D	78.4257	1
**4628**	381	D	72.474	1
**4628**	382	D	72.2187	1
**4628**	386	D	59.7222	1
**4629**	305	A	79.6249	1
**4629**	319	A	61.0082	1
**4629**	320	A	78.2286	-1
**4629**	321	A	24.0896	1
**4629**	351	A	7.882	1
**4629**	357	A	70.9861	1
**4629**	370	A	74.3461	1
**4629**	372	A	79.5058	0
**4629**	373	A	69.0642	1
**4629**	381	A	70.6656	1
**4629**	382	A	51.9617	1
**4629**	386	A	79.4396	1
**4629**	305	B	64.3152	1
**4629**	319	B	79.6092	1
**4629**	320	B	79.6036	-1
**4629**	321	B	79.4501	0
**4629**	351	B	21.347	-1
**4629**	357	B	79.5971	1
**4629**	370	B	69.5406	1
**4629**	372	B	79.5154	0
**4629**	373	B	50.0388	1
**4629**	381	B	79.5068	1
**4629**	382	B	51.4909	1
**4629**	386	B	79.5897	1
**4629**	305	C	79.463	1
**4629**	319	C	28.625	0

**4629**	320	C	71.981	-1
**4629**	321	C	72.0713	0
**4629**	351	C	7.8754	1
**4629**	357	C	70.0855	1
**4629**	370	C	79.5871	1
**4629**	372	C	71.3224	0
**4629**	373	C	76.0866	1
**4629**	381	C	70.4151	1
**4629**	382	C	29.1125	0
**4629**	386	C	58.4156	1
**4629**	305	D	60.9493	1
**4629**	319	D	66.6491	1
**4629**	320	D	78.195	-1
**4629**	321	D	68.4157	0
**4629**	351	D	18.4241	1
**4629**	357	D	66.3116	1
**4629**	370	D	79.548	1
**4629**	372	D	79.6641	0
**4629**	373	D	57.8682	1
**4629**	381	D	47.4625	0
**4629**	382	D	71.753	1
**4629**	386	D	59.3934	1
**4630**	305	A	43.5458	0
**4630**	319	A	32.1621	0
**4630**	320	A	73.7452	-1
**4630**	321	A	62.8997	0
**4630**	351	A	3.629	1
**4630**	357	A	63.5714	1
**4630**	370	A	74.0219	1
**4630**	372	A	54.6816	0
**4630**	373	A	36.0847	0
**4630**	381	A	71.4866	1
**4630**	382	A	66.8542	1
**4630**	386	A	74.7306	1

**4630**	305	B	12.0667	-1
**4630**	319	B	13.6039	-1
**4630**	320	B	68.9696	-1
**4630**	321	B	64.4517	0
**4630**	351	B	18.2975	1
**4630**	357	B	33.2974	0
**4630**	370	B	79.5975	1
**4630**	372	B	79.5553	0
**4630**	373	B	56.4908	1
**4630**	381	B	79.5834	1
**4630**	382	B	70.6733	1
**4630**	386	B	61.2135	1
**4630**	305	C	64.0811	1
**4630**	319	C	31.9574	0
**4630**	320	C	62.0658	-1
**4630**	321	C	49.2838	1
**4630**	351	C	2.8873	1
**4630**	357	C	70.5099	1
**4630**	370	C	70.7976	1
**4630**	372	C	73.2473	0
**4630**	373	C	64.4286	1
**4630**	381	C	79.5145	1
**4630**	382	C	72.0224	1
**4630**	386	C	33.4799	0
**4630**	305	D	56.1735	1
**4630**	319	D	64.5567	1
**4630**	320	D	51.0411	-1
**4630**	321	D	76.2799	0
**4630**	351	D	8.027	1
**4630**	357	D	62.8513	1
**4630**	370	D	58.5484	1
**4630**	372	D	71.4681	0
**4630**	373	D	55.9731	1
**4630**	381	D	79.4991	1

**4630**	382	D	49.1021	0
**4630**	386	D	73.1333	1
**4631**	305	A	46.8715	0
**4631**	319	A	45.1788	0
**4631**	320	A	79.4343	-1
**4631**	321	A	78.1252	0
**4631**	351	A	10.2128	1
**4631**	357	A	74.1618	1
**4631**	370	A	43.3625	0
**4631**	372	A	79.597	0
**4631**	373	A	56.955	1
**4631**	381	A	79.6503	1
**4631**	382	A	59.754	1
**4631**	386	A	78.4977	1
**4631**	305	B	74.8345	1
**4631**	319	B	59.0317	1
**4631**	320	B	70.1521	-1
**4631**	321	B	69.2978	0
**4631**	351	B	13.1037	1
**4631**	357	B	79.4386	1
**4631**	370	B	79.502	1
**4631**	372	B	79.4907	0
**4631**	373	B	62.1696	1
**4631**	381	B	70.8606	1
**4631**	382	B	27.5406	0
**4631**	386	B	71.4033	1
**4631**	305	C	64.8269	1
**4631**	319	C	72.8427	1
**4631**	320	C	63.1569	-1
**4631**	321	C	79.5693	0
**4631**	351	C	7.8817	1
**4631**	357	C	72.8435	1
**4631**	370	C	70.996	1
**4631**	372	C	79.5074	0

**4631**	373	C	63.2079	1
**4631**	381	C	73.1446	1
**4631**	382	C	72.4386	1
**4631**	386	C	36.3295	0
**4631**	305	D	42.8315	0
**4631**	319	D	65.0559	1
**4631**	320	D	79.6466	-1
**4631**	321	D	49.2191	1
**4631**	351	D	19.1133	1
**4631**	357	D	49.1995	0
**4631**	370	D	78.2541	1
**4631**	372	D	78.0155	0
**4631**	373	D	79.4472	1
**4631**	381	D	75.2245	1
**4631**	382	D	69.6622	1
**4631**	386	D	71.1822	1
**4632**	305	A	74.028	1
**4632**	319	A	71.4055	1
**4632**	320	A	72.0692	-1
**4632**	321	A	70.5291	0
**4632**	351	A	4.1059	1
**4632**	357	A	79.5332	1
**4632**	370	A	65.6838	1
**4632**	372	A	27.5967	1
**4632**	373	A	61.4292	1
**4632**	381	A	79.5567	1
**4632**	382	A	28.285	0
**4632**	386	A	79.5702	1
**4632**	305	B	79.4231	1
**4632**	319	B	75.8047	1
**4632**	320	B	76.1593	-1
**4632**	321	B	79.4874	0
**4632**	351	B	12.5932	1
**4632**	357	B	51.7122	1

**4632**	370	B	45.7266	0
**4632**	372	B	79.5652	0
**4632**	373	B	67.3378	1
**4632**	381	B	79.5674	1
**4632**	382	B	49.8633	0
**4632**	386	B	79.5295	1
**4632**	305	C	69.987	1
**4632**	319	C	79.5442	1
**4632**	320	C	58.4032	-1
**4632**	321	C	72.7025	0
**4632**	351	C	11.5723	1
**4632**	357	C	79.5944	1
**4632**	370	C	67.5278	1
**4632**	372	C	79.574	0
**4632**	373	C	70.7	1
**4632**	381	C	78.287	1
**4632**	382	C	77.857	1
**4632**	386	C	20.735	0
**4632**	305	D	79.4953	1
**4632**	319	D	79.5909	1
**4632**	320	D	79.5261	-1
**4632**	321	D	76.8767	0
**4632**	351	D	14.2947	1
**4632**	357	D	70.7163	1
**4632**	370	D	53.2188	1
**4632**	372	D	79.5778	0
**4632**	373	D	58.6336	1
**4632**	381	D	79.5735	1
**4632**	382	D	79.5389	1
**4632**	386	D	79.5465	1
**4633**	305	A	79.5892	1
**4633**	319	A	72.3046	1
**4633**	320	A	64.8082	-1
**4633**	321	A	74.6053	0

**4633**	351	A	14.3926	1
**4633**	357	A	62.0039	1
**4633**	370	A	79.4499	1
**4633**	372	A	73.3003	0
**4633**	373	A	59.9992	1
**4633**	381	A	79.5209	1
**4633**	382	A	66.7815	1
**4633**	386	A	56.6349	1
**4633**	305	B	64.0958	1
**4633**	319	B	79.5145	1
**4633**	320	B	53.6386	-1
**4633**	321	B	79.6156	0
**4633**	351	B	10.2438	1
**4633**	357	B	63.5819	1
**4633**	370	B	78.1369	1
**4633**	372	B	75.9729	0
**4633**	373	B	72.8377	1
**4633**	381	B	64.2022	1
**4633**	382	B	78.8151	1
**4633**	386	B	68.3492	1
**4633**	305	C	69.0259	1
**4633**	319	C	74.3009	1
**4633**	320	C	74.1961	-1
**4633**	321	C	79.4473	0
**4633**	351	C	9.9383	1
**4633**	357	C	79.5949	1
**4633**	370	C	60.7564	1
**4633**	372	C	68.5174	0
**4633**	373	C	79.4925	1
**4633**	381	C	71.1216	1
**4633**	382	C	78.0733	1
**4633**	386	C	15.8292	-1
**4633**	305	D	79.5108	1
**4633**	319	D	79.5207	1

**4633**	320	D	79.5788	-1
**4633**	321	D	79.5066	0
**4633**	351	D	7.5113	1
**4633**	357	D	71.3302	1
**4633**	370	D	78.3566	1
**4633**	372	D	59.3728	0
**4633**	373	D	57.3209	1
**4633**	381	D	77.2939	1
**4633**	382	D	62.3412	1
**4633**	386	D	79.5057	1
**4634**	305	A	79.6304	1
**4634**	319	A	41.2551	0
**4634**	320	A	79.5836	-1
**4634**	321	A	75.5593	0
**4634**	351	A	8.069	1
**4634**	357	A	32.7577	0
**4634**	370	A	79.5218	1
**4634**	372	A	61.4859	0
**4634**	373	A	79.481	1
**4634**	381	A	74.2136	1
**4634**	382	A	74.9014	1
**4634**	386	A	51.1121	1
**4634**	305	B	79.6005	1
**4634**	319	B	72.6812	1
**4634**	320	B	79.6386	-1
**4634**	321	B	79.5102	0
**4634**	351	B	15.8091	1
**4634**	357	B	74.3711	1
**4634**	370	B	61.607	1
**4634**	372	B	45.6175	1
**4634**	373	B	73.4268	1
**4634**	381	B	61.3936	1
**4634**	382	B	62.1348	1
**4634**	386	B	49.9431	0

**4634**	305	C	60.1537	1
**4634**	319	C	77.1453	1
**4634**	320	C	77.8587	-1
**4634**	321	C	79.6467	0
**4634**	351	C	10.6001	1
**4634**	357	C	79.5425	1
**4634**	370	C	79.5665	1
**4634**	372	C	79.4691	0
**4634**	373	C	73.9382	1
**4634**	381	C	42.4816	0
**4634**	382	C	65.8073	1
**4634**	386	C	44.4214	0
**4634**	305	D	72.5161	1
**4634**	319	D	79.6852	1
**4634**	320	D	37.8746	-1
**4634**	321	D	79.5301	0
**4634**	351	D	8.2746	1
**4634**	357	D	79.2972	1
**4634**	370	D	40.657	0
**4634**	372	D	69.7393	0
**4634**	373	D	73.8284	1
**4634**	381	D	50.0694	1
**4634**	382	D	79.6559	1
**4634**	386	D	77.2486	1
**4635**	305	A	77.0281	1
**4635**	319	A	75.8462	1
**4635**	320	A	79.5701	-1
**4635**	321	A	79.5509	0
**4635**	351	A	4.9785	1
**4635**	357	A	79.5008	1
**4635**	370	A	28.9227	0
**4635**	372	A	67.3358	0
**4635**	373	A	79.5806	1
**4635**	381	A	79.5941	1

**4635**	382	A	66.9738	1
**4635**	386	A	79.5332	1
**4635**	305	B	79.5217	1
**4635**	319	B	79.5837	1
**4635**	320	B	72.3662	-1
**4635**	321	B	79.5622	0
**4635**	351	B	5.8517	1
**4635**	357	B	79.6505	1
**4635**	370	B	32.7569	0
**4635**	372	B	72.3077	0
**4635**	373	B	71.2277	1
**4635**	381	B	72.9436	1
**4635**	382	B	75.2892	1
**4635**	386	B	79.5505	1
**4635**	305	C	59.9339	1
**4635**	319	C	33.7827	0
**4635**	320	C	76.0728	-1
**4635**	321	C	78.4457	0
**4635**	351	C	1.0675	1
**4635**	357	C	79.4967	1
**4635**	370	C	62.1933	1
**4635**	372	C	60.5735	0
**4635**	373	C	33.9712	0
**4635**	381	C	60.9449	1
**4635**	382	C	74.0885	1
**4635**	386	C	17.9892	-1
**4635**	305	D	65.4608	1
**4635**	319	D	79.5369	1
**4635**	320	D	73.37	-1
**4635**	321	D	77.7387	0
**4635**	351	D	4.1302	1
**4635**	357	D	79.2008	1
**4635**	370	D	44.8019	0
**4635**	372	D	53.0315	0

**4635**	373	D	73.9893	1
**4635**	381	D	54.2072	1
**4635**	382	D	71.4598	1
**4635**	386	D	75.5983	1
**4636**	305	A	65.9264	1
**4636**	319	A	78.0173	1
**4636**	320	A	79.2288	-1
**4636**	321	A	59.9415	0
**4636**	351	A	7.6757	1
**4636**	357	A	73.0065	1
**4636**	370	A	79.5656	1
**4636**	372	A	74.8771	0
**4636**	373	A	79.6613	1
**4636**	381	A	79.5312	1
**4636**	382	A	68.2514	1
**4636**	386	A	47.3791	0
**4636**	305	B	74.8516	1
**4636**	319	B	61.0456	1
**4636**	320	B	73.1273	-1
**4636**	321	B	79.5032	0
**4636**	351	B	16.6202	1
**4636**	357	B	70.8931	1
**4636**	370	B	21.7153	0
**4636**	372	B	61.4413	0
**4636**	373	B	39.2434	0
**4636**	381	B	79.6374	1
**4636**	382	B	75.9939	1
**4636**	386	B	41.7985	0
**4636**	305	C	74.629	1
**4636**	319	C	79.565	1
**4636**	320	C	48.2317	-1
**4636**	321	C	45.6458	1
**4636**	351	C	0.6249	1
**4636**	357	C	56.3171	1

**4636**	370	C	58.7654	1
**4636**	372	C	79.6027	0
**4636**	373	C	47.2779	0
**4636**	381	C	79.5756	1
**4636**	382	C	66.2114	1
**4636**	386	C	31.214	0
**4636**	305	D	56.5717	1
**4636**	319	D	79.5386	1
**4636**	320	D	72.6693	-1
**4636**	321	D	79.5118	0
**4636**	351	D	10.1085	1
**4636**	357	D	58.8344	1
**4636**	370	D	64.1126	1
**4636**	372	D	54.1978	0
**4636**	373	D	79.4127	1
**4636**	381	D	73.4221	1
**4636**	382	D	51.7929	1
**4636**	386	D	74.3453	1
**4637**	305	A	69.0906	1
**4637**	319	A	61.8475	1
**4637**	320	A	79.5449	-1
**4637**	321	A	79.6076	0
**4637**	351	A	8.8873	1
**4637**	357	A	79.5669	1
**4637**	370	A	74.1355	1
**4637**	372	A	79.6031	0
**4637**	373	A	63.3673	1
**4637**	381	A	79.6177	1
**4637**	382	A	69.4342	1
**4637**	386	A	72.4146	1
**4637**	305	B	47.3449	0
**4637**	319	B	79.5398	1
**4637**	320	B	79.561	-1
**4637**	321	B	79.6178	0

**4637**	351	B	17.1845	1
**4637**	357	B	66.3189	1
**4637**	370	B	71.9549	1
**4637**	372	B	79.6177	0
**4637**	373	B	79.5793	1
**4637**	381	B	72.5108	1
**4637**	382	B	37.9295	0
**4637**	386	B	79.539	1
**4637**	305	C	79.6133	1
**4637**	319	C	71.7793	1
**4637**	320	C	79.5984	-1
**4637**	321	C	26.828	1
**4637**	351	C	5.3927	1
**4637**	357	C	16.1596	-1
**4637**	370	C	74.5331	1
**4637**	372	C	72.7323	0
**4637**	373	C	79.6062	1
**4637**	381	C	56.4236	1
**4637**	382	C	34.2648	0
**4637**	386	C	32.1698	0
**4637**	305	D	46.2927	0
**4637**	319	D	48.8845	0
**4637**	320	D	79.5302	-1
**4637**	321	D	41.5461	1
**4637**	351	D	11.792	1
**4637**	357	D	79.535	1
**4637**	370	D	79.5606	1
**4637**	372	D	47.9284	1
**4637**	373	D	79.5527	1
**4637**	381	D	67.275	1
**4637**	382	D	60.7602	1
**4637**	386	D	46.6365	0
**4638**	305	A	58.4562	1
**4638**	319	A	64.9487	1

**4638**	320	A	79.6211	-1
**4638**	321	A	76.2687	0
**4638**	351	A	34.0118	-1
**4638**	357	A	73.6671	1
**4638**	370	A	60.9784	1
**4638**	372	A	34.4005	1
**4638**	373	A	53.422	1
**4638**	381	A	74.9616	1
**4638**	382	A	74.6579	1
**4638**	386	A	60.8109	1
**4638**	305	B	51.0145	1
**4638**	319	B	79.553	1
**4638**	320	B	63.5574	-1
**4638**	321	B	77.2965	0
**4638**	351	B	18.0751	1
**4638**	357	B	63.9558	1
**4638**	370	B	62.8726	1
**4638**	372	B	79.5324	0
**4638**	373	B	76.0884	1
**4638**	381	B	74.5325	1
**4638**	382	B	46.057	0
**4638**	386	B	75.4974	1
**4638**	305	C	54.4142	1
**4638**	319	C	64.9686	1
**4638**	320	C	79.5525	-1
**4638**	321	C	78.4825	0
**4638**	351	C	7.2279	1
**4638**	357	C	31.97	0
**4638**	370	C	79.5169	1
**4638**	372	C	71.6235	0
**4638**	373	C	75.4827	1
**4638**	381	C	20.5064	0
**4638**	382	C	58.5906	1
**4638**	386	C	18.7159	-1

**4638**	305	D	33.161	0
**4638**	319	D	50.5804	1
**4638**	320	D	59.9889	-1
**4638**	321	D	70.3312	0
**4638**	351	D	16.1264	1
**4638**	357	D	79.4992	1
**4638**	370	D	32.4963	0
**4638**	372	D	72.0026	0
**4638**	373	D	73.785	1
**4638**	381	D	49.0682	0
**4638**	382	D	68.1033	1
**4638**	386	D	74.5691	1
**4639**	305	A	72.8444	1
**4639**	319	A	73.5102	1
**4639**	320	A	73.3951	-1
**4639**	321	A	79.5473	0
**4639**	351	A	7.6331	1
**4639**	357	A	79.5823	1
**4639**	370	A	54.8302	1
**4639**	372	A	65.6584	0
**4639**	373	A	70.347	1
**4639**	381	A	72.9958	1
**4639**	382	A	56.3828	1
**4639**	386	A	72.0868	1
**4639**	305	B	74.0113	1
**4639**	319	B	76.9706	1
**4639**	320	B	67.5444	-1
**4639**	321	B	79.7889	0
**4639**	351	B	11.7217	1
**4639**	357	B	72.2806	1
**4639**	370	B	73.4613	1
**4639**	372	B	79.5782	0
**4639**	373	B	79.5189	1
**4639**	381	B	79.5483	1

**4639**	382	B	52.4687	1
**4639**	386	B	56.1515	1
**4639**	305	C	72.4393	1
**4639**	319	C	71.0563	1
**4639**	320	C	53.3436	-1
**4639**	321	C	76.5372	0
**4639**	351	C	5.9635	1
**4639**	357	C	75.5894	1
**4639**	370	C	38.9237	0
**4639**	372	C	55.7247	0
**4639**	373	C	48.6823	0
**4639**	381	C	79.5543	1
**4639**	382	C	79.537	1
**4639**	386	C	36.9075	0
**4639**	305	D	61.8274	1
**4639**	319	D	79.561	1
**4639**	320	D	79.5939	-1
**4639**	321	D	79.5525	0
**4639**	351	D	10.5808	1
**4639**	357	D	79.5882	1
**4639**	370	D	72.7602	1
**4639**	372	D	72.6533	0
**4639**	373	D	79.5569	1
**4639**	381	D	67.1487	1
**4639**	382	D	55.8187	1
**4639**	386	D	71.7862	1
**4640**	305	A	50.2264	1
**4640**	319	A	33.9949	0
**4640**	320	A	79.5554	-1
**4640**	321	A	68.2515	0
**4640**	351	A	7.9828	1
**4640**	357	A	79.551	1
**4640**	370	A	77.9415	1
**4640**	372	A	79.563	0

**4640**	373	A	56.9362	1
**4640**	381	A	73.6898	1
**4640**	382	A	79.557	1
**4640**	386	A	71.2036	1
**4640**	305	B	79.5835	1
**4640**	319	B	44.1915	0
**4640**	320	B	79.5598	-1
**4640**	321	B	70.1623	0
**4640**	351	B	17.0259	1
**4640**	357	B	51.6564	1
**4640**	370	B	79.1695	1
**4640**	372	B	79.6001	0
**4640**	373	B	12.567	-1
**4640**	381	B	69.8318	1
**4640**	382	B	35.36	0
**4640**	386	B	47.7243	0
**4640**	305	C	38.3311	0
**4640**	319	C	79.5473	1
**4640**	320	C	71.882	-1
**4640**	321	C	59.4396	0
**4640**	351	C	7.5509	1
**4640**	357	C	74.5908	1
**4640**	370	C	73.4474	1
**4640**	372	C	79.5492	0
**4640**	373	C	74.5773	1
**4640**	381	C	78.2208	1
**4640**	382	C	63.3263	1
**4640**	386	C	63.0489	1
**4640**	305	D	79.5578	1
**4640**	319	D	72.0807	1
**4640**	320	D	72.7684	-1
**4640**	321	D	77.5781	0
**4640**	351	D	14.9589	1
**4640**	357	D	72.7725	1

**4640**	370	D	63.5025	1
**4640**	372	D	79.5547	0
**4640**	373	D	56.726	1
**4640**	381	D	79.5452	1
**4640**	382	D	78.3051	1
**4640**	386	D	44.008	0
**4641**	305	A	79.59	1
**4641**	319	A	79.5754	1
**4641**	320	A	79.6234	-1
**4641**	321	A	72.0152	0
**4641**	351	A	4.6009	1
**4641**	357	A	76.7127	1
**4641**	370	A	79.535	1
**4641**	372	A	79.628	0
**4641**	373	A	78.1506	1
**4641**	381	A	79.5687	1
**4641**	382	A	79.6139	1
**4641**	386	A	79.6629	1
**4641**	305	B	67.0268	1
**4641**	319	B	72.5286	1
**4641**	320	B	57.3629	-1
**4641**	321	B	67.5123	0
**4641**	351	B	6.8262	1
**4641**	357	B	69.2261	1
**4641**	370	B	65.2763	1
**4641**	372	B	79.6087	0
**4641**	373	B	63.9482	1
**4641**	381	B	75.8103	1
**4641**	382	B	7.2431	-1
**4641**	386	B	58.7446	1
**4641**	305	C	74.4308	1
**4641**	319	C	65.5042	1
**4641**	320	C	79.4949	-1
**4641**	321	C	57.732	0

**4641**	351	C	1.1748	1
**4641**	357	C	79.5052	1
**4641**	370	C	66.639	1
**4641**	372	C	25.4623	1
**4641**	373	C	26.8578	0
**4641**	381	C	56.9374	1
**4641**	382	C	74.0674	1
**4641**	386	C	50.467	1
**4641**	305	D	79.507	1
**4641**	319	D	71.8603	1
**4641**	320	D	60.1486	-1
**4641**	321	D	56.736	0
**4641**	351	D	13.6441	1
**4641**	357	D	79.6894	1
**4641**	370	D	75.327	1
**4641**	372	D	55.3948	0
**4641**	373	D	56.8565	1
**4641**	381	D	74.626	1
**4641**	382	D	79.418	1
**4641**	386	D	73.6032	1
**4642**	305	A	53.2668	1
**4642**	319	A	72.8934	1
**4642**	320	A	71.804	-1
**4642**	321	A	77.3753	0
**4642**	351	A	6.6675	1
**4642**	357	A	79.501	1
**4642**	370	A	74.554	1
**4642**	372	A	72.9929	0
**4642**	373	A	79.6752	1
**4642**	381	A	49.9358	0
**4642**	382	A	22.6186	0
**4642**	386	A	31.1157	0
**4642**	305	B	79.5334	1
**4642**	319	B	76.2737	1

**4642**	320	B	79.5559	-1
**4642**	321	B	79.5454	0
**4642**	351	B	8.3029	1
**4642**	357	B	27.2029	0
**4642**	370	B	76.6229	1
**4642**	372	B	79.6085	0
**4642**	373	B	79.6396	1
**4642**	381	B	79.6706	1
**4642**	382	B	37.8962	0
**4642**	386	B	79.5469	1
**4642**	305	C	79.605	1
**4642**	319	C	78.8004	1
**4642**	320	C	79.0216	-1
**4642**	321	C	75.7553	0
**4642**	351	C	9.6884	1
**4642**	357	C	41.0047	0
**4642**	370	C	69.6362	1
**4642**	372	C	43.2217	1
**4642**	373	C	79.4712	1
**4642**	381	C	52.1706	1
**4642**	382	C	79.5752	1
**4642**	386	C	47.76	0
**4642**	305	D	53.5984	1
**4642**	319	D	68.0171	1
**4642**	320	D	79.509	-1
**4642**	321	D	71.1995	0
**4642**	351	D	15.7275	1
**4642**	357	D	79.6001	1
**4642**	370	D	73.9844	1
**4642**	372	D	72.1118	0
**4642**	373	D	48.4593	0
**4642**	381	D	76.9734	1
**4642**	382	D	78.7185	1
**4642**	386	D	43.1405	0

**4643**	305	A	79.6622	1
**4643**	319	A	75.4375	1
**4643**	320	A	79.5589	-1
**4643**	321	A	77.9381	0
**4643**	351	A	7.9796	1
**4643**	357	A	72.185	1
**4643**	370	A	79.5862	1
**4643**	372	A	51.3826	0
**4643**	373	A	50.7686	1
**4643**	381	A	79.4495	1
**4643**	382	A	79.618	1
**4643**	386	A	79.3412	1
**4643**	305	B	78.3986	1
**4643**	319	B	39.2761	0
**4643**	320	B	79.6436	-1
**4643**	321	B	71.7606	0
**4643**	351	B	15.4253	1
**4643**	357	B	79.5863	1
**4643**	370	B	79.6579	1
**4643**	372	B	61.1554	0
**4643**	373	B	41.4018	0
**4643**	381	B	65.9842	1
**4643**	382	B	69.734	1
**4643**	386	B	63.7848	1
**4643**	305	C	67.4645	1
**4643**	319	C	79.5991	1
**4643**	320	C	79.5702	-1
**4643**	321	C	47.7428	1
**4643**	351	C	5.1676	1
**4643**	357	C	79.5467	1
**4643**	370	C	78.9442	1
**4643**	372	C	73.166	0
**4643**	373	C	79.5198	1
**4643**	381	C	55.4564	1

**4643**	382	C	38.4503	0
**4643**	386	C	60.0751	1
**4643**	305	D	26.0368	0
**4643**	319	D	79.5656	1
**4643**	320	D	75.0764	-1
**4643**	321	D	73.0821	0
**4643**	351	D	10.9454	1
**4643**	357	D	27.7587	0
**4643**	370	D	79.5321	1
**4643**	372	D	79.5226	0
**4643**	373	D	58.7281	1
**4643**	381	D	79.6074	1
**4643**	382	D	79.5883	1
**4643**	386	D	73.1975	1
**4644**	305	A	65.1204	1
**4644**	319	A	79.6147	1
**4644**	320	A	47.6806	-1
**4644**	321	A	70.9296	0
**4644**	351	A	9.46	1
**4644**	357	A	79.5533	1
**4644**	370	A	67.3052	1
**4644**	372	A	51.624	0
**4644**	373	A	79.5238	1
**4644**	381	A	79.5217	1
**4644**	382	A	69.5135	1
**4644**	386	A	19.6071	-1
**4644**	305	B	43.1384	0
**4644**	319	B	53.069	1
**4644**	320	B	76.3641	-1
**4644**	321	B	79.4497	0
**4644**	351	B	11.8354	1
**4644**	357	B	73.4573	1
**4644**	370	B	71.8495	1
**4644**	372	B	72.7256	0

**4644**	373	B	62.431	1
**4644**	381	B	79.577	1
**4644**	382	B	43.1614	0
**4644**	386	B	54.9227	1
**4644**	305	C	55.133	1
**4644**	319	C	48.244	0
**4644**	320	C	79.5976	-1
**4644**	321	C	61.0839	0
**4644**	351	C	6.1811	1
**4644**	357	C	70.0817	1
**4644**	370	C	79.54	1
**4644**	372	C	69.4533	0
**4644**	373	C	22.8302	0
**4644**	381	C	49.6503	0
**4644**	382	C	79.5829	1
**4644**	386	C	28.9049	0
**4644**	305	D	67.5985	1
**4644**	319	D	59.5567	1
**4644**	320	D	79.5736	-1
**4644**	321	D	79.5758	0
**4644**	351	D	19.8747	1
**4644**	357	D	42.0811	0
**4644**	370	D	74.5141	1
**4644**	372	D	72.3252	0
**4644**	373	D	56.9083	1
**4644**	381	D	75.2731	1
**4644**	382	D	41.9856	0
**4644**	386	D	53.0577	1
**4645**	305	A	64.8824	1
**4645**	319	A	67.7382	1
**4645**	320	A	66.8262	-1
**4645**	321	A	46.0313	1
**4645**	351	A	2.1844	1
**4645**	357	A	79.6117	1

**4645**	370	A	51.6797	1
**4645**	372	A	75.2203	0
**4645**	373	A	79.5669	1
**4645**	381	A	46.7017	0
**4645**	382	A	79.5791	1
**4645**	386	A	79.6076	1
**4645**	305	B	79.6423	1
**4645**	319	B	75.2246	1
**4645**	320	B	79.6185	-1
**4645**	321	B	70.6643	0
**4645**	351	B	11.0608	1
**4645**	357	B	68.8808	1
**4645**	370	B	51.8198	1
**4645**	372	B	72.6611	0
**4645**	373	B	79.4526	1
**4645**	381	B	47.7964	0
**4645**	382	B	61.8512	1
**4645**	386	B	79.5564	1
**4645**	305	C	74.4476	1
**4645**	319	C	78.7801	1
**4645**	320	C	70.0237	-1
**4645**	321	C	33.4371	1
**4645**	351	C	9.8135	1
**4645**	357	C	78.4105	1
**4645**	370	C	50.2564	1
**4645**	372	C	79.5316	0
**4645**	373	C	72.2197	1
**4645**	381	C	79.5974	1
**4645**	382	C	65.6954	1
**4645**	386	C	25.9984	0
**4645**	305	D	70.9064	1
**4645**	319	D	79.5388	1
**4645**	320	D	79.5375	-1
**4645**	321	D	59.0647	0

**4645**	351	D	11.2534	1
**4645**	357	D	74.0383	1
**4645**	370	D	12.8498	-1
**4645**	372	D	79.5293	0
**4645**	373	D	79.4887	1
**4645**	381	D	79.5493	1
**4645**	382	D	79.4954	1
**4645**	386	D	33.461	0
**4646**	305	A	71.5275	1
**4646**	319	A	79.5944	1
**4646**	320	A	73.5331	-1
**4646**	321	A	45.2186	1
**4646**	351	A	8.8504	1
**4646**	357	A	79.4923	1
**4646**	370	A	54.4556	1
**4646**	372	A	57.4426	0
**4646**	373	A	75.2606	1
**4646**	381	A	79.632	1
**4646**	382	A	72.7834	1
**4646**	386	A	77.0776	1
**4646**	305	B	46.0893	0
**4646**	319	B	71.23	1
**4646**	320	B	72.4707	-1
**4646**	321	B	79.4697	0
**4646**	351	B	13.9575	1
**4646**	357	B	79.6052	1
**4646**	370	B	79.61	1
**4646**	372	B	56.643	0
**4646**	373	B	79.545	1
**4646**	381	B	76.985	1
**4646**	382	B	50.1081	1
**4646**	386	B	79.5985	1
**4646**	305	C	60.1618	1
**4646**	319	C	79.5909	1

**4646**	320	C	73.6448	-1
**4646**	321	C	46.9663	1
**4646**	351	C	4.9797	1
**4646**	357	C	79.5313	1
**4646**	370	C	79.5581	1
**4646**	372	C	70.5775	0
**4646**	373	C	79.6136	1
**4646**	381	C	43.2794	0
**4646**	382	C	55.1511	1
**4646**	386	C	40.1219	0
**4646**	305	D	70.9242	1
**4646**	319	D	77.0979	1
**4646**	320	D	24.1044	-1
**4646**	321	D	73.7375	0
**4646**	351	D	1.8933	1
**4646**	357	D	42.3238	0
**4646**	370	D	79.5462	1
**4646**	372	D	74.3848	0
**4646**	373	D	71.8611	1
**4646**	381	D	78.4292	1
**4646**	382	D	78.2926	1
**4646**	386	D	78.6799	1
**4647**	305	A	72.7262	1
**4647**	319	A	56.5774	1
**4647**	320	A	79.575	-1
**4647**	321	A	72.2179	0
**4647**	351	A	6.5218	1
**4647**	357	A	79.564	1
**4647**	370	A	79.542	1
**4647**	372	A	79.5702	0
**4647**	373	A	79.5737	1
**4647**	381	A	58.7933	1
**4647**	382	A	78.6426	1
**4647**	386	A	79.5613	1

**4647**	305	B	46.0079	0
**4647**	319	B	55.8645	1
**4647**	320	B	68.1388	-1
**4647**	321	B	70.1948	0
**4647**	351	B	20.7209	-1
**4647**	357	B	79.567	1
**4647**	370	B	70.3297	1
**4647**	372	B	70.2865	0
**4647**	373	B	63.7543	1
**4647**	381	B	73.409	1
**4647**	382	B	39.1576	0
**4647**	386	B	71.0873	1
**4647**	305	C	79.5924	1
**4647**	319	C	72.7487	1
**4647**	320	C	79.522	-1
**4647**	321	C	76.706	0
**4647**	351	C	5.3739	1
**4647**	357	C	79.5582	1
**4647**	370	C	79.6201	1
**4647**	372	C	72.4147	0
**4647**	373	C	79.4418	1
**4647**	381	C	45.344	0
**4647**	382	C	71.4603	1
**4647**	386	C	64.1403	1
**4647**	305	D	68.3696	1
**4647**	319	D	78.9494	1
**4647**	320	D	79.5933	-1
**4647**	321	D	75.4651	0
**4647**	351	D	12.1432	1
**4647**	357	D	74.8559	1
**4647**	370	D	79.2379	1
**4647**	372	D	78.5252	0
**4647**	373	D	71.0068	1
**4647**	381	D	79.5925	1

**4647**	382	D	65.5999	1
**4647**	386	D	74.7502	1
**4648**	305	A	66.5793	1
**4648**	319	A	42.0953	0
**4648**	320	A	79.5679	-1
**4648**	321	A	36.3959	1
**4648**	351	A	10.7346	1
**4648**	357	A	50.2491	1
**4648**	370	A	79.5591	1
**4648**	372	A	79.5531	0
**4648**	373	A	79.5467	1
**4648**	381	A	79.5502	1
**4648**	382	A	79.5632	1
**4648**	386	A	56.5501	1
**4648**	305	B	79.3796	1
**4648**	319	B	71.0715	1
**4648**	320	B	79.5282	-1
**4648**	321	B	79.6007	0
**4648**	351	B	13.457	1
**4648**	357	B	79.5369	1
**4648**	370	B	46.4223	0
**4648**	372	B	79.5231	0
**4648**	373	B	53.3312	1
**4648**	381	B	58.4357	1
**4648**	382	B	61.9997	1
**4648**	386	B	70.8231	1
**4648**	305	C	63.1389	1
**4648**	319	C	46.5192	0
**4648**	320	C	52.9977	-1
**4648**	321	C	79.6191	0
**4648**	351	C	2.948	1
**4648**	357	C	79.5482	1
**4648**	370	C	58.1714	1
**4648**	372	C	73.039	0

**4648**	373	C	73.0858	1
**4648**	381	C	62.1787	1
**4648**	382	C	71.8519	1
**4648**	386	C	40.587	0
**4648**	305	D	59.3874	1
**4648**	319	D	73.6161	1
**4648**	320	D	58.7885	-1
**4648**	321	D	79.5703	0
**4648**	351	D	0.1137	1
**4648**	357	D	72.5973	1
**4648**	370	D	42.7254	0
**4648**	372	D	79.5746	0
**4648**	373	D	76.0333	1
**4648**	381	D	79.5404	1
**4648**	382	D	79.543	1
**4648**	386	D	79.5692	1
**4649**	305	A	79.4621	1
**4649**	319	A	64.9214	1
**4649**	320	A	63.4613	-1
**4649**	321	A	71.359	0
**4649**	351	A	7.5487	1
**4649**	357	A	57.1413	1
**4649**	370	A	37.0859	0
**4649**	372	A	79.5308	0
**4649**	373	A	73.9619	1
**4649**	381	A	42.1259	0
**4649**	382	A	75.1412	1
**4649**	386	A	75.5872	1
**4649**	305	B	57.7762	1
**4649**	319	B	74.4347	1
**4649**	320	B	78.8595	-1
**4649**	321	B	57.2254	0
**4649**	351	B	14.5075	1
**4649**	357	B	69.7226	1

**4649**	370	B	79.1019	1
**4649**	372	B	72.3549	0
**4649**	373	B	76.5368	1
**4649**	381	B	62.5669	1
**4649**	382	B	16.9345	-1
**4649**	386	B	59.3944	1
**4649**	305	C	62.5626	1
**4649**	319	C	72.547	1
**4649**	320	C	29.1666	-1
**4649**	321	C	77.2419	0
**4649**	351	C	9.9374	1
**4649**	357	C	72.3092	1
**4649**	370	C	72.4184	1
**4649**	372	C	79.5815	0
**4649**	373	C	79.5158	1
**4649**	381	C	61.1121	1
**4649**	382	C	72.2994	1
**4649**	386	C	4.3723	-1
**4649**	305	D	70.8344	1
**4649**	319	D	13.9994	-1
**4649**	320	D	79.0569	-1
**4649**	321	D	25.704	1
**4649**	351	D	6.501	1
**4649**	357	D	39.0092	0
**4649**	370	D	72.9433	1
**4649**	372	D	79.5308	0
**4649**	373	D	48.3764	0
**4649**	381	D	75.5307	1
**4649**	382	D	62.008	1
**4649**	386	D	79.613	1
**4650**	305	A	69.7622	1
**4650**	319	A	63.9857	1
**4650**	320	A	79.7792	-1
**4650**	321	A	79.593	0

**4650**	351	A	20.8289	-1
**4650**	357	A	79.5882	1
**4650**	370	A	79.5124	1
**4650**	372	A	53.8043	0
**4650**	373	A	71.2043	1
**4650**	381	A	70.4775	1
**4650**	382	A	64.8136	1
**4650**	386	A	63.7985	1
**4650**	305	B	79.6562	1
**4650**	319	B	42.2758	0
**4650**	320	B	48.9425	-1
**4650**	321	B	47.8987	1
**4650**	351	B	25.479	-1
**4650**	357	B	67.3529	1
**4650**	370	B	79.6141	1
**4650**	372	B	79.5621	0
**4650**	373	B	79.5267	1
**4650**	381	B	29.7148	0
**4650**	382	B	53.5375	1
**4650**	386	B	73.9987	1
**4650**	305	C	79.6109	1
**4650**	319	C	79.53	1
**4650**	320	C	50.9188	-1
**4650**	321	C	77.6146	0
**4650**	351	C	2.3178	1
**4650**	357	C	78.2216	1
**4650**	370	C	79.4578	1
**4650**	372	C	78.5097	0
**4650**	373	C	72.3727	1
**4650**	381	C	71.2287	1
**4650**	382	C	49.9979	0
**4650**	386	C	44.254	0
**4650**	305	D	41.4732	0
**4650**	319	D	59.8867	1

**4650**	320	D	74.4526	-1
**4650**	321	D	79.6539	0
**4650**	351	D	9.0696	1
**4650**	357	D	75.6134	1
**4650**	370	D	79.5762	1
**4650**	372	D	79.6322	0
**4650**	373	D	79.243	1
**4650**	381	D	43.7556	0
**4650**	382	D	69.456	1
**4650**	386	D	51.4758	1
**4651**	305	A	53.3306	1
**4651**	319	A	78.7985	1
**4651**	320	A	79.4702	-1
**4651**	321	A	72.9576	0
**4651**	351	A	10.3204	1
**4651**	357	A	52.338	1
**4651**	370	A	30.9657	0
**4651**	372	A	79.6342	0
**4651**	373	A	72.9025	1
**4651**	381	A	73.4271	1
**4651**	382	A	54.9679	1
**4651**	386	A	29.5376	0
**4651**	305	B	50.0321	1
**4651**	319	B	79.6644	1
**4651**	320	B	79.6024	-1
**4651**	321	B	79.5106	0
**4651**	351	B	10.2539	1
**4651**	357	B	78.4751	1
**4651**	370	B	74.2425	1
**4651**	372	B	63.4195	0
**4651**	373	B	28.1238	0
**4651**	381	B	74.2583	1
**4651**	382	B	34.4326	0
**4651**	386	B	79.6673	1

**4651**	305	C	77.7111	1
**4651**	319	C	69.2908	1
**4651**	320	C	79.5902	-1
**4651**	321	C	49.9963	1
**4651**	351	C	11.5081	1
**4651**	357	C	77.016	1
**4651**	370	C	29.4623	0
**4651**	372	C	79.5777	0
**4651**	373	C	39.3197	0
**4651**	381	C	71.4794	1
**4651**	382	C	72.5553	1
**4651**	386	C	29.4048	0
**4651**	305	D	31.1919	0
**4651**	319	D	72.5317	1
**4651**	320	D	78.217	-1
**4651**	321	D	79.5503	0
**4651**	351	D	3.4869	1
**4651**	357	D	36.3692	0
**4651**	370	D	36.7871	0
**4651**	372	D	70.5974	0
**4651**	373	D	24.0944	0
**4651**	381	D	19.0174	-1
**4651**	382	D	79.5891	1
**4651**	386	D	66.2178	1
**4652**	305	A	24.1792	0
**4652**	319	A	29.3598	0
**4652**	320	A	53.5797	-1
**4652**	321	A	23.0277	1
**4652**	351	A	40.3076	-1
**4652**	357	A	79.5728	1
**4652**	370	A	74.4315	1
**4652**	372	A	67.2608	0
**4652**	373	A	75.0201	1
**4652**	381	A	46.0665	0

**4652**	382	A	77.6353	1
**4652**	386	A	73.8345	1
**4652**	305	B	79.5462	1
**4652**	319	B	79.5267	1
**4652**	320	B	79.5267	-1
**4652**	321	B	73.8582	0
**4652**	351	B	16.227	1
**4652**	357	B	79.5799	1
**4652**	370	B	49.1902	0
**4652**	372	B	31.0858	1
**4652**	373	B	54.0099	1
**4652**	381	B	79.5267	1
**4652**	382	B	36.1943	0
**4652**	386	B	79.4997	1
**4652**	305	C	65.3605	1
**4652**	319	C	66.1573	1
**4652**	320	C	64.8082	-1
**4652**	321	C	78.6903	0
**4652**	351	C	3.265	1
**4652**	357	C	73.8004	1
**4652**	370	C	78.0654	1
**4652**	372	C	79.6106	0
**4652**	373	C	79.5592	1
**4652**	381	C	79.588	1
**4652**	382	C	79.5269	1
**4652**	386	C	61.7143	1
**4652**	305	D	60.0558	1
**4652**	319	D	50.0016	1
**4652**	320	D	75.8011	-1
**4652**	321	D	79.5747	0
**4652**	351	D	13.8713	1
**4652**	357	D	79.5686	1
**4652**	370	D	77.5081	1
**4652**	372	D	79.1538	0

**4652**	373	D	79.5925	1
**4652**	381	D	45.2136	0
**4652**	382	D	79.611	1
**4652**	386	D	42.4018	0
**4653**	305	A	71.4588	1
**4653**	319	A	79.4927	1
**4653**	320	A	79.5079	-1
**4653**	321	A	79.4567	0
**4653**	351	A	1.4034	1
**4653**	357	A	78.9392	1
**4653**	370	A	64.1203	1
**4653**	372	A	68.5524	0
**4653**	373	A	79.4499	1
**4653**	381	A	71.7169	1
**4653**	382	A	48.4035	0
**4653**	386	A	44.6447	0
**4653**	305	B	50.8303	1
**4653**	319	B	79.5412	1
**4653**	320	B	60.2575	-1
**4653**	321	B	17.0867	0
**4653**	351	B	10.7047	1
**4653**	357	B	71.4549	1
**4653**	370	B	79.54	1
**4653**	372	B	61.8819	0
**4653**	373	B	79.6617	1
**4653**	381	B	23.607	0
**4653**	382	B	79.5434	1
**4653**	386	B	60.283	1
**4653**	305	C	61.3595	1
**4653**	319	C	79.8215	1
**4653**	320	C	74.029	-1
**4653**	321	C	79.5749	0
**4653**	351	C	8.3443	1
**4653**	357	C	79.5936	1

**4653**	370	C	79.5935	1
**4653**	372	C	62.5273	0
**4653**	373	C	72.4271	1
**4653**	381	C	79.4973	1
**4653**	382	C	79.5515	1
**4653**	386	C	31.5219	0
**4653**	305	D	72.5876	1
**4653**	319	D	59.215	1
**4653**	320	D	79.6452	-1
**4653**	321	D	79.465	0
**4653**	351	D	13.9047	1
**4653**	357	D	54.545	1
**4653**	370	D	74.8138	1
**4653**	372	D	79.517	0
**4653**	373	D	45.8936	0
**4653**	381	D	73.6339	1
**4653**	382	D	57.8016	1
**4653**	386	D	72.3141	1
**4654**	305	A	79.5532	1
**4654**	319	A	72.5491	1
**4654**	320	A	63.2788	-1
**4654**	321	A	68.6383	0
**4654**	351	A	9.4374	1
**4654**	357	A	51.4404	1
**4654**	370	A	61.4203	1
**4654**	372	A	66.6137	0
**4654**	373	A	40.4756	0
**4654**	381	A	66.0983	1
**4654**	382	A	31.6072	0
**4654**	386	A	63.6977	1
**4654**	305	B	79.5645	1
**4654**	319	B	79.4677	1
**4654**	320	B	43.5909	-1
**4654**	321	B	79.5218	0

**4654**	351	B	13.3504	1
**4654**	357	B	64.8733	1
**4654**	370	B	38.9115	0
**4654**	372	B	45.1317	1
**4654**	373	B	77.2042	1
**4654**	381	B	68.7367	1
**4654**	382	B	70.2304	1
**4654**	386	B	79.6329	1
**4654**	305	C	79.5961	1
**4654**	319	C	74.5424	1
**4654**	320	C	74.0642	-1
**4654**	321	C	61.9897	0
**4654**	351	C	1.4969	1
**4654**	357	C	54.2518	1
**4654**	370	C	78.4173	1
**4654**	372	C	67.8084	0
**4654**	373	C	79.3918	1
**4654**	381	C	78.1228	1
**4654**	382	C	79.5574	1
**4654**	386	C	67.6204	1
**4654**	305	D	75.9774	1
**4654**	319	D	79.6211	1
**4654**	320	D	73.7761	-1
**4654**	321	D	79.4664	0
**4654**	351	D	11.8575	1
**4654**	357	D	79.6337	1
**4654**	370	D	79.5705	1
**4654**	372	D	79.5012	0
**4654**	373	D	78.7984	1
**4654**	381	D	49.4476	0
**4654**	382	D	73.4508	1
**4654**	386	D	49.3651	0
**4655**	305	A	77.8501	1
**4655**	319	A	41.5215	0

**4655**	320	A	64.8044	-1
**4655**	321	A	69.9208	0
**4655**	351	A	10.7432	1
**4655**	357	A	79.3476	1
**4655**	370	A	79.3721	1
**4655**	372	A	79.5784	0
**4655**	373	A	79.5105	1
**4655**	381	A	79.4378	1
**4655**	382	A	61.423	1
**4655**	386	A	56.0304	1
**4655**	305	B	71.4511	1
**4655**	319	B	79.5806	1
**4655**	320	B	61.191	-1
**4655**	321	B	55.174	0
**4655**	351	B	14.523	1
**4655**	357	B	74.2914	1
**4655**	370	B	55.1027	1
**4655**	372	B	74.8274	0
**4655**	373	B	54.6951	1
**4655**	381	B	79.6083	1
**4655**	382	B	55.0949	1
**4655**	386	B	79.0471	1
**4655**	305	C	79.686	1
**4655**	319	C	66.8405	1
**4655**	320	C	72.0623	-1
**4655**	321	C	79.4456	0
**4655**	351	C	4.0561	1
**4655**	357	C	73.5576	1
**4655**	370	C	63.6929	1
**4655**	372	C	79.5399	0
**4655**	373	C	63.7374	1
**4655**	381	C	72.61	1
**4655**	382	C	60.5549	1
**4655**	386	C	61.7557	1

**4655**	305	D	45.255	0
**4655**	319	D	44.7395	0
**4655**	320	D	20.5742	-1
**4655**	321	D	65.1051	0
**4655**	351	D	19.3633	1
**4655**	357	D	71.8714	1
**4655**	370	D	77.572	1
**4655**	372	D	72.613	0
**4655**	373	D	79.5399	1
**4655**	381	D	79.5497	1
**4655**	382	D	66.2354	1
**4655**	386	D	70.8374	1
**4656**	305	A	69.1001	1
**4656**	319	A	45.5141	0
**4656**	320	A	79.6269	-1
**4656**	321	A	79.5568	0
**4656**	351	A	5.5698	1
**4656**	357	A	79.5796	1
**4656**	370	A	65.7471	1
**4656**	372	A	73.2971	0
**4656**	373	A	25.2162	0
**4656**	381	A	79.558	1
**4656**	382	A	77.0111	1
**4656**	386	A	15.9777	-1
**4656**	305	B	63.2238	1
**4656**	319	B	24.8223	0
**4656**	320	B	13.1234	1
**4656**	321	B	70.301	0
**4656**	351	B	14.8494	1
**4656**	357	B	62.9125	1
**4656**	370	B	79.5728	1
**4656**	372	B	79.6354	0
**4656**	373	B	47.8769	0
**4656**	381	B	70.5589	1

**4656**	382	B	33.2184	0
**4656**	386	B	79.5561	1
**4656**	305	C	75.6041	1
**4656**	319	C	65.2392	1
**4656**	320	C	79.7118	-1
**4656**	321	C	48.7905	1
**4656**	351	C	3.4979	1
**4656**	357	C	72.1683	1
**4656**	370	C	71.013	1
**4656**	372	C	60.3116	0
**4656**	373	C	70.6995	1
**4656**	381	C	70.6812	1
**4656**	382	C	76.0981	1
**4656**	386	C	54.5241	1
**4656**	305	D	34.9441	0
**4656**	319	D	73.5493	1
**4656**	320	D	79.5256	-1
**4656**	321	D	64.0621	0
**4656**	351	D	16.3007	1
**4656**	357	D	79.5753	1
**4656**	370	D	53.7079	1
**4656**	372	D	74.6051	0
**4656**	373	D	66.8813	1
**4656**	381	D	71.1199	1
**4656**	382	D	64.4993	1
**4656**	386	D	21.7889	0
**4657**	305	A	65.2868	1
**4657**	319	A	75.5476	1
**4657**	320	A	47.989	-1
**4657**	321	A	61.7973	0
**4657**	351	A	14.4491	1
**4657**	357	A	33.5201	0
**4657**	370	A	79.5445	1
**4657**	372	A	79.5038	0

**4657**	373	A	66.1749	1
**4657**	381	A	68.069	1
**4657**	382	A	59.8632	1
**4657**	386	A	79.4116	1
**4657**	305	B	56.6546	1
**4657**	319	B	76.7571	1
**4657**	320	B	63.5076	-1
**4657**	321	B	50.4847	0
**4657**	351	B	15.5757	1
**4657**	357	B	79.5655	1
**4657**	370	B	42.6195	0
**4657**	372	B	59.6665	0
**4657**	373	B	49.33	0
**4657**	381	B	57.2977	1
**4657**	382	B	35.9791	0
**4657**	386	B	37.2278	0
**4657**	305	C	78.0819	1
**4657**	319	C	51.4424	1
**4657**	320	C	59.2363	-1
**4657**	321	C	79.5226	0
**4657**	351	C	6.1842	1
**4657**	357	C	76.679	1
**4657**	370	C	77.2466	1
**4657**	372	C	79.5702	0
**4657**	373	C	46.7559	0
**4657**	381	C	79.4696	1
**4657**	382	C	36.5242	0
**4657**	386	C	51.5113	1
**4657**	305	D	69.4489	1
**4657**	319	D	53.6814	1
**4657**	320	D	64.3355	-1
**4657**	321	D	59.1302	0
**4657**	351	D	7.9616	1
**4657**	357	D	79.6418	1

**4657**	370	D	59.5381	1
**4657**	372	D	61.6051	0
**4657**	373	D	79.2168	1
**4657**	381	D	60.5899	1
**4657**	382	D	76.0901	1
**4657**	386	D	59.166	1
**4658**	305	A	55.8691	1
**4658**	319	A	49.7808	0
**4658**	320	A	79.6518	-1
**4658**	321	A	54.3371	0
**4658**	351	A	4.7865	1
**4658**	357	A	75.7125	1
**4658**	370	A	79.614	1
**4658**	372	A	64.3797	0
**4658**	373	A	59.5152	1
**4658**	381	A	35.5335	0
**4658**	382	A	70.558	1
**4658**	386	A	59.9493	1
**4658**	305	B	79.5677	1
**4658**	319	B	79.7546	1
**4658**	320	B	46.5646	-1
**4658**	321	B	78.4856	0
**4658**	351	B	24.087	-1
**4658**	357	B	74.657	1
**4658**	370	B	79.341	1
**4658**	372	B	79.5223	0
**4658**	373	B	42.103	0
**4658**	381	B	23.7252	0
**4658**	382	B	71.7625	1
**4658**	386	B	73.9983	1
**4658**	305	C	79.5773	1
**4658**	319	C	68.2315	1
**4658**	320	C	79.5613	-1
**4658**	321	C	79.6484	0

**4658**	351	C	4.9296	1
**4658**	357	C	79.44	1
**4658**	370	C	65.8205	1
**4658**	372	C	44.348	1
**4658**	373	C	68.3744	1
**4658**	381	C	13.975	-1
**4658**	382	C	79.743	1
**4658**	386	C	52.2606	1
**4658**	305	D	70.1178	1
**4658**	319	D	79.5971	1
**4658**	320	D	48.9499	-1
**4658**	321	D	69.0506	0
**4658**	351	D	7.9542	1
**4658**	357	D	72.5513	1
**4658**	370	D	79.6936	1
**4658**	372	D	79.4969	0
**4658**	373	D	57.9448	1
**4658**	381	D	24.2831	0
**4658**	382	D	74.4154	1
**4658**	386	D	69.0776	1
**4659**	305	A	70.4853	1
**4659**	319	A	79.5727	1
**4659**	320	A	71.437	-1
**4659**	321	A	16.9775	0
**4659**	351	A	36.0885	-1
**4659**	357	A	79.4032	1
**4659**	370	A	76.0204	1
**4659**	372	A	58.0826	0
**4659**	373	A	79.5714	1
**4659**	381	A	77.485	1
**4659**	382	A	51.6902	1
**4659**	386	A	73.0268	1
**4659**	305	B	68.2192	1
**4659**	319	B	68.386	1

**4659**	320	B	79.5102	-1
**4659**	321	B	79.7171	0
**4659**	351	B	13.2157	1
**4659**	357	B	78.6953	1
**4659**	370	B	64.3901	1
**4659**	372	B	73.9408	0
**4659**	373	B	79.6023	1
**4659**	381	B	37.3729	0
**4659**	382	B	46.4153	0
**4659**	386	B	47.805	0
**4659**	305	C	58.1176	1
**4659**	319	C	79.3293	1
**4659**	320	C	65.5678	-1
**4659**	321	C	79.4721	0
**4659**	351	C	5.5178	1
**4659**	357	C	76.1107	1
**4659**	370	C	79.4386	1
**4659**	372	C	63.0824	0
**4659**	373	C	69.5629	1
**4659**	381	C	79.5384	1
**4659**	382	C	27.4018	0
**4659**	386	C	18.0583	-1
**4659**	305	D	79.7297	1
**4659**	319	D	56.7941	1
**4659**	320	D	33.5774	-1
**4659**	321	D	79.4866	0
**4659**	351	D	19.6469	1
**4659**	357	D	67.4418	1
**4659**	370	D	70.0818	1
**4659**	372	D	44.397	1
**4659**	373	D	79.1532	1
**4659**	381	D	72.8958	1
**4659**	382	D	51.3845	1
**4659**	386	D	78.4113	1

**4660**	305	A	58.0742	1
**4660**	319	A	18.9905	-1
**4660**	320	A	51.3881	-1
**4660**	321	A	79.594	0
**4660**	351	A	10.7722	1
**4660**	357	A	79.5866	1
**4660**	370	A	70.4611	1
**4660**	372	A	70.2016	0
**4660**	373	A	79.5598	1
**4660**	381	A	79.5284	1
**4660**	382	A	57.134	1
**4660**	386	A	60.4241	1
**4660**	305	B	57.7672	1
**4660**	319	B	56.8756	1
**4660**	320	B	62.706	-1
**4660**	321	B	79.4609	0
**4660**	351	B	12.1767	1
**4660**	357	B	58.655	1
**4660**	370	B	77.5631	1
**4660**	372	B	53.6073	0
**4660**	373	B	52.6699	1
**4660**	381	B	75.7023	1
**4660**	382	B	29.9168	0
**4660**	386	B	23.5253	0
**4660**	305	C	32.2167	0
**4660**	319	C	68.9195	1
**4660**	320	C	22.5947	-1
**4660**	321	C	75.9086	0
**4660**	351	C	2.9725	1
**4660**	357	C	79.5197	1
**4660**	370	C	41.5388	0
**4660**	372	C	45.8163	1
**4660**	373	C	69.0695	1
**4660**	381	C	79.5307	1

**4660**	382	C	16.2049	-1
**4660**	386	C	40.6595	0
**4660**	305	D	58.4511	1
**4660**	319	D	73.0959	1
**4660**	320	D	79.5045	-1
**4660**	321	D	67.206	0
**4660**	351	D	4.8061	1
**4660**	357	D	74.4405	1
**4660**	370	D	73.7113	1
**4660**	372	D	69.7565	0
**4660**	373	D	73.0948	1
**4660**	381	D	42.2802	0
**4660**	382	D	79.6595	1
**4660**	386	D	60.4466	1
**4661**	305	A	79.6747	1
**4661**	319	A	44.1314	0
**4661**	320	A	68.8818	-1
**4661**	321	A	73.7714	0
**4661**	351	A	0.0285	1
**4661**	357	A	70.7074	1
**4661**	370	A	79.5119	1
**4661**	372	A	46.669	1
**4661**	373	A	79.5664	1
**4661**	381	A	79.5826	1
**4661**	382	A	72.7831	1
**4661**	386	A	74.1838	1
**4661**	305	B	73.4769	1
**4661**	319	B	57.1263	1
**4661**	320	B	33.1052	-1
**4661**	321	B	66.2511	0
**4661**	351	B	5.6546	1
**4661**	357	B	49.1952	0
**4661**	370	B	55.4847	1
**4661**	372	B	72.0987	0

**4661**	373	B	70.4485	1
**4661**	381	B	64.2098	1
**4661**	382	B	38.9608	0
**4661**	386	B	70.1635	1
**4661**	305	C	58.5214	1
**4661**	319	C	75.0715	1
**4661**	320	C	23.2712	-1
**4661**	321	C	79.4662	0
**4661**	351	C	9.2473	1
**4661**	357	C	52.8048	1
**4661**	370	C	76.792	1
**4661**	372	C	72.2086	0
**4661**	373	C	79.5708	1
**4661**	381	C	47.1298	0
**4661**	382	C	45.405	0
**4661**	386	C	41.8168	0
**4661**	305	D	79.7048	1
**4661**	319	D	70.6582	1
**4661**	320	D	62.6388	-1
**4661**	321	D	79.5895	0
**4661**	351	D	11.3882	1
**4661**	357	D	58.903	1
**4661**	370	D	73.7997	1
**4661**	372	D	72.3152	0
**4661**	373	D	56.4331	1
**4661**	381	D	58.9024	1
**4661**	382	D	31.9119	0
**4661**	386	D	69.5617	1
**4662**	305	A	79.5509	1
**4662**	319	A	41.5107	0
**4662**	320	A	79.5404	-1
**4662**	321	A	79.5279	0
**4662**	351	A	15.0032	1
**4662**	357	A	71.2215	1

**4662**	370	A	73.2904	1
**4662**	372	A	79.576	0
**4662**	373	A	78.4498	1
**4662**	381	A	67.5967	1
**4662**	382	A	79.5524	1
**4662**	386	A	42.5608	0
**4662**	305	B	73.2222	1
**4662**	319	B	58.363	1
**4662**	320	B	61.0367	-1
**4662**	321	B	68.1561	0
**4662**	351	B	19.5107	1
**4662**	357	B	79.5796	1
**4662**	370	B	46.4949	0
**4662**	372	B	79.5587	0
**4662**	373	B	52.2884	1
**4662**	381	B	77.8827	1
**4662**	382	B	33.4873	0
**4662**	386	B	72.1948	1
**4662**	305	C	59.1271	1
**4662**	319	C	79.5883	1
**4662**	320	C	74.1213	-1
**4662**	321	C	62.3346	0
**4662**	351	C	41.9665	-1
**4662**	357	C	53.3319	1
**4662**	370	C	73.051	1
**4662**	372	C	73.0247	0
**4662**	373	C	79.5551	1
**4662**	381	C	53.8063	1
**4662**	382	C	49.7148	0
**4662**	386	C	27.8358	0
**4662**	305	D	22.7701	0
**4662**	319	D	70.8981	1
**4662**	320	D	29.1096	-1
**4662**	321	D	48.246	1

**4662**	351	D	9.7371	1
**4662**	357	D	72.8081	1
**4662**	370	D	79.5304	1
**4662**	372	D	79.5679	0
**4662**	373	D	79.571	1
**4662**	381	D	75.8298	1
**4662**	382	D	52.0568	1
**4662**	386	D	41.7708	0
**4663**	305	A	79.5281	1
**4663**	319	A	60.8771	1
**4663**	320	A	62.0819	-1
**4663**	321	A	44.4968	1
**4663**	351	A	10.0504	1
**4663**	357	A	66.2304	1
**4663**	370	A	72.2386	1
**4663**	372	A	39.6992	1
**4663**	373	A	47.4647	0
**4663**	381	A	79.4956	1
**4663**	382	A	72.5912	1
**4663**	386	A	79.0631	1
**4663**	305	B	45.1798	0
**4663**	319	B	33.5476	0
**4663**	320	B	51.9686	-1
**4663**	321	B	67.1339	0
**4663**	351	B	30.355	-1
**4663**	357	B	42.7495	0
**4663**	370	B	79.6237	1
**4663**	372	B	66.093	0
**4663**	373	B	67.943	1
**4663**	381	B	61.167	1
**4663**	382	B	57.2101	1
**4663**	386	B	79.4914	1
**4663**	305	C	5.6161	-1
**4663**	319	C	72.5276	1

**4663**	320	C	67.9616	-1
**4663**	321	C	79.1005	0
**4663**	351	C	12.8089	1
**4663**	357	C	79.5573	1
**4663**	370	C	46.727	0
**4663**	372	C	79.4806	0
**4663**	373	C	52.5604	1
**4663**	381	C	60.1107	1
**4663**	382	C	1.3434	-1
**4663**	386	C	55.5922	1
**4663**	305	D	72.3122	1
**4663**	319	D	79.6541	1
**4663**	320	D	61.8851	-1
**4663**	321	D	79.5323	0
**4663**	351	D	10.3097	1
**4663**	357	D	64.1713	1
**4663**	370	D	78.9933	1
**4663**	372	D	79.556	0
**4663**	373	D	79.2828	1
**4663**	381	D	79.5556	1
**4663**	382	D	68.1562	1
**4663**	386	D	76.781	1
**4664**	305	A	50.612	1
**4664**	319	A	67.4198	1
**4664**	320	A	74.303	-1
**4664**	321	A	34.2796	1
**4664**	351	A	6.6289	1
**4664**	357	A	71.5022	1
**4664**	370	A	59.2611	1
**4664**	372	A	79.5615	0
**4664**	373	A	79.5817	1
**4664**	381	A	79.5057	1
**4664**	382	A	79.5764	1
**4664**	386	A	74.6423	1

**4664**	305	B	67.1007	1
**4664**	319	B	71.09	1
**4664**	320	B	64.8339	-1
**4664**	321	B	79.5359	0
**4664**	351	B	15.1762	1
**4664**	357	B	72.6397	1
**4664**	370	B	72.2663	1
**4664**	372	B	72.4739	0
**4664**	373	B	70.1895	1
**4664**	381	B	75.3154	1
**4664**	382	B	48.2342	0
**4664**	386	B	74.6158	1
**4664**	305	C	65.7525	1
**4664**	319	C	78.0393	1
**4664**	320	C	72.3231	-1
**4664**	321	C	53.5613	0
**4664**	351	C	0.9546	1
**4664**	357	C	79.5911	1
**4664**	370	C	29.5479	0
**4664**	372	C	79.5453	0
**4664**	373	C	79.5517	1
**4664**	381	C	78.9634	1
**4664**	382	C	59.1872	1
**4664**	386	C	64.8865	1
**4664**	305	D	47.3616	0
**4664**	319	D	67.7525	1
**4664**	320	D	34.368	-1
**4664**	321	D	36.2288	1
**4664**	351	D	11.6839	1
**4664**	357	D	79.5208	1
**4664**	370	D	79.4254	1
**4664**	372	D	74.3887	0
**4664**	373	D	77.0143	1
**4664**	381	D	55.3312	1

**4664**	382	D	79.5604	1
**4664**	386	D	37.6035	0
**4665**	305	A	79.5231	1
**4665**	319	A	25.6459	0
**4665**	320	A	79.5642	-1
**4665**	321	A	79.6164	0
**4665**	351	A	11.257	1
**4665**	357	A	57.7025	1
**4665**	370	A	71.6535	1
**4665**	372	A	75.3809	0
**4665**	373	A	23.6405	0
**4665**	381	A	79.5724	1
**4665**	382	A	68.8895	1
**4665**	386	A	78.3713	1
**4665**	305	B	66.1252	1
**4665**	319	B	62.6105	1
**4665**	320	B	9.8666	1
**4665**	321	B	55.152	0
**4665**	351	B	14.618	1
**4665**	357	B	63.0148	1
**4665**	370	B	49.6026	0
**4665**	372	B	64.965	0
**4665**	373	B	40.685	0
**4665**	381	B	79.5687	1
**4665**	382	B	11.5109	-1
**4665**	386	B	78.8232	1
**4665**	305	C	46.229	0
**4665**	319	C	79.5471	1
**4665**	320	C	15.1014	1
**4665**	321	C	76.4042	0
**4665**	351	C	4.8992	1
**4665**	357	C	25.9794	0
**4665**	370	C	77.5199	1
**4665**	372	C	79.5944	0

**4665**	373	C	79.611	1
**4665**	381	C	77.3066	1
**4665**	382	C	79.5476	1
**4665**	386	C	39.3661	0
**4665**	305	D	43.9885	0
**4665**	319	D	62.9564	1
**4665**	320	D	40.2105	-1
**4665**	321	D	72.3407	0
**4665**	351	D	14.0263	1
**4665**	357	D	79.5622	1
**4665**	370	D	71.8565	1
**4665**	372	D	79.599	0
**4665**	373	D	79.5717	1
**4665**	381	D	79.5447	1
**4665**	382	D	64.8379	1
**4665**	386	D	72.2524	1
**4666**	305	A	67.2634	1
**4666**	319	A	55.4392	1
**4666**	320	A	72.9402	-1
**4666**	321	A	73.6776	0
**4666**	351	A	5.558	1
**4666**	357	A	36.7073	0
**4666**	370	A	79.5454	1
**4666**	372	A	79.5492	0
**4666**	373	A	38.4684	0
**4666**	381	A	55.0804	1
**4666**	382	A	28.6439	0
**4666**	386	A	78.3914	1
**4666**	305	B	79.5769	1
**4666**	319	B	72.168	1
**4666**	320	B	33.9056	-1
**4666**	321	B	79.5507	0
**4666**	351	B	14.0119	1
**4666**	357	B	79.5532	1

**4666**	370	B	73.0345	1
**4666**	372	B	70.5047	0
**4666**	373	B	35.3532	0
**4666**	381	B	79.5644	1
**4666**	382	B	79.602	1
**4666**	386	B	79.1573	1
**4666**	305	C	69.0952	1
**4666**	319	C	68.2534	1
**4666**	320	C	79.5157	-1
**4666**	321	C	42.8091	1
**4666**	351	C	8.1049	1
**4666**	357	C	79.5658	1
**4666**	370	C	79.5919	1
**4666**	372	C	79.5677	0
**4666**	373	C	78.8199	1
**4666**	381	C	79.573	1
**4666**	382	C	48.2531	0
**4666**	386	C	72.2515	1
**4666**	305	D	34.8182	0
**4666**	319	D	66.9973	1
**4666**	320	D	79.5435	-1
**4666**	321	D	35.5961	1
**4666**	351	D	14.1111	1
**4666**	357	D	79.5589	1
**4666**	370	D	72.3301	1
**4666**	372	D	79.5515	0
**4666**	373	D	48.7261	0
**4666**	381	D	79.5495	1
**4666**	382	D	79.558	1
**4666**	386	D	17.4022	-1
**4667**	305	A	79.5049	1
**4667**	319	A	47.3389	0
**4667**	320	A	79.5442	-1
**4667**	321	A	29.7929	1

**4667**	351	A	6.3924	1
**4667**	357	A	62.1519	1
**4667**	370	A	20.2457	0
**4667**	372	A	72.2018	0
**4667**	373	A	34.6606	0
**4667**	381	A	75.3274	1
**4667**	382	A	44.549	0
**4667**	386	A	59.609	1
**4667**	305	B	79.079	1
**4667**	319	B	72.3981	1
**4667**	320	B	42.6517	-1
**4667**	321	B	79.6631	0
**4667**	351	B	18.5843	1
**4667**	357	B	79.5564	1
**4667**	370	B	79.4816	1
**4667**	372	B	79.3961	0
**4667**	373	B	70.4675	1
**4667**	381	B	79.6391	1
**4667**	382	B	27.561	0
**4667**	386	B	52.3457	1
**4667**	305	C	79.6475	1
**4667**	319	C	79.5197	1
**4667**	320	C	78.043	-1
**4667**	321	C	35.6117	1
**4667**	351	C	1.6335	1
**4667**	357	C	74.4527	1
**4667**	370	C	76.522	1
**4667**	372	C	79.5433	0
**4667**	373	C	79.537	1
**4667**	381	C	77.4608	1
**4667**	382	C	54.6293	1
**4667**	386	C	33.8818	0
**4667**	305	D	65.8371	1
**4667**	319	D	45.7634	0

**4667**	320	D	66.8204	-1
**4667**	321	D	79.6595	0
**4667**	351	D	1.4396	1
**4667**	357	D	14.6089	-1
**4667**	370	D	49.9563	0
**4667**	372	D	79.5841	0
**4667**	373	D	66.3712	1
**4667**	381	D	79.0092	1
**4667**	382	D	70.3744	1
**4667**	386	D	79.7253	1
**4668**	305	A	69.5848	1
**4668**	319	A	67.0827	1
**4668**	320	A	79.4961	-1
**4668**	321	A	78.695	0
**4668**	351	A	29.9208	-1
**4668**	357	A	79.0965	1
**4668**	370	A	73.6841	1
**4668**	372	A	79.4684	0
**4668**	373	A	46.8514	0
**4668**	381	A	18.0986	-1
**4668**	382	A	79.6324	1
**4668**	386	A	79.4996	1
**4668**	305	B	77.1734	1
**4668**	319	B	16.296	-1
**4668**	320	B	74.227	-1
**4668**	321	B	79.5997	0
**4668**	351	B	9.4921	1
**4668**	357	B	52.5505	1
**4668**	370	B	79.6304	1
**4668**	372	B	63.0714	0
**4668**	373	B	42.5239	0
**4668**	381	B	79.5324	1
**4668**	382	B	60.6925	1
**4668**	386	B	79.5817	1

**4668**	305	C	77.1069	1
**4668**	319	C	44.9145	0
**4668**	320	C	79.2384	-1
**4668**	321	C	53.1073	0
**4668**	351	C	1.8947	1
**4668**	357	C	79.6543	1
**4668**	370	C	56.577	1
**4668**	372	C	67.5435	0
**4668**	373	C	72.0321	1
**4668**	381	C	61.1853	1
**4668**	382	C	78.5924	1
**4668**	386	C	71.7552	1
**4668**	305	D	51.7926	1
**4668**	319	D	43.5349	0
**4668**	320	D	79.0553	-1
**4668**	321	D	74.5606	0
**4668**	351	D	18.5396	1
**4668**	357	D	79.4453	1
**4668**	370	D	78.589	1
**4668**	372	D	66.8248	0
**4668**	373	D	79.2997	1
**4668**	381	D	73.0928	1
**4668**	382	D	49.4097	0
**4668**	386	D	79.6505	1
**4669**	305	A	62.4598	1
**4669**	319	A	71.5639	1
**4669**	320	A	52.1325	-1
**4669**	321	A	38.4439	1
**4669**	351	A	6.698	1
**4669**	357	A	79.5471	1
**4669**	370	A	58.2801	1
**4669**	372	A	72.9466	0
**4669**	373	A	73.7676	1
**4669**	381	A	75.4415	1

**4669**	382	A	77.6831	1
**4669**	386	A	74.1589	1
**4669**	305	B	79.5253	1
**4669**	319	B	75.3918	1
**4669**	320	B	65.5924	-1
**4669**	321	B	79.5718	0
**4669**	351	B	8.9891	1
**4669**	357	B	73.3379	1
**4669**	370	B	79.6437	1
**4669**	372	B	73.8445	0
**4669**	373	B	58.8093	1
**4669**	381	B	57.3319	1
**4669**	382	B	70.6001	1
**4669**	386	B	65.6644	1
**4669**	305	C	70.5065	1
**4669**	319	C	42.3484	0
**4669**	320	C	79.6053	-1
**4669**	321	C	43.3103	1
**4669**	351	C	17.1781	1
**4669**	357	C	79.6259	1
**4669**	370	C	55.2004	1
**4669**	372	C	51.6357	0
**4669**	373	C	79.2934	1
**4669**	381	C	76.1817	1
**4669**	382	C	79.5277	1
**4669**	386	C	37.5966	0
**4669**	305	D	73.1295	1
**4669**	319	D	79.6301	1
**4669**	320	D	73.3305	-1
**4669**	321	D	71.6098	0
**4669**	351	D	3.6023	1
**4669**	357	D	55.6944	1
**4669**	370	D	73.7521	1
**4669**	372	D	75.3465	0

**4669**	373	D	79.5515	1
**4669**	381	D	79.5386	1
**4669**	382	D	76.9985	1
**4669**	386	D	57.9252	1
**4670**	305	A	68.2113	1
**4670**	319	A	72.7228	1
**4670**	320	A	79.4673	-1
**4670**	321	A	48.545	1
**4670**	351	A	20.2106	-1
**4670**	357	A	79.4171	1
**4670**	370	A	79.6367	1
**4670**	372	A	79.6568	0
**4670**	373	A	36.6068	0
**4670**	381	A	41.107	0
**4670**	382	A	78.0557	1
**4670**	386	A	79.5432	1
**4670**	305	B	74.6878	1
**4670**	319	B	79.3954	1
**4670**	320	B	79.6741	-1
**4670**	321	B	79.2472	0
**4670**	351	B	6.5109	1
**4670**	357	B	66.6335	1
**4670**	370	B	79.5591	1
**4670**	372	B	60.1579	0
**4670**	373	B	51.6345	1
**4670**	381	B	49.1147	0
**4670**	382	B	52.1042	1
**4670**	386	B	76.91	1
**4670**	305	C	79.5857	1
**4670**	319	C	66.3443	1
**4670**	320	C	73.1903	-1
**4670**	321	C	55.2575	0
**4670**	351	C	11.7552	1
**4670**	357	C	78.2765	1

**4670**	370	C	43.3871	0
**4670**	372	C	65.5711	0
**4670**	373	C	45.2475	0
**4670**	381	C	72.8136	1
**4670**	382	C	75.8596	1
**4670**	386	C	69.4289	1
**4670**	305	D	56.6285	1
**4670**	319	D	79.5728	1
**4670**	320	D	50.5811	-1
**4670**	321	D	79.5184	0
**4670**	351	D	12.1375	1
**4670**	357	D	79.5313	1
**4670**	370	D	73.0278	1
**4670**	372	D	61.5309	0
**4670**	373	D	68.1174	1
**4670**	381	D	41.5997	0
**4670**	382	D	59.0177	1
**4670**	386	D	68.1133	1
**4671**	305	A	79.4964	1
**4671**	319	A	79.1715	1
**4671**	320	A	79.5307	-1
**4671**	321	A	53.4596	0
**4671**	351	A	6.0754	1
**4671**	357	A	79.5067	1
**4671**	370	A	66.8527	1
**4671**	372	A	79.567	0
**4671**	373	A	46.0875	0
**4671**	381	A	79.5158	1
**4671**	382	A	78.9136	1
**4671**	386	A	74.2152	1
**4671**	305	B	72.0776	1
**4671**	319	B	68.9588	1
**4671**	320	B	68.7778	-1
**4671**	321	B	57.4722	0

**4671**	351	B	12.334	1
**4671**	357	B	75.579	1
**4671**	370	B	74.9301	1
**4671**	372	B	79.658	0
**4671**	373	B	79.5136	1
**4671**	381	B	79.6167	1
**4671**	382	B	76.6219	1
**4671**	386	B	43.9944	0
**4671**	305	C	78.9706	1
**4671**	319	C	79.5621	1
**4671**	320	C	76.518	-1
**4671**	321	C	79.5857	0
**4671**	351	C	8.8899	1
**4671**	357	C	75.1486	1
**4671**	370	C	73.1871	1
**4671**	372	C	79.6144	0
**4671**	373	C	71.7422	1
**4671**	381	C	75.1784	1
**4671**	382	C	66.4999	1
**4671**	386	C	53.5537	1
**4671**	305	D	47.0609	0
**4671**	319	D	68.1337	1
**4671**	320	D	49.1102	-1
**4671**	321	D	75.9083	0
**4671**	351	D	12.2667	1
**4671**	357	D	79.5787	1
**4671**	370	D	79.5616	1
**4671**	372	D	68.6847	0
**4671**	373	D	67.1065	1
**4671**	381	D	74.1669	1
**4671**	382	D	79.5501	1
**4671**	386	D	67.0194	1
**4672**	305	A	79.2833	1
**4672**	319	A	51.4259	1

**4672**	320	A	79.5398	-1
**4672**	321	A	18.9868	0
**4672**	351	A	3.949	1
**4672**	357	A	79.5512	1
**4672**	370	A	79.5643	1
**4672**	372	A	71.0561	0
**4672**	373	A	79.5765	1
**4672**	381	A	55.6129	1
**4672**	382	A	48.2317	0
**4672**	386	A	79.5678	1
**4672**	305	B	44.2517	0
**4672**	319	B	42.1518	0
**4672**	320	B	79.5842	-1
**4672**	321	B	72.0078	0
**4672**	351	B	16.7635	1
**4672**	357	B	41.0026	0
**4672**	370	B	69.9773	1
**4672**	372	B	66.7513	0
**4672**	373	B	53.7119	1
**4672**	381	B	72.9497	1
**4672**	382	B	71.4823	1
**4672**	386	B	50.9882	1
**4672**	305	C	71.9345	1
**4672**	319	C	27.1896	0
**4672**	320	C	70.9068	-1
**4672**	321	C	79.5514	0
**4672**	351	C	6.7126	1
**4672**	357	C	72.793	1
**4672**	370	C	58.1466	1
**4672**	372	C	79.5585	0
**4672**	373	C	72.2379	1
**4672**	381	C	79.5426	1
**4672**	382	C	67.7235	1
**4672**	386	C	34.5957	0

**4672**	305	D	79.5676	1
**4672**	319	D	47.7396	0
**4672**	320	D	79.534	-1
**4672**	321	D	77.0159	0
**4672**	351	D	13.5555	1
**4672**	357	D	27.8464	0
**4672**	370	D	53.6731	1
**4672**	372	D	71.9912	0
**4672**	373	D	79.5819	1
**4672**	381	D	73.6011	1
**4672**	382	D	73.2768	1
**4672**	386	D	72.081	1
**4673**	305	A	45.5871	0
**4673**	319	A	50.4384	1
**4673**	320	A	30.3827	-1
**4673**	321	A	43.8894	1
**4673**	351	A	17.2436	1
**4673**	357	A	79.3052	1
**4673**	370	A	66.3821	1
**4673**	372	A	61.5172	0
**4673**	373	A	43.5801	0
**4673**	381	A	73.9728	1
**4673**	382	A	55.4198	1
**4673**	386	A	30.8257	0
**4673**	305	B	72.849	1
**4673**	319	B	32.6664	0
**4673**	320	B	79.5648	-1
**4673**	321	B	61.0085	0
**4673**	351	B	20.5217	-1
**4673**	357	B	59.8189	1
**4673**	370	B	62.6172	1
**4673**	372	B	73.3607	0
**4673**	373	B	64.5835	1
**4673**	381	B	79.5974	1

**4673**	382	B	41.2107	0
**4673**	386	B	70.274	1
**4673**	305	C	71.9099	1
**4673**	319	C	70.5026	1
**4673**	320	C	63.3135	-1
**4673**	321	C	72.6344	0
**4673**	351	C	6.82	1
**4673**	357	C	64.2486	1
**4673**	370	C	59.1726	1
**4673**	372	C	73.2016	0
**4673**	373	C	50.6421	1
**4673**	381	C	26.6177	0
**4673**	382	C	73.568	1
**4673**	386	C	58.0543	1
**4673**	305	D	55.3117	1
**4673**	319	D	79.5964	1
**4673**	320	D	79.6246	-1
**4673**	321	D	79.5777	0
**4673**	351	D	6.7168	1
**4673**	357	D	49.5495	0
**4673**	370	D	72.6235	1
**4673**	372	D	73.0838	0
**4673**	373	D	79.5644	1
**4673**	381	D	64.8714	1
**4673**	382	D	74.9077	1
**4673**	386	D	78.9208	1
**4674**	305	A	61.1531	1
**4674**	319	A	79.4584	1
**4674**	320	A	77.0619	-1
**4674**	321	A	74.4357	0
**4674**	351	A	1.021	1
**4674**	357	A	69.3008	1
**4674**	370	A	50.7864	1
**4674**	372	A	64.5003	0

**4674**	373	A	68.4892	1
**4674**	381	A	62.1936	1
**4674**	382	A	78.1184	1
**4674**	386	A	79.6374	1
**4674**	305	B	56.6939	1
**4674**	319	B	44.6887	0
**4674**	320	B	79.4654	-1
**4674**	321	B	74.556	0
**4674**	351	B	20.5715	-1
**4674**	357	B	23.4779	0
**4674**	370	B	51.5701	1
**4674**	372	B	79.5871	0
**4674**	373	B	63.4082	1
**4674**	381	B	61.3188	1
**4674**	382	B	63.1783	1
**4674**	386	B	32.6654	0
**4674**	305	C	61.347	1
**4674**	319	C	55.0902	1
**4674**	320	C	79.5015	-1
**4674**	321	C	51.8465	0
**4674**	351	C	1.4808	1
**4674**	357	C	41.4333	0
**4674**	370	C	42.6621	0
**4674**	372	C	79.5853	0
**4674**	373	C	58.3072	1
**4674**	381	C	78.1253	1
**4674**	382	C	30.1772	0
**4674**	386	C	48.1783	0
**4674**	305	D	79.723	1
**4674**	319	D	79.6451	1
**4674**	320	D	79.6081	-1
**4674**	321	D	66.3804	0
**4674**	351	D	10.2755	1
**4674**	357	D	79.3225	1

**4674**	370	D	52.6587	1
**4674**	372	D	59.2793	0
**4674**	373	D	56.7418	1
**4674**	381	D	37.9225	0
**4674**	382	D	67.9153	1
**4674**	386	D	28.6396	0
**4675**	305	A	65.7481	1
**4675**	319	A	79.5157	1
**4675**	320	A	20.3148	-1
**4675**	321	A	76.3106	0
**4675**	351	A	25.0338	-1
**4675**	357	A	76.1467	1
**4675**	370	A	79.3814	1
**4675**	372	A	79.7626	0
**4675**	373	A	40.1098	0
**4675**	381	A	79.4601	1
**4675**	382	A	75.2228	1
**4675**	386	A	77.3666	1
**4675**	305	B	61.0895	1
**4675**	319	B	79.6501	1
**4675**	320	B	79.6224	-1
**4675**	321	B	79.5513	0
**4675**	351	B	12.6313	1
**4675**	357	B	71.8775	1
**4675**	370	B	65.1982	1
**4675**	372	B	79.5292	0
**4675**	373	B	54.953	1
**4675**	381	B	74.3455	1
**4675**	382	B	74.657	1
**4675**	386	B	64.3491	1
**4675**	305	C	55.2357	1
**4675**	319	C	79.6387	1
**4675**	320	C	76.4822	-1
**4675**	321	C	79.6028	0

**4675**	351	C	1.8113	1
**4675**	357	C	66.4822	1
**4675**	370	C	57.3915	1
**4675**	372	C	70.1218	0
**4675**	373	C	79.5378	1
**4675**	381	C	73.7671	1
**4675**	382	C	67.1407	1
**4675**	386	C	39.0416	0
**4675**	305	D	44.9037	0
**4675**	319	D	72.5872	1
**4675**	320	D	71.3848	-1
**4675**	321	D	51.556	0
**4675**	351	D	17.2244	1
**4675**	357	D	76.3984	1
**4675**	370	D	64.4813	1
**4675**	372	D	65.8502	0
**4675**	373	D	73.4243	1
**4675**	381	D	50.1438	1
**4675**	382	D	79.5818	1
**4675**	386	D	77.254	1
**4676**	305	A	72.9555	1
**4676**	319	A	40.7872	0
**4676**	320	A	79.528	-1
**4676**	321	A	67.6392	0
**4676**	351	A	9.5968	1
**4676**	357	A	72.4365	1
**4676**	370	A	79.552	1
**4676**	372	A	38.9177	1
**4676**	373	A	38.0969	0
**4676**	381	A	79.5578	1
**4676**	382	A	59.4569	1
**4676**	386	A	79.4516	1
**4676**	305	B	79.7455	1
**4676**	319	B	79.5355	1

**4676**	320	B	61.8534	-1
**4676**	321	B	75.2109	0
**4676**	351	B	20.4758	-1
**4676**	357	B	79.5503	1
**4676**	370	B	58.5049	1
**4676**	372	B	70.403	0
**4676**	373	B	58.4198	1
**4676**	381	B	46.8263	0
**4676**	382	B	41.7713	0
**4676**	386	B	79.5948	1
**4676**	305	C	77.425	1
**4676**	319	C	79.165	1
**4676**	320	C	73.2134	-1
**4676**	321	C	79.1234	0
**4676**	351	C	7.7425	1
**4676**	357	C	74.3383	1
**4676**	370	C	79.6101	1
**4676**	372	C	69.1929	0
**4676**	373	C	79.4059	1
**4676**	381	C	72.5173	1
**4676**	382	C	55.3521	1
**4676**	386	C	22.8636	0
**4676**	305	D	45.4562	0
**4676**	319	D	79.601	1
**4676**	320	D	74.3211	-1
**4676**	321	D	74.2038	0
**4676**	351	D	10.7919	1
**4676**	357	D	71.7183	1
**4676**	370	D	31.0234	0
**4676**	372	D	79.5525	0
**4676**	373	D	76.3568	1
**4676**	381	D	66.7239	1
**4676**	382	D	67.5172	1
**4676**	386	D	79.5223	1

**4677**	305	A	77.7306	1
**4677**	319	A	75.1013	1
**4677**	320	A	79.5936	-1
**4677**	321	A	70.7506	0
**4677**	351	A	0.2661	1
**4677**	357	A	74.0244	1
**4677**	370	A	54.2158	1
**4677**	372	A	61.0214	0
**4677**	373	A	79.5678	1
**4677**	381	A	79.5458	1
**4677**	382	A	36.8103	0
**4677**	386	A	63.9332	1
**4677**	305	B	79.5311	1
**4677**	319	B	79.5687	1
**4677**	320	B	35.2193	-1
**4677**	321	B	79.5479	0
**4677**	351	B	15.1812	1
**4677**	357	B	76.5426	1
**4677**	370	B	79.5474	1
**4677**	372	B	72.7894	0
**4677**	373	B	74.1065	1
**4677**	381	B	79.59	1
**4677**	382	B	36.9304	0
**4677**	386	B	55.9056	1
**4677**	305	C	79.1635	1
**4677**	319	C	74.4936	1
**4677**	320	C	73.8009	-1
**4677**	321	C	48.6589	1
**4677**	351	C	0.7455	1
**4677**	357	C	79.5858	1
**4677**	370	C	60.7662	1
**4677**	372	C	67.3581	0
**4677**	373	C	79.5203	1
**4677**	381	C	79.5512	1

**4677**	382	C	79.5579	1
**4677**	386	C	38.5956	0
**4677**	305	D	73.5897	1
**4677**	319	D	79.5818	1
**4677**	320	D	79.5592	-1
**4677**	321	D	70.7911	0
**4677**	351	D	8.1972	1
**4677**	357	D	74.2635	1
**4677**	370	D	75.0208	1
**4677**	372	D	79.5524	0
**4677**	373	D	72.9584	1
**4677**	381	D	69.9872	1
**4677**	382	D	71.9037	1
**4677**	386	D	78.6367	1
**4678**	305	A	57.1397	1
**4678**	319	A	79.5432	1
**4678**	320	A	53.3449	-1
**4678**	321	A	59.9097	0
**4678**	351	A	1.8197	1
**4678**	357	A	73.0862	1
**4678**	370	A	79.5726	1
**4678**	372	A	73.022	0
**4678**	373	A	73.0208	1
**4678**	381	A	34.7427	0
**4678**	382	A	33.7313	0
**4678**	386	A	74.7449	1
**4678**	305	B	72.9403	1
**4678**	319	B	75.8531	1
**4678**	320	B	22.5446	-1
**4678**	321	B	42.4911	1
**4678**	351	B	14.1596	1
**4678**	357	B	75.0987	1
**4678**	370	B	59.084	1
**4678**	372	B	36.7534	1

**4678**	373	B	79.5255	1
**4678**	381	B	79.5482	1
**4678**	382	B	44.6526	0
**4678**	386	B	43.9173	0
**4678**	305	C	71.7303	1
**4678**	319	C	79.5651	1
**4678**	320	C	71.3148	-1
**4678**	321	C	54.7899	0
**4678**	351	C	2.1708	1
**4678**	357	C	79.5523	1
**4678**	370	C	79.5228	1
**4678**	372	C	72.8804	0
**4678**	373	C	79.3922	1
**4678**	381	C	72.7804	1
**4678**	382	C	73.7995	1
**4678**	386	C	57.3595	1
**4678**	305	D	64.6802	1
**4678**	319	D	46.6851	0
**4678**	320	D	79.5493	-1
**4678**	321	D	71.7065	0
**4678**	351	D	13.7637	1
**4678**	357	D	79.5799	1
**4678**	370	D	76.8426	1
**4678**	372	D	79.5718	0
**4678**	373	D	63.1329	1
**4678**	381	D	21.7627	0
**4678**	382	D	79.0473	1
**4678**	386	D	59.8098	1
**4679**	305	A	79.5801	1
**4679**	319	A	74.8525	1
**4679**	320	A	71.8727	-1
**4679**	321	A	34.8518	1
**4679**	351	A	12.5837	1
**4679**	357	A	73.722	1

**4679**	370	A	79.5628	1
**4679**	372	A	74.6482	0
**4679**	373	A	68.0953	1
**4679**	381	A	79.5364	1
**4679**	382	A	77.2779	1
**4679**	386	A	79.5594	1
**4679**	305	B	46.711	0
**4679**	319	B	12.1681	-1
**4679**	320	B	59.6132	-1
**4679**	321	B	64.4222	0
**4679**	351	B	13.736	1
**4679**	357	B	79.5247	1
**4679**	370	B	79.4855	1
**4679**	372	B	79.0738	0
**4679**	373	B	71.3159	1
**4679**	381	B	79.514	1
**4679**	382	B	77.7855	1
**4679**	386	B	44.5344	0
**4679**	305	C	54.1991	1
**4679**	319	C	78.5669	1
**4679**	320	C	79.3594	-1
**4679**	321	C	78.6844	0
**4679**	351	C	13.7621	1
**4679**	357	C	74.0754	1
**4679**	370	C	79.5512	1
**4679**	372	C	35.1304	1
**4679**	373	C	57.9443	1
**4679**	381	C	63.5616	1
**4679**	382	C	79.5995	1
**4679**	386	C	15.6417	-1
**4679**	305	D	45.4849	0
**4679**	319	D	72.9163	1
**4679**	320	D	67.5896	-1
**4679**	321	D	79.5445	0

**4679**	351	D	13.7131	1
**4679**	357	D	79.5971	1
**4679**	370	D	60.1554	1
**4679**	372	D	54.1701	0
**4679**	373	D	77.7371	1
**4679**	381	D	52.4829	1
**4679**	382	D	73.121	1
**4679**	386	D	76.6927	1
**4680**	305	A	76.6371	1
**4680**	319	A	74.4402	1
**4680**	320	A	58.2122	-1
**4680**	321	A	76.36	0
**4680**	351	A	5.4754	1
**4680**	357	A	57.0511	1
**4680**	370	A	79.5372	1
**4680**	372	A	29.3844	1
**4680**	373	A	47.3942	0
**4680**	381	A	63.4049	1
**4680**	382	A	74.7085	1
**4680**	386	A	78.903	1
**4680**	305	B	72.5534	1
**4680**	319	B	79.5977	1
**4680**	320	B	16.5211	1
**4680**	321	B	77.9037	0
**4680**	351	B	1.9783	1
**4680**	357	B	54.8379	1
**4680**	370	B	74.6461	1
**4680**	372	B	79.5932	0
**4680**	373	B	57.7762	1
**4680**	381	B	79.5671	1
**4680**	382	B	37.3542	0
**4680**	386	B	79.6266	1
**4680**	305	C	67.8762	1
**4680**	319	C	79.8016	1

**4680**	320	C	79.4613	-1
**4680**	321	C	69.0031	0
**4680**	351	C	2.7701	1
**4680**	357	C	79.5504	1
**4680**	370	C	79.5598	1
**4680**	372	C	59.3536	0
**4680**	373	C	59.4659	1
**4680**	381	C	51.1145	1
**4680**	382	C	66.1039	1
**4680**	386	C	66.3404	1
**4680**	305	D	18.4121	-1
**4680**	319	D	73.0104	1
**4680**	320	D	67.1142	-1
**4680**	321	D	74.0911	0
**4680**	351	D	15.1022	1
**4680**	357	D	79.5383	1
**4680**	370	D	79.5009	1
**4680**	372	D	62.6151	0
**4680**	373	D	49.869	0
**4680**	381	D	73.5292	1
**4680**	382	D	68.2657	1
**4680**	386	D	40.7397	0
**4681**	305	A	76.0677	1
**4681**	319	A	79.2921	1
**4681**	320	A	74.8305	-1
**4681**	321	A	70.0751	0
**4681**	351	A	10.5247	1
**4681**	357	A	59.4291	1
**4681**	370	A	79.5189	1
**4681**	372	A	56.6938	0
**4681**	373	A	72.0484	1
**4681**	381	A	70.2525	1
**4681**	382	A	34.8061	0
**4681**	386	A	66.6581	1

**4681**	305	B	75.4611	1
**4681**	319	B	54.8501	1
**4681**	320	B	61.9149	-1
**4681**	321	B	32.8368	1
**4681**	351	B	16.4407	1
**4681**	357	B	40.5831	0
**4681**	370	B	32.3029	0
**4681**	372	B	67.862	0
**4681**	373	B	75.2944	1
**4681**	381	B	68.5839	1
**4681**	382	B	79.5019	1
**4681**	386	B	60.0456	1
**4681**	305	C	76.025	1
**4681**	319	C	53.8302	1
**4681**	320	C	65.2943	-1
**4681**	321	C	79.3944	0
**4681**	351	C	7.3445	1
**4681**	357	C	38.7899	0
**4681**	370	C	79.6006	1
**4681**	372	C	43.5651	1
**4681**	373	C	79.633	1
**4681**	381	C	30.9691	0
**4681**	382	C	52.5292	1
**4681**	386	C	38.7901	0
**4681**	305	D	79.4422	1
**4681**	319	D	79.6548	1
**4681**	320	D	75.1749	-1
**4681**	321	D	22.6746	1
**4681**	351	D	16.4907	1
**4681**	357	D	56.2421	1
**4681**	370	D	73.9076	1
**4681**	372	D	79.5134	0
**4681**	373	D	70.2114	1
**4681**	381	D	68.8914	1

**4681**	382	D	69.9796	1
**4681**	386	D	70.9476	1
**4682**	305	A	73.4968	1
**4682**	319	A	72.1077	1
**4682**	320	A	42.3435	-1
**4682**	321	A	44.1457	1
**4682**	351	A	31.7389	-1
**4682**	357	A	50.6454	1
**4682**	370	A	46.7266	0
**4682**	372	A	61.074	0
**4682**	373	A	23.6818	0
**4682**	381	A	27.9928	0
**4682**	382	A	60.7606	1
**4682**	386	A	58.6139	1
**4682**	305	B	71.0427	1
**4682**	319	B	40.3091	0
**4682**	320	B	72.8326	-1
**4682**	321	B	29.2486	1
**4682**	351	B	13.8982	1
**4682**	357	B	79.4789	1
**4682**	370	B	79.6706	1
**4682**	372	B	72.8169	0
**4682**	373	B	69.9817	1
**4682**	381	B	79.5239	1
**4682**	382	B	46.4323	0
**4682**	386	B	40.3271	0
**4682**	305	C	44.0421	0
**4682**	319	C	56.292	1
**4682**	320	C	72.4846	-1
**4682**	321	C	60.0777	0
**4682**	351	C	4.9312	1
**4682**	357	C	62.3411	1
**4682**	370	C	59.4629	1
**4682**	372	C	73.2673	0

**4682**	373	C	79.516	1
**4682**	381	C	57.9822	1
**4682**	382	C	62.451	1
**4682**	386	C	24.1851	0
**4682**	305	D	51.531	1
**4682**	319	D	72.2192	1
**4682**	320	D	31.4816	-1
**4682**	321	D	77.9911	0
**4682**	351	D	13.169	1
**4682**	357	D	79.5153	1
**4682**	370	D	66.9202	1
**4682**	372	D	79.6176	0
**4682**	373	D	59.0264	1
**4682**	381	D	38.221	0
**4682**	382	D	79.5989	1
**4682**	386	D	77.2554	1
**4683**	305	A	47.7144	0
**4683**	319	A	79.6392	1
**4683**	320	A	79.5857	-1
**4683**	321	A	78.9679	0
**4683**	351	A	2.0116	1
**4683**	357	A	74.849	1
**4683**	370	A	73.5207	1
**4683**	372	A	65.008	0
**4683**	373	A	66.2425	1
**4683**	381	A	79.5593	1
**4683**	382	A	61.7524	1
**4683**	386	A	25.9578	0
**4683**	305	B	79.6518	1
**4683**	319	B	38.778	0
**4683**	320	B	56.2028	-1
**4683**	321	B	72.8349	0
**4683**	351	B	18.4707	1
**4683**	357	B	61.9947	1

**4683**	370	B	44.843	0
**4683**	372	B	79.6433	0
**4683**	373	B	60.0925	1
**4683**	381	B	79.6373	1
**4683**	382	B	56.9738	1
**4683**	386	B	53.0053	1
**4683**	305	C	79.4853	1
**4683**	319	C	42.5207	0
**4683**	320	C	71.485	-1
**4683**	321	C	74.2801	0
**4683**	351	C	9.2912	1
**4683**	357	C	35.4023	0
**4683**	370	C	74.8458	1
**4683**	372	C	53.8461	0
**4683**	373	C	73.4382	1
**4683**	381	C	57.0229	1
**4683**	382	C	56.3858	1
**4683**	386	C	50.5134	1
**4683**	305	D	79.4422	1
**4683**	319	D	69.858	1
**4683**	320	D	79.5072	-1
**4683**	321	D	71.031	0
**4683**	351	D	41.0066	-1
**4683**	357	D	60.2095	1
**4683**	370	D	39.3075	0
**4683**	372	D	79.6247	0
**4683**	373	D	79.1642	1
**4683**	381	D	64.8347	1
**4683**	382	D	79.614	1
**4683**	386	D	79.6012	1
**4684**	305	A	68.1354	1
**4684**	319	A	68.1331	1
**4684**	320	A	71.7785	-1
**4684**	321	A	11.1982	0

**4684**	351	A	7.9815	1
**4684**	357	A	69.7078	1
**4684**	370	A	43.9369	0
**4684**	372	A	79.5792	0
**4684**	373	A	55.361	1
**4684**	381	A	57.9726	1
**4684**	382	A	72.8185	1
**4684**	386	A	53.2931	1
**4684**	305	B	68.1257	1
**4684**	319	B	72.6194	1
**4684**	320	B	71.2555	-1
**4684**	321	B	79.6913	0
**4684**	351	B	14.875	1
**4684**	357	B	54.5904	1
**4684**	370	B	54.0247	1
**4684**	372	B	53.6456	0
**4684**	373	B	70.5818	1
**4684**	381	B	72.5181	1
**4684**	382	B	71.6243	1
**4684**	386	B	79.539	1
**4684**	305	C	58.1694	1
**4684**	319	C	62.9443	1
**4684**	320	C	40.664	-1
**4684**	321	C	79.5753	0
**4684**	351	C	3.0986	1
**4684**	357	C	30.0136	0
**4684**	370	C	77.0732	1
**4684**	372	C	77.8451	0
**4684**	373	C	43.0944	0
**4684**	381	C	43.6386	0
**4684**	382	C	70.9463	1
**4684**	386	C	65.7973	1
**4684**	305	D	54.6929	1
**4684**	319	D	50.3059	1

**4684**	320	D	79.5592	-1
**4684**	321	D	79.5614	0
**4684**	351	D	14.1074	1
**4684**	357	D	79.5494	1
**4684**	370	D	58.7922	1
**4684**	372	D	79.5209	0
**4684**	373	D	79.5405	1
**4684**	381	D	75.3302	1
**4684**	382	D	79.6026	1
**4684**	386	D	79.5471	1
**4685**	305	A	68.5275	1
**4685**	319	A	10.2193	-1
**4685**	320	A	67.5726	-1
**4685**	321	A	75.1525	0
**4685**	351	A	5.2381	1
**4685**	357	A	66.5022	1
**4685**	370	A	79.6079	1
**4685**	372	A	54.7438	0
**4685**	373	A	36.3219	0
**4685**	381	A	79.5989	1
**4685**	382	A	65.1572	1
**4685**	386	A	79.5628	1
**4685**	305	B	79.567	1
**4685**	319	B	46.3599	0
**4685**	320	B	63.8481	-1
**4685**	321	B	48.2895	1
**4685**	351	B	22.1663	-1
**4685**	357	B	74.1156	1
**4685**	370	B	45.5343	0
**4685**	372	B	61.9311	0
**4685**	373	B	79.5386	1
**4685**	381	B	79.6737	1
**4685**	382	B	15.6867	-1
**4685**	386	B	62.6731	1

**4685**	305	C	51.4238	1
**4685**	319	C	79.1499	1
**4685**	320	C	71.446	-1
**4685**	321	C	58.6282	0
**4685**	351	C	6.4552	1
**4685**	357	C	64.32	1
**4685**	370	C	78.5621	1
**4685**	372	C	73.3904	0
**4685**	373	C	79.6595	1
**4685**	381	C	79.5549	1
**4685**	382	C	79.6048	1
**4685**	386	C	27.3697	0
**4685**	305	D	79.5453	1
**4685**	319	D	79.671	1
**4685**	320	D	44.3371	-1
**4685**	321	D	79.5204	0
**4685**	351	D	3.626	1
**4685**	357	D	65.0679	1
**4685**	370	D	79.5551	1
**4685**	372	D	75.2508	0
**4685**	373	D	75.384	1
**4685**	381	D	61.9844	1
**4685**	382	D	79.529	1
**4685**	386	D	63.6317	1
**4686**	305	A	62.5167	1
**4686**	319	A	79.289	1
**4686**	320	A	79.5731	-1
**4686**	321	A	25.7541	1
**4686**	351	A	13.6352	1
**4686**	357	A	67.6246	1
**4686**	370	A	59.6003	1
**4686**	372	A	77.2091	0
**4686**	373	A	79.5266	1
**4686**	381	A	79.4432	1

**4686**	382	A	56.5071	1
**4686**	386	A	79.5842	1
**4686**	305	B	79.5532	1
**4686**	319	B	73.2652	1
**4686**	320	B	25.4292	-1
**4686**	321	B	69.1338	0
**4686**	351	B	14.8542	1
**4686**	357	B	52.628	1
**4686**	370	B	31.1713	0
**4686**	372	B	79.5234	0
**4686**	373	B	79.2819	1
**4686**	381	B	57.1308	1
**4686**	382	B	44.3963	0
**4686**	386	B	56.8986	1
**4686**	305	C	79.5688	1
**4686**	319	C	40.134	0
**4686**	320	C	72.8945	-1
**4686**	321	C	79.538	0
**4686**	351	C	12.0076	1
**4686**	357	C	56.0163	1
**4686**	370	C	78.3754	1
**4686**	372	C	57.0019	0
**4686**	373	C	56.9815	1
**4686**	381	C	51.0138	1
**4686**	382	C	76.8104	1
**4686**	386	C	41.1375	0
**4686**	305	D	21.6331	0
**4686**	319	D	73.2902	1
**4686**	320	D	63.3007	-1
**4686**	321	D	67.0546	0
**4686**	351	D	0.0308	1
**4686**	357	D	79.5934	1
**4686**	370	D	75.4049	1
**4686**	372	D	73.0489	0

**4686**	373	D	24.4462	0
**4686**	381	D	75.5561	1
**4686**	382	D	79.5994	1
**4686**	386	D	79.5614	1
**4687**	305	A	59.7618	1
**4687**	319	A	78.485	1
**4687**	320	A	49.7552	-1
**4687**	321	A	47.0498	1
**4687**	351	A	0.6288	1
**4687**	357	A	79.5863	1
**4687**	370	A	79.6102	1
**4687**	372	A	79.6262	0
**4687**	373	A	53.9607	1
**4687**	381	A	63.357	1
**4687**	382	A	27.9187	0
**4687**	386	A	79.5384	1
**4687**	305	B	45.0439	0
**4687**	319	B	77.6662	1
**4687**	320	B	62.2432	-1
**4687**	321	B	51.7712	0
**4687**	351	B	19.9528	1
**4687**	357	B	57.9859	1
**4687**	370	B	58.1568	1
**4687**	372	B	79.501	0
**4687**	373	B	55.431	1
**4687**	381	B	79.6254	1
**4687**	382	B	30.4401	0
**4687**	386	B	79.4179	1
**4687**	305	C	79.5257	1
**4687**	319	C	61.731	1
**4687**	320	C	42.0319	-1
**4687**	321	C	70.1465	0
**4687**	351	C	2.1422	1
**4687**	357	C	46.4145	0

**4687**	370	C	79.5711	1
**4687**	372	C	59.9854	0
**4687**	373	C	79.4583	1
**4687**	381	C	60.5299	1
**4687**	382	C	79.4845	1
**4687**	386	C	64.0266	1
**4687**	305	D	68.4671	1
**4687**	319	D	73.0042	1
**4687**	320	D	56.1841	-1
**4687**	321	D	46.3816	1
**4687**	351	D	7.6573	1
**4687**	357	D	66.3572	1
**4687**	370	D	71.0481	1
**4687**	372	D	68.7073	0
**4687**	373	D	79.467	1
**4687**	381	D	73.0059	1
**4687**	382	D	79.5826	1
**4687**	386	D	55.8857	1
**4688**	305	A	68.922	1
**4688**	319	A	62.7947	1
**4688**	320	A	70.6399	-1
**4688**	321	A	56.7215	0
**4688**	351	A	3.3684	1
**4688**	357	A	59.6912	1
**4688**	370	A	79.5028	1
**4688**	372	A	79.6567	0
**4688**	373	A	68.4064	1
**4688**	381	A	68.649	1
**4688**	382	A	78.3712	1
**4688**	386	A	79.6075	1
**4688**	305	B	53.6421	1
**4688**	319	B	69.9276	1
**4688**	320	B	74.9832	-1
**4688**	321	B	79.6227	0

**4688**	351	B	13.1114	1
**4688**	357	B	71.8894	1
**4688**	370	B	79.4886	1
**4688**	372	B	72.6479	0
**4688**	373	B	68.7182	1
**4688**	381	B	79.6042	1
**4688**	382	B	65.9648	1
**4688**	386	B	52.996	1
**4688**	305	C	71.8607	1
**4688**	319	C	79.5244	1
**4688**	320	C	71.3961	-1
**4688**	321	C	65.7513	0
**4688**	351	C	0.3431	1
**4688**	357	C	74.0503	1
**4688**	370	C	79.4088	1
**4688**	372	C	79.6088	0
**4688**	373	C	74.4047	1
**4688**	381	C	69.6408	1
**4688**	382	C	68.1443	1
**4688**	386	C	65.5089	1
**4688**	305	D	61.143	1
**4688**	319	D	69.7642	1
**4688**	320	D	79.5003	-1
**4688**	321	D	44.1776	1
**4688**	351	D	9.7553	1
**4688**	357	D	72.6538	1
**4688**	370	D	73.8212	1
**4688**	372	D	71.9244	0
**4688**	373	D	65.2598	1
**4688**	381	D	48.9122	0
**4688**	382	D	79.5864	1
**4688**	386	D	74.2678	1
**4689**	305	A	75.9398	1
**4689**	319	A	49.2109	0

4689	320	A	62.2837	-1
4689	321	A	70.5731	0
4689	351	A	1.3149	1
4689	357	A	79.6647	1
4689	370	A	37.5267	0
4689	372	A	39.348	1
4689	373	A	68.0999	1
4689	381	A	42.8882	0
4689	382	A	73.6327	1
4689	386	A	79.5909	1
4689	305	B	79.4552	1
4689	319	B	79.1583	1
4689	320	B	79.547	-1
4689	321	B	71.361	0
4689	351	B	5.9695	1
4689	357	B	78.2401	1
4689	370	B	71.5579	1
4689	372	B	67.0692	0
4689	373	B	68.7678	1
4689	381	B	70.4522	1
4689	382	B	16.2035	-1
4689	386	B	79.351	1
4689	305	C	58.1575	1
4689	319	C	79.5155	1
4689	320	C	44.6757	-1
4689	321	C	62.0989	0
4689	351	C	7.2655	1
4689	357	C	79.5108	1
4689	370	C	64.7006	1
4689	372	C	79.4367	0
4689	373	C	78.3006	1
4689	381	C	41.5964	0
4689	382	C	63.2954	1
4689	386	C	8.988	-1

**4689**	305	D	73.9139	1
**4689**	319	D	66.7898	1
**4689**	320	D	79.5574	-1
**4689**	321	D	79.5647	0
**4689**	351	D	16.8647	1
**4689**	357	D	71.3551	1
**4689**	370	D	49.7485	0
**4689**	372	D	69.6625	0
**4689**	373	D	58.7406	1
**4689**	381	D	70.8347	1
**4689**	382	D	56.734	1
**4689**	386	D	79.4453	1
**4690**	305	A	73.3293	1
**4690**	319	A	64.3094	1
**4690**	320	A	79.5858	-1
**4690**	321	A	69.5621	0
**4690**	351	A	4.393	1
**4690**	357	A	72.3886	1
**4690**	370	A	77.0485	1
**4690**	372	A	70.6816	0
**4690**	373	A	72.3537	1
**4690**	381	A	79.5613	1
**4690**	382	A	73.8665	1
**4690**	386	A	70.1024	1
**4690**	305	B	51.4461	1
**4690**	319	B	71.8401	1
**4690**	320	B	79.5213	-1
**4690**	321	B	79.6434	0
**4690**	351	B	9.3651	1
**4690**	357	B	79.512	1
**4690**	370	B	47.6466	0
**4690**	372	B	79.5337	0
**4690**	373	B	52.4806	1
**4690**	381	B	79.5449	1

**4690**	382	B	71.3524	1
**4690**	386	B	77.0207	1
**4690**	305	C	79.5685	1
**4690**	319	C	72.5641	1
**4690**	320	C	61.7203	-1
**4690**	321	C	79.6152	0
**4690**	351	C	7.6598	1
**4690**	357	C	72.4336	1
**4690**	370	C	79.5255	1
**4690**	372	C	76.209	0
**4690**	373	C	67.134	1
**4690**	381	C	76.4416	1
**4690**	382	C	59.7615	1
**4690**	386	C	4.0121	-1
**4690**	305	D	60.7508	1
**4690**	319	D	72.9095	1
**4690**	320	D	79.5096	-1
**4690**	321	D	59.4075	0
**4690**	351	D	16.2293	1
**4690**	357	D	79.6083	1
**4690**	370	D	71.0001	1
**4690**	372	D	74.0083	0
**4690**	373	D	53.4761	1
**4690**	381	D	71.8689	1
**4690**	382	D	79.58	1
**4690**	386	D	79.5471	1
**4691**	305	A	77.9096	1
**4691**	319	A	51.4819	1
**4691**	320	A	79.4712	-1
**4691**	321	A	69.0819	0
**4691**	351	A	10.2453	1
**4691**	357	A	49.2534	0
**4691**	370	A	79.5741	1
**4691**	372	A	47.2067	1

**4691**	373	A	79.5949	1
**4691**	381	A	78.2328	1
**4691**	382	A	72.0976	1
**4691**	386	A	77.81	1
**4691**	305	B	79.5261	1
**4691**	319	B	79.5819	1
**4691**	320	B	20.58	-1
**4691**	321	B	27.3705	1
**4691**	351	B	17.8184	1
**4691**	357	B	55.9055	1
**4691**	370	B	52.9346	1
**4691**	372	B	66.2737	0
**4691**	373	B	37.4494	0
**4691**	381	B	45.8308	0
**4691**	382	B	16.6504	-1
**4691**	386	B	51.1963	1
**4691**	305	C	77.4914	1
**4691**	319	C	79.5775	1
**4691**	320	C	75.0528	-1
**4691**	321	C	74.0585	0
**4691**	351	C	1.2544	1
**4691**	357	C	79.5131	1
**4691**	370	C	78.7471	1
**4691**	372	C	79.4769	0
**4691**	373	C	42.3463	0
**4691**	381	C	36.4372	0
**4691**	382	C	48.6756	0
**4691**	386	C	39.358	0
**4691**	305	D	58.4842	1
**4691**	319	D	71.1948	1
**4691**	320	D	77.5931	-1
**4691**	321	D	32.4528	1
**4691**	351	D	11.6152	1
**4691**	357	D	79.5866	1

**4691**	370	D	69.8165	1
**4691**	372	D	59.5147	0
**4691**	373	D	38.7174	0
**4691**	381	D	51.7714	1
**4691**	382	D	79.5196	1
**4691**	386	D	73.6608	1
**4692**	305	A	36.8411	0
**4692**	319	A	39.9295	0
**4692**	320	A	79.5425	-1
**4692**	321	A	69.2279	0
**4692**	351	A	8.6387	1
**4692**	357	A	79.4686	1
**4692**	370	A	79.4698	1
**4692**	372	A	79.4993	0
**4692**	373	A	65.4069	1
**4692**	381	A	79.4875	1
**4692**	382	A	79.6807	1
**4692**	386	A	66.5047	1
**4692**	305	B	64.5078	1
**4692**	319	B	79.6271	1
**4692**	320	B	31.5623	-1
**4692**	321	B	69.0372	0
**4692**	351	B	16.6649	1
**4692**	357	B	72.5807	1
**4692**	370	B	19.3812	-1
**4692**	372	B	79.3474	0
**4692**	373	B	78.0649	1
**4692**	381	B	64.3458	1
**4692**	382	B	26.8355	0
**4692**	386	B	76.3097	1
**4692**	305	C	78.0527	1
**4692**	319	C	75.8215	1
**4692**	320	C	79.4745	-1
**4692**	321	C	61.4527	0

**4692**	351	C	9.8071	1
**4692**	357	C	64.3122	1
**4692**	370	C	64.2849	1
**4692**	372	C	63.7326	0
**4692**	373	C	65.9464	1
**4692**	381	C	74.5005	1
**4692**	382	C	72.1696	1
**4692**	386	C	55.3559	1
**4692**	305	D	63.3347	1
**4692**	319	D	45.4829	0
**4692**	320	D	79.4363	-1
**4692**	321	D	79.587	0
**4692**	351	D	13.5938	1
**4692**	357	D	65.272	1
**4692**	370	D	79.5707	1
**4692**	372	D	79.4598	0
**4692**	373	D	79.6231	1
**4692**	381	D	79.5926	1
**4692**	382	D	70.8638	1
**4692**	386	D	13.1439	-1
**4693**	305	A	72.9068	1
**4693**	319	A	25.0422	0
**4693**	320	A	68.314	-1
**4693**	321	A	79.5558	0
**4693**	351	A	4.4033	1
**4693**	357	A	71.446	1
**4693**	370	A	73.5898	1
**4693**	372	A	30.3468	1
**4693**	373	A	79.5828	1
**4693**	381	A	44.5779	0
**4693**	382	A	42.0233	0
**4693**	386	A	76.3954	1
**4693**	305	B	66.6687	1
**4693**	319	B	79.5121	1

**4693**	320	B	70.9078	-1
**4693**	321	B	62.2274	0
**4693**	351	B	12.6675	1
**4693**	357	B	54.9417	1
**4693**	370	B	46.3426	0
**4693**	372	B	79.5137	0
**4693**	373	B	79.5559	1
**4693**	381	B	76.7857	1
**4693**	382	B	49.057	0
**4693**	386	B	79.0746	1
**4693**	305	C	79.5388	1
**4693**	319	C	79.5789	1
**4693**	320	C	79.5158	-1
**4693**	321	C	73.3389	0
**4693**	351	C	8.4	1
**4693**	357	C	63.6273	1
**4693**	370	C	79.583	1
**4693**	372	C	79.5863	0
**4693**	373	C	78.9609	1
**4693**	381	C	57.877	1
**4693**	382	C	50.887	1
**4693**	386	C	41.7037	0
**4693**	305	D	46.2865	0
**4693**	319	D	73.1541	1
**4693**	320	D	29.876	-1
**4693**	321	D	58.6815	0
**4693**	351	D	17.986	1
**4693**	357	D	61.2277	1
**4693**	370	D	55.0553	1
**4693**	372	D	73.7491	0
**4693**	373	D	64.2813	1
**4693**	381	D	73.2745	1
**4693**	382	D	70.8258	1
**4693**	386	D	72.5086	1

**4694**	305	A	45.1453	0
**4694**	319	A	71.598	1
**4694**	320	A	48.0802	-1
**4694**	321	A	79.5287	0
**4694**	351	A	0.0	1
**4694**	357	A	79.6244	1
**4694**	370	A	56.2224	1
**4694**	372	A	25.4869	1
**4694**	373	A	73.5562	1
**4694**	381	A	25.6981	0
**4694**	382	A	74.6915	1
**4694**	386	A	75.1895	1
**4694**	305	B	79.5998	1
**4694**	319	B	54.3282	1
**4694**	320	B	68.6159	-1
**4694**	321	B	72.5704	0
**4694**	351	B	18.4234	1
**4694**	357	B	57.7464	1
**4694**	370	B	71.3797	1
**4694**	372	B	72.1652	0
**4694**	373	B	72.4044	1
**4694**	381	B	19.0753	-1
**4694**	382	B	75.068	1
**4694**	386	B	79.4009	1
**4694**	305	C	48.8271	0
**4694**	319	C	60.6899	1
**4694**	320	C	75.2567	-1
**4694**	321	C	72.9929	0
**4694**	351	C	16.9597	1
**4694**	357	C	37.5383	0
**4694**	370	C	54.9105	1
**4694**	372	C	79.6205	0
**4694**	373	C	40.502	0
**4694**	381	C	71.4646	1

**4694**	382	C	55.6157	1
**4694**	386	C	15.8411	-1
**4694**	305	D	69.7627	1
**4694**	319	D	68.8199	1
**4694**	320	D	79.3975	-1
**4694**	321	D	77.3125	0
**4694**	351	D	17.3616	1
**4694**	357	D	65.7699	1
**4694**	370	D	78.851	1
**4694**	372	D	68.376	0
**4694**	373	D	42.7646	0
**4694**	381	D	76.3168	1
**4694**	382	D	68.3054	1
**4694**	386	D	74.7219	1
**4695**	305	A	74.5827	1
**4695**	319	A	36.6299	0
**4695**	320	A	34.113	-1
**4695**	321	A	43.6458	1
**4695**	351	A	8.6227	1
**4695**	357	A	79.5711	1
**4695**	370	A	46.3747	0
**4695**	372	A	60.4895	0
**4695**	373	A	54.9282	1
**4695**	381	A	28.6203	0
**4695**	382	A	44.9395	0
**4695**	386	A	70.7718	1
**4695**	305	B	79.6018	1
**4695**	319	B	79.4334	1
**4695**	320	B	75.9182	-1
**4695**	321	B	79.626	0
**4695**	351	B	15.5922	1
**4695**	357	B	52.5301	1
**4695**	370	B	79.5226	1
**4695**	372	B	71.0169	0

**4695**	373	B	79.1714	1
**4695**	381	B	76.2201	1
**4695**	382	B	74.5723	1
**4695**	386	B	59.7596	1
**4695**	305	C	69.3602	1
**4695**	319	C	58.3697	1
**4695**	320	C	79.5039	-1
**4695**	321	C	54.2938	0
**4695**	351	C	17.3043	1
**4695**	357	C	63.4948	1
**4695**	370	C	79.5991	1
**4695**	372	C	79.4627	0
**4695**	373	C	71.0515	1
**4695**	381	C	62.9147	1
**4695**	382	C	18.1443	-1
**4695**	386	C	68.0414	1
**4695**	305	D	39.3429	0
**4695**	319	D	79.4755	1
**4695**	320	D	45.8444	-1
**4695**	321	D	58.7109	0
**4695**	351	D	5.9962	1
**4695**	357	D	77.9069	1
**4695**	370	D	78.8626	1
**4695**	372	D	37.856	1
**4695**	373	D	70.29	1
**4695**	381	D	76.9653	1
**4695**	382	D	70.332	1
**4695**	386	D	76.6449	1
**4696**	305	A	74.3052	1
**4696**	319	A	44.4836	0
**4696**	320	A	79.7219	-1
**4696**	321	A	79.0067	0
**4696**	351	A	10.0645	1
**4696**	357	A	76.0399	1

**4696**	370	A	71.7089	1
**4696**	372	A	72.7244	0
**4696**	373	A	22.6907	0
**4696**	381	A	79.4413	1
**4696**	382	A	58.8487	1
**4696**	386	A	71.4133	1
**4696**	305	B	70.7081	1
**4696**	319	B	60.008	1
**4696**	320	B	55.9123	-1
**4696**	321	B	71.4047	0
**4696**	351	B	17.3762	1
**4696**	357	B	79.5972	1
**4696**	370	B	72.1765	1
**4696**	372	B	79.7889	0
**4696**	373	B	59.2522	1
**4696**	381	B	79.5671	1
**4696**	382	B	50.3392	1
**4696**	386	B	36.7078	0
**4696**	305	C	60.175	1
**4696**	319	C	45.0634	0
**4696**	320	C	32.2386	-1
**4696**	321	C	54.7386	0
**4696**	351	C	0.0029	1
**4696**	357	C	65.9711	1
**4696**	370	C	79.2088	1
**4696**	372	C	35.1272	1
**4696**	373	C	73.7891	1
**4696**	381	C	49.6614	0
**4696**	382	C	71.165	1
**4696**	386	C	39.0657	0
**4696**	305	D	58.2765	1
**4696**	319	D	45.1856	0
**4696**	320	D	75.6917	-1
**4696**	321	D	47.5835	1

**4696**	351	D	17.1086	1
**4696**	357	D	55.4176	1
**4696**	370	D	54.3421	1
**4696**	372	D	64.2569	0
**4696**	373	D	71.6364	1
**4696**	381	D	79.4784	1
**4696**	382	D	73.9074	1
**4696**	386	D	72.6135	1
**4697**	305	A	79.6299	1
**4697**	319	A	63.8464	1
**4697**	320	A	79.5096	-1
**4697**	321	A	74.6601	0
**4697**	351	A	0.5371	1
**4697**	357	A	23.0303	0
**4697**	370	A	62.2274	1
**4697**	372	A	72.8726	0
**4697**	373	A	49.4065	0
**4697**	381	A	72.3295	1
**4697**	382	A	75.9312	1
**4697**	386	A	70.7922	1
**4697**	305	B	72.1622	1
**4697**	319	B	79.5267	1
**4697**	320	B	59.9169	-1
**4697**	321	B	33.9125	1
**4697**	351	B	15.7593	1
**4697**	357	B	79.3417	1
**4697**	370	B	76.0068	1
**4697**	372	B	70.4066	0
**4697**	373	B	79.6521	1
**4697**	381	B	46.4926	0
**4697**	382	B	35.0047	0
**4697**	386	B	62.6929	1
**4697**	305	C	79.539	1
**4697**	319	C	70.5806	1

**4697**	320	C	36.3223	-1
**4697**	321	C	59.1144	0
**4697**	351	C	4.3312	1
**4697**	357	C	68.103	1
**4697**	370	C	79.5104	1
**4697**	372	C	70.7713	0
**4697**	373	C	69.5169	1
**4697**	381	C	79.6392	1
**4697**	382	C	73.2707	1
**4697**	386	C	55.0274	1
**4697**	305	D	77.6867	1
**4697**	319	D	28.5591	0
**4697**	320	D	79.6446	-1
**4697**	321	D	44.4015	1
**4697**	351	D	14.6453	1
**4697**	357	D	62.5695	1
**4697**	370	D	38.3558	0
**4697**	372	D	79.5009	0
**4697**	373	D	79.4542	1
**4697**	381	D	63.3946	1
**4697**	382	D	59.1663	1
**4697**	386	D	63.948	1
**4698**	305	A	72.7483	1
**4698**	319	A	74.5778	1
**4698**	320	A	47.6816	-1
**4698**	321	A	46.3472	1
**4698**	351	A	13.0	1
**4698**	357	A	79.5678	1
**4698**	370	A	24.224	0
**4698**	372	A	62.2574	0
**4698**	373	A	69.6603	1
**4698**	381	A	79.6309	1
**4698**	382	A	79.5344	1
**4698**	386	A	51.5667	1

**4698**	305	B	73.56	1
**4698**	319	B	10.9465	-1
**4698**	320	B	39.0003	-1
**4698**	321	B	61.9029	0
**4698**	351	B	0.0722	1
**4698**	357	B	72.9777	1
**4698**	370	B	59.9291	1
**4698**	372	B	79.637	0
**4698**	373	B	53.7492	1
**4698**	381	B	79.362	1
**4698**	382	B	45.7844	0
**4698**	386	B	79.5988	1
**4698**	305	C	77.446	1
**4698**	319	C	75.2411	1
**4698**	320	C	49.2955	-1
**4698**	321	C	52.9847	0
**4698**	351	C	12.9133	1
**4698**	357	C	79.5762	1
**4698**	370	C	56.5808	1
**4698**	372	C	79.4674	0
**4698**	373	C	58.4567	1
**4698**	381	C	44.1088	0
**4698**	382	C	77.6689	1
**4698**	386	C	61.4611	1
**4698**	305	D	79.2541	1
**4698**	319	D	54.2818	1
**4698**	320	D	79.5205	-1
**4698**	321	D	77.9797	0
**4698**	351	D	11.1371	1
**4698**	357	D	35.553	0
**4698**	370	D	68.0103	1
**4698**	372	D	69.3726	0
**4698**	373	D	78.8247	1
**4698**	381	D	79.6064	1

**4698**	382	D	79.2835	1
**4698**	386	D	43.2733	0
**4699**	305	A	79.5423	1
**4699**	319	A	73.6559	1
**4699**	320	A	60.3074	-1
**4699**	321	A	79.5986	0
**4699**	351	A	0.3181	1
**4699**	357	A	79.529	1
**4699**	370	A	73.25	1
**4699**	372	A	26.7297	1
**4699**	373	A	70.2581	1
**4699**	381	A	14.9856	-1
**4699**	382	A	79.5807	1
**4699**	386	A	50.8294	1
**4699**	305	B	79.3211	1
**4699**	319	B	74.3595	1
**4699**	320	B	79.5656	-1
**4699**	321	B	51.681	0
**4699**	351	B	18.6316	1
**4699**	357	B	40.4652	0
**4699**	370	B	73.5412	1
**4699**	372	B	79.5249	0
**4699**	373	B	79.5071	1
**4699**	381	B	64.245	1
**4699**	382	B	72.6239	1
**4699**	386	B	51.6945	1
**4699**	305	C	41.8541	0
**4699**	319	C	47.4149	0
**4699**	320	C	58.4127	-1
**4699**	321	C	59.6417	0
**4699**	351	C	5.7694	1
**4699**	357	C	79.6162	1
**4699**	370	C	79.5529	1
**4699**	372	C	79.5856	0

**4699**	373	C	71.4428	1
**4699**	381	C	75.2401	1
**4699**	382	C	77.6014	1
**4699**	386	C	59.5016	1
**4699**	305	D	56.5279	1
**4699**	319	D	38.9971	0
**4699**	320	D	67.1711	-1
**4699**	321	D	69.6637	0
**4699**	351	D	6.4019	1
**4699**	357	D	39.0313	0
**4699**	370	D	74.4761	1
**4699**	372	D	79.5924	0
**4699**	373	D	79.5807	1
**4699**	381	D	70.2255	1
**4699**	382	D	61.3024	1
**4699**	386	D	78.4512	1
**4700**	305	A	79.571	1
**4700**	319	A	72.5741	1
**4700**	320	A	72.2312	-1
**4700**	321	A	79.5492	0
**4700**	351	A	8.9447	1
**4700**	357	A	67.0428	1
**4700**	370	A	79.626	1
**4700**	372	A	79.5778	0
**4700**	373	A	63.4773	1
**4700**	381	A	79.5724	1
**4700**	382	A	79.5773	1
**4700**	386	A	76.2661	1
**4700**	305	B	79.5848	1
**4700**	319	B	21.5721	0
**4700**	320	B	50.9955	-1
**4700**	321	B	79.5742	0
**4700**	351	B	14.9896	1
**4700**	357	B	79.5842	1

**4700**	370	B	79.3558	1
**4700**	372	B	65.7785	0
**4700**	373	B	45.0987	0
**4700**	381	B	74.094	1
**4700**	382	B	29.3993	0
**4700**	386	B	62.0624	1
**4700**	305	C	59.3972	1
**4700**	319	C	49.936	0
**4700**	320	C	79.5466	-1
**4700**	321	C	62.4249	0
**4700**	351	C	2.4563	1
**4700**	357	C	79.5695	1
**4700**	370	C	79.3372	1
**4700**	372	C	72.7307	0
**4700**	373	C	79.5492	1
**4700**	381	C	39.5846	0
**4700**	382	C	65.0268	1
**4700**	386	C	64.6784	1
**4700**	305	D	69.9412	1
**4700**	319	D	65.9366	1
**4700**	320	D	78.9739	-1
**4700**	321	D	78.3872	0
**4700**	351	D	15.3387	1
**4700**	357	D	63.189	1
**4700**	370	D	72.0549	1
**4700**	372	D	79.5555	0
**4700**	373	D	76.6258	1
**4700**	381	D	78.0743	1
**4700**	382	D	79.5916	1
**4700**	386	D	79.5665	1
**4701**	305	A	79.3945	1
**4701**	319	A	74.6137	1
**4701**	320	A	79.5407	-1
**4701**	321	A	57.4103	0

**4701**	351	A	7.7789	1
**4701**	357	A	79.5935	1
**4701**	370	A	79.6129	1
**4701**	372	A	28.1261	1
**4701**	373	A	61.9685	1
**4701**	381	A	73.8272	1
**4701**	382	A	75.0486	1
**4701**	386	A	79.5661	1
**4701**	305	B	79.6445	1
**4701**	319	B	32.844	0
**4701**	320	B	75.7982	-1
**4701**	321	B	72.3904	0
**4701**	351	B	3.5268	1
**4701**	357	B	78.6916	1
**4701**	370	B	68.9768	1
**4701**	372	B	78.9316	0
**4701**	373	B	63.7337	1
**4701**	381	B	79.5643	1
**4701**	382	B	52.7756	1
**4701**	386	B	79.6631	1
**4701**	305	C	54.4758	1
**4701**	319	C	79.4363	1
**4701**	320	C	48.8625	-1
**4701**	321	C	46.3412	1
**4701**	351	C	1.7809	1
**4701**	357	C	75.7444	1
**4701**	370	C	40.8201	0
**4701**	372	C	79.5979	0
**4701**	373	C	52.3303	1
**4701**	381	C	63.2751	1
**4701**	382	C	53.1837	1
**4701**	386	C	45.9588	0
**4701**	305	D	41.1666	0
**4701**	319	D	79.5354	1

**4701**	320	D	19.4941	1
**4701**	321	D	74.472	0
**4701**	351	D	11.8252	1
**4701**	357	D	70.2507	1
**4701**	370	D	34.0053	0
**4701**	372	D	79.5256	0
**4701**	373	D	15.8776	-1
**4701**	381	D	45.1969	0
**4701**	382	D	72.4244	1
**4701**	386	D	25.4805	0
**4702**	305	A	79.4702	1
**4702**	319	A	70.2516	1
**4702**	320	A	79.4887	-1
**4702**	321	A	37.3102	1
**4702**	351	A	12.7975	1
**4702**	357	A	70.8175	1
**4702**	370	A	62.6435	1
**4702**	372	A	74.7347	0
**4702**	373	A	79.6384	1
**4702**	381	A	79.5718	1
**4702**	382	A	72.3563	1
**4702**	386	A	77.9016	1
**4702**	305	B	79.6517	1
**4702**	319	B	78.0235	1
**4702**	320	B	79.5632	-1
**4702**	321	B	79.5063	0
**4702**	351	B	19.5887	1
**4702**	357	B	75.8963	1
**4702**	370	B	73.5169	1
**4702**	372	B	79.7235	0
**4702**	373	B	77.2129	1
**4702**	381	B	71.2702	1
**4702**	382	B	72.024	1
**4702**	386	B	30.2997	0

**4702**	305	C	79.5884	1
**4702**	319	C	79.4968	1
**4702**	320	C	52.2124	-1
**4702**	321	C	71.8704	0
**4702**	351	C	15.339	1
**4702**	357	C	79.3549	1
**4702**	370	C	63.1381	1
**4702**	372	C	72.7639	0
**4702**	373	C	79.4408	1
**4702**	381	C	55.5622	1
**4702**	382	C	79.5162	1
**4702**	386	C	60.6613	1
**4702**	305	D	73.5056	1
**4702**	319	D	65.3222	1
**4702**	320	D	79.6803	-1
**4702**	321	D	53.7578	0
**4702**	351	D	16.4539	1
**4702**	357	D	67.7115	1
**4702**	370	D	70.9192	1
**4702**	372	D	54.6751	0
**4702**	373	D	29.5373	0
**4702**	381	D	79.6064	1
**4702**	382	D	79.568	1
**4702**	386	D	79.4932	1
**4703**	305	A	79.3132	1
**4703**	319	A	78.5249	1
**4703**	320	A	58.3259	-1
**4703**	321	A	58.1443	0
**4703**	351	A	7.8616	1
**4703**	357	A	72.6404	1
**4703**	370	A	75.4881	1
**4703**	372	A	79.5442	0
**4703**	373	A	78.6895	1
**4703**	381	A	71.8146	1

**4703**	382	A	35.3419	0
**4703**	386	A	79.5943	1
**4703**	305	B	62.2237	1
**4703**	319	B	66.5537	1
**4703**	320	B	79.5158	-1
**4703**	321	B	73.5122	0
**4703**	351	B	13.1288	1
**4703**	357	B	79.5592	1
**4703**	370	B	66.064	1
**4703**	372	B	40.6343	1
**4703**	373	B	71.7539	1
**4703**	381	B	79.5114	1
**4703**	382	B	29.4991	0
**4703**	386	B	59.1197	1
**4703**	305	C	79.2721	1
**4703**	319	C	63.6355	1
**4703**	320	C	74.2316	-1
**4703**	321	C	58.1435	0
**4703**	351	C	9.4	1
**4703**	357	C	79.572	1
**4703**	370	C	78.5395	1
**4703**	372	C	79.5415	0
**4703**	373	C	79.3439	1
**4703**	381	C	72.5744	1
**4703**	382	C	79.5943	1
**4703**	386	C	48.8346	0
**4703**	305	D	61.7511	1
**4703**	319	D	56.1471	1
**4703**	320	D	78.2758	-1
**4703**	321	D	73.0391	0
**4703**	351	D	11.9378	1
**4703**	357	D	73.9145	1
**4703**	370	D	71.3623	1
**4703**	372	D	79.4933	0

**4703**	373	D	47.8577	0
**4703**	381	D	79.5834	1
**4703**	382	D	79.5349	1
**4703**	386	D	79.523	1
**4704**	305	A	19.5609	-1
**4704**	319	A	78.3471	1
**4704**	320	A	56.7945	-1
**4704**	321	A	49.0084	1
**4704**	351	A	12.426	1
**4704**	357	A	72.3188	1
**4704**	370	A	54.539	1
**4704**	372	A	51.679	0
**4704**	373	A	20.4146	0
**4704**	381	A	79.4634	1
**4704**	382	A	74.663	1
**4704**	386	A	79.2812	1
**4704**	305	B	79.5942	1
**4704**	319	B	64.0842	1
**4704**	320	B	62.1797	-1
**4704**	321	B	78.0365	0
**4704**	351	B	13.7474	1
**4704**	357	B	59.5567	1
**4704**	370	B	78.452	1
**4704**	372	B	79.5604	0
**4704**	373	B	79.5419	1
**4704**	381	B	70.1067	1
**4704**	382	B	64.9058	1
**4704**	386	B	60.6011	1
**4704**	305	C	33.2862	0
**4704**	319	C	72.3842	1
**4704**	320	C	79.604	-1
**4704**	321	C	79.612	0
**4704**	351	C	5.7289	1
**4704**	357	C	53.8946	1

**4704**	370	C	75.4062	1
**4704**	372	C	47.5687	1
**4704**	373	C	54.0201	1
**4704**	381	C	79.5094	1
**4704**	382	C	54.9148	1
**4704**	386	C	45.6756	0
**4704**	305	D	20.8793	0
**4704**	319	D	68.1981	1
**4704**	320	D	67.2494	-1
**4704**	321	D	42.4309	1
**4704**	351	D	12.3713	1
**4704**	357	D	78.297	1
**4704**	370	D	71.8363	1
**4704**	372	D	68.7245	0
**4704**	373	D	55.1675	1
**4704**	381	D	79.5511	1
**4704**	382	D	70.9559	1
**4704**	386	D	79.668	1
**4705**	305	A	79.6013	1
**4705**	319	A	68.9312	1
**4705**	320	A	72.9297	-1
**4705**	321	A	78.1173	0
**4705**	351	A	40.9555	-1
**4705**	357	A	68.2302	1
**4705**	370	A	44.078	0
**4705**	372	A	79.5072	0
**4705**	373	A	63.3604	1
**4705**	381	A	79.5263	1
**4705**	382	A	42.5983	0
**4705**	386	A	73.2036	1
**4705**	305	B	68.8548	1
**4705**	319	B	75.4155	1
**4705**	320	B	68.326	-1
**4705**	321	B	46.6727	1

**4705**	351	B	5.483	1
**4705**	357	B	43.5382	0
**4705**	370	B	73.8591	1
**4705**	372	B	57.4291	0
**4705**	373	B	69.9171	1
**4705**	381	B	52.9164	1
**4705**	382	B	70.7013	1
**4705**	386	B	72.6414	1
**4705**	305	C	68.9682	1
**4705**	319	C	60.2415	1
**4705**	320	C	78.1696	-1
**4705**	321	C	69.0696	0
**4705**	351	C	15.9495	1
**4705**	357	C	57.775	1
**4705**	370	C	71.2933	1
**4705**	372	C	79.5648	0
**4705**	373	C	79.5979	1
**4705**	381	C	79.557	1
**4705**	382	C	78.279	1
**4705**	386	C	23.2518	0
**4705**	305	D	35.757	0
**4705**	319	D	79.5296	1
**4705**	320	D	79.5913	-1
**4705**	321	D	72.648	0
**4705**	351	D	3.1454	1
**4705**	357	D	79.6174	1
**4705**	370	D	72.8115	1
**4705**	372	D	79.6303	0
**4705**	373	D	72.3522	1
**4705**	381	D	72.3668	1
**4705**	382	D	78.2319	1
**4705**	386	D	79.489	1
**4706**	305	A	77.0178	1
**4706**	319	A	29.3804	0

**4706**	320	A	56.7471	-1
**4706**	321	A	65.8285	0
**4706**	351	A	1.6256	1
**4706**	357	A	79.5926	1
**4706**	370	A	30.6933	0
**4706**	372	A	63.3582	0
**4706**	373	A	56.5745	1
**4706**	381	A	30.5576	0
**4706**	382	A	73.9723	1
**4706**	386	A	64.2593	1
**4706**	305	B	76.207	1
**4706**	319	B	79.6108	1
**4706**	320	B	79.5287	-1
**4706**	321	B	52.6766	0
**4706**	351	B	15.638	1
**4706**	357	B	74.8788	1
**4706**	370	B	55.2822	1
**4706**	372	B	68.4284	0
**4706**	373	B	68.208	1
**4706**	381	B	73.7644	1
**4706**	382	B	58.9259	1
**4706**	386	B	39.6605	0
**4706**	305	C	76.0413	1
**4706**	319	C	66.1606	1
**4706**	320	C	75.6268	-1
**4706**	321	C	78.8363	0
**4706**	351	C	9.2023	1
**4706**	357	C	72.173	1
**4706**	370	C	94.3875	1
**4706**	372	C	64.9307	0
**4706**	373	C	70.4432	1
**4706**	381	C	37.5892	0
**4706**	382	C	58.6256	1
**4706**	386	C	45.0196	0

**4706**	305	D	75.5745	1
**4706**	319	D	77.1129	1
**4706**	320	D	29.1998	-1
**4706**	321	D	69.0824	0
**4706**	351	D	7.9471	1
**4706**	357	D	79.5407	1
**4706**	370	D	5.4716	-1
**4706**	372	D	74.6288	0
**4706**	373	D	70.1666	1
**4706**	381	D	60.0717	1
**4706**	382	D	73.6201	1
**4706**	386	D	79.4471	1
**4707**	305	A	79.2578	1
**4707**	319	A	79.4613	1
**4707**	320	A	71.661	-1
**4707**	321	A	62.7413	0
**4707**	351	A	13.1203	1
**4707**	357	A	38.5891	0
**4707**	370	A	42.4414	0
**4707**	372	A	79.5091	0
**4707**	373	A	74.4482	1
**4707**	381	A	76.1817	1
**4707**	382	A	68.6008	1
**4707**	386	A	79.6178	1
**4707**	305	B	70.5855	1
**4707**	319	B	79.4683	1
**4707**	320	B	66.701	-1
**4707**	321	B	72.1127	0
**4707**	351	B	16.2844	1
**4707**	357	B	45.2917	0
**4707**	370	B	69.1493	1
**4707**	372	B	54.7304	0
**4707**	373	B	79.4988	1
**4707**	381	B	57.8511	1

**4707**	382	B	47.0736	0
**4707**	386	B	69.2476	1
**4707**	305	C	57.4015	1
**4707**	319	C	72.7983	1
**4707**	320	C	46.2434	-1
**4707**	321	C	79.5505	0
**4707**	351	C	10.0124	1
**4707**	357	C	72.7811	1
**4707**	370	C	79.5239	1
**4707**	372	C	79.5032	0
**4707**	373	C	58.4225	1
**4707**	381	C	65.327	1
**4707**	382	C	79.5471	1
**4707**	386	C	20.042	0
**4707**	305	D	79.6114	1
**4707**	319	D	41.7531	0
**4707**	320	D	50.1522	-1
**4707**	321	D	79.6049	0
**4707**	351	D	9.0217	1
**4707**	357	D	79.5758	1
**4707**	370	D	79.5865	1
**4707**	372	D	79.5524	0
**4707**	373	D	64.9001	1
**4707**	381	D	79.6121	1
**4707**	382	D	50.5419	1
**4707**	386	D	47.752	0
**4708**	305	A	73.7806	1
**4708**	319	A	57.6618	1
**4708**	320	A	75.4093	-1
**4708**	321	A	60.2641	0
**4708**	351	A	10.0704	1
**4708**	357	A	36.4081	0
**4708**	370	A	72.6505	1
**4708**	372	A	41.7541	1

**4708**	373	A	77.8771	1
**4708**	381	A	79.5095	1
**4708**	382	A	69.0304	1
**4708**	386	A	79.5746	1
**4708**	305	B	79.531	1
**4708**	319	B	68.9618	1
**4708**	320	B	35.9227	-1
**4708**	321	B	71.5221	0
**4708**	351	B	5.1619	1
**4708**	357	B	25.0538	0
**4708**	370	B	70.6277	1
**4708**	372	B	79.6089	0
**4708**	373	B	64.9156	1
**4708**	381	B	56.8169	1
**4708**	382	B	43.3652	0
**4708**	386	B	55.796	1
**4708**	305	C	75.0565	1
**4708**	319	C	76.7304	1
**4708**	320	C	75.1392	-1
**4708**	321	C	35.6274	1
**4708**	351	C	39.8095	-1
**4708**	357	C	72.969	1
**4708**	370	C	78.6344	1
**4708**	372	C	73.606	0
**4708**	373	C	55.5048	1
**4708**	381	C	79.5691	1
**4708**	382	C	53.0293	1
**4708**	386	C	4.2831	-1
**4708**	305	D	79.4227	1
**4708**	319	D	78.9144	1
**4708**	320	D	79.6362	-1
**4708**	321	D	53.6123	0
**4708**	351	D	10.7492	1
**4708**	357	D	70.8616	1

**4708**	370	D	51.5237	1
**4708**	372	D	56.1656	0
**4708**	373	D	32.1736	0
**4708**	381	D	60.7711	1
**4708**	382	D	51.4043	1
**4708**	386	D	60.5103	1
**4709**	305	A	25.524	0
**4709**	319	A	51.667	1
**4709**	320	A	73.557	-1
**4709**	321	A	79.6669	0
**4709**	351	A	0.3119	1
**4709**	357	A	79.5225	1
**4709**	370	A	79.497	1
**4709**	372	A	69.4727	0
**4709**	373	A	56.3212	1
**4709**	381	A	79.5929	1
**4709**	382	A	18.5047	-1
**4709**	386	A	36.6212	0
**4709**	305	B	61.2175	1
**4709**	319	B	56.3852	1
**4709**	320	B	71.9226	-1
**4709**	321	B	79.508	0
**4709**	351	B	10.3704	1
**4709**	357	B	72.6962	1
**4709**	370	B	57.5975	1
**4709**	372	B	76.4189	0
**4709**	373	B	79.5631	1
**4709**	381	B	52.7576	1
**4709**	382	B	68.6453	1
**4709**	386	B	79.4924	1
**4709**	305	C	61.0134	1
**4709**	319	C	79.4468	1
**4709**	320	C	72.8457	-1
**4709**	321	C	73.4087	0

**4709**	351	C	15.9253	1
**4709**	357	C	55.9876	1
**4709**	370	C	73.6576	1
**4709**	372	C	79.5453	0
**4709**	373	C	79.6239	1
**4709**	381	C	79.5812	1
**4709**	382	C	59.4604	1
**4709**	386	C	46.5847	0
**4709**	305	D	79.5794	1
**4709**	319	D	35.4811	0
**4709**	320	D	48.8014	-1
**4709**	321	D	45.3721	1
**4709**	351	D	0.8993	1
**4709**	357	D	73.3418	1
**4709**	370	D	79.5327	1
**4709**	372	D	72.0741	0
**4709**	373	D	74.3009	1
**4709**	381	D	60.6649	1
**4709**	382	D	75.1962	1
**4709**	386	D	75.4905	1
**4710**	305	A	79.528	1
**4710**	319	A	75.5266	1
**4710**	320	A	79.5786	-1
**4710**	321	A	48.0834	1
**4710**	351	A	8.1119	1
**4710**	357	A	79.326	1
**4710**	370	A	68.0262	1
**4710**	372	A	65.0912	0
**4710**	373	A	79.5895	1
**4710**	381	A	36.4401	0
**4710**	382	A	62.5929	1
**4710**	386	A	79.4775	1
**4710**	305	B	24.6734	0
**4710**	319	B	73.3179	1

**4710**	320	B	62.169	-1
**4710**	321	B	74.9146	0
**4710**	351	B	2.5522	1
**4710**	357	B	79.4927	1
**4710**	370	B	76.3698	1
**4710**	372	B	72.4672	0
**4710**	373	B	46.5061	0
**4710**	381	B	73.306	1
**4710**	382	B	73.8582	1
**4710**	386	B	64.973	1
**4710**	305	C	66.0363	1
**4710**	319	C	71.842	1
**4710**	320	C	79.5795	-1
**4710**	321	C	71.6758	0
**4710**	351	C	6.3557	1
**4710**	357	C	71.8049	1
**4710**	370	C	59.1391	1
**4710**	372	C	59.2159	0
**4710**	373	C	57.5859	1
**4710**	381	C	74.9349	1
**4710**	382	C	79.4741	1
**4710**	386	C	26.9179	0
**4710**	305	D	53.3758	1
**4710**	319	D	79.6196	1
**4710**	320	D	79.548	-1
**4710**	321	D	66.16	0
**4710**	351	D	14.2091	1
**4710**	357	D	79.5981	1
**4710**	370	D	78.7228	1
**4710**	372	D	73.8238	0
**4710**	373	D	74.183	1
**4710**	381	D	79.6247	1
**4710**	382	D	78.9595	1
**4710**	386	D	58.0787	1

**4711**	305	A	75.3427	1
**4711**	319	A	62.6709	1
**4711**	320	A	78.6349	-1
**4711**	321	A	70.6659	0
**4711**	351	A	13.3974	1
**4711**	357	A	76.7883	1
**4711**	370	A	79.5359	1
**4711**	372	A	47.4215	1
**4711**	373	A	69.9922	1
**4711**	381	A	73.888	1
**4711**	382	A	74.2854	1
**4711**	386	A	67.6244	1
**4711**	305	B	79.5284	1
**4711**	319	B	72.2777	1
**4711**	320	B	46.2522	-1
**4711**	321	B	74.0246	0
**4711**	351	B	12.2231	1
**4711**	357	B	79.5083	1
**4711**	370	B	70.9441	1
**4711**	372	B	65.2281	0
**4711**	373	B	71.6883	1
**4711**	381	B	79.5444	1
**4711**	382	B	77.9926	1
**4711**	386	B	62.376	1
**4711**	305	C	79.4052	1
**4711**	319	C	74.1247	1
**4711**	320	C	79.6315	-1
**4711**	321	C	62.7174	0
**4711**	351	C	6.9312	1
**4711**	357	C	73.2758	1
**4711**	370	C	46.7468	0
**4711**	372	C	79.0421	0
**4711**	373	C	58.5037	1
**4711**	381	C	74.6222	1

**4711**	382	C	49.3512	0
**4711**	386	C	53.8976	1
**4711**	305	D	72.8115	1
**4711**	319	D	79.5968	1
**4711**	320	D	54.65	-1
**4711**	321	D	79.2649	0
**4711**	351	D	11.3411	1
**4711**	357	D	67.507	1
**4711**	370	D	45.1259	0
**4711**	372	D	62.4246	0
**4711**	373	D	79.6008	1
**4711**	381	D	60.3181	1
**4711**	382	D	71.5125	1
**4711**	386	D	75.2602	1
**4712**	305	A	52.4315	1
**4712**	319	A	77.0428	1
**4712**	320	A	69.9914	-1
**4712**	321	A	79.6026	0
**4712**	351	A	19.3007	1
**4712**	357	A	35.5335	0
**4712**	370	A	79.1233	1
**4712**	372	A	57.0113	0
**4712**	373	A	79.6004	1
**4712**	381	A	53.8544	1
**4712**	382	A	49.565	0
**4712**	386	A	79.5024	1
**4712**	305	B	65.3785	1
**4712**	319	B	65.6781	1
**4712**	320	B	78.4408	-1
**4712**	321	B	58.4179	0
**4712**	351	B	11.0297	1
**4712**	357	B	52.9082	1
**4712**	370	B	79.5052	1
**4712**	372	B	79.5572	0

**4712**	373	B	74.2673	1
**4712**	381	B	79.5986	1
**4712**	382	B	68.33	1
**4712**	386	B	76.6279	1
**4712**	305	C	68.8981	1
**4712**	319	C	50.3982	1
**4712**	320	C	69.6107	-1
**4712**	321	C	12.7732	0
**4712**	351	C	3.2543	1
**4712**	357	C	36.5263	0
**4712**	370	C	71.2956	1
**4712**	372	C	79.6397	0
**4712**	373	C	55.8128	1
**4712**	381	C	27.514	0
**4712**	382	C	79.573	1
**4712**	386	C	31.563	0
**4712**	305	D	79.5704	1
**4712**	319	D	54.4958	1
**4712**	320	D	79.5099	-1
**4712**	321	D	79.5164	0
**4712**	351	D	4.5373	1
**4712**	357	D	79.5894	1
**4712**	370	D	61.2045	1
**4712**	372	D	75.9755	0
**4712**	373	D	71.9179	1
**4712**	381	D	72.8454	1
**4712**	382	D	50.808	1
**4712**	386	D	79.1074	1
**4713**	305	A	60.6993	1
**4713**	319	A	54.9328	1
**4713**	320	A	65.2473	-1
**4713**	321	A	79.574	0
**4713**	351	A	5.2162	1
**4713**	357	A	71.8151	1

**4713**	370	A	56.0325	1
**4713**	372	A	79.5648	0
**4713**	373	A	73.0534	1
**4713**	381	A	72.9199	1
**4713**	382	A	72.02	1
**4713**	386	A	53.0801	1
**4713**	305	B	79.3162	1
**4713**	319	B	60.2612	1
**4713**	320	B	69.4233	-1
**4713**	321	B	79.5183	0
**4713**	351	B	11.9145	1
**4713**	357	B	70.9244	1
**4713**	370	B	58.2546	1
**4713**	372	B	79.5592	0
**4713**	373	B	39.9767	0
**4713**	381	B	79.5515	1
**4713**	382	B	78.6028	1
**4713**	386	B	59.8747	1
**4713**	305	C	57.8446	1
**4713**	319	C	79.5591	1
**4713**	320	C	33.6134	-1
**4713**	321	C	76.0723	0
**4713**	351	C	3.7663	1
**4713**	357	C	70.9018	1
**4713**	370	C	79.4372	1
**4713**	372	C	26.8626	1
**4713**	373	C	74.0277	1
**4713**	381	C	79.6022	1
**4713**	382	C	79.5509	1
**4713**	386	C	4.6385	-1
**4713**	305	D	79.5348	1
**4713**	319	D	73.2211	1
**4713**	320	D	73.0849	-1
**4713**	321	D	79.5689	0

**4713**	351	D	16.3033	1
**4713**	357	D	76.9644	1
**4713**	370	D	79.5767	1
**4713**	372	D	79.6071	0
**4713**	373	D	79.5241	1
**4713**	381	D	62.2401	1
**4713**	382	D	79.5381	1
**4713**	386	D	51.0928	1
**4714**	305	A	67.0635	1
**4714**	319	A	61.3423	1
**4714**	320	A	79.4443	-1
**4714**	321	A	48.7578	1
**4714**	351	A	10.93	1
**4714**	357	A	71.2774	1
**4714**	370	A	79.5614	1
**4714**	372	A	79.5049	0
**4714**	373	A	35.1028	0
**4714**	381	A	72.1642	1
**4714**	382	A	20.4482	0
**4714**	386	A	47.3727	0
**4714**	305	B	79.6246	1
**4714**	319	B	79.2473	1
**4714**	320	B	42.7521	-1
**4714**	321	B	70.8555	0
**4714**	351	B	15.1142	1
**4714**	357	B	79.4305	1
**4714**	370	B	79.6347	1
**4714**	372	B	73.787	0
**4714**	373	B	75.4007	1
**4714**	381	B	79.5648	1
**4714**	382	B	35.9621	0
**4714**	386	B	41.1439	0
**4714**	305	C	60.2443	1
**4714**	319	C	79.5258	1

**4714**	320	C	79.586	-1
**4714**	321	C	72.2428	0
**4714**	351	C	6.579	1
**4714**	357	C	64.5239	1
**4714**	370	C	68.2171	1
**4714**	372	C	79.6611	0
**4714**	373	C	55.3287	1
**4714**	381	C	7.9398	-1
**4714**	382	C	77.5202	1
**4714**	386	C	58.81	1
**4714**	305	D	79.548	1
**4714**	319	D	79.5386	1
**4714**	320	D	61.034	-1
**4714**	321	D	79.5277	0
**4714**	351	D	3.7252	1
**4714**	357	D	46.9046	0
**4714**	370	D	62.983	1
**4714**	372	D	79.5037	0
**4714**	373	D	72.2964	1
**4714**	381	D	30.6321	0
**4714**	382	D	64.4741	1
**4714**	386	D	79.5746	1
**4715**	305	A	46.7766	0
**4715**	319	A	78.8764	1
**4715**	320	A	72.5542	-1
**4715**	321	A	72.4154	0
**4715**	351	A	6.9777	1
**4715**	357	A	51.064	1
**4715**	370	A	41.9361	0
**4715**	372	A	60.6292	0
**4715**	373	A	64.1579	1
**4715**	381	A	26.4552	0
**4715**	382	A	41.1308	0
**4715**	386	A	63.9156	1

**4715**	305	B	79.5807	1
**4715**	319	B	68.3157	1
**4715**	320	B	79.5834	-1
**4715**	321	B	79.5357	0
**4715**	351	B	8.8254	1
**4715**	357	B	35.9837	0
**4715**	370	B	79.5561	1
**4715**	372	B	72.6578	0
**4715**	373	B	79.5168	1
**4715**	381	B	72.5703	1
**4715**	382	B	17.24	-1
**4715**	386	B	67.7193	1
**4715**	305	C	74.6824	1
**4715**	319	C	65.3739	1
**4715**	320	C	62.5458	-1
**4715**	321	C	72.9863	0
**4715**	351	C	1.5313	1
**4715**	357	C	79.6057	1
**4715**	370	C	79.4886	1
**4715**	372	C	79.5117	0
**4715**	373	C	67.3729	1
**4715**	381	C	79.5883	1
**4715**	382	C	43.5141	0
**4715**	386	C	18.2333	-1
**4715**	305	D	77.6202	1
**4715**	319	D	73.0002	1
**4715**	320	D	74.8312	-1
**4715**	321	D	70.3566	0
**4715**	351	D	10.6698	1
**4715**	357	D	79.5463	1
**4715**	370	D	78.0109	1
**4715**	372	D	68.2824	0
**4715**	373	D	27.0236	0
**4715**	381	D	79.5779	1

**4715**	382	D	74.1024	1
**4715**	386	D	79.0441	1
**4716**	305	A	51.2926	1
**4716**	319	A	76.4569	1
**4716**	320	A	78.6448	-1
**4716**	321	A	61.9641	0
**4716**	351	A	9.0852	1
**4716**	357	A	78.3901	1
**4716**	370	A	74.4524	1
**4716**	372	A	79.3035	0
**4716**	373	A	45.258	0
**4716**	381	A	70.3303	1
**4716**	382	A	37.2228	0
**4716**	386	A	72.5994	1
**4716**	305	B	79.5624	1
**4716**	319	B	36.7557	0
**4716**	320	B	79.5753	-1
**4716**	321	B	72.1937	0
**4716**	351	B	15.8665	1
**4716**	357	B	77.7474	1
**4716**	370	B	60.2196	1
**4716**	372	B	74.1278	0
**4716**	373	B	79.5812	1
**4716**	381	B	68.5557	1
**4716**	382	B	56.3059	1
**4716**	386	B	79.4717	1
**4716**	305	C	76.9179	1
**4716**	319	C	79.533	1
**4716**	320	C	48.0525	-1
**4716**	321	C	55.2344	0
**4716**	351	C	35.8071	-1
**4716**	357	C	79.5134	1
**4716**	370	C	79.4987	1
**4716**	372	C	79.5837	0

**4716**	373	C	74.7247	1
**4716**	381	C	69.9683	1
**4716**	382	C	57.1763	1
**4716**	386	C	59.9426	1
**4716**	305	D	59.3904	1
**4716**	319	D	36.6312	0
**4716**	320	D	79.6041	-1
**4716**	321	D	48.791	1
**4716**	351	D	14.2503	1
**4716**	357	D	76.7739	1
**4716**	370	D	76.424	1
**4716**	372	D	73.0365	0
**4716**	373	D	44.4311	0
**4716**	381	D	79.5715	1
**4716**	382	D	73.8438	1
**4716**	386	D	73.5812	1
**4717**	305	A	79.567	1
**4717**	319	A	79.5234	1
**4717**	320	A	79.5822	-1
**4717**	321	A	62.8273	0
**4717**	351	A	9.6435	1
**4717**	357	A	50.5752	1
**4717**	370	A	78.371	1
**4717**	372	A	79.5671	0
**4717**	373	A	59.27	1
**4717**	381	A	23.2742	0
**4717**	382	A	73.6786	1
**4717**	386	A	72.5881	1
**4717**	305	B	76.138	1
**4717**	319	B	79.6766	1
**4717**	320	B	71.8706	-1
**4717**	321	B	62.6543	0
**4717**	351	B	14.1267	1
**4717**	357	B	60.6596	1

**4717**	370	B	72.78	1
**4717**	372	B	67.1405	0
**4717**	373	B	53.4238	1
**4717**	381	B	79.581	1
**4717**	382	B	79.6272	1
**4717**	386	B	79.5635	1
**4717**	305	C	79.5228	1
**4717**	319	C	79.5282	1
**4717**	320	C	79.5388	-1
**4717**	321	C	53.0946	0
**4717**	351	C	5.9313	1
**4717**	357	C	64.9962	1
**4717**	370	C	79.5364	1
**4717**	372	C	67.6917	0
**4717**	373	C	75.6921	1
**4717**	381	C	45.8606	0
**4717**	382	C	76.8698	1
**4717**	386	C	50.0439	1
**4717**	305	D	59.9429	1
**4717**	319	D	79.5769	1
**4717**	320	D	79.5777	-1
**4717**	321	D	72.7656	0
**4717**	351	D	11.0347	1
**4717**	357	D	79.5517	1
**4717**	370	D	56.9193	1
**4717**	372	D	78.0284	0
**4717**	373	D	79.5597	1
**4717**	381	D	79.5835	1
**4717**	382	D	49.5224	0
**4717**	386	D	13.384	-1
**4718**	305	A	79.5791	1
**4718**	319	A	56.7057	1
**4718**	320	A	65.3774	-1
**4718**	321	A	66.2858	0

**4718**	351	A	6.9238	1
**4718**	357	A	62.6423	1
**4718**	370	A	71.9209	1
**4718**	372	A	79.5026	0
**4718**	373	A	79.5504	1
**4718**	381	A	69.4944	1
**4718**	382	A	74.8365	1
**4718**	386	A	56.1854	1
**4718**	305	B	79.6574	1
**4718**	319	B	51.987	1
**4718**	320	B	78.5894	-1
**4718**	321	B	52.3293	0
**4718**	351	B	37.7321	-1
**4718**	357	B	70.5099	1
**4718**	370	B	79.6369	1
**4718**	372	B	72.7853	0
**4718**	373	B	69.8669	1
**4718**	381	B	66.6201	1
**4718**	382	B	24.4304	0
**4718**	386	B	79.5511	1
**4718**	305	C	72.3949	1
**4718**	319	C	69.4575	1
**4718**	320	C	79.5779	-1
**4718**	321	C	73.6397	0
**4718**	351	C	5.6493	1
**4718**	357	C	63.1778	1
**4718**	370	C	75.8853	1
**4718**	372	C	53.345	0
**4718**	373	C	74.673	1
**4718**	381	C	11.7887	-1
**4718**	382	C	45.7297	0
**4718**	386	C	66.5089	1
**4718**	305	D	39.6403	0
**4718**	319	D	79.4544	1

**4718**	320	D	79.6171	-1
**4718**	321	D	79.5657	0
**4718**	351	D	12.5095	1
**4718**	357	D	66.7646	1
**4718**	370	D	19.6052	-1
**4718**	372	D	79.5877	0
**4718**	373	D	72.9278	1
**4718**	381	D	79.5167	1
**4718**	382	D	79.5567	1
**4718**	386	D	79.5457	1
**4719**	305	A	70.6578	1
**4719**	319	A	70.746	1
**4719**	320	A	78.0024	-1
**4719**	321	A	54.5564	0
**4719**	351	A	4.82	1
**4719**	357	A	79.5844	1
**4719**	370	A	79.5299	1
**4719**	372	A	71.8468	0
**4719**	373	A	74.291	1
**4719**	381	A	79.5755	1
**4719**	382	A	70.971	1
**4719**	386	A	79.5694	1
**4719**	305	B	49.5845	0
**4719**	319	B	79.5429	1
**4719**	320	B	79.5871	-1
**4719**	321	B	79.5412	0
**4719**	351	B	13.4997	1
**4719**	357	B	73.7456	1
**4719**	370	B	79.4202	1
**4719**	372	B	79.5724	0
**4719**	373	B	33.9509	0
**4719**	381	B	71.939	1
**4719**	382	B	74.3653	1
**4719**	386	B	79.5382	1

**4719**	305	C	64.7908	1
**4719**	319	C	36.3263	0
**4719**	320	C	61.598	-1
**4719**	321	C	79.6124	0
**4719**	351	C	1.2136	1
**4719**	357	C	79.5442	1
**4719**	370	C	79.5048	1
**4719**	372	C	79.5603	0
**4719**	373	C	79.53	1
**4719**	381	C	73.1248	1
**4719**	382	C	66.2486	1
**4719**	386	C	8.3308	-1
**4719**	305	D	56.6315	1
**4719**	319	D	72.9511	1
**4719**	320	D	79.5542	-1
**4719**	321	D	73.498	0
**4719**	351	D	10.8092	1
**4719**	357	D	79.5499	1
**4719**	370	D	68.4548	1
**4719**	372	D	69.8346	0
**4719**	373	D	76.7559	1
**4719**	381	D	54.1822	1
**4719**	382	D	69.3702	1
**4719**	386	D	35.2337	0
**4720**	305	A	79.502	1
**4720**	319	A	54.6274	1
**4720**	320	A	72.8928	-1
**4720**	321	A	79.634	0
**4720**	351	A	12.0492	1
**4720**	357	A	79.5591	1
**4720**	370	A	73.6536	1
**4720**	372	A	77.4862	0
**4720**	373	A	58.9076	1
**4720**	381	A	65.2956	1

**4720**	382	A	79.6069	1
**4720**	386	A	79.468	1
**4720**	305	B	60.1443	1
**4720**	319	B	40.1385	0
**4720**	320	B	40.4122	-1
**4720**	321	B	79.5899	0
**4720**	351	B	10.8306	1
**4720**	357	B	79.5559	1
**4720**	370	B	79.5308	1
**4720**	372	B	79.5604	0
**4720**	373	B	64.59	1
**4720**	381	B	79.4776	1
**4720**	382	B	30.9658	0
**4720**	386	B	63.5839	1
**4720**	305	C	73.7403	1
**4720**	319	C	69.9175	1
**4720**	320	C	79.5753	-1
**4720**	321	C	48.8744	1
**4720**	351	C	7.4521	1
**4720**	357	C	79.5685	1
**4720**	370	C	66.2715	1
**4720**	372	C	73.6234	0
**4720**	373	C	79.5649	1
**4720**	381	C	79.5507	1
**4720**	382	C	79.2235	1
**4720**	386	C	16.8765	-1
**4720**	305	D	51.8162	1
**4720**	319	D	72.8157	1
**4720**	320	D	79.5694	-1
**4720**	321	D	71.3812	0
**4720**	351	D	13.4513	1
**4720**	357	D	79.5946	1
**4720**	370	D	48.771	0
**4720**	372	D	68.135	0

**4720**	373	D	75.4014	1
**4720**	381	D	79.5148	1
**4720**	382	D	79.5554	1
**4720**	386	D	46.8808	0
**4721**	305	A	73.3128	1
**4721**	319	A	46.4697	0
**4721**	320	A	55.7737	-1
**4721**	321	A	79.5448	0
**4721**	351	A	4.6347	1
**4721**	357	A	79.4955	1
**4721**	370	A	79.5261	1
**4721**	372	A	62.603	0
**4721**	373	A	79.4517	1
**4721**	381	A	71.5427	1
**4721**	382	A	31.764	0
**4721**	386	A	79.4867	1
**4721**	305	B	79.3879	1
**4721**	319	B	24.0809	0
**4721**	320	B	35.474	-1
**4721**	321	B	73.7242	0
**4721**	351	B	5.9928	1
**4721**	357	B	71.4392	1
**4721**	370	B	72.9262	1
**4721**	372	B	72.1639	0
**4721**	373	B	79.663	1
**4721**	381	B	30.9315	0
**4721**	382	B	51.6998	1
**4721**	386	B	79.5354	1
**4721**	305	C	40.2955	0
**4721**	319	C	9.6461	-1
**4721**	320	C	70.9253	-1
**4721**	321	C	69.1639	0
**4721**	351	C	3.2593	1
**4721**	357	C	68.8645	1

**4721**	370	C	72.6814	1
**4721**	372	C	79.5344	0
**4721**	373	C	75.2877	1
**4721**	381	C	75.0227	1
**4721**	382	C	21.148	0
**4721**	386	C	16.9508	-1
**4721**	305	D	49.2057	0
**4721**	319	D	73.0133	1
**4721**	320	D	79.4834	-1
**4721**	321	D	64.3031	0
**4721**	351	D	12.9424	1
**4721**	357	D	54.6456	1
**4721**	370	D	70.5488	1
**4721**	372	D	68.8471	0
**4721**	373	D	78.7917	1
**4721**	381	D	50.1279	1
**4721**	382	D	79.5632	1
**4721**	386	D	68.1779	1
**4722**	305	A	65.6808	1
**4722**	319	A	56.0748	1
**4722**	320	A	57.3177	-1
**4722**	321	A	79.5628	0
**4722**	351	A	11.642	1
**4722**	357	A	71.0367	1
**4722**	370	A	79.6248	1
**4722**	372	A	55.1411	0
**4722**	373	A	24.3431	0
**4722**	381	A	64.1529	1
**4722**	382	A	77.3585	1
**4722**	386	A	79.6029	1
**4722**	305	B	71.9145	1
**4722**	319	B	69.6728	1
**4722**	320	B	54.3265	-1
**4722**	321	B	50.8867	0

**4722**	351	B	2.0702	1
**4722**	357	B	73.8485	1
**4722**	370	B	57.7653	1
**4722**	372	B	44.6231	1
**4722**	373	B	73.0429	1
**4722**	381	B	79.5829	1
**4722**	382	B	69.3661	1
**4722**	386	B	37.874	0
**4722**	305	C	74.6801	1
**4722**	319	C	74.3141	1
**4722**	320	C	79.564	-1
**4722**	321	C	79.5582	0
**4722**	351	C	8.4401	1
**4722**	357	C	67.6118	1
**4722**	370	C	79.5708	1
**4722**	372	C	72.922	0
**4722**	373	C	79.5159	1
**4722**	381	C	70.2645	1
**4722**	382	C	79.5591	1
**4722**	386	C	39.3173	0
**4722**	305	D	48.8424	0
**4722**	319	D	79.5242	1
**4722**	320	D	31.3315	-1
**4722**	321	D	78.484	0
**4722**	351	D	13.498	1
**4722**	357	D	79.5545	1
**4722**	370	D	79.551	1
**4722**	372	D	79.566	0
**4722**	373	D	55.8992	1
**4722**	381	D	25.781	0
**4722**	382	D	72.9621	1
**4722**	386	D	79.5849	1
**4723**	305	A	69.7427	1
**4723**	319	A	79.4657	1

**4723**	320	A	79.531	-1
**4723**	321	A	63.3852	0
**4723**	351	A	8.2341	1
**4723**	357	A	68.6555	1
**4723**	370	A	76.9538	1
**4723**	372	A	13.878	0
**4723**	373	A	62.1035	1
**4723**	381	A	45.1784	0
**4723**	382	A	73.0163	1
**4723**	386	A	74.6962	1
**4723**	305	B	72.792	1
**4723**	319	B	71.6561	1
**4723**	320	B	31.9923	-1
**4723**	321	B	79.6028	0
**4723**	351	B	6.539	1
**4723**	357	B	79.5366	1
**4723**	370	B	74.2234	1
**4723**	372	B	79.6348	0
**4723**	373	B	46.7069	0
**4723**	381	B	79.59	1
**4723**	382	B	34.3026	0
**4723**	386	B	45.5678	0
**4723**	305	C	73.9344	1
**4723**	319	C	79.6974	1
**4723**	320	C	61.1025	-1
**4723**	321	C	69.938	0
**4723**	351	C	8.5541	1
**4723**	357	C	68.1159	1
**4723**	370	C	72.9827	1
**4723**	372	C	56.9211	0
**4723**	373	C	79.4624	1
**4723**	381	C	79.6097	1
**4723**	382	C	76.3184	1
**4723**	386	C	39.5491	0

**4723**	305	D	34.6977	0
**4723**	319	D	46.3564	0
**4723**	320	D	71.1845	-1
**4723**	321	D	79.5599	0
**4723**	351	D	37.5611	-1
**4723**	357	D	63.4055	1
**4723**	370	D	46.3538	0
**4723**	372	D	79.5263	0
**4723**	373	D	70.9625	1
**4723**	381	D	43.087	0
**4723**	382	D	79.5223	1
**4723**	386	D	37.0206	0
**4724**	305	A	61.1008	1
**4724**	319	A	18.1431	-1
**4724**	320	A	77.2435	-1
**4724**	321	A	61.2694	0
**4724**	351	A	8.8227	1
**4724**	357	A	50.9733	1
**4724**	370	A	76.1102	1
**4724**	372	A	79.6311	0
**4724**	373	A	79.5729	1
**4724**	381	A	73.1601	1
**4724**	382	A	71.5353	1
**4724**	386	A	79.5023	1
**4724**	305	B	56.1645	1
**4724**	319	B	72.7493	1
**4724**	320	B	76.8631	-1
**4724**	321	B	51.966	0
**4724**	351	B	0.5058	1
**4724**	357	B	66.7974	1
**4724**	370	B	64.8381	1
**4724**	372	B	25.8364	1
**4724**	373	B	52.7985	1
**4724**	381	B	56.2829	1

**4724**	382	B	79.5881	1
**4724**	386	B	79.6783	1
**4724**	305	C	71.2875	1
**4724**	319	C	47.9834	0
**4724**	320	C	79.5466	-1
**4724**	321	C	79.5849	0
**4724**	351	C	42.1763	-1
**4724**	357	C	40.0976	0
**4724**	370	C	69.4949	1
**4724**	372	C	78.8062	0
**4724**	373	C	57.3498	1
**4724**	381	C	78.1392	1
**4724**	382	C	21.9899	0
**4724**	386	C	20.4945	0
**4724**	305	D	77.4947	1
**4724**	319	D	71.6625	1
**4724**	320	D	66.6156	-1
**4724**	321	D	48.6401	1
**4724**	351	D	17.8293	1
**4724**	357	D	44.3854	0
**4724**	370	D	59.6752	1
**4724**	372	D	67.8423	0
**4724**	373	D	24.8766	0
**4724**	381	D	79.4615	1
**4724**	382	D	70.6864	1
**4724**	386	D	44.0249	0
**4725**	305	A	77.3788	1
**4725**	319	A	73.6551	1
**4725**	320	A	70.0813	-1
**4725**	321	A	78.8605	0
**4725**	351	A	11.5051	1
**4725**	357	A	64.4291	1
**4725**	370	A	77.1568	1
**4725**	372	A	79.533	0

**4725**	373	A	79.5875	1
**4725**	381	A	79.5236	1
**4725**	382	A	62.1817	1
**4725**	386	A	77.4194	1
**4725**	305	B	79.5448	1
**4725**	319	B	57.4507	1
**4725**	320	B	66.3042	-1
**4725**	321	B	75.7212	0
**4725**	351	B	1.1208	1
**4725**	357	B	69.1445	1
**4725**	370	B	79.5055	1
**4725**	372	B	69.7098	0
**4725**	373	B	60.8863	1
**4725**	381	B	70.0463	1
**4725**	382	B	20.9437	0
**4725**	386	B	78.1839	1
**4725**	305	C	79.7153	1
**4725**	319	C	67.4824	1
**4725**	320	C	79.444	-1
**4725**	321	C	35.6245	1
**4725**	351	C	7.9804	1
**4725**	357	C	57.831	1
**4725**	370	C	48.3539	0
**4725**	372	C	76.5636	0
**4725**	373	C	79.4879	1
**4725**	381	C	65.5771	1
**4725**	382	C	61.6159	1
**4725**	386	C	22.4377	0
**4725**	305	D	78.3637	1
**4725**	319	D	60.1569	1
**4725**	320	D	69.4465	-1
**4725**	321	D	73.8557	0
**4725**	351	D	20.2485	-1
**4725**	357	D	21.0174	0

**4725**	370	D	55.8644	1
**4725**	372	D	72.4282	0
**4725**	373	D	63.1451	1
**4725**	381	D	55.6632	1
**4725**	382	D	79.4961	1
**4725**	386	D	73.8205	1
**4726**	305	A	27.9122	0
**4726**	319	A	63.4745	1
**4726**	320	A	56.8351	-1
**4726**	321	A	66.4184	0
**4726**	351	A	9.7561	1
**4726**	357	A	71.6276	1
**4726**	370	A	79.5637	1
**4726**	372	A	78.2864	0
**4726**	373	A	42.7432	0
**4726**	381	A	68.573	1
**4726**	382	A	79.5649	1
**4726**	386	A	78.3904	1
**4726**	305	B	79.5733	1
**4726**	319	B	68.8185	1
**4726**	320	B	79.5531	-1
**4726**	321	B	68.3641	0
**4726**	351	B	13.0164	1
**4726**	357	B	59.2588	1
**4726**	370	B	79.5632	1
**4726**	372	B	73.7269	0
**4726**	373	B	77.848	1
**4726**	381	B	56.9465	1
**4726**	382	B	36.8854	0
**4726**	386	B	79.4761	1
**4726**	305	C	60.4677	1
**4726**	319	C	72.0649	1
**4726**	320	C	70.3966	-1
**4726**	321	C	63.1587	0

**4726**	351	C	7.755	1
**4726**	357	C	70.062	1
**4726**	370	C	57.0291	1
**4726**	372	C	74.6419	0
**4726**	373	C	79.0318	1
**4726**	381	C	79.1094	1
**4726**	382	C	67.0512	1
**4726**	386	C	39.4624	0
**4726**	305	D	79.5429	1
**4726**	319	D	79.3655	1
**4726**	320	D	79.574	-1
**4726**	321	D	79.5383	0
**4726**	351	D	10.2413	1
**4726**	357	D	28.665	0
**4726**	370	D	65.7913	1
**4726**	372	D	72.7088	0
**4726**	373	D	79.5519	1
**4726**	381	D	36.0563	0
**4726**	382	D	79.5918	1
**4726**	386	D	79.5847	1
**4727**	305	A	79.0244	1
**4727**	319	A	60.4346	1
**4727**	320	A	49.7056	-1
**4727**	321	A	72.931	0
**4727**	351	A	8.2161	1
**4727**	357	A	75.9915	1
**4727**	370	A	79.6079	1
**4727**	372	A	54.7412	0
**4727**	373	A	20.4146	0
**4727**	381	A	68.4682	1
**4727**	382	A	65.2474	1
**4727**	386	A	53.1586	1
**4727**	305	B	65.7386	1
**4727**	319	B	71.9076	1

**4727**	320	B	79.4226	-1
**4727**	321	B	59.1941	0
**4727**	351	B	27.9192	-1
**4727**	357	B	79.5475	1
**4727**	370	B	66.4523	1
**4727**	372	B	79.5902	0
**4727**	373	B	79.5686	1
**4727**	381	B	55.2436	1
**4727**	382	B	19.158	-1
**4727**	386	B	57.6027	1
**4727**	305	C	75.3903	1
**4727**	319	C	79.6472	1
**4727**	320	C	44.3372	-1
**4727**	321	C	79.5299	0
**4727**	351	C	0.0517	1
**4727**	357	C	79.5324	1
**4727**	370	C	79.5919	1
**4727**	372	C	74.854	0
**4727**	373	C	79.1073	1
**4727**	381	C	72.3576	1
**4727**	382	C	64.328	1
**4727**	386	C	39.6238	0
**4727**	305	D	32.7719	0
**4727**	319	D	79.6673	1
**4727**	320	D	79.6068	-1
**4727**	321	D	79.5154	0
**4727**	351	D	1.4672	1
**4727**	357	D	77.5108	1
**4727**	370	D	71.1024	1
**4727**	372	D	68.9623	0
**4727**	373	D	79.5841	1
**4727**	381	D	73.1089	1
**4727**	382	D	69.5109	1
**4727**	386	D	51.8151	1

**4728**	305	A	79.5986	1
**4728**	319	A	75.0811	1
**4728**	320	A	33.5756	-1
**4728**	321	A	75.5407	0
**4728**	351	A	5.0777	1
**4728**	357	A	68.5895	1
**4728**	370	A	33.9217	0
**4728**	372	A	76.1087	0
**4728**	373	A	79.5441	1
**4728**	381	A	79.4844	1
**4728**	382	A	21.3543	0
**4728**	386	A	70.9893	1
**4728**	305	B	72.8352	1
**4728**	319	B	59.7097	1
**4728**	320	B	67.8305	-1
**4728**	321	B	64.8543	0
**4728**	351	B	37.8291	-1
**4728**	357	B	79.5223	1
**4728**	370	B	53.3985	1
**4728**	372	B	77.1771	0
**4728**	373	B	78.9133	1
**4728**	381	B	79.6624	1
**4728**	382	B	29.1666	0
**4728**	386	B	73.7156	1
**4728**	305	C	65.3762	1
**4728**	319	C	52.8179	1
**4728**	320	C	79.6764	-1
**4728**	321	C	79.4764	0
**4728**	351	C	4.7667	1
**4728**	357	C	62.0363	1
**4728**	370	C	60.9435	1
**4728**	372	C	79.5927	0
**4728**	373	C	78.0817	1
**4728**	381	C	71.2892	1

**4728**	382	C	64.1163	1
**4728**	386	C	55.9557	1
**4728**	305	D	40.5838	0
**4728**	319	D	42.9084	0
**4728**	320	D	76.2602	-1
**4728**	321	D	79.5473	0
**4728**	351	D	4.0715	1
**4728**	357	D	43.2369	0
**4728**	370	D	66.0208	1
**4728**	372	D	59.3433	0
**4728**	373	D	76.0562	1
**4728**	381	D	48.4188	0
**4728**	382	D	54.3619	1
**4728**	386	D	62.7369	1
**4729**	305	A	77.3935	1
**4729**	319	A	79.6518	1
**4729**	320	A	79.6004	-1
**4729**	321	A	79.2531	0
**4729**	351	A	11.1924	1
**4729**	357	A	37.4691	0
**4729**	370	A	73.7967	1
**4729**	372	A	12.1802	0
**4729**	373	A	51.7337	1
**4729**	381	A	62.7727	1
**4729**	382	A	79.4933	1
**4729**	386	A	53.4052	1
**4729**	305	B	79.5866	1
**4729**	319	B	25.4654	0
**4729**	320	B	79.3445	-1
**4729**	321	B	76.0193	0
**4729**	351	B	24.4727	-1
**4729**	357	B	67.4751	1
**4729**	370	B	79.5383	1
**4729**	372	B	61.0855	0

**4729**	373	B	31.3309	0
**4729**	381	B	68.4239	1
**4729**	382	B	68.6538	1
**4729**	386	B	73.3179	1
**4729**	305	C	47.9165	0
**4729**	319	C	79.4806	1
**4729**	320	C	65.5457	-1
**4729**	321	C	69.1075	0
**4729**	351	C	2.5439	1
**4729**	357	C	74.9253	1
**4729**	370	C	67.0076	1
**4729**	372	C	62.6312	0
**4729**	373	C	79.2489	1
**4729**	381	C	58.7137	1
**4729**	382	C	79.4075	1
**4729**	386	C	63.5827	1
**4729**	305	D	78.2211	1
**4729**	319	D	79.7341	1
**4729**	320	D	70.7245	-1
**4729**	321	D	62.4204	0
**4729**	351	D	26.3853	-1
**4729**	357	D	19.566	-1
**4729**	370	D	55.3892	1
**4729**	372	D	51.7292	0
**4729**	373	D	58.396	1
**4729**	381	D	34.2169	0
**4729**	382	D	49.204	0
**4729**	386	D	72.9724	1
**4730**	305	A	51.3735	1
**4730**	319	A	47.5932	0
**4730**	320	A	52.1663	-1
**4730**	321	A	72.4266	0
**4730**	351	A	15.451	1
**4730**	357	A	79.5289	1

**4730**	370	A	79.4674	1
**4730**	372	A	72.9054	0
**4730**	373	A	73.773	1
**4730**	381	A	61.6045	1
**4730**	382	A	53.4142	1
**4730**	386	A	25.8127	0
**4730**	305	B	70.9668	1
**4730**	319	B	78.7914	1
**4730**	320	B	40.0039	-1
**4730**	321	B	78.7692	0
**4730**	351	B	16.4578	1
**4730**	357	B	70.5465	1
**4730**	370	B	62.4486	1
**4730**	372	B	72.6816	0
**4730**	373	B	78.2424	1
**4730**	381	B	79.6312	1
**4730**	382	B	79.6498	1
**4730**	386	B	75.0108	1
**4730**	305	C	48.141	0
**4730**	319	C	16.728	-1
**4730**	320	C	61.5248	-1
**4730**	321	C	78.8531	0
**4730**	351	C	1.2099	1
**4730**	357	C	34.2667	0
**4730**	370	C	70.765	1
**4730**	372	C	65.2872	0
**4730**	373	C	67.8775	1
**4730**	381	C	75.1821	1
**4730**	382	C	62.3374	1
**4730**	386	C	39.5229	0
**4730**	305	D	79.5723	1
**4730**	319	D	79.6303	1
**4730**	320	D	79.4509	-1
**4730**	321	D	79.5908	0

**4730**	351	D	18.3252	1
**4730**	357	D	72.1928	1
**4730**	370	D	71.2109	1
**4730**	372	D	79.4497	0
**4730**	373	D	79.563	1
**4730**	381	D	50.3394	1
**4730**	382	D	79.6109	1
**4730**	386	D	79.6025	1
**4731**	305	A	75.2304	1
**4731**	319	A	41.7827	0
**4731**	320	A	70.6379	-1
**4731**	321	A	72.2699	0
**4731**	351	A	8.1419	1
**4731**	357	A	74.869	1
**4731**	370	A	79.4713	1
**4731**	372	A	79.4987	0
**4731**	373	A	76.4048	1
**4731**	381	A	76.2356	1
**4731**	382	A	79.6277	1
**4731**	386	A	74.5187	1
**4731**	305	B	57.3138	1
**4731**	319	B	79.577	1
**4731**	320	B	79.4811	-1
**4731**	321	B	79.6178	0
**4731**	351	B	16.0387	1
**4731**	357	B	68.5928	1
**4731**	370	B	75.6201	1
**4731**	372	B	76.7915	0
**4731**	373	B	78.2281	1
**4731**	381	B	48.314	0
**4731**	382	B	47.39	0
**4731**	386	B	65.2692	1
**4731**	305	C	79.5453	1
**4731**	319	C	79.266	1

**4731**	320	C	72.2346	-1
**4731**	321	C	61.2096	0
**4731**	351	C	8.6863	1
**4731**	357	C	79.4944	1
**4731**	370	C	79.4756	1
**4731**	372	C	69.8568	0
**4731**	373	C	75.6708	1
**4731**	381	C	76.2308	1
**4731**	382	C	79.4781	1
**4731**	386	C	59.4294	1
**4731**	305	D	57.7543	1
**4731**	319	D	55.7096	1
**4731**	320	D	23.0812	-1
**4731**	321	D	58.6861	0
**4731**	351	D	5.2967	1
**4731**	357	D	79.5193	1
**4731**	370	D	66.8695	1
**4731**	372	D	79.6061	0
**4731**	373	D	79.5357	1
**4731**	381	D	79.53	1
**4731**	382	D	45.9048	0
**4731**	386	D	79.5468	1
**4732**	305	A	71.2452	1
**4732**	319	A	79.6055	1
**4732**	320	A	74.258	-1
**4732**	321	A	79.4681	0
**4732**	351	A	9.0211	1
**4732**	357	A	77.2208	1
**4732**	370	A	63.905	1
**4732**	372	A	79.6196	0
**4732**	373	A	42.2812	0
**4732**	381	A	79.5802	1
**4732**	382	A	24.3794	0
**4732**	386	A	76.1751	1

**4732**	305	B	71.8839	1
**4732**	319	B	79.6223	1
**4732**	320	B	71.2684	-1
**4732**	321	B	79.6433	0
**4732**	351	B	5.8768	1
**4732**	357	B	72.6299	1
**4732**	370	B	75.3021	1
**4732**	372	B	73.9875	0
**4732**	373	B	71.7868	1
**4732**	381	B	34.0278	0
**4732**	382	B	61.4107	1
**4732**	386	B	72.599	1
**4732**	305	C	68.1155	1
**4732**	319	C	64.785	1
**4732**	320	C	73.8879	-1
**4732**	321	C	73.2284	0
**4732**	351	C	10.8602	1
**4732**	357	C	79.5065	1
**4732**	370	C	73.1834	1
**4732**	372	C	73.6814	0
**4732**	373	C	64.4117	1
**4732**	381	C	75.4758	1
**4732**	382	C	29.2396	0
**4732**	386	C	43.6875	0
**4732**	305	D	1.4785	-1
**4732**	319	D	77.8271	1
**4732**	320	D	44.7711	-1
**4732**	321	D	79.5791	0
**4732**	351	D	3.3451	1
**4732**	357	D	32.4442	0
**4732**	370	D	78.8869	1
**4732**	372	D	71.8821	0
**4732**	373	D	55.7489	1
**4732**	381	D	76.1135	1

**4732**	382	D	65.2532	1
**4732**	386	D	63.5595	1
**4733**	305	A	38.7672	0
**4733**	319	A	79.423	1
**4733**	320	A	70.3515	-1
**4733**	321	A	73.9825	0
**4733**	351	A	0.0	1
**4733**	357	A	72.2773	1
**4733**	370	A	69.19	1
**4733**	372	A	60.9704	0
**4733**	373	A	60.4667	1
**4733**	381	A	72.7483	1
**4733**	382	A	71.8816	1
**4733**	386	A	73.4106	1
**4733**	305	B	79.584	1
**4733**	319	B	70.7225	1
**4733**	320	B	79.4358	-1
**4733**	321	B	65.7353	0
**4733**	351	B	23.5047	-1
**4733**	357	B	48.2407	0
**4733**	370	B	77.8716	1
**4733**	372	B	79.4532	0
**4733**	373	B	79.2691	1
**4733**	381	B	33.8348	0
**4733**	382	B	30.8759	0
**4733**	386	B	57.5734	1
**4733**	305	C	68.9155	1
**4733**	319	C	73.5854	1
**4733**	320	C	79.5832	-1
**4733**	321	C	71.5829	0
**4733**	351	C	2.5105	1
**4733**	357	C	50.8536	1
**4733**	370	C	68.2707	1
**4733**	372	C	74.7771	0

**4733**	373	C	45.121	0
**4733**	381	C	79.5321	1
**4733**	382	C	50.7007	1
**4733**	386	C	56.5443	1
**4733**	305	D	72.7038	1
**4733**	319	D	51.4951	1
**4733**	320	D	79.1891	-1
**4733**	321	D	67.1165	0
**4733**	351	D	10.2724	1
**4733**	357	D	79.569	1
**4733**	370	D	79.5628	1
**4733**	372	D	79.5791	0
**4733**	373	D	44.3625	0
**4733**	381	D	70.1856	1
**4733**	382	D	79.5231	1
**4733**	386	D	79.4842	1
**4734**	305	A	65.5107	1
**4734**	319	A	73.8596	1
**4734**	320	A	79.5673	-1
**4734**	321	A	59.7776	0
**4734**	351	A	35.5484	-1
**4734**	357	A	37.4678	0
**4734**	370	A	74.0494	1
**4734**	372	A	72.9559	0
**4734**	373	A	72.969	1
**4734**	381	A	54.5874	1
**4734**	382	A	78.7552	1
**4734**	386	A	69.9032	1
**4734**	305	B	72.4094	1
**4734**	319	B	74.7471	1
**4734**	320	B	73.1889	-1
**4734**	321	B	79.5953	0
**4734**	351	B	10.5595	1
**4734**	357	B	55.7434	1

**4734**	370	B	79.5412	1
**4734**	372	B	79.5268	0
**4734**	373	B	66.0288	1
**4734**	381	B	43.1351	0
**4734**	382	B	54.9312	1
**4734**	386	B	79.5767	1
**4734**	305	C	74.2661	1
**4734**	319	C	65.3498	1
**4734**	320	C	72.3771	-1
**4734**	321	C	47.7265	1
**4734**	351	C	1.0865	1
**4734**	357	C	72.8632	1
**4734**	370	C	58.0795	1
**4734**	372	C	78.9989	0
**4734**	373	C	56.5074	1
**4734**	381	C	79.2991	1
**4734**	382	C	71.1461	1
**4734**	386	C	29.0975	0
**4734**	305	D	32.2784	0
**4734**	319	D	79.5333	1
**4734**	320	D	79.5359	-1
**4734**	321	D	62.3477	0
**4734**	351	D	12.8266	1
**4734**	357	D	41.0644	0
**4734**	370	D	16.9446	-1
**4734**	372	D	63.0368	0
**4734**	373	D	79.5489	1
**4734**	381	D	79.5652	1
**4734**	382	D	78.6486	1
**4734**	386	D	37.6086	0
**4735**	305	A	63.3078	1
**4735**	319	A	64.0992	1
**4735**	320	A	79.6119	-1
**4735**	321	A	64.3468	0

**4735**	351	A	6.8642	1
**4735**	357	A	79.5698	1
**4735**	370	A	75.5763	1
**4735**	372	A	79.6425	0
**4735**	373	A	71.7084	1
**4735**	381	A	79.1148	1
**4735**	382	A	74.4526	1
**4735**	386	A	79.5891	1
**4735**	305	B	79.5556	1
**4735**	319	B	67.9745	1
**4735**	320	B	51.636	-1
**4735**	321	B	79.1629	0
**4735**	351	B	14.6019	1
**4735**	357	B	79.5529	1
**4735**	370	B	63.6689	1
**4735**	372	B	57.5791	0
**4735**	373	B	79.5256	1
**4735**	381	B	79.5974	1
**4735**	382	B	77.7763	1
**4735**	386	B	47.2612	0
**4735**	305	C	72.8695	1
**4735**	319	C	41.7502	0
**4735**	320	C	70.5313	-1
**4735**	321	C	52.7879	0
**4735**	351	C	5.1129	1
**4735**	357	C	79.5549	1
**4735**	370	C	54.258	1
**4735**	372	C	79.5696	0
**4735**	373	C	79.6053	1
**4735**	381	C	72.6843	1
**4735**	382	C	55.9333	1
**4735**	386	C	40.8634	0
**4735**	305	D	79.7671	1
**4735**	319	D	79.4799	1

**4735**	320	D	52.733	-1
**4735**	321	D	70.8501	0
**4735**	351	D	14.4962	1
**4735**	357	D	79.5812	1
**4735**	370	D	65.1646	1
**4735**	372	D	73.188	0
**4735**	373	D	50.1205	1
**4735**	381	D	79.5617	1
**4735**	382	D	73.1143	1
**4735**	386	D	79.3172	1
**4736**	305	A	39.205	0
**4736**	319	A	79.5469	1
**4736**	320	A	65.9134	-1
**4736**	321	A	52.7047	0
**4736**	351	A	12.8881	1
**4736**	357	A	74.3041	1
**4736**	370	A	75.7995	1
**4736**	372	A	71.2973	0
**4736**	373	A	45.057	0
**4736**	381	A	79.5618	1
**4736**	382	A	68.737	1
**4736**	386	A	78.7336	1
**4736**	305	B	79.5589	1
**4736**	319	B	29.9376	0
**4736**	320	B	79.5615	-1
**4736**	321	B	74.2354	0
**4736**	351	B	10.4023	1
**4736**	357	B	73.3255	1
**4736**	370	B	47.7083	0
**4736**	372	B	74.545	0
**4736**	373	B	63.1353	1
**4736**	381	B	19.1574	-1
**4736**	382	B	73.428	1
**4736**	386	B	79.4042	1

**4736**	305	C	68.1719	1
**4736**	319	C	76.3648	1
**4736**	320	C	53.2515	-1
**4736**	321	C	29.7617	1
**4736**	351	C	6.2268	1
**4736**	357	C	52.4563	1
**4736**	370	C	60.7262	1
**4736**	372	C	69.952	0
**4736**	373	C	75.1001	1
**4736**	381	C	73.033	1
**4736**	382	C	71.5791	1
**4736**	386	C	30.8868	0
**4736**	305	D	38.0812	0
**4736**	319	D	72.5536	1
**4736**	320	D	45.9548	-1
**4736**	321	D	75.0275	0
**4736**	351	D	3.8094	1
**4736**	357	D	79.431	1
**4736**	370	D	75.0395	1
**4736**	372	D	75.6592	0
**4736**	373	D	79.5969	1
**4736**	381	D	79.3826	1
**4736**	382	D	79.6502	1
**4736**	386	D	71.9498	1
**4737**	305	A	71.5894	1
**4737**	319	A	71.7142	1
**4737**	320	A	79.5577	-1
**4737**	321	A	67.8683	0
**4737**	351	A	0.3703	1
**4737**	357	A	57.3926	1
**4737**	370	A	67.9972	1
**4737**	372	A	71.1685	0
**4737**	373	A	64.1491	1
**4737**	381	A	27.0798	0

**4737**	382	A	67.4412	1
**4737**	386	A	78.9148	1
**4737**	305	B	72.4132	1
**4737**	319	B	79.5916	1
**4737**	320	B	79.448	-1
**4737**	321	B	75.9377	0
**4737**	351	B	9.6395	1
**4737**	357	B	56.5561	1
**4737**	370	B	71.873	1
**4737**	372	B	79.5398	0
**4737**	373	B	79.5368	1
**4737**	381	B	79.644	1
**4737**	382	B	49.8573	0
**4737**	386	B	60.8982	1
**4737**	305	C	25.8671	0
**4737**	319	C	58.9927	1
**4737**	320	C	79.7071	-1
**4737**	321	C	79.51	0
**4737**	351	C	8.2904	1
**4737**	357	C	71.4208	1
**4737**	370	C	52.9908	1
**4737**	372	C	79.1796	0
**4737**	373	C	79.7651	1
**4737**	381	C	72.97	1
**4737**	382	C	60.3208	1
**4737**	386	C	41.2993	0
**4737**	305	D	13.487	-1
**4737**	319	D	44.9033	0
**4737**	320	D	57.3961	-1
**4737**	321	D	79.7957	0
**4737**	351	D	8.5711	1
**4737**	357	D	79.5446	1
**4737**	370	D	76.081	1
**4737**	372	D	79.6114	0

**4737**	373	D	79.5467	1
**4737**	381	D	72.7249	1
**4737**	382	D	79.4387	1
**4737**	386	D	79.4959	1
**4738**	305	A	67.513	1
**4738**	319	A	41.8216	0
**4738**	320	A	79.5682	-1
**4738**	321	A	79.5643	0
**4738**	351	A	8.7684	1
**4738**	357	A	60.652	1
**4738**	370	A	48.541	0
**4738**	372	A	72.1963	0
**4738**	373	A	72.3686	1
**4738**	381	A	79.6227	1
**4738**	382	A	65.1298	1
**4738**	386	A	73.7992	1
**4738**	305	B	79.5639	1
**4738**	319	B	79.5679	1
**4738**	320	B	79.5662	-1
**4738**	321	B	72.1646	0
**4738**	351	B	16.4969	1
**4738**	357	B	29.9247	0
**4738**	370	B	53.8436	1
**4738**	372	B	72.3483	0
**4738**	373	B	38.0692	0
**4738**	381	B	79.5096	1
**4738**	382	B	24.3339	0
**4738**	386	B	41.6147	0
**4738**	305	C	78.684	1
**4738**	319	C	71.9321	1
**4738**	320	C	79.5476	-1
**4738**	321	C	79.58	0
**4738**	351	C	0.9033	1
**4738**	357	C	32.5006	0

**4738**	370	C	72.3426	1
**4738**	372	C	79.5665	0
**4738**	373	C	79.5637	1
**4738**	381	C	79.5405	1
**4738**	382	C	12.8689	-1
**4738**	386	C	36.7547	0
**4738**	305	D	79.5072	1
**4738**	319	D	79.4285	1
**4738**	320	D	44.2681	-1
**4738**	321	D	66.5371	0
**4738**	351	D	11.0363	1
**4738**	357	D	79.5897	1
**4738**	370	D	72.3917	1
**4738**	372	D	55.4576	0
**4738**	373	D	74.9709	1
**4738**	381	D	69.9526	1
**4738**	382	D	43.9582	0
**4738**	386	D	79.5562	1
**4739**	305	A	74.6798	1
**4739**	319	A	79.3574	1
**4739**	320	A	79.6347	-1
**4739**	321	A	42.7634	1
**4739**	351	A	6.6506	1
**4739**	357	A	45.2322	0
**4739**	370	A	71.4556	1
**4739**	372	A	75.8899	0
**4739**	373	A	74.7569	1
**4739**	381	A	61.9263	1
**4739**	382	A	77.3411	1
**4739**	386	A	79.4581	1
**4739**	305	B	75.2267	1
**4739**	319	B	79.365	1
**4739**	320	B	59.1907	-1
**4739**	321	B	59.8049	0

**4739**	351	B	18.2548	1
**4739**	357	B	79.415	1
**4739**	370	B	69.9921	1
**4739**	372	B	58.0494	0
**4739**	373	B	79.0642	1
**4739**	381	B	79.7488	1
**4739**	382	B	64.191	1
**4739**	386	B	79.7487	1
**4739**	305	C	79.5661	1
**4739**	319	C	71.0349	1
**4739**	320	C	51.4235	-1
**4739**	321	C	79.717	0
**4739**	351	C	3.8321	1
**4739**	357	C	57.6471	1
**4739**	370	C	79.5679	1
**4739**	372	C	63.7762	0
**4739**	373	C	25.6947	0
**4739**	381	C	71.4129	1
**4739**	382	C	79.5367	1
**4739**	386	C	63.9898	1
**4739**	305	D	71.0735	1
**4739**	319	D	69.6471	1
**4739**	320	D	79.414	-1
**4739**	321	D	79.7506	0
**4739**	351	D	14.0566	1
**4739**	357	D	71.4037	1
**4739**	370	D	78.1762	1
**4739**	372	D	79.4675	0
**4739**	373	D	50.2239	1
**4739**	381	D	29.6764	0
**4739**	382	D	75.0518	1
**4739**	386	D	66.2878	1
**4740**	305	A	79.5989	1
**4740**	319	A	41.4019	0

**4740**	320	A	72.5262	-1
**4740**	321	A	56.6953	0
**4740**	351	A	5.7199	1
**4740**	357	A	64.0556	1
**4740**	370	A	72.0911	1
**4740**	372	A	33.9633	1
**4740**	373	A	79.2763	1
**4740**	381	A	79.5617	1
**4740**	382	A	79.5516	1
**4740**	386	A	72.8846	1
**4740**	305	B	40.9468	0
**4740**	319	B	46.467	0
**4740**	320	B	69.5676	-1
**4740**	321	B	73.1136	0
**4740**	351	B	14.3429	1
**4740**	357	B	56.9024	1
**4740**	370	B	79.5712	1
**4740**	372	B	72.5787	0
**4740**	373	B	21.6054	0
**4740**	381	B	67.8671	1
**4740**	382	B	71.2382	1
**4740**	386	B	57.0713	1
**4740**	305	C	73.1192	1
**4740**	319	C	75.6594	1
**4740**	320	C	52.7042	-1
**4740**	321	C	40.7188	1
**4740**	351	C	12.3648	1
**4740**	357	C	48.6508	0
**4740**	370	C	79.5908	1
**4740**	372	C	68.3145	0
**4740**	373	C	79.5674	1
**4740**	381	C	79.6018	1
**4740**	382	C	61.8197	1
**4740**	386	C	51.535	1

**4740**	305	D	45.5629	0
**4740**	319	D	77.7071	1
**4740**	320	D	48.483	-1
**4740**	321	D	79.5355	0
**4740**	351	D	11.0691	1
**4740**	357	D	72.8399	1
**4740**	370	D	70.4467	1
**4740**	372	D	79.4488	0
**4740**	373	D	56.0314	1
**4740**	381	D	57.4187	1
**4740**	382	D	78.0077	1
**4740**	386	D	72.6618	1
**4741**	305	A	79.6048	1
**4741**	319	A	58.1911	1
**4741**	320	A	79.5945	-1
**4741**	321	A	76.6131	0
**4741**	351	A	6.3318	1
**4741**	357	A	29.6859	0
**4741**	370	A	76.0712	1
**4741**	372	A	79.5383	0
**4741**	373	A	69.641	1
**4741**	381	A	71.1629	1
**4741**	382	A	37.7325	0
**4741**	386	A	60.5407	1
**4741**	305	B	69.8721	1
**4741**	319	B	71.9858	1
**4741**	320	B	79.4859	-1
**4741**	321	B	79.6151	0
**4741**	351	B	10.4886	1
**4741**	357	B	79.5671	1
**4741**	370	B	66.7639	1
**4741**	372	B	78.0601	0
**4741**	373	B	67.3236	1
**4741**	381	B	77.3254	1

**4741**	382	B	26.5114	0
**4741**	386	B	79.6097	1
**4741**	305	C	56.6729	1
**4741**	319	C	79.6108	1
**4741**	320	C	75.0138	-1
**4741**	321	C	61.9037	0
**4741**	351	C	17.7963	1
**4741**	357	C	73.3539	1
**4741**	370	C	73.0893	1
**4741**	372	C	79.472	0
**4741**	373	C	72.6541	1
**4741**	381	C	54.9586	1
**4741**	382	C	74.8979	1
**4741**	386	C	56.0952	1
**4741**	305	D	59.6609	1
**4741**	319	D	79.5387	1
**4741**	320	D	72.385	-1
**4741**	321	D	79.5997	0
**4741**	351	D	9.1053	1
**4741**	357	D	79.5894	1
**4741**	370	D	32.9887	0
**4741**	372	D	62.1844	0
**4741**	373	D	58.4991	1
**4741**	381	D	65.3794	1
**4741**	382	D	50.4708	1
**4741**	386	D	78.0201	1
**4742**	305	A	72.9702	1
**4742**	319	A	44.0757	0
**4742**	320	A	79.5623	-1
**4742**	321	A	41.727	1
**4742**	351	A	8.5557	1
**4742**	357	A	79.5744	1
**4742**	370	A	71.9892	1
**4742**	372	A	46.8072	1

**4742**	373	A	64.5845	1
**4742**	381	A	73.129	1
**4742**	382	A	79.5352	1
**4742**	386	A	79.4822	1
**4742**	305	B	79.5633	1
**4742**	319	B	79.5862	1
**4742**	320	B	79.5527	-1
**4742**	321	B	70.6382	0
**4742**	351	B	14.8847	1
**4742**	357	B	37.9787	0
**4742**	370	B	72.5081	1
**4742**	372	B	79.5407	0
**4742**	373	B	79.5685	1
**4742**	381	B	79.1418	1
**4742**	382	B	44.4267	0
**4742**	386	B	79.5773	1
**4742**	305	C	73.3923	1
**4742**	319	C	44.5906	0
**4742**	320	C	22.2762	-1
**4742**	321	C	78.8824	0
**4742**	351	C	5.8262	1
**4742**	357	C	41.9626	0
**4742**	370	C	31.739	0
**4742**	372	C	79.5389	0
**4742**	373	C	72.7786	1
**4742**	381	C	79.5468	1
**4742**	382	C	78.5274	1
**4742**	386	C	36.5411	0
**4742**	305	D	79.5582	1
**4742**	319	D	73.1002	1
**4742**	320	D	48.5837	-1
**4742**	321	D	40.7498	1
**4742**	351	D	13.563	1
**4742**	357	D	63.9706	1

**4742**	370	D	50.6317	1
**4742**	372	D	51.9476	0
**4742**	373	D	75.4574	1
**4742**	381	D	61.7252	1
**4742**	382	D	76.2367	1
**4742**	386	D	79.5522	1
**4743**	305	A	58.1423	1
**4743**	319	A	79.2325	1
**4743**	320	A	30.0567	-1
**4743**	321	A	79.5134	0
**4743**	351	A	4.0507	1
**4743**	357	A	79.5796	1
**4743**	370	A	79.5799	1
**4743**	372	A	66.3915	0
**4743**	373	A	49.7004	0
**4743**	381	A	79.6766	1
**4743**	382	A	68.9805	1
**4743**	386	A	31.8882	0
**4743**	305	B	44.3846	0
**4743**	319	B	65.8554	1
**4743**	320	B	46.0973	-1
**4743**	321	B	32.9589	1
**4743**	351	B	7.8284	1
**4743**	357	B	59.1783	1
**4743**	370	B	50.0797	1
**4743**	372	B	69.3524	0
**4743**	373	B	47.9436	0
**4743**	381	B	79.5211	1
**4743**	382	B	34.4671	0
**4743**	386	B	79.4684	1
**4743**	305	C	79.5262	1
**4743**	319	C	67.7241	1
**4743**	320	C	79.5482	-1
**4743**	321	C	33.5429	1

**4743**	351	C	8.7885	1
**4743**	357	C	79.5617	1
**4743**	370	C	79.6729	1
**4743**	372	C	76.1473	0
**4743**	373	C	61.4542	1
**4743**	381	C	79.3298	1
**4743**	382	C	71.905	1
**4743**	386	C	35.552	0
**4743**	305	D	39.615	0
**4743**	319	D	79.5697	1
**4743**	320	D	50.9757	-1
**4743**	321	D	73.1769	0
**4743**	351	D	16.6138	1
**4743**	357	D	38.7106	0
**4743**	370	D	79.5395	1
**4743**	372	D	79.5384	0
**4743**	373	D	73.7846	1
**4743**	381	D	79.5581	1
**4743**	382	D	78.7017	1
**4743**	386	D	79.5794	1
**4744**	305	A	46.0351	0
**4744**	319	A	55.2171	1
**4744**	320	A	79.5508	-1
**4744**	321	A	71.278	0
**4744**	351	A	7.0741	1
**4744**	357	A	65.5198	1
**4744**	370	A	79.5406	1
**4744**	372	A	79.575	0
**4744**	373	A	79.4422	1
**4744**	381	A	79.5291	1
**4744**	382	A	72.9007	1
**4744**	386	A	79.5583	1
**4744**	305	B	61.6254	1
**4744**	319	B	79.6646	1

**4744**	320	B	79.6206	-1
**4744**	321	B	79.5732	0
**4744**	351	B	1.1178	1
**4744**	357	B	72.7012	1
**4744**	370	B	51.6548	1
**4744**	372	B	79.5378	0
**4744**	373	B	79.5173	1
**4744**	381	B	79.5928	1
**4744**	382	B	30.7335	0
**4744**	386	B	79.6357	1
**4744**	305	C	62.9088	1
**4744**	319	C	76.8647	1
**4744**	320	C	47.5004	-1
**4744**	321	C	52.7963	0
**4744**	351	C	6.864	1
**4744**	357	C	73.9073	1
**4744**	370	C	30.3927	0
**4744**	372	C	42.5498	1
**4744**	373	C	79.5327	1
**4744**	381	C	73.7918	1
**4744**	382	C	79.5674	1
**4744**	386	C	21.0054	0
**4744**	305	D	68.1103	1
**4744**	319	D	79.5961	1
**4744**	320	D	52.7828	-1
**4744**	321	D	76.9276	0
**4744**	351	D	11.2494	1
**4744**	357	D	62.9632	1
**4744**	370	D	76.2302	1
**4744**	372	D	76.8166	0
**4744**	373	D	77.8266	1
**4744**	381	D	79.5598	1
**4744**	382	D	79.5504	1
**4744**	386	D	79.5902	1

**4745**	305	A	79.5275	1
**4745**	319	A	79.5358	1
**4745**	320	A	52.9541	-1
**4745**	321	A	79.4526	0
**4745**	351	A	10.13	1
**4745**	357	A	32.733	0
**4745**	370	A	78.7081	1
**4745**	372	A	79.6827	0
**4745**	373	A	32.9082	0
**4745**	381	A	47.9927	0
**4745**	382	A	29.4643	0
**4745**	386	A	79.5823	1
**4745**	305	B	79.4815	1
**4745**	319	B	79.5346	1
**4745**	320	B	74.5872	-1
**4745**	321	B	79.5323	0
**4745**	351	B	21.4245	-1
**4745**	357	B	75.6096	1
**4745**	370	B	79.5922	1
**4745**	372	B	72.7767	0
**4745**	373	B	61.2219	1
**4745**	381	B	40.2083	0
**4745**	382	B	51.0175	1
**4745**	386	B	79.5514	1
**4745**	305	C	65.9204	1
**4745**	319	C	79.6021	1
**4745**	320	C	79.3732	-1
**4745**	321	C	68.4522	0
**4745**	351	C	33.2506	-1
**4745**	357	C	79.5312	1
**4745**	370	C	79.5384	1
**4745**	372	C	79.665	0
**4745**	373	C	44.7946	0
**4745**	381	C	78.9855	1

**4745**	382	C	48.2794	0
**4745**	386	C	50.122	1
**4745**	305	D	49.6902	0
**4745**	319	D	79.5665	1
**4745**	320	D	75.1667	-1
**4745**	321	D	69.9525	0
**4745**	351	D	13.1493	1
**4745**	357	D	63.7807	1
**4745**	370	D	68.2133	1
**4745**	372	D	75.8255	0
**4745**	373	D	70.1254	1
**4745**	381	D	78.1503	1
**4745**	382	D	73.1294	1
**4745**	386	D	15.8263	-1
**4746**	305	A	73.0419	1
**4746**	319	A	35.1152	0
**4746**	320	A	79.5838	-1
**4746**	321	A	61.6048	0
**4746**	351	A	2.9773	1
**4746**	357	A	56.2764	1
**4746**	370	A	76.9418	1
**4746**	372	A	27.9684	1
**4746**	373	A	60.8792	1
**4746**	381	A	79.5602	1
**4746**	382	A	77.378	1
**4746**	386	A	73.6218	1
**4746**	305	B	72.7335	1
**4746**	319	B	64.3307	1
**4746**	320	B	69.4814	-1
**4746**	321	B	79.6037	0
**4746**	351	B	9.8087	1
**4746**	357	B	72.2824	1
**4746**	370	B	77.8879	1
**4746**	372	B	67.6001	0

**4746**	373	B	71.944	1
**4746**	381	B	76.0755	1
**4746**	382	B	52.969	1
**4746**	386	B	57.5953	1
**4746**	305	C	36.8667	0
**4746**	319	C	79.5465	1
**4746**	320	C	55.3071	-1
**4746**	321	C	59.6993	0
**4746**	351	C	1.9058	1
**4746**	357	C	70.8478	1
**4746**	370	C	69.5475	1
**4746**	372	C	53.967	0
**4746**	373	C	79.5208	1
**4746**	381	C	79.5787	1
**4746**	382	C	54.5099	1
**4746**	386	C	37.0556	0
**4746**	305	D	22.0057	0
**4746**	319	D	65.1209	1
**4746**	320	D	53.7154	-1
**4746**	321	D	79.0553	0
**4746**	351	D	19.9038	1
**4746**	357	D	74.4502	1
**4746**	370	D	79.4079	1
**4746**	372	D	79.5592	0
**4746**	373	D	79.5625	1
**4746**	381	D	73.8034	1
**4746**	382	D	46.6258	0
**4746**	386	D	61.0839	1
**4747**	305	A	79.5707	1
**4747**	319	A	78.898	1
**4747**	320	A	79.5162	-1
**4747**	321	A	78.0532	0
**4747**	351	A	8.54	1
**4747**	357	A	79.5564	1

**4747**	370	A	69.6728	1
**4747**	372	A	63.4126	0
**4747**	373	A	78.067	1
**4747**	381	A	79.479	1
**4747**	382	A	38.0815	0
**4747**	386	A	79.4889	1
**4747**	305	B	71.1505	1
**4747**	319	B	70.3707	1
**4747**	320	B	79.6307	-1
**4747**	321	B	70.3703	0
**4747**	351	B	27.9062	-1
**4747**	357	B	57.0109	1
**4747**	370	B	57.118	1
**4747**	372	B	79.5527	0
**4747**	373	B	28.1774	0
**4747**	381	B	75.368	1
**4747**	382	B	70.2626	1
**4747**	386	B	58.5324	1
**4747**	305	C	57.1545	1
**4747**	319	C	47.2443	0
**4747**	320	C	68.8314	-1
**4747**	321	C	49.9154	1
**4747**	351	C	8.7537	1
**4747**	357	C	73.9393	1
**4747**	370	C	64.4269	1
**4747**	372	C	79.5204	0
**4747**	373	C	79.5161	1
**4747**	381	C	79.4608	1
**4747**	382	C	41.4295	0
**4747**	386	C	37.7439	0
**4747**	305	D	64.3173	1
**4747**	319	D	71.9556	1
**4747**	320	D	79.6172	-1
**4747**	321	D	79.5992	0

**4747**	351	D	17.7677	1
**4747**	357	D	56.3408	1
**4747**	370	D	46.3348	0
**4747**	372	D	79.6296	0
**4747**	373	D	60.4764	1
**4747**	381	D	46.9842	0
**4747**	382	D	79.2531	1
**4747**	386	D	72.2292	1
**4748**	305	A	73.0297	1
**4748**	319	A	67.8381	1
**4748**	320	A	60.7752	-1
**4748**	321	A	74.293	0
**4748**	351	A	11.8343	1
**4748**	357	A	45.2114	0
**4748**	370	A	74.3703	1
**4748**	372	A	50.0428	0
**4748**	373	A	79.5087	1
**4748**	381	A	79.111	1
**4748**	382	A	79.5758	1
**4748**	386	A	79.6228	1
**4748**	305	B	79.534	1
**4748**	319	B	79.566	1
**4748**	320	B	79.4324	-1
**4748**	321	B	76.6481	0
**4748**	351	B	14.2199	1
**4748**	357	B	76.7592	1
**4748**	370	B	60.6422	1
**4748**	372	B	79.5547	0
**4748**	373	B	68.0179	1
**4748**	381	B	79.5256	1
**4748**	382	B	57.2824	1
**4748**	386	B	71.4129	1
**4748**	305	C	55.5268	1
**4748**	319	C	73.4569	1

**4748**	320	C	78.6815	-1
**4748**	321	C	73.2785	0
**4748**	351	C	11.2336	1
**4748**	357	C	79.6158	1
**4748**	370	C	50.7281	1
**4748**	372	C	79.609	0
**4748**	373	C	73.1185	1
**4748**	381	C	67.6984	1
**4748**	382	C	79.6104	1
**4748**	386	C	41.2819	0
**4748**	305	D	77.918	1
**4748**	319	D	79.601	1
**4748**	320	D	79.6118	-1
**4748**	321	D	71.0111	0
**4748**	351	D	7.4036	1
**4748**	357	D	79.5405	1
**4748**	370	D	33.6975	0
**4748**	372	D	79.5237	0
**4748**	373	D	67.4058	1
**4748**	381	D	79.4731	1
**4748**	382	D	78.5353	1
**4748**	386	D	79.6026	1
**4749**	305	A	32.6914	0
**4749**	319	A	22.7624	0
**4749**	320	A	67.2108	-1
**4749**	321	A	49.7444	1
**4749**	351	A	5.5686	1
**4749**	357	A	79.5634	1
**4749**	370	A	79.6265	1
**4749**	372	A	78.7941	0
**4749**	373	A	77.6929	1
**4749**	381	A	79.5343	1
**4749**	382	A	79.5576	1
**4749**	386	A	71.5267	1

**4749**	305	B	70.3047	1
**4749**	319	B	56.6983	1
**4749**	320	B	75.7978	-1
**4749**	321	B	71.7073	0
**4749**	351	B	14.9535	1
**4749**	357	B	55.3698	1
**4749**	370	B	41.4473	0
**4749**	372	B	74.4078	0
**4749**	373	B	69.7719	1
**4749**	381	B	66.6606	1
**4749**	382	B	59.4383	1
**4749**	386	B	75.5881	1
**4749**	305	C	65.2939	1
**4749**	319	C	63.6562	1
**4749**	320	C	48.0605	-1
**4749**	321	C	61.1472	0
**4749**	351	C	10.9456	1
**4749**	357	C	66.1425	1
**4749**	370	C	34.6619	0
**4749**	372	C	79.5339	0
**4749**	373	C	79.394	1
**4749**	381	C	76.8029	1
**4749**	382	C	79.0346	1
**4749**	386	C	68.5155	1
**4749**	305	D	79.4631	1
**4749**	319	D	64.8694	1
**4749**	320	D	74.4549	-1
**4749**	321	D	79.5202	0
**4749**	351	D	11.9567	1
**4749**	357	D	79.6026	1
**4749**	370	D	69.0364	1
**4749**	372	D	78.3893	0
**4749**	373	D	77.3174	1
**4749**	381	D	78.5321	1

**4749**	382	D	64.6251	1
**4749**	386	D	79.0386	1
**4750**	305	A	70.9032	1
**4750**	319	A	30.9626	0
**4750**	320	A	53.176	-1
**4750**	321	A	61.1974	0
**4750**	351	A	30.3648	-1
**4750**	357	A	56.893	1
**4750**	370	A	48.8862	0
**4750**	372	A	20.9256	1
**4750**	373	A	73.8218	1
**4750**	381	A	78.9029	1
**4750**	382	A	76.4114	1
**4750**	386	A	45.6765	0
**4750**	305	B	44.2776	0
**4750**	319	B	71.6779	1
**4750**	320	B	54.8425	-1
**4750**	321	B	67.1463	0
**4750**	351	B	17.2008	1
**4750**	357	B	61.7261	1
**4750**	370	B	74.6028	1
**4750**	372	B	72.5223	0
**4750**	373	B	77.712	1
**4750**	381	B	79.5791	1
**4750**	382	B	78.7592	1
**4750**	386	B	54.1004	1
**4750**	305	C	73.703	1
**4750**	319	C	79.3791	1
**4750**	320	C	64.6468	-1
**4750**	321	C	48.1148	1
**4750**	351	C	2.6362	1
**4750**	357	C	72.5681	1
**4750**	370	C	79.5354	1
**4750**	372	C	49.9043	1

**4750**	373	C	72.0596	1
**4750**	381	C	50.7946	1
**4750**	382	C	70.692	1
**4750**	386	C	35.5497	0
**4750**	305	D	67.8172	1
**4750**	319	D	79.5969	1
**4750**	320	D	79.5945	-1
**4750**	321	D	73.2535	0
**4750**	351	D	10.1829	1
**4750**	357	D	79.0888	1
**4750**	370	D	77.7078	1
**4750**	372	D	73.3378	0
**4750**	373	D	79.5876	1
**4750**	381	D	79.5637	1
**4750**	382	D	63.0964	1
**4750**	386	D	79.5668	1
**4751**	305	A	72.8152	1
**4751**	319	A	73.242	1
**4751**	320	A	66.0494	-1
**4751**	321	A	77.9172	0
**4751**	351	A	4.9473	1
**4751**	357	A	76.6787	1
**4751**	370	A	79.5828	1
**4751**	372	A	79.8097	0
**4751**	373	A	45.91	0
**4751**	381	A	42.4796	0
**4751**	382	A	77.7041	1
**4751**	386	A	68.8602	1
**4751**	305	B	72.9474	1
**4751**	319	B	72.9517	1
**4751**	320	B	73.5316	-1
**4751**	321	B	79.4375	0
**4751**	351	B	16.2193	1
**4751**	357	B	71.014	1

**4751**	370	B	66.3808	1
**4751**	372	B	71.4976	0
**4751**	373	B	77.1766	1
**4751**	381	B	74.4899	1
**4751**	382	B	79.6384	1
**4751**	386	B	79.5229	1
**4751**	305	C	28.2535	0
**4751**	319	C	72.9844	1
**4751**	320	C	47.1444	-1
**4751**	321	C	79.5869	0
**4751**	351	C	6.6572	1
**4751**	357	C	26.584	0
**4751**	370	C	32.3757	0
**4751**	372	C	64.0565	0
**4751**	373	C	49.2999	0
**4751**	381	C	77.7195	1
**4751**	382	C	58.1978	1
**4751**	386	C	28.4395	0
**4751**	305	D	79.6483	1
**4751**	319	D	77.1365	1
**4751**	320	D	75.6225	-1
**4751**	321	D	79.6432	0
**4751**	351	D	10.668	1
**4751**	357	D	69.681	1
**4751**	370	D	60.2652	1
**4751**	372	D	72.5399	0
**4751**	373	D	79.7677	1
**4751**	381	D	72.5738	1
**4751**	382	D	79.5316	1
**4751**	386	D	65.1386	1
**4752**	305	A	52.1869	1
**4752**	319	A	50.7668	1
**4752**	320	A	46.0719	-1
**4752**	321	A	65.5803	0

**4752**	351	A	2.7668	1
**4752**	357	A	70.1191	1
**4752**	370	A	59.3646	1
**4752**	372	A	49.0376	1
**4752**	373	A	77.8361	1
**4752**	381	A	64.7768	1
**4752**	382	A	41.7556	0
**4752**	386	A	73.7704	1
**4752**	305	B	60.4786	1
**4752**	319	B	70.8286	1
**4752**	320	B	56.4784	-1
**4752**	321	B	62.3589	0
**4752**	351	B	13.8142	1
**4752**	357	B	68.9408	1
**4752**	370	B	74.8746	1
**4752**	372	B	41.7789	1
**4752**	373	B	73.104	1
**4752**	381	B	79.5327	1
**4752**	382	B	79.5938	1
**4752**	386	B	69.8077	1
**4752**	305	C	41.2513	0
**4752**	319	C	69.7182	1
**4752**	320	C	79.4695	-1
**4752**	321	C	51.2526	0
**4752**	351	C	25.7856	-1
**4752**	357	C	73.1967	1
**4752**	370	C	67.9708	1
**4752**	372	C	69.5882	0
**4752**	373	C	49.1663	0
**4752**	381	C	67.3231	1
**4752**	382	C	20.8477	0
**4752**	386	C	54.0587	1
**4752**	305	D	79.6062	1
**4752**	319	D	53.9595	1

**4752**	320	D	79.5715	-1
**4752**	321	D	61.1447	0
**4752**	351	D	6.4737	1
**4752**	357	D	54.4854	1
**4752**	370	D	41.5506	0
**4752**	372	D	79.6286	0
**4752**	373	D	75.9094	1
**4752**	381	D	72.3225	1
**4752**	382	D	62.2418	1
**4752**	386	D	78.981	1
**4753**	305	A	75.3184	1
**4753**	319	A	79.5857	1
**4753**	320	A	76.7418	-1
**4753**	321	A	79.507	0
**4753**	351	A	3.5422	1
**4753**	357	A	79.3093	1
**4753**	370	A	78.9317	1
**4753**	372	A	75.1168	0
**4753**	373	A	79.5429	1
**4753**	381	A	59.2072	1
**4753**	382	A	69.3025	1
**4753**	386	A	63.3472	1
**4753**	305	B	67.2904	1
**4753**	319	B	48.0463	0
**4753**	320	B	79.5081	-1
**4753**	321	B	68.4103	0
**4753**	351	B	15.2071	1
**4753**	357	B	64.5005	1
**4753**	370	B	11.8086	-1
**4753**	372	B	79.5922	0
**4753**	373	B	58.435	1
**4753**	381	B	59.1183	1
**4753**	382	B	38.5063	0
**4753**	386	B	31.8458	0

**4753**	305	C	38.8018	0
**4753**	319	C	74.8137	1
**4753**	320	C	71.6854	-1
**4753**	321	C	79.5156	0
**4753**	351	C	2.5899	1
**4753**	357	C	75.0496	1
**4753**	370	C	73.0602	1
**4753**	372	C	75.1447	0
**4753**	373	C	79.542	1
**4753**	381	C	79.5883	1
**4753**	382	C	51.0681	1
**4753**	386	C	57.5027	1
**4753**	305	D	41.5711	0
**4753**	319	D	73.9342	1
**4753**	320	D	69.4707	-1
**4753**	321	D	39.8558	1
**4753**	351	D	15.4611	1
**4753**	357	D	69.7514	1
**4753**	370	D	78.4108	1
**4753**	372	D	79.6741	0
**4753**	373	D	64.5719	1
**4753**	381	D	61.0518	1
**4753**	382	D	79.4824	1
**4753**	386	D	79.5165	1
**4754**	305	A	79.5209	1
**4754**	319	A	70.0499	1
**4754**	320	A	79.4696	-1
**4754**	321	A	66.9229	0
**4754**	351	A	11.1508	1
**4754**	357	A	55.7567	1
**4754**	370	A	60.0213	1
**4754**	372	A	48.2365	1
**4754**	373	A	78.7524	1
**4754**	381	A	79.5024	1

**4754**	382	A	79.5934	1
**4754**	386	A	61.9988	1
**4754**	305	B	76.9673	1
**4754**	319	B	79.6392	1
**4754**	320	B	60.7564	-1
**4754**	321	B	63.0695	0
**4754**	351	B	4.0214	1
**4754**	357	B	48.5207	0
**4754**	370	B	77.0971	1
**4754**	372	B	70.7451	0
**4754**	373	B	30.9959	0
**4754**	381	B	63.4331	1
**4754**	382	B	66.8447	1
**4754**	386	B	36.0392	0
**4754**	305	C	43.4584	0
**4754**	319	C	69.7761	1
**4754**	320	C	79.541	-1
**4754**	321	C	33.4212	1
**4754**	351	C	12.1157	1
**4754**	357	C	73.4602	1
**4754**	370	C	47.0082	0
**4754**	372	C	69.6287	0
**4754**	373	C	42.8968	0
**4754**	381	C	62.6983	1
**4754**	382	C	26.9177	0
**4754**	386	C	9.7245	-1
**4754**	305	D	75.9923	1
**4754**	319	D	59.7996	1
**4754**	320	D	79.569	-1
**4754**	321	D	67.8616	0
**4754**	351	D	10.5752	1
**4754**	357	D	71.7412	1
**4754**	370	D	58.6553	1
**4754**	372	D	70.6861	0

**4754**	373	D	63.5278	1
**4754**	381	D	49.2666	0
**4754**	382	D	47.0114	0
**4754**	386	D	78.8713	1
**4755**	305	A	72.6007	1
**4755**	319	A	72.4032	1
**4755**	320	A	54.2791	-1
**4755**	321	A	75.2528	0
**4755**	351	A	6.0053	1
**4755**	357	A	68.3421	1
**4755**	370	A	79.4853	1
**4755**	372	A	79.6154	0
**4755**	373	A	71.7616	1
**4755**	381	A	79.5824	1
**4755**	382	A	75.1041	1
**4755**	386	A	79.4549	1
**4755**	305	B	70.1541	1
**4755**	319	B	79.5551	1
**4755**	320	B	54.9449	-1
**4755**	321	B	74.3226	0
**4755**	351	B	23.0853	-1
**4755**	357	B	79.5582	1
**4755**	370	B	79.5082	1
**4755**	372	B	79.5356	0
**4755**	373	B	78.0133	1
**4755**	381	B	65.7373	1
**4755**	382	B	74.3529	1
**4755**	386	B	62.2386	1
**4755**	305	C	70.057	1
**4755**	319	C	79.5428	1
**4755**	320	C	70.7134	-1
**4755**	321	C	72.0786	0
**4755**	351	C	12.6281	1
**4755**	357	C	71.6486	1

**4755**	370	C	73.1925	1
**4755**	372	C	79.6158	0
**4755**	373	C	79.5652	1
**4755**	381	C	78.3966	1
**4755**	382	C	79.5072	1
**4755**	386	C	61.9419	1
**4755**	305	D	57.3506	1
**4755**	319	D	60.8803	1
**4755**	320	D	36.2752	-1
**4755**	321	D	64.6722	0
**4755**	351	D	13.9554	1
**4755**	357	D	55.5395	1
**4755**	370	D	70.6269	1
**4755**	372	D	79.5987	0
**4755**	373	D	67.4932	1
**4755**	381	D	61.4442	1
**4755**	382	D	52.6796	1
**4755**	386	D	79.5507	1
**4756**	305	A	66.5166	1
**4756**	319	A	79.4904	1
**4756**	320	A	79.7358	-1
**4756**	321	A	79.7069	0
**4756**	351	A	6.0475	1
**4756**	357	A	55.1486	1
**4756**	370	A	76.1577	1
**4756**	372	A	72.5483	0
**4756**	373	A	31.0708	0
**4756**	381	A	74.4556	1
**4756**	382	A	63.6462	1
**4756**	386	A	61.5119	1
**4756**	305	B	79.5527	1
**4756**	319	B	57.1255	1
**4756**	320	B	63.9064	-1
**4756**	321	B	74.2244	0

**4756**	351	B	17.5689	1
**4756**	357	B	68.0434	1
**4756**	370	B	68.2525	1
**4756**	372	B	71.3988	0
**4756**	373	B	47.5631	0
**4756**	381	B	64.1779	1
**4756**	382	B	79.6695	1
**4756**	386	B	79.7685	1
**4756**	305	C	13.7623	-1
**4756**	319	C	54.9443	1
**4756**	320	C	70.3174	-1
**4756**	321	C	34.2198	1
**4756**	351	C	6.7324	1
**4756**	357	C	61.4187	1
**4756**	370	C	37.1698	0
**4756**	372	C	67.7307	0
**4756**	373	C	65.2263	1
**4756**	381	C	56.9026	1
**4756**	382	C	73.6869	1
**4756**	386	C	46.0257	0
**4756**	305	D	79.5117	1
**4756**	319	D	79.7679	1
**4756**	320	D	79.6612	-1
**4756**	321	D	37.8165	1
**4756**	351	D	10.1817	1
**4756**	357	D	79.5114	1
**4756**	370	D	76.8314	1
**4756**	372	D	79.6012	0
**4756**	373	D	74.2871	1
**4756**	381	D	63.6337	1
**4756**	382	D	79.453	1
**4756**	386	D	76.6683	1
**4757**	305	A	20.6366	0
**4757**	319	A	79.2608	1

**4757**	320	A	79.464	-1
**4757**	321	A	78.6563	0
**4757**	351	A	12.7009	1
**4757**	357	A	72.851	1
**4757**	370	A	56.7412	1
**4757**	372	A	42.2031	1
**4757**	373	A	69.9908	1
**4757**	381	A	68.057	1
**4757**	382	A	71.8101	1
**4757**	386	A	71.6515	1
**4757**	305	B	79.556	1
**4757**	319	B	76.0433	1
**4757**	320	B	73.5865	-1
**4757**	321	B	79.5413	0
**4757**	351	B	13.2451	1
**4757**	357	B	59.8802	1
**4757**	370	B	77.2188	1
**4757**	372	B	64.3972	0
**4757**	373	B	48.2298	0
**4757**	381	B	72.6615	1
**4757**	382	B	45.413	0
**4757**	386	B	71.2173	1
**4757**	305	C	56.1123	1
**4757**	319	C	78.7317	1
**4757**	320	C	71.8669	-1
**4757**	321	C	61.1973	0
**4757**	351	C	14.2278	1
**4757**	357	C	73.6495	1
**4757**	370	C	79.529	1
**4757**	372	C	60.4833	0
**4757**	373	C	74.6361	1
**4757**	381	C	79.514	1
**4757**	382	C	55.4456	1
**4757**	386	C	44.8538	0

**4757**	305	D	70.3285	1
**4757**	319	D	62.4155	1
**4757**	320	D	72.1449	-1
**4757**	321	D	74.3103	0
**4757**	351	D	0.0	1
**4757**	357	D	39.999	0
**4757**	370	D	59.7888	1
**4757**	372	D	79.4501	0
**4757**	373	D	59.3483	1
**4757**	381	D	79.2782	1
**4757**	382	D	70.535	1
**4757**	386	D	32.1098	0
**4758**	305	A	56.5677	1
**4758**	319	A	77.2859	1
**4758**	320	A	67.6581	-1
**4758**	321	A	57.3199	0
**4758**	351	A	3.0938	1
**4758**	357	A	50.3338	1
**4758**	370	A	79.6951	1
**4758**	372	A	79.6287	0
**4758**	373	A	75.7378	1
**4758**	381	A	62.6858	1
**4758**	382	A	79.5954	1
**4758**	386	A	58.7044	1
**4758**	305	B	48.9887	0
**4758**	319	B	77.9605	1
**4758**	320	B	73.6831	-1
**4758**	321	B	58.1058	0
**4758**	351	B	7.8669	1
**4758**	357	B	55.5764	1
**4758**	370	B	72.5189	1
**4758**	372	B	79.6306	0
**4758**	373	B	40.8206	0
**4758**	381	B	75.3061	1

**4758**	382	B	20.4595	0
**4758**	386	B	71.813	1
**4758**	305	C	31.3074	0
**4758**	319	C	31.1445	0
**4758**	320	C	59.6604	-1
**4758**	321	C	68.9021	0
**4758**	351	C	5.1579	1
**4758**	357	C	48.6984	0
**4758**	370	C	66.2191	1
**4758**	372	C	71.4124	0
**4758**	373	C	64.9168	1
**4758**	381	C	29.9353	0
**4758**	382	C	79.5524	1
**4758**	386	C	34.0473	0
**4758**	305	D	48.4521	0
**4758**	319	D	53.0536	1
**4758**	320	D	75.3697	-1
**4758**	321	D	67.9029	0
**4758**	351	D	16.2001	1
**4758**	357	D	46.7778	0
**4758**	370	D	43.9994	0
**4758**	372	D	76.8937	0
**4758**	373	D	62.9441	1
**4758**	381	D	56.0976	1
**4758**	382	D	79.5343	1
**4758**	386	D	73.9584	1
**4759**	305	A	75.4553	1
**4759**	319	A	71.1985	1
**4759**	320	A	75.0288	-1
**4759**	321	A	57.0323	0
**4759**	351	A	9.0832	1
**4759**	357	A	66.251	1
**4759**	370	A	79.6295	1
**4759**	372	A	60.9297	0

**4759**	373	A	71.3537	1
**4759**	381	A	66.5954	1
**4759**	382	A	46.0206	0
**4759**	386	A	36.1233	0
**4759**	305	B	79.6447	1
**4759**	319	B	79.4781	1
**4759**	320	B	67.6773	-1
**4759**	321	B	52.9265	0
**4759**	351	B	23.7205	-1
**4759**	357	B	50.5018	1
**4759**	370	B	54.0903	1
**4759**	372	B	79.5909	0
**4759**	373	B	50.2012	1
**4759**	381	B	28.6005	0
**4759**	382	B	24.1376	0
**4759**	386	B	78.9433	1
**4759**	305	C	70.473	1
**4759**	319	C	40.4456	0
**4759**	320	C	57.0464	-1
**4759**	321	C	52.9679	0
**4759**	351	C	19.8184	1
**4759**	357	C	78.4697	1
**4759**	370	C	72.2101	1
**4759**	372	C	79.3866	0
**4759**	373	C	72.0964	1
**4759**	381	C	24.2702	0
**4759**	382	C	78.9967	1
**4759**	386	C	58.0924	1
**4759**	305	D	53.9251	1
**4759**	319	D	60.1997	1
**4759**	320	D	79.5808	-1
**4759**	321	D	28.7822	1
**4759**	351	D	8.9649	1
**4759**	357	D	75.189	1

**4759**	370	D	42.3936	0
**4759**	372	D	79.5684	0
**4759**	373	D	79.5984	1
**4759**	381	D	51.4072	1
**4759**	382	D	74.1758	1
**4759**	386	D	75.6764	1
**4760**	305	A	75.0559	1
**4760**	319	A	55.4835	1
**4760**	320	A	73.4065	-1
**4760**	321	A	79.407	0
**4760**	351	A	17.2843	1
**4760**	357	A	63.2441	1
**4760**	370	A	79.603	1
**4760**	372	A	57.806	0
**4760**	373	A	16.7436	-1
**4760**	381	A	54.036	1
**4760**	382	A	23.3471	0
**4760**	386	A	55.1653	1
**4760**	305	B	62.055	1
**4760**	319	B	69.6162	1
**4760**	320	B	67.7357	-1
**4760**	321	B	73.194	0
**4760**	351	B	17.8738	1
**4760**	357	B	37.9622	0
**4760**	370	B	62.3978	1
**4760**	372	B	73.5323	0
**4760**	373	B	79.3612	1
**4760**	381	B	79.4883	1
**4760**	382	B	76.824	1
**4760**	386	B	69.1592	1
**4760**	305	C	64.7764	1
**4760**	319	C	78.0072	1
**4760**	320	C	53.1065	-1
**4760**	321	C	66.7963	0

**4760**	351	C	2.3017	1
**4760**	357	C	79.4486	1
**4760**	370	C	78.4698	1
**4760**	372	C	63.4915	0
**4760**	373	C	74.0743	1
**4760**	381	C	29.0691	0
**4760**	382	C	74.629	1
**4760**	386	C	45.172	0
**4760**	305	D	61.6154	1
**4760**	319	D	74.0011	1
**4760**	320	D	38.2117	-1
**4760**	321	D	79.6073	0
**4760**	351	D	23.2293	-1
**4760**	357	D	79.5217	1
**4760**	370	D	56.6403	1
**4760**	372	D	33.5276	1
**4760**	373	D	47.0347	0
**4760**	381	D	71.6294	1
**4760**	382	D	68.4447	1
**4760**	386	D	54.9159	1
**4761**	305	A	6.2764	-1
**4761**	319	A	64.4015	1
**4761**	320	A	73.1734	-1
**4761**	321	A	79.5229	0
**4761**	351	A	3.8365	1
**4761**	357	A	57.1242	1
**4761**	370	A	71.7884	1
**4761**	372	A	79.6308	0
**4761**	373	A	38.5849	0
**4761**	381	A	79.4732	1
**4761**	382	A	74.336	1
**4761**	386	A	72.9832	1
**4761**	305	B	79.5994	1
**4761**	319	B	73.0446	1

**4761**	320	B	71.1927	-1
**4761**	321	B	72.4117	0
**4761**	351	B	8.8185	1
**4761**	357	B	33.0804	0
**4761**	370	B	65.9807	1
**4761**	372	B	71.7412	0
**4761**	373	B	72.564	1
**4761**	381	B	30.3334	0
**4761**	382	B	28.0161	0
**4761**	386	B	79.5613	1
**4761**	305	C	75.5226	1
**4761**	319	C	79.5903	1
**4761**	320	C	79.5552	-1
**4761**	321	C	79.48	0
**4761**	351	C	11.0866	1
**4761**	357	C	70.2919	1
**4761**	370	C	79.5746	1
**4761**	372	C	49.4116	1
**4761**	373	C	69.7397	1
**4761**	381	C	79.4559	1
**4761**	382	C	22.8367	0
**4761**	386	C	41.0377	0
**4761**	305	D	74.0271	1
**4761**	319	D	73.1675	1
**4761**	320	D	79.4551	-1
**4761**	321	D	69.683	0
**4761**	351	D	15.9929	1
**4761**	357	D	60.8579	1
**4761**	370	D	79.6458	1
**4761**	372	D	75.5758	0
**4761**	373	D	72.282	1
**4761**	381	D	51.3177	1
**4761**	382	D	79.5481	1
**4761**	386	D	79.5494	1

**4762**	305	A	44.7736	0
**4762**	319	A	38.6488	0
**4762**	320	A	56.9722	-1
**4762**	321	A	67.6587	0
**4762**	351	A	8.1073	1
**4762**	357	A	56.3634	1
**4762**	370	A	55.6804	1
**4762**	372	A	79.5568	0
**4762**	373	A	11.8719	-1
**4762**	381	A	79.1101	1
**4762**	382	A	79.5543	1
**4762**	386	A	43.6471	0
**4762**	305	B	79.7277	1
**4762**	319	B	79.6354	1
**4762**	320	B	60.7334	-1
**4762**	321	B	72.4178	0
**4762**	351	B	10.4128	1
**4762**	357	B	74.3679	1
**4762**	370	B	74.6792	1
**4762**	372	B	71.4094	0
**4762**	373	B	71.1572	1
**4762**	381	B	79.573	1
**4762**	382	B	79.6426	1
**4762**	386	B	59.6984	1
**4762**	305	C	79.6228	1
**4762**	319	C	49.7144	0
**4762**	320	C	79.5803	-1
**4762**	321	C	73.1154	0
**4762**	351	C	3.3245	1
**4762**	357	C	60.6521	1
**4762**	370	C	77.3904	1
**4762**	372	C	79.5428	0
**4762**	373	C	41.2615	0
**4762**	381	C	79.5885	1

**4762**	382	C	44.1927	0
**4762**	386	C	43.101	0
**4762**	305	D	32.0278	0
**4762**	319	D	79.5441	1
**4762**	320	D	72.7082	-1
**4762**	321	D	60.0426	0
**4762**	351	D	8.2776	1
**4762**	357	D	57.7101	1
**4762**	370	D	74.0288	1
**4762**	372	D	79.566	0
**4762**	373	D	79.5471	1
**4762**	381	D	72.349	1
**4762**	382	D	76.1782	1
**4762**	386	D	79.5545	1
**4763**	305	A	43.8353	0
**4763**	319	A	66.7945	1
**4763**	320	A	46.9889	-1
**4763**	321	A	31.2744	1
**4763**	351	A	9.9227	1
**4763**	357	A	63.7252	1
**4763**	370	A	77.2239	1
**4763**	372	A	71.5379	0
**4763**	373	A	71.0879	1
**4763**	381	A	52.0871	1
**4763**	382	A	46.6843	0
**4763**	386	A	29.3654	0
**4763**	305	B	69.9053	1
**4763**	319	B	79.5345	1
**4763**	320	B	25.291	-1
**4763**	321	B	79.4769	0
**4763**	351	B	30.5391	-1
**4763**	357	B	70.5284	1
**4763**	370	B	79.6095	1
**4763**	372	B	56.4604	0

**4763**	373	B	66.7261	1
**4763**	381	B	60.0184	1
**4763**	382	B	75.1131	1
**4763**	386	B	66.4182	1
**4763**	305	C	79.4318	1
**4763**	319	C	79.3668	1
**4763**	320	C	53.2611	-1
**4763**	321	C	79.5134	0
**4763**	351	C	4.5147	1
**4763**	357	C	77.1597	1
**4763**	370	C	79.5309	1
**4763**	372	C	79.6157	0
**4763**	373	C	61.2555	1
**4763**	381	C	69.8166	1
**4763**	382	C	47.8637	0
**4763**	386	C	35.1896	0
**4763**	305	D	51.3234	1
**4763**	319	D	42.288	0
**4763**	320	D	73.1832	-1
**4763**	321	D	74.3889	0
**4763**	351	D	9.8855	1
**4763**	357	D	79.6181	1
**4763**	370	D	51.4502	1
**4763**	372	D	79.3863	0
**4763**	373	D	79.5516	1
**4763**	381	D	74.2931	1
**4763**	382	D	75.1424	1
**4763**	386	D	65.6883	1
**4764**	305	A	49.9878	0
**4764**	319	A	71.8676	1
**4764**	320	A	79.5917	-1
**4764**	321	A	63.7691	0
**4764**	351	A	0.0036	1
**4764**	357	A	72.4508	1

**4764**	370	A	79.5414	1
**4764**	372	A	79.6203	0
**4764**	373	A	72.4428	1
**4764**	381	A	61.7405	1
**4764**	382	A	71.1242	1
**4764**	386	A	39.3845	0
**4764**	305	B	79.5561	1
**4764**	319	B	75.0475	1
**4764**	320	B	27.3075	-1
**4764**	321	B	79.5442	0
**4764**	351	B	6.005	1
**4764**	357	B	73.6134	1
**4764**	370	B	79.5476	1
**4764**	372	B	72.2751	0
**4764**	373	B	52.7851	1
**4764**	381	B	67.4462	1
**4764**	382	B	27.8895	0
**4764**	386	B	50.297	1
**4764**	305	C	54.2619	1
**4764**	319	C	65.6102	1
**4764**	320	C	79.7201	-1
**4764**	321	C	78.3615	0
**4764**	351	C	6.528	1
**4764**	357	C	63.4421	1
**4764**	370	C	77.6055	1
**4764**	372	C	79.2849	0
**4764**	373	C	72.8995	1
**4764**	381	C	79.5185	1
**4764**	382	C	66.043	1
**4764**	386	C	40.292	0
**4764**	305	D	79.5909	1
**4764**	319	D	59.2614	1
**4764**	320	D	79.5721	-1
**4764**	321	D	79.5773	0

**4764**	351	D	16.9815	1
**4764**	357	D	59.4427	1
**4764**	370	D	73.1693	1
**4764**	372	D	79.5289	0
**4764**	373	D	63.7884	1
**4764**	381	D	42.9926	0
**4764**	382	D	67.2571	1
**4764**	386	D	69.4459	1
**4765**	305	A	61.4609	1
**4765**	319	A	54.9258	1
**4765**	320	A	73.5709	-1
**4765**	321	A	76.2234	0
**4765**	351	A	9.0415	1
**4765**	357	A	79.5443	1
**4765**	370	A	74.3033	1
**4765**	372	A	75.7312	0
**4765**	373	A	40.2379	0
**4765**	381	A	79.5473	1
**4765**	382	A	33.9594	0
**4765**	386	A	71.2973	1
**4765**	305	B	79.7043	1
**4765**	319	B	72.3593	1
**4765**	320	B	43.9473	-1
**4765**	321	B	46.2183	1
**4765**	351	B	12.8869	1
**4765**	357	B	41.3841	0
**4765**	370	B	79.5645	1
**4765**	372	B	60.9326	0
**4765**	373	B	69.8253	1
**4765**	381	B	79.385	1
**4765**	382	B	62.2062	1
**4765**	386	B	62.7893	1
**4765**	305	C	79.5226	1
**4765**	319	C	69.0957	1

**4765**	320	C	79.3935	-1
**4765**	321	C	42.4627	1
**4765**	351	C	8.7893	1
**4765**	357	C	72.5039	1
**4765**	370	C	56.084	1
**4765**	372	C	71.4186	0
**4765**	373	C	79.6847	1
**4765**	381	C	51.2372	1
**4765**	382	C	35.7625	0
**4765**	386	C	42.6729	0
**4765**	305	D	79.5642	1
**4765**	319	D	53.555	1
**4765**	320	D	79.5732	-1
**4765**	321	D	62.423	0
**4765**	351	D	13.3985	1
**4765**	357	D	79.5009	1
**4765**	370	D	79.6225	1
**4765**	372	D	72.6469	0
**4765**	373	D	79.4938	1
**4765**	381	D	32.4481	0
**4765**	382	D	74.6032	1
**4765**	386	D	70.9597	1
**4766**	305	A	75.4999	1
**4766**	319	A	77.9022	1
**4766**	320	A	79.5589	-1
**4766**	321	A	73.4751	0
**4766**	351	A	6.484	1
**4766**	357	A	79.6108	1
**4766**	370	A	72.2599	1
**4766**	372	A	27.822	1
**4766**	373	A	79.5561	1
**4766**	381	A	63.8863	1
**4766**	382	A	79.2535	1
**4766**	386	A	54.8146	1

**4766**	305	B	58.5127	1
**4766**	319	B	55.2258	1
**4766**	320	B	78.8183	-1
**4766**	321	B	76.6539	0
**4766**	351	B	15.1726	1
**4766**	357	B	71.7997	1
**4766**	370	B	60.9306	1
**4766**	372	B	66.2621	0
**4766**	373	B	59.36	1
**4766**	381	B	65.4016	1
**4766**	382	B	30.8106	0
**4766**	386	B	79.5193	1
**4766**	305	C	74.2738	1
**4766**	319	C	77.6419	1
**4766**	320	C	79.5553	-1
**4766**	321	C	79.6366	0
**4766**	351	C	12.0573	1
**4766**	357	C	72.7274	1
**4766**	370	C	75.6366	1
**4766**	372	C	79.4615	0
**4766**	373	C	54.6032	1
**4766**	381	C	41.871	0
**4766**	382	C	73.9859	1
**4766**	386	C	48.0018	0
**4766**	305	D	76.4933	1
**4766**	319	D	60.121	1
**4766**	320	D	67.9997	-1
**4766**	321	D	71.4745	0
**4766**	351	D	9.8018	1
**4766**	357	D	48.902	0
**4766**	370	D	71.0972	1
**4766**	372	D	79.5954	0
**4766**	373	D	79.3371	1
**4766**	381	D	79.5349	1

**4766**	382	D	71.4136	1
**4766**	386	D	79.5192	1
**4767**	305	A	71.0518	1
**4767**	319	A	69.1797	1
**4767**	320	A	74.289	-1
**4767**	321	A	79.5228	0
**4767**	351	A	6.7337	1
**4767**	357	A	61.8281	1
**4767**	370	A	47.9948	0
**4767**	372	A	46.6748	1
**4767**	373	A	58.5415	1
**4767**	381	A	72.4218	1
**4767**	382	A	44.3258	0
**4767**	386	A	60.5607	1
**4767**	305	B	79.546	1
**4767**	319	B	79.5945	1
**4767**	320	B	68.8499	-1
**4767**	321	B	65.1823	0
**4767**	351	B	17.1343	1
**4767**	357	B	79.5797	1
**4767**	370	B	79.5483	1
**4767**	372	B	79.4479	0
**4767**	373	B	79.5828	1
**4767**	381	B	76.0959	1
**4767**	382	B	67.2679	1
**4767**	386	B	79.5701	1
**4767**	305	C	78.3098	1
**4767**	319	C	70.9675	1
**4767**	320	C	79.5586	-1
**4767**	321	C	76.7656	0
**4767**	351	C	6.3475	1
**4767**	357	C	79.5682	1
**4767**	370	C	79.5588	1
**4767**	372	C	52.123	0

**4767**	373	C	79.5483	1
**4767**	381	C	48.162	0
**4767**	382	C	58.7656	1
**4767**	386	C	44.8522	0
**4767**	305	D	70.0621	1
**4767**	319	D	75.1852	1
**4767**	320	D	67.6916	-1
**4767**	321	D	37.5982	1
**4767**	351	D	6.8306	1
**4767**	357	D	79.3127	1
**4767**	370	D	79.5729	1
**4767**	372	D	79.3018	0
**4767**	373	D	36.4352	0
**4767**	381	D	79.5018	1
**4767**	382	D	26.6693	0
**4767**	386	D	77.2733	1
**4768**	305	A	70.7349	1
**4768**	319	A	79.5727	1
**4768**	320	A	25.3397	-1
**4768**	321	A	60.61	0
**4768**	351	A	8.4539	1
**4768**	357	A	60.5343	1
**4768**	370	A	59.6491	1
**4768**	372	A	76.789	0
**4768**	373	A	72.8128	1
**4768**	381	A	48.3755	0
**4768**	382	A	79.5312	1
**4768**	386	A	57.946	1
**4768**	305	B	51.6444	1
**4768**	319	B	79.5624	1
**4768**	320	B	56.2005	-1
**4768**	321	B	74.5516	0
**4768**	351	B	15.5843	1
**4768**	357	B	61.5966	1

**4768**	370	B	79.4187	1
**4768**	372	B	79.5705	0
**4768**	373	B	51.7563	1
**4768**	381	B	63.9903	1
**4768**	382	B	66.5865	1
**4768**	386	B	66.4869	1
**4768**	305	C	74.6469	1
**4768**	319	C	64.6111	1
**4768**	320	C	67.4294	-1
**4768**	321	C	76.7217	0
**4768**	351	C	1.2731	1
**4768**	357	C	79.5192	1
**4768**	370	C	63.3072	1
**4768**	372	C	73.4778	0
**4768**	373	C	25.608	0
**4768**	381	C	78.5494	1
**4768**	382	C	79.5774	1
**4768**	386	C	53.5538	1
**4768**	305	D	54.3807	1
**4768**	319	D	78.339	1
**4768**	320	D	41.2093	-1
**4768**	321	D	35.4313	1
**4768**	351	D	10.2864	1
**4768**	357	D	79.555	1
**4768**	370	D	40.5495	0
**4768**	372	D	79.4692	0
**4768**	373	D	79.5556	1
**4768**	381	D	76.4777	1
**4768**	382	D	79.5779	1
**4768**	386	D	79.5728	1
**4769**	305	A	76.6029	1
**4769**	319	A	33.2997	0
**4769**	320	A	42.5534	-1
**4769**	321	A	51.4252	0

**4769**	351	A	1.3433	1
**4769**	357	A	73.2624	1
**4769**	370	A	76.0217	1
**4769**	372	A	33.2837	1
**4769**	373	A	60.879	1
**4769**	381	A	79.5654	1
**4769**	382	A	38.5354	0
**4769**	386	A	57.1952	1
**4769**	305	B	79.5303	1
**4769**	319	B	75.3164	1
**4769**	320	B	23.9957	-1
**4769**	321	B	51.783	0
**4769**	351	B	2.1288	1
**4769**	357	B	47.3669	0
**4769**	370	B	61.3977	1
**4769**	372	B	79.5843	0
**4769**	373	B	59.7588	1
**4769**	381	B	72.4726	1
**4769**	382	B	71.4651	1
**4769**	386	B	79.6516	1
**4769**	305	C	79.5705	1
**4769**	319	C	32.1001	0
**4769**	320	C	70.6354	-1
**4769**	321	C	65.2702	0
**4769**	351	C	17.1141	1
**4769**	357	C	76.3784	1
**4769**	370	C	43.2977	0
**4769**	372	C	59.0403	0
**4769**	373	C	70.3461	1
**4769**	381	C	38.1282	0
**4769**	382	C	36.2574	0
**4769**	386	C	47.6365	0
**4769**	305	D	71.1475	1
**4769**	319	D	68.5186	1

**4769**	320	D	78.0829	-1
**4769**	321	D	68.9381	0
**4769**	351	D	26.5951	-1
**4769**	357	D	39.5566	0
**4769**	370	D	49.9743	0
**4769**	372	D	79.6807	0
**4769**	373	D	66.4328	1
**4769**	381	D	63.8014	1
**4769**	382	D	63.154	1
**4769**	386	D	79.5476	1
**4770**	305	A	57.3272	1
**4770**	319	A	60.9203	1
**4770**	320	A	57.9123	-1
**4770**	321	A	78.5898	0
**4770**	351	A	2.9985	1
**4770**	357	A	46.3281	0
**4770**	370	A	79.5268	1
**4770**	372	A	79.5137	0
**4770**	373	A	73.7463	1
**4770**	381	A	63.9376	1
**4770**	382	A	69.9479	1
**4770**	386	A	72.02	1
**4770**	305	B	78.3704	1
**4770**	319	B	71.6753	1
**4770**	320	B	79.077	-1
**4770**	321	B	78.8164	0
**4770**	351	B	16.6837	1
**4770**	357	B	74.949	1
**4770**	370	B	53.2616	1
**4770**	372	B	69.5707	0
**4770**	373	B	78.7393	1
**4770**	381	B	79.5812	1
**4770**	382	B	52.41	1
**4770**	386	B	79.0098	1

**4770**	305	C	74.5418	1
**4770**	319	C	70.6501	1
**4770**	320	C	66.9866	-1
**4770**	321	C	30.5882	1
**4770**	351	C	4.5253	1
**4770**	357	C	55.4454	1
**4770**	370	C	49.2739	0
**4770**	372	C	72.1914	0
**4770**	373	C	65.6488	1
**4770**	381	C	59.8793	1
**4770**	382	C	43.1205	0
**4770**	386	C	37.428	0
**4770**	305	D	70.5156	1
**4770**	319	D	38.8292	0
**4770**	320	D	67.5528	-1
**4770**	321	D	79.5944	0
**4770**	351	D	16.247	1
**4770**	357	D	79.5455	1
**4770**	370	D	16.8181	-1
**4770**	372	D	72.6851	0
**4770**	373	D	59.7174	1
**4770**	381	D	66.6418	1
**4770**	382	D	72.2955	1
**4770**	386	D	49.2194	0
**4771**	305	A	75.8391	1
**4771**	319	A	59.9024	1
**4771**	320	A	52.7268	-1
**4771**	321	A	77.1382	0
**4771**	351	A	0.1615	1
**4771**	357	A	58.1635	1
**4771**	370	A	71.7951	1
**4771**	372	A	79.5335	0
**4771**	373	A	79.4973	1
**4771**	381	A	79.6148	1

**4771**	382	A	49.1386	0
**4771**	386	A	69.7282	1
**4771**	305	B	79.5526	1
**4771**	319	B	73.5308	1
**4771**	320	B	46.7925	-1
**4771**	321	B	72.3548	0
**4771**	351	B	16.7818	1
**4771**	357	B	60.6705	1
**4771**	370	B	79.527	1
**4771**	372	B	79.5265	0
**4771**	373	B	78.0175	1
**4771**	381	B	51.3329	1
**4771**	382	B	64.6249	1
**4771**	386	B	57.2932	1
**4771**	305	C	72.2962	1
**4771**	319	C	51.1197	1
**4771**	320	C	66.5736	-1
**4771**	321	C	71.4818	0
**4771**	351	C	7.6936	1
**4771**	357	C	79.5398	1
**4771**	370	C	51.2418	1
**4771**	372	C	34.016	1
**4771**	373	C	79.3081	1
**4771**	381	C	79.6064	1
**4771**	382	C	56.8557	1
**4771**	386	C	51.8372	1
**4771**	305	D	79.6123	1
**4771**	319	D	59.8826	1
**4771**	320	D	74.8073	-1
**4771**	321	D	73.0165	0
**4771**	351	D	14.6541	1
**4771**	357	D	79.4975	1
**4771**	370	D	31.6571	0
**4771**	372	D	64.5153	0

**4771**	373	D	79.5479	1
**4771**	381	D	72.3567	1
**4771**	382	D	79.4996	1
**4771**	386	D	55.0276	1
**4772**	305	A	60.1309	1
**4772**	319	A	73.9287	1
**4772**	320	A	49.4503	-1
**4772**	321	A	33.785	1
**4772**	351	A	9.8433	1
**4772**	357	A	58.2209	1
**4772**	370	A	79.6796	1
**4772**	372	A	73.8848	0
**4772**	373	A	16.1045	-1
**4772**	381	A	79.5216	1
**4772**	382	A	79.5927	1
**4772**	386	A	72.7516	1
**4772**	305	B	79.4986	1
**4772**	319	B	79.5382	1
**4772**	320	B	79.5704	-1
**4772**	321	B	50.2413	0
**4772**	351	B	22.5533	-1
**4772**	357	B	74.6666	1
**4772**	370	B	51.6012	1
**4772**	372	B	47.7612	1
**4772**	373	B	79.4251	1
**4772**	381	B	64.5406	1
**4772**	382	B	67.9124	1
**4772**	386	B	79.6474	1
**4772**	305	C	36.9268	0
**4772**	319	C	79.6314	1
**4772**	320	C	64.2641	-1
**4772**	321	C	79.6575	0
**4772**	351	C	2.2169	1
**4772**	357	C	79.4892	1

**4772**	370	C	79.5705	1
**4772**	372	C	66.9183	0
**4772**	373	C	72.1868	1
**4772**	381	C	64.3331	1
**4772**	382	C	46.4254	0
**4772**	386	C	67.5845	1
**4772**	305	D	13.6455	-1
**4772**	319	D	73.9336	1
**4772**	320	D	79.6276	-1
**4772**	321	D	48.677	1
**4772**	351	D	2.5128	1
**4772**	357	D	75.6189	1
**4772**	370	D	59.1896	1
**4772**	372	D	73.2303	0
**4772**	373	D	79.5365	1
**4772**	381	D	73.2373	1
**4772**	382	D	67.1767	1
**4772**	386	D	78.9261	1
**4773**	305	A	74.8411	1
**4773**	319	A	72.0287	1
**4773**	320	A	38.8846	-1
**4773**	321	A	52.7864	0
**4773**	351	A	16.0228	1
**4773**	357	A	79.4461	1
**4773**	370	A	79.7133	1
**4773**	372	A	73.3263	0
**4773**	373	A	65.4469	1
**4773**	381	A	60.8691	1
**4773**	382	A	65.6334	1
**4773**	386	A	68.0926	1
**4773**	305	B	72.981	1
**4773**	319	B	58.797	1
**4773**	320	B	79.544	-1
**4773**	321	B	74.1293	0

**4773**	351	B	6.1851	1
**4773**	357	B	79.6696	1
**4773**	370	B	79.5515	1
**4773**	372	B	79.1696	0
**4773**	373	B	62.2997	1
**4773**	381	B	69.1022	1
**4773**	382	B	8.2968	-1
**4773**	386	B	51.2077	1
**4773**	305	C	79.5372	1
**4773**	319	C	31.4087	0
**4773**	320	C	55.6977	-1
**4773**	321	C	74.642	0
**4773**	351	C	15.6927	1
**4773**	357	C	59.8071	1
**4773**	370	C	74.3662	1
**4773**	372	C	79.4718	0
**4773**	373	C	77.2862	1
**4773**	381	C	79.3892	1
**4773**	382	C	72.6932	1
**4773**	386	C	31.0787	0
**4773**	305	D	58.3814	1
**4773**	319	D	62.0313	1
**4773**	320	D	79.6196	-1
**4773**	321	D	79.6775	0
**4773**	351	D	16.952	1
**4773**	357	D	57.6069	1
**4773**	370	D	44.1889	0
**4773**	372	D	58.9617	0
**4773**	373	D	74.9435	1
**4773**	381	D	47.4017	0
**4773**	382	D	66.7801	1
**4773**	386	D	62.8001	1
**4774**	305	A	79.5614	1
**4774**	319	A	45.8525	0

**4774**	320	A	75.0568	-1
**4774**	321	A	69.8861	0
**4774**	351	A	19.1096	1
**4774**	357	A	79.5306	1
**4774**	370	A	52.1895	1
**4774**	372	A	42.1848	1
**4774**	373	A	18.4938	-1
**4774**	381	A	59.838	1
**4774**	382	A	79.5868	1
**4774**	386	A	73.0385	1
**4774**	305	B	62.5158	1
**4774**	319	B	79.4476	1
**4774**	320	B	73.5045	-1
**4774**	321	B	62.3188	0
**4774**	351	B	4.6159	1
**4774**	357	B	79.6293	1
**4774**	370	B	76.3853	1
**4774**	372	B	55.2971	0
**4774**	373	B	79.2598	1
**4774**	381	B	79.5482	1
**4774**	382	B	79.6149	1
**4774**	386	B	65.8529	1
**4774**	305	C	74.2496	1
**4774**	319	C	61.6345	1
**4774**	320	C	73.5312	-1
**4774**	321	C	61.1266	0
**4774**	351	C	14.0965	1
**4774**	357	C	54.2036	1
**4774**	370	C	67.7656	1
**4774**	372	C	78.5847	0
**4774**	373	C	79.5466	1
**4774**	381	C	69.9515	1
**4774**	382	C	79.6628	1
**4774**	386	C	32.2125	0

**4774**	305	D	44.9884	0
**4774**	319	D	50.3564	1
**4774**	320	D	79.4925	-1
**4774**	321	D	79.7064	0
**4774**	351	D	10.1382	1
**4774**	357	D	68.3246	1
**4774**	370	D	75.3293	1
**4774**	372	D	73.1659	0
**4774**	373	D	79.6483	1
**4774**	381	D	79.5575	1
**4774**	382	D	71.3363	1
**4774**	386	D	79.5979	1
**4775**	305	A	62.2226	1
**4775**	319	A	79.534	1
**4775**	320	A	57.2679	-1
**4775**	321	A	64.1748	0
**4775**	351	A	3.5237	1
**4775**	357	A	79.5768	1
**4775**	370	A	79.5881	1
**4775**	372	A	79.5704	0
**4775**	373	A	65.7558	1
**4775**	381	A	43.8648	0
**4775**	382	A	61.2298	1
**4775**	386	A	74.4015	1
**4775**	305	B	79.5237	1
**4775**	319	B	72.5437	1
**4775**	320	B	63.305	-1
**4775**	321	B	76.6442	0
**4775**	351	B	16.7844	1
**4775**	357	B	79.5381	1
**4775**	370	B	49.6989	0
**4775**	372	B	73.7189	0
**4775**	373	B	53.0695	1
**4775**	381	B	79.5687	1

**4775**	382	B	49.0679	0
**4775**	386	B	68.5422	1
**4775**	305	C	79.5347	1
**4775**	319	C	79.5915	1
**4775**	320	C	58.8857	-1
**4775**	321	C	70.9084	0
**4775**	351	C	41.1569	-1
**4775**	357	C	55.0597	1
**4775**	370	C	71.5552	1
**4775**	372	C	62.6898	0
**4775**	373	C	62.3722	1
**4775**	381	C	51.5106	1
**4775**	382	C	39.6679	0
**4775**	386	C	17.6062	-1
**4775**	305	D	73.0238	1
**4775**	319	D	78.3672	1
**4775**	320	D	79.574	-1
**4775**	321	D	73.3669	0
**4775**	351	D	17.3975	1
**4775**	357	D	73.6376	1
**4775**	370	D	76.3703	1
**4775**	372	D	79.5485	0
**4775**	373	D	67.9044	1
**4775**	381	D	71.7664	1
**4775**	382	D	78.2545	1
**4775**	386	D	37.1816	0
**4776**	305	A	78.7517	1
**4776**	319	A	64.3912	1
**4776**	320	A	72.2903	-1
**4776**	321	A	67.2012	0
**4776**	351	A	20.7362	-1
**4776**	357	A	74.0812	1
**4776**	370	A	73.7211	1
**4776**	372	A	46.9055	1

**4776**	373	A	69.8739	1
**4776**	381	A	79.5428	1
**4776**	382	A	36.6039	0
**4776**	386	A	76.6331	1
**4776**	305	B	72.3426	1
**4776**	319	B	79.636	1
**4776**	320	B	74.0468	-1
**4776**	321	B	65.2698	0
**4776**	351	B	16.0538	1
**4776**	357	B	72.6532	1
**4776**	370	B	46.7911	0
**4776**	372	B	79.6208	0
**4776**	373	B	38.928	0
**4776**	381	B	67.8005	1
**4776**	382	B	57.9915	1
**4776**	386	B	68.4547	1
**4776**	305	C	79.5103	1
**4776**	319	C	66.484	1
**4776**	320	C	72.2731	-1
**4776**	321	C	78.2027	0
**4776**	351	C	8.9925	1
**4776**	357	C	77.9607	1
**4776**	370	C	60.4662	1
**4776**	372	C	60.6239	0
**4776**	373	C	71.4747	1
**4776**	381	C	70.9278	1
**4776**	382	C	49.8941	0
**4776**	386	C	35.9255	0
**4776**	305	D	75.3672	1
**4776**	319	D	77.0312	1
**4776**	320	D	79.5395	-1
**4776**	321	D	79.6129	0
**4776**	351	D	9.8722	1
**4776**	357	D	79.4763	1

**4776**	370	D	22.3275	0
**4776**	372	D	70.7074	0
**4776**	373	D	73.5623	1
**4776**	381	D	58.7222	1
**4776**	382	D	72.5836	1
**4776**	386	D	60.8931	1
**4777**	305	A	67.6399	1
**4777**	319	A	72.9652	1
**4777**	320	A	76.5944	-1
**4777**	321	A	63.465	0
**4777**	351	A	12.721	1
**4777**	357	A	69.495	1
**4777**	370	A	73.6467	1
**4777**	372	A	75.2966	0
**4777**	373	A	42.0946	0
**4777**	381	A	62.4805	1
**4777**	382	A	24.5101	0
**4777**	386	A	78.4671	1
**4777**	305	B	76.5292	1
**4777**	319	B	74.6802	1
**4777**	320	B	79.6303	-1
**4777**	321	B	71.5043	0
**4777**	351	B	6.9993	1
**4777**	357	B	79.5615	1
**4777**	370	B	79.5618	1
**4777**	372	B	59.1088	0
**4777**	373	B	72.82	1
**4777**	381	B	54.9361	1
**4777**	382	B	66.4355	1
**4777**	386	B	79.578	1
**4777**	305	C	42.8133	0
**4777**	319	C	79.5138	1
**4777**	320	C	73.9547	-1
**4777**	321	C	69.2	0

**4777**	351	C	2.4659	1
**4777**	357	C	79.5952	1
**4777**	370	C	54.0688	1
**4777**	372	C	46.0531	1
**4777**	373	C	71.5126	1
**4777**	381	C	79.537	1
**4777**	382	C	79.5927	1
**4777**	386	C	43.936	0
**4777**	305	D	77.0621	1
**4777**	319	D	73.6148	1
**4777**	320	D	39.5098	-1
**4777**	321	D	79.6378	0
**4777**	351	D	10.9889	1
**4777**	357	D	38.9363	0
**4777**	370	D	33.5834	0
**4777**	372	D	66.0843	0
**4777**	373	D	73.1622	1
**4777**	381	D	79.589	1
**4777**	382	D	79.5848	1
**4777**	386	D	79.0414	1
**4778**	305	A	79.5421	1
**4778**	319	A	73.0459	1
**4778**	320	A	73.7423	-1
**4778**	321	A	74.4527	0
**4778**	351	A	7.346	1
**4778**	357	A	79.5469	1
**4778**	370	A	79.6181	1
**4778**	372	A	68.1385	0
**4778**	373	A	72.2461	1
**4778**	381	A	74.106	1
**4778**	382	A	72.126	1
**4778**	386	A	79.5453	1
**4778**	305	B	79.4178	1
**4778**	319	B	78.9295	1

**4778**	320	B	68.6385	-1
**4778**	321	B	79.5689	0
**4778**	351	B	5.863	1
**4778**	357	B	79.5414	1
**4778**	370	B	79.5591	1
**4778**	372	B	70.0129	0
**4778**	373	B	64.1447	1
**4778**	381	B	74.7309	1
**4778**	382	B	77.6477	1
**4778**	386	B	74.2774	1
**4778**	305	C	76.7272	1
**4778**	319	C	59.9839	1
**4778**	320	C	55.4184	-1
**4778**	321	C	79.3802	0
**4778**	351	C	13.6723	1
**4778**	357	C	72.2933	1
**4778**	370	C	67.7932	1
**4778**	372	C	61.5892	0
**4778**	373	C	36.4139	0
**4778**	381	C	40.075	0
**4778**	382	C	79.5802	1
**4778**	386	C	42.7587	0
**4778**	305	D	45.4158	0
**4778**	319	D	79.5695	1
**4778**	320	D	36.8553	-1
**4778**	321	D	79.4981	0
**4778**	351	D	14.3425	1
**4778**	357	D	79.5779	1
**4778**	370	D	79.5485	1
**4778**	372	D	79.5569	0
**4778**	373	D	79.5555	1
**4778**	381	D	60.3057	1
**4778**	382	D	79.5486	1
**4778**	386	D	79.4497	1

**4779**	305	A	72.3327	1
**4779**	319	A	72.7162	1
**4779**	320	A	79.5772	-1
**4779**	321	A	66.9832	0
**4779**	351	A	6.8682	1
**4779**	357	A	71.2864	1
**4779**	370	A	40.3429	0
**4779**	372	A	79.6061	0
**4779**	373	A	67.3313	1
**4779**	381	A	48.71	0
**4779**	382	A	52.8032	1
**4779**	386	A	49.5538	0
**4779**	305	B	79.6178	1
**4779**	319	B	79.58	1
**4779**	320	B	12.7268	1
**4779**	321	B	79.5535	0
**4779**	351	B	14.7006	1
**4779**	357	B	79.536	1
**4779**	370	B	79.5229	1
**4779**	372	B	67.2199	0
**4779**	373	B	79.5308	1
**4779**	381	B	79.5579	1
**4779**	382	B	32.1867	0
**4779**	386	B	79.6151	1
**4779**	305	C	69.9801	1
**4779**	319	C	66.0725	1
**4779**	320	C	60.6152	-1
**4779**	321	C	76.1913	0
**4779**	351	C	5.9445	1
**4779**	357	C	29.6527	0
**4779**	370	C	66.2655	1
**4779**	372	C	79.5833	0
**4779**	373	C	57.6093	1
**4779**	381	C	79.5945	1

**4779**	382	C	43.3462	0
**4779**	386	C	38.9156	0
**4779**	305	D	52.8524	1
**4779**	319	D	78.0729	1
**4779**	320	D	58.8427	-1
**4779**	321	D	75.3557	0
**4779**	351	D	12.5754	1
**4779**	357	D	79.5834	1
**4779**	370	D	77.3546	1
**4779**	372	D	67.075	0
**4779**	373	D	64.8592	1
**4779**	381	D	61.6418	1
**4779**	382	D	78.9875	1
**4779**	386	D	79.5659	1
**4780**	305	A	71.7245	1
**4780**	319	A	75.576	1
**4780**	320	A	79.6006	-1
**4780**	321	A	61.7658	0
**4780**	351	A	2.7528	1
**4780**	357	A	79.5413	1
**4780**	370	A	79.5926	1
**4780**	372	A	71.5783	0
**4780**	373	A	70.063	1
**4780**	381	A	52.5563	1
**4780**	382	A	57.5546	1
**4780**	386	A	79.4983	1
**4780**	305	B	79.1762	1
**4780**	319	B	60.5713	1
**4780**	320	B	79.5045	-1
**4780**	321	B	60.764	0
**4780**	351	B	5.6885	1
**4780**	357	B	73.7411	1
**4780**	370	B	79.683	1
**4780**	372	B	79.6364	0

**4780**	373	B	35.5178	0
**4780**	381	B	72.0671	1
**4780**	382	B	11.5161	-1
**4780**	386	B	72.0216	1
**4780**	305	C	65.5029	1
**4780**	319	C	79.5983	1
**4780**	320	C	79.5709	-1
**4780**	321	C	72.9194	0
**4780**	351	C	12.62	1
**4780**	357	C	60.3015	1
**4780**	370	C	51.9925	1
**4780**	372	C	57.2679	0
**4780**	373	C	42.869	0
**4780**	381	C	79.5344	1
**4780**	382	C	59.9788	1
**4780**	386	C	26.0569	0
**4780**	305	D	63.6904	1
**4780**	319	D	63.6345	1
**4780**	320	D	33.9759	-1
**4780**	321	D	76.2873	0
**4780**	351	D	11.0376	1
**4780**	357	D	76.2651	1
**4780**	370	D	72.9272	1
**4780**	372	D	54.7526	0
**4780**	373	D	77.0093	1
**4780**	381	D	79.6184	1
**4780**	382	D	33.0628	0
**4780**	386	D	56.4483	1
**4781**	305	A	78.5713	1
**4781**	319	A	74.1744	1
**4781**	320	A	72.4942	-1
**4781**	321	A	68.2134	0
**4781**	351	A	3.2727	1
**4781**	357	A	52.3868	1

**4781**	370	A	70.9576	1
**4781**	372	A	58.547	0
**4781**	373	A	69.4371	1
**4781**	381	A	79.5825	1
**4781**	382	A	74.0997	1
**4781**	386	A	35.0716	0
**4781**	305	B	42.8924	0
**4781**	319	B	61.9056	1
**4781**	320	B	18.5627	1
**4781**	321	B	79.4897	0
**4781**	351	B	10.7983	1
**4781**	357	B	56.2151	1
**4781**	370	B	79.6055	1
**4781**	372	B	20.0358	1
**4781**	373	B	71.0405	1
**4781**	381	B	62.2469	1
**4781**	382	B	68.0095	1
**4781**	386	B	79.4704	1
**4781**	305	C	79.2349	1
**4781**	319	C	79.5627	1
**4781**	320	C	67.8974	-1
**4781**	321	C	76.2475	0
**4781**	351	C	6.3095	1
**4781**	357	C	65.8313	1
**4781**	370	C	62.7288	1
**4781**	372	C	75.7067	0
**4781**	373	C	71.4831	1
**4781**	381	C	79.5867	1
**4781**	382	C	71.5971	1
**4781**	386	C	24.9624	0
**4781**	305	D	61.1869	1
**4781**	319	D	43.5482	0
**4781**	320	D	79.6109	-1
**4781**	321	D	51.9568	0

**4781**	351	D	15.652	1
**4781**	357	D	31.149	0
**4781**	370	D	72.1552	1
**4781**	372	D	52.2389	0
**4781**	373	D	78.2211	1
**4781**	381	D	79.5838	1
**4781**	382	D	71.8354	1
**4781**	386	D	60.7059	1
**4782**	305	A	63.7874	1
**4782**	319	A	59.0556	1
**4782**	320	A	58.9566	-1
**4782**	321	A	46.1584	1
**4782**	351	A	1.551	1
**4782**	357	A	64.2565	1
**4782**	370	A	72.4308	1
**4782**	372	A	51.1915	0
**4782**	373	A	73.2196	1
**4782**	381	A	69.011	1
**4782**	382	A	79.5358	1
**4782**	386	A	72.9667	1
**4782**	305	B	72.4038	1
**4782**	319	B	79.6448	1
**4782**	320	B	79.538	-1
**4782**	321	B	77.436	0
**4782**	351	B	12.2345	1
**4782**	357	B	61.5529	1
**4782**	370	B	72.9739	1
**4782**	372	B	73.2481	0
**4782**	373	B	79.5787	1
**4782**	381	B	70.7009	1
**4782**	382	B	79.6425	1
**4782**	386	B	79.5883	1
**4782**	305	C	58.2762	1
**4782**	319	C	79.5588	1

**4782**	320	C	46.469	-1
**4782**	321	C	79.4281	0
**4782**	351	C	1.2949	1
**4782**	357	C	68.9656	1
**4782**	370	C	79.3815	1
**4782**	372	C	65.1924	0
**4782**	373	C	73.6859	1
**4782**	381	C	49.8007	0
**4782**	382	C	60.4823	1
**4782**	386	C	49.127	0
**4782**	305	D	75.7197	1
**4782**	319	D	79.5771	1
**4782**	320	D	72.7803	-1
**4782**	321	D	67.0473	0
**4782**	351	D	13.7256	1
**4782**	357	D	79.5551	1
**4782**	370	D	57.7485	1
**4782**	372	D	73.2663	0
**4782**	373	D	77.2934	1
**4782**	381	D	79.5729	1
**4782**	382	D	79.5586	1
**4782**	386	D	79.5812	1
**4783**	305	A	79.5439	1
**4783**	319	A	55.5489	1
**4783**	320	A	79.5212	-1
**4783**	321	A	35.8734	1
**4783**	351	A	15.2037	1
**4783**	357	A	24.5936	0
**4783**	370	A	72.7839	1
**4783**	372	A	73.0834	0
**4783**	373	A	64.6275	1
**4783**	381	A	79.5621	1
**4783**	382	A	77.7702	1
**4783**	386	A	76.1727	1

**4783**	305	B	66.5888	1
**4783**	319	B	79.4374	1
**4783**	320	B	79.504	-1
**4783**	321	B	52.9593	0
**4783**	351	B	19.5918	1
**4783**	357	B	75.0244	1
**4783**	370	B	79.566	1
**4783**	372	B	60.7501	0
**4783**	373	B	53.8321	1
**4783**	381	B	70.0083	1
**4783**	382	B	76.4587	1
**4783**	386	B	46.3395	0
**4783**	305	C	74.0071	1
**4783**	319	C	79.5575	1
**4783**	320	C	72.4383	-1
**4783**	321	C	47.6239	1
**4783**	351	C	5.0676	1
**4783**	357	C	49.0393	0
**4783**	370	C	55.4771	1
**4783**	372	C	72.8739	0
**4783**	373	C	36.2565	0
**4783**	381	C	79.5653	1
**4783**	382	C	79.1903	1
**4783**	386	C	49.7296	0
**4783**	305	D	71.751	1
**4783**	319	D	79.5646	1
**4783**	320	D	79.5756	-1
**4783**	321	D	72.664	0
**4783**	351	D	12.1629	1
**4783**	357	D	72.984	1
**4783**	370	D	73.0323	1
**4783**	372	D	62.6884	0
**4783**	373	D	79.5307	1
**4783**	381	D	64.2728	1

**4783**	382	D	44.2205	0
**4783**	386	D	73.3724	1
**4784**	305	A	75.6534	1
**4784**	319	A	44.9015	0
**4784**	320	A	49.3218	-1
**4784**	321	A	13.4254	0
**4784**	351	A	9.1744	1
**4784**	357	A	52.0438	1
**4784**	370	A	67.1752	1
**4784**	372	A	79.4685	0
**4784**	373	A	58.0505	1
**4784**	381	A	73.8606	1
**4784**	382	A	64.5921	1
**4784**	386	A	78.6612	1
**4784**	305	B	74.5304	1
**4784**	319	B	72.8362	1
**4784**	320	B	74.8956	-1
**4784**	321	B	67.1338	0
**4784**	351	B	21.1627	-1
**4784**	357	B	79.5727	1
**4784**	370	B	73.2601	1
**4784**	372	B	65.9459	0
**4784**	373	B	78.1748	1
**4784**	381	B	79.5348	1
**4784**	382	B	74.2124	1
**4784**	386	B	79.5879	1
**4784**	305	C	79.5969	1
**4784**	319	C	77.6574	1
**4784**	320	C	59.7373	-1
**4784**	321	C	72.4411	0
**4784**	351	C	6.2497	1
**4784**	357	C	64.9897	1
**4784**	370	C	65.7407	1
**4784**	372	C	75.6795	0

**4784**	373	C	79.51	1
**4784**	381	C	70.9774	1
**4784**	382	C	20.0452	0
**4784**	386	C	56.9806	1
**4784**	305	D	48.9171	0
**4784**	319	D	72.5496	1
**4784**	320	D	79.5524	-1
**4784**	321	D	79.6006	0
**4784**	351	D	7.4453	1
**4784**	357	D	79.6506	1
**4784**	370	D	32.0563	0
**4784**	372	D	71.5473	0
**4784**	373	D	78.8069	1
**4784**	381	D	79.5353	1
**4784**	382	D	64.4952	1
**4784**	386	D	36.9474	0
**4785**	305	A	78.8123	1
**4785**	319	A	74.274	1
**4785**	320	A	54.3276	-1
**4785**	321	A	76.8111	0
**4785**	351	A	8.2091	1
**4785**	357	A	79.566	1
**4785**	370	A	65.4611	1
**4785**	372	A	79.5979	0
**4785**	373	A	6.0571	-1
**4785**	381	A	79.5695	1
**4785**	382	A	79.6007	1
**4785**	386	A	79.5528	1
**4785**	305	B	69.6236	1
**4785**	319	B	79.4207	1
**4785**	320	B	60.5133	-1
**4785**	321	B	66.0859	0
**4785**	351	B	15.3092	1
**4785**	357	B	61.5142	1

**4785**	370	B	22.2817	0
**4785**	372	B	79.5682	0
**4785**	373	B	49.4275	0
**4785**	381	B	72.7962	1
**4785**	382	B	40.3601	0
**4785**	386	B	79.4556	1
**4785**	305	C	78.9808	1
**4785**	319	C	76.0432	1
**4785**	320	C	74.2346	-1
**4785**	321	C	54.0445	0
**4785**	351	C	2.0164	1
**4785**	357	C	57.6847	1
**4785**	370	C	60.2411	1
**4785**	372	C	79.5636	0
**4785**	373	C	79.6126	1
**4785**	381	C	73.8025	1
**4785**	382	C	79.4935	1
**4785**	386	C	53.7013	1
**4785**	305	D	1.324	-1
**4785**	319	D	74.4553	1
**4785**	320	D	36.1326	-1
**4785**	321	D	67.3799	0
**4785**	351	D	14.922	1
**4785**	357	D	79.5648	1
**4785**	370	D	48.6914	0
**4785**	372	D	79.5683	0
**4785**	373	D	79.1141	1
**4785**	381	D	79.5924	1
**4785**	382	D	56.7676	1
**4785**	386	D	31.4514	0
**4786**	305	A	56.1163	1
**4786**	319	A	62.4585	1
**4786**	320	A	79.6302	-1
**4786**	321	A	76.7563	0

**4786**	351	A	11.3577	1
**4786**	357	A	57.913	1
**4786**	370	A	78.3911	1
**4786**	372	A	79.552	0
**4786**	373	A	21.4417	0
**4786**	381	A	76.8386	1
**4786**	382	A	41.7073	0
**4786**	386	A	69.5679	1
**4786**	305	B	79.6994	1
**4786**	319	B	70.2376	1
**4786**	320	B	77.8402	-1
**4786**	321	B	74.9686	0
**4786**	351	B	22.029	-1
**4786**	357	B	79.5585	1
**4786**	370	B	79.2565	1
**4786**	372	B	79.5654	0
**4786**	373	B	68.2161	1
**4786**	381	B	79.5374	1
**4786**	382	B	35.4424	0
**4786**	386	B	78.8677	1
**4786**	305	C	58.7218	1
**4786**	319	C	70.0674	1
**4786**	320	C	67.8603	-1
**4786**	321	C	61.7072	0
**4786**	351	C	37.9414	-1
**4786**	357	C	62.9704	1
**4786**	370	C	47.268	0
**4786**	372	C	46.7991	1
**4786**	373	C	66.9935	1
**4786**	381	C	33.5469	0
**4786**	382	C	79.5749	1
**4786**	386	C	53.7013	1
**4786**	305	D	52.4117	1
**4786**	319	D	69.8579	1

**4786**	320	D	79.6549	-1
**4786**	321	D	56.1031	0
**4786**	351	D	6.741	1
**4786**	357	D	49.7609	0
**4786**	370	D	72.0413	1
**4786**	372	D	79.6209	0
**4786**	373	D	61.1056	1
**4786**	381	D	79.533	1
**4786**	382	D	77.7251	1
**4786**	386	D	79.5799	1
**4787**	305	A	69.5958	1
**4787**	319	A	67.1777	1
**4787**	320	A	79.6091	-1
**4787**	321	A	73.0174	0
**4787**	351	A	13.0717	1
**4787**	357	A	79.5614	1
**4787**	370	A	79.5426	1
**4787**	372	A	76.7197	0
**4787**	373	A	55.7389	1
**4787**	381	A	79.5682	1
**4787**	382	A	54.2512	1
**4787**	386	A	60.7572	1
**4787**	305	B	73.0106	1
**4787**	319	B	79.5868	1
**4787**	320	B	22.3246	-1
**4787**	321	B	69.3626	0
**4787**	351	B	15.9224	1
**4787**	357	B	43.1302	0
**4787**	370	B	71.1719	1
**4787**	372	B	72.0009	0
**4787**	373	B	67.5337	1
**4787**	381	B	72.805	1
**4787**	382	B	59.3335	1
**4787**	386	B	76.5667	1

**4787**	305	C	72.3128	1
**4787**	319	C	79.5729	1
**4787**	320	C	63.724	-1
**4787**	321	C	70.1371	0
**4787**	351	C	1.5932	1
**4787**	357	C	60.8855	1
**4787**	370	C	63.7585	1
**4787**	372	C	40.6619	1
**4787**	373	C	67.9548	1
**4787**	381	C	55.0714	1
**4787**	382	C	62.6551	1
**4787**	386	C	43.8854	0
**4787**	305	D	71.9059	1
**4787**	319	D	79.559	1
**4787**	320	D	69.9994	-1
**4787**	321	D	35.2449	1
**4787**	351	D	18.2018	1
**4787**	357	D	65.0199	1
**4787**	370	D	40.4902	0
**4787**	372	D	79.4799	0
**4787**	373	D	76.9715	1
**4787**	381	D	28.9428	0
**4787**	382	D	74.7212	1
**4787**	386	D	23.308	0
**4788**	305	A	36.3145	0
**4788**	319	A	79.1634	1
**4788**	320	A	60.3122	-1
**4788**	321	A	79.5482	0
**4788**	351	A	7.4566	1
**4788**	357	A	79.4839	1
**4788**	370	A	51.2997	1
**4788**	372	A	71.9464	0
**4788**	373	A	41.0808	0
**4788**	381	A	79.4806	1

**4788**	382	A	79.6729	1
**4788**	386	A	68.0118	1
**4788**	305	B	59.3939	1
**4788**	319	B	60.5681	1
**4788**	320	B	79.545	-1
**4788**	321	B	79.5595	0
**4788**	351	B	1.2827	1
**4788**	357	B	40.9773	0
**4788**	370	B	79.5149	1
**4788**	372	B	72.2623	0
**4788**	373	B	54.1414	1
**4788**	381	B	75.2512	1
**4788**	382	B	78.6063	1
**4788**	386	B	79.6647	1
**4788**	305	C	79.6895	1
**4788**	319	C	79.6979	1
**4788**	320	C	64.1807	-1
**4788**	321	C	73.6919	0
**4788**	351	C	4.1912	1
**4788**	357	C	79.6868	1
**4788**	370	C	65.7727	1
**4788**	372	C	79.4823	0
**4788**	373	C	61.4486	1
**4788**	381	C	54.0419	1
**4788**	382	C	55.1707	1
**4788**	386	C	71.2051	1
**4788**	305	D	71.159	1
**4788**	319	D	69.44	1
**4788**	320	D	79.5441	-1
**4788**	321	D	79.4834	0
**4788**	351	D	13.7997	1
**4788**	357	D	51.8896	1
**4788**	370	D	63.7424	1
**4788**	372	D	79.5039	0

**4788**	373	D	79.4909	1
**4788**	381	D	69.0198	1
**4788**	382	D	79.5822	1
**4788**	386	D	79.4709	1
**4789**	305	A	69.6092	1
**4789**	319	A	37.2173	0
**4789**	320	A	70.4619	-1
**4789**	321	A	21.995	1
**4789**	351	A	0.0	1
**4789**	357	A	76.3941	1
**4789**	370	A	74.8225	1
**4789**	372	A	74.2762	0
**4789**	373	A	79.6949	1
**4789**	381	A	71.7288	1
**4789**	382	A	20.5183	0
**4789**	386	A	71.3913	1
**4789**	305	B	47.9712	0
**4789**	319	B	70.3662	1
**4789**	320	B	79.7931	-1
**4789**	321	B	14.7899	0
**4789**	351	B	23.9468	-1
**4789**	357	B	39.2007	0
**4789**	370	B	79.6162	1
**4789**	372	B	65.1201	0
**4789**	373	B	77.0683	1
**4789**	381	B	79.588	1
**4789**	382	B	26.4565	0
**4789**	386	B	56.7775	1
**4789**	305	C	72.6703	1
**4789**	319	C	67.9056	1
**4789**	320	C	51.8226	-1
**4789**	321	C	36.5532	1
**4789**	351	C	11.9557	1
**4789**	357	C	79.6777	1

**4789**	370	C	78.1662	1
**4789**	372	C	79.5674	0
**4789**	373	C	79.6118	1
**4789**	381	C	73.1492	1
**4789**	382	C	56.2049	1
**4789**	386	C	51.1844	1
**4789**	305	D	75.9763	1
**4789**	319	D	73.299	1
**4789**	320	D	57.8515	-1
**4789**	321	D	76.6951	0
**4789**	351	D	24.4138	-1
**4789**	357	D	79.5911	1
**4789**	370	D	69.2236	1
**4789**	372	D	79.4457	0
**4789**	373	D	79.8599	1
**4789**	381	D	73.3868	1
**4789**	382	D	79.7449	1
**4789**	386	D	79.5048	1
**4790**	305	A	79.531	1
**4790**	319	A	73.0341	1
**4790**	320	A	61.1118	-1
**4790**	321	A	17.8304	0
**4790**	351	A	2.1988	1
**4790**	357	A	79.6127	1
**4790**	370	A	72.259	1
**4790**	372	A	70.2003	0
**4790**	373	A	57.4637	1
**4790**	381	A	63.1257	1
**4790**	382	A	64.3077	1
**4790**	386	A	74.5691	1
**4790**	305	B	66.0854	1
**4790**	319	B	73.4667	1
**4790**	320	B	75.7106	-1
**4790**	321	B	74.994	0

**4790**	351	B	17.6501	1
**4790**	357	B	73.3772	1
**4790**	370	B	79.5837	1
**4790**	372	B	53.6458	0
**4790**	373	B	28.6626	0
**4790**	381	B	54.205	1
**4790**	382	B	19.1256	-1
**4790**	386	B	68.7445	1
**4790**	305	C	65.6731	1
**4790**	319	C	79.6454	1
**4790**	320	C	60.7357	-1
**4790**	321	C	74.4942	0
**4790**	351	C	10.0221	1
**4790**	357	C	79.4971	1
**4790**	370	C	79.4482	1
**4790**	372	C	68.7705	0
**4790**	373	C	79.6796	1
**4790**	381	C	65.92	1
**4790**	382	C	54.5489	1
**4790**	386	C	72.0536	1
**4790**	305	D	73.5411	1
**4790**	319	D	64.8765	1
**4790**	320	D	56.6071	-1
**4790**	321	D	56.2449	0
**4790**	351	D	42.2739	-1
**4790**	357	D	65.4522	1
**4790**	370	D	59.1964	1
**4790**	372	D	79.4357	0
**4790**	373	D	79.3881	1
**4790**	381	D	48.0526	0
**4790**	382	D	77.588	1
**4790**	386	D	55.3934	1
**4791**	305	A	70.4925	1
**4791**	319	A	14.7022	-1

**4791**	320	A	71.9635	-1
**4791**	321	A	79.5395	0
**4791**	351	A	24.9948	-1
**4791**	357	A	79.5861	1
**4791**	370	A	72.1619	1
**4791**	372	A	42.5688	1
**4791**	373	A	79.5683	1
**4791**	381	A	79.6018	1
**4791**	382	A	78.7945	1
**4791**	386	A	79.6265	1
**4791**	305	B	72.4757	1
**4791**	319	B	67.3134	1
**4791**	320	B	77.7797	-1
**4791**	321	B	79.6131	0
**4791**	351	B	22.1756	-1
**4791**	357	B	73.8813	1
**4791**	370	B	40.7023	0
**4791**	372	B	68.1439	0
**4791**	373	B	77.9478	1
**4791**	381	B	57.0159	1
**4791**	382	B	58.7935	1
**4791**	386	B	54.2056	1
**4791**	305	C	12.7602	-1
**4791**	319	C	28.3478	0
**4791**	320	C	58.8896	-1
**4791**	321	C	69.239	0
**4791**	351	C	12.8512	1
**4791**	357	C	73.9673	1
**4791**	370	C	59.7157	1
**4791**	372	C	79.5614	0
**4791**	373	C	79.5898	1
**4791**	381	C	79.5105	1
**4791**	382	C	79.4931	1
**4791**	386	C	29.2161	0

**4791**	305	D	71.4096	1
**4791**	319	D	79.5459	1
**4791**	320	D	65.4688	-1
**4791**	321	D	49.2075	1
**4791**	351	D	15.5764	1
**4791**	357	D	67.5313	1
**4791**	370	D	79.5992	1
**4791**	372	D	79.5465	0
**4791**	373	D	56.3995	1
**4791**	381	D	16.4699	-1
**4791**	382	D	79.5761	1
**4791**	386	D	70.151	1
**4792**	305	A	74.607	1
**4792**	319	A	63.9237	1
**4792**	320	A	73.1312	-1
**4792**	321	A	49.2624	1
**4792**	351	A	3.4415	1
**4792**	357	A	72.8286	1
**4792**	370	A	55.0226	1
**4792**	372	A	26.7845	1
**4792**	373	A	79.6038	1
**4792**	381	A	79.5673	1
**4792**	382	A	79.5768	1
**4792**	386	A	79.5795	1
**4792**	305	B	79.5783	1
**4792**	319	B	79.6029	1
**4792**	320	B	34.3929	-1
**4792**	321	B	68.4191	0
**4792**	351	B	10.2002	1
**4792**	357	B	79.5811	1
**4792**	370	B	79.5724	1
**4792**	372	B	79.5693	0
**4792**	373	B	78.2842	1
**4792**	381	B	69.8861	1

**4792**	382	B	44.1471	0
**4792**	386	B	67.39	1
**4792**	305	C	40.3505	0
**4792**	319	C	79.3235	1
**4792**	320	C	56.3056	-1
**4792**	321	C	60.6911	0
**4792**	351	C	5.7755	1
**4792**	357	C	79.5774	1
**4792**	370	C	72.9849	1
**4792**	372	C	72.2565	0
**4792**	373	C	75.1427	1
**4792**	381	C	75.1735	1
**4792**	382	C	62.999	1
**4792**	386	C	65.3206	1
**4792**	305	D	79.0438	1
**4792**	319	D	79.5648	1
**4792**	320	D	79.5459	-1
**4792**	321	D	47.4886	1
**4792**	351	D	8.8256	1
**4792**	357	D	79.6061	1
**4792**	370	D	90.6203	1
**4792**	372	D	79.5463	0
**4792**	373	D	70.8916	1
**4792**	381	D	73.0992	1
**4792**	382	D	79.5399	1
**4792**	386	D	77.7779	1
**4793**	305	A	79.5843	1
**4793**	319	A	71.6256	1
**4793**	320	A	79.5085	-1
**4793**	321	A	77.7772	0
**4793**	351	A	6.8801	1
**4793**	357	A	74.1559	1
**4793**	370	A	72.1194	1
**4793**	372	A	23.4194	1

**4793**	373	A	79.4196	1
**4793**	381	A	71.2273	1
**4793**	382	A	73.718	1
**4793**	386	A	37.5825	0
**4793**	305	B	79.5586	1
**4793**	319	B	79.5866	1
**4793**	320	B	79.5513	-1
**4793**	321	B	79.6175	0
**4793**	351	B	12.5926	1
**4793**	357	B	38.4876	0
**4793**	370	B	24.093	0
**4793**	372	B	79.6147	0
**4793**	373	B	54.8294	1
**4793**	381	B	48.2975	0
**4793**	382	B	78.8729	1
**4793**	386	B	46.2351	0
**4793**	305	C	71.6105	1
**4793**	319	C	66.404	1
**4793**	320	C	79.5581	-1
**4793**	321	C	79.6196	0
**4793**	351	C	1.1268	1
**4793**	357	C	53.6888	1
**4793**	370	C	48.341	0
**4793**	372	C	79.577	0
**4793**	373	C	60.4686	1
**4793**	381	C	72.6236	1
**4793**	382	C	60.8513	1
**4793**	386	C	56.8895	1
**4793**	305	D	73.6121	1
**4793**	319	D	44.4111	0
**4793**	320	D	76.5526	-1
**4793**	321	D	64.1402	0
**4793**	351	D	13.6202	1
**4793**	357	D	72.9725	1

**4793**	370	D	73.1601	1
**4793**	372	D	74.8286	0
**4793**	373	D	53.1225	1
**4793**	381	D	68.9058	1
**4793**	382	D	71.8802	1
**4793**	386	D	54.4947	1
**4794**	305	A	75.3696	1
**4794**	319	A	73.6902	1
**4794**	320	A	68.8625	-1
**4794**	321	A	72.7214	0
**4794**	351	A	5.6704	1
**4794**	357	A	68.9194	1
**4794**	370	A	79.5006	1
**4794**	372	A	73.4594	0
**4794**	373	A	67.3898	1
**4794**	381	A	53.4974	1
**4794**	382	A	79.4733	1
**4794**	386	A	79.6043	1
**4794**	305	B	64.0261	1
**4794**	319	B	79.4979	1
**4794**	320	B	20.432	-1
**4794**	321	B	70.9145	0
**4794**	351	B	18.9671	1
**4794**	357	B	68.6146	1
**4794**	370	B	79.5453	1
**4794**	372	B	71.8441	0
**4794**	373	B	77.0341	1
**4794**	381	B	79.549	1
**4794**	382	B	65.2308	1
**4794**	386	B	79.514	1
**4794**	305	C	65.4459	1
**4794**	319	C	63.9483	1
**4794**	320	C	79.5452	-1
**4794**	321	C	69.9036	0

**4794**	351	C	3.5681	1
**4794**	357	C	79.6322	1
**4794**	370	C	79.6721	1
**4794**	372	C	50.3949	0
**4794**	373	C	79.5992	1
**4794**	381	C	79.529	1
**4794**	382	C	42.6749	0
**4794**	386	C	13.4445	-1
**4794**	305	D	72.2249	1
**4794**	319	D	65.3193	1
**4794**	320	D	70.6157	-1
**4794**	321	D	58.6842	0
**4794**	351	D	20.465	-1
**4794**	357	D	48.0813	0
**4794**	370	D	76.3716	1
**4794**	372	D	57.9931	0
**4794**	373	D	78.3801	1
**4794**	381	D	79.528	1
**4794**	382	D	65.7958	1
**4794**	386	D	74.6814	1
**4795**	305	A	79.5509	1
**4795**	319	A	74.1827	1
**4795**	320	A	62.4978	-1
**4795**	321	A	72.5747	0
**4795**	351	A	8.8827	1
**4795**	357	A	74.3916	1
**4795**	370	A	56.9736	1
**4795**	372	A	47.4656	1
**4795**	373	A	72.541	1
**4795**	381	A	51.9592	1
**4795**	382	A	66.6687	1
**4795**	386	A	54.8849	1
**4795**	305	B	56.394	1
**4795**	319	B	79.601	1

**4795**	320	B	69.818	-1
**4795**	321	B	77.0451	0
**4795**	351	B	17.7552	1
**4795**	357	B	79.5932	1
**4795**	370	B	56.7311	1
**4795**	372	B	79.5828	0
**4795**	373	B	45.141	0
**4795**	381	B	66.4455	1
**4795**	382	B	58.7172	1
**4795**	386	B	79.5943	1
**4795**	305	C	27.5858	0
**4795**	319	C	32.6223	0
**4795**	320	C	79.5019	-1
**4795**	321	C	29.9275	1
**4795**	351	C	11.6251	1
**4795**	357	C	64.9448	1
**4795**	370	C	71.5619	1
**4795**	372	C	79.7159	0
**4795**	373	C	44.9921	0
**4795**	381	C	58.7188	1
**4795**	382	C	66.7547	1
**4795**	386	C	70.7723	1
**4795**	305	D	58.5557	1
**4795**	319	D	74.38	1
**4795**	320	D	66.4249	-1
**4795**	321	D	62.9707	0
**4795**	351	D	17.266	1
**4795**	357	D	40.5694	0
**4795**	370	D	70.4032	1
**4795**	372	D	59.6396	0
**4795**	373	D	79.285	1
**4795**	381	D	79.4961	1
**4795**	382	D	79.6164	1
**4795**	386	D	79.4555	1

**4796**	305	A	74.9536	1
**4796**	319	A	58.1552	1
**4796**	320	A	45.0176	-1
**4796**	321	A	72.857	0
**4796**	351	A	9.0155	1
**4796**	357	A	73.6787	1
**4796**	370	A	79.5564	1
**4796**	372	A	79.6147	0
**4796**	373	A	21.125	0
**4796**	381	A	79.579	1
**4796**	382	A	27.8086	0
**4796**	386	A	35.8688	0
**4796**	305	B	79.6675	1
**4796**	319	B	69.9709	1
**4796**	320	B	62.5115	-1
**4796**	321	B	79.6191	0
**4796**	351	B	0.3902	1
**4796**	357	B	65.9369	1
**4796**	370	B	79.4835	1
**4796**	372	B	76.3289	0
**4796**	373	B	27.3292	0
**4796**	381	B	74.5499	1
**4796**	382	B	33.1822	0
**4796**	386	B	39.8963	0
**4796**	305	C	71.3051	1
**4796**	319	C	73.2446	1
**4796**	320	C	76.6379	-1
**4796**	321	C	69.5749	0
**4796**	351	C	0.9326	1
**4796**	357	C	73.296	1
**4796**	370	C	74.0349	1
**4796**	372	C	79.6331	0
**4796**	373	C	40.939	0
**4796**	381	C	70.49	1

**4796**	382	C	68.1587	1
**4796**	386	C	48.2455	0
**4796**	305	D	71.227	1
**4796**	319	D	79.5596	1
**4796**	320	D	63.5521	-1
**4796**	321	D	79.6274	0
**4796**	351	D	10.9874	1
**4796**	357	D	49.9788	0
**4796**	370	D	63.991	1
**4796**	372	D	70.2719	0
**4796**	373	D	56.4199	1
**4796**	381	D	79.5104	1
**4796**	382	D	79.5074	1
**4796**	386	D	72.277	1
**4797**	305	A	79.1453	1
**4797**	319	A	69.7234	1
**4797**	320	A	48.1069	-1
**4797**	321	A	56.9559	0
**4797**	351	A	8.21	1
**4797**	357	A	79.6835	1
**4797**	370	A	75.1223	1
**4797**	372	A	19.8819	0
**4797**	373	A	79.5805	1
**4797**	381	A	78.1964	1
**4797**	382	A	79.5026	1
**4797**	386	A	68.4962	1
**4797**	305	B	79.492	1
**4797**	319	B	61.9117	1
**4797**	320	B	79.5474	-1
**4797**	321	B	74.7254	0
**4797**	351	B	19.9449	1
**4797**	357	B	79.6033	1
**4797**	370	B	79.5175	1
**4797**	372	B	79.4899	0

**4797**	373	B	42.9028	0
**4797**	381	B	76.1288	1
**4797**	382	B	18.92	-1
**4797**	386	B	77.6597	1
**4797**	305	C	69.4792	1
**4797**	319	C	79.4234	1
**4797**	320	C	79.5998	-1
**4797**	321	C	79.5389	0
**4797**	351	C	11.4805	1
**4797**	357	C	79.4445	1
**4797**	370	C	51.3438	1
**4797**	372	C	77.8233	0
**4797**	373	C	66.6706	1
**4797**	381	C	45.3547	0
**4797**	382	C	79.5725	1
**4797**	386	C	65.6453	1
**4797**	305	D	79.5347	1
**4797**	319	D	78.2272	1
**4797**	320	D	79.6607	-1
**4797**	321	D	79.4683	0
**4797**	351	D	6.09	1
**4797**	357	D	77.7741	1
**4797**	370	D	45.8596	0
**4797**	372	D	79.4807	0
**4797**	373	D	73.1019	1
**4797**	381	D	73.1543	1
**4797**	382	D	72.8994	1
**4797**	386	D	79.5165	1
**4798**	305	A	78.5403	1
**4798**	319	A	73.7723	1
**4798**	320	A	62.4663	-1
**4798**	321	A	45.9603	1
**4798**	351	A	1.0517	1
**4798**	357	A	79.5537	1

**4798**	370	A	79.5765	1
**4798**	372	A	62.1464	0
**4798**	373	A	12.4235	-1
**4798**	381	A	61.6678	1
**4798**	382	A	70.1805	1
**4798**	386	A	79.596	1
**4798**	305	B	73.0965	1
**4798**	319	B	66.966	1
**4798**	320	B	72.143	-1
**4798**	321	B	79.5672	0
**4798**	351	B	9.7756	1
**4798**	357	B	79.3801	1
**4798**	370	B	72.819	1
**4798**	372	B	62.5798	0
**4798**	373	B	54.2942	1
**4798**	381	B	45.7827	0
**4798**	382	B	74.149	1
**4798**	386	B	74.0412	1
**4798**	305	C	62.9528	1
**4798**	319	C	58.3868	1
**4798**	320	C	65.5604	-1
**4798**	321	C	73.2875	0
**4798**	351	C	2.1802	1
**4798**	357	C	79.5079	1
**4798**	370	C	40.8709	0
**4798**	372	C	72.6721	0
**4798**	373	C	76.5956	1
**4798**	381	C	79.6002	1
**4798**	382	C	52.1113	1
**4798**	386	C	37.8015	0
**4798**	305	D	45.5766	0
**4798**	319	D	73.271	1
**4798**	320	D	79.5913	-1
**4798**	321	D	79.6318	0

**4798**	351	D	25.9896	-1
**4798**	357	D	57.8832	1
**4798**	370	D	30.7217	0
**4798**	372	D	72.1843	0
**4798**	373	D	79.5574	1
**4798**	381	D	67.2985	1
**4798**	382	D	9.1889	-1
**4798**	386	D	75.0417	1
**4799**	305	A	57.4512	1
**4799**	319	A	78.2881	1
**4799**	320	A	53.8978	-1
**4799**	321	A	79.5531	0
**4799**	351	A	15.3927	1
**4799**	357	A	79.4559	1
**4799**	370	A	60.5214	1
**4799**	372	A	69.1869	0
**4799**	373	A	71.6009	1
**4799**	381	A	71.579	1
**4799**	382	A	72.032	1
**4799**	386	A	79.5171	1
**4799**	305	B	75.0263	1
**4799**	319	B	73.6328	1
**4799**	320	B	79.5685	-1
**4799**	321	B	79.4708	0
**4799**	351	B	34.2914	-1
**4799**	357	B	30.7525	0
**4799**	370	B	53.6344	1
**4799**	372	B	47.9506	1
**4799**	373	B	72.1057	1
**4799**	381	B	50.0197	1
**4799**	382	B	79.5592	1
**4799**	386	B	79.5931	1
**4799**	305	C	32.5725	0
**4799**	319	C	78.1962	1

**4799**	320	C	73.6797	-1
**4799**	321	C	66.2122	0
**4799**	351	C	4.5051	1
**4799**	357	C	37.6977	0
**4799**	370	C	61.7906	1
**4799**	372	C	79.6326	0
**4799**	373	C	79.57	1
**4799**	381	C	53.0231	1
**4799**	382	C	79.6504	1
**4799**	386	C	46.934	0
**4799**	305	D	79.7163	1
**4799**	319	D	22.5566	0
**4799**	320	D	79.3433	-1
**4799**	321	D	79.5471	0
**4799**	351	D	7.9675	1
**4799**	357	D	79.5206	1
**4799**	370	D	68.6064	1
**4799**	372	D	73.1694	0
**4799**	373	D	79.485	1
**4799**	381	D	71.3025	1
**4799**	382	D	79.5235	1
**4799**	386	D	79.5351	1
**4800**	305	A	79.5976	1
**4800**	319	A	79.5175	1
**4800**	320	A	74.7693	-1
**4800**	321	A	49.4269	1
**4800**	351	A	5.5285	1
**4800**	357	A	53.6594	1
**4800**	370	A	79.5605	1
**4800**	372	A	72.8302	0
**4800**	373	A	56.5146	1
**4800**	381	A	15.3796	-1
**4800**	382	A	28.0546	0
**4800**	386	A	58.2102	1

**4800**	305	B	72.5909	1
**4800**	319	B	79.5213	1
**4800**	320	B	71.8828	-1
**4800**	321	B	79.5912	0
**4800**	351	B	12.6248	1
**4800**	357	B	78.6252	1
**4800**	370	B	79.5671	1
**4800**	372	B	79.5565	0
**4800**	373	B	71.9465	1
**4800**	381	B	72.9978	1
**4800**	382	B	79.6429	1
**4800**	386	B	61.5764	1
**4800**	305	C	70.5299	1
**4800**	319	C	79.4927	1
**4800**	320	C	65.2593	-1
**4800**	321	C	79.6198	0
**4800**	351	C	2.5769	1
**4800**	357	C	79.4953	1
**4800**	370	C	48.1364	0
**4800**	372	C	74.7117	0
**4800**	373	C	57.2045	1
**4800**	381	C	79.5254	1
**4800**	382	C	33.0411	0
**4800**	386	C	40.5228	0
**4800**	305	D	65.6955	1
**4800**	319	D	79.5346	1
**4800**	320	D	42.2809	-1
**4800**	321	D	79.576	0
**4800**	351	D	9.3673	1
**4800**	357	D	73.2962	1
**4800**	370	D	26.9938	0
**4800**	372	D	73.303	0
**4800**	373	D	52.8655	1
**4800**	381	D	79.5436	1

**4800**	382	D	79.6069	1
**4800**	386	D	78.6508	1
**4801**	305	A	71.9934	1
**4801**	319	A	51.2922	1
**4801**	320	A	45.4314	-1
**4801**	321	A	79.5778	0
**4801**	351	A	1.3882	1
**4801**	357	A	79.5298	1
**4801**	370	A	72.6254	1
**4801**	372	A	71.8056	0
**4801**	373	A	69.7105	1
**4801**	381	A	74.3019	1
**4801**	382	A	70.0079	1
**4801**	386	A	39.3681	0
**4801**	305	B	72.2451	1
**4801**	319	B	75.5321	1
**4801**	320	B	79.6095	-1
**4801**	321	B	79.5345	0
**4801**	351	B	21.2726	-1
**4801**	357	B	49.6611	0
**4801**	370	B	43.7077	0
**4801**	372	B	79.764	0
**4801**	373	B	46.9653	0
**4801**	381	B	63.8029	1
**4801**	382	B	60.099	1
**4801**	386	B	59.3702	1
**4801**	305	C	73.2676	1
**4801**	319	C	39.4014	0
**4801**	320	C	48.1534	-1
**4801**	321	C	59.9677	0
**4801**	351	C	10.1625	1
**4801**	357	C	79.6296	1
**4801**	370	C	70.51	1
**4801**	372	C	72.1256	0

**4801**	373	C	58.006	1
**4801**	381	C	59.2029	1
**4801**	382	C	62.6823	1
**4801**	386	C	58.8927	1
**4801**	305	D	65.8684	1
**4801**	319	D	73.8061	1
**4801**	320	D	61.7089	-1
**4801**	321	D	61.7791	0
**4801**	351	D	14.7761	1
**4801**	357	D	79.4882	1
**4801**	370	D	21.0227	0
**4801**	372	D	75.5639	0
**4801**	373	D	60.1564	1
**4801**	381	D	74.4106	1
**4801**	382	D	74.8755	1
**4801**	386	D	72.2553	1
**4802**	305	A	68.7514	1
**4802**	319	A	49.9915	0
**4802**	320	A	79.5962	-1
**4802**	321	A	58.7392	0
**4802**	351	A	4.7027	1
**4802**	357	A	72.538	1
**4802**	370	A	57.7661	1
**4802**	372	A	49.3337	1
**4802**	373	A	79.5269	1
**4802**	381	A	62.8073	1
**4802**	382	A	76.6902	1
**4802**	386	A	63.7015	1
**4802**	305	B	69.2075	1
**4802**	319	B	79.582	1
**4802**	320	B	60.1858	-1
**4802**	321	B	74.4889	0
**4802**	351	B	25.1156	-1
**4802**	357	B	79.5907	1

**4802**	370	B	50.5402	1
**4802**	372	B	79.6261	0
**4802**	373	B	66.9205	1
**4802**	381	B	59.7734	1
**4802**	382	B	76.4435	1
**4802**	386	B	71.6516	1
**4802**	305	C	79.6561	1
**4802**	319	C	67.5412	1
**4802**	320	C	79.6603	-1
**4802**	321	C	32.4856	1
**4802**	351	C	4.0367	1
**4802**	357	C	72.3318	1
**4802**	370	C	46.8541	0
**4802**	372	C	70.4394	0
**4802**	373	C	65.0092	1
**4802**	381	C	79.4563	1
**4802**	382	C	70.3059	1
**4802**	386	C	45.7859	0
**4802**	305	D	65.9059	1
**4802**	319	D	58.7714	1
**4802**	320	D	74.5799	-1
**4802**	321	D	79.5077	0
**4802**	351	D	9.7306	1
**4802**	357	D	79.5127	1
**4802**	370	D	46.0227	0
**4802**	372	D	73.481	0
**4802**	373	D	25.4983	0
**4802**	381	D	72.4038	1
**4802**	382	D	65.6911	1
**4802**	386	D	79.6312	1
**4803**	305	A	8.9454	-1
**4803**	319	A	79.4885	1
**4803**	320	A	58.2005	-1
**4803**	321	A	79.6257	0

**4803**	351	A	17.4803	1
**4803**	357	A	57.9165	1
**4803**	370	A	71.6894	1
**4803**	372	A	79.5222	0
**4803**	373	A	72.2864	1
**4803**	381	A	74.1821	1
**4803**	382	A	75.9095	1
**4803**	386	A	31.1306	0
**4803**	305	B	74.7145	1
**4803**	319	B	79.4047	1
**4803**	320	B	77.2591	-1
**4803**	321	B	79.5347	0
**4803**	351	B	19.5294	1
**4803**	357	B	61.3421	1
**4803**	370	B	36.9102	0
**4803**	372	B	73.7276	0
**4803**	373	B	49.9986	0
**4803**	381	B	34.3885	0
**4803**	382	B	57.8845	1
**4803**	386	B	61.6108	1
**4803**	305	C	53.9958	1
**4803**	319	C	58.2197	1
**4803**	320	C	79.6146	-1
**4803**	321	C	79.4979	0
**4803**	351	C	4.3871	1
**4803**	357	C	79.5455	1
**4803**	370	C	79.1758	1
**4803**	372	C	66.9217	0
**4803**	373	C	42.4183	0
**4803**	381	C	79.4906	1
**4803**	382	C	74.6363	1
**4803**	386	C	50.1205	1
**4803**	305	D	40.9553	0
**4803**	319	D	79.5826	1

**4803**	320	D	79.5673	-1
**4803**	321	D	79.5245	0
**4803**	351	D	2.8923	1
**4803**	357	D	34.3552	0
**4803**	370	D	68.8006	1
**4803**	372	D	48.8149	1
**4803**	373	D	79.5225	1
**4803**	381	D	79.4473	1
**4803**	382	D	78.0386	1
**4803**	386	D	54.5721	1
**4804**	305	A	79.6017	1
**4804**	319	A	68.2893	1
**4804**	320	A	61.2067	-1
**4804**	321	A	56.2927	0
**4804**	351	A	0.6415	1
**4804**	357	A	51.2458	1
**4804**	370	A	77.7209	1
**4804**	372	A	75.4478	0
**4804**	373	A	47.8462	0
**4804**	381	A	78.3811	1
**4804**	382	A	55.579	1
**4804**	386	A	61.1036	1
**4804**	305	B	79.531	1
**4804**	319	B	18.7698	-1
**4804**	320	B	29.2552	-1
**4804**	321	B	40.9594	1
**4804**	351	B	2.1427	1
**4804**	357	B	79.4765	1
**4804**	370	B	75.3968	1
**4804**	372	B	35.237	1
**4804**	373	B	39.5377	0
**4804**	381	B	71.6029	1
**4804**	382	B	30.7121	0
**4804**	386	B	67.7993	1

**4804**	305	C	42.8086	0
**4804**	319	C	72.1389	1
**4804**	320	C	79.3425	-1
**4804**	321	C	73.8323	0
**4804**	351	C	2.0408	1
**4804**	357	C	79.5348	1
**4804**	370	C	74.7197	1
**4804**	372	C	79.5193	0
**4804**	373	C	69.5466	1
**4804**	381	C	37.3899	0
**4804**	382	C	28.7862	0
**4804**	386	C	59.9116	1
**4804**	305	D	79.4629	1
**4804**	319	D	64.244	1
**4804**	320	D	68.3145	-1
**4804**	321	D	70.6631	0
**4804**	351	D	7.0948	1
**4804**	357	D	33.4751	0
**4804**	370	D	79.6039	1
**4804**	372	D	51.1195	0
**4804**	373	D	77.7914	1
**4804**	381	D	69.0667	1
**4804**	382	D	68.3595	1
**4804**	386	D	44.1192	0
**4805**	305	A	46.2597	0
**4805**	319	A	72.4212	1
**4805**	320	A	79.5475	-1
**4805**	321	A	79.637	0
**4805**	351	A	0.8058	1
**4805**	357	A	79.4226	1
**4805**	370	A	74.0682	1
**4805**	372	A	77.288	0
**4805**	373	A	64.0167	1
**4805**	381	A	73.919	1

**4805**	382	A	52.6262	1
**4805**	386	A	79.5648	1
**4805**	305	B	77.8421	1
**4805**	319	B	79.5923	1
**4805**	320	B	64.7809	-1
**4805**	321	B	79.5563	0
**4805**	351	B	8.0944	1
**4805**	357	B	72.917	1
**4805**	370	B	68.244	1
**4805**	372	B	53.0746	0
**4805**	373	B	61.0783	1
**4805**	381	B	25.6773	0
**4805**	382	B	59.6549	1
**4805**	386	B	53.7235	1
**4805**	305	C	65.6277	1
**4805**	319	C	79.5685	1
**4805**	320	C	20.1771	-1
**4805**	321	C	69.2699	0
**4805**	351	C	7.3946	1
**4805**	357	C	79.6535	1
**4805**	370	C	51.7398	1
**4805**	372	C	76.036	0
**4805**	373	C	69.0439	1
**4805**	381	C	60.2375	1
**4805**	382	C	63.0786	1
**4805**	386	C	54.6055	1
**4805**	305	D	70.6322	1
**4805**	319	D	79.5596	1
**4805**	320	D	73.014	-1
**4805**	321	D	66.644	0
**4805**	351	D	4.1122	1
**4805**	357	D	72.1887	1
**4805**	370	D	70.0263	1
**4805**	372	D	58.5468	0

**4805**	373	D	78.0286	1
**4805**	381	D	79.5337	1
**4805**	382	D	79.6289	1
**4805**	386	D	79.5124	1
**4806**	305	A	79.5675	1
**4806**	319	A	60.9555	1
**4806**	320	A	79.5619	-1
**4806**	321	A	39.4773	1
**4806**	351	A	21.3784	-1
**4806**	357	A	64.3851	1
**4806**	370	A	77.3194	1
**4806**	372	A	23.2749	1
**4806**	373	A	48.4999	0
**4806**	381	A	79.57	1
**4806**	382	A	79.5345	1
**4806**	386	A	73.6344	1
**4806**	305	B	79.5293	1
**4806**	319	B	79.5374	1
**4806**	320	B	72.8611	-1
**4806**	321	B	79.5874	0
**4806**	351	B	15.7061	1
**4806**	357	B	70.6241	1
**4806**	370	B	61.6726	1
**4806**	372	B	79.5637	0
**4806**	373	B	61.8204	1
**4806**	381	B	42.9094	0
**4806**	382	B	32.8583	0
**4806**	386	B	55.7375	1
**4806**	305	C	59.101	1
**4806**	319	C	49.3489	0
**4806**	320	C	22.1193	-1
**4806**	321	C	51.6937	0
**4806**	351	C	2.9672	1
**4806**	357	C	79.583	1

**4806**	370	C	76.7617	1
**4806**	372	C	51.2024	0
**4806**	373	C	79.5633	1
**4806**	381	C	66.924	1
**4806**	382	C	79.5441	1
**4806**	386	C	19.3952	-1
**4806**	305	D	79.5379	1
**4806**	319	D	79.5317	1
**4806**	320	D	72.8707	-1
**4806**	321	D	73.7157	0
**4806**	351	D	12.0025	1
**4806**	357	D	72.5152	1
**4806**	370	D	78.3813	1
**4806**	372	D	79.374	0
**4806**	373	D	79.5338	1
**4806**	381	D	79.5598	1
**4806**	382	D	66.6979	1
**4806**	386	D	53.3586	1
**4807**	305	A	78.6952	1
**4807**	319	A	72.1319	1
**4807**	320	A	72.6234	-1
**4807**	321	A	70.8881	0
**4807**	351	A	2.5699	1
**4807**	357	A	54.391	1
**4807**	370	A	35.8428	0
**4807**	372	A	41.8476	1
**4807**	373	A	64.6568	1
**4807**	381	A	70.8189	1
**4807**	382	A	69.1051	1
**4807**	386	A	29.7593	0
**4807**	305	B	37.5288	0
**4807**	319	B	68.5775	1
**4807**	320	B	70.7571	-1
**4807**	321	B	42.0908	1

**4807**	351	B	15.0611	1
**4807**	357	B	79.5845	1
**4807**	370	B	68.0779	1
**4807**	372	B	79.5717	0
**4807**	373	B	69.9912	1
**4807**	381	B	79.5809	1
**4807**	382	B	14.8041	-1
**4807**	386	B	53.4573	1
**4807**	305	C	79.6312	1
**4807**	319	C	73.126	1
**4807**	320	C	36.8557	-1
**4807**	321	C	55.2065	0
**4807**	351	C	9.5355	1
**4807**	357	C	71.3729	1
**4807**	370	C	79.6025	1
**4807**	372	C	60.0057	0
**4807**	373	C	79.5624	1
**4807**	381	C	77.8986	1
**4807**	382	C	48.6886	0
**4807**	386	C	37.898	0
**4807**	305	D	6.0194	-1
**4807**	319	D	79.443	1
**4807**	320	D	62.9385	-1
**4807**	321	D	74.4093	0
**4807**	351	D	11.8036	1
**4807**	357	D	79.593	1
**4807**	370	D	73.3179	1
**4807**	372	D	22.2384	1
**4807**	373	D	79.5719	1
**4807**	381	D	38.1748	0
**4807**	382	D	41.0218	0
**4807**	386	D	79.5786	1
**4808**	305	A	79.0738	1
**4808**	319	A	69.467	1

4808	320	A	64.5884	-1
4808	321	A	41.924	1
4808	351	A	5.2613	1
4808	357	A	70.2478	1
4808	370	A	71.2613	1
4808	372	A	79.5252	0
4808	373	A	57.4321	1
4808	381	A	78.8933	1
4808	382	A	79.5371	1
4808	386	A	59.5062	1
4808	305	B	79.5553	1
4808	319	B	70.1178	1
4808	320	B	77.8332	-1
4808	321	B	73.4856	0
4808	351	B	11.4026	1
4808	357	B	79.4494	1
4808	370	B	63.1228	1
4808	372	B	78.4669	0
4808	373	B	62.5445	1
4808	381	B	79.3831	1
4808	382	B	40.8202	0
4808	386	B	79.4778	1
4808	305	C	35.2688	0
4808	319	C	65.6626	1
4808	320	C	63.0694	-1
4808	321	C	53.0118	0
4808	351	C	7.5054	1
4808	357	C	75.5879	1
4808	370	C	66.9622	1
4808	372	C	79.483	0
4808	373	C	62.011	1
4808	381	C	79.5737	1
4808	382	C	48.2795	0
4808	386	C	41.5969	0

**4808**	305	D	59.5161	1
**4808**	319	D	52.5676	1
**4808**	320	D	66.44	-1
**4808**	321	D	65.1269	0
**4808**	351	D	15.6494	1
**4808**	357	D	79.5574	1
**4808**	370	D	42.5921	0
**4808**	372	D	79.5958	0
**4808**	373	D	71.5269	1
**4808**	381	D	79.0538	1
**4808**	382	D	79.5981	1
**4808**	386	D	79.4354	1
**4809**	305	A	79.5788	1
**4809**	319	A	69.1464	1
**4809**	320	A	72.5792	-1
**4809**	321	A	67.8413	0
**4809**	351	A	5.7806	1
**4809**	357	A	72.596	1
**4809**	370	A	45.8772	0
**4809**	372	A	53.2115	0
**4809**	373	A	45.7312	0
**4809**	381	A	79.5417	1
**4809**	382	A	72.9632	1
**4809**	386	A	74.3545	1
**4809**	305	B	79.515	1
**4809**	319	B	79.5628	1
**4809**	320	B	79.5376	-1
**4809**	321	B	30.7515	1
**4809**	351	B	10.5004	1
**4809**	357	B	72.3134	1
**4809**	370	B	60.3249	1
**4809**	372	B	79.5267	0
**4809**	373	B	72.6612	1
**4809**	381	B	79.5497	1

**4809**	382	B	77.175	1
**4809**	386	B	59.0293	1
**4809**	305	C	59.6419	1
**4809**	319	C	62.3692	1
**4809**	320	C	61.3324	-1
**4809**	321	C	60.4827	0
**4809**	351	C	12.972	1
**4809**	357	C	54.2761	1
**4809**	370	C	79.5466	1
**4809**	372	C	74.6031	0
**4809**	373	C	72.4453	1
**4809**	381	C	69.0788	1
**4809**	382	C	79.5819	1
**4809**	386	C	31.3232	0
**4809**	305	D	62.7937	1
**4809**	319	D	75.2042	1
**4809**	320	D	59.5651	-1
**4809**	321	D	79.4559	0
**4809**	351	D	7.1619	1
**4809**	357	D	74.7054	1
**4809**	370	D	38.2365	0
**4809**	372	D	72.3169	0
**4809**	373	D	69.6785	1
**4809**	381	D	37.3515	0
**4809**	382	D	61.9871	1
**4809**	386	D	59.4955	1
**4810**	305	A	54.039	1
**4810**	319	A	65.9427	1
**4810**	320	A	79.5435	-1
**4810**	321	A	65.5858	0
**4810**	351	A	1.2955	1
**4810**	357	A	79.5001	1
**4810**	370	A	66.7851	1
**4810**	372	A	79.7013	0

**4810**	373	A	62.6172	1
**4810**	381	A	67.5532	1
**4810**	382	A	68.4653	1
**4810**	386	A	76.1996	1
**4810**	305	B	79.4991	1
**4810**	319	B	79.6684	1
**4810**	320	B	78.2089	-1
**4810**	321	B	75.8367	0
**4810**	351	B	16.9461	1
**4810**	357	B	66.9729	1
**4810**	370	B	63.9791	1
**4810**	372	B	67.9364	0
**4810**	373	B	72.596	1
**4810**	381	B	76.5636	1
**4810**	382	B	31.038	0
**4810**	386	B	68.504	1
**4810**	305	C	75.3766	1
**4810**	319	C	79.6092	1
**4810**	320	C	62.9132	-1
**4810**	321	C	77.0615	0
**4810**	351	C	5.7819	1
**4810**	357	C	79.5427	1
**4810**	370	C	70.451	1
**4810**	372	C	79.3474	0
**4810**	373	C	71.0003	1
**4810**	381	C	72.5111	1
**4810**	382	C	24.3631	0
**4810**	386	C	48.6384	0
**4810**	305	D	76.2184	1
**4810**	319	D	79.5213	1
**4810**	320	D	69.2569	-1
**4810**	321	D	53.783	0
**4810**	351	D	4.9546	1
**4810**	357	D	79.5895	1

**4810**	370	D	55.2777	1
**4810**	372	D	53.549	0
**4810**	373	D	74.7698	1
**4810**	381	D	77.1363	1
**4810**	382	D	45.7205	0
**4810**	386	D	75.2392	1
**4811**	305	A	71.185	1
**4811**	319	A	71.3565	1
**4811**	320	A	79.6193	-1
**4811**	321	A	7.1995	0
**4811**	351	A	3.2527	1
**4811**	357	A	58.5694	1
**4811**	370	A	57.5929	1
**4811**	372	A	75.7526	0
**4811**	373	A	76.9483	1
**4811**	381	A	79.4543	1
**4811**	382	A	50.6766	1
**4811**	386	A	56.8735	1
**4811**	305	B	71.5478	1
**4811**	319	B	62.7803	1
**4811**	320	B	76.4511	-1
**4811**	321	B	76.8077	0
**4811**	351	B	8.3645	1
**4811**	357	B	73.3944	1
**4811**	370	B	64.8705	1
**4811**	372	B	79.2727	0
**4811**	373	B	79.5857	1
**4811**	381	B	75.1906	1
**4811**	382	B	26.169	0
**4811**	386	B	65.3053	1
**4811**	305	C	71.3816	1
**4811**	319	C	64.0876	1
**4811**	320	C	79.7118	-1
**4811**	321	C	79.5825	0

**4811**	351	C	0.0285	1
**4811**	357	C	78.2459	1
**4811**	370	C	79.5136	1
**4811**	372	C	79.3128	0
**4811**	373	C	75.3095	1
**4811**	381	C	57.472	1
**4811**	382	C	79.5987	1
**4811**	386	C	45.4704	0
**4811**	305	D	55.4574	1
**4811**	319	D	75.2887	1
**4811**	320	D	75.1135	-1
**4811**	321	D	79.6437	0
**4811**	351	D	12.6669	1
**4811**	357	D	43.8224	0
**4811**	370	D	67.1356	1
**4811**	372	D	79.5622	0
**4811**	373	D	59.6993	1
**4811**	381	D	71.2059	1
**4811**	382	D	68.7964	1
**4811**	386	D	57.8339	1
**4812**	305	A	54.4029	1
**4812**	319	A	79.4221	1
**4812**	320	A	59.4917	-1
**4812**	321	A	72.5805	0
**4812**	351	A	31.8092	-1
**4812**	357	A	74.7692	1
**4812**	370	A	79.5621	1
**4812**	372	A	79.5242	0
**4812**	373	A	66.06	1
**4812**	381	A	60.7084	1
**4812**	382	A	63.6101	1
**4812**	386	A	72.0649	1
**4812**	305	B	78.9909	1
**4812**	319	B	72.1769	1

**4812**	320	B	58.6704	-1
**4812**	321	B	72.8899	0
**4812**	351	B	22.7365	-1
**4812**	357	B	77.3698	1
**4812**	370	B	64.0096	1
**4812**	372	B	65.1301	0
**4812**	373	B	78.957	1
**4812**	381	B	79.6177	1
**4812**	382	B	33.9947	0
**4812**	386	B	75.7985	1
**4812**	305	C	45.1195	0
**4812**	319	C	58.7193	1
**4812**	320	C	46.2943	-1
**4812**	321	C	70.8938	0
**4812**	351	C	19.3244	1
**4812**	357	C	79.5764	1
**4812**	370	C	78.9258	1
**4812**	372	C	71.0005	0
**4812**	373	C	79.6098	1
**4812**	381	C	36.8877	0
**4812**	382	C	79.5783	1
**4812**	386	C	49.564	0
**4812**	305	D	79.577	1
**4812**	319	D	47.3342	0
**4812**	320	D	56.3356	-1
**4812**	321	D	51.1257	0
**4812**	351	D	26.7762	-1
**4812**	357	D	79.5335	1
**4812**	370	D	79.5481	1
**4812**	372	D	66.3108	0
**4812**	373	D	79.576	1
**4812**	381	D	78.6912	1
**4812**	382	D	74.8537	1
**4812**	386	D	73.0545	1

**4813**	305	A	73.0384	1
**4813**	319	A	78.1066	1
**4813**	320	A	29.7859	-1
**4813**	321	A	77.2773	0
**4813**	351	A	16.256	1
**4813**	357	A	79.5609	1
**4813**	370	A	70.3386	1
**4813**	372	A	34.1775	1
**4813**	373	A	79.5947	1
**4813**	381	A	79.4593	1
**4813**	382	A	69.4124	1
**4813**	386	A	75.027	1
**4813**	305	B	71.7085	1
**4813**	319	B	79.6995	1
**4813**	320	B	79.507	-1
**4813**	321	B	79.5404	0
**4813**	351	B	3.8166	1
**4813**	357	B	26.5403	0
**4813**	370	B	70.5432	1
**4813**	372	B	56.018	0
**4813**	373	B	79.5161	1
**4813**	381	B	79.546	1
**4813**	382	B	63.6603	1
**4813**	386	B	79.5988	1
**4813**	305	C	57.9842	1
**4813**	319	C	75.5557	1
**4813**	320	C	79.5847	-1
**4813**	321	C	79.5224	0
**4813**	351	C	6.6293	1
**4813**	357	C	75.0338	1
**4813**	370	C	79.5708	1
**4813**	372	C	20.171	1
**4813**	373	C	67.5449	1
**4813**	381	C	62.5811	1

**4813**	382	C	79.5033	1
**4813**	386	C	37.1295	0
**4813**	305	D	79.5932	1
**4813**	319	D	79.5248	1
**4813**	320	D	71.3608	-1
**4813**	321	D	62.561	0
**4813**	351	D	10.5253	1
**4813**	357	D	45.1945	0
**4813**	370	D	79.5248	1
**4813**	372	D	79.5761	0
**4813**	373	D	75.9151	1
**4813**	381	D	56.7202	1
**4813**	382	D	79.5576	1
**4813**	386	D	79.5991	1
**4814**	305	A	79.4983	1
**4814**	319	A	52.6731	1
**4814**	320	A	67.524	-1
**4814**	321	A	46.0925	1
**4814**	351	A	8.4244	1
**4814**	357	A	74.5818	1
**4814**	370	A	51.2729	1
**4814**	372	A	77.5304	0
**4814**	373	A	77.0182	1
**4814**	381	A	79.6559	1
**4814**	382	A	79.6849	1
**4814**	386	A	73.15	1
**4814**	305	B	73.5234	1
**4814**	319	B	71.9792	1
**4814**	320	B	79.5535	-1
**4814**	321	B	73.6783	0
**4814**	351	B	5.5355	1
**4814**	357	B	72.3771	1
**4814**	370	B	39.4563	0
**4814**	372	B	55.8041	0

**4814**	373	B	74.2019	1
**4814**	381	B	60.3619	1
**4814**	382	B	79.5336	1
**4814**	386	B	56.5626	1
**4814**	305	C	62.7629	1
**4814**	319	C	76.9687	1
**4814**	320	C	78.849	-1
**4814**	321	C	79.8202	0
**4814**	351	C	6.9194	1
**4814**	357	C	48.8582	0
**4814**	370	C	77.3851	1
**4814**	372	C	73.0208	0
**4814**	373	C	79.552	1
**4814**	381	C	70.7996	1
**4814**	382	C	72.8288	1
**4814**	386	C	38.1325	0
**4814**	305	D	58.0814	1
**4814**	319	D	62.8295	1
**4814**	320	D	74.8842	-1
**4814**	321	D	51.5861	0
**4814**	351	D	22.3135	-1
**4814**	357	D	69.9978	1
**4814**	370	D	74.6247	1
**4814**	372	D	35.9342	1
**4814**	373	D	64.9294	1
**4814**	381	D	66.6045	1
**4814**	382	D	74.2096	1
**4814**	386	D	73.9059	1
**4815**	305	A	78.6936	1
**4815**	319	A	24.2483	0
**4815**	320	A	72.5939	-1
**4815**	321	A	64.6666	0
**4815**	351	A	9.9639	1
**4815**	357	A	76.2081	1

**4815**	370	A	54.899	1
**4815**	372	A	71.2789	0
**4815**	373	A	52.3842	1
**4815**	381	A	71.47	1
**4815**	382	A	70.2852	1
**4815**	386	A	73.1701	1
**4815**	305	B	74.9659	1
**4815**	319	B	69.8911	1
**4815**	320	B	63.7641	-1
**4815**	321	B	51.9312	0
**4815**	351	B	17.6715	1
**4815**	357	B	78.8993	1
**4815**	370	B	63.6756	1
**4815**	372	B	79.4536	0
**4815**	373	B	79.4582	1
**4815**	381	B	79.5856	1
**4815**	382	B	28.3653	0
**4815**	386	B	79.5211	1
**4815**	305	C	66.575	1
**4815**	319	C	77.4824	1
**4815**	320	C	66.1912	-1
**4815**	321	C	75.2515	0
**4815**	351	C	11.5044	1
**4815**	357	C	50.9441	1
**4815**	370	C	59.6986	1
**4815**	372	C	77.4943	0
**4815**	373	C	44.4557	0
**4815**	381	C	40.7936	0
**4815**	382	C	39.6981	0
**4815**	386	C	55.2476	1
**4815**	305	D	44.3711	0
**4815**	319	D	62.8362	1
**4815**	320	D	77.8175	-1
**4815**	321	D	50.05	0

**4815**	351	D	17.7349	1
**4815**	357	D	78.6684	1
**4815**	370	D	77.5567	1
**4815**	372	D	73.4788	0
**4815**	373	D	68.111	1
**4815**	381	D	51.11	1
**4815**	382	D	79.5266	1
**4815**	386	D	79.6773	1
**4816**	305	A	72.6244	1
**4816**	319	A	79.4787	1
**4816**	320	A	79.5653	-1
**4816**	321	A	64.2596	0
**4816**	351	A	8.3439	1
**4816**	357	A	43.159	0
**4816**	370	A	79.4532	1
**4816**	372	A	58.955	0
**4816**	373	A	78.6152	1
**4816**	381	A	69.4468	1
**4816**	382	A	70.6214	1
**4816**	386	A	74.9913	1
**4816**	305	B	75.1703	1
**4816**	319	B	44.4071	0
**4816**	320	B	37.54	-1
**4816**	321	B	71.9834	0
**4816**	351	B	6.3036	1
**4816**	357	B	73.0832	1
**4816**	370	B	71.5718	1
**4816**	372	B	79.5969	0
**4816**	373	B	62.8656	1
**4816**	381	B	79.6249	1
**4816**	382	B	68.4735	1
**4816**	386	B	56.9024	1
**4816**	305	C	64.8728	1
**4816**	319	C	31.4896	0

**4816**	320	C	73.3615	-1
**4816**	321	C	73.0574	0
**4816**	351	C	5.4942	1
**4816**	357	C	75.6542	1
**4816**	370	C	74.2175	1
**4816**	372	C	79.5187	0
**4816**	373	C	79.5353	1
**4816**	381	C	73.8417	1
**4816**	382	C	78.1029	1
**4816**	386	C	40.9939	0
**4816**	305	D	69.7033	1
**4816**	319	D	79.5007	1
**4816**	320	D	75.8458	-1
**4816**	321	D	46.7027	1
**4816**	351	D	14.0502	1
**4816**	357	D	74.2061	1
**4816**	370	D	39.2319	0
**4816**	372	D	79.5938	0
**4816**	373	D	79.5115	1
**4816**	381	D	60.1274	1
**4816**	382	D	79.5512	1
**4816**	386	D	79.5116	1
**4817**	305	A	68.0392	1
**4817**	319	A	7.6167	-1
**4817**	320	A	79.4597	-1
**4817**	321	A	35.2028	1
**4817**	351	A	9.8556	1
**4817**	357	A	79.5368	1
**4817**	370	A	79.6272	1
**4817**	372	A	79.6023	0
**4817**	373	A	68.0438	1
**4817**	381	A	69.5234	1
**4817**	382	A	79.5539	1
**4817**	386	A	66.18	1

**4817**	305	B	31.2399	0
**4817**	319	B	71.7325	1
**4817**	320	B	17.6377	1
**4817**	321	B	79.5558	0
**4817**	351	B	0.0012	1
**4817**	357	B	47.2248	0
**4817**	370	B	71.2483	1
**4817**	372	B	79.5697	0
**4817**	373	B	58.6685	1
**4817**	381	B	79.5721	1
**4817**	382	B	79.6081	1
**4817**	386	B	79.568	1
**4817**	305	C	55.2029	1
**4817**	319	C	65.7757	1
**4817**	320	C	43.4301	-1
**4817**	321	C	79.5433	0
**4817**	351	C	1.8512	1
**4817**	357	C	71.722	1
**4817**	370	C	79.5403	1
**4817**	372	C	78.3256	0
**4817**	373	C	79.5623	1
**4817**	381	C	72.5652	1
**4817**	382	C	73.5734	1
**4817**	386	C	43.5681	0
**4817**	305	D	68.8447	1
**4817**	319	D	65.1126	1
**4817**	320	D	10.3864	1
**4817**	321	D	70.3169	0
**4817**	351	D	9.4925	1
**4817**	357	D	42.2657	0
**4817**	370	D	79.5114	1
**4817**	372	D	61.35	0
**4817**	373	D	41.5333	0
**4817**	381	D	78.8108	1

**4817**	382	D	53.3866	1
**4817**	386	D	44.0477	0
**4818**	305	A	67.5517	1
**4818**	319	A	58.349	1
**4818**	320	A	79.5368	-1
**4818**	321	A	70.4077	0
**4818**	351	A	8.1729	1
**4818**	357	A	79.5435	1
**4818**	370	A	41.0358	0
**4818**	372	A	49.4183	1
**4818**	373	A	77.446	1
**4818**	381	A	71.5441	1
**4818**	382	A	50.9613	1
**4818**	386	A	55.8324	1
**4818**	305	B	62.8288	1
**4818**	319	B	60.4357	1
**4818**	320	B	53.9843	-1
**4818**	321	B	79.5114	0
**4818**	351	B	1.692	1
**4818**	357	B	17.4491	-1
**4818**	370	B	67.3314	1
**4818**	372	B	60.092	0
**4818**	373	B	68.9452	1
**4818**	381	B	22.0743	0
**4818**	382	B	20.7185	0
**4818**	386	B	72.3806	1
**4818**	305	C	64.7419	1
**4818**	319	C	76.0778	1
**4818**	320	C	77.4115	-1
**4818**	321	C	21.6651	1
**4818**	351	C	37.6782	-1
**4818**	357	C	57.6475	1
**4818**	370	C	79.612	1
**4818**	372	C	63.1469	0

**4818**	373	C	71.3668	1
**4818**	381	C	67.5084	1
**4818**	382	C	37.1061	0
**4818**	386	C	51.8884	1
**4818**	305	D	31.9445	0
**4818**	319	D	45.9931	0
**4818**	320	D	68.6501	-1
**4818**	321	D	43.8435	1
**4818**	351	D	21.7041	-1
**4818**	357	D	69.9513	1
**4818**	370	D	73.5314	1
**4818**	372	D	79.5154	0
**4818**	373	D	79.5745	1
**4818**	381	D	74.3997	1
**4818**	382	D	79.6081	1
**4818**	386	D	79.275	1
**4819**	305	A	44.3377	0
**4819**	319	A	54.013	1
**4819**	320	A	79.5677	-1
**4819**	321	A	71.0055	0
**4819**	351	A	4.8514	1
**4819**	357	A	74.6123	1
**4819**	370	A	79.4216	1
**4819**	372	A	79.5893	0
**4819**	373	A	79.5775	1
**4819**	381	A	79.5274	1
**4819**	382	A	56.5092	1
**4819**	386	A	40.4295	0
**4819**	305	B	64.6183	1
**4819**	319	B	65.1996	1
**4819**	320	B	52.2767	-1
**4819**	321	B	34.3327	1
**4819**	351	B	13.4973	1
**4819**	357	B	39.4443	0

**4819**	370	B	74.6834	1
**4819**	372	B	79.5659	0
**4819**	373	B	79.5209	1
**4819**	381	B	79.5637	1
**4819**	382	B	74.1784	1
**4819**	386	B	63.3255	1
**4819**	305	C	79.523	1
**4819**	319	C	79.5656	1
**4819**	320	C	42.0511	-1
**4819**	321	C	53.7354	0
**4819**	351	C	6.527	1
**4819**	357	C	79.5634	1
**4819**	370	C	34.8525	0
**4819**	372	C	79.5416	0
**4819**	373	C	47.737	0
**4819**	381	C	79.5747	1
**4819**	382	C	47.9926	0
**4819**	386	C	48.2711	0
**4819**	305	D	52.8871	1
**4819**	319	D	49.7844	0
**4819**	320	D	65.6964	-1
**4819**	321	D	79.652	0
**4819**	351	D	14.4926	1
**4819**	357	D	66.7565	1
**4819**	370	D	74.6513	1
**4819**	372	D	79.5777	0
**4819**	373	D	79.5515	1
**4819**	381	D	73.111	1
**4819**	382	D	60.0712	1
**4819**	386	D	79.5364	1
**4820**	305	A	73.9464	1
**4820**	319	A	69.5474	1
**4820**	320	A	79.5217	-1
**4820**	321	A	59.7561	0

**4820**	351	A	12.4179	1
**4820**	357	A	79.5575	1
**4820**	370	A	38.4981	0
**4820**	372	A	73.5806	0
**4820**	373	A	72.2843	1
**4820**	381	A	78.0325	1
**4820**	382	A	63.1109	1
**4820**	386	A	77.6208	1
**4820**	305	B	71.1647	1
**4820**	319	B	72.8324	1
**4820**	320	B	74.0286	-1
**4820**	321	B	69.5156	0
**4820**	351	B	30.5101	-1
**4820**	357	B	46.0633	0
**4820**	370	B	73.1027	1
**4820**	372	B	44.4553	1
**4820**	373	B	57.0073	1
**4820**	381	B	23.7276	0
**4820**	382	B	46.3878	0
**4820**	386	B	71.0983	1
**4820**	305	C	76.7603	1
**4820**	319	C	79.5675	1
**4820**	320	C	67.7675	-1
**4820**	321	C	78.4345	0
**4820**	351	C	0.1884	1
**4820**	357	C	50.9042	1
**4820**	370	C	79.5457	1
**4820**	372	C	70.6759	0
**4820**	373	C	71.1875	1
**4820**	381	C	73.0055	1
**4820**	382	C	72.3281	1
**4820**	386	C	46.5108	0
**4820**	305	D	72.3169	1
**4820**	319	D	73.9937	1

**4820**	320	D	79.62	-1
**4820**	321	D	78.5115	0
**4820**	351	D	8.5749	1
**4820**	357	D	64.3994	1
**4820**	370	D	23.195	0
**4820**	372	D	60.3124	0
**4820**	373	D	56.4683	1
**4820**	381	D	71.1766	1
**4820**	382	D	79.4058	1
**4820**	386	D	65.4627	1
**4821**	305	A	56.2397	1
**4821**	319	A	41.3086	0
**4821**	320	A	72.088	-1
**4821**	321	A	74.0018	0
**4821**	351	A	7.2808	1
**4821**	357	A	79.5493	1
**4821**	370	A	35.1292	0
**4821**	372	A	79.4932	0
**4821**	373	A	78.6978	1
**4821**	381	A	79.5811	1
**4821**	382	A	43.9123	0
**4821**	386	A	56.5713	1
**4821**	305	B	40.8972	0
**4821**	319	B	77.5226	1
**4821**	320	B	79.5617	-1
**4821**	321	B	73.3432	0
**4821**	351	B	16.506	1
**4821**	357	B	72.1146	1
**4821**	370	B	49.9396	0
**4821**	372	B	59.8673	0
**4821**	373	B	43.3924	0
**4821**	381	B	79.5916	1
**4821**	382	B	76.4701	1
**4821**	386	B	64.1278	1

**4821**	305	C	47.9682	0
**4821**	319	C	51.1073	1
**4821**	320	C	59.5145	-1
**4821**	321	C	69.2832	0
**4821**	351	C	4.4144	1
**4821**	357	C	77.4422	1
**4821**	370	C	65.4699	1
**4821**	372	C	79.5482	0
**4821**	373	C	79.5639	1
**4821**	381	C	57.328	1
**4821**	382	C	79.5459	1
**4821**	386	C	20.3367	0
**4821**	305	D	70.265	1
**4821**	319	D	47.111	0
**4821**	320	D	74.5194	-1
**4821**	321	D	72.9977	0
**4821**	351	D	9.7002	1
**4821**	357	D	56.1446	1
**4821**	370	D	74.6307	1
**4821**	372	D	55.9952	0
**4821**	373	D	48.9111	0
**4821**	381	D	79.5501	1
**4821**	382	D	79.547	1
**4821**	386	D	65.5598	1
**4822**	305	A	46.6255	0
**4822**	319	A	57.0217	1
**4822**	320	A	73.2178	-1
**4822**	321	A	59.3269	0
**4822**	351	A	4.2224	1
**4822**	357	A	56.4483	1
**4822**	370	A	78.0309	1
**4822**	372	A	64.3433	0
**4822**	373	A	68.7335	1
**4822**	381	A	73.5854	1

**4822**	382	A	50.5692	1
**4822**	386	A	79.5267	1
**4822**	305	B	72.4742	1
**4822**	319	B	72.8563	1
**4822**	320	B	28.1351	-1
**4822**	321	B	62.0142	0
**4822**	351	B	15.5413	1
**4822**	357	B	72.325	1
**4822**	370	B	54.532	1
**4822**	372	B	32.8328	1
**4822**	373	B	39.2066	0
**4822**	381	B	79.6302	1
**4822**	382	B	36.4296	0
**4822**	386	B	58.0914	1
**4822**	305	C	67.0065	1
**4822**	319	C	73.0205	1
**4822**	320	C	73.3256	-1
**4822**	321	C	79.6421	0
**4822**	351	C	2.5471	1
**4822**	357	C	72.6167	1
**4822**	370	C	76.0638	1
**4822**	372	C	67.5248	0
**4822**	373	C	73.7531	1
**4822**	381	C	79.5705	1
**4822**	382	C	64.0069	1
**4822**	386	C	41.404	0
**4822**	305	D	66.5221	1
**4822**	319	D	79.5671	1
**4822**	320	D	72.9946	-1
**4822**	321	D	79.5521	0
**4822**	351	D	17.0757	1
**4822**	357	D	79.5942	1
**4822**	370	D	79.5615	1
**4822**	372	D	79.5404	0

**4822**	373	D	72.3569	1
**4822**	381	D	79.5586	1
**4822**	382	D	79.5311	1
**4822**	386	D	77.0755	1
**4823**	305	A	61.5724	1
**4823**	319	A	79.4656	1
**4823**	320	A	76.1295	-1
**4823**	321	A	49.562	1
**4823**	351	A	2.8079	1
**4823**	357	A	75.7019	1
**4823**	370	A	70.769	1
**4823**	372	A	75.6139	0
**4823**	373	A	47.4057	0
**4823**	381	A	64.8355	1
**4823**	382	A	61.7028	1
**4823**	386	A	79.3894	1
**4823**	305	B	64.3776	1
**4823**	319	B	72.4056	1
**4823**	320	B	71.3073	-1
**4823**	321	B	79.6715	0
**4823**	351	B	11.9014	1
**4823**	357	B	73.3614	1
**4823**	370	B	64.3123	1
**4823**	372	B	62.1569	0
**4823**	373	B	71.3727	1
**4823**	381	B	58.009	1
**4823**	382	B	55.0273	1
**4823**	386	B	76.7845	1
**4823**	305	C	40.3741	0
**4823**	319	C	69.7531	1
**4823**	320	C	79.5503	-1
**4823**	321	C	73.7978	0
**4823**	351	C	5.9604	1
**4823**	357	C	72.625	1

**4823**	370	C	59.5556	1
**4823**	372	C	73.4205	0
**4823**	373	C	72.5523	1
**4823**	381	C	79.6543	1
**4823**	382	C	61.1663	1
**4823**	386	C	47.6126	0
**4823**	305	D	62.8811	1
**4823**	319	D	79.0036	1
**4823**	320	D	79.4834	-1
**4823**	321	D	77.9319	0
**4823**	351	D	0.4535	1
**4823**	357	D	79.528	1
**4823**	370	D	60.1467	1
**4823**	372	D	79.144	0
**4823**	373	D	73.2945	1
**4823**	381	D	79.5639	1
**4823**	382	D	74.8318	1
**4823**	386	D	57.2998	1
**4824**	305	A	65.2015	1
**4824**	319	A	79.54	1
**4824**	320	A	78.8016	-1
**4824**	321	A	64.946	0
**4824**	351	A	0.4359	1
**4824**	357	A	65.0951	1
**4824**	370	A	51.0728	1
**4824**	372	A	79.5948	0
**4824**	373	A	10.8437	-1
**4824**	381	A	73.7323	1
**4824**	382	A	75.3878	1
**4824**	386	A	79.6159	1
**4824**	305	B	79.4682	1
**4824**	319	B	75.62	1
**4824**	320	B	66.2221	-1
**4824**	321	B	79.5651	0

**4824**	351	B	14.918	1
**4824**	357	B	79.6003	1
**4824**	370	B	64.963	1
**4824**	372	B	48.9521	1
**4824**	373	B	62.2643	1
**4824**	381	B	14.5577	-1
**4824**	382	B	32.2302	0
**4824**	386	B	79.7076	1
**4824**	305	C	65.2201	1
**4824**	319	C	74.1993	1
**4824**	320	C	50.2858	-1
**4824**	321	C	77.244	0
**4824**	351	C	7.7376	1
**4824**	357	C	79.4864	1
**4824**	370	C	72.0535	1
**4824**	372	C	78.4231	0
**4824**	373	C	63.0526	1
**4824**	381	C	33.3526	0
**4824**	382	C	62.3001	1
**4824**	386	C	21.7847	0
**4824**	305	D	23.9479	0
**4824**	319	D	79.7299	1
**4824**	320	D	78.2744	-1
**4824**	321	D	78.747	0
**4824**	351	D	21.8587	-1
**4824**	357	D	27.5489	0
**4824**	370	D	79.5815	1
**4824**	372	D	75.5935	0
**4824**	373	D	71.3747	1
**4824**	381	D	78.9021	1
**4824**	382	D	71.742	1
**4824**	386	D	79.6019	1
**4825**	305	A	79.633	1
**4825**	319	A	70.9075	1

**4825**	320	A	35.0968	-1
**4825**	321	A	57.003	0
**4825**	351	A	5.1842	1
**4825**	357	A	65.4094	1
**4825**	370	A	48.9245	0
**4825**	372	A	61.7492	0
**4825**	373	A	71.6574	1
**4825**	381	A	26.0851	0
**4825**	382	A	77.6407	1
**4825**	386	A	79.6552	1
**4825**	305	B	79.5973	1
**4825**	319	B	72.8424	1
**4825**	320	B	79.6174	-1
**4825**	321	B	71.7483	0
**4825**	351	B	18.6132	1
**4825**	357	B	79.6813	1
**4825**	370	B	73.1815	1
**4825**	372	B	73.095	0
**4825**	373	B	59.9759	1
**4825**	381	B	79.6681	1
**4825**	382	B	60.7093	1
**4825**	386	B	79.5474	1
**4825**	305	C	55.2266	1
**4825**	319	C	37.7909	0
**4825**	320	C	69.0272	-1
**4825**	321	C	77.6903	0
**4825**	351	C	2.9025	1
**4825**	357	C	70.1029	1
**4825**	370	C	76.8263	1
**4825**	372	C	79.7672	0
**4825**	373	C	52.2444	1
**4825**	381	C	36.2036	0
**4825**	382	C	73.3275	1
**4825**	386	C	55.5642	1

**4825**	305	D	79.546	1
**4825**	319	D	69.8723	1
**4825**	320	D	74.8112	-1
**4825**	321	D	79.6542	0
**4825**	351	D	15.1796	1
**4825**	357	D	69.9534	1
**4825**	370	D	42.0719	0
**4825**	372	D	52.4525	0
**4825**	373	D	10.1596	-1
**4825**	381	D	56.7653	1
**4825**	382	D	79.513	1
**4825**	386	D	63.1678	1
**4826**	305	A	62.1293	1
**4826**	319	A	79.5417	1
**4826**	320	A	72.5389	-1
**4826**	321	A	79.6627	0
**4826**	351	A	6.3205	1
**4826**	357	A	79.4961	1
**4826**	370	A	79.4952	1
**4826**	372	A	71.2556	0
**4826**	373	A	79.5848	1
**4826**	381	A	30.5796	0
**4826**	382	A	79.4705	1
**4826**	386	A	79.5643	1
**4826**	305	B	72.6507	1
**4826**	319	B	79.5607	1
**4826**	320	B	54.5316	-1
**4826**	321	B	75.0858	0
**4826**	351	B	15.4053	1
**4826**	357	B	52.228	1
**4826**	370	B	79.5802	1
**4826**	372	B	73.9716	0
**4826**	373	B	74.652	1
**4826**	381	B	77.8168	1

**4826**	382	B	36.8308	0
**4826**	386	B	79.6764	1
**4826**	305	C	67.9965	1
**4826**	319	C	40.925	0
**4826**	320	C	79.6568	-1
**4826**	321	C	79.1703	0
**4826**	351	C	5.9041	1
**4826**	357	C	49.1909	0
**4826**	370	C	77.8042	1
**4826**	372	C	79.5521	0
**4826**	373	C	79.5847	1
**4826**	381	C	26.6939	0
**4826**	382	C	79.4843	1
**4826**	386	C	13.1161	-1
**4826**	305	D	74.9519	1
**4826**	319	D	79.6022	1
**4826**	320	D	52.3024	-1
**4826**	321	D	57.0153	0
**4826**	351	D	13.8782	1
**4826**	357	D	57.8413	1
**4826**	370	D	69.5678	1
**4826**	372	D	73.3689	0
**4826**	373	D	39.2849	0
**4826**	381	D	45.9348	0
**4826**	382	D	76.6901	1
**4826**	386	D	27.8709	0
**4827**	305	A	79.3518	1
**4827**	319	A	72.6321	1
**4827**	320	A	79.3959	-1
**4827**	321	A	69.4011	0
**4827**	351	A	20.7139	-1
**4827**	357	A	65.0876	1
**4827**	370	A	79.4865	1
**4827**	372	A	77.5263	0

**4827**	373	A	79.6151	1
**4827**	381	A	79.7977	1
**4827**	382	A	79.0121	1
**4827**	386	A	49.7093	0
**4827**	305	B	68.461	1
**4827**	319	B	72.7106	1
**4827**	320	B	78.2802	-1
**4827**	321	B	73.5429	0
**4827**	351	B	14.7149	1
**4827**	357	B	67.7562	1
**4827**	370	B	73.1682	1
**4827**	372	B	79.4577	0
**4827**	373	B	37.5614	0
**4827**	381	B	72.7397	1
**4827**	382	B	79.6865	1
**4827**	386	B	76.6399	1
**4827**	305	C	79.2457	1
**4827**	319	C	65.0111	1
**4827**	320	C	66.036	-1
**4827**	321	C	58.1769	0
**4827**	351	C	2.3761	1
**4827**	357	C	79.3278	1
**4827**	370	C	76.1346	1
**4827**	372	C	56.7979	0
**4827**	373	C	75.2328	1
**4827**	381	C	11.4678	-1
**4827**	382	C	64.9746	1
**4827**	386	C	55.1636	1
**4827**	305	D	79.5918	1
**4827**	319	D	61.2153	1
**4827**	320	D	76.9556	-1
**4827**	321	D	69.549	0
**4827**	351	D	6.1321	1
**4827**	357	D	79.3541	1

**4827**	370	D	62.3355	1
**4827**	372	D	79.4262	0
**4827**	373	D	44.3959	0
**4827**	381	D	61.6166	1
**4827**	382	D	67.9029	1
**4827**	386	D	44.4199	0
**4828**	305	A	34.5199	0
**4828**	319	A	79.542	1
**4828**	320	A	64.2944	-1
**4828**	321	A	68.4119	0
**4828**	351	A	14.828	1
**4828**	357	A	56.7342	1
**4828**	370	A	79.6641	1
**4828**	372	A	76.4477	0
**4828**	373	A	79.5516	1
**4828**	381	A	61.5419	1
**4828**	382	A	79.0288	1
**4828**	386	A	61.5696	1
**4828**	305	B	66.6572	1
**4828**	319	B	70.6275	1
**4828**	320	B	79.5462	-1
**4828**	321	B	79.5875	0
**4828**	351	B	1.1506	1
**4828**	357	B	56.6624	1
**4828**	370	B	54.7033	1
**4828**	372	B	47.7755	1
**4828**	373	B	79.4581	1
**4828**	381	B	55.9474	1
**4828**	382	B	32.0692	0
**4828**	386	B	38.3387	0
**4828**	305	C	45.7092	0
**4828**	319	C	48.5316	0
**4828**	320	C	79.6011	-1
**4828**	321	C	79.5403	0

**4828**	351	C	1.847	1
**4828**	357	C	63.1817	1
**4828**	370	C	73.4165	1
**4828**	372	C	79.6849	0
**4828**	373	C	72.044	1
**4828**	381	C	79.371	1
**4828**	382	C	69.7392	1
**4828**	386	C	55.8255	1
**4828**	305	D	79.781	1
**4828**	319	D	67.3412	1
**4828**	320	D	46.904	-1
**4828**	321	D	72.8246	0
**4828**	351	D	1.5255	1
**4828**	357	D	41.5022	0
**4828**	370	D	77.7971	1
**4828**	372	D	72.2826	0
**4828**	373	D	71.4225	1
**4828**	381	D	34.6868	0
**4828**	382	D	73.4093	1
**4828**	386	D	71.3722	1
**4829**	305	A	67.7795	1
**4829**	319	A	79.5323	1
**4829**	320	A	72.3909	-1
**4829**	321	A	64.147	0
**4829**	351	A	4.6268	1
**4829**	357	A	77.5529	1
**4829**	370	A	79.5922	1
**4829**	372	A	61.9912	0
**4829**	373	A	74.5965	1
**4829**	381	A	59.3714	1
**4829**	382	A	28.2647	0
**4829**	386	A	79.5568	1
**4829**	305	B	37.235	0
**4829**	319	B	78.5127	1

**4829**	320	B	72.2738	-1
**4829**	321	B	66.732	0
**4829**	351	B	8.028	1
**4829**	357	B	75.3309	1
**4829**	370	B	66.0447	1
**4829**	372	B	73.0113	0
**4829**	373	B	59.4966	1
**4829**	381	B	71.7471	1
**4829**	382	B	57.5131	1
**4829**	386	B	69.2631	1
**4829**	305	C	41.4458	0
**4829**	319	C	70.564	1
**4829**	320	C	66.0804	-1
**4829**	321	C	64.4612	0
**4829**	351	C	15.5417	1
**4829**	357	C	79.6274	1
**4829**	370	C	79.6018	1
**4829**	372	C	56.1098	0
**4829**	373	C	79.5967	1
**4829**	381	C	33.2679	0
**4829**	382	C	57.3566	1
**4829**	386	C	49.8096	0
**4829**	305	D	64.1517	1
**4829**	319	D	79.5435	1
**4829**	320	D	66.4246	-1
**4829**	321	D	62.3326	0
**4829**	351	D	12.8579	1
**4829**	357	D	77.947	1
**4829**	370	D	79.5828	1
**4829**	372	D	79.6173	0
**4829**	373	D	79.6544	1
**4829**	381	D	21.9815	0
**4829**	382	D	79.5265	1
**4829**	386	D	74.6849	1

**4830**	305	A	60.542	1
**4830**	319	A	78.7304	1
**4830**	320	A	69.3634	-1
**4830**	321	A	72.1025	0
**4830**	351	A	3.0859	1
**4830**	357	A	64.1128	1
**4830**	370	A	79.5628	1
**4830**	372	A	24.1121	1
**4830**	373	A	79.5544	1
**4830**	381	A	46.2593	0
**4830**	382	A	72.3807	1
**4830**	386	A	61.5632	1
**4830**	305	B	73.132	1
**4830**	319	B	37.67	0
**4830**	320	B	61.8353	-1
**4830**	321	B	79.25	0
**4830**	351	B	16.7275	1
**4830**	357	B	40.8198	0
**4830**	370	B	79.4884	1
**4830**	372	B	76.088	0
**4830**	373	B	69.4676	1
**4830**	381	B	61.8751	1
**4830**	382	B	24.6242	0
**4830**	386	B	50.9967	1
**4830**	305	C	79.6018	1
**4830**	319	C	76.3066	1
**4830**	320	C	79.5763	-1
**4830**	321	C	36.7966	1
**4830**	351	C	4.294	1
**4830**	357	C	78.3167	1
**4830**	370	C	38.6874	0
**4830**	372	C	64.9986	0
**4830**	373	C	79.5404	1
**4830**	381	C	79.7546	1

**4830**	382	C	34.6613	0
**4830**	386	C	50.9338	1
**4830**	305	D	71.3068	1
**4830**	319	D	56.3407	1
**4830**	320	D	42.7601	-1
**4830**	321	D	79.5965	0
**4830**	351	D	12.4	1
**4830**	357	D	79.6223	1
**4830**	370	D	61.4197	1
**4830**	372	D	79.5576	0
**4830**	373	D	54.7646	1
**4830**	381	D	77.7898	1
**4830**	382	D	79.5331	1
**4830**	386	D	79.4896	1
**4831**	305	A	54.4579	1
**4831**	319	A	63.1616	1
**4831**	320	A	66.4817	-1
**4831**	321	A	38.9396	1
**4831**	351	A	6.9445	1
**4831**	357	A	51.6291	1
**4831**	370	A	79.472	1
**4831**	372	A	79.536	0
**4831**	373	A	39.5811	0
**4831**	381	A	79.6346	1
**4831**	382	A	72.5335	1
**4831**	386	A	65.8931	1
**4831**	305	B	72.1738	1
**4831**	319	B	26.8805	0
**4831**	320	B	79.568	-1
**4831**	321	B	72.5342	0
**4831**	351	B	0.2376	1
**4831**	357	B	27.2252	0
**4831**	370	B	53.1252	1
**4831**	372	B	79.6186	0

**4831**	373	B	79.5122	1
**4831**	381	B	53.3536	1
**4831**	382	B	71.8892	1
**4831**	386	B	79.6381	1
**4831**	305	C	21.1944	0
**4831**	319	C	46.4411	0
**4831**	320	C	75.4868	-1
**4831**	321	C	72.6855	0
**4831**	351	C	7.7884	1
**4831**	357	C	56.7759	1
**4831**	370	C	76.7954	1
**4831**	372	C	79.5626	0
**4831**	373	C	79.5227	1
**4831**	381	C	45.2648	0
**4831**	382	C	55.8164	1
**4831**	386	C	44.6569	0
**4831**	305	D	39.8076	0
**4831**	319	D	78.3906	1
**4831**	320	D	72.064	-1
**4831**	321	D	79.5244	0
**4831**	351	D	17.5588	1
**4831**	357	D	79.5653	1
**4831**	370	D	54.0429	1
**4831**	372	D	77.6622	0
**4831**	373	D	67.326	1
**4831**	381	D	79.5973	1
**4831**	382	D	74.639	1
**4831**	386	D	72.4449	1
**4832**	305	A	79.6034	1
**4832**	319	A	79.5091	1
**4832**	320	A	41.1317	-1
**4832**	321	A	63.5585	0
**4832**	351	A	15.3671	1
**4832**	357	A	64.1741	1

**4832**	370	A	73.2493	1
**4832**	372	A	74.3705	0
**4832**	373	A	11.6307	-1
**4832**	381	A	73.2401	1
**4832**	382	A	47.6127	0
**4832**	386	A	71.6688	1
**4832**	305	B	46.7981	0
**4832**	319	B	79.5571	1
**4832**	320	B	79.5607	-1
**4832**	321	B	79.6651	0
**4832**	351	B	11.6787	1
**4832**	357	B	57.9216	1
**4832**	370	B	73.3677	1
**4832**	372	B	52.58	0
**4832**	373	B	53.2719	1
**4832**	381	B	71.4701	1
**4832**	382	B	18.8554	-1
**4832**	386	B	41.9073	0
**4832**	305	C	69.6753	1
**4832**	319	C	46.7858	0
**4832**	320	C	71.4344	-1
**4832**	321	C	58.2725	0
**4832**	351	C	6.2405	1
**4832**	357	C	79.6399	1
**4832**	370	C	52.0195	1
**4832**	372	C	22.2297	1
**4832**	373	C	24.3871	0
**4832**	381	C	34.577	0
**4832**	382	C	43.8613	0
**4832**	386	C	77.7352	1
**4832**	305	D	79.5055	1
**4832**	319	D	64.6968	1
**4832**	320	D	47.0572	-1
**4832**	321	D	33.7783	1

**4832**	351	D	10.7126	1
**4832**	357	D	68.9779	1
**4832**	370	D	64.309	1
**4832**	372	D	12.6505	0
**4832**	373	D	65.6628	1
**4832**	381	D	74.4385	1
**4832**	382	D	63.7629	1
**4832**	386	D	79.5014	1
**4833**	305	A	69.7388	1
**4833**	319	A	70.5953	1
**4833**	320	A	72.9922	-1
**4833**	321	A	34.7851	1
**4833**	351	A	13.6726	1
**4833**	357	A	77.6303	1
**4833**	370	A	79.577	1
**4833**	372	A	43.7746	1
**4833**	373	A	79.3158	1
**4833**	381	A	69.8675	1
**4833**	382	A	16.4112	-1
**4833**	386	A	59.1516	1
**4833**	305	B	79.6154	1
**4833**	319	B	70.4635	1
**4833**	320	B	79.5466	-1
**4833**	321	B	70.42	0
**4833**	351	B	9.3349	1
**4833**	357	B	72.8868	1
**4833**	370	B	75.8554	1
**4833**	372	B	77.0671	0
**4833**	373	B	79.5836	1
**4833**	381	B	50.5908	1
**4833**	382	B	61.0225	1
**4833**	386	B	75.452	1
**4833**	305	C	79.5053	1
**4833**	319	C	61.2944	1

**4833**	320	C	79.6123	-1
**4833**	321	C	68.985	0
**4833**	351	C	1.4954	1
**4833**	357	C	28.537	0
**4833**	370	C	61.3828	1
**4833**	372	C	65.1781	0
**4833**	373	C	45.988	0
**4833**	381	C	59.2723	1
**4833**	382	C	55.1949	1
**4833**	386	C	39.5542	0
**4833**	305	D	54.7703	1
**4833**	319	D	75.8288	1
**4833**	320	D	68.9729	-1
**4833**	321	D	57.5263	0
**4833**	351	D	12.7735	1
**4833**	357	D	68.7762	1
**4833**	370	D	64.4599	1
**4833**	372	D	73.4906	0
**4833**	373	D	73.8608	1
**4833**	381	D	69.7424	1
**4833**	382	D	73.7058	1
**4833**	386	D	75.9218	1
**4834**	305	A	47.4062	0
**4834**	319	A	79.6116	1
**4834**	320	A	79.5641	-1
**4834**	321	A	79.386	0
**4834**	351	A	7.7808	1
**4834**	357	A	79.5795	1
**4834**	370	A	79.5488	1
**4834**	372	A	45.4475	1
**4834**	373	A	71.2426	1
**4834**	381	A	78.5804	1
**4834**	382	A	73.5414	1
**4834**	386	A	79.495	1

**4834**	305	B	71.4034	1
**4834**	319	B	52.6117	1
**4834**	320	B	50.7736	-1
**4834**	321	B	63.3455	0
**4834**	351	B	10.8851	1
**4834**	357	B	79.6538	1
**4834**	370	B	67.561	1
**4834**	372	B	48.3031	1
**4834**	373	B	15.8323	-1
**4834**	381	B	79.581	1
**4834**	382	B	45.4049	0
**4834**	386	B	56.2643	1
**4834**	305	C	78.1736	1
**4834**	319	C	79.4287	1
**4834**	320	C	70.4743	-1
**4834**	321	C	79.5706	0
**4834**	351	C	8.6114	1
**4834**	357	C	79.4754	1
**4834**	370	C	79.4734	1
**4834**	372	C	79.4962	0
**4834**	373	C	28.1482	0
**4834**	381	C	31.9838	0
**4834**	382	C	65.5358	1
**4834**	386	C	57.9155	1
**4834**	305	D	73.1446	1
**4834**	319	D	79.6425	1
**4834**	320	D	64.7958	-1
**4834**	321	D	79.4811	0
**4834**	351	D	11.9457	1
**4834**	357	D	79.6806	1
**4834**	370	D	73.2482	1
**4834**	372	D	79.5557	0
**4834**	373	D	78.7027	1
**4834**	381	D	76.1625	1

**4834**	382	D	24.4957	0
**4834**	386	D	51.4807	1
**4835**	305	A	55.6442	1
**4835**	319	A	79.5435	1
**4835**	320	A	72.7405	-1
**4835**	321	A	52.2105	0
**4835**	351	A	3.9997	1
**4835**	357	A	79.5093	1
**4835**	370	A	79.606	1
**4835**	372	A	68.7569	0
**4835**	373	A	74.3913	1
**4835**	381	A	79.5332	1
**4835**	382	A	77.6125	1
**4835**	386	A	63.0484	1
**4835**	305	B	72.2954	1
**4835**	319	B	74.9819	1
**4835**	320	B	67.4749	-1
**4835**	321	B	73.6303	0
**4835**	351	B	7.1592	1
**4835**	357	B	79.4751	1
**4835**	370	B	55.1096	1
**4835**	372	B	79.4907	0
**4835**	373	B	35.919	0
**4835**	381	B	79.5041	1
**4835**	382	B	50.0861	1
**4835**	386	B	79.5947	1
**4835**	305	C	78.4187	1
**4835**	319	C	63.7895	1
**4835**	320	C	79.6477	-1
**4835**	321	C	72.7881	0
**4835**	351	C	5.1102	1
**4835**	357	C	69.1263	1
**4835**	370	C	53.8265	1
**4835**	372	C	79.5339	0

**4835**	373	C	79.563	1
**4835**	381	C	79.6336	1
**4835**	382	C	79.497	1
**4835**	386	C	63.3175	1
**4835**	305	D	73.6109	1
**4835**	319	D	79.5716	1
**4835**	320	D	67.076	-1
**4835**	321	D	79.5288	0
**4835**	351	D	17.0207	1
**4835**	357	D	79.6051	1
**4835**	370	D	18.8208	-1
**4835**	372	D	39.2749	1
**4835**	373	D	71.9111	1
**4835**	381	D	79.5224	1
**4835**	382	D	79.597	1
**4835**	386	D	79.2	1
**4836**	305	A	71.6212	1
**4836**	319	A	79.4355	1
**4836**	320	A	70.2669	-1
**4836**	321	A	79.3787	0
**4836**	351	A	24.1184	-1
**4836**	357	A	58.4462	1
**4836**	370	A	79.6191	1
**4836**	372	A	69.9403	0
**4836**	373	A	62.9924	1
**4836**	381	A	79.5852	1
**4836**	382	A	79.4193	1
**4836**	386	A	79.605	1
**4836**	305	B	75.684	1
**4836**	319	B	62.3448	1
**4836**	320	B	2.0041	1
**4836**	321	B	50.7572	0
**4836**	351	B	15.8058	1
**4836**	357	B	51.3378	1

**4836**	370	B	72.9601	1
**4836**	372	B	75.6918	0
**4836**	373	B	42.6799	0
**4836**	381	B	54.4016	1
**4836**	382	B	55.1157	1
**4836**	386	B	64.0752	1
**4836**	305	C	26.5326	0
**4836**	319	C	74.3031	1
**4836**	320	C	63.9616	-1
**4836**	321	C	69.6683	0
**4836**	351	C	14.0711	1
**4836**	357	C	66.1076	1
**4836**	370	C	51.4525	1
**4836**	372	C	44.7551	1
**4836**	373	C	67.7344	1
**4836**	381	C	13.3809	-1
**4836**	382	C	79.4279	1
**4836**	386	C	35.9341	0
**4836**	305	D	79.0634	1
**4836**	319	D	79.7861	1
**4836**	320	D	75.3265	-1
**4836**	321	D	79.5432	0
**4836**	351	D	4.2148	1
**4836**	357	D	68.087	1
**4836**	370	D	65.2774	1
**4836**	372	D	79.6989	0
**4836**	373	D	47.2736	0
**4836**	381	D	44.5946	0
**4836**	382	D	79.501	1
**4836**	386	D	79.6055	1
**4837**	305	A	28.2206	0
**4837**	319	A	70.588	1
**4837**	320	A	79.5605	-1
**4837**	321	A	69.7026	0

**4837**	351	A	6.6856	1
**4837**	357	A	79.5344	1
**4837**	370	A	53.579	1
**4837**	372	A	69.7815	0
**4837**	373	A	61.1429	1
**4837**	381	A	73.8023	1
**4837**	382	A	32.5591	0
**4837**	386	A	69.9867	1
**4837**	305	B	79.5411	1
**4837**	319	B	73.7941	1
**4837**	320	B	67.3736	-1
**4837**	321	B	79.5345	0
**4837**	351	B	14.6785	1
**4837**	357	B	62.4405	1
**4837**	370	B	69.3071	1
**4837**	372	B	66.2655	0
**4837**	373	B	64.3841	1
**4837**	381	B	79.5747	1
**4837**	382	B	52.9049	1
**4837**	386	B	42.2697	0
**4837**	305	C	65.7419	1
**4837**	319	C	64.8384	1
**4837**	320	C	79.5434	-1
**4837**	321	C	40.5127	1
**4837**	351	C	4.8686	1
**4837**	357	C	79.5701	1
**4837**	370	C	78.3452	1
**4837**	372	C	79.5912	0
**4837**	373	C	79.5333	1
**4837**	381	C	72.6757	1
**4837**	382	C	64.7272	1
**4837**	386	C	45.0707	0
**4837**	305	D	78.7969	1
**4837**	319	D	74.6239	1

**4837**	320	D	56.1947	-1
**4837**	321	D	17.2041	0
**4837**	351	D	12.9957	1
**4837**	357	D	40.8938	0
**4837**	370	D	69.6883	1
**4837**	372	D	49.9553	1
**4837**	373	D	71.2216	1
**4837**	381	D	78.4814	1
**4837**	382	D	79.5515	1
**4837**	386	D	54.1333	1
**4838**	305	A	72.3377	1
**4838**	319	A	62.6217	1
**4838**	320	A	79.559	-1
**4838**	321	A	64.1752	0
**4838**	351	A	9.2621	1
**4838**	357	A	49.1342	0
**4838**	370	A	50.8971	1
**4838**	372	A	79.5424	0
**4838**	373	A	78.1803	1
**4838**	381	A	51.7267	1
**4838**	382	A	45.52	0
**4838**	386	A	13.786	-1
**4838**	305	B	72.9598	1
**4838**	319	B	75.8304	1
**4838**	320	B	36.3072	-1
**4838**	321	B	73.2293	0
**4838**	351	B	13.5821	1
**4838**	357	B	48.3872	0
**4838**	370	B	79.5565	1
**4838**	372	B	56.6826	0
**4838**	373	B	69.6831	1
**4838**	381	B	79.5368	1
**4838**	382	B	78.0191	1
**4838**	386	B	79.4962	1

**4838**	305	C	48.9487	0
**4838**	319	C	35.5566	0
**4838**	320	C	20.0236	-1
**4838**	321	C	78.2566	0
**4838**	351	C	7.9109	1
**4838**	357	C	79.5825	1
**4838**	370	C	62.2062	1
**4838**	372	C	79.559	0
**4838**	373	C	65.4537	1
**4838**	381	C	79.549	1
**4838**	382	C	41.4884	0
**4838**	386	C	25.9632	0
**4838**	305	D	79.5641	1
**4838**	319	D	35.7834	0
**4838**	320	D	73.1452	-1
**4838**	321	D	73.1406	0
**4838**	351	D	13.6847	1
**4838**	357	D	72.6556	1
**4838**	370	D	79.5574	1
**4838**	372	D	41.0539	1
**4838**	373	D	63.2436	1
**4838**	381	D	76.9189	1
**4838**	382	D	79.5477	1
**4838**	386	D	78.9432	1
**4839**	305	A	59.3462	1
**4839**	319	A	60.6696	1
**4839**	320	A	79.5825	-1
**4839**	321	A	72.2542	0
**4839**	351	A	19.2217	1
**4839**	357	A	77.9254	1
**4839**	370	A	79.3697	1
**4839**	372	A	72.7453	0
**4839**	373	A	38.832	0
**4839**	381	A	73.1906	1

**4839**	382	A	74.0662	1
**4839**	386	A	46.4102	0
**4839**	305	B	79.5376	1
**4839**	319	B	29.9381	0
**4839**	320	B	79.4934	-1
**4839**	321	B	70.2338	0
**4839**	351	B	17.8241	1
**4839**	357	B	49.5462	0
**4839**	370	B	47.5315	0
**4839**	372	B	79.5425	0
**4839**	373	B	49.6828	0
**4839**	381	B	79.5562	1
**4839**	382	B	77.5447	1
**4839**	386	B	79.5963	1
**4839**	305	C	75.9314	1
**4839**	319	C	79.5948	1
**4839**	320	C	44.2049	-1
**4839**	321	C	48.9148	1
**4839**	351	C	7.0582	1
**4839**	357	C	64.1196	1
**4839**	370	C	76.6024	1
**4839**	372	C	54.9645	0
**4839**	373	C	56.3442	1
**4839**	381	C	78.7098	1
**4839**	382	C	73.0343	1
**4839**	386	C	41.3949	0
**4839**	305	D	47.1906	0
**4839**	319	D	79.6023	1
**4839**	320	D	29.3992	-1
**4839**	321	D	71.5142	0
**4839**	351	D	15.8514	1
**4839**	357	D	65.0898	1
**4839**	370	D	77.0883	1
**4839**	372	D	71.6714	0

**4839**	373	D	70.3695	1
**4839**	381	D	79.5628	1
**4839**	382	D	72.4835	1
**4839**	386	D	79.6258	1
**4840**	305	A	49.4832	0
**4840**	319	A	79.5376	1
**4840**	320	A	69.3653	-1
**4840**	321	A	79.5321	0
**4840**	351	A	13.4582	1
**4840**	357	A	78.402	1
**4840**	370	A	78.5272	1
**4840**	372	A	30.255	1
**4840**	373	A	79.5551	1
**4840**	381	A	79.3525	1
**4840**	382	A	72.0676	1
**4840**	386	A	76.8285	1
**4840**	305	B	67.5672	1
**4840**	319	B	19.1708	-1
**4840**	320	B	62.1869	-1
**4840**	321	B	79.5601	0
**4840**	351	B	13.1966	1
**4840**	357	B	79.5275	1
**4840**	370	B	79.6001	1
**4840**	372	B	79.5311	0
**4840**	373	B	79.5535	1
**4840**	381	B	79.4823	1
**4840**	382	B	50.976	1
**4840**	386	B	73.3975	1
**4840**	305	C	74.185	1
**4840**	319	C	79.3591	1
**4840**	320	C	79.6571	-1
**4840**	321	C	79.4927	0
**4840**	351	C	6.9584	1
**4840**	357	C	45.1922	0

**4840**	370	C	78.7286	1
**4840**	372	C	53.2396	0
**4840**	373	C	73.7702	1
**4840**	381	C	79.5747	1
**4840**	382	C	69.9768	1
**4840**	386	C	47.9396	0
**4840**	305	D	57.6442	1
**4840**	319	D	63.9951	1
**4840**	320	D	79.5467	-1
**4840**	321	D	77.078	0
**4840**	351	D	20.494	-1
**4840**	357	D	65.9331	1
**4840**	370	D	79.5592	1
**4840**	372	D	79.5956	0
**4840**	373	D	79.4783	1
**4840**	381	D	52.1515	1
**4840**	382	D	76.7043	1
**4840**	386	D	79.6507	1
**4841**	305	A	77.4637	1
**4841**	319	A	79.6207	1
**4841**	320	A	55.9409	-1
**4841**	321	A	47.0454	1
**4841**	351	A	11.3554	1
**4841**	357	A	60.6042	1
**4841**	370	A	66.6883	1
**4841**	372	A	79.6158	0
**4841**	373	A	79.6319	1
**4841**	381	A	39.6054	0
**4841**	382	A	79.3056	1
**4841**	386	A	43.281	0
**4841**	305	B	35.2991	0
**4841**	319	B	79.5354	1
**4841**	320	B	72.323	-1
**4841**	321	B	77.3683	0

**4841**	351	B	5.0852	1
**4841**	357	B	73.7812	1
**4841**	370	B	79.596	1
**4841**	372	B	73.2535	0
**4841**	373	B	79.5632	1
**4841**	381	B	60.2098	1
**4841**	382	B	79.5855	1
**4841**	386	B	71.8244	1
**4841**	305	C	72.6417	1
**4841**	319	C	69.8364	1
**4841**	320	C	11.5219	1
**4841**	321	C	79.6423	0
**4841**	351	C	12	1
**4841**	357	C	79.6016	1
**4841**	370	C	59.3664	1
**4841**	372	C	79.5785	0
**4841**	373	C	79.5574	1
**4841**	381	C	77.4557	1
**4841**	382	C	43.048	0
**4841**	386	C	33.8977	0
**4841**	305	D	52.6557	1
**4841**	319	D	75.7532	1
**4841**	320	D	48.8856	-1
**4841**	321	D	52.3552	0
**4841**	351	D	3.6806	1
**4841**	357	D	79.5782	1
**4841**	370	D	78.6275	1
**4841**	372	D	79.5638	0
**4841**	373	D	64.8719	1
**4841**	381	D	50.3512	1
**4841**	382	D	79.5625	1
**4841**	386	D	65.4027	1
**4842**	305	A	79.6056	1
**4842**	319	A	79.0733	1

**4842**	320	A	72.9624	-1
**4842**	321	A	78.578	0
**4842**	351	A	4.3954	1
**4842**	357	A	79.5188	1
**4842**	370	A	69.5971	1
**4842**	372	A	50.2278	0
**4842**	373	A	79.5503	1
**4842**	381	A	43.214	0
**4842**	382	A	79.2959	1
**4842**	386	A	77.7829	1
**4842**	305	B	59.0788	1
**4842**	319	B	79.5871	1
**4842**	320	B	79.5537	-1
**4842**	321	B	66.08	0
**4842**	351	B	12.6508	1
**4842**	357	B	79.6527	1
**4842**	370	B	79.5766	1
**4842**	372	B	76.9229	0
**4842**	373	B	71.1239	1
**4842**	381	B	40.8955	0
**4842**	382	B	48.561	0
**4842**	386	B	79.5402	1
**4842**	305	C	78.4604	1
**4842**	319	C	73.4611	1
**4842**	320	C	70.2228	-1
**4842**	321	C	75.6911	0
**4842**	351	C	7.3429	1
**4842**	357	C	70.2499	1
**4842**	370	C	45.3504	0
**4842**	372	C	75.4642	0
**4842**	373	C	76.4096	1
**4842**	381	C	79.4756	1
**4842**	382	C	50.7201	1
**4842**	386	C	70.5246	1

**4842**	305	D	66.1199	1
**4842**	319	D	53.5722	1
**4842**	320	D	79.551	-1
**4842**	321	D	35.0129	1
**4842**	351	D	15.1949	1
**4842**	357	D	79.5076	1
**4842**	370	D	70.5036	1
**4842**	372	D	72.5366	0
**4842**	373	D	79.5699	1
**4842**	381	D	54.4878	1
**4842**	382	D	79.5342	1
**4842**	386	D	72.614	1
**4843**	305	A	61.7319	1
**4843**	319	A	46.8557	0
**4843**	320	A	79.6681	-1
**4843**	321	A	49.0309	1
**4843**	351	A	11.5794	1
**4843**	357	A	79.5448	1
**4843**	370	A	79.4781	1
**4843**	372	A	72.3572	0
**4843**	373	A	76.7605	1
**4843**	381	A	79.6147	1
**4843**	382	A	48.7533	0
**4843**	386	A	74.9132	1
**4843**	305	B	79.3974	1
**4843**	319	B	76.0339	1
**4843**	320	B	64.6757	-1
**4843**	321	B	74.9084	0
**4843**	351	B	10.9975	1
**4843**	357	B	76.7119	1
**4843**	370	B	79.551	1
**4843**	372	B	79.6672	0
**4843**	373	B	60.4947	1
**4843**	381	B	79.5005	1

**4843**	382	B	70.1146	1
**4843**	386	B	78.9839	1
**4843**	305	C	79.4812	1
**4843**	319	C	73.4287	1
**4843**	320	C	79.435	-1
**4843**	321	C	66.3152	0
**4843**	351	C	8.1491	1
**4843**	357	C	68.7711	1
**4843**	370	C	72.0412	1
**4843**	372	C	67.0913	0
**4843**	373	C	68.6158	1
**4843**	381	C	79.4642	1
**4843**	382	C	48.1112	0
**4843**	386	C	65.0247	1
**4843**	305	D	78.0962	1
**4843**	319	D	49.1272	0
**4843**	320	D	70.7754	-1
**4843**	321	D	79.6648	0
**4843**	351	D	12.4171	1
**4843**	357	D	67.1946	1
**4843**	370	D	63.0327	1
**4843**	372	D	79.3664	0
**4843**	373	D	67.9609	1
**4843**	381	D	74.7898	1
**4843**	382	D	79.4587	1
**4843**	386	D	67.4623	1
**4844**	305	A	79.5313	1
**4844**	319	A	76.8251	1
**4844**	320	A	79.6352	-1
**4844**	321	A	73.1307	0
**4844**	351	A	7.2051	1
**4844**	357	A	70.8149	1
**4844**	370	A	49.6105	0
**4844**	372	A	74.0325	0

**4844**	373	A	79.471	1
**4844**	381	A	67.4488	1
**4844**	382	A	48.769	0
**4844**	386	A	79.5834	1
**4844**	305	B	79.2631	1
**4844**	319	B	72.893	1
**4844**	320	B	79.4839	-1
**4844**	321	B	58.707	0
**4844**	351	B	13.5356	1
**4844**	357	B	70.801	1
**4844**	370	B	12.9351	-1
**4844**	372	B	79.566	0
**4844**	373	B	76.0648	1
**4844**	381	B	74.2157	1
**4844**	382	B	23.7417	0
**4844**	386	B	42.2514	0
**4844**	305	C	47.0631	0
**4844**	319	C	79.4989	1
**4844**	320	C	67.083	-1
**4844**	321	C	75.2089	0
**4844**	351	C	39.7811	-1
**4844**	357	C	47.402	0
**4844**	370	C	71.8096	1
**4844**	372	C	79.1139	0
**4844**	373	C	73.5867	1
**4844**	381	C	55.3584	1
**4844**	382	C	71.5625	1
**4844**	386	C	50.1127	1
**4844**	305	D	70.9733	1
**4844**	319	D	35.8123	0
**4844**	320	D	79.665	-1
**4844**	321	D	70.1455	0
**4844**	351	D	31.4364	-1
**4844**	357	D	73.1327	1

**4844**	370	D	77.7948	1
**4844**	372	D	79.6003	0
**4844**	373	D	57.3401	1
**4844**	381	D	79.6155	1
**4844**	382	D	74.4096	1
**4844**	386	D	70.3473	1
**4845**	305	A	48.539	0
**4845**	319	A	71.7802	1
**4845**	320	A	70.4545	-1
**4845**	321	A	71.2313	0
**4845**	351	A	3.4976	1
**4845**	357	A	70.0284	1
**4845**	370	A	73.4881	1
**4845**	372	A	79.5486	0
**4845**	373	A	64.5979	1
**4845**	381	A	79.5856	1
**4845**	382	A	79.5948	1
**4845**	386	A	79.5572	1
**4845**	305	B	72.8718	1
**4845**	319	B	30.0571	0
**4845**	320	B	58.3651	-1
**4845**	321	B	68.5132	0
**4845**	351	B	15.3965	1
**4845**	357	B	53.6443	1
**4845**	370	B	79.5844	1
**4845**	372	B	71.0789	0
**4845**	373	B	71.5345	1
**4845**	381	B	73.8365	1
**4845**	382	B	70.8881	1
**4845**	386	B	79.5731	1
**4845**	305	C	79.5782	1
**4845**	319	C	72.6185	1
**4845**	320	C	56.3447	-1
**4845**	321	C	79.3134	0

**4845**	351	C	4.2335	1
**4845**	357	C	72.8154	1
**4845**	370	C	79.5489	1
**4845**	372	C	61.3049	0
**4845**	373	C	58.5579	1
**4845**	381	C	79.5606	1
**4845**	382	C	79.5617	1
**4845**	386	C	54.629	1
**4845**	305	D	44.1676	0
**4845**	319	D	64.7426	1
**4845**	320	D	79.5765	-1
**4845**	321	D	72.7508	0
**4845**	351	D	17.5622	1
**4845**	357	D	47.224	0
**4845**	370	D	79.3409	1
**4845**	372	D	29.4186	1
**4845**	373	D	79.5195	1
**4845**	381	D	58.1932	1
**4845**	382	D	74.5047	1
**4845**	386	D	79.5604	1
**4846**	305	A	66.744	1
**4846**	319	A	68.067	1
**4846**	320	A	32.3417	-1
**4846**	321	A	70.155	0
**4846**	351	A	5.5672	1
**4846**	357	A	78.1245	1
**4846**	370	A	73.3616	1
**4846**	372	A	40.7412	1
**4846**	373	A	34.9197	0
**4846**	381	A	71.7432	1
**4846**	382	A	77.1532	1
**4846**	386	A	78.0637	1
**4846**	305	B	75.2839	1
**4846**	319	B	76.928	1

**4846**	320	B	27.7808	-1
**4846**	321	B	79.5211	0
**4846**	351	B	20.0439	-1
**4846**	357	B	65.1297	1
**4846**	370	B	79.5424	1
**4846**	372	B	74.4767	0
**4846**	373	B	63.5608	1
**4846**	381	B	79.5536	1
**4846**	382	B	45.4025	0
**4846**	386	B	31.3002	0
**4846**	305	C	49.9367	0
**4846**	319	C	74.174	1
**4846**	320	C	74.81	-1
**4846**	321	C	71.1291	0
**4846**	351	C	12.9014	1
**4846**	357	C	75.4918	1
**4846**	370	C	51.5946	1
**4846**	372	C	69.6079	0
**4846**	373	C	64.9772	1
**4846**	381	C	73.4002	1
**4846**	382	C	44.9756	0
**4846**	386	C	56.0254	1
**4846**	305	D	43.6463	0
**4846**	319	D	64.3091	1
**4846**	320	D	79.3365	-1
**4846**	321	D	79.5617	0
**4846**	351	D	13.9239	1
**4846**	357	D	38.4908	0
**4846**	370	D	76.4555	1
**4846**	372	D	79.5439	0
**4846**	373	D	79.5686	1
**4846**	381	D	79.5702	1
**4846**	382	D	64.8296	1
**4846**	386	D	79.5663	1

**4847**	305	A	71.2782	1
**4847**	319	A	41.9857	0
**4847**	320	A	79.5994	-1
**4847**	321	A	63.8542	0
**4847**	351	A	12.9361	1
**4847**	357	A	73.3652	1
**4847**	370	A	75.6053	1
**4847**	372	A	75.98	0
**4847**	373	A	79.6532	1
**4847**	381	A	72.1632	1
**4847**	382	A	72.0707	1
**4847**	386	A	47.4221	0
**4847**	305	B	68.4366	1
**4847**	319	B	60.7037	1
**4847**	320	B	44.4232	-1
**4847**	321	B	15.0715	0
**4847**	351	B	7.62	1
**4847**	357	B	71.7516	1
**4847**	370	B	78.1185	1
**4847**	372	B	57.9864	0
**4847**	373	B	66.6756	1
**4847**	381	B	79.5668	1
**4847**	382	B	61.4818	1
**4847**	386	B	68.0384	1
**4847**	305	C	77.8388	1
**4847**	319	C	72.6833	1
**4847**	320	C	58.6	-1
**4847**	321	C	72.5416	0
**4847**	351	C	17.9284	1
**4847**	357	C	51.489	1
**4847**	370	C	69.7994	1
**4847**	372	C	71.1945	0
**4847**	373	C	78.7216	1
**4847**	381	C	79.4769	1

**4847**	382	C	66.1912	1
**4847**	386	C	58.2706	1
**4847**	305	D	68.8282	1
**4847**	319	D	75.9455	1
**4847**	320	D	51.2143	-1
**4847**	321	D	79.4526	0
**4847**	351	D	7.1119	1
**4847**	357	D	50.9433	1
**4847**	370	D	51.8985	1
**4847**	372	D	79.3554	0
**4847**	373	D	67.8372	1
**4847**	381	D	57.973	1
**4847**	382	D	71.4456	1
**4847**	386	D	79.5309	1
**4848**	305	A	71.9959	1
**4848**	319	A	79.5368	1
**4848**	320	A	37.4707	-1
**4848**	321	A	27.5857	1
**4848**	351	A	2.3118	1
**4848**	357	A	79.5465	1
**4848**	370	A	58.1056	1
**4848**	372	A	72.4712	0
**4848**	373	A	76.0229	1
**4848**	381	A	79.4912	1
**4848**	382	A	48.1972	0
**4848**	386	A	58.3242	1
**4848**	305	B	52.7217	1
**4848**	319	B	67.0876	1
**4848**	320	B	71.3752	-1
**4848**	321	B	66.3775	0
**4848**	351	B	0.7373	1
**4848**	357	B	68.8361	1
**4848**	370	B	69.4611	1
**4848**	372	B	79.309	0

**4848**	373	B	64.0653	1
**4848**	381	B	67.6233	1
**4848**	382	B	71.311	1
**4848**	386	B	78.1411	1
**4848**	305	C	75.3738	1
**4848**	319	C	79.5744	1
**4848**	320	C	42.2182	-1
**4848**	321	C	79.6049	0
**4848**	351	C	2.2087	1
**4848**	357	C	79.5289	1
**4848**	370	C	79.5014	1
**4848**	372	C	79.5535	0
**4848**	373	C	76.1485	1
**4848**	381	C	79.6529	1
**4848**	382	C	77.0708	1
**4848**	386	C	48.7179	0
**4848**	305	D	15.3888	-1
**4848**	319	D	60.0166	1
**4848**	320	D	54.249	-1
**4848**	321	D	72.2586	0
**4848**	351	D	7.5199	1
**4848**	357	D	52.1828	1
**4848**	370	D	74.5381	1
**4848**	372	D	79.5069	0
**4848**	373	D	53.0635	1
**4848**	381	D	71.5132	1
**4848**	382	D	60.123	1
**4848**	386	D	73.171	1
**4849**	305	A	61.1287	1
**4849**	319	A	64.1724	1
**4849**	320	A	74.9549	-1
**4849**	321	A	56.6354	0
**4849**	351	A	35.5362	-1
**4849**	357	A	79.5378	1

**4849**	370	A	73.9732	1
**4849**	372	A	79.5992	0
**4849**	373	A	79.5389	1
**4849**	381	A	79.6435	1
**4849**	382	A	75.4303	1
**4849**	386	A	79.5417	1
**4849**	305	B	57.0931	1
**4849**	319	B	74.5364	1
**4849**	320	B	77.369	-1
**4849**	321	B	71.0166	0
**4849**	351	B	22.9725	-1
**4849**	357	B	67.349	1
**4849**	370	B	79.5566	1
**4849**	372	B	72.5539	0
**4849**	373	B	72.3978	1
**4849**	381	B	77.2183	1
**4849**	382	B	54.1342	1
**4849**	386	B	54.9966	1
**4849**	305	C	79.5603	1
**4849**	319	C	70.8546	1
**4849**	320	C	72.8424	-1
**4849**	321	C	78.6876	0
**4849**	351	C	8.7827	1
**4849**	357	C	73.2964	1
**4849**	370	C	72.2131	1
**4849**	372	C	73.4858	0
**4849**	373	C	45.423	0
**4849**	381	C	79.5681	1
**4849**	382	C	73.0382	1
**4849**	386	C	50.4392	1
**4849**	305	D	15.1585	-1
**4849**	319	D	49.0694	0
**4849**	320	D	79.4933	-1
**4849**	321	D	51.1777	0

**4849**	351	D	13.7432	1
**4849**	357	D	79.5819	1
**4849**	370	D	78.6889	1
**4849**	372	D	76.8862	0
**4849**	373	D	79.5052	1
**4849**	381	D	58.1042	1
**4849**	382	D	72.773	1
**4849**	386	D	77.3961	1
**4850**	305	A	76.8576	1
**4850**	319	A	54.1096	1
**4850**	320	A	78.357	-1
**4850**	321	A	29.5015	1
**4850**	351	A	10.703	1
**4850**	357	A	79.5101	1
**4850**	370	A	47.8538	0
**4850**	372	A	12.4286	0
**4850**	373	A	79.2821	1
**4850**	381	A	63.8701	1
**4850**	382	A	66.2627	1
**4850**	386	A	79.6629	1
**4850**	305	B	79.467	1
**4850**	319	B	79.497	1
**4850**	320	B	49.8009	-1
**4850**	321	B	79.6523	0
**4850**	351	B	14.2101	1
**4850**	357	B	79.6663	1
**4850**	370	B	58.7045	1
**4850**	372	B	74.3545	0
**4850**	373	B	57.3088	1
**4850**	381	B	63.7626	1
**4850**	382	B	41.0908	0
**4850**	386	B	71.9081	1
**4850**	305	C	33.5251	0
**4850**	319	C	74.3094	1

**4850**	320	C	71.0948	-1
**4850**	321	C	79.497	0
**4850**	351	C	6.6406	1
**4850**	357	C	79.4063	1
**4850**	370	C	76.2413	1
**4850**	372	C	54.4653	0
**4850**	373	C	68.9169	1
**4850**	381	C	46.2643	0
**4850**	382	C	70.7544	1
**4850**	386	C	51.4641	1
**4850**	305	D	25.5816	0
**4850**	319	D	76.7168	1
**4850**	320	D	44.6958	-1
**4850**	321	D	59.9149	0
**4850**	351	D	7.3068	1
**4850**	357	D	79.5689	1
**4850**	370	D	79.5927	1
**4850**	372	D	72.4121	0
**4850**	373	D	79.5785	1
**4850**	381	D	79.763	1
**4850**	382	D	76.2981	1
**4850**	386	D	59.0492	1
**4851**	305	A	79.5701	1
**4851**	319	A	38.9856	0
**4851**	320	A	61.9929	-1
**4851**	321	A	68.5886	0
**4851**	351	A	8.1112	1
**4851**	357	A	79.5732	1
**4851**	370	A	65.5989	1
**4851**	372	A	53.3497	0
**4851**	373	A	75.0961	1
**4851**	381	A	79.5538	1
**4851**	382	A	78.2522	1
**4851**	386	A	72.3073	1

**4851**	305	B	73.1133	1
**4851**	319	B	79.5723	1
**4851**	320	B	79.5373	-1
**4851**	321	B	42.1959	1
**4851**	351	B	19.9862	1
**4851**	357	B	79.55	1
**4851**	370	B	79.5224	1
**4851**	372	B	73.2482	0
**4851**	373	B	36.6644	0
**4851**	381	B	64.4993	1
**4851**	382	B	32.2665	0
**4851**	386	B	72.5686	1
**4851**	305	C	66.4927	1
**4851**	319	C	78.7261	1
**4851**	320	C	68.7817	-1
**4851**	321	C	55.6644	0
**4851**	351	C	4.211	1
**4851**	357	C	73.472	1
**4851**	370	C	76.4349	1
**4851**	372	C	74.7476	0
**4851**	373	C	79.5595	1
**4851**	381	C	69.7269	1
**4851**	382	C	78.2347	1
**4851**	386	C	49.8978	0
**4851**	305	D	79.5455	1
**4851**	319	D	62.0311	1
**4851**	320	D	58.2662	-1
**4851**	321	D	74.1476	0
**4851**	351	D	14.7857	1
**4851**	357	D	69.9893	1
**4851**	370	D	69.5866	1
**4851**	372	D	73.7332	0
**4851**	373	D	77.0535	1
**4851**	381	D	73.0239	1

**4851**	382	D	79.5642	1
**4851**	386	D	74.0384	1
**4852**	305	A	69.6958	1
**4852**	319	A	74.1147	1
**4852**	320	A	47.6532	-1
**4852**	321	A	35.4904	1
**4852**	351	A	3.0071	1
**4852**	357	A	78.3404	1
**4852**	370	A	79.5462	1
**4852**	372	A	62.2425	0
**4852**	373	A	79.5192	1
**4852**	381	A	45.0128	0
**4852**	382	A	68.6311	1
**4852**	386	A	56.315	1
**4852**	305	B	79.6213	1
**4852**	319	B	79.6184	1
**4852**	320	B	75.127	-1
**4852**	321	B	73.7335	0
**4852**	351	B	10.3	1
**4852**	357	B	76.1085	1
**4852**	370	B	45.9161	0
**4852**	372	B	79.1503	0
**4852**	373	B	73.0594	1
**4852**	381	B	73.8434	1
**4852**	382	B	78.1116	1
**4852**	386	B	79.4498	1
**4852**	305	C	75.6111	1
**4852**	319	C	73.4252	1
**4852**	320	C	79.5217	-1
**4852**	321	C	69.4486	0
**4852**	351	C	1.8312	1
**4852**	357	C	55.9055	1
**4852**	370	C	50.6953	1
**4852**	372	C	79.6056	0

**4852**	373	C	51.7928	1
**4852**	381	C	79.083	1
**4852**	382	C	60.0072	1
**4852**	386	C	50.0617	1
**4852**	305	D	36.5253	0
**4852**	319	D	79.1237	1
**4852**	320	D	79.5843	-1
**4852**	321	D	79.5844	0
**4852**	351	D	16.1263	1
**4852**	357	D	79.5545	1
**4852**	370	D	79.5581	1
**4852**	372	D	79.4949	0
**4852**	373	D	73.7331	1
**4852**	381	D	32.0233	0
**4852**	382	D	79.4884	1
**4852**	386	D	43.7211	0
**4853**	305	A	78.5228	1
**4853**	319	A	77.2751	1
**4853**	320	A	79.6443	-1
**4853**	321	A	62.5275	0
**4853**	351	A	11.2006	1
**4853**	357	A	73.3918	1
**4853**	370	A	70.0615	1
**4853**	372	A	50.8868	0
**4853**	373	A	68.1351	1
**4853**	381	A	47.3081	0
**4853**	382	A	76.5201	1
**4853**	386	A	64.2135	1
**4853**	305	B	75.2764	1
**4853**	319	B	73.0729	1
**4853**	320	B	79.59	-1
**4853**	321	B	53.1465	0
**4853**	351	B	11.322	1
**4853**	357	B	62.1843	1

**4853**	370	B	79.5985	1
**4853**	372	B	68.9537	0
**4853**	373	B	60.7254	1
**4853**	381	B	79.5032	1
**4853**	382	B	69.5605	1
**4853**	386	B	74.3112	1
**4853**	305	C	72.4822	1
**4853**	319	C	79.5842	1
**4853**	320	C	65.3983	-1
**4853**	321	C	75.6842	0
**4853**	351	C	7.3573	1
**4853**	357	C	45.6985	0
**4853**	370	C	70.512	1
**4853**	372	C	79.6083	0
**4853**	373	C	79.5154	1
**4853**	381	C	77.5325	1
**4853**	382	C	56.6849	1
**4853**	386	C	39.9525	0
**4853**	305	D	73.3519	1
**4853**	319	D	79.5147	1
**4853**	320	D	65.0095	-1
**4853**	321	D	77.9635	0
**4853**	351	D	3.4618	1
**4853**	357	D	74.693	1
**4853**	370	D	79.6304	1
**4853**	372	D	79.5945	0
**4853**	373	D	76.355	1
**4853**	381	D	47.7159	0
**4853**	382	D	29.4201	0
**4853**	386	D	76.8708	1
**4854**	305	A	72.2127	1
**4854**	319	A	75.0811	1
**4854**	320	A	79.4829	-1
**4854**	321	A	77.2305	0

**4854**	351	A	4.1752	1
**4854**	357	A	60.5375	1
**4854**	370	A	79.5124	1
**4854**	372	A	64.5056	0
**4854**	373	A	72.4129	1
**4854**	381	A	79.6237	1
**4854**	382	A	79.6434	1
**4854**	386	A	79.0858	1
**4854**	305	B	79.6105	1
**4854**	319	B	72.8008	1
**4854**	320	B	65.1327	-1
**4854**	321	B	73.9424	0
**4854**	351	B	13.8447	1
**4854**	357	B	48.3021	0
**4854**	370	B	73.0836	1
**4854**	372	B	79.0225	0
**4854**	373	B	35.0356	0
**4854**	381	B	59.5941	1
**4854**	382	B	69.1438	1
**4854**	386	B	79.6151	1
**4854**	305	C	24.1594	0
**4854**	319	C	79.1298	1
**4854**	320	C	50.0181	-1
**4854**	321	C	40.2195	1
**4854**	351	C	3.8988	1
**4854**	357	C	79.4598	1
**4854**	370	C	79.4267	1
**4854**	372	C	57.1445	0
**4854**	373	C	66.1535	1
**4854**	381	C	34.5264	0
**4854**	382	C	65.7997	1
**4854**	386	C	27.8546	0
**4854**	305	D	36.0426	0
**4854**	319	D	42.6263	0

**4854**	320	D	78.7955	-1
**4854**	321	D	70.2018	0
**4854**	351	D	13.7126	1
**4854**	357	D	79.5568	1
**4854**	370	D	79.2876	1
**4854**	372	D	79.5515	0
**4854**	373	D	62.0508	1
**4854**	381	D	72.2428	1
**4854**	382	D	78.5782	1
**4854**	386	D	52.3282	1
**4855**	305	A	76.0837	1
**4855**	319	A	58.7073	1
**4855**	320	A	79.5055	-1
**4855**	321	A	76.9255	0
**4855**	351	A	10.6184	1
**4855**	357	A	57.9396	1
**4855**	370	A	41.6081	0
**4855**	372	A	79.5936	0
**4855**	373	A	62.3253	1
**4855**	381	A	79.5266	1
**4855**	382	A	75.3999	1
**4855**	386	A	64.3743	1
**4855**	305	B	43.6134	0
**4855**	319	B	75.775	1
**4855**	320	B	46.0427	-1
**4855**	321	B	79.5881	0
**4855**	351	B	5.4869	1
**4855**	357	B	46.2323	0
**4855**	370	B	79.548	1
**4855**	372	B	79.6805	0
**4855**	373	B	79.2962	1
**4855**	381	B	72.8971	1
**4855**	382	B	74.4364	1
**4855**	386	B	79.5692	1

**4855**	305	C	79.5402	1
**4855**	319	C	79.6577	1
**4855**	320	C	79.5662	-1
**4855**	321	C	74.5827	0
**4855**	351	C	0.0	1
**4855**	357	C	74.2656	1
**4855**	370	C	79.6681	1
**4855**	372	C	62.408	0
**4855**	373	C	61.6773	1
**4855**	381	C	70.8162	1
**4855**	382	C	50.1925	1
**4855**	386	C	63.1599	1
**4855**	305	D	44.1551	0
**4855**	319	D	8.6457	-1
**4855**	320	D	73.8296	-1
**4855**	321	D	79.5776	0
**4855**	351	D	14.8882	1
**4855**	357	D	79.5474	1
**4855**	370	D	43.4952	0
**4855**	372	D	79.5322	0
**4855**	373	D	79.5752	1
**4855**	381	D	79.3453	1
**4855**	382	D	74.3705	1
**4855**	386	D	79.589	1
**4856**	305	A	39.7907	0
**4856**	319	A	35.4507	0
**4856**	320	A	79.7192	-1
**4856**	321	A	71.8714	0
**4856**	351	A	8.5692	1
**4856**	357	A	79.5254	1
**4856**	370	A	79.5574	1
**4856**	372	A	79.4935	0
**4856**	373	A	53.4744	1
**4856**	381	A	57.7327	1

**4856**	382	A	24.535	0
**4856**	386	A	68.388	1
**4856**	305	B	79.5847	1
**4856**	319	B	57.9015	1
**4856**	320	B	61.8497	-1
**4856**	321	B	73.3952	0
**4856**	351	B	20.0595	-1
**4856**	357	B	44.0283	0
**4856**	370	B	74.6102	1
**4856**	372	B	76.3947	0
**4856**	373	B	60.271	1
**4856**	381	B	34.9237	0
**4856**	382	B	63.6233	1
**4856**	386	B	78.9528	1
**4856**	305	C	74.4952	1
**4856**	319	C	72.9862	1
**4856**	320	C	79.5477	-1
**4856**	321	C	23.0895	1
**4856**	351	C	3.1594	1
**4856**	357	C	79.5873	1
**4856**	370	C	79.544	1
**4856**	372	C	44.048	1
**4856**	373	C	44.99	0
**4856**	381	C	74.5053	1
**4856**	382	C	50.2964	1
**4856**	386	C	58.8651	1
**4856**	305	D	53.5917	1
**4856**	319	D	73.2405	1
**4856**	320	D	36.5452	-1
**4856**	321	D	41.6518	1
**4856**	351	D	11.1566	1
**4856**	357	D	66.888	1
**4856**	370	D	70.8944	1
**4856**	372	D	79.6145	0

**4856**	373	D	40.9732	0
**4856**	381	D	56.1422	1
**4856**	382	D	79.4906	1
**4856**	386	D	78.322	1
**4857**	305	A	67.1288	1
**4857**	319	A	53.3317	1
**4857**	320	A	79.6267	-1
**4857**	321	A	48.6982	1
**4857**	351	A	11.2624	1
**4857**	357	A	69.9344	1
**4857**	370	A	76.8577	1
**4857**	372	A	74.2728	0
**4857**	373	A	37.7519	0
**4857**	381	A	79.6045	1
**4857**	382	A	70.0848	1
**4857**	386	A	71.8809	1
**4857**	305	B	47.7889	0
**4857**	319	B	74.6814	1
**4857**	320	B	27.9328	-1
**4857**	321	B	63.0076	0
**4857**	351	B	12.6844	1
**4857**	357	B	38.47	0
**4857**	370	B	79.6583	1
**4857**	372	B	79.5335	0
**4857**	373	B	79.5474	1
**4857**	381	B	30.6859	0
**4857**	382	B	33.7975	0
**4857**	386	B	79.3286	1
**4857**	305	C	79.5464	1
**4857**	319	C	56.0246	1
**4857**	320	C	69.5186	-1
**4857**	321	C	50.4762	0
**4857**	351	C	8.0563	1
**4857**	357	C	51.7547	1

**4857**	370	C	73.6207	1
**4857**	372	C	71.9372	0
**4857**	373	C	74.724	1
**4857**	381	C	68.4986	1
**4857**	382	C	79.2898	1
**4857**	386	C	63.5759	1
**4857**	305	D	70.7404	1
**4857**	319	D	79.5398	1
**4857**	320	D	79.5357	-1
**4857**	321	D	79.5884	0
**4857**	351	D	8.9199	1
**4857**	357	D	72.6053	1
**4857**	370	D	79.4606	1
**4857**	372	D	79.3839	0
**4857**	373	D	64.3032	1
**4857**	381	D	79.5653	1
**4857**	382	D	79.657	1
**4857**	386	D	70.981	1
**4858**	305	A	67.4386	1
**4858**	319	A	18.8333	-1
**4858**	320	A	79.3173	-1
**4858**	321	A	73.6937	0
**4858**	351	A	8.4058	1
**4858**	357	A	74.296	1
**4858**	370	A	56.9958	1
**4858**	372	A	49.61	1
**4858**	373	A	79.5076	1
**4858**	381	A	58.4251	1
**4858**	382	A	79.5682	1
**4858**	386	A	73.9798	1
**4858**	305	B	79.5309	1
**4858**	319	B	36.2537	0
**4858**	320	B	79.5339	-1
**4858**	321	B	73.0322	0

**4858**	351	B	7.9065	1
**4858**	357	B	79.2821	1
**4858**	370	B	57.9498	1
**4858**	372	B	79.5433	0
**4858**	373	B	51.6959	1
**4858**	381	B	50.1853	1
**4858**	382	B	42.9434	0
**4858**	386	B	70.7795	1
**4858**	305	C	73.8065	1
**4858**	319	C	49.1628	0
**4858**	320	C	73.7281	-1
**4858**	321	C	57.6463	0
**4858**	351	C	4.3963	1
**4858**	357	C	74.388	1
**4858**	370	C	67.4305	1
**4858**	372	C	79.5963	0
**4858**	373	C	79.553	1
**4858**	381	C	32.4387	0
**4858**	382	C	68.3872	1
**4858**	386	C	65.0348	1
**4858**	305	D	79.5499	1
**4858**	319	D	79.5115	1
**4858**	320	D	75.6982	-1
**4858**	321	D	79.5781	0
**4858**	351	D	8.4514	1
**4858**	357	D	53.3347	1
**4858**	370	D	48.6896	0
**4858**	372	D	70.6263	0
**4858**	373	D	79.5191	1
**4858**	381	D	15.7364	-1
**4858**	382	D	79.5159	1
**4858**	386	D	41.3781	0
**4859**	305	A	78.3019	1
**4859**	319	A	54.1916	1

**4859**	320	A	79.783	-1
**4859**	321	A	79.7113	0
**4859**	351	A	9.4407	1
**4859**	357	A	41.6664	0
**4859**	370	A	79.5768	1
**4859**	372	A	79.3944	0
**4859**	373	A	70.1291	1
**4859**	381	A	55.374	1
**4859**	382	A	79.4858	1
**4859**	386	A	74.426	1
**4859**	305	B	71.8277	1
**4859**	319	B	68.5907	1
**4859**	320	B	72.3961	-1
**4859**	321	B	79.4226	0
**4859**	351	B	22.983	-1
**4859**	357	B	79.737	1
**4859**	370	B	75.6439	1
**4859**	372	B	78.9568	0
**4859**	373	B	44.6989	0
**4859**	381	B	79.6347	1
**4859**	382	B	67.5381	1
**4859**	386	B	71.2005	1
**4859**	305	C	70.6365	1
**4859**	319	C	79.594	1
**4859**	320	C	79.2475	-1
**4859**	321	C	78.0316	0
**4859**	351	C	4.807	1
**4859**	357	C	79.5531	1
**4859**	370	C	78.9824	1
**4859**	372	C	79.5999	0
**4859**	373	C	65.3207	1
**4859**	381	C	79.4515	1
**4859**	382	C	58.3807	1
**4859**	386	C	39.4693	0

**4859**	305	D	79.745	1
**4859**	319	D	74.6819	1
**4859**	320	D	68.1392	-1
**4859**	321	D	66.0593	0
**4859**	351	D	20.1197	-1
**4859**	357	D	74.9485	1
**4859**	370	D	79.5462	1
**4859**	372	D	79.7565	0
**4859**	373	D	79.3097	1
**4859**	381	D	38.8601	0
**4859**	382	D	79.5226	1
**4859**	386	D	63.2185	1
**4860**	305	A	79.5611	1
**4860**	319	A	63.732	1
**4860**	320	A	79.5627	-1
**4860**	321	A	67.6154	0
**4860**	351	A	5.105	1
**4860**	357	A	79.6029	1
**4860**	370	A	72.9855	1
**4860**	372	A	36.8985	1
**4860**	373	A	65.4439	1
**4860**	381	A	60.6391	1
**4860**	382	A	71.7487	1
**4860**	386	A	50.0646	1
**4860**	305	B	70.7893	1
**4860**	319	B	65.0868	1
**4860**	320	B	42.5714	-1
**4860**	321	B	67.547	0
**4860**	351	B	15.6128	1
**4860**	357	B	36.8354	0
**4860**	370	B	79.5391	1
**4860**	372	B	65.431	0
**4860**	373	B	63.7815	1
**4860**	381	B	19.2137	-1

**4860**	382	B	71.9327	1
**4860**	386	B	73.7455	1
**4860**	305	C	53.4359	1
**4860**	319	C	77.6503	1
**4860**	320	C	79.1252	-1
**4860**	321	C	34.8653	1
**4860**	351	C	8.4484	1
**4860**	357	C	79.5294	1
**4860**	370	C	73.8312	1
**4860**	372	C	58.1808	0
**4860**	373	C	78.6604	1
**4860**	381	C	42.4173	0
**4860**	382	C	49.9655	0
**4860**	386	C	56.0779	1
**4860**	305	D	59.5886	1
**4860**	319	D	33.6801	0
**4860**	320	D	73.0247	-1
**4860**	321	D	55.4965	0
**4860**	351	D	1.5969	1
**4860**	357	D	46.4377	0
**4860**	370	D	73.7631	1
**4860**	372	D	77.3023	0
**4860**	373	D	66.0505	1
**4860**	381	D	72.1849	1
**4860**	382	D	79.5939	1
**4860**	386	D	79.5567	1
**4861**	305	A	79.554	1
**4861**	319	A	63.7117	1
**4861**	320	A	79.5632	-1
**4861**	321	A	60.8013	0
**4861**	351	A	8.0161	1
**4861**	357	A	72.6148	1
**4861**	370	A	74.8583	1
**4861**	372	A	72.1129	0

**4861**	373	A	46.4385	0
**4861**	381	A	79.534	1
**4861**	382	A	66.7701	1
**4861**	386	A	79.54	1
**4861**	305	B	45.2409	0
**4861**	319	B	52.8595	1
**4861**	320	B	25.5216	-1
**4861**	321	B	71.1466	0
**4861**	351	B	17.5295	1
**4861**	357	B	50.738	1
**4861**	370	B	73.9272	1
**4861**	372	B	79.5737	0
**4861**	373	B	27.5968	0
**4861**	381	B	79.5868	1
**4861**	382	B	34.8826	0
**4861**	386	B	77.8377	1
**4861**	305	C	79.548	1
**4861**	319	C	53.4375	1
**4861**	320	C	37.7005	-1
**4861**	321	C	47.5595	1
**4861**	351	C	8.5318	1
**4861**	357	C	79.5586	1
**4861**	370	C	25.8187	0
**4861**	372	C	77.91	0
**4861**	373	C	69.24	1
**4861**	381	C	79.5626	1
**4861**	382	C	72.3966	1
**4861**	386	C	41.6333	0
**4861**	305	D	79.9272	1
**4861**	319	D	61.3939	1
**4861**	320	D	65.514	-1
**4861**	321	D	63.4994	0
**4861**	351	D	15.2209	1
**4861**	357	D	58.9338	1

**4861**	370	D	79.5508	1
**4861**	372	D	79.5034	0
**4861**	373	D	79.5813	1
**4861**	381	D	34.0901	0
**4861**	382	D	79.5564	1
**4861**	386	D	79.5446	1
**4862**	305	A	73.2583	1
**4862**	319	A	43.5714	0
**4862**	320	A	70.5605	-1
**4862**	321	A	78.2647	0
**4862**	351	A	14.611	1
**4862**	357	A	52.9974	1
**4862**	370	A	69.7667	1
**4862**	372	A	71.7805	0
**4862**	373	A	73.2072	1
**4862**	381	A	79.5674	1
**4862**	382	A	65.3255	1
**4862**	386	A	67.8274	1
**4862**	305	B	54.0103	1
**4862**	319	B	79.5951	1
**4862**	320	B	59.8919	-1
**4862**	321	B	66.1989	0
**4862**	351	B	18.0162	1
**4862**	357	B	79.5834	1
**4862**	370	B	73.7806	1
**4862**	372	B	79.5302	0
**4862**	373	B	57.3418	1
**4862**	381	B	79.4084	1
**4862**	382	B	38.8553	0
**4862**	386	B	51.3562	1
**4862**	305	C	75.6433	1
**4862**	319	C	58.8785	1
**4862**	320	C	23.908	-1
**4862**	321	C	43.2423	1

**4862**	351	C	4.3229	1
**4862**	357	C	70.7037	1
**4862**	370	C	57.9238	1
**4862**	372	C	79.5531	0
**4862**	373	C	79.5441	1
**4862**	381	C	60.2462	1
**4862**	382	C	65.6574	1
**4862**	386	C	23.6974	0
**4862**	305	D	19.3739	-1
**4862**	319	D	79.5617	1
**4862**	320	D	47.1623	-1
**4862**	321	D	52.1733	0
**4862**	351	D	12.0796	1
**4862**	357	D	74.7528	1
**4862**	370	D	79.5787	1
**4862**	372	D	76.7919	0
**4862**	373	D	47.1483	0
**4862**	381	D	79.5889	1
**4862**	382	D	69.2489	1
**4862**	386	D	79.5937	1
**4863**	305	A	70.3475	1
**4863**	319	A	69.5163	1
**4863**	320	A	60.4064	-1
**4863**	321	A	26.4906	1
**4863**	351	A	7.6766	1
**4863**	357	A	79.5284	1
**4863**	370	A	64.3932	1
**4863**	372	A	79.5361	0
**4863**	373	A	25.532	0
**4863**	381	A	42.3189	0
**4863**	382	A	42.3601	0
**4863**	386	A	69.755	1
**4863**	305	B	68.9105	1
**4863**	319	B	73.6814	1

**4863**	320	B	79.7835	-1
**4863**	321	B	68.238	0
**4863**	351	B	0.1458	1
**4863**	357	B	52.4192	1
**4863**	370	B	69.7492	1
**4863**	372	B	75.5895	0
**4863**	373	B	50.4582	1
**4863**	381	B	79.4026	1
**4863**	382	B	24.1839	0
**4863**	386	B	57.4359	1
**4863**	305	C	58.3525	1
**4863**	319	C	58.305	1
**4863**	320	C	74.5594	-1
**4863**	321	C	46.7244	1
**4863**	351	C	18.2818	1
**4863**	357	C	47.6759	0
**4863**	370	C	75.0664	1
**4863**	372	C	78.9425	0
**4863**	373	C	79.1633	1
**4863**	381	C	78.7963	1
**4863**	382	C	76.4632	1
**4863**	386	C	51.4494	1
**4863**	305	D	56.5425	1
**4863**	319	D	53.5338	1
**4863**	320	D	47.3446	-1
**4863**	321	D	5.1939	0
**4863**	351	D	24.953	-1
**4863**	357	D	72.1821	1
**4863**	370	D	72.5452	1
**4863**	372	D	79.7014	0
**4863**	373	D	37.8809	0
**4863**	381	D	58.3963	1
**4863**	382	D	52.5986	1
**4863**	386	D	79.4616	1

**4864**	305	A	69.4184	1
**4864**	319	A	73.4973	1
**4864**	320	A	70.4658	-1
**4864**	321	A	68.9408	0
**4864**	351	A	9.2996	1
**4864**	357	A	70.4544	1
**4864**	370	A	45.6245	0
**4864**	372	A	67.0168	0
**4864**	373	A	79.5043	1
**4864**	381	A	67.7907	1
**4864**	382	A	19.2624	-1
**4864**	386	A	79.5913	1
**4864**	305	B	59.37	1
**4864**	319	B	33.7702	0
**4864**	320	B	79.6372	-1
**4864**	321	B	72.6549	0
**4864**	351	B	20.7187	-1
**4864**	357	B	79.5851	1
**4864**	370	B	79.507	1
**4864**	372	B	38.834	1
**4864**	373	B	68.2238	1
**4864**	381	B	62.1396	1
**4864**	382	B	44.5125	0
**4864**	386	B	66.5637	1
**4864**	305	C	60.7997	1
**4864**	319	C	75.0109	1
**4864**	320	C	79.4732	-1
**4864**	321	C	33.9355	1
**4864**	351	C	7.8572	1
**4864**	357	C	58.2674	1
**4864**	370	C	70.1932	1
**4864**	372	C	59.8405	0
**4864**	373	C	79.5875	1
**4864**	381	C	21.8305	0

**4864**	382	C	66.6782	1
**4864**	386	C	58.4993	1
**4864**	305	D	70.4618	1
**4864**	319	D	33.8532	0
**4864**	320	D	79.6399	-1
**4864**	321	D	79.5667	0
**4864**	351	D	9.4655	1
**4864**	357	D	79.5229	1
**4864**	370	D	64.322	1
**4864**	372	D	73.0928	0
**4864**	373	D	35.5052	0
**4864**	381	D	31.7797	0
**4864**	382	D	57.7468	1
**4864**	386	D	40.3758	0
**4865**	305	A	33.5122	0
**4865**	319	A	73.5959	1
**4865**	320	A	41.1607	-1
**4865**	321	A	79.5493	0
**4865**	351	A	2.1227	1
**4865**	357	A	46.6355	0
**4865**	370	A	74.9848	1
**4865**	372	A	65.7206	0
**4865**	373	A	23.4486	0
**4865**	381	A	79.5988	1
**4865**	382	A	79.5565	1
**4865**	386	A	72.6427	1
**4865**	305	B	79.5566	1
**4865**	319	B	67.9393	1
**4865**	320	B	44.9805	-1
**4865**	321	B	79.6041	0
**4865**	351	B	15.5215	1
**4865**	357	B	79.4596	1
**4865**	370	B	79.5253	1
**4865**	372	B	79.5388	0

**4865**	373	B	28.9488	0
**4865**	381	B	61.5038	1
**4865**	382	B	78.4595	1
**4865**	386	B	79.556	1
**4865**	305	C	78.9535	1
**4865**	319	C	79.5695	1
**4865**	320	C	33.6446	-1
**4865**	321	C	42.0257	1
**4865**	351	C	4.4693	1
**4865**	357	C	74.5959	1
**4865**	370	C	79.5691	1
**4865**	372	C	72.0343	0
**4865**	373	C	79.5546	1
**4865**	381	C	79.5737	1
**4865**	382	C	40.5685	0
**4865**	386	C	19.592	-1
**4865**	305	D	68.3029	1
**4865**	319	D	60.093	1
**4865**	320	D	79.5437	-1
**4865**	321	D	26.2062	1
**4865**	351	D	12.6392	1
**4865**	357	D	79.5565	1
**4865**	370	D	72.3243	1
**4865**	372	D	30.4878	1
**4865**	373	D	69.016	1
**4865**	381	D	71.3706	1
**4865**	382	D	72.971	1
**4865**	386	D	12.2504	-1
**4866**	305	A	45.7066	0
**4866**	319	A	72.2717	1
**4866**	320	A	79.5705	-1
**4866**	321	A	53.2623	0
**4866**	351	A	0.0482	1
**4866**	357	A	79.5827	1

**4866**	370	A	72.373	1
**4866**	372	A	79.6083	0
**4866**	373	A	63.413	1
**4866**	381	A	59.684	1
**4866**	382	A	79.5635	1
**4866**	386	A	78.8762	1
**4866**	305	B	79.5569	1
**4866**	319	B	64.1975	1
**4866**	320	B	67.9288	-1
**4866**	321	B	44.7617	1
**4866**	351	B	1.4078	1
**4866**	357	B	45.7226	0
**4866**	370	B	73.0422	1
**4866**	372	B	79.5211	0
**4866**	373	B	63.2717	1
**4866**	381	B	79.5487	1
**4866**	382	B	49.7979	0
**4866**	386	B	79.5682	1
**4866**	305	C	51.6554	1
**4866**	319	C	79.55	1
**4866**	320	C	79.5843	-1
**4866**	321	C	55.4401	0
**4866**	351	C	6.9657	1
**4866**	357	C	56.1197	1
**4866**	370	C	57.8092	1
**4866**	372	C	79.5608	0
**4866**	373	C	68.1314	1
**4866**	381	C	79.5731	1
**4866**	382	C	67.446	1
**4866**	386	C	48.882	0
**4866**	305	D	60.558	1
**4866**	319	D	39.6228	0
**4866**	320	D	70.1946	-1
**4866**	321	D	79.5946	0

**4866**	351	D	7.8701	1
**4866**	357	D	46.4974	0
**4866**	370	D	79.6139	1
**4866**	372	D	72.762	0
**4866**	373	D	79.6102	1
**4866**	381	D	79.5768	1
**4866**	382	D	45.8521	0
**4866**	386	D	41.526	0
**4867**	305	A	72.8201	1
**4867**	319	A	70.5359	1
**4867**	320	A	79.5991	-1
**4867**	321	A	71.6673	0
**4867**	351	A	9.4013	1
**4867**	357	A	79.5777	1
**4867**	370	A	78.1669	1
**4867**	372	A	73.2731	0
**4867**	373	A	79.523	1
**4867**	381	A	57.7799	1
**4867**	382	A	24.4237	0
**4867**	386	A	73.7193	1
**4867**	305	B	79.6485	1
**4867**	319	B	79.3572	1
**4867**	320	B	65.2751	-1
**4867**	321	B	67.2866	0
**4867**	351	B	16.8895	1
**4867**	357	B	60.0878	1
**4867**	370	B	39.0555	0
**4867**	372	B	79.5816	0
**4867**	373	B	60.2184	1
**4867**	381	B	65.3939	1
**4867**	382	B	48.8923	0
**4867**	386	B	79.5463	1
**4867**	305	C	79.1814	1
**4867**	319	C	79.5174	1

**4867**	320	C	69.2033	-1
**4867**	321	C	42.9038	1
**4867**	351	C	5.5457	1
**4867**	357	C	72.8939	1
**4867**	370	C	78.1573	1
**4867**	372	C	79.5931	0
**4867**	373	C	44.5577	0
**4867**	381	C	79.5096	1
**4867**	382	C	57.2256	1
**4867**	386	C	45.4807	0
**4867**	305	D	77.379	1
**4867**	319	D	73.6289	1
**4867**	320	D	43.1147	-1
**4867**	321	D	76.3331	0
**4867**	351	D	14.6311	1
**4867**	357	D	57.5838	1
**4867**	370	D	78.8357	1
**4867**	372	D	74.8783	0
**4867**	373	D	76.2914	1
**4867**	381	D	30.0279	0
**4867**	382	D	70.9135	1
**4867**	386	D	79.5993	1
**4868**	305	A	72.8281	1
**4868**	319	A	18.1361	-1
**4868**	320	A	56.571	-1
**4868**	321	A	79.5111	0
**4868**	351	A	7.0599	1
**4868**	357	A	78.7381	1
**4868**	370	A	72.2269	1
**4868**	372	A	79.5192	0
**4868**	373	A	79.5228	1
**4868**	381	A	79.5753	1
**4868**	382	A	72.1625	1
**4868**	386	A	38.6692	0

**4868**	305	B	71.8889	1
**4868**	319	B	79.5441	1
**4868**	320	B	61.9318	-1
**4868**	321	B	79.5604	0
**4868**	351	B	44.0584	-1
**4868**	357	B	79.597	1
**4868**	370	B	79.5949	1
**4868**	372	B	50.9569	0
**4868**	373	B	59.403	1
**4868**	381	B	55.8915	1
**4868**	382	B	79.507	1
**4868**	386	B	79.6907	1
**4868**	305	C	73.8611	1
**4868**	319	C	79.5556	1
**4868**	320	C	79.476	-1
**4868**	321	C	74.8485	0
**4868**	351	C	39.522	-1
**4868**	357	C	33.896	0
**4868**	370	C	79.5974	1
**4868**	372	C	79.4666	0
**4868**	373	C	72.0791	1
**4868**	381	C	44.0373	0
**4868**	382	C	63.9035	1
**4868**	386	C	59.6449	1
**4868**	305	D	79.5577	1
**4868**	319	D	79.5923	1
**4868**	320	D	68.7092	-1
**4868**	321	D	79.5022	0
**4868**	351	D	15.5368	1
**4868**	357	D	71.1954	1
**4868**	370	D	44.5566	0
**4868**	372	D	77.6576	0
**4868**	373	D	78.7371	1
**4868**	381	D	72.4238	1

**4868**	382	D	41.698	0
**4868**	386	D	45.2483	0
**4869**	305	A	57.6448	1
**4869**	319	A	52.9927	1
**4869**	320	A	68.5011	-1
**4869**	321	A	76.9547	0
**4869**	351	A	5.9921	1
**4869**	357	A	53.424	1
**4869**	370	A	73.2924	1
**4869**	372	A	45.6309	1
**4869**	373	A	79.4869	1
**4869**	381	A	60.845	1
**4869**	382	A	46.9238	0
**4869**	386	A	79.5837	1
**4869**	305	B	49.7875	0
**4869**	319	B	72.8785	1
**4869**	320	B	69.489	-1
**4869**	321	B	47.3598	1
**4869**	351	B	19.418	1
**4869**	357	B	79.5355	1
**4869**	370	B	38.4493	0
**4869**	372	B	74.7333	0
**4869**	373	B	64.6649	1
**4869**	381	B	35.5101	0
**4869**	382	B	34.3581	0
**4869**	386	B	43.5988	0
**4869**	305	C	79.3162	1
**4869**	319	C	79.5964	1
**4869**	320	C	72.8786	-1
**4869**	321	C	79.5686	0
**4869**	351	C	0.7771	1
**4869**	357	C	56.8631	1
**4869**	370	C	66.877	1
**4869**	372	C	79.5526	0

**4869**	373	C	79.5685	1
**4869**	381	C	72.8234	1
**4869**	382	C	65.5963	1
**4869**	386	C	28.7442	0
**4869**	305	D	72.6556	1
**4869**	319	D	79.4565	1
**4869**	320	D	61.0828	-1
**4869**	321	D	65.0831	0
**4869**	351	D	12.7694	1
**4869**	357	D	79.5493	1
**4869**	370	D	73.5615	1
**4869**	372	D	79.4904	0
**4869**	373	D	73.4619	1
**4869**	381	D	79.5538	1
**4869**	382	D	58.7442	1
**4869**	386	D	71.3156	1
**4870**	305	A	79.5006	1
**4870**	319	A	60.8411	1
**4870**	320	A	45.3837	-1
**4870**	321	A	79.4866	0
**4870**	351	A	9.3757	1
**4870**	357	A	79.5841	1
**4870**	370	A	79.5549	1
**4870**	372	A	79.5769	0
**4870**	373	A	79.5492	1
**4870**	381	A	79.5676	1
**4870**	382	A	61.8259	1
**4870**	386	A	79.5517	1
**4870**	305	B	57.6565	1
**4870**	319	B	73.9078	1
**4870**	320	B	79.6086	-1
**4870**	321	B	37.2476	1
**4870**	351	B	12.6729	1
**4870**	357	B	51.1484	1

**4870**	370	B	50.3296	1
**4870**	372	B	64.2906	0
**4870**	373	B	57.1354	1
**4870**	381	B	67.0026	1
**4870**	382	B	55.0614	1
**4870**	386	B	32.5601	0
**4870**	305	C	62.773	1
**4870**	319	C	70.7723	1
**4870**	320	C	54.3857	-1
**4870**	321	C	71.8121	0
**4870**	351	C	13.2638	1
**4870**	357	C	79.5996	1
**4870**	370	C	79.4399	1
**4870**	372	C	73.6043	0
**4870**	373	C	41.8116	0
**4870**	381	C	79.5708	1
**4870**	382	C	79.5518	1
**4870**	386	C	26.7367	0
**4870**	305	D	43.1527	0
**4870**	319	D	71.0907	1
**4870**	320	D	76.0172	-1
**4870**	321	D	55.061	0
**4870**	351	D	11.9427	1
**4870**	357	D	77.9574	1
**4870**	370	D	79.5764	1
**4870**	372	D	79.5525	0
**4870**	373	D	56.5109	1
**4870**	381	D	79.54	1
**4870**	382	D	79.5254	1
**4870**	386	D	72.8329	1
**4871**	305	A	74.0669	1
**4871**	319	A	77.389	1
**4871**	320	A	18.5704	1
**4871**	321	A	74.6192	0

**4871**	351	A	11.1389	1
**4871**	357	A	61.8996	1
**4871**	370	A	68.6371	1
**4871**	372	A	69.62	0
**4871**	373	A	79.7238	1
**4871**	381	A	67.1825	1
**4871**	382	A	69.6878	1
**4871**	386	A	53.9048	1
**4871**	305	B	72.1171	1
**4871**	319	B	59.7354	1
**4871**	320	B	79.5993	-1
**4871**	321	B	70.2656	0
**4871**	351	B	6.0893	1
**4871**	357	B	78.9927	1
**4871**	370	B	79.5549	1
**4871**	372	B	20.8706	1
**4871**	373	B	71.1608	1
**4871**	381	B	47.494	0
**4871**	382	B	71.8026	1
**4871**	386	B	50.2736	1
**4871**	305	C	58.0325	1
**4871**	319	C	79.8331	1
**4871**	320	C	77.0144	-1
**4871**	321	C	74.7912	0
**4871**	351	C	15.665	1
**4871**	357	C	72.2037	1
**4871**	370	C	86.6037	1
**4871**	372	C	51.4157	0
**4871**	373	C	79.3886	1
**4871**	381	C	56.2693	1
**4871**	382	C	59.767	1
**4871**	386	C	51.6224	1
**4871**	305	D	72.3692	1
**4871**	319	D	66.1362	1

**4871**	320	D	56.4547	-1
**4871**	321	D	42.7246	1
**4871**	351	D	9.9588	1
**4871**	357	D	73.7291	1
**4871**	370	D	40.4016	0
**4871**	372	D	78.2261	0
**4871**	373	D	79.4473	1
**4871**	381	D	37.219	0
**4871**	382	D	61.5169	1
**4871**	386	D	75.5345	1
**4872**	305	A	66.052	1
**4872**	319	A	72.2043	1
**4872**	320	A	65.7557	-1
**4872**	321	A	72.1615	0
**4872**	351	A	1.9245	1
**4872**	357	A	67.3671	1
**4872**	370	A	35.8561	0
**4872**	372	A	32.1794	1
**4872**	373	A	79.6083	1
**4872**	381	A	79.5726	1
**4872**	382	A	66.2254	1
**4872**	386	A	79.6408	1
**4872**	305	B	79.2352	1
**4872**	319	B	66.9982	1
**4872**	320	B	79.5392	-1
**4872**	321	B	79.633	0
**4872**	351	B	7.7014	1
**4872**	357	B	58.8252	1
**4872**	370	B	70.7039	1
**4872**	372	B	49.4283	1
**4872**	373	B	50.05	1
**4872**	381	B	65.6972	1
**4872**	382	B	72.9643	1
**4872**	386	B	52.7953	1

**4872**	305	C	57.9064	1
**4872**	319	C	79.6857	1
**4872**	320	C	79.6297	-1
**4872**	321	C	70.2463	0
**4872**	351	C	3.3868	1
**4872**	357	C	40.4839	0
**4872**	370	C	56.3742	1
**4872**	372	C	71.0115	0
**4872**	373	C	79.5846	1
**4872**	381	C	76.5694	1
**4872**	382	C	65.3817	1
**4872**	386	C	52.5765	1
**4872**	305	D	75.3977	1
**4872**	319	D	63.0839	1
**4872**	320	D	69.075	-1
**4872**	321	D	66.8447	0
**4872**	351	D	12.3908	1
**4872**	357	D	79.6342	1
**4872**	370	D	54.9617	1
**4872**	372	D	79.3927	0
**4872**	373	D	78.6935	1
**4872**	381	D	79.5873	1
**4872**	382	D	79.6026	1
**4872**	386	D	71.8974	1
**4873**	305	A	72.1178	1
**4873**	319	A	59.1673	1
**4873**	320	A	73.4027	-1
**4873**	321	A	72.8956	0
**4873**	351	A	9.3791	1
**4873**	357	A	41.5649	0
**4873**	370	A	37.8137	0
**4873**	372	A	16.7833	0
**4873**	373	A	79.3995	1
**4873**	381	A	79.5797	1

**4873**	382	A	79.6125	1
**4873**	386	A	54.2271	1
**4873**	305	B	67.4499	1
**4873**	319	B	79.1487	1
**4873**	320	B	72.0687	-1
**4873**	321	B	77.5181	0
**4873**	351	B	14.4883	1
**4873**	357	B	78.926	1
**4873**	370	B	28.698	0
**4873**	372	B	74.1222	0
**4873**	373	B	74.6106	1
**4873**	381	B	79.5521	1
**4873**	382	B	78.1242	1
**4873**	386	B	65.1459	1
**4873**	305	C	79.4418	1
**4873**	319	C	79.5733	1
**4873**	320	C	71.9254	-1
**4873**	321	C	72.0302	0
**4873**	351	C	5.6809	1
**4873**	357	C	74.8311	1
**4873**	370	C	66.885	1
**4873**	372	C	79.5441	0
**4873**	373	C	72.5345	1
**4873**	381	C	78.9986	1
**4873**	382	C	79.5355	1
**4873**	386	C	57.7541	1
**4873**	305	D	76.2098	1
**4873**	319	D	79.1219	1
**4873**	320	D	73.1339	-1
**4873**	321	D	67.1061	0
**4873**	351	D	10.715	1
**4873**	357	D	38.3363	0
**4873**	370	D	68.0463	1
**4873**	372	D	67.5174	0

**4873**	373	D	12.7472	-1
**4873**	381	D	79.5542	1
**4873**	382	D	79.5515	1
**4873**	386	D	79.5382	1
**4874**	305	A	79.6336	1
**4874**	319	A	57.1062	1
**4874**	320	A	75.3228	-1
**4874**	321	A	78.5165	0
**4874**	351	A	2.5918	1
**4874**	357	A	79.5323	1
**4874**	370	A	62.8041	1
**4874**	372	A	65.4887	0
**4874**	373	A	79.525	1
**4874**	381	A	79.6155	1
**4874**	382	A	67.8397	1
**4874**	386	A	73.4193	1
**4874**	305	B	62.946	1
**4874**	319	B	75.5724	1
**4874**	320	B	79.5447	-1
**4874**	321	B	31.9414	1
**4874**	351	B	14.2768	1
**4874**	357	B	74.3321	1
**4874**	370	B	79.4918	1
**4874**	372	B	63.0349	0
**4874**	373	B	74.4378	1
**4874**	381	B	64.074	1
**4874**	382	B	45.7674	0
**4874**	386	B	77.7706	1
**4874**	305	C	56.1202	1
**4874**	319	C	79.3095	1
**4874**	320	C	79.5833	-1
**4874**	321	C	61.8374	0
**4874**	351	C	14.2577	1
**4874**	357	C	71.1549	1

**4874**	370	C	79.4688	1
**4874**	372	C	79.5998	0
**4874**	373	C	72.1086	1
**4874**	381	C	72.2181	1
**4874**	382	C	75.871	1
**4874**	386	C	24.8205	0
**4874**	305	D	79.4805	1
**4874**	319	D	70.3289	1
**4874**	320	D	75.2992	-1
**4874**	321	D	48.9606	1
**4874**	351	D	11.8606	1
**4874**	357	D	64.4265	1
**4874**	370	D	43.8878	0
**4874**	372	D	58.3948	0
**4874**	373	D	71.7004	1
**4874**	381	D	79.5426	1
**4874**	382	D	76.51	1
**4874**	386	D	72.3271	1
**4875**	305	A	78.8178	1
**4875**	319	A	75.7888	1
**4875**	320	A	79.5323	-1
**4875**	321	A	31.325	1
**4875**	351	A	15.6062	1
**4875**	357	A	74.5073	1
**4875**	370	A	51.815	1
**4875**	372	A	61.8819	0
**4875**	373	A	39.1229	0
**4875**	381	A	79.6069	1
**4875**	382	A	73.0116	1
**4875**	386	A	79.5521	1
**4875**	305	B	61.2583	1
**4875**	319	B	55.4046	1
**4875**	320	B	79.656	-1
**4875**	321	B	79.5812	0

**4875**	351	B	15.8397	1
**4875**	357	B	44.4708	0
**4875**	370	B	62.2681	1
**4875**	372	B	79.3639	0
**4875**	373	B	47.3774	0
**4875**	381	B	78.249	1
**4875**	382	B	64.5924	1
**4875**	386	B	62.7298	1
**4875**	305	C	79.5187	1
**4875**	319	C	64.363	1
**4875**	320	C	33.0581	-1
**4875**	321	C	61.4698	0
**4875**	351	C	10.674	1
**4875**	357	C	68.7886	1
**4875**	370	C	66.1029	1
**4875**	372	C	79.6024	0
**4875**	373	C	66.1807	1
**4875**	381	C	40.0751	0
**4875**	382	C	79.5656	1
**4875**	386	C	50.144	1
**4875**	305	D	41.6602	0
**4875**	319	D	74.9785	1
**4875**	320	D	79.5414	-1
**4875**	321	D	79.5685	0
**4875**	351	D	12.6785	1
**4875**	357	D	70.7283	1
**4875**	370	D	47.1037	0
**4875**	372	D	79.551	0
**4875**	373	D	79.4992	1
**4875**	381	D	79.5977	1
**4875**	382	D	56.9868	1
**4875**	386	D	74.1045	1
**4876**	305	A	48.668	0
**4876**	319	A	27.8154	0

**4876**	320	A	75.2883	-1
**4876**	321	A	79.5872	0
**4876**	351	A	11.1992	1
**4876**	357	A	72.3154	1
**4876**	370	A	51.0094	1
**4876**	372	A	45.556	1
**4876**	373	A	79.558	1
**4876**	381	A	34.8943	0
**4876**	382	A	58.6151	1
**4876**	386	A	70.7921	1
**4876**	305	B	67.0632	1
**4876**	319	B	73.1878	1
**4876**	320	B	57.2047	-1
**4876**	321	B	79.558	0
**4876**	351	B	9.7149	1
**4876**	357	B	53.1785	1
**4876**	370	B	60.1043	1
**4876**	372	B	79.5146	0
**4876**	373	B	79.3169	1
**4876**	381	B	27.562	0
**4876**	382	B	54.8362	1
**4876**	386	B	53.5672	1
**4876**	305	C	50.3656	1
**4876**	319	C	79.5793	1
**4876**	320	C	73.5039	-1
**4876**	321	C	73.7539	0
**4876**	351	C	9.4911	1
**4876**	357	C	50.1504	1
**4876**	370	C	79.5539	1
**4876**	372	C	59.7528	0
**4876**	373	C	53.7305	1
**4876**	381	C	72.6566	1
**4876**	382	C	79.5117	1
**4876**	386	C	59.2858	1

**4876**	305	D	71.0486	1
**4876**	319	D	63.719	1
**4876**	320	D	75.9147	-1
**4876**	321	D	79.5247	0
**4876**	351	D	7.9767	1
**4876**	357	D	31.0303	0
**4876**	370	D	78.4543	1
**4876**	372	D	79.5362	0
**4876**	373	D	49.2508	0
**4876**	381	D	60.6472	1
**4876**	382	D	79.5754	1
**4876**	386	D	51.7838	1
**4877**	305	A	77.7236	1
**4877**	319	A	79.5491	1
**4877**	320	A	79.5023	-1
**4877**	321	A	74.362	0
**4877**	351	A	12.5678	1
**4877**	357	A	22.2279	0
**4877**	370	A	79.0994	1
**4877**	372	A	79.5512	0
**4877**	373	A	42.3526	0
**4877**	381	A	68.266	1
**4877**	382	A	79.4808	1
**4877**	386	A	79.5255	1
**4877**	305	B	79.5799	1
**4877**	319	B	43.9498	0
**4877**	320	B	79.5678	-1
**4877**	321	B	45.8038	1
**4877**	351	B	25.8016	-1
**4877**	357	B	62.4158	1
**4877**	370	B	78.8811	1
**4877**	372	B	72.0467	0
**4877**	373	B	66.5974	1
**4877**	381	B	67.8001	1

**4877**	382	B	24.5795	0
**4877**	386	B	77.7352	1
**4877**	305	C	65.9181	1
**4877**	319	C	74.3978	1
**4877**	320	C	68.7133	-1
**4877**	321	C	58.3896	0
**4877**	351	C	0.3689	1
**4877**	357	C	74.5782	1
**4877**	370	C	64.9618	1
**4877**	372	C	79.5906	0
**4877**	373	C	72.0022	1
**4877**	381	C	79.4845	1
**4877**	382	C	74.61	1
**4877**	386	C	51.6446	1
**4877**	305	D	54.8631	1
**4877**	319	D	68.8627	1
**4877**	320	D	77.9111	-1
**4877**	321	D	61.5845	0
**4877**	351	D	17.974	1
**4877**	357	D	65.6417	1
**4877**	370	D	75.6965	1
**4877**	372	D	66.7245	0
**4877**	373	D	58.3126	1
**4877**	381	D	79.6005	1
**4877**	382	D	66.1922	1
**4877**	386	D	79.6147	1
**4878**	305	A	35.3348	0
**4878**	319	A	68.5136	1
**4878**	320	A	35.8808	-1
**4878**	321	A	79.4991	0
**4878**	351	A	20.8658	-1
**4878**	357	A	65.9342	1
**4878**	370	A	43.2299	0
**4878**	372	A	79.5578	0

**4878**	373	A	79.5423	1
**4878**	381	A	79.5715	1
**4878**	382	A	73.2325	1
**4878**	386	A	79.5657	1
**4878**	305	B	79.473	1
**4878**	319	B	72.8644	1
**4878**	320	B	76.3024	-1
**4878**	321	B	71.506	0
**4878**	351	B	14.7837	1
**4878**	357	B	78.136	1
**4878**	370	B	79.5866	1
**4878**	372	B	79.5464	0
**4878**	373	B	77.8701	1
**4878**	381	B	14.2542	-1
**4878**	382	B	54.5308	1
**4878**	386	B	65.4991	1
**4878**	305	C	79.5892	1
**4878**	319	C	53.5229	1
**4878**	320	C	79.4793	-1
**4878**	321	C	78.0098	0
**4878**	351	C	7.2498	1
**4878**	357	C	31.1909	0
**4878**	370	C	71.0982	1
**4878**	372	C	53.5403	0
**4878**	373	C	72.6645	1
**4878**	381	C	34.9879	0
**4878**	382	C	73.5591	1
**4878**	386	C	20.5836	0
**4878**	305	D	58.8998	1
**4878**	319	D	79.661	1
**4878**	320	D	79.5106	-1
**4878**	321	D	61.369	0
**4878**	351	D	13.5433	1
**4878**	357	D	69.1784	1

**4878**	370	D	79.6998	1
**4878**	372	D	76.7163	0
**4878**	373	D	79.5666	1
**4878**	381	D	79.5532	1
**4878**	382	D	55.4175	1
**4878**	386	D	79.5408	1
**4879**	305	A	40.1323	0
**4879**	319	A	79.6606	1
**4879**	320	A	54.8216	-1
**4879**	321	A	79.6436	0
**4879**	351	A	6.3832	1
**4879**	357	A	21.0487	0
**4879**	370	A	72.8899	1
**4879**	372	A	79.4986	0
**4879**	373	A	79.6533	1
**4879**	381	A	63.3715	1
**4879**	382	A	41.3615	0
**4879**	386	A	72.2038	1
**4879**	305	B	72.0739	1
**4879**	319	B	78.1926	1
**4879**	320	B	73.0046	-1
**4879**	321	B	21.4214	1
**4879**	351	B	15.5468	1
**4879**	357	B	76.382	1
**4879**	370	B	61.3458	1
**4879**	372	B	79.6364	0
**4879**	373	B	50.6003	1
**4879**	381	B	79.4851	1
**4879**	382	B	79.5118	1
**4879**	386	B	58.3245	1
**4879**	305	C	71.4232	1
**4879**	319	C	58.389	1
**4879**	320	C	28.9693	-1
**4879**	321	C	55.0559	0

**4879**	351	C	4.1297	1
**4879**	357	C	61.7575	1
**4879**	370	C	79.526	1
**4879**	372	C	54.1367	0
**4879**	373	C	79.7504	1
**4879**	381	C	79.7618	1
**4879**	382	C	79.4153	1
**4879**	386	C	44.4043	0
**4879**	305	D	79.6012	1
**4879**	319	D	71.203	1
**4879**	320	D	71.5696	-1
**4879**	321	D	72.556	0
**4879**	351	D	29.8306	-1
**4879**	357	D	55.9895	1
**4879**	370	D	79.623	1
**4879**	372	D	54.3425	0
**4879**	373	D	72.8233	1
**4879**	381	D	50.1134	1
**4879**	382	D	66.8	1
**4879**	386	D	22.5958	0
**4880**	305	A	75.087	1
**4880**	319	A	14.3769	-1
**4880**	320	A	79.5687	-1
**4880**	321	A	71.2775	0
**4880**	351	A	7.0736	1
**4880**	357	A	23.2287	0
**4880**	370	A	79.5633	1
**4880**	372	A	74.9178	0
**4880**	373	A	31.9526	0
**4880**	381	A	79.5641	1
**4880**	382	A	69.3068	1
**4880**	386	A	13.4331	-1
**4880**	305	B	79.5783	1
**4880**	319	B	75.2638	1

**4880**	320	B	44.6199	-1
**4880**	321	B	77.4759	0
**4880**	351	B	10.9309	1
**4880**	357	B	79.7035	1
**4880**	370	B	71.6132	1
**4880**	372	B	58.9926	0
**4880**	373	B	62.0317	1
**4880**	381	B	55.1711	1
**4880**	382	B	59.7538	1
**4880**	386	B	37.3082	0
**4880**	305	C	79.4018	1
**4880**	319	C	79.5706	1
**4880**	320	C	73.1632	-1
**4880**	321	C	79.5475	0
**4880**	351	C	5.8029	1
**4880**	357	C	64.8113	1
**4880**	370	C	68.6309	1
**4880**	372	C	60.643	0
**4880**	373	C	79.5788	1
**4880**	381	C	72.6646	1
**4880**	382	C	23.5309	0
**4880**	386	C	48.941	0
**4880**	305	D	12.3727	-1
**4880**	319	D	75.6056	1
**4880**	320	D	48.9658	-1
**4880**	321	D	77.4602	0
**4880**	351	D	18.9532	1
**4880**	357	D	52.3039	1
**4880**	370	D	74.5536	1
**4880**	372	D	64.8231	0
**4880**	373	D	21.4634	0
**4880**	381	D	62.8245	1
**4880**	382	D	79.5646	1
**4880**	386	D	79.5744	1

**4881**	305	A	77.8436	1
**4881**	319	A	75.2501	1
**4881**	320	A	72.0049	-1
**4881**	321	A	71.3246	0
**4881**	351	A	4.5932	1
**4881**	357	A	79.5799	1
**4881**	370	A	79.4503	1
**4881**	372	A	79.5031	0
**4881**	373	A	63.3042	1
**4881**	381	A	50.2111	1
**4881**	382	A	73.3044	1
**4881**	386	A	42.2308	0
**4881**	305	B	79.5735	1
**4881**	319	B	79.6132	1
**4881**	320	B	48.7579	-1
**4881**	321	B	78.6821	0
**4881**	351	B	12.7466	1
**4881**	357	B	67.5485	1
**4881**	370	B	43.0327	0
**4881**	372	B	63.5906	0
**4881**	373	B	70.7673	1
**4881**	381	B	55.6921	1
**4881**	382	B	30.5947	0
**4881**	386	B	79.5939	1
**4881**	305	C	78.2711	1
**4881**	319	C	66.8452	1
**4881**	320	C	72.2106	-1
**4881**	321	C	64.0109	0
**4881**	351	C	1.0638	1
**4881**	357	C	79.5176	1
**4881**	370	C	52.3554	1
**4881**	372	C	73.9436	0
**4881**	373	C	79.4707	1
**4881**	381	C	72.3376	1

**4881**	382	C	41.7581	0
**4881**	386	C	39.8698	0
**4881**	305	D	52.8293	1
**4881**	319	D	79.5652	1
**4881**	320	D	71.147	-1
**4881**	321	D	72.3925	0
**4881**	351	D	13.5124	1
**4881**	357	D	59.6423	1
**4881**	370	D	62.8261	1
**4881**	372	D	37.9922	1
**4881**	373	D	45.6345	0
**4881**	381	D	77.8561	1
**4881**	382	D	46.9134	0
**4881**	386	D	76.7206	1
**4882**	305	A	79.5268	1
**4882**	319	A	79.5035	1
**4882**	320	A	56.7014	-1
**4882**	321	A	79.5358	0
**4882**	351	A	324	1
**4882**	357	A	79.5803	1
**4882**	370	A	79.5962	1
**4882**	372	A	79.5258	0
**4882**	373	A	21.2299	0
**4882**	381	A	79.5878	1
**4882**	382	A	57.6081	1
**4882**	386	A	79.543	1
**4882**	305	B	28.5558	0
**4882**	319	B	64.7092	1
**4882**	320	B	31.2899	-1
**4882**	321	B	79.4793	0
**4882**	351	B	10.633	1
**4882**	357	B	79.5552	1
**4882**	370	B	73.0649	1
**4882**	372	B	67.132	0

**4882**	373	B	72.0829	1
**4882**	381	B	70.2884	1
**4882**	382	B	39.3702	0
**4882**	386	B	79.6111	1
**4882**	305	C	63.933	1
**4882**	319	C	54.7019	1
**4882**	320	C	24.8386	-1
**4882**	321	C	79.5305	0
**4882**	351	C	9.4038	1
**4882**	357	C	67.3905	1
**4882**	370	C	77.5911	1
**4882**	372	C	79.5555	0
**4882**	373	C	79.5784	1
**4882**	381	C	48.5592	0
**4882**	382	C	42.5834	0
**4882**	386	C	62.3449	1
**4882**	305	D	42.3286	0
**4882**	319	D	79.5854	1
**4882**	320	D	75.6678	-1
**4882**	321	D	65.7542	0
**4882**	351	D	9.8321	1
**4882**	357	D	73.3198	1
**4882**	370	D	59.1934	1
**4882**	372	D	49.0141	1
**4882**	373	D	45.3088	0
**4882**	381	D	29.6571	0
**4882**	382	D	79.5719	1
**4882**	386	D	79.5637	1
**4883**	305	A	60.7247	1
**4883**	319	A	60.4241	1
**4883**	320	A	79.5829	-1
**4883**	321	A	66.0881	0
**4883**	351	A	9.1996	1
**4883**	357	A	71.2821	1

**4883**	370	A	64.9191	1
**4883**	372	A	54.13	0
**4883**	373	A	79.6437	1
**4883**	381	A	75.4937	1
**4883**	382	A	52.8476	1
**4883**	386	A	59.7349	1
**4883**	305	B	79.513	1
**4883**	319	B	72.6778	1
**4883**	320	B	30.9223	-1
**4883**	321	B	73.7831	0
**4883**	351	B	0.0	1
**4883**	357	B	65.2269	1
**4883**	370	B	77.6685	1
**4883**	372	B	79.5311	0
**4883**	373	B	56.5749	1
**4883**	381	B	67.8273	1
**4883**	382	B	75.5585	1
**4883**	386	B	38.9295	0
**4883**	305	C	79.0306	1
**4883**	319	C	79.5035	1
**4883**	320	C	37.7662	-1
**4883**	321	C	79.4803	0
**4883**	351	C	30.5845	-1
**4883**	357	C	79.4867	1
**4883**	370	C	59.8976	1
**4883**	372	C	71.5632	0
**4883**	373	C	59.059	1
**4883**	381	C	60.4112	1
**4883**	382	C	79.6	1
**4883**	386	C	22.9912	0
**4883**	305	D	13.479	-1
**4883**	319	D	76.3477	1
**4883**	320	D	79.5303	-1
**4883**	321	D	79.6358	0

**4883**	351	D	3.2789	1
**4883**	357	D	71.5589	1
**4883**	370	D	65.6249	1
**4883**	372	D	79.6753	0
**4883**	373	D	69.0247	1
**4883**	381	D	57.2098	1
**4883**	382	D	65.0674	1
**4883**	386	D	79.4487	1
**4884**	305	A	79.064	1
**4884**	319	A	71.5472	1
**4884**	320	A	79.4649	-1
**4884**	321	A	78.9894	0
**4884**	351	A	13.47	1
**4884**	357	A	79.4816	1
**4884**	370	A	79.4989	1
**4884**	372	A	45.2857	1
**4884**	373	A	79.3189	1
**4884**	381	A	71.7923	1
**4884**	382	A	59.3044	1
**4884**	386	A	58.6981	1
**4884**	305	B	66.4236	1
**4884**	319	B	70.2499	1
**4884**	320	B	55.4595	-1
**4884**	321	B	79.5568	0
**4884**	351	B	11.671	1
**4884**	357	B	67.5226	1
**4884**	370	B	64.9154	1
**4884**	372	B	79.4556	0
**4884**	373	B	79.6669	1
**4884**	381	B	67.8327	1
**4884**	382	B	23.8865	0
**4884**	386	B	79.6288	1
**4884**	305	C	79.6917	1
**4884**	319	C	74.7355	1

**4884**	320	C	57.8661	-1
**4884**	321	C	62.7642	0
**4884**	351	C	3.2044	1
**4884**	357	C	79.6424	1
**4884**	370	C	68.0237	1
**4884**	372	C	79.6701	0
**4884**	373	C	79.6615	1
**4884**	381	C	66.4566	1
**4884**	382	C	71.9289	1
**4884**	386	C	51.2516	1
**4884**	305	D	57.5965	1
**4884**	319	D	72.4979	1
**4884**	320	D	78.5956	-1
**4884**	321	D	73.6348	0
**4884**	351	D	13.0642	1
**4884**	357	D	79.4368	1
**4884**	370	D	62.2913	1
**4884**	372	D	64.5043	0
**4884**	373	D	67.1304	1
**4884**	381	D	79.5205	1
**4884**	382	D	79.4899	1
**4884**	386	D	79.6181	1
**4885**	305	A	74.7108	1
**4885**	319	A	71.1101	1
**4885**	320	A	62.3832	-1
**4885**	321	A	45.8808	1
**4885**	351	A	10.3376	1
**4885**	357	A	54.7209	1
**4885**	370	A	72.5014	1
**4885**	372	A	28.4784	1
**4885**	373	A	51.3079	1
**4885**	381	A	72.6402	1
**4885**	382	A	71.6758	1
**4885**	386	A	79.5569	1

**4885**	305	B	71.0196	1
**4885**	319	B	71.3352	1
**4885**	320	B	42.2654	-1
**4885**	321	B	79.5835	0
**4885**	351	B	12.8927	1
**4885**	357	B	70.4377	1
**4885**	370	B	79.527	1
**4885**	372	B	79.5594	0
**4885**	373	B	79.5737	1
**4885**	381	B	67.3748	1
**4885**	382	B	64.7731	1
**4885**	386	B	64.9336	1
**4885**	305	C	52.5166	1
**4885**	319	C	71.112	1
**4885**	320	C	57.8028	-1
**4885**	321	C	30.3398	1
**4885**	351	C	3.5577	1
**4885**	357	C	55.0261	1
**4885**	370	C	73.6674	1
**4885**	372	C	79.5438	0
**4885**	373	C	64.5486	1
**4885**	381	C	57.7074	1
**4885**	382	C	79.5462	1
**4885**	386	C	39.0918	0
**4885**	305	D	37.6029	0
**4885**	319	D	68.3101	1
**4885**	320	D	56.4044	-1
**4885**	321	D	79.5526	0
**4885**	351	D	11.9932	1
**4885**	357	D	71.2714	1
**4885**	370	D	31.2882	0
**4885**	372	D	66.2085	0
**4885**	373	D	79.5687	1
**4885**	381	D	59.3322	1

**4885**	382	D	68.7853	1
**4885**	386	D	79.4439	1
**4886**	305	A	79.5256	1
**4886**	319	A	48.9112	0
**4886**	320	A	56.849	-1
**4886**	321	A	42.3615	1
**4886**	351	A	7.9435	1
**4886**	357	A	29.9914	0
**4886**	370	A	70.4475	1
**4886**	372	A	44.4285	1
**4886**	373	A	79.5895	1
**4886**	381	A	56.9367	1
**4886**	382	A	64.1712	1
**4886**	386	A	79.5791	1
**4886**	305	B	79.5247	1
**4886**	319	B	57.6805	1
**4886**	320	B	44.8128	-1
**4886**	321	B	79.6049	0
**4886**	351	B	13.8527	1
**4886**	357	B	73.9065	1
**4886**	370	B	64.7524	1
**4886**	372	B	79.5291	0
**4886**	373	B	68.7018	1
**4886**	381	B	79.5719	1
**4886**	382	B	71.5733	1
**4886**	386	B	79.358	1
**4886**	305	C	61.7307	1
**4886**	319	C	79.5596	1
**4886**	320	C	79.5222	-1
**4886**	321	C	79.586	0
**4886**	351	C	1.3486	1
**4886**	357	C	72.6651	1
**4886**	370	C	67.1491	1
**4886**	372	C	79.6291	0

**4886**	373	C	78.5577	1
**4886**	381	C	78.4056	1
**4886**	382	C	63.4996	1
**4886**	386	C	25.721	0
**4886**	305	D	68.9062	1
**4886**	319	D	47.514	0
**4886**	320	D	69.8926	-1
**4886**	321	D	43.6773	1
**4886**	351	D	12.2805	1
**4886**	357	D	79.5295	1
**4886**	370	D	63.0274	1
**4886**	372	D	76.7742	0
**4886**	373	D	79.5501	1
**4886**	381	D	79.5147	1
**4886**	382	D	70.1094	1
**4886**	386	D	75.8131	1
**4887**	305	A	79.59	1
**4887**	319	A	65.8466	1
**4887**	320	A	74.1005	-1
**4887**	321	A	49.2332	1
**4887**	351	A	16.1132	1
**4887**	357	A	79.5961	1
**4887**	370	A	63.4459	1
**4887**	372	A	79.5636	0
**4887**	373	A	79.5554	1
**4887**	381	A	60.3656	1
**4887**	382	A	79.5674	1
**4887**	386	A	51.3194	1
**4887**	305	B	79.0	1
**4887**	319	B	71.4728	1
**4887**	320	B	67.9587	-1
**4887**	321	B	76.5355	0
**4887**	351	B	10.5166	1
**4887**	357	B	79.5995	1

**4887**	370	B	53.7802	1
**4887**	372	B	78.8441	0
**4887**	373	B	79.5959	1
**4887**	381	B	40.3303	0
**4887**	382	B	55.5812	1
**4887**	386	B	44.7801	0
**4887**	305	C	50.2643	1
**4887**	319	C	72.1455	1
**4887**	320	C	70.5755	-1
**4887**	321	C	27.1713	1
**4887**	351	C	2.1936	1
**4887**	357	C	72.8488	1
**4887**	370	C	33.4023	0
**4887**	372	C	15.1748	0
**4887**	373	C	79.6011	1
**4887**	381	C	72.4116	1
**4887**	382	C	79.1171	1
**4887**	386	C	63.3685	1
**4887**	305	D	72.7248	1
**4887**	319	D	79.5905	1
**4887**	320	D	37.7905	-1
**4887**	321	D	75.3474	0
**4887**	351	D	6.194	1
**4887**	357	D	71.3568	1
**4887**	370	D	79.6296	1
**4887**	372	D	64.5869	0
**4887**	373	D	69.9758	1
**4887**	381	D	61.6967	1
**4887**	382	D	42.465	0
**4887**	386	D	79.4863	1
**4888**	305	A	69.8649	1
**4888**	319	A	79.5577	1
**4888**	320	A	74.9657	-1
**4888**	321	A	63.5083	0

4888	351	A	1.1969	1
4888	357	A	71.7061	1
4888	370	A	69.4133	1
4888	372	A	51.3869	0
4888	373	A	79.6495	1
4888	381	A	79.5781	1
4888	382	A	73.6855	1
4888	386	A	73.5027	1
4888	305	B	60.5273	1
4888	319	B	78.2618	1
4888	320	B	79.5384	-1
4888	321	B	48.1118	1
4888	351	B	3.9769	1
4888	357	B	78.8646	1
4888	370	B	38.6163	0
4888	372	B	41.222	1
4888	373	B	45.3181	0
4888	381	B	79.5404	1
4888	382	B	68.5644	1
4888	386	B	73.5766	1
4888	305	C	74.8081	1
4888	319	C	70.2164	1
4888	320	C	71.877	-1
4888	321	C	59.9579	0
4888	351	C	7.9634	1
4888	357	C	79.4412	1
4888	370	C	75.8194	1
4888	372	C	73.5698	0
4888	373	C	67.2169	1
4888	381	C	56.2096	1
4888	382	C	63.8778	1
4888	386	C	27.85	0
4888	305	D	62.531	1
4888	319	D	79.6751	1

**4888**	320	D	75.9771	-1
**4888**	321	D	59.2785	0
**4888**	351	D	6.8157	1
**4888**	357	D	74.5646	1
**4888**	370	D	79.5934	1
**4888**	372	D	49.0817	1
**4888**	373	D	79.5206	1
**4888**	381	D	16.439	-1
**4888**	382	D	73.371	1
**4888**	386	D	79.6196	1
**4889**	305	A	71.5196	1
**4889**	319	A	49.7886	0
**4889**	320	A	72.2929	-1
**4889**	321	A	67.2185	0
**4889**	351	A	15.5657	1
**4889**	357	A	79.437	1
**4889**	370	A	31.8705	0
**4889**	372	A	16.711	0
**4889**	373	A	76.9114	1
**4889**	381	A	79.4907	1
**4889**	382	A	65.708	1
**4889**	386	A	79.2033	1
**4889**	305	B	72.7555	1
**4889**	319	B	79.5859	1
**4889**	320	B	63.5688	-1
**4889**	321	B	43.2689	1
**4889**	351	B	26.9164	-1
**4889**	357	B	46.7559	0
**4889**	370	B	79.5519	1
**4889**	372	B	79.5112	0
**4889**	373	B	47.2752	0
**4889**	381	B	73.5245	1
**4889**	382	B	32.1367	0
**4889**	386	B	72.1996	1

**4889**	305	C	69.4016	1
**4889**	319	C	69.6883	1
**4889**	320	C	75.0896	-1
**4889**	321	C	45.7776	1
**4889**	351	C	7.6487	1
**4889**	357	C	56.0952	1
**4889**	370	C	79.5701	1
**4889**	372	C	56.841	0
**4889**	373	C	73.816	1
**4889**	381	C	79.0409	1
**4889**	382	C	63.6606	1
**4889**	386	C	63.6038	1
**4889**	305	D	79.581	1
**4889**	319	D	78.477	1
**4889**	320	D	79.5446	-1
**4889**	321	D	79.5725	0
**4889**	351	D	9.4892	1
**4889**	357	D	50.5582	1
**4889**	370	D	79.2452	1
**4889**	372	D	57.9568	0
**4889**	373	D	65.0697	1
**4889**	381	D	43.7254	0
**4889**	382	D	78.0239	1
**4889**	386	D	72.8748	1
**4890**	305	A	75.5614	1
**4890**	319	A	79.5329	1
**4890**	320	A	72.4664	-1
**4890**	321	A	34.4177	1
**4890**	351	A	5.2914	1
**4890**	357	A	58.8536	1
**4890**	370	A	73.6984	1
**4890**	372	A	67.9384	0
**4890**	373	A	77.8582	1
**4890**	381	A	79.472	1

**4890**	382	A	47.4693	0
**4890**	386	A	54.0579	1
**4890**	305	B	79.5081	1
**4890**	319	B	56.8847	1
**4890**	320	B	74.3086	-1
**4890**	321	B	79.6041	0
**4890**	351	B	15.1293	1
**4890**	357	B	73.0535	1
**4890**	370	B	79.1792	1
**4890**	372	B	73.9353	0
**4890**	373	B	24.5818	0
**4890**	381	B	79.4445	1
**4890**	382	B	56.9007	1
**4890**	386	B	55.32	1
**4890**	305	C	79.5594	1
**4890**	319	C	71.806	1
**4890**	320	C	79.5209	-1
**4890**	321	C	79.6337	0
**4890**	351	C	12.51	1
**4890**	357	C	79.5796	1
**4890**	370	C	45.1843	0
**4890**	372	C	58.6015	0
**4890**	373	C	79.6008	1
**4890**	381	C	76.8719	1
**4890**	382	C	79.4367	1
**4890**	386	C	48.6513	0
**4890**	305	D	42.6872	0
**4890**	319	D	44.7525	0
**4890**	320	D	78.1611	-1
**4890**	321	D	79.5937	0
**4890**	351	D	14.9502	1
**4890**	357	D	79.5209	1
**4890**	370	D	79.6185	1
**4890**	372	D	69.3637	0

**4890**	373	D	73.1086	1
**4890**	381	D	79.5742	1
**4890**	382	D	68.3988	1
**4890**	386	D	79.597	1
**4891**	305	A	66.1395	1
**4891**	319	A	79.5326	1
**4891**	320	A	49.527	-1
**4891**	321	A	79.5421	0
**4891**	351	A	0.3559	1
**4891**	357	A	71.4719	1
**4891**	370	A	69.3157	1
**4891**	372	A	38.6662	1
**4891**	373	A	74.8537	1
**4891**	381	A	79.6447	1
**4891**	382	A	64.5369	1
**4891**	386	A	38.8088	0
**4891**	305	B	49.8489	0
**4891**	319	B	17.0362	-1
**4891**	320	B	79.647	-1
**4891**	321	B	46.8288	1
**4891**	351	B	11.2802	1
**4891**	357	B	59.6793	1
**4891**	370	B	61.9712	1
**4891**	372	B	27.1649	1
**4891**	373	B	65.7003	1
**4891**	381	B	79.599	1
**4891**	382	B	24.2341	0
**4891**	386	B	65.9601	1
**4891**	305	C	34.0927	0
**4891**	319	C	79.5903	1
**4891**	320	C	79.5661	-1
**4891**	321	C	70.3657	0
**4891**	351	C	8.4856	1
**4891**	357	C	40.1013	0

**4891**	370	C	61.6824	1
**4891**	372	C	79.6064	0
**4891**	373	C	79.5568	1
**4891**	381	C	77.3194	1
**4891**	382	C	28.5761	0
**4891**	386	C	48.423	0
**4891**	305	D	77.9122	1
**4891**	319	D	64.5875	1
**4891**	320	D	79.6961	-1
**4891**	321	D	71.6505	0
**4891**	351	D	5.2389	1
**4891**	357	D	48.5369	0
**4891**	370	D	68.4875	1
**4891**	372	D	67.9768	0
**4891**	373	D	54.5251	1
**4891**	381	D	43.0491	0
**4891**	382	D	79.5379	1
**4891**	386	D	79.5553	1
**4892**	305	A	77.3886	1
**4892**	319	A	70.2878	1
**4892**	320	A	79.5973	-1
**4892**	321	A	69.9919	0
**4892**	351	A	40.5951	-1
**4892**	357	A	48.5431	0
**4892**	370	A	79.5929	1
**4892**	372	A	45.7528	1
**4892**	373	A	79.6045	1
**4892**	381	A	79.5082	1
**4892**	382	A	71.2407	1
**4892**	386	A	75.8645	1
**4892**	305	B	79.5844	1
**4892**	319	B	79.5447	1
**4892**	320	B	79.5426	-1
**4892**	321	B	67.1353	0

**4892**	351	B	11.2405	1
**4892**	357	B	79.5765	1
**4892**	370	B	77.8452	1
**4892**	372	B	79.5069	0
**4892**	373	B	58.5445	1
**4892**	381	B	79.5466	1
**4892**	382	B	71.98	1
**4892**	386	B	41.1938	0
**4892**	305	C	79.5781	1
**4892**	319	C	79.5748	1
**4892**	320	C	79.5248	-1
**4892**	321	C	79.5181	0
**4892**	351	C	6.1972	1
**4892**	357	C	68.1092	1
**4892**	370	C	78.752	1
**4892**	372	C	79.5802	0
**4892**	373	C	79.5737	1
**4892**	381	C	78.8256	1
**4892**	382	C	25.8146	0
**4892**	386	C	65.1415	1
**4892**	305	D	74.8246	1
**4892**	319	D	28.8319	0
**4892**	320	D	79.8062	-1
**4892**	321	D	79.5638	0
**4892**	351	D	14.3287	1
**4892**	357	D	79.5999	1
**4892**	370	D	79.2446	1
**4892**	372	D	53.6032	0
**4892**	373	D	79.5542	1
**4892**	381	D	66.2791	1
**4892**	382	D	79.5581	1
**4892**	386	D	51.2866	1
**4893**	305	A	79.5979	1
**4893**	319	A	28.296	0

**4893**	320	A	79.5276	-1
**4893**	321	A	72.4957	0
**4893**	351	A	8.6564	1
**4893**	357	A	54.4888	1
**4893**	370	A	79.2112	1
**4893**	372	A	79.624	0
**4893**	373	A	75.3623	1
**4893**	381	A	79.6769	1
**4893**	382	A	59.499	1
**4893**	386	A	46.0055	0
**4893**	305	B	23.1046	0
**4893**	319	B	66.5378	1
**4893**	320	B	71.4537	-1
**4893**	321	B	59.1017	0
**4893**	351	B	12.7894	1
**4893**	357	B	62.0813	1
**4893**	370	B	79.603	1
**4893**	372	B	79.5036	0
**4893**	373	B	79.2809	1
**4893**	381	B	73.5447	1
**4893**	382	B	49.6393	0
**4893**	386	B	79.5882	1
**4893**	305	C	79.4875	1
**4893**	319	C	79.5864	1
**4893**	320	C	76.3939	-1
**4893**	321	C	43.9239	1
**4893**	351	C	15.1421	1
**4893**	357	C	77.9116	1
**4893**	370	C	70.809	1
**4893**	372	C	53.6758	0
**4893**	373	C	79.3716	1
**4893**	381	C	75.221	1
**4893**	382	C	12.7218	-1
**4893**	386	C	56.5674	1

**4893**	305	D	50.5037	1
**4893**	319	D	79.3857	1
**4893**	320	D	69.4829	-1
**4893**	321	D	79.5229	0
**4893**	351	D	11.8615	1
**4893**	357	D	64.112	1
**4893**	370	D	79.6101	1
**4893**	372	D	71.3054	0
**4893**	373	D	71.8871	1
**4893**	381	D	43.2815	0
**4893**	382	D	76.2709	1
**4893**	386	D	79.5138	1
**4894**	305	A	66.5194	1
**4894**	319	A	63.9219	1
**4894**	320	A	50.7869	-1
**4894**	321	A	69.2237	0
**4894**	351	A	12.7187	1
**4894**	357	A	66.003	1
**4894**	370	A	31.9369	0
**4894**	372	A	15.307	0
**4894**	373	A	78.6066	1
**4894**	381	A	72.2021	1
**4894**	382	A	62.3222	1
**4894**	386	A	72.7879	1
**4894**	305	B	44.7758	0
**4894**	319	B	79.4821	1
**4894**	320	B	43.0105	-1
**4894**	321	B	73.3369	0
**4894**	351	B	0.0	1
**4894**	357	B	62.7967	1
**4894**	370	B	45.8699	0
**4894**	372	B	53.3999	0
**4894**	373	B	33.9721	0
**4894**	381	B	79.596	1

**4894**	382	B	34.3994	0
**4894**	386	B	79.6003	1
**4894**	305	C	70.0408	1
**4894**	319	C	44.3855	0
**4894**	320	C	79.5864	-1
**4894**	321	C	79.5708	0
**4894**	351	C	8.4756	1
**4894**	357	C	75.7607	1
**4894**	370	C	77.9716	1
**4894**	372	C	45.6555	1
**4894**	373	C	48.8149	0
**4894**	381	C	52.4569	1
**4894**	382	C	79.5157	1
**4894**	386	C	4.7798	-1
**4894**	305	D	63.2249	1
**4894**	319	D	43.2568	0
**4894**	320	D	79.6051	-1
**4894**	321	D	62.1165	0
**4894**	351	D	9.9361	1
**4894**	357	D	68.6339	1
**4894**	370	D	66.0136	1
**4894**	372	D	78.9064	0
**4894**	373	D	36.6557	0
**4894**	381	D	50.2634	1
**4894**	382	D	73.1481	1
**4894**	386	D	79.6399	1
**4895**	305	A	52.5627	1
**4895**	319	A	25.5599	0
**4895**	320	A	79.5685	-1
**4895**	321	A	67.0255	0
**4895**	351	A	2.432	1
**4895**	357	A	22.8236	0
**4895**	370	A	15.7023	-1
**4895**	372	A	12.2151	0

**4895**	373	A	79.5152	1
**4895**	381	A	70.8124	1
**4895**	382	A	79.5809	1
**4895**	386	A	63.6854	1
**4895**	305	B	79.2648	1
**4895**	319	B	65.5972	1
**4895**	320	B	67.6471	-1
**4895**	321	B	72.5397	0
**4895**	351	B	16.3393	1
**4895**	357	B	68.9441	1
**4895**	370	B	79.6453	1
**4895**	372	B	79.5799	0
**4895**	373	B	44.6165	0
**4895**	381	B	37.5443	0
**4895**	382	B	34.32	0
**4895**	386	B	43.2519	0
**4895**	305	C	61.9852	1
**4895**	319	C	67.749	1
**4895**	320	C	60.8114	-1
**4895**	321	C	54.1805	0
**4895**	351	C	4.7012	1
**4895**	357	C	79.4223	1
**4895**	370	C	64.1021	1
**4895**	372	C	79.5647	0
**4895**	373	C	44.3772	0
**4895**	381	C	33.8859	0
**4895**	382	C	53.3078	1
**4895**	386	C	66.2009	1
**4895**	305	D	54.3617	1
**4895**	319	D	79.6126	1
**4895**	320	D	47.1736	-1
**4895**	321	D	64.2956	0
**4895**	351	D	9.7981	1
**4895**	357	D	71.043	1

**4895**	370	D	74.298	1
**4895**	372	D	79.5455	0
**4895**	373	D	74.1584	1
**4895**	381	D	49.3553	0
**4895**	382	D	79.5654	1
**4895**	386	D	79.5059	1
**4896**	305	A	79.5274	1
**4896**	319	A	47.5428	0
**4896**	320	A	79.5331	-1
**4896**	321	A	66.3638	0
**4896**	351	A	12.2427	1
**4896**	357	A	41.0152	0
**4896**	370	A	50.4261	1
**4896**	372	A	46.5417	1
**4896**	373	A	79.5617	1
**4896**	381	A	55.8544	1
**4896**	382	A	69.3321	1
**4896**	386	A	74.0195	1
**4896**	305	B	79.4814	1
**4896**	319	B	79.5568	1
**4896**	320	B	49.4091	-1
**4896**	321	B	47.6664	1
**4896**	351	B	22.0892	-1
**4896**	357	B	17.567	-1
**4896**	370	B	63.934	1
**4896**	372	B	47.433	1
**4896**	373	B	33.0875	0
**4896**	381	B	62.7366	1
**4896**	382	B	48.6716	0
**4896**	386	B	77.5575	1
**4896**	305	C	79.5334	1
**4896**	319	C	79.5966	1
**4896**	320	C	67.7695	-1
**4896**	321	C	79.5406	0

**4896**	351	C	2.5365	1
**4896**	357	C	37.7973	0
**4896**	370	C	79.6174	1
**4896**	372	C	69.7348	0
**4896**	373	C	61.8096	1
**4896**	381	C	74.7019	1
**4896**	382	C	64.3528	1
**4896**	386	C	41.7015	0
**4896**	305	D	73.1349	1
**4896**	319	D	69.2189	1
**4896**	320	D	79.5421	-1
**4896**	321	D	79.5466	0
**4896**	351	D	3.4998	1
**4896**	357	D	51.0931	1
**4896**	370	D	56.7065	1
**4896**	372	D	58.5825	0
**4896**	373	D	79.5563	1
**4896**	381	D	79.5847	1
**4896**	382	D	79.4857	1
**4896**	386	D	79.5511	1
**4897**	305	A	49.167	0
**4897**	319	A	79.5035	1
**4897**	320	A	50.6126	-1
**4897**	321	A	70.5284	0
**4897**	351	A	0.0	1
**4897**	357	A	64.8391	1
**4897**	370	A	78.2323	1
**4897**	372	A	68.309	0
**4897**	373	A	79.5776	1
**4897**	381	A	49.0375	0
**4897**	382	A	42.7113	0
**4897**	386	A	49.9645	0
**4897**	305	B	79.5557	1
**4897**	319	B	79.601	1

**4897**	320	B	79.5816	-1
**4897**	321	B	35.0801	1
**4897**	351	B	10.405	1
**4897**	357	B	79.5463	1
**4897**	370	B	33.1994	0
**4897**	372	B	62.6603	0
**4897**	373	B	78.7171	1
**4897**	381	B	31.5017	0
**4897**	382	B	53.3177	1
**4897**	386	B	43.3923	0
**4897**	305	C	69.9605	1
**4897**	319	C	34.069	0
**4897**	320	C	62.6129	-1
**4897**	321	C	79.5613	0
**4897**	351	C	7.2565	1
**4897**	357	C	55.7044	1
**4897**	370	C	79.5584	1
**4897**	372	C	62.9745	0
**4897**	373	C	79.5596	1
**4897**	381	C	56.6802	1
**4897**	382	C	52.5224	1
**4897**	386	C	55.8073	1
**4897**	305	D	25.6589	0
**4897**	319	D	61.5867	1
**4897**	320	D	79.5881	-1
**4897**	321	D	67.7451	0
**4897**	351	D	13.9526	1
**4897**	357	D	79.5884	1
**4897**	370	D	79.5643	1
**4897**	372	D	69.4617	0
**4897**	373	D	47.2722	0
**4897**	381	D	79.5885	1
**4897**	382	D	50.7498	1
**4897**	386	D	72.6783	1

**4898**	305	A	77.5995	1
**4898**	319	A	37.9784	0
**4898**	320	A	71.2182	-1
**4898**	321	A	51.6884	0
**4898**	351	A	33.5583	-1
**4898**	357	A	79.5543	1
**4898**	370	A	79.4807	1
**4898**	372	A	79.6172	0
**4898**	373	A	77.79	1
**4898**	381	A	79.2656	1
**4898**	382	A	50.9161	1
**4898**	386	A	52.4395	1
**4898**	305	B	72.7926	1
**4898**	319	B	79.5676	1
**4898**	320	B	75.4005	-1
**4898**	321	B	65.5139	0
**4898**	351	B	44.7984	-1
**4898**	357	B	44.0136	0
**4898**	370	B	75.5348	1
**4898**	372	B	60.9943	0
**4898**	373	B	70.0667	1
**4898**	381	B	69.8596	1
**4898**	382	B	35.0909	0
**4898**	386	B	79.6878	1
**4898**	305	C	75.5074	1
**4898**	319	C	39.9414	0
**4898**	320	C	50.6574	-1
**4898**	321	C	65.8071	0
**4898**	351	C	1.1349	1
**4898**	357	C	79.5808	1
**4898**	370	C	78.126	1
**4898**	372	C	37.2007	1
**4898**	373	C	72.6681	1
**4898**	381	C	63.2479	1

**4898**	382	C	67.8108	1
**4898**	386	C	44.768	0
**4898**	305	D	79.4206	1
**4898**	319	D	67.3326	1
**4898**	320	D	64.983	-1
**4898**	321	D	79.614	0
**4898**	351	D	14.7305	1
**4898**	357	D	67.0223	1
**4898**	370	D	72.2069	1
**4898**	372	D	79.5989	0
**4898**	373	D	79.5207	1
**4898**	381	D	35.5879	0
**4898**	382	D	64.5966	1
**4898**	386	D	78.636	1
**4899**	305	A	77.2267	1
**4899**	319	A	56.9456	1
**4899**	320	A	79.5607	-1
**4899**	321	A	35.2772	1
**4899**	351	A	6.8144	1
**4899**	357	A	62.4062	1
**4899**	370	A	79.5231	1
**4899**	372	A	79.4803	0
**4899**	373	A	79.5975	1
**4899**	381	A	77.1307	1
**4899**	382	A	11.2248	-1
**4899**	386	A	79.5706	1
**4899**	305	B	23.9175	0
**4899**	319	B	67.4227	1
**4899**	320	B	65.1549	-1
**4899**	321	B	72.6042	0
**4899**	351	B	18.2701	1
**4899**	357	B	79.5546	1
**4899**	370	B	79.566	1
**4899**	372	B	79.1161	0

**4899**	373	B	31.7371	0
**4899**	381	B	55.1971	1
**4899**	382	B	32.7735	0
**4899**	386	B	60.9602	1
**4899**	305	C	61.5974	1
**4899**	319	C	79.5736	1
**4899**	320	C	58.4318	-1
**4899**	321	C	79.5345	0
**4899**	351	C	1.8762	1
**4899**	357	C	69.449	1
**4899**	370	C	77.9853	1
**4899**	372	C	71.1961	0
**4899**	373	C	62.4663	1
**4899**	381	C	14.8024	-1
**4899**	382	C	73.0748	1
**4899**	386	C	48.532	0
**4899**	305	D	79.0577	1
**4899**	319	D	52.3636	1
**4899**	320	D	79.4881	-1
**4899**	321	D	79.6302	0
**4899**	351	D	11.384	1
**4899**	357	D	74.5848	1
**4899**	370	D	32.7818	0
**4899**	372	D	79.5513	0
**4899**	373	D	54.0851	1
**4899**	381	D	51.9875	1
**4899**	382	D	68.0098	1
**4899**	386	D	79.5479	1
**4900**	305	A	40.4044	0
**4900**	319	A	74.1102	1
**4900**	320	A	79.4637	-1
**4900**	321	A	76.1591	0
**4900**	351	A	3.024	1
**4900**	357	A	60.7817	1

**4900**	370	A	77.3253	1
**4900**	372	A	79.6202	0
**4900**	373	A	76.4286	1
**4900**	381	A	32.7257	0
**4900**	382	A	35.9932	0
**4900**	386	A	68.4378	1
**4900**	305	B	79.511	1
**4900**	319	B	79.5194	1
**4900**	320	B	71.0152	-1
**4900**	321	B	74.429	0
**4900**	351	B	15.1097	1
**4900**	357	B	79.5848	1
**4900**	370	B	79.6122	1
**4900**	372	B	78.724	0
**4900**	373	B	73.3585	1
**4900**	381	B	15.5125	-1
**4900**	382	B	51.1508	1
**4900**	386	B	39.3194	0
**4900**	305	C	71.9031	1
**4900**	319	C	64.0698	1
**4900**	320	C	79.571	-1
**4900**	321	C	73.4028	0
**4900**	351	C	7.3291	1
**4900**	357	C	55.7092	1
**4900**	370	C	64.4558	1
**4900**	372	C	67.9124	0
**4900**	373	C	79.6083	1
**4900**	381	C	63.5711	1
**4900**	382	C	65.6985	1
**4900**	386	C	76.2137	1
**4900**	305	D	79.5072	1
**4900**	319	D	21.3435	0
**4900**	320	D	79.609	-1
**4900**	321	D	52.2671	0

**4900**	351	D	15.7879	1
**4900**	357	D	79.5437	1
**4900**	370	D	45.7584	0
**4900**	372	D	79.63	0
**4900**	373	D	62.1414	1
**4900**	381	D	79.5578	1
**4900**	382	D	74.4323	1
**4900**	386	D	79.5634	1
**4901**	305	A	50.0272	1
**4901**	319	A	65.1377	1
**4901**	320	A	79.532	-1
**4901**	321	A	52.6646	0
**4901**	351	A	3.2967	1
**4901**	357	A	69.4706	1
**4901**	370	A	79.5885	1
**4901**	372	A	74.4112	0
**4901**	373	A	79.5409	1
**4901**	381	A	40.9662	0
**4901**	382	A	65.2919	1
**4901**	386	A	71.4228	1
**4901**	305	B	77.1967	1
**4901**	319	B	73.107	1
**4901**	320	B	50.0155	-1
**4901**	321	B	79.6176	0
**4901**	351	B	5.2637	1
**4901**	357	B	79.5666	1
**4901**	370	B	58.032	1
**4901**	372	B	55.3568	0
**4901**	373	B	79.5938	1
**4901**	381	B	79.5923	1
**4901**	382	B	46.3129	0
**4901**	386	B	49.7433	0
**4901**	305	C	73.7153	1
**4901**	319	C	67.1219	1

**4901**	320	C	49.7589	-1
**4901**	321	C	76.93	0
**4901**	351	C	3.2987	1
**4901**	357	C	71.7638	1
**4901**	370	C	53.4352	1
**4901**	372	C	79.6128	0
**4901**	373	C	58.0357	1
**4901**	381	C	72.826	1
**4901**	382	C	61.5374	1
**4901**	386	C	47.1843	0
**4901**	305	D	72.3981	1
**4901**	319	D	75.137	1
**4901**	320	D	79.5072	-1
**4901**	321	D	74.0483	0
**4901**	351	D	18.6806	1
**4901**	357	D	79.5813	1
**4901**	370	D	61.5732	1
**4901**	372	D	79.5622	0
**4901**	373	D	70.2431	1
**4901**	381	D	70.508	1
**4901**	382	D	79.5744	1
**4901**	386	D	70.3465	1
**4902**	305	A	79.5682	1
**4902**	319	A	47.2311	0
**4902**	320	A	79.5783	-1
**4902**	321	A	63.1422	0
**4902**	351	A	5.8875	1
**4902**	357	A	77.521	1
**4902**	370	A	57.6632	1
**4902**	372	A	66.7053	0
**4902**	373	A	21.601	0
**4902**	381	A	70.7971	1
**4902**	382	A	70.1189	1
**4902**	386	A	79.6877	1

**4902**	305	B	73.2751	1
**4902**	319	B	69.2915	1
**4902**	320	B	71.4912	-1
**4902**	321	B	79.5993	0
**4902**	351	B	20.2669	-1
**4902**	357	B	64.4421	1
**4902**	370	B	77.1179	1
**4902**	372	B	79.7511	0
**4902**	373	B	65.3911	1
**4902**	381	B	71.9555	1
**4902**	382	B	51.9984	1
**4902**	386	B	75.0731	1
**4902**	305	C	76.1325	1
**4902**	319	C	73.8151	1
**4902**	320	C	79.6729	-1
**4902**	321	C	70.8462	0
**4902**	351	C	10.5175	1
**4902**	357	C	79.5162	1
**4902**	370	C	79.539	1
**4902**	372	C	73.6799	0
**4902**	373	C	78.3698	1
**4902**	381	C	76.2141	1
**4902**	382	C	76.5985	1
**4902**	386	C	32.7218	0
**4902**	305	D	73.7673	1
**4902**	319	D	44.6132	0
**4902**	320	D	79.3296	-1
**4902**	321	D	68.059	0
**4902**	351	D	6.4134	1
**4902**	357	D	56.7362	1
**4902**	370	D	75.3098	1
**4902**	372	D	79.7492	0
**4902**	373	D	79.6414	1
**4902**	381	D	48.8975	0

**4902**	382	D	79.5344	1
**4902**	386	D	57.519	1
**4903**	305	A	31.5371	0
**4903**	319	A	79.5817	1
**4903**	320	A	77.9344	-1
**4903**	321	A	67.9313	0
**4903**	351	A	6.2356	1
**4903**	357	A	74.0648	1
**4903**	370	A	45.3257	0
**4903**	372	A	48.4886	1
**4903**	373	A	79.5995	1
**4903**	381	A	55.7086	1
**4903**	382	A	66.3993	1
**4903**	386	A	79.5949	1
**4903**	305	B	60.7864	1
**4903**	319	B	61.1555	1
**4903**	320	B	43.5164	-1
**4903**	321	B	79.4843	0
**4903**	351	B	14.932	1
**4903**	357	B	19.4861	-1
**4903**	370	B	71.812	1
**4903**	372	B	73.3013	0
**4903**	373	B	79.523	1
**4903**	381	B	69.4738	1
**4903**	382	B	50.8132	1
**4903**	386	B	73.2524	1
**4903**	305	C	61.8576	1
**4903**	319	C	21.272	0
**4903**	320	C	79.4885	-1
**4903**	321	C	44.7757	1
**4903**	351	C	14.9805	1
**4903**	357	C	75.1567	1
**4903**	370	C	63.827	1
**4903**	372	C	79.5332	0

**4903**	373	C	62.8072	1
**4903**	381	C	79.5027	1
**4903**	382	C	79.5516	1
**4903**	386	C	25.8719	0
**4903**	305	D	17.3483	-1
**4903**	319	D	40.6686	0
**4903**	320	D	74.9454	-1
**4903**	321	D	44.0338	1
**4903**	351	D	15.0256	1
**4903**	357	D	79.3357	1
**4903**	370	D	79.1168	1
**4903**	372	D	79.55	0
**4903**	373	D	79.5801	1
**4903**	381	D	79.5486	1
**4903**	382	D	56.1491	1
**4903**	386	D	79.4799	1
**4904**	305	A	71.6066	1
**4904**	319	A	60.4319	1
**4904**	320	A	79.5559	-1
**4904**	321	A	76.6312	0
**4904**	351	A	4.1378	1
**4904**	357	A	79.5642	1
**4904**	370	A	43.5989	0
**4904**	372	A	37.9524	1
**4904**	373	A	78.1929	1
**4904**	381	A	79.5697	1
**4904**	382	A	79.6204	1
**4904**	386	A	73.5226	1
**4904**	305	B	75.9331	1
**4904**	319	B	73.7444	1
**4904**	320	B	56.7995	-1
**4904**	321	B	79.5975	0
**4904**	351	B	6.7146	1
**4904**	357	B	67.6692	1

**4904**	370	B	79.6062	1
**4904**	372	B	73.1106	0
**4904**	373	B	60.292	1
**4904**	381	B	44.5819	0
**4904**	382	B	36.29	0
**4904**	386	B	63.9007	1
**4904**	305	C	58.5515	1
**4904**	319	C	75.1077	1
**4904**	320	C	55.7885	-1
**4904**	321	C	39.6393	1
**4904**	351	C	0.0048	1
**4904**	357	C	66.7539	1
**4904**	370	C	61.9049	1
**4904**	372	C	78.654	0
**4904**	373	C	79.5338	1
**4904**	381	C	73.8882	1
**4904**	382	C	56.236	1
**4904**	386	C	34.0652	0
**4904**	305	D	79.5533	1
**4904**	319	D	35.1418	0
**4904**	320	D	75.6618	-1
**4904**	321	D	60.6231	0
**4904**	351	D	15.3777	1
**4904**	357	D	71.7021	1
**4904**	370	D	75.6635	1
**4904**	372	D	79.5726	0
**4904**	373	D	71.9021	1
**4904**	381	D	71.0539	1
**4904**	382	D	79.5628	1
**4904**	386	D	78.862	1
**4905**	305	A	77.7331	1
**4905**	319	A	69.5817	1
**4905**	320	A	78.9268	-1
**4905**	321	A	30.5486	1

**4905**	351	A	18.1622	1
**4905**	357	A	75.1118	1
**4905**	370	A	64.6878	1
**4905**	372	A	54.2561	0
**4905**	373	A	79.6028	1
**4905**	381	A	73.0298	1
**4905**	382	A	78.6025	1
**4905**	386	A	79.2381	1
**4905**	305	B	65.0704	1
**4905**	319	B	70.6772	1
**4905**	320	B	56.2108	-1
**4905**	321	B	79.4773	0
**4905**	351	B	10.7641	1
**4905**	357	B	55.9192	1
**4905**	370	B	64.0143	1
**4905**	372	B	68.2058	0
**4905**	373	B	77.695	1
**4905**	381	B	79.5409	1
**4905**	382	B	38.6133	0
**4905**	386	B	32.5188	0
**4905**	305	C	71.887	1
**4905**	319	C	79.7863	1
**4905**	320	C	71.6884	-1
**4905**	321	C	79.2938	0
**4905**	351	C	19.4426	1
**4905**	357	C	71.6375	1
**4905**	370	C	62.4936	1
**4905**	372	C	74.2143	0
**4905**	373	C	49.0173	0
**4905**	381	C	68.4525	1
**4905**	382	C	73.6757	1
**4905**	386	C	60.0801	1
**4905**	305	D	53.1565	1
**4905**	319	D	72.8084	1

**4905**	320	D	73.9741	-1
**4905**	321	D	79.582	0
**4905**	351	D	5.6276	1
**4905**	357	D	70.8336	1
**4905**	370	D	35.3242	0
**4905**	372	D	35.2888	1
**4905**	373	D	63.2945	1
**4905**	381	D	69.1131	1
**4905**	382	D	79.5434	1
**4905**	386	D	79.5169	1
**4906**	305	A	65.9635	1
**4906**	319	A	79.3918	1
**4906**	320	A	71.9153	-1
**4906**	321	A	74.2871	0
**4906**	351	A	12.7419	1
**4906**	357	A	72.9532	1
**4906**	370	A	79.5078	1
**4906**	372	A	79.4846	0
**4906**	373	A	72.3529	1
**4906**	381	A	54.8363	1
**4906**	382	A	58.8719	1
**4906**	386	A	64.697	1
**4906**	305	B	52.7077	1
**4906**	319	B	78.3669	1
**4906**	320	B	45.8532	-1
**4906**	321	B	52.8746	0
**4906**	351	B	15.9821	1
**4906**	357	B	70.5031	1
**4906**	370	B	13.4882	-1
**4906**	372	B	79.6682	0
**4906**	373	B	79.5694	1
**4906**	381	B	64.4714	1
**4906**	382	B	18.7651	-1
**4906**	386	B	64.1336	1

**4906**	305	C	68.492	1
**4906**	319	C	74.7865	1
**4906**	320	C	77.2409	-1
**4906**	321	C	63.2205	0
**4906**	351	C	0.5547	1
**4906**	357	C	74.2953	1
**4906**	370	C	43.2671	0
**4906**	372	C	54.6443	0
**4906**	373	C	62.2139	1
**4906**	381	C	64.346	1
**4906**	382	C	61.6289	1
**4906**	386	C	67.5586	1
**4906**	305	D	57.4711	1
**4906**	319	D	56.679	1
**4906**	320	D	64.0011	-1
**4906**	321	D	40.5749	1
**4906**	351	D	15.8032	1
**4906**	357	D	59.9115	1
**4906**	370	D	52.3965	1
**4906**	372	D	79.5443	0
**4906**	373	D	76.3461	1
**4906**	381	D	32.7107	0
**4906**	382	D	79.6529	1
**4906**	386	D	45.4998	0
**4907**	305	A	34.2904	0
**4907**	319	A	42.3091	0
**4907**	320	A	79.049	-1
**4907**	321	A	35.2495	1
**4907**	351	A	3.2845	1
**4907**	357	A	25.8615	0
**4907**	370	A	79.5855	1
**4907**	372	A	62.6002	0
**4907**	373	A	79.5724	1
**4907**	381	A	59.0152	1

**4907**	382	A	67.1975	1
**4907**	386	A	79.5223	1
**4907**	305	B	77.832	1
**4907**	319	B	33.088	0
**4907**	320	B	56.824	-1
**4907**	321	B	70.1069	0
**4907**	351	B	13.4347	1
**4907**	357	B	74.3684	1
**4907**	370	B	51.6809	1
**4907**	372	B	74.1472	0
**4907**	373	B	73.5606	1
**4907**	381	B	79.5653	1
**4907**	382	B	60.503	1
**4907**	386	B	72.9718	1
**4907**	305	C	73.6658	1
**4907**	319	C	56.5683	1
**4907**	320	C	38.567	-1
**4907**	321	C	75.2313	0
**4907**	351	C	4.6309	1
**4907**	357	C	74.3994	1
**4907**	370	C	69.8227	1
**4907**	372	C	79.6691	0
**4907**	373	C	78.4424	1
**4907**	381	C	79.5822	1
**4907**	382	C	71.2627	1
**4907**	386	C	47.6225	0
**4907**	305	D	79.6214	1
**4907**	319	D	72.7576	1
**4907**	320	D	79.5973	-1
**4907**	321	D	73.6098	0
**4907**	351	D	38.0841	-1
**4907**	357	D	78.1376	1
**4907**	370	D	79.5982	1
**4907**	372	D	67.0979	0

**4907**	373	D	79.4849	1
**4907**	381	D	74.544	1
**4907**	382	D	72.5436	1
**4907**	386	D	72.5161	1
**4908**	305	A	45.7022	0
**4908**	319	A	50.9504	1
**4908**	320	A	79.5653	-1
**4908**	321	A	37.7116	1
**4908**	351	A	0.2156	1
**4908**	357	A	58.0524	1
**4908**	370	A	79.4505	1
**4908**	372	A	79.5635	0
**4908**	373	A	72.2695	1
**4908**	381	A	73.1488	1
**4908**	382	A	23.2357	0
**4908**	386	A	79.5653	1
**4908**	305	B	78.8985	1
**4908**	319	B	37.2458	0
**4908**	320	B	33.4115	-1
**4908**	321	B	79.4626	0
**4908**	351	B	44.0313	-1
**4908**	357	B	79.4033	1
**4908**	370	B	33.1766	0
**4908**	372	B	69.4736	0
**4908**	373	B	79.5231	1
**4908**	381	B	39.662	0
**4908**	382	B	38.9605	0
**4908**	386	B	61.6977	1
**4908**	305	C	71.3109	1
**4908**	319	C	68.9421	1
**4908**	320	C	79.5678	-1
**4908**	321	C	44.4616	1
**4908**	351	C	4.7139	1
**4908**	357	C	76.6636	1

**4908**	370	C	70.9203	1
**4908**	372	C	72.7713	0
**4908**	373	C	60.3418	1
**4908**	381	C	79.6385	1
**4908**	382	C	79.5466	1
**4908**	386	C	13.5447	-1
**4908**	305	D	47.6808	0
**4908**	319	D	79.5132	1
**4908**	320	D	60.2514	-1
**4908**	321	D	48.9297	1
**4908**	351	D	4.6657	1
**4908**	357	D	79.6026	1
**4908**	370	D	74.2419	1
**4908**	372	D	72.6395	0
**4908**	373	D	61.7685	1
**4908**	381	D	64.3857	1
**4908**	382	D	60.951	1
**4908**	386	D	79.5281	1
**4909**	305	A	79.5416	1
**4909**	319	A	73.706	1
**4909**	320	A	79.5709	-1
**4909**	321	A	40.2766	1
**4909**	351	A	16.4246	1
**4909**	357	A	65.1333	1
**4909**	370	A	54.8874	1
**4909**	372	A	75.6181	0
**4909**	373	A	61.7682	1
**4909**	381	A	61.0745	1
**4909**	382	A	64.7352	1
**4909**	386	A	79.5724	1
**4909**	305	B	52.6446	1
**4909**	319	B	77.4951	1
**4909**	320	B	35.9029	-1
**4909**	321	B	72.89	0

**4909**	351	B	10.2418	1
**4909**	357	B	79.5394	1
**4909**	370	B	65.7829	1
**4909**	372	B	72.5898	0
**4909**	373	B	69.8807	1
**4909**	381	B	63.0368	1
**4909**	382	B	68.3251	1
**4909**	386	B	79.6254	1
**4909**	305	C	47.7755	0
**4909**	319	C	37.8914	0
**4909**	320	C	74.7573	-1
**4909**	321	C	79.5653	0
**4909**	351	C	5.7664	1
**4909**	357	C	79.2261	1
**4909**	370	C	79.562	1
**4909**	372	C	65.6556	0
**4909**	373	C	79.547	1
**4909**	381	C	72.7855	1
**4909**	382	C	48.3323	0
**4909**	386	C	36.41	0
**4909**	305	D	27.6473	0
**4909**	319	D	78.0015	1
**4909**	320	D	60.9602	-1
**4909**	321	D	49.4323	1
**4909**	351	D	4.1914	1
**4909**	357	D	55.8467	1
**4909**	370	D	71.6003	1
**4909**	372	D	57.9325	0
**4909**	373	D	72.2585	1
**4909**	381	D	72.3906	1
**4909**	382	D	79.5598	1
**4909**	386	D	79.582	1
**4910**	305	A	59.9845	1
**4910**	319	A	71.4618	1

**4910**	320	A	79.5381	-1
**4910**	321	A	79.3872	0
**4910**	351	A	0.7707	1
**4910**	357	A	79.5341	1
**4910**	370	A	72.3135	1
**4910**	372	A	71.8728	0
**4910**	373	A	59.0397	1
**4910**	381	A	79.4927	1
**4910**	382	A	78.652	1
**4910**	386	A	27.4885	0
**4910**	305	B	74.1531	1
**4910**	319	B	79.6013	1
**4910**	320	B	60.3273	-1
**4910**	321	B	64.5072	0
**4910**	351	B	7.1449	1
**4910**	357	B	62.936	1
**4910**	370	B	79.5656	1
**4910**	372	B	79.5508	0
**4910**	373	B	45.6631	0
**4910**	381	B	79.4237	1
**4910**	382	B	79.517	1
**4910**	386	B	47.7374	0
**4910**	305	C	50.6394	1
**4910**	319	C	79.3985	1
**4910**	320	C	79.5653	-1
**4910**	321	C	79.5993	0
**4910**	351	C	7.8496	1
**4910**	357	C	68.2317	1
**4910**	370	C	58.1632	1
**4910**	372	C	79.6318	0
**4910**	373	C	73.8545	1
**4910**	381	C	78.2164	1
**4910**	382	C	50.5634	1
**4910**	386	C	55.3198	1

**4910**	305	D	79.4751	1
**4910**	319	D	78.5887	1
**4910**	320	D	79.5955	-1
**4910**	321	D	75.3848	0
**4910**	351	D	43.7653	-1
**4910**	357	D	79.4555	1
**4910**	370	D	71.8395	1
**4910**	372	D	79.6725	0
**4910**	373	D	79.4876	1
**4910**	381	D	74.0927	1
**4910**	382	D	56.5849	1
**4910**	386	D	79.6413	1
**4911**	305	A	62.5443	1
**4911**	319	A	79.6676	1
**4911**	320	A	79.5972	-1
**4911**	321	A	79.4968	0
**4911**	351	A	4.1476	1
**4911**	357	A	79.5486	1
**4911**	370	A	30.5219	0
**4911**	372	A	34.3145	1
**4911**	373	A	75.7974	1
**4911**	381	A	79.6333	1
**4911**	382	A	63.9764	1
**4911**	386	A	68.2548	1
**4911**	305	B	71.8458	1
**4911**	319	B	76.7954	1
**4911**	320	B	71.6821	-1
**4911**	321	B	71.6757	0
**4911**	351	B	12.1897	1
**4911**	357	B	65.7638	1
**4911**	370	B	67.0696	1
**4911**	372	B	71.8077	0
**4911**	373	B	61.3554	1
**4911**	381	B	59.3675	1

**4911**	382	B	52.0024	1
**4911**	386	B	62.0083	1
**4911**	305	C	57.7864	1
**4911**	319	C	31.7728	0
**4911**	320	C	75.1128	-1
**4911**	321	C	71.0188	0
**4911**	351	C	10.1963	1
**4911**	357	C	72.5886	1
**4911**	370	C	79.6451	1
**4911**	372	C	72.2138	0
**4911**	373	C	70.7218	1
**4911**	381	C	79.3755	1
**4911**	382	C	70.9173	1
**4911**	386	C	48.1254	0
**4911**	305	D	70.9062	1
**4911**	319	D	79.5328	1
**4911**	320	D	74.9072	-1
**4911**	321	D	42.8548	1
**4911**	351	D	29.5839	-1
**4911**	357	D	62.0955	1
**4911**	370	D	67.3212	1
**4911**	372	D	45.862	1
**4911**	373	D	50.0629	1
**4911**	381	D	73.2437	1
**4911**	382	D	79.5845	1
**4911**	386	D	29.6051	0
**4912**	305	A	27.3716	0
**4912**	319	A	79.53	1
**4912**	320	A	66.9323	-1
**4912**	321	A	63.9679	0
**4912**	351	A	3.7595	1
**4912**	357	A	79.5481	1
**4912**	370	A	78.1838	1
**4912**	372	A	70.9154	0

**4912**	373	A	73.163	1
**4912**	381	A	77.8193	1
**4912**	382	A	79.5513	1
**4912**	386	A	76.7579	1
**4912**	305	B	73.3955	1
**4912**	319	B	67.1315	1
**4912**	320	B	68.0939	-1
**4912**	321	B	79.573	0
**4912**	351	B	11.7402	1
**4912**	357	B	79.56	1
**4912**	370	B	79.5633	1
**4912**	372	B	79.5548	0
**4912**	373	B	79.555	1
**4912**	381	B	34.3037	0
**4912**	382	B	55.1001	1
**4912**	386	B	69.8637	1
**4912**	305	C	39.0719	0
**4912**	319	C	45.3247	0
**4912**	320	C	41.8817	-1
**4912**	321	C	49.8662	1
**4912**	351	C	4.305	1
**4912**	357	C	79.5752	1
**4912**	370	C	35.6996	0
**4912**	372	C	79.5493	0
**4912**	373	C	73.5326	1
**4912**	381	C	79.5597	1
**4912**	382	C	79.564	1
**4912**	386	C	44.3885	0
**4912**	305	D	75.1763	1
**4912**	319	D	41.6853	0
**4912**	320	D	32.3245	-1
**4912**	321	D	49.3599	1
**4912**	351	D	5.2197	1
**4912**	357	D	79.5479	1

**4912**	370	D	72.6949	1
**4912**	372	D	79.5656	0
**4912**	373	D	73.6938	1
**4912**	381	D	79.4642	1
**4912**	382	D	79.5568	1
**4912**	386	D	36.009	0
**4913**	305	A	74.3641	1
**4913**	319	A	74.7119	1
**4913**	320	A	54.155	-1
**4913**	321	A	57.3204	0
**4913**	351	A	10.5147	1
**4913**	357	A	71.961	1
**4913**	370	A	27.4717	0
**4913**	372	A	42.8426	1
**4913**	373	A	45.9387	0
**4913**	381	A	74.733	1
**4913**	382	A	78.9842	1
**4913**	386	A	49.9886	0
**4913**	305	B	56.6883	1
**4913**	319	B	72.431	1
**4913**	320	B	79.4419	-1
**4913**	321	B	47.7377	1
**4913**	351	B	9.8124	1
**4913**	357	B	66.8675	1
**4913**	370	B	72.604	1
**4913**	372	B	72.33	0
**4913**	373	B	60.1884	1
**4913**	381	B	43.8012	0
**4913**	382	B	34.5582	0
**4913**	386	B	55.0315	1
**4913**	305	C	63.3508	1
**4913**	319	C	79.5321	1
**4913**	320	C	79.4248	-1
**4913**	321	C	54.445	0

**4913**	351	C	2.572	1
**4913**	357	C	26.9243	0
**4913**	370	C	73.3388	1
**4913**	372	C	53.7417	0
**4913**	373	C	70.8131	1
**4913**	381	C	79.611	1
**4913**	382	C	75.6134	1
**4913**	386	C	59.7	1
**4913**	305	D	33.3034	0
**4913**	319	D	68.9189	1
**4913**	320	D	72.0617	-1
**4913**	321	D	56.6146	0
**4913**	351	D	10.9464	1
**4913**	357	D	79.4262	1
**4913**	370	D	69.8678	1
**4913**	372	D	60.8103	0
**4913**	373	D	79.691	1
**4913**	381	D	62.3467	1
**4913**	382	D	65.6288	1
**4913**	386	D	19.9696	-1
**4914**	305	A	63.0842	1
**4914**	319	A	48.8575	0
**4914**	320	A	71.4308	-1
**4914**	321	A	75.6153	0
**4914**	351	A	9.6688	1
**4914**	357	A	59.7923	1
**4914**	370	A	46.1954	0
**4914**	372	A	79.5386	0
**4914**	373	A	58.1695	1
**4914**	381	A	79.6027	1
**4914**	382	A	72.6337	1
**4914**	386	A	79.5281	1
**4914**	305	B	76.6246	1
**4914**	319	B	79.4773	1

**4914**	320	B	79.481	-1
**4914**	321	B	66.163	0
**4914**	351	B	8.9462	1
**4914**	357	B	73.5241	1
**4914**	370	B	61.8836	1
**4914**	372	B	18.3031	0
**4914**	373	B	50.9219	1
**4914**	381	B	79.6442	1
**4914**	382	B	25.025	0
**4914**	386	B	57.9403	1
**4914**	305	C	68.3148	1
**4914**	319	C	79.5986	1
**4914**	320	C	64.6155	-1
**4914**	321	C	68.0958	0
**4914**	351	C	11.8162	1
**4914**	357	C	73.5665	1
**4914**	370	C	78.9972	1
**4914**	372	C	79.5559	0
**4914**	373	C	33.2931	0
**4914**	381	C	75.9254	1
**4914**	382	C	79.551	1
**4914**	386	C	37.819	0
**4914**	305	D	25.5416	0
**4914**	319	D	79.6335	1
**4914**	320	D	56.001	-1
**4914**	321	D	40.3175	1
**4914**	351	D	14.68	1
**4914**	357	D	79.4487	1
**4914**	370	D	42.011	0
**4914**	372	D	72.9349	0
**4914**	373	D	42.0151	0
**4914**	381	D	65.7352	1
**4914**	382	D	47.0003	0
**4914**	386	D	51.0361	1

**4915**	305	A	63.4588	1
**4915**	319	A	41.1681	0
**4915**	320	A	73.7483	-1
**4915**	321	A	65.994	0
**4915**	351	A	3.4966	1
**4915**	357	A	44.8123	0
**4915**	370	A	34.7414	0
**4915**	372	A	67.7378	0
**4915**	373	A	64.3701	1
**4915**	381	A	48.4871	0
**4915**	382	A	79.6182	1
**4915**	386	A	64.6283	1
**4915**	305	B	78.7596	1
**4915**	319	B	79.5599	1
**4915**	320	B	57.501	-1
**4915**	321	B	76.5403	0
**4915**	351	B	16.4378	1
**4915**	357	B	75.6355	1
**4915**	370	B	72.7715	1
**4915**	372	B	71.4596	0
**4915**	373	B	78.9232	1
**4915**	381	B	74.3408	1
**4915**	382	B	75.1252	1
**4915**	386	B	79.6654	1
**4915**	305	C	64.584	1
**4915**	319	C	58.5372	1
**4915**	320	C	79.4853	-1
**4915**	321	C	73.7666	0
**4915**	351	C	8.7238	1
**4915**	357	C	79.6146	1
**4915**	370	C	77.5787	1
**4915**	372	C	79.5648	0
**4915**	373	C	79.6057	1
**4915**	381	C	63.2825	1

**4915**	382	C	59.018	1
**4915**	386	C	39.9552	0
**4915**	305	D	73.7738	1
**4915**	319	D	69.1252	1
**4915**	320	D	79.538	-1
**4915**	321	D	70.7204	0
**4915**	351	D	12.1491	1
**4915**	357	D	65.0933	1
**4915**	370	D	52.513	1
**4915**	372	D	70.7501	0
**4915**	373	D	79.4862	1
**4915**	381	D	78.2008	1
**4915**	382	D	79.5121	1
**4915**	386	D	79.6743	1
**4916**	305	A	79.6159	1
**4916**	319	A	25.1334	0
**4916**	320	A	66.9828	-1
**4916**	321	A	78.6002	0
**4916**	351	A	6.3637	1
**4916**	357	A	22.5185	0
**4916**	370	A	52.2004	1
**4916**	372	A	53.1709	0
**4916**	373	A	79.4398	1
**4916**	381	A	53.7323	1
**4916**	382	A	52.7599	1
**4916**	386	A	79.5104	1
**4916**	305	B	41.7195	0
**4916**	319	B	79.699	1
**4916**	320	B	79.424	-1
**4916**	321	B	71.8043	0
**4916**	351	B	13.0542	1
**4916**	357	B	76.4792	1
**4916**	370	B	76.4591	1
**4916**	372	B	75.0909	0

**4916**	373	B	44.2684	0
**4916**	381	B	47.2543	0
**4916**	382	B	65.0289	1
**4916**	386	B	63.0953	1
**4916**	305	C	46.6116	0
**4916**	319	C	54.2075	1
**4916**	320	C	72.4504	-1
**4916**	321	C	33.9197	1
**4916**	351	C	1.159	1
**4916**	357	C	41.4684	0
**4916**	370	C	67.9312	1
**4916**	372	C	46.3208	1
**4916**	373	C	65.5976	1
**4916**	381	C	59.4242	1
**4916**	382	C	43.1666	0
**4916**	386	C	60.0497	1
**4916**	305	D	36.3235	0
**4916**	319	D	79.5054	1
**4916**	320	D	76.7988	-1
**4916**	321	D	79.3695	0
**4916**	351	D	21.8716	-1
**4916**	357	D	73.8584	1
**4916**	370	D	79.4987	1
**4916**	372	D	56.8806	0
**4916**	373	D	67.0164	1
**4916**	381	D	37.3929	0
**4916**	382	D	79.3394	1
**4916**	386	D	79.555	1
**4917**	305	A	71.1078	1
**4917**	319	A	35.1978	0
**4917**	320	A	79.5418	-1
**4917**	321	A	32.199	1
**4917**	351	A	0.1807	1
**4917**	357	A	79.5517	1

**4917**	370	A	79.5281	1
**4917**	372	A	79.5672	0
**4917**	373	A	71.4289	1
**4917**	381	A	79.5797	1
**4917**	382	A	66.3475	1
**4917**	386	A	53.064	1
**4917**	305	B	61.9112	1
**4917**	319	B	79.5787	1
**4917**	320	B	77.5816	-1
**4917**	321	B	58.519	0
**4917**	351	B	11.6102	1
**4917**	357	B	43.0089	0
**4917**	370	B	79.5541	1
**4917**	372	B	43.1089	1
**4917**	373	B	44.971	0
**4917**	381	B	19.9728	-1
**4917**	382	B	18.3909	-1
**4917**	386	B	57.2291	1
**4917**	305	C	55.8194	1
**4917**	319	C	40.1779	0
**4917**	320	C	34.1936	-1
**4917**	321	C	45.8892	1
**4917**	351	C	8.7711	1
**4917**	357	C	66.0394	1
**4917**	370	C	37.2715	0
**4917**	372	C	79.5577	0
**4917**	373	C	58.2986	1
**4917**	381	C	79.5932	1
**4917**	382	C	60.9052	1
**4917**	386	C	66.1027	1
**4917**	305	D	79.4855	1
**4917**	319	D	60.7995	1
**4917**	320	D	47.582	-1
**4917**	321	D	79.5525	0

**4917**	351	D	8.2029	1
**4917**	357	D	44.4697	0
**4917**	370	D	34.3273	0
**4917**	372	D	27.0087	1
**4917**	373	D	72.9038	1
**4917**	381	D	74.0176	1
**4917**	382	D	66.8398	1
**4917**	386	D	78.3879	1
**4918**	305	A	78.6546	1
**4918**	319	A	17.9572	-1
**4918**	320	A	79.584	-1
**4918**	321	A	79.5484	0
**4918**	351	A	7.855	1
**4918**	357	A	79.5267	1
**4918**	370	A	73.774	1
**4918**	372	A	79.588	0
**4918**	373	A	74.3012	1
**4918**	381	A	40.4241	0
**4918**	382	A	49.2035	0
**4918**	386	A	78.6704	1
**4918**	305	B	79.5903	1
**4918**	319	B	75.2587	1
**4918**	320	B	33.2089	-1
**4918**	321	B	69.1712	0
**4918**	351	B	11.204	1
**4918**	357	B	79.5845	1
**4918**	370	B	79.5465	1
**4918**	372	B	79.5485	0
**4918**	373	B	70.9889	1
**4918**	381	B	79.3845	1
**4918**	382	B	41.6313	0
**4918**	386	B	63.5588	1
**4918**	305	C	73.8803	1
**4918**	319	C	55.038	1

**4918**	320	C	54.9417	-1
**4918**	321	C	73.1952	0
**4918**	351	C	9.4031	1
**4918**	357	C	29.4903	0
**4918**	370	C	79.5404	1
**4918**	372	C	48.9634	1
**4918**	373	C	79.5336	1
**4918**	381	C	73.8943	1
**4918**	382	C	62.8446	1
**4918**	386	C	4.1858	-1
**4918**	305	D	37.9401	0
**4918**	319	D	70.528	1
**4918**	320	D	79.5573	-1
**4918**	321	D	79.545	0
**4918**	351	D	9.8882	1
**4918**	357	D	62.8941	1
**4918**	370	D	79.5701	1
**4918**	372	D	77.2506	0
**4918**	373	D	79.5636	1
**4918**	381	D	79.5799	1
**4918**	382	D	79.5812	1
**4918**	386	D	79.5409	1
**4919**	305	A	72.4806	1
**4919**	319	A	30.8375	0
**4919**	320	A	79.2038	-1
**4919**	321	A	64.0847	0
**4919**	351	A	8.446	1
**4919**	357	A	71.7437	1
**4919**	370	A	79.2785	1
**4919**	372	A	59.7726	0
**4919**	373	A	72.1437	1
**4919**	381	A	32.0888	0
**4919**	382	A	67.0823	1
**4919**	386	A	71.9559	1

**4919**	305	B	74.0829	1
**4919**	319	B	79.6204	1
**4919**	320	B	62.2697	-1
**4919**	321	B	79.7964	0
**4919**	351	B	14.0194	1
**4919**	357	B	79.573	1
**4919**	370	B	71.6324	1
**4919**	372	B	60.0315	0
**4919**	373	B	78.006	1
**4919**	381	B	79.4086	1
**4919**	382	B	51.5103	1
**4919**	386	B	59.4913	1
**4919**	305	C	60.4048	1
**4919**	319	C	79.5127	1
**4919**	320	C	70.4593	-1
**4919**	321	C	79.5638	0
**4919**	351	C	9.4463	1
**4919**	357	C	79.1757	1
**4919**	370	C	72.0024	1
**4919**	372	C	78.86	0
**4919**	373	C	71.7042	1
**4919**	381	C	79.5891	1
**4919**	382	C	11.4649	-1
**4919**	386	C	39.0341	0
**4919**	305	D	79.127	1
**4919**	319	D	52.6111	1
**4919**	320	D	48.1051	-1
**4919**	321	D	52.7706	0
**4919**	351	D	10.1671	1
**4919**	357	D	79.0192	1
**4919**	370	D	65.2644	1
**4919**	372	D	51.6722	0
**4919**	373	D	60.3786	1
**4919**	381	D	56.1063	1

**4919**	382	D	57.0904	1
**4919**	386	D	52.385	1
**4920**	305	A	25.5234	0
**4920**	319	A	42.7636	0
**4920**	320	A	79.513	-1
**4920**	321	A	70.7343	0
**4920**	351	A	18.3575	1
**4920**	357	A	70.9268	1
**4920**	370	A	79.5936	1
**4920**	372	A	79.6836	0
**4920**	373	A	48.0286	0
**4920**	381	A	79.6368	1
**4920**	382	A	72.5993	1
**4920**	386	A	79.5042	1
**4920**	305	B	73.5655	1
**4920**	319	B	55.4349	1
**4920**	320	B	79.5498	-1
**4920**	321	B	79.5645	0
**4920**	351	B	8.0308	1
**4920**	357	B	79.5306	1
**4920**	370	B	33.3262	0
**4920**	372	B	64.5299	0
**4920**	373	B	75.194	1
**4920**	381	B	79.3404	1
**4920**	382	B	71.829	1
**4920**	386	B	62.0969	1
**4920**	305	C	79.5593	1
**4920**	319	C	74.5465	1
**4920**	320	C	21.7921	-1
**4920**	321	C	40.1697	1
**4920**	351	C	2.4609	1
**4920**	357	C	72.563	1
**4920**	370	C	79.5374	1
**4920**	372	C	79.6495	0

**4920**	373	C	79.5159	1
**4920**	381	C	65.7585	1
**4920**	382	C	72.6672	1
**4920**	386	C	28.1613	0
**4920**	305	D	79.5864	1
**4920**	319	D	42.6723	0
**4920**	320	D	74.8804	-1
**4920**	321	D	79.5217	0
**4920**	351	D	30.0862	-1
**4920**	357	D	77.9843	1
**4920**	370	D	62.4336	1
**4920**	372	D	59.8978	0
**4920**	373	D	27.1822	0
**4920**	381	D	61.9795	1
**4920**	382	D	70.9802	1
**4920**	386	D	79.5822	1
**4921**	305	A	73.3093	1
**4921**	319	A	68.3844	1
**4921**	320	A	63.461	-1
**4921**	321	A	57.3363	0
**4921**	351	A	4.6748	1
**4921**	357	A	69.863	1
**4921**	370	A	58.1314	1
**4921**	372	A	73.2801	0
**4921**	373	A	77.7046	1
**4921**	381	A	38.5982	0
**4921**	382	A	73.9431	1
**4921**	386	A	79.5236	1
**4921**	305	B	60.7548	1
**4921**	319	B	79.5337	1
**4921**	320	B	60.1027	-1
**4921**	321	B	69.9153	0
**4921**	351	B	8.5587	1
**4921**	357	B	79.5354	1

**4921**	370	B	79.086	1
**4921**	372	B	77.3695	0
**4921**	373	B	53.2372	1
**4921**	381	B	67.8935	1
**4921**	382	B	6.5667	-1
**4921**	386	B	47.6185	0
**4921**	305	C	69.2693	1
**4921**	319	C	69.331	1
**4921**	320	C	79.2881	-1
**4921**	321	C	67.1435	0
**4921**	351	C	9.6015	1
**4921**	357	C	37.07	0
**4921**	370	C	70.894	1
**4921**	372	C	64.6521	0
**4921**	373	C	70.7688	1
**4921**	381	C	79.7263	1
**4921**	382	C	72.847	1
**4921**	386	C	67.3399	1
**4921**	305	D	52.1656	1
**4921**	319	D	79.7063	1
**4921**	320	D	78.5213	-1
**4921**	321	D	64.1066	0
**4921**	351	D	13.0834	1
**4921**	357	D	79.5745	1
**4921**	370	D	64.5328	1
**4921**	372	D	77.683	0
**4921**	373	D	40.3557	0
**4921**	381	D	72.7803	1
**4921**	382	D	75.628	1
**4921**	386	D	72.2694	1
**4922**	305	A	26.3793	0
**4922**	319	A	45.0514	0
**4922**	320	A	79.5657	-1
**4922**	321	A	70.2368	0

**4922**	351	A	10.7273	1
**4922**	357	A	74.9273	1
**4922**	370	A	77.4013	1
**4922**	372	A	79.0248	0
**4922**	373	A	72.3084	1
**4922**	381	A	50.2665	1
**4922**	382	A	61.6116	1
**4922**	386	A	79.5125	1
**4922**	305	B	67.4393	1
**4922**	319	B	39.9962	0
**4922**	320	B	44.8529	-1
**4922**	321	B	64.7527	0
**4922**	351	B	14.8344	1
**4922**	357	B	36.0789	0
**4922**	370	B	38.4265	0
**4922**	372	B	71.6721	0
**4922**	373	B	61.2245	1
**4922**	381	B	61.3693	1
**4922**	382	B	76.8127	1
**4922**	386	B	79.6085	1
**4922**	305	C	65.3738	1
**4922**	319	C	79.6384	1
**4922**	320	C	58.6821	-1
**4922**	321	C	79.5926	0
**4922**	351	C	6.6581	1
**4922**	357	C	44.7615	0
**4922**	370	C	60.6792	1
**4922**	372	C	79.5474	0
**4922**	373	C	76.8985	1
**4922**	381	C	58.0105	1
**4922**	382	C	74.1847	1
**4922**	386	C	45.0362	0
**4922**	305	D	72.5143	1
**4922**	319	D	44.1091	0

**4922**	320	D	69.9952	-1
**4922**	321	D	67.9602	0
**4922**	351	D	12.3025	1
**4922**	357	D	70.8828	1
**4922**	370	D	68.0932	1
**4922**	372	D	79.5899	0
**4922**	373	D	47.334	0
**4922**	381	D	79.5593	1
**4922**	382	D	79.467	1
**4922**	386	D	75.1268	1
**4923**	305	A	69.2848	1
**4923**	319	A	65.1656	1
**4923**	320	A	79.5231	-1
**4923**	321	A	73.4135	0
**4923**	351	A	0.4025	1
**4923**	357	A	58.1495	1
**4923**	370	A	79.0069	1
**4923**	372	A	73.5003	0
**4923**	373	A	79.3333	1
**4923**	381	A	79.5722	1
**4923**	382	A	64.2793	1
**4923**	386	A	79.5344	1
**4923**	305	B	68.0958	1
**4923**	319	B	72.3449	1
**4923**	320	B	36.7247	-1
**4923**	321	B	47.8219	1
**4923**	351	B	12.7095	1
**4923**	357	B	79.283	1
**4923**	370	B	77.3917	1
**4923**	372	B	63.361	0
**4923**	373	B	65.222	1
**4923**	381	B	79.5494	1
**4923**	382	B	18.0808	-1
**4923**	386	B	47.311	0

**4923**	305	C	47.5655	0
**4923**	319	C	76.0187	1
**4923**	320	C	67.6714	-1
**4923**	321	C	79.6156	0
**4923**	351	C	6.1712	1
**4923**	357	C	57.2336	1
**4923**	370	C	79.0111	1
**4923**	372	C	79.6065	0
**4923**	373	C	79.5142	1
**4923**	381	C	54.5256	1
**4923**	382	C	74.4834	1
**4923**	386	C	65.9574	1
**4923**	305	D	61.4568	1
**4923**	319	D	79.5273	1
**4923**	320	D	72.7852	-1
**4923**	321	D	61.3735	0
**4923**	351	D	7.6259	1
**4923**	357	D	45.2798	0
**4923**	370	D	76.019	1
**4923**	372	D	79.5237	0
**4923**	373	D	74.7851	1
**4923**	381	D	72.0954	1
**4923**	382	D	71.5151	1
**4923**	386	D	79.6262	1
**4924**	305	A	52.8478	1
**4924**	319	A	71.9327	1
**4924**	320	A	79.4735	-1
**4924**	321	A	78.8419	0
**4924**	351	A	12.709	1
**4924**	357	A	78.173	1
**4924**	370	A	53.2895	1
**4924**	372	A	71.1042	0
**4924**	373	A	79.6058	1
**4924**	381	A	59.4821	1

**4924**	382	A	79.5149	1
**4924**	386	A	78.4869	1
**4924**	305	B	79.5919	1
**4924**	319	B	34.1035	0
**4924**	320	B	74.8886	-1
**4924**	321	B	53.5991	0
**4924**	351	B	4.7243	1
**4924**	357	B	75.9922	1
**4924**	370	B	53.5538	1
**4924**	372	B	79.703	0
**4924**	373	B	56.6718	1
**4924**	381	B	45.5169	0
**4924**	382	B	42.8349	0
**4924**	386	B	61.0335	1
**4924**	305	C	79.6092	1
**4924**	319	C	25.6156	0
**4924**	320	C	71.3487	-1
**4924**	321	C	63.1154	0
**4924**	351	C	1.9125	1
**4924**	357	C	73.082	1
**4924**	370	C	79.6629	1
**4924**	372	C	79.2945	0
**4924**	373	C	79.672	1
**4924**	381	C	68.5504	1
**4924**	382	C	38.0194	0
**4924**	386	C	7.3825	-1
**4924**	305	D	64.0811	1
**4924**	319	D	66.0721	1
**4924**	320	D	79.1749	-1
**4924**	321	D	79.0985	0
**4924**	351	D	6.2048	1
**4924**	357	D	74.4271	1
**4924**	370	D	43.9019	0
**4924**	372	D	79.6421	0

**4924**	373	D	64.7068	1
**4924**	381	D	78.134	1
**4924**	382	D	79.521	1
**4924**	386	D	66.9852	1
**4925**	305	A	55.6261	1
**4925**	319	A	58.4221	1
**4925**	320	A	79.6112	-1
**4925**	321	A	55.438	0
**4925**	351	A	0.0	1
**4925**	357	A	71.6247	1
**4925**	370	A	79.6687	1
**4925**	372	A	79.5497	0
**4925**	373	A	79.6133	1
**4925**	381	A	71.6517	1
**4925**	382	A	75.4108	1
**4925**	386	A	32.8308	0
**4925**	305	B	73.7187	1
**4925**	319	B	75.3183	1
**4925**	320	B	74.0999	-1
**4925**	321	B	76.5713	0
**4925**	351	B	7.1376	1
**4925**	357	B	79.5356	1
**4925**	370	B	79.0756	1
**4925**	372	B	31.458	1
**4925**	373	B	52.2106	1
**4925**	381	B	72.5385	1
**4925**	382	B	76.1054	1
**4925**	386	B	48.3536	0
**4925**	305	C	79.5878	1
**4925**	319	C	79.6142	1
**4925**	320	C	42.2469	-1
**4925**	321	C	79.5531	0
**4925**	351	C	8.2725	1
**4925**	357	C	79.6088	1

**4925**	370	C	59.8551	1
**4925**	372	C	77.5244	0
**4925**	373	C	71.6919	1
**4925**	381	C	41.9878	0
**4925**	382	C	27.2315	0
**4925**	386	C	42.0161	0
**4925**	305	D	64.0221	1
**4925**	319	D	79.577	1
**4925**	320	D	75.8037	-1
**4925**	321	D	79.6209	0
**4925**	351	D	4.9856	1
**4925**	357	D	71.2502	1
**4925**	370	D	71.3665	1
**4925**	372	D	79.5812	0
**4925**	373	D	24.0415	0
**4925**	381	D	60.632	1
**4925**	382	D	43.2537	0
**4925**	386	D	62.658	1
**4926**	305	A	57.3933	1
**4926**	319	A	79.5697	1
**4926**	320	A	78.402	-1
**4926**	321	A	75.8636	0
**4926**	351	A	12.1665	1
**4926**	357	A	25.2272	0
**4926**	370	A	72.3422	1
**4926**	372	A	75.7125	0
**4926**	373	A	79.5461	1
**4926**	381	A	58.6401	1
**4926**	382	A	56.2691	1
**4926**	386	A	62.4367	1
**4926**	305	B	61.1979	1
**4926**	319	B	62.3386	1
**4926**	320	B	34.6171	-1
**4926**	321	B	63.4566	0

**4926**	351	B	14.0574	1
**4926**	357	B	54.4611	1
**4926**	370	B	79.5676	1
**4926**	372	B	73.3692	0
**4926**	373	B	38.088	0
**4926**	381	B	79.5697	1
**4926**	382	B	79.5887	1
**4926**	386	B	67.6581	1
**4926**	305	C	51.4059	1
**4926**	319	C	59.3408	1
**4926**	320	C	79.5864	-1
**4926**	321	C	79.5461	0
**4926**	351	C	3.0186	1
**4926**	357	C	72.951	1
**4926**	370	C	69.6736	1
**4926**	372	C	79.3126	0
**4926**	373	C	48.6909	0
**4926**	381	C	66.7996	1
**4926**	382	C	31.2815	0
**4926**	386	C	37.5152	0
**4926**	305	D	77.8786	1
**4926**	319	D	72.4302	1
**4926**	320	D	79.5551	-1
**4926**	321	D	79.5553	0
**4926**	351	D	11.9045	1
**4926**	357	D	50.6627	1
**4926**	370	D	75.5518	1
**4926**	372	D	65.8156	0
**4926**	373	D	50.7839	1
**4926**	381	D	78.7748	1
**4926**	382	D	72.9333	1
**4926**	386	D	79.554	1
**4927**	305	A	57.0571	1
**4927**	319	A	79.6218	1

**4927**	320	A	60.6519	-1
**4927**	321	A	53.4686	0
**4927**	351	A	12.0892	1
**4927**	357	A	69.9536	1
**4927**	370	A	49.1222	0
**4927**	372	A	54.3224	0
**4927**	373	A	38.961	0
**4927**	381	A	54.3854	1
**4927**	382	A	38.428	0
**4927**	386	A	73.3566	1
**4927**	305	B	79.827	1
**4927**	319	B	63.7938	1
**4927**	320	B	69.2547	-1
**4927**	321	B	47.3648	1
**4927**	351	B	23.4599	-1
**4927**	357	B	71.8814	1
**4927**	370	B	79.6566	1
**4927**	372	B	79.6484	0
**4927**	373	B	56.1218	1
**4927**	381	B	63.8893	1
**4927**	382	B	37.657	0
**4927**	386	B	79.5343	1
**4927**	305	C	79.601	1
**4927**	319	C	70.7679	1
**4927**	320	C	76.052	-1
**4927**	321	C	50.5427	0
**4927**	351	C	8.8497	1
**4927**	357	C	37.7464	0
**4927**	370	C	35.1331	0
**4927**	372	C	79.5889	0
**4927**	373	C	66.4169	1
**4927**	381	C	63.3227	1
**4927**	382	C	67.9931	1
**4927**	386	C	51.6153	1

**4927**	305	D	19.5102	-1
**4927**	319	D	79.5001	1
**4927**	320	D	79.5604	-1
**4927**	321	D	68.1055	0
**4927**	351	D	6.0034	1
**4927**	357	D	79.4541	1
**4927**	370	D	27.5547	0
**4927**	372	D	71.1592	0
**4927**	373	D	79.4487	1
**4927**	381	D	30.631	0
**4927**	382	D	64.1708	1
**4927**	386	D	79.03	1
**4928**	305	A	31.7918	0
**4928**	319	A	70.9653	1
**4928**	320	A	79.55	-1
**4928**	321	A	72.4028	0
**4928**	351	A	10.2323	1
**4928**	357	A	61.3318	1
**4928**	370	A	79.5681	1
**4928**	372	A	79.569	0
**4928**	373	A	79.5775	1
**4928**	381	A	79.5406	1
**4928**	382	A	79.5831	1
**4928**	386	A	73.0954	1
**4928**	305	B	71.9395	1
**4928**	319	B	21.3739	0
**4928**	320	B	43.3067	-1
**4928**	321	B	55.861	0
**4928**	351	B	11.7005	1
**4928**	357	B	73.7534	1
**4928**	370	B	57.8816	1
**4928**	372	B	72.871	0
**4928**	373	B	39.7161	0
**4928**	381	B	72.4953	1

**4928**	382	B	25.7744	0
**4928**	386	B	38.5161	0
**4928**	305	C	43.9579	0
**4928**	319	C	79.5469	1
**4928**	320	C	79.6995	-1
**4928**	321	C	73.6728	0
**4928**	351	C	8.1255	1
**4928**	357	C	46.8123	0
**4928**	370	C	69.1259	1
**4928**	372	C	66.7272	0
**4928**	373	C	79.6496	1
**4928**	381	C	51.2635	1
**4928**	382	C	14.6636	-1
**4928**	386	C	32.2459	0
**4928**	305	D	74.3038	1
**4928**	319	D	72.84	1
**4928**	320	D	66.2216	-1
**4928**	321	D	53.6693	0
**4928**	351	D	14.5136	1
**4928**	357	D	53.3105	1
**4928**	370	D	79.0124	1
**4928**	372	D	77.1337	0
**4928**	373	D	79.543	1
**4928**	381	D	72.1028	1
**4928**	382	D	79.2434	1
**4928**	386	D	79.5879	1
**4929**	305	A	72.9024	1
**4929**	319	A	72.8073	1
**4929**	320	A	73.1605	-1
**4929**	321	A	56.1583	0
**4929**	351	A	41.5487	-1
**4929**	357	A	79.5173	1
**4929**	370	A	79.6032	1
**4929**	372	A	33.1357	1

**4929**	373	A	71.2797	1
**4929**	381	A	79.2705	1
**4929**	382	A	79.5652	1
**4929**	386	A	79.5567	1
**4929**	305	B	79.5401	1
**4929**	319	B	70.461	1
**4929**	320	B	56.5611	-1
**4929**	321	B	79.3229	0
**4929**	351	B	21.4358	-1
**4929**	357	B	56.9744	1
**4929**	370	B	79.0473	1
**4929**	372	B	72.4508	0
**4929**	373	B	49.9092	0
**4929**	381	B	79.592	1
**4929**	382	B	26.633	0
**4929**	386	B	36.1451	0
**4929**	305	C	63.762	1
**4929**	319	C	79.6174	1
**4929**	320	C	55.7961	-1
**4929**	321	C	79.3546	0
**4929**	351	C	11.9963	1
**4929**	357	C	72.0994	1
**4929**	370	C	79.115	1
**4929**	372	C	54.1237	0
**4929**	373	C	59.7776	1
**4929**	381	C	79.5932	1
**4929**	382	C	75.1429	1
**4929**	386	C	44.3316	0
**4929**	305	D	64.6452	1
**4929**	319	D	79.5075	1
**4929**	320	D	69.2772	-1
**4929**	321	D	79.4967	0
**4929**	351	D	12.118	1
**4929**	357	D	78.7971	1

**4929**	370	D	72.8946	1
**4929**	372	D	66.0589	0
**4929**	373	D	63.0134	1
**4929**	381	D	79.634	1
**4929**	382	D	69.8028	1
**4929**	386	D	79.4984	1
**4930**	305	A	63.5444	1
**4930**	319	A	70.7468	1
**4930**	320	A	70.1207	-1
**4930**	321	A	74.1344	0
**4930**	351	A	32.933	-1
**4930**	357	A	79.5213	1
**4930**	370	A	38.1586	0
**4930**	372	A	49.0105	1
**4930**	373	A	66.9544	1
**4930**	381	A	30.3778	0
**4930**	382	A	56.9326	1
**4930**	386	A	52.173	1
**4930**	305	B	79.4159	1
**4930**	319	B	75.069	1
**4930**	320	B	51.384	-1
**4930**	321	B	70.3938	0
**4930**	351	B	15.3969	1
**4930**	357	B	79.5128	1
**4930**	370	B	65.3922	1
**4930**	372	B	79.4833	0
**4930**	373	B	62.5783	1
**4930**	381	B	79.5206	1
**4930**	382	B	23.2419	0
**4930**	386	B	45.649	0
**4930**	305	C	79.4832	1
**4930**	319	C	53.8811	1
**4930**	320	C	40.9159	-1
**4930**	321	C	56.3809	0

**4930**	351	C	18.1563	1
**4930**	357	C	76.6459	1
**4930**	370	C	74.5675	1
**4930**	372	C	42.1956	1
**4930**	373	C	57.6315	1
**4930**	381	C	79.6015	1
**4930**	382	C	20.401	0
**4930**	386	C	28.9539	0
**4930**	305	D	79.3701	1
**4930**	319	D	75.5414	1
**4930**	320	D	79.3698	-1
**4930**	321	D	79.5148	0
**4930**	351	D	14.5415	1
**4930**	357	D	16.9436	-1
**4930**	370	D	77.1382	1
**4930**	372	D	79.5258	0
**4930**	373	D	79.5141	1
**4930**	381	D	40.3674	0
**4930**	382	D	64.0551	1
**4930**	386	D	46.8611	0
**4931**	305	A	36.3085	0
**4931**	319	A	72.3887	1
**4931**	320	A	73.4256	-1
**4931**	321	A	76.4531	0
**4931**	351	A	34.9437	-1
**4931**	357	A	73.8643	1
**4931**	370	A	49.924	0
**4931**	372	A	79.5489	0
**4931**	373	A	60.5631	1
**4931**	381	A	79.5411	1
**4931**	382	A	79.5595	1
**4931**	386	A	36.0947	0
**4931**	305	B	64.1124	1
**4931**	319	B	78.4123	1

**4931**	320	B	52.5443	-1
**4931**	321	B	60.0362	0
**4931**	351	B	8.1579	1
**4931**	357	B	79.3573	1
**4931**	370	B	79.5665	1
**4931**	372	B	79.4799	0
**4931**	373	B	71.7653	1
**4931**	381	B	43.6699	0
**4931**	382	B	44.2532	0
**4931**	386	B	65.9455	1
**4931**	305	C	72.4027	1
**4931**	319	C	74.4094	1
**4931**	320	C	79.5395	-1
**4931**	321	C	79.6189	0
**4931**	351	C	394	1
**4931**	357	C	55.4185	1
**4931**	370	C	79.5534	1
**4931**	372	C	79.5681	0
**4931**	373	C	61.0376	1
**4931**	381	C	72.5425	1
**4931**	382	C	2.4433	-1
**4931**	386	C	42.3772	0
**4931**	305	D	50.6587	1
**4931**	319	D	74.2847	1
**4931**	320	D	73.108	-1
**4931**	321	D	73.0367	0
**4931**	351	D	18.6121	1
**4931**	357	D	57.8176	1
**4931**	370	D	79.3719	1
**4931**	372	D	74.4299	0
**4931**	373	D	79.5807	1
**4931**	381	D	79.5367	1
**4931**	382	D	79.5308	1
**4931**	386	D	72.5921	1

**4932**	305	A	79.5093	1
**4932**	319	A	57.6766	1
**4932**	320	A	65.3687	-1
**4932**	321	A	33.0392	1
**4932**	351	A	7.8324	1
**4932**	357	A	79.5837	1
**4932**	370	A	68.3887	1
**4932**	372	A	79.6539	0
**4932**	373	A	73.9666	1
**4932**	381	A	79.3685	1
**4932**	382	A	48.3609	0
**4932**	386	A	31.1436	0
**4932**	305	B	66.5236	1
**4932**	319	B	79.6808	1
**4932**	320	B	79.4189	-1
**4932**	321	B	75.6871	0
**4932**	351	B	13.4223	1
**4932**	357	B	18.432	-1
**4932**	370	B	79.5899	1
**4932**	372	B	79.5142	0
**4932**	373	B	79.457	1
**4932**	381	B	79.6167	1
**4932**	382	B	43.3176	0
**4932**	386	B	45.6958	0
**4932**	305	C	34.235	0
**4932**	319	C	69.5265	1
**4932**	320	C	79.6099	-1
**4932**	321	C	79.6117	0
**4932**	351	C	1.3095	1
**4932**	357	C	79.5411	1
**4932**	370	C	57.9527	1
**4932**	372	C	55.7401	0
**4932**	373	C	79.5877	1
**4932**	381	C	79.5742	1

**4932**	382	C	65.0253	1
**4932**	386	C	17.2055	-1
**4932**	305	D	57.8774	1
**4932**	319	D	65.9608	1
**4932**	320	D	56.2508	-1
**4932**	321	D	79.5816	0
**4932**	351	D	9.8764	1
**4932**	357	D	72.3819	1
**4932**	370	D	74.5432	1
**4932**	372	D	79.5655	0
**4932**	373	D	79.4575	1
**4932**	381	D	29.7978	0
**4932**	382	D	59.329	1
**4932**	386	D	79.5477	1
**4933**	305	A	66.5157	1
**4933**	319	A	74.1473	1
**4933**	320	A	50.9935	-1
**4933**	321	A	68.8689	0
**4933**	351	A	18.2136	1
**4933**	357	A	72.6631	1
**4933**	370	A	69.0984	1
**4933**	372	A	79.485	0
**4933**	373	A	74.6217	1
**4933**	381	A	79.5432	1
**4933**	382	A	38.1102	0
**4933**	386	A	73.6812	1
**4933**	305	B	64.7601	1
**4933**	319	B	48.2268	0
**4933**	320	B	71.532	-1
**4933**	321	B	48.2069	1
**4933**	351	B	15.9822	1
**4933**	357	B	43.5561	0
**4933**	370	B	57.1788	1
**4933**	372	B	73.1529	0

**4933**	373	B	69.5513	1
**4933**	381	B	55.8373	1
**4933**	382	B	21.7418	0
**4933**	386	B	49.3126	0
**4933**	305	C	76.5107	1
**4933**	319	C	79.6585	1
**4933**	320	C	79.5504	-1
**4933**	321	C	36.3531	1
**4933**	351	C	7.7594	1
**4933**	357	C	66.2424	1
**4933**	370	C	70.9031	1
**4933**	372	C	79.6267	0
**4933**	373	C	79.6197	1
**4933**	381	C	79.6303	1
**4933**	382	C	29.4249	0
**4933**	386	C	47.4871	0
**4933**	305	D	53.0231	1
**4933**	319	D	79.6006	1
**4933**	320	D	76.7041	-1
**4933**	321	D	45.2514	1
**4933**	351	D	7.2079	1
**4933**	357	D	72.7637	1
**4933**	370	D	74.2906	1
**4933**	372	D	70.8919	0
**4933**	373	D	23.0927	0
**4933**	381	D	79.6193	1
**4933**	382	D	72.0542	1
**4933**	386	D	79.5188	1
**4934**	305	A	30.2258	0
**4934**	319	A	35.708	0
**4934**	320	A	79.6013	-1
**4934**	321	A	47.6887	1
**4934**	351	A	2.343	1
**4934**	357	A	79.5771	1

**4934**	370	A	57.4891	1
**4934**	372	A	41.2863	1
**4934**	373	A	74.4684	1
**4934**	381	A	78.0995	1
**4934**	382	A	79.4554	1
**4934**	386	A	47.383	0
**4934**	305	B	56.9089	1
**4934**	319	B	70.7274	1
**4934**	320	B	65.259	-1
**4934**	321	B	74.1938	0
**4934**	351	B	15.754	1
**4934**	357	B	79.6061	1
**4934**	370	B	60.5064	1
**4934**	372	B	77.6052	0
**4934**	373	B	76.2584	1
**4934**	381	B	79.6025	1
**4934**	382	B	79.2376	1
**4934**	386	B	48.7738	0
**4934**	305	C	69.0049	1
**4934**	319	C	64.3654	1
**4934**	320	C	79.586	-1
**4934**	321	C	58.9645	0
**4934**	351	C	5.9403	1
**4934**	357	C	56.7133	1
**4934**	370	C	29.2744	0
**4934**	372	C	22.4613	1
**4934**	373	C	72.4325	1
**4934**	381	C	79.685	1
**4934**	382	C	62.3053	1
**4934**	386	C	18.1457	-1
**4934**	305	D	55.0906	1
**4934**	319	D	57.6672	1
**4934**	320	D	74.2515	-1
**4934**	321	D	76.0779	0

**4934**	351	D	41.0416	-1
**4934**	357	D	37.24	0
**4934**	370	D	34.8794	0
**4934**	372	D	79.6033	0
**4934**	373	D	79.6359	1
**4934**	381	D	75.0628	1
**4934**	382	D	79.5255	1
**4934**	386	D	51.1247	1
**4935**	305	A	64.6677	1
**4935**	319	A	77.5372	1
**4935**	320	A	79.4816	-1
**4935**	321	A	46.9368	1
**4935**	351	A	1.1559	1
**4935**	357	A	79.6102	1
**4935**	370	A	79.633	1
**4935**	372	A	57.7331	0
**4935**	373	A	55.9171	1
**4935**	381	A	79.6102	1
**4935**	382	A	72.7387	1
**4935**	386	A	79.5471	1
**4935**	305	B	79.3984	1
**4935**	319	B	72.3537	1
**4935**	320	B	73.7941	-1
**4935**	321	B	79.6049	0
**4935**	351	B	56.2257	-1
**4935**	357	B	72.9278	1
**4935**	370	B	52.0326	1
**4935**	372	B	61.3843	0
**4935**	373	B	19.4192	-1
**4935**	381	B	58.9083	1
**4935**	382	B	42.7917	0
**4935**	386	B	79.6839	1
**4935**	305	C	79.5995	1
**4935**	319	C	79.5612	1

**4935**	320	C	75.5942	-1
**4935**	321	C	72.3644	0
**4935**	351	C	0.0211	1
**4935**	357	C	60.7974	1
**4935**	370	C	79.5931	1
**4935**	372	C	69.8399	0
**4935**	373	C	79.3764	1
**4935**	381	C	76.5909	1
**4935**	382	C	39.3641	0
**4935**	386	C	54.6324	1
**4935**	305	D	79.4476	1
**4935**	319	D	79.4216	1
**4935**	320	D	53.6008	-1
**4935**	321	D	27.7115	1
**4935**	351	D	42.2759	-1
**4935**	357	D	79.5164	1
**4935**	370	D	13.6443	-1
**4935**	372	D	62.1749	0
**4935**	373	D	46.8575	0
**4935**	381	D	69.5576	1
**4935**	382	D	73.2351	1
**4935**	386	D	64.1418	1
**4936**	305	A	75.788	1
**4936**	319	A	73.1653	1
**4936**	320	A	74.2594	-1
**4936**	321	A	62.4644	0
**4936**	351	A	10.9154	1
**4936**	357	A	79.612	1
**4936**	370	A	79.5394	1
**4936**	372	A	73.1255	0
**4936**	373	A	78.8047	1
**4936**	381	A	38.0859	0
**4936**	382	A	75.0632	1
**4936**	386	A	38.3451	0

**4936**	305	B	79.5397	1
**4936**	319	B	74.6292	1
**4936**	320	B	79.5584	-1
**4936**	321	B	35.7803	1
**4936**	351	B	15.8799	1
**4936**	357	B	79.5371	1
**4936**	370	B	79.5559	1
**4936**	372	B	79.5622	0
**4936**	373	B	71.2254	1
**4936**	381	B	74.2989	1
**4936**	382	B	53.6671	1
**4936**	386	B	50.4047	1
**4936**	305	C	48.8373	0
**4936**	319	C	34.3682	0
**4936**	320	C	69.6839	-1
**4936**	321	C	74.4841	0
**4936**	351	C	6.5411	1
**4936**	357	C	75.6847	1
**4936**	370	C	73.1919	1
**4936**	372	C	72.3627	0
**4936**	373	C	79.472	1
**4936**	381	C	79.5523	1
**4936**	382	C	79.5656	1
**4936**	386	C	41.419	0
**4936**	305	D	69.0141	1
**4936**	319	D	36.6571	0
**4936**	320	D	22.8479	-1
**4936**	321	D	68.7508	0
**4936**	351	D	15.1834	1
**4936**	357	D	79.5276	1
**4936**	370	D	73.4473	1
**4936**	372	D	79.5838	0
**4936**	373	D	75.136	1
**4936**	381	D	58.5581	1

**4936**	382	D	79.5676	1
**4936**	386	D	19.861	-1
**4937**	305	A	68.4454	1
**4937**	319	A	72.4252	1
**4937**	320	A	69.9247	-1
**4937**	321	A	64.2369	0
**4937**	351	A	4.5862	1
**4937**	357	A	79.4394	1
**4937**	370	A	20.7263	0
**4937**	372	A	49.288	1
**4937**	373	A	73.3709	1
**4937**	381	A	74.0578	1
**4937**	382	A	51.3506	1
**4937**	386	A	79.4955	1
**4937**	305	B	79.5449	1
**4937**	319	B	63.5269	1
**4937**	320	B	48.5505	-1
**4937**	321	B	78.9242	0
**4937**	351	B	4.4359	1
**4937**	357	B	57.7386	1
**4937**	370	B	72.5498	1
**4937**	372	B	76.498	0
**4937**	373	B	42.5929	0
**4937**	381	B	79.6182	1
**4937**	382	B	54.0262	1
**4937**	386	B	59.2108	1
**4937**	305	C	66.4356	1
**4937**	319	C	79.6194	1
**4937**	320	C	68.9632	-1
**4937**	321	C	52.1872	0
**4937**	351	C	39.5309	-1
**4937**	357	C	79.4863	1
**4937**	370	C	71.4919	1
**4937**	372	C	79.5277	0

**4937**	373	C	76.083	1
**4937**	381	C	79.5096	1
**4937**	382	C	45.5125	0
**4937**	386	C	37.0551	0
**4937**	305	D	28.5017	0
**4937**	319	D	70.3831	1
**4937**	320	D	78.4207	-1
**4937**	321	D	74.1266	0
**4937**	351	D	41.1606	-1
**4937**	357	D	77.8897	1
**4937**	370	D	74.3695	1
**4937**	372	D	79.4435	0
**4937**	373	D	23.4739	0
**4937**	381	D	79.5441	1
**4937**	382	D	76.2523	1
**4937**	386	D	76.2406	1
**4938**	305	A	54.9744	1
**4938**	319	A	64.9494	1
**4938**	320	A	50.9732	-1
**4938**	321	A	39.1592	1
**4938**	351	A	2.3436	1
**4938**	357	A	61.3224	1
**4938**	370	A	72.5097	1
**4938**	372	A	78.2949	0
**4938**	373	A	59.9412	1
**4938**	381	A	79.5544	1
**4938**	382	A	54.0683	1
**4938**	386	A	69.9683	1
**4938**	305	B	72.5764	1
**4938**	319	B	44.7118	0
**4938**	320	B	21.4085	-1
**4938**	321	B	41.6221	1
**4938**	351	B	14.1306	1
**4938**	357	B	79.5625	1

**4938**	370	B	44.6283	0
**4938**	372	B	32.1282	1
**4938**	373	B	56.7169	1
**4938**	381	B	62.638	1
**4938**	382	B	47.3366	0
**4938**	386	B	79.6504	1
**4938**	305	C	69.6091	1
**4938**	319	C	73.9394	1
**4938**	320	C	68.4639	-1
**4938**	321	C	53.2731	0
**4938**	351	C	7.1564	1
**4938**	357	C	73.4416	1
**4938**	370	C	79.6085	1
**4938**	372	C	71.7633	0
**4938**	373	C	79.5979	1
**4938**	381	C	79.5801	1
**4938**	382	C	59.6979	1
**4938**	386	C	21.7583	0
**4938**	305	D	79.5596	1
**4938**	319	D	69.3712	1
**4938**	320	D	63.9815	-1
**4938**	321	D	79.5705	0
**4938**	351	D	10.5162	1
**4938**	357	D	73.5084	1
**4938**	370	D	60.7561	1
**4938**	372	D	73.091	0
**4938**	373	D	79.5518	1
**4938**	381	D	78.2456	1
**4938**	382	D	79.5366	1
**4938**	386	D	53.1082	1
**4939**	305	A	78.5558	1
**4939**	319	A	48.1214	0
**4939**	320	A	79.5961	-1
**4939**	321	A	75.5902	0

**4939**	351	A	4.8945	1
**4939**	357	A	69.0423	1
**4939**	370	A	79.6016	1
**4939**	372	A	79.5443	0
**4939**	373	A	77.3101	1
**4939**	381	A	39.3767	0
**4939**	382	A	72.2943	1
**4939**	386	A	79.5805	1
**4939**	305	B	68.9352	1
**4939**	319	B	79.6038	1
**4939**	320	B	51.8505	-1
**4939**	321	B	50.7211	0
**4939**	351	B	10.7279	1
**4939**	357	B	79.5642	1
**4939**	370	B	46.1509	0
**4939**	372	B	69.2084	0
**4939**	373	B	54.7117	1
**4939**	381	B	57.2516	1
**4939**	382	B	70.8817	1
**4939**	386	B	79.3379	1
**4939**	305	C	78.8137	1
**4939**	319	C	79.4418	1
**4939**	320	C	38.3034	-1
**4939**	321	C	72.8304	0
**4939**	351	C	7.2118	1
**4939**	357	C	75.9772	1
**4939**	370	C	78.5807	1
**4939**	372	C	79.5728	0
**4939**	373	C	66.0606	1
**4939**	381	C	44.7483	0
**4939**	382	C	45.244	0
**4939**	386	C	59.0762	1
**4939**	305	D	71.2813	1
**4939**	319	D	24.8359	0

**4939**	320	D	68.5978	-1
**4939**	321	D	79.5722	0
**4939**	351	D	11.3067	1
**4939**	357	D	72.8524	1
**4939**	370	D	55.8517	1
**4939**	372	D	51.1586	0
**4939**	373	D	25.2017	0
**4939**	381	D	64.7311	1
**4939**	382	D	78.7417	1
**4939**	386	D	14.1942	-1
**4940**	305	A	79.5301	1
**4940**	319	A	45.4428	0
**4940**	320	A	71.4881	-1
**4940**	321	A	19.9242	0
**4940**	351	A	3.9874	1
**4940**	357	A	73.773	1
**4940**	370	A	69.1812	1
**4940**	372	A	31.2563	1
**4940**	373	A	79.492	1
**4940**	381	A	40.5651	0
**4940**	382	A	55.6813	1
**4940**	386	A	7.2114	-1
**4940**	305	B	59.961	1
**4940**	319	B	52.5529	1
**4940**	320	B	79.5655	-1
**4940**	321	B	39.4084	1
**4940**	351	B	36.9176	-1
**4940**	357	B	78.3355	1
**4940**	370	B	79.6271	1
**4940**	372	B	73.7557	0
**4940**	373	B	73.4729	1
**4940**	381	B	75.41	1
**4940**	382	B	44.8393	0
**4940**	386	B	69.719	1

**4940**	305	C	75.0279	1
**4940**	319	C	69.2812	1
**4940**	320	C	76.8677	-1
**4940**	321	C	37.1115	1
**4940**	351	C	0.6292	1
**4940**	357	C	75.9373	1
**4940**	370	C	56.5576	1
**4940**	372	C	79.6637	0
**4940**	373	C	73.6351	1
**4940**	381	C	62.13	1
**4940**	382	C	34.6039	0
**4940**	386	C	56.7263	1
**4940**	305	D	70.0651	1
**4940**	319	D	67.1738	1
**4940**	320	D	27.0732	-1
**4940**	321	D	14.7538	0
**4940**	351	D	8.0116	1
**4940**	357	D	35.4885	0
**4940**	370	D	52.9492	1
**4940**	372	D	62.5623	0
**4940**	373	D	79.5232	1
**4940**	381	D	52.3423	1
**4940**	382	D	79.6003	1
**4940**	386	D	79.5515	1
**4941**	305	A	73.2024	1
**4941**	319	A	79.5818	1
**4941**	320	A	43.5694	-1
**4941**	321	A	79.4976	0
**4941**	351	A	7.1647	1
**4941**	357	A	79.6185	1
**4941**	370	A	67.5455	1
**4941**	372	A	79.611	0
**4941**	373	A	46.1052	0
**4941**	381	A	64.9821	1

**4941**	382	A	65.9319	1
**4941**	386	A	79.4662	1
**4941**	305	B	48.2293	0
**4941**	319	B	79.5967	1
**4941**	320	B	66.9394	-1
**4941**	321	B	70.2506	0
**4941**	351	B	10.6614	1
**4941**	357	B	73.2953	1
**4941**	370	B	79.5905	1
**4941**	372	B	77.8068	0
**4941**	373	B	55.8247	1
**4941**	381	B	66.0309	1
**4941**	382	B	74.8904	1
**4941**	386	B	52.6437	1
**4941**	305	C	74.8353	1
**4941**	319	C	79.3957	1
**4941**	320	C	52.8174	-1
**4941**	321	C	72.4667	0
**4941**	351	C	1.2159	1
**4941**	357	C	50.2117	1
**4941**	370	C	63.9646	1
**4941**	372	C	69.8605	0
**4941**	373	C	44.6404	0
**4941**	381	C	71.1401	1
**4941**	382	C	77.4822	1
**4941**	386	C	13.8461	-1
**4941**	305	D	63.5301	1
**4941**	319	D	79.5777	1
**4941**	320	D	68.8845	-1
**4941**	321	D	79.6278	0
**4941**	351	D	0.0042	1
**4941**	357	D	49.4789	0
**4941**	370	D	46.2884	0
**4941**	372	D	72.3472	0

**4941**	373	D	79.1998	1
**4941**	381	D	79.627	1
**4941**	382	D	79.5993	1
**4941**	386	D	79.6134	1
**4942**	305	A	76.2134	1
**4942**	319	A	77.7456	1
**4942**	320	A	51.248	-1
**4942**	321	A	57.8656	0
**4942**	351	A	9.0217	1
**4942**	357	A	70.051	1
**4942**	370	A	79.5772	1
**4942**	372	A	79.5748	0
**4942**	373	A	46.9883	0
**4942**	381	A	58.3528	1
**4942**	382	A	56.9567	1
**4942**	386	A	77.9541	1
**4942**	305	B	79.6318	1
**4942**	319	B	64.3732	1
**4942**	320	B	45.8774	-1
**4942**	321	B	79.6286	0
**4942**	351	B	14.6876	1
**4942**	357	B	79.6095	1
**4942**	370	B	50.2686	1
**4942**	372	B	79.4898	0
**4942**	373	B	50.6694	1
**4942**	381	B	35.7024	0
**4942**	382	B	65.608	1
**4942**	386	B	70.9331	1
**4942**	305	C	76.5248	1
**4942**	319	C	79.6334	1
**4942**	320	C	70.8971	-1
**4942**	321	C	77.8262	0
**4942**	351	C	3.7934	1
**4942**	357	C	71.7794	1

**4942**	370	C	51.6212	1
**4942**	372	C	33.6809	1
**4942**	373	C	79.595	1
**4942**	381	C	74.3264	1
**4942**	382	C	56.9425	1
**4942**	386	C	51.5377	1
**4942**	305	D	79.6603	1
**4942**	319	D	74.5344	1
**4942**	320	D	43.4564	-1
**4942**	321	D	79.6095	0
**4942**	351	D	10.8627	1
**4942**	357	D	79.6205	1
**4942**	370	D	54.4069	1
**4942**	372	D	72.5807	0
**4942**	373	D	75.2911	1
**4942**	381	D	79.4348	1
**4942**	382	D	79.5776	1
**4942**	386	D	79.5898	1
**4943**	305	A	72.2321	1
**4943**	319	A	59.1522	1
**4943**	320	A	73.1371	-1
**4943**	321	A	29.5882	1
**4943**	351	A	2.8761	1
**4943**	357	A	73.0684	1
**4943**	370	A	75.4165	1
**4943**	372	A	79.5221	0
**4943**	373	A	63.7024	1
**4943**	381	A	55.4193	1
**4943**	382	A	39.9617	0
**4943**	386	A	55.5216	1
**4943**	305	B	79.5219	1
**4943**	319	B	69.6019	1
**4943**	320	B	79.6585	-1
**4943**	321	B	79.5386	0

**4943**	351	B	21.2517	-1
**4943**	357	B	72.1695	1
**4943**	370	B	43.8019	0
**4943**	372	B	79.5485	0
**4943**	373	B	59.3455	1
**4943**	381	B	62.1638	1
**4943**	382	B	17.8405	-1
**4943**	386	B	38.6717	0
**4943**	305	C	44.9967	0
**4943**	319	C	79.4642	1
**4943**	320	C	58.227	-1
**4943**	321	C	77.3436	0
**4943**	351	C	6.4957	1
**4943**	357	C	75.2185	1
**4943**	370	C	75.0055	1
**4943**	372	C	46.4579	1
**4943**	373	C	64.9714	1
**4943**	381	C	65.6448	1
**4943**	382	C	66.3207	1
**4943**	386	C	42.3537	0
**4943**	305	D	67.5474	1
**4943**	319	D	79.5695	1
**4943**	320	D	70.4398	-1
**4943**	321	D	79.4809	0
**4943**	351	D	8.1246	1
**4943**	357	D	78.1803	1
**4943**	370	D	56.3726	1
**4943**	372	D	73.542	0
**4943**	373	D	74.6844	1
**4943**	381	D	46.7218	0
**4943**	382	D	67.6036	1
**4943**	386	D	74.0736	1
**4944**	305	A	73.0343	1
**4944**	319	A	79.5439	1

**4944**	320	A	79.6727	-1
**4944**	321	A	65.379	0
**4944**	351	A	12.7226	1
**4944**	357	A	43.6286	0
**4944**	370	A	58.5767	1
**4944**	372	A	27.3228	1
**4944**	373	A	71.6979	1
**4944**	381	A	79.6255	1
**4944**	382	A	79.4567	1
**4944**	386	A	79.6595	1
**4944**	305	B	79.4269	1
**4944**	319	B	75.487	1
**4944**	320	B	56.2043	-1
**4944**	321	B	79.5265	0
**4944**	351	B	12.2456	1
**4944**	357	B	79.5809	1
**4944**	370	B	50.2244	1
**4944**	372	B	58.2401	0
**4944**	373	B	37.271	0
**4944**	381	B	61.0536	1
**4944**	382	B	73.859	1
**4944**	386	B	79.4839	1
**4944**	305	C	75.9792	1
**4944**	319	C	70.7456	1
**4944**	320	C	70.5832	-1
**4944**	321	C	54.2451	0
**4944**	351	C	3.221	1
**4944**	357	C	69.0316	1
**4944**	370	C	73.5873	1
**4944**	372	C	79.6245	0
**4944**	373	C	79.4641	1
**4944**	381	C	52.9775	1
**4944**	382	C	79.4178	1
**4944**	386	C	47.0996	0

**4944**	305	D	72.794	1
**4944**	319	D	63.7793	1
**4944**	320	D	78.6961	-1
**4944**	321	D	72.4725	0
**4944**	351	D	4.1431	1
**4944**	357	D	67.0496	1
**4944**	370	D	78.9013	1
**4944**	372	D	79.6255	0
**4944**	373	D	79.5151	1
**4944**	381	D	72.6816	1
**4944**	382	D	75.877	1
**4944**	386	D	40.5592	0
**4945**	305	A	51.6168	1
**4945**	319	A	60.021	1
**4945**	320	A	79.4754	-1
**4945**	321	A	79.647	0
**4945**	351	A	6.4558	1
**4945**	357	A	49.4317	0
**4945**	370	A	73.0563	1
**4945**	372	A	59.306	0
**4945**	373	A	78.6643	1
**4945**	381	A	71.6892	1
**4945**	382	A	79.6318	1
**4945**	386	A	45.4686	0
**4945**	305	B	69.0851	1
**4945**	319	B	41.7747	0
**4945**	320	B	65.5901	-1
**4945**	321	B	41.9077	1
**4945**	351	B	4.8971	1
**4945**	357	B	79.5743	1
**4945**	370	B	25.0484	0
**4945**	372	B	46.3781	1
**4945**	373	B	47.4812	0
**4945**	381	B	66.5018	1

**4945**	382	B	35.6597	0
**4945**	386	B	52.2638	1
**4945**	305	C	59.1952	1
**4945**	319	C	79.7478	1
**4945**	320	C	69.5185	-1
**4945**	321	C	18.9489	0
**4945**	351	C	0.7882	1
**4945**	357	C	70.0599	1
**4945**	370	C	61.4625	1
**4945**	372	C	66.1971	0
**4945**	373	C	31.4067	0
**4945**	381	C	79.5969	1
**4945**	382	C	67.4115	1
**4945**	386	C	25.5509	0
**4945**	305	D	35.3057	0
**4945**	319	D	75.1869	1
**4945**	320	D	73.1266	-1
**4945**	321	D	47.9416	1
**4945**	351	D	10.7323	1
**4945**	357	D	73.0922	1
**4945**	370	D	73.6272	1
**4945**	372	D	74.3729	0
**4945**	373	D	39.651	0
**4945**	381	D	58.6163	1
**4945**	382	D	53.569	1
**4945**	386	D	78.2421	1
**4946**	305	A	67.0927	1
**4946**	319	A	19.7371	-1
**4946**	320	A	36.0811	-1
**4946**	321	A	79.5408	0
**4946**	351	A	12.861	1
**4946**	357	A	77.799	1
**4946**	370	A	66.689	1
**4946**	372	A	79.2346	0

**4946**	373	A	62.5425	1
**4946**	381	A	77.044	1
**4946**	382	A	40.6628	0
**4946**	386	A	62.443	1
**4946**	305	B	79.4623	1
**4946**	319	B	74.5088	1
**4946**	320	B	56.8715	-1
**4946**	321	B	68.7521	0
**4946**	351	B	7.9137	1
**4946**	357	B	44.4959	0
**4946**	370	B	79.4737	1
**4946**	372	B	77.5469	0
**4946**	373	B	73.9754	1
**4946**	381	B	79.5896	1
**4946**	382	B	41.9631	0
**4946**	386	B	79.5365	1
**4946**	305	C	77.1084	1
**4946**	319	C	46.4044	0
**4946**	320	C	66.2093	-1
**4946**	321	C	68.882	0
**4946**	351	C	3.6887	1
**4946**	357	C	52.9487	1
**4946**	370	C	79.499	1
**4946**	372	C	39.6957	1
**4946**	373	C	77.1368	1
**4946**	381	C	79.4308	1
**4946**	382	C	70.498	1
**4946**	386	C	67.3824	1
**4946**	305	D	74.8354	1
**4946**	319	D	41.2031	0
**4946**	320	D	79.4284	-1
**4946**	321	D	74.9917	0
**4946**	351	D	16.7986	1
**4946**	357	D	79.6582	1

**4946**	370	D	65.4305	1
**4946**	372	D	67.9321	0
**4946**	373	D	70.5677	1
**4946**	381	D	42.1432	0
**4946**	382	D	79.5722	1
**4946**	386	D	79.513	1
**4947**	305	A	78.4319	1
**4947**	319	A	62.7157	1
**4947**	320	A	79.4325	-1
**4947**	321	A	58.1928	0
**4947**	351	A	5.0955	1
**4947**	357	A	47.9228	0
**4947**	370	A	29.3296	0
**4947**	372	A	27.5488	1
**4947**	373	A	63.7402	1
**4947**	381	A	21.1935	0
**4947**	382	A	25.7423	0
**4947**	386	A	79.5198	1
**4947**	305	B	79.5109	1
**4947**	319	B	79.5911	1
**4947**	320	B	70.2309	-1
**4947**	321	B	79.5983	0
**4947**	351	B	18.2281	1
**4947**	357	B	79.4931	1
**4947**	370	B	58.1343	1
**4947**	372	B	79.5703	0
**4947**	373	B	77.0594	1
**4947**	381	B	30.8544	0
**4947**	382	B	75.5403	1
**4947**	386	B	60.3525	1
**4947**	305	C	75.4029	1
**4947**	319	C	66.3651	1
**4947**	320	C	71.4886	-1
**4947**	321	C	77.1118	0

**4947**	351	C	3.0378	1
**4947**	357	C	47.69	0
**4947**	370	C	67.3664	1
**4947**	372	C	62.8169	0
**4947**	373	C	59.1036	1
**4947**	381	C	71.7556	1
**4947**	382	C	79.5511	1
**4947**	386	C	9.3044	-1
**4947**	305	D	79.5121	1
**4947**	319	D	66.8736	1
**4947**	320	D	73.9242	-1
**4947**	321	D	59.8995	0
**4947**	351	D	11.039	1
**4947**	357	D	79.6578	1
**4947**	370	D	70.3546	1
**4947**	372	D	79.7238	0
**4947**	373	D	68.2144	1
**4947**	381	D	43.2646	0
**4947**	382	D	79.4894	1
**4947**	386	D	25.2815	0
**4948**	305	A	56.3514	1
**4948**	319	A	68.7673	1
**4948**	320	A	79.4825	-1
**4948**	321	A	79.4996	0
**4948**	351	A	1.9005	1
**4948**	357	A	42.6578	0
**4948**	370	A	79.5411	1
**4948**	372	A	66.0396	0
**4948**	373	A	47.2537	0
**4948**	381	A	76.5655	1
**4948**	382	A	56.4997	1
**4948**	386	A	79.0879	1
**4948**	305	B	79.5527	1
**4948**	319	B	79.5303	1

**4948**	320	B	77.5779	-1
**4948**	321	B	63.3838	0
**4948**	351	B	19.2636	1
**4948**	357	B	77.3895	1
**4948**	370	B	57.3423	1
**4948**	372	B	26.1256	1
**4948**	373	B	50.6312	1
**4948**	381	B	74.4305	1
**4948**	382	B	17.8658	-1
**4948**	386	B	67.4449	1
**4948**	305	C	79.5217	1
**4948**	319	C	79.646	1
**4948**	320	C	77.5214	-1
**4948**	321	C	78.7842	0
**4948**	351	C	15.0355	1
**4948**	357	C	79.6175	1
**4948**	370	C	63.2939	1
**4948**	372	C	76.316	0
**4948**	373	C	79.4192	1
**4948**	381	C	57.5676	1
**4948**	382	C	79.6281	1
**4948**	386	C	56.1074	1
**4948**	305	D	79.5675	1
**4948**	319	D	79.5345	1
**4948**	320	D	77.3227	-1
**4948**	321	D	78.1808	0
**4948**	351	D	9.408	1
**4948**	357	D	79.5867	1
**4948**	370	D	67.2443	1
**4948**	372	D	67.1887	0
**4948**	373	D	61.4478	1
**4948**	381	D	30.0596	0
**4948**	382	D	67.0853	1
**4948**	386	D	79.5549	1

**4949**	305	A	79.3858	1
**4949**	319	A	48.1471	0
**4949**	320	A	79.3633	-1
**4949**	321	A	55.538	0
**4949**	351	A	2.8941	1
**4949**	357	A	74.5808	1
**4949**	370	A	79.5288	1
**4949**	372	A	52.5914	0
**4949**	373	A	20.161	0
**4949**	381	A	79.565	1
**4949**	382	A	71.7575	1
**4949**	386	A	78.6434	1
**4949**	305	B	77.1076	1
**4949**	319	B	73.7863	1
**4949**	320	B	55.8591	-1
**4949**	321	B	79.4961	0
**4949**	351	B	8.5293	1
**4949**	357	B	79.55	1
**4949**	370	B	79.6435	1
**4949**	372	B	68.8007	0
**4949**	373	B	76.0874	1
**4949**	381	B	64.9895	1
**4949**	382	B	76.202	1
**4949**	386	B	54.257	1
**4949**	305	C	79.3601	1
**4949**	319	C	64.8041	1
**4949**	320	C	49.8435	-1
**4949**	321	C	26.7343	1
**4949**	351	C	12.9941	1
**4949**	357	C	73.9473	1
**4949**	370	C	75.6743	1
**4949**	372	C	67.0167	0
**4949**	373	C	71.437	1
**4949**	381	C	79.5345	1

**4949**	382	C	59.5726	1
**4949**	386	C	49.8597	0
**4949**	305	D	29.0737	0
**4949**	319	D	67.8293	1
**4949**	320	D	78.9802	-1
**4949**	321	D	53.2887	0
**4949**	351	D	12.0307	1
**4949**	357	D	37.9237	0
**4949**	370	D	15.9907	-1
**4949**	372	D	59.0738	0
**4949**	373	D	57.0341	1
**4949**	381	D	49.8899	0
**4949**	382	D	72.468	1
**4949**	386	D	74.0586	1
**4950**	305	A	67.7925	1
**4950**	319	A	77.001	1
**4950**	320	A	79.4896	-1
**4950**	321	A	50.009	0
**4950**	351	A	5.7	1
**4950**	357	A	39.5405	0
**4950**	370	A	72.8675	1
**4950**	372	A	24.1292	1
**4950**	373	A	65.661	1
**4950**	381	A	79.4107	1
**4950**	382	A	65.4829	1
**4950**	386	A	36.4792	0
**4950**	305	B	71.5439	1
**4950**	319	B	72.3295	1
**4950**	320	B	73.1764	-1
**4950**	321	B	79.5963	0
**4950**	351	B	3.7745	1
**4950**	357	B	72.6144	1
**4950**	370	B	71.7655	1
**4950**	372	B	66.6666	0

**4950**	373	B	59.7047	1
**4950**	381	B	60.1623	1
**4950**	382	B	69.5771	1
**4950**	386	B	62.3114	1
**4950**	305	C	56.0285	1
**4950**	319	C	39.2933	0
**4950**	320	C	72.2211	-1
**4950**	321	C	53.0163	0
**4950**	351	C	8.16	1
**4950**	357	C	71.4441	1
**4950**	370	C	67.3689	1
**4950**	372	C	79.4644	0
**4950**	373	C	79.531	1
**4950**	381	C	59.6106	1
**4950**	382	C	79.5539	1
**4950**	386	C	64.7191	1
**4950**	305	D	62.0211	1
**4950**	319	D	79.6152	1
**4950**	320	D	79.6819	-1
**4950**	321	D	79.5555	0
**4950**	351	D	7.5888	1
**4950**	357	D	57.5913	1
**4950**	370	D	62.9059	1
**4950**	372	D	79.6109	0
**4950**	373	D	79.5301	1
**4950**	381	D	76.0319	1
**4950**	382	D	79.5289	1
**4950**	386	D	60.8119	1
**4951**	305	A	50.5958	1
**4951**	319	A	33.1133	0
**4951**	320	A	31.61	-1
**4951**	321	A	63.2892	0
**4951**	351	A	8.4909	1
**4951**	357	A	55.1354	1

**4951**	370	A	79.5877	1
**4951**	372	A	75.017	0
**4951**	373	A	79.4986	1
**4951**	381	A	79.5948	1
**4951**	382	A	51.9406	1
**4951**	386	A	48.3644	0
**4951**	305	B	71.7457	1
**4951**	319	B	74.5398	1
**4951**	320	B	70.7853	-1
**4951**	321	B	41.636	1
**4951**	351	B	12.7657	1
**4951**	357	B	62.1129	1
**4951**	370	B	40.2329	0
**4951**	372	B	51.5996	0
**4951**	373	B	58.8825	1
**4951**	381	B	36.4898	0
**4951**	382	B	49.7366	0
**4951**	386	B	71.3975	1
**4951**	305	C	63.3565	1
**4951**	319	C	79.6171	1
**4951**	320	C	36.5252	-1
**4951**	321	C	64.1488	0
**4951**	351	C	3.2587	1
**4951**	357	C	73.8436	1
**4951**	370	C	67.1629	1
**4951**	372	C	79.5205	0
**4951**	373	C	75.4654	1
**4951**	381	C	76.3757	1
**4951**	382	C	77.5854	1
**4951**	386	C	57.0181	1
**4951**	305	D	72.6176	1
**4951**	319	D	77.4657	1
**4951**	320	D	52.2437	-1
**4951**	321	D	79.4141	0

**4951**	351	D	14.6602	1
**4951**	357	D	79.5986	1
**4951**	370	D	72.5347	1
**4951**	372	D	70.6079	0
**4951**	373	D	73.287	1
**4951**	381	D	79.442	1
**4951**	382	D	77.5347	1
**4951**	386	D	55.9701	1
**4952**	305	A	68.9171	1
**4952**	319	A	69.9512	1
**4952**	320	A	73.0071	-1
**4952**	321	A	59.8739	0
**4952**	351	A	2.7813	1
**4952**	357	A	34.2011	0
**4952**	370	A	79.5199	1
**4952**	372	A	38.8127	1
**4952**	373	A	57.6771	1
**4952**	381	A	70.764	1
**4952**	382	A	48.036	0
**4952**	386	A	71.5878	1
**4952**	305	B	79.5599	1
**4952**	319	B	68.5208	1
**4952**	320	B	68.0795	-1
**4952**	321	B	50.7811	0
**4952**	351	B	8.3786	1
**4952**	357	B	43.2336	0
**4952**	370	B	78.2348	1
**4952**	372	B	70.9245	0
**4952**	373	B	79.0996	1
**4952**	381	B	44.1078	0
**4952**	382	B	63.3947	1
**4952**	386	B	79.527	1
**4952**	305	C	79.4956	1
**4952**	319	C	79.6522	1

**4952**	320	C	64.2092	-1
**4952**	321	C	39.3728	1
**4952**	351	C	8.0638	1
**4952**	357	C	79.543	1
**4952**	370	C	67.9608	1
**4952**	372	C	79.5484	0
**4952**	373	C	57.1684	1
**4952**	381	C	72.456	1
**4952**	382	C	73.1662	1
**4952**	386	C	51.143	1
**4952**	305	D	79.6123	1
**4952**	319	D	79.3455	1
**4952**	320	D	52.4184	-1
**4952**	321	D	46.582	1
**4952**	351	D	13.9228	1
**4952**	357	D	61.7919	1
**4952**	370	D	78.9094	1
**4952**	372	D	73.1418	0
**4952**	373	D	77.4109	1
**4952**	381	D	79.6039	1
**4952**	382	D	79.556	1
**4952**	386	D	79.4882	1
**4953**	305	A	48.7435	0
**4953**	319	A	71.0902	1
**4953**	320	A	60.4448	-1
**4953**	321	A	69.2595	0
**4953**	351	A	6.004	1
**4953**	357	A	79.359	1
**4953**	370	A	23.457	0
**4953**	372	A	77.346	0
**4953**	373	A	79.2504	1
**4953**	381	A	35.8085	0
**4953**	382	A	15.9154	-1
**4953**	386	A	53.971	1

**4953**	305	B	79.2885	1
**4953**	319	B	72.7843	1
**4953**	320	B	79.5012	-1
**4953**	321	B	72.0722	0
**4953**	351	B	12.56	1
**4953**	357	B	79.6739	1
**4953**	370	B	79.4007	1
**4953**	372	B	79.2744	0
**4953**	373	B	58.4802	1
**4953**	381	B	79.5893	1
**4953**	382	B	75.3156	1
**4953**	386	B	78.7139	1
**4953**	305	C	24.6635	0
**4953**	319	C	52.9312	1
**4953**	320	C	58.8116	-1
**4953**	321	C	79.6353	0
**4953**	351	C	0.9088	1
**4953**	357	C	74.7931	1
**4953**	370	C	48.7969	0
**4953**	372	C	71.9843	0
**4953**	373	C	79.6458	1
**4953**	381	C	79.6026	1
**4953**	382	C	55.6295	1
**4953**	386	C	62.8308	1
**4953**	305	D	74.4616	1
**4953**	319	D	79.5588	1
**4953**	320	D	35.3549	-1
**4953**	321	D	79.1498	0
**4953**	351	D	43.2738	-1
**4953**	357	D	68.2498	1
**4953**	370	D	59.0607	1
**4953**	372	D	79.5273	0
**4953**	373	D	74.4562	1
**4953**	381	D	79.5523	1

**4953**	382	D	72.0154	1
**4953**	386	D	59.619	1
**4954**	305	A	56.5105	1
**4954**	319	A	79.5853	1
**4954**	320	A	40.9564	-1
**4954**	321	A	75.9863	0
**4954**	351	A	8.431	1
**4954**	357	A	51.4833	1
**4954**	370	A	27.6399	0
**4954**	372	A	29.8858	1
**4954**	373	A	57.8584	1
**4954**	381	A	64.4357	1
**4954**	382	A	36.4055	0
**4954**	386	A	72.2492	1
**4954**	305	B	64.0064	1
**4954**	319	B	77.5981	1
**4954**	320	B	72.2901	-1
**4954**	321	B	72.5775	0
**4954**	351	B	28.7794	-1
**4954**	357	B	75.0924	1
**4954**	370	B	63.3397	1
**4954**	372	B	79.5602	0
**4954**	373	B	38.5882	0
**4954**	381	B	66.9682	1
**4954**	382	B	79.6036	1
**4954**	386	B	79.6184	1
**4954**	305	C	71.7576	1
**4954**	319	C	73.1913	1
**4954**	320	C	60.5467	-1
**4954**	321	C	79.556	0
**4954**	351	C	4.9274	1
**4954**	357	C	78.2071	1
**4954**	370	C	64.2285	1
**4954**	372	C	79.5443	0

**4954**	373	C	79.5269	1
**4954**	381	C	65.0925	1
**4954**	382	C	55.5406	1
**4954**	386	C	52.9842	1
**4954**	305	D	62.7333	1
**4954**	319	D	17.6462	-1
**4954**	320	D	29.2628	-1
**4954**	321	D	75.1591	0
**4954**	351	D	25.0087	-1
**4954**	357	D	54.3306	1
**4954**	370	D	76.9286	1
**4954**	372	D	73.9196	0
**4954**	373	D	31.2515	0
**4954**	381	D	72.8659	1
**4954**	382	D	79.2164	1
**4954**	386	D	79.5668	1
**4955**	305	A	58.7005	1
**4955**	319	A	76.9273	1
**4955**	320	A	72.3911	-1
**4955**	321	A	79.5111	0
**4955**	351	A	17.3746	1
**4955**	357	A	52.1311	1
**4955**	370	A	79.5644	1
**4955**	372	A	77.7621	0
**4955**	373	A	40.5844	0
**4955**	381	A	79.3939	1
**4955**	382	A	79.5285	1
**4955**	386	A	23.3719	0
**4955**	305	B	55.9985	1
**4955**	319	B	75.8362	1
**4955**	320	B	56.292	-1
**4955**	321	B	79.6194	0
**4955**	351	B	7.8536	1
**4955**	357	B	78.7411	1

**4955**	370	B	48.6491	0
**4955**	372	B	79.6599	0
**4955**	373	B	43.3776	0
**4955**	381	B	79.5804	1
**4955**	382	B	61.6692	1
**4955**	386	B	57.4453	1
**4955**	305	C	62.1842	1
**4955**	319	C	34.7658	0
**4955**	320	C	45.6459	-1
**4955**	321	C	74.6994	0
**4955**	351	C	30.5556	-1
**4955**	357	C	69.2876	1
**4955**	370	C	79.576	1
**4955**	372	C	79.5798	0
**4955**	373	C	79.6174	1
**4955**	381	C	71.5344	1
**4955**	382	C	18.8701	-1
**4955**	386	C	31.1744	0
**4955**	305	D	53.8791	1
**4955**	319	D	79.4735	1
**4955**	320	D	75.7144	-1
**4955**	321	D	74.3474	0
**4955**	351	D	27.175	-1
**4955**	357	D	62.1647	1
**4955**	370	D	27.597	0
**4955**	372	D	58.0408	0
**4955**	373	D	61.2437	1
**4955**	381	D	47.5493	0
**4955**	382	D	79.4654	1
**4955**	386	D	63.9001	1
**4956**	305	A	79.4289	1
**4956**	319	A	36.6188	0
**4956**	320	A	79.5489	-1
**4956**	321	A	58.4806	0

**4956**	351	A	3.9002	1
**4956**	357	A	53.5245	1
**4956**	370	A	79.5542	1
**4956**	372	A	79.5616	0
**4956**	373	A	73.4791	1
**4956**	381	A	25.5941	0
**4956**	382	A	66.6101	1
**4956**	386	A	79.5493	1
**4956**	305	B	42.6263	0
**4956**	319	B	28.9205	0
**4956**	320	B	76.4155	-1
**4956**	321	B	53.6239	0
**4956**	351	B	12.9214	1
**4956**	357	B	47.8609	0
**4956**	370	B	79.6364	1
**4956**	372	B	79.5155	0
**4956**	373	B	44.9746	0
**4956**	381	B	43.3769	0
**4956**	382	B	76.5394	1
**4956**	386	B	31.6388	0
**4956**	305	C	38.6625	0
**4956**	319	C	79.5394	1
**4956**	320	C	67.4344	-1
**4956**	321	C	58.9325	0
**4956**	351	C	11.264	1
**4956**	357	C	28.5803	0
**4956**	370	C	79.5882	1
**4956**	372	C	79.5289	0
**4956**	373	C	77.9035	1
**4956**	381	C	40.9834	0
**4956**	382	C	68.5564	1
**4956**	386	C	56.1371	1
**4956**	305	D	45.7285	0
**4956**	319	D	79.5155	1

**4956**	320	D	72.1969	-1
**4956**	321	D	68.2215	0
**4956**	351	D	11.2091	1
**4956**	357	D	79.5948	1
**4956**	370	D	40.8044	0
**4956**	372	D	43.2679	1
**4956**	373	D	79.4538	1
**4956**	381	D	61.386	1
**4956**	382	D	79.5104	1
**4956**	386	D	79.5782	1
**4957**	305	A	27.6329	0
**4957**	319	A	66.9168	1
**4957**	320	A	57.2667	-1
**4957**	321	A	55.5644	0
**4957**	351	A	21.4871	-1
**4957**	357	A	52.2921	1
**4957**	370	A	61.9906	1
**4957**	372	A	72.785	0
**4957**	373	A	79.571	1
**4957**	381	A	79.6007	1
**4957**	382	A	79.528	1
**4957**	386	A	73.5759	1
**4957**	305	B	75.5685	1
**4957**	319	B	59.5525	1
**4957**	320	B	41.0918	-1
**4957**	321	B	69.8783	0
**4957**	351	B	12.7557	1
**4957**	357	B	79.5362	1
**4957**	370	B	79.5523	1
**4957**	372	B	79.5472	0
**4957**	373	B	40.0856	0
**4957**	381	B	79.5467	1
**4957**	382	B	59.0222	1
**4957**	386	B	53.2834	1

**4957**	305	C	19.3318	-1
**4957**	319	C	63.0735	1
**4957**	320	C	79.6625	-1
**4957**	321	C	79.4962	0
**4957**	351	C	2.9867	1
**4957**	357	C	73.8369	1
**4957**	370	C	51.0554	1
**4957**	372	C	68.7192	0
**4957**	373	C	72.2657	1
**4957**	381	C	79.21	1
**4957**	382	C	73.7802	1
**4957**	386	C	51.2112	1
**4957**	305	D	79.6156	1
**4957**	319	D	65.084	1
**4957**	320	D	65.662	-1
**4957**	321	D	52.5039	0
**4957**	351	D	8.1833	1
**4957**	357	D	73.8029	1
**4957**	370	D	79.3074	1
**4957**	372	D	72.8523	0
**4957**	373	D	62.7869	1
**4957**	381	D	51.055	1
**4957**	382	D	48.6212	0
**4957**	386	D	79.5176	1
**4958**	305	A	73.5196	1
**4958**	319	A	56.8158	1
**4958**	320	A	26.3821	-1
**4958**	321	A	62.1112	0
**4958**	351	A	3.8724	1
**4958**	357	A	68.1699	1
**4958**	370	A	79.5278	1
**4958**	372	A	64.3876	0
**4958**	373	A	78.9209	1
**4958**	381	A	75.1763	1

**4958**	382	A	44.5389	0
**4958**	386	A	77.1831	1
**4958**	305	B	79.4283	1
**4958**	319	B	71.8899	1
**4958**	320	B	70.4948	-1
**4958**	321	B	76.8259	0
**4958**	351	B	15.4428	1
**4958**	357	B	79.5338	1
**4958**	370	B	66.885	1
**4958**	372	B	60.2364	0
**4958**	373	B	24.7106	0
**4958**	381	B	79.5823	1
**4958**	382	B	64.1642	1
**4958**	386	B	79.5779	1
**4958**	305	C	70.5961	1
**4958**	319	C	76.0644	1
**4958**	320	C	79.5358	-1
**4958**	321	C	79.5428	0
**4958**	351	C	4.1106	1
**4958**	357	C	64.6509	1
**4958**	370	C	79.5663	1
**4958**	372	C	79.5808	0
**4958**	373	C	79.5944	1
**4958**	381	C	65.7214	1
**4958**	382	C	72.3172	1
**4958**	386	C	41.5829	0
**4958**	305	D	79.0294	1
**4958**	319	D	79.445	1
**4958**	320	D	79.5713	-1
**4958**	321	D	65.0717	0
**4958**	351	D	11.3745	1
**4958**	357	D	42.0533	0
**4958**	370	D	66.0725	1
**4958**	372	D	69.0953	0

**4958**	373	D	68.0197	1
**4958**	381	D	63.3339	1
**4958**	382	D	79.5496	1
**4958**	386	D	62.4634	1
**4959**	305	A	69.7118	1
**4959**	319	A	79.6701	1
**4959**	320	A	71.5002	-1
**4959**	321	A	58.9139	0
**4959**	351	A	6.5963	1
**4959**	357	A	79.595	1
**4959**	370	A	66.0434	1
**4959**	372	A	79.5747	0
**4959**	373	A	32.12	0
**4959**	381	A	79.5111	1
**4959**	382	A	74.0865	1
**4959**	386	A	25.1631	0
**4959**	305	B	62.3706	1
**4959**	319	B	79.523	1
**4959**	320	B	71.8091	-1
**4959**	321	B	79.5979	0
**4959**	351	B	11.2944	1
**4959**	357	B	74.0617	1
**4959**	370	B	44.133	0
**4959**	372	B	29.686	1
**4959**	373	B	37.0602	0
**4959**	381	B	79.6764	1
**4959**	382	B	36.2983	0
**4959**	386	B	57.075	1
**4959**	305	C	65.7825	1
**4959**	319	C	58.733	1
**4959**	320	C	66.6242	-1
**4959**	321	C	77.0962	0
**4959**	351	C	33.4716	-1
**4959**	357	C	61.436	1

**4959**	370	C	74.4859	1
**4959**	372	C	72.3545	0
**4959**	373	C	79.5687	1
**4959**	381	C	70.9671	1
**4959**	382	C	73.0007	1
**4959**	386	C	46.0786	0
**4959**	305	D	73.503	1
**4959**	319	D	51.6904	1
**4959**	320	D	79.5371	-1
**4959**	321	D	70.2474	0
**4959**	351	D	6.0253	1
**4959**	357	D	63.1191	1
**4959**	370	D	79.5357	1
**4959**	372	D	51.361	0
**4959**	373	D	65.4426	1
**4959**	381	D	65.7192	1
**4959**	382	D	76.498	1
**4959**	386	D	74.3942	1
**4960**	305	A	53.9372	1
**4960**	319	A	79.5472	1
**4960**	320	A	76.783	-1
**4960**	321	A	79.6145	0
**4960**	351	A	40.1767	-1
**4960**	357	A	60.3638	1
**4960**	370	A	74.698	1
**4960**	372	A	79.5629	0
**4960**	373	A	56.4539	1
**4960**	381	A	79.5417	1
**4960**	382	A	74.0096	1
**4960**	386	A	37.9753	0
**4960**	305	B	68.5829	1
**4960**	319	B	56.0502	1
**4960**	320	B	79.5058	-1
**4960**	321	B	74.0808	0

**4960**	351	B	20.7936	-1
**4960**	357	B	74.4769	1
**4960**	370	B	79.1975	1
**4960**	372	B	79.6086	0
**4960**	373	B	56.8342	1
**4960**	381	B	79.5549	1
**4960**	382	B	53.4534	1
**4960**	386	B	71.8007	1
**4960**	305	C	41.8208	0
**4960**	319	C	79.5502	1
**4960**	320	C	76.8176	-1
**4960**	321	C	52.9069	0
**4960**	351	C	10.6198	1
**4960**	357	C	79.535	1
**4960**	370	C	68.7503	1
**4960**	372	C	79.5908	0
**4960**	373	C	72.4231	1
**4960**	381	C	41.306	0
**4960**	382	C	73.15	1
**4960**	386	C	11.4645	-1
**4960**	305	D	39.1534	0
**4960**	319	D	43.2343	0
**4960**	320	D	79.5487	-1
**4960**	321	D	73.0881	0
**4960**	351	D	8.036	1
**4960**	357	D	79.5921	1
**4960**	370	D	38.9566	0
**4960**	372	D	79.5534	0
**4960**	373	D	71.2913	1
**4960**	381	D	55.072	1
**4960**	382	D	75.2434	1
**4960**	386	D	43.063	0
**4961**	305	A	39.4502	0
**4961**	319	A	26.3457	0

**4961**	320	A	57.7905	-1
**4961**	321	A	15.3783	0
**4961**	351	A	0.0	1
**4961**	357	A	79.2905	1
**4961**	370	A	77.57	1
**4961**	372	A	44.1422	1
**4961**	373	A	76.3642	1
**4961**	381	A	57.6812	1
**4961**	382	A	60.0313	1
**4961**	386	A	60.0753	1
**4961**	305	B	79.5274	1
**4961**	319	B	59.5533	1
**4961**	320	B	79.5366	-1
**4961**	321	B	67.9814	0
**4961**	351	B	17.493	1
**4961**	357	B	49.9427	0
**4961**	370	B	79.5864	1
**4961**	372	B	73.5935	0
**4961**	373	B	59.3032	1
**4961**	381	B	79.5646	1
**4961**	382	B	79.3913	1
**4961**	386	B	62.0504	1
**4961**	305	C	47.7883	0
**4961**	319	C	49.0838	0
**4961**	320	C	69.9501	-1
**4961**	321	C	47.1888	1
**4961**	351	C	4.2362	1
**4961**	357	C	71.221	1
**4961**	370	C	70.1649	1
**4961**	372	C	58.3058	0
**4961**	373	C	79.597	1
**4961**	381	C	79.5137	1
**4961**	382	C	79.5511	1
**4961**	386	C	40.1405	0

**4961**	305	D	68.4137	1
**4961**	319	D	75.1464	1
**4961**	320	D	79.5717	-1
**4961**	321	D	78.0686	0
**4961**	351	D	9.9113	1
**4961**	357	D	68.9005	1
**4961**	370	D	76.3852	1
**4961**	372	D	64.7098	0
**4961**	373	D	65.392	1
**4961**	381	D	79.5235	1
**4961**	382	D	49.937	0
**4961**	386	D	79.5476	1
**4962**	305	A	72.4891	1
**4962**	319	A	63.6168	1
**4962**	320	A	79.6012	-1
**4962**	321	A	79.5981	0
**4962**	351	A	11.0021	1
**4962**	357	A	74.9977	1
**4962**	370	A	78.7932	1
**4962**	372	A	56.8512	0
**4962**	373	A	71.8824	1
**4962**	381	A	79.5517	1
**4962**	382	A	79.5881	1
**4962**	386	A	76.2711	1
**4962**	305	B	44.9522	0
**4962**	319	B	52.227	1
**4962**	320	B	49.4918	-1
**4962**	321	B	74.7807	0
**4962**	351	B	14.2534	1
**4962**	357	B	73.5326	1
**4962**	370	B	73.556	1
**4962**	372	B	60.5424	0
**4962**	373	B	63.9247	1
**4962**	381	B	79.5614	1

**4962**	382	B	16.5133	-1
**4962**	386	B	50.2479	1
**4962**	305	C	68.0292	1
**4962**	319	C	73.2957	1
**4962**	320	C	79.5304	-1
**4962**	321	C	73.7947	0
**4962**	351	C	1.3831	1
**4962**	357	C	72.2062	1
**4962**	370	C	69.6887	1
**4962**	372	C	79.5532	0
**4962**	373	C	72.3517	1
**4962**	381	C	55.4695	1
**4962**	382	C	67.0388	1
**4962**	386	C	41.9528	0
**4962**	305	D	30.1184	0
**4962**	319	D	72.9092	1
**4962**	320	D	63.2075	-1
**4962**	321	D	79.5538	0
**4962**	351	D	11.8805	1
**4962**	357	D	71.0765	1
**4962**	370	D	33.5599	0
**4962**	372	D	79.5214	0
**4962**	373	D	49.4135	0
**4962**	381	D	79.43	1
**4962**	382	D	72.5029	1
**4962**	386	D	78.5934	1
**4963**	305	A	79.5076	1
**4963**	319	A	51.3526	1
**4963**	320	A	79.5662	-1
**4963**	321	A	72.0109	0
**4963**	351	A	8.9646	1
**4963**	357	A	77.0246	1
**4963**	370	A	79.526	1
**4963**	372	A	60.5821	0

**4963**	373	A	79.6232	1
**4963**	381	A	69.8902	1
**4963**	382	A	44.879	0
**4963**	386	A	70.9792	1
**4963**	305	B	79.4894	1
**4963**	319	B	42.5787	0
**4963**	320	B	77.7567	-1
**4963**	321	B	72.3798	0
**4963**	351	B	16.6666	1
**4963**	357	B	79.5456	1
**4963**	370	B	79.5339	1
**4963**	372	B	75.8791	0
**4963**	373	B	78.9024	1
**4963**	381	B	64.969	1
**4963**	382	B	53.5897	1
**4963**	386	B	79.5585	1
**4963**	305	C	76.0028	1
**4963**	319	C	78.8537	1
**4963**	320	C	62.1535	-1
**4963**	321	C	55.3831	0
**4963**	351	C	3.7961	1
**4963**	357	C	63.3585	1
**4963**	370	C	61.6822	1
**4963**	372	C	79.0872	0
**4963**	373	C	79.5121	1
**4963**	381	C	56.7153	1
**4963**	382	C	70.3538	1
**4963**	386	C	40.7084	0
**4963**	305	D	35.0649	0
**4963**	319	D	79.6359	1
**4963**	320	D	66.1615	-1
**4963**	321	D	76.427	0
**4963**	351	D	0.1406	1
**4963**	357	D	56.0825	1

**4963**	370	D	79.6008	1
**4963**	372	D	69.1332	0
**4963**	373	D	79.6063	1
**4963**	381	D	55.9417	1
**4963**	382	D	79.5371	1
**4963**	386	D	79.5492	1
**4964**	305	A	72.8908	1
**4964**	319	A	79.563	1
**4964**	320	A	58.7981	-1
**4964**	321	A	70.5133	0
**4964**	351	A	4.0246	1
**4964**	357	A	74.3355	1
**4964**	370	A	79.5601	1
**4964**	372	A	73.288	0
**4964**	373	A	46.1392	0
**4964**	381	A	72.2809	1
**4964**	382	A	43.2945	0
**4964**	386	A	35.0513	0
**4964**	305	B	79.5645	1
**4964**	319	B	77.7629	1
**4964**	320	B	71.3384	-1
**4964**	321	B	72.1694	0
**4964**	351	B	4.8175	1
**4964**	357	B	61.6676	1
**4964**	370	B	79.5783	1
**4964**	372	B	53.1499	0
**4964**	373	B	43.7975	0
**4964**	381	B	79.5682	1
**4964**	382	B	43.3442	0
**4964**	386	B	69.2174	1
**4964**	305	C	46.3595	0
**4964**	319	C	73.7644	1
**4964**	320	C	61.488	-1
**4964**	321	C	65.765	0

**4964**	351	C	4.6334	1
**4964**	357	C	79.552	1
**4964**	370	C	79.5012	1
**4964**	372	C	58.3227	0
**4964**	373	C	72.3836	1
**4964**	381	C	79.5481	1
**4964**	382	C	79.5242	1
**4964**	386	C	50.3605	1
**4964**	305	D	47.0537	0
**4964**	319	D	63.3079	1
**4964**	320	D	24.4577	-1
**4964**	321	D	59.7982	0
**4964**	351	D	14.6998	1
**4964**	357	D	71.2666	1
**4964**	370	D	75.2799	1
**4964**	372	D	41.7488	1
**4964**	373	D	71.3052	1
**4964**	381	D	46.9152	0
**4964**	382	D	79.5509	1
**4964**	386	D	65.6522	1
**4965**	305	A	67.1986	1
**4965**	319	A	43.3243	0
**4965**	320	A	71.5423	-1
**4965**	321	A	33.5779	1
**4965**	351	A	4.6977	1
**4965**	357	A	61.4235	1
**4965**	370	A	79.5599	1
**4965**	372	A	69.6489	0
**4965**	373	A	79.4845	1
**4965**	381	A	79.5951	1
**4965**	382	A	46.6607	0
**4965**	386	A	77.4509	1
**4965**	305	B	64.8827	1
**4965**	319	B	79.5609	1

**4965**	320	B	79.5801	-1
**4965**	321	B	58.3612	0
**4965**	351	B	10.7842	1
**4965**	357	B	72.6106	1
**4965**	370	B	42.2778	0
**4965**	372	B	79.5984	0
**4965**	373	B	68.4675	1
**4965**	381	B	54.4244	1
**4965**	382	B	78.5068	1
**4965**	386	B	73.1725	1
**4965**	305	C	79.5395	1
**4965**	319	C	77.4931	1
**4965**	320	C	79.5346	-1
**4965**	321	C	79.5203	0
**4965**	351	C	8.5451	1
**4965**	357	C	72.7083	1
**4965**	370	C	79.5814	1
**4965**	372	C	79.5816	0
**4965**	373	C	79.5538	1
**4965**	381	C	72.3467	1
**4965**	382	C	39.7156	0
**4965**	386	C	69.8339	1
**4965**	305	D	49.427	0
**4965**	319	D	79.5831	1
**4965**	320	D	64.3967	-1
**4965**	321	D	66.3457	0
**4965**	351	D	13.236	1
**4965**	357	D	54.1798	1
**4965**	370	D	77.3218	1
**4965**	372	D	68.1373	0
**4965**	373	D	79.3501	1
**4965**	381	D	77.1071	1
**4965**	382	D	79.5902	1
**4965**	386	D	77.3101	1

**4966**	305	A	62.4474	1
**4966**	319	A	79.5885	1
**4966**	320	A	71.8907	-1
**4966**	321	A	33.9773	1
**4966**	351	A	7.5445	1
**4966**	357	A	35.9983	0
**4966**	370	A	59.0525	1
**4966**	372	A	79.3549	0
**4966**	373	A	67.0789	1
**4966**	381	A	73.6465	1
**4966**	382	A	68.3618	1
**4966**	386	A	79.5842	1
**4966**	305	B	79.567	1
**4966**	319	B	72.1764	1
**4966**	320	B	69.1539	-1
**4966**	321	B	78.5424	0
**4966**	351	B	16.3277	1
**4966**	357	B	79.5765	1
**4966**	370	B	71.0839	1
**4966**	372	B	79.5599	0
**4966**	373	B	55.6371	1
**4966**	381	B	79.55	1
**4966**	382	B	53.1458	1
**4966**	386	B	49.3842	0
**4966**	305	C	77.9293	1
**4966**	319	C	79.5294	1
**4966**	320	C	46.6991	-1
**4966**	321	C	73.3441	0
**4966**	351	C	9.7995	1
**4966**	357	C	79.5786	1
**4966**	370	C	61.7619	1
**4966**	372	C	79.5644	0
**4966**	373	C	77.1398	1
**4966**	381	C	74.364	1

**4966**	382	C	39.567	0
**4966**	386	C	57.8138	1
**4966**	305	D	66.1479	1
**4966**	319	D	72.5426	1
**4966**	320	D	79.4674	-1
**4966**	321	D	74.1561	0
**4966**	351	D	13.909	1
**4966**	357	D	79.545	1
**4966**	370	D	79.5452	1
**4966**	372	D	62.8145	0
**4966**	373	D	49.5487	0
**4966**	381	D	74.7622	1
**4966**	382	D	33.8762	0
**4966**	386	D	79.1209	1
**4967**	305	A	70.2108	1
**4967**	319	A	67.0669	1
**4967**	320	A	79.566	-1
**4967**	321	A	31.728	1
**4967**	351	A	12.8002	1
**4967**	357	A	79.4958	1
**4967**	370	A	72.9682	1
**4967**	372	A	53.6731	0
**4967**	373	A	71.0827	1
**4967**	381	A	58.5911	1
**4967**	382	A	31.9192	0
**4967**	386	A	21.2108	0
**4967**	305	B	48.3276	0
**4967**	319	B	71.1463	1
**4967**	320	B	66.7965	-1
**4967**	321	B	79.4843	0
**4967**	351	B	41.69	-1
**4967**	357	B	64.6902	1
**4967**	370	B	79.6688	1
**4967**	372	B	74.0746	0

**4967**	373	B	79.0709	1
**4967**	381	B	79.5543	1
**4967**	382	B	37.0952	0
**4967**	386	B	64.2085	1
**4967**	305	C	79.7929	1
**4967**	319	C	79.5089	1
**4967**	320	C	70.908	-1
**4967**	321	C	76.3666	0
**4967**	351	C	2.3866	1
**4967**	357	C	64.141	1
**4967**	370	C	79.3291	1
**4967**	372	C	77.6994	0
**4967**	373	C	79.5735	1
**4967**	381	C	73.6913	1
**4967**	382	C	78.7424	1
**4967**	386	C	30.3229	0
**4967**	305	D	79.5643	1
**4967**	319	D	71.5664	1
**4967**	320	D	79.4472	-1
**4967**	321	D	79.5343	0
**4967**	351	D	18.1128	1
**4967**	357	D	75.9986	1
**4967**	370	D	74.9687	1
**4967**	372	D	79.489	0
**4967**	373	D	79.4599	1
**4967**	381	D	73.3588	1
**4967**	382	D	79.7266	1
**4967**	386	D	66.5448	1
**4968**	305	A	49.4554	0
**4968**	319	A	71.9791	1
**4968**	320	A	50.8665	-1
**4968**	321	A	72.7003	0
**4968**	351	A	8.806	1
**4968**	357	A	44.751	0

**4968**	370	A	73.3298	1
**4968**	372	A	79.5696	0
**4968**	373	A	79.5466	1
**4968**	381	A	79.4179	1
**4968**	382	A	72.4517	1
**4968**	386	A	74.3004	1
**4968**	305	B	72.2783	1
**4968**	319	B	69.535	1
**4968**	320	B	75.4237	-1
**4968**	321	B	26.9138	1
**4968**	351	B	15.9862	1
**4968**	357	B	23.1597	0
**4968**	370	B	35.7551	0
**4968**	372	B	75.99	0
**4968**	373	B	69.8288	1
**4968**	381	B	74.303	1
**4968**	382	B	78.4204	1
**4968**	386	B	79.6003	1
**4968**	305	C	39.7528	0
**4968**	319	C	48.2907	0
**4968**	320	C	76.3993	-1
**4968**	321	C	70.7467	0
**4968**	351	C	2.1043	1
**4968**	357	C	68.5344	1
**4968**	370	C	79.2675	1
**4968**	372	C	74.7636	0
**4968**	373	C	62.9962	1
**4968**	381	C	34.3801	0
**4968**	382	C	67.6174	1
**4968**	386	C	61.6754	1
**4968**	305	D	39.4135	0
**4968**	319	D	79.5466	1
**4968**	320	D	63.0719	-1
**4968**	321	D	79.5554	0

**4968**	351	D	12.3848	1
**4968**	357	D	44.0726	0
**4968**	370	D	78.7221	1
**4968**	372	D	72.4944	0
**4968**	373	D	67.1774	1
**4968**	381	D	79.5768	1
**4968**	382	D	65.7264	1
**4968**	386	D	45.6166	0
**4969**	305	A	64.8363	1
**4969**	319	A	72.5251	1
**4969**	320	A	72.4954	-1
**4969**	321	A	79.5811	0
**4969**	351	A	2.4223	1
**4969**	357	A	68.5126	1
**4969**	370	A	79.5231	1
**4969**	372	A	62.4212	0
**4969**	373	A	76.3368	1
**4969**	381	A	69.763	1
**4969**	382	A	65.1791	1
**4969**	386	A	79.5861	1
**4969**	305	B	79.5805	1
**4969**	319	B	30.3258	0
**4969**	320	B	71.419	-1
**4969**	321	B	56.4188	0
**4969**	351	B	13.8657	1
**4969**	357	B	53.4213	1
**4969**	370	B	26.0648	0
**4969**	372	B	27.2861	1
**4969**	373	B	79.5759	1
**4969**	381	B	67.7083	1
**4969**	382	B	38.2103	0
**4969**	386	B	57.7657	1
**4969**	305	C	65.4649	1
**4969**	319	C	72.2288	1

**4969**	320	C	70.965	-1
**4969**	321	C	72.0803	0
**4969**	351	C	18.6571	1
**4969**	357	C	65.5829	1
**4969**	370	C	62.1661	1
**4969**	372	C	69.7729	0
**4969**	373	C	52.3196	1
**4969**	381	C	74.1517	1
**4969**	382	C	78.6902	1
**4969**	386	C	36.3919	0
**4969**	305	D	62.1191	1
**4969**	319	D	67.163	1
**4969**	320	D	72.1982	-1
**4969**	321	D	41.161	1
**4969**	351	D	20.353	-1
**4969**	357	D	67.5876	1
**4969**	370	D	79.5414	1
**4969**	372	D	79.5771	0
**4969**	373	D	79.5845	1
**4969**	381	D	66.4597	1
**4969**	382	D	79.5562	1
**4969**	386	D	6.781	-1
**4970**	305	A	53.3935	1
**4970**	319	A	59.4312	1
**4970**	320	A	79.5537	-1
**4970**	321	A	77.1147	0
**4970**	351	A	8.1139	1
**4970**	357	A	79.4703	1
**4970**	370	A	72.1043	1
**4970**	372	A	57.4569	0
**4970**	373	A	73.4519	1
**4970**	381	A	74.361	1
**4970**	382	A	39.7882	0
**4970**	386	A	78.9331	1

**4970**	305	B	79.557	1
**4970**	319	B	79.6002	1
**4970**	320	B	72.5451	-1
**4970**	321	B	69.0706	0
**4970**	351	B	20.945	-1
**4970**	357	B	58.8638	1
**4970**	370	B	73.4144	1
**4970**	372	B	69.563	0
**4970**	373	B	75.2835	1
**4970**	381	B	72.702	1
**4970**	382	B	55.7433	1
**4970**	386	B	33.9908	0
**4970**	305	C	50.9312	1
**4970**	319	C	67.5798	1
**4970**	320	C	36.563	-1
**4970**	321	C	79.539	0
**4970**	351	C	4.3209	1
**4970**	357	C	72.5699	1
**4970**	370	C	79.5599	1
**4970**	372	C	71.6083	0
**4970**	373	C	79.5672	1
**4970**	381	C	67.719	1
**4970**	382	C	79.5986	1
**4970**	386	C	60.0427	1
**4970**	305	D	58.9419	1
**4970**	319	D	79.5513	1
**4970**	320	D	20.665	-1
**4970**	321	D	79.4967	0
**4970**	351	D	12.4036	1
**4970**	357	D	79.6044	1
**4970**	370	D	41.406	0
**4970**	372	D	79.5484	0
**4970**	373	D	76.6325	1
**4970**	381	D	58.5133	1

**4970**	382	D	64.9481	1
**4970**	386	D	25.6932	0
**4971**	305	A	67.6395	1
**4971**	319	A	25.434	0
**4971**	320	A	74.5227	-1
**4971**	321	A	79.074	0
**4971**	351	A	13.6603	1
**4971**	357	A	36.8653	0
**4971**	370	A	46.4605	0
**4971**	372	A	72.0087	0
**4971**	373	A	79.5658	1
**4971**	381	A	40.5884	0
**4971**	382	A	79.6726	1
**4971**	386	A	79.6622	1
**4971**	305	B	79.4427	1
**4971**	319	B	70.8389	1
**4971**	320	B	75.0072	-1
**4971**	321	B	78.968	0
**4971**	351	B	15.4827	1
**4971**	357	B	76.5337	1
**4971**	370	B	79.6645	1
**4971**	372	B	51.1001	0
**4971**	373	B	72.9197	1
**4971**	381	B	63.5059	1
**4971**	382	B	61.8215	1
**4971**	386	B	79.5234	1
**4971**	305	C	78.513	1
**4971**	319	C	74.6061	1
**4971**	320	C	73.097	-1
**4971**	321	C	71.684	0
**4971**	351	C	8.2212	1
**4971**	357	C	42.5232	0
**4971**	370	C	67.4777	1
**4971**	372	C	47.9954	1

**4971**	373	C	79.6748	1
**4971**	381	C	71.4152	1
**4971**	382	C	76.3189	1
**4971**	386	C	56.3366	1
**4971**	305	D	67.4942	1
**4971**	319	D	61.8361	1
**4971**	320	D	74.7235	-1
**4971**	321	D	71.1824	0
**4971**	351	D	13.5844	1
**4971**	357	D	62.1147	1
**4971**	370	D	76.2156	1
**4971**	372	D	79.5377	0
**4971**	373	D	47.6823	0
**4971**	381	D	79.5858	1
**4971**	382	D	62.322	1
**4971**	386	D	70.8015	1
**4972**	305	A	76.3398	1
**4972**	319	A	72.5523	1
**4972**	320	A	75.7886	-1
**4972**	321	A	19.1952	0
**4972**	351	A	5.5728	1
**4972**	357	A	79.6008	1
**4972**	370	A	73.3035	1
**4972**	372	A	54.4275	0
**4972**	373	A	79.6518	1
**4972**	381	A	73.2979	1
**4972**	382	A	79.5535	1
**4972**	386	A	63.5081	1
**4972**	305	B	57.6163	1
**4972**	319	B	72.2984	1
**4972**	320	B	53.1331	-1
**4972**	321	B	79.555	0
**4972**	351	B	13.834	1
**4972**	357	B	60.2517	1

**4972**	370	B	79.5771	1
**4972**	372	B	77.7209	0
**4972**	373	B	36.0029	0
**4972**	381	B	76.6235	1
**4972**	382	B	33.1283	0
**4972**	386	B	49.7474	0
**4972**	305	C	47.0187	0
**4972**	319	C	51.0021	1
**4972**	320	C	73.5637	-1
**4972**	321	C	74.405	0
**4972**	351	C	876	1
**4972**	357	C	76.5842	1
**4972**	370	C	52.0892	1
**4972**	372	C	79.5965	0
**4972**	373	C	79.2161	1
**4972**	381	C	76.3321	1
**4972**	382	C	66.8658	1
**4972**	386	C	38.1474	0
**4972**	305	D	67.0874	1
**4972**	319	D	74.5903	1
**4972**	320	D	60.4425	-1
**4972**	321	D	41.9412	1
**4972**	351	D	11.8368	1
**4972**	357	D	70.6312	1
**4972**	370	D	78.5289	1
**4972**	372	D	17.1241	0
**4972**	373	D	8.6489	-1
**4972**	381	D	74.4834	1
**4972**	382	D	79.5155	1
**4972**	386	D	71.5484	1
**4973**	305	A	79.6863	1
**4973**	319	A	78.5537	1
**4973**	320	A	78.109	-1
**4973**	321	A	79.8047	0

**4973**	351	A	7.405	1
**4973**	357	A	79.5166	1
**4973**	370	A	79.5834	1
**4973**	372	A	35.1758	1
**4973**	373	A	79.3618	1
**4973**	381	A	57.5145	1
**4973**	382	A	69.4701	1
**4973**	386	A	73.4851	1
**4973**	305	B	79.4837	1
**4973**	319	B	79.5579	1
**4973**	320	B	77.7067	-1
**4973**	321	B	56.2938	0
**4973**	351	B	19.5067	1
**4973**	357	B	64.2848	1
**4973**	370	B	79.5303	1
**4973**	372	B	36.1093	1
**4973**	373	B	77.5989	1
**4973**	381	B	79.5296	1
**4973**	382	B	52.9647	1
**4973**	386	B	53.5343	1
**4973**	305	C	79.6666	1
**4973**	319	C	63.6775	1
**4973**	320	C	39.7849	-1
**4973**	321	C	79.4685	0
**4973**	351	C	1.3913	1
**4973**	357	C	71.1074	1
**4973**	370	C	36.2344	0
**4973**	372	C	73.1063	0
**4973**	373	C	43.2673	0
**4973**	381	C	51.4873	1
**4973**	382	C	63.6283	1
**4973**	386	C	53.3975	1
**4973**	305	D	69.9464	1
**4973**	319	D	46.8001	0

**4973**	320	D	73.383	-1
**4973**	321	D	49.7277	1
**4973**	351	D	9.8886	1
**4973**	357	D	79.5176	1
**4973**	370	D	41.7937	0
**4973**	372	D	79.5879	0
**4973**	373	D	79.5795	1
**4973**	381	D	70.9187	1
**4973**	382	D	54.3072	1
**4973**	386	D	79.5093	1
**4974**	305	A	61.7323	1
**4974**	319	A	24.0976	0
**4974**	320	A	79.5986	-1
**4974**	321	A	72.3026	0
**4974**	351	A	2.2905	1
**4974**	357	A	67.6058	1
**4974**	370	A	71.0301	1
**4974**	372	A	79.4869	0
**4974**	373	A	70.681	1
**4974**	381	A	67.3607	1
**4974**	382	A	41.9083	0
**4974**	386	A	50.3496	1
**4974**	305	B	79.5566	1
**4974**	319	B	46.5694	0
**4974**	320	B	58.2695	-1
**4974**	321	B	49.4444	1
**4974**	351	B	22.3008	-1
**4974**	357	B	68.1442	1
**4974**	370	B	78.8651	1
**4974**	372	B	64.217	0
**4974**	373	B	59.3025	1
**4974**	381	B	79.5207	1
**4974**	382	B	53.442	1
**4974**	386	B	48.9552	0

**4974**	305	C	79.5505	1
**4974**	319	C	72.2917	1
**4974**	320	C	65.1246	-1
**4974**	321	C	69.1191	0
**4974**	351	C	27.282	-1
**4974**	357	C	67.3703	1
**4974**	370	C	74.339	1
**4974**	372	C	79.495	0
**4974**	373	C	79.6168	1
**4974**	381	C	79.6601	1
**4974**	382	C	79.5026	1
**4974**	386	C	48.2812	0
**4974**	305	D	63.1106	1
**4974**	319	D	55.6602	1
**4974**	320	D	73.3435	-1
**4974**	321	D	79.4657	0
**4974**	351	D	13.7993	1
**4974**	357	D	53.2367	1
**4974**	370	D	76.8229	1
**4974**	372	D	55.3201	0
**4974**	373	D	34.8291	0
**4974**	381	D	79.7031	1
**4974**	382	D	61.5693	1
**4974**	386	D	79.5613	1
**4975**	305	A	60.4885	1
**4975**	319	A	74.4686	1
**4975**	320	A	79.5634	-1
**4975**	321	A	79.1744	0
**4975**	351	A	11.7364	1
**4975**	357	A	55.94	1
**4975**	370	A	79.6412	1
**4975**	372	A	74.5878	0
**4975**	373	A	59.3165	1
**4975**	381	A	79.5269	1

**4975**	382	A	79.6391	1
**4975**	386	A	72.6872	1
**4975**	305	B	60.2497	1
**4975**	319	B	30.376	0
**4975**	320	B	57.2924	-1
**4975**	321	B	74.1266	0
**4975**	351	B	6.4288	1
**4975**	357	B	79.6154	1
**4975**	370	B	60.6908	1
**4975**	372	B	79.499	0
**4975**	373	B	52.2445	1
**4975**	381	B	79.6042	1
**4975**	382	B	22.0081	0
**4975**	386	B	79.4888	1
**4975**	305	C	77.2013	1
**4975**	319	C	68.6412	1
**4975**	320	C	65.0725	-1
**4975**	321	C	70.893	0
**4975**	351	C	15.1873	1
**4975**	357	C	40.5747	0
**4975**	370	C	46.0304	0
**4975**	372	C	79.5409	0
**4975**	373	C	74.327	1
**4975**	381	C	79.458	1
**4975**	382	C	26.3934	0
**4975**	386	C	48.5609	0
**4975**	305	D	74.1261	1
**4975**	319	D	61.1375	1
**4975**	320	D	79.5516	-1
**4975**	321	D	65.825	0
**4975**	351	D	11.5964	1
**4975**	357	D	70.3605	1
**4975**	370	D	46.398	0
**4975**	372	D	66.2628	0

**4975**	373	D	42.8235	0
**4975**	381	D	77.5264	1
**4975**	382	D	66.3319	1
**4975**	386	D	69.2683	1
**4976**	305	A	79.5559	1
**4976**	319	A	22.5514	0
**4976**	320	A	79.5742	-1
**4976**	321	A	57.1237	0
**4976**	351	A	10.4723	1
**4976**	357	A	55.0277	1
**4976**	370	A	62.4339	1
**4976**	372	A	43.1305	1
**4976**	373	A	45.9541	0
**4976**	381	A	65.9531	1
**4976**	382	A	75.9802	1
**4976**	386	A	79.5816	1
**4976**	305	B	73.773	1
**4976**	319	B	63.5019	1
**4976**	320	B	29.6308	-1
**4976**	321	B	64.4061	0
**4976**	351	B	16.0179	1
**4976**	357	B	53.7033	1
**4976**	370	B	35.564	0
**4976**	372	B	76.2448	0
**4976**	373	B	32.8726	0
**4976**	381	B	79.3357	1
**4976**	382	B	69.4381	1
**4976**	386	B	78.8078	1
**4976**	305	C	48.5098	0
**4976**	319	C	79.565	1
**4976**	320	C	53.2031	-1
**4976**	321	C	72.9597	0
**4976**	351	C	15.0334	1
**4976**	357	C	73.4061	1

**4976**	370	C	79.5885	1
**4976**	372	C	74.1321	0
**4976**	373	C	63.0474	1
**4976**	381	C	79.5379	1
**4976**	382	C	74.9857	1
**4976**	386	C	64.1338	1
**4976**	305	D	66.1016	1
**4976**	319	D	62.9644	1
**4976**	320	D	76.5238	-1
**4976**	321	D	29.9859	1
**4976**	351	D	10.6169	1
**4976**	357	D	79.5233	1
**4976**	370	D	78.9726	1
**4976**	372	D	79.5716	0
**4976**	373	D	79.5762	1
**4976**	381	D	76.2324	1
**4976**	382	D	63.4296	1
**4976**	386	D	77.5924	1
**4977**	305	A	74.1878	1
**4977**	319	A	48.4462	0
**4977**	320	A	66.4301	-1
**4977**	321	A	79.6729	0
**4977**	351	A	10.3083	1
**4977**	357	A	55.4948	1
**4977**	370	A	79.4987	1
**4977**	372	A	43.6527	1
**4977**	373	A	79.6312	1
**4977**	381	A	65.4571	1
**4977**	382	A	68.9696	1
**4977**	386	A	75.5068	1
**4977**	305	B	79.6674	1
**4977**	319	B	68.9235	1
**4977**	320	B	51.1156	-1
**4977**	321	B	24.3042	1

**4977**	351	B	14.8138	1
**4977**	357	B	71.2332	1
**4977**	370	B	46.563	0
**4977**	372	B	72.6618	0
**4977**	373	B	56.4512	1
**4977**	381	B	74.293	1
**4977**	382	B	51.4098	1
**4977**	386	B	79.5362	1
**4977**	305	C	79.1064	1
**4977**	319	C	72.5827	1
**4977**	320	C	43.0275	-1
**4977**	321	C	62.1435	0
**4977**	351	C	29.0706	-1
**4977**	357	C	69.4235	1
**4977**	370	C	50.4304	1
**4977**	372	C	54.8097	0
**4977**	373	C	67.8073	1
**4977**	381	C	78.1822	1
**4977**	382	C	79.0628	1
**4977**	386	C	52.4258	1
**4977**	305	D	49.4649	0
**4977**	319	D	74.0878	1
**4977**	320	D	56.238	-1
**4977**	321	D	64.1529	0
**4977**	351	D	7.9129	1
**4977**	357	D	79.3153	1
**4977**	370	D	65.9087	1
**4977**	372	D	79.5391	0
**4977**	373	D	55.8491	1
**4977**	381	D	69.8042	1
**4977**	382	D	71.2576	1
**4977**	386	D	79.4271	1
**4978**	305	A	58.6504	1
**4978**	319	A	74.6061	1

**4978**	320	A	72.9295	-1
**4978**	321	A	54.7203	0
**4978**	351	A	11.5573	1
**4978**	357	A	72.1885	1
**4978**	370	A	72.4065	1
**4978**	372	A	39.9241	1
**4978**	373	A	46.3625	0
**4978**	381	A	54.6915	1
**4978**	382	A	73.2942	1
**4978**	386	A	50.7882	1
**4978**	305	B	58.7914	1
**4978**	319	B	62.7186	1
**4978**	320	B	75.0543	-1
**4978**	321	B	43.0735	1
**4978**	351	B	13.9361	1
**4978**	357	B	69.5122	1
**4978**	370	B	78.0158	1
**4978**	372	B	76.4025	0
**4978**	373	B	77.7838	1
**4978**	381	B	73.0616	1
**4978**	382	B	75.2334	1
**4978**	386	B	58.7485	1
**4978**	305	C	61.1006	1
**4978**	319	C	70.9903	1
**4978**	320	C	50.6182	-1
**4978**	321	C	68.58	0
**4978**	351	C	4.8951	1
**4978**	357	C	76.3712	1
**4978**	370	C	79.6212	1
**4978**	372	C	79.5756	0
**4978**	373	C	79.6349	1
**4978**	381	C	74.9059	1
**4978**	382	C	54.4209	1
**4978**	386	C	9.8501	-1

**4978**	305	D	67.1719	1
**4978**	319	D	61.8259	1
**4978**	320	D	79.5456	-1
**4978**	321	D	76.7724	0
**4978**	351	D	10.1066	1
**4978**	357	D	79.5701	1
**4978**	370	D	54.9694	1
**4978**	372	D	79.5237	0
**4978**	373	D	79.6111	1
**4978**	381	D	77.0626	1
**4978**	382	D	78.9266	1
**4978**	386	D	73.1434	1
**4979**	305	A	55.1931	1
**4979**	319	A	79.3791	1
**4979**	320	A	73.155	-1
**4979**	321	A	40.8725	1
**4979**	351	A	4.5711	1
**4979**	357	A	75.4268	1
**4979**	370	A	59.2932	1
**4979**	372	A	52.2603	0
**4979**	373	A	8.4102	-1
**4979**	381	A	79.5242	1
**4979**	382	A	44.6539	0
**4979**	386	A	62.2209	1
**4979**	305	B	79.4612	1
**4979**	319	B	78.8281	1
**4979**	320	B	53.2525	-1
**4979**	321	B	79.5766	0
**4979**	351	B	24.6739	-1
**4979**	357	B	49.2525	0
**4979**	370	B	72.1595	1
**4979**	372	B	66.7564	0
**4979**	373	B	74.8036	1
**4979**	381	B	5.7107	-1

**4979**	382	B	41.0657	0
**4979**	386	B	79.6363	1
**4979**	305	C	72.8631	1
**4979**	319	C	33.5137	0
**4979**	320	C	59.5674	-1
**4979**	321	C	68.5757	0
**4979**	351	C	5.9086	1
**4979**	357	C	58.9599	1
**4979**	370	C	72.4187	1
**4979**	372	C	79.57	0
**4979**	373	C	52.9938	1
**4979**	381	C	69.5441	1
**4979**	382	C	59.5703	1
**4979**	386	C	60.5843	1
**4979**	305	D	67.7745	1
**4979**	319	D	79.3675	1
**4979**	320	D	66.3179	-1
**4979**	321	D	66.8931	0
**4979**	351	D	7.4337	1
**4979**	357	D	67.9007	1
**4979**	370	D	53.1365	1
**4979**	372	D	51.024	0
**4979**	373	D	79.5256	1
**4979**	381	D	58.7747	1
**4979**	382	D	63.3072	1
**4979**	386	D	79.5661	1
**4980**	305	A	48.0515	0
**4980**	319	A	79.5883	1
**4980**	320	A	66.4763	-1
**4980**	321	A	79.5805	0
**4980**	351	A	22.6482	-1
**4980**	357	A	21.5897	0
**4980**	370	A	50.4236	1
**4980**	372	A	48.9997	1

**4980**	373	A	46.84	0
**4980**	381	A	64.6535	1
**4980**	382	A	69.4332	1
**4980**	386	A	61.9889	1
**4980**	305	B	79.6331	1
**4980**	319	B	31.1459	0
**4980**	320	B	62.5638	-1
**4980**	321	B	72.2298	0
**4980**	351	B	0.0	1
**4980**	357	B	76.5194	1
**4980**	370	B	36.9791	0
**4980**	372	B	79.598	0
**4980**	373	B	58.9047	1
**4980**	381	B	79.5997	1
**4980**	382	B	17.9745	-1
**4980**	386	B	79.6648	1
**4980**	305	C	73.4237	1
**4980**	319	C	61.5383	1
**4980**	320	C	70.8583	-1
**4980**	321	C	79.5572	0
**4980**	351	C	13.6302	1
**4980**	357	C	71.3586	1
**4980**	370	C	79.5285	1
**4980**	372	C	79.6123	0
**4980**	373	C	65.0663	1
**4980**	381	C	79.6703	1
**4980**	382	C	33.2914	0
**4980**	386	C	52.5614	1
**4980**	305	D	79.5539	1
**4980**	319	D	72.5115	1
**4980**	320	D	44.5891	-1
**4980**	321	D	72.5419	0
**4980**	351	D	10.8213	1
**4980**	357	D	44.6077	0

**4980**	370	D	49.1463	0
**4980**	372	D	69.7591	0
**4980**	373	D	72.8117	1
**4980**	381	D	61.6392	1
**4980**	382	D	58.7756	1
**4980**	386	D	51.0908	1
**4981**	305	A	60.8542	1
**4981**	319	A	48.6678	0
**4981**	320	A	79.5257	-1
**4981**	321	A	65.1591	0
**4981**	351	A	8.6102	1
**4981**	357	A	71.1579	1
**4981**	370	A	32.741	0
**4981**	372	A	71.3217	0
**4981**	373	A	78.4471	1
**4981**	381	A	79.5941	1
**4981**	382	A	79.5399	1
**4981**	386	A	79.5417	1
**4981**	305	B	79.5021	1
**4981**	319	B	65.3603	1
**4981**	320	B	55.0988	-1
**4981**	321	B	71.3872	0
**4981**	351	B	20.854	-1
**4981**	357	B	78.1275	1
**4981**	370	B	77.9718	1
**4981**	372	B	71.6023	0
**4981**	373	B	79.4041	1
**4981**	381	B	21.2986	0
**4981**	382	B	66.071	1
**4981**	386	B	79.6009	1
**4981**	305	C	79.5449	1
**4981**	319	C	72.3994	1
**4981**	320	C	71.9509	-1
**4981**	321	C	79.276	0

**4981**	351	C	3.4223	1
**4981**	357	C	40.0116	0
**4981**	370	C	79.0952	1
**4981**	372	C	79.6021	0
**4981**	373	C	59.9422	1
**4981**	381	C	79.0395	1
**4981**	382	C	65.0392	1
**4981**	386	C	34.8414	0
**4981**	305	D	65.9682	1
**4981**	319	D	71.7009	1
**4981**	320	D	67.7488	-1
**4981**	321	D	56.9851	0
**4981**	351	D	10.8266	1
**4981**	357	D	79.4629	1
**4981**	370	D	79.4352	1
**4981**	372	D	79.5298	0
**4981**	373	D	79.5379	1
**4981**	381	D	62.7776	1
**4981**	382	D	79.5775	1
**4981**	386	D	79.5171	1
**4982**	305	A	79.5481	1
**4982**	319	A	50.6016	1
**4982**	320	A	79.5742	-1
**4982**	321	A	66.0463	0
**4982**	351	A	3.2771	1
**4982**	357	A	42.2034	0
**4982**	370	A	79.5977	1
**4982**	372	A	52.8373	0
**4982**	373	A	71.7465	1
**4982**	381	A	74.7579	1
**4982**	382	A	22.1171	0
**4982**	386	A	79.7376	1
**4982**	305	B	77.2109	1
**4982**	319	B	69.8894	1

**4982**	320	B	60.0668	-1
**4982**	321	B	79.5776	0
**4982**	351	B	23.7299	-1
**4982**	357	B	66.3987	1
**4982**	370	B	79.6252	1
**4982**	372	B	79.5609	0
**4982**	373	B	31.5745	0
**4982**	381	B	70.7026	1
**4982**	382	B	75.1178	1
**4982**	386	B	58.8194	1
**4982**	305	C	70.2223	1
**4982**	319	C	65.9252	1
**4982**	320	C	70.3679	-1
**4982**	321	C	59.8089	0
**4982**	351	C	7.8159	1
**4982**	357	C	79.6301	1
**4982**	370	C	66.7895	1
**4982**	372	C	79.5437	0
**4982**	373	C	79.5669	1
**4982**	381	C	76.7472	1
**4982**	382	C	26.3533	0
**4982**	386	C	65.7666	1
**4982**	305	D	79.5843	1
**4982**	319	D	22.262	0
**4982**	320	D	34.6745	-1
**4982**	321	D	74.1717	0
**4982**	351	D	3.6818	1
**4982**	357	D	79.5932	1
**4982**	370	D	74.4637	1
**4982**	372	D	61.3066	0
**4982**	373	D	75.4869	1
**4982**	381	D	68.9736	1
**4982**	382	D	76.5737	1
**4982**	386	D	52.5066	1

**4983**	305	A	65.815	1
**4983**	319	A	16.5926	-1
**4983**	320	A	62.1604	-1
**4983**	321	A	40.9825	1
**4983**	351	A	25.9241	-1
**4983**	357	A	72.7515	1
**4983**	370	A	79.4558	1
**4983**	372	A	79.5468	0
**4983**	373	A	56.7587	1
**4983**	381	A	79.4891	1
**4983**	382	A	42.4465	0
**4983**	386	A	79.5511	1
**4983**	305	B	79.4182	1
**4983**	319	B	44.491	0
**4983**	320	B	72.2169	-1
**4983**	321	B	70.3472	0
**4983**	351	B	14.1216	1
**4983**	357	B	79.5087	1
**4983**	370	B	79.5291	1
**4983**	372	B	79.6191	0
**4983**	373	B	39.0708	0
**4983**	381	B	73.9294	1
**4983**	382	B	43.2396	0
**4983**	386	B	44.8427	0
**4983**	305	C	75.691	1
**4983**	319	C	72.1629	1
**4983**	320	C	50.8021	-1
**4983**	321	C	51.8354	0
**4983**	351	C	15.4992	1
**4983**	357	C	75.3593	1
**4983**	370	C	75.7422	1
**4983**	372	C	74.8484	0
**4983**	373	C	73.3249	1
**4983**	381	C	53.6158	1

**4983**	382	C	60.5818	1
**4983**	386	C	37.8574	0
**4983**	305	D	16.6933	-1
**4983**	319	D	69.9345	1
**4983**	320	D	79.5065	-1
**4983**	321	D	51.5327	0
**4983**	351	D	49.755	-1
**4983**	357	D	79.1748	1
**4983**	370	D	46.2258	0
**4983**	372	D	79.5589	0
**4983**	373	D	79.5509	1
**4983**	381	D	63.8259	1
**4983**	382	D	69.6722	1
**4983**	386	D	64.4238	1
**4984**	305	A	79.5335	1
**4984**	319	A	69.7419	1
**4984**	320	A	69.1332	-1
**4984**	321	A	79.516	0
**4984**	351	A	5.4967	1
**4984**	357	A	79.5074	1
**4984**	370	A	62.6938	1
**4984**	372	A	72.7559	0
**4984**	373	A	76.526	1
**4984**	381	A	45.2763	0
**4984**	382	A	62.5822	1
**4984**	386	A	41.568	0
**4984**	305	B	74.6336	1
**4984**	319	B	34.7031	0
**4984**	320	B	76.2951	-1
**4984**	321	B	79.6224	0
**4984**	351	B	11.3568	1
**4984**	357	B	78.3192	1
**4984**	370	B	39.5973	0
**4984**	372	B	79.6121	0

**4984**	373	B	64.2582	1
**4984**	381	B	79.528	1
**4984**	382	B	33.066	0
**4984**	386	B	72.8578	1
**4984**	305	C	74.2903	1
**4984**	319	C	79.525	1
**4984**	320	C	79.5383	-1
**4984**	321	C	79.5415	0
**4984**	351	C	6.4021	1
**4984**	357	C	23.5336	0
**4984**	370	C	71.3136	1
**4984**	372	C	79.5752	0
**4984**	373	C	79.54	1
**4984**	381	C	79.5488	1
**4984**	382	C	70.0991	1
**4984**	386	C	52.8782	1
**4984**	305	D	58.392	1
**4984**	319	D	79.5543	1
**4984**	320	D	79.1723	-1
**4984**	321	D	50.95	0
**4984**	351	D	13.2501	1
**4984**	357	D	52.7852	1
**4984**	370	D	74.1137	1
**4984**	372	D	79.6025	0
**4984**	373	D	52.8601	1
**4984**	381	D	77.5767	1
**4984**	382	D	79.5347	1
**4984**	386	D	35.3305	0
**4985**	305	A	52.5799	1
**4985**	319	A	79.4332	1
**4985**	320	A	78.6766	-1
**4985**	321	A	70.8593	0
**4985**	351	A	8.8099	1
**4985**	357	A	72.5104	1

**4985**	370	A	79.6521	1
**4985**	372	A	53.3082	0
**4985**	373	A	74.2907	1
**4985**	381	A	70.4054	1
**4985**	382	A	79.4857	1
**4985**	386	A	45.2622	0
**4985**	305	B	48.4013	0
**4985**	319	B	74.2466	1
**4985**	320	B	35.9526	-1
**4985**	321	B	71.641	0
**4985**	351	B	8.0149	1
**4985**	357	B	11.8629	-1
**4985**	370	B	61.0373	1
**4985**	372	B	40.3679	1
**4985**	373	B	40.7071	0
**4985**	381	B	59.4095	1
**4985**	382	B	45.5832	0
**4985**	386	B	55.5135	1
**4985**	305	C	79.2724	1
**4985**	319	C	79.6607	1
**4985**	320	C	52.636	-1
**4985**	321	C	62.4518	0
**4985**	351	C	1.5613	1
**4985**	357	C	79.5479	1
**4985**	370	C	76.0317	1
**4985**	372	C	66.0218	0
**4985**	373	C	42.0013	0
**4985**	381	C	9.3145	-1
**4985**	382	C	72.2501	1
**4985**	386	C	53.9435	1
**4985**	305	D	42.753	0
**4985**	319	D	71.6426	1
**4985**	320	D	79.5683	-1
**4985**	321	D	68.8443	0

**4985**	351	D	5.1943	1
**4985**	357	D	45.1894	0
**4985**	370	D	73.9426	1
**4985**	372	D	79.6762	0
**4985**	373	D	79.522	1
**4985**	381	D	79.5958	1
**4985**	382	D	59.57	1
**4985**	386	D	66.8861	1
**4986**	305	A	70.1703	1
**4986**	319	A	71.218	1
**4986**	320	A	79.4565	-1
**4986**	321	A	59.5704	0
**4986**	351	A	3.0402	1
**4986**	357	A	72.4333	1
**4986**	370	A	72.665	1
**4986**	372	A	79.6148	0
**4986**	373	A	79.5043	1
**4986**	381	A	77.0049	1
**4986**	382	A	72.3804	1
**4986**	386	A	66.9973	1
**4986**	305	B	62.8698	1
**4986**	319	B	76.568	1
**4986**	320	B	79.569	-1
**4986**	321	B	67.7094	0
**4986**	351	B	10.4488	1
**4986**	357	B	54.9454	1
**4986**	370	B	79.0504	1
**4986**	372	B	79.6061	0
**4986**	373	B	73.2179	1
**4986**	381	B	58.0839	1
**4986**	382	B	34.4041	0
**4986**	386	B	70.2536	1
**4986**	305	C	63.3996	1
**4986**	319	C	75.4051	1

**4986**	320	C	79.5601	-1
**4986**	321	C	79.6371	0
**4986**	351	C	5.766	1
**4986**	357	C	40.3833	0
**4986**	370	C	33.0009	0
**4986**	372	C	49.1679	1
**4986**	373	C	54.8338	1
**4986**	381	C	79.5069	1
**4986**	382	C	72.0142	1
**4986**	386	C	57.5058	1
**4986**	305	D	79.5703	1
**4986**	319	D	45.5247	0
**4986**	320	D	59.993	-1
**4986**	321	D	67.9131	0
**4986**	351	D	13.9444	1
**4986**	357	D	79.5996	1
**4986**	370	D	54.2794	1
**4986**	372	D	79.4847	0
**4986**	373	D	55.0022	1
**4986**	381	D	64.6393	1
**4986**	382	D	79.5152	1
**4986**	386	D	70.7018	1
**4987**	305	A	35.1928	0
**4987**	319	A	69.9218	1
**4987**	320	A	38.7576	-1
**4987**	321	A	64.2289	0
**4987**	351	A	6.0883	1
**4987**	357	A	76.5267	1
**4987**	370	A	78.5699	1
**4987**	372	A	79.6431	0
**4987**	373	A	79.5189	1
**4987**	381	A	72.187	1
**4987**	382	A	57.2556	1
**4987**	386	A	76.8312	1

**4987**	305	B	75.7306	1
**4987**	319	B	47.5615	0
**4987**	320	B	75.2739	-1
**4987**	321	B	59.2563	0
**4987**	351	B	1.4715	1
**4987**	357	B	79.631	1
**4987**	370	B	72.455	1
**4987**	372	B	73.9878	0
**4987**	373	B	60.4037	1
**4987**	381	B	54.3677	1
**4987**	382	B	76.9786	1
**4987**	386	B	72.7322	1
**4987**	305	C	47.5872	0
**4987**	319	C	79.5384	1
**4987**	320	C	66.1382	-1
**4987**	321	C	79.6478	0
**4987**	351	C	1.8471	1
**4987**	357	C	55.5155	1
**4987**	370	C	43.6117	0
**4987**	372	C	71.9681	0
**4987**	373	C	57.2698	1
**4987**	381	C	72.9978	1
**4987**	382	C	64.5423	1
**4987**	386	C	68.2315	1
**4987**	305	D	66.5028	1
**4987**	319	D	77.4662	1
**4987**	320	D	79.6069	-1
**4987**	321	D	79.5711	0
**4987**	351	D	17.0586	1
**4987**	357	D	58.1458	1
**4987**	370	D	63.5357	1
**4987**	372	D	24.7269	1
**4987**	373	D	66.4858	1
**4987**	381	D	79.5015	1

4987	382	D	59.7396	1
4987	386	D	71.3353	1
4988	305	A	79.5354	1
4988	319	A	51.6768	1
4988	320	A	79.5408	-1
4988	321	A	38.3026	1
4988	351	A	11.6441	1
4988	357	A	39.8259	0
4988	370	A	79.5972	1
4988	372	A	64.2925	0
4988	373	A	47.1734	0
4988	381	A	79.5853	1
4988	382	A	62.8894	1
4988	386	A	23.0695	0
4988	305	B	79.2139	1
4988	319	B	42.223	0
4988	320	B	51.721	-1
4988	321	B	74.4386	0
4988	351	B	10.0318	1
4988	357	B	40.931	0
4988	370	B	58.0633	1
4988	372	B	57.7826	0
4988	373	B	57.8912	1
4988	381	B	79.5743	1
4988	382	B	6.4121	-1
4988	386	B	58.6872	1
4988	305	C	72.6282	1
4988	319	C	78.9493	1
4988	320	C	71.9248	-1
4988	321	C	79.5387	0
4988	351	C	1.5623	1
4988	357	C	25.4426	0
4988	370	C	79.4733	1
4988	372	C	79.5368	0

**4988**	373	C	57.0039	1
**4988**	381	C	74.7976	1
**4988**	382	C	62.8968	1
**4988**	386	C	70.736	1
**4988**	305	D	25.3729	0
**4988**	319	D	57.908	1
**4988**	320	D	78.5605	-1
**4988**	321	D	75.8751	0
**4988**	351	D	14.5885	1
**4988**	357	D	79.3718	1
**4988**	370	D	69.9467	1
**4988**	372	D	79.4412	0
**4988**	373	D	68.9875	1
**4988**	381	D	75.4473	1
**4988**	382	D	60.8549	1
**4988**	386	D	77.0241	1
**4989**	305	A	43.5677	0
**4989**	319	A	68.7025	1
**4989**	320	A	79.5947	-1
**4989**	321	A	71.0558	0
**4989**	351	A	11.8214	1
**4989**	357	A	69.6734	1
**4989**	370	A	57.8269	1
**4989**	372	A	79.4829	0
**4989**	373	A	79.4871	1
**4989**	381	A	51.8133	1
**4989**	382	A	59.5692	1
**4989**	386	A	48.3741	0
**4989**	305	B	75.2855	1
**4989**	319	B	71.6273	1
**4989**	320	B	59.913	-1
**4989**	321	B	24.5617	1
**4989**	351	B	5.9165	1
**4989**	357	B	79.5685	1

**4989**	370	B	79.6425	1
**4989**	372	B	74.4566	0
**4989**	373	B	76.4446	1
**4989**	381	B	47.1437	0
**4989**	382	B	32.3235	0
**4989**	386	B	79.5706	1
**4989**	305	C	77.4788	1
**4989**	319	C	22.1748	0
**4989**	320	C	72.7498	-1
**4989**	321	C	76.1737	0
**4989**	351	C	0.7294	1
**4989**	357	C	74.2797	1
**4989**	370	C	79.55	1
**4989**	372	C	73.3789	0
**4989**	373	C	76.4334	1
**4989**	381	C	71.7695	1
**4989**	382	C	66.6407	1
**4989**	386	C	27.854	0
**4989**	305	D	77.7719	1
**4989**	319	D	73.2085	1
**4989**	320	D	25.9608	-1
**4989**	321	D	66.8342	0
**4989**	351	D	8.2164	1
**4989**	357	D	70.5621	1
**4989**	370	D	79.6314	1
**4989**	372	D	74.1624	0
**4989**	373	D	68.4829	1
**4989**	381	D	31.2344	0
**4989**	382	D	68.5198	1
**4989**	386	D	72.4307	1
**4990**	305	A	79.5171	1
**4990**	319	A	60.0851	1
**4990**	320	A	73.1018	-1
**4990**	321	A	78.7137	0

**4990**	351	A	5.2742	1
**4990**	357	A	72.267	1
**4990**	370	A	72.296	1
**4990**	372	A	59.0014	0
**4990**	373	A	49.6581	0
**4990**	381	A	66.0135	1
**4990**	382	A	66.3487	1
**4990**	386	A	36.7419	0
**4990**	305	B	62.1007	1
**4990**	319	B	74.0116	1
**4990**	320	B	79.7029	-1
**4990**	321	B	60.8603	0
**4990**	351	B	11.606	1
**4990**	357	B	79.5887	1
**4990**	370	B	71.434	1
**4990**	372	B	76.3566	0
**4990**	373	B	76.057	1
**4990**	381	B	79.6141	1
**4990**	382	B	43.5612	0
**4990**	386	B	29.1666	0
**4990**	305	C	68.9701	1
**4990**	319	C	78.661	1
**4990**	320	C	79.4574	-1
**4990**	321	C	79.5943	0
**4990**	351	C	3.289	1
**4990**	357	C	79.5073	1
**4990**	370	C	63.6166	1
**4990**	372	C	79.6047	0
**4990**	373	C	71.0634	1
**4990**	381	C	79.3189	1
**4990**	382	C	62.6181	1
**4990**	386	C	20.4793	0
**4990**	305	D	72.4172	1
**4990**	319	D	71.1924	1

**4990**	320	D	79.6088	-1
**4990**	321	D	64.7546	0
**4990**	351	D	15.5872	1
**4990**	357	D	64.3261	1
**4990**	370	D	77.8341	1
**4990**	372	D	66.0532	0
**4990**	373	D	45.1096	0
**4990**	381	D	48.6602	0
**4990**	382	D	74.6864	1
**4990**	386	D	72.7621	1
**4991**	305	A	70.3095	1
**4991**	319	A	77.9497	1
**4991**	320	A	72.865	-1
**4991**	321	A	79.5721	0
**4991**	351	A	6.0425	1
**4991**	357	A	79.578	1
**4991**	370	A	57.667	1
**4991**	372	A	6.8423	0
**4991**	373	A	72.4766	1
**4991**	381	A	73.674	1
**4991**	382	A	73.6253	1
**4991**	386	A	79.6008	1
**4991**	305	B	79.5796	1
**4991**	319	B	77.3212	1
**4991**	320	B	75.7466	-1
**4991**	321	B	52.6303	0
**4991**	351	B	14.1646	1
**4991**	357	B	79.5418	1
**4991**	370	B	66.0214	1
**4991**	372	B	75.1107	0
**4991**	373	B	44.2875	0
**4991**	381	B	69.8412	1
**4991**	382	B	25.1036	0
**4991**	386	B	74.0349	1

**4991**	305	C	73.03	1
**4991**	319	C	56.4281	1
**4991**	320	C	79.5387	-1
**4991**	321	C	30.7772	1
**4991**	351	C	7.6392	1
**4991**	357	C	79.5844	1
**4991**	370	C	72.9762	1
**4991**	372	C	79.5651	0
**4991**	373	C	60.5244	1
**4991**	381	C	75.445	1
**4991**	382	C	79.5323	1
**4991**	386	C	62.281	1
**4991**	305	D	41.038	0
**4991**	319	D	48.1689	0
**4991**	320	D	72.4969	-1
**4991**	321	D	49.0124	1
**4991**	351	D	13.8563	1
**4991**	357	D	19.9466	-1
**4991**	370	D	37.0053	0
**4991**	372	D	77.8343	0
**4991**	373	D	73.4589	1
**4991**	381	D	74.5907	1
**4991**	382	D	79.5201	1
**4991**	386	D	68.4265	1
**4992**	305	A	79.5619	1
**4992**	319	A	67.2766	1
**4992**	320	A	69.6356	-1
**4992**	321	A	31.3497	1
**4992**	351	A	6.4799	1
**4992**	357	A	72.7155	1
**4992**	370	A	70.1874	1
**4992**	372	A	79.6135	0
**4992**	373	A	79.5403	1
**4992**	381	A	79.4828	1

**4992**	382	A	62.6478	1
**4992**	386	A	79.4871	1
**4992**	305	B	79.5578	1
**4992**	319	B	73.0028	1
**4992**	320	B	72.0941	-1
**4992**	321	B	75.0931	0
**4992**	351	B	9.6376	1
**4992**	357	B	56.0896	1
**4992**	370	B	79.5699	1
**4992**	372	B	55.964	0
**4992**	373	B	54.2955	1
**4992**	381	B	21.159	0
**4992**	382	B	45.7419	0
**4992**	386	B	79.5447	1
**4992**	305	C	79.5501	1
**4992**	319	C	78.7152	1
**4992**	320	C	71.245	-1
**4992**	321	C	71.6068	0
**4992**	351	C	1.7627	1
**4992**	357	C	78.5829	1
**4992**	370	C	78.8458	1
**4992**	372	C	69.1528	0
**4992**	373	C	63.4451	1
**4992**	381	C	78.9205	1
**4992**	382	C	70.5169	1
**4992**	386	C	38.7668	0
**4992**	305	D	74.3323	1
**4992**	319	D	79.534	1
**4992**	320	D	79.5037	-1
**4992**	321	D	70.7522	0
**4992**	351	D	3.0691	1
**4992**	357	D	55.9579	1
**4992**	370	D	73.1263	1
**4992**	372	D	79.5412	0

**4992**	373	D	79.5776	1
**4992**	381	D	79.5737	1
**4992**	382	D	63.6119	1
**4992**	386	D	78.5796	1
**4993**	305	A	79.5791	1
**4993**	319	A	56.0753	1
**4993**	320	A	54.4648	-1
**4993**	321	A	44.1286	1
**4993**	351	A	39.7235	-1
**4993**	357	A	79.5559	1
**4993**	370	A	74.4708	1
**4993**	372	A	78.5822	0
**4993**	373	A	79.6044	1
**4993**	381	A	70.4099	1
**4993**	382	A	50.8964	1
**4993**	386	A	77.7205	1
**4993**	305	B	50.6369	1
**4993**	319	B	79.5618	1
**4993**	320	B	79.5877	-1
**4993**	321	B	79.1466	0
**4993**	351	B	8.4463	1
**4993**	357	B	72.0073	1
**4993**	370	B	79.5695	1
**4993**	372	B	44.2284	1
**4993**	373	B	68.4083	1
**4993**	381	B	79.5767	1
**4993**	382	B	47.8603	0
**4993**	386	B	79.4736	1
**4993**	305	C	64.8002	1
**4993**	319	C	76.5146	1
**4993**	320	C	37.8227	-1
**4993**	321	C	74.3485	0
**4993**	351	C	11.9217	1
**4993**	357	C	53.0176	1

**4993**	370	C	79.5466	1
**4993**	372	C	79.5545	0
**4993**	373	C	79.5904	1
**4993**	381	C	48.16	0
**4993**	382	C	50.9229	1
**4993**	386	C	52.8039	1
**4993**	305	D	79.5723	1
**4993**	319	D	57.171	1
**4993**	320	D	78.4333	-1
**4993**	321	D	55.7436	0
**4993**	351	D	7.2384	1
**4993**	357	D	38.2498	0
**4993**	370	D	33.7362	0
**4993**	372	D	79.597	0
**4993**	373	D	60.8744	1
**4993**	381	D	36.1851	0
**4993**	382	D	79.5564	1
**4993**	386	D	79.5876	1
**4994**	305	A	57.2431	1
**4994**	319	A	51.3728	1
**4994**	320	A	64.3224	-1
**4994**	321	A	79.4955	0
**4994**	351	A	8.1632	1
**4994**	357	A	73.2301	1
**4994**	370	A	79.5531	1
**4994**	372	A	79.4475	0
**4994**	373	A	51.4071	1
**4994**	381	A	69.2999	1
**4994**	382	A	69.0967	1
**4994**	386	A	69.4404	1
**4994**	305	B	74.3872	1
**4994**	319	B	66.7793	1
**4994**	320	B	64.4991	-1
**4994**	321	B	40.2906	1

**4994**	351	B	10.7617	1
**4994**	357	B	75.172	1
**4994**	370	B	59.5261	1
**4994**	372	B	75.3323	0
**4994**	373	B	77.5632	1
**4994**	381	B	79.5832	1
**4994**	382	B	38.281	0
**4994**	386	B	45.5315	0
**4994**	305	C	65.9115	1
**4994**	319	C	78.4723	1
**4994**	320	C	56.1386	-1
**4994**	321	C	71.8041	0
**4994**	351	C	8.843	1
**4994**	357	C	61.3423	1
**4994**	370	C	75.7313	1
**4994**	372	C	79.5892	0
**4994**	373	C	73.5065	1
**4994**	381	C	69.3258	1
**4994**	382	C	79.6066	1
**4994**	386	C	45.2083	0
**4994**	305	D	43.6284	0
**4994**	319	D	78.5305	1
**4994**	320	D	79.6178	-1
**4994**	321	D	79.5758	0
**4994**	351	D	9.7347	1
**4994**	357	D	40.5029	0
**4994**	370	D	37.4346	0
**4994**	372	D	77.981	0
**4994**	373	D	75.7114	1
**4994**	381	D	23.963	0
**4994**	382	D	79.557	1
**4994**	386	D	73.258	1
**4995**	305	A	64.6271	1
**4995**	319	A	47.2508	0

**4995**	320	A	62.6266	-1
**4995**	321	A	60.8326	0
**4995**	351	A	9.9657	1
**4995**	357	A	79.2786	1
**4995**	370	A	65.2834	1
**4995**	372	A	27.5665	1
**4995**	373	A	69.5407	1
**4995**	381	A	79.5592	1
**4995**	382	A	68.6496	1
**4995**	386	A	79.5394	1
**4995**	305	B	79.56	1
**4995**	319	B	79.55	1
**4995**	320	B	72.4479	-1
**4995**	321	B	48.0456	1
**4995**	351	B	8.653	1
**4995**	357	B	79.5637	1
**4995**	370	B	79.5532	1
**4995**	372	B	79.5587	0
**4995**	373	B	61.7498	1
**4995**	381	B	79.5991	1
**4995**	382	B	74.3809	1
**4995**	386	B	71.5199	1
**4995**	305	C	37.5429	0
**4995**	319	C	79.5805	1
**4995**	320	C	79.5875	-1
**4995**	321	C	79.5799	0
**4995**	351	C	12.6456	1
**4995**	357	C	64.21	1
**4995**	370	C	79.5647	1
**4995**	372	C	79.5571	0
**4995**	373	C	56.4557	1
**4995**	381	C	79.5701	1
**4995**	382	C	59.9528	1
**4995**	386	C	57.9476	1

**4995**	305	D	62.9599	1
**4995**	319	D	73.1525	1
**4995**	320	D	38.8988	-1
**4995**	321	D	46.6048	1
**4995**	351	D	7.233	1
**4995**	357	D	70.7751	1
**4995**	370	D	79.578	1
**4995**	372	D	71.4433	0
**4995**	373	D	35.5517	0
**4995**	381	D	36.8785	0
**4995**	382	D	78.3407	1
**4995**	386	D	43.9238	0
**4996**	305	A	67.9051	1
**4996**	319	A	72.3158	1
**4996**	320	A	78.0669	-1
**4996**	321	A	35.801	1
**4996**	351	A	3.815	1
**4996**	357	A	40.8991	0
**4996**	370	A	54.0182	1
**4996**	372	A	39.2906	1
**4996**	373	A	79.2256	1
**4996**	381	A	79.5368	1
**4996**	382	A	75.5338	1
**4996**	386	A	77.7861	1
**4996**	305	B	66.092	1
**4996**	319	B	61.2246	1
**4996**	320	B	49.7415	-1
**4996**	321	B	79.4706	0
**4996**	351	B	17.8925	1
**4996**	357	B	72.7715	1
**4996**	370	B	43.9302	0
**4996**	372	B	71.3828	0
**4996**	373	B	56.7105	1
**4996**	381	B	79.5822	1

**4996**	382	B	63.9457	1
**4996**	386	B	79.4738	1
**4996**	305	C	73.9444	1
**4996**	319	C	71.6146	1
**4996**	320	C	61.2499	-1
**4996**	321	C	75.52	0
**4996**	351	C	0.5167	1
**4996**	357	C	34.3735	0
**4996**	370	C	75.8329	1
**4996**	372	C	73.6743	0
**4996**	373	C	63.8951	1
**4996**	381	C	79.5781	1
**4996**	382	C	54.0906	1
**4996**	386	C	57.7371	1
**4996**	305	D	79.5234	1
**4996**	319	D	46.6063	0
**4996**	320	D	74.6393	-1
**4996**	321	D	79.6178	0
**4996**	351	D	15.8625	1
**4996**	357	D	72.8145	1
**4996**	370	D	37.4378	0
**4996**	372	D	70.8282	0
**4996**	373	D	78.0354	1
**4996**	381	D	64.1938	1
**4996**	382	D	76.2453	1
**4996**	386	D	79.5497	1
**4997**	305	A	79.5655	1
**4997**	319	A	67.5981	1
**4997**	320	A	52.0527	-1
**4997**	321	A	46.0224	1
**4997**	351	A	11.3626	1
**4997**	357	A	70.029	1
**4997**	370	A	64.7409	1
**4997**	372	A	61.8428	0

**4997**	373	A	79.514	1
**4997**	381	A	65.626	1
**4997**	382	A	67.2441	1
**4997**	386	A	69.7663	1
**4997**	305	B	79.5542	1
**4997**	319	B	76.0232	1
**4997**	320	B	79.545	-1
**4997**	321	B	73.4437	0
**4997**	351	B	10.6911	1
**4997**	357	B	37.2487	0
**4997**	370	B	79.5226	1
**4997**	372	B	73.1815	0
**4997**	373	B	58.9469	1
**4997**	381	B	65.831	1
**4997**	382	B	52.7361	1
**4997**	386	B	79.6045	1
**4997**	305	C	51.8908	1
**4997**	319	C	69.046	1
**4997**	320	C	72.0835	-1
**4997**	321	C	79.5852	0
**4997**	351	C	10.0331	1
**4997**	357	C	72.1863	1
**4997**	370	C	79.5237	1
**4997**	372	C	79.4762	0
**4997**	373	C	57.914	1
**4997**	381	C	79.5542	1
**4997**	382	C	73.8502	1
**4997**	386	C	55.4153	1
**4997**	305	D	79.4026	1
**4997**	319	D	78.6633	1
**4997**	320	D	79.563	-1
**4997**	321	D	57.6118	0
**4997**	351	D	13.654	1
**4997**	357	D	73.1492	1

**4997**	370	D	71.6864	1
**4997**	372	D	75.1388	0
**4997**	373	D	70.3972	1
**4997**	381	D	79.5134	1
**4997**	382	D	58.4903	1
**4997**	386	D	73.0479	1
**4998**	305	A	79.5579	1
**4998**	319	A	72.5394	1
**4998**	320	A	79.6529	-1
**4998**	321	A	79.6254	0
**4998**	351	A	10.5978	1
**4998**	357	A	62.677	1
**4998**	370	A	79.6971	1
**4998**	372	A	71.9178	0
**4998**	373	A	79.4608	1
**4998**	381	A	60.287	1
**4998**	382	A	70.4845	1
**4998**	386	A	62.6993	1
**4998**	305	B	58.3368	1
**4998**	319	B	54.8445	1
**4998**	320	B	79.4473	-1
**4998**	321	B	71.7549	0
**4998**	351	B	0.0	1
**4998**	357	B	79.5449	1
**4998**	370	B	61.9038	1
**4998**	372	B	78.0863	0
**4998**	373	B	73.1367	1
**4998**	381	B	58.1684	1
**4998**	382	B	51.7078	1
**4998**	386	B	51.0654	1
**4998**	305	C	79.5507	1
**4998**	319	C	71.5776	1
**4998**	320	C	60.1451	-1
**4998**	321	C	79.6642	0

**4998**	351	C	16.226	1
**4998**	357	C	56.1785	1
**4998**	370	C	78.4467	1
**4998**	372	C	59.4343	0
**4998**	373	C	78.5559	1
**4998**	381	C	75.3758	1
**4998**	382	C	79.4462	1
**4998**	386	C	33.2111	0
**4998**	305	D	48.8233	0
**4998**	319	D	51.7718	1
**4998**	320	D	62.0331	-1
**4998**	321	D	54.7972	0
**4998**	351	D	14.1675	1
**4998**	357	D	36.0368	0
**4998**	370	D	69.5952	1
**4998**	372	D	62.6789	0
**4998**	373	D	70.8419	1
**4998**	381	D	25.2354	0
**4998**	382	D	79.6208	1
**4998**	386	D	79.6077	1
**4999**	305	A	55.8865	1
**4999**	319	A	60.0788	1
**4999**	320	A	71.1633	-1
**4999**	321	A	72.1424	0
**4999**	351	A	1.0785	1
**4999**	357	A	68.0863	1
**4999**	370	A	79.5737	1
**4999**	372	A	74.02	0
**4999**	373	A	73.3613	1
**4999**	381	A	72.2547	1
**4999**	382	A	78.7799	1
**4999**	386	A	79.6309	1
**4999**	305	B	55.0873	1
**4999**	319	B	67.7831	1

**4999**	320	B	46.4004	-1
**4999**	321	B	67.3434	0
**4999**	351	B	6.581	1
**4999**	357	B	75.3321	1
**4999**	370	B	39.695	0
**4999**	372	B	68.8041	0
**4999**	373	B	61.0287	1
**4999**	381	B	12.4244	-1
**4999**	382	B	35.8723	0
**4999**	386	B	37.0798	0
**4999**	305	C	77.1549	1
**4999**	319	C	78.3919	1
**4999**	320	C	35.865	-1
**4999**	321	C	72.6167	0
**4999**	351	C	11.4114	1
**4999**	357	C	46.4565	0
**4999**	370	C	79.6118	1
**4999**	372	C	72.6881	0
**4999**	373	C	79.7275	1
**4999**	381	C	77.8672	1
**4999**	382	C	51.8996	1
**4999**	386	C	56.9837	1
**4999**	305	D	76.8042	1
**4999**	319	D	49.611	0
**4999**	320	D	79.6059	-1
**4999**	321	D	69.1687	0
**4999**	351	D	13.2209	1
**4999**	357	D	71.8438	1
**4999**	370	D	68.4896	1
**4999**	372	D	75.316	0
**4999**	373	D	45.0266	0
**4999**	381	D	49.4427	0
**4999**	382	D	71.1049	1
**4999**	386	D	79.5944	1

**5000**	305	A	67.3978	1
**5000**	319	A	79.4247	1
**5000**	320	A	73.43	-1
**5000**	321	A	73.1006	0
**5000**	351	A	12.8106	1
**5000**	357	A	71.9695	1
**5000**	370	A	79.57	1
**5000**	372	A	79.5875	0
**5000**	373	A	79.5375	1
**5000**	381	A	71.5333	1
**5000**	382	A	33.1978	0
**5000**	386	A	79.5029	1
**5000**	305	B	79.53	1
**5000**	319	B	66.2143	1
**5000**	320	B	70.4274	-1
**5000**	321	B	40.0992	1
**5000**	351	B	16.4361	1
**5000**	357	B	79.4928	1
**5000**	370	B	71.7712	1
**5000**	372	B	70.4572	0
**5000**	373	B	44.1701	0
**5000**	381	B	65.6004	1
**5000**	382	B	79.162	1
**5000**	386	B	79.6237	1
**5000**	305	C	44.398	0
**5000**	319	C	62.0566	1
**5000**	320	C	47.1537	-1
**5000**	321	C	73.9344	0
**5000**	351	C	8.2409	1
**5000**	357	C	79.6005	1
**5000**	370	C	51.373	1
**5000**	372	C	79.5567	0
**5000**	373	C	45.1496	0
**5000**	381	C	77.4368	1

**5000**	382	C	55.4291	1
**5000**	386	C	34.9739	0
**5000**	305	D	74.0528	1
**5000**	319	D	52.9101	1
**5000**	320	D	79.6088	-1
**5000**	321	D	79.5928	0
**5000**	351	D	8.4889	1
**5000**	357	D	78.9471	1
**5000**	370	D	79.594	1
**5000**	372	D	79.4757	0
**5000**	373	D	79.5472	1
**5000**	381	D	75.066	1
**5000**	382	D	76.6188	1
**5000**	386	D	79.6267	1
**5001**	305	A	14.4792	-1
**5001**	319	A	63.0205	1
**5001**	320	A	79.199	-1
**5001**	321	A	38.0036	1
**5001**	351	A	15.6951	1
**5001**	357	A	73.2707	1
**5001**	370	A	79.5795	1
**5001**	372	A	65.1089	0
**5001**	373	A	79.0649	1
**5001**	381	A	68.2559	1
**5001**	382	A	42.3065	0
**5001**	386	A	79.5792	1
**5001**	305	B	71.8169	1
**5001**	319	B	57.5734	1
**5001**	320	B	40.9083	-1
**5001**	321	B	35.4126	1
**5001**	351	B	16.9243	1
**5001**	357	B	79.5436	1
**5001**	370	B	72.3473	1
**5001**	372	B	48.0072	1

**5001**	373	B	59.994	1
**5001**	381	B	75.638	1
**5001**	382	B	54.1835	1
**5001**	386	B	48.0875	0
**5001**	305	C	40.2474	0
**5001**	319	C	78.9538	1
**5001**	320	C	18.3607	1
**5001**	321	C	79.6077	0
**5001**	351	C	2.4498	1
**5001**	357	C	73.9619	1
**5001**	370	C	68.4938	1
**5001**	372	C	78.9809	0
**5001**	373	C	71.5613	1
**5001**	381	C	74.2216	1
**5001**	382	C	66.7273	1
**5001**	386	C	30.6162	0
**5001**	305	D	74.863	1
**5001**	319	D	71.9258	1
**5001**	320	D	58.7547	-1
**5001**	321	D	41.9307	1
**5001**	351	D	18.0329	1
**5001**	357	D	35.831	0
**5001**	370	D	77.0784	1
**5001**	372	D	79.5884	0
**5001**	373	D	79.5299	1
**5001**	381	D	72.619	1
**5001**	382	D	52.6644	1
**5001**	386	D	76.4815	1
**5002**	305	A	79.5725	1
**5002**	319	A	67.8843	1
**5002**	320	A	79.4575	-1
**5002**	321	A	72.6037	0
**5002**	351	A	15.6465	1
**5002**	357	A	43.2236	0

**5002**	370	A	54.5911	1
**5002**	372	A	50.3287	0
**5002**	373	A	79.6712	1
**5002**	381	A	51.583	1
**5002**	382	A	79.6031	1
**5002**	386	A	62.904	1
**5002**	305	B	73.2787	1
**5002**	319	B	74.9831	1
**5002**	320	B	77.0631	-1
**5002**	321	B	75.1827	0
**5002**	351	B	1.6949	1
**5002**	357	B	62.5146	1
**5002**	370	B	79.5771	1
**5002**	372	B	75.7736	0
**5002**	373	B	46.9629	0
**5002**	381	B	75.8278	1
**5002**	382	B	40.9911	0
**5002**	386	B	47.3318	0
**5002**	305	C	69.6863	1
**5002**	319	C	31.075	0
**5002**	320	C	39.6142	-1
**5002**	321	C	68.0698	0
**5002**	351	C	26.6621	-1
**5002**	357	C	74.2237	1
**5002**	370	C	51.2474	1
**5002**	372	C	79.4928	0
**5002**	373	C	79.4579	1
**5002**	381	C	70.3472	1
**5002**	382	C	77.6464	1
**5002**	386	C	59.527	1
**5002**	305	D	61.3204	1
**5002**	319	D	70.8398	1
**5002**	320	D	70.3789	-1
**5002**	321	D	51.9226	0

**5002**	351	D	9.4569	1
**5002**	357	D	48.7081	0
**5002**	370	D	79.4301	1
**5002**	372	D	70.6011	0
**5002**	373	D	61.6513	1
**5002**	381	D	69.1037	1
**5002**	382	D	73.1015	1
**5002**	386	D	79.5582	1
**5003**	305	A	74.9056	1
**5003**	319	A	50.7555	1
**5003**	320	A	48.4187	-1
**5003**	321	A	66.5017	0
**5003**	351	A	18.9355	1
**5003**	357	A	79.3873	1
**5003**	370	A	79.5064	1
**5003**	372	A	79.5485	0
**5003**	373	A	31.9827	0
**5003**	381	A	67.1818	1
**5003**	382	A	56.7089	1
**5003**	386	A	76.9173	1
**5003**	305	B	73.6347	1
**5003**	319	B	27.6473	0
**5003**	320	B	54.2689	-1
**5003**	321	B	73.5768	0
**5003**	351	B	22.7675	-1
**5003**	357	B	73.5624	1
**5003**	370	B	72.2295	1
**5003**	372	B	79.5295	0
**5003**	373	B	79.6826	1
**5003**	381	B	74.0535	1
**5003**	382	B	59.1679	1
**5003**	386	B	79.589	1
**5003**	305	C	79.5211	1
**5003**	319	C	48.444	0

**5003**	320	C	79.4208	-1
**5003**	321	C	79.4659	0
**5003**	351	C	10.391	1
**5003**	357	C	62.5722	1
**5003**	370	C	78.9058	1
**5003**	372	C	71.2125	0
**5003**	373	C	63.1798	1
**5003**	381	C	79.3411	1
**5003**	382	C	54.6331	1
**5003**	386	C	56.0158	1
**5003**	305	D	72.2866	1
**5003**	319	D	79.5742	1
**5003**	320	D	57.3167	-1
**5003**	321	D	79.627	0
**5003**	351	D	7.0329	1
**5003**	357	D	59.8681	1
**5003**	370	D	66.4905	1
**5003**	372	D	79.5528	0
**5003**	373	D	78.9015	1
**5003**	381	D	79.4663	1
**5003**	382	D	73.7617	1
**5003**	386	D	45.9634	0
**5004**	305	A	78.9178	1
**5004**	319	A	77.8533	1
**5004**	320	A	79.0997	-1
**5004**	321	A	79.4337	0
**5004**	351	A	11.143	1
**5004**	357	A	67.4108	1
**5004**	370	A	74.61	1
**5004**	372	A	79.5896	0
**5004**	373	A	53.2433	1
**5004**	381	A	79.5478	1
**5004**	382	A	62.9218	1
**5004**	386	A	76.4091	1

**5004**	305	B	79.5571	1
**5004**	319	B	62.9229	1
**5004**	320	B	73.8229	-1
**5004**	321	B	78.4591	0
**5004**	351	B	14.7991	1
**5004**	357	B	73.5076	1
**5004**	370	B	66.7709	1
**5004**	372	B	72.2194	0
**5004**	373	B	79.5281	1
**5004**	381	B	78.622	1
**5004**	382	B	31.9549	0
**5004**	386	B	79.6211	1
**5004**	305	C	53.4569	1
**5004**	319	C	64.7927	1
**5004**	320	C	26.6268	-1
**5004**	321	C	67.5514	0
**5004**	351	C	10.4227	1
**5004**	357	C	72.1482	1
**5004**	370	C	70.168	1
**5004**	372	C	67.1974	0
**5004**	373	C	61.8265	1
**5004**	381	C	50.1429	1
**5004**	382	C	56.549	1
**5004**	386	C	63.2674	1
**5004**	305	D	79.5387	1
**5004**	319	D	46.1323	0
**5004**	320	D	37.8939	-1
**5004**	321	D	59.738	0
**5004**	351	D	13.6513	1
**5004**	357	D	62.5501	1
**5004**	370	D	79.5333	1
**5004**	372	D	55.914	0
**5004**	373	D	72.6686	1
**5004**	381	D	72.2663	1

**5004**	382	D	79.5574	1
**5004**	386	D	75.5957	1
**5005**	305	A	65.2628	1
**5005**	319	A	6.6611	-1
**5005**	320	A	74.5582	-1
**5005**	321	A	41.7763	1
**5005**	351	A	9.4056	1
**5005**	357	A	46.782	0
**5005**	370	A	78.9375	1
**5005**	372	A	23.5785	1
**5005**	373	A	77.575	1
**5005**	381	A	72.3519	1
**5005**	382	A	79.4565	1
**5005**	386	A	33.5065	0
**5005**	305	B	74.4737	1
**5005**	319	B	77.1103	1
**5005**	320	B	75.1217	-1
**5005**	321	B	79.6368	0
**5005**	351	B	10.5276	1
**5005**	357	B	67.6596	1
**5005**	370	B	67.3959	1
**5005**	372	B	75.1523	0
**5005**	373	B	79.4014	1
**5005**	381	B	73.0168	1
**5005**	382	B	35.3518	0
**5005**	386	B	63.5254	1
**5005**	305	C	67.5385	1
**5005**	319	C	37.3786	0
**5005**	320	C	47.4372	-1
**5005**	321	C	79.5132	0
**5005**	351	C	16.2209	1
**5005**	357	C	60.7667	1
**5005**	370	C	70.1556	1
**5005**	372	C	78.6226	0

**5005**	373	C	58.9031	1
**5005**	381	C	43.8351	0
**5005**	382	C	57.0029	1
**5005**	386	C	50.9025	1
**5005**	305	D	64.6655	1
**5005**	319	D	65.1399	1
**5005**	320	D	62.985	-1
**5005**	321	D	41.2345	1
**5005**	351	D	19.8432	1
**5005**	357	D	69.8389	1
**5005**	370	D	74.9374	1
**5005**	372	D	56.9361	0
**5005**	373	D	63.5494	1
**5005**	381	D	79.3614	1
**5005**	382	D	73.8748	1
**5005**	386	D	56.4974	1
**5006**	305	A	79.6053	1
**5006**	319	A	79.5535	1
**5006**	320	A	79.5597	-1
**5006**	321	A	58.8435	0
**5006**	351	A	0.6882	1
**5006**	357	A	79.5519	1
**5006**	370	A	79.4784	1
**5006**	372	A	65.0745	0
**5006**	373	A	26.9578	0
**5006**	381	A	72.3597	1
**5006**	382	A	20.414	0
**5006**	386	A	79.6507	1
**5006**	305	B	71.8308	1
**5006**	319	B	72.5977	1
**5006**	320	B	73.7684	-1
**5006**	321	B	65.6037	0
**5006**	351	B	16.5543	1
**5006**	357	B	51.355	1

**5006**	370	B	54.6744	1
**5006**	372	B	57.6103	0
**5006**	373	B	60.6335	1
**5006**	381	B	79.5182	1
**5006**	382	B	66.3046	1
**5006**	386	B	79.5589	1
**5006**	305	C	79.5884	1
**5006**	319	C	65.4934	1
**5006**	320	C	79.5842	-1
**5006**	321	C	49.6828	1
**5006**	351	C	1.1958	1
**5006**	357	C	55.4583	1
**5006**	370	C	52.2136	1
**5006**	372	C	53.7784	0
**5006**	373	C	72.7292	1
**5006**	381	C	79.4842	1
**5006**	382	C	72.601	1
**5006**	386	C	47.2007	0
**5006**	305	D	65.1984	1
**5006**	319	D	56.0063	1
**5006**	320	D	70.5501	-1
**5006**	321	D	63.2497	0
**5006**	351	D	19.244	1
**5006**	357	D	79.4994	1
**5006**	370	D	53.4843	1
**5006**	372	D	79.5463	0
**5006**	373	D	65.5848	1
**5006**	381	D	79.5648	1
**5006**	382	D	74.5418	1
**5006**	386	D	50.0613	1
**5007**	305	A	68.4494	1
**5007**	319	A	29.4175	0
**5007**	320	A	74.4334	-1
**5007**	321	A	77.6566	0

**5007**	351	A	9.115	1
**5007**	357	A	42.234	0
**5007**	370	A	54.4665	1
**5007**	372	A	62.0696	0
**5007**	373	A	75.5387	1
**5007**	381	A	75.3062	1
**5007**	382	A	78.6595	1
**5007**	386	A	67.7009	1
**5007**	305	B	75.3975	1
**5007**	319	B	79.5171	1
**5007**	320	B	62.7776	-1
**5007**	321	B	34.3962	1
**5007**	351	B	20.3279	-1
**5007**	357	B	79.4987	1
**5007**	370	B	41.4958	0
**5007**	372	B	79.6134	0
**5007**	373	B	50.8297	1
**5007**	381	B	67.128	1
**5007**	382	B	74.8436	1
**5007**	386	B	79.4661	1
**5007**	305	C	73.348	1
**5007**	319	C	73.1876	1
**5007**	320	C	64.1057	-1
**5007**	321	C	39.9995	1
**5007**	351	C	3.7598	1
**5007**	357	C	79.436	1
**5007**	370	C	63.4427	1
**5007**	372	C	79.6359	0
**5007**	373	C	37.8471	0
**5007**	381	C	69.2977	1
**5007**	382	C	63.8472	1
**5007**	386	C	56.7257	1
**5007**	305	D	52.5479	1
**5007**	319	D	79.582	1

**5007**	320	D	63.1695	-1
**5007**	321	D	79.523	0
**5007**	351	D	7.6122	1
**5007**	357	D	57.9543	1
**5007**	370	D	79.5602	1
**5007**	372	D	76.0943	0
**5007**	373	D	77.1727	1
**5007**	381	D	79.5779	1
**5007**	382	D	79.3984	1
**5007**	386	D	26.4705	0
**5008**	305	A	45.0333	0
**5008**	319	A	71.6991	1
**5008**	320	A	79.5513	-1
**5008**	321	A	70.1726	0
**5008**	351	A	4.1428	1
**5008**	357	A	79.5372	1
**5008**	370	A	76.3631	1
**5008**	372	A	40.2035	1
**5008**	373	A	79.5773	1
**5008**	381	A	68.2714	1
**5008**	382	A	45.484	0
**5008**	386	A	59.9395	1
**5008**	305	B	71.8501	1
**5008**	319	B	79.6555	1
**5008**	320	B	71.2442	-1
**5008**	321	B	79.5866	0
**5008**	351	B	13.5337	1
**5008**	357	B	73.0373	1
**5008**	370	B	78.2238	1
**5008**	372	B	70.7892	0
**5008**	373	B	53.0422	1
**5008**	381	B	72.9638	1
**5008**	382	B	33.8898	0
**5008**	386	B	62.0549	1

**5008**	305	C	77.3713	1
**5008**	319	C	72.6481	1
**5008**	320	C	79.539	-1
**5008**	321	C	79.6029	0
**5008**	351	C	3.3648	1
**5008**	357	C	65.4967	1
**5008**	370	C	29.7963	0
**5008**	372	C	79.4637	0
**5008**	373	C	79.6373	1
**5008**	381	C	70.8256	1
**5008**	382	C	69.5739	1
**5008**	386	C	8.1383	-1
**5008**	305	D	28.1122	0
**5008**	319	D	61.4741	1
**5008**	320	D	71.7879	-1
**5008**	321	D	74.7805	0
**5008**	351	D	8.1172	1
**5008**	357	D	66.6312	1
**5008**	370	D	79.5865	1
**5008**	372	D	79.6482	0
**5008**	373	D	63.4922	1
**5008**	381	D	61.1741	1
**5008**	382	D	53.6794	1
**5008**	386	D	79.5538	1
**5009**	305	A	72.2899	1
**5009**	319	A	40.4086	0
**5009**	320	A	69.2544	-1
**5009**	321	A	61.5452	0
**5009**	351	A	13.5941	1
**5009**	357	A	59.7329	1
**5009**	370	A	26.6525	0
**5009**	372	A	77.6697	0
**5009**	373	A	79.5586	1
**5009**	381	A	79.5901	1

**5009**	382	A	79.5227	1
**5009**	386	A	78.3002	1
**5009**	305	B	70.1467	1
**5009**	319	B	52.4831	1
**5009**	320	B	57.5373	-1
**5009**	321	B	62.9186	0
**5009**	351	B	10.3579	1
**5009**	357	B	65.525	1
**5009**	370	B	51.367	1
**5009**	372	B	79.5345	0
**5009**	373	B	26.7508	0
**5009**	381	B	77.7366	1
**5009**	382	B	66.5758	1
**5009**	386	B	79.5535	1
**5009**	305	C	71.8914	1
**5009**	319	C	79.4366	1
**5009**	320	C	74.6002	-1
**5009**	321	C	70.7944	0
**5009**	351	C	3.5744	1
**5009**	357	C	74.6437	1
**5009**	370	C	62.7629	1
**5009**	372	C	26.9062	1
**5009**	373	C	79.535	1
**5009**	381	C	79.527	1
**5009**	382	C	68.1932	1
**5009**	386	C	34.0715	0
**5009**	305	D	64.1695	1
**5009**	319	D	48.0234	0
**5009**	320	D	72.4763	-1
**5009**	321	D	51.978	0
**5009**	351	D	17.069	1
**5009**	357	D	62.0984	1
**5009**	370	D	75.562	1
**5009**	372	D	57.1957	0

**5009**	373	D	77.7884	1
**5009**	381	D	52.2933	1
**5009**	382	D	76.2324	1
**5009**	386	D	74.2987	1
**5010**	305	A	72.3447	1
**5010**	319	A	69.8758	1
**5010**	320	A	58.1608	-1
**5010**	321	A	70.9769	0
**5010**	351	A	3.1924	1
**5010**	357	A	79.6107	1
**5010**	370	A	79.4745	1
**5010**	372	A	79.662	0
**5010**	373	A	62.6457	1
**5010**	381	A	79.5554	1
**5010**	382	A	67.5584	1
**5010**	386	A	79.5194	1
**5010**	305	B	74.6859	1
**5010**	319	B	59.7534	1
**5010**	320	B	44.6896	-1
**5010**	321	B	64.1261	0
**5010**	351	B	10.5259	1
**5010**	357	B	79.5	1
**5010**	370	B	79.4456	1
**5010**	372	B	79.655	0
**5010**	373	B	77.5216	1
**5010**	381	B	55.4451	1
**5010**	382	B	74.5223	1
**5010**	386	B	61.723	1
**5010**	305	C	34.1576	0
**5010**	319	C	79.5419	1
**5010**	320	C	79.4794	-1
**5010**	321	C	79.5009	0
**5010**	351	C	2.0094	1
**5010**	357	C	58.6725	1

**5010**	370	C	79.4847	1
**5010**	372	C	74.4161	0
**5010**	373	C	73.5117	1
**5010**	381	C	79.5001	1
**5010**	382	C	49.423	0
**5010**	386	C	67.6246	1
**5010**	305	D	68.2249	1
**5010**	319	D	56.2828	1
**5010**	320	D	62.1236	-1
**5010**	321	D	79.4148	0
**5010**	351	D	15.0576	1
**5010**	357	D	62.7927	1
**5010**	370	D	79.5401	1
**5010**	372	D	75.3362	0
**5010**	373	D	29.1963	0
**5010**	381	D	39.3454	0
**5010**	382	D	70.3854	1
**5010**	386	D	79.591	1
**5011**	305	A	75.7257	1
**5011**	319	A	58.5633	1
**5011**	320	A	79.4752	-1
**5011**	321	A	77.7326	0
**5011**	351	A	36.2896	-1
**5011**	357	A	36.7734	0
**5011**	370	A	79.5287	1
**5011**	372	A	79.6289	0
**5011**	373	A	75.5947	1
**5011**	381	A	79.127	1
**5011**	382	A	51.2751	1
**5011**	386	A	41.073	0
**5011**	305	B	49.0684	0
**5011**	319	B	75.8349	1
**5011**	320	B	62.073	-1
**5011**	321	B	67.3054	0

**5011**	351	B	15.8523	1
**5011**	357	B	59.1722	1
**5011**	370	B	79.5282	1
**5011**	372	B	72.9381	0
**5011**	373	B	78.3548	1
**5011**	381	B	68.821	1
**5011**	382	B	62.0728	1
**5011**	386	B	62.6623	1
**5011**	305	C	75.898	1
**5011**	319	C	74.2369	1
**5011**	320	C	71.4802	-1
**5011**	321	C	39.8984	1
**5011**	351	C	8.923	1
**5011**	357	C	79.5768	1
**5011**	370	C	65.4452	1
**5011**	372	C	79.5571	0
**5011**	373	C	59.2299	1
**5011**	381	C	79.5955	1
**5011**	382	C	43.9883	0
**5011**	386	C	49.7936	0
**5011**	305	D	58.6852	1
**5011**	319	D	79.5702	1
**5011**	320	D	47.2473	-1
**5011**	321	D	74.8436	0
**5011**	351	D	13.4657	1
**5011**	357	D	78.2089	1
**5011**	370	D	36.2553	0
**5011**	372	D	70.129	0
**5011**	373	D	79.5611	1
**5011**	381	D	77.4712	1
**5011**	382	D	69.4971	1
**5011**	386	D	66.43	1
**5012**	305	A	79.5316	1
**5012**	319	A	79.5727	1

**5012**	320	A	74.0817	-1
**5012**	321	A	55.1729	0
**5012**	351	A	6.4509	1
**5012**	357	A	49.5943	0
**5012**	370	A	59.4514	1
**5012**	372	A	77.9991	0
**5012**	373	A	74.4154	1
**5012**	381	A	79.2073	1
**5012**	382	A	60.7314	1
**5012**	386	A	45.4264	0
**5012**	305	B	64.8715	1
**5012**	319	B	77.4957	1
**5012**	320	B	79.5797	-1
**5012**	321	B	74.7271	0
**5012**	351	B	13.7493	1
**5012**	357	B	41.5304	0
**5012**	370	B	62.3691	1
**5012**	372	B	79.6409	0
**5012**	373	B	79.3062	1
**5012**	381	B	79.5476	1
**5012**	382	B	73.4678	1
**5012**	386	B	79.5315	1
**5012**	305	C	79.5568	1
**5012**	319	C	79.5709	1
**5012**	320	C	72.1154	-1
**5012**	321	C	79.5926	0
**5012**	351	C	5.591	1
**5012**	357	C	74.2963	1
**5012**	370	C	72.2961	1
**5012**	372	C	60.5377	0
**5012**	373	C	60.8539	1
**5012**	381	C	59.292	1
**5012**	382	C	77.0865	1
**5012**	386	C	59.6921	1

**5012**	305	D	58.0758	1
**5012**	319	D	51.1368	1
**5012**	320	D	70.797	-1
**5012**	321	D	62.1679	0
**5012**	351	D	13.7058	1
**5012**	357	D	79.2564	1
**5012**	370	D	69.2552	1
**5012**	372	D	71.3106	0
**5012**	373	D	50.1784	1
**5012**	381	D	64.8972	1
**5012**	382	D	77.164	1
**5012**	386	D	72.0568	1
**5013**	305	A	66.6283	1
**5013**	319	A	62.0146	1
**5013**	320	A	77.6933	-1
**5013**	321	A	69.8262	0
**5013**	351	A	7.12	1
**5013**	357	A	63.7669	1
**5013**	370	A	72.1682	1
**5013**	372	A	64.5544	0
**5013**	373	A	79.6802	1
**5013**	381	A	79.5167	1
**5013**	382	A	79.6028	1
**5013**	386	A	73.0062	1
**5013**	305	B	75.7229	1
**5013**	319	B	79.5795	1
**5013**	320	B	51.1776	-1
**5013**	321	B	79.6635	0
**5013**	351	B	15.7377	1
**5013**	357	B	60.657	1
**5013**	370	B	79.6747	1
**5013**	372	B	79.4689	0
**5013**	373	B	46.5485	0
**5013**	381	B	41.316	0

**5013**	382	B	79.4733	1
**5013**	386	B	72.6006	1
**5013**	305	C	62.3286	1
**5013**	319	C	64.3157	1
**5013**	320	C	75.2826	-1
**5013**	321	C	79.6977	0
**5013**	351	C	4.9571	1
**5013**	357	C	77.4209	1
**5013**	370	C	79.6923	1
**5013**	372	C	79.5954	0
**5013**	373	C	59.0636	1
**5013**	381	C	49.2203	0
**5013**	382	C	68.5399	1
**5013**	386	C	50.7342	1
**5013**	305	D	73.5949	1
**5013**	319	D	72.4237	1
**5013**	320	D	36.4361	-1
**5013**	321	D	60.979	0
**5013**	351	D	9.2781	1
**5013**	357	D	79.6572	1
**5013**	370	D	49.9441	0
**5013**	372	D	72.6073	0
**5013**	373	D	47.3361	0
**5013**	381	D	79.7565	1
**5013**	382	D	38.1679	0
**5013**	386	D	79.6235	1
**5014**	305	A	75.1513	1
**5014**	319	A	77.106	1
**5014**	320	A	79.5537	-1
**5014**	321	A	69.403	0
**5014**	351	A	11.5425	1
**5014**	357	A	72.0602	1
**5014**	370	A	32.4418	0
**5014**	372	A	73.4278	0

**5014**	373	A	76.2826	1
**5014**	381	A	75.425	1
**5014**	382	A	32.284	0
**5014**	386	A	43.7646	0
**5014**	305	B	42.1303	0
**5014**	319	B	50.1649	1
**5014**	320	B	46.3213	-1
**5014**	321	B	68.1534	0
**5014**	351	B	12.5495	1
**5014**	357	B	54.6241	1
**5014**	370	B	79.5497	1
**5014**	372	B	64.0098	0
**5014**	373	B	63.5186	1
**5014**	381	B	55.027	1
**5014**	382	B	44.6594	0
**5014**	386	B	60.7602	1
**5014**	305	C	69.3827	1
**5014**	319	C	71.0807	1
**5014**	320	C	79.5381	-1
**5014**	321	C	79.5298	0
**5014**	351	C	3.3099	1
**5014**	357	C	79.4191	1
**5014**	370	C	68.6882	1
**5014**	372	C	73.6523	0
**5014**	373	C	79.5275	1
**5014**	381	C	79.5578	1
**5014**	382	C	67.0416	1
**5014**	386	C	51.5784	1
**5014**	305	D	29.6394	0
**5014**	319	D	71.9149	1
**5014**	320	D	42.3736	-1
**5014**	321	D	79.5549	0
**5014**	351	D	16.6329	1
**5014**	357	D	26.6398	0

**5014**	370	D	22.338	0
**5014**	372	D	79.5653	0
**5014**	373	D	79.2902	1
**5014**	381	D	79.5616	1
**5014**	382	D	32.3244	0
**5014**	386	D	72.1671	1
**5015**	305	A	79.5767	1
**5015**	319	A	53.0438	1
**5015**	320	A	17.6233	1
**5015**	321	A	36.4939	1
**5015**	351	A	10.3533	1
**5015**	357	A	67.394	1
**5015**	370	A	79.5497	1
**5015**	372	A	70.9886	0
**5015**	373	A	57.9208	1
**5015**	381	A	62.5971	1
**5015**	382	A	33.9714	0
**5015**	386	A	74.1453	1
**5015**	305	B	71.7003	1
**5015**	319	B	54.6027	1
**5015**	320	B	31.3759	-1
**5015**	321	B	74.7768	0
**5015**	351	B	3.2557	1
**5015**	357	B	79.5555	1
**5015**	370	B	79.5049	1
**5015**	372	B	72.0929	0
**5015**	373	B	76.4323	1
**5015**	381	B	74.2678	1
**5015**	382	B	34.4306	0
**5015**	386	B	59.2249	1
**5015**	305	C	79.5089	1
**5015**	319	C	79.4607	1
**5015**	320	C	28.8122	-1
**5015**	321	C	79.1396	0

**5015**	351	C	5.9914	1
**5015**	357	C	46.9624	0
**5015**	370	C	60.0199	1
**5015**	372	C	79.5712	0
**5015**	373	C	44.0522	0
**5015**	381	C	71.6761	1
**5015**	382	C	45.5483	0
**5015**	386	C	36.9718	0
**5015**	305	D	63.733	1
**5015**	319	D	48.8879	0
**5015**	320	D	66.2807	-1
**5015**	321	D	37.7721	1
**5015**	351	D	10.7427	1
**5015**	357	D	79.5383	1
**5015**	370	D	72.8056	1
**5015**	372	D	79.5635	0
**5015**	373	D	68.12	1
**5015**	381	D	66.2777	1
**5015**	382	D	73.1542	1
**5015**	386	D	79.5761	1
**5016**	305	A	35.626	0
**5016**	319	A	79.5384	1
**5016**	320	A	79.6255	-1
**5016**	321	A	79.4681	0
**5016**	351	A	14.0496	1
**5016**	357	A	79.587	1
**5016**	370	A	59.7329	1
**5016**	372	A	30.3203	1
**5016**	373	A	79.6071	1
**5016**	381	A	79.4904	1
**5016**	382	A	43.1038	0
**5016**	386	A	79.6131	1
**5016**	305	B	70.1777	1
**5016**	319	B	74.6614	1

**5016**	320	B	79.4713	-1
**5016**	321	B	72.0545	0
**5016**	351	B	19.9873	1
**5016**	357	B	79.3933	1
**5016**	370	B	70.865	1
**5016**	372	B	77.4142	0
**5016**	373	B	68.766	1
**5016**	381	B	66.9168	1
**5016**	382	B	70.2862	1
**5016**	386	B	42.7708	0
**5016**	305	C	79.5445	1
**5016**	319	C	79.5016	1
**5016**	320	C	71.6168	-1
**5016**	321	C	63.6033	0
**5016**	351	C	1.7172	1
**5016**	357	C	49.1455	0
**5016**	370	C	79.5657	1
**5016**	372	C	79.581	0
**5016**	373	C	79.4844	1
**5016**	381	C	76.5537	1
**5016**	382	C	63.3295	1
**5016**	386	C	58.4438	1
**5016**	305	D	67.5316	1
**5016**	319	D	64.2232	1
**5016**	320	D	65.1121	-1
**5016**	321	D	79.5526	0
**5016**	351	D	18.9491	1
**5016**	357	D	71.5454	1
**5016**	370	D	57.2728	1
**5016**	372	D	79.5333	0
**5016**	373	D	79.5564	1
**5016**	381	D	56.0555	1
**5016**	382	D	79.6547	1
**5016**	386	D	77.8755	1

**5017**	305	A	65.0979	1
**5017**	319	A	7.4371	-1
**5017**	320	A	24.3371	-1
**5017**	321	A	77.4622	0
**5017**	351	A	1.2063	1
**5017**	357	A	22.5876	0
**5017**	370	A	49.0537	0
**5017**	372	A	79.7696	0
**5017**	373	A	79.6161	1
**5017**	381	A	79.5758	1
**5017**	382	A	79.4377	1
**5017**	386	A	79.5517	1
**5017**	305	B	77.154	1
**5017**	319	B	70.2262	1
**5017**	320	B	63.0309	-1
**5017**	321	B	79.5218	0
**5017**	351	B	20.6815	-1
**5017**	357	B	78.0514	1
**5017**	370	B	72.3872	1
**5017**	372	B	79.3568	0
**5017**	373	B	38.0459	0
**5017**	381	B	79.5188	1
**5017**	382	B	39.6194	0
**5017**	386	B	38.0649	0
**5017**	305	C	73.3714	1
**5017**	319	C	79.4329	1
**5017**	320	C	43.3745	-1
**5017**	321	C	68.5892	0
**5017**	351	C	11.0928	1
**5017**	357	C	67.1396	1
**5017**	370	C	78.0626	1
**5017**	372	C	66.6521	0
**5017**	373	C	63.4477	1
**5017**	381	C	79.569	1

**5017**	382	C	8.0654	-1
**5017**	386	C	46.7115	0
**5017**	305	D	79.3287	1
**5017**	319	D	68.4019	1
**5017**	320	D	43.1512	-1
**5017**	321	D	77.3904	0
**5017**	351	D	28.612	-1
**5017**	357	D	79.2976	1
**5017**	370	D	79.6245	1
**5017**	372	D	79.1786	0
**5017**	373	D	66.3072	1
**5017**	381	D	79.4251	1
**5017**	382	D	79.4844	1
**5017**	386	D	77.1252	1
**5018**	305	A	78.198	1
**5018**	319	A	61.0492	1
**5018**	320	A	41.3223	-1
**5018**	321	A	52.272	0
**5018**	351	A	1.2185	1
**5018**	357	A	44.4794	0
**5018**	370	A	79.5885	1
**5018**	372	A	22.4879	1
**5018**	373	A	78.6516	1
**5018**	381	A	65.0534	1
**5018**	382	A	76.8491	1
**5018**	386	A	79.5245	1
**5018**	305	B	75.4518	1
**5018**	319	B	79.4981	1
**5018**	320	B	79.5419	-1
**5018**	321	B	74.8007	0
**5018**	351	B	18.4984	1
**5018**	357	B	79.577	1
**5018**	370	B	33.0834	0
**5018**	372	B	68.5621	0

**5018**	373	B	79.511	1
**5018**	381	B	79.5902	1
**5018**	382	B	33.2382	0
**5018**	386	B	56.918	1
**5018**	305	C	79.5218	1
**5018**	319	C	75.5988	1
**5018**	320	C	61.1741	-1
**5018**	321	C	52.158	0
**5018**	351	C	10.4467	1
**5018**	357	C	79.5683	1
**5018**	370	C	79.5175	1
**5018**	372	C	79.5479	0
**5018**	373	C	73.5705	1
**5018**	381	C	79.5712	1
**5018**	382	C	46.3484	0
**5018**	386	C	28.2412	0
**5018**	305	D	67.0407	1
**5018**	319	D	30.9012	0
**5018**	320	D	75.1473	-1
**5018**	321	D	79.6064	0
**5018**	351	D	11.1235	1
**5018**	357	D	56.3017	1
**5018**	370	D	79.637	1
**5018**	372	D	79.5579	0
**5018**	373	D	66.5598	1
**5018**	381	D	69.2485	1
**5018**	382	D	73.289	1
**5018**	386	D	43.893	0
**5019**	305	A	48.8754	0
**5019**	319	A	47.6027	0
**5019**	320	A	79.5545	-1
**5019**	321	A	74.8437	0
**5019**	351	A	9.4019	1
**5019**	357	A	21.2864	0

**5019**	370	A	55.6945	1
**5019**	372	A	63.9414	0
**5019**	373	A	70.4048	1
**5019**	381	A	53.4051	1
**5019**	382	A	22.5649	0
**5019**	386	A	79.6245	1
**5019**	305	B	79.5997	1
**5019**	319	B	45.4838	0
**5019**	320	B	74.2095	-1
**5019**	321	B	74.4817	0
**5019**	351	B	18.7999	1
**5019**	357	B	51.3918	1
**5019**	370	B	58.6977	1
**5019**	372	B	64.2211	0
**5019**	373	B	17.2136	-1
**5019**	381	B	78.8801	1
**5019**	382	B	73.4593	1
**5019**	386	B	69.3009	1
**5019**	305	C	50.1019	1
**5019**	319	C	79.5761	1
**5019**	320	C	73.4077	-1
**5019**	321	C	48.1088	1
**5019**	351	C	9.8371	1
**5019**	357	C	50.6759	1
**5019**	370	C	46.8962	0
**5019**	372	C	74.8078	0
**5019**	373	C	71.8841	1
**5019**	381	C	79.5786	1
**5019**	382	C	32.3023	0
**5019**	386	C	19.8095	-1
**5019**	305	D	79.5063	1
**5019**	319	D	77.1814	1
**5019**	320	D	79.5197	-1
**5019**	321	D	66.0377	0

**5019**	351	D	13.1966	1
**5019**	357	D	42.9614	0
**5019**	370	D	78.095	1
**5019**	372	D	79.4907	0
**5019**	373	D	47.3929	0
**5019**	381	D	58.6449	1
**5019**	382	D	68.8677	1
**5019**	386	D	74.03	1
**5020**	305	A	79.5574	1
**5020**	319	A	79.0615	1
**5020**	320	A	75.4258	-1
**5020**	321	A	70.9233	0
**5020**	351	A	7.5113	1
**5020**	357	A	75.1158	1
**5020**	370	A	28.0006	0
**5020**	372	A	71.4519	0
**5020**	373	A	65.0304	1
**5020**	381	A	79.4037	1
**5020**	382	A	15.2701	-1
**5020**	386	A	59.2111	1
**5020**	305	B	71.3658	1
**5020**	319	B	72.7652	1
**5020**	320	B	71.1764	-1
**5020**	321	B	37.9661	1
**5020**	351	B	0.5422	1
**5020**	357	B	37.0511	0
**5020**	370	B	79.5136	1
**5020**	372	B	79.5345	0
**5020**	373	B	50.4906	1
**5020**	381	B	79.5952	1
**5020**	382	B	48.1101	0
**5020**	386	B	67.2678	1
**5020**	305	C	72.8956	1
**5020**	319	C	25.761	0

**5020**	320	C	72.7743	-1
**5020**	321	C	79.6496	0
**5020**	351	C	3.1326	1
**5020**	357	C	60.9566	1
**5020**	370	C	79.647	1
**5020**	372	C	73.7411	0
**5020**	373	C	79.6339	1
**5020**	381	C	68.577	1
**5020**	382	C	79.4706	1
**5020**	386	C	53.5261	1
**5020**	305	D	79.437	1
**5020**	319	D	45.1276	0
**5020**	320	D	78.2811	-1
**5020**	321	D	43.6998	1
**5020**	351	D	15.7857	1
**5020**	357	D	35.7416	0
**5020**	370	D	77.8316	1
**5020**	372	D	52.891	0
**5020**	373	D	39.7302	0
**5020**	381	D	79.5718	1
**5020**	382	D	65.1054	1
**5020**	386	D	64.8359	1
**5021**	305	A	35.2081	0
**5021**	319	A	75.8119	1
**5021**	320	A	63.3735	-1
**5021**	321	A	71.2208	0
**5021**	351	A	8.3967	1
**5021**	357	A	79.5612	1
**5021**	370	A	79.6139	1
**5021**	372	A	74.4002	0
**5021**	373	A	65.5997	1
**5021**	381	A	78.4008	1
**5021**	382	A	69.3107	1
**5021**	386	A	79.3361	1

**5021**	305	B	66.0724	1
**5021**	319	B	63.114	1
**5021**	320	B	74.9077	-1
**5021**	321	B	65.8641	0
**5021**	351	B	13.4704	1
**5021**	357	B	47.9788	0
**5021**	370	B	51.6107	1
**5021**	372	B	79.5103	0
**5021**	373	B	71.0242	1
**5021**	381	B	79.564	1
**5021**	382	B	40.7977	0
**5021**	386	B	76.04	1
**5021**	305	C	38.0104	0
**5021**	319	C	72.9383	1
**5021**	320	C	79.4002	-1
**5021**	321	C	79.5883	0
**5021**	351	C	9.1118	1
**5021**	357	C	79.4764	1
**5021**	370	C	46.8036	0
**5021**	372	C	70.2765	0
**5021**	373	C	60.3138	1
**5021**	381	C	36.0743	0
**5021**	382	C	77.4003	1
**5021**	386	C	57.0631	1
**5021**	305	D	79.5485	1
**5021**	319	D	40.0117	0
**5021**	320	D	79.5342	-1
**5021**	321	D	77.988	0
**5021**	351	D	19.606	1
**5021**	357	D	46.6648	0
**5021**	370	D	76.1171	1
**5021**	372	D	79.6572	0
**5021**	373	D	56.1019	1
**5021**	381	D	63.645	1

**5021**	382	D	79.3827	1
**5021**	386	D	63.8112	1
**5022**	305	A	79.607	1
**5022**	319	A	79.4988	1
**5022**	320	A	51.0771	-1
**5022**	321	A	73.258	0
**5022**	351	A	11.4433	1
**5022**	357	A	79.5414	1
**5022**	370	A	79.6383	1
**5022**	372	A	72.525	0
**5022**	373	A	73.2679	1
**5022**	381	A	71.5675	1
**5022**	382	A	77.8189	1
**5022**	386	A	79.4143	1
**5022**	305	B	79.4289	1
**5022**	319	B	72.2577	1
**5022**	320	B	71.2358	-1
**5022**	321	B	79.1975	0
**5022**	351	B	15.4367	1
**5022**	357	B	53.0259	1
**5022**	370	B	42.4879	0
**5022**	372	B	79.6213	0
**5022**	373	B	58.9544	1
**5022**	381	B	50.7651	1
**5022**	382	B	35.5821	0
**5022**	386	B	79.5726	1
**5022**	305	C	47.3681	0
**5022**	319	C	76.4547	1
**5022**	320	C	35.1331	-1
**5022**	321	C	68.2516	0
**5022**	351	C	7.8551	1
**5022**	357	C	46.5738	0
**5022**	370	C	70.4956	1
**5022**	372	C	63.877	0

**5022**	373	C	48.8788	0
**5022**	381	C	68.1426	1
**5022**	382	C	62.2592	1
**5022**	386	C	49.1556	0
**5022**	305	D	46.5213	0
**5022**	319	D	77.503	1
**5022**	320	D	79.5021	-1
**5022**	321	D	61.6708	0
**5022**	351	D	1.7594	1
**5022**	357	D	65.5865	1
**5022**	370	D	46.4663	0
**5022**	372	D	61.1699	0
**5022**	373	D	72.722	1
**5022**	381	D	79.567	1
**5022**	382	D	75.005	1
**5022**	386	D	79.5782	1
**5023**	305	A	79.5689	1
**5023**	319	A	77.1486	1
**5023**	320	A	73.5925	-1
**5023**	321	A	56.2702	0
**5023**	351	A	1.8406	1
**5023**	357	A	79.5368	1
**5023**	370	A	45.6272	0
**5023**	372	A	76.387	0
**5023**	373	A	48.1145	0
**5023**	381	A	73.4177	1
**5023**	382	A	71.7146	1
**5023**	386	A	17.5721	-1
**5023**	305	B	54.9262	1
**5023**	319	B	58.0353	1
**5023**	320	B	74.4384	-1
**5023**	321	B	42.4533	1
**5023**	351	B	11.7274	1
**5023**	357	B	79.555	1

**5023**	370	B	79.5734	1
**5023**	372	B	61.8401	0
**5023**	373	B	76.7281	1
**5023**	381	B	59.6212	1
**5023**	382	B	45.7772	0
**5023**	386	B	50.0647	1
**5023**	305	C	36.1553	0
**5023**	319	C	72.199	1
**5023**	320	C	79.5882	-1
**5023**	321	C	79.5772	0
**5023**	351	C	7.5495	1
**5023**	357	C	70.8369	1
**5023**	370	C	56.9626	1
**5023**	372	C	72.8586	0
**5023**	373	C	26.1947	0
**5023**	381	C	79.5725	1
**5023**	382	C	54.7492	1
**5023**	386	C	37.1667	0
**5023**	305	D	79.5773	1
**5023**	319	D	79.422	1
**5023**	320	D	74.7238	-1
**5023**	321	D	73.7975	0
**5023**	351	D	6.449	1
**5023**	357	D	72.2007	1
**5023**	370	D	76.1454	1
**5023**	372	D	79.562	0
**5023**	373	D	61.2414	1
**5023**	381	D	57.3761	1
**5023**	382	D	79.5555	1
**5023**	386	D	79.5296	1
**5024**	305	A	48.9205	0
**5024**	319	A	65.7739	1
**5024**	320	A	71.7945	-1
**5024**	321	A	37.203	1

**5024**	351	A	11.7603	1
**5024**	357	A	79.6187	1
**5024**	370	A	74.5503	1
**5024**	372	A	73.4983	0
**5024**	373	A	48.7847	0
**5024**	381	A	67.2363	1
**5024**	382	A	79.5155	1
**5024**	386	A	70.88	1
**5024**	305	B	79.496	1
**5024**	319	B	79.4256	1
**5024**	320	B	50.1172	-1
**5024**	321	B	58.8114	0
**5024**	351	B	13.5104	1
**5024**	357	B	14.423	-1
**5024**	370	B	53.5266	1
**5024**	372	B	74.1843	0
**5024**	373	B	78.6297	1
**5024**	381	B	74.8092	1
**5024**	382	B	69.2457	1
**5024**	386	B	74.3966	1
**5024**	305	C	79.6105	1
**5024**	319	C	65.6539	1
**5024**	320	C	79.5081	-1
**5024**	321	C	29.6842	1
**5024**	351	C	16.4477	1
**5024**	357	C	58.8603	1
**5024**	370	C	74.586	1
**5024**	372	C	74.6213	0
**5024**	373	C	79.3989	1
**5024**	381	C	72.833	1
**5024**	382	C	43.1219	0
**5024**	386	C	30.9715	0
**5024**	305	D	79.4518	1
**5024**	319	D	68.4681	1

**5024**	320	D	79.4469	-1
**5024**	321	D	74.3608	0
**5024**	351	D	18.4876	1
**5024**	357	D	75.3261	1
**5024**	370	D	72.855	1
**5024**	372	D	57.7272	0
**5024**	373	D	73.0494	1
**5024**	381	D	66.9488	1
**5024**	382	D	72.8693	1
**5024**	386	D	76.813	1
**5025**	305	A	40.7492	0
**5025**	319	A	79.6365	1
**5025**	320	A	56.4122	-1
**5025**	321	A	50.6077	0
**5025**	351	A	16.8505	1
**5025**	357	A	74.9653	1
**5025**	370	A	54.2077	1
**5025**	372	A	36.7637	1
**5025**	373	A	66.4368	1
**5025**	381	A	39.2617	0
**5025**	382	A	46.4056	0
**5025**	386	A	73.401	1
**5025**	305	B	70.839	1
**5025**	319	B	79.4574	1
**5025**	320	B	29.0872	-1
**5025**	321	B	61.9547	0
**5025**	351	B	11.295	1
**5025**	357	B	69.211	1
**5025**	370	B	74.4205	1
**5025**	372	B	79.2847	0
**5025**	373	B	79.5217	1
**5025**	381	B	66.5415	1
**5025**	382	B	75.8239	1
**5025**	386	B	79.6915	1

**5025**	305	C	72.4168	1
**5025**	319	C	41.6756	0
**5025**	320	C	79.6911	-1
**5025**	321	C	38.3846	1
**5025**	351	C	4.9583	1
**5025**	357	C	79.6748	1
**5025**	370	C	59.1346	1
**5025**	372	C	79.5616	0
**5025**	373	C	62.677	1
**5025**	381	C	55.2516	1
**5025**	382	C	68.7262	1
**5025**	386	C	44.4791	0
**5025**	305	D	30.2583	0
**5025**	319	D	79.5231	1
**5025**	320	D	67.6458	-1
**5025**	321	D	79.4457	0
**5025**	351	D	11.7189	1
**5025**	357	D	74.6283	1
**5025**	370	D	71.6178	1
**5025**	372	D	77.0719	0
**5025**	373	D	75.2479	1
**5025**	381	D	79.6462	1
**5025**	382	D	65.974	1
**5025**	386	D	67.5901	1
**5026**	305	A	74.8233	1
**5026**	319	A	79.5015	1
**5026**	320	A	79.59	-1
**5026**	321	A	73.7601	0
**5026**	351	A	2.8482	1
**5026**	357	A	79.5754	1
**5026**	370	A	79.5792	1
**5026**	372	A	57.0966	0
**5026**	373	A	79.6145	1
**5026**	381	A	34.2961	0

**5026**	382	A	64.3693	1
**5026**	386	A	67.7994	1
**5026**	305	B	79.602	1
**5026**	319	B	79.6171	1
**5026**	320	B	79.5555	-1
**5026**	321	B	79.6152	0
**5026**	351	B	12.3771	1
**5026**	357	B	66.8584	1
**5026**	370	B	30.0113	0
**5026**	372	B	79.5272	0
**5026**	373	B	56.8452	1
**5026**	381	B	79.5615	1
**5026**	382	B	75.81	1
**5026**	386	B	70.3156	1
**5026**	305	C	72.6745	1
**5026**	319	C	79.5064	1
**5026**	320	C	66.1308	-1
**5026**	321	C	75.3771	0
**5026**	351	C	3.7495	1
**5026**	357	C	79.517	1
**5026**	370	C	79.5859	1
**5026**	372	C	77.9121	0
**5026**	373	C	79.5681	1
**5026**	381	C	79.5074	1
**5026**	382	C	70.0327	1
**5026**	386	C	65.0108	1
**5026**	305	D	60.8357	1
**5026**	319	D	47.6373	0
**5026**	320	D	79.4869	-1
**5026**	321	D	51.5411	0
**5026**	351	D	18.2296	1
**5026**	357	D	44.4884	0
**5026**	370	D	46.6345	0
**5026**	372	D	79.4418	0

**5026**	373	D	79.5973	1
**5026**	381	D	76.7266	1
**5026**	382	D	69.0392	1
**5026**	386	D	79.5553	1
**5027**	305	A	70.9701	1
**5027**	319	A	74.1166	1
**5027**	320	A	79.5734	-1
**5027**	321	A	79.5258	0
**5027**	351	A	7.1222	1
**5027**	357	A	56.2345	1
**5027**	370	A	68.7538	1
**5027**	372	A	73.2648	0
**5027**	373	A	75.7045	1
**5027**	381	A	79.5605	1
**5027**	382	A	72.7246	1
**5027**	386	A	74.0783	1
**5027**	305	B	79.5831	1
**5027**	319	B	79.5648	1
**5027**	320	B	54.9581	-1
**5027**	321	B	79.5726	0
**5027**	351	B	18.8703	1
**5027**	357	B	79.5601	1
**5027**	370	B	72.5615	1
**5027**	372	B	79.5594	0
**5027**	373	B	12.4331	-1
**5027**	381	B	76.1749	1
**5027**	382	B	73.8568	1
**5027**	386	B	62.8627	1
**5027**	305	C	77.8696	1
**5027**	319	C	68.9216	1
**5027**	320	C	79.5414	-1
**5027**	321	C	75.8902	0
**5027**	351	C	15.9793	1
**5027**	357	C	77.2313	1

**5027**	370	C	79.6061	1
**5027**	372	C	79.3893	0
**5027**	373	C	79.4023	1
**5027**	381	C	79.5419	1
**5027**	382	C	77.688	1
**5027**	386	C	61.357	1
**5027**	305	D	22.3776	0
**5027**	319	D	34.3426	0
**5027**	320	D	70.0535	-1
**5027**	321	D	74.9912	0
**5027**	351	D	19.1311	1
**5027**	357	D	62.9275	1
**5027**	370	D	79.5987	1
**5027**	372	D	58.1137	0
**5027**	373	D	79.5035	1
**5027**	381	D	79.5407	1
**5027**	382	D	79.5423	1
**5027**	386	D	72.7081	1
**5028**	305	A	53.8526	1
**5028**	319	A	67.9886	1
**5028**	320	A	70.9929	-1
**5028**	321	A	79.5559	0
**5028**	351	A	6.0062	1
**5028**	357	A	73.0166	1
**5028**	370	A	63.2526	1
**5028**	372	A	53.9719	0
**5028**	373	A	27.9202	0
**5028**	381	A	78.2232	1
**5028**	382	A	79.5246	1
**5028**	386	A	68.9942	1
**5028**	305	B	67.5327	1
**5028**	319	B	66.473	1
**5028**	320	B	46.9482	-1
**5028**	321	B	74.3055	0

**5028**	351	B	22.8084	-1
**5028**	357	B	79.5293	1
**5028**	370	B	71.376	1
**5028**	372	B	79.6377	0
**5028**	373	B	58.1245	1
**5028**	381	B	79.3901	1
**5028**	382	B	20.608	0
**5028**	386	B	78.8365	1
**5028**	305	C	79.567	1
**5028**	319	C	67.8446	1
**5028**	320	C	73.4654	-1
**5028**	321	C	75.1894	0
**5028**	351	C	6.4153	1
**5028**	357	C	79.5918	1
**5028**	370	C	68.3842	1
**5028**	372	C	73.7063	0
**5028**	373	C	74.2931	1
**5028**	381	C	33.5263	0
**5028**	382	C	56.6201	1
**5028**	386	C	29.9497	0
**5028**	305	D	59.62	1
**5028**	319	D	77.5011	1
**5028**	320	D	56.4933	-1
**5028**	321	D	79.5778	0
**5028**	351	D	9.4046	1
**5028**	357	D	79.5662	1
**5028**	370	D	79.5879	1
**5028**	372	D	71.6609	0
**5028**	373	D	54.847	1
**5028**	381	D	79.5696	1
**5028**	382	D	71.4177	1
**5028**	386	D	79.6098	1
**5029**	305	A	35.4952	0
**5029**	319	A	77.7062	1

**5029**	320	A	43.3568	-1
**5029**	321	A	74.8172	0
**5029**	351	A	9.3344	1
**5029**	357	A	24.4084	0
**5029**	370	A	35.0674	0
**5029**	372	A	25.3269	1
**5029**	373	A	79.5724	1
**5029**	381	A	59.5727	1
**5029**	382	A	72.9251	1
**5029**	386	A	48.6642	0
**5029**	305	B	63.0908	1
**5029**	319	B	79.5483	1
**5029**	320	B	60.4526	-1
**5029**	321	B	37.3399	1
**5029**	351	B	14.1247	1
**5029**	357	B	62.7124	1
**5029**	370	B	73.2607	1
**5029**	372	B	71.7619	0
**5029**	373	B	60.7228	1
**5029**	381	B	72.6577	1
**5029**	382	B	19.1647	-1
**5029**	386	B	66.374	1
**5029**	305	C	78.9741	1
**5029**	319	C	79.5746	1
**5029**	320	C	79.5693	-1
**5029**	321	C	79.4805	0
**5029**	351	C	7.2342	1
**5029**	357	C	79.5608	1
**5029**	370	C	73.0984	1
**5029**	372	C	72.9122	0
**5029**	373	C	79.192	1
**5029**	381	C	55.2493	1
**5029**	382	C	46.1459	0
**5029**	386	C	52.2536	1

**5029**	305	D	61.8761	1
**5029**	319	D	79.5472	1
**5029**	320	D	47.8353	-1
**5029**	321	D	79.5831	0
**5029**	351	D	15.0596	1
**5029**	357	D	62.7471	1
**5029**	370	D	64.0524	1
**5029**	372	D	63.1413	0
**5029**	373	D	79.5416	1
**5029**	381	D	79.5562	1
**5029**	382	D	79.5482	1
**5029**	386	D	77.9123	1
**5030**	305	A	65.3978	1
**5030**	319	A	58.6433	1
**5030**	320	A	79.4071	-1
**5030**	321	A	32.7409	1
**5030**	351	A	19.5327	1
**5030**	357	A	75.7405	1
**5030**	370	A	8.8715	-1
**5030**	372	A	70.4628	0
**5030**	373	A	73.1226	1
**5030**	381	A	63.494	1
**5030**	382	A	66.3899	1
**5030**	386	A	35.9588	0
**5030**	305	B	73.3454	1
**5030**	319	B	79.5572	1
**5030**	320	B	71.1808	-1
**5030**	321	B	62.9967	0
**5030**	351	B	7.3245	1
**5030**	357	B	35.3816	0
**5030**	370	B	50.0229	1
**5030**	372	B	79.6718	0
**5030**	373	B	78.9595	1
**5030**	381	B	58.7496	1

**5030**	382	B	73.6797	1
**5030**	386	B	73.8883	1
**5030**	305	C	73.7906	1
**5030**	319	C	79.6787	1
**5030**	320	C	76.0059	-1
**5030**	321	C	75.7928	0
**5030**	351	C	19.3009	1
**5030**	357	C	71.1899	1
**5030**	370	C	79.5269	1
**5030**	372	C	79.6309	0
**5030**	373	C	72.1698	1
**5030**	381	C	52.02	1
**5030**	382	C	37.4851	0
**5030**	386	C	64.0157	1
**5030**	305	D	79.3711	1
**5030**	319	D	42.0018	0
**5030**	320	D	79.632	-1
**5030**	321	D	76.5508	0
**5030**	351	D	13.1096	1
**5030**	357	D	58.4173	1
**5030**	370	D	51.2978	1
**5030**	372	D	79.6107	0
**5030**	373	D	56.8473	1
**5030**	381	D	75.1857	1
**5030**	382	D	79.6213	1
**5030**	386	D	79.5122	1
**5031**	305	A	38.5978	0
**5031**	319	A	54.6365	1
**5031**	320	A	76.2823	-1
**5031**	321	A	71.0049	0
**5031**	351	A	10.202	1
**5031**	357	A	79.5702	1
**5031**	370	A	49.1175	0
**5031**	372	A	71.016	0

**5031**	373	A	71.6601	1
**5031**	381	A	68.2561	1
**5031**	382	A	45.6806	0
**5031**	386	A	79.652	1
**5031**	305	B	20.9595	0
**5031**	319	B	19.0364	-1
**5031**	320	B	71.9339	-1
**5031**	321	B	79.6271	0
**5031**	351	B	3.7282	1
**5031**	357	B	79.5828	1
**5031**	370	B	78.5337	1
**5031**	372	B	79.5677	0
**5031**	373	B	72.0888	1
**5031**	381	B	79.6863	1
**5031**	382	B	57.627	1
**5031**	386	B	33.9178	0
**5031**	305	C	59.2248	1
**5031**	319	C	63.2123	1
**5031**	320	C	63.8095	-1
**5031**	321	C	26.6256	1
**5031**	351	C	2.1231	1
**5031**	357	C	79.6794	1
**5031**	370	C	70.6561	1
**5031**	372	C	79.5648	0
**5031**	373	C	56.3463	1
**5031**	381	C	71.2941	1
**5031**	382	C	44.7101	0
**5031**	386	C	44.5911	0
**5031**	305	D	71.6913	1
**5031**	319	D	74.9729	1
**5031**	320	D	50.8511	-1
**5031**	321	D	73.8386	0
**5031**	351	D	9.2138	1
**5031**	357	D	69.4804	1

**5031**	370	D	79.5562	1
**5031**	372	D	54.2152	0
**5031**	373	D	56.6383	1
**5031**	381	D	25.7254	0
**5031**	382	D	67.4355	1
**5031**	386	D	46.3895	0
**5032**	305	A	79.3088	1
**5032**	319	A	55.9057	1
**5032**	320	A	74.0262	-1
**5032**	321	A	79.6001	0
**5032**	351	A	9.7571	1
**5032**	357	A	79.539	1
**5032**	370	A	74.2363	1
**5032**	372	A	73.9306	0
**5032**	373	A	76.3437	1
**5032**	381	A	79.4979	1
**5032**	382	A	67.2824	1
**5032**	386	A	56.6985	1
**5032**	305	B	37.9455	0
**5032**	319	B	72.1263	1
**5032**	320	B	79.6024	-1
**5032**	321	B	39.2238	1
**5032**	351	B	13.4831	1
**5032**	357	B	79.6603	1
**5032**	370	B	79.5953	1
**5032**	372	B	75.4345	0
**5032**	373	B	43.1185	0
**5032**	381	B	71.7638	1
**5032**	382	B	39.9168	0
**5032**	386	B	52.8151	1
**5032**	305	C	72.4102	1
**5032**	319	C	79.488	1
**5032**	320	C	79.559	-1
**5032**	321	C	79.5889	0

**5032**	351	C	1.6834	1
**5032**	357	C	79.5498	1
**5032**	370	C	67.1665	1
**5032**	372	C	72.7524	0
**5032**	373	C	79.6793	1
**5032**	381	C	61.4364	1
**5032**	382	C	71.1306	1
**5032**	386	C	28.3468	0
**5032**	305	D	24.6283	0
**5032**	319	D	71.5589	1
**5032**	320	D	79.5577	-1
**5032**	321	D	57.6679	0
**5032**	351	D	8.8577	1
**5032**	357	D	53.9127	1
**5032**	370	D	75.6124	1
**5032**	372	D	49.5035	1
**5032**	373	D	73.5031	1
**5032**	381	D	48.4181	0
**5032**	382	D	50.6414	1
**5032**	386	D	79.5081	1
**5033**	305	A	49.126	0
**5033**	319	A	16.0786	-1
**5033**	320	A	58.8485	-1
**5033**	321	A	75.8131	0
**5033**	351	A	0.0	1
**5033**	357	A	69.7669	1
**5033**	370	A	79.5819	1
**5033**	372	A	74.3849	0
**5033**	373	A	78.7463	1
**5033**	381	A	79.5443	1
**5033**	382	A	36.5487	0
**5033**	386	A	65.1322	1
**5033**	305	B	62.9967	1
**5033**	319	B	62.0531	1

**5033**	320	B	79.5637	-1
**5033**	321	B	64.5898	0
**5033**	351	B	9.1669	1
**5033**	357	B	59.5627	1
**5033**	370	B	29.9699	0
**5033**	372	B	79.5247	0
**5033**	373	B	66.4626	1
**5033**	381	B	47.3141	0
**5033**	382	B	73.909	1
**5033**	386	B	64.6227	1
**5033**	305	C	79.7934	1
**5033**	319	C	70.3066	1
**5033**	320	C	65.1381	-1
**5033**	321	C	57.1089	0
**5033**	351	C	5.0037	1
**5033**	357	C	73.6877	1
**5033**	370	C	62.1191	1
**5033**	372	C	77.1475	0
**5033**	373	C	72.5586	1
**5033**	381	C	79.5648	1
**5033**	382	C	78.9656	1
**5033**	386	C	51.8977	1
**5033**	305	D	53.0817	1
**5033**	319	D	71.1459	1
**5033**	320	D	79.5254	-1
**5033**	321	D	79.5639	0
**5033**	351	D	14.5743	1
**5033**	357	D	70.6348	1
**5033**	370	D	47.2568	0
**5033**	372	D	79.5193	0
**5033**	373	D	48.0427	0
**5033**	381	D	79.5413	1
**5033**	382	D	72.4137	1
**5033**	386	D	79.5555	1

**5034**	305	A	79.231	1
**5034**	319	A	71.1027	1
**5034**	320	A	79.6018	-1
**5034**	321	A	68.953	0
**5034**	351	A	5.1404	1
**5034**	357	A	74.1723	1
**5034**	370	A	79.61	1
**5034**	372	A	79.5619	0
**5034**	373	A	34.8807	0
**5034**	381	A	54.0686	1
**5034**	382	A	76.4034	1
**5034**	386	A	53.4587	1
**5034**	305	B	47.9122	0
**5034**	319	B	72.0933	1
**5034**	320	B	64.3371	-1
**5034**	321	B	24.3567	1
**5034**	351	B	16.6992	1
**5034**	357	B	65.0415	1
**5034**	370	B	72.664	1
**5034**	372	B	73.7535	0
**5034**	373	B	77.95	1
**5034**	381	B	79.5491	1
**5034**	382	B	4.7423	-1
**5034**	386	B	79.5477	1
**5034**	305	C	61.4397	1
**5034**	319	C	79.6511	1
**5034**	320	C	62.2146	-1
**5034**	321	C	79.6578	0
**5034**	351	C	5.5372	1
**5034**	357	C	68.6521	1
**5034**	370	C	56.3857	1
**5034**	372	C	44.4607	1
**5034**	373	C	65.9391	1
**5034**	381	C	59.9182	1

**5034**	382	C	79.6155	1
**5034**	386	C	59.709	1
**5034**	305	D	71.7974	1
**5034**	319	D	36.0669	0
**5034**	320	D	26.5579	-1
**5034**	321	D	67.5337	0
**5034**	351	D	11.0733	1
**5034**	357	D	52.3156	1
**5034**	370	D	79.6222	1
**5034**	372	D	79.693	0
**5034**	373	D	58.9308	1
**5034**	381	D	67.0053	1
**5034**	382	D	27.0403	0
**5034**	386	D	74.8599	1
**5035**	305	A	47.7041	0
**5035**	319	A	79.5484	1
**5035**	320	A	79.5164	-1
**5035**	321	A	79.6155	0
**5035**	351	A	10.6884	1
**5035**	357	A	62.7219	1
**5035**	370	A	70.32	1
**5035**	372	A	57.8956	0
**5035**	373	A	57.5178	1
**5035**	381	A	63.7453	1
**5035**	382	A	68.0218	1
**5035**	386	A	70.2551	1
**5035**	305	B	79.5631	1
**5035**	319	B	79.3223	1
**5035**	320	B	39.9778	-1
**5035**	321	B	79.6076	0
**5035**	351	B	17.8367	1
**5035**	357	B	79.5351	1
**5035**	370	B	38.7443	0
**5035**	372	B	79.4821	0

**5035**	373	B	75.8589	1
**5035**	381	B	79.6081	1
**5035**	382	B	44.0647	0
**5035**	386	B	79.5951	1
**5035**	305	C	71.2308	1
**5035**	319	C	79.5568	1
**5035**	320	C	79.5934	-1
**5035**	321	C	47.3357	1
**5035**	351	C	10.9812	1
**5035**	357	C	46.8705	0
**5035**	370	C	79.1445	1
**5035**	372	C	79.708	0
**5035**	373	C	79.5179	1
**5035**	381	C	76.7005	1
**5035**	382	C	43.8517	0
**5035**	386	C	44.2726	0
**5035**	305	D	59.6913	1
**5035**	319	D	48.3021	0
**5035**	320	D	79.5046	-1
**5035**	321	D	79.6066	0
**5035**	351	D	8.2215	1
**5035**	357	D	79.5567	1
**5035**	370	D	69.0334	1
**5035**	372	D	64.4109	0
**5035**	373	D	68.9208	1
**5035**	381	D	78.9807	1
**5035**	382	D	78.0647	1
**5035**	386	D	79.584	1
**5036**	305	A	79.6614	1
**5036**	319	A	71.3648	1
**5036**	320	A	79.6222	-1
**5036**	321	A	58.7834	0
**5036**	351	A	3.5674	1
**5036**	357	A	79.4525	1

**5036**	370	A	54.9388	1
**5036**	372	A	69.7087	0
**5036**	373	A	75.7483	1
**5036**	381	A	79.4883	1
**5036**	382	A	79.6486	1
**5036**	386	A	61.3721	1
**5036**	305	B	71.6293	1
**5036**	319	B	44.7831	0
**5036**	320	B	73.5518	-1
**5036**	321	B	48.8927	1
**5036**	351	B	12.5271	1
**5036**	357	B	79.6178	1
**5036**	370	B	37.1973	0
**5036**	372	B	49.2192	1
**5036**	373	B	72.4071	1
**5036**	381	B	79.4403	1
**5036**	382	B	68.944	1
**5036**	386	B	59.79	1
**5036**	305	C	47.8097	0
**5036**	319	C	38.1101	0
**5036**	320	C	79.5699	-1
**5036**	321	C	58.4139	0
**5036**	351	C	10.108	1
**5036**	357	C	32.5828	0
**5036**	370	C	79.4561	1
**5036**	372	C	64.9321	0
**5036**	373	C	52.0784	1
**5036**	381	C	79.5633	1
**5036**	382	C	55.7855	1
**5036**	386	C	58.5186	1
**5036**	305	D	79.78	1
**5036**	319	D	54.9413	1
**5036**	320	D	79.6542	-1
**5036**	321	D	53.2657	0

**5036**	351	D	17.1242	1
**5036**	357	D	68.259	1
**5036**	370	D	64.1958	1
**5036**	372	D	73.0814	0
**5036**	373	D	72.473	1
**5036**	381	D	74.7565	1
**5036**	382	D	61.384	1
**5036**	386	D	79.5571	1
**5037**	305	A	61.5012	1
**5037**	319	A	64.8834	1
**5037**	320	A	63.8029	-1
**5037**	321	A	46.319	1
**5037**	351	A	76	1
**5037**	357	A	62.0215	1
**5037**	370	A	65.5354	1
**5037**	372	A	56.3311	0
**5037**	373	A	74.3899	1
**5037**	381	A	74.2754	1
**5037**	382	A	38.3881	0
**5037**	386	A	53.9594	1
**5037**	305	B	71.2842	1
**5037**	319	B	79.4273	1
**5037**	320	B	54.3289	-1
**5037**	321	B	51.4092	0
**5037**	351	B	3.9239	1
**5037**	357	B	75.3237	1
**5037**	370	B	56.1347	1
**5037**	372	B	52.5904	0
**5037**	373	B	65.7515	1
**5037**	381	B	65.3634	1
**5037**	382	B	74.0189	1
**5037**	386	B	79.5151	1
**5037**	305	C	78.5834	1
**5037**	319	C	79.5091	1

**5037**	320	C	74.2048	-1
**5037**	321	C	57.7027	0
**5037**	351	C	8.2975	1
**5037**	357	C	79.561	1
**5037**	370	C	71.2484	1
**5037**	372	C	62.5973	0
**5037**	373	C	64.6211	1
**5037**	381	C	37.7226	0
**5037**	382	C	43.7746	0
**5037**	386	C	53.9845	1
**5037**	305	D	44.4509	0
**5037**	319	D	73.3905	1
**5037**	320	D	38.1526	-1
**5037**	321	D	67.6126	0
**5037**	351	D	11.1203	1
**5037**	357	D	79.6865	1
**5037**	370	D	58.945	1
**5037**	372	D	79.5637	0
**5037**	373	D	72.3649	1
**5037**	381	D	69.9442	1
**5037**	382	D	78.1355	1
**5037**	386	D	5.1487	-1
**5038**	305	A	58.9026	1
**5038**	319	A	44.8675	0
**5038**	320	A	79.0041	-1
**5038**	321	A	39.6151	1
**5038**	351	A	2.2277	1
**5038**	357	A	53.3429	1
**5038**	370	A	79.5691	1
**5038**	372	A	73.1412	0
**5038**	373	A	74.4241	1
**5038**	381	A	71.0409	1
**5038**	382	A	79.5274	1
**5038**	386	A	73.8124	1

5038	305	B	79.5905	1
5038	319	B	77.9865	1
5038	320	B	15.102	1
5038	321	B	79.5793	0
5038	351	B	16.8297	1
5038	357	B	74.7085	1
5038	370	B	67.7552	1
5038	372	B	79.6484	0
5038	373	B	58.0101	1
5038	381	B	73.2233	1
5038	382	B	42.0918	0
5038	386	B	49.5228	0
5038	305	C	69.8066	1
5038	319	C	70.8296	1
5038	320	C	79.5244	-1
5038	321	C	79.6121	0
5038	351	C	15.4201	1
5038	357	C	79.6233	1
5038	370	C	63.477	1
5038	372	C	79.5747	0
5038	373	C	53.6893	1
5038	381	C	78.8229	1
5038	382	C	56.2892	1
5038	386	C	46.3665	0
5038	305	D	23.0344	0
5038	319	D	70.2453	1
5038	320	D	79.5333	-1
5038	321	D	66.2595	0
5038	351	D	2.207	1
5038	357	D	76.9559	1
5038	370	D	67.6799	1
5038	372	D	64.0531	0
5038	373	D	60.9759	1
5038	381	D	79.4696	1

**5038**	382	D	68.625	1
**5038**	386	D	79.6304	1
**5039**	305	A	79.3445	1
**5039**	319	A	64.5779	1
**5039**	320	A	68.3048	-1
**5039**	321	A	66.6323	0
**5039**	351	A	0.5894	1
**5039**	357	A	79.6718	1
**5039**	370	A	76.0292	1
**5039**	372	A	66.5692	0
**5039**	373	A	79.537	1
**5039**	381	A	79.532	1
**5039**	382	A	54.0996	1
**5039**	386	A	79.5994	1
**5039**	305	B	64.6338	1
**5039**	319	B	77.3311	1
**5039**	320	B	73.6883	-1
**5039**	321	B	47.2781	1
**5039**	351	B	17.4353	1
**5039**	357	B	72.2079	1
**5039**	370	B	79.5996	1
**5039**	372	B	72.6098	0
**5039**	373	B	79.5963	1
**5039**	381	B	68.8691	1
**5039**	382	B	66.023	1
**5039**	386	B	79.5766	1
**5039**	305	C	79.0761	1
**5039**	319	C	70.8482	1
**5039**	320	C	71.8382	-1
**5039**	321	C	62.2862	0
**5039**	351	C	915	1
**5039**	357	C	79.4551	1
**5039**	370	C	79.6145	1
**5039**	372	C	79.5795	0

**5039**	373	C	79.6461	1
**5039**	381	C	79.6114	1
**5039**	382	C	73.2701	1
**5039**	386	C	51.1042	1
**5039**	305	D	69.9582	1
**5039**	319	D	79.6087	1
**5039**	320	D	71.7839	-1
**5039**	321	D	79.5418	0
**5039**	351	D	0.0	1
**5039**	357	D	30.7856	0
**5039**	370	D	67.5389	1
**5039**	372	D	79.56	0
**5039**	373	D	79.5475	1
**5039**	381	D	66.22	1
**5039**	382	D	66.0718	1
**5039**	386	D	79.625	1
**5040**	305	A	50.1749	1
**5040**	319	A	79.6144	1
**5040**	320	A	71.8686	-1
**5040**	321	A	79.6673	0
**5040**	351	A	5.1857	1
**5040**	357	A	79.4778	1
**5040**	370	A	53.4828	1
**5040**	372	A	46.5963	1
**5040**	373	A	59.7419	1
**5040**	381	A	72.3124	1
**5040**	382	A	78.2385	1
**5040**	386	A	79.4429	1
**5040**	305	B	78.7131	1
**5040**	319	B	71.9337	1
**5040**	320	B	65.4357	-1
**5040**	321	B	57.8429	0
**5040**	351	B	28.531	-1
**5040**	357	B	72.936	1

**5040**	370	B	76.3944	1
**5040**	372	B	79.5266	0
**5040**	373	B	79.5379	1
**5040**	381	B	79.6607	1
**5040**	382	B	44.1214	0
**5040**	386	B	50.5923	1
**5040**	305	C	62.0222	1
**5040**	319	C	79.5806	1
**5040**	320	C	37.927	-1
**5040**	321	C	60.2773	0
**5040**	351	C	12.3807	1
**5040**	357	C	73.6441	1
**5040**	370	C	61.4995	1
**5040**	372	C	72.2721	0
**5040**	373	C	62.3061	1
**5040**	381	C	45.9856	0
**5040**	382	C	79.2089	1
**5040**	386	C	62.2933	1
**5040**	305	D	16.8614	-1
**5040**	319	D	72.1492	1
**5040**	320	D	50.6215	-1
**5040**	321	D	64.6696	0
**5040**	351	D	12.3255	1
**5040**	357	D	79.6252	1
**5040**	370	D	49.1721	0
**5040**	372	D	73.6947	0
**5040**	373	D	79.6272	1
**5040**	381	D	45.9151	0
**5040**	382	D	79.5717	1
**5040**	386	D	72.4859	1
**5041**	305	A	78.955	1
**5041**	319	A	77.6647	1
**5041**	320	A	74.1759	-1
**5041**	321	A	65.2678	0

**5041**	351	A	24.0599	-1
**5041**	357	A	41.4557	0
**5041**	370	A	49.0582	0
**5041**	372	A	60.7612	0
**5041**	373	A	71.667	1
**5041**	381	A	79.5418	1
**5041**	382	A	65.3792	1
**5041**	386	A	79.6224	1
**5041**	305	B	72.2429	1
**5041**	319	B	79.5189	1
**5041**	320	B	78.7476	-1
**5041**	321	B	37.0189	1
**5041**	351	B	12.9271	1
**5041**	357	B	65.9566	1
**5041**	370	B	75.8036	1
**5041**	372	B	67.9022	0
**5041**	373	B	73.7226	1
**5041**	381	B	75.4752	1
**5041**	382	B	79.3556	1
**5041**	386	B	79.5312	1
**5041**	305	C	79.4666	1
**5041**	319	C	73.5024	1
**5041**	320	C	68.4788	-1
**5041**	321	C	12.2088	0
**5041**	351	C	1.7421	1
**5041**	357	C	66.9837	1
**5041**	370	C	79.5369	1
**5041**	372	C	39.224	1
**5041**	373	C	49.4473	0
**5041**	381	C	73.8702	1
**5041**	382	C	79.455	1
**5041**	386	C	64.7165	1
**5041**	305	D	73.4295	1
**5041**	319	D	30.3597	0

**5041**	320	D	43.1254	-1
**5041**	321	D	49.6422	1
**5041**	351	D	25.7937	-1
**5041**	357	D	79.701	1
**5041**	370	D	72.6852	1
**5041**	372	D	78.1776	0
**5041**	373	D	57.0207	1
**5041**	381	D	79.5502	1
**5041**	382	D	79.5379	1
**5041**	386	D	79.6906	1
**5042**	305	A	75.7121	1
**5042**	319	A	69.4112	1
**5042**	320	A	47.561	-1
**5042**	321	A	55.3962	0
**5042**	351	A	5.5668	1
**5042**	357	A	50.3331	1
**5042**	370	A	51.4286	1
**5042**	372	A	71.4276	0
**5042**	373	A	73.6766	1
**5042**	381	A	79.5331	1
**5042**	382	A	60.8215	1
**5042**	386	A	70.3212	1
**5042**	305	B	64.2208	1
**5042**	319	B	79.6849	1
**5042**	320	B	49.2945	-1
**5042**	321	B	71.3985	0
**5042**	351	B	0.2101	1
**5042**	357	B	68.084	1
**5042**	370	B	72.8362	1
**5042**	372	B	79.596	0
**5042**	373	B	73.7999	1
**5042**	381	B	79.4933	1
**5042**	382	B	27.0879	0
**5042**	386	B	79.6316	1

**5042**	305	C	55.2762	1
**5042**	319	C	57.3093	1
**5042**	320	C	79.1378	-1
**5042**	321	C	73.1754	0
**5042**	351	C	7.5497	1
**5042**	357	C	79.6262	1
**5042**	370	C	71.0931	1
**5042**	372	C	79.5434	0
**5042**	373	C	79.5262	1
**5042**	381	C	79.648	1
**5042**	382	C	77.8039	1
**5042**	386	C	63.6022	1
**5042**	305	D	45.3801	0
**5042**	319	D	79.6045	1
**5042**	320	D	79.5147	-1
**5042**	321	D	66.8717	0
**5042**	351	D	15.4579	1
**5042**	357	D	74.3029	1
**5042**	370	D	79.6266	1
**5042**	372	D	79.077	0
**5042**	373	D	30.2407	0
**5042**	381	D	79.5614	1
**5042**	382	D	72.3663	1
**5042**	386	D	79.5052	1
**5043**	305	A	60.0478	1
**5043**	319	A	79.5772	1
**5043**	320	A	71.4294	-1
**5043**	321	A	79.6025	0
**5043**	351	A	19.723	1
**5043**	357	A	79.6074	1
**5043**	370	A	61.962	1
**5043**	372	A	71.9523	0
**5043**	373	A	72.0902	1
**5043**	381	A	62.5784	1

**5043**	382	A	79.3484	1
**5043**	386	A	73.7532	1
**5043**	305	B	72.4588	1
**5043**	319	B	74.4034	1
**5043**	320	B	79.5875	-1
**5043**	321	B	66.3006	0
**5043**	351	B	12.2701	1
**5043**	357	B	79.6329	1
**5043**	370	B	78.849	1
**5043**	372	B	36.8525	1
**5043**	373	B	56.231	1
**5043**	381	B	72.6163	1
**5043**	382	B	78.4475	1
**5043**	386	B	47.1259	0
**5043**	305	C	74.1804	1
**5043**	319	C	76.4689	1
**5043**	320	C	57.6104	-1
**5043**	321	C	79.5891	0
**5043**	351	C	6.2602	1
**5043**	357	C	66.0813	1
**5043**	370	C	47.6744	0
**5043**	372	C	72.4481	0
**5043**	373	C	79.4869	1
**5043**	381	C	79.4513	1
**5043**	382	C	58.231	1
**5043**	386	C	49.3336	0
**5043**	305	D	68.9459	1
**5043**	319	D	48.6551	0
**5043**	320	D	73.9986	-1
**5043**	321	D	76.857	0
**5043**	351	D	3.7875	1
**5043**	357	D	74.8113	1
**5043**	370	D	69.9666	1
**5043**	372	D	67.177	0

**5043**	373	D	79.4695	1
**5043**	381	D	72.1899	1
**5043**	382	D	69.2367	1
**5043**	386	D	67.4303	1
**5044**	305	A	66.787	1
**5044**	319	A	39.4685	0
**5044**	320	A	47.0025	-1
**5044**	321	A	32.8935	1
**5044**	351	A	10.0409	1
**5044**	357	A	79.5405	1
**5044**	370	A	64.8672	1
**5044**	372	A	68.0534	0
**5044**	373	A	75.4678	1
**5044**	381	A	76.2637	1
**5044**	382	A	79.2738	1
**5044**	386	A	52.4075	1
**5044**	305	B	78.695	1
**5044**	319	B	57.3634	1
**5044**	320	B	36.6719	-1
**5044**	321	B	79.6438	0
**5044**	351	B	20.6217	-1
**5044**	357	B	21.1347	0
**5044**	370	B	70.3203	1
**5044**	372	B	79.5671	0
**5044**	373	B	74.4504	1
**5044**	381	B	74.793	1
**5044**	382	B	20.4452	0
**5044**	386	B	72.6397	1
**5044**	305	C	73.4025	1
**5044**	319	C	74.8232	1
**5044**	320	C	51.0862	-1
**5044**	321	C	41.6393	1
**5044**	351	C	5.6406	1
**5044**	357	C	74.8657	1

**5044**	370	C	68.0997	1
**5044**	372	C	53.3196	0
**5044**	373	C	52.2986	1
**5044**	381	C	79.4848	1
**5044**	382	C	41.2609	0
**5044**	386	C	35.2644	0
**5044**	305	D	51.4552	1
**5044**	319	D	51.2137	1
**5044**	320	D	79.561	-1
**5044**	321	D	79.6776	0
**5044**	351	D	2.7895	1
**5044**	357	D	42.7076	0
**5044**	370	D	79.5959	1
**5044**	372	D	29.7437	1
**5044**	373	D	75.5728	1
**5044**	381	D	52.0554	1
**5044**	382	D	52.9657	1
**5044**	386	D	79.3704	1
**5045**	305	A	79.5026	1
**5045**	319	A	65.6967	1
**5045**	320	A	58.5854	-1
**5045**	321	A	71.7511	0
**5045**	351	A	2.9648	1
**5045**	357	A	46.398	0
**5045**	370	A	37.5822	0
**5045**	372	A	53.811	0
**5045**	373	A	74.3028	1
**5045**	381	A	79.5889	1
**5045**	382	A	79.5711	1
**5045**	386	A	44.2568	0
**5045**	305	B	22.975	0
**5045**	319	B	79.6881	1
**5045**	320	B	56.7729	-1
**5045**	321	B	72.0745	0

**5045**	351	B	11.5741	1
**5045**	357	B	72.4549	1
**5045**	370	B	79.4824	1
**5045**	372	B	79.535	0
**5045**	373	B	72.1	1
**5045**	381	B	55.2221	1
**5045**	382	B	48.7747	0
**5045**	386	B	61.2278	1
**5045**	305	C	52.5111	1
**5045**	319	C	64.8849	1
**5045**	320	C	79.5598	-1
**5045**	321	C	75.0416	0
**5045**	351	C	12.5124	1
**5045**	357	C	45.7032	0
**5045**	370	C	71.5159	1
**5045**	372	C	79.5594	0
**5045**	373	C	79.5836	1
**5045**	381	C	66.26	1
**5045**	382	C	71.3667	1
**5045**	386	C	49.589	0
**5045**	305	D	70.8359	1
**5045**	319	D	32.745	0
**5045**	320	D	40.8744	-1
**5045**	321	D	52.8536	0
**5045**	351	D	8.4354	1
**5045**	357	D	67.4027	1
**5045**	370	D	71.4769	1
**5045**	372	D	44.5624	1
**5045**	373	D	74.7154	1
**5045**	381	D	23.2508	0
**5045**	382	D	78.4117	1
**5045**	386	D	79.5473	1
**5046**	305	A	44.2333	0
**5046**	319	A	68.701	1

**5046**	320	A	75.5286	-1
**5046**	321	A	57.1373	0
**5046**	351	A	13.1712	1
**5046**	357	A	77.4719	1
**5046**	370	A	66.4439	1
**5046**	372	A	79.6805	0
**5046**	373	A	79.4879	1
**5046**	381	A	74.2656	1
**5046**	382	A	72.5509	1
**5046**	386	A	24.751	0
**5046**	305	B	73.6101	1
**5046**	319	B	79.5838	1
**5046**	320	B	56.0527	-1
**5046**	321	B	71.1285	0
**5046**	351	B	9.6163	1
**5046**	357	B	72.4772	1
**5046**	370	B	55.4502	1
**5046**	372	B	29.634	1
**5046**	373	B	61.9609	1
**5046**	381	B	61.2074	1
**5046**	382	B	34.7174	0
**5046**	386	B	79.3907	1
**5046**	305	C	79.6753	1
**5046**	319	C	49.9643	0
**5046**	320	C	79.7542	-1
**5046**	321	C	79.5612	0
**5046**	351	C	5.4776	1
**5046**	357	C	79.5814	1
**5046**	370	C	79.4495	1
**5046**	372	C	71.1861	0
**5046**	373	C	77.3418	1
**5046**	381	C	69.7781	1
**5046**	382	C	21.8742	0
**5046**	386	C	39.3084	0

**5046**	305	D	75.6998	1
**5046**	319	D	79.6168	1
**5046**	320	D	52.4146	-1
**5046**	321	D	79.4595	0
**5046**	351	D	14.7907	1
**5046**	357	D	69.6718	1
**5046**	370	D	27.6589	0
**5046**	372	D	79.5782	0
**5046**	373	D	50.1659	1
**5046**	381	D	56.7295	1
**5046**	382	D	76.4419	1
**5046**	386	D	79.5459	1
**5047**	305	A	79.5808	1
**5047**	319	A	76.9643	1
**5047**	320	A	69.1127	-1
**5047**	321	A	77.7622	0
**5047**	351	A	5.6218	1
**5047**	357	A	74.015	1
**5047**	370	A	19.4121	-1
**5047**	372	A	50.7598	0
**5047**	373	A	77.4593	1
**5047**	381	A	79.5349	1
**5047**	382	A	75.6592	1
**5047**	386	A	73.4609	1
**5047**	305	B	75.6772	1
**5047**	319	B	73.62	1
**5047**	320	B	43.1231	-1
**5047**	321	B	72.5507	0
**5047**	351	B	12.311	1
**5047**	357	B	74.7293	1
**5047**	370	B	54.8487	1
**5047**	372	B	79.5395	0
**5047**	373	B	55.9154	1
**5047**	381	B	66.3546	1

**5047**	382	B	64.1769	1
**5047**	386	B	57.2729	1
**5047**	305	C	71.5295	1
**5047**	319	C	74.6837	1
**5047**	320	C	74.1928	-1
**5047**	321	C	54.2453	0
**5047**	351	C	4.7632	1
**5047**	357	C	63.3751	1
**5047**	370	C	40.6792	0
**5047**	372	C	79.54	0
**5047**	373	C	79.5536	1
**5047**	381	C	63.1471	1
**5047**	382	C	74.5289	1
**5047**	386	C	45.3507	0
**5047**	305	D	71.2184	1
**5047**	319	D	79.5198	1
**5047**	320	D	71.18	-1
**5047**	321	D	79.5477	0
**5047**	351	D	10.4714	1
**5047**	357	D	63.536	1
**5047**	370	D	79.031	1
**5047**	372	D	58.5341	0
**5047**	373	D	76.9746	1
**5047**	381	D	57.4081	1
**5047**	382	D	70.0349	1
**5047**	386	D	78.7477	1
**5048**	305	A	58.5338	1
**5048**	319	A	74.4042	1
**5048**	320	A	79.5392	-1
**5048**	321	A	75.6034	0
**5048**	351	A	12.1636	1
**5048**	357	A	57.2055	1
**5048**	370	A	79.5691	1
**5048**	372	A	73.8995	0

**5048**	373	A	69.7553	1
**5048**	381	A	37.1028	0
**5048**	382	A	72.8384	1
**5048**	386	A	77.4276	1
**5048**	305	B	79.5426	1
**5048**	319	B	30.5865	0
**5048**	320	B	25.8513	-1
**5048**	321	B	79.55	0
**5048**	351	B	17.354	1
**5048**	357	B	36.4131	0
**5048**	370	B	46.6125	0
**5048**	372	B	47.1054	1
**5048**	373	B	67.3584	1
**5048**	381	B	79.3131	1
**5048**	382	B	53.9726	1
**5048**	386	B	79.6079	1
**5048**	305	C	56.6957	1
**5048**	319	C	32.2208	0
**5048**	320	C	64.321	-1
**5048**	321	C	52.964	0
**5048**	351	C	5.9096	1
**5048**	357	C	73.5054	1
**5048**	370	C	70.6158	1
**5048**	372	C	79.5601	0
**5048**	373	C	79.5495	1
**5048**	381	C	51.4266	1
**5048**	382	C	46.9482	0
**5048**	386	C	13.8806	-1
**5048**	305	D	68.312	1
**5048**	319	D	52.6174	1
**5048**	320	D	67.9826	-1
**5048**	321	D	79.5757	0
**5048**	351	D	7.7939	1
**5048**	357	D	72.6834	1

**5048**	370	D	53.3332	1
**5048**	372	D	79.5429	0
**5048**	373	D	79.5663	1
**5048**	381	D	24.2091	0
**5048**	382	D	66.8699	1
**5048**	386	D	30.0437	0
**5049**	305	A	79.5885	1
**5049**	319	A	61.7777	1
**5049**	320	A	64.6609	-1
**5049**	321	A	66.491	0
**5049**	351	A	10.1668	1
**5049**	357	A	79.4002	1
**5049**	370	A	34.7335	0
**5049**	372	A	59.1691	0
**5049**	373	A	58.2222	1
**5049**	381	A	61.9926	1
**5049**	382	A	22.5919	0
**5049**	386	A	79.7102	1
**5049**	305	B	79.4894	1
**5049**	319	B	74.8004	1
**5049**	320	B	79.5918	-1
**5049**	321	B	75.8517	0
**5049**	351	B	9.4557	1
**5049**	357	B	71.9121	1
**5049**	370	B	79.5447	1
**5049**	372	B	64.7262	0
**5049**	373	B	62.4294	1
**5049**	381	B	70.0171	1
**5049**	382	B	79.5494	1
**5049**	386	B	79.51	1
**5049**	305	C	79.3239	1
**5049**	319	C	66.4386	1
**5049**	320	C	60.3687	-1
**5049**	321	C	51.2498	0

**5049**	351	C	17.2124	1
**5049**	357	C	79.5672	1
**5049**	370	C	72.7971	1
**5049**	372	C	78.5592	0
**5049**	373	C	75.7381	1
**5049**	381	C	79.5082	1
**5049**	382	C	41.8141	0
**5049**	386	C	42.1213	0
**5049**	305	D	36.1257	0
**5049**	319	D	42.3162	0
**5049**	320	D	68.333	-1
**5049**	321	D	79.6925	0
**5049**	351	D	12.1932	1
**5049**	357	D	69.6732	1
**5049**	370	D	60.3348	1
**5049**	372	D	79.5273	0
**5049**	373	D	52.5127	1
**5049**	381	D	57.1896	1
**5049**	382	D	66.1354	1
**5049**	386	D	79.4235	1
**5050**	305	A	79.5254	1
**5050**	319	A	79.6494	1
**5050**	320	A	64.2122	-1
**5050**	321	A	66.1121	0
**5050**	351	A	1.1951	1
**5050**	357	A	79.5431	1
**5050**	370	A	71.6156	1
**5050**	372	A	34.4501	1
**5050**	373	A	45.8295	0
**5050**	381	A	78.2612	1
**5050**	382	A	79.6279	1
**5050**	386	A	79.5393	1
**5050**	305	B	65.4194	1
**5050**	319	B	64.5376	1

**5050**	320	B	75.6531	-1
**5050**	321	B	79.6113	0
**5050**	351	B	17.5577	1
**5050**	357	B	72.8943	1
**5050**	370	B	79.5881	1
**5050**	372	B	79.5652	0
**5050**	373	B	78.7862	1
**5050**	381	B	79.5238	1
**5050**	382	B	36.5808	0
**5050**	386	B	68.2411	1
**5050**	305	C	44.7017	0
**5050**	319	C	78.9848	1
**5050**	320	C	56.9777	-1
**5050**	321	C	72.985	0
**5050**	351	C	1.6187	1
**5050**	357	C	79.6348	1
**5050**	370	C	79.2073	1
**5050**	372	C	74.5114	0
**5050**	373	C	28.5018	0
**5050**	381	C	73.6245	1
**5050**	382	C	76.6071	1
**5050**	386	C	68.8164	1
**5050**	305	D	47.8422	0
**5050**	319	D	79.4778	1
**5050**	320	D	78.8704	-1
**5050**	321	D	79.6001	0
**5050**	351	D	10.7609	1
**5050**	357	D	73.4024	1
**5050**	370	D	73.4865	1
**5050**	372	D	79.5677	0
**5050**	373	D	60.3678	1
**5050**	381	D	79.5224	1
**5050**	382	D	72.1668	1
**5050**	386	D	72.3005	1

**5051**	305	A	50.9741	1
**5051**	319	A	70.9177	1
**5051**	320	A	60.492	-1
**5051**	321	A	25.7015	1
**5051**	351	A	5.0972	1
**5051**	357	A	79.5927	1
**5051**	370	A	71.8052	1
**5051**	372	A	12.0141	0
**5051**	373	A	51.0332	1
**5051**	381	A	79.5374	1
**5051**	382	A	68.6394	1
**5051**	386	A	79.5722	1
**5051**	305	B	70.1703	1
**5051**	319	B	79.4544	1
**5051**	320	B	79.5768	-1
**5051**	321	B	79.5554	0
**5051**	351	B	17.1686	1
**5051**	357	B	72.8722	1
**5051**	370	B	53.7908	1
**5051**	372	B	79.5518	0
**5051**	373	B	44.5744	0
**5051**	381	B	79.5775	1
**5051**	382	B	45.6637	0
**5051**	386	B	50.1303	1
**5051**	305	C	71.8236	1
**5051**	319	C	46.0967	0
**5051**	320	C	44.857	-1
**5051**	321	C	57.6547	0
**5051**	351	C	4.2494	1
**5051**	357	C	79.5524	1
**5051**	370	C	76.0701	1
**5051**	372	C	79.5166	0
**5051**	373	C	79.5506	1
**5051**	381	C	79.1774	1

**5051**	382	C	40.701	0
**5051**	386	C	38.7784	0
**5051**	305	D	43.5803	0
**5051**	319	D	79.5505	1
**5051**	320	D	37.6477	-1
**5051**	321	D	70.3609	0
**5051**	351	D	9.888	1
**5051**	357	D	58.6625	1
**5051**	370	D	72.0794	1
**5051**	372	D	79.5759	0
**5051**	373	D	79.5618	1
**5051**	381	D	78.9487	1
**5051**	382	D	73.3891	1
**5051**	386	D	79.5792	1
**5052**	305	A	74.4631	1
**5052**	319	A	76.2202	1
**5052**	320	A	79.5673	-1
**5052**	321	A	79.4978	0
**5052**	351	A	17.3885	1
**5052**	357	A	40.6173	0
**5052**	370	A	41.9116	0
**5052**	372	A	65.031	0
**5052**	373	A	19.2187	-1
**5052**	381	A	53.7516	1
**5052**	382	A	35.909	0
**5052**	386	A	69.8332	1
**5052**	305	B	33.2035	0
**5052**	319	B	68.9172	1
**5052**	320	B	74.8118	-1
**5052**	321	B	75.1645	0
**5052**	351	B	28.1549	-1
**5052**	357	B	70.6559	1
**5052**	370	B	62.2981	1
**5052**	372	B	79.4859	0

**5052**	373	B	74.4869	1
**5052**	381	B	79.0545	1
**5052**	382	B	72.1042	1
**5052**	386	B	66.1718	1
**5052**	305	C	71.2818	1
**5052**	319	C	16.5906	-1
**5052**	320	C	79.5782	-1
**5052**	321	C	34.1789	1
**5052**	351	C	3.9533	1
**5052**	357	C	34.9295	0
**5052**	370	C	79.4725	1
**5052**	372	C	79.5083	0
**5052**	373	C	77.7533	1
**5052**	381	C	40.2432	0
**5052**	382	C	38.8106	0
**5052**	386	C	39.2256	0
**5052**	305	D	69.5131	1
**5052**	319	D	79.6449	1
**5052**	320	D	72.1567	-1
**5052**	321	D	56.9705	0
**5052**	351	D	19.2587	1
**5052**	357	D	79.5959	1
**5052**	370	D	72.686	1
**5052**	372	D	68.6623	0
**5052**	373	D	79.5112	1
**5052**	381	D	48.6932	0
**5052**	382	D	74.517	1
**5052**	386	D	74.0058	1
**5053**	305	A	63.43	1
**5053**	319	A	79.635	1
**5053**	320	A	74.5317	-1
**5053**	321	A	57.9494	0
**5053**	351	A	2.6707	1
**5053**	357	A	79.3611	1

**5053**	370	A	64.3474	1
**5053**	372	A	79.3851	0
**5053**	373	A	61.4401	1
**5053**	381	A	76.5969	1
**5053**	382	A	79.38	1
**5053**	386	A	52.2113	1
**5053**	305	B	79.5659	1
**5053**	319	B	18.4769	-1
**5053**	320	B	75.1589	-1
**5053**	321	B	55.5423	0
**5053**	351	B	0.0	1
**5053**	357	B	78.7713	1
**5053**	370	B	68.0081	1
**5053**	372	B	24.9162	1
**5053**	373	B	79.4474	1
**5053**	381	B	79.6369	1
**5053**	382	B	21.2595	0
**5053**	386	B	53.6324	1
**5053**	305	C	59.778	1
**5053**	319	C	56.7676	1
**5053**	320	C	77.27	-1
**5053**	321	C	46.8363	1
**5053**	351	C	10.8724	1
**5053**	357	C	79.4571	1
**5053**	370	C	73.4213	1
**5053**	372	C	79.6941	0
**5053**	373	C	79.419	1
**5053**	381	C	76.7111	1
**5053**	382	C	48.4735	0
**5053**	386	C	38.3622	0
**5053**	305	D	72.2233	1
**5053**	319	D	45.0756	0
**5053**	320	D	77.6888	-1
**5053**	321	D	76.5216	0

**5053**	351	D	10.3832	1
**5053**	357	D	75.4021	1
**5053**	370	D	71.1359	1
**5053**	372	D	79.6181	0
**5053**	373	D	71.7727	1
**5053**	381	D	79.5512	1
**5053**	382	D	73.685	1
**5053**	386	D	56.4723	1
**5054**	305	A	78.349	1
**5054**	319	A	48.6979	0
**5054**	320	A	43.8727	-1
**5054**	321	A	76.8225	0
**5054**	351	A	6.0167	1
**5054**	357	A	77.417	1
**5054**	370	A	79.5887	1
**5054**	372	A	77.333	0
**5054**	373	A	57.1078	1
**5054**	381	A	52.8172	1
**5054**	382	A	64.1491	1
**5054**	386	A	75.7104	1
**5054**	305	B	79.5313	1
**5054**	319	B	79.6041	1
**5054**	320	B	68.4816	-1
**5054**	321	B	79.5501	0
**5054**	351	B	12.2323	1
**5054**	357	B	74.328	1
**5054**	370	B	22.8342	0
**5054**	372	B	79.4859	0
**5054**	373	B	78.2328	1
**5054**	381	B	79.4838	1
**5054**	382	B	45.191	0
**5054**	386	B	46.9314	0
**5054**	305	C	73.6461	1
**5054**	319	C	79.5431	1

**5054**	320	C	71.9859	-1
**5054**	321	C	73.1708	0
**5054**	351	C	15.6592	1
**5054**	357	C	79.6059	1
**5054**	370	C	71.4442	1
**5054**	372	C	71.3072	0
**5054**	373	C	78.7948	1
**5054**	381	C	44.2647	0
**5054**	382	C	79.618	1
**5054**	386	C	70.0608	1
**5054**	305	D	68.1955	1
**5054**	319	D	62.2787	1
**5054**	320	D	24.9409	-1
**5054**	321	D	79.5626	0
**5054**	351	D	13.5651	1
**5054**	357	D	73.0694	1
**5054**	370	D	73.8514	1
**5054**	372	D	77.7353	0
**5054**	373	D	72.6725	1
**5054**	381	D	55.6592	1
**5054**	382	D	60.4757	1
**5054**	386	D	69.4416	1
**5055**	305	A	65.5	1
**5055**	319	A	72.2326	1
**5055**	320	A	71.876	-1
**5055**	321	A	66.7964	0
**5055**	351	A	5.2534	1
**5055**	357	A	54.8618	1
**5055**	370	A	65.2238	1
**5055**	372	A	78.3895	0
**5055**	373	A	65.9683	1
**5055**	381	A	25.9393	0
**5055**	382	A	73.8723	1
**5055**	386	A	72.5388	1

**5055**	305	B	79.7026	1
**5055**	319	B	32.3145	0
**5055**	320	B	65.389	-1
**5055**	321	B	38.8336	1
**5055**	351	B	13.4926	1
**5055**	357	B	63.6721	1
**5055**	370	B	62.914	1
**5055**	372	B	77.0207	0
**5055**	373	B	65.598	1
**5055**	381	B	49.0064	0
**5055**	382	B	76.4881	1
**5055**	386	B	52.4514	1
**5055**	305	C	72.7682	1
**5055**	319	C	76.7383	1
**5055**	320	C	79.6318	-1
**5055**	321	C	60.5888	0
**5055**	351	C	0.0087	1
**5055**	357	C	72.1628	1
**5055**	370	C	79.576	1
**5055**	372	C	79.5971	0
**5055**	373	C	72.8838	1
**5055**	381	C	79.5627	1
**5055**	382	C	76.0971	1
**5055**	386	C	64.2654	1
**5055**	305	D	15.5248	-1
**5055**	319	D	79.5373	1
**5055**	320	D	74.1268	-1
**5055**	321	D	78.9961	0
**5055**	351	D	18.9949	1
**5055**	357	D	41.5586	0
**5055**	370	D	69.8108	1
**5055**	372	D	73.1025	0
**5055**	373	D	56.298	1
**5055**	381	D	78.615	1

**5055**	382	D	41.6603	0
**5055**	386	D	64.8385	1
**5056**	305	A	78.8265	1
**5056**	319	A	74.2249	1
**5056**	320	A	73.1627	-1
**5056**	321	A	79.5443	0
**5056**	351	A	22.863	-1
**5056**	357	A	69.4687	1
**5056**	370	A	37.8992	0
**5056**	372	A	79.5474	0
**5056**	373	A	79.6082	1
**5056**	381	A	69.9444	1
**5056**	382	A	35.2342	0
**5056**	386	A	51.4169	1
**5056**	305	B	75.9297	1
**5056**	319	B	76.6915	1
**5056**	320	B	29.4287	-1
**5056**	321	B	47.0638	1
**5056**	351	B	8.7108	1
**5056**	357	B	68.7454	1
**5056**	370	B	73.2636	1
**5056**	372	B	71.6511	0
**5056**	373	B	37.5828	0
**5056**	381	B	79.5372	1
**5056**	382	B	40.637	0
**5056**	386	B	73.4208	1
**5056**	305	C	79.5014	1
**5056**	319	C	79.4428	1
**5056**	320	C	70.0177	-1
**5056**	321	C	79.6112	0
**5056**	351	C	3.4535	1
**5056**	357	C	33.4352	0
**5056**	370	C	79.4262	1
**5056**	372	C	79.5579	0

**5056**	373	C	78.3499	1
**5056**	381	C	62.0709	1
**5056**	382	C	35.2908	0
**5056**	386	C	43.6777	0
**5056**	305	D	38.2772	0
**5056**	319	D	67.4944	1
**5056**	320	D	79.5847	-1
**5056**	321	D	79.5451	0
**5056**	351	D	10.5001	1
**5056**	357	D	73.809	1
**5056**	370	D	78.8255	1
**5056**	372	D	79.5968	0
**5056**	373	D	9.3557	-1
**5056**	381	D	63.5534	1
**5056**	382	D	63.9796	1
**5056**	386	D	45.5938	0
**5057**	305	A	34.6039	0
**5057**	319	A	79.5346	1
**5057**	320	A	79.5733	-1
**5057**	321	A	21.4068	1
**5057**	351	A	8.8716	1
**5057**	357	A	79.5431	1
**5057**	370	A	34.8953	0
**5057**	372	A	49.1109	1
**5057**	373	A	73.5664	1
**5057**	381	A	69.3549	1
**5057**	382	A	60.0937	1
**5057**	386	A	44.1964	0
**5057**	305	B	72.3546	1
**5057**	319	B	47.8903	0
**5057**	320	B	22.3648	-1
**5057**	321	B	29.1182	1
**5057**	351	B	9.7574	1
**5057**	357	B	79.556	1

**5057**	370	B	46.5887	0
**5057**	372	B	66.364	0
**5057**	373	B	53.9495	1
**5057**	381	B	44.0461	0
**5057**	382	B	9.5168	-1
**5057**	386	B	69.7806	1
**5057**	305	C	76.5162	1
**5057**	319	C	63.1713	1
**5057**	320	C	40.2784	-1
**5057**	321	C	37.9847	1
**5057**	351	C	4.3093	1
**5057**	357	C	53.7343	1
**5057**	370	C	79.5626	1
**5057**	372	C	79.5686	0
**5057**	373	C	71.6677	1
**5057**	381	C	23.4697	0
**5057**	382	C	72.3276	1
**5057**	386	C	53.1523	1
**5057**	305	D	38.2041	0
**5057**	319	D	73.2112	1
**5057**	320	D	78.2716	-1
**5057**	321	D	67.7751	0
**5057**	351	D	12.8398	1
**5057**	357	D	46.9241	0
**5057**	370	D	79.6102	1
**5057**	372	D	68.9568	0
**5057**	373	D	71.7011	1
**5057**	381	D	26.3756	0
**5057**	382	D	74.3603	1
**5057**	386	D	73.2899	1
**5058**	305	A	56.0175	1
**5058**	319	A	40.4506	0
**5058**	320	A	50.2023	-1
**5058**	321	A	79.1883	0

**5058**	351	A	0.0858	1
**5058**	357	A	77.7727	1
**5058**	370	A	79.5964	1
**5058**	372	A	64.3768	0
**5058**	373	A	75.3281	1
**5058**	381	A	79.7288	1
**5058**	382	A	79.6274	1
**5058**	386	A	79.4897	1
**5058**	305	B	79.5862	1
**5058**	319	B	76.9832	1
**5058**	320	B	79.5915	-1
**5058**	321	B	61.6399	0
**5058**	351	B	17.4779	1
**5058**	357	B	78.2973	1
**5058**	370	B	52.6319	1
**5058**	372	B	79.5714	0
**5058**	373	B	79.1282	1
**5058**	381	B	71.1444	1
**5058**	382	B	67.9075	1
**5058**	386	B	67.4938	1
**5058**	305	C	72.5074	1
**5058**	319	C	50.5177	1
**5058**	320	C	79.7038	-1
**5058**	321	C	79.5307	0
**5058**	351	C	9.775	1
**5058**	357	C	64.1325	1
**5058**	370	C	68.4082	1
**5058**	372	C	79.4729	0
**5058**	373	C	76.8748	1
**5058**	381	C	73.6339	1
**5058**	382	C	54.1345	1
**5058**	386	C	64.3356	1
**5058**	305	D	69.5177	1
**5058**	319	D	50.4131	1

**5058**	320	D	79.561	-1
**5058**	321	D	79.6487	0
**5058**	351	D	5.788	1
**5058**	357	D	60.9766	1
**5058**	370	D	13.9516	-1
**5058**	372	D	79.3735	0
**5058**	373	D	44.5313	0
**5058**	381	D	64.1873	1
**5058**	382	D	48.3143	0
**5058**	386	D	66.8193	1
**5059**	305	A	79.5812	1
**5059**	319	A	36.5623	0
**5059**	320	A	73.2877	-1
**5059**	321	A	64.8297	0
**5059**	351	A	15.0127	1
**5059**	357	A	70.1137	1
**5059**	370	A	72.888	1
**5059**	372	A	79.5062	0
**5059**	373	A	63.6708	1
**5059**	381	A	79.5224	1
**5059**	382	A	36.0777	0
**5059**	386	A	52.8021	1
**5059**	305	B	57.2007	1
**5059**	319	B	77.2573	1
**5059**	320	B	73.3803	-1
**5059**	321	B	65.1275	0
**5059**	351	B	16.4248	1
**5059**	357	B	69.6595	1
**5059**	370	B	67.3201	1
**5059**	372	B	79.6392	0
**5059**	373	B	70.461	1
**5059**	381	B	76.433	1
**5059**	382	B	25.1309	0
**5059**	386	B	79.6162	1

**5059**	305	C	79.5361	1
**5059**	319	C	55.9552	1
**5059**	320	C	79.6668	-1
**5059**	321	C	79.6159	0
**5059**	351	C	12.5735	1
**5059**	357	C	72.625	1
**5059**	370	C	79.393	1
**5059**	372	C	68.5652	0
**5059**	373	C	79.5872	1
**5059**	381	C	73.8516	1
**5059**	382	C	79.4555	1
**5059**	386	C	39.7821	0
**5059**	305	D	79.5695	1
**5059**	319	D	79.6033	1
**5059**	320	D	79.5321	-1
**5059**	321	D	70.4269	0
**5059**	351	D	8.9943	1
**5059**	357	D	62.6378	1
**5059**	370	D	58.1103	1
**5059**	372	D	79.5857	0
**5059**	373	D	67.8009	1
**5059**	381	D	61.7538	1
**5059**	382	D	78.7501	1
**5059**	386	D	78.7512	1
**5060**	305	A	6.3101	-1
**5060**	319	A	75.1283	1
**5060**	320	A	72.8374	-1
**5060**	321	A	72.876	0
**5060**	351	A	7.6219	1
**5060**	357	A	74.7657	1
**5060**	370	A	62.8015	1
**5060**	372	A	67.8542	0
**5060**	373	A	79.157	1
**5060**	381	A	74.4229	1

**5060**	382	A	36.4966	0
**5060**	386	A	71.9035	1
**5060**	305	B	73.3487	1
**5060**	319	B	69.5645	1
**5060**	320	B	52.8043	-1
**5060**	321	B	53.3085	0
**5060**	351	B	14.7339	1
**5060**	357	B	79.5651	1
**5060**	370	B	46.0221	0
**5060**	372	B	79.591	0
**5060**	373	B	52.5041	1
**5060**	381	B	34.5906	0
**5060**	382	B	51.8355	1
**5060**	386	B	34.1727	0
**5060**	305	C	75.5957	1
**5060**	319	C	56.3065	1
**5060**	320	C	73.032	-1
**5060**	321	C	60.4289	0
**5060**	351	C	2.1077	1
**5060**	357	C	58.8695	1
**5060**	370	C	66.8766	1
**5060**	372	C	79.5517	0
**5060**	373	C	79.5494	1
**5060**	381	C	52.4091	1
**5060**	382	C	53.7834	1
**5060**	386	C	25.6881	0
**5060**	305	D	57.793	1
**5060**	319	D	34.8817	0
**5060**	320	D	42.6373	-1
**5060**	321	D	36.0272	1
**5060**	351	D	12.981	1
**5060**	357	D	51.9711	1
**5060**	370	D	31.2297	0
**5060**	372	D	79.1404	0

**5060**	373	D	77.4688	1
**5060**	381	D	73.9176	1
**5060**	382	D	59.83	1
**5060**	386	D	79.5724	1
**5061**	305	A	63.0526	1
**5061**	319	A	58.9629	1
**5061**	320	A	49.2967	-1
**5061**	321	A	74.9308	0
**5061**	351	A	11.0985	1
**5061**	357	A	79.5156	1
**5061**	370	A	62.9127	1
**5061**	372	A	57.2921	0
**5061**	373	A	70.1403	1
**5061**	381	A	64.8681	1
**5061**	382	A	73.6312	1
**5061**	386	A	65.7034	1
**5061**	305	B	78.6227	1
**5061**	319	B	72.8868	1
**5061**	320	B	59.7596	-1
**5061**	321	B	69.0512	0
**5061**	351	B	13.7906	1
**5061**	357	B	79.6256	1
**5061**	370	B	79.0685	1
**5061**	372	B	68.0221	0
**5061**	373	B	39.2681	0
**5061**	381	B	64.1079	1
**5061**	382	B	54.5102	1
**5061**	386	B	78.683	1
**5061**	305	C	71.3697	1
**5061**	319	C	79.5842	1
**5061**	320	C	15.329	1
**5061**	321	C	79.4812	0
**5061**	351	C	18.8747	1
**5061**	357	C	71.6764	1

**5061**	370	C	68.8923	1
**5061**	372	C	57.3738	0
**5061**	373	C	76.3227	1
**5061**	381	C	79.4734	1
**5061**	382	C	77.8018	1
**5061**	386	C	48.8587	0
**5061**	305	D	46.417	0
**5061**	319	D	79.4262	1
**5061**	320	D	59.2637	-1
**5061**	321	D	79.4999	0
**5061**	351	D	12.6273	1
**5061**	357	D	75.8088	1
**5061**	370	D	57.276	1
**5061**	372	D	61.5855	0
**5061**	373	D	73.8387	1
**5061**	381	D	73.1192	1
**5061**	382	D	79.4373	1
**5061**	386	D	60.8638	1
**5062**	305	A	79.5335	1
**5062**	319	A	74.8243	1
**5062**	320	A	79.5888	-1
**5062**	321	A	71.1949	0
**5062**	351	A	8.5727	1
**5062**	357	A	78.0589	1
**5062**	370	A	33.6653	0
**5062**	372	A	61.9791	0
**5062**	373	A	73.1494	1
**5062**	381	A	79.5505	1
**5062**	382	A	51.6826	1
**5062**	386	A	60.494	1
**5062**	305	B	78.362	1
**5062**	319	B	79.6686	1
**5062**	320	B	60.3782	-1
**5062**	321	B	79.3954	0

**5062**	351	B	542	1
**5062**	357	B	79.5557	1
**5062**	370	B	79.5702	1
**5062**	372	B	57.3974	0
**5062**	373	B	45.3141	0
**5062**	381	B	72.3714	1
**5062**	382	B	51.7703	1
**5062**	386	B	68.2401	1
**5062**	305	C	46.1663	0
**5062**	319	C	79.2291	1
**5062**	320	C	79.563	-1
**5062**	321	C	65.9045	0
**5062**	351	C	14.0533	1
**5062**	357	C	72.5928	1
**5062**	370	C	68.3075	1
**5062**	372	C	79.0653	0
**5062**	373	C	79.5829	1
**5062**	381	C	72.2099	1
**5062**	382	C	75.897	1
**5062**	386	C	24.259	0
**5062**	305	D	5.9769	-1
**5062**	319	D	72.5252	1
**5062**	320	D	79.5968	-1
**5062**	321	D	72.7419	0
**5062**	351	D	11.6944	1
**5062**	357	D	60.1246	1
**5062**	370	D	35.9915	0
**5062**	372	D	79.5335	0
**5062**	373	D	52.7224	1
**5062**	381	D	72.842	1
**5062**	382	D	28.3091	0
**5062**	386	D	65.9545	1
**5063**	305	A	63.2532	1
**5063**	319	A	64.7479	1

**5063**	320	A	79.5344	-1
**5063**	321	A	74.0546	0
**5063**	351	A	29.5591	-1
**5063**	357	A	79.4599	1
**5063**	370	A	71.7685	1
**5063**	372	A	15.5034	0
**5063**	373	A	79.5914	1
**5063**	381	A	71.9025	1
**5063**	382	A	79.4985	1
**5063**	386	A	73.2788	1
**5063**	305	B	79.5872	1
**5063**	319	B	79.7065	1
**5063**	320	B	58.2592	-1
**5063**	321	B	74.2383	0
**5063**	351	B	16.8319	1
**5063**	357	B	71.6628	1
**5063**	370	B	68.0269	1
**5063**	372	B	65.1057	0
**5063**	373	B	70.229	1
**5063**	381	B	52.1496	1
**5063**	382	B	76.8669	1
**5063**	386	B	65.5054	1
**5063**	305	C	79.6127	1
**5063**	319	C	66.6618	1
**5063**	320	C	79.5873	-1
**5063**	321	C	55.7033	0
**5063**	351	C	7.1776	1
**5063**	357	C	79.5302	1
**5063**	370	C	78.2511	1
**5063**	372	C	75.2884	0
**5063**	373	C	79.5841	1
**5063**	381	C	59.807	1
**5063**	382	C	68.4622	1
**5063**	386	C	29.0115	0

**5063**	305	D	70.229	1
**5063**	319	D	79.4998	1
**5063**	320	D	79.5422	-1
**5063**	321	D	60.7142	0
**5063**	351	D	16.9121	1
**5063**	357	D	79.561	1
**5063**	370	D	79.6956	1
**5063**	372	D	79.6381	0
**5063**	373	D	71.5208	1
**5063**	381	D	58.8872	1
**5063**	382	D	60.7287	1
**5063**	386	D	79.6143	1
**5064**	305	A	61.7465	1
**5064**	319	A	65.5916	1
**5064**	320	A	79.5242	-1
**5064**	321	A	78.9776	0
**5064**	351	A	5.8686	1
**5064**	357	A	71.8186	1
**5064**	370	A	76.7753	1
**5064**	372	A	78.2569	0
**5064**	373	A	73.7891	1
**5064**	381	A	65.4373	1
**5064**	382	A	64.1601	1
**5064**	386	A	72.9172	1
**5064**	305	B	90.4407	1
**5064**	319	B	70.5189	1
**5064**	320	B	74.8345	-1
**5064**	321	B	75.1089	0
**5064**	351	B	19.1815	1
**5064**	357	B	76.3129	1
**5064**	370	B	73.734	1
**5064**	372	B	79.6177	0
**5064**	373	B	79.2974	1
**5064**	381	B	24.856	0

**5064**	382	B	68.7142	1
**5064**	386	B	79.5441	1
**5064**	305	C	79.2431	1
**5064**	319	C	79.0933	1
**5064**	320	C	72.1715	-1
**5064**	321	C	75.5615	0
**5064**	351	C	32.8668	-1
**5064**	357	C	72.5906	1
**5064**	370	C	73.7611	1
**5064**	372	C	76.6165	0
**5064**	373	C	70.7224	1
**5064**	381	C	55.9768	1
**5064**	382	C	76.5174	1
**5064**	386	C	64.2403	1
**5064**	305	D	58.9646	1
**5064**	319	D	73.2774	1
**5064**	320	D	79.3795	-1
**5064**	321	D	66.3494	0
**5064**	351	D	20.8527	-1
**5064**	357	D	51.2227	1
**5064**	370	D	76.9393	1
**5064**	372	D	72.2138	0
**5064**	373	D	63.7944	1
**5064**	381	D	51.6916	1
**5064**	382	D	76.345	1
**5064**	386	D	31.5349	0
**5065**	305	A	71.1782	1
**5065**	319	A	69.0079	1
**5065**	320	A	79.5562	-1
**5065**	321	A	78.9371	0
**5065**	351	A	5.6455	1
**5065**	357	A	79.5304	1
**5065**	370	A	35.8526	0
**5065**	372	A	79.5877	0

**5065**	373	A	79.4487	1
**5065**	381	A	79.5928	1
**5065**	382	A	73.8055	1
**5065**	386	A	79.5319	1
**5065**	305	B	72.0727	1
**5065**	319	B	39.3731	0
**5065**	320	B	37.6029	-1
**5065**	321	B	57.6891	0
**5065**	351	B	17.2492	1
**5065**	357	B	70.9444	1
**5065**	370	B	69.3592	1
**5065**	372	B	72.5083	0
**5065**	373	B	37.3192	0
**5065**	381	B	74.0724	1
**5065**	382	B	45.01	0
**5065**	386	B	64.2935	1
**5065**	305	C	79.3713	1
**5065**	319	C	70.1279	1
**5065**	320	C	60.9621	-1
**5065**	321	C	61.7242	0
**5065**	351	C	6.3942	1
**5065**	357	C	79.5725	1
**5065**	370	C	73.3551	1
**5065**	372	C	75.9797	0
**5065**	373	C	76.1198	1
**5065**	381	C	73.8863	1
**5065**	382	C	71.6893	1
**5065**	386	C	43.5683	0
**5065**	305	D	71.3421	1
**5065**	319	D	76.9306	1
**5065**	320	D	75.5405	-1
**5065**	321	D	79.5805	0
**5065**	351	D	6.4691	1
**5065**	357	D	79.5429	1

**5065**	370	D	68.2177	1
**5065**	372	D	79.5983	0
**5065**	373	D	72.3636	1
**5065**	381	D	64.034	1
**5065**	382	D	79.5377	1
**5065**	386	D	55.3939	1
**5066**	305	A	60.2202	1
**5066**	319	A	69.0445	1
**5066**	320	A	71.3113	-1
**5066**	321	A	33.1534	1
**5066**	351	A	4.7422	1
**5066**	357	A	65.1384	1
**5066**	370	A	79.5732	1
**5066**	372	A	79.6962	0
**5066**	373	A	28.2943	0
**5066**	381	A	79.6203	1
**5066**	382	A	38.3443	0
**5066**	386	A	76.8136	1
**5066**	305	B	62.5428	1
**5066**	319	B	79.5932	1
**5066**	320	B	70.4121	-1
**5066**	321	B	72.5448	0
**5066**	351	B	14.5745	1
**5066**	357	B	42.1513	0
**5066**	370	B	70.6683	1
**5066**	372	B	79.5786	0
**5066**	373	B	65.8413	1
**5066**	381	B	79.7105	1
**5066**	382	B	50.2253	1
**5066**	386	B	44.8847	0
**5066**	305	C	62.5076	1
**5066**	319	C	79.4324	1
**5066**	320	C	73.0105	-1
**5066**	321	C	71.3703	0

**5066**	351	C	1.4317	1
**5066**	357	C	62.4943	1
**5066**	370	C	79.6712	1
**5066**	372	C	79.6179	0
**5066**	373	C	73.3637	1
**5066**	381	C	79.4768	1
**5066**	382	C	62.8095	1
**5066**	386	C	36.1259	0
**5066**	305	D	79.5299	1
**5066**	319	D	45.746	0
**5066**	320	D	79.5257	-1
**5066**	321	D	79.5287	0
**5066**	351	D	17.6847	1
**5066**	357	D	79.5082	1
**5066**	370	D	51.8689	1
**5066**	372	D	79.6136	0
**5066**	373	D	44.5889	0
**5066**	381	D	61.7329	1
**5066**	382	D	79.6643	1
**5066**	386	D	43.9416	0
**5067**	305	A	52.1373	1
**5067**	319	A	65.868	1
**5067**	320	A	79.4892	-1
**5067**	321	A	68.0018	0
**5067**	351	A	6.7069	1
**5067**	357	A	70.2919	1
**5067**	370	A	14.1368	-1
**5067**	372	A	61.3376	0
**5067**	373	A	72.1872	1
**5067**	381	A	57.6192	1
**5067**	382	A	79.4476	1
**5067**	386	A	73.355	1
**5067**	305	B	65.2099	1
**5067**	319	B	79.6846	1

**5067**	320	B	68.1531	-1
**5067**	321	B	72.0935	0
**5067**	351	B	25.891	-1
**5067**	357	B	37.674	0
**5067**	370	B	79.5749	1
**5067**	372	B	72.0565	0
**5067**	373	B	66.8476	1
**5067**	381	B	79.5009	1
**5067**	382	B	36.6276	0
**5067**	386	B	66.4925	1
**5067**	305	C	79.6047	1
**5067**	319	C	79.5377	1
**5067**	320	C	59.0814	-1
**5067**	321	C	79.6645	0
**5067**	351	C	4.8104	1
**5067**	357	C	69.9969	1
**5067**	370	C	78.8763	1
**5067**	372	C	63.9942	0
**5067**	373	C	79.5442	1
**5067**	381	C	35.0756	0
**5067**	382	C	66.5338	1
**5067**	386	C	46.4144	0
**5067**	305	D	33.4998	0
**5067**	319	D	27.5733	0
**5067**	320	D	60.3597	-1
**5067**	321	D	45.1725	1
**5067**	351	D	18.7963	1
**5067**	357	D	72.0276	1
**5067**	370	D	73.7141	1
**5067**	372	D	73.2725	0
**5067**	373	D	51.9146	1
**5067**	381	D	79.5998	1
**5067**	382	D	79.5791	1
**5067**	386	D	79.615	1

**5068**	305	A	55.7412	1
**5068**	319	A	59.6775	1
**5068**	320	A	69.0744	-1
**5068**	321	A	79.5304	0
**5068**	351	A	6.3439	1
**5068**	357	A	74.4312	1
**5068**	370	A	75.8672	1
**5068**	372	A	79.5294	0
**5068**	373	A	63.105	1
**5068**	381	A	79.5092	1
**5068**	382	A	79.6533	1
**5068**	386	A	55.7076	1
**5068**	305	B	76.5083	1
**5068**	319	B	71.694	1
**5068**	320	B	61.6963	-1
**5068**	321	B	79.499	0
**5068**	351	B	12.9824	1
**5068**	357	B	62.946	1
**5068**	370	B	38.0534	0
**5068**	372	B	62.6665	0
**5068**	373	B	59.4794	1
**5068**	381	B	74.5151	1
**5068**	382	B	52.8646	1
**5068**	386	B	44.5022	0
**5068**	305	C	64.464	1
**5068**	319	C	79.6029	1
**5068**	320	C	75.1075	-1
**5068**	321	C	74.2784	0
**5068**	351	C	5.1441	1
**5068**	357	C	50.101	1
**5068**	370	C	50.8099	1
**5068**	372	C	70.0737	0
**5068**	373	C	72.2307	1
**5068**	381	C	79.6994	1

**5068**	382	C	73.2161	1
**5068**	386	C	60.0526	1
**5068**	305	D	67.8721	1
**5068**	319	D	48.5823	0
**5068**	320	D	73.1163	-1
**5068**	321	D	61.4075	0
**5068**	351	D	12.0617	1
**5068**	357	D	73.283	1
**5068**	370	D	79.3193	1
**5068**	372	D	72.5047	0
**5068**	373	D	69.0993	1
**5068**	381	D	60.3067	1
**5068**	382	D	66.5506	1
**5068**	386	D	75.0909	1
**5069**	305	A	39.0168	0
**5069**	319	A	56.2095	1
**5069**	320	A	79.6342	-1
**5069**	321	A	76.1026	0
**5069**	351	A	11.3541	1
**5069**	357	A	73.9696	1
**5069**	370	A	79.5054	1
**5069**	372	A	62.1638	0
**5069**	373	A	77.7234	1
**5069**	381	A	69.069	1
**5069**	382	A	75.5667	1
**5069**	386	A	79.5705	1
**5069**	305	B	79.5268	1
**5069**	319	B	74.0092	1
**5069**	320	B	72.0082	-1
**5069**	321	B	64.1174	0
**5069**	351	B	11.9531	1
**5069**	357	B	50.161	1
**5069**	370	B	79.5061	1
**5069**	372	B	79.5366	0

**5069**	373	B	68.3624	1
**5069**	381	B	79.5416	1
**5069**	382	B	79.0818	1
**5069**	386	B	79.0084	1
**5069**	305	C	79.5617	1
**5069**	319	C	68.6475	1
**5069**	320	C	79.546	-1
**5069**	321	C	72.7293	0
**5069**	351	C	9.5065	1
**5069**	357	C	79.6339	1
**5069**	370	C	67.815	1
**5069**	372	C	72.4979	0
**5069**	373	C	58.1937	1
**5069**	381	C	79.5298	1
**5069**	382	C	76.3182	1
**5069**	386	C	40.6012	0
**5069**	305	D	51.5022	1
**5069**	319	D	75.0318	1
**5069**	320	D	61.5738	-1
**5069**	321	D	79.5272	0
**5069**	351	D	18.3338	1
**5069**	357	D	73.1798	1
**5069**	370	D	74.3629	1
**5069**	372	D	77.0094	0
**5069**	373	D	62.4479	1
**5069**	381	D	78.8473	1
**5069**	382	D	45.4768	0
**5069**	386	D	65.3071	1
**5070**	305	A	65.9595	1
**5070**	319	A	54.1302	1
**5070**	320	A	69.5289	-1
**5070**	321	A	76.95	0
**5070**	351	A	10.7101	1
**5070**	357	A	69.6561	1

**5070**	370	A	64.5585	1
**5070**	372	A	64.1189	0
**5070**	373	A	61.1872	1
**5070**	381	A	79.5924	1
**5070**	382	A	70.2245	1
**5070**	386	A	52.6585	1
**5070**	305	B	79.5091	1
**5070**	319	B	67.7449	1
**5070**	320	B	38.2717	-1
**5070**	321	B	40.8811	1
**5070**	351	B	19.684	1
**5070**	357	B	34.2282	0
**5070**	370	B	79.601	1
**5070**	372	B	79.6086	0
**5070**	373	B	51.2757	1
**5070**	381	B	39.8021	0
**5070**	382	B	44.846	0
**5070**	386	B	63.2815	1
**5070**	305	C	46.4937	0
**5070**	319	C	79.5713	1
**5070**	320	C	72.2848	-1
**5070**	321	C	79.6175	0
**5070**	351	C	13.0224	1
**5070**	357	C	78.0682	1
**5070**	370	C	77.2149	1
**5070**	372	C	79.5854	0
**5070**	373	C	79.5368	1
**5070**	381	C	79.6558	1
**5070**	382	C	60.9517	1
**5070**	386	C	55.9296	1
**5070**	305	D	47.913	0
**5070**	319	D	44.0803	0
**5070**	320	D	72.1924	-1
**5070**	321	D	79.6225	0

**5070**	351	D	1.9413	1
**5070**	357	D	74.0919	1
**5070**	370	D	72.6892	1
**5070**	372	D	79.6045	0
**5070**	373	D	79.4904	1
**5070**	381	D	66.4631	1
**5070**	382	D	72.669	1
**5070**	386	D	77.8684	1
**5071**	305	A	54.7978	1
**5071**	319	A	54.1678	1
**5071**	320	A	50.4996	-1
**5071**	321	A	74.2589	0
**5071**	351	A	9.0297	1
**5071**	357	A	47.4466	0
**5071**	370	A	79.6187	1
**5071**	372	A	77.581	0
**5071**	373	A	74.2767	1
**5071**	381	A	17.3402	-1
**5071**	382	A	69.8307	1
**5071**	386	A	34.5571	0
**5071**	305	B	79.5766	1
**5071**	319	B	73.7883	1
**5071**	320	B	78.8768	-1
**5071**	321	B	79.4618	0
**5071**	351	B	14.9377	1
**5071**	357	B	49.8651	0
**5071**	370	B	79.5238	1
**5071**	372	B	79.5992	0
**5071**	373	B	57.3383	1
**5071**	381	B	77.8252	1
**5071**	382	B	57.9972	1
**5071**	386	B	71.0721	1
**5071**	305	C	73.0814	1
**5071**	319	C	31.891	0

**5071**	320	C	20.1457	-1
**5071**	321	C	60.5162	0
**5071**	351	C	9.8866	1
**5071**	357	C	73.0162	1
**5071**	370	C	12.2831	-1
**5071**	372	C	71.4794	0
**5071**	373	C	73.2764	1
**5071**	381	C	79.6535	1
**5071**	382	C	79.63	1
**5071**	386	C	65.4152	1
**5071**	305	D	53.0555	1
**5071**	319	D	28.1728	0
**5071**	320	D	46.6961	-1
**5071**	321	D	73.1278	0
**5071**	351	D	14.9802	1
**5071**	357	D	75.4132	1
**5071**	370	D	77.1626	1
**5071**	372	D	67.0441	0
**5071**	373	D	50.0932	1
**5071**	381	D	79.4865	1
**5071**	382	D	79.6832	1
**5071**	386	D	78.5497	1
**5072**	305	A	75.5913	1
**5072**	319	A	12.3478	-1
**5072**	320	A	79.6441	-1
**5072**	321	A	64.6943	0
**5072**	351	A	1.9118	1
**5072**	357	A	72.5674	1
**5072**	370	A	66.0941	1
**5072**	372	A	79.6157	0
**5072**	373	A	71.8187	1
**5072**	381	A	68.4477	1
**5072**	382	A	64.6551	1
**5072**	386	A	76.0744	1

**5072**	305	B	74.4433	1
**5072**	319	B	79.3143	1
**5072**	320	B	79.4986	-1
**5072**	321	B	66.0411	0
**5072**	351	B	17.9657	1
**5072**	357	B	64.1226	1
**5072**	370	B	79.5473	1
**5072**	372	B	70.3538	0
**5072**	373	B	60.8473	1
**5072**	381	B	79.4605	1
**5072**	382	B	77.311	1
**5072**	386	B	79.6748	1
**5072**	305	C	71.4672	1
**5072**	319	C	49.736	0
**5072**	320	C	48.8592	-1
**5072**	321	C	67.5778	0
**5072**	351	C	1.0864	1
**5072**	357	C	79.4699	1
**5072**	370	C	65.5949	1
**5072**	372	C	47.9731	1
**5072**	373	C	79.6755	1
**5072**	381	C	48.1144	0
**5072**	382	C	72.5783	1
**5072**	386	C	51.3769	1
**5072**	305	D	79.514	1
**5072**	319	D	74.7495	1
**5072**	320	D	74.4585	-1
**5072**	321	D	79.6788	0
**5072**	351	D	7.8002	1
**5072**	357	D	47.4511	0
**5072**	370	D	79.5763	1
**5072**	372	D	79.6463	0
**5072**	373	D	69.6025	1
**5072**	381	D	79.6085	1

**5072**	382	D	61.304	1
**5072**	386	D	52.5349	1
**5073**	305	A	70.5823	1
**5073**	319	A	28.8571	0
**5073**	320	A	79.5335	-1
**5073**	321	A	50.2488	0
**5073**	351	A	1.2431	1
**5073**	357	A	69.155	1
**5073**	370	A	51.8507	1
**5073**	372	A	79.6231	0
**5073**	373	A	79.5246	1
**5073**	381	A	79.6498	1
**5073**	382	A	75.2851	1
**5073**	386	A	52.8835	1
**5073**	305	B	73.7877	1
**5073**	319	B	79.4541	1
**5073**	320	B	79.5354	-1
**5073**	321	B	79.5089	0
**5073**	351	B	20.7319	-1
**5073**	357	B	39.8616	0
**5073**	370	B	79.571	1
**5073**	372	B	70.9694	0
**5073**	373	B	70.177	1
**5073**	381	B	79.5239	1
**5073**	382	B	75.5185	1
**5073**	386	B	79.5989	1
**5073**	305	C	77.6742	1
**5073**	319	C	58.4232	1
**5073**	320	C	72.4106	-1
**5073**	321	C	78.7995	0
**5073**	351	C	1.4808	1
**5073**	357	C	79.1166	1
**5073**	370	C	41.4896	0
**5073**	372	C	72.1746	0

**5073**	373	C	56.6417	1
**5073**	381	C	79.4732	1
**5073**	382	C	56.5525	1
**5073**	386	C	24.6161	0
**5073**	305	D	61.4423	1
**5073**	319	D	79.5811	1
**5073**	320	D	79.5586	-1
**5073**	321	D	57.1441	0
**5073**	351	D	16.4859	1
**5073**	357	D	72.5887	1
**5073**	370	D	61.2654	1
**5073**	372	D	79.5567	0
**5073**	373	D	79.6282	1
**5073**	381	D	71.5393	1
**5073**	382	D	58.9446	1
**5073**	386	D	71.1169	1
**5074**	305	A	79.6373	1
**5074**	319	A	52.6545	1
**5074**	320	A	79.533	-1
**5074**	321	A	73.4353	0
**5074**	351	A	5.8759	1
**5074**	357	A	79.5523	1
**5074**	370	A	64.9355	1
**5074**	372	A	47.1368	1
**5074**	373	A	41.8542	0
**5074**	381	A	34.2092	0
**5074**	382	A	69.2317	1
**5074**	386	A	68.9565	1
**5074**	305	B	74.0371	1
**5074**	319	B	79.599	1
**5074**	320	B	73.3399	-1
**5074**	321	B	74.9655	0
**5074**	351	B	15.5284	1
**5074**	357	B	58.4504	1

**5074**	370	B	77.8604	1
**5074**	372	B	73.1708	0
**5074**	373	B	79.5332	1
**5074**	381	B	79.5514	1
**5074**	382	B	40.6526	0
**5074**	386	B	52.1178	1
**5074**	305	C	40.737	0
**5074**	319	C	48.0915	0
**5074**	320	C	79.3605	-1
**5074**	321	C	49.9161	1
**5074**	351	C	7.2791	1
**5074**	357	C	72.6621	1
**5074**	370	C	79.5246	1
**5074**	372	C	79.5633	0
**5074**	373	C	65.2088	1
**5074**	381	C	64.5668	1
**5074**	382	C	39.9197	0
**5074**	386	C	64.8482	1
**5074**	305	D	79.588	1
**5074**	319	D	63.9481	1
**5074**	320	D	77.2871	-1
**5074**	321	D	79.5606	0
**5074**	351	D	7.2505	1
**5074**	357	D	44.9861	0
**5074**	370	D	79.4465	1
**5074**	372	D	58.2353	0
**5074**	373	D	52.8739	1
**5074**	381	D	79.5442	1
**5074**	382	D	55.9085	1
**5074**	386	D	33.8741	0
**5075**	305	A	73.4727	1
**5075**	319	A	65.3558	1
**5075**	320	A	74.0709	-1
**5075**	321	A	64.8479	0

**5075**	351	A	9.3316	1
**5075**	357	A	60.0042	1
**5075**	370	A	61.2689	1
**5075**	372	A	60.0855	0
**5075**	373	A	47.8253	0
**5075**	381	A	72.2748	1
**5075**	382	A	79.5989	1
**5075**	386	A	73.223	1
**5075**	305	B	63.3439	1
**5075**	319	B	39.3215	0
**5075**	320	B	59.8083	-1
**5075**	321	B	49.775	1
**5075**	351	B	2.1658	1
**5075**	357	B	36.3914	0
**5075**	370	B	79.5319	1
**5075**	372	B	42.8179	1
**5075**	373	B	68.0811	1
**5075**	381	B	73.6267	1
**5075**	382	B	79.6604	1
**5075**	386	B	37.775	0
**5075**	305	C	60.6291	1
**5075**	319	C	70.9941	1
**5075**	320	C	72.5121	-1
**5075**	321	C	79.6772	0
**5075**	351	C	3.7385	1
**5075**	357	C	73.3229	1
**5075**	370	C	64.6788	1
**5075**	372	C	56.0506	0
**5075**	373	C	79.4966	1
**5075**	381	C	79.4508	1
**5075**	382	C	59.2478	1
**5075**	386	C	28.2502	0
**5075**	305	D	61.0816	1
**5075**	319	D	70.1848	1

**5075**	320	D	79.5004	-1
**5075**	321	D	79.6534	0
**5075**	351	D	32.6566	-1
**5075**	357	D	63.9862	1
**5075**	370	D	72.2458	1
**5075**	372	D	64.0158	0
**5075**	373	D	79.5846	1
**5075**	381	D	71.0811	1
**5075**	382	D	53.173	1
**5075**	386	D	79.0537	1
**5076**	305	A	64.398	1
**5076**	319	A	37.7219	0
**5076**	320	A	64.5483	-1
**5076**	321	A	46.8233	1
**5076**	351	A	10.6847	1
**5076**	357	A	75.1278	1
**5076**	370	A	42.4402	0
**5076**	372	A	71.0703	0
**5076**	373	A	79.5924	1
**5076**	381	A	26.7255	0
**5076**	382	A	79.5913	1
**5076**	386	A	72.3148	1
**5076**	305	B	79.5431	1
**5076**	319	B	79.5528	1
**5076**	320	B	75.2925	-1
**5076**	321	B	68.3171	0
**5076**	351	B	14.0515	1
**5076**	357	B	54.5119	1
**5076**	370	B	57.9999	1
**5076**	372	B	79.5008	0
**5076**	373	B	36.9955	0
**5076**	381	B	27.0615	0
**5076**	382	B	52.1308	1
**5076**	386	B	79.5493	1

**5076**	305	C	79.618	1
**5076**	319	C	68.775	1
**5076**	320	C	71.9817	-1
**5076**	321	C	69.8702	0
**5076**	351	C	17.9348	1
**5076**	357	C	73.7567	1
**5076**	370	C	79.0851	1
**5076**	372	C	79.4766	0
**5076**	373	C	79.6085	1
**5076**	381	C	75.5736	1
**5076**	382	C	47.3159	0
**5076**	386	C	48.2558	0
**5076**	305	D	43.4164	0
**5076**	319	D	79.5426	1
**5076**	320	D	79.6123	-1
**5076**	321	D	74.025	0
**5076**	351	D	7.6148	1
**5076**	357	D	77.2676	1
**5076**	370	D	67.8766	1
**5076**	372	D	61.909	0
**5076**	373	D	79.5705	1
**5076**	381	D	78.8383	1
**5076**	382	D	73.5054	1
**5076**	386	D	79.5696	1
**5077**	305	A	73.7217	1
**5077**	319	A	8.0773	-1
**5077**	320	A	74.4295	-1
**5077**	321	A	79.5554	0
**5077**	351	A	3.9251	1
**5077**	357	A	53.4534	1
**5077**	370	A	78.2773	1
**5077**	372	A	63.6272	0
**5077**	373	A	25.2503	0
**5077**	381	A	58.2479	1

**5077**	382	A	40.4054	0
**5077**	386	A	33.7218	0
**5077**	305	B	61.1957	1
**5077**	319	B	74.8828	1
**5077**	320	B	74.3806	-1
**5077**	321	B	77.0382	0
**5077**	351	B	7.1946	1
**5077**	357	B	72.5777	1
**5077**	370	B	72.5772	1
**5077**	372	B	79.6243	0
**5077**	373	B	73.1033	1
**5077**	381	B	74.2465	1
**5077**	382	B	37.964	0
**5077**	386	B	79.6061	1
**5077**	305	C	66.0909	1
**5077**	319	C	79.6492	1
**5077**	320	C	72.1859	-1
**5077**	321	C	69.4753	0
**5077**	351	C	6.1697	1
**5077**	357	C	74.6587	1
**5077**	370	C	69.6868	1
**5077**	372	C	71.0165	0
**5077**	373	C	38.7642	0
**5077**	381	C	51.7488	1
**5077**	382	C	51.8899	1
**5077**	386	C	48.5872	0
**5077**	305	D	69.9701	1
**5077**	319	D	27.5384	0
**5077**	320	D	79.5583	-1
**5077**	321	D	73.988	0
**5077**	351	D	8.6637	1
**5077**	357	D	79.5289	1
**5077**	370	D	72.9624	1
**5077**	372	D	72.2466	0

**5077**	373	D	79.5236	1
**5077**	381	D	62.1338	1
**5077**	382	D	66.5172	1
**5077**	386	D	23.8437	0
**5078**	305	A	59.0619	1
**5078**	319	A	77.5658	1
**5078**	320	A	66.9872	-1
**5078**	321	A	56.2354	0
**5078**	351	A	13.7645	1
**5078**	357	A	72.8486	1
**5078**	370	A	72.8861	1
**5078**	372	A	71.341	0
**5078**	373	A	60.2525	1
**5078**	381	A	79.5709	1
**5078**	382	A	70.5565	1
**5078**	386	A	52.5288	1
**5078**	305	B	72.2826	1
**5078**	319	B	79.5435	1
**5078**	320	B	59.3037	-1
**5078**	321	B	79.5645	0
**5078**	351	B	21.2809	-1
**5078**	357	B	47.1757	0
**5078**	370	B	79.5396	1
**5078**	372	B	72.9979	0
**5078**	373	B	67.9851	1
**5078**	381	B	79.5887	1
**5078**	382	B	79.6555	1
**5078**	386	B	64.4776	1
**5078**	305	C	26.8377	0
**5078**	319	C	72.7653	1
**5078**	320	C	23.2925	-1
**5078**	321	C	40.1939	1
**5078**	351	C	4.9703	1
**5078**	357	C	59.3056	1

**5078**	370	C	75.6697	1
**5078**	372	C	79.4963	0
**5078**	373	C	66.2771	1
**5078**	381	C	79.5664	1
**5078**	382	C	79.5542	1
**5078**	386	C	60.5253	1
**5078**	305	D	79.5732	1
**5078**	319	D	79.5755	1
**5078**	320	D	72.52	-1
**5078**	321	D	47.7386	1
**5078**	351	D	10.6087	1
**5078**	357	D	42.6613	0
**5078**	370	D	65.4304	1
**5078**	372	D	79.6157	0
**5078**	373	D	25.14	0
**5078**	381	D	79.5485	1
**5078**	382	D	77.9168	1
**5078**	386	D	79.5629	1
**5079**	305	A	67.9862	1
**5079**	319	A	59.2232	1
**5079**	320	A	70.3294	-1
**5079**	321	A	70.5561	0
**5079**	351	A	11.0481	1
**5079**	357	A	76.8244	1
**5079**	370	A	73.3464	1
**5079**	372	A	64.8588	0
**5079**	373	A	40.2868	0
**5079**	381	A	56.0665	1
**5079**	382	A	77.6722	1
**5079**	386	A	69.4678	1
**5079**	305	B	79.589	1
**5079**	319	B	64.0165	1
**5079**	320	B	79.4499	-1
**5079**	321	B	72.1863	0

**5079**	351	B	5.1076	1
**5079**	357	B	79.5608	1
**5079**	370	B	67.8674	1
**5079**	372	B	72.3335	0
**5079**	373	B	79.6042	1
**5079**	381	B	73.9149	1
**5079**	382	B	56.3754	1
**5079**	386	B	35.1588	0
**5079**	305	C	57.2433	1
**5079**	319	C	79.5848	1
**5079**	320	C	73.8288	-1
**5079**	321	C	79.551	0
**5079**	351	C	2.1013	1
**5079**	357	C	62.2148	1
**5079**	370	C	79.5152	1
**5079**	372	C	52.778	0
**5079**	373	C	30.3532	0
**5079**	381	C	72.0692	1
**5079**	382	C	41.2434	0
**5079**	386	C	23.1794	0
**5079**	305	D	51.2494	1
**5079**	319	D	79.4054	1
**5079**	320	D	79.5895	-1
**5079**	321	D	79.4052	0
**5079**	351	D	11.4621	1
**5079**	357	D	73.8902	1
**5079**	370	D	79.6005	1
**5079**	372	D	50.6418	0
**5079**	373	D	79.4964	1
**5079**	381	D	79.4041	1
**5079**	382	D	79.6573	1
**5079**	386	D	79.6775	1
**5080**	305	A	29.8486	0
**5080**	319	A	72.5695	1

**5080**	320	A	79.5722	-1
**5080**	321	A	62.377	0
**5080**	351	A	5.8114	1
**5080**	357	A	66.5777	1
**5080**	370	A	6.7231	-1
**5080**	372	A	64.0452	0
**5080**	373	A	79.5768	1
**5080**	381	A	79.5469	1
**5080**	382	A	57.8003	1
**5080**	386	A	43.5116	0
**5080**	305	B	51.9148	1
**5080**	319	B	49.501	0
**5080**	320	B	43.1365	-1
**5080**	321	B	79.7239	0
**5080**	351	B	14.9421	1
**5080**	357	B	78.8304	1
**5080**	370	B	76.7708	1
**5080**	372	B	73.1911	0
**5080**	373	B	40.7922	0
**5080**	381	B	79.5985	1
**5080**	382	B	33.533	0
**5080**	386	B	43.5194	0
**5080**	305	C	60.6169	1
**5080**	319	C	76.5074	1
**5080**	320	C	72.8231	-1
**5080**	321	C	79.5839	0
**5080**	351	C	6.6017	1
**5080**	357	C	73.6674	1
**5080**	370	C	79.5819	1
**5080**	372	C	47.2925	1
**5080**	373	C	79.0658	1
**5080**	381	C	64.9672	1
**5080**	382	C	79.5227	1
**5080**	386	C	53.9984	1

**5080**	305	D	53.4318	1
**5080**	319	D	35.5976	0
**5080**	320	D	55.4472	-1
**5080**	321	D	61.5008	0
**5080**	351	D	14.159	1
**5080**	357	D	72.5648	1
**5080**	370	D	79.3011	1
**5080**	372	D	78.585	0
**5080**	373	D	65.6674	1
**5080**	381	D	61.9737	1
**5080**	382	D	79.5576	1
**5080**	386	D	79.5502	1
**5081**	305	A	33.8274	0
**5081**	319	A	61.1348	1
**5081**	320	A	69.4038	-1
**5081**	321	A	64.8379	0
**5081**	351	A	16.8493	1
**5081**	357	A	67.6154	1
**5081**	370	A	70.2869	1
**5081**	372	A	42.1642	1
**5081**	373	A	79.5786	1
**5081**	381	A	68.6759	1
**5081**	382	A	24.7753	0
**5081**	386	A	74.8512	1
**5081**	305	B	72.024	1
**5081**	319	B	70.918	1
**5081**	320	B	65.0319	-1
**5081**	321	B	79.5896	0
**5081**	351	B	15.4489	1
**5081**	357	B	79.5391	1
**5081**	370	B	48.3535	0
**5081**	372	B	70.668	0
**5081**	373	B	59.2607	1
**5081**	381	B	78.3732	1

**5081**	382	B	23.9227	0
**5081**	386	B	78.5676	1
**5081**	305	C	72.8024	1
**5081**	319	C	38.2782	0
**5081**	320	C	29.6474	-1
**5081**	321	C	79.5752	0
**5081**	351	C	16.1559	1
**5081**	357	C	52.7436	1
**5081**	370	C	56.0586	1
**5081**	372	C	72.7151	0
**5081**	373	C	71.0171	1
**5081**	381	C	35.0565	0
**5081**	382	C	37.3831	0
**5081**	386	C	15.0463	-1
**5081**	305	D	79.5379	1
**5081**	319	D	41.5343	0
**5081**	320	D	41.591	-1
**5081**	321	D	65.3281	0
**5081**	351	D	14.8615	1
**5081**	357	D	56.7332	1
**5081**	370	D	79.3705	1
**5081**	372	D	71.6385	0
**5081**	373	D	79.5806	1
**5081**	381	D	79.4612	1
**5081**	382	D	79.5125	1
**5081**	386	D	74.0152	1
**5082**	305	A	56.0483	1
**5082**	319	A	74.2086	1
**5082**	320	A	79.4539	-1
**5082**	321	A	79.5915	0
**5082**	351	A	4.7132	1
**5082**	357	A	70.3117	1
**5082**	370	A	72.5868	1
**5082**	372	A	79.4332	0

**5082**	373	A	45.8335	0
**5082**	381	A	79.5531	1
**5082**	382	A	64.5177	1
**5082**	386	A	79.5957	1
**5082**	305	B	79.6355	1
**5082**	319	B	75.9051	1
**5082**	320	B	79.5053	-1
**5082**	321	B	68.7975	0
**5082**	351	B	21.7168	-1
**5082**	357	B	67.7986	1
**5082**	370	B	73.0467	1
**5082**	372	B	67.8738	0
**5082**	373	B	42.7263	0
**5082**	381	B	63.5398	1
**5082**	382	B	36.477	0
**5082**	386	B	73.5167	1
**5082**	305	C	79.5725	1
**5082**	319	C	79.51	1
**5082**	320	C	79.1121	-1
**5082**	321	C	66.431	0
**5082**	351	C	16.0776	1
**5082**	357	C	74.1272	1
**5082**	370	C	79.56	1
**5082**	372	C	79.6925	0
**5082**	373	C	76.577	1
**5082**	381	C	66.613	1
[truncated: 374,595 more chars]
